# Supplementary figures and images for: Identification of Avramr1 from Phytophthora infestans using long read and cDNA pathogen‐enrichment sequencing (PenSeq)
Source: Mol Plant Pathol. 2020 Sep 15;21(11):1502–12. doi: 10.1111/mpp.12987 (PMC7548994; doi:10.1111/mpp.12987)

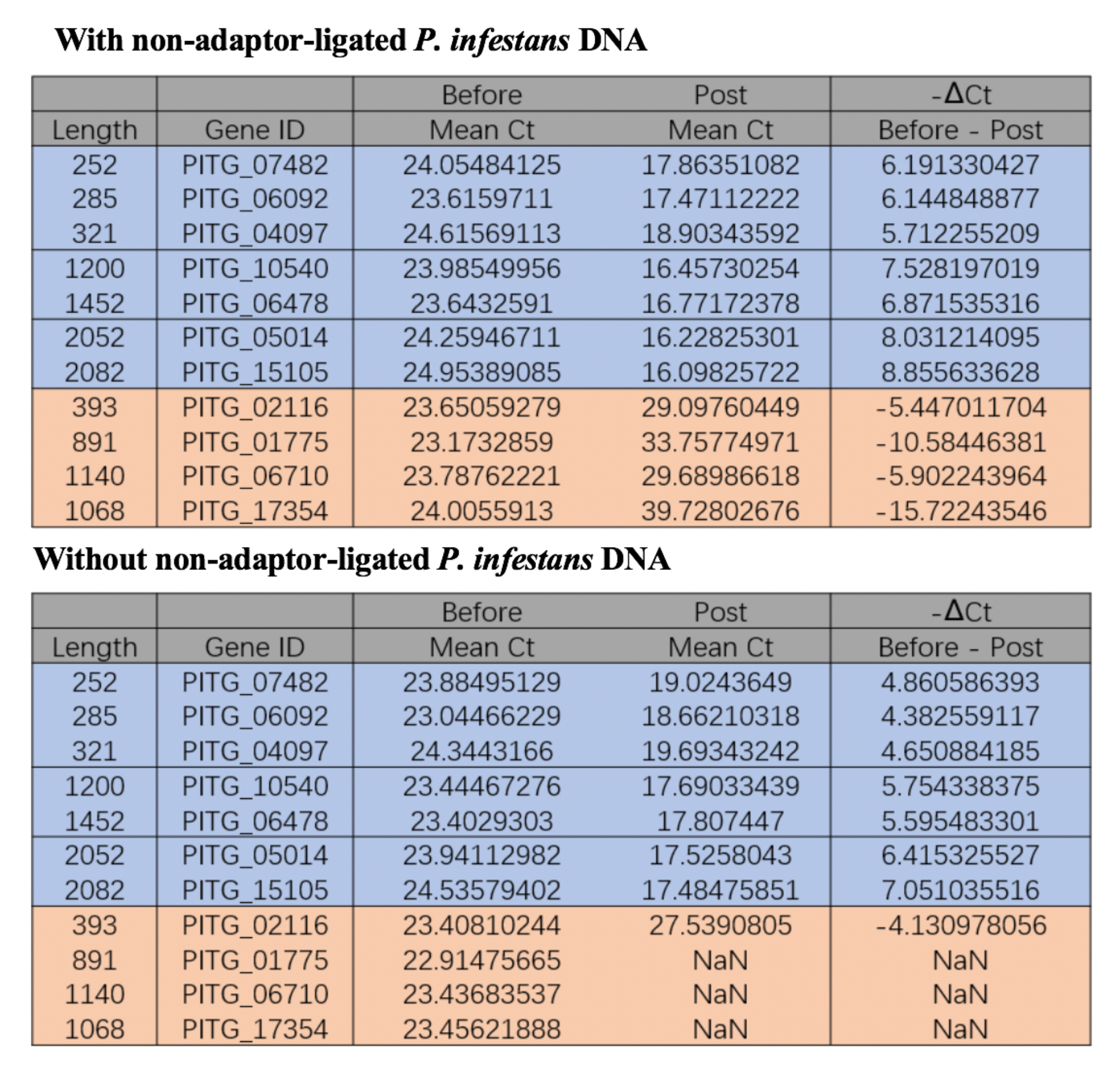

Supplement: Supplementary file 1 — FIGURE S1 Enrichment efficiency with/without non‐adaptor‐ligated DNA [file MPP-21-1502-s001.tif]

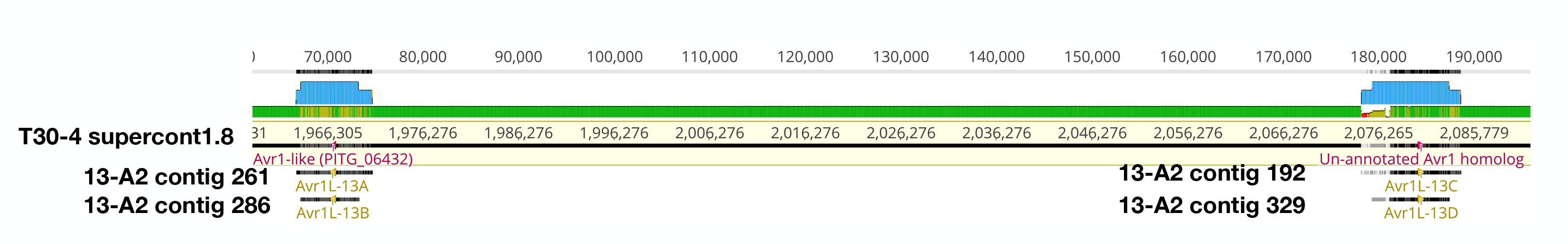

Supplement: Supplementary file 2 — FIGURE S2 Comparison of EU_13_A2 Avr1 contigs and the T30‐4 reference genome [file MPP-21-1502-s002.tif]
