# Supplementary material for: Identification of Avramr1 from Phytophthora infestans using long read and cDNA pathogen‐enrichment sequencing (PenSeq)
Source: Mol Plant Pathol. 2020 Sep 15;21(11):1502–12. doi: 10.1111/mpp.12987 (PMC7548994; doi:10.1111/mpp.12987)
Supplement: Supplementary file 6 — NOTES S1 PacBio PenSeq contigs of EU_13_A2 [file MPP-21-1502-s006.docx]

>Contig_6

AGAGACAAATTCAGCGTAAGCGGGCTTAAGTTTTATATCCCTACAAATGTGCTCTTTAGGACAAATAAAAATTGTGCTTAGCACCAAACGTTTTTCCATGCAAATTTAATACCTTTTCTATCGACTTGGTGGTTAGCATTATTATGTTCAATGGTTGCACTGGACTGTGGATCGGTAATTATGAACGGGCGGGGGTTCACATTTACTTGACAAAGTCAGGTAAATGTGAACCAGCGGTTCAGAATTAATTGACACAGTCAGGTAAATGTGAGCCAGCGGCTCAAAATTAATTGACAAAGTCAGGTAAATGTGAACCAGCGGTTCAGAATTATTTGACAAACTCAGGTAATTATGAGCGTTTCGCCCGTTTTGTGCAGCGAAAAGTGCAGACGTACGTAAAAGAGGTGAAATCCCACGTCCTTTCTTTAAGTGTTTGCTAGTGGTCAGAAAAGTGTATGCCAGCGGTCAGTTCGAGGCATAAGTGTTTGGTCGTGTCAAATTATGTCTACCGTTCACCTAAAAGTGTTTATTTTTGGCCAGAAAAGCGTATGCTCTCGTACCAGACTCCCTGATTTACTAAAAAAATTGAAAAAAAAAATTGATTAGGAGGGTGTGGACGAGAAAAAAAAAACGTACTTACGTAAAACAAAATCGTACCTATGGGGAAAGTGGTGAGTTCATGCTGTTATGTTTCGRCTGAATGATAACAATCGGATGCCTACGACAAAAAGACGGCTACAGTGTTTCTTATCACTAATCAGCTAGCGCTCATTTGATTTTTTGGTCTTAAGATTGAACTTTTGATTAAATGTGGCCCTTTATTTCCGGACAGAGGAAGTCATTAATTTTGCCCGCCCATCCTGCTTATATATTCGGGACGGAGGAAGTACCAATACATTATTGGCCATTTGCAACCCGGGTTGTATTTCTTGAAATCCGAAGCTACGATTGGCTGTCTTCTCGGTATCAGCCAGATCTGAGGTCGATACCGTGCCTAGATGTGGAGTGCCGTTTGCAACTATCGTTGCATGTACGGTAGCGTGAACGGTCGACTTTTGCAACCCTAGTATACAAATTTCAAGTCAAGTTACCTATAGTGAAGCTAAGTCACGGTTGCAAACGAGAGGGTACACAGCTCGTATACAAAAACTAAGTCATGGTTGCAACGTGAAAGATTCAGTTTGAAAATACAATCCACCACAATGAGCACTAGCCGCTACAAGGCAGAGCTCGTAAAGTTCATGTCGTTCAAGGACGACAAGGAGTACACTGCTAGCCACGAATTCACGCCAGCGGACCTCCTCAGTATCACGCCTGGACTGCTGTGCCGCTGGATGAACACGCGGGCCTACGGAGATTCAGAGCCAAGCGAAGACATGAGGCCTGTTCACCTTCGGTCGAGCACGCTGGAGTTCGCCAAGAAAGCCATCTCAGCGTACATGCCTAGGATCAACGCACCATGGGACCCYGTGGCCATGCAAGGCAATCCAACACGCTCCGATGATGTCAACAAGCTCATAAAAAGAGTCAAGCGCTTTGAAGTTCGTCGGGAAGGTGCTGAGTCAAAAGCTCGTCGCTCTTTTGAATTTGATGAATTCATGAACGTGTTGACGCTGGTAAGATCGCTGCATTCACGCTCTGATGAACAACTCATGGTTAGCAGTGTTTTGACACTGCAGTGGCATATTGTTGCTCGCATCGACGACATGATGAAGCTTCAATTTAACAATTTCACTCACAACACTCAGTACCCGTCTACTATTCTATGTCAAATGCGATGGTCGAAAAATATATCCGAAGAGAGAGACGCTCCGGAGCAGATTGTGGTTGGTAGTATGGATCCCAGAATGTGTCCCCTGCTCAATCTTGCAGTATACATCGAGGCAACGGTGAATGTGGCAAGATCTTCTTTCTTATTTGGAAATCCAAACGATAAAGATCGAGTGGTGAGGCGTTTTCTAGCTGATACAATTAAAAAATCGGAATTTAAGTCGTTGAAGACGGGAAAGCTGGGAACGCACAGCTTCCGTAAAGGTGCTGCTACTTATGCGACTCGTAGTGGTGTATCTAAAGACTTTGTCAATCGGCGAGGACGGTGGAGAACTCGCAAAGGCGTCGTCGACGTGTATATCGACAACACTCAGCCTTATCCGGACGCATGCACCGCCGCAGTCCTTGCTGGTCCAGCTGGACCTTGCTTTTACTCGCTGAAAGAGGGCATGCGGTGTGTCACTACACCACTTCTCGTCGACGAGATTGCTCCAACAATTAAACAGGTCATGGGAGAGCCAATAGCAAAAACATTGGCACAGGTGTTACTGTGGGCTGCGCTGGAGACGGATTCCAGCTTTAATTATTGTCTTCTTCCGGAAAAGCTGAAAAAAAGAATTTTACGGGCTTACATTAACGCCGGTGGAAGTACGAATTTGAATCCGATTCAAAGACAAGAATTTTATGTCTTGGGGGACGGGTCTCAGCTCAATCTCGTCGTTATTGACAAAACGCAAGAAGTGGAATCTGGTGTTGGTAGTATTGTTTCTGCAAGAAGTGCATTGGTAGCGGGCTCCAATGGACAGGGGACTCAACGTGAAATTGCTGCTGTACAATCTCAAATAGCGTCTGGCCGTCGCTACATGGCAGAGGTTATGAATGAAGTACTGCGGTCCCGAAGCGAGTCACATCGAGAGATGCAAAAGATACAAGCTATCTTGAGACGGATAGCCATGCAGCCGTTCACACCTCGTACTACTGATGGGCACGGAGTACCGTATCCACCAACAAACACTGCTCAAGGTGGATTTCGTGGACAAAACGCGGCACGTCTTTCAAAGAGACCTAAAGACCTGTATGAACTTTGGCACGAGTATCAGCTCGGATCCGGTGGGCTAAAACCAGCCAAGGAGTTTACATCCATTGAGCGAGGCGCAAACAAGTTCGCCTACTCCAGGCGGAAAGTCTTCTGGGATGTTATTTCTCAATTGGTTCGCTCTGGACACACTAGCGACTCTGCAATAGATAGAGTGTATCAAACGTACGGTAGGAATCTCTCTGTGTCTAGTATTTTAGTTAAACTTCGTACAGATCGTAGACGTGGAGGACATCCAAGTCTGCGATTGTAGCACTTCAACACGGTTCAATTCAGCCAATCAAACCTTATCCTTCGAAATATTAGCCTATCAAAACTATGGTTGCAATCCAAGTTGCATAAATGACTAATATTTATACAGTACTGTACGAAGTAATCACTGAGTACAGCGGGACACTTGCCGTAGTCACAAATCTTGTTTGAACGTGCCAACAGATCTATCCAGCTGTATTTTAATTAATAATGGCCGCACACCGCTCACTCTGATATTTCCCCGTTCCAGCACTCTTATGACATGCAGTGAGCAATTTACTTCGGAGCGAACCCCGGACACACCCTGATGATGCGACTCTACCTTACTGCGCTGTTAAGTGCAATTTCAGCTCTACTAGCGCCGGGTGGAAGTGCCCCAGTGTCTGCTCTTCCAGACTTTCCGGCTGGCTACTTACCATGTAATGAACTACGTACATTAACAAACGCACCTGAAGAATCTCCATCCCACAGGCGATTGAGAATTTCCGATACACATGATGACGAAGACAGAATAAAAAGTATCAGCATTGAAAAACTATCGGGGTTGATTAAGACTGGAGTATCGAGGATACATGGATACCTATATTTAGGACCGTCAGCAACTAGAGAACAACCAGCAGATGAGATTCTTCGAATGTTCAAGCTTGAGGATGGAATAGAGAAGGCCTTGGCTAGTTCTAACTTGAAGACCATGGAAACTTATGTGAAGGAACTGCGCACCAAGAACCGAAAGAGCACGACGTCAGTGCTTGGAATACTCACGAATCACTACGGGGACGACGCAGTAGCCAGTGCACTTGTGACTGCACCGCATAATACCATTATGAAAGACATGGAGGATACGATATGGCGATTACGAAACACACAGCTTTCAGCTTGGCTGAGTAGCGACAAGTCTGTCGACGATGTTTTCAACCTGCTAAAGCTCCGTCAAGATGGCTACCTAGCTCTCGCCAGTCCAAAGTTGGAGGTGCTGGACGACTACATAAAGCTGATTATCCGCTCCAAATCCAGCCAAGAAACGTTGCGTGATGTGTTAACGAGGGGATTTGGAGAGCGGAGATTGGCCAGACTGCTAGTCCGTGCGAAGCAAGATGACCGAACAAAAGAACTGGCGACGGCACTGCAAAATGCGATTTTAAACAAGTGGGTTACAGACAAGTTGCAGCCGGTCAACGTCCTACAACGACTGAGATTGGATAGAGGTGTCACCAAAGCTATGACAGACTTGAACCGAGACACTTTGACGAGGTACATCTCGCTGTTTAAAACACATAATCCAAGCAGTAAAACGTCATTCATTGGTACGCTTTCCGCGCATTATGGAGACGATGCAGTTGCGAAAGCACTCGTGACGGCGTCGTCGGATGCGAGTACAAAAGAAGGCGCGATTCAGCTACGGAGTGAGCAGCTGACTGACTGGCTGAACAACGAGAAGACTGTCGACGAAGTTTTCAAGCTGCTAAAGCTTCGCGATGACGGAGAAGTCGGTCTAATTAGTCATAAGTTGGAGGCTCTAAAAGATTACATCAAGCTATTCAACCGCGAAAGAACAGGAGATGAGACTTTACTCAAGACGTTGACGACTGGATTCGGCGGAGAAAGTGGATTCTCGAACATTTTACTAGCAGCAAAGGCCGATCGACGTACAAACACAGTGGCTATGTCATTGCAAAGCGAGCTACTTCATCAGTGGCTTAAGAGCGGATTGCAGCCGGGAAGCGTCTTGAAGAAGCTCAAATTGGACCGTGGGATAACAGAAGCACTCTCCGATGGAAACATCCACACTTTGACAGCATACATTTCGTTGTATAGCACACAGAATCCAAGTAATGCAGTATCGCTGATTAAGATACTATCCGCGCATTATGGAGACGATGTTGCCAAGGCACTTGCCATGGACGATTTTGCCACGACTGAGCTGGCGTCCAATCTGCTGACACAGCAGTTGCAGCTGTGGCTGAAATCTGTTGGAGACGTTTTCGCGATACTGAATGTTGGACACCTCGATTTCTTGTCCATGAAGAGTCAGAAGTTGCAGATTTTGGACAGCTACTTGAAGATGTATAACGCCAAAAATCCGCTTGACGCCAAGAGTATGTTCGCGGTAGTAAGAAAGGGCTTTGGCGGTGATGCCGGGCTTGCACGTGTGATTGGTAAGGCGCTTGTAACCTCGCAAAATGAGCCGAAGATGGCTCTCAAATACCAGAATGAGCTATTCAACCAGTGGTTCAATAGAAACATTGAGCCCAAGAACGTTTACGTAGAGGTCCTCAAGATCAAGAAGCGCTCTGCAGACTTTACAGCAAAGGGGGTCGCTAAACGATACAAGAACTATTACAAGAAACGGGTGGGGGAGGTTATAACCTTTAACAATCCAAGGCGGTCTTAAGAAAAAAAAAGCGTTATTAGATGTTTCAATCCTAATTCCAACGGAGCGTGCATAATCTGCATTGTTTTACGATCAGCGTGGAAATTAAATTATGTTTGCAAGTTGGGAGGAGTCGACGTCCTGTTAGCCAAAATTGCTAAAAATGGAAGAGACAGACGTTCGTATGACGCGTATCTTCACTACCAAACCTTTTCGGATGAATGTCTTAATCATATCATGTAAAACTGTCTTGACTGAGAAAAAGTGACGATAAAAAGCGCTTCTGTCGCTTGTATCCTATGCAAAATACGGTTAACCGTGAAGAATTGCGGTGCGCCACCAATAACGTGTGTTTTACCGCCGCGCCTTTTTCCTGCTGACCATGCCCAAAACCATCTTGCGCCGCAGCGGCTTATCTGTCGGCAGAAAATTCGATGGTTAAGTGGCAAGATATCGTACTTAAAAAAATCATATTACCGGTAGTCAGAATCACATGAACGTAAGCCACTCTAAAAGCCTACAGCTCATGGTCTCTTCCAGCTATTCGATGATCGGTGGGCTGGGATGAATAGTTGGTATCAGCGCAAAATAAAATGGAGTAGGGTCTTCGCTTGTGACCACTCCGGTCTACAAACGCCTTCAATCGTTGAAAGCCCCGAAACGACGAGGAACTATCTTCTCGTTGTAGTATACTGTGTATCGGCTTACAATCATATCATCCAAATGACTAGCAGAAGCCTCTTCAACATCGAGGAATCTGGAGTAAATGCTTTTTGGTTCTGCCTTGCTGCGGAACCAGCGCTTAAACAGCAACCTTTCGTACTCAGCACCAAAAGAGACGAGGGAGTCGATGTTGTGAACGCCTATCGACTCAATTGCACCAAAAACCGCGCGTGCAAATTTGCCTTCGCCACCAAATCCATCGCTTAGCACCGTGAGAATGTCGGTCTTGTATCCGGGATTAATGACGTTGATCCTCTTAACGTACTGCTCCAAGAGATGGAGCTTCGTACTTGCCACGGATACAAACTGGTTCTTGTTCGGGATGTTAAGGTTTAACTTAAGCAGTCTGAAGATGGTGTCGGGCAGGCCTCTGCGATTTGAATTATCATATTGAATAATATCGCATATTGGGAATGCAATTATGTATTGAGCAGCGAGTAAAATTGCAAGCATTTTTTAGCATTTTATCAGTTATTCTAGGTTAATACTGTCCACCAAAACTACTGGTTGCCACCACCTCAAAAGAGACTTTACGAGCCTTGTGCAATCTCCTCAGCTCATTCGACCTTTTGCAGTCATCCCAACAACTCTTCAGCCCCCTTTCGCCCAAGTACCCCATGAAATTTACAAACAAGGACATCTAAACGCGTATCTGGCTGATCATGAACCCATGTTGGCCAAGATACACGCGCTCATGAAACACCTGAGCACCATCAAGTGTCGTGCTGCCCTTCGCAAGGTGACCTCTCTAGCGCCCGTCATGCCCAACGCGACTCGTTGGTCGAGTACCTACAGCAGTACGACAAGATTTGTAGCGCTCTTCTCGCGTTGTACCACGCTACGGTGGCTAAGCATGACATCGCGCGCTTTCTACTGACGCCAGAGGAAACCGAAGCCGCTCGCTTCCTTCTCAAGTCGCTGCACGAGCTGAATGAGGTGAGTAAGACGCTACAAGACTCAACTCTAACAGTGGTGGGTGCACGACGTGCCTTTGATGCAGTGTTGCGCAAGTACCCCCGCATGAAGACCCGGCTTGCAAGTGACGCCTCCGTCGTGAACAATCCCGAACTGGAGAGCGGCATCGTGAAGATCATTGGCGGTGTCGGCTGAATGCACGCGAACAAGCGGCGTGTATTCATCTCAAGCGCAGCAGCGACGACACTGTGGTAAACCCAGCAGTTTCCACGTCATTTCTCGCGTCGGCCTTCAAGAAGGCCCCTGTGGCGCGCTCCCCATCGCAATACTTTCCTCTCGAGTGGGTTCCACCCACGTCGAACGAGTGCGAGCGTTTCTTCAGTCAAGCCAAGCTGGTTCTGACCGACCTAAGGAAAGCCATGGACCCTAACACTCTTGAACTGCTTATGTTTCTATCCTACAACAAGAAGGATTTAGTGTTAAAGCTATCAGGCAGAGTATGGGTAGTCAACTTCGGGAATAATGCCATTATTATTGTCTTGGCGTAATAGCAGCCGAATATATTGAGTATGGCATATTGGGGAACCCTGGTGTCGGGAGATTTGCCGCTTTTCAACCACGCATCCATTTGCTCTCTTAAGCTCTAAAGCCGTCGCCTTCGTGGCCTCATGAGACCTCGCAGCGCTGAGCGTCTCCGCCACTTCACCGTCCCCATAATTCCTGATAAATGTCTCGATCAATGACGTTTTGGTACGGGGATCCTTGCGTTGTACATCAAGATAAAAAGTGTGATAAGTTGGGTCCAGCAGAGCTTCATCCATGTACTCGTTCAGTTTGAGCATCTTCAGCACATCTACAGGTCGCGACTTACTCGAAAGCCATTTATTCAGTAGCGCGTTCTCCATCGCATTCGCCAGCTCACTCGTCCGGGGATCTTTCTTCGCACGAATAAGCAGATTGGCCAGGTTGTGCTCCCCGTCGAAACCTTTCGTCAAGACTTGAATCAACTTTACTTGTCCTGATTTTTCACGGTTGAACATCTTCATGTAGTAATCCAATGCTTCTAGCTTCGGACTTCCGAGAGCACTGTATCCGTCTTTCTGTAGCTTTAACAGCTTGAATACGTCGTCGACCGATTCATCATTCTTCAGCCAACCCGACAGCTGAGCATTTCGCAGCTGCAGAATTGTTTGAAGCTCACTTTCACTCTTTTTCGTCCTCTACGCGGATACGAGTACCGCCGCCACTGCATCATCTCCATAGTATACCGAAAACACTCCAAGTACCGACATTTTGTTCTTTCGTTTGTATTTGTTGACTGACCTTAGATAAGTTTCCATTCTCTTGACATCAGGACTACTCAGAGCCCTGTCCAAGCCGTTTTCTAGCCTGAATAGCTTGAGGATATCTTCTGCGCTCTGGCTGATTGGTAACCAGTACCCCAAGTTCAAATTTTCACCAATCTTTACTGCTCCCGTCTTGATCAGCCCTGCCAGTTTTCCGATCTCACCGCTTATCATCCTGTCTTCTTTTTCTTCGCGGAATTTCTCAAAAGTCGTCTCGCAGACGAATCTTCTGGCTTGATTGAACGCATTTGAGGTAAGTTCAAACCCTGCGCTTCCGAGACGACAGTCGTAAAACCCCAATTGGCACATACCAGCAAAATTGAAGCACCACACAATAGCGGTTTCAAGCAGAGCCACATGGTTACAGGCTTGAAAATTTGCGACTGGTTAACCCCCAGGAGAGCAAGCACTTTCGACCATTCTACAAATATTTTTGTCTCGTCCATGGAAGTGATACGAGTTCTTTTCTGTAATCAACGCTTGTTGCAGAATGGCGTCAGAAGAACATCTGTTTACGACAACACATCAGATTTTTAAGATCTAAAACCGACCACAGTACAAGTAATTTATTTTAAACGACGAGGCGAAAAGAATCTACATTTTATCGCAGAAACTGAATTTAAAGTACATACATGTCACAGACGTGTGTTTTTACACTTAATTGTGTATCACAGGATTTTTCACATAAAATGCGTGTACCACGTACACTAGCACAGTTTGGGACTTTCGTGAAGATCTTGTGGTCTTGCAGAATGGGTAGGAGAACGCTGGCACGAGAGCTCGTTCACAGTAGGCTGGAGCACCTCGCCGATGTGAAGAAAAACCTAGTAAGGGTGCGAGTGAATCTGGATAAGATGTTGGCTGTTTAACTGGAGATTCA

>Contig_7

GCCGCTGAAGCGTTTCACGCAGTACAGCTTGGTAGTTCGACGCCTCGAGGTGTTGGAGCACATGGTCACGGAAAATACCACGTGTGCTCCTCGCTCCGAGTAGTCGATGACCAATGTAGGAACCACCCCGAGGCTGGCTTCTCCATCGCTCACCCAGCGATTCCTGGGAGGCGCGTGTTCGTGTCCTCGATCGGGATGTTGTTTAATGCGGCAGGCGCTCTCGATCTGGAACGGTTATTCGCGTATGTCCTCTCCCCTCTTGCCGGTGAACTTGGGTAGATTGGTTGGCACTCGCGTGCGGTCTGCCATAACTTGATTAAGCCGGTCGATCTCAACTTGCAGCTGGGCGTTCGTCGCCATGGGCTCCGTTTGTCAGGTCACTGGGACCAATAGGTGCTACCAGTATTTCACAAAGGAACCTGCTTTAAGGGAGGGCGCCAAAAAAGGGAAACTGCCGCCTCCACAGTTTAAGACGGATGGAAGCTTCTAGAAGCTTCCACAGTCGCATGTGTTCAATTTAATAGCATCTTACTATGTCGTATTAGATCACAGTTATTTCTAGACTACATATAATACTAATCACTCACATTATTGATACAGTATCTGTGTCATGTGTGCAGCAGTGGTGTTACCTACGGCTCTCGTCGATACAGAGAGCCCAAAGTATGCACTTATTGACATTCGAAACGCTCTTTTTTGAAACGATCACTGCATTTGATTGTAATTTCTTGGTAGTCAATTTAATTTTTGCGAAGACTAAAAAAGCGGTCGTTATTGTCAGATGTCACAAACTTTCTTCTCTTCTCACAGATTTTGCGTGAATGGCGAAGATGACACTCAGCTCTGGCGAGGTGAGAAGAGCATTTACTGTCACCATTGACACCGTGGAAATCCAGTCCGTAGACAAGCTATGAGAAGAGATCAAGTCGCAAAGCGAAGGCATTTAGACTCCAGCATGTCTCTTGCAGCTGCTCTCGGTAAAGAAACTAGCCGGACAGTGGCTTATTACTGACAATGCAGACTTTGTGACGCTTGATAGGCACGGAGACCCGGTGGGCTTTGACGAGATCCATCCGTCGTTGCTAATAATTGACGACCTGGTTTTTGGAACAAGGTTGCAACCATCCGAAAGCGATATCCACGTGCTGGCTAAGTTTCGCCCAAACGGCCTTGTCTGGGTAATACTCATGCACTTGAGACATTTCCTCAGATTGATACCAAGGCTGGAGATTACGTGGCGTTGCCAAACATTACTCAAGGTTTGTAGATTATCTTCGGGTGAGGACATGATGCTGTATTGTCGTCCTCAAGTGCGTTAGATATGGACGTTTCTGAGTGAGAATGTGGTTAAAAAGGAAAAACAGGATTTATTGCGGGGCCACTGGCAGACAAATCTGCGTCTATTTTAAATTTTGCAGCTTCTTTGGATCGTCAAATTTGTCTGGGTTCGTTGCAGTATTTTGGAAATATCTTTCGTGGATATCGGAACAATGGAACATGCGAAATCTGGGCGTGCCTGGATGGAGTTAACCGTACTAACCAGAGAAAACTGTATCAAAACTCGAGAAGGACGACAGATTTGGTCATTTGCAAGCTCGCTTGGGAAGTTCAATGAAGTGCATGATACCCGTGCCGAAAATGAGTGCTTCTTCTTCTATTCATGGACACAAGACGATTGCCACCAACCAAGAATTCGGTGATAAGGTTGCGTCGAAGTTTGACACAAAAGTTCCTGTTCTGTGAATGACAGCGACAGCGACGAATAATTCAGTGAGGATGAAAAGAAAGATCGCGTTGATTCATGCTCCAGTACACTACCAAGAAGGTGAAAGGATGTTGGAGCGTGTCGACTCATGCTCCAGTACACTACCAAGAAGGTAAAAGGATGTTGGAAACGGGAGCGGAAGCCGCTGTCAAGATGGATATGGTCAAATACTTTGATGGCAATAGTCACATTGGTGTGATCAATCATCTATATGGTATTTCCTGTTAGCTCATACACTGCGTCTCTTTTGCGGAGAAGAAGTCATCAGGGGTTGATGAAGCGTGCCAACTAATTCCTAAGTGGATGGGTTTGTAATTGAATAACTTCAGCTCTCCTGACGATCCAGGTTTAAGGTATGCTTGCTTTGAGAAGCACAACGTTAGGAGAGAGTAGCGACCCCAGTTATGCGTTATTTCGAGCCCACCTTTACGGCAAGGAGTCTTTAGCTACTAAGCTTGTTTTGACACATCTCACTTTCGCGACGTGAATCTCCCATGAGATTATTCGTGTATTTCCAATCTGTAACAGCAGTACGAGAAAAAGATCATGGCTGATTATGGCAAGTGCGTTGCGCCAATGCCATTTTCACACGTCACAGATACTTTTCGCAGTTATGTAACGTACACATTCCTCTCTTACAACCCCCAAGTCTAAATTTCCCCCCGGCGCCCATATTCATCTCATTATCGTATCAATCGCCAAGGACGGAAGAATCTCCACACAAGAACCACCAAGATCCTCCTCCCGACACTTAAAGAGCCACAATGTTGCTAAATAAGCTCATGTTCTGGCTCATGATAACAGAGGCCGTCGTGTGTCTGCTGCTGAGCCTCCCGTTCGGCCAGTGGATCGCGCACGCCGTCATCACCTTCCTAGCGAAGACTCTAAAGGACACCCCAGCCAGTACAGTCGCCACCGTGGTGCTCTCCATAATTTCGCTGCTCTTTATTTGTACGTACACGAGTATCTGTGTTTGCACAGCTGCATCAGCTTCAAAGATGCACTACAGCCCGAGATCTAGCCAACTCTCTCTAACTGTTTTTTTTTTCCTTCTAATTTTTACAGCGGATGTCATGACAGTTTACAAGCACCACTCTTCGGACKAGGTGTTGGGCGATGGCTTGCGTATCCGTCTGCTCACAGCGCAGCGGGACATGTACATCACGGGCTTCTGCCTCTTCCTGTTCCTCCTTCTACGCCTCGTATACATCACTTTAGCCACTAACCTGCGCTTGGAGAAGAACTTGGCAGCTATGAAGAAGCAGGCGGAGGGCGCTGCAGCTGGCTACAAGTCGCTATTAGCGGAGAATGAGTCATTTAAGATACAAACAGAGAAGCTGCACCAGATGTTTGGAGATGAAGAAGGAGAAGAGAAGAAGAAGAAAGTGGACGCTCTCGCCAGATTGGTGCAGGAGAATGCGGATCTGGAGCGCAAGATCGAGACCTTGGACGAGAAACTTAAGAAGGCTGAAGACCAGGTCGCTGCTGTGACTAAACAAGCGGAAGGACAGAGCTCGGCGTTCATGAAACTAATGGACGAGAAGAATGAGTCGGACAAGCAGCTTGAGACGGCCAAGGCGCAGGAAGAAGAGATCAAGCGCCAACGCGACGAGATCGCGAGTCTGAAGGCGGAATGCGACTCCTTGAAGACTCAGATCCAGGACTACGACTTTATGTTCGCCGAAGCTAAGAAGAAAGCTGAATAAGCTGAAGAGAATTAAAGACAGAAAAGCAGCGTGACATAATGAAGGAGAAACAAAAAATTTGTCTGAAACTGTTTTATGATTTTAGTAACACAGAAGAAACTACTACTGTAATACTACTGAACAGTTCGTAGTACTATATTAATATTTCTATTGCCCAAAGCTGATCGAGCTGAAAATTATGCTTCTACATGTACTACATGTATCAGCAGTACACAGTACACTACATGTACAGTACATCAACAATACGTACTTGTAATGTAATCAAAGTACGAAACTGTCATACTTTAAAGTATTAGTACAAGTTTAGTTTCTTAAGAAGCTGATGCAGGTATCAGATCTTATTTTTTAAGCATATGAGTGCTACTCATACATTCTTGTGTATTCCTATGCTATTCCAGCTATCTTATGCATTATTGTGCAAATACTGCAAGCGTTCTTGAATAATCTGCAGTCTCGAGCTTGCCGAACTGGAAGTGGATGATGACACTGACGACGCTGGAGAGTTGGGTGCTAGTGGCCTGGGTGAGTCATCAGTGCTCGTCAGTTTGAAAGAATTTGGGCGAGACTTCCTGGTTTGTAGAGTGACAGCACTCTGGACATGGATCCGCATGGCCCACACTTTCCCAGTACCACCATCACAACTTGATGTGCTTCCGCGGTTTTCAGTATAACGTGTTAACACACGCACATGTAGCGCTCGGTTCTGCTTCAAGATCGGTATCAGGAGCAACAGAAACGCATCTTTCACCTCCGGCGTCATATCTTCCAGCTCCAGTGTACAACTCTCCGGAGGAAGTTCCAGGACACGGTTCGACGACGCTCCTCGAAGTGGCGGGACGCCAGGATACGCTTCCATCCGTGCCAACCAGCGTCTCACTCCCAACTGCTTTGTCCGGTCACTGGTCCAATTCTGTAAAATGAGTGCTGTCTCTCGAGAAGTGGTAGTAAAAGTGGGTGTGGGCATGGACCCATTTCCACTTTGGTCACAACCATCCACGCCACACTCGTACCATTGCGCGAGAGCATCCAGCACGCGTTTACGGAAACCTTGGACCGCTTTTAAAGTCACAACAGCGTCGTCTTGCTGGACCTGAGTCTTCAACACGGCAGCAAAGCGTACCAGCTTTCATAACCAACACCGGCTGGGTCTTGTCGCTAACTGTCTTGTCTTCAAAACGCTCACATAGCGTTGGAAGAAAAGTCGCATGGCTCTTGCACAAGGCTAACACGTCTTGTAATGCACTACTCAGTGCTTCAAGATATGGGAAGAAGTCTTCCAGCTTGGTTATTGCAGAGTGTAATATAACCCACGCCTTGGAAGAGGGCAGCGCGATGATTTCGGCGTCGTTAAGACCATGGAAAGTTCTCAACTCACCGCTTGGATTGCTTTCGTACACCTTGTGGTCTTCAGATGATAATAGTTGAGTCTCTAGACGCTCACAACGCGCTTGATACTGGCTTAAACGCTGCGAATGGTCCCCAGTTTTCTGCTTCATCATCGACTGGAAATGAAGAAGCTCTTCGGTCTTGACTTTCGATGTCTGTCGAGCTTCACGAACGTCTTGTTGAAGCGCCTTCTTCTCCATAGCTAGCACTCGAGCTGTCTTTTCATGTTCAGCAGCAAGTTGGCAAGTCTGCGCGAGTTTTTCGTCGAATAGTGTCACGTTTAGCTGTAACTTGGCCTCCGTATCCGTGAGTTGCGTCTGAACTCGTGCCAGCTCGTTTTCCACGTCACGCAGAGAACTTACGTGCTGTTCGTGGTCTTGTTCTTGAACAAGTAGCTTCTGCGAGGCCTGAGCAAGTTGTTGCTCGTATTGCTTCACTCCATCAGCCAATTTGGTCTCCGCTATCGTGAGTTTGGCCTGCACGTCCGCGAAATTAGTCTCCAAGTCTCGCAGTGTCGTCGCCTGTTGCTCCTGCTGCCTCAATTTGTCCAAAACCGCTTGAAACTTGACGTCTTGCTCTTTCAATTCGATCGCTCGGCTAGTTGTCGCTGCTGTGAGTTGTTTATGAGTCGTTTCCAGCTCATTTTCCAGTTTGGCCACCTTGAACGTGTATGTTTCTTGAATATCTGCTAGTTTCGCTTCATACTCCTTCGTTTCAGCACTTCGTTTGGCCTCAGAAGCCGCATCTTGGGTCTTAAACATCTCACACGTATTCTCCAAGTCTCTCAGCGTCGTTATATATCGTTCTTGTCTCTCTTTTTGTTCTTGAATAGTTTTCTCGTGCACAGTGAGCTCCTCGTCCTTCTCCTGCACTTGTTGTTGCAATATTCTACTCGTCTGGGCATGCGTCGCTAGGTTCTTGTGGAGTGTATGGTTCTCCTCCTCCAACGTCTTCTTGTCCTGCTTCAAGATCTCCACTTGAGTCTCCAAGAACTCATACCTCTCTTTAGCTGCTTGTTGTTCTAATAGCGCATCAGTCGCCCTGCTCTTCTCTGCCTGGAGCTGGTGCTGTGTGTCCTCCAGCGTCGTCTCCAGCTCGCCCTTCTGCAGCGCAAAAGCTGCCTGTTGCGCCAGCATCGTCTGCGTCTGCTGCTCGACGGTGGCGGTCAGACTGTGGAGCTTGTGAAGCAGCGCGAGCTTCTCCACTTCCTCGACTTCTTGTTGATTGTCTTCAACTTCGGGAGTTGCCTCCTTCTTTTGACAATTATTGTGCAATAATTGTATTTCCATTTCCGCCAATCGACTTTCGTAAATTGCAGCGAGACGCCATAATTGCGCATTGGCGTCCTCGTGTTGCATCTGTTGCGTCTCGCGACTCTCGCGAGCTTGTGCAGCGCTGGGAGGCGGCCCGTGGGTCTGCAGGTGGGACAGTGAGGCCTCTAGAGCACGTTTATCGCGGCGGGTCTTCACAAGGTCTGCCCGAAGCAGCTTGAGTGCTGCATATGCGTCGCGAAGATCCTTCTCCATGGTCAGACTGATAATGACGCCAGAGGGCTGGTGAGGTTGAGTTTTACTTGAAGTTTGTATGGTGTGGGATAAAGAAGTTTCCATAATTATAAAAAAGGTATTTGACGGGATAACGTGAAGCACCCGATAATGCTTGTGCGCCCAACAATTTCACTGTGGGTCGCACTACAACGTGCGCAGCCATCTTTGAAGCCAAACACTTGTGTTCTCGCCCAGTGATTGTCGCTGGTTATGCTGGACGCAAGCGCCGTGAGAGCTTCTGAGAAGCTATTATCTATTTTGACAACGGCAGTCTATCTTCCACATTGCAATTTTTATGAATGTCGCGAGCTTGTCGAGGCTGTCGACGAATGATTCCAGATATCGCAATGTGGAAGCTTACACCATGTACGTCGCAGAGCAACATGGTGGAAATGCTTTGCAGGATAAAGTAAAACCATTGTTCCACAACAAAGATCTTGTCGTTGTAATTGCAGCCGCTTCGACAGCTTTTTAGTAATGCATGCAGCTAGATTTACTAGAAGTCGATCGACATACCTGTTCACATTGTCATCTATTCCGTTGTGACCCGGGTCCGTGAGCTGTCAAGATAAACGAGAAACATAAACATCTCCCGAAAGGAAGATGGTCAGCATGGAAATTGAAGCGTGGTGTAAAGGGATCTGGCCAAACTGAAAGTGTATAATAATTCAAGCGCCACAGTCATTCGGTAAACAAGCCTCTGAGCCTGCACAGTTGCAATAATTGTACTTCATTTTTTTCACTTTGATAATATCTAAAGTAATTGCGTTTTTGCAAAGAGCCGTTTTTTTGCGGTGTTTTTCGGGTCAAAACGGGGTTTAATTTTCCTCGGACAACACTCCATTATACGAGCTTTTTGTGACCTTGCCGTGCTTTGGGTTACATGTATCCATTCAATTGATACAGGATCTATCCCGGATTTAGGATGGCTTCACAATAAGCTCACAAAGTCTGTAAGCTTCTTGTGAAGCCATTTAAAATCCGGTGTAGACGCAATGAAAAATGAAACGAAGTTTTTCATCAGAAGAAAAACAATAGTGCGGTTCAGTTTTGGTGCTGTAAAAAGGGTTCCTACCAATGTTAAATATCTTAGAAACATCACACTTGGCTCTCCAGACGGCTGCGCACGCCGAAGTAAGTATCGTAGCAATTGTTGGACTGCAATAGAAAACTTCGCTAATCTTGCGATGGGATATTAATCCCTAACACTTTCGACCAGCTAAAGTCAGGAATCTTTAACCTTTTGCAGCGGCTCAACTTGCAGCGTATGGGTCCTTCTTGGCGAACAATATTGTCACGATAATGCTGTTCCTCCCCAGTACTCAGCGAAATACGCCGTGAAGCTTTCCAAATTGCGGTACCTGGCATCACTCTTTGGGAGTTCCGCGAAGTTGATTGGATACGGTGAGCCAAATATTTTTTCGAGCTGCGATGGGGTTAGCCAATCTGCGTTTGTAAACCTTTTTGCGAGTTTCTTCAAATCTGGGATGTCCTTTAACGACTGAAGAAATATTCCAAATCGCGGCAAGGAAGCAAACTCATGTAAAAAGTCGAACACATGCACGTCTGTGAACGAGTCGCCTCCATTCACAGCTCTGTATGCTTTAATGTATCTGAGCCACTGAATAAACAGGGGATTACCGTCGAGAGCGTACAGAGGTTCATCCTCAAGCCGCAAGATATTGAAAACTACTTCGGGGGTTTCTCGGAATTTTAACCATGCCTCATTCATCAGTCTGTGACTAGACGCCGAGCTCAAGATCAGGTACGCCTGCATATCATCGGCGATATTCTTGATGTCCGGATAATGTCGAAGCGATTGAAACAGAGACACCAGTTCTTCTTCCGAATTCAGTTTCCGCACCAAATCAAATAGCTTGTCGTCACTGAACGAGGCCTTGTCTTGTAACTTAGCTCTGTATCGATTCACGTACAGAAGCCACCAAACAAATTCCTTGTCACCCTCCAGCTTACCGCCAGTTTTCACAGTACGTAAAAGCTTGAAAATTTGCATCGGATTCCTCGTGTTCTTCAGCGCCCACTTGGATGCCAGATTACCAAGCCATGCTATCCCAGAAACCCCTGCTCTCTCTTCGCTATTATCATCAATATCATCGTGCTTAGATGCGTCGCTGAACCTTAGAAACCTTGTTGTGGAATACGATCTCTTCGTTGCAGGGATGGACGTTGACCTGGTGTGGCTCAGGCCCTTGAGCTGGTTGGTGCTTATCAATGAGTTGAGCGAGGAGCAAAGCGAAATCCGTACGATGAATGTGATCAGCACGACATTGAAGAACATTTCTTCTCCTCACTTTTCAATAGTGAAGAGCTACTTTAAATCCCACTCTTATTACTGATTACGTTTTTTAGTTTTAAGATTACGGACTTCGATTACTCACTTGCTAAGAAATGAGCGTCAATAAAAGTGTCGTTGCCGTTCTGTCGATGTGCAATTCAGATGAGAATTTATATTCCACCTCTCCAAAAAGGTGTCCATATTATATTTTAAAAGTCTTGTGATGAGTATTTCCTTAATACTTCCCAAGAGGTAACATTCGTAAACGGAACGATATTACTTTCGAGTGCTTTTTTTTTTAGGAAATCGAGTGCACCGTTAGTTTTTTGTTCGAAAGGTGTGAAACCGAGTGTCCCTTGTTCAACTTGAGGGAATGACCGAGTGCACTGCTGAGGTTAAGGCTTGAGCACGGAAGAAGATGCGTCAAGGCTCTGCCTTCATTAGTTCAGACGTAGCTAATGTAAGCTACATGTTGCAGCAACGATAAGCAGGCGTTCGTCTACAAAGCGATCCACGAGCTAGGGTTAGTGTGCTTTCATCGCCAACTTCGCTCGCCAGCTACGCCAGCCCACAGTGTAAGAGTTCCTTTCGTTCAAGCGCTTCTCCGTCAACACGATAAGAGCAAATTCAACGCTCGCCGTACGTTCACCGTAACACTCTAAGTTGCTTCTACTTCATACTCATCTATCTACCGTGTGCAATTCTCATTGTGTTCAGGGCAATGGTGGAGAGCTCCACGGGAGAGCATAGCCCGAGTCGTCCATCGACCAGAGGACGGTACCAACGCATCTCGGTTCATGAAACGATGCAAAGCGCGCAAGTCGCGATATAGATGGCTTGTCGACTTCGAGGGAGGCAAGCAAGTCTTCCTAGAGTGCCAGGAATTGGTGCAGCTGATCGTAGAATCAAGACGTACTGGCCTCGATGTTACGAAGAGTGCTGCCGCCCACGCAAGCACACACGAATCGCATCCCCGCAGTGTCGACCGTGACGCCGACGACGATCAGTACTTGGACAGCTAAGACGATCAGGAGTGACAGTTTGCTTTGAGCATGTGGGCGTAGGACTGGACTAGCGGAGCTCAGCGTACCTTGTGTAGGAAGTACCAGGATCCAAGGCTCATTCATGTTTTCTTCTCACAAATTTATGTATTTTTCTCCTCTTACATCCTGGTGAGCTCTGCATCCGGCCCCAAGGACAGTGTAGTAAATTCAAACAAGCATAAACCGGACAAGACAAGTATAGAGCCGACGATATCGCCATGTGTTATCTGCACTCGGCATTCCGCCATTTCGTGGCAAAAATGTCTGCACTCGAAAACCCCTCAACGTGCACTCGAATACCATCCGCGCCCTTCGTAAACTGACCTCTTTTAAAATAGAAGACTGAGTGTAGAACTATAAATCGTCTTACGCTGAAAGTGTTTAGGATTGCGCGATACACGTGCCGCAAATCTACGTAGACAATTGGGTCGCCTTGCATTGAGCCTGCATCACATGTTGAGGCTGATAATACAACTTAACTGACGAGTGACGCCCCGCCAGAGCCAGCTTTTCAGCTCATAGTAGGGGTATAAACTTCTTTTAGTAAATGCGTTTAAATTCATATTCTGGATGTAAAAGGCTACACGGCAACTCAATTGTAATTCCTTCACCCAAATTATCTCCTATATGCGTGATTTATCCTATATTTCAGCAGGCTGGTTCTCATGAGCATTCCTCAAAATCTGTCGGACATCGTACCCATCTGATTGGTTCGCGTCGGACATTCTGTCTGACATTTTCTGNAAAAAAAAACTGTTTTTCCCTTAGCTTGTGGACGCCTTCCGGCAGTCGTCCTCCATGGCCTAAATGGCTCAGTGGCGAGCAGTGTCAGCGCGCCGCGCTGTCATCGGCGCACATTACCGCTATGAAGAGCAACTATCAGATTGAGTGTGCTTAGCTATGATATCAAATAGCATACTGATATCTTAGATGTCTCGTACGTGTATATTGAGTTGTCTGTCATAGCTTTTCAATGTGCGAGCACTGTTGTGTCAACTTATAAGAACCATTTGTGAAAATGAATAATGCCAGCAAGACACGGCAAGCATTTGCCAAACTCAACACGGACTGTATGACGTTCTCTAGGTAGCCTATCAAACTTCATGAAAATAGCGATGACTGACGAATGTCCGACGAATTCCGTCTAAGAATCATTAATTTAAGCAAAATGACTGTATCTTTTTAAACGTTGTATTAAATGAAAGGGTGAAATTCCTTGCTAGGCGCTCGAAGCGGCAGACAGTGCTGCGTAGGGATCGACATCCGTGAACAGCGTTTTCAATTTATTCAACAAAGGCGTACCACCTCGGCTTTCAGCGAAGTACATGGTGTAAGCTTCCAAATTGCGGTATTTGGGATCACTTCTCGCGACTCTGGTTGTCTCTAGAAGACCCAAGAGCTCAGATGGGGTAAGCTGGATCTCGATCATCCATTTCCGATAGAGGTGGGTCTGTAAGCTTAGTGCGAGTTTCTCCAAATCTGGGAAGTTCTGCAACGATTGAATTAAAAAATTTAAATTGTGGTACAGTCGAGAAACGCTTAAATAGTGTTTCCAAATCCGTCCGTGTACAGCCCTGTACATCGTAACATATCTGAGCCACTGAATAAAGAGGGGGTTATTCTCTAGCCGACTGATGCTGTTATCGCCAAGTCGCAAGATTTTGAAAACTTCGCCGGGAGTTTCTCGGGACATTAACCATGCCTCATTGATCAGTCTGTGACTAGACGCAGAGCTCAAAATCATGGACGCTTGCATATCGCTAGCCATCTTCGTAATGTCCGGGTATTGTCGAAGTGATTGGAACAGGGACACGAGTTCTTCTTCTGCTCTCGTTTTCATTAGAAGGCTGAGCAGCTCATCCTCACCGAACTTGACTCGTCGTGTCGCCTTGTATCGATTTACGTACTGGAGCCACCGAATGAATTCCTTGTTGCCTTCCAATTTGCCGCCAGTTTTCACAACATGTAAGCGCTGGAATGCATCCATTGGATCCCTCGTATTCTTCAACGCCCACTTGTACGCAAGATCGTCAATCATGGCTATCCCAGAAATTCCAGCTCTTTCGTCCGTATCATGACCGATGTTGTCTTGCTTAGGTGCGTCGTAGGATCTTAAGAACCTTTTTGGGGTCTTAATTGTTGAGATGAAAGGTGGACTGGGGAGAGAATTGGTGCTGGTGTGGCTTCGTTCCTTTAACGTATTGCCACTCCAATTGAGCGAGAGACACTTACAAATTCCAGCTAGGAAAATGATCAGAACGAAGCAGGAGCGCATAGTGGCTTTACAGTTTAAATAAGTGTTGGGCTACAGGAAAGTGAAATTGTGATATTGCTGTGTATCATGGGCCATGACTCAGGGGTCTTTCAAAGTCGTTTTACCGTAAACATAAATGAATCAGGTAGATTTGCCAAACGCCTTTTTTTCTGAATGCAATCAAGTAGAGCTCGGAGAATTTTAATTTTATGTTGAATTGGATGAGCCCTTTGTCTAAGGAAATAGAATCAGGCACTGTATTCACAGAATTTTAACGCTATCAGGATGCCAGATTACGGACCTCGCTTGTAATCATTACGGCATTGATTAGTGCTCAAATGCCAACATCATCAAAAATGTCGTGATGGTGCTGCTGATGTGCAATTCAGATGAGTCTGCATCAAAGCATCTGCTAAGAACCTTTGAGTTTCAAAGTGCAGAGAAATACTCTAGGACGATTTAAATATTTTCACGGATAAAATTCTGAGAATTTAGTCAATAGTTGGCAATTTGAGGAAATTGAATTCTGGTATGGATTTGGCACACGCGCTGAGGGCATTTAGTCAAGGCTCGTGTCTAAGAGAACTTAATTATTTAGAGAACCCTGCCATTGCCAAGCAGTCGTTGGATCGTTTAGTCTACATGCTTGGCTTAAGCCTGGGCCGGACATTGATACTTGTATGTACGCAACAGCCATTTCGCTGGGCGCTATTAATTACTTGCTTAATTCACTTTTGGGCGCCTTGAACACATTAACATTCCGCGTGTACTGGTTTCCGGAGTTGAAACAGGAAAAACACCGCCAAGGCAGGTGGCGAACCTGCCGGAACGGATAGCCTAGTGTGCAATTGCTATTCTGGTTACTGGTAAAATATAGAGTGATAGTCAGGAACAACTTGAGGCTGCCCTTCTTTCTCAAGCACATAGAGGTGCAGTTAGGGGACATACAAGGCCTAAAGTAAATCCACAAGGACAACTGGTTGCTAGCTAAAAGAATCTGTTATATAAATGACGTGAGGAAACGTCACTGACCAAGATGTACCTGGCCCGCCTCGTGTACTCCAGGGTCACTACAGATGGAGGAAGCTTGCTTGATTCACCAATCCACCACACTGTCCCTAAAGCGAAGTTTTCGCCGCTGAAGTACGCCTTCTCTACTAAACACCCAGTCAGCAGTCATATCAAGCACCATGTAGGTACTTCCTGGCCTCCTGTGAGTCTAGTAAACCCGTTCCTCTCCTTCAGTGTCTGCGGGAATGTGCTGCATCCTGCTGGGTTCTGGCTGCGGATTGGTCGGCACCAGCAGTCCGCGAGCACGTACATCGACCGACACGATCTCCATCGGCGGCATCCGTTGGAGATAAGCGCTGCCCTGAAGATAGTCGACTTCTTTGTCGATCCTGTGCTGCTGAGAAGGGGTATCAGTCGTAGACAAATTTGATTGTTGGTTTTCAAACCCAGGTCGGTTTACTAATGTTTGAAGCACTGCATCAGCTGAACCACCTCTGTTAGGCTCTTTAGATTCGACAGCCGAAATACCAGGGGCGACCGGGCGCGTTGAAGGTTGTCCATTGCGGTTGGCTTTGCGACGTCTATTGCATCGTCCAAGCCTTCCAATTGTTGAGGCCTCGGCAGCTTTTGGCTGCTGCAATTCCACACGGGTCACTCGCTGCCAGTGTTCTTGCAAATCTTAGTCAGCGGTGCTAACGTATTGTTGTATCAATGTTGCAACATCACGAGCTAGGTTGGTGAGCGGCTCGTACGAGGCCTCGATTGACTCAAGTCCTTCCACTGCACGAGAATTTCGAAGTCATTAATGCTACTATTCCACCGATTCCTTTGAGCTTATCCACAGCGAGAATAACACCTTGAGACGAAATGTGCTCCAAAAGCTCTTTAGTGACGTTAAAACTGGAATCCGCGTAAAATTTGAGCCTGGACATCCAGCTCTGTACCCGTGATCAGATGTTGAGCGAGGAAAGAGCGTGCATCTGCTCGCACAATACGATAAGGTCCGACTCAAGTTACCAGGCGTTTGCTTCCGCTTTTTCATCAACTCGGGAGAGTAACACATAGTCCTCTTCAGTAAAGTTGACAATATTTCCCCCTCTTTCTCGCTCTTTGTTCAGCAACTGCTGCTTCAAGCGCTGATCCTCTAAATCCTAGTGCATGGTGTGAATACTGTTCCGCAACTTACCGAGATAATCTTCCATTTTGTCACTAACAGGAACCTTCACCAAATCTCCACTGCTGGGCATGTAAAATTCACGAAGAGGAGATGGACACGGCAATCCTGTGAAAAACATCAAAGGAGCGCTGTTCCCAAGTGAAGGTATCGCAGTATGATTGAGGCTTGACTGTACCATGGGCACCAGGTACACTCAATCTTGATGGTTAATCTTGTACTCGAGAATCATCGCTCTGATCACTTGCAGAACGTCTCGATTAACGCGCTCGATAGAGCCGTTTATCCAGGGACAGTAAGCAGGAATAAATTGATGTTGCACCCTAAGTCTTCGAGACAGCTCCTTCACCACTTCATTCTAGAAGTGCGTTCCGTTGTCGCTTACCCAAAGAGATGCTAAGCGCGCGTACCACGCAAGAAGCGCTTCCACAGCAACACTACTGCCGGCTGTGTCTACCACAACCAATCCACAACAATGTGACGCATGATCCTTGAGAGCCAAAAATGTACTCTGAGTCACCGAAACTATTTCCTATGAAGAGAAAATCGAAATGAAGAACACCGTTGCGCTCCGAACACTCGATCGTTTCACTCCAAGTCCTAGTAATAATCCTCCCACTTTCGAATGCAGCCACAGCCGGCACTGCCGAATGAAGGTGGAAACCAAAGAGTTGAGAGAATCGATCAAAAAGATTCGGCGAAGATGCTTGATCATGGCAGATCGACTACGGTGGCCTCGGGCACCACAATGCGCAATAACGAAAAATCTCTAAATCAGCTCAACAGCCTCCGACGGTATAAAAAACGGCTGTCGAGAGTAAAAAAGCCGTCGGCATTCTGCAGCTAGAAATGGGGTGCGTGTTAATTGCTAATACAGACCGATGGAGGATCACACAGCGACTGCGCAGCAGCAGTATCCTCGAAAGTGGGCCAGATAAACCCCTCGTCATCGAGCGGGCGGAGGGTCGAGATGGTCGGCTCCGTTGTCGCCAGAGCCGTCACCTCATCCGTAAGGATATGGCATGGATCGACGAGAATCTATGCTAGAAGGATCGTGTCGGACACCGTCCGCGCTTATAATTCTACCACACCACTTAACTTCACGCTGGTAGAGGTTGGCTTTGTTTGCACTGCGTTACAGGCCGAAATCATTCATTAGCGAGAATAGCTCAGCAAGCTTCTCCAAATACTCATCAATGTCGTCGGCGTAGAGTAGCAGGTCGTCAATCCACACAAGAAGGTGTTTGTATAGAAGACTAGGAAAGCACTACTCCATGGTTTTTTGGAAGTAAATGGCTGGATTCTCACAACCTTGTGGGACTCGACGTGGCGTGAAATGTTTCTTGTCAATCATATAGGACAGCCACACCTGGCA

>Contig_8

CATTTTCAYCGTACGGTTATGCTGTATCATACGAGTTATGTGAGCAATGAAGCATTTGTTTTACATTTTATTGCTCTAATACAGGTTAAAGCACAACATAAAGCAGGATAAGTCCACCCTTATCACTTTACAGACAGTTTAAGCCGCTGAGTCGTGTGGCTGCTCTTTGACGGGTGTCTCTTGCTTCAGACCTAGCAGTTGCATCCACTTGCCCGAGGCCTTCGCTTCCTGCACCGCTGCCAGTCTCTGAGCCAGCACCTTAGATGGGTTCGCCCATTCGCCCCAGAACACAGTCATACCTCCTGAGCCCTTGGATTCCTGTTCGTTCAGCGCCTTGGTAATACGCTGAGCCAGTAACTGCGACGGATATTCGATCAGCTGGTATGTTGTCGTAGCCAGCATACACAGCATTGCAAAGCGCGAGAATAATCTGCAGTAGTAATTCGGCTGCGTGTTCACTGACTCGTTGTATAGCACAAAGCTATGCAGCAGATAGACCGAGAAGCTAATCTTCCCCCAATAACGCAGAAAGCTCCACTCGAGAATCGATGATAGCGGGGACGGCAGCAGCATTTCGCAGACAAATACTGTGGTCACCAGCACGCTAATGAACGGGAAGCCAGGCGTCTTAGGTGCCGTGTTCTCGTGGACCCAGATGAAGAACAGACCACTGAAGGCAATGCTCAGAAACGCCGCTAATGCCGAGTATCCTATTAAACGCAGGATAAGCTTATGGATAAGACGGAACTGGAAGCCACTAGCCTTAATCCATGTATCCAGCTTAACGAAGAGCACTGCAGCCATTGATCCTGCCAGGAACGTCGGGAGATGAGGACGCATTACCGTGTGACTTGTGCGATACTCGGTCCACCCTTGCGTGATGACCCAGTAGTACGCCGGGATAAACGGTACCCACCAGAGCTTACGCAGCATAAGCACACCCAATACAAACACTGGGATAAAGAAGTAGTACGAGATCTCAAGCGGCAGCGTCCAGAAGACGAAGTAACGGTAGTCGAACTCAAAGGTCAGAGTCCTGAACAGGTCGTACGTCTCTGGCTGCTTAATGAGGAAGTAGCGCTTCTTGTCCTCGTCGTCCATGAACCACAATGCGATGCATGTGAGTGCAAAAAGTGGGTACACTCGGCAGAATCGCTTGGAGAAATAGTCGGCCAAGGCGAAGATCCATTTGCGCAGTCCCGCACCTTGATCAAGCATACGGATGCTCTTCTTCATAAAGAGCCACGTGAGGAGAAATGACGACAAGACGAAGAACGAGTCAACCGCCACGGCGCCCAGGTTTAAATCCTGCATGTACTCGTGCGAGTGTTGCGTGACGACTAATAGCGCGGCTAGACCACGTATACCATCCAGGAATAAAACCTTGGTGGGTGGAGCGGCCGCCACTGCCGCCTTCTTGGCCTTCACGGGCTTCTTCTCCTTGTCGTTGTCCTGCTCGTCCTGAGCCAGTAGCGGTTGGTCCTCCGGCGCAGTCTGCACGTTCACAACCGTATCGGCTTCCGTAGTGATAGTGGAGGTGTCATGCTCCGATGGTGCGTGCGTGCGCAGCATTGTCGGCAATGAGGTTCACGAAGAGTTTTCCACCGCTAGACAATTGCCAAGTGGGGCACGTTGCTTTGCTGTTCGGCATGAGGTGGCTTCAGTAAACGGCTATATCGGGATTTGGCGTGTTGTGGCTGGCCCCTTACAAAAACCAAGTGATGTTGTCTTTACCACGGCGTGTGTTTTCGGAAGTATAACCTGTGCAAAGTGATTGCGGTAGTGGAGAGCTTCAACTAAAGCCGCATGTTTGACGTATCTTATGTATACATTTACTTCGGAACTCGATTGTGTATATGTACTACAAAGTGATAGCGGAAATTGCTAAAACCAGACGGTGTTTTCTCGCTGCGTGCGTTAAGATCCAAGCTCGCGGTACTGCATCTGCTGCATCTGCTGCAGAGCTATGGCTGCATCGAGCAAATGCGAGAGCACAATGTTGCATCCTTCATCACTTAAGCTGGACATTTTAATTTGGTTTCAGTATGCGTGCGACCGTTGGACTGAGATCTTTCCAAGCTGGCTGCAAGACGCACGCAACACTTCAGCATTGACCTCTTTCCCTCCCCCGATGCCACCATCAGGATGTCGATTGGAGCCCTAGTACAGTAGTGGTTCACTACGCATGATCGCAATTTAACTCTCACTTGAGGGTTGCCATCTACTGTAGTGCCGTTCACGACTCGTGATTCACGATTGACAATTAGTCGAATCTGGACACGCCATTCGTGATGTGTCAGGATGTCAGGTACCGGCTCAAGGTATGTAGAGGCGTCGACAGCTTTCTCGTTATTGCTGACAAAGATCGACAACTATACTACGTTTACAACACACTTTCACTTATGCTTTCGACTCGCGTGGCTTACTCTTCGCCAACGTTGTCGACCTGCTTCGTATCTTGTGCGCCACGCTTGGATGACAAGTAGTAACCCCACCGGAACCTTTCTTTTCCTGCTCCTCAAGCCCCTTAGTGGTTTGTTGCGACTGCAATTGCAACGGATACTCGACCACATGATCGAATATGGTAGACAGCAGTAGAACCAACGCAAGGCGAGAAAACACCTTATCGTTCGGCTGAGAACCGACCTCGTGTGCAAACAACACGCCGAAAAGCTGACCTTCCCCCAATAACATAGTACATCCCACTCGAAGATCCTTGACACACAGGACGGTACAATCATCTCGCAAAAATCATCGCCAACGGCAATCAAAAAGCTATTTATTGGTGCCGGGTTTTCATGCACCCAATGAAAAAACAAGCTGTGGAAGATTAAACTGAGAAACACTGCGACCGTAGTGTACTCGACGATGCAGATCACGACTACGTGCCCCTTTCGGAACTCAGAGGCGCTAGTTTTAACCCACGAGTCGATTTATATGAAAATGACCGCATACAGTGACTCGGCCAGAAATGTCGGGAAATGTGGACGCAACTCCATGTGGCTCGACCAAAATTGTACCAGCCTTCGTAGATAACCCACCAACACGCGGGGAAGAACGGTAGGAACCAGAAACGACGCAATGCGAGGATACCGATTACAAGCAAAGGGATGAAGAAATAGTACGCGACCTCGAGAGGTAGCGTCCAAAATACAAAGTAGCGGTAGTCGGCTTCAAGCAGCAGTACCTTGTACACAACGAAATCTTCTGGTTTCTGAGCTACATAGTAGAGCTTCTTTGCTTCAGCAGGAAATTACCGATCACAAAACTTGTCAACGCAAAAAGTGGGTGCACTCGACAGAAACGCTTGGAGAAATAGTCGTTCAGAGCGAATACCCATTTGCGGATACTCACCTTGTGTCAGTAAGCGAATGCTCTTTTTCATAATGAGCGAAGTCAATAGGAAGGGCGATACCACGAAGAACGTGTCGACTACGATGGCTCCAAGGTCGATGTCTGCCATGTATTCCTTCGAGTGTGACGTGCATACTATCAATGCTGTGAGGCCACGAACACCATCCAGGAACAGGATTTTGTGGGACGCTGGTCGATCTGGACTTTGCTGTGCTGTTGTGTGCCTCTTCATTCTTGTGCTGCTCGGACGGTGATTCCATGATGGACAAAGCGATGTCCGGAGATGAGAAAGCTTTGCATTGCGCAGCATCGCGTCTGAAGTAGATGCAAAAGTAGCTGAGCGGTAGCCTCTCTTCGAGTCGATAGATTCTCGACCTCGATTCGAGGCTCGAAGGTGTCCGTGGTTGCCATTTGGACCAACATGTCCGCTGACTTGATTCCCCGGCGACGGAAGGATTGGACAAAAAAACTGTTCACTATGGAGGTATCATAGTACTGTTGACTTTGTATACTAGCTTAGATGAGGTATCTATCACCTGGCCCACCTATGGTACTTCTACCATTTTCCTCGTTCACAATGAAGCACTGAGAATAGACGATAAGTATGCAACTAACACGCATCAATAATCTTATTATGGGCACAAAGCTACACCGAAAATTATAGATTTAGAACTCACGCTTGACTGTATTTCAAGGGGCTAAACTTGCAGATGAAGAGATATGGAAATAGTACCATTAATGGTGTGTTCTATCCTAAGAGGAGGACGAAGAGTTATTATTCAACCCATAGCTAACGGATTCTACTGACGCAAGAACACGTCTGTGCGGGTCCAAAATTGGGAAGCACGAGGAATATCTCGATACAGAGGAGGAGTTGAATCTCGCTCTCTTATTTCCTTCACATCGCAATTTACCAGCATCCTGCAAGCTCCCCCCCCTCCCCCCAACTTTCAGAGCTCCACGTGTCTCAAACACCTGAAACATGACGAAATCTACTTCGCGACAGAAGACCAACAACGCCAACACTAGCGCTGGAAAGAACGCGACCACCGCCCCCACAACACCTGCGAACCCGGCCAGCACTAAAATTACCAACAAAAACACGAACGTCACCCAGCATGCCAACAAGAACACGGGCAAAATGTTCGCACTCACTGAGGTGACTACGGCAAGCGTGCAACAAGCTCGCCAGATCCGCAAGACGGACTTCGAGCGCTTCCAGAAATTCCACAACTTCTTCATGGCTAATGAAGAACCGACTGCACGTGTGATAAACGACAAGGCTAACGCCAAGCGTGAGATAGTGGACAAGTGGATTGGTGGCGCTGTGAGCCCGAAGACGCTCGCGTGTATCCAGGAGATCCAGGAGACTCAATCGGGACTTCTGGAACTGCTCATCGTGTCAGCAGCACTCACCAAATACGGCGAGAAGGAGCTGGCTCGTAGAGACAGTCTAGGCGAGATGGACTACGTGGTAGGTGCTCGCTTCGGCACGAATGGGCTGCGCTCGAAGCAGCACGCACCACACAGTGTAGGCTTGAATTTCGTGCTGCGGTTGGTGCTCGGTCTGGCACCGGAGAAGCGTAGCTCGTGTGAAGTCTTACGTGAGATCCTGGGTCACGAGAGCAACGGACGCCTTGCATTAATCCCAACAGAAAGTGGTCTGCAGCCGTATTCTGATGCTACTGCTTCATACTCGGATGACTGGGCCGAGCTTAAGCCAGATCAGCTCCGTCAGATGGCAAGTCAGACGCAGTCCGACAAGTTCCAGGACACCATGCAGCAGCTCAGTGGTGAGCACCTGTACGATGCGATCCGTACACAGCTGAAGCAGACTGGCGGTGCGTCAGTGTGGGATGCTACTAAGGACAAGTCTGCACTCAAGGTGAAGAACATCCGTCCGCGCATGGGGTCGGCTGACAAGAATGCAAAGTCGAAAAAGGAGGCCGATCGTCAGCAGAAGCAGCCTGTGAAGATAAATGAAGCTTCCCAAGTGAAGAAACATCTTCCGGTTGAGGTTGAGAAGGTGACGCTGGTTCCTCCACCGGCCCCGGTGATCATGAAATCGACTACCGATGCAGCTACCAAGAAAGAGGGTGGAAAGAAGAAGGGGAAGAAGGGGAAGAAAGCGGACGCGGAGAAGAATGTCGCCCCTGTTAAGGACGCCACTGCGGTGAAGAACATTGCGACTACCAGCCAGAAAGCTGGTGCCATCAAGAGCAGCGCGCGTGACGGTCGGGCGGTTGTCTCCAAGGCCCTCTAGACGTGCATATTTGGTGATGCACTCATCTTTCATCTCAAGAGATTTATTAATAAACTTTTTTTAAATATAATTTGTTTTCTCGTAACGAGACCTGCACAGTATCGTACCGTATTGTAAAACCCGTGAAATCGAAAGAAAGAATCAGTGCACATTTCTCTCAGGTTTCACGAATACACTTTCATTCTTGAATTTGCATCCAGATGCATCTCTTCTCTCTGACAGCCGTAGCTTTCGTCATCGCCAGTTTATCTGTCGACGCATCAGTCGCGAAAGATCCACGAGGGCACGCTCCCAACAGGACTGAAGTCGATACCGTAAATGCGAGTTCAAGCACGAGGCTTTTGCGAAAAAATAGTACTGTTGATCTAGTCGGCGAGGAGAGAGCACCCAGCGTCGTAGAAAATATCAAGGCGTTGGTCAAGTCTTCAGCGGTGACTCCAGCGAAGCTTCAGCAATGGCTAGACGAGCGACTACCTGCGGGGCTAGTGTTCAAGAACATGAACCTTGACGAACCAAAGATCTTCTCTTTGTTGCATGAACCCAACTTTGTTAAGTGGGTCCAGTACGCCGACGACTTGAGTGCCAAGTCATCTCATAAAGAATCGTCAGTGATCTCCACCCTGACGTCATTGCACGGCGACAAAGTTGTCTACGACACAATTCAAGCTGCTAAAAAGTATCCACAGCTGAGTGAACTCGCCCTTAAATTGGAAAAGGACCAGATACGCTTCTGGATTGCCACTCGAAAAGACCCGTCGGTGTTTTTTGAAGCCCTCAACCTTAACTGGGTAGGGACATCCATCTTCTCAAAACCTGAATTCTCCGCTTGGCTCAAGTACGTGGACGATGTAAACGCAAGACATCCCAAGAAAACCCCATTTTCGATTATTCCTACGCTCAAGCAACATGTAGCTCAAAGTGACGAAGCCGACACAGACGTACTTCTTAAACTGATTGCGAACGGGAAAGCAACGGCTGAAACCAAAACTGTCGCCAACAAGGTAGATAGTGCATTGTTTGACTTCTGGCTCAGCAAGCGAGAAACACCCGACAAAGTTATGGACGCGTTCAAACATGGCAGTACAGCTCAAGCTTTCTTGGGGAGTTCACGGTGGAAAGAGTGGGAACGGTACTTGAGCGTTTACAACGCGAGATACCCTGAAAAGAAGACCACCGTGATAGAAACGTTAACGCGGAAGTACGGAGATGCACAATTACTCGACACGCTTATCACCGCGAGCTCGAAAGGTGAGACGAAAACGCTTGCAGCCAAGCTGCAGGCACAGCAGTTCGATAGGTGGATGAGCCTTAAAGAGTCTCCTCTCGACGTCTACAACAGGCTACGGCCTTCATATGGGGATAGGAGCTTCTTCGACGAGCCACAACTCAATGTGTGGGTCTCGTACATGAATGTGTTCGTCGACAAGAACCCCAGCAAGGTGGACAAAATGTTCTTGGAGTTAGGTGATACCTTTGGGAACATGCATCTCTTTCGAGTCCTCGGAGAAGCCAAAAAGTTCCCCAACATGGAAAGCACTACAGCCAAGCTGCAGATGGAGAAGGCTTCGACTCTTTTTGCCAGCGGAAAATCCCCGGAGGATATATTCAGGGTGCTAGCGCTTGACAAGGTCGGAAATGATATACTCAGCAACACGCTGTTCCACAAGTGGCTGGCATATCTGCAGAAATTCAACAAAGAGCACGAACAGCCAAGGATCGTGGTTTGACATGCTACGTATTATTTACCAAATGTTCGGTCTCGAAAGGATTATCGAGACGGGAAGAAAAAATCCAATCACAAGAGGGATGGCTGAAAAAGTGGAGGATGCGTATCACAACTACTGGTTGGATATTAAGATGGAGCCTAAGACAGCCTTCCGCTCCCTGCATCTCGACGAAAGCGGTGAGAAGCTCCTTGCCGACCCAAAATTCAACACGTGGGTGCAGTACCTGAAAACCTTCATCGACCGATATCCTAATGAGAAGACGACAGTCATCGACGGGCTCAGGGATAACTATCATGACATAGCTCTACTCCGAATGTTTTCAGCCGCGAAGAATGATCCCAGCACGGAGAAACTCGCTACTGATCTACAGAGTGCGCTAATCCTCAAGTGGCAAGATGCGAAGAAGACACCAGAAGAACTAAAGAGAGTGTTTGTTGGTGTGCCAACCTCTGGTGAAATAATCGATCGGTACGACAAGCTAATATCGGCGACGAGAGCCACCTTATAGTGTATTTGACCGAAGCGGTTATCGTGGAGAGCGAGAAGGAATCTAATGTAGCCCTTGATTTTGACTTTTTGGTAAGCTTTGAGTCGCATGAGCAGAACTGGTTCCTTCTGACTCGACGATACTACAGGAGGTGGCAGTGGTTACCGATCTCCATCTTCGATCGACGTGCCCTCCAGCCACTCGAAATATACAAGCAGAAGCGGCTATCAATTGTGATTTTCTCTGGGTGGAACCTCGTAGAAGTGCGTCCTTTAATTCTTGGGGGGGGCATAAATGCCAAGTTGTACCTGTGTTTTCATAATGGACCTTGCATATACAGTCTCCGTGTACGATTACGCGCCGTAAGCTACCAACTTCACGTAATATTTTACACTACGGCTGTGGGGGCATTCTTGATCGTCGAGCAACTCACGATAACAATTCCACGAAAGCTGGCAATTAATATCCTATGTATCATCCTTGTATGAACAATAACAGTTAGCTTTCAGAGCCATCAAACGTCAAATACTGTACGCAGGGCTATTCTGTGATGACATAAAGCTAAATCTTAATTTGAATAAATTTACATTGTGAAGTCCTCGTTAATATTCGTTTTGAGCTGCTGCCATCATATGCCGCGTATATTACCGATATAATCGAGTTACGTCTGTTGGTGCTGTAGTCGAGGCCATATTCGTACTGTGCCGCCAGTCGTGTGACTAGCAGCAGGGCTGCTCAAAATTAATCAATAGACCAAACATTACTATTTTGAAACTAATAGCACTCATAATTGTGGGTTTGCTACAATAAAAACTTTCCCTAATTGTTTTGGATTGAATTGGGATTTGAAGGGCGCTTAAAGTCGTCGTTCGTACAATCAAAAAGTGAATGTTGCTTTATTTTTTTAAATGTCAATCGTATTATACTTCATTTGCTGCTCTCTAACGAAAAAAGACATCATGTGCATATAAATTACATTCGCAATGGACTGACAGCGTTTATCTGATATTTGGCGAGCTATGAACGTGCGCAACTACAAGTAGCTGTACTAGATTACATTTCTTACACGTAAAAAAGTACCACCTTCTGCCATTAGCTGTGTAGCTTATTTTGTATTTGCCGACGTTTATAGACGCTAAAAATGGCAACCCGTTCTATAAATCGATGTACCAGCATGCGGTGCCTGATAAATTCACTCCTACTTGATTGTACAAAAGACTATACGAGCGAGGTTGGTGAAATTCCCACTCGATGATAAATTTAATTGTAGATATGCTTGATTTTCTGCTATTTGTTTAGACCTCTTGGTAAAAAAAATAAACGTGGTACATGTATTAATAATGTCACGGCCCATGATAGCCAATAGGTCGCTCATACATGTACCAAGAAAACGTACAGACTCTTAAGAGTCAGGAAGTGTTTCTGTGCATTTTTTGTAGATATGTCTGGTCAGGGCTAGGTTTAAAGAGGCAAGGATTTGGGAAGGTGAATTCGATCTTTTAAACTTAAGTTTTAATCCTGCATTTTCAGGCTAGAGGCAGGTGTAATTAGGCGCAAGTCGAAGCTAGAGTAGACCTACCATGCGTTTGTACGTATAGCTGGAATCACAAGACTCTTGGCGTTTATCGGGATTATAACTACCGTGACACCCATCCCATCGGGGACGGGAAGCAGTACGGTGTTTTTTTTTCACTCATTGACGTGACCTGGTCCTGGCCTGGCCCCGGACGATGATGTATATGAGCAAAAAAGAAGAAACAATTTATCCCAATCAACCGTAATCAATCATATGTTCAGTACGGGTACCCGAAGAAGTCCAGAATTAAGTTAAAAGATTTTTAAATTAAAACAAGCACTTTTCAATCTGTCTTGATATCAAAGTTAAACGATTAATAAAACAAATGCTCTTATTGTAAAAAAGCTCAGTCAAATCGCTACTAGCAGAGTAGACAGCATCGTTGTCGTGCTTGGGCTGCCACATATTAACGTCTTTTCGTATATTCCGTTCTGTTTGTCCTTGGCGCTTTGAAGCCCCTCCTGTGCCTGCTTTGCAGCCGCAGCATCATCCTACTGCTTACATGGAAAAGTATACCCCTCATAGTATGAGGGGTATACTTTTTTGCGAGCAAGAGCTACATCCATCAAACATAACACCGAAACACTCGCCCATCTCCTCGGCGATGGTGTTGCCGACACGTTCAGCGACTCGTTGCATGTAGGTCTTCAGCGTCTCCGCTGAGGTAGGCTTCATGCGAACTAACTGTCTCGTTAGTGGGTTCTCAACCTCGCACAGAGGGAGATTCCGCTCGACAATCCAGCGTAGCCAGTCGTACATGTGAGTCGTCGTTTCGTCCACGAATCCAAAAACCTCGAGGGAGGTCAGGTTTCGGCGCTGAAACTCAGCATACTCTTCGCCGTGAGTGGGATGCTTCGAGTTCAGGTGAGAGATCAGGTTTGTGTAGCCTGGGCGCTGGGCTTGCTTGCGTGGCTGTTCGCAGATGTTGCACCGGTAGAGCCCAGGTTCGAGAACGGTGTAGAAGAAAGCGGCGAGTTACTGGGACGACATGATGTAAATTAAGAATGACCGTCTTCTCAAAGATTGAAAGTAAACTAGGCGCTGAAAAATGGCTGGTGGAGAGGAATGGGTGGCTGCAAGCCACTCGAACGGTAAGCCTTTATATTTGTATTATTCCGTCCGTATCACACTATATTATTATCCCGAAAAAAACAATAATCCGTAGTATAATAAAAATAGGTAGTAATTGCGGTACTGATATTATTTTCTTGGAGGCCTGCTCATACATGTCTAATTAGAGATAAGGGCGAGAAAGGTCTCTACAGACGTGGTAAACACAAACCTCTCTAGTTGGTTTGACAGCGTTGCGTTAACTGCTACGAACCGTCG

>Contig_9

CTACTTTCTTACGAGCACGCCATCCTCGACCCATGGTCTGGATCCTCGTTGCAGCAACGTCGCGCATTCGATCTAGACGCCGTCGAATGAACGCAGCACGTCCGAGACGACCTCGGTATCTGCGTTGAATGATCACCGCTGCCTTGGTTAGATGCTGGGTGCTTCGAAGCATTGCAAGTTCACGACGAGCCTTGCGTCCTCGATACATGGCCTGAAGACGACTAGCAACGACGTTTTTCTTCCACTGATCGCGCATCTGTTGGACTCGGTTTCGGTCGATATACCCTCGTGCCAGAGCTTGGATACGCGTTGCCATGGCTTGACGATGTTTGTCGATTCGCTTGAGTTTCAAGAACTTTCGTGCGATACCTTTACGGAACATCCGCTGCATCTCAATCGCCGCACTTGTCTGCTTTGCAATGATCTTCTGTCTCCTCACAAGAGAAGCAAACATACGACCACGATAGACGCGGTACGAGCGTTGAATGACTTGAATGGCTTGCAACTCAAGTGCCGAGCGCTCCAGAGCCTTCTCGTGAGCTACATCGGACCACCACTTCGTCCACGCTTGACGCATTTTGCGTTGCGAATTATTCACTATGAACGCATCGAGTTTCTTACTCCCTTTGTACAGTAAATACGCTTGAAGCTTTTCCTGCTGCTTCTCACACGCGACAGCCTGTTTCCAGCGCTCCAAGGCCAGACCTTGGAGGTTGTACAGTAATCGACGGAATATATTCTCCACTTTGTCTCGCCCCCAACGTTTAAAGTAGAGCGTCGTACGTAGCTCCGATGCAGGACAGATCTTCTGTGCCCAGCAAATGTCGTCTTTCACTAACCGCGTGAGATGCTGCACGTTGTGTAGCGCAGACTCCAATTCTTTGCGTAATGCACCATTATCTGCTGCATCACGTGTTGCTGTGTTTTGCGTTGAGCGACCAACACGCTTTCGTTTGTTTACACGCAGCTGTTGACGTTTTTTATTGCTTCGTGCTAGACCAGGGAGTCTCCGTTCTTCAAGAGATGATTGAGTCGGCTGGCGAGGTACGGCACTGCCGCTTTGTCCGTTGAGCTCGGCTTCGAGGGCTTCGATTTCCTCCAGTGACATTCCCAGCGTCTCACTTGCTTGTTTCCGATCGACGTCTTGTTTGATTACTTGTATCTCTTTAGCTGCCATTGCACTCGCTGTACTTGCCACATTTACGGAAGGTAGTCGGAAAACTGGAAGAACGTATTGTTGCCGTTGTCCTGGTACCATTTGAGCATCCAATCCAACTTCAAATGGCGTTTCTCCTGTCGATGTCAACACTGGCAGCATATCCAGCGCTGTTCGAGCGTTTGGAGATATCGATGACGTTGGACCTGGTGCTGCATGAGCCTTTTTGTGTCGCAAAAACGCCTTGGATCGTTGATTTGGAGAGAGTTTCAGCGTCTTGGAATGCATAGCTTGTTGCAACTCCATTCGTTGCTGAAATTTCGAGCTTGTTGTGCCCATTGCTAGCTGAAAGCGCTGTTCGAGTTCTTCGACACTGGGGATAACTCCGTCGCGAGCATTGTTCAAAGCGGTCGATACTGCATCCGGGTGTTGTCGCTGTAGACAAGCCACTTTAAGCCTCCACTCGGCTTGTAACTTGGAGCGTGTACGCTCTAACTCTTCCAGAAGCCGTAATTCATGGTTGGTAAGACGATGCAATGCATCCGTATGTGGTGTCAATACATCAGTCTGGCTGTCTCCATCTAGGGTGGACCCATGTTTCGTAGCTGGAGGACAGGTAACCTTAGTTACTGCTGCGCTGGTTCTCTTCCCACCCGAGAGATTCCCACCAAACAACAGCATCACCACTGTGAAGGCAACTTTAAAAATCCTGTTTGGCAACTGTGGCAACTCATTCACATTCTAAATATAGGGTCGTAAATGGGCACTTTCACCGTAACAGCTACTTGAAGTAGCACCATTTTGAAACAATAGATCGGCAATACGTCACCAAAACTCGTCAAATTGACCACTTAAAAGAATTTCCTCTGCTCCACTCTGCCCCTCTGTCCCTACTTTCATAATTACGAGCTGACTGTGAGCACGTGCCTATATAAAGCCAAGTTTTGGCACGTTGCGCACTTGTCGCTATGCTCATCCAGTCAGAACTTGAAGTGAATGAGCATGAGTGTCGCCGCCATGCTGAGGAACACATGCGAGAGCGCGTTGTGGTTGAATTTGTCCTTGCTGGGAGCCCATACGAGGCCCTGGATAAGGCCACCCAGAAGCTTAACGCCGTACACGAGCAGTTCGTTACTCGTGTGGCTCCACTCCCAGTTCCACGTCATGAAGCAAGCGATGGGGCCAAAGAGCATCACGGGGATGAAGCCGGCGAAGAGGAACACCGCGTGCATGCTGACACTGGCCCAGACAGCCAACACCACGTACAACACGGCAGCCAGTACAATGATAGAGTAAGCCCAGGCCTCTGGCAGGTGTCGACTCGCGGCCAGAGACAGCAGCGCAAAGTTGGTGAGGGTGGAACAGGCCAGCACAACGCGCCACAGAGCTTCGATCATCTTGTCCACATTAGCACGACTCAGGTTGACACCGAAGACACGCGCTGTCATCACCAGATGCTTCTCTTGCTTGTGCGCGATGTCGCGCATGGCCTCGAACGCAACATGGTGGAGTAGGCCACCAAGCGCCGCCGAGATGCCTAGCGCCATGAAGTACATGATATACCAGGCCAGATCGCCCTTATCTGTGGCAGCGAAAAGTCCAATGTACACGGCGAAGGCGAAGCACTGCACGGCCAGCAGGTAGTCAGTGATGAAGCAAGCTGGCTGCGCATCCCACTCCTCGCCCGTCGCGTCCCGATAGGCCCAGAGCGACCAATTCTCGAGCTCTGCCACGGCCTGGCGACACGCCTGCGCTACGGTGGACGCGGCGGTGGCAGTAGATGCTGAGCACTTGCGCTGCGTGGCCGATGTCTGCTGCGTCAACACGTCCGAGCTCATAAAGACTGGTACCGACTGGTATTTGATAACTGGCGGATTGCGAACTGAACAATGCACCGCCGCCCGTTTCGAATGTCTCTATAAGTGGCGGTGCCTGTCTCGCATTGGCTAAAACCTTTTTAATAGAAGCTTGCTATTGGTCAGAATCTTTTCATTATCTTGAGATACTTCTATTAATAGCACCCACGTGCAATTAACTTCCGTGCATATTCTTAAGCGGATAATAAGATCAGGGCAAACTGCCCACTGGGCAGTGCTCAGTGCAATGCGGCATTCATGCTGCGGTTTGTCGCTGGTGACACGTGCTTGCAGTGATGTAGGAGCGTTTTTGGTGTACCGGACTCGCGGTAACGGTGCAAGGTTGAAGCCAAAACGAATCTCAAGATCCGACAGATTTGCTCATGGGTCCTGATTGCACCGTGGATGGTCAGCAGCGCTCATCAATGTAATCGTCGCTCAATGAGCTTTTGTTTGCTTGTGAGCAACAGCGATAAATATCGTACACGGGCTTGCTGTGTTCTTGCAGAGTACGGTGTACTGTTCTCGCGTGGTGCTCCTCGAAAAATGCGCTTCTTTCTGCTTATACATCCGACTGTGCTTACCGTATTCAGACGAGAAAAATGAAGGCACCGCACAGTCTGCAAAAGCTATACAATAATTTACTGCGGGCTTCACCTCGCTTACAGTTATCAGCTACGGCCGAATCTGTTGGTAGCTAACAAACTTGCGGCGTCGCTGCAATCGAGCGAGTTGCACACAGCAAACGAATGCCACCACGGACAGTGCGATGGCCACGACGAAAACGGGTGCAAAACATTGTGTACCGAAACACTTCTCGACACCATCAACAGTACGTGTTGCTGTTGGGTGCTGGTACACGTACGACGCCAGCGGGCTGAAGAAGAGCGGGTACCCGACACAATTAGCGATACTAATGAGGCCAAAGTTTTTGCCAAAGTGCTGCAAACCAAACTCCTCGCGAACGACTACCGGAAAGGTGCCGAACATGACGCCCTCTGAGAACCCAGCCATTGCCACAGGCAGTACGAGCCACGTCGGTGACACGGACAGGAAAACCACCTGCGTGACAGCCGTGAGAACCACCGAGAGTGCAGCAAAATATGCACGTGGATACTTCGCCAACAGATGATCCGAGACTGCTCCCGTTACAAGGCGACCGAGTGTATTCACGATGGAGAACAGTGCCACCATGAAGGGGACTTGATGTAGAGGACCACCCAAAGCTTCCACGATGAACGACACGTTGGACATGACGAGCAAGCCGGCGCCGATGACGATCATCACTGGAATGAAGAGCATCCAGAACCGCATATCTGTTAGCAACGTCACGCCCGTGATGTCGTCAGGTGTCTGGCTGTCTGCAGACCCATTCACGTCTCCACTCGATTCCATGCTAGTGGCCCCAAGACCAACCTCAATGCTTCCAGATCTCCTTTTCGTGTCCTTTTCTTCTTCATGCCGTGGACGGGCAAATACAAACCAACCAAACACGCAAGCAGCTAGTAGGTAGTTTCCGACAAATAGGAAGTAGCCCGGTACGTCGGTGCTGTGGAACCCCTCATGGAAGACGAACGCAAAGAAGGCACCGCCACAGCTGTATGACGACGTAAGCGTTGCTATGACCTTGCCACGATTGGCATCTCCGAACAAAGATTCATTGGGAACGATAGACGAGAAGACGCAAAAGGAGCTGAGGAGTCCGAACAACATCATGGCACAGCCGATACCCAGTGGACTAACGCATTCCGGTGCGAATTGCAGCGCAGTAAACATAGCGAGATGGACAACCAGCATGGCGCTCCCACTTAAAAGAACGCTCGTGTACGGCCCCAGTCGGTCGAAGATGACGCCAGGAATGTACGACATGTACGTGCCCAGCATGGTCATAGCGGAGACCGTGGAGATCCCAGCTTGTGTGAAGTGCAGAAGCGTCTTCAGCTGTGCATTCCACGCTGATAGCGCGTAGGTCGAGCCCACCCCGAGCATGAGCAACACGCCGGCAAGCAGGCTGCACGACCGGCGGAGCTGCAACGGCGACAACGCCATCAAGACCCGGTAATGATAAATGGTAGGATTGGTACACAGGTACCTCTGCCGCAGAATGAACATTGCCGAGCCAGCTTAAGTTGCAGTATCTTTTGCTGTATTTTACGATAAGCGAACATAATGCTGACGCGATGCAAATTTAAGACTCGCTACCAGCGACACCACACTCAATACCACCACTAGTAGGAACACAGGCTGGAAACACTCATCTCCAAGACACTTCTGGACGCCATTCACCGGCATGGCCGATAAACTGTACACGAATGAACCTATGGGGTTGTAAAACAACGGAAAGCCTACAGCATTTGCGACGCTGATGAGGCCAAAGTTCTTTCCGAAGTGGCGCGCACCGAACGTCTCGCGCGTTAGCACGGGAAAAGCGGCGAACATGACGCCGTCAGCGATGCCTGAGAGCGTAATGGGAACGACGAGATACGCCCTCGGAATAACCAGGAAAAGTGTGTGGATGGCACCCACAAGGACCGACGCTAGACTTACAAAGTAGATCCGAGGAAAACGATGCAACACACTGTCGGAAACGACACCAGCAACAACACGACCGCAGCAGTTGCCTACAGAGAAGAGCGCTACCATTGTGGAGACCTGCTCCATCGGACCGCCAAGAGACTCGACAATAAACGCAATGTTTGCCATGACAAACAGTGAAGATCCCACCAATATGAATACGGTAGAAAACAACAACCAGAAGCGCGAGTCGGTTAGAATATCGCGACCGGTGATGTCAGGTTGAGCCACTGCGAAGAGCTGGCAATGTTCTTCAACGTCAACACTACATGAAGACTTCAAAGCCGGTGTCTCTGGCGGCATGAATTCGTCCAATGCGTGAATGGGCGGAGCAGCCGCAATAGTGTGAGTTGCATCGTCTTTGGCAGGTCGATATAGCGTAACCCATGCTAGCAAGAACACTGTCAACATCAAGCACCCCATGAAGCGAAAGTACAGAGGTACATTTCCATCGAAAAAGTGCTTGTAAAGCTCAGCGAACAGCGCACCACCGGCACTGAAGGCTGCGAATTCCAACGCCATTACCTTGCCGCGATTTTCAGGTCCAAAGAGATCTTCGTTCGGCCCCAATGCCGCAAACACGCCGAAGTTGGTGGCTTGACCGAGCACTGCGAAGGCGATACCCACGCCCAGCGGCGTCATGGCCGCCGGAAATGCCGTCATTCCAGCGTAGAGCCCAATGTAGATACCAGCAAGCAAACATCCGCCCAATAAAACGCTGCGATGGGCGCCGATGTGGTCGAAAACGAACCCGGGACTCACCGAGAAGTAGGCACCAAAAGAGGCCATAGAGCAAACGAAAGAGATACCCGCCTCGGAGAAACCCGGCAACGCCTGCAGCTGTGTATTCCAGACCGAGATTGAATGATTCGAGCCCACGCTCCCCATGAGCAGCAGACCGGCTAGTACGCTAGCAAGGCGGCGGCATTGGTCCCAGCTGCATGACGCCGGCGTCATGACAAGAAATGAGTGAACAAAACATGATGCATGCTATCTGATTGGTTGAAAACAAGAAGTTTGTTCCTTAGCACCAATTTTCAACAAGTATAAATACCGGTTTTGAAATTTTAATGATTAAATAAAAAGTAGTACAGTAGTGTATCGAAATTGCGACGAAGGTCGTGTTACTGTGCATACCTATTGTAGCTGTGTATACAGCTGGGACTGTACAGTTGCAGATGGAAGTATACGCCCGTTAGTGTATAGTGCTAGCAAAGGGGGTTGTCACCTTCGAGGTTTCCTCCTTAATCCCAAAGTGCTCCGTGGGAAGACGTTCTCTAGTTGCTTTCCGTCATCTAAGCTGCTCCTTGCCCTACTGATTGCCTGCGCAACGTGCCAGGTTATGAGCCCAAGGCTTTGAAGCTTTACAGAGTACAATCTTTGAGTTTCGTGGGTATACAATGGCTACGTCAAGTAGGGCGGCGTCAGCTGCGGGCAGTTCGGCTGCCATGACGGCTAGTGGAGACCTTGTTACATCTTCTGAATACGAATGGGTATACTGGCGATGGATTTACGTTTCTAGGGTCAACTGGAAATGGGGGAGTGGTGAATAGTGGGGTAAACGCTGCTAACGGTGCACGTGCGGTGGCTTCGACTCCGTCTGCGGGGATTCCACCGATGTTCGCAACCTCAGCGTGGCCAACGGTCCCATCGATGAGATCGACAGGATTTGCGTCGGCAATGTCTATTGAAGGTGAGGCGAGGAAGGTGGAGGAGCTTGAGTAGAAGGAAGAAAAAAGAGATGTGATTGAATTTTATGAGTCTGTCTTCAGAGAGTTTCCTGGCACCATTGATTTATCTTTCCTGTAAAAGGTGATGTATCGACTTATGCAGGATTGGGTCCAGAACATATTTTGTATGGTAAGACATTCTGTACAATAACTTGTACGCTATGTCACCAATTAGGACACATCATTGACAGCCTCTCTGTGTCCCAAGTTGGGGGCAGTTCTTATCCCAGATGGTGCAGCTGATTTGCATTCAAGACGTTGAGGACTTTGACTTATATTACTACTTCGAGGATAATCGACTGTTTCTTGCGGTAGTGAGTTGCTAGAAAGTATGTCTATACCTTTCCGAAGGTCGCAAAGTTCTTTACCGTCGTGCAGTTTAAGTATCAGTGAAATACGCTTGCTTTCTCTTCCGTATCGTTCTCGTTGAGTGCATTAAGTGTTCATTTCCTGATCTTTCAACTTTTGCACAGGTGCGGTTTTCTTCTGATTTGCATGCAAGTATTGAAGGTGTTGATCAAGTGCAATCCTCAAGGGAGCGAGACTTAGTGCGATGCGCGCCTGACGCTATAGTCTGGGCTAGCTCTGAGTGAACTTGTTTCACTCGGTAGCGAATCGTCTCCACTGGTCTCAATAACCTTGGGTTTTGTCATATTGATTTACTGCAATCAATATTTTGCTGCTATGGTTCATGTGATTTGCACAGTACTAATATACTATCTTTTGTATACACAGATGCCTGTCAGTCTTCGCACATCAATTTTGTGCCAACATCAAGAGATGCTGTCGGACCGGTACTCAATGTGCGGTCTCTTATCGATCAGCAACGCTCTTCAAACTAGGGATTTTATCACCCCAGCCATGTTGCGGCCTATTTTGGCTCGGTTGGTAGAGGTGAGCCCAAGTGTGGATCATGGACATCCACGCTACGGTGCCTACACTATGGATGCGTTGCAGAAGGGCTTGCAGAAGCGGCGCAAACAGCTGATTTATCTGAACACAAAGTCGGGGTTCAAGAGCAGACCCAAGCGACCTGCCTCCATCGTGCGGAGTCGAAAATCGGCTCTGCTGATTATAGGTAGGCGGCCTGGGCAACGAGCAGGTACATGGCATTGTATCGCTCGTGCCTGGGTTGGTAACAAGTTTGTGTTCATCGATTCCGACACCTATCACTTCTTCCCTAACCGGGAGGAGCTTTTGACAAGTTTCTTTGAGACAGTAGATGGTGTGTACTCAATTGAGGACATTCCTCGTGCTAATCTTTAAGATTTAAATAATACGTATTTACTTTTAACAAAACTTGTTGCTGTATAAATTTTACATATCGATTAGTATTTTTCTTTTCCTTTTTTTTCTTTTTCTTATTTTTGTATAAGTGTCTTACATAGTGACCCTCTTAGATTTATAAAAAGGGGTTTTGATAGTGCTTTTGTAAAAATGTCTGCTACCATTTCTTTGGTGTTGCAGTACATAATTTCAAATTCTTTTCGCTCTACTTTTTCTTGTACAAAGTGATACCGAATGTCAATGTGCTTGCTTCTGCCATGCAGTTCTGGGTTGTAGGAAAGTTTAATACAACTTTGATTGTCCTCAAATAGGGTGATAGCATGTGAGCTTTCGTGTTTAAACTCTACTAAAAGAAGCTTTAAGAAAATAACTTCTTGCATGCAGTGACATAGGGCTATGTATTCTGCCTCTGTTGTGGACAGAACTACGGTGTGGTGCTTTCTCGATTGCCATGAAATTGGGCTTCCACACATATATGCGATATAGCCTCCCACAGATCGTCGTGTCTCTGGGCAATTGGCGTAGTCTGCGTCGGAGTATGCAGTGAGATAATGCTTTGACTTATACTGCTCTAGAGTTTGCTTTCCACCAAGGGTGATGCCAAGATTGCGCGTACCGTTTAAGTACTTCAATCCTCTCTTAACGGCATTCCAGTGAGGTTTTCCTGGGTTCACCAGGAATTGACTGACTTCTCGTAGAAAATATGCGAGATCAGGTCTAGTTCCCATCATTAAGTACATGAAGCTACCAACTGCCTCTCTGTACGGGATCGCTTTCATCTCATGTTTTTCTTCTGGCGTTTGGGGGCACATGGCCTCCGTCAGCTTCACACTGGGATCAGCGGGTGTACCAACCGGGTGTGCATCAAGATGAGAGAAGCGATCCAGGACTTTGGTAGCGTACTTCTCTTGGTGGATGAAAATGGTTCGGTCCGTACGGTTGCGCACGATTGACCAGCCTAAGATGTATTGAGCTTCACCGAGATCAGACATTTTGAAGCGCTGCTTGAGAGCGTTCTTTATGTTGTTAATGATATCAAGCGTAGGGGCTATGATGAGGAGGTCGTCCACATAGACTAKAATGTAGCATGTAGCTCCATCCACAACTCCAATGAAAACGCATTGGTCTTTGATGAGTTTGTGGAATTTCATGGTGAGAAGAAAAGAGCACAGGGTAAGATACCAGATCCGAGGTGCCTGCTTGAGACCATATAGGCTCTTCAAAAGCTTGCAGACTAAGTGCTCTTTTCCACGAACAGTAAACCCTTCAGGTTGCAGCATGTAAATTTCCTCATCGAGGTATCCGTTCAGGAAAGCGGTTTTAACATCCATTTGATGGATCTCAAGGTCAAGGAGTGCTGCAATCGTCAGTAAAAGTCGCAGCACTTCCATGCGGATCACTGGAGAGAATATCTCATCGTAGTCAACGCCATACTTTGCAAAAAACCTTTATTACCAGGCGAGCTTTATAGCGGTCAATACTGCCGTCTCCATTGTATTTTACAACGAATACCCATCGACACTGTAGGGCCCTTCTCCCTGTCGGTAGTTTTGTGAGGACCCACGTTTTGTTGTCAATCAATGATTTGTATTCACAAATGGCTGCCTTCTCCAGTGTTGTGCTTCTGGTAGTTTCTGGGATTCTAGGAACGTCTTTGGTTCAGATATGTGTCTGATAGCCAATAGAGCGTAGATATGAGTGAGGTGCTCTTTTCGTTCGTAATGTCGAGTGGATCATCAAGTAGACACGAGTGTTCATTCTCGTTCTGAGCGTTCGTTTTGTGTTTGACTTTCTTCATCTTTACTACTACTTCCGCGGCAAGCGCATTGACGCTGCGTTTTAGTAGTGAGGCTATTGGAGGTAGCAATTGTGGTGTTGGTAGCGACGGTGATAGCGTCGGTGCGGGGCCATTTTGGTCCGAGATTGCGGGTGTATTGAGGAATTCCAAGTGTGGGAACTCGTCTTCTCTAAACACAACATCCCTGCTGTAGATAACACGGTTTTCTTTCGTGTCAAGGAGTCTATATCCCTTTTTGTTTGATGGGAGTCCTAAGTACATGCACTTCTTAGCACGTGGTTCTAGTTTTGTGCGGTGTATCTCAGGGATGTGAGCGTACGCAGTACACCCAAACACTTTGATGTAGTGCGCGGATGGTTTTCGCTTGAACCATTTCTCGTATGGGGTGACCTTATCGGTTACCGAGCTGGGAGTAATGTTCAGAAGGTCAGCAACGTGTTGACGCATTCTCCCCAAAGAACCTGCGGTAGGTTACCATCAATGAGGAAAGCCCTGGTTCGCTCGAGTAGTGTACGCATGCGCCTCTCTGCAACTCCATTTTGCTGGGGCGTGTACGCATTTGTAAATTGTGCGTGCGTATTGTACTTCTGAAAAATCATTTTTCCAAGTTTCTCATATTCTCGGGCATTGTCGCTTTGAAGATGTCTAACTTCTTCATCGTGTATGTTCTCATTGAACTGGAGTTCGCCTACATCAGCGCGAAAAACGTTTTGTGCTTTCTTGCGAAAATCTTCATAGCATTCGTACAGCTCAGACTTTCGCTTGATGATGTAGATGAACATGTACCGTGAGTAGTCATTCTATAAGGTCAGAAAATATTGACACCCCGAGAGTGTGGGGACGGGTAATGGACCAGCCATATCAGAATGAACTTTTTCAAGAGGGAACTTTGCCCGTTCAACGTACGTTTATTGAATGGGTTACGATGGGCCTTGGCGTATGTGCAGGAAGAGCAAAAGTAGTCTTTCTTGGAATCAAAGTCCTTCAGGCCCATGTCCATGACAGTGTTGGCTTTAGCCAGATCCTGCATAATGCGAAGTTAGGATGACCCATTCGTTTATGCCAAAGTATAACGTTATCAGGCTTACCTGAAGTGGCCACATGGGCTACCTGACTTGAGGTTGCCGGGCTCGCTTGAAACTGGTAGAGTCGAGTCCCTTCACCAATTTTGGCTTCAAATTTGGTTCGGCTAGCGAAGAACAGAACGCACTTCTTGGCGTTGGGGAAGTTGATTTTGAAATCATCTTCGACGGCTTGGCGCACGGAGAGTAGGTTGAACTTCAGCTGGGGCGCATATAGCACGTTGGCCAAGGATATTTCTCGTTCCTTTCCCTTCGTGTCCTTGATTGTCATCTTGACATCACCAGTTCCTTTTATCGGAATCTGGTGATTGCCGCCGACAGTGATTGACTTGCCAGCACTTGGGATCAGCTTCTCAAACCACTTCGGATTGGACGTAACGTGGCGAGTGCAGCCTGAGTCGACAGTCCACACTGGGTCATGCTCCGCATCTTGGGCGGTGAGACTTACAGCCTCTGTTTCTTGTCTGAGGTCCAGAGTGGTGATGGCTATGATTCCATACTCGTTGTCGGAGTCGGTGTCCTTGCGGCGACCGTGGAATTTATTGTGACTCTTGGATTTTTCAGATGTATTCTTCTGGTTGCCGTTCTGGCTTCTGTTTCGATTGCTGTTCCGCTTGCTCTTGTAGTCATTCCGATTTTGGCTTCGTGACTTGCCGCCGAAACCGTCCTTGTAAGGATGCTGTCGAGCTTGATTGGACGAAGGTTTCAGCTTAAAGTTGGCGGGAAGCACCGTTCCTGCCTTTACCTGACCATTTTGTAGGTGGCGCTGTAGAACATAGCAGTCGACAGTATCGTGGTTGGTGCGCAGGCAGTATGTACAGCTCATCGTTGAGATGAGAGCCTTGCTCTCTTGATAGGGAGCTTGAACCAAGGTTGGTTCAGGTGCTAAAGCCTGTAGGGCCTGCTCGTTTCGGGTTTCCCTCGATTCGGGTGTTCCTTGTTTGAGCGTGTAGCGGTTTTTCGCCAGTTCATGCTGCACCTTGGCCTCGATGTGACGCTTTAGCTCTTCGTAGGGAATGAATTTCCTGCTACCCTTCCAGACCGATAGTTGCTGCTTCCATTTTCCAGGTAGCGCATGGTACAAGTAGAGTGACTTCTGTTCGTCACTGAGCACACTGTTTGTGGCTTCGGAGGCAACCTTCATGGCAGATTCGATGTCAATTATGAACCCCGTAAGGTCTGAACCTTCTTCATACCTCAGTGCCATCAGGTATGATAGCACATGATACGGGTCGCCGTGGATGGCAGCACCTTCGTACTTGCTACAGATCGTCTGGAAGATCTCAAACGCAGTAGTCTTGTCCTTGACCATGAGAACATGTTGGTCATCGATGGTCTTGATAAGGAAAGCTTTTGCTTTTGCCTCGTTGAGTCGAAGCTTTTTCGAGCTCAGCCTCTGGCTTTTAGCCCTCAGCTTCTCTGCTCGCTTCAGTTCATCCCGCTTCTGAGCAGTGAATGACTGGATAACGGGGGGATTCTCCTGTCCCATTTCCACATCGCCGTCGTCTCCGGCTGTGGGTGCATCCGAACTTGCGGAGCTGGCATCAGAGGAGGACTCGCTCGGTGAGGAGCCCACTATTTCGTCGGCCTTGGGAGCACCGGCAGCATCAAGCGCGGCGGACATGTCGTTCATGTCGGACAGTGCCGGGTTGAGCTCTTCATCGGAGTCGAACTCGTAGTCGGAGTCACCGGTGTAGTCGATACGCTCGACGTAGCCGAGAAGGTTCTTTCCTTCGAGGGCGGCAGTGACGCGGGTCTTCCAGATGATGAAGTTCCGGCCATTGAGGCGCGGAAACTCGCGGTCGTCAGCGGTGGAGGCTTGCGTTGAGCTCATAACCTATTGAAGGTGAGGCGAGGAAGGTGGAGGAGCTTGAGTAGAAGGAAGAAAAAAGAGATGTGATTGAATTTTATGAGTCTGTCTTCAGAGAGTTTCCTGGCACCATTGATTTATCTTTCCTGTAAAAGGTGATGTATCGACTTATGCAGGATTGGGTCCAGAACATATTTTGTATGGTAAGACATTCTGTACAATAACTTGTACGCTATGTCACCAATTAGGACACATCATTGACATTGTGCTTTGCTTCAACTGGGTATGATTTACCGTAGAGTGCATGATAGCGAACATATCTAGGGTTGTAAGACTGCACGAAGAAGTCAGGCGTCATTCTTCAATCTCATTCCACAATTTGCCTTGCATCGCAATAGTCTTCGCCCACGACGAAATAACAACCAGAAATCGCGAATGCGTCTGTCCTTCATCATCTTTGCCATCTCCCTTCTCGCAGGCGGTAGCGGAGCAGCTGAGGCCCTCCACCCTGCGTCGGATGTCTTGACATTGCGAGGTACAAACCAAGGCGCAAGCACCGGCAAGAGATCGTTGCGCTACGACAATAACGCGGAGCGCGCTGGCGAGGAGGACGATGAAGAAAGGGCTTTCCCAGGTGCAGAGGAGCTTTCTAGGCTCGCGAACCTAGCCCATACGAGCAAGGCTAATTCTCTTGGGACATCGCTGAAGAATTTTTTTAAGCAACTAGATAAAGCTAACGTCAACCCGTCCAACATTCATAAGTACGGCTTCTCAGGCGAAGAATTCGGTCAACTCCGTAAACGGTTCGGAACGTGGTACAGACACTACAAGGACATTGAGTAGTGCGCTCGCGTTCTGCTTCAATTCGTGAGTGCACAACCGCGTTCTGCCAGCTTGTAACCATCGAATAGAAGCACATCACCCAATTGGGGCTTCGTACCTTCATCATGTTTTCATGTACTTTTGATCAACCTTAGACTTCTAAGATCTTAAATGTTTTTTACTATAGCGGCGCATCGTTTGAAGGCCACCAGTAGTGGTTTCATGTGGGTATTTGCTTTAATGCACGTCGTGAATAGAAACGTTACGGTCTGCAAGGCTCGCCTTGGCGTAATACTAAAGTAGTACTATAATGTAGTACCTCCGTGCGATAAATATGCACTTCCAAAAGGATTTTTGGAAACGCTATGCCTCGTTCTTATTCGGGCTTTAAGGTTTGCGCAGGGCTATTGTCCTTGATAGTAGTCGAGTGATGTTGGTCTGCTACGACAGACAAAAATACGTTTAGACTGCGAGTGTGCTTGTTCCACGTAGCTGCTGACGTTCTTGCTCCGCCGTTTTAGCTAGTTCTTGAGGCTTACTTCTGGGCGTCGGATTTCGACGCCGACAAATGCCAACACACACATGCACGAATGCCGCAGAGACGTCCAGCTTCTATATATGCTTCCACTTGCTTCCCAGCGCAGCAGGGCGCTATGCGCAAGGCGGTCTTTACGCTCCCACACAACGCTAAATCACAAGACTGGCAGGTCCTCCGCGCCTTCACTCACGCATGGGCAACGACTTCATAATGGGACGACATCTAAGCGCTAATACTGCTATTCGCCAGGCCTTCTCTTTTTTTATTAAGCAATCTCGTGCATTCCAGCGCGTACCAACACACCTTCCAATGCCAATAGAAATTGTTAGAATCGACGCAGCAAAGACTCCGATGCCCTGCCGGCTCACCTGGGCATGCCGCCCCCCCTCCTTGTCCATAAATACACACATTTGGCAGTGGGGGCTCTTCATTTGCCACCACAGGCGCTCTCTTCTCTGCTTCTTCTGTCCTGCTATTGACCTTGTGAAGTAAGCGCAATGGGCGGGAGATTCGAACAGATAATAGTTTTGCAGTATCTTTAGAAATGGGCTCATGTGTATGTACACGAGATTAGCACAATTAGAGGGATATTCAATTTACACGGACTGTAATATGATGGACAAGGTGCATTATCTCCATCATGTACTAAGTATATTTTTTGGTAAATAGAGATCTAWCTAAAAAAAGTGTGGGGAAGTCAGAACTCGTTCGTATAATTTAAACAATGTCAAAACGCCAGATTTGTCCCGCAATCTAAAAAAAAAAAGACGCAATGTAAAGGTTGGTTCGCAATCCTGACAGATGGATACGAGGAGAGCAGCTGTCTATCGCTGTAAGCCTGTCACTGACAGTGTCACTATAATAGACACTGTCAGCGGTGCGTGGAGAGCAGAGCTAACCAATCCAGCAGAGGCGACTTGACGCGTAAATAAATGAATGAAACCTGGTTGCAGAGGTGTAGAAATTCTCTCTATGCTCCCTGACGGTCCGTTTTAGTTGATAATCACCCCTTTTGCCCTGTTTTATTCCTGAACAGAAAGGCTCAACAATTAGTGAGGTGTTTCCATGCTAAAGCGTGGACTGCAGTGGTGGCGCTGACGTATTATATTCATGTCAATTGTGCAGCCTCCAGTATGTTTTCTGTATGGCTGTAACAATGTTATGCAACGAATGAAAGAATTCTAATTATACAGCCCCCCCCCCCAANAAAAAAAAAAAACGTTTTTGTACACGAACAGACGATGTACTGTAGCGCACGAATATAATAACTAAGATTAAGATTATAAGATTAAAATTAAGCATACAACCGCATCGGCAGCAATTACCACCGACATAAGCATTCGCCCATTGACTGCAACATACCTTGAAACGCGCGGGAGTTGCTACCACCGCTAAAGGGACCGAGTCATTGACACACTCTTAGTAACAAGTGTACTTTTAGGTCGATCAGCAGAGCTACGAAGGGCTTCAACCTTTTTAGCTATAGGCCAAATTTCTATCTTACCATCGAAGTACTGCTTTTTGTGGAAATCGTATCGGTGCCACGCAGCTGCAGCCAAAAACATGTTTTACCGATGTAGCTTTTGTTCTGACATGACCGATATGGAAGGTACTCATTTACAGTCAGGTAATACTTCGTGGCACCTTTATAAACGTTAAACCACTTTTCATTGGTATGCACGGTGTCATACTGTACATGTGATGCATTCTTTACATCGTGGCACCTAAACGTCTTAAAACACAATATCTCTATTAATTTGAGAAATTAAATAGTATACATTTTACTTAACGTAGCGGCAAGTCAACGTGACTTGAAGACGCTTGATCTTGTGGCTATCCGTAAGTGTAGGCTTTACACGAAAAGAAGCGCGGTGTAGTGTTCCGTCGGCAAGGTGGCACTGGCGTGTACTTCTTGGTATTCCGGTTGCAGCGGCGAGCGGGCGCTGAGTTTGACGTTGTATCTCAAAACGTGACTGAGTGCGTATAAGCCTCTGCCAACCATGAAATTAGTGGTCTGCATATAATTATAAAAATGAAAGCCAATGAAATTGAGTATTCGTTTAGGTGCGACTTATAAAAAAACCAATCGTCATATTGGAGAAGTACCAGTTAAAGTTGTCCAATCGTAAAGCATCTAGAAGCTTACAAACACTTTTCAAGCTCACCAAATAGTATCAGTAACATTGAAATGTTTTCATTCTATTCTCTGATATCCAGTAATCCCAATGGGTATCTCTATCCAATTACATTCTAAAATATCCTGTAATTGGTTTAGTCTATTAAATTGTAAAGGGATACAGAAGTGGCGATTCTTCAGACGAAAAACGCCGCCAAATTTATTAACCAGGTAAGCAAACAAAGAGACAAAGCCGTGGTGCCACCTAGAACAGCTTTGGATTTTACCTTCAAAGTTGTTCTTTTAACATGGATGGGATTTTGTTTATCAAAAATTAACTCCATCCATGGAAGAGGCTTCATTAATCGTCATAACTCGTGTCGAGACTTCGTAACTAGAAGAGGTAACGATAAAGTAGCGGCGTGTGATAGATCATAAATGAAATTGAAGTCTTTCAATTGCTCAGTAAGCTCACGTTTGCAGAGCCGACCGCTGTTTGATACTCGCTCAAGCGTCGACTCCAGTGATGTAGATGAGGTCACAAAGCACCCAGAAGGAGGGCAACAACGTCACACCACTGCCGCCGAGCAAGTCGATGCAGAGATGTCACCACCACCCGGCAGGAAAGGCTGTCGGTTTCAGTTCTATTATTTACGGTCCTTATCGCAGGCGTGAAGATACGAGATCTTGGGGCTAGCAGCCTCGCAAAAGTGAGAGCTAAAGACACGACGAAATGACAAATAACGATTTTGCTAGTTTCTGTTCTTTGTTTATATCCTAGAAAAAGGCGCTTGTAGGATAACTTTGAGGGTGTTATATTAATAGAGGCGTTTATTGCTTTTATTATTTGATGCTAGTTTGCCATTCAAGGTAGCATCGTTTGGCTGCGTGTA

>Contig_12

AACTCGCGCACTGCACCACAGAAGTGTTGTTTCTACGGCAGCTATCAAAGGAGCTTCGATATGAGCAGGCGGGGACAGTGGTGCAAGAAGATAACTAAGCCTGCATTGGCATCGTGGAAAAGCCTGCCCAACACACCAGGGTGAAGCACATCGATGTGAGATACCACTTCATCCGGGTGTACGTGCAGCTCGATGAGGTGGTGCTGCAGTACGTCGCGAGAAAGGAGAATAAGGCTTACACGATGACCAACGGTCTGCCTCATGAGCAGTTTNGAGTATATCCGGGGATATTAAGTCGGTAGGGTAACACCCGGTGTCAGCGCTTTAGCACAAAGAAAATGTAGCAGGCTAAACCTTCATGGTGCCCACGTAATTCACAGAAGTCTGCACGAAGTGTAGCCGTCAATGACCGAGAAGTGATGCAACGATAGCAGACACCTAGGCGTGAGTTAACGAAGTCACAGGATCATGGAAGCTTGGAGCAAGGCAACGCGCAGGGTCAAAGGCAATGCAGTGGCACTGAGGCTCGTACTGCGGAGGGGGGTACTGAATTGTCCTTACGTCTAAGCTGCCTGTGAGCGCTTGCGCAACGCGGGAAAGGAGCTTGGCGTTGGTGCGCATGTGTTAGACAAATTCATTGGCTAACAGGGTTTCATGGAGTAGGATAGGTTGTTTTGATCAATTCAGCCAGATTGCTGCAGACAATTCTCATCACTTTAGGTTTTGCACAATAAGCGACAGGCAACAACTTCAGCTCCCTCATTCGGTCCTTCTTCACTTCAAGTTACTCACTAAATTCATTAAAGTCTTTGTTATATAGCTCTCTGTCTAGCTTGGAGATTGCGACAAGTACAGATGGCCACCAGGTCCTACCCTGTCCCCAAGACATCAACACGCCCCAAGTGTGAGTGCGTATTACCCCTTGAAAGCGTTTCGAGAGCGTTCATTGTGCTCCAGTCTTACGTTGCATACAGCGATGCCCTCATACGTAACAACGGGAGCTAGAGTCGATCGCTGTGTTGAGCACCAGGCCTGTACTCGTTTCTTTCGAATTATCTGTCTCGATTCCGCAGCTACGGACTGTAATGGGCCTATTCGGCGCCCACTACTTCGGCTTAAACGTTAGCGCACGCGTGAGGAAACGTTAAGGTGCGCTTCTCTTAGATTCGCTCCGAACGTATAGATTTTACTCAAATCAGGAAAGCAAAGCCGATTAACCGTAAACTTTAGAATTGTTTATTTGTTTGCTAGGGCTTTCCTCCTTATGTGATAATTGAAATGAAAGGTATTTGCCAACAAATGTGACAGAATCCTACTACTATAATTGGTTGAAGATGTTGAGTTTTAAGCAATGAGAGAACCAAAAATGCATATAGAGCAGTAGTCTGCCAAATGTAATTATTGTTGTATCGCACACCAACGTTCTTCGTAATCAAGTGGTTACATGTAGTGCAGCCCTGTTACACGGACAATGGGAATAAACAGAGCGGTCAGCACGGAGAGCTCCAAACACGCCGCAAGCGATCTGGAATACAGACAGCATTCAAGGTGAAGTTGATGCTGCACTCACTCCATATTCTGGATCTATCAAGGAGCTTCAAATTATAAAACGAAAATATGCGGACCAACCTCTGCTTTTCACGAATTTGTCAAATGAGAAAAGCAGGAAGGCTATTTTGAGGTGTCTATTAGCTACGCAACGAAAGGTTGAAAGCTTTGCCAGTCTTCTCGAGTGGCACGTAATCGGAGCAGCACCACAAAAAATATTATTTGCGTCCTCGGACTTCAGACGATAAACCGATGAAGCATGATTTGCTCTAAGGGCCAGCTGAATACTGAAATGAAACTATCGTTTTGGACACCATGTGATGGAAAGCTGCAACACGGAGGAGGAGTTTGGGATCATCATGACATCTCGTCGTATTTTTAGAAATGTCCAGACCGTTCTACATACAGTTGTATATTAGCTGAAGTATATTCTCGTACAGGTCTTCCGAGGGTCTGCGATCCTGGTAAGAAGTCTGGATATAATGTCCAAAAAGTTACTATGTATAAAAGAGCTAAGAACTGCAAATCGGCGGGCCCACCAGCGTGCTAAATTAAAAAGGTTACCACTGGTAGCTCCAATGCCATGTATCCCCGCTATTTGGTCAGAGTAAATACTGTCGATGTGACAACATCATCTGCGGAAACGAGAGTTTCGTTGAAAACGTTTTAAACAGCCTGTTCTACAGTATATGGAGAGGCGTCAAACATGAGTTCTGAGCAAAATGGATGCTCTAGAACAGCTGTTGTTGCTGTTTTAGTTATTCATAGAGACGCGTAGCTATATCAAACCATTGTGGAGGCGCGCCCAGCGCCGAGCTCACCAGCGAGCGAAACATCGAATTCTCCATTAAATCCTGTCTCAATGTCGCGAGCGATGCGAGCTCACGGCTCTTAGCATCATATAGCAAAGTCGTACATCGGAACAGCTCTGGATTGCAAAGAGACGAAGGCTAGTGGTACAGTGGTTCAGCGCGGGTCATACTTCCAGGACCGTTGTTTAAGCGCAAAGCATTGAAACATACTTACTTTGAGGGGAAATTACTGACTTCTGAGCAATGCCAATTGAAGGAGGATTAATGAGCAGATTGACCAGAAATATTACAACGTTAAAGTTGTAGTACTAAAAATGGTAACCTCACGACAAGTTCGTGAAGGTGGTCGATAACGAAAGCTCTCAATTCTCTATAATACTTATGCTGTAGCTATGACCTGTGTGTATTATTTGAATTGCTGTGCCGCTGTCCGCACACAAGGTTTTGCACATCAGAAAAAAAATGAAATCGAGAAAAAATGTGGGTACGACCAGTGTATTCGTACTGTTATTTGGCATTAATAAGCTCAAACTCATGCGGCCTCCACATTATGTCATTGTTTATTATGGGCAACAAAGCCGATGTTGCTGCTACGAAGCTGGCGCAGGGGATTATTTGCCACTGTAGCCACGTGTTTAAGTAAGTCAAATTCAAAACGAAGAAAGCACTCCTGCTTTCTCAGCCCCCGCAAAGCTCTAGTTGTCTCCATGTTCTGCTACAAAAAGATCGTGAAAAGGTGGCGATGGCCTCTTCGAAGCTTGCACTACAAGAAAAATTGTAGTTACTAGTTGGACGAACTATTTGTTTGTATTTTAAAGTGGTATAAAGCAGGAAAGAAACGTCCAAAAGACATCTTTCAAAAGGTAGTATTCTCGTACTTACTCGTTTCTAGAAAACGCTCTTTTGTAGCATGATTGCAACTTTGTCAAGCAAATTTTCACAGTCGACGCTTAGTCTATATCGACTGGTACGTCTTTTTCGACAGTCTACGACCGCAGATTTTGGCCGCTCTTCAATTGTGGGTGCCTTTCATTCCAAAATCTGGAGTATTGGGCCAACAGCTAAACATTCCCAGTATCCAGAAGGACATCGCCCTTGCGAGCTATCAACGTCTCTTTCATCGATTTTGGGGTATAACCCATTTTTTCGATTTCTTTATAGAGGTCATGGTTGCGCTGGTTGAGTAATTGGAGAGCTGCCTCGTCATCGATCTTGGATAGATCAACCATCGACTTGCTCTTTGTCAAAAGAGGCTCCTTCAGAGATAATGTCTTCTGTGAAAGCACCTTCTCCAGTCCCATGCCCCTCTCCTCCTCATCCACCTTTCCACTATCGGCCATCTTGGCATCCTTCATTTTCTCTTGAGTTCTCAAAAATCGCTCGCCACCAATCGTAGATATTATGCTCACAGAGTCCGTCTTGCCTGGGTAAAGGACGTTCGACTGACTCGTGACAGATGAGCCTTCAGCGAAGGTTGCAATGGCAGTAACAGCCGTCAGGATGTAGCAAAGGCGCATCTTGTGCGTTGGGGCCGGTGGCGAAGGGAGGAGGGGGGGTGGTCTTGTGCCTTGTGAGCAGATTTTGGAAGTGAAGGTATTGCCGAATGGTGGGGCTGATGCGATTGTAGTATGTATAGCAGCTGCCGATCAAAAAATGAGGGTAGATTCCCAAATGGTACGTATGCGGAACTGATCGGATTAGAGATCAGGATTTGAGACCTGAATTATGAACAGACTGAAACCAAAGAAAAACACTTTTAAAGGGGAGAAGATGAAGCATTTACCGAGGTCTTCTGGTTGCTACACGGACATATCTACAACGTAGCACTAGTGAGTGTACTACCGTCCGCTGCGGGTGTAGCAACTGCCTCCAGTTCCTCCAGTGGAGGATCCACGTCGCTGTCAACGTCAGAGGGAATGTCCACAACCTGTGGTAGCTGAATGACCTCGCGAGGAACACGGCGGGCTCGCCCACGAGGAACATCAGCAACGCGGCGTACGTACTCAGCTGATATAGCCTTGATCACCGTCACAAATGTCGCCATTGAAGGATGCGGGGTTGGGAAACGTGTATTAAGCTCCCTGTTAAACCTCTCCAGGGGATTATTCGTTCGTGCGACCAACTCGTTGTTCATGCCGAATACGTTCCAGACATCGATGGTATACTGCTCCAGCCAAGTGCGGTTGAAATACCCCCAAAAATCGGCCCATTTTTCAGTAGAGTAGGTCACGCCAGCTTGAGCACAGCGCAACTTGATCTCACGCTTTACCCACTTAATACCTCGCTCGATGTGAGCAGGATCAATCACCGTCAGGGTGTCTAGGACACCACGTGTCATTGCAATCAGACACTCTTCCTCCGGAATAGCATAGCGCTTCATAGCCCGTCGGAGTGCTTGTTTCAGATGAAACAAGCACCCCAACACAATTGCGTTCGGAAACTGTGTTTGTAGTGCGTCCAACAGTGGCGCTTCGAAGTCACAAACAATTTCGGCCGGTTCGAGTTGCTGGTCCGTTGATTGTACGATAAAATGAATCATATCCCAGTAGGCGTCGCCAGTACGCGAGGTGCTCAGAATGTAGAACACCGGCACGAACACTCCAGAGGCTCGGTCGTGGACCATGAACACAACGCACTGTGCGTAACCCGGCGGGACACACCGAAAAGTGCCATCCACAAACAACGTGGTACCATGATAGCGTAGTAGCGCGACCAGAGATGGATGCGCCCACCCAAGCAAGCGGGAAGGCTTGTTCAAATCGTTGCGGTTAATGGTGACGTAGTGAAACTGGAAAAAGGAAAGCTCCTCGTCTAGTGCCAATGAAAGCGGAGGAATTTCAATAGAGCCGTGAACATTGCTAGAGTAATGCGCGCTTCTTGCTCGGTGTACTCGTCGGACAACTTGTTGCTCTGACAACCCACGAACAACGTTGGGATTGTCAGCTGAGTAGAACTGGCGTCGCAATTCTTCCCACACTTGGCGCACAGGCCAAGCGACGTGCTCAATCGCAAGAAGGTCGGCTTGAGCTTTCATCTGGTCCTCAACGTTGATGACGACGTTGGCAATGGCTTCGATGCGGCATGTGTGCAGCTTTACAGCAGCATACGCCATCGATGCAATAGTAAACTCCAGCTTTCCTCTGCAACCTTGGCGAAACTTAGAGCATCTGTACACAATTTTCCTTGAGGACGACCATGCCTTGCAGTACTTATAGCCCTTGTAGAAGCATGTCGTCCGCTCACCACGTCGTGGCACAGACCCCACATCATTTGCGAGTTGCTGTCGAGCTTGCTCGAACAGCAACTCAGTGCTAACGACGTCCTCGACCGGCTCAGCTGGCTCCACTCCGCCATCCTCCGGCGCGCCCTCAGCCGGCTCAGCAGGCTCCACCTCGGCATCCACCGACGCGCCCTCGTTTGCCTCTCGATTCATCTTCTCCACTGACCAACGCACGAGCAGCCTCGAGGCTGAAGAAGTGCGTGAACTTACCAATCATTTTCAGATCCCATGTCAGATCTCCAGTGAGATAAATATCTGATCTAGAATCTGATCAGTTCCGTATACGTACCTTTTCGGAGCTAACCAAAATGAGAGCCTGTGCTTTTGGTTGTTTACGCATTTTCAAGATTATTTCAGCGAAACTCATGTACAGTAATTGGTCAGACTCTGTACGATAATGACAGCTTCGCATGGCTATTTTAAATTCGTGAGTGGGAGCCACACTCATCATCGAGTGGAAGAACTGGATTGCTGGCTACACTCAATTCGTATTTTACGAAGCTTTGCGAGCTTCTGTTCGGGTAGTTGCGCGAAATGTCGTGGATCAAGTTGTCGCGTGAGTAGATCATAGCAATTCTCATCTGCAATCGTGCGCTTCCGTTTTTCTGGACTACTTTGAATTTTCGCCTCGCGTAACATAAATCGGTTTTTGGTTTAGTTTAGGCTAAAACTTTATTATATCGTTTTATTACAACTTTGTAAGAACATCGCTCTAGGTGTTGCAAAAAAATAACGTAGCTAAAACCGAGAACACGTTACATGTAGTTGTTAGATCTATTTTTGCCAAGGTCAAAAGCAGATAAAGATTTGTAGAGGCAGCATGTCACATGCGTGGGGCTGGCATGGTAGCACACCTGTCGACGCATTCCCGCCACACGAGTTCCCTTGCTGTATTCATTCACCATTTCGCCTTCGAATACACAAACCTACGCCAAGCCGCCACATCATGCGATTGACGTCAATTCTTCTTCTCGCTGCGACAGTATCTTCTCTCCTTGCGGGCGACTGCACAGCACAGACAACGACAAAACTGTCAGTCACGGCGATGATGCTCCCTGAACAGACATACCCCACAAATGCTGGAAACACCGTCGAAGGCAAACGGTTTCTGCGCAGTCATAAAGTCGCGGTGTCTTCGGAAGATGTCGACGTCAAAGAAACTGCTGACGAAGAAGAAAGATTGAGTGGTCAGAAAATCGATGACTTAATGGCCGGTACAGCCGCCACTTAGTTTAAGCATTGGTTCGGCAATGGCAAGTCTGCGTCATCAATCTGGGATAAGTTGAAAGTCGACGACCACCCAGCCCGCCGCAAAATTTATGACTGGTACGTTAGCTACATCTACCGACACGGATAACTGTGAGGGTTGCTGTGCTTTACTTCATCTGAGACTATAAATGTGATACTTTCACGACGTACTGGTAGCTCAAGACTGTTGAAATGAGATTTTCTCAAGAAAACCCGAAGATCCCTGCGTCTGACCATATTTGTGCCAATCTTCAGTTTATCCACCTTTTTAAGCTCCCAAAGCAATGCTTGACTCCAAGTTTTATTTGTAGGAAAGAGAAGTGAACATTGCCGTCCCAGTCAGATTCATCACCCCTCCTCCAAGTATCTTATACCGCCGGCAGAGTTTGGGAAAGTTAAGTCATGATGTTATTGGCGGGAAGGAGGCTGGCCAGTAAATATCTGGGAAATTTATCCTGGCTATAGTAGTACGTACATTGTAACGAAACAGCAAGCATTTAATTAGCAGCAGCCGCTGCATATCCAAATGTCGTACATGCACAGAACTGGTTTGTGGTGGTTCTTGTCCGTGTCTGCGACTATTGCCTGGTATTACGGCCACAACAAAATTCTACACCATTTCAGGTCCGTTCTCCCCAGAGGACGGACTCAATACAACCACCTCCTGGGTAGTGATTTTATTAATCGCTTACTAAATTCATTAAAAACCACTTATTATATACGATACCTTAATTAATTAACGTAAATGCTATCGTCCATGCGCTGAGTATATGAATTGTCCAGGTGGCCATCCATGTATTGTCCAGGCCGTAAAATTACCGACCTGAAAATTATAAATTACGGGTTGTGTAAAATACATGTATTATGCATGTAAAATGTCCATGCTTCTCAACGATTTTGAGGGCTACCACAGCCACTCACTTCACTTTCGATAAGCCGGAGCTTCCGGTACCGTCGTCGCTGTGCACGTCGTTGCCGCCCTCCCCCGCTCCGATATGTCGGATAGCAAGTCTGAAACTCCCCCACTGGAGCCTCCCGCGCCCTCTTCACTGCCGACGCCGCCATCACCGGCCTCAGAAACCGCGACAGGTCAGCCAGAGGTGCGTTGCATAACTACCTGATCTCATACGTATGTCGGTTTTGCTAACTACGTGCCCTCAACAGGCTAGCTCGAGCGAAGAGGCGTCAGATGCTGCTCTTTCCGCTCCTCCGAACTCTGCAGAAGGCTCTGAGAACTCCGACGAGTCGGGCACCGACAATGCGGAACGTCCATGCAGCGTTTAGGTTTCAGATTGTCCAGTGCCTGTTTGTGCCCCGGAAGCCCTTCACTCAAGCTGGGCCGACTGGGCGGTCTATTTGAAAAAGTAAGCATCTTCAACGCGGCAGATCATCCGAGTTTTCATTACTGTGAGCTGCGACTTGCGGAACAAGCGGTTAGCTAAAACTGTCGCTGCACGAGAAGGTCTTGACGTACCATACGTTCCGACCCAGTGGGCAACGTACCAGCGCACATATATTTGTACGCATGGGTGGCGGACGAAGGATCGGAGCACAGGTGTGCGGCCGAAGCAGTTTGTTCGTGGCACTGGGTGTCCTTTTCGGTTCGTGGTTCAAGTCGTGGAAGAGGAAGGCCAGTGGCTGCTTCGAGTGAAGAACGGGGTTTACCACCACAACCACGTCGTGGGTACCGCCCACTTCAAAACTTACGCAGAGAACCGAAAGTTGGAGGATTCTGAGACAAGGTCCACCGTGCGCGAAATGGTGAAGTACGGTCGCAAGCGCGCTCGTATTTACGAATACCTGCTCGACGAAGGGGAAAGCCTGGTTAAGAAAGACGTGGACAACATTGTCCAGCGTGTCAAGGCCGAGTTTCGAGGCGGACTCGACGATGATGATACCTGCGCACAAGCACTTAACGCGTTTGCAGAGATGGATGGCAACGTGGCGACTGTGGACGAGACTGACGCTGACAACTCCGGTGTCATGAGCATGACTTTGAGGCACCAGCGAGACATGACCGTACGCTTCCCCGAGTTGCTGCTAGTGGATTGTACTCACAAGACCAACCGGTAGGTTATATCTATGTATTGTCTAGGTATTGTCCATCTATTGATCTTTATCTCATATCAATCTGCTCGTGTGTTCTAGGTACAACTACCAACTGTGTACTTTGATGGTCATTGACGATAACGGTCGTGGGCAGTCAATTCAACATTCGCTCATCGAACGAAATGCCGACTGGCACATGTCACGTGTTTTAGATCACCTGTTGCGGGTAGATGATAAGATCGGCGAAAAGGTGCAAGTTATCGTGGTCGATAAAGACTTGCACGAGATTTGCGTACTGAAGTAGTACTTTCCCAAGGCGCGTGTGTTGATCTGCTTCTATAATGAGCAAGGTGTCAAATTCTGCACTTGGCTGCTTCTAAACGTGATAAGCACCATACCTTCAGTGCAACATCCTGTGTTGGAAAACATGGTAAACCAAGTGGCGAAATCGAACCCACAGAGCACAATCGTGATCGAGGCCACTGAGTATCACTACGCGATGCTATACCGCTTGAAGCCACCATCCTGGGCGAACGACGCCATGATACGTGCTTTCTGCACAAGACTGTGTGCAACAAACCCTACTGCTCGTGTTCTGGGGATTGAGAGCACGGTGACGGGGCGAAATGCCAAGGGCATGAATGAATCCCTCAAGAAAAAAGCGCAAGAGCTGGTGGGAGAGGCAGACCTCCTTATGATCCCGGTTAACGTTGGAAATTCGCACTGGTGCGGAATTGCAGTAGATGTGAAGCGAACTCGAGTCCTTTATTATGACTCCATGAGCCAGAGGACTTACAAGACCGTGCTGGATCGACTGTCCTGGGATTTGGCGAAAACCCTGAGCGACGACTATGAGATTGTATCCATCAACGCGCCAATTCAGACGGACGGCCACAACTGTGGTTTCTTCGTAATGCTACGACTCTGGCGAATGGTAGACAATAGCATTGCGCTGGGTGTAACTCCAAAAGGTCTCACGATATTGCGCTTTCACATGCTTCAGCTAGTGCTCCATGGTAGATCTAACTAACCCATCTAATTTGAGTCTAAATACACGGAGGATATCTGACCAATGCATGGACTCGATACATGGACTATAATACTAACGTTACCTGTGTTCGTGTTCATGCCTAATGCACGGGTGATTTATGGGTTATACGTGGACTCAATACATGGACTACAAAATTGACAATGCAATTTAGCAGCAACTTGATGCGGTGAAAAATTGATCGGCGGTAAATACTTGATTTCAGTATTGATATAAATTACGCAGAACGTAATTTATGGACAACACCTGGCGTGGCTGCATGGGCAGTAGCATCTGCCATTAATTAATTGTATCAATCTAACAACATTTTTACTTTTTATTTAATTACTAATAAAAACGTGCTTTATGATTGATAAGCAAAAGCCTTTATGTTTTGATAATTTATTTGGAAAGGGCATTATAATTTGAGAGAAATGGTCAGTTTTAGTCAATCCAAGAAAGCCATTACCCACCATTTCTTTTTACTGCTCGATCTGCAGCACGTGCGACACGTCTGGGAACTCAGTGTGTGAAGCCAATTTCATACAACACGTAGTTACTCCGACGGGTTTTCCTATTAGTACGAATAAGGGAGGATCCTCCTCAGGAGGGGGTGATAGTAGACTCGCTCGGCAACACTAATCCATGCCTATCCGTGTGCGAAGCACGCGGGCTTAAGCACAGAACAAATCATAGCTATAATAAACCTCGCTTGGCCCTACGGGGTTAATGCCCAGGAAGGTCAGGATTAGGATTGAGGAAGGTGATAGAATTCCGCCAACTCCTCCCCTTTTGGAGATTGCAGTCTCCAGACACCCGTTTCTCATGTTGTAACAATAGTCTGCCTTTCGTGTATTCACTCGTTTTCCTCCCAGATCTAGCTTACAATAAAGAGCTGCTTGCGTATCGGAAAACAAACTTTTAAGTAAGGATGTTATAAATTTTCGTACGCAGTAAAATCAGACGTATTTCGATGGTCATGGCATCCACCGAAAAAAAATTCAGCGAAAGATGTCTGAACAAGCGCTCCGTTGGACACTCCCATCGACGGGCGCTCCTGAAGATGSTATTGTTGTAAGTCCTGGTAGCATGTTCTTGAGCGGTAAGATCAACTCAAGACTGCGAGCCTTCTGCGTAATTTTTGCGCAGCTTTTACATTACTTTTCACCGTGTGCCATACACTGTATGGTGCGAAATTCTGGGTTGTCCTCAGACACGAGATGATTTGAGATAAGTCGAAGGTAGATTGTCCAATGGGCAATGGGGGCAGTCGAGACTGGGGCCAAATTTGAAAAGCCGCCGTTGACAATCGGGTGTGCACATTCTTGTCCGTTAGTGACACTGCAGTCAAAATGCGGGCACTTTTAGTATCGATTGATGGAAGAGCGCGATATTATTTAGGAAAGGGGCGATAAATGAACCGGTCGAATAATACGAAAGAGCGTCAGCACCAGAAGCATGTATTGTAAACGATATCCGAGAGATTTCCTAATTCATACTTAAGTACTACAGAAAAGGTGCTAGAGTGATATTGACAAACATTTTGTACAAAAACAAGATTATAGCCTGGTATCCAGCAACAAATTCAAGAACAGATCACACAACACAACAACTTCAATAATTTGATGTCTGAAGTAGAAGATTTCGTATACGGTACCATCCACGTTGAGTGTAGGGGTGGAGATAGAAAGGTAAGCACTTCAAAGCCGTGGTCATCAAGTTTCGGATGACCATGACCGTCCATACGGTTGGCCCGAAAATATCGATCGGCTGGCCGAAAATTACGTGTAATGTATGTATTAAAGAAAAAAGGCAAAACATAATTGTCTTGGTCAATATTTCGGCTCAAAATAACCTGGCACGCCTCGTTTGCGCGATCCGTAGCGAAGTAAAGGGAAACATGCAGAGCAAAATATCACACCTTTTATCTCGTGAACGCGGTCAACGTAGAGTCGGACCAATTGCATTAGTTTCGATAGAATAATCTTGAAATGTGTAAACGACTAGGCTCTCATTTTTGTACGGGCAGGATATACAGTATTACATACGCATCAGCCCCAAGTGATTGGCATACGCGCACCAAGGGGACTAAATTGTACCTTTTTAAAACATTGTGAATGTAAAGTTGCCGGTCTGTCTTCTCCGACGGCCATCCCATAAAAAATAACATAACTACGACCTTTTTTAATATGTTGGATTACGGGACAATCATCGTTTCTGGTCGGTTAAATATGTTTTTAAAGATGTTTCTATAATTGAGCCCCCGCCCATTCGCTGAAACGTTCATTTCGAAATCTGCAAAGACCCTNCCCCCCCGACCCCCTTCGCCACCGGCCCAAACGTGCAAGATGCGCCTTTGGTACATCCTGACGGCTGTTACTACCATTGCGTCCTTCGCTGAAGGCTTATCTGTCACGAGTCAGTCGAACGTCCTTTACCCAGGCAAGACGGACTCTGTGAACAACATAATATCTACGATTGGTGGCGAGCGATTTTTAAGAACTCAAGAGGAAATGAAGGACGCCAAGATGGCCGATAGTTGGAAGGTGGATGAGGAGGAGATGGGCATGGAACTGGAGAAGGTGCTTTCACAGAAGACATTATCTCTGAAGGAGCCTCTTTTGACAAAGAGCAAGTCGATGGTTGATCTATCCAAGATCGATGACGAGGCAGCTCTCCAATTACTCAACCAGCGCAACCATGACCACTCTAAAGAAATCGAAAAAATGGGTTATACCCCAAAATCGATGAAAGAGACGTTGATAGCTCGCAAGGGCGATGTCCTTCTGGATACTGGGAATGTTTACCTGTTGGCCCAATACTCCAGATTTTAGAATGAAAGGCACCCACAATTGAAGAGCGGCCAAAATCTGCGGTCGTAGACTGTCGAAAAAGACGTACCAGTCGATATAGACTAAGCGTCGACTGTGAAAATTTGCTTGACAAAGTTGCAATCATGCTCCAAAAGAGCGTATTTCTAGAAACGAGTAAGTACGAGAATACTACCTTTTGAAAGATGTCTTTTGGACGTTTCTTTCCTGCTTTATACCACTTTAAAATACAAACCAATAGTTCGTCCAACTAGTAACTACAATTTTTTTTGTAGTGCAAGCTTCGGAGAGGCCATCGCCACCTTTTCACGATCTTTTTGTAGCAGAGCATGGAGACAACTAGAGGCTGAGAAAGCAGAAATGCTTTCTTCGTTTTGAATTTGATACCTTGTGCTACTTAAACACGTGGCTACAGTGGCAAATAACCCGTTGCGCCAGCTTCGTCGCAGCAACATCGGCTTTGTTGCCCCTAAGAAACGAGCGTAACACTACCAGGATAGTTAGCAACTTTGAAGCAGACGCAATTCTATCTTCTAACACTAGTGTAGGCATGGATCACATACAAAAAAGAGATTTGTCGAGTTGGACAAAATTTACGGCCACCATACTGTTACGAGAACCGTCCATGGCTCTGGGACTTGCAGATGAACGTTCCGCTGAAGACTGCCAACGCACAGAATCAGCGCTGAAAGTCGCGCGCGTTTGGTTCTTGAGCATCTGCATCGAAACTTGGGGATACGTCGGCTGTATTCAACTGCTCGAGAACTGTGCTCATCGACGTCGTCTCATGTGGATGGGTGGACAGCCTGAACGCTCCTGACGCCAAGTCATACAACGGCCAAGTCATAGATATCCTCTCCAAGCTACATCGCGAGACACGGATGCGTACGCTCATAAGGTTCAGAGTGCTCTTAAACCGTTTCGCATCGACGAGGGAGGCTTGACGACGCATTTGCACCGGACTTGCCCAATGACTAGTCGAAGGTCGTTTACGTCTATGAGTCACGTACTCTTGTATTTCAGTCCCTATGACGTTTCTTTCCGCCTAGAACACTCGGTATGGAGGTTGGTATATCGGCGGATGGTCGCCATTATCGACAGAAAATACAAGTAGGTTAGTACGCCCAGGACGCAACGACGTCTCGACGAGTATGTCGCCGAATGGTTGGAGGAACTCCGTGGCCAGCCCCTTCTTTTCGATACCAAGCTTGAACTCTTCTAAGGCGACTTCGAGTTCCTCGTTGAACACTCGGTCTAGTGTAGGCATGGATTCCGTCGCATCTACCGCTTCGTCACTAATATCTCCAATAACGGAGTGTATCCATTAGTCTTCCGCTTCACGGCCATTTGTTACGGCCCAGCTTGACTTATCTGACCTGCGTTCTATGTCCAGGAAAGGTTTTAGTTAAAACGCTGACTCGGGGATCGCAGTAATACCAGCCAATTGAGTACTCCTCTTGTTGACCGCGTCCTTAATACAGGGGCTGCACGCTTCCACGATCACTCGGTCATCATGTAGCGCAAACAGTGTTCTGCCGACTTCAACTTAGGCACCTAGAGCAGTAAGTCCTTCCACCAGAACGGTAGTTCCGTTAACCACTTGTACGTCAGCCACTACAAGCGCTTGTCCTCCCGGACGGATTTTGACTTAAGACGAGATACGAGGCATGTAAGACTCCTCCACTCGAGGGGATCCGATGGGGAGCACCTCGCCCGTGTCTTTCAACGTCACAGTGCTCTCAGCTAGCTCCACCACTATCCGAAAGGCGTTGGGTGTATTAGTGCCTAGGATAGCGTCGACGTGTAGCTTATGTACGACGGCAAAATGTATTCTTCTCTCAGCAGACCCCAAAGTCACCGGCAACCCGATAGCGCCGTCGATAACGAGTGTCGGCCCGGATATACCGTTCAGACTATCAGAGTAGGGCAGCAGAGGGGGCGTTAGCTAGGCCAAGACGCTTCAGAATACGTCAGTGCACTAAACACACTACAGCTCCGGCGTCAATGAGAAGTTCCGCAGGGCGATTATCAATATATCTTCAAGGCAGTGCTTCCACATCTTCTACTGATCCGATCACACCCTTGCCGTTGGGACGACCTCAAATTTTTCCGTCAGTGGAATTGTTCCGGGTAGGACTCGGTAACTCTACTAGACCGGGTTAGTGTCTGGGTTTGGGACCGTCGGCGACGACGTTCACACAGTCTACTCTCCCCATCTCGTAATGAACAATTTTCGCCCAGGGCTTCCGTGCGACCGCTAAACAGCGATGGCCAGTCTAAACTGACCTCCTTACGCTTAAGAAAGCGCTCGGTGCTATGACTCCTCAAATGGCAGAGTCTACACGGTGAGCCCCCCTCGAGAATTTTTAAGCGTAGCTTATCAGTGCTCTGTAATCTTCTTTGCAACGGCCCCCCCCTAGTCGAGAATCCGATGGCTCGACGAGACGCTCGAGTAGTTTACTAGTCCTGGTTAAAAAAACTCAGGGGCCCATATGGGATCGTATAATCGTCTCTTAACGCCTCCACTTGCAACCCTTCGTCACACCCTTTACCCAGGATGCCGTTGTAAGAAGCTTTAGCTTTCATAAGAGACTACAAGGCTCACCATATCACACCATATTATGGTTCAGGGGCGATCCCGCGAGAAACAATGAAGTCGTACTCGGCCCTGCTAAGGTGAGATGACTTTTCGGCGAGCCTTCCAGAGCCAGAGCAATGTTGTCCTCACAACCGAGTGGCCCAAATACATAATTCAACACTACGAGGAGGTATTTTTAACTCCGGGAAGTGTACTACCTTCTTAGGTAAATCCTTCCATGA

>Contig_15

GATGTCATTGAAGCGTTGAAGCACTCAATTCATTACGCACCTCGGGATACGGAACAAGGATTGCATTACTGTTTGATGGTTTGAGGAGTCTTCTTGGTGAAAGTTTTGTAGTAGAGTGGTGGAAGCAAAAAGAAGAGTCGATTGCCGTCTGACCCAGTTTCGCCAAATCATCCGCCGCCCCGCCCCTTGATGTCATGCGTTTTTCCCACAGTCCATCTGGAATCCACCGAACTGCGCCATCCACGCATCAATAGCAGTATAGTTCGATGTCCGAAGTACGTACAGCGATTTGCATGTTGACCGACTTCCACACGCTCCGCTACTTGCCGAGGCTGCGATGACCGTGGAATGTCGATTGTACCATCAGCTGGAGTGCGTTTCCTCTTTCTAGAAAACAGCTCTCGATACACGAAAGTCCCACCCTGCTCCAGCCGATCCATAGAATAAAACTCAAAGATGTATCCACACAGTGATTGTGCTAGCGGGTTTCCATCTGACGAACCGAGAAGGCTTTGCAGCTTCGCGCGGTCAAATCGCCACTTTGTTCGAACAATCAACTGCATCGCCAAATCTGATGCATAGACGACTTTGTATTCTCGATACGGTTCTTGGCTGCGAATGTGAATCAAAGTCTGAACGATCGTGGTCTTCGAGTTGACCCCAGAGTGTATAGACACCAACATAATGCAATCATCGAAGGAGAACAACTGCTGCATGCGGACATCAGCAAAGCTTGCGGGTTTGGTTCCAATGTCTTGCAACACCAGGCGAGGTACACCACCCAGCAATCAAATCGATTCTCCCACACAGCTGCTGCATGTGGATACATGGGAGCAATTGTCGCCAGTTCTTCTTTCGACCACAGTGGCATGTAGAAAACATCGGCAGTAGGAGGCTGCTTCTTGAACTCACGATGCAGTCTCGTCGCGGTGTGCTTGCGAGAAGAACTGAGCATAGCCGGTAGGGAAGCCCAGGGATACTTGTTGGGTCCATCGAATCAAACAAGGCCACCACAGATCAGGCGCCAAAACTGTACATTAAACTTGCTTGGCAACGCCAGACAGATCAGCATCGTCGATCCATCGAAGTAAAGAGCTTATTGTTGTCAAAAAAACAGCACGCCGCTTCTTCTCTTGATCAATCTTCACAGGACATAGTAGACGAACACCGACTTTCCAACCCCAGGCGTGCCCGTGACGACAGCATACTTGACCATGCCCACATTTGGATTGAACAGCGCTTGTTGGCGATCACTTGGTACGAAGAGCGAATGTAGAGCTTCTGACGTCTCGTTAGCGGCAGCAAACGACGAAAACTCGAGCCAATCACCAACTTGGTCAACTGTCGGGAATTGTAAGAAGAATGGATCACTGCAGTGGTCGAAAAACACCCGTCTAGAGAACCGAGGACCCTTGCTCCGGAACCACCACCAGCACGTGAATTTGCTCACTGGATGGAAGCTCCATGTTGTTCTTCGTGTAAAACCACGTTGTTACGGTCTTTGAGCCCAGAATCGGTTCTCCGGCCGTCAGCGCATGGATGTAGTCATCAGATTCTCCTTCCACCAGCTTCAGAGCAGCCCGATGTTCGTCTGGGAGCCACTTAAGATTTTCCTCCTTCATCTCCTCCGCAGAAGGGCGGTAAACAGGGACGACATCTTTGCCGCTCTCATCTTCGACGGACTGCTTCGCCAGAAGAGCTGCAGCTCGTCCGCAGGAAACTGATACATCTTCTTCGAGTTGATCGCATCCTTCAACTTGCTCACCTTCGCGCCTCGTCAATCTCCACATCGAACGAGCTTCCTGCCTGACCGACAATCGCGCACTGCAGGGAAACCACCACCATCTTTACTCGAGACGGCAGGTTCTGGAATGAGGAAGAAGGGGAGGGAGAATTTCTGGCGTACTTAAGCGGAAAGAAAGCGCGTGACATAGATTTTCACCAATGATGAGACGCCACGTCGCGCTGGTCCGAGCTAATCCTTGAGGTAAAAATCTGCAGCGTATTGCTTTTATCAATGGCACGTGTTGCTGCAACCGCCTGGAAGCCGTGAGACGGGACAAAAGTACCCGACCAACGCCCGACTGCACCGGCGGGTACCCGACTAGTCAAAAGCCAGTTTGCCACCCTTTTTTCGCGACAGCGTATATACGGATATTATCCCGGCCAAAAAGGCTTGGACAAAAATGGTAATATATTTTATCTTAAATTAGAGGAATGTAACTCCGCAACCGTAGAGCCGATTGTCCCCTTAATAGATAGCTTGGTGTCTATTTATTCTCGTAAGAACTTCACAGCATCTAATAATGTTCACTACTTTTCAAATGGTCGCCGGGTAAGAAATTGACTGCTGCTGAGAAGCACTTGGTTGTGAAGACCTACGAGTTCCTCCGCCAAAAGAAGGCCTCTGCACCGCATCTATGGAAAGGAGGAGTTCGTTATCATGTCAAAGAGTGTTTAGGCTTTGCTACGGGCACCATTTCAGCTATATGGGCGCACTGGGAGCAGCACCACGACAAGAATTTCACCTCGGTAGGCTGTACTTCATATCATTTCAGTTATTATTGATATTTATCATTTCAGTGGTCGACAAATTAATTATGACAGATGGAAGGTCCAAAACCATCTGGGAGGCCTCCGAAACATGGAGAAAGCATACAGCCTCGTCGGTGTCAAGAATGAACAAGAGCGGAAAACCTGTTGCAGCACCACAGTTAGCACAGCGTCTTAAAGAAGAGCACAACATTGTGGTATCAGTGCGCTCACTACGTCGCGTGCTTAGGAGGATGGGAATGCGATTTATAAAAGGAAGGTCTCGCAATATTATGGCAGAAACGACGGCAAATGTTGCGTTCAGGGCGCAATATTTGAGAAAAAAAGTTTTAAATCTGAACAGACGCAATTTTCCAGTGCGCCCCGAGGTTTATCTCGACGAGACTTTTTGTAACCTGCACCATGTCGCCAATTTATCGTGGGTTGATGAGGACAAGCTGCGGTATACGAAGAGTGGTCGAGGTCCACGGTGAGTGTTTTATTTTGTTTTTGTAAATTAAAACCTTAACGTTATTTGTATTAAGAATTTATAACAATTATTTAATTTTTTGTTTTTAAAGGTTTTGCATTATAGCCGCTGGTATACTTCGGCGAGAAAAGAATGGATTAGTTGGTGAATTGGTCAAAGAATCGAAAGAGGTGTGGCAATCCAACTTAAAACGTAAGAACGATGACGATTATCATGGCAACTTCAACATCGAGCAGTTTAAGAAGTGGTTCACGAATCTGAGTGCTATTTTGGCGTCCAAGTATGGGCCATGTAACATACACATGGATGGGGCCAGCTATCACAAACGCCAAACGAACCCCACTCCACAATCCAGCATCAGATATGCCTGAGCTACGCTGTAAAATCTACGAAGGTTTTGCATCGCTCACGTCAGATACGTGGGTAAATGCATACACACACGCACAGAAATACGAGACAAAATATCTGCAGCAAGGTGATGAATGCGAAATTGCATCGGAACCAGAGGGCAGCAACGAGGACACGGAAGAGGATGACGCCGTCGATGACAATGAAGCTGTAGCCGATTAGGAATATGAACGGGTGACCCGTTGTATGTTCTTTACACTCTAGATATTCTTTTGACGGGTTCTTTTTAACAAGTTTATAATTTTCTAACAATCGTCAAATTGCGGTAATATTTGGAATTTATCCATCCGGTTCTGAAAATCCCCACCTGTTATCCCGGCTCTGCCAATGAATACGCATGCATGTTAGTACGTGCCAAGCCCACAATTGCAAACAATTCTGCAAGATATAGAGCCTTTTTTAAACGGCCGATTGGGAGATCAATGGTTGCAGATACGCAAAAATAGACCTCAAAATCGGAATCTACGCCCGATTTTACCCCTTATATCTAACTATCGTTATGGGTTTTGTGAAGGGAAGGGTTACCTTTTTTGGACCAAAGTTTGTCCAAGGTTTGGTGGCCGGGATAATAATAGATTCTCTCACCTGGGAAGTGCAATGCGCGGCTCGCTGGTGAGCTCGGCGCTGGGCCCGCCTCCGCAAATACCGCGCGGCCCTGCTTCGCGGGCTCCCCGCGCGGGTTTGCTGCGGCTACGCGACCTCGCACCCGCTCGCCTTAGTTGGCGCCATAGCTGCGTCGCCCCTCCCCGGAAGGTGCTATTATGCGTTTGAGGGTGCTGCACACCGTTTCGAACGGTGTTTTTGCAATTTTGTCGCATTTTGTTCCGGCCTGCGTGTTCTTCCAGTTTGTTTGGCGTTTGAAGTGCTTGTCGTTGTTTCATTGTGTACGTTATAAATAGCAATGACAGTACTGGAGTATTAATACCTGTAGCTGTCCATCATGGACACTCCATCGGACTCCGATGGTCCGATTCGGAGAGCCATTGCGCAAGCCGGAGTGGCGGGGATTCCTTCTCCACCTTCGCATATTCATAGAAGGGGGAGGGTAATCTTGATCCGGTGCAAACAGCGGCGATTACTGTGTGTAGTATGGGAGTCAACTGACATTGACTCAAATTGACTCAGATAAAGTGCAACCACAAGCCAAACTCCTCGATGTCTGCACCTCCCGATGATAGCACGCATGAATTGAAAGCGCTCTGCGCATCCTGCTCAAGAGCGTAGGCAAATCGCGCTCAATACTTGAGCCAAGTGGGAAACCGACGGCTAAACAAATCGGACTTCACTTCAACGTGCTTTTGGTTGTCACAAACAGCTGGGGCGACAGCGCAGTTACGGTATCAAGACCGGTGCAGAAAGGTTGAGTGTCCTATGAATAACGCAATGTTGGGCATGCTTTCTGGACAAGCTGTAAGATTTTATTGAGAAGAAGAAACACGCTGCAACCATCAAGCATGGAATGTACAATTGCTTAAGCACCACTACAGCTAAATTAATGTACATGTTACATGAACAGTGTACAGATCTGTATAGTGTCGCAGCATGTCATGTCTGTGACACCTCTGTTTCGGAAGATGCACACTGTATGTACATACACGCGTTCTCCAGTCGTGCATGCTGATCTCTATGCATGTGGGTTCGACTGTGCTTCTCCACGTCTGTAAAACCGCCGCACCAACAGCGCTCTCTGGCCATCGACTTCATACCTTAACGCCACCAACGAGCGGGCGTTGCTCTATCTCGTGACGAGATTCTCGTCCAGCGCGGACGTCCCATCTTATCTAGCCAGCTTCGAAAATGTGCAGACACTGCGGAAGCGTTGCACCGCGCCTTATCTTCGAGATTTGCACGTCCTCTTCCAGAACACCTTCCAGAATGTCGACCGAGACTACATGTACACGAATATGTACAACTACCGCACCCCAACCGACCAACAAGCACGACAAGCTGCGTGCTCGCCGCAAATGACGACTAGCAAATTGCTAACGGCCATTGCTAAGCAATGGCCGTTGGTCTCCGCTGCACGAATTAAAGCCTCTTGCGTGAGTGAGGGTCTCGTCCGTCGAAACGCGAATCAGATCGCGTCATTAGAGAGATTAACCAATCAAATTGAAGGCCCATCACGTTACCTTTTGAGATCTCGCACGTGCGTTTCGCAGGCTGCGCGGTGAAGTGCCGCATCCATCATCTTTAGCCCATCCAAAGGCCCTTTAAAAACCGACGTTATCATTTCCAAATTGGCTCTCTAGGCCTCACAACACCTCCAACTACCACAGACGGGAGTTAACATAACCTCCCGTACTTTATCAACTAAGAACTTCAACTCGCATACTTTCCGACTGCTATGCCAGGCTACGAGAGTGGGGCGTCCTTGCCCTCACGCTCACTGCAGGATAACGGCAACTACGCCAGACTGGAGTCGCCTCGTGCGTCCTCCCACTCGCTCGAGTCGGCAGCCCACCATGTCGTGGACGTGTCCAGCGGCGCGCGCTCCTTCTCAGCCGCTGATCTGTACCACGTCGACGCCAAGATGGCAACCAACAGCAAATCGGTTGCTGGAGGATCCAGTACTCCGCTACCTGATCACCGTTCGCGATTCTTGAATGACGGAGAGCTTCTCCTTGCTGCCAATCCCGTGCCCACGCGCGAGTCCTTCGCGACACTTAATGAGACCGACGAGAAGACGTGGCACCAGCGATCTACCGAGCTGATCACTAAGATGCTAAACACGAACATCGAGCGGGGACTAAATGTCATGGACGTGGAGCGTCGGATACTGCGTTACGGTGTCAATGCACTGGAAGAAGAAGCTAAAACGCCAGTCAGCGTGATCTTCCTGCTGCAGTTCTACAATCTAATCATCGCCATGCTGCTCTTCGCTGCGTTGGCGTCTTTGGCCTTGCAAGAATGGGTTGAAGGTATCGCTATCCTCGCCATTGTCACGCTGAACGCAGGCGTGGCGACGTACCAAGAACACTCCGCCAGTAACGCGTTAGCCGCGCTTGCCAGTCTGTCCAGCCCGCAGTCGCTTGTCATCCGTGACGGGATGCAACAAGTGGTGGACAGCAAGCAGCTGGTGCCTGGAGACATCGTGATCCTCGTCACAGGCGACGTTGTGCCTGCAGACATCCGACTGTTCACTAGTGTCGATCTCAAGTGTAACGAGATGCTGCTGACGGGCGAATCGGAGGATGTCCCCAAGAAATACAACGCTCCGATCCACCCCGCTGGAGCCGGCAAACCTGCCAAGTTGACAGCGAGTAACATGGTCTTCTCGTCCACGACGATCACCGCCGGCAACGCTCGTGGTATTGTAGTGGAGACTGGTATGAACACGCGTGTGGGCTCCATTGCAGCTCTACTACAAGCCAAGAGCGGCACGGATGCGTCTGCAGAGAAGAAGTGGATCCGTAACCCGCTTGGTGACTGCATCGCCAAGCACCGTCCGAAGCTCACGCCTCTGCAGCGAGCTCTACACCACACTGGGTACGTCATGGGTCTTATCGCGGTGGGTGTGGCCATCCTCGTGTTTATCGTCGGTATGATCCGCGGCAATGAAGACCCTCGTCACCCGGACAGACCCACGTACTTGACTATGATTATGGCTGCTGTGTCTGTGGCCGTGAGTGCTGTTCCCGAGGGTCTACCGATGGTCGTGACTATCTGTCTGTCGTCCGGTACGGCCGAGATGGTCAAGAAGAATGTGCTTGTACGTAAACTCGCGTCAGTGGAGACGCTAGGCGCAGCGTCGGTCATCTGTACCGACAAGACGGGAACATTGACGGAAGGCAAGATGACTGCAGTGAAGCTCTGGGGCGATTTCCGCGAGTATTCAATCACTGGCAAGGGGTTCACACCTGAAGGCTCTATCCTCGCTTCGGACGGTAGCAGTCAAGGCGAACCGGAGGCCGGAAATGTGCAAGTTCGCGCGACTTTGATGGCCTCGGTGCTGTGCAGTAACACGCAACTCAAGCAAGTGGAAGGGGACGACGGCGAGACTCCACGTTGGCTTCCGTTTGGCAACTCGTCCGAGGCGCCTTTGGTCGTTGCAGCCGCTAAGGCAGGGATCTGGGAAGATAGTCTGCTGGAAGACTACCCTCGGCTAGTGGAAGTACCGTTCAGCTCATCACGTAAGATGATGGTCACAGTGAACGCCCTACCTGTGGTGAATGGCATGGCGATGTTCGATACGTTGGCGCTTCCAGGTGACCAGCCGCCCAAGCTGGTGGCGAACGTGAAGGGCGCACCGAACTACATTTTACGCAACTGCACCCAGTACTGCCGGAAGGATGGCACGTTCGAGACGTTGAACAACGTGCAGCGTCAGGAGATTCTCGAGGCGGTGGACGCGCTATCGTCGCAGGCGCTGCGTGTTCTTGCTGTGGCTATCCAGCCGATGCACGAATTGCCGTTCGGGGAAGACTGCGACGATGTGGACGAGAAGTTTGAAGCCTTGTCCAAGCCGTTGGTGTTCCTCGGACTGGTGGCGTCTATCGACCCGGAACGTGACGGCGTGCGCGATGCCATTGCCACTGCACGAGCTGCTTCCATCCGTACGGTGATGATCACTGGCGACTACCTCGCTACGGCTGTTGCCATCGCCAAGAACATTGATTTGCTACAAGTGGGCGCGGACCCGGAGGCTCAAGCGACAGACTGCACGCAGCTTCGTCCGAATGGAGACGTGTACTTGCCCCCCGCTGACATCGACGAGATCACGTCACGCACGTTGGTGTTCGCTCGTGCCAAGCCCGAAGACAAGATCGAGATCGTGAAATCTCTTCAACGTCAAGGACTCATCGCTGCTATGACTGGAGACGGTGTCAACGACGCACCAGCGTTGAAGGAGGCGGATATCGGTGTGGCCATGGGGATTTCAGGCACGGAAGTGGCCAAGGGAGCGTCCGACATGATCTTGATGGACGACAACTTCTGCAGTATCGTCACGGCTGTGGAGAAGGGCCGCGTCATCTACGCCAACATCCAGAAGTTTGTCATGTTCTTGCTGTCCACGAACATCGGAGAGATCATCCTGATCTTCATCTCGGTGGCTGGCGGGTTCCCGCTTCCTCTCGAGGCGCTGCACATTCTGCTGCTCAACCTGTTCACGGACGGTATGCCTGCTGTCGCTCTAAGCTTAGAGAAGGGCGACCCACACATCATGGCAGACAAACCACGTCACAAGCAGACTTCGCTGATCCACGGTCGACTGTGGCTGCTCGTGCTCTTCAACGCGTTCCTGCTTCTCGCTGGAGCTATGACCACTTTTCTCTTGGGACTGTACTGGAACTTCGGCGTGTTGCTGACGGACGACATCTACAATACTGGCGGCGGTCCAGATGGGACGGACTTCACGGACGTGACGTGTCGTCGATGGGAAGGCATCGACGATGGCTGGAAAGTGTACGGCAACTGTGCTGCTCAGTACTCGGACGGCTCGTACATTTTCGGCGAGGAAGTGGCCGGTATGACGTCGTATGAGAACTCGACTGTTTACTGTGAAGGAGGCGACTACGACTGCGTCCCAGCGGGTCTGGGACGAGCTCAGACGATGGTGTTCTTGGGTTTGGCCTTCACGGAGGTGCTGCGAGCCTACACGGTGCGTCACTTCACGGAGCCTGTGTTCGCACGGATGTTGTCGAATGGCTACATGCAGTTGGCGGCGTGTATGTCGGTGATTTTGACGGTGCTCGTGAGCAACGTCCCTGTTATCATGGACGACATCTTTGGATTCGAGTACATTCCGTGGTATCAGTGGCTTGTGGTCGGAGCTGTTGCAGTCAACAACGCGTTCTGGGGTGAGATCCTGAAGGCCTATTTGCGACGGAAGGACCGGGCGCAGGCTCGCTGGGACCACATGAAGAGCGGGTTCGAGGAGATTTTGCTCGAGATCCGGCATGTGCGCCATCATGTTGAGAGGCTTGAAGCGGGTGGAGCTCATCGTGGACTCAAGCGTGAGTAAAAGGCGACAATGTCACCTTGGGAGCGAAGGTGACGAAAGTGTGTATTGCGTATGGATCGACGTGCGTTAAGGCAGCTAAGACTAGGAAAAACTGCCGCGTGAGTTCGTTCTCGAGCCCCAAGAGCAGAGACCGAGTGTAGTTAGCTTTGCGGTTAATGAATTTATCAACATGAATGCTCATTGATGAATGCTTATTGAAACACAGTTAAATGGAGATATTGTAGGCGTCCTAGATGCTGAAGTACAAATCTCTGCCGTCTAGGAGACTGTACTCACGATATTGCTGTGGCTACAAAAATGCTACAATGTTACATGTAAAATGCTCAGGCTGGATCTGAGTCTGAATAGCTGATTTGGGCTTTATCACTTATGCGAGGGCGGAGGTTACGGACTGGGCTTTATCACTTATGGGAGGGCGGAGGGCTCAATTTGGGCCGGGTTTGCTGATTGCGCTGCGACGGGTTCTTTCGAGAATACAAACTGGCAGTATACGGATCAGCAGCAAGAAAAATGTAGACCAAGATAAAAGAGGGATCGGCTGGACCTCATCTTTTATAGTGAATAATGGTGGTAATGCTTGCGTTTACTATGGCAAGTCATTACTATCAAAATATGCTGCGAAAGGTGAAACTCAAAACTTCATACGAAAAGACATTGTGTGAATCCGTCAAAGTGAGTACGATCGACGAGCGCGTAGCCCCGAACATGCACGAAACCCGGATGGGGTGGCTGCAGAGCGGTGGTGGATAAGTGGTTGGTCACCGTCCATTCGCCTTCATCCGCCGTTTGTTTATTCGGCCTCATTCGCGAATCTGCCTCGGAGACAACAAGCGCCTCGAAATGCTACCACTACCGTGCTTGAGATTTGGCGCCCATGTAGTCATATCTCGATCTTGTGAGATTGACACCTCGGTAGTCTCGCCTTCTGTGCGGACGCAAACTCGAAGCTCACACAATTTATATACACCGACTTCAACATTGACAGTCATCCATTCGTCCACCCAGCTGTGGGCTGAAGATTGAGACTAGCCCCAACACAGCCGAAGTCCGCCCCCAAGCGTGTTTTGTGTCAAGATCCTGTACGAGCGCTACACGACACTCAAGTCTCCCTCGGTTGACTCCTGAAACCAGGAGCACGGATCAAGCTCGCCCAAGTTGTGTATCAACTAGAGCTAGGCCAAGTTGCTGTCAAGGCTCCAGTCCACGACGCCAATGCGTCTACCATGCCAGAGCTGCCACGTCAGCTGTCGCTAGTCTGATCAAGATGCCTGGATGCAATAAGCCCTCAACATGGATCAAGCCACCGTTTCTGGCACCATGAGGTCCATTGAGAAGCCATTCAAAGGAGTGGAAGCTGTGTGAGCCTCCACATATATGCTCGTCCTCGACCTCATCTGGAAGCCACCTTATGAATGTGCAGGCTAGCGTGGGGGCTGTTCGCGGTTGCAATTGCGTCTACAGCAGTGATTGCGGTAGCACCAAGCTGCAGGCTTTGCTCAAGATCACACTAGTTCCTGCCACTGATGATGTGATCCCTGCCACTGATTATGTGTTGGCGGTGCTGTGTTGTGACTCGTACACCTTCTATCAGTGTCGACCCAAGCCGAGGACGGTCATTTTCCCAAGTGCCATCCAGAGTGTACGCAAACATTACCGCAGCTCCTGGCTGTTGATGTCATTGCTGCCGCTTCAAAGTCTTTGCCGTTGCCGCCACCTATGCTACCACGCCCAGGCTGTTGGTGCTGTCGGTATCAACGGTACCAAGCTCACGTCGTCGGTGACACAGACCCCAATGCCACCCCCAAGCGATTGATGTTGTCGGTGCCAGCGCTGCCAATTTCATGCCATCGCTGCTGGCGCCATCTCTACCACGTCATCGGCTCTACCGGCACTAGTACTTCCACTCTCAAGTCGTTGGTGTTGCCGTCACTAAATCTTCCACTTTCATGTTGTCTGTGTTATCGGTGTCAACGCTACCAGCTCTATGCCGTCGGCGTTGCTACGAGTAATGCGGTCGCTTTCAAGTCCAGCCCCGATGTCGCTCTCCCAAGCTGCCGGCGTTGCCAGGACCAATGCTGTCACGTTCAAGTTATTGTCGCCAGTGTTGTCGGCACCAGCGTTCTACTCCAAGGCGTCGCTGTTTTCGACAAACTAGCAGCTTCACGTCGTCGGTACTGTCGCCGACAGCTTGCCGGTGCCATGCATATTCTGCTGGTCGCGCAATACTGCCCACGTCGATGGCGGCGATTCTGTTGCCACCAAGCACTACCATTTGTAACGAGATACACTGGTCGGCGTTGCTGCCGCTACTGTGAGAACGACGTGTGTTGGTGAGTCATTGTTGCGGAGTAAGGGCTTTCGAGAGGAGATGGTCGTCCACATACAAAACAGACACCGCGCATCGCCATGCTTGTGTCACGTTAGTGTCACACTCTCACCGTCTCGCGCATGTGTAGGCAGCCTTGCACTTTGCAGAGGACAAGATACAGCAGCTTGCTGAAGCGCGGCGTTCGTGTGATACACGTATGGCACCACGACGGAATGCCGTAATCTGCGTGCGCGTTGTGTCGTGGAGTCTAGATACACAAGTTTGCGTCGCTGACGCTCAACTATTGCGTCTGTACAACGGCAGGTAGCAGGCTGCACGCTACGAGTTTTACGGCGGTGGTCGTGTTACCAGAAGTGTTGAGCACTTCAATCGCTCTTCGCCGGCTGTCTGCTGTTGCTGCAGTAGATGACGGTATGCTCGCGGTGGTAAGGCGGTGCGCTGCATAATCGTCGTCACAAATGGCTCAAGCGCTGTTGCCGCTCCTTTTTCTGATAACTCACGGGAGATGCGACTCTTCGTTCTGTGGTCCACATAATCTGGGAAAATGACTTCTCAACGATGTGGTCCACAAAATGTGATTATAGAAACTGCACAAAAGAACCAGAATTGCCTTTCATGCTGACGAGAGGTCTGCTACATCGTCCCTGAAGCGCGAATGCACCCATCTCCGAGACCATGAAACTCAGGAGAAGCTGAACAAACAAGTCTCGTCCTTAGGGGTAACTTTGCGGGGAAAGAGTGTTGAAAGTGCGCTCGAGCTTTCGGCTGCAGTTGGATGTCGAGAGATCTTTGCATCTTCACTCGTCTGCAGTCGCTTGTCATCCGTGACGGGATGCAACAAGTGGTGGACAGCAAGCAGCTGGTGCCTGGAGACATCGTGATCCTCGTCACAGGCGACGTTGTGCCTGCTGACATTCGGCTGTTCACAAGTGTCGATCTCAAGTGTAACGAGATGCTGCTGACGGGCGAATCGGAGGATGTTCCCAAGAAATACAACGCTCCGATCCACCCCGCTGGAGCCGGCAAACCTGCCAAGTTGACAGCGAGTAACATGGTCTTCTCGTCCACGACGATCACCGCCGGCAACGCTCGTGGTATTGTAGTGGAGACTGGTATGAACACGCGTGTGGGCTCCATTGCAGCTCTACTACAAGCCAAGAGCGGCACGGATGCGTCTGCAGAGAAGAAGTGGATCCGTAACCCGCTTGGTGACTGCATCGCCAAGCACCGTTCGAAGCTCACGCCTCTGCAGCGAGCTCTACACCACACTGGGTACGTCATGGGTCTCATCGCGGTGGGTGTGGCCATCCTCGTGTTTATCGTCGGTATGATCCGCGGCAATGAAGACCCTCATCACCCGGACAGACCCACGTACTTGACTATGATTATGGCTGCTGTGTCTGTGGCCGTGAGTGCTGTTCCCGAGGGTCTACCGATGGTCGTGACTATCTGTCTGTCGTCCGGTACGGCCGAGATGGTCAAGAAGAATGTGCTTGTACGTAAACTCGCGTCAGTGGAGACGCTAGGCGAAGCGTCGGTCATCTGTACCGACAAGACGGGTACATTGACGGAAGGCAAGATGACTGCAGTGAAGCTCTGGGGCGATTTCCGCGAGTATTCAATTACTGGCAAGGGGTTCACACCTGAAGGCTCTATCCTCGCTTCGGACGGTAGCAGTCAAGGCGAACCGGAGGCCGGCAATGTGCAAGTTCGCGCGACTTTGATGGCCTCGGTGCTGTGCAGTAACACGCAACTCAAGCAAGTGGAAGGGGACGACGGCGAGACTCCACGTTGGCTTCCGTTTGGCAACTCGTCCGAGGCGCCTTTGGTCGTTGCAGCCGCTAAGGCAGGCATCTGGGAAGATAGTCTGCTGGAAGACTACCCTCGGCTAGTGGAAGTACCGTTCAGCTCATCACGTAAGATGATGGTCACAGTGAACGCCCTACCTGTGGTGAATGGCATGGCGATGTTCGATACGTTGGCGCTTCCAGGTGACCAGCCGCCCAAGCTGGTGGCGAACGTGAAGGGCGCACCGAACTACATTCTACGCAACTGCACCCAGTACTGCCGGAAGGATGGCACGTTTGAGACGTTGAACAATGTGCAGCGTCAGGNAGATTCTCGAGGCGGTGGACGCGCTATCGTCGCAGGCGCTGCGTGTTCTTGCTGTGGCTATCCAGCCGATGCACGAATTGCCGTTCGGGGAAGACTGCGACGATGTGGACGAGAAGTTTGAAGCCTTGTCCAAGCCGTTAGTGTTTNCTCGGACTGGTGGCGTCTATCGACCCGGAACGTGACGGCGTGCGCGATGCCATTGCCACTGCACGAGCTGCTTCCATCCGTACGGTGATGATCACTGGCGACTACCTCGCTACGGCTGTTGCCATCGCCAAGAACATTGATTTGCTACAAGTGGGCGCGGACCCGGAGGCTCAAGCGACAGACTGCACGCAGCTTCGTCCGAATGGAGACGTGTACTTGCCCCCCGCTGACATCGACGAGATCACGTCACGCACGTTGGTGTTCGCTCGTGCCAAGCCCGAAGACAAGATCGAGATCGTGAAATCTCTTCAACGTCAAGGACTCATCGCTGCTATGACTGGAGATGGTGTCAACGACGCACCAGCGTTGAAGGAGGCGGATATCGGTGTGGCCATGGGGATTTCAGGCACGGAAGTGGCCAAGGGAGCGTCCGACATGATCTTGATGGACGACAACTTCTGCAGTATCGTCACGGCTGTGGAGAAGGGCCGCGTCATCTACGCCAACATCCAGAAGTTTGTCATGTTCTTGCTGTCCACGAACATCGGAGAGATCATCCTGATCTTCATCTCGGTGGCTGGCGGGTTCCCGCTTCCTCTCGAGGCGCTGCACATTCTGCTGCTCAACCTGTTCACGGACGGTATGCCTGCTGTCGCTCTAAGCTTAGAGAAGGGCGACCCACACATCATGGCAGACAAACCACGTCACAAGCAGACTTCGCTGATCCACGGTCGACTGTGGCTGCTCGTGCTCTTCAACGCGTTCCTGCTTCTCGCTGGAGCTATGACCACTTTTCTCTTGGGACTGTACTGGAACTTCGGCGTGTTGCTGACGGACGACATCTACAATACTGGCGGCGGTCCAGATGGGACGGACTTCACGGACGTGACGTGTCGTCGATGGGAAGGCATCGACGATGGCTGGAAAGTGTACGGCAACTGTGCTGCTCAGTACTCGGACGGCTCGTACATTTTCGGCGAGGAAGTGGCCGGTATGACGTCGTTCGAGAACTCAACTGTTTACTGTGAAGGAGGCGACTACGACTGCGTCCCAGCGGGTCTGGGACGAGCTCAGACGATGGTGTTCTTGGGTTTGGCCTTCACGGAGGTGCTGCGAGCCTACACGGTGCGTCACTTCACGGAGCCTGTGTTCGCACGGATGTTGTCGAATGGCTACATGCAGTTGGCGGCGTGTATGTCGGTGATTTTGACGGTGCTCGTGAGCAACGTCCCTGTTATCATGGACGACATCTTTGGATTCGAGTACATTCCGTGGTATCAGTGGCTTGTGGTCGGAGCTGTTGCAGTCAACAACGCGTTCTGGGGTGAGATCCTGAAGGCCTATTTGCGACGGAAGGACCGGGCGCAGGCTCGCTGGGACCACATGAAAAGCGGGTTCGAGGAGATTTTGCTCGAGATCCGGCATGTGCGCCATCATGTTGAGAGGCTTGAAGCGGGTGGAGCTCATCGTGGACTCAAGCGTGAGTAAAAGGCGACAATGTCACCTTGGGAGCGAAGGTGACGAAAGTGTATATTGCGTATGGATCGACGTGCGTTAAGGCAGCTAAGACTAGGAAAAACTGCCGCGTGAGTTCGTTCTCGAGCCCCAAGAGCAGAGGCCGAGTGTAGTTAGCTTTGCGGTTAATGAATTTATCAACATGAATGCTCATTGATGAATGCTTATTGAAACACAGTTAAATGGAGATATTGTAGGCGTCCTAGATGCTGAAGTACAAATCTCTGCCGTCTAGGAGACTGTACTCACGATATTGCTGTGGCTACAAAAATGCTACAATGTTACATGTAAAATGCTCAGGCTGGATCTGAGTCTGAATAGCTGATTTGGGCTTTATCACTTATGCGAGGGCGGAGGTGGTACGGACTGGGCTTTATCACTTATGGGAGGGCGGAGGGCTYAATTTGGGCCGGGTTTGCTGAACGGGTTCTTTCGAGAATACAAACTGGCAGTATACGGATCAGCAGCAATAGACCAGGATAAAAGAGGGATCGGCTGGACCTCATATAGTGAATAATGGTGGTAATGCTTGCGTTTACTATGGCAAGCCATTACTATCAAAATATACTGCGAAAGGTAAAACTTCAAACTTCAAACGATAAGACATTGGGGTGAGCCCGTCAAAGTGAGTACGATCGACGAGCGCGTAGCTGACAACGGCCCCGGACATGCACGAAACCCGGATGGGGTGGCTGCAGAGCGGTGGTGGATAAGTGGTTGGTCACCGTCCATTCGCCTTCATCCGCCGTTTAATTATTCGGCCTCATTCGCGAATCTTCCTCGGAGACAACAAGCGCATTGAAATGCTACCACTACCGTGCTTGAGATTTGGCGCCCATGTAGTCACATCTCGATCTTGTGAGATTGACACCTCGGTAGTCTCGCCTTCTGTGCGGACGCAAACTCGAAGCTCACACAATTTACATACACCGACTTCAACATTGGCAGTCGTCCATTCGTCCACCCAGCTGTGGGCTGAAGATTGGGACTAGCCCCAACACAGCCGAAGTCCGCCCCCAAGCGTGTTTTGTGTCAAGATCCTGTACGAGCGCTACACGACACTCAAGTCTCCCTCGGTTGACTCCTGAAACCAGGAGCACGGATCAAGCTCGCCCAAGTTGTGTATCAACTAGAGCTAGGCCAAGTTGCTGTCAAGGCTCCAGTCCACGACGCCAATGCGTCTACCATGCCAGAGCTGCCACGTCAGCTGTCGCTAGTCTGATCAAGATGCCTGGATGCAACAAGCCCTCAACATGGATCAAGCCACCGTTTCTGGCACCATGAGGTCCATTGAGAAGCCATTCAAAGGAGTGGAAGCTGTGTGAGCCTCCACATATATGCTCGTCCTCGACCTCATCTGGAAGCCACCTTATGAATGTGCAGGCTAGCGTGGGGGCTGTTCGCGGTTGCAATTGCGTCTACAGCAGTGATTGCGGTAGCACCAAGCTGCAGGCTTTGCTCAAGATCACACTAGTTCCTGCCACTGATGATGTGATCCCTGCCACTGATTATGTGTTGGCGGTGCTGTGTTGTGACTCGTACACCTTCTATCAGTGTCGACCCAAGCCGAGGACGGTCATTTTCCCAAGTGCCATCCAGAGTGTACGCAAACATTACCGCAGCTCCTGGCTGTTGATGTCATTGCTGCCGCTTCAAAGTCTTTGCCGTTGCCGCCACCTATGCTACCACGCCCAGGCTGTTGGTGCTGTCGGTATCAACGGTACCAAGCTCACGTCGTCGGTGACACAGACCCCAATGCCACCCCCAAGCGATTGATGTTGTCGGTGCCAGCGCTGCCAATTTCATGCCATCGCTGCTGGCGCCATCTCTACCACGTCATCGGCTCTACCGGCACTAGTACTTCCACTCTCAAGTCGTTGGTGTTGCCGTCACTAAATCTTCCACTTTCATGTTGTCTGTGTTATCGGTGTCAACGCTACCAGCTCTATGCCGTCGGCGTTGCTACGAGTAATGCGGTCGCTTTCAAGTCCAGCCCCGATGTCGCTCTCCCAAGCTGCCGGCGTTGCCAGGACCAATGCTGTCACGTTCAAGTTATTGTCGCCAGTGTTGTCGGCACCAGCGTTCTACTCCAAGGCGTCGCTGTTTTCGACAAACTAGCAGCTTCACGTCGTCGGTACTGTCGCCGACAGCTTGCCGGTGCCATGCATATTCTGCTGGTCGCGCAATACTGCCCACGTCGATGGCGGCGATTCTGTTGCCACCAAGCACTACCATTTGTAACGAGATACACTGGTCGGCGTTGCTGCCGCTACTGTGAGAACGACGTGTGTTGGTGAGTCATTGTTGCGGAGTAAGGGCTTTCGAGAGGAGATGGTCGTCCACATACATAACAGACACCGCGCATCGCCATGCTTGTGTCACGTTAGTGTCACACTCTCACCGTCTCGCGCATGTGTAGGCAGCCTTGCACTTTGCAGAGGACAAGATACAGCAGCTTGCTGAAGCGCGGCGTTCGTGTGATACACGTATGGCACCACGACGGAATGCCGTAATCTGCGTGCGCGTTGTGTCGTGGAGTCTAGATACACAAGTTTGCGTCGCTGACGCTCAACTATTGCGTCTGTACAACGGCAGGTAGCAGGCTGCACGCTACGAGTTTTACGGCGGTGGTCGTGTTACCAGAAGTGTTGAGCACTTCAATCGCTCTTCGCCGGCTGTCTGCTGTTGCTGCAGTAGATGACGGTATGCTCGCGGTGGTAAGGCGGTGCGCTGCATAATCGTCGTCACAAATGGCTCAAGCGCTGTTGCCGCTCCTTTTTCTGATAACTCACGGGAGATGCGACTCTTCGTTCTGTGGTCCACATAATCTGGGAAAATGACTTCTCAACGATGTGGTCCACAAAATGTGATTATAGAAATTGCACAAAAGAACCAGAATTGCCTTTCATGCTGACGAGAGGTCTGCTACATCGTCCCTGAAGCGCGAATGCACCCATCTCCGAGACCATGAAACTCAGGAGAAGCTGAACAAACAAGTCTCGTCCTTAGGGGTAACTTTGCGGGGAAAGAGTGTTGAAAGTGCGCTCGAGCTTTCGGCTGCAGTTGGATGTCGAGAGATCTTTGCATCTTCACTCGTCTGCAGTCGCTTGTCATCCGTGACGGGATGCAACAAGTGGTAGACAGCAAGCAGCTGGTGCCTGGAGACATCGTGATCCTCGTCACAGGCGACGTTGTGCCTGCTGACATTCGGCTGTTCACAAGTGTCGATCTCAAGTGTAACGAGATGCTGCTGACGGGCGAATCGGAGGATGTCCCCAAGAAATACAACGCTCCGATCCACCCCGCTGGAGCCGGCAAACCTGCCAAGTTGACAGCGAGTAACATGGTCTTCTCGTCCACGACGATCACCGCCGGCAACGCTCGTGGTATTGTAGTGGAGACTGGTATGAACACGCGGGTGGGCTCCATTGCAGCTCTACTACAAGCCAAGAGCGGCACGGATGCGTCTGCAGAGAAGAAGTGGATCCGTAACCCGCTTGGTGACTGCATCGCCAAGCACCGTTCGAAGCTCACGCCTCTGCAGCGAGCTCTACACCACACTGGGTACGTCATGGGCCTCATCGCGGTGGGTGTGGCCATCCTCGTGTTTATCGTCGGTATGATCCGCGGCAATGAAGATCCTCGTCACCCGGACAGACCCACGTACTTGACTATGATTATGGCTGCTGTGTCTGTGGCCGTGAGTGCTGTTCCCGAGGGTCTACCGATGGTCGTGACTATCTGTCTGTCGTCCGGTACGGCCGAGATGGTCAAGAAGAATGTGCTTGTACGTAAACTCGCGTCAGTGGAGACGCTAGGCGAAGCGTCAGTCATCTGTACCGACAAGACGGGTACATTGACGGAAGGCAAGATGACTGCAGTGAAGCTCTGGGGCGATTTCCGCGAGTATTCAATCACTGGCAAGGGGTTCACACCTGAAGGCTCTATCCTCGCTTCGGACGGTAGCAGTCAAGGCGAACCGGAGGCCGGCAATGTGCAAGTTCGCGCGACTTTGATGGCCTCGGTGCTGTGCAGTAACACGCAACTCAAGCAAGTGGAAGGGGACGACGGCGAGACTCCACGTTGGCTTCCGTTTGGCAACTCGTCCGAAGCGCCTTTGGTCGTTGCAGCCGCCAAGGCAGGCATCTGGGAAGATAGTCTGCTGGAAGACTACCCTCGGCTAGTGGAAGTACCGTTCAGCTCATCACGTAAGATGATGGTCACAGTGAACGCCCTACCTGTGGTGAATGGCATGGCGATGTTCGATACGTTGGCGCTTCCAGGTGACCAGCCGCCCAAGCTGGTGGCGAACGTGAAGGGCGCACCGAACTACATTTTACGCAACTGCACCCAGTACTGCCGGAAGGATGGCACGTTCGAGACGTTGAACAACGTACAGCGTCAGGAGATTCTCGAGGCGGTGGACGCGCTATCGTCGCAGGCGCTGCGTGTTCTTGCTGTGGCTATCCAGCCGATGCACGAATTGCCGTTCGGGGAAGACTGCGACGATGTGGACGAGAAGTTTGAAGCCTTGTCCAAGCCGTTAGTGTTTCTCGGACTGGTGGCGTCTATCGACCCGGAACGTGACGGCGTGCGCGATGCCATTGCCACTGCACGAGCTGCTTCCATCCGTACGGTGATGATCACTGGCGACTACCTCGCTACGGCTGTTGCCATCGCCAAGAACATTGATTTGCTACAAGTGGGCGCGGACCCGGAGGCTCAAGCAACAGACTGCACGCAGCTTCGTCCGAATGGAGACGTGTACTTGCCCCCCGCTGACATCGACGAGATCACGTCACGCACGTTGGTGTTCGCTCGTGCCAAGCCCGAAGACAAGATCGAGATCGTGAAATCTCTTCAACGTCAAGGACTCATCGCTGCTATGACTGGAGATGGTGTCAACGACGCACCAGCGTTGAAGGAGGCGGATATCGGTGTGGCCATGGGGATTTCAGGCACGGAAGTGGCCAAGGGAGCGTCCGACATGATCTTGATGGACGACAACTTCTGCAGTATCGTCACGGCTGTGGAGAAGGGCCGCGTCATCTACGCCAACATCCAGAAGTTTGTCATGTTCTTGCTGTCCACGAACATCGGAGAGATCATCCTGATCTTCATCTCGGTTGCTGGCGGGTTCCCGCTTCCTCTCGAGGCGCTGCACATTCTGCTGCTCAACCTGTTCACGGACGGTATGCCTGCTGTCGCGCTAAGCTTGGAGAAGGGCGACCCACACATCATGGCAGACAAACCACGTCACAAGCAGACTTCGCTGATCCACGGTCGACTGTGGCTGCTCGTGCTCTTCAACGCGTTCCTGCTTCTCGCTGGAGCTATGACCACGTTCCTCTTGGGACTGTACTGGAACTTCGGCGTGTTGCTGACGGACGACATCTACAATACTGGCGGCGGTCCAGATGGGACGGACTTCACGGACGTGACGTGTCGTCGATGGGAAGGCATCGACGATGGCTGGAAAGTGTACGGCAACTGTGCTGCTCAGTACTCGGACGGCTCGTACATTTTCGGCGAGGAAGTGGCCGGTATGACGTCGTTCGAGAACTCAACTGTTTACTGTGAAGGAGGCGACTACGACTGCGTCCCAGCGGGTCTGGGACGAGCTCAGACGATGGTGTTCTTGGGTTTGGCCTTCACGGAGGTGCTGCGAGCTTACACGGTGCGTCACTTCACGGAGCCTGTGTTTGCACGGATGTTGTCGAATGGCTACATGCAGTTGGCGGCGTGTATGTCGGTGATTTTGACGGTGCTCGTGAGCAACGTCCCTGTTATCATGGACGACATCTTTGGATTCGAGTACATTCCGTGGTATCAGTGGCTTGTGGTCGGAGCTGTTGCAGTCAACAACGCGTTCTGGGGTGAGATTCTGAAGGCCTATTTGCGACGGAAGGACCGGGCGCAGGCTCGCTGGGACCACATGAAGAGCGGGTTCGAGGAGATTTTGCTCGAGATTCGGCATGTGCGTCATCATGTTGAGAGGCTTGAAGCGGGTGGAGCTCATCGTGGACTTAAGCGAGAGTAAAAGGCGACTATGTCACCTTGGGAGCGAAGGTGACGAAAGTGTGTATTGCGTATGGATCGACGTGCGCTAAGGCAGGAAAAACTGCCGTTCTCGAGCCCCAAGAGCAGAGGCCGAGTGTAGTAAGCTTTGCGGTTAATGGATTTATCAACATGAATGCTTATTGAAACACAGTTAAATGCAGATATTGTAGGCGTCCTAGATGCTGAAGTACATGTGTATTTGGTAACTCAGGGAGTTACCAGAGAAAATCTCTGCTGTCTAGGAGACTGTACTCACGATATTGCTGTGGTTACAAAAATGCTACAGTATTACATGTAAAATGCTCAGGCTGGATCTGAGTCTGAATAGCTGATTCGGGCTTTATCACTTATGCGAGGGCGGAGGTGGGCTTTATCACTTATGGGAGGGCGGAGGGCTCAATTTGGGCCGGGTTTGTTGATTGCGCTGCGACGGGTTCTTTCGAGAATACAAACTGGCAGTATACGGATCAGCAGCAAGAAAAATGTAGACCAAGATAAAAGAGGGATCGGCTGGACCTCATCTTTTATAGTGAATAATGGTGGTAATGCTTGCGTTTACTATGGCAAGTCATTACTATCAAAATATGCTGCGAAAGGTGAAACTCAAAACTTCATACGAAAAGACATTGTGTGAATCCGTCAAAGTGAGTACGATCGACGAGCGCGTAGCCCCGAACATGCACGAAACCCGGATGGGGTGGCTGCAGAGCGGTGGTGGATAAGTGGTTGGTCACCGTCCATTCGCCTTCATCCGCCGTTTGTTTATTCGGCCTCATTCGCGAATCTGCCTCGGAGACAACAAGCGCCTCGAAATGCTATCACTACCGTGCTTGAGATTTGGCGCCCATGTAGTCATATCTCGATCTTGTGAGATTGACACCTCGGTAGTCTCGCCTACTTCTGTGCGAGATCGGAAGAGCGTCGTGTAGGGAAAGAGTGTAGCCTCCGGTATCTCGGTGGTCGCCGTATCATTCAGCAGAAGACGGCATACGAGATTCCATTGGGTGACTGGAGTTCAGACGTGTGCTCTTCCGATCTAATTTTTTATTGGAAATGTACGTTATTTTCGTGCAAAAGTACTACAGTACTACGTATAACGTACTGCTACAAGTATTTCTTCTTAAAGCATACATACCGCGCTGACTTGTATGCTATCCAAAGTGTTCTGGTATCATAAGTGAAGTTTTTACTGAGCTAATCAGCTGTTAATTCACCACTTTTGTACATAAGTGAAGTTTATACTGAACTAATCAGCTGTTAATTCACCACTTTGTACACCTGCAAGGCAGTATTCTCCCCCGGCGTCTGAGGCTACTTCGTCACCCTCCTTAACGACGCTTGAGGGCTTCTCTTTCATTTTCTCCAGCTTCGCCTTCTTAGCCGCCGCAATCAGTTTGCTTATGTCTTTATCCGCCGGCCTCTTGAAGATTTTGTTACTACTCTGGACTATGACTCTCACAACATCTTCGCCCCGGCCGCCCCGGGCGACTTCTTTGCCTGCGACCCGAAGGGATCTTTTGGCAGAAGTGTCTGATAAGAGCCGGGTCTCGTCAAGATTCGGGAGCGCTGCAGAAGCGCTGCTGCGACACAAAATCGCAAAGGCAAGAGCCACGTAGAATATACTGCGCATGACTTCAAAGAGGAATACCACGGCAAAGAAGGTCTTCTGGTCTTGCGAGGAAAACGAGAAATGGCTGAAGCATTCTTATCAGTAATACGTACAGGCTGAACCGGTGGGCGATGTACCTACCTGCAACTCCCCAGTACCGGTACTGATGCTATTCATGGCATTGTTACCCAGAGAAAGATGTTACATGTATACTTTACCGTATACGAGATCCCCTACTTCAGAGAGATCTCCAAATTCATACTGTAGAAAAGGTGCTGAAGTGATATTGACAAACATTTTCAATACAAAACAAGATTACGGCTTAGTATCCAGCAACAAAATCAAGAACAGGCCACACAGCTCTACAACTTCAAAAATTTGATGTCTGAAGTAGGAGATCTGGTATACGGTAAGTACATGTGTACTTAAGTACTGAACACAGAGCTGTTATCCTTCGTTTGCTCAGTTAAATCATATTGGTTCTCCAAGCCGTAATACGTATTCTTTTATTAGCAAAAAAGCTCACTTATTCCACAATGCAATATGAAGAGACATCAACTGGATGATCAGGGATGTGTAAAGTGTACGGACCGGTAGCCGGTACATGAACCAGACACCAGCTTGATGATCAGGGATGTGTAGAAGTCTACCGACCGGGATAACCTGTCGGTAGAGGTGTAAATGTTTGCTAGAAAGGCGAACTGAACCGATACCGATACCGATGCTGTGCTGTTGAGTGGGCACCGTCGTATATAAAACAGCCATGAGTTATTTGATGATTTTTGCATTTCGCCGTCCAAATTTTTTTCTTACATATCTCCGACCAAAATGTTTCAAGCGACATGATAGTGCTAGTGTTGCACGGGAGTTGCCTGAGTTTATTGATGAATGGAAATACATGAAGAGCGCAGTTCTGGATGCTTATAAGCTAAATTGGAGTAGGTGCATAAATCCATAGAAAGTCGTTTTAGAAAATTTGGAAATGAAATATGAAACAAGCCGTATCAAATCGGTCGTTGTTCGTTATAAGCAGACGAGGAAAGTATTGGTTCCGTTCAGTTGGTGAAAGTTTCTGTCCGTCCGTCTATACGGTATAAGGTAAAACCCAACCCCCTGGTTTACGACCAATCGCCAACTGTCTTATACTGTACATATGTATTCCTATGTTAAGTATACAGTCAATATGACGTGTGGAGGTTGACGATTTGCTACGTTTATTGAAACCGTAATCCTCCGGATCTTTGAAATAAATTTTTCAGGAACGTGCCTCAATCATAATACTAAATCTTAATTGTTGTTAAAAAACGGTTGGAGTTCGGTATCGTAAATCACCGTCAGGTTATGGACCAGAGCTGCATAAAGTCCAGAGCAAGGTGTGGACTTAAGCTTTTTATCGTACTGTATTAATAGGATGGCGCTCACCCGGACTCCGGGCACTACTAAAATGGGGTATCGATGAGATAACTAGCGTTGAGCCTTTACGTAAATTGTAATGCTTTTACATTGTTCGTGTACTCGTGTTTTGTATCCATGCAAATGATAAGGCGAATAGTTTTATATATTTGATCGAATAGCATCCATTCGACCCAAATCCCTTCTAACGATCTTAATTTGAAGTTCCAAAGCCTTTGAGGAGATCGATGGAATGGTAACATTGAAATGCGTTTTAAATGTATATAGGAGTCCGTGTCTCCCGCTACAGGTTTCTACTCATCGAAGGCCAGCATACGACCAGCGGTGAACACCGCGTTACGTAAATCTCAGACGCCTTTAAAATGGCGGTGAAAACGGTAGCCATTTGGAGACCCATTGTGCTGCTTTTTACTTTGGAGCTGGATAAAGGGCAAGGGTGTAAAAGGAGGGATTATGTAGCCTCTCTGCTCATGCGAAACGGAGTTTAATGAAACCGTGCTAGTGCTTTAAAAATGTAAGCGCTTCAGAGATTGGCTTGAGCATGACCTAACTAGCTAAACTCAGAGGCGTGTTGGTGCTCCCTTGGTCTTGCCGGGCGCTAATTTGATGATAATGTTGCCACCCTCGGCTGCTGACTTCCAGGATAGCACAATTCTCCAAATACGATGTATCTCCTACATACAACTTTGACGCTATTGCCACAAATTGTGGCTCACAATCGGTGGCTCTCGGCAATGCCGAGTAGGCCTATTGTACTCTTGCCATCCGATTTGAGCCCTTAGCACCAAATTTCAGAGCTCATCCCGGGTAGCCTAGCACGGTTTTGAAATATAATTATAATGGTTCGAGCTCGAATTCCTAACGAACACCTGGGATTTCTGATTCTACAGTGCGATAGATACTTCAGGCGCGCTGCCAAACCAGAATTGAATTGCATCACGCTATAGGGCAATCAAGGTCACAGCGGTAAGCCACCACAGAGCAGCTAGAGAACACGTATTGGCTGAAACGTTGCATAGAATCTTGGTTGAAATTGCTAAAAGCATTAGCTCTGAGTCTATAATGGCTTATGCGTCTGGTCCTGTGATTTCGGAAGACCTGCAGATGGCTGGTTCCTTGTGAAAAAATAACCACTACCCAACTCACAGAAGAATAACGAGACGGGTGGTTGAACAGCATCCAAGAGTTATATTTTCAACGCGGAATACATGGTCGTAGGCGATGACAACCTCCACGACTGTTTTCTGAACAGTAATCGACTCAAGAACAACAAAACCTCTCTCGGAGGTATAATCAAGATTCAGAGGAGGTTGAAAATATGCAGCTTCGCTACCTATAGATTCACGGCGTGAAAATGCTAATGCAAATTCCGTTCGTAAACGACTGTGACAATCCCTCGTGGAGCACCGACGAGATCAAAACGATTCACAGAATCTTTAAGTGCGACTGTAAGCAATTCTATTAAACGGGCTGGATCCGTGCGTATTTATTGACATGTCTATACCTAGTAGACACGTTAGCTTTACATGTCATTCTACGAAGCCTACCGGCTCAAACGGTATAGGGAGACCTCGAAAAGAAAGAAGGTGCCTGGATCGTGACAGACCTCGAAAGGCGCAGTATGCGATAGACTCGCTGACCAAGCGCTTAGTAAATAGGCCTGCTGATGATCTCAAAGTCTTCTATTAGCGACAATGCATAAGAAAGAGCTTAATCACGTTCGGAAATTAAGCCGCCTTTCACGAGAGGCGGTAAACGGTACTGGAACACCGAATACGAGGAGTACAATGATGCCAGCACTTTGGGGATTGAAGAGCTGGCAAGAGTGATTATCTACTCGTACCAAATGGAACACAACATTGCCTTCAGCTAGAAGTACACTACGATGGAGATCTGCAGTCGCGTGTATTTTCCATATTTCGTCTAAAAATGACTAGCAGAAAACTAAATAATCAAATACAGTAAAACCTTGCATGGAAACACTGCGCATTTCAAGCTACATACAATTAGGCCCCTCGATATACAATTAATCCGACATATGGAAAAAGCCACAATGTTATAGAAAGAATCCGAATTGACACGACATAGAAAATAGCCAAAAAATATTATAAGAAATTCTCCCCCGAGCAATTACTTTAAGATATATCTTCTTAGTTCAGCATCATGGCACATCAAAATGTCAATATTCACGAGCAATAGGGTGGACGTTGCTTAATTTGCTAATCTCTTTCTCTCCAATATGTAAGGCATTGCTTCAGCAGCATTTCACTGACCATTGGCGATGAAAGTGCATCATTGTGTAAATCTCAATAAAGCAACTGAAATTCCTCATTGTTAATTCACTTGGGTCGGACAAAAGATTCGTACCGCCTCTCAATCAGAGGGCGAGAGGGGGGGAGGGGGTAATTTAATGGCGGCGCCAAATAAAATCCATTTATATCGCGTTTATAGTAGGTGCCTGGATTAACGTTGCAGGATGAGGATGAACAGAGAATTTGGTTTCGTTGGTATCGTTAATTTGCAGGTGATGATACTCGAAAGGCCACGATCATTCTGTGCGGTCAAGCAAATACATGATGTGAGGGCTCCACATGCTATTTAACCGATGTCTGCGCATTAACAGGGCAAATGTTTGTGACGAGGACTCGTATCACTAACATTCTTGATATCTGCGTACCTCTCCCGCCCGGAAGGTCCGGGTATCCAATACCGATTTCACTGGTGTTTACCTTTTGAAATTTAGAAATGTTGTGAAATCTCACCAACGAGTCAATAGAAATATATGCATGTTAGTAAGAAAGACCTTAAAAATAAACTACAAGTACAAATATTCATCGAGTTAACATGTAAATTGGTGCGATGCACCGCCTGAAATGCACTAAATTGACAATTAATTTTATGTAAATTTTCACAGGTGCACGTACAGTTGACGCAGTTCCTCACCAAGGTTTAAAATGTATTTCTACTTGAAGTAATTGCGTAAACCATTGTATAGCGTTTCTTGTATATTTTGGTATACCGAAATTTACAAGTATAGCCCTTTCTTGAGGTCGCAGCTGCAGACTCTCTAGCTGCATGGGCTACCTTGCCCACCTCTTAGACTTAGTCGCCTGATCTTTCGCTTTTTTTGCCGCCTCAAGGAGTTTGCTTATCTTGATATCCGGCTTCTTGACGATCTTATTAGTAGTATGGGCTATGGTTTTCCAGACACCACCGTATCCGTCTCCCCGGCCGCTCTGGATAACTTCTTGGGCCTCGACCCGAAGCAATCTTCTGGCCACAACATCAGGAGATGCCTTCGACAAAAGACGAGACTCGTCTGGGTTCGGGAATGCTGCGATGGCGCTGCCCCGAGCTAAACTGCAAAGGCAAGAACAGCGTAGAGAAAACTGCGCATGGCTGTAAAGGGGGGAAGGCTTAAAAGAAGGGTCTTCTGGGCTTGGGAGGAAAACGCGAAGTAAGCTTCGGCCGTTTTGCAATTCGTTCCAGACTGAACCGTTGGACGGTGTCTCTCTAAGAAAGTCTCTCAGTAGTGGTAACATTTTTTTGCGCTAGACTTAGATAAAAGGCAAGGTCTCATTGGCAATGCAATGCGTCGTTGCATATGGTAACCCCGACGGGACGTTCTCCAAAGCTGCAACGCTCAGCGTAGTACGTAGAGAAACGTTTGTTGATGCGTTGAGCACTGTAGAAGTACGGTACAGACGATCCTTTCTGTATTAATATCCTTAATAGTACCCTTCAAATAAAGAGTGTGCCTGTTTACAAACTTAGGATAAGTGGCACCTATCCTCTCATTTGATATGAAAATGACGGGTATTGTTATTAATAGGTGATTAACTATTAATTATATCCGCTCCGTAAAGTCGATTGACATTTGCTGTTTGCTCCTTGATTTTCCCGCAATAAGAGGTGGCAAACGCTCAGATCCATCCACATTGCATGTAAATGCAAAAGTGAAGCGTTTATTTTAATAATTTTAGCTGGCCCACCTGATTCGAGAAAGCGGACCGAACGGTGCGAGTTAGAGCTTCAAATGGAATGGCTTCGAGTAGGTATTGATGCTTCATGATTTTTTAATCATTGTTTGTGATAAAGCGCGCTGTAGCAGAAACACGTTTTAAACACCACAGCGCAAGCTGGACCTGCTTCGATATTGGATTTCGGCTTTATGGCGGCAGATCTGCCGTCGCGTTAGCCTGCTTTGCGGCGTATAGTGGGCGAAGGTGTACGCGGTATAGCAGTACTGGGCGGGGAATTAATAAACGACAATGCTAGTCGCCAAATATCAAGCCACTTTGCGGTTAACGAACGCAACGACGGCCCCTCGGTGTCGGGTTACGTGATATTGGGGACAGCTCTTTATAGTGATTCCTCCATATAATAATAGAATCCCCGACGCTATCAATATAGAGAGGATCGGCTGTAAAGTATTGAGTTCCATCTCCATTTAATAATACTGTCTCGTCTCGCTACTATGGAAGCTCCACCGGAACAGACCCTGTCTTATCCTGTACAGTAAATGTAGTACCGTACCAAACATAAAATCTACTTCAAAGTAATATTCAAGCCGAGCATGCGGNTTTTTTTAGCACGTGTGGCGGCACTCGACTGAGAATTAACGTGTTTGTACCGGTATTAAATCCGAGATAATTGCTGCAATCCAAAGCTAGAGAGATGCGCGTAATAAATAAGGAAAATATAAGTGGCTTCCTAAAATTGGACACTCATAAATCAGCGCCCAAACTAGTGCTGGGGTTGTATATTTATAAAAAATCAGGGGAACTTATAATTTCATTTAAATTATCCATATTGCGTCAGGGATCCGGAAACGTTTTGGACAAATGCATTTGTATTTAAAAGTAAAATTATTCATTACTATCCCGATAATAAAATAGTATATTCTTTGCCATACTTTGTATGCTTGGCAGCACTGATGAGGTATCTGCTGGTGTATTTCCGGTCCTCTGTCTTGTGGTACGTAGTAGAAGCTTTAAGCTGATTGTTAAACATTACACTGTTGAAGTCTGCACCACGTCACAGGATTGTGTGGTACACATAAAAGGCAACTTGCTGTGTCATCGAACATTCGGAGTCATTCCAATCTTCTAGAATTCGAGTGTTGCAGTAGGCAGTGCAGGAAGGATGTAGAGAGCTAGTTTAGTTTGATTCTCACTTTTCATTTTACGCTGCATCGGTAACAAGAAACTCAAGTCGTTTTTGGTTATGAGCCCAATAACGCCGTTGGAGTTTAACGACATCTTGCGAGAGGACAACTACTTCCTATGGGAATTCAACTGCCGCATGACGCTCGCGAGGAAGGACCTGCTAGAGCATGTACAAGATCTTACGGGGATGAAGCCAGCAGATGCAGCGAAGCGGGAGACTGCTGAGTGGAAAGCCGCCGACTTTAAGGCGCTGGCTGTGATCTCCAAGCTGTTGAGCCCAGTCTATCAGACGATGATTCGAGAATGTTCGTCTGCTAAAGAAGCGTGGAAGACGCTACGTGATTTTTTTGTCAAGAAGAATTTGCACAACCGAGTGATGCTTCGGAAGCAGCTGCATGAATTTCAAATGGCCCCATGATCGGATTTGCTGGATCACCTGTTAAAGTTTGACGACCTCTGTTTACGTCTATCTGCTGTTGGTGACGATTTAAGTGATGATGAGAAGTTGGTGATCCTCCTGGGGAGCTTGTCGAGTGAATATGACGCGATGGTGAAGATTATTGAGGCTCATTCGTCGGTAACGTTGATTGATGCGAAAGAGATGTTGCGTTGCGAGTTTGAGACGCTGCGGAAGCGTGACAAGCAAGATGAAGCGTTTAATGCAGTGTCGCAAGATGCACATGTTGGCAGACAAGGTAACAATGGGCGTAGTAAGTGGCCACAGTCAGGGATTCGTGGTCGTG

>Contig_21

CTTTGAATGTTGTTAGTACAGCTGATTTGCTGCATGATGCCGCGGGAGATGGGGCCACACGTCCAGTCTTGCGGCAAGCGGAACCAAGTTGCGGATCCATTAATGCTGAACGTCATTTCTCATTTATCGCTCTAGAGCTTACGAGAAACAAGCCCAAGCCTAGCTGCAGATTCCGTCACTCATGCATCTCCGTTTCGTGCTACTGACGATTGTCGCCACTCTTGTAGCGACATGTTGTAGCGAAGCTCGCTCGCTCCGGCAAGGGGCCTCCACAAAAACGACCGGTTTGGCCTCCGAACATGATCCAGAGTCGGAGGAGAGGGTGTTTAGAGTCCGCGACGTTGCATCTGGAGCTATCACAAAAGCTGAAAGCGCACCTCTCAAGACGTCGAAGTGGGATAAAGTCCTCAGCAAGTTGGCCAAGAAAATGCTTCCGGGTGCGAACGAGTACAAATTGGTGTACAATAACGGTCGGTGGAAGCCGGAGTATTACTTCTGAGTTTCGCCAGTCCTTAGAATGGCGGTGCTCATACATGTACATGCACTAAAAAGCACATACAAATCAAATTGAGAGCCACTTGTCATCACTCTTAATGCTTGCACCAGAATTCGTTACTACAGTATTTGTCAACAACTACTATAATAATCGACAACAATGCTATTGTTACGTTTTAGAATAAGTTCTGAGCACTTTCCTAATATTTTTTTTTTAATTACCGTGAGGCAAAAACTACTACTCTTCAAAAGGCTGCGACTACCATAGCTATAAGCAATGTTGCTCACTCACTCAATCATTCGTCTCGACGAGCCCAAATCGCGACTTTGCTGCTTAAGAACTACGGAGACTTTATGACACAGGAAGGCCATACCACCGCCTTCACAGGTCGATTTGACCACAACTGGATTTGCTTTCAATACTGCACGTCACGTACCAACTCATCGGCCAGGTCAACGGGAAAAACCCACCAATACCATGAACTTCAACAGCTTCTTCGTCGCTATGATGCCCGTCCTCGGTGCTGCCACCGCCGCCACAGCCGACCAACCTGTCGTCAAACCCCAGCCGAAGCATTCGAACTAGTTGAAGCGTCCACACTACCCGACGGCGTCACGTTCACAGGGGTGACGGAGAAGATCGATCCTCCTGCGAATGCCGCTGCAATGATCAAAAGGAGGCCCATGACGACAAGGATGACGAGAAGGAGGCAATACGGATGGGGGCTCGGTGGAGGCATAGTTTTTAGTTTTGGTGAGGTTACATTGATGTCACTTAGTTAGACTAACGACTTCCTATGCCCGTCTAAGTGGAGGCATAGGTGGTCTTGGCAGCTGGGAACTTGGTGGCTGGGGTGACTGGGGCGGTGGGGTGGCTATGGACCCCACCGCTTCGAATTCATGTGCGGCGGTGTCCCTGGCTGGGCCTATCCGTTGGGATACTGAAAACAGGTTTGGTGCGGGTCTGTACAGCGGCGGGTGCGGTCTTGGCATTCCTTTCGGTGGTCTCTACTACTGTTAATTCTTTGAGCTGTGGGCCTACGGAGTTAACACCTTAACAAAATAGTAGTGTGCTGGCGTTCCTACTACCAAAATTTGCTTCGTCTACGACTTCGAATGAACAGGTTATCTTTTTCATGGGCTTTGAGCACCTTTCGAGGTTACACGTACAAATTCATCAGTATTATCGTTGTTAACGACGAAGGGCTCAACGTTAAGGCAAATTAAGTTTGAATGAAGAGACCTGCCAACCATTAAGTGTAACCAGATTACATATATATAGAAATTTTGTAGTGATTGAAAAAACATTTAGATAAGTACTGCTTCAAACATTTTTTTTGCAGTGTTATATTCCCAATAGGAGCTCTGTACAAATATCAACTTGCGATTTGAACTGCATGCGAGGGATAATAGAATACAGTACATTATTGCAGCGATCTAGAATATCGTCGAGGTTGCGCTGCAGAGTCCTTCCAAGCAAAAGCTGATGGCATGAGAGGGGGGAGGGGGGCGCCAACTCTTAGACTTTGAAAAAGGGTTCCCGGGCCCCTCAACAACCCCATCCAAACCCCTTTCCGGGTGTATTACGTGAACAACTTACTTTTGCGCGCTGTGGTACACTCTCTTGGATTACGCGTTTTGTCGACTATGCAGACTCTTCCGATGTGTGATAGTGTTGTACGTGTTCTGCGCATGGTGGGTAATTATTTTTGATTATGCGCGTTGCCGATTGTCCAAAGGTTTCACAATCGTCGCGTGCATGTCCATTGTGTTTTTCTACCTGTACATAAATCTTAACTAAGCCCTTGGTCCAAGGAGTGTTGAGTTCCCTGCGATAATCCACAATAAATTACAGGTGCAGGTATTGATACATGTTTTATTATAATACATGTGCACCATTTCTAAATCAAATCGAGTATTTCCCAGATCAGCAACAGTACAGCCGATCTTAGCCGGCACGAAAAAGTTAGCTTTATTAGAGAAGGAGATAATACTTTGCACCTATAAGATCGGAAGAGCGTCGTGTAGGGAAAGAGTGTAGCCTCCGGTATCTCGGTGGTCGCCGTATCATTAATGATACGGCGACCACCGAGATACCGGAGGCTACACTCTTTCCCTACACGACGCTCTTCCGATCTATTGGGCTGGCTGCAAATTCGACCACCAGCTCCGCCGTGAAATTTAGAGATATCTAGCAATACCAGTGGCCATGTTTCCGCTGTTCAAGTGCGCTAGCGCGTCTGGCTTCTTTAAAGTTCATACTGCGAGCAAGGTGAAGTATCGAGTATGACTTTGCTCGATGTTGGCTCGCTGAAGGCGATCGTCAGGGTGCGATCCGATTCGCATACGTTCGTGAAACGCGGCAAAAAGCTGCCGAGAAGGTTTTTTAAAACCTTTTTCGGGGGTCAAGACCAATTGGTACGGATACGAATTATGTGTTTTATCGTTTTTTCTACTTCTAATTTGCTGAATGTGGATGGATGTTAATTGCCCGTCTTTAGGAAAATACTTCGAAGTAACAAGGATTTTGTCGGAAACTCCGGAAAGCGCGAGTTTCTTATATTTTACGTGTACTGAGGATTTAAGCTGTCTTAGCAACAAAGCTGTAAAAGGTTGGCCGAGTAAGTAGCGAGCGTCCGGTTTGCAGGTGTTGCCCGTGATCGCACCGGTCGCAACAGCCGCCATGCAAACTCCGATCCGCTTCGCATGTTGCTCTCGATGGCGTGCGTCGAGCTCTTTGTGGATGCACAACTTTTAAATGGCAAGCTACATATACCCATTACGGGTGGCCACCAGTCTTACAATTGAAGTTACAAGGGGGTGCTCTCTGGTCTCGCGTAGCTTGTCCGTTTTGCCTTGAACCTCAACGCATCATTCCTCAACTTGAGCTCGACAGCTCAATCTACCCAGAGACGCGCCGACGCTGATGCCTGTCGATGCTGTGAAGGCGTCGAAATGGTCTGCAAGAGCTAACTCCATTTGCTCCTAAGTGAAATAGCGAAACTGTGATCCGACCTTCAACTGCCTAGATACCTTCGACGTGCAGCACCCTCGAATCTGGACTGAGTCCGATGCTGCTTCGACGTCTGGCTCTGGTGATAGCCCCATGGCCTCATCAAATGGTTCGTCGGCTACCCAGAATACTGGAGGAGGCGACTTTCTTGCGCCGTTCTCGACAGAAGAACCGTCGACAACTCTCTTCCCTCGTAGTGCACTTGGCGAGATGGCTCCGATCATTAACGTTGCGGAAGGTCTGCTGGGCAAGCCACTACCAACGAACAAATGGTGGGGTAACATTATCCACACTACCGCTGAAGAGATGGATACGAAAGCCAACCCTGGCTGGTCCAACCCGTACGCCGTCAAGCTGCCGAAGGAAGCTCCGTACGGCATCCAAGCTTGTTACTCGTACAACTACCGTCAGCTGTCCGCTCTCACCGATGGCGTCGCGGAGTTTTATCTGCACGACTTTGTCAACGACCTCACGTTGTCGGCCACGGAATTTGCCGGTGAAGCCAAGCCCACGTATGAGATTTACGCCTTCAGCGACTTTGGCATCAACGTTCGAGCCTGTCTCGAGAACAAGGTGCAGTGCTTGGACTCTGCTCTTGTGCACGGCATGGCCTTCATCACAGCCACGTACGACCGTCTGACCGCTCGGATCGAGTCGGAATACACCATGGAGATCGTGGACAAGTCGGTGCCAGGCAAGTACATCCTTCAGCTTGGTGGGAACCAGACGTGGGTCGTGTACACTGGTAACAACGGCAGCTTTGCGCTCGCCGAGTCGGGCAAAGCTCTCGTTTCCAGTGGGTTGTTCAGTGGCACCGTGCGTGTTGCCATTCTACCATCGAAAAAGGCCACTACCGTCTACGATAAGTACCGGGCGTGTCACGTCCGTGGTGGGAACGTGTCTGTGGAGTCTCGTACTCAATACTCGTTAAACTGGGAAACGGTGGGGAAGAGCTGTAAGACCAACGGGATGCTTCACTTCGCACTGCCTCACCACCTCCCAGCGTTGAAAGGAGCCATCACTGCTAAAAGCCCCAAAGCGATTGTCCTTAACTCGGCCACTCGAGGCAAAATGGTCGCCCAAGTGGCCACGACTGGAAAATGGGTCCTTTCGGAGCCGGAAGATGAGTTGGAGGTGGACTTCTACCCAACCAGTAAACCGTCTGCCGAGGTCGTAGCAAAAGTGGGTCTTCTCCAGACTCTGCAGGCCGATATCGCTGACCACTGGGCGCTCAACAAGACGAGTTGGTACTTTAACGGCAAGCAGTACCAGAAGTACGCGTCGGTGTGTCTCATGGCTGCAGACTCGGCCATCGTAGGCAAGGACAAGAAGCTGCTCAGCGCATGCCTGACGAAGCTCGAGAAGCTCCTTGTGCCGTTCCTGGACAACACGCTGGACCCTCCTCTCCACTACGAGACGTCGTACGGCGGTCTCGTCAGCAGTCAGAGCTTCACGGTCCAAGACGTGAACGCAGACTTCGGGAACAGTGTCTACAACGACCACCACTATCATTACGGCTTCTGGGTCACCGCGTCTGCCATGCTCAAGAGTCTACACCCCAAGTGGGAGCGCATCAAAGAGCTGGACAGGATGATCTGGATGACGCTACGCGACGTGGCCAACCCCAGCACAGACGACCCTTTCTTCCCTCGGTTCCGCCACTTCTCCTTCTACCTCGGCCACTCGTACTCGCACGGTGTCACGCCCATGCTCGACGGTAAAGACGAAGAGAGTACGTCTGAAGATGTGAATTTCTACTACGGTATGAAGCTCTGGGGTCAGGTGTCCAGCAACAAGGCCGTCGAGGATCTAGGCAGTCTCATGCTACGTCTCAACGCCCGCGCTATCCGCACCTACTTCCTCATGACGTCGGACAACACCATCCACCCTACTGAGTTCGTACCCAACCACGTGACGGGCATCTTCTTCGACAACAAGGCGGCGTATGCGACCTGGTTCAGTGGCGAGAAGTACGCCATCCACGGCATCCAGATGATCCCAGTGTCGCCGATCAACGCTATGGTGCGCACGACAAAGTTCGTCCAGCAGGAATGGGACGATATCCTCTCCAAGCAGCCAATCGTCACGGAATCCAACACCACCAACGCGTGGTTATCGCTGCTTCTCGTCAACCAGGCTGCTGTGGATCAGGAGGACGCGCTGACCAAGCTACAGGAAGCCACAATGGACGATGGTCTCACCCGCTCTTGGGCTCTATACAACGCAGCTTCCCGTCCTCACAACGCCAAGCAGGTGGATGTAGCTTAGAAAAACGTACAAACTACGTAGATGTTGCACTTGTGTCGCTCGAATGAACTAGTTTTGCGTCGGTTTTGAGGCTTTCATGGCTCCACATGATCATTTGCCGAGGATTTCTCTGTTCGAAGCGTCAAGTCATTACGATCTCCATCTAGCAAGCGTACTAGACGTTGGGCTTGGACCGAGATGTTACCCAGGAAAAAGTGAAGAAGACGTATCGAAAGCTGCCACTGCAGTTCCACCTGGACAAGGTTTCGGATCCAGCAGCAACAGCAAAGATTCTTGTTTGCTTTCTCAGCTTTGTCTCTTTGTTTGTACATTGATACTAGTAGTACGACGTGCCGGAGCTCAACAAATCTAGTCCGACTTCCTTGGCGGTCACACTGTCATGTGGAAGGCCGTACCGCTCGCAAACTTCCTTCATGTGAGTTATCTGAAGCTGAAAATATTATTATGCATAGTATTTAGTAACTCACTATCCGAGTGGAACCGAAAAAGCTGTAGACGAATGTATTCGACACTGTAACACATTTGAGGTAGTTGGCATTCACACACCGCGTCTCCTTCTGAAGATGTTCACGCTGCTGCTATGACGTCGTTGGTCACACCAGTGAACCCATGCTCAAGAACAATATTCAAAGGACCTCACGCTTTTTAAAAACCTCCTTGGTGACCTCACAACGGCGGCTACGACTTCACGCTGACTGTCAGCATTCATTTGGCATCGCTCTCTATTGTATCATGCACTAGTATAACTTGTAGTAGTGAGCGCAGACGCGTACGGTGGCATTGCATTGTTGCACACATTGATGCTGACAAAGAAGAATTATTTGGATGTGATAGATAATATCAAATTATCCATTTTTGGATTGTAACGTAATAGTTGGTGTGGTACGCAAACCTTTGCAAAATTGAAGCTGCCTAGCCAATCATAGCACACGACGTTTCGCTACATGAGCAAGTTCGGCTGCTTGGGGCATCTGTACCGCTCTTTTCTTAGGACTGTATCTGCTTAAACAGCATTACGATTGCGCGGGTAGAAGTACTCACGCAGTATTGAAGGCATGGAGTGTAAGCCCCACACACCACAAAGGCCATCCGATTGCTTCGACCCACGACGGTTCATGGTCAATTGGACCGCTCAACAGCATCGAAAAGAGGCATTCACGGTCCTGCAGCTCCGTCTCCTCTGCCATCGACATTCATGCGGAGAGTGGACTCTCCCGTTTCGGTCGCTGCANTTTTGCTGTCGCACCACTCGAATGGGGCAGCTCATTCTGCAATTGGCCACGAATTGCTGGAGAACTCACCAACCTATCGTCGTTCCTCACGCAAACACGACTCGTCGACCGACGTTGATTATGTTGAGCCTCTTTCAGCCGGTGTAACACACCCGGTCCCACGCATCAGAGACCAGGCATGTCGCCGCCGCCGACATTAGCTCACCATTCCGAGTAGCAGCTGGTACGGTGAATATGCTCGGTCAGAGTTCATACCCGACAGGGCTTTCATCGTGAAAGCTTATACACAGCTCTACTGGGTCGGCTCTGTTCGGGGTATGGTGTCCCATCCAGCCTGCCGATGAGATCATGTGGGGTCAACTTGACGGCTACCACATCGCCCTACCGCACCAATCGCGCCAACAGGAGTCATGCACGTCCAAGGTCACTTCCTACACTGCAGTGACGGCCGCCACCATCAACAGTAGAATCAGGGCAGCTAAGATATACTCATCAATGCTAGTATCATATCTGGATAGAGGCCTGGTAGCAATCGACTTGTAGCTCAGTATCTCCACTACGGCAACACTTACGACATCCCATTACACGCCAGCACTCGACAACCCCACTACCCGCCAGAGTTCGACACCACCCACTTCCCACCAGAGCGCCACACTACCCGCTGCCCGCCAGAGCTCGTCTCCACCCACTGCCCGCCAGACCTGAGCAGATCCAAGAAGATCAGCGGCAACACAGCGGGACTTCACACGCGAGAAGAACTTCGTCAATGCAGTGATGCATCGAGTGCCCAGTTCATCCGTCCGCGAAAGCTGTCCCGCTCCCTCACTGTGGCATCGCCACGCGTCTTTATATCGATATGGACATACGAGAAGTCTCACCACCGGCGAATGCCCTTCGACAACACGCTCTACTGCGAGCTTGCTTAAAATCCCGCGCAGCACAGGACCCAGTGGCCAGACACGATAAGTGCGCGTGGCACTGCGCCGCAGTAGCTCGTGGCTCTGGCGTGAGCGACATCAACCTCGGCGTCGCTGCAGCAGCCACAGTGGCACTGTCAGTGCCCAAGCTCAGCTAGCGTCGGTGCTCAGTACAGCCAGACCAAATCGGCATGGGCCGAGATCGGCATCAAGGTGCTCAAGGCATTGCACGTTGACTTCACCCCAATTGCTACAACTCACCGCCGTACTACCCCACTGTGTACTACACGCGCATGAGTGAGTACCCGCACAGCCCGCACATTGTCACCAATCCGAGCTCGGGATCCACGAAGGGCCACATCCTACAGAAACCGATGCAGCCATGGGGGGGGGCTTTCGCAGTGCATGTTGCACGTCAGCGCGCATAATGTCCAGCGTACCTGTCGGGTACTCTCACGCGACAAAACCACATCGCGCGTGAAAACGTGTTTGTAGCGGTCATGTTGAAGTGTCAAGATCGTGTCTCCATTTTGGGCAATTTTCTAGTCCCGACTTCATTTTAAGTACGACCTTTTTTGCCAGCCGAGATGGACTACTTTTTGGGTCCCAGAGAATTTGAGATCAACCATCTTGCCGCTGCGAGCGAGTAGAGGAGACCCATATTTGCTTGGTGGACGTCGTCAAACTCACCATACGGTGATTTGCGAGTCTGCTGATGGCCAATGACAGCAGCTGTTCTGCGCGGAGACTATCTCAAGATGATATTTTGTGCTTTGGGCAGATCTGCGGTGCTGAATCGGGTAGCTCTGCGTTTTTGTGCTCTTCACCACTGCGTGTGACAAGGCTTTCGGCGACACAAGGTGCAGCACATGTTTAGGCTGGTTTGCATAGTTGGACTGGTTGCAGATTCGACCGCCAGCTCCGCCGTAAAATCCAGAGATTTCTAGCAATACCAGTGGCCACGTTTTCCGCTGTTCAAATGCGCTAGCGCGTCTGGCTTCTTAAATGTTCATACTGCGAGCAAGGTGAAATATCGAGTATAATTTTGCTCGATGTTGGCTCGCTGAAGGCGATCGTCAGGGTGCGATCCGATGCGCATACGCTCGTGAAACGCGGCAAAAAGCTGCCTGAGAAGGTTTCCAAAACTTTTTTCGGGGGTCAAGACCAATTGGTACGGATACGAATTATGTGATTTAATTGTTTTTTTACTTCTAATTTGCTGAATGTGGATGTTAATTGCCCGTCTTTAGGAAAATACTTCGAAGTAACAAGGATTTTGTCGGAAACTCCGGAAAGCGCGATTTCCTTATATTTTACGTGTACTGAGGATTTAAGCTGTCTTAGCAACAAAGCTGTAAAAGGTTGGCCGAGTAAGTAGCGAGCGTCCGGTTTGCAGGTGTTGCCCGTGATCGCAGCGGTCGCAGCAGCCGCCATGCAAACTCCGATCCGCTTCGCATGTTGCTCTCGATGGCGTGCGTCGAGCTCTTTGTGGATGCACAACTTTTGAATGGCAAGCTACATGTACCCATTACGGGTGGCCACCAGTCTTACAATTGAAGTTACAAGGGGGTGCTCTCTGGTCTCGCGTAGCTTGTCCGTTTTGCCCTGAACCTCAACGCATCATTCCTCAACTTGAGCTCGCCGACGCTGATGCCTGTCGATGCTGTGAAGGCGTCAAAATGAGCTAACTCCATTTGCTCCTAAGTGAAATGGCGAAACTGTGAGCCGAACTTCAACTGCCTAGATGCCTTCGACGTGCAGCACCCTCGAATCTGGACTGAGTCGCCTTCTTTAGTATCGTCGCGTCCACTTGCAAGACATCACTTGGTGCCGTCATCATGACGCTGTAACAAACGACACTCTAGTTTCTCTTTAAATTATGTCGCCGCCAGTGCTACGTCTGGATCCGAGGACAGCGCAAGTGCTGACACTTCAACTTCTAGTTCGACTGACACCCTGTCCGCTTCAGGATCCGATGCTGCTTCGACGTCTGGMTCTGGTGATAGCCCCATGGCCTCATCAACCGGTTCGTCGGCTACCCAGAATACTGGAGGAGGCGACTTTCTTGCGCCCTTCTCGACTGAAGAACCGTCGACAACTCTCTTCCCTCGCAGTGCACTTGGTGAGATGGCTCCGATCATTAACGTTGCGGAAGGTCTGCTGGGCAAGCCACTTCCAACGAACAAATGGTGGGGTAACATCATCCACACTACCGCTGAAGAGATGAATACGAAAGCCAACCCTGGCTGGTCCAACCCGTACGCCGTCAAGCTGCCGAAGGAAGCTCCGTACGGCATCCAAGCTTGTTACTCGTACAACTACCGGCAGTTGTCCGCTATCACCGATGGCGTCGCGGAGCTTTATCTGCACGACTTTGTCAACGACCTCACGCTGTCGGCCACGGAATTTGCCGGTGAAGCCAAGCCCACGTATGAGATTTACGCCTTCAGCGACTTTGGCATCAACGTTCGAGCCTGTCTCGAGAACAAGGAGCAGTGCTTGGACTCTGCTCTTGTGCACGGCATGGCCTTCATCACAGCCACGTACGACCGTCTGACCGCTCGGATCGAGTCGGAATACACCATGGAGATCGTGGACAAGTCGGTGCCAGGCAAGTACATCCTTCAGCTTGGTGGGAACCAGACGTGGGTCGTGTACACTGGTAACAACGGCAGCTTTGCGCTCGACGAGTCGGGCAAAGCTCTCGTTTCCAGTGGGTTGTTCAGTGGCACCGTGCGTGTTGCCATTCTACCATCGAAAAAGGCCACTACCGTCTACGATAAGTACCGAGCGTGTCACGTCCGTGGTGGCCACGTGTCTGTGGAGTCTCGTACTCAATACYCGGTAAACTGGGAAACGGTGGGGAAGAGCTGTAAGACCAACGGGATGCTTCACTTCGCACTGCCTCACCACCTCCCAGCGTTGAAAGGAGCCATCACTGCTAAAAGCCCCAAAGCGATTGTCCTTAATTCGGCCACTCGAGGCAAAATGGTCGCCCAAGTAGCCACGACTGGAAAGTGGGTCCTTTCGGAGCCTGAAGATGAGTTGGAGGTGGACTTCTACCCAACCAGTAAACCGTCTGCCGAGGTTGTAGCAAAAGTGGGTCTTCTCCAGACTCTGCAGGCCGATATCGCTGACCACTGGGCGCTCAACAAGACGAGTTGGTACTTTAACGGCAAGCAGTACCAGAAGTACGCGTCGGTGTGTCTCATGGCTGCAGACTCGGCCATCGTAGGCAAGGACAAGAAGCTGCTCAGCGCATGCCTGACGAAGCTCGAGAAGCTCCTTGTGCCGTTCCTGGACAACACGCTGGACCCTCCTCTCCACTACGAAGACGTCGTACGGCGGTCTCGTCAGCAGTCAGAGCTTCACGGTCCAAGACGTGAACGCAGACTTCGGGAACAGTGTCTACAACGACCACCACTATCATTACGGCTTCTGGGTCACCGCGTCTGCCATGCTCAAGAGTCTACACCCCAAGTGGGAGCGCATCAAAGAGCTGGACAGGATGATCTGGATGACGCTACGCGACGTGGCCAACCCCAGCACAGACGACCCTTTCTTCCCTCGGTTCCGCCACTTCTCCTTCTACCTCGGCCACTCGTACTCGCACGGTGTCACGCCCATGCTCGACGGTAAAGACGAAGAGAGTACGTCTGAAGATGTGAATTTCTACTACGGTATGAAGCTCTGGCAACAAGGCCGTCGAGGATCTAGGCAGTCTCATGCTACGTCTCAACGCCCGCGCTATCCGCACCTACTTCCTCATGACGTCGGACAACACCATCCACCCTACTGAGTTCGTACCCAACCACGTGACGGGCATCTTCTTCGACAACAAGGCGGCGTATGCGACCTGGTTCAGTGGCGAGAAGTACGCCATCCACGGCATCCAGATGATCCCAGTGTCGCCGATCAACGCTATGGTGCGCACGACAAAGTTCGTCCAGCAGGAATGGGACGATATCCTCTCCAAGCAGCCAATCGTCACGGAATCCAACACCACCAACGCGTGGTTATCGCTGCTTCTCGTCAACCAGGCTGCTGTGGATCAGGAGGACGCGCTGACCAAGCTACAGGAAGCCACAATGGACGATGGTCTCACCCGCTCTTGGGCTCTATACAACGCAGCTTCCCGTCCTCACAACGCCAAGCAGGTGGATGTAGCTTAGAAAAACGTACAAACTACGTAGATGTTGCACTTGTGTCGCTCGAATGAACTAGTTTTGCGTCGGTTTTGAGGCTTTCATGGCTCCACATGATCATTTGCCGAGGATTTCTCTGTTCGAAGCATCAAGTCATTACGATCTCCATCTAGCAAGCGTACTAGACGTTGGGCTTGGACCGAGATGTTACCCAGGAAAAAGTGAAGAAGACGTATCGAAAGCTGCCACTGCAGTTCCACCTGGACAAGGTTTCGGATCCAGCAGCAACAGCAAAGATTCTTGTTTGCTTTCTCAGCTTTGTCTCTTTGTTTGTACATTGATACTAGTAGTACGACGTGCCGGAGCTCAACAAATCTAGTCCGACTTCCTTGGCGGTCACACTGTCATGTGGAAGGCCGTACCGCTCGCAAACTTCCTTCATGTGAGTTATCTGAAGCTGAAAATATTATTATGCATAGTATGTTCAAACGGTTAGTAACTCACTATCCGAGTGGAACCGAAAAAGCTGTAGACGAATGTATTCGACACTGTAACACATTTGAGGTAGTTGGCATTCACACACCGCGTCTCCTTCTGAAGATGTTCACGCTGCTGCTATGACGTCGTTGGTCACACCAGTGAACCCATGCTCAAGAACAATATTCAAAGGACCTCACGCTTTTTAAAAACCTCCTTGGTGACCTCACAACGGCGGCTACGACTTCACGCTGACTGTCAGCATTCATTTGGCATCGCTCTCTATTGTATCATGCACTAGTATAACTTGTAGTAGTGAGCGCAGACGCGTACGGTGGCATTGCATTGTTGCACACATTGATGCTGACAAAGAAGAATTATTTGGATGTGATAGATAATATCAAATTATCCATTTTTGGATTGTAACGTAATAGTTGGTGTGGTACGCAAACCTTTGCAAAATTGAAGCTGCCTAGCCAATCACAGCACACGACGTTTCGCTACATGAGCAAGTTCGGCTTTGGGGCATCTGTACCGCTCTTTTCTTAGGACTGTATCTGCTTAAACAGCATTACGATTGCGCGGGTAGAAGTACTCACGCAGTATTGAAGGCATGGAGTGTAAGCCCCACACACCACAAAGGCCATCCGATTGCTTCGACCCACGACGGTTCATGGTCAATTGGACCGCTCAACAGCATCGAAAAGAGGCATTCACGGTCCTGCAGCTCCGTCTCCTCTGCCATCGACATTCATGCGGAGAGTGGACTCTCCCGTTTCGGTCGCTGCGTTTTGCTGTCGCACCACTCGAATGGGGCAGCTCATTCTGCAATTGGCCACGAATTGCTGGAGAACTCACCAACCTATCATCGCTCCTCACGCAAACACGACTCGTCGACCGACGTTGATTATGTTGAGCCTCTTTCAGCCGGTGTAACACACCCGGCCCCACGCATCAGAGACCAGGCATGTCGCCGCCGCCGACATTAGCTCACCATACCGAGTAGCTGCTGGTACGGTGAATATGCTCGGTCAGAGCTCATACCCGACAGGGCTTTCATCGTGAAAGCTTATACACAGCTCTACTGGGTCGGCTCTGCTCGGGGTATGGTGTCCCATCAAGCCTGCCGATGAGATCATGTGGGGTCAACTTGACGGCTGCCACATCGCCCTACCGCACCAATCGCGCCAACAGGAGTCATGCACGACCAAGGTCACTCCCTACACTGCAGTGACGGCCGCCACCATCAACAGTAGAATCAGGCAGCTAAAAAATACTCATTAATGCTTGTATCATATCTGGATAAAGGCCTGGTGGCAATCGACTTGTAGCTCAGTATCTCCACTACGGCAACACTTACGACATCCCATTACATGCCGGCGCTCGACAGACCCACTACCCGCCAGAGTTTGTCACCACCCACTTCCCACCAGAGCTCAGCAGATCCGAGAAGATCAGCGGTAACACAGCGGGATTGCACCCGCGAGGAGAACTTCGTCAATGCAGTGATGCATCGAGTGCCGAGTCCATCCGTCCGTGAAAGCTGTCCCACCCCCTCACTGTGGCATTGCCACGCGTCTCTATATCGGTACGGACATACGAGAGGTCCCACCACCGGTCAATGTCCTTCAACAACACGCTCTACTGCAAGCTTGCTCAAAAGCCGCGCAGCGTAGGATCCAGAGGTCAGACACGATAAGTGCGCGTGGCACTTTGCCACTGCGCCGCAGCTGCTCGTGGCTTTTGCTTGAGCGGCAGCAGTCTTGGCGTCGCTGCAACAGCCACAGTGCCCAAGCTCAGCTAGCGTCGGTGCTAAGTACAGTCATCACAATCCGAATGGTATTGAAAGAGCTGCATACGAATGCATTCGACACTATTTTCCTTTGGTACTTCATCATATTGTTTGGGGATGCACAATCAAGCTGAGATTTTATTTATCGGCTCGGTCTCTCGAGCGCTTGCGCTCGTGCTGCTCCAAGTGGAAGACCGAGCGAATTTGGCTTTTGGGATGATTGACCCATGCGTTTTGCGAGCTGAAGCGTTGCCGCTGGTGCTTCCTCTATGAATGCTTGATACTCGTCCGACGGCCATCGTCCATGCACACGAATTGAGTCCATATCAACTCCGGCGGCATGAAGCGCCGATGC

>Contig_22

TTGGGTTTAAAATGCCTTAGTGAAATCAAGAATGTTGTATTTTAAGGCGACAGCTGTCAAAATCTGTCGGCGCAGCAATCTTCCGTGCCAACGAAAATTCAGCATCCAGGTGACTGCAGCTCAATACAAGGATAACTACGAAGAAAAGATGGCTCCTCCCGTATTAACCATAGTGGGTGGGGACGTAAACAGTGTAACGTCAAGTTTTTCGTCCGAAAGTTCCCGTTAACGTTTTTATGACGTAAAATTGGCATCCGAAAAGTATTCATTGTGGTAGATATTATTATATTTTACCGGATACACGTGACTTGTGGAGTAGAGCGTGATACCTTCCAGGCACCGAGTTCATCGCCTGATGTATAACTACCGTTGCAGACGCACTTTATCGGTGTGCGTGTTCAATGTGTGGTAAGCCAGCGCGTGCAAAGGTGTAGACACGCGAATGGGAATTGGGACCTTCCTGCCAAGACCTTAAATCTCGGCTACTGCGTGTCCACGAAACCTAATAACATTACGTAGGGCGTTCTGGACCCGTGCGAGACATTGGGCGACAAGGAAGCCTACAACAGATCGGCAACAAGCTGTCCGATGCTTCTCGTACCTAACTTTCTTTAAGTCTGCTATCGGCACTATGCATTGTATAGCGTGCAGAGAAGANCCCCCCCCTCCAAAGCGCTTTTAGCTTAAACCACTATCGGTTTCGACCTGTTTAATAGTCCAACGTGGCCATGATTGGCCGTTCTATGGTCATTTAGCCGAAATCTGTCCACTGCGGGAATCGGCACGTATAATTAAAATCGTATTAATAATCGCTAATAACGGAAAATTCATGTAGACTAAATTATCTTAGTCATTTAAGAGAACCATTTACGGCATTTTTGAAAAGCTAAAACTCGTTTAAAAATGCTTTTTTCCAGGGCCAAAAATGCTCTTCAAAAGTACGATGGAGTTTGTCCTAGAATGGCTTTTTCTTGGCGCGATGNTTTTGGGACTGCTCTCAATTAAATGAAACAGCTGGCGGTGACCCCCACTCCCAGTACCAAACTTGGATTAATTACTTTTATCGGAAGTTGCATCTGAAGTGTTGGACCAGGCCTTCCGATAATACAACTGCAATGAGATTCTTGCTTTTTTGTGAAATCAGTACTTGCAGGGAAACATTTGATATTGACAAATACGTTATTGTTTTGATAAGAGCAATATCACACCACTACTTCCAAGATACATTGCTGGTTGGTGAAGGGGTTGTCAAAGGCATGGCGAATTGGATAATGAGGAGGACTGAGGTGGATGCACCTCCTAGGACTTGCCAATCAACCAAGCAGCGGTTGGCTCTCAGTTTTGCTTAGCTGGGATTGAGTAGTTTTGGGCTAAAAATAATTTATGACGATACCAGAAATATCTTACTGAAAGTTTTCATAAGGTTGTCAGGCCCTTATTAATAGCGCTAAGGACTGTAATAAAGAAGTACAGTAGAAGGAATTTAGCCGGGAAGGCGTAAACCTCAGGTGCGTGCTTCGATTAACACATACCGATGGGTACTATCCTATAGCAGAGCTGTGGTCTTGCGCAAGTTCCACCTGTGCAGGACATGCAATATACTACTACAGTGAGCAAAGTGTCCATTTAAAAATATTCATCTTTGTCCAAGCTTTCGTGAAACTTAGGTCTATCTCTCCTTTTTTTTCAGTCGTGTACAGGCATTCCGATAATACAATTGCAATCATATTGAAGGTCCATGCCAAAATAAGCTATTGCTACAAAAACTACAGCTTAAGAAATAAATAATTGACATCAATAGTAGTGTATTGGAAAATTAACTACTTAGATGTTTGACACTGCACATTACTTTGCGTTCACGATAGCTGCTACAGTGTTTGCGTCCCAGAAATGCCGATTCATCTTCAGGAACAACACGGTTTCAAGGTGAATAGGCAGCATACGCTGGCGATGGTGGAAAAGAGTATGTTTCGCCACCGCTCCACGGTGTTTGACGTCGGCGGGATGAAAAGCACACCATCAAATATCTTCTTTGCACCGCGCCGCTTTTTTCTAGTCGCCAAGACTCTGTCGGCAAAGCCCTGTGGGCGTCCGTGCCTGGTGTCCGGTGTCGTAATTGCAGGTACCGTCTCAAATGGCTGCAACATCAGCTCCTGGTCGTTCGTCAGCTCGTCCACGGCGCCTGACAGGGCTACAACGCACGCTCGCTCGAAGTCCACGTATTTGCGTCGGCAGCAAGATACTTTTTCACAACGGAATGCTTCTCGATGAGCGCATCAAAGATATCGCGCACATCGAGAAGCGTTACGTTTTCGTCTTGAAGCTTCATGGTGGCCGACTGGAAGTCGCCAAGCTGGCAGTAAAAAAGTAGCTGCAGACTTGCTTGGTCGTGAAGGTGTTGGCGAGAGCCATGGTGAGATCAAAAATGATAGACGAGCGCTTCAGGTTCGCGTTTTGAGCGACCGTTGCTAACAGCCATTCGATTCTGTCTGGAGACCGAGCTCCGTATTTACGCAAAACAAAGCAGAAAAAACTAGATTTTACGAAAAAATAAAGTACTTTGCTGTAAATACAAAATAACTAATTGGACAAAGTCATCCAATTTGTAAAAATCAATAATTAAGTGTAAACTATTGTATTGAAGCAGCAATACAATGATTATTTCGGAATGCCTGGTCGTGTACTTCCCACAATGTGATAGTAGAGCGAAGCCATGGGCTCGGCTTTTCGTCGTATTCTCAGGTCGCTTTCTACGTCGGTGAAGCTGCATTAATCTGGTTTCGTCCAGCCTGCGGACAGGTCAACGTCTAGGATTGATGGAGGTGTCAATATGAGGAGGAGCCATCTTTTTTTTGTAGTAGTCCTTGTATCGAGCTGCAGTCGCGCGCTCCAAATTGGAAGCTGTATTTTCGCTGGAACGGAAGATTGTACCGTAAATGTCACTAGGCTCAGTTTCGCGCTTGAACCATCGCCCAAACAACATTCGCTGGTACTCGGTAGCAATCGAGATCTCATCGAGGTCCGCATGGCCGTTTCGTATTAAGTGCTCCCTTGCCCTTGTTGTCTGGATGGCGAGTTCGTCCTCACCACCAAAGCCTTTGCTAACGGCCCTAAGCATACCCTCATTGTACAGGAGATTTTTGTTGTTGAGAGTCGTGAGGTAGCCACTCAATGTATCAAACTTCGGATTCGTGGGAGATAACAAATCATTGAAGTGAATATCCAGTAGTCTGAAGACACTGTTCGCGGATATTCCACCTTCCTTCCACTTGAGAAACTGTTGCTTCTGTAGAGTTATCACCAAATTCATCGTCGCAGCATCGTCAGGCTTGGCAAAAGTGAGCACCGTCGCCACCGCCTTATTCCCGTATTGCGCAGCAAAGCTGTCAATCAATGAAGCCTTGCTGTTTGGGTTCTTTGCGTTGTACATGGAGATAAACGCCCCAAAAGTGTGTACATTCCGGTCCAATAGAGCATCCAAACCACGGTTCAGTCTTAGCTTCTTGTAGATGTGTTTCGGACGCAGCAAGTCCACTCGAACCCACTTGTTAATTAGCGTGTTCTGCAACGCCGACGCCAGTTCGCGAGTTTTGACATCCTCTCTCGCTCGAGCCAAAATTGTTGCCAGTTGCTCTTCTCCACCAAACCCCTTCGTTAAGGTCTTGAGCAAAGAATCTTTCGTGGATTTCGTGCTAATAACGAGCTTCATGTAGTCGTCCAAAACCTCCATCTTCGGACTCGCAAGAGCTAGATACCCATCGCTACGAAGCTTGAGCAAGGTGAAGACATCGTCGACAGATTTTCTCTTTTGCAGCCACGATAGTAGCTGGGCATGCCGCAACCGCTGTATCGTGTTCAATTCATCGCCTGTCCTCTGCGCTGTCACTAGTGCGTTCGCAACGGCGTCATCTCCGTAGTGCGCCATGAACATCCCAATCACGGATGCCTTATTGTTATTATTCTGCGTGTTGAGCTCCTTGACGTAGTTCTCCAGAGCCTTCAAGTTGGGACTAGCCAGAGCATCGTCCATCCCATTCGTGAGCTTGAACCTTCTCAAAATCGGCTCTGCAGGTGGGTCCTTTGAGATCCAGGACCCCAAGTTGATCTTTGATACACCCGCCTTCGCTAGGTCTGTGAGCTTTTCAAGCCCAGCGCTGATCACTCTGTTCTCGCCATTCTCTGTATTCTGCGTGGACACTCTCAAGAAGCGTGGTAGCGAGTTGTTTTCTGGTTTGTCGATCGGTAGTGGAAGCCTTGTGGATAAATGGCTCCCCGTCGTTTTGGAGGCGGTAGGCACAGAGATCAGCAAAGCTGCAATCGCACACAGAATCGTACCCGAGTAAAGCCGCATGGCCTTTTCAGTGGTTCGAGAGAATTGCCAAGTGGCAAAAAATTGACTCAGCCTTCGGATACGGCTTACGGTACAAAAGGGAGTTTTCAAGATTTACATGTACTGTACATGAACATGTATTAGTACTGCCTATTAATACATGCCAGTGATTAGTAATCGACATATACGGTACAGTACATGCATCTTCCAATACGCTGTGCAGATGGAATGACGCTCGGTTTTATGGCGTAGATCGAGCATGGCCAGCGCGTTCACGGTACTCCTAGCTACCATTATAAGTGCTCCCATCTTTGTGACTGCCTACTTTATGGTATCATGAATCTCCGCCAGTACGATATGTTTGCTTGCACAGCGCAGTACGGAACGAAATACACCACCAGGTTTGTGGTGCTCCTCGCACCGTACACTGTACCGTATATTTACAGTTCCAGTACAATATACTGTACTGGATGAGTAACTGCAAGTGCCCCGTAATCTTCACTTGCAGTAAGCAAAACAATCCCTAACAGTACATATCTTGGCTCCGGTATTGTACTGTGAAATTTGTTCATTGTTCTTCGTTAAAAAGTGTGTAATTAAGTATCACTTCGAGGTAAAATATTACAGTACTGCACCCCGTTTTCGCGGGGGTGGACCATATAGGATTTGCAAATAAATCATATTTAAGCTTACAAGAGATTGCTTATTTGGATTCGGGATCATCACATCCGGAAGTTTATCAAAGGTCCAAAACGGGGTGCAGTACTGTATTAATATAAGTTGGCAATATATATCGAGGTGCACAAAGCAATTTCCCAGCAGCGGTACCGCCAATACTTTTGGCTTGCTAAATTCGTGTACATTGCCGGTACAGTATTCACGGGCAATTCTATACAGTACTGTTACTGTTTTCCATCTGTCCAAAGCTGATCTCTTGAGATACTGACAAATGGAGGTGACAGCCAAGCGCCAAGTGCTTATACACGCACTATTAATACGTTTTGCTGGGCCCCTCTCTCCAACCACTTCGCAATTCGACCCAGAATCCAGAAGATCCAGGATTTTACTTTGACGATGCATCGCTACTCTATCATTTTACTGGCTACTGCTGTCCTCCTTGCGCATACCACCGAATGTCGACAACGCATCTAAAACACCTTGTTTTGCAGGATAACACTTAAGCTTTGTTTTCACTTGGTATTTGTAAGAGCAGCCGGGGGGGGATTACCCGGCTCTCCTGTGGAAAGGTTTTATTTAATGTAAAGAGGTCGTTACCAGCCGCGCTTCGCTTGGCTGGGGCTCGCTTCGCTCGCGATTAATAGATTCTCTCACCTGGGAAGTGCAATGCGCGGCTCGCTGGTGAGCTCGGCGCTGGGCCCGCCTCCGCAAATACCGCGCGGCCCTGCTTCGCGGGCTCCCCGCGCGGGTTTGCTGCGGCTACGCGACCTCGCACCCGCTCGCCTTAGTTGGCGCCATACAGCTGCGTCGCCCCTCCCCGGAAGGTGCTATTATGCGTTTGAGGGTGCTGCACACCGTTTCGAACGGTGTTTTTGCAATTTTGTCCCGAATGTCTGGGCGGCTGTTGCACTGAGGAAGATTCACCCGCTAGCATCAAGTATTTGCTTCAATTTGAGAAAAATGGTGTAGGCTGATGAGAGCTCACAGAAGGAACGAGGCGGTCTAAGTGATCTGATTCGAGCGGGTACATCAAGGGTGGACGAACTCATCACACCCAAAACGTTGGAGAAAGATGAGCAACTTGGAGACGATATGGTAGCTTCTCTAAAAGCTCGCAGCTGAAATAAATGCACTACGTACAATCGTAACAAGGACTTAAGCGATCAAGTCTCATTGATCAAGCCACTTCTAGATAGGTGCGGGGACGACACAGTCGCAAGGACTCTCATGGCATTGGAGAAAACGACGGAAAGTATGACTCCGAATTTTAATGGAACCCTTTATTATCAGTGATAAGTTATCACGACAACAACGCCCTTCACCTCTTCACCTGATTATGGACTCTGTGAGTAAATTTCACTTCGTTGAGTGGACGGCGAACAAGCACTCAGCTCGACTTTTACGGCATCAGGTGGGATAGACAGCCGTCGGGATAAAATGGTGGCGCCCGTGAGTCCCAGCGAGACGCGACCGCTGCTGCTCCAACCAGAGCACTAGAGTCTCGTAGCCATCGCCGATAGCCACGAGCTCAAGCCTCAAGAGCCGGAACCTAATGCGACCGAAGAACTGCAGACGCTTCTGGCACTCGTCTACCCAGTCGTACGCAATAACACAAAGGGATGTGTCACACATCCCTTTGTGCTATTGGCGAAGACGCGGCCGCATCGAGTGCAGGTAAAGCCAGTCATGGCACTTGCAGCTCTTTCGCTCTTACTTTATTTTTATAAACCCAGAAAACCCAGCCTAAAAAAAGAATAGGTGGGTGGGTCGTTCAATATACCCAATACCCAAAAATTCCAAAACCCATTTTGCGAACCCTGGATGTGTGTCATAACAACAGTGTGCTTATCTCGCTGCGGTTAATTAACAGGTGGGCACGACAGCGCTCGAGTTCCTGCCAGGACTCACGTGCATCGTCTTGGCGGGACACATGGACTCACCGTACACACAGCAGTACGTTGACGCCGCCACGCTCTCGACCATGGTAAGAGCTCAACAAATATCGACTCCAGTTAAGATGTTCGTTAAAAACATGCTGCTAACTGTTACATGCATAGCTATTGAGCCTTACCAATTATTCAATCTGTTTCGGTCTCTCGTCGGCACTGGATACGCTCTGTTCCTAAGCGTATGGCGCCAAATGATACGACAAGATCGGCGTGTACTTTCAAGCTGGCGTTTTGGTACTAGCAGCTGTGTTTGGACCTATTTTCCTGCTAAATTGGTACACGGAGAGCGTGGTGGTGTCCTTGGGGCAAGACGAGGAGATCTCAAAGCTGGCTCAGACGTTCTCGAGGTGGATGTTGGTGGGGATGCCGTTCGTGGTGCTATACRAACTTGTACGTAAGGTTCTGCAGGCGCAGAATATCATGAAGMCGCTGGTTACTATCGCGACRATCGGCAACGTTGTTAACATCATAACCGGTTATGTGTTAGCTTATCATACGCCCATGGGMTTCGATGGCATCGCCTTGTCGAGATCTCTGGGAAATATTACTCTGCCAGTACTATTAATTCCATACTTTTACTACCACCCAGATCACTTGAATCAGTGGTGGAGTGGCTGGAACCTCAAGGAAGCGCTAGCACACGTGGGGCTCTTCATGCGCCTGGGCATTCCTGGCTGCTTGATGATGACGATGGAGTGGGTCGCGTTCGAGCTGCTGACGTTGATGGCTGGCGTGCTGCCTAACGCTGTGGTGTCCATGAGTGCCCATTCGGTGCTAGTGAGCATTAACAGTATCATTTACATGATATTTGCTGGTCTGGCGGTGGCAGTCAACATCCGCGTTGGCAACTGCCTTGGAGCCAATTTGCCTGAGCTAGCGAAGACTTCGTGTACGGTGTCGCTGACTCTCACTCTGGCCATTTCGCTGTCGTTTATTGCGTTCTTGTACGCGACGCGGTGGACGCTTCCGAGTCTTCTTCTCAATGACCACGAGAGCATCGTATGGGCGGCGAGCGCACTGGCAGTCTGGGCTCCGTTTGAGATCCTGGACGGACAGAATACCGTGCTGCAAGGTGTGTTTCGTGGCGCTGGGAAGCAAACTGTTGGTGCTACTATCAATGCAGTGGTCTACTACATCTTCGGGACGCCACTTGCTGCTTTGCTGGGCTTCTACTAAGGTTTTAGTGTTAAAGGTCTTTGGGTGGGATTTGGGCTGGGAATCCTGGTGTCGGCTTCCTGTCTCTATTTCCTGCTGTTCAAGCGCTGGACGTAGGAGGAGCTTGCACACGACGCACAAAAGCGCACGTCGGTGTGATATTCAATGGATGTTCGGCTGAAACGAGTACGTTTAACTTTTTGAGAGCAGGATCAATTAAGGTAGCAGGACTATGGGATACCGTGTCCGTCACACCCCTCTTCTTTTAGTTGAGATTTCTCACTTGGGTTTAAAATGCCTTAGTGAAATCAAGAATGTTGTATTTTAAGGCGACAGCTGTCAAAATCTGTCGGCGCAGCAATCTTCCGTGCCAACGAAAATTCAGCATCCAGGTGACTGCAGCTCAATACAAGGATAACTACGAAGAAAAGATGGCTCCTCCCGTATTAACCATAGTGGTGGGGACGTAAACAGTGTAACGTCAAGTTTTCCGTCCGAAAGTTCCCGTTAACGTTTTTATGACGTAAAATTGGCATCCGAAAAGTATTCATTGTGGTAGATATTATTATATTTTACCGGATACACGTGACTTGTGGAGTAGAGCGTGACACCTTCCAGGCACCGAGTTCATCGCCTGATGTATAACTACCGTTGCAGACGCACTTTATTGGTGTGCGTGTTCAATGTGTGGTAAGCCAGCGCGTGCAAAGGTGTAGACAAGCGAATGGGAATTGGGACCTTCCTGCCAAGACCTTAAATCTCGGCTACTGCGTGTCCACGAAACCTAATAACATTACGTAGGGCGTTCTGGACCCGTGCGAGACATTGGGCGACAAGGAAGCCTACAACAGATCGGCAACAAGCTGTCCGATGCTTCTCGTACCTAACTTTCTTTAAGTCTGCTATCGGCACTATGCATTGTATAGCGTGCAGAGAAGANNCCCCCCCCNCCTCCAAAGCGCTTTTAGCTTAAACCACTATCGGTTTCGACCTGTTTAATAGATTCCAACGTGGCCATGATTGGCCGTTCTATGGTCATTTAGCCGAAATCTGTCCACTGCGGGAATCGGCACGTATAATTAAAATCGTATTAATAATCGCTAATAACGGAAAATTCATGTAGACTAAATTATCTTAGTCATTTAAGAGAACCATTTACGGCATTTTTGAAAAGCTAAAACTCGTTTAAAAATGCTTTTTTCCAGGGCCAAAAATGCTCTTCAAAAGTACGATGGAGTTTGTCCTAGAATGGCTTTTTCTTGGCGCGATGTTTTGGGACTGCTCTCAATTAAATGAAACAGTTGGCGGTGACCCCCACTCCCAGTACCAAACTTGGATTAATTACTTTTATCGGAAGTTGCATCTGAAGTGTTGGACCAGGCCTTCCGATAATACAACTGCAATGAGATTCTTGCTTTTTTGTGAAATCAGTACTTGCAGGGAAACATTTGATATTGACAAATACGTTATTGTTTTGATAAGAGCAATATCACACCACTACTTCCAAGATACATTGCTGGTTGGTGAAGGGGTTGTCAAAGGCATGGCAATTGGATAATGAGGAGGACTGAGGTGGATGCACCTCCTAGGACTTGCCAATTAACCAAGCAGCGGTTGGCTCTCAGTTTTGCTTAGCTGGGATTGAGTAGTTTTGGGCTAAAAATAATTTATGACGATACCAGAAATATCTTACTGAAAGTTTTCATAAGGTTGTCAGGCCCTTATTAATAGCGCTAAGGACTGTAATAAAGAAGTACAGTAGAAGGAATTTAGCCGGGAAGGCGTAAACCTCAGGTGCGTGCTTCGATTAACACATACCGATGGATACTATCCTATAGCAGAGCTGTGGTCTTGCGCAAGCTCCACCTGTGCAGGACATGCAATATACTACTACAGTGAGCAAAGTGTCCATTTAAAAATATTCATCTTTGTCCAAGCTTTCGTGAAACTTAGGTCTATCTCTACNTTTTTTTTTTCAGTCGTGTTACTTCCCACAATGTGATAGTAGAGCGAAGCCATGGGCTCGGCTTTTCGTCGTATTCTCAGGTCGCTTTCTACGTCGGTGAAGCTGCATTAATCTGGTTTCGTCCAGCCAGATCCAGCCTGCGGACAGGTCAACGTCTAGGATTGATGGAGGTGTCGATACGAGGAGGAGCCATATGTTCTTTGTAGTAGTCCTTGTATCGAGCTGCAGTCGCGCGCTCCAAATTGGAAGCTGTATTTTCGCTGGAACGGAAGATTGTACCGTAAATGTCACTAGGCTCAGTTTCGCGCTTGAACCATCGCCCAAACAACATTCGCTGGTACTCGGTAGCAATCGAGATCTCATCGAGGTCCGCATAGCCGTTTCGTATTAAGTGCTCCCTTGCCCTTGCTGTCTGAATGGCGAGCTCGTCCTCACCACCAAAGCCTTTGCTAACGGCCCTAAGCATACCCTCATTGTACAGGAGATTTTTGTTGCTGAGAGTCGCGAGGTAGCCACTCAATGTATCAAACTTCGGATTCGTGGGAGATAACAAATCATTGAAGTTAATATCCAGTAGTCTGAAGACGCTGTTCGCGGATATTCCGCCTTCCTTCCACTTGAGAAACTGTTGCCTCTGTAAAGTTATCACCAAATTCATCGTCGCAGCATCGTCAGGCTTGGCAAAAGTGAGCGCCGTCGCCACCGCCTTATTCCCGTATTGCGCAACAAAGCTGTCAATCAATGAAGCCTTGCTGTTTGGGTTCTTTGCGTTGTACATGGAGATAAACGCCCCAAAATTGTGTACATTCCGGTCCAATAGAGCATCCAAACCACGGTTCAGTCTCAACTTCTTGTAGATGTGTTTCGGACGCAACAATTCCACTCGAACCCACTTGTTAATTAACGCGTTCTGCAACGCCGACGCCAGTTCGCGAGTTTTGACATCCTCTCTCGCTCGAGCCAAAATCGTTGCCAGTTGCTCTTCTCCACCAAACCCCTTCGTTAAGGTCTTGAGCAAAGAATCTTTCGTGGATTTCGTGCTAGTAACGAGCTTTATGTAGTCGTCCAAAACCTCCATCTTCGGACTCGCGAGAGCTAGATACCCATCGCTACGAAGCTTGAGCAAGGTGAAGACATCGTCGACAGATTTTCTCTTTTGCAGCCACGATAGTAGCTGGGCATGCCGCAACCGCTGTATCGTGTTCATTTCGCCGCCTGCCCTCTGCGCTGTCATTAGTGCGTTCGCAACGGCGTCATCCCCGTAGTGCGCCATGAACATCCCAATCACGGATGCCTTATTGTTATTATTCTGCGTGTTGAGCTCCTTGACGTAGTTCTCCAGAGCCTTCAAGTTGGGACTAGCCAGAGCATCGTCCATCCCATTCGTGAGCTTGAACCTTCTCAAAATCGGCTCTGCAGGTGGGTCCTTTGAGATCCAGGACCCCAAGTTGATCTTTGATACACCCGCCTTCGCTAGGTCTGTGAGCTTTTCAAGCCCAGCGCTGATCACTCTGTTCTCGCCATTCTCTGTATTCTGCGTGGACACTCTCAAGAAGCGTGGTAGCGAGTTGTTTTCTGGTTTGTCGATCGGTAGTGGAAGCCTTGTGGATAAATGGCTCCCCGTCGTTTTGGAGGCGGTAGACACAGAGATCAGTAAAGTTGCAATCGCACACAGAATCGTACCCGAGTAAAGCCGCATGGCCTGTGTAGGCTTTTCAGTGGTTCGAGAGAATTGCCAAGTGGCAAAAAATTGACTCAGCCTTCACGGCTTACGGTACAAAAGGGAGTTTTCAAGATTTACATGTACTGTACATGAACATGTATTAGTACTGCCTATTAATACATGCCAGTGATTAGTAATCGACAAATACTGTACAGTACATGCATCTTCCAATACGCTGTGCAGATGGAATGACGCTCGGTTTTATGGCGTAGATCGAGCATGGCCAGCGCGTTCACGGTACTCCTAACTACCATTATAAGTGCTCCCATCTTTGTGACTGCCTACTTTATGGTATCATGAATCTCCGCCAGTACGATATGTTTGCTTGCACAGCGCAGTACGGAACGAAATACACCACCAGGTTTGTGGTGCTCCTCGCACCGTACACTGTACCGTATATTTACAGTTCCAGTACAATATACTGTACTGGATGAGTAACTGCAAGTGCCCCGTAATCTTCACTTGCAGTAAGCAAAACAATCCCTAACAGTACATATCTTGGCTCCGGTATTGTACTGTTCGTTAAAAAGTGTGTAATTAAGTATCACTTCGAGGTAAAATATTACAGTACTGCACCCCGTTTTCGCGGGGGTGGACCATATAGGATTTGCAAATAAATCATATTTAAGCTTACAAGAGATTGCTTATTTGGATTCGGGATCATCACATCCGGAAGTTTATCAAAGGTCCAAAACGGGGTGCAGTACTGTATTAATATAAGCTGTCAATATATATNNNGGTGCACAAAGCAATTTCCCAGCAGCGGTACCGCCAATACTTTTGGCTTGCTAAATTCGTGTACATTGCCGGTACAGTATTCACGGGCAATTCTATACAGTACTGTTACTGTTTTCCATCTGTCCAAAGCTGATCTCTTGAGATACTGACAAATGGAGGTGACAGCCAAGCGCCAAGTGCTTATACACGCACTATTAATACGTTTTGCTGGGCCCCTCTCTCCAACCACTTCGCAATTCGACCCAGAATCCAGAAGATCCAGGATTTTACTTTGACGATGCATCGCTACTCTATCATTTTACTGGCTACTGCTGTCCTCCTTGCGCATACCACCGAATGTCGACAACGCATCTAAAACACCTTGTTTTGCAGGATAACACTTAAGCTTTGTTTTCACTTGGTATTTGTAAGAGCAGCCGGGGGGGGATTACCCGGCTCTCCTGTGGAAAGGTTTTATTTAATGTAAAGAGGTCGTTACCAGCCGCGCTTCGCTTGGCTGGGGCTCGCTTCGCTCGCGATTAATAGATTCTCTCACCTGGGAAGTGCAATGCGCGGCTCGCTGGTGAGCTCGGCGCTGGGCCCGCCTCCGCAAATACCGCGCGGCCCTGCTTCGCGGGCTCCCCGCGCGGGTTTGCTGCGGCTACGCGACCTCGCACCCGCTCGCCTTAGTTGGCGCCATAGCTGCGTCGCCCCTCCCCGGAAGGTGCTATTATGCGTTTGAGGGTGCTGCACACCGTTTCGAACGGTGTTTTTGCAATTTTGTCCCGAATGTCTGGGCGGCTGTTGCATCTATGTTGACTGAGGAAGATTCACCCGCTAGCATCAAGTATTTGCTTCAATTTGAGAAAAATGGTGTAGGCTGATGAGAGCTCACAGAAGGAACGAGGCGGTCTAAGTGATCTGATTCGAGCGGGTACATCAAGGGTGGACGAACTCATCACACCCAAAACGTTGGAGAAAGATGAGCAACTTGGAGACGATATGGTAGCTTCTCTAAAAGCTCGCAGCTGAAGAAACTGCACTACGTACAATCGTAACAAGGACTTAAGCGATCAAGTCTCATTGATCAAGCCACTTCTAGATAGGTGCGGGGACGACACAGTCGCAAGGACTCTCATGGCATTGGAGAAAACGACGGAAAGTATGACTCCGAATTTTAATGGAACCCTTTATTATCAGTGATAAGTTATCACGACAACAACGCCCTTCACCTCTTCACCTGATTATGGACTCTGTGAATAAATTTCACTTCGTTGAGTGGACGGCGAACAAGCACTCAGCTCGACTTTTACGGCATCAGGTGGGATAGACAGCCGTCGGGATAAAATGGTGGCGCCCGTGAGTCCCAGCGAGACGCGACCGCTGCTGCTCCAACCAGAGCACTAGAGTCTCGTAGCCATCGCCGATAGCCACGAGCTCAAGCCAAGAGCCGGAACCTAATGCGACCGAAGAACTGCAGACGCTTCTGGCACTCGTCTACCCAGTCGTACGCAATAACACAAAGGGATGTGTCACACATCCCTTTGTGTTATTGGCGAAGACGCGGCCGCATCGAGTGCAGGTAAAGCCAGTCATGGCACTTGCAGCTCTTTCGCTCTTACTTTATTTTTATAAACCCAGAAAACCCAGCCTAAAAAAAGAATAGGTGGGTGGGTCGTTCAATATACCCAATACCCAAAAATTCCAAAACCTGGATGTGTGTCATAACAACAGTGTGCTTATCTCGCTGCGGTTAATTAACAGGTGGGCACGACAGCGCTCGAGTTCCTGCCAGGACTCACGTGCATCGTCTTGGCGGGACACATGGACTCGCCGTACACACAGCAGTACGTTGACGCCGCCACGCTCTCGACCATGGTAAGAGCTCAACAAATATCGACTCCAGTTAAGATGTTCGTTAAAAACATGCTGCTAACTGCTACATGCATAGCTATTGAACCTTACCAATTATTCAATATGTTTCGGTCTCTCGTCGGCACTGGATACGCTCTGTTCCCAAGCGTATGGCGCCAAACGATACGACAAGATCGGCGTGTACTTCCAAGCTGGCGTTTTGGTACTAGCAGCTGTGTTTGGACCTATTTTCCTGCTAAATTGGTACACGGAGAGCGTGGTGGTGTCCTTGGGGCAAGACGAGGAGATCTCAAAGCTGGCTCAGACGTTCTCGAGGTGGATGTTGGTGGGRATGCCGTTCGTGGTGCTATACAAACTTGTACGTAAGGTTCTGCAGGCGCAGAATATCATGAAGACGCTGGTTACTATCGCGACGATCGGCAACGTTGTTAACATCATAACCGGTTATGTGTTAGCTTATCATACGCCCATGGGCTTCGATGGCCGCCTTGTCGAGATCTCTGGGAAATATTACTCTGCCAGTACTATTAATTCCATACTTTTACTACCACCCAGATCACTTGAATCAGTGGTGGAGTGGCTGGAACCTCAAGGAAGCGCTAGCACACGTGGGGCTCTTCATGCGCCTGGGCATTCCTGGCTGCTTGATGATGACGATGGAGTGGGTCGCGTTCGAGCTGCTGACGTTGATGGCTGGCGTGCTGCCTAACGCTGTGGTGTCCATGAGTGCCCGTTCGGTGCTAGTGAGCATTAACAGTATCATTTACATGATATTTGCTGGTCTGGCGGTGGCAGTCAACATCCGCGTTGGCAACTGCCTTGGAGCCAATTTGCCTGAGCTAGCGAAGACTTCGTGTACGGTGTCGCTGACTCTCACTCTGGCCATTTCGCTCAGGCAAGGGAAAAAATCAATTTGCATCGATTTTTTTCGATAATCCTCCATTGCAGTATGCATTGCAGTTGGCTCTTCAGCCTGAAGTTCAGGTGCAGTTTGGTATTGCAATTTTGGGGAGGCATGCAATCGGGTATTTTAACCCCTTGGTTCCCAACGTCATAAGTTACGACGTAACGGAATTGTTCGTCGTAACTTACGACGAAAATCTTTGAACAATAGAAATTGGCTCGGAGGCAAAAATCAATACTTTAATCAAGTTATTTTCCACTCGAGATAAATATGAGCTCAT

>Contig_23

TTTCGGAAGTTGCAACAACGAATGCGGCCTACCGCTTTTCTAGGAGTCTTCTAAACAATTTAAAAAGAGCTTGTAGCGGTTTCAAGCTAATTGCATTAAAAATGAAAGAGCTAACTATTTTTAAATAAATGTGCTACAATCTTTTTTTGGTTGTATCTTCTCTCGTGGCCAGGACAACATTGCCTGTGACTTGARGTTCCCTCATATCAACGCCCTGAGTTGGTCCGGTCATTGAAGTATGAACGTGTCTCAAATCTGATTTCCGATTTGGGACAGACCCAACAACTGCCTCAGTCGTTGCGACTTGTAAATGGGCACCGGAGCCTGCATCGTAAGGTGCTGCCTCAACGGATGNMACCTCGCGGTGCATGCGCGACACTGGCAGGGCCATTGCTCTTTGTCGCATTGCGTCCGATTGTGTACAAGTGTATACTTCTCCCAGCTGATCACTGTATCATGCCGTGCGAGTCACGGCATGCCCAATACTAAGTCAAACTTTTCGCCCAGCTCTAGGACGATAATATTTTCCACAAACACTCGGTGTTTGTACGAGAAGCGCGCGCGCTTTACGCGCTTCTCCGTTCTTACGGTGGCACCTGCTGCCAACCGCACTTCCAACACATTTCGAGGTGTTTCAGCTCCTCGTAGTTTAGCTTCGGGAGACTTTCCAGTCGAGCAAAATTGCTCGACGCACCTGAGTCAACCAAAGCACGAAGCACTGATCTCGGGGTTGCTTCAGCTGGTTATTGCGCACTTTATCTTGGACGTGTTGATGTCGATGTCAAAATCAAATGAGGACTGATGTGCAGCTCGGTTACACTACGGCTTTGGCGCTCAAGTTTTTCAAAAGAAAAGAATGTCGTTCCACAACATCTTTTAAACGAACGACAGCGCTGCACTAAGATGATTATCGTCCGAATTGTTTTACTACCAGTAGAAGTTTTAGTACTACTCGAAGACGACGTAAGTTACTGAAGTACTCAGCACATAAATTGAAGAAGATTTTTAAACTACATATTACTATTGTCGTTTGTAGAAATATAATACTGCTGGATTTTACCATTTGCTGCCTTACTGACAATCCCTGCCCACGGCACAATACAGTAGAGCATTCTGTCAACGTTCCGTGAACCCGATTGGGTAGTAAGATCTGCAACCTGCTAATCTACCAATGCCACGATCATACAGTACATGTATCACCGAAATCGCCCTACATGTACAGTACGCATTTGTTTATTGTATTTTTCCTCATAAATAACTCCTATACCAAACTTGCCGTTACCCCGATATATTCTAGTACAGTACTGCACCCCGTTTTCGCGCCCATGGGCCTATGATAAACTTCCGGATGTGATGATCCTGAATCCAAATGAACGAAATATTAGAGCTCATTCGTACATACAGAGTATGTGTAACAGTACCATTCAAGAATTCTGGAACGCTGTAGTACAATTCTGGAATGGAGTTTACACATGTGGATAGGAAACATCTGATGATTTGATCGCTATATATTTTCATAGTTTGCATGCGAAGAGATAGTAGCCATAATTCGCCCTATCTGTTTGCTGAGATAATGCAAGATGTAAAATGCAGTTCCTCGGTCATTTCCGAATTTTCTATCTACATATAAAGCCACTATGGTACGCTCCCAAGACCGCCAAGACCGCGCCGCTGAGACGCTCAACGAGGCCGACTTTCGTGCCTCCACTGCCGAGTTATTTTAGCGGGAGGAACGTCGCAAGGCAGAGGTCGCTCAAATCGTCGTACATAGCCGCAGAGACGACAATGACGAAGGGGACAGCTCCGGCGTGGGAGCTTTGCCGTCCGTCTTTGACTTCTGCTTACAAGCTGAAGGGCCCGATGGGGTGCTCAAGCTCACCAACTTTGCGCCAGAGGAGCTCGACCATGTTTGGGCAGCTGTGTACCCTCATTTGCAGGGGCAATGGAACGTGGGCCGCGGTAAAAAATGCCGCTACGCTGCGCGCGACGTTTTCTTTATGACACTGAGTTCGCTCAAACATCTTGGAAAAAGGGACACCGTTGCTCGAGTTTTCAGAATCCCGCCATCCACCTTTCAGAAAATGATCCGCAAGTTTATGGATATGCTGTCTCCTATTCTCTACGAAATGTACGTAGAGAAAGCGAATGATCAGTGGACACTGGGGAAGATTGTACGATCAGGGCATGCATTTAAGGACTTTCCGTATGCTCGATATGCTACGGACGTTACCTTTCAGCATGCTAACAAACCCAGCGGTAACATGAGCGAGATCTTACGCTATTACAGTGGCAAACACCACTTGAATGGCTACAAAATGGAGGTGTCGGTGCTGCCGAACGGTGTCGCGATTAATTGCATGGAGCACACCGGTGGGAGCACGCATGACGCTGAGATTTTTCGCAGGAGCGCAGCATTTCATTCGCGAGCTCTTCACAAACACTCTAGTGATGCCAATGTGCGAGACGAAGGAAGACTACAAGACAAGTATCCGAAAGAATGGGCGTTTGCTAAAATCTGATTTAATTTGCAAATCCTATATGGTCTACCCCCGCGAAAACGAGGTGCAGTACTGTACTGATCATCGAGTTCCTATCTTGAAACAAAATGTGCTAAATCGGACCCAATTACCGTAGCCGCTGTCAGTCGGTCCCCGAGATAAAACAGCAAATGGAGCGGTAATTTTTTAAGAAATAAGATAAACCTTTTAAAAATAATTATCGCCCGCAGTGATCATACATCCCATCATTTTTCGGTATCTTAGCCTCGTTTTCGTCTCAGAAGCGAACCGTACCACATCGAATAGATGGTTGCCACAGCGTCACTTCGTCATTCTCTCTCTGAGCCAAGCGAAGCTCGGAGTGAGCTCCCCAGGTGCCGCCATGCGAGTCCCCAGGGCCATACTACTGACAGTTGTCTTGATGGCAATATCCGATACTGTCTCCTCAGCTGCGAAGTCCCACTTGACCACACCATGTCTAACACGGCACGATACGAAGAGGTTTCTAAGGGCTCACAATACCGAGGATAGAGGGATCAGTACCCCCAATGTCGAGATGCTGCAGGGGTGGCTCAAGAAAGGTCTGCTCTCCGACGAAGCCGTTGGCCTGTTATCACTCGGTCACAAGGCCGACGATTTACTTAGCGGTTCGCTATTGAGCGCTTGGGTCAGCTACATAAAAGTCTTCAATAAAGAACACCCTACAGAGAAGATGAAGACGATCTCGGCGCTCACCGCTCGCTTCGGAGACGAAGCTCTGTCCACGATGATTGAAACAGCTAAAAGGGTCCCGAAGACGGAGGACGTCGCTACTAAAATGCAAGCCAAGCAGATCCAGAACTGGATGACGCTTGGTAAAACCCCGGACGACGTTTTTACGCTGCTGAAACTCAATACCGCCAAGTCGCTTTTATTCGATCAGCCTCCAGTCAACACTTGGCTACAGTATATGGACGATTTCAGCAAGGCTAAACCTGAAGCACAGTTCTCTACGATCACGACATTGAGGAAATTGTACACCGACGATGTATTAGCCAAGATGATCATTGTGGCTGGTAAGAACGCGAAAACTGCGGAGGCTGGTAAAAACGTGGAAACGGCGTTGTTACGCACCTGGTTTAACGAAATGAAGACCCCGACAGATATCCTAAGGCTGTTAAATGCTCGCGAGACTGGGCAGAGTCGAAAATTCTTCGCGTCTATATGGACGAAATACGACGATTTATTCCAAAAAGTGGATCCCAAATTCAAGACCGACATGCTCAAGGACTGGCTGAAGAAGGGGTTGATCACTGACGAGACGTTCCGAATGCTAACGCTGGGCAACGCGGCTGACGAGTTCCTCAACGGCTCGTTGCTAAGCGCTTGGGCCACGTACATCAAGGTGTTTAACCAGGAGAGTCCTACGCAGCAACTGAGTTTACTCGCGACACTCACCGCTCGATTCGGTGACGAAGCTGTGTCGACGATGGTTGAAACAGCCAAGAGAGTGCCCACGACAAAGGACGTCGCCAATCGAGTTCAAGCGGAGCAGATTCAGCACTGGATGACGCTTGGTAAAACCCCGGACGACGTTTTTACGCTGCTGAAACTCAATACCGCCAAGTCGCTTTTCTTCGATCAGCCTCCAGTCAACACTTGGCTACAGTATATGGACAATTTCAGCAAGGCTAACCCTGGAGCACAGTTCTCTACCATCGCGACATTGAGGAAATTGTACACCGACGATGTATTAGCCAAGATGATCATTGTGGCTGGTAAAAACGCGAAAACTGCGGAGGCTGGTAAAAACGTGGAAACGGCGTTGTTACGCACCTGGTTTAACGAAATGAAGACCCCGACAGACGTCATACGTCTGCTAGGTCTTCGCACGCCCGGCCAAACGTCTGTAGCCCCAGTTTTGACCAAGTACATTGCGTTATTCAACAAGGTGGATCCCCGATTTAAGACTGAAATGCTCCAGAATTGGCTAAAAAGAGGTTTAATCACTGACGAAACCTTCCGACTGCTCACATTGGGCAACGCGGCTGACGAGCTCCTCAACGGCTCCATGCTAAGCGCTTGGGCCACGTACATCAAGGTGTTCAACCAGGAGAATCCAACGCAGCCAATGAGCCTACTCGCGTCGCTCACCGCTCGATTCGGTGACGAAGCTGCGTCAACGATGCTCGAAGCAGCTAGGAAGACGCCTACGACGAAACGTCTTGCTTCGAGTATCCAGAGAGAGCAGAGTCGACATTGGCTCAGCGTCAAGAAACATCCGGACGACATCTTCGTCCTACTGAAGCTCAATACCGCGACCTCTCGGCTGTTTGACCAGCCTCAACTGAACACGTGGGTGAGGTATGTGGACGCTTTCAATGAGGCCAACCCGACGAGTACAACGACGTTATTGTCCACCTTGCGGACACGATACAAGGAGGACACGTTGGCTCAAATGCTCGTCGTGGCGAGGACCAAAGGGGGCTCCGTGGGGCAAACCGCGACTCGAATTCAGGCGGAACAAACGAAACTTTGGCTGAAAAGTAACAAAACGCCGGGAGAGGTGTTCGAAATGTTACAATTGAAGAAATTGGGCACCAACTTCCTTAGTCACCCAATTTTCAATGCATGGGTGAAATACACGGACGACTACCGCAAGAAAAACCTAGGGACATATCGCTCTGCACTGACCACGTTGAGAAAAACCCACAGTGACGAAACGCTGGCGAAATTGTTCATTGAGGCGAGTAAAGTGGCGAAAACGGCGAAAATGGGGAAACGTCTGCATGCTGAGCTACTACGCGAATGGTCCCTCACTGGAGCGACACCCGTGCGGGTCTTTTTGCGTCTGAACCTCGGCAAAATGGACCCAAAGGTGTTTGAAAGTCCGCTGTACTCTATGTGGACGAGCTACATTTCCATGTTCAAAAAGGTGAACCCCACGTTCAAGGACGATCCAGTGAAGATGCTGGTATCGATCTATGGTCACAGAGACCTGACGGCCTTGCTCCTTGCGGCGGAGAAAGCTCCGAGTACTAAGGATATCGCCATTAAGTTGCAGAAGGAGCTGCTCGAGCTCTGGCAAGCAGCCAAGATGGACCCATCACGCGTCTACAGTGCACTGCATGTGGAACGTGAAGCCAAGAATTCACCCATTAGAATGTTCTGGAGTGAGTACGTCAAGGCCTTTAGGAACTCGAATTAAATAGTACTAATATAAAAATTAATACAAAGAAAGTATTACTGAAGTACTATTACTACTTAGTAATCTTTTTACTAACTAATCTTGAGAAAGCCCAGTGCTCTGGGGAGGCTTGATAAGTTTCGTAGCGCCGAAATTAGATTCAATCTCGGCTTGAGTCGGTGTAGGCTTCCACTTTCTTCAAGCCAAGCGCTCGACGAGCGTCGTGCGTTGACGAGGACTCGGTAGCCATGGGAGCAGCTGCGTCCATGGACGACCCCGACGCGTCCGTGGTTTTTAGAGAGGTTTGTATTCTTGTAAACTTTATTTTTTTAGCTAGTATTCTAACATTATTGTTGCAGACGAAAGAGGAATATGAGCGGAGAGCTGCTGCGGGTGACTCGAAGGAAGAAATTTTCTCCAGTCTTCGAGATATCGTTGCCACCCGTTTAGGAGGCACACACCCACTCCTCCACACGTCACAGAGTCTGGAATCCTTTCTCGTCCAAGATTTAGCAATATCTGAGGTAAAAAAATCTACGTGTTTTTTATCATCGGCTACTTGGTTGTCCACAACTCACCTCTCTTTTATTGTTTTAGTCTGGAGATCTAGAGAAACGTTCCAGCTCGTTGCTGGATGCTGCTACGTTAGCTGCAGTAACGACCTCGTCCGAGAGCAAAGAATCGACTATCGAGACCAGTATCGCACTAGCCAAGACGATACCCGACGACGACGACGTCCAATTGAATCCATTCCATGACGACCATGAAGTCGAAACGCACATCGATGAGCAGGGAATCCTCCGAGCAACTGTTGAAAAAGGCTTTGGACGGGCTGTGGGTTCGTCTCGGGCTGCACTTGATCTGGTGCAAAACCTCGCTCCTGTATTTACAACTGACGTACTAACTGACCCAACAACGCTCAAGATGATCCTCAAGTACGCAGAAGCCAACAAAGTGCCCAGCGGAGCGGATATGCTCCGATTTTGGGTCGAAATCGACGAACTGCAGCACCTTCCGTCCCATTCGTACACGCACCGTCGACTACGCAAGATTTACGACAAGTTTTTGTCCCCTGAAGCGCCTTCGCCTGTGTGTGTCACGGCGCAGATGCTGCAGGATATCGAGAAAGCCCTCGAGGGAGATAATATCTCCGCTGGCATCTACGCCGGTGCACAGCAAATCTGCTATATCGCGCTGGAAAAATCCGTGTATCCGCGATTCCGAGACAGCAAATTGTTCCGTAAGATGCAGGATTTCTGCGCTCCGGTCGTTCCTAACGCTGGCGCGTCATCTAACATTGGCTCGAGCAATGGACCTACTTTGTTAGCTGCTGCTTCGGCTACAGGAACGGTGGCTGCCAACATCACGGATAATATGGAAGATGCTGAGGATTATTCGCTGTTGGGTATTCTAGCACATCCAGCTAAGCTGCGCTTCCTCAAGACGTTCTGTATGGAAGCATTGGCGCTGGAAAATCTGCTTTTCTACCTGGAAGTCGAAGATTGCAAGAGATTGCCGAATCTGTCGTTCGTGGTCAACAAGACGCGAAAAATCTACGATCGCTACTGCTCTCCATCGTCCAAGAACTTTATTGTCGGGCTGGGAGACAAAGACGCGCTGAAAGAAATCCACGACGTGGTGGAGAACAAAGGAGCTCTCGTGCCAAAGTTATTCTACGAGGTCCAGATAGGCGTATTTAACCGGATTAGCGACGATATCTGGCCTGGGTTCTGTCGCTCCCAGGAATATCTGGATCATTCGAAAGAGGTCCAACCGGACGCGAAACATCTGGCACGACGCGGCAATCGCTTCGAAGAGAGCGAGGCGGTGCAAAAGAAACTGGAAGGCTTGGCAGAGCTCCAGCTCATCGACGCGGCCATGCATTATCCGGTCGAGAAGCTCATCCCTATCTCAGTTCCAGATTCTATCCAAGGGGCAGCTCGACGAAAATCCATTCAAAAACTCGAAGAGGAGCAGACTCTCACGCCTGAGCAGGAGCTGAAGCTGCTGTTGGGCGACCCATTCGCCAAGAAATATCTAAAGCTCTTCATGACTCGACGAGGCGTGGATTCTTTGCTTGCCTTCTGCGAAGAAGTGGAAGATTTTAAGCTGCTACCAGGTATCGAGTTTCTCCAACATTCCGCTAAGAAAATCTACCGCAAGTACATTATCCCCAGTGCTCGACTACAGGTGGATATGAGTAAAACGATGCGAGAAGAGATTTTTACGCGACTCGCGAATCCAAGCGTGGATATGTTCAAGAAGATCGCGAACCGGGTACGTCACGGGATGCTGCAGGACTCGCTGCCTCGCTTCGTCAAGTCAAATTACTACAAGGATCTACGTCGGGATAGTAAAGCTACGCCTGCCGATCCCCACTTGGCCACAGTCGACCAAGCAGCAAAAGCAGGAAAGCTCGAGCTGTGCCACTTGGATGTCTTTCTAACATATCCAGGATGTATGCAAGCCTTCCGGAAGTTTCTGGATTTTCAACATTGTTCCGAGAATCTAATGTTGTGGGAGGAGATTGAACACTACCGAAGACTGCCGAGCTACCAGATCGTCCTGCGTTCAGCCAAGAAAATCTACGACAAATATCTGAATCCGAATAATCCACGATCGCAGATTCCGTTGGCTCCAGCACTTCTTCAACGTGTAGAGACGCAACTGGAAGTGGCGAGTCGAACGACGTTCGATGAGGTCGAGAACGAGTGCTACGACCACATGCGCAACGTCGTGATGCCGGATTTCCTGGATTCTCGGATTTTTATGGCCCTAGTGGGGACGTGGGCCCCGGTTCACGAAGATTATCCAGCCGAGATGCTACGTGGAGAACTGGAAATGGCGTTTCTCTGCGTCATCGCTTCCATCTGGTTCAAGAAGCTCGATGGAATGTCTCGAGACTCGACGGTCAGCAACTTTGACAAAGGACGCAACGGAGCGACCCCCACTCTAGGCACGACAAATAGCGGCCGAGTCACAACGGATAATCCAGAAATCTATTACCGATTCCAGATTTTAAAAAATCTGAATATATTTGAAAAAGTCTAAATCGCTATGCAACTACTATTACCCGACTTAACAGAGAAATCTTTAAACCTTCATAGACTCCTACGATCCCTATGCGAACAGCACATGCAGCGCCGCTTGTTGCGTGGCCGGCAATGAGGCCCACGC

>Contig_26

CTTAGTGTTTCTCGTTTGATCTCCGTCATCCTGCCTACCGCGTTTGTCTTGGTTCTCTTTCGCGGTCACTGCCATTGCCGCTCGGTGCTCTCGACGAGCTTGTTTGGCGGATTTAGCACTGTCCCCTTGCTGCTTACTTGGAGCTGCGTATATTGCATCTGACTGATACTCGTCTGGGACGTAAGGCCTGATAGTGCGATGCACTTGGTCAATTTTTTCTGCATTTCGCGGAGGCGGCGGCATCGTCTCCAGGTAGCTGCTGAACATGCGCGTCACCTTGTGGCGTTCGGTGAGATGGCCATCTAGCTTCCTACGCAGATCCTTCTCCACTGCAATGCTGGTGGGGCCCTTTTTGCAGATCACAACCCCCGGTTTCGTGAAATCTATTGAAAATATTTGAAACTGCTGCATGCCAAGCAATTTCTTGTAGAGCTCTGTGAGTAGTGGCTTGTAGCTCTTGAAGAACCCATCGTCCCGTGAAATGTGCACAGTTTCATTGGATGAAGCGGCTTCTTTTACTTCTCGGACAATCTGACCCAAAGTCCAGCAGTCTGTGCGGGCGAGGTGCTTGCGAATATGACCAAAGCCACGGTCACATGAATTCTTAGTGTGTCCTTTCACGAAGAACTTATAATCCACGCTCTCAAAAACCTCCATGTGGGCTTGTGCTAGCAGAAACTTGATCACGTAGTTATTTTTATTCTGGCCAGAGCAGTTGTCAGCGTATATCACCAATCGTTTTTTGCCAGCAGGAACCAACACATTGTCGATAAAGTGCTGCAGCGTGGAGCTGATTTGCTCGCTTCCCTTCCCACCGACAGACTCATCGTAGATGTAGTTCGGTTGCTTGCCTTCATTCTCGTAGTATATCCCAAACACACTGACAGAGACCAACGAGCAGAAGTACCATTGCGACGGCGTAGATGTGACGGACGGAGCAGTCAGATTCTGAGAGTAATCCATAACGATTACTGCGACGTCCTTGCCCTCTGTTGTCTTTACGAGAGCCTTATCCCTGTTATATTCCTGTCTACAAATACACCAGCAAAATAAAATGATATTAGTGTCCACCTYGGAAATTGCTAGTAAAAAAAATGATACTCATATCCCAGATCCAAAATTACCTCGAATCCTGATTTCCTCCACGTATGTGTGCATTTCATCATAGATCGCATCCCACGTGAGCGTCGGCGGTAGCAAAGTGTACATCTCAGAGCTGTATTACTTTTTCACTGTGCCATTCGCCGTCTTCTGCAGCCGAACACGCACTGGTACTACTTCCCCGACCTCCGCAGAGAACTCTTTAAACCACTTCACCATCCACACGAGATCGATGACAGAAGCGTGCTTGTTCAGTGTGTTCCCGTGCGCCTTAGCTGCAATGTTTCCTTCGCGCACACGCTTCTTGTATCGCTGGATAGTCAAAGGAGTCACCCCGAGACATTCTGCAAACATGGTCCGGCAAACTCTCCCAACCAGTGGCAGGTAGTAGTGAAATTTCTCGCGTTTTCCGTCTCCTCGGTGCCTCTCCACAGTATCCGTCTGCATGAGAACGCCCAGCATCGTGTCAACTTCTCTGATCTGGTCATCTGTGAGATTGAACACACGATCCACTCAAGTTCTTGGGCTTTTCCCTGAAGACATCGGCGATCACACTTGTCGGCCTGAATTAGACCCGCCACTGTTTGTTGCAGACTCACCTCAATCAAATTGATACTTATGTCCTCCTCCTCTTCAGCTGCGTCGCTTACACCACTCTCACCAGTGCTTGCCTCCTCGTCTTCCTCTTCATCTTCATTGTCGTCACTCCAATCTTCGTCGTCACTGTCGTCAGCTTCCTCATCGCTCTCCTGTTCGTCTTCACTCCCTTCATCTGCTTCATCGCTGGCAGGCGACCCTTCCTGCTNGGGGTACTGTAGCGGAGGGCAAAGCGACACGAGGAGCAGTAGGTTGAGCGATACTTGTTAGGTCGGCGGTGGCCGTGTTTGTGTGCGCCCTGGTGTCGGCTGGTTGCTTCGTGGCACGGTGGGGGCCGTGCTCTCCCGCATTGACCAGGACAGGGGGAGCAGGGGGCGGCACTGGAACAGCGACGGCAGGAGCGGGTGCCGTCGTGTTGCTTTCGGTCTCGGGGCACTGCATTCTCGAGGCTGGAGTGCGCTGTTCGGGAATGATACACCTGCTTCAAATTTCTAAAAAACTTTGACGCTTGTTTTTTCAGCTACTCGATAAAGTGCGAAAACACGTGGGCGATCTTTAGACGCGAATATCTCGATAACTCCAAATGGTTGATTAGTATCATTATGTCTCGCGCAGTCTCATATTCGGTCCTTTACAAAAAACTATGTAAAATAAAAATGTGTGATTACGGCTATTGCTCGACGTTGTTGCCACAATACCGGTTTCCCAAGCGAACGGCCGTGTGCGCAGCTTGTTGTAGGTTTTGGGTTGCTACACAAAATTAGTAGCTAAAACTTAAGATTTAGGTAAATTCACTGATACTTAAGTTGTTTATTTTAGCCAATAAAAAAGTGGACACGGCGGAATTCGAACCCGCGTCCAGTATTCTCGTGATCAAACCAACTGCCACATCCAGCAGCTAACCGGAATAGTTTGTTGAGCATCCGAGGTGACGGTCCCGGCCTACTTCAACGACTCGCAGCGTCAGGCGACTACAGATGCTGGAGCTATTGCTGGTTTGAACGTGCTGCGTATCATTAATGAGCCGACGGCTGCTGCGATCGCCTATGGGCTCGACAAGAAAGGAGGCGAGCACAACGTGCTGATCTTCGATCTCGGAGGCGGCACGTTCGACGTCTCACTGCTAACTATTGAAGACGGGATCTTCGAGGTCAAGGCGACGGCGGGTGACACGCACTTGGGCGGTGAGGACTTCGACAACCGTCTGGTCGAGTTCTTCGTGACCGAGTTCAAGCGTAAGCACCGCAAGGATATGACGTCCAACCAGCGAGCTCTGCGTCGTCTTCGTACGGCGTGCGAACGCGCCAAGCGGACGCTGTTCACTTCGGCGCAGGCGTACATCGAGATCGACTCGCTGTATGACGGCGTGGACTTCAACTCGACCATCACGCGCGCGCGGTTCGAAGACTTGTGCAGCGATTATTTCCGCAAGACCATGGATCCTGTTGCGCAGGTTTTACGAGACTCTAAACTGTCCAAGAACCAGGTGAACGAGATCGTCCTGGTCGGAGGCTCGACGCGCATCCCGAAGGTGCAGCAGTTGCTGAAGGATTTCTTCAACGGGTAGGAGCTGTTCAAGTCCATCAACCCGGACGAAGCCGTCGCGTACGGCGCCACCGTGCAGGCTGCGATTCTGAGTGGCAACGAGTCGTCTTCGAAGCTGCAGGACCTGTTGCTACTGGACGTCGCGCCGTTGTCGCTGGGCCTGGAGACAGCCGGTGGCGTCATGACTAAGCTCATCTTCGTCAGCAGCCACTTCGAGTGCCTTAAACTTCATCATTGGCGAAGGTGACAGGACAGCCGGCTTTGCGCATTGAATTTATCCACAAGACGATAGCACTCTCAGCCTCACTGGAAAGTATCGTTCCTTGTTCACTACACCGAAGATTCTTGTGATGGCCATGTCCACCTTCGCAATCGGCTTGTATCTGCGCTGCCTGCTTTTTCCACTTCGATATCTGATTTTGCTTGCGCTTTCTGTCTGAGCTAGATAAGGGCGGAAAGAGGTGAGCTATTGCTTCATTCACAGTATGCTTAGATTCCATATATTGTAGTATGGTACGCTTATGAGCATAGTCTACAGCAATACGAGTAAACCTCTTTGGCCTTCTGCCTGTTCCAGATGAGCGTGGCCTCCCCCTGTCCTAAGCGAAGTGCGAATTTTTATTTTAGCCAATCACATTTCTTCATTTAAGAATTATGTCCACAGCTAAAAATACTCTCACAACCAGTTAATTATGAGATTGTTCCTGCCACACATTTGGCTTTGTCACATGCTGAACACACCATAGGGAAGGCCGAAAGAGAAAAACAAGTACGAGCAAATCCTAAGTCAAATCACTATGTATATGAGATTCGTCAAAACCTCTAGAGAATTATTCAAATCGTCCATTAAGATCTTCTAAAAGAAAAATAGAAAATCCAGACTCGGTCAAATTTAATATCCTTCTCTCCCCAATAAATTAATAAATATTGCTATCATGACCACAAGAGTCCTAATCTGGAAATCGAGTAGCTTAGTTTGTGCGTCGCAAAATTTAAATGAGTGGTGTTTATTTTTCTCGCATTTGATGTATGGTTAAAACAGATATACGAAAAACGAATTGTTTAACAGAGATAAACGCGACATACAGCAATCAGTTTAAAAACCGTGTGGAAACTTTGGTTTCGTTTCTTGAAATATTAAAAATTCTATCGCCCATGTTTTTGAAAGAAAAACCAACCAAATCAAATTTTGTCATCACAATTCGAGTCAAACGCTCGCCGACGGCGCACCATCTTCGACAAGCGCTCATGTAGCCTGGTGTTTGTACTGTATTACCCCTTCATATCTGGGATACAAATGTTGGAGGGATGATTTGCGCAGAGTTGTTAGGGCAGTACAGAACGGAGTGAGGACAAAAGTAGGCAGCCATAGTGGAGAGGAAGTCTTGAGTGTAGAAGGAGAAGACGCTTGTGTTGGACTTGGTAAAAGGAGAAGACACTTGTGTTGGACTTGGTAAAAGAAGAGGTGATGAATTATAGAGTCTGGTGTTCTGGTGGGAGTTACTCTCCACCAACCAGTCCAGTTATGTACGATTTCTCTACATGAAATAGTACAGTTATATTCCACTAGATAGTTATCATCACATCGGAGATGCTTACTTCGACAACCGATAATCTAAAGTGCACTGGTGCAGAAAATTGACAATTACAAAGTTAACAAATAAAGGCACGAGTTGCAGTGCCCGGCTAAGAATCAACGACCTTAGTAAACCCAAGACAACGGCTCATCACCATCACAATCCTTAAGATAAAGAGGACGGAAGTCACTTGCATCTTTATGGTTTGCTCCGCTACGCGTGGCAAATGCAGAGCTGCGCTCCTCCGATAAAAAGATTCTCTTAGCTGGAGAGTGCACCGCTCGACTCGCTGGAAAGCTTGGTGCTGGGCCCGCCTCCGGAAAAAACTCACGGCCTTGCTTCGCGGGCACTCCGCGCGGGTTTGCTGCGGCTACAGTTGGTCTTCTTCGCTTTGATTTTAACTCATTTTCACCCCCTCCTCCCGCAGCCGGGCACGATAGTCACTTTCAATAATAAATGCATTCTGGAGGTTGCACCCGGACATGAAAAAACATGTGCTGGGGAGTGCAATGGGTCCGTTTTCTGAGCTGAAAAGTATTAGGAAAGGCGCGGCGCTCCCTAGATTATCTACAGTATCTATTAGATTACCGGTACAGTACATGTACCCCCTTTATCTGGCGTTTGACAAATCATGGTTCCCTTTCTTACGGTACTGAGTACCGGTACCGGTACAAGAAAACTATACGGATTACGGTGGTGGAAGGTACAATCAGCCTGCAAGAAATGAGGACCAGTAGTCTACTGGCAGTAGACAAATTTTCACAAAGCTGCTAGACCCAGGGTCACGAATATGCACGCGTACTCGGCGGCTGTACTGATGGGTCTCCTAATGGTTGCCGAGGGTGCCTATGTATCTACGACCGCTTGTGCACGCTCGGCGCCGAATCCCACGATCCAACGCTATGAGCCTGAAAGCATCCCAACCAGATTGGCTCCCAATAGGCTACTGAGAGAACCTGAGACAACCGAAGCGAGCAACGAGGACCGAGTTGTAAGTATCCATGCTGGCATTGAAAAACTCTCGGACCTGATAAAGACTGGAGTATCAAAGGTTCATGGATACCTGAACTTGGGACCGTCAGCAACCAGAGACCAACCGGCAGATGAAATTCTTCGCATCTACAAGCTAGATGATGGAATCGAAAAGGCTCTGGTCAGTCCAAACTTGAAGGCTATGGAAAGCCACGTGAAGGAGTTAAGCACGAAAAACCGAAAGAGCGAAGCGTCGGTGATTGGAATTCTCACGAGCCATTACGGGGACGATGCAGTGGCGAAAGCCCTTGTGACTGCACAGAAAACTGTACAATCGGATGATGATGTGAAAACGATATGGCGATTGCGAAACGCGCAGCTTTCCAGCTGGTTTAGCAGCGATAAATCTGTCGATGATGTGTTCACTTTGTTGAAGCTTCGACATGATGATTACTTAGCCCTTGCCAGTCCGAAGATGGAGGTCTTGGATGATTATATGAAGCTGATCAACCGCGTGACATCAGGTCAAGAGACTTTGCTAAACGTGTTAACAAAGGGCTTCGGTGGAGAACAGACCATGGCAAAACTACTTCTTCGTGGGAAGGAAGAGCCCCAGACCCGCGAGCTGGCGACGGCGTTGCAAAATGCGCTGCTGAACAAGTGGGTCACTGATAAATTTCAACCGGAAAGTGTCTTGAAGAAGCTCAAACTGGACCGAGACCTGATGAACGCTCTCTCGGATCCAACCCGACACACTTTGACTAGTTACATCGCGGTCTTCAACACTAGGAATCCAGGGAAAAAGGCATCGTTTATCGGCACACTTTCTGCTCATTACGGAGATGAGATGGTGGCGAATGTACTCATCGCGGCCTCGAGGAACGGCAACACAAGGAGAATGGCCAACCAACTGCGAACCGATCAGTTGTCAGACTGGTTGAACAACCAAAAGTCTGCTGACGAAGTCTTCAGCCTTCTAAAGCTGCGGGCAGATCTTCCGAACATAGATGGAGCTCTCGCCAGTGGCAAATTGAAGTTACTGGAGGACTACATCAAGCTATTTAACCGTGAGAAGGCTGGAGACGAAACGTTGCTCAAAACGTTAACAACCGGCTTCGATGGAGAAAGTAACTTGGCAAAAGCGTTGCTGACTGCTGAGATCAACCCACACTCGAATAAAATGGTTGTTAAGTTACAGGGTGAGCTGTTGAACCAATGGCTCCTGAAGGGATTGAAGCCGGAAAGTGTCCTCAAGAATCTTGGACTGGACCGTGGGATGAAAGAGGTACTATCAGACCCGAATCGGCACTTTTTGACCAAGTACATATGGGAGTACAACTCAAGAAATCGATTCGACAGAACGTCATTGATTTGGACTCTTTCTGCGCATTACGGAGACGACGTGGTAGCGAGAGCTCTTGCTGTTGCGAAGGGCGACTCTGGCCTGGCACGGACGGCTGCAATTCTGCAGAGGCAACAGCTGGAAGGGTGGCTCAGTAGTGGGAAATCTGCAGACGATGTCTTCACGCTACTGAGAATTGGAGCCGACGATTTCTTACCCTTGAATAGCCAGAATTTGGAGACACTGGAAGATTTTGTCTGGCTATTGAACCTCAAAAATCGCCGCATCCAGACGAATATATTCACAGTGGTAGAAAACAAATTCGGTGGAGATGTTCAGCTGGCACGAGCGGTCGTTAAAGCCCTGAATGAAGCAGACGAGAGAGGGCTGCGCGATTCTGTCGGTATTGCGTCAAGATACCGGAGCAAGCTGTTTGGTCGATGGTTCGACAAAAGTATCGAGCCAAAAGATGTTTACGCGATGATCCTCAAAGTAAACGGAGCTTCTGCAGATGCTTTGGAGAAGTCGATTGTTTCCCGATACACGGCATTCTACAAAAAGAGGCTGGCTAAGGCTTTCACCTTCGACAGTCCTAGACGGCTGTAGCTTTGCTTTTTAACCAATTTAAAAAATATCGCCTGTCATGCTCCTATACTTGGAGAAACAATGGAGAGCTCGACGTGCCTTCTTTTAAGAAGTACCTTGTAAGTACCCAAACGGTAATCCAAGGGTCTCTATGTAGATCCTGCAATCATGCTAACCGGGCTAATAGAATACACTATGCATAAGGCGTTCCGCCAAAATGCCAGGAAGTTAACCAGATCGATGCACTGTCAAAGCTACATAGCTACTATTGCTTGTAGTTGTCGCTCAGCCACACAAGTACTTTCGTCAATTGACACATTTAACTTTTCATGTGTCCTCGTTCAATTGCACGGGCTCGTACACTGAGGCTAGACCACATTCATGAACGCCTGCATTTGCGCTAGAGTGAGCTCCTGTTGCTTCATTTATACGTTATTATGCTACTTTATGCCGCCGACTAGCAAAGCTGAGGCGTAATAATAAATATGGCAACAACACAATACATAGCACATATAAAGTGCTTTTTTCGTGTATGCCTAGAGCTCTGTACTACATCATGAGGTTGCTTGTGCAGATAGCGCTTGTCCACAGTCTTCACCAAATCCTACTAGTCACTACATGTATTCCAAAGCAATCTCTTACAAAAAACTATGGTGATTGCGGAAACAATCGACGAAAACAAAAGAGTTGAGTTTTACGGTGATGTATATAGTAAACACAATTGCGAATACATGAGGTAGATAGGTACTGTAGTACTTGACAAAGTTACTCCAAGCCCCACAGTTCGACAGCCAGCTTCACTCTACCCATGCGACTCTCCTCGACTGGGCTGCTAGCAATTACAGCTCTTGTTGCATCTAGCGGCATTGCCTCGATGTCTACTATCGCCAACATGGCGGTGCCTGACAAACTGTATAATCTGTCTAGTACAATTTCCACCAGGTCGTTGAGAGATTCTGAGCCAAAAGGCGGAAGAAACGAGGACCGAATGAATGCCGGTATCGAAAAACTCTGGGGCCTGATCAAAGCGGGGACATCCAAGATCAAGGAGTACGGGCAGTTCGGGACACTAGAGTGGTTTAGCAGAAAGCTACGAACGTGTGACGCCCGGTTTTTTTTGTGAGGATTGCCAGAGACGAGCGTCACACGTTCGTTCAAGTGATGTAGCTACGACTGATAAGCTTTCTACAAGTCGTATACAAATGTATATCTATGCACTTTAACATCCGAATACTGTATACTTGAGTACATTTTGTGCCCATTATAGCACTGCCGTTGGTTGCGCACGGGTCGGGCGATGCCACGCACCGTGCTATCAATGAAGCCGATACACCTGTCGATAGGACTGCCGCGTGCAAGAAGCTTGCTGCACTTAGCGTAGATTAGGTCCACCATGAAGCGAAAGATCAGGCACAGCGCACTGGGCGACCTGCCGAACATCACAGCAAGATCCCCGTACCGTCCAGGGTACGCGAGCTGGCGCAGCAACACACAGAACCCCTCGACTTCACCAACGCGAAGGCGGATCGGTAGCTTGTAGTCTTCCTCAAGCCGCAGCGCCTTGTGCAGCCGAATTACGTCACGCTTCCAGAATCGGAAATTAAAACGACAGTCTCCGTCGCTTAATGCGTTGAAATTGAAGTTGCCCTTCAGTGTGAGGACTGGTCGTAGAATAGGAGATCTGGCGAAGAGCACAGCGGCGTCCTCGACGAGGACGAGGTTCAGAGCGGTCGCTGCGAGGACTAAGCTCTCCTCGAGCATCTTGACTGGCTTGTGTGCTTTGCAAATGTAGTATACACCAAGGACGGATAAGAGCTCGCGTTCGCTCTGCAAAATGCTTCGCTGGCAAACCAACGTGTGCGGCAGTGTGATTAGGGTTGTGTGATTGGCGCTAGTCTACAACTCACACGAGTTCCTGCTGCTAAACCACTCTACTGGGATCTAAAGACCAACCTGCAGCGGAGATTTTGAAATTTTTCAATCTTGCGGATGGTATGGACAATGCTCTGGCTAGTTCCAACTTGAAAGTTATGGAAAACTACGTGAAGAATCTAAATTCGAAAAACCGAAACAGCAAGGCATCGATGATCGGGGTTTTCACGACGCACTACGGAGACGAAGCTGTGGCGAGCGCTCTAGTGGCTGCGGAGAAAAACGCGAAAACGACAGAAGCAGCTAACACGATAAAGCAGTTGCGAAAGGATCAATTATCAGCTTGGCTGACCAGTAAAGTATCCATCGACGATGTCTTCCATTTGCTCAAATTTCGCGATGATGGCTACGCAGTTCTTGCTAGCTCAAAGATGGAAGTACTGGACGACTACATGAGGCTATTTAATCGAGAGAAATCAGGCCATGAAACTCTGTTGGACGTGCTTACGAAAGGTTACGTTGGGGAAGGATATCTGACGAAGGTGCTTGAGCGTGGGAAGAAAGATGCGAGTACGAGTGAGTTGGCGACTGCGCTGGAGAACGCGCTTTTTAACAAGTGGTCAGTGGAAAATTTACGGTCGGAAGATGTCCTGAAAAAGCTAGGACTCGGCCGCGACATGAAGAAGGTTCTCTTCGATTCAAATCGAGACATTCTAACGAGGTACATTTCCATGTACAATGCGAAGAACGCAGAGAGCATGACGTCGTTGATTCAAACTCTTTCGACCCATTATGGAGACGGCATCGTTGCAGGCACATTGGTGATTGCGTCGTGGGATAAAAACACAGAGACACTCGCGAGACAGCTACGGAGTGACCAACTAACTGAGTGGTTGACGAGTCAGACATCTGTCGCCGAAGTTTTTCCGCGACTAAAGCTCGGGGATGACCGCTACCCGGATCTCATCGATCCAAAGGTGGAGGTACTGGACGACTATATTAAACTGTTTAACCGAGAGAAATCAGGTCAAGAAACGTGCTCAATGCCTTTACAGAGGGCTTCGGTGGAGACGACATTTTTTTGAGAGTGCTGGCGGCTTGGAAGGAAGAACCTCACACCCGCGATCTTGGAACTGCATTGCAGGACACCCTTTTCAAAAAGTGGGTCGCTGATGATTTGCAACCGGTAACTGCACTGAAGAAGCTCAGACTGGATCGTGACATGATGAGCGTATTATCGGATCCAAACCGACACACTTTAGCCAGGTACATATCAATGTACAATGAATGGAATCCGACCAGCAAGGCCTCGTTGGTTGGAGCGCTTTCCTCTCATTATGGAGATGGTGTTGTGGCTTACATCAGGGCATGGGAAATACAATACCCATTGTTATTTTGCGTGAATCACATCCGTAATAAAGACTGCAATACCTGGCAACACAATAATAATCACTGCAATAATCACAATATATTGCTATCACTTAGTCTTCGATGCAATCGAGAAGGGTGGAGCAGTTCCACATACGGCGATTAGCACGAAGAAACATGGCCGTTTCAAAATTAGCTGGTAAAGTAGATGAACGTAGCGATGTAAAAACAGGCTTACACTCAGAGAAAAGTCTCTCGCACGTGTTGCTTGTCGGAGGAGCGCAGAGCAGGAGAGGATCATACTCGACAGACGCCTGCGATGCAAGCCGCGGCTTCTTGGCTTGCCGGAGGATGGAGGTCGCGAAGTCCGCCGCAGTCGGCACTTCCTCTTGTGCGGTAGACGGAGATAAAATAAAACTGTCGACTGCATGTAATTCATCCGCGGAGAGGGGGTAGGTCGTTTTGCAGCTTTACTATTGCAGACTCGAAATGGGGCGAGTGAACAATCTCAGCTGTTGGCGCTAAGTAGTTAGCCATGACAGGATACTTAGCAGCACACGCAYCAAACAACAGGCGCACATCGGCAAGCGTGCATGACTCCGCTTGGATTTTCACACACACACTGTCCAGCTCACGAAGCTTGTCCAATACACCTCTAACGCGGCGGTGCGCATGTCCACGAGGCACAAACTCTTCAACAGCCTCCACCGTCAGGATCGCATCACGGATCTTGCAATATCGGTCAATCATCTGGAATGTAGACGACCAGCGCGTAGTGTTTGCCTTAACAGGCTTGTAGTTGGTCGCCTTTGCCAGTGCAGTAGCATTTTTGACATGACGCAGCTGAATCATCAGGTTTTGAATCAGATCTATCTGAGTCTGATAATCCTGTAGAAATCGATTAACCGCGAGGTTAAATCGATGACTTGCACAGCCAATGAGGGGACGCCCATTCGTGTAGCCATACTCTGATTGGTTGCACAATTGTCCCCCACAACATAGCGCACCATCTGCAGGTTCTTCTCGTACACGCTCAGTACGGTGGATATATGATCCAGGTGGGCTTCAGCTGTCTGGCCGTCTTCCATTGGCGAAAGCGCAAGAAGGCGCTGGTGGCGCACACCATTGTAGACGTAGATCGCGTAGATGCCGAGGAAGTGAAGTGAGTGAATAGTCCAACCGTCGAACATTAAAGCAAAGGAAGTTCCCATCTCCTTTGCAATGATTGCTCCAACGGCTAGAGCCACATGACGCATGTACCGCTTCAGTGTCTTGGTAGTCGTAGGCTTCATTGTAACTACAGCACGCGTAGCCTTGTTTTCCACTTCAGTGATTGGTAGATTTCTCTCGATCACCCATACCATCCATTGGTAGATATTGCGCGTCACATCGTCCACAAAACCAAACGTGGTAAGTGCTGGCGTCTGCGTGGCATGAAGTTCTGCAAACCCAGCAGCATACCCAGCGTGCTTGCTGTTAAGGTGGCTGAGGAGGTTGCTGTACCCCGTGCCGGGCGTCTGCTTCCGAGGAAGCCCACAGGATTTACACTCAAAGCGACCTTCACCGAGGTCGCTGAAGTAAAATGCGCAGAGTTGACGGGAGGTCATCGTGGGTAGCGTGGTGGGTAGCGTGGCTAAGAGTGGTGGCTTGTTCTCTAAAGAGAAAATTGAGAAGTGGGGAGTGTGTGAATCAAAGCGCGCAGAGTGTATTTGCAAGGCAGTAGAGAACTGTTGGTTGTCAGCGCTCCTGGAAATGGTTGGAAGCGTGTAAAATATTGTATTATTGTGTACAGTTATGTATTATATTTATCTTGGAAAAATCAATAGTAAGTTATTCCGTAAATATAATACCCGTAATATTGTTATTATACTATTTTTCCCATGCCCTGGCGTGGCCATGTCGTCGTATAAAGACACAAAAACGGTCGCGCAGGAGCTACGGAGTGAGCTCCTCACTAACTGGCTTAACAATGAGAGATCTGTCGACGACGTTTTCAGACTGCTAAACCTGCGTGGTGACGGGTTCATGGCTTTAATGAGCCCTAAGCTGGGCGCCTTGAAAGATTACATTAAGGCATTTAACCGTGAGAAATCTGCTGATGAATCTCTACTCAAGGTTATGACAAACGGCTTCGGTGGAGAAAGAGAATACGCAAAGTTTTTGGTGAAAGCACAGTTGGAAGTAGTCCCAGGAAGCGCGGCCAGATCGTTTCAGACGGCACTGTTTAACCAATGGCTTCAGCGCAATATTGACCCAGAAATGCTCTTGAAGAACCTCCGACTGGACCGTGGTATGGAGGACGTCCTTGCCGACACAAACCTGGTCACGTTGAGCACGTACATTCCCGTGTACAACGCAAGGAATCCAAACCGTAAGACATCGCTGATCGGGACACTGTCTGCCCATTACGGAGATAAAGAAGTGGTGAATGCGCTTTATATTAACAGAGACGACTCACGCATTGACAGTCTAGTTGTAAACCTGCAGAGAAAGCAGTTGGCCTGGTGGTTAGAAAACGAGAAATCTGCGGACGACGTTTTCTCGCTACTGAAGATTGAATCGGATGATTTCTCGCCCACGAAGGGCCAAACTTTTGCGGCATTGGGAGAATTTGTCGCTCTATTGAACGCCATAAATCCCCAGCATC

>Contig_28

CTTATGGGACTGGTTATATTGTCGCTGGAGCTCTTCGCTTTGGACCGCTGGATTGTACCCAAAATTGCAGTTGTGGATCGAGTTATCTTCGGTCGTCGTGTCGAAGTGAGCACTTTGTCAGCCTTGGTCTCGTGTCTCTGGACTATTTTCCTCTGGGATTGCAAGCTCATGTGGTTTCTCTCTGCTGCACTTCGTCGTCAACGTCAACGTCGCTTAAGTGCGGTTGGCGCTAGTGAACTAAATGATGACGATGATGAAGGAGAGAGGCTGCTGTTGATCGGCAAAGTTCAGTACTTTTACGAGTTAGCATCGCGCACACGGTCTCGAACACGTTGGCTGAAAGGTGTTCAGATGCTTCGGATCTCACAAGCTATCCAGCGACGTCGAACAAGGAAAAAGTTAACTGTGGAGAACCGGTAAAGACAAATTATTATCGTTATAAGGAGTAATCTGAGCAGGGCGACGGATGAGTACATTGAAGTGGAGGTTATGAGTACCTTTCCGAACGATGATGCAACGTGCTACGTTGCATTTACGTCGGACTAATCATTTCGGTCCAACTGTAAGCGTGCAAAGCAAAAAAAGAAATGTCTTTCAACGGAGCCATGATGGTGAACTATGATCAGTCCACTTCAACATGATGAGTATGAGCCACTCGATGAGTCATGTTGGGGTATACTAGGACGGTTTAACAAGCGATACTGCAATGCGCCCTCTCTCCACCGGAACAGCTCATCAAGAAGCGATACCGGATGGCAACTAAAGCCTTGGCCATTTTCAAACGTGCCGCCTGGTGCTCAAACCGCTTCATCGACTCATCAAGGCAAGACTACGATGTCGTCCTTCCGACTTCTCCAGTCGTTGCTGTTTATCCTGTTGCTTATCGAGCTCCCCGGACACGCTACAGCGACGTATAACATCGGTGTGGGCAAGAGCGACATCACTGGACCTGCAGCCCAAGTGGTAATGATGGGGTTCGCAAGCTCCGACGAAAGCTCCGCTGGGATCCTTAATTGTCTGTACGCCCGAGCATTCCTCATCCAAGACGCTATTACCAGGAAGCGTGTCATGTTTGTCCACTGCGACTTGCACTCCGTCATGCAACTAGTACATCAAGAAGTTCTAGCTCAGCTCGCTACCAAATACAAGGGACTCTACACCGAACAGAACGTCATTCTACATGCTACGCACACCCACGCGGGGCCAGGAGGGACCGCAAGATATTTCCTCTACGACGTCTCCAGCTTTGGATACGTCAGTGAAAACTTCGACAAGATCGTCAGTGGTATCGTGAGCGCCATTGACGCTGCACACAACTCCATCCAGTCCGGTACCATCCGATGGAACAAGGGAGAAGTCTCTAAAGGAGTCAAGAACCGCTCTCCCGAGGCCTACCTGGCCAACCCGTCGTCTGAACGTGCACAATACAGCAGTGACGTCGACTCGACAATGCGGGCACTACATTTCTTCAGTAGTTCCGGTAAGCTTCGAGGTGTGCTGGCGTTCTACCCCGTCCATCCTACCAGTCTTAGTGTACAAAACCTTCTCATCAGTGGAGACAACAAGGGATACGCCGAGCTCCTTCTTGAAGATGAACTAGACGACGTCATCGTTGGGATCGGCATTACTAACGCTGGTGACGTGAGTCCGAACCTCATCGATAATGGAGACGGGACCTTTAGTGGCGAGGGCAATACAACGATCGAGTCTGCCGAGATTATGGGCAAACGGCAGTACGATACGCTGTCATCGCTGATCAAAGGAACCTCTGAGCTCGTCCATGGCTCCACCGTCGCTAATCTTTCTTACGTCGACTTCTCTAATGTAAAACTCTCCGGCGCCACACCAAACACAGCAGACCCGTACGCTAATAGAACGTGTCCTGCGGTCATCGGACAGAACTTTGCTGCTGGCAGAGAAGACGGTCGAGTGCTCAGTATGTTCACAGAGGGAAACCGTAAGGCCAACGCACTCTTCCGGACCCTCGGTGGTGGCACCACAATGGGTGAAGGACTGCCAGAACGTCAACAAAGTGCCACTACTGGCAGTCGGACTCATGGATCCTGTGCCGTGGGTACTCAACATTCTACCCGTACAGATCGCTAAGATCGGCCAATTCGCTATCGCAGTCACCACGTTTGAGACTACCGGAAGACGTATCCGAAGCACAGTCAAGAAGGCACTTACAGGTGCTGGAGTCACGGAGGTTGAGTTGGCTGCCATCAGCAATGCTTACGCGCAGTATATGACCACCAAAGAGGAATACCGTACGCAGCACTACGAAGGCGCGTCGACGCTTTTCGGTCCCAACCAGCTGGCTGCAGTGCAACAGGAGCTCACTAGAGTCGCCACGTCCGTCGCTGATTCGTCCGTACCGCTGGACGTGGGTCCCACACCGTTGCAGATCAACCGCAACTCGCTCATCACGTTGCAGACTGGTGTGGTGATGGACTCAGCTCCGCTTCTGCGCTCCTTTAGTGATGTACGCACACAACCGTCGAGTTCGTACTCCGTGGGGAGTGTCGCTTCAGCGGTATTCGCCGGCGCGCACCCGAAGAACGCACTCACGCTGGTCTCATCGTTCTGTGACGTGCAGAAACTTGGATCGAATGGCGCGTATACAACCGTCATGACCGATGCGCATTGGGATTTGCGCTACCGCTGGGAGCGCTATCTCATTGCTGAGAGCAAGAACACTTGTGAGTGGAACATCCGCAAAGGTGGACGCACTAGTGTGGCAGGCACGTATCGCTTCGTGCACCGCGGGTATTCGAAAAACCTGCTTGGTGCATTGGCGGCGTATGAAGGTACGTCTAGCACCTTCATCGTGACGGTTTAAAGCAGCGGCACATCAACGTTCCAACGAGTTGATTAGTTCATTTTCAAATAGCGTCATCAACTTGTCCAAATAATGCCCAAATCGCTTAGGAAGATTAACGTTGTCTCAATACCGGTAAACTGTCCAAAAACAAGTATCGAATTAATTATGTAGTTTTATTTCTGGCGACCGGGAGGGAGTGTGGCAAACCCGCGCGAGATTTTGTGTAGGCGGGTTCAAAACCGAGCTTACCAGCCAGCCATCGATTGTACTCTCCTTAAAAAGAGCATTTAATCGCGAGTGCCGCGCTGTGCGAAATCATGCTGAAATAATAATAATTAAAAAATGCTTTAAAATCACGAAATCCAAATTTAAATGTACCGTGTTTTCAAAGGCACCCCGTTTTTATTTCGTTGAATGAAACGCCATCAAATCTAGTGTTCATCTAGACTTCATAACTGCTGCCAGAGCATCGACAGGGTCATCGATTTTGAAATATGCTCTCACTTTCTGAAGCAAAGGTTGTCCGCCCTTGTTTTCAGCATAGAAAAGCATATAACTCTCCCAAGTGCCGTATCTGGGATCATCCTTCGGCAGGTTCAAAACTTTTTCGTTGAAAGGACTTGCCAGCCATTTTGAAGCCTCATCTGGATTTATCTTCGCACTTATCCAATTCTGGAATAGGGAAGACTGCATGTTTTCCGCTTGTTTCTTCAAATCTGGAACTTCCTTAAGAGATTGAAACTGTGTTGCAAATATTCAATCTGATTTCAATGGGTCGGACTTCGTCAAGAATTGCAAGGTTTGCTCATCCGAAAACGCACTCTTCTTAATGCGGTTACGAAACAGTTGGGTATACCTGAGCCACTGAATCATGTCACTGTCGTCTCTGGCCCGACTTTCCGCCACACGTAAGACCTTGAACATTTCCTCGGGTGGCTCTCGAAACTTTAGCCAAACGTCATTCATAGTTTTGCGACTGGTTTTAAATTTCCAAAACATAAATCTCTGCATGGTTTCAGCGTTATTCTTCATGTTCGGGACCTTCCGAAGTGAATAGAAAAGGGTTGTCAATTCACGTTCCGGCATTGTCTTTTGCAATAACGAGAACATATCAATGTCTCCGAACATAACCGTCCCTCTCTTCGCTCTGTACGCCTCCACGTACTTCAACCATTGGTGGAAAACGGGCGTGTCATCCAACTTCGAGCCAGCTTCTTTTAGGCGTAGCACTTCGAAGGCTGTCTTTGAACTTGTTTCTTTCAGTAGTGTTTTGGACACCCACGCTCCAGATAGTGACTCCTGGATATTCGACGCAAACTTACTCAGCCCTGGAGTTTTCTTTAGAGATTTCAGTAGTGTCATCAGTTCCTGGTTGCTGTGAGTCTCTAGTAACAGGTAGTAGACATCCAAATTCAAAATCGCCTTCTCGCCATGTTTAGTTTGGTAAGGCGCTACGAATCTGAACCATTGAAGAAGATTCGGATTGTCGTCAAGCTTGGCTGCGCTCTCTCCAAGACGTAAGGTGCTAAACAGGTCTTTAGGGTGCACCTTGGACTGCACCCACGTTCCGATTTGCACTTTATCAGGTGACAAACTGGACTTGAACATGGATTGTATCAGATCGTCGACCTTTGATAACCAGGGTACATTGACTCTGTCCTCGCCATCTTCACGTAAAGTCGATGTTTGGAAGGATCTTAACTGTCTTTCCGTCGCTATGCTGATGTGAGACTTGGAGACAATGGAAGTTCTTTTCACCGAGTACGCTTGAGTAGTATGTTCTTGAGCAAGTGCTCCGGCTAACAAGGCTACAACAGTAACGAGCATTAAACCCGCGATGCCCATGGTTGTTAAGAGCAACTTGCTCTTGATGAATCGCCAAATGACCGAGGTGCACCAATTATTAAAATGGCAGCTAATAATAAGGAGATTCGCATTCGCTAACACTTCTATATTAATAATGTCGAAATCCTGATCATCACTGATCGCGTGACTAATCTCACACGATCATTTAGAAAGTCGCAAATCTGACTTACCTAAAAGAGATCATTAAGTACGAAATACGTATATTACCAAAACGGCAAATTTGGTCGGAAATTCACAGAGGCAAACGTCGCATTGCATGTAAATGCAACAGTGACGTTTTGTCTTACCTTTTTTTCCTACCAACCAGTCTTTGTGAGCTCTGAATCTAACTTTGAGTAGCTGTTCTTGCTCCAGGGTGTTTCTAATTGTTGCATGTGATCAAGCGCATTGTACCCCTCAGTGGACGAAGGTGTGTAGTGTTGGGTGAAGAAATTTGATAGATGCCATCGCCGTTGGTAGCTGATGCCACACCACTTGAGCAAACCGGCAAAAATTGAGCCACTTTGTGGTTAAGGAACGGCAACAACGACCTGGTAAATCGGGTCCAGCTCCTAATACTGATTCCTCTCAATAATACTGTACTAATAAAATGCGGCTCCCCGATACATGGTCGAGCGAGGCACTTCATTACATGTATCAATCCCAACCGACTATATTATTAAAGTACCTAATTCCCATCCACAGTGGCGGCATTGGTTTCAATACTTTCCGTCGGACTCGCTGCTAGAGGTTCTCCGTCCCGACTTCCACACGCGTAGGCCTTGCGAACGAGTCGCATCTCCTCTGCCATTACTTGAGACGCTACGGCTGAAAACTCTTTCTTCACAGGGTCAATGAAAAGTTTCTAAGTCAGCAGCACACAAAAAACAACAACAACAAAAATCTTAGCAAACGGGAACCGTTGAACGCCGTCGACTCTTAGAAAGTACCTTAATGCGATCATCGAAGCGAATGATCGACTGGAAGAGGTTTAAGTTCTCAGACGCACTGTTATCCTCGACGAGTTTGCTGTAAATGAGCGCTAGAATATCGCACAAAGACAGCAAAATCTCTCGGTAGTCCAGTGGAAAAGGCTATAAAACACGCAGAATATTAATACTCATAAATAAATATTCCAATAAAAAAAAACCTACAATCGGAGGCGTCAATAGTCGTCGATAAACTGGCCGCTGGTTCCACTTGTGCATAGTGGGCCGGAATGGCTCATCGGCTGAGTATTGCGGACTATTCTCCGTGTCCAGAGGCTCGTGTAGCGTGCTTGACGTCGCCACCATCATTTTCTATATAACCATAACGTTAATATCTCCTCACTACAATTAAAAAAAAAATGGTTTTTTACTTACCATTCCTTGCACCGTCGAGGACGAGAAGTAAAATTCCAGTTCTTCCAGTAATTGGCAGCAGTCCATAGCCAGTAGTTCCGGGTGGGTCGATTCCCCCACCAATCGCCCTAATCCGATCCCCAAGTAGAAAAACTGCGCACCACGTCGAATACTTTGCTTGTACCCCACATGATTCATCACTTGCTGCTCTCTTAGTAGAGCCACATTTAACCAGTAGTTGGAGCCTTCATGCATCGCTTTAAGGTAGTTCCAGTCCGAGCGACGGTGTTGCACTAAATCCTCCAGGATCCTCATTCTCTGCTGAGCTGTCACTTGTCGTAGGACTGCTGCGGCCTCTGCAGCAACCGCTGCAGCCTCTGCAGCAACCGCTGCAGCCTCTTCAGCTGCTTTGTCAGCGCTACTCGAGCGATCTTCCACGTCGTCAGCGTTGCTTACACTGGAGCTTTGGGACGCGAATAGTAGCGATCCGAAGGACGATGACGCGCTGGCTAGCGACGATGTGAGGCCTGAGGTCAAAGAGCCGGCAGCAGAGGCCACAGAGCCCAAAGGATCGGCGATGGCGGCGTCGATTGAGCTCGGAGAGGTCGTCCCCGCCTCGCTTTGGGTCGATGTAGAGGAAGGAGACGATGTGGCTGTTGTGTTGCTCTTACTACTGTGGCCTAGCGGCGACAGTACTTTACGCCCGCTTTTTGCGAGCTTAGAGGCCATGGAACGCGCAGCGGATGAAGCTTTGGCGGCGGATCCAGGCAGCTTGGAGTTAGAGGATGTGCGCTCCGGAGTCTCTTCATTGGTGACATTCGATGCACTGTGCTCGTTGTCGGTGGCGTCGGCTTTTCCTGGGTAGTCAGGGAGAGCCGAGGGAGGAGGAGGAAGCGATGCCAGCAGCTCCTGCTCGCTTAAAGCGGGCGCGTCTCCGGAGGCGTCCATTCCAGATAAAAATCAAGAGCTCGTTTTCTACTTTTTCTTGGGAGTGCTCAAAACACGAAAAGGCACCCAATTGTTTTTTTTTGTGATCGTTTTGAGAAATAATTAAAGTGACCTATTAAAAACAGGCGCGCATGTACAGTACCGTATTCCTTCTGTGATTAACGACAACCAATTTTTAAAGGGAACCGTTGGAGGCCAGGATTGTTTAATTCGGGAAATGTCTGTAAGATAAATTTACTTCCGTTCTGCGCTATTTCCGATCAAGATAAATTACGCGATTGTCTTTCATTTTGGTTTCTGTGGCTAGACGTGAGAAGATGTCGAGGACGAGGAAGGAGACATGCGCACTCGGGACGGTTCTCCCCGCTGCAGAGGCCCCGGCCTCTCAGACATCACGCGAGCTGGTGATACGGAGACGCCAAGCGCTGAAGAAGGGTCGGTTGCACAGAGAGATATAGAAACCGTATCTGGAAATGACGTGGGGAGCGAGGGTGAATTGAAAGTGGAGGTTGATGATGATGGCGTGGATGTAAAGAATGATGACGTGATGTAAAGAATGATGACGTGGATGTGGATGTTGAGAATAATGGCAAAGACATGGAGGTGGAGAATGAGGATGAGGACGTGGACGTGGAGAATGAAAGTGGTGATGGCAAAGCATCTATGACCGTGGAGGCGATGGGGCCCCTCCATTATATAAGTAGGTAGATTTCTTTACATAAGTAGGTGCATCAGCACACGACTAGCGTGTGCTGAAAAGCTGTCATGCCATTGGCTGATATATGGGGAGCCAGACCCATTATTAGCCAATTGGACTATGACGTG

>Contig_29

CACAGTAACAACAGGAAGTAAAAATGGACCTAGCCCACCCAAACAGCGCACCAGCCACCGAGCAGCTGCCGCTGTAGCCAACGCGTTCATGGCTGCACAAGCGCAAGAAGAAGCGGCTCCCTTGGGCGTCGTCATGGCTAAAGCTTTCAGTGCTAAACCTGCAAAACATAATACAATAGAGTCCAGCAGTGCTCCAGCTCAAGAGCAGCACAATAACTTGGGTATGTTGCTCTGTGCCGCCCTGGGGGGCGAAGACACGAAGAAACGAGCCACCGAGTCGCTCCCTCCAGCTCCAAAGCGAGCCAAAGTCTCGAGTAAGTCCAACAAGGTGGAGGTCAGAGGCAAGAAGCGACGCCAAGCGCCAGTATACGACGCAGCTCCCATGGAGGCGTCGCCCATCATCACAGACCTCCCGCCACTGCCCTTCACGACGCCGGATGTAGCCAAGAAGAAGTATAAGATTCGAAAGGTCGCGCCGCTCAAAGAGAGCGACAGCGAGGACTCTCGCCACTGCGAGTTCTGCAGGAAACGAGCCAACATCTGCGTGCTGCTGCACTGCCACGCGTGTCGCCGCGCGTATCATGCCGGCTGCTTCGTGCACGCCTTCAAGGCGCTCGTGGACGCGGAGACGCCCATTCTGGACCAAATGGAGCGTCTGCAGCTGGAAGCGCCCGAGAGACGAGGCAACATTTTCCGCTGTGCGTCGTGCAAAGCGGCCTTTCTGGACTTCTACGAGAGCGACGGCTACCTCTGGCACTGCGACTGCCCCACGTGTGTGGAGCCCGACAAGAAGATTTTCTACCGACAGCGGAAACTGGTGCAGATGATGAACGAGATGGAGCTCGAGAAGCAACTCAAGAAGGAGAAAAAGGGACAAGGCAAAGCTGCAACAGCCAAAACGTCGACGTCGACGCCTCGTGGGACCAATTTATCGTCCCGAAGTCGACGCACAAGAGGCGGCAGCGCTGCGTTGGACGAGCAGGTTGTCGCCTCTCGAGCGTCTCTAGAAGCTGTGGAGAAGGAGAGCAACGACGAGAAGACGAAGGAGCTGAAAGTGGACGAAGGTGGACAGGAAGCCGAGACGCTGCGTCTCCCAGCTGAGACTGAAGACGCTTTTGCGCTGTCCGGCGACGCGCTGGTTCAAGCTGTGCGCGTGGCACACGACGACAGGAGCGGGGCCTGGACGTTCCCAGTCATGAGCTCGCGCAGCTCGAGTCTCCACGCGTCTGGCATCATGAAAACGGGCACTTGCCGGTGGTTTGTCAAGAAAGTGGCCTCGGTGCAGTGCGACTGCTGCCACCGACTGTTTCGCTTCCCAGAGTTTGTGCACCACACGGACAGCGCGTTAGTGCGAGACGCCAAGTGCGCGGACGAAGACCCGATGCCGTTTCTATTCGTCGAGCATTGCGACAACACGCAGCATTCGCCTCTCGAGGAGTTCCAGGCTGCGCTACGCAGTTGGGTGGGGCGTCAGAGTGCGAGCAACACGCCGACTAAGGCGCCTAACAGTCGGAACTTAAATCCGGACGCACGCATTGACACACCGACGACGCCAGAGCCAGAAGTGGACGCAGCCATGTCCAGACTACGCACGCTGGCGCTGTTCAAGCGTCGGAAGCACCGGAGCGACAAAGTGTCTACAGCCGCGCGTCTGACTGACCAAGAGCCGATGGATTTCGTGGCTCAAGTCGTGTGTCTGTCGCCAAAGTACGTCATGAACATGGCGGACGGAGCTCTTGCCGACCGTGTGGTGCGGTCCAGAACAGCCGTTCCGGACGGCTCGTTCCCGCGCAAAGCCGGCTGGCTGACGTTCAATAGCAACGGCCTCAAGGCGCGCCAGATCACGTGCGTCTGCTGCGAGAAGAGATTCGTCTGCGAAGAGTTCGTCAACCACGCTGGCATCTCGCAGACGGAGCTGAAGAAGACGCCCCGCAAACTTTTGTACGTCGTGGAGCGTCAGGACGAGTCGGCGTTGATGCCGTACATGACGTTTGCAAAAGACCTGGAAGCTGCAGCCACCAATAAGGTGCTGGATGCGCTGCTCGATGGGCTGCAGCCGCCACCTCCGTCCCCTCGACCACTCGAGCTATGACAAGACTAAGGATTAAGATTTTACTTTAAGAAATGCACACTGACTTTGGGTTGTGTACAGCCGGTTTCGTACCAAAGTCTACGAAAGCATTTTTTGTTCTTTCACTAGGGTGTTTATTAAGAAAAAAAAAGAAGTTGAATTGACTGGTAATTATAACGAAGCGGGCTCTATAGAATGCAAGAGCGCACCCAAAAACACTGCTACTTCCGATGCTGTGCGCGTTGGGCGTTTGGATTCCTCTCGTTGAATATCTTAATATATATTCGTCTTCTTCACCAGTCATCGATAACTTTTAAAAGCGCAGAAACATCGGACTTACTTGGTCACTTGGGCAATGACCGTCTACTGTAGGCCAACGGACTTCTCCATCGATTCAACTTTTCTTTTTTGCAAACTTGTCGGCACTTACAAGATCTTTTGACACTTCCGCTTCTGTACATACATGTAAGCATCTGGAGCACCGGCGCTTCGGGATTTTCAAAATGAACTGGGAGGTATACGACGAGAAAATCCTCAAACGACCGCTCTTAAACGCATCAGCTACATTTTCCTAGCTCTAGTCTCGTCGGGCTGGAGCTTATTCTTTAACCATTTCTTGACAACGGCGGCCATTTTCTCGAAACTGGTACTTGGACTGTGTCTTACAGCGGGTAATCCTGATCGATTTAAAACCCTCGATTAATAAAACGGCCTCTATTTATTAAAAATCGTGCATCAGGCTAAGTGAAAAGGACTAAAGTCCACTTTTTTTAAAAAAAATACTTCTGTACACCTCATACTGCACTACACCCGGGCAAGACCATCTCGTTAGGAGAAAAATTAATATCGCAAGTCGCTACGGAGACGATGCCGTGATACAAGTACCGAACGAGAATAGGGACAAATTTAAAAAATACGGCGTTGACAAACCTAGATTCAAGGCTATAATACGGGATGGACATTCCATCTGCAAAGCGCAAGTAGTTAATGCGGTACAACCGTGGTCGAAAAGTGCTATTGAACGAAGTAATAGGAAACATTGGTGCAAGAATATTACCGGTATTGCTATCTGTAGGAATAGATATCATTGGTACCAGAATATTACCGGTATTATTTTTGTTTGTTTTCAATGATCCGCTTTTTTGTGTAACACAAATTGTGTTCGTTGCCAGCGAATCGTCGATACTGTACAGGTAAATCATGATCGGAGGTACCATCCCAAATCGATCTTGCTACCCTGGCTCCGTACATGAACCAGTACCGGTACCATACACATGAAATGATTATGGTGCGGAACCAGGGTATTCAAAGCAAGATAGATTTGGAATACATGTACCAGGTACAGTAACGAGCCTCATTTCTTGAAAGATTCTATGGCCGGGCCATAGCATCTTTCAAGAAAAAGTCCTACGGTACTACAACGTCTGGCTGGCTCTCAACTTCTGGTCCTATCTGAAAACCTTACCAACCATTGGTTGGTACAGTAAGGTTTTCAATTATTTATTGTATTAAAGCATCATTTTGTTGGGTTTTGATTTGCTTTTAGGCATACTTCATCTGGTACAGCTGGTCGTAGTTTTCATACATCTTCCTGATCGCATTGTCCTTCGACGTCCCCTCCACACGAAGCCATTGATAAACATTGGTGGGGCTTTCTCTGTTGTACATCCAACGGAGCGTGAAAGCATCCAGTAAGTCAGTAGCCATTGTCTTCGTGCTGGAGACCTCGCTTGCCTCAACGAGCATCTTTGCTAGAACACCATCGTCTTTGTAGTGCTCCTTCAAGATCGCGAGCATGTTCACTTTCTGCTTCGGTTTTCTTCGCCTGAAGTAGTCGACGTACTGCCTCCAAGTATCGAGAATTGGACTATCAAAAAGCCTTTCCCCTGTTTTGTCGAGCTGCAGTCGTTTGAAGACGTAACTCGGGGCATTTCCTTGCTTATACCAGTTTTCAAGTAGCTCACGCTGCAATTGACTCGCCAAGCTCTCTGTGCTCGGGTTCATGCTCGCTAGAGCAAGTATACTTGCCAGCTTATCATTCGGGTAATTCTTCGTCAAGGTCGAAATCACGGGGAATGTTTCCTTGTGATACATCAAATTGTAGTAGTCCGTGTATTTCGTCCATGCAACAAAGAGCGGGCTCTCCAGGAGAGCATCTCCGCCCATGTCCAGCTTTAGCAGCTTGAAGATAGCGTTGGGGGATTTCTGTATTGACAGCCACGCGTTCGTCAGGTCAGCCTGGACTCGCACTGCAATTTTCTCCGTAGCTGGAACCTTCTGCGCAGCGATCAGCATATCCACCAGAGTTTGCTCGTCGTATTTCTTTAACATTGAAAACAGAGTCGTCTTTTGCTCAGGGTGAGCCTTGTTGAAAGCGTCCACATACTTCGCCCAAGTAACCACCTGAGGCTGCTTGAACAGCTTATCTCCCGCGTTGTTAAGCTTCAGTAGCTTGAACACGACGTCTGGGGACTTCTCGTAAGCGATCCAACGTTGAGTTTGCTCGGTCTGTAATCGCTTCGCGATAGTCGCTGTGGCTGGGACCTGCTTCGCTGCTTCGATTATCTTGGCTACACCGTCGTCGCCGTAATGCGAAGTGAGAGTCTTGATTAATGTTGTTTGCTGCTTCGGGTTCTCCGTATTAAATTTTTTCATGAACTTGATCCAAGCTTGCAACTGAGGGCTTGCTAAGAGAGTTTCGGCTGCATCATCGAGTGTGAGCAGCTTGAAGACGTCATCTGCGTTCTTTCCTCCATTAAGCCACGACATGAGCTGTTCATTGGTAGTTGACTTCAGCGAGTTGGATAGTGATTCTACACTGACACCGGATACCCCCCTCTCCTCGTCTAAGTCGTAGTATGAGGTCTTGCCCGCCCTCAAAAGCCTCTTGGTGGAGATGTGGTGACTATCAATACTTGAGAGGGGATTCGGCGTGACCATCGAGTATTTCGCTAGACACGAAGTTGAGAGTGAGATGGCAGCCAGAAGCAGCAGTCGCGTCTTGAGATGTAACATGTCTGCGCTCGAGCGCAAGGAATGGGAAATGCGAAGCTAGAGAGAGAGAGAGACACCGCGACCACAAAACAATTGTCTCTGACAGAAACTGTAGACAGTATGGACAGAAACTGGAGACAGTATGGACAGTACATTCTGGACAGAAACTGTAGACAGTATGGATATCACAGTACATTCTATACCGGTATTTGAACTGTGTATCTTTTCACGGTATAAAGAATGGCTCATTGTTTAATTCGTTTTAACCACTTGATTTAAGAATGGTTTCGCATCTCAAGGCCAATCAAAAAACGACTTAAGTTTCTTGTTACCGATGCAGCGTAAAATGAAAAGTGAGAATCAAACTAAACTAGCCCTCTACATCCTTCCTGCACTGCCTACTGCAACACTCGAATTCTAGAAGATTGGAATGACTCCGAATGTTCGATGACACAGCAAGTTCCCTTTATGTGCACCACACAATCCTGTGACGTGGTGCAGACTTCAACACTCCCCCTCAACTTGCTCTTCTTGCACTTGGTTGTTCACAACTCCGCTCGATCTCACGAGAGCAATAAACTGAGGAGTCGGTAGGGGCTTCGTCATGTAGTCGGCCAGCTGGAGCTTGGATGGCACGTGCTCGAACTTAATTGTCTGCTTTTGAACGTGATCACGGACGAAGTGTACACGAAGATCAATGTGTTTGGCACGAGGAGTGTACCCGCTGTTGGTAGCCATGCTTATAGCAGCCTTGTTGTCAACTAGCACCGTCGTAGCGCTATGGATCTTGACTGTCATCTCTGTCAGTAGCTGCCGCGCCCATAACACCTCTTGAACCGTGACGGCGACTGCCATATACTCAGCCTCAGCTGATGACAGCGCGACTGTAGCTTGTTTCTTGGCCTTGAACAAAACTGGTCCACCACAAATTTGAACCAGCACTCTAGAAGTTGAACGGCGCGTAGCTAAATCACTGCCCCAGTCAGCATCGCTAAATGCGACCATTTTAAACTCGGTAGCACGCATGTACTTGATTCCAGTATAAGAGGTCCCCTTGAGGTAACGCAGCACTCGCACCGCAGCTCTCCAGTGCATTTCGCACGGATTTTCAAGATGCTGAGAAAGAATACTGACAGCGATGCAAATATCGGGTCTAGTTGCATTTGCAACGTACAGTAACGAGCCGACAAGCTCCCGATACGGGGTTTTTGAGTCCAAACGAGGGTGTTCGTCACTGGCACGTAAGTCTTGACCGACTACACTTGGGTTTCGAACTGGGTATGCACTCGCTTGCCCAAAACGCTCAAGCATCCGCAAAATATACTGCGACTGGCCCACGAACAGCCCCCCCTTCTTTAGGTCATAGTGAAGCTCCATCCCAAGGATGTATTTTGCATCTCCTAGTGACTTCAGCCGGAAATGCGTACTTAAATTCGCAGCAATACTTGACGCCTCCTCTGCTTTAGCACAACCAATAAGCAGATCGTCCAAGTACAAAACGACAAACACCGGACCGTGTGAGCCAACACGAACAAAAAGACACGGCTCTGATCTGCACTGCTTGAATTCCATTGCGAGAAACACTTGACGAATCGTCTTGTGCCAAACTGCTGCTGCCTGTTTTAAGCCGTAAAGGCTGCGACGTAAACGACATACCATGCCATCTTGAGCTCGAATCCCTTCCGGTGGCACCATGTACACTACTTCTTCTAGATTGCCGTTTAAGAATGCTGTCTCCACATCGTATTGCTTCACCACGAAACCAAGCTGGCAACAAACCGCCAAAAATATGCGTACAGTGTTCAAATTTGCAACAGGCGAATAGGTCGACGTGTAATCCACCAAACGTCTGAAAACAACCGAGAGCAACCAGTCTCGCCTTGTATCGGATAATGTTGCCATGTTCATCTCTTTTGAGTGCAAACACCCACTTATGGCCAATGACTCGCACACCTGGGGGGCGATACACCAAGTCCCAAGTGTGGTTTCTCGCATGAGCACGAATTTCAGCACGGCAAGCCTCCTTCCACTTGGCGGCATCAGGTGACTTCATCGCCTCCATAAACGAATTGGGAACTGACTCAACAGCAGAGAGGGCGATTTCGTATCCATCATCAATTCTTGGCCGTTTAGAGTCAGGTTCAGCAGTACTTTCATCCGACGAGTAGCCGTTTCCACGTTCATTGTCTCCATTTTCCAGTAGTAGCGACGCAGCATCATCTTGACTGTTCCGGGCTTGGCTCGGAGGTAACGCCAGATACCTGGACGGTGTTCTCGAGCTGGCTGACGGAGGTCCAGCCTCTAGAAGGCGGGGTTGGAACTGATTCGAAATACGAGCTTGGCGTGGTCTAGTCGGGCCAAATACCAGATTATCGCTCAACGAAGTATCCGGTCGACGCACTGCTTGAGATAGTGTTGGTACTACCGCCGTGGACTCTAGCGATGACGGCTGCGCGAGTTGACGAGTAGAAACAACACTTGAACTTAAATGTGATGGTAGCATCGGTGCGTGTCTTTCATCTCCCCCTCTACGAGACAACTCGTGTCCGCAACGCTGCACTTGAAGTGACATATCCGGCGCGTCGTCAACCTCCATATCCACCGTAGCATTATTATCCTCCTCCATGTCAACATCAGTCTCACCATCAGTATTAGCAGGAGGTGAAGACTGTCGCTGCAACGCATTTGCATCATCGTCAATCACCATTGGAGCTTGTTGTGTCGTGTGAATCACAGTGTGATAGCTATCAACTGGGCGTTCATCCAGTGCAACCGTCCTCGTCGTCACTAGTCGCTCATCCTCACAGTCCCAGACTCGATACGCTTTTGATCCTGGCGCATATCCGAGGAAAATGCAGCGATGCGCTTTAGCGTCCCATTTGCTCCGACGTGACTTGTCAACGTGTACAAAACCTTTCGCTCCAAATACGCGGAGATGGCTGAGATCGGGCTTGACACCGGTGAAAACTTCAAGTGGAGTGCTCTGTGGCCGTGCGGTGTTAGGAACACGGTTCACAATGTATGCTGCCGTACATACCGCTTCGCCCCACCATCCTCGATCGAGACCGCGATGATAAACCATTGACCGGGCCATTTCAACAAGAGTGCGATTCTTTCTTTCTGCAAGTCCATTTTGCTGTGGGGTGTGCGGTGTCGATGTTTGATGGACGATACCGTAAGCCACAAAGAGTTCCTCAAAGCGCTTGTTCACGTATTCTCCACCATTATCGCTTCTCAGGCAGCGAATCTTGCAACCAGTCTGCGTCTCCACTAACTGCTTGTACTCTTGAAATCGCNAAAAACACCTCCGACTTCGCCTTCATCAGGTAAATGTGCACGAACCTCGAATAGTCATCGACAAAAGTCACCACGTACCGGGCTCCCCCTTTCGAAAGAGGCTTCATGGGGCCCATGACATCCGAGTGAACAATGTCGAGTAAGCCTTGGGTCTTCACTGTGCTGCCAGATTGTCTTGAGAACGGATTCACAGTCATTTTTCCACACGAGCACCCAACGCATGGCTCTCCGCTAGTCACATCACTCACCGTAAAAGAAGGAATCCCGCTTGCAGCTCGCGACGCCAGAACGAGCTTCTTCTTGGATACATGGCCATCATGCCACNAGTTCTTCAGTAGGCACCAACGCAGATGCAGCAATTTCCGCTGCTTCATCTTCGGGTTTAATCAATGTCACGCTCAACACAAATAACTTTCCACACTTTCTCACGTGTGCAACCACCTTGTCACCAAAACGAATGCTACAGCCATCTCCATCAAAACTAATCGCTGCTCCTTTTGCCACAAGAGATGGAATTGAGAGCAAGCGCCTGTCTAAATCAGGCACATAGAGAACTTCAGTCACCTTGATCACATCCATTTCCAACAGTAAAACGAACCACTCCAGTCCCAACAGCACGCAGTCGTTGGCCACTTGCGACCACAATATCCGTCGGTTCTTGCAATGCACGGAGCTCATCAAAATCAGAACGATCATGCGTCATATGCGAACTAGCTCCACTATCCAAAATCCAGGCAGGTGAGGTTTCCGTCGACGCAGCAAATACAAATTCATCACGGTCATCACTATTGGCGAGTCGCGGGCATTCACTACGCCTGTGTCCATACTGGTGACACTCAAAACACTGACCCGAGAAACCTGAGCCTCCGCGACCCCCACGTCCTCGTTGACTTCCGTCGCCACCACGACCACCAGCACGTAGACCACCGGCTCCTCGACTACCGGCTCCTCGACTACCGGAACCTCGACCACCGGAACCTCGACCACCGGAACCTCGACCACGACCACGAATCCCTGACTGTGGCCACTTACTACGCCCATTGTTTCCTTGTCTGCCAACATGAGCATCTTGCTACACTGCATTAAACGCTTCCTCTTGCTTGTCACGCTTCCGCAGCGTCTCAAACTCGCGGCGCAACATCTCTTTCGCATCAATCAACGTTACCGACGAATGAGCCTCAATAATTTTCACCATCGCGTCATATTTC

>Contig_32

CAAAATCACTCGGGATTTAACAATCTTGCATCTTGTTTCGTCCTTATTTAAAGGACAAATAATTTTGTTGGGGTTAAGGGACATAGACTTTATTAAATTAGTTTTATCGCAGCCTTTTATGTTAAGTGACCCAAAATTGGTTGCCCACCCCTTTTAATAAGTGTGACATAGAGCAAGGTGATTTAGATCGGATGTGTAACTACCGGTGCAAGGTTTGCCAAACCTAAGTCGTACACATACTTGTTATTATTGTAGCTGCGCAATAAGGGTTTTGAACTTGCGGTCTCGTAAACCGGTAAATGAGATTCTTTCTCTTGGTTGTGTTCCGGACCAGACATTTCTGGTGTGGGACATATTGATAGCTGCAGCTTACATGAAAGGGTTTTTTATGCGAACGTGAGTGGGGCATTGCCGTTTCGTGGAATAACATCTTGTTCTCTGAGATGAAAAGCTTTAATTGAAAACATACGGTACAATATAAACCATGAACTTCATGATGAACCGACACTTTAATACATGTACAGTACAGATTAAAAACGTCGTGTGTTCGTTGGTCCAACGATTTGATCGCATCTGGTCTGCGGAACTTTGTCAAGAAAAGGTCCTTCATGCTTACACTGGGTGGTCCACAAACATTTTTGAGCATAAAAACTGAAATAAGACATGGTTCTCTATGATGAAGGATTTATTTAGCAGCGCAGTGTTCATCGTAGATTTTCCCCTATCCGTATATACGTCGCAGCTCATATTAGTAATATTTTGCCTTTCTACACATGTATTTTGACTACTGTAAAACATTTGTGCATGCGGACGCACAAGATTGCATACAAATGAAAAACCTCATGCTTTGAAGCTTACCTTTTCTCTACTCCTATGCAAAATGCAAATCTTCCTGCCGATCCACTACTGCTGATCATCTGTCTCGTTAATGAACTGGTTGATGACATCGTCAGATTCGTTGACGGTCTCTTCAACCAGTTGCAGTCGCTCCAACGCTTCAACGAGTTGGTTTTCGTGCTCAGTTGCCGGACTTACCGCAAGCTGGCGTTTAAAGCCGCTTTGAATCACCGAAGCGCCGAGATCGTTCCACGCTGCCGTAGCCCAGTAGATAGCATCGTTTCTAGAGGGAGGATTAAGCTGAAATTTGCCGCCTGAATTTCCTTGACGGAATTGTTGTAGCTGCTCCGACACGTGCTGAACCCAGCGCTTTCGAAGGCGTTCTTTAAGTGGTCGAAACCAGGATATATCAGCTGGTTGGCACACAGAAGTTAATCCAGGAGGAACTTTAAGTAGGTGCACATTTAGCAGCTCTGCATGCTCAAGCACTTCATCAGTCCAATGAGCCGAGAAATCATCCCAAAGCAACAGTACCGGCTGTGCCTCATCTCGATTTGCGAAATGGTAGTCCAGAAAAGCGACGGATAAGCCTGAGTTCCACCACGCAGAGCTGTTGCCATAGATTTGCAGAGCAGCATCACTCTGAATTGTTTGCATTGTTTTCCACAAGGAGTCTCCAAAGCCATGTTGGGCCACGTAGTTGTAGGCCGCTTTGTCGGGGTTGCGCGAGGGCTCCGTCTTAACCACGAAAAAAGGGTCATACTGTTTTCCAGTCGAATCTGCCAGAAACATGCCTGTCAGGCGTTCCTTGTCCTTTCCACCACATCTGACCCACACTGTATTGACACCTTTTCTGTCCACCGTCTGCTTGGGCAGATACTCGAAGCAAATGCCGCTTTGGTCTGCATTGTAGAATTTATCTACACCAAGATCATTTTTTGCTTGCTCTACCTCTTGCCAAAAGTTGTTGGCAGCGTCTTCCATAGCAGCAGGGGTTTTCTGGCCTTGACGCGTTTTTGTTCTCAAAGACAAGCCATGACGAGACAGAAACCCCTGCTGCCAGCACCAACTCCCAGTGAAAACACCATCTGCTACGCCTTCGTCACGGGCAACTTCCTGAGCTTGAAGTTGAAGCATTAGACGGGACACAGGGATACCCTCACCACGTAGCGAATTAATCCACTCAACAATGCTTTCTTCTCCAGACTTGCTGATTGTTGTTGCTGTTCCTGGAGCTCTGTAAGACCTCAAATTTGCCGTAGCGGTGGAGGCAACCATGGCTTCAATGTGTGTACGCTGCTTCGACCACTCGTTGATCCGCTTGCGCTTTGATACGCGTTTTGCTGGGATGAGTTGGCAGTAAAATCTATCGAGAGTAGCATCCATGTCATCATTGTTGTCACGAAGGAAATTTATTACGTCCAGCTTTTTAGTGTAGCTCTCGCACTCGCGTTTGTAGCCGTTTGGCTTTCGCCCAGCGCCACCAGGAGCGCGGCTCCGCCCTCTTGCCATGCTGGCACTTGCAGGCTCTGAATGTGAAAGGCTGGTTGTCTGCTTGCTAGTAGTTTCACCGCGGCTTTATCCTGTGCATAAATAGTAGAGTGGGTAAAAAAAGGTAGATCCGCATTGACAAATGCTCCATAGTAAGGTTTTTTCAAATAGCTACATCATTATGATTGGCTAATTCTGGCGTCCGGATGGACAAAAGGTCCGGATGCACAAATGTTTTACAGTATTACAGTACAGAATGATACAAGTTATTTTTAGCCTTTTAATAAATTAATTTATTAAGAGTACGCCATGGAACAAATCCCTCGATCTGGTCCGATACTTAAAAACAATCAGCAATTTGTTTAATCAGGTGTAAGAAGTTTATTTTCAAATTAGGCATGAATACTTTATGCGAGTGGCAACAAACTTTTCCACTATACATTACATAGTGAAAAAGTTTAATATACACCTCTAGGTCCGACAAACACTTGAATATGGTATAGTCGTGACACACAATTTATCCTACAGCCAAATATACTTTTAAGCTGCAGAGGAGGCTTTTACGAGGACTTACGAGGTGTACAGTAGATGCAGATACGATAGAGTCTATCATAGACTGGTGAAGCCTCCCAATCACAAAATGATCCTAATACCCTAGGACGCAATTCGACGGTGTTACAGCCACACTTTTGTAACATTTGCTGCGTAGAGCTGCCTTGTCCACAAGACAAAGTACGTGCTTATTTTAATTAAAAGGCACTCGTTATTTCGCCCGACTGAGGTAATATAGCATCGACTTTTATACGTGTACAGATAGTATAGTACGGTTAAATTGGAGTAAAGGTAGGACACTAGCGTGGTCATTATATCCAATCAAAACTATGGCGCATTTTTCCGTAGCATCTCTTAAAAGTACACTATGGAGGATACTGGTCAGTGCTGGCGTATTTTTCCGTGGCAAGCGACGCCAAGTAAGCGTGGCGTATCAGGACGGGGGGTACTGGCTGTCTAGTTATGAGGGAAGCAAAAATGGCGGTTCGCTACTCGCTTTTTATATCCAGGTGTATCTCCGTGAATTGGGCATCACTCGTTAACTTTCGAAATGGGTAATTAATCAAGTCCAATGGGTCAATTCTTTCTTCTTGAAATAAAGGACTGTAGTGCAGTGCATGTATTTTTGCTCTGATTATCTCCGTCAATTGGAGTAAACTGGAGCGCATTCCGCAATTCTTGAACCAGCCCTCAGTAGCCACCTGTTGATATTAATAGAGATGCGTTTCTTCCCAGCCGTACTCCTGGCAACTGGGGGTGTTCTCCTGACAAATGCTACGTGTGCGTCCTTCGACTCGGAGTTTGAGCTAGCCACCGCCGACCATCCGACTGTGTTCCGTTCCCTCGCCTACCACCATAACAGCGTCGCTTCTAAACGGCTGTTGCGACGTTACGATGTTGACAACGAAGGGAGAACCGTCGGGGGCGGCGCCAAGATCAAGAGCCTTTGGTTGAAGGTCCGTGCGTATTTACTAAATCGTAAGAAAGATGAGGCTGACATGGCAGCGAAATTACAACTCGGCGGTATTGACCACGCCTTGTCAAGCTCAAAACTGGAGCAATTGACCAAGGAAGTCCAAGTGTTTAACAAAAAGTCCATGGCCAAAGTCACGGTGATTGGGACGCTCAGGACCCTCTACGGAGATATCGATCTGGCAAAGGGGCTCAAGGCTGCCGAAAGGGAGGCGTCGCCCACACTTCTAGAACAACTCAAAGCGTTGAGGCAGGACCTACAGTCGAAGTGGCTGAACCGCGGCATCTCCGCGGACGTTATTTTCAAGCAACTGGGGATTCGCGAAGAAAAGTATCAAATGTTTTTCAGTGGGAAACTGGACATTCTGGAAGCCTATATCAAACTCATCAACCAGAACAAAAAGAAGGGCGATCCGGTATCTCTGGTGAGCATCTTGAGTAAGGGATTTGGCGGGGAGGACAAGCTGGTGGCGCTCGTGACTTCTGCAAAAAAAAGTAGCATGACGGGGAAAAAAGCCGACGAATTGGAGACTGCTTTACTCAATAAGTGGCTGAGAGAAGATACCTTACCGAAAGACGTCTTTGTATGGCTGAAGCTTTCCGACGACGTGGACGACGCTTTCTCCCCTCAAAACTTGAACAAGTTCGCAGCGTACATCGACAATTTTAACACGAGGAGGCCCAATCATCAACAGTCAGCGATTGCTATCTACACGAGCAGTTTTGGAGACGCTGCTGTCGTAAACAAGCTCATCTCGGCAGTGGACGACGGGGCGACAAGAAGTATCGCGAACAAGCTACAGGAGGCGCAGTTTGAGAGCTGGGTCAGCCGCAGATTGGGTTTTGGCCAAGTCGAAACGATACTGAAGATTGACAGCTCTGGCGATGCAGTAGTTACTCGCCGAAAGCTAGGCTTGTTGGTCAAGTATATTACGCAAATGATGGATGGAGATGAACGTTTAATCAGGACGTTGACGGAGCAGCTTGGAGGGAGAGACAAACTGGCGTTGGTGTTGGAGAAAGCAAGTGAGTCCACGGCCGCCTCTGCGCTGCAGAAGAAGCAATTTGCGTCATTGAAAGACGAACGTATTACACCGGAAGTCATCGTCTCCTTTTTATTTAAAAAGGCTCAAACAACTACGACAGCCGAGAAGGCGATTGTGGCCAAGTTCAATCTGTTCTTCATTGAGACAAGTGGGTGATGGAGCGAAGGCGTTCAGGATAGAGTATCTTACTGGGTCATAGGAAGTATACAGGACTACGTAAATTTCACGCATACTTAAGCACACGGAAATGTAGTGTTCATGTAGCACTGCTACCTTTGTTATTGGTTCTCTTTTACTTCAAGTATAAGACTTACTATTAATCGTTTTAATACATGTACCTTTAAAACCCATTCACATGTAAAATAACCAAAACCCAGTAGAGGATCGTTGCATTTTTCTATGAGCTGTCTGTTGTAGCTCTCCACACTATGAATCTCGTGGGGCCACCAACGGCAAAGGCTCTTTTTGTAGCGGTCAGCATCGTAAGGCGACAGGATATATCGCTCGAGTGTCCAGACAAGCTTTATTTCGAGCCTGGCAGGAGCGATGCAATGCTTGAGCGATTCCATTGAATACCTTTTCCAGCTAAGGGGTTTGCAGCAAATGCGTCATTTGGTTATACGGTGTCGTACAGAGCAGTAACATGTGCTTCGTTTTAAACATCCCAGTTTGTACATAACGAAGTTGTGTTTTACGTAGACGTAAACACTGGAAAATGTTTTGGTCAGTGCTGGCAATCGCTACTCGTGATTGTTCAGCACGTTTGTTGACGCGTCCAAATGCTTATGCCAAATGCCAGCATCACGCAGCGGAGAAGGAAGAAGTCTCACGCCCTGCAGCTAAATCAGCGCGGGTGGCGGATCATCTCACTCTTCAAAACATTCTCAGCTTCACAAGCTCACCAACANCCCCCCCCCTCACCCTCACTTCAACCATGGCCTCCACCAAGAAGACAGCTCTCGTCACCGGCAGCACGCGCGGTATTTTGTCGAGCACTACGTCAAGGCTGGCTGGAACGTCATTGGCACGGCTCGCGCCAATAGCAACACGGAAAAGGTACAAAAAAATGTTGCTAAGAGACGTTGGGGTTATCAGTACTAGTTGCTCATCTTTACTGTCATTGTGCAGCTGAAGTCGCTTGCCCCGTTCAAGATCATTGCAATGGATACGAGCGACGAGGTCTCCATCCTCGAGGCAGCTCGTCAGCTGGAGGGGCAGCCCATCGATCTGCTCATCAACAATTCCGGTATTGGTATCCCAAGCGAGTTCGATACTGGCACCAAGGACGCCCTTATGCGCCAATTCGAGGTGAATGCCGTCGGGCCATTCCTCGTGACCAGATCTTTGCTACCCAACCTGGAGCTGGCAGCAAAGGACAATGGTAGTGCCTTCGTTGTGCAGCTCTCGTCATTCCTCGGCAGCATCGGCAGCTACACAAATGACACTGTCGATTTCTCCAAGCAGGCTGGCTACGGCTACTCGTCCTCCAAGACTGCGCTCAACATGATCACGCGAGGGCTTGCGTTCGACCTGCGCTCAAGTGGCGTCGTCGTCGTGTCGGTGCATCCAGGATACGTGGACACGGACATGACCCAGGGCAAGGCGACGCTGAAGCCAGCGGATAGTGTGGCGGCCATGACCGGCCTCATCGCCAAGCTTGGCTCTCAAAGTACGGGCAAGTTCTTCAACGTGGACCCGCAGATCCCCGTGGTGGAGCTGCCGTGGTAACTTGGTATGGGTTACTGCGTGAGACAGAAAAATACGAGTACGGTAGCTGCTAGGACGTTGGAGTTCTGGCTAGGTTACATTCGTGCGTCTCACCCGAAACAGGCTTAAAATTCATTAGTATTTCTTTCAACCATGTTTTAATTTACAACAGTGAAAGAGCCATGATCAAGGAGTCCTAGATCAAAGCAGTCGTCACACCTAGACTAGGACAGCGATTTGATAAAAAGTTGCTCATCAACTTCCAGAAGGATTATTACTGTGGGATAAGTTCTATTGCTGTGACGGGGTCACACAGCACCTTGCACAGAGAAGTGCTTAAGCGTATTAACTACCCGAGTGAACGTTAGTCCACGTGTTCCCGGCCTCAAAGGGAACGGTTTGCAGAGTAGATTTAAGACTGATGTATTTATAAGTAAGATAAGCTAGGAAGAGAATGCATTTGTACGAAGAGGCTTCCCTTGTATCCCTTCCAGTTGTTACGAAAAAGATGAGGAGGGCGCCTCGCGGGCTTGACATGCCTGCTGGCACGGTAGGGCAGGAGCTTGCTGCAGTGAAGGTTTTCAATGAATTCCTGGTATCAACAGATATAAGCAGTGACGCCCTAGACAAACTGCACTCCTCGTTATCCAATGCAACTAACATGCCGATGCAAAACTTCTACTCTTTACTAACAGCCTTTGGAATCTTTTTACAGACAAAGAAAAGTGGAAAAGCTCGTGCTGCTGATGAATTCCTAGCAAAGGCAACAGCCTTGGGCTACTTCTCTCAAATTATGAACCTATTACGCGAACGATACAGTGGATCTCTCTCTCGGATGCCAAGCGAGTTGCTAGAATTTAAGACCAAATGGCTAGTGCTATCGAAGGACGGAATCTCCGTTCCAATGTGCAGAGCAATGGTTTTCCGGGCTGTACTTTATCGGATCTCTGTGTGTTGGTGGAACATCTTATTGTTCATGCTGATGCGACCACGAGAATCAAGTGTGTTCATGAAGCAGCTATGTTAGCTATGATGTGGCATACATTTGAACGAGCCATCGATACGTGTTTCGCCCGGAAGCAACAAATTTCGATCTCTGCATCTGGAGAGCTGTTTTCGCATATCGCTCGCTTGAAGACATCAGTTGTCCAAGGTGTATCTATTTACAAGTCCGCAGAGAGATGACAACAGTGCATGTTGCATGCGTTTGGTATGCTATTTATCTGCTACGATGATCCATCCGAATACCTCTTCCCACTAGTGCCCCGTTGCGCGGTGTCGGGCCTTCCAGGAGGCCACACGTACACACAAGAAGAAGCTGTGATCTTTTGGGAGAGCTTGCATGACAACATTGAAAAGGAGACCCAGCCGCACCTAAACGGGAGAGAAAGCGGCCAAACATAGCGATTCATATTACCAAAGTCATCCGCGATTACATCCGAAACATGCCTCCCAATGTGCACCAGACCGTGACGCCGAACATGAGCAGCCACTCGCTGCGTCGAGGTGCCGCTGCGTACGCAAATGCGTCCCCGAAGTTGAAAATCCAGTGGATATCGACTAGAGATGCTTGGTTGTTGGTGTCACTTACGAAGGTCTTTGCCTACATAGGAACAATCACGCGTGAGGATCAAAGTGTTGCAAAAGTACTGGCAGGATACGAGGCTCCAGACTTACCTTTCTCAACCCCCACTGTTAGTGATCTGCAGCAGTGCCTTTCGACAGCGGAGTTTGGGCAGCTCGTGACGCTGTGCAATGGATTATTGCGGCACGTGCTGGGCTCGCTTGACCCCCGACTGAATGTCGCTAGCGATGTCGTGAATGCTACGTATGCAGCGCTGCTAATTCACATGGGCATAGTGTAAGATACCACAGCAAATAGTGTTACCACTCCGGCGTTTGTGTCACGCTACTGTGGTGTAGTGATTTCTCCGTAGTTGCGGATTATCACCCGTCGACAAAGACGTGACACCGCCCAAGATGTTCAAGCGACGAACCCTTGGTCAGGTGATGTACGGAGACGCCAAGGTGCAAGGGACTCGACGGCGTCACTCTCGATCTTTCAACAAAGTACCGGATACGGTCATATGTCATTGAGTCCAGGCTGTCCTTATTATTAATAATGGACTGGCCGGATTCGCATTCAGCGGACCATCTACGGTACG

>Contig_33

CCGACGCCAAAAAATACGATTCTATTACGGAATGTATCGAGTTGCTCGGACTGGAAGATATCGCCGACCAGATCATCCGAGGCAGCTCGGTGGAGCAGATGAAGCGCTTGACTATCGGCGTGGAGCTGGCTGCACAGCCCAGTGTCATTTTCCTGGATGAACCCACGAGCGGATTGGACGCTCGCTCGGCAAAAAATCATCATGGACGGCGTCCGCAAGGTGGCCGACTCCGGACGGACCCTCATCTGTACGATCCACCAACCTTCGGGCTGAAGTGCTCTACCTGTTCGACCGACTTTTGTTACTACAACGTGGTGGACAGACGGCCGTTTTACGGAGATCTGGGCGAGAATTGCCGGAATTTGATCGATACTTTGAGAACATCCCGGGTGTGGCTCCGCTTTCGGTTGGGTACAACCCGGCGACGTGGATGTTGGAGTGTATTGGCGCAGGTGTCGACATGGAACCGAAGACTTGATGGACTTTGTGAGCTACTTTAAGAACAGTCCGTACACCAGCAGCTGAAGACGAATATGGCCAAGGAAGGCATCATGACGCCGTCTCCGGAACTTCCCGAAATGGTCTTTGGTAAGAAACGTGCTGCGGACTCGAAGACCCAAGCGAGGTTTGTGATTTGGCGCTTCTTCCAAGATGTATTGGCGTACACCGAGCTATACGTTGACGAGGATGTACTTGTCTATCTTCCTGGCCATGCTTTTCGGATTGATCTTCGTGACCAATGACGATTACGCATCGTATTCCGGACTCAACTCCGGAGTCGGAATGGTCTTTATGTCCGGATTCTTCAGYTCTATGGCAGTGTTCCAGAGTGTCATGCCGTTGACGTGTCTGGAGCGAGAATCATTCTACCGTGAGCGTGCATCGCAGACGTACAATGCCTTCTGGTACTTCATGGCGTCGACGCTAGCCGAGATCCCGTACTGCTTTGTGAGCTCTCTGATCTTCACTGCCATCTTCTACTACTTCGTGGGCTTCACTGGCTTCGCTACCTCGGTCGTATTCTGGCTTGCATCGGCGCTTCTTGTGCTCATGTTCGTGTATCTGGGCCAGTTCTTCGCGTACGCCATGCCGTCCGAAGAAGTCGCACAGATCATCGGAATTTTGTTCAATAGCGTCCTAATGATGTTTATCGGATTCAGTCCACCCGCGTACGCTATTCCGTCGGGCTATACGTGGCTGTACGACATCTGTCCGTTCAAGTTCCCCATCGCCATCCTGGTCGCGCTGGTGTTCGCGGACTGCGACGAAGAACCGACTTGGAACGAAACTTGGCAGACGTACGAGAACGTGAACTCGCAGCTTGGCTGTCAGCCCATGTTGGATGCTCCGGAGACGGTGGGCCACATCACGATCAAGGGATACACGGAGGAATACTTCGGTATGAAGCACCACCAGATCGCCCGGAACTTCGGGATCACGATCGGAATCATTGTGCTCTTCCGTATCTGGGCTGCTCTGGCGCTGCGTTTCATCAACCACCAGAAGAAGTAGGCTCTCCTGTGTAGATATCAAGTTCGTATGAATACATGTGCAATGCCAATATAAACAGAAATATTTTGCATCTAAAGTACTTTGCGATAAAGAAACCAGAAGTGCATTCACGAACAAGTGGCCCAGTTCTCCAAGTCCAACGATAACTTCATTCTCGCACATGATTGCGACCTCCTTATCGACGCTATGCTGACGCAAAGTCGCTTGATATTAGAAACATGTTCGCGATTTATGAGTTTCAGTAATCAGCTCTTTAGTAAACCCGCCGTTTCTGGAGAGCAGCCTAAAGGTGAAAACCAATAGTGCTCGGATCATTCGTCCTATGGTGYTGTCAAGCGCAGATTGTAAAGTGAATTTTGAAGTGTGCTTTCTTGCCGCCATGCCRTACTATATAGCCCAACAGATTGAGCTTTCAATAGGATACCTCCTCGTCAATGGTATAACGAAGGACCCCACTGATGCAAATAGCTCCGCACATTTGCTACTTTTCCTTGTTGCTTTGACGGAGAAGTGGAAAAGGAATGGAGGTTTCTTGATTCACCGAGCTATGTCAATATATCCAGTACTTGGGTGACATGCTTTAAATTTAATTGTTTTCTAATTCTGCACAAGCGTCGGCAAACTTTTTGACAGATTAACAGGAGGCACGGGTGACCCCTTCACAAATAATAAATATCAGATAATGAGCTGTTATCGTAATGAATAGATACTTCAATAAACGTAACACCCCTTATAATGAAAATTACAGGATTAAATGTTGTTCTCCAGCTTCGCTCAAGGTTACACCATGCTGAATTTTGTGCATATAGTCCAATAGGAGCCTTTCATCCAGCACTGCTTGAGCAGCGTCAGGAACAGTGTGGTTGTCAAGTGGTCGCTCTTAAGCTAGCGTGGGTCAGCTGCTCGAGCTGAGGCGATCGAAATCACGCCCTTTACCGATGAGCTCCCATTTTTTGGTTAGTCATTATTCTGTTTTAGCAACTACCGGTAGCAACTATTATGATGACGAATCCGTGATTTGTTGTGTGCTACATGTAAGAACTTTCATTCTCTATTCCGCGTAAAAAACTCTTTATGCGTGAACACCTCGTTTCAAACATGCGACGTCTGTGGTGATGTTGCTTGCCAAAGTACCAGTACCAGCAGTTGTGAAGTAATCTTAGTATTCGCTCCAGTCTCGTAGTAGCTGAGCGCATTAATTCTTTGAATTTTAATTGTCCAAGTCCGACATTAACCGAAATCCAATCCGCTACTCGTTTCGGTTGCCCATTCCTACCCATGACGGAAACAGCAGAACGAGAACACATTAAATGTGTAAAATGAAGAAAAAAAACAAGAAAGCGTTTATTTTGGAGCAGTTGTGAACGCTGATAAATCAAGGAAAATAATTATTTGAATACCCGAATCAAATCCGAACAACTGCCAAATCGACCGGGAATATCCTCTACTTGTAGGTCGGGTACCCGATTGCGAGCTCTGCAGCTCACCATCCGGCCCCGATCATCTTCTCCCTCGTGCACACCGTAGTGGCCTTTGGAGCAAAATTGATTTGAAGTAATACACTTTGGTTAAAATACATAATATATAATATTGACACTACTAACAGGGCTACTTGCTTTTTACGTAGGGGAACTTGTAAGAAGCCATTTGTTTCGATAATTTTCTAAGCAATCGCATGGAAAATCGTGCCCCATACAGATCCTTCCTTAGCTCTGAAATAAATTAGGTACAGATAATAGAAGAGGGTGTACTTTATTGAAGTATTTTGTAATGTGGATTTGTTGAAATTTGTTTGTTACTTAGAAACTTGATCTTTCTGCAGGTAGCCGATGTCAAACAAACGTGAATAATTCCAAGGCCACTAGCTACTAAATGCCAAGCACTAGCATTTTCTCCGCCAGTTTGCGAAGTCGAAAGCTACAATATACATTTGCCCACAATAATGCCCCCGTAATTTCGTAGCAAGTCTGTCATTCGGGCAATATCAAGGGTCTCTCTTTGCTACTTGCCTCAATACGCCTTAAAATGGTCGCTTATACTTGTTTTTATTTTTTTATCATACCAAAATATGTTTTCATGTTTTATTTTTTTTGTGGCTAACACTTGGCTGCCTGTTATCACAAATGCACTACCCAAGATGCGCGCCCCTGAAACTTCTTGTGCATTGNTTTTTTTTTCCAGCTAATTCATGTCAAATTGACGTTTTCGAGCTGCGGGTCTCTCTTCGGTAAATCTCCACGCTTGACTAGCGCGTTTTTCTTCTCCGCCGCCTCCAGAATCTTTACCATTATTGGGCGCCTGCCAACCAGTTTTTTCGCGTCGCTAATCGGGACCTATAGTGGTTCACACGCACGTAAGCTTCCCAATAATGCAAAAACTATTTTTTGCCGCATATCTACTCACATCAATTCTGTAGCGGGAGCTTTCGGCCCAATCGTCAAAGCGTTGGACATCATCCATCGTGTAGGCAAATAATCCATATCCTTTGTCGCCGTCCTTGTACGTAAACTTGCCTAGGTCGTGATTCAGACGCGCTTGAATCTGCAGAACGACGATTAGTAAAACAAAATTATAAATATACAAAAAACATTGCAAAAAAATAGACGTACCCCTGCTTCCTCTATAACCTCGCGCAATGCGGCTTTCTCAATGCTCTCGCCATGGTCCCAACCTCCCTTTGGAAGGATCCAATCGTTCGGTTTTTTCGAGCTTGAAATGAGGAGGACGTTATCTCCATCACCGATCACAGCGGCAGACAGCAGCCGATTATTCTCCTTGTCGACGAGCTGGCCAGCACGTGCTACATGGGCTTCTAGATATTTCTGAGGAGCAATTTTCATGCCCTCAAAGGGCTCGATCTTTTTTCGCATTCCACTGCCAGTTTTCTTGTCAAACACGCGATTATCTTTCAGCCTTTGTTCGTCCACTTTCGCCAGCTGTCTCAGTGTTGTAAGGCTTAGCTTGTCGGCAACCGAATACTTTCCGGAGAGATGCTTCACGAGACCTGCATGCTCCATCTTCTCGACTGCTGTCTTCGTTGTCGGTGCTACGTGCACTTTACCCAGCTTACCAGCAACGTCGTCCATCTTCCCCGTTATCCCCAACGCGTCGTCTACTTTCGTAATCAGGTCATCCACCTTGTCAAGACCACCTCTATCTTCATCATCGGACTCGATGTCCGCCTCCTCTTCGAGGTAATGTCGACGCAGGAACCGGTGGACTTCGGCAGCAATAGGGAATCGCTCGGTCTTCGTGATACCGACGTGCTCCGAGGAAGTAGCCGACGCAGATGAAATCAGGACAAAGAGAATCAATAAAACTCGCATCTTTTGTGGTGGAGATTGGTGTGGTGTCTTGAGGTTGCGAGAGCAGATTGCGAAGTGTGGATCGACCGTAAATGTTTTCACTTCATTCATTGAAAGCGCTGGCTGGTGGCGATCCAGATCGTTTTAAGTTTTCATGAAGCATGTAATCACACGAAGATCGCAAACAAGATGGCTAGTCCAAGCACACTACCGTACTAAATGATGCGCTTAGTGACTGAACATGAAAGGGCTTGGGATGTGCTTCACAATGTCGAAGCACGATCGTCCTTGTCAGCCAACCAAACAACGCGATGTTAAAGCTGCCAGCCGGTATCCTGCATCAGAATAAACCTTTCGAGGAGTTGTTGTCACAGCCGGCAGACTGCAATTTGCAATTTGCAACCGGATGGCATCGTAAAACTACTAAATAGTGGTTATGAAGATGCAAACCATTTTCAAAATAGTTAAATTTTGAAGTACACGTATCAATAAGAAACATGTTTCATTACACACTGAAGTCGCGCGCTGTTATAAATAATAAAGCACATAGTCATTCACAGTAGTTATTGTGAAGAAGCAGCCGTCTGAACCAGTTTTGGTTTTGCTTAGCACGACAGAACGGTTACTAGAGCATCTACAGTATCATGAAAAAACCAGAAACCAGCTGTTCAAATTAATCGTACCTTTTGTGCAAGATTATCCTAGAGACGTGCACACCCCAAGAGCATTCACACAGATCGAGTCTTTTCTGCTTGGCTACACGAGTTCCTTCTGTAGTTTACACCTGTTCAGTGCTTCTTTTCACCACTCTCTTTGAAAACGCGATCAAGATATTATAATGCTGAAAAACTGGTATGTGATCGTGCGTAGTGTCCTCTGCTATAGTCAATAGTTTTGACAGAAAATAATGATATACATGTAGCTGCTGTTTTTGAGCCGTAGTTTGCAACTCCGATTTTTCTACGCCATCCATTGCCAATCAGATATCCCCATCAGGTTGCCTACAGCAAGAAGCTATTACGAGGTGTGTTACAAGATCGGAACGTTGACTCGCGCTATTGATCGAAATGTACTGTATCGCCTATAAATTATGACAAAAAGCTTTCTTTTGCTCTAATTCACGAGGAACCGAATGGCGCAAAGGAACGAGATAGCTCACTCGTTACCATACTATAACATAATTTTGCTTTTGCAAGTCATATTTAGCCGCCCTCTGTGACTAATGATGGCCTCGACACGACACCGGAGAATAGTTAAAATTATGCTTACTTAATTATTAAATCTCGAAGATTTGTCACCACAAAAATGGTTTTGTGGAAAATTATCATCTTCAAGTCATTCCATTCGATTCTATTATCTAGTGACACTTGGGAGTACTCAGCTGTACTTCTTTTTGGGCTTTGAGGAGCCTGATCGACTGATTATTATGTTTTAAAAGCATTGGGATTTTTTTTCCTNTTTTTACGACTCAGTTCCTCTGACTATTTCCCAGGCTCATTACTGCTGTTACTTTGTGATAAATAGTAGTAGTATTGTAGTTGTAATGGAAGTAGTAAAAAAAACTGAGGAACCAAGTGTCAGACAATTAAAAAATCTGATCACGTTTATCTTAAAGCTTGTCGATATTTAGAGAAATAGGCTTTATTCGGAAGGGTGCTTTATTCGGGCGGATCCGCTTGTGTTTTATAGTAGTATTGCAGTAGCCCATAATACTGATTTATATCCACTGATTTAGACCTGCGTAAGTGCATAAACCAGCTGAATATCATGCTAGAGCGAAGTCCAATATTTTGGCAGCTTTTTTTTTTAAATGTCCAGATGAAAAAATCGACGACTTTTATGTAGCATCATATTAAATCATGTTGTTGCAAAGGCTGTTTCATTTGCACTTCATCGTTTCCCGCGCAGCACGAGGAAGTGCTACCTTCTGCACGTTTTACTTCCGAGGTGTATAATTTTAAGCGATCATAGTCCGCTACATGTGCACATGTCCGACTTGGACCATTTATTCTACTCTAAGTCCATTCAATTCAACCACTATACCCATTTATTGGTACATTTGGCAACATTCTACCCATGTCCTTCATCAAGATAGCGTCTACAAAAGTGTGGTGGGTGCACTTTCGACACAACTCCGACGTCCACCTCCGCGAAAATGTTGTTGGTTGCATGAAGAAGGGACGACAAGCAATGATCCTGAATCTTCATTTCCTTTGAAATACTCATAATGTTGCTGCGGTTTGAAGACATGTACATCTTTACGACAGCCTTGTACGCTGTAAAACAGCTCGTCGTTCCGTTCAACATAGGCGAGTACCGCATTAAAATACTCTGCCAAATACATGTCTGGTTTTGGCTGCTCGTTCTGCATTAAGTATTTTGGAAGATCGCTTGGTAAAAAGTGTAAGCGTCATGGCCCTGTTAAGCAGAAGCTGGGATCAGGAGCCTGAGCAAAACGATCAAAGCTCCGGTTTCTACCTTCGCGATCTTTGGGAGTGTCTCTAATAGCTGGGTTCAGACCACTTTCATATTGCTAGTCGTCTTTATGAGGCAGACCACTTATTCTCTGTCCATCCACACGTTTTCAGCGGCTAGCTAACTGAAAAAAGCAGTCGGTCTCTTCTAGAAGCACTGTACTACCGACGCGTAGCTACCTGCGGACGCTGTGTTCATGCTGGTGTAAGACAACCTGTCGTGGATATGAATTTCTTGACGTTAGGGCCGTCTCATCAGCCCTTAGTTAACATACCCCCGTAGCGACACCAAGTGGTATTTTGTGACCAATCCACGCTGGATGTAGGCTTGAGCATAGTCAGCGATTATTGATCTAATGTCTCAGCACCTTCATTCCGTGACGGCCGCAGCATCTTTTGCACGTCTCCTGCTGCATTTGTGGGGTTTCTCGTGGAACGTTTTGTGCCATTTGTGACCAGGTTCAGTGAAAAAAAGGTGGTATTGAAAACGTTTTTCCCCGAAACCTGGAAAAATGTCGCAATTTGTTTTGGGCTCCAGGTTTCAAGCACTAGCTGAACAGTCCCATTGTATACCAACATCATTAAATCGAGCATTCACCACAAGGCTACGGTTTTTGAGCTCTTGAGACGTCTCACGCAGGTATCGAACGATGTCTAGAACAGGCTGTAAGCAAATTAAATAACGACATCGTATGAATCCAAGGCAGGAAATTGATTGTGCGGCTGCAGAAG

>Contig_34

GTCCAACTCGAGCGATGCTTCCAGCACAGACAAGGACTGGACCACGTCTGAATGTGTAGTTCCGGTCAAGAACCAAGGCCAGTGTGGTTCCTGCTGGGCCTTCGCAGCTGTGGCAGCTCTCGAGAGCGCCATTTGTCTGTCTGGCCAGCCTCTGACGCCCCTGTCGGAGCAACAAGTGGTGGATTGCGACGAGGCTTCGTACGCTTGTCAGGGAGGCTTCCCTGGCGATGCGTTGACTTTCATTCAACAGTCAGGCGGTGTCTGTACTGAAGAGGCTTACCCGTATGTATCCGGTGACTCGGGCGACCGCGATACTTGCAAGTCATCTTGCACTCGTGAAGCTGTGACTATCCGCAAGGTGGTGGGCGTTCCTGAGAGTGACACTGGGTTGGTCCAAGCAATCAACACTCAACCAGTTGCTGTGGGTGTCGCAGCAGGAAACCCCACGTGGAAGCAGTACAAGAGCGGAATTGTGTCGTCCTGCACGACGTCAGAGCTCGACCACGCTGTGCTGGCAGTGGGCTACTCACCGTCCTACTTCAAGATCAAGAACTCGTGGAGCACGCAGTGGGGCGAGGAAGGCTACATGCGACTCAAGCGTGGTGCCGGCACTAGCAGCGCCGGCACGTGCGGCATCATCGGCCCCAAGTCCGTCTACCCTCAACTGTAGACGAATGGAATGTAGCCAACTTCAAGGTATGCACACGTTTTAACCGCTAAAACCATCATTCTTGTTTTGGATTTTTTATCTTGACTTTTGTAAGAGCGATCTTAAAGAGACGCGATCTTTGCCAAACGGTCTCTTACCATGTATACACATTTGCCATTCGTCGCTTAGGATGGCGACTAACTCACGCCCCGCATGGCGCTGGGCATCGCTGCGACTAGTGAGTTTACTAGTGCTGCTAGTGGCTCTGGTGGTCGCCCAGGTGACTGAAAACCCGCCTTCAGAGCATAAGGGAGCCCCAGAGCACGAAGCTAAAGAAGGAGAAGGCGAGGGGGAGCCGGACAGTGGTCGAGTGGGCGTCGTGGCGGCAGTGACCATCTCGACGCTTGTAGCCATCTCTATCTTGTTCGAAGTCTGTACAGAGGAGCTGCGGGAACACACAGACGAGCTCAACATGCCGTTCGTCAACACGGTCTTCGGAGAGCTCACGACGCTAGGTTTTATCGGKTTACTGCTCTTTGTTGTCACCAAGATCGAGGTGCTACCGTGGCTCTCCAGGGTTATACTAGGAGGCAGTGCGGAGCTGCAGGAGATCATCGAGAAGCTGCACATGGCGTTGTTTCTCTTTATCGTCATCTTCTTGGTGCTCTGTCTCGGATTGCTACGTCTCGGGATGCACGTGCAGCACGAGTGGCGCGAGTTTGAGCGCAGTTGCGCTGACATTCCCTCTGTCCTCTCAGAGTACGCTCTCGCTACGGAGCCTCCTAAGACGTGGATCCATCGTTTGTCGTGGCGTCGAGCGACCACAGCAAGGAAAGCCCAGCGAGAAGTGGTCTACCTTGCTCTACGACGACGATTCATGGACTACCGCTCCAATCATCCGGATGAAGAAACAGCACGACTCTTAGCAAAGGAATTCCAGCTTCAAGGAGACGATTCTCGGTTCCCATTCAATGAATATCTCTCTATCATCTCTGGCGAAGTCATGGGCCGACTGATTCAGATCGACATGGCTACATGGCTCGCCCTCGACGTCGTCTTGGTGGTTCTTCTTGGTCTCTGCTGGCACGCAGGACCTCGCGGAGAAGTCGCGATCCTCCTGATCGCTGGCTTTTCCCTCATTGCCCTGAACGATTTCGTGTACCGACGAGTGAATGCGATGCGGTGTTTACTGACACCAACGCGACTACAGCACGACGCAGAGAGACTGCGTCGTAAGGCTGCGTGGCGTTGCCAGCACGGGTTATCGCCGCTCGCAGAGCCAGGAACCTCTCCTCATCCCAATGAGAAAACATGGCTACTAGTCGATGCAGAGGAAGCCGGACGCGACTATGGCGACCCGGACGGATGGGTCCCTCCCTACGTGGATCTGCTCCCCAACGGCGGACGGGATCTCCCCGAGAAGGAACTTAAACGACGTCAACGAAGCCTCATCGGTGCTGGATGGGGCAATGGAGTCGTCCTGGCACTCTTCTCTACCAGGATGGTGTTCCTGCTCACAGCGCTGCATCTCTCAACGTTCTTACTGCGTGAGACGTACCAGATCTCGGAGCTTTTCGGTGACCACCCCATCTTCGTAGCTCTACTCTGTTTCCTCTTCTTAGTTCCTAGTATCGCAGTGCCCTTCATGTCCGCACGCATCGCACGCGACGGCCTCTTAGCATTCAACGTGGAGCACATGAAAGTGTCCCAAGTCATCGTCCAAGTGACGCGGCTACTGCGTGCTCGACAGACGTTGAGAACGCTCCGCTTTGTAGCTGAAATGAAGATTCATCTTCGAGAAAATGTCCGGCGCAATCACGAACAATTGAGGGAAAATATCACCTTGATGCCGGAGGTTCCCATGCCCGTCAGTCGTCGACGTTCCAGTATCATGCCTATCGACGCTAGCGCAACATTCAAAGCTGCTCGTCGTCGCTCCTCGACGCATCTCGACCCGACCGTCACTGCCGGAGTGCTGCGCTCTATCCAGCAAGTCCCTGTCCCGCTCCAAGTGTCTCCAAAGGCGACAATCGAGCCGCCGTTGTCTCCGTTGGCAGCATACCTTACACCGAGAGAGAAACGAGGAGACGCGTACCGTCGAGAGATGGAGAGACGGGAAATTCACACGATTTTCTGTCTCTTTGACGTGGACGGCTCCGGGTCGGTATCGAGAGACGAGATGGCCAGTCTCTTACTAGCTATTACCCACGATCTGGACGATATGCAGCTTAACCGGTTAATGACGGATCTGGTAGCGGAGGAACTGGAGGGCGACAGTGATGCAGTTAAAGAAGAGGTAATGGTTGAAGGGCCTCAGGAAATCACGTTCGAGGCCTTCTACAAGTGGTGCAGCGCGAGGATCCAAGAGAGTCGCCACTCGAAAGAAGAACTCGTGGAAGAGATTTTCCGGATGGTGGACGCCGATGGCAGCGGAACTATCTCAGTGGACGAGTTCGTGTCCATTTTTAAGACGTTGGGCCAAGCGCTAGACCATGACGACGTGCGCGAACTGGTTTACCAGATGGACCGCAATGGCGACGGCAAGATCGACCTCGAAGAGTTCAGTAAGATGCTACAGAAGCACGAGGTGTAGCTATACGAAATTAATACAGTTACCAGTATTAGGTTCGTATCGATAGAACGTATTACAGTTTTTAGTTACTGTTCGCCCCCAGCGATATTTCCTGGCGTTCGCGGACAAGAAGCAATACAGGATCTCAGTTAAGTTAGTTAGCTTTTACTTCACGTACATGAACGTCAAGCTTACATGACCGTATTTCGGAATTTTGCAATGAAAATGATCACATTGTTATCGGTTCTTCATGGTCGATGCGTCCCTCTCCACCTCTCATCGCGTTAGGCGAAAAGCTCGTCTAATATGCACCGGTCTCAAACAGGAACTTTAGAATGACTGGAGCTGAGCATATCGACATGGCATATTCGGTGACGCTTTCCAAATCCCGATCCCCCGCTTCAGCCCTCGTTAAAAAATAATTGCTACATGTAGGAAAAATGATTGTAGGGTATCTAAACTATCTAAGTCCAAGTGTAGCCGTCAAAAAGCAAATTTTCTTTTTACTCATGCTGAAGATTCCGCTACCATTATCTTCTCTCCTTTCATTTGCCAGACAAACGCTGCTCCAATGTCTGAGCAATCCAATAGTTTCTGTCGTCTTGTTTCGCTGTTGCGATCATCTTCCCGAGCCCCGCATCATCAATACCAGCCTTTTTGAACCACGAGACCAGCAGCTCATAAGGGTCGTTTCTTTTCATCTTCCACACATAATGAAACCAAATCTCCAAAATCGGATTTTTTAAAAGAGTAGTGCCTGCGTCATTCAATTTCAGCAGCTGGAACACCTCCATCTCAGTAACGTGATTCTTTCGCCAAGCAGTGCTCAGTTCCTTCTCGAGCTGCCCGCTAATGCGTTTGTTGACACCGCTCTGTGATGAAACAATCATTTTCGCCACGGTTACGTCGTCGTATTGCTCGCGTAGCTTTAAAAGCAATGCCTCGATGTAATCGGTACTTGGACTCTTTACATAACTCAGCCATGCCGGTAACCCCGGATTATTCAAGACGTTTTGGCCTCCTGCGTCAAGCTGGAGAATAGCGTAAACATCATCCGCACTTTTTCCACTTTCCTTCCAACTTTTCTGCTGAATTTCTTTTAGCTGATATACCACCGTGTCTCTGCTTGTTCGCTTCGCAGTAGCCAGCATGCGCGCCAGTTCGTCATCGCCATAGTGAGCCGCCATCGTCTTGAAAATTGCTCGATCTGCTGGCAAGCGTCCTCTCTTGCAGATTTTGGCCACATAGTGCGCCCAGTCAGTAAATTCGCTACTCGAGAATACGTCGGTTTTTACTTGTTTGACCCGAAGCACTTCAAAGCGGTTATTGACCTTGAGCTGCTTGTCTAGACTAAATGTCAGTTTAGCGTTGTGAGCTAGATCTTTCAACGCCTTCACAACAGAGAATACTCTGTCTTCCGAGGCGTAGTAGGCATCAGCTGTCGTCGTCCTCAAACGTCGATAGGCTTGGATGTCAGCCAAATCAGCTCCAGCCTTGGCATCAGCTCCCTTAGTGATTGCAAGGAAGAACAATACAGCAATCGAAGTCAAGAGGGCGCCCCAGCGAGCACTACTTAAACGCTTGAACGTGATCATTATCCGTGGCTTCTGCGCTTTATGAATCAAAGTAAGAGCAGTTCACTACTTATGTGATTGGCGACGCTCGGCCATTAATCCAGCAGCCTATCAAATTGATTTAAATTTCGTGTCGTCGCGCTTTATGTGCTTGGCGACCAATATACATGTAATTTTGAAACATGTAATATAGTTTTTCTATGCAGGTATCTTTTATGCTGGCAGAAAGCTCAAGAATTTGACTGTCGCCAATCACATAAGGAACACTGTTCACCGCTGGGTCTTGTTTATGATGTGCGGTGTTCAGTACTGTACGGTACTATTAAGTAAGATACAGACAGAACATGCCAAACAAACACATGTATTTACTGCTTCAGCACACTTGATTTGACTTATGTATACTTTTGAACTTCTGAGTGTCGCAATAAGACATAATCGACGATTAGAGGTCACAGCAGAAAAGAGACCAGCCGCGCTTGCCCCTTCGGCGCCCCCGCGGTTCGCTGCGCTCGCAATATGGAGATAGAATGTTCTGGAAAGTGTAGTGCTCGGCTCGCTGGTGAGCTCAGCGCTGGGCCCGCCTCCGCAAATACCGCGCGGCCCTGCTTCGCGGGCTCTCCGCGCGGGTTTGCTGCGGCTACGCGCCTCGCACCCGCTCACCTTAGTTGGCGCCGCGGCTATGGCACCCACGTCGCCCAGCCCCGTGTGTGTTATCCCAGCGATGGACGTGTTTCTCCTTTTTTCTCTAAGATACAGTGCTATCATAATGCGGACATGTTGAAGTTATACTTCGCAGTCGGTACAATGTACTGTACATGTACCGGTAGTATCAAATTTAGGGATAGGGCTACGACGCATGATTTTGATCGAGATATTTTCATAAATTAGTACATGTAAATTCTTTCAAATATTTTGCATGCTAATATACATCCAAAAATTATCCATTTCGACGGCTACAGTCTGTAACGAAACTAAAAGCCTATTCCAATCTTCAGCAAATCATCTTTACAGTTCTATATTGAAAATTGTAAAGGCCGTAATCTATTTCGATCCCAATTGTCCGCGCTTACACTTGTCTTTGGTATTGTTAGTGCATCTTCCGTCGATAATATCGACGGAAGGTATGGCCCAGTGATATATATATAGTACACATATAATGTGTAGGCTATTTGGGCGTACGCTAGCTATTTCCGGGTAAATTGTGCGTGGTGAATTCCGTGCAAGAAGGTGCTCTTCGGCTGGTCGGTCGGGAACTTTTGTATCTTTTTGAAACTGCCTCCTCGGTCGACCATAAGAAGCTGGGGTTTTCCGCCAATCACGAATCGCGGAAAATTATTCAGATACAGCTGAGAATTGTTCGTTACCTATGTCAAAACAAGGATGGTGGGATAGGTTTTAATATTAGCCTAAGTTAAAAAAGTATATATTTTGAAAAGAATTACTTTTTTTAGCTTAGTTCAGTTATTAAAACCTCTAGTTGTCCCATTTTTTAAGAGAGTGCAGTCCAGACGACCGACCAAATTTTGAAGGCTAATATATTCACCCTCACTGATCTAATTGAATCGTCAGTCGTATTTTGAGCCCCCAAAACAATTTATAAGCCTACCTCCGAATTTTACAACGAAATTTAATTTCATTTGGACAGGCAAGATAAATCCGGGCTAATTTGGCGGTCAAACGATGTCAGATGTCTACAATTATGCCTACATTTTGTAGAACAAGAGTGAGTGAGGACCTTGTTGCACGAAAATGGTGTTAAAAGGTCATTTAGTCGAGCATGGTCTAAATAAATGATTATTTATTTATTATATTGTTTAATATATTAAAAAATAAATTAAAGTGTAGCCGTAACTTTTAACTTTTAAAATTTTTGTGGCTTCTAAAGACGAACGACTGTGAAATATATTCGTTCTCATTGAGGGTAGCCAACTACGATCATAAGAGGGCACAAACGTGATCCAAAGCTTACTGTAAGTGCAAATGCAAAAAGGTCAATGAAGAAGACGTAAGGCACATTACGGTGCCAAGATTCCTGTTTATTCCCGAGCTATGTGGTCACTCGAATTATTCGAACATAGCGTATGTACCCGAAGATAGTGTGGTGTAGTGATTTCTCCGTGGTTGCGGATTATCACCCGTCGACAAAGACGTGACACCGCCCAAGTTGTTCAAGCGACGAACCCTTGGTCGGGTGATGTACGGAGACGCCAAGGTGCAAGGGACTCGACGGCGTCACCTCCGAGTTTTCAACAAATATCGGATACGGCCCTATGTCACTGAATCCAGGCTGTCCTTATTATTAATAATGGACTGGCCGGATTCGCATTCAGCGGACCATCTACGGTACGAACCCTCAGTGGCGGTTGGGATGAGTTCGCGGAATACTCAGCGACCTTTGGAAGACACGTTAACCGTCTGGGACAGGGCGCGGACCGTTAAAGATGCTCATGCGACAGTGCAGACGACGCTTGGCATGCAAGAAGATCCATAACGGATCATTGTTCACTTGATATTTGTTTACTTGATTAGGACTTCTTATTAGGAATAGACCCGAAAGGGTAAGCTTAAATACAGAACAGAATTTCCACTCAAGCACACAGAGCGACGTCCGGCTTTAAGGTAAGCGTCAGGTCAAGCTTGTTTGAGTGTGTTATGCCTGTCGGTGAACTCGAAGCTCTGTAGCGAGGGTTCACCCGGCAAGGCCAAGAGACTTTGTGTATTGGCCGATTATTATCGGGAGGTTTTACGTGACGGCTTATTGTTGCCTATTTGCACGACTGGGAGTGCTTGAATTGCCTCCCTTTTATTTCTTCCAGCCGAGTGTTGAACACTCGGTGATCTCGTAAAAGGCTTACCCGTGCCTGGTACCCTTCCGCTGTTGGGACCACGAGGATTCAAAACACCTGCTAGCTTTGCTCGTGCTAGTAGCGTGTTCTTAGTTTTACTGGTTGGCCTATATAAATAGTTTACAAGGTCCAGTTTGTGGCGGTGCAAGGGCAGAACTGGGGTCTTCAGGTCCGCCCGAACGATCGTTTACTATCCTAGTAGACGTATTGTTGTGGGAACGTCCTAGCTACATTTGTAGCTAGAGTTCACGTGGGAATTCCAACACCACAAGTGGTGCCGCGACCCAGGAACAGATTGTGACCATTGTGAGCTGTTTGTATGGCCTTCCAGTTTACGAGATACCAAGAGAAGAAGAAGAACAAGAAAAAGAAAACAAGAAACATGACGAAAGGACAACCAACGGTACCAGTTACGCCTGCCAAGTCTGGCGGGGCTCGCAACGAGAGTTCAAGCCCGGCTGCGCGCTTTGCAGCCGCCACCGAGCTTTCAGTGAGCTTCGACGACGAAGCGGACATCGGTCCTGACGCCCACGATTATGAAGATTACGAAGATAAGACGCCGGCTCCAGAGCCAGGCGCAGCGACGCCGGAGACGGCCACGCTATCATCGGGGCTGAGCGAGGCGACCAAGTCCCTTTCGAGGAACCTCGCCGACGAGCTCGATGATGTTGCGGGACCGGAACCCGCCTATGATGATTATGACGATGGGACCGAGGGTTTTAAACCTTCGGTGGCGAGTGCAAAGCATTCAGCACTGCTGAAGGCACCTCGCCCACCTATCAACGGTGATACACCAGCCGCCAAGCGCGTTTTCGACCGCGTACAAGCGCTGATGCAGAATGATGAGTGGATGCAGCTGTTCAAGCCCATCCCCAAGAAAAAAGCCGACTGGCCTGCGCTTGCGCAGGAACTTCAATACCCAGTCAACTCGGTTTCGACTAGTCAAGTTGCCGAGGATTCAGTTTCGCTACTGTTGTCGCTGGGGTTCGAGAACGAGACGTACCCATCGACGATGTCGCTCCAGGATTGGAGTCCACATGAAGCCGGAGCTGCTCTCCAGAAATGGAAGAAGAAGCTTCGGAAGGCGTTTGGCGCCGTTGGCATCAATGCCGGAAAGCAGCCTGTGAGTCGACCGATGATTCAAGATGAAGATCCGTCTAAGATTCCACTGCCGCCGACGCCCCAACGACTCGTTCGTACTCCTCCGACCAAGCGTGGAGCTGAGCGTGGTGCCTTTGGCACTACCGAAGCTTCACCGTACTTTCAAGACTCGCACATGGTTACGCCACGTTCGGAGAACCGTATGCAACGTATTTACGCAAGTAACGAGGGTTCGTTCGGTCGCCGAGGCGGTGCGCGAGCTCCCAAAGGTGGTGCCGGACGTCGTCAAGTCGACAGCGATGGTTCTTCCAGTGACGATGACATTTTGGTGCGTACATTCGACGAGGAAGATCCTCAACTCGAGTTGATGCGTCAGATGAATGAGCTAGCGACGCAGAACGACGTGGACCCAACACCGAGGATTGAAATGGCTTCGCACCGACCGCTGGATCGCATCACGCCGTTCTCAGGCTCTCGCAACAAGAGCGAGAACTGTATACAGTGGCTCCGTGGGTTCGTCTATGAAATGACTGGAACCCACACAGATGCCGACAAATGGTGCATTCCATTTGAGCTAAGTCTCCGGGACGGAGCCATTCATTGGTTCCGTCAACTTCCGAAGAAGACGAAGCGCAAGTGGAAGCTGCTGAGCGACGCGTTCATCCGCTACTACTGCTCGCAGATAACGCAGACGGCACTGTCGCGTTACTACTCCGCCAAGCGGGAACGTAATGAACATCTGTGCGACTACATAAACAGGCTGAATGGCTATGCTCGGACCGCTCGTCTACACTTCGAGAAGAGTGGTCGCGACGCGAAAGAACACGTACAGCAGTTCCTGGCAACATGCGATGATGACAAGCTGGCGGAGAGTCTCTACCACGCTCGAGTCGCCGACATTCACGAGCTCGAAGAGATCATCGAAGATGTTCTCAAGGGCAAGGAGAGAATGGCCAAGCGTGACAGTGAGTACCGATACTCCAAGAGTCGTGATACGCGACGCGACGATGGATTCGCCCGCAACAACCGGCGTGATGACCGTCGCCGCGATGACAACCGCAACAAACCAAGAGTGACGCTCATTGAAGCCACAGTGGATGACCTACTAGCTGAATTAGAAGGTCGTGAAGCTGCTCAGGGAGGCACCGAGTATTCAAACGAAGATCCGAACGCTTACAACGATGTCCAAGACGAGTACAACGACTTTCAGTCCGATAATACGGATGGTGATTTCTCATTGGATGAAAACGACCGATACCTCGCCGCTGCTAACGAAAATGAGCGCCGTGCAGCTGCTGAAGGCACCTACGCAC

>Contig_35

TGCCAACAAGCGGAGGAGGGCGCTCCTCGCCGTTCTTCCACAAGTCATGCCAAATTAAGCAGTCAATCCCACCAAATCATAGCTTGCAGTTTAGGGCAGCATTTGGCAACTGCGACGGTAACGGCCCCCTTGGAAGCTCCTGCGGTCGTGGAACAGCGTTCGTAACGAGCACTGGGGGGAAGCTCTTCTTATTCGTTTGTATTATGCCGACGGTGTACAGCAGTTGTAACGAAAGTTGAGCCGACGTGTAAAACCGATCGGTGATGACTGCGTGATCGATGCCTTCCTCTATAGGGGGAAGGACTTTGGTCATGTTCCTCAAGACTGCGCTCGGTCCAAAATTTGGGTCAGCAGAGAGCTGCTTTGGAGCCCCACCACCAAGCTCATCCGTGTGCTGATCAGCTCCGCATAATTCTCCAAGTCTTTCATGGCGTATATACAGCATAAATACATATTAGAAAATAAGATTATACAACATTTGGAACTTAGAAAAAAAAACGCTGTCCTTCACAGGATAGCGCTGATACTTACCGAAGACAATACGCGGTTTCTGCGCAGCACATCATGAAGACATTCGTACCCATTTGTAAGGCTTATCTTTCATAAACTGGCGGGCCACATTGTTGCGGCTACGTGACGAGATCATGACTTCATCGAACGACATGACAGGCGGGGTGTGGTACCTGCTGTTGCAGCGTGTCCACCACAAATCGCACCTTCCAGGCTCGATCCGTCTCCGCCCTCGAGTCGGCGTTGTCCGTAAAGTACAGGCTCTGCATTATCCTACCGAAACGCGCCTTTGACGTGTACTGCCCGAACGTCCATTTTGGGATAGCACCAAYATCAGGCTTGGCCCACTGATCAGCGAATCGTCGCTTGTGAGGGCAAAGCATTCTCGCCACAAATAATCCAATGCAATGCAATACTTCCTTCGCCTTGATTTGTCTTTTTGTTTCACCGAGCAGTACTTCATCCCTGGACGTATCCTCACCTTCACCGAAGCGTTTTTAATGCATTGTATCCACGCGCTCGTTCAAATGCTGGCTGTAGTATCGATTGCTCTCTGTAGCAACTCGCTCCATTCAAAGCTCCATCACTTTCATCTGAAGGAGTTTGGTCAGGCTTCTCTTCAGGTTCTTCATAATCACCCTCTGGTGTTAGCGATGCAGTCGCATCAATGACACCTGACAGCGTCTTCAAATTCGTCTCCGTTTCGATATACCACTCGTCTGAGTCAGCACCGTCCCATCCATCAAGCTCGGAATTCAGTGCCTCGTCGTCAGAAGTAGCATCCGCGCGCGAAGCCTCATCTGGTCCGTGCTCGGCGGATTCGACAGTGTTGCTTCTATAACATTTCACATAGTAATGCGTTAATATACAACATAATCGTTTTCAAATATGTCCTACATAGAACATGATATAGCAACTTACCCATCTGGAGAGTCAGGAGCCTGTACTTGCGCTGTAGAAGCATTCGTTACAACAGGTTGTTGTGATTGCTCAGGTAGGCCCACCGACCACTCATCAGAGGTGTTTAAGTCATCTACTTCCGCGGTGGTATCCACCACGTGAGAACCGGAGTCCTCGCCGACCCGGCGCCGGGGACGAGAGGCTGCAAATTGAAATGCACAAAAAAATCAACATTATGTTAGTCAACACAAGTAGTATATGCTCCATGATCGCGTGACTAACCTTGTCGTGTCGCTCTTTTTGCATTTTTCTGCACCCGTGGCAGTCGTCGTCTCCGACAAGCCAGCAGTCTCCACTGATCTTTTTCTTTCAGATCAGCAAGGGGCGCGTTTACGTGTGTCGGCTCCTCGTCGTTCCTGCGCTCTCGCTTTTTCGCTTTCGAAGCGTATCGAAGTGCGGCTGCTGATTCAGCTCGCACCAGCGCCCTGGACTCCTAAGCTTTTTTTTCGAGTTAGGGCAGAGTGCTGTCGCTATGATATCTTCTGTTTGACCGGGCTAGGATAATATAGCATGAATATAACATCAGTTCAGTCCTCACACGTTGTATATACTTCATGAACACTACATACCTCTTTCGCGTTGATGTCTTGGTCTTGCAGGACGGACCGCCCTTCTTCTTCGCACCCATCTTCTTCTTCGCCGCGGCTTTTTCAGCCAGGCGGTCCGCCATGCGCTGCTTTGCGGCCTCGATGTATGAGGCGGTCTTTCACGTGGGAGGGCGCTGTATTCCTGACGCGGGTACGCGCTCCGGCCCCTCAGCGTTCGTGTCCCTCGTCTCGTCTCCGAAGTCGGCATCATTTTGCCTCCCACCACTCAAAAACGCTCGGTGCTCTGCCAGCAGGCGGTGCCTGTCGGCAGTACTCTTTCCCTCAAGGCGCGTTCGGAACGCTTCAGCCTTGCGGCGCGTCTCCCTTCGCGGCGCCATAATCGTTTTATTTATTTTTTCGAATGAGGGGGAGACTCCACAGACACAAAACTATTCGTACTTAGCAACAAGCTGGCTTTCCTGCCTAGGCCGTCTTTGTCATTTTTGCTCGATTTTTGTAAATATTTCTGCTACACCTGTGCATCCCATGTGGAGATAGACGAGTCGCTCTATTTATGTACATATTGTCGTTTACAACATATGAGTACGGGGGGGGGTAACTGTAATAGAAGTCGCTCGGGCTCCTTCTTTTGCAGATTAGGTGTGAAACAAAGACTTCATATAAAACATAAATGCTAATTATGCTGCATTTATGTTTTATATGAAGGCTTTGTTAGACCATTACTTATCAGAAAGTATTACATGTAAGGCTCAGCCAATTGGAATACCTATATTTATTCTTTCTATACCGGTAATTTTATGATACACAAAAACCTAAGCAGAATCATTGAAAGCACTTCTTTTAGTGATAATCCACTTACGTACGATGTAGGCCCAATAAAGTTTCTTTTTAAAATAACAGAAAATTTGCATGCGAGTGGCTCATTGAATTTGACACCAAATATTTTTTCTCGCTCAGGTACAGTAAAAGTTTCCTACATTGCTTACAGCTTCTACCTTGTGAAATTGTTCCCTGTTGCCAACATGTGTCGGCAAAGGATTTTTTTTAAAGTTTTTTTGCGTTTCCTTCACATCTATTAATAGGAACAACACTTTGGACCTTCTCCCACTGCAGCTTCATTTCAATATTTTAAATGTCAAATTACTTGGTGGCTGCTTCAAGGATCCGAAAGACACGAACGTCTTGCCATTTTGAACAGCTGCATGACCTCAGAGGTATCATCTAACTCAAAGGACAATTTGCAGCTGGAGTTCTTAGTGTCGAGCACGTTCATGCACAATTATATGGCGATACTGAAACCGCTTTAAAGACCTGATACGCGTAAAAATGCAGCATCTATTCAATTGCAATTATCAAGAATCACATTCGATAGCTCTACTCTGTGGAATAGATTATGGCGTAGATGACGCCACCGCCAGTAGCTTGATGTACCGGTCGAGCATTTCATCAGCGGCTGGCACACCAACAAACACTCTCTTTAGTTCTTCTGGTGTCTTCTTCGCATCTTGCCACTTGAGGATTAGCGCACTCTGTAGATCAGTAGCGAGTTTCTCCGTGCTGGGATCATTCTTCGCGGCTGAAAACATTCGGAGTAGAGCTATGTCATGAGAGTTATCCCTGAGCCCGTCGATGACTGTCGTCTTCTCATTAGGATATCGGTCGTTGAAGGTTTTCAGGTACTGCACCCACGTGTTGAATTTTGGGTCGGCAAGGAGCTTCTCACCGCTTTCGTCGAGATGCAGGGAGCGGAAGGCTGTCTTAGGCTCCATCTTAATATCCAACCAGTAGTTGTGATACGCATTCTCCACTTTTTCAGCCATCAATCTTGTGAGTGGATTTTTCCTTCCTGTCTCGATAATCCTTTCGACGCCGAACGGTTGGTAACTAATACGGAGCATGTCAAACCACGATTCTTGATTGTTTGGGTGCTCTTTGTTGAATTTCTGCAGATATGCCAGCCACTTGTGGAACAGCGTGTTGCTGAGAATATCATCTCCGACATTGTCAAGTGCTAGCACCTTGAATATACCCTCCGGGGATTTTCCGCTGGCAAAAAGAGTCGAAGCCTTCTCCATCTGCAGCTTGGTTGCAGTGCTTTCCAAATTGGGGAACTTTTTGGCTTCTCCAAGGACTCGAAAGAGACGCATGTCCCCAAAGGTGTCACCTAACTCCAAGAACATTTTGTCCACCTTGCTGGGGTTCTTGTCGACGAACACATTCATGTAGGAGACCCACACATTGAGTTGCGGCTCGTTGAAGAAGGCGGTATCCCCATATGAAGACCGTAGCCTGTTGTAGACGTCGAGGGGAGACTCTTTAAGGTTCATCCACCTATCGAACTGCTGTGCCTGCAACTTGGCTGCAAGAGTTTTCGTCTCACCTTTCGAGCTCGCGCCGATAAGCGTGTCGAGTAATTGTGCATCTCCATACTTCCGCGTTAACGTTTCTATCGCTGTGGCCTTCTTTTCAGGGTATCTCGCATTGTAAGCGCTCAAGTACCTTTCCCACTCTTTCCACCGTGGACTCCCCAAGAAAGCTTGAGTCGTAGTGCCATATTTGAACGCATCCATAACTTTGTCGGGCGTTTCTCGACTGTTGAGCCAGAAGTCAAACAGTGCACTCTCTACCTTGTTGGCGACAGTTTTGGCCTCTGTCGTTGCTTTCCCGTTCGCAATCAGTTTAAGGAGTACGTCTGTGCCGGCTTCGTCACCTCGAGAAAAACGTTGCTTGAGCGTAGGAATAATCGACAATGGGGCTTCCTTGGGATGTCTTGCGTTTACATCGTCCACGTACTTGAGCCAAGCGGAAAATTCAGGTTTTGGGAAGATGGATATCCCTGCCCAGTTAAGGTTGAGGGCCTCAAAAACCACCGAGGGGTCTTTTCGAGTGGCAATCCAGAAGCGTATCTGGTCCTTTTCCAATTTAAGGGCGAGTTCACTCAGTTGTGGATACAGTTTAGCAGCTTGAATTGTGTCGTAGACAACTTTGTCGCCGTGCAATGATGTCAGGGTGGAGATCACTGAAGATTCTTTATGAGATGACTTGGCACTCAAGTCGTCGGCGTACTGAACCCACTTAGCAAAGTTGGGTTCATGCAACAAAGAGAAGATATTTGGTTCGTCAAGGTTCATGTTCTTGAACACTAGCCCCGCAGGTAGTCGCTCGTCTAGCCATTGCTGAAGCTTCGCTGGAGTCACCGCTGAAGACTTGACCAACGCCTTGATATTTTCTACGATGCTGGGTGCTCTCTCCTCGCCGACTAGATCAACAGTACTATTTTTTCGTAAAAGCCTCGTGCTTGAACTCGCGTTTACGGTATCGACTTCAGTCCTGTTGGGAGCGTGTCCTCGTGGATCTTTCGCGACTGATGCGTCGACAGATACACTGGTAATGACGAAAGCTACGGCTGTCAGAAAGAAGAGATGCATCGGGATGCAAATTCAAGAATGAAAGTGTTTTCGTGCAACCTGAGGGTTTACAATACGGTACGGTACTGTGCAATGAAATGTACAGTACATTGGTATTAATAAAATGCTTTACGCTTACGTACGCCGGTAAACATAAATGCAAAGAAAGGTTAGTCTGGTCCGAAAATGGTTATGTTTACGACTTTGTTCATAAACGACTCACGTCGTCTTGCAATCATGAACACTACTTGATGCATGGCAATGGATACGGATACTTATGTGCAACAAAGGCGTGCAATCGCCCAGAACAGCTCTGGATTTTAAATCGGATTGCAAAACGACTGGCCGCCAGAAGTAGAACTTGCAAATTAATGCGACAGTCATTGATACAAAGATGTTGCGACAGAATCAATTCATCCTATAATAGTACAGAAGTCCCTCAAAGTAAGCGTCACCTACTTACGTAATACCCCGCCATATCTGCTACGGTACAAATAGGTTCAACTGGCCAATCACAAAAATGCACTGATTTTGGATGAACTGCAGCGGCGGGAAAGATCTCCCATCATTTCCAGACCGGTCCGGCCTCTTCGCTAACTAAACCATTGAAAGACTGAGAATGGAGATTAAATGTACGAGGTAACTCATGCTCCCGCTACGTACGCCTCCCTCCATGCACACTATCACTCTTCCAAGCGACCATGACCGCCAGCCCCATGCCGCGCCGGATCCGCCTTAATATGGAGCAGAAGATCGCCATGTGCAAGTATGCGAAGGATATGCGCGCCAAAGGCAAACTGTCCAACACACAGCTCGCAGCGTGGGCAACACGTGCGTTCAAGCTGGAAAATTCTCTTTCACGCGATGCCGCTCGCAATGTTATCGCTGGGGAGTTTAAGTGGGCCGCTATGGGGCCCTGTCAGCTACAGCGCAAGCAGCTCGTCAGCCCCCAAATCCAGGAGACGGACGCTCGCATTATGAAGGTCTTTAAGGCCATGGACGGCAAGGTGGAGGCTATCACAGGCAAGGTAATAAAGGCCATTGCTCTTCGCGTGAACGCGTATGGCGAGAACACCACGTTGAAGCTCTCTAAGGGCTGGCTGTATAGATTGCAAGAACGTCACGGAATCTCCAAGAAGCGGAAGCACGGCGAAGCTGCGTCAGTTGACCAAGAGGCAGCTGAAGCCGGACGTGTGAAACTACGAAAGCTGACGGACTCGTACGCCCGGTCCGAGATCTGCAACGTGGATGAGACCTCGTTCTTCTTTCGGAGCGAGGCTAAGTACACTCTCACGCAGAGGAAGGTGATCTCTGGACGCAAAGACCCCAAACATCGGCTGACACTGGCTCTAGATATCTACACCGCTCTTTGTTGAAGTAAGGTGGCCTACGACGAGATGTCGCCCACAACCATTCGGAACTGCTAGCGGCACGCAGGCATTCTGTCGAGATCGCCTCGCTTCTTAACTAAATATCAGTACTTTTTAAACATCTGTTTTATCACCCTCTAAGATCGTGTCTCGGTCCCCCCTACGCTTACTTTGAGGGACTTCTGTACATGCAAACGCAGCGTGCAGCTTCATGAACACCTACGGACGGCGTCGGAGCCATCCTAGCAGTGCTAGCAGTGTTAGCAAAGGCCACGGGCTATGGCCGCAGGCCAATGGCCGGGCCTGTCTATTGGCCTAAACCCATGTCGAAAGGATTAAGGCACTCGTTATTTTGCCCGACTGAGGTAACATATCATCGACGTTTATACGTGTACAGATTGTGTAGTACGGTTAAATTGGAGCAAAGGTAGGACACTAGCGCCGTCTGCAGCGTGGGCCCGCCCCTCTCTTTGCTTGCTTGCTGCGCTGCGCTCGGGGGGCGCCGCTGCGCGGCCTGCCCCACCCAGCCTCCAGGTCCTCGACTTAGCAAGGCGCTGTGTCTGGGATTAGATATTAAGTCCAAGCTATTAATAGTGTTGCCAAGACCGTCGGCTAAGTCCATTTTATGCATGTTGGGAGTACAATTGTATAGTCGGTTAGCCAGTCGGGCAAAATACTAGGATGAAATAACTCGTTTCAACCCTACGATTTAAATGTCAAGCGCACTAAGGGCGATACGTTAATTCATATCTTATATTAATAAATCACAGCGCCTTCAGCTCAACGCCTGCCGCACTTACAACGCCAGTGTGTTCGCGGCATGATACTCAAGCTGCTAGAGCTGGTCGTCGCCCCAATGGACAAAGAGAACGACTACGTCGGTACCTCGCCGGATGGTACCGTCTTCACGACTCGCTCGCTGTGTTGATGGTCCGCGAGACTGCTCATACAGAAGTGGAGGGAGCCACCCAGCCAATCTGGCGGCGAGGTCATCTCGGTCTCCTGTCCCGAGAACGACAGCATCAAGATCGTCGAAGTCGAGCGTCAGACGTGGCGTTTTGGGCGCCAAGACTGAGGCTGGGCAGTCGGCTTTATCACAGGGAGTGAATGCTGCGGTCGTTGCCAGCAGCTCTAAGTACGGCATGGTCAATTCCATTATGAAAATCTCTAGTATCGGAAGCATATTCCCAGTTGCCGTGCGTGTTGCGGTGTAGAACGCATCACCAGCAACACTTGGATAATCGCAGTTGCTGCAGGCGTATCCGTCGAATCGAGTGAGTCATCGGCTGCGTCCGTGTTCACTGCCTCATTTGCGCGATCGAACCTGATGCGGCGATTGTCACTTGATCAACGCTAACCTTCGTGATGGTGGAGCTCATAGCCCAATGCCGCACTTGGGCTGGCAGTTTGATCCACCACGGTTTCTGTTGCTGAAGGTGTGTCTTTGCCCGGGCTAGTTGATGGCGCCACTGTCGTGACCGGGAGAGCTTAAACGCGTTGCAGCTGTGACGAAGGGGGTGGAGGTAACGGGAGGAGCGGATGTATTGGTGTTGGAGATTAAGTAGGGCTGCCGAGACGTCAAAGGTAGGCCAGGTCGCGTACAGCTCGACACAGCATACATCTTGCTGTCTCCGAAGAAGCTGCGCTGGACACGAACGTAACCATCTTCACCACATGAAGTATCCCCACTGCTTCTTAATGCGCCAATACAGAATGCTATCTTTCCTCTCACCAAACCCACTCCAAGTCCCCATGTCTTATCGCAGCTCGCACCCGTAAGCACGCCACCTGAGTATTAATAGTACTGGAAAGCAGTCGACCGCTGTACAGGAAGATCGACATTGACTGCGCAAGGCATCCTCAACCGCGCCAGGATCTTCGCGAACGGGTTCGTAACCGGTCACACCAACTTTAAATTCTGCGGAGAAATTCGGCAAATATTGCCATATTGCCGTTCTATAAAGCAAACGGATACGCGTCCAGCGCGCACATTTTTCCATTGCGCTCGCTAGCGATCCAGCACGCAGCGTAATCTCATCAACAGCCACTGTAGGAGCCCGAAGACGATGTTGTTGCGCTGGATCCCGCCGAGCTGGAACATGATAGCAACGTAATCGTCTCGTAACATCCCAGTGACCCCACGTTCACTTCATTAGAGGAACTCACACTGCCAACAGCAGCGGTCTTGGACGACGGAGAGAAGGGCCCGGCCATGCTAGAGACGCTCAAGTGTTGCTGTTGTTACTAATACACAGATCCCTAGAGGCGAGTACACTGAAATGGACATAATATACGTCTTCGATTGCTACATACGTACATCCCCAGTGCAGATGATGTGAAGAAGTCCATTGAGCTCGCTGTTGACGCCATGATGGACTACCTCGCAGGTTGTAATGCACTTGAGACGTTCTTGCTCGATATATCGTTGGCGAGCAAAGGGTAGAACATAGAGATCCTGGTAATGCTAGGAGAGGCTGGCGCCAACCTGCGC

>Contig_36

GGTTTTCTGTCCAATCAGGCTCACGGAACGTTGACATAATGCTAACCTGCAGATGAGTTGAAAAACAAACCTTTTTGAGTGGTCGCAGGTAACGGCTACCGACTTCCACTCGGCTACCATAACCGACTCCCAGTTTGCAGGGTCAGTGCAGGTGTGAAGATACAGGAATCCTATTGGCTGGTGAAACAGAACAAAACCCGGGTTATTAATTCTCGGAACACCAAAACCGTCAAATCGAGAAACGAAATGTAAAGGTTGCATCCCAATTTACATGATTACCGTTCTTTGTAAAAAAATCTCGTCATGTTTTCCAAAAACTCCACATGTTCGCGAAGTGTTCTGCCACGCAATTAAAGGGATATGGGTCTACAAAGAAGGAAATTAGGCTATTGATTATGGTACGTTTAGTATTAATAGTAATCCCTACACCTGCCGCCTCTGCAGCCATCGCAGCTTGCAGCTGCTCCGCTCTGATCTCAGATAGAGCTCTGCCTGCTCCATCACCTGCTTGCGTTCGGCTGCCTCAATCTCCTTGCGGAAGTTCAAAAACTGCGCCTTGTCCGGGTAAAACCCCAACGCCCTATCAACGACCTCGTTCGCTTCGTTAAAGCGACGCTCCATGAGCCGCAGCTTGGCCATCGCGTACAGCGCCTTGGTGGACGTCTCCTCTACAGCCAATGCCTTGGCTGCGTACTCGTTGATTAGCGATCGATCCTTCATTTTGATGCCACAGATCGCGACGTTATTGCACAGCGTCACGTATCTTTCTTGCATGTCTAGATCGTCCCGCTCCGCCGCAGGGACAGTTTTGCCGTAGTATTCACTCTGCTGGTGCTTTAGCACGGCCTCCAGGCTCTTTAAGAAGGCGTTCTTTGCCGCATAATAGTTCTTTTCCTTGAATTTCGACTTGCCATCGCTTTGCGCTCGTCCGCCTCCTCCAGAAACTCAGGAGGCGTCACATCGTCCGGATTCTGCAGCGTCATGGCTGCAAGACGCTCTTCTGCTGCTCGAGAATACCACTGGTCCAGGTCTTTACCCTGCGCGTGCGAATGCGGCTCAGAGTCCCGCATCTGTAAGAGTGTTTGTCGCACTTGTGGGGACAGAGATTAAATTATTGGTTCCTGAAGTTTAGTACTTAAAGTAGCAAACAAGCCTAAGATGATTTTACCTTTCACCTCTCCCTTGTAATAGGGATTCCGAATTACCTAGAATAACTGTTATGAGGTGCTTAATTATGGGAATGTCACTATTGTGTATCATATCCGAAGAAGGCAAGCAAGGACACTTGCTGTTTGCACCCTTCCTTCCTGCTTAGCTCATATCGTAGCTATCTCTGCATCGATCCAAGTTTTTGATCACCTCTACCGACACACCCGTTGAATCTGCTCAAATAATTAGCTTTGCGACCCACTTTAGGTGTTTCATAATATTCGTAAGAATTGAGCTCTGCATAACCATTATCTGCTTCACTGAGCATGAGCATGATGTGAGCTGACTTCGATTCGACTGGCGTCTTGATGGCACCAGCTTTGAAAAATGAAGAAAATCGCAAGGAATGTACTTGTAATGGATGATAGCGTGCTGGCTTCTGTAAATAGACCATTTTGAAGGTACACATAGCTGAGCCACCTCACAACAAGAATACCCTTATGTAGGCATAAAGTGTTGGTTGGTTCCCGGCGCTACGACTACGTAAGGGCTCACCTGTATTTTTTATCGTGCTGGACTGTACACCTAGAAGTTAGTAATCATGTGTACCGGTACAGTATGCTTAACTGTAAGTATCATTCTGGATATTCGCCTAATAATGTGTTCTCCAAATAAATGGTTACCTTCTAGTATGTTCTTCTTGGGTGGAGACAGCTACACCAAGAGGTCGTTATCTACCGAGTGAGGTCGATGATCGACAAAGTGTTCAATGTGAACACGTACAAACTGAGTATTAGGACCAGCGTAAGTCTGAAAACCGCATAAAAAAAGGTCACTAATTGCTCCAGTTACACAAAAGAGCGTCCTCGATGATGCCGACCTCTGTCAATTTAGAGATACAAGCCCGGCTCCTATTACAGTAATACTCTTAATAAGGGAGCCGAATATACCGGGCTCTCGACTTGAAAATATAAAAGCGTGCTCCGGCTCGGTTCGTCCATCCCATGATGATTACACACTGTGCTTCGTTTAAAAGGTACTACAGAAGTGCTATTACATGTGCCATAATAATACCCACCGGTATTAAATTGATACCGGTGTGTATTCTTTACTATTACATGTAATAGGTAAGAACTGTACATGTATTTGATTCCAAGATTTAAGATGCGATTGTGGAGATTGTGAAACAAGTGACGACCTCCCGGAGCCTGTCAACTTCACAATTTGCTGTCCAACGCTCAAGTATGCGATTCTATTCGGTGTTGCTGACTATTGTGACTCTCATTGCCAGTACTTATGATGCAAAAGTAAACGCTTCAGGCATCCAGGCTATTGCTGTCAGTAGCATTTCCCACGATGCTCCTGCTGCGAGGATGCTACGAGCTGACCATGCCGATGAGAGAGGAATAAGCGTACCCAGTGCCTCAAAGATCGTCGAATGGATGCTTACTCCGAGAAAGCTCACATTTCTCGAAAACCGCAAGGTCCAGAAGTGGGTAGACAAACAGAAGACGCAGGAGTATGTCTTTACGAAGCTGGGCCTTAACAGCGGACTTGATAAGGCTCTCTCCAACCCGAAGCTCCATGTTTATGCTGCCTACATCGATCGTTTCAACGTGAAAAACCCCTCAAACAAAGTGGCATTACTCGACAAGTTTAGTGAAAAGTACACAGACGAAGGGGTAGCCAAGATGGTAGAAATGGGAATACGATCCTCAAACTTGGAGACGGAGAATTTCGCGTCGAGACTGTGGAGGGAACTGCTGAATAAGTGGATGGGCAATGCCGAATCTGCCGAGGGAGTTTTCAAGATTCTAAAGCTTGACGAAGTGGGAGGTGGCATTTTCGCGACGCCGCTGTTTAATACTTGGTACGCTTTCATAAAAGAGGGTTATACGCGGCAGGCAGAGGATGTCGTGCTTCGAGTCTTGTCGGACAGGTATGGCTATGACGGGCTATCGAGGATTTTCTTCCGTGGGCAGCGTAACTTTGATCTTGTGGGCGATCTCCCCATCAAATTGGAGACAAGAATGGTGAATAATTGGCTGAACAAAGATGTATCTCCTGACAAAGTGTTCAAGCTGTTGAAGCTGGATGAGGGACTGGATAAGCTTCTGACCAACTCGAATATGCAGGTGTGGGAGAGCTACATGATGAAATATAATTTGATGCCCGATGTGGAGCCAACGACGATGATGCAGACGATCACGCGTTTCTACAATTTCAAAGAATTGTCATCTATGCTCGAGAACGCGAAAATGGTGCCTGAACTGAATAAAGTTGCAGAGAGATGGCAACATGAACTACGTGTTCATTATTTGAGAGCCCCCAAGATGAAGAAAGAAGGATGAAAACGTCGAGCAGAGTTGTACTGAGGGTTAGCTTGCTTTCCCTGCCGCCAAGGCATGACTCAAATACGGGTTAACTAACAAAGACAAGATAATGCATGTACAGCAGATCCGTTGTGCTGGAGAGGGGAGTAAGAACGGAAGAAGATGATACTTCAGTACGTCCTAAACGTCCTACTGCTACTGTAGTATTTGTCTAAATAAAACGATTTATTTTTGAGGTCCAATTACCAGTTATGCTTTTAAGTAAGAATCCTCGTATGTGTGATTGGTCAATGTCCCTTTTGAGATCTATATGATTGGTTGAAATATGTACAAGATGATTTGTATGCATTTAATTATAATTTGTCCTAGCGCTGCGAATGAAGAAGGCTAACCGCGCTTTCGTTGAAGCAGTACAACAGAAGTTGTCACGACTTTAGAGACCTACTATTTTTTTTTCGGACCGGACAAGTATGTCGATAGCTGCATGAAAATCTCATATTAAAGACAAGTTTAAATATTTAAAAAAATAGGACATTCTAAACTCGCTTTGGTCCCGAGCCTCAGCATGCCTTTTCAGCAGGTGATCACACGCAGCGTAAGTCTAGACAAACGGATCAGTGAGGGACTATTAAAAACATTACAGCTGGCGTGCTTACTCATTTGGTGATAGTAGGGATCAACTAGATCGTACGCAGGTAAAAAAAAATATTTTATCCGAGTCTAAATTCGCACTATGCCCAATTTTGCACACACTTGACAAGTGTACGATTCAGCTAACCGGTCCGTTGGACCGGAAGAGGAACAAGAGGTTTTCGACGCGCAGAAGAAACAACGGCATTTACCACGTCCCGGAGCAGAATGAAGAGGAGCACGGTTACCCAGGACTTCGAGTCCCTGCACAGCGAAGATCGAGCCAGGCCCAGCATTATGACTCTTGACCCCCTGACGTCCATGACGCGCTCCGAGGAACTCCGCAACCAGGATGTACGGCTCGAGTGCCCGCCTAAGCTGGATGACGGCGAGTGGGACGGCTTCATTTACTCGCGGCTTCAGTAGCCTCGTCATATCAAGCCCATCCACCCAAGCGCCTTTGAGCTCGATGATTCTTCCTGACGCCCCGGTTGGTTGGGTGAGCTGATGCTCGATGCTCCATAATTTGATGCCGTGCCAACATGCCTCGATGCCTGGCAGCCACACCGGTGATTATCCCATTCCCCCGCATCGGATGACCGAGGACCTCAGCCAGGAGCTATAAGACCGATCTCGTCGGTTACTGTTCTTGTTTATCCGGTTCAGAGTGTGTTAAATATCCTGCGAGGTACGAGCTTACCACGAAGCTCATCTGGACGTCTATCTCGTCGGACAAGTTGTTGCCTATTTCCATTCGCGGGCTGCCAGCTGTAGTACGCCGACCGGTGCTGGCCTGCAGCGAGCTTGTGACTTCATGTGTATTGCGGATCTGCGCTCTCGGGTGGATATACCAGCGAATCATGTGGCATTCCTTCCACCTCTGAATGGGGCCTCCGAATAACCCGTGACGAGATCAGCGTCGGCAGGTGTCACGGCGTTTCCGTTGATGCCCAGCCATCACGACGCCTAACGCCCTTTTGATCTTGATGGTCGAATTAATTATATCGGACAAGCCGGAACCTGAATTAATTCGGTTTTACTAACATTGGAACCTTTATCCCGCCATCAACTATTAACTTTAGTGAAGGAGAACTTCGTGTAATTTCTGAATAGGATACACATTTGTCCTCTGAATTACCCTATCCAGTACATGAGCCAGCTCGATCCTCAACTCGCATGGATGATGAGGTTAACATCGATTGAGCGTCATATTTCAACCTTCTTGCTATCGGCCTATCTCGTAGCCAGCCCCTTTCCGGTGGAACACCCGACTTTTCTAGATTCGGCGACCGTACTGCCATAGAGGTCTTCCTGTGAACGGCCCCTTCCGTCCAGACGGTGATCGAGAACACAGAGCCACGCCCTTTGCTCGAATCTGTAACATCGCTCGAGCCGACACCTAGTGTTAGTGTAACGGACGCGAGCTCTGGCGAACCTGTCTCTACGGTCGTCCGTTTTGACCGCGGCAGTACAACGGGCCCTACACAGACCAAGTTTCCATGTCTACTTCTACCCTGAATCCGACGGATGAACACCTTGAAGCAATTGCAGCGCTAGTAATAGCCACCCAAGCGGACCTCTTTTGGCAGGATGCAGCTCAACAAGCATTCCAGGAGCAGCTCCTGCAGCGCCTGGCGCTACGCAACGATGTACCATCGCCTAGACTTACCGAAAACCAGGTGTCCGATATCCGAAACAACGGTTTGTAAAACACAACTACTATTAATAGTCTTCTGCTCGTCTCCCAGGTGTCACGCGGTAAGGTTGGGCTGTCCGGGTCGTGTATCCTGGTAAGATAGGTCACTACCTCAGCTCCAAGTTTTGCGAAAGAGTGAGCGTGTTTGCAACTGCGAAAGTTGCATCGCTTTTGAATCTGCAACCGCAGGCACAGCGCCTTGTCCTCGTGTCTGGAAAGATCGGAAGAGCGTCGTGTAGGGAAAGAGTGTAGCCAGCTGGATCTCGGTGGTCGCCGTATCATTAATGATACGGCGACCACCGAGATACCGGAGGCTACACTCTTTCCCTACACGACGCTCTTCCGATCTAGCTTTCATTGAGGAAATCTACCGGACGGTTAAGGCATCCTCCGTGTTTCATGACAGCTTCGCTGGGAAGAAGGTCATTATTGTCCTGGATAATGCCCCCGCTCACCGCCAAACCGAAGAACGTGTCGAAGCACGTGACGACCTCGTACTCCTACGACTCGGACCCTACTCACCGATGTGCAACCCGATTGAGGGTTGCTTCTCCGTTTTAAAAGCTCGAATCAAGGGATACCTGGCCTTGTACCGCGAAGAGATCTGCGACCGGAGTAACATGGTGGAAGAAGACGGCACCCCAATAACGATCAAGGAGCGCACTATGCGCTTCTTAGAGAAGGCTGCAGAATCGAGTATGAAGTATATAACACCTACGTTAGTGACGAAGATGGAGCTTCACGCTCGCGATGCGGTGAACGCGGCCGAGGAGATGAAAGACATGGTCTATGGGAAGTAGCTCACTGTGTCGTCTCGGTTTGAATGAGATTATTCTCTGATTCACTGGTAATTTTGATCCCGTAATCAGATATTTGTAATAATCAAAAACATATTGTCCACTTTGCTTGACATTATTCTCTGTTTGGACGGGACTGACTAAATGACTCTTTGTCTGTACTCTATGTATACATACTATGTATACTTGGCAATACATACCCTGTATATATTGCCAGCTCGGGCCGGCAATACTGAACTTCAATCATCACTCGATTGGTAGGCAGCAGCAACCCTGCCACTGCCAATCCGTAGATCAGTACTGCCGCCCACTGTAGTGGCTGGATGAGGCTACATGCCCCCAGCCCACTACAGACCCTCTTCTTAAAGACTCATCCAGCCACGTGGTATGATCTTCTCCTTGGTCTTGCACTTTCATGTCCACGCACTCGCGCTTCTAGACACTGCCATGCTCGTGACCTCTAATGGCAGGCACACACGCACCTCCACAACATCCCCAGGGTGAGATGCTCAGGTGTCAGGAACTCTCTGACACCACGTACCTTGGAATTGACGCCTCCGAGCTCGATGATCAAATGCCTGGCACACCCGCGCCCACAAGCACTGACACGACCCGACCTTGGCATCCACCCGGGCATGACGCTCCAGCATCCAGCACACTCGCGCCTTCCCGACACTGTTTCCTCGACGGCACCCACGATGTTCCGGTGTCGGCACACTCGCGCTTCTCAACCGTCACGCCTCAGATGCCTGCCGATTCTTGCGCTTCCCGGGGCTTGTTGTTTGAGTCCCGACGAGCGCGTTGGACGCCTTCACGTACTTGACCTTGGCATCCCCGTCCTCGGCTTCCAACGCTCCAGCGTCCTTGTACTCACGCCGTCGGACTCACGTTCTTGGGTCCAAGACGATCGCGCCCTCCGACACCTTAGACACCTCGACGCACTCGGACTCAGCATCCTCAGTGTCCGGAACGCTCACGCCCTCCGACCCCATGACTTTGGCACCACCTTCGTCGTCCGCCACACGCCATCTTGGATCTTGAACCTCTTCACTGACCCGGGCACTAAAGTTAACTTTGTAGTAGGTCGTGGAAGTAACACACAACTGGGGGAGCTGGCACTCCCTAAAGCTTCTGCCTAGTGTATTGCTCTCCACTAAGCAAGTTCTAGACTGACTAACTGACTCTTTGTCTGTACTCTGTGTATACATACTATGTATACTTGGCATACCCTGTATATATTGCCAGCTCGGGCCGGCAATACTGAACTTCAATCATCACTCGATGGTTGGCAGCAGCAACCCTGCCACTGCCAATCCGAAGATCAGTACTGCCGCCCACTGTAGTGGCTGATGCCCCCAGCCCACTCTACACAGCGTAATGATTTGATGGACGAGAGTACCGGTATTTAAATTAAGCCGAAGCCTCAGTGAGCTTTTCATTCAGCAATTTGCTACGAAGCCCTCACTTTTCGTTTTTGTCGGTGCGATGCCCCTCAAACTTGCCTTAACACCACGCTTAAGCTACTTCATCTTGCTGGCGGCCCTCGCTCTGCTGGTATGCATCCACAGTGGATCTAAGGTGGCTACGGCATCCAGCGTAATCGACGCCAGAAATGATGCTTTGGATCGCGAGGTGGGCGGTCAGATCCACCGATACTTGCGTGAGAGCAAGTGGACAGCTGAACAGGAAGACGGCAGCCCCGAGGCGAGGGCGATTGGAGTTAAGCTGTCAGCTGCTACGGTGCTTCAAAAGCTGATTAATTCGCAGCCAACGAAAAAACTTTCAACAAGAGCCATGGAAATTCTTACATCCAACAATCAAAGAGCTACCGACAAACTGTTTGCGAGTCTCGGGGTCTGGGAGGTGGAATCGAAGCTGTTTGCGTCTACTCCGTATCAGAAGTGGGCTGCATCCGTGATGAAATCCTACAAGAAGAACCCTGAAAAAGGCCAAGCAGCGATATTCTCTACACTTGTACGTCACCACGGCGATGACATTCTGGCCAAACTGGTATCAGAAGCACAGCCAACCGCAGCGGCGATAAGGACAGCCAAGAAAATTGAATCGATGCAGATAGCCAATTGGGTCACAAACAGAAAAACGGAGGAGGACATCTACAAGCTTTTAAAGCTGGACGCGGACAAGGAGGGTCTTCTGAGAAATCCGTTGTTGAATACGTGGGTTTCGTTTGTCAAAAAGCTTGATAACGAGGATCCGTACAATTTGTTGCTGCTCAAGTTGACAAAAACCAATGATGAAGAAGCACTAGCTCACATGCTAATTGCAGCAAAGGGCGATAGCCTTACAAGTAGTGTGGTTCGTAATCTGGAGAATGCACTGCTTACAGCCTGGCTGAGGGATGGTAAAACGACAGGTGACGTCGCCAAGCTTCTCCGACTAAACGCAGACAAGGGAGAAAGTTTTTTGCGGAGCGCGGCTCTGCCCACGTGGTTGTCGTACATTCGACAACGCAACAAAGATCCCAACAAAGTTTTATTCTTGGAGCTGCAGAATCGATTTAGCGACGCAGAGTTAGCCAGAGTGTTAGTCGCGGCGTCGAGGGACAAAAACGTGAAGATCAATGTTACTGCTCTACAAAAGTTACAGCTCAGTAAATGGCATGGAAGGGGGGACACGGCAGATGACATTTTCAGACATCTTGGACTAAGCAAGGAGGGTGAAGAACTCCTAGAGAGCTCAGTGTTTAATACTTGGGTGTCCTACGTGAAGTTAGTTGACTATACAAATACTGATGCCTTGGTGTTCTCAGTGATGAAGAAGCACTACAGCGACGAGATCCTCGCAAAAATGATTGCTCAAGCAAAAACAAGTGTATTAACGAGAGCCCTGGCTTCAAAATTAGCGGCGGAAATGTGGCGGAGCCCAGGGAAATCTGCGGATGATATTTTTAAGTTCTTTCAACTTGACAAAACAGGTGATGACCTGTTCGAGGCCCCGATGTTTGACGCTTGGATCTTGTATGTTGAAAGACTCAACAAGTACGAGAAACATCCAGATAAATTCGCTCTTTTTACGGAGCTCGAAAAACGCTTCGATTACGTGGACCTCGCACGAATGCTTAGCCATGCTAAGATCCAAGCCGAGATGAAAGGACATTCTGTAGAGCGTCTTTTCAGTTTGCGGAACCAGCAGTTCGATCAGTGGATGAACCAAAAGAGGTTGGATCCAGGAAGAGTCGCTGCACTGGTAGCCGAGCAACCGCGTGACATAAGAAACAACGGCGTCGTCCTCGGCTTCTATGACTTCTACAAGGCTAATGGTGGATCGCCGTTGTACTAATAGGGCGCCGAGGAGTTTATGGCTTAGTAGAGGTAGAACACTTCTAACAATGTTACACCTACTCCTCGAATTAGGAGCTTTAACTGTTTATCCTGGCAGTGTATTCGCACACGGATCATAAACACCAGATACAGCTCTATTAATACCTTTATCATGGGTCGGGGTTATTAATAAAGCCCGCGTGCTTCGCACATGGAGCGTAAACACAGGTAGCGTCCTATAATACCACGTGTATAAATCCTTGAGCACCCTGTAAACTAAGCTCAAAACCTCAGAGTGCGAACCCTGGCCCCTCTACCTCCCGCGGGGCTTCGGCGGCGTTGCTGCTATGGTTTTCTCTTGCAAACAAAGTCGCGCCTTCGGCCTGGATTTGTTTAAGGTTTGCTAAAGCTACATGTAGACTTACAAGGCTAAGAAAATGAACTAAGGAAATCATATCAACGAGCACACAGTAAATCTATTTTTGAAGCTGAAAAGAATTTATACTTTTTTAACGCTTGTCACTTTTTTTAAAGAGAATACCTTAATAAAAAGTATTCTCTGAGATTTATTTCAGAGTTTTACAACAATATGATTTATTGTGAAAACCGAGCACTATGTCCATTCCAAATCCCAAGTAAAACCGAATTTAAGTGATCAGGGCAAGATATTAAAAGGCGCCTACATTTACTAAATTGTGGTGTATATCGGTATAGAGAGCTATTAATCGTAGTTAATGCCAAGAGGCCAGAGGAGCCAGCAGTTTTAAGCGATCGAGCTATAGCCATGTACTCAGCAGACTTTCGATGGAGGGCCATCACGCTACACTATGCGTACAGTGTTCCGTGCGAGCAGGTGGGCCGCATCTTTGGGGTGTCGGGCCGCACAGTACGTCGCTGGTACAAAGAGTTCAAGTCAAGTGGCCACGTAATACCAGACAGTCGAGACTCCAGCAATGTCCGTGATCCTGAAGTATTGTCGTTTGTAAGTAAGTACGTTAAGGAGCACCCATGCTTTTACGTAGAAGAACTCCAAGCGGCACTACGCATCAAATTCGGCGCAGACAAGACAGGACTATCAGCGTCAAGCATCCTACGGCTTCTAAAGTTTGAGCTGGGGCTGAGCAGAAAGGTGCTAGAGCGCAGAGCCCGCGAGGCAGTGCCTGATGAGATTGAAGCATTCATGTGGAAGATTCGATGCTGGTACAGCTATCCCGAGCAGCTTATTTTCGTGGACGAAACGTCTAAGAACGGGCTTGACAGCGCACGGCGGTACGCGTGGGCAAAGCGTGGAGAGCGAGCGATCGTTCGGGTGCCTTTCGCACGTGGAGAGCGAGTCTCCATTCTGGCAGCCTGTGATGTGAGCGGATTTGTGGGGTGGCGCACAACACGAGGGACGTTTGATCGGCTTGGTTTCCACCGTGCATTTGTTGAGTGTGTGCTCCCTCGTCTGAACCCCTGGCCCCTACCCAGATCCGTCGTTGTAATGGATAACGCGTGCATTCATATGTACCCGGAACTAGAGGAAGCCATACATTCTGTGGGAGCCCTGCTGCTGTTTCTACCTCCGTACTGCCCTCAGTTTAATCCTATCGAGGTGATGTTTGGGCAGTTAAAACGCTGGCTAGTAAGACATGCTAACCTTGCATTCGCGCTTTACCCGGAGGAGGTGTTGGAGGTGGCTATGGCGGCGTGCGTGAAGGACGAGA

>Contig_38

GCACTAGGATTTAGAGGATCAGCGCTTGAAGCAGCAGTTGCTGAACAAAGAGCGAGAAAGAGGGGGAAATATTGTCAACTTTACTGAAGAGGACTATGTGCTACGCTCCTGAGTTGATGAAAAAGCGGAAGCAAACGCCTGGTAATTTGGGTCGGACCTTATCGTATTGTGCGAGCAGATGCACACTCTTTCCTCGCTCAACATCTGATCACGGGTACAGAGCTGGATGTCCAGGCTCAAATTTTACGCGGATTCCAGTTTTAGCGTCACTGAAGAGCTTTGGAGCACATTTCGTCTCAAGGTGTTATTCTCGCTGTGGATAAGCTCAAAGGAATCGGTGGAATAGTAGCATTAATGACTTCGAAATTCTCGTGCAGTGGAAGGACTTGAGTCAATCGAGGCCTCGTACGAGCCGCTCACCAACCTAGCTCGTGATGTTGCAACATTGATACAACAATACGTTAGCACCGCTGACCAAGATTTGCAAGAACACTGGCAGCGAGTGACCCGTGTGGAATTGCAGCAGCCAAAAGCTGCCGAGGCCCCAACAACTGGAAGGCTTGGACGATGCAATAGACGTCGCAAAGCCAACCGCAATGGACAACCTTCAACGCGCCCGGTCGCCCCTGGTATTTCGGCTGTCGAATCTCAAGAGCCTAACAGAGGTGGTTCAGCTGATGCAGTGCTTCAAACATTAGTAAACCGACCTGGGTTTGAAAACCAACAATCAAAGATATATTTGTCTACGACTGATACCCCTTCTCAGCAGCACAGGATCGACAAAGAAGTCGACTATCTTCAGGGCAAGCGCTTATCTCCAACGGATGCCGCCGATGGAGATCGTGTCGGTCGATGTACGTGCTCGCGGACTGCTGGTGCCGACCAATCCGCAGCCAGAACCCAGCAGGATGCAGCACATTCCCGCAGACACTGAAGGAGAGGAACGGGTTTACTAGACTCACAGGAGGCCAGGCAGTACCTACATGGTGCTTGATATGACTGCTGACTGGGTGTTTAGTAGAGAAGGCGTACTTCAGCGGCGAAAACTTCGCTTTAGGGACAGTGGAGCACATGGTCACCACGTGTGCTCCTCGCTCCGAGTAGTCGATGACCAATGTAGGAACCACCCCGAGGCTGGCTTCTCCATCGCTCACGTAGCGATTCCTGGGAGGCGCGTGTTCGTGTCCTCGATCGGGATGTTATTAATGCGGCAGGCGCTCTCGATCTGGAACGGGCCATTCGCGTAAGTCCTCTCCCCTCTTGCCGGTGAACTTGGGTAGCTTGGTCGGCACTCGCGTGCGGTCTGCCATAACTTGATTAAGCCGGTCGATCTCAACTTGCAGCTGGGCGTTCGTCGCCATGGGCTCCGTTTGTCAGGTCACTGGGACCAATAGGTGCTACCAGTATTTCAAAAGGAACCTGCTTTTAGGGGGGGCGCCAAAAAAAGGGAAACTGCCGCCTCCACAGTTTAAGACGGATGTAGAAGCTTCCACAGTCGCATGTGTTCAATTTAATAGCATCTACTATGTCGTATTAGATCACAGTTATTTCTAGACTACATATAATACTAATCACTCACATTATTGATACAGTATCTGTGTCATGTGTGCAGCAGTGGTGTTACCTACGGCTCTCGTCGATACAGAGAGCCCAAAGTATGCACTTATTGACATTCGAAACGCTCTTTTTTGAAACGATCACTGCATTTGATTGTAAATTTCTTGGTAGTCAATTTAATTTTTGCGAAGACTAAAAAAGCGGTCTTATTGTCAGATGTCACAAACTTTCTTCTCTTCTCACAGATTTTGCGTGAATGGCGAAGATGACACTCAGCTCTGGCGAGGTGAGAAGAGCATTTACTGTCACCATTGACACCGTGGAAATCCAGTCCGTAGACAAGCTATGAGAAGAGATCAAGTCGCAAAGCGAAGGCATTTAGACTCCAGCATGTCTCTTGCAGCTGCTCTCGGTAAAGAAACCGAGGGTATAACAAAGTCGTGCTTCACCCGAAGAAGCTCTGAAAACGCGGATATTGCAAGTGCAACAGACTGGTCATATTTTAAATCATAATCTCAGATTTTACTCGATCCCGACGATGATGTTCGCTTGCTGATTCTACATTTAATGTAAGTACAGCATGAGGCCAAGTTGGTGAGGAGCCACATGCGAAGCAACCTTTTCGACCTTCGCTTGCTCGTTTTGGCCTACGGCGCTTATCGTCGGATAAGGGTACGTACAAGTAGCAGGTATCGTATTTTTATGGCCTTGCGCCCAAAACTACCCTTATGCCCGCATTTACCCACCATTCCGACAGGTATTTCTTCGTGTATTATTCGTGTAACATCCAATTTATAATAGGCGCCGAAGGATTCTGAAAACGCTGTGATAGAGAAATTAAAGCGCTATAATAGATGGGGTGTGGCCTAAAATTCCAGTCTGCGCCTCCACAGTTTCTGCCGCGGTCCTAAACGAAGTCCTCAAACACCAGTGCGTAGTAGGCGCGGTGTGGTGGATTGCTGAATCAAGCAAGCTTCCTCCATCTGTAGTGACCCTGGAGTACACGAGGCGGGCCAGGTACATCTTGGTCAGTGACGTTTCCTCACGTCATTTATATAACAGATTCTTTTAGCTAGCATCCAGTTGTCCTTGTGGATTTACTTTAGGCCTTGTATGTCCCCTAACTGCACCTCTATGTGCTTGAGAAAGAAGGGCAGCCTCAAGTTGTTCCTGACTATCACTCTACATTTCACCAGTAACCAGAATAGCAGTTGCACACTAGGCTATCCGTTCCGGCAGGTTCGCCACATGCCTTGGCGGTGTTTCCCCTGTTTCAACTCCGGGAACCAGTACACGCGGAATGTTAATGTGTTCAAGGCGCCCAAAAGTGAATTAAGCAAGTAATTAATAGCGCCCAGCGAAATGGCTGTTGCGTACATACAAGTATCAATGTCCGGCCCAGGCTTAAGCCAAGCATGTAGACTAAACGATCCAACGACTGCTTGGCAATGGCAGGATCCTCTAAATAATTAAGTTCTCTTAGACACGAGCCTTGACTAAATGCCCTCAGCGCGTGTGCCAAATCCATACCAGAATTCAATTTCTTCAAATTGCCAACTATTGACTAAATTCTCAGAATTTTATCCGTGAAAATATTTAAATCGTCCTAGAGTATTTCTCTGCACTTTGAAACTCAAAGGTTCTTAGCAGATGCTTTGATGCAGACTCATCTGAATTGCACATCAGCAGCACCATCACGACATTTTTGATGATGTTGGCATTTGAGCACTAATCAATGCCGTAATGATTACAAGCGAGGTCCGTAATCTGGCATCCTGATAGCGTTAAAATTCTGTGAATACAGTGGCTGATTCTATTTCCTTAGACAAAGGGCTCATCCAATTCAACATAAATTAAAATTCTCCGAGCTCTACTTGATTGCATTCAGAAAAAAGGCGTTTGGCAAATCTGCCTGATTCATTTATGTTTACGGTAAAACGACTTTGAAAGACCCCTGAGTCATGGCCCATGATACACAGCAATATCACAATTTCACTTTCCTGTAGCCCAACACTTATTTAAACTGTAAAGCCACTATGCGCTTCTGCTTCGTTCTGATCATTTTCCTAGCTGGAATTTGTAAGTGTCTCTTGCTCAATTGGAGTGGCAATACGTTAAAGGAACGAAGCCACACCAGCACCAATTCTCTCCCCAGTCCACCTTTCATCTCAACAATTAAGACCCCAAAAAGGTTCTTAAGATCCTACGACGCACCTAAGCAAGACAACATCGGTCATGATACGGACGAAAGAGCTGGAATTTCTGGGATAGCCATGATTGACGATCTTGCGTACAAGTGGGCGTTAAAGAATACGAGGGATCCAATGGATGCATTCCAGCGCTTACATGTTGTGAAAACTGGCGGCAAATTGGAAGGCAACAAGGAATTCATTCGGTGGCTCCAGTACGTAAATCGATACAAGGCGACACGACGAGTCAAGTTCGGTGAGGATGAGCTGCTCAGCCTGCTAATGAAACGAGAGCAGAAGAAGAACTCGTGTCCCTGTTCCAATCACTTCGACAATACCCGGACATTACAAAGATGGCTAACGATATGCAAGCGTCCATGATTTTGAGCTCTGCGTCTAGTCACAGACTGATCAATGAGGCATGGTTAATGTCCCGAGAAACTCCCGGCGAAGTTTTCAAAATCTTGCGACTTGGAGATAACAGCATCAGTCGGCTAGACAATAACCCCCTCTTTATTCAGTGGCTCAGATATGTTACGATGTACAGGGCTGTACACGGAGGTGACCCATTTGCGAATTTGGAAGCACTATTTAAGCGTTTCTCGACTGTACCACAATTTGGAACTCTCATTCAATCGTTGCAGAACATCCCAGATTTGGAGAAACTCGCACTAAGCTTACAGACCCACCTCTATCGGAAATGGATGATCGAGATCCAGCTTACCCCATCTGAGCTCTTGGGTCTTCTAGAGACAACCAGAGTCGCGAGAAGTGATTTTAAATACCGCAATTTGGAAGCTTACACCATGTACTTCGCTGAAAGCCGAGGTGGTACGCCTTTGTTGAATAAATTGAAAACGCTGTTCACGGATGGCGATCCCTACGCAGCACTGTCTGCCGCTTCGAGCGCCTAGCAAGGAACTTCACCCTTTCATTTAATACAACGTTTAAAAAGATACAGTCATTTTGCTTAAATTAATGATTCTTAGACGGAATTCGTCGGACATCCGTCAGTCATCGCTATTTTCATGAAGTTTGATAGACTACCTAGAGAACGTCATACAGTCCGTGTTGAGTTTGGCAAATGCTTGCCGTGTCTTGCTGGCATTATTCATTTTCACAAATGGTTCTTAAAAGTTGACACAACAGTGCTCGCACATTGAAAAGCTATGACAGACAACTCAATATACACGTACGAGACATCTAAGATATCAGTATGCTATTTGATATCATAGCTAAGCACACTCAATCTGATAGTTGCTCTTCATAGCGGTAATGTGCGCCGATGACAGCGCGGCGCGCTGACACTGCTCGCCACTGAGCCATTTAGGCCATGGAGGACGACTGCCGGAAGGCGTCCACAAGCTAAGGGAAAAACAGTTTTTTTTTTTCAGAAAATGTCAGACAGAATGTCCGACGCGAACCAATCAGATGGGTACGATGTCCGACAGATTTTGAGGAATGCTCATGAGAACCAGCCTGCTGAAATATAGGATAAATCACGCATATAGGAGATAATTTGGGTGAAGGAATTACAATTGAGTTGCCGTGTAGCCTTTTACATCCAGAATATGAATTTAAACGCATTTACTAAAAGAAGTTTATACCCCTACTATGAGCTGAAAAGCTGGCTCTGGCGGGGCGTCACTCGTCCGTTAAGTTGTATTATCAACCTCAACATGTGATGCAGGCTCAATGCAAGGCGACCCAATTGTCTACGTAGATTTGCGGCACGTGTATCGCGCAATCCTAAACACTTTCAGCGTAAGACGATTTATAGTTCTACACTCAGTCTTCTATTTTAAAAGAGGTCAGTTTACGAAGGGCGCGGATGGTATTCGAGTGCACGTTGAGGGGTTTTCGAGTGCAGACATTTTTGCCACGAAATGGCGGAATGCCGAGTGCAGATAACACATGGCGATATCGTCGGCTCTATACTTGTCTTGTCCGGTTTATGCTTGTTTGAATTTACTACACTGTCCTTGGGGCCGGATGCAGAGCTCACCAGGATGTAAGAGGAGAAAAATACATAAATTTGTGAGAAGAAAACATGAATGAGCCTTGGAACCTGGTTTACTTCCTACACAAGGTACGCTGAGCTCCGCTAGTCCAGTTCTACGCCCACATGCTCAATGCCAACTGTCACTCCTGATCGTCTTAGCTGTCCAAGTACTGATCGTCGTCGGCGTCACGGTCGACACTGCGGGGATGCGATTCGTGTGCGCTTGCGTGGGCGGCAGCACTCTTCGTAACATCGAGGCCAGTACGTCTTGATTCTACGATCAGCTGCACCAATTCCTGGCACTCTAGGAAGACTTGCTTGCCTCCCTCGAAGTCGACAAGCCATCTATATCGCGACTTGCGCGCTTTGCATCGTTCATGAACCGAGATGCGTTGGTACCGTCCTCCGGTCGATGGACGACTCGGGCTATGCTCTCCCGTGGAGCTCTCCACCATTGCCCTGAACACAATGAGAATTGCACACGGTAGGATAGATGAGTATGAAGTAGAAGCAACTTAGAGTATTACGGTGAACGTACGGCGAGCGTTGAATTTGCTCTTATCGTGTTGACGGAGAAGCGCTTGAACGAAAGGAACTCTTACACTGTGGGTTGGCGTAGCTGGCGAGCGAAGTTGGCGATGGAAGCACACTAACCCTAGCTCGTGGATCGCTTTGTAGACGAACGCCTGCTTATCGTTGCTGCAACATGTAGCTTACATTAGCTACGTCTAAACTAATGAAGGCAGAGCCTTGACGCATCTTCTTCCGTGCTCAAGCCTTAACCTCAGCAGTGCACTCGGTCATTCCCTCAAGTTGAACAAGGGACACTCGGTTTCACACCTTTCGAACAAAAAACTAACGGTGCACTCGATTTCCTAAAAAAAAAAGCACTCGAAAGTAATATCGTTCCGTTTACGAATGTTACCTCTTGGGAAGTATTAAGGAAATACTCATCACAAGACTTTTAAAATATAATAATGGACACCTTTTTGGAGAGGTGGAATATAAATTCTCTTCTGAATTGCACATCGACAGAACGGCAACGACACTTTTGTTGACGCTCATTTCTTAGCAAGTGAGTAATCGAAGTCCGTAATCTTCAAACTAAAAAACGTAATCAGTAATAAGAGTGGGATTTAAAGTAGCTCTTCACTATTGAGAAGAAATGTTCTTCAATGTCGTGCTGATCACATTCATCGTACGGATTTCGCTTTGCTCCTCGTTCAACTCATTGATAAGCACCAACCAGCTCAAGGGCCTGAGCCACACCAGGTCAACGTCCATCCCTGCAACGAAGAGATCGTATTCCACAACAAGGTTTCTAAGGTTCAGCGACGCATCTAAGCACGATGATATTGATGATAATAGCAAAGAGAGAGCAGGGGTTTCTGGGATAGCATGGCTTGGTGATCTGGCATCCAAGTGGGCGCTGAAGAACACGAGGAATCCGATGCAAATTTTCAAGCTTTTACGTACTGTGAAAACTGGCGGTAAGCTGGAGGGTGACAAGGAATTTGTTTGGTGGCTTCTGTACGTGAATCGATACAGAGCTAAGTTACAAGACAAGGCCTCGTTCAGTGACGACAAGCTATTTGATTTGGTGCGGAAACTGAATTCGGAAGAGTAACTGGTGTCTCTGTTTCAATCGCTTCGACATTATCCGGACATCAAGAATATCGCCGATGATATGCAGGCGTACCTGATCTTGAGCTCGGCGTCTAGTCACTGATGAATAATGAGGCATGGTTAAAATTCCGAGAAACCCCCGAAGAAGTTTTCAATATCTTGCGGCTTGAGGATGAACCTCTGTACGCTCTCGACGGTAATCCCCTGTTTATTCAGTGGCTCAGATACATTAAAGCATACAGAGCTGTGAATGGAGGCCACTCGTTCACAGACGTGCATGTGTTCGACTTTTTACATGAGTTTGCTTCTTTGCCGCGATTTGGAATATTTCTTCAGTCGTTAAAGGACATCCCAGATTTGGAGAAACTCGCAAAAAAGTTTACAAACGCAGGGTTGAGGTCGATTGGCTCACCCCATCGCAGCTCGAAAAAATATTTGGCTCACCGTATCCAATCAACTTCGCGGAACTCCCAAAGAGTGATGCCAGGTACCGCAATTTGGAAAGCTTCACGGTGTATTTCGGTGAGTACTGGGAAGGAACAGCATTATCGGATAAAGTGACAATATTGTTCGCCAAGAACGACCTATACGCTGCAATTCGAGCCGCTTCAAAAGGTTAAAGATTCCTGACTCTAGCTGGCCGAAAGTGTTAGGGATTAATAACCCATCGCAAGATTAGCGAAGTTTTCTATTGCAGTCCAACAATTGCTACGATACTTACTTCGGCGTGCGCAGCCATCTGGAGAGCCAAGTGTGAGGTTATCTAAGATATTTAACATTGGTAGGAACCCTTTTTACAGCACCAAAAAAATGAACCGCACTATTGTTTTTCTTCTGATGAAAAACATGTATCTTCGTTCCATTTTTCATTGCGTCTGTATTACACCGGATTTTAAATGGCTTCACAAGAAGCTTACAGACTTTGTGAGCTTATTGCCTAAATCCGGGATATCCTGTATCAATTGAATGGATACATGTAACCCAAAGCACGGCAAGGTCACAAAAAGCTCGTATGATGGAGTGTTGTCCGAGGAAAATTAAACCCCGTCCCGACACGAAAACACCGCAAAAAAACAGCTCTTTGCAAACGCAATTACTTTAGATATTATCAAAGTGATCCAGCATTAAAAAATGAAGTACAATTATTGCAACTGTGCAGTTGTTTACCGAATGACTGTGGCGCTTGAATTATTATTCACTTTCAGTTTGGCCAGAAATCCTTAACACCACGCTTCAATTTCCATGCTGACCATCTTCCTTTCGGGAGATGTTTCTGTTTCTCGTTTAGCTTGACAGCTCACGGACCCGGGTCACAACGGAATAGGAACAGGTATGTCGATCGACTTCTAGTAAATCTAGCTGCATGCATTACTAAAAAGCTGTCGAACGGCTGCAATTGCAACGTCAAGATCTCTGTTGTGGAACAATGATTTTACTTTATCCTGCAAAGCATTTCCACTATGTTGCTCTGCGACGTACATGGTGTAAGCTTCCACATTATCTGGAATCATTCGTCGACAGCTCGCGACATTCATGTGGCAGATTGACTGCCGTTTTCAAAATAGAAAATAGCTTCTCAGAAGTTCTCACGGCGCTTGCGTCCAGCATAACCAGCGACAATATCTTCAACGATGGCTGCGCATGACCCACGGTGAAATTGTTGGGCGCAAAAGCATCGTCGGGTACACGTAATTCCGTCAAATACCTTTTTTATACTTCAAGTAATTATGGAAACTTCTTTATCCCACACCATACAAACGTCAAGTAAAACTCAACCTCTGGCGTCATTATCAGTCTGACCATGGAGAAGGATCTTCGCGACGCATATGCAGCACTCAAGCTGCTTCGGGCAGACCTTGTGAAGACCCGCCGCGATAAACGTGCTCTAGAGGCCTCACTGACCCACCTGCAGACCCACGGGCCGCCTCCCAGCGCTGCACAAGCTCGCGAGAGTCGCGAGACGCAACAGATGCAACACGAGGACGCCAATGCGCAATTATGGCGTCTCGCTGCAATTTACGAAAGTCGATTGGCGGAAATGGAAATACAATTATTGCACAATAATTGTCAAAAGAAGGAGGCAACACCCGAAGTTGAAGACAATCAACAAGAAGTCGAGGAAGTGGAGAAGCTCGCGCTGCTTCACAAGCTCCACAGTCTGACCGCCACCGTCGAGCAGCAGACGCAGACGATGCTGGCGCAACAGGCAGCTTTTGCGCTGCAGAAGGGCGAGCTGGAGACGACGCTGGAGGACACGCAGCACCAGCTCCAGACAGAGAAGAGCAGGGCGACTGATGCGCTATTAGAACAACAAGCAGCTAAAGAGAGGTATGAGTTCTTGGAGACTCAAGTGGAGATCTTGAAGCAGGACAAGAAGACGTTGGAGGAGGAGAACTATACACTCCACAAGAACCTAGCGACACATGCCCAGACGAGTAGAATATTGCAACAACAAGTGCAGGAGAAGGACGAGGAGCTAACTCTGCACGAGAAAACTATTCAAGAACAAGAAGAGAGACAAGAACGATACATAACGATGCTGAGAGAGTTGGAGAACACGTGTGAGACGTTTAAGACCCACGATGCGGCTTCTGAGGCCAAACGAAGTGCTGAAACGAAGGAGTATGAAGCGAAACTAGCAGGTATTCAAGAAACGTACACATTCCAGGTGGCAAAGCTGGAAAGTGAGCTGAGAACGATTAAAAAACAACTGACAGCAGCGACAACTAGCCGAGCGATCGAATTGAAAGAGCACGACGTCAAGTTTCAAGCGGTTTCGGACAAATTGAGGCAACAGGAGCAACAGGCGACGACACTGCGAGACTTGGAGACTAATTTCGCGGACGTGCAGGCCAAACTCACTATAGCGGAGACCAAATTGGCTGATGGAGTGAAGCAATACGAGCAACAACTTGCTCAGGCCTCGCAGAAGCTACTTGATCAAGAACAAGACCACGAACAGCACGTTAGTTCTCTACGTGACGTGGAAAACGAGCTGGCACGAGTTCAGACGCAACTCACGGATACGGAGGCCAAGTTACAGCTAAACGTGACCATATTCGACGAAAAACTCGCGCAGACTTGCCAACTTGCTGCTGAACATGAAAAGACAGCTCGAGTGCTAGCTATGGAGAAGAAGGCGCTTCAACAAGACGTTCGTGAAGCTCGACAGACATCGAAAGTCAAGACCGAAGAGCTTCTTCATTTCCAGTCGATGATGAAGCAGAAAACTGGGGACCATTCGCAGCGTTTAAGCCAGTATCAAGCGCGTTGTGAGCGTCTAGAGACTCAACTATTATCATCTGAAGACCACAAGGTGTACGAAAGCAATCCAAGCGGTGAGTTGAGAGCTTTCCATGGTCTTAACGACGCCGAAATCATCGCGCTGCCCTCTTCCAAGGCGTGGGTTATATTACACTCTGCAATAACCAAGCTGGAAGACTTCTTCCCATATCTTGAAGCACTGAGTAGTGCATTACAAGACGTGTTAGCCTTGTGCAAGAGCCATGCGACTTTTCTTCCAACGCTATGTGAGCGTCTCGAAGACAAGACAGTTAGCGACAAGACCCAGCCGGTCTTGGTTATGGCGTTAAAGCTGGTACGCTTTGCTGTCGTGTTGAAGACTCAGGTCCAGCAAGACGACGCTGTTGTGACTTTAAAAGCAGTTCAAGGTTTCCGTAAACGCGTGCTGGATGCTCTCGCGCAATGGTACGAGTGTGGCGTGGATGCTTGCGACCAAAGTGGAAATGGGTCCATGCCCACACCCACTTTTACTACCACTTCTCGAGAGACAGCACTCATTTTACAGAATTGGACCAGTGACCGGACAAAGCAGTTGGGAGTGAGACGCTGGTTGGCACGGATGGAAGCGTATCCTGGCGTCCCGCCACTTCGAGGAGCGTCGTCAAACCGTGTTCTGGAACTCCCTGCGGAGGGTTGTACACTGGAGCTGGAAGATATGACGCCGGAGGTGAAAGATGCGTTTCTGTTGCTCCTGATACCGATCTTGAAGCAGAACCGAGCGCTACATGTGCGTGTGTTTACACGTTATACTGAAAACCGCGGAAGCACATCAAGTTGTGATGGTGGTACTGGGAAAGTGTGGGCCATGCGGATCCATGTCCAGAGTGCTGTCACTCTACAAACAGGAAGTCTCGCCCAACTTCTTTCAAACTGACGAGCACTGATGACTCACCCAGGCCACTAGCACC

>Contig_41

TTGAGGGAATGATTTGCGCAAAGTTGTTGGGACAGTACAGTATGGAGTGAGGACAAAAGTAGAGTGGTGTAGGCGGCGATAGTGGAGAAGAAGTCAAGGAGAAGACGGTAAAAGGAAAAGTGATGAATTAGAGAGTCTGTTGGTCTGGAGAGTAGTTATTCTCCAACAACCAGTCCAGTTATATATATGTCTCTTTATGAAATAGTACAGCTATAAAGACAGTGGTGACGTGGTGTAAACGCCATCCTTGTTTAAATCTAAAGAGCCAGGGTGTGGATACATGCACCGTAAAACTGTAAATAGTGTAGGGAAACAAACTTTATTGCCACGACCTTTACATTATACACTGGAAATGTCTTATAGCATTGCTGTAGGTCTATATCCAATCTTACGGAGAAAACGATAATTGAATAGCCCATGCCAGCGTGAACATGGGCGTTTCAGCATCATTTTTGTTGGAGTAAGCGTCTCCGATGTGATGTTGACTATCTAGTGGAATATTTTACTTTTTAWTTTTGTATCATTATGCGTTGACATGTGAATACCGGTAGAGATAATTCACTTTGAAGTGCGCAGGGGTTATACCAGCGTGTTACTAGGTCGTGGAAGTAACACACAACTGGGGTAGCTGGCACTCCCTAAAGCTTCTGCCGAGTGTATTGCTCTCCACTAAGCAAGTTCCACACCTTTGCCCGTCCAAACAGAGAATAATTTCAAGCAAAGTGGACAATTTGGTTTTTATAGTACAAATTTTAACTTTAGTAAAAGTAATCAACAAACAAACAGAGAAAGATCTCATCCAAAGCGAGACAAGGAAAGGCGATGGCTGGAAATCATTCGTGAAGGGGTGGTGAGGGTGCGTTGGCGCCAAAATGCGCGCGAAAACCTCGCCTGAAGCAAACCATCGCCTGAAGCAGTTCACTTTTCAATTCACCTCTTCAGACGAATGCCCTTCCAACCACTCCTCCGCAAGCACTCCCCCGCTGAGAAGCTCCGTGTCCTAGCTGCCCATCGCGCGGGACGCGCTGACTGGCTTCAGGTGGCCGCCAATAACGGCATATCGCGTGCGGTGGCATACCGCACTGTAGCCACAGAACGCGTAGAAGATCTACCGCGCGGTGGAGCTCGTGGTAGAGCTGTGAAGATGACACCAGAAGCAAAGTCCAAGCTGGAGGAATATCTTGACGACAATTGCACCTTCACCCTGGAAGCTATGCGCACGATGCTCTTTTTAGATACGAACGTGCGGGTCTCCACTTCAACGATCAGCCGCCACTTGCTTGGCATGCTGTTCACCGTCAAACAAACTCGCATAGAGCCCATGACATGCAACAACGAGATCAACAAGACCAAGCGGCAGAAGTTTGCAAAATCGCTGAAGGACCATCAAAAGAACGGTGACTGTATAATCTATTTTGATGAGACTAACTACAACGTTTACTGTACGCGAGGTCGAGGACGTGCACGGAGAGGTGAGCGTGCAACGCTCGTGATGCCGCCGTCCAAGGGTGCAAACTTGCAAGTCCAGTGCGCTGTAAGCTCGGCCATGGGTGTGGTTCTCCACCGTCTGGAGAGGGGAAGCATCCGAATGGAGCAGAATGCAGCTTTCATTGAGGAAATCTACCGGACGGTTAAGGCATCCTCCGTGTTTCATGACAGCTTCGCTGGGAAGAAGGTCATTATTGTCCTGGATAATGCCCCCGCTCACCGCCAAACCGAAGAACGTGTCGAAGCACGTGACGACCTCGTACTCCTACGACTCGGACCCTACTCACCGATGTGCAACCCGATTGAGGGTTGCTTCTCCGTTTTAAAAGCTCGAATCAAGGGATACCTGGCCTTGTACCGCGAAGAGATCTGCGACCGGAGTAACATGGTGGAAGAAGACGGCACCCCAATAACGATCAAGGAGCGCACTATGCGCTTCTTAGAGAAGGCTGCAGAATCGAGTATGAAGTATATAACACCTACGTTAGTGACGAAGATGGAGCTTCACGCTCGCGATGCGGTGAACGCGGCCGAGGAGATGAAAGACATGGTCTATGGGAAGTAGCTCACTGTGTCGTCTCGGTTTGAATGAGATTATTCTCTGATTCACTGGTAATTTTGATCCCGTAATCAGATATTTGTAATAATCAAAAACATATTGTCCACTTTGCTTGACATTATTCTCTGTTTGGACGGGACTGACTAAATGACTCTTTGTCTGTACTCTATGTATACATACTATGTATACTTGGCAATACATACCCTGTATATATTGCCAGCTCGGGCCGGCAATACTGAACTTCAATCATCACTCGATTGGTAGGCAGCAGCAACCCTGCCACTGCCAATCCGTAGATCAGTACTGCCGCCCACTGTAGTGGCTGGATGAGGCTACATGCCCCCAGCCCACTACAGACCCTCTTCTTAAAGACTCATCCAGCCACGTGGTATGATCTTCTCCTTGGTCTTGCACTTTCATGTCCACGCACTCGCGCTTCTAGACACTGCCATGCTCGTGACCTCTAATGGCAGGCACACACGCACCTCCACAACATCCCCAGGGTGAGATGCTCAGGTGTCAGGAACTCTCTGACACCACGTACCTTGGAATTGACGCCTCCGAGCTCGATGATCAAATGCCTGGCACACCCGCGCCCACAAGCACTGACACGACCCGACCTTGGCATCCACCCGGGCATGACGCTCCAGCATCCAGCACACTCGCGCCTTCCCGACACTGTTTCCTCGACGGCACCCACGATGTTCCGGTGTCGGCACACTCGCGCTTCTCAACCGTCACGCCTCAGATGCCTGCCGATTCTTGCGCTTCCCGGGGCTTGTTGTTTGAGTCCCGACGAGCGCGTTGGACGCCTTCACGTACTTGACCTTGGCATCCCCGTCCTCGGCTTCCAACGCTCCAGCGTCCTTGTACTCACGCCGTCGGACTCACGTTCTTGGGTCCAAGACGATCGCGCCCTCCGACACCTTAGACACCTCGACGCACTCGGACTCAGCATCCTCAGTGTCCGGAACGCTCACGCCCTCCGACCCCATGACTTTGGCACCACCTTCGTCGTCCGCCACACGCCATCTTGGATCTTGAACCTCTTCACTGACCCGGGCACTAAAGTTAACTTTGTAGTAGGTCGTGGAAGTAACACACAACTGGGGGAGCTGGCACTCCCTAAAGCTTCTGCCTAGTGTATTGCTCTCCACTAAGCAAGTTCTAGACTGACTAACTGACTCTTTGTCTGTACTCTGTGTATACATACTATGTATACTTGGCATACCCTGTATATATTGCCAGCTCGGGCCGGCAATACTGAACTTCAATCATCACTCGATGGTTGGCAGCAGCAACCCTGCCACTGCCAATCCGAAGATCAGTACTGCCGCCCACTGTAGTGGCTGATGCCCCCAGCCCACTCTACACAGCGTAATGATTTGATGGACGAGAGTACCGGTATTTAAATTAAGCCGAAGCCTCAGTGAGCTTTTCATTCAGCAATTTGCTACGAAGCCCTCACTTTTCGTTTTTGTCGGTGCGATGCCCCTCAAACTTGCCTTAACACCACGCTTAAGCTACTTCATCTTGCTGGCGGCCCTCGCTCTGCTGGTATGCATCCACAGTGGATCTAAGGTGGCTACGGCATCCAGCGTAATCGACGCCAGAAATGATGCTTTGGATCGCGAGGTGGGCGGTCAGATCCACCGATACTTGCGTGAGAGCAAGTGGACAGCTGAACAGGAAGACGGCAGCCCCGAGGCGAGGGCGATTGGAGTTAAGCTGTCAGCTGCTACGGTGCTTCAAAAGCTGATTAATTCGCAGCCAACGAAAAAACTTTCAACAAGAGCCATGGAAATTCTTACATCCAACAATCAAAGAGCTACCGACAAACTGTTTGCGAGTCTCGGGGTCTGGGAGGTGGAATCGAAGCTGTTTGCGTCTACTCCGTATCAGAAGTGGGCTGCATCCGTGATGAAATCCTACAAGAAGAACCCTGAAAAAGGCCAAGCAGCGATATTCTCTACACTTGTACGTCACCACGGCGATGACATTCTGGCCAAACTGGTATCAGAAGCACAGCCAACCGCAGCGGCGATAAGGACAGCCAAGAAAATTGAATCGATGCAGATAGCCAATTGGGTCACAAACAGAAAAACGGAGGAGGACATCTACAAGCTTTTAAAGCTGGACGCGGACAAGGAGGGTCTTCTGAGAAATCCGTTGTTGAATACGTGGGTTTCGTTTGTCAAAAAGCTTGATAACGAGGATCCGTACAATTTGTTGCTGCTCAAGTTGACAAAAACCAATGATGAAGAAGCACTAGCTCACATGCTAATTGCAGCAAAGGGCGATAGCCTTACAAGTAGTGTGGTTCGTAATCTGGAGAATGCACTGCTTACAGCCTGGCTGAGGGATGGTAAAACGACAGGTGACGTCGCCAAGCTTCTCCGACTAAACGCAGACAAGGGAGAAAGTTTTTTGCGGAGCGCGGCTCTGCCCACGTGGTTGTCGTACATTCGACAACGCAACAAAGATCCCAACAAAGTTTTATTCTTGGAGCTGCAGAATCGATTTAGCGACGCAGAGTTAGCCAGAGTGTTAGTCGCGGCGTCGAGGGACAAAAACGTGAAGATCAATGTTACTGCTCTACAAAAGTTACAGCTCAGTAAATGGCATGGAAGGGGGGACACGGCAGATGACATTTTCAGACATCTTGGACTAAGCAAGGAGGGTGAAGAACTCCTAGAGAGCTCAGTGTTTAATACTTGGGTGTCCTACGTGAAGTTAGTTGACTATACAAATACTGATGCCTTGGTGTTCTCAGTGATGAAGAAGCACTACAGCGACGAGATCCTCGCAAAAATGATTGCTCAAGCAAAAACAAGTGTATTAACGAGAGCCCTGGCTTCAAAATTAGCGGCGGAAATGTGGCGGAGCCCAGGGAAATCTGCGGATGATATTTTTAAGTTCTTTCAACTTGACAAAACAGGTGATGACCTGTTCGAGGCCCCGATGTTTGACGCTTGGATCTTGTATGTTGAAAGACTCAACAAGTACGAGAAACATCCAGATAAATTCGCTCTTTTTACGGAGCTCGAAAAACGCTTCGATTACGTGGACCTCGCACGAATGCTTAGCCATGCTAAGATCCAAGCCGAGATGAAAGGACATTCTGTAGAGCGTCTTTTCAGTTTGCGGAACCAGCAGTTCGATCAGTGGATGAACCAAAAGAGGTTGGATCCAGGAAGAGTCGCTGCACTGGTAGCCGAGCAACCGCGTGACATAAGAAACAACGGCGTCGTCCTCGGCTTCTATGACTTCTACAAGGCTAATGGTGGATCGCCGTTGTACTAATAGGGCGCCGAGGAGTTTATGGCTTAGTAGAGGTAGAACACTTCTAACAATGTTACACCTACTCCTCGAATTAGGAGCTTTAACTGTTTATCCTGGCAGTGTATTCGCACACGGATCATAAACACCAGATACAGCTCTATTAATACCTTTATCATGGGTCGGGGTTATTAATAAAGCCCGCGTGCTTCGCACATGGAGCGTAAACACAGGTAGCGTCCTATAATACCACGTGTATAAATCCTTGAGCACCCTGTAAACTAAGCTCAAAACCTCAGAGTGCGAACCCTGGCCCCTCTACCTCCCGCGGGGCTTCGGCGGCGTTGCTGCTATGGTTTTCTCTTGCAAACAAAGTCGCGCCTTCGGCCTGGATTTGTTTAAGGTTTGCTAAAGCTACATGTAGACTTACAAGGCTAAGAAAATGAACTAAGGAAATCATATCAACGAGCACACAGTAAATCTATTTTTGAAGCTGAAAAGAATTTATACTTTTTTAACGCTTGTCACTTTTTTTAAAGAGAATACCTTAATAAAAAGTATTCTCTGAGATTTATTTCAGAGTTTTACAACAATATGATTTACCTCTCTAATATGACGGTTTCTGGTTTTGATCGATTGCTCGAGAACTTCCAAAACCTGTATAAAACGGTTTTATCACAAAGTTTTGATGACCCCTTCTTTAATCACACTTTTATCACGGTGATGCGTTTTTATACCGGTTTTATCAAAACCGTTCTGCCAAAACCCTACATTAAAATTTATTTCGTTCCGAATAGAGTGAGGTTGATGTATCTAACATATTGATTCATTATTAACGGACGATCTTTACTAATTTAAATGGGACAAAAAACTGATAAATTTGTCTATTTTTTAAGATACCACCACGATGGCGCGCTGCCAGCGACCCCTTCCCTGCAACTCACGGCTTTCTCTCGCCTGATTCAAACGAGAGAGGGGCCCATGCGCGGCGCCAGCGTTCTTCTCGCGCTATGCGCGACGTCGCAAACGCGACACAATCTCGAAAAGTGTGTTAAACGGCTTCGTAAACGGCTGCATGAAGAGAGGAGGCGTGTAGAGTGGGAACGGTTGCTGCTCATGCGGTATTACGTGACCCTCGACTGTCTAAAACACCCCGAATAGTCGAATTGGATGGATTTTTGGCGTCGGGGCACCGAGTAGAATATGCTCAACAAAACTAGCCTATCAAGGTAGTGACTTACGACGATGTTTGTATGTGCCAGAGCAATGATTTGTTTTAACCTGTTATTTTTTTTTATTTATTTATGTGTGCCAGGGGCGCGTTTCAGCAGCTCTTGGAGCGATTCTCCCAATTCTACATCATTCCAGTATACAATCCAGCAGGCGGTCGGCCACGGCGGCTCCAGCACCACCACCAGGTGTCGAGCGTCGTTGTTCTTGTGCTCACCCTCAACGGTAGCACTGCGCGAGTAAGGGCTTCTCTTGTCGATCATAGTAACATTGACGGATAAACCAGGGTCTTCTCGTGTTCTCCGAACCCCGGCAACTCACCGCCCTCGTGTTGATCAGCAGGACGCGCCTCTGCCTTCTCTTCTCGGAGTTTGCTGCCAGGACAGGAATCTTTATCACCACATCTACCGAATTCCTCGATGAGTTGGCCCCCCGTGGTACATCAGCAAAGGGGGCCAACTCATCGATGAAGACCCTTCAGACACACATGCATATCATTGGTTAAAACTGCACGGATCTTCTGACCAGTGTCGGCTGCAAGTCTGTGATTGGACGTTAATCGGGTNGGGGGGGGCCAACTCATTGAGGAAGTCTCTTCAAATGTTGCCCATCATTTAGTATAGGAAATCCGGGTGGGGGGGCAACTCTTCGGTGAAATTTGAGGGGGCCAACTCATCAAAGAATTCGGTAGTGATCTCGCTCGGCCTGAAGACCAAATAGTACGGAAAAATAATGTATCAGTATAAAAGAAACGCACAGCAATACGCACTCACCGGCCTGTAGTGAAGCCTCGTCACAGCTGCTTCATAAGGTTAGCACATATGATGGATGATCCAGATCTACGATGGTACGTACCGCAACCATGCAACGCCGACGTCTCGACGAGCCTTATGCGGCAGGCAGCAAATTCGTAAATGAAACGATCCTTAGTGGGCGCGAATTTTCGCGCCTTTGAAGCTGTCGAAGATCTCATCTTCAATTTCAAAATTTTTGCT

>Contig_45

ACAATCGAGTTTTCACTCGTCGGATTTGCAGTAGTCAACATGTGTACTAGATCATCGTCGCCGTAGTGAGACCCAAACGTGTTGAACATCGCATCGTCAGCAGAGGACTGACTATTGTTCTAGATTTTGAGTACATGACGAGCCCATTCATCGAATGCTTCACTCAAGAAGCAATCGGACTTTGCTTTATCAAGCCTCAACTTCGGAAAGCTTGGAGTAGCCATGGTGAGCTGGTTATCCGTTTTGAAGATTGATTTCGCGCAGTCTCGTAGCTTCTGAAAGCAAATCTTTGGAACCAGCTCTGTCATCTTCGTAATACTGGGACTAACTCTATCTTCTGAGGCATTACTGTTGGCGCCCTAGTGCGCCGATGGTACGAACGTGATTCACACTGGGAGCCATGAGTCCAGCACTAGCCAACATCAAAGCCGAACCTAAATAGAGCAGAAACGAAATTGTTACCAAGAGCATTCCACGACGGAGCTCCATTTTGCGTTTGAAAGCGTTATTATTTGTGTACTTGAAAGAAAACTGCGAAGTGGGCTCTTTTGAAGTTGAATAGGACAGCTTCATCTGTAAAGGTTGCTCTTAACCATTGAAGGGCATATCTTGCGGCTGCTTCCTGAGTGTTTATGCGGCTGCCTTTGTTGATGACGTTCGAGGATGCGATACCAATTGCTAATGGAATCGGAGCTGGTACGTTGTTTCTATCGTATGATGGTACCGATGGATGCGCTAAACATCAGACATTTGTTCTCTGACTGTTCGGTTTGCTTCGATGTGCATTCTTTCTTTAACCGGTTTTAAATTTTTTACTATTATGTGCGTCAAGAAATCAGACGTCGAGAGAGAGAGAGAGCTGATGGTGAAGATTAATAAGTCAAGTAGTACGCAATAAAGCTGTACCGTGGTAAGCCTCAATGGCTCCTTTTTCCTGTACATGTATCTCTCGAATCTTCTCGCCACAAGTTGGCGTAAGCTACCATACTTCGAAAAGAATTAATAATACTTACGAAAGCGATAGAGCTCCTTCCAATTTGCAACGTGTCTGGGTCGCCGGTCAAAGCGCTATAAGCATGCCATAGCGACTAATCATTGCCACTGACACAGGTTGCTGGGAGGGGGGGGATCTGACACCTCCATATCCTTTAATATAGAGACGTTTTACAGTTATTTTGTTCATTTTAAATGTGTCCTTATCCACGGTGAATATGAAAACGTAGCATTTTGTCAGCTTAAAGGTAGGGACAGACTAGATTCTTATCTGTGGGTTTGGCAAGACGTTTTTAAAGTGGGCCTGACAAAAACTGGCTTATAAGGTGATTAAAAAGATGTGTGATTTTACCGTTTTATGTAGGGTTAGGAAGGCACCCTGTTGCCTATTAATCTAGCCAATCTAATTTCGTTAATATCCAAATAATGGTAGATCATTTCGGCTCGATTAATACATGTATCTTAAGATTTCTTGCTAAATATTCGAAAAAATCATGAGGTACATTTCATTACCTGGTATATGTGTTTTTGGCCAATTTTACAAGGGCTAGGTTTAAAGGTACGTTTTAGCTTATTTTGTAAACTTATGCAAAGATTCTCTGAGTTGACACGAGCTTCGATTAGAGTGAAGTCCGTTCAAGTGTTTCAATCACGTTGCTACTAAAAAATCTGATATTACTTGTTTGATGAAATTATCATGCGATTGAGATCTTTTTTTGAAAGGCCCAGCAAAAACAGAGCAGAACATGACCAATGAATAATCAATCGTCTGAGTGTACAGTAAAACTTATGTGCAAACGGCCGTTCGACAGTTAAGAACTACCAAGCAAAAACAAGTATTTTGTGTACTCTGATTGTTAATTGAAAACGCCACTTTTGGGCGGTGTTCATTGGCTAGAAATTAGTCTAGGATAAATTATGAGCGGTCCGAATGCACATAAGTTTTACGGTATCTGAATGATTAGAGCTATGTTCATTTATTTTGCTTATTCGTTTTAAGACTTAAACAAATGTTGGATAAAATACTGTATTATAGTACAGTAAAATACAACAAAAACTGTAGCCAGAAGATAGGGGTCTGGTAGTTCTTCGCAGAAATTCTGACTGTACATGTATCTATATTGCATGTATGCCCCAATCCTATTAGTAGTGATACGTTTCGATTCAAACACAAGTTGCTAAAAATCAAGATCAGTGGTTTATTCAATTTTAAGAAAACATAGCGGATATTCTAGGATCGAAGAAATTTGGGGTGTGCTTCCTACCTCTAAATCTACCAGCTACTGTCGACCCATGCGCAGAATGTCGAATATCCCTTCACCGAAAAAAGTATTGAAATCGCTCATAACTCTGTCGCTTTCAGAGTTGCGATCACTTGGACTTAGCATGCTGCTAAAGGTTTTGGGGTTCTTTTTTTCACCCTCCCACTTAGAGAATTGGCTCCGCTGTAAATCACGAAGAAACCGTTTCGTAGGTTCGTCAGTCGCAGTCCGCTTTGAAGCTGCAAGCATCTGAGCCAAACGAACTTCGCCGAAATGTTTCTCCATCTTTGTGATGACTGCTTTTTCATTGTATTGTTTCATCGTCTTCATGTAGGTGTACCACGTTTTGAAAGCGACGCTTTCAACTACGTCGCTTTTCTTAACGTCCAGTTTCAAGAGATCAAAAACATCGTCTGCGCTTCGACCGCTGGCTCCCCAAACCTGCAGCTCCAATTTCGTTGCGGTTGCCTTGGTTCTACCGACCGTCTTGGCTCTGGTAATAATCTTCGTCAATTGCTCCTCACCAAGTTGTGTCTTTAGTACCGAATACATTCTATTGTCAGCGTCCCCTTTCAAGTTTTTCAGATATGCGACCCAAGTTCTCCACAGCGGATCTTCAAGCACTTGGTCTCCTGCCTTGTTAAGCTCCAGGAGCTTGAACAGCTCTTCGTCACTTTTTCCGGCCTTGAGCCAACTCTCGATCTGTGTTCTCTGCATGTTCTGGACAATGATTATCGAGTTTTGATCCGACTTCGCAGAAGCAATCACCCTTGCCACGGAAGCGTCAGTGTGGTGTGCCTTAAACTTCAGTAGTAGCAGCTCATAAGGATTCTGCTTGAGCGAGTAGACGTAGGAGATCCAAATCGGGAGCGTTGGATTCTTCAGAAGAGAAGCTCCTTCCTCCTTTAACTTGAGAATATTCGAAGCGTCCGTGGCGGTTTTCCCACTGCTTTGCCATTCTCGAACAGTTTATTCGTCATGTCAGTCCTGATAAACGACACGACCTTGTCATCTTTAGCAGTAATAATCATCTTCGCTAGTCCCTCATCATCGTAGCGTGCAGTCAGCTGAATAGCTCTTCGTACGGAGATTTACCTCCGACCCTGTGGCCGTAAGAAATCCAAGTGCTCAGCACGGGGCTCTTCAGTAGATTCACCTCGTCAATATCTAGTTTCAGAAGCTTGAACACGTCGTCTGTAGTCCGTGCTTCAGTCTGCCATTTAGTCAGCAATGAGGACATAAGTTTATTCTCCAAGCTAGGATCAATCGTCTGGGAGCTCTTTGCAGTAATCACCATGCTCGCGAGAACATCATCACCGTAGCGAGACGAAAGAGTCGAGACCATGGCAAAGTCTCCAGCCTCGGCGTTTTTCTTATACGACTTAGCCACAGACGCGGCCTCATTGTTTGAACTGCGAGCTCTCAAAGATGTTCTGCTCTACTTTGCCGACCTTTAATTTCTTAAACAGCTTGTCCGTGGCCTGCTGGTTGTTCGAAGTCATCTTGGTMTTTGCGTTGCTCGCCAGCTTTTGCAGCTTCTGAGTTCCAGTTGTAGCTACATCTTTAAGCTTCGTGAGACCTAGAGCATCGAATGCGCGATCTTCGGTGGTGAGGTCTGGTGCTCTCAAATACCGTCTGACACGGGTGTCGAAGCTGTTGGCAGATATTTTCATACTGTTCAACTCGGAGGGAATGGATCCGGCACTGACACACACCAGTAGTGCAACCAATGTCAACACAACGGCGTGGTAGCTCTGGTTGGCTCTAGGATTCATTCTTGAAGTGGTCGAAAGTGTGCTCTGGAGTTGATCGTGAAACGGAAAGGGGGAGTTGAAAGCATGATAATCATCATGCGGGGATTTACAGTAGGCAATAAGAGACACAGCAACTTTTATTAACCCCTTCAAACCGGCTGATTTTTCCTTTCCTTAAATTGCATATGCAAGATAGTTTGACTTGGTGTTTGTATACTTTTACTTGTTACAGAGATGGTCGACGAACCGAGCGTTGTAATAACCGGTGCAAACTGCATATTAACGTGCATACACGGCTGCAAGTTTAGGTTCTAAACTGCATTATTCATTCCATTTCCGTAACATAGATGCTGACTTCCGGTCGTCTGGTAGTACAGCGATAGGCGGGTGCAGAAACGGTTGCAAGTTTAGAGATATTTGAATGTCGAATTTTAGAAACTGTCCAATTGCGTACATATATTGAAGTTTTCCCATCCAAGTGGCGAGCAATCAAAAGTTCAGTTATTATGGCTCATACAAATATGAAGCCTATAATATAATCTGAGCATGGGCTTCCGGCTCTGGCCTCACGGACTGCATAGTAAGCAAAATGAATCGACGGTTGCACTTTATTCTCAACCTGCAAGAGATATAGAAAGACAATTTTCACTGCAGCCATTACTTCGCAAAAACACAAATACTGATGATAGAATGCATATTCACCCTTTGGTATCCTGCATATGTATGATACTTAATCAGCAGCATCTCTCAGTCTTTTGCGTGATCCACCACCTTTCGTTGGAGTTGCTGGGCGCATACGGATGCGGCGTTCTTAGTTCGAAGGTCGCAGCTTTCTTTCACTCCATTCCTTGTGCCACACGTCAAAGCAGGTTGTCAAGACTCCGCGAATCACATGCCTCGGGTGCATACACAGGTAGATAGGCGCATTACCATCGCACTCGGAGCAGTAGTACGCCGTCGTGTGGGGCTTCGCGCCCTGCATCAGAGAGCAAACTTTACACGCACGCTGACGCCGCTTCGGCTGCGTATCTCAGGTCCTCCATTCATCCACCAACTGTGCTACATGAGTAGAAGCGTGCCCCGCCACACCAGCATCTTGCTGAATCTGCTGCGGCTTGGAAGCATGGCTTCATCAAATGAAAGCTCTGCAGGTGGCACGTATCCTTCGCGAAACGTCCGCTGCAGCACTTCAACAACCTTGCGGATCTTCCAGGCTCGATCCGTGGTCGCTCTTGGGTCGTCGTTATCATTGAAGTGTAGATTTCTGCAGATATCCATGAATCGATCTCGTGCCATCACAGAACTGAACGCTCCACGTGGAATCGCCCCCTCGTCGGTGGTTTTCCAGTGGTTTGCCAGCTTTTCTCGGTTTGGGCAAATTGTTCGCGCGATCAGTAGTCCCACGAAGCGACACAGCTCGTGTGGTTTGATTGGAGGAACTGCTTGAAGCTCTTGTAGCACATCGCGCCGTGTCTTCTTCTTCGTGGCAGGATTCGCTTGACAGCGTCGCTTGTGGCGCTTATAGGCATCGTCAACACGAGCGTGGAGCATATCTCTTTGGTACTTGTTTGAACAGACAGCAACGTGCTGCCACAATGCAACCGGCATGAAGTAAAAGAACAACGCCATAGGCGAGTTTCCACGATGCAGTACTTCAGGAGTGGGTCCAGAATAGCCTTGGTGAAGTCCCGGGTAGCTACTAGCGCCTTCAACAGGAACGTAAGGCGTCTGTAACTCCTCATACACATCCGGTTCTTGAATTGGGCCCCATCCATTGTCGAAAACTCTCTCAGTGTTCTGTCCAGCAAGTTACCCGCCAACACAGCGTCGGTGCCTCCAAATCCATCCAGAAATTCTTTTGCCAGCGCAATATCCATCTCGTCTCGTTCAGGGTTTCGGCACCGATCCATCGGCCGCACTGGCATTTGATACCCAACCACTGCAATCAGACTGGGTATCTGTGACAACGTCGTCCCTTGCCGCTTCATCGCCAGATTCAAGTGCATCATAGTTGGGGTCTCTTCCGCCCGGGGCGTCCACATTCAACCGGTCAGTTGTTTCTGCAAGTATATCTTCGTAACACAAGCATAAAAATCGTTATCATGAATAATCAGTATAAGGTATCATGAATATGCAGCATACGCACTGAATCGCATTGTAAGACATACCCGTTTCGTTTGTCTCTTCAACAGACTCAACGCTGGAATACCCTTCGTCCCCCGATTCAACATTTTCTTCGTCACCTCCATGAGGACCTGGCAACACGATGGGGGTTTGAGCGACGACTTGAGTAACGGGTTCAGCTACTGGCACTGGCGCTTTTTCTTCATCAGATATGGCGTCGTCAACTATAGCGCATTCACAAGCGTTAGACTGCATCAATATGCATGTGGTCGATGAAATATTCAGGATAGCGTACGGGCTTCTTCGGGCGGATGTACTGCATGAGCTAATGCATAATGCTAGTTAGTTATCCTGCATATCCTGCATATCATATCATGAATATGCAACTGGTACTCACATGAGCGGCGACGTTTCTGCTTGTCCGTGGCAGCCGGCTTGGGCACTTTTCGAGTGCGTTTCACTTGCGTTTATCGCGTCAAAGGCTGCTGAGTACCTGAAGAAGGTTGAAGTGGAGACGTGGGTTGGGTGGTGGCCGCTTGAGCGGGTGGTGATCTTGTTGTGACAGCAGGATCAGCAGGAGGTGCTGGTGGCGTCTGGAATGGTCCAGAACCGGTAGACGAAGGCTTTTTGGAAGCTGTAACATCATACAAACAATTGGTTAGACTGTGTAGCCAGCAATAGCCACCATCTCGCATACCACCACTGTAGTACTCCAACAGCTCTTCCTCCCCAAGAAAAAAGTCGATTCCTTTGTGCCGTTAACATGTCCGCCAGGGCGAACGTACTTCCATCTCGGATCTAAGTCCTTAGCTCGCGGTCTTACAAATGTCCATCCCTGCTTCTTCAGCTCCTTCCACGCCACACCAAACGGGATGTTTTGACTGCTCACGTACTTGCCTCGGACAGCAACCATTTCATGCTGAGAGCCTGGCTCCGTTCTTTTCCAGCGCGAATTCTCGAATGAGTGGCTGGAGAGGGAGCGAAAAAATACCTTTGGAAAATAATGTAAAACTTATGTTTACCACATAAACTTTTATATCTTATATTCAAATTTAGAGAAATATTATCTGTCTCGTCGCTTGTTATGTGTATCTTCTATGCTACCACACGTACAGAGAGCCTGCACTTGTAGGGTTCTTTTTGACTTAGATTTTGTATAGCCGAGGATTTGAAAAAAAAGTATCTATACAAACTTTAAGTCATATTTGGGCTTACCTGCATTATGCAGGTTAAGCATTTTTGAAAAGCTGCATATGCAGGCTCCCGGTTTCAAGGGGTTAGTAAGTATTAATAGTCGGTGCACTTGGATTTGTAATCTATCAAGCGATGGGTACAGTACATGTACAATAGATGTTTTCAAAAGTTGGAAGTGGCTTGATTACGGACATGCTCTGCTAGCCATGGATCGACATCAAAGATGACTTGTGTTAGCCAGGTTGCTCTATACAGTAGATGTCACCAGACTGGTGGCTTAAGTAGCAGCTTAAATTAGAATCGAAATCAAAATTGGGCTCCCGGTGTTATTACATGTGGATATGTAATTATTAATTGTTTTGCAGAAACACTTTTTCTCTCTCTTTTTTATAGTGCTATAACAGACTTTTAAGACAGCTAAAACAGACTTTTTTTAATTTTGTTCTTAAATTAAAACGAATTTAATTATGACTCATTAACACCTTGCGTCCGGACGGTACTTACCCAGAACGCGTAGCGCATGTCGTAAGATATGGCATAGCGATGTCACTTCGTATTTGTATGAGTGCCCGTTTAGCCTGAGACTAGCTATATTAATTCGCAAAAGGATTCCTTTTTAATGTCGTAACTCTGACGGCATTGAGCGACTGTCTGACTGCTCCATGTCGTTTGTGCCACCAACATATGGTTTAATTGCTTCACCGACTGTCCACTCACTTCAAGAAATGCCAGAAAGAAGGTGCGTTGGTTCAAGAAACTGTGCATTTCAAACAAATCACTTATCAACTGACACGCAGATCACCCTCACCCGTCAGCGCTACCATCCTCGCTCTTTCCGCCGTTGCCGCTGTAGGTACCCGCCATCGCACACAAGCCGAAGTTCTCGTCG

>Contig_47

CTTTCGTAGCCCGTGCTTTTCAACGTTTTGGGGTGCCAATTGTATTCCGTCATCTGTACGCTCCCGTCATGTTCTTGCCTTTCCAAGTTGTCATGGTAGTGTGCTCGTCCGTGGATCGTGCTTATCAGAATCATTAGGTGCCTTCATTGATTTAGTTCTCTTTCATGGTTCGTGGCGTCCTTCATGTCCTTCGTTCTATCGCATCATGGTACAGTCTAGCTCGGTGCTCTCCGTGTTTCGTATTCTTATTTGATCATGCGCCTCTACGCTAATTAAATCACAAACACTGCTCCCAGTGCACTGCATATACGGCTACGTATCGTGTTGGAAAACGCGCACATTCTGTAAGGGTCGCACACTACGCGCAAATACAACGAAACACGCTCTTTTTTTCGCGGCGGAGGAGTGGTGTGTTTGGGTAGGTAGTGTGTGGAGCCATTATAGTCGTATTTGCAAGCAATTCATATTTGTAATAAAATTGTGTTTCTTAAGCGTTAGAAATAATCAATATTTTAATTTAATTTAGCACTGATTTAATAATTATTAGTACGACTACTACAAATAATTTGAAAGCAAAATATGAAAGAAATTTAGAATAGTACTACGCAAAACAATCTTGTAAATAAAATTATGAAAAACGTGCTTCAAATCTATAAATATCAAATTTGCTAGCTATACGTACTTAAATGACTATTTAAGACGAATCGTGCGTCACCAGGTCCTACAATTTTTACTACCAGCTCCTACGTCAATATAGTACGTGTATGCACGTCGGCGAAGCCTACCAATGCAAACTAACCCGCACACGCGCAGCGCCCTTTGCGTGGCCTGGCTGAAAATGGTGCAAAATGAAATTTTAACGGTGTATTTAAGTATGATGTGTAGACCACATATAATATGTACGCCACATATTGCAATTTTAGCAAAACGACGCCGCCTTTGCTACGCTCGGCAGCTATGTAACTCTAAACAGACGGACACCAGGGACCTTTTGTCAAAAGGTCCCTGATCAAAAGGCTTTTAATGCTTTTTTGATACACTGTATGTGCTGCCATTTCTGGGAGGTGCAACAGAGCTTCAGAATTTCAGGTGAAAACATATGAGGATTTTAAGAAGGTCAATCTGGCTCGGATTTGACTTTCTCTTTCCTTTACTGTACATGTACTGTACATGGACAATAAAAAAAGTACTTCACATCATGCAAAAATGACGCCAACTACTTTTGTAAATTGCACGTGTTGCTGTGCATAAGTAAATCCCCAGATAACGGCTCATCACCATCAAAATCCTCAGGAAGTCGCTTGCATCTTCAGAATCTGCTCCGCAAATGCAGTCGCCACGCTCCTGCGATAAAAAGATTCTCTTACCTGGAGAGTGCTGTGTTCGGCTCGCAGGAAAGCGGCGCTGGGCCCGCCTTTGCAAATACCGCACGGCCCCTGCTTCGCGGGCACCCCGAGCGGGTTTGCTCGCATCCGCTCTCATAAGATGGTCTTCTCCCTTTTGATTTGCACTCAATTTCACCCTGTCCTTCTCCAGCCGAGCACCATAGTCACTTGCCTTATAAAATTCTACTAGTAGATACAAATATAAATACATGAGCACTAGTACATGTAGCTCTGCTGTCATCACCTCAGTCGAGAGGGGCTTTACCGTAGTCTCCTTCACTTAAGCTTCGTAAAACACTTACAAGAGAGCCATTCGTGGTAGGATTTAAAACAGGATCTGAGTGTGCAAAAAAGTCAATGCGGCGCACATGTACGGTTCCGGTACATGTAGTTTTTTTTTAATAACCAGTGATGCAGTACAGTACTTGTACAGTGTCTCTTGCTCCCTTCATCATTTGGCGAACACCACAGAATTACTCGGATCATGAGGCTTTACTCGGCTGCGCTGCTATCTACGATTGCAGCCTTCCTGTCGTCGTCCTCGGTGGCTGGGGCGACTGCTGACTTACAAACGACGAATTGTATTCGTACACTGTCCGATGCGCCTGCTAATAGTGTGACGAAAAGGCGATTGAGAGCATCCGAGACAGGGACAAACGACAACGAAGACAGAGTGTTGAACGCTGCTATTGAAAAGCTCACGGGTCTGGCGAAGGCGGGGGCCTTGAAGATCAGTAACATGGAGTGGAAGTTTATGCTGACGGGGGAAGGTGGGGCTGATAAGATTTTAAAGTGGTTTGACCTCGATAGAGGAATGAAGAGGGCTCTGGCCAGTCCGAACTTGAAGGTTTTGGAAAGCTACGTGAGAGCAATGAACGGCAAAAACAAAATATCAGTTATCGGAATATTCTCGACGCATTATGGAGACGACCTCGTCGCAAAGTCTCTCGTGACCATGGAGAGCAAGGCTAAGACGCCAGAAGCGGTGAATACTATAAAGAATTTACGCAAGGATCAACTATCGGCCTGGATGAATAGTGARAGGTCTGTCGACGATGTTTTCAACCTGCTAAAGCTTCGTGAAGATGGCTACAAAGCTCTTGCCAGTCCGAAGATGGAGGTATTGGATGACTACATGAAAATGGTGATCCAGACTAAAGCAGGCAAGGAAACGTTGCTCCAGACCTTAACTAAGGGCTTTGATGGCGAAGAGAAGTTGGCGAGGCTGCTGGTGCGTGCGAAGGAGCATTCAAAGTCGAAGGAACTGGCGACGGCCTTGCAGAACGCACTCGTGAAGAAGTGGATCGAAGCGGACAACATGACACCGGATAGTGTCGCTCATATGCTTCAACTGGATCGTAATCTGGATGCGCTAGTGAACCCAAACGTACACACTTTGGGGGCGTTTATCTCGGTTTACAACGCCAGGAATCCAGCCAGCAAGGCGTCGTTGATTGGGAGGTTCACTACGCAGTACGGGGACGATGTGGTGGCACTGGATCTGGTGTATGCGAGATCTAAATCTGCGAAAAGACCTGTGGCCATATTTATGCAGCAGCAACAGTTCCAGGCCTGGCAAAAGAGCAAGAAATCGGCGGTTGATGTTTTCAAGCAATTGGATATTACACCCACGGACTTCGAACCCGTAGTGAGCCCAAAGATGGAGGTACTGAGCGGATATATCAACGCTCTTAATGCCGCCAATCGAGACAAAACCGATATGATCACGGTGCTCATCCACGGGGCCGACGGGGAAGGCCCTCTTGCACGTGGGGTCGTTACGGCTCTTTGGAATGCAGCGTCACAGGATCGGGTTAAAGCCGTCATCTCCACAGCAGCTGAGTACGAAAAGTTGCTGCATAAGCGGTGGTATAGGAGCAAGATTGAGCCGCCAAGAATTTATACGGATATTCTCAAAGTACAGGAGACTTCTGCTATCGGTCTGGACCAGCTGATCGTAGCCCGATACGCGAGCTACTACAGCGATAAGATCGCTGCTGCTCGTGCTACTCCTTCGATGGAGAATGCCATTCGTCCTAGGCGCTCTTAGTTGCCTCGACCAGTATGTCGGCTGGAAAGGTGGAGTCAAACATGTTTTGCTCATGTAAGGTAGCAATGCCGTCGGCGATCTTGCGTTAGACTCTGTGCGACACTGCTGCCTTCTACTGCCTAATTGTAGTTAGTAATATCCAAGCGATCGTTCAACATGTAGCCGAAAGCCAACAACGCTGAGAAAATAAATTGGATAATCGATAAGTGAATACTACGACGAAAAGAAAGAAGCTTCTCGTCATCACTTTTGAATCCCTCGACGCTAAATCAGCCGTTGAATTTCGGCTTTGCGGCGAGTTCGATTTGGTTCATTCGTATCGGAAGAAAATATTGCCCAGACCAGACCTTTGTCATCTGAGTAGTACTTGCTCACCATATCACTGTATTGCTACATATCAATAATAAAAAACAAAATATGCGATACTGTCCTATTGGTTCGGGCTAATACCGGTAGTACTATTTTGCTTATTTGTGTTAATCCCGCACAGGCCCTTCAAGAGCAAGCATGCTGCTGCCCTATCGACACCTATTTTTCGTACTATACGAGAGCCTCCTTAGCGGGTCCCTTGCATAAAAATATGGTCATCTGACATGCATTAATAAAAATTAACTCATATCGTAAGTTTTTTTTGCTAATCTATGAAGAATGTTTTGGTGAAACGTTATTGCAAATTAATGATTCTACATCTGGTCCGAAATGATGGATAATATGCTACAGGATGGTATATGAAAAATAGGTTGAAAGCAACTCGTTTTGTTTTGTTAAATCGATCAGACCATCTTTAACAGGTATTACTTATTCTTAGACTCAAAGAAGATGAGACTTAGACGGCCAGCCTCACTAAGCTATGTCTTGGCTCTGTCGTACTTCTTTCGCATTCTAGTACTTGAGGAAGAAGACCTCTCCTCCGCAGCTTATCTTTTCTAAGAAGTGGCAAGGTAGATTAATTCCCACCCTGTTGTTGAAGCACGCATAGCATACAGCTCTGCAAAGTCAGCAAAATGTTGTCCAGTGTGTCGGCACTCATGTCTCCGTACGCATCTTGAGCAGCCTTGATGAGATTGTCGATTTCACGGATGGGTATGCGGTGTTGGATGCTTTGATATTCATTGGAGACCAAGGTCCAGCACGTTAAGGTCCGGTGAATTGGCAGGCTGGCACAGCTGACGGAAGCACCACCCACCTTCACACCCCGCATCAGCAATGTCAGGGTGTTGGAAAGACACGTGGGTCTTAGCGTTATCCTGTTGGATAAGGATAGGGCGCCGTCGGTCTCCACAAGGCCGACGCTTCTTAATAGCAGGAGTAGCCAATCGAATAAGAAAGTCCTAGTAAACAGCTCGATTCACCACTTCAATATTGCACGTACACACAGATCCTTTGGGGCGGTTACAGCCGCTTATCTAGGTTATGTACGCCTACGTCGGTTGCCACAGTCCCAGCTTTTCGTTTCAACTTACAATGCGAGTCATATCTAATTACACAGAGTTTAAAGGTTAATTTGTTTGTATAACATATAAAAAGTAATGGGTATTACCTCGGTCGGGCAACGTCTGTCAGGAACATGGTCTTCGGAATAAACGTCTTGTGCGGTGTGGCGGGGTTTCGCCAGGAAACAGTAAGTAGCTTCGCTTATCCCTGTCCTCAAAGAACCTTTTTTCATCTACGTGGACCACGTCGTCCAGCGGCTCGAACTCAAGCGTGTCGTCGTTAGTAAAGGTGAGCACATGCTCCACCCTATCCAGCTTGTTTTTTGGTGTCAATGTCGACCGGATGCACGCAACCTTCTTTACCAGCTCGCCCGCAGCCACAAGCAAGCGTATGATGCGTCGCGAAACGCCAGTTAGCCCGGCAGCTCGACGTTCAACCGAGCGATCTTYTATCGGAATCTCTTGCAGTCGCGATGCATTTCGGCCTGCCCTTCCTCTTTCTACCGGAGCTCTCCTTGATACAGCTTTGCCACTCCCCTCCTTCTACCCCTGCGGTGACGCTGATGCAATAGCGGGTCCAGATTTTGCTGATAGTGGACGGGCTGCGCTTAAAGTCTCTGGCGACGGCCGCCATTGCTCAATGAGGTAGATCTCCGTCAATACTGAGTTTTAGGAGCTCGTCGATGATGCAAAGGCGCTGGTAATCGGTCTGGTTGGTCATAGAGAAGTCGAAGAGCACCGATAGAGCGTAGTGCTAGCTGAGCGAGTACTTGGAAAATGAGGAAGAAGGTGGACAGCTATTCAAATAATTGTAGCTCGATCCCCAGTACGCTTTCAGCGCCAATCAAACCACCAGCCAATAGCTGGCAAATACACGCTCCAGTTGAACCAAATAGATTTTGATAACAATGGCTGGAATTGGTTTTAATTACATTTAAATTAAATATCCGTCATTTTGGATCAGAGGTAGTACGTCGTTTTATACGACAAAAAAAGAACTTTAGTTTCGGATAATAACGTAAGGCTTTGAATAGCGAATATCTGGATGAAATATGAATAAATGGCAAGTGAATTACCGATCGTTATTCCAGTACTCTATCCAGTGGAATAACGCGACCCTTCCATTATACTGGAATATATATTTCTGAACGAAGAAAATTATTATTCCAAACCATGAGTTATTTTCTAATGAATGCGTGACAAGACGTTATGCATTATATAAGTGGCTAAAAGAAAGGCATTTTTCTTCAATCCAGCATATCCCCTTGTTCGGAGTGACATTACCCCCCGCCGTACTTACAGAAGTTTTGCCACCCGGTTTTGAAGAGTTCTCCTCTTTTTTTCTAACCATGTTGTATTTTAGAAAGAATTGATAAATATACAACATGCTAAAAACAAATTACTCTTGGGTTTGTATTGAATACCAGAAGTAAGGGACCGCTAAAGCAGCCCGGGAAGATCTCCAGACAAGTGATATTTTGATTGCAAACTTCCTAGAAGTTTCTGTTCCTCCACGTTGTCAAATACACCCACGCTTCGGTGTCGAGCTCAAACTGGGTATATCCTATACATGTTTGTAGTGTCCGTTTTATTAAAAATGAAGCACTGCACACAAATTGTTTTGGCTATGTATGTTGTCAAC

>Contig_50

ACGGAAAGAGACAAGACAGACGGCGGGAAGCGACGGAACGTTACGGCTAAGTGTCAAAAGGAACGAGGACTACGAACGGCTACAGCGGGAGGGTCTCGGAGCTACTTGACGGCCTTCTTCTGGCTGTCCTCATCTTCATCCTTGTCGCTTTCCTCGTCGTCATCCTCGTGCGCGACGAATTCCTCCTCTTCCTGCTCGTCTTCGTCGCCATCGACCACGGCTTCCTTCTTTTCGTCTTCTCCATCGTCTTCGAGCTCGGACCAATCGTCTCGATGACGTTCCGTACTGGACGGAGGACGAGCAGTGGGGACCTTGTACATTCCCGCGGCGAGCTGAGCCATGATGTCGTCTTGAGCCAACTTAACAGTGTGACGATTAACGAGCGACAACCACGGCTCCTTGTTCTTGGCGCCTGTCCAAGTGGTGCTTGGCCTCCTACAGGACGTGGTGTTGCGACGAACTCGAGCCAGAGCAGCGAGGGAGAGTCGATTCTTCTCGTCAGCGGCTGGGTTTAGCGCGTCCGTAGACTCGAGCAAAGAGCGCATGTCGGGATACCCGTAGGTGTCGATAGCCGACGGGTCCAGCACGTTGGCGAGCGTGGCGTCGTATCGTTCCTTATCCCTCTGTTTCAAGGACGCTAAGTCCTCGGTGTTGGGTCCAGTGTAGGAACTACCGTCGCGTGTGGCTCTGCCGGGCTGGCCTCCGAGCCAGTACATCTCGGAACGCTTCTCCAGCATATCGAGGAAGGCATAGAGCCCCACGTCTCGGTGCAAAGACTTGTGAAGACCCAGCGAGCTTGGACGGCGCGCATCTTCAGCTTACGACGCTGGGTCATGGCAGCTGCCGTGGCCAGAGGATCGTGGAGAGCCCACTCCCAGAAAGCTCGACGGTGCGTCATCTAGAAGCGCCAGTGCGCGCGATACACGTGTTAAAACTGATGGAGTCGTACCAAATGGGACGTCCAGTCCAGGGGAACGCAAGACGTCAGGGCTTCTTACCGAGAGCCTCTGCCTTGGCCAAGACGGCGGCGATTGCACGGCGAACTGGGCTGGAGCGGCTTGGCTCACGTCGAGGAGATGGCCAAACTCGTTGGACGGGACATGTGGTTTCTTGGGCTTGCTGACGACGAGAGTCTTCTTCGCGGACGCCTTAGGAGGGGGGGGGTCGTCACTGCCGGCCTTGCGTTTACCACGCGCTGGCGACTTGGGCGACTTGGACGGCTTCTTAGAGGGCGACTTGGACTGTTTGCTGGACAAAGTTGTCGACGCCGAAGCGGAGCTGAAGAGGGAGCGGAGTCAGGCAGCGCGGGGGTGGCAACGACGATTCCTTGGGGAGAAGCGCCGGTCCAACCATTCAGGATGTCCTCGGCGAGCTGGCCCAGGAACTGAAGACCTGACGACATGCTGTTAGCGCCCAGACGCGTGCGCAGCTCGGTCCAAAACGGCTGCTGGAAGGGCCGCGCGGTGTCCCCAGTGGTGCCCTGCTCCTCATGGTCGAGGGCGGCGTTGATGAACGACCGGAAGCACCTCAGCTCGGCGGTGATAAGTCGCGTGGACATGGCGAGATGGCAAAATGCGAAATGAGAGAGATTTGAAAGCTGTAAAGACTCGCGTCTTTCTTTTGATGGCTGCCAAGAGGCACGCGAGAAGGTTCGACAGCTGGGACGTGGCGTTGATTGAGGGACGCAATGATGCTTGGTGACTTGGGCGGACAAGGTGTCGGTTGAAGAACCGTCCTAACGGCTAACGGTCTGGAGAAGCTGCGGTCGTGACGGCGTCACTTGACGATTAGTGGCGATGTTGGGAGTGTTTGTGGAATGGGCGGAGCTAGTGAACGTGGTGACGGAGATTGAAGTGGAGGACGATATTAGGGTCGTCGGCAGCGCTGTAAGGGGATACGTGGTTTGGATCGCAACGACTCCTCTTCGGAACGTGTCGTGGGCGTTCCTCGACGGGACGATCGAGAGCGTACGTGCCATCTACTTCTTCTGGTACACGTGCTGTGACCACTACCTGGTGGCGGCCCGAAATGCCCATGCCGCTGCTGCGCCTCCTCCGGAGAACGCGTGCGCGCTAGTGAAGCTGCTGTTTTTGGTTCCTATCGCGGTGCTACAGGACTACGAGTCGCGGTTTGGAATTACAGGTCGTATGTGTCTTGAGTGGTGTGAAGCGTTCCACCATTTGTGCTGTATATACGTTCCTGATGCGAGCCTACTGCTATGGAGCACGTCCTGCCCGTACGCACGACCGCGAGCGAAGCTGTGGTAGCTGTTGTGGATAGAGTTGGAGATTTTATCGCTGGGAGCTACTTGCTGACGTCTCTACTCCTGTCGATCCTAATCTACTTGCCCCGCCTGGTGTACGAGAGTGTCGATGGAGTGCGTGTGGCTGGATGGGGGTGGCATTGGTTGTTTGGTGGTGGACGTCTACCTGGAGCTCGATATTGGCTGCTGTCACTGGAATTTGGCTGTGTTGGTGGTCGGCTGCTATAGTTATGCGATGTGCTATTACGAAGGGCTCAGGCCGAGTGCCGCGGACCAGAATGGCAGGGCTGTGAGACGCAAGTGGGAAGGACTTGCTGGGCGCATCGGGCGTCGTGAGGAAAGAGAGCGTGCTAGTGTGGAGCGACTGCGAGGAACACAAGCCGAGGGAATGTTGTCTGGAGCAAGGAACGAGAGTCAAACTGCGAGTGGAACGTCACGTGGGAACGTTCCAGATTTGGCTGCGGAGCACACCTTGCGACAGGCTCTGGGAGATGTGGCTCGTCGAGGACTCCGAGCATCAGTCGGGGAGAATTGTCACGATGTACAGTTAGATGGTGGATTAACGATACTGGCTGGCTGCCTGCAGAAGGTCTACTTACTCCAGACTCTCCGGACCACGTCCAAGCAAGCGGGACCTGCGGCCAAGACAGTGACGTTCCAAGTGTGAAGTCGTGAAGCAGCTGGTCGTGGCGAGAGTTAGAGCTGGACCGCCACACACGAGGGATCTGCTGAAAGGTAAGGAGCTTTAAAACTCTTAGCGAGGGAAAATGAGGAGGTCAGTCGTGGATTTCCTTCTAAAAAACAACACAAGAATATATCAGCTTTTACATTACTATACCAGTAGTGTCGCTATTACTACTACCTCCTACTTCGGCCCAAGATACACGACCCCGATTAGAGGAGGACCGCGACAATTTGCAAAAGCTCATTGCTAGACCGCCAGCCTTCAGCTTTTAATATTATTAACAGCCGTTTTATTTTTGTTTCCTTTCTCCCTACGAGGGCGGCTGGTTTACTATTGGTTTTACAGAATTATAATGTGCGTTAGTTATAGGAGGCACAAGAAAGGGCCAGCAAGCCGGTCCAAAAAATCTTTGTTTTTAAGAGCGACGGATCAAATTGAAGAGTACCTTTTTTCTGGCACTTGGAAAATGGTAACCCTTTCTTGTGCCTCCTATAACTGACGCACATTATAATTATGTAAAACTGAAACAAAAAGAAAAAACGGCTGTTGATATTATTAAAAGTTGGAGGCTGGCGGTGCTTTTGCTTCGTCTGCAACAGCTGCACCGCACGGAACTCACAACGTAATCTAAAAAAATCATAATCGACTCGTGTAAACAGCTTCCCGCATGATAAGGAAACCTACGGACTTTTAAGTGCAGTGGAAAAGTAAAATTCATGCTCACTTCGCATCTTACCGGTACTGCCAAGCGCACTTGTCTACAATGATTCTATGGGTGTCATCCAGCCGTATAAAGAGCGTTTACAGCGGTCCGCTTTTGGCTACTGTTACTCTGTTGATCTTCGTTGGATTGGCGTTAGGAGCAGCCAATGTGAAGGTGGAGGGTACTGATGTGGACCTGAAGAATAACATCCGACTTGATCGGCGGTTGAGGACGCTGATTGCTAATTCTAACTACAGCCCCGAAGACAGAGGCGTCTCCAGTATAAAAGAGCTGGCTCAAACAATTAGTGTGGATAAACTTGCTAAGAGTATTAAGACCTTGGTGGCACCTGACAGCCAGACCAAGATCGCCAAACGCTTTAATAAGATCAAGGGTAAAGGCGTGGAGTCCAATGTGCTCTTGAGCGACAAGTTTGATGGCTCGGCTAGCTACGTACTCCTCAAGGTACTCAAGGGTGAGCAGGCAGCAGCAGACAAAGCGATATTCGCGACGCTGGCAACGCACTATGGTGATGCCTTCCTGCCACACCTGTTGGAGGCAAAGCGGATGGGTAAACACTCGATAGTCAGTCGGTTGGAAACGATTCAGCGTAAAAACTGGATGGGCGATGGAAAAAGTGTGGACGACGTCTACAGAATACTAAAACTCGACGAAGAAGGCCAAAATATTCTCAAGAGTCCAGCCTTAAGCACGTGGATGAGCTATGCATCGAAGCTGAAGGAAGATCCGCTCAACATGCTACTGCTCAAACTACGAGCACAATACGACGACGCAGTAGTACTAAAAATGATTGCGATGTCAAAGGATAAATCCGATGACGACACGTTTAGGAAACTTGAAGGTGCACTGATGGGGAAATGGCGACAAGACGGTAAAACTGCGGACGATGTGTTCCGGCTGCTGAAGCTGGATGAGGAAGGTTCAGATCTTCTAAGAAATCCGTTGCTAAAAGCTTGGGTGTCTTACGCGGGTGGTCGGCTCAGAGCTGACCCGTATACGCATATGATTTCAGTTCTCAGACAAAAAAAGTGTCAATGATGCGAAGTTAGCGTGGATGATCGGTACAGCAAAGCAAGACCCCAGTGCAACGTGGATTGCCGGCAACATGGAGTCTAGGCTGATCAAACAATGGTCGGGCGAGAGAAAAAGTGGTGAGGACGTCTTCACGCTGATCGGACTCCATAAGGAGAGAGACCAGTTGTTTAAGAGCCCCGTATGGAGTACTTGGGAAGCCTACCTGAAAAAAATTGAAAAAGAACCGTACAAAGCCATGTACGCAGTATTGGGGGCACGATTCGACGATTCACAGTTGGCGGCTCTTGTTTCGAGTTCGAAAGAGGCCGAGAAAGTTATTCTGAACGTTTGGAGCGCGAATGGAAAAGCCGCAGACGAAGTTTTTGACCTCCTGAAGTTGAGCGAGCAGGAGAGCAAGATGTTCGAGAGTCCGGTATTTGGCACCTGGGTCACCTATGTCGACAAGTTGAACAAACTCAGGCAATACCCAGATGAGTTTGAAGCTGTCAAGCTGTCGGAAAAACGACTAGGCAGCTCTCCCCTGGCACTTGAACTCGCTTATTACAAGTATAAGGTCTATACTGCAAGTGCACAACAAGTGATCAAGAGGTTGCAAGCAATGCAATTCCAAAATTGGCTGGCCCAACACGAAAATCGATTCACCCCCGTTGAAGCTATGGCATTATCGATCCATCCAGCGGTGAAATCAGAATTCCTTCACTACTTCACAGGTATGTTGGAACACGGTGGCTTCGAAGCAACTCGCAAGCACGCTGTCAGATAAGAAACCGTGCTGATTCGAGCGGTGTAGGCAATATGGTTTGGTCGTTAATGCCATGAACCAGGCCAATTCCTGAAGAACAATTCCTTATGTAGGCTGTCATCTGTGGCCAATGTCGCGAGAGTAAGGCTGATACTTCCTGGCTGCTTAAACAATGCTTCAGTAATCGGTAAGCAGTACTGCATGCACGTATTTGATCTTTGTTTATCGCACTGCTGTAGTAATTATTAAACTTCTTGCGTAGTAGTATTATAAAACGCTACTGATATACTATAGGCTAAGGAATAATGTACTATTAATAAGTAGCGTATTCTTCCGAACAATAACAATACTGTGGACCGGCAGCTTTAAGTTAAGTCGTGAGATGTAGTGGCCTATCCGGACGAGTTTTGCACCGACTACCGCTGCGGCGTCTACACTCCACGCTGTCACTGGTGGCATCGAGCTGATCATGCCGAGTAGTTGAAGCTTATCGTCGTCTATGACTGCTGGCTAATACATGTAGAGAGGCGTCCTCGGATAAATGCTCGATTATTTTTGAGGATTTGCAATTATGAGCGCCCGACGACATGATAGGCTGGAACAAAACAATGTTACTCTAGTCGTACGAAGCCGTGTTTCACTGAGAAGCTCTGCATTACACTACTATGTGTAGGGTGGGTGCTTCACTATTGGCTTCACCGTATTGTAACGGACGTCAGTAAGGAGCGTCACATTCTCAGTGCCAGAAAAAAAGTACTTGTCAATTTGATCTGCTCAAACTTTACCAGGATATTAGTTCCTATGCGCTCCTTGTTGCCGCACTTTACAGTATACTATTCCTGCAGTCATGTTTACCGTAGTAAATACGCGTCGCACTGCGGCCACAAGAAGGATATTTAGCAGCCGCGGTAAATCTGTTCGGGCAACATTCACGTGATTAAATTACTTCGGCTACCGGTAGTCGTAATTTACGGGATGTTGGGTAGTATCACTGATGCAATCATTGACTAAAGTCAATGTGTTGTTTCTGATACAAGCTGAAATGACACGTTGTGCAGGCTCAGACAGTAAACCCATTATATTGGGTATGACAATCCAATGTAGATCTTGTACTCATGTAGCAGGCGTATCAAGTGCTGTAGTAAATTTATTAAAGTTTTGAGCTTGAAGCGTTAGTTTGGGATCTGTTAAGAGTAGTTTTGGGTCAGAATCACTCGCCTTCTCAACTCTGGATATAATCCTGAGAGCTCGATACGATACGATGTTGCCAAATGGGCGGGATGACCTCCAAAGGCCTGCACGCTCTGCTTTTATCTAATATTCCCTTGAGCATAAGCGACTAGGAGTTTGGACTCGCATCTGAAAATGAGGACAACACGCACTTTAAGGATGGGTCTAACGTCGCAGGTCAGGTCTGGTTAAACTGAACATTTTCATTCATTCAGCTCTGAACATGGATCCAAAAATGGTTGTCAGCACCTTCAATTGGGTACATGTAATCTAATCTATCTGACACGGCTGAAAGTGGTTGGCGAAAGCTTTGTTTGACGCAATCGCCCGTAACATTTGCTCGTCTGTCTAGAGCTCACTCACTTTAGCTAAACCTAAAGAGCTTTCAAACTAAAAATGCCGACTTCCAACGTTGCCTATCGTTTCGGTGCACAAATTCGACGGAGCCCTTGAAAGACAATGAAGTCCTTATCGAACTCCGTGGAGCTGCTCTCAACTACCTTGAGATCTCGTCATCGCCAACTCCACCTACCCCAGTTCATTGACGGAGCACGTCGATCCATGCTCCGATGGGGCTAAAGTAGCAACAGTACTGGTCGACAGCATTCTAGTGGGTGACCGTGTCATCACCAACTTTGACGTTGCTAGCTTAAACAACCCCGCTCTTAGCGATGGATCGCATTTAGGAGGTACATTAGGTGGTATGTTGCGTCAGTACGCTACGGTGCCAGCTCAAGCTGCCCCAAGGACTGTAAGCTAGACTTCGTGCAGCTCGCCTTTCTCGTGTGTGTGACTAGTCTAACTCCACTGCGTTCTGGTGGTTCTCCGAGGCACGGGTGGCGTCTCCATCTTCGGCTTGCGGCTACTTCAGCCTCGGACGAGAACTCAAGTTCGTGAAGGTAAACGCGTCGACTTCGTGATCGAGACTGGAGGCTCTGGTAGAGCATCAGCGGCATTAAACTGGGTGGTCATCGGCTTCCTGTCTCGCGCAAGCCCGAGTCGCAGGCATGGCGTTAGGCAATTGATCTATCATTAGCATTCAGATCGGCAGCAAGCAACTCACGGAAGAGTTGGTACGTGTGGTCACAAGCCAAAAACATCAAGCCGCATTTTTGCAAGACGTTCGGGCTCGACGAGGAAGAACTGTACGAACAGCGTTTTAAGTATCTGGAGGTAGCTAGTCACATTGGAAAGATCAGCAACACCATCATAGACTCATAAAGCCCCTAAAGCTTTCCTCTTCTAAGTCGATTACACGCATCCTTTTTCGCTTATAAATCCTCATCGTCGAGCCCGAGGAAGTCGGCAAGTCCACCATTGGACAGTTTGTTCTCGGTTTACTTTTCTTGCTCACGTCTATTCTGCTTCCTAGAAG

>Contig_55

AACTGAGCTTAAAAAAAAAGCGGAACAGGAAGAGGCCCGCTGGAAAGGAAATTTTGTGCTTGCAAGAATCAGAGAAGAGCGCGAAAAGAAATGGTCGGAAGAGGTAGCACTCCGCCGAAGCGAAATGAAACTCCATAAAGAGTATCTCACAATACGGAAGCATTAAATAAACATCCAGCCGATTCTTGATCCAAACACTCGAGCCATCGTTTGATGAATAAATAAGGAGCGCCGTTTAAAAAAGAAACTAACGCCTTAATTACGAGCTGTAATTCACATAATCACCAATCGCCTAGCAACTCGGATACGATGCTAGATATTCCGTCGAAGGTAGACGGGTTACAGCCGAAGTACCTAGTGTTGTTGTTTCCCCCGTTGGCGATCTTTACACAATTCGAGAATACGACTCCAGAGCGCTGCCGTCGGTGTGGTAGCAATGCGCAGATGTCACTTGTAATCAAGAAAGGAGTAGTACTTCGTCGTATAAAAATAGGACCATTCTCCTGATACTCGCACGGCTCCCTTCTGTGGTCTGTTTACTCCTCCACCTTTGTGCGCATAGCAAAATACGGCAGATTGGTCGAATGGTTGCGTGGGTGAATATCAATATACCCTCGATACTTCCAGGCTCTTTTGCGCTGAAGCACCATTGCGATCCATGTTCTTGATGATACGGGTTTCATCAAGTAACAAACGCCAGCTAGGTTTCCGTCCAATAACTCAACGACAAAGTTGCGGATGCTGGCGCTCTTCCGAACATTCCCGCTATGTCAATCCAGCGCTTTGTATATATCAATCGATACAGCAACTTGATTTTAAACTTGCTGCTATGTCTGAAATACACGCAGCAAATAACAGTATCGATTAGACGACGTGATTTTAGTTGACGGTGTTTCTGAAGATCTCTAATAAGGCGAACCTCACGTACGACTGGCGTGCGCGTATGTGCTTTAAATTTGGAATTCATGCTGGAGAAATAGTGCGTTTTGGCCATGTGGTCGTGTCTAGTGCTAGATTCAGGCGGACTTGGTAAGCGCAAGAGAGGCGGGAGACCCGAAAGTGAATTTGGAGAAATTTCCGAGCAAAAGACGACATACTACTTCGTAAAGGCAATAAAGTGTTATGCTAAATTTATTTTTGAAACGCTTTTTTACTTCATATATTTTCAGTATTCGCCCTAAAACCGAAAATTTAAAAAATATAAATAAGTGTTTAAAAGTACTATTTACTTAATAGTTTATTTGTCAAAATAAAATAATTTAAATAAAGGATTCATTAGGGTTTCGGAAAATGAGCACAAGTCGCTATATACCATGGCAAGTAAAAAAAGCTCCATCCTTCGTGTATATTTTACTTTGCGTAAAGAGCTCTTCGAGGTCGATCTTACCGTCGCCATTGCGGTCCATCTGGGAAACCACCTCACGCCCGTCGTCATAGTCTAGCGCTTGGCCCAAGGTCTTAAAAATGGACACGAACTCGACCATCGAGATAATCCTGCCGCCATCAGCGTCCACCAACCGGAAAATCTCTTCCACGAGTTTTTCCTTCGAGTAGTGACTCTCTTGGATCCTTGCGCTGCACCACTTGCAGAAGGCCTCGAACGTGATTTCCTAAGCCCTTCAGCCATTGCGTCCTCCCCTCTACCAACATCGCTTAGAAGAAGCGAGGGGGCGTAGACTAATAACACATTGACGTTAGTTTTTTTCGAGCTCACCTACAGCTGAAGGTAGACGGACAATGGACCGATGATGCCGCACGTACCGGTGCTCCTTGTGCCGCTGCCACACTTGAGTCGGCTGTAGCCATTTTCACTCAACTGCTGGTCCCACGAGTCCATGATCTTCAAGTAAGACGGCGAGTTGCCCACAAACACCCCCGCATGTTCAAACTGCGTCGTCGAGCAGTTGGACATGATGCCGCTTTTGCACTGCTTAACCGGTACAGTAACAATCTAGCAAAGGGAATTCTGAATGAACACAATGCAATGTTGTATTTTTAACGAAATTTTCCATCAATTGGGAAAATAAAATATCCACCAGCTCTATCTATCCAGGATAGTGCTAGCCATTTGTGTGTGGGTTTCTGGGGACCCCCCCCTTCGTGCTCAGTATCACGTTGGGACTGAAATAAGGGTCGTACTCTGGCATGACATGTGGAGCTACCTTAGCCACCCACATGCATTTATTTCGATGTTATAAGAAGTACACAAAAGTTGTGGTTGACAAGTGAAAATGCGTAAAGGTAAGAGGTTAAACTGTGCCTTATCTTCAGTCTCAATCGTCACAGAAGCGCTATGCACCAATCACGGCCGCGGATTCGCCGCATTATACTTCTTCATGAACAAGGTAGTACTTCCTTCCGTTCTTGTTGCTCATGTCACCGCCCACACTAGTCAATTTCTCCTTAATTTGGGCCGGCGTGTATTTACGATCGAGCCAAGACTCCGCCCTTTTAGACCACCTTGCAGTCTATGCGGCAAGCAGACAAGTGTAAGCGCGGACAATTGGGATCGAAATAGATTACGGCCTTTACAATTTTCAATATAGAACTGTAAAGATGATTTGCTGAAGATTGGAATAGGCTTTTAGTTTCGTTACAGACTGTAGCCGTCGAAATGGATAATTTTTGGATGTATATTAGCATGCAAAATATTTGAAAGAATTTACATGTACTAATTTATGAAAATATCTCGATCAAAATCATGCGTCGTAGCCCTATCCCTAAATTTGATACTACCGGTACATGTACAGTACATTGTACCGACTGCGAAGTATAACTTCAACATGTCCGCATTATGATAGCACTGTATCTTAGAGAAAAAAGGAGAAACGCGTCCATCGCTGGGATAACACACACGGGGCTGGGCTACGTGGGTGCCATAGCCGCGGCGCCAACTAAGGTGAGCGGGTGCGAGGCGGGCCCAGCGCTGAGCTCACCAGCGAGCCGAGCACTACACTTTCCAGAACATTCTATCTCCATATTGCGAGCGCAGCGAACCGCGGGGGCGCCGAAGGGGCAGCCAAGCGAAGCGCGGCTGGTCTCTTTTCTGCTGTGACCTCTAATCGTCGATTATGTCTTATTGCGACACTCAGTATACATAAGTCAAATCAAATGATTTGCTGTGTGCTGAAACAGTAAATACATGTGTTTGGCATGTTCTGTCTCTATCTTACTTAATAGTACCGTACAGTACTGAACACCGCACATCATAAACAAGACCCAGCGGTGAACAGTGTTCCTTATGTGATTGGCGACAGTCAAATTCTTGAGGTGGTCTAGCCTTTCTGCCAGCATAAAAGATACCTGCATAGAAAAACTATATTACATGTTTCAAAATTACATGTATATTGGTCGCCAAGCACATAAAGCGCGACGGCACGAAATTTAAATCAATTTGATAGGCTGCTGGATTAATGGCCGAGCGTCGCCAATCACATAAGTAGTGAACTGCTCTTACTTTGATTCATAAAGCGCAGAAGCCACGGATAATGATCACGTTCAAGCGTTTAAGTAGTGCTCGCTGGGGCGCCCTCTTGACTTCGATTGCTGTATTGTTCTTCCTTGCAATCACTAAGGGAGCTGATGCCAAGGCTGGAGCTGATTTGGCTGACATCCAAGCCTATCGACGTTTGAGGACGACGACAGCTGATGCCTACTACGCCTCGGAAGACAGAGTATTCTCTGTTGTGAAGGCGTTGAAAGATCTAGCTCACAACGCGAAACTGACATTTAGTCTAGACAAGCAGCTCAAGGTCAATAACCGCTTTGAAGTGCTTCGGGTCAAACAAGTAAAAACCGACGTATTCTCGAGTAGCGAATTTACTGACTGGGCGCACTATGTGGCCAAAATCTGCAAGAGAGGACGCTTGCCAGCAGATCGAGCAATTTTCAAGACGATGGCGGCTCACTATGGCGATGACGAACTGGCGCGCATGCTGGCTACTGCGAAGCGAACAAGCAGAGACACGGTGGTATATCAGCTAAAAGAAATTCAGCAGAAAAGTTGGAAGGAAAGTGGAAAAAGTGCGGATGATGTTTACGCTATTCTCCAGCTTGACGCAGGAGGCCAAAACGTCTTGAATAATCCGGGGTTACCGGCATGGCTGAGTTATGTAAAGAGTCCAAGTACCGATTACATCGAGGCATTGCTTTTAAAGCTACGCGAGCAATACGACGACGTAACCGTGGCGAAAATGATTGTTTCATCACAGAGCGGTGTCAACAAACGCATTAGCGGGCAGCTCGAGAAGGAACTGAGCACTGCTTGGCGAAAGAATCACATTACTGAGATGGAGGTGTTCCAGCTGCTGAAATTGAATGACGCAGGCACTACTCTTTTAAAAAATCCGATTTTGGAGATTTGGTTTCATTATGTGTGGAAGATGAAAAGAAACGACCCTTATGAGCTGCTGGTCTCGTGGTTCAAAAAGGCTGGTATTGATGATGCGGGGCTCGGGAAGATGATCGCAACAGCGAAACAAGACGACAGAAACTATTGGATTGCTCAGACATTGGAGCAGCGTTTGTCTGGCAAATGAAAGGAGAGAAGATAATGGTAGCGGAATCTTCAGCATGAGTAAAAAGAAAATTTTCTTTTTGACGGCTACACTTGGACTTAGATAGTTAGATACCTTACAATCATTCTATTGGTTCAAATACGTTTCAGGCTGAAAATACTAACGGGTTTTTTTTTTTGCTAAATGAAACGTACCAATCTATACGCAGGCATGTATTTTACGATAGTGTTGTTGCCGGTACGGGAGTAGTAAAATGACTTTACAGTTAAATTTGCTTGCTCGCTTAAGGTATCCCCTCAGACATAGAAGATCGGACGATATCTACGAATAAAATATTAGATTGGATTCAGATAATAATTTAAGTATTTTTTTAGTATATAGATGCACAAATTCTGTTCAGCCATTGGATTCAGGATTTCTGACGGAGTTACATTTAGGTTTGGCTTTTATTAAATTTAAGCTGGTTGGCTTTGAGTTGTCATCTCATGCCGGTGACAGAGCTCGACCAGTTTCTTCTCTAATTCGTCTTGCTGACGAGATCGCACTACAGAGAGTTAGTGCGCCAAATATAGCGAGCATTTACGTAGTACTTCGTATTAGTACTTCACAATAAGAAGACTTCCAAAGGCTGTCAAGACTGTTTCTACGTGCCCCTAGTACGTGGTTACGTCATTTCTTATCATCTTAAAGTGCTGGCATTAGTGTAATTAAGAACAACATTTTGTATAATCTCCAAGTGCTCACATAAGCGTAGTAGTAATAGCAAGTCAGCTCGAAAGCTAGCTACCGACAACAAGACTCCGGCATACCCCCGATGCAGCAAGTATCCATTGTAGATCTCATCGAAATTCTTGCCGTTAGCCTTGCGACTCGCCCGTTTCAAGAACTCATCAATTTTAGACAAAGAATACTGGTTATGTTCCCACCACTGGAATGCAGCTTTCGGCAACCGAGGATTGCCCATGAGATCTTCGGCCAATTGTTTAGCGTGTCTCCCTTCTTTAGTTCAGCCAAGTTAAAATTCGGGGCTCGCTTCTCAGCCACTGCTTCATTCTCTTGAGGCGTGCGAAGAAATATTCCTCTGGCTGAAACGTGAGAGTGAGCTGGTAATTCATTCATCAATATCTTGGTTTGGTCGAACTCTGCTACACTGCAGGACGCGAAGCTAATAGCAATTACCGGAGTGAACACCATACTTGCCAGGCGCATGGTATGGAAAATTGAAGGGTACAGAGCTTTAAGTCGCACTCTGCGAAATGAGTAGCGGTGCTTCTTGAGCGAGAAGGGACTTCACAACCCTTGACTACTACACCTTTGTGCACCCAACCTCTTAGAAAACGGCAGTCAAACTCGTCTCAAAAAATGGCAAATAGGAAAATTACGGTAAGAGTCCCTAATTAATATTCAATGTTGAAAAAACTGCAATCAACCTGGTACATGTACAAGATTTGCGCGACTGGTTCGCGCCAAAACGATAGGCTCTGTTTGACTCGCGCCAAAGCTACCTGCGTCTTGATGCTCCTTTAATTTCACGGCGAACACACGTCGTTTCTCAGTTGCTATCGCTATTACACGCTGTGGGCTCGACTCTCACTTGTTGTACGGTAAACAGCATGCCATTCAGGTGCCGGCTAATCGTCGACGTGTCCACCTTAACGCCACAGGCCAGAAACAACATTGTCCTCATAGTGTCCAGCGTATAGGTACAGTTATCATTCAGGTACTCCTCCAGGAGGACCTTAGCTTCAGGCGTCATCTTGACGTGCTTCGCGCCTTCCCTAGGGAGATTGTCCACCAGTCGCGCAAATCTTGTACGAGGTTGATTGCAGTTTTTTCAACATTGAATATGAAACTGATTAGGGGCTCCTACCGTAATTTTCCTATTTGCCAATTTTTAAGTTACGAGTTTGACTGCCGTTTTCGACAGGTGTAGTACAAACATCGCTCTCTATTTCGGTACTTGTATAAATTAATACCCTGAGCAAAGCGATGCAGCTGTTTTAAGGGGGGAGTTTCTGGCGGGAAATGCTCAGCTCCAGTCATTCTAAGGAGCAGAACCGTGTTAATGTCGAACAGTGCGTAATTTACGTCGGCCCATACCCATGGGAGCCTGGATGAAGACAAAACAGAAGCGCGATTTCACCCAGAAGGCGAGCGACTGCCCCAGCATGACGCAAGAAGAGCTGGCGGCATGGGCAAAAGCGACGTTCAAGCTGAAGCGAGTGCCGGCGCAAACTACTGTTTCTGATATCCTTCAAGACGCCTCCAAGATAGTGAACAGCAGTTTCTTCCGCCCAACACTACTGCGTTCCTACAACCTATGGATGCTGGCATCATTGCATCATTTAAACTGGAGTACCGAAAGAAGCAGTTGCGGTGGGTTTTCGACAAGATCAAGGACGGTGTTGAGATTGAAAGGAAGGCATATGCTGTGGATCAGCGTACTATCGAGAACTGTTTTCGCCACACAGGCATTACTTTTAATGCCGTAAATGAGAGAAGTAAATCAACCGAGCAATTTAGCTACGGGCCCGATGTAGACGTAGAAGCTGTCATTATCTTAGCCTCACAGCTGAGCTTGTAACTTTTTACTATATCAAATTCAAGGAGATAGTCGGACCACGATACCGGAAGTGAGAGCGATATTTGCATTGGCAGAAAATAGTACTCGTATCATTAGTGGTTATCATGAAAAGAAGTGAGATTTCAGCAGCCAATCAGATCAAACCTACCTCCCCCATCTTTTTCAGCGCTCACCTTCCTCTGAGATAAGAAGATTTGACGTAATTGGTGAAAAATCGGTCACTACAAATACCTACTTCCGGTATGTCGGTGGGCATCTTTGCATGTTAGAACTATAGCGTAGAAGGAAGAGCCTTCAGTGCCATTGATATATCAACCAATCAGAGTACTGCAGCCATTTTTCACAGTTAAAAAGTGAAGATTTGATATACAGTTAAAAAGTGAAGATTTGATTTGACTGGCTGCTGAAACATATAAAGATTTCAACCGCTCCGGTGCCGGAAGGGGAGCCGTTTCCTTATAAGTCGAGGCCCCACTGTGTTAATAGCAAGGATAGTCATCTGGGCCTAAACAATCCCCTTGGTACATGTAGTACAATGCGAGAAGCCGCTCATTATTGAAGACAAAGGCACCGGGGTTGGCATCCGCAGCAGTGGTATCAGCTAGCGCAAGCTTTGGATGCTTATAGTTATCACAAGGTCATAATATCTCCAGCTGATTACTCGTCTACGCAACCTTTTCCAGAAAACGCTCAGCAAGTTGGCACAATAGCGATTCTTGTCGCTTTCGCTTGAACCCACTTATTTTGGCGGTATGGCGTTTTTTCTCGATGCGCATGAAGCAGTTGCCGTCTCTCTCGGCTGCATTCTTTTATTGCTTGTCTGTCCCA

>Contig_58

GTTGATTTTGAAATCATCTTCGACGGCTTGGCGCACGGAGAGTAGGTTGAACTTCAGCTGGGGCGCTTATAGCACGTTGGCCAAGGATATTTCTCGTTCCTTTCCCTTCGTGTCCTTGATTGTCATCTTGACATCACCAGTTCCTTTTATCGGAATCTGGTGATTGCCGCCGACAGTGATTGACTTGCCAGCACTTGGAATCAGCTTCTCAAACCACTTCGGATTGGACGTAGCGTGGCGAGTGCAGCCTGAGTCGACAGTCCACACTGGGTCATGCTCCGCATCTTGGGCGGTGAGACTTACAGCCTCTGTTTCTTGTCTGAGGTCCAGAGTGGTGATGGCTATGATTCCATACTCGTTGTCGGAGTCGGTGCCCTTGCGGCGACCGTGATATTTATTGTGACTCTTGGATTTTCCAGATGTATTCTTCTGGTTGCCGTTCTGGCTTCTATTTCGATTGCTGTTCCGCTTGCTCTTGTAGTCATTCCGATTTTGGTTTCGTGACTTGCCGCCGAAACCGTCCTTGTAAGGATGCTGTCGAGCTTGATTGGACGAAGGTTTCAGCTTAAAGTTGGCGGGAAGCACCGTTCCTGCCTTTACCTGACCATTTTGTAGGTGGCGCTGTAGAACATAGCAGTCGACAGTATCGTGGTTGGTGCGCAGGCAGTATGTACAGCTCATCGTTGAGATGAGAGCCTTGCTCTCTTGATAGGGAGTTTGAACCAAGGTTGGTTCAGGTGCTAAAGCCTGTAGGGCCTGCTCGTTTCGGGTTTCCCTCGATTCGGGTGTTCCTTGTTTGAGCGTGTAGCGGTTTTTCGCCAGTTCATGCTGCACCTTGGCCTCGATGTGACGCTTTAGCTCTTCGTAGGGAATGAATTTCCTGCTACCCTTCCAGACCGATAGTTGCTGCTTCCATTTTACAGGTAGCGCATGGTACAAGTAGAGTGACTTCTGTTCGTCACTGAGCACACTGTTTGTGGCATCGGAGGCAACCTTCATGGCAGATTCGATGTCAATTATGAACCCCGTAAGGTCTGAACCTTCTTCATACCTCAGTGCCATCAGGTATGATAGCACATGATACGGGCCGCCGTGGATGGCAGCACCTTCGTACTTGCTACAGATCGTCTGAAAGATCTCAAACGCAGTAGTCTCGTCCTTGACCATGAGAACATGTTGGTCATCGATGGTCTTGATAAGGAAAGCTTTTGCTTTTGCCTCGTTGAGTCGAAGCTTTTTCGAGCTCAGCCTCTGGCTTTTAGCCTTCAGCTTCTCTGCTCGCTTCAGTTCATCCCGCTTCTGAGCAGTGAATGACTGGATAACGGGGGGATTCTCCTGCCCCATTTCCACATCGCCGTCGTCTCCGGCTGTGGGTGCATCCGARCTTGCGGAGCTGGCATCAGAGGAGGACTCGCTCGGTGAGGAGCCCACCATTTCGTCGGCCTTGGGAGCACCGGCAGCATCAAGCGCGGCGGTCATGTCGTTCATGTCGGACAGTGCCGGGTTGAGTTCTTCATCGGAGTCGAACTCGTAGTCGGAGTCACCGGTGTAGTCGATACGCTCGACGTAGCCGAGAAGGTTCTTTCCTTCGAGGGCGGCAGTGACGCGGGTCTTCCAGATGATGAAGTTCCGGCCATTGAGGCGCGGAAACTCGCGGTCGTCAGCGGTGGAGGCTTGCGTTGGGCTCATAACCTATTGAAGGTGAGGCGAGGAAGGTGGAGGAGCTTGAGTAGAAGGAAGAAAAAAGAGATGTGATTGATTTTTATGAGTCTGTCTTCAGAGAGTTTCCTGGCACCATTGATTTATCTTTCCTGTAAAAGGTGATGTATCGACTTATGCAGGATTGGGTCCAGAACATATTTTGTATGGTAAGACATTTTGTACAATAACTTGTACGCTATGTCACCAATTAGGACACATCATTGACACATCCTATTTTTTGTGTTCGCTAAAAAATATAGAGTGCTTTGGATGGCTCTCTAGCGGGATGGGATGCCTGCGATTTCGCGAATCCGCAGGACTTCCAACCCGGTGTTATAGACAGGTGGATCGTGTCGGTGGTGTGTCACACACACTGTGTGCTGCTGTGGAATAGACGATTGGACTTGAGATAAGCCCAAGACCGTGCCACACAACGTCCCAGTGGGACGAGAAGGGGTGTCAACCTTACATGTCATCCCAATATCCCGCAAACTAGATCGCTGATTAGTCCATCCCTCTTTTGGGACGATATAAGACTGAGCCTAACGCCTCTCCCTGCGAGAACGGGTGCAGTACAGAACGCTACTGCGACATTCGCATTACATTAAAAATACAGACAGTACATTTTCTTTTAATACCGGTACGGATGCACCAGTACGTTTTATTAATAGAGAGAGCAAGTTCGAATCGAGCTTCGATGAACATGTATTTGTGATGAAAAAGGACGCAATAATTACATAAAGACTCTTTATTGTTTACGCCTCTTTATTAAAGAGGCGTAAACAGTTTTAAAAACAGCTTTCAGATACGGTGTTATTAATAAGCAGATTTTATTTTTTGAAATCTGTCAGTGGAATTTGGAAGCATTGCCTCTACTCCTGAACATGTCCACAAACTGGAAGTCGTGAGCTCTGGTTTTGGCGATGAGGCCAAGAGAAAGTGAACCGAAGATCTTATCCGAAGATTACCAGATGGTATTGCAGTACACCTATTGCAGGTACCGGTACATGTACATATTTTCACTACAGTAAGACGGGTGTCACATTAATTACTGAGGGGTGCCTCACTCCGCAATTGGACATCTCGAGTAATCACCATGACTATCAAGCCCAAGCGCAAGTTGTTTTTTTTATCAAGTACTGCTGGTGACTGTAGCTCTATTGATCTGTGCCAGTAAGGCATTGACGGCCATCGATTCCAAGAATCTCCCCACCCTCGACAGAATCAGAGCCGACCGGCGATTGAGGGCGACGGTAGATGGTTACGACTACAACTACGAGTCCGAAGACAGAGCCTTCACCGGCATTACGAAGCTAAAAGAATTTGCTCAGGCTGGGACGAAGAAGCTGCAGAAGGCCGTTGATACTGCCAAGACGAAGCTGACGTCCAAGCCCACTATTGACCAGCGCTTTAAGCAGTTCAAAGTCGATCAAGTCGAGTCCAACGTTTTCGAAAGCATGCAATTTAATGCCTGGGCCAAGTCTGTTGCAAAAACGACCAAGAACAACCAGGACGCCACCGACGCTGCAATGCTCGCAACGTTGGCGACTCATTACGGCGATGAGACTCTTGCCCGTATGCTAGATGCCGCAAAACAGGTGTCAAGTACAAAATCGACGGCCACTAGACTGGAAAATGCGCAGATAAGCAAGTGGGTGGATGACGGGGACGCACAACTCAACAAATGGCTTGCTGATGGAGTAAGTGCGGACAGCGTTTATAAGCTTCTGCGGATTGATGCTGAAGGCAGTAATCTACTGAAAAGCCCCAAGGTGAACATGTGGATGAGCTACCTGACAAAGTTGAACAAGGACCCATACGACGTCCTACTTTACAAGGTGAGAGCGCACTATGACGACGTGGGACTAGCGAAAATGTTTGTTCTGTCCAAGAAAGACTCTTCAACAAAAGTGCTGGCCGAGAAGCTCGAGACGCTGCAGCTCGAGAAGTGGATGAACAATAAAAACAGCGCGGCTGACGTCTTCCGGATCCTGAAGTTAAACCAAGAAAGCACGACACTTCTAAAAAATCCAGTGCTAACCACATGGGTCGCATACGTTGAAAAGCTGCAAAAGAATCCCTACGAAATGCTGTTTTCAGCGATCAAGGCAAAGGGCTTCGACGACGTAGAGTTGGCGAGGCTCATCACCGCAGCAAAGCAAGACCTCCATACAGGGACTGTTGTCGCGAAACTGGAGAAAGTGCAGCTCCAGAAATGGGCCACGGATGGGAAGACCAGTGGAGACCTCTTTAAGTACCTCGGACTGTACAAAGCGGGTGACAAGTTTCTTGATAGTCCAGTGCTGAACAATTGGTTCTCTTACATGGAAATGCTGAGGAAGGACCCCTACACGATGCTAGTCCATACAATCAGGAAGTCAGGCTTGGACGAAGTAGACTTGGCGAGGCTTGTTAACAAGGCCAAGCAAGACACCAATTCGAAAACCATGGCTGCGAATGTGGAGAAGATGCAACTTGGTAAATGGTCGGTAGATTCGAAAACCAGCGACGATGTCTTTAAACTCCTTCGACTCGACAAAGAAGGAGACAAGGTGTTCGAGAGCCCAGTGTGGAGCACTTGGACCGCATATCTGAACAAGGTGGAAATTGACCCTGACGCAGATCTGGTCATGTACACAGTACTGAGGAACAAATTTGGTGACGAAGGGTTGGCAAATCTGGTTGCGAAAGCGAAGCAAGTAGCGAACACCAAAGAGACCGCTGAGAAGCTGCAGCTGGAGATTTGGCGGGTCGGTCAGAAAAGCTCAGATGACATTTTCAATCTTCTCAAGTTGAACGAAATGGGTACAAAGCTGTTCGAAAACCCGGGGCCATTAAGGACGTGGATAGCTTATGTGAACAGGGTGAATAGCTTCAAGAGAAATAGAGTGAAGGTGTTCCAGCCGATCATTCAATTGGAGAAACGCTTTGGAGAAGAGGAGCTAGCGGTGCTGTTGGTCAACTCGAAAGCGAAACACTATTTGACCAAGGCTGGTATAGCCGAAGATTTGCAAGAGTGGCAATTCAAGAAATGGATGGTTCACAAGACAAACGTCGATAAGATGTTCCCTTTTGAAGACCATACCAGCATACGAATTAAATGGGAGTACAAGCAGTTCTACAAGGAGAATGCCGATTCATTGATCATTTAGACACGATACAAGGCAAAGTAGTTCTCTTAGCGAAAAGTGGTTCACCGCTAGCTTGACAGTACTTTAGAAAGGATTCCAGGTTCGGATAATAAACGGGATCCATAAAGATAGATAGTTTTAGATTGAACAATAAATGAACCCATATGTGTTACTGCTGTAAACCATTGTTTTCTAAGATAGCAAAATCCGATCCTACTAAGCTGGCGGACGTGGCGCCGACAGGGGCGTAGGATACGATTTGAAATGGAGCGAACAAAACATGAAAGGTGCTAATTATAGTTGGCTAAGTAGATAGTTTTAGATTGAACAAAAAGAACTCATACGTGTTACTGCTGTAAGCCAGAGTTGCTTAGATAGCAAAATCCGATACTACATAATGGGTGGACGTGTCGCCGACAAGGGCGTAGGATACGATTTTAAATTGAGCGGTAAGACAACAACAAAACACGAGAGGTGTTAATTATAGTTGGCTAAGTACATGTAGATTTCATAAAAAAAGCTAATAAATTTGGATCCTAAATGACATTAACCATCCTGGACTTGCGACGCATTAACCAGATGCTTTAATCATAACTGGCACAGATTAGAGTGTCTAATGATTCATTCTAAATCAGCACAAAATGAGCGCTAAATGTATTAAAATGAGCAAAAATTGAATATTTGTTGAGTTTGGTATGAGAAGTGATCAATCTTTAGCTACTTACAAGATTAAACATGCAGTACAAGCGGGTAAGTAGTGCAAGCTGACACGCCATTACTATCTTTTGCGCTGATGTGAGCTAAGTTTCATGGCTCAGAAAGTACGACGCTAAACTAAACATATTGTGTAGCTTTTCTTATAAATGTATTTGGAGTGCAGTGGTTTAGGGTGGAAGATGAAGATACAATGATAGCCAACAATGATAAAGAGGTTGTAGTAAGAAGCTTGAATCAGGATCATTAGAATAATTATGCTTAATCAGGTAAGCATGATGCTGACCCAGATTACTTTATGAAATTAAAAATATCGAGGCGGCATAGGGGGCCTAACTTTTGTAATACTTATCCCGCCGAAGCGTAGCGGAGGCGGGAAGGCCACAACAAGTCTAACTAAACCTAAACTACCGCTGCGTAGCGGCGGGTAGCAGTGTAAATAACGCCACAACATCCAAAATTTAGAGTTATTAAAACAGCATTAAATTGCGTGTGCACCACACACGGGTGTGTTTCTTACACTAAACCGTGTGGAACACATTTTTTCACACTTAGCAAATATTTCAAAGATCCAACATCGCAGCAAGGATGGACGGAATGTCGCTCGCTCGGGAGCTGGTGCGCGCGCCCATCTACGAGCTCATGGACATGGAGAACACGCTAGAAAAGATCGCGCAAGCCTCGAGAAGATACTAATAGCTCAGATGGAGTTACTGTCACGCATTGAGGTGAGCGAGGAGAGCATCTACGTGCTGGCGTCTGAGATTGCCAACGCTGGTGCGAGTCATGACGCCCACCTATTCTTCGGAGTTTTGCTAGAAATGGCAGTTAACTGATTGGATTGGTAGTACAGTAGTATGCTAACGGTTTTGTATTAACGGATAGCGCAGCTACAGCTACCTACTTCAGTTGCTTGAGCAGTTTCTCATGAATAGACTTCGTCTTCTCTGCAGTGCGACGGGCCATACGGCTCTTTCTCGATTTACAGAGCATTTGCAGCGGGGTGCCGTAGCTTTAGCGTCAGAGGAAGACGTTCGACTCAAAGAGTCACTAATACTAAGTTCCTTATCCAAAGGAACTTACAGCCCACGACCAGGGCTCAAAGTTAATACCTTAACTTGTATGCTTCAAAACCAGAAGTGTTTTATGTACTATTATTGCGGCCAAGCCGCCTAGGGGTAAATATGTCTGTTTAACTATCTAGCATAGTTAAGCCTAATCAAATGCATAACGTGTGGCTTTCAGCTAACGTACTGAGAGACTATGTTAACTTTCCGCCTTATCTACTTAGATTTTGTTAACGCTACGGGAACTCCGTACCCCTGTGTGTATCACACCATGTAACCCCGCTACAGTATGAACCACAGCATTCGAAGGCGCATAGCTTCCAAATCCCGCTCGGAAATGTTTCTGCATTGCCAGCGAAGATTCACGGTCACATAGCTGTAAGGCTGGGCCACAACAAACACGCCACAGCTTGAGTAGTCGGGCTGCTGTGGAACTTCAATCCACTCAATAGGGTTCATGCATACTTTGACCACAGGTGCCGATGCCTTACGTCATCTCGCCACAAAATCACAAAGCCCTTCCATAACTGAGCTACCATCGCCGTCCTTAACACCGTTCCGCACCACCTTCATGTCCCCATGATAACCTTCGTCAAACAGGGGCTCATACAGGTAGGGATGCACATGTAATGTGTCCTTGGCTTCGACGTAGCGTAGCGTAGCTATTACGACCCCCCCGTGGCTTTGGTTCAGGTTGACAGGCTGAATCATGGACTTGGTAGAAGTGATAGACTTGTTTGGTGTCTTTGGCCATCCGACAGCTGCCGACAGGACGTAGCAATCCCGCACCGTGCTTCCAATGGTTTCCAAACAGAAGTCAATTGGGCTGTCATTCAGCCATCCTCTACAGAGTCCACCCACAACGTTTTCGAAGGCGATGCAGGCCTTACCCGACCGTGTCGTGTATTCAGAACTCGGTCGACTGGATGCAACCTTTGAAATCACTTCGTTTGTCATGGTGTAATATCGCTACTTTACAGCACCAACGGATAGCCCACTCGCTTGCTCCTGACTTCACAGCAAATAGATCCACATCGGAGTTTACCTTCCGCCAGTTTTTCTCCAACCATTTCCGGTCTTCGAACCATGCTTGTACTTTGGTCTTAACCGCGTAGAATGTAATTGCAGCCCCCACGTATTGTCTAGTGCAAATCACAGCTCACAGTTATAGCAACCACAGTATGACGACCATAAAAAGCAACCATACGTGAATGACAACCTTACCCGTTGAGCTTCCAATGTGTGGTGTCGAAAGAAGCTACGAGATCGAGCACGATTTCTTGTTTCTTGATGCGGTCGTCGAGTGGATTTAGGTGACGTTGCTCGCCTTGGCTGTCTTGATTGAAGCTTTTATTGACCGCG

>Contig_62

AACCTACCATGTCAGCACTTGATGTATTTTGCTGAGCATGTCCATCGTTTCAAGTATCTAGCTGCCATGACTGTTCACCGCACGCTGGGATATGATGGCGATGGTGGAGCTGGAGAAGGATTTCAGAGATGGTATATCATCGCTTCAAGCTGTGGAGGCCTCGATGAAAGTAGCAGGTGTAGCTTATGAACATGATGGTTTGACTCAAAAAGGAAAGCGGGATCGGATGCGGTCGAAACACAACTTGATTCGGTCGAGATGATCGAAGCGACCTATCATTATATTCCGCAACACAAGAAGCAACCCATGACTCAACTGAAGAAAATCATGATCAAATTGGCGGTGGAGCCCAGCGTAAGCCTAAACGCCGTGTCGTATATGTAGAGATGCGGCGCCGAGAGCGTGCCGACGTTGTTGTTCTGTCATCAGAAGAGAAATACTGCTATGCAAAAGCTGTGTTTGAACCTGTGATGGAACATCTAGCGTAATTGTCGAGTCCAGCATTCTACTCCGCCCTGAAGGCCCGGAAAACAATAGTTAACAAAGGTCTACACGATGATGGATCAGCCGAAGAGCAAACATCAGACGCTACAACAGTGTCAGATGAAGACGGATCAGGAGAAGACTTGGATGCTATTGACACTACGTCTGACATAACACCAGCAGATCTCATTGAAACTATGAATTTCATTCGTGAAATGGAGAAACGTGAGTTCGATCTGCAAAAACAACCTACAAGGGCCGGGTGATCGCAGAGAAGTGAGCTTGGTAGCTCAACCTCACGGAATGAAACAGTTCCATCCAGTGACATTGACTTCGCCACTTTTTTACTTCGGCGAATCCTGGCGTGACGCCCTGATTAACGAAGTATTACCCGACAAAAGCCGCGGTCACGGTNAAAATCTACGGAAGCACCACCCAGTGTGAAAAGCGATTACCCCCAAAAGCTGCAATAAATGCAGAAGCGACCAAGAAGATCGAAAAAAGTTGGAACACAAGGAGGTCGTCTGTAAGTGCTACGTATTCCAAAACCCAAACCACAAAGCAACCAGAAAAAAAAAACACTGAAGCAAGCTCGGCTGGAAAAACTCGCAAAGCCTAATAAGCTAGCCGTTATTACGCTACCGGACAAACATGTGCCATCTGTGTCTCGCGTCATCGTGTGGGCAACAAATACTTCCGACCGGAATCATGTCAGCGAGATCTTGGCAGGTTACCCAGCGATCTTGGACGACGATTTCATGAATGCACGTGTTGCACACAGCTGTCGAGAGTCTGTGTCTCCCAACGACTACGTCTATAACTTTGTGATTCCTAAACCGCTAGTAATCAAGCTGAAAGCGTTCATTGAAGCAGAACGGAAGAAGCGACCCAGGTCGAAGTGTTTCAACCCCGTGGTTGAGCACCAGGACAGCAACACCGAAGCAATTATTGCATATTTTCCTGGTGGAACGCCACGATTTACAAGGTACGCTTTTGTTGTGTTGCTTGTTGTGTTGCCCGGTGTGTTGCTCGGTGTGTTGATATGACTGACTTGCTTCTACAATGTTGTTGGCTGCTGTACAAATCCGTTACAACAGTGAAGCCGTCTTCAAAATGTCGGAGTTTTACAACGTGGTAAAAACAGCTAGCGCATGGAGAGCTGGCATGGAATGGCTGCAAACGACAATATCCTTGCAAACCCGAGTTGTTTAAAGCGGAGACTGATTCGGACGACATATTCTTACTGTCGGCGGGGAAAAACATCAGGAGCTGGCAAACGAAGTGGTGGTACAGCTAGAGGGTGCTTGTTTAAGTTCTAATTTCCGACTATCCAGTGGTGAAGGCGCCGTTAAGGTCGACACGCTCGTTGGAATGCTTGCGCGTGACATAATGCTGTCTGATGGCATTATCAATTTCAGTGTTCGATGCATCTGTGATGCGCTGGGAGATTGTTACGCATTGGATTCATTTTCTCCAACTATGGGCTGTCCGAAACCACCACAGTCACGGATTTTAAGCTTCCACTACCTTGTGTTGCCTCTGCATTTAAGCAACATCCATTGGGGTGTTGTCATTGTCGCTATTGCTTACAGAAGATAAGATCCGTGCTTTACACCCTACTACTACGAGCCAATGTGCGGATCTTCGTATAGCGATACGATGGAAGCGACTTACACGTCTACGGTTGTGCCATTTTTGAAAGAGTGGCATGATAAAACCATGCCGAATGAAGATTACCCAGTGGAGAACCGTAAAGTATGGTTAATGTCGCCAAAGCAGCCCGATGGAACTTCGTGTGGTGTCTTGACTATAGCCCAAGTGTTTTCCGTGTTAAAGGGCAGCTTCCCGCTTGCACAAGGTGTCGTTACAAAGGACGATATCGCAATTATGCGGCTACGTATCATGTGGAGGATAGTTATGCAACCAGACGTTAACACTGGAGCAAACCAAATTGCTAAGGAGATAGAGGCGGCTGACTTGGAGTTACTTGCGACCATAAAACATTAGCACGAGCAGTTGCCTTCGCCAAATGGTATCGACTTTTTAAGTAGTTACGTTGCCTCGTCTTCTTCCTGTGCGTTTTGTATTATGCTGATTTAAATATGCTCTAATAGAGTTTCGCAGCACTGTACGAGAGCCACTGAGAACGACATCACGTAGCCTGTTTCATGTGTAGGTATTCCAGCAGCGGCGGCAAGGCGGATAGTCTGTTCCTACTCGTGAGCATCATCAGAGCCAACTCGTCTCGTTCATTCATAAGCTCTAGATAATACTGTGAGATTTTCTCCAGCTTTTCTTCTATCAGCTTGATATTCTGGAGGCGAGAGCGCAGTATTTGCTTGCAAACCATAGCCTTTCGAGTGCGCGAGAGAGTGGTGCCTTCATTACTTCGTGACTGTAGCGTTGGGGGGGGGGGGCAGTGGCGGTGGCAGTTGATCGGAGCAAATGCCGTCGAAGTGTTTGCCGCTGCCTTGGTCCATTTTTTGTGTGCGCCAACAGCCGCTATTCATCTTGGGCAACTGTGGTATTTGTCAAATGGCTTATGGAACTTTCACAGTTCCGGTATTTTATTTCTCAAAAGAAGAAAGCCCTAAAATAAATTACGCCTTGTGTTGCTTCTGGGGTTGCTTCTTGTGTACGGGAGGCTAATAGGTTGCTGCAAGACTGTCGTTTTGTCGTAATGAGGTTGCAGTAACGGGACTCAGAAAATCTTGTCCAACGTTCAATGACGAGTACAATAACTATCCTATTTGGAACGTCACCATCGTCCAGGAGCAAACCAAAGGCGAAGTTTGGAGTCTTGACTCTTTCAGTAGTAATTGGGGGAGTCGGACATTGCAACAAAAAAAAGGGCTCAAAATTTTCTCCGGGTTACGCTTTTTGACAATATCCTCGAAGTAAGCCACCTCCGGAGGTGCCTTATGGGCTAGCCCTTTTATTTCCTAAATAATTCTCCTTCCCATGGAAATGAAGGAATGTGCGCGCCTGTGTCGGCGCCCCAGCAGTGTATGTGCGGGTCGTTGCTTTAAAATGCAAATAGTCTCTTTTAAGTGCGAATTTGAGCTTACTCAGTAAATGTGCACTCCCCCGCTTCAAATTGGATACGTCTATGCTTATTTTTTTGGACCATTTTTTAATGTCGACTCCCCCTAATAATATATATCACAAGCGAGCTGATAATCGAAGTACCACGCTTGATAGTGTACAAACGGAGTCTATTTACGGTAGGCTCGAAGTGTATGCAGCATTAAAGCGTTTGTAGTTTTCGACGATTTCCTTTTCGAGCTGTGTGGTTCGTTGACTGGTAGCGCTTCCGGGTACAGTTCTAGACGAAACTAGGAGGTGCGCGACAGCACTTGGCGTCTTCTCCTCACTTGCCCACTTGCCAAACTGCAAGATCTGGAAATCATTAGCCATTTTCTTAGAGCTGTCGATCAACAAACCCTTGTGGGCAATGCGAGCCAGATCTCCGTCCTTATAGTGCTTGGACAGTGTTGATAGCAAGGTCGTGGATTCGTCTGCGAAGTCCTTGTTAAATCGCTTCATGAAGGTGATCCACGTGCTCAACTGTGTCCTAGCGAAAAGGTCGTCCCCTGCGAGATGGAGTTTAAGCAGTTTAAACACTTCATCGGTCGACTGTCCTGTGGCCATCCAATTGTTCACTTGTGCGGCCTCAAGTTTATTCGCAATGCTCTTCGTCTTGCGGCTCATCTTCGCCGCCGCGAGCATCTCAGCTAATACGTCGTCACCGTAGCGAAGCGTTAACACGGAGGCCATTGTTTGTTCCGGATTGGTCTTGTCGAATTTGCTCACATAAGTAACCCAAGTTTTCCATCGTGGGTTCTTGAACAAATTGGACCCTGCTTCGTGAAGTTTCAGTAGGTTGAAGACTTCTTTCGGTGACTTTTTCGTTATTAACCACACTGGAAGTTGGGCGTACCCCGTGAGCTTTGATGTCACAGACTTAGCTGCGTTTGTTAATGCGGACGTTGGGAGCGCTCGACCTTCTTTGTCGTCTTCGATTGTCAGCGCAGCAGCTCGAAGCCGTCTCCTGGTGAGACCGCGGGAATCGCGTATGGCTGTCGTCGACGCCGCAGCTGCCGATAATACCGCCGCAAGAATCAAAATCGCGAAGAATGTTCGCCTCATTTACCTAATTTAAATTCCGAAGTGGTGCTGATGCACAGGACAGGTACGAATTGTTGGTGCACTGCTGAGGTTGCATGACTGTACCGGTACTGTAAATCCAGTTCGAGCCCGGAAGCATAAATTTTGATGTGCTAGTACATGTACAGAGACATGAAATCCGCTCAGTTTTACTCACTATTAGTACATGAAGAGTATACCCCTGAAACATCCACCACATGGAGGTTAAAGCCTCGCTTGTATCTAGTCATGATCGGGACTAGGTTTGCATCCGCGTGTTCCGCACAGGCTCTGAAACCGCAGGCCGCCTGCCAGGCGTGCTCGCTGCGCTCGCATTAGATACCCTGCGCGCGTGGTTCAAGTTTGGTTTTATTAATAACCCTTCGGTGTGGGGTCTAGCAAAAAAGCTTCACTTGCTACACGTATATTACTTCAAAGCGCTGCATTACGATGATTCATGTATTATTAATAGCAGGGTGCACTAATACACGTACAACACATTGTGCTGCAGCAGAGTGCTGTACCTCGTCAGTGGTGAATCGTGATCATCTCAAATTGAGATACAATTGAGCCATCGAATATTGATAATTATAAACACATTAGGGTACGGTACTGTTTCTGTAATTTTTAGCAAAGCAGAAGAGTCTAGTAACGCGGGAACCAACCTAGCAAAGGGTATTCTTGGCTCGGCCAGGTGTATTTCCGAAATGGTACACTGTACATTTACAGAGCACGCTACCATCCATTCCATTCCTTGCGTTTGCGGTCCTCTCGGGTCTCCCCCATTCTTCGATTTTCCAAGCTATTGCCACCAAAGGAATCTAAGTCAACCCGCTACATGCTCACCAATGTTCAGTGTTGTGCCGCAATATCATTCAGTTTGTATGCAGAGAAATGAAGTAAAATGATAGGTCAATAATGGTGTAGCTCTCTGCTATTGGTTGCGATGGCAGCAACTCGTCCTAGCCATTCAAGAAGAAAGAGGCCGCGTGCCCCATCACTAAGATAGGTGAAGCCGAGGGATGTAATGGAGAGATGGCCGATGCCATCTCACTTCCAAAACTGACGGCCTAGATCAACAACTACCTACAGCTGTACGCAACATTTCAAGTACTCCGATGTCCACCGATGCTAAGTCTGGCGGTGGACTCGAGAAGTTTAAGGGCAAGTCGTACACAATGTGGAAAGACAAGCTTTTGACGCACGTCAATCAACTCGACTATCAGTACCAACGCAAGCAGCTGGAAAAGGGCCAGCCTGAAGCGAAGGTGCTCATGGCGGACTTCCTGCGGGGATGTCCGGACAAGCCACCATCACCGACCAATACTATGGATGAGCAAGAGGCGCTCTCTATCAGATGGGACATGATGCACTGGATGAGAGGAAGAGGAGACCTTCAGAACCTCCTCAACCAAGTCCTCCCAGACTTCTTCCTAAACACGTTGCCGGACGTGGTGTCCTCAATGGACCCCAGCGAAGTGATTCGGCTCCTGGAAAAGGATTTCGGTCAGGGTGACGCGGCTGGCCTCATCGACTTGATGCGATCCTGGGCTAAGTTGACACGCGGCCCATGGCGCGATTTAAGGTCGCTGTTCGCGCAACTGAAGAAGGCTAAGAACGAAATCAATAGAAAGACAAAGAAGCTGTTCGATGAGGAAATGGTGACAGAGTCCTGGGTGTGTGTCGAGGTCCTCTCACAGCTGCCTAGCGAGTTCTGGGCCTCTTCGATCTCGCTGAAGAAGGGCGATTTCAATATCGATCAGGTCGAGAGCGCACTGCGCAAGATATTTGGAGACAAGTCCAAGAAAGAAGTGGGTCTTATGGATAAGTCCCACCCGATCACCATCAACAACGTGCGGGTGAATCGTGGGCAAAAGCGAAAAATGGGTGGCAATGAGGGTGGCAAGTGCTTCTACTGCCTACAGACGGGACACTTCAAGACGAATTGCCCGACTATGGCTGCCGACAGGGACCCCAACCGTTCAGGTGGACCCCTCTTCCGCACCGACGTTAACACGGCACCAGGTGCAAAGAAGGCCAAAAAGGGCAGGACGACCGCTATCAACACGATCACAGCGGTTGTGAAGGACGGCAAGACGCACGTCAACAAGGACGGAAAGACGCTGTTGGAGGAGTGCATGGAGGATGAGGCTATGGACGACATTGAGAGTTTAAACCCAGCCCTCCATGAGGATTTTGAGGACATGGGTAACTCAACCCAGACCCCAAATGAGGCTCTCCAAGATATGGACCAACTCGAAGATGAGGTACGTAAACAAAATGCATCGTACTTAAAAACGTTAAAGCGGTTAACCGTAACCGATGATATGTGGGTGGTCGACACTGGTGCAGGCCGAGCTATAACATCAGACAGAAGCTGGTTTACTGGGAAGCTCCGACCTGGCCAGAATACCGTGTTCACCTATGGTAATGGAACTGTTTCACACAGCTCTCTTAATGGATCAATAAAACTTAGCGTTTTGACTCCAAAAGGAAGATTAAGTGATATATCATTGACAGATATTTCGTTTGACCGACAGTGTGATAGCAACTTACTCAGTTCATATTACCTTGCTCGGCACGGTTATAGACACCTACAGTCTAAATCAGGTGATTTTCTGTTCTTTTTAGGAAAGAACTTTAAATTGCTGTTTGCAGCTGTGGCAATAGGCGAAGTCTACTACTTACCGAGCGCGAAACCAGCTAGAAAGAGGTGCTTTTAATGCTCAACTAAGCAAAGTTGGAGACATACTAAAGGAATGGCATCTGAGATTAGGCCATGTGGGCAAAGAACGCCTAATTAGAAGCATGAGCAATCAGAAACTAAAGGGTTACCTAACCTCTCTTACTCAGAACTCAAGAAAGTTTCATTCTTTTGTAGTACATGCGCCTCAATGAAGGATCGCAGAATGTCCTACAGGAATTTGATAGGAAACAAAATCAACAGAGCCATTGCACACTCTGCACATGGACTCTACTGGAAGGTTGAGGGTTAATGGACTGTATGGTTCCTTCGGTTATCGGTATGCCCTTGCAGTGGTAGATGATGCCACTGCCTATAAGTGGTATTTTGCAGTAAAATCCTTGAAAGAGGTTCGAGGCAAAATCCCGGACCCTCTTAAAACAGTTGGAAGTACAGTTTCCCTTATTTAAGGTTCGCAGAATTAGGACAGACGGTGGGACTGAGTTTCTAAAACAGTGAGGTTTTTTAAACTGTGTTCTAAACTCGGTCTAGATTTCCAAAGCTCTAACGTCGAATCCCAAGAAAGAAAATGGGAGTGCTGAAAGAGCACATCAAACCATGATGGCTGGTGTGCGCTGTGCACTTCGAGGGGCCAATATGCCAGCAAAGTGGTGGCCCGAAGCTCTACTCTATATGTCGATATTACAAACCGACTACCTATGGCGAGGCTCGGGATGAAAACCCCGTATGAGCTGTTACATCGGAAACAACCCAGTGGTTTAGCATTTAGGATCTGGGGCTCAAGTGTTTCGCACATGTTCCTAAATCAAAAAGGAAGGACCCAAAGCTGGGTGATAGGGCAATTGAGTGCAAGCTTTTGGGACTTCACCAAAATACAAAGGGTACCGTCTTCTTGATGTGAAGGCGAACAAGTATCTTATAGCGAGAGATGTGAAGTTAG

>Contig_65

CCTAGTCGTAGCAACAGAGGATTAGTGTAATTTACAGCTTCGCTCTTCTGGGTCAAGCGAAACCCCCACTTGCAGCACATAGCTTCGTCGACCACTAGTAAAAGCATGATTTCCTTTTTCACAGTATTGGTATTGATACTACACAGATCGACAGCCAGTTTCTCCTATTAATTCTTTACACCCGAGGTAGATTCTTTAAATATTACAGTCCTTATCTACCTAAGACTACTATCCAGTACAGGAAATGCTTCACCGTGGCGTATCATGTGAGCGCTTGCTTCACATCAGCTACCCCCTGCGACCGTGTGACCCAGTCGTTTTGCAGCTACTAATTCTTACTTAATCAAATCGAAAGTTGCCGACGCGCCGGGGTATGAACCCGCAACCTCTCGATCAGAAGTTCAGTGCTTAACCACTGCACCACCTGGCCGGGCATCTAGTTTTCTCCTGTAGCATCACGTAGTGCTCGAGTCTACGTCAAAAATTCCTCCCTCTTGCATTTTCAAGACACAGCACGGAGCACATTCATAGGGGCCCAAATCCTCCTTCTCGAATTCTAACAGCCGAGCTTAAGGTTGTGGTCTGCCATACCCTTGATCGTGGACATGCGCACGTACCAAAGGCGGTTATGGAGTAGCTTCGTTGCATGGCCTTGTCCCAACAACCAACGCTTTCTTAAAATAAAGCGGTGTCTGCAAACGGTACAACCCGCGATCCAACTCGAACTTGAGCTTCAAAGCCCTTGGTCAGATACGCCGTACGCTCGGCGCGGGAATATGAGAGTCAAAAGTCACACGTAAGCTACATGAACGTTTGGCCGATCAGATTGAGCAGGGATGACTTGGAAAAGCTCATGTCAAAGAGGAGGCGCTTCTCTTCCTGACCGGTTGCCCATTGCGTACATGCATATTGACCGCTCCAACCGTCGCGTACTCTAGAGTCCCATTACCAAACGCCAATACAGACGTATCGTCATAATGAAGCTCCGTGAATAGCGTCGAATGACCAGCTACACTCACTTGAGCGCCCGAATCGAGGATCCACTCTTTAGCTAGTACTCTTATCAGCAGTGTTGCCTCGGGCCACAAGAACCGAGCCAGCTTCGTCCAAAACATGAAATGCGCAGGAGCTCTCTACAATCACTACAAGTTGCATCGAACGCTCGCCTGACTCTCCGCCCTTACTACGCGCATTGCTCCTATACTTAGGGCACTCTTCATCACCTCGCCAGAGACCGATTGCGCCACATGCGTGGCCCTTTGTATTTGCCTTGCGGCGCAAACTATTTCCCGACAGCCCTATGGTAGCACTACGTCGCTTTAAACGGAGCGGTGGGTGATGAGCTGCGAATTTGACTGTAGCGTCTGGTGCCGCTCGATTTACTGCCATCATGCGAACGACGCCCTCCGCGACTCTTATTTCCGATAGCGCTTACGACTTGGCGCTTCATCCCTTGACCGCCACTGCTACCGCGATCGCCCCGCTGTCGACCGGTTTCCTGGTGTGCGTGCCCAGTGAGTAATTGCCACTCATTTGCCTCTTCCCATGTAATACACTCGTCTCTACGGAGTCGCATCTCTTGCTCACGCACCAAATCAGGATACACTCCGATTACATTTGGTATCAGTAAAGACGCAGTTTCATCTTCTGGTAATGACTGACCCAGTCGGAGCTTAAAGTCGTCAATGTCCTTGAAAGAACCGTCGACATTTATATCGCTTTGAAGACGGCGATTGAGCCCGTCTCACTTTAGAAGCACCACATTTTCTCCCGGATTGTTGCCGTAATGATCATCAAGAGTGTCAATAAATGTGCCGGGCCCACAAGATTGATGTACTCCCCCGAGCCACCCTTGTACGTCTGGACGCATCGATGTTACCAGCATGAGCTGCGCCAACACGCTGGCGAAAACACGGACGCTGGTCTTTGGTATCTTGATCACTAATGATTTCTTCGCCAGCTAACGGCGCCAACAAGTTGGAGATAACACATATTTCGAGATGCCACGACTTGTAATTTTCGTCAGAGAGCTTGTTGCTCAGGTCTACGTGGCGGTAGTTTGCAAACCGAGGGCAACTATGCTTACCAGTGCGCACTTCGGCGGGTGATACCGACACAGACCCAACCGCTTCTTGAACGGCGGCAGCAGCACCAGTAGCAGCCTTCGCTCCCTCCATTACAGTATATTACTAGCCGTTCGCCAGCGCGCGCACACGAGCTGTTGTAGTAGATGCAACGCCACGACTCTCACGGCGAAGTGCGTACGGTGTTGAGACAAAGGTGTCTCGCCGGGAGCGGCTGGTTGCGGCAAATTCCTGGATAAGCCTTTCCTGGGGAGCCGGTGTTGTCGCCACCATGTCCACGGCTCCACTTTGATTGGTCCAAAACGCTTGCGATCGATTCAAAATTAGTCAGTAGCCTATCGTGGTGCGGCTTGTCGCGTGCTAGCTGCGTTGTGTCGGATGAAGACGGCGGAGTCTGTACACAATGATCGCAGTCTTGTTGCACAGGGTGCTACCTCGTTGGGCTCATAACCTGAAAGTTGTCAGTAGACTGCCCTACACTTCAGGTATGTATGTAAATAGAAGAACATGACTATTTGTAGCGAGCAAAGTGAAAAAGGCTGGTGTCCTCACTATTTGGAGAAAACACAAGTGTAGATACTAACCAAAACCCCTGTTTATTTCGATAAAAAGCGCCGAGCCAGCATTAGCCCAGACTGATGTCCTGACTTGGCAATTTTAGACGAGCTTAGTTTCTTATGGAAGGTACTGGGTCGTAGAACCTAGCCAACCGCCTCTGAAATACGTTTAGACCATAGTTTTACGGTAGCGAGTCGAACAACTTGTCGGATCAGGCACTTAGTCGATTTTGGTCGCCTTCCGAGCGTTTTGCACGATTAGGAGCTTTCAATACTTATTTGCGGCATTGATTAAACAAGATTTGACAAAAACTAATATACCGGTAAAATACTACAACGTAAATTGTTCTTTTTGTCTCAATTAGGTATGCTTTACACAATGAGAGACTGTGCAGAGTATAATAGATTAAACAGTGCTGCTTATATTATAGGGACACTACATTTTTCAAACTGTACTCACAATACGAGGAGTTAGATGGTGCAGCTTACTCCAAGAAACACTTGCCTAATGTCTAACACGGCAACCATTTTTAAAAAAATGTACGTATTGCAAATTTTAAAATGAAATTGCCATTTTAGGGGGTGTTCTTATCTATGGGGACACCACCATGAATGGCGTTCGGACTTCGGCCAATCTCTCGTGCTATCCAGTACTTTCCTTTTCCATCCTTAAAGTACACTTTTACCCGTCCTATCTCCTCCTCAGGCAGTTTCTTGCCTCTTGGTATCGTGGAGGTGCCTGCGGATTCCGCGAGGAAGAAATGTACAGTTTGTAGCTTTATGCGTAATTTCAGCCAATCAAAACCAATAGTCGCTGAAGTATCCACACAAAAAGCAAGGACCAACCATCATGGTTACCATGAGCCATGTTTCTGCTGCTTATGGCAGCCTTCTTTACTTGGGTTTCAGCACAACCACCCCCTAGCTAAGCCGACAGGTCGAAGATGATTGATCCCGATGTGCTCATGAAGACCGCGTCACTGTCAAAAACCACAATTGCTACTTCATCCAACCGATTTTTAAGGCTCCTCGACACAGAAGTCCAAGATACAGTCCGTGGTGATAATGATGTAGACCGCGAGGACAGAGGGAATACGCCGTCGATATCCAAGGTCGACGATTTGATACACAAAGTATTTAAATCTAATCCGGAGAAAGCGCAAATCGAGGCATGGATGAAGTCTCGAGTGCACCCTCAAGCGCTTTTTGCTACTTTGCGTCCTGGAAAGAGTACGACGAAGCTGGACAATGACCCTGATCTTCTCCTTTGGTTTAAGCTCGTTGCTGCATTTAGAGCTAAGAAAGGCAACAAGGCGTTTTCGGATCTCGATCTCTACCACTTACTACTGAAGAAAAACAGTGCCGAAGAACTGAACATACTTTTCGAATCTTTCCTGAAGACTGGGGGCTTGAAGGATCTTGGCAAAAGTATGCAGAAATCTCTATCTGGTTCTTGGATGTCAAAAGCCATAAAGCACGAAACAAGCCCAACAATCGTTTACGATACCTTACGTCTTCGAGAGGCTGGCACGAAATTGGGTGATTCGCCAATCTTTCATCAGTGGCTCTCTTACGTGCAGAAGTATCGGGCGCAGGAGCGGAATCACTGGTTCGGAGACCGCGAAATGCTCGACCTGTTTCGAAAGACCATGCCGGAAGACGACGTAGTAACGCTGCTTCATTTGCTTCAAAACGTCCCGGGCATGAAAAACCATGGCGATGCGATGCAGCGGCTCATGTTTTTATCGTCTAAAACCAGCCGAAAAACGATGAGCGACGTGTGGCTAAAATACGACGTATCGCCCGAAGAAGTTTGGAAGATTTTGCGCTTGGCAGAGACCAATATGGACGCTTTAAACATTAACGCAATGTTTCATTCAGTGGTTCAGGTACGTCAAATTGTACAGGATCCACACAAACAAGAGCGTATACACCAGCGACAAAATTGTGCGCTTCCTGACAGAAAGCAAACCGTTGCGGTCAGACTGGAAATTTGCAACATTTTTTCAATCACTCAAGGACGTCCCTGATTTGAAGTTGTTTGCTGAGAACATGCAAACTAACCTATTCCAAAAATGTCTGCAACTGGAGTGGGACCCGAAGGCTGTCTCGAGTATGCTGGCAATTCCCTATCCTACTAGTGCTCTGCATCTGCCGAAGAGTGATCCTATTTACAAGACTTGGGAGGCATACACTCTTTATTTTTCAGAAAGGAAGGGCGGGGTGTTGTTGCTGAATAAAGTGAAGACACTTCTCGACAACGACAACCCGATAGGTGCACTTACCCAGCTATGAAAGCTCAGCGATCTGCTGTTCCGTTACTAGTGAGATCTTTTCAAGAAAATAAAAAATGACTCGAGGAATCTCTGGAGGTTGTTTCACAGTTACTGCAACGGATACGGGAACTTGATTAATAGGGTCTTTTACAATACCGACAATGCTCTTGCTGTGTTTACCAGGGCAACAGACTTCCATTTTTTTTTAAAGAAGCTGNTTTTTTTTTCTGAAGCAACCAGAAAGTTCGGGCATCGTTAGGTGAGCTGTTTTCGGCATCTAAACAGCAAGTTCTTGAGTGATCTCTGTGAGCATACGACCAACTGCAAAGGCTTTCCCGGGATTAAATCGAGGGTGGGCGGCGTCTTAATGAATGTCCCAGACAGACCAAAAAATGGTCTCTTTCGAGATGTTTAAATGAAAACAAAGCTGGTGGTTTAAACCTAGTTCTGGGATAATGACACCAGCATACTTCATACAGGGTTTTACTGACTTCGCATTACGCTATACGTAACTTCAAAGGAGCTATATACCTCGCAGAAGAAAATACGCCTTAACGACTCTTAAATGGCCTACTGTACTTGTCCAAAAAACGCAGTTTAGGACTTGTTTACGCGTATTATATGACATCTGCAGTGTGTTATTCGACAGTCATTCCGCACGTCGTTGCCCGTATTGTATTGTGACTATAGTCGATAGGTTATCAAGTTTTAGAACTACTAGCCACTTCGTAAGGACACAAGACCAATCATGGGCTGCCCGAGATTGTGTTTACTTCCCGTAGGTTTGATCGAGGGTTACTACCTCAAATGCGATCCAAACCTCTGTCCACGGACAATACTTCGAAAACTTTAACTTTCTCCAACAGGTTGAGATCCCATATACGGTGTTGTGAACACTCTGGCAGTAAGGGTTTTCACCTTTTCACTCGTAGCGAGCTCTCGATAGAGAACTTGTCATCTTGCAGTGCGTTATTCGATAACTTTGCGTCCATTCCGCATGTCACCACCGGCATTCGGAGTGAGGTTATTATGTTTAAAACACACAAGCCACTTCGTAATACGACCACAAGCACACAAGACGATCCATGAGCTACCTGAGATTGCTATTTCCAGTCATCGTTGTCTTCTTGGGGAGAGCTTCACCGCATGCCCCCCCCCCCNGTGACCAAGCCTACGAGTTCAAGTTGACTAGTCCCAATGTGATCGTCGACACCAAGTCTTTCTCTGCGGGCAACAGGTTCCTAAAATCTCACGACGCAGAAATCAGGAAACCAATCGATGTTCCGGACACTGCTGAACGGGTTAATACATGTACGACCGCGTTATCGGTAGTCAATGACCTGGTGTACAAAATGACCAAAACGAGCCCAGTGGTCGGGAAACTAAAATCGATTATCACCGTCCAGAAAGGCTTTTTCAAGTTTTACCTCTTGGAATGGGTACAGCAAAATTGCCGAAGCTCCTGCTTTGGTTCAAACACGTGGCCGCCTTAAGAGTTAAAAAAGGCGATAAGGCGTTCTCAGATTTGGATATATACTGCTTGCTGCTGAAGACAAACAGTGCGGAGGAACTGACAGCACTCTTCCAAAGTCTTCAGAAAACACCAGGTTTCGAAAAGCTAGCCAAGAGTATCCAGAATTCGCTCTACTACCAGGGCATGGGAAATACAATACCCAGTGTTATTTTGCGTGAATCACATCCGTAATAAAGACTGCAATACCTGGCAACACAATAATAATCACTGCAATAATCACAATATATTGCGATCACTTAGTCTTCGATGCAACCGAGAAGGGTGGAGCAGTTCCACATACGGCGATTAGCACGAAGAAACATGTAACGGGGTACGTAGCACTAAGTGTACCCTGGTTACATGAAGTGTGACACTTCACACTTCAGGAGTATGACATGTCATACTCCGGGATATTTGGTACAAGGTACCAAGGTCATTCAAAACCAACCATTGGTTAAATGACATTGCATTTCTATAGGTAACTGATAGTTTGTATTAACCTCAAACTTAACCTACCTATAAATATACTCAGTTTACAATCTACCTTATTTAGGTCACTTCGCTCTCTTTAATTAAAGAGGGGACCTGTGAACTGCCGTTCACTGAGAGTTTGTATACTCTCGGAAGATAACTTCCTCTAGCGCAAGGTGCTGTGTGACCCCGTCACACTTGGTAGCACTTGTAGTCAATCCACGCCATCGCGGGTAGACTACCTGAACCTCCGAAGTTCGTTGCCATGGATAGGAACGACTTCTCCCATATGAACGACTCACAGTTCGAGTCGGTTCGGAAGATGGCAGGCATCTTCGGGATGGAGGCCCTGCAGAGCCTGGTGGTATCAACACCGGCTGAGCAGCTCGAGCGCGTCAATGCATTCGACGCGTACGAGCAGGGCCTCATCGCGCATGTGCGGGAGAACCTGCAGGCTCCGGCGGCGGAAGCAAACCCGCCTCACCCGAAGCCAATACGGCTGAAGGTAAAGCCGTATGAAGGGAAGGAAGGCGAGAACCTTCACTTCTGGGTCCGAGAAGTAGAGCTCGCGATGGACGCGGCTCTGATCTCGACCGAGCAACTGCGTGTTGCTTTTGCACTCTCCAACCTTAGCGGTCGTGCTAAGAGTTGGGTGTACACTCGGGAAGCGACATCCCCTGGATGTTTCACATCCTRGGCTCAGCTGTGCGAACAGCTCCGAGCCGCGTTCCTCCCGGCGAACTACGAGTACCGCCAGCGTTCGCGCTTCCTCGCGTGTAAACAAGGAAGACGCGAACTGCACGAGTATGTGCAAGAGATACGGAAGCTCACGGCGTCCCTAGTGGGAAACCCACTCAATGAACACATCAAGATGACTGTGTTCATGGACGGCCTTCGAGTGGGCCCCGCACGCACTCAGCTGTTCCGGGTGCAGGCAAGCACCCTTGAAGAGGCGATTCAAGTGGCTCTTCAAGAAGAATACAGCCATCGCCAGGCGCGTACGCCGGCCTCAGCGTGGCCTGGGAGTTCCTTGTCGGCTCCCGGTCGAACAAGCCCCAACGGACCAGTTCCCATGGAACTCGGGTCCGCCGAACTGCGTGACATCCGCTGCTTCGGCTGTGGGAAGATGGGCCACTTCAAGCGCGACTGTCCCGCGGAAAGACTCCGCAGACGGTCCTTCCCCAAGCCCGTCCTCAAGGGTCGCTGGCAGAAGCCAGGATCCCGGGGACAGGGGAACGCAGGGAACCAGTAGGGGCGGGGCGCCCTACTGGGGAAGAACTAAGTCTTCGTGATGGGTGCGCGGGTGGCGCGTACCACGGGGACTTAGCCCCTGAGAGTCTTCGCACTCTCGAATCGGCGAAGAGCAGCGGCGGGTTGCTCGTAGTACACGGATGTGTACGGGGGGTACGATCACCCCTTTAGAATCTTGATTGACTCTGGGGCGTCGAAGAATTTCGCACGCCGCCAGACGGTTGCTCGTAACAGCAACAAGTTGTCCGAAGCTTTGCGAGAAAGCAAGGGCAACGGGACGGCGTCAGTGCGACTGGCCGACGGGAAGGTCGTCACTGTCCCCAACGTCCAAGTGGATCTGGCTGTCAAGTTTGAAGACTTTGACAGCCTCGAACAGTTCACTGTTCTCGATATGGATCCATATGATATGATCCTGGGCATGCCCTGGCTTGAGAAGCACGAGCCCTGGATCGACTGGCGAGGCAAGGCAATCGGTGCCAGCCGCCCTGCGCAGTCCGACAGAGCATTGGTGAGTCATGTTCCCACCTCTGTCAGGACCCGGGGCGCCCGCGAGGGCTGCCAGGGTACCAAAGCATCCGGCAGATGCTTGGGAGTCGTCGACGTATATGACGACTCCGAGGATGTTTTGATGGTGGCCGCGCCCAGAAGAGGCGCCGGCCAAGTGGGTAACTTAGGTCCACAGGCGGGTAACTTAGTTCCGCAGGCAGCTGCAGCTGTCATGAACACTGAGAACAGCGTGTTCAGAGTGGGTAACAGAGTTCCACTCACGGTCGCCCAGACCATAACTGAAGAGGAAGACGTCGGATACGCTTCCTGTGTGAGTAACACAGTGCCACACGCGGTCGCCCAGACCTTAACTGAAGAGGAAGGCGTTGAACACGCTTCCTGTGTGGGTAACACAGTGCCACACGCGGTCGCCCAGACCTTAACTGAAGAGGAAGGCGTTGAACACGCTTCCTGTGTGGGTAACACAGTGCCACACGCGGTCGCCCAGACCTTAACTGAAGAGGAAGGCGTTGAACACGCTTCCTGTGTGGGTACAACAGTACCACACGAGGCCACGAGTGCCTCGGATGTCGTAGCGACGACATCCCTAGACGTGGGCAGCAAAGCTCCCCGTCACAGAAGCCGTCGGCAAAGACGCCGCCGCCGCCTCAAGTCGGATCCGAACTTCGTTCCGATCGACGTCGCTTCAAGCGACTCGGAGTCCAGGGCCCGGGCTCCTCCGACAAGGCCGGTGGAAGAGTGTTATCACATCTTCGATAGCGAGACGGGTTTACCCGTCAAGGCATCTGGTGTCCACTTGGAACCGTTGCCAGAGGTTGCAGAGATACTGAACCTCGAGGAAATGACGGCTGAGTCTTTCCTGGCCAACCTCAAGGCTGGAGAGATCGCTGAGATGGTACTCATGAGACCTGAGACCACTCAGGAGGAGCTGAAYTCCTCCTCTGTCTTGGACGAGAACGTCCTGGAAGACATGAACAAGCAGCGTCAGGCGCGCTTGGGTTCTGAGRTTCTCAAGAACCCCAAGGATCCGGTGTATCCTTTGGTGACGGAGTATGCGGACGTGGTGAATAAACACCCACCGACCCAACTCCCACCGGATCGAGGCGTACGGCACGAGATTGATCTCGTGCCCGGCACAAAGTATTGTGTCACGAGGCAATGGCCTCTCCCGCGCGAACAGTGCGAGGTCATTGATGCCTTCTTCGCCGCGAAAGCGAAGGCAGGCATGGTGCGGGAGTCGAAATCCCCGCACTCGACGCCGACTTTCTGCGTTCGTAAGCCGAACGGAAAGTGGCGTCTGGTTCACGCTTATAACAAGCTGAACAACGCAACGGTGCCGGCCCAAACACCGATCCCACGAAAGGATGTGTTGTTGAACAACATGGCCGGGAGCGTACTGTACAGTGCGCTGGATTTGGTAGACGGATACTACCAAATCCTGATGCGAGAGAGTGACATCCCGCTCACCGCGGTAAGCACTCCAAGCGGCATGCTTTGGGAGTGGCTCGTCATGCCGCAGGGCTTGTCTAACGCCCCGGCGACGTTCAATCGCCTGGTGACGCAATTGTTCCGCCCTTTGCGTACCTTCGTACAGACGTACTTCGACGACATTTTCGTCCATAGCCGTGCCGAGGGAGGGCAAGACGGCCATGGAGGTGCATCTAGGACACCTCCGTCGAGTGTTGGACGTGATGCGAGCGAACAAGCTCTACGCCAACATCGACAAGTGCGTTTTCGCGTCACCAGAGATCAAAGTTCTCGGTTGCTTCGTAAGCAACGTGGGCGTTCGGGCTGACCCAGACAAGGTCAAGGCCATAGCAGCATGGCCGACGCCCCGATCCCAGAAGGATCTTCGAAAGTGGTTGGGTTTGGCCAACTACCTTCACAAGTACAGCGCTGGGTACGCGGGCTTCGCTAAGCCCTTGTCGAGTCTCCTTCAGAAGGACGTCGACTGGCGCTGGGAGCAAGAGCACCAGGATGCTTTTGACAGCATCAAGGCCAGTCTCCAAGAGGCCCCTGTCCTGGCTCTGCCGGACGAGACGAAGCCGTTTAGCGTCGTCTGCGATGCCTCCGATTACGCCATCGGGTGTGCATTTCTGCAGGACGACACGGCTGGACAGGAACGAGTCATCTCATTCCAGTCCAGGCAGCTCAAAGCCGCCGAGCGGAACTACCCTGTCCATGACAAGGAGCTCCTGGCAATGAAATATGCCCTTGTGAAGTTCCGTGTTCACCTTCTGGGCTCTACGCCCTTTGTGATCTACACAGACCACGCGTCCTTGAGGACGGCGACTAACTCGCCTCACCTCTCCCAACGGATGGCGAGGGTGGCTGTCCTTCTTCGCGGAATATAACTTCCGCGTCGAGTACAAGCCGGGCAAGCTCAACGTGCTTGCGGACGCCCTCTCTAGGCGTCCCGACTATGAGTTGGCCCACATCACGCGGGTGACAACGGATCTATATGATCGGATACGCATGGCGTATCGGGACGATGAGGCTGTCGCGCCTATCGTCAAGTTCCTTAAGGCCGGAACGAGCGCGAAGTCCGACTGGCTCTCGTCGCGACAGCGCTCACGGCTCCACCGATATGAGTTGAAGGAGGGCCTCCTCTATTATCGGGTGGAGCCCACTGATCCTCCACGGGTGGTCGTACCCAACGACGAGGATCTGAAGTTCGACATCCTCGCGGAGGCGCATGACGCGCCCTCCAGCGGCCACTTGGGCCGCGAGAAGACCTTTCTGTCGGTCTCTCAGTCCTTCTGGTGGACCCACATGTACAAGTGGGTCGCCCGGTACGTCAAGACATGTGAAACATGTCAGCGGGTTAAACCCGCAGGGCACTCTTCGGCTCCACTACAGAGCCTGCCCGTACCGGCTGATTGTTGGAAGTCGATGAGTCTTGACTTCATCTTTGGGCTTCCGCCGACGGACACGGCAACACCGGAATTTTGGTGTTCGTGTGTCGGCTGAGCAAGATGGTACATCTTGCTCCAGTTCCCGTCACTGTGACGGGCGAACAAACCGCAAGGTTSTTCGTCGATGGCGTGTTCCGCTATCACGGTCTCCCGGAGACGATTGTCTCGGACCGAGACCCGCGGTTCACTGCTGCGTTTTGGAAAACGCTGTTCCCTTTACTGGGAACTCGTCTGCAGATGTCCACTGCGGATCATCCGCAGACAGACGGCCAAACAGAGCGCGTCAATCGAGTGCTCGAGGACACTCTGCGCAGTGTGTGCGCGGCAGCGCCACGTACGTGGTCGACGCAGCTTCCAGTTGTTGAGTTTGCGCTCAACAACGCGGTTGCACGCGTCCACGGGGTTCACGCCCGTTCTACTTGAACGGCTTTCCGCCACCCCAGAGTGCCTCTCACTCTTCGAGGAAACACTGACTCCTCTATTCTAAGTGGGGGAGAGGCTCGGAAAGCCCTTTCCTCCCAGGTCTCGGATGTGAGGCAGTTTCGCTGCGGAAACAGGTGGAATCCTTTATGGATACCAAGCTGAACATTGTCAGCAGGGTCCGCGATGCCATGGCAGCGGCCCAGGATAGACAAAAGGAGTACTCAGACAAACATGGAAGAGGAAACTTAAGTGTATTTAAAGTGGGGGACTTAGTGCTATTAGACACGAGGAACCTACCGCTAGACACTGTCAGCGTCTGTCGGCAGCAACAAGCTGAAGCACCGCTTCAATCAGCGACCTTTTGCAGTCCTGGCCAGACATGGTGCGTTCGTACACCATCGACCTTCCAAAGTCAATGACGACGCACCCGACGTTCTATGTCGGACGTCTTAAGCGGTACTTTGACCCGCAGGGGGTTGCTGACTCGCAGACCCCCCTTGACCCAGCTCCTCGAAGCGAGGGCCGGTCACAGTCGTCGACGAGGGCGTCGGAGCAGGCACCCAAGCCTCGGTCGGGACGGCAAGGCTGCGGTCAAGACCGTACGCCGGAGGGGCTCCATACGAACGGACCCCGGCGCCATACGCCGAGTACGCTGAGTACGCCGGGTACGCACCATACGCGTACGTCTGGGAGTGTGCCGCAGGCGGCTCCGGACGTGGCAACAAAGCCTCGATCCGTCGGATCATGCGAAAGGCCTCGTCCAGATCGGACCGAAGAGGCCCGGCGGTCCACCGCAGGTGCAGGCTCGCGACCTCTCGCTCCACCAGGTCCAGGCGACCCGCAAGACGAGCGAGCTCGCCCTGATTCCCGGCCTGGGCGGCAGCATTACCGCCAGTCCCATGGCCAAGTGCAATCTCAAGCACGAGGAGACGTTGACGGAGGCGGTCAACCTCAGGGTCCCGTGTCGGACCCGCAGGTCGGAGCACTCGAACGAGAGTGGCTCTCGGGAGGCGCCGAGTACTCGTACTCGTACGAGGGCACCTCCGCCCCTGCTTGACCGGAATGGAGAAGTCCACTTCCACGTGGAGCGGATCCTCCAGGAGCAACGGTGTCGCGGGAAGGCGACAGCTCCTCGTGAAGTGGAGGGGGTATGCCCACTCCGAGAACTCGTGGGAGCCGATTGAACGGCTCCCTGATTGACTGCCCGAAGGCCTGTCGCAATCTGGGAACAGAAGCGGCGGCAGCTTCATAAGTAGTCCGGGGCTTCTTGCCCGAACGGGGTACGGATGATGAATACATACCGTACCCGCGCTCATCGTGAGCGCTAGAAGCCGCGCGCTTCAGGATGTCCCTTGGCAGCATACGCTTGCTGATCAAGTGGGCAAACCTCACACGAAGATCACGCTGGTCCAGGATGTGGGACAGCTGCTGATCTTCATGGAGTTCCCATAACTCCGTGGGCGAGTACTTGAGAGTCTCCACCCACGCCCAGAACATGTCCTCACAAGAACTCCATGTGGGACAGTTCTCGGGCATAACCCAGGGTATCCCCAAACAGGATAGAGACCACCCCGGGTCTCGGGCTTCTTACGACAAGTCGTGAAGTCCTTAAGGGGAATGCGTTGTGGCGCGAGAAGCACACACCGAGCAGGCCTCCACGTTGTCGACGAACAACTGTTTCTTTCTGAACAAGGGCTCGTTCATAGATGCCCCTAGAAGTGTGGCCGCCCACAAGGCCACGAGGAGCGACGAGAAGCTCTAAGAAGAGGTCAGCCCCAGAGCTCTCCTCGGGAGGCCAATACCCTCTTGAGTTGATGGCAGCTCCCTGGTTGACGGGTGCCGCCACCGAACCACCACGCACACGCTGGGCAGCTTGGAAATCCTCCTGCTGCTGGTCGAGGTCATTCGAGACCTCCCGGGGCTCAACAATGGCACCCTCCTCTTCCTCTTCTTCCTCCTTCTCCTCCTCTTCATCCGAGGAGTCGAAGAGACTCTGATAGGAGCGTCGAGTCTCCTTCTCCTCGCGAGTGGAGACTTAGAGGCGGTACGCTTCTTGAGGGCTTGGCGCCTTCGGCTTTAGAGGCCTTAGCGCGCGCAAGACCCTCGGCAAGCGAGAGACGTTTGCGAGGACTCCCTTCCTCATCGCCACCGGCTTTGGGAGTGCCCTTAGCCTTAACGGCCGGACTCTCAGAGCGCACAGAGGTCCTGGGACTGTGATCACCGTGATCACTAGCGGACGACTGCACGCTGTCTTCTTTGTGCTCACTGGGCACTGGGCTGTGCCCTCCCACAGAGCCCGCAGGAGCATCATGGGTGCTCTCGAACTCGGCATCGTCCTCACTGCCCGAGTTGAACTCATGTTCTACGAACCGAGGGTTCGGACGATTGTCTGAAGACGAGGGCGACGGACTGCGGCGCCTGCTTGCGCGCTTGTCCGCAGCCGCGTCACCCAGAGGATCCGATCGCCTCGGAGGCCACCTGAGTAGCCTCCTTGAGTGCAGAGCCTTTGCCGTGAGGCTTAGTGCTCTTGGCCTGCGCCTTACCGGCTGCAGCCTTGGAGCGTGTCTTAGCACCGCTCTTAGCAGTGCCACGAGGCATGTTAACGGTCGAAGTGGAGACCTTGACGAAGAGCGCACAGGTTGCTGCTCGAGGCTCTAACTGTAAAGTGAGCCTCCGCCGCGCTCGCGCGGCCGAGTCGATGGTTTTCGACTTTGAGAATGAGAGTTTGTGACGAACATCTCTTCATTCTGATTGGTCGGTAAACCGAACCTAAGTGGGGGAGGATGTAACGGGGTACGTAGCACTAAGTGTACCCTGGTTACATGAAGTGTGACACTTCACACTTCAGGAGTATGACATGTCATACTCCGGGATATTTGGTACAAGGTACCAAGGTCATTCAAAACCAACCATTGGTTAAATGACATTGCATTTCTATAGGTAACTGATAGTTTGTATTAACCACAAACTTAACCTACCTATAAATATACTCAGTTTACAATCTACCTTATTTAGGTCACTTCGCTCTCTTTAATTAAAGAGGGGACCTGTGAACTGCCGTTCACTGAGAGTTTGTATACTCTCGGAAGATAACTTCCTCTAGCGCAAGGTGCTGTGTGACCCCGTCACAACACCCCTGCTGGCACAATAGGGCAAGAGGTTGCGGTGGAGAAGGTGTTTGAGGAGCTCCTCACTTCAATAGGATTATCGAAAGTCGATTTAGATGACATACATAAAGGTCATAGAGAATTAGCTAGTCTCCCGACCCAGAACATACTCATTGCTAACAGCTTTTTCGATTTACATACAGACGAAGAAAAGCAACAAGGCGAGAGCGGCGGATGGACTACTAGCAAAATCGACAGCGCTAGGGTATTTTTCACAAGTTGTGAACATGCTTCGCGAGCGCTACACCAACTCTCTTGGCGACTCTAAGAGAATTGCCAAGATTCGGGACAAAATGGCTAGTTACATAGAAGAGAGGAATTTGTGCGCAAATGTAAAACCAATGACGGTCCTGCGTGTAACGTGGATGATCTAAGTGTACTCGTTGGGTATCTAATTGCGAAAGCTGACGTGGCTACCGGTTTAAAGTCTGTACATGATGCGGCTCTATTGACTATGATGTGGCATACATTTGGGAGAGCTGTCGATACCTGCTTTGCACGTAAGCAACAGCTTTCGATTTCAGCTTCTGACGATCTTTTCCTACGCATTGCCCGGATCAAAACTTCTATGGTTCAAGGCGTCTCAATATTTAAGTCCCCGGAGAGATGGCAGCAGTGTATGCTTCACGCATTCGGGTTGCTGTTTATCTGCTACGATGAACCCTTTGAATATTTGTTTTCGTTGGCACCTAATTCCGCAGAGTCCGACCTTCCTGGGAATAAACCGTATTCACAAGAGGAAGCTGTTTTGTACTGGGGTCAACTATTAGAAGACGATAACAGCACGTCAGAGCCGCCCCAAAAGCGAGAGAGGAAGAGACCAAATATCGTAAGTTATTTTACCGAGGTCATTAGAGATTCGCTGAAGTCGATGCCTCCTGCTCTCAGCCAAACGGTGACGCTGAATATGACCAGCCACTCCATACCGCTGCGTACGCCAATGCGTAGCCGAAGCTGTCGATTTTAGTGAATTTCTACTCGCGGGGCTTGGCTGTTGGAGTCACTAACCAAGACGTTTGCCTACATTGGGACGACAACAAAGGAGGATCAGAGCGTAGCCAAAGTGTTAGCAGGGTACCAAGCTCCAGACCTACCTGTGGTAACGCCGACAATTCGTGACCTGCAGCAGCGTTTGACGGTACTGGAGTTTGGTCAGCTTGTAACCCTTTGAGACAAGCTGTACCGCCACGTACTTGGTTTCCAAGACGCTCGATTTAATGTCGCATTGGACGTCGCTGATTGCACGTTTGCATCGCTGCTAATGCACATGGATGAGGTTCGCATCGACATCGAAAACACCACCAGCACCTCGGTCCCGTCGCGCTACATGTACGAGCTGCATCGCGGTATCGCCTCTTCCAACGCTCGTCTTGGCTCCTGCATCCCGATACGAACATGCTGTGAGTGGGGGGCGTACTTGAGAGCTCACTGGAAGACGGTAAACCACGTGCCGCTTAGCAATGCTGTCGCTGGTAACGACACGCTGCTATCATCCACTCTTAACAGCATGTTAGCCCAGCTTGTGAACATCAGCGAGCGACTAGGTCGCCTTGAAGACTCGCAAGCCCAGATGCAGAGCCAAGCCTTAGGGAGTACTCAGACGCATCAGCAGTCGCCACAGCTGCCTACTGCTGGTGAGGTTACCGTCCCAGCTTTAGCGTCAGCCTCCACCCTTGCTGGATGCCTGCTCAACTGGTACACTAACCACATATGGCAAACGGTTAAAGGTAAAAGGAACAAAACCAGCGTGCCGAAGCAAAGGCAGCAGTGAACATAATGATGGTACTGTACCAGAAGCCATACTAAGTCCCAGAGCAGCCTCCACGAGCCGCGCAGCTTACAAGGTCTGGAAGCACGTAGTCTGGGAGCTGGCACTCAAGCTGGATACAGCCGCAAACCAGCGTCTCCACGCCTTCGACCAAAGGAATTCAACTCGCAAATCTTCTCCACTTCGCAAGAATTGGCGCCTGCTGCGTACAGCAGTTGACTTGGATTAACAAGCCGTTGGGCAGCACAGTACAGCAAACATAAGCACGGGCAAACAGCACTCACCAGCATCAACACCATTAAACGGCAGTTCACCACTGTAGATAGCTTAGCAATCAGTACAGCCTACATTACAAAGCATCACACACCACACACAATGCACACAACATGTTACAACACACAGTACACATCATAGCAGCACACGCACAGCACAACACAACACTGCAGCATACGCACAACACACAGCACAGCGCGCCCAATCCAGCAATACACATGCAAATGCACATTTCAGCATGTACATATATATACCGCATCACACGCGTCGCTCCATAGCACTCCACACAGCACCAGCTATTACAAACACATCATAGAGTACAACACAAACACAATCCACGCACCACAGCTTACAACTATCTGCCTCCGGCTAGTTTCGAACCAGCGGCCTGGATACGTAGTCTCCCGCTCTACCGCTGAGCTACGGGGGCGTTGGGGTCGGAGAAGGCACGCACTGTGGTCTGGAGCTGGAGCTGTGGCTGGAGGCTTGAAGGGTACGCCTGAAAATCGCTGAATGAACAGCGAAGAAGACTGTCGCAAGGTAGCCATCTATTTGCAATTCATTGAATTGCCATTTAAAGCCTATCCACTGTCATCCGGAAATAATAATCTGACCGTGATTGGCCGGATTCCATCCGGCATGACTCAGTCAGTCCGTACGGAGACCATCCGGACTGGTGTCCGGAATGGCCCCCACAAGCAACATTTACCTGGGTGACATCACTTGTGCAGAGGATATCAGAAATACTTAACCATTATCCTTGCTTAAAGGGAAGTTGGGTGTCAGATACTAGCTGAAATGACAGATTATGCAAGTTTAGAGAAGAAAACTCACTAAATTAAGTATGACAATCTAGTGTAGATCTTATACAACAAGCGTATCAAGTGCTGAAGCATTATTATTAAAGTTTGAGCTGAAAACGTTATCTGAAAATAAGAAGTTAGAGTTTTGAGTCGCCTCCTAGAACTCTGGATATAAAATCCAGAGAACATCGATAACTATCTGTATTGACGATTTCGAACGGCTTGTACGCTACTCTCCATAGTTGTAAGTGTTCCAAGCTATCAATCTTTGGCCAGAGATATTGTGTTGCCTCTTACGAAGCATATCTAGCTCCTCTGTATTCACTAGGGCTGCAGCATCGGTTAGGAATTGGTTCGGTAGCAGAGGTTGGAAAATAAACTATGCTTTTAAATTTTCGTTTTCAAGCTCAGCAGTATACATTGCAATACAGGACTAATCAATAATATTGTTTTGACCAATTATTGCAAAAAATAAGTTAGCCACACTTTGATATTTACCTAAATTTATATGTCAGTAAATGGCAGATGCGTAAGATCACAGTAAATTACGACGAGCCGAAGTAATTCAATCAGCGTGAATGTTGTCCGAGCAGACTTCAGCTAACACTGAGCGCAGATGATGCCTTCAGCAATGATGTCAGCATACCATACCACCAATATCCCTCATGTGACCGCAGTGCGACTCGCACAGTGTGATCGCGTATTTATTAGTACTATATTACGGTATACTGGAGTGCAGAAGTGGTATACAGTATTGTGCGTCAATAAGGAACTCATGGGAACTACTATCCTCGTAAGCATGATACAGAACAAATTGACAAGTATATGCACTTTGAGTATACTGAAAAGTGCGGCAATAAGGAACGCATGGGAACTTTTACAAAACATGTTGTAATACCGTATTCCTTATGTGATTGGCGACAGTCAAATTCTTGAGGTGGTCCAGCCTTTCTGCCAGCATAAAAGATACCTGCATAAAAAAAACTATATAACATGCTCAAAATTATATTGGTCGCCAAGCACATAAAGCGCGACGACACGAAATTTGAATCAATTTGATTGGCTGCTGGATTTGTCGCTAATGACCGAGCGTCGCCAATCACATACGGAATACGGGTACATGTATCCTAGTTGAAAAGTACCTTTTTCTGACACTTTGAGTACGATATACAATATATGGTACTGTAAA

>Contig_66

GCCGTCAAGAACAAAGATTCGAGCGTTCAAATCCAGCGGCTCTGTGGGAAGCATACGTCGCAGACAGTGAGTACTGACCACCATAACTCCGAAGACAGACATTTGAGTTCCACATTTGGCGGCACTTAAAAGCAAGTCCGGGAACTTGAAGAAAACCGCAACGGCGTTAAACATTCCGAAAGAACGGAGTGATGTCAGCGTTCTTTTTCAGAGTTTCGCTTTGGATGATAAAATCGAAACTCTGTGAGAGCGACCAGTACAACACATGGGCTTTGGCTGTGGCCAGAGCGTACGAGGGCAACAAAAAGGCAGGTCAGGAGACAATGTTTGCAACACTGAGAGCGAATTATGGCTCTGGCCAAGTTTCTAGTTGAAGGCGCACAAAGCCCAAAAGGACCGCCAGCAAGTTTGGAATGATCCAACGCGACTTCTGGCCAAAAGAAGAGAGAGCGCCAGATGATATCTATCTTTTGAAGAATCAACGCTAAACACGTGGATCTCGTATGTTTCTATGCTTGGTAAAGAGAACCCATACAAGCTATTGGCTCTGAAGCTCGCCGAACGACTATAATTTAGCTTCAGCTCCAGTTGCAGCAAAGACAGATAGCAGCGCAATAGCGTCGCCACTAAGTTGGAGCTAGCGCAGTTTTCGCTTTGAAGGAGCGCAGGAAAAACTTCCGACGATGTCTTCAAGATACTACGACTTTACAAGGACAAAAACTTGAAGTAGTAATTATTAAACGCGAGCATGACTCCAGCGTTCCTAGGGTCTGCTGGGTATTTGTTTAAGCTCTCGTACAGGATTGCTGGGGTCCATCGCTTCACCTTTACCCACTGCTTGAGCTGCTGGTCATAGTAATTTTTCAGTACCGATAACATCACCGTGTATTGGGTTGATAGGAAAACCACTTTCCTCGCATCGGGTTTTCGGAGAAATTCTTGCCACTTTCCCTCAGTTTGAGAAGATTAAAAACGTATTATCTACGTCCGACCAAGAGGTGAAATGAGCCTATTTTTAAGCCCATCCACATCTCAGACCTCATTCTGGGCCACGCCTTAAAGTCGCCAATCAGCTTGGCGAGCTCTTCCTCGCTGACCTGTGCCTTCGGTTTTTTGAAGAGAAAGTCGTAGGGGCTCTCCTTGCGCGCCTCCACACGAATACCATGTGGTCGAATTTTTATTGTGGACACGCGACGTATTCAAGCCTTTAATCTAATCTGGCATATGATACGCCAGATTAGATGAAAAGGCTTTAGAGCAATTCGTGATTCGGTAGGAGAGAATGCGTCCTACTAGGAGTCCTAAAGTGTCCTACTGTCCTAAAACCTGGGATAAAAAACTAGCAATTCGGCTCATAAACAAACCCATATTTAGGACAACACAATGAGTTTAGGACGAAACAAGCCAGGGTCCGCGTGTCCTAATAGGACACTCGACAACTGGACACTCAGTCTGTCCTATTAGGACAGACTGCAACTCAGACCACTGTCCTATTAGGACAACATAAAGCCCAACATGCACTGTCCTCAAGCCATATCCTCAACTGGCCTAGCTAGAAACAATAAGCATTTTAAAGCCCCTCTCTCCAAGAGCAGGTTGTGCGCGAAAAGCACCTCAACAAAACTTCTCTACTACAAAATGTGTGACAATATTTTTAAAGTGTTTCAAAGGATGATAGTCATTGAAGGTAATAATAGCCGCTAATAACGGTTGAGCGACTTCAACATGCACATAGCAAACGACGGTTAATAATCAGATCGTGACGACATANGGGGGGGGGGCTCAAAAAGCAGCAAAGTACTCGCAAGGGCTACTTGGCTGCAAGTCGATCCGAAGACGCGGTTAGCAAGCGATGGGGGTCTTGGTTAGCGCGGTAGTGTGCTCGGCCTGGTTCGTCGATTTGTCTGGCGTCGTCGGGGTGTCGACAACAGGTGCGTTTGTGGCAGGCGAGTCCGGGCTGGGAGCCGACGTCGATGGGCAGTTGGACGGCACGTAGACTACCAGGTCGGCCTTCAAGTTGGAACCGACACCGACCGGCAGTTGGCACTCCTCCAGGTTCATGGCCTTCACGTCAGCGAGCAGGGTGTTGCAGGCAGTCGAGGCGCACATCTTGTCAATGGTGGCCTGGTCCGGGATCTCAGTGGTCCGTGCCGCACTGTTGAATGTTCGCGCTCGTGAGCAGAGCGGACAAGACACTGCTTCCGCAGGTGGCAGCAGAGGCCAGATAGGCGAGCGCCCTAGCTGCAGAAGGAGTAGCCGCAATGGTCCATGATGCTTGGCTGCAGTAGTGGAGATTCAGAAATGGTTGGTTTGCCCCAGGACAATAAGTAAAGCTAATGTGAGTTTTAAGTAACTCTTACGTAAATTTACGTAGATAGTCCGGAAAAAGTTAATATTTGAGGTTTAGGACAATCTGGAGGACACAAATTATAATATTCTGTAACGAAGGATTAGTCGCTTTTTAGGACACATCTCCAAAATCTCCCTGTAAAATTTAGGACAAATAGGACAGTTTAGGACGTGTCTTAGGACGCATTATCCTACACCCGTGATTCGACTTTCCTTGCGATCAAGTCTCTTTTGGACTTTATCAGAATAATTGAAATTTGACGCCTACATGAGCCGGAAGCTCTCTTAGATATCACACCGAGAAATAGCTCCGCGTTTGCTGTGTTTATCTTACACGAAACTTGTCTTTATTCTTCTACAAATGTCTTTTTTGTATAACGTTCTCGAGATAACCAAACTCTTCGTATATTGGGTGTCCTCCTCCGTAACAGGGATAATGGATCTTCTAACTGCTATTTTTAGCGCTGTAATTTTACTATTAAGAAAAGTTCTGACAAGACAAATGGGGAATGGACACATAAACGGACACGATACCGGTAAAGTCAATGATAATATTAGCTTCAGTAACGTAAAAAAGCAATTGACACTGTGAATCTTACATGAACTTTTTCATTACCTTTTGGTCAAGGATAGAGACACCAAACAAGAACTCGTAAGAAACTATATGTTGGGCACGAAACAACGAACACTTTTATTTCACTCTACGCACTGTAAAAATGATATTGGGGATGAATGCCATGTCGCGCAAATCCTACCAGCAGCGGCAGCAACATTACCGCCAAGGTAGCGCCCTGCATGATTTAGGGAGCACCAACACGGCTCTGAGTATAGTTAGTTAGATCATGCCCAAGCCAACCTCTGAAGCGCTTACAATTTAAGGCACTTGCACAGATTCATTAAACTCCGTTTCGGATGAACTGTATCACCCCTCCTTTACACCCTAGCCCTGTTTGGTGCCTTTATCTACCTGTAGTTCCTTAACTTTTTGATCCAGTAGACACGACCTCCAAAATAGCCTACAGTCCCCAATGAGTATATGGTATTTGTCTTTTTGAACACGAATAAGAAGCAATTCAGTACCAGTGTCAGCAGTTGTAGTTAGTACTTGACACGCAACAAAAGAAGCACAAGTAGTGATGGTTATGACGTATCCTTGCTAAAATGATCAACCACATTCTCAGTTACGCCTCAACGTACACAAATTGCGTGACAGAAGCCATCATGGCAGACACCGTATTGGCACTTAGGTCTCCTAAGCACTTGTCTTCTTCTTGTAGTAGTCTTCGTATCTCTTCCAAACGGCGTAGTTCGGGTTATTGACGACGATGGAGCGGTCCATGCCTTCGAAGTATTTCTTGTAGACGCTCTGAGGTGTCCTCCCCATCATGTACCAGTACAGAAACTGGGTATTCTTCAGAAACGACCGGTCTTCTTCTTGGGCGGACAAATTGCCATGGCGAGCACCATCATCTCTTAAAAGCCTTTTGCCAGCGACAACATTCTGATCAGTTATGTTTGAAGCTTGTGCTAGAACCACGAGCTTGGTTTCTTCGGAATTTACAGAGGGGACAACAATTTCACCACTTGCAAGGATGGCGACTGTAGTGACCGAAATGGTGTAACTAAAACGCATGACAAGTAGCAAACTGTCGTCGGGGCTTGAAAGTGACTTGCGAACTGAGCGACGAATTTGATAGAGAAATAAGTTGACCAGCAAAGCGGGCAGTAGGTGCTAGTCTTGTACGATGGCAATGCCCCAAGATAGCACTTAAAAGGCATGCAAGTACAGGAAAATCAAGTGGCGACTCACGTTGGTTTATGTTTGTAAATCGGTTCGTAATCGATGTTCCTGTTAAAAAAATTGTTGCGTCGGACAATATGCTACAGTACAATCAGCTAATAGTTTTAAGAATCTACTTCAATGTTAGCCTTGAGCCGCTAAAATACTGCTATAAGCTCGGTACATGAACCATTGTAAGCAATTAGCGCATCATTTATTAAAACACATGGTTACGGATCACCAATAATCTATTGGGCGGTGTTCAGTCAGCCTAGTACTTATGCCCTCGGTACCAATATTGCTGCACCAATAATCTATTTTGGTCTAGCCAGTTCTCTAGCCCAATTATCTATTTTTTGCTTCGATTAGCAATAGATAAAAATGCAATATTCAGCATCCCTGGACAGGTACCAACAGCCAAGACAGCTCGTGTTCCGAGTGGAAGTGGGGAACTTACGGTCACTCCTCTGCTCAATCAGCTACCCGTCTGAATCAGACTCTGATAGACAGAGTGATGGCGAACCGACTGCTATTCTCCTTTTCGTTTTGATTACGTCACTACTCTGTCCAGTTGATCACAACACTTCCCTGCATGCATCGTCCCTTCGTTAATGACACTGTGGCATTGTTATATCGATAGAGATGTATATTTCCGTCTCTTTTTTATTTATCGCCTACGTATTAATAGGTACAGTATCGAATTTCCCAGTCCACCCACATGGCGAACAATTTCCATTTCTAAATCGACCTTTAAGACAACAAACAGCATGTTGTCGCTTAAGACACTGTGAAGGGTATTAGTATCAGCGGTGAGGCCAAGTCGGAATCTGAAGTACGAGATGACGCCGACTCTCGTCGCTACCTCAAAGCTGCCACTGACTCATCTGGCCCAGCGACAGAAGCGAGAGGGATAAGTATCAAGATGCCTAGCTTCAATGGAATCAAGTCACTGTTCTCCAAGACTTCAAACCTTGGCGCTGGAATGAAACATAGCCCGGAAGTTGCCACGGCACTCAAAAACCCGAAAGTCAATCAAGCATTCAAGGAAGTGGTAAAGCAACCAGGTATTGTCCAAAGCTTCAGGAATAACCTGAAGCTGAACAGCCTTGCGTCGCTCCTACGAAGAAGACCAGCTCAGTTCACGTCGTGTAGCTTTGACAAAGCATCTCCTGCGCCTGCTTCGCAGCACCATCGTCTACTTTGTTTTGCCATACTCCAATGTGGTGCGTACAGCCTGGCAAGCGCACAGCAATAAGCGTGTGAAGTTGACATCAGAACATGAAAGTACTCCGACTTTGAAGCTCACAATTATCGAGTTTATCGTAGGTACTAAGGTCATTCTTGCTACCTGCTATATTCTTGTTTGCCAGTAGAAGAGCAACTCATGCGCAAGTACACTCCTTATCAGTCTCACAGCCAGAAAATGACCTGCTCAGCGGCTTCATCCATCATCCTTTTGCTGAGACAGCAGTGGCGGGTGTTAGCACAGTCGACAGCACAATGGAAATTGTAAACAGAGACTCCATTCTCCTCCAGCAACATTACAATGTAGATGATTTAGTATAATATACGCAGTTTTACGTATCTTCACTTTCAGAAAACACGCACCTAATCGCAAGGAAAGTGCTATTGTGAATTCCGACCAAATTTTGCCATTTGCAAATTTATTCATCAAATTGTGTCCTAAAGTGGCTGCGCAATGTCATTAGCAGAAGTTGCAGTCGTGGACGCGGACGTCTCGTCCTCGTCGCCACTTTCACTCTTCGTGCACTGTACTCGTCTCGCTGCAGCTTCCGCTTCGACGGCTGCTATCCGCTTGCAGCTGCTGGACCCTCCAACTTTATACGAGGCCGAAGTCTCTTCCCGTCACAAGCCACGCGCGCTCGACTGCTCCGGTGTCGAGTATGTGGCGGCGGTGGAGACAGCGCTAAGTTTAATGGCAGACACAAAGCCTCGCTTCGAGCTCCGATGGTCAAGACAGAAGCGCACACTGACGCTAATGGAGCGCTCCGAGTTCGCCATGAAATTCTGCGCCATCGAGTTCCAAGCCACGGAAAGTGGTGACAAATGGCGTATGTTACTGCATCAAGTGGCAACACAGCAGCAAAAAGAAAGAGAATTAATTGACAATAAACGTAGCAGAGTGGCACAGCTCGAGACGCTGCTGGAGCAGAAGGAGAAGCTTCTGGAAACGGCATTGGCAGCAAAGCAGAGCACGGAGGATTGTCTGATCCAGGGCTTCTGCGCCGTGTTAAATGCCAAGAAGGACGAGATCAGGAGACTGCAGGACGAGGTAGAAACAGCGCAGAGATATGGCATTACTCCAATGGTCAAGAAGAAGCCAGTGGTGAAGAGAACGAGGAAGGCGACGGGGGCCAAGTTAAAGAGGAAGGAGGAGAGCGATGAGTCTAGTAAAGAAGAGAGTGATGAAGATATGGGTACAGAGGAGGAAGAGGAGGAGTCTAAGCGAACGAAGAGAGATGCTATTGACGCGTACAGTCACCTGCCCGCTAATCTGAAGCCGAGTTCGGTGCAGGTTAGCTCGGCGGAAGATCTGCTGTCGAGTATGGACGACATTATCAAGAACGAAGAGGAGGTGGATGAAGCTACGCAGCGAGGAGATAAGATCCAGGTGAAGTCGAAGCTAGTGGCAGCGTCACAAGAGTCGAGACCCCTCGTGAAAAATGAGGCCACGAGACCAAATCCAGTGAAGCCAACGCCACCTCCTCAGGTCGACGAGACGATGGACTCAGAAGAGGAAGATCTCCTTGACATGTTATCGTAAGTAGCCGCTGGTCTGCGTAAGTACTAAACATATCTTATTCGATTATTCAGTTACACGCACACTTGAGGATCAAACAGCCTAAGCCGTCTACACTAAAGTTTTTTTCTAAGCCCATTTCTGCGAGACTATAAAAAACAACGGCGTACGGCAACTAGATCCAAACTTTGATTTTGGTTCACAGAAGCAGTGATGGGTAAAAATGTATTTCAAGTATTGGCATTGATCAATACTATTATACTTGCAATGCCATACTGCAAGCATTTACAATACTGCACTACGTACTGCTGGAGCGCTGTCACGGTGTACAGTTAGATGGTGGATTAACGATGCTGGCTGACTGCCTGCAGAAGGTCTACTCACTCCAGACTCTCCGGACCACGTCCAAGCAAGCGGGGCCTGCGGCCAAGACAGTGACGTTCCAAGTTTGAAGTCGCGAAGCAGCTGGTCGTGGCGGGAATTGGAGCTGGACCGCCACACACGAGGGATCTGCTAAAAGGTAAGAAGCTCTAAAAACTCTTAGCGAGGTGAAATGAGGAAGTCAGTCGTGGATTTCCTTATAACAAGCAGCACAAGAATATATCAACTTTTACATTACTATCACAGTAGTGTCGCTAATACTACTACCTCTTACGTCGGCCCAAGATACTCACGACCCCGATTAGAGGAAGACCGCGACACTTTGGTGGAGAAGCAGGGTACCTTCTAGCCGAATTTCGTAGGAGTACAAGCGTAAGAAGAAACCTTGGCAACGGAGTGCCAAGGAAGAAGGAGAGAGAAGTGGAAAAAGGATGAGTGCTCAAGGATGTCCAGCAAGGGACTTGGAGCCAGTGAAGGGGTAGGGGGATGGGCTCCTCTGTTGGAAACGAAGCCCGTGGAGATCCTCGAGGAGGAAGAGGAAGCGCGCGCCGAGAGAGCTGCTGACGGCGGCACGCCGCAGAGACTTCAAGGAAGGGAGCGCCAGGGAGATGGCGGGGAACGTGGCACGCCGCAGCGAGCTGATCACGAGACGGGAGGGCACGAGACACGAGCGGTCCACGTGGGAGGCGAGGACATGTTCGACGTGGCGTCTCCGTCTCCGGGAACGAGAAGGACGGTTGAGTCTGTGTCTGGAACGCCTCAGGGTGGACCGATGTCCAACACAGTCCTTGAGCAGTGGGCACGTCAACCAGCGCCCAGCGAAGGAACGACGCCTGCAAGGACGACGACGGGTACGCTCTTGGACGCTTCGGTGATGGACTCGGGAGGTCGCCGCGAGGTGGGGCTCGCCTTGAATCGGAGTCGTCCCTCCTTGCAGCCGATGGACAACTCTAGAAGGGACGTGCCCATGGTCCTGTCAGTGTACGGACAGCCCAGCAATAACGTACCGGGCCAACAAGGAAGCCGACGTGCCCCAGGGGCTGCACATCTTGTTGCTGGAACACCTACGCGAGCGTACGTACCTGGTGGAAGTCGAGGAGCGACGCCTGTTCAATCGATAAGAACGCCGACGAGAGGAAGTGGAACGCCGACGACGGACCCAGCCTTACAGGCTCTCATCATGGACGGTCTCGCCCAATTGCTACAGAGGGCTGGGGTGACGAACGGAACGCCGGTGCCGTCACCAAGACCTCAAGCTACAGGACACGTGCCACGTACTGTGCCGACACCGACCCGACCAGTGACGTCTTCTGTGATGGGGTACCAAGAACCTATGACCCCGAGGATGAGGATGGAAACGCCGGTTTCGACACCTAGCCGAATGCCGATGGCTCTTCCTTCGTATGGACGCGTGTCAATTCCAATGGCTAGTTCCACGTGGAACGGTGGAGGTGGTGGAACGCCCCAAAGGAGTGCGTTTCACCCTCCCTTCCAACCGCCAATGACGTCGACACCTGGGTGGGCTGCTGCTGGTACGATACCCGCGTGGTCTGCAGCGTCTGCACGACAGACACCTGCCAGCCCCAGAAGCGGAACGTACGGATTGCCGTTCCAGAGAACGTCTCCTATGAATGTTGCAACGTTCCAGGGACGAGCAGGAGCGCTAGGACAAGAAGGGAACGTGGGTGCTCAAGGAGGCAGGTACGCACCTAGCGCAGTACCACCGCAAGGGCAAGTGACGAGTCCGTACTACGACCAACGCGTCCGCAATGGCTTGCACAGGTCAGGGAGAGGTGCTCAGCGGGACGTTCCAATTATTGGTATCCCAACGGAATTGCGGAACGCGGTGAAAGTTTTCGTTCCATTTTACTCAGAGACGGCCACGTACGAAAGGGCGGCCGCGATATGGCTTTCCTTTGAGAAGTGTACGCTCGGTATGGACGACCAAATGCGGTTGACAGCCTTCGAGCAGGTTCTGAAGGACAAGTTGGGTCAGGAATGGTGGTACAATGCGAGGATCAACGATTTTGAGACTCTGCGCGGGAGATTCCACAACCGCTTCATCTTTCAGACGCCAGCTCAGCTGTGGGGCCGAATCAAGGCCGCTAAGAGGAATTACGGCGAATCTGCGGAAGAGTGGGGCGACAGGATCATCACGATGTGCGAGTACCTAAACTATCACGAGCCTCGTATGCAGTACGAGTTCTTCTTAGTACTTGACTCGCAACAAAAGAAGCAAACGTAGTGATGGTTATGACGTATCCTTGCTAAAATGATCAACTACATTCTCAGTTACGCCTCAACGTACACAAATTGCGTGACAGAAGCCATCATGGCAGACACCGTATTGGCACTTAGGTCTCCTAAGCACTTGTCTTCTTCTTGTAGTAGTCTTCGTATCTCTTCCAAACGGCGTAGTTCGGGTTATTGACGACGATGGAGCGGTCCATGCCTTCGAAGTATTTCTTGTAGACGCTCTGAGGTGTCCCCCCCATCATGTACCAGTACAAAAACTGGGTATTCTCCAGAAACGACCGGTCTTCTTCTTGGGCGGACAAATTGCCATGGCGAGCACCATCATCTCTTAAAAGCCTTTTGCCAGCGACAACATTCTGATCAGTTATGCTCGACGCTTGTGCTAGAACCACGAGCTTGGTTTCTTCGGAATTTACAGAGGGGACAACAATTTCACCACTTGTGAGGAAGACGACTGCAGTGACGGAAATGATGTAGCTAAAACGCATGACAAGTACCAAACTGTCGTCGGGGCTGGAAAGTGATTTGCGAACTGAGCGACGAATTTGATTGAAAAATAAGTTGACAAGCAAAGAGGGCAGTATATGCAAGTCTTGTAAGATGGTAATGCCCCACGATAACAATTATTATACATGTAAAAGGCATGGAAGTACAGGAAAATCAAGTGGGACTGTGGCGATTTAGGTTTATGGTTGTAAATCGGTTCGTAATCGATATTCATGCAGTTAATACTGTAAAACCTCTAATTGTGCCCCTGTAGTATGTACCCCACCCTCAAACACTCCAGGGGCACAAATCAATGTCGAGTCGTATTGAGGTTGAAGTTCTGCCAGGCCCTCCTACATTTAAGTTCATCTATGATTAGCTCAACTTCGTCGGGAAGGCTATTGGCTTCTTCGTCTTGGTCAGAATGCAACCTCGTTACCGGTACCGCCTTGGTTCGCAGTGGATAAGTCCAANGGGGGGGGGGTTAAGGGAGTCCAACTTGCTCAAAATATAGGGGGGGGCTGGCTATCTTAGGGAAAGGCACAATTATAGGTTCAGTAGTTTGTAATTAACCCTTTTAAAGCGGGCTTTTATTCTGTATTATACAGAATAAAGGCTTATCTTGATTAAACTATTGACAGAAGAATGCAACAATTTATCTGGTGTTAGCTGTCTCGCGAATCATGTGAGGTGGACACTACGAAAAAGTGCAAATACATAATTTATACGGACAAAAACTTCACAATGGATCCACCATACGCTAATATATGAGGTTTTGATTGGCTGCTGCAGAATAAAAACAGATCGCAGTGTCTTCTGTAAGGTATTTCCCCTCCAAAACGATTAGTGAGATTTGCGGCTTCGCGCCATTTTGGATGGCCGCCCGAGCTTCATTGCTACGCGGCAGAAGTTCGCTCACCAAATTTTTGGCCAGTTCTAGACTAGCAAGAGTAGAAGAAGAACTAGGCTTGAGCTGACCGAAACGTAGAAGAAGAACTAGAGTAGAAGTTAGTCTAGACTAGCAAGAGTCGAAGAAGAACTAGGCTTGAACTGGCCGAAATGTTGGGTGAGCGAACAGTCTATTGATTTGACTACTAGCTAGCTAGCTAACTACTGTCTTCTTCCTCTTCGCCTGCGCGCTTACGCTTGCGAAAGCGTATGCGCTTCTCCAGGTGTGCAGGAATTGCAGCTCCGTTTCACCAGACCTAATGCCAAATCTGGCTGCACGTGAGCGTGTTGCCGCTATCCTCTCTCCGAACCCGATTACACAGAGGTACGTAGCCGTCGTGTGATTGAGTGCACTGGGGGCAGAAGTAGCTGCTTTTAAAGCTTTTCGTTTGGTCGCCATTGCCGAGCATACCTTGAAAAAGTGCTGACGACGTTTGTTCTTGCCATTTCCATCATACCTGTCGTTCGTATTCTGAAGGGCGTGTGGCTGCGTTCGAATTGGCTCCGTAATCAAGTCCTCTGCGTTGGTGTTTGCACGCAATGTGGCTTCTGTTGTTGCTAAAAGCTCCGTTTGCAGTCTTCGCATGTATTCTGCGTGCGTCGGAACGTGTTCTCCCTTTTTCTTCAAGATGCGTTTGTGCAGGATGAACCCATTGACAATCGCCATGTCAACAAACCCCAAGAATAACTGGCGGTAATATTTTTTGAAAGCCACACATCGCTGTATCGAATCTCGCTGCAGTCGCAGCTGGTCGTGCTGGTCGACACCCCCCCATACCAGTATGATAATCGGCAACCAGCTGGGGACATGGTACGTCTGCACGAACAATCTCACCCTTCACTCGGCGTCTCACACTAGTCATCGTCGCTGCACATCCAGTTGCAATCATAGACACAGGTTTCGAGCCTATCCAGGACACAGCAATGAGCTCAGGGAAGTCTTTAGTGCGATACGTCCCTCGGGGCATACGCTTGGGCCGTGTTTTCTGTGTGGATGCAAAGCTGACTGGCCATCCCAATCTGTCGTTGCGGTGCGTTCCCACGTAGTAGTAACCCCTCCGAAGTAAATCCAGTACCAGTACACACGAGCTGTAGAAGTTGCCAGCGACAACAAGTCGCTTTGCTGGTTGCCCTTCGAGGACTTTGTTCACGTTTCGTATAGCAGCCTTCTGTGCCGTTCCATTTGCTGCCGCTGGATTGTTAGCCGCGCCGAGATAAACTTCAATTCTGTAGCATACAGAAGAAGACACAGCATATTTAAGGTGAGTTCACCCGTGTTCTGTAGAAAGAGGAATTGGCAATCTGTATTATACTGAGGAACACCCCGATACTACTGATACTACTAGCAATTCTGTATCAGCGTGCCGAGAACAGTAGCCAACCTCCGCGCAACACGTCATGTAAACCTTAGTCCCGTACTTGTGAGGTTTGTCCTTGTTGTAGACGCGCACCGGATTGAACTTGCTCCTGTTTGAAATAATGCCTTCGTCAAAACTGATTCGCGACCCCAACCGGAAGCCACGCCGAAATGTTTTCTCGAGTACCTGCAGTACCGGTCTGACTTTCCATGCTTTGTCCTTCGACATCTGCAGCGTTGTTACTAGAAAAATGCAGGTAACGAGTGACCATCTTGAATCTGTCTCTCTTCATATACGAAAACGTTTCACGAGGGACTGCGCTATCTTCTCGAGTAGACCAGTCTTGTTTTTGGCACAAAGGGTTCGAGCAACGAGGAGACCAATCACGTGGATGATTTCGTGAACCTGGATAGGTTTTACTTGCCGTAGCTTCTCTACAATCTCTTGAACAGAGTCGATCTTCTTTTGGGGGATCCTTTGTTTGTGCTTGAAGCTGCTTCTCTCGTTGCTGACGTGCGACAAGTGGGATGCAAGCCTAGCGATACAAATTCGTCTCCTTCTGAATCACAAAACCAGAGCTTCTTGGGAAGAAAGTAGAAAACATTCCTTATGAGGACTCTGCAAATGCTGCTGCAGATCTAGTTGGCCCCCAGTTTCCGTCGTACAACTGCGACGGTTTCAAGAGTCAAATCACCTGTTTTTAATATAATACAGAGTTAGAAAAGAATCAAAAGAGTTATTCTGTAGACGACCGACTTACCAGAATGTAGCTCGTCATAAATATTTCACCCAGAAGTTCTCAAGCGCCGCATGTCTTCGTCGGCTTGCACAAATAGCTCACTGGCCTCCGTACAGTCGCTGTCAGACTCTACTTCGCTTTCGTAGTCCTCTCGTGGCGACACGGGCACATCCAGCTCATCATCCACCGGCGCACGACGGCGGGCCAACTCATACGCCGCATTCTTTCCCATGGGGATCCTTGCTGGGAGTGATGGCAAAGGTGAGAGCTTGAGCAACCCCTGACGACGTCCAAATATCATGAGCTCTTGTTCGCCTGCACATACCTTTAAGTACTGTGAGTATATATACCAAGCTGTCTGCAGAACGTACCAACGAAGTATTCAACCCCGCGCTTCTCTGCATCCAAGCGGCCAGTCACACTGGGCATCACGTACGTGAAGTCCGCACTCAGTCCCTTAGGCTTCCTCGCTTTCCATCCTGCCTTGGATAGCTCGCGCCATAACGGCTTGAACGGTTTCTCGCTGGGAGCGTTCATTCTCGAATAAATCTGAAACCTATGGCTACGCAGAAAAATAGCAATAAAACTGATTTTTCAAAACCTCCAGTCATCTTATACTGTCTTTTCTATCCAGCTTGAATTATCCCCGGCTATCTTCCCTTCGAAGATCCTTCAAGATCGAACTACCCATCTCTACCCCTTACAAACACAAGTATCCGAGGAATTACGACGACTACAAGTTTGCTGGAAATTATGTCCTGTCATAATTTTTATTCTGTAATATACAGAATAGCGCTGTAAAGGGGTTAAATCTACTTCAACGCTGAAGTTGAGCCGCAAAAACACTGCTACAAGCTCGGTGCCATTGTCTTACCATAAACTTTGCAATTTAACATTTGTTAAAAGGCATGGTTATGAGTGATGACGAACCGACTGTTTTTCTCCTTTTCGTTTTGATTACGTCACTNACTTTGACCAGTTGATCACGACACTTCCCTCCACGCATCGTTAACGACGCTGTAGCATTGCGTTGCATGTATTAACTGGTACGTAGTAATGAATTTTGTGGATGGATTGACAAACGCCACATATATCGATAGTGACGAAAGTTTCCGTCTCTTTTATTAATCGCCTACGTAGGTATCGACTTTCCCAGTC

>Contig_67

GATCGAGAATGAAAACTGTAAGCGGTTTGCAGTTGTTTGAAGTCGATAGAGCGAAATCAAGGTATTGCTCTTGTTCTCTGCCGATAGCCTCGTCCAGATCGTGCGTCTCATCAGCCTGCAGCCTGTGAAGCTAGATTAATGCGCTCTGCAGTGCCAACACACAGCACCAACCTTGCCGTCACTCTCACAATTGTCCCAGACTCACTGTTGATTAGTATTAATTCAGCTAGTCGAACTTCAACGACATATTATTGGTGGATTGGAGCCGATGACGCAATCTTAGAGAAATTTAATATCGTTGTAGCGAATATTAAGTTGATATTTAATAGTATTATGTATTTAAATAAAAAATACTAGGTAATTTTTCGTATATAAACTCGTCTATTATACCAGGTGATACTATCACTATATTGACATTAACTTCACCGATTTTGGAGTTACAGTCAGGGGTTAATGGTAACTCCAAAATCGGTGAAGTTAATATTGTGAAAATATCACCTGTAGACCAGCTAATATAAAAATATCATCTAATATTTTCGTCTGAGACAATATAATATTATTAAATATCAAATTAATATTAGCTAATACGATATTATATTTTTCAATGATTGCGTAATCGGGCTACATTAACCAATCAGATGTCGTAAACGCTCGACTAGCTGAAGTATTACTAATCAACCGTGAGTACCGGGACAATTGTGAGTGACGGCAAGGTTGGCGTTGTGTGTTGGCACTGCAGAGCGCATTAATCTAGCGCCACAGGCAGCAGGTTTATTAGACGCACGATGTCGACGAGGAGATCGGTAGAGAACAAGAACAAATGTCTTGATTTCGCTCTATCAACGTTAAACAGCTGCAAGCGTTTGCAGTTTTCCATCTCGATCGCCTTTGTCTGTGTCACAGTGAAATACTAGAGCAAATACTCGTCAGTCTTAAAGCCTACTGCTTTTCTAGAACCTGATCGAAAAAGTTATTTGGCTCCGCTCGGTTCACAGAGGTAAGCTGGCAAGAAACGTACTTAAGTGGGAAAATATCATAAAATATCATGTAGTATCTAATATTTTTCAGTTCTCCGAGCTAATATAAAAATATTATGTTAGCTTTTGCAAAAATAAGTTACTATTAATATTTAATATCGAAAATAGTACTATTACTATCAAAAATATCATATGATATTTGGTTACCATTAGCCCCTGGTTACAGTACCATTATTAATAACCCCTGACTGTACGTCATTGTTTACGTACGTTGGTAACCATACATGTATTCATTGTTACGTACGTAATCCTGTAATGCGAACGAGTCGGTCGCCACGACTCGTGAGCGTGGGCCACGAGAGAGTCTCTCGGAGAGACCAATACAGAAACGCACCCACTGTACGTTTTCGTATTTCCGAAATACTAGTAACGGTGCACCCAAACTATGCATCTGAATGGCGGTGTACTGTTGGGAATCGCCGTACTGCTCGCACGGATCATCGCAATCACGGGGACTGAACTCTCCACCAACAGATTCTTGAGGATCCGCAACATCGCCAAGGAGAACGATGAAGAGAGGATGACCAGCATGAATTCAGTCCCGGTGGTTGAGAAGATATCTCGTTCTGTGAGCTTCAATGCATTCCACAAGATTGACCCAGACGTGGCGGTGTCCCAGCTAGTCCGTGCAGCACAGAATTCCCCGAAATCTGAGAAGATAGCTACATCCTTCCAAAATAAACAGCTTCACGCCTGGTTGGACGGTGGAGTGCCCCCACAAACAGTCTTCCAGTTCTTAGCGCTCGACACACAGGGAGATAACTTGTTTGCCAGTCCACAATTCAAGGCCTGGTTGGAGTACTCAATGAATTTCAAGAAAGCGAATCCTCGCACAAATACGATACCCGTTATTGATACACTTGCAGTTTATTACAGTGATGAGGCTTTAGTGCGAATTATTAAGGCGTCCAAGGAAGCCGCACACACGAAAATCAGGTCCGATTACTTTGAGAAAGCTCTGTTCGCTAAATGGGTGGAAAACAAGAAAACCTCAGCGTACATCTCGAACATGATGGGCAGAGAAATGACCCCAGACGAAGCCTTCAAATTGCTCACGCTCGATAAGGCAGGGGATACAACCTTCACGGACTCAGGGTTTGGCACTTGGCTCACGTTCACGAAAGTTTTCAAGAAAGAGCATCCGAAACTTAAGACCAATCCAGCTATTGATACACTCACGACTTACCTTGACGACCAGGTGCTATCCAAATTGATCAAACGGGCAGAGCAGAGCCCAGGGATGGAGGAAATGGCTACCTATGTCAGGAATGCTCTTCTTGACAAATGGGTAACGGATGGAAAAGTGCCAGCATTCGTGGTTAAGAAACTTGGGACATCGAAAGAAAGAACAGCAGAGCTGTTGCGTACATTCCTCGACAAAATTAAATCAAGGCCGCCTTTTAAGGCTGAAGCAAATGTTACGAGAAAAAGAAAACGGTACGCGAACGTGGTTTACAATTAGCCGAAGCCTCATGTAAGCTCCACTACTCGTAGTATCTTGCACTAGCTACAATTGGCATTAAACATTTCTCGCCAGTACATCTTCTAAATGATAAACCCTTGACTCGCCGTTTTTTGCGCCTATACCTTGGCGTACTACATGTACTGTACTGTAGTAAGTCGAACGTGCACACCTGTTTTGGCACCAAAACCAATCTATCAGTTCCTATCAGCAATGAAAATAGGCCAGCTGGTGCTTTTTTTTCGTAAAATAATTATGAGGATATTTAGAAACCGTTGGAGGCCAGGATTATTAGGCCCTTGTTCAGGTAGAGAACCTGAGTTTCAAAGTAGCATTTACTGGTCAAAATTATGCTAGTACACTGTACCACTAGGGGCAGTTCACGTTTGTTCTGGACACACCTCCACCAAGTGTTCTAGTCGCAGCACACCTGTGCCATGGACGACGTCACAGCTGCAGTATTAGCTGTAGTGTTGGGAGCTGAGTGATGGTCGCTCCTCGGCTACGCCGAGTGGTGACCATTGTGATGAAGGAAGTAGTCGTAACAACAGTAAGTTGCAGGGACTGAAAGTCCAGCAGGGGAGAAAACCGAGTGCTGCTGCTTGCTCTTGACGGCGCAAGTTGTCGAGTGTGTAGCAGCACACGGAAAAACCCCGGTAACATATATTGGTAGGATTAGCAGTAGGATGGGGCTGGCGGCTCGCCAGCCAATGGTCCGTCGAAGTCCGGCTTCTGGACGCGAGCATCGCGTCCTGGAGGCACCTGAGCCTCAGGTTTGAGCTGGACGCAGTCCTGGCTCTTCCTCCACACTTCACACTCCCACTTGATCTAGTCGAAGCGCAGCTTCAACATGATCACCTTGTAGAGCTCACCACGATGTTCTTGGCATCTTCTAGCTGCGCTGCAGCACGACGCTTCGTCCTTGGTAGCGCTCTTCCTCCTTCTTGTCCTTCGCATGCATGAACGGGTAGTAGTAGCTTGCATTCACGCATGGCGATGATGTTTGGCTCCCACTGAGCGAGCTGAGCCTTGCTGACGGTCGATGCGCAGCACCTTCATCCCATCACGGCAATGTTCGAACTTGTCCTTCGGCAATGGCTTCGTCATCGCGTCGGCGATCATGTCGTCGGTGCCAACATGGCGAATCCGTAGCTGCTCGTTCTCCACGAGGTGGCGCACCAAGTGGAACTTGTTCATAATGTGCTTGTTCTTGCTGTGCTTGCCAGGCTTGGCAGTCAGATAGATGCACGACTTGTTATCGCCGATAATCTCCGGAGTCGCGAACTCCCAGCATAGTTCTTCACAGAGTCCACGTAGCCACTGTAGATCTCTAGTGCCTTCATTCATGGCAATATACTCTGCTTCCGTTGTGCTCTGTGCGTTAATCTCTTGCTTTCTTGATCCGTACGAAACCACGTTACCATTGACGAACGTCACGAACCCGCTAACACTCTTTCGGTCATCAGGGTCATTAGCGTAGTCAGCATCGGTGTAACACGTCAGCTTGACGTCGTTCCCAATAACAATGTCCATCACTAAGCCGTGGTCTTGAGTACCACTCAGGTAGCGTAGCACCCTCTTTCCCATAGCGTAGTGTGTGTGGTCGAAGCAGGACAGGTACTTGCTGAGATTACGCACAGCGTGCGCAATGTCCGGCCTTGAGGCTGAGACCAGATATTGTAGTGCACCAACGAGCTCTCGGTACGGGGGCGGGGAGTCATCTTTCGCATTTGTGACCACCTCAGCGCGCAGCGCTTCCGGTGTTGCAACCGCGTTGCAACCCTCCATGTGAAATCGCTTTAGCACCTCCGTAATATGTTGCCGCTGGCAAAATACCGCATGTCGACGCTCGCTGTCGATCAGTATCTCCACGCCTAGTAGATACTTGACTGGTCTGAGAGATGTGAGTTCATACTCCTCACTGAGTTGCATAGCAACCTTAGCRCAAAGGTCACTAGGGCCGAGGAGCAGTAGGTCGTCGACGTACACCGTGAGCAACATGGTGACTTCTCCTCCCACTTTCCTGGCGTAGAGTCCGTAGTCAGAGTCGAGGCGCTCGAGTCCGATCTTCGTCAGGAACTTGTGCAATGTCTTGTTCCAAACGCGAGGCGCTTGCTTTAGCCCATACAAACTCTTCTGTAGCTTGCATACATAGCGCGTAGAGTCTTCTCCGAAGCCTGGAGGCTGCTCCATGTAGACCTCCTCGTCGAGRTCACCGTACAAGNNAACGCGGTCTTGACGTCGAACTGCAAGATGGCCCATCCGCGNTTGCAGCGCGTATACGATCACGCATCTGATCGTCTCAAATCTGATAACAGGTGCGTATGTTTCACTGTAGTCGATGCCCATGCGTTGTAGAAAGCCTCTGATCACTAGTCGAGCTTYATATCGCTTAATCCGGCCCGTAGAGTCTCTCTTGAGCGCGTACACCCACTTGCACGAAATGACACGCTTGCCCTTGGCCTCCGTGCGTCGCACGAGCACCCAAGTTTCGTGCTGCTGCTGTGAGTCCATCTCAACTTGCATCGCTGCTTTCCACTCCTTCCACTTCTTTGACCTTGCCGCATGCTTGTGACTAATGGGGATCTGCACATCCTCGACGTTCTGCTTCGTCACATTCGTCACTACGTAGTCCTTGAGTCGGGCGGATGGTTGTCGCTTCCGCTTCGGTCGCGACGTAGCTGCACTGTCGTCTGGCGACGACTTCTGCGTCGTGCATGGCTCCCAGTTCTCGTCTTCGTGAGCATCACGTCGACTGTCCTTGGCCTTCGAGGCTCCCACTCGATTTGCACATGCTTTCGCYTTATTCTTGCCYTTTGTGTTCTTCYGCTTGGAACCTCCCACTGAAGCTGCTGGCTTCTTAGCGTCCTCGCTTGGTAGCTGTGGGTCATCACTTCGAGTCGCAGACTCGGCTTCAACTTCAACACCTTGGTCATGGCTCTCACCAGCAGTGGCCAGCTTCGCCATCTCCGCTGCTCGCCTTTCTGCAAGCTCTGCAGGAAGCTGCAGGTACGTAGACATTGTCGTCCGGATCGGCACGATCGGGATCTCCGCAGGTAAGTCGTAGTCTCCGTAAGCGTAGGTGTTTGCAAGAAGTTGCTCAATATACACAGTGTTYACCGTGTACTTCTCATGTGCCTTCATGATTCCATCTCTGCACGTCGTCACTGATCCTGTGCGCAGGTTCAGCAGCTTATAGCCCTTGGTCTTCTCGCTGTAGCCAAGTAGTAAGCACATCTGCGTCCTTGGAGTGAGCTTCTCCTTTCTCTGCCGAGAATCATCAGGGATGTGCGCGTGCACCAAGCACCCCCACGTGCGCAGCACGCTGAGGTCTGGCCGCTCTCCGTACAGGGCCTCGTACGGGGACTGCTGGTCCTTCAACACCGGCGTCGGCGTCTTGTTGAGCGTCTCAATTGTATGTAGGAAGGCCTCTCCCCATAATGAAACAGGCATTTGCGTAGCTTGTAAYATAGATCTGACCTGCCGCATGGCGGTCTGGTTCGTGCGCTCCACGATAGCGTTCTCCTGCGGGCTGTAGGGATTGGATGAACGCGTGTCGATGCCGAGGTCCTTGCARAAGGTCTCGAGCTGCATGTTGACGAACTCGCCACCACCATCGGAGAACAGCRTCTTGATGGTCGCCTTCGGGAACTGGTTCTGGATCCAGCGAATCTGCTTCTGAATCAGCTCCGTGGCTTCGTCTTTGTAGGCTAATAGATATCCCCACTTGTATCTCGTAGCTTCGTCGATAATCAATAGAAACTGGTTTGATTTCGATATTGTATCCACTCCAACACTGCATAGGTCGACACAAAGCTTGTGCAAGGGCTTTGCAGAGCGAGACGGCTTGCGCTTGTAGCTCATGCGCTTCGTCTTTGACTCTAAGCATGGAATGCATTCATAGAGAGCAAAATTCTTCGACTGCATGTTGATGCCAAAGTTATAGGTTCCTGCCATAGTCTTGATAGTCTCCATGTTTACGTGGCCCATGCGGTTGTGCAGCAACGTCATGGGGTTCTCAGCTTCACTGGCCTTGACGCTCATCACGTTCTTGCTCTGACGTGGCGTCCACATGCGGTACATCCCGCGGATGTACTGGAAGCGCAGCTTCAATGTGTCCTTGACCAGCCACGTAGTCTTCTGATCATGCGAGATGCGCACCTTGAAGTTTCCTTTGAATTGCATGTAGGTCTGCGAGATCAGGTTGTAGGCAGCCGACTTGGCGTAGCTCACGTCATCAAGGAGGCGCTCCTCGATCTTGCCCGTGTGCTCGTTCAGCACTTGCATAAGCACTGTGCCCACAGTTGCGCGCTCGGTTGTGCCGTTGCCAAAGACTAGCTCTGCAACTGAGTCTTCGTGTAGCTCCGTAAACAAGGTAAGGTCGCTCGAGACGTTGGTCTGCGCGCCGCTATCCAACACCCACTCCACCATGTCCTGGTCCTTGTCCTTGTCCTTGTAGTTGAAGTTGTCACGCACCGCGAGTACATGCTCTTGTCCTTCAGCTTGAGTCGGTGCAGCCGACTTGGCTGTCTTCACAGTAACCACATCGATCATCCTGCGCGGCGGGTTGCTCGAGTTGTTGTTGTTGTTATTGTTGYCACGCTGGCGGCACTCAGGATCGCCACTCCAATGTCCTTGCTCTCCGCACGTGTTGCACTTCGTCTTCGCCTTTCGCTTGGCGATGTCATCCCTCGCACTGGATGAGCTCTTGCTCTTCTGCCGCTTGTTCCGCCCACGCGGCGATGAGCTGCGGCTGCGACGATTGTTGCGGTTGTTGTTGCCTCGTGCTTGGCTGCTGCTCGAGTTTCGACGTCCTCTTCCGTCGTTGTGTCCGTCGCCACGGCCACCAACAAATGCAACATCTCGCTGGACGGCACGTCTACCTCCACCACGTCCTTGCTGTAGCTCAGTCTCTTGGCGTGAGTGGTCAGCGAGTCGAAGGCGCTCGATCGCTTGCTCCCACGTAATGGACTCATCACGTCCAATACGTACTTCATGCTCGTTTACCACCGCTGGGTACACACCCACTGTATTCGACAATAGGATAGAGACGGCTTCCTGCTCAGGCAGCTGCTTATTCGCGCGGCGAAGTCTCTGTTGGTAGTCGTCGATGTCACGAATGTAATCGTCAAGGTCGTTCGTCACGTTGCAGGCGACGATTCAGCATATCTCGCTTGAGAAACACGCTGTTTACTCCAGGATGGTTATCAAAATGATCTTCCAGTGTTCGCCACAGTAGGATAGGATCAGCACGTTGAATAACACTTGTAAATCGCATCTGTAGCTCTTGACTAAGTCCCATCGTAAGTTCACGTTGAGCAACACGCAGTCGTTGCTTGAAACAGCCGCGCTGTCCTTCGTTACGTTGGTCTGCCATCGTTTCGCGCTCCATGATGGGGTCCAAGAGGTGTGCGACGTCGAGAATGGAGCAAATCTCGACGCGCCACGTCTGGTAGTTGGTATCGGTAAGTCTTGCGTTCAACTGCATCGTGTCCGCTCTGGTTCATGCACCGCGTGCATCTGTGGTCTCCAACCATGGGTTCGTCCGTAGCGGAGTCGGTCCCGGCCTCGGTCTGGACTGGCTGCGCTGCAGCCTCGGACGTTCTTGGGTTTCTTGGCGGCTCGCTCTGACGCGTGCTGGCGTCGGCGGTAAGTTCCTCTGTGCTTGGGTTTCCTCGGCGGGGCCCGTACGGCGTCGGCACTCGCTGCGGTTGTGCTCGAGCCGCTGAGAATTCTTGAATGAGCTGCCTTTGTGGGCGTCGGGTGGATGCTTCCACTGGAATGTTCTGCACTTGCTGCTGATTGGTCAGTGTAGTCATAACGGTACCGAATTGGGCCAATAAGAGCTCGAGTTGTTGCTCAACTCGGGTTGATGACGCTTGTGACGTCGCATGTGTCCTCGTCTGACGTTGTCTCGTCTGCTCTTGCAGGCAAACTGCAACTCGTCGGACGCGCGCGAGCTCTGCGTACTCCGGACTGGGCTTCCAGCTCTCCTTCTTGGGCTCATAACCTGAGTGATGGTCGCTCCTCGGCTACGCCGAGTGGTGACCATTGTGATGAAGGAAGTAGTCGTAACAACAGTAAGTTGCAGGGACTGAAAGTCCAGCAGGGGAGAAAACCGAGTGCTGCTGCTTGCTCTTGACGGCGCAAGTTGTCGAGTGTGTAGCAGCACACGGAAAAACCCCGGTAACATATATTGGTAGGATTAGCAGTAGGATGGGGCTGGCGGCTCGCCAGCCAATGGTCCGTCGAAGTCCGGCTTCTGGACGCGAGCATCGCGTCCTGGAGGCACCTGAGCCTCAGGTTTGAGCTGGACGCAGTCCTGGCTCTTCCTCCACACTTCACACTTAAGTCGTGTACGAATACGACTCCGTGTATATCGTGGTAGAATGCTCCGCGGCTGAGCTCGTACTGGCGATGACCTCCAACATCCAGGAATTCCACAAAAACTTCTCGTCCTGCCGGTCCCAACGACGTGAGTAGCACGTGGACATCGCATCCAGTGGTCCACAAGCTCGACGGCTTGTCGTTAGAGAGCGGGGTGGGCGAGTATAAGCGGCAAATGGCACGCAGCAACGCCGTCTTCCCCACTCCGGATTCTCCAAGAACAAGAACACGAACACTTGGAGTTTCCATTTCAATTATGGGCACTTTACAAACAAACGACATTATAATTAACCAATCAGGAGAGTGAAATGAGTCATTATCATAGTTTTGATACCATTTTTGCAGATTATCATGGCGGAGGAGTGGGCGTACGAGGAAGCCTCGGACGAGGAAAAGCTGCAGATTGCGCAGCGTTTCTTGCTGGCTAGTCCACCTGGACAGGTCCACGAGGTTCTGCGTGACGTGGCCAAGCTCGTCCCGGCGCATGTGCTACCTGATGCGGCGCTTCGAGGGGCGCTGCATGCCTACAACGTTAAGAACTGCGTGCCCGTAGACGTCCCCGATGCTGATTACAAGGTCTACTAGACTCTGTTGCTGCGAGCATTACCTCTATATAAGCCCTGAAATATATATTACATGATAGATCTTGATCTGTGAAGAGGGAGAAGTGGACGCTGCGCACTACGTGGACCCCATCGGCAACCGCGTGCTTGGATTTGACCATATCAAGCAGCAAATTGTACCGGACGATGTCGCTGAGATTCCAGAGGACAAAGTCACGGACTTTGAGAAGGATCGGTAAGAAAAAATTTGGCGTATTATGAAATAAATAATTTATATATTGAATAATTGCCAGTCAGGAGGTGCAGAAGACGTTGCAATCGTACTTGCAGTTCGAATATATGCACGGGTAAGCAACATTACTGCTACAAAGAGGTGATGTATAAATGTTTAATATTTACTGTTGATGTTGCAGTGGAAGTGCCGGTGTGTACATTGTGGGTACCAAATTGGTGGTGAATTTGTGCACGGAGCGTATCAATTTGCGCAACTATTGGGGAGGACGCTGGAAATCGCGATGGGAAGTGGATCTGACAGCCAATCCGGCCAAGATTAAGGGCAACATACAGCTGCATGTTCACTACTTTGAGAACGGCAACTTGCAACTGCAAAATTCCAAGGATATCGACGAGGAGATCACGGTGCAACGTCCTGGCGGTCTGGGTGACGCCATTCTGCGCGTTATGAAGGAGGCAGAGGACGATCTGCAGAGCAACCTCGAAGACATGTACATCAACATGTCCGAGGAAACCTTCAAGGAGATGCGTCGCGTCATGCCAGGTTCGTTATTGTCAAAATTGAAAATCTCAAGTGTCAATGCTAATGTCTCATTATTTCCTTAGTGACCCAAACGAAGATGGAGTGGAGTTTGCATGCCCACCGCACGGCCAAGGACCTTGGACGCAAATAACACGAGAGAAACAACTTCTAGAAAAGTTGCATTTCGCAGTCAACACAAGTGAAAATCAAAAAGAAAAGTTGCGTATCACGTAAACCTGGTTTTATGTTGCCTCTAGTGCCTCCAGCAACTCTCCTTCTCCTGTTTGGAAGTTGGCAAGTTGCCACGACAGTTTCCAAATGTGCACCACACCTTCAGCGTCCCCTGCCGCCAAGAAGTTCCTCTGTCGAGGATTAAAGTCGAGCGCAAACATCGGGGCGGCAACTGCAGTCTTTGACGTTGTGATTGTACTGCCAGTCTTTGCCCCTGAAGTTGATCCCGTGATGGAATCTTTACCGCTAAGAACCAACACGGGACTGACGCGATCAGCTTTGATGTCGAAGACATAGACGTTCCCGTCTTCACTGGCAGCAGCGAACACCATCGGACGTGTACGAGACCACTCGGCCGCATAGAGATACGCTGATGACGGCGATACTTCAAAAGATAATAGCAATTCACGCTGCATTGTTGAGTATACACGCGTGGTTCCATCAGCTGATGCAGTGAGGAAAATACTTTTCCGGAAAGGTGAGAAGCTCGCGTCATATACGGGAGCGGTATGAGCTTCGAAGACAAAGTCCATGGCACTCGGGAATATTATTGTAGGGTCGAGTCTGGCTTCATACACCGTTGCCAGTGTCACTTCTTTACTGCGTTTTACAGACGCATACGCCTCCACTTGTCGACGTGCCGCAGGGATCTTTGAAGTTGGGAGTTTACTCACAAGACGTGCAGCTGTAGCTGTCCACTTCTTGTCCCCTTTAAAGTCCGACGAACGAACATTTGTGGCAGCTTTCGCGAAACATCTAGCGATGACGCCACCTTCTGAGCCAATGACGAAAGCGCGACTGGCTTTGTCCGTGCTACTGAAGGCTAATGCCTTACCACCGATCACCGGCGAGCGTGTGTCATCGTTGTTGCTTTTACTTCCAGAACCCCCGATGCCTTGGGGTAAATGCATGACGTAACCTTCAACAGGGAACGCAAGTTTGTCTTTAACTCGCCAAAAGAGAATTTTACCATCGCCACTCACACTTGCAATCTACAGGAAAATGGAGTTAGTTGCTGGTAAAAGGGAAAGAGAGTTTAAATCTTACGTTGTAGTCGGCGGTTTGGATGTCGTAGACCCAGGCCACTTTGGTGACTGGTTCGCGGTGGAAATAGTCTCCGATACCTGACGAGTAGAATTTATACTCAGCARGTTCCATATCCCACACGAAGACTTCTCCATTGAAAGAGCCAGCAGCAACAACGGAAGGATTTTGGGGGTGAAACGCCACACACATGAGTCCACTCTGTAGGAAGCGGCAATTTCAGATCGGAATTGTAAAAATCGGGACTAGAATCCGAAACGTACCGATGTTTCTAGGACAAGGGAGGGTTTCTGTTGGTTAAGGTCACTTTGGAAGACATTCCATAAACAGAGGGCCGATCGATAGTTGCACCACCCGGAGTGGTCGAAGCGTCCATACGCAACTGCGATGACCGACCCCGTTGCATTCCAGGACACACCCGTGCACGATAATCTCAATGAAGATAAAGATCCTGTTGTGGTTGTCGTTGCAGTGCCTTGTTGCGGCTGGAAGTACGTGTCGTAGTCGAATTGCAGTCGAAAGAGTTTGGTAATATCCTTCTCCTCTGACTCTTCCAAGTATCGTTCTAAACCTGCATCAACATATCAGTACCAAGATTCTGGGAGAGGAATTAAACTGTGGCGTACCGCTGAAGCAGGAGCTGGTGGCTCCATTCTTGGCCATTTCTCGTAGCATGAGTCCTCCAACGTTTTGCAAGCACTCAGCCACTTGTCGTTGGGTTTCTTCTTCAGTATTAATTGTTGCATCTTTGCCTTTGCCTCTGGCTGGTGGTTGTTGGGTTGGACCCGTTTGAGTGGAGCTCGAGACGATCTCGCTCTCCGTCTGAGCTTCACAGTCGAACATGTCGATTTCAATCGTTTGGGCAAATACTTCGGCCACTGTCTTCGTTCGCTTGGCCGCGAAGCTCGCACTGCCCAGTGTCACGTCCTGGAACATGTTGCCTCGTTGTCTTAGCAAGATGCCAAATATCGCGTCTTTTCATGCCAAAAAGTATCTTTTAGAAAAAAAATACCTTTTGAAAAATGGTGTTCATTGCCACTCAATTGAGAAATTCACCACTTGCAAATTTGTGGCACCGGTTCATGGAAAACGCGCATGTAGTTTTGATTATCGTTTTGTGCACATATCTGGTGTCAAAATTCACAGCCTACCACGACGGAGTGGCCGCCGCCTCGCCGCCGCAACCTTCGCCACAGCCGACACCGCTCGAAGAAATAACCTTTGGCGCCTTGCCACGCATGGCGGCACGGAACAGATCTGCCTTGTGCTGCTTGAAGAGCGTCTTCAGACCGCCTCCGTTGAGGAATGCCTCCTCAAGTTGCGTCAACGAGAAGGCCACCTGGTGGTCCTTGACACTCACCACTCGGTTACGCAGATCCACCGTAACTTCCTCACCTTCAGCGGCCAGTTCGTGGAACTTCTCGTCCGTCACGACAATGCCCAACAGAGCGTTGTTGGGCTGGTTACGAGCGTAAATGTAAGCGAATGACTTGGCAATCACGGCCTGGACGCCGACAGCCTTAAGGCAAATCGCGGCCTCTTCACGAGACGAACCACTACCGAATGCCGTACCGGCCACAATGATGTTTAAGCCCTCTTTCACCTTCTGCGGGAACTCGGGACGCACATAGGCAAACGACTTGGCAGCGAGCACATCCAGCTCTGTCTCACACTCAGCAGGCCACAAAGGCGACGACTTGTTAAGACCCATAAACTGGGCCGGGATCACAGCGTCTGTGTCGATGTTGTCGCCGAACTTTTGCACTTTGCCACTGAATTTAGGTGGCAAAGCAGCTTCCAGTTCGGCAACAGCAGCCTGTTCCTTGGCCTGCTGGACGGTCTCTTCATCGTTACCCGAAGCGAGCACGGGTTTGGGCTCCGTCACTGTGTACGACTTAGTCGTTTTGGCCGGTGATGGCATGGACGAGTCCGGCTCCACCCAGTTACGGTAGCGGTCAAAGTCGTCCTTGTTGATCTTGTCCAAGTACGGCAGAGGGTCCACCACCTCCATATCGAAGCTGGAGGCAGCCACTACCGCCGCCGAGCTTAAGTTACCGATCGATCCTTTACCCATACGGTTCCGGAAGTTTCAGTTCTGACTGGAAAGCCACACTTCACCTTCGCCGGCAACATCGGCACCGATGCCGACACAGAAACTGCAACTCGGCACGCCGACAGTGAAGCCTGCTTCTTCGTAGATACGGACCAGACCAAGTTCTTTTAGACGGTCGACAATCTCCAGACTGCCGGGAGTAACACGACGGTTGCCGTTAGCCGAGGGACGTTTTCCCTCCAACATGGCTTGTTGCAACACCATTGCACCTAGAATCAGATCTTCCTGCGTGGTAGTGCAGGCACCAATGAAACACCCATCCAGCTTCATTCCGAGTTTCTCCTGGACACTCACACAGTTGTCGGGCGATGGGTACAGAGCGATCAACGGCTTGACATCAGCCAGGTTGATCACATGGTGGGCGGCGTAGTTGCATCCCTCGTCCGGCTCAAAGAACAGAGCTTCGTCGAAGTGAGACTCGCGTGCTTCGAGTACACGTTGCGTGATGGTATCGGCCTGGAACACGCCGGCAATGCCACCGAACTCGGTACTCATGTTCGCGACGGCAAAGCGAGCGTCGTCGGACAGGTACTTGAGCCCTGCGCCGCCGTAGTACACAGCACGCTCAAACGCGACGGTGTTGCGCTTCAATTCTCCAAGGATGTGAAGAATCACGTCCTTACCACCGATACCGAAGCTCGGAGCGCCGACAAACTCAATGTAGCACACTTCCGGCACCTTGAACCACGTCTGACCCGTGACTAGTGGCATAACGACGTCGCCAGCGCCCAAACCGGCAGCAAATGCGCCCATTCCACCGGCACTGCATGTGTGGCTGTCAGCGCCAATGACGATCTGGCCGGGTTGCGCACGCTTACGAGCAAACTCGGTGTGCATGATGGTAGTGTTGGCAGGCTGGAAGTCCGTGAGCCCATGGGTCTTGGCGAAGTTGGTAGACATTTCAATAAGCGCCTTCTGCTTAGGCAAGTGGTTCACACGCGGGTCCACCGTGTGGTCCAGAGCCAGCCAGAAGCGGTCGGGACGGTGGAGAGGCGGTCGGCCGATGGCCTCGTGCATAGTGTCCATGGCTTGGAAGGTCAGTTCACTGGCAATAGTCCAGTCAGCCTTGACACACAGCACGTCGCCCACGCGCACGTAAGGCTTGGAGAGGCCTACAGCATGGTGGCACAGGATCTTCTCGATCACGTTCATCGGGGGAGCAGACGCCATTGTCACGGACACGCAAAGTCGAGGAATGAAGCGAGTCCGGTGGTGCGTTGCAAAAATGGCAAAGGTGGATGGGCGACAAAAATGAGATAATTGATACCCGTTATGTTGTTTACTGTTATAAATCGGAAATAGTGCTGCTAACAAAAGATTAAAATTCATCTGAGGCTGTTATGTACCGTATCCTGCGATAGTACATCGAATGACAAACATATATTTTAAAAGGTGTTTGCGCATTCGTGGCTCCTGCGGTTCATTAGATGAGCTCGCTCGACTCAGCGCACACTTTACAAGGTTCTTCATCATCAGGCAATGCTCAAGCAACGACAATGTCCATGTTGATGCAGCTACATGTAAAGAGTAATCAAGCTGAGTTCGCCACCCTGGCAACTCCTACGGCTCGTCAGCGGGGTAAACAAGTCCAAGGTCCTTGCGGATTTTGTCCATGATGTTCATGATCCCCAGCGACTCAGCATGTGAGTACTCAATGCATTCGATCTCGTTGCGTTGGATAGCCTCCGTGGCAGCTTCAGCTCCGTAGAGGAAGCCCGAGTGATGGTCACTTGGTGTCGGCCACGGAAAGCGCGACGTCTTCTCCGTCTCCTTGCCGCTTTCGTCGTACGTGGCGACCGAGATCTCCATCGACGCGTGTGCCGGCGGGTGGATAAAGATCCGCCCTTTAGTGCCAGTGATCGTCACCGTCTCACTGATCTTCGCGAGTGTCGAGTACTCAAGCGTAGCGAAGCGCTGACCACTGTACTCGAGCGTGATGGAACTGTACACGTCCACTCCTTCGTCATTGAGTTTCCCAGCCGACGAGATCTTCTCGGGTTTAAACCCAAACACCATCGTGGCAGTTGCCAACACGTAGATACCAATGTCCAATAACCCACCGCCGCCCAGTTCTTTCTTCCAGATACGGTCATTGTCGGCTTTGAATGCCACTCCGAAGGCCGAGTGCATGTGATGCACGTCGCCAATGCCGCCTTTATCGATGACTTGGCGCACAAAGCGAACTGCGGGGAAGAACCGCGCCCACATGCCTTCCATGAAGAAGAGGCGTTTCTGTTGCGCTAGTGTGATGACTGTTTCGGCCTCTTTCGCGCTCATGGCCATGGGTTTTTCCACCAGAACATGTTTGCCATGGTTCAATGCCAATAATGCAAGCTCACAATGCGTCGGGTGGAGTGTGCCAATGTACACTATATCCACCTCGGGGTCGTTTCGTACGACTCGTACGCGTTAGAAATGCCTAAACACAAGTTGCAGCAAAAGTTGGTTAATTATCACGATGAAAAAGAAAAGAAAACTAACTCATACCGTGTTTGTTGGCAAACTCTTGCGCTTTATCCAGTGAACGAGCTGCGCACGCGTGGAAAATGGCGGTTTTAAGTGGCTTTACATTGCTGGCGAACGTGTGGGAGATGCGTCCGCAGCCGAGAATGCCCCAGCGCAAAGGAGTGTTGGTAGCCATGGTTGTGGCAAGTGCAACAAAATGAAGAATCAAGTGATAATTTGCCTCTGACAAAGTCAAAGGGCAAAAAAGTGTTATCAGTTGCCAAGCATATCCTGAATTGTGGTTGAAGTTTTGAGCTACGTGGCACTAATCAGCAAAAATGTTTCCTAGTTTGCGTCAGTAGGGTACACAAGCCCGATTTCCTTACGGATCTTGTCCATAATGGTCATGATACCAAGCGATTCATCCAGCGAGTACTCTTTCGCTTCGAGTTCCTTGGACTGAATAGCCTTCGTGACCGCTTCGCCTTCATACAAAAAGCAAGAGTATCCAGCGTCTGGCGTAGGCCACGGAAACTTCGTCGTTGCCGATTCTTCTTTGCCTGGTCCAGAGCTGCGAATGACGGAGACTTCAGAAGCGACATGAGCTGGCGTGTGGATGAGGATGCGTCCAGTAGTTCCAGAGATCGTGACGGTCTCACTGAGTTGCACCAGCATAGAGTACTCGATGGTAGCGAAACGATTGTCGTTGTAACGCAACGTCACCGAGTTGTGTACGTCTACACCGCCATCGTTGAGTGTACCGGCCGTCGTCACCTTCTCAGGCTCCGTACCGAACACCATGGTCGCGAAGGCAAGAGGATAAATGCCGATATCGAGCAAACCTCCTCCTCCCAACTCATTTTTCCACTGACGCGCTTCGTCTTTGGGGAACGGGTAGCCAATCTCGGCGTGAACGTGGCGAACTTCTCCAATTTCGTTGTCGTCCAGTAATTTGCGAACGAAACGAATGCTCGGGAAGAAA

>Contig_68

ACGAAGATCGGACAGATCAAGTCCAGGAACCTCCAAACTGGCGCCAGCAGCAGCAGCCTTGTGAGGTTCGTGCATTGGAATGACGCCGTTCCGACGAATGAGCCGCTTCTTCCGCAAGATCACTGACCGCTGCTCAGCGATCACCAACAGAGCGTGTGGCAAGGGTTGGGTGCCCGTTGAGCTGTACAGCTGCACCGATTCCTGCAGCTGCGCGAAATGCTTAGAATTAATGCCTTGGAGAGTAACAAACGCCGCACCCCTCTCGGAAATAGATCGGAGGAGGACTGCGATGTCGGCATCTCGATCCAAGACTTGCTGATGATGGAGCTGGGCCTGACTCAACGCAGCTTCAACCTTGTCTTGCTCAGCTTCGATAGCCCGAGCAAGTTTCGCTTCACTCAGGGCTACAGCTTTTCGCAGTGAAGCAAGGAGATTAGCCGACTGCGTGGCAACAGCCTCCAATCGAGACACCTGTTGTCGCGCCGATGAGAGCTGAGTGGACTTGTCAACATTGATGAGGGCAAGAGTGTCCAATCGCCGAGTCAATGCGCGGTTCTCTTCTTGAACAGCTTGAATGTCGGCCCTGAGATCACCAATGATCTGGGGATAATCAAGAGCGGCGTAGCTGCCTTCCAAGATGTCCAATAGTTCCCCCACGGAAGAAGCCTACAGGATCGCTCGCTGCTGCACGGGATCAGAAGGCTCCACGAGACGAATGAATCCGTAGGCCATGAGCCTCGCCAAGAAGGTCAATTCTTCTCGACGCTCGTATTCCTGCTCAGTAACCCTGGTCATGAGAGAGTCAAGACTCACTAGAGCAGCCGAGCTTGACTGACTGTTGGCGCCCGAAGTGGTTGTCGCCCGGCAAGCTCGGTGGACCAGGAGGGCGCGCGCGAGGTGACTCCGCCGACTCCACGTCAGACTCATGAAAGTTGTCTGGTCGACCAACCCAGCTTCATGCCGGTCGCTGACCCCACGAGTACCGACGGGTCGAGCTATCGACTCAGCTGGCGCGCCTTCACCTCGACGAAGAGCTGGAAGAGACGGATGGTGCCGCGAATTGTGCTGGAGTAACATGCGAGGAGTAAGGAGTTGCCAAGTGAGCGACAGCGGAAGCAACAAACGTACCGAAGAAGGTGGTGGAGAGCCGCGAGACGACATCAGCCGAGTATGGACCAACGGTGGCAGAACCCAATGAAGGATTAAGACGCTCACCAGTTCGGCTCGTATCATAACCGGGGAAGTTGTGGTGTAGGGATCCCTACACCACAACCTCCCCGGTTATTATACCCAGATCTTAGGAACAAAGAATCACGGTAAGTACTCAGCGATCTGTCCAGATGATGCTATCCGGATAGCCGCTGAGCTCAACGGTCGCCTGAACCCAGCGGAAGGGTCGGTAGAAATCCGAAACGACTACATTTTATGAGAAGCATAGGATTAAATAGGGGGAGCCGAAAAGGCTCCCGGAAGATAACAGAGCAGAATTTTCCTAGTCCAAGACACGTTGTACACTTTCCGGTAAGTCCCTGCATAGGGCTGGTTTATGTCTGCCGTTGATACCCGAGAGGCATAGCTTGTAAGGTATTGACTGGTAGCGCCAAGAGATTAGGTCGGTCTGGGTAGGACAAAGTCTCGCTACGCGCGAAAGAAAGCCCCGCATCCTCAGCGGGCCGGTACGTCGGATAGCCCGGGTGCCGAAACACTTCTAGCTGAGCAGCCTCTGCGATCGGTGACCTGTAAAAGCGCTCGCGCGCCGTCGTCCATGATTTGGTAAAGGGCACAGGGTTTGTGAACGAGTTCATCCGAACCAAACTCTGTGCAGCCTCGGAAGTGCTCTGACTTGGTTAGTCGGAACCGAGTGGAAGCGAGCCACTCGTCTGTCCCGGAAGGGTCGAGCCGCGAGGGGACCACCCTCTGCGCAGGCCATACAACCGCCGACCTAACTCGGCGATTTGTTGTGGTTTGTGTAAGGAATCCATATCTGACGGTATTTGTGTGTTCTACTACACCACAGTGATCTTCCGGGGATCCAGAAACGCACCCTCTTCCTCATCCTCCTCCATTTCGCTCTCTTCATCCGAGGAACCAAAGATGTTCCGATAGGAGTACCCTGTCTCCTTCTCCTCATGGAGTGAAGACTTCGAAGTTGTACGCTTTTCAGAGGGTTTGGCGCCCTCGGCCTGAGTGGCCTTAGCACGCGCAAGACCCTCGGCAAGCGAGAGATGTTTGCGAGGTTTCTCCTCTTCGTCGTCGCCAGCTATGGGAGTGTCCTTAGCCTTGATGGCAGGACGCTCAGAGCATAAAGACGTAGTGGTACTGTGATCACCGTGATCACTAGGTAGCGACTGCACGCTGTCGTCATTGCTCTCGCTAGTCACTGGGGTGTGCCCTCCCACGGAGTCCACAGGAGCATGGTGAGTGCTCCCGCTCTTGGCCTCGTCCTCACTGCCCGAGTTGAACTCACGTTCGTCGAACCGAGGATTTGGACGGTTGTCTGGGGAGACCGAAGGTGACGGGATGCGGCGCTTGCTCGCATGCTTGCTCACAGCCGCGCTGAGCCGATCGCCTCCGAGGCCACCTTAGTAGCCTCCTGGTGTGCAGAGTCCTTGCCGTGAGGCTTAGCGCTCTTGGCTTGCGTATTTCCTGCAGGAGCCTTGGAGCATGTATTCGCACCGCTTTTTGCAGTGTCACGGGGTATGATAACGGTCGATGTGGAGACCAAGAAGAAGGGCGCTCAACCGCTGCTCGAGGCTCTAACTGTGAAGTGAGCGCTCGCGCAACCAAGCCGATGTATTTCGACTTTAAAAATGAAAGTTTGTGACGTACAATTCTTTATTCGGTTTATTCCGTAAATCGAACGTAAGTGGAAAAAGACGTAACGGGGTACGTATCTCCAAGTGTACCCCTAGTTACATGAAGGGTGACACGTCATACTTCGGGATATCTGGTACATGGTAACAGGGTCGTCCGAGGCTAACCATTGGCTGATGACATTGCTTTTCTATAGGTGACTTATAGTTTGTAAATTAAACTCAAGCTTAGTCTACCTTTAAATATACCGGTATCTCGTTTATAACTTACCTTAAATTGGTCACTTCGCTCAGGGGACCTGTGAGCTGCCGTTCACGGATCGTTTGTATACTCTCAGAAGCTAATTTCCTTTAGCGCATAATTTCCTCTAGCGCTAGGTGCTGTGTGAACCCGTCACAATTATTGTTTTAAAGGTCGAAGACCTTGAATGCAGTCTTGCTTGTCACAGTTAAGTTGGTGGCACAGTACCCACTGGGCATGCACCTGTTTAGCAGAAGGATCAGCAGCCTTCAAGAACATGAAAAATAGGAGGCGGAGCGCTGCAAAATAGTTTGGTAAGATGATTTATCTCCCGAGCGGAGGTAGTATTTCTTCTTCTTTATACTGTATTTCGCTTTTAGTTTCAGCATGTGCGTGGAGGCAATAGAATATCCGGTCCTTGCTTGTTTTCGTCTATAGACACATCCTTTTTAACGACAAGTCGCACACCTCATGAAGTTTAAACAGGGTTTACACAGAAACTTAAATGAAGAAGGACAATAACTACGGGCGAGCTAGATAGCGCTAATGATTCACTTTTGAAAACTAACATGTTTGAAGTGACCACTAACAGTTAACAATATATACTACTTCCATATCAGACAGTGGTTGATTAAATTAAACCACAATAACATTTCAGAGCTGTTTCGTGCACCGCGCAACTTCTTGAACTATCCGTCATATAACACACAATGCAGTGGCCCCCCAATCCTACGATAAAACTGAAGCAAGAAAAGTAAAGCAAGCAAGGTCGCATCAATGTTTGCGTTGTTAACTGGATAAGCGAATCACATCACACTGCAGGTCGAACCTTGACACGCTTTTCCTTCGTACTTCGTTACCTGGCACAAAACATACCAACCAGTCTGTGTCCCACCACGAGCTTATTTTGCGATTTTTTTTATTTATTTATAACCCAGTCTCATTGCCTCATCGGTCTTGTGCTTTACAATCTTCGCCCAATCGGGTGTTGATATGACAGTCTCCTTTAGTTTTTCCAAGCTTTTCTTGGGCGTGATTTTTTCCACTACAGCTTTCAATTTTTCTTTGAAGCCTTTGGCTCTCTCTTCCACGATCCCGCCTTCGTCGTTTCCGACACGACGCAGTAAACGCGGGTCCTGCACGTCGGTCGTGGTGATCATGTCAACACCATATTCATTCGAAGCCTTGCCCGCAACCGCAGAATTGGCATTACCAGTGGCGTGGAAAGTGACCGCGACTAGCACTGCAAGGATGCATGTGAGGCGCATTTTGTTGTCGTTTGGGTCAACAGCTAGGGCACTTGAGTCGAAATGAGAGAGTGCGAGCAGATTGCAGAATGAGGTGTCGGACGAAGACAGAGCCGTACTACGTGCTGGAGCACGAGGAAGTTCCACTTTGTACTGTTTTCTTCCAGTCTGTACAAATAACGCTGGGATATAGCTACCGATCCAGTACATATTTCCAGACAGTTATTGAACAAAAAACCAGTACACTTAAGTTCGCAGCTAATAATGGTTTTTGTAGCCCTATTCACTTATAAAAACTAATGTCTAGTCACTATTAGAAAATTGTGTTTAATACACAAGAAGAGCAATTCAATTCGCAATTCAAAAGTGCGACAGCAGACCTCCTCCAGTCATCCTCTCCGAGTTGCTTTCTTAAAATGGATGGCCACCGCAGCATTCTTGCAGCGACATCAGTGGCAGCACTTTTCTCATGCGTAGTACTACTATAGAAGCACTGAGCTACCACATTGATTTAAATCAAAGTCACGATTGGGCTTGTTCTCGATCACTAGATACTTCTCCGCCCAATATTCAAGCTCATCGTTCTCAAATGACGTATCAACCATGGCTGAACGCATGTCATTAATTGTTCGACCAGTTAACTCCACCAGCTGAGTTAGTGTTGCCAACTTCCGCTCAGACTTGAATGTCAATTCGAAATCTTGATCAAGTGTAGAAAGCTCAAACGTCTTCGAAGTGGCTGTTTTCCATGGCTCCTTGACTTCATAGTCATCCTTCTATTGCTTGTATGCAGCAGAATCACAACCAGCAGCAATCCCTTCAGTTTGCCTTCGTTCAGTGATGGATTATACTTGGAATCAAACCAACCATCAAATTCTTTGGTAGCATACTCCTTGTTGAAACGCTCTACTTTTTTGTTTGAGCACACAGAGATAGGTCTTGCGCACTAAATGCCAATCCGAATGAACTTGACAAGCAAAGAATCCAGCGAGATTCCACACGTTTTGGTTGTTGGAGAACAAATCGCCTCGAATCATCATGCACAATTGATACAATCGCTCATCGTAGGATTGAGTTGCAAGGAAGTATTCCACACCGCATTCAACGTTTGCTTGTTCAAATGATTCACGGTCTTCATTGGACTTAAAGAAGAAATTATCAAGCATCTGCGATTCATCCAGTTCATTAAATTGATATTGCAAAAGGTACGTGTATCCGTCGTTGCCAAAGTCAGGTGTGCCATCATCACTGTGCTTGTAGTTGATGGAATCCCCAACTGCGTGCATCTTCGACTTGATCCAATTACTTGGTGACAACACGCGCGAATCTCCCAACCTGGGTTCATCGTATTTATCAACAGATTTGATGTGCTGAATGCAATGCTCACCATAAATTTCTTCAATAGACTTGGTCATCGTTGGTTCAGCAACTGCAGGCACAACAGCTACAGATTCAGCAGCTACTAGTTCAGCAATCACTGGTTCAGCAACTATTAGTTTAGCAATCACTGGTTCAGCAACTACTGGCTGATACTTCGCGTGGTGTCGGCACCAATCGCCTTTTGAGCCGCACTGTTGGCTCGCCTTTGCGTGGTCTTCTGGGAAGATGTACTTGCACATTGTTATTTCTATATATTTATTTTTTATCGCTCAAGATGAACTCACTATTATTTAGTTTCTATATATTGAGGAGTATTTTTGTCAGAAATAAAAATATTATTTTAAAGTGATGTGATTGGCTAAAACTGTGCGGTGTGCTGCGTGAATGAGTGCTGCTGCTCAGTCGAGAACTTTCCGCACGAAGATGGCAGAGGTCGAGTTGGAGTGCGCGGTATACGGCGAAGGGACTGTGTTTCCCGTGAAGATAGCGAGCAACGCGAAAGTGAGCGCGCTGCAGAAGGCCATGTGAACGAGAAGAAAGATGTCGATGATCGCTTCGAAGTTGATCCAGCCAGATTGACGCTCTACTTGGCGCGGGAGAAGGGGGAAGCCACGTGGATGAAGCACGACCACACGGTGAAAGGCTTCTTGTGAGGTGGCATCAGCACCGAGTATGAAGAGATGCTTTCGTCGTGGATTCTCGACGAAGATTGCGGTCGTGGTGATCATGTCAACACCATATTCATTCGAAGCCTTGCCCGCAACCGCAGAATTGGCATTACCAGTGGCGTGGAAAGTGACCGCGACTAGCACTGCAAGGATGCATGTGAGGCGCATTTTTTTGTCGTTTGGGTCAACAGCTAGGGCACATGAGTCGAAATGAGAGAGTGCGAGCAGATTGCAGAATGAGGTGTCGGACGAAGACAGAGCCGTACTACGTGCTGGAGCACGAGGAAGTTCCACTTTGTACTGTTTTCTTCCAGTCTGTACAAATAACGCTGGGATATAGCTACCGATCCAGTACATATTTCCAGACAGTTATTGAACAAAAAACCAGTACACTTAAGTTTGCAGCTAATAATGGTTTTTGTAGTCCTATTCACTTATAAAAACTAATGTCTAGTCACTATTAGAAAATTGTGTTTAATACACAAAAAGAGCAATTCAATTCGCAATTCAAAAGTGCGACAGCAGACCTCCTCCAGTCATCCTCTCCGAGTTGCTTTCTTAAAATGGATGGCCACCGCAGCATTCTTGCAGCGACATCAGTGGCAGCACTTTTCTCATGCGTAGTACTACTATAGTAGCACTGAGCTACCACATTGATTTAAATCAAAGTCACGATTGGGCTTGTTCTCGATCACTAGATACTTCTCCGCCCAATATTCAAGCTCATCGTTCTCAAATGACGTATCAACCATGGCTGARCGCATGTCATTAATTGTTCGACCAGTTAACTCCACCAGCTGAGTTAGTGTTGCCAACTTCCGCTCAGACTTGAATGTCAATTCGAAATCTTGATCAAGTGTAGAAAGCTCAAACTTCTTCGAAGTAGCTGTTTTCCMTGGCTCCTTGACTTCATAGTCATCCTTCTATTGCTTGTATGCAGCAGAATCACAACCAGCAGCAATCCCTTCAGTTTGCCTTCGTTCAGTGATGGATTATACTTGGAATCAAACCAACCATCAAATTCTTTGGTAGCATACTCCTTGTTGAAACGCTCTACTTTTTTGTTTGAGCACACAGAGATAGGTCTTGCGCACTAAATGCCAATCCGAATGAACTTGACAAGCAAAGAATCCAGCGAGATTCCACACGTTTTAGTTGTTGGAGAACAAATCGSCTCGAATCATCATGCACAATTGATACAAATGCTCATCGTAGGATTGAGCTGCAAGGAAGTATTCCACACCGCATTCAACGTTTGCTTGTTCAAATGATTCACGGTCTTCATTGGACTTAAAGAAGAAATTATCAAGCATCTGCGATTCATCCAGTTCATTAAATTGATATTGCAAAAGGTACGTGTATCCGTCGTTGCCAAAGTCAGGTGTGCCATCATCACTGTGCTTGTAGTTGATGGAATCCCCAACTGCGTGCATCTTCGACTTGATCCAATTACTTGGTGACAACACGCGCGAATCTCCCAACCTGGGTTCATCGTATTTATCAACAGATTTGATGTGCTGAATGCAATGCTCACCATAAATTTCTTCAATAGACTTGGTCATCGTTGGTTCAGCAACTGCAGGCACAACAGCTACAGATTCAGCAGCTACTAGTTCAGCAATCACTGGTTCAGCAACTATTAGTTTAGCAATCACTGGTTCAGCAACTACTGGCTGATACTTCGCGTGGTGTCGGCACCAATCGCCTTTTGAGCCGCACTGTTGGCTCGCCTTTGCGTGGTCTTCTGGGAAGATGTACTTGCACATTGTTATTTCTATATATTTATTTTTTATCGCTCAAGATGAACTCACTATTATTTAGTTTCTATATATTGAGGAGTATTTTTGTCAGAAATAAAATAATTATTTTAAAGTGATGTGATTGGCTAAAACTGTGCGGTGTGCTGCGTGAATGAGTGCTGCTGCTCAGTCGAGAACTTTCCGCACGAAGATGGCAGAGGTCGAGTTGGAGTGCGCGGTATACGGCGAAGGGACTGTGTTTCCCGTGAAGATAGCGAGCAACGCGAAAGTGAGCGCGCTGCAGAAGGCCATGTGAACGAGAAGAAAGACGTCGATGATCGCTTCAAAGTTGATCCAGCCAGATTGACGCTCTACTTGGCGCGGAAGAAGGGGGAAGCCACGTGGATGAAGCACGACCACACGGTGAAAGGCTTCTTGTGAGGTGGCATCAGCACCGAGTATGAAGAGATGCTTTCGTCGTGGATTCTCGACGAAGATTGCGGTCGTGGTGATCATGTCAACACCATATTCATTCGAAGCTTTGCCCGCAACCGCAGAATTGGCATTACCAGTGGCGTGGAAAGTGACCGCGACTAGCACTGCAAGGATGCATGTGAGGCGCATTTTTTTGTCGTTTGGGTCAACAGCTAGGGCACTTGAGTCGAAATGAGAGAGTGCGAGCAGATTGCAGAATGAGGTGTCGGACGAAGACAGAGCCGTACTACGTGCTGGAGCACGAGGAAGTTCCACTTTGTACTGTTTTCTTCCAGTCTGTACAAATAACGATGGGATATAGCTACCGATCCAGTACATATTTCCAGACAGTTATTGAACAAAAAACCAGTACACTTAAGTTCGCAGCTAATAATGGTTTTTGTAGCCCTATTCACTTATAAAAACCAATGTCTAGTCACTATTAGAAAATTGTGTTTAATACACAAGAAGAGCAATTCAATTCGCAATTCAAAAGTGCGACAGCAGACCTCCTCCAGTCATCCTCTCCGAGTTGCTTTCTTAAAATGGATGGCCACCGCAGCATTCTTGCAGCGACATCAGTGGCAGCACTTTTCTCATGCGTAGTACTACTATAGTAGCACTGAGCTACCACATTGATTTAAATCAAAGTCACGATTGGGCTTGTTCTCGATCACTAGATACTTCTCCGCCCAATATTCAAGCTCATCGTTCTCAAATGACGTATCAACCATGGCTGAACGCATGTCATTAATTGTTCGACCAGTTAACTCCACCAGCTGAGTTAGTGTTGCCAACTTCCGCTCAGACTTGAATGTCAATTCGAAATCTTGATCAAGTGTAGAAAGCTCAAACGTCTTCGAAGTGGCTGTTTTCCATGGCTCCTTGACTTCATAGTCATCCTTCTATTGCTTGTATGCAGCAGAATCACAACCAGCAGCAATCCCTTCAGTTTGCCTTCGTTCAGTGATGGATTATACTTGGAATCAAACCAACCATCAAATTCTTTGGTAGCATACTCCTTGTTGAAACGCTCTACTTTTTTGTTTGAGCACACAGAGATAGGTCTTGCGCACTAAATGCCAATCCGAATGAACTTGACAAGCAAAGAATCCAGCGAGATTCCACACGTTTTGGTTGTTGGAGAACAAATCGCCTCGAATCATCATGCACAATTGATACAATCGCTCATCGTAGGATTGAGTTGCAAGGAAGTATTCCACACCGCATTCAACGTTTGCTTGTTCAAATGATTCACGGTCTTCATTGGACTTAAAGAAGAAATTATCAAGCATCTGCGATTCATCCAGTTCATTAAATTGATATTGCAAAAGGTACGTGTATCCGTCGTTGCCAAAGTCAGGTGTGCCATCATCACTGTGCTTGTAGTTGATGGAATCCCCAACTGCGTGCATCTTCGACTTGATCCAATTACTTGGTGACAACACGCGCGAATCTCCCAACCTGGGTTCATCGTATTTATCAACAGATTTGATGTGCTGAATGCAATGCTCACCATAAATTTCTTCAATAGACTTGGTCATCGTTGGTTCAGCAACTGCAGGCACAACAGCTACAGATTCAGCAGCTACTAGTTCAGCAATCACTGGTTCAGCAACTATTAGTTTAGCAATCACTGGTTCAGCAACTACTGGCTGATACTTCGCGTGGTGTCGGCACCAATCGCCTTTTGAGCCGCACTGTTGGCTCGCCTTTGCGTGGTCTTCTGGGAAGATGTACTTGCACATTGTTATTTCTATATATTTATTTTTTATCGCTCAAGATGAACTCACTATTATTTAGTTTCTATATATTGAGGAGTATTTTTGTCAGAAATAAAATAATTATTTTAAAGTGATGTGATTGGCTAAAACTGTGCGGTGTGCTGCGTGAATGAGTGCTGCTGCTCAGTCGAGAACTTTCCGCACGAAGATGGCAGAGGTCGAGTTGGAGTGCGCGGTATACGGCGAAGGGACTGTGTTTCCCGTGAAGATAGCGAGCAACGCGAAAGTGAGCGCGCTGCAGAAGGCCATGTGAACGAGAAGAAAGACGTCGATGATCGCTTCAAAGTTGATCCAGCCAGATTGACGCTCTACTTGGCGCGGAAGAAGGGGGAAGCCACGTGGATGAAGCACGACCACACGGTGAAAGGCTTCTTGTGAGGTGGCATCAGCACCGAGTATGAAGAGATGCTTTCGTCGTGGATTCTCGACGAAAATTGCGGTCGTGGTGATCATGTCAACACCATATTCATTCGAAGCCTTGCCCGCAACCGCAGAATTGGCATTACCAGTGGCGTGGAAAGTGACCGCGACTAGCACTGCAAGGATGCATGTGAGGCGCATTTTTTTGTCGTTTGGGTCAACAGCTAGGGCACTTGAGTCGAAATGAGAGAGTGCGAGCAGATTGCAGAATGAGGTGTCGGACGAAGACAGAGCCGTACTACGTGCTGGAGCACGAGGAAGTTCCACTTTGTACTGTTTTCTTCCAGTCTGTACAAATAACGATGGGATATAGCTACCGATCCAGTACATATTTCCAGACAGTTATTGAACAAAAAACCAGTACACTTAAGTTCGCAGCTAATAATGGTTTTTGTAGCCCTATTCACTTATAAAAACTAATGTCTAGTCACTATTAGAAAATTGTGTTTAATACACAAAAAGAGCAATTCAATTCGCAATTCAAAAGTGCGACAGCAGACCTCCTCCAGTCATCCTCTCCGAGTTGCTTTCTTAAAATGGATGGCCACCGCAGCATTCTTGCAGCGACATCAGTGGCAGCACTTTTCTCATGCGTAGTACTACTATAGTAGCACTGAGCTACCACATTGATTTAAATCAAAGTCACGATTGGGCTTGTTCTCGATCACTAGATACTTCTCCGCCCAATATTCAAGCTCATCGTTCTCAAATGACGTATCAACCATGGCTGAACGCATGTCATTAATTGTTCGACCAGTTAACTCCACCAGCTGAGTTAGTGTTGCCAACTTCCGCTCAGACTTGAATGTCAATTCGAAATCTTGATCAAGTGTAGAAAGCTCAAACGTCTTCGAAGTAGCTGTTTTCCATGGCTCCTTGACTTCATAGTCATCCTTCTATTGCTTGTATGCAGCAGAATCACAACCAGCAGCAATCCCTTTCAGTTTGCCTTCTTTCAGTGATGGATTATACTTGGAATCAAACCAACCATCAAATTCTTTGGTAGCATACTCCTTGTTGAAACGCTCTACTTTTTTGTTTGAGCACACAGAGATAGGTCTTGCGCACTAAATGCCAATCCGAATGAACTTGACAAGCAAAGAATCCAGCGAGATTCCACACGTTTTGGTTGTTGGAGAACAAATCGCCTCGAATCATCATGCACAATTGATACAAATGCTCATCGTAGGATTGAGCTGCAAGGAAGTATTCCACACCGCATTCAACGTTTGCTTGTTCAAATGATTCACGGTCTTCATTGGACTTAAAGAAGAAATTATCAAGCATCTGCGATTCATCCAGTTCATTAAATTGATATTGCAAAAGGTACGTGTATCCGTCGTTGCCAAAGTCAGGTGTGCCATCATCACTGTGCTTGTAGTTGATGGAATCCCCAACTGCGTGCATCTTCGACTTGATCCAATTACTTGGTGACAACACGCGCGAATCTCCCAGCCTGGGTTCATCGTATTTATCAACAGATTTGATGTGCTGAATGCAATGCTCACCATAAATTTCTTCAATAGACTTGGTCATCGTTGGTTCAGCAACTGCAGGCACAACAACTACAGGTTCAGCAGCTACTAGTTCAGCAATCACTGGTTCAGCAACTACTAGTTTAGCAATCACTGATTCAGCAACTACTGGCTGATACTTCGCGTGGTGTCGGCACCAATCGCCTTTTGAGCCGCACTGTTGGCTCGCCTTTGCGTGGTCTTCTGGGAAGATGTACTTGCACATTGTTATTTCTATATATTTATTTTTTATCGCTCAAGATGAACTCACTATTATTTAGTTTCTATATATTGAGGAGTATTTTTGTCAGAAATAAAATAATTATTTTAAAGTGATGTGATTGGCTAAAACTGAGCAGCAGCACTCTGAATGAGTGCTGCTGCTCAGTCGAGAACTTTCCGCACGAAGATGGCAGATGTCGAGTTGGAGTGCGCGGTATACGGCGAAGGGACTGTGTTTCCCGTGAAGATAGCGAGCAACGCGAAAGTGAGCGCGCTGCAGAAGGCCATGTGAACGAGAAGAAAGACGTCGATGATCGCTTCAAAGTTGATCCAGCCAGATTGACGCTCTACTTGGCGCGGAAGAAGGGGGAAGCCACGTGGATGAAGCACGACCACACGGTGAAAGGCTTCTTGTGAGGTGGCATCAGCACCGAGTATGAAGAGATGCTTTCGTCGTGGATTCTCGACGAAGATTGCGGTCGTGGTGATCATGTCAACACCATATTCATTCGAAGCTTTGCCCGCAACCGCAGAATTGGCATTACCAGTGGCGTGGAAAGTGACCGCGACTAGCACTGCAAGGATGCATGTGAGGCGCATTTTTTGTCGTTTGGGTCAACAGCTAGGGCACTTGAGTCGAAATGAGAGAGTGCGAGCAGATTGCAGAATGAGGTGTCGGACGAAGACAGAGCCGTACTACGTGCTGGAGCACGAGGAAGTTCCACTTTGTACTGTTTTCTTCCAGTCTGTACAAATAACGCTGGGATATAGCTACCGATCCAGTACATATTTCCAGACAGTTATTGAACAAAAAACCAGTACACTTAAGTTTGCAGCTAATAATGGTTTTTGTAGTCCTATTCACTTATAAAAACTAATGTCTAGTCACTATTAGAAAATTGTGTTTAATACACAAGAAGAGCAATTCAATTCGCAATTCAAAAGTGCGACAGCAGACCTCCTCCAGTCATCCTCTCCGAGTTGCTTTCTTAAAATGGATGGCCACCGCAGCATTCTTGCAGCGACATCAGTGGCAGCACTTTTCTCATGCGTAGTACTACTATAGTAGCACTGAGCTACCACATTGATTTAAATCAAAGTCACGATTGGGCTTGTTCTCGATCACTAGATACTTCTCCGCCCAATATTCAAGCTCATCGTTCTCAAATGACGTATCAACCATGGCTGAACGCATGTCATTAATTGTTCGACCAGTTAACTCCACCAGCTGAGTTAGTGTTGCCAACTTCCGCTCAGACTTGAATGTCAATTCGAAATCTTGATCAAGTGTAGAAAGCTCAAACGTCTTCGAAGTAGCTGTTTTCCATGGCTCCTTGACTTCATAGTCATCCTTCTATTGCTTGTATGCAGCAGAATCACAACCAGCAGCAATCCCTTTCAGTTTGCCTTCTTTCAGTGATGGATTATACTTGGAATCAAACCAACCATCAAATTCTTTGGTAGCATACTCCTTGTTGAAACGCTCTACTTTTTTGTTTGAGCACACAGAGATAGGTCTTGCGCACTAAATGCCAATCCGAATGAACTTGACAAGCAAAGAATCCAGCGAGATTCCACACGTTTTGGTTGTTGGAGAACAAATCGCCTCGAATCATCATGCACAATTGATACAAATGCTCATCGTAGGATTGAGCTGCAAGGAAGTATTCCACACCGCATTCAACGTTTGCTTGTTCAAATGATTCACGGTCTTCATTGGACTTAAAGAAGAAATTATCAAGCATCTGCGATTCATCCAGTTCATTAAATTGATATTGCAAAAGGTACGTGTATCCGTCGTTGCCAAAGTCAGGTGTGCCATCATCACTGTGCTTGTAGTTGATGGAATCCCCAACTGCGTGCATCTTCGACTTGATCCAATTACTTGGTGACAACACGCGCGAATCTCCCAGCCTGGGTTCATCGTATTTATCAACAGATTTGATGTGCTGAATGCAATGCTCACCATAAATTTCTTCAATAGACTTGGTCATCGTTGGTTCAGCAACTGCAGGCACAACAACTACAGGTTCAGCAGCTACTAGTTCAGCAATCACTGGTTCAGCAACTACTAGTTTAGCAATCACTGATTCAGCAACTACTGGCTGATACTTCGCGTGGTGTCGGCACCAATCGCCTTTTGAGCCGCACTGTTGGCTCGCCTTTGCGTGGTCTTCTGGGAAGATGTACTTGCACATTGTTATTTCTATATATTTATTTTTTATCGCTCAAGATGAACTCACTATTATTTAGTTTCTATATATTGAGGAGTATTTTTGTCAGAAATAAAATAATTATTTTAAAGTGATGTGATTGGCTAAAACTGAGCAGCAGCACTCTGAATGAGTGCTGCTGCTCAGTCGAGAACTTTCCGCACGAAGATGGCAGATGTCGAGTTGGAGTGCGCGGTATACGGCGAAGGGACTGTGTTTCCCGTGAAGATAGCGAGCAACGCGAAAGTGAGCGCGCTGCAGAAGGCCATGTGAACGAGAAGACAGATGTCGATGATCGCTTCAAAGTTGATCCAGCCAGATTGACGCTCTACTTGGCGCGGAAGAAGGGGGAAGCCACGTGGATGAAGCACGACCACACGGTGAAAGGCGTGGCATCAGCACCGAGTATGAAGAGATGCTTTCGTCGTGAATTCTCGACGAAGATTGCGGTCGTGGTGATCATGTCAACACCATATTCACTCGAAGCTTTGCCCGCAACCGCAGAATTGGCATTACCAGTGGCGTGGAAAGTGACCGCGACTAGCACTGCAAGGATGCATGTGAGGCGCATTTTTTTGTCGTTTGGGTCAACAGCTAGGGCACTTGAGTCGAAATGAGAGAGTGCGAGCAGATTGCAGAATGAGGTGTCGGACGAAGACAGAGCCGTACTACGTGCTGGAGCACGAGGAAGTTCCACTTTGTACTGTTTTCTTCCAGTCTGTACAAATAACGCTGGGATATAGCTACCGATCCAGTACATATTTCCAGACAGTTATTGAACAAAAAACCAGTACACTTAAGTTCGCAGCTAATAATGGTTTTTGTAGTCCTATTCACTTATAAAAACTAATGTCTAGTCACTATTAGAAAATTGTGTTTAATACACAAGAAGAGCAATTCAATTCGCAATTCAAAAGTGCGACAGCAGACCTCCTCCAGTCATCCTCTCCGAGTTGCTTTCTTAAAATGGATGGCCACCGCAGCATTCTTGCAGCGACATCAGTGGCAGCACTTTTCTCATGCGTAGTACTACTATAGAAGCACTGAGCTACCACATTGATTTAAATCAAAGTCACGATTGGGCTTGTTCTCGATCACTAGATACTTCTCCGCCCAATATTCAAGCTCATCGTTCTCAAATGACGTATCAACCATGGCTGAACGCATGTCATTAATTGTTCGACCAGTTAACTCCACCAGCTGAGTTAGTGTTGCCAACTTCCGCTCAGACTTGAATGTCAATTCGAAATCTTGATCAAGTGTAGAAAGCTCAAACGTCTTCGAAGTGGCTGTTTTCCATGGCTCCTTGACTTCATAGTCATCCTTCTATTGCTTGTATGCAGCAGAATCACAACCAGCAGCAATCCCTTCAGTTTGCCTTCGTTCAGTGATGGATTATACTTGGAATCAAACCAACCATCAAATTCTTTGGTAGCATACTCCTTGTTGAAACGCTCTACTTTTTTGTTTGAGCACACAGAGATAGGTCTTGCGCACTAAATGCCAATCCGAATGAACTTGACAAGCAAAGAATCCAGCGAGATTCCACACGTTTTGGTTGTTGGAGAACAAATCGCCTCGAATCATCATGCACAATTGATACAATCGCTCATCGTAGGATTGAGTTGCAAGGAAGTATTCCACACCGCATTCAACGTTTGCTTGTTCAAATGATTCACGGTCTTCATTGGACTTAAAGAAGAAATTATCAAGCATCTGCGATTCATCCAGCTCATTAAATTGATATTGCAAAAGGTACGTGTATCCGTCGTTGCCAAAGTCAGGTGTGCCATCATCACTGTGCTTGTAGTTGATGGAATCCCCAACTGCGTGCATCTTCGACTTGATCCAATTACTTGGTGACAACACGCGCGAATCTCCCAACTTGGGTTCATCGTATTTATCAACAGATTTGATGTGCTGAATGCAATGCTCACCATAAATTTCTTCAATAGACTTGGTCATCGTTGGTTCAGCAACTGCAGGCACAACAGCTACAGATTCAGCAGCTACTAGTTCAGCAATCACTGGTTCAGCAACTATTAGTTTAGCAATCACTGGTTCAGCAACTACTGGCTGATACTTCGCGTGGTGTCGGCACCAATCGCCTTTTGAGCCGCACTGTTGGCTCGCCTTTGCGTGGTCTTCTGGGAAGATGTACTTGCACATTGTTATTTCTATATATTTATTTTTTATCGCTCAAGATGAACTCACTATTATTTAGTTTCTATATATTGAGGAGTATTTTTGTCAGAAATAAAATAATTATTTTAAAGTGATGTGATTGGCTAAAACTGTGCGGTGTGCTGCGTGAATGAGTGCTGCTGCTCAGTCGAGAACTTTCCGCACGAAGATGGCAGAGGTCGAGTTGGAGTGCGCGGTATACGGCGAAGGGACTGTGTTTCCCGTGAAGATAGCGAGCAACGCGAAAGTGAGCGCGCTGCAGAAGGCCATGTGAACGAGAAGAAAGACGTCGATGATCGCTTCAAAGTTGATCCAGCCAGATTGACGCTCTACTTGGCGCGGAAGAAGGGGGAAGCCACGTGGATGAAGCACGACCACACGGTGAAAGGCTTCTTGTGAGGTGGCATCAGCACCGAGTATGAAGAGATGCTTTCGTCGTGGATTCTCGACGAAAATTGCGGTCGTGGTGATCATGTCAACACCATATTCATTCGAAGCCTTGCCCGCAACCGCAGAATTGGCATTACCAGTGGCGTGGAAAGTGACCGCGACTAGCACTGCAAGGATGCATGTGAGGCGCATTTTTTTGTCGTTTGGGTCAACAGCTAGGGCACTTGAGTCGAAATGAGAGAGTGCGAGCAGATTGCAGAATGAGGTGTCGGACGAAGACAGAGCCGTACTACGTGCTGGAGCACGAGGAAGTTCCACTTTGTACTGTTTTCTTCCAGTCTGTACAAATAACGATGGGATATAGCTACCGATCCAGTACATATTTCCAGACAGTTATTGAACAAAAAACCAGTACACTTAAGTTCGCAGCTAATAATGGTTTTTGTAGCCCTATTCACTTATAAAAACTAATGTCTAGTCACTATTAGAAAATTGTGTTTAATACACAAGAAGAGCAATTCAATTCGCAATTCAAAAGTGCGACAGCAGACCTCCTCCAGTCATCCTCTCCGAGTTGCTTTCTTAAAATGGATGGCCACCGCAGCATTCTTGCAGCGACATCAGTGGCAGCACTTTTCTCATGCGTAGTACTACTATAGAAGCACTGAGCTACCACATTGATTTAAATCAAAGTCACGATTGGGCTTGTTCTCGATCACTAGATACTTCTCCGCCCAATATTCAAGCTCATCGTTCTCAAATGACGTATCAACCATGGCTGAACGCATGTCATTAATTGTTCGACCAGTTAACTCCACCAGCTGAGTTAGTGTTGCCAACTTCCGCTCAGACTTGAATGTCAATTCGAAATCTTGATCAAGTGTAGAAAGCTCAAACGTCTTCGAAGTGGCTGTTTTCCATGGCTCCTTGACTTCATAGTCATCCTTCTATTGCTTGTATGCAGCAGAATCACAACCAGCAGCAATCCCTTCAGTTTGCCTTCGTTCAGTGATGGATTATACTTGGAATCAAACCAACCATCAAATTCTTTGGTAGCATACTCCTTGTTGAAACGCTCTACTTTTTTGTTTGAGCACACAGAGATAGGTCTTGCGCACTAAATGCCAATCCGAATGAACTTGACAAGCAAAGAATCCAGCGAGATTCCACACGTTTTGGTTGTTGGAGATCAAATCGCCTCGAATCATCATGCACAATTGATACAATCGCTCATCGTAGGATTGAGTTGCAAGGAAGTATTCCACACCGCATTCAACGTTTGCTTGTTCAAATGATTCACGGTCTTCATTGGACTTAAAGAAGAAATTATCAAGCATCTGCGATTCATCCAGCTCATTAAATTGATATTGCAAAAGGTACGTGTATCCGTCGTTGCCAAAGTCAGGTGTGCCATCATCACTGTGCTTGTAGTTGATGGAATCCCCAACTGCGTGCATCTTCGACTTGATCCAATTACTTGGTGACAACACGCGCGAATCTCCCAACCTGGGTTCATCGTATTTATCAACAGATTTGATGTGCTGAATGCAATGCTCACCATAAATTTCTTCAATAGACTTGGTCATCGTTGGTTCAGCAACTGCAGGCACAACAACTACAGGTTCAGCAGCTACTAGTTCAGCAACCACTGGTTCAGCAACTATTAGTTTAGCAATCACTGGTTCAGCAACTACTGGCTGATACTTCGCGTGGTGTCGGCACCAATCGCCTTTTGAGCCGCACTGTTGGCTCGCCTTTGCGTGGTCTTCTGGGAAGATGTACTTGCACATTGTTATTTCTATATATTTATTTTTTATCGCTCAAGATGAACTCACTATTATTTAGTTTCTATATATTGAGGAGTATTTTTGTCAGAAATAAAAATATTATTTTAAAGTGATGTGATTGGCTAAAACGGTGCGGTGTGCTGCGTGAATGAGTGCTGCTGCTCAGTCGAGAACTTTCCGCACGAAGATGGCAGAGGTCGAGTTGGAGTGCGCGGTATACGGCGAAGGGACTGTGTTTCCCGTGAAGATAGCGAGCAACGCGAAAGTGAGCGCGCTGCAGAAGGCCATGTGAACGAGAAGAAAGACGTCGATGATCGCTTCAAAGTTGATCCAGCCAGATTGACGCTCTACTTGGCGCGGAAGAAGGGGGAAGCCACGTGGATGAAGCACGACCACACGGTGAAAGGCTTCTTGTGAGGTGGCATCAGCACCGAGTATGAAGAGATGCTTTCGTCGTGGATTCTCGACGAATATTGCGGTCGTGATGATCAAGTCAACACCATATTCATTCGAAGCCTTGCCCGCAACCGCAGAATTGGCATTACCAGTGGCGTGGAAAGTGACCGCGACTAGCACTGCAAGGATGCATGTGATGCGCATTTTTTGTCGTTTGGGTCAGCAGTGTGAGCTATAAAACTTACCTGCGTGTCGAGCGCCAGTGCATTGCAGTCTGCCAGTGTCCGGTGCCCCCATCTACGAGCCACCGTCCACTGCCAGTGTCTGCAAAGACACATCCCAAGGACACTCATGCGCCTGGCAGTGCAGCTTGCGGCTGCACTACAACCGGCTACACTGAGTACACATGCGTGCAATGGCGAAGGACTATACCAAGGACTGTATGCACAAGGACATCGTTGGCTGTGCGCTGCCAAGATGGTGTACTCAAACGAGAGTGCATTTATCTAGAGTTTAGTATTGACTTATTTGCTATTTGTATACTAATCTACTTTTGCGGTACAGCGGTGTTCTGGCGGAGGCTGCTCAACACTTTGCGTTTTGACGCCTGCCGCTCATCAGAGAGCAGCAGAGAAGAGCAGCCTCGCCAGACACCGCAGTCCTGTCGCGGCCTTTTGCCCGGCACTTACGTGTTTACGTTACTTCCTGATTAGTTTCCTAAAGTTGACATTTAAGTGTATTTACATTTAAATGGCTCCTACTCTTAGAAGCGATCGACACATGGAGAACAATCCCCCCGTGACGAACCCTACGTCACCACTGGTTATGAGCCCAATCAGGGCGCAGTCTGCGACTGACGTGGCTGCTGGCGTTCCAGACACAAGTTCTGGAATTTCGACCAATCAAGATCAAGCTTCAACCCCATTGGCAGGAAATTCAAGCTTGAACAATGACGCAACACTGTCGCACAGTGATGCAGAACGACTAATTCGTTCATTGGCTGGAATCCCGGGTGGCCGCCCGTTATTCCAAATGTTACTCGAAAGTATGCATAAACTTTCGTCGACGAACACGGAAGAATCCGTGCCAGTTGACAATCCTCCCCAGTCGCCGCTAAACCAACAAAACGGCGACCGAGACCCTGACGATCCGTGGCGCCCTCTAGACAACATGAACCCTACTGGCCGCTACAACGACGGCGAGCTGGCTGAGTACACCAAGGTCCTTATCAACTCGCCTCCGATCAAGCTGCCCAAGCTACATCTGAAAGGCGACTACAAGGCCTGGAAGAGCGAGGTGCCGTTGCACTTCGAGCCGAAGATGCTGGGAGACATCACCTATGGCCCTGAGCGTTACGATGCAGTAGAAGGCATGCGTCGTGACAAATATCGCGTGTGGTATAAGTCGCGCAAGAACAAGGCATTCTCAGCACTCGCGTTGTCGCTGTCTGTTGACCTACGTACGACGTTCAAAATCGACGACATCCACGACAACATGGACGCCGCCGCTATGCTCTACGATGCGATTACGCAGCACTTTGAGGCTGGAGATGGTATTAATCCTGATTACCTACTGCAAGAGCTAGTGACGCGCCGGCTCAAGCCAAACGAGTCAGTATCGCAGTATGCTGACGACATTGCAAAGAAGGTGACACTTCTACATCAAGCTAATGGTGAGTTTGCTGAGTGGCAACATGCAAGCCTACTACTATCGAACAGCGTGGAAGCGTTTAAAGATCTCGCCCGTGAACACGGCGATTGGCTTAACAACCATGATCGTAAGACACTTACACTTGCTGAAGCTTTACAACGACTACGCGCTGCTGAACACCAGCGTGACCAGCTGCGTGCACAGACCAGACAACCGGTCGCGCATTCAATGCAAATTGCTCATGTCGGAACGGACCAAGGACAAGGTCAAGCCCGAGGACGCAAGCGCAACAAGCAACAGCGCAAGCGATCTAAGGGCGTTGCAGATAAGAAGCAGCGCACAAACTGCGCGAACTGTCAAGGAGAAGGACATTGGTATGCCGAGTGTACGGCTCTCACTGGCCAGCAACTGAAGCCAGAGCTCGCGAGAAAGCTGCAAGAGAGACAACTAAGGGCTCCCGTGTCGCTGGTGAACTCTGTACGTCGTGTGGATGCGGTAGCGTCGGAAGAAGATGGCCAAGGACTGTTTGCTGGCCTATCACTATGCGATGCGAATGCGATTGACCTTACGGATGGTGCTGCCCTATCGTCTACGGCTACGCAATCACCTGCATGGTCACTAACGTCTCCTGCAGAAAGTGAAGAAGGAGGAGAGCGTCGTGTTGTTGAAGGGGTGCGGGCAAGTACGCAACGCACTCAATCGTCGGTTGCGCAAGCAACGCAGCGACTGCAGCTGCAAGTTGGAGTTCCTGTTCAACACATACAAGTTGGAGGACAAGGACTACAACAACGACAATCAACCTCACTGCCTACACTGCAAGCACCAGCTGTAGCTGTGCAAACTGCCATACAATCTGCGATACAACCTGAAGTGCAACCTACTGGACAACCTGCTGTTCAACAGGGTGTACAAGCTACTGTACTGCAAAACATGCAACCTACTGTATCGAGTACTGCGCAGACTCAGTGGGAGGGCAATCGGTCTACAGCTGGATTGGTGCCTACGTGGGAATCAGTCATCTACTACTTGGGGCAAGGACTGTCGAGTAACTCGAAAGGGCCTACACTTGAGCCATGGCGCAATCAAGCTGAACCACAAGGGGGTTCGGCTGTGCCGGGGGATAGCTGTCGAGGTCCTGATGTCGGGTTTAACAGACGCCAAGAATCAGGGATGCGCAATGATTGCAGACCTAACTTGGAGAGTGCGTACGAGAGAGGACTCTGGAGTCAAGATGTTGGACACTTTGACCCTCCTATTCAAGGACGAGGACGAAGTCGTTCACCACCCCGATTCTTCAATCGAGAACGAACTCCACCAAGAGGAAATCCAGGGCGTTATCGATCTCCACCGCGTCAGCAGCAGTGGCGTAACGAGAGACCGAGTCGCTATGAGCCGCCACCTCAACGTGATGATCGACTGAGTCGCTATGGACCGTCGTCTCCGCAGCAGCAGTGGCGTGACAATCCTTCGAGAGGACGAGGACCTTCACCTCGACGTGACAATTTCCAAGGGCGGAACATGTCTCCGCGTCGATTTGAAGACCGTCGTGAGAACAGACCACCCAACTCTCGTCAAGTCAACGCTGTGAACCAGAACCAAGGTATGCAATCAGCTCAGTCTGAGTTTGTGGAGTGGATACTTGATTCAGGATCCCAGGCAAATGTCTGTGGTGACCTGTCATTGTTTACTACCATAAGGGAAGACCAAGCTAGTCGACTTGACTTTGCGAATGGCACAACTGAACACACTTCTGTTTGTGGGTCTGTGCTATTGCGTGTTGTTAACCAGGCAACTGGCCAGCTCGAGGACCGTCTCCTGGACGACGTGGTGTACACACCGAACGCGAAGGTGAACATCATTAGTTTGGGCTACCTACAAACGAAGGGCAACTTCAAGTTGACATGCTCAGAGGACCAACAGACTGCTTGGATCTCCAAGCCAAGCACATCACTCAAGTTCATGATGCGTGACAGTATCTACCGACTACGTGCAGAAAAAGTGACTGGAGTAATGGTGATGGCGGCTCTAAAAGAGGACATGGACAGCAAGAAAGCCATGGAGCTGCTTCACCAACGATTTGGTCATATGAGCATGGAGACGGTGAAAGTGTTGGCAAACAAACTCGATGTTGGAGTTAAGATGAATGCAAAAGGTCTGACTTCCTACGAGTGCGTTGCTTGTGCTGCCAGTAAGGCAAAACGAATGTCTTATAAACGGATTCCATCGCGGAAGTCCGAACCTCTGGAGACTCTCATGATGGACATTTGCTCTGTGAACGAACAGTCTGTGGATGGAGCTACAATGTTTCTCTTTATCATAGATGAGTCTACAAGGTACAAGTGGCCGTTCAGATCGGAAGAGCACACGTCTGAACTCCAGTCACCCAATGGAATCTCGTATGCCGTCTTCTGCTTGAATGATACGGCGACCACCGAGATACCGGAGGCTACACTCTTTCCCTACACGACGCTCTTCCGATCTAGTTCCTGCTGCTCTTCAAGGAGAGCAACAACATCAGCAACATCACATGGGTCAAGGATCCCAAGAACCCCGACGTACTGGTGCGTTGGCTTCAGTTACTCCACCTACTATTATGCACCAGTCTGCGTTAATACGGCACCGCTATGCAGGCGACGACCTGCTGTGAATTAGCTACTAACAAGTGTTCTGGTTGCTTTGGCAGATCCCAGGACGCAACCCCACCAAAAGACCCAAGCCACAGCCTGCAAAATGAGTCTGTTTGCTTGCTTGATCATGGAATTAATGGACATCTTATATGTACACCATACTTTTAATCTGTATCTTTGCTTGCTTTTGGATAAAGTGAGACAACCAAAGGACGAACTCGTCCAGGGGCCGAGCTTGGGGTTTGTGCCTTAGGCTTCGTTGCCTGGTTGGTATGCACCGGGTTTCTGATGCCTTGCAAGTAATGAGGGAGTCGATGCTCCGGACGAATATCCAAGCAGATCCCAGGCTTTCGTGCCAACGGGTTCTCTTTACGTTGACGCTGCTCGACCTGTTCAATCCACGTCGAAGCCCGTGCCTTGCGGAACGAAAACCTCGTAACGGAGGGTATTTCGGATGTGCACTTCCACTCATCGGCACTGCCCATTGATCGTGAGTCGGGCCGCCCTCCCCACTGCGAAAGTCGACCCAGAGCGAGTTCCTGATATGGGCGGCAAAATGAATACTACTGTTGTACTCTGCTACCGAAGACATGGAAAAGATACAAAACACTTACGCCACTTTGACGAGCAGGCGGATCTGTAAGTAGACCTTTCGGCTTCTTCATCACATTGTAGGAGCCAGGGCCAACACATTCGGGAGTGGGAGACTTCTGCGCGAGTCTCATTTCACTACTGTTAGGCACGCTGATTGTCATACAGTATTGACAATTTGTCATTAATACTTACATAGTTGAAGCGCTTGGTGGTACTGCACAAGCTGGTGCCCCGTAAAGAGCCTGTAAAAGTGTAAATACTGTCATTTGGTTATTGAGTTCATAGAGAGCTTAACGTACGATGAACGTCATAACCCATCGAGTTGTGGTTATTGCTGTAGATTTGGTCGGCACTAAGACACTCGTTCCGGAAGGCGGTTCCCGGCTTCACTGTTGTCTTTGGCAGTCGCAGATCGATGTGGGGACCGTGAGTTGTCCACATCCGTCGCGATTCCCGTGCATATACGCGTTGGTCTGGTACCATCCCGCCCTTCGTTAGGGCTGTGTTGGCGTATGGGTCGAAGAAGAAGGACTCGCGAAAGGGCTGGTATGAGGCCCTGGTGATCCCCAGCGTGCGTATCTGCGCGGAAGTCATTCTGGAAGGCAGGACGTCTGGTCCATCGGGGCCCGAAGGCAAAGCCCCGGGGCTGCACATGACCAATTGCGGTAAGAATCAGGGCAACTTCAACTTTACCACCGAAATAGACAACGCATAGGCATTCAAGACTGCAGGCACGTGGCACATGAAGTGAAACTTCGACTAGCACGTTAATCCATTGTATCCAAGTCATGTACTTCGAAGTAGCAAAAAAGCAGTTAAGCATGGTAATAAGGTCTCTGTTCTGTAGATGCCAAGCCACTAACCTCTACGAGTGTAGATAGCTAAACTACTCACGAGTTGCACAAAGGCGATCAGCAAGGAAGTCGTACCTACTACGACGTCTACATCATCTCATCCATCTCCTCGTCCTCATCGAACTCGCCCTCTTCCTCTGCAGTGGCGTCCTGGTACTGCTGGTACTCAGACACCAGATCGTTCATGTTGGACTCAGCCTCAGTGAACTCCATCTCGTCCATACCCTCACCGGTGTACCAGTGCAAGAAAGCCTTACGACGAAACATAGCCGTAAACTGTTCGGACACACGCTTGAACATCTCTTGGATAGCAGTAGAGTTACCAATGAACGTAGTGCTCATCTTCAGACCCTTGGGCGGGATGTCACACACGCTAGCCTTGATGTTGTTGGGGATCCACTCGACGAAGTATGACGAGTTCTTGTTCTGCACGTTCAGCATCTGCTCATCAACCTCCTTCGTGCTCATGCGTCCGCAGAACATACAAGCTGCAGTTAAATAGCGGCCGTGGCGAGGGTCGGCGGCACACATCATGTTCTTAGCATCGAACTGCTGCTGGGTCAGCTCGGGCACCGTCAGGGCGCGGTACTGCTGCGAGCCCGCGCGATGTCAGAGGAGCGAAACCAATCATAAAGAAGTGGAGACGCGGGAACGGGATCAGGTTCACGGCCAGCTTACGCAGGTCCGAGTTCAGCTGACCGGGGAAACGAAGGCACGTGGTAATACCGGACATGGCGGCACACACCAAGTGGTTCAGGTCACCATAAGTGGGGGTGGTGAGCTTCAATGTGCGGAAGCAAATGTCGTACAGGGCCTCATTGTCCAGGCACATGACCTCATCGGCGTTCTCGACAAGCTGGTGTACCGATAGCGTAGCGTTATAGGGCTCCACGACCGTGTCCGATACCTTGGGCGACGGGCAGACCGAGTATGTGCACATGATGCGATCGGGGTACTCCTCACGAATCTTCGAGATAAGAAGCGTACCCATACCGGAACCGGTACCGCCACCAAGCGAGTGCGTGATCTGGAAACCCTGAAGGCAATCAACAGCTCTCTGCCTCCTTGCGAACGACGTCAAGCACCGAGTCAATCAGCTCAGCGCCCCTCAGTGTAGTGTCCCTTGGCCCAGTTGTTACCAGCGCCAGTCTGTCCGAACACGAAATTGTCTGGGCGGAAAAGCTGACCGTAGGGGCCAGCGCGAACGGAGTCCATGGTACCCGGGCTCAAGATCCATGAGGATGGCGCGGGGCACGTAACGGCCGCCCGTGGCCTCGTTGTAGTACACATTAATGCGCTCCAGTTGCAGGTCTGAGTCGCCGTGGTACGAGCCCGTGGGGTCCACGCCGTGCTCGTCGGAGATGACCTCCCAGAACTTGGCACCGATCTGGTTACCACACTGACCACCCTGAATGTGAACGAGCTCTCTCATTTTATTGTCTGCGAGTAAATTGAGGCAACGAAAGCGTCAGCAAGAGTAACAACAGGCAAAAGATCGCGATACGCCTCCTTCCCCCCCCCCTTTCCCTTCGTATGGTGGGGGATTCCAGCTGCAGACGGAGGATTGAGGGGGCCCGCCTGAGTACCACATGAGGGAGGAAGGGGGAGGCGCTTATTAGCGTTGCTGGAATGTCATACCTGTTGCTTTACGTCGACGGTCGGGATTCCTGCGGCGGCTCTTGGCTGGCTGGCGCGGGCTCTGAGGAGAATTCGGACTTTTGGCGAATGAAGGAGTTTTGCGTTGCGGGCGGCGTCGGGACGCGCGGGAAAAATTACCGTTGGAGTTAAATATGGTCCAGCCCCCGTTTCAAAACGTCGGTGACCAACACTCCTTTCTCGTGCTTACAAATGCCAAGAAAGAGAGCAAGAAAAGGGCACCGAATCTGAAACCACTGCTGGTTTGAATTTCAGGACGGATGGCATGATTCAAGTCAAAGTCGGTTTAGCTTAGAGTAAAGGAGGGGTGAAAGAAAGGACGTGTCTTCTGTTTTGCTTTCTCAGAAATTCACGTAAAGAAAGCCACATACTGCAATATTCTCAGCTTATTACGTAAATTACCGATCCAAATGTAGGAGGCGAGCGCGTAACGGACACCACTTCCCAATGTCTCGAACCACCGACGCCTATTGTTATCACACTTTGACACAAAGCTTTGTGCGATTGTGTTGAGCCCTTTTGCTGCACTGTAGTGCAATTA

>Contig_70

CTTCCTTGAGCATAGTCGAAGATTGCTTCTTGTCAAAGTACTGCTTTTGACGCTCTTGGGCTGGACGAAGATGCATTTTGTCAAATCAATGATCTCGTGACGTTTGTCTTGGAACTGTTTCGCATATTCCACAACTCCACGCTAAATTTGAGTCACGGTACTTGGATTGCTCAAATGATTCAACGGATTCCGTTCTTTGCGTCCAGTATCAACTTCAAAGGTCAACATACCGGTTTATGCGCTGACGAGAGTAGCATGAGCATACTCAATCGTCCGAGATTACCCGACCAATCACGTCCGAGGTACAATATCAGAAGGCGTGCAGCTTCTTCCAAAGTCAGATTCAAACGTTCGGTTTGTCTATCGACATGGGCACGATAGGAAGTTGTCATGTGCAGACAAATTCCCATTAGCTTAGCCAAGGATGACCAGAACTTGGAGACGAACTTGGAATCTCGATCGCTGATGACGATCAGTGGTAAGCTGCGATGGCGAACAGCGCAATCAGAGAAGTGATACGCAGTTTTCTGTGCATCATCAGTCCGGCTGCACGTCCGATACTTCGGGTGCTTGGACAGCCGCGACAACAGTCAAGACTGCATCAAAACCATTGGAATCAGGTAATCCAGTGATAAAGTCCATAGAGACGACAGACCTGCATTCCTCAGAAACGGAATGGGAATCATCTCGCCATTGGCCGCTGGCTTACAGATCATTCGTGCTTTACCACGTATGCGATAATTCTAGCGAGCATTGCTGTGCTTCCAGCGAGTCCAAGTTTCACAACTTTGCACATAATCGCGAACGTCTTGGTCCAAGGTGGGCCAATGATACCATTGCGAAATGCGAAGGTACGCTCGATGAGCTCCAGGTTCAGCAGCGATATTGGGATATTGGAGTCATGGAAGTAAGAGATGATGGAAGTACGAAGATGATTGTCCCGAGTAAACGGAGCATAAAACGCATCGTCGTTTGATACCTTTGGAAAGGTAAACCATTTTGTTTGGCAAATGGTTGTTACGGATGCTTAAATCGATCTCCGTAATCTGGGCAATTGCGATATCCAGCAGCGAATTCTCTGCTAATCGAATCAGACCAACCTAGCGAGCTCCAATTTTGAGCTTGTTGGGTTACATAGGTAGCCACAATAATATTCTCAGCTGCAGCGACGGTACACGGCGTTTCCACTGGTACATGTCGGTAAGCTTGAGTTGAGGTCGGAATAGGGCGCGTGTAGTAACCAGCAGCAACATCCTCCCCACGAAATTGAGGGCGAGAGGGCGGTCGAACCAAATGTTGGTTAAGATTGCGGTAGTCGGAACAAGCGCGTCGATTCGTCAGGTTTCGAGCTGCATGGTGAGCTATAACCGGCGGAAGTCTATACGCCTGCGCCTCCCATACAAGGTGTTGCTTCAGCCGAAGCTTCTGTCCAAACCCTAGCTGTTTGGCGCTAGTCACTGGCAACCGAACTGGAAAGCCACTTGTGGTTCAGCCGAGAAAGTTCCAAAGTGACAGGTGGGCTAGCTGGAGCGCGACTCTTGGGGGGGAGTGAGTGGTTAATACTATAATCACTAGGAAGAAGTCGATAATTGAATACGATATAGTATTACATGGTAAGTCTCAATTAAATAGGATACAACATATGTAGGGCGGCTGTGGAAGCTTCTAAAAGCTTTCTTATGCCCTTCCCCAAGCTATGCTTCTTTTTGATAAAAGGAGATTTACTAATGATATTTTCTCAACTTATTTATAAAATCGCTTTCTTTCACCCTCCTCGAACTCCGTAACCATGTACTCCCTCCCCATTGACCACGTTTCGACCCTTATCTAGACTACGACATGGAGACCTTAGGAGCGTTGTACCGCTTCACGATGGATTCAATAGGAAATGATACAGCAATGGATACGAACGCCACTACCATATCCGATATATTGTACTTCAAACGTAGTCTCAAACCAAAATAGCTTTAGTATCGGCCACGGAATAGACATTTTAATCCTTTTCACCTCGATGTTTTAGCTTTTAAGTCTTATAAAGGCGTGGGCGCAGATCAAATTGCGTATACCCGGAGGAATAGGTGTGACGTCGTAGCTATTAAGTAGGACATGAGAGAAATCCTCGCTGATTTTCAGTAAGAAAGTCCACAATTTCTGCTTGAACCGGATTCCGCTCATGATGATAAGTTTAAACCAGCTTTGACACCCCACAACGCCACTCACTTATTAAGTCAACCGGTGTCGCCAGAGCACAATGTACGATCCGATAGGCCATACTAAAACGTACTGTGCAGGACCGTTTTAGTAGTGAACAGCACTCAGTCACTACCTCCCAAAACTAGTCTTCATGTGTTCACATAGTGCATTGATGCAATCTGGACGCTTTTGATAGCGACCGGAAGGTTAGAGAGCACGCAGCGGCCTTAATACTGAGCAAACAATGGTGATCCTGAGTTTTATGATTAAGCGACCATCCTATATAGAAAGTTTAATATAATGACGCAGGCTATTTGCTAGAATGTTGACTTCAGACGCATTTAAGTTCCATACATCAATTCATACGTAAGGCTCTTTCTATACAGACAGGTACCCAAACAAGTGCAGGAAACTTGTTTTTATCAATACTTCATCACTGTATCCATGATGTATTTGAAATAATCTCCCCAAATTGCTTTCTCCCGAGCATACCCATGAATTGTGTTACGATCAGCGTTGAGCACGTTCGTGAAAACCTCATCTGCTGTCTTGTACTTATCGACGACATACCACTTGTAGAACTGCGCCTTTTCCAACTTCTTTGCGACGTCGCGCGNAATTTCTGGACAGTTTCCCGACTAGGATCATTATTGCCACATTTTTCTCACCAAAGACATTTGTGAGTGTTGTTAGCATCGCGCCATAGGGGTCCTCCTTGTTGATCTTGACGGCGTGCTGGGACCAAACCTTGAAGTTTCGGCTTGACAACAACTTGGCCACCATCTTGGTCTCGACAAAGTCATGACTACTAATCCGCATATTACTGAGTTTTAGCGTTTCGAAAGCACTATCAAGTGCCTCGTCGGCCATAAGCTTTCCTTTCAGCTCCGTAGTTTTGGATGACGTAAATATTTTTGAGATTTTCTCCAAGCTTGGAACATTTATCGAAATACCCCTTTGTTCTTCTTGTTCTGTGCCTTCTTCCTTGCCACTACTGTGGGCCCTTAGAAGCCTCTTGGTAGTGACGCTGTCGACAACGCGAGTTGAACTGCGCGTGTGGGCACTCAGATTCAATTCCCTTGTAGCTGGGACCGGAGCAAAATATGCAAACAGTAGGAGAAAGACCAAACAGACAGCGTAGGGATACTTCATAATGAGGCTCGTAGGTCAGTTCTTGGCCAAAAGAATATTGAGAAATGATGACTGAACGGGTTCTGTCGGAAAGATTCCACCGGCCCATCCCGGATGTACCAGTATCCCAGCTTTGAAGAGAATAACGGTTAAAAATACTCCCGCTCTTTTAGTTGTATCGTAATCGTCGATCACAAGGCGGGAAATTATAAATCATCTCCTAGACAGCTACCATCAGGTTCCGATACGCGTTGAATCATCGTAATGGGACATTGTTTTTGAGAAGGAGGAAGAGCTAATTGAATTCTGCTGTGTACAATCCTGGTCTTCCCTAAAGTATTCACAGGTGTCGGTCTGGGGAGTGTAGGGTGTTTGTAGGGCACTCAAAGATTACTATTCTCAGGCATGTCCGCAGGGGGTATTTTCAAAATTGTGCCCCTTTGACCGAAAAGAAAAGGGGGGCGGCATTATACTGACATTTTGTAAGTGCATCAGGATTTAGCACACTTTTTCACATCCCAAGGTAATCAACCTGCATCCGCTTTGATTGGTAGAATTTCCTTCATTTTTCAAGGGGTACTTCGAGGTACTCGTACTTGTGAAAACTTCGAAATGGTGTTTTGTATTTTTTCTGAGCGCAGTGGACATGCCCGAATACAGAAGGATCCTTACAAAATATAATATTAACCCCTTGGTTCCCGACGTCGTAACTTACGACATGACTCTTTGTTTATGTCGTAACTTACGACATAATTGGAATAACATTAGAAGATGGCTCAAAAATGGAAATCCTTCACTTTAATCAAGTCATTTGAAATCGATAAAGTTCAACCGGTAAATGAGAAAAATCCAAAAACGAGCCAGGGATCAGGGACGCGCGGAAAAGATGGGATACGCGGGAAAGTATCCAGTTGCCACTCGGTGGCCCACAGCTCATTACAGAATTTGCTCGATGGCTCGCATCCGGCCTGGAAAACGAACCAAAACAGCCGCCGTTGCTGCGGTGGCGCGCAACATTGACTTTGGCCACTTTTGGCGACAACTTCGAGCTGTGGGATGGAAGTACAAGCGACCAACCGGGATACAAACTAAAGGGAAGTACGTAAGTGCAGATGGTTCAAAGGTGTTAGTTGGAGAGGAGGCTGTGGTGGCGTATGCATTAGAGACAGGAATATTGGCTGAACCGGAGAGCGCAGAGGAAGCCAACGCTGCAGACGATGAGGTCACTGTTGACGTAGTAGCCGACGATGACGAGAGCGCCGCTGCTTCTGCTACTGAAGACGCCGTCACTTCCAGTAAAGACGCCGCCGATAATTGTTTAGAGGACGTACGTGCGTCGCAGATCGACACAAGTGCTGAGCTGTCACAGCAGACCATGAATGATCTGTTTGGCACCCCAAGCAGCTGCAATAGCTCTGAAGTCGAGCTTTCACAGGCAGCAGTTACTCGGGCTTTTAATTTGTCGCCCGGAGAGCTTCGAGACGCAGCGACAAACCTGCAGTTTCTGTCATCTGGACCCGAGAGTGACGCTCAGTCGGATGTTGGCGCTGCTTCCCCGCCTGGCAGTCCAGTGCCCGCACAGCCTATAACTGTGCCGACCGCCCTACCTGTCACTGTGCCCGTCGCCCAACCCACACGTGAGCCTGACGCCCAACCCGTACGCAAGCACGCTTCCCAACCTGTTCGTGTGCCAGCTACGCCGACCTGTCCTGTCCTACGCCCCCGTCATCCTGTCAAGAAGGATGTAAATTTTGTGACTGACGACGAAGACCTGAGTGACTACGAGCGCTTTAGCTCTGGAGACAGTGATGACGACATTGAAGAAGACAATGATGACGATGACGGCAATGATTGTGAAGTACCTGAAGAATTGGATGAGCTGTCAGAAGATGACGCTGTGCCAATGGACGCAGCGTTCATTGACTCACTTCAAGTAGGCAGTGATGCCCTCAACAGTCAAGCCAAGCAGCAACGTGAGGACGCGTTACGTTCAATGGAGTGGTCTTCAGTCACTCACGAGTTTGAGGAGGGTGTCGAAGCGTACTCTGGTCTGAATATGGAAGAGGCACGACCAATTGCTGAGCTACTAAACGTTTGCCACTCTCCACTACTTACATTCTTTTACTTTATGCCCAAGTCGCTATGGGTCAAGATAGCTGCAGAGACGAATCGGTATGGTCTTCAGCAAGTCACCAGACGTGCAGAAAGAATTCATGCAAAGCAACACGATCGGAGAAAGGAGACGGTTAAGCAGATTAGTCGGCGTTTGAAGGCAAAGCCGGGATTCGAGACGCACGAGATCTTACACATGATAGGTCTCCTGATTGCGCGCATGCTCTGCCCGCAAAAGCGACGTTTTGCCGCTCACTGGTCGATGGTGGAGGATGGGGCCGTTCCTGCAGGTAATTTTGGAAGGTTCATGGGCCGGAATCGATGTCAAGACATGTTGCGCGACTTGCACTTTGTGGACAACGAGGCGGAGCGTACGCGAGACAGGCTCTGGAAGTTGCGAATGGTAGTCGAAACAATGCAGAATCGTTTCTTGATTGCGTGGACCCTCCCGGCAGTGTTCGCTTTTGACGAGGGTGTTCTGCCTTCTACTTCTAAAAGAAACACGACTAGAATGTCCATGCTTGATAAGCCTCATCGGTATGGCTCGAAAATGTTTATGGTGTGTGATTCAAGGACCGCGTACTGTCACAGGTTTGGTTTTTTTTACTTACGTCGTAAGTTATGACATTAGATTGGTTTCTCACCAATTATGTCGTATGTTACTAGATTTGAGCTGTATGCGGGCAAAAGAAGAGATGGAGAGCATTCAACATTTGACCATAAGACCGGTGCGGCTGCAGTCGTTAGAAATTTGAAGACAGTGCTTGGTCCCAACACCCGGCTTCCATGGCACGCAGTAGTTATCGACCGCTTCTATTCGTCAATTCTACTTGCTATCGAGCTACTGGGCATGCAAGTGTACGTGATAGGCACGATAATGACCAACCGGCTTGGATATGATGCAAATGTCAAAGAGAAACGCAAGTCACGTCCAGCAAGCGTTCCCCGAGGCATGTTCACGTTTTCTCGTTCTGTCGCCATTCCAAATATGGTTGCATTTCACTGGTGGGACCGGAAACCTGTGCATTACCTGTGCACAGGCTCGGCAATGACAGAGACGACTATTACACGAAACGTCAAGCGCGTTGGTACAATTACCGTACCCTGTCCGGCGGCTGTAACTGACTATCAACGTTGGATGGGCGGAGTTGATGTGCATGATCAACTCCGCCTACAAAAGTACTCTTTGCAGAAGTCTACGAAGTTCAAGAAATACTACAAAAGCCTTTTTTTAGGCTTCGTGGATTTGGCACTTGTAAATGCGTTTATTTCGCGCAAAGAGGCTGCACGTATCGCTGGAACGCCGGTGATGAAGCGAGGAGAATGGTTCGGAGTCCTGCAAAACCAATTACTGCAGCTGAAAGTGGAGGACTTCGCTGGTATTGTTGCAGCACCGACACCTAGCAGCCAGAAGCTGAAGCGCCTTCAACTACGTCTGACACATGCTGTCGAGCAGGCCGACGATTGGGTGACCGTTACGGGCATTCAGAAGCGTCGCCAGCGATCTTGCAAGGTGTGTGCCCTGTTGCGCACGGATCGAAAGAAGAAGTCGTTTGCTACAACTTACTTTTGCGAACGCTGTTCTATTGACGATGCAAAGTGCTGGCTGTGCAGCAAGATACGACGTCAGTACAAGGGTGTAGCTAAAACGTGCTTTGAGATCTGGCACGACGACTTCGACTGCGGCCAAAACATTCCTGCAACACTGGGAAAGAGAGTTGTCTTGCGACGCCCGGGGAAGAAGGCAGGTGTGCACAAGAAGACCCGTCGAGAGTTGCGACTTCGTGAAGATGAGGAAGACGGCGGTGATGAACATGAGAGTGAACAAGAGTAGGCTTGCCAGCGTGACATCAAAAGAGTGTGGGCGCTTGTCATGTCGTAATACACATTTCCTTGAGCATCTGTACTTTTTGTTCTGTCTATTCTACTGATCTGGATTCATAAAACAGCTCTGTGCACCACTTTTTCCCCGATGTGCAGCAGATCTTCCCGCTGTACTTTCTAATGTGGGGCAACTTACGACATACGGTGTTTTCTGCTAGCTACTAAGTAGTATAACTTACGACTCCTGAGAGAGCAACCTGTCAAAAACTTATACACCTATACGCACGTCGAACATATGCCACATACAAACAAGTAGTGACATGGACATGTCGTAAGTTACGACATTAATTATCTCGTTGTCGATACATTTCGCCGGGAACCAAGGGGTTAAACAAAGGTACGACTAGTGAAAGATGCTTGCTCTCTGTTACTAATTGTTTAAGACGGGACTACATGTATCCTTTTGTTAACCGTAAGCAATGCGACGAATCAAAACTGAAAACAACGTGGCGAATGAAAACTGTAAACAATGAGACGAATCAAAACTGAAATCAATGGGGGGAATGAAAGCTGTGAACAATTCCATAAACAAAATGGCACATGGTTCAGATCTATCAATTATCCAACAATAAAATGCTAAAAGTTATGTACAAGTTTCTAGCTTTCTGTGATTTTAAATTTTTTTCCCGGTCGAATTTTGTAATTCTGAAGGCAGATTGACTATGTATGTATGTATGTATGTGAAATTGAGAAAAAATGATATTTTAATCGACTACATTACTGTAAAATGTTGAGTTCGCAATGTCTATCCAAAACCAGTCATTCTGATTCGTATTGGTAAGACTTTCCCAACCAGCTCTTCTTGTGATTAATAGAAGTAGTCGACACCGATGGAGCGACGTTCAATGGCTTACTTAAAAGTATGAGTCACGTAGAGGATGTGCATGTAGTTTTTCGAGCTCAGCAAGGAGCCAACCTCGATCACCGTGTCGAAGGTGACTCCGCATTGAACCGTGCAGTTGAATTTGGTCATCTTAAAGTCGTCGATTAGGTATCGATATCAACGCCATGCGTTGATTACTTCCACGCTTGGTGATGCTTGCAAAGTACATTTTGTGAAGGAGGGGCCCCCACAAACGGATCGATTAAAGGAAACATCTCTCTGTCTTA

>Contig_72

GCCTAGAGTATTCTATGAAGGCGTGGAAGGTGCCCTATGTGGTTGGATGGGCGTTGCCTTAGCTACTCTAGTGGTGGACGTGATATTGGAATTCTCGGTCGCGAGTCTCATTGGGAACGTGTTCGTATTGGTACTGGGCTGTTATAGCCACGTTACGAGGTACTACTGGGCGTCGAGGGTGAAGACTGTCGACGGCTCTGGAATTGAGGCCGCACAAAAGGGTTGGGCTAAGCTTAAAAGTCGGAAGGAGCTACGAGAAAGGAGAGAGAGGGCTAGCCTCGACCATTTGCGTGCAGGTCCTAATGATACGACCGCTGAGGCGCAAGGAAATTCGGATTCGACGTCGATTGGAACGAGGTCGATCGTTCCAGATCTGCCGGTCGACTCGGCGATTGGACCTGCTGGGAAAGATGAGGCTCGGCGAGGACTCCGAGCGGCAGTCGGGGAGAAATGTCACGGTCTTCGGCTAGAGGAAGGACTCGCTATTCTGGACGACTGCCTACGGCAATTTAGCGAGCGGCGTAAGAAGAGTGTCAGGCCCGATCAGTCTCGTTCCCCGTCGCCGACTACCTCGTCCCTTGTCTAAAATGGAACGAAGGAGTTCAGACAGTGGTACGCTGACTCCGTTTACGAACGACAACTAGAGGTACTGGTGCTGGTAGGAGGTACTTTGGCAAGGTAGAGGGCTGGTGAGAGAGCTCTCAGTCGTGAATTTCCTTCTTTTACGAGAAGTGCTCAAGAAAAACAAATAAGATACAAGTATATTTTACACATCTAATACGTCTAAGTTCCTACGTCGGCTTTCACAAGATACACGACCCCGACAAGAGAAGGAGACCGCGACAATTGGCGATGGGCGGAATAAGAGCAGACAGTCATTGCAGACCACTGGACCAGTCGCCTGCAAAGACTGTCTGCCCTTATTCCAGTGACTGAAGGCAACCGACACTATAGTGGCTATTAATAGACTTCCTGATATTTGTAGGCGTCGCACAGCTTACGGATTGATGAGTATTATACTGTAGGAGGTGAATTAACTTAAGAATCGGGCATTACTATGGACTCGGAGTACATGTACCAGTCTTGCCACATCACACTTTAGACATTATCTTTATGATACGACTGGCTTAGATTCATTAAGGTTTGCGTACAATGCCAAGTAATGTTTGCAATTTTGAATTTTGTGCCAAGAGTCAGTTTTTAACCAATTACTACCGTTCCGAAGAATGGAGATCGTATCACTGGATTAAATAGGATTGCGGTGTCTACCCCATAGACGTCATCATTAGCTACATGTGATAGTACTGTACCGGTACAGTAGGAGCAACATACCGGTACCTAAATACCTGCTGCTCGAGAAATTATTTTTACAGCGGTAGACATTTATGTAATGACGTCGCGGAGGGATACCAATCATACGGTTAGAAAATGCTGGTGCGTTATGCGAACCTAATTTTTCGGCGACAGTATAAGTTTTTTTACTGGCCTCTGGGTACCTTTTCTGCTGGATTATGGTAGTGGCTTTACCATAAACCAATAGAAAAAGTCTGCCTATCCCATTACTTGTCGAACTGTATACACCTTTAAAATACAGTACAGTACAGTATATATTAATAGTATGGTTTGCTTCGCATGAACAAGTGACTCGGAACGTAATAGGTGGTTATAATAGGATAGTATGGACTTAGTTTCGTGAGCATTTATGTGCTGGTGTTCTTTTCATTTCTAACGACTCGACGAAACACATCGTTCATTTATCGCGAATGCCCACTGGGCATGCATAGGTACAGCCTGTCATATGAGTGCTCGGGGGAGGGGGCGGGGTTAATCCGTTCCGTGTCACTTCACGTTTTGTTCACCTGTACGCAAGACGATGGGCTGCCGGTATGCTGTGCTCGCATTAGCTGTAGCTTATTTCGCAGGCTCGATCGCAGCCAACGATTCGCAGATTGTCGCTGTTAAAGGCCCTGCTTCGATTCGATTTACACCTGCAATCCATGTCGTCCGTGGAAGGTTCTTGAGGGCTGCGAACACCGCTGACGAGCGCAACGAGGACAGGGGAATCAATCTCAAGTCAATGCCTGGCTTCGAAAAAATAGCAAGTCTGTTCACGAAGAAGAACACGCCAGGCCCGCTCTTAAGTTGGTTCGAAAAGAAGAAGTCACCGGACTACGTATTTCTTAAATTAAAGATTAACAAGGGCAAGCAACAGCTTTTTGACCACCCCGACTGGAACGTCTGGGTTCAGTACACGACCAGTGTGGTGAAGTCGGATCCGGAAGAAGCAATGATCGCCGCACTGAGGACACACTACACTGACGATATTCTGTCAAAGCTTCTCGAGTCGGCCAAGAACGTCCCGAAGACTAGTGGACTTGCCACCAAAATGCAAATGGAGCATTGGGTGGCCAGCAAGACACCGAGCCAAATGTTTCAGTTCCTTCGGCTTGATAAGGTCCGCAACGGAGTCCTCGACGACCCGACGCTTTCCATCTGGATTAACTACATGAAGCTGTACAATTCCAAGCCAGTGAACAAGAAGCAGCAAGTGACTTTAGTCAGCATGCTGACAACACACTACAAAGATCGAGGAGTGCTGGACATCATTGAAGCGGCGAAGAAAGTCCCGAAAACGGCTCCCGCTGCGAGACAATTGGAAATGGAACAAATCCAGTTTTGGTTAAAGAATGGCAAGTCACCGGACGAGCTACTCACGGTTTTGTCCCTCGATAAGGCCGGAAACCAGCTCCTCGCCAGTCCGCGATTCAAATTTTGGTCCAAGTACGTCGACAACTACAACAGAGACTTCCCCGACGAGGCTACAACCGTGATGGCGACTTTGCGGAACCAGCTCGGCGACGAAGACATCACGCCAATTCTAATAGCTGCAGGGAAAGTACCAAGCACCGAAAAGGCCGCCGCTAAACTGCAGGCCGAGCAGTTCAAAAGCTGGCTACGTGAAAACGAAGATCCGGCGAAAGTTTTCCAGCTGCTAAAGCTCGACAATTCAGCGGATGATCTTCTGGGCAGTCCACAGTTTAAACTTTGGGGGAAGTACGTGGAAGATCTCAACTTGAAGCCAGAACACAACGACCTTCAAGTCTCCATTATCACCATCTTGCGGAAAAACTATGGCGACGATGTGCTGGGGAACATGGTACTTGCTGGGAAGAAGGCTCCTAGTACGAGCTTTATGGCCCGACGACTTGAGGACGAGCTCTACAAGGGATGGATTGCCGCAGGCTCGTCACCCGACGGTGTCTTCAAGCACCTGAAATTCGACAAGGCGGGAGAAAACGTGATCCAAAGCCCGCTCTGGGGCCTGTATACAAAGTTTTTGGAGCACTATTACAAGTCGTTCCCGACGCCAATGATGTCGGCACTTGCGAAAGGCTACGATGGAGATGCGCTCGCGAAACTTCTCATTGCAGCGGAGAAAATCCCGACTTCGAACACGCTGGCAACGAAGCTACAAACTGGCCAAATTCAACGTTGGCTGGATGATAAGGACCAGCCAGGAAAGATATTCAAGGCGCTCTTGCTTGACGACATGGCGGACGACATTCTCACCAGCCCGCTGTTCAATACCTGGACAAGGTACTTGGATGAGTTCAACAAGAAGTTCCCTGATGAGAAAGTGTCCATGACGGACACGTTCCGCACCAGCCTGGACGATGAAACTTTGAAAAGTTTACTCATCACAGCAAAGGAACTTCCCGACATGAAAACGCTTTCGACCAAGCTACAGACAGTCCAGATTGAGCGTTGGTTGGCGAGTAAAACCTCTCCGGAGGATGCTTTCGCAGTACTCGCACTTAATAAAGCAGGGGGCAACGTTCTGTCGAAGCCATTGCTGAACACGTGGGCGGCGTATCTGGAGAGTTTCAACGCCAAGTTCCCAAGATCGCGAGTTTCAATGATTGACACATTTAGAGAATTTTTCGGCGACAAAGCGTTACTGACTACGCTAGCTGCGGCGAAGGAGGTTGAAAGCACCAAGAAAGTGGCGACGAGTTTGCAGGATTCGCTCCTTTCGAAGTGGGTACTGGCTAAGAAACCCCCAAGCGGCGTAGCTAAGCTGGTGGGGACGGATGAGGCGGGCGCGAAGCTACTGAAAACCTACACCACCAAGTATATGGAAAGATACGGGCAGTGATTCTCTAAGCTCGCAGCTAGAACGTAGGGTGAACCATAACTTACCGATATTAGACTTGCAACCACGAGCTTTGTTATTTAAAACGGTTGCTGATCTGTAAAATGGCAACTCAGATTTCAACAAAGGATAAATGCCAGCGCAATTTTAGATACATCCTAAAACTAGTTGGTCAAGGTTCACAATACCGGTTACTGTGTACTTATCAATGGGCCACTCCTCATTTGAGTGACAAGAAACCCCAAACATGAGCCGAGATCATCGTCTGTCACCCTCTTTATCCTCTGTTCTGTAGATGTTTCTTTTGACTAACCTCTGATCTCCCGCCATGCAGGTGACAAGGGCCGTAGCGAGCGCAGACGACACGCTAGGACGTCGTCAAGCCACAGATCCAGTCGTAGAGACGAAGAACGTTTGTCTAGACATGGGTCAAAACTGCCGAAGGGAGCTAAAAAGATCACAAAAGAGGACTATTTTCTGTGCCAAAAGCAGTTTCGCGTGTGGCTGGCACAATCCAGGTACGTGGCAGAGTCGGCGATTCTGCGACTGAGACCAAAAATGGATCTCATTTGTGATGATTATTTTGCAACGATAGGAACAAATATGTCGATGACCTGTCGACGGACGAGGCCATGGAGCTGTTCACGGACGAGTTTGCGAAGAAATGGAACCGTGGGAAGCTGTCCAAGATGTTCTACCAAGGCCTCCCCGACGCTGTGGTGGAGCAGACGAAGCGGACGCGTCATCAATGGGGTTTTGTGTCTAAACTGGGAGACAGGGAGAAGTTTGAGTTGGCAACAGCCAAAGATTCGGTGGATGTAGCCACAAAGAAGAAGAACTTGCTGGTGTCTGACGAGAAAGCGAAGGGGGGAAGAGAAGAAGAAGAAGGAGACAGGAGCAGGTCTAGACGTCGAGCTAAAGAGAACGGAGAGGGCGATAGAGAAGGAGACAGAAAACGTCGCAAGCTGGAGCGTAGAAGAGATCGAGAGTACCGAGACGTGGTGATGGATGAACTCGCGCCCAGAGCGACGGGAAGAGAAGCGCAGATCGAGAAGAGACGCCAAGTTGGAGACAAACTGCACGGAGCCGCGAGAGACCGGGAAGATACGCGAGATGGACTTGATCTAAGTGACGCCTTCTTGATGGGCGGAGGTGGAGGTGATGACGATCTGAAGCGCCGCATGGCTCAGAGGGATGTGGCACGACGTCGCAAACAGGAAGAGCAGCAGGACAAACTAGCGGGTCTGAAGGTACGAGGCTTTTTTGGAGAATATTGTAACGTGGGTTGCCGAGTTGATGAGGTTGTGCTTGATCGTGCTGCTAGGCGAAGGAGTCGGCGCGTATGGACAAGTTTTTGGAAGATATGGGCCTTGCTGGACCCAACGCGAATGGTGGCAAGCCCATGACCATTGCGCCTCGTCGATAGACACTCGCTGCATGGATTCTCGAAGGACGTAAAACCAAGGAAAATATCATCTGTGTAGTATTAGATACGCGGTAGTGTGGGTTGTCAGTAGCTTCGCGCTCGCTTCATCCGCCCCAAGCTGTTTGCGTACGACTGCTGGGATTTTCTTTGCAATAATCCATGTGTTGATCAGTGAGCTCTCAAAATCGGTGGCGATGTTCTTGGTGCTTTGAACCTCCTTCGCTGGGATCAGCATCTTCACGAGAGCTTTGTCTCCAAAGAATTCCCGGAAAGTGTCAACCATTGACACTTTCGATCTTGGGAACTCGGCGTTGAACTCCTCCAAATACTTGGCCCAAGTATTTAGCAACGGGCTGGACAGAACGTCGTCCCCCCGATCATCAAGCTTTTTTTAATATATCCGCCGGGGTCTTTTTACTCGTCAGCCAACGCTGGACCTGCTCACTCTGCAGCTTGGTCGTAAGCGTTTCCATCGTTGGAATCTCCTTTGTTTTGACCAACATACCCGCGATCGATTTGTCATCAAAGCGCTTCCGCAACGTATCGATCATCGAGACTTTGTCTTCCGGGAATTTGGCGTTGAACTCATCCAAGTACGTGGCCCAAGTGTTGAATAGCGGACTGGTGAGAGCGTCATCTGTTGCCTCGTTAAGCTTGAGGGCCATGAAGATCTTGGTCGGCCTGTAGCTTTGTAGCGAGCTTCTCCGTTTGTGGCACTTTCTTCGCTGCAATGAGAAGTGTTGCCAGTGCGTCGTCTTCGTAGTTTCTCGCAAATGCTGATATCATCGAAGTTTGCCTATCTGGAAACACTTCCTTGTGGTGCTTTGTATACATGCTCCAGAGAGGGCTGGCAAAAGCCTTTTCACCTGCCTCGTTCAGCTTGAGATTCTTAAAGACGACGTCTGGAGTATTCCCGTGCAGTATCCATCCTTTGAAGAGCTCGTCTTCCACTCGCTGAGCCATGTCCTTCGTGCCAGGAACCTTCATCCCCGCAAGAATCATTTTCGCCAGCACGTCGTCGTCATAGTTGTCCCGTAGAATGTTCCTGTAGCATACTATCCATCATTTCGGACCAGAGGTAGTAATAAGCGAGACTTGGCGGTCGTTGTTTTCCGCCTTCAGGTTGAGATTCTCCACATAAAATATTATTTGCCCCAGTACTTGAGCTGAGGGTTGACTAGAAAATCATCGCCCGCCTTGTCGAGTTGCAGCAATTGAAGACCTTATTTTTCTCCCCATTCAATCAACCCACCTATGGAAACTTCGAGCACCCTCCAAAAGGCTTTAATTCCACCACAAAAGGTGTACA

>Contig_73

TTTGGGCAGCTGACTGCCCCATTGTACAGAAGGCCCAGCTCTTTAGCTTCTTTAGCTCGGAGGCGTCAATTTGCCTGCTTGTGACTGTGTGGTTCTCCACTTGCATCCTCTCGCAGATGTTCCGGTGGACCAACCTAATGCCAATGCTACGCCGAGCGAATCCCAGAGTCACGCGGGCGCTCGCAGCCTAATACTACACCTTTGCCAGTCCAAATCGGCGACAGTGTAAAGCAAAGTGGAAACGTCCCGTAATTAATTTTTTTTAATTTTTTTTTGCAATTTATTTTTTAGAAAAAGCCTGTCATGCAAAGTTGATTTAGTGTAGTGCAAAACTTTACGTGCCGTACTCCATAGGTTCGCTACCGAATAGCTGCCGCCACTGACAACGCACAGTGGCGCGCCATCTTGTTAACCAAGCGCAGATCCAAACATGGCAAGCTATGCTCTGCAGCGTTTTCAAGCAGCTGCATCCGGCGCTCTGTTTCTTCTCCGTGTGGAGCAAGAAACATTACGTCATGACTTAAGGCAAGATAAGCCTTAATGCGTGCTTTTAATACACTATGTACATGTAAAACACCCTGCCGCACAGTAAAATAGACACAAATGTTAGTACATGTGCACGCTATAATTGTCTGTAAAACGTACCCTCAATCGGGGTTACACTGGGGGAGTATGGCGCCAACCGCAACAGCTCCAAGTCCTCCTGCTCAGTAATCAGATTCCTCAGTCTGGCTATGAGCAGGAGCGTTGTCTAAGACAATGACAAACCGTCTTGCCAGCGAAGTGGTCGCGGTACACGTCGTGGCGCTTGACCGCCTCGTACACCGCGTCCACAAACGCAGCATTGACCTCCATTTTAATGCTGCCTCTCTGTTTAGCGTAGTGCACCAGGCATCTTGCACGAGCGTGTATTGGCAGTTATCCTCCAGGTATTCTACGAGGGCCTCCTCCATCTCCGGCGTGCACTTAGTGCATGTCGCCCGCGCGCCCGCCTCTCTTCTTGATGTCCGGGGGTTCCTCGCTCGACGATAGTCCGAGCCGTTTGTAGCTGGAATTCCGTTGCAATCGGCTACTAAGGCCCAGTCCTGCCCAGCCTTGTGTGCGTCGAGAACGCGTGTCCGGGCCGCGAGGCCATGCTTTGGTTCTGTTGGCATGATGCCAGAAGAAGAGGTTTTGATGCCGACCACAGTTAGCTCTGCACTAAATTCGCCATTGCTCTAAAGTCAAATTTTGAAAATAAAAATTAAAAATTTCTTTGTCACTTAAGTTACTTCCACACCGCTTTTGGACTGCAAAGGTGTAGTAGTACGTAACTACTACTGTAGTAGTATTCCGCTTCCAAATGTAATAACGTTAAAAAGTTTAAAGATTCTCGTTTGAAGTTCGTGTATTTAGCGGAAGAGCCACTGTACTATTACTCGATTCAGAAATGAATTGGACACAACAAATTTTGATTTGGTACACAGTCAGCTGTGGACGGCGTCTGCCGCTAGTCCAGTCTCGCTGTGTGATTGGCATCGATCTCCATGGGCTTCTTTGCGTCTCCTTGGAGCGGCGGCTGTGGCTGCTGGCCTGAGCAATGCACACCGTGTCCACACCAGCTCTATCGCGCTGCAGACGCCGACTGTGATCCTGCAGCAGGTAGACTTTGAGGCGTCGATCGAGCTGCCGAGCTCAAGAATGGTTCGTGATTTTGTACAGTCTCCGTTGCGGGCTTGCAATGCATTACAACTAACATTTCTATAGGGTCTGTATACCCGAAGCCTTTGTGGTATCGCCGTAGAGGATTCGGACGGGTCTGTTGTGACCAATGGCACCGTCAAGACGCTCGATGTCGATGGCAATTTCAGGTCTCAGCTGTCGATCCCTATCAAGCAGTTGCAGTTGGACTCTTATGGAGAGCACAACCTCACTACAACCGTGTGGGAGGAACAGAAGATTGAGTCGCTAACGCAATCGCCTGAAGTGAATGAATCAATCGCCAACACCTCGGTGCTGCTTACGTCGACGGTACAACTCGATGAGGTGCAATTCGACGATGTGCTGCTGTTCCGTGCTACTCACACTACCGTAGTAGTTGTGTCACCGGGGTGGGTATCACTGCTACCACCACTGGTGACTCTGGTAATGTCGGCTGTGCTGGGCCAAGTAACAGTATCGCTTCTGGCCGGTATCTGGTGTGGCGCCATCATCGTCTCAAATGGAGACCCGTTCACGGCTTTCCTGCGCACATTCGACCAGTACTGGGGTGAATGCGTTTACAGTGGACGATCACGCTGGTGTGTTGCTATTCACGATTGTTCTTGGTGGGACTATTGGCGTGTGTTCAGAAGGGTGGAGGCGGCCACGGTTTAGCGCTGGTTGCCAAGAAGTTTATGACGTCGTCTCTGCGTATGCAACTGTCGACCTGGCTATTGTGTCTTGTCATCTTCTTCGATGACTACTCGTGTATCCTGATTGTTGGAAGCTCGCTACGTCAGGTGCTGAGTCAGACGGGTGTCAGTCGCGAGAAGTTTGCGGCTATTATTCACACTGTCGGCGTCTGCTTGCCATCCATGTGTAAGAACCTTGATCAGACCTACCGTTGCTGCTGTAGTACCTAACTAAGAATCGGAAGAGCGTCGTGTAGGGAAAAGTGTGCCTCCGGTATCTCGGTGGTCGCCGTATCATTAATGATACGGCGACCACCGAGATACCGGAGGCTACACTCTTTCCCTACACGACGCTCTTCCGATCTCAGCGCGCATAATGTCCAGCGTACCTGTCGGGTTACTCTCACGCGAACAAACACCACATCGCGCGTGAAAACGTGTTTGTAGCGGTCATGTTGAAGTGTCAAGATCGTGTCTCCATTTTGGGCAATTTTCTAGTCCCGACTTCATTTTAAGTACGACCTTTTTTGCCAGCCGAGATGGACTACTTTTTGGGTCCCAGAGAATTTGAGAATCAACCATCTTGCCGCTGCGAGCGAGTAGAGGAGACCCATATTTGCTTGGTGGACGTCGTCAAACTCACCATACGGTGATTTGCGAGTCTGCTGATGGCCAATGACAGCAGCTGTTCTGCGCGGAGACTATCTCAAGATGATATTTTGTGCTTTGGGCAGATCTGCGGTGCTGAATCGGGTAGCTCTGCGTTTTTTAGTGCCTCTTCAACCCACTGCGTGTGACAAGGCTTTCGGCGACACAAGGTGCAGCACATGTTTAGGCTGGTTTGCATAGTTGGACTGGTTGCAGATTCGACCGCCAGCTCCGCCGTAAAATCCAGAGATTTCTAGCAATACCAGTGGCCACGTTTTCCGCTGTTCAAATGCGCTAGCGCGTCTGGCTTCTTAAATGTTCATACTGCGAGCAAGGTGAAATATCGAGTATAATTTTGCTCGATGTTGGCTCGCTGAAGGCGATTCGTCAGGGTGCGATCCGATGCGCATACGCTCGTGAAACGCGGCAAAAAGCTGCCTGAGAAGGTTTCCAAAACTTTTTTCGGGGTCAAGACCAATTGGTACGGATACGAATTATGTGATTTAATTGTTTTTTTACTTCTAATTGCTGAATGTGGATGTTAATTGCCCGTCTTTAGGAAAATACTTCGAAAGTAACAAGGAGTTTTGTCGGAAACTCCGGAAAGCGCGATTTCCTTATATTTTACGTGTACTGAGGATTTAAGCTGTCTTAGCAACAAAGCTGTAAAAGGTTGGCCGAGTAAGTAGCGAGCGTCGGTTTGCAGGTGTTGCCCGTGATCGCAGCGGTCGCAGCAGCCGCCATGCAAACTCCGATCCGCTTCGCATGTTGCTCTCGATGGCGTGCGTCGAGCTCTTTGTGGATGCACAACTTTTGAATGGCAAGCTACATGTACCCATTACGGGTGGCCACCAGTCTTACAATTGAAGTTACAAGGGGGTGCTCTCTGGTCTCGCGTAGCTTGTCCGTTTTGCCCTGAACCTCAACGCATCATTCCTCAACTTGAGCTCGCCGACGCTGATGCCTGTCGATGCTGTGAAGGCGTCAAAATGAGCTAACTCCATTTGCTCCTAAGTGAAATGGCGAAACTGTGAGCCGAACTTCAACTGCCTAGATGCCTTCGACGTGCAGCACCCTCGAATCTGGACTGAGTCGCCTTCTTTAGTATCGTCGCGTCCACTTGCAAGACATCACTTGGTGCCGTCATCATGACGCTGTAACAAACGACAYTCTAGTTTCTCTTTAAATTATGTCGCCGCCAGTGCTACGTCTGGATCCGAGGACAGCGCAAGTGCTGACACTTCAACTTCTAGTTCGACTGACACCCTGTCCGCTTCAGGATCCGATGCTGCTTCGACGTCTGGCTCTGGTGATAGCCCCATGGCCTCATCAACCGGTTCGTCGGCTACCCAGAATACTGGAGGAGGCGACTTTCTTGCGCCCTTCTCGACTGAAGAACCGTCGACAACTCTCTTCCCTCGCAGTGCACTTGGTGAGATGGCTCCGATCATTAACGTTGCGGAAGGTCTGCTGGGCAAGCCACTTCCAACGAACAAATGGTGGGGTAACATCATCCACACTACCGCTGAAGAGATGAATACGAAAGCCAACCCTGGCTGGTCCAACCCGTACGCCGTCAAGCTGCCGAAGGAAGCTCCGTACGGCATCCAAGCTTGTTACTCGTACAACTACCGGCAGTTGTCCGCTATCACCGATGGCGTCGCGGAGCTTTATCTGCACGACTTTGTCAACGACCTCACGCTGTCGGCCACGGAATTTGCCGGTGAAGCCAAGCCCACGTATGAGATTTACGCCTTCAGCGACTTTGGCATCAACGTTCGAGCCTGTCTCGAGAACAAGGAGCAGTGCTTGGACTCTGCTCTTGTGCACGGCATGGCCTTCATCACAGCCACGTACGACCGTCTGACCGCTCGGATCGAGTCGGAATACACCATGGAGATCGTGGACAAGTCGGTGCCAGGCAAGTACATCCTTCAGCTTGGTGGGAACCAGACGTGAGTCGTGTACACTGGTAACAACGGCAGCTTTGCGCTCGACGAGTCGGGCAAAGCTCTCGTTTCCAGTGGGTTGTTCAGTGGCACCGTGCGTGTTGCCATTCTACCATCGAAAAAGGCCACTACCGTCTACGATAAGTACCGAGCGTGTCACGTCCGTGGTGGCCACGTGTCTGTGGAGTCTCGTACTCAATACTCGGTAAACTGGGAAACGGTGGGGAAGAGCTGTAAGACCAACGGGATGCTTCACTTCGCACTGCCTCACCACCTCCCAGCGTTGAAAGGAGCCATCACTGCTAAAAGCCCCAAAGCGATTGTCCTTAATTCGGCCACTCGAGGCAAAATGGTCGCCCAAGTAGCCACGACTGGAAAGTGGGTCCTTTCGGAGCCTGAAGATGAGTTGGAGGTGGACTTCTACCCAACCAGTAAACCGTCTGCCGAGGTTGTAGCAAAAGTGGGTCTTCTCCAGACTCTGCAGGCCGATATCGCTGACCACTGGGCGCTCAACAAGACGAGTTGGTACTTTAACGGCAAGCAGTACCAGAAGTACGCGTCGGTGTGTCTCATGGCTGCAGACTCGGCCATCGTAGGCAAGGACAAGAAGCTGCTCAGCGCATGCCTGACGAAGCTCGAGAAGCTCCTTGTGCCGTTCCTGGACAACACGCTGGACCCTCCTCTCCACTACGAGACGTCGTACGGCGGTCTCGTCAGCAGTCAGAGCTTCACGGTCCAAGACGTGAACGCAGACTTCGGGAACAGTGTCTACAACGACCACCACTATCATTACGGCTTCTGGGTCACCGCGTCTGCCATGCTCAAGAGTCTACACCCCAAGTGGGAGCGCATCAAAGAGCTGGACAGGATGATCTGGATGATGCTACGCGACGTGGCCAACCCCAGCACAGACGACCCTTTCTTCCCTCAGTTCCGCCACTTCTCCTTCTACCTCGGCCACTCGTACTCGCACGGTGTCACGCCCATGCTCGACGGTAAAGACGAAGAGAGTACGTCTGAAGATGTGAATTTCTACTACGGTATGAAGCTCTGGGGTCAGGTGTCCAGCAACAAGGCCGTCGAGGATCTAGGCAGTCTCATGCTACGTCTCAACGCCCGCGCTATCCGCACCTACTTCCTCATGACGTCGGACAACACCATCCACCCTACTGAGTTCGTACCCAACCACGTGACGGGCATCTTCTTCGACAACAAGGCGGCGTATGCGACCTGGTTCAGTGGCGAGAAGTACGCCATCCACGGCATCCAGATGATCCCAGTGTCGCCAATCAACGCTATGGTGCGCACGACAAAGTTCATCCAGCAGGAATGGGACGATATCCTCTCCAAGCAGCCAATCGTCACGGAATCCAACACCTTCAACGCGTGGTTATCGCTGCTTCTCGTCAACCAGGCTGCTGTGGATCAGGAGGACGCGCTGACCAAGCTACAGGAAGCCACAATGGACGATGGTCTCACCCGTTCTTGGGCTCTATACAACGCAGCTTCCCGTCCTCACAACGCCAAGCAGGTGGATGTAGCTTAGAAAAACGTACAAACTACGTAGATGTTGCACTTGTGTCGCTCGAATGAACTAGTTTTGCGTCGGTTTTGAGGCTTTCATGGCTCCACATGATCATTTGCCGAGGATTTCTCTGTTTGAAGCGTCAAGTCATTACGATCTCCATCTAGCAAGCGTACGAGACGTTGGGCTTGGACCGAGATGTTACCCAGGAAAAAGTGAAGAAGACGTATCGAAAGCTGCCACTGCAGTTCCACCTGGACAAGATTTCGGATCCACCAGCAACAGTAAAGATTCTTGTTTGCTTTCTCAGCTTTGTCTCTTTGTTTGTACATTGATACTAGTAGTACGACGTGCCGGAGCTCAACTAATCTAGTCCGACTTTCTTGGCGGTCACACTGTCATGTGGAAGGCCGTACCGCTCGCAAACTTCCTTCATGTGAGTTATCTGAAGCTGAAAAATATATTATGCAAAGTATGTTCAAGCGGTTAGTAACTCACTATCCGAGTGGAACCGAAAAAGCTGTAGACGAATGTATTCGACACTGTAACACATTTGAGGTAGTTGGCATTCACACACCGCGTCTCCTTCTGAAGATGTTCACGCTGCTGCTATGACGTCGTTGGTCACACCAGTGAACCCATGCTCAAGAACAATATTCAAAGGACCTCACGCTTTTTAAAAACCTCCTTGGTGACCTCACAACGGCGGCTACGACTTCACGCTGACTGTCAGCATTCATTTGGCATCGTTCACTATTGTATCATGCACTAGTATAACTTGTAGTAGTGAGCGCAGACGCGTACGGTGGCATTGCACTGTTGCACACATTGATGCTGACAAAGAAGAATTATTTGGATGTGATAGATAATATCTATTATCCATTTTTGGATTGTAGCGTAATAGTTGGTGTGGTACGCAAACCTTTGCAAAATTGAAGCTGCCTAGCCAATCACAGCACACGACGTTTCGCTACATGAGCAAGTTCGGCTGCTTGGGGCATCTGTACCGCTCTTTTCTTAGGACTGTATCTGCTTAAACAGCATTACGATTGCGCGGGTAGAAGTACTCACGCAGTATTGAAGGCATGGAGTGTAAGCCCCACACACCACAAAGGCCATCCGATTGCTTCGACCCACGACGGTTCATGGTCAATTGGACCGCTCAACAGCATCGAAAAGAGGCATTCACGGTCCTGCAGCTCCGTCTCCTCTGCCATCGACATTCATGCGGAGAGTGGACTCTCCCGTTTCGGTCGCTGCGTTTTGCTGCCGCGCCACTCGAATGGGGCAGCACATTCTGCAATTGGCCACGAATTGCTGGAGAACTCACCAACCTATCGTCGTTCCTCACGCAAACACGACTCGTCGACCGACGGTGATTATGTTGAGCCTCTTTCAGCCGGTGTAACACACCCGGCCCCACGCATCAGAGACCAGGCATGTCGCCGCCGCCGACATTAGCTCACCATACCGAGTAGCAGCTGGTACGGTGAATATGCTCGGTCAGAGTTCATACCCGACAGGGCTTTCATCGTGAAAGCTTATACACAGCTCTACTGGGTCGGCTCTGTTCGGGGTATGGTGTCCCATCCAGCCTGCCGATGAGATCATGTAGGATCAGCTTGACGGCTACCACATCGCCCTACCGCACCAATCGCGCCAACAGGAGTCATGCACGACCAAGGTCACTCCCTACACTGCAGTGACGGCCGCCACCATCAACAGTAGAATCAGGGCAGCTACCAAATACTCATTAATGCTTGTATCATATCTGGATAAAGGCCTGGTGGCAATCGACTTGTAGCTCAGTATCTCCACTACGGCAACACTTACGACATCCCATTACACGCCGGCGCTCGACAGACCCACTACCCGCCAGAGTTTGTCACCACCCACTTTTCACCAGAGCGCCACACTACCCGCTGCCCGCCAGAGCTCGTCTCCACCCACTGCCCACCAGACCTGAGCAGATCCAAGAAGATCAGCGGCAATACAGCGGGACTTCACACGCGAGAAGAACTTCGTCAGTGCAGTGATGCATCGAGTGCCCAGTTCATCCGTCCGCGAAAGCTGTCCCGCTCCCTCACTGTGGCATCGCCACGCATCTTTATATCGATATGGACATACGAGAAGTCTCACCACCGGCGAATGCCCTTCGACAACACGCTCTACTGCGAGCTTTTCCTTAAAATCCCGCGCAGCACAGGACCCAGTGGCCAGACACGATAAGTGCGCGTGGCACTGCGCCGCAGTAGCTCGTGGCTCTGGCGTGAGCGACATCAACCTCGGCGTCGCTGCAGCAGCCACAGTGGCACTGTCAGTGCCCAAGCTCAGCTAGCGTCGGTGCTCAGTACAGCCAGACCAAATCGGCATGGGCCGAGATCGGCATCAATGTGCTCAAGGCATTGCACGTTGACTTCACCCCAATTGCTACAACCCACCGCCGTACTACCCCACTGTGTACTACACGCGCATGAGTGAGTACCCGCACAGCCCGCACATTGTCACCAATCCGAACTCGGGATCTACGAAGGGCCACATCCTACAGAAACCGATGCAGCCATNGGGGGGGGGCTTTCGCAGTCAGCGCGCATAATGTCCAGCGTGCCTGTCGGATACTCTCACGCGACAGAACCACATCGCGCGTGAAAACGTGTTTGTAGCGGTCATGTTGAAGTGTCAAGATCGTGTCTCCATTTTGGGCAATTTTCTAGTCCCGACTTCATAAGTACGACCTTTTTTGCCAACCGAGATGGACTACTTTTTGGGTCCCAGAGAATTTGAGATCAGCCATCTTGCCGCTGCGAGCGAGTAGAGGAGACCCAAATTTGCTTGGTGGGCGTCGTTAAACTCACCATACGGCGATTTGCGAGTCTGCTGATCGCCAATGACAGCAGCTGTTCTGCGCGGAGACTATCTCCAGATGATATTTGGTGCTTTGGGCAGATCTGCGGTGGTAGTTCTGCGTTTTTGTGCTCTTCACCACTGCGTTTCGGCGACACAAGGTGCAGCACATGTTTAGGCTGATTTGCAAAATTGGGCTGGCTGCAAATTCGACCACCAGCTCCGCCGTGAAATTTAGAGATATCTAGCAATACCAGTGGCCATGTTTTCCGCTGTTCAAGTGCGCTAGCGCGTCTGGCTTCTTTAAAGTTCATACTGCGAGCAAGGTGAAGTATCGAGTATGACTTTGCTCGATGTTGGCTCGCTGAAGGCGATCGTCAGGGTGCGATCCGATTCGCATACGTTCGTGAAACGCGGCAAAAAGCTGCCGAGAAGGTTTTTTAAAACCTTTTTCGGGGGTCAAGACCAATTGGTACGGATACGAATTATGTGTTTTATCGTTTTTTCTACTTCTAATTTGCTGAATGTGGATGGATGTTAATTGCCCGTCTTTAGGAAAATACTTCGAAGTAACAAGGATTTTGTCGGAAACTCCGGAAAGCGCGAGTTTCTTATATTTTACGTGTACTGAGGATTTAAGCTGTCTTAGCAACAAAGCTGTAAAAGGTTGGCCGAGTAAGTAGCGAGCGTCCGGTTTGCAGGTGTTGCCCGTGATCGCACCGGTCGCAACAGCCGCCATGCAAACTCCGATCCGCTTCGCATGTTGCTCTCGATGGCGTGCGTCGAGCTCTTTGTGGATGCACAACTTTTAAATGGCAAGCTACATATACCCATTACGGGTGGCCACCAGTCTTACAATTGAAGTTACAAGGGGGTGCTCTCTGGCTTGTCCGTTTTGCCTTGAACCTCAACGCATCATTCCTCAACTTGAGCTCGACAGCTCAATCTACCCAGAGACGCGCCGACGCTGATGCCTGTCGATGCTGTGAAGGCGTCGAAATGGTCTGCAAGAGCTAACTCCATTTGCTCCTAAGTGAAATAGCGAAACTGTGATCCGACCTTCAACTGCCTAGATACCTTCGACGTGCAGCACCCTCGAATCTGGACTGAGTCCGATGCTGCTTCGACGTCTGGCTCTGGTGATAGCCCCATGGCCTCATCAAATGGTTCGTCGGCTACCCAGAATACTGGAGGAGGCGACTTTCTTGCGCCGTTCTCGACAGAAGAACCGTCGACAACTCTCTTCCCTCGTAGTGCACTTGGCGAGATGGCTCCGATCATTAACGTTGCGGAAGGTCTGCTGGGCAAGCCACTACCAACGAACAAATGGTGGGGTAACATTATCCACACTACCGCTGAAGAGATGGATACGAAAGCCAACCCTGGCTGGTCCAACCCGTACGCCGTCAAGCTGCCGAAGGAAGCTCCGTACGGCATCCAAGCTTGTTACTCGTACAACTACCGTCAGCTGTCCGCTCTCACCGATGGCGTCGCGGAGTTTTATCTGCACGACTTTGTCAACGACCTCACGTTGTCGGCCACGGAATTTGCCGGTGAAGCCAAGCCCACGTATGAGATTTACGCCTTCAGCGACTTTGGCATCAACGTTCGAGCCTGTCTCGAGAACAAGGTGCAGTGCTTGGACTCTGCTCTTGTGCACGGCATGGCCTTCATCACAGCCACGTACGACCGTCTGACCGCTCGGATCGAGTCGGAATACACCATGGAGATCGTGGACAAGTCGGTGCCAGGCAAGTACATCCTTCAGCTTGGTGGGAACCAGACGTGGGTCGTGTACACTGGTAACAACGGCAGCTTTGCGCTCGCCGAGTCGGGCAAAGCTCTCGTTTCCAGTGGGTTGTTCAGTGGCACCGTGCGTGTTGCCATTCTACCATCGAAAAAGGCCACTACCGTCTACGATAAGTACCGGGCGTGTCACGTCCGTGGTGGGAACGTGTCTGTGGAGTCTCGTACTCAATACTCGTTAAACTGGGAAACGGTGGGGAAGAGCTGTAAGACCAACGGGATGCTTCACTTCGCACTGCCTCACCACCTCCCAGCGTTGAAAGGAGCCATCACTGCTAAAAGCCCCAAAGCGATTGTCCTTAACTCGGCCACTCGAGGCAAAATGGTCGCCCAAGTGGCCACGACTGGAAAATGGGTCCTTTCGGAGCCGGAAGATGAGTTGGAGGTGGACTTCTACCCAACCAGTAAACCGTCTGCCGAGGTCGTAGCAAAAGTGGGTCTTCTCCAGACTCTGCAGGCCGATATCGCTGACCACTGGGCGCTCAACAAGACGAGTTGGTACTTTAACGGCAAGCAGTACCAGAAGTACGCGTCGGTATGTCTCATGGCTGCAGACTCGGCCATCGTGGGCAAGGACAAGAAGCTGCTCAGCGCATGCCTGACGAAGCTCGAGAAGCTCCTTGTGCCGTTCCTGGACAACACGCTGGACCCTCCTCTCCACTACGAGACGTCGTACGGCGGTCTCGTCAGCAGTCAGAGCTTCACGGTCAAAGACGTGAACGCAGACTTCGGGAACAGTGTCTACAACGACCACCACTATCATTACGGCTACTGGGTCACCGCGTCTGCCATGCTCAAGAGTCTACACCCCAAGTGGGAGCGCATCAAAGAGCTGGACAGGATGATCTGGATGATGCTACGCGACGTGGCCAACCCCAGCACAGACGACCCTTTCTTCCCTCAGTTCCGCCACTTCTCCTTCTACCTCGGCCACTCGTACTCGCACGGTGTCACGCCCATGCTCGACGGTAAAGACGAAGAGAGTACGTCTGAAGATGTGAATTTCTACTACGGTATGAAGCTCTGGGGTCAGGTGTCCAGCAACAAGGCCGTCGAGGATCTAGGCAGTCTCATGCTACGTCTCAACGCCCGCGCTATCCGCACCTACTTCCTCATGACGTCGGACAACACCATCCACCCTACTGAGTTCGTACCCAACCACGTGACGGGCATCTTCTTCGACAACAAGGCGGCGTATGCGACCTGGTTCAGTGGCGAGAAGTACGCCATCCACGGCATCCAGATGATCCCAGTGTCGCCAATCAACGCTATGGTGCGCACGACAAAGTTCATCCAGCAGGAATGGGACGATATCCTCTCCAAGCAGCCAATCGTCACGGAATCCAACACCTTCAACGCGTGGTTATCGCTGCTTCTCGTCAACCAGGCTGCTGTGGATCAGGAGGACGCGCTGACCAAGCTACAGGAAGCCACAATGGACGATGGTCTCACCCGTTCTTGGGCTCTATACAACGCAGCTTCCCGTCCTCACAACGCCAAGCAGGTGGATGTAGCTTAGAAAAACGTACAAACTACGTAGATGTTGCACTTGTGTCGCTCGAATGAACTAGTTTTGCGTCGGTTTTGAGGCTTTCATGGCTCCACATGATCATTTGCCGAGGATTTCTCTGTTTGAAGCGTCAAGTCATTACGATCTCCATCTAGCAAGCGTACGAGACGTTGGGCTTGGACCGAGATGTTACCCAGGAAAAAGTGAAGAAGACGTATCGAAAGCTGCCACTGCAGTTCCACCTGGACAAGATTTCGGATCCACCAGCAACAGTAAAGATTCTTGTTTGCTTTCTCAGCTTTGTCTCTTTGTTTGTACATTGATACTAGTAGTACGACGTGCCGGAGCTCAACTAATCTAGTCCGACTTTCTTGGCGGTCACACTGTCATGTGGAAGGCCGTACCGCTCGCAAACTTCCTTCATGTGAGTTATCTGAAGCTGAAAAATATATTATGCAAAGTATGTTCAAGCGGTTAGTAACTCACTATCCGAGTGGAACCGAAAAAGCTGTAGACGAATGTATTCGACACTGTAACACATTTGAGGTAGTTGGCATTCACACACCGCGTCTCCTTCTGAAGATGTTCACGCTGCTGCTATGACGTCGTTGGTCACACCAGTGAACCCATGCTCAAGAACAATATTCAAAGGACCTCACGCTTTTTAAAAACCTCCTTGGTGACCTCACAACGGCGGCTACGACTTCACGCTGACTGTCAGCATTCATTTGGCATCGTTCACTATTGTATCATGCACTAGTATAACTTGTAGTAGTGAGCGCAGACGCGTACGGTGGCATTGCACTGTTGCACACATTGATGCTGACAAAGAAGAATTATTTGGATGTGATAGATAATATCTATTATCCATTTTTGGATTGTAGCGTAATAGTTGGTGTGGTACGCAAACCTTTGCAAAATTGAAGCTGCCTAGCCAATCACAGCACACGACGTTTCGCTACATGAGCAAGTTCGGCTGCTTGGGGCATCTGTACCGCTCTTTTCTTAGGACTGTATCTGCTTAAACAGCATTACGATTGCGCGGGTAGAAGTACTCACGCAGTATTGAAGGCATGGAGTGTAAGCCCCACACACCACAAAGGCCATCCGATTGCTTCGACCCACGACGGTTCATGGTCAATTGGACCGCTCAACAGCATCGAAAAGAGGCATTCACGGTCCTGCAGCTCCGTCTCCTCTGCCATCGACATTCATGCGGAGAGTGGACTCTCCCGTTTCGGTCGCTGCGTTTTGCTGCCGCGCCACTCGAATGGGGCAGCACATTCTGCAATTGGCCACGAATTGCTGGAGAACTCACCAACCTATCGTCGTTCCTCACGCAAACACGACTCGTCGACCGACGGTGATTATGTTGAGCCTCTTTCAGCCGGTGTAACACACCCGGCCCCACGCATCAGAGACCAGGCATGTCGCCGCCGCCGACATTAGCTCACCATACCGAGTAGCAGCTGGTACGGTGAATATGCTCGGTCAGAGTTCATACCCGACAGGGCTTTCATCGTGAAAGCTTATACACAGCTCTACTGGGTCGGCTCTGTTCGGGGTATGGTGTCCCATCCAGCCTGCCGATGAGATCATGTAGGATCAGCTTGACGGCTACCACATCGCCCTACCGCACCAATCGCGCCAACAGGAGTCATGCACGACCAAGGTCACTCCCTACACTGCAGTGACGGCCGCCACCATCAACAGTAGAATCAGGGCAGCTACCAAATACTCATTAATGCTTGTATCATATCTGGATAAAGGCCTGGTGGCAATCGACTTGTAGCTCAGTATCTCCACTACGGCAACACTTACGACATCCCATTACACGCCGGCGCTCGACAGACCCACTACCCGCCAGAGTTTGTCACCACCCACTTTTCACCAGAGCGCCACACTACCCGCTGCCCGCCAGAGCTCGTCTCCACCCACTGCCCACCAGACCTGAGCAGATCCAAGAAGATCAGCGGCAATACAGCGGGACTTCACACGCGAGAAGAACTTCGTCAGTGCAGTGATGCATCGAGTGCCCAGTTCATCCGTCCGCGAAAGCTGTCCCGCTCCCTCACTGTGGCATCGCCACGCATCTTTATATCGATATGGACATACGAGAAGTCTCACCACCGGCGAATGCCCTTCGACAACACGCTCTACTGCGAGCTTTTCCTTAAAATCCCGCGCAGCACAGGACCCAGTGGCCAGACACGATAAGTGCGCGTGGCACTGCGCCGCAGTAGCTCGTGGCTCTGGCGTGAGCGACATCAACCTCGGCGTCGCTGCAGCAGCCACAGTGGCACTGTCAGTGCCCAAGCTCAGCTAGCGTCGGTGCTCAGTACAGCCAGACCAAATCGGCATGGGCCGAGATCGGCATCAATGTGCTCAAGGCATTGCACGTTGACTTCACCCCAATTGCTACAACCCACCGCCGTACTACCCCACTGTGTACTACACGCGCATGAGTGAGTACCCGCACAGCCCGCACATTGTCACCAATCCGAACTCGGGATCTACGAAGGGCCACATCCTACAGAAACCGATGCAGCCATGGGGGGGGGGCTTTCGCAGTCAGCGCGCATAATGTCCAGCGTGCCTGTCGGATACTCTCACGCGACAGAACCACATCGCGCGTGAAAACGTGTTTGTAGCGGTCATGTTGAAGTGTCAAGATCGTGTCTCCATTTTGGGCAATTTTCTAGTCCCGACTTCATAAGTACGACCTTTTTTGCCAACCGAGATGGACTACTTTTTGGGTCCCAGAGAATTTGAGATCAGCCATCTTGCCGCTGCGAGCGAGTAGAGGAGACCCAAATTTGCTTGGTGGGCGTCGTTAAACTCACCATACGGCGATTTGCGAGTCTGCTGATCGCCAATGACAGCAGCTGTTCTGCGCGGAGACTATCTCCAGATGATATTTGGTGCTTTGGGCAGATCTGCGGTGGTAGTTCTGCGTTTTTGTGCTCTTCACCACTGCGTTTCGGCGACACAAGGTGCAGCACATGTTTAGGCTGATTTGCAAAATTGGGCTGGCTGCAAATTCGACCACCAGCTCCGCCGTGAAATTTAGAGATATCTAGCAATACCAGTGGCCATGTTTTCCGCTGTTCAAGTGCGCTAGCGCGTCTGGCTTCTTTAAAGTTCATACTGCGAGCAAGGTGAAGTATCGAGTATGACTTTGCTCGATGTTGGCTCGCTGAAGGCGATCGTCAGGGTGCGATCCGATTCGCATACGTTCGTGAAACGCGGCAAAAAGCTGCCGAGAAGGTTTTTTAAAACCTTTTTCGGGGGTCAAGACCAATTGGTACGGATACGAATTATGTGTTTTATCGTTTTTTCTACTTCTAATTTGCTGAATGTGGATGGATGTTAATTGCCCGTCTTTAGGAAAATACTTCGAAGTAACAAGGATTTTGTCGGAAACTCCGGAAAGCGCGAGTTTCTTATATTTTACGTGTACTGAGGATTTAAGCTGTCTTAGCAACAAAGCTGTAAAAGGTTGGCCGAGTAAGTAGCGAGCGTCCGGTTTGCAGGTGTTGCCCGTGATCGCACCGGTCGCAACAGCCGCCATGCAAACTCCGATCCGCTTCGCATGTTGCTCTCGATGGCGTGCGTCGAGCTCTTTGTGGATGCACAACTTTTAAATGGCAAGCTACATATACCCATTACGGGTGGCCACCAGTCTTACAATTGAAGTTACAAGGGGGTGCTCTCTGGCTTGTCCGTTTTGCCTTGAACCTCAACGCATCATTCCTCAACTTGAGCTCGACAGCTCAATCTACCCAGAGACGCGCCGACGCTGATGCCTGTCGATGCTGTGAAGGCGTCGAAATGGTCTGCAAGAGCTAACTCCATTTGCTCCTAAGTGAAATAGCGAAACTGTGATCCGACCTTCAACTGCCTAGATACCTTCGACGTGCAGCACCCTCGAATCTGGACTGAGTCCGATGCTGCTTCGACGTCTGGCTCTGGTGATAGCCCCATGGCCTCATCAAATGGTTCGTCGGCTACCCAGAATACTGGAGGAGGCGACTTTCTTGCGCCGTTCTCGACAGAAGAACCGTCGACAACTCTCTTCCCTCGTAGTGCACTTGGCGAGATGGCTCCGATCATTAACGTTGCGGAAGGTCTGCTGGGCAAGCCACTACCAACGAACAAATGGTGGGGTAACATTATCCACACTACCGCTGAAGAGATGGATACGAAAGCCAACCCTGGCTGGTCCAACCCGTACGCCGTCAAGCTGCCGAAGGAAGCTCCGTACGGCATCCAAGCTTGTTACTCGTACAACTACCGTCAGCTGTCCGCTCTCACCGATGGCGTCGCGGAGTTTTATCTGCACGACTTTGTCAACGACCTCACGTTGTCGGCCACGGAATTTGCCGGTGAAGCCAAGCCCACGTATGAGATTTACGCCTTCAGCGACTTTGGCATCAACGTTCGAGCCTGTCTCGAGAACAAGGTGCAGTGCTTGGACTCTGCTCTTGTGCACGGCATGGCCTTCATCACAGCCACGTACGACCGTCTGACCGCTCGGATCGAGTCGGAATACACCATGGAGATCGTGGACAAGTCGGTGCCAGGCAAGTACATCCTTCAGCTTGGTGGGAACCAGACGTGGGTCGTGTACACTGGTAACAACGGCAGCTTTGCGCTCGCCGAGTCGGGCAAAGCTCTCGTTTCCAGTGGGTTGTTCAGTGGCACCGTGCGTGTTGCCATTCTACCATCGAAAAAGGCCACTACCGTCTACGATAAGTACCGGGCGTGTCACGTCCGTGGTGGGAACGTGTCTGTGGAGTCTCGTACTCAATACTCGTTAAACTGGGAAACGGTGGGGAAGAGCTGTAAGACCAACGGGATGCTTCACTTCGCACTGCCTCACCACCTCCCAGCGTTGAAAGGAGCCATCACTGCTAAAAGCCCCAAAGCGATTGTCCTTAACTCGGCCACTCGAGGCAAAATGGTCGCCCAAGTGGCCACGACTGGAAAATGGGTCCTTTCGGAGCCGGAAGATGAGTTGGAGGTGGACTTCTACCCAACCAGTAAACCGTCTGCCGAGGTCGTAGCAAAAGTGGGTCTTCTCCAGACTCTGCAGGCCGATATCGCTGACCACTGGGCGCTCAACAAGACGAGTTGGTACTTTAACGGCAAGCAGTACCAGAAGTACGCGTCGGTATGTCTCATGGCTGCAGACTCGGCCATCGTGGGCAAGGACAAGAAGCTGCTCAGCGCATGCCTGACGAAGCTCGAGAAGCTCCTTGTGCCGTTCCTGGACAACACGCTGGACCCTCCTCTCCACTACGAGACGTCGTACGGCGGTCTCGTCAGCAGTCAGAGCTTCACGGTCAAAGACGTGAACGCAGACTTCGGGAACAGTGTCTACAACGACCACCACTATCATTACGGCTACTGGGTCACCGCGTCTGCCATGCTCAAGAGTCTACACCCCAAGTGGGAGCGCATCAAAGAGCTGGACAGGATGATCTGGATGATGCTACGCGACGTGGCCAACCCCAGCACAGACGACCCTTTCTTCCCTCAGTTCCGCCACTTCTCCTTCTACCTCGGCCACTCGTACTCGCACGGTGTCACGCCCATGCTCGACGGTAAAGACGAAGAGAGTACGTCTGAAGATGTGAATTTCTACTACGGTATGAAGCTCTGGGGTCAGGTGTCCAGCAACAAGGCCGTCGAGGATCTAGGCAGTCTCATGCTACGTCTCAACGCCCGCGCTATCCGCACCTACTTCCTCATGACGTCGGACAACACCATCCACCCTACTGAGTTCGTACCCAACCACGTGACGGGCATCTTCTTCGACAACAAGGCGGCGTATGCGACCTGGTTCAGTGGCGAGAAGTACGCCATCCACGGCATCCAGATGATCCCAGTGTCGCCAATCAACGCTATGGTGCGCACGACAAAGTTCATCCAGCAGGAATGGGACGATATCCTCTCCAAGCAGCCAATCGTCACGGAATCCAACACCTTCAACGCGTGGTTATCGCTGCTTCTCGTCAACCAGGCTGCTGTGGATCAGGAGGACGCGCTGACCAAGCTACAGGAAGCCACAATGGACGATGGTCTCACCCGTTCTTGGGCTCTATACAACGCAGCTTCCCGTCCTCACAACGCCAAGCAGGTGGATGTAGCTTAGAAAAACGTACAAACTACGTAGATGTTGCACTTGTGTCGCTCGAATGAACTAGTTTTGCGTCGGTTTTGAGGCTTTCATGGCTCCACATGATCATTTGCCGAGGATTTCTCTGTTTGAAGCGTCAAGTCATTACGATCTCCATCTAGCAAGCGTACGAGACGTTGGGCTTGGACCGAGATGTTACCCAGGAAAAAGTGAAGAAGACGTATCGAAAGCTGCCACTGCAGTTCCACCTGGACAAGATTTCGGATCCACCAGCAACAGTAAAGATTCTTGTTTGCTTTCTCAGCTTTGTCTCTTTGTTTGTACATTGATACTAGTAGTACGACGTGCCGGAGCTCAACTAATCTAGTCCGACTTTCTTGGCGGTCACACTGTCATGTGGAAGGCCGTACCGCTCGCAAACTTCCTTCATGTGAGTTATCTGAAGCTGAAAAATATATTATGCAAAGTATGTTCAAGCGGTTAGTAACTCACTATCCGAGTGGAACCGAAAAAGCTGTAGACGAATGTATTCGACACTGTAACACATTTGAGGTAGTTGGCATTCACACACCGCGTCTCCTTCTGAAGATGTTCACGCTGCTGCTATGACGTCGTTGGTCACACCAGTGAACCCATGCTCAAGAACAATATTCAAAGGACCTCACGCTTTTTAAAAACCTCCTTGGTGACCTCACAACGGCGGCTACGACTTCACGCTGACTGTCAGCATTCATTTGGCATCGTTCACTATTGTATCATGCACTAGTATAACTTGTAGTAGTGAGCGCAGACGCGTACGGTGGCATTGCACTGTTGCACACATTGATGCTGACAAAGAAGAATTATTTGGATGTGATAGATAATATCTATTATCCATTTTTGGATTGTAGCGTAATAGTTGGTGTGGTACGCAAACCTTTGCAAAATTGAAGCTGCCTAGCCAATCACAGCACACGACGTTTCGCTACATGAGCAAGTTCGAGATCGGAAGAGCACACGTCTGAACTCCAGTCACCCTCCGGTATCTCGTATGCCGTCTTCTGCTTGAATGATACGGCGACCACCGAGATTCCATTGGCTACACTCTTTCCCTACACGACGCTCTTCCGATCTAAAGCAGCATGTACGCGTTAGCATTGGCGAGTGAGATAGCGTCACAAGGGCAGTGACTTAGGGAGAGTGCCAGCTCGTACTTGCTTGTCCTTGTGCCGGAGGCGCGCAAACTTGTGCGTGGGTGTAAGTAGTGGACGGGTCTGTCTGAGCGCACGCCGTATGTAGCCTTCCTAGATCAGCTGCTGAGCCAAATAGCAGACGCGCGCTGAAGCAGACGCTGGTTTTCGCGCTCATTGACAGGAACTGCAGCGATTCTCTCGCACAGCTCAGCTCGATTCACCTTCTTCCTACCACATGCGCTCTTCCTTGACTTGACCACGGCTGCTACTCGATCTCCCATCGAATTAATTCCACGCCTCCAAATTCGTTCGACAACTTGGCGGCCCACACGGAAGAACGCTGCAACACGAACGATCTCTCCATGTTTGAGYACTCCTCCCACGCTGCGAAGCAGTAAGGACTCCCAGACGGCCTGGCGCTCACTGTCTGTGATCTCCCCCAGGTGACAGCTTTCAAAGGAGCGGTAGCCAGTCTGGTCGGGAAAGCGCGGGAGATTCGAAATCCAAAAAGTGATCGGYATAGAGGGGGTATAGTTTTGGGTAGGCTGTAATATAATTTGAAATGGTTATCTTTTTCAGAACGGAGGGTAGTATTTTGTTGGGAAGGCTGTAATATAATTTGAAATGGTTATCTTTTTCAGAACGGAGGTAGTATTACTACATAGTATGAGTATAAAAGGATTCTAAACTGGATTTAAAACCCATACATTGATTCATACGTAATCCACTATATGTTAACCCAAAACAAGAGAAGTGCTGGAAACTTGGTTTATCAATATTTCATCACTGTGGTCGTGACGTACTTCGCGTAATCTCCCCAAATTTCTTTCTCCCGACCATACCCATGAATTCTATTTCGATCAGCTTTCAGCACGTTCGTGAAAACCTCATCTGCTGTCTTGTACTTATCAACGAAGTACCACTTGTAGAACTGGGCCTTTTCTAACTTCTTTGCGACGTCGCGCGAGTTTCTGGACAGGTTCCCGACTAGGATCATCATCGCCACATTTTTCTCACCAAAGACATTTGTGAGTGCTTTAAGCATCTCGCCATAGGGATCATCTTTGTTGATCTTGACGGCGTGCTGGGACCAAATCTTGAAATTGCGACTTGACAGAAATTTTGCCACCATCTTGGGATCGACAGAGCCATCCTTGCCAATCCGCATTGTACCAAGTTTTAGCGTCTTGAAAGCACTCCCAAGGGCCTCGTCAGCCTTAAGCATTCCTTGCAGCTCCGTAGTTTTGGCTGACGTAACTTTTTTAAAGATTTTCTCCAGGCTTGAAAAATTTATCGAAATTGCCCTTTCCTCTTCTTCTTTTTGTTCTTCCTTGTCACTACTGTGGGCCCTTAGAAGCCTCTCAGTCGTGGCGTAGTCGACAACGCGAGTTGAATCGCTAGGGGCGGCCCTCATATTCAATTCCCTTGTAGCGGGGGGCAGGAGCAAAAAATGCAAGCAGCAGGAGTAAGACCAAATTGACAGCGTAGAGGGACTTCATCATCAGGTTCGTAGGTGAGTTGTTGGCCACAAGACAATTGAGAAGTGATAAGTGAACGGGTTCTACCGGAAACCTTCCACCGGTCCACCCCGTTCGTACGGGTATCCCAGCTCTTAAGCTACACAGACAGGGACGGTGAAAAAAAGCTACCGCTCTCTAAACTAGTTCAACGCTGGATAGACCTTGCGCATGTAAGTATCGCTCGGGTGTGGGGGACGAAGCGATTGATTGGTCACACCATCCGTCTAAGTGCGTCAACAACCAAGATGGACGATGCAACCGCTATCGGTATTGTGGTTTTTATCAATTATGCCGCTAAATAAAACGAACAGAAGCTAAGGGTAATCGGATGGGAGGCGAAAATCACAGACCCCTAAGGCCGTGTTCGATTTAAGCTAAAACCAGCTGTAATTGGTATTATAGAGCTGGAATGTCTCAACCAGCTTTATTCCGCTAAGAAACACAAAAAATGACATACAGCAGGATAGCTCCAAACGACTGGTTTCAAAATTAAGCTTTGGGTTGAATTTTGAATCGTCCTGCTTTGCGCCTTCGAATCCAGTCTTTTCTGATGGAACCACGATCACTTATTTTATCTTTATTTTTCTTAACAAAACGACTGTTCTACAGCTCTAGCTTATTTTCGAACAAGCTGGATTTGCCCCTGCAAGCTGAACACGGCCTAAATTTATTCACGATCGCCCATATTGAACTTAAGCTGTTTGCAAATGTTTAATCAACGGATATTTTTCCAAATACAGGCACAGGCGGGTTTCCTGTTACAAGCTAAATCTACAGTACCGAAACTGAGGTAGAGAAGTCCCCCTGAATAAGAGTAGCATCTACTTAATATACCCCTTTATATCGGATCATTTGGATCGGGGTACTTTTGACAAGAGCACAGATCGCTGTGGATTCCCTATGCCAAATTAATCCGACGACCTTTACGCTTACTGAAAAGGACTTCTGTGTCACCCATAATACTTCAAAGTATTAGTCGTGGCGTTGATATCTACTTGACTGAACAAAGTTCCGGGTGTATTTTGTACAAAAGGGAAATTTACATCATCACTTATGCACGCCAGTTTCACAGGAAACTATATCTTAGCTTAATGTGTAAGGCTCGAAGAATAACATTTACAGTAGTTAAGAGATAGCAGGATTAGCTTCGAAAAATTCTGAAAATAAAAAAAACTGAGAACCTTATCACTGATCGCATTAAAGATCAATGCGTACGTCGTATGAGTAGAAAACAGGCTACGATTTTTGGGTTAGCATCAGAGTGGTGAATTCACAGCAGGTCTCATTTACGGTAATATCTCCCATGGTCGCTATGTTGTAACCGTATATACAGTGCATTGTACTGTACACTAAAGGTAAAACATATGGATTGTAAGTGCTACTACGATATTAATAGCCTCCTCCACAAGAGCCCCCCCCCGCCCGTGGAGTAGTATTCAAAGCCACTCACTTTGAAATTTGCTCTCCACCTCTCAGGCTGCGATATACAC

>Contig_74
[truncated: 4,899,653 more chars]
