# Supplementary material for: Identification of Avramr1 from Phytophthora infestans using long read and cDNA pathogen‐enrichment sequencing (PenSeq)
Source: Mol Plant Pathol. 2020 Sep 15;21(11):1502–12. doi: 10.1111/mpp.12987 (PMC7548994; doi:10.1111/mpp.12987)
Supplement: Supplementary file 7 — NOTES S2 PacBio PenSeq contigs of EC1_A1 [file MPP-21-1502-s007.docx]

>Contig_4

AAAAAACCATGGAGTAGTGCTTTCCTAGTCTTCTATACAAACACCTTCTTGTGTGGATTGACGACCTGCTACTCTACGCCGACGACATTGATGAGTATTTGGAGAAGCTTGCTGAGCTATTCTCGCTAATGAATGATTTCGGCCTGTAACGCAGTGCAAACAAAGCCAACCTCTACCAGCGTGAAGTTAAGTGGTGTGGTAGAATTATAAGCGCGGACGGTGTCCGACACGATCCTTCTAGCATAGATTCTCGTCGATCCATGCCATATCCTTACGGATGAGGTGACGGCTCTGGCGACAACGGAGCCGACCATCTCGACCCTCCGCCCGCTCGATGACGAGGGGTTTATCTGGCCCACTTTCGAGGATACTGCTGCTGCGCAGTCGCTGTGTGATCCTCCATCGGTCTGTATTAGCAATTAACACGCACCCCATTTCTAGCTGCAGAATGCCGACGGCTTTTTTACTCTCGACAGCCGTTTTTTATACCGTCGGAGGCTGTTGAGCTGATTTAGAGATTTTTCGTTATTGCGCATTGTGGTGCCCGAGGCCACCGTAGTCGATCTGCCATGATCAAGCATCTTCGCCGAATCTTTTTGATCGATTCTCTCAACTCTTTGGTTTCCACCTTCATTCGGCAGTGCCGGCTGTGGCTGCATTCGAAAGTGGGAGGATTATTACTAGGACTTGGAGTGAAACGATCGAGTGTTCGGAGCGCAACGGTGTTCTTCATTTCGATTTTCTCTTCATAGGAAATAGTTTCGGTGACTCAGAGTACATTTTTGGCTCTCAAGGATCATGCGTCACATTGTTGTGGATTGGTTGTGGTAGACACAGCCGGCAGTAGTGTTGCTGTGGAAGCGCTTCTTGCGTGGTACGCGCGCTTAGCATCTCTTTGGGTAAGCGACAACGGAACGCACTTCTAGAATGAAGTGGTGAAGGAGCTGTCTCGAAGACTTAGGGTGCAACATCAATTTATTCCTGCTTACTGTCCCTGGATAAACGGCTCTATCGAGCGCGTTAATCGAGACGTTCTGCAAGTGATCAGAGCGATGATTCTCGAGTACAAGATTAACCATCAAGATTGAGTGTACCTGGTGCCCATGGTACAGTCAAGCCTCAATCATACTGCGATACCTTCACTTGGGAACAGCGCTCCTTTGATGTTTTTCACAGGATTGCCGTGTCCATCTCCTCTTCGTGAATTTTACATGCCCAGCAGTGGAGATTTGGTGAAGGTTCCTGTTAGTGACAAAATGGAAGATTATCTCGGTAAGTTGCGGAACAGTATTCACACCATGCACTAGGATTTAGAGGATCAGCGCTTGAAGCAGCAGTTGCTGAACAAAGAGCGAGAAAGAGGGGGAAATATTGTCAACTTTACTGAAGAGGACTATGTGTTACTCTCCCGAGTTGATGAAAAAGCGGAAGCAAACGCCTGGTAACTTGAGTCGGACCTTATCGTATTGTGCGAGCAGATGCACGCTCTTTCCTCGCTCAACATCTGATCACGGGTACAGAGCTGGATGTCCAGGCTCAAATTTTACGCGGATTCCAGTTTTAACGTCACTAAAGAGCTTTTGGAGCACATTTCGTCTCAAGGTGTTATTCTCGCTGTGGATAAGCTCAAAGGAATCGGTGGAATAGTAGCATTAATGACTTCGAAATTCTCGTGCAGTGGAAGGACTTGAGTCAATCGAGGCCTCGTACGAGCCGCTCACCAACCTAGCTCGTGATGTTGCAACATTGATACAACAATACGTTAGCACCGCTGACTAAGATTTGCAAGAACACTGGCAGCGAGTGACCCGTGTGGAATTGCAGCAGCCAAAAGCTGCCGAGGCCTCAACAATTGGAAGGCTTGGACGATGCAATAGACGTCGCAAAGCCAACCGCAATGGACAACCTTCAACGCGCCCGGTCGCCCCTGGTATTTCGGCTGTCGAATCTAAAGAGCCTAACAGAGGTGGTTCAGCTGATGCAGTGCTTCAAACATTAGTAAACCGACCTGGGTTTGAAAACCAACAATCAAATTTGTCTACGACTGATACCCCTTCTCAGCAGCACAGGATCGACAAAGAAGTCGACTATCTTCAGGGCAGCGCTTATCTCCAACGGATGCCGCCGATGGAGATCGTGTCGGTCGATGTACGTGCTCGCGGACTGCTGGTGCCGACCAATCCGCAGCCAGAACCCAGCAGGATGCAGCACATTCCCGCAGACACTGAAGGAGAGGAACGGGTTTACTAGACTCACAGGAGGCCAGGAAGTACCTACATGGTGCTTGATATGACTGCTGACTGGGTGTTTAGTAGAGAAGGCGTACTTCAGCGGCGAAAACTTCGCTTTAGGGACAGTGTGGTGGATTGGTGAATCAAGCAAGCTTCCTCCATCTGTAGTGACCCTGGAGTACACGAGGCGGGCCAGGTACATCTTGGTCAGTGACGTTTCCTCACGTCATTTATATAACAGATTCTTTTAGCTAGCAACCAGTTGTCCTTGTGGATTTACTTTAGGCCTTGTATGTCCCCTAACTGCACCTCTATGTGCTTGAGAAAGAAGGGCAGCCTCAAGTTGTTCCTGACTATCACTCTATATTTTACCAGTAACCAGAATAGCAATTGCACACTAGGCTATCCGTTCCGGCAGGTTCGCCACCTGCCTTGGCGGTGTTTTTCCTGTTTCAACTCCGGAAACCAGTACACGCGGAATGTTAATGTGTTCAAGGCGCCCAAAAGTGAATTAAGCAAGTAATTAATAGCGCCCAGCGAAATGGCTGTTGCGTACATACAAGTATCAATGTCCGGCCCAGGCTTAAGCCAAGCATGTAGACTAAACGATCCAACGACTGCTTGGCAATGGCAGGGTTCTCTAAATAATTAAGTTCTCTTAGACACGAGCCTTGACTAAATGCCCTCAGCGCGTGTGCCAAATCCATACCAGAATTCAATTTCCTCAAATTGCCAACTATTGACTAAATTCTCAGAATTTTATCCGTGAAAATATTTAAATCGTCCTAGAGTATTTCTCTGCACTTTGAAACTCAAAGGTTCTTAGCAGATGCTTTGATGCAGACTCATCTGAATTGCACATCAGCAGCACCATCACGACATTTTTGATGATGTTGGCATTTGAGCACTAATCAATGCCGTAATGATTACAAGCGAGGTCCGTAATCTGGCATCCTGATAGCGTTAAAATTCTGTGAATACAGTGCCTGATTCTATTTCCTTAGACAAAGGGCTCATCCAATTCAACATAAAATTAAAATTCTCCGAGCTCTACTTGATTGCATTCAGAAAAAAAGGCGTTTGGCAAATCTACCTGATTCATTTATGTTTACGGTAAAACGACTTTGAAAGACCCCTGAGTCATGGCCCATGATACACAGCAATATCACAATTTCACTTTCCTGTAGCCCAACACTTATTTAAACTGTAAAGCCACTATGCGCTCCTGCTTCGTTCTGATCATTTTCCTAGCTGGAATTTGTAAGTGTCTCTCGCTCAATTGGAGTGGCAATACGTTAAAGGAACGAAGCCACACCAGCACCAATTCTCTCCCCAGTCCACCTTTCATCTCAACAATTAAGACCCCAAAAAGGTTCTTAAGATCCTACGACGCACCTAAGCAAGACAACATCGGTCATGATACGGACGAAAGAGCTGGAATTTCTGGGATAGCCATGATTGACGATCTTGCGTACAAGTGGGCGTTGAAGAATACGAGGGATCCAATGGATGCATTCCAGCGCTTACATGTTGTGAAAACTGGCGGCAAATTGGAAGGCAACAAGGAATTCATTCGGTGGCTCCAGTACGTAAATCGATACAAGGCGACACGACGAGTCAAGTTCGGTGAGGATGAGCTGCTCAGCCTTCTAATGAAAACGAGAGCAGAAGAAGAACTCGTGTCCCTGTTCCAATCACTTCGACAATACCCGGACATTACGAAGATGGCTAGCGATATGCAAGCGTCCATGATTTTGAGCTCTGCGTCTAGTCACAGACTGATCAATGAGGCATGGTTAATGTCCCGAGAAACTCCCGGCGAAGTTTTCAAAATCTTGCGACTTGGCGATAACAGCATCAGTCGGCTAGAGAATAACCCCCTCTTTATTCAGTGGCTCAGATATGTTACGATGTACAGGGCTGTACACGGACGGATTTGGAAACACTATTTAAGCGTTTCTCGACTGTACCACAATTTAAATTTTTTAATTCAATCGTTGCAGAACTTCCCAGATTTGGAGAAACTCGCACTAAGCTTACAGACCCACCTCTATCGGAAATGGATGATCGAGATCCAGCTTACCCCATCTGAGCTCTTGGGTCTTCTAGAGACAACCAGAGTCGCGAGAAGTGATCCCAAATACCGCAATTTGGAAGCTTACACCATGTACTTCGCTGAAAGCCGAGGTGGTACGCCTTTGTTGAATAAATTGAAAACGCTGTTCACGGATGTCGATCCCTACGCAGCACTGTCTGCCGCTTCGAGCGCCTAGCAAGGAATTTCACCCTTTCATTTAATACAACGTTTAAAAAGATACAGTCATTTTGCTTAAATTAATGATTCTTAGACGGAATTCGTCGGACATTCGTCAGTCATCGCTATTTTCATGAAGTTTGATAGGCTACCTAGAGAACGTCATACAGTCCGTGTTGAGTTTGGCAAATGCTTGCCGTGTCTTGCTGGCATTATTCATTTTCACAAATGGTTCTTATAAGTTGACACAACAGTGCTCGCACATTGAAAAGCTATGACAGACAACTCAATATACACGTACGAGACATCTAAGATATCAGTATGCTATTTGATATCATAGCTAAGCACACTCAATCTGATAGTTGCTCTTCATAGCGGTAATGTGCGCCGATGACAGCGCGGCGCGCTGACACTGCTCGCCACTGAGCCATTTAGGCCATGGAGGACGACTGCCGGAAGGCGTCCACAAGCTAAGGGAAAAACAGNTTTTTTTTTCAGAAAATGTCAGACAGAATGTCCGACGCGAACCAATCAGATGGGTACGATGTCCGACAGATTTTGAGGAATGCTCATGAGAACCAGCCTGCTGAAATATAGGATAAATCACGCATATAGGAGATAATTTGGGTGAAGGAATTACAATTGAGTTGCCGTGTAGCCTTTTACATCCAGAATATGAATTTAAACGCATTTACTAAAAGAAGTTTATACCCCTACTATGAGCTGAAAAGCTGGCTCTGGCGGGGCGTCACTCGTCAGTTAAGTTGTATTATCAGCCTCAACATGTGATGCAGGCTCAATGCAAGGCGACCCAATTGTCTACGTAGATTTGCGGCACGTGTATCGCGCAATCCTAAACACTTTCAGCGTAAGACGATTTATAGTTCTACACTCAGTCTTCTATTTTAAAAGAGGTCAGTTTACGAAGGGCGCGGATGGTATTCGAGTGCACGTTGAGGGGTTTTCGAGTGCAGACATTTTTGCCACGAAATGGCGGAATGCCGAGTGCAGATAACACATGGCGATATCGTCGGCTCTATACTTGTCTTGTCCGGTTTATGCTTGTTTGAATTTACTACACTGTCCTTGGGGCCGGATGCAGAGCTCACCAGGATGTAAGAGGAGAAAAATACATAAATTTGTGAGAAGAAAACATGAATGAGCCTTGGATCCTGGTACTTCCTACACAAGGTACGCTGAGCTCCGCTAGTCCAGTCCTACGCCCACATGCTCAAAGCAAACTGTCACTCCTGATCGTCTTAGCTGTCCAAGTACTGATCGTCGTCGGCGTCACGGTCGACACTGCGGGGATGCGATTCGTGTGTGCTTGCGTGGGCGGCAGCACTCTTCGTAACATCGAGGCCAGTACGTCTTGATTCTACGATCAGCTGCACCAATTCCTGGCACTCTAGGAAGACTTGCTTGCCTCCCTCGAAGTCGACAAGCCATCTATATCGCGACTTGCGCGCTTTGCATCGTTTCATGAACCGAGATGCGTTGGTACCGTCCTCTGGTCGATGGACGACTCGGGCTATGCTCTCCCGTGGAGCTCTCCACCATTGCCCTGAACACAATGAGAATTGCACACGGTAGATAGATGAGTATGAAGTAGAAGCAACTTAGAGTGTTACGGTGAACGTACGGCGAGCGTTGAATTTGCTCTTATCGTGTTGACGGAGAAGCGCTTGAACGAAAGGAACTCTTACACTGTGGGCTGGCGTAGCTGGCGAGCGAAGTTGGCGATGAAAGCACACTAACCCTAGCTCGTGGATCGCTTTGTAGACGAACGCCTGCTTATCGTTGCTGCAACATGTAGCTTACATTAGCTACGTCTGAACTAATGAAGGCAGAGCCTTGACGCATCTTCTTCCGTGCTCAAGCCTTAACCTCAGCAGTGCACTCGGTCATTCCCTCAAGTTGAACAAGGGACACTCGGTTTCACACCTTTCGAACAAAAAACTAACGGTGCACTCGATTTCCTAAAAAAAAAAGCACTCGAAAGTAATATCGTTCCGTTTACGAATGTTACCTCTTGGGAAGTATTAAGGAAATACTCATCACAAGACTTTTAAAATATAATATGGACACCTTTTTGGAGAGGTGGAATATAAATTCTCATCTGAATTGCACATCGACAGAACGGCAACGACACTTTTATTGACGCTCATTTCTTAGCAAGTGAGTAATCGAAGTCCGTAATCTTAAAACTAAAAAACGTAATCAGTAATAAGAGTGGGATTTAAAGTAGCTCTTCACTATTGAAAAGTGAGGAGAAGAAATGTTCTTCAATGTCGTGCTGATCACATTCATCGTACGGATTTCGCTTTGCTCCTCGCTCAACTCATTGATAAGCACCAACCAGCTCAAGGGCCTGAGCCACACCAGGTCAACGTCCATCCCTGCAACGAAGAGATCGTATTCCACAACAAGGTTTCTAAGGTTCAGCGACGCATCTAAGCACGATGATATTGATGATAATAGCGAAGAGAGAGCAGGGGTTTCTGGGATAGCATGGCTTGGTAATCTGGCATCCAAGTGGGCGCTGAAGAACACGAGGAATCCGATGCAAATTTTCAAGCTTTTACGTACTGTGAAAACTGGCGGTAAGCTGGAGGGTGACAAGGAATTTGTTTGGTGGCTTCTGTACGTGAATCGATACAGAGCTAAGTTACAAGACAAGGCCTCGTTCAGTGACGACAAGCTATTTGATTTGGTGCGGAAACTGAATTCGGAAGAAGAACTGGTGTCTCTGTTTCAATCGCTTCGACATTATCCGGACATCAAGAATATCGCCGATGATATGCAGGCGTACCTGATCTTGAGCTCGGCGTCTAGTCACAGACTGATGAATGAGGCATGGTTAAAATTCCGAGAAACCCCCGAAGTAGTTTTCAATATCTTGCGGCTTGAGGATGAACCTCTGTACGCTCTCGACGGTAATCCCCTGTTTATTCAGTGGCTCAGATACATTAAAGCATACAGAGCTGTGAATGGAGGCGACTCGTTCACAGACGTGCATGTGTTCGACTTTTTACATGAGTTTGCTTCCTTGCCGCGATTTGGAATATTTCTTCAGTCGTTAAAGGACATCCCAGATTTGAAGAAACTCGCAAAAAGGTTTACAAACGCAGATTGGCTAACCCCATCGCAGCTCGAAAAAATATTTGGCTCACCGTATCCAATCAACTTCGCGGAACTCCCAAAGAGTGATGCCAGGTACCGCAATTTGGAAAGCTTCACGGCGTATTTCGCTGAGTACTGGGGAGGAACAGCATTATCGTGACAATATTGTTCGCCAAGAAGGACCCATACGCTGCAAGTTGAGCCGCTGCAAAAGGTTAAAGATTCCTGACTTTAGCTGGTCGAAAGTGTTAGGGATTAATATCCCATCGCAAGATTAGCGAAGTTTTCTATTGCAGTCCAACAATTGCTACGATACTTACTTCGGCGTGCGCAGCCGTCTGGAGAGCCAAGTGTGATGTTTCTAAGATATTTAACATTGGTAGGAACCCTTTTTACAGCACCAAAACTGAACCGCACTATTGTTTTTCTTCTGATGAAAAACTTCGTTTCATTTTTCATTGCGTCTACACCGGATTTTAAATGGCTTCACAAGAAGCTTACAGACTTTGTGAGCTTATTGTGAAGCCATCCTAAATCCGGGATAGATCCTGTATCAATTGAATGGATACATGTAACCCAAAGCACGGCAAGGTCACAAAAAGCTCGTATAATGGAGTGTTGTCCGAGGAAAATTAAACCCCGTTTTGACCCGAAAAACACCGCAAAAAAACGGCTCTTTGCAAAAACGCAATTACTTTAGATATTATCAAAGTGAAAAAAATGAAGTACAATTATTGCAACTGTGCAGGCTCAGAGGCTTGTTTACCGAATGACTGTGGCGCTTGAATTATTATACACTTTCAGTTTGGCCAGATCCCTTTACACCACGCTTCAATTTCCATGCTGACCATCTTCCTTTCGGGAGATGTTTATGTTTCTCGTTTATCTTGACAGCTCACGGACCCGGGTCACAACGGAATAGATGACAATGTGAACAGGTATGTCGATCGACTTCTACTGCATGCATTACTAAAAAGCTGTCGAAGCGGCTGCAATTACAACGACAAGATCTTTGTTGTGGAACAATGGTTTTACTTTATCCTGCAAAGCATTTCCACCATGTTGCTCTGCGACGTACATGGTGTAAGCTTCCACATTGCGATATCTGGAATCATTCGTCGACAGCCTCGACAAGCTCGCGACATTCATAAAAATTGCAATGTGGAAGATAGACTGCCGTTGTCAAAATAGATAATAGCTTCTCAGAAGCTCTCACGGCGCTTGCGTCCAGCATAACCAGCGACAATCACTGGGCGAGAACACAAGTGTTTGGCTTCAAAGATGGCTGCGCACGTTGTAGTGCGACCCACAGTGAAATTGTTGGGCGCACAAGCATTATCGGGTGCTTCACGTTATCCCGTCAAATACCTTTTTTATAATTATGGAAACTTCTTTATCCCACACCATACAAACTTCAAGTAAAACTCAACCTCACCAGCCCTCTGGCGTCATTATCAGTCTGACCATGGAGAAGGATCTTCGCGACGCATATGCAGCACTCAAGCTGCTTCGGGCAGACCTTGTGAAGACCCGCCGCGATAAACGTGCTCTAGAGGCCTCACTGTCCCACCTGCAGACCCACGGGCCGCCTCCCAGCGCTGCACAAGCTCGCGAGAGTCGCGAGACGCAACAGATGCAACACGAGGACGCCAATGCGCAATTATGGCGTCTCGCTGCAATTTACGAAAGTCGATTGGCGGAAATGGAAATACAATTATTGCACAATAATTGTCAAAAGAAGGAGGCAACTCCCGAAGTTGAAGACAATCAACAAGAAGTCGAGGAAGTGGAGAAGCTCGCGCTGCTTCACAAGCTCCACAGTCTGACCGCCACCGTCGAGCAGCAGACGCAGACGATGCTGGCGCAACAGGCAGCTTTTGCGCTGCAGAAGGGCGAGCTGGAGACGACGCTGGAGGACACACAGCACCAGCTCCAGGCAGAGAAGAGCAGGGCGACTGATGCGCTATTAGAACAACAAGCAGCTAAAGAGAGGTATGAGTTCTTGGAGACTCAAGTGGAGATCTTGAAGCAGGACAAGAAGACGTTGGAGGAGGAGAACCATACACTCCACAAGAACCTAGCGACGCATGCCCAGACGAGTAGAATATTGCAACAACAAGTGCAGGAGAAGGACGAGGAGCTCACTGTGCACGAGAAAACTATTCAAGAACAAAAAGAGAGACAAGAACGATATATAACGACGCTGAGAGACTTGGAGAATACGTGTGAGATGTTTAAGACCCAAGATGCGGCTTCTGAGGCCAAACGAAGTGCTGAAACGAAGGAGTATGAAGCGAAACTAGCAGATATTCAAGAAACATACACGTTCAAGGTGGCCAAACTGGAAAATGAGCTGGAAACGACTCATAAACAACTCACAGCAGCGACAACTAGCCGAGCGATCGAATTGAAAGAGCAAGACGTCAAGTTTCAAGCGGTTTTGGACAAATTGAGGCAGCAGGAGCAACAGGCGACGACACTGCGAGACTTGGAGACTAATTTCGCGGACGTGCAGGCCAAACTCACGATAGCGGAGACCAAATTGGCTGATGGAGTGAAGCAATACGAGCAACAACTTGCTCAGGCCTCGCAGAAGCTACTTGTTCAAGAACAAGACCACGAACAGCACGTAAGTTCTCTGCGTGACGTGGAAAACGAGCTGGCACGAGTTCAGACGCAACTCACGGATACGGAGGCCAAGTTACAGCTAAACGTGACACTATTCGACGAAAAACTCGCGCAGACTTGCCAACTTGCTGCTGAACATGAAAAGACAGCTCGAGTGCTAGCTATGGAGAAGAAGGCGCTTCAACAAGACGTTCGTGAAGCTCGACAGACATCGAAAGTCAAGACCGAAGAGCTTCTTCATTTCCAGTCGATGATGAAGCAGAAAACTGGGGACCATTCGCAGCGTTTAAGCCAGTATCAAGCGCGTTGTGAGCGTCTAGAGACTCAACTATTATCATCTGAAGACCACAAGGTGTACGAAAGCAATCCAAGCGGTGAGTTGAGAACTTTCCATGGTCTTAACGACGCCGAAATCATCGCGCTGCCCTCTTCCAAGGCGTGGGTTATATTACACTCTGCAATAACCAAGCTGGAAGACTTCTTCCCATATCTTGAAGCACTGAGTAGTGCATTACAAGACGTGTTAGCCTTGTGCAAGAGCCATGCGACTTTTCTTCCAACGCTATGTGAGCGTTTTGAAGACAAGACAGTTAGCGACAAGACCCAGCCGGTGTTGGTTATGGCGTTAAAGCTGGTACGCTTTGCTGCCGTGTTGAAGACTCAGGTCCAGCAAGACGACGCTGTTGTGACTTTAAAAGCGGTCCAAGGTTTCCGTAAACGCGTGCTGGATGCTCTCGCGCAATGGTACGAGTGTGGCGTGGATGGTTGTGACCAAAGTGGAAATGGGTCCATGCCCACACCCACTTTTACTACCACTTCTCGAGAGACAGCACTCATTTTACAGAATTGGACCAGTGACCGGACAAAGCAGTTGGGAGTGAGACGCTGGTTGGCACGGATGGAAGCGTATCCTGGCGTCCCGCCACTTCGAGGAGCGTCGTCG

>Contig_5

GGTGATTCCATGATGGACAAAGCGATGTCCGGAGATGAGAAAGCTTTGCATTGCGCAGCATCGCGTCTGAAGTAGATGCAAAAGTAGCTGAGCGGTAGCCTCTCTTCGAGTCGATAGATTCTCGACCTCGATTCGAGGCTCGAAGGTGTCCGTGGTTGCCATTTGGACCAACATGTCCGCTGACTTGATTCCCCGGCGACGGAAGGATTGGACAAAAAAACTGTTCACTATGGAGGTATCATAGTACTGTTGACTTTGTATACTAGCTTAGATGAGGTATCTATCACCTGGCCCACCTATGGTACTTCTACCATTTTCCTCGTTCACAATGAAGCACTGAGAATAGACGATAAGTATGCAACTAACACGCATCAATAATCTTATTATGGGCACAAAGCTACACCGAAAATTATAGATTTAGAACTCACGCTTGACTGTATTTCAAGGGGCTAAACTTGCAGATGAAGAGATATGGAAATAGTACCATTAATGGTGTGTTCTATCCTAAGAGGAGGACGAAGAGTTATTATTCAACCCATAGCTAACGGATTCTACTGACGCAAGAACACGTCTGTGCGGGTCCAAAATTGGGAAGCACGAGGAATATCTCGATACAGAGGAGGAGTTGAATCTCGCTCTCTTATTTCCTTCACATCGCAATTTACCAGCATCCTGCAAGCTCCCCCCCCTCCCCCCAACTTTCAGAGCTCCACGTGTCTCAAACACCTGAAACATGACGAAATCTACTTCGCGACAGAAGACCAACAACGCCAACACTAGCGCTGGAAAGAACGCGACCACCGCCCCCACAACACCTGCGAACCCGGCCAGCACTAAAATTACCAACAAAAACACGAACGTCACCCAGCATGCCAACAAGAACACGGGCAAAATGTTCGCACTCACTGAGGTGACTACGGCAAGCGTGCAACAAGCTCGCCAGATCCGCAAGACGGACTTCGAGCGCTTCCAGAAATTCCACAACTTCTTCATGGCTAATGAAGAACCGACTGCACGTGTGATAAACGACAAGGCTAACGCCAAGCGTGAGATAGTGGACAAGTGGATTGGTGGCGCTGTGAGCCCGAAGACGCTCGCGTGTATCCAGGAGATCCAGGAGACTCAATCGGGACTTCTGGAACTGCTCATCGTGTCAGCAGCACTCACCAAATACGGCGAGAAGGAGCTGGCTCGTAGAGACAGTCTAGGCGAGATGGACTACGTGGTAGGTGCTCGCTTCGGCACGAATGGGCTGCGCTCGAAGCAGCACGCACCACACAGTGTAGGCTTGAATTTCGTGCTGCGGTTGGTGCTCGGTCTGGCACCGGAGAAGCGTAGCTCGTGTGAAGTCTTACGTGAGATCCTGGGTCACGAGAGCAACGGACGCCTTGCATTAATCCCAACAGAAAGTGGTCTGCAGCCGTATTCTGATGCTACTGCTTCATACTCGGATGACTGGGCCGAGCTTAAGCCAGATCAGCTCCGTCAGATGGCAAGTCAGACGCAGTCCGACAAGTTCCAGGACACCATGCAGCAGCTCAGTGGTGAGCACCTGTACGATGCGATCCGTACACAGCTGAAGCAGACTGGCGGTGCGTCAGTGTGGGATGCTACTAAGGACAAGTCTGCACTCAAGGTGAAGAACATCCGTCCGCGCATGGGGTCGGCTGACAAGAATGCAAAGTCGAAAAAGGAGGCCGATCGTCAGCAGAAGCAGCCTGTGAAGATAAATGAAGCTTCCCAAGTGAAGAAACATCTTCCGGTTGAGGTTGAGAAGGTGACGCTGGTTCCTCCACCGGCCCCGGTGATCATGAAATCGACTACCGATGCAGCTACCAAGAAAGAGGGTGGAAAGAAGAAGGGGAAGAAGGGGAAGAAAGCGGACGCGGAGAAGAATGTCGCCCCTGTTAAGGACGCCACTGCGGTGAAGAACATTGCGACTACCAGCCAGAAAGCTGGTGCCATCAAGAGCAGCGCGCGTGACGGTCGGGCGGTTGTCTCCAAGGCCCTCTAGACGTGCATATTTGGTGATGCACTCATCTTTCATCTCAAGAGATTTATTAATAAACTTTTTTTAAATATAATTTGTTTTCTCGTAACGAGACCTGCACAGTATCGTACCGTATTGTAAAACCCGTGAAATCGAAAGAAAGAATCAGTGCACATTTCTCTCAGGTTTCACGAATACACTTTCATTCTTGAATTTGCATCCAGATGCATCTCTTCTCTCTGACAGCCGTAGCTTTCGTCATCGCCAGTTTATCTGTCGACGCATCAGTCGCGAAAGATCCACGAGGGCACGCTCCCAACAGGACTGAAGTCGATACCGTAAATGCGAGTTCAAGCACGAGGCTTTTGCGAAAAAATAGTACTGTTGATCTAGTCGGCGAGGAGAGAGCACCCAGCGTCGTAGAAAATATCAAGGCGTTGGTCAAGTCTTCAGCGGTGACTCCAGCGAAGCTTCAGCAATGGCTAGACGAGCGACTACCTGCGGGGCTAGTGTTCAAGAACATGAACCTTGACGAACCAAAGATCTTCTCTTTGTTGCATGAACCCAACTTTGTTAAGTGGGTCCAGTACGCCGACGACTTGAGTGCCAAGTCATCTCATAAAGAATCGTCAGTGATCTCCACCCTGACGTCATTGCACGGCGACAAAGTTGTCTACGACACAATTCAAGCTGCTAAAAAGTATCCACAGCTGAGTGAACTCGCCCTTAAATTGGAAAAGGACCAGATACGCTTCTGGATTGCCACTCGAAAAGACCCGTCGGTGTTTTTTGAAGCCCTCAACCTTAACTGGGTAGGGACATCCATCTTCTCAAAACCTGAATTCTCCGCTTGGCTCAAGTACGTGGACGATGTAAACGCAAGACATCCCAAGAAAACCCCATTTTCGATTATTCCTACGCTCAAGCAACATGTAGCTCAAAGTGACGAAGCCGACACAGACGTACTTCTTAAACTGATTGCGAACGGGAAAGCAACGGCTGAAACCAAAACTGTCGCCAACAAGGTAGATAGTGCATTGTTTGACTTCTGGCTCAGCAAGCGAGAAACACCCGACAAAGTTATGGACGCGTTCAAACATGGCAGTACAGCTCAAGCTTTCTTGGGGAGTTCACGGTGGAAAGAGTGGGAACGGTACTTGAGCGTTTACAACGCGAGATACCCTGAAAAGAAGACCACCGTGATAGAAACGTTAACGCGGAAGTACGGAGATGCACAATTACTCGACACGCTTATCACCGCGAGCTCGAAAGGTGAGACGAAAACGCTTGCAGCCAAGCTGCAGGCACAGCAGTTCGATAGGTGGATGAGCCTTAAAGAGTCTCCTCTCGACGTCTACAACAGGCTACGGCCTTCATATGGGGATAGGAGCTTCTTCGACGAGCCACAACTCAATGTGTGGGTCTCGTACATGAATGTGTTCGTCGACAAGAACCCCAGCAAGGTGGACAAAATGTTCTTGGAGTTAGGTGATACCTTTGGGAACATGCATCTCTTTCGAGTCCTCGGAGAAGCCAAAAAGTTCCCCAACATGGAAAGCACTACAGCCAAGCTGCAGATGGAGAAGGCTTCGACTCTTTTTGCCAGCGGAAAATCCCCGGAGGATATATTCAGGGTGCTAGCGCTTGACAAGGTCGGAAATGATATACTCAGCAACACGCTGTTCCACAAGTGGCTGGCATATCTGCAGAAATTCAACAAAGAGCACGAACAGCCAAGGATCGTGGTTTGACATGCTACGTATTATTTACCAAATGTTCGGTCTCGAAAGGATTATCGAGACGGGAAGAAAAAATCCAATCACAAGAGGGATGGCTGAAAAAGTGGAGGATGCGTATCACAACTACTGGTTGGATATTAAGATGGAGCCTAAGACAGCCTTCCGCTCCCTGCATCTCGACGAAAGCGGTGAGAAGCTCCTTGCCGACCCAAAATTCAACACGTGGGTGCAGTACCTGAAAACCTTCATCGACCGATATCCTAATGAGAAGACGACAGTCATCGACGGGCTCAGGGATAACTATCATGACATAGCTCTACTCCGAATGTTTTCAGCCGCGAAGAATGATCCCAGCACGGAGAAACTCGCTACTGATCTACAGAGTGCGCTAATCCTCAAGTGGCAAGATGCGAAGAAGACACCAGAAGAACTAAAGAGAGTGTTTGTTGGTGTGCCAACCTCTGGTGAAATAATCGATCGGTACGACAAGCTAATATCGGCGACGAGAGCCACCTTATAGTGTATTTGACCGAAGCGGTTATCGTGGAGAGCGAGAAGGAATCTAATGTAGCCCTTGATTTTGACTTTTTGGTAAGCTTTGAGTCGCATGAGCAGAACTGGTTCCTTCTGACTCGACGATACTACAGGAGGTGGCAGTGGTTACCGATCTCCATCTTCGATCGACGTGCCCTCCAGCCACTCGAAATATACAAGCAGAAGCGGCTATCAATTGTGATTTTCTCTGGGTGGAACCTCGTAGAAGTGCGTCCTTTAATTCTTGGGGGGGGCATAAATGCCAAGTTGTACCTGTGTTTTCATAATGGACCTTGCATATACAGTCTCCGTGTACGATTACGCGCCGTAAGCTACCAACTTCACGTAATATTTTACACTACGGCTGTGGGGGCATTCTTGATCGTCGAGCAACTCACGATAACAATTCCACGAAAGCTGGCAATTAATATCCTATGTATCATCCTTGTATGAACAATAACAGTTAGCTTTCAGAGCCATCAAACGTCAAATACTGTACGCAGGGCTATTCTGTGATGACATAAAGCTAAATCTTAATTTGAATAAATTTACATTGTGAAGTCCTCGTTAATATTCGTTTTGAGCTGCTGCCATCATATGCCGCGTATATTACCGATATAATCGAGTTACGTCTGTTGGTGCTGTAGTCGAGGCCATATTCGTACTGTGCCGCCAGTCGTGTGACTAGCAGCAGGGCTGCTCAAAATTAATCAATAGACCAAACATTACTATTTTGAAACTAATAGCACTCATAATTGTGGGTTTGCTACAATAAAAACTTTCCCTAATTGTTTTGGATTGAATTGGGATTTGAAGGGCGCTTAAAGTCGTCGTTCGTACAATCAAAAAGTGAATGTTGCTTTATTTTTTTAAATGTCAATCGTATTATACTTCATTTGCTGCTCTCTAACGAAAAAAGACATCATGTGCATATAAATTACATTCGCAATGGACTGACAGCGTTTATCTGATATTTGGCGAGCTATGAACGTGCGCAACTACAAGTAGCTGTACTAGATTACATTTCTTACACGTAAAAAAGTACCACCTTCTGCCATTAGCTGTGTAGCTTATTTTGTATTTGCCGACGTTTATAGACGCTAAAAATGGCAACCCGTTCTATAAATCGATGTACCAGCATGCGGTGCCTGATAAATTCACTCCTACTTGATTGTACAAAAGACTATACGAGCGAGGTTGGTGAAATTCCCACTCGATGATAAATTTAATTGTAGATATGCTTGATTTTCTGCTATTTGTTTAGACCTCTTGGTAAAAAAAATAAACGTGGTACATGTATTAATAATGTCACGGCCCATGATAGCCAATAGGTCGCTCATACATGTACCAAGAAAACGTACAGACTCTTAAGAGTCAGGAAGTGTTTCTGTGCATTTTTTGTAGATATGTCTGGTCAGGGCTAGGTTTAAAGAGGCAAGGATTTGGGAAGGTGAATYCGATCTTTTAAACTTAAGTTTTAATCCTGCATTTTCAGGCTAGAGGCAGGTGTAATTAGGCGCAAGTCGAAGCTAGAGTAGACCTACCATGCGTTTGTACGTATAGCTGGAATCACAAGACTCTTGGCGTTTATCGGGATTATAACTACCGTGACACCCATCCCATCGGGGACGGGAAGCAGTACGGTGNTTTTTTTTTCACTCATTGACGTGACCTGGTCCTGGCCTGGCCCCGGACGATGATGTATATGAGCAAAAAAGAAGAAACAATTTATCCCAATCAACCGTAATCAATCATATGTTCAGTACGGGTACCCGAAGAAGTCCAGAATTAAGTTAAAAGGTTTTTAAATTAAAACAAGCACTTTTCAATCTGTCTTGATATCAAAGTTAAACGATTAATAAAACAAATGCTCTTATTGTAAAAAAGCTCAGTCAAATCGCTACTAGCAGAGTAGACAGCATCGTTGTCGTGCTTGGGCTGCCACATATTAACGTCTTTTCGTATATTCCGTTCTGTTTGTCCTTGGCGCTTTGAAGCCCCTCCTGTGCCTGCTTTGCAGCCGCAGCATCATCCTACTGCTTACATGGAAAAGTATACCCCTCATAGTATGAGGGGTATACTTTTTTGCGAGCAAGAGCTACATCCATCAAACATAACACCGAAACACTCGCCCATCTCCTCGGCGATGGTGTTGCCGACACGTTCAGCGACTCGTTGCATGTAGGTCTTCAGCGTCTCCGCTGAGGTAGGCTTCATGCGAACTAACTGTCTCGTTAGTGGGTTCTCAACCTCGCACAGAGGGAGATTCCGCTCGACAATCCAGCGTAGCCAGTCGTACATGTGAGTCGTCGTTTCGTCCACGAATCCAAAAACCTCGAGGGAGGTCAGGTTTCGGCGCTGAAACTCAGCATACTCTTCGCCGTGAGTGGGATGCTTCGAGTTCAGGTGAGAGATCAGGTTTGTGTAGCCTGGGCGCTGGGCTTGCTTGCGTGGCTGTTCGCAGATGTTGCACCGGTAGAGCCCAGGTTCGAGAACGGTGTAGAAGAAAGCGGCGAGTTACTGGGACGACATGATGTAAATTAAGAATGACCGTCTTCTCAAAGATTGAAAGTAAACTAGGCGCTGAAAAA

>Contig_11

ACTCAGTCTACAAGACTAGTGCCTCAGCCGCCGTAGGGAACCGTAAAAAATCGTCCTCTGTCAGTCTCGGCACACGTAACATACCGTTGATGATCGACCGCTACTTACCCGTCCGTGCCCCTTGCAATTGAGCTCCTGCGTTTGAAGGTGTATGTTATTGGCACGATCATGACCCGACCGCCTTGGGTTCAACAAGAATTTCAAGACTACAAGGAAGACACGCCCAGGTAGTTACCCCACGGTACATTCTCATTCTCGTGCTCAAATCGCAATTCCGACAATGACTTCGTGCGTTTGGTGGGACCGGAAGCCCGTATATTGTCTCTGCACGAGCTCCGGTATAACGGCTTCGACAATTGGGCGAAAGTATAAGCGTATCGGTGCGGTCCGAGTGCAGTGCCCCTTTGCTGTGAGCGACTACGTGAACTGGATGAGCGGAGTTGATGTCCTCGAGTCGACTAGTCGTTTCGCTGCAAATGTCCGGAGTATTAAAAAACAACCTGTCTTAGGCTACCCGGACATGGGCATAGTAAACGCGTATATGACCAGCAACGGGGGGGGGGGCTAAGATCAAGGGGATGATTGCAATGAAACGCTCAAAGCGACTTTTCATTCTCCAAAAATCTGTTTCTTGAACTCAAGCCGAAGATTTTGCAGGAGTAGAGGCAACGCCGCCCGCAAGCGTGCCAGAAGTGTCAGAGCATTCCAGTTCGACTCACGCACGCCACGGCCAGCAGTCCGAAGCCTGGGTCATTTGTGACTGGGGTCCCAGATGCGCCGGCAGAGATCGTGCAAGATCTGCGCCCTTGCGTACTGGAAAGAAGAAGTCATTCGCAACTACGTTCTTTGCAAACGATGCTTGGTTGACAATGCCAGGTGTTGGCTGTGCAACCGGCTTCGACGGGAACACAAGCGCATAGCTAAGACATGCTTCGACATCTGGCATAATGACTTCGACGCTGCCAATCCATTCCACTAAAAACCGGGGAAGCGCATCGGTCTACCGTCGACCCGGCCAGAAAGCTGTTCCAAGAAGAAGAGTCGACGAGAATACTTCGGCTTGTGTGCGATGGCGGTACCTACAGCGGCAACGGCGGAAAGAGCGAGAATGGTAGCGCTGACGGGTGAGGGTGATCTGCGTGTCAGTTGATAAGTGATTTGTTTGAAATGCACAGTTTCCTTGAACAACAACGCACCTTCTTTCTGGCATTTCTTGAAGTGAGTGGACAGTCGGTGAAGGCAATTAAACCATATGTTGGTGGCACAAACGACATGGAGCAGTCAGACAGATCGCTCAATGCCGTCAGAGTTACGACATTAAAAAGGAATCCTTTTGCGAATTAATATAGCTAGTCTCAGGCTAATACGGGCACTCATACAAATACGAAGTGACATCGCTATGCCGTATCTTACGACATGCGCTACGCGTTCTGGGTAAGTACCGTCCGGACGCAAGGTGTTAATGAGTCATAATTAAATTCGTTTTAATTTAAGAACAAAATTAAAAAAAGTCTGTTTTAGCTGTCTTAAAAGTCTGTTATAGCACTATAAAAAAGAGAGAGAAAAAGTGTTTCTGCAAAACAATTAATAATTACATATCCACATGTAATAACACCGGGAGCCCAATTTTGATTTCGATTCTAATTTAAGCTGCTACTTAAGCCACCAGTCTGGTGACATCTACTGTATAGAGCAACCTGGCTAACACAAGTCATCTTTGATGTCGATCCATGGCTAGCAGAGCATGTCCGTAATCAAGCCACTTCCAACTTTTGAAAACATCTATTGTACATGTACTGTACCCATCGCTTGATAGATTACAAATCCAAGTGCACCGACTATTAATACTTACTAACCCCTTGAAACCGGGAGCCTGCATATGCAGCTTTTCAAAAATGCTTAACCTGCATAATGCAGGTAAGCCCAAATATGACTTAAAGTTTGTATAGATACTTTTTTTTCAAATCCTCGGCTATACAAAATCTAAGTCAAAAAGAACCCTACAAGTGCAGGCTCTCTGTACGTGTGGTAGCATAGAAGATACACATAACAAGCGACGAGACAGATAATATTTCTCTAAATTTGAATATAAGATATAAAAGTTTATGTGGTAAACATAAGTTTTACATTATTTTCCAAAGGTATTTTTTCGCTCCCTCTCCAGCCACTCATTCGAGAATTCGCGCTGGAAAAGAACGGAGCCAGGCTCTCAGCATGAAATGGTTGCTGTCCGAGGCAAGTACGTGAGCAGTCAAAACATCCCGTTTGGTGTGGCGTGGAAGGAGCTGAAGAAGCAGGGATGGACATTTGTAAGACCGCGAGCTAAGGACTTAGATCCGAGATGGAAGTACGTTCGCCCTGGCGGACATGTTAACGGCACAAAGGAATCGACTTTTTTCTTGGGGAGGAAGAGCTGTTGGAGTACTACAGTGGTGGTATGCGAGATGGTGGCTATTGCTGGCTACACAGTCTAACCAATTGTTTGTATGATGTTACAGCTTCCAAAAAGCCTTCGTCTACCGGTTCTGGACCATTCCAGACGCCWCCAGCACCTCCTGCTGATCCTGCTGTCACAACAAGATCACCACCCGCTCAAGCGGCCACCACCCAACCCACGTCTCCACTTCAACCTTCTTCAGGTACTCAGCAGCCTTTGACGCGATAAACGCAAGTGAAACGCACTCGAAAAGTGCCCAAGCCGGCTGCCACGGACAAGCAGAAACGTCGCCGCTCATGTGAGTACCAGTTGCATATTCATGATATGATATGCAGGATATGCAGGATAACTAACTAGCATTATGCATTAGCTCATGCAGTACATCCGCCCGAAGAAGCCCGTACGCTATCCTGAATATTTCATCGACCACATGCATATTGATGCAGTCTAACGCTTGTGAATGCGCTATAGTTGACGACGCCATATCTGATGAAGAAAAAGCGCCAGTGCCAGTAGCTGAACCCGTTACTCAAGTCGTCGCTCAAACCCCCATCGTGTTGCCAGGTCCTCATGGAGGTGACGAAGAAAATGTTGAATCGGGGGACGAAGGGTATTCCAGCGTTGAGTCTGTTGAAGAGACAAACGAAACGGGTATGTCTTACAATGCGATTCAGTGCGTATGCTGCATATTCATGATACCTTATACTGATTATTCATGATAACGATTTTTATGCTTGTGTTACGAAGATATACTTGCAGAAACAACTGACCGGTTGAATGTGGACGCCCCGGGCGGAAGAGACCCCAACTATGATGCACTTGAATCTGGCGATGAAGCGGCAAGGGACGACGTTGTCACAGATACCCAGTCTGATTGCAGWGGTTGGGTATCAAATGCCAGTGCGGCCGATGGATCGGTGCCGAAACCCTGAACGAGACGAGATGGATATTGCGCTGGCAAAAGAATTTCTGGATGGATTTGGAGGCACCGACGCTGTGTTGGCGGGTAACTTGCTGGACAGAACACTGAGAGAGTTTTCGACAATGGATGGGGCCCAATTCAAGAACCGGATGTGTATGAGGAGTTACAGACGCCTTACGTTCCTGTTGAAGGCGCTAGTAGCTACCCGGGACTTCACCAAGGCTATTCTGGACCCACTCCTGAAGTACTGCATCGTGGAAACTCGCCTATGGCGTTGTTCTTTTACTTCATGCCGGTTGCATTGTGGCAGCACGTTGCTGTCTGTTCAAACAAGTACCAAAGAGATATGCTCCACGCTCGTGTTGACGATGCCTATAAGCGCCACAAGCGACGCTGTCAAGCGAATCCTGCCACGAAGAAGAAGACACGGCGCGATGTGCTACAAGAGCTTCAAGCAGTTCCTCCAATCAAACCACACGAGCTGTGTCGCTTCGTGGGACTACTGATCGCGCGAACAATTTGCCCAAACCGAGAAAAGCTGGCAAACCACTGGAAAACCACCGACGAGGGGGCGATTCCACGTGGAGCGTTCAGTTCTGTGATGGCACGAGATCGATTCATGGATATCTGCAGAAATCTACACTTCAATGATAACGACGACCCAAGAGCGACCACGGATCGAGCCTGGAAGATCCGCAAGGTTGTTGAAGTGCTGCAGCGGACGTTTCGCGAAGGATACGTGCCACCTGCAGAGCTTTCATTTGATGAAGCCATGCTTCCAAGCCGCAGCAGATTCAGCAAGATGCTGGTGTGGCGGGGCACGCTTCTACTCATGTAGCACAGTTGGTGGATGAATGGAGGACCTGAGATACGCAGCCGAAGCGGCGTCAGCGTGCGTGTAAAGTTTGCTCTCTGATGCAGGGCGCGAAGCCCCACACGACGGCGTACTACTGCTCCGAGTGCGATGGTAATGCGCCTATCTACCTGTGTATGCACCCGAGGCATGTGATTCGCGGAGTCTTGACAACCTGCTTTGACGTGTGGCACAAGGAATGGAGTGAAAGAAAGCTGCGACCTTCGAACTAAGAACGCCGCATCCGTATGCGCCCAGCAACTCCAACGAAAGGTGGTGGATCACGCAAAAGACTGAGAGATGCTGCTGATTAAGTATCATACATATGCAGGATACCAAAGGGTGAATATGCATTCTATCATCAGTATTTGTGTTTTTGCGAAGTAATGGCTGCAGTGAAAATTGTCTTTCTATATCTCTTGCAGGTTGAGAATAAAGTGCAACCGTCGATTCATTTTGCTTACTATGCAGTCCGTGAGGCCAGAGCCGGAAGCCCATGCTCAGATTATATTATAGGCTTCATATTTGTATGAGCCATAATAACTGAACTTTTGATTGCTCGCCACTTGGATGGGAAAACTTCAATATATGTACGCAATTGGACAGTTTCTAAAATTCGACATTCAAATATCTCTAAACTTGCAACCGTTTCTGCACCCGCCTATCGCTGTACTACCAGACGACCGGAAGTCAGCATCTATGTTACGGAAATGGAATGAATAATGCAGTTTAGAACCTAAACTTGCAGCCGTGTNATGCACGTTAATATGCAGTTTGCACCGGTTATTACAACGCTCGGTTCGTCGACCATCTCTGTAACAAGTAAAAGTATACAAACACCAAGTCAAACTATCTTGCATATGCAATTTAAGGAAAGGAAAAATCAGCCGGTTTGAAGGGGTTAATAAAAGTTGCTGTGTCTCTTATTGCCTACTGTAAATCCCCGCATGATGATTATCATGCTTTCAACTCCCCCTTTCCGTTTCACGATCAACTCCAGAGCACACTTTCGACCACTTCAAGAATGAATCCTAGAGCCAACCAGAGCTACCACGCCGTTGTGTTGACATTGGTTGCACTACTGGTGTGTGTCAGTGCCGGATCCATTCCCTCCGAGTTGAACAGTATGAAAATATCTGCCAACAGCTTCGACACCCGTGTCAGACGGTATTTGAGAGCACCAGACCTCACCACCGAAGATCGCGCATTCGATGCTCTAGGTCTCACGAAGCTTAAAGATGTAGCTACAACTGGAACTCAGAAGCTGCAAAAGCTGGCGAGCAACGCAAAGACCAAGATGACTTCGAACAACCAGCAGGCCACGGACAAGCTGTTTAAGAAATTAAAGGTCGGCAAAGTAGAGCAGAACATCTTTGAGAGCTCGCAGTTCAAACAATGGGCCGCGTCTGTGGCTAAGTCGTATAAGAAAAACGCCGAGGCTGGAGACTTTGCCATGGTCTCGACTCTTTCGTCTCGCTACGGTGATGATGTTCTCGCGAGCATGGTGATTACTGCAAAGAGCTCCCAGACGATTGATCCTAGCTTGGAGAATAAACTTATGTCCTCATTGCTGACTAAATGGCAGACTGAAGCACGGACTACAGACGACGTGTTCAAGCTTCTGAAACTAGATATTGACGAGGTGAATCTACTGAAGAGCCCCGTGCTGAGCACTTGGATTTCTTACGGCCACAGGGTCGGAGGTAAATCTCCGTACGAAGAGCTATTCAGCTGACTGCACGCTACGATGATGAGGGACTAGCGAAGATGATTATTACTGCTAAAGATGACAAGGTCGTGTCGTTTATCAGGACTGACATGACGAATAAACTGTTCGAGAATGGCAAAGCAGTGGGAAAACCGCCACGGACGCTTCGAATATTCTCAAGTTAAAGGAGGAAGGAGCTTCTCTTCTGAAGAATCCAACGCTCCCGATTTGGATCTCCTACGTCTACTCGCTCAAGCAGAATCCTTATGAGCTGCTACTACTGAAGTTTAAGGCACACCACACTGACGCTTCCGTGGCAAGGGTGATTGCTTCTGCGAAGTCGGATCAAAACTCGATAATCATTGTCCAGAACATGCAGAGAACACAGATCGAGAGTTGGCTCAAGGCCGGAAAAAGTGACGAAGAGCTGTTCAAGCTCCTGGAGCTTAACAAGGCAGGAGACCAAGTGCTTGAAGATCCGCTGTGGAGAACTTGGGTCGCATATCTGAAAAACTTGAAAGGGGACGCTGACAATAGAATGTATTCGGTACTAAAGACACAACTTGGTGAGGAGCAATTGACGAAGATTATTACCAGAGCCAAGACGGTCGGTAGAACCAAGGCAACCGCAACGAAATTGGAGCTGCAGGTTTGGGGAGCCAGCGGTCGAAGCGCAGACGATGTTTTTGATCTCTTGAAACTGGACGTTAAGAAAAGCGACGTAGTTGAAAGCGTCGCTTTCAAAACGTGGTACACCTACATGAAGACGATGAAACAATACAATGAAAAAGCAGTCATCACAAAGATGGAGAAACATTTCGGCGAAGTTCGTTTGGCTCAGATGCTTGCAGCTTCAAAGCGGACTGCGACTGACGAACCTACGAAACGGTTTCTTCGTGATTTACAGCGGAGCCAATTCTCTAAGTGGGAGGGTGAAAAAAAGAACCCCAAAACCTTTAGCAGCATGCTAAGTCCAAGTGATCGCAACTCTGAAAGCGACAGAGTTATGAGCGATTTCAATACTTTTTTCGGTGAAGGGATATTCGACATTCTGCGCATGGGTCGACAGTAGCTGGTAGATTTAGAGGTAGGAAGCACACCCCAAATTTCTTCGATCCTAGAATATCCGCTATGTTTTCTTAAAATTGAATAAACCACTGATCTTGATTTTTAGCAACTTGTGTTTGAATCGAAACGTATCACTACTAATAGGATTGGGGCATACATGCAATATAGATACATGTACAGTCAGAATTTCTGCGAAGAACTACCAGACCCCTATCTTCTGGCTACAGTTTTTGTTGTATTTTACTGTACTATAATACAGTATTTTATCCAACATTTGTTTAAGTCTTAAAACGAATAAGCAAAATAAATGAACATAGCTCTAATCATTCAGATACCGTAAAACTTATGTGCATTCGGACCGCTCATAATTTATCCTAGACTAATTTCTAGCCAATGAACACCGCCCAAAAGTGGCGTTTTCAATTAACAATCAGAGTACACAAAATACTTGTTTTTGCTTGGTAGTTCTTAACTGTCGAACGGCCGTTTGCACATAAGTTTTACTGTACACTCAGACGATTGATTATTCATTGGTCATGTTCTGCTCTGTTTTTGCTGGGCCTTTCAAAAAAAGATCTCAATCGCATGATAATTTCATCAAACAAGTAATATCAGATTTTTTAGTAGCAACGTGATTGAAACACTTGAACGGACTTCACTCTAATCGAAGCTCGTGTCAACTCAGAGAATCTTTGCATAAGTTTACAAAAWAAGCTAAAACGTACCTTTAAACCTAGCCCTTGTAAAATTGGCCAAAAACACATATACCAGGTAATGAAATGTACCTCATGATTTTTTCGAATATTTAGCAAGAAATCTTAAGATACATGTATTAATCGAGCCGAAATGATCTACCATTATTTGGATATTAACGAAATTAGATTGGCTAGATTAATAGGCAACAGGGTGCCTTCCTAACCCTACATAAAACGGTAAAATCACACATCTTTTTAATCACCTTATAAGCCAGTTTTTGTCAGGCCCACTTTAAAAACGTCTTGCCAAACCCACAGATAAGAATCTAGTCTGTCCCTACCTTTAAGCTGACAAAATGCTACGTTTTCATATTCACCGTGGATAAGGACACATTTAAAATGAACAAAATAACTGTAAAACGTCTCTATATTAAAGGATATGGAGGTGTCAGATCCCCCCCCTCCCAGCAACCTGTGTCAGTGGCAATGATTAGTCGCTATGGCATGCTTATAGCGCTTTGACCGGCGACCCAGACACGTTGCAAATTGGAAGGAGCTCTATCGCTTTCGTAAGTATTATTAATTCTTTTCGAAGTATGGTAGCTTACGCCAACTTGTGGCGAGAAGATTCGAGAGATACATGTACAGGAAAAAGGAGCCATTGAGGCTTACCACGGTACAGCTTTATTGCGTACTACTTGACTTATTAATCTTCACCATCAGCTCTCTCTCTCTCTCGACGTCTGATTTCTTGACGCACATAATAGTAAAAAATTTAAAACCGGTTAAAGAAAGAATGCACATCGAAGCAAACCGAACAGTCAGAGAACAAATGTCTGATGTTTAGCGCATCCATCGGTACCATCATACGATAGAAACAACGTACCAGCTCCGATTCCATTAGCAATTGGTATCGCATCCTCGAACGTCATCAACAAAGGCAGCCGCATAAACACTCAGGAAGCAGCCGCAAGATATGCCCTTCAATGGTTAAGAGCAACCTTTACAGATGAAGCTGTCCTATTCAACTTCAAAAGAGCCCACTTCGCAGTTTTCTTTCAAGTACACAAATAATAACGCTTTCAAACGCAAAATGGAGCTCCGTCGTGGAATGCTCTTGGTAACAATTTCGTTTCTGCTCTATTTAGGTTCGGCTTTGATGTTGGCTAGTGCTGGACTCATGGCTCCCAGTGTGAATCACGTTCGTACCATCGGCGCACTAGGGCGCCAACAGTAATGCCTCAGAAGATAGAGTTAGTCCCAGTATTACGAAGATGACAGAGCTGGTTCCAAAGATTTGCTTTCAGAAGCTACGAGACTGCGCGAAATCAATCTTCAAAACGGATAACCAGCTCACCATGGCTACTCCAAGCTTTCCGAAGTTGAGGCTTGATAAAGCAAAGTCCGATTGCTTCTTGAGTGAAGCATTCGATGAATGGGCTCGTCATGTACTCAAAATCTAGAACAATAGTCAGTCCTCTGCTGACGATGCGATGTTCAACACGTTTGGGTCTCACTACGGCGACGATGATCTAGTACACATGTTGACTACTGCAAATCCGACGAGTGAAAACTCGATTGTCACGCGGTTGAGAGAGATGGAAAACTGCAGGGAAAAGTATGAATGTAGTAATACATACGATCAGAAGACTTCAGCATTGGGCATGTGGATTAACAATGCCTTCAACACGCTTGAGAAACCAGTAGACGAAATTGTGAGAGCACTTAAAGCACACAACGACGATGTACTCGTAGCGAAATTGATGGCTCTGTCAAGAAACGACGTCAGCAGCGGCATGGCTTGGAAATTGGAGAGGTCGCTGACGGAAACGTGGCAAAGAAACGAGAGTGTAACTGGAGTGTTTTAGCTACTCAGATTCAACGAAGAAGGCACGTCGCGTTTTACAAATCCTGCGTTGGGGATTTGGGTGTCCTACGCGATGAGGAAGAGCCAGGATCCCTTTGAGTTTGTTGTCAGCCATTGGAAAGAAAGGCATCGATGATGCGTAATTAGCGAGGATGCTCGGCACGGCAACTAAAAGACGTCTCGAAGACCCTACTAGCAATGGGTGGGTTGCCGGGAATGCCGAGTTCCATTTGCTTAATACATGGTAACAAGAGGAAAAGAGCGGCGAGCAGGTCTCCACAATGGTTGGACTTGACAGGGAAGGGGGGTCGTGCTGCTGCAAAGTGTTGTAATTCTCATTCGATATGAGCGCTAATAATTGAGTGCAGCTTACGATTACGGGGGTTGCGTTTGGGAACAGATATAGGTAGGTACCTTTGAAATCCACTTCAACCCTGACACAATATGTAGTTTTAGCTCTCAGCAATTTGACATTTTATTCGCACGCTTAGGATACCATGATACTATCTGATAAGTACTTATCACACTCATCCTAGCCAGTCCGCGTAAAAGCAGCCATAATGCTGCGTAGAACACCCAATCGAATTTGTCCTAGCAGCTTGGATTTTGATGCCTACAGAATTCATTTTGTCATATCAGACGCTCAGAAGGACACAATTTGACTTTTTATTGTTAAGAGCGATTCCAGTTTACTACCGGTAAGTTAGTAGCTAATCGCAAAAAATATTTTCGTCAATATTTACAAATATACTTTAAAGTTAGTTTTGGGTTTTCCACGTACGATTTTATTTTTTTTTATTTTTTGAGCAGACGTACAGCCAAAAGGTGAAGCTCATCAAAACAAAAAATTACAGTCGTTGCCGTCTCTTACTTTGGCCGGAGAAGTGGACTGCAAAGAGGCTCAACAAGACGATGAGCCTTAAACACAAGAAGCCGTTCTAGCTGCTGGAAATTATTAAAACTAAACACATTTCTGAAGTTGAGCGACGTGACAGCGTTGCCATGGGAAGATTAAGCGTTGGAAATCCGAGATAAGTATTATCGGCTTTGCCTTACGCTGAAACAAATTGGTCAAACTTTAAAATACACTTAGACACGATTAAATTCAGAAGCATTGCAATATGCAGCGCTCCGACCCAATACTTAGCTAACCTAACGCCTTCTTATAAATACTGCCACATCCGTCTTCGAGATTGCCTCAGTCAAAGGTCTGTAGCACCAATTGCA

>Contig_14

TTGCGTCCGATTGTGTACAAGTGTATACTTCTCCCAGCTGATCACTGTATCATGCCGTGCGAGTCACGGCATGCCCAATACTAAGTCAACTTTTCGCCCAGCTCTAGGACGATAATATTTTCCACAAACACTCGGTGTTTGTACGAGAAGCAGCGCGCGCTTTACGCGCTTCTCCGTTCTTACGGTGGCACCTGCTGCCAACCGCACTTCCAACACATTTCGAGGTGTTTCAGCTCCTCGTAGTTTAGCTTCGGGAGACTTTCCAGTCGAGCAAAATTGCTCGACGCACCTGAGGTCAACCAAAGCACGAAGCACTGATCTCGGGGTTTGCTTCAGCTGGTTATTGCGCACTTTATCTTGGACGTGTTGATGTCGATGTCAAAATCAAATGAGGACTGATGTGCAGCTCGGTTACACTACGGCTTTGGCGCTCAAGTTTTTCAAAAGAAAAGAATGTCGTTCCACAACATCTTTTAAACGAACGACAGCGCTGCACTAAGATGATTATCGTCCGAATTGTTTTACTACCAGTAGAAGTTTTAGTACTACTCGAAGACGACGTAAGTTACTGAAGTACTCAGCACATAAATTGAAGAAGATTTTTAAACTACATATTACTATTGTCGTTTGTAGAAATATAATACTGCTGGATTTTACCATTTGCTGCCTTACTGACAATCCCTGCCCACGGCACAATACAGTAGAGCATTCTGTCAACGTTCCGTGAACCCGATTGGGTAGTAAGATCTGCAACCTGCTAATCTACCAATGCCACGATCATACAGTACATGTATCACCGAAAKCGCCCTACATGTACAGTACGCATTTGTTTATTGTATTTTTCCTCATAAATAACTCCTATACCAAACTTGCCGTTACCCCGATATATTCTAGTACAGTACTGCACCCCGTTTTCGCGCCCATGGGCCTATGATAAACTTCCGGATGTGATGATCCTGAATCCAAATGAACGAAATATTAGAGCTCATTCGTACATACAGAGTATGTGTAACAGTACCATTCAAGAATTCTGGAACGCTGTAGTACAATTCTGGAATGGAGTTTACACATGTGGATAGGAAACATCTGATGATTTGATCGCTATATATTTTCATAGTTTGCATGCGAAGAGATAGTAGCCATAATTCGCCCTATCTGTTTGCTGAGATAATGCAAGATGTAAAATGCAGTTCCTSGGTCATTTCCGAATTTTCTATCTACATATAAAGCCACTATGGTACGCTCCCAAGACCGCCAAGACCGCGCCGCTGAGACGCTCAACGAGGCCGACTTTCGTGCCTCCACTGCCGAGTTATTTCAGCGGGAGGAACGTCGCAAGGCAGAGGTCGCTCAAATCGTCGTACATAGCCGCAGAGACGACAATGACGAAGGGGACAGCTCCGGCGTGGGAGCTTTGCCGTCCGTCTTTGACTTCTGCTTACAAGCTGAAGGGCCCGATGGGGTGCTCAAGCTCACCAACTTTGCGCCAGAGGAGCTCGACCATGTTTGGGCAGCTGTGTACCCTCATTTGCAGGGGCAATGGAACGTGGGCCGCGGTAAAAAATGCCGCTACGCTGCGCGCGACGTTTTCTTTATGACACTGAGTTCGCTCAAACATCTTGGAAAAAGGGACACCGTTGCTCGAGTTTTCAGAATCCCGCCATCCACCTTTCAGAAAATGATCCGCAAGTTTATGGATATGCTGTCTCCTATTCTCTACGAAATGTACGTAGAGAAAGCGAATGATCAGTGGACACTGGGGAAGATTGTACGATCAGGGCATGCATTTAAGGACTTTCCGTATGCTCGATATGCTACGGACGTTACCTTTCAGCATGCTAACAAACCCAGCGGTAACATGAGCGAGATCTTACGCTATTACAGTGGCAAACACCACTTGAATGGCTACAAAATGGAGGTGTCGGTGCTGCCGAACGGTGTCGCGATTAATTGCATGGAGCACACCGGTGGGAGCACGCATGACGCTGAGATTTTTCGCAGGAGCGCAGCATTTCATTCGCGAGCTCTTCACAAACACTCTAGTGATGCCAATGTGCGAGACGAAGGAAGACTACAAGACAAGTATCCGAAAGAATGGGCGTTTGCTAAAATCTGATTTAATTTGCAAATCCTATATGGTCTACCCCCGCGAAAACGAGGTGCAGTACTGTACTGATCATCGAGTTCCTATCTTGAAACAAAATGTGCTAAATCGGACCCAATTACCGTAGCCGCTGTCAGTCGGTCCCCGAGATAAAACAGCAAATGGAGCGGTAATTTTTTAAGAAATAAGATAAACCTTTTAAAAATAATTATCGCCCGCAGTGATCATACATCCCATCATTTTTCGGTATCTTAGCCTCGTTTTCGTCTCAGAAGCGAACCGTACCACATCGAATAGATGGTTGCCACAGCGTCACTTCGTCATTCTCTCTCTGAGCCAAGCGAAGCTCGGAGTGAGCTCCCCAGGTGCCGCCATGCGAGTCCCCAGGGCCATACTACTGACAGTTGTCTTGATGGCAATATCCGATACTGTCTCCTCAGCTGCGAAGTCCCACTTGACCACACCATGTCTAACACGGCACGATACGAAGAGGTTTCTGAGGGCTCACAATACCGAGGATAGAGGGATCAGTACCCCCAATGTCGAGATGCTGCAGGGGTGGCTCAAGAAAGGTCTGCTCTCCGACGAAGCCGTTGGCCTGTTATCACTCGGTCACAAGGCCGACGATTTACTTAGCGGTTCGCTATTGAGCGCTTGGGTCAGCTACATAAAAGTCTTCAATAAAGAACACCCTACAGAGAAGATGAAGACGATCTCGGCGCTCACCGCTCGCTTCGGAGACGAAGCTCTGTCCACGATGATTGAAACAGCTAAAAGGGTCCCGAAGACGGAGGACGTCGCTACTAAAATGCAAGCCAAGCAGATCCAGAACTGGATGACGCTTGGTAAAACCCCGGACGACGTTTTTACGCTGCTGAAACTCAATACCGCCAAGTCGCTTTTATTCGATCAGCCTCCAGTCAACACTTGGCTACAGTATATGGACGATTTCAGCAAGGCTAAACCTGAAGCACAGTTCTCTACGATCACGACATTGAGGAAATTGTACACCGACGATGTATTAGCCAAGATGATCATTGTGGCTGGTAAGAACGCGAAAACTGCGGAGGCTGGTAAAAACGTGGAAACGGCGTTGTTACGCACCTGGTTTAACGAAATGAAGACCCCGACAGATATCCTAAGGCTGTTAAATGCTCGCGAGACTGGGCAGAGTCGAAAATTCTTCGCGTCTATATGGACGAAATACGACGATTTATTCCAAAAAGTGGATCCCAAATTCAAGACCGACATGCTCAAGGACTGGCTGAAGAAGGGGTTGATCACTGACGAGACGTTCCGAATGCTAACGCTGGGCAACGCGGCTGACGAGTTCCTCAACGGCTCGTTGCTAAGCGCTTGGGCCACGTACATCAAGGTGTTTAACCAGGAGAGTCCTACGCAGCAACTGAGTTTACTCGCGACACTCACCGCTCGATTCGGTGACGAAGCTGTGTCGACGATGGTTGAAACAGCCAAGAGAGTGCCCACGACAAAGGACGTCGCCAATCGAGTTCAAGCGGAGCAGATTCAGCACTGGATGACGCTTGGTAAAACCCCGGACGACGTTTTTACGCTGCTGAAACTCAATACCGCCAAGTCGCTTTTCTTCGATCAGCCTCCAGTCAACACTTGGCTACAGTATATGGACAATTTCAGCAAGGCTAACCCTGGAGCACAGTTCTCTACCATCGCGACATTGAGGAAATTGTACACCGACGATGTATTAGCCAAGATGATCATTGTGGCTGGTAAAAACGCGAAAACTGCGGAGGCTGGTAAAAACGTGGAAACGGCGTTGTTACGCACCTGGTTTAACGAAATGAAGACCCCGACAGACGTCATACGTCTGCTAGGTCTTCGCACGCCCGGCCAAACGTCTGTAGCCCCAGTTTTGACCAAGTACATTGCGTTATTCAACAAGGTGGATCCCCGATTTAAGACTGAAATGCTCCAGAATTGGCTAAAAAGAGGTTTAATCACTGACGAAACCTTCCGACTGCTCACATTGGGCAACGCGGCTGACGAGCTCCTCAACGGCTCCATGCTAAGCGCTTGGGCCACGTACATCAAGGTGTTCAACCAGGAGAATCCAACGCAGCCAATGAGCCTACTCGCGTCGCTCACCGCTCGATTCGGTGACGAAGCTGCGTCAACGATGCTCGAAGCAGCTAGGAAGACGCCTACGACGAAACGTCTTGCTTCGAGTATCCAGAGAGAGCAGAGTCGACATTGGCTCAGCGTCAAGAAACATCCGGACGACATCTTCGTCCTACTGAAGCTCAATACCGCGACCTCTCGGCTGTTTGACCAGCCTCAACTGAACACGTGGGTGAGGTATGTGGACGCTTTCAATGAGGCCAACCCGACGAGTACAACGACGTTATTGTCCACCTTGCGGACACGATACAAGGAGGACACGTTGGCTCAAATGCTCGTCGTGGCGAGGACCAAAGGGGGCTCCGTGGGGCAAACCGCGACTCGAATTCAGGCGGAACAAACGAAACTTTGGCTGAAAAGTAACAAAACGCCGGGAGAGGTGTTCGAAATGTTACAATTGAAGAAATTGGGCACCAACTTCCTTAGTCACCCAATTTTCAATGCATGGGTGAAATACACGGACGACTACCGCAAGAAAAACCTAGGGACATATCGCTCTGCACTGACCACGTTGAGAAAAACCCACAGTGACGAAACGCTGGCGAAATTGTTCATTGAGGCGAGTAAAGTGGCGAAAACGGCGAAAATGGGGAAACGTCTGCATGCTGAGCTACTACGCGAATGGTCCCTCACTGGAGCGACACCCGTGCGGGTCTTTTTGCGTCTGAACCTCGGCAAAATGGACCCAAAGGTGTTTGAAAGTCCGCTGTACTCTATGTGGACGAGCTACATTTCCATGTTCAAAAAGGTGAACCCCACGTTCAAGGACGATCCAGTGAAGATGCTGGTATCGATCTATGGTCACAGAGACCTGACGGCCTTGCTCCTTGCGGCGGAGAAAGCTCCGAGTACTAAGGATATCGCCATTAAGTTGCAGAAGGAGCTGCTCGAGCTCTGGCAAGCAGCCAAGATGGACCCATCACGCGTCTACAGTGCACTGCATGTGGAACGTGAAGCCAAGAATTCACCCATTAGAATGTTCTGGAGTGAGTACGTCAAGGCCTTTAGGAACTCGAATTAAATAGTACTAATATAAAAATTAATACAAAGAAAGTATTACTGAAGTACTATTACTACTTAGTAATCTTTTTACTAACTAATCTTGAGAAAGCCCAGTGCTCTGGGGAGGCTTGATAAGTTTCGTAGCGCCGAAATTAGATTCAATCTCGGCTTGAGTCGGTGCTAGGCTTCCACTTTCTTCAAGCCAAGCGCTCGACGAGCGTCGTGCGTTGACGAGGACTCGGTAGCCATGGGAGCAGCTGCGTCCATGGACGACCCCGACGCGTCCGTGGTTTTTAGAGAGGTTTGTRTTCTTGTAAACTTTATTTTTTTARCTAGTATTCTAACATTATTGTTGCAGACGAAAGAGGAATATGAGCGGAGAGYTGCTGCGGGTGACTCGAAGGAAGAAATTTTCTCCAGTCTTCGAGATATCGTTGCCACCCGTTTAGGAGGCACACACCCACTCCTCCACACGTCACAGAGTCTGGAATCCTTTCTCGTCCAAGATTTAGCAATATCTGAGGTAAAAAAATCTACGTGTTTTTTATCATCGGCTACTTGGTTGTCCACAACTCACCTCTCTTTTATTGTTTTAGTCTGGAGATCTAGAGAAACGTTCCAGCTCGTTGCTGGATGCTGCTACGTTAGCTGCAGTAACGACCTCGTCCGAGAGCAAAGAATCGACTATCGAGACCAGTATCGCACTAGCCAAGACGATACCCGACGACGACGACGTCCAATTGAATCCATTCCATGACGACCATGAAGTCGAAACGCACATCGATGAGCAGGGAATCCTCCGAGCAACTGTTGAAAAAGGCTTTGGACGGGCTGTGGGTTCGTCTCGGGCTGCACTTGATCTGGTGCAAAACCTCGCTCCTGTATTTACAACTGACGTACTAACTGACCCAACAACGCTCAAGATGATCCTCAAGTACGCAGAAGCCAACAAAGTGCCCAGCGGAGCGGATATGCTCCGATTTTGGGTCGAAATCGACGAACTGCAGCACCTTCCGTCCCATTCGTACACGCACCGTCGACTACGCAAGATTTACGACAAGTTTTTGTCCCCTGAAGCGCCTTCGCCTGTGTGTGTCACGGCGCAGATGCTGCAGGATATCGAGAAAGCCCTCGAGGGAGATAATATCTCCGCTGGCATCTACGCCGGTGCACAGCAAATCTGCTATATCGCGCTGGAAAAATCCGTGTATCCGCGATTCCGAGACAGCAAATTGTTCCGTAAGATGCAGGATTTCTGCGCTCCGGTCGTTCCTAACGCTGGCGCGTCATCTAACATTGGCTCGAGCAATGGACCTACTTTGTTAGCTGCTGCTTCGGCTACAGGAACGGTGGCTGCCAACATCACGGATAATATGGAAGATGCTGAGGATTATTCGCTGTTGGGTATTCTAGCACATCCAGCTAAGCTGCGCTTCCTCAAGACGTTCTGTATGGAAGCATTGGCGCTGGAAAATCTGCTTTTCTACCTGGAAGTCGAAGATTGCAAGAGATTGCCGAATCTGTCGTTCGTGGTCAACAAGACGCGAAAAATCTACGATCGCTACTGCTCTCCATCGTCCAAGAACTTTATTGTCGGGCTGGGAGACAAAGACGCGCTGAAAGAAATCCACGACGTGGTGGAGAACAAAGGAGCTCTCGTGCCAAAGTTATTCTACGAGGTCCAGATAGGCGTATTTAACCGGATTAGCGACGATATCTGGCCTGGGTTCTGTCGCTCCCAGGAATATCTGGATCATTCGAAAGAGGTCCAACCGGACGCGAAACATCTGGCACGACGCGGCAATCGCTTCGAAGAGAGCGAGGCGGTGCAAAAGAAACTGGAAGGCTTGGCAGAGCTCCAGCTCATCGACGCGGCCATGCATTATCCGGTCGAGAAGCTCATCCCTATCTCAGTTCCAGATTCTATCCAAGGGGCAGCTCGACGAAAATCCATTCAAAAACTCGAAGAGGAGCAGACTCTCACGCCTGAGCAGGAGCTGAAGCTGCTGTTGGGCGACCCATTCGCCAAGAAATATCTAAAGCTCTTCATGACTCGACGAGGCGTGGATTCTTTGCTTGCCTTCTGCGAAGAAGTGGAAGATTTTAAGCTGCTACCAGGTATCGAGTTTCTCCAACATTCCGCTAAGAAAATCTACCGCAAGTACATTATCCCCAGTGCTCGACTACAGGTGGATATGAGTAAAACGATGCGAGAAGAGATTTTTACGCGACTCGCGAATCCAAGCGTGGATATGTTCAAGAAGATCGCGAACCGGGTACGTCACGGGATGCTGCAGGACTCGCTGCCTCGCTTCGTCAAGTCAAATTACTACAAGGATCTACGTCGGGATAGTAAAGCTACGCCTGCCGATCCCCACTTGGCCACAGTCGACCAAGCAGCAAAAGCAGGAAAGCTCGAGCTGTGCCACTTGGATGTCTTTCTAACATATCCAGGATGTATGCAAGCCTTCCGGAAGTTTCTGGATTTTCAACATTGTTCCGAGAATCTAATGTTGTGGGAGGAGATTGAACACTACCGAAGACTGCCGAGCTACCAGATCGTCCTGCGTTCAGCCAAGAAAATCTACGACAAATATCTGAATCCGAATAATCCACGATCGCAGATTCCGTTSGCTCCAGCACTTCTTCAACGTGTAGAGACGCAACTGGAAGTGGCGAGTCGAACGACGTTCGATGAGGTCGAGAACGAGTGCTACGACCACATGCGCAACGTCGTGATGCCGGATTTCCTGGATTCTCGGATTTTTATGGCCCTAGTGGGGACGTGGGCCAYGGTTCACGAAGATTATCCAGCCGAGATGCTACGTGGAGAACTGGAAATGGCGTTTCTGCGTCATCGCTTCCATCTGGTTCAAGAAGCTCGAGGAATGTCTCGAGACTCGACGGTCAGCAACTTTGACAAAGGACGCAACGGAGCGACCCCCACTCTAGGCACGACAAATAGCGGCCGAGTCACAAACGGATAATCCAGAAATCTATTACCGATTCCAGATTTTAAAAATCTGAATATATTTGAAAAAGTCTAAATCGCTATGCAACTACTATTACCCGACTTAACAGAGAAATCTTTAAACCTTCATAGACTCCTACGATCCCTATGCGAACAGCACATGCAGCGCCGCTTGTTGCGTGGCCGGCAACGAGGCCCACGCTAGAGTCATAACTGGTTCAGGCACCGTAGAGCGGAGTCCTGCGAGTGTAGACGCGAGTTCTTGACGAAGAGCGGCGGCCTCCGTCTCGCCCACCGCCTCGCTCATCTTCCGCAGGAATTTGTCCTCGGCCACAGCCGCTAAGACGGCCACCACCTTGCCTAGATGTCTGTAGTCTGGCGCGCCCAACACAAGCGGATGTCG

>Contig_20

CGAAAAGTGCAGACGTACGTAAAAGAGGTGAAATCCCACGTCCTTTCTTTAAGTGTTTGCTAGTGGTCAGAAAAGTGTATGCCAGCGGTCAGTTCGAGGCATAAGTGTTTGGTCGTGTCAAATTATGTCTACCGTTCACCTAAAAGTGTTTATTTTTGGCCAGAAAAGCGTATGCTCTCGTACCAGACTCCCTGATTTACTAAAAAAAATGAAAAAAAAAATTGATTAGGAGGGTGTGGACGAGAAAAAAAAACGTACTTACGTAAAACAAAATCGTACCTATGGGGAAAGTGGTGAGTTCATGCTGTTATGTTTCGGCTGAATGATAACAATCGGATGCCTACGACAAAAAGACGGCTACAGTGTTTCTTATCACTAATCAGCTAGCGCTCATTTGATTTTTTGGTCTTAAGATTGAACTTTTGATTAAATGTGGCCCTTTATTTCCGGACAGAGGAAGTCATTAATTTTGCCCGCCCATCCTGCTTATATATTCGGGACGGAGGAAGTACCAATACATTATTGGCCATTTGCAACCCGGGTTGTATTTCTTGAAATCCGAAGCTACGATTGGCTGTCTTCTCGGTATCAGCCAGATCTGAGGTCGATACCGTGCCTAGATGTGGAGTGCCGTTTGCAACTATCGTTGCATGTACGGTAGCGTGAACGGTCGACTTTTGCAACCCTAGTATACAAATTTCAAGTCAAGTTACCTATAGTGAAGCTAAGTCACGGTTGCAAACGAGAGGGTACACAGCTCGTATACAAAAACTAAGTCATGGTTGCAACGTGAAAGATTCAGTTTGAAAATACAATCCACCACAATGAGCACTAGCCGCTACAAGGCAGAGCTCGTAAAGTTCATGTCGTTCAAGGACGACAAGGAGTACACTGCTAGCCACGAATTCACGCCAGCGGACCTCCTCAGTATCACGCCTGGACTGCTGTGCCGCTGGATGAACACGCGGGCCTACGGAGATTCAGAGCCAAGCGAAGACATGAGGCCTGTTCACCTTCGGTCGAGCACGCTGGAGTTCGCCAAGAAAGCCATCTCAGCGTACATGCCTAGGATCAACGCACCATGGGACCCCGTGGCCATGCAAGGCAATCCAACACGCTCCGATGATGTCAACAAGCTCATAAAAAGAGTCAAGCGCTTTGAAGTTCGTCGGGAAGGTGCTGAGTCAAAAGCTCGTCGCTCTTTTGAATTTGATGAATTCATGAACGTGTTGACGCTGGTAAGATCGCTGCATTCACGCTCTGATGAACAACTCATGGTTAGCAGTGTTTTGACACTGCAGTGGCATATTGTTGCTCGCATCGACGACATGATGAAGCTTCAATTTAACAATTTCACTCACAACACTCAGTACCCGTCTACTATTCTATGTCAAATGCGATGGTCGAAAAATATATCCGAAGAGAGAGACGCTCCGGAGCAGATTGTGGTTGGTAGTATGGATCCCAGAATGTGTCCCCTGCTCAATCTTGCAGTATACATCGAGGCAACGGTGAATGTGGCAAGATCTTCTTTCTTATTTGGAAATCCAAACGATAAAGATCGAGTGGTGAGGCGTTTTCTAGCTGATACAATTAAAAAATCGGAATTTAAGTCGTTGAAGACGGGAAAGCTGGGAACGCACAGCTTCCGTAAAGGTGCTGCTACTTATGCGACTCGTAGTGGTGTATCTAAAGACTTTGTCAATCGGCGAGGACGGTGGAGAACTCGCAAAGGCGTCGTCGACGTGTATATCGACAACACTCAGCCTTATCCGGACGCATGCACCGCCGCAGTCCTTGCTGGTCCAGCTGGACCTTGCTTTTACTCGCTGAAAGAGGGCATGCGGTGTGTCACTACACCACTTCTCGTCGACGAGATTGCTCCAACAATTAAACAGGTCATGGGAGAGCCAATAGCAAAAACATTGGCACAGGTGTTACTGTGGGCTGCGCTGGAGACGGATTCCAGCTTTAATTATTGTCTTCTTCCGGAAAAGCTGAAAAAAAGAATTTTACGGGCTTACATTAACGCCGGTGGAAGTACGAATTTGAATCCGATTCAAAGACAAGAATTTTATGTCTTGGGGGACGGGTCTCAGCTCAATCTCGTCGTTATTGACAAAACGCAAGAAGTGGAATCTGGTGTTGGTAGTATTGTTTCTGCAAGAAGTGCATTGGTAGCGGGCTCCAATGGACAGGGGACTCAACGTGAAATTGCTGCTGTACAATCTCAAATAGCGTCTGGCCGTCGCTACATGGCAGAGGTTATGAATGAAGTACTGCGGTCCCGAAGCGAGTCACATCGAGAGATGCAAAAGATACAAGCTATCTTGAGACGGATAGCCATGCAGCCGTTCACACCTCGTACTACTGATGGGCACGGAGTACCGTATCCACCAACAAACACTGCTCAAGGTGGATTTCGTGGACAAAACGCGGCACGTCTTTCAAAGAGACCTAAAGACCTGTATGAACTTTGGCACGAGTATCAGCTCGGATCCGGTGGGCTAAAACCAGCCAAGGAGTTTACATCCATTGAGCGAGGCGCAAACAAGTTCGCCTACTCCAGGCGGAAAGTCTTCTGGGATGTTATTTCTCAATTGGTTCGCTCTGGACACACTAGCGACTCTGCAATAGATAGAGTGTATCAAACGTACGGTAGGAATCTCTCTGTGTCTAGTATTTTAGTTAAACTTCGTACAGATCGTAGACGTGGAGGACATCCAAGTCTGCGATTGTAGCACTTCAACACGGTTCAATTCAGCCAATCAAACCTTATCCTTCGAAATATTAGCCTATCAAAACTATGGTTGCAATCCAAGTTGCATAAATGACTAATATTTATACAGTACTGTACGAAGTAATCACTGAGTACAGCGGGACACTTGCCGTAGTCACAAATCTTGTTTGAACGTGCCAACAGATCTATCCAGCTGTATTTTAATTAATAATGGCCGCACACCGCTCACTCTGATATTTCCCCGTTCCAGCACTCTTATGACATGCAGTGAGCAATTTACTTCGGAGCGAACCCCGGACACACCCTGATGATGCGACTCTACCTTACTGCGCTGTTAAGTGCAATTTCAGCTCTACTAGCGCCGGGTGGAAGTGCCCCAGTGTCTGCTCTTCCAGACTTTCCGGCTGGCTACTTACCATGTAATGAACTACGTACATTAACAAACGCACCTGAAGAATCTCCATCCCACAGGCGATTGAGAATTTCCGATACACATGATGACGAAGACAGAATAAAAAGTATCAGCATTGAAAAACTATCGGGGTTGATTAAGACTGGAGTATCGAGGATACATGGATACCTATATTTAGGACCGTCAGCAACTAGAGAACAACCAGCAGATGAGATTCTTCGAATGTTCAAGCTTGAGGATGGAATAGAGAAGGCCTTGGCTAGTTCTAACTTGAAGACCATGGAAACTTATGTGAAGGAACTGCGCACCAAGAACCGAAAGAGCACGACGTCAGTGCTTGGAATACTCACGAATCACTACGGGGACGACGCAGTAGCCAGTGCACTTGTGACTGCACCGCATAATACCATTATGAAAGACATGGAGGATACGATATGGCGATTACGAAACACACAGCTTTCAGCTTGGCTGAGTAGCGACAAGTCTGTCGACGATGTTTTCAACCTGCTAAAGCTCCGTCAAGATGGCTACCTAGCTCTCGCCAGTCCAAAGTTGGAGGTGCTGGACGACTACATAAAGCTGATTATCCGCTCCAAATCCAGCCAAGAAACGTTGCGTGATGTGTTAACGAGGGGATTTGGAGAGCGRAGATTGGCCAGACTGCTAGTCCGTGCGAAGCAAGATGACCGAACAAAAGAACTGGCGACGGCACTGCAAAATGCGATTTTAAACAAGTGGGTTACAGACAAGTTGCAGCCGGTCAACGTCCTACAACGACTGAGATTGGATAGAGGTGTCACCAAAGCTATGACAGACTTGAACCGAGACACTTTGACGAGGTACATCTCGCTGTTTAAAACACATAATCCAAGCAGTAAAACGTCATTCATTGGTACGCTTTCCGCGCATTATGGAGACGATGCAGTTGCGAAAGCACTCGTGACGGCGTCGTCGGATGCGAGTACAAAAGAAGGCGCGATTCAGCTACGGAGTGAGCAGCTGACTGACTGGCTGAACAACGAGAAGACTGTCGACGAAGTTTTCAAGCTGCTAAAGCTTCGCGATGACGGAGAAGTCGGTCTAATTAGTCATAAGTTGGAGGCTCTAAAAGATTACATCAAGCTATTCAACCGCGAAAGAACAGGAGATGAGACTTTACTCAAGACGTTGACGACTGGATTCGGCGGAGAAAGTGGATTCTCGAACATTCTACTAGCAGCAAAGGCCGATCGACGTACAAACACAGTGGCTATGTCATTGCAAAGCGAGCTACTTCATCAGTGGCTTAAGAGCGGATTGCAGCCGGGAAGCGTCTTGAAGAAGCTCAAATTGGACCGTGGGATAACAGAAGCACTCTCCGATGGAAACATCCACACTTTGACAGCATACATTTCGTTGTATAGCACACAGAATCCAAGTAATGCAGTATCGCTGATTAAGATACTATCCGCGCATTATGGAGACGATGTTGCCAAGGCACTTGCCATGGACGATTTTGCCACGACTGAGCTGGCGTCCAATCTGCTGACACAGCAGTTGCAGCTGTGGCTGAAATCTGTTGGAGACGTTTTCGCGATACTGAATGTTGGACACCTCGATTTCTTGTCCATGAAGAGTCAGAAGTTGCAGATTTTGGACAGCTACTTGAAGATGTATAACGCCAAAAATCCGCTTGACGCCAAGAGTATGTTCGCGGTAGTAAGAAAGGGCTTTGGCGGTGATGCCGGGCTTGCACGTGTGATTGGTAAGGCGCTTGTAACCTCGCAAAATGAGCCGAAGATGGCTCTCAAATACCAGAATGAGCTATTCAACCAGTGGTTCAATAGAAACATTGAGCCCAAGAACGTTTACGTAGAGGTCCTCAAGATCAAGAAGCGCTCTGCAGACTTTACAGCAAAGGGGGTCGCTAAACGATRCAAGAACTATTACAAGAAACGGGTGGGGGAGGTTATAACCTTTAACAATCCAAGGCGGTCTTAAGNAAAAAAAAAGCGTTATTAGATGTTTCAATCCTAATTCCAACGGAGCGTGCATAATCTGCATTGTTTTACGATCAGCGTGGAAATTAAATTATGTTTGCAAGTTGGGAGGAGTCGACGTCCTGTTAGCCAAAATTGCTAAAAATGGAAGAGACAGACGTTCGTATGACGCGTATCTTCACTACCAAACCTTTTCGGATGAATGTCTTAATCATATCATGTAAAACTGTCTTGACTGAGAAAAAGTGACGATAAAAAGCGCTTCTGTCGCTTGTATCCTATGCAAAATACGGTTAACCGTGAAGAATTGCGGTGCGCCACCAATAACGTGTGTTTTACCGCCGCGCCTTTTTCCTGCTGACCATGCCCAAAACCATCTTGCGCCGCAGCGGCTTATCTGTCGGCAGAAAATTCGATGGTTAAGTGGCAAGATATCGTACTTNAAAAAAATCATATTACCGGTAGTCAGAATCACATGAACGTAAGCCACTCTAAAAGCCTACAGCTCATGGTCTCTTCCAGCTATTCGATGATCGGTGGGCTGGGATGAATAGTTGGTATCAGCGCAAAATAAAATGGAGTAGGGTCTTCGCTTGTGACCACTCCGGTCTACAAACGCCTTCRATCGTTGAAAGCCCCGAAACGACGAGGAACTATCTTCTCGTTGTAGTATACTGTGTATCGGCTTACAATCATATCATCCAAATGACTAGCAGAAGCCTCTTCAACATCGAGGAATCTGGAGTAAATGCTTTTTGGTTCTGCCTTGCTGCGGAACCAGCGCTTAAACAGCAACCTTTCGTACTCAGCACCAAAAGAGACGAGGGAGTCGATGTTGTGAACGCCTATCGACTCAATTGCACCAAAAACCGCGCGTGCAAATTTGCCTTCGCCACCAAATCCATCGCTTAGCACCGTGAGAATGTCGGTCTTGTATCCGGGATTAATGACGTTGATCCTCTTAACGTACTGCTCCAAGAGATGGAGCTTCGTACTTGCCACGGATACAAACTGGTTCTTGTTCGGGATGTTAAGGTTTAACTTAAGCAGTCTGAAGATGGTGTCGGGCAGGCCTCTGCGATTTGAATTATCATATTGAATAATATCGCATATTGGGAATGCAATTATGTATTGAGCAGCGAGTAAAATTGCAAGCATTTTTTAGCATTTTATCAGTTATTCTAGGTTAATACTGTCCACCAAAACTACTGGTTGCCACCACCTCAAAAGAGACTTTACGAGCCTTGTGCAATCTCCTCAGCTCATTCGACCTTTTGCAGTCATCCCAACAACTCTTCAGCCCCCTTTCGCCCAAGTACCCCATGAAATTTACAAACAAGGACATCTNAAACGCGTATCTGGCTGATCATGAACCCATGTTGGCCAAGATACACGCGCTCATGAAACACCTGAGCACCATCAAGTGTCGTGCTGCCCTTCGCAAGGTGACCTCTCTAGCGCCCGTCATGCCCAACGCGACTCGTTGGTCGAGTACCTACAGCAGTACGACAAGATTTGTAGCGCTCTTCTCGCGTTGTACCACGCTACGGTGGCTAAGCATGACATCGCGCGCTTTCTACTGACGCCAGAGGAAACCGAAGCCGCTCGCTTCCTTCTCAAGTCGCTGCACGAGCTGAATGAGGTGAGTAAGACGCTACAAGACTCAACTCTAACAGTGGTGGGTGCACGACGTGCCTTTGATGCAGTGTTGCGCAAGTACCCCCGCATGAAGACCCGGCTTGCAAGTGACGCCTCCGTCGTGAACAATCCCGAACTGGAGAGCGGCATCGTGAAGATCATTGGCGGTGTCGGCTGAATGCACGCGAACAAGCGGCGTGTATTCATCTCAAGCGCAGCAGCGACGACACTGTGGTAAACCCAGCAGTTTCCACGTCATTTCTCGCGTCGGCCTTCAAGAAGGCCCCTGTGGCGCGCTCCCCATCGCAATACTTTCCTCTCGAGTGGGTTCCACCCACGTCGAACGAGTGCGAGCGTTTCTTCAGTCAAGCCAAGCTGGTTCTGACCGACCTAAGGAAAGCCATGGACCCTAACACTCTTGAACTGCTTATGTTTCTATCCTACAACAAGAAGGATTTAGTGTTAAAGCTATCAGGCAGAGTATGGGTAGTCAACTTCGGGAATAATGCCATTATTATTGTCTTGGCGTAATAGCAGCCGAATATATTGAGTATGGCATATTGGGGAACCCTGGTGTCGGGAGATTTGCCGCTTTTCAACCACGCATCCATTTGCTCTCTTAAGCTCTAAAGCCGTCGCCTTCGTGGCCTCATGAGACCTCGCAGCGCTGAGCGTCTCCGCCACTTCACCGTCCCCATAATTCCTGATAAATGTCTCGATCAATGACGTTTTGGTACGGGGATCCTTGCGTTGTACATCAAGATAAAAAGTGTGATAAGTTGGGTCCAGCAGAGCTTCATCCATGTACTCGTTCAGTTTGAGCATCTTCAGCACATCTACAGGTCGCGACTTACTCGAAAGCCATTTATTCAGTAGCGCGTTCTCCATCGCATTCGCCAGCTCACTCGTCCGGGGATCTTTCTTCGCACGAATAAGCAGATTGGCCAGGTTGTGCTCCCCGTCGAAACCTTTCGTCAAGACTTGAATCAACTTTACTTGTCCTGATTTTTCACGGTTGAACATCTTCATGTAGTAATCCAATGCTTCTAGCTTCGGACTTCCGAGAGCACTGTATCCGTCTTTCTGTAGCTTTAACAGCTTGAATACGTCGTCGACCGATTCATCATTCTTCAGCCAACCCGACAGCTGAGCATTTCGCAGCTGCAGAATTGTTTGAAGCTCACTTTCACTCTTTTTCGTCCTCTACGCGGATACGAGTACCGCCGCCACTGCATCATCTCCATAGTATACCGAAAACACTCCAAGTACCGACATTTTGTTCTTTCGTTTGTATTTGTTGACTGACCTTAGATAAGTTTCCATTCTCTTGACATCAGGACTACTCAGAGCCCTGTCCAAGCCGTTTTCTAGCCTGAATAGCTTGAGGATATCTTCTGCGCTCTGGCTGATTGGTAACCAGTACCCCAAGTTCAAATTTTCACCAATCTTTACTGCTCCCGTCTTGATCAGCCCTGCCAGTTTTCCGATCTCACCGCTTATCATCCTGTCTTCTTTTTCTTCGCGGAATTTCTCAAAAGTCGTCTCGCAGACGAATCTTCTGGCTTGATTGAACGCATTTGAGGTAAGTTCAAACCCTGCGCTTCCGAGACGACAGTCGTAAAACCCCAATTGGCACATACCAGCAAAATTGAAGCACCACACAATAGCGGTTTCAAGCAGAGCCACATGGTTACAGGCTTGAAAATTTGCGACTGGTTAACCCCCAGGAGAGCAAGCACTTTCGACCATTCTACAAATATTTTTGTCTCGTCCATGGAAGTGATACGAGTTCTTTTCTGTAATCAACGCTTGTTGCAGAATGGCGTCAGAAGAACATCTGTTTACGACAACACATCAGATTTTTAAGATCTAAAACCGACCACAGTACAAGTAATTTATTTTAAACGACGAGGCGAAAAGAATCTACATTTTATCGCAGAAACTGAATTTAAAGTACATACATGTCACAGACGTGTGTTTTTACACTTAATTGTGTATCACAGGATTTTTCACATAAAATGCGTGTACCACGTACACTAGCACAGTTTGGGACTTTCGTGAAGATCTTGTGGTCTTGCAGAATGGGTAGGAGAACGCTGGCACGAGAGCTCGTTCACAGTAGGCTGGAGCACCTCGCCGATGTGAAGAAAAACCTAGTAAGGGTGCGAGTGAATCTGGATAAGATGTTGGCTGTTTAACTGGAGATTCAAGCTGCTATTGAGGAGGATGAAGAGCGCAGCTACGAAATAGCAGCACGTCTAGGAGTCGGAGGGTCTCAATTTAGTGGCGTTCTATCATTCGAAGTGTTGCTGGAGATGGCAATTAACAGGCTGAAAGCTTAGCATACACGTTCAGCTGTATTAAATTTTTAATACAGGTCTGATTTCCTACTTGAGCTCGTCCTGTAGGCTCTCATGAATCTTACTTACTTTATCAGCAGTAGCATTGGACGCTTTGCGCTCATTCGAATTACACGATGGTCCATAGCATCCGTAAGCGCATTACCTTCACATCGTTCTTTGAGACGTTGTACTTCTGCCGTTCCACGTTTCCAGTAAGGTAATTGTGTGCCTGGGCTACCACCAAGACACCACAGCTAGCAAAGTCGGGCTGCTGTGGGGCATCAACCCACTCAATTGGGTCGAAAACAACTTTGCTTCATGGCATCGAAGCCTAATGCCACCGCGCTACAAAGTCCACTAGCCCTTCTTTTACCTTGGTTTTGGTGCCATTCTCCTCCAAACTATAGCTTTCATTCCACACTGTCTCCATTTCGTCATGGTATTCGTCGTCAAAAAGAGGTTCATACATGTAAGCATGCGCATGTAGCGGGATTGTAGCTCCATTGGCACCATTGCGGCGCAAACCAACAATAATGATCCCCAGTGATTCTCGTTCAAATGTACTGGGTGGACGATGAAATCGGTGTCTTGCAGCGAGTTTCTTGGGGTTGTCGGCCACCTAAGCGACCACGAGAAAGACGATAGCACGTAGCACTTACCCACTGAAGCTACAATAGTTTCCAAGCAAAATTCGATTGGGCTGTCATTAAGCCACCCACGACATAGCCCACCCACAATGTTATCAAAGCTGATCATTGCTTTCCGTGAGAGCGTTCTAAATTCGATGAGCAGACGACTAAAGGTGAACTTGGAAAAAACCTCGTTCGCGACTCCTGGTGACGAGAACAGATAGCATCGACTGATATGCCTGCCGTCTTCGGCTCATTCGCGAACGAGTTGACATCCTATTCAACAAATCGCCAGTTTTGCTCCAGCCACTTGTGATCTTCCAGCCATCCCCCCCCTTGGCCTTAACGGCGTAGAATCCTGTGAGTGCGAGCACAAATAGGCTAAAAGCATAAATATGTTAAATCAACCATACTAAAACCACCTCATTGCAGCAACCATAATATTTAAGCAGCCTTACCTGCTAAATTTGTGCAGTTTCGGATCAATCGAAGCGACAATATCGAGTAGGACGCCTTGATTCTTGACACGCTCGTCCAGCTCAGGGGGTTGATGCTGTGTATTTCTGGCTTCGCTTGTTGCAGCCTGCATTGAACGGGTGAGGTCTGCCGGAATTACAAATGGGTGCATGTAATCCTTGGTTGGTCGTGCGTCCCA

>Contig_21

ACTTACTGTATAGATAATCTAATGCAGAGCACATGTATCAAGACCTGAAAATACGGCTACAGTACTACGCTGCAACTTGATTCATTAATAATAGCCGTATTTTGGTCAATTCACTGTTTGCATCACCCAACTTTTGCTTGCACTCGCAAAGATTTGCAGCAAGAGAGCGACATAAAGCAGCGATGAAGTGGTCTTCAACACAAAGTGCGAGCCAAAGAGGTCTACATGTACAGTACAATCTACGATTAATCGCGAAAGCTGAAGCATTTTGTGACAACGCATCTAAAACAACCTTGTTTTGCAGGATAACACTTAAGCTTTGTTTCACTTGGTATTTGTAAGAGCAGCCGGGGGGGATTACCCGGCTCTCCTGTGGAAAGGCATTATTTAATGTAAAGAGGGTCGTTATTAATACCAGCCGCGCTTCGCTTGGCTGGGGCTCGCTTCGGTCGCGATTATTATCCCGGCCAAAAAGGCTTGGACAAAAATGGTAATATATTTTATCTTAAATTAGAGGAATGTAACTCCGCAACCGTAGAGCCGATTTCGTTGTGTGTCCCCTTAATAGATAGCTTGGTGTCTATTTATTCTCGTAAGAACTTCACAGCATCTAATAATGTTCACTACTTTTCAAATGGTCGCCGGTAAGAAATTGACTGCTGCTGAGAAGTACTTGGTTGTGAAGACCTACGAGTTCCTCCGCCAAAAGAAGGCCTCTGCACCGCANTCTATGGAAAGGAGGAGTTCGTTATCATGTCAAAGAGTGTTTAGGCTTTGCTACGGGCACCATTTCAGCTATATGGGCGCACTGGGAGCAGCACCACGACAAGAATTTCACCTCGGTAGGCTGTACTTCATATCATTTCAGTTATTATTGATATTTATCATTTCAGTGGTCGACAAATTAATTATGACAGATGGAAGGTCCAAAACCATCTGGGAGGCCTCCGAAACATGGAGAAAGCATACAGCCTCGTCGGTGTCAAGAATGAACAAGAGCGGAAAACCTGTTGCAGCACCACAGTTAGCACAGCGTCTTAAAGAAGAGCACAACATTGTGGTATCAGTGCGCTCACTACGTCGCGTGCTTAGGAGGATGGGAATGCGATTTATAAAAGGAAGGTCTCGCAATATTATGGCAGAAACGACGGCAAATGTTGCGTTCAGGGCGCAATATTTGAGAAAAAAAGTTTTAAACCTGAACAGACGCAATTTTCCAGTGCGCCCCGAGGTTTATCTCGACGAGACTTTTTGTAACCTGCACCATGTCGCCAATTTATCGTGGGTTGATGAGGACAAGCTGCGGTATACGAAGAGTGGTCGAGGTCCACGGTGAGTGTTTTATTTTGTTTTTGTAAATTAAAACCTTAACGTTATTTGTATTAAGAATTTATAACAATTATTTAATTTTTTGTTTTTAAAGGTTTTGCATTATAGCCGCTGGTATACTTCGGCGAGAAAAGAATGGATTAGTTGGTGAATTGGTCAAAGAATCGAAAGAGGTGTGGCAATCCAACTTAAAACGTAAGAACGATGACGATTATCATGGCAACTTCAACATCGAGCAGTTTAAGAAGTGGTTCACGAATCTGAGTGCTATTTTGGCGTCCAAGTATGGGCCATGTAACATACACATGGATGGGGCCAGCTATCACAAACGCCAAACGAACCCCACTCCACAATCCAGCATCAGATATGCCTGAGCTACGCTGTAAAATCTACGAAGGTTTTGCATCGCTCACGTCAGATACGTGGGTAAATGCATACACACACGCACAGAAATACGAGACAAAATATCTGCAGCAAGGTGATGAATGCGAAATTGCATCGGAACCAGAGGGCAGCAACGAGGACACGGAAGAGGATGACGCCGTCGATGACAATGAAGCTGTAGCCGATTAGGAATATGAACGGGTGACCCGTTGTATGTTCTTTACACTCTAGATATTCTTTTGACGGGTTCTTTTTAACAAGTTTATAATTTTCTAACAATCGTCAAATTGCGGTAATATTTGGAATTTATCCATCCGGTTCTGAAAATCCCCACCTGTTATCCCGGCTCTGCCAATGAATACGCATGCATGTTAGTACGTGCCAAGCCCACAATTGCAAACAATTCTGCAAGATATAGAGCCTTTTTTAAACGGCCGATTGGGAGATCAATGGTTGYAGATACGCAAAAATAGACCTCAAAATCGGAATCTACGCCCGATTTTACCCCTTATATCTAACTATCGTTATGGGTTTTGTGAAGGGAAGGGTTACCTTTTTTGGACCAAAGTTTGTCCAAGGTTTGGTGGCCGGGATAATAATAGATTCTCTCACCTGGGAAGTGCAATGCGCGGCTCGCTGGTGAGCTCGGCGCTGGGCCCGCCTCCGCAAATACCGCGCGGCCCTGCTTCGCGGGCTCCCCGCGCGGGTTTGCTGCGGCTACGCGACCTCGCACCCGCTCGCCTTAGTTGGCGCCATAGCTGCGTCGCCCCTCCCCGGAAGGTGCTATTATGCGTTTGAGGGTGCTGCACACCGTTTCGAACGGTGTTTTTGCAATTTTGTCGCATTTTGTTCCGGCCTGCGTGTTCTTCCAGTTTGTTTGGCGTTTGAAGTGCTTGTCGTTGTTTCATTGTGTACGTTATAAATAGCAATGACAGTACTGGAGTATTAATACCTGTAGCTGTCCATCATGGACACTCCATCGGACTCCGATGGTCCGATTCGGAGAGCCATTGCGCAAGCCGGAGTGGCGGGGATTCCTTCTCCACCTTCGCATATTCATAGAAGGGGGAGGGTAATCTTGATCCGGTGCAAACAGCGGCGATTACTGTGTGTAGTATGGGAGTCAACTGACATTGACTCAAATTGACTCAGATAAAGTGCAACCACAAGCCAAACTCCTCGATGTCTGCACCTCCCGATGATAGCACGCATGAATTGAAAGCGCTCTGCGCATCCTGCTCAAGAGCGTAGGCAAATCGCGCTCAATACTTGAGCCAAGTGGGAAACCGACGGCTAAACAAATCGGACTTCACTTCAACGTGCTTTTGGTTGTCACAAACAGCTGGGGCGACAGCGCAGTTACGGTATCAAGACCGGTGCAGAAAGGTTGAGTGTCCTATGAATAACGCAATGTTGGGCATGCTTTCTGGACAAGCTGTAAGATTTTATTGAGAAGAAGAAACACGCTGCAACCATCAAGCATGGAATGTACAATTGCTTAAGCACCACTACAGCTAAATTAATGTACATGTTACATGAACAGTGTACAGATCTGTATAGTGTCGCAGCATGTCATGTCTGTGACACCTCTGTTTCGGAAGATGCACACTGTATGTACATACACGCGTTCTCCAGTCGTGCATGCTGATCTCTATGCATGTGGGTTCGACTGTGCTTCTCCACGTCTGTAAAACCGCCGCACCAACAGCGCTCTCTGGCCATCGACTTCATACCTTAACGCCACCAACGAGCGGGCGTTGCTCTATCTCGTGACGAGATTCTCGTCCAGCGCGGACGTCCCATCTTATCTAGCCAGCTTCGAAAATGTGCAGACACTGCGGAAGCGTTGCACCGCGCCTTATCTTCGAGATTTGCACGTCCTCTTCCAGAACACCTTCCAGAATGTCGACCGAGACTACATGTACACGAATATGTACAACTACCGCACCCCAACCGACCAACAAGCACGACAAGCTGCGTGCTCGCCGCAAATGACGACTAGCAAATTGCTAACGGCCATTGCTAAGCAATGGCCGTTGGTCTCCGCTGCACGAATTAAAGCCTCTTGCGTGAGTGAGGGTCTCGTCCGTCGAAACGCGAATCAGATCGCGTCATTAGAGAGATTAACCAATCAAATTGAAGGCCCATCACGTTACCTTTTGAGATCTCGCACGTGCGTTTCGCAGGCTGCGCGGTGAAGTGCCGCATCCATCATCTTTAGCCCATCCAAAGGCCCTTTAAAAACCGACGTTATCATTTCCAAATTGGCTCTCTAGGCCTCACAACACCTCCAACTACCACAGACGGGAGTTAACATAACCTCCCGTACTTTATCAACTAAGAACTTCAACTCGCATACTTTCCGACTGCTATGCCAGGCTACGAGAGTGGGGCGTCCTTGCCCTCACGCTCACTGCAGGATAACGGCAACTACGCCAGACTGGAGTCGCCTCGTGCGTCCTCCCACTCGCTCGAGTCGGCAGCCCACCATGTCGTGGACGTGTCCAGCGGCGCGCGCTCCTTCTCAGCCGCTGATCTGTACCACGTCGACGCCAAGATGGCAACCAACAGCAAATCGGTTGCTGGAGGATCCAGTACTCCGCTACCTGATCACCGTTCGCGATTCTTGAATGACGGAGAGCTTCTCCTTGCTGCCAATCCCGTGCCCACGCGCGAGTCCTTCGCGACACTTAATGAGACCGACGAGAAGACGTGGCACCAGCGATCTACCGAGCTGATCACTAAGATGCTAAACACGAACATCGAGCGGGGACTAAATGTCATGGACGTGGAGCGTCGGATACTGCGTTACGGTGTCAATGCACTGGAAGAAGAAGCTAAAACGCCAGTCAGCGTGATCTTCCTGCTGCAGTTCTACAATCTAATCATCGCCATGCTGCTCTTCGCTGCGTTGGCGTCTTTGGCCTTGCAAGAATGGGTTGAAGGTATCGCTATCCTCGCCATTGTCACGCTGAACGCAGGCGTGGCGACGTACCAAGAACACTCCGCCAGTAACGCGTTAGCCGCGCTTGCCAGTCTGTCCAGCCCGCAGTCGCTTGTCATCCGTGACGGGATGCAACAAGTGGTGGACAGCAAGCAGCTGGTGCCTGGAGACATCGTGATCCTCGTCACAGGCGACGTTGTGCCTGCAGACATCCGACTGTTCACTAGTGTCGATCTCAAGTGTAACGAGATGCTGCTGACGGGCGAATCGGAGGATGTCCCCAAGAAATACAACGCTCCGATCCACCCCGCTGGAGCCGGCAAACCTGCCAAGTTGACAGCGAGTAACATGGTCTTCTCGTCCACGACGATCACCGCCGGCAACGCTCGTGGTATTGTAGTGGAGACTGGTATGAACACGCGTGTGGGCTCCATTGCAGCTCTACTACAAGCCAAGAGCGGCACGGATGCGTCTGCAGAGAAGAAGTGGATCCGTAACCCGCTTGGTGACTGCATCGCCAAGCACCGTCCGAAGCTCACGCCTCTGCAGCGAGCTCTACACCACACTGGGTACGTCATGGGTCTTATCGCGGTGGGTGTGGCCATCCTCGTGTTTATCGTCGGTATGATCCGCGGCAATGAAGACCCTCGTCACCCGGACAGACCCACGTACTTGACTATGATTATGGCTGCTGTGTCTGTGGCCGTGAGTGCTGTTCCCGAGGGTCTACCGATGGTCGTGACTATCTGTCTGTCGTCCGGTACGGCCGAGATGGTCAAGAAGAATGTGCTTGTACGTAAACTCGCGTCAGTGGAGACGCTAGGCGCAGCGTCGGTCATCTGTACCGACAAGACGGGAACATTGACGGAAGGCAAGATGACTGCAGTGAAGCTCTGGGGCGATTTCCGCGAGTATTCAATCACTGGCAAGGGGTTCACACCTGAAGGCTCTATCCTCGCTTCGGACGGTAGCAGTCAAGGCGAACCGGAGGCCGGAAATGTGCAAGTTCGCGCGACTTTGATGGCCTCGGTGCTGTGCAGTAACACGCAACTCAAGCAAGTGGAAGGGGACGACGGCGAGACTCCACGTTGGCTTCCGTTTGGCAACTCGTCCGAGGCGCCTTTGGTCGTTGCAGCCGCTAAGGCAGGGATCTGGGAAGATAGTCTGCTGGAAGACTACCCTCGGCTAGTGGAAGTACCGTTCAGCTCATCACGTAAGATGATGGTCACAGTGAACGCCCTACCTGTGGTGAATGGCATGGCGATGTTCGATACGTTGGCGCTTCCAGGTGACCAGCCGCCCAAGCTGGTGGCGAACGTGAAGGGCGCACCGAACTACATTTTACGCAACTGCACCCAGTACTGCCGGAAGGATGGCACGTTCGAGACGTTGAACAACGTGCAGCGTCAGGAGATTCTCGAGGCGGTGGACGCGCTATCGTCGCAGGCGCTGCGTGTTCTTGCTGTGGCTATCCAGCCGATGCACGAATTGCCGTTCGGGGAAGACTGCGACGATGTGGACGAGAAGTTTGAAGCCTTGTCCAAGCCGTTGGTGTTCCTCGGACTGGTGGCGTCTATCGACCCGGAACGTGACGGCGTGCGCGATGCCATTGCCACTGCACGAGCTGCTTCCATCCGTACGGTGATGATCACTGGCGACTACCTCGCTACGGCTGTTGCTATCGCCAAGAACATTGATTTGCTACAAGTGGGCGCGGACCCGGAGGCTCAAGCAACAGACTGCACGCAGCTTCGTCCGAATGGAGACGTGTACTTGCCCCCCGCTGACATCGACGAGATCACGTCACGCACGTTGGTGTTTGCTCGTGCCAAGCCCGAAGACAAGATCGAGATCGTGAAATCTCTTCAACGTCAAGGACTCATCGCTGCTATGACTGGAGATGGTGTCAACGACGCACCAGCGTTGAAGGAGGCGGATATCGGTGTGGCCATGGGGATTTCAGGCACGGAAGTGGCCAAGGGAGCGTCCGACATGATCTTGATGGACGACAACTTCTGCAGTATCGTCACGGCTGTGGAGAAGGGCCGCGTCATCTACGCCAACATCCAGAAGTTTGTCATGTTCTTGCTGTCCACGAACATCGGAGAGATCATCCTGATCTTCATCTCGGTGGCTGGCGGGTTCCCGCTTCCTCTCGAGGCGCTGCACATTCTGCTGCTCAACCTGTTCACGGACGGTATGCCTGCTGTCGCTCTAAGCTTAGAGAAGGGCGACCCACACATCATGGCAGACAAACCACGTCACAAGCAGACTTCGCTGATCCACGGTCGACTGTGGCTGCTCGTGCTCTTCAACGCGTTCCTGCTTCTCGCTGGAGCTATGACCACGTTCCTCTTGGGACTGTACTGGAACTTCGGCGTGTTGCTGACGGACGACATCTACAATACTGGCGGCGGTCCAGATGGGACGGACTTCACGGACGTGACGTGTCGTCGATGGGAAGGCATCGACGATGGCTGGAAAGTGTACGGCAACTGTGCTGCTCAGTACTCGGACGGCTCGTACATTTTCGGCGAGGAAGTGGCCGGTATGACGTCGTTCGAGAACTCAACTGTTTACTGTGAAGGAGGCGACTACGACTGCGTCCCAGCGGGTCTGGGACGAGCTCAGACGATGGTGTTCTTGGGTTTGGCCTTCACGGAGGTGCTGCGAGCCTACACGGTGCGTCACTTCACGGAGCCTGTGTTCGCACGAATGTTGTCGAATGGCTACATGCAGTTGGCGGCGTGTATGTCGGTGATTTTGACGGTGCTCGTGAGCAACGTCCCTGTCATCATGGACGACATCTTTGGATTCGAGTACATTCCGTGGTACCAGTGGCTTGTGGTTGGAGCTGTTGCAGTCAACAACGCGTTCTGGGGTGAGATCCTGAAGGCCTATTTGCGACGGAAGGACCGGGCGCAGGCTCGCTGGGACCACATGAAGAGCGGGTTCGAGGAGATTTTGCTCGAGATTCGGCATGTGCGTCATCATGTTGAGAGGCTTGAAGAGGGTGGAGCTCATCGTGGACTTAAGCGAGAGTAAAAGGCGACTATGTCACCTTGGGAGCGAAGGTGACGAAAGTGTGTATTGCGTATGGATCGACGTGCGCTAAGGCAGGAAAAACTGCCGTTCTCGAGCCCCAAGAGCAGAGGCCGAGTGTAGTAAGCTTTGCGGTTAATGGATTTATCAACATGAATGCTTATTGAAACACAGTTAAATGCAGATATTGTAGGCGTCCTAGATGCTGAAGTACATGTGTATTTGGTAACTCAGGGAGTTACCAGAGAAAATCTCTGCTGTCTAGGAGACTGTACTCACGATATTGCTGTGGTTACAAAAATGCTACAGTATTACATGTAAAATGCTCAGGCTGGATCTGAGTCTGAATAGCTGATTCGGGCTTTATCACTTATGCGAGGGCGGAGGTGGGCTTTATCACTTATGGGAGGGCGGAGGGCTCAATTTGGGCCGGGTTTGTTGATTGCGCTGCGACGGGTTCTTTCGAGAATACAAACTGGCAGTATACGGATCAGCAGCAAGAAAAATGTAGACCAAGATAAAAGAGGGATCGGCTGGACCTCATCTTTTATAGTGAATAATGGTGGTAATGCTTGCGTTTACTATGGCAAGTCATTACTATCAAAATATGCTGCGAAAGGTGAAACTCAAAACTTCATACGAAAAGACATTGTGTGAATCCGTCAAAGTGAGTACGATCGACGAGCGCGTAGCCCCGAACATGCACGAAACCCGGATGGGGTGGCTGCAGAGCGGTGGTGGATAAGTGGTTGGTCACCGTCCATTCGCCTTCATCCGCCGTTTGTTTATTCGGCCTCATTCGCGAATCTGCCTCGGAGACAACAAGCGCCTCGAAATGCTACCACTACCGTGCTTGAGATTTGGCGCCCATGTAGTCATATCTCGATCTTGTGAGATTGACACCTCGGTAGTCTCGCCTACTTCTGTGCGTCGAAGCTCACACAATTTACATACACCGACTTCAGCATTGGCAGTCGTCCATTCGTCCACCCAGCTGTGGGCTGAAGATTGGGACTAGCCCCAACACAGCCGAAGTCCGCCCCCAAGCGTGTTTTGTGTCAAGATCCTGCACGAAGCGCTACACGACACTCAAGTCTCCCTCGGTTGACTCCTGAAACCAGGAGCACGGATCAAGCTCGCTCAAGTTGTGTATCAACTAGAGCTAGGCCAAGTTGCTGTCAAGGCTCTCAGTCCACGACGCCAATGCGTCTACCATGCCAGAGCTGCCACGTCAGCTGTCGCTAGTCTGATCAAGATGCCTGGATGCAACAAGCCCTCAACATGGATCAAGCCACCGTTTCTGGCACCATGAGGTCCATTGAGAAGCCATTCAAAGGAGTGGAAGCTGTGTGAGCCTCCACATATATGCTCGTCCTCGACCTCATCTGGAAGCCACCTTATGAATGTGCAGGCTAGCGTGGGGGCTGTTCGCGGTTGCAATTGCGTCTACAGCAGTGATTGCGGTAGCACCAAGCTGCAGGCTTTGCTCAAGATCACACTTGTTCCTGCCACTGATGATGTGATCCCTGCCACTGATTATGTGTTGGCGGTGCTGTGTTGTGACTCGTACACCTTCTATCAGTGTCGACCCAAGCCGAGGACGGTCATTTTCCCAAGTGCCATCCAGAGTGTACGCAAACATTACCGCAGCTCCTGGCTGTTGATGTCATTGCTGCCGCTTCAAAGTCTTTGCCGTTGCCGCCACCTATGCTACCACGCCCAGGCTGTTGGTGCTGTCGGTATCAACGGTACCAAGCTCACGTCGTCGGTGACACAGACCCCAATGCCACCCCCAAGCGATTGATGTTGTCGGTGCCAGCGCTGCCAATTTCATGCCATCGCTGCTGGCGCCATCTCTACCGCGTCATCGGCTCTACCGGCACTAGTACTTCCACTCTCAAGTCGTACGTTCATGTTGTCTGTGTTATCGGTGTCAACGCTACCAGCTCTATGTCGTCGGCGTTGCTACGAGTAATGCGGTCGCTTTCAAGTCCAGCCCCGATGTCGCTCTCCCAAGCTGCCGGCGTTGCCAGGACCAATGCTGTCACGTTCAAGTTATTGTCGCCAGTGTTGTCGGCACCAGCGTTCTACTCCAACGCGTCGCTGTTTTCGACAAACTAGCAGCTTCAACGTCGTCGGTACTGTCGCCGACAGCTTGCCGGTGCCATGCATATTCTGCTGGTCGCGCAATACTGCCCACGTCGATGGCGGCGATTCTGTTGCCACCAAGCACTACCATTTGTAACGAGATACACTGGTCGGCATTGCTGTCGCTACTGTGGGAACGACGTGTGTTGGTGAGTCATTGTTGCGGAGTATGGGCTTTCGAGAGGAGATGGTCGTCCACATACAAAACAGACACCGCGCATCGCCATGCTTGTGTCACGTTAGTGTCACACTCTCACCGTCTCGCGCATGTGTAGGCAGCCTTGCACTTTGCAGAGGACAAGATACAGCAGCTTGCTGAAGTGCGGCGTTCGTGTGATACACGTATGGCCCCACGACGGAATGCCGTAATCYGCGTGCGCGTTGTGTCGTGGAGTCTAGATACACAAGTTTGCGTCGCTGACGCTCAACTATTGCGTCTGTACAACGGCAGGTAGCAGGCTGCACGCTACGAGTTTTGCGGCGGTTGTCGTGTTACCAGAAGTGTTGAGCACTTCAATCGCTCTTCGCCGACTGTCTGCTGTTGCTGCAGTATATGACGGTACGCTCGCGGTGGTAAGGCGGTGCGCTGCATAATCGTCGTCACAAATGGCTCAAGCGCTGTTGCCGCTCCTTTTTCTGATAACTCACGGGAGATGCGACTCTTCGTTCTGTGGTCCACATAATCTGGGAAAATGACTTCTCAGCGATGTGGTCCACATAATGTGATTATCGAAATTGCACAAAAGAACCAGAATTACCTTCCATGCTGACGAGAGGTCTGTTACATCGTCCCTGAAGCGCGAATGCCCCCATCTCCGAGACCATGAAACTCAGGAGAAGCTGACCAAACAAGTCTCGTCCTTGGCGGGGAAAGAGTGTTGAAAGTGCGCTCGAGCTTTCGGCTGCAGTTGGATGTCGAGAGATCTTTGCATCTTCACTCGTCTGCAGTCGCTTGTCATCCGTGACGGGATGCAACAAGTGGTGGACAGATCCTCGTCACAGGCGACGGTGTGCCTGCTGACATTCGGCTGTTCACTAGTGTCGATCTCAAGTGTAACGAGATGCTGCTCACGGGCGACTCGGAGGATGTCCCCAAGAAATACAACGCTCCGATCCACCCCGCTGGAGCCGGCAAACCTGCCAAGTTGACAGCGAGTAACATGGTCTTCTCGTCCACGACGATCACCGCCGGCAACGCTCGTGGTATTGTAGTGGAGACTGGTATGAACACGCGGGTGGGCTCCATTGCAGCTCTACTACAAGCCAAGAGCGGCACGGATGCATCAGCAGAGAAGAAGTGGATCCGTAACCCTCTTGGTGACTGCATCGCCAAGCACCGTCCGAAGCTCACGCCTCTGCAGCGAGCTCTACACCACACTGGGTACGTCATGGGTCTCATCGCGGTGGGTGTGGCCATCCTCGTGTTTATCGTCGGTATGATCCGCGGCAATGAAGATCCTCGTCACCCGGACAGACCCACGTACTTGACTATGATTATGGCTGCTGTGTCTGTGGCCGTGAGTGCTGTTCCCGAGGGTCTACCGATGGTCGTGACTATCTGTCTGTCGTCCGGTACGGCCGAGATGGTCAAGAAGAATGTGCTTGTACGTAAACTCGCGTCAGTGGAGACGCTAGGCGCAGCGTCAGTCATCTGTACCGACAAGACGGGTACATTGACGGAAGGCAAGATGACTGCAGTGAAGCTCTGGGGCGATTTCCGCGAGTATTYAATCACTGGCAAGGGGTTCACACCTGAAGGCTCTATCCTCGCTTCGGACGGTAGCAGTCAAGGCGAACCGGAGGCCGGCAATGTGCAAGTTCGCGCGACTTTGATGGCCTCGGTGCTGTGCAGTAACACGCAACTCAAGCAAGTGGAAGGGGACGACGGCGAGACTCCACGTTGGCTTCCGTTTGGCAACTCGTCCGAGNGCGCCTTTGGTCGTTGCAGCCGCCAAGGCAGGCATCTGGGAAGATAGTCTGCTGGAAGACTATCCTCGGCTAGTGGAAGTACCGTTCAGCTCATCACGTAAGATGATGGTCACAGTGAACGCCCTACCTGTGGTGAATGGCATGGCGATGTTCGATACGTTGGCGCTTCCAGGTGACCAGCCGCCCAAGCTGGTGGCGAACGTGAAGGGCGCACCGAACTACATTTTACGCAACTGCACCCAGTACNGGCCGGAAGGATGGCACGTTCGAGACGTTGAACAACGTGNCAGCGTCAGGAGATTCTCGAGGCGGTGGACGCGCTATCGTCGCAGGCGCTGCGTGTTCTTGCTGTGGCTATCCAGCCGATGCACGAATTGCCGTTCGGGGAAGACTGCGACGATGTGGACGAGAAGTTTGAAGCCTTGTCCAAGCCGTTAGGTGTTCCTCGGACTGGTGGCGTCTATCGACCCGGAACGTGACGGCGTGCGCGATGCCATTGCCACTGCACGAGCTGCTTCCATCCGTACGGTGATGATCACTGGCGACTACCTCGCTACGGCTGTTGCCATCGCCAAGAACATTGATTTGCTACAAGTGGGCGCGGACCCGGAGGCTCAAGCAACNAGACTGCACGCAGCTTCGTCCGAATGGAGAYGTSTACTTGCCCCCCGCTGACATCGACGAGATCACGTCACGCACGTTGGTGTTCGCTCGTGCCAAGCCCGAAGACAAGATCGAGATCGTGAAATCTCTTCAACGTCAAGGACTCATCGCTGCTATGACTGGAGATGGTGTCAACGACGCACCAGCGTTGAAGGAGGCGGATATCGGTGTGGCCATGGGGATTTCAGGCACGGAAGTGGCCAAGGGAGCGTCCGACATGATCTTGATGGACGACAACTTCTGCAGTATCGTCACGGCTGTGGAGAAGGGCCGCGTCATCTACGCCAACATCCAGAAGTTTGTCATGTTCTTGCTGTCCACGAACATCGGAGAGATCATCCTGATCTTCATCTCGGTTGCTGGCGGGTTCCCGCTTCCTCTCGAGGCGCTGCACATTCTGCTGCTCAACCTGTTCACGGACGGTATGCCTGCTGTCGCGCTAAGCTTGGAGAAGGGCGACCCACACATCATGGCAGACAAACCACGTCACAAGCAGACTTCGCTGATCCACGGTCGACTGTGGCTGCTCGTGCTCTTCAACGCGTTCCTGCTTCTCGCTGGAGCTATGACCACGTTCCTCTTGGGACTGTACTGGAACTTCGGCGTGTTGCTGACGGACGACATCTACAATACTGGCGGCGGTCCAGATGGGACGGACTTCACGGACGTGACGTGTCGTCGATGGGAAGGCATCGACGATGGCTGGAAAGTGTACGGCAACTGTGCTGCTCAGTACTCGGACGGCTCGTACATTTTCGGCGAGGAAGTGGCCGGTATGACGTCGTTCGAGAACTCAACTGTTTACTGTGAAGGAGGCGACTACGACTGCGTCCCAGCGGGTCTGGGACGAGCTCAGACGATGGTGTTCTTGGGTTTGGCCTTCACGGAGGTGCTGCGAGCTTACACGGTGCGTCACTTCACGGAGCCTGTGTTTGCACGGATGTTGTCGAATGGCTACATGCAGTTGGCGGCGTGTATGTCGGTGATTTTGACGGTGCTCGTGAGCAACGTCCCTGTTATCATGGACGACATCTTTGGATTCGAGTACATTCCGTGGTATCAGTGGCTTGTGGTCGGAGCTGTTGCAGTCAACAACGCGTTCTGGGGTGAGATTCTGAAGGCCTATTTGCGACGGAAGGACCGGGCGCAGGCTCGCTGGGACCACATGAAGAGCGGGTTCGAGGAGATTTTGCTCGAGATTCGGCATGTGCGTCATCATGTTGAGAGGCTTGAAGCGGGTGGAGCTCATCGTGGACTTAAGCGAGAGTAAAAGGCGACTATGTCACCTTGGGAGCGAAGGTGACGAAAGTGTGTATTGCGTATGGATCGACGTGCGCTAAGGCAGGAAAAACTGCCGTTCTCGAGCCCCAAGAGCAGAGGCCGAGTGTAGTAAGCTTTGCGGTTAATGGATTTATCAACATGAATGCTTATTGAAACACAGTTAAATGCAGATATTGTAGGCGTCCTAGATGCTGAAGTACATGTGTATTTGGTAACTCAGGGAGTTACCAGAGAAAATCTCTGCTGTCTAGGAGACTGTACTCACGATATTGCTGTGGTTACAAAAATGCTACAGTATTACATGTAAAATGCTCAGGCTGGATCTGAGTCTGAATAGCTGATTCGGGCTTTATCACTTATGCGAGGGCGGAGGTGGGCTTTATCACTTATGGGAGGGCGGAGGGCTCAATTTGGGCCGGGTTTGTTGATTGCGCTGCGACGGGTTCTTTCGAGAATACAAACTGGCAGTATACGGATCAGCAGCAAGAAAAATGTAGACCAAGATAAAAGAGGGATCGGCTGGACCTCATCTTTTATAGTGAATAATGGTGGTAATGCTTGCGTTTACTATGGCAAGTCATTACTATCAAAATATGCTGCGAAAGGTGAAACTCAAAACTTCATACGAAAAGACATTGTGTGAATCCGTCAAAGTGAGTACGATCGACGAGCGCGTAGCCCCGAACATGCACGAAACCCGGATGGGGTGGCTGCAGAGCGGTGGTGGATAAGTGGTTGGTCACCGTCCATTCGCCTTCATCCGCCGTTTGTTTATTCGGCCTCATTCGCGAATCTGCCTCGGAGACAACAAGCGCCTCGAAATGCTACCACTACCGTGCTTGAGATTTGGCGCCCATGTAGTCATATCTCGATCTTGTGAGATTGACACCTCGGTAGTCTCGCCTACTTCTGTGCGTCGAAGCTCACACAATTTACATACACCGACTTCAGCATTGGCAGTCGTCCATTCGTCCACCCAGCTGTGGGCTGAAGATTGGGACTAGCCCCAACACAGCCGAAGTCCGCCCCCAAGCGTGTTTTGTGTCAAGATCCTGCACGAAGCGCTACACGACACTCAAGTCTCCCTCGGTTGACTCCTGAAACCAGGAGCACGGATCAAGCTCGCTCAAGTTGTGTATCAACTAGAGCTAGGCCAAGTTGCTGTCAAGGCTCCAGTCCACGACGCCAATGCGTCTACCATGCCAGAGCTGCCACGTCAGCTGTCGCTAGTCTGATCAAGATGCCTGGATGCAACAAGCCCTCAACATGGATCAAGCCACCGTTTCTGGCACCATGAGGTCCATTGAGAAGCCATTCAAAGGAGTGGAAGCTGTGTGAGCCTCCACATATATGCTCGTCCTCGACCTCATCTGGAAGCCACCTTATGAATGTGCAGGCTAGCGTGGGGGCTGTTCGCGGTTGCAATTGCGTCTACAGCAGTGATTGCGGTAGCACCAAGCTGCAGGCTTTGCTCAAGATCACACTTGTTCCTGCCACTGATGATGTGATCCCTGCCACTGATTATGTGTTGGCGGTGCTGTGTTGTGACTCGTACACCTTCTATCAGTGTCGACCCAAGCCGAGGACGGTCATTTTCCCAAGTGCCATCCAGAGTGTACGCAAACATTACCGCAGCTCCTGGCTGTTGATGTCATTGCTGCCGCTTCAAAGTCTTTGCCGTTGTCCGCCACCTATGCTACCACGCCCAGGCTGTTGGTGCTGTCGGTATCAACGGTACCAAGCTCACGTCGTCGGTGACACAGACCCCAATGCCACCCCCAAGCGATTGATGTTGTCGGTGCCAGCGCTGCCAATTTCATGCCATCGCTGCTGGCGCCATCTCTACCGCGTCATCGGCTCTACCGGCACTAGTACTTCCACTCTCAAGTCGTACGTTCATGTTGTCTGTNNGTTATCGGTGTCAACGCTACCAGCTCTATGTCGTCGGCGTTGCTACGAGTAATGCGGTCGCTTTCAAGTCCAGCCCCGATGTCGCTCTCCCAAGCTGCCGGCGTTGCCAGGACCAATGCTGTCACGTTCAAGTTATTGTCGCCAGTGTTGTCGGCACCAGCGTTCTACTCCAACGCGTCGCTGTTTTCGACAAACTAGCAGCTTCAACGTCGTCGGTACTGTCGCCGACAGCTTGCCGGTGCCATGCATATTCTGCTGGTCGCGCAATACTGCCCACGTCGATGGCGGCGATTCTGTTGCCACCAAGCACTACCATTTGTAACGAGATAACACTGGTCGGCAATTGCTGTCGCTACTGTGGGAACGACGTGTGTTGGTGAGTCATTGTTGCGGAGTATGGGCTTTCGAGAGGAGATGGTCGTCCACATACAAAACAGACACCGCGCATCGCCATGCTTGTGTCACGTTAGTGTCACACTCTCACCGTCTCGCGCATGTGTAGGCAGCCTTGCACTTTGCAGAGGACAAGATACAGCAGCTTGCTGAAGTGCGGCGTTCGTGTGATACACGTATGGCCCCACGACGGAATGCCGTAATCCGCGTGCGCGTTGTGTCGTGGAGTCTAGATACACAAGTTTGCGTCGCTGACGCTCAACTATTGCGTCTGTACAACGGCAGGTAGCAGGCTGCACGCTACGAGTTTTGCGGCGGTTGTCGTGTTACCAGAAGTGTTGAGCACTTCAATCGCTCTTCGCCGACTGTCTGCTGTTGCTGCAGTATATGACGGTACGCTCGCGGTGGTAAGGCGGTGCGCTGCATAATCGTCGTCACAAATGGCTCAAGCGCTGTTGCCGCTCCTTTTTCTGATAACTCACGGGAGATGCGACTCTTCGTTCTGTGGTCCACATAATCTGGGAAAATGACTTCTCAGCGATGTGGTCCACATAATGTGATTATAGAAATTGCACAAAAGAACCAGAATTGCCTTTCATGCTGACGAGAGGTCTGCTACATCGTCCCTGAAGCGCGAATGCACCCATCTCCGAGACCATGAAACTCAGGAGAAGCTGAACAAACAAGTCTCGTCCTTAGGGGTAACTTTGCGGGGAAAGAGTGTTGAAAGTGCGCTCGAGCTTTCGGCTGCAGTTGGATGTCGAGAGATCTTTGCATCTTCACTCGTCTGCAGTCGCTTGTCATCCGTGACGGGATGCAACAAGTGGTGGACAGCAAGCAGCTGGTGCCTGGAGACATCGTGATCCTCGTCACAGGCGACGTTGTGCCTGCTGACATTCGGCTGTTCACTAGTGTCGATCTCAAGTGTAACGAGATGCTGCTGACGGGCGAATCGGAGGATGTCCCCAAGAAATACAACGCTCCGATCCACCCCGCTGGAGCCGGCAAACCTGCCAAGTTGACAGCGAGTAACATGGTCTTCTCGTCCACGACGATCACCGCCGGCAACGCTCGTGGTATTGTAGTGGAGACTGGTATGAACACGCGTGTGGGCTCCATTGCAGCTCTACTACAAGCCAAGAGCGGCACGGATGCGTCTGCAGAGAAGAAGTGGATCCGTAACCCGCTTGGTGACTGCATCGCCAAGCACCGTTCGAAGCTCACGCCTCTGCAGCGAGCTCTACACCACACTGGGTACGTCATGGGTCTCATCGCGGTGGGTGTGGCCATCCTCGTGTTTATCGTCGGTATGATCCGCGGCAATGAAGATCCTCGTCACCCGGACAGACCCACGTACTTGACTATGATTATGGCTGCTGTGTCTGTGGCCGTGAGTGCTGTTCCCGAGGGTCTACCGATGGTCGTGACTATCTGTCTGTCGTCCGGTACGGCCGAGATGGTCAAGAAGAATGTGCTTGTACGTAAACTCGCGTCAGTGGAGACGCTAGGCGCAGCGTCAGTCATCTGTACCGACAAGACGGGTACATTGACGGAAGGCAAGATGACTGCAGTGAAGCTCTGTGGCGATTTCCGCGAGTATTCAATCACTGGCAAGGGGTTCACACCTGAAGGCTCTATCCTCGCTTCGGACGGTAGCAGTCAAGGCGAACCGGAGGCCGGCAATGTGCAAGTTCGCGCGACTTTGATGGCCTCGGTGCTGTGCAGTAACACGCAACTCAAGCAAGTGGAAGGGGACGACGGCGAGACTCCACGTTGGCTTCCGTTTGGCAACTCGTCCGAAGCGCCTTTGGGTCGTTGCAGCCGCCAAGGCAGGCATCTGGGAAGATAGTCTGCTGGAAGACTACCCTCGGCTAGTGGAAGTACCGTTCAGCTCATCACGTAAGATGATGGTCACAGTGAACGCCCTACCTGTGGTGAATGGCATGGCGATGTTCGATACGTTGGCGCTTCCAGGTGACCAGCCGCCCAAGCTGGTGGCGAACGTGAAGGGCGCACCGAACTACATTTTACGCAACTGCACCCAGTACTGCCGGAAGGATGGCACGTTCGAGACGTTGAACAACGTACAGCGTCAGGAGATTCTCGAGGCGGTGGACGCGCTATCGTCGCAGGCRCTGCGTGTTCTTGCTGTGGCTATCCAGCCGATGCACGAATTGCCGTTCGGGGAAGACTGCGACGATGTGGACGAGAAGTTTGAAGCCTTGTCCAAGCCGTTAGTGTTTCTCGGACTGGTGGCGTCTATCGACCCGGAACGTGACGGCGTGCGCGATGCCATTGCCACTGCACGAGCTGCTTCCATCCGTACGGTGATGATCACTGGCGACTACCTCGCTACGGCTGTTGCCATCGCCAAGAACATTGATTTGCTACAAGTGGGCGCGGACCCGGAGGCTCAAGCAACAGACTGCACGCAGCTTCGTCCGAATGGAGACGTGTACTTGCCCCCCCGCTGACATCGACGAGATCACGTCACGCACGTTGGTGTTCGCTCGTGCCAAGCCCGAAGACAAGATCGAGATCGTGAAATCTTCACTCTCAACGTCAAGGACTCATCGCTGCTATGACTGGAGATGGTGTCAACGACGCACCAGCGTTGAAGGAGGCGGATATCGGTGTGGCCATGGGGATTTCAGGCACGGAAGTGGCCAAGGGAGCGTCCGACATGATCTTGATGGACGACAACTTCTGCAGTATCGTCACGGCTGTGGAGAAGGGCCGCGTCATCTACGCCAACATCCAGAAGTTTGTCATGTTCTTGCTGTCCACGAACATCGGAGAGATCATCCTGATCTTCATCTCGGTTGCTGGCGGGTTCCCGCTTCCTCTCGAGGCGCTGCACATTCTGCTGCTCAACCTGTTCACGGACGGTATGCTTGCTGTCGCGCTAAGCTTGGAGAAGGGCGACCCACACATCATGGCAGACAAACCACGTCACAAGCAGACTTCGCTGATCCACGGGTCGACTGTGCTGCTCGTGCTCTTCAACGCGTTCCTGCTTCTCGCTGGAGCTATGACCACGTTCCTCTTGGGACTGTACTGGAACTTCGGCGTGTTGCTGACGGACGACATCTACATACTGGCGGCGGTCCAGATGGGACGGACTTCACGGACGTGACGTGTCGTCGATGGGAAGGCATCGACGATGGCTGGAAAGTGTACGGCAACTGTGCTGCTCAGTACTCGGACGGCTCGTACATTTTCGGCGAGGAAGTGGCCGTATGACGTCGTTCGAGAACTCAACTGTTTACTGTGAAGGAGGCGACTACGACTGCGTCCCAGCGGGTCTGGGACGAGCTCAGACGATGGTGTTTCTTGGGTTTGGCCTTCACGGAGGTGCTGCGAGCTTACACGGTGCGTCACTTCACGGAGCCTGTGTTTGCACGGATGTTGTCGAATGGCTACATGCAGTTGGCGGCGTGTATGTCGGTGATTTTGACGGTGACTCGTGAGCAACGTCCCTGTTATCATGGACGACATCTTTGGATTCGAGTACATTCCGTGGTATCAGTGGCTTGTGGTCGGAGCTGTTGCAGTCAACAACGCGTTCTGGGGGTGAGATTCTGAAGGCCTATTTGCGACGGAAGGACCGGCGCAGGCTCGCTGGGACCACATGAGAGCGGGTTCGAGGAGATTTTGCTCGAGATTCGGCATGTGCGTCATCATGTTGAGAGGCTTGAAGCGGGTGGAGCCTCATCGTGGACTTAAGCGAGAGTAAAAGGCGACTATGTCACCTTGGGCGCGAAGGTGACGAAAGTGTGTATTGCGTATGGATTCGACGTGCGCTTAAGGCAGGAAAAAACTGCCGTTCTCGAGCCCCAAGAGCAGAGGCCGAGTGTAGTAAGCTTTGCGGTTAATGGATTTATCAACATGAATGCTTATTGAAACACAGTTAATGCAGATATTGTAGGGCGTCCTAGATGCTGAATGTACAATGTGTTATTTGGTAACTCAGGGAGTTACCAGAGAGAAATCTCTGCTGTCTAGGAGACTGTACTCACGATATTGCTGTGGTTACAAAAATGCTACAGTATTACATGTAAAATGCTCAGGCTGGATTCTGAGTCTGAATAGCTGATTCGGGCTTTATCACTTATGCGAGGGCGGAGGTGGGCTTTATCACTTATGGGAGGGCGGAGGGCTCAATTTGGGCCGGGTTTGTTGATTGCGCTGCGACGGGTTCTTTCGAGAATACAACTGGCAGTATACGATCAGCAGCAAGAAAAATGTAGACCAAGATAAAAGAGGGATCGGCTGGACCTCATCTTTATAGTGAATAATGGTGGTAATGCTTGCGTTTACTATGGCAAGTCATTACTATCAAATATGCTGCGAAAGGTG

>Contig_30

GAGTCGGACTGTGGCTCATACGATGTAGCGGGTAGCGATGAAGACTGTGGTTCGTACGATGTTGCTGGTGGAAGTGCCGATGAGGGCCAAGACACGAAGCAAACGAGCACAGGCGGTAACACTTACCAACAGGGTTCAACTACCACTGGTGGCAACACGTACCAGCAGGGCTCCACGAACACTGGTGGTAACTCGAACCAATGGTCGAACACTGGAGGTAACACAAATCAGTGGTCGAACAATGGTGGGAACTCAAACCAATGGACGAACACCGGCACTGGGTCTCAGCAGAATCCGTATCAGGGCACCGGCACGGGTTCGCAGAACAGTTTCGACTTCAACACATTCCAGGGCACGCCGAATGCTGGTACAGTGGCGCCGCAGTATATGTAGATGTCGGTTCCCTTTTTTGCGTAAGTGTAGCGTATAATACTTGTCTGAGAATGAGCAATAAACATTTGTTTGTGTACCAATTTGCTCTACATTACTCTTATCATCTCAACGTCATGTTCGTCGCAATACTACTACTGATATTGCAGAAATATTTTCATAAAATTAAATTAAAATTTCTGATAGTAGTATGAGAGCGAAAATGCGTGATTCATATTAATACCGTGGAACCCTTCCCCTTGCGTAGCGGTACATGTGACATTATAAAATCCAGATTTTCACATATTTATATAGTTAAATTTAAAATAAATACTAAATATTATCCAGCTATTGCACAAGTATTACTACAACGTTAATTAAAAAAAAATTGCCTAAAAATCTGTCGGGGGCTGGGGGGGGGATGGCATCGCTTCAATGCGGTCGATGAAAAGATACACAGACTAAAAAACGGAGCGCATATGCACTAAAGTGCGATATTTATCTCCTCCATTCAACAGCATGTACTGTAGCAAGTGCAATATCTCGCATATTGAGGTGCCTTATATTATATTATAGTCCTTATCGATCTGTACACCGTTCACTCTTGTTAGTCGTGCTTCGAATTAGTCATGAATCGTAATTATATGAGAAACTATGGTACACTAATCATGTATAGTCGTGCAATAATATTAATCGTGCCCAGCTCGCGGCGCCAGCCATCCCCCGACAAGTTGCCGTTGGACATCGACAGCCACAACTTATGAGAGTGCTCACTTTCCGAATTAGGATGAACGACACAGAGACACGACTGCAGCATGCTGTCGGGGTAAAAGGTTGGGGTCGCTGTGTAGCTTGGATGGAGGGCAACGCTGTCGAGGATGGAGAGAAGGGTCTCGACGGTCGTAGGGTCGATTAGTTCCCGTCCCTCTTCCAGGCCACCGACCGCCGCGTCAACCTTAACAAGGCGCGAGACTGGAGCGCAAAGCGTTCAGTCCTTCAAGCTGCGCTGGACGACGGCCAGCAGCGCCATGTATGCCAGGACGGCGAGCGGCAGAAGACACCAGTTTACGCACAAGGCTCTGGTCGGTCGTGGGCGCAGGCTCGATGGACACTGGGCCTGGCTCTATCCGCTGTTACTTGCAGAGTTTGAGCGGTTCGATGAGCGAGTTTAAAGCTTTCTTTCCGGCTCTTGATGGACATGGCAATGACCACGATCGAAGACAAGAAACCACGCTGCATACAACTCCGGGTACGGCTTTAGTGAAAAGAAGCTCTGTACACTGACCACGCCTCGACGAATCCAGGATCTCACCGACCGGCAAAATTTTGTGTACCGAAAGTTGAAAGGGAAGAAGCAGGTTGAGTGACAAAAAGATTGCACAAATCAACCAGTCTATAGCGTCACACCTTGGTATGCTGAAACGCGAATTTGATGACGGCCGGCTTGACCCTGACCAGCAGTGCAACATAGATGAATCACATTTTGTCATAGATCTGGATTACGGTAAGACATTGGACTTCCGCGGTGCCGACGGCGCAAAAACCGTGGTATTGTATCAGGTACCACATATTGCAATTTGTTACCCAAATACNTTTTTCTGCGCCAATTTAGTCTACTGATAAATATTAACTTATGTGAGTACGTCGAGAAGGAGTTACAAAGCGCGGATGATTGAAGGAAAGTCGAGACGCCATTCTGAAGCTAATAATCAACAATGTCAAATCAAGCTACCCTATCCAAGGCCTATCGGACACTGCTAAAATAGTTGCATACCGCTCGTCGCCCAGAGCCTTTATCAACAACAAACTGATGTAGGAGTGGCTATAGGACGCCGGCTTCTGGGGTCCATCAGGGCCTTTGGGCAAGAGCGTGTGCTGTTGGATGGACAGATCAAGTGGACACGCAGGTGATGACGCTGTGTCACTTGGAAAGGAGCTCAAGACTTGCAGCCGCTTCTTCCCTCCTAACTCTACAGATTTCGCCCAGCCAGTCGACCGGTTCCCTATACAAAGGGTTTAAAGTGCACTGGCGGCGGTTGTGTGAGGAAAGAAACATGGAAGCAATTCGGCGTGGCGATTGGATGCAGGGTGCCAAGTCGTCGGGGTCTCTTGCAAACCGAGGTAAAACGTTTTTCTAGATACTGCAGCAGAATGCGTCCGTCTAGTGAATGCAGAGATGGACAAGGAGGGAATTAGCTGGGCTAAGAAGTCGAGGCTGCTGTGNCGGCTTAGACGTAGATAGTGATGGTAGTGGCAGGTTAGGCAGCTCAGCAAGCCTTTGCGAGAGATTGTAGAAACGTATCGTGCTGAGTTGGATAAAGGCTACGAAGAGTGCGCAGCATCGGCGACTGTTTAGCAGTCCCTAAAAAAGACAGTCGATTTCACCATAATTAGTCATGCCCCCTATATAGTCGTGCACCTAATTGAACTCTTTATAAAATGGCACAATTATATTTCACTAGATAGTCATCACCACATCGGAGATGCTTACTGCAACAGCCTCTTACATCGAGGAATGTGTATGATGCTCTATCTGTCGGAAAGTATTTAATCCACCACATGTGGCCGTTTATCCTGTGTGTACATGAACATTCACAAGAAATAGATTTTGTTTTGATGTTGATGATATTTTTTCTTTGACTTCTATTGTCAGATTTGTCATTTTGGTTGCCTTTTTTCTGGCAGGGACTGTGTTGATTTCTTGTTTTCATTGTTATTTTGGCTACTCAGTAAAACCTTAGGGGATTGAACCTGTTCACCGTAGTGATTGCTGCTATTATCGTTTTAGCTATACGAATTTGTAGAAGCTACTTCAGGAGAGCCTTTAATTTTAAGGATCAAAACCTGTTAGCTGTTTTGTTGGCTACTTCAGGAATTCTTTGAGGATACACCTGTTCACCGTATTGATCTTTAAATATTTTGTGGTAGAGTTTAGCTTTGTTCATTTCTTAACTGCCATTGTTTTGCTCTTTATCTGTGGAATGTTTTTCACAATGTGTGGTTTTGTTTTTGTTTTTCGTTGCTGAGGTGTTATAGAATTGCCTCTTAATTCTTGAAATGGTTAACAACTTGTAACAGAGGTTTGTTTTTATCGAGCGTGATGCCACGCTTAGGTAAGACTACAGCTAGTATAAGAGTTTTTCACGAGAGAATTCAAAGCAATGTGTCATTGCAACTAAGGAAATGTTATTCGTATGCTGAACTCAGCGTAGTACGTATTGGACATCTAGCGCGGGAGATGCTAGCGGAGGTCTACCACGCGTATGCCTCAAGCATAAATTATTATACTGCCAATATTGTTTGAAGTAGTAGTGGAGATAGTAATACTACAGTATGATATTATCGTGGGCGATATTACAGCCCGCATGCTCTGCTTATAGCAGGCCAATGCGTGAATTCTGCCCATGTGCTCCGCTTATAGCGGGCTCAATATGCAGTGCTTATCATCGTGGGCGCAATTACAACCCAAATGCACCGCTTATAGCGGGATCATTACGCAGTGCGTATGGTGCGAAGTCCAGAATGATTTTCCTCTAGCTCGATACCGCGAGGCAAACGTTTTGCCTACCGCGCGCAGCTCGTTGGCTGGAGTACTTAGAGGGCAACATGGCCTTGAAGTGCCGGTGGCACATGGTAGACTCCCGTGCGTCCTCTTGTCGGCGTCGAATTCATGCCCTGCTGAGACAGGTGGTTCTCTGCGTTTGCGCCGCCCTTATTGGTAGGAAGCGTTGGGCTTATAACCTGCTGAAGGGATGATTTGCGCAGAGCTGTTAGGGCAGTACAGTATGGAGTGAGGACAAAAGTAGAGTGGTCTAGGCGGCGACAGTGGAGAGAAAGTCTTGAGTGTAGAAGAAGAAAGATGCTTGTGTGGTGAAGATGGTAAAAGAAGAAGTGATGAATTAGAGAGTCTGGTCTCCACCAACCAGTCCAGTTATATATGATTTCTATATATGAAATAGCACAATTAAATTTCACTTGATAGTCATCATCACATCGGAGATGAGTTCTATTAAATCGTCTAGTGGGTACGTTTATGCGGCACTCAAGTGAACAACAGCGAGCCTCTTTTTTTCACTGAAAACTCCCCGTCTCAGCACAAAGTGACAACTTTTGATGAATGTCTTGTGTAGCCTATTTAAAAGCGCGGGTACGGTAGCTTGGAAATTTGAAGACCAGTACCGGGTAAGAAACGGATATCGTGGCAGGCGCTACAGAATCCATCGATACGTGCTGCACAAAAAATATCAAGCGGCCTGAAACATAAATCGCTGATCTTATTCAGGCGCAGCACTTACATGTTAGTGTCCATGCTACCGCCACAAAAGCTCGCCACTTCTGCACGATATGTAGTACTTAAGCTCTTTGTATTCATCAGCAAACAGTTTTTGCATGATTCTTCTGTAAAGAGTAAACATATTTGCTTGGAGGAATTAATTGTCTGAGGCTCTCTCTAGTATAGTCTCAATTTTTAATATTAATATACACTGCATTACTTGGGAAGTACTGCACAGTAGCTCCACGAAAATGCGGTAAGTACAGTAAGTTGACAAAGGGAACATATACATAATGAGCATCGTCATCCATTGAGATGTAATCGTATCGAGGCGCGTAATCAAATACCGGGAGTTCGCACAACGATCGCCACAGTGATTTAACCCTTCCAACACGGGAGCGTGCCCCCGACGATAATACTTTCTTGTGCTCCACTTATCAGTTTGAATTTTCTCAAGCCTCCACTGAGCACCGATGTCCCGTTATTCAATGATGCTATTACTCCGGTTGGCCGTTTTGATCACTTTTGGGTTAAGTTGCGCTGTAGCCTTGGCCACCGACTCTAACAATGTGGTGAAATCTAATCCTACTATCAAGGACACTCAAGCCTTGCGATTTCTGCGTCGTTATGCTTTCGATGAGGAAGCTAACCGTGATAACGAGGATGAAGAGAGAGGTATCGGTGTCTCTAAACAGCTGGACGATGTGCTATTGAAGGCCGACGAGGTTATAGGAATATTGAAAAATGTTGTTAGAAAAGTTGGCGGTGCGCTCATCAAAAACTCCCGCGAGACCGACGAGTATTTACGTCTAACAAGGGCTGTATCCGGCAAGTATCCGACTGCAAAAGAGTTGAGCCTCTCTACATTGAGGCAGCTTAAAAAGATTGAAGCCGTGAGAAAGAAAGACATCGAAAAGGGTATCGATGTCAGCAAGGCAGCGACCGATGGCATACACAGGGATATTAAGCTCTTTGATGGGAATAAGAGGGTCCCTGACAAGTATATGGGGGCCCACGTGGGACGCGACCAGCAGCGTTTTACCGAAGCCGATACTCGGAGTCTGGTAGCCGGTGTGGTTACAAGGACAAACAAGAAAGGCGAGCAGGAAATTCTTCTGGTTTCGAGCTCGAAACCCACTAAGTATGAGTTTATGATATCGAAGGGGGTTGGGAAAAAGACGAGAGTGTTGAAATGGCAGCGCTGCGTGAGGTCATTGAAGAAGGAGGGGTACGTAGACCGCTCGTGGATATTGGGAGCAGTGACAATCTATACTAACTCGCGGTGATGCAGGTGAATGCAAACATTTTGCACGATCTGGGTACTTTTGAGCTCAAGGATGGAAAAGTTGCCAAGGCCTTCCTGATGAAAAGCGACACGATCTACGACGATTGGGCCGAGAGCATTCGTTATCGCTTATGGGTACGTGGTCCAAGGAGACTTTATCAGCTTAATCCGAACTAAGCGTATTACTGTATCCATTGATACTGAATCAGGTTTCGTACGATGACGCCATTAAGCTGCTGAAAAACCGCGAGCAGATGGTTGCGATGGTGAAAAAAGCCAAAGCCATGGCCAAGAAAGATTCCAACCCAAATTTCTCCTAATTCAAGCTAGACCTTGAATAAGTGGTTGGGCAGCTACAGAAAAGAGCTGGCGCAGAAAAACCTATACGAGCCACCAAGCGAACGCTGAAGAACCTAGCATAATTGGATGTCCATCCCANTTTTTTTTTTACAAAAATGGGAAATCTCTTATGAAGAAATTTTGTGCAAAAAGAGTGTATATTAACAGCAATTGGGTGATACAATTGTTGAGATTACATACACACAACAGAAATCGCATTGCTAATAAAACGAAATGCTCGTGACAGAAATAAAAAAACTAAGGTTAATCCAATAGGGGTCGTCAATGTTATCAGCAGTTAGATAGATAAAAGTGATACATAAAGCTCGCACATACAGGGACCAACGCAAACCCCTTTGAGAAAATTTCGCATTGACACAGTTGACACAAGAAAACTGTCAGAGTCGGGGTACCTGCTACTAATCTTCGACCGTGTTCTGCGTGTATTTACGAGAATTACATACGTGGCTAGTAATCTCGCTAGTAAGGGCACATAATTCACAACGGACACAAACTGAAAACAATTTGAAAAAGGGGCAGCTTAAGGGCTGTCCAAGAGGGTTCACTCTTTAAAGTCCATCGATTTGAATAGGGGCGGGTGCGTCGACCCGTCCAGCGAGCTTTACGCTTTGAACGTACTCATAATAGTCCCACCCGGTAGTACTCTGACATGAGGAGCGTAACAAAGGGTGCCCGCGAAGCAAGATCGCTCAGTATTTGCGTGCAGGCCAAAAGATAAGATTGTTTGGTAAGTCCAGGTTTCTTTAGTTCGTCTGCTCACAACAATGACGGGCAGTGTTTCCGTCGAAATTCGAATTTTAAGACAATCTCAAATGAGTTATTTAGCCTTTGAGCACTTGGCTCTCGCCTCTCGTGGTGGTCTCGAGGTTTTTCAAGTGAAGATTTCGGGTGCAAATGCAAAAATTCGAATTTAAAAAAATAATTGTGCGCAACATATTATTTCATCTATTTTTGTCGTTTGGGATTTTTTTTCTCTCTTTTTATCATTAACGGTACTATGGTGCGTGTGTTAAAATTGACAATGACGTATTCTCAGTTTTGTTGCTCGACACTTTTTTGTTTTGCCATTTCTTACTTTTACTATTCGAGCATAAAAACAAAGCAAACCCTATTCCCTAAAATACTCTAAACTTGCCACGTCATCGCCGCAGGACTAGGCTCAAGGAATACAGCTCGGCCAAGAGGGCTTAACATCAAGGTGCGAAGCTTCGCACGCTGTACTGAACACGACCAAATTGATGATAAGGGTATAAACTGCCCACAATAGCTTGATCATCGGATCTGTTACCGAATAGCTGATCTAAACACTGAGTGTCTTTTAAACCACGAGGTTTCCGGGTTGAGATGGACAGCTTATTATGAGATTGCACTCCCACACTTTTTGTTTCTGCCTTTATCGATACGAGCACATGATTCTCTGACATCACCGCCGACTTCGCACGCTATGGCCGTGTATTAAAGCGGTGCTGTTGATATGGGAACGGGGACCGTCTTGCTCTGCTTAGGTAGGTCCATTACAAGGTCTGAAAAGTTTCAATGTTGAAGATGCTGATACCTATCAATAGCATGCTCGGTGCAAGCCTTTTTTGCTACACGCTACAGGACTTATTGCGTGTTTATCGTCTGTTGGATTGTTAGCCCCGAAGATTACTTTACCCACTCGGTTGTACAAGACAATATATATTCCAACCTTCTTCAAGCAACATTGACTTGGTTAATAATTTTTGATGTTACCCATGGCCGCCACAGGCAACTTTCTTCTGTGTTTTTCTGATGTCCACCGGGGAATGTTGTAATCAATCTATTTTTAGTACCAGGTGGTGCATTTACTTACGAATGGGTACAAACGAATATGTTTAAAAAGATAGTACCGGTACCACATCGCCGCCAGTGAGCCACGGAGACGCGGGGAAGTGAGATTACAACGAAATTAGAGGCAACAGGTCTACCAAGTTATGAAGAAAGCGTTTTTTTCCAATATGAGTAAGAAGTAAGAATTTTCCCTTAGATCGAGTGTCATCCCTGGAGAAACCGTTGATTAAAACCCAGAGAGCTATTACTACACGTACGTAAAAAAAGACAATAACATGGTCTCGACCATTTCTTGACTTATTTCTTCATTTTCTGGCAGTGCATAGTAAGGCATCATTAAAACTGCTCACATTCTTTGCTTTAGAGCCACCAGACCATGGTACTTCGAAGTGTTAATTGCCGTGAATGATGCCCTCCAGCGTCACCATGCCCTGTCAACAATCACCGTGCTAGGACTATTTTCTATAGTTTTGTTTTTAGTTATAACAGCGGTGGATAGATCGCAAGTAAAGACAAAAAGTGAGGGCGAGAGGCCCCAAAAGGCTCTGCACTCTTATGTCTAAAACCAACGAGCCGTTCCAGACACGATTGACCTCTATTTCGCGGNCGAAACAAGTTCATCATCGAAACATTGTCGTGAATAACCTTCTTACTGTTTTTTTTAAGTTTTTGGATCCGTATTTCCGACATTTTCCGCCAAAACTACGCTCCTTCTAAATTTTATATGGAGCATAATTACATGTAGAAGTAAAAACATTTAGTATAAATACTGCTGAAAATTAGATTATCATGGATTTAAATCATGTAATTAGGCTGCTTTTTTCTTCAGTAAATTACATTTAGTCATCGCCAAGACATATACGCATATTATGTGGATATCTATCGAAGAATCGCCAGCTCAATTTAGCCTCATTTTAATGTGGTCCACATCTGAACTTTTAAAATTGGACCAAAGATTGGTCTATATCATATGATTGGCCAAACGTTTGAGTTTTGCATGATTTATCAAAATAAGTTCGAAAAGACGCTTTACTTGGACCAGTCTACTGGAACTCTACCAAAACCTACACAAATGCAATCACAAACACAAATTTAAAAGCGAAATGTGTTGAAATATATGTCGAATTTGTGATATGTCTGCTATCATACGAAAATCAACACTTTTAACGTTATGGGTACATGTACGCGGGCCATTCACCTTTTTTATGGCTAGGGCCCAGCGACTTGTGCTAGCGTGCGAGCACCGGAATGCGCGTTTAAAGAAGCAGGTGTTGTTGATTCTACGGCTGCGTTGTATGTACTCTTTTGATCGTAATGTTCTAACAGTTGCAGCGCTAATTGATCCTGCTAAATCGCCCTGGCACACGTTGTATGCTAGTTGAGATCGTGGGAGCTTCATATCAGTTGTCTCCCTTGACCCTGAGGCATTTGATTGCTTGCTACAGGTGTTTAGGGAGAATCTGCTCGACGAGAGATAAGAGGCCTGCAATAGTGAAAGCAGCGTTAAAATCAATCATGAGATCCTATTTGTGACGCACTACGTACGTTCAACCTGAGGCGTAGTGAAGCCCCGTCCTCTTCTAGCAGTACGGCGGCTCATCGTATGAGCTGTTGTGTCCCAAAGCAAAACTAGAAGTGGGCTTGACTGGATACCGATAGCTGTACCAGAAATGAGGTACGAACGATTTTGCGTCGACACATTTTGTGGAGACGAGAAAAACGCCTCAACATTTTACTACCGATTTGTGACAGCAAATACGTCACGGCTTTGTCAGACGCAAAAATCGAGTTTGTGTAGGTTTTGGTAGAGCTCCACTGAAGTCCCAAAATACGCTGGACATATATGACAAACAGGAACGTGTAGGTCTGCCTAAAATCTGGCTGCGGATATTCCTCATTTCTACGGTCCCTATGTACCCGCTGCCTCCACAGCATTACACACGACCTCATGCCAGGTTCTACTTGGCGTGGACTGCATTCCAATACGAAAGGAATAAGGGCTCATAGTAATGGGCTTCTTCCGGCGTAATACCACCTTCTTTTCTGCGATGTACCTTTCCAATGTTTTGGGAGTGAATCCCTGCTTATCCAACGTTTTAATCGACGTCATCAGTCGTGCCATCATGTCTTTGTGAGCATCATCAGCGCTCTTGCCAACGAGCAATGCGTTAATTGATTCGTCGATGTGGTCTGCTTGCCCCACTCCTTTGAACTTGGGTATATCGTTAACTGCGTCATCAACTTTCCTGACGGTCCAGATACTTTTTAGCTTCTCAGCAAACGCTTTAACAGCTGCCGAAAGATCTCTCTCTTCGTCAGTTGACTCTGTATCATGCTCGGTGTCATCGTGGACAGGCTCATGCTTTCTCAGTAGCCGCTGGGCATTGTTGGTGGCATGGCCAGCCACAGCAGGATCTTGAACGACCCCAGATCGAATCGCTGCGAGCGTATCAATCTCGACAGCACCACTACCATTGACAAGTATAGCGACAGTGACCTGGATAGCCAGCAGAACGATGAGTCCGCGCATCGAAAGTTGTGAATAGATCGTGCTCGACGGAGAAAAATGAAGAATGGTTCAGAATGAGTTAGTGTTCTTGGCACGTTTGTTGATTAGTCACGATTGGGTAAATCGTGCCTAATCAATCAGCCCGTATCTTACTTTAGGTAAAATCAAATATTACGAGCTTTCTCCAAAGAATAGCTTTAGATAGTATTTTAACCCTGTAGAAAAACCTCGGTTCGTAGTTAACAGCACATCTCATTTTAATGTGGTCCACATATGGACCTTTCATTGGACAAAACACTATTCTATATTATATGATTGGCCAAACGTTTTACGTTTTCCATGTGGCATCGAGAAATCTTAATATGACCAGCCCCAAAGTACGCTAGGCATATATGACATAGGAAAATGTTTCCGGAATAAATGGATACATTTTTTTGATTAATACGCTAAAAATTGAAGGTGCTGCCATAAAAACAAAACTAATGCTTTTCAAAATCGTCGACGCAAATTCAACCTTTGCATTTACAATTTTAGCTGTTATTGACAGCCAATTTTATCACGCGAAGCAGGATAATCTCAGGATTATGTTTAGGTATGGTAGCTCCCGAACCAAATATTCTGGAACCATTATGGTTCGAAATTCAAACTGTCGATTCTTAAGTGGTCCAAAGGGTCGTCTATGGATTTTCGCGAATCAAACAAAAAATAACGATAAAAAGAATTTTCTTGGTCTAAAACCCGGTCATTTTGCGCAACATATCGAATCGTGTACTGTGGCATGGAATACATTTATCTGTCAAACTGACATGGCTCGAGAGAACGCGTCCGCGTCGTGGGTATCGTGCGAGGTACGTTCCAGAAGTGGCACCAAGCTGAAGGTCAAATGGCAGGCGTTCCGGTTCAGGCGACTAGATTCACTTTCGAATTTGCGGAGTATGGGGACACTAACTATTTTCAACAAGCTTCACTATACCTTTCTTGAGCTCCCACTGCGCTACGAACATGTCTAGGCTTAGTGGACCGATTATGTGGATCAGTGTCGAGAGGAGATGGCTGACGTTTAACTGTCCCGAAACAGCAAAAGATGTCATTATTATAAATACTAGCTGAGAGGCCACTGCGTCAGTGTTTCGCTATTAGCAAGCGTTTATAGTAGATAGTTCCAATATCTGGCATCAGACCTTCGCGTGGGCCCATGACCCCGCAAGACATCATCTCCATTGCTCGACGACTCCGTCAGCGTAACAACTCTCGACTGTTGTTGCAATGCTTCTTTTTGTACTACTACGTTGACGGACACGCAATCGAAGAGTTTCGCGTCAACGGGTAGACGTCGATCAAGAAATTGAGCCTTTGCTGAATAGGAACATGTTTGATCGGACGTTTAGAATGTCTGCGGACTGTTTTTCCACTTGTTGAATCTATTAACACCTGCACGCGCAGTCGCCGCGCAACAATCGATAAATTCTAGCGGAGTGGAGCCGATATCGCCTGCAATAATGCTCATGAGAGCTTTGCGGTATTTGGCAGGAGCCTCTACCTTGTTTACATAGGGCGGTTGGGATCTGTGCTTCATCGTACTATCGAGTTATTGATCTTACTCTGTCTGGTATCCTATCTCTTCGCGAGTTGCGAATCGTATTCCCCGAATCTGATTCGGAGAGAGAGGTTGCGATGGGTGATTTCAAGGCAATCAGTTCTGGTGATGTGATGAGTGGATGTATCGGTTGTGTAGATATATGGCTTTCTAGGATTAAGACCCCAACTTATGCAGACGATGGTGACGTCGGTCTTGGACGCTACTATAGCGGCCATTATGGGTGCCCCGGCATTAATGTACAAGCTGTGTGTGATACACACTGCCGGTTCACCGCCATTAATGCAAGCTATTCTTGGAGCAAAAATGACTCGCATGCATTTCGTGATACCGGTAAGGCTCGAGTTCTTGTAACATCCTTCCACATCTCACGAAGTTTTGCGGCATCATGGGGGACTATAACGCCTAGGTTGCATTTGTCAAATGTGATGAGCGTCGAGCTGAGCTTTCTAAAATATGCCTGATCACTATGGAAGGCTGAATGAAAATTCGTTCAAAAAGCTGAGCTGGCGTGAGTTTGGTTAGAACCCAGATCTTGTCGGGTCGCGCTTCCGTCGATTTCATGCCGTCTAAGCAAACTTCTCGGAAAACATGACATTTACTAGCTGCATCATGCAGTGTCGGGAGCGTCGTTTCGACCTCGTCGTCGCTACCGCCTTCGATAGTCCGTCCTCAACTCCAAGAATCTGCATTTCGGAAAAAGCTTGTTTGTTCGCTGCATTAGCAGCTCTACGTAGCCAACCTTGTGATCGTTACAGTCGACACCCTTCTTGACGATGCGGAGCTGTCGTAAATATCACTCGGTTCGCAATTGCTTAGACCCCATAGGCCATCAATCTTGCCTCTGTGTCCTCCAACGTCATGGAGGCGTTAAAAAGACAATCTGTGATGGATTTCGGAATCCCTATCGATGTTGTTGACGTACCGACAGCTCCTAAATGCGGCGGCGATCTGGAACTACTGCAAGTCACATTGGAAGACATCGTGGAGGACTCAGATGCTTGGTATGACCTCGAGAAAGCTGTGAGCTGCTGGTGAGAAGCTGTATTGAAAAAAGTGGATCGATATTAAAAATTATGGTAACTAGCACTCTACCGCTGATAATCATAAGGTTTGTATTCCCTCCTATCCACCCTCCATCCACGTCCACCACCATGATATTCACCCGGTGGACGTGGATCAGATTCAATTAGCGAACCCACGTGGCGTCCACCACCGTATGTGCTCAAGCGATTCTAGCGGGCCACTAGAGACAAATCCACCGTAACGTTTTGGCTAATACCGGGCGTAAAAGAAGGAAGGCAAAAATTGTGGGAGCATGCTGAAAATAATTGGGAAACTAGCAATGCCATGTGGGTTATGGCGTATTGTGTTTATTTGCACAGCTAGAAAATAACCATATAAAGAGCCTATTGGGCAAAGACAAGCAGCCGTTTCTCAATCAACACTAGCTTGAAATGATGTTATTGCTACACCAGCATAAGGAGGAGGAATACTACGCCGTGTAAATTCCAGAAGATCAACGGAATACTTCGTATACCGAGGAGATGAGTGAGATACTGGGCATGACAATGCGATGGATAGCTGCTGTCTAAAACGACACAATCTGACGTTAATGTGGAGCTGGCGAAGGCGAGCAGCTACACTAGTGTAGACATTGGAGATACGACAGCTTCAACATGAATACATATTAGAAAAGGAGACCAAGCTGTTTGGTTGCGAGTCGAGCAGACTATTAACCTTCCGTGCCTACACGCGATAGTTAAGAAGCGGTCAGCAGAAGGCTGTTCGTGGTTTCGTACGCTGCAATTGCTCCAGGGCTGACCATGTTTTCCGAGTTCTATCTAGCTTTTGGATTAAATGCCTAGATTTATCCATAAATTAACCACGTTTGTTGGCTTTGACTGTATTTATCTTTCTAGATGATACGCAGCGACGATTGTCTACGCAATTGGGGCTTGTTAAGCCCCAATTGCCGCAAATCCAAAGTGAGTGAATTAGCGTTAATCAAAACTGTCCAATATGTATCTCTAAACACTATCTATATGTTGCTGTCCTCTCGTAAGTGTGGCGTAATCAACAATGCCATGTGCCTTCATACAATTATAGCGTTCCAACATGTGATTACCCAATGGCGTTCCAGAAGCAGCTCCACCCAATAGTGGAGCGGTTCAGTACCCGTTCCAACGGTCACGCCTAACCGTGCTGATCCAGTGCAGCTACAACACTCCCATTCATCGATACGGTTGAACTAGTGAGAACACTGCTTGTCTAAGAGCATGCAAATAGCAAAGTGAGATTAGGAATAGTCTTTGAAGCCAAGCCGACGCTCTGGACTCGGAATCTTCCAGCAAGTTGTCCGATTTCGGGATTTAGTACTACCGTCATCAACACTTGATAGCGCTAGAATGCATGTACCACTTGTACATCAATGACATTATGGAGTAATGCCGACCCCCCCCCCACACACACACACACAAGAGGTGCATGGAGTCCTTATTAAACCTTGACGCTTCTACGAGACCACAGAGGTCCCGCTTGCGCTGGAATTGTGTCCGGGCGAGGGCCTTCGCTAGTAAGTCCGCCGGCATGTCTCCAGTAGGAATGTACTTCACTGTTAGTTACCTTTGTGAATCAAGTCCTTCCCGGCGTGATACTTGACATCGACTGTTTTCTGCGTATCACCGAGGCCATTTCGTTGGAGACGCTTAATTGTGGATTGACTGTCCATGATAAGCTTAAACAGCAACTTCTTGTGAAGTACCTGGTTAGCGATCAGCTGGGCCATCAATACGTCCTCAACCCCAGCGTCTCCCGCCACGCACTCAGCCGCCGTACTGGACCTGCATACA

>Contig_34

TAGTCTACAGAACATAGTATTATCCAGGTACTAAGTAATTATGCTTGTGTATTATTCTGTATAGGGCCGAGATATACCAAGGAAAGCTCAACAGTGACTGTGCAGACCTGAACCAAGGCCCCAACGCTGTGATCCGTAATTTTGAAGAGGTGCCGGGACAGACTACCAAGGAAGCGTCTGATTATCACCGACCGTTTCTACTCAAGTGTGCTGCTGTCGTCTATCCTTCTACAACGTGGACTCTACCATGTCGGCACCATTCAAACGAATCGGCGAGGCTACTGTAAAGACATTCCGTACAAGCAAGCTAAACGACCAAAGACGATGGCGAGAGGGGTATACCGGATTGCACAGTCCAAGACTGAGCCGAATTTGATTGCAGTGTCATGGATGGACAACCGTCCTATACACTTCATTGCTACGGGGTGTAGTACCCGACCGACAACCCTGTCAAGACGCGCAGGTGCTGAAGTAGCTGACGTGCCAGCTCCTCAACTCGTGAAAGACTATCAAGATGGCATGGGAGGAGCAGATATTCATGATCAACTGCGCTTGCAAAGGTACTCGATTCAGGTATTACTTGCTCTTTTCTTATCCTGTATTATACATGACAATATAAATTAACAGTCGAATGTTTTCCTGCATACTACAGAGAGCGATGAAAATGAAGAAATATGACCACACAATCTTTCTTGGGTTAGTTGACATGGCGCTTACCAATGCATACATCATCTATCGACGTATTCAAGGCAATCTTGCTCCCAGTAAAGCACCCCCGACACATGCTGAGTTTCAGCGGCTGATGCAGACTGCACCACTCAACATTGGACCGGCTGACTTTGCAGGAGACTTGTCAGTTGAAGCATTGGCAGACAATCCGGTTTCCCGCTCGTCAGCTCCGCGCTACACGCTACCAGCACAGCACACGACTAAACAAGTGAGCGTATTTCGCACAACACGTGGAAAGAACGGGACCTACTCACAGAAGCGCCGTCAATACGGCTGCAAAGTGTGCTCGTTGCTGCGTCCGGGCAAGAACCCGTGGGAAACCACGTTTTACTGTGTGGAGTGCTCTGAAGTGAGGATGCGGAGCGTGGATGACGATAACGCCGTTGGAAGAGGGAAGGTGTATCTATGCCAAAAGGTGCGGCAACATGATCCTAGTGGACCTACAAACTCGGCCACCTGCAGCCAGATATGGCACGATCTATGGCGGGTAGGAGCGATAATTCCTTCCGGTCTCAAGGCTATTCGGCTGCGTGCTCCTACTAGAGCAGGTGCGCAACGTATAGACGACGAGCTGGAAGGTGACAGTTGTTGCGATGACGGTTCCAGCGTAGGGTCGGGAAATTCACGAAGCACAGCGAGCGCTACGAATGCAGGACGTTCTGCAGAGAATATTTGTCTATGAATAATCAAATATGGAATGAATGCAACACTTTGTGAGTGATGGTTTTTGTTGATATTTAGAAATTTTCCCTGTAGCCGATTTGTGCGATACAGGATATTGTGCCATATGTTAGAACCACCATTTTAACTCTTCCCCTGTACTGCACAGAGTAGATAACTGACAGTGATTGTGAGCTTGGAAAAACTCCGCAAATAATAAACCTAAGTCATTTTCTTACCCTGTAGAATACAGGGTAATTTCCGCCCGAAAACGCGGGGCTAGAGGGGTTAATTATGGGAATGTCACTATTGTGTATCATATCCGAAGAAGGCAAGCAAGGACACTTGCTGTTTGCACCCTTCCTTCCTGCTTAGCTCATATCGTAGCTATCTCTGCATCGATCCAAGTTTTTGATCACCTCTACCGACACACCCGTTGAATCTGCTCAAATAATTAGCTTTGCGACCCACTTTAGGTGTTCCATAATATTCGTAAGAATTGAGCTCTGCATAATCATTATCTGCTTCACTGAGCATGAGCATGATGTGAGCTGACTTCGATTTGACTGGCGTCTTGGCACCAGCTTTGAAAAATGAAGAAAATCGCAAGGAATGTACTTGTAATGGAGTGCTGGCTTCTGTAAATAGACCATTTTGAAGGTACAACTTAGCTGAGCCACCTCACAACAAGAATACCCTTATGTAGGCATAAAGTGTTGGTTGGTTCCCGGCGCTACGATTATCGTGCTGGACTGTACACTTAGAAGTTAGTAATCATGTGTACCGGTACAGTATGCTTAACTGTAAGTATCATTCTGGATATTCGCCTAATAATGTGTTCTCCAAATAAATGGTTACCTTCTAGTATATTCTTCTTGGGTGGAGACAGCTACACCAAGAGGTCGTTATCTACCGAGTGAGGTCGATGATCGACAAAGTGTTCAATGTGAACAAGTACAAACTGAGTATTAGGACCAGCGTAAGTCTGAAAACCGCATAAAAAAAGGTCACTAATTGCTCCAGTTACACAAAAGAGCGTCCTCGATGATGCCGACCTCTGTCAATTTAAAGATACAAGCCCGGCTCCTATTACAGTAATACTCTTAATAAGGGAGCCGAATATACCGACTTGAAAATATAAAAGCGTGCTCCGGCTCGGTTCGTCCATCCCATGATGATTACACACTGTGCTTCGTTTAAAAGGTACTACAGAAGTGCTATTACATGTACCATAATAATACCCACCGGTATTAAATTGATACCGGTGTGTATTCTTTACTATTAATAGGTAAGAACTGTACATGTATTTGATTCCAAGATTCAAGATGCGATTGTGGAGATTGTGAAACAAGTGACGACCTCCCGGAGCCTGTCAACTTCACAATTTGCTGTCCAACGCTCAAGTATGCGATTCTATTCGGTGTTGCTGACTATTGTGACTCTCATTGCCAGTACTTATGATGCAAAAGTAAACGCTTCAGGCATCCAGGCTATTGCTGTCAGTAGCATTTCCCACGATGCTCCTGCTGCGAGGATGCTACGAGCTGACCATGCCGATGAGAGAGGAATAAGCGTACCCAGTGCCTCAAAGATCGTCGAATGGATGCTTAGTCCGAAGGTAGCGAAAGAGCTCACATTTCTCGAAAACCGCAAGGTCCAGAAGTGGGTAGACAAACAGAAGACGCAGGAGTATGTCTTTACGAAGCTGGGCCTTAACAGCGGACTTGATAAGGCTCTCTCCAACCCGAAGCTCCATGTTTATGCTGCCTACATCGATCGTTTCAACGTGAAAAACCCCTCAAACAAAGTGGCATTACTCGACAAGTTTAGTGAAAAGTACACAGACGAAGGGGTAGCCAAGATGGTAGAAATGGGAATACGATCCTCAAACTTGGAGACGGAGAATTTCGCGTCGAGACTGTGGAGGGAACTGCTGGATAAGTGGATGGACAATGCCGAATCTGCCGAGGGAGTTTTCAAGATTCTAAAGCTTGACGAAGTGGGAGGTGGCATTTTCGCGACGCCGCTGTTTAATACTTGGTACACTTTCATAAAAGAGGGTTATACGCGGCAGGCAGAGGATATCGTGCTTCGAGTCTTGTCGGACAGGTATGGCTATGACGGGCTATCGAGGATTTTCTTCCGTGGGCAGCGTAACTTTGATCTTGTGGGCGATCTCCCCATTAAATTGGAGACAAGAATGGTGAATAATTGGCTGAACAAAGATGTATCTCCTGACAAAGTGTTCAAGCTGTTGAAGCTGGATGAGGGACTGGATAAGCTTCTGACCAACTCGAATATGCAGGTGTGGGAGAGCTACATGATGAAATATAATTTGAAGCCCGATGTGGAGCCAACGACGATGATGCAGACGATCACGCGCTTCTACAATTTCAAAGAATTGTCATCTATGCTCGAGAACGCGAAAATGGTGCCTGAACTGAATAAAGTTGCAGAGAGATGGCAACATGAACTACGTGTTCATTATTTGAGAGCCCCCAAGATGAAGAAAGAAGGATGAAAACGTCGAGCAGAGTTGTACTGAGGGTTAGCTTGCTTTCCCTGCCGCCAAGGCATGACTAAAATACGGGTTAACTAACAAAGACAAGATAATGCATGTACAGCAGATCCTTTGTGCTGGAGAGGGAATTAAAAACGGAAGAAGACAATACTTCAGTACGTCCTAAACGTCCTACTGCTACTGTAGTATTTGTCTAAATAAAACGTAAGGTACGATTTATTTTTGAGGTCCAATTACCAGTTATGCTTTTAAGTAAGAATCCTCGTATGTGTGATTGGTCAATGTCCCTTTTGAGATCTATATGATTGGTTGAAATGTGTCCAAGATAATTTGTATACAACCCCATTTAATTATAATTTGTCCTAGCGCTGCGAATGAAGAAGGCTAACCGCGCTTTCGTTGAAGCAGTACAACAGAAGTTGTCACGACTTTAGAGACCTACTATTTTTTTTTCGGACCGGACAAGTATGTCGATAGCTGCATGAAAAATCTCATATTACAGACAAGTTTAAATATTTAAAAAAAAGGACATTCTAAACTCGCTTTGGTCCCGAGCCTCAGCATGCCTTTTCAGCAGGTGGTCACACGCAGCGTAAGTCTAGACAAACGGATCAGTGAGGGACTATTAAAAACATTACAGCTGGCGTGCTTACTCATTTGGTGATAGTAGGGATCAACTAGATCGTACGCAGGTAAAAAAAAAATATTTTATCCGAGTCTAAATTCGCACTATGCCCAATTTTGCACACACTTGACAAGTGTACGATTCAGCTAACCGGTCCGTTGGACCGGAAGAGGAACAAGAGGTTTTCGACGCGCAGAAGAAACAACGGCATTTACCACGTCCCGGAGCAGAATGAAGACGAGCACGGTTACCCAGGACTTCGAGTCCCTGCACAGCGAAGATCGAGCCAGGCCCAGCATTATGACTCTTGACCCCCTGACGTCCATGACGCGCTCCGAGGAACTCCGCAACCAGGATGTACGGCTCGAGTGCCCGCCTAAGCTGGATGACGGCGAGTGGGACGGCTTCATTTACTCGCGGCTTCAGTAGCCTCGTCATATCAAGCCCATCCACCCAAGCGCCTTTGAGCTCGATGATTCTTCCTGACGCCCCGGTTGGTTGGGTGAGCTGATGCTCGATGCTCCATAATTTGATGCCGTGCCAACATGCCTCGATGTCTGGCAGCCACACCGGTGATTATCCCATTCCCCCGCATCGGATGACCGAGGACCTCAGCCAGGAGCTATAAGACCGATCTCTTCGGTTACTGTTCTTGTTTATCCGGTTCAGAGTGTGTTAAATATCCTGCGAGGTACGAGCTTACCACGAAGCTCATCTGGACGTTTTTCTCGTCGGACAAGTTGTTGCCTATTTCCATTCGCGGGCTGCCAGCTGTAGTACGCCGACCGGTGCTGGCCTGCAGCGAGCTTGTGACTTCATGTGTATTGCGGATCTGCGCTCTCGGGTGGATATACCAGCGAATCATGTGGCATTCCTTCCACCTCTGGATGGGGCCTCCGAATAACCCGTGACGAGATCAGCGTCGGCAGGTGTCACGAGCGTTTCCGTTGATGCCCAGTCATCACGACGCCTAACGCCCTTTTGATCTTGATGGTCGAATTAATTATATCGGACAAGCCGGAACCTGAATTAATTCGGTTTTACTACATTGAAACCTTTATCCCGCCAGGCCCGATTCAACTATTAACTTTAGTGAAGGAGAACTTCGTGTTATTTCTGAATACGGCCGCATGATACACATTTGTCCTCTGAATTACCCTATCAAGTACATGAGCCAGCTCGATCCTCAACTCGCATGGATGGTGAGGTTAACATCGATTGAGCGTCATATTTCAACCTTCCCTATCTCGTAGCCAGCCCCTTTCCGGTGGAACACCCGACATTTCTAGATTCGGCGACCGTACTGCCATACATGTAGAGGTCTTCCTGTGAACGGCCCCTTCCGTCCAGACGGTGATCGAGAACACAGAGCCACGCCCTTTGCTCGAATCTGTAACATCGCTCGAGCCGACACCTAGCGTTAGTGTAACGGACGCGAGCTCTGGCGAACCTGTCTCTACGGTCGTCCGTTTTGACCGCGGCAGTACAACGGGCCCTACACAGACCAAGTTTCCATGTCTACTTCTACCCTGAATCCGACGGATGAAGACCTTGAAGCAATTGCAGCGCTAGTGATAGCCACCGAAGCGGACCTCTTTTGGCAGGCCGCAGCTCAACAAGCATTCCAAGAGCAGCTCCTGCAGCGCCTGGCGCTACGCAACGATGTACCATCGCCTAGACTTACCGAAAACCAGGTGTCCGATATCCGAAACAACGGTTTGTAAAACACAACTACTAGTCTACTGCTCGTCTCCCAGGTGTCACGCGGTAAGGTTGGGATGTCCGGGTCGTGTATCCTGGTAAGATAGGTCACTACCTCAGCTCCAAGTTTTGCGAAAGAGTGAGCGTGTTTGCAACTGCGAAAGTTGCATCGCTTTTGAGTCTGCAACCGCAGGCACAGCGCCTTGTCCTCGTGTCTGGAAATCTATCGGATGATGTTGTCAGGAACCGACGACGATTGCTGACACCGTGAACGCGAACCCCGCTGCAGACCACCACGGAGTCGAGAGCTAGCAGGTCGATTTGAGGTGTGGTCGCTTCTCAAAGAAAGTGGGGTTTCAAATTGAGCCCACGTCGACAACAGGCCTCGAGCAATGAAATGTTGAATCAGGCGAATATGGTCAGGTAAATGCGGAGCTATACTTGCGCGAGTTTAGTATTTGGCAGCGCACCGCGGGTTGATCTAAAAAAACAAATGCGTAAAACGTGCGAATGTAGGTTCGCCATGGCGCGTACAAGGCGTCGTGGGGCGTCATCGAGCACCCCGATAATTGTAATCGACGTGGGCTGCCGCAAATACGCTCACCACTGGTTCAGGATACCATTCCGTGGTCACTTCACCGAAGGCAGTGTTATTGTTGACGTAAGTCATCGAAGGACTTGATTAGGCTACCAGGGGGGGCAGGAGCGAATGTGAAGACGGTGCGCATGTCGTCATCCCGGGCCGGCGGAGGAAGGATGTGATCTAACGTCTCCCTTTGAAATTCACGGCAAATATTATTT

>Contig_36

GGTTTCCGACGCCAAAAAATACGATTCTGTTACGGAATGTATCGAGCTACTCGGACTGGAAGATATCGCCGACCAGATCATCCGAGGCAGCTCGGTGGAGCAGATGAAGCGCTTGACTATCGGCGTGGAGCTGGCTGCACAGCCCAGTGTCATTTTCCTGGATGAACCCACGAGCGGATTGGACGCTCGCTCGGCAAAAATCATCATGGACGGCGTCCGGAAGGTGGCCGACTCCGGACGGACCATCATCTGTACGATCCACCAACCTTCGGCTGAAGTGTTCTACCTGTTCGACCGACTTTTGTTACTACAACGTGGTGACAGACGGCGTTTTACGGAGATCTGGGCGAGAATTGCCGGAATTTGATCGATTACTTTGAGAACATCCCGGGTGTGGCTCCGCTTTCGGTTGGGTACAACCCGGCGACGTGGATGTTGGAGTGTATTGGCGCAGGTGTCGGACATGGAACCGAAGACTTGATGGACTTTGTGAGCTACTTTAAGAACAGTCCGTACAACCAGCAGCTGAAGACGAATATGGCCAAGGAAGGCATCATAACGCCGTCTCCGGAACTTCCCGAAATGGTCTTTGGTAAGAAACGTGCTGCGGACTCGAAGACCCAAGCGAGGTTTGTGATTTGGCGCTTCTTCCAGATGTATTGGCGTACACCGAGCTATACGTTGACGAGGATGTACTTGTCTATCTTCCTGGCCATGCTTTTCGGATTGATCTTCGTGACCAATGACGATTACGCATCGTATTCCGGACTCAACTCCGGAGTCGGAATGGTCTTTATGTCCGGATTCTTCAGTTCTATGGCAGTGTTCCAGAGTGTCATGCCGTTGACGTGTCTGGAGCGAGAATCATTCTACCGTGAGCGTGCATCGCAGACGTACAATGCCTTCTGGTACTTCATGGCGTCGACGCTAGCCGAGATCCCGTACTGCTTTGTGAGCTCTCTGATCTTCACTGCCATCTTCTACTACTTCGTGGGCTTCACTGGCTTCGCTACCTCGGTCGTATTCTGGCTTGCATCGGCGCTTCTTGTGCTCATGTTCGTGTATCTGGGCCAGTTCTTCGCGTACGCCATGCCGTCCGAAGAAGTCGCACAGATCATCGGAATTTTGTTCAATAGCGTCCTAATGATGTTTATCGGATTCAGTCCACCCGCGTACGCTATTCCGTCGGGCTATACGTGGCTGTACGACATCTGTCCGTTCAAGTTCCCCATCGCCATCCTGGTCGCGCTGGTGTTCGCGGACTGCGACGAAGAACCGACTTGGAACGAAACTTGGCAGACGTACGAGAACGTGAACTCGCAGCTTGGCTGTCAGCCCATGTTGGATGCTCCGGAGACGGTGGGCCACATCACGATCAAGGGATACACGGAGGAATACTTCGGTATGAAGCACCACCAGATCGCCCGGAACTTCGGGATCACGATCGGAATCATTGTGCTCTTCCGTATCTGGGCTGCTCTGGCGCTGCGTTTCATCAACCACCAGAAGAAGTAGGCTCTCCTGTGTAGATATCAAGTTCGTATGAATACATGTGCAATGCCAATATAAACAGAAATATTTTGCATCTAAAGTACTTTGCGATAAAGAAACCAGAAGTGCATTCACGAACAAGTGGCCCAGTTCTCCAAGTCCAACGATAACTTCATTCTCGCACATGATTGCGACCTCCTTATCGACGCTATGCTGACGCAAAGTCGCTTGATATTAGAAACATGTTCGCGATTTATGAGTTTCAGTAATCAGCTCTTTAGTAAACCCGCCGTTTCTGGAGAGCAGCCTAAAGGTGAAAACCAATAGTGCTCGGATCATTCGTCCTATGGTGTTGTCAAGCGCAGATTGTAAAGTGAATTTTGAAGTGTGCTTTCTTGCCGCCATGCCATACTATATAGCCCAACAGATTGAGCTTTCAATAGGATACCTCCTCGTCAATGGTATAACGAAGGACCCCACTGATGCAAATAGCTCCGCACATTTGCTACTTTTCCTTGTTGCTTTGACGGAGAAGTGGAAAAGGAATGGAGGTTTCTTGATTCACCGAGCTATGTCAATATATCCAGTACTTGGGTGACATGCTTTAAATTTAATTGTTTTCTAATTCTGCACAAGCGTCGGCAAACTTTTTGACAGATTAACAGGAGGCACGGGTGACCCCTTCACAAATAATAAATATCAGATAATGAGCTGTTATCGTAATGAATAGATACTTCAATAAACGTAACACCCCTTATAATGAAAATTACAGGATTAAATGTTGTTCTCCAGCTTCGCTCAAGGTTACACCATGCTGAATTTTGTGCATATAGTCCAATAGGAGCCTTTCATCCAGCACTGCTTGAGCAGCGTCAGGAACAGTGTGGTTGTCAAGTGGTCGCTCTTAAGCTAGCGTGGGTCAGCTGCTCGAGCTGAGGCGATCGAAATCACGCCCTTTACCGATGAGCTCCCATTTTTTGGTTAGTCATTATTCTGTTTTAGCAACTACCGGTAGCAACTATTATGATGACGAATCCGTGATTTGTTGTGTGCTACATGTAAGAACTTTCATTCTCTATTCCGCGTAAAAAACTCTTTATGCGTGAACACCTCGTTTCAAACATGCGACGTCTGTGGTGATGTTGCTTGCCAAAGTACCAGTACCAGCAGTTGTGAAGTAATCTTAGTATTCGCTCCAGTCTCGTAGTAGCTGAGCGCATTAATTCTTTGAATTTTAATTGTCCAAGTCCGACATTAACCGAAATCCAATCCGCTACTCGTTTCGGTTGCCCATTCCTACCCATGACGGAAACAGCAGAACGAGAACACATTAAATGTGTAAAATGAAGNAAAAAAAACAAGAAAGCGTTTATTTTGGAGCAGTTGTGAACGCTGATAAATCAAGGAAAATAATTATTTGAATACCCGAATCAAATCCGAACAACTGCCAAATCGACCGGGAATATCCTCTACTTGTAGGTCGGGTACCCGATTGCGAGCTCTGCAGCTCACCATCCGGCCCCGATCATCTTCTCCCTCGTGCACACCGTAGTGGCCTTTGGAGCAAAATTGATTTGAAGTAATACACTTTGGTTAAAATACATAATATATAATATTGACACTACTAACAGGGCTACTTGCTTTTTACGTAGGGGAACTTGTAAGAAGCCATTTGTTTCGATAATTTTCTAAGCAATCGCATGGAAAATCGTGCCCCATACAGATCCTTCCTTAGCTCTGAAATAAATTAGGTACAGATAATAGAAGAGGGTGTACTTTATTGAAGTATTTTGTAATGTGGATTTGTTGAAATTTGTTTGTTACTTAGAAACTTGATCTTTCTGCAGGTAGCCGATGTCAAACAAACGTGAATAATTCCAAGGCCACTAGCTACTAAATGCCAAGCACTAGCATTTTCTCCGCCAGTTTGCGAAGTCGAAAGCTACAATATACATTTGCCCACAATAATGCCCCCGTAATTTCGTAGCAAGTCTGTCATTCGGGCAATATCAAGGGTCTCTCTTTGCTACTTGCCTCAATACGCCTTAAAATGGTCGCTTATACTTGTTTTTATTTTTTTATCATACCAAAATATGTTTTCATGTTTTATTTTTTTTGTGGCTAACACTTGGCTGCCTGTTATCACAAATGCACTACCCAAGATGCGCGCCCCTGAAACTTCTTGTGCATTGNTTTTTTTTTCCAGCTAATTCATGTCAAATTGACGTTTTCGAGCTGCGGGTCTCTCTTCGGTAAATCTCCACGCTTGACTAGCGCGTTTTTCTTCTCCGCCGCCTCCAGAATCTTTACCATTATTGGGCGCCTGCCAACCAGTTTTTTCGCGTCGCTAATCGGGACCTATAGTGGTTCACACGCACGTAAGCTTCCCAATAATGCAAAAACTATTTTTTGCCGCATATCTACTCACATCAATTCTGTAGCGGGAGCTTTCGGCCCAATCGTCAAAGCGTTGGACATCATCCATCGTGTAGGCAAATAATCCATATCCTTTGTCGCCGTCCTTGTACGTAAACTTGCCTAGGTCGTGATTCAGACGCGCTTGAATCTGCAGAACGACGATTAGTAAAACAAAATTATAAATATACAAAAAACATTGCAAAAAAATAGACGTACCCCTGCTTCCTCTATAACCTCGCGCAATGCGGCTTTCTCAATGCTCTCGCCATGGTCCCAACCTCCCTTTGGAAGGATCCAATCGTTCGGTTTTTTCGAGCTTGAAATGAGGAGGACGTTATCTCCATCACCGATCACAGCGGCAGACAGCAGCCGATTATTCTCCTTGTCGACGAGCTGGCCAGCACGTGCTACATGGGCTTCTAGATATTTCTGAGGAGCAATTTTCATGCCCTCAAAGGGCTCGATCTTTTTTCGCATTCCACTGCCAGTTTTCTTGTCAAACACGCGATTATCTTTCAGCCTTTGTTCGTCCACTTTCGCCAGCTGTCTCAGTGTTGTAAGGCTTAGCTTGTCGGCAACCGAATACTTTCCGGAGAGATGCTTCACGAGACCTGCATGCTCCATCTTCTCGACTGCTGTCTTCGTTGTCGGTGCTACGTGCACTTTACCCAGCTTACCAGCAACGTCGTCCATCTTCCCCGTTATCCCCAACGCGTCGTCTACTTTCGTAATCAGGTCATCCACCTTGTCAAGACCACCTCTATCTTCATCATCGGACTCGATGTCCGCCTCCTCTTCGAGGTAATGTCGACGCAGGAACCGGTGGACTTCGGCAGCAATAGGGAATCGCTCGGTCTTCGTGATACCGACGTGCTCCGAGGAAGTAGCCGACGCAGATGAAATCAGGACAAAGAGAATCAATAAAACTCGCATCTTTTGTGGTGGAGATTGGTGTGGTGTCTTGAGGTTGCGAGAGCAGATTGCGAAGTGTGGATCGACCGTAAATGTTTTCACTTCATTCATTGAAAGCGCTGGCTGGTGGCGATCCAGATCGTTTTAAGTTTTCATGAAGCATGTAATCACACGAAGATCGCAAACAAGATGGCTAGTCCAAGCACACTACCGTACTAAATGATGCGCTTAGTGACTGAACATGAAAGGGCTTGGGATGTGCTTCACAATGTCGAAGCACGATCGTCCTTGTCAGCCAACCAAACAACGCGATGTTAAAGCTGCCAGCCGGTATCCTGCATCAGAATAAACCTTTCGAGGAGTTGTTGTCACAGCCGGCAGACTGCAATTTGCAATTTGCAACCGGATGGCATCGTAAAACTACTAAATAGTGGTTATGAAGATGCAAACCATTTTCAAAATAGTTAAATTTTGAAGTACACGTATCAATAAGAAACATGTTTCATTACACACTGAAGTCGCGCGCTGTTATAAATAATAAAGCACATAGTCATTCACAGTAGTTATTGTGAAGAAGCAGCCGTCTGAACCAGTTTTGGTTTTGCTTAGCACGACAGAACGGTTACTAGAGCATCTACAGTATCATGAAAAAACCAGAAACCAGCTGTTCAAATTAATCGTACCTTTTGTGCAAGATTATCCTAGAGACGTGCACACCCCAAGAGCATTCACACAGATCGAGTCTTTTCTGCTTGGCTACACGAGTTCCTTCTGTAGTTTACACCTGTTCAGTGCTTCTTTTCACCACTCTCTTTGAAAACGCGATCAAGATATTATAATGCTGAAAAACTGGTATGTGATCGTGCGTAGTGTCCTCTGCTATAGTCAATAGTTTTGACAGAAAATAATGATATACATGTAGCTGCTGTTTTTGAGCCGTAGTTTGCAACTCCGATTTTTCTACGCCATCCATTGCCAATCAGATATCCCCATCAGGTTGCCTACAGCAAGAAGCTATTACGAGGTGTGTTACAAGATCGGAACGTTGACTCGCGCTATTGATCGAAATGTACTGTATCGCCTATAAATTATGACAAAAAGCTTTCTTTTGCTCTAATTCACGAGGAACCGAATGGCGCAAAGGAACGAGATAGCTCACTCGTTACCATACTATAACATAATTTTGCTTTTGCAAGTCATATTTAGCCGCCCTCTGTGACTAATGATGGCCTCGACACGACACCGGAGAATAGTTAAAATTATGCTTACTTAATTATTAAATCTCGAAGATTTGTCACCACAAAAATGGTTTTGTGGAAAATTATCATCTTCAAGTCATTCCATTCGATTCTATTATCTAGTGACACTTGGGAGTACTCAGCTGTACTTCTTTTTGGGCTTTGAGGAGCCTGATCGACTGATTATTATGTTTTAAAAGCATTGGGANTTTTTTTTCCTTTTTTACGACTCAGTTCCTCTGACTATTTCCCAGGCTCATTACTGCTGTTACTTTGTGATAAATAGTAGTAGTATTGTAGTTGTAATGGAAGTAGTAAAAAAAACTGAGGAACCAAGTGTCAGACAATTAAAAAATCTGATCACGTTTATCTTAAAGCTTGTCGATATTTAGAGAAATAGGCTTTATTCGGAAGGGTGCTTTATTCGGGCGGATCCGCTTGTGTTTTATAGTAGTATTGCAGTAGCCCATAATACTGATTTATATCCACTGATTTAGACCTGCGTAAGTGCATAAACCAGCTGAATATCATGCTAGAGCGAAGTCCAATATTTTGGCAGCTTTTTTTTTTTAAATGTCCAGATGAAAAAATCGACGACTTTTATGTAGCATCATATTAAATCATGTTGTTGCAAAGGCTGTTTCATTTGCACTTCATCGTTTCCCGCGCAGCACGAGGAAGTGCTACCTTCTGCACGTTTTACTTCCGAGGTGTATAATTTTAAGCGATCATAGTCCGCTACATGTGCACATGTCCGACTTGGACCATTTATTCTACTCTAAGTCCATTCAATTCAACCACTATACCCATTTATTGGTACATTTGGCAACATTCTACCCATGTCCTTCATCAAGATAGCGTCTACAAAAGTGTGGTGGGTGCACTTTCGACACAACTCCGACGTCCACCTCCGCGAAAATGTTGTTGGTTGCATGAAGAAGGGACGACAAGCAATGATCCTGAATCTTCATTTCCTTTGAAATACTCATAATGTTGCTGCGGTTTGAAGACATGTACATCTTTACGACAGCCTTGTACGCTGTAAAACAGCTCGTCGTTCCGTTCAACATAGGCGAGTACCGCATTAAAATACTCTGCCAAATACATGTCTGGTTTTGGCTGCTCGTTCTGCATTAAGTATTTTGGAAGATCGCTTGGTAAAAAGTGTAAGCGTCATGGCCCTGTTAAGCAGAAGCTGGGATCAGGAGCCTGAGCAAAACGATCAAAGCTCCGGTTTCTACCTTCGCGATCTTTGGGAGTGTCTCTAATAGCTGGGTTCAGACCACTTTCATATTGCTAGTCGTCTTTATGAGGCAGACCACTTATTCTCTGTCCATCCACACGTTTTCAGCGGCTAGCTAACTGAAAAAAGCAGTCGGTCTCTTCTAGAAGCACTGTACTACCGACGCGTAGCTACCTGCGGACGCTGTGTTCATGCTGGTGTAAGACAACCTGTCGTGGATATGAATTTCTTGACGTTAGGGCCGTCTCATCAGCCCTTAGTTAACATACCCCCGTAGCGACACCAAGTGGTATTTTGTGACCAATCCACGCTGGATGTAGGCTTGAGCATAGTCAGCGATTATTGATCTAATGTCTCAGCACCTTCATTCCGTGACGGCCGCAGCATCTTTTGCAC

>Contig_38

CCTAACGGCCTATAGATTGCAAAGTGTTTGAGCAGATATTACTCTACCATGATTGATGTTAAAGCAGACTTAAAATATGTCCGTGTGCGCCCGGACTGGGGCTTTGAGTTGGTGCGCTATTGGGGGCCTTACAAAACCTGTATAAAAAGTTTAATCATTGGGGTTAGGGTGGTTTAAAAGGTGATAAAAACTTAGTGATAAAATGGAAAATTTTGATGAGACCAAAAAGTCGTCTCATCAAACTTCGCGTTTAATCACCTTTTATCCTCTTTAATCACGCACCCTAATTTAGCAGCTGATAAATAAGTGACTTGCGTATTGTATATGATTAAGCGACTCCCCTAACTAGCAAAAATTTTGAAATTGAAGATGAGATCTTCGACAGCTTCAAAGGCGCGAAAATTCGCGCCCACTAAGGATCGTTTCATTTACGAATTTGCTGCCTGCCGCATAAGGCTCGTCGAGACGTCGGCGTTGCATGGTTGCGGTACGTACCATCGTAGATCTGGATCATCCATCATATGTGCTAACCTTATGAAGCAGCTGTGACGAGGCTTCACTACAGGCCGGTGAGTGCGTATTGCTGTGCGTTTCTTTTATACTGATACATTATTTTTCCGTACTATTTGGTCTTCAGGCCGAGCGAGATCACTACCGAATTCTTTGATGAGTTGGCCCCCTCAAATTTCACCGAAGAGTTGNCCCCCCCACCCGGATTTCCTATACTAAATGATGGGCAACATTTGAAGAGACTTCCTCAATGAGTTGCCCCCCCCCGATTAACGTCCAATCACAGACTTGCAGCCGACACTGGTCAGAAGATCCGTGCAGTTTTAACCAATGATATGCATGTGTGTCTGAAGGGTCTTCATCGATGAGTTGGCCCCCTTTGCTGATGTACCACNGGGGGGCCAACTCATCGAGGAATTCGGTAGATGTGGTGATAAAGATTCCTGTCCTGGCAGCAAACTCCGAGAAGAGAAGGCAGAGGCGCGTCCTGCTGATCAACACGAGGGCGGTGAGTTGCCGGGGTTCGGAGAACGAGAAGACCCTGGTTTATCCGTCAATGTTACTATGATCGACAAGAGAAGCCCTTACTCGCGCAGTGCTACCGTTGAGGGTGAGCACAAGAACAACGACGCTCGACACCTGGTGGTGGTGCTGGAGCCGCCGTGGCCGACCGCCTGCTGGATTGTATACTGGAATGATGTAGAATTGGGAGAATCGCTCCAAGAGCTGCTGAAACGCGCCCCTGCCACACATAAATAAATAAATAAAAATAACAGGTTAAAAAAAATCATTGCTCTGGCACATACAAACATCGTCGTAAGTTACTACCTTGATAGGCTAGTTTTGCTGAGCATATTCTACTCGGTGCCCCGACGCCAAAAATCCATCCAATTCGACTATTCGGGGTGTTTTAGACAGTCGAGGGTCACGTAATACCGCATGAGCAGCAACCGTTCCCACTCTACACGCCTCCTCTCTTCATGCAGCCGTTTACGAAGCCGTTTAACACACTTTTCGAGATTGTGTCGCGTTTGCGACGTCGCGCATAGCGCGAGAAGAACGCCGGCGCCGCGCATGGGCCCCTCTCTCGTTTGAATCAGGCGAGAGAAAGCCGTGAGTTGCAGGGAAGGGGTCGCTGGCAGCGCGCCATCGTGGTGGTATCTTAAAAAATAGACAAATTTATCAGTTTTTTGTCCCATTTAAATTAGTAAAGATCGTCCGTTAATAATGAATCAATATGTTAGATACATCAACCTCACTCTATTCGGAACGAAATAAATTTTAATGTAGGGTTTTGGCAGAACGGTTTTGATAAAACCGGTATAAAAACGCATCACCGTGATAAAAGTGTGATTAAAGAAGGAGTCATCAAAACTTTGTGATAAAACCGTTTTATACAGGTTTTGGAAGGCACCCATTCGTTAATGGGCGAGTTCTGGTTCTCGAGCAATCGATCAAAACCAGAAACCGTCATATTAGAGAGGTAAATCATATTGTTGTAAAACTCTGAAATAAATCTCAGAGAATACTTTTTATTAAGGTATTCTCTTTAAAAAAAGTGACAAGCGTTAAAAAAGTATAAATTCTTTTCAGCTTCAAAAATAGATTTACTGTGTGCTCGTTGATATGATTTCCTTAGTTCATTTTCTTAGCCTTGTAAGTCTACATGTAGCTTTAGCAAACCTTAAACAAATCCAGGCCGAAGGCGCGACTTTGTTTGCAAGAGAAAACCATAGCAGCAACGCCGCCGAAGCCCCGCGGGAGGTAGAGGGGCCAGGGTTCGCACTCTGAGGTTTTGAGCTTAGTTTACAGGGTGCTCAAGGATTTATACACGTGGTATTATAGGACGCTACCTGTGTTTACGCTCCATGTGCGAAGCACGCGGGCTTTATTAATAACCCCGACCCATGATAAAGGTATTAATAGAGCTGTATCTGGTGTTTATGATCCGTGTGCGAATACACTGCCAGGATAAACAGTTAAAGCTCCTAATTCGAGGAGTAGGTGTAACATTGTTAGAAGTGTTCTACCTCTACTAAGCCATAAACTCCTCGGCGCCCTATTAGTACAACGGCGATCCACCATTAGCCTTGTAGAAGTCATAGAAGCCGAGGACGACGCCGTTGTTTCTTATGTCACGCGGTTGCTCGGCTACCAGTGCAGCGACTCTTCCTGGATCCAACCTCTTTTGGTTCATCCACTGATCGAACTGCTGGTTCCGCAAACTGAAAAGACGCTCTACAGAATGTCCTTTCATCTCGGCTTGGATCTTAGCATGGCTAAGCATTCGTGCGAGGTCCACGTAATCGAAGCGTTTWTCGAGCTCCGTAAAAAGAGCGAATTTATCTGGATGTTTCTCGTACTTGTTGAGTCTTTCAACATACAAGATCCAAGCGTCAAACATCGGGGCCTCGAACAGGTCATCACCTGTTTTGTCAAGTTGAAAGAACTTAAAAATATCATCCGCAGATTTCCCTGGGCTCCGCCACATTTCCGCCGCTAATTTTGAAGCCAGGGCTCTCGTTAATACACTTGTTTTTGCTTGAGCAATCATTTTTGCGAGGATCTCGTCGCTGTAGTGCTTCTTCATCACTGAGAACACCAAGGCATCAGTATTTGTATAGTCAACTAACTTCACGTAGGACACCCAAGTATTAAACACTGAGCTCTCTAGGAGTTCTTCACCCTCCTTGCTTAGTCCAAGATGTCTGAAAATGTCATCTGCCGTGTCCCCCCTTCCATGCCATTTACTGAGCTGTAACTTTTGTAGAGCAGTAACATTGATCTTCACGTTTTTGTCCCTCGACGCCGCGACTAACACTCTGGCTAACTCTGCGTCGCTAAATCGATTCTGCAGCTCCAAGAATAAAACTTTGTTGGGATCTTTGTTGCGTTGTCGAATGTACGACAACCACGTGGGCAGAGCCGCGCTCCGCAAAAAACTTTCTCCCTTGTCTGCGTTTAGTCGGAGAAGCTTGGCGACGTCACCTGTCGTTTTACCATCCCTCAGCCAGGCTGTAAGCAGTGCATTCTCCAGATTACGAACCACACTACTTGTAAGGCTATCGCCCTTTGCTGCAATTAGCATGTGAGCTAGTGCTTCTTCATCATTGGTTTTTGTCAACTTGAGCAGCAACAAATTGTACGGATCCTCGTTATCAAGCTTTTTGACAAACGAAACCCACGTATTCAACAACGGATTTCTCAGAAGACCCTCCTTGTCCGCGTCCAGCTTTAAAAGCTTGTAGATGTCCTCCTCCGTTTTTCTGTTTGTGACCCAATTGGCTATCTGCATCGATTCAATTTTCTTGGCTGTCCTTATCGCCGCTGCGGTTGGCTGTGCTTCTGATACCAGTTTGGCCAGAATGTCATCGCCGTGGTGACGTACAAGTGTAGAGAATATCGCTGCTTGGCCTTTTTCAGGGTTCTTCTTGTAGGATTTCATCACGGATGCAGCCCACTTCTGATACGGAGTAGACGCAAACAGCTTCGATTCCACCTCCCAGACCCCGAGACTCGCAAACAGTTTGTCGGTAGCTCTTTGATTGTTGGATGTAAGAATTTCCATGGCTCTTGTTGAAAGTTTTTTCGTTGGCTGCGAATTAATCAGCTTTTGAAGCACCGTAGCAGCTGACAGCTTAACTCCAATCGCCCTCGCCTCGGGGCTGCCGTCTTCCTGTTCAGCTGTCCACTTGCTCTCACGCAAGTATCGGTGGATCTGACCGCCCACCTCGCGATCCAAAGCATCATTTCTGGCGTCGATTACGCTGGATGCCGTAGCCACCTTAGATCCACTGTGGATGCATACCAGCAGAGCGAGGGCCGCCAGCAAGATGAAGTAGCTTAAGCGTGGTGTTAAGGCAAGTTTGAGGGGCATCGCACCGACAAAAACGAAAAGTGAGGGCTTCGTAGCAAATTGCTGAATGAAAAGCTCACTGAGGCTTCGGCTTAATTTAAATACCGGTACTCTCGTCCATCAAATCATTACGCTGTGTAGAGTGGGCTGGGGGCATCAGCCACTACAGTGGGCGGCAGTACTGATCTTCGGATTGGCAGTGGCAGGGTTGCTGCTGCCAACCATCGAGTGATGATTGAAGTTCAGTATTGCCGGCCCGAGCTGGCAATATATACAGGGTATGCCAAGTATACATAGTATGTATACACAGAGTACAGACAAAGAGTCAGTTAGTCAGTCTAGAACTTGCTTAGTGGAGAGCAATACACTAGGCAGAAGCTTTAGGGAGTGCCAGCTCCCCCAGTTGTGTGTTACTTCCACGACCTACTACAAAGTTAACTTTAGTGCCCGGGTCAGTGAAGAGGTTCAAGATCCAAGATGGCGTGTGGCGGACGACGAAGGTGGTGCCAAAGTCATGGGGTCGGAGGGCGTGAGCGTTCCGGACACTGAGGATGCTGAGTCCGAGTGCGTCGAGGTGTCTAAGGTGTCGGAGGGCGCGATCGTCTTGGACCCAAGAACGTGAGTCCGACGGCGTGAGTACAAGGACGCTGGAGCGTTGGAAGCCGAGGACGGGGATGCCAAGGTCAAGTACGTGAAGGCGTCCAACGCGCTCGTCGGGACTCAAACAACAAGCCCCGGGAAGCGCAAGAATCGGCAGGCATCTGAGGCGTGACGGTTGAGAAGCGCGAGTGTGCCGACACCGGAACATCGTGGGTGCCGTCGAGGAAACAGTGTCGGGAAGGCGCGAGTGTGCTGGATGCTGGAGCGTCATGCCCGGGTGGATGCCAAGGTCGGGTCGTGTCAGTGCTTGTGGGCGCGGGTGTGCCAGGCATTTGATCATCGAGCTCGGAGGCGTCAATTCCAAGGTACGTGGTGTCAGAGAGTTCCTGACACCTGAGCATCTCACCCTGGGGATGTTGTGGAGGTGCGTGTGTGCCTGCCATTAGAGGTCACGAGCATGGCAGTGTCTAGAAGCGCGAGTGCGTGGACATGAAAGTGCAAGACCAAGGAGAAGATCATACCACGTGGCTGGATGAGTCTTTAAGAAGAGGGTCTGTAGTGGGCTGGGGGCATGTAGCCTCATCCAGCCACTACAGTGGGCGGCAGTACTGATCTACGGATTGGCAGTGGCAGGGTTGCTGCTGCCTACCAATCGAGTGATGATTGAAGTTCAGTATTGCCGGCCCGAGCTGGCAATATATACAGGGTATGTATTGCCAAGTATACATAGTATGTATACATAGAGTACAGACAAAGAGTCATTTAGTCAGTCCCGTCCAAACAGAGAATAATGTCAAGCAAAGTGGACAATATGTTTTTGATTATTACAAATATCTGATTACGGGATCAAAATTACCAGTGAATCAGAGAATAATCTCATTCAAACCGAGACGACACAGTGAGCTACTTCCCATAGACCATGTCTTTCATCTCCTCGGCCGCGTTCACCGCATCGCGAGCGTGAAGCTCCATCTTCGTCACTAACGTAGGTGTTATATACTTCATACTCGATTCTGCAGCCTTCTCTAAGAAGCGCATAGTGCGCTCCTTGATCGTTATTGGGGTGCCGTCTTCTTCCACCATGTTACTCCGGTCGCAGATCTCTTCGCGGTACAAGGCCAGGTATCCCTTGATTCGAGCTTTTAAAACGGAGAAGCAACCCTCAATCGGGTTGCACATCGGTGAGTAGGGTCCGAGTCGTAGGAGTACGAGGTCGTCACGTGCTTCGACACGTTCTTCGGTTTGGCGGTGAGCGGGGGCATTATCCAGGACAATAATGACCTTCTTCCCAGCGAAGCTGTCATGAAACACGGAGGATGCCTTAACCGTCCGGTAGATTTCCTCAATGAAAGCTGCATTCTGCTCCATTCGGATGCTTCCCCTCTCCAGACGGTGGAGAACCACACCCATGGCCGAGCTTACAGCGCACTGGACTTGCAAGTTTGCACCCTTGGACGGCGGCATCACGAGCGTTGCACGCTCACCTCTCCGTGCACGTCCTCGACCTCGCGTACAGTAAACGTTGTAGTTAGTCTCATCAAAATAGATTATACAGTCACCGTTCTTTTGATGGTCCTTCAGCGATTTTGCAAACTTCTGCCGCTTGGTCTTGTTGATCTCGTTGTTGCATGTCATGGGCTCTATGCGAGTTTGTTTGACGGTGAACAGCATGCCAAGCAAGTGGCGGCTGATCGTTGAAGTGGAGACCCGCACGTTCGTATCTAAAAAGAGCATCGTGCGCATAGCTTCCAGGGTGAAGGTGCAATTGTCGTCAAGATATTCCTCCAGCTTGGACTTTGCTTCTGGTGTCATCTTCACAGCTCTACCACGAGCTCCACCGCGCGGTAGATCTTCTACGCGTTCTGTGGCTACAGTGCGGTATGCCACCGCACGCGATATGCCGTTATTGGCGGCCACCTGAAGCCAGTCAGCGCGTCCCGCGCGATGGGCAGCTAGGACACGGAGCTTCTCAGCGGGGGAGTGCTTGCGGAGGAGTGGTTGGAAGGGCATTCGTCTGAAGAGGTGAATTGAAAAGTGAACTGCTTCAGGCGATGGTTTGCTTCAGGCGAGGTTTTCGCGCGCATTTTGGCGCCAACGCACCCTCACCACCCCTTCACGAATGATTTCCAGCCATCGCCTTTCCTTGTCTCGCTTTGGATGAGATCTTTCTCTGTTTGTTTGTTGATTACTTTTACTAAAGTTAAAATTTGTACTATAAAAACCAAATTGTCCACTTTGCTTGAAATTATTCTCTGTTTGGACGGGCAAAGGTGTGGAACTTGCTTAGTGGAGAGCAATACACTCGGCAGAAGCTTTAGGGAGTGCCAGCTACCCCAGTTGTGTGTTACTTCCACGACCTAGTAACACGCTGGTATAACCCCTGCGCACTTCAAAGTGAATTATCTCTACCGGTATTCACATGTCAACGCATAATGATACAAATATAAAAAAGTAAAATATTCCACTAGATAGTCAACATCACATCGGAGACGCTTACTCCAACAAAAATGATGCTGAAACGCCCATGTTCACGCTGGCATGGGCTATTCAATTATCGTTTTCTCCGTAAGATTGGATATAGACCTACAGCAATGCTATAAGACATTTCCAGTGTATAATGTAAAGGTCGTGGCAATAAAATT

>Contig_39

ACAGGCCTCCTTGTGTGTGATGTACGCGTTTACAAGCGCTAAGTCAAACAGACCTAAGAATAGACCCTTATAGTACTTTCGGAACCTGAAAGAAGTCTGGACGGAGAACGTTTGAAGGCGGAGTTGATCATGCACATCAACTACGCCCATCCAGCGCTGGTAATCGGTCACCAGTTTGGGACAGCTGAACGTCGACTGCCCCACCGTCTTCAGGTTTCTCTTGATGGTATCTTCGGCCATTGACACCCCCAGTGGCCAGGTAGTGCAACCGGCTTGCGGTCCCACCAGTGGCATGCAACCATGGTTGGGCACCGCCACCGACCTTGAGTACGTGAATGACCCCGCGCTCTACTGTGGTTGGTCGTGTTTCTCCTTCGCTCAACGACGTGCTTGTCGAATCCCAGCCTTGACGTCATGATTGTTCCAAATCATGTAGACCGACATCGAAAGTAGTTGAATGGCGAGAGCAACGGAGGTGTAGAATCTGTCGACAACAATGACACGAAATCCGGGGTCGTTCTCATCCAACACAGCCTTCAAATTTCGAATTACAGCGGCTGCTCCTGTCTTGTGGTCAAACGCCTGACTTGCACTTTCATGGTTTTGACGCTTCCCTACATATATCTCGAATCTAAAGTCGTAAGTGACAGCATAATGCAAATGTCAGACCATCAAGTCATAACTTACGACTTTGCAACACTTATAAAAAAATGCGCACCTGTGGCAGTAAGCAGACACTGCGTCGCACGTCATGAACATCTTGGTGCCATAACGGTGCGGCTTGTCCGGCAAAAACATACGGGTACTGTTACGCTTGGACGTTTGAGGAAGTACACCTTCGTCGAAAGAGATGATTGCAGCCACTATCCACGCAGCTAGGAATGTCATTTGAAGTACGTCAACAACAGCTCGCAGTTTCCACAACTTGTCTCTCTTGTCAGCTGTGTCATTGTTGCAGAAATGTAGATCTCGTAGGATAGAAGTACAGCGATTTCGAGAAATGAAATTGCCAAACCGTCCCGCCGAAAGCGCGCCATCATCGGTCATAGCCCAGTGATCTGAAAACGGACGGGTAGGGCTGAGCATATGAGCTACAAGTAGACCCATGACACAAAGAATCTCGCGCGGTACGTAATCAGGTTCGACTCGTATGCGGCGGCGAACATCCCGTAGCGTTTGACCTGGTACGCTGCGTCCTTCACGAACGAGACGTGCACGCTCTTGGCGCGCCTTTTCCTCGAGGTGTGACCTTTTGTATCTGTTTGTCTCGCTCGTGATGAATGTCCACAGGTTCACCGGCATAAAATAGAATAGCAATTCCAATGGAGAATCCTTTTTAGCACGAAGTGCTTCCACAGGCCTAGCCGCTTGATCCTTCATACCTTTGAACGCGCTATTGTCGGTTTCATACTCGGAGGATGCAGGAGTCCACGCCATCGAACGAAGTGTAATCTGGTTCATGTCTTCGATAGATAGAGAACCCCCCAAGCAATCAATAAATGCTGCATCCATAAGAGGCGCCTCTTCTTCGTCAGATGACACGTCGTCACCAATTTGTCTTTGAGCAATATCACTATCATCATCTGATCCAAAGCCATCCGACGCCGAGTCCATGCTCTCATACTCGTCTGCATTGTCCTCGTCCTTCATGATGTTCACGTCCTCTTGTAGCAAGCCACGATCTTCTCTCGAGCTTGTTCGCTCATCGTTTCGTCTCGAGTTTGTTGGCGTCACAGGCTCGACGACAGCACCATTTTGACCGGCGCATAGTGACAGCGCTACATCAGGTTCATCACCGCTCATAGCTCCACAAAATATCTCGTCTATGGTGTTTGCTGCAAGCGCAATACTTGTGTCTATCTGTGACGCCCTTGAGCTCTCATCCTCTATGTGCGGACTTGTGGCGCATAGCGGAAGTTCTGTAGGTGTCTCGCTGTCCTCGCTATCGGATCCAAACAATGCTTCAACGGTGTTCGCTGAGAGAGCAACACTCGTGTCAATCTGAGACGGTGTTACCCTCTCACACTCTGCAGGAACTTCTGGCTCACCTTCTTCACTCTCGTCGTCTCTGTCGTCGCCTGCACCTCCCTCGTCAGCGCCAATGATGCCCTGTTCAAACGCATACTTTACTACCGCATGTTCTCCTTCGCATACACAGCACAGAGCACGTATTAGACACACTCACAATAGACTAACCACATCAGACTTACCGACAAACATGTTCACGCCCTCGATGTGTAGCTCCTTTGCAGCGTTCGGTGGGTAGTACGAGTGCAAATTGGACAGCCGCGTTGGCCTCTTTGACGTCCATCCGACACGCACGAGCTGACGCCATAGATGACCAAAATCCACATCTCGCACGTCGATTGCTGAGAGCGCCTCTGCTTGAGCTTTTGGCCGCTTGCTTGTTCTGACCGGCATCTTTGCTTGAGCCCGCAAATTCTGTACTGGTGGAGAACATCCACCGTCCACCAAACGTACGGTCCCCAGATAGTGGGACAGTGAATCCACCTACAGCCTTTTTCTCCCCTGTCGCCCACTACCTTTCCAAGCTTATATTTATTTTGCAGAGAAACAGACTTGATTAAAGTATGTGATCGTCGCATCTGAGCCATTTCCTGTTTATCTTGACAGTTAAGTCGTAACTTACGACTTAAGGTTTTCACTACGTCGTAAGTTACGACGTTGGTAACCAAGGGGTTAATAGTCCCTCACTGATCCGTTTGTCTAGACTTACGCTGCGTGTGATCACCTGCTGAAAAGGCATGCTGAGGCTCGGGACCAAAGCGAGTTTAGAATGTCCTATTTTTTTTAAATATTTAAACTTGTCTGTAATATGAGATTTTTCATGCAGCTATCGACATACTTGTCCGGTCCGAAAAAAAAATAGTAGGTCTCTAAAGTCGTGACAACTTCTGTTGCAACGAAAGCGCGGTTAGCCTTCTTCATTCGCAGCGCTATTAATAGGACAAATTTTAATTAAATGGGGTTGCATACAAATCATCTTGTACATATTTCAACCAATCATATAGATCTCAAAAGGGACATTGACCAATCACACATACGAGGATTCTTACTTAAAAGCATAACTGGTAATTGGACCTCAAAAATAAATCGTTTTATTTAGACAAATACTACAGTAGCAGTAGGACGTTTAGGACGTACTGAAGTATCATCTTCTTCCGTTCTTACTCCCCTCTCCAGCACAACGGATCTGCTGTACATGCATTATCTTGTCTTTGTTAGTTAACCCGTATTTGAGTCATGCCTTGGCGGCAGGGAAAGCAAGCTAACCCTCAGTACAACTCTGCTCGACGTTTTCATCCTTCTTTCTTCATCTTGGGGGCTCTCAAATAATGAACACGTAGTTCATGTTGCCATCTCTCTGCAACTTTATTCAATTCAGGCACCATTTTCGCGTTCTCGAGCATAGATGACAATTCTTTGAAATTGTAGAAACGCGTGATCGTCTGCATCATCGTCGTTGGCTCCACATCGGGCATCAAATTATATTTCATCATGTAGCTCTCCCACACCTGCATATTCGAGTTGGTCAGAAGCTTATCCAGTCCCTCATCCAGCTTCAACAGCTTGAACACTTTGTCAGGAGATACATCTTTGTTCAGCCAATTATTCACCATTCTTGTCTCCAATTTGATGGGGAGATCGCCCACAAGATCAAAGTTACGCTGCCCACGGAAGAAAATCCTCGATAGCCCGTCATAGCCATACCTGTCCGACAAGACTCGAAGCACGATATCCTCTGCCTGCCGCGTATAACCCTCTTTTATGAAAGTGTACCAAGTATTAAACAGCGGCGTCGCGAAAATGCCACCTCCCACTTCGTCAAGCTTTAGAATCTTGAAAACTCCCTCGGCAGATTCGGCATTGTCCATCCACTTATTCAGCAGTTCCCTCCACAGTCTCGACGCGAAATTCTCCGTCTCCAAGTTTGAGGATCGTATTCCCATTTCTACCATCTTGGCTACCCCTTCGTCTGTGTACTTTTCACTAAACTTGTCGAGTAATGCCACTTTGTTTGAGGGGTTTTTCACGTTGAAACGATCGATGTAGGCAGCATAAACATGGAGCTTCGGGTTGGAGAGAGCCTTATCAAGTCCGCTGTTAAGGCCCAGCTTCGTAAAGACATACTCCTGCGTCTTCTGTTTGTCTACCCACTTCTGGACCTTGCGGTTTTCGAGAAATGTGAGCTCTTTCGCTACCTTCGGACTAAGCATCCATTCGACGATCTTTGAGGCACTGGGTACGCTTATTCCTCTCTCATCGGCATGGTCAGCTCGTAGCATCCTCGCAGCAGGAGCATCGTGGGAAATGCTACTGACAGCAATAGCCTGGATGCCTGAAGCGTTTACTTTTGCATCATAAGTACTGGCAATGAGAGTCACAATAGTCAGCAACACCGAATAGAATCGCATACTTGAGCGTTGGACAGCAAATTGTGAAGTTGACAGGCTCCGGGAGGTCGTCACTTGTTTCACAATCTCCACAATCGCATCTTGAATCTTGGAATCAAATACATGTACAGTTCTTACCTATTAATAGTAAAGAATACACACCGGTATCAATTTAATACCGGTGGGTATTATTATGGTACATGTAATAGCACTTCTGTAGTACCTTTTAAACGAAGCACAGTGTGTAATCATCATGGGATGGACGAACCGAGCCGGAGCACGCTTTTATATTTTCAAGTCGGTATATTCGGCTCCCTTATTAAGAGTATTACTGTAATAGGAGCCGGGCTTGTATCTTTAAATTGACAGAGGTCGGCATCATCGAGGACGCTCTTTTGTGTAACTGGAGCAATTAGTGACCTTTTTTTATGCGGTTTTCAGACTTACGCTGGTCCTAATACTCAGTTTGTACTTGTTCACATTGAACACTTTGTCGATCATCGACCTCACTCGGTAGATAACGACCTCTTGGTGTAGCTGTCTCCACCCAAGAAGAATATACTAGAAGGTAACCATTTATTTTGGAGAACACATTATTAGGCGAATATCCAGAATGATACTTACAGTTATTAATAAGCATACTGCTACACATGATTACTAACTTCTAAGTGTACAGTCCAGCACGATAAAAAATACAGGTGAGCCCTTACGTAGTCGGAACCAACCAACACTTTATGCCTACATAAGGGTATTCTTGTTGTGAGGTGGCTCAGCTATGTGTACCTTCAAAATGGTCTATTTACATAAGCCAGCACGCTATCATCCATTACAAGAGTCAGCTCACATCATGCTCATGCTTAGTGAAGCAGATAATGATTATGCAGAGCTCAATTCTTACGAATATTATGAAACACCTAAAGTGGGTCGCAAAGCTAATTATTTGAGCAGATTCAACGGGTGTGTCGGTAGAGGTGATCAAAAACTTGGATCGATGCAGAGATAGCTACGATATGAGCTAAACAGGAAGGAAGGGTGCAAACAGCAAGTGTCCTTGCTTGCCTTCTTCGGATATGATACACAATAGTGACATTCCCATAATTAAGCACCTCATAACAGTTATTCTAGGTAATTCGGAATCCCTATTACAAGGGAGAGGTGACAGGTAAAATCATCTTACTACTTTAAGTACTAAACTTCAGGAACCAATAATTTAATCTCTGTCCGCGGGTATTCCAAGACCCACAAGTGCGACAAACACTCTTACAGATGCGGGACTCTGAGCCGCATTCGCACGCGCAGGGTAAAGACCTGGACCAGTGGTATTCTCGAGCAGCAGAAGAGCGTCTTGCAGCCATGACGCTGCAGAATCCGGACGATGTGACGCCTCCTGAGTTTCTATTATGCGGCAAAGAACGCCTTCTTAAAGAGCCTGGAGGCCGTGCTAAAGCACCAGCAGAGTGAATACTACGGCAAAACTGTCCCTGCGGCCGGAGCGGGACGATCTAGACATGCAAGAAAGATACGTGACGCTGTGCAATAACGTCGCGATCTGTGGCATCAAAATGAAGGATCGATCGCTAATCAACGAGTACGCAGCCAAGGCATTGGCTGTAGAGGAGACGTCCACTAAGGCGCTGTACGCGATGGCCAAGCTGCGGCTCATGGAGCGTCGCTTTAACGAAGCGAACGAGGTCGTTGATAGGGCGTTGGGGTTTTACCCGGACGAGGCGCAGTTTTTGAACTTCCGCAAGGAGATTGAGGCAGCCGAGTGATGGAGCAGGTATCTGAGATCAGAGCGGAGCTGCGATGGCTGCAGAGGCGGCAGGTGTAGGGATTACTATTAATACTAAACGTACCATAATCAATAGCCTAATCTCCTTCTTTGTAGACCCATATCCCTTTAATTGCGTGGCAGAACACTTCGCGAACATGTGGAGTTTTTGGAAAACATGACGAGATTTTTTTACAAAGAACGGTAATCATATGCAACCTTTACATTTCGTTTCTCGATTTGACGGTTTTGGTGTTCCGAGAATTAATAACCCGGGTTTTGTTCTGTTTCACCAGCCAATAGGATTCCTGTATCTTCACACCTGCACTGACCCTGCAAACTGGGAGTCGGTTATGGTAGCCGAGTGGACGGTAGCCGTTACCTGCGACTAGAGCTGTCGACGTCTGGTGTATTCAAACTTCGACACCCACCAATGGGTCACTCTTTCAGATTAAGGTGCTGCTGTTTGTGGATAGCAACCTCACCAAATCTGGCGTTTGTGTTGAACAACTGTTCATCTTTCAATGGAGGATGACAATGGAGGACCTCCATGACCGTTCGAAGCTTCCAATCTATCTACTGGTACAGACCCTCAGAGGAACGTCCGATGCGCACGTCGCACAGCGGCAAGCAGGGACTGACAAAAGCTGCAGTACTCGGACAATCACGGAGTCGTTACGGCAACCGTAAATCTGAGACATGTTGGGTCGCTCTAGAGATTTATGGTTGCTTTAAGCGTTTTTTAATTACCAGGCGTAATTTAAATTTACGTACTTTTCAACGTCTTAAGTTCGAACGACTCGAGCCGCTCATTTCCAAAGCGGCGGGCATTAGAAGCAAGCGGGGGTCGAGCTCTACGACGGCAAGGAGCGGCGTGAATCTAATGCAGGAGACTCATGTGACTGGTACGTTACCCCTTCATTAATATTGCAGCTCATGTTGCGGTGACTGATGTATGCAGAGGTACTTACAATGTGCGTGTATGCGTGCACCAGGGTGCACGTTGCGTGATCAAAGCAAGCACAGCCTGAACTAGATGCTGGTGACGCTGGGCATTGAGAAGGATGACGACAGCGATGAAGACTTCGTCCTGGACTACATGGAGGAAGATAATGAAGAAGAAGAAGACCTCCCCTTCTCGGTGTCTGACGCTGAAATCACGCAAAAAGCCGGTAAGACGTTCGGATTCGTAATTTGTCACGTGCACTTGGTTAACGTTGAACTCGTTTTCTATCTATATCACGTGTAGCTCCCCAACGACGGAAACACCAGAAACAGAATCGCAATGCCGAAAGTGTGTCGCAGTCGACGCGCACGATCGACACCCAGCCAAGACAGGGCAACCAGAAACGCGCGACCGACACACAGCGTACGCACATTCCGATAATGCTGCTTAAAATAATGTTGCTGAATGATAATAACTCGCATGTCTGTCTTTATTGTTAAAAGATGATGGGCCATTACCAAAGCAACAAAAGAAGGACACGTTGTACGAACCA

>Contig_44

CGGCCAGCGAAGAAACATCTGCGGCCAGCGAAGACACATCTGCGGCCAGTGAGGAAACCGGAAGCTTGTCGGAATGGACGACTACACCGGTGAAGGGCAAGAAGACTCCGTCCACCGAGTCAGCGTCCGCCTCGACGTCGGCTTCTGCGTCGGCATCGACTGCAGCATCGGATGCGTTGGAAGCCGAGCCGGCCAGCGAGGACGCGTCTGCGGCCAGCGAAGACACATCTGCGGCCAGCGAAGACACATCTGCGGCCAGCGAAGAAACATCTGCGGCCAGCGAAGACACATCTGCGGCCAGTGAGGAAACCGGAAGCTTGTCGGAATGGACGACTACACCGGTGAAGGGCAAGAAGACTCCGTCCACCGAGTCAGCGTCCGCCTCGACGTCGGCTTCTGCGTCGGCATCGACTGCAGCATCGGATGCGTCAGAAGCCGAGCCGGCCAGCGAGGACGCGTCTGCGGCCAGCGAAGACACATCTGCGGCCAGTGAGGAAACCGGAAGCTTGTCGGAATGGACGACTACACCGGTGAAGGGCAAGAAGACTCCGTCCACCGAGTCAGCGTCCGCCTCGACGTCGGCTTCTACGTCGGCATCGACTGCAGCATCGGATGGGTCGGAAGCCGAGCCGGCCAGCGAGGACGCGTCTGCGGCCAGCGAAGACACATCTGCGGCCAGTGAGGAAACCGGAAGCTTGTCGGAATGGACGACTACACCGGTGAAGGGCAAGAAGACTCCGTCCACCGAGTCAGCGTCCGCCTCGACGTCGGCTTCTACGTCGGCATCGACTGCAGCATCGGATGCGTCGGAAGCCGAGCCGGCCAGCGAGGACGCGTCTGCGGCCAGCGAAGACACATCTGCGGCCAGTGAGGAAACCGGAAGCTTGTCGGAATGGACGACTACACCGGTGAAGGGCAAGAAGACTCCGTCCACCGAGTCAGCGTCCGCCTCGACGTCGGCTTCTACGTCGGCATCGACTGCAGCATCGGATGCGTTGGAAGCCGAGCCGGCCAGCGAGGACGCGTCTGCGGCCAGCGAAGACACATCTGCGGCCAGTGAGGAAACCGGAAGCTCGTCGGAATGGACGACTACACCGGTGAAGGGCAAGAAGACTCCGTTCACCGAGTCAGCGTCCGCCTCGACGTCGGCATCATCATCGTTGGATGCCTCATTGTTTGCTTCTGAAACTACTGATAGCCATGTTGACGACTCAAGCTACCCATTGGAAGGTAGTCAAAACTCTGATTCTCCGATTCAAATGGCGGATGAACCCATTTCGACGAAAGCTCAACGTATGCGAGGCTCAACCAAGTTTGCGAAGCCGTCACAATCTCTCCGTGGGAGTACATTCAGCTCGTCTGACTCGGATAATACCCCTTTCCAGCAATCCTAAACCATGAAGATGAACTGAAGGAGTGGGTGTCGACGTAAGACTCCCACGATGCGTTCTCTGATGAAAAAGGCGTAACCAATACACTGCGTATGCCTACTTGTTTCACCGAAATATCTTCCGACCATAAAGCACATGAACTTAGCGCGTCAATGTCTTACCAACGGGTGATAGCATTTCGCCTCACTCTCCTAATTCACTCGATCTCCATTAAAGAGTTTCTCAGCTTCCTTTGTGACCTGTACTACACAATAGTCAACGGGGCCTGTTTTGCCACAGCTTCTCGCATGCGAGATGGAATAGGCTCCACCCCGCCAATATTCCGCACCCAATAGTCCGCCTTTGATGTCGGCATTTGTAATCCACCCCAATACTTTGCCTTTGATAGCAGCATTTGTATCGGTATCATCACCCTTGAGTAAAGTCTGCTGAAAAGCGCGCTCATAGATCTCGTTCCGACGCAAGAAAGTACGTGGCGAGCACAAAAGCCCAGCGGACATGACCAACTTTACTCGTACACACGAGGCCCGAGATATCCAATGACTCCTCCAGAAACCACGTGCGCACCACTGAGGTCACATTATTGATTACAAAGCGTTCACCACGACTTACAGCTCTACGAAAATCCCCTGGAACACGCAACAAGTGCTCCACCATACGACAACAAACGGCATTGCAGTCTTGACACACGTGTGTGGCGTTCTGGCGAGAGCAGCGATGGTCTCCCACGACTGCCCTACTCCAGATCGCCAGAGGCAATGCGCATCAGCGATCCGTTGGCTTCACTGGCTGCGCAACTACCGGTAGCACAGCAGTTTGTATCACAACAGGCTGTCAAATTTAAATAGTTCACGCACGTATTAATACCGGAAGTTTTTATTTGTGTTAAAACTAAAACCAGTTTATTACTTTTTTTAATTTTTGACCATTTTAAAGTTTTTTACATCATTTTTATTATTAAACTCAGCTTCTTAAAAATGTACTWAAAAAATATATATGTTTGCTCTTGATACACAAGATGGCGTACGACAACTGAGCCGCACAATCATTATACTTTTAGTATGAGCAGGTTCATTTGCTTGTAACAATACCTACATGTATAATAGCTCGATCTTGACACTCATGGGCTCTTACTTCTCGAAGTACATGTTTTATAACTTTGTATTGCGATGGCTTTGCAATTTTTAAAAAAATAAAAAAAATTCCAGACCGGTATTTGATTTCGCTGCTTGTACGTGTCGCGAAAAAAAAAATCCGCGAGAGGAACTTGGATGGTGTAGGCCCTACACCATCCAAGTTTCTCTCGCGAAACCCTAAACCCTACACTGTAGGATACAAATACGGTGAAGGCAATTTTTCTCCAGATGGGAAATGGAAGATACTATTATATTCGTCAATGAACGTTGGACCAGATTATTTTGACATGCCGTTTGAAAAGGCGAAACTTCTCGAGCTGGCACCAAAATAGAACAAGGAGCCTACGTAAATGTTCCTTAGTATCTCGTGCATCTAGTGAGCTATTTGCGTCCTTACTAATATGTCTGAGCCAAGCACCTCGCAGCAAGAGCTGGCACCAGAAGGATTACGTAGTACGGTGCTTAGACTTCATGTTCAAAGCTAAGGAAGATGGCCTCTTCAGAGGGCAGCGAACCCGGGACCTGGTTGCTGAGTGTCTACAGATAGCACACGGCACTGTTACGACTGTGACGAGCACCTACAGAGCCTACAATGAGACCAAATTTGAGGTGCACATCAGCCATTTAGCGGCATGTTAACGTTAACCTCTATTATTTGATATGTGAAACAACGCGGAAGGAAGCGTGATTACGACCCAGCAGAGCGAAGGATAAGGTGCCTGTTTAAATAAATATTTGTTTTTTCCTTGCACTCAAATCGTTACAATCGTTATCGGATTTGCTGCTGTTCAGTTGCGTACTTTCTTGTACTTTCTTGCGCAAATCCATGTGGTTTTGGGAATTGCCCAATCAAATGACCGCCTCAAAGGCAACCCGTTTCCCGTAAATCTGGGGCATCTGGAAAGAGTTTAAAAATAGTCTTGTAGCGCAAAAACGACTGCGAATCAGCAAATTCTGACCACATTTTTGAAATCTACGCCCGATTTTACCCCTTATATCTAGCTGTTGCTATAGGATTTGGGTTGCTGAATCTTCATAACAGATGGCTACATTTTTGTCCAAGGACTTGTGACGAGTACAATAACGTTGCAGTCGTGGCAGCGGAAGGACAGTGGCGTAAATTACGGCCCCTACAAATAACAAATATGCCAGTCATTTTACAATTTGCTGAGCGCAAGTGCCACCTTTCAACAGGTCCAAGCATATTCCCCAAATGAAACTTGCCGTCGGCCTCGTGCTCGCAGCCGTCGCTGCTGTCGCCGTGAGCGCTGGCGACCCGGCGACACTGTCGACAAAGGAAGAAGCCACGAAAACCACGAAAACGACCAAGTCGGAGAAAACTGGTACCGCTCCTAGCAGCGGCATCGACAGCTTGTTCGCGGATGGCTCCAACGGTGTTATTGGGGATGAGAGTGCAGCTGGTGTGAAAACAGACCCTAAGCTGCTCAAATCGATTGGTATCATGGGCAAGGATGGCTCCGGGGGATTAAACTTAGGTCCTGGCCCTGCGGTGTACCCTAGCGACTCAGGCTCGTGGGAATGGAGCTGGGACTCGATCGAAGGCTCCGGCAGCTCCCATTACGACTGTGATACGGAGTGCCCGGACGACTTCAACCCTGTTTGCGGCTCCGACCATGTCACTTACACGAACGACTGCGCGTTTACAGTTGCTCAGTGTAACGCGACCGAACTAGTCGTGGCAAACTCTGGTGAGTGCGCAAAAAGCTCTTCTGGCAGCAACGCTGAGTCCTGCCCTGATGCCTGTACCATGGAATACTCGCCCGTGACCGATGAGAATGGTAAAAAGTACTCCAACGAGTGCGCTATGCGGCTAGCCAAATGCAAGGGTGAGGCTGGCGAAGAGAAGAAAATTGTCACTTTTGCAGCTCTTGACGAAACTACTGATGAGAAGGGAGCCAAGGAGACCCCTGCCCCGGCCTCCAAGACCACGAAGGCTACCAAGTCTGAGAAGACTGGCACCGCTTCCGGCAGCGGTATTGGTGCGCTATTCGAGGATGGATCGAATGGTGTTATCGGCAGCAAAGACAAGTCAGGTTCAGCGGATCCCACCCTGGTAAAGGGTACTAAGGAGACCCCTGCTCCGGCCTCCAAGATCACAAAGGCTACCAAGGCCGAAAAGACTGGCACCGCTTCCGGCAGCGGTATTGGTGCGCTATTCGAGGATGGATCGAACGGTGTTATCGGCAGCGAAGACAAGTCGGGTTCGGCGGATCCCACCCTGGTAAAGGGTACCAAGGAGACCCCTGCTCCGGCCTCCAAGACTACGAAGGCTACCAAGTCTGAAAAGACTGGCACCGCTTCCGGCAGCGGTATTGGTGCGCTATTCGAGGATGGGTCGAACGGTGTTATCGGCAGCGAAGACAAGTCGGGTTCAGCGGACCCCACCCTGGTAAAGGGTACCAAGGAGACCCCTGCTCCGGCCTCCAAGACCACGAAGGCTACCAAGTCTGAGAAGACTGGCACCGCTTCCGGCAGCGGTATTGGTGCGCTATTCGAGGATGGATCGAATGGTGTTATCGGCAGCGAAGACAAGTCAGGTTCAGCGGATCCCACCCTGGTAAAGGGTACTAAGGAGACCCCTGCTCCGGCCTCCAAGATCACAAAGGCTACCAAGGCCGAAAAGACTGGCACCGCTTCCGGCAGCGGTATTGGTGCGCTATTCGAGGATGGATCGAACGGTGTTATCGGCAGCGAAGACAAGTCGGGTTCGGCGGATCCCACCCTGGTAAAGGGTACCAAGGAGACCCCTGCTCCGGCCTCCAAGACTACGAAGGCTACCAAGTCTGAAAAGACTGGCACCGCTTCCGGCAGCGGTATTGGTGCGCTATTCGAGGATGGATCGAATGGTGTTATCGGCAGCGAAGACAAGTCAGGTTCAGCGGATCCCACCCTGGTAAAGGGTACTAAGGAGACCCCTGCTCTGGCCTCCAAGATCACAAAGGCTACCAAGGCCGAAAAGACTGGCACCGCTTCCGGCAGCGGTATTGGTGCGCTATTCGAGGATGGATCGAACGGTGTTATCGGCAGCGAAGACAAGTCGGGTTCAGCGGACCCCACCCTGGTAAAGGGTACCAAGGAGACCCCTGCTCCGGCCTCCAAGACCACGAAGGCTACCAAGTCTGAGAAGACTGGCACCGCTTCCGGCAGCGGTATTGGTGCGCTATTCGAGGATGGGTCGAACGGTGTTATCGGCAGCAAAGACAAGTCAGGTTCAGCGGATCCCACCCTGGTAAAGGGTACTAAGGAGACCCCTGCTCCGGCCTCCAAGATCACAAAGGCTACCAAGGCCGAAAAGACTGGCACCGCTTCCGGCAGCGGTATTGGTGCGCTATTCGAGGATGGATCGAACGGTGTTATCGGCAGCGAAGACAAGTCGGGTTCGGCGGATCCCACCCTGGTAAAGGGTACCAAGGAGACCCCTGCTCCGGCCTCCAAGACTACGAAGGCTACCAAGTCTGAAAAGACTGGCACCGCTTCCGGCAGCGGTATTGGTGCGCTATTCGAGGATGGGTCGAACGGTGTTATCGGCAGCGAAGACAAGTCGGGTTCAGCGGACCCCACCCTGGTAAAGGGTACCAAGGAGACCCCTGCTCCGGCCTCCAAGACCACGAAGGCTACCAAGTCTGAGAAGACTGGCACCGCTTCCGGCAGCGGTATTGGTGCGCTATTCGAGGATGGATCGAATGGTGTTATCGGCAGCGAAGACAAGTCAGGTTCAGCGGATCCCACCCTGGTAAAGGGTACTAAGGAGACCCCTGCTCCGGCCTCCAAGATCACAAAGGCTACCAAGGCCGAAAAGACTGGCACCGCTTCCGGCAGCGGTATTGGTGCGCTATTCGAGGATGGATCGAACGGTGTTATCGGCAGCGAAGACAAGTCGGGTTCGGCGGATCCCACCCTGGTAAAGGGTACCAAGGAGACCCCTGCTCCGGCCTCCAAGACTACGAAGGCTACCAAGTCTGAAAAGACTGGCACCGCTTCCGGCAGCGGTATTGGTGCGCTATTCGAGGATGGATCGAATGGTGTTATCGGCAGCGAAGACAAGTCAGGTTCAGCGGATCCCACCCTGGTAAAGGGTACTAAGGAGACCCCTGCTCCGGCCTCCAAGATCACAAAGGCTACCAAGGCCGAAAAGACTGGCACCGCTTCCGGCAGCGGTATTGGTGCGCTATTCGAGGATGGATCGAACGGTGTTATCGGCAGCGAAGACAAGTCGGGTTCAGCGGACCCCACCCTGGTAAAGGGTACCAAGGAGACCCCTGCTCCGGCCTCCAAGACCACGAAGGCTACCAAGTCTGAGAAGACTGGCACCGCTTCCGGCAGCGGTATTGGTGCGCTATTCGAGGATGGATCGAATGGTGTTATCGGCAGCGAAGACAAGTCAGGTTCAGCGGATCCCACCCTGGTAAAGGGTACTAAGGAGACCCCTGCTCTGGCCTCCAAGATCACAAAGGCTACCAAGGCCGAAAAGACTGGCACCGCTTCCGGCAGCGGTATTGGTGCGCTATTCGAGGATGGATCGAACGGTGTTATCGGCAGCGAAGACAAGTCGGGTTCAGCGGACCCCACCCTGGTAAAGGGTACCAAGGAGACCCCTGCTCCGGCCTCCAAGACTACGAAGGCTACCAAGTCTGAAAAGACTGGCACCGCTTCCGGCAGCGGTATTGGTGCGCTATTCGAGGATGGATCGAATGGTGTTATCGGCAGCGAAGACAAGTCAGGTTCAGCGGATCCCACCCTGGTAAAGGGTACTAAGGAGACCCCTGCTCCGGCCTCCAAGATCACAAAGGCTACCAAGGCCGAAAAGACTGGCACCGCTTCCGGCAGCGGTATTGGTGCGCTATTCGAGGATGGATCGAACGGTGTTATCGGCAGCGAAGACAAGTCGGGTTCGGCGGATCCCACCCTGGTAAAGGGTACCAAGGAGACCCCTGCTCCGGCCTCCAAGACTACGAAGGCTACCAAGTCTGAAAAGACTGGCACCGCTTCCGGCAGCGGTATTGGTGCGCTATTCGAGGATGGGTCGAACGGTGTTATCGGCAGCGAAGACAAGTCGGGTTCAGCGGACCCCACCCTGGTAAAGGGTACCAAGGAGACCCCTGCTCCGGCCTCCAAGACCACGAAGGCTACCAAGTCTGAGAAGACTGGCACCGCTTCCGGCAGCGGTATTGGTGCGCTATTCGAGGATGGATCGAACGGTGTTATCGGCAGTGAGTCTGCCTCTGCTGATCCTACTCCTGTGAAGGGCGTGAAGCCCGTTGCTGAGAAGAATGTTCCTGCCTCAAAGACTACGAAGATTATGTCGTAAATTCTGATGTAGACAGGCAAGCACTGCAGGCGTTCGAAGCGAACCGTGTCCTGCACGTATTGGTATCTAATCGTATTTGTTTGGTTTGAGACTCTGTCTTTACGACGATCTCTGATAATTATCTTTCCTGCCTTACTGTTTGCTGCGAGACAGCGAGGGGGTCATGATATTTTATACGGCTGGATCCAGAACACACACTTCGCGCATTTCGTCAGAATGTAAATACCGGTAGATCGCCTGTGGACATCACATGCCAGTTTTCTACTGATACGAATACCGGTAAATTGACTTTGAAGCACTTTCAGATAAATAGTACTAATAGATAATTAAAAACATATATTGTGGCTTGCAGGTTTTTTGGCAAAACGGATGGTAAGATATCCGATATATCGGGTCCGATATATTTTGCAGCTCCGAAGTGGAAGAGCATGTCTCCTGTCTTGCTACGATTTACACAGATCGTTCTAGCCCGAATAATCAAATAAAGTCCGTTTTGCTGGTTTAAATCGGCAAAAATGAGTGGTCGTTGCTTTGCTTTGAGTTTGCTGCATTTTTTCGCCTATTAATTCACTGTCGTATGACTTACTCCGACGACTTTCGGTGGCGTGCAGTCGCTCTCCTTCACGTCTACAATATTCCTGTCACGCATGTGTCAGAGCTGCTAGGTCCAAAGCTACGGTCTATTCGACGCTGGTACGCTCTTTTCTTAAGTGATGGCATTGTCAATGACAAGCCTGAACAAAAAGAGAAGAGGACCAAATGGCCACCAGAGGTACTTCAGTTTGTGCAAGCGTATGTGGATGACCACCCAACGTTTTACATTGAGGAGCTACGAGACAGCATCTCCATACAATTTCCTCTTCTTAGAACGATTTCAACCTCTACTATTTGTCGTGCTCTCAGCTTTGATTTAAATCTCAGCCGCAAGGTGTTGTCGAAGGCGGCACGCGAAGCAGTTCCAGCCGAAATTCGTATCTACAAGGAAAAGCTGTTACCACTTTACTCGTACGCTGAGCAATTAATTTTTCTTGATGAAACTTCCAAGGATGGACGCCACGCTTATCGACGCTACGCGTGGTCCCGACGAAACACGAAAGCTATCGTGAGGTTGCCATTTCGACGGGGAAAGAGACTCTCGATTTTGGCTGCACTCGACGTCTCCGGGTTTATTGCATGGGAATGGACAGAAGGAACGTTTACAAGAGGAAAATTTCACGAGGCTTTTTTAAAGCATGTCATCCCGCTGCTAAACCCGTGGCCACCTTCAAGATCAATTGTCGTAATGGATAATGCGAAAATCCACGTTTATACTGAGCTTCAAGCTGCGGTTCATGCGTGCGGTGCTCGCCTGATCTTTCTTCAACCATACTGTCCGCAGCTAAACCCAATCGAGCAAGGTTTCGGCCTTTTAAAACGATGGATTCAACGCCACGCGAATCTTGTATTTCCTCTCTACCCAGAATTGGTATTGGATGGAGCCATGAACAAATGCACCCAGCGGGAACGAGACATTTTTTTTGGGTCTTTCCAGCACTGTGGCTACAACAGTTTCTCGCTGCGAGACAAAGTATTTGAGGGTTTAATCGATAGCTGCAAGAAACACGAGTAAAGTGTAATACATTCAGGCGAGCAAGTGATCTAAATCAATCCTAAATCATAGAGTTCCATGGCTTCTCCTTCATTGTCTTCTTCTTCATGCTCCTCTTCTTCTTTGTGCTCAAAGTCTTCTAGGTTTGGCTGTGATGGAGCTGTTGGCGTGAACTCGTGATAGATTCCAGACGTAAAAATGTCGTGGTCTGGTAAGCCAATAGCTTTCCTCAGTGAAACCACATCAACTTCTACAGGAATCCACACTCCACTCCATTTCAGTTTGATGATGGTTGTTTGAACTAGCGCTTGTTCTTCAACCTGTATGCGCTGGGTTGTTAATGCTTCCCGCTGTCTATCTAATTCCATCGCTTGAGCAGTAGTATTGTCGTCCGGAACGTGAAAGACAGCGCTGTCTGGACGACGCGCATTGACGATGTCAAGATGATGTCCGGTAATAGCACCAAACGTAACTGCGTTGGTAGCTTCGTGAGCTAAACGCTGCAAGCGTGCTCGCTGTATGCGCTTCTCGGTAGCTCGCTGAAAGTCAGCCGATGCAGCAATCGGTGTAGCGTTTTTTACATACAGGAACGCCAAGAATCTGAATCCACTTCCTGGGGATCTGTCACTCGACTTTAAAAGCTGCCACCGCCGGCCCAAAGACTCCACGAAATTTGAAGTCGACAATCGCATGTATTTGTCTTGATTTTCGTTGCTCGATCGCTTCAGGTAAATGTCACTGTTCTCCCGCAATTGTAGTTGATTCTTGCGCTTCAGTACGTCACCCACTTGCTGCTAAAGCATTAAACTTCATAATGCACTTTGCGCGAAACACATCGTAACCTTCGGCTATGTCAAACATCACATCTTGCTTCCAACCGCCTCGAGCACTACCGTGTGCTTCGCCAATTCGGACAGTTAAACAGGCAGTTAAAACTCTTGGTAAATTACCTTCGGGTTCACTGGTAGAAATCGCAGCTTGATTCTCTCTTGCATCCATGACAAAACGCACACACTAGCAATTGCAGACAACTCGACTTGTGGTGACAATTGTGTTTTGGAAATGCCACTTCCAGTATATTAAAAACTAATCTGGCACAAGTGCACCGTACATTTAATTAGTATATACTCAAGTAAAGCGGCATAGAGGACATTTGATGGAACTTACATGCTATCTCGCCGCGTGGTTGATGCATCTCGTTGTCTGTTAGCAACCATGATACCACACCCACACACGCTCTACCAAACAAGAACGTCACATGTCTTCGCCACCTATGCCGCCACATGAACAACGCAAAGACATTAGCCAGTCATTTGTAATTTGCTGCATATAAGACGCTAGTACCTGTTTGTCTGCCTTTCCCTCCAGTCTATTGCCCTTGTTTGTCAGTCTAGCCTTCTTATACTACAAAAGAACACAAACACGAGAGGATATTAGCAGCTAGGCAGATCATACAGGGGAGGTAGTGAGGCTTTACCTGCCGGAGCCTCGTGTCTTGCTGGTGAGGGCTGCGGACGGTTGGCTGTGACTTTGGGGGGAGGTCGGATTAACGAAGTTTCAGCAGTGGCAGAACGGTCGCTTTCTCTCAGAAATAAATAAACCTATCCCTCGCACAGACCGATTTATTTTGTCGCACCTCTACGATATCCGACGTCAAAACGATGGGAAAAAAAATTGCAAGCCACAA

>Contig_45

GATTTTCTCAGCAACATGCTCGTGCAAGACTTGAGGGCCTTCAGCCATCAGTGCATCTGCTGAGTCGTACCCAAGTTTCTTGCATTCCGCCATCTTGATCGAGTCTTGGTCGGAAGCAAGGAACTTCGAGAATGAGCTGTTCGAGATGATTTTTAGCATAAAAACGAGGCAACAACAGCGTACATTATGTGCCAGGCTGGAGGGAGACACATAGACGACGAGAACAATCAGGCAGTGCGAAAAGAACTAAGTACGAGCGTATAACGTGTCCATGATCAGATAGTAATCCTTGTGCTATTCTTGTACGCGATTGGCGAGCCAGCGATGATTACTTTGGACAATGATTTGCAACTGGTTGCACTTGCCATCAGCATTTGGCTGATGATGCCAAGCTAGCGAAAAGGGGATACCGGTAGATGGCGTGCACTCATTTGCCTATCTTTCGTCGTTCACCAGAATATCAACAATAATTATACTAGTAATAAGGATCGCGTTGGATTATGGTACGCCGATGTAATGAGGTTGTCTATTTTTTTTAAAAGGTTTTACATGTATTTTTACCAAGGATGATGCACTTATGCCGTTGCAACCAAACAACGCGTGGCTCTCGATTCGAGACACACTCATGTTCACGTTACACCCCTTTCATCGGACTGAATTATCAGCAGTAAACGACCAATCCGGCATAAGACTTGTTGCTGATCGACTATTAGATGCAGCTCTTCATACGCCACACGCACGATGCATGTTGGCATCATCGACATCGTGAGAGTATTATTTGGGTCATCGATACGAGCTACAGCAATACTAAAGTCGACTCACAGAAGCAATCTGATTCCAGTTTCTTAACTGGGTTTACTAACACTAAGCGAAAACCAATTTATGGTACATTTTATCTGTTTCATTTCCGCGTAGTATTTTTATTTTATAATCAGATATTTTGCTTTGTATTTAAGAGAATCTTTTTTAAAAGAGCTGAAAAGCAATTATTTGGTTTTCAAAATTATTTCATTTGCGTGAAAAAGATCTATCCATGGCCGGCCATGGTCGCCGCAAGAAGCGTCGTGTGACTGCGCCGACGCCACAAGACGNANAGAAGCAGCTCGCCTCAGGTTCCTCGAAGCTCTAGAAGCGCAGCGAGCCAATGCTCGGAGCCAAGAGACCAATAAATCCGCTGAAACTCGGGGTGTACCAACCCCAGCACCTTCACTACCTGGTTTTTATTACGACGAGGACAAACGGCGCTATTTTCGAGCATCTCCAGCATCTGAGCGCCAACGCCGCGATGAAATGGAGCTGCAGCGACTGCAAAATCAGCAACACGCACAGCAGAATCCCACAGTTACAGTGAAAACACGTCGTGGGCTTGGAGTTCGTGGCCACTCTTGGGTGGACTACATGGCCAGGCGACAAGCGGACTATGCGTGGAGTGCCCAGCGTCGTGACAGTCGGGAATTGATACCCAAAATGCTTTCAGGGTTCCTGGTATTTTGGGTGTATATTTATTTTTTATTTGGAAATTAGCCGAAAAGTAATTGTTGTGACRTGGATTACCAGTCTTCGCAGGTGGTGGAGTCCCAATGCATGAACCAAAACGGTCGACTCACAGCGTTGGCACTCCATCCTCGAGCCAGCGATCTTGGAGCAGTTGGAGGTACGTTTGGGAGAGTTGGGGTCGTTTWAAATAGTAAATAACTTGCCCGGTTTTGCTGTTCTACTGTAAAGCGAGCAATGGGTCGTTGAAGATCTTGGGTGTGCAGAGGATGACAYCAACACCAGCTCAGCGTGCTCTTACCATCTGCAATTTCCATGTTCCAGCAKTTATTACGTCATTGCAATGGCGTCCAGTCCAGGAAATCGGTATTCTCGTGTGCCATCTCGGACCTGATCGTAGAAGCACGACACAGTCGCCGTCTGGTAGCGTGTGCTTTATACGCGTGGGGGCCCAGCAACCTCGAGGAGTCGCATTGGTGTCAATAGCACACAAAAAGGTAAAGYTCGAAGTGGGATCTTGTGTTTACTATTATTTTATAACATTATTTTTTATGATAACTAGGAAATCGATTTTGCTGATCCATGGACAGCGAAATGGTTGGGGTTATGAGTGCTAGCCTGCTTAAAGTCCTATGTTGAACTTGCTCTAACCATTTGTGTGCTACTTGTGGTCACTAGGAATCCCACCGACCCTAGCAAGTTCAGCGTCGGATATGGCGGGTACGAGCACTAACGAAAATATTTAAAATCTAGAGTATACTCACTGAGTTTGTGGATTTTCAGGTCGTCGAAGGCTGCCTACGTGGATATAGCAGCCGAAGCCCGTATTCTTCAACGTGCGCCGACTGGGGCTATAACTAGCGATGTGCACGCTCAGAGTTTCTATTCTACGGTTCGTGTTATGCTGCTTTAATCTGCTTGTGCTGTTTATAATTACTAGTTTGCTAATTTTTGAACTATGCAGGGCAATGTTGTGTTGAATGGGACGAAGAGCGGAGGTCTATGGGGTTGGGATCTTCGTGCACCTAGCCGAGCGCTCGAATGGGAAGGAGAAGCTACGCCAGACCGACCTGCAGGCGCCGTCCTCGACATTCACATGCTGAGCGACTGTCGTAGAGCTGTAGTGCAGCGATCAAATGGAGAGCTTCGGGTTGTGGATCTACGGACGTCGAAGCCGGTGGTGGAGCTCATGCCAGGTGCAGCGAAACGATATCTGCCAAATCTTCGCTGTGCTATTGACAAATACGAGTCCGTTGTGGTGGCTGGAGGTGATGCACGTCGGCCATTGGCAGTCAACAGCTTTAATCTCCAAGACGGGCGTTGTGTATCGTCACTTGAGGTGAAGGATCCCCCACTACTGCAGAAGCGCTCGACTATGTTGCAACAGGTGCAACTGAAGTCTGGCCACTACGGAACTCGCTACGAGAATACACCTGAGATTTGGGCCTTATCTCGCAACGAGCTCTACATTTGCAGTGGACGAGTCGATAAATGATCATGCGATGAATATTGAGCCTAGCGTTAAGGTTTTTTTAAAGAACATAAATTTTTCTCCTTGCGGAAATGCATCTTTACTGGTATTAACAATGTACAGCACAATAAAAACACGTCAAGCTTGTAGCTGAAGGTACCTTATTTTTCAACGCCTTCATAAACTACTCCGTACTGTACACAATTGCATGCCCCCCCCCCCCCACCACCACCCTACGCTTGCGGAGCAACTAGTGACTTTTTTTACTGGTAAAATCCCCGTTAAGGATAAGCATCAAACTAAAGTAGTTGTAATTCTTGTGGACTTCATTACTTACTACTAGTATGTAGCAGATAAGACAGACCGTCGGCTGGGAGACCTAGCGCCTCGGATCATTGAAGGTGAAGACAGGAGGAGGAGCCATCTTCTTGTTGTAGTATGACGTGTATCGGGTAACAACAGCAATGTCTAGAGCGCCAGCAGACGCTGCGTCCACCTTGAGGAACTTCGTGTAAATGCTTTCTGGCTCGATATCGTTTCTGAACCAGCGCTTAAACAGCGCCTTTTGATACACAGTGCCAAGATAGGTGGCAGTGTCCACATTGTACATTTCGAGCTTCTCCATCATCGCAACGATCGTTCGTGCCAGTCCGCCTTCTCCACCGAAGCTGTTGCTGACCACCGTGAACACATCAGTGTTCTCTCGGGGGTTCACAGTCTTGAGAGCCGTGATGTATTCGCTCAAGACCTCCAACTTTGGGCTAAGTTGTTCTAAAAACTCATTGGAGTTAATCTTTAGCAACTTCAAAACATCGTCGGCTGATTTTTTGCTCGTGAGCCACTGGTTGAACTGCTGAATCTGCATACTCGAAGCCAGGTCTTTCGTGGCATCATTAGACCTAAATTTAAAGAGTGTCTTCGCCACCACATCGTCCCCGTAATGGGCGGAAATCGTCCCAATCAATGATGCCTTGCTACTTGGGTTCCTTGAGTTGTACTTCAAGATGAACGCGGCTAATGTGGGTAGGGTTTGGCCGAATAGGGTATCTTCCAAACTGCTGTCCACACTAAGTCTCTTCAGCACGCTTTCAGGCTGCATTTTGCTTGCCATCCACTTGTTGAGTAGCGCATTTTGCAACGCTGCCGCCAGTTCGCTTGTACTGGCATCGTCTTTTGCGATCAGCAGTATCTTGGCCAGTTTATCTTCTCCACCAAAACCCTTCGTTAAAGTCTGGAGTACCGTTTCCTTGCCTGAATAAGTGCGGATAACAAGTTTCAAGTAGTCATCCAGTACCTCCATCTTCGGACTCGCAAGAGCTTGGTACCCATCCTCGCGAAGTCTTAGCAGCGTGAAAACATCATCAACAGATTTCTCGCTGGCCAACCAATTCGATAGCTGGTCTTTTCGTAATTGACGAATCGTATTGAATGCACCCTTTGTCTGAGCATCCTTTTGTGCAGTCACGAGTGCTTTTGCCACTTCGTCATCTCCGTAGTGTCGTGTGAATACTCCAATCACTGACATTTGGCTGTTTTGGTTCCTCGTGTTCACTTCTCTCACGTAGCGCTCCAAACTCTTCAATCTAGGGCTGAACAGAGCTGATTTTATGCCATCCTTTAGCTGAAACACACTTAGAATTTCGTCGGCCGATTGCTCCCCTGCAAGTAAGGACTTCCAGTCCACATCCCCATGGACCTTTGATGCCCCCGCCATCACCACACTCTTCAGCTTTTCAATAACAGCATTCATCATTCTGTCTTCACTTCTTTTGTCATTTGCAACAGAGACTCTTAGTAGCCTACTGGACGAGATACCAACTTGATTACCAGGCAACATTCGGAATTTCGCTGGTGAATTAGCAGTCGTTGTTTTCGACAAGCCACACACCGCGGCATTTTCGACGGAAGACAGGAGGGCTGCCATTATAGCTAACAGCGTCGCCGAATAAAGTTGCATGTTTCGGCGAGCTAGTAGCCCGGAAATCGCAAAATACATGCTATTATGTAGCTCTTAATTGTCGGATTTTGCCTACTCATTAATGACAATAGAAATAGCCAATCAGGACATGTCATGTGCTTGCTGACAGCATAGTAAGTGCAAGATTACACCTGATCAATTTTCGATTGTATGTTTTTTGTTTAGCGATAGACAGCATTCACATCAAGAGTGCATCACAGATGGAATACGGTAATTTGTTACATGTATAAGACATGATGTATTTGCCATACCGGTATTTGGTATAGAGACTGCAAATCTTTAGATCAACTGGATAGAGCCAGGTATCGAAATCGCGCGAGTTAAGCCGAATTGACCCTCGATCCAACTTAACTCGCGCGCGTAAATCTGAATCAGCCCTCGACTCAACTTAACTCGCGCGAGTAAACGTGAGTCTTATGAATCCACATCAGCTGTTAGCAATCAATCCTACTTAACTTTTCTTGCTACACTGAGTTTTAAATGTGGCTAAGACAGCCAATTCGTGGATTAGTGTCGTAGATGCTTCATTTCAAACGCAGCTATTTCCTGCTCAAAGTGAAGTTGTGCATGCGTTTCAGGAGCAGTGGCAGTGCATATTTGCAGTTCGCCATGCACCTGTGTGGGATGATGCCGCACTGGGAGGGGGGGGGGTAGATAATGAATGAAGCGGAATGCTGTGGAGCTTGCAGTATATGGATAAATGTACAGGATATCCTTTTGTGAACACCAGCTTACTTAATATTATAAAAATACATGTTACAATGGGGCTGGCTCTGCACTTGTACTGTCCCATCCCATAACCCATATGGGGGCGATCTGTGAATAAGTTTGGGGAATCTGCGATGACAACGCATCCAAAAAAAAGATCGCACCTCAGCTTTGCTTTGACTTGGTATTTCCGGGAGGTATTGTCCGGCTCTCCTGTGGAAAGGCATTATTTTATGTAAGAGGTCGTTACCAGCCGCGCTTCGCTTGGCTGTAGCACAGCAGTAGCAGTGCATATTTGCGGTTCGCCATGCACCTGTGTGAGACGATGCCGCACTGGGAGGANGGGGGGGGGGGGTAGACAATGAATGAAGCGGAATGCTGCGTAGATTGCAGTAATACATGTATACTATTTGATATCCTTTTGTGAACACCAGCTTACTTAATACATGTTACAATGGGGCTGGCTCTGCACTGGTACTGTCCAATCGCATAACCCATATGGGGTGGGGGGTCTGTGATGACAACGCATCCAAAACACCTTGTTTTGCAGGATCGCACCTCAGCTTTGTTTTGACTTGGCATTTGTAAGAGCAGCCGGGAGGTATTACCCGGCGAAGGCCTTAAATAATGTAAGAGAAAACATTATACAGTAAACACACGACCAACACAACAATGCCGGGAAGATTAGACCACAAAAAGACAGAAATCAACGGCCTTTTTAGTAGCTCAGCGCTTGTACCACAGGGCTCATCGCGGCGCATACTGTACCTTGGACGGCACAAAAGTACCACATAAGTAATCGTAAAAAGTAAGAGTGGAGTAAATGAACTGTAATGAAACTTATAATTAAAGTAACGTTAGGATCGTACTACTTTTTGCAAACGACCTGCATATCTACTCCTCGTATCCTCCAACGGTGTCAGCACCGAGGACTCCGTCTCTGCAGTCCAGAAGTCGCTGATGCGCGATGCCCGGCTTGAGTCTGGAGGAACCCCCTTCAGAAGAGTTATGCGCAGGAGACCTGATCGAGTACTACAGTTGTCGTTTTGTGTGCGGCGATTCTCGTGGCCTTCTCGAGTCTGTCGTCTTGGCTGTCGACGACAGCCCAGACAACCTTCTACCCGTGAGTCTGGATACCGGGGAGGCGTTGCCCCTTTCAAACTTTATGCGTCGTCGACGGGACAGTGCAGGCAACGATCTCAGCATCGAGGAAGTCAAGTGGCGGAAATTGAGAACGTACAAGCTAAAGCCAAGAAAGGTCCGGCACCCTACCCAAGCCAGCGCACTCAGAAAAGCTTTAAGTGCTGCAGTCGATGCAGTGCGTGAAGATCTGCGATCCGAGTCTGCGGAAACATCTTATCGACACTCCCTACGAGGCGGCGAAATCAATTTAGCACGTAGTAACCGATTATCTAATCATGAAACAGCCCCAGCTCTCTGGAAACTGTCATCAATACGCAAACGAAATGTGGAGCAGCTGCAAAAGGTAGTGAAGAGCACACTGTATGCGAGGAATTTCAAGCTGGACTTGGTTGACCCGACGTATAGCCAAATCGATGAAATCTTCGGCGGTGATGATGCAGCGGTTTCCCCTGCTTCGCACACCACGTGTAAAGAAGGTCTGGAGTTGGAGAAGACGGGTGAGAGAATAATCGAGAACGCGGAAAGCGTTAAAGATAGTGTTGACGGGGATTTAGCCGATGCCGAACTTCTTGCAGCCGATACCTACATCAGTTTCGTTCCTACACGTTGGATGCGTGAGAAGACCCGCCATCCAGCAAAGAAAAGAAGCGGTACGTGGTCTGTGCCTCGCTCACGCAAAAGAAGACACCAAACGAAGTGCGCAATCACACGCAGTGGATCCCAGATCTACCATACCCACTCTTTTAAGGCTCAGAAAGCGAAGGCCGTGTTGCGGCTTCCTGGGGTTAAATCTAGGCTGCGCAATTTGCATGTTCGTCGACCAATTTACGTCGAGGCTACTGAAGACGACTCCCACACCCCGTCAGAAGTCCAATGGCCCGAGGGAGTTGGAAAAAATCAACGAATGTTTGAGCCCAACAGGGATAACGTTCAAGGATATATGCGATTGCGGCCTCTGTGGTTGTATGGGGGATTACTTCATGGACAGTTGCACAAATGTATTCGCGGCAGTGTACTGTACACCTGATTCGTGCAAGTTGAACACAATTTGTAGCAACTCACCTAAAACTCGATGCACCCTGAAATTGTACGACACTGGCCGTGTAGGCCTTGGTGTGTTCACGACCACCGAGCTAGACGTTGTGGATGTAGTTGGTGAGTACGTTGGCATGCTGTACGAATACGAAGGCCTAAGAAAAGGGCAACCAAATTAAGCGCTGAAACCAAACGGTGGCTACACCATGTTGTACAACAACCTGTCATCTACCGGCAAATACGTGTACGTGGAGGCACTACAATATGGTTCGATAACCAGGTTTATAAGTCACGTGCGTGAGCCCAATACTGCCTTCGTCGAAATGCAGAACAGATCACACGTAAAGGCGTTAGTGACGATGACAAAAGACGTGAAAGCAGGCGCCCAGATAACGGTGAGCTATGGATCGCAGCGGTGGTTCAAGTGCGCGTGATGTTTGCTGGTTCCAACACGATAACGAGATTGAGGGCGACAAGTCTAGCTAGAAATATACCGATTTTGTTCCATAAAAAAGAAATCGGAGGTTTCCTCGTATGAATTGAACGCATTAAGACATGTAGAGTAGAAAAATACCAATGGTAGTAAACGACGTGGAGACCTACGACTCTGGACGCATGTAGACTACAT

>Contig_47

TTTGCTCTGTTTGCATTCCCGAGGGGGGAAAATTATCCCAAGACCGTGGGGAGAACTGATAAAGTGCAACGAGCGAAACAAGGTCCTTCATTTTGATTACCTATATCTGGGCCCGAGCTACGGAACGAGTTGCTACATTCTGGCACTTAAAAGACCACGCAACCCACTACTGCGAACTAGTGGTGGCGGACTCAGCGGAAAGTCCTGTTGTGGTGGAAGCTTTACTAGCCTGGCACTCTCGCTATGGAATACCGCCGTCCTGAGTATCAGATCAAGGGACACACTTCAAGAACGAAGTGGTGGCTGAGCTGAGTCGAAGACTCCGCACGCAGCAGACGTTTACGCCGGCATACAGTCCATGGGTGAATGGTTCAATCGAGCAAATTAACCGTGACATTCTCCAGGTCATTCGTGCGATGACGCTGGAATATCGGATTAGTCACAAAGATTGGGTGTATTTGGTGCCATTAGTGCAGTCAAACCTGAACCACACTGCTGTGCCTTCCTTAGGCAACCATTCGCCATTGGAGCTGTTCACGGGATTGGAAAACCCGACGCCGCTGAGCGAGTTCTACCTGCCTGACAGACGCCGCCTGCAAACAATCCCTGCTAGTGCTGAGGTAGACGGCTACCTGGAGAACTTGAGGGGGAGCATAAGGTCTATGCACTGTGCGGTGAAGGACAGACGAGAGAAACAAAGGCTCCTGAATAAGAAACGTGAACGTGGTGAGAACATGGTAAACTTCACCATCGGTGATTACGTGTTAAGGTCCCGAGTGGACGAGAAGCACGGCAACAAGTTACAGGTGATGTGGGTTGGTCAGTACAAGGTAACGAGAGCCGACGCCCATTCATTTCGTGTGAAGCACCTCGTGACTGGTGATGAACAAGATGTTCATGCATCAAGATTAAAGTTCTACTCCAACGATAGCCTGGAGGTGACGGACGAGCTCTTGGAGCATGTGTCCTCACAAGGTATCGTGTTGGCAGTGGAAGGACTAAAGGGACACAAGTCGAATAGTGACATCAATGACTTTGAAATCCTGGTAAGCTGGAAAGGGCTACAACCTATCGAGGATTCGTATGAACCCATGTGTGCTTTGGGCAAGGAGATCCGAGTATTGGTTGGGAACTATGTGTCAAGTACCGATGACCAGCGGCTTCGTGAGCACTGGCAACGGCTTCAAGGAGGACGTGAAGAGGGTCTAGTGCACGCGGATCGCCAGTTAATACTGAAAGTGCGGCCGAACACGGACTCCGAAGCAACACCGTCGACTTGTGACACGCGGGGGCCTCAGGGTAAAGCCCAGCAGGCACGTCCTCGTCTCACTGGACAGAAGCGACGACTCAGAACCGCTAGCAGACGCAAGCGGAGAAGTAATCGAGCGACTGAGGAAGAACCTCAGGCCGACTCACAACAGACATCGGGGCAGACTGCAGACGGGGATGGTGAGCTGCACCTGGGTGCCGAGGTCCAAGAGCCTTCACTTCCTCGAGGTCGCCGAGTACGAGCTGAGATGCGGGTTGGGACGCCTTCACGTATTCAGCCACGCCGTTCAAGTCGCCGTATGGACCGGGGCCAGGGCAGTTAGTTGTTTTGGTGAACACTACTGTGACGGTGATCGGTAAGACGAGAACAGGTGAAACCGAGCTTCGGGGGCTGTGTAGTGAACTGTGGGTTTCCACCTCGGTTTCTCTTGCACTTAGAGCCGGAGCTGGGGGTGTCCACCCAAATGTGTCTACAGCCCAGTGGCTAGAAGACAATGGGTGGAAATCTGGTGGTGTGTCTGACGCCTGTCGGCTGACGATATCACAGACACTTGGACAGCCAGTGTCCTTGTGTATTATACTAGGATTTAGTAAGGCCTACTGCATGTTTCCTTTCATGTGGATGCATGTCCATTTCTGACTGCTGCCTGTCGCTCTGAACAAGTGGCAGCAGCATTGAGGAGGAACGGGCTGGCTAGCGCCAGACCCATGATCCTCCCCACTCTTCGATTTGCTAGGAACTAGCTAAATACATTCGGCCTATACTTCAGTGCGGGCTGGCCCCCGCCTTGCTGGTGTTCCTGCTTCGCTTCAAATCCCGGGCTACACTACAGAATGTTTTGAAAAGTGAGATGATCCGCCACCCGCGCTGATTTAGCTGCAGGGCGTGAGATTTCTTCCTTCTCCGCTGCGTGATGCTGGCATTTTGCATAAGCATTTGGACGCGTCAACAAACGTGCTGAACAATCACGGAAAGCGATTGCCAGCACTGACCATCATTTTCCAGTGTTTACGTCTACGTAAACACAACTTCGTTATGTACAAAGTGGGTTGTTCAAAACGAAGCACATGCACTGCACTGTACGACACCGTAAGACCGGATGACACTTTTGCTGCAAACTCCTATACTGGAAAAGGGATTCAATGGAATCGCTCAAGCATTGCATCGTTTCTGCCAGGCTCGAAATAAAGCTTGTCTGGACACTCGAGCGATATATCCTGTCGCCTTACGATGCTGACCGCTACAAAAAGAGCATTTGCCGTTGGTGGCCCCACGAGATTCATAGTGTGGAGAGCTACAACAGACAGCTCATAGAAAAATGTAACGATCCTCTACTGGGTTTTGGTTATTTTACATGTGAATGGGTTTTAAAGGTACATGTATTAAAACGATTAATAGTAAGTCTTATACTTGAAGTAAAAGAGAACCAATAACAAAGGTAGCAGTGCTACATGAACACTACATTTCCGTGTGCTTGAGTATGCGTGAAATTTACGTAGTCCTGTATACTTCCTATGACCCAGTAAGATACTCTATCCTGAACGCCTTCGCTCCATCACCCACTTGTCTCAATGAAGAACAGATTGAACTTGGCCACAATCGCCTTCTCGGCTGTCGTAGTTGTTTGAGCCTTTTTAAATAAAAAGGAGACGATGACTTCCGGTGTAATACGTTCGTCTTTCAATGACGCAAATTGCTTCTTCTGCAGCGCTGAGGCGGCCGTGGACTCACTTGCTTTCTCCAACACCAACGCCAGTTTATCTCTCCCTCCAAGCTGCTCCGTCAGCGTCCTGATTAAACGTTCATCTCCATCCATCATTTGCGTAATATACTTGACCAACAAGCCTAGCTTTCGGCGAGTAACTACTGCATCGCCAGAGCTGTCAATCTTCAGTATCGTTTCGACTTGGCCAAAACCCAATCTGCGGATGACCCAGCTCTCAAACTGCGCCTCCTGTAGCTTGTTCGCGATACTTCTTGTCGCCCCGTCGTCCACTGCCGAGATGAGCTTGTTTACGACAGCAGCGTCTCCAAAACTGCTCGTGTAGATAGCAATCGCTGACTGTTGATGATTGGGCCTCCTCGTGTTAAAATTGTCGATGTACGTTGCGAACTTGTTCAAGTTTTGAGGGGAGAAAGCGTCGTCCACGTCGTCGGAAAGCTTCAGCCATACAAAGACGTCTTTCGGTAAGGTATCTTCTCTCAGCCACTTATTGAGTAAAGCAGTCTCCAATTCGTCGGCTTTTTTCCCCGTCATGCTACTTTTTTTTGCAGAAGTCACGAGCGCCACCAGCTTGTCCTCCCCGCCAAATCCCTTACTCAAGATGCTCACCAGAGATACCGGATCGCCCTTCTTTTTGTTCTGGTTGATGAGTTTGATATAGGCTTCCAGAATGTCCAGTTTCCCACTGAAAAACATTTGATACTTTTCTTCGCGAATCCCCAGTTGCTTGAAAATAACGTCCGCGGAGATGCCGCGGTTCAGCCACTTCGACTGTAGGTCCTGCCTCAACGCTTTGAGTTGTTCTAGAAGTGTGGGCGACGCCTCCCTTTCGGCAGCCTTGGGCCCCTTTGCCAGAGCGATATCTCCGTAGAGGGTCCTGAGCGTCCCAATCACCGTGACTATGGCCATGGACTTTTTGTTAAACACTTGGACTTCCTTGGTCAATTGCTCCAGTTTCGAGCTTGACAAGGCGTGGTCAATACCGCCGAGTTGTAATTTCGCTGCCATGTCAACCTCATCTTTCTTACGATTAAGTAAATACGCACGGACCTTCGACCAAAGGCTCTTGATCTTGGCGCCGCCCCCGACGGTTCTCCCTTCGTTGTCAACATCGTAACGTCGCAACAGCCGTTTAGAAGCGACGCTGTTATGGTGGTAGGCGAGGGAACGGAGCACAGTCGGATGGTCGGCGGTGGCTAGCTCAAACTCCGAGTCAAAGGACGCACACGTAGCATTTGTCAGGAGAACACACCCAGTTGCCAGGAGTACGGCTGGTAAGAAACGCATCTCTACTGAGGGCTGGTTCAAGAATTACGGAATGCGCTCCAGTTTACTCCAATTGGCGGAGATAATCAGAGCAAAAATACATGCACTGCACTAATCACTCCTGTCCTTTATTTCAAGAAGAAAGAATTGACCCATTGGACTTTGATTAATTAGAGTGATGCCCAATTCACGGAGATACACCTGGATATAAAAAGCGAGTAGCGAACCGCCATTTTTGCTTCCCTCATAACTCATTTAGACAGCCAGTACCCCCCGTCCTGATACGCCACGCTTACTTGGCGTCGCTTGCCACGGAAAAATACGCCAGCACTGACCAGTATCCTCCATAGTGTACTTTGAAAGAGATGCTACGGAAAAATGCGCCATAGTTAAGATTGGATATAATGACCACGAGAGATTTGCAAATTTCAAAGAAGGAACGTATTTTTACACAGAAGACTACCGGTACAATGATTGCCAAAACAGCAGACACATCATAAAGATCATCTTGAAACCATTTTTAATTAAAATAAGCACGTACTTTGTCTTGTGGACAAGGCAGCTCTACGCAGCAAATGTTACAAAAGTGTGGCTGTAACACCGTCGAATTGCGTCCTAGGGTATTAGGATCATTTTGTGATTGGGAGGCTTCACCAGTCTATGATAGACTCTATCGTATCTGCATCTACACCTCGTAAGTCCTCGTAAAAGCCTCCTCTGCAGCTTAAAAGTATATTCGGCTGTAGGATAAATTGTGTGTCACGACTATACCATATTCAAGTGTTTGTCGGACCTAGAGGTGTATATTAAACTTTTTCACTATGTAATGTATAGTGGAAAAGTTTGTTGCCACTCGCATAAAGTATTCATGCCTAATTTGAAAATAAACTTCTTACACCTGATTAAACAAATTGCTGATTGTTTTTAAGTATCGGACCAGATCGAGGGATTTGTTGCATGGCGTACGACACTCTTAATACATTAATTTATTAAAAGGCTAGAAATAACTTGTATCATTCTGTACTGTAATAGTCAAAATACATTGTAGAAAGGCAAAATATTACTACTATGAGCTGCGACGTATATACGGATAGGGGAAAATCTACGATGAACACTGCGCTGCTAAATACTGTACTGTAAATCCTTCATCATAGAGAACCATGTCTTATTTCAGTTTTTATGCTTAAAAATGTTTGTGGACCACCCAGTGTAAGCATGAAGGACCTTTTCTTGACAAAGTTCCGCAGACCAGATGCGATCAAATCGTTGGACCAACGAACACACGACGTTTTTAATACTGTACATGTATTAAAGTGTCGGTTCATCATGAAGTTCATGGTTTATATTGTACCGTATGTTTTCAATTAAAGCTTTTCATCTCAGAGAGCAAGATGTTATTCCACGAAACGGCAATGCCCCACTCACGTTCGCATAAAAAACCCTTTCACGTAAGCTGCAGCTATTAATATGTCCCACACCAGAAATGTCTGGTCCGGAACACAACCAAGAGAAAGAATCTCATTTACCGGTTTACGAGACCGCAAGCTCAAAACCCTTATTGCGCAGCTACAATAATAACAAGTATGTGTACGACTTAGGTTTGGCAAGCCTTGCACCGGTAGTTACACATCCGATCTAAATCACCTTGCTCTATGTCACACTTATTAAAAGGGGTGGGCAACCAATTTTGGGTCACTTAACATAAAAGGCTGCGATAAAACTAATTTAATAAAGTCTATGTCCCTTAACCCCAACAAAATCATTTGTCCTTAAAATAAGGACGAAACAAGATGCAAGATTGTTAAATCCCGAGTGATTTTGTCCTTAAGACCTCGATTATAAGGACAAAAACTAAGCGCGGCAAGCGCAATTGCCTCTGTCTGATATGGCCACTTTGTATGGAATTGAAATTATTCTTGAGTATACTATCGATTTTGATGACTTTTGTCCTAAACTTAAGGACAAAACACTCTCCGGGAAGTCATTCCAGACACCTTACGGCATAGCAGCTTATGCAACAACGCGTAGGTTTATCGAAGAGTTGTATTGTGCTTTGCACGCATTGTGGCTAAACCAGCCCATAACTCGTTGTTTTAGCAAATCATCAAGATCACGACAACATAGACTTACGTGCTCATCTCTTCATATTAAAATTGAATAGCCTGCTTTCACGATGAGTTAAGGTTGTATTGGTGCAGCTTTCGTTGAATGGCTGCGGTAAAAACGACAAACCAACGTCATTCCTCATTCTAGCGCTTTCTCGTAGACTCTGTCATCTTAGCAATGACAATCTCAATCGACATGAGATGGCGATCTATCGTCCTAACCTATATCTACGACATCGATTTAAGTGTGGTGGCCTCTGTAATGGGGGTGTCGACGCGGTCAATCTCTCGATGGGGACTCTTATTTCGCAGGCGTGGAAATGTGATACCAAACGCTCCTATTGTGCGGAAGACGAGATGGCCGCCGGAATGCATCAAGTTCGTACAACGAAATTTGCTTGTTTAGTTACTGCTACTATTTTGTTATTGTGCTGCGTCTTCATCGTCATCAATATTTTCAGTAGGATACGTCGTGTCGAGGTCTGCCCGAAAAGGCCAACGAAATTTGCTTGTTTAGTTACTGCTACTATTTTGTTATTGTGCTGCGTCTTCATCGTCATCAATATTTTCAGTAGGATACGTCGTGTCGAGGTCTGC

>Contig_50

TTGGAATTCTCGGTCGCGAGTCTCATTGGGAACGTGTTCGTATTGGTAGTGGGCTGCTATAGCCACGTTACGAGGTACTACTGGTCGTCGAGGGTGAAGACTGTCGACGGCTCTGGAATTGAGGCCGCACAAAAGGGTTGGGCTAAGCTTGAAAGTCGGAAGGAGCTACGAGAAAGGAGAGAGAGGGCTAGCCTCGACCATTTGCGTGCAGGTCCTAATGATACGACCGCTGAGGCGCAAGGAAATTCGGATTCGACGTCGATTGGAACGAGGTCGATCGTTCCAGATCTGCCGGTCGACTCGGCGATTGGACCTGCTGGGAAAGATGAGGCTCGGCGAGGACTCCGAGCGGCAGTCGGGGAGAAATGTCACGGTCTTCGGCTAGAGGAAGGACTCGCTATTCTGGACGACTGCCTACGGCAATTTAGCGAGCGGCGTAAGAAGAGTGTCAGGCCCGATCAGTCTCGTTCCCCGTCGCCGACTACCTCGTCCCTTGTCTAAAATGGAACGAAGGAGTTCAGACAGTGGTACGCTGACTCCGTTTACGAACGACAACTAGAGGTACTGGTGCTGGTAGGAGGTACTTTGGCAAGGTAGAGGGCTGGTGAGAGAGCTCTCAGTCGTGAATTTCCTTCTTTTACGAGAAGTGCTCAAGAAAAACAAATAAGAAACAAGTATATTTTACACATCTAATACGTCTAAGTTCCTACGTCGGCTTTCACAAGATACACGACCCCGACAAGAGAAGGAGACCGCGACAATTGGCGATGGGCGGAATAAGAGCAGACAGTCATTGCAGACCACTGGACCAGTCGCCTGCATTGCCCTTATTCCAGTGACTGGAGGCAACCGACACTACAGTGGCTATTAATAGACTTCCTGATATTTGTAGGCGTCGCACAGCTTACGGATTGATGAGTATTATACTGTAGGAGGTGAATTAACTTAAGAATCGGGCATTACTATGGACTCGGAGTACATGTACCAGTCTTGCCACATCACACTTTAGACATTATCTTTATGATACGACTGGCTTAGATTCATTAAGGTTTGCGTACAATGCCAAGTAATGTTTGCAATTTTGAAACTTGTGCCAAGAGTCAGTTTTTAACCAATTACTACCGYTCCGAAGAATGGAGATCGTATCACTGGATTAAATAGGATTGCGGTGTCTACCCCATAGACGTCATCATTAGCTACATGTGATAGTACTGTATTGGTACAGTAGGAGCCACATACCGGTACCTAAATACCTGCTGCTCGAGAAATTATTTTTACAGCGATAGACATAATGCGTCGCGGGGGGATTGCCATCTCTACCAATCATACGGTTAGAAAATSCTGGTGCGTTATGCGAGCCTAATTTTTCGGCGACAGTATAAGTTTTTTACTGGCCTCTGGGTACCTTTTCTGCTGGATTATGGTAGTGGCTTTACCATAAACCAATAGAAAAAGTCTGCCTATCCCTCAGCACATCCCATTACTTGTCGAACTGTACTTGTATTATACACCTTTAAAATACAGTACAGTACAGTATATATTAATAGTATGGTTTGCTTCGCATGAACAAGTGACTCGGAACGTAATARRTGGTTATAACAGGATAGTATGGACTTAGTTTCGTGAGCATTTATGTGCTGATGTTCTTTTCATTTCTAACGACTCGACGAAACACATCGTTCATTTATCGCGAATGCCCACTGGGCATGCATAGGTACAGCCTGTCATATGAGTGCTCGGGGGAGGGGGCGGGGTTAAYTCATTCCGTGTCACTTCACGTTTTGTTCACCTGTACGCAAGACGATGGGCTGCCGGTATGCTGTGCTCGCATTAGCTGTAGCTTATTTCGCAGGCTCGATCGCAGCCAACRATTCGCAGATTGTCGCTGTCAAAGGCCCTGCTTCGATTCGATTTACACCTGCAATCCATGTCGTCCGTGGAAGGTTCTTGAGGGCTGCGAACACCGCTGACGAGCGCAACGAGGACAGGGGAATCAATCTCAAGTCAATGCCTGGCTTCGGAAAAATAGCAAGTCTGTTCACGAAGAAGAACACGCCAGGCCCGCTCTTAAGTTGGTTCGAAAAGAAGAAGTCACCGGACTACGTATTTCTTAAATTAAAGATTAACAAGGGCAAGCAACAGCTTTTTGACCACCCCGACTGGAACGTCTGGGTTCAGTACACGACCAGTGTGGTGAAGTCGGATCCGGAAGAAGCAATGATCGCCGCACTGAGGACACACTACACTGACGATATTCTGTCAAAGCTTCTCGAGTCGGCCAAGAACGTCCCGAAGACTAGTGGACTTGCCACCAAAATGCAAATGGAGCATTGGGTGGCCAGCAAGACACCGAGCCAAATGTTTCAGTTCCTTCGGCTTGATAAGGTCCGCAACGGAGTCCTCGACGACCCGACGCTTTCCATCTGGATTAACTACATGAAGCTGTACAATTCCAAGCCAGTGAACAAGAAGCAGCAAGTGACTTTAGTCAGCATGCTGACAACACACTACAAAGATCGAGGAGTGCTGGACATCATTGAAGCGGCGAAGAAAGTCCCGAAAACGGCTCCCGCTGCGAGACAATTGGAAATGGAACAAATCCAGTTTTGGTTAAAGAATGGCAAGTCACCGGACGAGCTACTCACGGTTTTGTCCCTCGATAAGGCCGGAAACCAGCTCCTCGCCAGTCCGCGATTCAAATTTTGGTCCAAGTACGTCGACAACTACAACAGAGACTTCCCCGACGAGGCTACAACCGTGATGGCGACTTTGCGGAACCAGCTCGGCGACGAAGACATCACGCCAATTTTAATAGCTGCAGGGAAAGTACCAAGCACCGAAAAGGCCGCCGCTAAACTGCAGGCCGAGCAGTTCAAAAGCTGGCTACGTGAAAACGAAGATCCGGCGAAAGTTTTCCAGCTGCTAAAGCTCGACAATTCAGCGGATGATCTTCTGGGCAGTCCACAGTTTAAACTTTGGGGGAAGTACGTGGAAGATCTCAACTTGAAGCCAGAACACAACGACCTTCAAGTCTCCATTATCACCATCTTGCGGAAAAACTATGGCGACGATGTGCTGGGGAACATGGTACTTGCTGGGAAGAAGGCTCCTAGTACGAGCTTTATGGCCCGACGACTTGAGGACGAGCTCTACAAGGGATGGATTGCCGCAGGCTCGTCACCCGACGGTGTCTTCAAGCACCTGAAATTCGACAAGGCGGGAGAAAACGTGATCCAACGCCCGCTCTGGGGCCTGTATACAAAGTTCTTGGAGCACTATTACAAGTCGTTCCCGACGCCAATGATGTCGGCACTTGCGAAAGGCTACGATGGAGATGCGCTCGCGAAACTTCTCATTGCAGCGGAGAAAATCCCGACTTCGAACACGCTGGCAACGAAGCTACAAACTGGCCAAATTCAACGTTGGCTGGATGATAAGGACCAGCCAGGAAAGATATTCAAGGCGCTCTTGCTTGACGACATGGCGGACGACATTCTCACCAGCCCGCTGTTCAATACCTGGACAAGGTACTTGGATGAGTTCAACAAGAAGTTCCCTGATGAGAAAGTGTCCATGACGGACACGTTCCGCACCAGCCTGGACGATGAAACTTTAAAAAGTTTACTCATCACAGCAAAGGAACTTCCCGACATGAAAACGCTTTCGACCAAGCTACAGACAGTCCAGATTGAGCGTTGGTTGGCGAGTAAAACCTCTCCGGAGGATGCTTTCGCAGTACTCGCACTTAATAAAGCAGGGGGCAACGTTCTGTCGAAGCCATTGCTGAACACGTGGGCGGCGTATCTGGAGAGTTTCAACGCCAAGTTCCCAAGATCGCGAGTTTCAATGATTGACACATTTAGAGAATTTTTCGGCGACAAAGCGTTACTGACTACGCTAGCTGCGGCGAAGGAGGTTGAAAGCACCAAGAAAGTGGCGACGAGTTTGCAGGATTCGCTCCTTTCGAAGTGGGTACTGGCTAAGAAACCCCCAAGCGGCGTAGCTAAGCTGGTGGGGACGGATGAGGCGGGCGCGAAGCTACTGAAAACCTACACCACCAAGTATATGGAAAGATACGGGCAGTGATACTCTAGGCTCGCAGCTAGAACCTAGGGTGAACCATAACCTACCGATATTAGACTTGCAACCACGAGCTTTGTTATTTAAAACGGTTGCTGATCTGTAAAATGGCAACTCAGATTTCAACAAAAGGATAAATGCCAGCGCAATTTTAGATGGATCTAGCCCGACTGGGTGTGAAAGAGTCAGGCTACATTGCCCGACCCAACCCGACGGATGCCCGACTGACCCCGACTAGATAAAGGTTCAGTTGTGCAATCACAGGAAACGCACTCCAAGTATACGTGTCCAGCGCGTATACACGGATATTAAGACTCTGAATCTGACGAAGAGGGCAAAGCATTCAATGTACTCCGTTCACAGCGGATCCAACTTAGCACTCAAAAACGTTTTTCTAAAGTAAAACGATTGCTAACTTGTAAGGCTTACACCCACATGCAGAGGTATCGATAGCGCAAATACTGTAGTTCCTTTCTGTTCAGGTAGCTAAGTCCGTCGGCCCCAGCAAACACTTCAAACGCCAGCAAGACATACATCCCGCAGTTAAAACTGTCGACCTGAACGCCTAGATTTCCACGATAGGGAGAGACACGATACTTTCGTGGGGCGGAATTTGGCAGCTGGGTCACTATCTTCTCAGTAAGCGCTCGGACTCCAATCATGTATGTAGAAGCCATTGGGTCGTACACGATTGCATCTGTGCGCTCCACATCCACCATAATACCACACCAATGGTTCTTGTTGCAGCATACAGGGATCAGAATTTTCTCCGTCGACAAGTTCTTGAACATGTCCGCGAATACCGTCATCGGGACTGAGACAGCACCATGTTCCATTGGACCGATAACATTTGTATCCACAGTTATAATACTGGCTTTGGACTCGAAGAGCGAGGACATAACAAAGCTAATAGTATCGTCACTTAGTTGAGTTTTCCCAATGAGGTCCAGTATTTCCGCCTTAACTCACATTAAGGAAAGTTAGCATGAGCTGAAACGATTTTGTACAACGACAAAATACATACCTTTGATGACAGCAGGGGGATAGCCTTCAGTTTTGCTGGAAGAATGGACTGCTCTGTGACTTGAAACGACTGCGGCATGGTGACATCGAAATCAATACTCTCCGCCCAACTCAGACCTCTCTCGATAAGTTTAATGGATTTTGTAGCTGTGTGCCATTTCTTCATGATACCCACCGTTCGTGTTGAATACAAACCAATCCCAGCAATATCCAGCACCACACCCTGTTCGCGTAGTCCTTTGTATTTTGCTTGCAGTTTTGCGACTTTTCCACTGCATGTTCGAATTTGTTCGGGACGTAAAATTCTCGTTATAGAGTCCGATTGTATAACCAGCTTCGTTGGTGGTAGGCTGGATACGGTTCGAGCTGTAGGCGCCTTAAACTTGAAGCTTGTGTCAACCATCTTGAACTTTGTGAGAACAGCAGCCGCGGATTCGTATGTAGCGTCGATAGATAACACACTTTCAACTGTGGCAAGGCTTAAATTTGCCGCGTAAAGTTCCGCGTCTTCTTTCGCCATATCAACAGCTTTTTTGCGGGCTGCTTTCTTGGCTTTGGGGGTTAACTTTGGCCTTCCTCGTGACCTCGGAGGACTTGCGATTTCAAACGGCGTGTCCTCTGCAGCTGCCTCTAGGGGCGCGTTTAGTGGAATTTCATCGTTAATTAGTAAAGCTCCACAATCTCCACGTGAACTCTCATTGCCTATACCGCTCGATTCAATCGTTTGTGAAGACTCTCCAAGCTCTCCACAATGGCCCGACCGGCCCGACTGACCCGACTCATCCGACTGCCCCGACTGACCAGACCGCTCTGACAAGTTTGCTATGTGATTATCCTCCTCTCTTGCCGTTGCGAAAGCTGTGACTTCTTCGTCTGGCTCGGAAACGCATGTAGCTGGTACAGATGTGTCTGGTAAAAGCTCCTCCGGATCGGGCTCGCCCACGGCTCAGGTAGGATCTTCGAACTCACCTCTCTTGAAACGACCTGCCACTTCTTGAAGGTACTGCATGGCATCTTGATACTGTGCCATACCCAGTCCAGCCATAGTGTCGCAAATTTCGGATGCAATAGGTAAGGCTTCTCTATACTTGCGATTTGAATCCCAAGGTCGATTTCGAGCAGGCAGAACATTCTTCACTTCGAACGGCTTCGGTGTAGAGACGTGATTACTGGTACCGTTGTTTTCAATTGCCGACCGCACGGTAGATATCAGCCATCTCTTGTTTAGCGTGAGGGTCGGAATAACAGTTTCTTGGCCCAAGGCTTTGCGGAGGTAAAATACGTGGCGGCATGGCAGCAATCGCGTGCACATGAACAGGCAAGAGCACGTCCAGTCGCACTTTGTAACGGAGTATTCTGTGTCGACCTCATCCAGTGCATCG

>Contig_53

TTAGTTTCAACTGGGGCTAAAGATGACCTACGCTGGTTCGAGTTAGTCTTATCAACAGGGGCTCTTAATATGCCATCCCTCTCGCTCGTTTTGTACGGACGACATGAACCCGATTACCACATCCAAATGGATGCTAGTGATCGCGGTTTGTGCGCACTTTTTCCAGCAAGACGGCAGTTCTTACAAGTGGAGTTTAATTATGAGAAACTTCGACTTATTGAAGAATTCAACAGATCAGGGGAGGGAGATTTCGGGATCAATGTCCGTGAATTGATGAGTGCTGTGTTTGCTGCATTGGTGTGGGGGCATCTGTGGAGTCGTCCGAGTCAAGATAGTGACGCTCATGTCCGATTCTGGATTGATAACATAGCAGCTGTTTCGTGGAGTAACCGCAGAGCAAGTCGCAATCGTTTCGCGCAAATGCTCCTGCGCATTCTAAGTCTGTCCGAAGTAAAGCATGGATTTTATTCCACCGCCGAACACGTGGCTGGTGTAGACAACGATGTGGCCGACGCAGGCAGCAGGGTGTGGCAGTCACCACTACTGGCGACAAAATTTTCTAACATGTCTTGTGGTTGGCAACAGGCTCAGATCCCTCACGACTCGCGAAATCTCTCGCGTCTTTGGGAGCATTACTCCGCGCAGGGGCTTTAGCACAGTCGTCGAGGAAACATTACACAAGAGCGTGGTCTCAATGGACGTATTGGTGTAAAATAATGGGACTTAACCCGTGGCTCAGTAAGGACTCGATAGACAAGAACGCGGAGCAATTGGGTGCGTTCGCTGTTTATCTCTGGCAGTACGGAATGAATCACTCTCAGCAAGGAAACACATATTCAACGATTTGTTCGAAATTGTGTGCGGTACGCTGGTTTCATCGCAATACGGCAGGATATGACCCTGGTGTGAATGCCAGTCATGCGATTTTACTTCGAGGAATTCGGCGATTTACCAATCCTGTCGTTAAGCAGCAGCCTCTGACAGCACGCCTGCTTCGTCTCATTTTCGAAGAACTTGACCTCACGCAGACTCGACACCAACTTTTATGGGGTGGTCTTCTACTCGGATATTTCTTCCTGCTGAGACGTTCTGAATATCTATTCATTGGGCGGCAATTTCACAACTATATTCTTCGACTCGAGGCAATTAAGTTTTACGACAATCACGAACGTTTAGTAGCAATTAAGCGAGCCATGGTAGTTGGCATCTCTCTCAATGGAGCCAAAAACAACCAATTCGGCCGTGAGGAAATGCGTTACCACAGCAAGTCAAAGGATCCGCTGATCTGTCCGGTTCGAGCAGCGCGATGGGTCCATAAAGCTGCAGCAGTGCTGGGCACGAGTGCAAACGATCCAGCCTTGTCAGTGAGAAAGGGTGGAATCACATCAACGGAGATCGCTGCGATCATTAAAAATGCGGCAAGAAAAGCCGGCTTTGATCCGGCACGATTTTCCACTCATTCTGCTCGAATAGGAGGCGCCACAGCGTTGCTCAACGCGGGCGCCGATCGGCTTGTCATCTAACTTATGGGTCGTTGGTAGTCCAACGCGTTCGAGGGGTATCCAGTGCTTTCCGCCAAGGGGTCTGCGGAACTTTCACGCTACATGTGCTAACAAAAAAGGCGACCACACCCACGGCTCAACTCGCGGCAATCTCATGATGGGGCCACCGCCACGCCTCGCCCAGGTGTGGCAAAATGCAACTCAATCAATTTGCCCCAACGTGACATTGTTGGAAATTGCTGGTTGCGGAGGCAACGGCAACATGAGATGTGCAAGTTGCCGACGAAGGCGTATTTTCCCGATGTCGCAGTTAGTTATATTTAGTTCTGTAGGAGCACGAGAAGCTCAGTTGCTTCCAGCTCACTTTCGCTTTCTTTTCGCCGCATTGACGGTCGTCTAGATCATGTGTACAAGGCAGGTAGGTGGTATAAGAGAGCACACTGCGTGCGAGGAGTCTACTTGGTATAGACACACAATCGACAGGGACAAGGTCTTTTTGGACGCCATGATGACCAGCATTTTGCTAGAGTGCGGAGGCCCATATGTACCGGTATGCTTACATTGTAGGTTCTTGCAAAGGCCAAACTCTAAGTAACATGTGCCTCCAATGACCGTCACGCAGCTGACACGGTCATGACTGCTCTTAACTGGATGCTGGGCCAATGCAGACGGCCTGGAGCTGGTCGATATTGCAGCCAGCACGCAAGAGTGCGCTAGAATCCTCAGCTAAGCAACTTGATGCTTTCAACGATCCTACTTTCCGCTCAAAACTCTACAATCTGAAAAGTTAGAAAACTATAAGAACACTCGTGGCTCAAAAGAAAACTCTGACTCTTCAAGAAAATATAGATAACCCAAGAAAATTTTGTTCCAAGGTCCTACTGTCGCGATAATTTAACTTCAAGTCGTAGACTGGGGAGATTTCGATAGTATCACAACAGAAAGTAAACTGCAATAGCTCATATCTAGCGAGAGCTGAATCGCCCAGGTACTCATTGCTTGCACGATGCTCTAGCGACTCTCGTTGAAAATTACGCTCGAAGCTTGGAGGGAGAGACAAACTGGCGTTGGTGTCATCACAACAGAAAGTAAACTGCAATAGCTCATATCTAGCGAGAACTGAATCACCCAGGTACTCATTGCTTGCACGATGCTCCAGCGACTCTCGTTGAAAATTACGCTCGAAGCTTGGAGGGAGAGACAAACTGGCGTTGGTGTTGGAGAAAGCAAGTGAGTCCACGGCCGCCTCAGCGCTGCAGAAGAAGCAATTTGCGTCATTGAAAGACGAACGTATTACACCGGAAGTCATCGTCTCCTTTTTATTTAAAAAGGCTCAAACAACTACGAGAGCCGAGAAGGCGATTGTGGCCAAGTTTAATCTGTTCTTCATTAAGACGAGTGGGTGATGGAGCGAAGGCGTTCAGGATAGGCTTCGATGAGTATCTTACTGGGTCATAGAAAGTATACAGGACTATTTTACGCATACTCGAGCACACGGAAATGTAGTGTTCATGTAGCACTGTTACGTTCGTTATTGGTATTCTCTTTAATGAGACTTACTATTAGTCGTATTAATACATGTACCATTAAAACCCATTCACACGTAAAACAACCAAAACCCAGTAGAGGATCGTTGCATTTTTCTATGAGCTGTCTGTTGTAGCTCTCCACACTATGAATCTCGTGGGGCCACTAACGGCAAAGGCGAAGCAGTACTCTTATTTGTAGCGGTCAGCATCGTAAGGCGACAGGATTTATCGCTCGAGTGTCCAGACAAGCTTTGTTTCGAGCCTGGCAGGAGCGATGCAATGCTTGAGCGATTCCATTGAATACCTTTTTTAGCTAAGGGGTTTGCAGCAAATGCGTCATTTGGTTATACGGTGTCGTACAGAACAGTGCATGTGCTTCGTTTTGAACATCCCAGTTTGTACATAACGAAGTTGTGTTTACGTAGACGTAAACACTGGAAAATGTTTTGGTCAGTGCTGGCAATCTTATGCCAAATGCCAGCATCACGCAGCGGAGAAGATAGAAGTCTCACGCCCTGCAGCTAAATCAGCGCGGGTGGCGGATCATATCACTCTTCAAAACATTCTCAGCTTCACAAGCTCACCAACANCCCCCCCCCCCAGGGCATGGGAAATACAATACCCAGTGTTATTTTGCGTGAATCACATCCGTAATAAAGACTGCAATACCTGGCAACACAATAATAATCACTGCAATAATAAAAATATATTGCTATCACTTAGTCTTCGATGCAACCGAGAAGGGTGGAGCAGCTCCACATACGGCGATTAGCACGAAGAAACATGACCGTTTCAAAATTAGCTGGTAAAAGTAGATGAACGTAGCGATGTAAAAACAAGCTTACACTCAGAGAAAAGTCTCTCGCACGTGTTGCTTGTCGGAGGAGCGCAGAGCAGGAGAGGATCATACTCGACAGACGCCTGCGATGCAAGCCGCGGCTTCTTGGCTTGCCGGAGGATGGAGGTCGCGAAGTCCGCCGCAGTCGGCACTTCCTCTTGTGCGGTAGACGGAGATAAAATAAAACTGTCGACTGCATGTAATTCATCCGCGGAGAGGGGTAGGTCGTTTTGCAGCTTCACTATTGCAGACTCGAAATGGGGCGAGTGAACAATCTCAGCTGTTGGCGCTAAGTAGTTAGCCATGACAGGATATTTTAGCAGTAAACGCATCAAACAACAGGCGCACCTCGGCAAGCGTGCATGACTCCGCTTGGATTTTCACACACACACTGTCCAGCACACGAAGCTTGTCCAATACACCTCTAACGCGGCGGTGCGCATGTCCACAAAGCACAAACTCTTCAACAGCCTCCACCGTCAGGATCGCATCACGGATCTTGCAATATCGGTCAATCATCTGGAATGTAGACGACCAGCGCGTAGTGTTTGCCTTAACAGGCTTGTAGTTGGTCGCCTTTGCCAGTGCAGTAGCATTTTTGACATGACGCAGCTGAATCATCAGGTTTTGAATCAGATCTATCTAAGTCTGATAATCCTGTAGAAATCGATTAACCGCGAGGTTAAATCGATGACTTGCACAGCCAATGAGGGGGACGCCCATTCGTGTAGCCATACTCTGATTGGTTGCACAATTGTCCCCCACAACATAGCGCACCATCTGCAGGTTCTTCTCGTACACGCTAAGTACGGTGGATATATGATCCAGGTGGGCTTCAGCTGTCTGGTCGTCTTCCATTGGCGAAAGCGCAAGAAGGCGCTGGTGGCGCACACCATTGTAGACGTAGATCGCGTAGATGCCGAGGAAGTGAAGTGAGTGACTAGTCCAACCGTCGAACATTAAAGCAAAGAAAGTTCCCATCTCCTTTGCAATGATTGCTCCAACGGCTAGAGCCACATGACGCATGTACCGCTTCAGTGTCTTGGTAGTCGTAGGCTTTATTGCAACTACAGCACGCGTAGCCTTGTTTTCCACTTCAGTGATTGGTAGATTTCTCTCGATCACCCATACCATCCCTTGGTAGATATTGCGCGTCACATCGTCCACAAAACCAAACGATGGTAAGTGCTGGCGTCTGCGTGGCATGAAGTTCTGCAAACCCAGCAGCATACCCAGCGTGCTTGCTGTTAAGGTGGCTGAGGAGGTTGCTGTACCCCGTGCCGGGCGTCTGCTTCCGAGGAAGCCCACAGGATTTACACTCAAAGCGACCTTCACCGAGGTCGCTGAAGTAAAATGCGCAGAGTTGACGGGAGGTCATCGTGGGTAGCGTGGTGGGTAGCGTGGCTAAGAGTGGTGGCTTGTTCTCTAAAGAGAAAATTGAGAAGTGGGGAGTGTGTGAATCAAAGCGCGCAGAGTGTATTTGCAAGGCAGTAGAGAACTGTTGGTTGTCAGCGCTCCTGGAAATGGTTGGAAGCGTGTAAAATATTGTATTATTGTGTACAGTTATGTATTATATTTATCTTGAAAAAATCAATATTAAGAATTCCGTAAATATAATACCCGTAATATTGTTATTATACTATTTTTCCCATGCCCTGCCCCCCTCACTTCAACCATGGCCTCCACCAAGAAGACAGCTCTCGTCATCGGCAGCACGCGCGGATTTTTGTCGAGCACTACGTCAAGGCTGGCTGGAACGTCATTGGCACGGCTCGCGCCAATAGCAACACGGAAAAGGTACAAAAAAATGTTGCTAAGAGACGTTGGGGTTATCAGTACTAGTTGCTCATCTTTACTGTCATTGTGCAGCTGAAGTCGCTTGCCCCGTTCAAGATCATTGCAATGGATACGAGCGACGAGATCTCCATCCTCGAGGCAGCTCGTCAGCTGGAGGGGCAGCCCATCGATCTGCTCATCAACAATGCCGGTATTGGTATCCCAAGCGAGTTCGATACTGGCACCAAGGACGCCCTTATGCGCCATTCGAGGTGAATGCCGTCGGGCCATTCCTCGTGACCAGATCTTTGCTACCCAACATGGAGCTGGCAGCAAAGGCCAATGGTAGTGCCTTCGTTGTGCAGCTCTCGTCATTCCTTGGCAGCATCGGCAGCTACACAAATGACACTGTCGATTTCTTCAAGCAGGCTGGCTACGGCTACTCGTCCTCCAAGACTGCGCTCAACATGATCACGCGAGGGCTTGCATTCGACCTGCGCTCAAGTGGCGTCGTGGTCGTGTCGGTGCATCCAGGATACGTGGACACGGACATGACCCAGGGCAAGGCGACGCTGAAGCCAGCGGATAGTGTGGCGGCCATGACCGGCCTCATCGCCAAGCTTGGCTCTCAAAGTACGGGCAAGTTCTTCAACCTGGACCCGCAGATCCCCGTGGTGGAGCTGCCGTGGTAACTTGGTATGGGTACTGCGTGAGACAGGAAAATACGAGTACGGTAACTGCTAGGACGTTGGAGTTCTGGAAGAGTGATTAGTGCAGTGCATGTATTTTTGTTCTGATTATCTCCGTCAATTGGAGTAAACTGGAGCGCATTCCGCAATTCTTGAACCAGCCCTCAGTAGCCACCTGTTGATATTAATAGAGATGCGTTTCTTCCCAGCCGTACTCCTGGCAACTGGGTGTGTTCTCCTGACAAATGCTACGTGTGCGTCCTTTGACTCGGAGTTTGAGCTAGCCACCGCCGACCATCCGACTGTGCTCCGTTCCCTCGCCTACCACCATAACAGCGTCGCTTCTAAACGGCTGTTGCGACGTTACGATGTTGACAACGAAGGGAGAACCGTCGGGGGCGGCGCCAAGATCAAGAGCCTTTGGTCGAAGGTCCGTGCGTATTTACTTAATCGTAAGAAAGATGAGGTTGACATGGCAGCGAAATTACAACTCGGCGGTATTGACCACGCCTTGTCAAGCTCGAAACTGGAGCAATTGACCAAGGAAGTCCAAGTGTTTAACAAAAAGTCCATGGCCATAGTCACGGTGATTGGGACGCTCAGGACCCTCTACGGAGATATCGCTCTGGCAAAGGGGCTCAAGGCTGCCGAAAGGGAGGCGTCACCCACACTTCTAGAGCAACTCAAAGCGTTGAGGCAGGACCTACAGTCGAAGTGGCTGAACCGCGGCATCTCCGCGGACGTTATTTTCAAGCAACTGGGGATTCGCGAAGAAAAGTATCAAATGTTTTTCAGTGGGAAACTGGACATTCTGGAAGCCTATATCAAACTCATCAACCAGAACAAAAAGAAGGGCGATCCGGTATCTCTGGTGAGCATCTTGAGTAAGGGATTTGGCGGGGAGGACAAGCTGGTGGCGCTCGTGACTTCTGCAAAAAAAAGTAGCATGACGGGGAAAAAAGCCGACGAATTGGAGACTGCTTTACTCAATAAGTGGCTGAGAGAAGATACCTTACCGAAAGACGTCTTTGTATGGCTGAAGCTTTCCGACGACGTGGACAACGCTTTCTCCCCTCAAAACTTGAACAAGTTCGCAACGTACATCGACAATTTTAACACGAGGAGGCCCAATCATCAACAGTCAGCGATTGCTATCTACACGAGCAGTTTTGGAGACGCTGCTGTCGTAAACAAGCTCATCTCGGCAGTGGACGACGGGGCGACAAGAAGTATCGCGAACAAGCTACAGGAGGCGCAGTTTGAGAGCTGGGTCAGCCGCAGATTGGGTTTTGGCCAAGTCGAAACGATACTGAAGATTGACAGCTCTGGCGATGCAGTAGTTACTCGCCGAAAGCTAGGCTTGTTGGTCAAGTATATTACGCAAATGATGAACGTTTAATCATGACGTTGACGGAGCAGAGCTGTAATTACAGTAATTAATCGAGAATCACCCACCGTTCGCCCAACATTGTGGTACATTTCGGCTGGGTGAATTCATGCAACCAGTGTGGAAATAAATAAAAAAGATAAATATAATCCATGGCAAAGATCGTTTGGTTAAAACGAAAAGTTTAAATCAACCGACTAAACCCACGCAGTGCAGAAATCCTTTATCACTTCCACCATAGCTGCAACATCACGTAGAGGCACTCAAAACCCTTTCCTAGTCTAGTGATTCATTGGCAAGGATCGTTTGGTTAAAACGAAAAGTTTAAATCAACCGACTAAACCCAAGCAATGCAGGGAATCCTTTATCACTTCCAATGATAGCTGCAACATCATAGAGGCACTCAAAACCCTTTCCTAGTCTAGTCTCCTTTTCGTACTCCCATCCGAAGAAAGAAGTTTCAGTACCTACTTTCTCTCGTCCTCTACAAACCGAACTACGTGATGACGACCGTAGATTTCTCTTCATCCTCATCCGCCGCAATTTGCTGGTGCTGAGCAACACGCACATCAGCGAGCTTTGTATTGAATTGACAAGCCTCCTCGCGAAAGACACCCAAGCCGGTAAAGTGCGCAATTCGAACGATGTCACCCGGAGAAGGAAGTCCTTTACTTTCTTCTACCACATCCCATACGGTAGCCGTCATTGGCTCTTTGTCAGTATCAAAGTGGCGGTCTTCTTCGTACTTGTAGTTCTATCTCACATCATGCAAATTAGACAATAGTGTTATATTACATCATTAAAAATACCATACCTCAAGAAGGTCGGCCAACGAGTCGTCAGCAACAGCGTCGTAAAGATGCAGATGAGCAATATGTCTGCCTCTTTCAGTGGGGTGGATAGACCCCCACAAGAACAATCCATAGATGACGACTATTTCATTGGGATCTTTAAATGCTTTGGCGTACGTCCGGAGTTCTAGAAAGCAGATTGTGTTAATATTAGCGGTTAAGTCGTACAACATACCATANCCCCCCCCCAAACCTTGAGCAGTTGTAATAACTTTCTCCGTCATCGCGATACCAAGTTCGGGTTTCACGGCAGCGAGATCAGTATGGATGGTATCGTAGAAACAATGTTCTCTGCCGCGACGTGCAGCTCACTTCTAGTTTAAAAACGGATTTGGATGAAAGTCATGATCAACACAATGTTCCGAGTGATTGTACAACTGAGGGCAACGCAGAGGAATCAGCGATGACCACCGAATAAGATACAGCGCATTGGCGATTTTATGCCCCCCGCACCATCGATATTCGGCGCAAATGATGAAGATGAGCTTTGTTCCCAAACAGCAGTTCCCCTAGGCACTGTCTATGCCGGCGAACACGCCAACGCGCAAGCCCGAACAATTCTGAAGGATACCTCTGATGTTGTGGTCTGGCGATCATCAGAGATATTGTCAGATTTTACTCCGGCTTACTGGGCTTACACGTTTCGTGAACTTTTTACCTATAGTCGTGGCGGACTCGATGAGCCAAGAGCCGTACGAATCGGTGTTGACGAATATGTGCGCTATTGTTTGCGGCTATCGCAGCAAAAACATGCTCATCATCCATCATTCATGCTTATTGCTTTTGACATACTGGCGCGTCACCACGTACGTGCCGTTTATCTACGCGCTAAACTAGCGCCTCATGTGGCAAGCAGCTCCGCAAATGTTGATCGCTCCGAGTTACTCAGTCACTTGTAATATCGGGATGCCCGCCTAAGAGCCATAAATAAAAAAAAATGGGTACCAGATCCTCCACCATCCTCTAAAGCCGTCCAAGATCTTTTCTCTGGCATTTCGACGGGCCTTAAAGCTCGTTGGGGTCGAACGAAGAGCGCGGAGATGCTTGAAGCAAAGTTATGTCGCTGCAGTTGGAAGCGGGGCAGCCATCCATTTTCTTTACGCTGTCGCCTAGTGCTAGTAGCACATATCAAATTGCAAGGCCCCCTTTGAGGGAATCATTGTCGGGTGAAATGCTTGGCGAGAGCTCGAATATCACTGGATGAGTGCCTGGAGCTTACGCAATCCCGCCTCAGCATGATTGCAAAAGAAAACCCTACAGCTTGTGCAAGGTTCGCTTTGCTTTCGACATCTAAAAATTATGATCTCTTTTTACAAGCTCTTATCAATTTGTTTTGGTATGCACAGATACTTTGATCACCTCATGAACAATTTTATTGATGTAGTTATCAATTGGGATCAAGAAAAACATACTTTCAAGCCAGGTTATGGGCGGACACGGGCTTTCTTTGCTGCGACAGAATCCCAA

>Contig_54

CTTGTCGTAGCTCGATTTATTAGCCACTCTAACTCGATCTATCCCTTTTTATAACCTCGATAAATAAAATGACTTCAGTTTATTAAAATCGAGCACCCACCCTACCGGTAGTGATAAGCTTTGCAAAGCACAATATTGTGCTTTGCAGACGGAATGTTCATCCGTTTTTTTAGCCTAGATCGAGCATGATCAGAGCATAAACCCACGCGTAAAAATGGGATGGGAAGAGGCAAGCATCGATTTGTTTTATCTGTGATCGAATTACCATTTCAAAACGGTAAAAATGATCAATTTACGGGATATCGATGTGGATTAGGTGTTAGATCGATCTGGAGCGGTCGATTTGGCTATCGCAGCTTGATTTACATGTATCAGCCATTCTAGCTTGATCGATTTGTACTTTTTATAGACCTTGATTTAATAAAACGACCTCTATATATTATAAATCGAGCATCAGGCTAAGTGAAAAGGACAAACGCATTGAAATGCACAATATATGTTGGGACTTTAGGTTCTGCAATGGTACTGTCCAATCGCAAAATCCATATGGAGTCCTATACCGGAAAGGGCGATCTGTTCTCGAGATTTTATATCCAAGGTTCGAGATCCAAAACTACCTATACGCATATCCCAAACGAACGTTTTCAGCACAAAACTTAAAAAAATTTACGTCAGCAATTGACTTGTCGTACCCAAAATAGTGGGCTTCTTCTGTAAACGTATAGCCTGTTACTTCAGCTTGTATTAGAAACATCATTTTTACTTTAATTAAGAAGAATGTTAATATATACCGGTAACTGAATATCCTCTGTGTTGCCTGTGTCGGCAAAAACATGCAGAAATTAGGGATCATCCTCAAGCAGCTGCATGTACATCACCCACTCGCTGGGCTTCCCTAGTCGATGCGCGCAGTCGCGATCAAAACCTGAAGGAGAGTTAGGCTGCATCCTGGTCGCTTGACGAAAGGGGTTAGCTATTTTTGGGGTCGTTTACGGGTAGGGCCTGATAATTGGGTCAGGCGGGTTTCCTGACGGCCCTACCAAACACAGGACCAATTGCTAAGCTACATATTGAAGTGAAAGATATGAAGCTAAGCAGTTGGGTTAAAATGCTTAGACCACGATGGTCTGCGCGGCACGAGGTTGGTCGGCTTCAGTGTCGCATTCATCGGCATCACTGTCATTGCTGATCACATCTGCGCCCAAATTCGTAGCAGCAGCGTCCTCTTCGCTGCTAGTACCAGAGACGCCCTCGTTGTCATCATCACTGTCGACATCTACAGCCTCGTCATCTTCTTCACTGCTCGATTCTTCTTCTTATGTAGGAGGATCCTGTTGGAAATGGAACCTGACCCAATAATCAGGCCTGACCTGTATACGACCCCGTTAAAATTCTACCACGAAAGGTACACTTCATTTTATAAAGCAACCTTATTGTGCTGGTGGGGGATCATCATACGAAAAAGTATTGATATGCGCTCAATGGAGGTTATAAAAAGAAAAGTAAAACTCAATTAGAAGACGCCGAAAAGAGCATTCCTTGCATGATACAGATTCAGAGAGCTGGGCTGCATCGTGTCTGTGGCACGTCATGTACGGTACAACACGTGAGCTGGACACGTACATGTATTATATTAGCAATAATATAGGTTTAGCAGATCATGATACGATTGGATCAACGCGCTCACCAAGTGCAGCATCGAGTGGGGGTGCCTACACCTTCCAGTACGTCAGAGAGAAGCGCTTCAGCTAGACTACTCTTGTTAATGATGCCGTTCGGTTATTGATTTAGGAATGGGTATGCCATTTGTCGCTTCCAGATAGTTGATTTAAAAATCTATGGAGATCGGGTCCCCAGATCGATCGAGGGTAGAATACCACGATATCGTTTTACGTAATGCTGATTCAGTATTAATAAAACGAAAATGACTATGAGTACAAAACATACGGAGTGATGACCATTCTTTGATTTTACCGGTACTCAGATTTTGTGTACTACCGGAACATGTAATATGGTCCTATTAATACAGCTCGTAGTTCGAGGAAAAAAAAAACCAAGTCTCAATGCGATTAGCACTGCCGGACGATTGTCACGGCTTTTTCTTTAAAAAACAATGAGCGTGCGGTTGTGTACATGGACTTGGTTGCTATTCGCTTAAATCTACCGGTAATAAATCTAGACTCACAGCCAGTTCAATAGCATACAAAACTCAATCGTGAGCTTTTTTTGTTAGAATGTAGCATGTACATCCGGTCAACACGTCCGTCCGGTCGTTCTGTGGTAATCACAGTGTGATTATCATCCCCCTGTAAGGGCTCTGGGAAGAACTATGGAGCTCGAGCTTAGGGAGCTCGAGGTACCCAAATTTAAAAATATTGCTATTTGATGTACTCAAATGCATTTTGTGTTTTAAACATAGTTGTCAGGTTGGACTTTAATTGAGCTGCCACAATATCAGCACAGCTACATTTTGCAGCACGTCCGAGATGACAGTGATGATTACAAGAAGTTCTCCATTGGGGAAGCTTCGGGATTAAAAAAATATGCTTATCACTTCTCTTCTTCAAAGTCAGGTTTAATTACAGTCATTTAGCTGTCCTAACGACCACATTAATACTTGTCACTACTCCAAAGCAGGACATTTTTTTTAAAACTTTTTTTGGCTGCATGAATTCCATTCATTTATTTAATGAGCTTGGGAGGTAGACTATTCTATAAAATGCAATACGCTTCGACTCGATGCTGCGGCATTGCTGTATACCAGACCTGTTCTCCGCATAAGTGCCCCTCCTCGTCCACGGACCACTTCGTAGGTTTAGATAGCACTAGAGTAGTCTATTATTCTTTCTTGTGGTATTTTGTGTATCTTAGCAGTCTATGAGGGTATGCAGCATTTGTTACGTAGTCGATGAGCACTTTGCGTCATTGGAGAGGTCAGACCATTCTTCTTCAGAGGTTAGTGGATGAACGGGATAATACTACTTAAGTTTCTACCAGTGCTGCTGTGGCTGATGAAGGAATAACCCTGACAAACACCATTAACCCTTAGACAAACACCATTTACAAAAGAAAATCAAGGAAAGCTCCAGATTTCCTACAGAGCTATAGGTATACAAGAGCGAAGCGCTTTATAAATGCTTGCTACGGTGCAGGATATCCTAAGGCGCTCCGCCGAAAAATCTAGGAAGCGCACTATCAAAGGCCCATGGCTACTTGTAAGTCAGCTAAACTAAACACTACACAATTAAGTTTTCATGTGTCAGCGTACGATTTGTTGGTCTAGCTCGCATTAAAAAACGCCTACACTTGCTCTAGAATGGGCAATTGTCGCCTCATTCAGGGTGATCGATGTATCACTCTTATTAAGCCACCGCCTAGCAAAGCTGAGGCGTAATATGGCTTCGTGATATATAACAATACATCTCCGCTGTAACTACCTGAGCGAGGCATACCGTATTCTCTTGGTCCAGCTCACGACATTGCAATTCTATGAGGGTCTACAGGCTGTAGTACATCGAGACCTTTGGATCATTTTTTGAGTACATCAAAAAGCACTATTTCTAAATTTGGGTACCTCGAGCTCCCTAAGCTCGAGCTCCATAGTTTTTCCCAGGGCTCTACGATAGAACCCCACCCCCGGATATACTATCCGGGGTGGGCTTATCTTGAGATGGGATCGCCAAAGCGCCCCGAGCTTTGGCTTAAAGTTTAAGCGCCCTACAAGCTAGCCCCCTGGATGCGGAAGGATCTCTACTTTGTGTGAGGTCAGAATCCAAGTCACTCCACCAACTCAAGGCCTCTAGAATGCGTTTTAAACGCAAGAAAACTTAATTATACTAAATAGGATTTGACACCGAATTTGCCTTCCAAACGAGGCAGGGAGCCACTCTCATTTTGATAAATACGAGCTTCGTTGTACATGTAGGCTACATGATGTAGCACCCTCGGGATAATTGTCTCACCAGAGAAAGAACGCTTACTGTTCTTCCCGCCTTTGGCTTTGCTGCTGGCACGGTATGATACCATAGAACTTCTATTAGAAGGCCATACATGAATACTGTACCTATCTTCTATGTAGCCGGTAAAGACCCCAAAACTTATTCACAAACCCGTGTTTCATGGAGAAGCTCTGGATTATACTAATGTGTAGGGCTTAGCCACAAGTAAAAGGTACTTTTCAATTTGTTCTATATAATGTTTACAGTAACATCAGCTCCCATGCTTCCCTTATTGATGCATTACTGTATACTACTACTGCAGTCCTGTATACCGAAGTAAATACGCGATGACACTGTGCGGGTCGCACTGCGACCACAATAGAGATATTGGCGGTATAGTATGTCCTACGCATGTGCTGCACCTACTCCACAGCCGCATCATCTGCGTGGAAGCTGAAGTAATTTACAGTGATGTTACCAACTTAACGCGAGTGTATTTACCTAAGGGTTGATGCTTAAAAGTTCAATTATTTTAGTAAGCTTTAAAAAGAGCTTACAAAATAAACCTTCTCTCGAATTTTCACTTATAATAATACAGTACCAACACAGCTCATGATAATCTGGCTAGTTACGTGAAAAGTTCGTTGTAACCATTGCCCCCGACCTCGGTCGAGGTGTACCCGCTTGTCATTTCGTAATTCATTCTTCAGACACCAGGACTATATCTACAGAACTCAGCAGAAGTCCCTTGCGATGATGCATCTCTTTTCAGTCGTATTGCTGGTTGTTGCCGCATCTCTAGCGTGCGCCGCAGAGGACACAGCCTCGATGAGGTTCCCACGCGGTTATCTAGATGAGAAAGACAGTGCTCCTGCTCAAAGATTCTTGAGGGTCTACGACAACGAGAATGACGAGGAGAGAGTTCCTGTTGGTAACGTCTTAAATTCTATTATGACCAGTACATTGAATGCCGTCGAGTCCGCTAAGCTGAAGGTGTTTTTGCTCCAGAAAAGTAGTGGAGTTGACGTTTTGAACAGCTTAAAACTCGGGGACGATGCGGCAGCTGCTCTGAAAAACTCAAAGCTGGAGACTTTGAATAAGTACGTCACGAGGTTTAACCAGGAGAATCCGGACAAGACAATCTCACTGGTTGGGACCCTCACGACCCGCTACGGAGACGATGCTTTGGCGAAAGCGTTAGTAAGTGCCCAAACGCATGCAAAGTCTTCAGCTGAGATGGTGACGATGGCGAAGAAGCTGCGAGCTGAACAACTGAGTGCCTGGCTGGGCGGCGGTAAATCTGTCGAAGATGTTTACGCTTTGCTCAAGCTTGATGAAGACGGGTATCTAGCGCTCACAAGTAGAAAGTTGGATGTACTGGATGATTACATTGTGAAGATCAACAGCGAGAAAAACGGCCAGGAAACTTTGCTCAAGACCTTGACGAAGGAGTTTGGCGGTGAGAAAAAATTGAGGGCGATTCTAAACATAGCAGGACTGAGCTACTTCACACATGTGAAGGCTTTCGAGCTAAAGAAGCTCATAGAGTGGAAAAACAGAAACTCGGAACCAGCAGCTGTCATGAAGCTGCTAAATCTTGATAACGATGTGGGTAAGGCTTTAAAAAGCACAGAACTGAGAAGGCTTGATGAGTACATAATCGACTTCAACCTCAAGAACCCAAACAATCAGGCGACGCTGCTTAGGTACGCTCACGAAGAAATACGGCGAGTCTGGTGTGGCAAAGGCGATTGTGTCTGCAGTCAAGGATGACAATATGCTCGCTAAACGCCTGCAGAACCAACAGTTTGAGGATTGGTTGAAAAAGGACATGTCTGTGGACCAAGTTTTCAACGTTCTAGATTTTAAGAGCGCTGGTATTGGAGCTGTTATCAGCCGAAACGTGGACACTTTGRWCAAGTACGTCACGCTATACAACAGAAAGACATCAGCAGACGAAACGTTGGTAGGCGCATTCGTGAAGGCTTTCGGCAAAAAGCAGTTGGGCAATATGCTGCAACGTTTTCCCGATACAGATAAATACTCAGCAAAGTTACGCACGCAATTCAGGGAGCTAAACTGAGGCGGTTAAGCAAACGCAGCAGACGAAAGTAAGCAACGTACAGAAATGGATGATTCATGGCTCAACATCACTTCTGGTTTTTCTAACTAACATCTTGCGCCCAATTAATTATTTTTCTTTTTAGCTTCCATCGAGGAGGTAGGTCAAGTATGTAAGGACTCATCATCAATGTTCCACTCATTTCCGTGAAAATGCAATATTTAAAAGTCGATCAATTTGGACTAAAGAATCCTACTACGTAGTACTAAAGTAGTAGTTCTTGGTTCCTATTAATAAAATCATATTTGTATTTCACTACGCGCATTCGTCCATGTTATGTTGCATGACGCGGCGATTTTGCTCGACGTTGCTGAGAAAATACCCTAATGCTTGTGTCATTGGCAGTGCCTGCTGTCACACCTGATAAACCGCCTATGACATATTAATTGAACAATATAAACATCTGTACGTGAGGAATGTAAGGCCGTGTTCGATTTAAGCTAAAACCAGCTGTAATTGGTATTAAAGAGCTGGAATGTCTCAACCAGCTTTATTCCGCTAAGAAACACAAAAAATGACATCCAGCAGGATAGCTCCAAACGACTGGTTTCAAAATTTAGCTCTGGGTTGAATTTTGAATCCTCCTGCTTTGCGCCTTCAAATCCAGTCTTTTTCTGATGGAACACGATCACTTATTTTATCTTTATTTTACTTAACAAAACGACTGTTCTACAGCTCTAGCTTATTTTCGAGCAAGCTGGATTTGCCTCTGCAAGCCGAACACGGCCTAACAGAATTATAAAAAATTGATTTTTTTTTGAATGAACGTTTGCCCGTGTGGTAGAAAATATAGAGTACGAGCGAATCGCGCCTACTTTATCTTATCAGCATTTCTTAAATTGTCGCGTTAAAAACCGAAACCTCCGCGGATGTAGCGACCTGGTTTGGGTCTGGGTTTTAAGCGTCCCCATCAGCTCAATTTACTAGTCATCGTCTTCATCAACCAAACTATCCACTACATCACCGCCGCTACCTACTTTTGAGAGTAATGAACATTTTTCGAGCTCTGCAACAACTTCATTAACCTCCTGCTGAATGTCCGGGCTGTCTTCAGCGTCAGCAGCATCGTCACCGTCGCCAATTTGACATTTCGTAAAGCCATTCCGTATCATCGATGCCGGAATAGCATTCCAAGCGCTAGCAATCCATTCGGTGATTGTGCTACGGCTTGGTGCCATAAGATCAAACGGAACAGATGCCTGTTCTACGTTAGCGTTGTGCTTGGCCAGTTGATCTCGAAGGTAGGTGGTCCAGTGGCGTCAAAGTCGGTCCTTTATAGGCTTGCTCCACCGCAGCTCTGACGCTAGTGCTGTGAGAGAACTGTAGAACTCTCCGTTTAACCTTTTTGTGTATGCTTTACGTTCAAAACGAACGGCTTGACGGCCGTTTCCACGAGCGCGTAGGCAGCCTACGTGCGCTCGCAAAGTGGTCGGAGTCAAACAGGTTGCGAATTCGACAATGCCATTGCGTGCTGTCCAGCAGTAACTACGTTTCTCTTGGTGTTCATTTACGCTCTTGTGGATTTAAATATTTAATTTTTACCGTAATGTGTTTTGGTAAATACGATGTGCCGAAAAAATCAACTTCCGGGGCTACCGCCTATGACATCCTACCGCTAATGACATAAGCATTAGGGTAGTACGAGCAAATCCTGGTGCTTTACTTCAAAATTAAATGATACTTTAGTTTTTGACCTGTTATAAATGTTGCTTAAATCACAAGAAAATGAAAATAGTTTAAAAACTCGAAAAATATCCGACTAACGCATCTACTGCGCGTTTCACATCCACGTGCTGTCAACAATTTTTGTATATTTCTCCCGCAGGAGCAACAGTATTTTGTCGCTAGTAATATTAGTGGCGCCAACGTATCTTACTAAAAAAATAATTAAAGGTCGAAGTCAGTGAGGTTTGGTCTGTTGAATTGCTGCTTGAGCTCTTGGAGGCAAATGCCGAAGTGAGTATATAGCTCTAGCGGAAACTCACCAACATGTCTTTCTAAACACAATCCCGCTGCTCTTCCCTTTGTTGTCTTCTTGTACGTGTTTGTGTAAGCACCGCTCCTGTCATACCTCAACCATCCATCCCGTAACTTCTGGCTAGTGTCCGAACACATCCCTTTCTTGTTGGGACGTAATCTGATCTGGAGCAGAACGGGGCAGACCGTAATCCGATCCGCTAGCCGGACTGTAACCGAATCGTGCGATGACAGCGTACTTCAACACTCCCATCCGCCGTTACTGCACGAGGACCTTCTCCTTAATACCGCTTGCGCCACGCAGATACTTCTGTGTCTTGGTCCCGATCCCTTTGTTAGCATGTCGAAAACTTGACGCATGGTATCAACACAGTCCAGTTCGACCGTTCCCCGTTCTACGTTCTCGCGCGTGATGTGGTGTCTGATGTCCACGTGGTAAGTCCGCGCGGTATATCCAGCGCTCCGAGCCAACGTAATTGAACCCTGATTATTTCCTCAGACAAGTGTCGCACCTCTTGTTCGTGACCCATGTCCTCAAACATAGCTCGCGTCTACAGCACTACTTGCGTACACAGGCTCAGACCCATGTACTCCGGCTCAGACGAGCTAAGCGCGTCCGTCCTTTGGAACTTGAGCACCACCGATCCATCTCCGATCATCACCATGACCCCGGACACCGAGCGCCTATCGTTCGCCAGTCGGTGTTCAAGAACGCGAATCTCTCGATCTTATCTGGCCCACAGGCTTCAGTCCCGCGATATTTTGTTCTGGCAATTTCAGTCCCCCGGTTCCGTCCTGCCATTTCCTGTCCTGGTATGGCCCTGTCCAGGTATACAATACTGTGCTCACGCGGCGGCTTTTAGTACGTCAACACTCGGATAGCTGCCCGCCTATGCTGAATTCAAGAGTTCTCGAGGAACCGTGACAGCTACGTCACCTCGTACGCAATGTCCGGCCGAGTACATGCTGTAATGTAAGGTAGACAACCAATTACGGACCGGGGAGGTTTGGACCGCATTTCCGTCCGTACTTCTTCTATTTCAGGGACTGCAGCTTGGTCAACGTCAGATTTACTACACACGGATTGTATACAGCCTTTGCGTTCTGCTTGCCGAATTGCTATGCACATCATCGATGTATCGCGAATGCTTAATCATGAGCGTCCGTTTCGTTCTGTTGTGGTGGATCTCCATCCCGAGAATGTATCTGGCCGTGCCTAACTCCTTCATCTTTGAAACGGGGAATTTAGTGGCATCTTTCCTTCTTGAATCTCGTCATGGGTATTGGTAGCAATGATCATTTCTTCCACAAACAAGCACACGTTGGTGAGCTCGTTTCTGAAAACGCTTCACGTAGAAACACTGTTCTGCGCCCAGCATTTAAGCCCGTTCTTCGGCAAAACGTTGATGGATCATCCTATTTTACGAACTTGCGGCTTGCTTCAGATCGTAAATAGCCTTGTTCAATAAACAATCGGGGTCGCATGCTCCTTTAGCGCACAATGGGATTCTTCACGTAAACGCGGTCTTTCAGCACGCTGTTAAAACACCGCTGTGGCGCGTGCCATTGCTCCATTACGTATCCGACCGAAACCCATACGGGAGGTACCGTTTGAATCGAGTTCATGATTGCTACATGCGAGTAAGTTTCAAAGAGGTCTATCTCGA

>Contig_55

TCACCAAGTTTGCGCCAGAAGAGTTCGACCATGTTTGGGCAGCTGTGTACCCTCATTTGCAGGGGAAATGGAACGTGGGCCGCGGTAAAAAATGCCGCTACGCTGCGCGCGACATTTTCTTTATGACGCTGAGTTCGCTCAAACATCTTGGAAAATGGGACACCGTTGCTCGAGTTTTCAGAATCCCGCCATCCACCTTTCAGAAAATGATCCGCAAGTTTATGGATATACTGTCTTCTATTCTCTACGAAATGTACGTAGAGAATGCGAATGACCAGTGGACACTGGGGAAGATTGTACGATCAGGGCATGCGTTGAAGTACTTCCCGTATGCTCGATATGCTACGGACGTTACCTTTCAGCATGCTAACAAACCCAGTGGTAGCATGAGCGAGAGCTTACGCTATTACAGTGGCAAACACCACTTGTATGGCTACAAAGTGGAGGTGTCGGTGCTGCCGAACGGTGTCGCGATTNAATTGCACAATGCACACCGGTGGGAGTACGCATGACGCTGAGATTTTTCGCAGGAACGCAGCATTTCATTCGCGAGCTCTTCACAAACACTCTAGTGATGCCAATGTGCGAGACGAAGGGAGACTACAAGACAAGTATCCGAAAGAATGGGCGGTGTTAGTCGATAAGGGCTACCAGGGTATGGCGCGTGAGTACCGAGCAATTCACCCCATTAAAAGCGGGCGTCTGCAGCCGCTCTCGCTCGAGGACGCCAGCTTTAACGACGAGCTCGCGCATGACCGTGTCATTGTTGAGAATTTCTTCGGTAGATTGAAGTCTCTGTGGGGAATTTGTTCCGACAAGTGGAAGTTTGACGAGACCTCTTACGACCTATACTTTCGGGCTTGTGTCGCTCTTACAAATGTCAATATTCGATTGAGGCCCTCTTCGGAACGATGACGGAGATGACAACACCAAGTACGACACTCGTTTGCGCGAAATTGGAGCAAGTACACTCGAGAAGCAAACACGAAAGCGGAAGCGCTATCAAGCCAATCGACAGGCAAGGCTTCGTACGGCATACCGTCGGCGGACTTGTCTCTCGTGCTCCATTTATATGGGAGTTGAAGAAGACTATAGCGACGACGATACACAGTTGTGATATTGTCACCTATAAAAGCAAGATACGGAGTTGTATTTGTGAAGTTAGTACGAGTCAGCGTAATTACTGGAGCATTCGTTCCGATAGACTACCATATTCGCTAAGTGCAGATCAAAGCCAAATACTACCTTGAATCTAGCGATATACCAATCTAAAATACGAATTTGCTACGAATTTTAGAAATTGAACAATATTCTGTCATATTCCAAGTAAGTAATTTTGAGCCCTATTGACCGCTCGGGACCAACCTCCACATCTACATCGATATCCTGTAAATCGGCCCTTTTTGCCGTTTTGAAATGGTAATTCGGTAACAGATAAAACAAATCGATGCCTGCCTCTTCCCATCTCATTTTTACGCGTGGTTTTTTATGCTCCGACCATAGGTTTTAAACGGATGGACTGCAAAGCACAATACAGTAAGTAACCTACCGGTATCCTATCGTAGGCACAGTACAGTACTGCACCCCGTTTTCGCGGGGGTGGACCATATAGGATTTGCAAATAAATCAGATTTTTGCTTACAAAGTTACGTTCATTTGGATTCAGGATCATCACATCCAGACGTTTATCAAAGGTCCAAGGGCGCGAAAGCGGGGTGCAGCACTGTACCGGTACTTAAGAACATGAAGTGTTTACGTGGTCGGGGAAGGCTCTCGCAAACATGGCAGCTGGGCTGCCGTACAGTACCGGTAGCCTACAATTTGTAGAATCGTGCAATGTTTTCGGCTTCGCAGATTGCTTGGCAAGTAATCATTTTCAAGGATTTCCTCATACAGTTCGAAACAGAAGTTTGATCGGCTGTTTCATACCGGAGGAATCCTTGACAAACTTGTTAACTTTTAAACGCACTTGATTTGAATGTTTATCATTTTCTTTGAATCTACTTCGATATTGAGCACCTTTGCAGGATTCTTAGCCTCTGTAATATACAGTACATTTTTCTGCCGAATTCGTAAGGTGGAAACAATAAAGACTGCGACGTTGAAAAATTCGTTCAGGTCTCACGTAGATTCAAATGAATTGTGTTATCTAAACTCTTGTATTCTGATTGTGCGCGTTTACAAGTAGACGACACTTGTCTCGCATCTTTCTTTACAGTACAAATCGCTGCCGCAGGCACGCATTACTTCTTCAAGGTACATTGCTGGTCGGATTAGGCTGTACAGTACATGCTTAAAGTACATGATGTACTGTATCTATTTTAAACAGGCCTGGGATCGGGCTGAGCCTATAAGACTGAGCTTTTAGGACGATATAAGACTGAGTCACTGACTCAGTCCAAGCTGACCCAGCCTCTCACTGAGAGGCTGAGAGTCTCAGTCACTGAAAGGCTGAGTCAATGAACTTAGGCTTGGTCGACAACTTGAACTCCACCCTATTTTCAGCTGAGCAAATTATTAGATTTTAATGAAAGCCGAAGAAGATAGCGCTAGCTCCTTTCCTTGTAGGAGGTGCTGATGAAGTCCAGATGTCAGCTACCCAACAGTAGAACACTGCATCGAAGAGCCACTGCAATATTTGCTACAAACTTTTATGAGCTTAACACTTAACTAGCATTTTGACAGCTAAAAAGAAAATTGAAGATGTAAATTAGTACTAAGTCCAAGCAATGGCTATTGGTGCGGATCCTCTAGAGCACATTATGAAAGTTTACGGAGCTGCTCAAAAGAGTTTTAAATGGACTCGCGTAGTACCAAACATGAACGTGAAGTTAAGAAGGTCATAACTCTGTAAATTTAGGAGCGTATTCAAAGAACATAATATGTTCTTTGAATACGCTACTAAAAAAACAGTACGGTATATTATACTGTACTGTTTTTTTTTAAACTTAAAATACATGAGCCGGTACAGGCATGTACTTTGAATACTGTACGTGTTCAGAAAACGTTGCGGGACGAGTACAATGATGACGATGCTTCTGCAACACATGTATTCCCAGCAGACATGGCTTCACAGCAGTGTCACTGGCTTTGGCACTATAATACCAGGCTGGTAGAAGCCATAGTTATGGATATGGCTGGTTACCAGCCATATCCATAGCTATGGCTTCGACCCAGTTATCAATTTCCTCACCATACACCCATCGAGCGTTCTTGCACGTACTGACTACCGGTAGCTTTACGCTAGGAAGTTTGTCATGTCAACTGCGAGGACCCAGCCCTCCGTCACGAAAATACTGGCTACAGTAATTATCCTGGCATGCATCAACGTGGTCTCCACAGCTTCGACGTCCAAGCTGGTCAAAATAGGGAGTTTTGCGGCCAATTCTGACCTGAACGAGGTTTCTCATCGACGCTTCTTGCGAACGTCCGTTGCCGAAGAGAGAAAACTTGCTTTAAGCTTTCCATGGCTTGGACAAGCCGTAAGTGGTACACAGTCCTGGGCAGCGACGTTGCTTCAGACACTCCAACAAAAGTGGTCACAGATGAGAATGAAATCTCCGAATGATATGTTCAAGAAGCTGAAACTTGACAATACGGGCGACCAACTGTTCAGCAGCCCAAGATTTTCCAAGTGGCTCAGCTACGTCAGGACAAACAGTAAGACAAACCCCGACATGGCAATCTTTTCGACATTGGCATATCATTACAGCGACGAAGCTTTAGTTAAATTGCTTGATGCAGCCAAGAAGGTCGACAGCACAAAAGTTCTCGCTACTAAACTGGAGGGCCTTCAGTTCACAAATTGGGTCCACGCTAGGGAATCCCCTGAATACGTTTTCAAGGTTCTGGCACTCGATCGGATGGGGTCAAACACCTTTACCAGTCCTCAGTTTTCCAAATGGCTGTCATATATGAATAAAGCCGAGACGAGCGACCCGGAGATGGCCATTTACAGAGTATTGGGAACATACCATAGCGATGACGTCCTGGTAAAGATGTTCGCTGCTGCAAAACAAGCTGAAAGTACACGAGCCCTTGCCTCATCACTGGAAAAAGTTCAATTTGAAAACTGGGCTCGCGGTGGTGAATCTCCTAGCCACGTTTTCAAAGCTTTGGCGCTTGATCAGATGGATACACAAATTTTTGCAAGCCCGCAGTTTTCTAAGTGGACTTCATTTGTTTCTAAAGCAAACACGAAGAACCCGGATGTAGCCATGTACACAACACTGGGAACCTTCTACAGCGACGATATTCTGGCGAAGATGTTTGCAGCAGGTAAACAAGTCGACAGCACGAAAGGTCTTGCCACTAGACTAGAAGGAATCCAGCTGGCAAACTGGGAGAACGCTGGTAAATCAGCTGAGAGCGCCTTTAAAACGCTGAAGCTCGACACTTTGCCTGGGAGCCAGCTATTCGAGAGTCAGTTCATCAATACGTGGGCCTCTTTTGTGACCAGGACGCACAAGGATCCAGATGCAATTATGGTTGCGCTGTTAAAAGATCGATATGGTGATGAGACTCTCGCGAAGATGATTGCGGCGGCCACCAAGACTGAAAGGACGGAGAAGCTAGCTGTGGATTTACGCTCTGCACAGTTTAAGACGTGGTTCAGCCAAGGCAAAACCCCCGAGAATGTCAACACTCTGTTCAAGGTAGCAGCTAATTCTGACGACCTGACGAAGAAAATATCACGAGAATACGATATATTCTTTAGCAAAAGCAAGGTGGCTTTCAATAGACCGGCGAACAGACCTGCACGGAATGGGATTTATATAGCAGGATAATCTCTTTCAGAATAAACTCCAAAAGTTTCAAACTAAGTGTTTTAATTGAGAACTTAATTCTTTAACATGGGAAATCTACACGACGAGAAATCATGGTACCGAAATATGGGTACGTAGTAAATTAAGCCTTTTTTGGCATTGGATGGTCAAATATTTCGATAAGTTAATCAGTGAAAAAAACTGAAACCAAGAAAACATTTGACTAGCTACCCCGACAACGACTGAGGCCTATAATAAGGTACCAGACCCCCCCACGTTGCATAGATTTTCATACTGTTATCCGCTTAAGAAAATCTTAGCGAAACGAGCGACTTTAAGCGAGCTTAAATTTGTCTGACCTCCGAAAACTGCTTCTTTTCGCCTGGTTGGGAAAGTTTTCTTATGCTATTAAGAAGTGGCCTGCGCAAGTACAACATTCTCTTACTCGTTTTAATTAAAAATTGAGGGCATTTCTTACTTGCGCAGGCCTGTTAGTACTACTGCTACAAGCATTAATAATAATATGTACTAGTGCTCTGAAGCTAACGTTTTGGGTCGTACAACTAGTTTGACCTGATATTAGTTAAGATCGAAATTATTTATCCATCTCACTGTGGGTGTACAAACCAATACGACAAAAGTTTTTAGACTTTCTTTTTTAGCCTCACAGCTGTATCAAATCGACGGCGCTGAAAGTTCTTAGATACAGTGCTGGCACCAGCTACTGTATAGGAACTATACCATTTATTAGATTTTGGCCACCACAATTCTTCAAATGTGATCAAAATTGGCCAATCGACGCGTCGGCGAAAAATACCGCCCTGGCGTGGTGGCATGTCAGCCGGATTACGGCCGTTGATCAAGCTATGGTCACGACAGCGTGCTGGCATGAACCAATGGCGAGAGTGAAAGCTGTAAGGCACGCGTCATAGTGCCTTTCGATGAAGAGGAGGCTTACGCTACTGCGCTGCATTGGACTTGGAGCACGCTGCATGAATGACGAGTGGACACGGGCACGTGTCCTCGACTGCATAATCGCTGAGCCTGGATGAAGACACAAGGAGAGTTAGTTCAGGTCAGCGAGTTTGTTTGTTTGCTTTTCTTTCGTTAATAAACTTTCCTCATCGGAAGAAAACCCCGGGTCTCGCGACAAAGTAGCTTTGCGCTCTATGAAGTGTGAGTTTGTTGATCAACCTTTCTGGACGCAGTCTACCGTCCAGATCGTCATCTCCTCCACGCGCTACTGGATATTGCGCGCCCAAGTGCGATCGGCTTCGACTGACTTAAGCTCGACTTAAGTTCGGTTGATGTGTTAAGTTTGAACAACTTCAATATGATGGGCTTCCCTGTTGACGCTGACGGAGACGTAGAGATGTCAATCCCTCAACCGATCTTTGAGGTCATTAAGGCTCCAGAGCTGACCAGCTGGGAGCACGCTGCTCTGATTGAGTGCCATCGTGAGTGGGAGCGTTACGTGGAAAAGATTCGCCACCGATGCTCCACCACGGGTGAGACGTACGACAATGTTGTTGCAACTGTCAGGGGCTCGGTGAGACGGCAGACGCTTAATAACCTGGCTAAGTACGTGCTCAAGAAGCCTATTGCGTTGGTAACCGACGCGGACATCATGAGTGTTGTCGAAGCACGCTGTCGTAAGCTCAAAAACGAGTTCGTTCCTGACGTCTCGTCGCTGTTCCGTGCGAGCCTAAGGATGAACATGACAATTGATGACTGCGATGCACTCATCTTCTGCTACTATAAAGATTTTAACGGGATTGTGGAGGACAATGGGCTACAAGGGCTTATTGGGAACGAGAATGAAGCGGACGCAGGCTACAAGAGTAGAATGAAGGCCTGTTGCCGACTAGTGGTTGAAAACTTGCAGCCCCCTGTCCTCAAGGCTCAGATCGGTCGCCGTATCGATTTAGAGAGGCGTGACTGCAAATCTGATGATGTCGCCCTTTTCGACCTGATTCTGGAGCATGCAAAGGTACAACAGCGGTTTCATCGAATTTCACAGGATTATGCGGGAAAGCAGGATTCTAAAACTATCAAGCCAGAAAAGAAACCACAGCGTGGTGCTCCGACCAAGCCCACGTCAGCACTCTCGCCGGCGCCCACGACGACCACTACTGCGACGGGACCTCGCCCTACGCGCTCTCCTCCTCGTGATGGCTGTCTGTTCTGCAAGGGAGAACACTGGCTTAATGACTGCCCCACAGGCACGGACGCACAGCGCGAAGAGGCCGTGAAGAAGTTTCGCGCGGCTAAAGAGCGGCGTTCGGGGCCGGTGCGGTCAAAGGCCGCCAGCTATGCGACGCCTGCCGGCTCTGTACGAATCAACGAGCTCCTAGATGTTCCCTATACACCCGATACCGGCGCTGACAAGAGCGTGGTTCCGGAGAAAATAATGGCTTTACTCCTGGCAGTGCAATCTACGCTAGAGACTACCTCACTAAGTACGCCTATTGAAACTGTTATGGCTGACGGCAGGGTCCAATTGTTCAAACAAGAGGTGAAATTGGATCTCGAGCTAACTACAGTGGCCAGGTTGGTCTCACTGCGCTCAGTACCGTGCTGGGTTCTATCTGGTGAAGGTGATGAGTTTCTATTAGGCCGGAATGTCCTGAAGGGGCTCGGCATCGACGTGGAGCAACAGCTCGCTCAACTGGCTGGATCCCCACTGCCGGAGGCACAACCTGACGAATTTCCTGTAGGTGCTGAGTTTTCGGGCCTAAATAGACCAATCGACTCGCCGGAATCTCTTCTCGACCGAGCGGTGGCCAATGGGCTGCCGAGTGAACATGGCGGTACTGTTAGTGACTTGCTGGATGAGTTTTCAGACGTCTGGCGTGATGCAGTTGGTCCTGATCCCCCTGCCAATGTGGAGCCTCTACGCGTGTCGCTGAAGGTGGACGCAACGCCCTACAGGAGCCCTCCACGCAAGTATGCGCCTTTGCAAGCCCAGTTCATCCGTGATTATGTGCAATATTTGGTTGATAATGGGCTGGTAGAGCAGAATAACCCATCTCGCTGGGCAAGTGCTGTAGTGCCCGTGCGCAAGCGGGGGACCAAGGACGAGTTCCGGGTGACCATTGACTATAGGGTCGTCAACAGTATGACAGTCCCCATTACTGGTACGATGCCTAGTGTCGCCACTACGACCGACACCTTCAACGGTAAGAAGTTCTTCGGACGTTTTGACTTCACTAAGGGATTCTGGCAATTACCTCCACATGAAGAAAGTCGAGAGATATTTTCTTTTATTACGCCCGATGGTGTATTTACACCTGATCGAGTCCACCAAGGAGCGATAGACTCCGCCTTGCATTTCCAGAGCCAAGTCCAGACCGAACTAGCCCCGCTAATACCACATTCGGCCCTTGTGTGGGCCGACGACGTAATTTTATTCGCACCAACAATACATGACTTCCTACAGACGCTTCGAAAGTTCTTCGAGATCGTCAGTGCA

>Contig_57

AGCATCGCCGCGGATCGGACCTAAGACTCGAGCGAAGGCTCGGGGGCCCACAAAGCTCTCTAAGACGACTGAAATTCAGACGAAAACGTCTGTGGCCCAAAACCGATCTGCTGAATTCCAGACTGCCTCAAATCGACTTGGAGGAGTGGATCTAACCCCGTTCCGCAACGAAACGGGCTCCAAGACCACCATGAAAGGAGACAAGGAGATCATGGAGGCAAGTTTCGCGAAATCAAAGCGGATCCTAGCCGCTAAAGCGATTACGGCGCTTAAATCCAAGCTGGACTAACTAGAAACAGCCTCGAAAAAGAGCGGAGGGAGTATGCTAGAAACGATTTTGCTACTTCGCGAAGAGACCGAGCGGAAGGCGGAAATCCGCCGTGCGGAAGAGGATCAACGACGTCGTGACGATGCTGCAACCCAGGAGGCACGTCGCCTCGCGGACAAGACGGAAGCTGAAGAGCACCGTCGTCAAGACAAGATCGAGATGGACGAACGAGCTCGCCGTGACAAGGAAGAAGCCAGAGCTCGCACGCAGGAGCTCATCTTGCTCACCACGAGCATCAACAAGAAGCCCTAAGGGCCATCGGAAACAGAAGAAGGCGCCAAAAGACCGGCACCTTCGAAGTGACGTCGTTGTTACCTGTAGGATCTCTACAACGCATTAAAACATAATTTTGGATGTCAAATTACTAGCATGGTCACGTATCGAAACAAAATAAAATCCAAAAACCTGAGAAGGTTTACTAAGAAGCAGATTTCAGTCTAGATACCAATCATGCTCAAAGTAAAGTAAACAAATCGGCCTAACAAAGCTACCCCTTCATCCATAACACCCGAGAAAGTCGTACGTATTTGCGAGATCCCAACAGTCCGTACCCGGTAGTTGGTAAGGCGAAACAAATTGTCCAGCCTCATACCGCGAAGCTGTGGATCGTATGGGAGCGGCAAACTAAGCCGGGGGTATACCTTCTGCATACTCCCCATGCCCCATTCGGCTGCTTGACGCACCGAAGTTATAGCATTATGAAGGGTGCGTGCTTGACTGCGCAGGGCAGGGTGAATCCGCTCTAAGTCGCCGTCCTTCAGCGGCGTTAAGATGCGTCCGACCATCGCTGTAGAGCACGGAAAGGCAGAATCGAACACGACATTATTTCTCGCATCTGAGCACAACGCCGGGTCCAGGAGCTTCGACCAAAATTCCATGGAGGTGTCCGAGTCGTTCCAGGACCCGGGACAGTTGTTCTTGCACCAGATTACACAGCCATCAGCCGCGAAGCAAATGACCCCCGTAACCAATACTGTATGCAGCCACCCGCTGTACATCGCGTTCTGTAGATCAGCGTTGGATGGCTGCTGCATCTACTTTGATAGCATTGGTAAAATATTCGAATTAATACACTTTCTAAATGCAAACATGTACGCACTCGTAGGTTTTTACCGTCGATAAACCCGAACGTGTGATGAAGAAGGGTTTAACGGGCTTCTACCAGCCTCGACAGCTTGACTTGATGGCCTGGTGACGGCCAGGATATGCGCGCAGGAGCGAAATCATGTAGCGCCTTGCTCAACGACTCCTCCACTTTCCGTAGTGTCCTCGATAGCGTGCTGGGAGGTGCTCCAAAGAGCATACAGAGCGTCGTTCGCTCCATCGAGCTAGTGTAGAGCACTAATACTAAGCCCAACACCTGTTGGAGGTTACGGAACTTTGGCGGGCGTCCACGTACTCTTGGCCCCGGTAAACTGTAGAATTGTGAAAAACGCTGTAAGAGCTGAGAGAAAGCAGACCTGTTGGTACATGGCCTGTCAGCATCCAAGTCAAGAACAGCATCCAGACGAGCAATAATAGGCGACCTCGTTAAACTGGTGGCGTTAAGAAAGTTTAGATCACTCCCGTTCTCGTATAGAGCCATCCAGGACGCGTTGCTCGGGGAATCCAAACAATACCCGGTCAAATAGTGGCGGCTGCGCATGGCGGCTCGCCAGGCCTCCTCGATTAGGAGGCCGAAGACCTCTCGGCGCACTTCGTGCAGCTCCTGCCTCTCCCGGTCGATGGCCTCGGCCACAACAAGCATTGCTTCCGTGTCTTCGCTCTCCATGCTTTCCCGGACGTGCGTAAATTGCGAAGTGACGTACAAGAAGAGCTTCCCCTGCCTGTGTCCCATTCGAAGTGTCGGGTACGCTTCCATTTAATGAGTTCGGTCTGTGTAAGATCTAGGTAGAGACATATAAACAGGACAGAGGTCTTTGAGATATGCCTCATTTATGGTATTTGTGGTGGACGTTTTGATACTTAAGCTCAGCATACATGTAAAGTATGACAGGCTTATCTGCTGCAAATATGACACGTGCTTATGCTATTTATGGAAGGCACCCACTACTGGCACAATACCTGATACGAAACTTTAAGGACAAGAAGAACTAGTAATTAATTTGATGTCAGTAAACAGCAACCAGCAGCGTTTATATAATTCTTCCTGCATTGAGAGCACATGCGATTTGTATGTGTTCTCGATGCAGTCGAAGTCGGCRCTGAAATATTATGTACCAAAACTGTTACTAGTCTAGTTAAGATTTAGTCGGTCACAGGTCCATCAACAAGTTGGTTGCCGAAAATACAAAACGACAGTAATCCATCTAAACGCTATCATGGTATCTGAAAATAATCAATATTATACGTGTTTTGGAGAATGCGATGCTCCATTGCATTTTCACAAGTATTCCTGACATAGAGTCGAAGTTAGTGGAGGTCTACACGTGTATACATGTAATTGAGGCTGCACGTAGGTGAAGAAGCGTTGAATGACCTGGTGTTTCTCAGTTTAGCAAATGATTACTTATCCTGGAGTCCAGGAGAAACTTTCTTACTTAAATCCATATAGACGGTTTCAGTATGATAGGCCTGTGTGGCTCCACATTAGACCTGAAAACGCACAGCAAAGTACATCTGCATGTTATGCTTCTGTAAGCCGGCATGCTTCCTCACCTGGCGCATTATACAGACTTTCTAACTCAACCAACGACTAAAGACGACCTGGGTAAGACGATGTGCTTATGCCTGATAGAGAAGTCGTCCACGGGTTAAATCAGGTGTGAAAGGTCTTACTCAATTATGCATTTCGGTGTCTCGCAAGCCGTTCTCAGCAGTGTTTTTGCATATTATGTCCTGTTATTGGAGGAACCAATGTATTTCTGAAAAACGGTTTGTACTTTCCACCGTCGAGACTACGTGGGGACTCTTACGCCGACTGCTTTCAAGTAGTCAGTGTACATTTCCAATGTCTTAGCCGTCAGGGCATCGTCGACATCTGGCATTTCTTTCAGTATCTTGGCCAGTTCGTCGGGACTCTTGTTCGCATCGTCAGCCTGCTTGAACAGATTATTCAGCTTGTCATCTATAATAGTCGAAATCCTCTTGAGCTCCCCAAAGTCGTCAATTTTCTCGAGATCCGAAAACGAGCTCTTCTTCATCAGCTTGTTAACAAAGTTGTTAGTCATCGCTTGCTTAACAAGCTCCAGAAAGTTTATCTCTTCGTTGTCATCTTCGTCGATGCTGCCGCCTCTGAGAAACCGGGACTGGTCGCTTACACTTTGGTCAGCGCCAACGAAGTGAATGAAGCCCAGTGACAGTACACCGGTTAGCGCAACATCGTTAGCGAAGTAGGGGGTAGAGGTGACGTGGCGGGCGAATAGGGTCGACGCAGCGGCCAGCAAGACGTAAGTTAGGCGCATTGCTGGTGTCTGGATGCTTTAGGTGTCGAAAGCGAGTCAAATTGCGAAGTGAGAATTGGTATCCAAACCACACAAGAAACGCGTGGCCAGAATTCCTATAACAAAGTGGTTTGGGATGTGTATTATCTGTCGATGGTGAGAAATAGAGCAATTCGGAAAGAGGTATGTTGTTTATAAAGAATGTTGTATGGTGGTCGTATTGTACAGAACGCGAACAATTATACATCCGGGTAATTAGGATTCCCTCTCTTCAGTCAAAAGGGACCGTTTTGAAAATACCGTCTGCGGGCATGCCTGCAAATTAAGCTAGAATTACGAAATCCAAATGGTTATACTTAATTCTTGTAAGGGCATCAAGATTAAAGGTAATCTCTTATAACAACATATTAATACATCCGCTTTGATAGGGCATAATTTCCTTCATTTAAGAAGGAGTACCTTTGAGCGCTTCAAAAGGTATTTGTCTCTTTTCTGAACGCAGTTCCCTGCGGGCATGCCTGCTGATCCAAAACGTTTTCTGCGACCCGTAGTAATCACAAATAGTTCAGGAAAGATTACCAGGATATTAGAAATATGTAGTTTTAACCGTACCGGGCGACGTCCAACCACCTCATTTCTCATTCCACAATTTATCCACCAGTCCACAAGAAAAATTCCGGCCATGCGTCTTTTCAACTTCACTGTTGCGGCTCTCGCGGCTGTCCTCATTGCAAGTGGCGCAGCAGTGTCAAAGACTGATCAGACCTCACGTGTTCTCGCCCGGAATGACAAGCGGTTTCTCCGAATCTACCAAGCGATGGATGGCAAAAACAAAATTACGTAATACGACAATGAAGAGAGAAATATTAACTTATTCAGCGCATTGAAACTGTCTGATATGCAGCACGATGGAATTTTTCGATTCCTCATGTTTGGAAAATGGAAAAGCCAAGTATACTCCGCGGATGACATCGCAGAGCACATTCCGGCGAGCCTCCTCGAGAAATACCAAGCGTACAGGAGTATCCATGGCTAAGAGGCGGTTTCAAGCTACCAGAGAATAGAGCTCGGCCACAACGACACTCGAATGAGGTCGCGATACTGGCACCCGCCTTGATGCTATCGAAAATATGTCTCGGGCGCGGTATTGCAGAGGAAAGACTTTACTCAAATAAAACTCGTGTATGGTTCGTTAGACTTTCTCAAACGACCACTGCATTTCTTGTCCGACTCCAACGGAGGCCCACCCACTCTTAAAATCGGTAATTTGTACGTAGTAATACGTTAGCCTGGACGCTCGGACGCTCATCTGGAGAGTGTGGCCGTTTGACAGAGCTCCAATTAGTAGAGTGTTTTTGCGCTAGGTTTGAGAGAAACTAGTAGACCAGAGGTTCCAAGAAAATAATACTACTAGAAATATTTTATAATATTTCCGTGAAACAACATATTACGAATCGGTGAAACTTAAATTTTATAGGCAATATTTCCAAATTTCGTAGAAATATTGGGAAGACTGTACAGTTCACGACTCACATTAATAGTACTAGCTGCCCTCATACGTACCAGATACCTTCTACTGACATTCATTCCTCCTACGTCAAACTGCGTGGAGAGAATATTCTCTCAGGCTTTCTCATCATCCCACTCGGATTAAATTAAGATGCTGCTCTTTTTTAAGACAACAGGCGCTTTTGGTCGGCGAAAACGGTAAGTAAAGTTGTGATTCGTCGATCCCCGTAGTGCTTTTTGCCGAATATAATATTGATATTTTATTGTGGAAGTTTTTTTTTATAATTGACTGCTCGACGACCTTGAATCCCAAACAATTATCTGTTTGCGTTGATCTCTTAATAACATGATAGTTGAACAAACTGGACCCCTGCTTATGTAGCCCTAACGCATGCGTACACATCCAAGGCTCTATTTAAGAGCTTGAAAGTATCAAGGGAGACTGGGTGATGGATTCATCTTCTGGGCTCCCGATTATATCATGGCGCTATTAATAATTTCTTACGAAATCATTCATGTATTTATTTTGACTTCATTTATGAAATTAATAGCTTTTTTGCTAATAGCGATCTCTTATAATTTAAGAAAATCATTATGTACGAGCCACATTGCATCTTAACTTACATACATCGAGAGTTCCCAAACCAACTACGCGCACAAGCCGGTGAAACGATCACTTTTCATTTTGGACAAACTACAAACGATAAGCACGAAAGCAGTTCAGACTGTCGATGAAAATGGTGTTACTTACCTCTGATTGTGCTCTTATTTATCACTTGATCACTGTAGATCCCATAATTTACGGTTTGTCGCAAGCTGCCTCATGCGAGGGACAAACGTGCGAGGATTTCTGATATAACAATGACATAATCAGGCAGACTCTCTTCCGGTTAGCTCTCAGTCTTACATTTCCCACCTTCTACTCAAGTGGAAGCCAAGTCAATAAAGAAACTTATCATCACTTTAAGTGCTCTTGTCTACCGTTCCGGCCACTGAACGAGACCGTACTCTCGAGCGATGAAGATCGACGTTGGAAACACCACCATAATTTTCCATGTACTACTTTGCGCTGTGGGGGGACGATGCAATGCCATAATCGGGCCCGCTGCAGGTTATAACGTCGAGGTGGCATTGACCAGGATCCAGGGGCAGCATCATAAGCAAAGTCAACGTGTAAGCGTCTCGCTATTGCAGTGACAACGGAGATCAGGATAATTTTGTAACAAGTCTCATGACGCCTGATTGCCGGCGGGTCGTAGCGCTTGACAGTGCTGCGGTGCTCTGTCACGGGCAGAGTTCGACTAGATACAATACACAACACCTGTAGGCCACCGCTAAGTAGCCGGAACGGACTCGTTCCTATTTATTGACTCGTTCTTACATACTTATTTTCGTCCAAATCATCCTTCGTCTCATAGTCCTTCTTCTAGTACTCGTCCTTTCGGTTGTTGTCATCGAGCTCGTACCCGTCCAACTTGCTCTTCGTACCGTACATCGTACTCGTCCAGCTTGCGCTAACCACCAAACTCGTGTTGACTCAACTCATAACTCGTCACTTTCACTACAAAGACTTCTCGTTCTTGTTCGCTCCGCTATAGGGCAGCGACGTCTCATCACGGGTACATGTCGATTCACCACGCGTCCGTCATTCCCCAACAGCTCATCTCATTTGGTGCGTCTTACTGCTCAGTCCCTCTCAAAGGCTGTGCCTCACTGCACAGACTTCTCAACATCATATGCTTCGTCACTCACGACAAAGACTTCTCGGCCGTCATAACTAAGTCACGCAGCTCCCCATAATTCAGCTAGTACTCCACCGAATCCGTACGAGGTCTTCTCTCAGTAGAGCTGTCGACGTCAGTTATATTTGAAGATTTACACCTCGACGTCCATAAATGCAAACGACGTAAAACAATACACCCCTTCGACACCCACTTATGGATTAAGGTGCTGCGGCTCACCAAATATGGCGTTGTTGTTCATCCTTTATGACCGTTTGAAACTTGAAATTCTTCTTTTGGTACAAGCCCGCAGAGGAACGCCCGATGCGCACGTCGCACAACAGTAAATAGGGATTGGCAGAATCTGTAGTGCCCAGACATCAATTATTGCTCCGCACTGGTTCCGTGCATACGAGTGCTTTCTGTCATTAGGTCGATGTTCGGATGACCGCGCAGTGCTGGCAGTGGAAAGATACGACGTCTTTACAGCGGACATGTTTAAAAGTGTCTAAGTGTCCATTGGATTTTGAAGGAACAAACAGACGTCTGTTCAAAAGGTACAGTGTACTT

>Contig_60

CATTGACGCATTCTTCCGTGCGAAGCACGAGGCAGGGATGTTTGCATGAGAGTAAGTCTCCTCACTCGACACCCACGTTCTGTATCAGAAAGGCGAACGGCAAGTGGTGCACTGTTCATGCTTTTAGCAAGCTAAATGCAGCCTATATCACGGCGCAAACGCCGATTCCTCGCGAGGATGTTCTACAGAACGACATGGTAGGCTGTACGTTGTACAACGCTTTCGATTTGGTCGATGGTTACTACCAATTGCTCATGAGAGCGAGCAATATTCCGCTAACAGCGGTGAGTACCTCGAGCGGTATGCTCTGGGCATCTCTGATCATGCCCCAGGGGCTGTCGAACGTACCAGCAACGTTCAATCGTCTTGTGACGAAGTTGTTTAGGCCCCACCGGGCGTACGCTCAAACGTACTTTGACGATATATTCGTCCATAGTCGTGTTGAGCACGAAAAGTCAGACGTGGAGGGCCACATTGACCACTTGCGAGTGGTGCTCGAGTGCATGTGCACTAATAAGTTGTGTGCCAACTTGGACAAGGGCGGCTTTGACTCGGAGGAAATAACATTTCTCGGGTGCTTCATTGGGAAGCGAGGCTTACGGGCAGATCCTGCCAAAGTCAAGGCCATTGGGGACTGGTCGATTCCCAAAAACCAAAAAAGGATTTCGCAAGTGGCTAGGCTTAGCCAATTACTTGCACAGGTACAGCGCGGACTAYGATGAGACGGCTCGGCCATTATCAGATCTCCTTACCAAGGACGCGTCATGGTGCTGGATCGTCGAGCACGACGGAGCCTTTGAGGCTGTGAAGGAGAGCCTTCTCCAGGCTCCGATCCTTGCGCTACCGGACCCGGATAGTCCGTTTAGCGTCGTCTGTGACGCCTCCGAATTTGCAATCGGTTGTGCTTTACTACTAGCAGACGCCGAAGGGCGTGAACGCGTGATCGCGTTTGAGTCTCGCCAGCTCAAAGCTGCAGAGAAGAACTATCCAGTTCATGACAAAGAGCTATTTGCAATGAAGTATGCTCTAGTCAAATTCAGGGTACACCTGCTAGGCTCTAAGCCTTTTGTGATCTTTATTGATAGAAAGCCACGATGGCTGCTGTACGCTACCACTCACGCGTAACCGAACACTCGAGGCAACTTGCAGTCTCAAGATCGATACCAGCAGATCATCGTCCAAACGTCGCTGAGTCACGTGCTACCGTGCCGATTGAAGGCTAGGTCATTGGAGTCTGTCCACATAGTACCCCAGACTATAATTTTCTGTGAGTACTACCAGACAGATTTGTGTCTAAACGCAAAGCGTAGCTGCTTTCGCGATTTCTATCGGAAAGAAGTGTGAGCGTTGCAAATCACAGTGATTAATACTTCGGTTTATTATGCCAAAACGTGTGTGATAAATAACACATTTTTAGTGGAATAACAACAACAGCTTCGCTTAGTGGGCTCATGCACCCTTATCGTTACCGCAACGGTCATCTCAAAAAAAACAGCCTAGTTACTAAAGCGTAGTAAGGCACCGGAGGCAACTTGTCAGCTAACCGTCATGTCACGGCACAGATTCGGCATTACAGGATCAATCTTCAGTGTAGCTTAGGCAGCATTAAGGGCAGTGGTTGCTGAGAATCGGCTCTGGTTGTGCTTGTTATTGCTGAAGTTTAAGAGAGAGAGAGAGAGAGACGAGCGTTGCTGTCTCCCACGAGAGAGACTAGGCCCAGGAATGAAGAAGAATGCGGTGCTGCTGCGTGAAGGTGGCTCTTATCCTTACGCAAGTCTTTTCATAGTCTCTTACACGATGTTATAAGAACGTGTCRCTTAGAAGAAGAGCAAGATGCACTAAGAAGTAATTGGAAATTTGTATGTGTGTGYGTGACTTGCCTGTGCGCTCTACTCGAGCTTTGTTTTGAACCTGAGTTAGAATAAAGGTGAGGGTGGATCAGCCATGGCCGCCTCGATCAGTGACCTACTGCGTACGCTAAAATAATACGAACTGCGAGGCATTTTAAACATTGCCGAGACAGCTTCGTTGTTCTTCTTGCGAGCCTCGCCAGAAAGCACGTGCAACACAGACGGTTGCATCCAATATTAATTGGGATGTCAAGAAAGTTGGAGGTTGAAAAAGAATACTGCAGAACAGACTACTTCGGACGTGCTGCACAATACTGACTTTTCATAATTAAGAACCCCAGAGCAGTCATTTGTGGTAGTTTTGTATTTCATATTACGAGGCTTGTAAACTGCTTGACTTCGGAGCCCGAAATTTTATTCCTCTCGGAGGGTATTCCAAGAGTATGATTTTTTGCCGAAGTCCATAAAAGTAATGATTTCTCATATTTAGGAGCGTCCAGTCGGTCTACAGAGAGCGCCTCAAAGGGAAGAAATTGACAAGTAAAGCTGCTTCTTAAGAAGCGCGACAGCACGGCAATGGCAAAACTACCTCGACTTTGCCGGATTTCAAGCTACGTCGATCATGAATTCACCGAAAGGATTGCCAGTATTTTTTGAAATGTATGCATACAAAGCTTAATGAAAGTCGGTTTCGATCATAAAAAAGTACCGGGCATTCTTCCAGTGGTCAATTTTCTGAGGTAGTGCTTCATCCTCGGGCTAACACGTTTTGAGGACAGCACTATAGAGATTATTTATCGAGTGTGAGGACCACTCTACTTGTAAATAGTCAAGTGATACAACACAACGGTGTCGCGTCCTTGATGCGAAGCATGCTACCTACACTGCTTCAAATACATCGTCGATCGAAAGTGCAAACGTCCTATATAATCTAACTTTTCATGTGCTCTAGCAAACATTTGTTTTAGATCCAAAATGAATCTGTCGCACTTTTTGATCATCATATAAAATGATAGAGCATCATTTATGGAAGGGACCTGTAGTGCTTCAGTATGACGTTGTCCAAAGTTGCGGTGTTAGTGCGATCAGAACTATTGTGACCAGCGAGCTCGCAAATATTTTTTCAGTCTGTCCTGAATGGGTCATTCAAGTACTGTGATAAATGATTTGGTTACAACCAAGCAAAATCCCAGAATTAGGCAACCCAAGCACGTCTTACGACGAGTTAAAATTATTATATCCAGTGGTACGATAGTTGGGAGCACACATTATGTGGTAAAAAGTTTGATTTATGTATGTTCAGCATGGTGCTGATGCCACTTCGTCTGATTCCTTAGCTATGGTGTGGAAAAGCACACGATGTTTAACCTGACCGTACATCAATCTCTTCTTGGAGACCATAAGTTCAACAGCTCGCCAGCGCCGTGTGCGTTATACCGAAGCGCCACCCAAGTCCTATACCCGTCATCGGCCTTTAATATTTCATCCAGGATTGCCTTAAGCAATGATCTGAGGTCTGCATTCTCCACCATGTATTTAATCTTAGCCATTAGTTCACCAAGACCTCGCTCCTCCGCTCCGGTTTCTCCATCCTTTCCGTGTACTCGTAAATATCTTTTCGGGGTCCCAGCGTCGCTGCGCACTTCGTCAAGCGTGTGGACGAGCTCTGGCGATCCCATTGCTGACAGCGTGATTGGATTGAAGTCTCCAGTCGCGTTACAGATCGCAAAGTAGAGGGTAGCCATCGCAACAGACAGCATGAATGAGATACGCATGCTGCGAGAGAATATTTGTGGAACTAAAGCGCTTTAACCTTGGCGAACGAATAATGTCATCACTTTTCACCCAGACCGGTGGTGGTGCGCGTCTAGATTTCGGTGCGGTATTCACTACGATCCGCGATACGTACCGACAGCAGCCCACATATTTGTATTACACTTGGAAAACAGTAATCGCAGGACCTGTTTATCGAGGACTGCACCACCGATAATTTTTGATGTGGCAAAGCACGATTCCTGGGCTTTCCGCCCGAAGCTACTTTCTCTACGCTCCAATGCTATACTGTACCACAGACTGTACAGCAATGTACGTAGTCCGATGATGTACACGTTGCCCTTATTTGACTACTACAATTCCAGACGTGCCGAGTGCCGACAGCAAAATGTGCACGAGCCCTCCGCAGCAACAAGAAATAAGGCGTACGTCTGAGATCAATTGGCAATTTGTTTATGCACGTGAGCGCCTTCTGTGGAACGTTGTGTGATCATTTCGGGTGGTGGTGGTGATAGTAGGGGGGGGGGGGTACTGGATCTGGACTGCATATACCTACTGTACATGTATTGGCATCGCTATTTATTACTTTGAAAGTTCGCACGTTGTCAATGATGTGTCCTAATTGGTGACATAGCGTACAAGTTATTGTACAGAATGTCTTACCATACAAAATATGTTCTGGACCCAATCCTGCATAAGTCGATACATCACCTTTTACAGGAAAGATAAATCAATGGTGCCAGGAAACTCTCTGAAGACAGACTCATAAAATTCAATCACATCTCTTTTTTCTTCCTTCTACTCAAGCTCCTCCACCTTCCTCGCCTCACCTTCAATAGGTTATGAGCTCAACGCAAGCCTCCACCGCTGACGACCGCGAGTTTCCGCGCCTCAATGGCCGGAACTTCATCATCTGGAAGACCCGCGTCACTGCCGCCCTCGAAGGAAAGAACCTTCTCGGCTACGTCGAGCGTATCGACTACACCGGTGACTCCGACTACGAGTTCGACTCCGATGAAGAGCTCAACCCGGCACTGTCCGACATGAACGACATGTCCGCCGCGCTTGATGCTGCCGGTGCTCCCAAGGCCGACGAAATAGTGGGCTCCTCACCGAGCGAGTCCTCCTCTGATGCCAGCTCCGCAAGTTCGGATGCACCCACAGCCGGAGACGACGGCGATGTGGAAATGGGACAGGAGAATCCCCCCGTTATCCAGTCATTCACTGCTCAGAAGCGGGATGAACTGAAGCGAGCAGAGAAGCTGAGGGCTAAAAGCCAGAGGCTGAGCTCGAAAAAGCTTCGACTCAACGAGGCAAAAGCAAAAGCTTTCCTTATCAAGACCATCGATGACCAACATGTTCTCATGGTCAAGGACAAGACTACTGCGTTTGAGATCTTCCAGACGATCTGTAGCAAGTACGAAGGTGCTGCCATCCACGGCGACCCGTATCATGTGCTATCATACCTGATGGCACTGAGGTATGAAGAAGGTTCAGACCTTACGGGGTTCATAATTGACATCGAATCTGCCATGAAGGTTGCCTCCGAAGCCACAAACAGTGTGCTCAGTGACGAACAGAAGTCACTCTACTTGTACCATGCGCTACCTGGAAAATGGAAGCAGCAACTATCGGTCTGGAAGGGTAGCAGGAAATTCATTCCCTACGAAGAGCTAAAGCGTCACATCGAGGCCAAGGTGCAGCATGAACTGGCGAAAAACCGCTACACGCTCAAACAAGGAACACCCGAATCGAGGGAAACCCGAAACGAGCAGGCCCTACAGGCTTTAGCACCTGAACCAACCTTGGTTCAAGCTCCCTATCAAGAGAGCAAGGCTCTCATCTCAACGATGAGCTGTACATACTGCCTGCGCACCAACCACGATACTGTCGACTGCTATGTTCTACAGCGCCACCTACAAAATGGTCAGGTAAAGGCAGGAACGGTGCTTCCCGCCAACTTTAAGCTGAAACCTTCGTCCAATCAAGCTCGACAGCATCCTTACAAGGACGGTTTCGGCGGCAAGTCACGAAGCCAAAATCGGAATGACTACAAGAGCAAGCGGAACAGCAATCGAAACAGAAGCCAGAACGGCAACCAGAAGAATACATCTGAAAAATCCAAGAGTCACAATAAATTCCACGGTCGCCGCAAGGACACCGACTCCGACAACGAGTATGGAATCATAGCCATCACCACTCTGGACCTCAGACAAGAAACAGAGGCTGTAAGTCTCACCGCCCAAGATGCGGAGCATGACCCAGTGTGGACTGTCGACTCAGGCTGCACTCGCCACGTTACGTCCAATCCGAAGTGGTTTGAGAAGCTGATCCCAAGTGCTGGCAAGTCAATCACTGTCGGCGGCAATCACCAGATTCCGATAAAAGGAACTGGTGATGTCAAGATGACAATCAAGGACACGAAGGGAAAGGAACGAGAAATATCCTTGGCCAACGTGCTATATGCGCCCCAGCTGAAGTTCAACCTACTCTCCGTGCGCCAAGCCGTCGAAGATGATTTCAAAATCAACTTCCCCAACGCCAAGAAGTGCGTTCTGTTCTTCGCTAGCCGAACCAAATTTGAAGCCAAAATTGGTGAAGGGACTCGACTCTACCAGTTTCAAGCGAGCCCGGCAACCTCAAGTCAGGTAGCCCATGTGGCCACTTCAGGTAAGCCTGATAATGTTATACTTTGGCATAAACGAATGGGTCATCCTAACTTTCGCATTATGCAGGATCTGGCTAAAGCCAACACTGTCATGGACATGGGCCTGAAGGACTTTGATTCCAAGAAAGACTACTTTTGCTCTTCCTGCACATACGCCAAGGCCCATCGTAACCCATTCAATAAACGTACCGTTGAACGGGCAAAGTTCCCTCTTGAAAAAGTTCATTCTGATATGGCTGGTCCATTACCCGTCCCCACACTCTCGGGGTGTCAATATTTTCTGACCTTTATAGATGACTACTCACGGTACATGTTCATCTACATCATCAAGCGAAAGTCTGAGCTGTACGAATGCTATGAAGATTTTCGCAAGAAAGCACAAAACGTTTTTCGCGCTGATGTAGGCGAACTCCAGTTCAATGAGAACATACACGATGAAGAAGTTAGACATCTTCAAAGCGACAATGCCCGAGAATATGAGAAACTTGGAAAAATGATTTTTCAGAAGTACAATACGCACGCACAATTTACAAATGCGTACACGCCCCAGCAAAATGGAGTTGCAGAGAGGCGCATGCGTACACTACTCGAGCGAACCAGGGCTTTCCTCATTGATGGTAACCTACCGCAGGTTCTTTGGGGAGAATGCGTCAAACACGTTGCTGACCTTCTGAACATTACTCCCAGCTCGGTAACCGATAAGGTCACCCCATACGAGAAATGGTTCAAGCGAAAACCATCCGCGCACTACATCAAAGTGTTTGGGTGTACTGCGTACGCTCACATCCCTGAGATACACCGCACAAAACTAGAACCACGTGCTAAGAAGTGCATGTACTTAGGACTCCCATCAAACAAAAAGGGATATAGACTCCTTGACACGAAAGAAAACCGTGTTATCTACAGCAGGGATGTTGTGTTTAGAGAAGACGAGTTCCCACACTTGGAATTCCTCAATACACCCGCAATCTCGGACCAAAATGGCCCCGCACCGACGCTATCACCGTCGCTACCAACACCACAATTGCTACCTCCAATAGCCTCACTACTAAAACGCAGCGTCAATGCGCTTGCCGCGGAAGTAGTAGTAAAGATGAAGAAAGTCAAACACAAAACGAACGCTCAGAACGAGGATGAACACTCGTGTCTACTTGATGATCCACTCGACATTAACGAACGAAAAGAGCACCTCACTCATATCTACGCTCTATTGGCTATCAGACACATATCTGAACCAAAGACGTTCCTAGAATCCCAGAAACTACCAGAAGCACAACACTGGGAGAAGGCAGCCATTTGTGAATACAAATCATTGATTGACAACAAAACGTGGGTCCTCACAAAACTACCGACAGGGAGAAGGGCCCTACAGTGTCGATGGGTATTCGTTGTAAAATACAATGGAGACGGCAGTATTGACCGCTATAAAGCTCGCCTGGTAATAAAAGGTTTTTTGCAAAAGTATGGCGTTGACTACGATGAGATATTCTCTCCAGTGATCCGCATGGAAGTGCTGCGACTTTTACTGACGATTGCAGCACTCCTTGACCTTGAGATCCATCAAATGGATGTTAAAACCGCTTTCCTGAACGGATACCTCGATGAGGAAATTTACATGCTGCAACCTGAAGGGTTTACTGTTCGTGGAAAAGAGCACTTAGTCTGCAAGCTTTTGAAGAGCCTATATGGTCTCAAGCAGGCACCTCGGATCTGGTATCTTACCCTGTGCTCTTTTCTTCTCACCATGAAATTCCACAAACTCATCAAAGACCAATGCGTTTTCATTGGAGTTGTGGATGGAGCTACATGCTACATTCTAGTCTATGTAAACGACCTCCTCATCATAGCCCCTACGCTTGATATCATTAACAACATAAAGAACGCTCTCAAGCAGCGCTTCAAAATGTCTGATCTCGGTGAAGCTCAATACATCTTAGGCTGGTCAATCGTGCGCAACCGTACGGACCGAACCATTTTCATCCACCAAGAGAAGTACGCTACCAAAGTCCTGGATCGCTTCTCTCATCTTGATGCACACCCGGTTGGTACACCCGCTGATCCCAGTGTGAAGCTGACGGAGGCCATGTGCCCCCAAACGCCAGAAGAAAAACATGAGATGAAAGCGATCCCGTACAGAGAGGCAGTTGGTAGCTTCATGTACTTAATGATGGGAACTAGACCTGATCTCGCATATTTTCTACGAGAAGTCAGTCAATTCCTGGTGAACCCAGGAAAACCTCACTGGAATGCCGTTAAGAGAGGATTGAAGTACTTAAACGGTACGCGCAATCTTGGCATCACCCTTGGTGGAAAGCAAAATCTAGAGCAGTATAAGTCAAAGCATTATCTCACTGCATACTCCGACGCAGACTACGCCAATTGCCCAGAGACACGACGATCTGTGGGAGGCTATATCGCATATATGTATGGAAGCCCAATTTCATGGCAATCGAGAAAGCACCACACCGTAGTTCTGTCCACAACAGAGGCAGAATACATAGCCCTATGTCACTGCATGCAAGAAGTTATTTTCTTAAAGCTTCTTTTAGTAGAGCTTAAACACGAAAGCTCACATGCTATCACCCTATTTGAGGACAATCAAAGTTGTATTAAACTTTCCTACAACCCAGAACTGCATGGCAGAAGCAAGCACATTGACATTCGGTATCACTTTGTACAAGAAAAAGTAGAGCGAAAAGAATTTGAAATTATGTACTGCAACACCAAAGAAATGGTAGCAGACATTTTTACAAAAGCACTATCAAAACCCCTTTTTATAAATCTAAGAGGGTCACTATGTAAGACACTTATACAAAAATAAGAAAAAGAAAAAAAAGGAAAAGAAAAATACTAATCGATATGTAAAATTTATACAGCAACAAGTTTTGTTAAAAGTAAATACGTATTATTTAAATCTTAAAGATTAGCACGAGGAATGTCCTCAATTGAGTACACACCATCTACTGTCTCAAAGGAACTTGTCAAAAGCTCCTCCCGGTTAGGGAAGAAGTGATAGGTGTCGGAATCGATGAACACAAACTTGTTACCAACCCAGGCACGAGCGATACAATGCCATGTACCTGCTCGTTGCCCAGGCCGCCTACCTATAATCAGCAGAGCCGATTTTCGACTCCGCACGATGGAGGCAGGTCGCTTGGGTCTGCTCTTGAACCCCGACTTTGTGTTCAGATAAATCAGCTGTTTGCGCCGCTTCTGCAAGCCCTTCTGCAACGCATCCATAGTGTAGGCACCGTAGCGTGAATGTCCATGATCCACACTTGGGCTCACCTCTACCAACCGAGCCAAAATAGGCCGCAACATGGCTGGGGTGATAAAATCCCTAGTTTGAAGAGCGTTGCTGATCGATAAGAGACCGCACATTGAGTACCGGTCCGACAGCATCTCTTGATGTTGGCACAAAATTGATGTGCGAAGACTGACAGGCATCTGTGTATACAAAAGATAGTATATTAGTACTGTGCAAATCACATGAACCATAGCAGCAAAATATTGATTGCAGTAAATCAATATGACAAAACCCAAGGTTATTGAGACCAGTGGAGACGATTCGCTACCGAGTGAAACAAGTTCACTCAGAGCTAGCCCAGACTATAGCGTCAGGCGCGCATCGCACTAAGTCTCGCTCCCTTGAGGATTGCACTTGATCAACACCTTCAATACTTGCATGCAAATCAGAAGAAAACCGCACCTGTGCAAAAGTTGAAAGATCAGGAAATGAACACTTAATGCACTCAACGAGAACGATACGGAAGAGAAAGCAAGCGTATTTCACTGATACTTAAACTGCACGACGGTAAAGAACTTTGCGACCTTCGGAAAGGTATAGACATACTTTCTAGCAACTCACTACCGCAAGAAACAGTCGATTATCCTCGAAGTAGTAATTTAAGTCAAAGTCCTCAACGTCTTGAATGCAAATCAGCTGCACCATCTGGGATAAGAACTGCCCCCAACTTGGGACACAGAGAGGCTGTCAATGATGTGTCCTAATTGGTGACATAGCGTACAAGTTATTGTACAGAATGTCTTACCATACAAAATATGTTCTGGACCCAATCCTGCATAAGTCGATACATCACCTTTTACAGGAAAGATAAATCAATGGTGCCAGGAAACTCTCTGAAGACAGACTCATAAAATTCAATCACATCTCTTTTTTCTTCCTTCTACTCAAGCTCCTCCACCTTCCTCGCCTCACCTTCAATAGACATTGCCGACGCAAATCCTGTCGATCTCATCGATGGGACCGTTGGCCACGCTGAGGTTGCGAACATCGGTGGAATCCCCGCAGACGGAGTCGAAGCCACCGCACGTGCACCGTTAGCAGCGTTTACCCCACTATTCACCACTCCCCCATTTCCAGTTGACCCTAGAAACGTAAATCCATCGCCAGTATACCCATTCGTATTCAGAAGATGTAACAAGGTCTCCACTAGCCGTCATGGCAGCCGAACTGCCCGCAGCTGACGCCGCCCTACTTGACGTAGCCATTGTATACCCACGAAACTCAAAGATTGTACTCTGTAAAGCTTCAAAGCCTTGGGCTCATAACCTGGCACGTTGCGCAGGCAATCAGTAGGGCAAGGAGCAGCTTAGATGACGGAAAGCAACTAGAGAACGTCTTCCCACGGAGCACTTTGGGATTAAGGAGGAAACCTCGAAGGTGACAACCCCCTTTGCTAGCACTATACACTAACGGGCGTATACTTCCATCTGCAACTGTACAGTCCCAGCTGTATACACAGCTACAATAGGTATGCACAGTAACACGACCTTCGTCGCAATTTCGATACACTACTGTACTACTTTTTATTTAATCATTAAAATTTCAAAACCGGTATTTATACTTGTTGAAAATTGGTGCTAAGGAACAAACTTCTTGTTTTCAACCAATCAGATAGCATGCATCATGTTTTGTTCACTCATTTCTTGTCCTGGGACCAATGCCGCCGCCTTGCTAGCGTACTAGCCGGTCTGCTGCTCATGGGGAGCGTGGGCTCGAATCATTCAATCTCGGTCTGGAATACACAGCTGCAGGCGTTGCCGGGTTTCTCCGAGGCGGGTATCTCTTTCGTTTGTTCTATGGCCTCTTTTGGTGCCTACTTCTCGGTGAGTCCCGGGTTCGTTTTCGACCACATCGGCGCCCATCGCAGCGTTTTATTGGGCGGATGTTTGCTTGCTGGTATCTACATTGGGCTCTACGCTGGAATGACGGCATTTCCGGCGGCCATGACGCCGCTGGGCGTGGGTATCGCCTTCGCAGTGCTCGGTCAAGCCACCAACTTCGGCGTGTTTGCGGCATTGGGGCCGAACGAAGATCTCTTTGGACCTGAAAATCGCGGCAAGGTAATGGCTTTGGAATTCGCAGCCTTCAGTGCCGGTGGTGCGCTGTTCGCTGAGCTTTACAAGCACTTTTTCGATGGAAATGTACCTCTGTACTTTCGCTTCATGGGGTGCTTGATGTTGACAGTGTTCTTGCTAGCATGGGTTACGCTATATCGACCTGCCAAAGACGATGCAACTCACACTATTGCGGCTGCTCCGCCCATTCACGCGTTGGACGAATTCATGCCGCCAGAGACACCGGCTTTGAAGTCTTCATGTAGTGTTGACGTTGAAGAACATTGCCAGCTCTTCGCAGTGGCTCAACCTGACACCACCGGTCGCGATATTCTAACCGACTCGCGCTTCTGGTTGTTGTTTTCTACCGTATTCATATTGGTGGGATCTTCACTGTTTGTCATGGCAAACATTGCGTTTATTGTCGAGTCTCTTGGCGGTCCGATGGAGCAGGTCTCGACAATGGTAGCGCTCTTCTCTGTAGGCAACTGCTGCGGTCGTGTTGTTGCTGGTGTCATTTCCGACAGTGTGTTGCATCGTTTTCCTCGGATCTACTTTGTAAGTCTAGCGTCGGTCCTTGTGGGTGCCATCCACACACTTTTCCTGGTTATTCCGAGGGCGTATCTCGTCGTTCCCATTACGCTCTCAGGCATCGCTGACGGCGTCATGTTCGCCGCTTTTCCCGTGCTAACGCGCGAGACGTTCGGTGCGCGCCACTTCGGAAAGAACTTTGGCCTCATCAGCGTCGCAAATGCTGTAGGCTTTCCGTTGTTTTACAACCCCATAGGTTCATTCGTGTACAGTTTATCGGCCATGCCGGTGAATGGCGTCCAGAAGTGTCTTGGAGATGAGTGTTTCCAGCCTGTGCTCCTACTAGTGGTGGTATTGAGTGTGGTGTCGCTGGTAGCGAGTCTTAAATTCGCATCGCGTCAGCATTATGTTCGCTTATCGTAAAATACAGATACTGCAACTTAAGCTGGCTCGGCAATGTTCATTCTGCGGCAGAGGTACCTGTGTACCAATCCTACCATTGATCATTACCGGGTCTTGATGGCGTTGTCGCCGTTGCAGCTCCGCCGGTCGTGCAGCCTGCTTGCCGGCGTGTTGCTCATGCTCGGGGTGGGCTCGACCTACGCGCTATCAGCGTGGAATGCACAGCTGAAGACGCTTCTGCACTTCACACAAGCTGGGATCTCCACGGTCTCCGCTATGACCATGCTGGGCACGTACATGTCGTACATTCCTGGCGTCATCTTCGACCGACTGGGGCCGTACACGAGCGTTCTTTTAAGTGGGAGCGCCATGCTGGTTGTCCATCTCGCTATGTTTACTGCGCTGCAATTCGCACCGGAATGCGTTAGTCCACTGGGTATCGGCTGTGCCATGATGTTGTTCGGACTCCTCAGCTCCTTTTGCGTCTTCTCGTCTATCGTTCCCAATGAATCTTTGTTCGGAGATGCCAATCGTGGCAAGGTCATAGCAACGCTTACGTCGTCATACAGCTGTGGCGGTGCCTTCTTTGCGTTCGTCTTCCATGAGGGGTTCCACAGCACCGACGTACCGGGCTACTTCCTATTTGTCGGAAACTACCTACTAGCTGCTTGCGTGTTTGGTTGGTTTGTATTTGCCCGTCCACGGCATGAAGAAGAAAAGGACACGAAAAGGAGATCTAGAAGCATTGAGGTTGGTGGGGCCACTAGCATGGAATCGAGTGGAGACGTGAATGGGTCTGCAGACAGCCAGACACCTGACGACATCACGGGCGTGGCGTTGCTAACAGATATGCGGTTCTGGATGCTCTTCATTCCAGTGATGATCGTCATCGGCGCCGGCTTGCTCGTCATGTCCAACGTGTCGTTCATCGTGGAATCTTTGGGTGGTCCTCTACATCAAGTCCCCTTCATGGTGGCACTGTTCTCCATCGTGAATACACTCGGTCGCCTTGTAACGGGAGCAGTCTCGGATCATCTGTTGGCGAAGTATCCACGTGCATATTTTGCTGCACTCTCGGTGGTTCTCACGGCTGTCACGCAGGTGGTTTTCCTGTCCGTGTCACCGACGTGGCTCGTACTGCCTGTGGCAATGGCTGGGTTCTCAGAGGGCGTCATGTTCGGCACCTTTCCGGTAGTCGTTCGCGAGGAGTTTGGTTTGCAGCACTTTGGCAAAAACTTTGGCCTCATTAGTATCGCTAATTGTGTCGGGTACCCGCTCTTCTTCAGCCCGCTGGCGTCGTACGTATACCAGCACTCGACAGCAACACGTACTGTTGATGGTGTCGAGAAGTGTTTCGGTACACAATGTTTTGCACCCGTTTTCGTCGTGGCCATCGCACTGTCCGTGGTGGCATTCGTTTGCTGTGTGCAACTCGCTCGATTGCAGCGACGCCGCAAGTTTTTTAGCTACCAACAGATTCGGCCGTAGCTGATAACTGTAAGCGAGGTGAAGCCCGCAGTAAATTATTGTATAGCTTTTGCAGACTGTGCGGTGCCTTCATTTTTCTCGTCTGAATACGGTAAGCACAGTCGGATGTATAAGCAGAAAGAAGCGCATTTTTCGAGGAGCACCACGCGAGTACAGTACACCGTACTCTGCAAGAACACAGCAAGCCCGTGTACGATATTTATCGCTGTTGCTCACAAGCAAACAAAAGCTCATTGAGCGACGATTACATTGATGAGCGCTGCTGACCATCCACGGTGCAATCAGGACCCATGAGCAAATCTGTCGGATCTTGAGATTCGTTTTGGCTTCAACCTTGCACCGTTACCGCGAGTCCGGTACACCAAAAACGCTTTTACATCACTGCAAGCACGTGTCACCAACGACAAACCGCAGCATGAATGCCGCATTGCACTGGGCACTGCCCAGTGGTATCTATATGCACGGAAGTTAATTGCACGTGGGTGCTATTAATAGAAGTATCTCAAGATAATGAAAAGATTCTGACCAATAGCAAGCTTCTATTAAAAAGGTTTTAGCCAATGCGAGACAGGCACCGCCACTTATAGAGACATTCGAGCGGTGCATTGTTCAGTTCGCAATCCGCCAGTGATCAAATACCAGTCGGTATGAGCTCGGACGTGTTGACGCAGCAGACATCGGCCACGCAGCGCAAGTGCTCAGCATCTACTGCCACCGCCGCGTCCACCGTAGCGCAGGCGTGTCGCCAGGCCGTGGCAGAGCTCGAGAATTGGTCGCTCTGGGCCTATCGGGACGCGACGGGCGAGGAGTGGGATGCGCAGCCAGCTTGCTTCATCACTGACTACCTGCTGGCCGTGCAGTGCTTCGCCTTCGCCGTGTACATTGGACTTTTCGCTGCCACAGACAAGGGCGATCTGGCCTGGTATATCATGTACTTCATGGCGCTAGGCATCTCGGCGGCGCTTGGTGGCCTACTCCACCATGTTGCGTTCGAGGCCATGCGCGACATCGCGCACAAGCAAGAGAAGCATCTGGTGATGACAGCGCGTGTCTTCGGTGTCAACCTGAGTCGTGCTAATGTGGACAAGATGATCGAAGCTCTGTGGCGCGTTGTGCTGGCCTGTTCCACCCTCACCAACTTTGCGCTGCTGTCTCTGGCCGCGAGTCGACACCTGCCAGAGGCCTGGGCTTACTCTATCATTGTACTGGCTGCCGTGTTGTACGTGGTGCTGGCTGTGTGGGCCAGTGTCAGCATGCACGCGGTGTTCCTCTTCGCCGGCTTTATCCCCGTGATGCTCTTTGGCCCCATCGCCTGCTTCATGACGTGGAACTGGGAGTGGAGCCACACGAGTAACGAACTGCTCGTGTACGGCGTTAAGCTTCTGGGTGGCCTTATCCAGGGCCTCGTATGGGCTCCCAGCAAGGACAAATTCAACCACAACGCGCTCTCGCATGTGTTCCTCAGCATGGCGGCGACACTCATGCTCATTCACTTCAAGTTCTGACTGGATGAGCATAGCGACAAGTGCGCAACGTGCCAAAACTTGGTTTTATATAGGCACGTGCTCACAGTCAGCTCGTAATTATGAAAGTAGGGACAGAGGAGCAGAGTGGAGCAGAGGAAATCCTTCATGAAGCTTTTAAGTGGTCAATTTGACGAGTTTTGGTGACGTATTACATCTATTGTTTCAAAATGGTGCTACTTCAAGTAGCTGTTACGGTGAAAGTGCCCATTTACGACCCTATATTTAGAATGTGAATGAGTTGCCACAGTTGCCAAACAGGATTCTTAAAGTTGCCTTCACAGTGGTGATGCTGTTGTTTGGTGGGAATCTCTCGGGCGGGAAGAGAACTAGCGCAGCAGTAACTAAGGCTACCGGTCCTCCAGCTACGAAACATGGGTCCACCCTAGATGGAGACAGTCAGACTGATGTATTGACACCACATACGGATGCATTGCATCGTCTTACCAACCATGAATTACGGCTTCTGGAAGAGTTAGAGCGTACACGCTCCAAGTTACAAGCCGAGTGGAGGCTTAAAGTGGCTTGTCTACAGCGACAACACCCGGATGCAGTATCGACCGCTTTGAACAATGCTCGCGACGGAGTTATCCCCAGTGTCGAAGAACTCGAACAGCGCTTTCAGCTAGCAATGGGCACAACAAGCACGAAATTTCAGCAACGAATGGAGTTGCAACAAGCTATGCATTCCAAGACGCTGAAACTCTCTCCAAATCAACGATCCAAGGCGTTTTTGCGACACAAAAAGGCTCATGCAGCACCAGGTCCAACGTCATCGATATCTCCAAACGCTCGAACAGCGCTGGATATGCTGCCAGTGCTGACATCGACAGGAGAAACGCCATTTGAAGCTGGATTGGATGCTCAAATGGTATCAGGACAACGGCAACAATACGTTCTTCCGGTTTTCCGACTACCTTCCGTAAATGTGGCAAGTACAGCAAGTGCAATGGCAGCTAAAGAGATACAAGTAATCAAACAAGACGTCGATCGGAAACAAGCAAGTGAGACGCTGGAATGTCATTGGAGGA

>Contig_61

TAAACCAATTAGAATAGTAGGATTCGGTCAACTATTCGTTGATTAATACCTTTCATTTAATTAAGAGAAAAATCCAAACAAGCAAATAAACAGTTCTCATTTTCCGGAAATCGGCTTGGCTTTCCTATTTAAGTTTATCTATACTTTCGGACAAATCTAAGTGAAACACAGCTACCGCTTCCTCATACACGTGCGCTAACGTGTAAGTCGAAGTAGTGGGCTGAAGTGGGACCATTTCACCTGGTAGCACTACTTTGTAGTCTGTCCAACGACCGATAGTTCGTATGGAATACGAACACGGGACGGAGAACCAGCTCATGGACGGTAATCCGTCCATGCGTAGCTTAAGTGCTTCTGCCGAACACATTGTGCGTGCAGACTGTCCCCATCTGGCAGAACCAGAGTGGAAAGCTCTCCGAAGACTGTCAGCAGTCATTGGAGAGGCAGCCGTAGCGACAATGCTACGTACGCTGTCACCGACAGAGCAACACGGTGCTGCTGCTACGAAAGTGTCGCCCGAGACGACACTGCGCATGCAGTTGTTGAAACAGCACGTGAGCAGTTATGTGGGGAAAGAGGGCGAGCATCTTCTCTGATGGCTTGTCGAAGTCGACACTTCCATAACGGCTCGTCATATCACTGACGAACCGTCGAAGGTTGCGCTCGCCATATCATGTCTTGGCGGACGAGCGAGGAGCTGGGCGTATGGCCGCCGGCTCACAGATCATACGTGCTTTAGCATGTTTGCGCTATTCAAGGAGGAGCTCAGACAAGTGCCCGAGCCCCATCAGAATGAGTTCCGGTCGAGACTCGATCTGCTGCATGGTAAGCATGACATTCACGGTCTCGAGCATCGTGACCAATCCGATCGACGAGGCAACAAAGGTCGTCACGTTTATAAAGGGGCTGAAGGATTGGTCGGTAAAGACATTTCTCTTCCGATAATACCCAAGTACTTTTCGACGCTGCGATTGCTGTTGCAATACAAGAGGAGTTTAGCCTGAAGCAGGCTAAACTGCATACGAACGTGCCGAGGATGGCACGTCCGATGACGAGAGCGGGAGGCTCAGAGCCTATGGACCTCACGATTGCTTCTGCTGTTGGACGCCAACAGCCTAAAGGGGAGCAAGTGCGGTGTTACGGATGTGAATACAATGGGCACTCTGCACGTGAGCGCATCGACGGTCATTGAAGTATGAACGTGTCTCAAATCTGATTTCCGATTTGAAACAGACCCAACGGCTGCCTCAGTCGCTGCGACTTGTGAGTGGGCACCGGAGGCTGCATCGTAAGGTACTGCCTCGACGGTGGGTTCGGATGCACCTCGCGGTGCATGCGCGACACTGACAGGGCCATCGCTCTCTATCGCATTGTGTCCGAAGTGTACAGCTGTACGCTTCCCCCAGTTAATCACTGGATCATGCCGTGCAACCCACAGCATGCCCAATACCAAGTCAAACTTATTGTCCAGATCCAGGACGATAAGATCTTCCACAAACACCCTGCGTTTGTATGCGTAGCAAACGCGTAGTACGTGCTTCTCCGTTCTTACGGTGGCACCTGTTGCCAGTCGGACCTTGTCAACCAAAGAGTCTGCTCTCTTCGACGACACTTTCAGGGTGACAAGATTTGGCTAGTCATATTGCTTTTTGCATCGAGAATCTCTCCCGCTGGGGGCTGCAGGCCTCCGTGTCGCGTGGCTGTTGTAGACACGCGCGACTGCAATATCAGTAGGGCGCCCCGCGCCTACTGGTTCTGTACGTTTGTTTTATTGCACGCGGCCATGCCCGGCATCGCCACGTTGCCCCCTGGCCGAGTGCACAGGCGGCGCGTAGTACCAACGACTTGGCTGCTACACCCTCTGCCGATGTGCTTGATGGTAGCACAAGAGAAGATCAGATGAGCTGCCAGGGTATGGGCTCCGGCAAGTGGGTTTCATAAACGTCCCAAAGCCGAGGTCTCGAGGGAGGCAACGATCCAATCCGAAGCAGCTAAGACAGACAAATCTTACCTCAGCTCATACTCGTCTCATCGTGCACAAATACTCTAGCGATCTCACCGTACAGCTTGACGAGCTTGTCGTGTGGGCACGCAACACAGAGAAAGTAAGAGCTGTGGCGGGCATTATAGACAAACATCCTGTCCATTTAGATGATGCGTACCTCAGGGCTCGCACAATCGAATGCAGCCGGGAAGCTGTACGATCGACAACGTACATGCACCAATTTGTAATCCCAGAAGACATCACGCGGTCAATAAAAGCTTCAATCAAGACAGCCAAGGCGAGCAACGTCACCTAAATCCACTCGACGACCGCATCAAGAAACAAGAAATCGTGCTCGATCTCGTAGCTTCTTTCGACACCACACATTGGAAGCTCAACGGGTAAGGTTGTCATTCACGTATGGTTGCTTTTTATGGTCGTCATACTGTGGTTGCTATAACTGTGAGCTGTGATTTGCACTAGACAATACGTGGGGGCTGCAATTACATTCTACGCGGTTAAGACCAAAGTACAAGCATGGTTCGAAGACCGGAAATGGTTGGAGAAAAACTGGCGGAAGGTAAACTCCGATGTGGATCTATTTGCTGTGAAGTCAGGAGCAAGCGAGTGGGCTATCCGTTGGTGCTGTAAAGTAGCGATATTACACCATGACAAACGAAGTGATTTCAAAGGTTGCATCCAGTCGACCGAGTTCTGAATACACGACACGGTCGGGTAAGGCCTGCATCGCCTTCGAAAACGTTGTGGGTGGACTCTGTAGAGGATGGCTGAATGACAGCCCAATTGACTTCTGTTTGGAAACCATTGGAAGCACGGTGCGGGATTGCTATGTCCTGTCGGCAGCTGTCGGATGGCCAAAGACACCAAACAAGTCTATCACTTCTACCAAGTCCATGATTCAGCCTGTCAACCTGAACCAAAGCCACGGGGGGGTCGTAATAGCTACGCTACGCTACGTCGAAGCCAAGGACACATTACATGTGCATCCCTACCCCTGTTTGACGAAGGTTATCATGGGGACATGAAGGTGGTGCGGAACGGTGTTAAGGACGGCGATGGTAGCTCAGTTATGGAAGGGCTTTGTGATTTTGTGGCGAGATGACGTAAGGCATCGGCACCTGTGGTCAAAGTATGCATGAACCCTATTGAGTGGATTGAAGTTCCACAGCAGCCCGACTACTCAAGCTGTGGCGTGTTTGTTGTGGCCCAGCCTTACAGCTATGTGACCGTGAATCTTCGCTGGCAATGCAGAAACATTTCCGAGCGGGATTTGGAAGCTATGCGCCTCCGAATGCTGTGGTTCATACTGTAGCGGGGTTACATGGTGTGATACACACAGGGGTACGGAGTTCCCGTAGCGTTAACAAAATCTAAGTAGATAAGGCGGAAAGTTAACATAGTCTCTCAGTACGTTAGCTGAAAGCCACACGTTATGCATTTGATTAGGCTTAACTATGCTAGATAGTTAAACAGACATATTTACCCCTAGGCGGCTTGGCCGCAATAATAGTACATAAAACACTTCTGGTTTTGAAGCATACAAGTTAAGGTATTAACTTTGAGCCCTGGTCGTGGGCTGTAAGTTCCTTTGGATAAGGAACTTAGTATTAGTGACTCTTTGAGTCGAACGTCTTCCTCTGACGCTAAAGCTACGGCACCCCGCTGCAAATGCTCTGTAAATCGAGAAAGAGCCGTATGGCCCGTCGCACTGCAGAGAAGACGAAGTCTATTCATGAGAAACTGCTCAAGCAACTGAAGTAGGTAGCTGTAGCTGCGCTATCCGTTAATACAAAACCGTTAGCATACTACTGTACTACCAATCCAATCAGTTAACTGCCATTTCTAGCAAAACTCCGAAGAATAGGTGGGCGTCATGACTCGCACCAGCGTTGGCAATCTCAGACGCCAGCACGTAGATGCTCTCCTCGCTCACCTCAATGCGTGACAGTAACTCCATCTGAGCTATTAGTATCTTCTCGAGGCTTGCGCGATCTTTTCTAGCGTGTTCTCCATGTCCATGAGCTCGTAGATGGGCGCGCGCACCAGCTCCCGAGCGAGCGACATTCCGTCCATCCTTGCTGCGATGTTGGATCTTTGAAATATTTGCTAAGTGTGAAAAAATGTGTTCCACACGGTTTAGTGTAAGAAACACACCCGTGTGTGGTGCACACGCAATTTAATGCTGTTTTAATAACTCTAAATTTTGGATGTTGTGGCGTTATTTACACTGCTACCCGCCGCTACGCAGCGGTAGTTTAGGTTTAGTTAGACTTGTTGTGGCCTTCCCGCCTCCGCTACGCTTCGGCGGGATAAGTATTACAAAAGTTAGGCCCCCTATGCCGCCTCGATATTTTTAATTTCATAAAGTAATCTGGGTCAGCATCATGCTTACCTGATTAAGCATAATTATTCTAATGATCCTGATTCAAGCTTCTTACTACAACCTCTTTATCATTGTTGGCTATCATTGTATCTTCATCTTCCACCCTAAACCACTGCACTCCAAATACATTTATAAGAAAAGCTACACAATATGTTTAGTTTAGCGTCGTACTTTCTGAGCCATGAAACTTAGCTCACATCAGCGCAAAAGATAGTAATGGCGTGTCAGCTTGCACTACTTACCCGCTTGTACTGCATGTTTAATCTTGTAAGTAGCTAAAGATTGATCACTTCTCATACCAAACTCAACAAATATTCAATTTTTGCTCATTTTAATACATTTAGCGCTCATTTTGTGCTGATTTAGAATGAATCATTAGACACTCTAATCTGTGCCAGTTATGATTAAAGCATCTGGTTAATGCGTCGCAAGTCCAGGATGGTTAATGTCATTTAGGATCCAAATTTATTAGCTTTTTTTATGAAATCTACATGTACTTAGCCAACTATAATTAACACCTCTCGTGTTTTGTTGTTGTCTTACCGCTCAATTTAAAATCGTATCCTACGCCCTTGTCGGCGACACGTCCACCCATTATGTAGTATCGGATTTTGCTATCTAAGCAACTCTGGCTTACAGCAGTAACACGTATGAGTTCTTTTTGTTCAATCTAAAACTATCTACTTAGCCAACTATAATTAGCACCTTTCATGTTTTGTTCGCTCCATTTCAAATCGTATCCTACGCCCCTGTCGGCGCCACGTCCGCCAGCTTAGTAGGATCGGATTTTGCTATCTTAGAAAACAATGGTTTACAGCAGTAACACATATGGGTTCATTTTGTTCAATCTAAAACTATCTATCTTTATGGATCCCGTTTATTATCCGAACCTGGAATCCTTTCTAAAGTACTGTCAAGCTAGCGGTGAACCACTTTTCGCTAAGAGAACTACTTTGCCTTGTATCGTGTCTAAATGATCAATGAATCGGCATTCTCCTTGTAGAACTGCTTGTACTCCCATTTAATTCGTATGCTGGTATGGTCTTCAAAAGGGAACATCTTATCGACGTTTGTCTTGTGAACCATCCATTTCTTGAATTGCCACTCTTGCAAATCTTCGGCTATACCAGCCTTGGTCAAATAGTGTTTCGCTTTCGAGTTGACCAACAGCACCGCTAGCTCCTCTTCTCCAAAGCGTTTCTCCAATTGAATGATCGGCTGGAACACCTTCACTCTATTTCTCTTGAAGCTATTCACCCTGTTCACATAAGCTATCCACGTCCTTAATGGCCCCGGGTTTTCGAACAGCTTTGTACCCATTTCGTTCAACTTGAGAAGATTGAAAATGTCATCTGAGCTTTTCTGACCGACCCGCCAAATCTCCAGCTGCAGCTTCTCAGCGGTCTCTTTGGTGTTCGCTACTTGCTTCGCTTTCGCAACCAGATTTGCCAACCCTTCGTCACCAAATTTGTTCCTCAGTACTGTGTACATGACCAGATCTGCGTCAGGGTCAATTTCCACCTTGTTCAGATATGCGGTCCAAGTGCTCCACACTGGGCTCTCGAACACCTTGTCTCCTTCTTTGTCGAGTCGAAGGAGTTTAAAGACATCGTCGCTGGTTTTCGAATCTACCGACCATTTACCAAGTTGCATCTTCTCCACATTCGCAGCCATGGTTTTCGAATTGGTGTCTTGCTTGGCCTTGTTAACAAGCCTCGCCAAGTCTACTTCGTCCAAGCCTGACTTCCTGATTGTATGGACTAGCATCGTGTAGGGGTCCTTCCTCAGCATTTCCATGTAAGAGAACCAATTGTTCAGCACTGGACTATCAAGAAACTTGTCACCCGCTTTGTACAGTCCGAGGTACTTAAAGAGGTCTCCACTGGTCTTCCCATCCGTGGCCCATTTCTGGAGCTGCACTTTCTCCAGTTTCGCGACAACAGTCCCTGTATGGAGGTCTTGCTTTGCTGCGGTGATGAGCCTCGCCAACTCTACGTCGTCGAAGCCCTTTGCCTTGATCGCTGAAAACAGCATTTCGTAGGGATTCTTTTGCAGCTTTTCAACGTATGCGACCCATGTGGTTAGCACTGGATTTTTTAGAAGTGTCGTGCTTTCTTGGTTTAACTTCAGGATCCGGAAGACGTCAGCCGCGCTGTTTTTATTGTTCATCCACTTCTCGAGCTGCAGCGTCTCGAGCTTCTCGGCCAGCACTTTTGTTGAAGAGTCTTTCTTGGACAGAACAAACATTTTCGCTAGTCCCACGTCGTCATAGTGCGCTCTCACCTTGTAAAGTAGGACGTCGTATGGGTCCTTGTTCAACTTTGTCAGGTAGCTCATCCACATGTTCACCTTGGGGCTTTTCAGTAGATTACTGCCTTCAGCATCAATCCGCAGAAGCTTATAAACGCTGTCCGCACTTACTCCATCAGCAAGCCATTTGTTGAGTTGTGCGTCCCCGTCATCCACCCACTTGCTTATCTGCGCATTTTCCAGTCTAGTGGCCGTCGATTTTGTACTTGACACCTGTTTTGCGGCATCTAGCATACGGGCAAGAGTCTCATCGCCGTAATGAGTCGCCAACGTCGCGAGCATTGCAGCGTCGGTGGCGTCCTGGTTGTTCTTGGTCGTTTTTGCAACAGACTTGGCCCAGGCATTAAATTGCATGCTTTCGAAAACGTTGGACTCGACTTGATCGACTTTGAACTGCTTAAAGCGCTGGTCAATAGTGGGCTTGGACGTCAGCTTCGTCTTGGCAGTATCAACGGCCTTCTGCAGTTTCTTCGTCCCAGCCTGAGCAAATTCTTTTAGCTTCGTTATGCCGGTAAAGGCTCTGTCTTCGGACTCGTAGTTGTAGTCGTAACCATCTACCGTCGCCCTCAATCGCCGGTCGGCTCTGATTCTGTCGAGGGTGGGGAGATTCTTGGAATCGATGGCCGTCAATGCCTTACTGGCACAGATCAATAGAGCTACAGTCACCAGCAGTACTTGATAAAAAAACAACTTGCGCTTGGGCTTGATAGTCATGCTGATTACTCGAGATGTCCAATTGCGGAGTGAGGCACCCCTCAGTAATTAATGTGACACCTGTCTTACTGTAGTGAAAATATGTACATGTACCGGTACCTGCAATAGGTGTACTGCAATACCATCTGGTAATCTTCGGATAAGATCTTCGGCTCACTTTCTCTTGGCCTCATCGCCAAAACCAGAGCTCACGACTTCCAGTTTGTGGACATGTTCAGGAGTAGAGGCAATGCTTCCAAATTCCACTGACAGATTTCAAAAAATAAAATCTGCTTAACACCGTATCTGAAAGCTGTTTTTAAAACTGTTTACGCCTCTTTAATAAAGAGGCGTAAACAATAAAGAGTCTTTATGTAATTATTGCGTCCTTTTTCATCACAAATACATGTTCATCGAAGCTCGATTCGAACTTGCTCTCTCTATTAATAAAACGTACTGGTGCATCCGTACCGGTATTAAAAGAAAATGTACTGTCTGTATTTTTAATGTAATGCGAATGTCGCAGTAGCGTTCTGTACTGCACCCGTTCTCGCAGGGAGAGGCGTTAGGCTCAGTCTTATATCGTCCCAAAAGAGGGATGGACTAATCAGCGATCTAGTTTGCGGGATATTGGGATGACATGTAAGGTTGACACCCCTTCTCGTCCCACTGGGACGTTGTGTGGCACGGTCTTGGGCTTATCTCAAGTCCAATCGTCTATTCCACAGCAGCACACAGTGTGTGTGACACACCACCGACACGATCCACCTGTCTATAACACCGGGTTGGAAGTCCTGCGGATTCGCGAAATCGCAGGCATCCCATCCCGCTAGAGAGCCATCCAAAGCACTCTATATTTTTTAGCGAACACAAAAAATAGGATGTGTCAATGATGTGTCCTAATTGGTGACATAGCGTACAAGTTATTGTACAAAATGTCTTACCATACAAAATATGTTCTGGACCCAATCCTGCATAAGTCGATACATCACCTTTTACAGGAAAGATAAATCAATGGTGCCAGGAAACTCTCTGAAGACAGACTCATAAAAATCAATCACATCTCTTTTTTCTTCCTTCTACTCAAGCTCCTCCACCTTCCTCGCCTCACCTTCAATAGGTTATGAGCCCAACGCAAGCCTCCACCGCTGACGACCGCGAGTTTCCGCGCCTCAATGGCCGGAACTTCATCATCTGGAAGACCCGCGTCACTGCCGCCCTCGAAGGAAAGAACCTTCTCGGCTACGTCGAGCGTATCGACTACACCGGTGACTCCGACTACGAGTTCGACTCCGATGAAGAACTCAACCCCGGCACTGTCCGACATGAACGACATGACCGCCGCGCTTGATGCTGCCGGTGCTCCCAAGGCCGACGAAATGGTGGGCTCCTCACCGAGCGAGTCCTCCTCTGATGCCAGCTCCGCAAGTTCGGATGCACCCACAGCCGGAGACGACGGCGATGTGGAAATGGGGCAGGAGAATCCCCCCGTTATCCAGTCATTCACTGCTCAGAAGCGGGATGAACTGAAGCGAGCAGAGAAGCTGAAGGCTAAAAGCCAGAGGCTGAGCTCGAAAAAGCTTCGACTCAACGAGGCAAAAGCAAAAGCTTTCCTTATCAAGACCATCGATGACCAACATGTTCTCATGGTCAAGGACGAGACTACTGCGTTTGAGATCTTTCAGACGATCTGTAGCAAGTACGAAGGTGCTGCCATCCACGGCGGCCCGTATCATGTGCTATCATACCTGATGGCACTGAGGTATGAAGAAGGTTCAGACCTTACGGGGTTCATAATTGACATCGAATCTGCCATGAAGGTTGCCTCCGATGCCACAAACAGTGTGCTCAGTGACGAACAGAAGTCACTCTACTTGTACCATGCGCTACCTGTAAAATGGAAGCAGCAACTATCGGTCTGGAAGGGTAGCGGGAAATTCATTCCCTACGAAGAGCTAAAGCGTCACATCGAGGCCAAGGTGCAGCATGAACTGGCGAAAAACCGCTACACGCTCAAACAAGGAGCACCCGAATCGAGGGAAACCCGAAACGAGCAGGCCCTACAGGCTTTAGCACCTGAACCAACCTTGGCTCAAGCTCCCTATCAAGAGAGCAAGGCTCTCATCTCAACGATGAGCTGTACATACTGCCTGCGCACCAACCACGATACTGTCGACTGCTATGTTCTACAGCGCCACCTACAAAATGGTCAGGTAAAGGCAGGAACGGCGCTTCCCGCCAACTTTAAGCTGAAACCTTCGTCCAATCAAGCTCGACAGCATCCTTACAAGGACGGTTTCGGCGGCAAGTCACGAAACCAAAATCGGAATGACTACAAGAGCAAGCGGAACAGCAGTCGAAATAGAAGCCAGAACGGCAACCAGAAGAATACATCTGGAAAATCCAAGAGTCACAATAAATATCACGGTCGCCGCAAGGACACCGACTCCGACAACGAGTATGGAATCATAGCCATCACCACTCTGGACCTCAGACAAGAAACAGAGGCTGTAAGTCTCACCGCCCAAGATGCGGAGCATGACCCAGTGTGGACTGTCGACTCAGGCTGCACTCGCCACGCTACGTCCAATCCGAAGTGGTTTGAGAAGCTGATTCCAAGTGCTGGCAAGTCAATCACTGTCGGCGGCAATCACCAGATTCCGATAAAAGGAACTGGTGATGTCAAGATGACAATCAAGGACACGAAGGGAAAGGAACGAGAAATATCTTTGGCCAACGTGCTATAAGCGCCCCAGCTGAAGTTCAACCTACTCTCCGTGCGCCAAGCCGTCGAAGATGATTTCAAAATCAACTTCCCCAACGCAAAGAAGTGCGTTCTGTTCTTCGCTAGCCGAACCA

>Contig_63

CGATTCTGTGCCAGATGTGATTAAAGTTTTGAATAATGTGTCGCATGATGTCTTGCTGTTGTTGTTGTTGACGGAAAAGCTTTTTTTTTGATATTTTAACAATAATTACAGGATGGTTACACGTCATATAGGCTCTAAGTTTTACGTTTCTGAATGGGGTCTACTTAACCAAATATAATTCACACCTCTCGTGTTTTGTTGTTGTCTTGCCGCTCCATTTAAAATCGTATCCTACGCCGCTGTCGTCGACACGTCCGCCAGTAAAGTAGTATCGGATTTTGCTGTCTAAACAACTCTTAGAAAACAACGGCTTACAGCAGTAACACATACGAGTTCATTTTGTTCATCTATCTTTATGGATCAGGTGGCAACGTTTATTAGCACAACGTTCTTTTTACGGCCAGTTCTTTTGACCAGCTTCTGTTGTACATGTACTACGATTCATCAGCTCAGCCAGTTACCTCACTCTCAGATAATTTATTAATATTTCTACATCAAAACCTTGAAGTGCAGGTGATTCTAGGTGGAAGATGTAGGTACAATGAAAGCTAACAATCATAAAGAGACTTTGTCAAGACCTCTAGAGTAATCTTCATAAGCATCGTAAGTATGATGCTAGGGACTCAGTCTTTTGTAGTACTGTAATACTTGCACGGCTTACTGGGAAATTGTAGCGACTGGTCTGAATGGAACCCAGATTTATTAAATTTTAAGAAGTCGATTTAGGCACATGGGGATTCAGAGTTTTTGGAATAATTATCCCGCCGAAGCGAAGCGGAGGCGGAAAGACAAACATATAGAAAAGAACCTAAACTACCGCCGCGTAGCTGCGGCGCTCGGCGAGTAGTGGTACTATTAATACATGTACGTAGTAACTCTAATTATATTTACTCTTGAGTCAAGAGACTTGACTACCCGTTTTGGGCAATTTACGACAATGTAAATGTAAATGGGTTTAGAAAATTGCCCAAAACGGGTAGTCATAATACATGTACAAGACTCACCATCCAGTGATGGAGCCAGCGATGCCCATCATTTGCTTCATTCACTCCACCTTTCGCTGACCAAACGCAAGGTGCAATCCGTAATGCGCCCACTTTACGTCGTGGTCCTGGTTGTCGCTGCTCTCCCCGCTGTAAACGCAGGAGCGCACACAAACTACCAGCGTACAATCGCGACCGGATGGCAAGAAGTCCCCTCCAGTCTTCATGAGCTCACCACCGATACATTGAGCACAGACAACAGTGTGCACGAAAGCAGAGATACCACTCGATCCGCCGTCGAAATGTTCCGCAACGCCCTGAAGTCGTCAGTGCCCGATAGTCAACTCGAAATGTGGTTTAAACACGCAGTCTCAACCGACGACGCGCTTAAACTCTTCTTACGGGACAACGCAGCTGACGACCTCTTAACCGTTTGGCTTAGCTACATGAAACGTTTTTACAATGCGGAATTGGCTAAAAAGACGAGTTTAATCGCGACCTTAACTGCCCACTACGGGGACGACGGAATGGCAAAGCTAATCGAAACAGCCAAGCAAATGCCATCCACAGCAAAGACCGCTAGGCGTCTGGAGAGCGAACTAACGCAGCTGTGGCTGGGTCGAAAAAAATCCGTGGACGACGTGTTTAAACTGCTGAAACTTGACGAAACAGGAGATCAGCTCTTCACGCAGCCTCAAATCGTCGCGTGGGCCAAGTACGTGGACGATTTCAACAAGGCCAATCCGGACAAACACGTGACGTTATTTTCGTATTTAAAAGGCCTCTACACTCAGGAAGAAACTCTTGTCAAGATGCTGATTACAGCGAAAAAGTCGCCCATGACGGAGAAAATTGCAGTCCGAATACAAGCCGAACAGAGCAAAAACTGGCTCAGCAACAAGAAATCTCCAGGCGTCATCTTCACGTTGTTAAACCTTAATGAAGACGGGATATCTCTAGTCAAAAGTCCTCTATTCCAGAGCTGGGTCAAGTACACGGACGAGTTTAACATGGTCAATCCAGAGAATACAGTGGCCGTGATCTCGGTCCTAAAAGCTCACTACAGCGACGACGTGTTGGCACGAATGAGTCTATCAGCGGACAAGACGCCGAGCACGCAGAGCGTTGCGAACCTTCTGCGCTCCGAGCTGCAACGCGAGTGGTACGCGACCTTAAAATCTGGCGACGTCGTGTTTAAAACGCTGAAACTCGACAAATCGGGGTCGAAAGTGTTCGAACGGTCCCTTTTCCCTCTTTGGAAGGATTACATGCAGTTTGTGAGCTTGAAGGACCCGAGGATTAAGGTGAGTTATATAACCCCGCTCATAAAGGTATACGGCGAGAAGAAACTGGCTCAGATCCTCATTGCAGCCGAAAAAGTCCCAAGCACTAAGAAATTCGCTACTGAACTACTCGATAAGCTTTTCTATCGCTGGCTGGACGAGAGGAAAAGCCCGACTCTGGTTTTCTCGCTGCTGCGTGTGGATGGAGCAGCAAAGCACGACGCGAGCAGCTTGATTTATAAAAAATACGCTGAGGCTTTTGCCGGCCTACCCTAAGCTACCTAACTGTAACAACAATATGTTGGTGTATTTTTTTCTAGTCAAGATAGTGCGTATGCCTTAAAATATTTAAAGTATAGCTGTCTGTATGGATCTTTCGAGTCTTCGACTCGTTCCAGCCGCAACAGAGCATATATGTCCATCGGGATCCTCTCATCCTTCAGCCAGACCTTGACTAGGTCATCTTCTAGTTCTTTAGCCATCTTCTTGGTATTCGGGTTCTTGCTGCCCGCTCGGATCACTTTTACGAGGTTCTTTTCGCCATACACATCTAGCAAATTCGTGAGAAAACTCGGTTTATATTTTGGCTCTGCAGCCCCGAAATACGTCGCGTACTTGCTCCAAACATGAAACAGCGGACGGTCAAACGCGTTCACTCCTTCGCTGTATAAATTGAGCGTCGTGAACACATGTTGAGGCGAATATTTGTGGATGTACCAGTTCCTCAACTGCTCAGTTTCGAGCCGTTTCGCGATGTTTGCAGTACTTGGCGAGCTAAGTGCTTGAAGGATCATCTTTGTCAGGGTTTCGTCACCATATTTCGCCGTTAACGTCGCGATTGTAGTGAGCTCTGTTCCGTAGTAAATCTTCCGGAAGTCGTCGGTATACTGGACCCAAGCGCGAAAAAGAGGGTTCTGGATGATGTCATGTCCTTCCTTATCAAGTCGAAGCAGTTTGAAGACGTCCCCCGGCTCCTTCTGAGTGTTCAGCCATAGCTTCGTTTGGACAGCTTGCACTTGTACTGCAATTTTCTCGGTGCTCGGAACATTCTTCGCCGCGATGAGCATTTGTAACAGAGTTTCGTCATTTAAGCGCGTAGCTAGCGTAGAGAACAGAGTAACTGGTTTGTCCGGGTTCACCTTATTGAAATCACCCACATATTTCGCCCACGAGACCATCTCAGGGTGGGTGAAAACCTTCTGTCCGGCCTTGTCAAGTTTCAACAATGTAAACACATCGTCCGGGGATTTCCCTTGATTAAGCCACAGCTGAAGCTGCTCGGTCTGTAGTCGCTTGGCGAAAGTCGCTGTGTCCGGCACTTGCTTTGCTGCTACGACCATCTTGGCCAGAGCTTCGTCGCCGTAGTGAGTTGTGAGGGTTCTGATCAGCGACGTTTGTTTTTTAGGATTTGCCTGGTTGAACAGCTTCATGTACGTGATCCACTCTGTTAACTTCGAGTTGGCCAGAAGATCGTCCGCGGCGACGTCGAGTGTAAGAAGCTTAAAAACGTCGTCGGTAGGTTTTCCCTTTGCGAGCCATTCGTCAAGTTGAGTGCTTGCGGATTTGAGTGAGTTGACAACTGTTTCCGCTGCAGGAGCACTTACACCTCNCTTCTTCATCGTGAGCTCTCGTAGACTCGTACAGTCGCAGTAACCTCGGGACACTATCCATGGGAGTGATTGGACGTTCCGGTATCTCGGTTGTTTTCGGGCTCCAGTCAGCATGATCAGCGAATGTGATAGCTACCAGCAGTACGTATAAGACCCAGTGACAACGCATTTTTTTCGGGTAGGGAGCTAAGCGGGAAGTGGTCGAGACCAACCGCAGCGAACGACAAAACGCAATGCCCGCATTACAGCAATCGGTTGCGGTACTGTGCACTTGAATCACTGGATGGTGAGATATACATGTACAGCACAATGTCGTGAGCGTACATACATGCACAGTGCATTGCACTCAGTGGGTTGCACTGCACCGCTGATCCGACGGTATCAAAGTCGCATTATTAATAGACGGTGCATTCAAAAGTTTTGCATCAAAACGCTTCAGTGATTTGATCCCACGAACATGTTTGCCACGTGCTGTCTGTCATATTTTAATACCTATTTAAAGTAGAGAGTGTCTATATTGACCGACTGAGTTAACCTTCCCCTACAAATTTTTGACGCGCTCCCATCCGTACGATGTATACAGTGTGTACCATGTATACCATTCAGCCTGACAGCCACTAAATTTCGAACAGCGTAACGATCGTAAGGCTGTAGGAGGGGGAAACGGATCTCCGCGTCCGAGCCGTCCAGAAGAGGCCGCTAACTGGGGCTGTCGCGCGCGAGATCGACGAGAGCGACAGCAGCGATTCTGAGCCGCCCCGAGAAGACTCCCAGGACGACGTCTGGCTCCCAACACCCGCTGCGAGCGGTGCTGGTTCGGGTGATGGAGAGTCTTCCGAGGCTTTCAGTGTGTCGGATGTAACCGATTTAACCAGGAGCACTAGCGAGGGTGGTCCCCCCACACGCGTGGTGGCATTACCACTCAACACGGTGGAGTTCGCCTCGTGGGAGGCCTTCGACGAGTACCTGGCTGCGTATGAAGCCCAAACGTTTCAAGTATGCGAGATTCATTCAAGATTTAATAAACGGATGTCTGACCCCGCTGTCCTACTAATTTTGTGCTATCTGTCTTGTAGATATTCAGGGCGCGCTCTGATACCAGCGTGGCAGAGCGCAATGCCAGGATTGACAAGTCACAGTCAAAAGCAGCTAAAATACCAGCATCGTGGGAACAGTATTCGAGGACGTTTGTACGCACTCAGGRAAGTACAAGTCTGTAGCTACGAAGCGGCCACGCCAAGAGACTCGAGCACAGGGCTGCAAGGCGCAGGTACATATATGTCGGCCTGGTCGGGACTGGTCGGGTCCAGTCGGGGTCAATCGGGTAGTTCACTGACTGACAGTGGTTATATATGCTCTACAGATTAATGTCTGCGTCCAGGTAGTTGATACAAAGAATACGACGTTTGCTGTGCGGATCACTCGTTGCCGACCGGAGCACAATCATCGTCTGACAGGCCTGGCTTTCTCTTCACATCCATCGAAACGCATTATCCTCGACGAGTCAACGCTAACTACAGTTGATCTACTTCGAAAAAAAAGGTGTCAAGAAGTCTGGTATCATCAGCCACCTAATGAAGCACACCGACAGTAACCCCACCCCAAAAGATGTGCAAAACCTCGTCCAGAAACTAAAGGCTCGCGAACAACGCGATGGGCCTTCAAGCGCTGACAAGCGGCTTAAGAAGTGGATGAAGCAATTCGGTGATGTGCCAGGGAACGTTGGTCGGGTTTTCTTGGACGACGTTGGTGGCAAGGTAGAGTGAATGCTTTTATCAGGTCTAATGTCAATTCATGAAGTTAACATTTGTTGTTTTGTGTGCTATTGGATACGCAGAAAGTTGCGACTTGCATCACATTGCAAACAAAGCACATGCGAACACTATTCGACCAATTTCCTGAAGTTCTGATGATTGACGCAACGCACGGGACGAACCGGTCTAAGTATAAAGCTTTTTCATTCATGGCACACGATACGTTCGGCAAGGGCCAGTTTGTGCAGCACGCGCTGCTTCAAAATGAGCGCTATGAAACCCTGTTGACAGCGATAGAGGAATTCAAAGCGAACAATCCTGCGTGGTCAAGACTTCAATGTGTGCTGGTGGATAAGGATTTCACAGAACTGTCCGTACTTGAAAAGGCATTTCCTGGGGTGACAGTCTTACTTTGCCAATTCCACGTGCTGAAGTACCTACGGGAAGAGATTGCTTCAGCTGACTATGGATTTTCGAGGTCTCAGAAAGAGCAACTTGGCGGTGTTGTGAGCTTACTTGTCTACGCAAAAACGGAAGCAGAGTACGACAAACGCTTCCAGTACATGGTGCATCTTGCGTCGATTGGTTATTCCGGTCCAGTTGCGAATGATCGATCTGGTATCGTAGTCGGTGTGGAGTCGGGCTCAGTCGGTGTGGAGTCGGGCTCAGTCGGTGTGGAGTCGGGCTCAGTCGGTGTAGAAGTTACAGATCATGAACTGGAAGATGTTTCTTCTCGCATTGATGAACATCCTTTTGTGAAGTACTTTCGAAAGAATTGGGACAGCTGTCGTGAGCTCTGGTGTGCACATAGGCGCCAGAATACCGTCACACTCGGCAATAATACCAACAATCGCCTAGAAGCCTCTTGGAAGCAGCTTAAAGAGTGGGTGGATTCGTTTATGGGAGTGGATGAGTGCATCGCTTCGACCATGTACTATCAAACGCTACAAGAGCGATTGTTCATTGCAGAAGTATACAAGAATGTGACCGTCCAGCATCCTGATTACGACAGCGAGATGACACTGGTGGCGAATATTGTCAGTGAGCATGCTTGTGAACTTATATTCGGGCAGTATTCGTATGCTATTGGATCTGCGTCGTACACGTTTTATGAGGGATGCCCAGACGTGTATTTTATCAAGAGTATATCTACAGCTGATGACGCGCTGGACGAGATGAACGCCGAGTATTTAGTTACTAAGCGCGAATGGAAGTGCTCCTGTCTATTCATGTGCACGAGATTGCTACCCTGTCGACACGTTTTCTACCTCCGAAAGGCTCTTGGTCAGGAAGGAGACAGTCATCCCCATACTACTGTTAAACAGGCGGTGGCTGTTGTCTTCTGTGCGTGCGGCGATTAAAAATTCAAGTGTCGGCTCTGACACAGCGGAGCCAAAGTCTTTTGAGGTAAAAAATGTGATATCCTCACAGGCGAGACCGTGGGACTCGAACCACAAATATCGTGAAGCACTGCAATTTGCATCCCAAATTTGTGACACCATGGCGGGACGCGGCATGACGCAGTATAAAGAAGCGATGCAGTATCTTCAAAGTGTGACTGTTCAAGAGAGGCAAATTCGAGGATCCCGGTGTGCCAATTTTACCACCAGCTGAATCTGAGCGTCACGGTTCGACTAGCAACTCGCGCGATGATGCGCTGTCGCCGCCACTACTTACTGGTCACGATCCGACTGCACGGACGACTCA

>Contig_64

GGAGAACGCTTGCTGGAGCTAGACTAATATTTTAACCGCCCTATTGCCCACAGCTCAACCCGATAGAGCCGTGCTTTGGCCTTCTAAAACGTTGGCTTCAAAGGCGTGCTAATCTCGTTTTCCATTGTAGAAGCTTAAAAAGGTATAAAAGGTGGAGTTACTACTGGGTAAACGTACCAGGCGCGAAGGGTGATCATTCCTTGTCGTTTGAGCTCATTGGATGATTTTTCAGTGAAAAGGCAGGTGAGGAGCAGAGCAAAACACTAATAAAGTGACTGTACACACGTTAGTGTATGAGTAATCCCTCTATGGGGGGGGCAGCAGCACCAAAATAGTGGCGGATTGCAAGCACCATCTGTATGGTAGTGTATCGCTTTAAAAACGAACACATGCAAGATGTGAAGGAACTTCACACCAACTCAAACGATAAAAACACTACGTTCACTGAGATAGCTCAGTGTCATACAATCGCAACTACACTAGTAATGGCACTTAGATCCCTATACGTGTAGTTGTGACTTAGAACGAAAAGAACGTAGCTATCTTCAACCGCTGAAGATCTGCATTTGCGAATTAAATGTAAATGTATTGTATGGAAGCATTGTCCACGTGTGGCCGCACTGGTTGTAAAGGTTCAACTGTCGCTGACAATTCGGTTCGAATAGCTTCATCTGAAAAAAAACTGGATGAATCTGCTCTTGGCTGCATCACGGCCGTCGTTGCAGCCGACAATGACTACAAGATGAGGGACGGAGTGGAGGTTGTGGTCAACATGGCCCGACTTTCTTTGTCACTAACAGTGTCAGATCTCGAATCCAGATCAGGTTCTGGATGTTGCAATGCGAGCGTGTACGCGTCTCGAAGATAAAGGTTGTTTTGGCACTTTTCGTCACTGTGGCTACATGGCTACTGGAATAAGAGACCAAGTGTTTGACGATCTGATTCGAAGAAGTGCAGACGATGAGTAGCTTAATTTTGTAATGATGAATATTATTCGATACATGCATCTCGTACTGCACTAAGCTCGCGTTTTCTTCTAGTTCATGGGCTCGTCATCGGCATGGTCCTCGTCGTTCATCATTCCATTGGTTGGCTGAGTGGGTGTGAATTCATGGTAGATACCGCGAGAGAATATATCGTGGTCTGGCAGTCGTAATGCACGCCGAAGTGACAGAATGTCGACCTCAAGAGGCATCCATAAACCATTGACCCTAATGCTCACAATTGCTGTATCCGGTATGGCCTGTTCATCGGCTTCCTGCTGCTGCAGAGCCTCTCGCTATCGGTCTAATTCCATTGATTGTGCAGTTGTGTTATCCTCAGGAACTTCAAATGAAGCCGACTCTGGTCTTCTTGCATGCACAACATCAAGATGGTGCGAAGTGATTGCTCCAAACTGCACTGCATTAGCTGTCTCGTAAGCTATCCTTTGTACTCGAGCATTGGCAATGCGACGCGCGGTAGCACGGTAATATTGCTCTGGTTGAGCCGCCCGCTTGACGTAGAGGAACGTCTTAAAGCGAAAATCTCCAATGTGGCGGAGATCATCGGTTGAGAGTAGTTACCATCGGTGTTGAAGTGTTGTCAAAAAGCTATCAGACGTCAGCTCGATATACTTGGCTTGAGTATCGTTCCTCGCACGCCGCAAGTAAACGGATGAGTCGTCTGGTAGTACGAGAGGCTTTTTTGATACTTCAGGAGACGCCACGAGCGCAGTATATTTTGCAATACACTTCTGCTTAAAAACAGCATAGCCTTCAGAAACTTCGAAATCGACATCACTCTTCCAGCCACCACGAGCGCGGCCAAGCGGTTCTCCCACTCGTATAGTCAGTATTGCATCAGCTCGACTTGGCAACGGCGGAGGGCCAACATCGGGCGGCCCGAGAGCATCGACTGCCCATGGAGCGGAGGACGACGTAGCCCCCGTAGATGCACCGACACCAGGCACGATACTGCTGTCGTTCGATCCATCGAAACCGCTAGTCGACATGGCAGCAAAACTCTAAATGTTCACCTGCTAGGCGATACGAATTCAAGTTGTCCATACAAATTATTGCTGTGCTTGCTCGTCCTCAGTATAAGGCTTTATTGCAGACCAATATGATGTCCGTAGTGTATGAGGGCCTACGCTTCTTATCGAGGCATTAGACCCGAATCATAATTAGCACAGCGATCCTTCAAGCCTGCTACTCAATAGCCGACGACTAACAATGCAGCTGCAACTTACCTGTAAATTTACCGACAAGATTGATAAACTTCCTATGCATTCTGTGCGCCATTATGAGCAGTACGAAGTCGGTCAGTTAGCATTGATCAAAAGCCGTAAAGAGACCGTACCTGTGCGCTAGCGTGCATGCCTCGTGAGCACGGCGTCCCAAGCAGCTTCACGCATATTTCCTTCTCCTGCGGCTGCCAACCCTCGATGTCCGTGGGCTCCTCGGGTCTTCTCTGGCCTTCGTCTTATCCAGCATTCTGTACAGACTAGGTGTAGTTATAAATGTGCATTTCCCGCCAAGTAATGCGCTTATAGCTTACCGACGGCGTGCGCCCCTGGTTACCTCGCTGCCTGCTCCGCTGCTTTGCTGTCTCCGTGGCCAGTGAGGATGTCACTCGGCTGGCCCTCCAAAATGATTCAACCTATCCCTATAAGAGGTCAGTTTTACAAATCGGACCCCCTCAGAGATGCGTAGCTAAAACGGGCGGCAAAAAGGCTGAAAATTACAATACATGTACTACGAGCCGCTAATGGAGCGTGCTGCGCGCTCGCTTCAGCCTCATCTGGGATATTGTTGCGCACCACACTGCATCCATCGCTTTCCTACGGGTGGAAATTGATTAAGAACGTGTTGAATCGTCGCCATCTACGAAAAGTCCGTGGACAAGGCGGTACGAATTCGAGTGCCTTGGGCGCATCGCCACCCGAGGAAATGTAGGTCATGTTTGCTTCGTCTGTCTGTTGGTTAAGCATTCCACGCATCCCTACCTACACCAAACGTCGCCTCGCACGTCTACTCGAGCGATTGATGCCAGCGTACGCCCAGGCATGGCCCGTGGCTGGCCAACGATAGGTATCAGCTTGTGCAATTTTATTCCCGGGATTAAAGTTATTCTGACTTTGAAAGTCAGAGTAAAGATAATTCGAGAATAACCTCTAAGCCAATGAAATTAAAGCATTTGTCAAAAAGGGCTAGGCTAGAATATCTTCATTTATTTTTATTCTACTTAATCTCAAATGAAACAGCCCCTAAGGGCTGTCGAACATGGGCGTTCGCTTATTAATAGGACAGGGGCGATGCGGTGTTTGTGTGAAAACCCGATTGCGGGGAAGTGTTTATGAGATTAAATGTTTACGTGTCATAATAATGTATGACAAAATTATCGTAATACAGCTCGATTTCATGGCCACAAGACACATTGATTTCACAACTGTCTGGACCAGCAACTATGCGGCTTCGTTGCTTTCTGGTATTAATTTGTGTGCCTATCATCTTCTCGATCACATCCATTGATGCTTCAAGTGCCAAAGTACCTCGCTCACCGTTTTCTAGCGGTAACGATGTTCACAAGGCCGCAGAGAGATTCTTGCGAGTTAGCCACGCCACTGATAATGATGGCGACGAGGAGAGGGCAGGAGGGGGTCTTTCTGTGCCTATCGTAGAGAAAGTAAAGACGCTGGTCACGCCACTGGTTGTGACGCCAGATTTCATTAGAAGATGGCTCAAGAAAGAGAAATCAGCTGCATCGGCATTCCAGCGCATGCATCTCCAGGATGCCGGAGATGATCTCTTTCTGAACGAACAGTTCTTCAAGTGGGTCTCGTACGTAGATGAGCTCAAAGTGGTCACCAAGAATGAAAATATTAACCCAACCTCGACACTAGCTGCCAAGTACGGCGACGATTCCCTCTGCAAACTCATTGAAAAGGCCAGGGAGAGCACGAACGTGAAAACGAAGGAATTGGCAACGCATCTTCAAGCAGAGCAAATGCGGTACTGGATAGATACAAGAAAGGATCCTAGCGACGTCTTTCATCTATTCCAGCCCAGGATCAAGTTAAGTCAGGATATCTTCAGCAACCCTGAATTCAGATCTTGGGTCAAGTATGTGGACGATTTGAACGCAAAGTATCCAGAGCACACTGTGTCAATGGTTCCAGCATTACGGAAATATGCCACGGATGGTGATCTGCTCCAGATAACTTTGAAAGCGAAGAAGATTGATGCAACCAAAAGCATCGCAAAGAGGGTGGAGGACGATGTGCTTCAGTTCTGGTTGACTAGTCGAAAAACACCGGATGAGGCCCTGGTTGGTCTCAAACTTGACGTGTTGTACTTATTAGAAAACCCGATATGGAGCACGTGGACCAAGTACCTAAAACAAATTCAACAAAAGGTACCCCCCCCCAACGAGGCAACCAGCACGGTCGCCACATTGACTCGGAGGTTTGGCGATAAAGAAGTGGCGACATTCTTATAGCGTCGAAAGCGAAAGGTATGACGAAAAAGAGGGCCACGGAACTGGAGTCTGAACTGTTTCAGATGTGGCTGAGAAATGGTGAGTCTGTTCAAAAAGTCTTCGTCAAGCTAGAGCTACACGAAGCAGATTTGTATCGCACGCAGCTCCTCAAGACTTGGGTCTCGTACATGGATACTGTCGCCACTAACGATCGAGGAAAAATACCAAAGATATTCGCGACATTAAAGAAGGATTACGAAAAGAATCCCGGCCCCTTACTCCATATACTCGATGCTGCAATGAAGTACCCCAGCATGGAAAAGGCAGCGACGGAATTGCGAGATAGGACGCTTACTGGTATTGTTGCGCTTTGGAAATCTCATGGCGAGGTATTTAAGCTGCTAGGGCTTCACAAAGTCCCGGAAAGTGATCTCGTCAGCAACTCCATGTACAAATGGTGGATGCGATATGTGAAGAATATGGAGAACTTAAGTTCGACTTGAATCATGATTTGAACGGGATGGCGCCAAATCACGAAGGAAGGAAAAGCGAGGCGATCGGCTACCACTACCACTACACTAATCGTACCTGGTACCACAAGTTTACACAAAAAAGCTTTAAAAGGTTAATTTTTGCTTCTTCATACTAGTACTGCACAAAACTCGACAGCTGCAGACAATCCTCTCGGCAGAAATATCCCAAAACGACACTCCATAAAATTTCGGTTTCCGAATGGCTTCCAAGGTTAGTGATTTGAAAAGCTGAATTATCACTAAACAAAGGTCATTAACTAAGGAAAGGAATCATACACGGAAGGATGGTCAATGCAATTCAGCAGTGCTTGCAACCCGTATATCTGTAGGTGCTGTGTGCTGAAGAGGCGATTGGGTGACGTCATGCGTATCAACACTTCGCCCACTCCCTCAGTAAAGACTTGCTCGGAGCAAGTTGCTCCACGGCAGCAGCTGCTACTGTAGCAGAGGCGTCATGAAGCTAAGAGCTTGCTTTGCAATGTACATGCACTGCATAAATGACTGGATGGCTACATTCGTTTGCATGTACATGTAACTTACGTTTCGCTCACCTCAGCTCTTCGTACTCTGGGGGTTGTTGTGCGCCAGTAGGTCCGAGGCTAGCTTGGCCAGCGTCTCCATCGTCGCGTAGTCCTTGGTGGAGATGTTGTTGTGCGCAACTCCGTCTGCTGCACACAGGGATCCTACCACTTCTCCGTTCGGGGCTCGCAGCGGGAAACCAGCGTAGAACTTGACGCCCAGGTCTTCCACGCACGGCATCTGAGCGAAACGCATATCGCGCTGCGGGTTTTTAACCACTAACGGTTTGTCCGCGTACACGGCGTGCATGCACATGTTCTCGTTACGAGGAAGGTGATGCGCTTCCTCGGGTAGCTTGTAGTTGCCGATAGCGTGGAACTGCGCATCGTCTACCATCGAGACGAAGCCGATGGGACAGGCGAGGCGTTCGGCCGCCACCTGCGCGATCATATTCAGCGCCGAGCGGTCGTAGTCTGACCGCAACGCGCCAGATTGCTGGATAAAGTCGACGCGTCGGGCGTCCTTCTGTCCATCGGGCATAGGGGCTAACGGGTGGTCTTCATTCGTGACTTTGGAGGTATCGAACTCGTACTTGTAGTCTCTGGTGCGATCGCTTACGTCACAGGCATCAACGGTGTATTTGAGCCGTGACCTGGCCAGCTCGTGGGTCACGTGGTTCTCCACACGCTTCCGCACGCGTTGAGACTGCGTTTTGGTAGTTTTCTTACTCTGCCTCTTAAGGCGTCGAGTACATCCATTACTAGATAGTTTCACGAGCTTACGGCTCGAGTCCGAGGAAGAATTGGAGCTTGCTGGGCTCCCTACAACTCGACCAAGCATCTCCAGTGCCCGACTGCGTGTCTCCGGATCTTCGGAACAAAGCTGACCGGGTAGATCGTCCATATCAGACTCAGAAGACGTGTCGTCTACATTACCGTTCGAGCCCAAGCTCTCCCAGACGTCGGGAGGCGCTTCGACGAGGATTGGACCCAAAGCAGGCAACAGATCTTCGTCGTCGAACTTACACGAGTCAACACGTACGACGCACCGAACACAGCAGCGGTTCTTACGAACTTCTCCGATACGAGCTTCCACCTCGTACAGCTGAGAGCAGTCTCCGCATACCATGTGGCCGCAGAGTTGGCAGTAGAAGTCACGACGGAAGAAGTGGAAGCTCTTGCTGCAGATCGAGCAGCGTTTCTCCAGAAACGGGTCTGCGTATTCCTTGGGGAAATAGATGAAAGACTGGAAGCCAAACCGCCGGCGACGGATCACCCGTTCAAACTGCGCGACCGACTCGGTCAACACCCTCATCACATGACGCGCCTCCGGGTTCATCAGTGCTGTACGGCGGCGGCTCAAGTCGGACGCGTGAATTCTCGCAGCCGCCTCACGCTTCTTGGCTACGGACGCTGCCCGGTTCTCGCCGGTTTCCTCTCGGAAGGCCGATGACGGCCGGTCCGAGGCGTACGCATGGGCAAAAATGCGCGTCGTCTGGCGACTACTACCTCCAGGACGTGGCGTGAACTGCAGATCAAACCCGACAGCAATGTGGTCGAGTTGACGGCGCAGCGAAGAGCGGTTCGAGCTAGGAAGCACCTGGTCGTGGACAGATTTGGGCACTGTCTTCATCACGTGCACGGCGCGTTTCTTGTCTGGATACTGGTGCGTACAGGTCGAGAACAAGAGTTGTTGCGTGTGTTTTATGACGAGAGGGTAGTAGCAAACCAGCGCTAGGACGCAACGTGGCCGACGCGACAGTGATGACGCTTTGCTGCGGTGTGTCGTCGTTTCCATCTTCCACGGGAACACGAGCTTGCTCCTGCGAGTTAGTGTCGAACGCCACGCGTTGGCGGAACAGTACTTCGCCCTTCTTAAACCTCTTGCCGTGCAAAGCTTTCATCGTTGTATCATACGCATTCGAGTCTTGATGCAGCAACACACTCAGTGCCTCGTT

>Contig_65

AAAAGTTGTGCATCCACAAAGAGCTCGACGCACGCCATCGAGAGCACATGCGAAGCGGATCGGAGTTTGCATGGCGGCTGCTGCGACCGCTGCGATCACGGGCAACACCTGCAAACCGGACGCTCGCTACTTACTCGGCCAACCTTTTACAGCTTTGTTGCTAAGACAGCTTAAATCCTCAGTACACGTAAAATATAAGGAAATCGCGCTTTCCGGAGTTTCCGACAAAATCCTTGTTACTTCGAAGTATTTTCCTAAGACGGGCAATTAACATCCACATTCAGCAAATTAGAAGTAAAAAAACAATTAAATCACATAATTCGTATCCGTACCAATTGGTCTGACCCCCGAAAAAAGTTTGGAAACCTTCTCAGGCCAGCTTTTTGCCGCGTTTCACGAGCGTATGCGCATCGGATCGCACCCTGACGATCGCCTTCAGCGAGCCAACATCGAGCAAAATTATACTCGATATTTCACCTTGCTCGCAGTATGAACATTTAAGAAGCCAGACGCGCTAGCGCATTTGAACAGCGGAAAACGTGGCCACTGGTATTGCTAGAAATCTCTGGATTTTACGGCGGAGCTGGCGGTCGAATCTGCAACCAGTCCAACTATGCAAACCAGCCTAAACATGTGCTGCACCTTGTGTCGCCGAAAGCCTTGTCACACGCAGTGGTGAAGAGCACAAAAACGCAGAGCTACCCGATTCAGCACCGCAGATCTGCCCAAAGCACAAAATATCATCTTGAGATAGTCTCCGCGCAGAACAGCTGCTGTCATTGGCCATCAGCAGACTCGCAAATCACCGTATGGTGAGTTTGACGACGTCCACCAAGCAAATATGGGTCTCCTCTACTCGCTCGCAGCGGCAAGATGGTTGATCTCAAATTCTCTGGGACCCAAAAGTAGTCCATCTCGGCTGGCAAAAAAGGTTCGTACTTAAAATGAAGTCGGGACTAGAAAATTGCCCAAAATGGAGACACGATCTTGACACTTCAACATGACCGCTACAAACACGTTTTCACGCGCGATGTGGTTTTGTCGCGTGAGAGTACCCGACAGGTACGCTGGACATTATGCGCGCTGACGTGCAACATGCACTGCGAAAGCCCCCCCCCATGGCTGCATCGGTTTCTGTAGGATGTGGCCCTTCGTGGATCCCGAGCTCGGATTGGTGACAATGTGCGGGCTGTGCAGGTACTCACTCATGCGCGTGTAGTACACAGTGGGGTAGTACGGCGGTGAGTTGTAGCAATTGGGGTGAAGTCAACGTGCAATGCCTTGAGCACCTTGATGCCGATCTCGGCCCATGCCGATTTGGTCTGGCTGTACTGAGCACCGACGCTAGCTGAGCTTGGGCACTGACAGTGCCACTGTGGCTGCTGCAGCGACGCCGAGGTTGATGTCGCTCACGCCAGAGCCACGAGCTACTGCGGCGCAGTGCCACGCGCACTTATCGTGTCTGGCCACTGGGTCCTGTGCTGCGCGGGATTTTAAGCAAGCTCGCAGTAGAGCGTGTTGTCGAAGGGCATTCGCCGGTGGTGAGACTTCTCGTATGTCCATATCGATATAAAGACGCGTGGCGATGCCACAGTGAGGGAGCGGGACAGCTTTCGCGGACGGATGAACTGGGCACTCGATGCATCACTGCATTGACGAAGTTCTTCTCGCGTGTGAAGTCCCGCTGTGTTGCCGCTGATCTTCTTGGATCTGCTCAGGTCTGGCGGGCAGTGGGTGGAGACGAGCTCTGGCGGGCAGCGGGTAGTGTGGCGCTCTGGTGGGAAGTGGGTGGTGTCGAACTCTGGCGGGTAGTGGGGTTGTCGAGTGCTGGCGTGTAATGGGATGTCGTAAGTGTTGCCGTAGTGGAGATACTGAGCTACAAGTCGATTGCTACCAGGCCTCTATCCAGATATGATACTAGCATTGATGAGTATATCTTAGCTGCCCTGATTCTACTGTTGATGGTGGCGGCCGTCACTGCAGTGTAGGAAGTGACCTTGGACGTGCATGACTCCTGTTGGCGCGATTGGTGCGGTAGGGCGATGTGGTAGCCGTCAAGTTGACCCCACATGATCTCATCGGCAGGCTGGATGGGACACCATACCCCGAACAGAGCCGACCCAGTAGAGCTGTGTATAAGCTTTCACGATGAAAGCCCTGTCGGGTATGAACTCTGACCGAGCATATTCACCGTACCAGCTGCTACTCGGAATGGTGAGCTAATGTCGGCGGCGGCGACATGCCTGGTCTCTGATGCGTGGGACCGGGTGTGTTACACCGGCTGAAAGAGGCTCAACATAATCAACGTCGGTCGACGAGTCGTGTTTGCGTGAGGAACGACGATAGGTTGGTGAGTTCTCCAGCAATTCGTGGCCAATTGCAGAATGAGCTGCCCCATTCGAGTGGTGCGACAGCAAAACGCAGCGACCGAAACGGGAGAGTCCACTCTCCGCATGAATGTCGATGGCAGAGGAGACGGAGCTGCAGGACCGTGAATGCCTCTTTTCGATGCTGTTGAGCGGTCCAATTGACCATGAACCGTCGTGGGTCGAAGCAATCGGATGGCCTTTGTGGTGTGTGGGGCTTACACTCCATGCCTTCAATACTGCGTGAGTACTTCTACCCGCGCAATCGTAATGCTGTTTAAGCAGATACAGTCCTAAGAAAAGAGCGGTACAGATGCCCCAAGCAGCCGAACTTGCTCATGTAGCGAAACGTCGTGTGCTATGATTGGCTAGGCAGCTTCAATTTTGCAAAGGTTTGCGTACCACACCAACTATTACGTTACAATCCAAAAATGGATAATTTGATATTATCTATCACATCCAAATAATTCTTCTTTGTCAGCATCAATGTGTGCAACAATGCAATGCCACCGTACGCGTCTGCGCTCACTACTACAAGTTATACTAGTGCATGATACAATAGAGAGCGATGCCAAATGAATGCTGACAGTCAGCGTGAAGTCGTAGCCGCCGTTGTGAGGTCACCAAGGAGGTTTTTAAAAAGCGTGAGGTCCTTTGAATATTGTTCTTGAGCATGGGTTCACTGGTGTGACCAACGACGTCATAGCAGCAGCGTGAACATCTTCAGAAGGAGACGCGGTGTGTGAATGCCAACTACCTCAAATGTGTTACAGTGTCGAATACATTCGTCTACAGCTTTTTCGGTTCCACTCGGATAGTGAGTTACTAAATACTATGCATAATAATATTTTCAGCTTCAGATAACTCACATGAAGGAAGTTTGCGAGCGGTACGGCCTTCCACATGACAGTGTGACCGCCAAGGAAGTCGGACTAGATTTGTTGAGCTCCGGCACGTCGTACTACTAGTATCAATGTACAAACAAAGAGACAAAGCTGAGAAAGCAAACAAGAATCTTTGCTGTTGCTGCTGGATCCGAAACCTTGTCCAGGTGGAACTGCAGTGGCAGCTTTCGATACGTCTTCTTCACTTTTTCCTGGGTAACATCTCGGTCCAAGCCCAACGTCTAGTACGCTTGCTAGATGGAGATCGTAATGACTTGACGCTTCGAACAGAGAAATCCTCGGCAAATGATCATGTGGAGCCATGAAAGCCTCAAAACCGACGCAAAACTAGTTCATTCGAGCGACACAAGTGCAACATCTACGTAGTTTGTACGTTTTTCTAAGCTACATCCACCTGCTTGGCGTTGTGAGGACGGGAAGCTGCGTTGTATAGAGCCCAAGAGCGGGTGAGACCATCGTCCATTGTGGCTTCCTGTAGCTTGGTCAGCGCGTCCTCCTGATCCACAGCAGCCTGGTTGACGAGAAGCAGCGATAACCACGCGTTGGTGGTGTTGGATTCCGTGACGATTGGCTGCTTGGAGAGGATATCGTCCCATTCCTGCTGGACGAACTTTGTCGTGCGCACCATAGCGTTGATCGGCGACACTGGGATCATCTGGATGCCGTGGATGGCGTACTTCTCGCCACTGAACCAGGTCGCATACGCCGCCTTGTTGTCGAAGAAGATGCCCGTCACGTGGTTGGGTACGAACTCAGTAGGGTGGATGGTGTTGTCCGACGTCATGAGGAAGTAGGTGCGGATAGCGCGGGCGTTGAGACGTAGCATGAGACTGCCTAGATCCTCGACGGCCTTGTTGCTGGACACCTGACCCCAGAGCTTCATACCGTAGTAGAAATTCACATCTTCAGACGTACTCTCTTCGTCTTTACCGTCGAGCATGGGCGTGACACCGTGCGAGTACGAGTGGCCGAGGTAGAAGGAGAAGTGGCGGAACCGAGGGAAGAAAGGGTCGTCTGTGCTGGGGTTGGCCACGTCGCGTAGCGTCATCCAGATCATCCTGTCCAGCTCTTTGATGCGCTCCCACTTGGGGTGTAGACTCTTGAGCATGGCAGACGCGGTGACCCAGAAGCCGTAATGATAGTGGTGGTCGTTGTAGACACTGTTCCCGAAGTCTGCGTTCACGTCTTGGACCGTGAAGCTCTGACTGCTGACGAGACCGCCGTACGACGTCTCGTAGTGGAGAGGAGGGTCCAGCGTGTTGTCCAGGAACGGCACAAGGAGCTTCTCGAGCTTCGTCAGGCATGCGCTGAGCAGCTTCTTGTCCTTGCCTACGATGGCCGAGTCTGCAGCCATGAGACACACCGACGCGTACTTCTGGTACTGCTTGCCGTTAAAGTACCAACTCGTCTTGTTGAGCGCCCAGTGGTCAGCGATATCGGCCTGCAGAGTCTGGAGAAGACCCACTTTTGCTACAACCTCGGCAGACGGTTTACTGGTTGGGTAGAAGTCCACCTCCAACTCATCTTCAGGCTCCGAAAGGACCCACTTTCCAGTCGTGGCTACTTGGGCGACCATTTTGCCTCGAGTGGCCGAATTAAGGACAATCGCTTTGGGGCTTTTAGCAGTGATGGCTCCTTTCAACGCTGGGAGGTGGTGAGGCAGTGCGAAGTGAAGCATCCCGTTGGTCTTACAGCTCTTCCCCACCGTTTCCCAGTTTACCGGGTATTGAGTACGAGACTCCACAGACACGTGGCCACCACGGACGTGACACGCTCGGTACTTATCGTAGACGGTAGTGGCCTTTTTCGATGGTAGAATGGCAACACGCACGGTGCCACTGAACAACCCACTGGAAACGAGAGCTTTGCCCGACTCGTCGAGCGCAAAGCTGCCGTTGTTACCAGTGTACACGACCCACGTCTGGTTCCCACCAAGCTGAAGGATGTACTTGCCTGGCACCGACTTGTCCACGATCTCCATGGTGTATTCCGACTCGATCCGAGCGGTCAGACGGTCGTACGTGGCTGTGATGAAGGCCATGCCGTGCACAAGAGCAGAGTCCAAGCACTGCTCCTTGTTCTCGAGACAGGCTCGAACGTTGATGCCAAAGTCGCTGAAGGCGTAAATCTCATACGTGGGCTTGGCTTCACCGGCAAATTCCGTGGCCGACAGCGTGAGGTCGTTGACAAAGTCGTGCAGATAAAGCTCCGCGACGCCATCGGTGATAGCGGACAACTGCCGGTAGTTGTACGAGTAACAAGCTTGGATGCCGTACGGAGCTTCCTTCGGCAGCTTGACGGCGTACGGGTTGGACCAGCCAGGGTTGGCTTTCGTATTCATCTCTTCAGCGGTAGTGTGGATGATGTTACCCCACCATTTGTTCGTTGGAAGTGGCTTGCCCAGCAGACCTTCCGCAACGTTAATGATCGGAGCCATCTCACCAAGTGCACTGCGAGGGAAGAGAGTTGTCGACGGTTCTTCAGTCGAGAAGGGCGCAAGAAAGTCGCCTCCTCCAGTATTCTGGGTAGCCGACGAACCGGTTGATGAGGCCATGGGGCTATCACCAGAGCCAGACGTCGAAGCAGCATCGGATCCTGAAGCGGACAGGGTGTCAGTCGAACTAGAAGTTGAAGTGTCAGCACTTGCGCTGTCCTCGGATCCAGACGTAGCACTGGCGGCGACATAATTTAAAGAGAAACTAGAGTGTCGTTTGTTACAGCGTCATGATGACGGCACCAAGTGATGTCTTGCAAGTGGACGCGACGATACTAAAGAAGGCGACTCAGTCCAGATTCGAGGGTGCTGCACGTCGAAGGCATCTAGGCAGTTGAAGTTCGGCTCACAGTTTCGCCATTTCACTTAGGAGCAAATGGAGTTAGCTCATTTTGACGCCTTCACAGCATCGACAGGCATCAGCGTCGGCGAGCTCAAGTTGAGGAATGATGCGTTGAGGTTCAGGGCAAAACGGACAAGCTACGCGAGACCAGAGAGCACCCCCTTGTAACTTCAATTGTAAGACTGGTGGCCACCCGTAATGGGTACATGTAGCTTGCCATTCAAAAGTTGTGCATCCACAAAGAGCTCGACGCACGCCATCGAGAGCAACATGCGAAGCGGATCGGAGTTTGCATGGCGGCTGCTGCGACCGCTGCGATCACGGGCAACACCTGCAAACCGGACGCTCGCTACTTACTCGGCCAACCTTTTACAGCTTTGTTGCTAAGACAGCTTAAATCCTCAGTACACGTAAAATATAAGGAAATCGCGCTTTCCGGAGTTTCCGACAAAATCCTTGTTACTTCGAAGTATTTTCCTAAAGACGGGCAATTAACATCCACATTCAGCAAATTAGAAGTAAAAAAACAATTAAATCACATAATTCGTATCCGTACCAATTGGTCTTGACCCCCGAAAAAAGTTTTGGAAACCTTCTCAGGCAGCTTTTTGCCGCGTTTCACGAGCGTATGCGCATCGGATCGCACCCTGACGATCGCCTTCAGCGAGCCAACATCGAGCAAAATTATACTCGATATTTCACCTTGCTCGCAGTATGAACATTTAAGAAGCCAGACGCGCTAGCGCATTTGAACAGCGGAAAACGTGGCCACTGGTATTGCTAGAAATCTCTGGATTTTACGGCGGAGCTGGCGGTCGAATCTGCAACCAGTCCAACTATGCAAACCAGCCTAAACATGTGCTGCACCTTGTGTCGCCGAAAGCCTTGTCACACGCAGTGGTGAAGAGCACAAAAACGCAGAGCTACCCGATTCAGCACCGCAGATCTGCCCAAAGCACAAAATATCATCTTGAGATAGTCTCCGCGCAGAACAGCTGCTGTCATTGGCCATCAGCAGACTCGCAAATCACCGTATGGTGAGTTTGACGACGTCCACCAAGCAAATATGGGTCTCCTCTACTCGCTCGCAGCGGCAAGATGGTTGATCTCAAATTCTCTGGGACCCAAAAAGTAGTCCATCTCGGCTGGCAAAAAAGGTCGTACTTAAAATGAAGTCGGGACTAGAAAATTGCCCAAAATGGAGACACGATCTTGACACTTCAACATGACCGCTACAAACACGTTTTCACGCGCGATGTGGTTTTGTCGCGTGAGAGTACCCGACAGGTACGCTGGACATTATGCGCGCTGACGTGCAACATGCACTGCGAAAGNCCCCCCCCCCATGGCTGCATCGGTTTCTGTAGGATGTGGCCCTTCGTGGATCCCGAGCTCGGATTGGTGACAATGTGCGGGCTGTGCGGGTACTCACTCATGCGCGTGTAGTACACAGTGGGGTAGTACGGCGGTGAGTTGTAGCAATTGGGGTGAAGTCAACGTGCAATGCCTTGAGCACCTTGATGCCGATCTCGGCCCATGCCGATTTGGTCTGGCTGTACTGAGCACCGACGCTAGCTGAGCTTGGGCACTGACAGTGCCACTGTGGCTGCTGCAGCGACGCCGAGGTTGATGTCGCTCACGCCAGAGCCACGAGCTACTGCGGCGCAGTGCCACGCGCACTTATCGTGTCTGGCCACTGGGTCCTGTGCTGCGCGGGATTTTAAGCAAGCTCGCAGTAGAGCGTGTTGTCGAAGGGCATTCGCCGGTGGTGAGACTTCTCGTATGTCCATATCGATATAAAGACGCGTGGCGATGCCACAGTGAGGGAGCGGGACAGCTTTCGCGGACGGATGAACTGGGCACTCGATGCATCACTGCATTGACGAAGTTCTTCTCGCGTGTGAAGTCCCGCTGTGTTGCCGCTGATCTTCTTGGATCTGCTCAGGTCTGGCGGGCAGTGGGTGGAGACGAGCTCTGGCGGGCAGCGGGTAGTGTGGCGCTCTGGTGGGAAGTGGGTGGTGTCGAACTCTGGCGGGTAGTGGGGTTGTCGAGTGCTGGCGTGTAATGGGATGTCGTAAGTGTTGCCGTAGTGGAGATACTGAGCTACAAGTCGATTGCTACCAGGCCTCTATCCAGATATGATACTAGCATTGATGAGTATATCTTAGCTGCCCTGATTCTACTGTTGATGGTGGCGGCCGTCACTGCAGTGTAGGAAGTGACCTTGGACGTGCATGACTCCTGTTGGCGCGATTGGTGCGGTAGGGCGATGTGGTAGCCGTCAAGTTGACCCCACATGATCTCATCGGCAGGCTGGATGGGACACCATACCCCGAACAGAGCCGACCCAGTAGAGCTGTGTATAAGCTTTCACGATGAAAGCCCTGTCGGGTATGAACTCTGACCGAGCATATTCACCGTACCAGCTGCTACTCGGAATGGTGAGCTAATGTCGGCGGCGGCGACATGCCTGGTCTCTGATGCGTGGGACCGGGTGTGTTACACCGGCTGAAAGAGGCTCAACATAATCAACGTCGGTCGACGAGTCGTGTTTGCGTGAGGAACGACGATAGGTTGGTGAGTTCTCCAGCAATTCGTGGCCAATTGCAGAATGAGCTGCCCCATTCGAGTGGTGCGACAGCAAAAACGCAGCGACCGAAACGGGAGAGTCCACTCTCCGCATGAATGTCGATGGCAGAGGAGACGGAGCTGCAGGACCGTGAATGCCTCTTTTCGATGCTGTTGAGCGGTCCAATTGACCATGAACCGTCGTGGGTCGAAGCAATCGGATGGCCTTTGTGGTGTGTGGGGCTTACACTCCATGCCTTCAATACTGCGTGAGTACTTCTACCCGCGCAATCGTAATGCTGTTTAAGCAGATACAGTCCTAAGAAAAGAGCGGTACAGATGCCCAGATCGGAAGAGCGTCGTGTAGGGAAAGAGTGTAGCCAATGGAATCTCGGTGGTCGCCGTATCATTAATGATACGGCGACCACCGAGATAGAGTTCTCTACACTCTTTCCCTACACGACGCTCTTCCGATCTCGGTAGTCTTACGATGGCGGATGTCAGGTGAAGCGAGCTCGTCCTTCAAGTGGAAAAGAAGCGGTATCATGTACGAAATGAATGGAAAGTGCTAGATCTCGCTTATATTTAGCTCTCAGTCTTATATTTTCTACCTTCTATGCAAGTTGAAGCTAAACCAATAAGGAAACTTATATTTTTAAGTGTTTCTTATTCCTGCCACTGAACGACCCTAGGAGAAAGACGGTATTCGACAGTCGCCTCATATTACCGGTATGTTGGAAAGAACGAAACTACTTTATGGGCGAATGGGTTGCCTACAGATTGTAGTATAGAGTTCCTATAACAGCAACCGCTACAACAACAACGCTTGCTAACCGAATTTTACATAGCGTTTAATTCTAAGTACACTCCTGGACACAGTTATACGGCCACCTCGTCGAGTGCCCATAACTCAGTCAATTTTTATTATGTGACGGTGCGGTTTTCACACAAGCTGCAAACGCGCTTCAAAACAGCAAAGATGTGTTAACGCTCGCGTTTTTGCACTTTATAGGGGGGCTCACATACAAAAAAAAGATTTGTCGAACTGGACACAATTTACGGCCACCATCCGACCAATACCAAAACTTGTATAAAATAAAACAGGATAATTTTTAGCTAGTGTCATCCGCATTTTTTTAAACCTAGTATATAATATGGGAAACACCGTGTAGTCGTCAGGATCTTACATTTAAAAAATCTCCATCAATATCGATCAAGCAGAGGTGCGAGTGCAACACGTTACATATAATACAATTTGGTACAGCCTCAACATGTAGCTACTTTGAGCTTTCGATCCCTTGTGTCCATTTAATCGCCTACAGAAACTCAGCCCCTAGAGATTTCTAGTCAGCAAAAATGACACATGTTTATTTTATATAGGTTTTTGTATTGGGCGGATGGTGGCCGTAAATTTTGTCCAGCTCGACAAATCTCTTTTATGTATGTGATCCATGCCTACACTAGTGTTAGAAGATAGAATTGCGTCTGCTTCAAAGTTGCTAACTATCCTGGTAGTGCTACGGTCGTTTCTTAGGGGCAACAAAGCCGATGTTGCTGCGACGAAGCTGGCGCAACGGGTTTATTTGCCACTGTAGCCACGTGTTTAAGTAGCACAAGGTATCAAATTCAAAACGAAGAAAGCATTCCTGCTTTCTCAGCCTCTAGTTGTCTCCATTCTCTGCTACAAAAAGGTCGTGAAAAGGTGGCGATGGCCTCTCCGAAGCTTGCACTACAAAAAAAATTGTAGTTACTAGTTGGACGAACTATTGGTTTGTATTTTAAAGTGGTATAAAGCAGGAAAGTAACGTCCAAAAGACATCTTTCAAAAGGTAGTATTCTCGTACTTACTCGTTTCTAGAAAACGCTCTTTTGTAGCATGATTGCAACTTTGTCAAGCAATTTTTCACAGTCGACGCTTAGTCTATATCGACTGGTACGTCTTTTTCGACAGTCTACGACCGCAGATTTTGGCCCGCTCTTCAATTGTGGGTGCCTTTCCTTCCAAAATCTGAAGTATTGGGGTCCACAGGAAAATATCCTCAATATCCAGATAGGCATTGCCCTTGCGAGCTATCATCGTCTTTTTCATTAAATTTGGGGTATAACCCATTTTTTCGATTTCTTTAAAGAGGTCATGGTTGCGCTGGTTGAGTAATTGGAGAGCTGCCTCGTCATCGATCTTGGATAGATCAACCATCGACTTGCTCTTTGTCAAAAGAGGCTCCTTCAGAGATAATGTCTTCTGTGAAAGCACCTTCTCCAGTCTCATGCCCCTCTCCTCCTCATCCACCTTCCAACTATCGGCCATCTTTGGCATCCTTCATTTTTTCAGTTCTTAAAAAATCGCCTCACCACCAATCGTAGATATTATGTTGTTCACAGAGTCCGTCTTGCCTGGGTAAAGGACGTTCGACTGGACTCGTGACAGATAAGCCTTCAGCGAAGGACGCAATGGCAGTAACAGCCGTCAGGATGTACCAAGGCGCATCTTGCACGTTTGGGCCGGTGGCGAAGGGGGTCGGGGGAGGAGGGTCTTGTGCCTTGGTGTGCAGATTTCGAAATGAACGTTTCAGAGAATGGGCGGGGGCTCAATTATAGAAACATCTTGAAAAACATATTTAACCGACCAGAAACGATGATTGTCCCGTAATCCAACATATTAAAAAAGGTCGTACTTATGTTATTTTTTATGGGATGGCCGTCGGAGAAGACAGACCGCAACAACTTTACATTCACAATGTTTTAAAAAGGTACAATTTAGGTCCCCTTGGTGCGTGTATGCCAATCACTTGGGCCTGATGCGTATGTAATACTGTATATCCTGCCCGTACAAAAATGAGAGCCTAGTCGTTTACACATTTCAAGATTATTCTATCGAAACTAATGCAATTGGTCCGACTCTACGTTGACCGCGTTCACGAGATAAAAAGGTGTGATATTTTGTTCTGCAT

>Contig_67

TGTACGACGCATCAAATCGCGAGCTCGTGCTGGATACTGTGGCGGCGTGTCTGACCAAGCGTTCTGCTGTGGAAGCCGCTGCACAATACGGAGCTCATAAAGTCGCGTGGAGACAAACGGTTTGGTTGCGTCTGGCGGCGCTGATGACGGTGGAGAAGTGCTACTAGGCCAACTTCTCCGGTAAGGCTGGCGAGCACTTCAGTGCGGTTAACTCTGGCGTCGAGACGCTCGTTGAAGCCGTGATGACCGCGGTTGCAATTACGTAGCGGGAGTCCGTGTGGTCGTCCTGGCCTCGACCTGCCGTAACTTCGATGCTAAAATTTCTGGCCGCATCGTGTACAGCTTCGACGAGGACCAAGCGAAGTGCAACATGAGAATGGTTATCATCGGCCGTTGACTGAGTGGTCGTCGGATGCTGCGATTGAGGATGTTGAAGGCTGTCATCATTCAGCCCGCGTAATTTACGCATTTGCCTCAGGCGCAGAGGACAACTAGGCCGCGTCGGTCCATCAATTCATGCGTGTGATGCGCACCGGGATCGTGAAGGATACCATGAAAATTGGTTGCCTTCTGCGTATGATCACTTAAAGTATACACGTATTATATGAAACGATGCACTTATAAAATAAAAAAGAAATGACTATCCTAAGCTCCTACGTGGGCCTGCTAGATACACGACCCCGACCTAGGAGGGATTACCGCGACAATTTGGTGGACAAGCAGGGTATCTTCCCGAAGAATCTTAGGAACGTAAGCGCGGAGTGAGCCCCCCTTGGTAATAGAGCGCCAAGGCGGAGGAATAAGGAAGTGGTAGCGCTATGAGTGCCCACGGTTGGTCCGGTGAGGGACACGGCGGTGAGACAAGAGAAGAAGGCGGAGGATGACCGTCTCCATTACTACATGCATTTGAGTCGGTGCTCCAAGCCGAGACTTACAAAGAGAACAAGTGCGCACCAAGTATCTCCGTCATTGACCCGCAGGGAGGGACTCGCGTTGGGGAAAGTAGATATGTTCTCGCGGAGGCTGGAATGTCTTGTTCAGTCGACTCTGGAGACCGGAGAGACGCTGAGGAGGAAGCATTTAGCTTCGATTTTGTATAGCCAAATCATCAATTACTACAGCAATTTAGCAAAGTGGTGATTGGCTACTGTACTGTCAACGATAATATTCTTAGGTCTTCGTCGCTCGATATGCAATTTTAACATTAAATTTAGCAYGTACACATTACTCTGTTGTCGACTACATATGCTCTACGTAACTGGAATGCCTCAGTATTTCTACTCATACTGTCGATCCATAGGCGGGCACAGCTTCTGGATCGTCATTGCGGCTCACGACACAATCTACATGATCCCACAACCAGAGTACAAATAAGTCTGTGCTGCAAAACGATTTGTGAAACTTAACAGACTTGCTGAGTGGGCACTGACCATCTTTGTATTTGACGTCGTTTAAACATTACCTAGCCTGATTATAATGATCCGACTTCCTATAGCTCTGTAGTGACGACTACAATCATCATAGCAATAACTCCACTTGTGACTGCTTCTTGGTGCTATGGAGTCGTCATTGCCCGCTGTATATAAACCTCAACATATCAAGTCAGATCCAAGTCACATCAGTACACGCTTTTCCACAACCAACTGAATATAATCCACATTACTCCGACTCATCATTTTTAAAAGGGTATTCGATTCTGACCAACCGCAGCCCATTCATTCACAAGCCACGAAGCCATGGGAGCAGACGTCGAAGACGTCGAGTCTACAACGCTGCGCCGAGGGGGAACCTACACGCTTGAAGACCTCCTCATAGTTCTCAAAAGAGTTACGAAAGGGGAGAAATATGCTGTTGTGTCGCGCATGACCAACATCCCGTTGAGAACGTTGCTCAAGAAGGCGAAAGAGCAGGGAGAAGGGAAGGATTTGACGGGTAGTCGTCGTGGGACCAAGCCGACGTTGCCTACGGAGCTAGAAGATGACCTGGTGGAATGGGTGTGTGCTATGCAGCGAGTTGGCTTACCTGTTGGGCCGTCTGCCATATTGGCCAAGGCTAACGAAGTTTACATTGCAACGCTGCCCATGCCAACTCGCTCGACAGAACCAGCGACGCCGCTCACGGCGGGCTGGTACCGACGTTTCTTAGGGAGGCACCCACAGCTGATACCTCGGGTCGCTCAGCAGATCGCTTGCGTCCGCAATAACGTTGAGGCAGGGGCTGTGCGTGGCCTCTTCTACACTATGGCGCAGCCTGTTATCGAGCATAAGCGGGATAAGAGTCGCGTTTTCAACATGGACGAAACGGCGTTTATGCCGAAGGCATCTACGCGGAAGGTTGTGGCGTTGAAAGGGTCGTCCAATGTGTGGACACAAGAGTCTAAGGCAAATTTTCACATGACCGTGGTAGCTGCTGTGAATGCGGCACGCGACGCAGTACCTCCCTTGCTTATTTTGCCGGGGCAGAGGATTTATCGAGAGGAAATGGCTGCCATATCCATTGACAACACAACTGTGACTGGTGCACCCAAAGGTTTCAGTAATACCGGTATATTTAAGCTCTAGATGAAGACGTTTGACTCGTATTTGACAAGCCACAACGTGAAGCGTTCCGTGGTGCTGGTCCTGGACAACAGTTCGACTCATGTCGAAATCGGTAAGAGCTTTTATATTTTATTGGAATTATTTTAATTATTAACAAGGATTTTGTAAAAAATCAATTTTGACGGGTTAATTTATTTCAGGTGAAGTTTCCGTTTGGTCACACAGAGGAATTTTGCTCGTCGGCCTCCCTCCAAATGCCACTCACCTCTATCAGCCTCTTGACGTGGCTGTTTTCAAGCCTTTCAGGAGCATGGTGAAATCTACTTTACAAGCCAAAATGCTGCACAGTTCGGACTGTACTATTGCAAAATCGGATGCCATCGAGATAGCGTGCCATGCGTATCGTAACGCGGTGATGACAAGCCCTGAAAACGCTGTAAATGGCTTCAGAACCACGGGAATCTATCCACCATCCTTGCCTAGTATGCAGGCTCGTTTACGACTGTTTGCGAGCGGTGGAGCACGCGGAGACATTGGAAGGGCGGCATGGCTCAAGCGAAAGCGTGAAATTCAAGAAGATATACGTAGCAGCATATTACAGCTGCCACCAGCACCTCGCACTAAAGGCAAACGCTCACGACGCACCGTGGACTTTGTAGGGCGTCTTATAACCAAGGAGATGCTGCTTCAGGGTAAGCTTTAGTCTCGAAATGACAAAAGCTGGTACTTTCGCAGTAATTGGCAAAAAAAATAAAAGAGACACCTGTGTGTATTGAACTCAGGCACTGAATGTTTTGAACTGAGATTTGCCCGGTCCTATGTGCAAGTCGGCTCCGGGGCGGTTGTATCGAGCTCCTGCGGTGGGGAGAACGAACCTGAAATGGTGTAGACTAAATAAGCCGGGGGTGTTTATGATTTGCAGGACCGTGACATCCGTCCTGAAATTATCTGTGAGAACACATATTTCAGATTTACAATGATAGCATATTTAATACCAGGTATAGTGTTGCTGATCGCGCCGATGCAGTGGCACCTGTATCACCCTGACTAAAAAAACTCGATTCCTCATTGTCCAATTTACCCGCCCTCCGAAGAATAAGCATGCCCTCCTTTTTGTGTTGGCCATCTTTCTTATTGGTATTGGCATGTGCTTCGCCAATCAGCAAACCGACCCGGGTCACACCACTTCGGTCCTCTCCCTGGGACACCTCTCTTCATTGAAACCAAGAGCTCAATAACCTCAAGCAGATCCTTGCGATCCCACGACACGTATAAGCAAGTCAATAAAATCAGCAACGAGGAAAAGATCGAGGAGGACAGAGCTGACATTCCTGGGATGACAAAAATCGAAGAACTAGTCCCTAAGCTTGCTTTGAAGCTGAAGGTAGACCCAACAAGGTTTTTAAGAATTTACACGTTGTGAAATCTGGTGCTAAGCTAGATGGCAACAGAAGTTTTCTTGTCTGGCTTCTGTACGTGAAGCAGTACCGAATTAAACGAGGAGGCGACTTTTTTATGGCGACGACGTTCTGTTAGACTTACTGAGGAAAACGAAATCGGAAGAAGAACTGGTGCGACTATTTCAATCGCTTCGGCAAAATCCAGCCTTCAAGGACACCGCGGAGAAGATGCAGATGGACATGGTTTTGAGCTCTGCATCTAGTCATAGACTAGTAAATGCGGCGTGGCTGAATTCCCGAGAAACTCCCGAAGAGGTTTTTAAAATATTGCGGCTGAAAGGTGAAGCTTTCGAGAGTCTTAACAGGAGCCATCTGTTCATCCAGTGGCTCAGATATACCAAGTCGTACAGGGCAGTGGTGGATGACTTAATTAACGACGTCCAAACTCTCAACCTCCTACTGAAGGCAACGCCGTCCTATAACAAATAAACGTTTGGAGCACTCATTCATTCAATCAAGGACATCCCGGATTTGGAGCAGCTCGCAAAAAACTTACAGAAGCATCTTTATGAAAAATGGTTGATCGATTACAAGTTAACTCCATTAAGAATGGTCGAAGATCGTGTAGGCGCCCCGTTTTCAATTACGTCCGGGAAACTACTGGAGACAGACCCCAGCTATCGCAACACGGAAGCTTACACAATGTATTTCTTAGAACACCGAGGTGGGCCAGTTTTGTTGGACAACGTGAAAGCGTTGTTCGCCCAGAACTATCCCTACTCCGCAATTTCTGCAGCATCGAAAGCTTAGGGTTCTGACTGTACAGCACTTCTAACGTAGAAAACCGAAACTAAAGATTGCCACCACATTTTTACTCTATTTTTTTAAAAGTAGTTTGGCTACGTGCTATCGGCATTTAAAATTAGCGCGTCAAGAAAATTGATGTCTAATTTTAACCCCAACCCGACGTGCTGCATAATGTAGTTTTTTTTATAAACTTTATGCTACGTAAGAGCAATTTAGACTTAAAATTTGTAAAGAGCGTATTTTCCCTCGCCAGCGGGTATACAAACTTTAAGATAAGCGATCTTAACAAAATGATCTTTTTCTGCTACATAACCAAAATACGATCAGTCTTCTGGTATGAGAAAGTCGCTCATATTTAAAAAAATTTGAGACAAACGATTTTGATGCGGATCAGCAAATTTGACAGGTAACGGCTGACAAGCCACTCGCCGACGGAGGGATTTCTTTTTCATTCGAGATTTTGCGCGCCTAGGACACAAGTGGAGACCCAAGATGGTGCGAGCCCGACACAAACAAGTTCGTGACGCCGACGTGACGTCGAGTGACCTTTCCTGGCGGTCGGTGTGGCGTGAGCTGAGAGCCCAAGACTGGACTAACAAGCGTCCGTCAAGCTGTGGCCTTGACAACAGATATCGCTACGTCCGTCCCGGATGCAACCCCGACGGAGTCGAAGGAGTCGACGTTTTCCTGGGAGAAAAAGCAGTGCTGGAGTATTGTGCGAACCGTAAGGTACATGAGTGATGTGATTATTAATTGTTGACTAACGTTTCATGTTTACGTATACAGAGCTACGTACTCAAGCGCGAACATACAGTCTGCCTACCCGTGGTGATGCTGATCTAGAGCGCACTGCTCAAGTCGCTCGCGAAACCTACGGCCACCTATTTGAAGACACTATTGGTCGCAACGAAGCTGAGAGCGTTCCACAGCCTTCGTAACCATCTCAAACGCTTCCACCACCTCGAAGTGCACCTCCTAATGTCCCTGCAAGTGCCCCAACAACCCCACCGTCTAAAGTCTTGGAGCTGGGCGACGGTCTTCGCGCCCTACGAGGCGCTCATTGGCTGATACTGGGAGTGCTATGGCGGCCACACCTCTACCACACACGACACTGATTGCTTCGTTACCCCATGTCAGCGTTGGTAGTCCGGACGACTTGGGCGACGACTCCGAGTTTGATGCCGGGGAGACTGTGGACGATGTGTCGGTGGAATCGGTCGAGGAAGGAAGCGATTTCCAGCCTGGTACGTTTTATATTATGTCGCATCTTGTGCTGCTTATAGTGATGCATCTTGTGGTGCATTTCACTGATTTATGTATGGTTATTTGCTTTATAGATGGTAGTGTTGGAGATGGAGTTGACGATGAAGATAAAGATGGAGGAGTGAGCAGTGAGCTACTTGTTGATAAAGACGACTCGCTCAATACGGTGTGTGATGGAGACGACGCTGCCGAGTATACTGCCATGGAATCTGGTGATGATGCTGCAAAAGACGATTTGGTTATGGATGAAGACAGTGGAGACGATGACGAATGCGCTGATACGTACATTCCACCTGATGAAGACGAATCTGAAGCTTCTGAGACTGAGATTGCCGCTGAAGTTCTGCTCGCGGAGCGATTCTTGAATAGTTTTGGAGGAGAGGACGCAGTGTTAGCTGGTAACCTCAAGAGCGATGTTCTACGTGAAGTGGCTGCGACTGGTTGGGAAGATGTGGAGGAACCAGATACGTTTGACTACATGAACACGCCGTACGAGCCTGTGGACAACACACGAAGCTACCCTGGGCTACGGCAAGGGTATTCGGGCCCTACAGCTGATGCACTGCGCAATGCTGACTCTCCGCTTGCGCTTTTCTTTTTGCTTTCTACCAGTAGTGCTGTGGCAGCACATTGCGGTGTGCTCAAACGAGTATGGGCGTGAAATGGCACCCCTTCGTGTTGATAAACAGTCCAAACGCTACCGTAGCAAGTGCCAGCTCAATTCTCACTACCAAAGCAAACCAAGCGAGACATCCAGACATAGCTAGAAGGCGCGAAGCCGATACAACCTCACGAGCTTTGTCGCTATATCGGCCTCCTACCGCGAAAAACTTGCAAACCATTGGGATACTACTGATAAAGGCGGTATTCCACGTGGAACGTTTGGATTCGTGCTCCGTCGCGATAGATTGATGGAGATTTTTCGAAACCTACATTTCAATACAAGCTCTGATCCGAGGGCGGTCACAGATAGAGCATGGTAGATTCGCAAAGTTGTTGAAGTTACCAAGGACATTTGAGCGAGGGTATGTGGCTCCAGCGCACTTGGCGTTTGATGAAGCGATGATTCCTAGTCGCTCGTCGTTCAACAAGATGCGTGTCTATATGAAAGCCAAACCCCACAAGTGGGGCACGAAGCTCTTTATGCTGTGCAGTGCGGTGACAGCCTACTGCATCAGGTGTTTTTTTTTTGTTCTAAGTTTTTCTTATGCTGAATAATGCAGACTAAAGCTGGATATTTTGCTATGTGTATGCTGTATTTATACAGGTTCGAGGTTTACTGCGGTAAGAAACAGCACTCCAGTGATGCACACAAGACAGATATGAAGTCACGGCCTGCTGCAGTAGTCCGCAATCTTCTTGCTGTGTTTGGGCCAGATTCTCGGATGAATGGGATGAGACTTGTTGTAATGGATCGGTTCTATACGTCGGTGACCTTGGCAATTCAGTTGCTGCTCATGGGATTCTACTGTGTTGGGACTATTATAATGAACCGGTTGGGCTATTGCAAAGGAGTCGTTGAAAAAAAGAAGTCTAGACCTGCTACTATTGCTCGCGGCTTTTTCAAAGTTTCAAGATCAAGGTTAGTGCCAAACATGACTGCTTTTAGCTGGTGGAACGCGCGGTCGGTTCACTTCCTGCACTGGCGGGAGTCTAGAGATGGATCGTGTTGCTCGCCAAGAATTCGCAGATAAAGTCGAAGTTCCTTGCCCACGCGTTGTGAAGGACTACCACGCCTTCATGGGGGGCGTGGATGCTCACGGCCAATTACGTTTGCAGCGGTACTCTATTCAGCGGGCCGTGCGGCTCAAGAAGTACTACAAGTCGCTGGTGTTGGGATTAATTGATTTAGCAATCGTGAATGGCTACATAGTCCACAAAGCCTACTACAAGCAGAATTCGTTTCGCCCATTGACGCAGGTGAAGTACATGAAGAAGCTACATTTGCTGCTGTCGCAGCTGCGCGCGTCTGGCATGTATGAAGGCAACACGTTTGGTACACACGAACCCTCACAAGGCGCTTCAGTTCCGGGATCTGGAGGTATGGAAACTACTCACGCTGTCCGCAAGCTTGATGAGTGGCGCAATACGAGGGCGCAGCCGAAGCGCCGCCAGCGCGCATGTAACGTCTGCTCGCTTCTCCGTGTTGCGGACAAACCCGCGACNAACAACATCGTTCTTTTGTCAAGAATGCAGCAGCCCGGGGCCAAAGCTCCTGTGTAAGAAGCCGCGCCGACAGATAGGTGGAGTAGCTATGACATGCTGGGATGTCTGACACCGAGAGTGGATGAACGGCAAGTTGGTTCCGGTCGACACTGGTAGAAATATCAGAGTGCGTCGCAGACACGTCAACTGGAGCTCCGTCAACGCCTGAAACGCCTGTCACGCCTATCACACCACTGGATCCGGCTAGTAGAGGAAAGCGACGTAGGACGGACAATTAGCTGGTAAACTGCATTGTTGTGCTTGGTGTAACTTAAATAAAAGCTTTGAACAGAATATATGCAGTCTAAGAGATCAGGATGATTGCTTGCGCGAACGTCCCGCACAAGGTATTGCTTGTTCATCTGACACTGTGCCTAATCCATCAGATTCTTTTGTCAGTTGATCTTCTAACATGATTTGAGTAAATGTACCTCGAGCTGTCGCACCGCATTATCTGATGGCGTTACATTCTGTGGGTGACCCCCCACCAGACACTATGACACTTTGTATGAAAG

>Contig_68

TAGTTCGCGACAAAGTGCAGCGAAGCTCAGTTTCAGGCGATATCGAGACATGCTTTTGTCACTCTCTATTGATCAGATCAAGGGAATTGCGGAAGCACAGCGTGTACGGCATTGTGGGAGGATGTTGAGGCAATGGGTTGGGCGGCAGCTTTTCCCAAGCAGCCCAACTAAAAATGATGCAGGGCGAGCCAACTCTTACGCTGGTGGCTGTTTCACTAGTCACGCTCAATGTCACCATGTTGAATATTTCGCTTCATTTTTACGTTAGCTTTTCGAACTAGACACAAAATAACGCCTCCATGGCGAATTTTGTTTTAACCACTTCTTTTTTTCTTATTTGGGCAAGAAAGGCTTAGTCTCTCCACCATGCAGCATAAAGTTGTTGTTGTCGGCGTAAAACACATAGTGCTGCAAGATGAACACCACGTCCACAATAATACTCACAAAGCCCAGCGAGAACTTGACTGGGTCCCCCGTCATGGCTGTCCAGTCGTTCGTCGCTGCAGAGTCCAGCAGCTGCTGCCCAATGGACAGCAACCCTCCAGCAATATCCAGCAGCACGTTCCAGATTGTCCACCCCACCGTCGACTTGCGCTGGTAATTCAGCGCAATCTGCGGGAGACACTTCACCAGCGTCGTCATGAGCTTCACGTAGCTCAGCAGATACAATAAGTTCAACGTATTGATGAGCGAATCTTCGTTATTTCCCGTGAACAAGACTGCAAGCCCGAAGACAACAGCGGCGACGATAGTCGCGCCGGTCCACAACATCGTGGGCTTGCTGACCACCTGTCCGCCTCGCGGGTATATTGCGCACTGGAAGAGCGATACGGCCACCAGAACCGCCGCATGCAGAGAGAAGAAGACGTCGTTGAGCTCCACGGCGTTGCGGTGGCCGTCGTGGCGCCGCATGTACTGCTCTTGAACGCTCTCGCTGTAGTAGAAGGCCACGTTAAAGATGGAGTAGCACGTGAAGCCCAGCATATTGAGCACCGTGTAGTCCAGCGACAACCCCACGACGCTGCGGCGTTGGCGGTTCAGGAACACTTGCGGGTAGAAGCTCACGCTCCAGCAGAAGAAATAGATCCAGCCAATGATGCTGGAGACGCGGTTCCACGGCTTGGGGATGTGCGCATTGGCGTCGAGCGAGAGACCAAAGAAAAGCCCGAACAGAAGAATTAGGATGGAGATGACGACGACCCGCTGGTTGACGGGCAGCTCCATGAAGGAAAACCCCATGAGTGTCGAAAGGAACGAAGAAGGCGTTTTGATTCCCAATCGACCGCACTTGAAGTGATGAAATGTGACAAAGATGAACCGGTATGAAGCGATTCATCGCGGCGAATCAGACAGTGCAATTCGATCGACGTGCGGTTTTACCAGCGAATCAGATGTCGCCGAAAATATGACAGTTTTAAGCTCAAAACCGGTAAAAGCTCACAATCGCAAAAAAATGACAGTTTTGCACTCAAAACCAGAACAAGCTCAAATTTACGAAAAAAAGGACAGTTTTGCACTCAAAACCGGTACAAGTTCACATTTACGACGGTATTTAACTTTTTGTACCGCTTTTCCGAGCTCCAGCTGTTGTAATAAATAATAAAAAGAGTATTTGTGATGATCAAGATTAATACAATACAAGTAGCGCTAGTACTAATAAATGTACTACGTAGTAGTATCTGGCGGCAAGACGATACCCGACATGCGTAAAACAATACCGACTCTTCTTCGAAACTACAACCAGGACTTAAGGCTTCCGCATCACATATCAAAGGACGTATTACAAAGTTTTGGTTTTCAGTGTACACAGGCAGCTTCACACAAAAAAGAAATAAATGTTGTAGGTAATTCAGGCTGTACCTTTCATCATCATTATACGTTACACCTTTGATCATCATCTTACGTTACACCTTTCATCAGCATCATACTGGGCGTACTATTATTAATAATCGTATTCGCGTTTCATCACCGATTTGCCAGCAGATTTAGCGATACTGGACTCCTATTCCTAGTCTTTCCTTTGATTAAATGATGGTAGAGACTGTGAGCTTGATCCTTGCGAATAACGTTAATTGCGTATCTATAGAAGATATTTTGTCTTGTGATAGTTCAGCTGGATTTCTAGGCCATTCCACGATGCATGTACTGTTGATTTTTTTTGGGCTGCTCAAAATTTAAATAATATCAATACAAATGTTTTTTTTTAATTATTAAAATAAATTTGATAACTAACATGTAAACTGCTATTAATAGCTCTATTCACACTATAGATTTTACGCAGGCACAATCACTGCTTGTGCCTGCAGAACCTCAGAAATGGAACTGTACTTCGATGCAAGATGGGATTGTTGGGATTGTCGAGCGAGCAGTTGTTTGCTTTATGTTGCTTTTATAGCGAGCTTCTCAAATTGTAATTTGCCACCCAACGCGCGGAGCATGCGATTCTTCGTGGTATTACTGGCTACCCTATTTGCAGGAACTTCTGCTTTATCCGCGGCTATGGAAGCAAATGTCGGCCTGCCAGTCGATTTTTTAAGAAACAACCCTCGAGATGCTGCTCCTACCAGGAAGCTACGAAGTGACAGAACGGAGGACAGCGAACAGCGAGCAATCAGCTTCAACGCTCCCACACCATCAAAGTTTGTCGAATGGTTGTTTTCACCCAAGGTGGCAGCGGCGAAGAAGCTCAATTTAGCCGAAAAAATCAGGATCGAAAAGTGGATGTACAAGCAGAAGAACTCGGAATACGTCTTTAACAAGCTAGGTCTTAGCGGCGGGCTGGATGGGATTCTCACCAACCCGAAGCTACATCTCTACGCAGCCTATATCGACCGGTTCAACAAGCAACACTCCACGAATAAGGTCTCAATGCTTGACATGTTCACGACGACTTATGGAACTGACGGTGTAATCAAACTTCTTGGAATGGGAACAAGCGACGCAACTACTATGAAATTTACATCGAGACTGCGGACGGAGCTCGCAACTAGTTGGGTCACGTCGGGGAAAACCGCAGACGAGATTTTCACGCTGCTAAAGCTGGACAGGGGGGCATATAAAATCTTCACGACTCCGCCGATGAACAAGGAAGTCACCAACTCAAAGCTTTATCTGTACGCACGCTACCTTGATATATTTAACAAGGAAAAACCCAAGGAGCAGGTTTCAATGCTCGACATGCTCAGTAAGACCTACGGTGAGGACGATGTGGCGAAGATGGTTGAACTGGGGTTGAAAAACCCCAAAATGGAAAAAGTCTCATCATTATTGCGGGGAGATCTGTTAGGTAAGTGGCATAACAGTGAAGAACCCGCGGAGAATGTTTTCAAGATTCTAAAGCTTGACGAAGCGGGATATGACCTCTTCGCGTCTCCGCAGTTGAATACATGGTATGTCCATATCAAAAGCAGTTATGATCGAGCACCCGAAGATGTTATGCTGGATGTGTTGGTTGGACATTATGGTTACGACGGCCTATCAAAGATTTTTATACTTGGAGAGCAAAGGATAGATCTTTTTAAGAAGCTTCCTATGGATTTGGAGAACATTATGGTAAAACGGTGGCTGACAGAAAGCAAATCATCGAACCAAGTTTTCGAGCTATTGAAGCTGAATAGAGGACTGGACGGTCTTCTGATTAACCCCAACTTGAGAATGTGGGAGAGCTACAGGATGAAGCTGAGCTCACAAACACCTGAGAAGGTTCCTCCGGCGATAAGCACGGTCCTGACATTCTACGGTGTCAAGGATCTGTCCGCTATGCTCGAGAAAGCGATCAACGTTCCTGCAACTGCGAGTATTGCAGCGAAATGGCAGCATGAGCTGGCTAATAAGATCAAGCGATAGCAATTTAGAATTGTTTGGTTTTATACTGTAGATAGGCAAATACAGTTTATTGCTATCAAAAGTGCATTTATTTCTCTAGACTTTACTGGCTAAACTCGAGTTTGATGTACTCTTAAGTACTGAAAAGAGGCTTGCATGGCACTTTCTGCTCTCCCTGAAAACCCTCNTTTTTTTTTTGCTTCTCGGCGTTAGTGTTCTCATGGCGTATGGGAGTTATACGGTCGGTGCTCGAGAGAAATGAGCGTAGCAATGTACAGTATTACCTTTTAATATCAGAAAGCTAAGGTATTCTGCATGTAGCTCAAAAGAGTGGGTAAAATATTTGAAATGGTAAAATAAACCAAATTGTAGATATTGAATACTGTATGAGCCGGAACGGTAAAATATTATTGCTACAGTATTAGCCATGTACCCACTTCAACGTTGGTTTCATATGGCTACATCCCATTTGAGTTCTCAGCCAATCTTCGTTCTTCTTTCACATTTTCCCCTCCAATCACATCTCGTTATTTCGTGGAAGATATTCTCGCAAGCGTCGCGACACGCAGTTAGAAACAGTCGCATTCCCCAGTACGCCGCCTCATCTCGAAGGCCATGGGCGACGCTGCGGAGCTCAAGAAGAAATGCGAGAGCCTCAAGGAGACGATTGAGAAGACGCGTGAAGCCAAGAGTGATGGCGGCTTCCAGAGCGCCAATGCCAGCTCCGGCGCCAAGGCGATCCTGGCCCCACCCAAATGTCGACGACTATTGAAAGGCCACTTTGGCAAGATCTACGCCATGCAATGGGGCGGAGACAGCTCGAGTCTTGTGTCGGCGTCACAGGACGGCAAGCTCATCGTGTGGAACGCTCAGACCACCAACAAGATCCAGGCCATCCCTCTACGCTCCAGTTGGGTCATGACCTGTGCCTTCGAGCAGAAGCAGCGCAACATGGTCGCCTGTGGCGGGTTGGACAACTTGTGCAGCATTTTCCACCTATCACAGGCGCAGGTGATGCGCGCCACCAAGGAACTAGCCGCCCATGATGGCTACCTGAGCTGTTGCCGATTCGTCGACGAAGCGAACATTGTCACTAGCTCGGGGGACTCCAACTGCATCTTGTGGGACGTCGAAAGTGGCGAAGTAAAGACCACATTCCGCGAACATTCGGGCGACGTCATGTCCGTGAGCATTAACCCACATAACCCCAGTATGTTTATTTCGGGCTCGTGTGATTCGACCGCCAAGGTCTGGGATATCAGAACGGGCAAGACCACGCACACGTTCCAGGGCCACGAGTCGGATATTAACTCGGTCGATTTCTTCCCGAGTGGCAACGCTCTGGGTACCGGCTCGGACGACTCGAGTTGCCGTCTCTTTGATCTTCGAGCGTATGGAGAGCTCAACAACTTCAGTAACGACAAGATTCTATGCGGCATCACGTCCGTGAGCTTCTCTAAGTCGGGTCGCTTCCTCTTCGCGGGCTACGACGACTACAATTGCTACTGCTGGGACGTGCTCAGCACTTCCGGAGCGCATATCTACCAGCTCGCGGGACACGAGAACCGTGTGTCTTGCCTCGGAGTTAACCCGGCAGGTCAGGCACTGTGCACAGGCAGCTGGGACACGCTACTCAAGATCTGGGCCTAATATCTGGCAAAAAAAAGAAGGTGTCATAGGCAACTTCACCTGCAGCATTTTATATCAGATTCTACCTGCATTTTTCAGTTTTTTTTTATTTTGGTCTATTTCAAGAAAGTGGATATAATCTAACTTGTTAGAGCGTGTGCTTAAATGTAAGGTACCCAAGAAATTGTCGGAGCTCGTGCCACTCGATACGTCGCGTGGCTTGACCAGGTCGTGGCGAGACTTGAAATGATTGAGAATTATGCCGCGTTTTCTCAGTTGATCCCAGGTCGATTCTGGTTTAGGCGGCGCGTTTATAGGGTCTAAATAGTCTATAAAATGGAAACTAGACCAAGTTGAACTTCTGGTAGAGTTAGTCTGGCAGACGTCTGACGTTGCGAAGAGAAGGTGGATCCGGTTGGCGAATGATTGGCTTCCGGTGTGTTTATTGGCCTCAAGACCATACGAAACCCACTCCATCACGTTGAGAGATCCCTATCTGACGCGTTCATGAATTTCTCGACCATTTTTGAGCTCGGTTTCGACCCAAACATAAGCCAGATCAGAATATTTAGCTCTTTGATATTGATTGAGTGTTCTCTTTCGCAAGGAACTTCATTGTCTGCAGTTAGAAATCGATCTCGTGTTTTCCAGCTGCGTTTCCTATTCGGAGCACTGGATTTTCTGGGGGGTTTTCGGTTTGGCAAAATATTAGAGTGCTGCAGATAGTTTCTTCAGCATCAAGTCGTACTGCGTTTGGTCTCGGAAAGCTCTCTTGTAAGTAAATCAAACTCGACGATTATCTCTTCAAACAGATAATTTTATGTAGGGGGGTTGATTTTTAATTGGCCAGAAGCCTCTCCATTATATTGTAGTGTATGTATCCTAGCGCAATTTGAGGTATTGTAATGGAATAGTATACCGCACGTAGTATATTACTTGGTAGAATCCATTAGTACTAGCTCAAAATATAGTACTTTAGCGAGCTTGTTAACAATTTATGTGTTCGTCGTATTATATTGTGTGTTGCAACTCTCGTAGTGACAAAGCACCTCCTGTGGCTATTTCGCAACCGCAGCATCAACTTTGGTTGCCATACTCCGGCTTGGTGCAGACAATACGGAAAGCGCACGGGGCTGGAGAAAACAAACCTAAATATGCTCTAAAATGCACTAATTGCACTAAAAGATACTGATACAAGCTAATTGAAGCTGCCGCAACAACGGCCTCCCGATGGAGGTGAGCGGGTGCGCTGTTGCGTAGCCGCAGCAAATTTGCGCGGGGTGCTCGCGTGCTGAATGGTCAGCTTAAGCTAAAGCTTAGCGCTTAACGCTTTTATCTATTCTCTTAATGCTCTTATAGACTGATGCGCTCAGTCGTAGGAAAAAGGAGAAGTGAGCTTCCATTAGCACTTAGACCACCATCGCGCGAGCGATGCCGCCTGCAAAGATCTTTCCCTGTGCAGGCTGTTTTAATTTTACTGATTCGCTACGTATGTGTTTCTCATGCCGGCTTCACCTATCCTTATTTGTGGAGCCGCCTATCTAACCAGTGGACACTTCCCACGT

>Contig_71

GCACTCAGTTTGAGCCTGAAATCATTCATCAGCGCGAATAGTTCACCGAGTGTCTCTAAATATGTATCAACGTTCTCTGCATACAGTAGTAGATCGTGAATCCATACATACAAGTGCTTGCATATAGTCAATTTGGCAAAGCAATGCTCCATAATCCTCTGAGAATGCAAAGCCGCGTCACCGCAACCTTGCGAAACACGGGGTGGATGTGTAGACCTTCGAGTCTGTCATGTAGCTTAGGATATTTGGACTCGCCTTGGCTCGAGGGGGCTACCAAAAACCCTTGATAAAATCAAATAGGCCAAAGGAAGTCAATTCACACACGAGCTTCGTTATTATTTGCTGATTTGGCATGACAACAGCAATCGGCTCTGTTTCCGCGTTACCGGTCTATAGTCACTGGTTTGTTGAAACTCGTTCTTTCCCGGATTTTGAATCGTTAGGTCAGGGCAAGTCACACTACTTGAACCGCTCTCGAAATCCCATTCGAGACGAGCTAGTTCTTCACTAAAGTCATACAGAAATTTCCATATATGCGGCGGATACACCCTCGGCTTGCACCTGCAAGGTGTAGCACTCTTTTTCAACCGATATTCCAAAGGCAGGACCATGTCAGGTGGGTCTGGGTTCTCCGCACGTCATACATGTAAATAATTGTCATCAACGTGTTCTCTCTGGTCACAGGAAGCCGGCTAACAAGCGCTGTCGCGATCATACCCCCACAGCTTGACGAACCGCTTCACCAGTTGAATAATATGCTTTAAAGTGGCCTCAACATTAATTAAGGGGTCGTCATCCGCTGAGGTCGCTCGCGCCAAGTGCTCCAGCTGGCGATCGATGAGGATACCCAAATCAGTAAGAATGTCTTCGCCTCAGATAAATTACTCCGCACCTTCGTCAATCACTAACACTACACACGGATTGACGGGTTTTACAGACCCAGCAGCTGTATGTAGCATTATTTGTAGTAGAACTGACCGATACACCAAAATCGGGTGCTTCAGATCGTTTCACAGACTATCGACGTCTTCAGCTGGTGGGTTCCACAAGTTCATCCATCTTCGTTAGCTCGCTCAGCTTTCGAGCACTGATGATGCTCTTCTCCGCCCCGGGATCAATGCAGTAGGGCCTGGACAGCCTATCGTCCAACACGATAGTCTTGTCGTGCTGCGAGAGACAGTCTTTTATGCGCATCAATCTTCACGCGTCGATCTTAGCTCGCTTATTGTCTCGGCGCTCATGCATCTAGCGGAGGAGCTCCTTCTTCTCATCAATGATGACAGCTGGGCATTATACTAACCAACGATCGCATTTACACTTAAGACAGCCACCAGTCGGAGCGGTGCTCTCTTTTGGGCAGATGCCTTTTTCTTGGTATGTTTAATGCTAGTGCTACTGCTCTTTTCAACTGGTTGGTCGGCAAGCTCGACCTTCCGATACTTTGAAGGAGGCCGGCTGTTTTTACTGTCGGCCGGCTTTTCTTGTCGATTGGATCCACTCATATCTCTACCATGAAAGCACTTGCGCCGCTGAAAATCACGGTACTGGTCGAAAGCCTTTTCATATATCAGTTTAATCAGCTCCTTCTCATTTTCCTAGGCCGTTCGTCCTAAGTACCTAGCGATTGCAGTCACCTCCTCATGAAGGCTGTGAGGCTAAAGTGATTCAACAAGTAGCTTGCATTTAAGCGGCACACCTTCTTTTCCATTAAAAAGGCCTACTAGGGCAGCGAAGCTACTTCATACCACGACATCAACAGAGATGATCAGATTGTTTTTTTTACATACCAGCTCCTAGACGAACGGCACTACAGACTACACTCCGCGACATAAGTTTTGTGACACCTATACTTTTGGCTGTATCTCGGCTTCTAGGTCGAGTTAGACGTCGCTATTTAGATATGTTCCACCTATCAGAGAGAGCGAAAGGAGGCTTGTGACGCCGATCTAGATTTCGTGACGAAGAACGTGACCGCATGAAGATACTCCACGAGGCAGGCTTGAGTGTCAGCGCGATCGCTCAACGGCTTGAGCGGTCGGTGAAAGCGTCAGCTACGCCCTGAGGGCACCTACTACTAAAGCCAAAAGAGGGGGCGTCCGGCAAGCCTAACAGATCGCCAAGTCCGCCAGATCGTCTGCAGCGCCGCAACAGCAGACTACTCGGCGTCCGAGTTGAAGGTCACGTACGGTTTGGCATGCTCGGTGCGTAAGATCCAGAGACTTCTGTCTCGGGTAGACTATCTAGTCTACTCTAAAATGGATCGTACGCTACCTCTAATAAAGGAGCAAAAGGAGGCGCGCCTAGAGTTTGCCGAGAGACTCTCGACGGCTGCGCAGAAGGAGGTGGTTTGGCCCATGATCATCTTCTCAGATAAAAAAGTTTAATCTCGACGGCCTAGATGGGTACAAGCACTGTTCGCGTGACATGAGGCGTCCTCCCCGTCGTTACATGTCTCGCCAAAATGGGGGAGGCTCGCTAATGGTTTGAGGAGCTTTCGATGTCAAGGACAAGTCGGAGTTGGTGGTTCTGGATGGGCGCCAGAACTCGGGCCACTACATCTATACGGGGTCGGAGCATATGCTCCCATTTGCCCACAAGAACTACGGCACGGATTTTATATTTATGCAAGATAACACTTCTATCCATGCCTCGTACGCAACGACCGAGTTTTTCACGGAGACTGGGGTGCGGTTGCTTGACTGGCCAGCTCGCTCACCCGATCTCAATCCGATTGAGAACGTGTGGGCAATTTTGGATAGGAAGGTCTACAGTCATGGAAAGCAGTACAACTCTGTAGCTGAACTCAGTGTTGCTGGTATGGAGGCGTAGGATTCAGTGACGATGAAAGCGCAAGTCAATACGATAAGCCTCATTAGCTATTTCACAGTCAATTAGCCCCTAGTTGTTTTAGTGGGAAGATCAGCTATTTAGACCAAAGCAGTTACAGCAGGCATAATGACATGGACATAAGTATCTGAACCAAGCCAAATCATTGGGTTTAAGGACTGGAGAAAAGATTAATTTTACGCATTTCGGTAGAAGTTCCTGCACCTGGCCCAAATCGGCCGAACGTCAAGTGCTAAGGAGAAAAAGCACGTTTTTCACGCGTAGTTTTAATCTGAGCCTGCTTTTTCACACTGTATCTTCATCCGTGTCACGCGTACACTATACGTTAGTTAATAACGCGTCTGTGTGTCGATCTATTCGCCGTAGCAATTTTTGTGAAAGCGATATTAAAAAGAAGAAAGTAATATGTATTCATTAAACCTTCTCGTATCCATTTCGTTCCGTCTCCGCCGCTTACGAACTAGCTGCAGGACCACCTGTCAAATATACCAAATTGATTGCTTTAGCTAAGTGACGTAGCTAAAGCAATCAACTTAGCCTAAGTCCTTACGCTGACTTGGCAATAATCACCGTAGATACTGTAACTAAAACTAGATGATGGGCGCTTTTCAGCCCACTTCAATTCTGTAGCACAAAACAGTGCACTTCTAATGCTCCGGCAATACGGTTTAAGTAAGTCACAAAAATGTGGACAATAAGCCCGTCTGTCATTGCTTAAAAAAATGAAGAATGCATTAAAAGTTTTTATTTAGGCGAGCTTTTAATACTGGAGTCAATTTCCTTAAAATAGACGAGTGGGATTATTGACACTGAAAATTAGTCCGAGGTACGTGTAATGAATCCTTTCATCGAGATACTTGATTTTGTCCGATCATACGTGATGTTCAGAGGACGCAGACTTCACTCCTGAAATACCGGTAAGCACAGCAAAGGCGCACTCGAGCCTGTGAGGTTAAAGCCCAGGAAAGGAGGTGATATAGCCCCGTTAATTTTGTGAAATTTTACAGAATCTTTGGTCCGTAACCGAATTTATACTTGTTGCCTAGGTACATCTGGATAATAATCGAAAACCATGGTATATAAAAATGTATTTCTTTTCATTCGCGGAGTGATTTGTCAAATCATTGGACATATACTACCAGCTTTAGCTACAGATTCAACGTGATAGTTTGGACACATCGTGGACAAGATATCCAGTGCAGAGTTAGGAAAATATAATAAGTTAATACATTTTTGTTTTTATGCTCAGTTCTTAAATAATATGGTATTTTGACCAATTATTGTAAGAAATAAATTAGCCTTTTCTACTTATGTCACTAAGTATTGAGAACGTATTTTGAATATTCATAAAGTATTGGGAGGAAGATGGTGCAAAAATAGGTTATTCCGTTGTACCATACTTGTTTTACTAATATTATTTTCTGCAACCTCTGATCCAGTGCCTACTTTTATTAACTGTACACGGCAATTGATTTGTCACAAGATAAGATCGCGCACTTGAAGCTCCACGTGACTAATCTTGGGTACTTCACTGTTGGACGAGCGGTGTTGACCATTGGTCCATCACTGGAAGTGGGGCTTGTCACTTTTCAATTTGCCAGCAAGCCTCGACAGTGTCTTCATCATGCATTTGCTACACCAGGCTATGCTGCTGTTTGCAGCTTTCTTCTTCTTAAATGTTGATGCTGAATCGAAGGTCCGAAGCTTGAATCGCGCAGTGTCGAAGAATCCGATCAACCGCTACCTGAGGGTTGTGAGTGACGACGAAAGAGGATATATCAGCTTTCTCGGCCTCGAGAAAACACAGGAATGGATTAATTCTCTGGTGAAGAAAATAATCGCGAGCCCAAAGCCGACCCAGGATAATATTCCCGACGAAACGCTATCACATGTGCTCAATGCAGTAAGCCCACTAAAGAATAGCATTTCAGCTGCTACCAAGCATCAAAGTCCCCAGATCCAGATGTGGCTCAACGAAGGGAAATCAACAGGAGAAGTGTTTAAGCTGCTTGAATTGAAAAAAATCACGTTCCCAAACTTCAAAATAGTTGATGGCACGCTGTTCGATCAACCAAGCTTTCACACTTGGGTGCAGTACGTAGACGATTTCAACGCAAAGAACCCTATGAAGCAAGAATCGATGACCCCGTCTTTATTGACTGTTTACAGCGACGCAGGCCTGTCAGAAGCCCTTGCGCTGGCGAAAAAAGGCCCGACCACTGAGGCTCTCGCCACTAAACTAAGGAAGGAACACATTCAGTGGTGGCTGAAAGACGACAAAACCCCAGAGACTGTTTTCAAATTGTTTATGCTCGACGAGAAAGTGGATGATCTACTGACGAATCCGCAGTTTGTCGCTTGGACCAAGTACATAAATCACTTTAATGTCAAGCACCCGGGAAAGGAAACTTCGATGGTATTTCCGATTGCAGCTCATTACGGCGACGATGCTTTGTTTGGAGTACTGGAAGCTGCGAAGAAAGTTCAATCGACTAAAGAACTAGCGTCAAAGCTGCAGGTGGAGCAGATAAAAAAGCTGCTGAGCAGCAATGAATCTCCCACGTATGTCTTCAAAGCGTTCAATCTTGACAAGACAGGAGACACAGTTCTAGACAGCCCGTTGTTCAAGACTTGGCTCGACTACATGAAGACATTCAACGACCAAAACCCCCGGAAAATAGAGAGTATGCTGACTTCAATTCATAGATATTATGATCAAGATAATGGTGTGGCGAAGATAGTTGACGAAGCGATGAAGAACCCGAGAACAGAAACGCTTGCGAAGGAGCTACAGGCAGAACGATACAATCGTTGGCTGGCCGGCGAGAATTCTCCTGAAAGCGCTTTCAACGCGTTTGTATTGCACAGGCCTGGAGCGAGTGATGATATTCATTTCAAGAAACATCCTGGTGGCACCCTTCACTTTACGCAAATCGACAATGTATCAGACGACCTCCTTTCCAGTGCAGATTTCACGTTTTGGAAGCAGTATTTAGATGATTTCAACACCATGTACCCCGACAACCAAGCGTCTATGCTCAAGATTCTGCAAACATTCTACACTGACGAGGCTCTAAAGAAACTGCTCGATACGGCGCGCAAAAATCCTCGTACGCAGGAGTTAGCATCGAATTTGGAGGCCGCCATGTCTAAAGTGTGAGCTCGTGTGAAGAAACCCTAGCGCAGTTTGCAGCTCGTTAGCGAAGCTGGTCATCAGCTGCTGATTTACCCTATGCAGCGAAGATTCGAGTACATTATTTCGATATGGGCAGTCGAGCTCGTATGACTGCAATCTAAAACACGTTTTTAAGCCAAACTCGTAAGATCGTGTAAGAGGAAACCGTAATTCCTATAGGTACATGACGACGCGTACCACGTAGCTTTCCAATGTTTCATTTTAGCGAACAGAGATACGTGGTATGTAACCTCTACCGCCTGCTAAGATCTATGGTTAGGCGAATAGTTACCGCGACCCATTAAGCCACATACATTATCTGCACGACTCCAAACAGTATTCAAGAGTTGACCAGAATGTTCTATCTTCGGCGTGTTAGATTTAGTATGACATGCCGATGCACCAAAGCAGTCGTAGAAACACGACCACATTCGCTTTGACTGGTTATTCTTGTTGGTGTCAGATTTTTCTTTGTTGGTGTTGTTGGTATTCACTCGATTTCGCAGAAGTAGCTAATTCCGTGCCATAAACGGTAAAGCTCTGTTTTTTTTACTTACAATCAATTAACCCCTCATTCACCATTGTCGGGGATCCGACAAACAGGTGAGATTGGGCTTCACCACGGCCGGGTATAAGTTTGCCCCCGGATGGGTACAGGAAGGCAACTCGTAAAGTCGGTTGGTTTCGAGTGACCGAATCAAGCAGGGAATGGGAATTATGAAAGCTTTTRCTATTGTTTTCTTTCGTTTTTTATCGCAACGCGCAACCTGTACGAGCGACGTATGCTTTCGCAGATTTTTATGGCATACAATCTGAAAATATAACCCATATAGCATGTTATTATTGTAAACATATGTTAAATAGAATCATTGGCTATCTAGTCGAAAATGGCGACTGGCTAAGGCCGTCCGAGGGTTGGTTTCTAGGGGGGTTGTGACCTGCGCCGTTCGTGGTAAATTACCACATCAGGAGAGAGCGGTCCGGACCGGTCCATTCGCAAAGACTTGATAAGACGCCAAAAGATGATCAGGATGCACACAGGGGAGGACAAGTGGAGACTGGTAGCAGTGCTAGTCGCTTGCCCCAGCGACAAGGTATCCAGGCGAGGTGTGACTGCGTTGAAACTGCTGTGGTGCCGTGTCTCAGAGATCGAGGAGCCAACCCAAGCTTGTCAAGATGCAAATCCTGCTGGGAGATCTGCTTGAGCTGCACGAAGATATCACTGCACGTCTCTCTGAAGACACGAACGAATCAGTGCAGACGTCAACTATAGGCGTCGAGCTGCGTCAGAGAGTGTGGGAGCCATTTTCAAAGGTTCTGAGTACTACTATTAGCAATCACCAAGTGTAGTAAGTAGAAGGACGGTTTTAGTTTAGAAATGAATGTGTATTGACAGAGATGTTATCCTCAACTAAATGCTTTCTTGACCATCTTCCTGATCTCCACCATTTTTGCCCATAACGCAGCGTCTTCCGCTTCATCTGGCGTGACTCCATCATGAAGAGTTGCCCACAACAGCCGAAGTCGTATAAGCTCTAAGTCGCGGTTCAAAACTCTTTTGGCAGCCTTTAGTGAGCGCTCACCTGAGATGTAGCTGTAGGCTTGAGCCGCACACAGAGCCTCGCAGCTGACTGAGTCGGGCGTGTCATATTCACTGCCAAGACAATCGGAAACTCCACTTCCGTAGCTGAAGTTTAATTACGCTGTGTACAACCGCCGTTTTTGTGTTTCTTTACACGCATGTTGGAAGCCAGGTTGCAGAACGTTTCGCGGTCCACATCTCGCTTAAACCACGCTTCAAGGGGAGGTAGTGTGAATGACGTTTACTTTGTCTGACACATTGCCAGATTCGAATCCACACCGAGTGGATCGTAAAACGTTGCCCAGATTTTGCGCTTGGACGGCCCCGTGTCCGACATTTATGCCTCTACAATCTTGATAATCCAATGCTTTAAAACGATCATGTGTACCGGTACCAGCACAAACTTGCAGCTAGAGAGTGGATAATCCAGAAAAAAACTTCTCGTCCGTCACAGATAAAGAGTCACC

>Contig_73

ATCCCATGACTAATTCAGCGAAAGAAGACTTCCGTTCGAGAACGGACCCAACAACTGATAGGTGGTTCACTTGTAGCATGACTAACAGTAGTGGTGATTGGCTGAACAGCTTCGACCCGCGCGGAATGAGCCGAGCCCAATATCGGCTCACTTGAAGTAGCCAATCAGCAAACTCTATTTGTAAATGCGAGCTTGGTTCCTACTCTATCCAAAATTTTAGGGGAGCGGGGCTCTTGAAGATACAGACAATCTACTCGGTAGCAAGCCTTAGACCCGAAATACAATTCTGAACAGGGGGCGTCGGAATTGGGATATGGTACGAAACATGTGACCACTTGGACGACGACATAATGGCCAGACATCTCCCAAACCATCAAGTAAACCAATACTGAAGTCATTCTGATGCAACAACCAGGTCAGAACTCGCTCTGAGTTTTGCTTCGTGTAAGAATCCGAGGAAGGTGAAGATCGTTGTGCAGCACAAGAAGGCATTCACTTTTCTCTTTCCAATCACCCTTCTCCAGTGGACACAATGGACGCTCATGGGTCAGATCTACCAAGTATTTACCAGCGTCCGTACTTTGACGGCATCGTTCAGCTGCCGGTGCTGCCATCGCATCTGGCCGGGGAGTAACATCCAGCGATGCCATCTTTTACATACTGGCCCAGCCACCTCGTCCGTCTGCAACACCCGATTCCGCTCCGAACTCTACTGAATGGATTCAAACAATTCCAGTATCTACAGCTACAGCTAACCCGTCGACTCCGGTATATATTACCGTGAACGGAAAACGCAAAGCAGGCGCCAAATCCAGCGCTGGGCAACAAGGAAAAAAGAAGAATTCCTCCAAAAAGGGAACAACGCCATCCAAGCGCTTCGTCTGGACGAATAAGATGATCGAAGAGCTCCTCGACATGCGCTTCAAAGACGATGACGCTAGGCTTCGTGTCCAAAATGCCGATACTAACCGAAAAAAAGCTCTCGCATGGCAATTTTTTGCCAATCGATTATCGGAGAAACTGGAGGTGGTGCTCTCAAGTGATCAGGTACTTTACAAACTATTTTTTTTCTCCAGTTCCTAACATTTTTTGCAATTCTAGGTTGCGAACAAGTACAAAATACTCAAGTGCGAGTACAGACAAGGAAAGGAGGCTAGAACAGACACTGGCAACAATGGAAATGAAGATGAGAGTGAGATCTGGGGTATTCTGAACAGCGCTTTCGCTGGTCGTACTGGAATTGGTGGAGCAACTCTGGCGGACGCAGATAACGATTCGGATTGTGTTGAAAAAAGAAAAGTCTTTCTCATCCTCGGACAGCCGCCAAAAAGCGGCTACATCCATTGCACGTCTGGCTGCTGCCATGAAAGACGGCATGACAGCTATCGCCGCATCGATGGGGACTGACGATAAGCTCGTTGAAGTGCTCCGTGATGTCCGGGCCGCTCAAGAAGCCTCCCGGATTTACAGGCGCGCCAGTTGACACTGCTTGAACATCTTGTCTCCGAGATGTCCCCACCCAATTAAATTGTAGTCGAGGAATAGCTTTAGCTTCAGCCTAGCGTTCTACATTGCATTAAATGATAGTGGGTCATTGAGGATAGACATTGGCAATGTAACTGTAAATTGCATACTTCCTAAAAAGTACCAAGATCCTCCATAATATTTTTCTGTTTCGCTAGACCCTGCTCATGGCTCACTTCATCTTCTCCAGAAAGAGAGGACAACATACTTGGTGGAGCTCGATCGGGGTCATCTCCTACCACCGTGTAGCTGTCTTTAAGACCAATCATCAAATTATGTAAAACTAAGCAGCTTACAATAACATTCGTGACACGAGCTGGGGATTTTTGGGATATCTTGCCCAGAAGTATGCGGTAGCGATTCTTGATGATGCTAAATGCTCGTTTGACATAATTCGGGTATGGGATAGCTTGTAGCTGTAGCGCCGGCTCAGGGCACTGGACGATGCCTCTTCTTCGTCAAAAGGAGTAAGAAGATGCCGAAATATCTTGTATCCAGCATCGCCGAGAAGATGCATTCCACTAGGCACAGAAATTTTATTGCGCAGACCCGACTGATTCCACAAAGCTTGATCGTTGATAGACCCAGCCCGAATACAATAAGAACGAAATCGAGTCTCATGATCCACTACGGTCTGCATATTCACCGCAGGAAATTTTTTCTACAATACCAGCCTTCGTGCTCCACAGGTCGGCTAATTCGCATCAAGCAGCCATCCACTGCACCAATGACGTCAGGAAAGCCTGCGATTCGCTCAAATCCATCGGTAACTTTCTTGATTTTGTTGGGCATTGTGATTTCTTCCTTTGCCATTTTCGTCAAAACCGACAGCACTTGGCAAATGTATACGACAGCACTTGGCAAATGTATACGACAGCACTGGTTTTTGACATCCCCATGACAGTACCAGCACTGTCAATCGTTCCACCGGTAGATAGATAAATGATCGTCACAGCAACACGTTTGGTGATGTCAAACTTGACATTGTGATGGATACGCTCTCTCCAGTTGATCCTGACAAGAGCCACAACCGCAAGAAATGTTCGCTTGTCACAACGCATCTTCGAGTGAAACCATTTTGTTTTCTTAGTCTGCATTGCTCTATCAAAAGCTGTAGCTGCAAACGTGTTGACACTGGAACGGGTCTCTCCACCGAGACGACGACGATCGCTACACAGAGCGGACACGGCTGTACAAACGCACTGCAACATGCAGAGAACGGCGAGTTTAGCTTTGGTGCCCATTACGAAGCGACGTGACTTGACAGGGGATTAGTGCTGAGGTCTACCGGAGGCAAACAGAAAAATGAGCTATTCCTAAAGTCGGCACGGGTATACATCGGCAGAGACGCCAAACTCGTTCCCACTTCGGAATAGGTCGCGGAGGTATTCTGAGAACGCCATACAGAACGGTATAATTTGAAAACGCGTTCCGAAATCTGTTTCTATTTTCAGCCAATGACCGAGCAGAATTTCTTGGAACGAGTTTCGGAATTTCAACTTAGACCAGGGCCTAAGGGATTCACATCATGCGTCTGGCAATTATTCTGTCTGCTACGGCTGTCGCCATTTCTTTTGCAACCTGCAGTGCAATAGACCAAACCAAGGTCCTAATGTATGGGACTCCAGCTCACTACATACACGATTCTGCCGGCAGAAGACTTCTTCGCAAGAACGGAGAGAACAAAGAAACATCTGAGGAGCGAGCCCCAATTTCAATTTGGCGAATTTTAGTGAGGAGATGTTTAAGGTGGCTGCGTTGACGAATAAAGCAGATGCCAAAAATCTGGCTAAACAACTCATGGGTAATGGTGAGCTGGCGGATGCTGCATACATTTGGTGGCAGAACAAGGGGGCTCCCCGCAAACCCGCGCGGGGAGCCCCCTTGTTCTGCCACCAAATGTATTGGGCTTTTGAGCGTGCTGCACACCGTTTCAAACNGGTGTTTTTGCAATTTTGTCTGGTGGACCGTGATGATCAGTGATGATAACAAAGTACTGTACCAGAAGTTCGTAATCGATTAGTACCTGTACAGTAATCCCCGTCTAATCCTGAAAGGTGGGGCCTGCGAGGTTGGATGATTTGCACAGGTACATGAACTGTACAGTACAGCACCCCGTTTTCGCGCCCTTAGACATTTAATAAACGTTCGGATGTAATGGAGTCTGATTCCAAATGAACTTTAGTTTGTCAGCTAAAATCTGATTTATTTGCAAATCCCATATGTTCCACCCCGCGAAAACGGGGTGTAGTACTGTATCACAATGTGATTGCGAAGTGTATTGTATTAATCTAGACCCCATCCATTTTTTTTNAATGATGCGACACTTTCCCGCATTCACAACTTCAGCTCGGCAGAACTATCCATACAATACATTGTCGCAATCTCAGCAGAATTTCCATAATACAGTACATAGGTTCTGTAAAAATAAATAAAATTTAAGTACTGTTAGCAGTACATATTAAGTGCGCTGCGAACTTAAGATTCACTATCTACGGCTGAAGGCTGTTAATCCGCGCGCTACGTCGACTAGGATTTGTCTTCTTTCGCGTATGGCGGTTTCGTCCTCTGGTAGACTGATACAGCCAGACGAAGTTGATAGTCTCCTTTTTGAAAATCACCTTCAGCTTCCAGGCGAGGTTGTGAGCAGGTTTCCAGGTGAGTTCGTGACAGTCAACAGATTGGATAGGAAACAAATTAAAAGCATTCACCTAGTGTTTTGCGTACACTTTTCGACGTCAAACTATCGTTCTTCTCTACCTCTTCTTGCTCGGTTACTGGCAATAGGCCACCGCCGCTGCGCATCAAATTGATCAAATTTCTGTCTTCTTCGGTGCTTGGTGAGCGGTTCGTAAAGTTGGGATTGATAGTGAGCCGGTAGAAAGTTGGCGAGACGTGGGGATGGGGTGGAGCAGCAGTGGAAAATCTTCACTCTGCGTCCGTGGATGGTTCCGTATCCTAAGCGAATATTCGAGCTTATGGATCCGGCCGTCGAGCCTGCGCTTTATATTACTGACATGTTTCTTCCTCTTATTAGCGGGTAACACTCATTTTCCTTTTCCGATTTTCTTTTATAAATTACATGTTATAGCAGAAGATTGGACAATGTTTCTGTCCTTTTCGGCAGGCTCATATTAAGAAATAACAAAAACTCTAACCCAGTAGTTCTTTTGACCGATACGCTGTCAAGCTGCAGCCGATTGAGGATACGTGAAAGCGTAACGCTGAAGAAGGAGATACATCTCCACAAATCCATCGTTAATATAAAAATGCTAATCGGTGAATGTGCATTCTATTTTACAAATAAACAGTTAGAAAAGATGAAGTGAAATTAAACCAATCCAGACTCATTTTTAATCAGGGTGAGCCGACATATTTTTAGATGAAAGTTATGCAAGTGTGGCTTAATTTCTGCAAGAAAAGGCTAGATTCTTTCAGCTTCCTACGTCAGAGCAGTTCGCGCTTGTATTCGTTATGTTTCGGCTAGGGTCCTGGCTTAGCCCTACGCTTACCGATACGTGCTGGTTCCACCTCAACAAAGACTTCAGGGCAATCCTCACGAAGAATTTCTTCAACCCAAATAAGTTCTGTAAATGATGATAGCATCTCTACCTCACAAGGTGCTGAGCACGACTCTAACGGATATGCCGCACCAAAAACGCATTCCAGGTAATAGTGAAGCTTAATGAGTAGCGTTGGCGACGCCTAGACTTAGGTAATTACTCGCGCTACGCATCGCCATGCGCTGGAACACTGTCTCGACACATGAGTCCACGATGCGAATGTTCCCGTTNCACCGCTAAGAGGCCGCTGTCGACGCGATACATCAGTGGGTAGACTCGCTCGAGTCCCCCCACACGTTTTACAATAACTTGCAGACCTGGAGCGTATCGTCACTCAGCTGCACATTCATTGATTTGTTTCTGCATTCCGAGCACTTTGTTCCGCAATATATGTGGCCCGGCCCCTTCAGTCTCGGGGCAAACGCCTCCGCACTTAGCCTAAACAGTGGCCTGTTGTGGCTAGAGCAAGGTCACATAGAAAACCTTTTTTCCGGAAGGTTGAGCGTGTACGCCAGCCCTTCTTGCTCACGACCACGAGGGTCGCTCATTACCACTGCCACGCGTACCATAGAAATTAAGTCGTGGCACGGTGTGGTTAAGAATAGAATCGCCCTGTCATTGTGCCATTATAAATATCTGCAGAGTATACCTTAATGGAGATATTAGCGTGGCGCATAGCAATTGCATTTTTGCCCTGGCACTTGCAGATGTCATCTGTGGCACTTCCTGTCAATTCATTCACTTCAAACTCCCCTAGCACTCGTTTATTCAATTAAACCACAGCATTTCGTGGTTAGACCCTGGTTGAGGTGGAAGAATGTGGTCTGTGATAGAATTTATGGCATATTATTGGCCAACATAAAGTGAGTGGGGTGTTCATAGACAGTACTTTTGATTTGGTTTGTGTTCTGAATGACTTGTTACCACAATACCTACAGGAGGTGCTCTTGGGGCNNAAAAAAAAAAAAACACCCATGGTAATCACGAAAAATCTACCCAATCGCTGATTTTACTGCAATATTGTTTTACAAAGCATACTGCCAGGCCTTAAATATGACCCAGATCTTGTGTTAAATTGCTACAAAGCTTAATGAGAGAGAGGGTTAAAAATATCCAAAAACGGGTGTAGCAGTAGACTGTATTAAAATCTACTGTTCTTAGTACTTTCTGCTATTAATAAATGGGAAGAAGCTCTTTCCATTTTACTGTATTTTGTATTAATATTAATATTAATACAGCTGTACCATCTCCAATGTACCTGTGGTACCCATGGTGGTCCTTGATGATTGGTGATGATTACAAAATACCAGGTCGTTCGTAATCAATTAACAGTAATCCCCGCCTATTTAATAATCCGGAAAGGTGGAACCATTGATTCGCACAGATACATGTACAATTGTATCACAACAGGAACAGGGATACAGTACAGTAATGTGCCTCAACAGCGAGCACCGCACATTCACTAAAAGGTAAAGGTATATTGGATACAATGGGTTCATACGTTTATAAGTACAGATCCGAGTTGACCACTTTCCTGAGCATATTAAATGTCGAGTGTTCGCTTTTGAGGCACATAACTGTATTACATTGGTTAAAAACGTGTTTCGAAGGTGCATATTCAGGGGGCAGAACTGTATTCACATAATAAAAGGTCCATCTTATTAAGGCGCGAATGGATGGCGTGCCACTGGCACTTCGTCCMTTATTCGCCCTTTTGCGCTGCCACTTGTCCACTTCGACTTTTACCGTGGACGCAAGACCATCACCAAGCAACCTAGCGTGACCATGAGCAAGCTGTTCTACGCGTTTGCCGTGCTCGCAGTACATGTACTTACTAGTAGCCCGACAACTGCAGCATCTAACCTACCCGTTAATCTGGTAACCGGCCCTGCATTCACGACACCGGCTGGTGCTGCCCATAGAAGATTTCTTCGATCCATCCACGAGGGAGAGGACAGCTTGAAGCCATCAGCGTTTTCTGAAGAACGCACAGCACTGAGAGCAATGGCTTATCTCTTGAAACCGAAGCAGAAGAAGCTCGCTGCGGCTCTAAAAAAATCGACCTCGAAACACAAACTCAGTGTTGCTCCGGAGAAAATGAAATCAATTCAGGTTCTAGGAGATCCAAAGAATCCGGAAAGAGAGTGGTTCAAGCGGCTTTATAACAGCAAAAGGGGGGACCCACAAACGTTGAGGAAACTTGGACAGTTCAAGACCGAAGCGCAGCTTTCGCGGTATATCAAATTCTACGATGACATGTTAGCGAAAGCCAAGAATGTCCGAGTTCAGACGAAATAACTCGCCAACGAAGTATGGTTGCAGGAGGTCGTTGGTCGTTGTCTAGCTACGAATACAATTGTCGAGCAATAATCGCTAAATACATTCCGTATTAGTCGTTTCACCACCGTCATGTTCTGTCACACCAGCATTCGATGCTTAGGGCAGGCGATTGGATCTCAACACTCCACGGCCACTTACAGTAGCACTCACAATGGTATTAGAGGTCGTGGACTGTCACCAGAGGTCGGAAAATGCTAAAGTGGATCAGGGCCACATAATGCCGTTGTAAATACTCCAATATAAGCCATTCTTATAATAAATTTTGCCGTAATTTAAATATATTGAAACATTTTGAAACTAGCTATTGCTCTGCTAGCACCGGCAGTACTGGGAATCGGTGCGTCCATATTTTCGTAGGGTGAAGCAGCTTAAGCTGCTACCAATCTTCCGCAACTCTGGCTCGCTGGCGCAGAAAGTGAAGGAAATAGTCTAGCGCTTGCTTTGGTAATCTAGTGAGTCTTCGCGGCACAAAAACTGAATTGCGGATCTGCACCACGAACAGAGATCTACCTTGCCGAGCTCAAGAAACTTGAATACGTGTAGCACGCGTCCAAGTACCACGATATCACCCATTTTGCTACTTTTACCATCGAGCGACCAAGTTGCAATGGCAAGTGCACCACAAAAGAACCAACAAAAAACTTTAAGAAACACTTGATTTAGTTTAATGATCGAAATTTGAAAAAATACCAAAGTAGAATAAGCAGGCCAAATCAGTNAAAAAAGGGGGTTTCTGTTAAAAATAGAGATGTCAATTTTGCGTGGATCAAAAAAAATATGTGTCGTACTGTACTGATGATAATCTACTCTGGCGTATCCTCTAGGGTATTTTCTTGAAAAGAACGCATTTAACTGGTTGGGGGATCATTAACACGATGACAATCTTTGTGAAGCAAAAAATCGGAAGTATATTTATTGAGGAATTCGTAGTTCTCAGCGCGCGGTCCTGATAAAACGTTTCCGACGTTTCTGCTTACAGCACCATAAAGTACTTACTTTAAATCTAAAAAAATGAATAAAAATGCTGACAACTCTGGACGAGTGCTAAAGCTTTGGGCGAACCCAAAGATTGTGGATCGCGCTCTCACAGCTGATTGCTGTGCGTCGCTTGACCTTGACAGTCAAGCAAGTATCTGTCTATCATGCTTTTTTCGATCGCCTTCGGGTTGCATAGCTGATGTCTTCGCTCTTCCATAACAACTGACGGTGCTTCAGTGGATACCGTCGACGATCGGTTCCTACGGCTGCACTGAACGAGCTCGTCCCAGTTCTTTATATTATACTTTTTATTTCGACCAATAAAGTAGCGGTATGTTTTTTTAAATATTTTTGAACGTTGATTTTGTGATTAATAATAGAAACTTTAGATGGTCCTTCGCTTTTAAAACATCAGTGAAACATCTATCGTATCTAATATTTGAAGCAAAATCGTGCCTTGCACGAAGAAGCTCTGCTATATAAAAAGCATAAATTTGTGATTGAGAGGGCTGGATAAGCGTGGAGTTGGTCACATTCTGCTTCCAGGCAGCTAAGACGTTCTGTTTCCCTTAGCGCGGCCTACGAAGGCTATGATCCTGTAAAAAATAAGGCGTAAGTGGCTCGACGAGGAGCATGCATCGCATTGACTTGACTCTGGTTTCAGTCACGTAAAAGGAAGATCGTGAGGCTGACGACGGGTAAGATATCGTAGCGGCCGATAACTTGACTCTGGTTTCAGTCACGTAAAAGGAAGATCGTGAGGCTGACGACGGGTGCGATGTCGTAGCGGCCGATAACTTGACTCTGGTTTCAGTCACGTAAAAGGAAGATCGTGAGGCTGACGACGGGTGCGATGTCGTAGCGGCCGACAACCACCTTTTTAACCGTAATACCCTGGCACGTTTCGGTCGGTTACCCCTTCACTCCCGAATTTCGGGACACCGAACACGTTATAAACGGTAATTCAATGTCGCAACATACTTCAAAGCTTTGTACTTCTTTTCATAATGAAGATGTATAAGTTTTCAACAGGTTTTTCAGCAATGTCGTCACTTATGCAGCATAAATAAGCTTCTTCTGCTCTCAMTGTCGTAACTTGCCCCCCCCCCCCCCGATCAAGAAAAGAAAGACCTACACAGCTGTACAATGCATCCTACCATACAAACAAAGAATGCGCATCAATGCAAGAGCTATACATTATGGCCTGTATAGAGCAATCAGACATTCTGGACGATGCTCATTACACCATTACAGATGCGCACGCACACTAATTATACCTCATCAGTTTCGCATCCCTCACCTTCTGCATCGTCCTCTTGGAGCTCCAGTTCTCGGCGCGTTTTCTTCCGCTCTCCAGCCACTTCTCCAGGTCGGCGAAGTACATCGTTTTTGCACAATGTTACTGGAATGCTTTGACCACACTTAAAGTCTTCGTGTCAGATCGCGAAGCACGTTTCGGCGACACATTTTGATTTATGGCGGATTTTGTTGCAGAGCCAGCATTTCACCGAGTCAACAAAGCGGCGATGACAGTAAAACGTGGTTGCATACGACTTCTTCTTCGCGTTGCCTCGCAACAGCGCGCACACCTTACAGGATCACTAGCGGCGCTTCTGTACGCCACTAACCATCACCAAATCTTCTTGTTTTTCGAGTGCATGTGACATCCTAACGGGTATCCGTCTGTGCTTCTGGGGTTGACAGGCGTTGTCACATCCACACCGATAAAGTCTGCTGATTTCTACTGAAGCAGTTGGTTCTGCAGGGTACAAAACAAGTCATTACGAGACATCGCAGTTGTGTCAGCCAATCGTGCCGTTTCCTTGTGCGATATATACGCATTCACTAGCGCTAAATCCACAAACCCCAAAAGCAAACACTTGTAATACCTAATGAACCTCATATATGTCTGCAATGCAAATCTCTGGAGGCGAAGGTGGTCATGGACGTCCATCCAGCGCTGATAATCATTGACGGCTGCCGGACATGGACCGTTTTCCGCCCAACCCTCTTTAAATTTCTATGGATCGAGGAGATTGTCATAGCGGGGCCGTTGCACAAGTAGTGCGTCGCTTTGCAATTTCCACCAAAAGAACGCAGCCATACTTGGAACCGCCGTCGAGCGGGCGAGCAGGAAGGAGCCTCGAGGAATAGCCGCCGGTCTATTTTTCCGTAGCGATCTGACTTGTTTGCCAAATCCACATCTATCAGACATAATAGTCCCAATTACGTAAACATTCATACTCAGCAACTCGGCGGCGTGTAGAATCGAAGAATGGAACCGACCCACAATTACTAGATGTCACGGGTGACGCTGGTTTTACTCCAGAACCATCTTCATGTTTCGGACAATTGCTGCAGCTCCTATCTTGTGATTAATGGAAGGGTATCTGCCTTCTCCCGCGTTGAGTTTTCCAGCATACATCTCAAATATAATAAGTGACGACATATTTTATTTCCAAATCCGTCATTTACGACATTAGCAAAACAATCAAATGAGCCTGTGGCAGTAGGCGCTTCTTGAATCACTCAGATTATAAAGTTTTTGACCCGTACCGATGAGGCTTATCGGGCATGAACATGGGAGATGTTGTGCGCCTATCAATGGAAGGCAGCACCCAAAACGCGAGCTGACCTCTTCTTCGTTAGGCTAACTAAAAAGCGATTTCTTTTACCACTGGCGGTTCAATTTGCTTAATGCGAAAGGCTACTGCGAGCGTATTATCGCGGCTCGCCAGCGCTTCGAGACCTTTTTGAAACGGCATCTAGTCGATCGCCTCCATATTGACGCTATGGAAGCGGGAATCCCCTGTGCAAGGCCACCTAAAACTGCGTGTTCGCACTGCCGCCTCTGGGCAGTGCGCCGTTCCGAGCGCGATGTTCTTCAACTTCAGCGTTAGTAAACGCAGCCATTGGGTACACTTCCGTACGTGTATTTGCTTCTCTGCGAGGCCTCCGTGCTCGAACCGATGAGGCTTATCGGTCATGCACATGCTAGTAATTTTGCGCTTAGAAGTGGAAGGCAACACCCAGAACGCCAGCTGCCATCTTCTTCGTCAATCTAAATAGAAAGCGATTTCTTTACCAATAGTCGTTCAATTTGTTCAATTGTCGGTGATATTTAAATCTAGGAGGCCTTGCTCACGATCATGATCTGTAGCACAATCCCAGCGAGGATTCTGCGGTAGCAGGCTTTCATGACGATACTGCTTGGACGCGTCAATCCAACTCCTTTTTAACACCTTAATCAGGTATAACATTTTACGAATTACTGGCGCAACATTCTACACTAAAATCCGCGGACGTATCGGATACGACGACGACACAGGTAGCATTTATTCCTTATAAAGCGACAACGGGTACTAGATTATGGGCATTTTTGGGCTCCTGACGTCCGTATTTTTCTTGCAGAACATGCTCGTATTGGTAAATACTTAAATCAGGGTATCGAAGATGGGAAGAATGGGAGCTAACTCGGATCTTAGCTCTAAAAACAAATTTTTAGAGAACAGCAATGAAACATCTATCGTAACATTTTTTAAAAGCAATGAAGCATTTATCGAATATTAATCATACAGATTAAATCAGTACTTTTCAGATTTTTATTTTATAGCCTACACCGACTCACTGGCTACGAGCTCGGATAGCATCATATTGCCGCCACTGATGGTAGAGTATGGCCCCTACCGACTTGCATGGAATGCACATCCAGCTAAGGCGGAGTTTTGAGACGTCTCTGGTTTCTACCTCGGGGGCGGCAGCTATTATTATAAACAAAATCAAATATTTGCCATTGCAACTTGGTCGCTCGATGGTAAAAGTAGCAAAATGGGTGATATCGTGGTACTTGGACGCGTGCTACACGTATTCAAGTTTCTTGAGCTTGGCAAGGTAGATCTCTGTTCGTGGTGCAGATCCGCAATTCAGTTTTTGTGCCGCGAAGACTCACTAGATTACCAAAGCAAGCGCTAGACTATTTCCTTCACTTTCTGCGCCAGCGAGCCAGAGTTGCGGAAGATTGGTAGCAGCTTAAGCTGCTTCACCCTACGAAAATATGGACGCACCGATTCCCAGTACTGCCGGTGCTAGCAGAGCAATAGCTAGTTTCAAAATGTTTCAATATATTTAAATTGCGGCAAAATTTATTATAAGAATGGCTTATATTGGAGTATTTACAACGGCATTATGTGGCCCTGATCCACTTTAGCATTTTCCGACCTCTGGTGACAGTCCACGACCTCTAATACCATTGTGAGTGCTACTGTAAGTGGCCGTGGAGTGTTGAGATCCAATCGCCTGCCCTAAGCATCGAATGCTGGTGTGACAGAACATGACGGTGGTGAAACGACTAATACGGAATGTATTTAGCGATTATTGCTCGACAATTGTATTCGTAGCTAGACAACGACCAACGACCTCCTGCAACCATACTTCGTTGGCGAGTTATTTCGTCTGAACTCGGACATTCTTGGCTTTCGCTAACATGTCATCGTAGAATTTGATATACCGCGAAAGCTGCGCTTCGGTCTTGAACTGTCCAAGTTTCCTCAACGTTTGTGGGTCCCCCCTTTTGCTGTTATAAAGCCGCTTGAACCACTCTCTTTCCGGATTCTTTGGATCTCCTAGAACCTGAATTGATTTCATTTTCTCCGGAGCAACACTGAGTTTGTGTTTCGAGGTCGATTTTTTTAGAGCCGCAGCGAGCTTCTTCTGCTTCGGTTTCAAGAGATAAGCCATTGCTCTCAGTGCTGTGCGTTCTTCAGAAAACGCTGATGGCTTCAAGCTGTCCTCTCCCTCGTGGATGGATCGAAGAAATCTTCTATGGGCAGCACCAGCCGGTGTCGTGAATGCAGGGCCGGTTACCAGATTAACGGGTAGGTTAGATGCTGCAGTTGTCGGGCTACTAGTAAGTACATGTACTGCGAGCACGGCAAACGCGTAGAACAGCTTGCTCATGGTCACGCTAGGTTGCTTGGTGATGGTCTTGCGTCCACGGTAAAAGTCGAAGTGGACAAGTGGCAGCGCAAAAGGGCGAATAATGGACGAAGTGCCAGTGGCACGCCATCCATTCGCGCCTTAATAAGATGGACCTTTTATTATGTGAATACAGTTCTGCCCCACCTTCGAAACACGTTTTTAACCAATGTAATACAGTTATGTGCCTCAAAAGCGAACACTCGACATTTAATATGCTCAGGAAAGTGGTCAACTCGGATCTGTACTTATAAACGTATGAACCCATTGTATCCAATATACCTTTACCTTTTAGTGAATGTGCGGTGCTCGCTGTTGAGGCACATTACTGTACTGTATCCCTGTTCCTGTTGTGATACAATTGTACATGTATCTGTGCGAATCAATGGTTCCACCTTTCCGGATTATTAAATAGGCGGGGATTACTGTTAATTGATTACGAACGACCTGGTATTTTGTAATCATCACCAATCATCAAGGACCACCATGGTACAATGGAGATGGTACAGCTGTATTAATATTAATATTAATACAAAATACAGTAAAATGGAAAGAGCTTCTTCCCATTTATTAATAGCAGAAAGTACTAAGAACAGTAGTTTTTAATACAGTCTACTGCTACACCCGTTTTTGGATATTTTTAACCCTCTCTCTCATTAAGCTTTGTAGCAATTTAACACAAGATCTGGGTCATATTTAAGGCCTGGCAGTATGCTTTGTAAAACAATATTGCAGTAAAATCAGCGATTGGGTAGATTTTTCGTGATTACCATGGGTGTTTTCTTTTTTTGCCCCAAGAGCACCTTATGTAGGTATTATGGTAACAAGTCATTCAGAACACAAACCAAATCAAAAGTACTGTCTATGAACACCCCACTCACTTTATGTTAGCCAATAATATGCCATAAATTCTATCACAGACCACATTCTTCCACCTGAACCAGGGTCTAACCACGAAATGCTGTGGTATAATTGAATAAACGAGTGCTAGGGGAGTTTGAAGTGAATGAATTGACAGGAAGTGCCACAGATGACATCTGCAAGTGCCAGGGCAAAAATGCAATTGCTATGCGCCACGCTAATATCTCCATTAAGGTATACTCTGCAGATATTTATAATGGCACAATGACAGGGCGATTCTATTCTTAACCACACCGTGCCACGACTTAATTTCTATGGTACGCGTGGCAGTGGTAATGAGCGACCCTCGTGGTCGTGAGCAAGAAGGGCTGGCGTACACGCTCAACCTTCCGAAAAAAAGGTTTTCTATGTGACCTTGCTCTAGCCACAACAGGCCACTGTTTAGGCTAAGTGCGGAGGCGTTTGCCCCGGGACTGAAGGGGCCGGGCCACATATATTGCGGAACAAAGTGCTTGGAATGCAGAAACAAATCAATGAATGTGCAGCTGAGTGACGATACGCTCCAGGTCTGCAAGTTATTGTAAAACGTGTGGGGGGACTCGAGCGAGTCTACCCACTGATGTATCGCGTCGACAGCGGCCTCTTAGCGGTGAACGGGAACATTCGCATCGTGGACTCATGTGTCGAGACAGTGTTCCAGCGCATGGCGATGCGTAGCGCGAGTAATTAGCTAAGTCTAGGCGTCGCCAACGCTACTCAATGAGCTTCACTATTACCTGGAATGCGTTTTTGGTGCGGCATATCCGTTAGAGTCGTGCTCAGCACCTTGTGAGGTAGAGATGCTATCATCATTTACAGAACTTATTTGGGTTGAAGAAACCCTTCGTGAGGATTGCCCTGAAGTCTTTGACGCATATGGCCTGGCCATCCACTATCGACTCGTCATCATGGCTTGTGAAGTTGTCAGTAATGCAAGGTGGCTAAACGTTGATTGCTGATATCCGACTCTGGTAAATGCCTGGAGCAGCCGGACTGAGACCAGCAGTGCTCGCAGTGGAAGTAGGAGGCAGTAGGTGCTCGCGGTGGTGGGGACGTAAACCCACAGGAGGCTGACGCTTTGCTTTTGCTGGGCACGGTCCAAGCGACTCGATAAACGTGTGTTTGTTTCGGAGAAACTTGCTCAGAACGGGAGAAGGGGCGCTCTAGCAGAGCATGTGGTTTGTCCCAGCCGGGATCGAGCCGCATACCTTTGGCCTCCAAACCCAATGCTTTATCAGTGAGCTCTCTCACTGCGTACGGTGGTTATACTTTGTGGACTCGAGTGGGATTGAACCACTGACATGTCACTTTCGAAGCCAGCGATCTACCATTGCGATTTAAAGATGATCTTCTTAAGCTTGACCGTTTACGGATCCCTGCCCATAGTATGCGAGAGCTGTGCCCGAGTGGGATCGAACCACCGACCTACCGCTTAAGAAGCGAGTGATC

>Contig_75

CAGCGATTCAGCGGAAGTCGGTCTTCTCCGGCACCGGAGAAGGTGGACGTCTCTAGCTTCACGACGTCATTCGTGGAGATACGCGAGGGAGTTGACACTCGCGTTCCATGAACTCTTGTGCCGCAACGCACTGCTCGTCGGGGGTCGCTGAGCGCAGCAGCGGCGTGACGACGGCTTCTCCGGACACCGCCGCGAGGCGGTAGAGAGCTTCCCACTCGATCCGAGTAAGATGGAGGAAAGCAGCATGGTCGAGGTCTTGGCTCATGTCGAACTTGGTCGTTGGAAGGACTACGAAGTGCTACCAGGTGTAACGAGCCAGGTATTTACTCCTTTACTTACTTACACTATTGTGTTGAGGAAGCTCGCAATAGGACGACTGAGCTAAGGAAAAATCAACTCTGACTTTATTTTAGGACCAAGGCCACAGCCCCTGGCCACTCCAATACACGTCATAGTCCAATTGGCTAATAATGGGTCTGGCTCCCCATATATCAGCCAATGGCATGACAGCTTTTCAGCACACGCTAGTCGTGTGCTGATGCACCTACTTATGTAAAGAAATCTACCTACTTATGTAATGGAGGGGCCCATCGCCTCCACGGTCATAGATGCTTCGCCATCACCACTTTCATTCTCCACGTCCACGTCCTCATCCTCATTCTCCACCTCCATGTCTTTGCCATTATTCTCAACATCCACATCCACGCCATCATTCTTTACATCCACGCCATCATCATCAACCTCCACTTCCAATTGACCCTCGCTCCCCACGTCATTTCCAGATACGGTTTCTATCTCTCTCTGTGCAACCGACCCTTCTTCAGCGCTTGGCGTCTCCGTATCACCAGCTCGCGTGATGTCTGAGAGGCCGGGGCCTCTGTAGCGGGGAGAACCGTCCTGAGTGCGCATGTCTCCTTCCTCGTCCTCGACGTCTTCTCACGTCTAGCCTCAGAAATGAAAGACAATCGCGTAATTTATCTCGATCGGAAATAGCGGAGAGCGGAACTTACAGACATTTCCCGAACTAAAATCCTGGTTCCCTTTAAAAATTGGCTGTCGTTAATCACAGAAGGAATACGGTACTGTACATGCGCGCCTGTTTATAATATATCACTTTAATTATTTCTCAAAACGATCACAAAAAAAACAATTGGGTGCTTTTTGGTGTTTTGAGCATTCCCAAGAAAAAGTAGAAAACGAGCTGCTTGATTTTTATCTGGAATGGACGCCTCCGGAGACGCGCCCGCTTTAAGCGAGCAGGAGCTGCTGGCATCGCTTCCTCCTCCTCCCTCGGCTCTCCCTGACTACCCAGGAAAAGCCGACGCCACCGACAACGAGCACAGTGCATCGAATGTCACCAATGAAGAGACTCCGGAGCGCACATCCTCTAACTCCAAGCTGCCTGGATCCGCCGCCAAAGCTTCATCCGCTGCGCGTTCCATGGCCTCTAAGCTCGCAAAAAGCGGGCGTAAAGTACTGTCGCCGCTAGGCCACAGTAGTAAGAGCAACACAACAGCCACATCGTCTCCTTCCTCTACATCGACCCAAAGCGAGGCGGGGACGACCTCTCCGAGCTCAATCGACGCCGCCATCGCCGATCCTTTGGGCTCCGTGGCCTCTGCGGCCGGCTCTTTGACCTCAGGCCTCACATCGTCTCTAGCCAGCGCGTCATCGTCCTTCGGATCGCTACTATTCGCGTCCCAAAGCTCCAGTGCTAGCAACGCTGACGACGTGGAAGATCGCTCGAGTAGCGCTGATAAAGCAGCTGAAGAGGCTGCAGCGGTTGCTGCAGAGGCTGCAGCGGTTGCTGCAGAGGCCGCAGCAGTCCTACGACAAGTGACAGCTCAGCAGAGAATGAGGATCCTGGAGGATTTAGTGCAACACCGTCGCTCGGACTGGAACTACCTTAAAGCGATGCATGAAGGCTCCAACTACTGGTTAAATGTGGCTCTACTAAGAGAGCAGCAAGTGATGAATCATGTGGGGTACAAGCAAAGTATCCGACGTGGTGCGCAGTTTTTCTACTTGGGGATTGGATTAGGGCGATTGGTGGGGGAATCGACCCACCCGGAACTACTGGCTATGGACTGCTGCCAATTACTGGAAGAACTGGAATTTTACTTCTCGTCCTCGACGGTGCAAGGAATGGTAAGTAAAAAANCCATTTTTTTTTTAATTGTAGTGAGGAGATATTAACGTTATGGTTATATAGAAAATGATGGTGGCGACGTCAAGCACGCTACACGAGCCTCTGGACACGGAGAATAGTCCGCAATACTCAGCCGATGAGCCATTCCGGCCCACTATGCACAAGTGGAACCAGCGGCCAGTTTATCGACGACTATTGACGCCTCCGATTGTAGGTTTTTTTTTATTGGAATATTTATTTATGAGTATTAATATTCTGCGTGTTTTATAGCCTTTTCCACTGGACTACCGAGAGATTTTGCTGTCTTTGTGCGATATTCTAGCGCTCATTTACAGCAAACTCGTCGAGGATAACAGTGCGTCTGAGAACTTAAACCTCTTCCAGTCGATCATTCGCTTCGATGATCGCATTAAGGTACTTTCTAAGAGTCGACGGCGTTCAACGGTTCCCGTTTGCTAAGATTTTTGTTGTTGTTGTTTTTTGTGTGCTGCTGACTTAGAAACTTTTCATTGACCCTGTGAAGAAAGAGTTTTCAGCCGTAGCGTCTCAAGTAATGGCAGAGGAGATGCGACTCGTTCGCAAGGCCTACGCGTGTGGAAGTCGGGACGGAGAACCTCTAGCAGCGAGTCCGACGGAAAGTATTGAAACCAATGCCGCCACTGTGGATGGGAATTAGGTACTTTAATAATATAGTCGGTTGGGATTGATACATGTAATGAAGTGCCTCGCTCGACCATGTATCGGGGAGCCGCATTTTATTAGTACAGTATTATTGAGAGGAATCAGTATTAGGAGCTGGACCCGATTTACCAGGTCGTTGTTGCCGTTCCTTAACCACAAAGTGGCTCAATTTTTGCCGGTTTGCTCAAGTGGTTAGCATCAGCTACCAACGGCGATGGCATCTATCAAATTTCTTCACCCAACACTACACACCTTCGTCCACTGAGGGGTACAATGCGCTTGATCACATGCAACAATTAGAAACACCCTGGAGCAAGAACAGCTACTCAAAGTTAGATTCAGAGCTCACAAAGACTGGTTGGTAGGAAAAAAAGGTAAGACAAAACGTCACTGTTGCATTTACATGCAATGCGACGTTTGCCTCTGTGAATTTCCGACCAAATTTGCCGTTTTGGTAATATACGTATTTCGTACTTAATGATCTCTTTTAGGTAAGTCAGATTTGCGACTTTCTAAATGATCGTGTGAGATTAGTCACGCGATCAGTGATGATCAGGATTTCGACATTATTAATATAGAAGTGTTAGCGAATGCGAATCTCCTTATTATTAGCTGCCATTTTAATAATTGGTGCACCTCGGTCATTTGGCGAATCATCAAGAGCAAGTTGCTCTTAACAACCATGGGCATCGCGGGTTTAATGCTCGTTACTGTTGTAGCCTTGTTAGCCGGAGCACTTGCTCAAGAACATACTACTCAAGCGTACTCGGTGAAAAGAACTTCCATTGTCTCCAAGTCTCACATCAGCATAGCGACGGAAAGACAGTTAAGATCCTTCCAAACATCGACTTTACGTGAAGATGGCGAGGACAGAGTCAATGTACCCTGGTTATCAAAGGTCGACGATCTGATACAATCTATGTTCAAGTCCAGTTTGTCACCTGATAAAGTGCAAATCGGAACGTGGGTGCAGTCCAAGGTGCACCCTAAAGACCTGTTTAGCACCTTACGTCTTGGAGAGAGCGCAGCCAAGCTTGACGACAATCCGAATCTTCTTCAATGGTTCAGATTCGTAGCGCCTTACCAAACTAAACATGGCGAGAAGGCGATTTTGAATTTGGATGTCTACTACCTGTTACTAGAGACTCACAGCAACCAGGAACTGATGACACTACTGAAATCTCTAAAGAAAACTCCAGGGCTGAGTAAGTTTGCGTCGAATATCCAGGAGTCACTATCTGGAGCGTGGGTGTCCAAAACACTACTGAAAGAAACAAGTTCAAAGACAGCCTTCGAAGTGCTACGCCTAAAAGAAGCTGGCTCGAAGTTGGATGACACGCCCGTTTTCCACCAATGGTTGAAGTACGTGGAGGCGTACAGAGCGAAGAGAGGGACGGTTATGTTCGGAGACATTGATATGTTCTCGTTATTGCAAAAGACAATGCCGGAACGTGAATTGACAACCCTTTTCTATTCACTTCGGAAGGTCCCGAACATGAAGAATAACGCTGAAACCATGCAGAGATTTATGTTTTGGAAATCTAAAACCAGTCGCAAAACTATGAATGACGTTTGGCTAAAGTTTCGAGAGCCACCCGAGGAAATGTTCAAGGTCTTACGTGTGGCGGAAAGTCGGGCCAGAGACGACAGTGACATGATTCAGTGGCTCAGGTATACCCAACTGTTTCGTAACCGCATTAAGAAGAGTGCGTTTTCGGATGAGCAAACCTTGCAATTCTTGACGAAGTCCGACCCATTGAAATCAGATTGAATATTTGCAACACAGTTTCAATCTCTTAAGGAAGTTCCAGATTTGAAGAAACAAGCGGAAAACATGCCGTCTTCCCTATTCCAGAATTGGATAAGTGCGAAGATAAAACCAGATGAGGCTTCAAAATGGCTGGCAAGTCCTTTCAACGAAAAAGTTTTGAACCTGCCGAAGGATGATCCCAGATACGGCACTTGGGAGAGTTATATGCTTTTCTATGCTGAAAACAAGGGCGGACAACCTTTGCTTCAGAAAGTGAGAGCATATTTCAAAAACGATGACCCTGTCGATGCGCTGGCAGCAGTTATGAAGTCTAGATGAACACTAGATTTGATGGCGTTTCATTCAACGAAATAAAAACGGGGTGCCTTTGAAAACACGGTACATTTAAATTTGGATTTCGTGATTTTAAAGCATTTTTTAATTATTATTATTTCAGCATAATTTCGCACAGCGCGGCACTCGCGATTAAATGCTCTTTTTAAGGAGAGTACAATCGATGGCTGGCTGGTAAGCTCGGTTTTGAACCCGCCTACACAAAATCTCTTGCCACACTCCCTCCCGGTCGCCAGAAATAAAACTACATAATTAATTCGATACTTGTTTTTGGACAGTTTACCGGTATTGAGACAACCTAAGCGATTTGGGCATTATTTGGACAAGTTAATGACGCTATTTGAAAATGAACTAATCAACTCGTTGGAACGTTGATGTGCCGCTGCTTTAAACCGTCACGATGAAGGTGCTAGACGTACCTTCATACGCCGCCAATGCACCAAGCAGGTTTTTCGAATACCCGCGGTGCACGAAGCGATACGTGCCTGCCACACTAGTGCGTCCACCTTTGCGGATGTTCCACTCACAAGTGTTCTTGCTCTCAGCAATGAGATAACGCTCCCAGCGGTAGCGCAAATCCCAATGCGCATCGGTCATGACGGTTGTATACGCGCCATTCGATCCAAGTTTCTGCACGTCACAGAACGATGAGACCAGCGTGAGTGCGTTCTTCGGGTGCGCGCCGGCGAATACCGCTGAAGCGACACTCCCCACGGAGTACGAACTCGACGGTTGTGTGCGTACATCACTAAAGGAGCGCAGAAGCGGAGCTGAGTCCATCACCACACCAGTCTGCAACGTGATGAGCGAGTTGCGGTTGATCTGCAACGGTGTGGGACCCACGTCCAGCGGTACGGACGAATCAGCGACGGACGTGGCGACTCTAGTGAGCTCCTGTTGCACTGCAGCCAGCTGGTTGGGACCGAAAAGCGTCGACGCGCCTTCGTAGTGCTGCGTACGGTATTCCTCTTTGGTGGTCATATACTGCGCGTAAGCATTGCTGATGGCAGCCAACTCAACCTCCGTGACTCCAGCACCTGTAAGTGCCTTCTTGACTGTGCTTCGGATACGTCTTCCGGTAGTCTCAAACGTGGTGACTGCGATAGCGAATTGGCCGATCTTAGCGATCTGTACGGGTAGAATGTTGGGTACCCACGGCACAAGATCCATGAGTCCGACTGCCAGTAGTGGCACTTTGTTGACGTTCTGGCAGTCCTTCACCCATTGTGGTGCCTCTTTGACGACACCACCGAGGGTCCGGAAGAGTGCGTTGGCCTTGAGGTTTCCCTCTGTGAACATACTGAGCACTCGACCGTCTTCTGTGCCAGCAGCAAAGTTCTGTCCGATGACAGCAGGACACGTTCTATTAGCGTACGGGTCTGCTATGTTTGGTGTGACGCCGGTGAGTGCTACATTAGAGAAGTCGACGTAAGAAAGATTAGCGACGATGGAGCCATGGACGAGCTCAGAGGTTCCTTTGATCAGCGATGACAGCGTATCGTACTGCCGTTTGCCCATAATCTCGGCAGACTCGATCGTTGTATTGCCCTCGCCACTAAAGGTCCCGTCTCCATTATCGATGAGGTTCGGACTCACGTCACCAGCGTTAGTAATGCCGATCCCAACGATGACGTCGTCTAGTTCATCTTCAAGAAGGAGCTCGGCGTATCCCTTGTTGTCTCCACTGATGAGAAGGTTTTGTACACTAAGACTGGTAGGGTGGACGGGGTAGAACGCCAGCACACCTCGAAGCTTACCGGAACTACTGAAGAAATGTAGTGCCCGCATTGTCGTGTCGACGTCACTGCTGTAGTGTGCACGTTCAGTCGACGGGTTGGCCAGGTAGGCCTCGGGAGAGCGGTTCTTGCCTCCTTTAGAGACTTCTCCCTTGTTCCATCGGATGGTACCGGTCTGGATGGAGTTGTGTGCAGCGTCGATGGCGCTCACGATACCACTGACGATCTTGTCGAAGTTTTCACTGACGTATCCAAAGCTGGAGACGTCGTAGAGGAAGTATCCTGCGGTCCCTCCTGGCCCCGCGTGGGTGTGCGTAGCATGTAGAATGACGTTCTGTTCGGTGTAGAGTCCCTTGTATTTGGTAGCGAGCTGTGCTAGAACTTCTTGATGTACTAGTTGCATGACGGAGTGCAAGTCGCAGTGGACAAACATGACACGCTTCCTGGTATCAGCGTCTTGGATGAGGAATGCTCGGGCGTACAGACAATTAAGGATCCCAGCGGAGCTTTCGTCGGAGCTTGCGAACCCCATCATTACCACTTGGGCTGCAGGTCCAGTGATGTCGCTCTTGCCCACACCGATGTTATACGTCGCTGTAGCGTGCCCGGGGAGCTCGATAAGCAACAGGATAAACAGCAACGACTGGAGAAGTCGGAAGGACGACATCGTAGTCTTGCCTTGATGAGTCGATGAAGCGGTTTGAGCACCAGGCGGCACGTTTGAAAATGGCCAAGGCTTTAGTTGCCATCCGGTATCGCTTCTTGATGAGCTGTTCCGGTGGAGAGAGGGCGCATTGCAGTATCGCTTGTTAAACCGTCCTAGTATACCCCAACATGACTCATCGAGTGGCTCATACTCATCATGTTGAAGTGGACTGATCATAGTTCACCATCATAGCTCCGCTTGAAGACTTTTCTTTGCTTTGCACGCTTACAGTTGGACCGAAATGATTAGTCCGACGTAAATGCAACGTAGCACGTTGCATCATCTTTCGAAAAAGTACTCATAACCTCCACTTCAATGTACCCATCCATCGCCCTGCTCGATTACACTCCTTATAAGCGAAAATAATTTGTCTTTACCGGTTCTCCACAGTTAACTTTTTCCTTGTTCGACGTCGCTGGATAACTTGTGAGATCCGAAGCATCTGAACACCTTTCAGCCAACGTGTTCGAGACCGTGTGCGGGATGCTAACTCGTAAAAGTACTGAACTTTTCCTATCAAGAGCAGCCTCTCTCCTTCATCATCGTCATCATTTAGTTCACTAGCGCCAACCGCACTTAAGCGACGTTGACGTTGACGACGAAGTGCAGCAGAGAGAAACCACATGAGCTTGCAATCCCAGAGGAAAATAGTCCAGAGACGCGAGACCAAGGCTGACAAAGTGCTCACTTCGACACGACGACCGAAGATAACTCGATCCACAACTGCAATTTTGGGTACAATCCAGCGGTCCAAAGCGAAGAGCTCCAGCGACAATATAACCAGTCCCATAAGCTCGAAAGTCAGCACGGATAGCATAAGAACAGAGCGTCGAAAAGCCAATAAACGCATCGTACTCGAAGGCAAAGCGTCCAGCGTAATGGCCCACTGCATCCAAAGCCACGCGCCCCACACCCACACAACACGACGATCCCAAGTCAAAAATTGACTCAAAGCTAACGACGCTACTGTGAGCTTCGTGGACAAAAAGAGGAAGTTAAACGACGAGTATAATCTTTTGAGTAGTTGCCGTTGATATGAAGCAAAGAATACGCCACAAAAGACCAGCGAAGTGACGAACGCTACGCCTTGAGTACGTGCTAACCCTGGTCGTATATGATCCAGATTCTGGTCTTTAGAAATCCATGCAACCAGAGCACCCAGCACCAAAACACCCATCCCAATGTAGCCTAAAGCATGAAAGAT

>Contig_76

TCAACTACGCGGGTAGCTTGACAACCCCGCCGTGCACCGAGTTTGTGGATTGGTGGTTGATTCGCAAGCCTGCTCGGATATCCTCAGCTCAATATGATCGCATCAAAGCGAATCTTGCTGAGCTTGACGTAACCGTCAGTGGAAAGAACGCACGCCCCATTCAACCACTTTACGAACGCACTGTCACTTTGTACAGCTAAGCGAACTGTCAGTTTAACTCCTGCATAGAAACAAGCGCCTGTTAATAAAAAAAACTAGCGTAACTTTGCTCAGTTTTAAAATGACCTTCGTCGCAGCTTGTGGAATCCCGAAAAATGGATCCAACGGATTAATACGGTACTTACTTGCTTAACATTCGTAAAACAATCAAAAGTACAGTGCATCAGTTTAGTATCCCTACAGTTCGCTGAGAGATCTGTACTGATTATGTATGTATGATTTGTAGTGGCGAAATGCGAGCGAATGCCAGTGCAGTTAATTTGTACAAAATGTCAATGCAGTAAACAAGTAAGTTGGGTCATGCAGTAATGGAGCTAGAACAAACACAGCAACCGCTGCAATTGGCTCATTGTATCTTTAAACTAGCTGGCATCTATCTAGTTAATTGGTTTGCGGACTTTTGGTAGCAGTTCGGTAACCGGTCTGGCTAGCCAGCACTAACTGCACAATTTTTTTTAAATATAGTTATCAAGGATAAAAATATGGATACAATATTTTAATTTGTCTTATCCTTTTATCTCTCTCTTACAATTTGAAAGGGCGCAAACCATTCTTAATACAAGGCGGAGGGTACTAAACGGTGGTCTTCTGTTTTGCGTTTGGGTAATGAGCCAGCAACGATTGCTGGAGAACTGGATGACTGTTTTGGAGGCACGTACATGGGCGATTCCCCATAGGAGCTTATGCACTTTAGCACACACCCGCTTCCTTTGTTGGTCACGTTTTCGTGTCACTGGAAGAATGTTTATCCGAACCACTGTCTTCCAAAGCTAGTATTGGCTGTTCCTTCCGTCCAGAGGCTCCGAAAATGTTATTTTTATTGAGACTGGCAAAACAGCAATAAAAATAATTTTTAGTGCTTGGAGCACCACGGATGCAAAAATTATTGGTGTTATTGTATGCAATATACAGTGGGCTGATACTCGTCTAGTTGGTGGGAGCCTCACCACGCACAACCTTTCGGACTGTGGCAGCGTTCCAGTAGGCTCGGTTCAGCGACCAACCATCAAAATGAGAGGGCAAGAGCGCGGCAGATCGATTGTAGATTGCGGTTTAGCTGGACCGCTGGTCCGTAGGGCTCCATCGTGCCCGGTTAAAGGTTACTGCCTGGAGAGAGCGCAGAATTAATTATTTGATTTTGAAGTGGGAACTCAAAAATAAATTATTTTAATACTGCAAACTAAAAATATATTTATTAAAAATAACATTTTAGGACCCTGCTTCCGTCACTGTGAAAACGGCAACATCGTCGAACGTCCTTACTGTGATGGGGGAGGGGCTAGACTACACTGTGGGGCATCGACACCGACGTCATAGATTAACCGGAGGCGGCGTGCACTTAATCCACCACATGAATGNTTTTTTTTTTTTTGGGGGGGGGGGCTGGGGGAAGGGGTGTTTCTAACGTGTGCTATCCGATTTACAAGGCTTAGACCGATAAAAAGATAAAAAAAAGACAATATATCCTTATTTTTATCCTGATAAAACCTTTCGGTTTTTTTTTATGTAAGGCTTCATAATATGATATTATCAGCATTTAATAATCACTGACGACGCATTCACGTACTGACTTATAAGTTCGTTTCTTTTCATATGCATTACGCAAGATTACTCCTGGCCTACCAGGTATTTTCCTTTCCTTTGTCCACAAATCCCGATTGTATCTCGGTACCCCATTGACCTTCGTGTGAGCGGATATATTTTACCGAAGGGGCTGCCATTTGCGGGACAAAGTGAAACATGGTGATATGAGTTATTAACAAGAGTTAAACTAATTAATTGATTTGCCCTGCCACGATACGCGTGCATCATCTCTTTGTCTGATCTTATGACGTATTGTGTATTGCAGGTGGAATGTCAATAAACCACATAAAATCAACGAATTTCACTTTCTGTCGCAATCGGATCGAGTTTAACGAACAATTTCATCCGGACCGGTGTACTCTCCCGGAACATTTGCTCCGGGTTGCCTATAAGTCAGTTAATGGCTCCTGCAGGAACATCACTGTGATCGTCATCAAGATTTACATTGAAACCCGTACCCCGAGTACTACTGTACAATGAGGTTCATGATGAGCAGTCAGGGTATCTCCAATCCCATGATCCATGAGGCTCATAGATTCCGGGATTGGATCTGAAGTGCAATTGCGCAGTCAGCACTGCTGATCCAGTTGTCTGGATTAGAGTGTAGTGGGCTGTCAAGTCTAGACAAACCCACTTTGCATTCTACTGACAAGCACACACTTGCGCAGATATGATGCGCTTGCTCGGCATCTTCTTCTCGGTCGTATGTCTCGTCGCTGAACTTGCTTCTATCTCAGCACATGTCTACTCTGACAAAGCCGAGATCGCTATCTTCAGCCAACACGAGCCTGTCGCGTTGAAGAAAACATCAACTTCACCAAGGTTATTAAGATCGACGACGAACGAAGTTAAACAAAATATTGTCTCCGGAGAAGAGAGGGCTACCACATCAAGTATTTCTCGTTTTGGCGATGAGGTCTTGATCAAGTTAATGAAGAAAGTGAGCATGAACCCTACAAGTGTTTTCAACCGCTTACACTTCGGAGAAGCCACCACTAAATTCACCAGCAGCAGCAAGGCTTTCATTGACTGGCTTCAGTACGCGAACAAGTTCATGGCGACTAAAGGGACTGAGCAGTTCTCCACGCACCATTTATTCAATCTGCTTTGGCATAGTCGCCAATCGAACGAAGACCTGATAAATCTCTTCCAGACACTTAGCCGTGTTGAGGGCATGAAGAACCTTGCCAACACGTTGCAGCTATACATGTTCCGCGCAGCGAAATCCAGTCACAAAATGATGAATACGGTCTGGTTGCAGGCCCTAGAAACTCCGTCCGAAGTATTTACCACGCTACACCTCGCAGACAACGTCTCAGACGATCTCTATCGACCAGAGCTAATCGCATGGCTTCGGTACTCCTGCGACTACAGCAATGATGTAAAGAAGACGCTTTCCGCAAAGGAGACCTTAAATCTCTTGATGAAAGAACCTCACCACAAAGAGACAGATTTTGGTGTGCTATTCCAATCGCTCGCAATGGACAAAGCAGTCAGAACGGACACTGGTGTGGCACAACTTCTTGAGAAGCTACAATCTCGACTCTTTAAGAATTGGATCAATGCCAAGATAACGCCGGATAAACCCGGGGTCATGATAGCGAGCCCAGTTACTGAGAATTGGAGGCCTGTTCTCACTCTGCTGGTTACCGAACCCAGGTATATACTTCTAGAAGCGTAAATATTGCGGTATGCGGCAAACCGTGGCGATCAAATACTGAACAGTGTAAAAAATCTATTAGCTGAAAACAAGCCGGTGGATGCTTTTACACTGTAAGTTACAGCGGCCATGAAGATCTAGTAAGGATCGGTATTGATTTCTATTCTGCACGGTTTTTTTGCTGCTTCACCGTTCTATTGTAATACGTAGCACTAAATTAATAATATCATCTATTGTGCTATAGTAAAACCAAAAATCAATTTGGATCGTCTTGTCTTATTACCCATGCATGTGATTATGCAGTCTCCGATCTCAAAACGAGGTGGGTTTGCGTGCGAAACTTGCAGTTCAAGGTCGTGGAGTGCATTATCAAGAGTTGAYGATGCGCCCTACCACAGCAGGGTCATTTCTAAATTGCCCCATTTAGCAGCAGGGGCTCTAAGCACCTCTAATGAACGAGTTACCACCCGATAGTGGAGATGCACTAATGGCACTAGAAGCGATCAACCTCCCCAAGAACATCGCCACGAATATTGAGGCCGCAATCGGGCAGCCACTTCCTAAGATTGAGGTGCGCTTCCACAACGTCGCCGTCTCAGCTGACGTCGTGGTCAAGGACGAGACCAACCTCAAGACTGAGCTTCCTACGCTCGTGAATGTAACTAGGACGGGTCTAGCTAAGATGGTTGCTAAGACTCACATTGTGAAAAAGGAGATTCTACGGAGCGTGAGCGGTGTTCTGTAGCCAGGCACCATGACATTGGTGTTGGGCCAGCCTGGCTCCGGTAAGTCGTCACTGCTCAAGATCTTGAGTGGGAGATTCCCAACTAGCAAGAGCATTTCAGTCAATGGCCAGGTGACTTACAACGGCACGTCCCAACAAGAGCTTTGTAGACGACTGCCGCAGCTTGTGTCCTTCGTGGACCAACACGACAAACACTTTCCCACTCTCACCGTCAAGGAGACGCTTGAGTTCGCTAACGTGTGCTCGGGAGATACGTTGTCCAAGACAGAGGAGCAGCTGTACTCGAATGGAAAACCCGAGCAGAATCAATCGGCACTCGACTTGCTGCGAGCCATGTACAAACATTATCCGGGCGTGATCATCCGTCAGCTTGGTCTTGAAAACTGCCAGAACACCATCCTAGGCAACGCCATGTTGCGTGGCATATCGGGTGGCGAACGTAAGCGCGTGACGACAGGCGAGATGATGTTCGGCAACAAATTTGTGCTCATGATGGACGAGATCAGCACGGGACTGGACAGCGCCGCGACGTTCGACCTCATCTCGACACAGCGCAGTCAGGCCAAGACGCTGAACAAGACGGTGGCCATCTCGCTACTGCAACCGTCTCCAGAAGTGTTCGCGCTGTTTGACGACGTGATCTTGCTGAACGACGGGTACGTGGTGTATCACGGTCCTCGCTCAGAGGCATTAAACCACTTCGAGAGTCTGGGCTTCAGGTGTCTTGCGAATCGTGACGTGGCAGATTTCTTGTTGGATTTAGGTACGAACAAGCAACAGCAGTACGAGGTGGGGGGCTGTCCAGGCTCCGCATTCGAGCTTGCCGATGCGTTCAGGCGATCGCCTATCTGCAACCGAATGAGGATCGAGCTCAACTCCCCGCTAGATCCTTCGCTGCTCGTCGATAAGAAGAAATTCATGAATCCTATTCCGACGACACTTACTCGGCGTCAAATGACTGTAACACTTCGCAATAAGGCCTTGATAAAAAGTCGTCTGTTGATGTCGGTATTGCTGGGCCTTCTGAACGGTAGTACATTCTACCAGCTCTACGCATCGGACGTTCAGGTGGTCATTGGCATAATCTACGTGGCAATCAATTTCGTGACAGTCGGACAGTCTTCGCAGATGCCGATCTTCATGGACCTTCGTGACGTGTTTAACAAACAGCGTGGAGCCAATTTTCACCGAACTTCTTCTTTTGTTCTCGCAGCGTCAGTGAGTCAGGTCCCACTGGCGCTAATGGAGACAATGATCTTTGGCTCGATCATCTACTGGATGTGTGGGTTTGTCTCGACTGTGTCTGGGTTCGTATTGTTTGAGTTGGTGATGTTTCTGACGAGTATGGTCTTTGTAGCGTGGTTCTTCTTGCTTGCGTCCGTACTCCCAGATATGAATGTGGCAGGCCCGGTGAGCCAACTCTCGTTGTATTTCATGACCCYCTTTGGTGGTTTTGTGATCACGAGAGGCCAGATGCCACACTATTTGGTCTGGATGTACTGGATTAGTCCCCAGGCTTGGAGTCTACGCGCAGCAACGGTGAATCAGTACACAGACTCGAGTTTTAACGTCTGTGTATACGAGGGTGTCGACTATTGCCAGACTTATGGGAGCACCATGAGTGAGTACTCGTTAAACTCCTTCGATGTACCTACCCAGCGAATTTGGTTGTGGCTGGGAATCGCCGATATGATTGGAATGTACGTTATGTTTATGGTGATTGCTTGGGCCATTCTCGAGTACAGACGTACAGCAGAGCCTGTTAACACACCATTTACAGTGGAGAATGTCGGGTCAATGTCTGATGGCTACGCGCTTGTGAGCTCCCCGAAGGCTGCTGAGCAACACAAACAATCGAATAGTGACAGCTCGATCCCTATTGCCCAGCCAGCTGATAGGAACTTTGTTCCGGTCACACTGGCGTTCAAAAACTTGTGGTACTCGGTCCCTGATCCAGCGGGTTCCAAAACCACAATCGACCTGCTTAAAGGTGTCAGCGGATTCGAACTTCCCGGAACAATTATAGCGCTTATGGGATCTTCGGTGCTGGTAAGACGACACTTATGGACGTGATCGCTGGGAGGAAGACTGGAGGACAGATCCGTGGTGACATTCTGCTCAATCGCCACCCAGCTACCGACCTTGCTATCCGTCGAGCCACCGGTTACTGCGAGCAGATGGACATCCATTCGGGGGCGTCTACCTTCCGTGAGGCACTTACGTTTAGTGCCTTCCTGCGTCAAGGTGCTGAGGTGCCAGATGCCCAGAAATACGACTCCGTCAATGAATGTCTTGACCTGCTTGACCTCATCCCATCGCTGATCAGATTATTCGTGGTAGCTCCACGGAACAGATGACGCGACTGACTATCGGTGTGGAGTTGGCAGCCCAGCCCAGTGTGTTGTTCCTGGACGAACCGACGAGTGGTCTGGACGCGCGTTTTGCCAAGCTCATCATGGATGGCGTTCGCAAGGTGGCAGACACGGGTCGAACTATCGTGTGCACAATTCACCAGCCTTCAGCAGTTGTGTTCAGGGTGTTTGATAGCTTGTTGTTGCTTAAGCGCGGAGGCGAGATGGTGTTCTTCGGCGATCTTGGAGAGAAGGCGTCGAACTTGGTGACGTACTTTGAGTCGATGGACGGTGTCTCCACGCTGGAAGTTGGAAACAATCCTGCGACGTGGATGTTGGAGGTGATCGGTGCTGGCGTGGGGAACGATAGTAGCGCCAAGATGGATTTTGTGGAGATATTCAAGTCGAGCGAGCAATACCGCTGGCTGGAGACCAATCTAGATTGCGAGGGGATCACGCGCCCGTCGCCCTCTACACCAGCCATAACATTTGGAAATAAGCGAGCTGCGAGCAGCTGGACGCAGGCAAAGTTCTTGACTAAGCGATGGTTTGACATGTACTGGAGAACGCCATCATTCAACTTGACGCGAGTTATCGTAGCTGTGGTCTTGGCTGTGTCACTAGGTGTTTCGTACTTGGATACGGAATATGATTCCTATCAGGGCGTCAACTCGGGCATGGGTATGGTCTACATGGCTGCGGTTAACATGACCATCATCACTTTCAACGGCTCTCTGCCTATCACCTGCAAAGAGCAAGCGTCTTACTATCGCGAGCGAGCCTCAGAAACCTTCAACGCATTTTGGTACTTTATCGGTGCGACTTTGGTCGAGATTCCGTATTGTTTTGGCACAACACTGCTTTTCATGGCGATTTTCTATCCTATGGCGGGCTTTACGGGCGTCGCTGCATTCATGACCTTCTGGCTCAATCTTTCGTTGATTGTGCTGCTAATGTCGTACTTCGGACAGTTCCTGGCTTTCCTTCTACCAAGTCTGGAAGTGGCATCCGTTTTTATGATAATTGTCAACATTATCTGCACTCTATTCGCGGGCTTTAACCCTCCGGCTGTATCGATTCCACGTGGCTACAAGTGGCTACATCCCAAACAAGTACGCTTTCTCCAGTCTCGCAGCCATCGTGTTCGGCGACTGTTCTAGTGATGGAGACGGCAGTCAACGAGGCTGCCAGCAGATGACAGGTACGCCTCCAGGTTTGCCCGACGGAATGACGCTGAAAGAGTATTTGGAAACGAATTTCCTCATTAAAGACAGCGACATCTGGAGCAATGTCGGCATTTTGATAATTTGGTTCGTTTACTCTCACTACTGGCGCTACGATACGTGAATCATCAAAGGAAGTAAGAAGAAGAAGGGGCATCAATGGCAAAAATAATGAAGATACTGCTTTACATTGCACTAAGAAAACAAATTCGAGCGAGCTTGGCAATGGCTACATTGCAAAAACCGAAATTCTGAGTGGGGAGTTCGTCCGGAAATCGAGTAGATGGTAGTGAAGATATGAATAGGCTACTAAGCATGAGCCTAATTATCAACTGCAAGAAGTTTTCAAGCTCTCAGTGTACAAGCAAGACACAAATTTGCGAGCTCCTGCTCTGAAGATAGCCGACAAATTATCACTCAAAAAATATTTACATTTATAATAAAATGATAAATTCCTGGCTTGAAAAGTATAAATATTAAGTGTCAGTGTCATGTCACTTTGAACTTACGTTTTGTCACTTTGATTTTACCTGTCGCCATTTTGAAGTTGAAAAGGTGTAGATTTGGTTTTTTGCATTACCGTATTCCTTATGTGATTGGCGACAGTCAAATTCTGCCAGCATAACAGATACCTGCATAGAAAAACTATATTACATGTTTAAAATTATATTGGTCGCCAAGCTTGTCGCTAATGACCGAGCGTCGCCAATCACATAAGGAATACGGTATCAGGGCAGCGATATAGATCAGAAATCATAGATATTTTGCTCCTGTGCACTATATTTTAGAGATTGCATATGACACTTATGGGCGGCGTCCAATGGTGAAGCAACCGCCCTACACACAGTAGTGTAATCCAGAGCTTCTCCGTGAAACACGGCATTGTCATCAGTGCAATTCAGCACAACCGCGGACACTTTACGTAGCCGCCATTGCGGCCTTAAGTGCATCTACGGGCTTGTTCTGCTCAAACAATTTCTGCACTCTGGCCAACAGATCACCCCCTTCGTTTGCTGCATACTTTAAAGTATACTCTTCCACAAGCTTAAATTTGGGATCTGTTATAGGCAGGCTGAAAACACGCTCCCAGTTAGTTGTCGCCGAGCTGACAACCCTTCCTGCGATTAAGTCGGGTGTCATTTTGCTGTCCATCCAGTTCTTGAAAAGTTGAGCTTGTAGTTTTTCAACAAGCTTCATCACACCAGCGTCCTTCTGGATCGAAGATTCTTTTTTTAGTGACAAGAACAGCAGTCCGAAGTCCGTGTCTCTCTTGTGGGGTGCCTTCATCAAGAGATCCAATGTGTCCTTGGTGGAAAAGCCCACACTCTTGCTGTAGTCCTTAGAATACCTTAGCCACCCAATCAGGTTTGGGCGATTAAAATTTTCGAAAGTATTGTCTGCAAGACGTAACGTGGTAAATACTTCGGCAGGAGTTTCAAAGGCCTGCAACCACACCGTATTCATCACGTTGCGGGTGGATTTTGACGCTTGGAACATGTACAGCTGCATCGTGTGTGCGAGATCCTTCATGCCCTCAACACGACTGAGCGACTGGAAGAGCTGTACCAGGTCTCCCTTCGACTCAGCATTTCTCCATAAGAGGTTGAATAAGTGATGCGTGGAGAATTGAACTGTTCCTTTGGTCGCCATGAACTTGTTCACATATTGAAGCCACGCGACGAACGCCTAGTTGTTGCTGCTAAGCTTGGCGCCAGCCTCTCCAAAACGTAACCGACTGAAAATGCTCGATGGATTCATGCTCATCTTCTCCATCAACTTAGCAGCCACCTTATCTGCAATGTGTTGAACACTTGACGTGCCAGCTCTCTCTTCTCTGATCTTGTCATTTCTAGCTTCGCTTGTCGATCTTAAATGTCTTTCTGCAGTAGGTGCGCGTCGGTTGATGTAGGCCTTCTCAGAGTCGACAAGTGCCGAGCCAGCATCGAATCCAGCAAGAAGGAACAAGATCGAAAAGATAACACAGACCAAACGCATGGCTTCCGTTCGTCCGGCTGGGTGTTGGAGAGCAGAATTTGAAGTGGCCAGTGCTGTGCAGTGCACAGGCACTACAGTACATGCACCTGCCCGGCAGCACCACCTCACATCATATATCCAATCCCATGATACAGGGCTCTCTGGTCGCAACATGTTGCAATTTCAAATCTTGATGACGACAGTTGTGACTTTCCCGTGGCAGAAACTTTTGATAAAGCAATCTGCCTAAATGTTCTGGTCTGAGGGTGGAGTAGAGTTAATAGAGCCGATTTCGAAACCTGCCTAGTGTTGAAAAGTGGCTTTCCGGCGGGACACATGTATCACTACAGTACAGTAGAAGACCAGCTATGCGCAGCGTAGCGGAGGCCTGCAATGGTCGCGTAGTCTGGAGTGGGTCCACCAAGAAGGAGCTTAGAATGTACATATTTATCGTGGCCTTGCATGCGATGATCGAAGCGTAGTATCCGTACTACGCTTCGGTCGTCGCCGACGAACTACTACGAAGCCGTGCTAGTCGAATAATCTGCTGCACATACAGTGCTGGTTTGAATTTTTGTCGTGGCCGCAAACCGATCTCAACAGTAACCCGCAATATAAGTAATTCTGCAGAAATGAAAGATGCCAATACCCTATCAATATATTGTTGTCCTCAATTCGCTCATTCAACTAAAAACATATCCCTCACAAATCACTATTCAAGGTCACGTTTTCACGAATAGCACTTTCTTAAGTACGCTTTCTGAAATATAACCATTATTTTCAGAAGGCAAGTTTNAAAAAAAAATATTGGGCTATGATATGCCATAATAATCCCATTCTTACATGTAATGGAGTATGGCTAGTTTTGTTTTCCGATATTGTAAAATTCGACCAAATTTGCCGTTTTGGCTCTACTACTAATATTACAGTGTTCAGTGGGTCACTTGCTTACAAATTTCTTGTTAATTACAAACGTGCCCAGATCGTGCTTAAGATATCAAGTCTATTGCAGATTTGGGATGTTGCATTCGCGTTCAACCGCGCACGGAAATTTTGAGTAGAGCGCTTAATATTAGGTCGAGACACCCGCTTGATCTTGCATACGAAAAGAAGGTACGTCTACATATAGACGGTACTCAGTTATGATTGAACATTCAGGCGCTATTGAGGCAGCGTGTAGTCAAAGAGTTGTAGCTACACCCCGCTACAGTCTGGAATCAATTGTTTTTTCCAGCCAGGTCTGCAGATATAATTTTTCCTTGACAGGTTTCATAGCCACCATGTCTGGGGTCTGTGAAAAGAGGGCTCTAATTCTGCCTATAGAGGATATATTAGGTAGTAATTCTACTAAAAATTCTCTGCTCTGCTCTATCAGAGCAGTCTTCATCCTTCCCTTAGATTTGTGTCATACTAAGTTCTTTTCAATTTTATTATATGTTATGGAGCTACCTTCACTAAATAGATATAAGGAGTCATCTGTGTTGACCGATAGGGACAAATATCACTACATGTAGTTGCTTTGTACCCGGCTAAATCAGGTATTAATAGGGCCCAATTTCGTTAGACTATTAAAAAAAAGACTATTAACACCTTGCGTCCGGACGGTACTTGCCCAGAGCGCGTAAGCGCATGTCGTAAGATACGGTATATCAATGTTACTACGTATTTGTATGGGTGCACTTTTTGACGTGAGGTTAGGTGCATTAATTTTCAACAGGTTTTTCTTTCAATGTCGTAACTCTGACGGGATTCAGCGACAGTATTGCCTGCTCAATGTCGTAACTGACTGCAAGTGCAGTCAACAGATGGCTAAATTGCTTCACAGGCAGTCCACTTAAATCAAGAAACGCAAGGACGAAGGTACGATGGTTCAAGAAGCTGTGTATTTGAAACAAATCACTAATCAACTGACACGCAACCAAGCTCACTTGTCATCACTACCATTCTCGTTGTCACCACCGTTGCCGTCGTCGCTGCCAGCATCGTGCCCAAGTCGAAGTTCCCGTCGCGTCTTCTTTCTTTGGCCAGCTTTCTGGCCGGGCCGACGGAGCACAATGCGCTTCCCTAAGTTTGGTGGGATAGCTTGCCCGGCGTCGAAGTCATCATGCCAGATATCATAGCATGTCTTCGCAGCGCCCTTGTATTCCCGTCGAATACGGTTGCACAACCAGCATTTCGCATTATCAACGGAGCAGCGCTCGCAAAAGAACGTTGTTGCGAAGGACTTCTTCTTTTCAGTACGCAAGAGCGCGCAGACCTTGCACGATCTCTGCCGGCGTTTCTGGGCCCCAGTAACGGTAACCCAATCTTCAGACTGCTGCAGCGCGTGCGTGAGGCGTACTGGGGTGCGCTGGCGCTTTTGGCTCGCAGGAGGCGGTGTTGCCTCCACTCCAGCAAAATCTTCGGCTTTGAGTTGGAGAAGTTGATTTTGAAGGACGCAAAACCACTCTGAGCGCTTCATAGCAGCCATCCCCTTGATCTTGGCCCCCTCTTTGTGCGTAAGATACGCATTTACCATGACCATGTCCAGCAAACCTAAGAATAGGCTCTTATAATATTTCGTGAACCTCGTCGACATTTGCAGTGAATACGACTGGAGACGGAGTTGATCGTGGACATCAACTCCGCTCATCCAGTTCTGGTAGTCATTTACAGCAGAGGGGCACGGCACTCGAACCGCACCGATACGTTTTATTTTTCGCTCAATTGTCGACGCCGCCATAATGGAGCCCGTACAGAGATAATAGACGGGCTTCCGGTCCCACCAAACACAGGAAATCATGGTCGGGATGGCGATAGAGCGAGAGAACGAGAATGTACCGTGGGTTATGCTCACTGGGCGTGTCTTGCTCGTAGCCCTGAGATTCGGGCTGAATCCAAGCCGGTTAGTCATGATCGTGCCAATAACATACACCTTCATACTCAGGAGCTCAATCGCAAGGAGCACGGACGAGTAGTAGCGATCGATTACGACGGTATGCCACTTGTGACGAGACTGGGCGAGAACAATCTTCAAATTCCGGACCACGGCTGCAGCTCCAGTCTTAAAGTCAACTGAGGTGTCAGGTCCACCAGCAGATGCACGCTTACCCACATAAACTTCGAACCTAGATAGTTACGACATCCACTGTTGTCAGGCAAACACAAAGTAGATAGTTACGACATACAAATTTTAAACGTACCTGTGGCAGTATGCCGTCTGCGCGTCACAGACCATAAATAGTTTGGACCCGTAGCGGTGAGGCTTGTCGGCCATGAACATCCGGGTCGTGTTCCGCTTCGACGTGGACGGCAGTATACCCTCATCAAACGAAAAAATAGCAGAAAGAGACCACCCCAGCAAAAAGCGCTCTTGGACCTTGTCAACAACGGGGCGAAGTTTCCAGAGTTTATCGTTACCGTGGTCAATCGTGTTGTCTACGAAGTGCAAGTCTCGCATGATACTCTGGCACCTGTCACGCGTCATGAACCGCCCGAAAAGACCGGCGGGAACAGCACCGTCTTCCACCATACTCCAGTGCGCACCAAATCGTTTCTTTTGAGGGCAGAGCATCCGTGCAACGAGGAGTCCAACAACGTGAAGAATCTCGTGTGTAGAGTATGCTGCCTTAGCCTTCAAACGACGTGTAATCTGCTTGATAGTTTCACGCCGAAGGTCAGTCTGCCGTGCCTGAATGTTACGGGCGCGCTTGCCAGCTTGCTGTAGACCATATCGGTTCGTTTCAGCCGCGATAGAAACCCACAGAGATTTCGGCATGAAATAAAAGAAGGTGAGGATTGGCGATCTCCATAATTGCAGCAGGTCGGGCACAGGCCTTGCTTCTTCGGTGCCAAGTCCAGGGTACGCAGCAGCATCTGTCTCGTACTCCGAAGACACTGGCGTCCACTGCATTCCTCGTAGTGTCTGCTCTCTCTCCGTTGCAGCGGTTCTGCTAAGTGCGTTATTGCCCACCTGTAGTGCCGCAATGAAAGCCTTGTCCATTTCAACTGCGTCACTGTCGGACAGGACGTCGTCATCTTCCTCGGACATCCCGGCTCCGGAATCGCTGTTAATATCACTCATGCCGTCGTCGTCGCTATCACCCGAGCTGTAGTTCTCGTAGTCACTCGGCTTCTCCCCATCCAGCAAAACATTCACGTCCTCTTTGCCTGATAACGGCGTAAGTGTCCGTCGTCGAGGTTGAGACGCAGCGGCTTCTTCTTCGTCTACATCACCCTCGCTCTCTAATCCAGAAGCCTCCGAAAGTAAATGCAGTCCAGCAGCGGCCTCGCGGGCACCGCCGTCTTGTAGATYTCCTGAGGATAGATTGAATGCTCTGCTGACTGCAGTCTGCGACAGCTCGATGTCATCATCACTTGGGGTGCCGAACATTCGATCAATGGTATGCTGCGAAAGAAGGACACTCGTGTCGATCTGGGAAGGCCGAACGACGTCTTCCCCCTCGTGTTGTTGCTCTTCACCACTACTACCAGCTCCTGCTTGGTCTAGTTCCCCACCTTCTTCATCTCGGACTTGAGCATCAGCATCCGCCTCTTCCAGCAAGCCACTGTCGAACGCGTAGTCCACGACTGATTTCTCCCCCACGAAGACGTGCGTGTTGTCAGGACTCGAGTAGCTCCACTCAGTCTGGAGGCCACTTGGACGCTTGGACTTCCACCCCGCCGCCCGAAGCTGCCGCCATAAGTGGCCAAAGTCAATATCGTGTGTAGCGGCAGCCGGCGCGGCGGGCTTCTCGCGTCGACTATTCCGTATGCGCACCATCCAGCAAAATGTCGAATGAGTGGCTGTTCACGACGTGGCTGATCCCCTTCCCCTGGCCTGGTTTTGACTTGTAGCAAAATGACCTCTCGCAGATTATATTTTTGTGGTAACTTGATTAAAGTATTGAAAAAACTTTTCCGAGCCATTTGCTAATGACCTTCGTGCAATGTCGTAATATACGACGTAACAAAAATAATATGTCGTCTTATACGACGTTCGTAAGCAAGGTGTTAACAAGATGCAAGTGAAGTTACACAAAATGACCGCTGTAAAGCGCCGCAATGGCAGGTTTCATCCAATCATATATATTTGAACCCACCTAAAATCTTCTCCTTTTTGTTTGTGCGCATGTTTGACGTGTGATGGTTTTGGTGTTTTATTCGAAGCCGGTAGCGCAATAA

>Contig_77

ATCGAGAGCTCAAAGTAGTTGCATGGGGGTGTACACAATACTGTTATATGTAACGTGTTGTACTCGCACCTCTGCTTGATCGATATTGATTAATATATGATTAATATCGATCGATTGAATCAATATCGATCAAGCAGAGGTGCGAGTACAACACGTTACATATAACAGTATTGTGTACACCCCCATGCAACTACTTTGAGCTCTCGATCCCTAGTGTCCATTTAATCGCCCTCAGAAACTCAAACCATACAAATTTCTAGTCAGCCAAAATGACATATGTTTTATTGTATATAGATTTTTGTATTGGGCCGATGGTGGCCGTAAATTGTGTCCAGCCTGACAAATCTCTTTTTTGTATGTGAGCCTCCCTATAAAGTGCAAAACCGCGAGCGTTAGCACATCCTTGCTATTTTGAAGCATGTTTTGCAACTTGTGTGAAAACCGCACCGTCAAATATTAAAAATTGACTGAGTTATGGAGGTGGCCGTATAATTGTGTCCAGGAGTGTAATAGTAGTAATAGTTAACAACTAATGCGGTTGAATGAGATTCAGACTAACTCGGGAAATTTAATGGGTGAGGGCAATCAGTTTAACTCGCGCGAGCTAATTTGAATCGAGGTTCACCTCATCTTAACTCGCGCGAGTTAATTTAAGTCGAGGACATTAACGCTAATGTGAGAATCGGTGAAATCATCAAAATGGTCGCATCATTGACTTTTAATTCGTTTTTGCGTGACGAACTTGAGGTCGATACGAGCTTACATCTAGCTTGTTCTTCATGTACTCGACAGGGGTTTGATCTACTTATAAATGGTAGAAAGTAGGTGGCTTCTTCTTCTTGTTTTGTTTCGACAGAAAGTTGAGATACGTAGAGTACTTCATGTACTCGTCCGTTTGCATGAGCCGCTGGAGAACGGTGTTCGAGCTTTGAGCACGTACAACATCGTCAGCAATGCCTAAGTGCTTAGCAAAATCGTCCAAAGAATTCCCCGCTTCAAATAACCGCTTCATCTCGGCTTTAGATAGAGACCTCTCCTCATCGGGTTCCTGATCCTCCACAGGGGTCTGGTGAGACCGCAATTGACGTTGGTGCTGCTCATTTGGTGAAGAGAGCATAGCTTGGTTCGAGTTCACGAGCGCGTTGCTAGAAACGAAGAGAGTTGCCATCGTGGCCGCGAACACGTAAGGAAGACGCATGTGTATAGCGCAGCTTGAGAGGTGGAGAGCAAATCTCAAAGTGAGTGGCTTTGAAAACTACTCCACGGGCGGGGGGGGCTCTTGTGGAGAAGACTATCGTAGTAATCGACACCCTGCACTTACAATCCATTTGTTTTACCTTTAGTATACAGTACAATGCACTGTACATAAGGTACAACATAGCGAGCATGGGAGATATCAAGTGAGACCTGCTGTAAATTCACATTTAGGTAGAGGCTGAGCCTCTGATGCTACCCAAAATCGTAGCCTGTTTTCTACTCATGTCGTCGATCAGTGATAAGGTTCAGTTTTTTTATATTCAGAATTTTTCGAAGGTAATCCTGCTATCCCTTAGCTACTGTAAATGGTTTTCCAAACATTAAGCTAAGATGTAGTTTCCTACGAAACTGGCGTGCATAACTGATGATGTAGATTCCCCTTTTGTACGACCACCCGGAACTTTGTTCAGTCAAGTAGATATCAACGCCACGACTAATACTTTGAAGTATTATGGTGACACAGAAGTCCCCTTCAGTAAGCATAAAGGTCGTCTGATTAATTTGGATATGGAATCCACAGCGATCTTGTGCTCTTGTCAAAAGTACCTCAATCCAAATGATCCGATATAAAGGAGTATATTAAGTACATGCTACTCTTATTCAAGGGACTTCTTTACCTCACTTTGGTACTGTAGATTTAGCTAAACCCGCCTGTGCCTGCATTTGGAAATATATCCAGTTAATTAAACATTTGCAAACAACTTAAGTTCAATATGGGCGATCTGTGAATAAATTTAGGGGTCTGTGATTTTCGCCTCCCATCCGATTACCCTTAGCTTCTGTTCGTTTTATTTAGCGGCAAAATTGATGAAAACCGCAATACCGGTAGCGGCTTACATCGTCCATCGTGGTTGTTGACGCACTCAGACGGTTGGTGTGACCAATCAATCGCTTCGTATTTCAAAAGAGATGGTTTGATTAGTCACGGGGATTCGACTCTAAGTAATCGGACTGCCTTAGTGATACTCAATTGTGCTTTGGAAATGGAGTGCCAGCGTGGTCAGCGCATAAAACCAGGCGAACAATAAAACACGTAAATGTATTAATGCATGTAGGTCGAATCAAATTTACGCAACACTGCCAAAACGGATGTCGATATTTAGATCCAACTAAATCCAGAACTTCATGCTTTTCATTAGAATTGTGAAAAACAGTACATATTTCGTACTGCGTACATTACATAAAAGTGAGCGTTTCAGGGTCTGTCGTGTGTCAAAGCTTAATATTTTTATGGCCCAATCTATCCTTCGAGTCAGGTTTGAGGTCGGCTACGAGTCAGAGTACACATCCGAGGTTCTTACACTACTGTAGTTTCGGATACGATCAGCCGTCACCTGGATTATCTTTATAATACTTCGGTCAAATAGCAACCCCCACACCCGAGTGATACTTACATGCGCAAGGTCTATCCAGCGTTGAAGCCGGTTGAAGCGAAGCATCCTCCTAGCGTATTGACAATCTAACAAGCAGCAAGAGTCAATCTTCGGATCTGTCGAAGTAAGCATCTCCGATGTGATGATGACTATCTAGTGGAATATAACTGTACTATTTCATATAGAGAAATCGTATATAACTGGACTGGTTGGTGGAGAGTAACTCCCCTCCAGAACACCAGATTCCATTATTCATCACTTCTTCTTGTACCTAGTCCACCACAAGCTCTTCTCCTTTTACCATGCCAGAGTACAGATGACGGATAGGAGGCGAGCTGGACGTGGCACTCCAAAATGTTATAAGCCCGACGCTTCCTACCAATAAGGGCGGCTCAAACGAAGAGCGTCACCTGGCTTAACAGGGCATGAACTCGACGCCGACAAGAGGACGCACGGGAGTCTACCATGTGCCACCGGCACCTCAAGGCCATGCTGCCCTCTATGCCAGCTAACGTGCTGCGCACGTTAAGCAGGACGTCTTCCCCGCGATGTTGAGCTAGAGGAATATCTCCCTACACTACAAACCATACGCACTGCGTATTGATCCCGCTATAAGCGGCGCATTTGGGTTGTAAATGCGCCCACAATGATAAGCATTGCATATTGAGCACGCTATAAGCGGAGTACGTGGGCAGAAATGTCGCCCACGCATTGGCCCGCTACAAGCGGAGCATACGGGCTGTAATATCGCCCACTATAATACGCACTGCGTAACGAGCCTGCTATCAGCAGCTGTATTATCGGCCGATAATGTTGGGATTACCATACATGTATTACTATCTCACTACTACTACAAACAATAATCCAGTATACTACCATTGAGTGTAAGGATAAAAGCAAGCCAGCGTGCCTCTGCATATTCAGGCAAAGTGCCATTTACTGTATGATCCAACAATCAAAAGTTTTGATTCAAAAATTAAAGAAGATAATCCAAAGTAGGCAAGCCAACGAGCCACTGCCATCGATGTTGCTGCATACTTGTATTGTTCACGAAGAAAACATAGAGAAGAGAAATCAAAGTCCAGGCAAGCCACTGAGCCGCTGTACAATCCTAAAGAAAAAGAAATAAAGAGTGAAAATACACTACACACACGAAAACGTCAACTTGACAATGCAAACACAAATGATGCCTACTGCCCAATTCAACCAAGTGAAAAAAAACTGTATTGTACTCTGAGTTAAACGACCTCAAGAAACTAATCTGAAAAGATAAGCCAAATAAAAATGAGGAACTGGAAAATAACTTAGTCAAGGTGAAAATGAAAGGCAAGAACAAAAATGTACAAGCCTCTGCCGTAAAATGTCTCGACAGATAATGAATGGCATACTACCAAGACATTCAGCTTCCTGAACAGTAAAGATGTGCTAACAATGCCTCCATCTACGCTTACCGAGCAATAACACTTTTTTTGCATGAGCAATGTCTCTGTCAATTGACTAAATAGTGTGCATGCTTCTCACTACTTCGAAGTATAATAAAATCCGGAATACAACTCGAAGAGCCACTAGGGAGAACAATGCCCCAAAGATGTGAGCTATAATAGGTAAAATGTGAGTGAAGTGTGTTCCTACTGAGAATGTTGCTTTGGTGCAAGCATTGTTACCTAGCTGCTCAATACATATTTATGCTACATTCACAAAAGAAATATAATGAACAAAAACAAATATGCAATTTGCATGATGCAAATACAAAATTGCAAATTTCACAAAGACTGCCTAGCTTGTACGACNAAAAAAAAACGCTGAGAGAGAAATACTTTAACAAATGTATGAAACTGGGATGCATCAATGCCAAAAACTATTCATGCATTCGCCAAACTTTAACATTTATTGTTACTGGCAAAAGTCTCAAACATTTCCATAACCGTACCAAAAGAATGAATAAAAAATAAAAATTATTCAGCACTTTATGACTACAAGACAAGCCTACAAAAGCCTCTGTCTACAGAAAGCAAACTATAATTTTACTGCATCAATTTCATCTACAATGAATTCGATAGTTTGCAAATCTATCGCTAGCGCTTGTGAAGTAAAATAAAAATTATTATAATTCCAAACGAGAAACAGCCTCTAGGGAGAAAAAGAACCCCAACGACTAGAGCCTGCGCACTTACTGCAAATATGATGCGTACGCTACCGATGACACTGTTTGATGTTATTTGTGCTTTTAAAAGATGTAGCCTTTCTTAAAAAATATTTGAAAACTATAAGCTCTCAATAACAATAGTCAGAACAATTTTTCAATGACTGCAACACCTATATCAACCATTTCATGAAATTGGGAGGCAATTCTATAAGACCTCAGCAGCGAAAAACAAAAACCAAACCACACATTGCGAAGAAATCCACATATGAAAGAGCAAAACAATGGCAGTTTAAAAAATGAACAAAGCTAAAACGCTTACACATTATATATAAAGATAAATACGATGAACAGGTGTATCCTCAAAGGTTTCCTGAGTAGCCAACAACAGAGCGAACAGGTTTTGATCCTTAAAATTAAAGGTTTCCCTGAGGTAGCTTCTACAAATTCGTAAAGCTAAAACGATAACAGCAACAATTCAGCGAACAGGTTTTGATCCTTAAAATTAAAGGTTTCCCTGAAGTAGCTGCTACAAATTCGTATAACTAAAACGATAACAACAACAATCACTACGGTGAGCAGGTTTAATCCTCTAAGGTTTTCCTGAGTAGCCAAAATAACAATGAAAACAAGAAATCAGAACAGTTATTGCCAAATCAAAACGGCACAAGGTGACAAATCTGACAATGAAAGTCAAAGGAAAAAGATCATCAATATCAAAACAAGATCTACTTCTTTTTAATGCTCATGTACGTACAGGACTGTTATAAACAGCCACCTGTGGTACATTAAATACTATCCGACAGTTAGAGCATCATACACATTTCTCGATGTGGGAAGCTGTCGAAGTAAGCATCTCCGATGTGATGATGAATATCTAGTGACATATAACTGTACTATTTCATATAGAGAAATCGTATATAACTGGACTGGTTGGAACTTCCCTCCAGAACACCAGACTCTATAATTCATCACTTCTTCTTTTACCAAGTCCACCACAAGCTCTTCTCCTTTTACCAAGTCCAACACAAGCGTCTTCTCCTTCTACACTCAAGACTTCCTCTCCACTATGGCCACCCTACTTTTGTCCTCACTCCGTACTGTACTGCCCTAACAGCTCTGCGCAAATCATCCCTCCAACAGGATCCATGCCATTAATAAGAATGATACAGTAATGCTAGTTTAGAGAGCGGGAGCTTTTTTTCACCGCCCCTGTCTGTGTAAACTTAAAGCTGGGATACCCGTACGGACGGGGCGGGCCGGTGGAAGCTTTCCGGTAGAACCCGTTCACTTATCACTTCTCAATTTTCTTGTGGCCAACAACTCACCTACGAACCTGATTATGAAGTCCCTCCACGCTGTCAATTTGGTCTTACTCCTGCTGCTTGCATGTTTTGCTCCTGCCCCCGCTACAAGGGAATTGAATCTGAGGGCCGCCGCTAGCGATTCAACTCGCGTTGTCGACAACGCCACTACTGAGCGGCTTCTAAGAGCCCACAGTAGTGGCAAGGAAGAACAAAAAGAGGAAGAGGAAAGGGCAATCTCGATAAATTTTCCAAGTCTGGAGAAGATCTTAAAAAACGTTACGTCAGGCAAATCTACGGAGCTGCAAGGAATGCTTAAGGCTGACGAGGCCCTTGGGAGTGCTTTCAAGACGCTAAAACTTAGTACAATGCGGATTGGCAAGGATGATACCAAGATGGTGGCAAAATTTCTGTCAAGCCGCAATTTCAAGATTTGGTTCCAGCACGCCGTCAAGATCAACAAAGATGATCCCTATGGCGAGATGCTTAAAGCACTCACAAATGTCTTTGGTGAGAAAAATGTGGCGATGATGATCCTAGTCGGGAACCTGTCCAGAAACTCGCGCGACGTCGCAAAGAAGTTAGAAAAGGCCCAGTTCTACAAGTGGTACTTCGTCGATAAGTACAAGACAGCAGATGAGGTTTTCACGAACGTGCTGAAAGCTGATCGAAATAGAATTCATGGGTATGGTCGGGAGAAAGAAATTTGGGGAGATTACGCGAAGTACGTCACGACCACAGTGATGAAATATTGATAAACTAAGTTTCCAGCACTTCTCTTGTTTTGGGCTAACATATATACTATACGTAATCCACACTATATAGTGGATTACGTATGAATCAATGTATGGGTTTTAAATCCAGTTTAGAATCCTTTTATACTCATACTATGTATTAATACTAGTTTAAAGTAGAAGTAAAATGTTTCTAGTTCATATTGATATGATGTTAGTAGATATTGAGTATTTCAATGCTTTTGACCAGCCTGACGCTCAATTTATAATAAATAGAGGTCGTTTTATTAGATCGAGGTCGATAAAAATACAAATCGATCAATCTTTAACCCCTTGGTTCCCGGCGAAATGTACCGAAAACGAGATAAATAATGTCGTAACTTACGACATGTCCATGTCACTACTTGTTTGTATGTGGCATATGTTCGACGTGCGTATAGGTGTATAAGTTTTTGACAGGTTGCTCTCTCAGAAGTCGTAAGTTATACTACTTAGTAGCTAGCAGAAAACACTGTATGTCGTAAGTTGCCCCACATTAGGAAGTACAGCGGGAAGATCTGCTGCACATCGGGGAAAAAGTGGTGCACAGAGCTGTTTTATGAATCCAGATCAGTAGAATAGACAGAACAAAAAGTACAGATGCTCAAGGAAATGTGTATTACGACATGACAAGCGCCCACACTCTTTTGATGTCACGCTGGCAAGCCTACTCTTGTTCACTCTCATGTTCATCACCGCCGTCTTCCTCATCTTCACGAAGTCGCAACTCTCGACGGGTCTTCTTGGGCACACCTACCTTCTTCCCCGGGCGTCGCAAGACAACTCTCTTTCCCAGTGTTGCAGGAATGTTTTGGCCGCAGTCGAAGTCGTCGTGCCAGATCTCAAAGCACGTTTTAGCTACACCCTTGTACTGACGTCGTATCTTGCTGCACAGCCAGCACTTTGCATCGTCAATAGAACAGCATTCGCAAAAGTAAGTTGTAGCAAACGACTTCTTCTTTCGATCCGTGCGCAACAGGGCACACACCTTGCAAGATCGCTGGCGACGCTTCTGAATGCCCGTAACGGTCACCCAGTCGTCGGCCTGCTCGACAGCATGTGTCAGACGTAGTTGGGGGCGCTTCCGCTTCTGGCTGCTAGGTGTCGGTGTTGCAACAATACCAGCGAAGTCCTCCACTTTCAGCTGCAGTAATTGGTTTTGCAGGACTCCGAACCATTCTCCTCGCTTCATCACCGGCGTTCCAGCGATACGTGCAGCCTCTTTGTGCGAAATAAACGCATTTACAAGTGCCAAATCCACGAAGCCTAAAAAAAGGCTTTTGTAGTATTTCTTGAACTTCGTAGACTTCTGCAAAGAATACTTTTGTAGGCGGAGTTGATCATGCACATCAACTCCGCCCATCCAACGTTGATAGTCGGTTACAGCCGCCGGGCAGGGCACGGTAATTGTACCAACGCGCTTGACGTTTCGTGTAATAGTCGTCTCTGTCATTGCCGAGCCTGTGCACAGGTAATGCACAGGTTTCCGGTCCCACCAGTGAAATGCAACCATATTTGGAATGGCGACAGAACGAGAAAACGTGAACATGCCTCGGGGAACGCTTGCTGGACGTAACTTGCGTTTCTCTTTGACATTTGCATCATATCCAAGCCGGTTGGTCATTATCGTGCCTATCACGTACACTTGCTTGCCCAGTAGCTCGATAGCAAGTAGAATTGACGAATAGAAGCGGTCGATAACTACTGCGTGCCATGGAAGCCGGGTGTTGGGACCAAGCACTGTCTTCAAATTTCTAACGACTGCAGCCGCACCGGTCTTATGGTCAAATGTTGAATGCTCTCCATCTCTCCTTTTGCCCGCATACAGCTCAAATCTAGTAACATACGACATAATTGGTGAGAAACCAATCTAATGTCATAACTTACGACTTAAGTNAAAAAAAAAAACCTGTGGCAGTACGCGGTCCTTGAATCACACACCATAAACATTTTCGAGCCATACCGATGAGACTTATCGGGCATGAACATTCTAGTCGTGTTTCTTTTAGAAGTAGAAGGCAGAACACCCTCGTCAAAAGCGAACACTGCCGGGAGGGTCCACGCAATCAAGAAACGATTCTGCATTGTCTCACCATTCGCAACTTCCAGAGCCTGTCTCGCGTACGCTCCGATGCCCTCAACAGTCAAGCCAAGCAGCAACGTGAGGACGCGTTACGTTCAATGGAGTGGTCTCCAGTCACTCACGAGTTTGAGGAGGGTGTCGAAGCGTACTCTGGTCTGAATATGGAAGAGGCACGACCGGTCGCTGAGCTACTAAACGTTTGCCACTCTTCACTACTTACATTCTTTTACTTTATGCCCAAGTCGCTATGGGTCAAGATAGCTGCAGAGACGAATCGGTATGGTCTTCAGCAAGTCACCAGACGTGCAGAAAGAATTCATGCAAAGCAACACGATCGGAGAAAGGAGACGGTTAAGCAGATTAGTCGGCGTTTGAAGGCAAAGCCGGGATACGAGACGCACGAGATCTTACACGTGATAGGTCTCCTGATTGCGCGCATGCTCTGCCCGCAAAAGCGACGTTTTGCCGCTCACTGGTCGATGGTGGAGGATGGGGCCGTTCCTGCAGGTAATTTTGGAAGGTTCATGGGCCGGAATCGATGTCAAGACATGTTGCGCGACTTGCACTTTGTGGACAACGAGGCGCTGCCTACTYGAAGTGAGTCAATGAACGCTGCGTCCATTGGCACAGCGTCATCTTCTGACAGCTCATCCAATTCTTCAGGTACTTCACAATCATTGCCATCATCGTCATCATTGTCTTCTTCAATGTCGTCATCACTGTCTCCAGAGCTAAAGTTCTCGTAGTCACTCAGGTCTTCGTCGTCAGTCACAAAATTTACATCCTTCTTGACAGGATGACGGGGGCGTAGGACAGGACAGGTCGGCGTAGCTGGCACACGAACAGGTTGGGAAGCGTGCTTGCGTACGGGTTGGGCGTCAGACTCACGTGTAGGTTGGGCGACGGGCACAGTGACAGGTAGGGCGGTTGGCACAGTTATAGGCTGTGCGGGCACTGGACTGTCAGGCGGGGAAGCAGCGCAACATCCGACTGAGCGTCACTCTCGGGTCCAGATGACAGAAACTGCAGGTTTGTCGCTGTGTCTCGAAGCTCTCCGGGCGACAAATTAAAAGCCCGAGTAACTGCTGCCTGTGAAAGCTCGACTTCAGAGCTATTGCAGCTGCTTGGGGTGCCAAACAGATCATTCATGGTCTGCTGTGACAGCTCAGCACTTGTGTCGATCTGCGACGCACGTACGTCCTCTAAACAATTATCGGCGGCGTCTTTACTGGAAGTGACGGCGTCTTCAGTAGCAGAGGCAGCGGCGCTCTCGTCATCGTCGGCTACTACGTCAACAGCGACCTCATCGTCTGCAGCGTTGGCTTCCTCTGCGCTCTCCGGTTCATCCAATATTCCTGTCTCTAATGCATACGACACCACAGCCTCCTCTCCAACTAACACCTTTGAGCCATCTGCACTTACGTACTTCCCTTTAGTTTGTATCCCGGTTGGTCGCTTGTACTTCCATCCCACAGCTCGAAGTTGTCGCCAAAAGTGGCCAAAGTCAATGTTGCGCGCCACCGCAGCAACGGCGGCTGTTTTGGTTCGTTTTCCAGGCCGGATGCGAGCCATCGAGCAAATTCTGTAATGGGCTGTGGGCCACTGAGTGGCAACTGGATACTTTCCCGCGTATCCCATCTTTTCCGCGCGTCCCTGATCCCTGGCTCGTTTTTGGATTTTTCTCATTTACCGGTTGAACTTTATCGATTTCAAATGACTTGATTAAAGTAAAGGATTTCCATTTTTGAGCCATTTTCTAATGTTATTCCAATTATGTCGTAAGTTACGACATAAAAAAAGAGTCATGTCGTAAGTTACGACGTCGGGAACCAAGGGGTTAAAAGACTGACTGATAAATCAAACTACGCCAGCCAAGTCGATTGCACGGGATCGATCAACCACCGAATCCACATCGATCTCCTGTGAATCGGCCCTCTTTCAATTTTGTAATGGTAAGAGCCTTGTCCAATAGAAAATCGATATTTTTTCTTACATATTTTTTTCCTTTGAAGTAGCACTACGGTATGGAAAACGAGGTGCCGAGATTACCTCTATTTAAAGCGGCAAAACTAATAGGTGCGGCACCTATTCTTTCGGACAACGACGGCTTATTTGATGGGATGGGAATAAGGATGCAGAGAAGGATGAAGTTGCTGTGGGCAGGAAAGGTGCCTGCGACGGCGACCTGCGCTCGGAAATACCCACAGTGTCGTTGGGGGGTATAAGAACCAACAGAGAGCGCCTACACTCTGCATGTGGAAGTAGGATTCAGCACCTTGTCGAAGCTGTAATGCTAGATCGACGCTGTACTACAGCGTCGATCAGCGTCGATCCTCATAAATATTACACTACAGCGGCTGTAGCGGTCTTTTCAGTCCACACACATGCTCGCAAGACGGAAACGCTGTGTTGAGAACACGTTTATGTCATCATTTGGCTGTAAATTATGAATGGCGTTACAGCAACCGCAATACAACACTACGACAAGAATTAGAGCAACCTTATCGCCTAAAGCAACCCTGTTCCTGGGTAATTTATTTTAAATGACACCAGCGTCAGTTTAAATAACGGGACCTTCACTCTAGATTTAGAGTTCAAATTCACACGTCACACACATATTACACAAAACTGTGTGACCCACATCGTGAGCACGTTGTGATTTTTCAGGTGCACCAGAACAACAGACCCACTCCAAGGATGGACTCGAGAACTCTAGCCCGGGAGCTGCTACGCGCGAGGATTAACGAGCTCGCGGACTTGAAGTCAAAGCTGGAGAGTATGACCACAAGCCTGGAACAGCTATTAAGACTTCAACAGGACTTTCAGTCGCGAGTTGAAGAAAGCGAAGAGCGCAATTATGAGCTGGCAGCAACGTTAGATGGCGAAGGTAATGCTCCAAATGCCC

>Contig_79

GTACATACGACAGGTTTTGCGTAACTAAATGTAGATAATCAATCATCTGCGCAGCTCGCCTGGCTGTTTGCAATCAATCGCTGTGTTTCGTTCTTTGCAGAAATGTTTACCTTACGTCTAATCGCGAGGGAGAGAGGCGGTCTGATCGTGCGACAGGTCCAGTCAGTGTCGCAGGTCGGTCAATATTTGGCAAATCAGATGGCGAGCTAATTGTGTGACGCTTTTGGATATATTTGTAATTTTGGCATAGCTAGGTCGAGTCAGAAAGATAGGAGCGGTCGGTAGGATGGGAGAAAACTGTATTAGCCGGGCCGGATGTAGCCATGCGAAAAGGCAGGTCAAAGCAGGAAGACAGGGAGTGCGACGGACGGACAGACAGACCGACAGGTTACAAGGTTAGGGAGCGGGAAATACTTTACTTTCAATCAATCATCTGCGCAGCTCGCCTGGCTGTTTGCAACCAATCGCTGTGTTTCGTTCTTAGCAGAAATGTTTACCTTACGTCTAATCGCGAGGGAGAGAGGCGGTCTGATCGTGCGACAGATCCAGACAGTGTCCGACACAAATATTGACCGACCTGCGACACTGTGTCGCAGGTCGGTCAATATTTGGCAAATCAGATGGCGAGCTAATTGTGTGACGCTTTTGGATATATTTGTAATTTTGGCATAGTTAGGTCGAGTCAGAAAGATAGGCGCGGTCGGTAGGATGGGAGGAAATGTTTACCTTACGTCTAATCGCGAGGGAGAGAGGCGGTCTGATCGTGCGACAGATCCAGTCAGTGTCGCAGGTCGGTCAATATTTGGCAAATTAGATGGCGATCTAATTGTGTGACGCTTTTGGATATATTTGTAGTTTTTACATAGTAAGGTCGAGTCAGAAAGATAGGCGCGGTCGGTAGGATGGGACGTAGCTGTATTAGACAGGCCGGGTGTAGCCATGCGGAAAGGCAGGTCAAAGCAGTAAGACAGGGAGTGCGACGGACGGATTGACAGACGGACGGACATATGTACCGGTACAGACAGACCGACAGGCTACAGTACAAGGTTAGAAGCGGGGAGATACTTTGCTTTCCTCGATTTTCTTACTCACTTCGATTTTCCCCTCCTGGTACCGAGCAACCCATCATATCGATGCGCGTGCTGTGTTTGGCGCTGATGGCGACGGCCACTGTTCTAGTACAGAGTCCTGCGTCCGGTTTGACCACGACTGTGGCTGACACGGCCCAGACGGCAACCAGCATTCTAACTCCTGTTCTAGCTGGGGAGCCGAACAAACACGTTGCAACGCGATCTTTGAGAACGCATCCGATAGACGACAGCGACGATGGCGAAGAGCGACTGCTTAATGGTATGACAGATTTTTTCAAGTACCACGCTGGAAAGATGAGTCCCGAGCAGCTTTACAAGTACTTAAACTTAAAAGGACTTGGTCAAGAAGCCTACAAACACAAGAACTACGCTAGTTACATTAAGAAGTCGAAGAAGTGGTGGAAGAACCAGTAAGACCGTAGTTTCAAGAGTTTTGAAGGATGGTTTATCAATTGAGTTGAAGCAATTGAAGACGCTAAGAAAAAATGCTTAGTTCGGTTCGTTTTGTCACTGGACTTCAGTGCATTCCTCGTGTGCTTGGGGTTTTGCTTTTGTTTGTGGGGATGTAGATTTCTTGAGTGTTAAAAAAGAATTACAATTTTTAGTACAAGTACTACAGTAGTAAGCTGCACGAATATCCCTCCTGGGAGAGGATACTAATTGAAAGCGGGTCATAAAAAAATTAACTCCTCGTAGCGTCAGAGTTCTTGACAATATATCGTCACATACCCCCCCCTCTCCATATATCGGTAAGAAAAACGAGGTATTTCTCGATAGTAGTATAATTTACGTGATTTTAATTCGCAACCTACACGCTAAATCATACTAGCGCCATGCTCCACACGTAGAATCTCTGAACGACATCACCACCCTCTCCGCGCATTGAGGAAATCAGAATTGAATTTTGGACGAGATTAAATAAAAATGCGTACTTAAATGAATCCTCTGCTAGTAATAAATTTAAATCCCAATAACATATACATGTGCTGCCTTTAAGGTCACGTCCGTCCGCGAAGCACACTCTCCACGAGCTCCTGTTTAAATTTGCTCCAATGTGTATCTCGGACTAACTGCTCTAACAGATTCGAGTTTCTTCTCCCTCCAGGTGGACCTCGTAAACGTGATACCAGCGACAAGTCTGTCTTGTACTTATGTTTGTTCATCAAGGCAATGTCCTGTAAATTACGATGTAGTTGCGAACGGCTATCAGCAGAGAATCCCTGCATGATATCTGCAGCCGACAAGAGCAGAAGCTCTACTGTAGAGACCGATATCAACGCTGCCTTGGCGCAAGATATTCCATGAATCTGCTGTTTTTCTTGTCGAGACTGGCGCGGTGGAACCCAGGATGATGACGCACAAAAACATTCTCCACGTCGCAGTGCCAGCGCAACCGGTTGCGTTGGCGTTGTCCCCCTTCCAGTCCCCATACAGTTGATAGCGACACTTGTTCGCCTGGTATTGGTTGATTTTGGTCGTGTAGTGGCAGCTCGCAGAAGCTCCCGCTGCTTGGACTTCACGTAATTTTGTGGAAGAAATCCATGATGCGCCTCATTAATACGTAAAAAGCTCAGAGGATCCCCTGCAGTCTCAAGTTGTGTGTAAAATAGTCGAGCGCTTTGAGATTCAGATAGACGTCCACTAAGTGCGAATGATATGGACATATCAGGTGGTGTTCGTAATCCACAAGGCGAAAATTTGCAGAGTTCGCCTTCTCGAACCAGTATCAGCCCATTCACAGACTGGCCTTCACAGCACACGACTTTGGAGCCGATGACCTTGACAAATGTAGACTGATACAAGAATTTTTTCTGTAAGAAAAAAATATTGATTAGACTAATAGCGGACGTACTCGGTTTGCAAGGTTGCGGATATCTACTACGGGGATATTATTGAACGCTGGGGCCATGGAGAGGAATCGAGCGGCGCGGTCTCGCTAGGAAAATAAAACGATATTTAAAAAATATATTTACTATTGTAGGATTGAGACTACTTACCTCGACTTCGTGTTGTGCACGGTCAATTCGACTCCACTGATGCCACGATAAAGCCCCTATCACACACGTTGTTGTCGTAATTGCTGAATACGCTCTACAGTAATATTTGATTAGCAGTCAAATATTTTTAATAGCGATTGTTTCTTACCGTGGAGCGTTTGTAATCCTCCCTTGGTGACCAAAAATATCTCCGTGCTCAAGCTCACAGAGCTCTAATTGCGTTTGCTTCCAGTCCGAATTAACGGGTATTTGATAAGTATTCCATGGAGGTTCCCACGTGACGTCGGACGCACCCATTTCAGCTTCATCCAGCTCTTCCTGCACTTTAAAGCGCTCTTCAGAGCTCTCTAGGTACTCTGCCGACAGCTGAAACATTCTACCAGCAGTCTTTGAGACAGATACTCTTCCTTGAAAAATAAAATACAATGACTTATCATGTGGAGCTTCAACACCATTCTGTTCCATAAAAATTGGTGTTCGGGCTGGAAGAGCTCCATCGGGACTTAGCACAAGATCACGACATAAAAGCTTGCAAACTTCCGGATTCAACGCCGCGCAAACTAAGAATCTCTTCTGCATAAAATCGACGAGGTATTGAAGGTCACTGTCGCTTCTTTCAGACGGTGGAAGTCGAGCAATCTCCAGACTGCGCTGAAGCGTCCAAGCTGTCAGTGCATCTTCAGTCTCATCAGGTTCGCTTTGACACTCGTTTGTGTCTTGGTTTGTGTCAGGTTCGATCTCCAATCCACGTGCTATTTCTTCGTTATGTACACTTTGTTGAGGGATTCTTTTCTTCGGAGATGTGGCACGCTTCACGGTGTTGTACAGGTGCTCAGCAGTGAACATCACACTCTCGTCTGCCAAGGTAGTAGAGCCCCCTGAGTTCTTTTGAACAAGGTCGTAATCTTGTGCGGCCTTCTCGAAATCTCCATTCTTACGGTACGCTGCTGCTCGCTGTCGATAAAATGCACTTGGGGGTGATCCTCTATTGGACCCTTTTTTGTCTTTGCTGACACTAATCGCACGTGAAAAATAGTCGACAGCTTGAGCTACTTTATCGACACGCATACACAAATTGCCCATATTATAGTACGCAAAGGCATGTTGTCCACCGGAAAGTCTCTGAACTTGCTTGTAAGCTGCTAACGCGGGTCTGATTTGACCTGTTCGTTCTAAACACACAGCGAGCTGGAATTTCGCCTCAATTTCACTCGGTTTTGCAGTCGATAAACTCGTGTAAAACTTGATAGCTCGGTCATAATCTCCATTTGCTCGTGCCTGGAAAGCTCTCGAGCGCAGACGTTCTGTAGCATTCATCGCGGCTGTGGAGACGACACGAGCTCGGGACAATAACTCCATAGTTCTAGTTTCTACTCCATCACGATTGGGCTTACCATCCCGACCAAGTTGCCCTCGAGACTCCACACTTCGACCAATAAGCGGGTTCACATACTGTTCCTGGTTTCGTTCGCGTCGAATGGCCTCTTGTATCGCTATAGTGTCACTTCTCGTCGTAAAATGTTCCTCATGTTCATGATGACGGATTTGAGTCTTCGAGGTTCGACTGGTAGCAGGTCGTTTGGGTAGTTTGATGCCCTCCGATGGAGCAGATACTCGAGCGGTAGAAGGCCTTAAAGGTCGTTGCTGATTGGTCTGCTGTGTGTTAGTCGAGCCCATTGTGCGGTTGCTGGATAGGACAATTCCTGTCGAAGTCTGCGATGTGAAGTAGCCGAAGACCCCTGTGCAGACCTCACGGTTCCATCGGGATATGTCCTCGTTGATGGGGTACATCCTATAGATTCTGATGCTAACGGAGGTCCTTGCATTTTCGCTTGCCACAGCACGTGCTCTTTTTTTCTTGATGAGCATTCACTATTATTAAAAGGTCTTGACGTTTATAAATACCTGACAACACTGCACCGATCAACCAAATTGATGATATGGCTTCTTCTATTCCAACAGTAGGCGACCAGGATGACGACTTGGAGAACGCAGTATTTCGCGAGAAGCAAGGGATAGGAAGCGATCTTCCTTCACCAACTTTTTCGCGTTTATTATGCTGTCTACGAAGTCGCTGTCTTCGTGGAATCTGTGTGGGTTTTCCGTGTGTGGTATTTTTACTACTATTTTTAAGTCTTCCCTCCAGTACGGCGACAAGTACCAGACGAGTTTTCTTGATCCTAGCTGCCGTCCCACTGATTATTTACATTGTCGTTGGCATATCCACTGTGGTACTCGCGTATTGCACTCAAGTTTCGCTCGAAACGCAATATGCTCAGGCAGCTCATCAATACTTGAACACTAAAACTCAACAGAGTAAAATCGCTGTTACTACAGGGCAGAAACAACAGAGCAACAACTTTCTTGGTCGTTTCGCACTTTTTCTTGAGGCTGCGCGGTCGGGCGATGCAAAGACGGCGCAATTGTGTTTGAGAAATGAGCAAATCGTGGACGAAGTGGACAATCTTGGGCGAACAGCTCTTCACTGGGCAGCGCTGTCAGGATCAGACGAGGTATTATCTCTTCTCTTGCGGAATGGAGCATCGATTGATCAACAGGACTCACTGGACGGGCTTACGGCGCTACACTACGCAGCTTTTTATGGTCATATCAAGAGCACAAGACTATTGGTCTGTGCTGGTGCCTCAATGACCATCATGGACAATCGACGAATGAATCCACTGCAACTCACCGAAATGGCGAGTCTCAAGCTAGCGAGTGTTCAACCTACGCATCAGATGATCATTAAATACCTGCGAATTGCCATGAAGGACGACGTCACATCACCACTCGAACATGTTGCGGGACTCACTGTTTTGCAACTTGTTGAACGACAAGCGCATAATCTCCCGCAGATGGAGTGACTAAGCGTAACTTTGAATTGATACTTCGAAGTAGTCAGCTTATAAAACCTAGTATCACTACGTACCTCGGTATTCGAATTTAAACAGACACGTACAGTTTTAATACTACTACTACTACTAGTAGTAGTACAAGTTTAGCATTTAACATGAGTTAGTAGCTCAATTAAAAATGCAAATATCATTGATATTTCCCCCACTTCAGTCCGTCACCCACTGCACACTCCACAGTCTTGCTATCTCGAAGACTCACGATGCTCGTCTGGCTGCGTAGTGTTTTGACTTCTATCTGGTCGATTGCGTCCTCGCTGTGACACAATGAGTGTCTTTGCGTCAACGCCCATGCTTCCAAATCGTTTTTATTCGTCTCTCATGGTTTTAGTCCTTCAATTGCTCGCTATTGACGCTTGGAACCTCTTGCTGACTCTTGTCGATATCGCTCAGAGACAACTTGTGAGCAAGAATAAACGTGACTTTAGTGTTTGTTCCGCCATTCTGCCGACTTCTTCAAGTACTTCTGGAAGTTCGAGCTATCGCGAGCGCTTTGGCCCAGTCCATCTAAGCCCAAGTATGTGTGTAATTGCTTGGGATTCATCTTGCCCCTCTTGTATTTGATCCAATTTGTAACACGACTAAGCCACCGCTCTTCGTCATGGTCCTTGTGCTCAACATTGCCACCACTCGAGCGACCTGTCCTTAGAGCTCGCATGGCAAGGTCTTTGTCCTGTACTCCTGCGAGCAATGCGGACGAAATATCGGTTGTCGTCTGCGTCGTGTCGGCGTTAGCCTTCGTCGTCAATGCAAAGGCATGACTGTACAAGAGCGCAGCGGTCGTTGATATTAACGTTATGTACAGGACACGCATCGCACTTTAGATAGTTGTGCCTAGTGGTTGCGTCCGGCGAGGGAAAAGGTAAAGTGGGGAATCGCATCAAAGAGAGGTTAGACGACACGAAGTGCAGAGTATGGATGAAATATCCAGTTTATTTATACTTACAGAAAAACATAAGCTGTGTGATGACGAGCTCGCTTATAATAATGCAAAGCCAATTTGCAGGACCTGGGACCAAACATAAGTGGTATGATGACGAGCTCGTTTACTGTGTGATGACGAGATGAGTTATCTACTTGCGTCTAGTGGTTTACGCTCAGGCAGTCCGTAATTATTCGATCAAGGTTTTTGGGGGGATCACTGCTCTTCGAAGACCTCTCGTGCGTTGGTGAGAGAGCCTACTAAATCTAAGTGCGGAGGTGTAGAGTACATGTATTGTACCAGATGACAGCGTCGACACAGCAGCGACCAACACCGACAACCGGGCCGACACTAGCAGCGACCGTCACGAGGAGCGACACTGGATCAAGCTGCGTCAATAGTAGGACTGCACAATCAGCTACGTCAGCAGCGGCACCAACTTACATAATCTGCGGCGTAAGTAGCGGTCATGCTTAAACAAAGGGGGAAGACGCTACCGCAGTCCTGTAGTGAAGGACTAGGATGGTAAGCAGTGCTCTTACAATAAAATAAAAAATTGTAATGGGTTTCACAGAAGCACGTAATAATCATTGCAATATACCGATCCACAATAATAAATTATTCAACTATTTTGCTGTATTGCACGGGTCAGGACGGGGCAGTGACCCTATCACGCCACTGGCTTGGGTTCGTAGCAGTGTTGGCTGGTTTTTGGTTTTCGGATTTAATGGTTTTAAATGGTTTTGGTGTAGAAAACACCAGTGTAAAACCAGTGCACCTGGAGGTTGGTGGTGATTCACTGGACGGAGTGATCCGGTCTCCACCGTCAAAGTAATACTGATACCACATACAACAAGTCTCTATTTCTGAAATACGCACGCTCGACAAGTGTATTTAGCTATCGCACATCGAACACTACACCGTACCGAAGCATGGGCCACCCATCGTGCCAAGAGCGCGACGAGTTCACAGACTTGCATGAAGACGCCGGTAACAACCGGACATACCACATTTGCAACCACTGCAACAATGCGTATCGTGCTTCGCGGCAGCCAGCTGAGGGTAGTTACCGCGCAACACCGCACGAGCCTCCTGTCCCTGAGCCGAAGAGACTTCGAGGGAGGCCGGAAAACTATAGAAGCCATCTCAAGAGCTGTAAATTCTATCAAGCCTATATTCAAACACGCAACTCTGTGGAAGCTGCTAGCCGTTCTTCCAGCTCAACAGCCTTTCAACCTAATAGCAGTTCGTCTGTTCACGACGATGCGTCCTCGACACGAAGTGAGAAAAGACAGAAGCGTATCGACCAGTTTTTTTCAGGACCATCGCATGACGCGGAGGGAGAGATCTTCATGAGATTACTAGTTGAGTTTCAAGCTGATAATCGGCTTCCTGACAGTTTTATTGAGCGCTTATCTACTGTACGTTTCTGCATCACGTAAGCAAGATGATCGCTGACATCTTCCCACTACCAACCCGCAAGAAGCTTGGTGGACCCATTCTAGACGCGTACGCTTTCGACTGCGAGAGCCAAGATAGCACGGCACTTCGTAATGTACAATATGCCAATGAAGGTGCGCGTGAATTTCTTAAGTGATGTTTGGCAGAACATTTCTCGCGCTCACTTGCTTGGTTGCCAATTGGATCTTTTTGGTTGTGGTATGACATACGCTTTGCTTCCTACGGGACCCAGGCATGACGCTATCGCAATTGCGGAACAAATGGAATGTGTGATGGAAAAGATGCAGAGTGATGGTTGGACAATTGGTGGCGTTGTTACCGATAATGCCGGACAATGCGCGCGGGCACGACGAATTTTAAGTCTACGGTGGCCGCATATCTCATTTCAGATCTGTTTTGCTCATGATCTAAACAATTTAGTCAAGGCAGTTCTAAAGTCAGATTTTTCTGAGGTCACCAAGCAAGCATCAGACGCGGTCAATGCTCTGAATGCGTCTTCCGCAAAGTGGTTAGTCGAAGCAAGGACGTGCATGGTGGATACTTATGGATATTTCCTGAATCTGAAACAATTATGTGAAACCCGTTGGAGCAGCATGCATGGATGTTTTGCATCGTTGTTGCGCGTTCAAAGTGCGCTTGAGTTACTGGAGTTTAAGTTTCGAGCTGGCGATGATTTTCCTGATGCTGCACGCTTTTTAGCAGAGGATCATTTTGGGAACGGCTTAAAGACGCTGAAGAGGTGGTGCGTCCGCTTGCATTTGCCTCTTTGAAGTTGCAGCGCGACGAGAACTCGCTCGCGGATGTTGTTGTGTGCTACATGGATATGATCAAAGGATTTTCGGTGCGTCCGTTTGGCACGCGCAACCTTATCAAGCAAGTGGAGCAACGATGGAAACAGTGTGAGCAGCCGCTCATGTTGCTCGCTCTATTTTTACATCCATTACACACCAATGCCGCTCAAAAGCTGTTAAACAAGACCCCGCTGACAACGATAGGCTCCCTGTGCCGCATCGGCGTTTACTATTATCGGCGATCCATGCGCGCGGACCCCGGCAACTTAGGAGAGGATATTTACAAGTGGCTGAAAGGAAAGCTAGCACTTCCACCACAGGGAGGGTTCAGTATAGTCAGTTCGTTCTGGGATTTCATGCGCGATGACATGCCTTTTAGCAAGTTGCCGATACTTCCGATGCAAGTGCTGTCTATTGTGGTCAACACAGCAACATGCGAGCGCTACTTCAGTGAGTTAGCGCTGATTCACACAGCAAAACGAAACAAATGTCGGTGGAGAAGGCTCGCAAAATCGCCGCTGTTCGGAAGAAGGTTCGTGAGTCGGATCAGCTTGATAGCATCTTGAAATCCCAGCGTATTAAAAAGCTGATCAACCTAACAGAGCACCCTCGACGCCAAGACGAGCGATTTGAATGCAAAAGTCGATACTGAAGACGAGTCAGACGACGAAACAGGAAGTGAGTTGGGGGGGGGGACGATGCTTTGAATACTGGGGTGCTGTTCTGGCAGAGCTAGATGGAGATGAAGACGTGCCGCCACCGAATCACAGGGCTGTGGTTGAGATCAGTGAGTTATCTGCCGATCGAGCGTCGTACTGTAGTGATTCTGACAGTAGATCGAGACCAATACTACCGGCAGACCGGGGCAGTGACGAAGCTGAGCGTGCGTTCAGAGACAAGATTATCAAAAT

>Contig_81

GGTAGTCTCGATGGAGGTTCTGATGACTCCCCCGACGACGTCGGCTACGGATACTTCAAGGAAACAAACGGGTGTTCGCATAGCGGGATTCATCTCGTGAATGACTCGACCGGTGAAGTACTGGGAGTGGGTACAGAAAATGTACGATGGACTGTACGATCGCTTCGATGGCAACATGGATCTGGTGGTACAGCAAATGCACTACGCTGAGTGCGAAGTGTTTAACCAGGTTTACCGCATCGAAGAGCTCTCTGACGACTTCGTCGAAAACTTTCACTTGGCTTCAACTCGTCCAATTTGTGGCAAGAAAGTTGAGGAGATCGAATCCGGCGACGTGGTCGTGGCTTGCAAGACCGACGAGTCAAGCCACAAGCCGTTGAGTTTGTGGACGACGATGCTATTGAAGACATGAAGAACAGCTACGTGCCCACTTCCACCGCGACGGAACAGTATATGGACACGAAATACTACGCTGACGCACAGGCGAGAGCACAGCAGACTGGTACCGCTGCAGCGAATAATGGATACGGCGTACCGTACTTAGCGCCCACACCAGCTCCCACGAACGCTGCATCCAGCCCTCACTCTCCTGACCATTTCAGCTCTCACAAACACTGCCAGTAGGAGAGCGTGCTGTACTTGTACTTGTAGCGACAAAATACGTCAACTTCGTTTTATCCTTGTGAACGTACACTGACTGTGAAAAAGGGGCAAGCGNTACACTGCNAATATAAATAAATAAAGTGGAAAATACCACAATAATGTATTGCTTCTGCTGTTGACTTTGCTTTAAAGAACTACGACAGCCAATAATAATCAGTGCAATACGACCAGCAGGGCTCGGAAAAAATTATTATAATATAATATCTGTGCAATTGTATTGACGGAATAATGGATTATTGGCAAATTGATCTTTCCGTAAAATATGCAATTGTGCCCGTAATAATTATATTAAATTTTTACCCAACGTAAAAAGCAACAGAGAGTAAAGTAGTGGTTGTCAAGCATGCAATCAAAACGAATGGTTTGTGTGAGTTTGCCGGAGGCATCAGGCTGAGCTTTCGTGCCTCTCATTTCTCATTTCGCCAGTGCCATGTCAACTTCTCGCGCCATCTCCAGCTTTTTCTTCACGGACTGCGGCAACGGGCAGTTTTCCTGCAAGCAGTGCGGCAAGGCTCGCAAGCAGACGCCGGGGACTGGCTACACAAATCTCATCAACCACCTCGCAACGAGCCACCCCGGCTACAAGAAGACTTACGACGAGAGCCACCGATCGTTCGGCCAGTCCCTCGAAGCACACGGCTTCATTGACCCGCGCACTATGGAGATCTTCAAGTGGATGGAGTGGGTTCTCGTCCGGAACATGCCCCTGAGTGTGGTGGACGACGCCATGACGCGTGCGCTGGCTGCAATCAAGCCAGTGTCTTCGAAGACGTTGGTCAAGTACATGCGTCACGTGGCGGGCAAGGTGGGAACGCGTATTGCGGCAGACATTGGCAACCAGTTTGACCTCATGTTTGACGGCTGGACTTCAGGGACCTACCATTTCATCGCTATCTACGGTGTCTTCACGAAGGACGGGGCGCTACATGAAGTTTTGCTCGGGCTGTCTTCTGCCGAGCACGGCCAGACCGCTGATGCTCACATCGACATGATTGATGCGGTGCTGGATGTCTACAAGAAGGATCGCAGTATGGTCCTGTTCATGGTTGCTGACAACTGCCCTACAAACCAAGCCGTCGCTACGCGCATGGGAGTACCGCTCGTAGGATGCGCAAGTCATCGCTTCAACCTCGCTGTCGGCCGGTACCTTGAAGACTACAAGCCGCTGATCGACCAAGTCCAAAGCTGTGTATTCAGCTGCGGCACGCCAACAATGCAGCGGACCTGGCTCGGTTTACGAAGTACAAGCCGCTGAAGGCAAACGCTACGCGCTGGTCATCCACTTACCAGATGCTGGTTCGCTATGTGGAGATCCGGGACGCTATCAAAATGGTGACAGCTGTGGAGGACCTCCTACCCCGACCAAGCACCCATCGGCAGATTGTCCAGCTTGTCACTAAGCTTGAGGCGCTAGACAGCGTCTGTGTCAAGCTACAGGCTGAAGAAGGTAACCTTGCCGATGTGCGACTCCTTTTCGCCGCTGTCATTTCCAAGTACCCAGGGACGGCACACCACCTTAGTGCATCGGCTCGAATCGTCCACTCGCCGGCCTTTGAGAATGCGGTTGTCAAGCTGCTGTCGGATCGACCGTTAAGCGCCGATGAAGAAGAAGCTGTGGCTCGTTTTGCTGGCCCTGCTGATCGCGAACCAGCAGCACCGAACACGAAGAAGAAAACAGATTTCGCGACGGAAACGCTTCGTCAAGCGAAGAGACCTCGCCGAGCCGCTGGAACCAAGTACATTGACGTGCTACAAAAGGTTCCTTCAACCAGCAATCGCTGTGAGCGCCTCTTCTCGCAGTGCAAGTTGGTGCTCAACCCTCTACGCTCCAGTCTGTTGCCAGCTAACTTCGAGATGTTGGTGTTCTTACGCGCTAACCGTGAGCTCTGAGACTTCACTTCTTTGTTGGGGTTCCATGAGTCGGCCGATGAAGACGCAGAGTAGATGAAAGTCACTCAAGAAGGATAACCTCTTATTAGTGATAATATGTCTTATCATTAAATATAGCAGGTATAAAACTTTCAGATACAAAATTCATATCCGCTGGTGGACTGCCTGCCGTCGCCGGCGCATTTTTTGTTTGCTAGAGTACCCTAAGCCCGGACAAAAGTCCCGGTGGTAGCCACCAGCTTCTCTTACAGGTGTGGGACTTGAACCCTGTGCAGTGGATTGGTAGGGTCATCCCAAACCACTGCACCGCTCTGACCCTACCAATTCCTGACAATAATTGCAGTACTTAATATTGTTTTTATCAATACGGAATAACGGATTTTTCTAAATCCAATACAATATTTTATTTAATTTTCCGACCAGGTTCAATAATCAATGGACCAATACCGTAGTATTTGGTTTCAATTTTATTTTCACCGATCCCAGGTATTTGTCAACTTTGTTTCTAAACTGATCCTCCCACACTTGCATCTAAACTCAAGAGGCGGAACAAGTACGCACGCAGTCTAAACACTAAATTTTGCGTCTGTTACCAAAGAGAAGACTAAATCCCCTCAAATCAAGCAAGTTCCTGACAAAAAGCTTTGCGCAAATCGCCCTCGTGTTTATTCCGGTTATTAAACGAGGCGGAGCGCCGCAATTTTTCTTAAGGTGCATTTGTTTCAGCACGAAAAATGATTTCGTCTGTTTTAAATGAGCTAAGTGCCGAGTAACTTGTTTTTCGCGAAACCCTGCGCTGATCTTACCATTGCGCAGTGTTCAAGAATGCAAACGCACGAGCAGTGTGCCACTTTTTTCATCCAATCTTCACTCAATCAAGAGTTCGGGCCTTTGGTACCAAAAGGTAACTGTAGTACTTGCCGATAGTTGTTGGGATGTCTTCTGGCGACATACTGTTGAGATTTAGTGCCGTCTTGACGAATGATTCAACTTTTTCCTTGGACTCTCCCGCACTGAACATGCGATTAGTCTTGCTTTTCAAAAAAATGACGTATAAGTCGAAGTATTGGCGCTCTAGGAGCGCTGCGGGCGACAGGTTGTCCAGTCTTAAGGCCGCTTTGACGTAATCGGCAGGTCTGTCGTCCAACCTCCATCTTGATATTCTTGCCGTCGCCTCTGCATAAGTTACGTCGTCGCTGATCACTGGTATGGGCGTTTTGTGTTTGACGTTATTCTTAAGCGCAGCGTCGTCAAAATGTCTAGCGTATCGCATGTACAATTGGAATGCATCCGTCGCGTGTGCGGCATATACGTCGTCGATTGTCTTGAGTTTTTCGAATCCCATTCGCCTCCAAATTTCAGAAGTTGAGGTCATTTTCTTGAGCGAGATATCCAAGAGAGCCTCTTGAAACTGCTTATTATGCTTGTAGTTCTTGTTACTCTTGAGCGTATGTCCCGATAGACTTTAAGGTCTAAATTCTCAATCACGAAATCGTCGGACTTGCCTGCCCGAGCCCACTGTAACATTTTACTTTTCATGCCACCAAACCTCCCAAAGTAGTCGGACAGCTTGCCACCGCCTCTCCCCTCGATAATATCTTCACCGCCAACCACACCATTTTCCTTATCGTTGAATCTCAGTGATCTCTTTGCGTTTGAAACGATCGCCGTGGTTGACGATTTCGTCAAGCCTGTTTTCCTTTGTCCGGAATGTGCTGCTGCTGACAGAGAATCAGTGCTGGCGAGGAATGCAAACACAGTCACCAGGAGAGCATGGAACCAATACATGATAACGCGGACTCACGCGGGAAAATTGAAAACTGAATCTGGTAGCATTTACGGAATCCGGTCCGTAATGAAAGTGTAGGGCAGACTCAGTTTAAAGACCTGTATTTTTGCGACGCTTGGGAGACCAGCATACTGCACAGAGTTGCTTACAGTACTACTTTGTAGTCGTCAGTAAAGGTGCGACTACCGACCAGACCAAATTTCTTATCTCATGATTTGTGCGCAGCTCGAATAGGGTAAAATGCCACGACAGTCTCGTGACGAGACATGATATGATGACGGAAAACCGACTGACTTCGAATCGAAGCTCGAAAGCGTAATCGCACAAGGGATTCTCGACTGAATTGTTTTTAAAATCCTTAAATGGTGGCCACGCCTGCCTCGCTCAGGCCTTCCGCACCAACTTTTCGTATTGGCACCGTGTTTCTCTTCTATTATAGAAACCTTTTACCTTTTCAAACAGGCTATTACTTGTTGAAATACTAATAACCTGTCTTCGACGCTCTAAAACATTCGAGCGTAGCCCATAAACTGAATACAAGACTATCTTTTAAAGCAAGCTTTACTGTCTGAATGAAAGTACGATACCCACTTCTACTCATATTGTGTGACGTAGCAAATTACCATAATCGGGTATCACAGTATATACGCATTGTACAAGAGGTGCTAAGGAACATTGATAATCGAGCCATACACTGTGCTTTCGTTCGATTTGCGGAAGCGAGACAAGCTGAACCCAATCTGGGCTGGCACCGCCATAGCATGGTTCAGTCTACGACTTACACCCTTGACTAATTATTCTTGCTTGAACTCCCCAACTCAGGGCTCTGTCTAGGGTTTTACTCCGATGATTTGCGACTTGGTGACCATGACATCCCTATCTTTAACCTCGAGCACCATCTATAAATACAGGGTTATGGAGGCTTTACCCCTTTTTATCTGAAAAAGTGCAAATATCTACTTCGAAGTAGTTTAATATGTCAAAATATAAGTTGAAAATGCCGGTCTGAAGAAGTCGATAAAAACGATATAGCCTGTAAAGCGGAAAAAACAGGTGATTAGACAAAGTCGCGCGCTGCATGAAGAAGCTCTGCAGTTTGAGGAACATCAACCACTGTCCAATCTATCCTGATATTGAAGCTAAATCTGAACCAATGATATTCATTTGCGATAAAACGTTCTGTCAATGCGCTACTGGTAGAGCAGACAGCATTAGTACAGTACTACACCCCGTTTTCGCGCCCTTGGACCTTTGATAAACTTCCGGATGTGATGATCCTGAGGGTGGCTCTTAACCACTGCCACGTGTGCCATGCCATGGCACTAGAAATTAGCCATGGCATGGCGTGGTTACGAATAGAATTGCCCTGCCACTGTGCCATCATGAAAACTATTTTTAGAACATACATTAATGAAAATAAAAGTAGCATGGCACATGGCAATTGCCTCTTGCCATGACACTTGCGAATGCCATCCGTGGCACTTCCTGTCATTTTATTTACTTTAAACTCTAGCCCTAGCAAGCGCTCATCCAATCGGATCACAGCATTTTGTGCCATGGCATGGTATGGCGTGGTTAAGAGGGACCCTGAATGCAAATAAACCAGATTTTGAAATAGGGGTGCAGTACTGTATCGTACTCGGACTGACACATAGTAATGCAAGTATACTTCGTATATTCCTTAGTACTTCGATGCATCTCCTGCGGTTGCAAAGCAGGCCTCAGAGTTGCCGGCTTCATAAAAAAAAAAAACGATTTGGATTTTAAATGTATCGGCTGAAATATTTTACTTTTAAAATAAATTACGAATCGTTGAAGTTTCGTATCAGTCCATGTTGGCTTAAAAAACCCGTTTACAATTACGCAGACAGCCCAACCATGAGTTTACACTGTCCTATCGCGAGGCGTGGGCTACTTCTTATCGTTGCTGCCCGAATCAAAGTTTGCCTTCAGACAAAAACTTGGATGTTAATTGTATTCTGGATAGCAATGATTTACATTACAAACAAAATGCAGGGAGGAATTGAAGATTGAAGTTCAGCTTGATTCAACCGATTGCGGTCACGACCAGAAATTCTCTAGGGACAGTCTTCACTCTGCAATTTGTTACCCAAGAAGCAACCACCCACGATGAGTTTGCGATGTCTCATTTTGACGCTTGGCTACTTATCTTTGCTGCTCGAAACAATACTTGCTATGGACTATCTTTTCCCAACTTAAGGGCTATGCCTCATGTAGCCGCCGCAGCACCTACAGCGAGCAGATCATTTAGGTCTACACCCCCGACGAATGAAACGCGTTTTGGCGACCAAGATAAAGAGGAACGAGCTCAAGGCAAAAGAAGATCGGCGTTTACGGAGAAGCTGACGTACAAGTTGGCGCTAAAAATGCAGGTTAAACCCGAAGACTTCTTCACGCGAATCCGTTTCTCCGAGACTGTCGGGATGCTTGACGACAGCACAGAGTTCATGAGCTGGCTTCAGTATGTACTTAGGTACAGAAGCAAAATGGGAGAAACCTCGTTCTCAGACGCCATATTTGCCGCCTTGCTACGAAATGCAAAACCGGATGAAGAGGTATTGGAGCTGCTTCAATCCCTCCGCCGAGTCGACGGCATGAAGGGTCTTGTTGATCGGCTGCAGACGCACTTGTTTGAAACGTATCCATCCATGCACAACTTGATGAACGGAGCGTGGCTGCATTCCCGGCAAAACCCCAGAGAGCTGTTCAACTTGGTACTTCCCCCAAACGGTTTCCAAGACCAGTATCTTATTCAATGGCTCAGATACACGGAGATGTACAAGACAGCGATGGGGGTCGATTCGTTTCCCGTATCCCAGATGAATGAGCTCTTTTTGAGGCATTGGTCGATTCCGAGTTTGGTTGAACGTCTCCAATCGATCAAGAATATCCCGGATTTGGAGAAGCTCGCCGAAAGTATGCAAGCTCAGGTGTTTCACGGATTATACGCGAAATCGACACCGAAAGATTTCCAAAATCGTTGGGTGTCACCGCTGGTGACAAAGAGTGATCCACTTTACGGCACGCTAAAGGAGTACACCTTGTGGTATGCAGCACAGCGAGGTGGGGAGGGCACGGTGGAGCGAGTGAAAACGTTATTCGGCAAAAACAAAATTAATGCTGCTCTTGATGCTGCTGCGAACGTACAGCGATGATTTCGTAAGTTCATTCTACCGCATGAGAAAATGACACCCAAAGCTAAAAATTAACGATGTTGATTAAAGCTGGCCTCGTCTTTTTCAAATATTTTCATTTTCTATTGAAGCCTCATTAACTGCTATGCGTCTGCTCGTGTACGAGGTAAATTTTAAAAGATCTTCTTTGGTGGCGTTTCATCACCAATTGTATGAGCAAATATTTCAATTGAAGTAAGAGAGCAGGGACGCGACTTCGTTAGCTCACGAAATGTTTTCATAAATCCTCGCAAGCTTTAGCAGCTTCGACGTCGACACCTGCGAGGGCTCGTAAAATTTTCTTTTTGATGCTGAATGTTGATCGAGATATCATTGATACTTCTCCATTTTAAGTGCGAATAGTACAGCGAACGTTGACGTAGCAGCAATAGATAATCATTGATTATTAACCATTCAGTTTTTAACGCTCCGAGCGGAATACTGGTCCGACGCCAATTGGTCTGCAAAGCTGATTCGAATCGAATATTTAGAGGCTAAACAAAAGGCTCAAGAAAGAAGAGACTGAAATCCACGATAAAAACTTTATCAATGCCGAATCTGTGCTGAAGCTATAGATGTCGACGTCGTCCACACCGATTTACATGTAGCTAAAACCCAAAAGACAAAAAAAGAGGCGCTTTTAGCATATTGATCGAGCGTCAATTGACAGCAGTGCGTGAGCACTCACACCGTGCGTCACAGGTGCTCGGAGGCTCTCCGCGTAGTTCGCCTGTCCCAAAGACGCAATGTAACCGTTGTTGATTCTAGAGATAGAGCTGGAGTATAGGACTTGAGCAGGAGTTCGATTACTTTAGAATATGAAGAAAAAAAGGTTCCAATCTCCAACCAGGAAATTGGCCGGGTGAAAACATTTCTAGCTACACTCCTGCACCAACGGATCCAGGTGACCTACAGCCATAAGAAAACACAGCACGAAAGTGTTCATTGCCAACTTCTGTACCAGCAGTATTTTGGTCATCACCGGACTCAATGTGTACCGTCACTGACGGTCCGTCAGTAAGGGTCCGACCACGGACCAGACCAAATTGCTTATCTCATGATATGTTTGCAGCTCGAACTGGGTAAAATGCCACGACAGTCTCGTGACGAAACATGATATGATGACGGGAAACCAACTGACTTCGATTCGCAGCTCGAAAGCGTAATCACAAGGGATTCTCGACTGTACTTTTTTTAAATTCTTAAATGGCGGCCATGCCTGCCTCGCTCTAGCCTTCCGCACCAACGTCTCGTATCGATACCGTGTTTCTTTTCTATTATAGCAACCTTTTACCATTTCAATCAGACTATTACTAGTTAAAATACTAATAACCTGTCTTCGACGCTCTAAAGTTTTCGATTGTAGGGAGACGTAACCCATTGTTTGGCGGCTGCTGAATACATGACTCTCTGTTTATATATCTGCCTGGTAAAACAATTGAGGAGAGAGTCTGATGAAACTTAATGAAAGTACTGTACCCACTTCCACTCATATAGTGTAGCGGAACACTCATACAGTGTAGTGTGACGTAGCAAATTACCAAAATCGGGTAACACAGTGTATGGACCTTTAGGACCTAAGCCTTAAAGTCCCTGGAAGGGCCTCTTACGTAAGCATACGTTTGGCTAAATATACATTTATAGACCGTTTGCCAGTCCCTGCTGTCGTGCCCTGTCTTTTGTTTTCCAAGAGTCCCAGTTGGAATTCTAACTCGACCAGAATGACATCACAATAGTAAATAGCCATAAATCATTATTCGAAATTGCGAAATACGGTAATCTTAATGGTCACATTTTTGAAGGCCCTGGCAGGTCTCCGCTCTAAAAGGGTTAAGGGGCATTGATGATCGAGTCATACACTGTGCTTTCGTTTGATTTGCGGAAGCGGGACAAGCTGAACCCAATCTGCACCGGCACGCCATAGCATGGTTCAGTCTACGACTTACACCCTTGACTAATTATTCTTGCTTGAATTCCCAAACTCAGGGCTCTGTCTAGGGTTTTACTCCGATGATTTGCAACTTGGTGACCATGACATCCCTATCTTAAACCTCGTGCCTTATATGTAGGTAAATTGTTCAATCCTGATACTGTTTGATTAGGCATCGTCAAGCGTAATTACATTTACCAGATCCTGGCTGGCTGATTACATTAAATGCAATTAAATTTTGGGACCTGGTATTCGAAAAATCAGATCACAAGTCTCCATTTTCAATTTCCTCCAGCGTGATGTCGGGGTGCGTGACGGTGGAGTAGCCTCCCGGTCTCCATTTCGCCCTCCATTAGTATGTGGCTCACCACTCGACAAGACGCAACGCTACACATAAAGGGTTTCGCGTTTGTACAGGACAAACAGGCCGGGCTGAGCTCGAAGCTGTCTGGGGGAGCAGCGAATATCTACGTGTGTTCCAGCAAAACCATGTGCTCATTTAAGATTCGTGTGCTTCGCTCGAAGAGCACATTGGCCAGCGACTTCTCCGTCAGCTTGCTCAACAGAGAGCGCTACGCTGCACAGGTTTCGCCAAGGCGACGGCAATCCAGATCACAAACATGAGACAAGCCCGCACCGCAGCCAAAAGCAGCCAAAATGTGTTTGCCAAGGATCTCATTGAAAAGGCCCAAAGAGTTGAGAGCGCGCAAGTGATCCAGCGCATGGGGTATCGTGTCCGGGACATGATAGGGAAGGAGATTTGGTTGCAACCAGTGCTCCTGTCAAATGTTGAGGTTGCAAGTATTTAAGAAGGCCAACCTCAGTGCCCATGATGCGTATGAAAACCTGTTCACGTTTGCACATATTAGCTTATTAATTGTTCTTATAACACCAGTGCGTTTGTCTTCTCCTTCCTACCTACTATCCATACGTAGTACATCTGTGCTCTAGAGACGTTTTCAGTAGTTTACTTCGCTTTCGTAGACCTCTCGTGGCGACACGGGCACATCCAGCTCATCATCGAAGACACAACCGATACTGTTGTTGTCATCTTCAGCTTCATCGCACACATTCACATCATCTACAGCATCTGACCCGAACAGCTTCTCCCCACGTAGCGCATCCATGAAGTTCTCTCCATCAAACTCATCAATCCCGTTGAAATCGTCGCTTTCACGCTTCGACTCGTCGGCACGCTCATGACCCGAGCGTGCATCGTATTCCGGATCCTCCATACTAGTAGTGGTGCTCCCACCTTCTTGTTCTGTATCTGGCTGAATTGCTTGAGCTTGAGTCGACGGTGGAGAAGCTGAAGTACGCGAAGGGAAACTCGAAGGAGGAGATGGAGGAGTAGGCGTCACCTCCAAAGTTGGTGCCGCTTGTTCTTCCGCTGGCAGCACTTCTTCAACCGGCGCACGACGGCGGGCCAGCTCATACGCCGCATTTTTTCCCATGGGGATCCTTGCTGGGAGTGATGACAAAGGTGAGAGCTCCAGCAACCCCTGACGACGTCCAAATGTCATGAGTTCTTGTTCACCTGCACATACCTTTCAGTAATGTGAGTATATATACCGAGCTGTCTGCAGAACGTACCAACGAAGTATTCAACTCCACGCTTCTCTG

>Contig_82

GCATTGCGTCCGATTGTGTACAAGTGTATACTTCTCCCAGCTGATCACTGTATCATGCCGTGCGAGTCACGGCATGCCCAATACTAAGTCAAACTTTTCGCCCAGCTCTAGGACGATAATATTTTCCACAAACACTCGGTGTTTGTACGAGAAGCGCGCGCGCTTTACGCGCTTCTCCGTTCTTACGGTGGCAACTGCTGCCAACCGCACTTCCAACACATTTCGAGGTGTTTCAACTCCTCGTAGTTTAGCTTCGGGAGACCTTCCAGTCGAGCAAAATTGCTCGACGCACCTGAGTCAACCGAAGCACGAAGCACTGATCTCGGGGTTGCTTCAGCTGGTTATTGCGCACTTTATCTTGGACGTGTTGGTGTCGATGTCAAAATCAAATGAGGACTGATGTGCAGCTCGGTTACACTACGGCTTTGGCGCTCAAATTTTTCAAAAGAAAAGAATGTCGTTCCACAACACCTTTTAAACGAACGACAGCGCTGCCCTAAGATGATTATCGTCCGAATTGATTTACTACCAGTAGAAGTTTTAGTACTACTCGAAGACGACGTAAGTTACTGAAGTACTCAGCACATAAATTGAAGAAGATTTTTAAACTACATATTACTATTGTCGTTTGTAGAAATATAATACTGCTGGATTTTACCATTTGCTGCCTTACTGACAATCCCTGCCCACGGCACAATACAGTAGAGCATTCTGTCAACGTTCCGTGAACCCGATTGGGTAGTAAGATCTGCAACCTGCTAATCTACCAATGCCACGATCATACAGTACATGTATCACCGAAATCGCCCTACATGTACAGTACGCATTTGTTTATTGTATTTTTCCTCATAAATAACTCCTATACCGAACTTGCCGTTACCCCGATATATTCTAGTACAGTACTGCACCCCGTTTTCGCGCCCATGGGCCTATGATAAACTTCCGGATATGATGATCCTGAATCCAAATGAACGAAATATTAGAGCTCATTCGTACATACAGAGTATGTGTAACAGTACCATTCAAGAATTCTGGAACGCTGTAGTACAATTCTGGAATGTAGTTTACACATGTGGATAGGAAACATCTGATGATTTGATCGCTATATATTTTCATAGTTTGCATGCGAAGAGATAGTAGCCATAATTCGCCCTATCTGTTTGCTGAGATAATGCAAGATGTAAAATGCAGTTCCTCGGTCATTTCCGAATTTTCTATCTACATATAAAGCCACCATGGTACGCTCCCAAGACCGCCAAGACCGCGCCGCTGAGACGCTCAACGAGGCCGACTTTCGTGCCTCCACTGCCAAGTTATTTCAGCGGGAGGAACGTCGCAAGGCAAAGGTCGCTCAAATCGTCGTACATAGCCGCAGAGACGACAATGACGAAGGGGACAGCTCCGGCGTGGGAACTTTGCCGTCCGTCTTTGACTTCTGCTTACAAGCTGAAGGGCCCGATGGGGTGCACAAGCTCACCAACTTTGCGCCAGAGGAGCTCGACCATGTTTGGGCAGCTGTGTACCCTCATTTGCAGGGGCAATGGAGCGTGGGCCGCGGTAAAAAATGCCGCTACGCTGCGCGCGACGTTTTCTTTATGACACTGAGTTCGCTCAAACATCTTGGAAAAAGGGACACCGTTGCTCGAGTTTTCAGAATCCCGCCATCCACCTTTCAGAAAATGATCCGCAAGTTTATGGATATACTGTCTCCTATTCTCTACGAAATGTACGTAGAGAAAGCAAATGATCAGTGGACACTGGGGAAGATTGTACGATCAGGGCATGCATTTAAGGACTTTCCGTATGCTCGATATGCTACGGACGTTACCTTTCAGCATGATAACAAACCCAGCGGTAACATGAGCGAGATCTTACGCTATTACAGTGGCAAACACCACTTGAATGGCTACAAAATGGAGGTGTCGGTGCTGCCGAACGGTGTCGCGATTAATTGCATGGAGCACACCGGTGGGAGCACGCATGACGCTGAGATTTTTCGCAGGAGCGCAGCATTTCATTCGCGAGCTCTTCACAAACACTCTAGTGATGCCAATGTGCGAGACGAAGGAAGACTACAAGACAAGTATCCGAAAGAATGGGCGTTTGCTAAAATCTGATATAATTTGCAAATCCTATATGGTCTACCCCCGCGAAAACGAGGTGCAGTACTGTACTGATCATCGAGTTCCTATCTTGAAACAAAATGTGCTAAATCGGACCCAATTACCGTAGCCGCTGTCCCTTTTTTTAAATCGGTCCCCGAGATAAAACAGCAAATGGAGCGGTAATTTTTTAAGAAATAAGATAAACCTTTTAAAAATAATTATCGCCCGCAGTGATCATTCATCCCATCATTTTTCTGTATCTTAGCCTCGTTTTCGTCTCAGAAGCGAACCGTACCACATCGAATAGATGGTTGCCACAGCGTCACTTCGTCATTCTCTCTCTGAGCCAAGCGAAGCTCGGAGTGAGCTCCCCAGGTGCCGCCATGCGAGTCCCCAGGGCAATACTACTGACAGTTGTCTTGATGGCAATATCCGATACTGTCTCCTCAGCTGCGAAGTCCCACTTGACCACACCATGTCTAACACGGCACGATACGAAGAGGTTTCTAAGGGCTCACAATACCGAGGATAGAGGGATCAGTACCCCCAATGTCGAGATGCTGCAGGGGTGGCTCAAGAAAGGTCTGCTCTCCGACGAAGCCGTTGGCCTGTTATCACTCGGTCACAAGGCCGACGATTTACTTAGCGGTTCGCTATTGAGCGCTTGGGTCAGCTACATTAAAGTCTTCAATAAAGAGAACCCTACAGAGAAGATGAAGACGATCTCAGCGCTCACCGCTCGCTTCGGAGACGAAGCTCTGTCCACGATGATTGAAACAGCTAAAAGGGTCCCGAAGACGGAGGACGTCGCTACTAAAATGCAAGCCAAGCAGATCCAGAACTGGATGACGCTTGGTAAAACCCCGGACGACGTTTTTACGCTGCTGAAACTCAATACCGCCAAGTCGCTTTTATTCGATCAGCCTCCAGTCAACACTTGGCTACAGTATATGGACGATTTCAGCAAGGCTAAACCTGAAGCACAGTTCTCTACCATCGCGACATTGAGGAAATTGTACACCGACGATGTATTAGCCAAGATGATCATTGTGGCTGGTAAAAACGCGAAAACTGCGGAGGCTGGTAAAAACGTGGAAACGGCGTTGTTACGCACCTGGTTTAACGAAATGAAGACCCCGACAGATATCCTAAGGCTGTTAAATGCTCGCGGGACTGGCCAGAGTCAAAAATTCTTCGCGTCTATATGGACGAAATACGACGATTTATTCCAAAAAGTGGATCCCAAATTCAAGACCGACATGCTCAAGGACTGGCTGAAGAAGGGGTTGATCACTGACGAGACGTTCCGAATGCTAACGCTGGGCAACGCGGCTGACGAGTTTCTCAACGGCTCGTTGCTAAGCGCTTGGGCCACGTACATCAAGGTGTTAAACCAGGAGAATCCTACGCAGCAACTGAGTTTACTCGCGACACTCACCGCTCGATTCGGTGACGAAGCTGTGTCGACGATGGTTGAAACAGCCAAGAGTGTGCCCACGACAAAGGACGTCGCCAATCGAGTTCAAGCGGAGCAGATTCAGCACTGGATGACGCTTGGTAAAACCCCGGACGACGTTTTTACGCTGCTGAAACTCAATACCGCCAAGTCGCTTTTCTTCGATCAGCCTCCAGTCAACACTTGGCTACAGTATATGGACAATTTCAGCAAGGCTAACCCTGGAGCACAGTTTTCTACCATCGCGACATTGAGGAAATTGTACACCGACGATGTATTAGCCAAGATGATCATTGTGGCTGGTAAAAACGCGAAAACTGCGGAGGCTGGTAAAAACGTGGAAACGGCGTTGTTACGCACCTGGTTTAACGAAATGAAGACCCCGACAGACGTCATACGTCTGCTAGGTCTTCGCACGCCCGGCCAAACGTCTGTAGCCCCAGTTTTGACCAAGTACATTGCGTTATTCAACAAGGTGGATCCCCGATTTAAGACTGAAATGCTCCAGAATTGGCTAAAAAGAGGTTTAATCACTGACGAAACCTTCCGATTGCTCACATTGGGCAACACGGCTGACGAGCTCCTCAACGGCTCCATGCTAAGCGCTTGGGCCACGTACATCAAGGTGTTCAACCAGGAGAATCCAACGCAGCCAATGAGCCTACTCGCGTCGCTCACCGCTCGATTCGGTGACGAAGCTGCGTCAACGATGCTCGAAGCAGCTAGGAAGACGCCTACGACGAAACGTCTTGCTTCGAGTATCCAGAGAGAGCAGAGTCGACATTGGCTTAGCGTCAAGATACATCCGGACGACATCTTCGTCCTACTGAAGCTCAATACCGCGACCTCTCGGCTGTTTGACCAGCCTCAACTGAACACGTGGGTGAGGTATGTGGACGCTTTCAATGAGGCCAACCCGACGAGTACAACGACCTTATTGTCCACCTTGCGGACACGATACAAGGAGGATGCGCTGGCTCAAATGCTCGTCGTGGCGAGGACCAAAGGGGGCTCCGTGGGGCAAACCGCGACTCGAATTCAGGCGGAACAAACGAAACTTTGGCTGAAAAGTAACAAAACGCCGGGAGAGGTGTTCGAAATGTTACAATTGAAGAAATTGGGCACCAACTTCCTTAGTCACCCGATTTTTAATGCATGGGAAAAATACACGGACGACTACCGCAAGAAAAACCTAGGGACATATCGCTCTGCACTGACCACGTTGAGAAAAACCCACAGTGACGAAACGCTGGCGAAATTGTTCATTGAGGCGAGTAAAGTGGCGAAAACGGCGAAAATGGGGAAACGTCTGCATGCTGAGCTACTACGCGAATGGTCCCTCACTGGAGCGACACCCGTGCGGGTCTTTTTGCGTCTGAACCTCGGCAAAATGGACCCAAAGGTGTTTGAAAGTCCGCTGTACTCTATGTGGACGAGCTACATTTCCATGTTCAAAAAGGTGAACCCCACGTTCAAGGACGATCCAGTGAAGATGCTGGTATCGATCTATGGTCACAGAGACCTGACGGCCTTGCTCCTTGCGGCGGAGAAAGCTCCGAGTACTAAGGATATCGCCATTAAGTTGCAGAAGGAGCTGCTCGAGCTCTGGCAAGCAGCCAAGATGGACCCATCACGCGTCTACAGTGCACTGCATGTGGAACGTGAAGCCAAGAATTCACCCATTAGAATGTTCTGGAGTGAGTACTTAGGAACTCGAATTAAATAGTACTAATATAAAAGTTAATACAAAGAAAGTATTACTGAAGTACTATTACTACTTAGTAATCTTTTTACTAACTAATCTTGAGAAAGCCCAGTGCTCTGGGGAGGCTTGATAAGTTTCGTAGCGCCGAAATTAGATTCAATCTCGGCTTGAGTCGGTGCAGGCTTCCACTTTCTTCAAGCCAAGCGCTCGACGAGCGTCGTGCGTTGACGAGGACTCGGTAGCCATGGGAGCAGCTGCGTCCATGGACGACCCCGACGCGTCCGTGGTTTTTAGAGAGGTTTGTGTTCTTGTAAACTTTATTTTTTTAACTAGTATTCTAACATTATTGTTGCAGACGAAAGAGGAATATGAGCGGAGAGTTGCTGCGGGTGACTCGAAGGAAGAAATTTTCTCCAGTCTTCGAGATATCGTTGCCACCCGTTTAGGAGGCACCCACCCACTCCTCCACACGTCACAGAGTCTGGAATCCTTTCTCGTCCAAGATTTAGCAATATCTGAGGTAAAAAAATCTACGTGTTTTTTATCATCGGCTACTTGGTTGTCCACAACTCACCTCTCTTTTATTGTTTTAGTCTGGAGATCTAGAGAAACGTTCCAGCTCGTTGCTGGATGCTGCTACGTTAGCTGCAGTGACCACCTCGTCCGAAAGCAAAGAATCGACTATCGAGACCAGTATCGCACTAGCCAAGACGATACCCGACGACGACGACGTCCAATTGAATCCATTCCATGACGACCATGAAGTCGAAACGCACATCGACGAGCAGGGAATCCTCCGAGCAACTGTTGAAAAAGGCTTTGGACGGGCTGTGGGTTCGTCTCGGGCTGCACTTGATCTGGTGCAAAACCTCGCTCCTGTGTTCACAACTGACGTATTAACTGACCCAACAACGCTCAAGATGATCCTCAAGTACGCAGAAGCCAACAAAGTGCCCAGCGGAGCGGATATGCTCCGATTTTGGGTCGAAATCGACGAACTGCAGCACCTTCCGTCCCATTCGTACACGCACCGTCGACTACGCAAGATTTACGACAAGTTTTTGTCCCCTGAAGCGCCTTCGCCTGTGTGTGTCACGGCGCAGATGCTGCAGGATATCGAGAAAGCCCTCGAAGGAGATAATATCTCCGCTGGAATCTACGCCGGTGCACAGCAAATCTGCTATATCGCGCTGGAAAAATCCGTGTATCCGCGATTCCGAGACAGTAAATTGTTCCGTAAGATGCAGGATTTCTGCGCTCCGGTCGTTCCTAACGCTGGCGCGTCATCTAACATTGGCTCGAGCAATGGACCTACTTTGTTAGCTGCTGCTTCGGCTACAGGAACGGTGGCTGCCAACATCACGGATAATATGGAAGATGCTGAGGATTATTCGCTGTTGGGTATTCTAGCACATCCAGCTAAGCTGCGCTTCCTCAAGACGTTCTGTATGGAAGCATTGGCGCTGGAAAATCTGCTTTTCTACCTGGAAGTCGAAGATTGCAAGAGATTGCCGAATCTGTCGTTCGTGGTCAACAAGACGCGAAAAATCTACGATCGCTACTGCTCTCCATCGTCCAAGAACTTTATTGTCGGGCTGGGAGACAAAGACGCGCTGAAAGAAATCCACGACGTGGTGGAGAACAAAGGAGCTCTCGTGCCAAAGTTATTCTATGAGGTCCAGATAGGCGTATTTAACCGGATTAGCGACGACATCTGGCCTGGATTCTGTCGCTCCCAGGAATATCTGGATCATTCGAAAGAGGTCCAACCGGACGCGAAACATCTGGCACGACGCGGCAATCGCTTCGAAGAGAGCGAGGCGGTGCAAAAGAAACTGGAAGGCTTGGCAGAGCTCCAGCTCATCGACGCGGCCATGCATTATCCGGTCGAGAAGCTCATCCCTATCTCAGTTCCAGATTCTATCCAAGGGGCAGCTCGACGAAAATCCATTCAAAAACTCGAAGAGGAGCAGACTCTCACGCCTGAGCAGGAGCTGAAGCTGCTGTTGGGCGACCCATTCGCCAAGAAATATCTAAAGCTCTTCATGACTCGACGAGGCGTGGATTCTTTGCTTGCCTTCTGCGAAGAAGTGGAAGATTTTAAGCTGCTACCAGGTATCGAGTTTCTCCAACATTCCGCTAAGAAAATCTACCGCAAGTACATTATCCCCAGTGCTCGACTACAGGTGGATATGAGTAAAACGATGCGAGAAGAGATTTTCACGCGACTCGCGAATCCAAGCGTGGATATGTTCAAGAAGATCGCGAACCGGGTACGTCACGGGATGCTGCAGGACTCGCTGCCTCGCTTCGTCAAGTCAAATTACTACAAGGATCTACGTCGGGATAGTAAAGCTACGCCTGCCGATCCCCACTTGGCCACAGTCGACCAAGCAGCAAAAGCAGGAAAGCTCGAGCTGTGCCACTTGGATGTTTTTCTAACATATCCAGGATGTATGCAAGCCTTCCGGAAGTTTCTGGATTTTCAACATTGTTCCGAGAATCTAATGTTGTGGGAGGAGATTGAACACTACCGAAGACTGCCGAGCTACCAGATCGTCCTGCGTTCAGCCAAGAAAATCTACGACAAATATCTGAATCCGAATAATCCACGATCGCAGATTCCGTTCGCTCCAGCACTTCTTCAACGTGTAGAGACGCAACTGGAAGTGGCGAGTCGAACGACGTTCGATGAGGTCGAGAACGAGTGCTACGACCACATGCGCAACGTCGTGATGCCGGATTTCCTGGATTCTCGGATTTTTATGGCCCTAGTGGGGACGTGGGCCACGGTTCACGAAGATTATCCAGCCGAGATGCTACGTGGAGAACTGGAAATGGCGTTTTCTGCGTCATCGCTTCCATCTGGTTCAAGAAGCTCGAGGAATGTCTCGAGACTCGACGGTCAGCAACTTTGACAAAGGACGCAACGGAGCGACCCCCACTCTAGGCACGACAAATAGCGGCCGAGTCACAAACGGATAATCCAGAAATCTAGTACCGATTCCAGATTTTAAAAATCTGAATATATTTGAAAAAGTCTAAATCGCTATGCAACTACTATTACCCGACTTAACAGAGAAATCTTTAAAACTTCATAGACTCCTACGATCCCTATGCGAACAGCACATGCAGCGCCGCTTGTTGCGTGGCCGGCAACGAGGCCCACGCTTGAGTCATAACTGGTTCAGGCACCGTAGAGCGGAGTCCTGCGAGTGTAGACGCGAGTTCTTGACGTAGAGCGGCGGCCTCCGTCTCGCCCACCGCCTTGCTCATCTTCCGCAGGAATTTGTCCTCGGCCACAGCCGCTAAGACGGCCACCACCTTGCCTAGATGTCTGTAGTCTGGCGCGCCCAGCACAAGCGGATGTCGGTCGTTCACGTAGCCTAAAAGACGACGAGACACCGCGAGAGATTCCTCCAGATCTCCACGAAGAGGAAGCAGCTCCAGCCACTGCGGGAAGAGCGTGGCGGCGTCTACTGCGCCGGCCTGGAACTCGCACAATTTGCCTAATGCGGCCACCGCGTTGTCGGTCGCGTTACGCTGCTCCGAGCTGTGTGCATTTGGGTGGGCCACTACGTTGTGAAGCAGCTGGAGACACCGATCGG

>Contig_85

TAGCTCACAATACGGAAGCATTAAATAAACATCCAGCCGATTCTTGATCCAAACACTCGAGCCATCGTTTGATGAATAAATAAGGAGCGCCGTTTAAAAAAGAAACTAACGCCTTAATTACGAGCTGTAATTCACATATCACCAATCGCCTAGCAACTCGGATACGATGCTAGATATTCCGTCGAAGGTAGACGGGTTACAGCCGAAGTACCTAGTGTTGTTGTTTCCCCCGTTGGCGATCTTTACACAATTCGAGAATACGACTCCAGAGCGCTGCCGTCGGTGTGGTAGCAATGCGCAGATGTCACTTGTAATCAAGAAAGGAGTAGTACTTCGTCGTATAAAAATAGGACCATTCTCCTGATACTCGCACGGCTCCCTTCTGTGGTCTGTTTACTCCTCCACCTTTGTGCGCATAGCAAAATACGGCAGATTGGTCGAATGGTTGCGTGGGTGAATATCAATATACCCTCGATACTTCCAGGCTCTTTTGCGCTGAAGCACCATTGCGATCCATGTTCTTGATGATACGGGTTTCATCAAGTAACAAACGCCAGCTAGGTTTCCGTCCAATAACTCAACGACAAAGTTGCGGATGCTGGCGCTCTTCCGAACATTCCCGCTATGTCAATCCAGCGCTTTGTATATATCAATCGATACAGCAACTTGATTTTAAACTTGCTGCTATGTCTGAAATACACGCAGCAAATAACAGTATCGATTAGACGACGTGATTTTAGTTGACGGTGTTTCTGAAGATCTCTAATAAGGCGAACCTCACGTACGACTGGCGTGCGCGTATGTGCTTTAAATTTGGAATTCATGCTGGAGAAATAGTGCGTTTTGGCCATGTGGTCGTGTCTAGTGCTAGATTCAGGCGGACTTGGTAAGCGCAAGAGAGGCGGGAGACCCGAAAGTGAATTTGGAGAAATTTCCGAGCAAAAGACGACATACTACTTCGTAAAGGCAATAAAGTGTTATGCTAAATTTATTTTTGAAACGCTTTTTTACTTCATATATTTTCAGTATTCGCCCTAAAACCGAAAATTTAAAAAATATAAATAAGTGTTTAAAAGTACTATTTACTTAATAGTTTATTTGTCAAAATAAAATAATTTAAATAAAGGATTCATTAGGGTTTCGGAAAATGAGCACAAGTCGCTATATACCATGGCAAGTAAAAAAAGCTCCATCCTTCGTGTATATTTTACTTTGCGTAAAGAGCTCTTCGAGGTCGATCTTACCGTCGCCATTGCGGTCCATCTGGGAAACCACCTCACGCCCGTCGTCATAGTCTAGCGCTTGGCCCAAGGTCTTAAAAATGGACACGAACTCGACCATCGAGATAATCCTGCCGCCATCAGCGTCCACCAACCGGAAAATCTCTTCCACGAGTTTTTCCTTCGAGTAGTGACTCTCTTGGATCCTTGCGCTGCACCACTTGCAGAAGGCCTCGAACGTGATTTCCTAAGCCCTTCAGCCATTGCGTCCTCCCCTCTACCAACATCGCTTAGAAGAAGCGAGGGGGCGTAGACTAATAACACATTGACGTTAGTTTTTTTCGAGCTCACCTACAGCTGAAGGTAGACGGACAATGGACCGATGATGCCGCACGTACCGGTGCTCCTTGTGCCGCTGCCACACTTGAGTCGGCTGTAGCCATTTTCACTCAACTGCTGGTCCCACGAGTCCATGATCTTCAAGTAAGACGGCGAGTTGCCCACAAACACCCCCGCATGTTCAAACTGCGTCGTCGAGCAGTTGGACATGATGCCGCTTTTGCACTGCTTAACCGGTACAGTAACAATCTAGCAAAGGGAATTCTGAATGAACACAATGCAATGTTGTATTTTTAACGAAATTTTCCATCAATTGGGAAAATAAAATATCCACCAGCTCTATCTATCCAGGATAGTGCTAGCCATTTGTGTGTGGGTTTCTGGGGACCCCCCCCTTCGTGCTCAGTATCACGTTGGGACTGAAATAAGGGTCGTACTCTGGCATGACATGTGGAGCTACCTTAGCCACCCACATGCATTTATTTCGATGTTATAAGAAGTACACAAAAGTTGTGGTTGACAAGTGAAAATGCGTAAAGGTAAGAGGTTAAACTGTGCCTTATCTTCAGTCTCAATCGTCACAGAAGCGCTATGCACCAATCACGGCCGCGGATTCGCCGCATTATACTTCTTCATGAACAAGGTAGTACTTCCTTCCGTTCTTGTTGCTCATGTCACCGCCCACACTAGTCAATTTCTCCTTAATTTGGGCCGGCGTGTATTTACGATCGAGCCAAGACTCCGCCCTTTTAGACCACCTTGCAGTCTATGCGGCAAGCAGACAAGTGTAAGCGCGGACAATTGGGATCGAAATAGATTACGGCCTTTACAATTTTCAATATAGAACTGTAAAGATGATTTGCTGAAGATTGGAATAGGCTTTTAGTTTCGTTACAGACTGTAGCCGTCGAAATGGATAATTTTTGGATGTATATTAGCATGCAAAATATTTGAAAGAATTTACATGTACTAATTTATGAAAATATCTCGATCAAAATCATGCGTCGTAGCCCTATCCCTAAATTTGATACTACCGGTACATGTACAGTACATTGTACCGACTGCGAAGTATAACTTCAACATGTCCGCATTATGATAGCACTGTATCTTAGAGAAAAAAGGAGAAACGCGTCCATCGCTGGGATAACACACACGGGGCTGGGCTACGTGGGTGCCATAGCCGCGGCGCCAACTAAGGTGAGCGGGTGCGAGGCGGGCCCAGCGCTGAGCTCACCAGCGAGCCGAGCACTACACTTTCCAGAACATTCTATCTCCATATTGCGAGCGCAGCGAACCGCGGGGGCGCCGAAGGGGCAGCCAAGCGAAGCGCGGCTGGTCTCTTTTCTGCTGTGACCTCTAATCGTCGATTATGTCTTATTGCGACACTCAGTATACATAAGTCAAATCAAATGATTTGCTGTGTGCTGAAACAGTAAATACATGTGTTTGGCATGTTCTGTCTCTATCTTACTTAATAGTACCGTACAGTACTGAACACCGCACATCATAAACAAGACCCAGCGGTGAACAGTGTTCCTTATGTGATTGGCGACAGTCAAATTCTTGAGGTGGTCTAGCCTTTCTGCCAGCATAAAAGATACCTGCATAGAAAAACTATATTACATGTTTCAAAATTACATGTATATTGGTCGCCAAGCACATAAAGCGCGACGGCACGAAATTTAAATCAATTTGATAGGCTGCTGGATTAATGGCCGAGCGTCGCCAATCACATAAGTAGTGAACTGCTCTTACTTTGATTCATAAAGCGCAGAAGCCACGGATAATGATCACGTTCAAGCGTTTAAGTAGTGCTCGCTGGGGCGCCCTCTTGACTTCGATTGCTGTATTGTTCTTCCTTGCAATCACTAAGGGAGCTGATGCCAAGGCTGGAGCTGATTTGGCTGACATCCAAGCCTATCGACGTTTGAGGACGACGACAGCTGATGCCTACTACGCCTCGGAAGACAGAGTATTCTCTGTTGTGAAGGCGTTGAAAGATCTAGCTCACAACGCGAAACTGACATTTAGTCTAGACAAGCAGCTCAAGGTCAATAACCGCTTTGAAGTGCTTCGGGTCAAACAAGTAAAAACCGACGTATTCTCGAGTAGCGAATTTACTGACTGGGCGCACTATGTGGCCAAAATCTGCAAGAGAGGACGCTTGCCAGCAGATCGAGCAATTTTCAAGACGATGGCGGCTCACTATGGCGATGACGAACTGGCGCGCATGCTGGCTACTGCGAAGCGAACAAGCAGAGACACGGTGGTAGATCAGCTAAAAGAAATTCAGCAGAAAAGTTGGAAGGAAAGTGGAAAAAGTGCGGATGATGTTTACGCTATTCTCCAGCTTGACGCAGGAGGCCAAAACGTCTTGAATAATCCGGGGTTACCGGCATGGCTGAGTTATGTAAAGAGTCCAAGTACCGATTACATCGAGGCATTGCTTTTAAAGCTACGCGAGCAATACGACGACGTAACCGTGGCGAAAATGATTGTTTCATCACAGAGCGGTGTCAACAAACGCATTAGCGGGCAGCTCGAGAAGGAACTGAGCACTGCTTGGCGAAAGAATCACATTACTGAGATGGAGGTGTTCCAGCTGCTGAAATTGAATGACGCAGGCACTACTCTTTTAAAAAATCCGATTTTGGAGATTTGGTTTCATTATGTGTGGAAGATGAAAAGAAACGACCCTTATGAGCTGCTGGTCTCGTGGTTCAAAAAGGCTGGTATTGATGATGCGGGGCTCGGGAAGATGATCGCAACAGCGAAACAAGACGACAGAAACTATTGGATTGCTCAGACATTGGAGCAGCGTTTGTCTGGCAAATGAAAGGAGAGAAGATAATGGTAGCGGAATCTTCAGCATGAGTAAAAAGAAAATTTTCTTTTTGACGGCTACACTTGGACTTAGATAGTTAGATACCTTACAATCATTCTATTGGTTCAAATACGTTTCAGGCTGAAAATACTAACGGGNTTTTTTTTTTGCTAAATGAAACGTACCAATCTATACGCAGGCATGTATTTTACGATAGTGTTGTTGCCGGTACGGGAGTAGTAAAATGACTTTACAGTTAAATTTGCTTGCTCGCTTAAGGTATCCCCTCAGACATAGAAGATCGGACGATATCTACGAATAAAATATTAGATTGGATTCAGATAATAATTTAAGTATTTTTTTAGTATATAGATGCACAAATTCTGTTCAGCCATTGGATTCAGGATTTCTGACGGAGTTACATTTAGGTTTGGCTTTTATTAAATTTAAGCTGGTTGGCTTTGAGTTGTCATCTCATGCCGGTGACAGAGCTCGACCAGTTTCTTCTCTAATTCGTCTTGCTGACGAGATCGCACTACAGAGAGTTAGTGCGCCAAATATAGCGAGCATTTACGTAGTACTTCGTATTAGTACTTCACAATAAGAAGACTTCCAAAGGCTGTCAAGACTGTTTCTACGTGCCCCTAGTACGTGGTTACGTCATTTCTTATCATCTTAAAGTGCTGGCATTAGTGTAATTAAGAACAACATTTTGTATAATCTCCAAGTGCTCACATAAGCGTAGTAGTAATAGCAAGTCAGCTCGAAAGCTAGCTACCGACAACAAGACTCCGGCATACCCCCGATGCAGCAAGTATCCATTGTAGATCTCATCGAAATTCTTGCCGTTAGCCTTGCGACTCGCCCGTTTCAAGAACTCATCAATTTTAGACAAAGAATACTGGTTATGTTCCCACCACTGGAATGCAGCTTTCGGCAACCGAGGATTGCCCATGAGATCTTCGGCCAATTGTTTAGCGTGTCTCCCTTCTTTAGTTCAGCCAAGTTAAAATTCGGGGCTCGCTTCTCAGCCACTGCTTCATTCTCTTGAGGCGTGCGAAGAAATATTCCTCTGGCTGAAACGTGAGAGTGAGCTGGTAATTCATTCATCAATATCTTGGTTTGGTCGAACTCTGCTACACTGCAGGACGCGAAGCTAATAGCAATTACCGGAGTGAACACCATACTTGCCAGGCGCATGGTATGGAAAATTGAAGGGTACAGAGCTTTAAGTCGCACTCTGCGAAATGAGTAGCGGTGCTTCTTGAGCGAGAAGGGACTTCACAACCCTTGACTACTACACCTTTGTGCACCCAACCTCTTAGAAAACGGCAGTCAAACTCGTCTCAAAAATTGGCAAATAGGAAAATTACGGTAAGAGTCCCTAATTAATATTCAATGTTGAAAAAACTGCAATCAACCTGGTACATGTACAAGATTTGCGCGACTGGTTCGCGCCAAAACGATAGGCTCTGTTTGACTCGCGCCAAAGCTACCTGCGTCTTGATGCTCCTTTAATTTCACGGCGAACACACGTCGTTTCTCAGTTGCTATCGCTATTACACGCTGTGGGCTCGACTCTCACTTGTTGTACGGTAAACAGCATGCCATTCAGGTGCCGGCTAATCGTCGACGTGTCCACCTTAACGCCACAGGCCAGAAACAACATTGTCCTCATAGTGTCCAGCGTATAGGTACAGTTATCATTCAGGTACTCCTCCAGGAGGACCTTAGCTTCAGGCGTCATCTTGACGTGCTTCGCGCCTTCCCTAGGGAGATTGTCCACCAGTCGCGCAAATCTTGTACGAGGTTGATTGCAGTTTTTTCAACATTGAATATGAAACTGATTAGGGGCTCCTACCGTAATTTTCCTATTTGCCAATTTTTAAGTTACGAGTTTGACTGCCGTTTTCGACAGGTGTAGTACAAACATCGCTCTCTATTTCGGTACTTGTATAAATTAATACCCTGAGCAAAGCGATGCAGCTGTTTTAAGGGGGGAGTTTCTGGCGGGAAATGCTCAGCTCCAGTCATTCTAAGGAGCAGAACCGTGTTAATGTCGAACAGTGCGTAATTTACGTCGGCCCATACCCATGGGAGCCTGGATGAAGACAAAACAGAAGCGCGATTTCACCCAGAAGGCGAGCGACTGCCCCAGCATGACGCAAGAAGAGCTGGCGGCATGGGCAAAAGCGACGTTCAAGCTGAAGCGAGTGCCGGCGCAAACTACTGTTTCTGATATCCTTCAAGACGCCTCCAAGATAGTGAACAGCAGTTTCTTCCGCCCAACACTACTGCGTTCCTACAACCTATGGATGCTGGCATCATTGCATCATTTAAACTGGAGTACCGAAAGAAGCAGTTGCGGTGGGTTTTCGACAAGATCAAGGACGGTGTTGAGATTGAAAGGAAGGCATATGCTGTGGATCAGCGTACTATCGAGAACTGTTTTCGCCACACAGGCATTACTTTTAATGCCGTAAATGAGAGAAGTAAATCAACCGAGCAATTTAGCTACGGGCCCGATGTAGACGTAGAAGCTGTCATTATCTTAGCCTCACAGCTGAGCTTGTAACTTTTTACTATATCAAATTCAAGGAGATAGTCGGACCACGATACCGGAAGTGAGAGCGATATTTGCATTGGCAGAAAATAGTACTCGTATCATTAGTGGTTATCATGAAAAGAAGTGAGATTTCAGCAGCCAATCAGATCAAACCTACCTCCCCCATCTTTTTCAGCGCTCACCTTCCTCTGAGATAAGAAGATTTGACGTAATTGGTGAAAAATCGGTCACTACAAATACCTACTTCCGGTATGTCGGTGGGCATCTTTGCATGTTAGAACTATAGCGTAGAAGGAAGAGCCTTCAGTGCCATTGATATATCAACCAATCAGAGTACTGCAGCCATTTTTCACAGTTAAAAAGTGAAGATTTGATATACAGTTAAAAAGTGAAGATTTGATTTGACTGGCTGCTGAAACATATAAAGATTTCAACCGCTCCGGTGCCGGAAGGGGAGCCGTTTCCTTATAAGTCGAGGCCCCACTGTGTTAATAGCAAGGATAGTCATCTGGGCCTAAACAATCCCCTTGGTACATGTAGTACAATGCGAGAAGCCGCTCATTATTGAAGACAAAGGCACCGGGGTTGGCATCCGCAGCAGTGGTATCAGCTAGCGCAAGCTTTGGATGCTTATAGTTATCACAAGGTCATAATATCTCCAGCTGATTACTCGTCTACGCAACCTTTTCCAGAAAACGCTCAGCAAGTTGGCACAATAGCGATTCTTGTCGCTTTCGCTTGAACCCACTTATTTTGGCGGTATGGCGTTTTTTCTCGATGCGCATGAAGCAGTTGCCGTCTCTCTCGGCTGCATTCTTTTATTGCTTGTCTGTCCCAGCAGCAACGCTGAATGTTACTTCATACAATATTTCTAAAATTAATAAAATTGTATTACTAGTACAGTAGCTACGGTATTTAGTCTCACTCATACTAATTTATGTGAGGTGCTGCAGACTCTGCTCACCTAAATTAGTATGAGTGTGGTTAATTATGCACTCTGACTATTTAAGGTGTGGTAGAATTAGTCACACTTAGACTAATTAATGTCTCACCTTAATAAGTCACACTAGTTTACGATTGTTGAGAAGCTGGTCTGGTTATTCTTTTGTTTTTTTGTGTGTTTGTTTGCCATATCCAATGGAACCGCTTGTCCAGCAAATTGATCTGGGAACTTCTTGATGATTACTTGAAGGTGGCTGAATAGCTGTTCAGTTCTCCATACACCATCAACATCCAGCGAGAGTCCGCATCGAATCATGGCTTTCCGAGCGTAGTTCACATTATTCTTGTCCCTTTGAGAGTTGACTTAGCGTACTGCTTTAGCAGCCAGGGACAGAAAAAAAATGCTTTCCTGGATTCTTAAGTTTTCCTTAACCTGTTGTGACGCTCAAGATGATGTTGACGTTGGCAACTCAGTCACGACCACATTGGAAATAGAGATTATGTCGCCTTCAGAGCTCTCTTCTGAGTCGTCATC

>Contig_87

CTGGTTCTTGATGCGACGAGTAGCTGATCGTTGAAAGTAGATACAGGGAAATTTTACGGGCGGTAATGGATTAAGACCTGAGACGAACGAGTAAAAGTTTGCATGAACAGTTCAACAATCAGTGAAGATTGAGGATATCTAGTTTCCGACGGGATCTCCATAAACACGACGAACTTTCGCAAGTGCAACAGCGAACTCATTTGCGTTTTGGCACCTTGAATTTTTCAAATAGCGTTTTTGATAGCTTGTCCATGTAAACTAAAATGTGTTTTATGTTCGCAGTAAGTGAACTGTACAAAAGCGTAAATTCACCAGAACGCCATGTCGAGCTCGAATTATCTCTACAGCCCCAAACCGTTTGCCTCTATCACAAAAGGCTGGTGTCTGATCCAATCCATTACCACCCATAAGTTTATGTATCTACTCACAGCACTCCTGAATGTATCGTCAAACAAAACGATATTTATGAACCTAAGTACGAATCGCGATTTAAGCACTCTAAACGAAAGCGGGGTGTCGCTTCGATCTTGTGAGCACTTGTAAGGTACTGAATGTTTAAATCTGAAGAAACTGTGGTCGCCCATTATCTCCGGTTATGGGCCATCGAAATGACGATACATTTAGGAGTGCTGTGAGCTCAATGTTTTTTTTNTTTTTTCAATTTTGAAGATCCAAGATTTTTTTATAAGCCCTTGGTAAACGCAACTGAATAAGGGTAGTTTCACAGCTTCATAGAAACTTAACATAGAATGTCACCGCTCTGGTTAAGTGCGAAAATCAATTGGCCAACTTTGAACTCGATTATCTCAATAACTCTAAATGGTATGTATTTAAAAAAAGTGATATAATCGTAAAACCATTACGTATTGCAACCGGTTTACATACAGGTGTTTCTAACTCTTTGTTTAACATTATTGGTTAAGAAAATCAAAGTAGTTTTGATATCTATTTCTATTTAGCTAAGACGCGCTGGTATTCACTTAACATTGCTGCAATTCACAACATGTTTCTTTAATAATTAAGCAATTTAAAGTTTTTTATTTCAATTTGACGGAGATAAGCACTTAACAGGTACTTCGTAAGATTGTAAAGTATTGTACGCCGATATACCTCGGGTTTTTTTTATACGATTCAATAAAAATGCTTACCAACACTATATAAGTACTGAAATTCTACCGGCTTTGTGTGACTTTCGTTTGCCCAACATTTTGAATTTCGGAATAAATATTCTATACCGGTACCATTGATGACCCTATTTACAAGGAGTTGCTATTGATATTAAGCTGCAGGCTTCATAGGACTGGTCTAAAGTTGCTGAATGTCAAAGGTTTTGCGGCACAGCTCTTCGAGCTGCGAATAATCATTGAAGTTTTAGGTCTGTTTTCCAGCACTGTACATGTACATTTGTGCGCCCAATTCAATCTCAAGATTGCCGACCCTTGATCGGCGATCAAAAAGCTGCGTTTTTCGACCACACAGTCGCAAAGCAAAGCAAACTTGCAAGCTCTGCGTCAGTATATACACTTACATCGGGCTCGCCTTTACCCTTATACGGATCCTCAAACCGACTGCGATTTTCTCAAATTTATAGTACTATCGCCAAACTAAGTAATGAAATACCTCCGGTAATTATAAACGTAGCTGAGCTTCTGCAACTGAAGGCATTCATAGTGCGTGGGCCCGAAGTGTACAATGGTGTATAATATGATTTCTGATTTGGTGCCAGCTTTTACACTGGTAATAAATATTTTCTTAGTTTAAATCATGTCCCCACCGCCTTTCCGCGGAGGACTTCTTTAATCAGCTTACAACAAACGTGTTCTTATTTATATAAATATACGGAGTGTTTATATGATAACATTACGCCAGCATTTTTGGCGGCTTGAGAGGTATTAATAACAGTAAACCTGTTGAAATTAGATTAATAAAAAAGTGAACAGACAATATTTAAATCTACCGTAATAGTGTAAACTATTCATGGAGTCAATCATCAGATTTACGCTTGGTCATCGTACAGGAGAGATGAACGGAATGGCAGCCAGAAAAACTGGAAATCTCTTCATATATTTTTTTTTACTCGGCTATGATAGCTAGCTCTTAGCCTTACCATCTCCGTCTCCCTAAAATCGACACATCCAGATTAAATGAGAGTGTATCCAATAGTTAAGGCTGAGAAGGGTATATACATAATATACATAGAATCAATACATAATAACTGAAGGGCACAATATTATAAATGCCTAATCTTTAAATTTGTGTTAGAATTTTTATATAAACCGATTCCACATCTTTTCCGCAATTGATTGAGACCGGATTTACGTCTAAGGGCGGCGTGCTATGCCAAAGATATAATTAATTAGAGGTCTCGACATACATGCATCGTGGCAGTTTTGGGCGGCGTCCATGGTGTGGGCTTTGCATACATACGGTGTATTGATACTATGTAAACTCCGTAAACAAATTGGTTATGATGGGAAATGACGAAAAAGGTGTACATAGGTAATTTCTAAATTGAAAAACTGCATCAGTATGCAGTTCTGTTGTGTTTTGTTTTTTAGTGATACTGTAGCACTGATCAAGTCAGCCAATAATTCAACTTATCTTGATATTTAGAGTAATGTATTAGGGTTTGAACTGATATTATACAATATGTGTATGGGCAGTGTTTGCCGGTCTCGGCTACGACAAATCGGAAAGCATTGACGCAGCGTCGCATTTGTCATTTGGAAACTTTCTCATCGGAGATCACTTGCCATACTTGTCGAGCCGCGAAGCGTTCAAGGACTTTCAGAATGGTATTCAAGCCGAATTCAGGAGTTTAATTCTCAATGGTAAAAGGGCTGGTCATACTCATTCGTTTAGCATGCTTTAACGCGGCCTCCGCGGATTCCTTGGTTAAAGGCAGCTCGCAGCAGCTCGCGGTGACTTTTGAACGGGATGGGCTTTCCTCTCGACGCTTTTTGAGAGCGCCTTTGAGCGAAGAGAGAAAGCTTGTTATAAACTTGGCGGGTTTTGGACAAGCTACAAGTGGTACGAAGTCCTGGGCAGCAAAAATCCTTCAGACGATGCAACACAAATGGTCACAGATGCGAAAGAAATCCGCGAACGATATGTTCATCAAGCTCAAACTCCACAAATCGGGCGACCAGCTGTTCAAGAGCCCAAGCTTTTCCAAGTGGCTCACTTACGTTCTAACAAGCAGTAAAACGAACTCCGACATAACAATCTTTTCCACGTTGGCATACTAACAAGAGGTTCGCTCCAGTTGTGGTATGACCAGTTGACGCATACGACGTGAGACTCAAATTTCTAGTTTTCCTTTCTCAGCTTGAAGTGTTGTTGTGGGCAGCCACACAGCATACATACTTAATATAACTTACTTCTTCTTTTGCCAAATAAGGCTCAGGTCCTCTGCAAGCCACGACCTACAGCTAACACCTTGCTGAGAGCCCCTCTGCGAGCCAAGGGTTGTCACTTTGAGATATCTCAAACCTTGTGGAGGCAAGGAAGGCAAGAAAGTAACAATGAGTGAAGAAGGTGCGGATGTGTCCAGAACAGTAACGTTCCAGGTTACGACACGAAAGAAGACTACGGGTCGCGAGCGTGAGTTTATCAAGATCAGTATTCCGCGTTTTAGTGGAGGCTCGGCTCAAGAGTGGCTGCGGTGGATACAGCAGTTCGAACATCTGAGTATCATGAAGAAGTGGACGTCTGAAAGAAGTCACAGCACTTGCATTTAGTGCTTGACGATGAAGCCTTGGATGCGTGGATTGACACAGCGGAAGATGTGGACATGACGGACAAGGATGCCTTTCAGAAGGCATACGAGTCGTGGGGTCACATGTTTGTACCTAATATGTACCACGAGCGGCTCGAAGAAGAGCTATTTCTATTTGCGAAGCGTCGCAATGAAACGGTCGCTGAATGCCATCAGCGAATGCGTCAGATTATTCGCATGCTTAAGAACCTACCAACCAATGCTCTGGAAATAGACGAGCAGTCAATGATCCGTACATTTAAGCGGGCCATGCCAAACGAGTGGAAGACCGCCTATGAGTTCAGCGGAGTTCACCTCACCACCATGCCAAAAGCCGTACAGTACTTCGAGAGACTCGAGCAGTCCGAGCGACGTCAAGGTAAAGAGCGCCATGCTGGAGGGTGGAAACAGCATGCCAAGCAGAATGGCAGTAAGCAGAATGGCGGTAAGCCGAATGGTGGGAAGGCCAATAGTGGGAAACACTTCGGGAAGAAGAACAAGAAGGAGTTTAACTCGTCCAAGCAGTTCCAGTCACGTGATAAGACCAACAGTGATAAGTGGTGTAGCTTACACAAGACAAGTTCGCATAGTGACAGCGAGTGTTTTACGCAGCAGAAGAATGCGTCGAGAGGTGGTACTAGTCAGTGGTCAGCAGCAAAGAAAACGGAGGAGGCGAAGATGACAAACGTCGAAGAAATGGCCTCGGATTACTCGTTCAGCGATGAAAGTGAGTGTAAGGTCATCAGCAGAATACTGAATGACAAAGAGCCAGGCAGGAAGCCGCCAATGCGTCTCCGTTTCCGACTGTGTAAGACATTGCAGGAGCGCGTGGCGTTAGTTGACACAGGTTGCGGAACAAGTTTCATCAATGCCGATCTATTAGCAGCTAATCAAGAACTTGGGTTCGAAATGACTCCCTTGGATCTGACTTATGAGCAAGTCGATGGGTATGTCAAAGCTTCAGGGTCTACGCCAGTTCAATTCCGGTTTATGGATTTGAATTATGGATCTCGCATTACGCACACATTTTCAGTCATTAACAACACCAGAGACGAGATGGTGATTGGACGGGATCTGTTGAGTGCACTCGGAGTAATTGTCAACTTCCGGGACGGAATGGTGGAGTGGAAAGGCAACACTGTGGCAGTGAACACTGGAGACAAGTCCAGGGTGAGCGATGAAAGAGGAGGCGAAGACGTGCAGGAGGAGGTGAAAGAAATCAACGACACGTCAGTGGAACCGAAGGATCTGGTGGGAACGGCAGATATTGACCAATCCACATACGAGCAGATTCTGGTGCTGTTAACGCAGTTTGAGCAGCTGTATAATGGACACTTAGGACGGATGACATTTCCAGATTATATTCTGCCTATGGCAGAACAGTACACGCCTGTCCAAGCTCGCCCATATTCGATTCCAAGGTCTGAAGAGAATGCAGCACGAAAAGAGATTCATCGACTCATGCAAGTCGACGTGTTGGAACAGATCTTTGGTAGCGAGTGGTCTTCCCCAGCGTTTTTCTTGAAGAAACCAGATGGACGTCTGAGATTGCTAACTGACTTCAGAGCTTTGAATAAGTTCCTGAAGCGTAACCCATATTACGTACCCCTAATTAGGGATGTTTTCGTTCGACTTGGTGGTGCAAAGTTCTTCTCAACACTGGATGCTAACATGGGATATTACGCTAGACAACTGGCTGAGGAGAGCAGGCCGTTTACTGCATTCTGTACTCCGTTTGGTAAATTTCAGTATAAGAGACTGCCGATGGGAATCTCAACGGCGCCAGACGAATACCAAGCGTGTATGTCAAGAATTTTAGGCGATTTGGACTTCGTTATCGTCTATCTAGATGATATTCTCATTTTCTCCAGAACGGCAGAAGACCATTTGGAACATCTACGAGTGGTGCTTTCACGTTTGCAGGAGTTCAACGTGACACTGAATGGTAAGAAATGCCGTATCTTCTGCTCTAGTGTGGAGTACTTGGGCTTTACCATTAATGCAGACGGGATCAAGCCCCAAGAGAAAAAGATTCAAGCCATTCTCCAAATCCAACGGCCAAAGAATAAGAAAGAGCTACGTCGGTTTATCGGTATGATAAACTATTACCGGGAAATGATACCGAGTAAGACAGCGATGTTGAAGCCGCTGACGAGATTAACGTCACCGAAGGTTGTATTTCAATGGACATCGAGCGAAGACGACGCTTTTGAAGGGATCAAGAAAGCTTTAGCAAGGGCCGTACTTCTGACCTTTCCCGATTTCTCGCAACCGTTCGAGATTTATGCAGATGCGAGTGGAAAGCAGTTGGGTGGATTGATCGAGCAACAAGGGAAGCTGATAGCGTCGTACTCTAGAAGCCTCACTACAGCACAACAGAATTATACAACGACGGAATTGGAGTTGTTGTCTGTGGTTGAGATCTTGAAAGAGTACAGATCAATGCTACTTGGATTCCCAATTGTGGTCCATACAGACCATAAGAACCTCATTTATCCAACGGAAAATTCCTTACGGGTCAAGCGCTGGAAATTACTTTTGTCGGAATACCATATCACGTGCAAGCACATTCCAGGGAGAGACAATATCGCTGCAGATGCGTTTTCTCGAATGACGTATGACACGAGTAAGAAGACAGGAAATGCTGAGCTAGAAGCCCTGTTAATCGATCCGGTTGAGTGTGTGCTTGACGGAAATTACATCAAGCAACACCAGGATGCAGACTCGACCACACAAAACATAGTGCAAACATGTATGAACGGCACCAACAATCCCGATTATCAGATCGAAAACATAATGGGTGCACGTCTTCTTAGTTGCCAGCATCGTGTTGTGATTCCAAAGGAATTACGTCAAGAATTGGTGGAATGGTATCACACTCAGTTGGTTCATCCAGGTGCGAAACGCCAATATCACACGATGAAGTCGTTTTACTATTGGTCCGGTATGGAGCGTACAATCGTAGATTTCGTGAAGAAGTGCTTAACATGTAAGCGTGCCAAGCTACACGGTGGGCCACAAGCGTATGGCAAACTACCACCGCGTCAAATGCATAACGTCGATCCATTTGATGTAGTGCATGTGGACACCATGGGACCGTATGGTAGTGATCGATACTACGCCTTAACCGTGATTGATGAAGCCACGAGATGGTTAGAAGTATCGATCCAAGAGAACAACAAAGGAAAAACCACTGCAGAGAACTTCGACGTGACGTGGTTATGTCGTTATCCAAGACCAAAGGAAGTTGTATTCGATCGAGGTAATGAGTTCAACACAACAGAGTTCCGTGAGATGCTTGCGAGCTATGCAGTCAAGCCGAAACCAATATCCTCGAAGAATCCTCAAGCCAATGCAATTTGTGAACGAGTACATTTGGTACTAAACAACTGTATTCGCTGTTATCCGGAGGCAGACTGGCGTCAAGTTATTCAATACGCTGCATTCGCAGTACGTGCAAGTTTTCACACGATTCTGGATACAACACCTGCACATCTGGTCTTTGGGCAGGACTTGATTACACGACAACTTCATGAGACGAATTGGAGTTATCTCACTAAGCGCCGATTTGAAGCCATATTGAGAGACAACGGGCACGAGAACACCAAGCGGCTGGAGCATTTCTATCACCCAGGAGATCAAGTAATGATTCGAGTAGCTGCAAAAGATCGCGGCAACAAACACCGTGAGGTGGCCAAAGGACCATACTCCATCGTGGAAGTGCACGGTAATGGCACCGTGACGCTGGATTATGGAGTGACGCAGGAGCGCGTCAACATTCGACGTCTCTTTCCGTGTTGAGCGGCAACTGGCATGGGGGTGAATAACAAGAGGTTCGCTCCAGTTGTGGTATGACCAGTTGACGCATACGACGTGAGACTCAAATTTCTAGTTTTCCTTTCTCAGCTTGAAGTGTTGTTGTGGGCAGCCACACAGCATACATACTTAATATAACTTACTTCTTCTTTTGCCAAATAAGGCTCAGGTCCTCTGCAAGCCACGACCTACAGCTAACACCTTGCTGAGAGCCCCTCTGCGAGCCAAGGGTTGTCAATACCATTACAGCGACGAAGTTTTGGTCAAGATGCTTGATGCGGCTAAGAAAGTCAGCAGTACGAAACATCTTGCTACTAAACTGGAAGGCCTTCAGTTCACGAATTGAGTCCACGCTAGAGAATCTCCCGACTACGTTTTCAAGGTGCTGGCGCTCGATCGGATGGGATCAAACACCTTTACCAGTCCTCAGTTCTCCAAGTGGTTGTCATTTATGAAAAAAGCCGAGACGAGCGACCCGGAGATGGCCGTTTTCAGAGTGTTGGGATCATAACATAACGATGACGTCCTGGTAAAGATGTTCGCTGCTGCAAAACAAGTTGAAAGCACACGAGCCCTTGGCTCAACACTGGAGAAAATTCAATTTAAAAATTGGGCTCGCGGTGGTGAATCTCCTAGCCACGTTTTCAAAGCTCTGGCGCTTGATCAGATGGATACACAAATATTTGCAAGCTCGCAGTTTTCCAAGTGGACTTCATTTGTTTCTAAAGCAAACACGAAGAACCCGGATGTAGCCATGTACACAACACTGGGAACTTTCTACAGCGACGATATTCTGGCGAAGATGTTTGCAGCAGGTAAACAAGTCGACAGCACGAAAGGTCTTGCCACTCGATTAGAAGGAATCCAGTTGACAAACAGGGATAACGCTGGAAAAACCGCTGAGAGCGTCTTTAAAACGCTGAAGCTCGACATGACTGGTAGTCGGCTATTCGACAGCCGGGTCATAAATACGTGGGCCTCTTTCGTGACCAGGACGCACAAGGATCCCGATACAATTATGTTTGCGCTGTTAAAAGAGAGATATGGGGATGAGACTCTCGCGAAGATGATTGCGACGGCCACCAAGACTGAAAGCACGGAGAAGCTAGCTGTGGGCTTACGCTCTGCGCAGTTTAAGACTTGGTTCAGCCAAGGCAAAACCCCCGAGAAGGTCAACACTATGTTTAAGGTATCCGCCAATTCTGACGACCTGATGAAGAAAATAGCGCGAGAATACGATATTTTCTATGGCAAAAGCAAGGTGGCCGAAACAGGAACTATCAACAGACCGGCAAACAGACCTGCGCGGAATGGAATTCATATCGGATAGTCTCTCGTCCAACGTGCGTCACGGAACTATTGTGCGCTCTACTCGATATTGCGTTCTTTGTTTAAACGCGCGCATATCTGTTATTGACTTGCTTTTAGTTTAAGGTCACTTAAGTACGATCTGGGTCGTCACTCATTGAATAAAACCAAAACGGCAAATTTGGTTGGAACTCAATCTCATGAAAAACATTTACCACGGGCCAAACTGGCGGTTTCAGACAAGACGTTATATTACCGCTCGGACAATACAATAAAAAAGTAATGTACCTTTGTGAAATCGGCCTTTTGCAACCATCGTTGTATATTCTTGCTTCTCATTGGCTGGCAAGTGATTTTTCCTGCGGTATCAGCAAGTTGTGGTGTAAGAACTACGGCAAGAAGCATGGTATCGTTTGCTAAATTGTTGCATAGAGCGTTTGTATTATCGCAAGCAAATGCAACCCCGCCAAAACAGAAATGAAATGACGTATTTTCCCTCACGGTTGCTCGCTGAACACAGAGAGATTTGGTGGCCGGCGCCCAATGCAAACATGACACCAAAACGCTACATAGCGATCCTAGTGGAGCTCATGAGCTTAATGATGGCTGGGCCTACACAACGGAGACGGTGTTCAGCAGCGAGGAGCTTCTTGGCATTAAGCCGGAGGATGTCTGCAGGTGGATGAACTTTCGTGCCTTCGGAGAAGCGTATCCAGGAGAAGATGCCAAGCCTGTTTGCGTTAGGTCCTCGACCCTTGCCTACGCAAAGAAAGCATTTTTAAGTTGTATGTCTCGTATTTCTGTTTCGTGGGACCCTATCTGAAAAGAAGGCAGCCCTACGTGCTCAGAGTTGGTAAACAAGGTCTTTAAGACAGTGAAACGCTTCGAGACTCGTCGAGAAGGGGTGCAGTCGGCGGCTCGTCGATAAGGGTGCGGTCGGCAGCTCGTCGTCCTACAGAGTACAAAGAGTTCCTCAACTTAATTGAGCTGGTACACGCTTCACAAGAAAACAACAAGCTAAAGTACGTCGTATGCCGTGTATTAACGCTACAGTGGCGATAACAAGAATCAACGACATAATGCAGCTCAAGTTTGACAGTTTCTCCCACAATGCTCAGCACCCTCGGACACTATTGTGTCAACTGCGGTGGTCGAAGAACATTTAGGAGGAGCGTGACGCTCCAGAGCAAATATTGTTGAGAGGCATGAAATCACGGGTGTGTGTCCTTATTAATCTAGCTATTCACGTCGAGATACTTGCTGATCGTGGCGATTCGCCTTTCCTTTTCGGGAATCCGCAGGATAAAGATCGAGTAGCTCGCCGCTTTCTACAAGAGATGTTTGACAAGGAGAAATTCTACAAGCTAAAGAAAGTGATTTAGGTACTCATAGTTTGCGTAAGGGAGCTGCGACGTATGATACGAGGCGTGGTTTACCAAATTACTTTATCAACCGACGCGGAAGGTGGCGCACGCAGAAAAGTGTTGTCGACGTGTATATCAACAATACACAGTCGTATCCGGGCGCTGCTGCAGCAGGTGTGTTGACGGAGCCCCAGGGACCATGCTTTTACGCCGCCAAGGATGGAATAGAAAGTACCACCGATGACCTACTCGTAAATTAGATTTGCCGTCATATCAAGATGAAAATGGGGAGAGTGATAGCGCGAGAGCTTGGTGTCGTGCTGTTGTGGGCTGCCCTGATCCCTGTTGGTACCTTCGACTACAATCTAGTTCCTGCACCACTTATCAGCGATCCGTCGCTGCTTACGCTAACGCTGGAGGCAACGTGGAAAAAAATCCGGTACAACGTGTACCTATTCAGGCTACTGGCAAAGGTGCCCAACTGCAGTTGATTGAGCTCCATGAAAGTAGCGCCAACATTTCTGTCGGCGAACCTTTTACACAACAGATCAGTCGCAGCGGTATGCCTATGGACAGCACTCGAAAAGTGTTTACAGCTCGTCACTCACAACTATTTAAACGCCAGATCGGCAGCGTGCTGAATGAAGTTCTCCGTCTGAGAACAGATATGCAGTGCGAGTATCCGAGAACACAGGCCGTGCTACGTCGAGTAGCACTGACGCCAGTGGCTCGCACAGTACAAACACCTGGTGCTACAACTCGTTCCTCTTGCGAAGAAATTATGGATTGTATTGTACGTCGCATATCTGCTACATTGCCAAAGTGACCACGAGATCTATTTGAGCGTTGGCACGAGTTTAAAAATGGATTAAATGGAGCAAAACCGGCAAAGGATTATACCAGAGCTGATCGAGGAGCCAATAAATTTGCGTTCTCTCGGCGTAAAGTGTTCTGGGACGTCGTTACGCATCTTATCCGAGCGGGGCTTACAATTGAAGCTGCAATTGATAAATTTACGACGTAGACGGGCACCAGCGTTCAGTAGCTGCAATCCTTGTTGCGCTTCGCTAGGACAAGAAGAAAGGTGGGCATTCATCGCTGTCTCTTTCCATGAAGGCAATCAGCACTGAACAAGCTGTCACGCTTAGCCCACGATCCGTCTCTATGTTTACATTTCAGTGCATGTGTATACGTGTTAGAGCCCTTGCAACATGAGTCGGTCTTCAGAGTACGGCGACAAGCTGGAACAGTCCTACAGCCACCCCGGTGCAACACGAACAAAGCTACTGATAGATTTCTTGCTGAATAGGCCGCTCTTCTCCGAGCTCACTGACATGTTTGAAAGCTTGACTAAGTCGCATTCATGTAGATTGTGTATAATCCCGTATAATCTGCCCCCTCCCAGCACGCACGCTCTTTATAATCTGCGTACAGCAGTCCAGCTAGTATCTCCACGGTGACAGGAGCTATATTTCCGTCTAGATGTGATCCGAAGCACTGGAGATCATGGTCCGAGGTTGTACGGTCCAAATTCGTCTGATCTAGTGTTGTGGTGGCCTCGTGTCCCCTCTTGCTTGCCCCGAGGTTTACTACACGTGTGCAGGATAAGATAGTTGTTTAACCCAATCAAATCACGGTTGCACTGTTGGTTGCACCCTTTGCCAGTATTATTGTAGTTTTGCACAGCAATAGTAAATAAATGAGCGAGGAGAAAGCAATAAAAATGAGCAATACGTATTGTATTGTGTTCCAAAATTGTAGGTGTCTTCATTACTACTTAGTAGTAGCATCTATTAATAATCCGTGCTCGAAAAAACTATATATAATAATAATATGCATTGATATTTTCTA

>Contig_89

AAACACTTTTATGAACAGACCTTACAAATTTCTATATTAAAAGAGTGATGGTCAAAATAAGTAGATGGTTGGCGGAAATGAGCGGGTAGCTAGACGCTATGGAGGCTGTGAGCCTTTCATTTTCTAATGGGCCACGGCAAGACCCTCTCCCAACAAGAGTACTGGTTTATCGTCGGTCTCCACGACGGAGGCGTCTCTCTGCACGAGATCGCTAGGAAGACAGGTCGGTCCCGTACGTCCGTCCGGAATGCAGTCAAGGCGGAGCGGGGACGGAAGGAAGACAGGGGTGGAGAGCGAAGGGCTGGGCGGCAACCGGCACTGACGGAACGAGAGCTGCGGCAGCTTGTGCGTGCCGCGGCGACAGGTGAGTTCTTTGCTGCGGAGCTCAAGACGAAGTTCGGCATCAAGGCGTCCGTGAGGACGATCCAGCGGGTTCTGCAGCGTGTGGACAATCTGGTTTGCACCAAGATGGACCGCACGCTTCCCCTCACAACAGCGCACAAGGCTGCCCGTATGCAATGGGCGGAAGATTACATACTGAAGCCAGGTAAGCCTCTCATATTAAAGTTTTGCTGCAATTTTGTACTTCCTCTGGTCGGAAATGATCACGCGAAGTTAGTATGATAGGTCTTTCAACTGTTCAATCCTCGATAGAAATTTGAAAAAACCTATCATACTAACTGACACGTCTTTCAACTGCTCTATCCTCGATAGGAATGCTCTTCAGTTTGTCACGTACTTCGTCTCGATTCTTCCGCTTTCTGCCGCTGTTCTACTTAAGCCGGTTAAGTCACTCACCTCTACGACACCAGCCGTCATTGCAGAGCTGTACAGCTCCAATTAACTTCACGGTGTCCGGAAGTCGGCTCATAAGAACATCAAACGCTTCACGGGGTAGTTCTCTGTTGTTCTCTGTTGTTGCTGATCTTTAGAAGCTCGTCAATGACGGCTTTTGCGCTCGTTGTCGATAAGGTTGGACTTCGGCATGTTTATAGAGGAAGAAACAGCGTAGCGCAGGCGTGGCGGGTCGCACTCCAGGCGAGGCTTGAAACCTTAAGAGCTAAAACTGAAATTGCAGAGGCTATAGAAGTCCGTTCTGCGCACAACCGGTGGCTTTAAAAAGAGGAGGTGCAGGGCTTCCGTTTGGTCCCATCAGATGCCGTATTGGATAAATAGAGCTGAAGCGATTGGTCATTTTGTCGGTTTCGCGTGATCATTTCCGACCAGAGGAAGTATTATAACAATATTTATTTTTTTAGAAACTTGGATTTACACTATTTTTTCAGATGAAAAGAAGTTTAATCTAGATGGTCCTGATGGATTCAAGTATTACTGGCGGGACATGCGCCAGCCTGCACAGTCTTATCAGCGTCGCCAAAATGGAGGAGGGAGCGTCATGGTTTGGGGCGCTTTTAGTGCAGCAGGTAAGAGTGAGCTGGCCATCTTGCGTGGACGTCAGAATTCAGGTGACTATATTTACACGTTATCTGAATATCTACTCCCTTTTGCTCACGCTAACTATGGTGTTGACTTTGTATTTCAGCAAGACAATGCTTCGATTCATGCCTCGCATGAAACGAAGCAATTCTTGCAGGAGATGCAACTCAACACCATGGCTTGGCCAGCTCGCTCGCCTGACTGCAACCCCATCGAGAACGTCTGGTCGGCGATGGCGGCCAAGGTGTATGCACATGGCCGCCAGTACCAAAACGTTGCTGATCTGGAGGCAGCAATCATGGCTGCATGGGACTCCATTCAGCTAGACTACTTGCTGAAGCTGATAGAGTCCATGCCTCGCCGCTGTCTAGCGGTGATCAAGAAGAAAGGTGGTCTGACTAAGTACTAATTTTTAATATAGAAATTTGTAAGGTCTATTCATAAAAGTGTTTTTTCGTCATAAAAAGGATGTGGTTNTTAATAATTTGACGCTGATGTCCAAACCTTGAATTTGTATACCAGCCCTGTCAGATACATAGAAATCTAAGTTGTTAACACAAGTGGCACCTCTGTAAGCCTAATAGAGAGAACATATCAAAGTAGCAGCTCTCAACTCAGGCTAGAAGCCGAGATATTGTCAGAATTGTCCCGCCAAATCGGGGGTCTTAATGATTTGGCGGGCACTGTATGACACGGATTCGCTCTATCTAGCAGTGTACACTAGGGAATCGTAGCAAAACACCTGCTTGTAATGCAGAGCGAACGCTCAAAAGCTACGACGCAGATAAAATATCAGCATCACTTCCTAATATTTTCTGCAAAACGACTTCTATGTCGTTAGTAGCTCTGCACGAAGAATTGAATACAAAACACCTGATTTGCACGACGATTTCCGTAGAAGTACAATTTCACATTTGCCACAGCAAACACTCAGGTGCTTCCTATTTTCAAACCTGCGGGTTCTTCGTTCGATGAGAAGTGGCACCAAGTTGGTGCGATGGGTTGGTGCCACCAACCCATCGACACTGTTAGTAAATGCAAGAGTCAGCTGTTTTTGTCTTTATTTCTTCCTGACACACGCTTTCTAGTAAGAGTACAAGCAAAATAGACCAGTCTCATTCTCATCCAAATTGCTTTTGCCTGATCCTATTGTACCCTCTTATTGGTACACGCTGGCTTGAGATAACTTTAGTTTGAGAAATTCCGTGGTCCCCGAAGCTATCACAACCGAGAGGATTGGCTGTGCAGCAATGCTGCCCGGCAAAGCAAAAGGCATCACGCCGGGCTTCCCAGTGCTCAAGATTTGAAGAGCCTTCGCCATCTGTAACTTTTTCTATTCGATGGCGCCAGTCGCGAAATCACCGTCAAGGGCTCAAGTCGGGCGTCCCCGTCGCGAGCCAAGAATGCGCCTCCCCTATATCCTGCTCGTTGCTTTGGTCACCTCCCTTCCCTCGCTCTCGGTTGAAGCCAATGCTGGAACGAGGACTCTGCGATCGCTCAAGACGAGCAACAGCGAAGACGCGGCCGAAGAAGAGCGCGTACTACCTGTCGTTCCCTTTCAGCACACTTTTAATTTTAACGCTATGGACGAGGTTCTTTTCAAGTTTATCAAGTTGCCGGAACAATTCGAACGAATGAGGACTCAGCCAGAGCGCCTACGAACAATTCTTAAAGACTGGTACGATACTCTCCAAAGCGTTGACGATATCGTCGCCTTCATGAAGAAGCAAAATTTGAGTCCGAAAGCCATTGAAGAGTTCAAGGGAGCGTACGAGGCATATATCAAGTATGCAAAGTCTATCAACGTGGATCCGGCTTTTTTAGCCCTTCATTAAATAATTGAAGCCGCTACCATTAGTAAATTGTTGGTATCGAAGAATAATGCCACCTCTGGGGCCATAATCGAGTGCATTTAACTCNTATTTGCCGAGTTTGTGTCATCGCACATGTCTTCATAATTCATCGTGAGTCTTCAGCATCTCCAGTTGACGTATTTTGTGCCAATGAGTCCGCATGTGGGTGTCCAACGTACTTGCCAGTGCCGTTCTTGGTACTCTACGGATCGAGAAGGTGGTCAAGAGTTACCTGAAGGAAAAGAATGCTGTCAAATACTATAATACAGCAGTATTGCACATGTTAAGCGTAGCCGAGAGCACGAGTAGGTAGTCTCACCCGCTATTTGGAAGTTGCCAAGACGGCCGTTTAAGCGGAAGAACACCTGGATTATTTTTTATCTAACGAGAAAGAGACATCCGTTCCGGGCAACAAGTGACAGCGAGAGTTTTTGAAAATCTGGAGTTGGTCAATTGTTAAACTTTATGATTAAGTGTTTTCGTGCAAGGAACTGAGCTAGCTGCGAGATACTTAGTAGTGTCAGTTTCGATATGGAACGACACCCTCTGCCGTGCAATAAACTTTTTTTGCTTTGGCTCTGAATGGGCCAAGCAAACAACTGAAAAGAAAAGATATTATTTTCTCCATACCACATATTCTGCCGGGCAGGGGCAGAGTATGCAATATTTCTCTTCTCTTTTAGTTGTTTGCTTGACCAGAATCGTTTCACGAGGCGTGTTTCCGTACCAGATATTCGGTGGGGCACGACATCAGGAGTATGAAGTGCAAACCTGGCCTTTTACTTCATATCACGAAGTATTTTCGATTCCTTGACAGCTATTCTCTAATGCTCGATGTCTTATGCTAGTAAGACGTGTTTTACACGGAGAAGCTCTGGATTCTTCAATAGTTATCAATAAATTGGGGCAAAGCCTCCCTTTAATAACACAACACTTTGTCCTCTGATAAAGTACAACATCTACTCTTTTTACAGACATTTTAGCGGTTTTAGTAAACGGGTAGGGCTCTTTTAAGAAATGGAACAGCAAAGGACCAAGTTTCACGAAAAAAAAACTATTTTTAAAGAACATGAAAACTGTTACGTGTGGGGTCTAAAACTGAAATGCGCAAACTTGCCCTTGACTAACAGTCAAAAAACAGAGGAGCCATTTTTGTTGATATTGTGAAGGGTCGCTTTGTATCAGGCAGTGTTAAATTACGTGACGAAGTCGTGCTTCGCTCTGAACTTTGATAGAATCTAAGCAAGTAACAATTAAAGGTTGAAGATGAAAGGCAGTTAAAATGACTGAGAAAATGTTCTCAAGACAGGGCAGCAAGTGGTGGCGACACGAGTTAAAAATGACTGCCTTTCCGATCGTAGAGCGGTGTATTGGTTCGGGTTGGGTCATTTTGTTTTCTGCACGCACTCCAAGGCATCAGCTCAGGACACAGCGGAAAGCTCCAGACCGAATCAAACGGGATCCAAGCGCCCCGCCATCAGCAAGAAAGCGAGAGGAAAAACCAACGTTCGGAAAAATCTACCTGTTAAGTACCGATAGTCAATCTAGTCTCTTCGTTATTTTTTAATCACAATACATGGCATTATTTAAGCGATTTATTAGATTTACACGATACTGGCTAAACACTAATGCATGCCGAATATCGTTGCTCCTTGTTGGTGTCTCACAAATGTAGCTAAACCCTTCTTCAAGAGCCTGTGAGTGCCTACAAACTTTAGTATAAGTTAAGAAGAACACAAATTGGTGTGTGAAAAATGAACCAGCCCAACTCCGTCGACTTCATTTGCGAGACACGAACAAGCAGCAAATGTATGTGGACTAGTACTGTAGTACAATTAGAAGGACCGTAAGCTTACAAGTATCGTGTAAGCTTTTAATCGCTTGAAATATAATCTCTATTCCCGTATTTTTCGACAGTCTCTGTGTTCGCGCCAAGCAATCTTCATCAGGAACCTGTGAGTGCCTACCAACATGCTAGTGCCTCGTTAGCCGTCGCTAAAATGTCGATGGAGCTGGTCTAGTTGATCCATATTAAAAAATTGGTACAGAGATCTTCAAACCATTCGTCTGCATTTACACGCGGTTCACACAGGATTAGTTTCTGCGAATTGTTCGTGCAACATTCAATAGTATTGAATGTTTAACCCTTCTAGCCCCTCTGTTTATGCTGTATTATACAGCAAAAGAAATAAGCTTTCCTATAGTATGACGGCGTACCTGTGTGAAATTTATCTGTTTGTAGCCCCCTTCTAGCAATCGAGCCGGATTCTAGTACAATTTATGATGCTGCCAGTGAATTCAAGAGTCCTAATTGGCTAGATTGATGGATACATTTTGTGTAAAATCGTGAAAAATGTCTGTAGCACTCGTTACCCTTTCATACTCGATGTACGGAAGTCGTCGTGGCGATTCGCGCCAAAACTGGTGGCGTGGAGAGCGCTGCAGCTACCCCGCGCTTTCCGCGGAGGAAACTTTTGCCCATTTGTCAAGCAAAGACTTGTGTAAGCAACAAATGGGCAAAGTTCCTCCACATTAGATCAGAGGTGAGCTACTCGTCATCTACTTCCTGCAGCTTTCTTTTCGTGAATCTAATTTTCTTCTCATGTCCGGC

>Contig_90

AACTCTATTAGAGTAAAATGGGGAGCGGGGTGACTTCGCTAACGTGCAGGCAACACAAAAGAAATCTGTCTTTGGACCTAGAGGTATAGATCCATCGAGTCCATGTATGGCCTGACTCCTGAGGCAATAAGAGCCGCTGGAGGTGCCGCTGGTTTGGTATGGAACTGATACAGATCCGTGTTGGTAGCCATGAGCGCTTTGATCTTGAAGCGCTGGTCAGTTTGCATAGAGCACTGTTTGCGGTCAAAGTTGAAGCAAAAGTCGTGCTTCACAGCTGCTGGAATTGAAAGCAAACTGAATTGGAGCTGGGGAGCATACAGGACGCCATGAAGGATAAGCTTCTGCATAGTGCCCTTGGAGTCAGTGACTTCCAGATCGACCCGTCCAATGCCTTCGATGGGGATTTGGTTATTACCACCAACCGTAATGGATCCACCACTGGAGGTGATGTCGGTAAACCACTGAGACTCATGGGTGACGTGTCGAGTGCAGCCAGAGTCGATTGTCCAGGTGGGATCGGGTTCTATCTTGGCTTGAGCAGTGAGGCTAAACGGGAGATTGACTGTGGTAGCAACAGCGATGAGACCGGTGTCACGACGTCCTTGGCGAGTTACCTTGCGGTTGTTTCCAGAATCACTGTCGTCGTCATCTTCCTCGTCATTGTCTGACTCAAGCGGGCGGTCCTTGTAGCGATCTTGACCGCATTGTTTGCCGCCATTTCCGTGCTTGTTCTTGCTACTACTGTTGTTGCCCTTGAAGTCGCGGTTGTTATTATTCTTGCCGTGTCGCCCATTGCCACCTTGTTTGAGTTCTGGTACGGGTGATCGCGTTTCGAGTTACCCTTGAAGGCGAAGTTAGCAGGTAGCACTGTCCCAGCCTTCACTCGTCCATCTCGCAAATCCTTTTGAAGGCCTCGGCATTGGCGAATGTTGTGGCGTGGGCGATCACAATAGGAGCAGACATTGGCGCTGTCACGGCCTTGAGCTTGTGACACAGGAGGTCCGGTAGCCACTAGAGCTCTCTCACCCTTGGTTGCAGAGGACTCTGGTGTGCCCTTGGCCAAGGTGTAGCGCTCTTGAGCTTGAAGATCGCGAACCTTGCCTTCAATGCTCTGCTTGAGGTCTTCATACGGGATATACTTCCGCTGGCCCTTCCAGATGCGGAGATCGTCCTTCCACGACTTGGGCATCGAGTGGAACAGGTAGATAGACTTCTGTCCCTTGGTCATCACCGATTCGGTGGCTTCTGACGCGGCTTTCATCGCGTTCTCAAGCTTGAGAAAGAACTCAGTTAAGTCGGAGCCTTCCTCATACTTGATCTCCATCAAGTAGTGTTGAATGAAGTACGGATCTCCATGAAAGGCTGCTCCCTCGTACGTCTGGCAGCTGTAGTTGAAAATCTCGTAGGATGTCGTGAGATTCTTGACGAGCCGCACGTGCATGTTGTCCATCGTTTTCATCAAGAAGGCTTTGGTCTTGGCTTCTTGTCGACGGCGTTCGCGCTGGCTAAGAAGCTGTCTCTTCTCTTTCTTGGCGCGTTTGCGTGCACGGCGAGCCTCGCGATGGTTAAATGGTCGAGCTACCGGGAGGTCCTTCCTCTTGATTGACGTATCGGAGCTGGCTCCAGAGTCTTCGTCCGAGTCCGAGGGCGGCTTGAGCTCGTCGTCGCTCTCGTCGTAGTCCACAGCGTCGGAGTCGATTTCGACGTTCTTGGTAGTTTTGGCATGCGGTTCATCGTTGGCATCAGACATATCGCTGCCACTGTCTTCACTCTCGTCTTCCGACACACCGTCGTAGTCGGGCTGCTTCACGTAGCCGAGTAGGTGCTTGCCATCTAGTGCTACACACACACGGGTCTTCCAGACGTCGAAATTCTCGGCTCCGATGAGACGTGGGAAGTCGTCGCTTGAGGCTTGGCTTGGGCTCATAACCTATAACTCGTATGACAGGTCGAGAAGAAGTAGGGAGAGGAAGAAGACGTTACTTGTAGGCGTCAAAAGACGAAGTGAGTACTTTATTAGTATTAGGAAGCCACCAATAGCTGTACACCAGCCATTAGTGTTTGTATAGTTTAAGCCAGTCATACCGACTTAAAATAGGACCTCCTAAATCTCATTATAATATTTTGGAGTGACTAGGTCAAGGTAATCAAACTGTTTAGAGGCTACTTCAACATTGTACAACCTAGGTAGAGATCTGCGCTCCGCCGTCCACGTGTAGAATAACACTGGTACGCTACGAAAAGAAGTAGATGCAATGTCCACGGCTAGCTAGTGCCACGTGCCACGAGTGACAAGCAGAGCATCACATGCTACTCATCCGCGAATACACTTGTCAACGTTGATTTCCACTTCGAGATCGCTGTGATGCTTCAGTTTAATTCTACACTCCTGGACACAATTATACGACCACCTCGTCGAGACAGAACATGCCAAACACATGCATTTACTGTTTCAGCACACAGCAAATCATTTGACTTGACTTCTGTAAGGTTTTGACCTTCTGAGTGTCGCAATAAGACATAATCGACGATTAGAGGTCACAGCAGAAAAGGGACCAGCCAAGCGAAGCGCGGCTGGGCACCCCTTCGGCGCCCCCGCGGCTCGCTGCGCTCGCAATATGAAGATAGAATGTTCTGGAAAGTGTAGTGCTTGGCACGCTGGTGAGCTCAGCGCTGGGCCCGCCTCGCACCCGCTCACCTTAGTTGGCGCCGCGGCTATGGCACCCACGTCGCCCAGCCCCGTGTGTGTTATCCCAGCGATGGGCGTGTGTTTCTCCTATTTTCTCCCTCGTCGAGTGCACATAACTCAGTCAAAATTTAGTTTTTGACGGTGCGGTTTTCACACAAGATGCAACACATGCTTCGAAATAGCAAGGATGTGCTAACGCTTGCGTTTTTGCACTTTATAGGGAGACTTACAAAAAAAGAGATTTGTCAAGGTGGTCACAATTTACGGCCATCATCGACCCAATACAAAAACTATGTAACATAAAACGTGTGTAAATTTTTGGCTGACTTGAAATTTGTAAGGACTGAGGATAGAGAGCTTAAAGTAGTTGCATGGGGGTGTAGACAATAATATTATATGTAACGAGTTGCACTCGCACCTCTGCTTGATCGATATTGATGAAGACTACCCAATCAATATGAAAATTTTTTTTTCTCAAATGTAAGATCGCGACGACTACACAACATGATTTGCTTTAGAAAGAACCATACTGATGACTAGCTCAACATTCTCCATCAATATCGGGGATGCATTGAGCGCTAAGGTTCACTTACTACGAAGCCTTAAGGAACAGAAGCCATTAGATAACGGACTCGAAGGTAAACCAGATCTTTATTGGAGACAACAGACAGCACTGTGCAGCCCAATCATTTTGTAAAAAGCGAACTTAGGGAGAAGCAGCAGCAAATTGCTTAGACGACGCAATACACAAGTCCTTGAACGTCATTATAACGTACGACTGAATGCATATCTTACAAGAAAAACTCACTTACCGTCTTGCTTTTCTATGGAGGGACAGTTATAAATCTGGAGAGCAGGATGCGACGTAACGTAGATCCGTTTTTCTACACTTAAAGCATGTTTTCATGTCATCTTGAATTATTAAAACTAATATGTTGTAAAGCAGTTGCAAATATCGGCCCCCGTACAGCAGCAGCGTTATCGGATGTCCTAGGAGACTGCTAAACGTTCTATAATACTCGAAGTGGTCTAGCTCCTTCAATAAGCAAGCGATAGTCAAAAGCGTAGTAGCGGAGCAATTACATGCTGAATGAAAAAAAAATGCTTACTTACTTGAATTTCGTGAATACACCGAATTGCGCGAGTTATTGTGAATCGTCACTGGGCTTAGATTAAATTGCGACAGTAGCGTCAACAGGTTTGTCGTTATGCCTGTTGGATAGTCAGTAAGTTCTCAACTATGGATGCGCAGCGCATCATGCGCTGGTCACCATGGACAGTGATTGGGGCTGGTCCATAATGACAAGAAACCGGTCAATGTGTTCTGCTGATATGGAAGAAAATTCCTATCAGCTCCTTTACATTAAATGATGTTAATATGGATAATCCAGAGAGTTCTTTATCACTTTAAAGAACGATTTACTATTGTAATAGTAAAAACCACAAAAGATACAATCACAAGCGAATGAGGCTAAAGGAGGCGCTGTCTAGAAATTAAAGAGTGAAATTGATTGAGTTAGAATCCTAGTACAGTACCCCCTTCTACATCTAACATGCAGCCCCTACTGTAGGGCCTCCCAACTACGKGCAGCCAGCGTTTTAGAAGCTAGCATTTATATCGAACAATATTTTTCTTGAAGGAAGATGGGTACTCGCACCTATCGCACTCTTCTTTATAGCCAGTGACCTTTTGTGCGGGGTCCCGTACATGGTAACAATGTCCAATTTAGGTCGCTGGAACAGGAAATTGCAAGTGTTTCTTAGCTCGCTCGGTTTATTTCATTGATAATGAAGCATGTGCTTAGAACAGACAGGTAGTCATTTAATTCTACCGCAGTCTATCGTCGTTTTACATGTTTATTGAGGCTCTGGTGCAGGCGACACATCTCTTATGGTACGTTAGATGGAACAGAAGAAGCACTGCATGGGGTGAACATCTCTAGCTGTATTAGACTACTGGTCGCTCAAACATCGTCACCTACAGCCTTGAATGTGGATTTCTTGACGATGGCAATGCTTATTTACTTGTGCGTTTACAGCGCTTGTAGACTCACTAAGCTTAACATGGTCTTGCACTTGTCGCAGATACATTATACGAACTCGTTTACCCAATCAGCTACACGAAATACCATCTCGTACAGTTTACCAGCGTTTTATCGTGTTGGGTCTACATTCTTCGTGTGATATTGCTGCGCTTAGCCAAGGTGTAAAAAAGTTTTATAAGCTCATCAACTACTACGTCTTCAACAGCAAGACACCATCATCGTCATGTTTAAAGCAACGCTGCAGCCCAGGTAACTGCTGTATTCAATAGTTACGAAAGATGATTGTTCACAATGAGCTTGTAGTCATCCGGTTACTTGTTGTGGTAGTACCAGCTGCGGTACAGCATACGGAGATTGTCGTACTCCTCGTCAGGAATTMTGTTACCGAGTTTGACAGGGCTGTAGCCGTATGCTTCCAATCTCTTAAAATAATTCAAGAGGCTTCCACTCACATTGTTGGCAGTTCGCTTCTGGGCTTTGTTTACATACACCGTTAGCTTGTCGACGTTGAAGAGGTTGGCAGCATTCGCCCTTTCCTCTTCATCGTCATCGTCGCGATAATCGTTGCTGTCGAAGCGCAAAAGCCTCTTCTCGCGCTCAGCACCAAGGTGAACTGAAACTTCTGGTGATGCATACTTCGCGATATTGGGCATGGGCTCGGAGGCTTCGGCTGCAGCATTAGCGCTTGCGATGATAGTGGTCACAGTAGCGACTACTAGATARASGCAGGACAGACGCATTGGTGAACGGCGGTGGCTCTTGATCCGAAATGAAGACAGTTGGGGCACAAAGTGATGGCTTTCGTACGACGCAGATTGCAGAGTGAAATTGTTAGCTGCTTTCGTGTGCTCAGCTACTTCGATGTCTAGTGATATCAATATTACAAAGTACAGGTGTCACAACGCAAGCCAGGTCTCTGTATGGGACAGTATGTGAAAAAGGCAGGGCATTGATTAGATACAGGTTACGATATCCTGAATATCCACTCGGTTTCTAAATCGGTTTATATATATTGCTTGTCTGGTCCTCTCTTTTAGACTGCTATGCTTTCTGTTCAGTAGTATATTAAACCACTTGAAAACTTCAAAAATTGGGAAGCGTTCGTCAGAAGCACGTTTCCCTGAACTATAATGTTACTGGTCGTAACGCTACACTTCACCGTCGTTGACTGTAAGCGATAGTGTTACGTAATTGCGCTGGTATTCTGGACAGTAGCAGAAAGCGAATAAAATCTTGTTACTTCCCTTACTACCTCGACCGCAACTACGGTTTCTTCTTGAGTATGCTTCTCTCTCAAAAGCAAAAGGCAAACTGTGGCCAGATCTCTTGTGTCCTTACAGCTAAACTTTTCCGTCCTTAGTCTTCGACCACCTTTCTCCTTCTGGGACTGTACGTGCTGTGCTTACTAAGTCGTACAGCTCACCCAATATCTTAACACGCCGCCCTCCGAGATAGCTCTCTCGGAACTAATCTTCATCTCTCTTCACTCTGCTCCCACACAGTACTTTCTCACGATACTTCTCTCTTTCGGTCTCACGGCGAGTCTTGCTGATCTTCGGTTTCATACCTATGATGGTCTTTGTCCTATCAAGCCGCGTCAACCCCATTGGTTTCGTCAGAAGGTCCGCAGGATTTTCGTCTCCTTTCACGTTCCATAGTTTAACACCTTTCTGTCGAACCATATCTCTCACGTAGTGTAGACGTAATTCAATATGACGTGTCKTTCTGCTGTACGTGGGGTGAGTAGCTAGTGAAATCGCTGCTTGGTTGTCAATTCCAAGTTTAAATGACAGAGTGCTCTTACCGACAATCTCCGACAAGATGTTCTTAATAGAAGCTCCTTCTTGACACGCCTCACAAGCCGCAGCATATTCTGCTTCTGCAGTGCTTAGCGCCACAATTGTTTGTCGTCGCGCAGCCCACGTCACCGGTCCTCCCGCCATCATCATCACATATCCAGTTACGCTTTTTCTCGTGTCCGGACAATTGCCCCAATCAGCATCAACGTATCCGTCGATTTCCAACTTCTCTGCAGCTTCATTGTCTTGGTTCTTAAAGAACACGCCATGGTCCGAAGTTGCAACTAGGTACCGTAAAATTCTTTTTAGTGCTCCTGCATGTGCTGCGTTCGGATGCTGTACATGACGGCTCAAATAACCAACAGGAAAAGCAAGATCCGGGCGAGTGCTTGTCGCCAAGTACATCAATGCTCCAATTACTTCACGATACGGCAAACCGGGTTCTTTATCACCTTCTTTCGCAGCTTCCAGCTTGACCGTAGTGTCCATTGGTGATCGGCAACCAACAGCGTCTGCAAACCCAAATCGCTTCAAGATTTCTTTCGCGTACTTTTTCTGGTGAAGTAAAACACCATCATCTTGCCATTCAACTTGTACTCCAAGATAATTTCCTAGCCGACCCAAGTCTTTGATGCCATACTTTGCATCAATGTCTGCGAAAAACGTTGTCTTCCAGCTCGGGTTGTTCGTAGCATAGATAAAATCATCCACGTAGATATGAAGGAGAGCAATATATTGCCCTTCAAATCGGAAATACATACAGGGTTCAGTCGTACTTCTCTCCATTCCTTTTTCACTCAGCCAACTGTGCAGCTCGTCGTACCATTCACGTCCACTTTCATGCAAACCATACAACGCATTCTTCACCTTGTAAACCCATCCTGGTTTGAGTGAGAATCCTATGATTCGTCGAATGAATGCGAGTTGTTTCTTCGGTGCATTCAGGTAGGCTGTGTTGATATCACCTTGGTGGGCAACCAATTGGTAAATTTGGCAAAGTGCAACAAACAATCTGAATGAAGCCATTCGCGCCACAGGTGAGAATGTATCCAATACACTGAAGTCAATACCTGGTACCTCTTTGTCTCCACGAGCACAGAGGCGAGAGCGATAGCGCAAAATATTTCCAAAATCATCAGTCTTGGGCTGAAATCGCCACATCGTCTCAAGTGTTTTCATCCCTATTGGGATGTCCGTCTCCGGAACCAGCTCAAGTACGTCTTTTTCAACCATTGCTGATACTTCTTTTTCCATTTCTTTCCACCATTCGGCTCTCTGGGGTGAGTTCATCGCCTGGGATACTGTTCTTGGGACTTTTAACTCCGAAATGTGCCATGTGCGTTGCTGTGGCTGCGTAGGCGTCTCTGTAATAAGTGCAATCGCGGTTAGCGCAGCATACACTTGCATCTCGTCAGCAGTTAAATCTTCTTCTTCAGGCTCCGCAAACTCCTTCATACCCCTCTTTAAGTGCTGTTTCTNTCTCCTTTTCAGCACCATCTTTACTTCCATCACTAAGCGTATTCTCATCCACGTCGTCGAGCAGCGATCTTGCTATTAATTCTGCGTATTCAAATTGCTCTTCAGCTTCTTGGTCAAGATCATCTGATTTTTCTTCCAAACTCGAGTCCACTGTCTCCTGACATGTGCTAGTATTTGGTTCACTATCATCGTCAACAGCAAGTCTGATGTGTTTCATACTGTCACTAACTCGTTTTCGACGATCCACTTCAGGATGCACCACCATGGTGTTTTCAAAGCTTGGCAGCGGTTTCCGTAGCCATGGCGGCTCTCTGACGCTTGGTCCATCAGAGCTTGAGCTATGTGGCTCGGTATTGACGGTATCATTGCTGCTATCTCCGTCACTCCCCCCTACGACTGGTAAGTCGTAATCATGACGGCCTGCGCTTGTTAGATCTGGTCGCTCAGCTAGCACCCAGTCTCTTACGCGGCGATCAAAATCAGTGTGGTGTCGATCTTTGTATAGAACTCGTTCGTTGGCAGTGATTTCTCCAGCAATTAATACTGAACCTTCTGCAGGAAAATAGACTTTGCATCCAAGAACTCCATCACGATAGCCGAGCACAAATCCAATCTTGCAGTTGTCGGCAAATTTGTGACGTGACGGCCCAACCTTCGTGTGTGCGTAGACTAGAGCTCCGAATTTCCTAATGTGGTGAATATCTGGGCGTCTTCCCCACATTCCTTCGTACGGCGTGCAGTTAATTGAACTTGAGAGTGATCGATTCTTAAGATACACTGCATAATACAATGCTTCGATCCAAAATGACGTCGGGAAGCCCGATTCCTTCATCATTGTCTTCATCATACCAATTATCGTCTGATGGGTACGCTCCACCATGTTCAGCCGAGATGTTTTTGGTGGTATGACGGTATGTATTATGCCGTGTTCCTGGTACCATGTTGTGATCTGTCCATTCTCGAACTCACCATCGCCGTCTGTAATGATCTCATTCACCTTCCACTCTGGTGATTGCCTTTCAGCCCAAACGACATATCGCTTCATCAGTGCATTAATTTCTTCCTTCTGCTTGGTCTGCACCGGATAAATCGTTGTAAACCTCGTGTGAGCGTCCATTATCACCAATACTGCTTTGAAACGCGTACAATTGTAGTGACTTGGCGGAAATAGCAGATCGGCAAACACTAACTGGTTTCGTCGTTTCACTCCACGATCTAGTTGTTTCAATGGTGTCTTTGCGCGTTGTTTTCCAAGTTGACAGGCTTCACACATGTCAAAGTCCCTCTTTCTTAACATCATCCCTTCGACCAAGTTTTGATCGGCCATTTGCTTCACAAATCGAGGGCAGATGTGGCCCAGTCGCTCGTGCCAGCTCTGCAAGTCTGCGACGCCATCACTAATTGCGTAGTTGATCATTGTCTTTGGCGCTGACTGGTTTTCTTTCGACTTTTTCCCTGGTAAGAATGAGTTGTCTGCTGTGAACGTCCACAGCTTGGACGGTTGTACTTGCACCTTAAGTGCTACTTCACCGTTCATATTCAACGTGTATTCTCTCGTCGAGTCGTCATATGAGGTTTGGAATCCTTGCTCCGTTGCCGCCGACTGGGAGAGCAAATTAGTCCGTTGTGGCGAATAGTATACGTTCTCGACTCGAAGAACTACTGGATTGTCGTTAACATTGACCCATAAGTCAATTGATCCCATGCCGATTGGAGTACTCTTGCCGTTGTAGCCATGAACTGTAGCTCTATCTTGTTCTTCTAGCGTTAGATCACGGTAGTTGACAAAGTATCTCTTATCTCCGGCCAAATGCTGTGTTGCTCCATTGTCGAACACCCAAGTCTCCGGCTGGTATTCAAGCTTCTGTTGGTACCTGGCAGTGCCTTTCTTCGACTGTGAAGCTTCTCGTGAGTAGGCGCGTCCCTCCTGCTTTTGCGCAGGTCCTTGTGGTTCGCGGCTAGCCACCCCCGACGAATGAGTGAAGTTGGCATGATTGCGGACGTTACCGACACTACTGCCATTTTGGCTCTTCGGCTCCGGCTTCATCTCTTCTGGGCAGTTTCTTCGAGTGTGCCCCGGCTGGTGACAGCCAAAGCAGCTCTTGTTCTCTCGATCTTCTTCATACTGCCGCTGCTGCTCGTCGGTTCTTCTCTTGAAGTACCCCGAGTACTTCTGACTGCCACCGCTGTTTATGGTAGACTTGTCCTTGTTACGATTCTGGTACTTTGGCATCGGCTTGCCACCGTTACTGCGGCCACTTTGCCGACCGCCTCCAGCTTCATCTCGTACATCGGGATTAATTCGAACTTGGGATTGTTTATCCACTAGCTCCTCCTTGTCCAGAACGAGGATCATTGACTTCACTTCATCTGGTGTCTTCATCGTGTGGAGGCCTCCAGTTAGCATGAGAGTTCTCAACTCTCTGTAAAATGGCAGCTGCGACACAGATCGAACCATGAAGTCGACCATCCAAGAGTCACTAACGTCTGCATCCAGTACAACCAACTGTTCGCGTAGCCCCATCATGTACAGTAAGTGGTTCTCAACGTCTGCACCTGGCCGACATGATGCACTTTCGAGCTTCTGACGAACGGCACGTTGTGTGTACAGTGTAGTCATCTCGTTTTCACGCCCTTCGAAGCGCGTAGCCAAGTAGTTCCACATGTCCGACCCGTAGTCAAACCGCATTACTTGCTGGGCGAGGGTGCACGATAGTGAACTTGCAATCAACTTGGCGAGTAACTGGTCTTGCCGCATAAATCGTTGCTCCTCATCGTTACCATTGGTGTTTCGGTACTCAACACTACCGTCACAGATGTCGTCCAAACGCCAGCCATTGTCCTCTACGTTGTAGCAAGTCAGGTATCTCATCATGGTGCGCTTGTAAAGAGGCCAATTGCGTCCATTCCAATTCACTGGACGACCATCTTTATTCTACTCTACCTCCGCTTTCTTGGCTTCTGAGTCTCCGTTTCCTCTGTTCCGCATCGTTGGGCTCATAACCCTGTTACGTAATTGCGCTGGTATTCTGGACAGTAGCAGAAAGCGAATAAAATCTTGTTATTTCCCTTACTACCAAACCATCCAGTGGAGAAGATGATTGTTCACAATGAGCTTGTAGTCATCCGGTTACTTGTTGTGGTAGTACCAGCTGCGGTACAGCATACGGAGATTGTCGTACTCCTCGTCAGGAATTATGTTACCGAGTTTGACAGGGTTGTAGCCGTATGCTTCCAATCTCTTAAAATAATTCAAGAGGCTTCCACTCACATTGTTGGCAGTTCGCTTCTGGGCTTTGTTTACATACACCGTTAGCTTGTCGACGTTGAAGAGGTTGGCAGCATTCGCCCTTTCCTCTTCATCGTCATCGTCGCGATAATCGTTGCTGTCGAAGCGCAAAAGCCTCTTCTCGCGCTCAGCACCAAGGTGAACTGAAACTTCTGGTGATGCATACTTCGCGATATTGGGCATGGGCTCGGAGGCTTCGGCTGCAGCATTAGCGCTTGCGATGATAGTGGTCACAGTAGCGACTACTAGATAGACGCAGGACAGACGCATTGGTGAACGGCGGTGGCTCTTGATCCGAAATGAAGACAGTTGGGGCACAAAGTGATGGCTTTCGTACGACGCAGATTGCGGAGTGAAATTGTTAGCTGCTTTCGTGTGCTCAGCTACTTCGATGTCTAGTGATATCAATATTACAAAGTACAGGTGTCACAACGCAAGCCAGGTCTCTGTATGGAACAGTATGTGAAAAAGGCAGGGCATTGATTAGATACAGGTTACGATATCCTGAATATCCACTCGGTTTCTAAATCGGTTTATATATATTGCTTGTCTGGTCCTCTCTTTTAGACTGCTATGCTTTCTGTTCAGTAGTATATTAAACCACTTGAAAACTTCAAAAATTGGGAAGCGTTCGTCAGAAGCACGTTTCCCTGAACTATAATGTTACTGGTCGTAACGCTACACTTCACCGTCGTTGACTGTAAGCGATAGTTGGGTATAAAATAAATGATTTTTCTCGAGGGTCTTAATTGAGAGTTGGTAGAATTTTCTGGAGATTTGTTGCTTGTCTCACGCGGGCAGTAATTTAGCTTATATTGGAAGCTGTATTAGGCTGTTTTCGTACTATTTGTCTCCGTAGACAGCAATGTCGTTAGTGTATCGCTGAGAACCTGTTTACTGACTGTTAGCTAGAGAGTCACGGAAGTAGCTGACATGCTGGTGGAGTTAGACTGGTTCATCGTCAATTTCGGAGCAGTTTGTCTTTCTCGTGTGTGTTTTCGTTTTGATGTGAGCCGTTTGTGTTTCGATCTAACGATTTGATCCAGAAATAGTGTACATTGTAAACTGGGGATCCTCACCCAATCTTGCTTAAGCCTAAGGCTTGCCGTGCCTTCGGTGGTCTATTACATTGTAATACTCCAGAGTTTTAATATTTCGTTTGGTTGGATGTCGTTAATGTCAACAGGCTTGTCGTTATGCGTGTCGGATCGCAACCAAATGCTGCACTATCCATGCGCTGGTTATCGGGATCTGCGTTATATCATTGGATTTGTTATGCCTGGTCCTTTTTGACGAGAGACCGGTCAATGTGTTGTTAGCATATTGAGCTGCAAAACAGAATATTGCAAGACGCTTCAACCACGTGGATTTACGTAACGTTTATGATGCCAGGTGAAGAATTGGTGCCTGTAGTTACATGTATCTGCTTCCATCGGCTACATGCAAGTTCATTCATTCATACTTACAATACAATCGTGTATTATTGGGATGACACTTTTGTAACGCCGAAAACTACGACTGTACTGATGCATGAGCGCTAAGGTTCACTTACTACGAAGTCTTAAGGAACAGAAGCCAATAGATAACGGACTCGAAGGCAAACCAGATCTTTATTGGAGACAACATACAACACTGTGCAGCCCAATCATTTTGTAAAAAGTGAACTAAGGAGAAGCAGCAGCAAATTGCTTAGACGACGCAATACACAAGTCATTAAGCGTCATTATAACGTACGACTGAATGCATATCTTACAAGAAAAACTCACTTACCGTCTTGCTTTTCTATGGAGTGACAGTTATGAATCTGGAAAGTAGGATGCGTAGATCCGTTTTTCTACACTTAAAGCATGTTTTCATGTCATCTTGAATTATTAAAACTAATATGTTGTAAAGCAGTTGCAAATATCTGCCCCCGTACAGCAGCAGCGTTATCGGATGTCCTAGGAGACTGCTAAACGTTCTATAATACTCGAAGTGGTCTAGCTCCTTCAATAAGCAAGCGATAGTCAAAAGCGTAGTAGCGGAGCAATTACATGCTGAATGNAAAAAAAATGCTTACTTACTTGAATTTCGTGAATACACCGAATTGCGCGAGTTATTGTGAATCGTCACTGGGCTTACATTAAATTGCGACAGTAGCGTCAACAGGTTTGTCGCTATGCCTGTTGGATAGTCAGTAAGTTCTCAACTATGGATGTGCAGCGCATCATGCGCTGGTCACCATGGACAGTGATTGGGGCTGGTCCATAATGACAAGAAACCGGTCAATGTGTTCTGCTGATATGGAAGAAAATTCCTATCAGCTCCTTTACATTAAATGATGTTAATATGGATAATCCAGAGAGTTCTTTATCACTTTAAAGAACGATTTACTATTGTAATAGTAAAAACCACAAAAGATACAATCACAAGCGAATGAGGCTAAAGGAGGCGCTGTCTAGAAATTAAAGAGTGAAATTGATTGAGTTAGAATCCTAGTACAGTACCCCCTTCTACATCTAACATGCAGCCCCTACTGTAGGGCCTCCCAACTACGGGCAGCCAGCGTTTTAGAAGCTAGCATTTATATCGAACAATATTTTTCTTGAAGGAAGATGGGTACTCGCACCTATCGCACTCTTCTTTATAGCCAGTGACCTTTTGTGCGGGGTCCCGTACAGCCTTAGTGGTAACAATGTCCAATTTAGGTCGCTGGAACAGGAAATTGCAAGTGTTTCTTAGCTCGCTCGGTTTATTTCATTGATAATGAAGCATGTGCTTAGAACAGACAGGTAGTCATTTAATTCTACCGCAGTCTATCGTCGTTTTACATGTTTATTGAGGCTCTGGTGCAGGCGACACATCTCTTATGGTACGTTAGATGGAACAGAAGAAGCACTGCATGGGGTGAACATCTCTAGCTGTATTAGACTACTGGTCGCTCAAACATCGTCACCTACAGCCTTGAATGTGGATTTCTTGACGATGGCAATGCTTATTTACTTGTGCGTTTACAGCGCTTGTAGACTCACTAAGCTTAACATGGTCTTGCACTTGTCGCAGATACATTATACGAACTCGTTTACCCAATCAGCTACACGAAATACCATCTCGTACAGTTTACCAGCGTTTTATCGTGTTGGGTCTACATTCTTCGTGTGATATTGCTGCGCTTAGCCAAGGTGTAAAAAAGTTTTATAAGCTCATCGCCTACTACGTCTTCAACAGCAAGACACCATCATCATCATGTATAAAGCAACGCTGCAGCCGAGGTAACTGCTGTATTCAATAGTTACGAAGCCAAACCATCCAGTGGAGAAGATGATTGTTCACAATGAGCTTGTAGTCATCCGGTTACTTGTTGTGGTAGTACCAGCTGCGGTACAGCATACGGAGATTGTCGTACTCCTCGTCAGGAATTATGTTACCGAGTTTGACAGGGTTGTAGCCGTATGCTTCCAATCTCTTAAAATAATTCAAGAGGCTTCCACTCACATTGTTGGCAGTTCGCTTCTGGGCTTTGTTTACATACACCGTTAGCTTGTCGACGTTGAAGAGGTTGGCAGCATTCGCCCTTTCCTCTTCATCGTCATCGTCGCGATAATCGTTGCTGTCGAAGCGCAAAAGCCTCTTCTCGCGCTCAGCACCAAGGTGAACTGAAACTTCTGGTGATGCATACTTCGCGATATTGGGCATGGGCTCGGAGGCTTCGGCTGCAGCATTAGCGCTTGCGATGATAGTGGTCACAGTAGCGACTACTAGATAGACGCAGGACAGACGCATTGGTGAACGGCGGTGGCTCTTGATCCGAAATGAAGACAGTTGGGGCACAAAGTGATGGCTTTCGTACGACGCAGATTGCAGAGTGAAATTGTTAGCTGCTTTCGTGTGCTCAGCTACTTCGATGTCTAATGATATCAATATTACAAAGTACAGGTGTCACAACGCAAGCCAGGTCTCTGTATGGGACAGTATGTGAAAAAGGCAGGGCATTGATTAGATACAGGTTACGATATCCTGAATATCCACTCGGTTTCTAAATCGGTTTATATATATTGCTTGTCTGGTCCTCTCTTTTAGACTGCTATGCTTTCTGTTCAGTAGTATATTAAACCACTTGAAAACTTCAAAAATTGGGAAGCGTTCGTCAGAAGCACGTTTCCCTGAACTATAATGTTACTGGTCGTAACGCTACACTTCACCGTCGTTGACTGTAAGCGATAGTTGGGTATAAAATAAATGATTTTTCTCGAGGGTCTTAATTGAGAGTTGGTAGAATTTTCTGGAGATTTGTTGCTTGTCTCACGCGGGCAGTAATTTAGCTTATATTGGAAGCTGTATTAGGCTGTTTTCGTACTATTTGTCTCCGTAGACAGCAATGTCGTTAGTGTATCGCTGAGAACCTGTTTACTGACTGTTAGCTAGAGAGTCACGGAAGTAGCTGACATGCTGGTGGAGTTAGACTGGTTCATCGTCAATTTCGGAGCAGTTTGTCTTTCTCGTGTGTGTTTTCGTTTTGATGTGAGCCGTTTGTGTTTCGATCTAACGATTTGATCCAGAAATAGTGTACATTGTAAACTGGGGATCCTCACCCAATCTTGCTTAAGCCTAAGGCTTGCCGTGCCTTCGGTGGTCTATTACATTGTAATACTCCAGAGTTTTAATATTTCGTTTGGTTGGATGTCGTTAATGTCAACAGGCTTGTCGTTATGCGTGTCGGATCGCAACCAAATGCTGCACTATCCATGCGCTGGTTATCGGGATCTGCGTTATATCATTGGATTTGTTATGCCTGGTCCTTTTTGACGAGAGACCGGTCAATGTGTTGTTAGCATATTGAGCTGCAAAACAGAATATTGCAAGACGCTTCAACCACGTGGATTTACGTAACGTTTATGATGCCAGGTGAAGAATTGGTGCCTGTAGTTACATGTATCTGCTTCCATCGGCTACATGCAAGTTCATTCATTCATAMTTACAATACARTCGTGTANCTATTGGGATGACACTTTTGTAACGCCGAAAACTACGACTGTACTGATGCATGAGCGCTAAGGTTCACTTACTACGAAGTCTTAAGGAACAGAAGCCAATAGATAACGGACTCGAAGGCAAACCAGATCTTTATTGGAGACAACATACAACACTGTGCAGCCCAATCATTTTGTAAAAAGTGAACTAAGGAGAAGCAGCAGCAAATTGCTTAGACGACGCAATACACAAGTCATTAAGCGTCATTATAACGTACGACTGAATGCATATCTTACAAGAAAAACTCACTTACCGTCTTGCTTTTCTATGGAGTGACAGTTATGAATCTGGAAAGTAGGATGCGTAGATCCGTTTTTCTACACTTAAAGCATGTTTTCATGTCATCTTGAATTATTAAAACTAATATGTTGTAAAGCAGTTGCAAATATCTGCCCCCGTACAGCAGCAGCGTTATCGGATGTCCTAGGAGACTGCTAAACGTTCTATAATACTCGAAGTGGTCTAGCTCCTTCAATAAGCAAGCGATAGTCAAAAGCGTAGTAGCGGAGCAATTACATGCTGAATGAAAAAAAAATGCTTACTTACTTGAATTTCGTGAATACACCGAATTGCGCGAGTTATTGTGAATCGTCACTGGGCTTACATTAAATTGCGACAGTAGCGTCAACAGGTTTGTCGTTATGCCTGTTGGATAGTCAGTAAGTTCTCAACTATGGATGTGCAGCGCATCATGCGCTGGTCACCATGGACAGTGATTGGGGCTGGTCCATAATGACAAGAAACCGGTCAATGTGTTCTGCTGATATGGAAGAAAATTCCTATCAGCTCCTTTACATTAAATGATGTTAATATGGATAATCCAGAGAGTTCTTTATCACTTTAAAGAACGATTTACTATTGTAATAGTAAAAACCACAAAAGATACAATCACAAGCGAATGAGGCTAAAGGAGGCGCTGTCTAGAAATTAAAGAGTGAAATTGATTGAGTTAGAATCCTAGTACAGTACCCCCTTCTACATCTAACATGCAGCCCCTACTGTAGGGCCTCCCAACTACGGGCAGCCAGCGTTTTAGAAGCTAGCATTTATATCGAACAATATTTTTCTTGAAGGAAGATGGGTACTCGCACCTATCGCACTCTTCTTTATAGCCAGTGACCTTTTGTGCGGGGTCCCGTACAGCCTTAGTGGTAACAATGTCCAATTTAGGTCGCTGGAACAGGAAATTGCAAGTGTTTCTTAGCTCGCTCGGTTTATTTCATTGATAATGAAGCATGTGCTTAGAACAGACAGGTAGTCATTTAATTCTACCGCAGTCTATCGTCGTTTTACATGTTTATTGAGGCTCTGGTGCAGGCGACACATCTCTTATGGTACGTTAGATGGAACAGAAGAAGCACTGCATGGGGTGAACATCTCTAGCTGTATTAGACTACTGGTCGCTCAAACATCGTCACCTACAGCCTTGAATGTGGATTTCTTGACGATGGCAATGCTTATTTACTTGTGCGTTTACAGCGCTTGTAGACTCACTAAGCTTAACATGGTCTTGCACTTGTCGCAGATACATTATACGAACTCGTTTACCCAATCAGCTACACGAAATACCATCTCGTACAGTTTACCAGCGTTTTATCGTGTTGGGTCTACATTCTTCGTGTGATATTGCTGCGCTTAGCCAAGGTGTAAAAAAGTTTTATAAGCTCATCGCCTACTACGTCTTCAACAGCAAGACACCATCATCATCATGTATAAAGCAACGCTGCAGCCGAGGTAACTGCTGTATTCAATAGTTACGAAGCCAAACCATCCAGTGGAGAAGATGATTGTTCACAATGAGCTTGTAGTCATCCGGTTACTTGTTGTGGTAGTACCAGCTGCGGTACAGCATACGGAGATTGTCGTACTCCTCGTCAGGAATTATGTTACCGAGTTTGACAGGGTTGTAGCCGTATGCTTCCAATCTCTTAAAATAATTCAAGAGGCTTCCACTCACATTGTTGGCAGTTCGCTTCTGGGCTTTGTTTACATACACCGTTAGCTTGTCGACGTTGAAGAGGTTGGCAGCATTCGCCCTTTCCTCTTCATCGTCATCGTCGCGATAATCGTTGCTGTCGAAGCGCAAAAGCCTCTTCTCGCGCTCAGCACCAAGGTGAACTGAAACTTCTGGTGATGCATACTTCGCGATATTGGGCATGGGCTCGGAGGCTTCGGCTGCAGCATTAGCGCTTGCGATGATAGTGGTCACAGTAGCGACTACTAGATAGACGCAGGACAGACGCATTGGTGAACGGCGGTGGCTCTTGATCCGAAATGAAGACAGTTGGGGCACAAAGTGATGGCTTTCGTACGACGCAGATTGCAGAGTGAAATTGTTAGCTGCTTTCGTGTGCTCAGCTACTTCGATGTCTAATGATATCAATATTACAAAGTACAGGTGTCACAACGCAAGCCAGGTCTCTGTATGGGACAGTATGTGAAAAAGGCAGGGCATTGATTAGATACAGGTTACGATATCCTGAATATCCACTCGGTTTCTAAATCGGTTTATATATATTGCTTGTCTGGTCCTCTCTTTTAGACTGCTATGCTTTCTGTTCAGTAGTATATTAAACCACTTGAAAACTTCAAAAATTGGGAAGCGTTCGTCAGAAGCACGTTTCCCTGAACTATAATGTTACTGGTCGTAACGCTACACTTCACCGTCGTTGACTGTAAGCGATAGTTGGGTATAAAATAAATGATTTTTCTCGAGGGTCTTAATTGAGAGTTGGTAGAATTTTCTGGAGATTTGTTGCTTGTCTCACGCGGGCAGTAATTTAGCTTATATTGGAAGCTGTATTAGGCTGTTTTCGTACTATTTGTCTCCGTAGACAGCAATGTCGTTAGTGTATCGCTGAGAACCTGTTTACTGACTGTTAGCTAGAGAGTCACGGAAGTAGCTGACATGCTGGTGGAGTTAGACTGGTTCATCGTCAATTTCGGAGCAGTTTGTCTTTCTCGTGTGTGTTTTCGTTTTGATGTGAGCCGTTTGTGTTTCGATCTAACGATTTGATCCAGAAATAGTGTACATTGTAAACTGGGGATCCTCACCCAATCTTGCTTAAGCCTAAGGCTTGCCGTGCCTTCGGTGGTCTATTACATTGTAATACTCCAGAGTTTTAATATTTCGTTTGGTTGGATGTCGTTAATGTCAACAGGCTTGTCGTTATGCGTGTCGGATCGCAACCAAATGCTGCACTATCCATGCGCTGGTTATCGGGATCTGCGTTATATCATTGGATTTGTTATGCCTGGTCCTTTTTGACGAGAGACCGGTCAATGTGTTGTTAGCATATTGAGCTGCAAAACAGAATATTGCAAGACGCTTCAACCACGTGGATTTACGTAACGTTTATGATGCCAGGTGAAGAATTGGTGCCTGTAGTTACATGTATCTGCTTCCATCGGCTACATGCAAGTTCATTCATTCATACTTACAATACARTCGTGTAYTATTGGGATGACACTTTTGTAACGCCGAAAACTACGACTGTACTGATGCATGAGCGCTAAGGTTCACTTACTACGAAGTCTTAAGGAACAGAAGCCAATAGATAACGGACTCGAAGGCAAACCAGATCTTTATTGGAGACAACATACAACACTGTGCAGCCCAATCATTTTGTAAAAAGTGAACTAAGGAGAAGCAGCAGCAAATTGCTTAGACGACGCAATACACAAGTCATTAAGCGTCATTATAACGTACGACTGAATGCATATCTTACAAGAAAAACTCACTTACCGTCTTGCTTTTCTATGGAGTGACAGTTATGAATCTGGAAAGTAGGATGCGTAGATCCGTTTTTCTACACTTAAAGCATGTTTTCATGTCATCTTGAATTATTAAAACTAATATGTTGTAAAGCAGTTGCAAATATCTGCCCCCGTACAGCAGCAGCGTTATCGGATGTCCTAGGAGACTGCTAAACGTTCTATAATACTCGAAGTGGTCTAGCTCCTTCAATAAGCAAGCGATAGTCAAAAGCGTAGTAGCGGAGCAATTACATGCTGAATGAAAAAAAAATGCTTACTTACTTGAATTTCGTGAATACACCGAATTGCGCGAGTTATTGTGAATCGTCACTGGGCTTACATTAAATTGCGACAGTAGCGTCAACAGGTTKGYSTTATGCCTGTTGGATAGTCAGTAAGTTCTCAACTATGGATGTGCAGCGCATCATGCGCTGGTCAACCATGGACAGTGATTGGGGCTGGTC

>Contig_92

GATGCAGTGCTGCGAAGTGTTAAAAGCTCGTTCGATGTCGATTTGCTGACTACGCTGTGCGAGGCGAATTGGGGTGTTTCCAAGAGCAGTCTTACCGATGAAATTCTGCTAGAGCAGATTCACGCAATCACGGACAGCTATCAGAACCAAGTTTTGCCCCCTGTCAATGAACTTTTCGCCGTTGACTGAAAATGAACATGACTAACTCCGACATCCAGTCTCGCGTGATTGATTATTTCTCTCGTGCAACTCGTTGATCAACAAGTATGGTTTTTACTAGTTTTTTCGATGGTGACAAGGGAGCTAAGAAGAAATATAAGCTTTTGGTTAATTCGTTGCCGGAAGATCTGAAAGTGAAAGTGAAGAACGAGATCTATAATCGTTGCCCAGAGGCAAGCACCAGTGTATTACGACTATCGAAACTTATCAACCAGCAGGCTCTCGAGCAAGTCATCGGGGACCGTGCCTTGAAGAGGATCGAAGGTGCAAAGCGCAAGCCGGTGCCGAGTGAGCAGCACCGAGACTTTCAGAGCAAGAAGCGTTCATTTAAGGGCCAGCAACACCAATCCGAAAAGCAGCCGAAGAAATTCACCTCGCGGGAGGTTTNAAACTGTTGTTGGAAGAAATGGTCAAACATCCAAGAAAGGAGTCCCACAGAAAGGATGCAGTAGTGCGCACTATTTGAGTACATGCCCCACCGCTACCCAAGACGACCGAGATCACATTGCGGCTCAACGGGACAAGAAAGGTGGGATGCTTCCCGCGGGGAGGCACCGCTCGCCTCCGACGATTAGCTGAATGCCTGCCAACACAGACCCGCTCGGTCGTGTTGGAAGACGCCTATACGGTGCCTTTCTGTGTGGACAGTGGTGTGGACAGGTCCGGAATGAGCATGAACGTACCGGTATATGAAGATTTTGTGCGAGTTTGTCCTGAAGCTCAAATGGTGAAACTTGAAGTGCCGTTCACTTGCAAAGGAGCTGCTGGAGACCTTATTGAAGTTAAAATGACTGTGAACTCGTAGTTTAAACTACGGACAGTGGTTGGTAGCGTTCGAATCACCGAGCCAGTTGAGTGCCTTATCATTCCTGGTGACGCGACCAAGGTCTTGCTGGGTAACGACGTGCTGACCATGCTTGGCATTGACGTGAACGAGCAGCTCGATTTGCTAGTGGCCAATGCTGTCCAAGGTAGCAAAGACGATGAATTTTATGATATGAATGAACCCGAGATCGGTACGAATGTGAAGCTGAGTGATGAGATTCGTGTCGCCTTGGAAAAATTAATGGAAAAGAAAAAAAGGGTTTTCCAAAGGAACTGTTGCCTCAGTTACGGCGTATTGCTACCCGTTTCGATATCTGGAAACTGAGGCACGAATGCCGCCGATGACAATTCGTCTCAAAAACTGGTGCAAAGCCATATCGCTACAAGGCAAGGCGACATAATATAACAAGAAACTGATTTTGTGTTTGTGTCAGCTATTTTTCTCCGTTGTGCCAGCAACGTCGAGTAAGTTTCTGTCCCAAATGTACCATAAATCGGACAAATACGCGCCACAAAAAAAAAATGCGATTTCGGGAAGCACAATAAGATATCAAAACAGTATATGCTAGATATAATACTGCTTGCTCCGCATGGCTTCATTCAATATCTACACCCCCTCTTCCCAGTGCGGCATCGTCTCACACAGATGCATGGCAAACCGCAAGAGTACACTGCTACTGCTTCGAAAACGCACGCCTAACTTCTCTTTGAGCAGGGAATGGCTGTGTTTGAAATAAATGCGAAAGAAGCATCTGCGGCACTTTTCCACGAAGTGGCTATCTAAGCCACATTTTAAGCTCAGTGTAGCAAGCAAACTTAAGAAAAATTAATAGCTAACAGCTAATGTGGATTAATGAGATTCAGATTAACTCGCGAGAGTTAATTTGAGGTGAGGGCAATCAGTTCAACTCGCGCGAGTTAATATAAGTCGAGGACATTAACGCTAATGTGAGTATCAGTGAAATCAACAAAATGGTCGCATCATTGCCTTTTAATTCTTTTTTGCGTTACGAACTTAAGATCGACACGAGCTCACATCTAGCTTGTTCTTCATGAACTCGACAGAATTTGCTCTGCTTATGATCCGTAGATAAGAGGTGGCCTCTTCTTCTTGTTTTGTTGTGACACAAAGTTGAGATACGTAGCGTACTTCATGTACTCGTCCGTTCGCATGAGCTGCTGGAGAACGGTACTCGAGGTTCGAGCACCTACAACATCGTCAGCAATGCCCAAGTACTTAGCAAAATCGTCCACAGAATATCCCGCTTTAAAAGACTGCCTCATCTCGGTAATAGATAGAGACCTCTCCTAATCAGGTTCCTGATCCTCCACAGGGGTATGGTGAGACCGCAATCGACGTTAGTGCTGCTCATTTGGTGAAGGGAGCATCGCTTGGTTTGAGTTCACGAGCGCGCTGCTAGAAACGAAGAGAGTTGCCACCGTGGCTGCGAACACGTAAGGAAGACGCATGTGTATATCGCAGCCTGAGAGGTGGAGAGCAAATTTCAAAGTGAGTGGCTTTGAATACTACTCCACGGGCGGGGGGGGGCTCTTGTGGAGGAGGCTATTAATATCGTAGTAGCACTTACAATCCATATGTTTTACCTTTAGTGTACAGTACAATGCACTGTATATACGGTTACAACATAGCGACCATGGGAGATATTACCGTAAATGAGACCTGCTGTGAATTCACACTCTGATGCTACCCAAAATCGTAGCCTGTTTTCTACTCATACGACGTACGCATTGATCTTTTAATGCGATCAGTGATAAGGTTCTCAGTTTTTTTTATTTTCAGAATTTTTCGAAGCTAATCCTGCTATCTCTTAACTACTGTAAATGTTTATTCTTCGAGCCTTACACATTAAGCTAAGATATAGTTTCCTGTGAAACTGGCGTGCATAAGTGATGATGTAAATTTCCCTTTTGTACAAAATACACCCGGAACTTTGTTCAGTCAAGTAGATATCAACGCCACGACTAATACTTTGAAGTATTATGGTGACACAGAAGTCCTTTTCAGTAAGCGTAAAGGTCGTCGGATTAATTTGCATAGGGAATCCACAGCGATCTTGTGCTCTTGTCAAAAGTANCCCCGATCCAAATGATCCGATATAAAGGGGTATATTAAGTAGATGCTACTCTTATTCAGGGGGACTTCTCTACCTCAGTTTCGGTACTGTAGATTTAGCTGTAACAGGAAACCCGCCTGTGCCTGTATTTGGAAAAATATCGAGTTGATTAAACATTTGCAAACAGCTTAAGTTCAATATGGGCGATCTGTGAATAAATTTAGGCCGTGTTCAGCTTGCAGGGGCAAATCCAGCTTGTTCGAAAATAAGCTAGAGCTGTAGAACAGTCGTTTTGTTAAGAAAAATAAAGATAAAATAAGTGATCGTGTTCCATCAGAAAAAGACTGGATTCGAAGGCGCAAAGCAGGACGATTCAAAATTCAACCCAAAGCTTAATTTTGAAACCAGTCGTTTGGAGCTATCCTGCTGTATGTCATTTTTTGTGTTTCTTAGCGGAATAAAGCTGGTTGAGACATTCCAGCTCTATAATACCAATTACAGCTGGTTTTAGCTTAAATCGAACACGGCCTTAGGGGTCTGTGATTTTCGCCTCCCATCCGATTACCCTTAGCTTCTGTTCGTTTTATTTAGCGGCATAATTGATAAAANACCACAATACCGATAGCGGTTGCATCGTCCATCTTGGTTGTTGACGCACTTAGACGGATGGTGTGACCAATCAATCGCTTCGTCCCCCACACCCGAGCGATACTTACATGCGCAAGGTCTATCCAGCGTTGAACTAGTTTAGAGAGCGGTAGCTTTTTTTTCACCGTCCCTGTCTGTGTAGCTTAAGAGCTGGGATACCCGTACGAACGGGGTGGACCGGTGGAAGGTTTCCGGTAGAACCCGTTCACTTATCACTTCTCAATTGTCTTGTGGCCAACAACTCACCTACGAACCTGATGATGAAGTCCCTCTACGCTGTCAATTTGGTCTTACTCCTGCTGCTTGCATTTTTTGCTCCTGCCCCCGCTACAAGGGAATTGAATATGAGGGCCGCCCCTAGCGATTCAACTCGCGTTGTCGACTACGCCACGACTGAGAGGCTTCTAAGGGCCCACAGTAGTGACAAGGAAGAACAAAAAGAAGAAGAGGAAAGGGCAATTTCGATAAATTTTTCAAGCCTGGAGAAAATCTTTAAAAAAGTTACGTCAGCCAAAACTACGGAGCTGCAAGGAATGCTTAAGGCTGACGAGGCCCTTGGGAGTGCTTTCAAGACGCTAAAACTTGGTACAATGCGGATTGGCAAGGATGGCTCTGTCGATCCCAAGATGGTGGCAAAATTTCTGTCAAGTCGCAATTTCAAGATTTGGTCCCAGCACGCCGTCAAGATCAACAAAGATGATCCCTATGGCGAGATGCTTAAAGCACTCACAAATGTCTTTGGTGAGAAAAATGTGGCGATGATGATCCTAGTCGGGAACCTGTCCAGAAACTCGCGCGACGTCGCAAAGAAGTTAGAAAAGGCCCAGTTCTACAAGTGGTACTTCGTTGATAAGTACAAGACAGCAGATGAGGTTTTCACGAACGTGCTGAAAGCTGATCGAAATAGAATTCATGGGTATGGTCGGGAGAAAGAAATTTGGGGAGATTACGCGAAGTACGTCACGACCACAGTGATGAAATATTGATAAACCAAGTTTCCAGCACTTCTCTTGTTTTGGGTTAACATATAGTGGATTACGTATGAATCAATGTATGGGTTTTAAATCCAGTTTAGAATCCTTTTATACTCATACTATGTAGTAATACTACCTCCGTTCTGAAAAAGATAACCATTTCAAATTATATTACAGCCTTCCCAACAAAATACTACCTCCGTTCTGAAAAAGATAACCATTTCAAATTATATTACAGCCTACCCAAAACTATACCCCCTCTATGCCGATCACTTTTTGGATTTCGAATCTCCCGCGCTTTCCCGACCAGACTGGCTCCCGCTCCCAGCTTTCATTTTGAAAGCTGTCACCTGGGGGAGATCACAGACAGTGAGCGCCAGGCCGTCTGGGAGTCCTTACTGCTTCGCAGCGTGGGAGGAGTGCTCAAACATGGAGAGATCGTTCGTGTTGCAGCGTTCTTCCGTGTGGGCCGCCAAGTTGTCGAACGAATTTGGAGGCGTGGAATTAATTCGATGGGAGATCGAGTAGCAGCCGTGGTCAAGTCAAGGAAGAGCGCATGTGGTAGGAAGAAGGTGAATCGAGCTGAGCTGTGCGAGAGAATCGCTGCAGTTCCTGTCAATGAGCGCGAAAACCAGCGTCTGCTTCAGCGCGCGTCTGCTATTTGGCTCAGCAGCTGATCTAGGAAGGCTACATACGGCGTGCGCTCAGACAGACCCGTCCACTACTTACACCCACGCACAAGTTTGCGCGCCTCCGGCACAAGGACAAGCAAGTACGAGCTGGCACTCTCCCTAAGTCACTGCCTTGTGACGCTATCTCACTCGCCAATGCTAACGCGTACATGCTGCTTTTTGCAGCGAGCAGCCCCCCCACCGCCTACCGCTGAAGTTCTAGAAGCCCCACTGAGCGCCCCTTCATAATGTGGCGATATAGTTGCCGTCTTAGATCCGTGTTTGTATCTGTAGTTGCACGGCGGAGCATGAACGCCTGTGCCTTGCTTAGTTTCTCACTGTCAGCTCTCGGTCATAAAGCTAGCTAGCCAGCCTCTCACAAATTAATAAAGGGTCAAATGAACGAGGCGGAGCGCTGCGCNAAAAAAAAACAAATGGTTATCTTTTTCCGAGCGGANGGTAGTAGTAGTACTACTGTACTACTACGTAGTACTACATGTACTACTAGTAGTAGTAGCAATTGAAGAAGCGATACTTAAGTAATGGAGACGTTCTCGTTCGGCCTAGAGGTCTACTACATCATACCAGAAGATGAATCCCGTTAAGATAGCACTTTAGACTTGTTCTTCTTCGTCCGAGTCGCCCTCGTTTTCGCTTACGATTTCGTTTCTACTACTACAGTAGTAGTACCATGTAAATATGTTCTGCTCTCTTTTCGACACCAGTCTAACGTGAGAGTATCGATGCGGTTGGATTTGTCTCTAAGAGCGCATCCAACGGATCGTAGCAGCGGGCAATACTCCGTCACTGCCTCCCAGAATGTATCTCCGTACATTTTGTTTTTTTATAGTGTATCTGTGAGTTGGATGCAGTCGACATCGACCCAAAGGCACATTAGTGCAGGACATGCCTTCAGCACAGCACGAGCTTCAGTAGCCAAGACGCCATACCTCGGAGGATGTTAGAATGCACCTTAATTGGTCAAATATGTAGCCAATAGAAAAGGTTCAAACATCCTACTTCTTAAGGAAACTTAATAAGTAAACACATTGCCGAGGGATGTTAATGGCGAGGAGGACGCGTGGCGCGTACGCTTGAAAAGGCCTCCTCACTTTGAAACTGACGGCCTATTACATAGAGCTTAATATTTCAGATACKTACGGAGACTAAGGCTCATGGTTAAAGGACACAATAGGAGTGCCCAGGTCCTGGGACTGGAAAATTTCCATGGGGAGGACTACACCATGTGGCGAGACAAGTGCTAACGCACATAGAGACGCTGTATGAGAAATACCAGCGAGGGTTGTTGGAAAAGGACCAGTTTGAGGCTACGGTTGTCATGATGGACTTCCTTGAGGGTACGCCTGAGAAACCCATCATTTCGGTGGAGGGAAATATTTCTCAAAAGGAGGCGAAGGCCAGGCGTTGGCGCCACCAACACTGGACTAGGGCTCGGTCCGAGCTGTTGAACTTATTCAACCAAGCGTTGCCTAACGTTTTCATGAGTGGGCTGCCGGATCAAGTGTCCAGAATGAATCCCTGTGACATATGGAAGGAGCTCGAGCAAAAGTACGGGCTCGGGGATGCAGGAGGCGTAATCGAGCTGCGCCGCCGGTGGGAGCGTCTTCTCGCAGCGAACTGGACCAATCTGGGGGCCCTATTCGCCCAGTTAAAGATGCTGCGTAACGACATTAACAGGAAGATGAGGGGTCTGGTTGGCAAGGACATGGTGACCGAGACCTGGCTGTGCATGGAAGTGTTGGCGTTACTACCTAGCGAGTTTTGGGGAGGCACAATCATCATGACTGAAGAGTGGTTTTCTATTGAAAACGTGGAGACGAGTCTTCGCCGCGTTTTCGGTGATCGATCGAGGAAGGAAGCCACGATGCTGACTGATAAGAGACGCCCAGTGACTGTCAATGCGGCAAGAAAGTTTACGGGGAAGAAAAGGAACCAAGGACGATCTCTGAAGTGGCAGTAAGCGAGTGTTATTACTGCTTTGAAGATGGCCACTGAAAGAAGGCCTGCCCAGTGTTAGCGGCTGACCGCAGCCCGAAAATGGCTGGAGGCAAGCTGCTTCGTTCAA

>Contig_94
[truncated: 4,534,372 more chars]
