# Supplementary material for: Identification of Avramr1 from Phytophthora infestans using long read and cDNA pathogen‐enrichment sequencing (PenSeq)
Source: Mol Plant Pathol. 2020 Sep 15;21(11):1502–12. doi: 10.1111/mpp.12987 (PMC7548994; doi:10.1111/mpp.12987)
Supplement: Supplementary file 8 — NOTES S3 PacBio PenSeq contigs of EU_6_A1 [file MPP-21-1502-s008.docx]

>Contig_6

CCCGTGTCCATCTCCTCTTCGTGAATTTTACATGCCCAGCAGTGGAGATTTGGGTGAAGGTTCCTGTTAGTGACAAAATGGAAGATTATCTCGGTAAGTTGCGGAACAGTATTCACACCATGCACTAGGATTTAGAGGATCAGCGCTTGAAGCAGCAGTTGCTGAACAAAGAGCGAGAAAGAGGGGGAAATATTGTCAACTTTACTGAAGAGGACTATGTGCTACGCTCCCGAGTTGATGAAAAAGCGGAAGCAAACGCCTGGTAATTTGGGTCGGACCTTATCGTATTGTGCGAGCAGATGCACACTCTTTCCTCGCTCAACATCTGATCACGGGTACAGAGCTGGATGTCCAGGCTCAAATTTTACGCGGATTCCAGTTTTAGCGTCACTGAAGAGCTTTTGGAGCACATTTCGTCTCAAGGTGTTATTCTCGCTGTGGATAAGCTCAAAGGAATCGGTGGAATAGTAGCATTAATGACTTCGAAATTCTCGTGCAGTGGAAGGACTTGAGTCAATCGAGGCCTCGTACGAGCCGCTCACCAACCTAGCTCGTGATGTTGCAACATTGATACAACAATACGTTAGCACCGCTGACCAAGATTTGCAAGAACACTGGCAGCGAGTGACCCGTGTGGAATTGCAGCAGCCAAAAGCTGCCGAGGCCTCAACAACTGGAAGGCTTGGACGATGCAATAGACGTCGCAAAGCCAACCGCAATGGACAACCTTCAACGCGCCCGGTCGCCCCTGGTATTTCGGCTGTCGAATCTCAAGAGCCTAACAGAGGTGGTTCAGCTGATGCAGTGCTTCAAACATTAGTAAACCGACCTGGGTTTGAAAACCAACAATCAAATTTGTCTACGACTGATACCCCTTCTCAGCAGCACAGGATCGACAAAGAAGTCGACTATCTTCAGGGCAGCGCTTATCTCCAACGGATGCCGCCGATGGAGATCGTGTCGGTCGATGTACGTGCTCGCGGACTGCTGGTGCCGACCAATCCGCAGCCAGAACCCAGCAGGATGCAGCACATTCCCGCAGACACTGAAGGAGAGGAACGGGTTTACTAGACTCACAGGAGGCCAGGCAGTACCTACATGGTGCTTGATATGACTGCTGACTGGGTGTTTAGTAGAGAAGGCGTACTTCAGCGGCGAAAACTTCGCTTTAGGGACAGTGGAGCACATGGTCACCACGTGTGCTCCTCGCTCCGAGTAGTCGATGACCAATGTAGGAACCACCCCGAGGCTGGCTTCTCCATCGCTCACGTAGCGATTCCTGGGAGGCGCGTGTTCGTGTCCTCGATCGGGATGTTATTAATGCGGCAGGCGCTCTCGATCTGGAACGGCCATTCGCGTAAGTCCTCTCCCCTCTTGCCGGTGAACTTGGGTAGCTTGGTCGGCACTCGCGTGCGGTCTGCCATAACTTGATTAAGCCGGTCGATCTCAACTTGCAGCTGGGCGTTCGTCGCCATGGGCTCCGTTTGTCAGGTCACTGGGACCAATAGGTGCTACCAGTATTTCAAAAGGAACCTGCTTTTAGGGAGGGCGCCAAAAAAAGGGAAACTGCCGCCTCCACAGTTTAAGACGGATGTAGAAGCTTCCACAGTCGCATGTGTTCAATTTAATAGCATCTTACTATGTCGTATTAGATCACAGTTATTTCTAGACTACATATAATACTAATCACTCACATTATTGATACAGTATCTGTGTCATGTGTGCAGCAGTGGTGTTACCTACGGCTCTCGTCGATACAGAGAGCCCAAAGTATGCACTTATTGACATTCGAAACGCTCTTTTTTGAAACGATCACTGCATTTGATTGTAAATTTCTTGGTAGTCAATTTAATTTTTGCGAAGACTAAAAAAGCGGTCTTATTGTCAGATGTCACAAACTTTCTTCTCTTCTCACAGATTTTGCGTGAATGGCGAAGATGACACTCAGCTCTGGCGAGGTGAGAAGAGCATTTACTGTCACCATTGACACCGTGGAAATCCAGTCCGTAGACAAGCTATGAGAAGAGATCAAGTCGCAAAGCGAAGGCATTTAGACTCCAGCATGTCTCTTGCAGCTGCTCTCGGTAAAGAAACCGAGGGTATAACAAAGTCGTGCTTCACCCGAAGAAGCTCTGAAAACGCGGATATTGCAAGTGCAACAGACTGGTCATATTTTAAATCATAATCTCAGATTTTACTCGATCCCGACGATGATGTTCGCTTGCTGATTCTACATTTAATGTAAGTACAGCATGAGGCCAAGTTGGTGAGGAGCCACATGCGAAGCAACCTTTTCGACCTTCGCTTGCTCGTTTTGGCCTACGGCGCTTATCGTCGGATAAGGGTACGTACAAGTAGCAGGTATCGTATTTTTATGGCCTTGCGCCCAAAACTACCCTTATGCCCGCATTTACCCACCATTCCGACAGGTATTTCTTCGTGTATTATTCGTGTAACATCCAATTTATAATAGGCGCCGAAGGATTCTGAAAACGCTGTGATAGAGAAATTAAAGCGCTATAATAGATGGGGTGTGGCCTAAAATTCCAGTCTGCGCCTCCACAGTTTCTGCCGCGGTCCTAAACGAAGTCCTCAAACACCAGTGCGTAGTAGGCGCGGTGTGGTGGATTGCTGAATCAAGCAAGCTTCCTCCATCTGTAGTGACCCTGGAGTACACGAGGCGGGCCAGGTACATCTTGGTCAGTGACGTTTCCTCACGTCATTTATATAACAGATTCTTTTAGCTAGCATCCAGTTGTCCTTGTGGATTTACTTTAGGCCTTGTATGTCCCCTAACTGCACCTCTATGTGCTTGAGAAAGAAGGGCAGCCTCAAGTTGTTCCTGACTATCACTCTACATTTCACCAGTAACCAGAATAGCAGTTGCACACTAGGCTATCCGTTCCGGCAGGTTCGCCACATGCCTTGGCGGTGTTTCCCCTGTTTCAACTCCGGGAACCAGTACACGCGGAATGTTAATGTGTTCAAGGCGCCCAAAAGTGAATTAAGCAAGTAATTAATAGCGCCCAGCGAAATGGCTGTTGCGTACATACAAGTATCAATGTCCGGCCCAGGCTTAAGCCAAGCATGTAGACTAAACGATCCAACGACTGCTTGGCAATGGCAGGATCCTCTAAATAATTAAGTTCTCTTAGACACGAGCCTTGACTAAATGCCCTCAGCGCGTGTGCCAAATCCATACCAGAATTCAATTTCTTCAAATTGCCAACTATTGACTAAATTCTCAGAATTTTATCCGTGAAAATATTTAAATCGTCCTAGAGTATTTCTCTGCACTTTGAAACTCAAAGGTTCTTAGCAGATGCTTTGATGCAGACTCATCTGAATTGCACATCAGCAGCACCATCACGACATTTTTGATGATGTTGGCATTTGAGCACTAATCAATGCCGTAATGATTACAAGCGAGGTCCGTAATCTGGCATCCTGATAGCGTTAAAATTCTGTGAATACAGTGGCTGATTCTATTTCCTTAGACAAAGGGCTCATCCAATTCAACATAAATTAAAATTCTCCGAGCTCTACTTGATTGCATTCAGAAAAAAGGCGTTTGGCAAATCTGCCTGATTCATTTATGTTTACGGTAAAACGACTTTGAAAGACCCCTGAGTCATGGCCCATGATACACAGCAATATCACAATTTCACTTTCCTGTAGCCCAACACTTATTTAAACTGTAAAGCCACTATGCGCTTCTGCTTCGTTCTGATCATTTTCCTAGCTGGAATTTGTAAGTGTCTCTTGCTCAATTGGAGTGGCAATACGTTAAAGGAACGAAGCCACACCAGCACCAATTCTCTCCCCAGTCCACCTTTCATCTCAACAATTAAGACCCCAAAAAGGTTCTTAAGATCCTACGACGCACCTAAGCAAGACAACATCGGTCATGATACGGACGAAAGAGCTGGAATTTCTGGGATAGCCATGATTGACGATCTTGCGTACAAGTGGGCGTTAAAGAATACGAGGGATCCAATGGATGCATTCCAGCGCTTACATGTTGTGAAAACTGGCGGCAAATTGGAAGGCAACAAGGAATTCATTCGGTGGCTCCAGTACGTAAATCGATACAAGGCGACACGACGAGTCAAGTTCGGTGAGGATGAGCTGCTCAGCCTGCTAATGAAACGAGAGCAGAAGAAGAACTCGTGTCCCTGTTCCAATCACTTCGACAATACCCGGACATTACAAAGATGGCTAACGATATGCAAGCGTCCATGATTTTGAGCTCTGCGTCTAGTCACAGACTGATCAATGAGGCATGGTTAATGTCCCGAGAAACTCCCGGCGAAGTTTTCAAAATCTTGCGACTTGGAGATAACAGCATCAGTCGGCTAGACAATAACCCCCTCTTTATTCAGTGGCTCAGATATGTTACGATGTACAGGGCTGTACACGGAGGTGACCCATTTGCGAATTTGGAAGCACTATTTAAGCGTTTCTCGACTGTACCACAATTTGGAACTCTCATTCAATCGTTGCAGAACATCCCAGATTTGGAGAAACTCGCACTAAGCTTACAGACCCACCTCTATCGGAAATGGATGATCGAGATCCAGCTTACCCCATCTGAGCTCTTGGGTCTTCTAGAGACAACCAGAGTCGCGAGAAGTGATTTTAAATACCGCAATTTGGAAGCTTACACCATGTACTTCGCTGAAAGCCGAGGTGGTACGCCTTTGTTGAATAAATTGAAAACGCTGTTCACGGATGGCGATCCCTACGCAGCACTGTCTGCCGCTTCGAGCGCCTAGCAAGGAACTTCACCCTTTCATTTAATACAACGTTTAAAAAGATACAGTCATTTTGCTTAAATTAATGATTCTTAGACGGAATTCGTCGGACATCCGTCAGTCATCGCTATTTTCATGAAGTTTGATAGACTACCTAGAGAACGTCATACAGTCCGTGTTGAGTTTGGCAAATGCTTGCCGTGTCTTGCTGGCATTATTCATTTTCACAAATGGTTCTTAAAAGTTGACACAACAGTGCTCGCACATTGAAAAGCTATGACAGACAACTCAATATACACGTACGAGACATCTAAGATATCAGTATGCTATTTGATATCATAGCTAAGCACACTCAATCTGATAGTTGCTCTTCATAGCGGTAATGTGCGCCGATGACAGCGCGGCGCGCTGACACTGCTCGCCACTGAGCCATTTAGGCCATGGAGGACGACTGCCGGAAGGCGTCCACAAGCTAAGGGAAAAACAGNTTTTTTTTTTCAGAAAATGTCAGACAGAATGTCCGACGCGAACCAATCAGATGGGTACGATGTCCGACAGATTTTGAGGAATGCTCATGAGAACCAGCCTGCTGAAATATAGGATAAATCACGCATATAGGAGATAATTTGGGTGAAGGAATTACAATTGAGTTGCCGTGTAGCCTTTTACATCCAGAATATGAATTTAAACGCATTTACTAAAAGAAGTTTATACCCCTACTATGAGCTGAAAAGCTGGCTCTGGCGGGGCGTCACTCGTCCGTTAAGTTGTATTATCAACCTCAACATGTGATGCAGGCTCAATGCAAGGCGACCCAATTGTCTACGTAGATTTGCGGCACGTGTATCGCGCAATCCTAAACACTTTCAGCGTAAGACGATTTATAGTTCTACACTCAGTCTTCTATTTTAAAAGAGGTCAGTTTACGAAGGGCGCGGATGGTATTCGAGTGCACGTTGAGGGGTTTTCGAGTGCAGACATTTTTGCCACGAAATGGCGGAATGCCGAGTGCAGATAACACATGGCGATATCGTCGGCTCTATACTTGTCTTGTCCGGTTTATGCTTGTTTGAATTTACTACACTGTCCTTGGGGCCGGATGCAGAGCTCACCAGGATGTAAGAGGAGAAAAATACATAAATTTGTGAGAAGAAAACATGAATGAGCCTTGGAACCTGGTTTACTTCCTACACAAGGTACGCTGAGCTCCGCTAGTCCAGTTCTACGCCCACATGCTCAATGCCAACTGTCACTCCTGATCGTCTTAGCTGTCCAAGTACTGATCGTCGTCGGCGTCACGGTCGACACTGCGGGGATGCGATTCGTGTGCGCTTGCGTGGGCGGCAGCACTCTTCGTAACATCGAGGCCAGTACGTCTTGATTCTACGATCAGCTGCACCAATTCCTGGCACTCTAGGAAGACTTGCTTGCCTCCCTCGAAGTCGACAAGCCATCTATATCGCGACTTGCGCGCTTTGCATCGTTCATGAACCGAGATGCGTTGGTACCGTCCTCCGGTCGATGGACGACTCGGGCTATGCTCTCCCGTGGAGCTCTCCACCATTGCCCTGAACACAATGAGAATTGCACACGGTAGGATAGATGAGTATGAAGTAGAAGCAACTTAGAGTATTACGGTGAACGTACGGCGAGCGTTGAATTTGCTCTTATCGTGTTGACGGAGAAGCGCTTGAACGAAAGGAACTCTTACACTGTGGGTTGGCGTAGCTGGCGAGCGAAGTTGGCGATGGAAGCACACTAACCCTAGCTCGTGGATCGCTTTGTAGACGAACGCCTGCTTATCGTTGCTGCAACATGTAGCTTACATTAGCTACGTCTAAACTAATGAAGGCAGAGCCTTGACGCATCTTCTTCCGTGCTCAAGCCTTAACCTCAGCAGTGCACTCGGTCATTCCCTCAAGTTGAACAAGGGACACTCGGTTTCACACCTTTCGAACAAAAAACTAACGGTGCACTCGATTTCCTAAAAAAAAAAGCACTCGAAAGTAATATCGTTCCGTTTACGAATGTTACCTCTTGGGAAGTATTAAGGAAATACTCATCACAAGACTTTTAAAATATAATAATGGACACCTTTTTGGAGAGGTGGAATATAAATTCTCTTCTGAATTGCACATCGACAGAACGGCAACGACACTTTTGTTGACGCTCATTTCTTAGCAAGTGAGTAATCGAAGTCCGTAATCTTCAAACTAAAAAACGTAATCAGTAATAAGAGTGGGATTTAAAGTAGCTCTTCACTATTGAGAAGAAATGTTCTTCAATGTCGTGCTGATCACATTCATCGTACGGATTTCGCTTTGCTCCTCGTTCAACTCATTGATAAGCACCAACCAGCTCAAGGGCCTGAGCCACACCAGGTCAACGTCCATCCCTGCAACGAAGAGATCGTATTCCACAACAAGGTTTCTAAGGTTCAGCGACGCATCTAAGCACGATGATATTGATGATAATAGCAAAGAGAGAGCAGGGGTTTCTGGGATAGCATGGCTTGGTGATCTGGCATCCAAGTGGGCGCTGAAGAACACGAGGAATCCGATGCAAATTTTCAAGCTTTTACGTACTGTGAAAACTGGCGGTAAGCTGGAGGGTGACAAGGAATTTGTTTGGTGGCTTCTGTACGTGAATCGATACAGAGCTAAGTTACAAGACAAGGCCTCGTTCAGTGACGACAAGCTATTTGATTTGGTGCGGAAACTGAATTCGGAAGAGTAACTGGTGTCTCTGTTTCAATCGCTTCGACATTATCCGGACATCAAGAATATCGCCGATGATATGCAGGCGTACCTGATCTTGAGCTCGGCGTCTAGTCACTGATGAATAATGAGGCATGGTTAAAATTCCGAGAAACCCCCGAAGAAGTTTTCAATATCTTGCGGCTTGAGGATGAACCTCTGTACGCTCTCGACGGTAATCCCCTGTTTATTCAGTGGCTCAGATACATTAAAGCATACAGAGCTGTGAATGGAGGCCACTCGTTCACAGACGTGCATGTGTTCGACTTTTTACATGAGTTTGCTTCTTTGCCGCGATTTGGAATATTTCTTCAGTCGTTAAAGGACATCCCAGATTTGGAGAAACTCGCAAAAAAGTTTACAAACGCAGGGTTGAGGTCGATTGGCTCACCCCATCGCAGCTCGAAAAAATATTTGGCTCACCGTATCCAATCAACTTCGCGGAACTCCCAAAGAGTGATGCCAGGTACCGCAATTTGGAAAGCTTCACGGTGTATTTCGGTGAGTACTGGGAAGGAACAGCATTATCGGATAAAGTGACAATATTGTTCGCCAAGAACGACCTATACGCTGCAATTCGAGCCGCTTCAAAAGGTTAAAGATTCCTGACTCTAGCTGGCCGAAAGTGTTAGGGATTAATAACCCATCGCAAGATTAGCGAAGTTTTCTATTGCAGTCCAACAATTGCTACGATACTTACTTCGGCGTGCGCAGCCATCTGGAGAGCCAAGTGTGAGGTTATCTAAGATATTTAACATTGGTAGGAACCCTTTTTACAGCACCAAAAAAATGAACCGCACTATTGTTTTTCTTCTGATGAAAAACATGTATCTTCGTTCCATTTTTCATTGCGTCTGTATTACACCGGATTTTAAATGGCTTCACAAGAAGCTTACAGACTTTGTGAGCTTATTGCCTAAATCCGGGATATCCTGTATCAATTGAATGGATACATGTAACCCAAAGCACGGCAAGGTCACAAAAAGCTCGTATGATGGAGTGTTGTCCGAGGAAAATTAAACCCCGTCCCGACACGAAAACACCGCAAAAAAACAGCTCTTTGCAAACGCAATTACTTTAGATATTATCAAAGTGATCCAGCATTAAAAAATGAAGTACAATTATTGCAACTGTGCAGTTGTTTACCGAATGACTGTGGCGCTTGAATTATTATTCACTTTCAGTTTGGCCAGAAATCCTTAACACCACGCTTCAATTTCCATGCTGACCATCTTCCTTTCGGGAGATGTTTCTGTTTCTCGTTTAGCTTGACAGCTCACGGACCCGGGTCACAACGGAATAGGAACAGGTATGTCGATCGACTTCTAGTAAATCTAGCTGCATGCATTACTAAAAAGCTGTCGAACGGCTGCAATTGCAACGTCAAGATCTCTGTTGTGGAACAATGATTTTACTTTATCCTGCAAAGCATTTCCACTATGTTGCTCTGCGACGTACATGGTGTAAGCTTCCACATTATCTGGAATCATTCGTCGACAGCTCGCGACATTCATGTGGCAGATTGACTGCCGTTTTCAAAATAGAAAATAGCTTCTCAGAAGTTCTCACGGCGCTTGCGTCCAGCATAACCAGCGACAATATCTTCAACGATGGCTGCGCATGACCCACGGTGAAATTGTTGGGCGCAAAAGCATCGTCGGGTACACGTAATTCCGTCAAATACCTTTTTTATACTTCAAGTAATTATGGAAACTTCTTTATCCCACACCATACAAACGTCAAGTAAAACTCAACCTCTGGCGTCATTATCAGTCTGACCATGGAGAAGGATCTTCGCGACGCATATGCAGCACTCAAGCTGCTTCGGGCAGACCTTGTGAAGACCCGCCGCGATAAACGTGCTCTAGAGGCCTCACTGACCCACCTGCAGACCCACGGGCCGCCTCCCAGCGCTGCACAAGCTCGCGAGAGTCGCGAGACGCAACAGATGCAACACGAGGACGCCAATGCGCAATTATGGCGTCTCGCTGCAATTTACGAAAGTCGATTGGCGGAAATGGAAATACAATTATTGCACAATAATTGTCAAAAGAAGGAGGCAACACCCGAAGTTGAAGACAATCAACAAGAAGTCGAGGAAGTGGAGAAGCTCGCGCTGCTTCACAAGCTCCACAGTCTGACCGCCACCGTCGAGCAGCAGACGCAGACGATGCTGGCGCAACAGGCAGCTTTTGCGCTGCAGAAGGGCGAGCTGGAGACGACGCTGGAGGACACGCAGCACCAGCTCCAGACAGAGAAGAGCAGGGCGACTGATGCGCTATTAGAACAACAAGCAGCTAAAGAGAGGTATGAGTTCTTGGAGACTCAAGTGGAGATCTTGAAGCAGGACAAGAAGACGTTGGAGGAGGAGAACTATACACTCCACAAGAACCTAGCGACACATGCCCAGACGAGTAGAATATTGCAACAACAAGTGCAGGAGAAGGACGAGGAGCTAACTCTGCACGAGAAAACTATTCAAGAACAAGAAGAGAGACAAGAACGATACATAACGATGCTGAGAGAGTTGGAGAACACGTGTGAGACGTTTAAGACCCACGATGCGGCTTCTGAGGCCAAACGAAGTGCTGAAACGAAGGAGTATGAAGCGAAACTAGCAGGTATTCAAGAAACGTACACATTCCAGGTGGCAAAGCTGGAAAGTGAGCTGAGAACGATTAAAAAACAACTGACAGCAGCGACAACTAGCCGAGCGATCGAATTGAAAGAGCACGACGTCAAGTTTCAAGCGGTTTCGGACAAATTGAGGCAACAGGAGCAACAGGCGACGACACTGCGAGACTTGGAGACTAATTTCGCGGACGTGCAGGCCAAACTCACTATAGCGGAGACCAAATTGGCTGATGGAGTGAAGCAATACGAGCAACAACTTGCTCAGGCCTCGCAGAAGCTACTTGATCAAGAACAAGACCACGAACAGCACGTTAGTTCTCTACGTGACGTGGAAAACGAGCTGGCACGAGTTCAGACGCAACTCACGGATACGGAGGCCAAGTTACAGCTAAACGTGACCATATTCGACGAAAAACTCGCGCAGACTTGCCAACTTGCTGCTGAACATGAAAAGACAGCTCGAGTGCTAGCTATGGAGAAGAAGGCGCTTCAACAAGACGTTCGTGAAGCTCGACAGACATCGAAAGTCAAGACCGAAGAGCTTCTTCATTTCCAGTCGATGATGAAGCAGAAAACTGGGGACCATTCGCAGCGTTTAAGCCAGTATCAAGCGCGTTGTGAGCGTCTAGAGACTCAACTATTATCATCTGAAGACCACAAGGTGTACGAAAGCAATCCAAGCGGTGAGTTGAGAGCTTTCCATGGTCTTAACGACGCCGAAATCATCGCGCTGCCCTCTTCCAAGGCGTGGGTTATATTACACTCTGCAATAACCAAGCTGGAAGACTTCTTCCCATATCTTGAAGCACTGAGTAGTGCATTACAAGACGTGTTAGCCTTGTGCAAGAGCCATGCGACTTTTCTTCCAACGCTATGTGAGCGTCCTCGAAGACAAGACAGTTAGCGACAAGACCCAGCCGGTCTTGGTTATGGCGTTAAAGCTGGTACGCTTTGCTGTCGTGTTGAAGACTCAGGTCCAGCAAGACGACGCTGTTGTGACTTTAAAAGCAGTTCAAGGTTTCCGTAAACGCGTGCTGGATGCTCTCGCGCAATGGTACGAGTGTGGCGTGGATGCTTGCGACCAAAGTGGAAATGGGTCCATGCCCACACCCACTTTTACTACCACTTCTCGAGAGACAGCACTCATTTTACAGAATTGGACCAGTGACCGGACAAAGCAGTTGGGAGTGAGACGCTGGTTGGCACGGATGGAAGCGTATCCTGGCGTCCCGCCACTTCGAGGAGCGTCGTCAAACCGTGTTCTGGAACTCCCTGCGGAGGGTTGTACACTGGAGCTGGAAGATATGACGCCGGAGGTGAAAGATGCGTTTTCTGTTGCTCCTGATACCGATCTTGAAGCAGAACCGAGCGCTACATGTGCGTGTGTTTACACGTTATACTGAAACCGCGGAAGCACATCAAGTTGTGATGGTGGTACTGGGAAAGTGTGGGCCATGCGGATCCATGTCCAGAGTGCTGTCACTCTACAAACCAGGAAGTCTCGCCCCAACTTCTTTCAAACTGACGAGCACTGATGACTCACCCAGGCCACTAAGCACCCAACTCTCCAGCGTCGTCAGTGTCATCATCCACTTCCAGTTCGGCAAGCTCGAGACTGCAGATTATTCAAGAACGCTTGCAGTATTTGCACAATAAAGCATAAGATAGCTGGAATAGCATAGGAATACACAAGAATGTTTGGAGTAGCACTCATATGCTTAAAAAATAAGATCTGATACCTGCATCAGCTTCTTAA

>Contig_7

TGAGGAGAAATGACGACAAGACGAAGAACGAGTCAACCGCCACGGCGCCCAGGTTTAAATCCTGCATGTACTCGTGCGAGTGTTGCGTGACGACTAATAGCGCGGCTAGACCACGTATACCATCCAGGAATAAAACCTTGGTGGGTGGAGCGGCCGCCACTGCCGCCTTCTTGGCCTTCACGGGCTTCTTCTCCTTGTCGTNTGTCCTGCTCGTCCTGAGCCAGTAGCGGTTGGTCCTCCGGCGCAGTCTGCACGTTCACAACCGTATCGGCTTCCGTAGTGATAGTGGAGGTGTCATGCTCCGATGGTGCGTGCGTGCGCAGCATTGTCGGCAATGAGGTTCACGAAGAGTTTTCCACCGCTAGACAATTGCCAAGTGGGGCACGTTGCTTTGCTGTTCGGCATGAGGTGGCTTCAGTAAACGGCTATATCGGGATTTGGCGTGTTGTGGCTGGCCCCTTACAAAAACCAAGTGATGTTGTCTTTACCACGGCGTGTGTTTTCGGAAGTATAACCTGTGCAAAGTGATTGCGGTAGTGGAGAGCTTCAACTAAAGCCGCATGTTTGACGTATCTTATGTATACATTTACTTCGGAACTCGATTGTGTATATGTACTACAAAGTGATAGCGGAAATTGCTAAAACCAGACGGTGTTTTCTCGCTGCGTGCGTTAAGATCCAAGCTCGCGGTACTGCATCTGCTGCATCTGCTGCAGAGCTATGGCTGCATCGAGCAAATGCGAGAGCACAATGTTGCATCCTTCATCACTTAAGCTGGACATTTTAATTTGGTTTCAGTATGCGTGCGACCGTTGGACTGAGATCTTTCCAAGCTGGCTGCAAGACGCACGCAACACTTCAGCATTGACCTCTTTCCCTCCCCCGATGCCACCATCAGGATGTCGATTGGAGCCCTAGTACAGTAGTGGTTCACTACGCATGATCGCAATTTAACTCTCACTTGAGGGTTGCCATCTACTGTAGTGCCGTTCACGACTCGTGATTCACGATTGACAATTAGTCGAATCTGGACACGCCATTCGTGATGTGTCAGGATGTCAGGTACCGGCTCAAGGTATGTAGAGGCGTCGACAGCTTTCTCGTTATTGCTGACAAAGATCGACAACTATACTACGTTTACAACACACTTTCACTTATGCTTTCGACTCGCGTGGCTTACTCTTCGCCAACGTTGTCGACCTGCTTCGTATCTTGTGCGCCACGCTTGGATGACAAGTAGTAACCCCACCGGAACCTTTCTTTTCCTGCTCCTCAAGCCCCTTAGTGGTTTGTTGCGACTGCAATTGCAACGGATACTCGACCACATGATCGAATATGGTAGACAGCAGTAGAACCAACGCAAGGCGAGAAAACACCTTATCGTTCGGCTGAGAACCGACCTCGTGTGCAAACAACACGCCGAAAAGCTGACCTTCCCCCAATAACATAGTACATCCCACTCGAAGATCCTTGACACACAGGACGGTACAATCATCTCGCAAAAATCATCGCCAACGGCAATCAAAAAGCTATTTATTGGTGCCGGGTTTTCATGCACCCAATGAAAAAACAAGCTGTGGAAGATTAAACTGAGAAACACTGCGACCGTAGTGTACTCGACGATGCAGATCACGACTACGTGCCCCTTTCGGAACTCAGAGGCGCTAGTTTTAACCCACGAGTCGATTTATATGAAAATGACCGCATACAGTGACTCGGCCAGAAATGTCGGGAAATGTGGACGCAACTCCATGTGGCTCGACCAAAATTGTACCAGCCTTCGTAGATAACCCACCAACACGCGGGGAAGAACGGTAGGAACCAGAAACGACGCAATGCGAGGATACCGATTACAAGCAAAGGGATGAAGAAATAGTACGCGACCTCGAGAGGTAGCGTCCAAAATACAAAGTAGCGGTAGTCGGCTTCAAGCAGCAGTACCTTGTACACAACGAAATCTTCTGGTTTCTGAGCTACATAGTAGAGCTTCTTTGCTTCAGCAGGAAATTACCGATCACAAAACTTGTCAACGCAAAAAGTGGGTGCACTCGACAGAAACGCTTGGAGAAATAGTCGTTCAGAGCGAATACCCATTTGCGGATACTCACCTTGTGTCAGTAAGCGAATGCTCTTTTTCATAATGAGCGAAGTCAATAGGAAGGGCGATACCACGAAGAACGTGTCGACTACGATGGCTCCAAGGTCGATGTCTGCCATGTATTCCTTCGAGTGTGACGTGCATACTATCAATGCTGTGAGGCCACGAACACCATCCAGGAACAGGATTTTGTGGGACGCTGGTCGATCTGGACTTTGCTGTGCTGTTGTGTGCCTCTTCATTCTTGTGCTGCTCGGACGGTGATTCCATGATGGACAAAGCGATGTCCGGAGATGAGAAAGCTTTGCATTGCGCAGCATCGCGTCTGAAGTAGATGCAAAAGTAGCTGAGCGGTAGCCTCTCTTCGAGTCGATAGATTCTCGACCTCGATTCGAGGCTCGAAGGTGTCCGTGGTTGCCATTTGGACCAACATGTCCGCTGACTTGATTCCCCGGCGACGGAAGGATTGGACAAAAAAACTGTTCACTATGGAGGTATCATAGTACTGTTGACTTTGTATACTAGCTTAGATGAGGTATCTATCACCTGGCCCACCTATGGTACTTCTACCATTTTCCTCGTTCACAATGAAGCACTGAGAATAGACGATAAGTATGCAACTAACACGCATCAATAATCTTATTATGGGCACAAAGCTACACCGAAAATTATAGATTTAGAACTCACGCTTGACTGTATTTCAAGGGGCTAAACTTGCAGATGAAGAGATATGGAAATAGTACCATTAATGGTGTGTTCTATCCTAAGAGGAGGACGAAGAGTTATTATTCAACCCATAGCTAACGGATTCTACTGACGCAAGAACACGTCTGTGCGGGTCCAAAATTGGGAAGCACGAGGAATATCTCGATACAGAGGAGGAGTTGAATCTCGCTCTCTTATTTCCTTCACATCGCAATTTACCAGCATCCTGCAAGCTCCCCCCCCNTCCCCCCAACTTTCAGAGCTCCACGTGTCTCAAACACCTGAAACATGACGAAATCTACTTCGCGACAGAAGACCAACAACGCCAACACTAGCGCTGGAAAGAACGCGACCACCGCCCCCACAACACCTGCGAACCTGGCCAGCACTAAAATTACCAACAAAAACACGAACGTCACCCAGCATGCCAACAAGAACACGGGCAAAATGTTCGCACTCACTGAGGTGACTACGGCAAGCGTGCAACAAGCTCGCCAGATCCGCAAGACGGACTTCGAGCGCTTCCAGAAATTCCACAACTTCTTCATGGCTAATGAAGAACCGACTGCACGTGTGATAAACGACAAGGCTAACGCCAAGCGTGAGATAGTGGACAAGTGGATTGGTGGCGCTGTGAGCCCGAAGACGCTCGCGTGTATCCAGGAGATCCAGGAGACTCAATCGGGACTTCTGGAACTGCTCATCGTGTCAGCAGCACTCACCAAATACGGCGAGAAGGAGCTGGCTCGTAGAGACAGTCTAGGCGAGATGGACTACGTGGTAGGTGCTCGCTTCGGCACGAATGGGCTGCGCTCGAAGCAGCACGCACCACACAGTGTAGGCTTGAATTTCGTGCTGCGGTTGGTGCTCGGTCTGGCACCGGAGAAGCGTAGCTCGTGTGAAGTCTTACGTGAGATCCTGGGTCACGAGAGCAACGGACGCCTTGCATTAATCCCAACAGAAAGTGGTCTGCAGCCGTATTCTGATGCTACTGCTTCATACTCGGATGACTGGGCCGAGCTTAAGCCAGATCAGCTCCGTCAGATGGCAAGTCAGACGCAGTCCGACAAGTTCCAGGACACCATGCAGCAGCTCAGTGGTGAGCACCTGTACGATGCGATCCGTACACAGCTGAAGCAGACTGGCGGTGCGTCAGTGTGGGATGCTACTAAGGACAAGTCTGCACTCAAGGTGAAGAACATCCGTCCGCGCATGGGGTCGGCTGACAAGAATGCAAAGTCGAAAAAGGAGGCCGATCGTCAGCAGAAGCAGCCTGTGAAGATAAATGAAGCTTCCCAAGTGAAGAAACATCTTCCGGTTGAGGTTGAGAAGGTGACGCTGGTTCCTCCACCGGCCCCGGTGATCATGAAATCGACTACCGATGCAGCTACCAAGAAAGAGGGTGGAAAGAAGAAGGGGAAGAAGGGGAAGAAAGCGGACGCGGAGAAGAATGTCGCCCCTGTTAAGGACGCCACTGCGGTGAAGAACATTGCGACTACCAGCCAGAAAGCTGGTGCCATCAAGAGCAGCGCGCGTGACGGTCGGGCGGTTGTCTCCAAGGCCCTCTAGACGTGCATATTTGGTGATGCACTCATCTTTCATCTCAAGAGATTTATTAATAAACTTTTTTTAAATATAATTTGTTTTCTCGTAACGAGACCTGCACAGTATCGTACCGTATTGTAAAACCCGTGAAATCGAAAGAAAGAATCAGTGCACATTTCTCTCAGGTTTCACGAATACACTTTCATTCTTGAATTTGCATCCAGATGCATCTCTTCTCTCTGACAGCCGTAGCTTTCGTCATCGCCAGTTTATCTGTCGACGCATCAGTCGCGAAAGATCCACGAGGGCACGCTCCCAACAGGACTGAAGTCGATACCGTAAATGCGAGTTCAAGCACGAGGCTTTTGCGAAAAAATAGTACTGTTGATCTAGTCGGCGAGGAGAGAGCACCCAGCGTCGTAGAAAATATCAAGGCGTTGGTCAAGTCTTCAGCGGTGACTCCAGCGAAGCTTCAGCAATGGCTAGACGAGCGACTACCTGCGGGGCTAGTGTTCAAGAACATGAACCTTGACGAACCAAAGATCTTCTCTTTGTTGCATGAACCCAACTTTGTTAAGTGGGTCCAGTACGCCGACGACTTGAGTGCCAAGTCATCTCATAAAGAATCGTCAGTGATCTCCACCCTGACGTCATTGCACGGCGACAAAGTTGTCTACGACACAATTCAAGCTGCTAAAAAGTATCCACAGCTGAGTGAACTCGCCCTTAAATTGGAAAAGGACCAGATACGCTTCTGGATTGCCACTCGAAAAGACCCGTCGGTGTTTTTTGAAGCCCTCAACCTTAACTGGGTAGGGACATCCATCTTCTCAAAACCTGAATTCTCCGCTTGGCTCAAGTACGTGGACGATGTAAACGCAAGACATCCCAAGAAAACCCCATTTTCGATTATTCCTACGCTCAAGCAACATGTAGCTCAAAGTGACGAAGCCGACACAGACGTACTTCTTAAACTGATTGCGAACGGGAAAGCAACGGCTGAAACCAAAACTGTCGCCAACAAGGTAGATAGTGCATTGTTTGACTTCTGGCTCAGCAAGCGAGAAACACCCGACAAAGTTATGGACGCGTTCAAACATGGCAGTACAGCTCAAGCTTTCTTGGGGAGTTCACGGTGGAAAGAGTGGGAACGGTACTTGAGCGTTTACAACGCGAGATACCCTGAAAAGAAGACCACCGTGATAGAAACGTTAACGCGGAAGTACGGAGATGCACAATTACTCGACACGCTTATCACCGCGAGCTCGAAAGGTGAGACGAAAACGCTTGCAGCCAAGCTGCAGGCACAGCAGTTCGATAGGTGGATGAGCCTTAAAGAGTCTCCTCTCGACGTCTACAACAGGCTACGGCCTTCATATGGGGATAGGAGCTTCTTCGACGAGCCACAACTCAATGTGTGGGTCTCGTACATGAATGTGTTCGTCGACAAGAACCCCAGCAAGGTGGACAAAATGTTCTTGGAGTTAGGTGATACCTTTGGGAACATGCATCTCTTTCGAGTCCTCGGAGAAGCCAAAAAGTTCCCCAACATGGAAAGCACTACAGCCAAGCTGCAGATGGAGAAGGCTTCGACTCTTTTTGCCAGCGGAAAATCCCCGGAGGATATATTCAGGGTGCTAGCGCTTGACAAGGTCGGAAATGATATACTCAGCAACACGCTGTTCCACAAGTGGCTGGCATATCTGCAGAAATTCAACAAAGAGCACGAACAGCCAAGGATCGTGGTTTGACATGCTACGTATTATTTACCAAATGTTCGGTCTCGAAAGGATTATCGAGACGGGAAGAAAAAATCCAATCACAAGAGGGATGGCTGAAAAAGTGGAGGATGCGTATCACAACTACTGGTTGGATATTAAGATGGAGCCTAAGACAGCCTTCCGCTCCCTGCATCTCGACGAAAGCGGTGAGAAGCTCCTTGCCGACCCAAAATTCAACACGTGGGTGCAGTACCTGAAAACCTTCATCGACCGATATCCTAATGAGAAGACGACAGTCATCGACGGGCTCAGGGATAACTATCATGACATAGCTCTACTCCGAATGTTTTCAGCCGCGAAGAATGATCCCAGCACGGAGAAACTCGCTACTGATCTACAGAGTGCGCTAATCCTCAAGTGGCAAGATGCGAAGAAGACACCAGAAGAACTAAAGAGAGTGTTTGTTGGTGTGCCAACCTCTGGTGAAATAATCGATCGGTACGACAAGCTAATATCGGCGACGAGAGCCACCTTATAGTGTATTTGACCGAAGCGGTTATCGTGGAGAGCGAGAAGGAATCTAATGTAGCCCTTGATTTTGACTTTTTGGTAAGCTTTGAGTCGCATGAGCAGAACTGGTTCCTTCTGACTCGACGATACTACAGGAGGTGGCAGTGGTTACCGATCTCCATCTTCGATCGACGTGCCCTCCAGCCACTCGAAATATACAAGCAGAAGCGGCTATCAATTGTGATTTTCTCTGGGTGGAACCTCGTAGAAGTGCGTCCTTTAATTCTTGGGGGGGGCATAAATGCCAAGTTGTACCTGTGTTTTCATAATGGACCTTGCATATACAGTCTCCGTGTACGATTACGCGCCGTAAGCTACCAACTTCACGTAATATTTTACACTACGGCTGTGGGGGCATTCTTGATCGTCGAGCAACTCACGATAACAATTCCACGAAAGCTGGCAATTAATATCCTATGTATCATCCTTGTATGAACAATAACAGTTAGCTTTCAGAGCCATCAAACGTCAAATACTGTACGCAGGGCTATTCTGTGATGACATAAAGCTAAATCTTAATTTGAATAAATTTACATTGTGAAGTCCTCGTTAATATTCGTTTTGAGCTGCTGCCATCATATGCCGCGTATATTACCGATATAATCGAGTTACGTCTGTTGGTGCTGTAGTCGAGGCCATATTCGTACTGTGCCGCCAGTCGTGTGACTAGCAGCAGGGCTGCTCAAAATTAATCAATAGACCAAACATTACTATTTTGAAACTAATAGCACTCATAATTGTGGGTTTGCTACAATAAAAACTTTCCCTAATTGTTTTGGATTGAATTGGGATTTGAAGGGCGCTTAAAGTCGTCGTTCGTACAATCAAAAAGTGAATGTTGCTTTATTTTTTTAAATGTCAATCGTATTATACTTCATTTGCTGCTCTCTAACGAAAAAAGACATCATGTGCATATAAATTACATTCGCAATGGACTGACAGCGTTTATCTGATATTTGGCGAGCTATGAACGTGCGCAACTACAAGTAGCTGTACTAGATTACATTTCTTACACGTAAAAAAGTACCACCTTCTGCCATTAGCTGTGTAGCTTATTTTGTATTTGCCGACGTTTATAGACGCTAAAAATGGCAACCCGTTCTATAAATCGATGTACCAGCATGCGGTGCCTGATAAATTCACTCCTACTTGATTGTACAAAAGACTATACGAGCGAGGTTGGTGAAATTCCCACTCGATGATAAATTTAATTGTAGATATGCTTGATTTTCTGCTATTTGTTTAGACCTCTTGGTAAAAAAAATAAACGTGGTACATGTATTAATAATGTCACGGCCCATGATAGCCAATAGGTCGCTCATACATGTACCAAGAAAACGTACAGACTCTTAAGAGTCAGGAAGTGTTTCTGTGCATTTTTTGTAGATATGTCTGGTCAGGGCTAGGTTTAAAGAGGCAAGGATTTGGGAAGGTGAATTCGATCTTTTAAACTTAAGTTTTAATCCTGCATTTTCAGGCTAGAGGCAGGTGTAATTAGGCGCAAGTCGAAGCTAGAGTAGACCTACCATGCGTTTGTACGTATAGCTGGAATCACAAGACTCTTGGCGTTTATCGGGATTATAACTACCGTGACACCCATCCCATCGGGGACGGGAAGCAGTACGGTGTTTTTTTTTCACTCATTGACGTGACCTGGTCCTGGCCTGGCCCCGGACGATGATGTATATGAGCAAAAAAGAAGAAACAATTTATCCCAATCAACCGTAATCAATCATATGTTCAGTACGGGTACCCGAAGAAGTCCAGAATTAAGTTAAAAGGTTTTTAAATTAAAACAAGCACTTTTCAATCTGTCTTGATATCAAAGTTAAACGATTAATAAAACAAATGCTCTTATTGTAAAAAAGCTCAGTCAAATCGCTACTAGCAGAGTAGACAGCATCGTTGTCGTGCTTGGGCTGCCACATATTAACGTCTTTTCGTATATTCCGTTCTGTTTGTCCTTGGCGCTTTGAAGCCCCTCCTGTGCCTGCTTTGCAGCCGCAGCATCATCCTACTGCTTACATGGAAAAGTATACCCCTCATAGTATGAGGGGTATACTTTTTTGCGAGCAAGAGCTACATCCATCAAACATAACACCGAAACACTCGCCCATCTCCTCGGCGATGGTGTTGCCGACACGTTCAGCGACTCGTTGCATGTAGGTCTTCAGCGTCTCCGCTGAGGTAGGCTTCATGYGAACTAACTGTCTCGTTAGTGGGTTCTCAACCTCGCACAGAGGGAGATTCCGCTCGACAATCCAGCGTAGCCAGTCGTACATGTGAGTCGTCGTTTCGTCCACGAATCCAAAAACCTCGAGGGAGGTCAGGTTTCGGCGCTGAAACTCAGCATACTCTTCGCCGTGAGTGGGATGCTTCGAGTTCAGGTGAGAGATCAGGTTTGTGTAGCCTGGGCGCTGGGCTTGCTTGCGTGGCTGTTCGCAGATGTTGCACACCGGTAGAGCCCAGGTTCGAGAACGGTGTAGAAGAAAGCGGCGAGATCGGAAGAGCGTCGTGTAGGGAAAGAGTGTAGCCTCCGGTATCTCGGTGGTCGCCGTATCATTAAGAAGGATGGATCCAGCAAAATATCCACGTCACACCAACCGTCCTGGATTGCTAACACGAGGATCACAAGGACTCTCTTCCAAGCTCGGCCCGCATGGCTACGCCGGTTACCACGCTGCTTGTTGAAGTTGGACAACCAGGTCGAACCCTGCAATTGATTCCGAGAGATGGGGAACCGGTGAGTGCCCTCCCATATAGCAACCCGGTGCTTGGAGGCGAAATCCCGGAACGCCCGAACGAATACCCCGAGTCGCCCACGCACATCACCCCTGAAGCTAACCGGATCCACCGGTGTGGTGACCAGTGTATCCCACGGGGCAGCACTTGTCAGTTCCTCACGAGGGGTCTGGTGATGAGCGAATCCACGTACCCGGCCCTCGGGATGGCGTCCGCTGTCCGAGGGAATATCCACTCAGCCCGAAAAGGCAACTCCGGAAACAGAACCGTGATCTTCATGGAGATGATCGCAACTCGGTTCAATTTCTTGGCTGACCAAGGCTCGATGTCTCTGGCAGCGAAGGCCACAGGAGCACGTCTACCAGGAATGAAAGCAGCATCCGGACCGAACGGA

>Contig_14

GCCGTGCGAGTCACGGCATGCCCAATACTAAGTCAAACTTTTCGCCCAGCTCTAGGACGATAATATTTTCCACAAACACTCGGTGTTTGTACGAGAAGCGCGCGCGCTTTACGCGCTTCTCCGTTCTTACGGTGGCACCTGCTGCCAACCGCACTTCCAACACATTTCGAGGTGTTTCAGCTCCTCGTAGTTTAGCTTCGGGAGACTTTCCAGTCGAGCAAAATTGCTCGACGCACCTGAGTCAACCAAAACACGAAGCACTGATCTCGGGGTTGCTTCAGCTGGTTATTGCGCACTTTATCTTGGACGTGTTGATGTCGATGTCAAAATCAAATGAGGACTGATGTGCAGCTCGGTTACACTACGGCTTTGGCGCTCAAGTTTTTCAAAAGAAAAGAATGTCGTTCCACAACATCTTTTTAAACGAACGACAGCGCTGCACTAAGATGATTATCGTCCGAATTGTTTTACTACCAGTAGAAGTTTTAGTACTACTCGAAGACGACGTAAGTTACTGAAGTACTCAGCACATAAATTGAAGAAGATTTTTAAACTACATATTACTATTGTCGTTTGTAGAAATATAATACTGCTGGATTTTACCATTTGCTGCCTTACTGACAATCCCTGCCCACGGCACAATACAGTAGAGCATTCTGTCAACGTTCCGTGAACCCGATTGGGTAGTAAGATCTGCAACCTGCTAATCTACCAATGCCACGATCATACAGTACATGTATCACCGAAATCGCCCTACATGTACAGTACGCATTTGTTTATTGTATTTTTCCTCATAAATAACTCCTATACCAAACTTGCCGTTACCCCGATATATTCTAGTACAGTACTGCACCCCGTTTTCGCGCCCATGGGCCTATGATAAACTTCCGGATGTGATGATCCTGAATCCAAATGAACGAAATATTAGAGCTCATTCGTACATACAGAGTATGTGTAACAGTACCATTCAAGAATTCTGGAACGCTGTAGTACAATTCTGGAATGGAGTTTACACATGTGGATAGGAAACATCTGATGATTTGATCGCTATATATTTTCATAGTTTGCATGCGAAGAGATAGTAGCCATAATTCGCCCTATCTGTTTGCTGAGATAATGCAAGATGTAAAATGCAGTTCCTCGGTCATTTCCGAATTTTCTATCTACATATAAAGCCACTATGGTACGCTCCCAAGACCGCCAAGACCGCGCCGCTGAGACGCTCAACGAGGCCGACTTTCGTGCCTCCACTGCCGAGTTATTTCAGCGGGAGGAACGTCGCAAGGCAGAGGTCGCTCAAATCGTCGTACATAGCCGCAGAGACGACAATGACGAAGGGGACAGCTCCGGCGTGGGAGCTTTGCCGTCCGTCTTTGACTTCTGCTTACAAGCTGAAGGGCCCGATGGGGTGCTCAAGCTCACCAACTTTGCGCCAGAGGAGCTCGACCATGTTTGGGCAGCTGTGTACCCTCATTTGCAGGGGCAATGGAACGTGGGCCGCGGTAAAAAATGCCGCTACGCTGCGCGCGACGTTTTCTTTATGACACTGAGTTCGCTCAAACATCTTGGAAAAAGGGACACCGTTGCTCGAGTTTTCAGAATCCCGCCATCCACCTTTCAGAAAATGATCCGCAAGTTTATGGATATGCTGTCTCCTATTCTCTACGAAATGTACGTAGAGAAAGCGAATGATCAGTGGACACTGGGGAAGATTGTACGATCAGGGCATGCATTTAAGGACTTTCCGTATGCTCGATATGCTACGGACGTTACCTTTCAGCATGCTAACAAACCCAGCGGTAACATGAGCGAGATCTTACGCTATTACAGTGGCAAACACCACTTGAATGGCTACAAAATGGAGGTGTCGGTGCTGCCGAACGGTGTCGCGATTAATTGCATGGAGCACACCGGTGGGAGCACGCATGACGCTGAGATTTTTCGCAGGAGCGCAGCATTTCATTCGCGAGCTCTTCACAAACACTCTAGTGATGCCAATGTGCGAGACGAAGGAAGACTACAAGACAAGTATCCGAAAGAATGGGCGTTTGCTAAAATCTGATTTAATTTGCAAATCCTATATGGTCTACCCCCGCGAAAACGAGGTGCAGTACTGTACTGATCATCGAGTTCCTATCTTGAAACAAAATGTGCTAAATCGGACCCAATTACCGTAGCCGCTGTCAGTCGGTCCCCGAGATAAAACAGCAAATGGAGCGGTAATTTTTTAAGAAATAAGATAAACCTTTTAAAAATAATTATCGCCCGCAGTGATCATACATCCCATCATTTTTCGGTATCTTAGCCTCGTTTTCGTCTCAGAAGCGAACCGTACCACATCGAATAGATGGTTGCCACAGCGTCACTTCGTCATTCTCTCTCTGAGCCAAGCGAAGCTCGGAGTGAGCTCCCCAGGTGCCGCCATGCGAGTCCCCAGGGCCATACTACTGACAGTTGTCTTGATGGCAATATCCGATACTGTCTCCTCAGCTGCGAAGTCCCACTTGACCACACCATGTCTAACACGGCACGATACGAAGAGGTTTCTGAGGGCTCACAATACCGAGGATAGAGGGATCAGTACCCCCAATGTCGAGATGCTGCAGGGGTGGCTCAAGAAAGGTCTGCTCTCCGACGAAGCCGTTGGCCTGTTATCACTCGGTCACAAGGCCGACGATTTACTTAGCGGTTCGCTATTGAGCGCTTGGGTCAGCTACATAAAAGTCTTCAATAAAGAACACCCTACAGAGAAGATGAAGACGATCTCGGCGCTCACCGCTCGCTTCGGAGACGAAGCTCTGTCCACGATGATTGAAACAGCTAAAAGGGTCCCGAAGACGGAGGACGTCGCTACTAAAATGCAAGCCAAGCAGATCCAGAACTGGATGACGCTTGGTAAAACCCCGGACGACGTTTTTACGCTGCTGAAACTCAATACCGCCAAGTCGCTTTTATTCGATCAGCCTCCAGTCAACACTTGGCTACAGTATATGGACGATTTCAGCAAGGCTAAACCTGAAGCACAGTTCTCTACGATCACGACATTGAGGAAATTGTACACCGACGATGTATTAGCCAAGATGATCATTGTGGCTGGTAAGAACGCGAAAACTGCGGAGGCTGGTAAAAACGTGGAAACGGCGTTGTTACGCACCTGGTTTAACGAAATGAAGACCCCGACAGATATCCTAAGGCTGTTAAATGCTCGCGGGACTGGGCAGAGTCAAAAATTCTTCGCGTCTATATGGACGAAATACGACGATTTATTCCAAAAAGTGGATCCCAAATTCAAGACCGACATGCTCAAGGACTGGCTGAAGAAGGGGTTGATCACTGACGAGACGTTCCGAATGCTAACGCTGGGCAACGCGGCTGACGAGTTTCTCAACGGCTCGTTGCTAAGCGCTTGGGCCACGTACATCAAGGTGTTTAACCAGGAGAATCCTACGCAGCAACTGAGTTTACTCGCGACACTCACCGCTCGATTCGGTGACGAAGCTGTGTCGACGATGGTTGAAACAGCCAAGAGAGTGCCCACGACAAAGGACGTCGCCAATCGAGTTCAAGCGGAGCAGATTCAGCACTGGATGACGCTTGGTAAAACCCCGGACGACGTTTTTACGCTGCTGAAACTCAATACCGCCAAGTCGCTTTTCTTCGATCAGCCTCCAGTCAACACTTGGCTACAGTATATGGACAATTTCAGCAAGGCTAACCCTGGAGCACAGTTCTCTACCATCGCGACATTGAGGAAATTGTACACCGACGATGTATTAGCCAAGATGATCATTGTGGCTGGTAAAAACGCGAAAACTGCGGAGGCTGGTAAAAACGTGGAAACGGCGTTGTTACGCACCTGGTTTAACGAAATGAAGACCCCGACAGACGTCATACGTCTGCTAGGTCTTCGCACGCCCGGCCAAACGTCTGTAGCCCCAGTTTTGACCAAGTACATTGCGTTATTCAACAAGGTGGATCCCCGATTTAAGACTGAAATGCTCCAGAATTGGCTAAAAAGAGGTTTAATCACTGACGAAACCTTCCGATTGCTCACATTGGGCAACGCGGCTGACGAGCTCCTCAACGGCTCCATGCTAAGCGCTTGGGCCACGTACATCAAGGTGTTCAACCAGGAGAATCCAACGCAGCCAATGAGCCTACTCGCGTCGCTCACCGCTCGATTCGGTGACGAAGCTGCGTCAACGATGCTCGAAGCAGCTAGGAAGACGCCTACGACGAAACGTCTTGCTTCGAGTATCCAGAGAGAGCAGAGTCGACATTGGCTCAGCGTCAAGAAACATCCGGACGACATCTTCGTCCTACTGAAGCTCAATACCGCGACCTCTCGGCTGTTTGACCAGCCTCAACTGAACACGTGGGTGAGGTATGTGGACGCTTTCAATGAGGCCAACCCGACGAGTACAACGACGTTATTGTCCACCTTGCGGACACGATACAAGGAGGACACGTTGGCTCAAATGCTCGTCGTGGCGAGGACCAAAGGGGGCTCCGTGGGGCAAACCGCGACTCGAATTCAGGCGGAACAAACGAAACTTTGGCTGAAAAGTAACAAAACGCCGGGAGAGGTGTTCGAAATGTTACAATTGAAGAAATTGGGCACCAACTTCCTTAGTCACCCAATTTTCAATGCATGGGTGAAATACACGGACGACTACCGCAAGAAAAACCTAGGGACATATCGCTCTGCACTGACCACGTTGAGAAAAACCCACAGTGACGAAACGCTGGCGAAATTGTTCATTGAGGCGAGTAAAGTGGCGAAAACGGCGAAAATGGGGAAACGTCTGCATGCTGAGCTACTACGCGAATGGTCCCTCACTGGAGCGACACCCGTGCGGGTCTTTTTGCGTCTGAACCTCGGCAAAATGGACCCAAAGGTGTTTGAAAGTCCGCTGTACTCTATGTGGACGAGCTACATTTCTATGTTCAAAAAGGTGAACCCCACGTTCAAGGACGATCCAGTGAAGATGCTGGTATCGATCTATGGTCACAGAGACCTGACGGCCTTGCTCCTTGCGGCGGAGAAAGCTCCGAGTACTAAGGATATCGCCATTAAGTTGCAGAAGGAGCTGCTCGAGCTCTGGCAAGCAGCCAAGATGGACCCATCACGCGTCTACAGTGCACTGCATGTGGAACGTGAAGCCAAGAATTCACCCATTAGAATGTTCTGGAGTGAGTACTAGTACGTCAAGGCCTTTAGGAACTCGAATTAAATAGTACTAATATAAAAATTAATACAAAGAAAGTATTACTGAAGTACTATTACTACTTAGTAATCTTTTTACTAACTAATCTTGAGAAAGCCCAGTGCTCTGGGGAGGCTTGATAAGTTTCGTAGCGCCGAAATTAGATTCAATCTCGGCTTGAGTCGGTGCAGGCTTCCACTTTCTTCAAGCCAAGCGCTCGACGAGCGTCGTGCGTTGACGAGGACTCGGTAGCCATGGGAGCAGCTGCGTCCATGGACGACCCCGACGCGTCCGTGGTTTTTAGAGAGGTTTGTATTCTTGTAAACTTTATTTTTTTAGCTAGTATTCTAACATTATTGTTGCAGACGAAAGAGGAATATGAGCGGAGAGCTGCTGCGGGTGACTCGAAGGAAGAAATTTTCTCCAGTCTTCGAGATATCGTTGCCACCCGTTTAGGAGGCACACACCCACTCCTCCACACGTCACAGAGTCTGGAATCCTTTCTCGTCCAAGATTTAGCAATATCTGAGGTAAAAAAATCTACGTGTTTTTTATCATCGGCTACTTGGTTGTCCACAACTCACCTCTCTTTTATTGTTTTAGTCTGGAGATCTAGAGAAACGTTCCAGCTCGTTGCTGGATGCTGCTACGTTAGCTGCAGTAACGACCTCGTCCGAGAGCAAAGAATCGACTATCGAGACCAGTATCGCACTAGCCAAGACGATACCCGACGACGACGACGTCCAATTGAATCCATTCCATGACGACCATGAAGTCGAAACGCACATCGATGAGCAGGGAATCCTCCGAGCAACTGTTGAAAAAGGCTTTGGACGGGCTGTGGGTTCGTCTCGGGCTGCACTTGATCTGGTGCAAAACCTCGCTCCTGTATTTACAACTGACGTACTAACTGACCCAACAACGCTCAAGATGATCCTCAAGTACGCAGAAGCCAACAAAGTGCCCAGCGGAGCGGATATGCTCCGATTTTGGGTCGAAATCGACGAACTGCAGCACCTTCCGTCCCATTCGTACACGCACCGTCGACTACGCAAGATTTACGACAAGTTTTTGTCCCCTGAAGCGCCTTCGCCTGTGTGTGTCACGGCGCAGATGCTGCAGGATATCGAGAAAGCCCTCGAGGGAGATAATATCTCCGCTGGCATCTACGCCGGTGCACAGCAAATCTGCTATATCGCGCTGGAAAAATCCGTGTATCCGCGATTCCGAGACAGCAAATTGTTCCGTAAGATGCAGGATTTCTGCGCTCCGGTCGTTCCTAACGCTGGCGCGTCATCTAACATTGGCTCGAGCAATGGACCTACTTTGTTAGCTGCTGCTTCGGCTACAGGAACGGTGGCTGCCAACATCACGGATAATATGGAAGATGCTGAGGATTATTCGCTGTTGGGTATTCTAGCACATCCAGCTAAGCTGCGCTTCCTCAAGACGTTCTGTATGGAAGCATTGGCGCTGGAAAATCTGCTTTTCTACCTGGAAGTCGAAGATTGCAAGAGATTGCCGAATCTGTCGTTCGTGGTCAACAAGACGCGAAAAATCTACGATCGCTACTGCTCTCCATCGTCCAAGAACTTTATTGTCGGGCTGGGAGACAAAGACGCGCTGAAAGAAATCCACGACGTGGTGGAGAACAAAGGAGCTCTCGTGCCAAAGTTATTCTACGAGGTCCAGATAGGCGTATTTAACCGGATTAGCGACGATATCTGGCCTGGGTTCTGTCGCTCCCAGGAATATCTGGATCATTCGAAAGAGGTCCAACCGGACGCGAAACATCTGGCACGACGCGGCAATCGCTTCGAAGAGAGCGAGGCGGTGCAAAAGAAACTGGAAGGCTTGGCAGAGCTCCAGCTCATCGACGCGGCCATGCATTATCCGGTCGAGAAGCTCATCCCTATCTCAGTTCCAGATTCTATCCAAGGGGCAGCTCGACGAAAATCCATTCAAAAACTCGAAGAGGAGCAGACTCTCACGCCTGAGCAGGAGCTGAAGCTGCTGTTGGGCGACCCATTCGCCAAGAAATATCTAAAGCTCTTCATGACTCGACGAGGCGTGGATTCTTTGCTTGCCTTCTGCGAAGAAGTGGAAGATTTTAAGCTGCTACCAGGTATCGAGTTTCTCCAACATTCCGCTAAGAAAATCTACCGCAAGTACATTATCCCCAGTGCTCGACTACAGGTGGATATGAGTAAAACGATGCGAGAAGAGATTTTTACGCGACTCGCGAATCCAAGCGTGGATATGTTCAAGAAGATCGCGAACCGGGTACGTCACGGGATGCTGCAGGACTCGCTGCCTCGCTTCGTCAAGTCAAATTACTACAAGGATCTACGTCGGGATAGTAAAGCTACGCCTGCCGATCCCCACTTGGCCACAGTGGACCAAGCAGCAAAAGCAGGAAAGCTCGAGCTGTGCCACTTGGATGTCTTTCTAACATATCCAGGATGTATGCAAGCCTTCCGGAAGTTTCTGGATTTTCAACATTGTTCCGAGAATCTAATGTTGTGGGAGG

>Contig_17

CGTCAATCCCATTATCACCTGACTTGCCGCTCGTGGAGCTCCTAAACGCAATACCGGCGACCTCTCGCCTTGCTACGCCTGCTCCTCCTCGTCGTACGGCTGCGGCCCCGACAATCTACGCACACGTCAACCGTGTGCTTGTTCGCGTCGCATTGCCGCCGGCGTGTCATCCTGCTCTCACGTCGCACTCCTTCCGTCGTGGTGGGGTCCAACATGTCAACGGCTGCGAGAAGATGACCGCGCGCCGGATCTTTAATCGCGGATCTTGGAACCTAAGCACCATAAACAAAGGGTCCAATTACATTTTCAACACAAGTAGAGAAGACCTCATGATTACCAAAGTATTGTGTGGATAGAAACCTCGCGGACCGTGCCCCTCTATAAAACCTGTCCGGCTTTGACGTAGACACCGTTGACGCGATTGCCGAGCTCCAACGAGGCTAATTCTCAGCTTGCTACCAGCTAAAGACGAAGAGCTTCAACATCAACAGGAAGGTTCTGGACGTCCTATCCGCGTGCGTCGTTAATCAATATCCCATAGTTAAGGGAGCAAAGGTCTTGAGTCCCAACAGTCCTGTAATGCAGCGACCGGTAACCTGTGTTACGCAGGCTGGACGTACGCTACCGGACCTTTAAGACTGGGCGACGCATCTTACAGGGGCGAATCATTCCTGTAAGGACAACAATGGCAGCGTCCACCTCACCTCCTTGCCTATCGAACCAAGCCAAAAACAGAAGAGCATCGACCACCAGGCCCGCTGTAATTAAAACCTTTATCGAGAACCCCAAGCGTCAAGACGGACGCATGGATATGCTTGAGGCTATGCTGAACGACGATCCTGTAGCCGAACGCGAAATAAAAGCATTTAAGATGAAGACGGCTCAGATGTGAGCATACTTAAGCTAAAACAGCGTAGAGGATCGGTTACCTACCTTCATGCGACTTGCTTTACCTGGTATGCGCAAGGGCCACACTGGTACGCTAAAGTACCGAAGCAGCAGCGCTCGAAGGTCCACTGCGTAGGCGCGCTAAATTCCAAGATCCAACGCCACCGTCAGTTGTTCAGACAGCTGCAATCCAAGGACAGACTCCTGGCTACACGCAGGAAGTTCTTGAGGGGGTTAGAAAGTTGGACAAATAAACTAGATTGGAATGTATTCACCCTTCCTCACAGCATCAAGGCATGTCAACCTCAAGACACGTATGCAGTCACTTTTCTTGCTGATCTGAGCTGTCCTCGACTTTTGAACTTCGCCACAACGCCTCTAATCCAAGCAGAGGCAATAATGATGACCCTATTTGAACCGGCATTCTCAAGACTGAAGCCTTGTGCTCTCCCAGTTAAGCTACACCGTTGGAAGGGGGGTCAGAGGCGACCTGGCGGCTTAGAAGACCGTGCATCTCGTACAGGGCATCAGCGTAGTTGCGTTAAGAAATGTAAAATTGATCAGCGGTCAGAAACGGATTGAATGAAAGTGAGAGGCTGCAAACCATTCGATTTCTGTATTCTAACGCAAATACAAGTGGTACGTTTTTACTGGCAACAAAATATGCAATATTAGCTATGATAACTGGCTGTCGTTAATCTTAATTTATCGAGCACATGCAAAATTATAAGGCCGATCGTGGCGCTTCTGACTCATTGTTAAATTTGCCAGCTCGTGAGCACGTGACTCTCATCCCAAAAATGCATAGAAAAAATAGTAATTTTCTCAGCCCAGAGGGCGTTTCAGGCCATTTCCTGCCGTCAACAGACAGTCTATTTGGCCACTCTTTGGTATTTTCCGTCATTTCACGTCGGATATGCACTCCCATAAACCTTTCACCCATCTGGGACAAACTGTGTACTATAAATTAGTCCGATAAGGTCATCCAGTCCGATAAATTTTGGTATGGGGAAGCACATATTGTGAGCCATAACTTGCGATTACATGTACAGAGAGTTAATTATGTGCGTTGAAGAAGCGGAATCAACTTTGCTAAGATTAGTTTTAGTCATCATACGTTTCTGACGAAACTACGCATAAATGTATATCTGTGAATCATCATTGTTGACGAAGCAATTTTTTGAATTTCTGCAAATTACCATTGCTGACGAAGCTATCCATGAATACCAGTATCTGTTAATAATCAACGCTGACGAAGCTCTTCATGGATATTTGTCGAGGGAAAATTGAAGGGGCAAATACCTGAAATATTTCGAGTAAGCGGACATGTCAGTTGGATCCCACATAAGTGTACCATATATATAAGTGGTAATACTAGTAAGCTGGTAAAATCATCAGCACAGTTGGCGATCTCGAGCCAATCCAAAACAATATGTTTCGCAAAGGCAACGCGTAACCATAAGTCCTATCAGCGCCTAAAATATACCAACTGCATGCAATGAGCACGCTAAATTAGTGAGGACTTGAAGGTGCCACACTTTCCTTTTCGACGGGTGGCTGGGAACGTTCAAGAAACGACATACAGAAGTCACCCTTAACTAGCGTAGGGGGTCAGCTGATAAATTTGCATAGGGAAATCAAAGCGATCTGGTGATTTGTCTGTAATTTCTTCAATTCCTATGATCAAGCGGTATATGAAGTGCAAGCTACGTTTAACAGGAGGACTTCTGTATACGAACGAATCAGGTTGTGTATTGACGAAGTGCTGTTGTGCCTTTCTCCTGTGCTCGACCGATACAAGGTACTAAACTTATACATGTAGGACGTTTTAATTATACTGAGACGGGACTGTTTCAGGAATGGCCGCAGATTTACACCAGATATAATTCATTATATTTGGTCCGGTAAGCTTGGAGCGTCCACGTTCTCAACACAGTATAGATACTGGAACCACCGAGATGTCTATTCTGAAACAAAAAGCCAACAAGTTACATTTATAGCAATATTTTATATTGCTAATATATAATCACCTGAAAATTAATGGTTTTACGAGTTGAAAGAATAATTATATTTAGATCAAACCTGCTCCTGAGTTTCATGCACGGTTATTATAAGATGGTGTGGGGAACGCAAACTAGACGTATAAATAATAACGCTTAATATATTGTGTAGTCTTTGACGAACTTCGAGCAGAGAATACGGTGTTACATTTTCTTGGGGCCAAAGTTCAGGTCCTTTATCAATGAGCTGACGAGCACTTTACTGTTTATATAAACTGACTGCCTAACATGGACGGCTGCGCAACTTAGTTAGCAACCAATTTAGCTGGGTTTTGCGACTTCAAAGAACTGAGTTACTGTATATTGTTGGAGGACACTGTAAAAGTGCTGGCAAACAGCGCTGGTTTGCGTGATGTTGGAGGACGGCAAACTTTTTATGGTCCTAAGTTGCTAAAATGACAAACATCGCTGGTTGGGCAACTTTGTTTAGTTGGCGTAATGTTGCAGCACAACATGCTTTTAACTTGCTACAATAACAAACACCGCTAGTTGCGCATCTTTGTCAAATAAATGAACACCTGAACATGTTCAGAGACAGTACCGAGACGATTAAAGTATTTTTATGAGCAATGGATTTCAAAAATACTCACTTCATAGTTTTAATTTAAATTTATAAGCTAACTTTGAGCATGCAAAAAAAAAGGAGAAATAACTAAGGCAGGATATTATGGGTAAATATAGCTTGACGATGCTGGAGATAACATATTTTGAATTTTCGCACAACCTCGAATACTCTGAGCGGTAAAGCAAACCTTTCAAACCTCCACCTACAAAAAACAAGTCACAAACCAATCGCGCATCTTGGTATAACATTTTGACATGTGACTCACGGTATATGCCATTCAGAATAATATGATATTGTTCATGGTCAGTCAGCTCATCGCCCTCCTCGACCATGCGCTTTGCCGAGGAACAATATATATCGGGTTACAGCGTTTGCTCGCTCGCTCGCATCGAACAGCCAGAGACTCGCTCTCGTAGCCGCTCTCAATCTACTTCGGTGTTGTTTTGAGCTGAACTGTATTACAAAAAACGATCGTTTATTTGGTCCCAAAATGCCTCTATATCGTGCAAAAAAGATTTATATTCGGGACATTTGGGATGTATAGTTGGCTCCAGTTAAGAGCTACTACTACTACTAAGTCGTAAATTAGAGCTGCCGCCACAGGCAACATTCTTCTGTGTTTTTGTGACACCCACCGGGCATTAATTATAAGAGGTCAAACGATCAAGTTTCGGTACGGAGTGGTCCATTAATTTATCGTTTAAAGATAGAATGCCACCATATTTTTTCTGTATGACCGTGTGTGTTTGGTGGTGGTGGTTGANGGGGGGGGGGGGCTAGATGATACCGTAGCCGTGAGGAAGGAAAAAATTGGTCACGCAAGAGGCGACGGCTGAGTGGCTTTTGAATCTAGCACCAAAGATAATGGAAGAACAAATGAAGTAGACCTGGGTCTTCATTATGCGGTCGAGTGCTACGGTAGCTGATCAAATTAGGCCACATCACGCCCTATTCCCTAAAGAATGCCTGAATTACCCACGTTGTGACTGCTGGTGTTAGCGAGCCAGCGGGTACCTTGACGACCGTCGGCGACGATTAGTTCTTAGTCCTTCATGATCGTCTCTCTCATTTTTTCGAGCCACGCCTGTAGCTTCATCGTTTCGAGGATAGACTTCTTGCGTTTTGCAGCTTCTTTTGCCTTTTTCACGGCTTCAACGGGATCCAGCTTCTTCGCAGCTTCCAAAATAGCGTTACCGATGGTCCTCTCTTCCTCACTGTCATCTCGATCGTTGTCGACACGCCGAAGGAATCTGCCGCCATCCAAAGGGGCTGACGCGACAACGTCAAACGACGCCCCATTCTCGGTAATTGGCTTGGGCTCCGTGACAGCAGGAAGCGCAGAGGTACCGAGGTGGAGAGTGGCTGCAGTCATCGTCGCTAGGATGTATGTCAGGCGCATAGTTACTGTTAGTTTTGAAGGGCTAAGACGAGAGTGTCGTTCGCTAAAGTGAGTCAGTTGTGGAATGCCAAGAGAATCGAGGAATGGAGCTCTCGGAGTTTATCGAGTTGGTTTGTCGAGAGCACTTGAGCTGACTCAAATACAGCGTTTCCAAGGCCAGCGTGCAATCGTCGATTGAGGAGAGTGGTCATCGATTGCCAATCGATGACCACTCTCCTGCCATTTTCACAAAAAAAGGCAAATATTTTAGGATTTGAAATCATTTGGTTCTATAATCAAATCAAACCTTTTGCTATAATTACACACTCGCAACGATTTTTGGAGCGGCCATCAAGAACATTCGCAATGGTGGCATACATACGTACGAGGAAAAAGAAGTCTAAGCACAAACCCACGAGTTTTTCGTGTCTAGACCATGATGCTAGATCACAGCTGTGATCTTGTGTTGGCTGTGAAAATCCGCGGCACTTTAAAATTGCTTATCCATAAAAATACTACAACCCCCCCCCCCCTCGTGTGTTGCACCCGAGGATGGGTACTGTATTACTTAAAATTGCAGTGGGCAAAGAACTATGTGCAGTAAACTTGCTATGCTCATCAAGATACAGTACCTCCGTATGTGATAGTACCGGTATGGAGCTTTAGTTGTTTACATTGGGAGCAGCAATACTCTGGATCAAATCTGAATTAATGACGAATGCAGTTACGAAGGGATGATTAAATACCTAATTCCAGAGAGTCTATGGTTTAGGTCAGTACAGATGTAGCCCATTTATTAAAAAATTACAGTTTACGGTGATCTAAATGGGCTAAATTCTGAAGTATTAAGTTCATGTATTTTATTCATCAAACACAACTGCATGATCAAGAGGCGTAAACAGCCAGATACGTAAGCAAGGGAAGAAGGAGAAGATGTGGCGTAGATGTGGCGTGCGAAAATAATACGTGTAGGAAAGTGAATCTGTCCATCAATTTGCATATCCAGTGTTCAACGGGTTGTAGAGTGGTTGTGTGTATATACTGTATATCCACAACTCACTTGAGATTTTTTTTACCAAGTGAACTTTGGCAATCAAAAGTGTCTGAGTTTTAATACACGACATAATAACTGTAGCATTTAAGTACCACTATTGTCTTATTAATTGCCCACAAGAACTTAACCCATGCAAATGTTTCGAATCGTAGCCACACACACATGCCTTGTCAAAATCGGTGGTGCGATGCTCACCCTCTTGAGTTGCTATTAATACTGTTGCGACAATGCCGCCTGTTTTACAATAGATCACCGTGTTGTTGTAGCGATCTTACTTGCGTTTCAATTTCGCTCGAATAGACGTGGGGCGGCAACACATTTTAGATAATTTTAAAACAGAGTAACCCCCTCATGCAGCTGCTGGAGGCCCTCGATCGTTTGTGCCCATTTAATCGGCCACAGAAATTAAACCTAAGCAGCAGAGAAGATTCGCATCCTGCGCGAATATAGACAAACTCAAGAAGCACCAGTAAATACATAATAAAATCTACGCAAATGAATGTTTCAGACCTGTCGACGTGCGTTAGATGATCTTTAAGGGACATTGACCGATCAGAAACACAATAACGCCAACATTTAGAAATGAATAGACGTGACAAAAAACCAGCATATCATGCCAGTCTACGCATTGTACGCTTATGCGTGCCGCTGAGACCGCAAGAACACAGTGCACCGCCGCTACTACAGGGGAGCGGCTTGCTTACGTGTTAAACCGATCGAGTTACGGCTGCGTGTTTTATCCAGCATTAAGTTTGCTCAATAGTTTTCATCCTTTACGGTATACTATTAGTTGCTACGGAAAAGCATGCTATAATTTTTTTGGATGAAACGAACACGAGAGATTGGCAATTTCCAAAGAAGAAGCGCATTTTTTAAGCGAAAGATGATACAAAACCTAGCAACATCTGAAATGCCACTTTGGACAAAGGGTCAAGGTACGACCAATACAATATGCGGAAATAGGTAGAGAGCTATTGCAAGCCAGAGTATGACCATCGACAGAGAATACACATCCCAAACCAGTTTGCTCTAAGAATTCTGGCCACGCGTTTGCTGTGCGGTTTAGACCTCAATTTCTCACTTCGCATTTTGACTCGCTATCTACACCTAAAGCATCCAGACACCAACAATGCGCCTAAATTACGTCTTACTGGCCGCTGCCTCGACCCTACTCGCCCGCCACGTCACCTCCACCCCGTACTCCGCTAATGATGTTGCGCTAACTGGTGTAATGTCGCTGGGCTTCATTCATTTTGTTGGCGCTGACCAAAGTGTAAGCGACCAGTCCCGGTTCCTGAGAGGCGGCAACATCGACGAATATGACAACGAAGAGAGAAGTTTTGTGGAGGTTGTTAAGCAAGCGATGATTAACAACTTTGTTAACAAGCTGATGAAGAAGAACTCGTTTTCGGATATCGAGAAAATTGACGATCTTGGGAAGCTCAAAAGGATTTCGGCTGCTGCAGATGACCAATTGAATTCGGCGTTCAAGCTGGCTGACGATGCGAAAATGAGTCCCGACGACCTGGCCAAGGTGCTGAAAGAAATGCCAGGTGCCGACGATGCCCTGACGGGTAAGGCAAAGGAAATGTACACTGAGTACTTGAAAACAGTTGGCGGAAGAGTGCACACGTAGTCTCGACGGCGAAAAGTACAAACCGTTTTTTAAAAAATGCATTGGTCCCTACAATAACAGGACATACATGTAATAGCAAAAACACTACTGAAAACGGCGTGCGCCACACAGAAATGCATAATTCAGGAAGACCTTTCCTACCTGATATAACCCGTGGACGACTTCTCTGTCAGGCACAGCAGCTTATCGTGTTACTCAGGTCGTTCGTTGAGTTAAAAATCGATATCAGGCGCCAGGTGAGGAAGTATGCCGGCTAACAGAAGCATAAAATACAGATGTACTTCGCTGTGCGTTTCCAGGTCTAACGTGGAGGCACACAGGCATATCATACTAAAGCTGTCTAAATGGGTTTAAGTAAGAAAGTATCTCCTGGACTCATGGACTCGTGGATAAGTAATCATTTGTTAAACTGCGGAACACCAGGTCATTCAATGCTTCTTCACCTACATGTACCTCAATTACATGTATACATGTGTAGACCTCCACTAATTTAGACTCCATGTCAGGAATACGTGTGAAAATGCAATGGAGCATCGCACTCTCCAAAAACGCATAAAAATGATCATCATTTTCCGATAACACGATCAGGGGTTAATGGTAACTCCAAAATCGGTGAAGTTAATATTGTGAAAATATCACCTGTAGACAGTTAATAAAAAAAATATCACCTAATATTTTCTTCTGAGACAATATGATATTATTAAATATCAACTTAATATTAGCTAATGCGATATTATATTTTTAAAGATTGCGTAGTCGGGATAAATTTACCAATCAGATGTCGGTAACTAGCTGAATTATTACTAATCAACCGTGAGGGAGGGACAATTCTGAGAGTGACGGCAAGGGTTGGCGTCGTGTGTTGGCACTGCTGAGCGCATTAATCTAGCGCCACAGGCAGCAGGTTTATTAGACGCACGATGTGGACGAGGAGATCGGTAGAGAACAAGAACAAATGTCTTGATTTCGGTCTATCAATCAAAGTTAAACACCTGCAAGCGTTTGCAGTTTTCCATCTCGATCGCCTTTGTCTGTGTCACAGTGAAATACTAGAGCAAATACTCGTCAGTCTTAAAGCCTACTGCTTTTCTAGAACCTGATCGAAAAAGTTATTTGGCTCCGCTCGGTTCACAGAGGGTAAGGTGGTAAGAAACGTACTTAAGTGGGAAAATATCATAAAATATCAAGTGTATCTAATATTTTTTATTTCTCCGAGTTAATATTAAAATACTATGCTAACTTTTGCAAAGAAGAGTTAATATTAATATTTAATATCGAAAATAGTACTATTACTATCAAAAATATCAAACGATATTTGGTTACCATTAGCCCTAGAGGTTAGATGGATTACGGTCTTTTTGTATTTTCGGGCAACAAACTTGTGGTGGACCATTGTGACAGATTAACTTTTTTTGGACTAGTTATAATTTTGGTACATAAATATATTTTAGTTCTGACTTCGACTGCATCAACAACACATACAGATCGTATGTGTTCTCGATGCAGGAATGCACTAGCTGCTCAAGTTCCGGGACATTTCCTTGAAAGTACTAGATGATTTATAAATATTTACCGACTAATATATAGGCCTTATTAATCCGTCGAAACCGGGTGCTGCATAATGCCGGAATACAATTTCAGTTTAACGACTTTTAGACTTAAAGTTTGTATGACGTGTTTTTTCGCTCGCCATAAGGCATACAATTTTTAAGACAACCAAAAATGTATGTTTCTGTGTGGGTCTTTTTTTTCTCAAGAGGTTAAACGGGGCAACATTTGTCTACCGGTAAATCTAAAGATCAACTTTTCGATGTTTGTGAGAATCTAGATGATTTGTTTCACCTACCGGAGGTAACGGGCTGATTCCACTCAGTGAGTGGTGCACAGCCAACGACGATCACCGCCTTCTCTCATTCGAGATTTTACGCCCCGAGGGCCGAAGACGCAGGAGGATGGTGGGAGTCCGACGCAAAGTGCTACTAGACTGCGAAGTTACCGCACGCGACTTATCTTTCAAGTCGGTGTGGAGAGAGCTGAAGAAGGACGGGTGGACACGAAAGCACCTTCGGGACGCAGTCTCGATGACCGCTACTTTTATATGCCTCCCGGTAAAAACGTGAAGGGGGGCGAAGGCGTCGATTACTTCCGTGGCGAACAAGCTGTATTGGAGCATTATGCAAAGGGTGTGTGTATAACTTGCAGTTGAATAGCTCCTTGCTATCTCTAATACCAAGTTTGTTGCTTGTAGAACTGCGACGCCATGCGGTAGGCGTCCAACCCAGTGTTCCTGGAGATGATCAACTAGCTGCCGCAGCTATCGTACGTGAAAACTACGCTGTTGATATCGAAGCGGCAGAAGCAAGCGCTCGAGCTCAGGCTACTTCTCAACGTGTCCCCACCAACTAGCCAACTAACGGTGTGCCTCCCGTCCCCGTACGTGAATCTACTACCAGGCCACATTCACCATCACAGCGAGCGCCGACGCGTCGCTCTTTGATGAATACTTCAAGTCCTATGGCAAACTGCCCAGTTAGAAGTACCTAGCCAACGTCTCCGCTCACCCCGACGTCACCACACGTTCAAGAGGCCAACTGTTCATCTGAAGAAGCAGAAGAATCTGATAATATCAGCGGCAGCAACTCCACACATCCAGCACAAACTCAAAC

>Contig_18

CGATCGCGAAAGCCTCGTAACTAAAGCAGCGCCAGCTGGTAACTAAATAATCAGCTGAGCACTCTGACATAGTTTCGGTGCTGCCAGAATTGTTCTTTTTCCTCTACCTCTAGCTAATAGTTTGCATACCAAATCAAGAAGAGAATCGTTGACCCATTCAAAATCGTCATCTGAAATGTGAGAGACATAGGTACCGTCCAAATTGTAGTCAGGATAGCCGGGNAAAAAAATCATAATCCATAGTGTCTGTTTTCTGGCTGACTTCGCTGAACGTCTCTCATTTATCACTCACTGCTGCTTCTGTCGTCATTGTCCTTCAAACTCGTGCTATCACTGTCATCCTCTCCACAATCTCTGTGACTTTCCATAACGGATGCAATAAGATGATTAAGATCGCTATCAATACCACGGAAGCCCAACGATCCGCAGTCCTTGGATGAGCTCTGTAAAGCGAATGCAGCAGTATATACACCAGGTCAATTCTGATCATCACTTGTCGCCGGAGTGAAAGGTTTCATGAGAGATGAGCTTGGCGTTGTGGAGTACAATCGGTCAATAACAACCCTTGTGTCGAGACGATGTTTCATCGACATGAGCGACCCCNAGAACTATTTCATATGAAGAATTCGTTTATAATTATATAGATCAAAACCTTCTCTTGTAACAGACTTGTAAACATTGACCCATGTTTTTGATAAAGGGTTGACTTTGCTTTGACCCAAAGGCCTTTAGAATAGTCATATTTTACCTGATTATCTTAAGTTGGGTCAATGTTGACCCAAACTAATCTAATTGGGTCACGATTGACCCTTGTTGCTCGACCTGGGTCAATTGTGACGGGCCTTTCTTTTGGGTAGTTACATACTCTCCAGATACCATGTCTATCACGAGAGCCCTCCCGACGAGGGACCTCTTTAGCGATCCCGCGGAAGGGTCGGTCGGGTGGAAACACAAGCGCGATGAGCCGTCCGGCACCAGAAAGCACCGCCGTGTGGTGGTTAAGAGCGCCTCTTGCAAAAAGCAGGCGAAGGTTCTTGCGTCGATTGACATGCGAAGCGATCCATAACGGATCGTAGCTCACTTGATATTAGTATACATGTTTAGGATTACTTATTAGGAATAGGCCCAAAGGGCAATACTAACACAGAACAGGATTTTCACTCAGGTGCACAAGCGACGTCCGGCTTCGAGGCAATCGTCTCGTCAAGTTTAATTGTATGTGTTATGCCTGTCGGTAGACTCGAAGCTTACTAGCGAGGATTTTTTCGGCAAGGCCAAGAGACTGTCTGTGTTTTGACCGATTATATATCGGAAGGTTTACGTGACGGCTTTATTTTGCGTATTTGTACGACTGGAATTGCGTGTTGCATCCCTCTTATTCTTCCAACGGAGCTCTAAACACTAACTGCCCTCGTTAAAGACTTGCCCGTGCCTGGTGCCGTTCTGCTGTTGGGACCACGAGGATCCAAAACACCTGCTTGCTTTGCTCGTGCTATTGTGTTATCATTTTCACTGGTTGGCCTATATAAGCTATTCGCAAGGTCCAATCTGCCCTGCTGCTTGGGCATATTTGTGGTCTTCAGGTCCGCTCGATATATCGCTTGCCGTTCTGGTAGACGTATTGGTACGGGAACAACCTATCTACACTTGTAGCTAGAGTTCACGAGGGATTTCCAACACACAGTGTTTTCTATAATGATGTCTCCATTGAGGTGTCCACTAGTATAGGCAACGTATCCGCTGTAGCTTTCTAGCGTTGTTGTGAAGTCTTTTAGCAATCAGGGTAAGTTTCTAGGAAAAGGGTGGAGGTGGGGGCGGCGCACGCGCTCACCCCCGCTGACAAAAATTCTACTGTGGAATTACTCAAAAGAGCATTCCTGAACCCTTACAGCACCGGAGTGCAAGGGGCCTGCTCCGGCTCATGTCAACGCCCTCATTGAGCAACGTCAAGCACCAGTCAGGTAAGTATGATCTGAATTAAGTTATTCCGGGTAATACTATAGCACTTAGCAGACCTCATGTACATATTCTGATTATTGTTATCCCAATTTAATTCTCATTACAGCTAATAATAACAAAACTCTTCATTAAAAATGACAAATGAGACTTGGTCTTAGAAAAGCTACGATGTACTAAAGCTGCGCAACAAGAACATTACTGTAGATCCATAAATCTACCCTTCTCTTCTATCAACTATAGCATAGTAGACTTGGCCTTGCTCTTTTTCTTCTTAGTGAGCTTCTTGACAGCCTTCTTGTGCTTCTTAGTCGCGAATCCTTCGTCCTCACTCTTCGCAACTGTCTTCTTGTCCACAAAACCGTCCCGAAGGTACGTTTTCAGCGCAAGGACCGTATCCTCCAACTGCTTGTGCGTATGCAGCGCCGAGATAAAGAAGCGCAGACGACATTTCCCTTCCTCCACCGCAGGGTACACGATCGGCTTGACATTGATCTTGTGCACCGCCAAGAACTCGGACGCTTTGGCAGTCGCAATAGTACTTCCGATCATCACCACGACCACTGGAGCACCACGGAAAGTGGTCTCCCCCATAGGAATGTTGTGTTCCTTACACAGATCATAGAAACATTTACTACGCTCCTGTAGAGTAGTCGTACGTGACGGACTCTGTGTCATCAGCTGCAACGATTTCAACGCAGCAGAGCCACACGCCGGCGCTAAGCCCACCGAGAACACGAAGCCTCCAGCACAATGCTTGAGGTATTTAACCAGCGCCTTCGAGCCAAGGATGAAGCCTCCAACGGACCCCAAGGCCTTGCTCATCGTACCCATACGTACATCAATGTCTTTCGGGTCCACCTCGGTGTGTTCACAGATACCGCGCCCAGTACCGCCCATCGTACCGAACGAATGCGCCTCGTCCATGAACAGTAACGCCTTGTACTTTTTCTTGATACGAATCATCTCTCGGACATCCGGGACGTCACCGTCCATGCTGTAGACCCCTTCAACGACAATAAGAACACGTCGATACTTGGTACGAAGCTTCGACAACATACGTTCCAGAGCCTTCGTGTCATTGTGCGGGAACGGCAAGATAGTGGCGCCACTGAGACGTTGGCCACTCACACACGAGTCGTGATTAAGCGCGTCACAGAGAATCAGATCACCTTTAGACACCAACGCATCGATCGTCGTGACATTGGACACCCAGCCGCCCACGAAGAGAACACTGGCTTCCGCCTTAAAGAACGTACACAGCGCCGTCTCCAAGTCCACATTCACCTGCGTCTGACCCACAATAGGCGACGAACTCATCGTCGTACCGTACTCGTCGATTCCTGCTTTTGAAGCCGCAGCAACCTCGGGGTCCGACGCGTTGCCGAGATAGTTGTACGTGTTGAAGTTCGTCATGCGACGCTTCGCCGGCGTCAGCGTCTCCAGAAACGGTACACGTAGTCCGGCACTCTCGAACTCTTTCATTTGTCCAAAAAGGCCCTGAACTTCGGGGAAATTTTCGATTTCAAAGCAACTCGAGTCGATCTCGGCAACTGTCGTGGCCGTCTGAGCACCAGCTTGGTCGTCACCTTCCTGGTCGCCCGCGAGGTCTCCCGTCGCCAATCCGGGGGCATTGGCGAGCTTCAACGGCGTGTCGTATTGGAAAAACGCCGAAGGAGACACAATGCAACCCAGAAACTCTCCTAGATCCGACGATAACCCCACAATCGCCACGGAATCCATACCAAACATGGCCCACGGTGTGTTTGGGTCGATCTCGGCAGCATTCGCAGTTGCTTTCTGGCCGTCAGACGACTCTCCTGTGGGCATCTCCATCTCCTGCGCCACATGTTCTAATAGCCACGCGAGGATCTCGTCCGACGTCTTCACCGCTCCAGAAGACGTAGGAATAGGCTGTTCCTTCGGCTTTCCAGCGCTAGAAGACAAACCTGCCTTCGCTCTGTACTCGAATAAAGGCTTAGCAAGTGTGCCATCCAAGAAATGAGCCTTGGAGGCACTTCGTTGGATCTTGCCACTCGTCGTCTTCGGGATCGTCTTCTGACGCAACAGAACGATCGCTTCGCACTTCAATTGGTGCTCCGACAGGACCGTCTTGATGATCTCCCGGCAAATCTCCTCGAGTGTCTCCTGCGAGGATCCGTTCTTGACCTCGGCGACCACGACAAGCGCTTCCTCGTCACCTTTTTCGATCGAAAAGGCCGCAGTGCATCCGGGACGAACATTTTCGTGCGCGTGTTCAACAGATGCTTCCACATCCTGAGGACATACATTCCGACCACGAATGATGATCAGGTCTTTGAGTCGACCTGTCACAAACAACTCGCCTTTACGAAGGAAACCCATGTCGCCAGTGCGTAAATACGTGTTGGTCGCGTCCTTCTCGTGTGCCACTTGTGCACGGAACATCTCCTGTGTATACTCCGGTCGGTTCCAGTAGCCAATTGCCACTGATGGTCCCTGCACCCATACTTCTCCGACTTGTAATTCGTCCAATGGCTGCTTGGTTTCGGGATCCACAATGGCGACGCTAAATGTGGGCATGGCTTTACCACAACCCACAAGTTGCATCACTTCGGGTTTCTTTCCCTTCTTGTTGGAGCTCACAACAGCAACTTTACGTTGCGTTTCCAACACGCGCTTGTTAACGTCCAATAGCGTTGGCTGCTGAGGCGGCTCTTGGCCCGTACACACCAGAGTGACTTCCGCCAATCCGTAGCCACAATTGAAAGTATTCGGATCGAATCCGGAGCCACTAAACTTGGACGTAAAGGCCTCCAGTGACTCGCGACGAATGGGTTCAGCCGCACAGATTGTCTGTTTGAGAGAACTCAGATCCATCTCTGCCGCCTGCTTGTCGCTAGTCTTTCTAGCTGCCAACGCATAGCCGAAATTCGGGGCACACACATGCGTAGCCTTGTATTTACTTGCAGTGCGCATCCACAACGCCGGGTCTTTAATGAAACTAATAGGAGACATACTAACACACCGCGCAGCTGTGACGCATGGAGTTATGATGAATCCGACCAGTCCCATGTCGTGGTAACTCGGCAGCCAACTGACCATCGTGTCGGTGGGTTCAATGCTCTCCCACGTCTTCAGTTGCGCTCGTAAGTTACCGTGAGAGATCATCACAGCCTTGGGAGCTGACGTGGACCCCGAGCTGTATTGAAAGAACGCCACGTCATTGGACGTTAATGACAGAGCATCTTCTTCGTCGTATTGCGCAACTAACGAGTCGGGAAGACTATTCGTAGTGATCCACTGCAAGTTGGCGGGCCACGACGTCCTGGACGTGGAGAAGTAGCCTTTCACTGTAGCCATCTTACTGGCAAGATGATACGTCGTGTTGGTGAGGACCACGGCAGCCCCCGAGTCCTCCACCAGACGGTTAAACTTAGGTAGATCTTTGGCCAGAGTGCCAGGGTACGGGGGGTACACGGGAATACCCACGACGCCGGCGTATAGGCAGCCCCAGAAGGCCAGAGCGAAGTCTAGACCTGGGGGGAAGCACAGCACCACCCGGTCACCCTTGACCACGTGCGCGTCACGTTGGAGCGCTGCGGCCACTTTGCGAGCCGCACGGTCCACATCTTCGAAGCTTAAGTTGACGGTCTCACGGCCCAAATCGTCCAGGAAGGTGTAGACGAGCTTGTGACGCCACTTGGTGGACGCAGCACGACGCTTAAGTTCCCCAACCACGGGCTCCGTGTTGATGGCGACGCCGTTAGCAACAACCACCGAATGTTTCGGTTGTGCGGCATCAAGCTTCTTGTAGCGACGCTTCTTGGGCGCGGGGGTTGAGGACTCGTCGGTCTCACGCTGGCGCTTTTTGTAGTGTGCACCCAAGGCCGCGACAGACGCAGCGGCAGCTAATGATGCTGCTGCCACCACCTCAACGGCCACGTCGCCCAGCAGGGAGGACATTAGGTCTGAAAGGCGTAGGGTGACTGGCGGACTGTGAGGGATCGGTGTAAAATACACACGATGTGGTACACTTAATTATGTCTTTTTAGCCAATGGGAAAGGAGCTGATCCCTTTATTTCCCTGTCTATTCACACTGCAGATTGGGAGGGGTATGTAGCCATGTGTCATAGGCACTTCAACATCGTTTTATAATCCTGTCCTCTCACCGCTTGTGCAGACGACTGAACGTCGGCATGTTGCCGCTACGAGCAGCTGGGGGTTGTGACGGCATGTGTCCTGGTCTTGACTGAGTCGATAATCGTGGCAAGGTGGCGCTGTTGCCAACCACCAAGCCTGGTCCACGCATCATGGCACTGAGTGGCGCTGCATTTCGTTGGATTTGGGGCAACTTGTTGGCGACCTGCCCAGCCACAGGACCCGACGTCGGTCCTCCACGTCGACTTAGTGGCTGCCGAGTTGACATCGACCTGACTGGAGCTGTAGAGCGAGCGAATGGCAGTCTGGTTCCGCCCAAGCCAGAGGTTTGTCGAGTATTAGCTCCAATTACCGCGCTCCGAGCAAAAGATCGCTGGTTTCGGGTGGCAAGTGTTGTTGTCGTACCAAAACGACCCGTAGATCTACTGTTAGTTGTGACTGGTAGACGCTGGAAAGCCTCTTTCCGATTCGTATACCCTTTCTTGATGTCATCTAGCTTTTGTAGATGGGTGTTCGCAATACGCTTCCCAGATGCAGCACCCACGGGGGCTCTTACACGCGGTTGTGGGGGTAGTCGCTGCTTCTGGTTTCTTTCCGTTGTTGACTTTGCCGATGCACCAGCCCGTACGTGCGATGTGGTGGCCTTCCGTTGGTTGGCAGCAGCGCGCTTCTTCTCAAGCCATCCTTCAATGACGACGTCCTGTTTGCGAGCTTGTCGTTGCGCTTCCTTCGCCTTCTCGTTCTTAAGGAATGTCCGTCGTTCACGTTCCGTCATGCGTCGAAGTTGACGCTGCTTGTTCTCAGGGAGACTCGTAAGGAACGTGTCTGTCTTCGATCTGGTCCTGTTCCTCGTTCCAGTATTGCTATTAAGCGCTCGCTTCTTCGCGTTCGCCGCTGCTGTCGACCCCTGTGCTCGTCGTGTTGACGTCACTGTTGTTTTATCTCGTGCACGCATTTTAGACGGCCCGGTGACAGCATACACCGTTCTGGAGGGCTCATGCAGCCCACCAGTAGCTCTCCTTTTAGCAAACCGGAGACCGCTACCAGTGGCGGATTGCTTGGACCCTGCTGTTCTACTTGAGGTGTTACCACCTCGTGCGTTGTCAAGATCATCCACTGCTAAGCCAGCATCTTCCAGATGCCTAATACGCTGCTCCAGCTCCATCAGTTTGTCAAGACTGGTGACTACGTTTGCGTTGTCTTGTGCAGCTTTGCCTCCTGCAGCCATCGCAGCGAGGTGATACACACTATTACTATTCCCACGGTCGCGTTTGTTAGGTGAGCTGGTTTCCTCGTCTTGATTCGATCCCTTCGCCTTTGACCGACTGGAATCGTTCCCACTGGCTTGAAGCTCCAGCATCTTTTCGCGATATTGTTGACGTCTTCGCTGCATATCCTCGCGTTGTGTCATCGACTCTTTGAGTTTTCGAAGATGCTGGATGTGGCGCTGTACATCGTCGTCGAACTTGGCCTCGTCACGTTGGCCTTTGGCTTCCACAGTGCGGAAAAACTCCGTGTTTACCTGCAAAGGCTTAAGTTGCTCTTCTTCGATAATCTGCTGGGCACGCTGCGCACGCTCTTCGCGCTCACCCTGAGTGAGCGCACTGGCGAATTGCGAGTCTTCCTGTTTGATCTTATTGGTCTTGATGATGGCAGCCTTGAGAGCTGCCTCGTTCGCTAGTTTGTGCCAATTTAGCAACTTCATTTCTTGCTGTAGTTCTTCTGTTTGTGAGATCGACATGGCGCTCGTCGCTCGTGGCTTCATCTTTCGGGTTGTATTCGCAGTAGAAATTGATGACTGTCGGGTCGCTTTCCCACTCTCATTTGGAGCTTCAAGACGCTCGTCAGCTTGACCATCGCCACTCTGGTATTCGTCTAGCTCACGCACCCAACCAGAGTAGTCTGCAGCTACCGACATGGCTCCGGTTGCCGCAACAGCGAGAAAAGCTTAACGAAAGAGTGTAAGGTACAAACACAGCGCCAGACTTACGTGGAAAGACATCGCTGTTTTTAGCCGAGGGTCTAACATGCAAGTGTAAATAGCCATCCAATCGCTTCGAGCTGCCATGAAGAAGAGGGTTACCCCAGGGTTACCCACCCGTCCACGTGGATTATTAAATAACCCATCCACCCCGTTTACCGGCTACTCATAAAAGAAACAATACTTCTATTCAAGCGACGTACCAAGCTACAGTGGTAGCTATAGTAGTACTACGCATACTCGGCACTCACTCATCGACGCTGAGTCGCGCATTACGCCTACGTGGACGCTCAGTTCGCAGCTTGCGTTGTCGCCCCCGCGTAGACTGGTACATCCAAATAGAATCAATAGTTTCTCGCTCAAACTCACGAGCCGCTTGCCACGGAATGTTGGCTACAGGCTCCCAGGTCAGTTCATGGTATTCTCCCCACTGCACCAGATAGAATGTAGCCTCATTAATCCAGCGCTTAGCCCACACCTTCTTGAGAGGCATACTCGTTCTCTGCAGTGCCACCACGTCTCGTAGCAAACAATCCTCCACCACGCCGTCTACGACTGGCCGAATCTCCGTGTCTTCCTCATCACCACTAGACGGGTCTACATGCTCGTGTTGCAATCGCTTCCTCCAAAACGAGCCGTCGAGGCACCTACCGTCTTACCCCGCATATAAGGCATATGTGTTCTAAATAAATTCGGGATTCGCTCGACATCTAGCTTAAACCTAATATTCTTGAGAGCTCCCAACCAACCACCCTTCTGAACAAACTTGTCGGTGCTGCTGTTACTTGGCTTTAATTCTCTCATTTAATAATTTACACAGTCATGAGTGCTGAGCGACGTAGCTATAGTATCCACGAGAAACTCGCTGTTCTAGCAGACTCGGATAGGCGTGAAAGGTCACGGCTTTGCTGCTCTTAGCAAGAAGCACAGCGTGGCGGTTTGAACCCACGCCGATCGCGTAGGAGGGTCAAGCCTAAACGACCAAGCCAGATTCCAGGACCCACAAACTTGATTCTTCAAAGTCAAACAATTTGTTCGTTGTACAACACATAGGCTTTCTAAACGAATATAACACACAAGCAGCGTTGTATTCATTTACAGCACCTATGCCTTATATGCGGGGTAAGACGGTAGGTCTGTATTGAATTGCGAATTGAATTGCTCTTTCTTTGTATTAAACACAATTTCCTAATAGTGACTAGACACTGTTTTTGTAAGTGAATAGGGCTACAAAAAACCATTATCCAACCACCCCAGCAACCTCTTCGACCCACCCAATGTCTTGAGAAAACCCGGGTTTTCCAGAACACTGACCACCAATAGATACTGTATCATGTCCCGGGAACCACTCGTCGTTTTTTAATTACGTACCAGTACATGTACCGGTACTCCGTAATTTACGCTAGCTAGTAAAGTATAGTTTTTNAACTGACATGACTGGATAGTGACAGAGTACTACCGAATGCGAAACTAAATTCGCCAGTATCAGTATAAATTGCTATTCCTGTAGTGGCAAATACATTTTAAAAATATATGTCAGCAGCCTACAAACATTTCTAAAGTTTGAATCATCGAGCGTTCTTCTCGAGCGATACTATAATTTGGGTCAGCACTACATATATGAATGGCAAGCAAAAGTCGAGCTAATGGACCGTTTGAGGTCGCCTGTTATCCAAATTAGCATTTTGTGCTGTTGCTTGACGCTGGAATTCTCGTGCACAAAGTTGCGTGCGATATGAGCCGTGCAGTATTTTACGTTAAGTCCGCGTGAAGCTAAGACGGACAGTGTTGTCTTCTGGTGGTACCCACGCAAAAGCGTGTACATGTTAGTATCCCGACCTACGAGGTGCATTTGCAATCCGCCGATAGTACTGGTATTTTGAATGGTAATGGTGCTGATGTCGGAGGTGGAGCCTCCATAACTGCTAGCGGAGGATTGAGCTGCAATGCTGGTGGGTGTTGGCGGATCGTCCGGCGTGTCATAGCTCACTTGTGTGAATAGTGAGAAGAAAGTGATCTCATTAACTTTTGCTCGGCCTTTTATACCCTTCCTAGAGCCTTTTTTATCGTAAACTATCACTCCGATTCCTGTCTAAGGGGAGATTTTTCCTGAGTCTTTTTACATCATAAACATAGCCCCCCCCCATCGCAGGTCGGAGTCGACGCATGTGGATGGTTTACTTATTAGCCTCGTTTGCTTACCACAACGGTGCCGTTGCTGCGACAGCATCGACCTCGTAAGGTCCGGCATAGATTAGGTCGATTTTCGCTAGCCGCGAGTCATTGCCTGATACCATGACCAGGTCGCTCGAGACATACTCGTGAGTATCACGTCCGGCATTCTCTCTGGTGCTAGCCGTAGCCACTTGAGTCATTTTCTGCTCTTGTTGGGCTTTCTAGTCGACTGCGTACGGTACATCGAGCTTTAAATCACGGCCAAATGCCGCTTTAGCCGGCTGCACCCCATCATGGTACGGTGCGATGCCCGCAGCGCGTACGCTACAGCTGACAGGCAGTTTTCCCAGTCAGCCTTGATTGTGATAGACTCAGAGCGCAATTTCTCATTAATACAGCGGTTTAACCTCTCGATTGTAGAATTAGCTTGCGGGTTGCGCACTGTAGTGGGTGCGCCTCAATCCAATAGGAGTCGAGGAGCTCACGAAATTCGCGCTTGAATTCACTGCCTGCGACAAAGATACAACGTTCGGTCGAGGGTACTTGTTGACCATAGTTTGGTCGAGACTTCGTGCTGATTCTGCTCCGGAGGAGTCGTTTTGTACGACAAGCTCAAACTATCGTATGTTTGTAGCCATAATCGAAACCTCGCCCGATAGGTCCGATTGAATCGATAGATAGTTCATTCCATGGTCGGACTTCGACCGTCTTTGACGGGATCTTACCACAAACCGTAGGGGAGATTTTGTGCAGCGAGCACACCCTTGCAGCGCTGGAACCATTTCTTAATATCACTTTCCATACCACACCGATGAAACACAGCCTGATCGACTTAATCATGATAGCCAATTCCGGGATAAATTAGCCATTCATGGTAGGCTCGCATAATGGCGGGTGCAGCGGCAGTGTAACAACGATTCTACCATCTTTCACGGTCCGTAAGTAGGTTAACTACACTAATTTCTCGCATCACCACGGCTTCTGTCGGCTTCAACTTTACTTTCACTTGTTCGTCCCTGATGAATTTGGCAGCCAGATAGAATAGCTCTGCATCTTCCCAGTTCTAGTAGCGACCACGTGAGCTGACTGTCCAGTCGTCTCTATCGGTAGTCTGGACAGCGCATCTGCTACTACATTATCCTTGCCCTGGATGTGGATCAGCTCGGGTCCGTACTCTTTAATCTCTAGTCTCCACCGAAGCATTGTTCAATTTAGTAAA

>Contig_19

GGACGGTGAATAACAAGCCCAAGACGTTCTCGACACCTGAGCCTAGTACGTTGCTATTACCAGGGCGAGGACCTCTTCCCATGGCTGCATCGCCCTTGATCACGCCATCATCAGCGCTCCAGGATCCTCGGAATTTCGGTGGTCGTGGGATTAGTATTCCGGGACAAGCACCTCGAATGTCAACAGGAGGCAACGGTATGCCCGGCTTTGACGACGGTCACGGCGGCGCAGCTCCTACCCCCAAGCGACGACGATGGTACCTGGGTATTCAGTCGAAAAAAGAGCCCGCTCACGTCATGTCGGAGGTGTACAAGGCGCTGTTTGTGCTCCACTTTGAGTGGAAAGTCGTGGCGCCGTACCGTGTAAAGTGTCGATGGCAATCGCCTGCAGGAGACCCCAATGGAGGGCGCCGTGATGAACGCGGAAGACGCGAAACTGCAACAACAGATGCAACGCATCAAGATCGGCTTGCAGCTGTACAAGGTGCAGCAACACATCTATCTCTTGGACTTCCAGCGTCTGGATGGCAATGCCTTCACGTACATGAACCTCTGTGCACGCATCATTACGGAGCTCAAGACGCTCTCGGGGATCCGACCRCTGGCTAGTGGCCACGACCCACGCTTTGGAGATGGCAACATGAACTCGGTGCACTTACACCAGCACAATCCACACGCTCAAGGCGCTCAGTGAACTTTGTGACTTGCAGAAACGTATGTTGTCATCTCTTGGCTACATGTTTCGCTTAAAACAACCTCCAGGACTTAATCATGTTACCTCTTTGGTTTTATACATTGCATTCTGCTAATGTGCGAGGTTCAAGATTGACTGCTAATAGGCCCAGACGGGAGCATTTGGCTTGACGGAATCGACACTCGTTCCCGTATAATTGGCCGTTGGAAGCGCAGACAGGACTGTACGTCTGTGTGCAGGTAGCTGGACATTGTGGCATGACGTTGACGTCTTCGCAGGACCCGGAACGGATCAGTATTAGACTCTGGTTGCAACGTTGTTGACGTCTGAAGTAACACAAGTTTTGCATTGTTCCTGCACTTGTACACACCGGATCTAGCACGGTAGAGCAGGTTGTACTTGTAGTTGCGAGGCAGTTGGTACTGGGACTTGTTAATGGAGGGTTATACTGCGTGTTAAGAGCCTTTTCAGCTGCACAAAGGCCACGGAAAATGACGTGTAGGTCGGTGTTGCCAGTCGAACAGCGAGCTTCTTTGAGGAAGCACGAGTTGGCGTACGAAACGTTGTCCGAGCCACAGACTGGAGAGCTTATGAGTTGACAATTGAGCTTGCATGTTGATGTGGGCGTGGTGATGATGGAGGATTGTTTTGGCTCGCAGACAGCTTCGGGTAGACAGGGGGCACTTGCACAAGGGGTCACGCCACGTAAGATACACGTTTGATCGTCGCCGCATCGACCTGGAGCGCACAATTGCGCGCAATACTCGGCGTTGTTGTACACGAAACACTGCTCGTACTGGCCACAGACACGTGCACAACTTGTTGGGAGTGTAGCTGCGTCAATTAGACGTAGCCACAGCAAGATCCATGTGATAATTAGTAAGAAATAACTCTTCATGATTCGATGCGACGTTTTAAGGTTTTCACAAATATTTCGCTTACCCTTTGAAAGTTTTGGACATTCTTTCCTATTTGTCGTAAATATTTCGTTTACCCAAAGAAATTGAAGTTAGCATGTTGTTGGACGCACAGAGTTGCTGCAGCATCTCGAGTTGTTGACGTTGCATGGCTTCCAGTCGCTTGTTGGACTCCATGATAGCGTCGAGTTTCGCCATGCTTTCATCCTTGTCACTCTGTTGTCGCCCCGGAAAGATGGAGCCAAGTCGAGTGTTGTCACTCGAGCAAGTAGAAACGTTGCCCTCTGTCTTTAGTCGCTTTGGCAACGTTAAATCTTCAGCTACAGACTCGCGTTTTAGTCGCTTCTCTTCGACTGCTGATAGCAGCTCCTGTACGTCTGTACTATCGTTTTCTTCCTTGAAAGCAACTTTCTCTTCCTCCGTCCCGAAATTTTCCTCTTTTCGGACAGTTTCTGGTGCTCGTCCGGATAACACTGGGTGTCTCTCTTTAAACCTACGCAGCCATCCATCCTTGAGTTTCTCGCCAGTTTCTCCCGTTAGTGCATAGTAATATTCGTTCGCTCTAGCTAGAATGTCCTTCTTGGTGACTGTTGTGCCCTGACTTTGCATGGCTAGAATCCAAGTTACCAGCTCCTGTTCCGGTTCTCCCTCTAGAAGAGGCCTCGGCCCTCGTCTCTTATCAACTTCCCCTCGTTGAACGCGTAACACTCGACGACGGATTGTGCGTTGATGTAATAGTGGGAAGCGCTGCGTCACTTCGCTCATAGGTCTCCCTGCTAGATACATCTCCACTGCTGCGTCCATGTGCTTCCTATTGTATTTCCTAGTCTTTGCAGGACCTTCTTCTGCATCATGTGTGGCCTCTGACTGCTTAGTGGCCTCAGGGTCGTCGGAATTCTCCTCTTTTTGCTCCAAAACAGGTAGAGTGGCGTCATTTAATGCAAATTTTACAGTAGAATCTTCGTTTGTAGCTGGCGCTGCGTTTTGGACCGCTGCTGCGCTCCCGGAGCGTAAACTGAGCTCGGGATGTCTCGCAATGAAGCGATTGCACCAGCCCATTCCCACCGTTTGGGATGGTTTTTGGTCCTGCGATGAATTTTTGTTCTGACGGAGCATTTCTTGAGCTTGCGCGATGACGTCGTCACGCGAAACGCGCTGTCCTGCGCTCTGTTTTGCTAGCACCCAATGCGCTACTTGCACCTCAGTTTCCATAGACAGCAGCGGCTTCGGCCCGCGTCGGCGCGCGGGGCTGTCAGCGGCAGCGGCATCCGTGGTCTCCTTGCGCGGCATGAAGAATGAAGTTAGAAATTGAATGGAATTTTTCAGACTAAATTTAGCTTCTTAACCTGATATTGTAAAATTTACCAGTATTCCCTAAAACGTTAACAAAAAAAATGTTGAAGCCTTTTTAAATTTGTAAAATATTCACTAAAAAGACACTGGAAAACCTACGTCGATTCTCACGCATGCAGTACAAAAATCTACGCGTTAAAGCTAGCCACTCGCTCACAGCAAGTAGCTTAAAAGTTGTACTTTTTGTTCTCCAGCACACGACGCAGCATCCAGTCGTCTGTGTCTTTCACACTCGCGGAAGTGAATGCACCCATCATGGAGATCGAGGACAACGTCGTTGGCACGCTGTTCCCTCGTCGTTGTCGAGATGTTTTGTTTTGTTTTGTTTGGTGAGGTGTATTGTGTTACCAGCTTGCTGCCTGAACTCTAATAACCATTCCCGACTAGCTTGTGTTGCACCGTCGAGTTGTCCTTGCTCCTCTCCTTCTGTGCTACTTCTTCCTTTTGTCTTCCGGGCTTGCTATCTGGTGCCTGGATCACGTTCGCTGCTCACTCCTTTCCTCCATTGGTTGACGTCCCCTTGTATCCACATCTCCGCCAATGTTCTCAGTTCGTGCTCCATCCCCTCCGCCTCCGCCGTGAACATCACGCTCAAAATCGTCGTCGAGATGTTCCAACATATCGTCGAGATCCACCTGCTGACCCCAGTAGGTCCCCCAGCCGCCGCCAAGACCTTGCGTGGGCCATAAAGAGATATCGAAAGGTTTGCCTTCACACCCGCTGGACGTCGACCATCCCCCGGGTGACCCATCCAACGATCGATAGCATTTGTCGGGACATTGAGGAGAACCGTCTTTGGCCAGGTCTCCGATGTAGAGCTTCCCCATTGAGTTATCCGACCAGCCTAATTCCGAGGCTTCCTTCGCAGCAAAAACCACAATATTCACCTTGATCTCGTCGTATGGCCAGCAGTTGTAGCCGATCAGCCAGCGATTCCACGCTGCGTGTCGACGAGTCAACACGGGCTGGAGCTTCTTGGCGATCTTCTTAGTTAGTTTGGTGCTGCTGCTGTCCCAGCGGATGCAGTAGTTGATGGTTCCTTTATTGGCCACAAGATGGTCTAAGATCCAGTTGTTGTACACCATAACTTCATCTTTGATTCGGTTGTCCCAGACCCACTGCAAGTCCTTGGGGGAGATAAAAGTGTCAGGATCGCCGACAATGCAACCCCCGGTCTTGGACCTGATGGTTCCGAACGTGGCGTGCTCGCGACTGCTAGTGTTACTAGATGTTTGGGCTGTCGGTCGTTGCTCCTGTTGACCTGTTTCAATCGTTGCCGATGTCGGTGTGCTAGACGATGAATCTGTCACAGAAATTTGTGTCTGTAACGAGTCCCCTTCTTCACCAACATTGCTGGAAGTCGTTGGCGTCTCATTCTTTGATGTCGCAGCAGGACGGTTCTCGAACTCCGCCGACGAGGTTGATGGTGGTAAACTGGGTTCACTAGACGTCGTCGGGGCCATCGTCGTGGGTGCGAAACCGGTGTAGATTCAGCCTCAGCCTCGTCCGACCCACTGCACTCCAGGCTACCAGTGAAGAGGTCCTCATCCAAACCACTGCATTCAAGACTTCCATCCATTCCCAGAGGTCCGTTCTCCTGATCACTGTATCCGCTCGGCGGTGTCCAGCTTTGATCACCCATTCCGCTAGAGAGGTCACTTGTCGGGTTTCACTGCCACTTGTCGGTTTCCACGCGGAGGGGGCGTCTGTCGACCAGCCCTGATAGCCACTTCCACTGGAAATCATCATGTTGTTTGACGACGCTGTCTGCATCCACTGGTTATCGTCCCCGAAGCCCCCAAAGCCTCCAGCCCCAGAAGAAGCGGTCGAGCCTTGTGGCTCGCCTCCTGGAGCAACAGTGCCAGCAGTTTGCGTGGCATCATTCTCGCCACCGAAGTCAATCGCAGACGCCGCTGCCAAGAAAGCCACGACGACGACAGCAGAGGCGTGGAAAAATCGAGTTAGCGGCATAGTGTCGAAGAAGCGATAATGAGCCGGTATACAAGTATATGTAGTTCGAAGTAGATTATCGAGGCTATCGCTTTAGGTCTGAGCCCACATCGCATGAGTGATCCAATCGATGCTGTGCTATTTTACATGTCCTAACGTCACCATCTCTACGGTGAAAGCAGAAAACTCCGGTATCGGGTATGTAAAAGCGCCCGCCGATATCAGATTGTTATTAAATCAGTAGAACTCTTGTGTTTTTAATTGGTTTCATGTAATTCGCACATGTGATCGTTCCTCAATACTAAAGAACTGTACAGTGAGCTCCCGGGATGGGAAATACGTTGATCGCAATTTCGTTTCTCGTGAGTACCGGCACACTCCTTTCACGACCAGCTACTGTATCGACGCTAGCTGTACTACTTGAAGTACTTATTGTACGCCGAACGGTGGTCGTCGCCGTGGTCTGAAGCGTCTTCAATCGAGCTTCCAGCTGAGAAACGTAGCGAATATTTTTGAGTCGTCAGCTCACGACATTTTTCGCCTTGAAACCAGCTGTCACAGCTTGCAAGACAGCCACATACCGCATGACATACAATTTTTGCGTGGTCAACAATCTGTAGCCCAGAATCTAAAGTGGAACAAACAACCAAACTCACGCGAGTGCGTCATATAACGTGCTTTTCGTAAAGGAGTTCTGCACAACAACCGCCGACCGCCTGTACAGGATCGTAAAACGCGGGCTTGTCGTGCAGTACACATACTGTACGAACAACTATTAAAGCTCAAACGCTCTGAATGTTAAGATCTCATCGACATGCCTTGAAACCACGCAATGCGTGTACTTATTTAACAACATAATTAATATCTGTAGTATATGTATACCTTACACATATCCTAAGATCTTATATGGCGGAGTTTGCATCAATAGGGTGAGCGGTTATCCATATTACCGGTACGCTAGCGGTTTAATACGTGTGTAAACGCTCTCCAGACACATATTATATGCAGACCCCCTCAAGGACGTATCTTACTTTTATCAGCCGATCTAATACTGAAGGAGCCTAGCTTGCCCACAGCAAGTCGAAGGGCTGCGGCCTAAATTGATGAAGCAACAGTGTTCACATGGCAAGAATAACAAAGATATCTGTAGTGTGTTCTGAAACAACAGCAGATGAAAAAGGCCCAATCTCTTGCACACGTCAGGAGGCCGTCTTCGGAGCTCGTGTGGCTGGAAACGCGGCGGGGCCAAAAGCTGTCTTCGACTCTATCAGCTTGCTCTCATTCTCGCTAGAACATCATCCGTTCTGGTGCATTATAACTGTAAATTGTTTTTGTTTTTCACTGAGCATTATTATAAATCTAATTTACATCATAAGCTTAATTATATAAAAATACTACACGCAACAAAGAAATCTCAGCGACTTTGTTAATGAACGTATGACACGAATCTGTTGTGCTTTATACTGTATAGTACAGTGTATTGCATTGAGGATGCCAGCCAAAACGCTAAGAATGCTCTACTATTATATACTGCAGTATCTGAAGAGCACTGTATCGCTTCATCTCGACATTACTTCATCTTAGTACGAGAATGTTCGATCACTCCTGCTGCTATCCGATAACTGCATTCATCAAATGCGAAATTCCGGTTCAAACAGCAAACGTTTTCTTCGCCACTTGGAGTGACCACATGGATAGAGGCGACCTCGCAGATGGCAAGTCGTTCTGTTGTTGCCATAAAGCGTTGAGGTCGAGCTCCACCGTATGGACTTCGTACAAACCAGACCACACACGTCTAACATCATTGAGGTGACACACCTAGCTTCAGTATTCAATTGTCCGCCATGGCCCCAAGTCTTCTATCGAACTTCTCTGCGGTCGTTCTCGCAACCACCTTGGCGACTGTGCCAATTGAGGCAGCGTCCACTAACAGCAGCGCCTCGCACGCACCGTTCGGAACTATTACGTCTAAGCCTGGCGAGTGCGTTGTCGGCAATCCCAACACGTATGTTTCTGCTGCTGACCTAACGTGGATCTGGAACAACCGCATGAAGAAAGATGTGGAGCCGTACAACAACTGGATCATGGACCACCTCGTCAAGAACAAAGGCTCTATCAACTACTACATCCGTTGGGATAGTAACAAGAAATTAACCAAGGAGATCGCTGCCAAGCTCCAGCCTATGTTGACCCGTCAACACGCGGCTTGGAACCGCTGGCTCATCGGCTATGATTGCTGGCCGTACAACGAGATCAAGGTCAACGTCGTGGGGATTGCCGTCAAGGACAAGTCGGTATTGGGCTGGAACGACGACTCACTCGGCAAGATTTACGTCGGTGACCTTGACGATGAGGGCTCTCCGCAGTGTCCCGAGAACTGCTACCGTGCTGCGGACAGCTACTCTGGCAAGTGGTCCGAGTCGAGTGGATGTGACGGCAAGCCATTCGACATTTCACTGTGGCCGACGCAAGGACTCGGTGGAGGCTGGGGAACGTACTAAGTGGTTCTTGACGACATGTTGAAGTACCTTGATGATGAAGAGCTCGAGATTGTCTCACACGAAATGGGCCACGGCTTCGGACTCGTGGACTTCTACCAGCAGCCCAAACCTGATAACTTCAAGCCTTGTATCATGGATGCCTTCACCTCCTCGTCTGTGAAGGACACGGATGGCTGGTTGCTGCGTCGCGTGTTGGAGAGCAAGAAGAAAAACTATGATTTTTAGGTTTGCTACAGAGCTTTCGTAACTTTCAGTAGATCATGTTGCTGAGGCATAGCGAAGTTCTAGATTCATGTAGGTCGCTTAAAATAATAAACTGAAGTGGGTGTTTTCTTTTACTAATCGTTAAAATATTGTAAGGGAAGCGAATAATCCATAAACCTTTGCACGGCGAAGGTGTGCGTACACAGAAGATATTGTTCGTGACGAAGCTTGCTGAGCCCGAGCGCACCACTGGAAACGACGACTGTAAGAAACTTGGTTCTTTTAAAACTCGCATTCACTGCACAGTGCAGGGCAATAATGCTGTCAGCATGGAAACTTTCTACCAGGAAATGGTCCTCATATCATAAAATCGACTTCGCACTGCCTTTCCGTGTTGCTGGTTGCGATGCAAGTTAAGAATCATGTGTCGCTGACAATGCCACAGTGGGCCGTTCGTGATGAAATTTACTTAACTACACCTGTATGTGCAACTTGACAATCGATCGACCATTCCGCTGGCCGTGCTGTAAGTAATAGCGTGGCCTCCACAAAAAAATCCGCGCCATATCGAATTGATTCGTACTCGTGACAGTGTACTCAACATGACTATCACGATCACGCAAGATCTGTCACCGATTGAGTCAAAAGCACTCGTACATGGATCTCTGTTGTGTTCACTCTTCGTTTGCCAGTTATGACCCCAAGTCTTCGTTCAAGCATCTTTACAGTCGCTCTCGCTTCGGCACTGATCGTCGCGCACAGTGAAGCCGCAACAAATGTCACTCACGCCCCGTTTGGAACCATCACGTGCAAGTCTGGAGAGTGTGTCATTGGCGATCCCGACTCGTACATATCTCCCAAGGACCTCCAATGGATCTGGGATAACCGTCTGAAGCACGACGCAACGACTTACAGCAACTGGATCATCGATGACCTCGTGGCCAACAATGGAGCCATCAACTACTGCGTCCGCTGGGATAGCACCACCACCCCACTGACCAAAGAGGTCGCAGCGAAGCTTGAGCCCATGTTGGCTCGTCAACACGCCGCGTGGAATCGCTGGTTGATTGGATACGATTGCTGGCCATTCGACGAGATCAAGGTTAAAGTCGTGGGCTTCGCTGCGAAGGACGCCTCGATCCTGGGCTGGAATGACGACTCACTAGGCAAGATCTACATCGGCGACCTCGATGAGAAGGGCTCACCTCAATGTCCGCACAATGCTACCGTGCAGTAGATGAATTCCTTAACGGATGGTCGGAATCTAGTGGATGTGAGACAGAACCATTCGACGTCTCGGTTTGGCCTACACAGGGCATTGGTGGCGGCCTGGGTAACTATTGGGGTCAGCAGGTGGATCTTGATAATATGCTGGAGCACATTGACGACGAAGAACTTGAGATCGTCTCGCATGAAATGGGTCATGGCTTCGCTCTTCCGGATTTCTACCAGGAGACCAAACCAGACAACTTTAAGCCATGCCTCATGGACGCAACCACATCGTCCAAGGTGACGGACACGGACGGTTGGATGCTGCGCCGCGTGCTCGAGTACAAGAAGCGCAACTACAACTTTTAGAAATGCAAGTAGGTGATGCATGTGTCATTTTCGCCCTCTTGAAGAAGACGAAATTTTTTTCTTGTGCTGGCCCCGATTTAAGGACAGGAATAAGTTTTGTGTTCTGAGTTACACTTGATATTGCCTAAAATTATGCAGCTTATGTTTACTAACTATTAGGTAGAACCCTGGGCGGCCTAACTTACGCCCTTAGAGAATTTAATATGTAATATTAAAATATGTATTATTTCTTTCAAGNTTTTTTTTTTAATAAAAAAAGCTTTTTTTAAGTTTTCCGCAATTACCGGTAGCCGGAAATAAAAAAACTGAAGCAATGCGAGGGTCATAAAAAATATGGTCTAGAGGGGACCAATTAATTTGACCAATGAGCGTGCACTAACATGTAAACCATATCAACTTTAAGAATCCAAAGTTGACTCAACATCTATTAGTGCAGTTTGGTGAGAGATGCGGGTGCCGAGTGCGCGGCGTTTCGTGACGGCGCTATGCGTGCTACTGCTAGCCGGCAAGGGCGCGCAGAAGCTGCGCCTATTTGTGGCGTCGATGCTGGCGAAGTTTCGCGCTCTCACGGCGACTGGATCACCCACCACACCGGCCTGGCGACTATTCAGTGTCTTCCTGGGCGATCTAAAGGACCACCGCGTTGGTAAAGTGCTGCTTGCAGGAGACCACTGCATCGTACAGGCTAAAGATGGCGCTGAATCTTACAAGTACGTAGTAAAAAGTATCTTTGGGGAAGATATAAAATCTGACAATCTGTGGTATTCGATGTGCCTTATAGAGTGCTGGTGCCGCCAAGGACGGAGAACACATACATTCTCGACGCGTTAGTGCGTTCAGGAGTGGCTTTCGGCTCCGTGCCACCATCTACAAGCAGCAAACTACTACCTGTAGCCATCGCCCTGATGCCCTTCCTATACTTGGGACTTACATACAAGATGCTGCGTGGGATGTACGGCCCCGACGCTGGAGCAGTGGGTAAAGATGGCACCAAGAAGGTGCGTAAACAGCAAGAAACCGAGCGCATCTCGTTCGATGACGTGGCCGGCATCGACGGCGCCAGGAAGGAGTTAGAGGAGGTTGTAGATTTCCTAAGACACCCGACCAGATACCACGCTATTGGAGCTAAAGTGCCTAAAGGAGTGCTACTCTGTGGGCCTTCAGGAACCGGTAAAACACTGCTGGCTCGAGCGGTGGCTTCAGAAGCCGGAGTAGCGTTCCTGTTCTGCTCAGCATCGGACTTTGTCGAGATGCTAGTAGGTCGAGGCGCTGCTCGAGTGCGAGATCTCTTCACCCAGGCCTCCCAGTATCCGCAATGTATCATTTTTATCGACGAGATTGACGCCTTAGCTAAAGCGCGTGGAGGTCTGAATTCGAATGACGAACGGGAGCAAACATTGAACCAATTATTGACCGAGATGGACGGCTTTGAAGGGAATGTGAACGGAGTTATCGTGATTGCCGCGACGAATCGCCCAGAGGTGCTGGACCCAGCGTTATGTCGTCCTGGCCGCTTCGATCGCCATGTGTATGTGGGGTTTCCGGATGCCAAGGGACGTCAAGAGATCCTAGAAGTGCATTGTCGTAACGTCAAACTTGATAGTGAAGTGGATCTTGCGGTCGTTGCAGAGCAGTGTGGACCCATGGGTCAACGCTCGGGGGCTCAGCTTGCCAGTCTCGTCAATGAAGCTGCGTTGCTAGCTGTACGTCATGGAGACACTAGTATCAAGTATACGCACTTTGAACAAGCAATGAACCGCGCCTTTGCCTCGCAGACACGTAGTATTGCGGGGTCGTCGTTCGAGTTCGCAATGGAGGAGGCGTAGATTGTCAGAGCTTATCAAAAATGCTTATTCGATCAAATGCAGTATAAGCGATTTCTTAAACGGTACTACGTGAAGAACGACGCTATTAAATGCGTTACTTGAAGCTGTAGCGATCTCGGATGCTCTCGTATGCGCGACGAAACATCCATCCGTCTGTATCCGTGATAGTCATCGAGCTACCGGCCATCATGATAGCCGGGGCCATGCCTTTCGCAGGCTTATCAGCGTCCTCATAGAAGTCCGGAAGACCGAAGCCATGACCGATTTCGTGAGCCACAATGTGGAGAATCTCCTCGTCTATGTTCTGTATCATGCTCTCGAGATTCACTTCCTGGCCCCAATCGTAGCCAAGGCCGCCTTCGAGACCTTGTTTCGGCCAAAGCGACAAGCCGAACGGCTCACCCTCACATGCACTCGTGTCCGACCAGGTTTTAGGACCGTTATCGAAGAAATCGTAGCAGGTTTGAGGGCATCGTGGAACACCCTCAGGGTCCAAGTCCCCGGCATAGATCTTACCGAGCGAGTCATCTTTCCAATCCAACAAAGACGCCTCCTTGACAGCAAAGCCAACCATGTTGACCTTAATCTCGTCGTACGGCCAGCAGCCGTAGCCGATCAGCCAGCGGTTCCATGCAGCGTATTGACGCTCCAACATGGCCTTGAACTTGGAGGCCACAGTCTTGGAAAGTTTCTGCGTACTGTCCCATCGCACACAGTAGTTGAGTGTACCCTTGTTCTTGACAATGTGGTCGAAGATCCAGTTCTTGTTGCTCGTCTGGTTAACACCCATGCGGTTCTCGAAAATCCAATTTACGTCTTTGGTGCTGATAGTGCTCGAGTCCGGATCACCGATGACGCACCCTCCGGATTTGGACGTCACCTTTCCGAATGGAGCATGGTCAGCGGAACTTGAAGACGTCTGCGTCGCGTCGGAGCCTCCTTGTGTCGGTTCCGGGTCTTCCTGCGTCGTTGAGGACGCTGTGTTAGAGGACTGAGCCGTGGTTGACGACGAGGCGGTATTTGAGGATGATTCCGTATTTGAGGATGACTCAGTGTTAGAAGAGGACGTCGTCGGAGGGATTTGAGGGCCTTGGTCACCTGTTCCACTAGACTCCATCGAGGTCGTAGGAGTAGGCTCTTCTATCCCTCCTGGTGCAGGCGTGTCTGGTTCGTCAGATTCACTAGAGGTAGCTGGAGCTGTTGTGGCGGTTGGGGTGGTGTTTGAGGATCCCTCTGTCGAAGTTGGTACAGTCAGCTCCTCTTCGTCGGATCCACTGCACTCCAGACTGTAGTCGTTGTCAGGGCTCTCCCCCTCGCCTGAATCACTGCACTCGAGACTCATATCGTAGCCAGGAGACTCACCGCCTTCGCTTGAGCCGCTGCAGTCAACGCCCAGAGTTTCGTCGTCGCCACTGGAGTCACCGGTTGAAGGCGGTTCGGCGTTAGATGAAGGCGGCGACCATCCTTGGTCTCCTGTTCCGCCGTCTTGGCCACTTGTCGCTTGGTTTCCGGTATCACTCGAGGTTGGTGAAGCCCAACCTTGATCACCACTTCCTGACGGTGGCGCCCAACCGCTGTTTCCGCTACCACTTGAGGCAGACGGAGTCCAACCTTGATCACCACTTTCCCCTGACGCCGGTGGGGCCCAACCGTTGTTTCCGCTACCACTTGAGGTGGGTGGCGTCCAGTCCTGGTTACCGGTGCCACTCGATTGTCCGCTGGTCGGTGTCCACACTGAAGGGGTGTCTGGCATCGTGGGCTGCGACCAAGTTTGGCTACTTCCACTTGTCTGGCCACTACCCGCCGAATAGACCGAAGGGGTGTCCGGCGTTGTCGGCTGAGACCAGCCTCCGCTGCCGCTTGAGGTGACCATTGGAGGCGTATTGGGGGCACTGTTTGAGGAGGCCTCATACTGCATTGGAGAATCGAAATTTCCGAAGCCACTCGCTCCAGTCGCGGCATTCGACGCAGGTGTTTCGCCTCCTGGAGCTACAGTTCCTGCAGTCTGACTGACATCATTCTGGCCGCTGAAGTCGATGGCGTAGGATGTAGCAGCTAGAGTAGCCGTAAGCAATACACGTCCTGGAAAGCGGATAAGTATCATCCCCAGACAGAGGCGATGATCTCTTGGCAACAACAAAAAAATTAATGGACTGAAGACTTGAATTTTACTGCGTTTACTGTCGTACGGTACGACTCGTACCTAATCACGGAAATCCCATCAATCCTAGTCGCTCTCGCTGTTCGTCACACGATTGGACGAGAAAGACTGGGCAAATTGTGAAGATAATTGTGGCTGCTGATAACCAGAGTTGAAAAGACTTATCTTCTCGGCGCCCACCTTTACAACAATCGGCCAGGATGGGCTGCCGCAGTGTTGCCAACACTAATCCAACACTGGTATATTGTGCCGCGGCTTTATTTTCGGCTATTCGATAAAGCCGAAATAAAGCCGATCGGCTTTATAAAAAGCCGCGGCTTTTGCATCCGGACGGCTGTAGAAGCGTCTGTCATTTATCCGGACGAATCCGGACAGTCACGGCAAACATTGTGCCTTCAATTCTTTGTCGTCATAGGGGATGAACTGCGCGCCACTATGGATGATCTGCAGATGATCGAGGCCGCCCAACAGAGCTGGACAAACTGCAGAGTGAATACGACCGTGATTTTGTGGATTTAAGAGGCGAAATTGAGAACGAACGACTGCGTCTGGAGGCTAAAATCCGAGAGAATTTGGCAGCTAAACGTCGGCAACGAGCAGCAGTGGAGAAGTCAAATGACAGTGGAATTASGWSKGAMRWSMKTSGWRTRGGGAAAGAGTGTAGCCAGCTGGATCTCGGTGGTCGCCGTATCATTCAAGCAGAAGACGGCATACGAGATTCCATTGGGTGACTGGAGTTCAGACGTGTGCTCTTCCGATCTAGGATTATTGAACGTCCTGTTCATATTTAATGTAGTTGAAGCATTCGTGAGATGGCAACATTTCCCAGCGTTTTCTAAACGCGTTGGGTCCATGCCTGCACGCATGTAAGCGCGGTCCATAAGGAGGGATGTCACAGCTGTCAGTCCTGTACTAAACCTCATAAGATTTTTTCTTTTAAATTAGGAATTTAAGCAAAAAATGTTGTCTCACTATTGTTAGCAAGCTAACCTTCAAGTGGGCCCACGGCCCAAACCTCCACGTCCCATAAAAAATAATATTTGTTGTGTGTTTCCATAAATTTACAGCTCTCCAATTTCCAAAAATAAATAATCTTGTGAGGAAATAGCTTATTGTTCGATAATGTTATTGACAATGCTGTTTTCGGAGGCCCTGCGGGATAAGGTGTACAATCCCTCAATTACCAATGCTAGCGTGGCCTCACGTTCTGTGCGGCCCAGTGCGTTGGACTGCGCCAGCCACTACATGTACAGGCACCCGATTCTGGATAATAGCACTTGAAAAGTAATAAAGTGTAGTGAGTCACAGTGTCCGAGCACAAGTCTTACGGCCTATTTTATATATAGGCCGTAAGACATGTGCAAGCTAGGCTATAAATTCAATGGGTTTATCATAAACTGTAGTATTGTCTAGAAGTTCAGCCATAGAAAAGCATTGACGGGGCGGGTAACCTGCACCAGAATAATACGGTCTCGTATTCAAGTTGCTTTATTTCTTCTGTCGCTACACAAACACTGAAAAATGGGACAAAGAGGGGAGTTCAAAACGAACTTCAGCTGTTGAACTACTTGTAGCTCTCTACTAGCGACAATCGTGTGGTCTTCGGGTGAGGCGTGCGTAAAATTCTACTTCTTCGTGGGTATTGTTTGGCGGTAATTGTCGTAACGCTTGACGATCATACTGACGTCATCGCCCGACTTGCCAAGCGAAGCGAAGTATTCTCGGACTTTGTCGGGGTGCATTTTCTTTCCGGCCCAAGAGGGGAGCGCAGCTGTAAACAGGAATTCATCGCTTCCGAACTGTTTGAACCACTTGTCAACGGCAGCTTTCCTTTTAGCCGCATCCTGGGCCTTCAGTGCTGCCTTTTGAGACGCCTTAAGTGCTTGTGCGTTTTTAAAGGCGCTTGTGCCCGGAACAATCTTCTTAATCGACGCGAAGTTAATTGTCCTTTCTTCTTGGTTGCCGGTTTCCACAGGCTGTGGCACAGCATTCAACCGCAACAATCTTGCTGTTTCGTGAGTACCTTCACTGCTCTTGGGAGGTCCGAGTGCTGCCGCGGAGACTCGTCCATCAGGAATGATCGCAAATGCTACAGCCACCAAAACAAAAGCTGCCAGTCGCATAGTTGGTGGAAAAGAGTAGTGGTGGGGTTAGTACCTTCGGGTAGAATTGCGAATGAAGTAAATAAAGGAAAAGGTGGATCACAAAGAGGCGAAACGCTCAAGCACCTGGATCGAAAGAGCCAGTAAACAGTCCGTGTGCTGTACCAATTCGTATTACAAACATCTAAGGATAATACATGTAGTACATAGTATGTACTACATTCAATAGACATTTAATATCCTTCGATGTGTCGTCTTGATATTAAATAGGCACTCGTTATTTTGCCCGACTGAGGTAACATAGCATCGCATTTTATACGTGTACAAATAGTATAGTACGGTTAAATTGGGGTAAAGGTAGGACACTAGCGCGTTTACACTCGTCTGCGGACTTATAGCCGTGGGCCCGCCCCTCTCTTCACTTGCTGCGCTGCGCTCGGGGGGCGCCGCTGCGCGGCCTGCCCCACCCCGGCCTCTAAGTCCTCGACTCGGCAATGCGCTGTGTCTGGGATTAGAAATTAATTCCGAGCGAGTGTTGCCAAGACCGTCGCTAAGGCCATTTTATGCATGTTGTGGGGGTATAATTGTATAGTCGGTTAGCCAGTCGGGCAAAATCCTCACTTCCTATTAAATAGCATGAAGAAGTATGTATTCGTATTAGTTACTACTAGTAATAGCAAGGGCTTGCGACACATCTAAGGATATTCGTAATATCATAATAAAAATGCAAACCTCTTCTGAAAATCGTCCAGTACTGTACGATAATAAACCAGTACAATGTACAGTACTGCATTCCGTTTCCGAGTCCTTGGACCTTTGATAAACTTCCGGATTTGATGATCCAAAATAAAACAAAACTCAGCTGATTAAGCTAAAATGTTATTTATTCGCAAAATTTATATAATACACCCCCGCGAAAACGGAGTGCAGTACTGTACATGTCTTTGCCCTGCAGAAGTTCTACTTAGATTGGGAGCTCGAGGAGCCCAAATTTAAAAGGGAGTGAGTGCTGCACCCACCTCGTGCACATGGGACAGTGCTGGAGGGCTTGAGATCCGCATCCGGCTTGCGGGTATACCTCGTGTAGCGTATACTTCCCCCCCCACTCATTTTTGCAGCGGGGGCAAATCAGCAGCAGCCAGGAAGCCATGGCGCCCGACCTGCGCGTCAAAGCGGCGGTGCGACCGCCGCGCCACGGAGGAGGGGCGAGTGATAGTGAAGAGGGGGTCGACAGCGACGAGGAGTGGTTCGACTCGTCAGCGGAGTCGTCGGGTACCGGCAGCTCCACATCCAAGCAGCGTCGATCGGATGTTTCCGACAGTCTCCAGACAATCCAGAGCGGTGAGCAGCCAGTGGTGATAGCTCCGTCACTCGAGAAGAAAGAGTTTAATTCCTGGGGTGCCTTAGACGCGTATCTAAAAGTCTATTCAGCCGAGACATACCAGGTAGCGTTTGATGTACTGCATATGGTAGCAGGAGCCGTATCGCATTATCTAACTGGTTTGTGCGTACAATTGCTGCGTACAGAGCTTCCGCGTTCGCACTAACAACAAGGTCGTGACGAGAAATAAGAAGATTCGAGACTCCGGGTCTACGAAGCCGCTTGTGCCCGAGGGGTGGACTCATTATTCGAAGACTTTCGTCTGCACCCACGCAGGGAAGTACAAGCCGCGTGGACAAGGCAAGAGAAAGCGGCAAGAGTCGAGAGCTCTCGAGTGTGACGCGCAGGTGTGTGACGCTCAGTCGGGCTCAGTCGGGCTCAGTCGGGCTCAGTTGGGCTCAGTTGGGCTCAGTTGGGCTCGGATGGACTGAAATGGATTTGAATGGTGCTGTTGGTTGGCCATGCGTGTGCTCACGTACACTTGTATCACTACGTAGATCAATGCCTGCGTACAAGTGACCGACTCGGCTGCAGCAGTTCCCACTTTTGTGCTGCGAATTACTGTTGCCCGTTTGGAGCATAACCATCCGCTCTCCAGACATACTTTCGACCATTATCCACACAGCCGGACAGCTGTTAAGTCTGAGCTGGCCGGGACTGTTAGCGAGCTTGTCAAAGCCGGAGCCAAGAAGAAACGCATCCTGCAGTTCATCCACGAGAATTCTAGCTGCTACCCAACGAGCCAGGACGTACACAATCTGGTGCGCAAGCTGAAGAAACAGACCCATACAGCTCAGACATCTGCAAAACGATTGAGGCAATGGATGACTGAGCTCACGCAAGAGCTAGGGAATGTTGGAGGCATTTTTGTCGACTCAATCCATGATAAGGTATGAACACTATTGCTGTTACTGTGTAGCCGTCGCTTGTTTAACTTTCTATGCTGATGTTAGACTGTAGCTACATGTATAACACTGCAGACAAAGCATATGCGGGAGCTCTTCGACCATTTCCCAGAGGTCGTGATGATCGACGCCACGCACGGTACAAACTTGTCAAAGTACAAGGTTTTCTCGATTATGGCACACGACGCATTTGGGAAGGGCCAGTTCGTACAGCATGCTGTTCTCCAGAACGAGCGTAACCAGACGCTTCTGACGGCCCTGGAGCAGTTCAAGCGCTACAATCCGGCGTGGACGCACATCAAATGCATACTGATCGACAAAGATTTCGGCGAGATCGGCGTGTTGAAGAAAACGTTTCCGGA

>Contig_25

AGAGCACTGATAAGCTACGCTTAAAAATTCTCGAGGGGGGCTCACCGTGTAGACTCTGCCATTTGAGGAGTCATAGCACCGAGCGCTTTCTTAAGCGTAAGGAGGTCAGTTTAGACTGGCCATCGCTGTTTAGCGGTCGCACGGAAGCCCTGGGCGAAAATTGTTCATTACGAGATGGGGAGAGTAGACTGTGTGAACGTCGTCGCCGACGGTCCCAAACCCAGACACTAACCCGGTCTAGTAGAGTTACCGAGTCCTACCCGGAACAATTCCACTGACGGAAAAATTTGAGGTCGTCCCAACGGCAAGGGTGTGATCGGATCAGTAGAAGATGTGGAAGCACTGCCTTGRRGATATATTGATAATCGCCCTGCGGAACTTCTCATTGACGCCGGAGCTGTAGTGTGTTTAGTGCACTGACGTATTCTGAAGCGTCTTGGCCTAGCTAACGCCCCCTCTGCTGCCCTACTCTGATAGTCTGAACGGTATATCCGGGCCGACACTCGTTATCGACGGCGCTATCGGGTTGCCGGTGACTTTGGGGTCTGCTGAGAGAAGAATACATTTTGCCGTCGTACATAAGCTACACGTCGACGCTATCCTAGGCACTAATACACCCAACGCCTTTCGGATAGTGGTGGAGCTAGCTGAGAGCACTGTGACGTTGAAAGACACGGGCGAGGTGCTCCCCATCGGATCCCCTCGAGTGGAGGAGTCTTACATGCCTCGTATCTCGTCTTAAGTCAAAATCCGTCCGGGAGGACAAGCGCTTGTAGTGGCTGACGTACAAGTGGTTAACGGAACTACCGTTCTGGTGGAAGGACTTACTGCTCTAGGTGCCTAAGTTGAAGTCGGCAGAACACTGTTTGCGCTACATGATGACCGAGTGATCGTGGAAGCGTGCAGCCCCTGTATTAAGGACGCGGTCAACAAGAGGAGTACTCAATTGGCTGGTATTACTGCGATCCCCGAGTCAGCGTTTTAACTAAAACCTTTCCTGGACATAGAACGCAGGTCAGATAAGTCAAGCTGGGCCGTAACAAATGGCCGTGAAGCGGAAGACTAATGGATACACTCCGTTATTGGAGATATTAGTGACGAAGCGGTAGATGCGACGGAATCCATGCCTACACTAGACCGAGTGTTCAACGAGGAACTCGAAGTCGCCTTAGAAGAGTTCAAGCTTGGTATCGAAAAGAAGGGGCTGGCCACGGAGTTCCTCCAACCATTCGGCGACATACTCGTCGAGACGTCGTTGCGTCCTGGGCGTACTAACCTACTTGTATTTTCTGTCGATAATGGCGACCATCCGCCGATATACCAACCTCCATACCGAGTGTTCTAGGCGGAAAGAAACGTCATAGGGACTGAAATACAAGAGTACGTGACTCATAGACGTAAACGACCTTCGACTAGTCATTGGGCAAGTCCGGTGCAAATGCGTCGTCAAGCCTCCCTCGTCGATGCGAAACGGTTTAAGAGCACTCTGAACCTTATGAGCGTACGCATCCGTGTCTCGCGATGTAGCTTGGAGAGGATATCTATGACTTGGCCGTTGTATGACTTGGCGTCAGGAGCGTTCAGGCTGTCCACCCATCCACATGAGACGACGTCGATGAGCACAGTTCTCGAGCAGTTGAATACAGCCGACGTATCCCCAAGTTTCGATGCAGATGCTCAAGAACCAAACGCGCGCGACTTTCAGCGCTGATTCTGTGCGTTGGCAGTCTTCAGCGGAACGTTCATCTGCAAGTCCCAGAGCCATGGACGGTTCTCGTAACAGTATGGTGGCCGTAAATTTTGTCCAACTCGACAAATCTCTTTTTTGTATGTGATCCATGCCTACACTAGTGTTAGAAGATAGAATTGCGTCTGCTTCAAAGTTGCTAACTATCCTGGTAGTGTTACGCTCGTTTCTTAGGGGCAACAAAGCCGATGTTGCTGCGACGAAGCTGGCGCAACGGGTTATTTGCCACTGTAGCCACGTGTTTAAGTAGCACAAGGTATCAAATTCAAAACGAAGAAAGCATTTCTGCTTTCTCAGCCTCTAGTTGTCTCCATGCTCTGCTACAAAAAGATCGTGAAAAGGTGGCGATGGCCTCTCCGAAGCTTGCACTACAAAAAAAATTGTAGTTACTAGTTGGACGAACTATTGGTTTGTATTTTAAAGTGGTATAAAGCAGGAAAGAAACGTCCAAAAGACATCTTTCAAAAGGTAGTATTCTCGTACTTACTCGTTTCTAGAAAACGCTCTTTTGGAGCATGATTGCAACTTTGTCAAGCAAATTTTCACAGTCGACGCTTAGTCTATATCGACTGGTACGTCTTTTTCGACAGTCTACGACCGCAGATTTTGGCCGCTCTTCAATTGTGGGTGCCTTTCATTCTAAAATCTGGAGTATTGGGCCAACAGGTAAACATTCCCAGTATCCAGAAGGACATCGCCCTTGCGAGCTATCAACGTCTCTTTCATCGATTTTGGGGTATAACCCATTTTTTCGATTTCTTTAGAGTGGTCATGGTTGCGCTGGTTGAGTAATTGGAGAGCTGCCTCGTCATCGATCTTGGATAGATCAACCATCGACTTGCTCTTTGTCAAAAGAGGCTCCTTCAGAGATAATGTCTTCTGTGAAAGCACCTTCTCCAGTTCCATGCCCATCTCCTCCTCATCCACCTTCCAACTATCGGCCATCTTGGCGTCCTTCATTTCCTCTTGAGTTCTTAAAAATCGCTCGCCACCAATCGTAGATATTATGTTGTTCACAGAGTCCGTCTTGCCTGGGTAAAGGACGTTCGACTGACTCGTGACAGATAAGCCTTCAGCGAAGGACGCAATGGTAGTAACAGCCGTCAGGATGTACCAAAGGCGCATCTTGCACGTTTGGGCCGGTGGCGAAGGGGGTCGGGGGGGAGGGTCTTTGCAGATTTCGAAATGAACGTTTCAGCGAATGGGCGGGGGCTCAATTATAGAAACATCTTTAAAAACATATTTAACCGACCAGAAACGATGATTGTCCCGTAATCCAACATATTAAAAAAGGTCGTAGTTATGTTATTTTTTATGGGATGGCCGTCGGAGAAGACAGACCGGCAACTTTACATTCACAATGTTTTAAAAAGGTACAATTTAGTCCCCTTGGTGCGCGTATGCCAATCACTTGGGGCTGATGCGTATGTAATACTGTATATCCTGCCCGTACAAAAATGAGAGCCTAGTCGTTTACACATTTCAAGATTATTCTATCGAAACTAATGCAATTGGTCCGACTCTACGTTGACCGCGTTCACGAGATAAAAGGTGTGATATTTTGCTCTGCATGTTTCCCTTTACTTCGCTACGGATCGCGCAAACGAGGCGTGCCAGGTTATTTTGAGCCGAAATATTGACCAAGACAATTATGTTTTGCCTTTTTTCTTTAATACATACATTACACGTAATTTTCGGCCAGCCGATCGATATTTTCGGGCCAACCGTATGGACGGTCATGGTCATCCGAAACTTGATGACCACGGCTTTGAAGTGCTTACCTTTCTATCTCCACCCCTACACTCAACGTGGATGGTACCGTATACGAAATCTTCTACTTCAGACATCAAATTATTGAAGTTGTTGTGTTGTGTGATCTGTTCTTGAATTTGTTGCTGGATACCAGGCTATAATCTTGTTTTTGTACAAAATGTTTGTCAATATCACTCTAGCACCTTTTCTGTAGTACTTAAGTATGAATTAGGAAATCTCTCGGATATCGTTTACAATACATGCTTCTGGTGCTGACGCTCTTTCGTATTATTCGACCGGTTCATTTATCGCCCCTTTCCTAAATAATATCGCGCTCTTCCATCAATCGATACTAAAAGTGCCCGCATTTTGACTGCAGTGTCACTAACGGACAAGAATGTGCACACCCGATTGTCAACGGCGGCTTTTCAAATTTGGCCCCAGTCTCGACTGCCCCCATTGCCCATTGGACAATCTACCTTCGACTTATCTCAAATCATCTCGTGTCTGAGGACAACCCAGAATTTCGCACCATACAGTGTATGGCACACGGTGAAAAGTAATGTAAAAGCTGCGCAAAAATTACGCAGAAGGCTCGCAGTCTTGAGTTGATCTTACCGCTCAAGAACATGCTACCAGGACTTACAACAATACCATCTTCAGGAGCGCCCGTCGATGGGAGTGTCCAACGGAGCGCTTGTTCAGACATCTTTCGCTGAATTTTTTTTCGGTGGATGCCATGACCATCGAAATACGTCTGATTTTACTGCGTACGAAAATTTATAACATCCTTACTTAAAAGTTTGTTTTCCGATACGCAAGCAGCTCTTTATTGTAAGCTAGATCTGGGAGGAAAACGAGTGAATACACGAAAGGCAGACTATTGTTACAACATGAGAAACGGGTGTCTGGAGACTGCAATCTCCAAAAGGGGAGGAGTTGGCGGAATTCTATCACCTTCCTCAATCCTAATCCTGACCTTCCTGGGCATTAACCCCGTAGGGCCAAGCGAGGTTTATTATAGCTATGATTTGTTCTGTGCTTAAGCCCGCGTGCTTCGCACACGGATAGGCATGGATTAGTGTTGCCGAGCGAGTCTACTATCACCCCCTCCTGAGGAGGATCCTCCCTTATTCGTACTAATAGGAAAACCCGTCGGAGTAACTACGTGTTGTATGAAATTGGCTTCACACACTGAGTTCCCAGACGTGTCGCACGTGCTGCAGATCGAGCAGTAAAAAGAAATGGTGGGTAATGGCTTTCTTGGATTGACTAAAACTGACCATTTCTCTCAAATTATAATGCCCTTTCCAAATAAATTATCAAAACATAAAGGCTTTTGCTTATCAATCATAAAGCACGTTTTTATTAGTAATTAAATAAAAAGTAAAAATGTTGTTAGATTGATACAATTAATTAATGGCAGATGCTACTGCCCATGCAGCCACGCCAGGTGTTGTCCATAAATTACGTTCTGCGTAATTTATATCAATACTGAAATCAAGTATTTACCGCCGATCAATTTTTCACCGCATCAAGTTGCTGCTAAATTGCATTGTCAATTTTGTAGTCCATGTATTGAGTCCACGTATAACCCATAAATCACCCGTGCATTAGGCATGAACACGAACACAGGTAACGTTAGTATTATAGTCCATGTATCGAGTCCATGCATTGGTCAGATATCCTCCGTGTATTTAGACTCAAATTAGATGGGTTAGTTAGATCTACCATGGAGCACTAGCTGAAGCATGTGAAAGCGCAATATCGTGAGACCTTTTGGAGTTACACCCAGCGCAATGCTATTGTCTACCATTCGCCAGAGTCGTAGCATTACGAAGAAACCACAGTTGTGGCCGTCCGTCTGAATTGGCGCGTTGATGGATACAATCTCATAGTCGTCGCTCAGGGTTTTCGCCAAATCCCAGGACAGTCGATCCAGCACGGTCTTGTAAGTCCTCTGGCTCATGGAGTCATAATAAAGGACTCGAGTTCGCTTCACATCTACTGCAATTCCGCACCAGTGCGAATTTCCAACGTTAACCGGGATCATAAGGAGGTCTGCCTCTCCCACCAGCTCTTGCGCTTTTTTCTTGAGGGATTCATTCATGCCCTTGGCATTTCGCCCCGTCACCGTGCTCTCAATCCCCAGAACACGAGCAGTAGGGTTTGTTGCACACAGTCTTGTGCAGAAAGCACGTATCATGGCGTCGTTCGCCCAGGATGGTGGCTTCAAGCGGTATAGCATCGCGTAGTGATACTCAGTGGCCTCGATCACGATTGTGCTCTGTGGGTTCGATTTCGCCACTTGGTTTACCATGTTTTCCAACACAGGATGTTGCACTGAAGGTATGGTGCTTATCACGTTTAGAAGCAGCCAAGTGCAGAATTTGACACCTTGCTCATTATAGAAGCAGATCAACACACGCGCCTTGGGAAAGTACTACTTCAGTACGCAAATCTCGTGCAAGTCTTTATCGACCACGATAACTTGCACCTTTTCGCCGATCTTATCATCTACCCGCAACAGGTGATCTAAAACACGTGACATGTGCCAGTCGGCATTTCGTTCGATGAGCGAATGTTGAATTGACTGCCCACGACCGTTATCGTCAATGACCATCAAAGTACACAGTTGGTAGTTGTACCTAGAACACACGAGCAGATTGATATGAGATAAAGATCAATAGATGGACAATACCTAGACAATACATAGATATAACCTACCGGTTGGTCTTGTGAGTACAATCCACTAGCAGCAACTCGGGGAAGCGTACGGTCATGTCTCGCTGGTGCCTCAAAGTCATGCTCATGACACCGGAGTTGTCAGCGTCAGTCTCGTCCACAGTCGCCACGTTGCCATCCATCTCTGCAAACGCGTTAAGTGCTTGTGCGCAGGTATCATCATCGTCGAGTCCGCCTCGAAACTCGGCCTTGACACGCTGGACAATGTTGTCCACGTCTTTCTTAACCAGGCTTTCCCCTTCGTCGAGCAGGTATTCGTAAATACGAGCGCGCTTGCGACCGTACTTCACCATTTCGCGCACGGTGGACCTTGTCTCAGAATCCTCCAACTTTCGGTTCTCTGCGTAAGTTTTGAAGTGGGCGGTACCCACGACGTGGTTGTGGTGGTAAACCCCGTTCTTCACTCGAAGCAGCCACTGGCCTTCCTCTTCCACGACTTGAACCACGAACCGAAAAGGACACCCAGTGCCACGAACAAACTGCTTCGGCCGCACACCTGTGCTCCGATCCTTCGTCCGCCACCCATGCGTACAAATATATGTGCGCTGGTACGTTGCCCACTGGGTCGGAACGTATGGTACGTCAAGACCTTCTCGTGCAGCGACAGTTTTAGCTAACCGCTTGTTCCGCAAGTCGCAGCTCACAGTAATGAAAACTCGGATGATCTGCCGCGTTGAAGATGCTTACTTTTTCAAATAGACCGCCCAGTCGGCCCAGCTTGAGTGAAGGGCTTCCGGGGCACAAACAGGCACTGGACAATCTGAAACCTAAACGCTGCATGGACGTTCCGCATTGTCGGTGCCCGACTCGTCGGAGTTCTCAGAGYCTTCTGCAGAGTTCGGAGGAGCGGAAAGAGCAGCATCTGACGCCTCTTCGCTCGAGCTAGCCTGTTGAGGGCACGTAGTTAGCAAAACCGACATACGTATGAGATCAGGTAGTTATGCAACGCACCTCTGGCTGACCTGTCGCGGTTTCTGAGGCCGGTGATGGCGGCGTCGGCAGTGAAGAGGGCGCGGGAGGCTCCAGTGGGGGAGTTTCAGACTTGCTATCCGACATATCGGAGCGGGGGAGGGCGGCAACGACGTGCACAGCGACGACGGTACCGGAAGCTCCGGCTTATCGAAAGTGAAGTGAGTGGCTGTGGTAGCCCTCAAAATCGTTGAGAAGCATGGACATTTTACATGCATAATACATGTATTTTACACAACCCGTAATTTATAATTTTCAGGTCGGTAATTTTACGGCCTGGACAATACATGGATGGCCACCTGGACAATTCATATACTCAGCGCATGGACGATAGCATTTACGTTAATTAATTAAGGTATCGTATATAATAAGTGGTTTTTAATGAATTTAGTAAGCGATTAATAAAATCACTACCCAGGAGGTGGTTGTATTGAGTCCGTCCTCTGGGGAGAACGGACCTGAAATGGTGTAGAATTTTGTTGTGGCCGTAATACCAGGCAATAGTCGCAGACACGGACAAGAACCACCACAAACCAGTTCTGTGCATGTACGACATTTGGATATGCAGCGGCTGCTGCTAATTAAATGCTTGCTGTTTCGTTACAATGTACGTACTACTATAGCCAGGATAAATTTCCCAGATATTTACTGGCCAGCCTCCTTCCCGCCAATAACATCATGACTTAACTTTCCCAAACTCTGCCGGCGGTATAAGATACTTGGAGGAGGGGTGATGAATCTGACTGGGACGGCAATGTTCACTTCTCTTTCCTACAAATAAAACTTGGAGTCAAGCATTGCTTTGGGAGCTTAAAAAGGTGGATAAACTGAAGATTGGCACAAATATGGTCAGACGCAGGGATCTTCGGGTTTTCTTGAGAAAATCTCATTTCAACAGTCTTGAGCTACCAGTACGTCGTGAAAGTATCACATTTATAGTCTCAGATGAAGTAAAGCACAGCAACCCTCACAGTTATCCGTGTCGGTAGATGTAGCTAACGTACCAGTCATAAATTTTGCGGCGGGCTGGGTGGTCGTCGACTTTCAACTTATCCCAGATTGATGACGCAGACTTGCCATTGCCGAACCAATGCTTAAACTAAGTGGCGGCTGTACCGGCCATTAAGTCATCGATTTTCTGACCACTCAATCTTTCTTCTTCGTCAGCAGTTTCTTTGACGTCGACATCTTCCGAAGACACCGCGACTTTATGACTGCGCAGAAACCGTTTGCCTTCGACGGTGTTTCCAGCATTTGTGGGGTATGTCTGTTCAGGGAGCATCATCGCCGTGACTGACAGTTTTGTCGTTGTCTGTGCTGTGCAGTCGCCCGCAAGGAGAGAAGATACTGTCGCAGCGAGAAGAAGAATTGACGTCAATCGCATGATGTGGCGGCTTGGCGTAGGTTTGTGTATTCGAAGGCGAAATGGTGAATGAATACAGCAAGGGAACTCGTGTGGCGGGAATGCGTCGACAGGTGTGCTACCATGCCAGCCCCACGCATGTGACATGCTGCCTCTACAAATCTTTATCTGCTTTTGACCTTGGCAAAAATAGATCTAACAACTACATGTAACGTGTTCTCGGTTTTAGCTACGTTATTTTTTTGCAACACCTAGAGCGATGTTCTTACAAAGTTGTAATAAAACGATATAATAAAGTTTTAGCCTAAACTAAACCAAAAACCGATTTATGTTACGCGAGGCGAAAATTCAAAGTAGTCCAGAAAAACGGAAGCGCACGATTGCAGATGAGAATTGCTATGATCTACTCACGCGACAACTTGATCCACGACATTTCGCGCAACTACCCGAACAGAAGCTCGCAAAGCTTCGTAAAATACGAATTGAGTGTAGCCAGCAATCCAGTTCTTCCACTCGATGATGAGTGTGGCTCCCACTCACGAATTTAAAATAGCCATGCGAAGCTGTCATTATCGTACAGAGTCTGACCAATTACTGTACATGAGTTTCGCTGAAATAATCTTGAAAATGCGTAAACAACCAAAAGCACAGGCTCTCATTTTGGTTAGCTCCGAAAAGGTACGTATACGGAACTGATCAGATTCTAGATCAGATATTTATCTCACTGGAGATCTGACATGGGATCTGAAAATGATTGGTAAGTTCACGCACTTCTTCAGCCTCGAGGCTGCTCGTGCGTTGGTCAGTGGAGAAGATGAATCGAGAGGCAAACGAGGGCGCGTCGGTGGATGCCGAGGTGGAGCCTGCTGAGCCGGCTGAGGGCGCGCCGGAGGATGGCGGAGTGGAGCCAGCTGAGCCGGTCGAGGACGTCGTTAGCACTGAGTTGCTGTTCGAGCAAGCTCGACAGCAACTCGCAAATGATGTGGGGTCTGTGCCACGACGTGGTGAGCGGACGACATGCTTCTACAAGGGCTATAAGTACTGCAAGGCATGGTCGTCCTCAAGGAAAATTGTGTACAGATGCTCTAAGTTTCGCCAAGGTTGCAGAGGAAAGCTGGAGTTTACTATTGCATCGATGGCGTATGCTGCTGTAAAGCTGCACACATGCCGCATCGAAGCCATTGCCAACGTCGTCATCAACGTTGAGGACCAGATGAAAGCTCAAGCCGACCTTCTTGCGATTGAGCACGTCGCTTGGCCTGTGCGCCAAGTGTGGGAAGAATTGCGACGCCAGTTCTACTCAGCTGACAATCCCAACGTTGTTCGTGGGTTGTCAGAGCAACAAGTTGTCCGACGAGTACACCGAGCAAGAAGCGCGCATTACTCTAGCAATGTTCACGGCTCTATTGAAATTCCTCCGCTTTCATTGGCACTAGACGAGGAGCTTTCCTTTTTCCAGTTTCACTACGTCACCATTAACCGCAACGATTTGAACAAGCCTTCCCGCTTGCTTGGGTGGGCGCATCCATCTCTGGTCGCGCTACTACGCTATCATGGTACCACGTTGTTTGTGGATGGCACTTTTCGGTGTGTCCCGCCGGGTTACGCACAGTGCGTTGTGTTCATGGTCCACGACCGAGCCTCTGGAGTGTTCGTGCCGGTGTTCTACATTCTGAGCACCTCGCGTACTGGCGACGCCTACTGGGATATGATTCATTTTATCGTACAATCAACGGACCAGCAACTCGAACCGGCCGAAATTGTTTGTGACTTCGAAGCGCCACTGTTGGACGCACTACAAACACAGTTTCCGAACGCAATTGTGTTGGGGTGCTTGTTTCATCTGAAACAAGCACTCCGACGGGCTATGAAGCGCTATGCTATTCCGGAGGAAGAGTGTCTGATTGCAATGACACGTGGTGTCCTAGACACCCTGACGGTGATTGATCCTGCTCACATCGAGCGAGGTATTAAGTGGGTAAAGCGTGAGATCAAGTTGCGCTGTGCTCAAGCTGGCGTGACCTACTCTACTGAAAAATGGGCCGATTTTTGGGGGTATTTCAACCGCACTTGGCTGGAGCAGTATACCATCGATGTCTGGAACGTATTCGGCATGAACAACGAGTTGGTCGCACGAACGAATAATCCCCTGGAGAGGTTTAACAGGGAGCTTAATACACGTTTCCCAACCCCGCATCCTTCAATGGCGACATTTGTGACGGTGATCAAGGCTATATCAGCTGAGTACGTACGCCGCGTTGCTGATGTTCCTCGTGGGCGAGCCCGCCGTGTTCCTCGCGAGGTCATTCAGCTACCACAGGTTGTGGACATTCCCTCTGACGTTGACAGCGACGTGGATCCTCCACTGGAGGAACTGGAGGCAGTTGCTACACCCGCAGCGGACGGTAGTACACTCACTAGTGCTACGTTGTAGATATGTCCGTGTAGCAACCAGAAGACCTCGGTAAATGCTTCATCTTCTCCCCTTTAAAAGTGTTTTTCTTTGGTTTCAGTCTGTTCATAATTCAGGTCTCAAATCCTGATCTCTAATCCGATCAGTTCCGCATACGTACCATTTGGGAATCTACCCTCATTTTTTGATCGGCAGCTGCTATACATACTACAATCGCATCAGCCCCACCATTCGGCAATACCTTCACTTCCAAAATCTGCTCACAAGGCACAAGACCACCCCCCCTCCTCCCTTCGCCACCGGCCCCAACGCACAAGATGCGCCTTTGCTACATCCTGACGGCTGTTACTGCCATTGCAACCTTCGCTGAAGGCTCATCTGTCACGAGTCAGTCGAACGTCCTTTACCCAGGCAAGACGGACTCTGTGAGCATAATATCTACGATTGGTGGCGAGCGATTTTTGAGAACTCAAGAGAAAATGAAGGATGCCAAGATGGCCGATAGTGGAAAGGTGGATGAGGAGGAGAGGGGCATGGGACTGGAGAAGGTGCTTTCACAGAAGACATTATCTCTGAAGGAGCCTCTTTTGACAAAGAGCAAGTCGATGGTTGATCTATCCAAGATCGATGACGAGGCAGCTCTCCAATTACTCAACCAGCGCAACCATGACCTCTATAAAGAAATCGAAAAAATGGGTTATACCCCAAAATCGATGAAAGAGACGTTGATAGCTCGCAAGGGCGATGTCCTTCTGGATACTGGGAATGTTTAGCTGTTGGCCCAATACTCCAGATTTTGGAATGAAAGGCACCCACAATTGAAGAGCGGCCAAAATCTGCGGTCGTAGACTGTCGAAAAAGACGTACCAGTCGATATAGACTAAGCGTCGACTGTGAAAATTTGCTTGACAAAGTTGCAATCATGCTACAAAAGAGCGTTTTCTAGAAACGAGTAAGTACGAGAATACTACCTTTTGAAAGATGTCTTTTGGACGTTTCTTTCCTGCTTTATACCACTTTAAAATACAAACAAATAGTTCGTCCAACTAGTAACTACAATTTTTCTTGTAGTGCAAGCTTCGAAGAGGCCATCGCCACCTTTTCACGATCTTTTTGTAGCAGAACATGGAGACAACTAGAGCTTTGCGGGGGCTGAGAAAGCAGGAGTGCTTTCTTCGTTTTGAATTTGACTTACTTAAACACGTGGCTACAGTGGCAAATAATCCCCTGCGCCAGCTTCGTAGCAGCAACATCGGCTTTGTTGCCCATAATAAACAATGACATAATGTGGAGGCCGCATGAGTTTGAGCTTATTAATGCCAAATAACAGTACGAATACACTGGTCGTACCCACATTTTTTCTCGATTTCATTTTTTTTCTGATGTGCAAAACCTTGTGTGCGGACAGCGGCACAGCAATTCAAATAATACACACAGGTCATAGCTACAGCATAAGTATTATAGAGAATTGAGAGCTTTCGTTATCGACCACCTTCACGAACTTGTCGTGAGGTTACCATTTTTAGTACTACAACTTTAACGTTGTAATATTTCTGGTCAATCTGCTCATTAATCCTCCTTCAATTGGCATTGCTCAGAAGTCAGTAATTTCCCCTCAAAGTAAGTATGTTTCAATGCTTTGCGCTTAAACAACGGTCCTGGAAGTATGACCCGCGCTGAACCACTGTACCACTAGCCTTCGTCTCTTTGCAATCCAGAGCTGTTCCGATGTACGACTTTGCTATATGATGCTAAGAGCCGTGAGCTCGCATCGCTCGCGACATTGAGACAGGATTTAATGGAGAATTCGATGTTTCGCTCGCTGGTGAGCTCGGCGCTGGGCGCGCCTCCACAATGGTTTGATATAGCTACGCGTCTCTATGAATAACTAAAACAGCAACAACAGCTGTTCTAGAGCATCCATTTTGCTCAGAACTCATGTTTGACGCCTCTCCATATACTGTAGAACAGGCTGTTTAAAACGTTTTCAACGAAACTCTCGTTTCCGCAGATGATGTTGTCACATCGACAGTATTTACTCTGACCAAATAGCGGGGATACATGGCATTGGAGCTACCAGTGGTAACCTTTTTAATTTAGCACGCTGGTGGGCCCGCCGATTTGCAGTTCTTAGCTCTTTTATACATAGTAACTTTTTGGACATTATATCCAGACTTCTTACCAGGATCGCAGACCCTCGGAAGACCTGTACGAGAATATACTTCAGCTAATATACAACTGTATGTAGAACGGTCTGGACATTTCTAAAAATACGACGAGATGTCATGATGATCCCAAACTCCTCCTCCGTGTTGCAGCTTTCCATCACATGGTGTCCAAAACGATAGTTTCATTTCAGTATTCAGCTGGCCCTTAGAGCAAATCATGCTTCATCGGTTTATCGTCTGAAGTCCGAGGACGCAAATAATATTTTTTGTGGTGCTGCTCCGATTACGTGCCACTCGAGAAGACTGGCAAAGCTTTCAACCTTTCGTTGCGTAGCTAATAGACACCTCAAAATAGCCTTCCTGCTTTTCTCATTTGACAAATTCGTGAAAAGCAGAGGTTGGTCCGCATATTTTCGTTTTATAATTTGAAGCTCCTTGATAGATCCAGAATATGGAGTGAGTGCAGCATCAACTTCACCTTGAATGCTGTCTGTATTCCAGATCGCTTGCGGCGTGTTTGGAGCTCTCCGTGCTGACCGCTCTGTTTATTCCCATTGTCCGTGTAACAGGGCTGCACTACATGTAACCACTTGATTACGAAGAACGTTGGTGTGCGATACAACAATAATTACATTTGGCAGACTACTGCTCTATATGCATTTTTGGTTCTCTCATTGCTTAAAACTCAACATCTTCAACCAATTATAGTAGTAGGATTCTGTCACATTTGTTGGCAAATACCTTTCATTTCAATTATCACATAAGGAGGAAAGCCCTAGCAAACAAATAAACAATTCTAAAGTTTACGGTTAATCGGCTTTGCTTTCCTGATTTGAGTAAAATCTATACGTTCGGAGCGAATCTAAGAGAAGCGCACCTTAACGTTTCCTCACGCGTGCGCTAACGTTTAAGCCGAAGTAGTGGGCGCCGAATAGGCCCATTACAGTCCGTAGCTGCGGAATCGAGACAGATAATTCGAAAGAAACGAGTACAGGCCTGGTGCTCAACACAGCGATCGACTCTAGCTCCCGTTGTTACGTATGAGGGCATCGCTGTATGCAACGTAAGACTGGAGCACAATGAACGCTCTCGAAACGCTTTCAAGGGGTAATACGCACTCACACTTGGGGCGTGTTGATGTCTTGGGGACAGGGTAGGACCTGGTGGCCATCTGTACTTGTCGCAATCTCCAAGCTAGACAGAGAGCTATATAACAAAGACTTTAATGAATTTAGTGAGTAACTTGAAGTGAAGAAGGACCGAATGAGGGAGCTGAAGTTGTTGCCTGTCGCTTATTGTGCAAAACCTAAAGTGATGAGAATTGTCTGCAGCAATCTGGCTGAATTGATCAAAACAACCTATCCTACTCCATGAAACCCTGTTAGCCAATGAATTTGTCTAACACATGCGCACCAACGCCAAGCTCCTTTCCCGCGTTGCGCAAGCGCTCACAGGCAGCTTAGACGTAAGGACAATTCAGTACCCCCCTCCGCAGTACGAGCCTCAGTGCCACTGCATTGCCTTTGACCCTGCGCGTTGCCTTGCTCCAAGCTTCCATGATCCTGTGACTTCGTTAACTCACGCCTAGGTGTCTGCTATCGTTGCATCACTTCTCGGTCATTGACGGCTACACTTCGTGCAGACTTCTGTGAATTACGTGGGCACCATGAAGGTTTAGCCTGCTACATTTTCTTTGTGCTAAAGCGCTGACACCGGGTGTTACCCTACCGACTTAATATCCCCGGATATACTCAAACTGCTCATGAGGCAGACCGTTGGTCATCGTGTAAGCCTTATTCTCCTTTCTCGCGACGTACTGCAGCACCACCTCATCGAGCTGCACGTACACCCGGATGAAGTGGTATCTCACATCGATGTGCTTCACCCTGGTGTTGTTGGGCAGG

>Contig_28

ATGGCTATGAGCAGCTACCACAGTAAGAGACCTGGAATATCCAGCAGCTCCAGTAACAACCGGGAACACGAACATGACGTCTCTTACGAAGCTATGAACAATTTGTCGACCAACAAGCTACATGCACCTAAATCTGGTGCGTCTCGGGTTAGAAACAACAATTCGAAGGATGTTGAGACTTCCCGTACTGAAAATGTAAGCAAAGTTGTCTGTTGTCATCACTTTGATAGCTAGGACACTAATGTTTCTTGTGATCTTTAGATTAAGAGCAGCCCAGAGAGCCCAGAGGAGCCTGAGGACAAAGTATCTGTTACAAAAACGATCTCACCGCAAAGTAGTCACGGTCACACAGTTAAGGAGAAATCTCGCGCTGTTCCATGCAGCAACGTAGACAACAGTAACAGCAGCATAGCGTCATCTTCATCGGGTGCTTTAACGACAAACACAGCAGCAGCTACCTCTTCACCGACGGCTCCACCACCTCCGAAAGTGACATTCCGTAAGGGCGAGAAGAGTTTTCTCGACTTCCACCAGCACTACGCCATCACGAGACACTTGGGAGAAGGCTCGTACTCGACGGTGAAGCAGGTGACGCACCGCAAGAAAGGAGGCTTTTATGCCTGCAAGATCGTGGACAAGTTGAGTCTCTCGGATGTGGATCGTGCAGCGCTTTCGCATGAAGTACGAGTGCTGTCGAGTGTCAGCCACGTGAACATCATGCGTCTGTATGAAGTTATTGAAGACGACGCCAAGTGCTATTTAGTCACGGAGCTGGCTGAAGGAGGCGATCTCTTCGACCGTATCGTTAAGCAAGGTAAATTCCCAGAGCGAGAGGCACAGAAGGTAGCAGCAGCGTTAGTGGAAGCGCTCCACTACTGCCACAAACACAGCATCATCCATCGCGATGTCAAACCGGAGAACGTGCTGCTATCTGGAGACGATGTCAAGCTGTGCGACTTCGGCTTTGCTCGGCAACTCGATCATCAAGAAGACCAGGCCTCAGACTCATGTGGAACACCTGGTTACGCCGCTCCCGAGATCCTAGACGGACGTTCGTATGGTCTGGAAGTCGACGTGTTCTCGCTGGGTGTCGTGACGTACATTATGCTGTGCGGATACCCTCCCTTCCCAATGAAACTTGCCCAGCTGCGTACACATCGCTTCAATGTGCGGTTTCCATCCAAGGACTGGGCTGCTATTCATCCTGATGTCAAGACCTTAATTTCTAAGATGTTACATGTCAACCCGAAGGAGAGACCGTCGATGGCAGTTCTTCGAACGCACCCGTGGATCCAGTTGGGTAGAGTCACGCTGGAGCGTCTGCGTAAGGAAAACGAGGAACGTCGACGTCTTGCTGATCTAACGCGACGTCAACACGCAGCTTCAGCGATCCGAAAGAAGCTCGTCATGGGCGGCTTCGAGGCTGTTAAATACGGTCGCAATGGTCTGCCACATCGCACTAAGCTACGTCTGTCAACTGACGGTAAAGTCATGAGCTGGCAACCAAAGCTACTGAAGCGAAGCCTGCTGCGGTATCAGAATGCACGCAGCTTTACAAGTATCTTTGGTATTGGCGGCAAGGAAAACAGTCAGCCAGACCTGCATGCTCCACAGTCAGAGCCGAAGCGAATCCCGACTGGCGGTACGCCTAACAACGCGCACGATGAGTCTGAGCACACTGCTAACGCTGCTTGCGTGTCGTCCCCTCATCCCACTGACAGCTCGACCAGCAGCACTTCCGACGCTATTAACGAGAAGCGTCTGTGGTGGCGTTCGCTACGTCGGGAGCGTGGTGCGAAGACTGAACGCACGGCCAGTGGTGGATTCACACTCAACCTTTCCGCTCGACAGACCGGCAGCACCCCGACATCGCCAATAACGCCCCCAGCAGCAAAGGTGCTTCGGATGCGAACACCTAGCTCCATCTCGGAGCAGCCTGAGACCCCTTCCGCGCTGTCTCCGGAGCGTCTGGACGACTCAATCAAACTACACGACATCCGCCAACTGCTTGCCGGTGATGACGCGCCGTTCTTCGCTGGCCACGCTATGAACTTGCCTAATTCCTCCAAACGCGCGGTGGATCCTGCTTGTGTGCTGTCCGTATGCACTCGATTCCGCGAACTTCATCTCGAATTCCCGAACGAAGGTATACGTGATGGATTCATCTATTTACTCCAGCAAGCCACGTTGCCATTGCAGCAGCGTGGCCCAGTTCAGTCTACACGAGTGGTGAAGGCGTCGTCAATGCCACCACCCGCACCAACCAACGTGTCCGACAGAAAACAAGATACTTTTGTGGTCACGACGCCACCCGATGAAGAGGCTAAGATCCAACCTGATGATCGTGAACATAGCAGTGAACCGGAAGTCGACGAAGAGGATGTGGAGACCGTTGCGAAGACAGCGAGGGCAGTGAACTCCAACCAGGACGAGTGACTTTCTCCTTTAATTATTATTCGCGGAGCAATTTCGATAATCCAATAATGGGAAAAAAGATCCTCATTACTTTGATTCTTGATAGCAAAGTTATCGATTACTACGTAGTAGAAATCCTTATTGATGAAGTCCTGATACATAGAATTGTGTTTTGTTACGATCTCTAATCCCCGAAAGATTTTGAAGCTTGACGCCGCACTCTTGAGGTTGGAGAGAATGCGTCCTCTTCTCCACCTGAATCCATGCAGACGCCACTGGAGCCTGCGCGTTGTCTTGAGTCGACGTGAAGATCTAGAACGTCACTTGAGTTTCGATGTCGTTCCATTGGCTTGCCTCGTGTTGACGGTAACCGGTAAATCGATTTTCGACGTGCGAGTACGATTCTGCGATTGAAGACGGACGAGCAAGCGAGGAAGACGGAGATGGCAGCCACAAAGAGCAGGTACGTCCGACGCGATACAAGCTTCGATGCGTGAGCCTTAATCATGAAAATGAGCGCAAGTTCTCCAATCTGACAGAGACCAACAGCAGCCATGAGAGCCTTGCGAGGACTGATACGAAAGAAGGTCATGACTCCAGTCATGGACACTGTCTTAATCCCCACGATCAGCAACACCATGGACAAGATCTCGCCAGCGTTCCGGATCAGGAACATTGGGTGCAGAATCATACCCACACAGGCGAAAAACATGCTGCCGAACAAGTTTTCCATCACCCGGATCGACATGAACGCAGCCTTGGCATTCCCAGACACGTGCACGAAAGCAAGACCAGCTAGAAAAGCCCCACACTCGAGTGAAAGACCAATCATCTCAGAGAAAAGTGCCATGAACAGACACACAGACACCACTCCTAACAGAACCAAAGGAGCGTTGTGTACTTCCTCCATGGCAGTCAAAAAGCGCAGCAGCTTCGGCACTACGTATTGGTGCAGACATACGGTCATGCTGACTACCACAGCGTAGGCCACAATAGTTCGTACCATGGCGACACCAATCCATCCGACTGAGCGTACGGAAAACCACTCGGGCGTGCCTAGAGCAAAGCTCATTAACAAATCTTGGATCGCGATAATCGACGTGACCGTCTACAATAAACGAGCAAACACCTTGCGTTAGTACCACAGCAAAGTAGCGAACTAAGACGCGAGTACTGTAAAACGCTTACCCGCCCGAAGGAAGAGTCCTGGATCCGATTCTCCCGAATGTGTTCGTAAAGTGGCGCAGTCGTCGAAAAGCACACACCCAAGCCAATACTCAACCCCTCAATAAACGAATGCGTCCATTCGATGTAGACCGCCACACCAGCAACCAGCAGCACAGTAGACAAGATATACGCCACGCCACCGACAAAATACTTCCTGAAGATATCCTCCGGCTGCTGCGGTGAGTACGCTGCTCCATGTCCAAATAGCAGGAATATAGAGCCGAAGAGTGAGATCGTCTCCACCTCCTTGTACAGTCTCACAACACCCAGACACGACGGTCCCACGAGCGCCCCACCCAGAACATACCCTGCATGAGGAGGGATATTAAAGTACGTAGCAATAATCCCTCCAATCGCCGAGACTCCTAGCAGCACAGCCACTTGCGCCAGCAGCTCCATATCGTAGTGTAACACAGCTGGATCCGCCTTCTTCTCCACCTCTTCCAGCTTCTTCAACTGCTCAGCATTCAGCCCCGTCGTGTTCTTCAAGTGTCCTGTCTCGTAGTCTACTTCTTTGATGCCATTCTTCTCCAGTTTCTGTTCTCTCTCCTCTTCTTCCTGATGAGCCAACACCTCTTTCAGCTTCTTGGACTGCAGCACCTCCTCCTGCTCGACGTGTTCGAGCTCCAACAACGTTCCGTTGAGACCTAAAGCGAGTCTCTTGAGGTTTCTCTGCACTAACTGTAGCTCTTCTCTCAAGTGCTGTCTCTCGCGAAGCTGGGCCTGCGTGTACTTGGCAAACTGGTCGCCTGTGTCCATTTCCTGCAGACTTTTCACTACCGAAAGCTCTAGTTGCTGCACAATGTGGATATGCTCCTCGACCTGGTCGATAGAAACGTCGGTAGCCGTCACCACGGCGTCTATAGTATCTAGGGATCCATGTGTGCTGGAATCCAGGCTCGAAAATGAAAGGGGGGCCATACGAGGCGCAGGTTTCTGGTCAGTGTTGCCAGATGATGAGGCCCTCGTCGCGTCCTGCGCCACTGTGGGCGCTGGAGATGCTGGGGAGGCGGCATGTTCAGGATATGATAGCACGTTGATTTGAGCTACAGCATACTGGTAAGGAGAGAGCGTGAACGCAAGCAGGAGCAGAGATAACGTGACGCCACTTCGAGTGTGCAGCATGGAGATGGCGGCTGACACGTTTTCGACAGGAATGGGTATAATTGCAAAAGCGAATTAATTAATTATGGGGAAGCGGACATAACGGATTTGCTGTTGCCATTTTGACCTATTCTTGCCTTACTTTGTTGAAGTCAGCGGCAGTTGAGCAGCCATAATTCCGGAAACTGCATCGATAGTATCGATTCGATACACCAAGACCTCTTTCTTAGCACCCACACGACGACCTTCCAGATGCATGTAGAATCTGCTCCCAATTACAAGCAGATGCCCCCTCCCTGCCGCCATTGAGTGTCAAACCTCCACCCTCAATGCGTTCGCGAATGCGAGAATTCACTGCGTACCGTTTATGAATTGCCTACCATGAACTGCTGGCCGTTGCCATTTCTCATTCTCCTCCAGGACAATGGCGTCTTTGTCTCCGTTCACTTCATGGCAAAGCGCGTTACTTCATACGCAAGAAGCTTGGACAGCATTGCAACCGAAGCTCCTCCCATACGCGAAGTCGAACGCGCTGACTGTGGCTCTGCAGATCCTGCTCGTCCTCATCGCACTCACGTGGCTGGCGAGTGTCTTGACACGCAACTACTCGTTCACTGACCGTCTTTGGTCTATCACTCCACCGATGTTCGCCTGGCACTTTGCCTTTCACGCGTACCTAAAAGACGGTGGCGTTTGGGATCAGCGACTGCTGCTGATGGCGGGTTTGACGACGTCGAGGGGCTGCAGACTCACGTTTAATTTCTGGCGCAAAGGGGGTTACAAACTTAGCGAGGAGGACTATCGTTGGGCTGTGGTGCGGCAGTACATGCACTGGACTCTGTTCGAAGTACTGAATCTTACGTTCATCGCCGGATATCAGCACCTGCTGCTGTTGCTGCTGGCTGTGCCTTCGTATGTCGTGTATTTCCACCGCCACGAGGAGTTGAACGAGATGGATGCTGTCGCGACTGTGGCATTCCTCTTGTTACTCGTGCTTGAGACCGTGGCTGACCAACAGCAGTGGATCTTTTACAGCATTAAATACGAGCTGATCGCACAGAAGAAGCAGCTTACTGGAGACTATAAGGCCGGCTTCAACCGCAGTGGTCTCTTCCGCTACAGTCGTCACCCGAATTTCTTCGGCGAGATGTCGCTGTGGTGGGCATTCTACCTCTTTAGCGTTGCAGCATCAAAGCAGGCGTTCAATCCGGCAAGCTTGGGCACGATCTTGCTGGCGTTGCTGTTTCAGGGATCCGCACCATTTACGGAGTACATCACGGCGTCAAAATATCCGCTGTACAAGCAGTACCAGCGTCGGGTGCCGATGTTTACTCCTTGGCTCCCGTCGACTGTACCAAGCTCTGACATCCACCTGGAGTAGTGTCAGAAGTTATGCTGCCGATGCAGTAATCCGAACGCGACCAGTTCAGGACCACTTTTCAGACGTCAAAGTCACGATGAAGAAGACCTATTTGATGGCACATCATACGGTGCAGAAGCGCTAGCTTAGTGCACTGATGCATTTTGGATCATTGTTATCGGGTGGGAACTCGAGAACGGATGGCCATTAACATTCTACCTCTCGTCGACGAACAATTCCAGACCACCTCCAAGTTGCGGGTTTAATAAGCTAAAAGGGACACACATTGCATTAGCCACATACATAAGCTCATGATCTCTACAGAAGCCTTAGCCTCGTACTACAGTACTAATTCGCAAGCGTATTCACCCGCTACTAATGTAAACACGCTTCCATAACCCCAACTTCGTTCCCAAAGGTACTCACTTACAAAATGCCCCAATCATCTTTGCTTTAACAAAGAGCATATCTGCCGTATTTCATAGCTCGGTGGCGTCTATTTGCAAGAATAAAATGTCAGTCGAGTGGTGACATTGCGCATCAAGTAGCCCTGAGCGAGCTTGCGCCTTGCCGTTTTTCAGAAGCGGTTCTTATATGGAAGTTTCTTAGGCGATGTCATTACTGAGCCACACACAGCCTCTTTTCCAGAAGTTCACCGTACGCTTAGTAAAACCTGGCGAAACAAGCGTCTATCTTTTCACTTTGTGGTGCATTAGTGTTCAAGCTCTGTAGTGTCTTTCGACCCTTGTGCTTTTTGCTTGCTTGAGAATTCGGAGGCTAAATGAATTCTGTTATTGATCTCTCAGCTCTCACTACTCTGTAGTAGTTTCTGTTCGTTTCCTAATTGTTGTCTTCCTCCTAATATGCATCATACATTGCCCGAATGTTGTCGACTTGCCCTCCGGTCGTTGCACCGCTCATCGGTTGCTATCAGGCTGCAACTGCTGTCCGTCGTTTGCTTGCGTTGCTGCGTTACGTCTGCTGAAATATTTTAAGCCACCACTACAAGCTCGCAGCTCACGGCTCTAGGTGGATGCGACTTGTCTAAAATTGGCTTTGTAAAGGACTTTGTGATGAGCAAGTGGTCCGGAATTGCTCGCGGTCCGGAACTGGACACCAACGGATCAAAAGCGCGTGCACGCGACGCCAATCTCTTGTTTAGCGTCAAGGCGGCTGCATTGCAACCAGCAAGTCTTTGCAGTGCAAGATACATGCACGGGACTCAGTGTTTTTTTACTGGCAGTAAAGCGGGTTTTCTCAGTGAATTTACCTAAATCTTAAGTTTTAGCTACTAATTTTGTGTAGCAACCCAGAACCTACAACAAGCTGCGCACACGGCCGTTCGCTTAGGACAGGGGGAAACCGGTACAGTAAACGCCCAACCCTGTGTGTTTCTGTTCGAGCAAATTTTTAGGTGGAAAAATGCGATATTTCAGCAGACTATCCTTTACCGGTAAGTACTGATGAGTGTTCATGCTTGGTAACCATGCTGCGATTACTCGACTCCCTCCACAATGTTGTCTAAGTCAACTTGTCTTGCACTGATGACTTGCAGCCTGCAGGTTACCATGGCTGACAACGTTAGTGCTATGGACGGCAAGTGCTCGGAGGGCGCGTCATTGAAAAGCTTTCACAGTGTGACACCGAGCTAGAAGTTCTAACTTAGAACCAATTCCTTTGATTTCTTTAGCCCATAGTTTCCCTTGTATCCGCAAAGATTGGTGAATCAGCCACAAATGCCACAAAAAGACAAACAGAAAAAAAACGCTCGTACGCGTGTGAAGCGTGATTCCCTCTCAACGGCGTTCTTCTGCGTTGAGAGGGAACCACACTGTCGGTTCTTAAACACTGTCGGTTCTTAAAAAAACTGACAGTGCGGTATATGATGCGGCTTCCATTGCGAACTTCGGCGTGCGCTAGTACTTCACAGTGCGAAGTACCAGCGCACGCCCCTTCAACGACTTAGAACGTAAGAAAATGCTAAAGCTCGCTAAAACGAAT

>Contig_32

TAGTTATTCTCCAACAACCAGTCCAGTTTATATATATGTCTCTTTATGAAATAGTACAGCTATAAAGACAGTGGTGACGTGGTGTAAACGCCATCCTTGTTTAAATCTAAAGAGCCAGGGTGTGGATACATGCACCGTAAAACTGTAAATAGTGTAGGGAACAATTTTATTGCCACGACCTTACATTATACACTGGAAATGTCTTATAGCATTGCTGTAGGTCTATATCCAATCTTACGGAGAAAACGATAATTGAATAGCCCATGCCAGCGTGAACATGGGCGTTTCAGCATCATTTTTGTTGGAGTAAGCGTCTCCGATGTGATGTTGACTATCTAGTGGAATATTTACTTTTTTATATTTGTATCATTATGCGTTGACATGTGAATACCGGTAGAGATAATTCACTTTGAAGTGCGCAGGGGTTATACCAGCGTGTTACTAGTCGTGGAAGTAACACACAACTGGGGTAGCTGGCACTCCCTAAAGCTTCTGCCGAGTGTATTGCTCTCCACTAAGCAAGTTCCACACCTTTGCCCGTCCAAACAGAGAATAATTTCAAGCAAAGTGGACAATTTGGTTTTTATAGTACAAATTTTAACTTTAGTAAAAGTAATCAACAAACAAACAGAGAAAGATCTCATCCAAAGCGAGACAAGGAAAGGCGATGGCTGGAAATCATTCGTGAAGGGGTGGNTGAGGGTGCGTTGGCGCCAAAATGCGCGCGAAAACCTCGCCTGAAGCAAACCATCGCCTGAAGCAGTTCACTTTTCAATTCACCTCTTCAGACGAATGCCCTTCCAACCACTCCTCCGCAAGCACTCCCCCGCTGAGAAGCTCCGTGTCCTAGCTGCCCATCGCGCGGGACGCGCTGACTGGCTTCAGGTGGCCGCCAATAACGGCATATCGCGTGCGGTGGCATACCGCACTGTAGCCACAGAACGCGTAGAAGATCTACCGCGCGGTGGAGCTCGTGGTAGAGCTGTGAAGATGACACCAGAAGCAAAGTCCAAGCTGGAGGAATATCTTGACGACAATTGCACCTTCACCCTGGAAGCTATGCGCACGATGCTCTTTTTAGATACGAACGTGCGGGTCTCCACTTCAACGATCAGCCGCCACTTGCTTGGCATGCTGTTCACCGTCAAACAAACTCGCATAGAGCCCATGACATGCAACAACGAGATCAACAAGACCAAGCGGCAGAAGTTTGCAAAATCGCTGAAGGACCATCAAAAGAACGGTGACTGTATAATCTATTTTGATGAGACTAACTACAACGTTTACTGTACGCGAGGTCGAGGACGTGCACGGAGAGGTGAGCGTGCAACGCTCGTGATGCCGCCGTCCAAGGGTGCAAACTTGCAAGTCCAGTGCGCTGTAAGCTCGGCCATGGGTGTGGTTCTCCACCGTCTGGAGAGGGGAAGCATCCGAATGGAGCAGAATGCAGCTTTCATTGAGGAAATCTACCGGACGGTTAAGGCATCCTCCGTGTTTCATGACAGCTTCGCTGGGAAGAAGGTCATTATTGTCCTGGATAATGCCCCCGCTCACCGCCAAACCGAAGAACGTGTCGAAGCACGTGACGACCTCGTACTCCTACGACTCGGACCCTACTCACCGATGTGCAACCCGATTGAGGGTTGCTTCTCCGTTTTAAAAGCTCGAATCAAGGGATACCTGGCCTTGTACCGCGAAGAGATCTGCGACCGGAGTAACATGGTGGAAGAAGACGGCACCCCAATAACGATCAAGGAGCGCACTATGCGCTTCTTAGAGAAGGCTGCAGAATCGAGTATGAAGTATATAACACCTACGTTAGTGACGAAGATGGAGCTTCACGCTCGCGATGCGGTGAACGCGGCCGAGGAGATGAAAGACATGGTCTATGGGAAGTAGCTCACTGTGTCGTCTCGGTTTGAATGAGATTATTCTCTGATTCACTGGTAATTTTGATCCCGTAATCAGATATTTGTAATAATCAAAAACATATTGTCCACTTTGCTTGACATTATTCTCTGTTTGGACGGGACTGACTAAATGACTCTTTGTCTGTACTCTATGTATACATACTATGTATACTTGGCAATACATACCCTGTATATATTGCCAGCTCGGGCCGGCAATACTGAACTTCAATCATCACTCGATTGGTAGGCAGCAGCAACCCTGCCACTGCCAATCCGTAGATCAGTACTGCCGCCCACTGTAGTGGCTGGATGAGGCTACATGCCCCCAGCCCACTACAGACCCTCTTCTTAAAGACTCATCCAGCCACGTGGTATGATCTTCTCCTTGGTCTTGCACTTTCATGTCCACGCACTCGCGCTTCTAGACACTGCCATGCTCGTGACCTCTAATGGCAGGCACACACGCACCTCCACAACATCCCCAGGGTGAGATGCTCAGGTGTCAGGAACTCTCTGACACCACGTACCTTGGAATTGACGCCTCCGAGCTCGATGATCAAATGCCTGGCACACCCGCGCCCACAAGCACTGACACGACCCGACCTTGGCATCCACCCGGGCATGACGCTCCAGCATCCAGCACACTCGCGCCTTCCCGACACTGTTTCCTCGACGGCACCCACGATGTTCCGGTGTCGGCACACTCGCGCTTCTCAACCGTCACGCCTCAGATGCCTGCCGATTCTTGCGCTTCCCGGGGCTTGTTGTTTGAGTCCCGACGAGCGCGTTGGACGCCTTCACGTACTTGACCTTGGCATCCCCGTCCTCGGCTTCCAACGCTCCAGCGTCCTTGTACTCACGCCGTCGGACTCACGTTCTTGGGTCCAAGACGATCGCGCCCTCCGACACCTTAGACACCTCGACGCACTCGGACTCAGCATCCTCAGTGTCCGGAACGCTCACGCCCTCCGACCCCATGACTTTGGCACCACCTTCGTCGTCCGCCACACGCCATCTTGGATCTTGAACCTCTTCACTGACCCGGGCACTAAAGTTAACTTTGTAGTAGGTCGTGGAAGTAACACACAACTGGGGGAGCTGGCACTCCCTAAAGCTTCTGCCTAGTGTATTGCTCTCCACTAAGCAAGTTCTAGACTGACTAACTGACTCTTTGTCTGTACTCTGTGTATACATACTATGTATACTTGGCATACCCTGTATATATTGCCAGCTCGGGCCGGCAATACTGAACTTCAATCATCACTCGATGGTTGGCAGCAGCAACCCTGCCACTGCCAATCCGAAGATCAGTACTGCCGCCCACTGTAGTGGCTGATGCCCCCAGCCCACTCTACACAGCGTAATGATTTGATGGACGAGAGTACCGGTATTTAAATTAAGCCGAAGCCTCAGTGAGCTTTTCATTCAGCAATTTGCTACGAAGCCCTCACTTTTCGTTTTTGTCGGTGCGATGCCCCTCAAACTTGCCTTAACACCACGCTTAAGCTACTTCATCTTGCTGGCGGCCCTCGCTCTGCTGGTATGCATCCACAGTGGATCTAAGGTGGCTACGGCATCCAGCGTAATCGACGCCAGAAATGATGCTTTGGATCGCGAGGTGGGCGGTCAGATCCACCGATACTTGCGTGAGAGCAAGTGGACAGCTGAACAGGAAGACGGCAGCCCCGAGGCGAGGGCGATTGGAGTTAAGCTGTCAGCTGCTACGGTGCTTCAAAAGCTGATTAATTCGCAGCCAACGAAAAAACTTTCAACAAGAGCCATGGAAATTCTTACATCCAACAATCAAAGAGCTACCGACAAACTGTTTGCGAGTCTCGGGGTCTGGGAGGTGGAATCGAAGCTGTTTGCGTCTACTCCGTATCAGAAGTGGGCTGCATCCGTGATGAAATCCTACAAGAAGAACCCTGAAAAAGGCCAAGCAGCGATATTCTCTACACTTGTACGTCACCACGGCGATGACATTCTGGCCAAACTGGTATCAGAAGCACAGCCAACCGCAGCGGCGATAAGGACAGCCAAGAAAATTGAATCGATGCAGATAGCCAATTGGGTCACAAACAGAAAAACGGAGGAGGACATCTACAAGCTTTTAAAGCTGGACGCGGACAAGGAGGGTCTTCTGAGAAATCCGTTGTTGAATACGTGGGTTTCGTTTGTCAAAAAGCTTGATAACGAGGATCCGTACAATTTGTTGCTGCTCAAGTTGACAAAAACCAATGATGAAGAAGCACTAGCTCACATGCTAATTGCAGCAAAGGGCGATAGCCTTACAAGTAGTGTGGTTCGTAATCTGGAGAATGCACTGCTTACAGCCTGGCTGAGGGATGGTAAAACGACAGGTGACGTCGCCAAGCTTCTCCGACTAAACGCAGACAAGGGAGAAAGTTTTTTGCGGAGCGCGGCTCTGCCCACGTGGTTGTCGTACATTCGACAACGCAACAAAGATCCCAACAAAGTTTTATTCTTGGAGCTGCAGAATCGATTTAGCGACGCAGAGTTAGCCAGAGTGTTAGTCGCGGCGTCGAGGGACAAAAACGTGAAGATCAATGTTACTGCTCTACAAAAGTTACAGCTCAGTAAATGGCATGGAAGGGGGGACACGGCAGATGACATTTTCAGACATCTTGGACTAAGCAAGGAGGGTGAAGAACTCCTAGAGAGCTCAGTGTTTAATACTTGGGTGTCCTACGTGAAGTTAGTTGACTATACAAATACTGATGCCTTGGTGTTCTCAGTGATGAAGAAGCACTACAGCGACGAGATCCTCGCAAAAATGATTGCTCAAGCAAAAACAAGTGTATTAACGAGAGCCCTGGCTTCAAAATTAGCGGCGGAAATGTGGCGGAGCCCAGGGAAATCTGCGGATGATATTTTTAAGTTCTTTCAACTTGACAAAACAGGTGATGACCTGTTCGAGGCCCCGATGTTTGACGCTTGGATCTTGTATGTTGAAAGACTCAACAAGTACGAGAAACATCCAGATAAATTCGCTCTTTTTACGGAGCTCGANAAAACGCTTCGATTACGTGGACCTCGCACGAATGCTTAGCCATGCTAAGATCCAAGCCGAGATGAAAGGACATTCTGTAGAGCGTCTTTTCAGTTTGCGGAACCAGCAGTTCGATCAGTGGATGAACCAAAAGAGGTTGGATCCAGGAAGAGTCGCTGCACTGGTAGCCGAGCAACCGCGTGACATAAGAAACAACGGCGTCGTCCTCGGCTTCTATGACTTCTACAAGGCTAATGGTGGATCGCCGTTGTACTAATAGGGCGCCGAGGAGTTTATGGCTTAGTAGAGGTAGAACACTTCTAACAATGTTACACCTACTCCTCGAATTAGGAGCTTTAACTGTTTATCCTGGCAGTGTATTCGCACACGGATCATAAACACCAGATACAGCTCTATTAATACCTTTATCATGGGTCGGGGTTATTAATAAAGCCCGCGTGCTTCGCACATGGAGCGTAAACACAGGTAGCGTCCTATAATACCACGTGTATAAATCCTTGAGCACCCTGTAAACTAAGCTCAAAACCTCAGAGTGCGAACCCTGGCCCCTCTACCTCCCGCGGGGCTTCGGCGGCGTTGCTGCTATGGTTTTCTCTTGCAAACAAAGTCGCGCCTTCGGCCTGGATTTGTTTAAGGTTTGCTAAAGCTACATGTAGACTTACAAGGCTAAGAAAATGAACTAAGGAAATCATATCAACGAGCACACAGTAAATCTATTTTTGAAGCTGAAAAGAATTTATACTTTTTTAACGCTTGTCACTTTTTTTAAAGAGAATACCTTAATAAAAAGTATTCTCTGAGATTTATTTCAGAGTTTTACAACAATATGATTTACCTCTCTAATATGACGGTTTCTGGTTTTGATCGATTGCTCGAGAACTTCCAAAACCTGTATAAAACGGTTTTATCACAAAGTTTTGATGACCCCTTCTTTAATCACACTTTTATCACGGTGATGCGTTTTTATACCGGTTTTATCAAAACCGTTCTGCCAAAACCCTACATTAAAATTTATTTCGTTCCGAATAGAGTGAGGTTGATGTATCTAACATATTGATTCATTATTAACGGACGATCTTTACTAATTTAAATGGGACAAAAAACTGATAAATTTGTCTATTTTTTAAGATACCACCACGATGGCGCGCTGCCAGCGACCCCTTCCCTGCAACTCACGGCTTTCTCTCGCCTGATTCAAACGAGAGAGGGGCCCATGCGCGGCGCCAGCGTTCTTCTCGCGCTATGCGCGACGTCGCAAACGCGACACAATCTCGAAAAGTGTGTTAAACGGCTTCGTAAACGGCTGCATGAAGAGAGGAGGCGTGTAGAGTGGGAACGGTTGCTGCTCATGCGGTATTACGTGACCCTCGACTGTCTAAAACACCCCGAATAGTCGAATTGGATGGATTTTTGGCGTCGGGGCACCGAGTAGAATATGCTCAACAAAACTAGCCTATCAAGGTAGTAACTTACGACGATGTTTGTATGTGCCAGAGCAATGATTTGTTTTAACCTGTTATTTTTTTTTATTTATTTATGTGTGCCAGGGGCGCGTTTCAGCAGCTCTTGGAGCGATTCTCCCAATTCTACATCATTCCAGTATACAATCCAGCAGGCGGTCGGCCACGGCGGCTCCAGCACCACCACCAGGTGTCGAGCGTCGTTGTTCTTGTGCTCACCCTCAACGGTAGCACTGCGCGAGTAAGGGCTTCTCTTGTCGATCATAGTAACATTGACGGATAAACCAGGGTCTTCTCGTGTTCTCCGAACCCCGGCAACTCACCGCCCTCGTGTTGATCAGCAGGACGCGCCTCTGCCTTCTCTTCTCGGAGTTTGCTGCCAGGACAGGAATCTTTATCACCACATCTACCGAATTCCTCGATGAGTTGGCCCCCCGTGGTACATCAGCAAAGGGGGCCAACTCATCGATGAAGACCCTTCAGACACACATGCATATCATTGGTTAAAACTGCACGGATCTTCTGACCAGTGTCGGCTGCAAGTCTGTGATTGGACGTTAATCGGGTGGGGGGGGCCAACTCATTGAGGAAGTCTCTTCAAATGTTGCCCATCATTTAGTATAGGAAATCCGGGTGGGGGGGCAACTCTTCGGTGAAATTTGAGGGGGCCAACTCATCAAAGAATTCGGTAGTGATCTCGCTCGGCCTGAAGACCAAATAGTACGGAAAAATAATGTATCAGTATAAAAGAAACGCACAGCAATACGCACTCACCGGCCTGTAGTGAAGCCTCGTCACAGCTGCTTCATAAGGTTAGCACATATGATGGATGATCCAGATCTACGATGGTACGTACCGCAACCATGCAACGCCGACGTCTCGACGAGCCTTATGCGGCAGGCAGCAAATTCGTAAATGAAACGATCCTTAGTGGGCGCGAATTTTCGCGCCTTTGAAGCTGTCGAAGATCTCATCTTCAATTTCAAAATTNTTGCTAGTTAGGGGAGTCGCTTAATCATATACAATACGCAAGTCACTTATTTATCAGCTGCTAAATTAGGGTGCGTGATTAAAGAGGATAAAAGGTGATTAAACGCGAAGTTTGATGAGACGACTTTTTGGTCTCATCAAAAATTTCCATTTTATCACTAAGTTTTAATCACCTTTTNAATCCACCCTAACCCCAATGATTAAACTTTTTATACAGGTTTTGTAAGGCCCCCAATAGCGCACCAACTCAAAGCCCCAGTCCGGGCGCACACGGACATATTTTAAGTCTGCTTTAACATCAATCATGGTAGAGTAATATCTGCTCAAACACTTTCCAATCTATAGGCCGTTAGGTTTTTGTCGCAGTTCAATCAATATTTAGCGATATTAAACCATATGTTAAGGGCAGGTAGACCATACAATTATAAGCCCAGTGCTACAGCCCATGTTTTTCATTAGGATTAGGGAACAGTAATCATAGATTTTGCCCATGATTACTATAATTTCTCCATGATTCCCCCGAAGTATATTACGGAGTGGGGTATTTTATTTTGAACATGTATTCCCCATGTTGGGTCACGGCCAGAACATGTTCGCACCACTACCCGATAATTGAAGACTGAACGCGATTTTCGCCTGTCGGACCCTCATACTGCATGAGCAATACTACCACGTGTCATAAATGATGTCCATGTATTTGCCATTAATAACTATGGTAAAACATATTTCCAGGACGATATATAATTTGAGGAGTGAGAGAATAGCTGGGGTGTCGGTCAGCTCTCCCCTCTTATCTTGCATGATCCAGAACTACTTAGTATAATATAGTGAAACGCGCAATGTGTATGCATCCATCGTGTTAGTGGTGCATCTTAGTCCTGCTCACCTTTACTCGCATCGCTAAAGTCCACCACGGTCCGAAGAACTTGTTGTCAGTCTTATCATCACCAACGTCTCGGGTTGACCCCCGCAATATCATCGGTGTTCTGAGCATTTGGTACGCCATCAAGCACAGCCTCGGTCATCATCACTCCCAGAAGATACTTGCAGCTCAGCGTCATCGAGCAGATTGTCCGCGTCGCTACT

>Contig_33

TACCTCACTGAGAAAAAAAGTTACTTATTAACCCCCCCCCCGTACTCACGGTTTTTTCGAGTACCGGTTATGAAGTATATACACCATGTTGTAGCTGTTTACAACATAAAAGAAAAAAGCGTACTAGATTTTAGTAATAACTTGCGATAAGTACGACCAACGTTTGTGTACGGCAAAAAGCTACCTAAAAGTTTTGACACGTCTATGTTTTGTCATGTAGCTAAGAACATCATCCGAAGGGCAACACGAACTGGACAGGGAGTAAATGCAGCATGATTTCTGCACCTTATATTTGGATAGAGCAAAACGCGAGCCGTGTTACCTGTCTTTGTAAAGGTAAACATCGATTACAACATGTAGAATAGATGCTATGCACTTCAATTGACCAAAACCTGGTTAGCAATCAAATGAACGTCCTACTTGGTGGGCCACATCATCTCTTAGATCACCTCAGTCTGGCGGCCTGGCACCACGCCTGGACCTTTGTCGTCTTCCTCCTCCTCCTCCTCCTTCTCCTCTACTTCCTCCTCTTCATCCTCCTCTTTCTCCTGCTCGTTTATCTCTTCTTCTTTCCCGTCACGTTTTAGCGAATGGCTTCGTTTTTTGCCGAGCTGGCGCATTTGGCAGCCTCTGCCAACAAGCGGAGGAGGGCGCTCCTCGCCGTTCTTCCACAAGTCATGCCAAATTAAGCAGTCAATCCCACCAAATCATAGCTTGCAGTTTAGGGCAGCATTTGGCAACTGCGACGGTAACGGCCCCCTTGGAAGCTCCTGCCGGTCGTGGAACAGCGTTCGTAACGAGCACTGGGGGGAAGCTCTTCTTATTCGTTTGTATTATGCCGACGGTGTACAGCAGTTGTAACGAAAGTTGAGCCGACGTGTAAAACCGATCGGTGATGACTGCGTGATCGATGCCTTCCTCTATAGGGGGAAGGACTTTGGTCATGTTCCTCAAGACTGCGCTCGGTCCAAAATTTGGGTCAGCAGAGAGCTGCTTTGGAGCCCCACCACCAAGCTCATCCGTGTGCTGATCAGCTCCGCATAATTCTCCAAGTCTTTCATGGCGTATATACAGCATAAATACATATTAGAAAATAAGATTATACAACATTTGGAACTTAGNAAAAAAAAAACGCTGTCCTTCACAGGATAGCGCTGATACTTACCGAAGACAATACGCGGTTTCTGCGCAGCACATCATGAAGACATTCGTACCCATTTGTAAGGCTTATCTTTCATAAACTGGCGGGCCACATTGTTGCGGCTACGTGACGAGATCATGACTTCATCGAACGACATGACAGGCNGGGGTGTGGTACCTGCTGTTGCAGCGTGTCCACCACAAATCGCACCTTCCAGGCTCGATCCGTCTCCGCCCTCGAGTCGGCGTTGTCCGTAAAGTACAGGCTCTGCATTATCCTACCGAAACGCGCCTTTGACGTGTACTGCCCGAACGTCCATTTTGGGATAGCACCAACATCAGGCTTGGCCCACTGATCAGCGAATCGTCGCTTGTGAGGGCAAAGCATTCTCGCCACAAATAATCCAATGCAATGCAATACTTCCTTCGCCTTGATTTGTCTTTTTGTTTCACCGAGCAGTACTTCATCCCTGGACGTATCCTCACCTTCACCGAAGCGTTTTTAATGCATTGTATCCACGCGCTCGTTCAAATGCTGGCTGTAGTATCGATTGCTCTCTGTAGCAACTCGCTCCATTCAAAGCTCCATCACTTTCATCTGAAGGAGTTTGGTCAGGCTTCTCTTCAGGTTCTTCATAATCACCCTCTGGTGTTAGCGATGCAGTCGCATCAATGACACCTGACAGCGTCTTCAAATTCGTCTCCGTTTCGATATACCACTCGTCTGAGTCAGCACCGTCCCATCCATCAAGCTCGGAATTCAGTGCCTCGTCGTCAGAAGTAGCATCCGCGCGCGAAGCCTCATCTGGTCCGTGCTCGGCGGATTCGACAGTGTTGCTTCTATAACATTTCACATAGTAATGCGTTAATATACAACATAATCGTTTTCAAATATGTCCTACATAGAACATGATATAGCAACTTACCCATCTGGAGAGTCAGGAGCCTGTACTTGCGCTGTAGAAGCATTCGTTACAACAGGTTGTTGTGATTGCTCAGGTAGGCCCACCGACCACTCATCAGAGGTGTTTAAGTCATCTACTTCCGCGGTGGTATCCACCACGTGAGAACCGGAGTCCTCGCCGACCCGGCGCCGGGGACGAGAGGCTGCAAATTGAAATGCACAAAAAAATCAACATTATGTTAGTCAACACAAGTAGTATATGCTCCATGATCGCGTGACTAACCTTGTCGTGTCGCTCTTTTTGCATTTTTCTGCACCCGTGGCAGTCGTCGTCTCCGACAAGCCAGCAGTCTCCACTGATCTTTTTCTTTCAGATCAGCAAGGGGCGCGTTTACGTGTGTCGGCTCCTCGTCGTTCCTGCGCTCTCGCTTTTTCGCTTTCGAAGCGTATCGAAGTGCGGCTGCTGATTCAGCTCGCACCAGCGCCCTGGACTCCTAAGCTTTTTTTTCGAGTTAGGGCAGAGTGCTGTCGCTATGATATCTTCTGTTTGACCGGGCTAGGATAATATAGCATGAATATAACATCAGTTCAGTCCTCACACGTTGTATATACTTCATGAACACTACATACCTCTTTCGCGTTGATGTCTTGGTCTTGCAGGACGGACCGCCCTTCTTCTTCGCACCCATCTTCTTCTTCGCCGCGGCTTTTTCAGCCAGGCGGTCCGCCATGCGCTGCTTTGCGGCCTCGATGTATGAGGCGGTCTTTCACGTGGGAGGGCGCTGTATTCCTGACGCGGGTACGCGCTCCGGCCCCTCAGCGTTCGTGTCCCTCGTCTCGTCTCCGAAGTCGGCATCATTTTGCCTCCCACCACTCAAAAACGCTCGGTGCTCTGCCAGCAGGCGGTGCCTGTCGGCAGTACTCTTTCCCTCAAGGCGCGTTCGGAACGCTTCAGCCTTGCGGCGCGTCTCCCTTCGCGGCGCCATAATCGTTTTATTTATTTTTTCGAATGAGGGGGAGACTCCACAGACACAAAACTATTCGTACTTAGCAACAAGCTGGCTTTCCTGCCTAGGCCGTCTTTGTCATTTTTGCTCGATTTTTGTAAATATTTCTGCTACACCTGTGCATCCCATGTGGAGATAGACGAGTCGCTCTATTTATGTACATATTGTCGTTTACAACATATGAGTACGGGGGGGGGTAACTGTAATAGAAGTCGCTCGGGCTCCTTCTTTTGCAGATTAGGTGTGAAACAAAGACTTCATATAAAACATAAATGCTAATTATGCTGCATTTATGTTTTATATGAAGGCTTTGTTAGACCATTACTTATCAGAAAGTATTACATGTAAGGCTCAGCCAATTGGAATACCTATATTTATTCTTTCTATACCGGTAATTTTATGATACACAAAAACCTAAGCAGAATCATTGAAAGCACTTCTTTTAGTGATAATCCACTTACGTACGATGTAGGCCCAATAAAGTTTCTTTTTAAAATAACAGAAAATTTGCATGCGAGTGGCTCATTGAATTTGACACCAAATATTTTTTCTCGCTCAGGTACAGTAAAAGTTTCCTACATTGCTTACAGCTTCTACCTTGTGAAATTGTTCCCTGTTGCCAACATGTGTCGGCAAAGGATTTTTTTTAAAGTTTTTTTGCGTTTCCTTCACATCTATTAATAGGAACAACACTTTGGACCTTCTCCCACTGCAGCTTCATTTCAATATTTTAAATGTCAAATTACTTGGTGGCTGCTTCAAGGATCCGAAAGACACGAACGTCTTGCCATTTTGAACAGCTGCATGACCTCAGAGGTATCATCTAACTCAAAGGACAATTTGCAGCTGGAGTTCTTAGTGTCGAGCACGTTCATGCACAATTATATGGCGATACTGAAACCGCTTTAAAGACCTGATACGCGTAAAAATGCAGCATCTATTCAATTGCAATTATCAAGAATCACATTCGATAGCTCTACTCTGTGGAATAGATTATGGCGTAGATGACGCCACCGCCAGTAGCTTGATGTACCGGTCGAGCATTTCATCAGCGGCTGGCACACCAACAAACACTCTCTTTAGTTCTTCTGGTGTCTTCTTCGCATCTTGCCACTTGAGGATTAGCGCACTCTGTAGATCAGTAGCGAGTTTCTCCGTGCTGGGATCATTCTTCGCGGCTGAAAACATTCGGAGTAGAGCTATGTCATGAGAGTTATCCCTGAGCCCGTCGATGACTGTCGTCTTCTCATTAGGATATCGGTCGTTGAAGGTTTTCAGGTACTGCACCCACGTGTTGAATTTTGGGTCGGCAAGGAGCTTCTCACCGCTTTCGTCGAGATGCAGGGAGCGGAAGGCTGTCTTAGGCTCCATCTTAATATCCAACCAGTAGTTGTGATACGCATTCTCCACTTTTTCAGCCATCAATCTTGTGAGTGGATTTTTCCTTCCTGTCTCGATAATCCTTTCGACGCCGAACGGTTGGTAACTAATACGGAGCATGTCAAACCACGATTCTTGATTGTTTGGGTGCTCTTTGTTGAATTTCTGCAGATATGCCAGCCACTTGTGGAACAGCGTGTTGCTGAGAATATCATCTCCGACATTGTCAAGTGCTAGCACCTTGAATATACCCTCCGGGGATTTTCCGCTGGCAAAAAGAGTCGAAGCCTTCTCCATCTGCAGCTTGGTTGCAGTGCTTTCCAAATTGGGGAACTTTTTGGCTTCTCCAAGGACTCGAAAGAGACGCATGTCCCCAAAGGTGTCACCTAACTCCAAGAACATTTTGTCCACCTTGCTGGGGTTCTTGTCGACGAACACATTCATGTAGGAGACCCACACATTGAGTTGCGGCTCGTTGAAGAAGGCGGTATCCCCATATGAAGACCGTAGCCTGTTGTAGACGTCGAGGGGAGACTCTTTAAGGTTCATCCACCTATCGAACTGCTGTGCCTGCAACTTGGCTGCAAGAGTTTTCGTCTCACCTTTCGAGCTCGCGCCGATAAGCGTGTCGAGTAATTGTGCATCTCCATACTTCCGCGTTAACGTTTCTATCGCTGTGGCCTTCTTTTCAGGGTATCTCGCATTGTAAGCGCTCAAGTACCTTTCCCACTCTTTCCACCGTGGACTCCCCAAGAAAGCTTGAGTCGTAGTGCCATATTTGAACGCATCCATAACTTTGTCGGGCGTTTCTCGACTGTTGAGCCAGAAGTCAAACAGTGCACTCTCTACCTTGTTGGCGACAGTTTTGGCCTCTGTCGTTGCTTTCCCGTTCGCAATCAGTTTAAGGAGTACGTCTGTGCCGGCTTCGTCACCTCGAGAAAAACGTTGCTTGAGCGTAGGAATAATCGACAATGGGGCTTCCTTGGGATGTCTTGCGTTTACATCGTCCACGTACTTGAGCCAAGCGGAAAATTCAGGTTTTGGGAAGATGGATATCCCTGCCCAGTTAAGGTTGAGGGCCTCAAAAACCACCGAGGGGTCTTTTCGAGTGGCAATCCAGAAGCGTATCTGGTCCTTTTCCAATTTAAGGGCGAGTTCACTCAGTTGTGGATACAGTTTAGCAGCTTGAATTGTGTCGTAGACAACTTTGTCGCCGTGCAATGATGTCAGGGTGGAGATCACTGAAGATTCTTTATGAGATGACTTGGCACTCAAGTCGTCGGCGTACTGAACCCACTTAGCAAAGTTGGGTTCATGCAACAAAGAGAAGATATTTGGTTCGTCAAGGTTCATGTTCTTGAACACTAGCCCCGCAGGTAGTCGCTCGTCTAGCCATTGCTGAAGCTTCGCTGGAGTCACCGCTGAAGACTTGACCAACGCCTTGATATTTTCTACGATGCTGGGTGCTCTCTCCTCGCCGACTAGATCAACAGTACTATTTTTTCGTAAAAGCCTCGTGCTTGAACTCGCGTTTACGGTATCGACTTCAGTCCTGTTGGGAGCGTGTCCTCGTGGATCTTTCGCGACTGATGCGTCGACAGATACACTGGTAATGACGAAAGCTACGGCTGTCAGAAAGAAGAGATGCATCGGGATGCAAATTCAAGAATGAAAGTGTTTTCGTGCAACCTGAGGGTTTACAATACGGTACGGTACTGTGCAATGAAATGTACAGTACATTGGTATTAATAAAATGCTTTACGCTTACGTACGCCGGTAAACATAAATGCAAAGAAAGGTTAGTCTGGTCCGAAAATGGTTATGTTTACGACTTTGTTCATAAACGACTCACGTCGTCTTGCAATCATGAACACTACTTGATGCATGGCAATGGATACGGATACTTATGTGCAACAAAGGCGTGCAATCGCCCAGAACAGCTCTGGATTTTAAATCGGATTGCAAAACGACTGGCCGCCAGAAGTAGAACTTGCAAATTAATGCGACAGTCATTGATACAAAGATGTTGCGACAGAATCAATTCATCCTATAATAGTACAGAAGTCCCTCAAAGTAAGCGTCACCTACTTACGTAATACCCCGCCATATCTGCTACGGTACAAATAGGTTCAACTGGCCAATCACAAAAATGCACTGATTTTGGATGAACTGCAGCGGCGGGAAAGATCTCCCATCATTTCCAGACCGGTCCGGCCTCTTCGCTAACTAAACCATTGAAAGACTGAGAATGGAGATTAAATGTACGAGGTAACTCATGCTCCCGCTACGTACGCCTCCCTCCATGCACACTATCACTCTTCCAAGCGACCATGACCGCCAGCCCCATGCCGCGCCGGATCCGCCTTAATATGGAGCAGAAGATCGCCATGTGCAAGTATGCGAAGGATATGCGCGCCAAAGGCAAACTGTCCAACACACAGCTCGCAGCGTGGGCAACACGTGCGTTCAAGCTGGAAAATTCTCTTTCACGCGATGCCGCTCGCAATGTTATCGCTGGGGAGTTTAAGTGGGCCGCTATGGGGCCCTGTCAGCTACAGCGCAAGCAGCTCGTCAGCCCCCAAATCCAGGAGACGGACGCTCGCATTATGAAGGTCTTTAAGGCCATGGACGGCAAGGTGGAGGCTATCACAGGCAAGGTAATAAAGGCCATTGCTCTTCGCGTGAACGCGTATGGCGAGAACACCACGTTGAAGCTCTCTAAGGGCTGGCTGTATAGATTGCAAGAACGTCACGGAATCTCCAAGAAGCGGAAGCACGGCGAAGCTGCGTCAGTTGACCAAGAGGCAGCTGAAGCCGGACGTGTGAAACTACGAAAGCTGACGGACTCGTACGCCCGGTCCGAGATCTGCAACGTGGATGAGACCTCGTTCTTCTTTCGGAGCGAGGCTAAGTACACTCTCACGCAGAGGAAGGTGATCTCTGGACGCAAAGACCCCAAACATCGGCTGACACTGGCTCTAGATATCTACACCGCTCTTTGTTGAAGTAAGGTGGCCTACGACGAGATGTCGCCCACAACCATTCGGAACTGCTAGCGGCACGCAGGCATTCTGTCGAGATCGCCTCGCTTCTTAACTAAATATCAGTACTTTTTAAACATCTGTTTTATCACCCTCTAAGATCGTGTCTCGGTCCCCCCTACGCTTACTTTGAGGGACTTCTGTACATGCAAACGCAGCGTGCAGCTTCATGAACACCTACGGACGGCGTCGGAGCCATCCTAGCAGTGCTAGCAGTGTTAGCAAAGGCCACGGGCTATGGCCGCAGGCCAATGGCCGGGCCTGTCTATTGGCCTAAACCCATGTCGAAAGGATTAAGGCACTCGTTATTTTGCCCGACTGAGGTAACATATCATCGACGTTTATACGTGTACAGATTGTGTAGTACGGTTAAATTGGAGCAAAGGTAGGACACTAGCGCCGTCTGCAGCGTGGGCCCGCCCCTCTCTTTGCTTGCTTGCTGCGCTGCGCTCGGGGGGCGCCGCTGCGCGGCCTGCCCCACCCAGCCTCCAGGTCCTCGACTTAGCAAGGCGCTGTGTCTGGGATTAGATATTAAGTCCAAGCTATTAATAGTGTTGCCAAGACCGTCGGCTAAGTCCATTTTATGCATGTTGGGAGTACAATTGTATAGTCGGTTAGCCAGTCGGGCAAAATACTAGGATGAAATAACTCGTTTCAACCCTACGATTTAAATGTCAAGCGCACTAAGGGCGATACGTTAATTCATATCTTATATTAATAAATCACAGCGCCTTCAGCTCAACGCCTGCCGCACTTACAACGCCAGTGTGTTCGCGGCATGATACTCAAGCTGCTAGAGCTGGTCGTCGCCCCAATGGACAAAGAGAACGACTACGTCGGTACCTCGCCGGATGGTACCGTCTTCACGACTCGCTCGCTGTGTTGATGGTCCGCGAGACTGCTCATACAGAAGTGGAGGGAGCCACCCAGCCAATCTGGCGGCGAGGTCATCTCGGTCTCCTGTCCCGAGAACGACAGCATCAAGATCGTCGAAGTCGAGCGTCAGACGTGGCGTTTTGGGCGCCAAGACTGAGGCTGGGCAGTCGGCTTTATCACAGGGAGTGAATGCTGCGGTCGTTGCCAGCAGCTCTAAGTACGGCATGGTCAATTCCATTATGAAAATCTCTAGTATCGGAAGCATATTCCCAGTTGCCCGTGCGTGTTGCGGTGTAGAACGCATCACCAGCAACACTTGGATAATCGCAGTTGCTGCAGGCGTATCCGTCGAATCGAGTGAGTCATCGGCTGCGTCCGTGTTCACTGCCTCATTTGCGCGATCGAACCTGATGCGGCGATTGTCACTTGATCAACGCTAACCTTCGTGATGGTGGAGCTCATAGCCCAATGCCGCACTTGGGCTGGCAGTTTGATCCACCACGGTTTCTGTTGCTGAAGGTGTGTCTTTGCCCGGGCTAGTTGATGGCGCCACTGTCGTGACCGGGAGAGCTTAAACGCGTTGCAGCTGTGACGAAGGGGGTGGAGGTAACGGGAGGAGCGGATGTATTGGTGTTGGAGATTAAGTAGGGCTGCCGAGACGTCAAAGGTAGGCCAGGTCGCGTACAGCTCGACACAGCAT

>Contig_34

CTTGCAAACCGAGGGTAAAACGTTTTTCTAGATACTGCAGCAGAATGCGTCCGTCTAGTGAATGCAGAGATGGACAAGGAGGGAATTAGCTGGGCTAAGAAGTCGAGGCTGCTGTGCGGCTTAGACGTAGATAGTGATGGTAGTGGCAGGTTAGGCAGCTCAGCAAGCCTTTGCGAGAGATTGTAGAAACGTATCGTGCTGAGTTGGATAAAGGCTACGAAGAGTGCGCAGCATCGGCGACTGTTTAGCAGTCCCTAAAAAAGACAGTCGATTTCACCATAATTAGTCATGCCCCCTATATAGTCGTGCACCTAATTGAACTCTTTATAAAATGGCACAATTATATTTCACTAGATAGTCATCACCACATCGGAGATGCTTACTGCAACAGCCTCTTACATCGAGGAATGTGTATGATGCTCTATCTGTCGGAAAGTATTTAATCCACCACATGTGGCCGTTTATCCTGTGTGTACATGAACATTCACAAGAAATAGATTTTGTTTTGATGTTGATGATATTTTTTCTTTGACTTCTATTGTCAGATTTGTCATTTTGGTTGCCTTTTTTCTGGCAGGGACTGTGTTGATTTCTTGTTTTCATTGTTATTTTGGCTACTCAGTAAAACCTTAGGGGATTGAACCTGTTCACCGTAGTGATTGCTGCTATTATCGTTTTAGCTATACGAATTTGTAGAAGCTACTTCAGGAGAGCCTTTAATTTTAAGGATCAAAACCTGTTAGCTGTTTTGTTGGCTACTTCAGGAATTCTTTGAGGATACACCTGTTCACCGTATTGATCTTTAAATATTTTGTGGTAGAGTTTAGCTTTGTTCATTTCTTAACTGCCATTGTTTTGCTCTTTATCTGTGGAATGTTTTTCACAATGTGTGGTTTTGTTTTTGTTTTTCGTTGCTGAGGTGTTATAGAATTGCCTCTTAATTCTTGAAATGGTTAACAACTTGTAACAGAGGTTTGTTTTTATCGAGCGTGATGCCACGCTTAGGTAAGACTACAGCTAGTATAAGAGTTTTTCACGAGAGAATTCAAAGCAATGTGTCATTGCAACTAAGGAAATGTTATTCGTATGCTGAACTCAGCGTAGTACGTATTGGACATCTAGCGCGGGAGATGCTAGCGGAGGTCTACCACGCGTATGCCTCAAGCATAAATTATTATACTGCCAATATTGTTTGAAGTAGTAGTGGAGATAGTAATACTACAGTATGATATTATCGTGGGCGATATTACAGCCCGCATGCTCTGCTTATAGCAGGCCAATGCGTGAATTCTGCCCATGTGCTCCGCTTATAGCGGGCTCAATATGCAGTGCTTATCATCGTGGGCGCAATTACAACCCAAATGCACCGCTTATAGCGGGATCATTACGCAGTGCGTATGGTGCGAAGTCCAGAATGATTTTCCTCTAGCTCGATACCGCGAGGCAAACGTTTTGCCTACCGCGCGCAGCTCGTTGGCTGGAGTACTTAGAGGGCAACATGGCCTTGAAGTGCCGGTGGCACATGGTAGACTCCCGTGCGTCCTCTTGTCGGCGTCGAATTCATGCCCTGCTGAGACAGGTGGTTCTCTGCGTTTGCGCCGCCCTTATTGGTAGGAAGCGTTGGGCTTATAACCTGCTGAAGGGATGATTTGCGCAGAGCTGTTAGGGCAGTACAGTATGGAGTGAGGACAAAAGTAGAGTGGTCTAGGCGGCGACAGTGGAGAGAAAGTCTTGAGTGTAGAAGAAGAAAGATGCTTGTGTGGTGAAGATGGTAAAAGAAGAAGTGATGAATTAGAGAGTCTGGTCTCCACCAACCAGTCCAGTTATATATGATTTCTATATATGAAATAGCACAATTAAATTTCACTTGATAGTCATCATCACATCGGAGATGAGTTCTATTAAATCGTCTAGTGGGTACGTTTATGCGGCACTCAAGTGAACAACAGCGAGCCTCTTTTTTTCACTGAAAACTCCCCGTCTCAGCACAAAGTGACAACTTTTGATGAATGTCTTGTGTAGCCTATTTAAAAGCGCGGGTACGGTAGCTTGGAAATTTGAAGACCAGTACCGGGTAAGAAACGGATATCGTGGCAGGCGCTACAGAATCCATCGATACGTGCTGCACAAAAAATATCAAGCGGCCTGAAACATAAATCGCTGATCTTATTCAGGCGCAGCACTTACATGTTAGTGTCCATGCTACCGCCACAAAAGCTCGCCACTTCTGCACGATATGTAGTACTTAAGCTCTTTGTATTCATCAGCAAACAGTTTTTGCATGATTCTTCTGTAAAGAGTAAACATATTTGCTTGGAGGAATTAATTGTCTGAGGCTCTCTCTAGTATAGTCTCAATTTTTAATATTAATATACACTGCATTACTTGGGAAGTACTGCACAGTAGCTCCACGAAAATGCGGTAAGTACAGTAAGTTGACAAAGGGAACATATACATAATGAGCATCGTCATCCATTGAGATGTAATCGTATCGAGGCGCGTAATCAAATACCGGGAGTTCGCACAACGATCGCCACAGTGATTTAACCCTTCCAACACGGGAGCGTGCCCCCGACGATAATACTTTCTTGTGCTCCACTTATCAGTTTGAATTTTCTCAAGCCTCCACTGAGCACCGATGTCCCGTTATTCAATGATGCTATTACTCCGGTTGGCCGTTTTGATCACTTTTGGGTTAAGTTGCGCTGTAGCCTTGGCCACCGACTCTAACAATGTGGTGAAATCTAATCCTACTATCAAGGACACTCAAGCCTTGCGATTTCTGCGTCGTTATGCTTTCGATGAGGAAGCTAACCGTGATAACGAGGATGAAGAGAGAGGTATCGGTGTCTCTAAACAGCTGGACGATGTGCTATTGAAGGCCGACGAGGTTATAGGAATATTGAAAAATGTTGTTAGAAAAGTTGGCGGTGCGCTCATCAAAAACTCCCGCGAGACCGACGAGTATTTACGTCTAACAAGGGCTGTATCCGGCAAGTATCCGACTGCAAAAGAGTTGAGCCTCTCTACATTGAGGCAGCTTAAAAAGATTGAAGCCGTGAGAAAGAAAGACATCGAAAAGGGTATCGATGTCAGCAAGGCAGCGACCGATGGCATACACAGGGATATTAAGCTCTTTGATGGGAATAAGAGGGTCCCTGACAAGTATATGGGGGCCCACGTGGGACGCGACCAGCAGCGTTTTACCGAAGCCGATACTCGGAGTCTGGTAGCCGGTGTGGTTACAAGGACAAACAAGAAAGGCGAGCAGGAAATTCTTCTGGTTTCGAGCTCGAAACCCACTAAGTATGAGTTTATGATATCGAAGGGGGTTGGGAAAAAGACGAGAGTGTTGAAATGGCAGCGCTGCGTGAGGTCATTGAAGAAGGAGGGGTACGTAGACCGCTCGTGGATATTGGGAGCAGTGACAATCTATACTAACTCGCGGTGATGCAGGTGAATGCAAACATTTTGCACGATCTGGGTACTTTTGAGCTCAAGGATGGAAAAGTTGCCAAGGCCTTCCTGATGAAAAGCGACACGATCTACGACGATTGGGCCGAGAGCATTCGTTATCGCTTATGGGTACGTGGTCCAAGGAGACTTTATCAGCTTAATCCGAACTAAGCGTATTACTGTATCCATTGATACTGAATCAGGTTTCGTACGATGACGCCATTAAGCTGCTGAAAAACCGCGAGCAGATGGTTGCGATGGTGAAAAAAGCCAAAGCCATGGCCAAGAAAGATTCCAACCCAAATTTCTCCTAATTCAAGCTAGACCTTGAATAAGTGGTTGGGCAGCTACAGAAAAGAGCTGGCGCAGAAAAACCTATACGAGCCACCAAGCGAACGCTGAAGAACCTAGCATAATTGGATGTCCATCCCATTTTTTTTTTACAAAAATGGGAAATCTCTTATGAAGAAATTTTGTGCAAAAAGAGTGTATATTAACAGCAATTGGGTGATACAATTGTTGAGATTACATACACACAACAGAAATCGCATTGCTAATAAAACGAAATGCTCGTGACAGAAATAAAAAAACTAAGGTTAATCCAATAGGGGTCGTCAATGTTATCAGCAGTTAGATAGATAAAAGTGATACATAAAGCTCGCACATACAGGGACCAACGCAAACCCCTTTGAGAAAATTTCGCATTGACACAGTTGACACAAGAAAACTGTCAGAGTCGGGGTACCTGCTACTAATCTTCGACCGTGTTCTGCGTGTATTTACGAGAATTACATACGTGGCTAGTAATCTCGCTAGTAAGGGCACATAATTCACAACGGACACAAACTGAAAACAATTTGAAAAAGGGGCAGCTTAAGGGCTGTCCAAGAGGGTTCACTCTTTAAAGTCCATCGATTTGAATAGGGGCGGGTGCGTCGACCCGTCCAGCGAGCTTTACGCTTTGAACGTACTCATAATAGTCCCACCCGGTAGTACTCTGACATGAGGAGCGTAACAAAGGGTGCCCGCGAAGCAAGATCGCTCAGTATTTGCGTGCAGGCCAAAAGATAAGATTGTTTGGTAAGTCCAGGTTTCTTTAGTTCGTCTGCTCACAACAATGACGGGCAGTGTTTCCGTCGAAATTCGAATTTTAAGACAATCTCAAATGAGTTATTTAGCCTTTGAGCACTTGGCTCTCGCCTCTCGTGGTGGTCTCGAGGTTTTTCAAGTGAAGATTTCGGGTGCAAATGCAAAAATTCGAATTTAAAAAAATAATTGTGCGCAACATATTATTTCATCTATTTTTGTCGTTTGGGATTTTTTTTCTCTCTTTTTATCATTAACGGTACTATGGTGCGTGTGTTAAAATTGACAATGACGTATTCTCAGTTTTGTTGCTCGACACTTTTTTGTTTTGCCATTTCTTACTTTTACTATTCGAGCATAAAAACAAAGCAAACCCTATTCCCTAAAATACTCTAAACTTGCCACGTCATCGCCGCAGGACTAGGCTCAAGGAATACAGCTCGGCCAAGAGGGCTTAACATCAAGGTGCGAAGCTTCGCACGCTGTACTGAACACGACCAAATTGATGATAAGGGTATAAACTGCCCACAATAGCTTGATCATCGGATCTGTTACCGAATAGCTGATCTAAACACTGAGTGTCTTTTAAACCACGAGGTTTCCGGGTTGAGATGGACAGCTTATTATGAGATTGCACTCCCACACTTTTTGTTTCTGCCTTTATCGATACGAGCACATGATTCTCTGACATCACCGCCGACTTCGCACGCTATGGCCGTGTATTAAAGCGGTGCTGTTGATATGGGAACGGGGACCGTCTTGCTCTGCTTAGGTAGGTCCATTACAAGGTCTGAAAAGTTTCAATGTTGAAGATGCTGATACCTATCAATAGCATGCTCGGTGCAAGCCTTTTTTGCTACACGCTACAGGACTTATTGCGTGTTTATCGTCTGTTGGATTGTTAGCCCCGAAGATTACTTTACCCACTCGGTTGTACAAGACAATATATATTCCAACCTTCTTCAAGCAACATTGACTTGGTTAATAATTTTTGATGTTACCCATGGCCGCCACAGGCAACTTTCTTCTGTGTTTTTCTGATGTCCACCGGGGAATGTTGTAATCAATCTATTTTTAGTACCAGGTGGTGCATTTACTTACGAATGGGTACAAACGAATATGTTTAAAAAGATAGTACCGGTACCACATCGCCGCCAGTGAGCCACGGAGACGCGGGGAAGTGAGATTACAACGAAATTAGAGGCAACAGGTCTACCAAGTTATGAAGAAAGCGTTTTTTTCCAATATGAGTAAGAAGTAAGAATTTTCCCTTAGATCGAGTGTCATCCCTGGAGAAACCGTTGATTAAAACCCAGAGAGCTATTACTACACGTACGTAAAAAAAGACAATAACATGGTCTCGACCATTTCTTGACTTATTTCTTCATTTTCTGGCAGTGCATAGTAAGGCATCATTAAAACTGCTCACATTCTTTGCTTTAGAGCCACCAGACCATGGTACTTCGAAGTGTTAATTGCCGTGAATGATGCCCTCCAGCGTCACCATGCCCTGTCAACAATCACCGTGCTAGGACTATTTTCTATAGTTTTGTTTTTAGTTATAACAGCGGTGGATAGATCGCAAGTAAAGACAAAAAGTGAGGGCGAGAGGCCCCAAAAGGCTCTGCACTCTTATGTCTAAAACCAACGAGCCGTTCCAGACACGATTGACCTCTATTTCGCGGCGAAACAAGTTCATCATCGAAACATTGTCGTGAATAACCTTCTTACTGTTTTTTTTAAGTTTTTGGATCCGTATTTCCGACATTTTCCGCCAAAACTACGCTCCTTCTAAATTTTATATGGAGCATAATTACATGTAGAAGTAAAAACATTTAGTATAAATACTGCTGAAAATTAGATTATCATGGATTTAAATCATGTAATTAGGCTGCTTTTTTCTTCAGTAAATTACATTTAGTCATCGCCAAGACATATACGCATATTATGTGGATATCTATCGAAGAATCGCCAGCTCAATTTAGCCTCATTTTAATGTGGTCCACATCTGAACTTTTAAAATTGGACCAAAGATTGGTCTATATCATATGATTGGCCAAACGTTTGAGTTTTGCATGATTTATCAAAATAAGTTCGAAAAGACGCTTTACTTGGACCAGTCTACTGGAACTCTACCAAAACCTACACAAATGCAATCACAAACACAAATTTAAAAGCGAAATGTGTTGAAATATATGTCGAATTTGTGATATGTCTGCTATCATACGAAAATCAACACTTTTAACGTTATGGGTACATGTACGCGGGCCATTCACCTTTTTTATGGCTAGGGCCCAGCGACTTGTGCTAGCGTGCGAGCACCGGAATGCGCGTTTAAAGAAGCAGGTGTTGTTGATTCTACGGCTGCGTTGTATGTACTCTTTTGATCGTAATGTTCTAACAGTTGCAGCGCTAATTGATCCTGCTAAATCGCCCTGGCACACGTTGTATGCTAGTTGAGATCGTGGGAGCTTCATATCAGTTGTCTCCCTTGACCCTGAGGCATTTGATTGCTTGCTACAGGTGTTTAGGGAGAATCTGCTCGACGAGAGATAAGAGGCCTGCAATAGTGAAAGCAGCGTTAAAATCAATCATGAGATCCTATTTGTGACGCACTACGTACGTTCAACCTGAGGCGTAGTGAAGCCCCGTCCTCTTCTAGCAGTACGGCGGCTCATCGTATGAGCTGTTGTGTCCCAAAGCAAAACTAGAAGTGGGCTTGACTGGATACCGATAGCTGTACCAGAAATGAGGTACGAACGATTTTGCGTCGACACATTTTGTGGAGACGAGAAAAACGCCTCAACATTTTACTACCGATTTGTGACAGCAAATACGTCACGGCTTTGTCAGACGCAAAAATCGAGTTTGTGTAGGTTTTGGTAGAGCTCCACTGAAGTCCCAAAATACGCTGGACATATATGACAAACAGGAACGTGTAGGTCTGCCTAAAATCTGGCTGCGGATATTCCTCATTTCTACGGTCCCTATGTACCCGCTGCCTCCACAGCATTACACACGACCTCATGCCAGGTTCTACTTGGCGTGGACTGCATTCCAATACGAAAGGAATAAGGGCTCATAGTAATGGGCTTCTTCCGGCGTAATACCACCTTCTTTTCTGCGATGTACCTTTCCAATGTTTTGGGAGTGAATCCCTGCTTATCCAACGTTTTAATCGACGTCATCAGTCGTGCCATCATGTCTTTGTGAGCATCATCAGCGCTCTTGCCAACGAGCAATGCGTTAATTGATTCGTCGATGTGGTCTGCTTGCCCCACTCCTTTGAACTTGGGTATATCGTTAACTGCGTCATCAACTTTCCTGACGGTCCAGATACTTTTTAGCTTCTCAGCAAACGCTTTAACAGCTGCCGAAAGATCTCTCTCTTCGTCAGTTGACTCTGTATCATGCTCGGTGTCATCGTGGACAGGCTCATGCTTTCTCAGTAGCCGCTGGGCATTGTTGGTGGCATGGCCAGCCACAGCAGGATCTTGAACGACCCCAGATCGAATCGCTGCGAGCGTATCAATCTCGACAGCACCACTACCATTGACAAGTATAGCGACAGTGACCTGGATAGCCAGCAGAACGATGAGTCCGCGCATCGAAAGTTGTGAATAGATCGTGCTCGACGGAGAAAAATGAAGAATGGTTCAGAATGAGTTAGTGTTCTTGGCACGTTTGTTGATTAGTCACGATTGGGTAAATCGTGCCTAATCAATCAGCCCGTATCTTACTTTAGGTAAAATCAAATATTACGAGCTTTCTCCAAAGAATAGCTTTAGATAGTATTTTAACCCTGTAGAAAAACCTCGGTTCGTAGTTAACAGCACATCTCATTTTAATGTGGTCCACATATGGACCTTTCATTGGACAAAACACTATTCTATATTATATGATTGGCCAAACGTTTTACGTTTTCCATGTGGCATCGAGAAATCTTAATATGACCAGCCCCAAAGTACGCTAGGCATATATGACATAGGAAAATGTTTCCGGAATAAATGGATACATTTTTTTGATTAATACGCTAAAAATTGAAGGTGCTGCCATAAAAACAAAACTAATGCTTTTCAAAATCGTCGACGCAAATTCAACCTTTGCATTTACAATTTTAGCTGTTATTGACAGCCAATTTTATCACGCGAAGCAGGATAATCTCAGGATTATGTTTAGGTATGGTAGCTCCCGAACCAAATATTCTGGAACCATTATGGTTCGAAATTCAAACTGTCGATTCTTAAGTGGTCCAAAGGGTCGTCTATGGATTTTCGCGAATCAAACAAAAAATAACGATAAAAAGAATTTTCTTGGTCTAAAACCCGGTCATTTTGCGCAACATATCGAATCGTGTACTGTGGCATGGAATACATTTATCTGTCAAACTGACATGGCTCGAGAGAACGCGTCCGCGTCGTGGGTATCGTGCGAGGTACGTTCCAGAAGTGGCACCAAGCTGAAGGTCAAATGGCAGGCGTTCCGGTTCAGGCGACTAGATTCACTTTCGAATTTGCGGAGTATGGGGACACTAACTATTTTCAACAAGCTTCACTATACCTTTCTTGAGCTCCCACTGCGCTACGAACATGTCTAGGCTTAGTGGACCGATTATGTGGATCAGTGTCGAGAGGAGATGGCTGACGTTTAACTGTCCCGAAACAGCAAAAGATGTCATTATTATAAATACTAGCTGAGAGGCCACTGCGTCAGTGTTTCGCTATTAGCAAGCGTTTATAGTAGATAGTTCCAATATCTGGCATCAGACCTTCGCGTGGGCCCATGACCCCGCAAGACATCATCTCCATTGCTCGACGACTCCGTCAGCGTAACAACTCTCGACTGTTGTTGCAATGCTTCTTTTTGTACTACTACGTTGACGGACACGCAATCGAAGAGTTTCGCGTCAATGGGTAGACGTCGATCAAGAAATTGAGCCTTTGCTGAATAGGAACATGTTTGATCGGACGTTTAGAATGTCTGCGGACTGTTTTTCCACTTGTTGAATCTATTAACACCTGCACGCGCAGTCGCCGCGCAACAATCGATAAATTCTAGCGGAGTGGAGCCGATATCGCCTGCAATAATGCTCATGAGAGCTTTGCGGTATTTGGCAGGAGCCTCTACCTTGTTTACATAGGACGGTTGGGATCTGTGCTTCATCGTACTATCGAGTTATTGATCTTACTCTGTCTGGTATCCTATCTCTTCGCGAGTTGCGAATCGTATTCCCCGAATCTGATTCGGAGAGAGAGGTTGCGATGGGTGATTTCAAGGCAATCAGTTCTGGTGATGTGATGAGTGGATGTATCGGTTGTGTAGATATATGGCTTTCTAGGATTAAGACCCCAACTTATGCAGACGATGGTGACGTCGGTCTTGGACGCTACTATAGCGGCCATTATGGGTGCCCCGGCATTAATGTACAAGCTGTGTGTGATACACACTGCCGGTTCACCGCCATTAATGCAAGCTATTCTTGGAGCAAAAATGACTCGCATGCATTTCGTGATACCGGTAAGGCTCGAGTTCTTGTAACATCCTTCCACATCTCACGAAGTTTTGCGGCATCATGGGGGACTATAACGCCTAGGTTGCATTTGTCAAATGTGATGAGCGTCGAGCTGAGCTTTCTAAAATATGCCTGATCACTATGGAAGGCTGAATGAAAATTCGTTCAAAAAGCTGAGCTGGCGTGAGTTTGGTTAGAACCCAGATCTTGTCGGGTCGCGCTTCCGTCGATTTCATGCCGTCTAAGCAAACTTCTCGGAAAACATGACATTTACTAGCTGCATCATGCAGTGTCGGGAGCGTCGTTTCGACCTCGTCGTCGCTACCGCCTTCGATAGTCCGTCCTCAACTCCAAGAATCTGCATTTCGGAAAAAGCTCGTTTGTTCGCTGCATTAGCAGCTCTACGTAGCCAACCTTGTGATCGTTACAGTCGACACCCTTCTTGACGATGCGGAGCTGTCGTAAATATCACTCGGTTCGCAATTGCTTAGACCCCATAGGCCATCAATCTTGCCTCTGTGTCCTCCAACGTCATGGAGGCGTTAAAAAGACAATCTGTGATGGATTTCGGAATCCCTATCGATGTTGTTGACGTACCGACAGCTCCTAAATGCGGCGGCGATCTGGAACTACTGCAAGTCACATTGGAAGACATCGTGGAGGACGAAGATGCTTGGTATGACCTCGAGAAAGCTGTGAGCTGCTGGTGAGAAGCTGTATTGAAAAAAGTGGATCGATATTAAAAATTATGGTAACTAGCACATAATCATAAGGTTTGTATTCCCTCCTATCCACCCTCCATCCACGTCCACCACCATGATATTCACCCGGTGGACGTGGATCAGATTCAATTAGCGAACCCACGTGGCGTCCACCACCGTATGTGCTCAAGCGATTCTAGCGGGCCACTAGAGACAAATCCACCGTAACGTTTGGCTAATACCGGGCGTAAAAGAAGGAAGGCAAAAATGGTGGGAGCATGCTGAAAATAATTGGGAAACTAGTGGGTTATGACGTATTGTGTTTATTTGCACAGCTAAAAAATAACCATATAAAGAGCCTATTGGGCAAAGACAAGCAGCCGTTTCTCAATCAACACTAGCTTGAAATGATGTTATTGCTACACCAGCATAAGGAGGAGGAATACTACGCCGTGGAAATTCCAGAAGATCAACGGAATACTTCGTATACCGAGGAGATGAGTGAGATACTGGGCATGACAATGCGGTGGATAGCTGCTGTCTAAAACGACACAATCTGACAGCTGGCGAAGGCGAGCAGCTACACTAGTGTAGACTTTGGAGATACGACAACCTTCAACATGAATACATGTTAGAAAAGGAGACCAAGCTGTTTGGTTGCGAGTCGAGCAGACTATTAACCTTCCGTGCCTACACGCGATAGTTAAGAAGCGGTCAGCAGAAGGCTGTTCGTGGTTTCGTACGCTGCATTGCTCCAGGGCTGACCATGTTTTCCGAGTTCTATCTAGCTTTTGGATTAAATGCCTAGATTTATTCATAAATTAACCACGTTTGTTGGCTTTGACTGTATTTATCTTTCTAGATGATACGCAGCGACGATTGTCTACGCAAAATTATTGGCTTGGGGCTTGTTGAATAAGACGCAAATCCAAAGTGAGTGAATTAGCGTTTATCAAAACTGTCCAATATGTATCTCTAAACACTATCTATATGTTGTTCCTCTTGCTGTCCTCTCGTAAGTGTGGCGTAATCAACAATGCCATGTGCCTTCATACAATTATAGCGTTCCAACATGTGATTACCCAATGGCGTTCCAGAAGCAGCTCCACCCAATAGTGGAGCGGTTCAGTACCCGTTCCAACGGTCACGCCTAACCGTGCTGATCCAGTGCAACTACAACACTCCCATTCATCGATACGGCTGAACTAGTGAGAACACTGCTTGTCTAAGAGCATGCAAATAGCAAAGTGAGATTAGGAATAGCCTTTGAAGCCAAGCCGACCCTCTGGACTCGGAATCTTCCAGCAAGTTGTCCGATTTCGGGATTTAGTACTACCGTCATCAACACTTGATAGCGCTAGAATGCATCTACCACTTGTACATCAATGACTTTATGGAGTAATTCCAACTCCCCCCCCCCCACACACACAAGAGGTGCATGGAGTCCTTATTAAACCTTGACGCTTCTACGG

>Contig_35

AAGCAGGGATGGACATTTGTAAGACCGCGAGCTAAGGACTTAGATCCGAGATGGAAGTACGTTCGCCCTGGCGGACATGTTAACGGCACAAAGGAATCGACTTTTTTCTTGGGGAGGAAGAGCTGTTGGAGTACTACAGTGGTGGTATGCGAGATGGTGGCTATTGCTGGCTACACAGTCTAACCAATTGTTTGTATGATGTTACAGCTTCCAAAAAGCCTTCGTCTACCGGTTCTGGACCATTCCAGACGCCTCCAGCACCTCCTGCTGATCCTGCTGTCACAACAAGATCACCACCCGCTCAAGCGGCCACCACCCAACCCACGTCTCCACTTCAACCTTCTTCAGGTACTCAGCAGCCTTTGACGCGATAAACGCAAGTGAAACGCACTCGAAAAGTGCCCAAGCCGGCTGCCACGGACAAGCAGAAACGTCGCCGCTCATGTGAGTACCAGTTGCATATTCATGATATGATATGCAGGATATGCAGGATAACTAACTAGCATTATGCATTAGCTCATGCAGTACATCCGCCCGAAGAAGCCCGTACGCTATCCTGAATATTTCATCGACCACATGCATATTGATGCAGTCTAACGCTTGTGAATGCGCTATAGTTGACGACGCCATATCTGATGAAGAAAAAGCGCCAGTGCCAGTAGCTGAACCCGTTACTCAAGTCGTCGCTCAAACCCCCATCGTGTTGCCAGGTCCTCATGGAGGTGACGAAGAAAATGTTGAATCGGGGGACGAAGGGTATTCCAGCGTTGAGTCTGTTGAAGAGACAAACGAAACGGGTATGTCTTACAATGCGATTCAGTGCGTATGCTGCATATTCATGATACCTTATACTGATTATTCATGATAACGATTTTTATGCTTGTGTTACGAAGATATACTTGCAGAAACAACTGACCGGTTGAATGTGGACGCCCCGGGCGGAAGAGACCCCAACTATGATGCACTTGAATCTGGCGATGAAGCGGCAAGGGACGACGTTGTCACAGATACCCAGTCTGATTGCAGWGGTTGGGTATCAAATGCCAGTGCGGCCGATGGATCGGTGCCGAAACCCTGAACGAGACGAGATGGATATTGCGCTGGCAAAAGAATTTCTGGATGGATTTGGAGGCACCGACGCTGTGTTGGCGGGTAACTTGCTGGACAGAACACTGAGAGAGTTTTCGACAATGGATGGGGCCCAATTCAAGAACCGGATGTGTATGAGGAGTTACAGACGCCTTACGTTCCTGTTGAAGGCGCTAGTAGCTACCCGGGACTTCACCAAGGCTATTCTGGACCCACTCCTGAAGTACTGCATCGTGGAAACTCGCCTATGGCGTTGTTCTTTTACTTCATGCCGGTTGCATTGTGGCAGCACGTTGCTGTCTGTTCAAACAAGTACCAAAGAGATATGCTCCACGCTCGTGTTGACGATGCCTATAAGCGCCACAAGCGACGCTGTCAAGCGAATCCTGCCACGAAGAAGAAGACACGGCGCGATGTGCTACAAGAGCTTCAAGCAGTTCCTCCAATCAAACCACACGAGCTGTGTCGCTTCGTGGGACTACTGATCGCGCGAACAATTTGCCCAAACCGAGAAAAGCTGGCAAACCACTGGAAAACCACCGACGAGGGGGCGATTCCACGTGGAGCGTTCAGTTCTGTGATGGCACGAGATCGATTCATGGATATCTGCAGAAATCTACACTTCAATGATAACGACGACCCAAGAGCGACCACGGATCGAGCCTGGAAGATCCGCAAGGTTGTTGAAGTGCTGCAGCGGACGTTTCGCGAAGGATACGTGCCACCTGCAGAGCTTTCATTTGATGAAGCCATGCTTCCAAGCCGCAGCAGATTCAGCAAGATGCTGGTGTGGCGGGGCACGCTTCTACTCATGTAGCACAGTTGGTGGATGAATGGAGGACCTGAGATACGCAGCCGAAGCGGCGTCAGCGTGCGTGTAAAGTTTGCTCTCTGATGCAGGGCGCGAAGCCCCACACGACGGCGTACTACTGCTCCGAGTGCGATGGTAATGCGCCTATCTACCTGTGTATGCACCCGAGGCATGTGATTCGCGGAGTCTTGACAACCTGCTTTGACGTGTGGCACAAGGAATGGAGTGAAAGAAAGCTGCGACCTTCGAACTAAGAACGCCGCATCCGTATGCGCCCAGCAACTCCAACGAAAGGTGGTGGATCACGCAAAAGACTGAGAGATGCTGCTGATTAAGTATCATACATATGCAGGATACCAAAGGGTGAATATGCATTCTATCATCAGTATTTGTGTTTTTGCGAAGTAATGGCTGCAGTGAAAATTGTCTTTCTATATCTCTTGCAGGTTGAGAATAAAGTGCAACCGTCGATTCATTTTGCTTACTATGCAGTCCGTGAGGCCAGAGCCGGAAGCCCATGCTCAGATTATATTATAGGCTTCATATTTGTATGAGCCATAATAACTGAACTTTTGATTGCTCGCCACTTGGATGGGAAAACTTCAATATATGTACGCAATTGGACAGTTTCTAAAATTCGACATTCAAATATCTCTAAACTTGCAACCGTTTCTGCACCCGCCTATCGCTGTACTACCAGACGACCGGAAGTCAGCATCTATGTTACGGAAATGGAATGAATAATGCAGTTTAGAACCTAAACTTGCAGCCGTGTNATGCACGTTAATATGCAGTTTGCACCGGTTATTACAACGCTCGGTTCGTCGACCATCTCTGTAACAAGTAAAAGTATACAAACACCAAGTCAAACTATCTTGCATATGCAATTTAAGGAAAGGAAAAATCAGCCGGTTTGAAGGGGTTAATAAAAGTTGCTGTGTCTCTTATTGCCTACTGTAAATCCCCGCATGATGATTATCATGCTTTCAACTCCCCCTTTCCGTTTCACGATCAACTCCAGAGCACACTTTCGACCACTTCAAGAATGAATCCTAGAGCCAACCAGAGCTACCACGCCGTTGTGTTGACATTGGTTGCACTACTGGTGTGTGTCAGTGCCGGATCCATTCCCTCCGAGTTGAACAGTATGAAAATATCTGCCAACAGCTTCGACACCCGTGTCAGACGGTATTTGAGAGCACCAGACCTCACCACCGAAGATCGCGCATTCGATGCTCTAGGTCTCACGAAGCTTAAAGATGTAGCTACAACTGGAACTCAGAAGCTGCAAAAGCTGGCGAGCAACGCAAAGACCAAGATGACTTCGAACAACCAGCAGGCCACGGACAAGCTGTTTAAGAAATTAAAGGTCGGCAAAGTAGAGCAGAACATCTTTGAGAGCTCGCAGTTCAAACAATGGGCCGCGTCTGTGGCTAAGTCGTATAAGAAAAACGCCGAGGCTGGAGACTTTGCCATGGTCTCGACTCTTTCGTCTCGCTACGGTGATGATGTTCTCGCGAGCATGGTGATTACTGCAAAGAGCTCCCAGACGATTGATCCTAGCTTGGAGAATAAACTTATGTCCTCATTGCTGACTAAATGGCAGACTGAAGCACGGACTACAGACGACGTGTTCAAGCTTCTGAAACTAGATATTGACGAGGTGAATCTACTGAAGAGCCCCGTGCTGAGCACTTGGATTTCTTACGGCCACAGGGTCGGAGGTAAATCTCCGTACGAAGAGCTATTCAGCTGACTGCACGCTACGATGATGAGGGACTAGCGAAGATGATTATTACTGCTAAAGATGACAAGGTCGTGTCGTTTATCAGGACTGACATGACGAATAAACTGTTCGAGAATGGCAAAGCAGTGGGAAAACCGCCACGGACGCTTCGAATATTCTCAAGTTAAAGGAGGAAGGAGCTTCTCTTCTGAAGAATCCAACGCTCCCGATTTGGATCTCCTACGTCTACTCGCTCAAGCAGAATCCTTATGAGCTGCTACTACTGAAGTTTAAGGCACACCACACTGACGCTTCCGTGGCAAGGGTGATTGCTTCTGCGAAGTCGGATCAAAACTCGATAATCATTGTCCAGAACATGCAGAGAACACAGATCGAGAGTTGGCTCAAGGCCGGAAAAAGTGACGAAGAGCTGTTCAAGCTCCTGGAGCTTAACAAGGCAGGAGACCAAGTGCTTGAAGATCCGCTGTGGAGAACTTGGGTCGCATATCTGAAAAACTTGAAAGGGGACGCTGACAATAGAATGTATTCGGTACTAAAGACACAACTTGGTGAGGAGCAATTGACGAAGATTATTACCAGAGCCAAGACGGTCGGTAGAACCAAGGCAACCGCAACGAAATTGGAGCTGCAGGTTTGGGGAGCCAGCGGTCGAAGCGCAGACGATGTTTTTGATCTCTTGAAACTGGACGTTAAGAAAAGCGACGTAGTTGAAAGCGTCGCTTTCAAAACGTGGTACACCTACATGAAGACGATGAAACAATACAATGAAAAAGCAGTCATCACAAAGATGGAGAAACATTTCGGCGAAGTTCGTTTGGCTCAGATGCTTGCAGCTTCAAAGCGGACTGCGACTGACGAACCTACGAAACGGTTTCTTCGTGATTTACAGCGGAGCCAATTCTCTAAGTGGGAGGGTGAAAAAAAGAACCCCAAAACCTTTAGCAGCATGCTAAGTCCAAGTGATCGCAACTCTGAAAGCGACAGAGTTATGAGCGATTTCAATACTTTTTTCGGTGAAGGGATATTCGACATTCTGCGCATGGGTCGACAGTAGCTGGTAGATTTAGAGGTAGGAAGCACACCCCAAATTTCTTCGATCCTAGAATATCCGCTATGTTTTCTTAAAATTGAATAAACCACTGATCTTGATTTTTAGCAACTTGTGTTTGAATCGAAACGTATCACTACTAATAGGATTGGGGCATACATGCAATATAGATACATGTACAGTCAGAATTTCTGCGAAGAACTACCAGACCCCTATCTTCTGGCTACAGTTTTTGTTGTATTTTACTGTACTATAATACAGTATTTTATCCAACATTTGTTTAAGTCTTAAAACGAATAAGCAAAATAAATGAACATAGCTCTAATCATTCAGATACCGTAAAACTTATGTGCATTCGGACCGCTCATAATTTATCCTAGACTAATTTCTAGCCAATGAACACCGCCCAAAAGTGGCGTTTTCAATTAACAATCAGAGTACACAAAATACTTGTTTTTGCTTGGTAGTTCTTAACTGTCGAACGGCCGTTTGCACATAAGTTTTACTGTACACTCAGACGATTGATTATTCATTGGTCATGTTCTGCTCTGTTTTTGCTGGGCCTTTCAAAAAAAGATCTCAATCGCATGATAATTTCATCAAACAAGTAATATCAGATTTTTTAGTAGCAACGTGATTGAAACACTTGAACGGACTTCACTCTAATCGAAGCTCGTGTCAACTCAGAGAATCTTTGCATAAGTTTACAAAATAAGCTAAAACGTACCTTTAAACCTAGCCCTTGTAAAATTGGCCAAAAACACATATACCAGGTAATGAAATGTACCTCATGATTTTTTCGAATATTTAGCAAGAAATCTTAAGATACATGTATTAATCGAGCCGAAATGATCTACCATTATTTGGATATTAACGAAATTAGATTGGCTAGATTAATAGGCAACAGGGTGCCTTCCTAACCCTACATAAAACGGTAAAATCACACATCTTTTTAATCACCTTATAAGCCAGTTTTTGTCAGGCCCACTTTAAAAACGTCTTGCCAAACCCACAGATAAGAATCTAGTCTGTCCCTACCTTTAAGCTGACAAAATGCTACGTTTTCATATTCACCGTGGATAAGGACACATTTAAAATGAACAAAATAACTGTAAAACGTCTCTATATTAAAGGATATGGAGGTGTCAGATCCCCCCCCTCCCAGCAACCTGTGTCAGTGGCAATGATTAGTCGCTATGGCATGCTTATAGCGCTTTGACCGGCGACCCAGACACGTTGCAAATTGGAAGGAGCTCTATCGCTTTCGTAAGTATTATTAATTCTTTTCGAAGTATGGTAGCTTACGCCAACTTGTGGCGAGAAGATTCGAGAGATACATGTACAGGAAAAAGGAGCCATTGAGGCTTACCACGGTACAGCTTTATTGCGTACTACTTGACTTATTAATCTTCACCATCAGCTCTCTCTCTCTCTCGACGTCTGATTTCTTGACGCACATAATAGTAAAAAATTTAAAACCGGTTAAAGAAAGAATGCACATCGAAGCAAACCGAACAGTCAGAGAACAAATGTCTGATGTTTAGCGCATCCATCGGTACCATCATACGATAGAAACAACGTACCAGCTCCGATTCCATTAGCAATTGGTATCGCATCCTCGAACGTCATCAACAAAGGCAGCCGCATAAACACTCAGGAAGCAGCCGCAAGATATGCCCTTCAATGGTTAAGAGCAACCTTTACAGATGAAGCTGTCCTATTCAACTTCAAAAGAGCCCACTTCGCAGTTTTCTTTCAAGTACACAAATAATAACGCTTTCAAACGCAAAATGGAGCTCCGTCGTGGAATGCTCTTGGTAACAATTTCGTTTCTGCTCTATTTAGGTTCGGCTTTGATGTTGGCTAGTGCTGGACTCATGGCTCCCAGTGTGAATCACGTTCGTACCATCGGCGCACTAGGGCGCCAACAGTAATGCCTCAGAAGATAGAGTTAGTCCCAGTATTACGAAGATGACAGAGCTGGTTCCAAAGATTTGCTTTCAGAAGCTACGAGACTGCGCGAAATCAATCTTCAAAACGGATAACCAGCTCACCATGGCTACTCCAAGCTTTCCGAAGTTGAGGCTTGATAAAGCAAAGTCCGATTGCTTCTTGAGTGAAGCATTCGATGAATGGGCTCGTCATGTACTCAAAATCTAGAACAATAGTCAGTCCTCTGCTGACGATGCGATGTTCAACACGTTTGGGTCTCACTACGGCGACGATGATCTAGTACACATGTTGACTACTGCAAATCCGACGAGTGAAAACTCGATTGTCACGCGGTTGAGAGAGATGGAAAACTGCAGGGAAAAGTATGAATGTAGTAATACATACGATCAGAAGACTTCAGCATTGGGCATGTGGATTAACAATGCCTTCAACACGCTTGAGAAACCAGTAGACGAAATTGTGAGAGCACTTAAAGCACACAACGACGATGTACTCGTAGCGAAATTGATGGCTCTGTCAAGAAACGACGTCAGCAGCGGCATGGCTTGGAAATTGGAGAGGTCGCTGACGGAAACGTGGCAAAGAAACGAGAGTGTAACTGGAGTGTTTTAGCTACTCAGATTCAACGAAGAAGGCACGTCGCGTTTTACAAATCCTGCGTTGGGGATTTGGGTGTCCTACGCGATGAGGAAGAGCCAGGATCCCTTTGAGTTTGTTGTCAGCCATTGGAAAGAAAGGCATCGATGATGCGTAATTAGCGAGGATGCTCGGCACGGCAACTAAAAGACGTCTCGAAGACCCTACTAGCAATGGGTGGGTTGCCGGGAATGCCGAGTTCCATTTGCTTAATACATGGTAACAAGAGGAAAAGAGCGGCGAGCAGGTCTCCACAATGGTTGGACTTGACAGGGAAGGGGGGTCGTGCTGCTGCAAAGTGTTGTAATTCTCATTCGATATGAGCGCTAATAATTGAGTGCAGCTTACGATTACGGGGGTTGCGTTTGGGAACAGATATAGGTAGGTACCTTTGAAATCCACTTCAACCCTGACACAATATGTAGTTTTAGCTCTCAGCAATTTGACATTTTATTCGCACGCTTAGGATACCATGATACTATCTGATAAGTACTTATCACACTCATCCTAGCCAGTCCGCGTAAAAGCAGCCATAATGCTGCGTAGAACACCCAATCGAATTTGTCCTAGCAGCTTGGATTTTGATGCCTACAGAATTCATTTTGTCATATCAGACGCTCAGAAGGACACAATTTGACTTTTTATTGTTAAGAGCGATTCCAGTTTACTACCGGTAAGTTAGTAGCTAATCGCAAAAAATATTTTCGTCAATATTTACAAATATACTTTAAGTTAGTTTTGGGTTTTCCACGTACGATTTTATTTTTTTTATTTTTGAGCAGACGTACAGCCAAAAGGTGAAGCTCATCAAAACAAAAAATTACAGTCGTTGCCGTCTCTTACTTTGGCCGGAGAAGTGGACTGCAAAGAGGCTCAACAAGACGATGAGCCTTAAACACAAGAAGCCGTTCTAGCTGCTGGAAATTATTAAAACTAAACACATTTCTGAAGTTGAGCGACGTGACAGCGTTGCCATGGGAAGATTAAGCGTTGGAAATCCGAGATAAGTATTATCGGCTTTGCCTTACGCTGAAACAAATTGGTCAAACTTTAAAATACACTTAGACACGATTAAATTCAGAAGCATTGCAATATGCAGCGCTCCGACCCAGTACTTAGCTAACCTAACG

>Contig_41

TTCGAGTCCGATTTTTTAGGAGCCGGGTGAGATGCTCTGGTAGCACTCCCCCCTCACGAGCTGTGCACTGTAATCGACTGACTCGACGACGAAGAGAACGTCAAGCTTTATCTGAAGCCATGTCTCCCACTGAAAATGTATCATGGTCCGTGGTGCATTGGTCGCCTGTCGATTCAAACACATTCACACCATAATCCCTCAAAGACGGCTACATTCTGTTGCTTTCGGGGCTGGGATAACTGTAAAAATCACACCCACGCTTTTAGCTGTAATTTCGAGACGGCTTAACTATATTGATACATTTTTTTGTCACAACGACTTCAGAAAGAAAATGTTGTCCCTTTCTGAAGTCACTGTGACAAAAAAAACAGTGACTTCAAAAAGTTGTCAGCATAGTTACGGCGTCCCGCTGTGGCTGGGCGGGGATCGGTGGGTATCTTACCGTGCGCAGCGGGACGCCGTAACTACTCCACCATGTTGACAACTTTTTGGCAATTATTCGCAAGTGCGAAGAATACAAGACAGCTGTGATCGTTTGTACTCTATCTCTACACATCACCCAGAAAAACCAACTTGTGAGCGGCACGCTCGGGCTTCAACTGACCACAGTCGTTAAAGTTCGACAAGCTGAAATATAACTATCGAGTCCAAGACAATGTGATTGGTGTGGTGTGCTGGCACTGTTGAGCGCATTTATCTGGCGTCGCAGTTTGCAGGTTTACGACTTCAAACAACTGCAACCATTTGCAGTTTTCCATTTCAATCGGCTTTGTCTGTGTCGTTGTGAAATACCAGAACAAATACGTATCAGTTTTAAAGCCTGCCATCACTAGTGCTGCTTTTAAGTCAATTGCTATCCCAGTACGGATGGGAGGCCGGTAAAAACGGTGTAAAATTCAATATTTTAAACTACCACGGTGTGTCCTCAAATACGTTCTGGAGTGCCCAGTTCTAGCTACTCTTTAAGAGCAGTTTTCTATCTAGCTCAAACTGCGAAATACTCATGGCTGAAATAGCTACTGTATCGATGTAAAGAGAGAGCGTGTCTTACCCGCAGCAGCGGGCCTGGCCGCCGGCCTCATATCGCAAGTAAGCGAATTGCAGCTTTTAAAATTATTTTTTTGGTAAGGATCCGGATTAATCCTTAGCGTGGAGAAGCAGCTTTGTCTAGTAAGGAGAGAAACTCCGGGCTCTCTTCGGCGCTGTTAGTCCCACAGATATGTTTGTTATTGTCAAAGTCTACTTTTGTAGACCCTGCGTGTCTTTTGGAACATGTGTGCTGCTGTCCATACACGCAGGGGTTAATGGTAACTCCAAAATCGGTGAAGTTAATATTATGAAAATATCACCTGTAGACCAGTTAATATAAAAATATCACCTAATATTTTCTTTTGAGACAATATGATATTATTAAATATCAACTTAATATTAGTTAATACGATATTATATTTTTCAATGATTGCGTAATCGGGCTATATTAACTAATCAGATGTCGTTAACGCTCGACTAGCTGAAGTATTACTAATCAACCGTGAGTACCGGGACAATTGTGAGTGACGGCAAGGTTGGCGTTGTGTGTTGGCACTGCTGAGCGCATTAATCTAGCGCCACAGGCAGCAGGTTTATTAGACGCACGATGTCGACGAGGAGATCGGAAGAGAACAAGAACAAATGTCTTGATTTCGCTCTATCAACGTTAAACAGCTGCAAGCGTTTGCAGTTTTCCATCTCGATCGCCTTTGTCTGTGTCACAGTGAAATACTAGAGCAAATACTCGTCAGTCTTAAAGCCTACTGCTTTTCTAGAACCTGATCGAAAAAGTTATTTGGCTCCGCTCGGTTCACAGAGGTAAGCTGGCAAGAAACGTACTTAAGTGGGAAAATATCATAAAATATCATGTAGTATCTAATATTTTTCAGTTCTCCGAGCTAATATAAAAATATTATGTTAGCTTTTGCAAAAATGAGTTACTATTAATATTTAATATCGAAAATAGTACTATTACTATCAAAAATATCATATGATATTTGGTTACCATTAGCCCCTGCATACACGTCCTCCCAGGATAAACACTTCATCCTTCTAATAATCACTGCTTTTTGATTACCAGCACGACAGATCGGGACGAAATTTAGTGACTAAACAAGATCAAGGAGTGGACACGATAGAGTGTAGACGTCCTAGAAATTCTAGTTAACAATGGTAGTCACTTTTCAATTTGTTGCCGAACAACACAAACGCTTCCTCAGTCTATTACACTTGACTACCCAGAAACTCGAGAATCAGCCATGCGTGTCTACTTTATCCTCATCCTGGCTGTTGCCACTGTGTCGGGCGCCACCTCCGAGATGCTGTCGTTCCAGAATGCCATTCAATCCTCAGGTGGTGCCAAAATAGAGACAAGCGCTAGGAGACTTCTCCGAGCAGAGTTGACGACAGATGAGACGTACCCGGAAGAGAGGACCTTTTGGGCCGCTGTCAAAAAGCTAAATGCGAAGACAAAAGATGTACTAAAAGACAAGTGGGATGCGGCATACGTGAAGTATCAGTCCGCTTTCATCTATAATGACGGCATTGGGCAATCAATAATGAAGAGAAATATCGACCCTGACCAGGTGTACAGATACCTGACGCTGCAAAAGCTTGATAATCGAGTTAGTTGGACGATCGGAGAGACTCCTCGGTATAGACTATGGAAGGCCTATTTGGAACTGTGGAAGAAAAACCATCCCAGATGGAAAAGCAAGCTGCAGACGGTGGACTAACCGAGATCTCTATCATTTCATTCGCATCGAATGCAGTGCTTCATTTAAATCCTACGCTGCGGATAATAGAGATGTCAAACTTTTCAAACCAACGAAATACTACCTCAGCTCGGAAAAAGATAACCATTTCAAACTACTACCTCCCATCGGAAAAAGATAACCATTTCAAACTACTACCTCCGCTCGGAAAAAGATAACCATTTGTTTTTTTTTTGCGCAGCGCTCCGCCTCGTTCATTTGAGCGTTTACTAATTTGTGAGAGGCTGGCTAGCTACAGCTGTAGCTTTATGACCGAGAGCTGACAGTGAGAAACTAAGCAAGGCACAGGCGTTCATGCTCCGCCGTGCAACTACAGATACAAAAACGGATCTAAGACGGCAACTACATCGCCACATTCTGAAGGGGCGCTCAGTGGGGCTTCTGGAACTTCAGCGGTAGGCGGTGGGGGGCTGCTCGCTGCAAGAAACAGCATGTACGCGTTAGCATTGGCGAGTGAGATAGCGTCACAAGGCAGTGACGTAGGGAGAGTGCCAGCTCGTACTTGCTTGTCCTTGTGCCGGAGGCGCGCCAACTCATGCGTGGGTGTAAGTAGTGGGCGGGTCTGTCTGAGCGCACGCCGTATGTAGCCTTCCTTGATCAGCTGCTGAACCAGGTAGCAGCTCATGTTGGACGCGCGCTGAAGCAGACGCTGGTTTTCGCGCTCATTGACAGGGACTGCAGCGATTCTCTCGCACAGCTCAGCTCGATCCACCTTCTTCCTACCACATGCGCTCTTCCTTGACTTGACCACGGCTGCTACTCGATCTCCGATCGAATTGATTCCACGCCTCCAAATTCGCTCGACAACTTGGCGGTCCAAGCTGCAACCCGAACGATCTCTCCATGTTTGAGCACTCCTCCCACGCTGCGAAGCAGTAAGGTCTCCCAGACGGCCTGGCGCTCACTGTCTGTGATCTCCCCCAGGTGCCAGCTTTCAAAATGAAAGGTGGGAGCGGGAGCCAGTCTGGTCGGGAAAGCGCGGGAAATTCGAAATCCAAAATGTGATCGGCATAGAGGGGGTATTGTTTTGGGTAGGCTGTAATATAATTTGAAATGGTTATCTTTTTCAGAACGGAGGTATTAATAGTATATTACAGCCTACCCAACAAAGTACCCTCTCTATGCCGATCTTTTTGTACCTCTACACATCACCCAGAAAAACCAAGCACGCTCGGGCTTCAATTGACCACAGAATGTAGTCTCTTCTAGCGGAAAATTGAATTGTGTTTTTTACTCGTAAACATACAAACATTCACGTTAGATGATGCATCTAACTAAAGTGCTTCATCAAAAGCTGATCTCGAATTTCTCAGGAGAAGCGTACGCGATTTCCAGTAGACGGCTTGCTAGGCTTGAACTTTTTCACCTTTGGGGATGTACGCTTGAACTCGTTCAGGTACTCTACCAGAAGCTTTTTATAGTGCGGGTTACGCTTGAAAACCATATTTTCTCGGATCTTACCGATGCTCTGGTGGTGGTTGTCCCAATTCTTAAACATTTTCATTTTGAACGCTTCGTCATTCATCATCCTGGATAGTTTCTCCATTTTGAGGTCATTCCTCCCAAGAATTTTGTTGAAAAGTCAGTGGCCAGCTCTCTCTTCATCAGCAGCAACGATCATTGCGCTGTCGACGTTTATTTTCTGGTTGCCGCGCAGAAGTCTCTTATCGTTACCACCTTGAGCCAAGCCCGCGACCGAGTAGCCAGCAGTCATCGAAGCGCTACAGGGACGAAGCAAAGCCGCGACAGTGAGCAACATGTAGACTAGATGCATTGCGTAGTAAGGCCACGCAATGCGTACAAGGCCTTTTTCCTTCAATGGCGAATTTTGAACTGGCAGTAGCATTATCCATATCCCTGGTCGCTTCGTATGGATTTCCACACAACGTTAAATTGTAAAGTTGGTATACAGTAGCTATTGCGATTTGGCTTACGTGCGGCAAGCAGCCAGTGACTGACAAAGAGAGAGATCGTGTTCAATGATCAATTATCACGATTACTTATTTAATTGCATACTTAAGCAAATACCCGTAGCCGAGCGTCGGAGCCTGTCCAGGCTAGACAGCCTTCGACACTGGACTCCAGCAGCGCCTAAACAGGGGTTCATGGTAACTCCAAAATCGGTGAAGTTAATATAGTGAAACTTTCACTTGGTAGACGATTTCATTTTAAAAAAATTACCTAATATTTTCTCCATTGACCTTTTTTTTTTTTACTATATATCAACTTATTAATAGTATTTGTTAATACTGTAAGAATCTACCAATCATTTGTCGTTAAAGTTCGACAAGCTGAAATATAACTATCGAGTCCAAGACAATGTGATTGGTATGGATGAATAACATTATCTCTCCGACGTATCTTACTTTTAGTATGTTGCGCACTAGCCAATGATGATCGTTTCTTATTCTTTATAGCCAATGAAAGCGATCTCCGTTGCATTGGGCGAATCAAATTGCTTCAAGAATACAATTATATCCAATTAAAACTATAGTTTAGGGTGCAGTCGGAATTCCGACGTATCTAGTCGGAGTTGACGACCAGATATGTGTATAGTAATACTTGCTAAAGGTTTTTGCCACGTATCACCTGCATATTTCTACCTCTCCGACCAAGAACAATGAGTCTGAACGACTTGAAGCCCGCCAACACAAAGCGCGCGCGTGCGACGGCTTTAAAAGCGTTTGAGCGCTTCCTGTCTTTAGAGAATACTACCATGGAGCACGTCGTGGCTTTGATCGTCGCAGACTCTAGTCGCGCGAGTAGTATACTAGTGACTCTAATGGACAAGTTTGGTGTGTACCTGGCATTCCACGCAGGAGCAAAGGGGCAGCCTCTGTCGCGCCACTCCGTCGCTCAATACTTCCGCCAGGTGAAATGCTGGCTTTTGGATGAGTATCCTGTTCAAGGTGGCGCTGTAGCACGACAGCTGCTCAGCATGGGGCGTACCCTTGAACAGCACTGCATCAAGCGTGAAAGTGGTGGGTTCGTCAAGAAGGCTATTGCGTGCACAAAAGAGGATCTCAAAAAGATGATACGCTACTTATATTCAACGGCTCGCTGTGCGTCGGACTACCAAGATGCTGCGTTGTTGTGCCTCCTGTGGTACCTCTTCGGCCGTGCATCAGACATTACGTTCGTCCGGAAGCAGCAGCTCTCCGTCGGAGCTGGAGGGGTTTTCTTTATCCGATTCATCCGCGTGAAGACGTCCGATGAGCAGTCACTGTCACTTTTTCCTGACGCTGACTTCATGACTTGTCCGCTGCTAGCGCTCGCGCTGGCCTTGAATACGCAGGAGTCTCCGTGCGCAGCGTTGCTAAACCATTTGCCGGTGAAGTCGAAGGACGTTCCTGTTGACCTAGCTGCCTCGATACCATTACTGGAACTTCTTGACGACAATAGTGCCCTGAATTCTTCTCAGACGTCGCCAGGTAGCACCACTGTTCACGCGACACCAACAGTTGGAATCCACGCTCTTGTCAACCGCGTGCTCGATCGCGTTGCTGGTCCTGCTGGAGTCGAAGTGTCGCTGACGTCACATTCGTTTCGCCGTGGCGGCGCGCAACACGCGAACGCAAACTCAGAGCTGGCAGCTCAATGGATCTTTGATCGCGGAGCTTGGAACTTAACTACGACTAACAAAGCCTTTGCGTACGTCTTCAACACCCCTAAAGAAGATCATCAAGGTGCTAAGGTGCTGAGTGGGATGACGCCAAAGCAAGCCTCTTCTCTCCCGTCCCTGGATTCCTTCGACGCTGCAACACTAGAAAAGGTTAGAGAGGTTAGCCGGAAGCTTTTCAACTCGAGCCATGATCTGGGTAACAAGGCCTTTAACGTCTGCGCTGTTGTGAGAGATGTGCTAACAGCAACCCTCCTCCTCCACTACCCGTCGTTAGTGAAGATCAACGCGAAGACACCTGCAATGAAGACAATTCACCTATGTGCGGAAGCCTCTGGAGTGACGACGACCAACCTGCTTGCATGGTCACAACGGCTCACAGCGTCAAATGAACACGCCAACACCACCGCCGAAGCCATGACTGAAGAAAATGTAAAGGCCAACGCTGCGTTGAATTATCAAACTGCCTTGATTGAAAGGTTAATTGAGGTCAACCGTAGCCTTGAAGCTCGAGTTAACTCGCTAGAAGCTGCAATGAACAACACCGAAGGGGAAACTTTAGCACGAGATAATCAAGCCAAGCGAGTAAGAGACGAAGATGAAATCGTACCAGTAAAGCGTCGCCGTCGATCCAAGACAATACCGCTTGTTGACACGTGGTTTGCGTGGTACACGGCTGTTCCGCGTGGATGGCAGAGTACTAATAAGCACAAGAAATCGATTTCCAAGCAGCTCGTGGCCTTCATGAGGCTCTTCATCGAAGACGGGTACGCGCTCGACGAGTTATCATCCAGCTACAAAGATACAGTACGTCAGCTCGGAGAGTTAGCGTCAAACAAAGTCACAGTGTTCCTAGGCGAGCAGGGCGTACCGTCAAAGGGATCCAACGCTGTGCTTAAGGTTCTTCGGCAGATGCACCGCGAAGGCAAGCTGAATAGTCTCATCAGTGCCTACCATCAGCGTTTTACCATCGGCAGAGTTGTAGACCCTACACCAACACAGCACCAAAGCATGCTCAAGCTGCTCCCGTAACACAATCCAATCTCTCTGTGTCCTTGTAGCTGGTTCTTATATCATGTCAATCATGTAGAGACCTGCAAGGCATTCGAGCATAATTTTTTTACCGATGGGTGAATTTGAACCACCTCCTTCCCTTTCGGGAGCTCTACCATTGAGCTACATCGGTGGGGGTTAGACAACAACAGCACAGGGATACTCATCTGGGTGCTCGACGGGGACTTAACGGTGATCCACTGTTCTTTGTGGCCGCGACATACCCTCTTAGGTGTACCGATGCGCGGTACGCCCCCCTCAATTCACCGTTCCAGGTGGTCAATCGAGCACTGAGCTTGCAGGGCAAAATGGCGCAGTCAATATTGCACCGGCGCCACTCGTTTACGAGGAGTAATTACGCTCAATGAACCAATTATAAAGTTTTGCGATACGTCGGATCGGAGTCGGAAGGTGCTTGGCTGGTCGGAGTCGTTATGATATGGTGTGCTGGCACTGTTGAGCGCATTTATCTAGCGTCGCAGTTTGCAGGTTTACGACTTCAAACAACTGCAACCATTTGCAGTTTTCCATTTCAATCGGCTTTGTCTGTGTCGCAGTGAAATACCAGAACAAATACGTATCAGTTTWAAAGCCTGCCATCACTAGTGGCTTTTAAGTACGGATGGGAGGCCGGTAAAAACGGTGTAAAATTCAATATTTTAAACTACCACGGTGTGTCCTCAAATACGCTCTGGAGTGCCCAGTTCTAGCTACTCTTTAAGAGCAGTTTGAAATACTCATGGCTGAAATAGGTACTGTATCGAGACATAGAGAGAGCGTGTCTCGGCGGGCTTACCCGCAGCAGCGGGCCTGGCCGCCGGCCTCATATCACAAGTAAGCGAATTGCAGCTTTTAAAATTATTTTTTGGTAAGGATCCAGATTAATCCTTAGCGTGGAGAAGCAGCTTTCTCTAGTAAGGAGAGAAACTCCGGGCTCTCTTCGGCGCTGTTAGTCCCACAGAGATGTTTGTTATTGTCAAAGTCTACTTTTGTAGATCCTGCGTGTCTTTTTGGAACATGTGTGCGGCTTCATCATTCTATAATCACTGCTTTTTGATTACCAGCACGACAGATCGGGACGAAATTTAGTGACTAAACAAGATCAAGGAGTGGACACGATAGAGTGTAGACGTCCTAGAAATTCTAGCTAACAATAGTCACTTTTCAATTTGTTGCCGAACAACACAAACGCTTCCTCAGTCTATTACACTTGACTACCCAGAAACTCGAGAATCAGCCATGCGTGTCTCTTTATCCTCATCCTGGCTGTTGCCACTGTGTCGGGCGCCACCTCCGAGATGCTGTCGTTCCAGAATGCCATTCAATCCTCAGGTGGTGCCAAAATAGAGACAAGCGCTGGGAGACTTCTCCGAGCAGAGTTGACGACAGATGAGACGTACCCGGAAGAGAGGACCTCTTTGGCCGCTGTCGAAAAGCTAAATGCGAAGACAAAAGATGTACTAAAAGACAAGTGGGATGCGGCAAACGTGAAGTATCAGTCCCCTTTCATCTATAATGACGGCATTGGGCAATCAATAATGAAGAGAAATATCGATCCTGACAAGGTGTTCAAATACCTGACGCAGCAAAAGCTTGATCGGACGATCGGAGAGAATCCTCAGTATGGACTATGGAAGGCCTATTTGGAACTGTGGAAGAAAACCCATCCCAGATGGAAAAGCAAGCTGCAGACGGTGGACTAACCGAGATCTCTATCATTTCATTCGCATCGAATGCAGTGCTTCATTTAAATCCTACGCTGCGGATAATAGAGATGTCAAACTTTTCAAACCAACGAAATACTACCTCCGCTCGGAAAAAGATAACCATTTCAAACTACTACCTCCCATCGGAAAAAGATAACCATTACAAACTACTACCTCCGCTCGGAAAAAGATAACCAATTTTTTTTTTTTTTGCGCAGCGCTCCGCCTCGTTCATTTGAGCGTTTATTAATTTGGCTAGCTAGCTTTATGACCGAGAGCTGACAGTGAGAAACTAAGCAAGGCACAGGCGTTCATGCTCCGCCGTGCAACTACAGATACAAAAACGGATCTAAGACGGCAACTACATCGCCACATTCTGAAGGGGCGCTCAGTGGGCCTTCTGGAACTTCAGCGGTAGGCGGTGGGGGGCTGCTCGCTGCAAGAAACAGCATGTACGCGTTAGCATTGGCGAGTGAGATAGCGTCACAAGGCAGTGACGTAGGGAGAGTGCCAGCTCGTACTTGCTTGTCCTTTGTGTCGGAGGCGCGCCAACTCATGCGTGGGTGTAAGTAGTGGGCGGGTCTGTCTGAGCGCACGCCGTATGTAGCCTTCCTTGATCAGCTGCTGAACCAGGTAGAGTGGTGAGGTAGGGTTCGTCACGGGGGGATTGTTCTCCATGTGTCGATCGCTTCTAAGAGTAGGAGCCATTTAAATGTAAATACACTTAAATGTCAACTTTAGGAAACTAATCAGGAAGTAACGTAAACACGTAAGTGCCGGGCAAAAGGCCGCGACAGGACTGCGGTGTCTGGCGAGGCTGCTCTTCTCTGCTGCTCTCTGATGAGCGGCAGGCGTCAAAACGCAAAGTGTTGAGCAGCCTCCGCCAGAACACCGCTGTACCGCAAAAGTAGATTAGTATACAAATAGCAAATAAGTCAATACTAAACTCTAGATAAATGCACTCTCGTTTGAGTACACCATCTTGGCAGCGCACAGCCAACGATGTCCTTGTGCATACAGTCCTTGGTATAGTCCTTCGCCATTGCACGCATGTGTACTCAGTGTAGCCGGTTGTAGTGCAGCCGCAAGCTGCACTGCCAGGCGCATGAGTGTCCTTGGGATGTGTCTTTGCAGACACTGGCAGTGGACGGTGGCTCGTAGATGGGGGCACCGGACACTGGCAGACTGCAATGCACTGGCGCTCGACACGCAGGTAAGTTTTATAGCTCACACTCCCACTTGATCTCGGCGACGCACTTGGAAGTCGACGCGATCACACACGCGCATCCTAGTTGCCGCCATAGTCCTAGTAGTAGCGTAGTCGCAGCTCAAAGACAATAGCAGTCCACTCAGTAACAGTACTCCCACTCGATCGCCCTCGTCTTCATCAATGTCGCTCAGGTACAGCCTCCGCATCAACAGCAGTAGTCAACGCAGTAGCAGTAGTCGTAGTTGTAGCCATGTCCTCGGATACGTTGTCATCGCTCGACACAATAGGTAAGACTTTCATAGCCTTGCGAAAGTGTGTGAACTTCACCACTGCAAGTGCCTTTGTCATTATATCGGCCACCATATCATCAGTTCCGATGTGTTGGGTTGTAAGTTGCTTCAACTCCACATGGCGTCTGATCATGTGGTACTTGTTATCGATATCTTTCGATTTCGAATGTTTGCCAGGTTTCGCAGTTAAGGCAATGGCTCCTTGATTGTCTCCCAGTAGTTGAGGTACTGGATGCTTCCAGCTCAACTCTTTGCATAGGCCAGCAAGTCACAACAAGTCCTTGGTCGCCTCTGCCATTGCTACGTACTCAGCTTCACAGGTACTAAGCGCGTTGATCTCTTGCTTGCGAGATGCGTAGGAGATGACGTTGTCGTCCAGCATAGTGATGTAGCCACTGATACTCCGTCGATCCACAGGGTCGTTCGCATAATCAGCATCAGAGTAGCCACTAACTTGCACATCAGTCTGTAGCTTCACATTCATCACTAGTCCGTAGTCACATGTAGCCTTCAGGTAACGCAGTACCCTCTTGGCTTGTGCGAAATGCATATGATCATAGCTCGCAAGATACTTGCCCAGGTGTCGGGTAGCATGAGCTATGTCAGGTCTCGATGCACTAACGAGGTACTGGAGAGMACCCACCAGTTCTCTGTAGGGAAGATACTCCTTCGAAGCAGGCACGTCTGTSTTCGAGGGCGTCGTAGCTTCTGGTGTAGCGCAGCCATTACAGTTCTCCATATGAAAACGCTTCAACACCTCCAGTACGTACTGCTTCTGGCAGTACACTATTTGCCGGCGCGGCCGGTCAATCAGGATCTCGACGCCTAAGAGATATTTGACTGTGCCCATCGTCGTGAGCTCGAATGTCTCTTGCAGTGAGGCAGCGATTGTGGCACACAGGTCACGTGAACCCATCAGCAGTAGATCGTCCACGTATACAGTCAATAGCAACTTGACTTCACCATCCTTCCTCAATGCATATAGGCCGTAGTCAGAGTCCGTCCTAGTAAACCCCATCGCAACAAGCTTCGTATGAAGCGTGCGATTCCAAACGTTCGGTGCTTGCTTCAGTCCGTACAGGCTCTTCAACAATCTGCAAATGGACCCAGGTCCATCCACCTGGAAGCCAGGAGGCTGCTCCATGAAGATAGTCTCGTCCAAGTCTCCATACAGAAAAGCCGTCTTCACGTCAAATTGCAACACCTCCCAACCTCTTTGTACAGCGTAGTAGATAGCCGCACGTATGGTTTCGAACCGGATCACAGGTGCATAGGTCTCAGAGTAGTTAATCCCAAGTTGTTGTTTGAATCCATGGATCACTAGTCGCGCCTTGAAACGTTTGATTCGTCCACGCTCATCTCGCTTCACAGCAAAGACCCACCTACACGTTATCACCTTCACTTTCTTGGCATCTTTGCGGGGTACAAGTTTCCAGGTCTTGTGACCCTTCAACGATTGAAGTTCCACCAACATTGCAGCTCTCCACTGCTGCCAGTACTTGCTTGCACGTGCTTGTTTGTACGTCGTCGGAATCTGCACATCCGTAGTCATAGCCTGCACTAGGCCTACCACGTAATCCGACAATCGAACGTTCGCCTTCTGCTTCCTTCTTGGTCGCTTTAGGCAGGGCTCTCTTGATGGAGATTGAATCTCGAAGCCACTTGTCTCCTTCTCCACCATTGCAGGTTTCTTCTTTGTTCTGCGTCTCTTGCGCTTCCTCACTTCCAAACCTTCCTCGGTTTCTTCAGTAGGATCCTGCAACACGGCTGGTGCTGCAACACCAGCAGTCTCCGCACTGTCCTTGCTCACAGGTGCGTCAAYGATTGACAAACTTCCACTTGAACTTAACTCAGTTTCTGCAGGTTCGTCAGCAAGTGGTACCGCCTCAAGTGGCTTCTCCGCACTAGAGATGCCGTCACTCCAACCTGGAAGGTACGTCTCCATGTTGGTCTTGATGCGCGCCACAGGTACAGTCTCGCGTAGAGTATGATCACCATCAAAGAATGCGTTCTCTAATAGATGCTTCATGTAGATGCCATCGGTTGTGAACTCTTCATGGAACTTCACATTCCCACTACGAGCTGTCACCACTTGAGCTGTCACCAGGTCAATGAACTTGTAGCCTAGGGTCGATTCGCTATAACCCAGTAGTAAACACAGTCTTGCCCTTGCTTCCAGCTTTTCTTTCGCTTGTCTTGACTCAGGGGGAATACGCACGTGTGCCAGACAGCCCCAAGTACGCAAATCATCCAGCATGGGTTCCGATTCGTACAGTTTCTGGTGCGGGGACACCAATCCTAAAGGCTTGGTGGGTAGCCAGTTTAACGTCGTCACCACATGCAGGAAGRCTTCTCCCCACAAGGAGTTGGGCATGTGTGTAGCCATCAGCATCGCTCTGATTCTTGGTAGAACCACACCATTAGCACGCTCCACAATGCCGTTCTCTTGAGGAGAATAGGCGTTCGTAAACTGCTGCTCAATCCCTTGGTCGACGCAGTAGTCACTTAGCTCCTTGCTTGCAAATTCACCTCCTTGGTCAGAGTGTAGTCGTCGCACCTTCTTCTCAGGGAAAACGAGTTCGCAGCCTGTTCAGTAGGACCTTGATGTGAAACGTTGCTTCACTCTTCTTCTTGAGTAGGAACGGCCACTTGTACCTTGTAGACTCATCTATGATAAAGAGAAACATTGTAGCTCCATCCACAGACTGTTCGTTCACAGAGCAAATGTCCATCATGAGAGTCTCCAGAGGTTCGGACTTCCGCGATGGAATCCGTTTATAAGACATTCGTTTTGCCTTACTGGCAGCACAAGCAACGCACTCGTAGGAAGTCAGACCTTTTGCATTCATCTTAACTCCAACATCGAGTTTGTTTGCCAACACTTTCACCGTCTCCATGCTCATATGACCAAATCGTTGGTGAAGCAGCTCCATGGCTTTCTTGCTGTCCATGTCCTCTTTTAGAGCCGCCATCACCATTACTCCAGTCACTTTTTCTGCACGTAGTCGGTAGATACTGTCACGCATCATGAACTTGAGTGATGTGCTTGGCTTGGAGATCCAAGCAGTCTGTTGGTCCTCTGAGCATGTCAACTTGAAGTTGCCCTTCGTTTGTAGGTAGCCCAAACTAATGATGTTCACCTTCGCGTTCGGTGTGTACACCACGTCGTCCAGGAGACGGTCCTCGAGCTGGCCAGTTGCCTGGTTAACAACACGCAATAGCACAGACCCACAAACAGAAGTGTGTTCAGTTGTGCCATTCGCAAAGTCAAGTCGACTAGCTTGGTCTTCCCTTATGGTAGTAAACAATGACAGGTCACCACAGACATTTGCCTGGGATCCTGAATCAAGTATCCACTCCACAAACTCAGACTGAGCTGATTGCATACCTTGGTTCTGGTTCACAGCGTTGACTTGACGAGAGTTGGGTGGTCTGTTCTCACGACGGTCTTCAAATCGACGCGGAGACATGTTCCGCCCTTGGAAATTGTCACGTCGAGGTGAAGGTCCTCGTCCTCTCGAAGGATTGTCACGCCACTGCTGCTGCGGAGACGACGGTCCATAGCGACTCAGTCGATCATCACGTTGAGGTGGCGGCTCATAGCGACTCGGTCTCTCGTTACGCCACTGCTGCTGACGCGGTGGAGATCGATAACGCCCTGGATTTCCTCTTGGTTCACACCCTCGCATTGACGGAGAACGGCCTCTTCCTCTTGGTGGAGTTCGTTCTCGATTGAATAATCGGGGTGGTGAACGACTTCGTCCTCGTCCTTGAATAGGAGGGTCAAAGTGTCCAACATCTTGACTCCAGAGTCCTCTCTCGTACGCACTCTCCAAGTTAGGTCTGCAATCATTGCGCATCCCTGATTCTTGGCGTCTGTTAAACCCGACATCAGGACCTCGACAGCTATCCCCCGGCACAGCCGAACCCCCTTGTGGTTCAGCTTGATTGCGCCATGGCTCAAGTGTAGGCCCTTTCGAGTTACTCGACAGTCCTTGCCCCAAGTAGTAGATGACTGATTCCCACGTAGGCACCAATCCAGCTGTAGACCGATTGCCCTCCCACTGAGTCTGCGCAGTACTCGATACAGTAGGTTGCATGTTTTGCAGTACAGTAGCTTGTACACCCTGTTGAACAGCAGGTTGTCCAGTAGGTTGCACTTCAGGTTGTATCGCAGATTGTATGGCAGTTTGCACAGCTACAGCTGGTGCTTGCAGTGTAGGCAGTGAGGTTGATTGTCGTTGTTGTAGTCCTTGTCCTCCAACTTGTATGTGTTGAACAGGAACTCCAACTTGCAGCTGCAGTCGCTGCGTTGCTTGCGCAACCGACGATTGAGTGCGTTGCGTACTTGCCCGCACCCCTTCAACAACACGACGCTCTCCTCCTTCTTCACTTTCTGCAGGAGACGTTAGTGACCATGCAGGTGATTGCGTAGCCGTAGACGATAGGGCAGCACCATCCGTAAGGTCAATCGCATTCGCATCGCATAGTGATAGGCCAGCAAACAGTCCTTGGCCATCTTCTTCCGACGCTACCGCATCCACACGACGTACAGAGTTCACCAGCGACACGGGAGCCCTTAGTTGTCTCTCTTGCAGCTTTCTCGCGAGCTCTGGCTTCAGTTGCTGGCCAGTGAGAGCCGTACACTCGGCATACCAATGTCCTTCTCCTTGACAGTTCGCGCAGTTTGTGCGCTGCTTCTTATCTGCAACGCCCTTAGATCGCTTGCGCTGTTGCTTGTTGCGCTTGCGTCCTCGGGCTTGACCTTGTCCTTGGTCCGTTCCGACATGAGCAATTTGCATTGAATGCGCGACCGGTTGTCTGGTCTGTGCACGCAGCTGGTCACGCTGGTGTTCAGCAGCGCGTAGTCGTTGTAAAGCTTCAGCAAGTGTAAGTGTCTTACGATCATGGTTGTTAAGCCAATCGCCGTGTTCACGGGCGAGATCTTTAAACGCTTCCACGCTGTTCGATAGTAGTAGGCTTGCATGTTGCCACTCAGCAAACTCACCATTAGCTTGATGTAGAAGTGTCACCTTCTTTGCAATGTCGTCAGCATACTGCGATACTGACTCGTTTGGCTTGAGCCGGCGCGTCACTAGCTCTTGCAGTAGGTAATCAGGATTAATACCATCTCCAGCCTCAAAGTGCTGCGTAATCGCATCGTAGAGCATAGCGGCGGCGTCCATGTTGTCGTGGATGTCGTCGATTTTGAACGTCGTACGTAGGTCAACAGACAGCGACAACGCGAGTGCTGAGAATGCCTTGTTCTTGCGCGACTTATACCACACGCGATATTTGTCACGACGCATGCCTTCTACTGCATCGTAACGCTCAGGGCCATAGGTGATGTCTCCCAGCATCTTCGGCTCGAAGTGCAACGGCACCTCGCTCTTCCAGGCCTTGTAGTCGCCTTTCAGATGTAGCTTGGGCAGCTTGATCGGAGGCGAGTTGATAAGGACCTTGGTGTACTCAGCCAGCTCGCCGTCGTTGTAGCGGCCAGTAGGGTTCATGTTGTCTAGAGGGCGCCACGGATCGTCAGGGTCTCGGTCGCCGTTTTGTTGGTTTAGCGGCGACTGGGGAGGATTGTCAACTGGCACGGATTCTTCCGTGTTCGTCGACGAAAGTTTATGCATACTTTCGAGTAACATTTGGAATAACGGGCGGCCACCCGGGATTCCAGCCAATGAACGAATTAGTCGTTCTGCATCACTGTGCGACAGTGTTGCGTCATTGTTCAAGCTTGAATTTCCTGCCAATGGGGTTGAAGCTTGATCTTGATTGGTCGAAATTCCAGAACTTGTGTCTGGAACGCCAGCAGCCACGTCAGTCGCAGACTGCGCCCTGATTGGGCTCATAACCAGTGGTGACGTAGGGTTCGTCACGGGGGGATTGTTCTCCATGTGTCGATCGCTTCTAAGAGTAGGAGCCATTTAAATGTAAATACACTTAAATGTCAACTTTAGGAAACTAATCAGGAAGTAACGTAAACACGTAAGTGCCGGGCAAAAGGCCGCGACAGGACTGCGGTGTCTGGCGAGGCTGCTCTTCTCTGCTGCTCTCTGATGAGCGGCAGGCGTCAAAACGCAAAGTGTTGAGCAGCCTCCGCCAGAACACCGCTGTACCGCAAAAGTAGATTAGTATACAAATAGCAAATAAGTCAATACTAAACTCTAGATAAATGCACTCTCGTTTGAGTACACCATCTTGGCAGCGCACAGCCAACGATGTCCTTGTGCATACAGTCCTTGGTATAGTCCTTCGCCATTGCACGCATGTGTACTCAGTGTAGCCGGTTGTAGTGCAGCCGCAAGCTGCACTGCCAGGCGCATGAGTGTCCTTGGGATGTGTCTTTGCAGACACTGGCAGTGGACGGTGGCTCGTAGATGGGGGCACCGGACACTGGCAGACTGCAATGCACTGGCGCTCGACACGCAGGTAAGTTTTATAGCTCACACTGCTGACCCAAACGACAAAAAATGCGCATCACATGCATCCTTGCAGTGCTAGTCGCGGTCACTTTCCACGCCACTGGTAATGCCAATTCTGCGGTTGCGGGCAAGGCTTCGAATGAATATGGTGTTGACTTGATCATCACGACCGCAATATTCGTCGAGAATCCACGACGAAAGCATCTCTTCATACTCGGTGCTGATGCCACCTCACAAGAAGCCTTTCACCGTGTGGTCGTGCTTCATCCACGTGGCTTCCCCCTTCTTCCGCGCCAAGTAGAGCGTCAATCTGGCTGGATCAACTTTGAAGCGATCATCGACGTCTTTCTTCTCGTTCACATGGCCTTCTGCAGCGCGCTCACTTTCGCGTTGCTCGCTATCTTCACGGGAAACACAGTCCCTTCGCCGTATACCGCGCACTCCAACTCGACCTCTGCCATCTTCGTGCGGAAAGTTCTCGACTGAGCAGCAGCACTCATTCACGCAGCACACCGCACCGTTTTAGCCAATCACATCACTTTAAAATAATATTTTTATTTCTGACAAAAATACTCCTCAATATATAGAAACTAAATAATAGTGAGTTCATCTTGAGCGATAAAAAATAAATATATAGAAATAACAATGTGCAAGTACATCTTCCCAGAAGACCACGCAAAGGCGAGCCAACAGTGCGGCTCAAAAGGCGATTGGTGCCGACACCACGCGAAGTATCAGCCAGTAGTTGCTGAACCAGTGATTGCTAAACTAATAGTTGCTGAACCAGTGGTTGCTGAACTAGTAGCTGCTGAACCTGTAGTTGTTGTGCCTGCAGTTGCTGAACCAACGATGACCAAGTCTATTGAAGAAATTTATGGTGAGCATTGCATTCAGCACATCAAATCTGTTGATAAATACGATGAACCCAGGTTGGGAGATTCGCGCGTGTTGTCACCAAGTAATTGGATCAAGTCGAAGATGCACGCAGTTGGGGATTCCATCAACTACAAGCACAGTGATGATGGCACACCTGACTTTGGCAACGACGGATACACGTACCTTTTGCAATATCAATTTAATGAGCTGGATGAATCGCAGATGCTTGATAATTTCTTCTTTAAGTCCAATGAAGACCGTGAATCATTTGAACAAGCAAACGTTGAATGCGGTGTGGAATACTTCCTTGCAACTCAATCCTACGATGAGCGATTGTATCAATTGTGCATGATGATTCGAGGCGATTTGATCTCCAACAACCAAAACGTGTGGAATCTCGCTGGATTCTTTGCTTGTCAAGTTCATTCGGATTGGCATTTAGTGCGCAAGACCTATCTCTGTGTGCTCAAACAAAAAAGTAGAGCGTTTCAACAAGGAGTATGCTACCAAAGAATTTGATGGTTGGTTTGATTCCAAGTATAATCCATCACTGAACGAAGGCAAACTGAAGGGATTGCTGCTGGTTGTGATTCTGCTGCATACAAGCAATAGAAGGATGACTATGAAGTCAAGGAGCCATGGAAAACAGCCACTTCGAAGACGTTTGAGCTTTCTACACTTGATCAAGATTTCGAATTGACATTCAAGTCTGAGCGGAAGTTGGCAACACTAACTCAGCTGGTGGAGTKAACTGGTCGAACAATTAATGACATGCGTTCAGCCATGGTTGATACGTCATTTGAGAACGATGAGCTTGAATATTGGGCGGAGAAGTATCTAGTGATCGAGAACAAGCCCAATCGTGACTTTGATTTAAATCAATGTGGTAGCTCAGTGCTTCTATAGTAGTACTACGCATGAGAAAAGTGCTGCCACTGATGTCGCTGCAAGAATGCTGCGGTGGCCATCCATTTTAAGAAAGCAACTCGGAGAGGATGACTGGAGGAGGTCTGCTGTCGCACTTTTGAATTGCGAATTGAATTGCTCTTCTTGTGTATTAAACACAATTTTCTAATAGTGACTAGACATTAGTTTTTATAAGTGAATAGGGCTACAAAAACCATTATTAGCTGCGAACTTAAGTGTACTGGTTTTTTGTTCAATAACTGTCTGGAAATATGTACTGGATCGGTAGCTATATCCCATCGTTATTTGTACAGACTGGAAGAAAACAGTACAAAGTGGAACTTCCTCGTGCTCCAGCACGTAGTACGGCTCTGTCTTCGTCCGACACCTCATTCTGCAATCTGCTCGCACTCTCTCATTTCGACTCAAGTGCCCTAGCTGTTGACCCAAACGACAAAAAAATGCGCCTCACATGCATCCTTGCAGTGCTAGTCGCGGTCACTTTCCACGCCACTGGTAATGCCAATTCTGCGGTTGCGGGCAAGGCTTCGAATGAATATGGTGTTGACATGATCACCACGACCGCAATTTTCGTCGAGAATCCACGACGAAAGCATCTCTTCATACTCGGTGCTGATGCCACCTCACAAGAAGCCTTTCACCGTGTGGTCGTGCTTCATCCACGTGGCTTCCCCCTTCTTCCGCGCCAAGTAGAGCGTCAATCTGGCTGGATCAACTTTGAAGCGATCATCGACGTCTTTCTTCTCGTTCACATGGCCTTCTGCAGCGCGCTCACTTTCGCGTTGCTCGCTATCTTCACGGGAAACACAGTCCCTTCGCCGTATACCGCGCACTCCAACTCGACCTCTGCCATCTTCGTGCGGAAAGTTCTCGACTGAGCAGCAGCACTCATTCACGCAGCACACCGCACAGTTTTAGCCAATCACATCACTTTAAAATAATTATTTTATTTCTGACAAAAATACTCCTCAATATATAGAAACTAAATAATAGTGAGTTCATCTTGAGCGATAAAAAATAAATATATAGAAATAACAATGTGCAAGTACATCTTCCCAGAAGACCACGCAAAGGCGAGCCAACAGTGCGGCTCAAAAGGCGATTGGTGCCGACACCACGCGAAGTATCAGCCAGTAGTTGCTGAACCAGTGATTGCTAAACTAATAGTTGCTGAACCAGTGATTGCTGAACTAGTAGCTGCTGAATCTGTAGCTGTTGTGCCTGCAGTTGCTGAACCAACGATGACCAAGTCTATTGAAGAAATTTATGGTGAGCATTGCATTCAGCACATCAAATCTGTTGATAAATACGATGAACCCAAGTTGGGAGATTCGCGCGTGTTGTCACCAAGTAATTGGATCAAGTCGAAGATGCACGCAGTTGGGGATTCCATCAACTACAAGCACAGTGATGATGGCACACCTGACTTTGGCAACGACGGATACACGTACCTTTTGCAATATCAATTTAATGAGCTGGATGAATCGCAGATGCTTGATAATTTCTTCTTTAAGTCCAATGAAGACCGTGAATCATTTGAACAAGCAAACGTTGAATGCGGTGTGGAATACTTCCTTGCAACTCAATCCTACGATGAGCGATTGTATCAATTGTGCATGATGATTCGAGGCGATTTGTTCTCCAACAACCAAAACGTGTGGAATCTCGCTGGATTCTTTGCTTGTCAAGTTCATTCGGATTGGCATTTAGTGCGCAAGACCTATCTCTGTGTGCTCAAACAAAAAAGTAGAGCGTTTCAACAAGGAGTATGCTACCAAAGAATTTGATGGTTGGTTTGATTCCAAGTATAATCCATCACTGAACGAAGGCAAACTGAAGGGATTGCTGCTGGTTGTGATTCTGCTGCATACAAGCAATAGAAGGATGACTATGAAGTCAAGGAGCCATGGAAAACAGCCACTTCGAAGACGTTTGAGCTTTCTACACTTGATCAAGATTTCGAATTGACATTCAAGTCTGAGCGGAAGTTGGCAACACTAACTCAGCTGGTGGAGTTAACTGGTCGAACAATTAATGACATGCGTTCAGCCATGGTTGATACGTCATTTGAGAACGATGAGCTTGAATATTGGGCGGAGAAGTATCTAGTGATCGAGAACAAGCCCAATCGTGACTTTGATTTAAATCAATGTGGTAGCTCAGTGCTTCTATAGTAGTACTACGCATGAGAAAAGTGCTGCCACTGATGTCGCTGCAAGAATGCTGCGGTGGCCATCCATTTTAAGAAAGCAACTCGGAGAGGATGACTGGAGGAGGTCTGCTGTCGCACTTTTGAATTGCGAATTGAATTGCTCTTCTTGTGTATTAAACACAATTTTCTAATAGTGACTAGACATTAGTTTTTATAAGTGAATAGGACTACAAAAACCATTATTAGCTGCGAACTTAAGTGTACTGGTTTTTTGTTCAATAACTGTCTGGAAATATGTACTGGATCGGTAGCTATATCCCAGCGTTATTTGTACAGACTGGAAGAAAACAGTACAAAGTGGAACTTCCTCGTGCTCCAGCACGTAGTACGGCTCTGTCTTCGTCCGACACCTCATTCTGCAATCTGCTCGCACTCTCTCATTTCGACTCAAGTGCCCTAGCTGTTGACCCAAACGACAAAAAAATGCGCCTCACATGCATCCTTGCAGTGCTAGTCGCGGTCACTTTCCACGCCACTGGTAATGCCAATTCTGCGGTTGCGGGCAAAGCTTCGAGTGAATATGGTGTTGACATGATCACCACGACCGCAATCTTCGTCGAGAATTCACGACGAAAGCATCTCTTCATACTCGGTGCTGATGCCACGCCTTTCACCGTGTGGTCGTGCTTCATCCACGTGGCTTCCCCCTTCTTCCGCGCCAAGTAGAGCGTCAATCTGGCTGGATCAACTTTGAAGCGATCATCGACATCTGTCTTCTCGTTCACATGGCCTTCTGCAGCGCGCTCACTTTCGCGTTGCTCGCTATCTTCACGGGAAACACAGTCTCTTCGCCGTATACCGCGCACTCCAACTCGACCTCTGCCATCTTCGTGCGGAAAGTTCTCGACTGAGCAGCAGCACTCATTCACGCAGCACACCGCACAGTTTTAGCCAATCACATCACTTTAGAATAATATTTTTATTTCTGACAAAATACTCCTCAATATATAGAAACTAAATAATAGTGAGTTCATCTTGAGCGATAAAAAATAAATATATAGAAATAACAATGTGCAAGTACATCTTCCCAGAAGACCACGCAAAGGCGAGCCAACAGTGCGGCTCAAAAGGCGATTGGTGCCGACACCACGCGAAGTATCAGCCAGTAGTTGCTGAATCAGTGATTGCTAAACTAGTAGTTGCTGAACCAGTGATTGCTGAACTAGTAGCTGCTGAACCTGTAGTTGTTGTGCCTGCAGTTGCTGAACCAACGATGACCAAGTCTATTGAAGAAATTTATGGTGAGCATTGCATTCAGCACATCAAATCTGTTGATAAATACGATGAACCCAGGCTGGGAGATTCGCGCGTGTTGTCACCAAGTAATTGGATCAAGTCGAAGATGCACGCAGTTGGGGATTCCATCAACTACAAGCACAGTGATGATGGCACACCTGACTTTGGCAACGACGGATACACGTACCTTTTGCAATATCAATTTAATGAACTGGATGAATCGCAGATGCTTGATAATTTCTTCTTTAAGTCCAATGAAGACCGTGAATCATTTGAACAAGCAAACGTTGAATGCGGTGTGGAATACTTCCTTGCAGCTCAATCCTACGATGAGCATTTGTATCAATTGTGCATGATGATTCGAGGCGATTTGTTCTCCAACAACCAAAACGTGTGGAATCTCGCTGGATTCTTTGCTTGTCAAGTTCATTCGGATTGGCATTTAGTGCGCAAGACCTATCTCTGTGTGCTCAAACAAAAAAGTAGAGCGTTTCAACAAGGAGTATGCTACCAAAGAATTTGATGGTTGGTTTGATTCCAAGTATAATCCATCACTGAAAGAAGGCAAACTGAAAGGGATTGCTGCTGGTTGTGATTCTGCTGCATACAAGCAATAGAAGGATGACTATGAAGTCAAGGAGCCATGGAAAACAGCTACTTCGAAGACGTTTGAGCTTTCTACACTTGATCAAGATTTCGAATTGACATTCAAGTCTGAGCGGAAGTTGGCAACACTAACTCAGCTGGTGGAGTTAACTGGTCGAACAATTAATGACATGCGTTCAGCCATGGTTGATACGTCATTTGAGAACGATGAGCTTGAATATTGGGCGGAGAAGTATCTAGTGATCGAGAACAAGCCCAATCGTGACTTTGATTTAAATCAATGTGGTAGCTCAGTGCTACTATAGTAGTACTACGCATGAGAAAAGTGCTGCCACTGATGTCGCTGCAAGAATGCTGCGGTGGCCATCCATTTTAAGAAAGCAACTCGGAGAGGATGACTGGAGGAGGTCTGCTGTCGCACTTTTGAATTGCGAATTGAATTGCTCTTCTTGTGTATTAAACACAATTTTCTAATAGTGACTAGACATTAGTTTTTATAAGTGAATAGGACTACAAAAACCATTATTAGCTGCAAACTTAAGTGTACTGGTTTTTTGTTCAATAACTGTCTGGAAATATGTACTGGATCGGTAGCTATATCCCAGCGTTATTTGTACAGACTGGAAGAAAACAGTACAAAGTGGAACTTCCTCGTGCTCCAGCACGTAGTACGGCTCTGTCTTCGTCCGACACCTCATTCTGCAATCTGCTCGCACTCTCTCATTTCGACTCAAGTGCCCGAGCTGTTGACCCAAACGACAAAAAATGCGTCTCACATGCATCCTTGCAGTGCTAGTCGCGGTCACTTTCCACGCCACTGGTAATGCCAATTCTGCGGTTGCGGGCAAAGCTTCGAATGAATATGGTGTTGACATGATCACCGCGACCGCAATTTTCGTCGAGAATCCACGACGAAAGCATCTCTTCATACTCGGTGCTGATGCCACCTCACAAGAAGCCTTTCACCGTGTGGTCGTGCTTCATCCACGTGGCTTCCCCCTTCTTCCGCGCCAAGTAGAGCGTCAACCTGGCTGGATCAACTTTGAAGCGATCATTGACGTCTTTCTTCTCGTTCACAATGGCCTTCTGCAGCGCGCTCACTTTCGCATTGCTCGCTATCTTCACGGGAAACACAGTCCCTTCGCCGTATACCGCGCACTCCAACTCGACCTCTGCCATCTTCGTGCGGAAAGTTCTCGACTGAGCAGCAGCACTCATTCACGCAGCACACCGCACCGTTTTAGCCAATCACATCACTTTAAAATAATATTTTTATTTCTGACAAAAATACTCCTCAATATATAGAAACTAAATAATAGTGAGTTCATCTTGAGCGATAAAAAATAAATATATAGAAATAACATGTGCAAGTACATCTTCCCAGAAGACCACGCAAAGGCGAGCCAACAGTGCGGCTCAAAAGGCGATTGGTGCCGACACCACGCGAAGTATCAGCCAGTAGTTGCTGAACCAGTGATTGCTAAACTAATAGTTGCTGAACCAGTGATTGCTGAACTAGTAGCTGCTGAATCTGTAGTTGTTGTGCCTGCAGTTGCNTGAACCAACGATGACCAAGTCTATTGAAGAAATTTATGGTGAGCATTGCATTCAGCACATCAAATCTGTTGATAAATACGATGAACCCAAGTTGGGAGATTCGCGCGTGTTGTCACCAAGTATTTGGATCAAGTCGAAGATGCACGTAGTTGGGGATTCCATCAACTACAAGCACAGTGATGATGGCACACCTGACTTTGGCAACGACGGATACACGTACCTTTTGCAATATCAATTTAATGAACTGGATAAATCGCAGATGCTTAATAATTTCTTCTTTAAGTCCAATGAAGACCGTGAATCATTTGACCAAGTCTATTGAAGAAATTTATGGTGAGCATTGCATTCAGCACATCAAATCTGTTGATAAATACGATGAACCCAAGTTGGGAGATTCGCGCGTGTTGTCACCAAGTATTTGGATCAAGTCGAAGATGCACGCAGTTGGGGATTACATCAACTACAAGCACAGTGATGATGGCACACCTGACTTTGGCAACGACGGATACACGTACCTTTTGCAATATCAATTTAATGAACTGGATAAATCGCAGATGCTTAATAATTTCTTCTTTAAGTCCAATGAAGACCGTGAATCATTTGAACAAGCAAACGTTGAATGCGGTGTGGAAGTACTTCCTTGCAACTCAATCCTACGATGAGCATTTGTATCAATTGTGCATGATGATTCGAGGCGATTGGTTCTCCAACAATTAAACGTGTGGAATCTCGCTGGATTCTTTGCTCGTCAACCTCATTCTGATTGGCATTTAGTGGCGCAAGACCTATCTCTGTGTGCTCAAACAAAAAAGTAGAGCGTTTCAACAAGGAGTATGCTACCAAAGAATTTGATGGTTGGTTTGATTCCAAGTATAATCCATCACTGAAAGAAGGCAAACTGAAAGGGATTGCTGCTGGTTGTGATTCTGCTGCATACAAGCAATAGAAGGATGACTATGAAGTCAAGGAGCCATGGAAAACAGCTACTTCGAAGACGTTTGAGCTTTCTACACTTGATCAAGATTTCGAATTGACATTCAAGTCTGAGCGGAAGTTGGCAACACTAACTCAGCTGGTGGAGTTAGCTGGTCGAACAATTAATGACATGCGTTCAGCCATGGTTTGATACGTCATTGAGAACGATGAGCTTGAATATTGGGCGGAGAAGTATCTAGTGATCGAGAACAAGCCCAATCGTGA

>Contig_42

ACAATTGACGATGTGACTCACCGATTACCTGCGGATAAAATAACACACGCGTTGTCAGTCCTGCCGCTAACGAACGTCGAGAAGTACGTGGCGGTGTGGGAGGAATACGGAGATGTCACCCGAGAACAACTTATGTTCCTAGAGCGATATGGAGACGCAAAAAATCAGATTGAGGATGTTGAACATACACTAGAATGGATTGAGGATGTAAAATGGAGGGCTACCGCTGTTGAAGAGGTTCCTGTCATGTTTTGGGAGCCACTTCAGGAGAGAAGGGTACGTTGACTTCTCGCACACGGTTACTGCTACGAGAAGTTATGCGAGATTGGATACGTGCTGACGTCAGTAATTTTTATCATAATACGTTTCGCAGGACTACATTCTACAGCAAATTAAGGAGTTACCCCTGCGTTTATTCTCCGTTGGAAAAGAGGCTATCGATGGATACCGTTTGCTCAGCTTTCGACGAAANCCAATGGCTCAGCACGACAGCCATGATAACAACGATGAAAGTGTTGGCTGAGAAGTACACTGACGTCGGTGTGGTGAGTCCCTCCTTTCGCGAGCCGAAGGAACCCGACCGAAAGAGAATAGTCGCAAATGCTTATAAGGCATTCACACCCACCAAGAGCAAGTTGATTGGGGCGCTAAACTACGATGGAGCACACTGGGTAGCCTTCTTCATTGACGTGGGGGATCGGGTCTGCATACTCTTTGATCCACTACAAGAGGATATGAACTACGCAAAAATTAAAGCAAGCATCAAGGAAGTGGTGGAACCTCTGTTGCCTGTAAATACCGAGCTGACGTACGACAACTACACATCGTGCTTTCAGCAGGACAGCGACAACTGTGGATTGTGGTGCCTCATTGTTCTTGAGCTAAGTTTAACTGGTATGCCATGGCACAAGGGATTGTACCAGCTGGTCCCGTATCTGCGACTGCGTTTCCTGAGCCTGTGCTTGGGCTACGTTGAGGAAAAGTGATAATGCTGAGTGTGGAATCTCGTATTGAATTGCGACTTAAACTTTTCATCGTACTGCATTCATGAATGCTTCCTTTTGCTTTTGTTTCACTAAAATGTCTGCATTTACTTGCTCAACCTGTACAGTAACGTTCGTTGAGATCTACATTAACTGAATTCGCAGAATTTGCTCTATGCATTCCTAAAGAAGACCATTCTGTTGGTATACGTTTTTTAATTGCGTGGCTGCTGTGAGTGGTCTAGCCGTAAGTGTATGCGCTGTTGTTTTAAGTGCTGTAGCCGTTGCTTCGTCTCGGTAATAATGAAAATGATATCGTGTTCTGTTTTATCGCGGTGCTGTGGTATCTGCTGCTACGGTGTTGTACTGTGAAGGTGTAACTGTGAGTTCTGCAGATTTCTCTATTTTTAGAGACGTGATGCTGTTAGAGTGCAGCCCTCTTTGGCAAAGCACGGCCAAGATAAAAAACACGATCTGCTGTTTCGTGTTTTTTCCGGTATCTCTAGCAAGAGCCGTCCTTACCGGTAGTGAAGTAGCTTCGGAGAATCTTGGCCATGGACTGTTTTTGGTCGGGGTACTTGGCGTTATAATCGTCAAGGTACTTAGCCCAAATCTGAAAATCGGGACTGGAAAGAAGATCATCCGGCACCTTGTCAAGATGTAGACGATAGAAGGTGCCGTCCGGACGTTCAACGATACGAATAATGTCATTCGCCCCAGGCTTCTCTAAGATGAACACGCTGAGCGCATCTTCCGGAGGTTTTTTTGGCACGCAGCCAACGCCTGTATCGTTGATTCTGCAGCTGCTTCGCGAGTTCTACTGTACTCGGATTCTTCATACCATCTGCGACTATCTTTGCCACACCACTGTCTCTAAAATGTTTGTGAATATGATCCAGCAAAGTTTCTGCGTCGATGTTCTTTTTGTTGAAGCTCTCGAGGTAGTTGAACCACGTTTTGAACATCGGAGTGCTTAAAAGATTGCTCGTGGAGATTGAACGCCTCGAAGACATACTCGGGAGAGCGATTGCTGTTCAGTAGCCTCTTAATCTGTTCAGCCTGCAATCTGGAGGCGGCATTCTCTGTTAATTGGACTTTCTTGGCCGCTTCAAGCATGGCAAATACAGCATCATCGCCGTAATGAGCTGCAATGGGTGGTTTCATCGACGCTCTCGAAGTAGGATACTTGAGATTGAAATCGTCTACGTACTTGGTCCAAGCGATGAATTGCGGGCTTGTTAGTAGTCCATCCACTTTGTCGTTAATCATGAACATCTCGAAGACGTATTTCGGAGTTTGGGCGTCGTCTAACCACCTCTGGATTTGCTCCTTCCTTACCTTCGTGGCAAGCGCATTTGTGGTTGAGTACGTCTCGGCCAGCTCAAGAGCCCTTGTCAGGCCTTCGTCGCTGTAAATAGTCCTCAACGTAGGGATCATGGACTTCTTCTTCGTAGGATTCTTTGCGTTGAAATCGTCTACGTAGCTCACCCAGGTGTTGAAGTACGGCTGACCAAAAACGGTGCCTTCGATCTTTTTAAAGTTAATCACCATAAACTTGTTCAGTTCAAGCAGCTGAAATACTTCACGTGTTGATTTCCCCTCGTCAAGCCACCTTTGGGTCTGCAGAGTCTTTATTAATAGTCTGCCAGCAGCTTCAACCGCCTTATGTACTGGCTTTGCTGCACCTAGCAGCTGCGATAGTTTTTCGTCCGGGACATCGCTTTGGGAATTTCGGTGTGATTTTCTTAGCCACATATTCGATCGTTTCTGTGATCTTTTCAAAGGTTGGAAATCCTCTCTCGTTGTCGTCCACAACTCTTAAGTGCCGTTTGATCAGATTCTCCTCGACTGGTGGAGTGAAGCGCGGGGTGGTTAGGTGCACCACATGGAAGGTCACACCCTGTGAGATAGAATCCACCTGAAAAGGGCGAAAGCCGCAAGCACCAACACCACACGATAGATAAAATGCATTCCGAAAGTCCTTCAAAGCTGGCGCAAATTACGAAGTGATAATTTACATGGATGTTACTGGTTCAGTGCTAGCAGCTTCGTCACGTTTGGACTTCAGAATGCTCTCGAGGAGGTTTACAGATAGTGGTAGGACAAGTCATCCTCATGTTCCTATTAATGTAACTCGAATACCGAATTTAAAATCGTGAAGGCAAATGTGCGAATCAAATATTCCTCGTACGTCATGAATCGTGCTTGATCCTGACTAGATTAGTGATTCTGGTCTTGAGCGTGACGAAACAAAGTTGACTTTTCATTCACGTTTGATCAATTTGCACCTAGATGTGTGATTACTAACGCCTTAGCGCTTTGGTACATTTTACGATCAAATGATCTGAGTGTGACGAATCAAAAATAAGACGCAATGATCATACATGCACTACTGATTTTAGTACAATAGTGGTTAATCCTGGTACGGTAGACTCGTGTGGTACAGTAGAGCCATGTGGGTGTTCTTTAAAACCACGAATGTCAGCTATTGAGCATCTTGATGTTACCGATCATGGATCGCAGGCACTTCGCGCCATTGACGATGCCGTTGGCGACTGCGGTGAAATGCCCGAGAAGTCCTAGAGTGGCCACAGATATCGCAGTCTGCACCACGTTCACATATTTCGCGTAAACTTCCATGCCGCAGTCGTCATTCTGGCGGATGCATTCTATACCACACTGGACGGGATACTGGAGTGGGCACTGCGTCCAACACATTTTCCAGTTGTAGTCGAAGGCAACGGGACAGCTGTCCATGATATGCGCTCTGCGGTTGCAAGCCCACTCGCCACGATCTGGCTGGTCGATGGTGGGGCCGACCCAGTTTCTGTAGCTCGATTCTACCGTACCGGTGGTTTTAGCGATCGATTCAATCGAGATGTGCTGTTTCCTTCGGCACTCGAGTTATGGGATCTTGAAGATGGAATACTCTCACTCTCGAGTTCATTTAACGCAGTAAATCGCTTAAAGAATAGAAAATTTATCGTGGATACCAATTCTTCATTCACGACAGCAGTAGCACTATTCGAAACTAATCTTTTATATTTACTTGTACTTTGGTAGAGGAAAATACTCCCGCGTTATCGTTTAAGACGACCATTGCACAGTGCTTCGCTAAAAAAAATTGAGACTTAATTTTGACAAGGGTACTTTTGTATTTTGAATCGGACACTGAAAAAAATAAGTAACCAATCGGGTACTTTTGAAACTTTACTTGTCAGTTATGGCTATGTTTGCAATATGTGATATATATTTATATATTTTTGTAATTCTAGAGATATCCATGTACCCCTCACGTGTAACCTAGGATACGAAGCGCGAGTATGGTGACAGCTTCATGTGAAGAAGACCACCGCGAGCAGCGAATTACGAGCGAAGGCGAAGCCGTAGCAGGCCACGACATGAACATCCTGCTGGCGTTTAAGATCCGTGTGCGTAGCACGCGGGGTTAAACCCAGTTGTACAAGTGGACAAGCGAAGGCGAAGCCGGAGCGGGCTTAGGGTTTTGTCGAAGCGAGCTCAGCGAGCAACTAAGGGTTTTTGGGTCCGCGCGTAGCGCGAGTTTGGTGACAGTTTCAAGTGGAGAAGACTACCTTGGTTTACACGACATGAACATCCTGCTGGCGTTTAAGATCCGTGTGCGTAGCACGCGGGGTTAAACCCAGTTGTACAAGTGGGCAAGCGAAGGCGAAGCCGGAGCGGGCTTAGGGTTTTGTCGAAGCGAGCTCAGCGAGCAACTAGGGGTTTTTGGGTCCGCGCGTAGCGCGAGTTTGGTGACAGTTTCAAGTGGAGAAGACTACCTTGGTTTACGTGCACAGAGCTGGAGGTAGGTTTGCGTACGTCGGTAAATAGAAGAAGGAGTGGATGGCACAAGTTAATGTTCGCCGGTATGAAGTTGTACGACGATAAGTTGACCGTCTTCTGGACTTGAGGACCACTTGCTGTTCATGTCGCACTTCAACGACCATCACTCTTGCCCGCATTATATTGATGTCGCCCCACCTGTTGTTACCATTTGCGTTNGGTTTGAATGATTATGTCATTCCTCCTTTCTTATGTGTTGCCTTTCAGTCTACAAATCGCCGGTACAACCACAATGGTGCAGACGAGAAAGCGCGCTCGGCTGGCTGCTGCTAGCGACAATGTAATGCACGAAGGAGGCGAAGACGCTTCAAGCAGCGACGACTCGAGCGCCCCAGCGGCAGTAGCATCACCAGTCGCGCACCTCACAGATTGCCAGCTCGGAGAAAGGCAACAGGATCTGACTGGTCTTGCTCTTGCTCAGACCTACCACGACAGTGACGATCTGCAGACTGCACGTGAAGACGACACAGCGCAGGATATCGGCAATAGTCACGCAGAGCGTCCAAAGCGGTTTCGCCGTGGCATTCCCAACCGAAAGTGGTCACTGCTGAAGACGTGCTGTACTTTCGATGAAGCCAACTCATTTCGCAAGAAGTTGGAGTTCAATTTTGTCATTGCTGGTCATTCTCGGACAATGTATGGTAATTCCAAAACCTACAGATGTAAAAGCCACGAGAATTGCAATGCTTTCGTTCGCATTCGGCCGCATCAGATGGAGTTCGTGTGTGAACTTTGTGGTGATCACGACGGAGGAGCAGAAGAAGTTCAAGCTTCGAGGGGTATTCAAAAGTGCTTCGATAAAGAGCTTGAGTTGATGTTTGCAAGAGGACAGACGCCACTTGGCGCTCTTGACCAGTTGACCCGCATTTATGGAGATGACAGTGAAAAGGAAAAACTGCTGCCTGATATCGGCCAAGTCCGCAATCGTAAAGCCGGATGAAAAAAAAGCAGCTCACGGGCAAATGGAAACAACTGGGCACACACACCGAGCTGTTCGAATGGGCAGCGTCACGATTGTGCACGACTAAAGACGAATTTGACGCTGTTGGCGAACGAGAACTTGTGGTACTTCAGGTTTTTGAGTCCAAGGAGGTGAACAAAACAAAAGAGGCTTCTTTTGGTATGGTCCTGAGCTGCAAGAAGCTTTTGCGTAATTTTCTTACGGCTGCTGCTTACAGAGACCCAGTTTCGATCTCTTGCGACGGCACTTATCGGTTAACAGACGCTGGTTGGACTTTGATTAATCTCGGTGTAGTAGGTGTGATTTACGATGAAAAGGAACGCCAGTTCCACCAGCGATTTTACCCCTGTGCGTTTGGATTTGTTCGTACGGAGTGTGAGCTGGCGTATGAAGCGTTATTCGAGTCTGTGAATCGCTGCGTCTATAACTGCCTGGGTATGGAGTTAAAGCCGACCGTGGGGGCCACAGACCATGCTGTCTGCATTGCAGCAGCTCTTGGAAAAGCATGGCCCGAGATGGTCTTTGTCACGTGTTGGCCTCATGTAAGCCGGAAGTGCCTCGCCACCGAAAATAAGAGCATGCTATCAGATAAAGAATTTCTTGTGTCTGTTGTGAAAACAGACATCAACAGCCTCCACAAATGCACAAGTTACAAGCACTTTAAAGCTCTTTCGGCAAAGCTGATGACAAAATGGCGCAACAAAGGAGAAGCCCGGTATGCCGATTGGTTCACAGACACCTATCTGACGGCTCCATTTGATTCCTGGTTCGTTGGAGCTTCGACACTACCTGGATCCCTGCCACAGCAGCAGGGAATTGAGTCATTTCACAAGAGTATAAAGGCTCATATGGGCCTTTACCTTCGTGCCGCTATGGTCTACTTCTTGGAGCTTTCGATGGGCCAAATTCTCCATTTCTGCTGTGCGCACAGGGCACCAGAGGAAATACGGCCTTTTGCTGAAGCACCTTTGCTTCACTCACAGCTTACGAAGGCCAAAGCAATTACACTGGATACCACGAAGTTCTACGTTGTGCAAACCCAATGTCTCCAGACTTTCTACATCAACCGAAAGAATCATTTTGATCAGCGTGTAACCAAAGCTCTGACAAAAAGGTACTCAAGCTTTTAAGTTCTACTTCTAGTACGGAAACTTTTACAAACTGAGATTTTGCTTGAAGGTACGACAAGGCACAAGACCATTCACTATATCGTGTCCGTGTAAGCGCCAAGATCAAGTTTGAAAAGCTGCGAGCTTTGCCAAAGGTGAACAGCGACACGGCTCAACTCTTCCGTAAAAAAATATATCTGTGAGTGCAAGGCTTACGTCATGTCGGGTTGGGTGTGCTCGCATGTACTGGCAGCAGCGGCTATCAATCAACACCTAAATATTGACGAAATTCAAGCTGGAATCCCCCACCGCCGTCTCCCTGGACGGCCACGCAAGGAGCTCAGCTGTCTTGTTCGAGATAGTCTCGAAGCTGCTGAAGGTGACGACACACTTTCGACTGGACCGACGGCTCGGCTTAAACGACATCTGGAAAAATACCCAAGCGACGCTATCGGATACAAAGCCTTGCATCGCTTTAAAACGTCGTTACCTGAATCACCAAAGGAGTTCCAGATCAGCTGCTTCAGTGGTGTAGTGACCTCCGTTCAGGCTGACAATAAGCCAGTTACTTGGACGATCACGTTTACCGATGCTGATAGTCATGACGTTGAAGTGGGTGAGCTCGTGACGGACATTACCATGGCTACCAGCTGCCACATTTAAAGGTTTGAAAATAATTTTACTGACTGCATTTGCTTTTATAATCGGTCGTTTTGCTGGTTCAACAGGTTAGTCGTCACTACATGAGGAACCCAGATCAGAATACACAAGTGCCCGCTCAAAAATGCACAACTAACCGCTTCAGAATGATTTTACTGACGGTTTTTTGTTTTGGATCGGCCACTTTTGCAGGTTCAGCAGGTTACTAGTAACCAGGAGGTTACCAACCCCAACATTCAGAACTTTTTGCTGGTTCAACAGGTTAGTCGTCACTACATGAGGAACCCAGATCAGAATACACAAGTGCCCGCTCAAAAATGCACAACTAACCGCTTCAGAATGATTTTACTGACGGNTTTTTTTGTTTTGGATCGGCCACTTTTGCAGGTTCAGCAGGTTACTAGTAACCAGGAGGTTACCAACCCCAACATTCAGAACTTTTTGCTGGTTCAACAGGTTAGTCGTCACTACATGAGGAACCCAGATCAGAATACACAAGTGCCCGCTCAAAAATGCACAACTAACCGCTTCAGGATGATTTTACTGACGGTTTTTTTGTTTTGGATCGGCCACTTTTGCAGGTTCAGCAGGTTACTAGTAACCAGGAGGTTACTAACCCCAACATTCAGAACTAGCCGATTGAAAACGAATATACTAGAGACCTTTCGCAGCTCCTTTTGAGTTTATTTTCAGCGAATCCCGGCTGCCACAGGATTGCCTAAATTGATTTGCATTCGAGAACTTTCGCAGCCACGCTTTCTATTTAGTTTCAGTCTCAATATTACAACTGCTAACTCACTGCCAGCTCTGCACCTTACGACATGGAAGAGAACGCGCCGTTGAGCTTGCCCCCTGCACAACAAAATGATCAGGAGAACCTCACAGTGGGTGGGACTTTCGTACAGCCCGCTGGTCCAGCTCGAAAGAACTTTACGACAGCCGATGATCTGATTTTACTGCGCGCTGTCAACACGATCAAGCCGTGGGAATCCGCGAAAGGTACAGCGAACGGTATAATGAAAAGCTTTGACAAGATTGCAGACATCTGCAATGAAACGGCTGGTTTCGTGCGAGACAAGCAAGGCCCAGCGCTGCGTACGCGATTTGATAAGCTTATTCGTCAGCATCGCGACGCACAAGTAGTTTCAAGGAGGTCGTCTGGAACGACGGAAGAGTACAACGAGAGAGACGTGCTCTTGCAAGACATTGTCACCCGTATGGACGACTGGAAGGAGCGTCAAGATAGCGAAAAGCAGCTCCAGCGTGCCAAAGATAATGGCATTGAGGCGTCGGGTGTCTTAATGCGTCGATTGGCAATGGGAGAGCTGGTGGAAGAGCTGCAATCCGACACAAATTCCAACAGTGATGAAGGCAACGAGGGACAAGACGAAGGTGCAAATGCAGGCACTTCCTTATGTGGTAGTTTGAAGCGTGGGAACAGGGCTAGCAGCGGTCGCGCTACCAAAGTGGGAAAGATAGAGCGAGTAGCAGCCGTGACTGATGCTATCGCGATGGCAATCACCGAGATGAATAAGGGGAACCCTGAGAAGTATGTGTATCTTAATAGTCGCTTACAATTCGAGAAGGACGAGGCCAAAAGAATGAGAGAGCACGAGTTAAAACTGGAACAGCAGCGTCAAGAAGCTGAGCGCGCACGTGATGAGCGTATGCAAGCTGCTGAACGTGACCGCGAGAAGTTTATGTTGCAGCTACTGGAAATAGCTCTCGGACGTGCAAAAGACAAACAGTAACGGATTATAAACAAACGACACGTTTTGTCATTCTTAATTTGCGTTAAGGTAGCTGTCTACTGATGGTGGAAGTAAATTAAAGTATATAGATATCTGGTTTCCTGTTGGTTGAAGACATGCATGAAGGTTAGATAGTAGCACTCCAACAACGAAAAGCTTTCCAACCGCGGTCTGTCGGGTTCGCAGCTTTTTATCCCAATTCAGGAATGACCATAGCATCTTGACACGCCCGAATCCCCACTCTACGGCCTCGCGAACACTGCTCATACGCGAATTGAAAAGTTTCATAGCTCCATTTGATCCAGGATTTTCGAATGGACAGCAAACGTACTGACTGCAGCCATACGCCGGATCTCCGTAAATTACTTTTCCATTGAAAAGCATGGATTCATCATTTTTAAAGACATCCAAAATACCACTCATGCTTAACATCGTCGAATCATGCCGCCTGCCTTCCACGGGACCGAATATGCTGACAATTATTCCATCTGGAGCTGTAACTGCTTGCCAGTTGAAGCAGTGGCGTCTCGGATGACCATTATACAGGGATCTTTGTAGGTTCTCGTATGGTGACGTCGGATTCCTACGAGCGCTTGGTCTGGATACATACTCCTTAGTTCCATCCACAAAACCCCAGCAACGTGCTAACGGGGAGTCCTTGGCTCTAATTGCGTCGCAATACCTCTCAATACGGGCTACAACAATACCATGGTTGAAATACAGGAGAGCCTTGTATTTGTTGTACAACGTATCCGCTAAGTGGCGACAGACGCGCGTGTACGCCCCAGGTGATCTGCCGAACTCATTAGCTACTGTATACAGCATCGATGGCTCCTTCAGCGTTTCTGCACAGCATAGCAAGCGCTTCAACGGTCGGAACAGCGTCACGCTCTGGGGTAATAATGACTGGTTTCGGTAAATGCATTTTGGTCGCTAGCAATCGTAGCTGAGGCATCGTAAAATTAAAGCGTGTCCGTGCCGTCACGTCGTCCATGGAGGGGTCGAAGTGGTTGCTTGTGTTGATCCGAGGAGGTGGGTCTCGAAGACGGAAGCAATGACTGGCGATGAGGAAGTGCATTGTGCGCAGGAAGCGCTTCCGATTACCCCGTGCAGCAAGCAACAAACAGCTAATGATGACAAGTGCTTGCTCCGCTGTGAGCTTCGGTGCTATGAAGACCATTGACGAGGTTGGATGGTGCTAGCTGTGAAGACGAAAGTGAGCTCAAATTCGAGTTGAGCAAACCGGCCAGTTAGCTCAAACGCAATTCTGTTTGAGTATCGTGTTTGAGAGGCTCACCCTAGAAACTCAAAAGAAGACTGAACTCAACAAAAACCAGGTTGCGAAAGGTCTCTACTACCCTCTTTTTCGAACTATGTTGGTCACTTTTGCCGGTTGAGCAGGTTACTAGTAACCAGGGTAACCAACTCATAAGTATCCGATCGAGCATATAATTTCGCAAGTATCCGATTCAAAATATAAAAGTACCCGACTCAAAATTTCCACCCAAGAAAATTAATTAGCGCACCATAACGTCAGTAGTGACCGAATCTACCATGTACTTCGTCACCAAATATGCCTGATAGCACTGCCATAACCGTCCTTAGATACGCCTTGAATTAACATAGTCATGGTACAAATGCAAGCCATGATTGTAAACTAAAAGACTTATTAAGACTAATCACTTTTTTCCGCAGCCTACAAGAGACTTGAATATACCCGCTTTCTTTTGCAACAACACAGTTGGCGTGTCGTATTCTACCACGTGGCCTGCATCCGATACCAGAATCTTGTCACTGTCCATGATCGTCTCGATACGGTGCGCAATGCACAGCACAGTGCTGTCACCCCCGCCAAAACTCTCCTTCATCGTCTGTTGGATCAACTTATCCGACTCCTGATCCACATTGGCTGTCGCTTCATCCATCACGATCACTTTACTGTCTCGGATCAGTGCACGAGCGATGCACAGTAATTGACGTTGCCCCACGGACAAGTTGTCCCCCTTCTCCGCTACCTCATAGTCGAGTCCTTTTCCCCACGATGACACCGTATCAGCCAAATGAACCTTGCGCAGAGCCTCCCACAACTCCGCATCGCTCTTGTCAGCAAACGGGTCTAGGTTCAAACGTAGTGACCCGCTGAAAAGCATCGGATCTTGTGGAATGATCGTCAGCTTTGATCTCAATTGGTGTACCGTAATGGTAGAGATATCCACTCCGTCAATGAGTACGCGACCGGACGTTGGGATCTCTACAACTCGGAAAAGTACACTCATAAGTTAGCTCTTTCCGGACCCGGTACGTCCACAGATACCAACCTTCTCCCCGCTAGCTACAGAAAAAGAAACACCTTTCAACACCAGTGGGAGGTCTTCTCGATACCTCATGGAGACGTTCTCGAATACGACGTCGCCAGTCTTCGGCCAAGCAGGAGATAGCGTAGATTTGTTGGAGGGTGTACGCTCGTACCCTTCTTCATCAAGTGATCCATAGTGAGCAATACGTTCGAAGCTCGTCATGCTACTTTCGACTTTAGTGGCAAGTGTAGTCATCCGTTGGAAACTGGATGACAACTGAGCTGCGTACGTCAACCCGAGACCTGCAACCGATGCACCCACAGTGGACTTGGCTGCAATCGACAGAACGGCCACAGCTCCAATGATCAACGATACGAGCCAGTCGGATCTCATGTCGAACCAGCGACCTGATACCTGCGCCAGGAAGAAAACCCTCATGTTTCTGTTGAGCAGTTCTTCGCATCGAGAGGCGAAGGCGTCCACCATTTTATACGACCGCACAGTCTCGATGCCCGAGATGGTCTCGGACATTAAGTTAAGGAAAGGTGAGCGGGTGACCCCATCCAGACGCTTCAACTCTCGAGCGGAAGCTCGGTAAACATTGGCAGCGAACCAGCATCCGTACCCGACTGGAGCGTACACGATCAGTACGAATGGGTTTACGAACGCACAGACGATAAATGAAGAAGCCACTTGGAAAATGCACAGCACCAGCCACATACCCCAGTACGGTAAAGGGTTGTCAACCTCGTCGAGATCTCTAGAAAACCGGTTCAAGATCCGACCGATGGGTGTGACATCGAAGAACGTGGTGATCGGAGCTGCCATCACCTTCTGCAGGTGTTTAAAGTGGATCTTCAGTGAACAGACCATGCAGATGGTCATGAAGAAGAGACACCGACCCAGGGACAGGAGAGCTGCTAGGACGACAATCCCGATATATACTGCTAGTGAGCTCATCTGGCTGAGCCCCAACGAATCTTGGGACCACTGACTCAAGAACCAATCACACCCGAACAAGACCACTTGAGCAATAGTAAACAACACGATCATGAGCCCAACAAGAACACGTCCATCCCATTCCGAGCTGGCGAGATAGTCGACGTACGATTGCAACTTGACAGCCCCCACAAGTCGCTCTTCTGTCGTCGTTAGTTTTCCAGAAGATGGTGCTTTAACTGCCTCATCATGAGTCACCACAAGGCTCGCCTGGGTGGATTTCCTGCCATCTTCGATACTGGAATCCTTATTTTGTGCTGGTTCTTCGATGCTGGAGCGACGTAGTCGTGGCGAAGAACGCAAGAGCGTGAACTGGTCTTGTAGCTCCGTCAACGTGCCATCCCCCACAATCTGCCCGTCAGACATGACCAACACTCGATCGGCGTGTTGTAACAAATGGTAGTGCGAGTTCAACACCAACAACCTCGTTGTTCGACTCGCTATCCCCATAAGACCGTCCGAGAATACTGCATTCGCCACATGCACGTCCAAAGCACTGAGAGGGTCATCCAGTACAACAAAATCGAACTTAGCAGCACGATACATGGCACGAGCCACAGCGATGCGAGCCTTCTGACCGCCACTCAAATTGATGCCACGCTCTCCGATCTCCGTTTGATCAAGTATCTTGAAGTCTGGAGCGAGTTGGCAAGCAGCGACCACATTTTGATAATGTTCCTTTTCGTAACTTTCCCCAAAGTTAATATATTCACGTACAGTTTGGTTCTGGATCCACGCTTGCTGACTGGCATAGGCAATGTTACCATTAACGAGACGCTTACCACGGGCTTGGAGCATCTCACCGAGGATAGCGTTAACAAGGCTGGACTTACCACTACCAATCGGAGCCGACTACGACAGTGAGAGAGCCAGGTTGGAGCGATACGCTGATGTTAGATAACGTTGAGTTGGTCAGC

>Contig_44

GTTGGAAGAAAAGTCGCATGGCTCTTGCACAAGGCTAACACGTCTTGTAATGCACTACTCAGTGCTTCAAGATATGGGAAGAAGTCTTCCAGCTTGGTTATTGCAGAGTGTAATATAACCCACGCCTTGGAAGAGGGCAGCGCGATGATTTCGGCGTCGTTAAGACCATGGAAAGTTCTCAACTCACCGCTTGGATTGCTTTCGTACACCTTGTGGTCTTCAGATGATAATAGTTGAGTCTCTAGACGCTCACAACGCGCTTGATACTGGCTTAAACGCTGCGAATGGTCCCCAGTTTTCTGCTTCATCATCGACTGGAAATGAAGAAGCTCTTCGGTCTTGACTTTCGATGTCTGTCGAGCTTCACGAACGTCTTGTTGAAGCGCCTTCTTCTCCATAGCTAGCACTCGAGCTGTCTTTTCATGTTCAGCAGCAAGTTGGCAAGTCTGCGCGAGTTTTTCGTCGAATAGTGTCACGTTTAGCTGTAACTTGGCCTCCGTATCCGTGAGTTGCGTCTGAACTCGTGCCAGCTCGTTTTCCACGTCACGCAGAGAACTTACGTGCTGTTCGTGGTCTTGTTCTTGAACAAGTAGCTTCTGCGAGGCCTGAGCAAGTTGTTGCTCGTATTGCTTCACTCCATCAGCCAATTTGGTCTCCGCTATCGTGAGTTTGGCCTGCACGTCCGCGAAATTAGTCTCCAAGTCTCGCAGTGTCGTCGCCTGTTGCTCCTGCTGCCTCAATTTGTCCAAAACCGCTTGAAACTTGACGTCTTGCTCTTTCAATTCGATCGCTCGGCTAGTTGTCGCTGCTGTGAGTTGTTTATGAGTCGTTTCCAGCTCATTTTCCAGTTTGGCCACCTTGAACGTGTATGTTTCTTGAATATCTGCTAGTTTCGCTTCATACTCCTTCGTTTCAGCACTTCGTTTGGCCTCAGAAGCCGCATCTTGGGTCTTAAACATCTCACACGTATTCTCCAAGTCTCTCAGCGTCGTTATATATCGTTCTTGTCTCTCTTTTTGTTCTTGAATAGTTTTCTCGTGCACAGTGAGCTCCTCGTCCTTCTCCTGCACTTGTTGTTGCAATATTCTACTCGTCTGGGCATGCGTCGCTAGGTTCTTGTGGAGTGTATGGTTCTCCTCCTCCAACGTCTTCTTGTCCTGCTTCAAGATCTCCACTTGAGTCTCCAAGAACTCATACCTCTCTTTAGCTGCTTGTTGTTCTAATAGCGCATCAGTCGCCCTGCTCTTCTCTGCCTGGAGCTGGTGCTGTGTGTCCTCCAGCGTCGTCTCCAGCTCGCCCTTCTGCAGCGCAAAAGCTGCCTGTTGCGCCAGCATCGTCTGCGTCTGCTGCTCGACGGTGGCGGTCAGACTGTGGAGCTTGTGAAGCAGCGCGAGCTTCTCCACTTCCTCGACTTCTTGTTGATTGTCTTCAACTTCGGGAGTTGCCTCCTTCTTTTGACAATTATTGTGCAATAATTGTATTTCCATTTCCGCCAATCGACTTTCGTAAATTGCAGCGAGACGCCATAATTGCGCATTGGCGTCCTCGTGTTGCATCTGTTGCGTCTCGCGACTCTCGCGAGCTTGTGCAGCGCTGGGAGGCGGCCCGTGGGTCTGCAGGTGGGACAGTGAGGCCTCTAGAGCACGTTTATCGCGGCGGGTCTTCACAAGGTCTGCCCGAAGCAGCTTGAGTGCTGCATATGCGTCGCGAAGATCCTTCTCCATGGTCAGACTGATAATGACGCCAGAGGGCTGGTGAGGTTGAGTTTTACTTGAAGTTTGTATGGTGTGGGATAAAGAAGTTTCCATAATTATAAAAAAGGTATTTGACGGGATAACGTGAAGCACCCGATAATGCTTGTGCGCCCAACAATTTCACTGTGGGTCGCACTACAACGTGCGCAGCCATCTTTGAAGCCAAACACTTGTGTTCTCGCCCAGTGATTGTCGCTGGTTATGCTGGACGCAAGCGCCGTGAGAGCTTCTGAGAAGCTATTATCTATTTTGACAACGGCAGTCTATCTTCCACATTGCAATTTTTATGAATGTCGCGAGCTTGTCGAGGCTGTCGACGAATGATTCCAGATATCGCAATGTGGAAGCTTACACCATGTACGTCGCAGAGCAACATGGTGGAAATGCTTTGCAGGATAAAGTAAAACCATTGTTCCACAACAAAGATCTTGTCGTTGTAATTGCAGCCGCTTCGACAGCTTTTTAGTAATGCATGCAGCTAGATTTACTAGAAGTCGATCGACATACCTGTTCACATTGTCATCTATTCCGTTGTGACCCGGGTCCGTGAGCTGTCAAGATAAACGAGAAACATAAACATCTCCCGAAAGGAAGATGGTCAGCATGGAAATTGAAGCGTGGTGTAAAGGGATCTGGCCAAACTGAAAGTGTATAATAATTCAAGCGCCACAGTCATTCGGTAAACAAGCCTCTGAGCCTGCACAGTTGCAATAATTGTACTTCATTTTTTTCACTTTGATAATATCTAAAGTAATTGCGTTTTTGCAAAGAGCCGTTTTTTTGCGGTGTTTTTCGGGTCAAAACGGGGTTTAATTTTCCTCGGACAACACTCCATTATACGAGCTTTTTGTGACCTTGCCGTGCTTTGGGTTACATGTATCCATTCAATTGATACAGGATCTATCCCGGATTTAGGATGGCTTCACAATAAGCTCACAAAGTCTGTAAGCTTCTTGTGAAGCCATTTAAAATCCGGTGTAGACGCAATGAAAAATGAAACGAAGTTTTTCATCAGAAGAAAAACAATAGTGCGGTTCAGTTTTGGTGCTGTAAAAAGGGTTCCTACCAATGTTAAATATCTTAGAAACATCACACTTGGCTCTCCAGACGGCTGCGCACGCCGAAGTAAGTATCGTAGCAATTGTTGGACTGCAATAGAAAACTTCGCTAATCTTGCGATGGGATATTAATCCCTAACACTTTCGACCAGCTAAAGTCAGGAATCTTTAACCTTTTGCAGCGGCTCAACTTGCAGCGTATGGGTCCTTCTTGGCGAACAATATTGTCACGATAATGCTGTTCCTCCCCAGTACTCAGCGAAATACGCCGTGAAGCTTTCCAAATTGCGGTACCTGGCATCACTCTTTGGGAGTTCCGCGAAGTTGATTGGATACGGTGAGCCAAATATTTTTTCGAGCTGCGATGGGGTTAGCCAATCTGCGTTTGTAAACCTTTTTGCGAGTTTCTTCAAATCTGGGATGTCCTTTAACGACTGAAGAAATATTCCAAATCGCGGCAAGGAAGCAAACTCATGTAAAAAGTCGAACACATGCACGTCTGTGAACGAGTCGCCTCCATTCACAGCTCTGTATGCTTTAATGTATCTGAGCCACTGAATAAACAGGGGATTACCGTCGAGAGCGTACAGAGGTTCATCCTCAAGCCGCAAGATATTGAAAACTACTTCGGGGGTTTCTCGGAATTTTAACCATGCCTCATTCATCAGTCTGTGACTAGACGCCGAGCTCAAGATCAGGTACGCCTGCATATCATCGGCGATATTCTTGATGTCCGGATAATGTCGAAGCGATTGAAACAGAGACACCAGTTCTTCTTCCGAATTCAGTTTCCGCACCAAATCAAATAGCTTGTCGTCACTGAACGAGGCCTTGTCTTGTAACTTAGCTCTGTATCGATTCACGTACAGAAGCCACCAAACAAATTCCTTGTCACCCTCCAGCTTACCGCCAGTTTTCACAGTACGTAAAAGCTTGAAAATTTGCATCGGATTCCTCGTGTTCTTCAGCGCCCACTTGGATGCCAGATTACCAAGCCATGCTATCCCAGAAACCCCTGCTCTCTCTTCGCTATTATCATCAATATCATCGTGCTTAGATGCGTCGCTGAACCTTAGAAACCTTGTTGTGGAATACGATCTCTTCGTTGCAGGGATGGACGTTGACCTGGTGTGGCTCAGGCCCTTGAGCTGGTTGGTGCTTATCAATGAGTTGAGCGAGGAGCAAAGCGAAATCCGTACGATGAATGTGATCAGCACGACATTGAAGAACATTTCTTCTCCTCACTTTTCAATAGTGAAGAGCTACTTTAAATCCCACTCTTATTACTGATTACGTTTTTTAGTTTTAAGATTACGGACTTCGATTACTCACTTGCTAAGAAATGAGCGTCAATAAAAGTGTCGTTGCCGTTCTGTCGATGTGCAATTCAGATGAGAATTTATATTCCACCTCTCCAAAAAGGTGTCCATATTATATTTTAAAAGTCTTGTGATGAGTATTTCCTTAATACTTCCCAAGAGGTAACATTCGTAAACGGAACGATATTACTTTCGAGTGCTTTTTTTTTTAGGAAATCGAGTGCACCGTTAGTTTTTTGTTCGAAAGGTGTGAAACCGAGTGTCCCTTGTTCAACTTGAGGGAATGACCGAGTGCACTGCTGAGGTTAAGGCTTGAGCACGGAAGAAGATGCGTCAAGGCTCTGCCTTCATTAGTTCAGACGTAGCTAATGTAAGCTACATGTTGCAGCAACGATAAGCAGGCGTTCGTCTACAAAGCGATCCACGAGCTAGGGTTAGTGTGCTTTCATCGCCAACTTCGCTCGCCAGCTACGCCAGCCCACAGTGTAAGAGTTCCTTTCGTTCAAGCGCTTCTCCGTCAACACGATAAGAGCAAATTCAACGCTCGCCGTACGTTCACCGTAACACTCTAAGTTGCTTCTACTTCATACTCATCTATCTACCGTGTGCAATTCTCATTGTGTTCAGGGCAATGGTGGAGAGCTCCACGGGAGAGCATAGCCCGAGTCGTCCATCGACCAGAGGACGGTACCAACGCATCTCGGTTCATGAAACGATGCAAAGCGCGCAAGTCGCGATATAGATGGCTTGTCGACTTCGAGGGAGGCAAGCAAGTCTTCCTAGAGTGCCAGGAATTGGTGCAGCTGATCGTAGAATCAAGACGTACTGGCCTCGATGTTACGAAGAGTGCTGCCGCCCACGCAAGCACACACGAATCGCATCCCCGCAGTGTCGACCGTGACGCCGACGACGATCAGTACTTGGACAGCTAAGACGATCAGGAGTGACAGTTTGCTTTGAGCATGTGGGCGTAGGACTGGACTAGCGGAGCTCAGCGTACCTTGTGTAGGAAGTACCAGGATCCAAGGCTCATTCATGTTTTCTTCTCACAAATTTATGTATTTTTCTCCTCTTACATCCTGGTGAGCTCTGCATCCGGCCCCAAGGACAGTGTAGTAAATTCAAACAAGCATAAACCGGACAAGACAAGTATAGAGCCGACGATATCGCCATGTGTTATCTGCACTCGGCATTCCGCCATTTCGTGGCAAAAATGTCTGCACTCGAAAACCCCTCAACGTGCACTCGAATACCATCCGCGCCCTTCGTAAACTGACCTCTTTTAAAATAGAAGACTGAGTGTAGAACTATAAATCGTCTTACGCTGAAAGTGTTTAGGATTGCGCGATACACGTGCCGCAAATCTACGTAGACAATTGGGTCGCCTTGCATTGAGCCTGCATCACATGTTGAGGCTGATAATACAACTTAACTGACGAGTGACGCCCCGCCAGAGCCAGCTTTTCAGCTCATAGTAGGGGTATAAACTTCTTTTAGTAAATGCGTTTAAATTCATATTCTGGATGTAAAAGGCTACACGGCAACTCAATTGTAATTCCTTCACCCAAATTATCTCCTATATGCGTGATTTATCCTATATTTCAGCAGGCTGGTTCTCATGAGCATTCCTCAAAATCTGTCGGACATCGTACCCATCTGATTGGTTCGCGTCGGACATTCTGTCTGACATTTTCTGAAAAAAAAACTGTTTTTCCCTTAGCTTGTGGACGCCTTCCGGCAGTCGTCCTCCATGGCCTAAATGGCTCAGTGGCGAGCAGTGTCAGCGCGCCGCGCTGTCATCGGCGCACATTACCGCTATGAAGAGCAACTATCAGATTGAGTGTGCTTAGCTATGATATCAAATAGCATACTGATATCTTAGATGTCTCGTACGTGTATATTGAGTTGTCTGTCATAGCTTTTCAATGTGCGAGCACTGTTGTGTCAACTTATAAGAACCATTTGTGAAAATGAATAATGCCAGCAAGACACGGCAAGCATTTGCCAAACTCAACACGGACTGTATGACGTTCTCTAGGTAGCCTATCAAACTTCATGAAAATAGCGATGACTGACGAATGTCCGACGAATTCCGTCTAAGAATCATTAATTTAAGCAAAATGACTGTATCTTTTTAAACGTTGTATTAAATGAAAGGGTGAAATTCCTTGCTAGGCGCTCGAAGCGGCAGACAGTGCTGCGTAGGGATCGACATCCGTGAACAGCGTTTTCAATTTATTCAACAAAGGCGTACCACCTCGGCTTTCAGCGAAGTACATGGTGTAAGCTTCCAAATTGCGGTATTTGGGATCACTTCTCGCGACTCTGGTTGTCTCTAGAAGACCCAAGAGCTCAGATGGGGTAAGCTGGATCTCGATCATCCATTTCCGATAGAGGTGGGTCTGTAAGCTTAGTGCGAGTTTCTCCAAATCTGGGAAGTTCTGCAACGATTGAATTAAAAAATTTAAATTGTGGTACAGTCGAGAAACGCTTAAATAGTGTTTCCAAATCCGTCCGTGTACAGCCCTGTACATCGTAACATATCTGAGCCACTGAATAAAGAGGGGGTTATTCTCTAGCCGACTGATGCTGTTATCGCCAAGTCGCAAGATTTTGAAAACTTCGCCGGGAGTTTCTCGGGACATTAACCATGCCTCATTGATCAGTCTGTGACTAGACGCAGAGCTCAAAATCATGGACGCTTGCATATCGCTAGCCATCTTCGTAATGTCCGGGTATTGTCGAAGTGATTGGAACAGGGACACGAGTTCTTCTTCTGCTCTCGTTTTCATTAGAAGGCTGAGCAGCTCATCCTCACCGAACTTGACTCGTCGTGTCGCCTTGTATCGATTTACGTACTGGAGCCACCGAATGAATTCCTTGTTGCCTTCCAATTTGCCGCCAGTTTTCACAACATGTAAGCGCTGGAATGCATCCATTGGATCCCTCGTATTCTTCAACGCCCACTTGTACGCAAGATCGTCAATCATGGCTATCCCAGAAATTCCAGCTCTTTCGTCCGTATCATGACCGATGTTGTCTTGCTTAGGTGCGTCGTAGGATCTTAAGAACCTTTTTGGGGTCTTAATTGTTGAGATGAAAGGTGGACTGGGGAGAGAATTGGTGCTGGTGTGGCTTCGTTCCTTTAACGTATTGCCACTCCAATTGAGCGAGAGACACTTACAAATTCCAGCTAGGAAAATGATCAGAACGAAGCAGGAGCGCATAGTGGCTTTACAGTTTAAATAAGTGTTGGGCTACAGGAAAGTGAAATTGTGATATTGCTGTGTATCATGGGCCATGACTCAGGGGTCTTTCAAAGTCGTTTTACCGTAAACATAAATGAATCAGGTAGATTTGCCAAACGCCTTTTTTTCTGAATGCAATCAAGTAGAGCTCGGAGAATTTTAATTTTATGTTGAATTGGATGAGCCCTTTGTCTAAGGAAATAGAATCAGGCACTGTATTCACAGAATTTTAACGCTATCAGGATGCCAGATTACGGACCTCGCTTGTAATCATTACGGCATTGATTAGTGCTCAAATGCCAACATCATCAAAAATGTCGTGATGGTGCTGCTGATGTGCAATTCAGATGAGTCTGCATCAAAGCATCTGCTAAGAACCTTTGAGTTTCAAAGTGCAGAGAAATACTCTAGGACGATTTAAATATTTTCACGGATAAAATTCTGAGAATTTAGTCAATAGTTGGCAATTTGAGGAAATTGAATTCTGGTATGGATTTGGCACACGCGCTGAGGGCATTTAGTCAAGGCTCGTGTCTAAGAGAACTTAATTATTTAGAGAACCCTGCCATTGCCAAGCAGTCGTTGGATCGTTTAGTCTACATGCTTGGCTTAAGCCTGGGCCGGACATTGATACTTGTATGTACGCAACAGCCATTTCGCTGGGCGCTATTAATTACTTGCTTAATTCACTTTTGGGCGCCTTGAACACATTAACATTCCGCGTGTACTGGTTTCCGGAGTTGAAACAGGAAAAACACCGCCAAGGCAGGTGGCGAACCTGCCGGAACGGATAGCCTAGTGTGCAATTGCTATTCTGGTTACTGGTAAAATATAGAGTGATAGTCAGGAACAACTTGAGGCTGCCCTTCTTTCTCAAGCACATAGAGGTGCAGTTAGGGGACATACAAGGCCTAAAGTAAATCCACAAGGACAACTGGTTGCTAGCTAAAAGAATCTGTTATATAAATGACGTGAGGAAACGTCACTGACCAAGATGTACCTGGCCCGCCTCGTGTACTCCAGGGTCACTACAGATGGAGGAAGCTTGCTTGATTCACCAATCCACCACACTGTCCCTAAAGCGAAGTTTTCGCCGCTGAAGTACGCCTTCTCTACTAAACACCCAGTCAGCAGTCATATCAAGCACCATGTAGGTACTTCCTGGCCTCCTGTGAGTCTAGTAAACCCGTTCCTCTCCTTCAGTGTCTGCGGGAATGTGCTGCATCCTGCTGGGTTCTGGCTGCGGATTGGTCGGCACCAGCAGTCCGCGAGCACGTACATCGACCGACACGATCTCCATCGGCGGCATCCGTTGGAGATAAGCGCTGCCCTGAAGATAGTCGACTTCTTTGTCGATCCTGTGCTGCTGAGAAGGGGTATCAGTCGTAGACAAATTTGATTGTTGGTTTTCAAACCCAGGTCGGTTTACTAATGTTTGAAGCACTGCATCAGCTGAACCACCTCTGTTAGGCTCTTTAGATTCGACAGCCGAAATACCAGGGGCGACCGGGCGCGTTGAAGGTTGTCCATTGCGGTTGGCTTTGCGACGTCTATTGCATCGTCCAAGCCTTCCAATTGTTGAGGCCTCGGCAGCTTTTGGCTGCTGCAATTCCACACGGGTCACTCGCTGCCAGTGTTCTTGCAAATCTTAGTCAGCGGTGCTAACGTATTGTTGTATCAATGTTGCAACATCACGAGCTAGGTTGGTGAGCGGCTCGTACGAGGCCTCGATTGACTCAAGTCCTTCCACTGCACGAGAATTTCGAAGTCATTAATGCTACTATTCCACCGATTCCTTTGAGCTTATCCACAGCGAGAATAACACCTTGAGACGAAATGTGCTCCAAAAGCTCTTTAGTGACGTTAAAACTGGAATCCGCGTAAAATTTGAGCCTGGACATCCAGCTCTGTACCCGTGATCAGATGTTGAGCGAGGAAAGAGCGTGCATCTGCTCGCACAATACGATAAGGTCCGACTCAAGTTACCAGGCGTTTGCTTCCGCTTTTTCATCAACTCGGGAGAGTAACACATAGTCCTCTTCAGTAAAGTTGACAATATTTCCCCCTCTTTCTCGCTCTTTGTTCAGCAACTGCTGCTTCAAGCGCTGATCCTCTAAATCCTAGTGCATGGTGTGAATACTGTTCCGCAACTTACCGAGATAATCTTCCATTTTGTCACTAACAGGAACCTTCACCAAATCTCCACTGCTGGGCATGTAAAATTCACGAAGAGGAGATGGACACGGCAATCCTGTGAAAAACATCAAAGGAGCGCTGTTCCCAAGTGAAGGTATCGCAGTATGATTGAGGCTTGACTGTACCATGGGCACCAGGTACACTCAATCTTGATGGTTAATCTTGTACTCGAGAATCATCGCTCTGATCACTTGCAGAACGTCTCGATTAACGCGCTCGATAGAGCCGTTTATCCAGGGACAGTAAGCAGGAATAAATTGATGTTGCACCCTAAGTCTTCGAGACAGCTCCTTCACCACTTCATTCTAGAAGTGCGTTCCGTTGTCGCTTACCCAAAGAGATGCTAAGCGCGCGTACCACGCAAGAAGCGCTTCCACAGCAACACTACTGCCGGCTGTGTCTACCACAACCAATCCACAACAATGTGACGCATGATCCTTGAGAGCCAAAAATGTACTCTGAGTCACCGAAACTATTTCCTATGAAGAGAAAATCGAAATGAAGAACACCGTTGCGCTCCGAACACTCGATCGTTTCACTCCAAGTCCTAGTAATAATCCTCCCACTTTCGAATGCAGCCACAGCCGGCACTGCCGAATGAAGGTGGAAACCAAAGAGTTGAGAGAATCGATCAAA

>Contig_46

GTCCCGAGTGCGCATGTCTCCTTCCTCGTCCTCGACATCTTCTCACGTCTAGCCACAGAAACCAAAATGAAAGACAATCGCGTAATTTATCTTGATCGGAAATAGCGCAGAACGGAAGTAAATTTATCTTACAGACATTTCCCGAATTAAACAATCCTGGCCTCCAACGGTTCCCTTTAAAAATTGGTTGTCGTTAATCACAGAAGGAATACGGTACTGTACATGCGCGCCTGTTTTTAATAGGTCACTTTAATTATTTCTCAAAACGATCACAAAAAAAAACAATTGGGTGCCTTTTCGTGTTTTGAGCACTCCCAAGAAAAAGTAGAAAACGAGCTCTTGATTTTTATCTGGAATGGACGCCTCCGGAGACGCGCCCGCTTTAAGCGAGCAGGAGCTGCTGGCATCGCTTCCTCCTCCTCCCTCGGCTCTCCCTGACTACCCAGGAAAAGCCGACGCCACCGACAACGAGCACAGTGCATCGAATGTCACCAATGAAGAGACTCCGGAGCGCACATCCTCTAACTCCAAGCTGCCTGGATCCGCCGCCAAAGCTTCATCCGCTGCGCGTTCCATGGCCTCTAAGCTCGCAAAAAGCGGGCGTAAAGTACTGTCGCCGCTAGGCCACAGTAGTAAGAGCAACACAACAGCCACATCGTCTCCTTCCTCTACATCGACCCAAAGCGAGGCGGGGACGACCTCTCYGAGCTCAATCGACGCCGCCATCGCCGATCCTTTGGGCTCTGTGGCCTCTGCTGCCGGCTCTTTGACCTCAGGCCTCACATCGTCGCTAGCCAGCGCGTCATCGTCCTTCGGATCGCTACTATTCGCGTCCCAAAGCTCCAGTGTAAGCAACGCTGACGACGTGGAAGATCGCTCGAGTAGCGCTGACAAAGCAGCTGAAGAGGCTGCAGCGGTTGCTGCAGAGGCTGCAGCGGTTGCTGCAGAGGCCGCAGCAGTCCTACGACAAGTGACAGCTCAGCAGAGAATGAGGATCCTGGAGGATTTAGTGCAACACCGTCGCTCGGACTGGAACTACCTTAAAGCGATGCATGAAGGCTCCAACTACTGGTTAAATGTGGCTCTACTAAGAGAGCAGCAAGTGATGAATCATGTGGGGTACAAGCAAAGTATTCGACGTGGTGCGCAGTTTTTCTACTTGGGGATCGGATTAGGGCGATTGGTGGGGGAATCGACCCACCCGGAACTACTGGCTATGGACTGCTGCCAATTACTGGAAGAACTGGAATTTTACTTCTCGTCCTCGACGGTGCAAGGAATGGTAAGTAAAAAACCATTTTTTTTTTAATTGTAGTGAGGAGATATTAACGTTATGGTTATATAGAAAATGATGGTGGCGACGTCAAGCACGCTACACGAGCCTCTGGACACGGAGAATAGTCCGCAATACTCAGCCGATGAGCCATTCCGGCCCACTATGCACAAGTGGAACCAGCGGCCAGTTTATCGACGACTATTGACGCCTCCGATTGTAGGTTTTTTTTTATTGGAATATTTATTTATGAGTATTAATATTCTGCGTGTTTTATAGCCTTTTCCACTGGACTACCGAGAGATTTTGCTGTCTTTGTGCGATATTCTAGCGCTCATTTACAGCAAACTCGTCGAGGATAACAGTGCGTCTGAGAACTTAAACCTCTTCCAGTCGATCATTCGCTTCGATGATCGCATTAAGGTACTTTCTAAGAGTCGACGGCGTTCAACGGTTCCCGTTTGCTAAGATTTTTGTTGTTGTTGTTTTTTGTGTGCTGCTGACTTAGAAACTTTTCATTGACCCTGTGAAGAAAGAGTTTTCAGCCGTAGCGTCTCAAGTAATGGCAGAGGAGATGCGACTCGTTCGCAAGGCCTACGCGTGTGGAAGTCGGGACGGAGAACCTCTAGCAGCGAGTCCGACGGAAAGTATTGAAACCAATGCCGCCACTGTGGATGGGAATTAGGTACTTTAATAATATAGTCGGTTGGGATTGATACATGTAATGAAGTGCCTCGCTCGACCATGTATCGGGGAGCCGCATTTTATTAGTACAGTATTATTGAGAGGAATCAGTATTAGGAGCTGGACCCGATTTACCAGGTCGTTGTTGCCGTTCCTTAACCACAAAGTGGCTCAATTTTTGCCGGTTTGCTCAAGTGGTGTGGCATCAGCTACCAACGGCGATGGCATCTATCAAATTTCTTCACCCAACACTACACACCTTCGTCCACTGAGGGGTACAATGCGCTTGATCACATGCAACAATTAGAAACACCCTGGAGCAAGAACAGCTACTCAAAGTTAGATTCAGAGCTCACAAAGACTGGTTGGTAGGAAAAAAAGGTAAGACAAAACGTCACTGTTGCATTTACATGCAATGCGACGTTTGCCTCTGTGAATTTCCGACCAAATTTGCCGTTTTGGTAATATACGTATTTCGTACTTAATGATCTCTTTTAGGTAAGTCAGATTTGCGACTTTCTAAATGATCGTGTGAGATTAGTCACGCGATCAGTGATGATCAGGATTTCGACATTATTAATATAGAAGTGTTAGCGAATGCGAATCTCCTTATTATTAGCTGCCATTTTAATAATTGGTGCACCTCGGTCATTTGGCGATTCATCAAGAGCAAGTTGCTCTTAACAACCATGGGCATCGCGGGTTTAATGCTCGTTACTGTTGTAGCCTTGTTAGCCGGAGCACTTGCTCAAGAACATACTACTCAAGCGTACTCGGTGAAAAGAACTTCCATTGTCTCCAAGTCTCACATCAGCATAGCGACGGAAAGACAGTTAAGATCCTTCCAAACATCGACTTTACGTGAAGATGGCGAGGACAGAGTCAATGTACCCTGGTTATCAAAGGTCGACGATCTGATACAATCCATGTTCAAGTCCAGTTTGTCACCTGATAAAGTGCAAATCGGAACGTGGGTGCAGTCCAAGGTGCACCCTAAAGACCTGTTTAGCACCTTACGTCTTGGAGAGAGCGCAGCCAAGCTTGACGACAATCCGAATCTTCTTCAATGGTTCAGATTCGTAGCGCCTTACCAAACTAAACATGGCGAGAAGGCGATTTTGAATTTGGATGTCTACTANCTGTTACTAGAGACTCACAGCAACCAGGAACTGATGACACTACTGAAATCTCTAAAGAAAACTCCAGGGCTGAGTAAGTTTGCGTCGAATATCCAGGAGTCACTATCTGGAGCGTGGGTGTCCAAAACACTACTGAAAGAAACAAGTTCAAAGACAGCCTTCGAAGTGCTACGCCTAAAAGAAGCTGGCTCGAAGTTGGATGACACGCCCGTTTTCCACCAATGGTTGAAGTACGTGGAGGCGTACAGAGCGAAGAGAGGGACGGTTATGTTCGGAGACATTGATATGTTCTCGTTATTGCAAAAGACAATGCCGGAACGTGAATTGACAACCCTTTTCTATTCACTTCGGAAGGTCCCGAACATGAAGAATAACGCTGAAACCATGCAGAGATTTATGTTTTGGAAATTTAAAACCAGTCGCAAAACTATGAATGACGTTTGGCTAAAGTTTCGAGAGCCACCCGAGGAAATGTTCAAGGTCTTACGTGTGGCGGAAAGTCGGGCCAGAGACGACAGTGACATGATTCAGTGGCTCAGGTATACCCAACTGTTTCGTAACCGCATTAAGAAGAGTGCGTTTTCGGATGAGCAAACCTTGCAATTCTTGACGAAGTCCGACCCATTGAAATCAGATTGAATATTTGCAACACAGTTTCAATCTCTTAAGGAAGTTCCAGATTTGAAGAAACAAGCGGAAAACATGCAGTCTTCCCTATTCCAGAATTGGATAAGTGCGAAGATAAATCCAGATGAGGCTTCAAAATGGCTGGCAAGTCCTTTCAACGAAAAAGTTTTGAACCTGCCGAAGGATGATCCCAGATACGGCACTTGGGAGAGTTATATGCTTTTCTATGCTGAAAACAAGGGCGGACAACCTTTGCTTCAGAAAGTGAGAGCATATTTCAAAATCGATGACCCTGTCGATGCTCTGGCAGCAGTTATGAAGTCTAGATGAACACTAGATTTGATGGCGTTTCATTCAACGAAATAAAAACGGGGTGCCTTTGAAAACACGGTACATTTAAATTTGGATTTCGTGATTTTAAAGCATTTTTTAATTATTATTATTTCAGCATGATTTCGCACAGCGCGGCACTCGCGATTAAATGCTCTTTTTAAGGAGAGTACAATCGATGGCTGGCTGGTAAGCTCGGTTTTGAACCCGCCTACACAAAATCTCGCGCGGGTTTGCCACACTCCCTCCCGGTCGCCAGAAATAAAACTACATAATTAATTCGATACTTGTTTTTGGACAGTTTACCGGTATTGAGACAACGTTAATCTTCCTAAGCGATTTGGGCATTATTTGGACAAGTTGATGACGCTATTTGAAAATGAACTAATCAACTCGTTGGAACGTTGATGTGCCGCTGCTTTAAACCGTCACGATGAAGGTGCTAGACGTACCTTCATACGCCGCCAATGCACCAAGCAGGTTTTTCGAATACCCGCGGTGCACGAAGCGATACGTGCCTGCCACACTAGTGCGTCCACCTTTGCGGATGTTCCACTCACAAGTGTTCTTGCTCTCAGCAATGAGATAGCGCTCCCAGCGGTAGCGCAAATCCCAATGCGCATCGGTCATGACGGTTGTATACGCGCCATTCGATCCAAGTTTCTGCACGTCACAGAACGATGAGACCAGCGTGAGTGCGTTCTTCGGGTGCGCGCCGGCGAATACCGCTGAAGCGACACTCCCCACGGAGTACGAACTCGACGGTTGTGTGCGTACATCACTAAAGGAGCGCAGAAGCGGAGCTGAGTCCATCACCACACCAGTCTGCAACGTGATGAGCGAGTTGCGGTTGATCTGCAACGGTGTGGGACCCACGTCCAGCGGTACGGACGAATCAGCGACGGACGTGGCGACTCTAGTGAGCTCCTGTTGCACTGCAGCCAGCTGGTTGGGACCGAAAAGCGTCGACGCGCCTTCGTAGTGCTGCGTACGGTATTCCTCTTTGGTGGTCATATACTGCGCGTAAGCATTGCTGATGGCAGCCAACTCAACCTCCGTGACTCCAGCACCTGTAAGTGCCTTCTTGACTGTGCTTCGGATACGTCTTCCGGTAGTCTCAAACGTGGTGACTGCGATAGCGAATTGGCCGATCTTAGCGATCTGTACGGGTAGAATGTTGAGTACCCACGGCACAGGATCCATGAGTCCGACTGCCAGTAGTGGCACTTTGTTGACGTTCTGGCAGTCCTTCACCCATTGTGGTGCCACCACCGAGGGTCCGGAAGAGTGCGTTGGCCTTACGGTTTCCCTCTGTGAACATACTGAGCACTCGACCGTCTTCTCTGCCAGCAGCAAAGTTCTGTCCGATGACCGCAGGACACGTTCTATTAGCGTACGGGTCTGCTGTGTTTGGTGTGGCGCCGGAGAGTTTTACATTAGAGAAGTCGACGTAAGAAAGATTAGCGACGGTGGAGCCATGGACGAGCTCAGAGGTTCCTTTGATCAGCGATGACAGCGTATCGTACTGCCGTTTGCCCATAATCTCGGCAGACTCGATCGTTGTATTGCCCTCGCCACTAAAGGTCCCGTCTCCATTATCGATGAGGTTCGGACTCACGTCACCAGCGTTAGTAATGCCGATCCCAACGATGACGTCGTCTAGTTCATCTTCAAGAAGGAGCTCGGCGTATCCCTTGTTGTCTCCACTGATGAGAAGGTTTTGTACACTAAGACTGGTAGGATGGACGGGGTAGAACGCCAGCACACCTCGAAGCTTACCGGAACTACTGAAGAAATGTAGTGCCCGCATTGTCGAGTCGACGTCACTGCTGTATTGTGCACGTTCAGACGACGGGTTGGCCAGGTAGGCCTCGGGAGAGCGGTTCTTGACTCCTTTAGAGACTTCTCCCTTGTTCCATCGGATGGTACCGGACTGGATGGAGTTGTGTGCAGCGTCAATGGCGCTCACGATACCACTGACGATCTTGTCGAAGTTTTCACTGACGTATCCAAAGCTGGAGACGTCGTAGAGGAAATATCTTGCGGTCCCTCCTGGCCCCGCGTGGGTGTGCGTAGCATGTAGAATGACGTTCTGTTCGGTGTAGAGTCCCTTGTATTTGGTAGCGAGCTGAGCTAGAACTTCTTGATGTACTAGTTGCATGACGGAGTGCAAGTCGCAGTGGACAAACATGACACGCTTCCTGGTAATAGCGTCTTGGATGAGGAATGCTCGGGCGTACAGACAATTAAGGATCCCAGCGGAGCTTTCGTCGGAGCTTGCGAACCCCATCATTACCACTTGGGCTGCAGGTCCAGTGATGTCGCTCTTGCCCACACCGATGTTATACGTCGCTGTAGCGTGTCCGGGGAGCTCGATAAGCAACAGGATAAACAGCAACGACTGGAGAAGTCGGAAGGACGACATCGTAGTCTTGCCTTGATGAGTCGATGAAGCGGTTTGAGCACCAGGCGGCACGTTTGAAAATGGCCAAGGCTTTAGTTGCCATCCGGTATCGCTTCTTGATGAGCTGTTCCGGTGGAGAGAGGGCGCATTGCAGTATCGCTTGTTAAACCGTCCTAGTATACCCCAACATGACTCATCGAGTGGCTCATACTCATCATGTTGAAGTGGACTGATCATAGTTCACCATCATGGCTCCGTTGAAAGACATTTCTTTTTTTGCTTTGCACGCTTACAGTTGGACCGAAATGATTAGTCCGACGTAAATGCAACGTAGCACGTTGCATCATCGTTCGGAAAGGTACTCATAACCTCCACTTCAATGTACTCATCCGTCGCCCTGCTCAGATTACTCCTTATAACGATAATAATTTGTCTTTACCGGTTCTCCACAGTTAACTTTTTCCTTGTTCGACGTCGCTGGATAGCTTGTGAGATCCGAAGCATCTGAACACCTTTCAGCCAACGTGTTCGAGACCGTGTGCGCGATGCTAACTCGTAAAAGTACTGAACTTTGCCGATCAACAGCAGCCTCTCTCCTTCATCATCGTCATCATTTAGTTCACTAGCGCCAACCGCACTTAAGCGACGTTGACGTTGACGACGAAGTGCAGCAGAGAGAAACCACATGAGCTTGCAATCCCAGAGGAAAATAGTCCAGAGACGCGAGACCAAGGCTGACAAAGTGCTCACTTCGACACGACGACCGAAGATAACTCGATCCACAACTGCAATTTTGGGTACAATCCAGCGGTCCAAAGCGAAGAGCTCCAGCGACAATATAACCAGTCCCATAAGCTCGAAAGTCAGCACGGATAGCATAAGAACAGAGCGTCGAAAAGCCAATAAACGCATCGTACTCGGAGGTAAAGCGTCCAGCGTAATGGCCCACTGCATCCAAAGCCACGCGCCCCACACCCACACAACACGACGATCCCAAGTCAAAAATTGACTCAAAGCTAACGACGCTACTGTGAGCTTCGTGGACAAAAAGAGGAAGTTAAACGACGAGTATAATCTTTTGAGTAGTTGCCGTTGATATGAAGCAAAGAATACGCCACAAAAGACCAGCGAAGTGACGAACGCTACGCCTTGAGTACGTGCTAACCCTGGTCGTATATGATCCAGATTCTGGTCTTTAGAAATCCATGCAACCAGAGCACCCAGCACCAAAACACCCATCCCAATGTAGCCTAAAGCATGAAAGATGTGTAGCAGTAGACAGCGACGACTGTGTGAGGCAAATGATCCTAAAAATTGGTCCAGAAACCCACCAAATCCTGGTACAAGTGTTTTAAAAGCATTGAAATCTGCTCCAACTGGTACAAATTGCAATTGGATGGTCTGGGCACGGTGTCGCTCTTGTACTCTGGGTCCAAATAAAGTCGGGTGGCGACAGGTGTACGATAACTTTCCAAAGGCTGGATACTTAGCACACTCCCTGCCCGAGAACGCGTCGATGGACCACTGGTACTGGGTATGGAGCTTGTACTCCTCGACCGCATGATGGTGTTCCGGATACCACTACTTTTGGGTTGTAGTCCGACCGTGCAGTAGTAGCTGACGCAGCGTCTTCGACTAGGGATATAGCTGGTCTCGGCATTGACATGACGTACTGTGGCCGAGTCTTGTTCACACATTGTGACATAATTACGATAGACTAGTCTCCCAAAGAGTAGTACCATGTTAAGTTGGGTATTCATTAGGGTATCCCGAATAGAAAACCCGTAT

>Contig_47

AATGAGCCGAGAAATCATCCCAAAGCAACAGTACCGGCTGTGCCTCATCTCGATTTGCGAAATGGTAGTCCAGAAAAGCGACGGATAAGCCTGAGTTCCACCACGCAGAGCTGTTGCCATAGATTTGCAGAGCAGCATCACTCTGAATTGTTTGCATTGTTTTCCACAAGGAGTCTCCAAAGCCATGTTGGGCCACGTAGTTGTAGGCCGCTTTGTCGGGGTTGCGCGAGGGCTCCGTCTTAACCACGAAAAAAGGGTCATACTGTTTTCCAGTCGAATCTGCCAGAAACATGCCTGTCAGGCGTTCCTTGTCCTTTCCACCACATCTGACCCACACTGTATTGACACCTTTTCTGTCCACCGTCTGCTTGGGCAGATACTCGAAGCAAATGCCGCTTTGGTCTGCATTGTAGAATTTATCTACACCAAGATCATTTTTTGCTTGCTCTACCTCTTGCCAAAAGTTGTTGGCAGCGTCTTCCATAGCAGCAGGGGTTTTCTGGCCTTGACGCGTTTTTGTTCTCAAAGACAAGCCATGACGAGACAGAAACCCCTGCTGCCAGCACCAACTCCCAGTGAAAACACCATCTGCTACGCCTTCGTCACGGGCAACTTCCTGAGCTTGAAGTTGAAGCATTAGACGGGACACAGGGATACCCTCACCACGTAGCGAATTAATCCACTCAACAATGCTTTCTTCTCCAGACTTGCTGATTGTTGTTGCTGTTCCTGGAGCTCTGTAAGACCTCAAATTTGCCGTAGCGGTGGAGGCAACCATGGCTTCAATGTGTGTACGCTGCTTCGACCACTCGTTGATCCGCTTGCGCTTTGATATATATACGCGTTTTGCTGGGATGAGTTGGCAGTAAAATCTATCGAGAGTAGCATCCATGTCATCATTGTTGTCACGAAGGAAATTTATTACGTCCAGCTTTTTAGTGTAGCTCTCGCACTCGCGTTTGTAGCCGTTTGGCTTTCGCCCAGCGCCACCAGGAGCGCGGCTCCGCCCTCTTGCCATGCTGGCACTTGCAGGCTCTGAATGTGAAAGGCTGGTTGTCTGCTTGCTAGTAGTTTCACCGCGGCTTTATCCTGTGCATAAATAGTAGAGTGGGTAAAAAAAGGTAGATCCGCATGGACAAATGCTCCATAGTAAGGTTTTTTCAAATAGCTACATCATTATGATTGGCTAATTCTGGCGTCCGGATGGACAAAAGGTCCGGATGCACAAATGTTTTACAGTATTACAGTACAGAATGATACAAGTTATTTTTAGCCTTTTAATAAATTAATTTATTAAGAGTACGCCATGGAACAAATCCCTCGATCTGGTCCGATACTTAAAAACAATCAGCAATTTGTTTAATCAGGTGTAAGAAGTTTATTTTCAAATTAGGCATGAATACTTTATGCGAGTGGCAACAAACTTTTCCACTATACATTACATAGTGAAAAAGTTTAATATACACCTCTAGGTCCGACAAACACTTGAATATGGTATAGTCGTGACACACAATTTATCCTACAGCCAAATATACTTTTAAGCTGCAGAGGAGGCTTTTACGAGGACTTACGAGGTGTACAGTAGATGCAGATACGATAGAGTCTATCATAGACTGGTGAAGCCTCCCAATCACAAAATGATCCTAATACCCTAGGACGCAATTCGACGGTGTTACAGCCACACTTTTGTAACATTTGCTGCGTAGAGCTGCCTTGTCCACAAGACAAAGTACGTGCTTATTTTAATTAAAAGGCACTCGTTATTTCGCCCGACTGAGGTAATATAGCATCGACTTTTATACGTGTACAGATAGTATAGTACGGTTAAATTGGAGTAAAGGTAGGACACTAGCGTGGTCATTATATCCAATCAAAACTATGGCGCATTTTTCCGTAGCATCTCTTAAAAGTACACTATGGAGGATACTGGTCAGTGCTGGCGTATTTTTCCGTGGCAAGCGACGCCAAGTAAGCGTGGCGTATCAGGACNGGGGGGTACTGGCTGTCTAGTTATGAGGGAAGCAAAAATGGCGGTTCGCTACTCGCTTTTTATATCCAGGTGTATCTCCGTGAATTGGGCATCACTCGTTAACTTTCGAAATGGGTAATTAATCAAGTCCAATGGGTCAATTCTTTCTTCTTGAAATAAAGGACTGTAGTGCAGTGCATGTATTTTTGCTCTGATTATCTCCGTCAATTGGAGTAAACTGGAGCGCATTCCGCAATTCTTGAACCAGCCCTCAGTAGCCACCTGTTGATATTAATAGAGATGCGTTTCTTCCCAGCCGTACTCCTGGCAACTGGGGGTGTTCTCCTGACAAATGCTACGTGTGCGTCCTTCGACTCGGAGTTTGAGCTAGCCACCGCCGACCATCCGACTGTGTTCCGTTCCCTCGCCTACCACCATAACAGCGTCGCTTCTAAACGGCTGTTGCGACGTTACGATGTTGACAACGAAGGGAGAACCGTCGGGGGCGGCGCCAAGATCAAGAGCCTTTGGTTGAAGGTCCGTGCGTATTTACTAAATCGTAAGAAAGATGAGGCTGACATGGCAGCGAAATTACAACTCGGCGGTATTGACCACGCCTTGTCAAGCTCAAAACTGGAGCAATTGACCAAGGAAGTCCAAGTGTTTAACAAAAAGTCCATGGCCAAAGTCACGGTGATTGGGACGCTCAGGACCCTCTACGGAGATATCGATCTGGCAAAGGGGCTCAAGGCTGCCGAAAGGGAGGCGTCGCCCACACTTCTAGAACAACTCAAAGCGTTGAGGCAGGACCTACAGTCGAAGTGGCTGAACCGCGGCATCTCCGCGGACGTTATTTTCAAGCAACTGGGGATTCGCGAAGAAAAGTATCAAATGTTTTTCAGTGGGAAACTGGACATTCTGGAAGCCTATATCAAACTCATCAACCAGAACAAAAAGAAGGGCGATCCGGTATCTCTGGTGAGCATCTTGAGTAAGGGATTTGGCGGGGAGGACAAGCTGGTGGCGCTCGTGACTTCTGCAAAAAAAAGTAGCATGACGGGGAAAAAAGCCGACGAATTGGAGACTGCTTTACTCAATAAGTGGCTGAGAGAAGATACCTTACCGAAAGACGTCTTTGTATGGCTGAAGCTTTCCGACGACGTGGACGACGCTTTCTCCCCTCAAAACTTGAACAAGTTCGCAGCGTACATCGACAATTTTAACACGAGGAGGCCCAATCATCAACAGTCAGCGATTGCTATCTACACGAGCAGTTTTGGAGACGCTGCTGTCGTAAACAAGCTCATCTCGGCAGTGGACGACGGGGCGACAAGAAGTATCGCGAACAAGCTACAGGAGGCGCAGTTTGAGAGCTGGGTCAGCCGCAGATTGGGTTTTGGCCAAGTCGAAACGATACTGAAGATTGACAGCTCTGGCGATGCAGTAGTTACTCGCCGAAAGCTAGGCTTGTTGGTCAAGTATATTACGCAAATGATGGATGGAGATGAACGTTTAATCAGGACGTTGACGGAGCAGCTTGGAGGGAGAGACAAACTGGCGTTGGTGTTGGAGAAAGCAAGTGAGTCCACGGCCGCCTCTGCGCTGCAGAAGAAGCAATTTGCGTCATTGAAAGACGAACGTATTACACCGGAAGTCATCGTCTCCTTTTTATTTAAAAAGGCTCAAACAACTACGACAGCCGAGAAGGCGATTGTGGCCAAGTTCAATCTGTTCTTCATTGAGACAAGTGGGTGATGGAGCGAAGGCGTTCAGGATAGAGTATCTTACTGGGTCATAGGAAGTATACAGGACTACGTAAATTTCACGCATACTTAAGCACACGGAAATGTAGTGTTCATGTAGCACTGCTACCTTTGTTATTGGTTCTCTTTTACTTCAAGTATAAGACTTACTATTAATCGTTTTAATACATGTACCTTTAAAACCCATTCACATGTAAAATAACCAAAACCCAGTAGAGGATCGTTGCATTTTTCTATGAGCTGTCTGTTGTAGCTCTCCACACTATGAATCTCGTGGGGCCACCAACGGCAAAGGCTCTTTTTGTAGCGGTCAGCATCGTAAGGCGACAGGATATATCGCTCGAGTGTCCAGACAAGCTTTATTTCGAGCCTGGCAGGAGCGATGCAATGCTTGAGCGATTCCATTGAATACCTTTTCCAGCTAAGGGGTTTGCAGCAAATGCGTCATTTGGTTATACGGTGTCGTACAGAGCAGTAACATGTGCTTCGTTTTAAACATCCCAGTTTGTACATAACGAAGTTGTGTTTTACGTAGACGTAAACACTGGAAAATGTTTTGGTCAGTGCTGGCAATCGCTACTCGTGATTGTTCAGCACGTTTGTTGACGCGTCCAAATGCTTATGCCAAATGCCAGCATCACGCAGCGGAGAAGGAAGAAGTCTCACGCCCTGCAGCTAAATCAGCGCGGGTGGCGGATCATCTCACTCTTCAAAACATTCTCAGCTTCACAAGCTCACCAACANCCCCCCCCCTCACCCTCACTTCAACCATGGCCTCCACCAAGAAGACAGCTCTCGTCACCGGCAGCACGCGCGGTATTTTGTCGAGCACTACGTCAAGGCTGGCTGGAACGTCATTGGCACGGCTCGCGCCAATAGCAACACGGAAAAGGTACAAAAAAATGTTGCTAAGAGACGTTGGGGTTATCAGTACTAGTTGCTCATCTTTACTGTCATTGTGCAGCTGAAGTCGCTTGCCCCGTTCAAGATCATTGCAATGGATACGAGCGACGAGGTCTCCATCCTCGAGGCAGCTCGTCAGCTGGAGGGGCAGCCCATCGATCTGCTCATCAACAATTCCGGTATTGGTATCCCAAGCGAGTTCGATACTGGCACCAAGGACGCCCTTATGCGCCAATTCGAGGTGAATGCCGTCGGGCCATTCCTCGTGACCAGATCTTTGCTACCCAACCTGGAGCTGGCAGCAAAGGACAATGGTAGTGCCTTCGTTGTGCAGCTCTCGTCATTCCTCGGCAGCATCGGCAGCTACACAAATGACACTGTCGATTTCTCCAAGCAGGCTGGCTACGGCTACTCGTCCTCCAAGACTGCGCTCAACATGATCACGCGAGGGCTTGCGTTCGACCTGCGCTCAAGTGGCGTCGTCGTCGTGTCGGTGCATCCAGGATACGTGGACACGGACATGACCCAGGGCAAGGCGACGCTGAAGCCAGCGGATAGTGTGGCGGCCATGACCGGCCTCATCGCCAAGCTTGGCTCTCAAAGTACGGGCAAGTTCTTCAACGTGGACCCGCAGATCCCCGTGGTGGAGCTGCCGTGGTAACTTGGTATGGGTTACTGCGTGAGACAGAAAAATACGAGTACGGTAGCTGCTAGGACGTTGGAGTTCTGGCTAGGTTACATTCGTGCGTCTCACCCGAAACAGGCTTAAAATTCATTAGTATTTCTTTCAACCATGTTTTAATTTACAACAGTGAAAGAGCCATGATCAAGGAGTCCTAGATCAAAGCAGTCGTCACACCTAGACTAGGACAGCGATTTGATAAAAAGTTGCTCATCAACTTCCAGAAGGATTATTACTGTGGGATAAGTTCTATTGCTGTGACGGGGTCACACAGCACCTTGCACAGAGAAGTGCTTAAGCGTATTAACTACCCGAGTGAACGTTAGTCCACGTGTTCCCGGCCTCAAAGGGAACGGTTTGCAGAGTAGATTTAAGACTGATGTATTTATAAGTAAGATAAGCTAGGAAGAGAATGCATTTGTACGAAGAGGCTTCCCTTGTATCCCTTCCAGTTGTTACGAAAAAGATGAGGAGGGCGCCTCGCGGGCTTGACATGCCTGCTGGCACGGTAGGGCAGGAGCTTGCTGCAGTGAAGGTTTTCAATGAATTCCTGGTATCAACAGATATAAGCAGTGACGCCCTAGACAAACTGCACTCCTCGTTATCCAATGCAACTAACATGCCGATGCAAAACTTCTACTCTTTACTAACAGCCTTTGGAATCTTTTTACAGACAAAGAAAAGTGGAAAAGCTCGTGCTGCTGATGAATTCCTAGCAAAGGCAACAGCCTTGGGCTACTTCTCTCAAATTATGAACCTATTACGCGAACGATACAGTGGATCTCTCTCTCGGATGCCAAGCGAGTTGCTAGAATTTAAGACCAAATGGCTAGTGCTATCGAAGGACGGAATCTCCGTTCCAATGTGCAGAGCAATGGTTTTCCGGGCTGTACTTTATCGGATCTCTGTGTGTTGGTGGAACATCTTATTGTTCATGCTGATGCGACCACGAGAATCAAGTGTGTTCATGAAGCAGCTATGTTAGCTATGATGTGGCATACATTTGAACGAGCCATCGATACGTGTTTCGCCCGGAAGCAACAAATTTCGATCTCTGCATCTGGAGAGCTGTTTTCGCATATCGCTCGCTTGAAGACATCAGTTGTCCAAGGTGTATCTATTTACAAGTCCGCAGAGAGATGACAACAGTGCATGTTGCATGCGTTTGGTATGCTATTTATCTGCTACGATGATCCATCCGAATACCTCTTCCCACTAGTGCCCCGTTGCGCGGTGTCGGGCCTTCCAGGAGGCCACACGTACACACAAGAAGAAGCTGTGATCTTTTGGGAGAGCTTGCATGACAACATTGAAAAGGAGACCCAGCCGCACCTAAACGGGAGAGAAAGCGGCCAAACATAGCGATTCATATTACCAAAGTCATCCGCGATTACATCCGAAACATGCCTCCCAATGTGCACCAGACCGTGACGCCGAACATGAGCAGCCACTCGCTGCGTCGAGGTGCCGCTGCGTACGCAAATGCGTCCCCGAAGTTGAAAATCCAGTGGATATCGACTAGAGATGCTTGGTTGTTGGTGTCACTTACGAAGGTCTTTGCCTACATAGGAACAATCACGCGTGAGGATCAAAGTGTTGCAAAAGTACTGGCAGGATACGAGGCTCCAGACTTACCTTTCTCAACCCCCACTGTTAGTGATCTGCAGCAGTGCCTTTCGACAGCGGAGTTTGGGCAGCTCGTGACGCTGTGCAATGGATTATTGCGGCACGTGCTGGGCTCGCTTGACCCCCGACTGAATGTCGCTAGCGATGTCGTGAATG

>Contig_48

TCCCACCAATTTAATAGAATTTCGTTTTTGTATGAAAATGCTATAATGATCCCATTGGCTGATGCTGTTTAGTGCTTCTTCCGATGATTTGATGACATTTTCTTCTGATTTGATACATATCGCCTTAATAAGCAGTAAAACCCCATCCAATCGAGCGCCTATTAGCGAGTGTTACATACGTCGATAGTAATTACGCTGATGTGTATACATGTACAAATCGAAATTAGCATTGGCAGAGACTATTGACACAAGAGAGTGTATATTTCCAAAAGTGGAGGACTTGGCGTGAATTCTAGCACCCTCCTAAGCCCAAACCAAGGTCTTCTTGGGCATTCAACCCTATAGGGCCGAGCGAGGCTTAGCCATGCCTGGTCCTGGGTTTAAGCCCGCGTGCGTCGCACACGGATCTCCACAGGCATGGATTCGTGTTGCCGAGCGAGTTGGCTTTTACCCCTCCTCCCTATTTCGTACTTTCTGGGGAGAACCCTACGTGGCAGACCTAGCACCATGGAGGCGTCTTCGTTTGGGTCTAGAAAAAGAAGCTAGCACTCTCATCACGACAGGCGTCGAGCCGGCACGAGGAAACATCGCTCAGATAGCGCCTAGAGACGACGGAGGTTTGGTAAGGGGCCCTATAATTCCTAGAGCACCGCTACATTGGCTGACATGCTGATGGCGATTGTACATTTCAGGACAAGGTTGACAACAGGGTGGAACGCATTGAACCTAATGTCGCTCCATTTGACGGCACATATGTAGTTTGCTGTGCCGAATATTCCTATGGATATGGAGATCGTTATGGATGACGGACACCAGGTAGCTTTGTTTTGGCAGCGATTGAAGCGGCAGTCTTCGTGTAACAACGCTGAAGCTGCAGCGGTAGACGCATCANCTCTCTCTTTCTTCGATCGCTTCTGCAGCCGCACAATCAATTTCCTGCCTTGGATTCATACGATGTCGTTATTTAATTTGCTTACAGCCTGTTCTAGACATCGTTCGATACCTGCGTGAGACGTCTCAAGAGCTCAAAAACCGTAGCCTTGTGGTGAATGCTCGATTTAATGATGTTGGTATACAATGGGACTGTTCAGCTAGTGCTTGAAACCTGGAGCCCAAAACAAATTGCGACATTTTTCCAGGTTTCGGGGAAAACGTTTTCAATACCACCTTTTTTTCACTGAACCTGGTCACAAATGGCACAAAACGTTCCACGAGAAACCCCACAAATGCAGCAGGAGACGTGCAAAAGATGCTGCGGCCGTCACGGAATGAAGGTGCTGAGACATTAGATCAATAATCGCTGACTATGCTCAAGCCTACATCCAGCGTGGATTGGTCACAAAATACCACTTGGTGTCGCTACGGGGGTATGTTAACTAAGGGCTTAACACAGCTGATGAGACGGCCCTAACGTCAAGAAATTCATATCCACGACAGGTTGTCTTACACCAGCATGAACACAGCGTCCGCAGGTAGCTACGCGTCGGTAGTACAGTGCTTCTAGAAGAGACCGACTGCTTTTTTCAGTTAGCTAGCCGCTGAAAACGTGTGGATGGACAGAGAATAAGTGGTCTGCCTCATAAAGACGACTAGCAATATGAAAGTGGTCTGAACCCAGCTATTAGAGACACTCCCAAAGATCGCGAAGGTAGAAACCGGAGCTTTGATCGTTTTGCTCAGGCTCCTGATCCCAGCTTCTGCTTAACAGGGCCATGACGCTTACACTTTTTACCAAGCGATCTTCCAAAATACTTAATGCAGAACGAGCAGCCAAAACCAGACATGTATTTGGCAGAGTATTTTAATGCGGTACTCGCCTATGTTGAACGGAACGACGAGCTGTTTTACAGCGTACAAGGCTGTCGTAAAGATGTACATGTCTTCAAACCGCAGCAACATTATGAGTATTTCAAAGGAAATGAAGATTCAGGATCATTGCTTGTCGTCCCTTCTTCATGCAACCAACAACATTTTCGCGGAGGTGGACGTCGGAGTTGTGTCGAAAGTGCACCCACCACACTTTTGTAGACGCTATCTTGATGAAGGACATGGGTAGAATGTTGCCAAATGTACCAATAAATGGGTATAGTGGTTGAATTGAATGGACTTAGAGTAGAATAAATGGTCCAAGTCGGACATGTGCACATGTAGCGGACTATGATCGCTTAAAATTATACACCTCGGAAGTAAAACGTGCAGAAGGTAGCACTTCCTCGTGCTGCGCGGGAAACGATGAAGTGCAAATGAAACAGCCTTTGCAACAACATGATTTAATATGATGCTACATAAAAGTCGTCGATTTTTTCATCTGGACATTTAAAAAAAAAAGCTGCCAAAATATTGGACTTCGCTCTAGCATGATATTCAGCTGGTTTATGCACTTACGCAGGTCTAAATCAGTGGATATAAATCAGTATTATGGGCTACTGCAATACTACTATAAAACACAAGCGGATCCGCCCGAATAAAGCACCCTTCCGAATAAAGCCTATTTCTCTAAATATCGACAAGCTTTAAGATAAACGTGATCAGATTTTTTAATTGTCTGACACTTGGTTCCTCAGTTTTTTTTACTACTTCCATTACAACTACAATACTACTACTATTTATCACAAAGTAACAGCAGTAATGAGCCTGGGAAATAGTCAGAGGAACTGAGTCGTAAAATAGGAAAAAAAATCCCAATGCTTTTAAAACATAATAATCAGTCGATCAGGCTCCTCAAAGCCCAAAAAGAAGTACAGCTGAGTACTCCCAAGTGTCACTAGATAATAGAATCGAATGGAATGACTTGAAGATGATAATTTTCCACAAAACCATTTTTGTGGTGACAAATCTTCGAGATTTAATAATTAAGTAAGCATAATTTTAACTATTCTCCGGTGTCGTGTCGAGGCCATCATTAGTCACAGAGGGCGGCTAAATATGACTTGCAAAAGCAAAATTATGTTATAGTATGGTAACGAGTGAGCTATCTCGTTCCTTTGCGCCATTCGGTTCCTCGTGAATTAGAGCAAAAGAAAGCTTTTTGTCATAATTTATAGGCGATACAGTACATTTCGATCAATAGCGCGAGTCAACGTTCCGATCTTGTAACACACCTCGTAATAGCTTCTTGCTGTAGGCAACCTGATGGGGATATCTGATTGGCAATGGATGGCGTAGAAAAATCGGAGTTGCAAACTACGGCTCAAAAACAGCAGCTACATGTATATCATTATTTTCTGTCAAAACTATTGACTATAGCAGAGGACACTACGCACGATCACATACCAGTTTTTCAGCATTATAATATCTTGATCGCGTTTTCAAAGAGAGTGGTGAAAAGAAGCACTGAACAGGTGTAAACTACAGAAGGAACTCGTGTAGCCAAGCAGAAAAGACTCGATCTGTGTGAATGCTCTTGGGGTGTGCACGTCTCTAGGATAATCTTGCACAAAAGGTACGATTAATTTGAACAGCTGGTTTCTGGTTTTTTCATGATACTGTAGATGCTCTAGTAACCGTTCTGTCGTGCTAAGCAAAACCAAAACTGGTTCAGACGGCTGCTTCTTCACAATAACTACTGTGAATGACTATGTGCTTTATTATTTATAACAGCGCGCGACTTCAGTGTGTAATGAAACATGTTTCTTATTGATACGTGTACTTCAAAATTTAACTATTTTGAAAATGGTTTGCATCTTCATAACCACTATTTAGTAGTTTTACGATGCCATCCGGTTGCAAATTGCAAATTGCAGTCTGCCGGCTGTGACAACAACTCCTCGAAAGGTTTATTCTGATGCAGGATACCGGCTGGCAGCTTTAACATCGCGTTGTTTGGTTGGCTGACAAGGACGATCGTGCTTCGACATTGTGAAGCACATCCCAAGCCCTTTCATGTTCAGTCACTAAGCGCATCATTTAGTACGGTAGTGTGCTTGGACTAGCCATCTTGTTTGCGATCTTCGTGTGATTACATGCTTCATGAAAACTTAAAACGATCTGGATCGCCACCAGCCAGCGCTTTCAATGAATGAAGTGAAAACATTTACGGTCGATCCACACTTCGCAATCTGCTCTCGCAACCTCAAGACACCACACCAATCTCCACCACAAAAGATGCGAGTTTTATTGATTCTCTTTGTCCTGATTTCATCTGCGTCGGCTACTTCCTCGGAGCACGTCGGTATCACGAAGACCGAGCGATTCCCTATTGCTGCCGAAGTCCACCGGTTCCTGCGTCGACATTACCTCGAAGAGGAGGCGGACATCGAGTCCGATGATGAAGATAGAGGTGGTCTTGACAAGGTGGATGACCTGATTACGAAAGTAGACGACGCGTTGGGGATAACGGGGAAGATGGACGACGTTGCTGGTAAGCTGGGTAAAGTGCACGTAGCACCGACAACGAAGACAGCAGTCGAGAAGATGGAGCATGCAGGTCTTGTGAAGCATCTCTCCGGAAAGTATTCGGTTGCCGACAAGCTAAGCCTTACAACACTGAGACAGCTGGCGAAAGTGGACGAACAAAGGCTGAAAGATAATCGCGTGTTTGACAAGAAAACTGGCAGTGGAATGCGAAAAAAGATCGAGCCCTTTGAGGGCATGAAAATTGCTCCTCAGAAATATCTAGAAGCCCATGTAGCACGTGCTGGCCAGCTCGTCGACAAGGAGAATAATCGGCTGCTGTCTGCCGCTGTGATCGGTGATGGAGATAACGTCCTCCTCATTTCAAGCTCGAAAAAACCGAACGATTGGATCCTTCCAAAGGGAGGTTGGGACCATGGCGAGAGCATTGAGAAAGCCGCATTGCGCGAGGTTATAGAGGAAGCAGGGGTACGTCTATTTTTTTGCAATGTTTTTTGTATATTTATAATTTTGTTTTACTAATCGTCGTTCTGCAGATTCAAGCGCGTCTGAATCACGACCTAGGCAAGTTTACGTACAAGGACGGCGACAAAGGATATGGATTATTTGCCTACACGATGGATGATGTCCAACGCTTTGACGATTGGGCCGAAAGCTCCCGCTACAGAATTGATGTGAGTAGATATGCGGCAAAAAATAGTTTTTGCATTATTGGGAAGCTTACGTGCGTGTGAACCACTATAGGTCCCGATTAGCGACGCGAAAAAACTGGTTGGCAGGCGCCCAATAATGGTAAAGATTCTGGAGGCGGCGGAGAAGAAAAACGCGCTAGTCAAGCGTGGAGATTTACCGAAGAGAGACCCGCAGCTCGAAAACGTCAATTTGACATGAATTAGCTGGAAAAAAAAACAATGCACAAGAAGTTTCAGGGGCGCGCATCTTGGGTAGTGCATTTGTGATAACAGGCAGCCAAGTGTTAGCCACAAAAAAAATAAAACATGAAAACATATTTTGGTATGATAAAAAAATAAAAACAAGTATAAGCGACCATTTTAAGGCGTATTGAGGCAAGTAGCAAAGAGAGACCCTTGATATTGCCCGAATGACAGACTTGCTACGAAATTACGGGGGCATTATTGTGGGCAAATGTATATTGTAGCTTTCGACTTCGCAAACTGGCGGAGAAAATGCTAGTGCTTGGCATTTAGTAGCTAGTGGCCTTGGAATTATTCACGTTTGTTTGACATCGGCTACCTGCAGAAAGATCAAGTTTCTAAGTAACAAACAAATTTCAACAAATCCACATTACAAAATACTTCAATAAAGTACACCCTCTTCTATTATCTGTACCTAATTTATTTCAGAGCTAAGGAAGGATCTGTATGGGGCACGATTTTCCATGCGATTGCTTAGAAAATTATCGAAACAAATGGCTTCTTACAAGTTCCCCTACGTAAAAAGCAAGTAGCCCTGTTAGTAGTGTCAATATTATATATTATGTATTTTAACCAAAGTGTATTACTTCAAATCAATTTTGCTCCAAAGGCCACTACGGTGTGCACGAGGGAGAAGATGATCGGGGCCGGATGGTGAGCTGCAGAGCTCGCAATCGGGTACCCGACCTACAAGTAGAGGATATTCCCGGTCGATTTGGCAGTTGTTCGGATTTGATTCGGGTATTCAAATAATTATTTTCCTTGATTTATCAGCGTTCACAACTGCTCCAAAATAAACGCTTTCTTGNTTTTTTTTTTCATTTTACACATTTAATGTGTTCTCGTTCTGCTGTTTCCGTCATGGGTAGGAATGGGCAACCGAAACGAGTAGCGGATTGGATTTCGGTTAATGTCGGACTTGGACAATTAAAATTCAAAGAATTAATGCGCTCAGCTACTACGAGACTGGAGCGAATACTAAGATTACTTCACAACTGCTGGTACTGGTACTTTGGCAAGCAACATCACCACAGACGTCGCATGTTTGAAACGAGGTGTTCACGCATAAAGAGTTTTTTACGCGGAATAGAGAATGAAAGTTCTTACATGTAGCACACAACAAATCACGGATTCGTCATCATAATAGTTGCTACCGGTAGTTGCTAAAACAGAATAATGACTAACCAAAAAATGGGAGCTCATCGGTAAAGGGCGTGATTTCGATCGCCTCAGCTCGAGCAGCTGACCCACGCTAGCTTAAGAGCGACCACTTGACAACCACACTGTTCCTGACGCTGCTCAAGCAGTGCTGGATGAAAGGCTCCTATTGGACTATATGCACAAAATTCAGCATGGTGTAACCTTGAGCGAAGCTGGAGAACAACATTTAATCCTGTAATTTTCATTATAAGGGGTGTTACGTTTATTGAAGTATCTATTCATTACGATAACAGCTCATTATCTGATATTTATTATTTGTGAAGGGGTCACCCGTGCCTCCTGTTAATCTGTCAAAAAGTTTGCCGACGCTTGTGCAGAATTAGAAAACAATTAAATTTAAAGCATGTCACCCAAGTACTGGATATATTGACATAGCTCGGTGTCAAGAAACCTCCATTCCTTTTCCACTTCTCCGTCAAAGCAACAAGGAAAAGTAGCAAATGTGCGGAGCTATTTGCATCAGTGGGGTCCTTCGTTATACCATTGACGAGGAGGTATCCTATTGAAAGCTCAATCTGTTGGGCTATATAGTACGGCATGGCGGCAAGAAAGCACACTTCAAAATTCACTTTACAATCTGCGCTTGACAGCACCATAGGACGAATGATCCGAGCACTATTGGTTTTCACCTTTAGGCTGCTCTCCAGAAACGGCGGGTTTACTAAAGAGCTGATTACTGAAACTCATAAATCGCGAACATGTTTCTAATATCAAGCGACTTTGCGTCAGCATAGCGTCGATAAGGAGGTCGCAATCATGTGCGAGAATGAAGTTATCGTTGGACTTGGAGAACTGGGCCACTTGTTCGTGAATGCACTTCTGGTTTCTTTATCGCAAAGTACTTTAGATGCAAAATATTTCTGTTTATATTGGCATTGCACATGTATTCATACGAACTTGATATCTACACAGGAGAACCTACTTCTTCTGGTGGTTGATGAAACGCAGCGCCAGAGCAGCCCAGATACGGAAGAGCACAATGATTCCGATCGTGATCCCGAAGTTCCGGGCGATCTGGTGGTGCTTCATACCGAAGTATTCCTCCGTGTATCCCTTGATCGTGATGTGGCCCACCGTCTCCGGAGCATCCAACATGGGCTGACAGCCAAGCTGCGAGTTCACGTTCTCGTACGTCTGCCAAGTTTCGTTCCAAGTCGGTTCATCGTCGCAGTCCGCGAACACCAGCGCGACCAGGATGGCGATGGGGAACTTGAACGGACAGATGTCGTACAGCCACGTATAGCCCGACGGAATAGCGTACGCGGGTGGACTGAATCCGATAAACATCATTAGGACGCTATTGAACAAAATTCCGATGATCTGTGCGACTTCTTCGGACGGCATGGCGTACGCRAAGAACTGGCCCAGATACACGAACATGAGCACAAGAAGCGCCGATGCAAGCCAGAATACGACCGAGGTAGCGAAGCCAGTGAAGCCCACGAAGTAGTAGAAGATGGCAGTGAAGATCAGAGAGCTCACAAAGCAGTACGGGATCTCGGCTAGCGTCGACGCCATGAAGTACCAGAAGGCATTGTACGTCTGCGATGCACGCTCACGGTAGAATGATTCTCGCTCCAGACACGTCAACGGCATGACACTCTGGAACACTGCCATAGAGCTGAAGAATCCGGACATAAAGACCATTCCGACTCCGGAGTTGAGTCCGGAATACGATGCGTAATCGTCATTGGTCACGAAGATCAATCCGAAAAGCATGGCCAGGAAGATAGACAAGTACATCCTCGTCAACGTATAGCTCGGTGTACGCCAATACATCTGGAAGAAGCGCCAAATCACAAACCTCGCTTGGGTCTTCGAGTCCGCAGCACGTTTCTTACCAAAGACCATTTCGGGAAGTTCCGGAGACGGCGTCATGATGCCTTCCTTGGCCATATTCGTCTTCAGCTGCTGGTTGTACGGACTGTTCTTAAAGTAGCTCACAAAGTCCATCAAGTCTTCGGTTCCATGTCCGACACCTGCGCCAATACACTCCAACATCCACGTCGCCGGGTTGTACCCAACCGAAAGCGGAGCCACACCCGGGATGTTCTCAAAGTAATCGATCAAATTCCGGCAATTCTCGCCCAGATCTCCGTAAAACGCCGTCTGTCCACCACGTTGTAGTAACAAAAGTCGGTCGAACAGGTAGAGCACTTCAGCCGAAGGTTGGTGGATCGTACAGATGAGGGTCCGTCCGGAGTCGGCCACCTTGCGGACGCCGTCCATGATGATTTTTGCCGAGCGAGCGTCCAATCCGCTCGTGGGTTCATCCAGGAAAATGACACTGGGCTGTGCAGCCAGCTCCACGCCGATAGTCAAGCGCTTCATCTGCTCCACCGAGCTGCCTCGGATGATCTGGTCGGCGATATCTTCCAGTCCGAGCAACTCGATACATTCCGTAATAGAATCGTATTTTTTGGCGTCGGAAACCAAGGCATCTTGACGTAAGAAGGAGCTGAAAGTCAGGGCTTCTCGGATCGTCGCCGCTTCGGAATRCACGTCCATTTGCTCACAGTACCCCGTACAGCGTCGAATAGCCAGATCCGAAGCCTCGTAGCCGTTCAACATGATCTTTCCCGTGATTTTGCCGCCAGTTTTACGACCAGCAATCACATCCATCAGCGTCGTTTTACCCGCTCCAGTAGAGCCCATCAATGCCGTGATGGAGCCAGGAACAGCGAACCCGTCGATACCCTTAAGTAATTCGAGCTGTTCCTTGGGATTCTTCGGATTCGGAACAAAGTAGTGCAAATCTTGGAACGCGACGGTAACCGGTACGAAGTTCTGCTCACGAGCGGCAACCGGGAGACCAATTAACACGTTCCCGGTCTTCCCCTTGGGCGTCTCAGCTAGAACGTACGAGCTCTCGTCTTCAATCGACTTAACCGACACATCGACATTTTCCGGAGTCTCGTACCGGATGAACTCCATCGCTAAGTACGATAAGAACATGAAGAACACGTACACGGCGATGAGGTAGACGAAGGCGTACGCGATGAACTTCTTCTCCGTCTCCATGCCGAACAGGTCCAAATAGTACTCGCCCATAGTCATCCCGTTATACTTCGTACAGTAGTCCACATCTCCATAGACGCAGACGTCAAAGTCATCGGACCGGTATTGGTTGATAGCCAAGGCCTTGAGAGCCCAAGCAATGGGACTGATCCAGTGCGCCCAGATGAGGTAATCCGGGATCTGAGCCTTGGTCACAATGAACCCAGCGAAGATAATAAACACCAGGATGGACGACATCCCCACCGGCATCACCACGTTGGCATCCGGTAGAGCGCCAGCGAGGAAGAAGAACCACATCCCCATCGCCAGATTGGACAAGAAGAGCACCAGTTCGAAGATGATGAACAGCTTGAAATCCGACGCGAAGCCACAGACCCAGTTACACGATGGATCCGAAGATGACGGTCTCGGTTAAAGCTAAGGGGATCTGACTTACGGTAGTTGCCAGGACATAGGAGCCCGTCCGGAAGAAGTTGGCACGACGGTGT

>Contig_51

ATCAGTACACGAAGAGTTCAGGAAACCATAAAGCTAGCGGCCACATTCAAATTAAGCTCCTATTTGCAAGGGATCTAGTAGAAAAAGGATGTTGGGCTGCCCAGTACTACCTACTTACATATATGTCCTAACTAAGGTAAGTAGTTTTACAACCCTGAGAGGCACAATTGGAGTGAAATACCTACTGACATCGATGAACACTGCATGGGACAGTTAGAATGAGTATTTAGATAATGATGTTAATGCCAGTGAGCCTACGAGTAAGTGGGAAGTGTCCACTGGTTAAAAAGGYGGCTAAACAAATAAGGATAGGTGAAGCCGGCATGAGAAACAAATACGTAGCGAATCAGTAAAATTAAAACAGCCTGCACAGGGAAAGATCTCTGCAGGCGGCACCGCTCGCGCGATGGTGGTCTAAGTGCTAATGAAAGCTCACTTCTCCTTTTTCCTACGACTGAGCGCATCAGTCTATAGGAGCATTAAGAGAATAGATAAAAGCGTTAAGCGCTAAGCTTTAGCTTAAGCTGACCATTCAACACGCGAACACCCCGCGCAGGTTTGCTGCGGCTACGCAACAGCGCACCCGCTCACCTCCATCGGGAGGCCGTTGTTGCGGCAGCTTCAATTAGCTTGTATCAGTATCTTTTAGTGCAATTAGTGCATATTTAGAGCATATTTAGGTTTGTTTTCTCCAGCCCCGTGCGCTTTCCGAATTGTCTGCACCAAGCCGGAGTATGGCAACCAAAGTTGATGCTGCGGTTGCGAAATAGCCACAGGAGGTGCTTTGTCACTACGAGAGTTGCAACACACAATATAATACGACGAACACATAAATTGTTAAAAAAGTTCGCTAAAGTACTATAGTTTGAGCTAGTACTAATGGATTCTACCAAGTAATATACTACGTGCGGTATACTATTCCATTACAATACCTCAAATTGCGCTAGGATACATACACTACAATATAATGGAGAGGCTTCTGGCCAATCAGAAATCAACCCCCCACATAAAATTACCTGTTTGAAGAGATAATCGTCGAGTTTGATATAGCGCTTTCCGAGACCAAACGCAGTACGACTTGATGCTGAAGAAACTATCTGCAGCACTCTAATATTTTGCCAAACCGAAAAAACCCCAGAAAATCCAGTGCTCCGAATAGGAAACGCAGCTGGAAAACACGAGATCGATTTCTAACTGCAGACAATGAAGTTCCTTGCTGCTGATGACGAAAGAGAACACTCAATCAATATCAAAGAGCTAAATATTCTGATCTGGCTTATGTTTGAGTCGAAACCGAGCTCAAAAATTGTAGAGAATTTCATGAAAGCGTCAGATAGGGATCTCTCAGCGTGACGGAGTGGGTCTCGTATGGTCTTGAGACCAATAAACACACCGGAAGCCAATCATTCGCCAACCGGATCCACCTTCTCTTCGCAACGTCAGACGTCTGCCAGACTAACTCTACCAGAAGTTCAACTTGGTCTAGTTTCCATTTTATAGACTATTTAGACCCTATAAACGCGCCGCCTAAACCAGAATCGACCTGGGATGAACTGAGAAAACGCGGCACAATTCTCAATCATTTCAAGTCTCGCCACGACCTGGTCGAGCCACGTGACGTATCGAGTGGCACGAGCTCCGACAATTTCTTGGGTACCTTACATTTAAGCACACGCTCTAACAAGTTAGATTACATATCCACTTTCTTGAAATAGACCAAAATCAAAAAAACTGAAAAATGCAGGTAGAATCTGATATAAAATGTTGCAGGTGAAGTTGCCTATGACACCTTATTTTTTTTGCCAGATATTAGGCCCAGATCTTGAGTAGCGTGTCCCAGCTGCCTGTGCACAGTGCCTGACCTGCCGGGTTAACTCCGAGGCAAGACACACGGTTCTCGTGTCCCGCGAGCTGGTAGATATGCGCTCCGGAAGTGCTGAGCACGTCCCAGCAGTAGCAATTGTAGTCGTCGTAGCCCGCGAAGAGGAAGCGACCCGACTTAGAGAAGCTCACGGACGTGATGCCGCATAGAATCTTGTCGTTACTGAAGTTGTTGAGCTCTCCATACGCTCGAAGATCAAAGAGACGGCAACTCGAGTCGTCCGAGCCGGTACCCAGAGCGTTGCCACTCGGGAAGAAATCGACCGAGTTAATATCCGACTCGTGGCCCTGGAACGTGTGCGTGGTCTTGCCCGTTCTGATATCCCAGACCTTGGCGGTCGAATCGCACGAGCCCGAAATAAACATACTGGGGTTATGTGGGTTAATGCTCACGGACATGACGTCGCCCGAATGTTCGCGGAATGTGGTCTTTACTTCGCCACTTTCGACGTCCCACAAGATGCAGTTGGAGTCCCCCGAGCTAGTGACAATGTTCGCTTCGTCGACGAATCGGCAACAGCTCAGGTAGCCATCATGAGCGGCTAGTTCCTTGGTGGCGCGCATCACCTGCGCCTGTGATAGGTGGAAAATGCTGCACAAGTTGTCCAACCCGCCACAGGCGACCATGTTGCGCTGTTTCTGCTCGAAGGCACAGGTCATGACCCAACTGGAGCGTAGAGGGATGGCCTGGATCTTGTTGGTGGTCTGAGCGTTCCACACGATGAGCTTGCCGTCCTGTGACGCCGACACGAGACTCGAGCTGTCTCCGCCCCATTGCATGGCGTAGATCTTGCCAAAGTGGCCTTTCAATAGTCGTCGACATTTGGGTGGGGCCAGGATCGCCTTGGCGCCGGAGCTGGCATTGGCGCTCTGGAAGCCGCCATCACTCTTGGCTTCACGCGTCTTCTCAATCGTCTCCTTGAGGCTCTCGCATTTCTTCTTGAGCTCCGCAGCGTCGCCCATGGCCTTCGAGATGAGGCGGCGTACTGGGGAATGCGACTGTTTCTAACTGCGTGTCGCGACGCTTGCGAGAATATCTTCCACGAAATAACGAGATGTGATTGGAGGGGCAAATATGAAAGAAGAACGAAGATTGGCTGAGAACTCAAATGGGATGTAGCCATATGAAACCAACGTTGAAGTGGGTACATGGCTAATACTGTAGCAATAATATTTTACCGTTCCGGCTCATACAGTATTCAATATCTACAATTTGGTTTATTTTACCATTTCAAATATTTTACCCACTCTTTTGAGCTACATGCAGAATACCTTAGCTTTCTGATATTAAAAGGTAATACATTGCTACGCTCATTTCTCTCGAGCACCGACCGTAYGATTCCCATACGCCATGAGAACACTAACGCCGAGAAGCAAAAAAAAAGAGGGTTTTCAGGGAGAGCAGAAAGTGCCATGCAAGCCTCTTTTCAGTACTTAAGAGTACATCTAACTCGAGTTTAGCCAGTAAAGTCTAGAGAAATAAATGCACTTTTGATAGCAATAAACTGTATTTGCCTATCTACAGTATAAAACCAAACAATTCTAAATTTCTATCGCTTGATCTTATTAGCCAGCTCATGCTGCCATTTCGCTGCAATACTCGCAGTTGCAGGAACGTTGATCGCTTTCTCGAGCATAGCGGACAGATCCTTGACACTGTAGAATTTCAGGACCGTGCTTATCGCCGGAGGAACCTTCTCAGGTGTTTGTGAGCTCAGCTTCATCCTGTAGCTCTCCCACATTCTCAAGTTGGGGTTAATCAGAAGACCGTCCAGTCCTCTATTCAGCTTCAATAGCTCGAAAACTTGGTTCGATGATTTGCTTTCTGTCAGCCACCGTTTTACCATAATGTTCTCCAAATCCATAGGAAGCTTCTTAAAAAGATCTATCCTTTGCTCTCCAAGTATAAAAATCTTTGATAGGCCGTCGTAACCATAATGTCCAACCAACACATCCAGCATAACATCTTCGGGTGCTCGATCATAACTGCTTTTGAGATGGACATACCATGTATTCAACTGCGGAGACGCGAAGAGGTCATATCCCGCTTCGTCAAGCTTTAGAATCTTGAAAACATTCTCCGCGGGTTCTTCACTGTTATGCCACTTACCTAAAAGATCTCCCCGCAATAATGATGAGACTTTTTCCATTTTGGGGTTTTTCAACCCCAGTTCAACCATCTTCGCCACATCGTCCTCACCGTAGGTCTTACTGAGCATGTCGAGCATTGAAACCTGCTCCTTAGGTTTTTCCTTGTTAAATATATCAAGGTAGCGTGCGTACAGATAAAGCTTTGAGTTGGTGACTTCCTTGTTCATCGGCGGAGTCGTGAAGATTTTATATGCCTCCCTGTCCAACTTTAGCAGTGTGAAAATCTCGTCTGCGGTTTTCCCCGACGTGACCCAACTAGTTGCGAGCTCCGTCCGCAGTCTCGATGTAAATTTCATAGTAGTTGCGTCGCTTGTTCCCATTCCAAGAAGTTTGATTACACCGTCAGTTCCATAAGTCGTCGTGAACATGTCAAGCATTGAGACCTTATTCGTGGAGTGTTGCTTGTTGAACCGGTCGATATAGGCTGCGTAGAGATGTAGCTTCGGGTTGGTGAGAATCCCATCCAGCCCGCCGCTAAGACCTAGCTTGTTAAAGACGTATTCCGAGTTCTTCTGCTTGTACATCCACTTTTCGATCCTGATTTTTTCGGCTAAATTGAGCTTCTTCGCCGCTGCCACCTTGGGTGAAAACAACCATTCGACAAACTTTGATGGTGTGGGAGCGTTGAAGCTGATTGCTCGCTGTTCGCTGTCCTCCGTTCTGTCACTTCGTAGCTTCCTGGTAGGAGCAGCATCTCGAGGGTTGTTTCTTAAAAAATCGACTGGCAGGCCGACATTTGCTTCCATAGCCGCGGATAAAGCAGAAGTTCCTGCAAATAGGGTAGCCAGTAATACCACGAAGAATCGCATGCTCCGCGCGTTGGGTGGCAAATTACAATTTGAGAAGCTCGCTATAAAAACAACATAAATCAAACAACTGCTCGCTCGACAATCCCAACAATCCCATCTTGCATCGAAGTACAGTTCCATTTCTGCGGTTCTGCAGGCACAAGCAGTGATTGTGCCTGCGTAAAATCTATGCACATTTAAAGTGTATGTACCGGTATGAGCTAGCAGTTTACATGTTAGTTATCAAATTTTATTAATAATTNAAAAAAAAAAAAAACATTTGTATTGTTATTATTTAAATTTTGAGCAGCCCAAAAAAAATCAACAGTACATGCATCGTGGAATGGCCTAGAAATCCAGCTGAACTATCACAGGACAAAATATCTTCTATAGATACGCAATTAACGTTATTCGCAAGGATCAAGCTCACAGTCTCTACCATCATTTAATCAAAGGAAAGACTAGGAATAGGAGTCCAGTATCGCTAAATCTGCTGGCAAATCGGTGATGAAACGCGAATACGATTATTAATAATAGTACGCCCAGTATGATGCTGATGAAAGGTGTAACGTAAGATGATGATCAAAGGTGTAAGTTATAATGATGATGAAAGGTACAGCCTGAATTACCTACAACATTTATTTCTTTTTTGTGTGAAGCTGCCTGTGTACACTGAAAACCAAAACTTTGTAATACGTCCTTTGATATGTGATGCGGAAGCCTTAAGTCCTGGTTGTAGTTTCGAAGAAGAGTCGGTATTGTTTTACGCATGTCGGGTATCGTCTTGCCGCCAGATACTACTACGTAGTACGTTTATTAGTACTCCGCTACTTGTATTGTATTAATCTTGATCATCACAAATACTCTTTTCATTATTTATTACAACAGCTGGAGCTCGGAAAAGCGGTAGAAAAAGTAAAATACCGTCGTAAATGTGAACTTGTACCGGTTTTGAGTGCAAAACTGTCCTTTTTTCGTAAATTTGAGCTTGCTCTGGTTTTGAGTGCAAAACTGTCATTTTTTTGCGATTGTGAGCTTGTACCGGTTTTGAGCTTAAAACTGTCATATTTTCGGCGACATCTGATTCGCTGACGTCGATCGAATTGCACTGTCTGATTCGCCGCGATGAATCGCTCATACCGGTTCATCTTTGTCACATTTCATCACTTCAAGTGAGGTCGATTGGGACTTCTTCGTTCCTTTCGACACTCATGGGGTTTTCCTTCATGGAGCTGCCCGTCAACCAGCGGGTCGTCGTCATCTCCATCCTAATTCTTCTGTTCGGGCTTTTCTTTGGTCTCTCGCTCGACGCCAATGCGCACATCCCCAAGCCGTGGAACCGCGTCTCCAGCATCATTGGCTGGATCTATTTCTTCTGCTGGAGCGTGAGCTTCTACCCGCAAGTGTTCCTGAACCGCCAGCGCCGCAGCGTCGTGGGGTTGTCGCTGGACTACACGGTGCTCAATATGCTGGGCTTCACGTGCTACTCCATCTTTAACGTGGCCTTCTACTACAGCAAGAGCGTTCAAGAGCAGTACATGCGGCGCCACGACGGCCACCGCAACGCCGTGGAGCTCAACGACGTCTTCTTCTCTCTGCATGCGGCGGTTCTGGTGGCCGTATCGCTCTTCCAGTGCGCAATATACCCGCGAGGCGGACAGGTGGTCAGCAAGCCCACGATCTTGTGGACCGGCGCGACTTTCGTCGCCGCTGTTGTCTTCGGGCTTGCAGTCTTGTTCACGGGAAATAACGAAGATTCGTTCATCAATACGTTGAACTTATTGTATCTGCTGAGCTACGTGAAGCTCATGACGACGCTGGTGAAGTGTCTCCCGCAGATTGCGCTGAATTACCAGCGCAAGTCGACGGTGGGGTGGACAATCTGGAACGTGCTGCTGGATATTGCTGGAGGGTTGTTGTCCATTGGGCAGCAGCTGCTGGACTCTGCAGCGACGAATGACTGGACAGCCATGACGGGGGACCCAGTCAAGTTCTCGCTGGGCTTTGTGAGTATTATTGTGGACGTGGTGTTCATCTTGCAGCACTATGTGTTTTACGCCGACAACAACAACTTTATGCTGCATGGTGGAGAGACCAAGCCTTTCTTGCCCAAATAAGTCTAAGAAAAAAAGAAGTGGTTAAAACAAAATTCGCCATGGAGGCGTTATTTTGTGTCTAGTTCGAAAAGCTAACGTAAAAATGAAGCGAAATATTCAACATGGTGACATTGAGCGTGRCAAGTGAAACAGCCACCAGCGTAAGAGTTGGCTCGCCCTGTATCATTTTTAGTTGGGCTGCTTGGRAAAAGCTGCCGCCCAACCCATTGCCTCAACATCCTCCCACAATGCCGTACACGCTGGGCTTCCGCAATTCCCTTGATCTGATCAATAGAGAGTGACAAAAGCATGTCTCGATATCGCCTGAAACTGAGCTTCGCTGCACTTTGTCGCGAACTAAAATCATCCTCGATTAGAAATCTTTTAGCGACTTRAATTAATTTTTACACAACTGAAGAGAAAACGAAGTAAGCAAGTCCGGAGAGGAGGTCATTGCGCCAGCACCCATCCATTCAAGCTGCACATGCACTGTAAAGAGCTCCATTATTGCAGCTGTCGAAGCTGGTTACCGTTGATTTTTAGATAAAGTTTTTACCCGAACGGCTCAAATTATTGTTTTTTTTTTTCACTCTAAATCTTCTGCAGAACATGC

>Contig_55

TTCGCTCTCTTCATCCGAGGAACCAAAGATGTTCCGATAGGAGTACCCTGTCTCCTTCTCCTCATGGAGTGAAGACTTCGAAGTTGTACGCTTTTCAGAGGGTTTGGCGCCCTCGGCCTGAGTGGCCTTAGCACGCGCAAGACCCTCGGCAAGCGAGAGATGTTTGCGAGGTTTCTCCTCTTCGTCGTCGCCAGCTATGGGAGTGTCCTTAGCCTTGATGGCAGGACGCTCAGAGCATAAAGACGTAGTGGTACTGTGATCACCGTGATCACTAGGTAGCGACTGCACGCTGTCGTCATTGCTCTCGCTAGTCACTGGGGTGTGCCCTCCCACGGAGTCCACAGGAGCATGGTGAGTGCTCCCGCTCTTGGCCTCGTCCTCACTGCCCGAGTTGAACTCACGTTCGTCGAACCGAGGATTTGGACGGTTGTCTGGGGAGACCGAAGGTGACGGGATGCGGCGCTTGCTCGCATGCTTGCTCACAGCCGCGCTGAGCCGATCGCCTCCGAGGCCACCTTAGTAGCCTCCTGGTGTGCAGAGTCCTTGCCGTGAGGCTTAGCGCTCTTGGCTTGCGTATTTCCTGCAGGAGCCTTGGAGCATGTATTCGCACCGCTTTTTGCAGTGTCACGGGGTATGATAACGGTCGATGTGGAGACCAAGAAGAAGGGCGCTCAACCGCTGCTCGAGGCTCTAACTGTGAAGTGAGCGCTCGCGCAACCAAGCCGATGTATTTCGACTTTAAAAATGAAAGTTTGTGACGTACAATTCTTTATTCGGTTTATTCCGTAAATCGAACGTAAGTGGAAAAAGACGTAACGGGGTACGTATCTCCAAGTGTACCCCTAGTTACATGAAGGGTGACACGTCATACTTCGGGATATCTGGTACATGGTAACAGGGTCGTCCGAGGCTAACCATTGGCTGATGACATTGCTTTTCTATAGGTGACTTATAGTTTGTAAATTAAACTCAAGCTTAGTCTACCTTTAAATATACCGGTATCTCGTTTATAACTTACCTTAAATTGGTCACTTCGCTCAGGGGACCTGTGAGCTGCCGTTCACGGATCGTTTGTATACTCTCAGAAGCTAATTTCCTTTAGCGCATAATTTCCTCTAGCGCTAGGTGCTGTGTGAACCCGTCACAATTATTGTTTTAAAGGTCGAAGACCTTGAATGCAGTCTTGCTTGTCACAGTTAAGTTGGTGGCACAGTACCCACTGGGCATGCACCTGTTTAGCAGAAGGATCAGCAGCCTTCAAGAACATGAAAAATAGGAGGCGGAGCGCTGCAAAATAGTTTGGTAAGATGATTTATCTCCCGAGCGGAGGTAGTATTTCTTCTTCTTTATACTGTATTTCGCTTTTAGTTTCAGCATGTGCGTGGAGGCAATAGAATATCCGGTCCTTGCTTGTTTTCGTCTATAGACACATCCTTTTTAACGACAAGTCGCACACCTCATGAAGTTTAAACAGGGTTTACACAGAAACTTAAATGAAGAAGGACAATAACTACGGGCGAGCTAGATAGCGCTAATGATTCACTTTTGAAAACTAACATGTTTGAAGTGACCACTAACAGTTAACAATATATACTACTTCCATATCAGACAGTGGTTGATTAAATTAAACCACAATAACATTTCAGAGCTGTTTCGTGCACCGCGCAACTTCTTGAACTATCCGTCATATAACACACAATGCAGTGGCCCCCCAATCCTACGATAAAACTGAAGCAAGAAAAGTAAAGCAAGCAAGGTCGCATCAATGTTTGCGTTGTTAACTGGATAAGCGAATCACATCACACTGCAGGTCGAACCTTGACACGCTTTTCCTTCGTACTTCGTTACCTGGCACAAAACATACCAACCAGTCTGTGTCCCACCACGAGCTTATTTTGCGATTTTTTTTATTTATTTATAACCCAGTCTCATTGCCTCATCGGTCTTGTGCTTTACAATCTTCGCCCAATCGGGTGTTGATATGACAGTCTCCTTTAGTTTTTCCAAGCTTTTCTTGGGCGTGATTTTTTCCACTACAGCTTTCAATTTTTCTTTGAAGCCTTTGGCTCTCTCTTCCACGATCCCGCCTTCGTCGTTTCCGACACGACGCAGTAAACGCGGGTCCTGCACGTCGGTCGTGGTGATCATGTCAACACCATATTCATTCGAAGCCTTGCCCGCAACCGCAGAATTGGCATTACCAGTGGCGTGGAAAGTGACCGCGACTAGCACTGCAAGGATGCATGTGAGGCGCATTTTGTTGTCGTTTGGGTCAACAGCTAGGGCACTTGAGTCGAAATGAGAGAGTGCGAGCAGATTGCAGAATGAGGTGTCGGACGAAGACAGAGCCGTACTACGTGCTGGAGCACGAGGAAGTTCCACTTTGTACTGTTTTCTTCCAGTCTGTACAAATAACGCTGGGATATAGCTACCGATCCAGTACATATTTCCAGACAGTTATTGAACAAAAAACCAGTACACTTAAGTTCGCAGCTAATAATGGTTTTTGTAGCCCTATTCACTTATAAAAACTAATGTCTAGTCACTATTAGAAAATTGTGTTTAATACACAAGAAGAGCAATTCAATTCGCAATTCAAAAGTGCGACAGCAGACCTCCTCCAGTCATCCTCTCCGAGTTGCTTTCTTAAAATGGATGGCCACCGCAGCATTCTTGCAGCGACATCAGTGGCAGCACTTTTCTCATGCGTAGTACTACTATAGAAGCACTGAGCTACCACATTGATTTAAATCAAAGTCACGATTGGGCTTGTTCTCGATCACTAGATACTTCTCCGCCCAATATTCAAGCTCATCGTTCTCAAATGACGTATCAACCATGGCTGAACGCATGTCATTAATTGTTCGACCAGTTAACTCCACCAGCTGAGTTAGTGTTGCCAACTTCCGCTCAGACTTGAATGTCAATTCGAAATCTTGATCAAGTGTAGAAAGCTCAAACGTCTTCGAAGTGGCTGTTTTCCATGGCTCCTTGACTTCATAGTCATCCTTCTATTGCTTGTATGCAGCAGAATCACAACCAGCAGCAATCCCTTCAGTTTGCCTTCGTTCAGTGATGGATTATACTTGGAATCAAACCAACCATCAAATTCTTTGGTAGCATACTCCTTGTTGAAACGCTCTACTTTTTTGTTTGAGCACACAGAGATAGGTCTTGCGCACTAAATGCCAATCCGAATGAACTTGACAAGCAAAGAATCCAGCGAGATTCCACACGTTTTGGTTGTTGGAGAACAAATCGCCTCGAATCATCATGCACAATTGATACAATCGCTCATCGTAGGATTGAGTTGCAAGGAAGTATTCCACACCGCATTCAACGTTTGCTTGTTCAAATGATTCACGGTCTTCATTGGACTTAAAGAAGAAATTATCAAGCATCTGCGATTCATCCAGTTCATTAAATTGATATTGCAAAAGGTACGTGTATCCGTCGTTGCCAAAGTCAGGTGTGCCATCATCACTGTGCTTGTAGTTGATGGAATCCCCAACTGCGTGCATCTTCGACTTGATCCAATTACTTGGTGACAACACGCGCGAATCTCCCAACCTGGGTTCATCGTATTTATCAACAGATTTGATGTGCTGAATGCAATGCTCACCATAAATTTCTTCAATAGACTTGGTCATCGTTGGTTCAGCAACTGCAGGCACAACAGCTACAGATTCAGCAGCTACTAGTTCAGCAATCACTGGTTCAGCAACTATTAGTTTAGCAATCACTGGTTCAGCAACTACTGGCTGATACTTCGCGTGGTGTCGGCACCAATCGCCTTTTGAGCCGCACTGTTGGCTCGCCTTTGCGTGGTCTTCTGGGAAGATGTACTTGCACATTGTTATTTCTATATATTTATTTTTTATCGCTCAAGATGAACTCACTATTATTTAGTTTCTATATATTGAGGAGTATTTTTGTCAGAAATAAAANATATTATTTTAAAGTGATGTGATTGGCTAAAACTGTGCGGTGTGCTGCGTGAATGAGTGCTGCTGCTCAGTCGAGAACTTTCCGCACGAAGATGGCAGAGGTCGAGTTGGAGTGCGCGGTATACGGCGAAGGGACTGTGTTTCCCGTGAAGATAGCGAGCAACGCGAAAGTGAGCGCGCTGCAGAAGGCCATGTGAACGAGAAGAAAGATGTCGATGATCGCTTCGAAGTTGATCCAGCCAGATTGACGCTCTACTTGGCGCGGGAGAAGGGGGAAGCCACGTGGATGAAGCACGACCACACGGTGAAAGGCTTCTTGTGAGGTGGCATCAGCACCGAGTATGAAGAGATGCTTTCGTCGTGGATTCTCGACGAAGATTGCGGTCGTGGTGATCATGTCAACACCATATTCATTCGAAGCCTTGCCCGCAACCGCAGAATTGGCATTACCAGTGGCGTGGAAAGTGACCGCGACTAGCACTGCAAGGATGCATGTGAGGCGCATTTTTTTGTCGTTTGGGTCAACAGCTAGGGCACATGAGTCGAAATGAGAGAGTGCGAGCAGATTGCAGAATGAGGTGTCGGACGAAGACAGAGCCGTACTACGTGCTGGAGCACGAGGAAGTTCCACTTTGTACTGTTTTCTTCCAGTCTGTACAAATAACGCTGGGATATAGCTACCGATCCAGTACATATTTCCAGACAGTTATTGAACAAAAAACCAGTACACTTAAGTTYGCAGCTAATAATGGTTTTTGTAGTCCTATTCACTTATAAAAACTAATGTCTAGTCACTATTAGAAAATTGTGTTTAATACACAAAAAGAGCAATTCAATTCGCAATTCAAAAGTGCGACAGCAGACCTCCTCCAGTCATCCTCTCCGAGTTGCTTTCTTAAAATGGATGGCCACCGCAGCATTCTTGCAGCGACATCAGTGGCAGCACTTTTCTCATGCGTAGTACTACTATAGTAGCACTGAGCTACCACATTGATTTAAATCAAAGTCACGATTGGGCTTGTTCTCGATCACTAGATACTTCTCCGCCCAATATTCAAGCTCATCGTTCTCAAATGACGTATCAACCATGGCTGAACGCATGTCATTAATTGTTCGACCAGTTAACTCCACCAGCTGAGTTAGTGTTGCCAACTTCCGCTCAGACTTGAATGTCAATTCGAAATCTTGATCAAGTGTAGAAAGCTCAAACGTCTTCGAAGTRGCTGTTTTCCATGGCTCCTTGACTTCATAGTCATCCTTCTATTGCTTGTATGCAGCAGAATCACAACCAGCAGCAATCCCTTCAGTTTGCCTTCKTTCAGTGATGGATTATACTTGGAATCAAACCAACCATCAAATTCTTTGGTAGCATACTCCTTGTTGAAACGCTCTACTTTTTTGTTTGAGCACACAGAGATAGGTCTTGCGCACTAAATGCCAATCCGAATGAACTTGACAAGCAAAGAATCCAGCGAGATTCCACACGTTTTAGTTGTTGGAGAACAAATCGCCTCGAATCATCATGCACAATTGATACAAATGCTCATCGTAGGATTGAGCTGCAAGGAAGTATTCCACACCGCATTCAACGTTTGCTTGTTCAAATGATTCACGGTCTTCATTGGACTTAAAGAAGAAATTATCAAGCATCTGCGATTCATCCAGTTCATTAAATTGATATTGCAAAAGGTACGTGTATCCGTCGTTGCCAAAGTCAGGTGTGCCATCATCACTGTGCTTGTAGTTGATGGAATCCCCAACTGCGTGCATCTTCGACTTGATCCAATTACTTGGTGACAACACGCGCGAATCTCCCAACCTGGGTTCATCGTATTTATCAACAGATTTGATGTGCTGAATGCAATGCTCACCATAAATTTCTTCAATAGACTTGGTCATCGTTGGTTCAGCAACTGCAGGCACAACAGCTACAGATTCAGCAGCTACTAGTTCAGCAATCACTGGTTCAGCAACTATTAGTTTAGCAATCACTGGTTCAGCAACTACTGGCTGATACTTCGCGTGGTGTCGGCACCAATCGCCTTTTGAGCCGCACTGTTGGCTCGCCTTTGCGTGGTCTTCTGGGAAGATGTACTTGCACATTGTTATTTCTATATATTTATTTTTTATCGCTCAAGATGAACTCACTATTATTTAGTTTCTATATATTGAGGAGTATTTTTGTCAGAAATAAAATAATTATTTTAAAGTGATGTGATTGGCTAAAACTGTGCGGTGTGCTGCGTGAATGAGTGCTGCTGCTCAGTCGAGAACTTTCCGCACGAAGATGGCAGAGGTCGAGTTGGAGTGCGCGGTATACGGCGAAGGGACTGTGTTTCCCGTGAAGATAGCGAGCAACGCGAAAGTGAGCGCGCTGCAGAAGGCCATGTGAACGAGAAGAAAGACGTCGATGATCGCTTCAAAGTTGATCCAGCCAGATTGACGCTCTACTTGGCGCGGAAGAAGGGGGAAGCCACGTGGATGAAGCACGACCACACGGTGAAAGGCTTCTTGTGATGGTGGCATCAGCACCGAGTATGAAGAGATGCTTTCGTCGTGGATTCTCGACGAAGATTGCGGTCGTGGTGATCATGTCAACACCATATTCATTCGAAGCNCTTGCCCGCAACCGCAGAATTGGCATTACCAGTGGCGTGGAAAGTGACCGCGACTAGCACTGCAAGGATGCATGTGAGGCGCATTTTNTTNGTCGTTTGGGTCAACAGCTAGGGCACTTGAGTCGAAATGAGAGAGTGCGAGCAGATTGCAGAATGAGGTGTCGGACGAAGACAGAGCCGTACTACGTGCTGGAGCACGAGGAAGTTCCACTTTGTACTGTTTTCTTCCAGTCTGTACAAATAACGATGGGATATAGCTACCGATCCAGTACATATTTCCAGACAGTTATTGAACAAAAAACCAGTACACTTAAGTTCGCAGCTAATAATGGTTTTTGTAGCCCTATTCACTTATAAAAACTAATGTCTAGTCACTATTAGAAAATTGTGTTTAATACACAAAAAGAGCAATTCAATTCGCAATTCAAAAGTGCGACAGCAGACCTCCTCCAGTCATCCTCTCCGAGTTGCTTTCTTAAAATGGATGGCCACCGCAGCATTCTTGCAGCGACATCAGTGGCAGCACTTTTCTCATGCGTAGTACTACTATAGTAGCACTGAGCTACCACATTGATTTAAATCAAAGTCACGATTGGGCTTGTTCTCGATCACTAGATACTTCTCCGCCCAATATTCAAGCTCATCGTTCTCAAATGACGTATCAACCATGGCTGAACGCATGTCATTAATTGTTCGACCAGTTAACTCCACCAGCTGAGTTAGTGTTGCCAACTTCCGCTCAGACTTGAATGTCAATTCGAAATCTTGATCAAGTGTAGAAAGCTCAAACGTCTTCGAAGTGGCTGTTTTCCATGGCTCCTTGACTTCATAGTCATCCTTCTATTGCTTGTATGCAGCAGAATCACAACCAGCAGCAATCCCTTCAGTTTGCCTTCGTTCAGTGATGGATTATACTTGGAATCAAACCAACCATCAAATTCTTTGGTAGCATACTCCTTGTTGAAACGCTCTACTTTTTTGTTTGAGCACACAGAGATAGGTCTTGCGCACTAAATGCCAATCCGAATGAACTTGACAAGCAAAGAATCCAGCGAGATTCCACACGTTTTGNGTTGTTGGAGAACAAATCGCCTCGAATCATCATGCACAATTGATACAATCGCTCATCGTAGGATTGAGYTGCAAGGAAGTATTCCACACCGCATTCAACGTTTGCTTGTTCAAATGATTCACGGTCTTCATTGGACTTAAAGAAGAAATTATCAAGCATCTGCGATTCATCCAGTTCATTAAATTGATATTGCAAAAGGTACGTGTATCCGTCGTTGCCAAAGTCAGGTGTGCCATCATCACTGTGCTTGTAGTTGATGGAATCCCCAACTGCGTGCATCTTCGACTTGATCCAATTACTTGGTGACAACACGCGCGAATCTCCCAACCTGGGTTCATCGTATTTATCAACAGATTTGATGTGCTGAATGCAATGCTCACCATAAATTTCTTCAATAGACTTGGTCATCGTTGGTTCAGCAACTGCAGGCACAACAGCTACAGATTCAGCAGCTACTAGTTCAGCAATCACTGGTTCAGCAACTATTAGTTTAGCAATCACTGGTTCAGCAACTACTGGCTGATACTTCGCGTGGTGTCGGCACCAATCGCCTTTTGAGCCGCACTGTTGGCTCGCCTTTGCGTGGTCTTCTGGGAAGATGTACTTGCACATTGTTATTTCTATATATTTATTTTTTATCGCTCAAGATGAACTCACTATTATTTAGTTTCTATATATTGAGGAGTATTTTTGTCAGAAATAAAATAATTATTTTAAAGTGATGTGATTGGCTAAAACTGTGCGGTGTGCTGCGTGAATGAGTGCTGCTGCTCAGTCGAGAACTTTCCGCACGAAGATGGCAGAGGTCGAGTTGGAGTGCGCGGTATACGGCGAAGGGACTGTGTTTCCCGTGAAGATAGCGAGCAACGCGAAAGTGAGCGCGCTGCAGAAGGCCATGTGAACGAGAAGAAAGACGTCGATGATCGCTTCRAAGTTGATCCAGCCAGATTGACGCTCTACTTGGCGCGGAAGAAGGGGGAAGCCACGTGGATGAAGCACGACCACACGGTGAAAGGCTTCTTGTGAGGTGGCATCAGCACCGAGTATGAAGAGATGCTTTCGTCGTGGATTCTCGACGAAAATTGCGGTCGTGGTGATCATGTCAACACCATATTCATTCGAAGCCTTGCCCGCAACCGCAGAATTGGCATTACCAGTGGCGTGGAAAGTGACCGCGACTAGCACTGCAAGGATGCATGTGAGGCGCATTTTTTTGTCGTTTGGGTCAACAGCTAGGGCACTTGAGTCGAAATGAGAGAGTGCGAGCAGATTGCAGAATGAGGTGTCGGACGAAGACAGAGCCGTACTACGTGCTGGAGCACGAGGAAGTTCCACTTTGTACTGTTTTCTTCCAGTCTGTACAAATAACGATGGGATATAGCTACCGATCCAGTACATATTTCCAGACAGTTATTGAACAAAAAACCAGTACACTTAAGTTCGCAGCTAATAATGGTTTTTGTAGCCCTATTCACTTATAAAAACTAATGTCTAGTCACTATTAGAAAATTGTGTTTAATACACAAAAAGAGCAATTCAATTCGCAATTCAAAAGTGCGACAGCAGACCTCCTCCAGTCATCCTCTCCGAGTTGCTTTCTTAAAATGGATGGCCACCGCAGCATTCTTGCAGCGACATCAGTGGCAGCACTTTTCTCATGCGTAGTACTACTATAGTAGCACTGAGCTACCACATTGATTTAAATCAAAGTCACGATTGGGCTTGTTCTCGATCACTAGATACTTCTCCGCCCAATATTCAAGCTCATCGTTCTCAAATGACGTATCAACCATGGCTGAACGCATGTCATTAATTGTTCGACCAGTTAACTCCACCAGCTGAGTTAGTGTTGCCAACTTCCGCTCAGACTTGAATGTCAATTCGAAATCTTGATCAAGTGTAGAAAGCTCAAACGTCTTCGAAGTAGCTGTTTTCCATGGCTCCTTGACTTCATAGTCATCCTTCTATTGCTTGTATGCAGCAGAATCACAACCAGCAGCAATCCCTTTCAGTTTGCCTTCTTTCAGTGATGGATTATACTTGGAATCAAACCAACCATCAAATTCTTTGGTAGCATACTCCTTGTTGAAACGCTCTACTTTTTTGTTTGAGCACACAGAGATAGGTCTTGCGCACTAAATGCCAATCCGAATGAACTTGACAAGCAAAGAATCCAGCGAGATTCCACACGTTTTGGTTGTTGGAGAACAAATCGCCTCGAATCATCATGCACAATTGATACAAATGCTCATCGTAGGATTGAGCTGCAAGGAAGTATTCCACACCGCATTCAACGTTTGCTTGTTCAAATGATTCACGGTCTTCATTGGACTTAAAGAAGAAATTATCAAGCATCTGCGATTCATCCAGTTCATTAAATTGATATTGCAAAAGGTACGTGTATCCGTCGTTGCCAAAGTCAGGTGTGCCATCATCACTGTGCTTGTAGTTGATGGAATCCCCAACTGCGTGCATCTTCGACTTGATCCAATTACTTGGTGACAACACGCGCGAATCTCCCAGCCTGGGTTCATCGTATTTATCAACAGATTTGATGTGCTGAATGCAATGCTCACCATAAATTTCTTCAATAGACTTGGTCATCGTTGGTTCAGCAACTGCAGGCACAACAACTACAGGTTCAGCAGCTACTAGTTCAGCAATCACTGGTTCAGCAACTACTAGTTTAGCAATCACTGATTCAGCAACTACTGGCTGATACTTCGCGTGGTGTCGGCACCAATCGCCTTTTGAGCCGCACTGTTGGCTCGCCTTTGCGTGGTCTTCTGGGAAGATGTACTTGCACATTGTTATTTCTATATATTTATTTTTTATCGCTCAAGATGAACTCACTATTATTTAGTTTCTATATATTGAGGAGTATTTTTGTCAGAAATAAAATAATTATTTTAAAGTGATGTGATTGGCTAAAACTGAGCAGCAGCACTCTGAATGAGTGCTGCTGCTCAGTCGAGAACTTTCCGCACGAAGATGGCAGATGTCGAGTTGGAGTGCGCGGTATACGGCGAAGGGACTGTGTTTCCCGTGAAGATAGCGAGCAACGCGAAAGTGAGCGCGCTGCAGAAGGCCATGTGAACGAGAAGAAAGACGTCGATGATCGCTTCAAAGTTGATCCAGCCAGATTGACGCTCTACTTGGCGCGGAAGAAGGGGGAAGCCACGTGGATGAAGCACGACCACACGGTGAAAGGCTTCTTGTGAGGTGGCATCAGCACCGAGTATGAAGAGATGCTTTCGTCGTGGATTCTCGACGAAGATTGCGGTCGTGGTGATCATGTCAACACCATATTCATTCGAAGCTTTGCCCGCAACCGCAGAATTGGCATTACCAGTGGCGTGGAAAGTGACCGCGACTAGCACTGCAAGGATGCATGTGAGGCGCATTTTTTGTCGTTTGGGTCAACAGCTAGGGCACTTGAGTCGAAATGAGAGAGTGCGAGCAGATTGCAGAATGAGGTGTCGGACGAAGACAGAGCCGTACTACGTGCTGGAGCACGAGGAAGTTCCACTTTGTACTGTTTTCTTCCAGTCTGTACAAATAACGCTGGGATATAGCTACCGATCCAGTACATATTTCCAGACAGTTATTGAACAAAAAACCAGTACACTTAAGTTTGCAGCTAATAATGGTTTTTGTAGTCCTATTCACTTATAAAAACTAATGTCTAGTCACTATTAGAAAATTGTGTTTAATACACAAGAAGAGCAATTCAATTCGCAATTCAAAAGTGCGACAGCAGACCTCCTCCAGTCATCCTCTCCGAGTTGCTTTCTTAAAATGGATGGCCACCGCAGCATTCTTGCAGCGACATCAGTGGCAGCACTTTTCTCATGCGTAGTACTACTATAGTAGCACTGAGCTACCACATTGATTTAAATCAAAGTCACGATTGGGCTTGTTCTCGATCACTAGATACTTCTCCGCCCAATATTCAAGCTCATCGTTCTCAAATGACGTATCAACCATGGCTGAACGCATGTCATTAATTGTTCGACCAGTTAACTCCACCAGCTGAGTTAGTGTTGCCAACTTCCGCTCAGACTTGAATGTCAATTCGAAATCTTGATCAAGTGTAGAAAGCTCAAACGTCTTCGAAGTAGCTGTTTTCCATGGCTCCTTGACTTCATAGTCATCCTTCTATTGCTTGTATGCAGCAGAATCACAACCAGCAGCAATCCCTTTCAGTTTGCCTTCTTTCAGTGATGGATTATACTTGGAATCAAACCAACCATCAAATTCTTTGGTAGCATACTCCTTGTTGAAACGCTCTACTTTTTTGTTTGAGCACACAGAGATAGGTCTTGCGCACTAAATGCCAATCCGAATGAACTTGACAAGCAAAGAATCCAGCGAGATTCCACACGTTTTGGTTGTTGGAGAACAAATCGCCTCGAATCATCATGCACAATTGATACAAATGCTCATCGTAGGATTGAGCTGCAAGGAAGTATTCCACACCGCATTCAACGTTTGCTTGTTCAAATGATTCACGGTCTTCATTGGACTTAAAGAAGAAATTATCAAGCATCTGCGATTCATCCAGTTCATTAAATTGATATTGCAAAAGGTACGTGTATCCGTCGTTGCCAAAGTCAGGTGTGCCATCATCACTGTGCTTGTAGTTGATGGAATCCCCAACTGCGTGCATCTTCGACTTGATCCAATTACTTGGTGACAACACGCGCGAATCTCCCAGCCTGGGTTCATCGTATTTATCAACAGATTTGATGTGCTGAATGCAATGCTCACCATAAATTTCTTCAATAGACTTGGTCATCGTTGGTTCAGCAACTGCAGGCACAACAACTACAGGTTCAGCAGCTACTAGTTCAGCAATCACTGGTTCAGCAACTACTAGTTTAGCAATCACTGATTCAGCAACTACTGGCTGATACTTCGCGTGGTGTCGGCACCAATCGCCTTTTGAGCCGCACTGTTGGCTCGCCTTTGCGTGGTCTTCTGGGAAGATGTACTTGCACATTGTTATTTCTATATATTTATTTTTTATCGCTCAAGATGAACTCACTATTATTTAGTTTCTATATATTGAGGAGTATTTTTGTCAGAAATAAAATAATTATTTTAAAGTGATGTGATTGGCTAAAACTGAGCAGCAGCACTCTGAATGAGTGCTGCTGCTCAGTCGAGAACTTTCCGCACGAAGATGGCAGATGTCGAGTTGGAGTGCGCGGTATACGGCGAAGGGACTGTGTTTCCCGTGAAGATAGCGAGCAACGCGAAAGTGAGCGCGCTGCAGAAGGCCATGTGAACGAGAAGACAGATGTCGATGATCGCTTCAAAGTTGATCCAGCCAGATTGACGCTCTACTTGGCGCGGAAGAAGGGGGAAGCCACGTGGATGAAGCACGACCACACGGTGAAAGGCGTGGCATCAGCACCGAGTATGAAGAGATGCTTTCGTCGTGAATTCTCGACGAAGATTGCGGTCGTGGTGATCATGTCAACACCATATTCACTCGAAGCTTTGCCCGCAACCGCAGAATTGGCATTACCAGTGGCGTGGAAAGTGACCGCGACTAGCACTGCAAGGATGCATGTGAGGCGCATTTTTTTGTCGTTTGGGTCAACAGCTAGGGCACTTGAGTCGAAATGAGAGAGTGCGAGCAGATTGCAGAATGAGGTGTCGGACGAAGACAGAGCCGTACTACGTGCTGGAGCACGAGGAAGTTCCACTTTGTACTGTTTTCTTCCAGTCTGTACAAATAACGCTGGGATATAGCTACCGATCCAGTACATATTTCCAGACAGTTATTGAACAAAAAACCAGTACACTTAAGTTCGCAGCTAATAATGGTTTTTGTAGTCCTATTCACTTATAAAAACTAATGTCTAGTCACTATTAGAAAATTGTGTTTAATACACAAGAAGAGCAATTCAATTCGCAATTCAAAAGTGCGACAGCAGACCTCCTCCAGTCATCCTCTCCGAGTTGCTTTCTTAAAATGGATGGCCACCGCAGCATTCTTGCAGCGACATCAGTGGCAGCACTTTTCTCATGCGTAGTACTACTATAGAAGCACTGAGCTACCACATTGATTTAAATCAAAGTCACGATTGGGCTTGTTCTCGATCACTAGATACTTCTCCGCCCAATATTCAAGCTCATCGTTCTCAAATGACGTATCAACCATGGCTGAACGCATGTCATTAATTGTTCGACCAGTTAACTCCACCAGCTGAGTTAGTGTTGCCAACTTCCGCTCAGACTTGAATGTCAATTCGAAATCTTGATCAAGTGTAGAAAGCTCAAACGTCTTCGAAGTGGCTGTTTTCCATGGCTCCTTGACTTCATAGTCATCCTTCTATTGCTTGTATGCAGCAGAATCACAACCAGCAGCAATCCCTTCAGTTTGCCTTCGTTCAGTGATGGATTATACTTGGAATCAAACCAACCATCAAATTCTTTGGTAGCATACTCCTTGTTGAAACGCTCTACTTTTTTGTTTGAGCACACAGAGATAGGTCTTGCGCACTAAATGCCAATCCGAATGAACTTGACAAGCAAAGAATCCAGCGAGATTCCACACGTTTTGGTTGTTGGAGAACAAATCGCCTCGAATCATCATGCACAATTGATACAATCGCTCATCGTAGGATTGAGTTGCAAGGAAGTATTCCACACCGCATTCAACGTTTGCTTGTTCAAATGATTCACGGTCTTCATTGGACTTAAAGAAGAAATTATCAAGCATCTGCGATTCATCCAGCTCATTAAATTGATATTGCAAAAGGTACGTGTATCCGTCGTTGCCAAAGTCAGGTGTGCCATCATCACTGTGCTTGTAGTTGATGGAATCCCCAACTGCGTGCATCTTCGACTTGATCCAATTACTTGGTGACAACACGCGCGAATCTCCCAACTTGGGTTCATCGTATTTATCAACAGATTTGATGTGCTGAATGCAATGCTCACCATAAATTTCTTCAATAGACTTGGTCATCGTTGGTTCAGCAACTGCAGGCACAACAGCTACAGATTCAGCAGCTACTAGTTCAGCAATCACTGGTTCAGCAACTATTAGTTTAGCAATCACTGGTTCAGCAACTACTGGCTGATACTTCGCGTGGTGTCGGCACCAATCGCCTTTTGAGCCGCACTGTTGGCTCGCCTTTGCGTGGTCTTCTGGGAAGATGTACTTGCACATTGTTATTTCTATATATTTATTTTTTATCGCTCAAGATGAACTCACTATTATTTAGTTTCTATATATTGAGGAGTATTTTTGTCAGAAATAAAATAATTATTTTAAAGTGATGTGATTGGCTAAAACTGTGCGGTGTGCTGCGTGAATGAGTGCTGCTGCTCAGTCGAGAACTTTCCGCACGAAGATGGCAGAGGTCGAGTTGGAGTGCGCGGTATACGGCGAAGGGACTGTGTTTCCCGTGAAGATAGCGAGCAACGCGAAAGTGAGCGCGCTGCAGAAGGCCATGTGAACGAGAAGAAAGACGTCGATGATCGCTTCAAAGTTGATCCAGCCAGATTGACGCTCTACTTGGCGCGGAAGAAGGGGGAAGCCACGTGGATGAAGCACGACCACACGGTGAAAGGCTTCTTGTGAGGTGGCATCAGCACCGAGTATGAAGAGATGCTTTCGTCGTGGATTCTCGACGAAAATTGCGGTCGTGGTGATCATGTCAACACCATATTCATTCGAAGCCTTGCCCGCAACCGCAGAATTGGCATTACCAGTGGCGTGGAAAGTGACCGCGACTAGCACTGCAAGGATGCATGTGAAGATCGGAAGAGCACACGTCTGAACTCCAGTCACCCAGCTGGATCTCGTATGCCGTCTTCTGCTTGAATGATACGGCGACCACCGAGATCCAGCTGGCTACACTCTTTCCCTACACGACGCTCTTCCGATCTTTGCATTAATACTACTGGCTTATTATAATAGCAACCTCCATCTTTTACCGGTATCTCAGTTTTGTTTGCAAGCTCTCAAATGTTTTAAGTAAAGACGGACGAGCTTGATTCGAGCTTACAGCTTACACAAGCGCTTTTGTTTTTCAACAACAAAGTTTTAGCCATGACTTTTTTCGTCAGGCAAATAAACTCGCTATTACATGTACGAGGTATGAATGCCATCATCATCTGTAAAATCCGTGCATGTCAGCTTTGAAACGAAATAGCGTATATGCACATACAAAGAGAAATGCTGCTTGCGTTTCAAGGGCAATCCCAATGAATACACACAGTCAAGCGTTTATTGCAGCTCGTAATTTATAATGAGATCCATCGCTACATGCCTACAGGATGCTAGATTCTGTGAGAGATTGAAGCCAATATATGTCTTCGCCTGAAGCTGTCGGGCGTGATGTCATAATACCGGTGTCGCTGGCTCGTTGGTGGAGGTCGAGCAGCTCGTCGCGTCCACCACGTACATACTGATGCCTTAGCTCGATGTTTTGATAAATAACGCTAGCTCAGATTGAATACATTCTTGTGCGTTTTTCACAAGTACACACTATCATGGATTCGTCTCAAAAGCCATCCTTTCAGAGGATGGAATACCCATTTCGTCCGCTCTGTCCACTCCTGTCTGCTTAAGTGCGACATCTCGATTAATGTGATATGATGCCGAAATTTTCAGAAAGGATGCATGTAATTAAAATGTTATTACACACTTATGGCCGGTAGCGTGTGCGATATCAGGCAGGAGTCAAGGCCCTCATGTGCCACACCTCATACACCATGCTTGGTTGGGTAACTATTTGCGCCTAGCTTCGAGGCGCTGGGTGCACATCAGCAACGACAGCATGGAATCAATCTATCAGGACGAGGCTGGTGACTGGCTCACAGACCCGCGACGGGTTGGCCAAACTGTACGCAGCGTACTGGCCAAAGCGTAATACGCTGGCTACGAATGAAACCACCGAGGCTTAACGCATCGGCTCATGGGTGGGCTGTCCCCTCGATCTTCACGAAGAGATAACGCGGGAGTTTTCCTGGTTTTGGAATTGTCACGTAGACACGTAGATCAATTGGTTGCTCTTTTGTAGGCTTTAGGGCTAACCCGTGCGACTCGAGTGTAGGTACATGACATGCACGATGATCTTGCGGATGCATCTTGCACGCGCAAAGCATAGCACAGCAACCAAGCTAGAAGCGAAAAATATATTGAAAATCAAGCACGTAAGCATGAATATCGGCGTAGACTACAGTACTGCCTTCGATGCGACATCCTAGGTCCTGGATGGCCTAAGGCCATGCGCTGCTGCTTCCTCCCTTTTTAGAAGGGATTCGACGTCGGCTGCTCAATCCAGACCCACAGCGTGATGAAGATGAGTCCAAAGGCAATCGAGATCGAGCCCACAACGTTACCCTGGTCCATCAGACTCTCGTAGCTCCAGCCAAAATATTCCTGGATGCTCATACGCACGCTCGGCGCAAGCTTTACGGCGATGAATAGACCCATCATGAGACTTGCGAAGAGCAGTGACGCGCCGTGCGACGTCTGGATGCTGTTGTACCCAGCCAGGAAACGCACAACCGACCACAGTACACCCACGACGGTTCCGAAGAAGATGATGACGTTCGGTAGCTCTGCGGTAGAGCCACGGCTGGCACCGCCCGTGTTGAACCACTTGGGCTCCTTGCCCGTGATCTTCCACCAGAACGCCTCCAGCACGGCCGTGCAGTTGGTGAACGCGTAGGCGAACCACACCTCCTGCGATCGAATGACATCGCTGTTGTCCACGGTACGGTTGCTCAGCGCCGACAACACGCCCTGTATGAGCAGCTTTGGCACCAATGCGTACACAAGACGCGCACCAGCCATGTAAATAGGAGCGTAGCCCGAGAATAGGAAGTAAAGCGTGATGTAGTAGAACAGGATAGCCGTGATCGAGCCAAGCGGGTAGAGCGTCGAGTTAAAGTAGAACACGCGACGCATGAACTTGTTCGACTTGTGGTACGACGGGATTTGCGCCTCCGGCATCTTCCACTCCGGGTCAAAGTACTGAGTCTTCTTGTTCATCAATGCGATCTGGAAGTTACCCTTGGCCCAGCGTTTACGTTGAGCCAACGAGCCAGCCACGGAGTCAGGAATCAGTCCTTCAGCCAGACGGATACGCTCTGACGGCTCGCCCTCGAAATCCTTACGGAAGTACTGAGCCTTCCAGCCCATGGAGCACAGCACCTGACCAGTGTAGCAATCTTCCGTCAGACTACCGTACTGGATTCCACCAATCGAGTCCAAAGCTTCACGACGGAACATAGCGTTCGTGCCGGCAAAAGACGCACAGTCGTAGCCATCTTGACCAGTCTGAATAGCATCGTAGAACGTGGCGTTACGGTGACCCATCGGGTCTCCAATCTGCAGCTGAATGCAATCGCGGAAGTACTGAGGCGTTTGAACAAAGGCCACCGCGTTGTCGCGCACGTCATCGGAGTAGTTGTGGAGCGATGAAACATCCTCCCCATCCGCCCCGTGGTTGCCACACTTGCGGCACACACCGTTCTTGGGCAGTTTGGCATCACAGTTCATGCAACGCATGTCGATCTTGCTGGCGCGAGTATCGCTCATAGTGCCGTTCACCTGGCGACGGTGGACGGCGCTCTGCACGTGCATGGCATTCTCGAAGCAGTCCTTGGAGCAGTAGCTGATCTGCTCCTCTGGCACGCCGGCAATCTGGCACGAGGCACAGCAAAGCTTGGCGACAGCGTTGCAGCCGATACCACAGCAAATGTACTTAGCCTTATCCTGGCGATCCTCGTCGTCAAAGAAGAAAGGAAGCGTAGCCAAAATGAACTTCGGGTGAGGCTGCATGTCCGTATCCAGGATGATAAGGTAGCGGCCATTGGCTCCTTCATTGTACAGGCAGTTGTTGATGTTACCGGCCTTCGAGTAGTGCGTTTCTGGTTTCACACGACCAATGAAGGTCACGTGCGGCAGACCGGGGTAGCGGTAGTCGTCACGGAACGAGCCCACGGCACAAATCAGCCACGGTTAACCACCTTCACACGGGATGGAGACGGCAGGTTGGCGGACGAGTGCAGCTTACGCCACGCATACACCTCCATATCCTCGTTGGGGTCGCACACACGGTCGTACATGAACTGAGCCACTTCTTGACGGAGGTCTCCCGCAGTTTCCAAGATGCCCTTGTTCAGCTCCACGGTCGGGACCGGGTTGCCCTTGGTCCACTTGGTCTTGCAGTAACCGTCATCACAGACCCAGATCTGCAACAGGTGCGGAGGGTACTGCAGGTTCATACAGGCCATCAACGTGTCGATCGTCTCTTCAGCCGGCTCAGAGTAGTGACACAACAGCACATCGACCTTGGGCCACAGATCATCCGGGACGGGCGGCTCGAACTCKTCGAAGTAGTGGGCGCCACGACGCACGGGCTTCCACATACCAAGATAGTAGAGAAACACCCAGAGCGAACGTCAGCACCTCAGCGAACAGCATGGCGAAGTACGAGGCCCATTGAGGAATAGGCGGATCGCGGAAGCAGATCACGTTTATCGACGTCAGCCACCTCCAGATACCAATCGGGTGGGGTGAACCAGCCACTAAACGGCTCGTTTGAGCAACGGAGCGTCTCCAATGACGTGCTTGTAATTGTGGGCACAGTCAACTTCCAGACGTGAGGCATCGCAAGGCTCCAAGCCGAGTCACGAACACCAAAACACGATAGCAAGAAGCTCTACAATGCCGGCGATCAGGAACAGGAAGTAGAAGAACCGGTATGTCTGGTTGCCCTTCATCAGGTACTTGCGGACATTAGGCAGCTTGCGCTCGATAATGTTGCCGGCACCGTTGGCCACAATGGCGCGTTCGACGCCCTGGAACCAATGCCGGTACTCCTCGTCCGTCCTCAGGACATAGCACCACACACGACGCTTAGTGTGCACCTCGAAACCCTTGTGAGGTAGATCGAGACGCGCACACGGACGCAGCTGCAGCGCGTCTTTCAGCTCGATGGCGCCCACAACCAGATCGGCGTTACGCGTGGTGAAATACTGGCGGCACTCCAGGTGCGAGTAGAAGTAGTAGATCTTAGCCTTCTCTAGCGCAAAGTAGCGGCGTTTCCAACTCTTGTGGATCGGTCCCCTTGATCGTGCGCTTACCCTGTTTGTGCATGTAGCCGTGCACCGAAATGATGTTGTCACGCTGACCGCCGAGACCCACGGCGTCCGAGACATTCATGGGAGGTAGTGCTGGACCGCGCGGATACCAACATAAACCCTGCTGGGGGGTCGGACGACCCTCCTGCGCGTAGAAGCCGGCGCCGCCGTCATTAGCTCCCGTGGCTGGCGTGCCGTGCAGCTCGTAGTCCTTCGTGCTTCATAAGCGACTGCTTGTCGGTTGCCGTACATGGTCGACACGCGTATTAGTACTACACAGCAGCCTCTCGATCTCTCGATCCACCTCGCTTAGAGTCAAGGCAGTGAGAAGGAGTGACTTCGCCTGTGTGTATGTGCTTTGGTCTCTCGTGAAGAAGCGCAACTGGCACTCGGGCCCGATTCCAGCCGGACGAAACCCCTACCAGGTCGTTACATAGTGAGCGCGAGTTATTTGCGCCGTCAAGTCCCTCTCTATGCCGACCTGTTGAAAACGCATTCGCACGTGTTTGCACAGAGTCACAGGGACAGTACGGTCCACTCATGGCGGACGCCAAAAGTACCCAATTGTGTGGCTCACATAAAATTATGATGACTTTGCTTTTCAGTGATGCAGTGATAAGATGTATTCGCTGTTGCGACTCTATGAAGCAGTGCTCGGCCTTTCCCGGAATAAGATCTATGGATTGTTTGATATCCCAGCAGAAACAAGCCTGTGAACCCATTTGCGAGAGATCCTTGCGCAGGATTCTTGGCACCCGGTTTCATTGCTTCAGCTTAGCTGAATCAGCTAGCGCATCGTTGGTGCGACTTCTTCTGTCAGCAGTTCAGAGCGAATCTCCGCTGACCGTCGAGCTGCGTGGCGCAATACGATTCGCGATTCACAAAGTATAACTTGTCCGTCCTTCTACCCGTTACGATGCTGTTACACTGCACTTACAGGAGTTGCAAACGCCTTCTTTCGTCAGTAGCGACTCGTTTCGGTTGGCAAGCACTGTCTGCTCTGGGTTCAAGTCGCGGCGTTGAGTTCGAACGTGGCGGACAAATCGTAAGGCTCGAACAGGTCAAACTTTGACCTGTCCGCCTTTGACTCAAAGGCTTCAAGAAGAAGAAGAAGAAGCCCAAATTGATGTTGAAGTCACAATCTAAGCACGAAGCGGACATCATATATGTAGTTAAGTAATGGAGATCGCTCGCAAACTGTATCTTCAGTTAAACAACAACTTGACTCAAGTCTTGAGTGAAGTAGTTTGTCGCAGTGGTGAGCAATCCTGTGGCGAAAGTTACAACTACTATTAGTAGTTGTTACAGCACTCAGCAGCGTGACTAACTGTGTATCGCGTGGATCTCAAGGCTTAGTAGACTACTAAGCCTAAGGCTTAAGTAGGCTACTAAGCCACGC

>Contig_61

GCTCTATGTGTAATTCGAAGGTTTAAACGAAAACCGTTTTTAGACCTCGAGAATCGCAGAGAGAGTCGTGGCAGTATCGATGAATCTTACTGAGCCCTCAATTGACGTCAATCGGTACAGCAAAGATCACGAGATCCTGCCAGCACGTACGACTACCGTGGCGCTTCACCGCAGCATCTGAATTATCGACAAACCGATACTTATGCAGTAAAATCTGTAATTACCTCTCAGAAAATATATTTAACCTCCTATATTGTATAGCGAAAGGTCACCCTCAACACGACAGGAGTCGCGCCACGCGGTTGCTTAGGTACGACACAGATTCGCGCCCACGGTACGCTGTACGATTCCTCGGTGCTGTAGTGCATCTCCACATCCTTCTCGAATTGTACCAGCGAAGAAGATGGCCTCCACATCCAAGCCTTGTCCGTGCACGAACGCCTCCTTCAGCTTGAGTACGCCGCTCCAGTGATTCTCCACACCCGCCGCCTCACATATGCGCACGTAGAAACGTCGCTCCCCCCAGTCCAAGCTGCCGTCGATGACAAGCGACACGATGAGCAGGAAAAAGAGGTTGCGAAGGTGGTACGACGAGAGCAAGAGCAACGATGACGCTGAAGCAGTTGGTGAGACGGATGGTGCCGCTGGATTGCTGTTGCCTAATAAACGAAGGTCGGTAATTAAGACGTCGATGTCGTCCAGTACGTGGGCTGGACATCGTTCCGAGGACTCTTGCAGACACGTGCAGCCGTCGTCGCTTACGGGCCACGCGTCTGCGTTGATCCAACGGTGTCGCAAATGTTCTACCATAATCTCCATGTTCGGGTGTACCATCCGCTTACACACGAGACAGCATCCCATGATACGCATGTAGTCGACTCGCTGAGCTGGTTTGAAGCACGCGTCCTCCAACAGAAAGCTCTCGAGGGCCTCGATCGCGCATGGTGGTCCAACGATATTAATTCGGCAGTTCCGCAGCACACGTCGTATTGTCCAGGCATTTAAGAGACCTTGGATCGGAAGTACAGTAGCGTTCAGCGCGGAGCGTGCGGCAGCGCGCCACAGAACACGCTTAATAAAGAGCTTGAATAGGAATTTAACCACATACCGTTTGCTTTTAAACACGAGATAAGTCATCAGCAACACGAGTCGCGGAGATCCACGCATTGGGTCCATTCCAAAAAGCGTATCTTTACGAYGTCCAACTTGTAAAGCTGCGCGTGCGACGGCGCGAGCGAGAAACTCACGCTCATGGTCTAGAGGGTATAAGATGAGACCGGCACTAACAGTCAGTCTGAACGCGCAAGCCACAGCCATCGTATAGAGCAACGTCACTTCAAGCAGTGACACAGCGATATTGATGAGCAACACGTGGCCATTCAAGCCCCACACTTCATCGCCCGCAAGATGAGAAGGTAACAAACTCTCTATGCCCATAGAAACGAGTCCACACGAGAGTCCTATCAAGAAACCCAGCGTTGTTCCTGACCATTCGATGTTGCGAAGCTGACACATAACCTCAGGAGAGAAGCCATATCGACGATCAGGTATGTTTTCCTGATTTTGAAGATGCAGACCACCATGGAAAGTAGGCTTTCTCTTAGCGGAAACCAAGAGAGATTGAGGAGGCGCGACAGCGTATTGTTCATCTACGGGCAGAGTCTGTGAAGCTCGTGCCCATGTTCCAAAACGAGCAGACAGACTGACCGCAAGCGTCACTCGCTTCACCCGTTTCACACGTTCCTTTGACACACGCGTGAGGTACTCGACACCTATACGTTCCATTCGAGAAATATAACTATTCGACGATTCGTTGGCCGTCTCGTCGGCTTCGGGGGCTTTGGGCCCCCGTTTCAAGCGCTTGCGCGTTGGTCGTCGCTGTTGTCTATCAGCCGAAGTATGTATAAAAGACGGTAACTCGGTAAAGAGATCAACGGAAAAATACTTGTGCAATTCGGAAGTGCCGGTACGCTGATCACCCCGTGAGTCCTGCCACGCCGCATACAAAAAGTCGCTCCATTCGATGCGCCCGTCACCATTGGGGTCCAGTCGCACCCAAATCGCATCAAGTTCTTCATCACTAAATACGAGCCCAAGCTTCTGTAGTGCGATTTCCAGCTCGGTTGATGAGATCTCGTGACTGCCGTCGGTATCGCAGCGATTGTATGCGTTTTTCAGCGCCTTACATCGAGCCACAAATCTTGTGGAAGGGAGTGCTCTTCGCTCTTGCGAACACCGTACAATAACATGTCGATACCTTTCCAAGAATGAGTCGTAGGTTATAGTCTGATCTCTTAGATCGCTTGTTGATATTCCAAGCACTGGAGATTCCGCTGTATGAGTTCCAAGAAGACAGACAACTCGTTCGTCATTATTGCGCCATCGTCGTCAGCGTCACAAGCGTGAAAGAGAGCGCGTAATCGACGCATATACTCTTCCCCAACTTCTTCATTGAGACTCAGAGGGTTCCTTGCATTCAGTCGCCTGGCTGCTACCGCTGCAAAAAGTGTCGCTTTTCGTCTCCTCAATGCCTCACTGGGTGGCCCATTCTCAATTTGCGCTCGCGGCCGCTTCTTCGAGCTTGCGGATAGTCGGTGCTTCGCCATCGAGTAATGTACAGCAGCATCGTTGGTAGTGGGTGAAGGGCATCTGACCTCCGTGAGCGACGTATCCGTGGCAGGCGTGACTTTTGATACCATCACAATGTGGCTAAAGAACAATCGGATGTATGGAGCATATTAATAGAGTCGATCTCTGAGTTAAAAAAAACTCTATTTTCCTTCCATTAAAACTTCAGCTCTAAAGTTTTTCATGGATAATACTGTGTAAAAAATAACCCTTTCAGGATTTTGCTGGTACGTTCCGGATCTGCCGGTTGTACGCGGTCATAAACTTGGAGAAGTCCTTATCTTTCTTAACACCCCTTTGCGTTTTCTGCAGGATAAACACACGCACCTGTTCCGGCTTCAATCCAGCATGGAGCCACAGTTCGGATTTCTCCATCTCAGACACCGGCTTCGAGATCTTGCTCCCAGGCTGGTTTCTCTTGTAGTTGACAGCGAACTTGAGATACTGCTCGGAATCCTTGCGCATCAGGATAGTACGAAGTGGCTGAGACGGGTCTTTCTTCAGGACCTCCACTCGAACTTGGGACGGCGTTAATCCTGCATAATGCCACAATTCGTACTTCTGCATTTGTGACGCAGGCTTTGGAATTTTAGTCCCTGGCTGATGTCGTTGGAATTCAACAATAAACTTGAGATATTGGTCGGAATATCTGCTTCTCAGAACATAGCCAAGCCCCTGTAGCGGCGTTCTCTTCGCACTGAGGGCGTTCACTGCACTCTGCACCTGGGACGGCGTTAATCCAGCATACAACCACAGCTCATATTTCTCCATCTGTGACGCCGGCTTTGGAATTTTACTTCCTGGTTTTCGCCCCCGATAGTCAACCATGAACTTAAGGTACTGGTCGGAATCCTTGCTTCTCAGAATATAGCCAAGTGCCTGCGACGACTTCTTGTTCACACCCAGGGCGGCGCTCACTTGACTTTGGACACGCCGCGGCGTCAATCCAGCCCTCAACCATTGCTGGTACTGCTCCAGCTTCATAGCCTCCCTAACCGCGCTGGGTCCGCCGGTCTCGACAACAGACCCCACCTTTCCGAGAAGCCCATTAATCATTGGAGCAAACATCTTTTCCTCTGATTCTTCAGCTCCATCGTTAGTTTGAACCGTGTTTCGCAGGAATCTCTTGCCAAAATCGTCTTCTCTTTGGAAATGTGGCGAGTGGACCACGAGTGGGGCGTCTATCGGTGAGTTCTCGGTGGTCTCCCTGGCTGCTGAGACGATGTCGGTGTCGGCAAAGAGCACTTTTCCAGTTGCCAACAGAGCGTAAGTTAGACGCATATTTGGTCTTAAGGTGTCCTGAACAAGAAATCGAAAGCTAACCGACAGTGTCCTCAGTTCAGACACAGTAGAGTCTCTCCTCTCTACTAGAGAGGCGGGTAGTGCAGATCACTGGCAAAATCCTGAGCTCCGGATAAGATTTGTATTCCTAACTAAGGGTATGTTTAGGTACACTAGTTTTTCCAAAAGTGATCATGTTTGATACGTAGGTCTACAGAGGTCTGTACATGTATGATACGCTGTTGGCGGAAGCAAGGCATAGGCAAGCGTACATCTCCACTAACAACAATCGTACTACCCAGAAATTTTTATTACACTGCTATGTCGGCCGAAACTCAAGCCTTGAGTTTTACCATTGTACTTCGTAATAACACTAATGTTTAGTGCATCAAACTCGAGCTGCGGACGTTATATTTCATTCCTTCTGCTGCTTTCAAGTGATCAATTCAATTTCAATTTCAAGTTAGGTATATTGTGTTCTGTTAGTGACCTAATGATCATCAATATCCGAGACTACCACCCATTCCGTTGCTAGTCTTTTTGTGCTTCGGTCTCTACTTTAGGATGACTAGTCCTTTCAAGTGATCAGCTTTACTAACAGTCAATGAGCTATGCCCAACGCACCAGCAAGACACCATACGATGCTACTCTATTGTACCAACTTTCTTCAGTTGAAATCGGCGTAATAAAGTATGACTATTTAGAGGTGCAATAATTTGATGAGCGGGCAAAGGACACACAGCTCCCTCATCAATTATAAGTACGGTAGTACAAGCCGGCGAAAGTGGGCTAAATTGCTTGCGTTATGTGATCCATTCGCAATCTCGCTGCAAAAGAAGCGGCTGAATAAACAGAAAAACGAATTCTGAAAAACAGGTGGGTGACAGGCGTTACTTCAGTAAATTGTTCCAGGTACGTGACCTCTAATGAACACTGTTTATTACCACGGAGACATCTTAAATGGTGGTTCCCACATTTCCCACAATAAAGCTTATGGTGGTATTCATGTGGTACTAGATGTGGGATAGTGTGGGTAAATCTGAAAATAGCTTATATGTTTTGAAACGTTTACTACGAATTAAATCGACTTTTGTCTGTGGAAACCAGTGATACTGTATCACGCTGCAAAGTTCGTAGAATGCCCTCGAGTGATTGCCGGTATTTATTGCTTTCCTTATACGTACAGGTCATGGGGCAAAAGATCAGCCCATTCACAGTTATCGTCGGACTCGATGAGTCAAGTCGATAGGCGGTACGCGAGCGCGGTGCCGGCCCGAGAGTTGAGTCGGCGAGGGCAGCCCTGGCTGTGACGGATTCACACAGCACCTTGCACAGAGGAAGTGCTTAAGGATATTTACTACCCGAGTGAACGTTAATTCTCGGCCTCAGAGAGACCGGTTTGCTACTTACAAGAGTAGATGTAAGACTGATGGATTTATAAGTAAGTTTAGCTTGGGTTCTAATTCAAACTACACCAATTCCAGTCCAAACTGGGACGCCCACCAGAGTTATCCGGGGCGCGCGAGAGTCGGGGAGTATATTACCTAAACCAGTAAAAAAAAACGACATTTATTTCACTTACACGTTCATTATGAAAAAAACTCCTTTTCAAAGACGATCAAATATAGCACATGCCTCAACCACCACGTCGTCATATCGCTGCGTCGCCGCTGCTCTCTCTCCAAAGCCGCAGCGTACTGCCACGGAGAAGGCGCACCGATGCGCTCTGAGCACTTACGTAGATGGGGAAGTTTGGCTTACAGTCGCCATGCACAGCGGAATGCTGCGCTCTGCAGCCTATCGTCTCTGCAAGTCTGGCGATCTTACTCCATCTAGGTGCGCGGCGGTGTGCGCGACGCCTCAACAAAGTGCACAGATCAAATGATCATTGCGATGGAGTCATCCCTGAAAGAAGACTGCACGCTCTCGTTAGAGCGACATAGCGAGCGAGTTGTTGACGTTTGCGGAACAGCTTATTAAAGGCATTGTGGGCAGCGACTAGATTGTGTATTACGACGAAACGATTTACAATCTTTTTTTGTATGCGCTCGCATGGACGCGCTGTGAAAGAAGAGCGCGCTGTCGTCAAGCGTCTCCCCTCGTAGGGCAAGAACTTGCAAGTTCAGTGCGCAGTGTCAACTGAAGATGGCCTCGTGCTGCACCGGGGAGCATCCGCATGGAAGAAAATGCGGAGTCTGCGCATTTACACCACCGTCAAAGGTTTGAGGAGCTTTTAGCAGTAAATTGGAGGGCGGAAATTTGTCATTGTGCTCGACAATGCTCCCGCCAACAATCTAACCGAGAGTCGGCTTGAAGAGGAGCTGGGAGAGCACGACAACACTATCTTGCTAAGGTTGGGGGCCTACTCCCCAAAGCTGAACCCAATTGAAGGTACGTTTTTGCCGGTCGCAATTACAATTATACTTTTTTGAAAAGTAACTTTATTTTTGGCTGTTACAATTGCGCAGGATGCTGCAGTGTCTTCAAGAGCAAAGTGAAGGCATATCTAGCGGCGTATTCTGGACGTTTGTTCAGCTGAGGCGAACACGACACGCTCACGGCGTCGCGGGCCACGCTGTTGACGGACGCTGCAGAGCCATCTGTCCACTGTATCCACCGCCATCTTGTCTTGGTGATGGCTTTACATTGTCAGCGGGCCGTTGCCGATGCACCGAGGCTCGAGGATATGTCTCCCCACGAGACGGACGGTGTCTTTCGATCGAAACTTTAGGTCTGAAGGCATTGGACAAAGCAGTTGCGAAATACTTAAGGTATTTAATTTTGTATCTCTATAGATAAAACTTTAGTAAAATTGCTCTAGCAACCAATTCTTTTCGCCAACTCACCAGTCTCTGATCGACAGCGACCACTCGCAGGCGCCCCCCCGTAGAGATTGTTTCGAAGCGTCTACATGGACGAGCACACGCGTGATGGTATTGCGAATTCTCAAGAAATTCATTAAGTGGTGACAGACATTATCACAGGCCACGCCGCTGCGCGCCCTTCCAACACCGTTAACCCCTTGGGACCCGTGGCCTGGACCGTACAGGTTACGTGAAAAAACTTAACGTGGACCGTACAAGTTAGATAATATTGTACTTAGTTTTTGTATCCGCTAATACAAACTTCATGTCAACCAGGGGT

>Contig_62

GATAGCCAGCTTGAATAAAACAATAGTCAATAAGCCTGTTGATATGCACCGACTCTGCACGGTTCGGCTAACCGGTGTGACGTTCCAAAATGGACCGGTCCCAAACCGGTTAATCTGGTGGATGGCCAGGAGCACTGAAGTTTAGAGGACAGATTTAATTGACTTTAATCTACTCACTTCGGCATTTGCCTTTAACAACAACAACAGGTTAACAGCCGCAAACGCTCAACTCTACTGGAACGTTCCAAAATATGACGACGACCAAGCGCGCCGAAGTCCTCCTAGGGAGTGGGAACTACTTCCACTGGGAGTACAATATGCGCATGACGCTAGCCAGGAAGGGCCTGCTTGCGCATATTCAAGCCGTCAAGCCGGAGAACGAGATCACCGAGGCGTGGCTCGTCAATGATGCCAAGGCGCTCGGCATCATCGCTCAGGGAGTGGAACTCCAGCATCAGACCAAGATTCGGTCGGCCACTCGTTCCATAGAAGCTTGGAGAACGCTGCAAGAGTTCTATAACCGTACCACGCTCCACAACCGCGTGACGATGACGCGTCGCCTCCGCGAGTTCAAGATGGAAAGTGGATCGACTATGGCAAAGCATCTGGATGCGTTTGATGAGCTGATCGTCGGGTTGCAGACGCTAGGAGAGCCCATGGACGAGGCACGCCAGCTGGTGGTGCTGCTGAGCAGCCTCCCCGATGAGTACGAGCTCATTTCGTCTATCGTGGAGAACGCCAAGGGTGTGACGCTCATCGAGGCCAAGGAGAAGCTGTTGAAGGAGTATGAGCGACTCGAGAAGAAGGAAACCACGGAGAAGGCGTTCCAAGTGAATGGGAACGCAGGCAAGTTCCGAAACGGCCGAGGGAACGGTCGAAAGTGGAACGCTCCGAAGCGGAACGTTGGTTTCAAGGGCAAGTGCTTCAACTGTGACCTCGTGGGCCACATGAAGCGGGACTGTCCTGCTCCGAAGAAGGGTAGCCAAGACGATGCAGTGTTTGCAGTTGGTGGGGAACGCTCGGCCGGGTGGCTAATTGATAGCGGTGCGACGTCGCACATGACCCCTCACCGGAACGACCTGTTTGAGTACAAGTCGTTGGACACTAGCATGGAGGTGACGATTGCAGACGGCAAGAAGCTGCACGTCGCAGGAACTGGAACGGTCAAGTTGACTGGTTTGGACGGTAGACGCATCCGTATGGTGGACGTGCTCCACATCCCCGGGCTCGATAGACGCTTATTTTCAGTCGGGAAGCTCGCGGAACGCGGTATGAGTGTGGAGTTCCAGCGTTTCAGTTGTGTAATTTGGGGACGGAACGCTGCAATAGCGTCAGGGAAGAAGGTCGGTAAGGCGCTCCTTTTGGACTGTCAGCAGGAGGAGGCCCGTTTCGTGGAATACGCCGGTCCTGACAGCGAGTGGGAGCTTTGGCCCGCTCGCATGGGTCACCCCAACAAGGACGCACTGACGAAGACCCAGCGTTCCACGACCGGTATCCCGCCAGTAAGAAGGGGGTGCGAGACTTTGTGCGGTGGATGCATGAAGGGAAAGCAGACCGTGACGGCGTTCCCGTCACGATCACTGACGAAGACAACGCGTGTGTTGGAACTAGTACACACAGACGTTATGGGACCAATGAGAACGCTCTCAAAGGGGGGTGCCAAGTACGTGCTGACCTTTGTAGACGACTACTCAAGGTATGTCGTGACATTCTTCATGAAGAGCAAGAGTGAGGTGGGCAGCAAGCTGAAGGAGTTCAAGGCGCTGTATGAAAATCAGTGGGGGGAACGATTGAAGTGCCTGCGTTCCGATAATGGAACGGAGTTTGTAAGCAAGACGGTATTGGAACTCTGTCAGCGGTATGGCATCGTGCATCAACGCAGCGTTCCATACAGCCCTCAGCAGAACGGTGTTGCGGAACGCATGAGCCGTACCATCATGGAGAAGGCACGAAGCATGCTACACTACAAGGCTATGTCGACGGAGTGGTGGGCTGAAGCGGTGAGTACTGCAGTGTACCTTATCAACCGTTCCACGAACACATTCAACTCGGATGTCACGCCATATGAGCTGGCGTTCCAGGTGAAGCCACGGATGGAACACCTACGTGTTTTTGGGTCACAAGGGTACGCTCATGTGGACGACGTGATGAGAACGAAGCTGGAACCCAAGAGCTTCAAGTGCACGTTCCTCGGGTACGCTGAGAACGTGAAAGGATATCGTGTGTACGACATGGATGCGTCGAAGGTCAAGGTGACACGGTCGGTCAAGCTAGACGAGCGTGAGGTGGGCGGAATCTATGACACGCAGTCGCCAGCTTCAGGAACGGTCATCMATGTGACGAGGAACGATGATACGATCGTTCCAATATCGGGAGTGGAACGTAAGCCTGCTCAGGATGAACCGATGGAAGAAGCAGAGGAGCCTGCTCAAGACATTGAGATGGACGACCTTGAACCGGAACGGAACGCAGAAATACAGCAGCTACCCGCGCCGGAGGAACCCAGGTTAAATGGGTTGGATTTGGCAACGTTCCATTCGCAGCCTCCATCGTTCCATGAGGACCGGATGATCTTCCATCCTGAAACGGAACGATCTACGCGCCCTCGTGAGCCATTACTTCTACTTGGGAATGGTGACGATGAAGATGTGGAGCGTGGAAGTGATGGACCGTCATCTCCTAAGCGTGCCAGAATCGACGAAGATGGTTTGCTCGCAGAGGCAGTGTTGGCGTACGCGGCCAGCATTGGTGGCGTTCCAGATACGCCTAACACGTACGCAGAAGCTATTGCCAGTAACGAAGCAGGAGAGTGGCGTCGTGCTATGCAGTCAGAGCTGAATTCGCATTCCCGGAACGGAACATGGACGCTCGTTCCACGTGGAACGACAACTCGTTCCATTGGATGTCACTGGGTGTTCACGAAGAAGCGTGACGAGAACGGCCGGGTTATCCGTTACAAGGCACGCCTAGTGGCGCAGGGGTTCAAGCAGAAGTTTGGAATAGACTTCTTTGAGACGTACTCTCCAGCTGCGAATATGAACTCGATTCGAGTTGTACTCGCTGTTTGCGTTACGTGCGGCTACATTATGGAGCAGCTGGATGCCGACACAGCGTTCCTAAACAGTAATTTGAAGGATTTGGTCCACATGGACGTTCCATTTGGCCTCGAGAATGCTGAAGGCATGAAGTGCAAGCTGCTAAAGGCCATCTACGGCTTGAAGCAAGCTGCAAGTGCATGGAACAAGACTATTTATCGCGTGTTCCTGCAGAATGGTTTCAAATGTTGCGGAGCCGACCAGTGTGTCTGCGTCAAGCGTTCCAAAAACGCATTCGTGTACGTATGTCTATATGTGGACGACATGATCATTGCGGCCAAGACGCGCGACGAAATTCGCGAGGTCAAGAACGCCCTGAAGAGCGCATTCAAGATGAAGGAGCTTGGCGAGGCCAAATTCATTTTAGGAATGGAGATTGACCACGATAGAGAATGTGGAACGTTGATGATCAAGCAAACTCGGTACATTGACGACATTGTGGAACGATTCAATCAACGGAACGCCAAGATGGTGGAGAACCCTTGTGCAGCAAACCTGAAGTTGTCCAAGATGATGTCGCCGACTACGGAAAAGGAACGTGCCGAAATGCGGTCACGACCGCATCGTTCCTTAATCGGGTGCATCATGTACATCACAACCTGTACTCGGCCGGACATTGCCTACGTTGTCACGTAGCTGTCACGCTTTCTAGAGAATCCTGGAACGCAACACTGGAAGGCAGAGATTCGTGTTCTGCAATATTTGAAGTCCACTCGCCATCATGGTATCGTTTACAAAGTGGAACGAGTGGATTTGGAACGCAAGCTGTCAAGGCAGAAGCGTTTACGGACGCAGACTGGGGAAGCAGCATTGACGACAGACGTTCAGTGTCTGATGTCATGATCATGATTGGGAACGCTCCGGTGGTGTTTAAGTCCAAGTACCAGAGGACGGTTGCACTTAGCTCAGCGGAAGCAGAGTACATGGCACTAAGCCTTTGTACTCAAGAGGTACTATGGACACGTGCCATGCTGAAGAACATGGGTCACGAGCAAGTGGGAGCGACGCAGGTCTGGGAAGACAATCAAGGTGCTATTGCACTTGCAAGCAACGCTGGATATCACGCGAGGACCAAGCACGTGGATATCCGTCACCACTTTATTCGAGAGAATGTGGAACGTTCCACAATCAAGGTGGCCTACATCGACACGAAGCAGCAGTTAGCTGACATGCTTACGAAAGCACTGGGAACGAAGTCGCTAGCGTTCCTTCGCGAAGCAAGTGGAATTAAGAAGAGGAACGACGTGCAGTAGCGGTGAGTGGGAGTGTTGATATGCACCGACCCTGCACGGTTCGGCTAACCGGTGTGACGTTCCAAAATGGACCGGTCCCAAACCGGTTAATCTGGTGGATGGCCAGGAGCACTGGGACAGATTTAATTGACTTTAATCTACTCACTTCGGCATTTGCCTTTACCAACAACAACAAAGCCATGCTTGTACGACTTAGCAAAAAAGAAAAAGTATGCGCAGCGACATCAGTGCCTATACCGAAAATGAGCTTTGAACTGCTGCTAAGGCTCTCCACCATTGCTGCTTCGTGGCACGTATTTCTAAGCGTAAGCTTAAGGCTATTAGTACCAGGATGCTGTAGTTAGTTTGTGCCAAAGATAGTGCAAACAGTACCTAGTATTTGCACTACCTAATCGGGAAAGCGCTTTTGCTCTTATATTTTACACATTCGAGATAATTTGACACCTGCTCTTACATGCCACAATACAGCTATGGCATTATTAGTTTTTCTTATCCCTTTCAGGCTGCAGTCTAGCAGGGCTCCGCGAAGGCCGGGAGCCGAGACTTACTCATAAGTAAAACTAAGTACTTCTGAGTAGTTTTACCCGGTTTAAAGTGAAGCTAATACTGGTCTTGAAATGCTGAAAGGTGGAGTAAAAATACTACTGATCTTACAAATACGCGATCTTAGGGCCGGAGGGGGGGGTACAACGACAATCTGTAGTAAATTACACCAGCGTCCCTTATTGAATTTGAGAACAGCCCCTTTTTTAATACTGGCTCTCTACCTACCTCTTCAATCAGATTAAGTGTATCACCACGCAGCCTGGATGTAGATTTACTATCTTTCAGTTTTTGATCGCGACTGCGCGCATCAGCTCCAGAGTTGGTTATACGATAAGCTATACGTAGCCTCATTTCACGATGGGCAACGTGAATTTTGCCTTACGGCTGATGCTTGAAATTCTCAAAGTGGCTTCTCCAAGTAAACATCTTTTCAGGAACTTTCAGGATATAACAACTGTTTTTAGGTAACAACACAGCTCATGATTTTTCCAGTGATCACGGTAGTAATTGCGGTAGCCAATTGTGTGAGCGCCAATCCCTCGTGCACTCCCGACCTCGTTCGACTCGCTTGTCATTTCGCAATTCATTATTCAGACATCAGGACCATTTCTACAGATCTCAGCAAAAGTCCCTTTCGATGATACGTCTCTTTGTAGTTGTATTGCTGATTTTTGCCGCATCTCTAGCGTGCGCCGCGAAGGAAACGACCTCGGTGAGGACCACACACAATTATCTTGATGAGAAAGATAGTGCTCCTGTTCAAAGGCTCTTGAGAGCCGATAATGACAAAGAGAGAGTCGCTGTAGAAAACGCCTTGAATTCAGTTATGACCAGTATATCGGAGGCCATCGACTCCGCTAAGCTGAAAGTGTTTTTGCTCAAGAAAAGTAGCGGGGACGACGTCATGAATAGCTTAAAGTTCGGGGACGATGCGGCAGCTGCTCTAGAACATTCTAAAATGAAGACTTTGAATACGTACGTCACGAAGAATCCGGACAAAACAATCTCACTGGTCGGGACCCTCACGACCCGCTACGGAGACGATGGCTTGGCGAGAGCGTTAGTAAAGGTCCAAACGCATGCAGATTCTTCAGCTGAGGCTGTGGCGCTGGCGAAGAAGCTGCGAGCTGAACAACTGAGTGCTTGGCTGGATGGTGGTAAATCCATCGATGATGCTTTCGCTTTGCTCAAGCTTCGTGGAGACGGGTATCTAGCGCTTACAAGTCGAAAGCTGGATGTACTGGATGATTTCATTGTGAAGATCAACAGCGAGAAAAACGGTCAGGAAACGTTGCTCAAGACCTTGACAAAGGGTTTTGGCGGTGAGGACAATTTGAAAAAAATTCTAGACAGAGCACAATACAATCACTATACACATGCGAAGGCCATCGAGCTTAAGAAGCTCAGAGAGTGGCAAGGCGACAATCTGGATCCAGCAAGTGTCATGAAGCTGCTAAATCTTGATAACGATGTGGGTAAGGCTTTGAAAGGCACAGAACTGAGAGGACTTGATGAGTATATCATCGATATCAACCTCAAGAGCGGAAACAATCAGGCGACGCTGCTCGGGGCGCTCACGAAGAAGTACGGTGACTCTGATGTGGCAAAGGCGATTGTGTCTGCAGTTAAGGATGACAATATGCTCGCTAAACGTTTGCAGAACCAACAGCTTGAGGGCTGGTTGAAAAAGGACATGTCCGTGGACCAAGTTTTCAACGTTCTAGATTTTAAGAGCGCTGGTATTGGAGCTGTTATCAGCCGAAACGTGGACACTTTGGACAAGTACATCATGTTATACAACAGAAAGACTTCAGCAGACGAAACGTTGGTAGGCACGTTTGTGAAGGCTTTCGGGGAAAAAAGGTTGAGCAACATGCTGCAGCAAATTCCCGCTTCGAATAAAAACTCAGCAAAGCTTCGTGCGCAGCTCGAGGAGCTCAAGCGACTCGGCTAAGCAAATGCAACAAACGAAAGTAAACAACGAAGCTGTGTCTGATGCAAATCAAATACAGAAACGGTCACTTTTTGGTTGGTCTACCTCCCGTTTTATTTTCAAAGATAAAAAACTTTTTCGTCAATTTATTAACTTTCAGAGAGAAGCGGTCGCTGCTCTCCTAGTTGCTCTCCTACTTCTAATTCAGATTCTTCAAACGCGAAAGTCTGGCATCTTGGAGCTCGAAGGCACGTCGGATCATTCCACATGACTCCAAGTTCGCCATTTATCCTTGCTAGCCCCAGCACAGTAAGCAGCATCATAGGTACAATGTACATAAGAGCAGGCTGTCCAGCCACAACGTCTCGAAGAACAACAGCCATGATATTGGCAGCCATTAAGCCGAGCGCATACGCACACGTTGCCATACAAAAGTACCCACGTGACAATGGCCGTCCACAATAATAATCGTATCGAATACAAAACGCGACTAGAAGGCCAGGGATTATGATATCTCCCAGTCCTAATAACGCCTCACCTCCATACACTGAGAATACCAGTGGGACACTCAACACCATCGGTGTCGGCTGCACAGTAACCTCCTCGCCATCTGCAACCGCACTTAACCGCGTACTCGCACCTCCAGACGCCACATCTACCATGACATTAGAGCCGAAAATTAGCGGCGAAATGTAGACGAAGAACACGTCGTAGATGAATGCTGCGGTCAGTAAACTTGTAGCCACTCGAAGATTCGGAAGCCGGACGACGTCGATGAATACGAGACCGACTGTCAGCGCCATGAAGTCCTGGATCGGCCAAACCCACGCCTGAGATCGAGCCAGAAACCAGCACACAGCCAACGCTGGAGCCGTCAACATCACTAAAAGCAGTACAAGAAACTGACAATTCCCACTATTTGGAGACACCATCTTCGCCACTAGAGGGAGCGTGAACACATGGGAGAGTGCTGCACTCGCAGCTAGAGCGAATAATACGCTCAGAATCAGAGCCAGATGCACGTAGTACAGTAGCAGCAGCAGACACGACGATCCTACTAGAAAGAATAATGCATGCTTGCTACTGAGCTCCAGTCTGTCGTCGTCGATACTCGCGTACTCATTGGCTGCTGTCGACTCCACTAACGTCGAGATGGAACTACTGGAAGGGTTCCGATCCAGCGTTCCGGTCAGTATTCGAGCCATTTGATCATACGAAAGTTGGCGCTCTTCACTGCACGAATAATACGCGGACACTACAACCGCAAACACCCCAAGCATCCACGTCAACAGCATCGACACATTCCACGGCGATTCCTCCGTGTTGAACGCCCATACCAGTACATCTGTGTCGTCGATAATACGCACTGCATCGTCGATTCTATTGCCGTCCTGGAACGAGACTAATAAAACGGGAATACTCACCACAAGACCCACAAATGTGCGCTCTTGCTTTACCGGCTGCATTTGTAGCCAATTACTGATAAAGCAACAGGCTTGGAAGCGGTCGGTTGACATATCCGCCGTCTTCCCCGTAAGTACACAAGTATTTGTCACACACGCTGGCGTGGAGGAGCATGTCCAGGCCGAAAAAGGGTCTGCGAGCTCCGGGAACACGGTGCTATTGGTCGTCACTGTCTCCTCTCCAAGCGTACAGTCATAGTACACACTGTGACTCAGCTCCGCAGCAAAGGCGCCCGCTATTGAGTCGCGAATAATAACTAGGGACGCGCCCGCCTGCTGTGCTGCTAGAGCTTGCTGCTCTACGGAGCATGTGACGCTGCGAGGGATCAGCAGAGCCGACTGCTCGGGTAACGCGACCATTTCTTCGTTCACGGGCTTCACTTTACTGCAGTCGGATGTCATCTGTGCCTGCACGAGTCTCCGCGATTGATCCGGCGTCCTGAATGCTTTGTTTACTAGCAATTGTCCGGTGGAGCGTGAATAGGAAGCAGAAGGCCAGGACACCTGCGGTAAAGCGGCTCCGAATGCTGCAGCTGAGGCGTGAAATTGCTGCGATGAGGTTGGCGATACAGTACGCAGCTGCAGCATGTCGATGCACTGCGCTACATCGAAAAGAAAGACCAGCAACAGCGCGATCAGTGGCACCATGGCAGCGGAACTTGTCGGCACCCGCGAAACTGACCTTTCGCACCGGAAAACGGAAACCTTTAATGCAGAACTGGGTATTTTTATTCTAAAAATGATTAACTTTGTAGGAAGGTCACTTTTGGTTGGTCAGTCGGATTTTTTTGTACCCTTTGAAATCACCCAATCAGACCCCCGGGATCGGATTGCGATCCCACACAACACCTTAAATAAATGGCACTTGCTATTCAACCCGACTGGTGTGGAAGAGACAGGCTAATGGTGCCCGACAGCACCCGATCAATGCCCGACCATGCCCGACACAGATTAAGTCAGTTGAGCAATCAAGCGTCCGTATACGTATATCCGGATGTGAAGACTTTGTGGTTTGAGCGTGCAAGTACGACCGGCATCATGTCATGAGCATGAGAAACTTTATTCAGTAGAGCTCAGTGGGACTGGTAAGTGCAGAATCAATATCAAAAACTTGTTGAAAGGGTTAGCTATAGACACATATATATGTATCTGTAGCGCAGATACTGTAACTCCTTCCTACTCAGCATGCACAGACCTTGGGCACCAGCGAATACCTCAAACGCTAGCAGAACATACATACCACAATTGTATGAATCTACTTGTACCCCTAGATCGCCCATGTAGTGGTGTACTCGGTATTTCCTTGGTGCGTAGTTTGGCAGTTGCATTGCCAGCTTTTCTGCGACCGCTCGGACTCTAACGACATAGCTCGAGCTCATTGGGTCATAAATGCACACATCATTCAGATTTAAGTTCACCATTACGCAGCACCAATGCGTGTTGCTGCAGCATATAGGGATCAGAATTTTCTCGTTCGTCTTGCCCGCGAAAATTGTAGCGAAGGCTCTGGTATTGA

>Contig_64

CTAAGGAGACCACTTTTTAACATCAAGATTTGTGACTGTACAGCTGTAGCTGTATCTGGTTCAATCGGAAGAGCCGGTAGATCAGCGTTTCTGCAAGTGTGCACTTTGAGAAACCATTACCGGTACTGTATCCTGATCTCGGATATCTGTGATTTCATAGGCATTCGTAGGTACCGTATGCCCTTCGACTCAAAATACCGACATTCCTATCTCATAGTTTGAGATAGGAATTTCGGTATTTAAAAGATCATTTTGGAGTCACTTAATGGCAATCGTTTAGCTGAATAGAAATGATAGGCCAATCGCAACTCGTCCGTCACTTTGATCGATCGGTGTACTGTACTGTAGTGTGTTTGTGCCTCGTCCAAATGTGTGTAATCATGTCATGATTCATGACACCAGCTCAGCCGCTCTCGTCCGTTTGTAATTCGCTTCCAATTTTTTGCACAAGAGCATAAATGCCATTCGTGTTCAGGGGTACTGTAGCACCCCCCTACTGTGGGCAGCCGCTACCCCTGGTCTTGTCGGAGACATGGTTTCGAAGTGATCGGATCTACCGGGTGTCAGCTTTAGCGTATTTCATTGTAGCCTTTAATAACAAGAGGACGTGTCCAATGACGTCCTCCCGTTTGTCATTCCTGTGCCGCTGTTGACGCGGTTGGTGTGCAGTGTTGCTGCTGACGCAGCTTGCCCTTGTAGCTTTTTCAAACGACTTCGACTTTATTAAGCTAAATATTCAATCCTTTCACAAGGTGAATCTGGTACTGTAAAGCACTGCGTTTTCCAATAATGTCTCATCGATTTTAAAGCTCTGATTAAACATGACCGAAGCATAAACTTAGCTTAAATGTGGTGGAAGAGATTTTGTTCTAATATTTGTTATCAAGCTTCCTCTTCGAAAAGTGTCGGCAGATAGACTCGAAGCCAACCTGTCTTCTACTAGACTTGGATAAATAAAGCGAGATCGATAAAATTGACAAGGCAAAAAGGACGGACACGCAAACTACTGGTTTTTAAATGTTTACGGGGGTATCTTGATGAGCAAAAACAAACTACTAATCATGCGGCTGGGAATTTGTAAAGTGCTTGCTACATGGACAGCCTCCGTGATGGGCTATGAGATTTGACGTGGGGCTGGTCCAGGCCGCGATGAAAGGCTATAGGAGGCGCACCATTTCCTAATTTTCTCGTCCAACGCCACATCATCGCTGTTTTCCTCTTATAATGCGTGCTTGTGCCATCCTGGTGGTCGCTGCTGCCGCTGTACTTACCGGTTCTACTGCTATATCCTCCACCGATGCTTTGGAGCTGGCTACGCTCTCCAAGACTGCCCAGGACGTCGAACTATCGTCCGTTGCTGCGCAGCCAAGAAGCAACATCCAGCGGCGTTTGAGAAAACATAAGACTGTCAACACCAATAGCGAAATGGAGTACGAATCGGAAGCGGAAGCTAGAGGCTTGGTGCCGGAGAAGCTGACCAACCTCGTGAACAAGTTCAAGAAGGTGGGGGGAGAAGTCATGCTCAAAACCAAGAGCTTGAACCAACTGAAAAAGCTTTCTGAAAAGATGGAGGAAAAAAGTTTGTATGCGCTTACTGATTTGGAGAAGAAGGGATACACTCCTGAAACGCTCAGGGACGCCATCAAGAATACCCCCGCCCAAGGGAATGAAGGATGCAGATGCCGATGAGCTAATAAAGTTTTACGATGAGTACTGGAAAATCTTCCATTAATGGAGGGGACTGAAAAAATGAGAAATTGTGGGCGATGTCGACATAGCGGATGGATCACTAGTTTGAAAAATTATGATACCCCAAATAACACGCTTCGTTCTTCATTCATGTACCGGATTACGTTTGCGTGAGGTACATCATTCGCATTCTTGTACAGTAGAAGTATTACGACATGTCGTCTTAGCCGACAAAATTGCTGAAACTATACTAGAGACGGACGCGCTTGAGCTGGAATCGAGTTTTCTTACCAAGGATAGAGTTGTGAAGCACGAGAAAGACAACAGAAAAGTCGCGTGGGCCAGTGTTGTCAGATCTGGTGTTGATATAGTCGATGATGGCACCAGCAAGAATCATGATAACTTACAGTTAAACAAGGTGGACGGGAATATATTTTGGGGGGTATATTACGGCTGTGGAAACATAAACGAATATTGGTTTTCTTTGACGATCAATAATAATTGCAATATTGAAAAGCTAGATGTTGGATATTCCAATAAATTGGCCATTTTATGAGAAGTTGACATTAATACTACATATAGTACTATTAATAGTACTTCAGGTAGAAAAATTCATGAACCTAAATGTCGATGAAGATGTCCTTAAGCTGTGAGCCGTCATATTTTAAATTTGACGGTGAGAATCCAAGAACCTGGATGTTATATTTCCAGGCTTTGTACTCGCTGGTATGTTGACAATACAGTAGTCATCAGCCGATATTCTCAGCTCTCATAAATGCTTGTCCTTTGCCTGCAATGAGAAAGTAAAAGGTTCTGCTGCGCGTGTGCTAGACCATTGGCGGTTACTGACGTACACCGGAAATGGACCCTTCTCTGTAGACTGCAGAGGCCCAAAAAATAGCAGCCATTGCGACCCTGAACTAAATTTGTGATTATTGCAATTCTTCCTTAATATTAGACCTGTAAGTGATCTGCAATAGACCCTTTGTACTTTTAAAGTATTCTGAAGTAGTAATTCTTGAAGAATGGATCCTGGTCCTTCCCGACAACACGTACGATGCTTGAGCTGCTAAAAGAACCTTGAGAACTGAGGTTGCTGTATATAGATCGGAAGAGCGTCGTGTAGGGAAAGAGTGTAGCCAGCTGGATCTCCGGTGGTCGCCGTATCATTCAAGCAGAAGACGGCATACGAGATCCAGCTGGGTGACTGGAGTTCAGACGTGTGCTCTTCCGATCTACCCCTGGCCTCGTGTGAACCACTCAAACACATGTTTATACCTGATTGGTCAATAAGTGAGTCTGGCTACTCACTATCAACCAATCAGATGATGACACCTTGGCACACGCTACCCGTGTGCCACTTTACCTACTTATGTAATAANCTAAAGAAATATCTACCTACTTATGTAATTGGAGTTGGGCCCATTACAGACAGCGTGCTATTCGCTTTATTTTTGTGACTTTATAACAGCGACCAGCAAGAAAATAACGCCGCGTGTGGGCTCATATTTGGGTTAGTTTTCGGAGTTTGTAGGTTTATTAGCGAAATTTTATAAAAATTACGGTAAATGCTCATTTCGATATGGCGATCCTATACATTGTGTGTAGCACTCGCTATTAATAATAGGTGCTCAATTACATAAGGTTTTACTGCTTGTTAAGTCGATATGTATCAAATCAGAGGGAAATGTCATCAAAACACTGGAAGAAACACGGTAACCAGCCAATGAGATCATTGTAATTTTTTGTGTATTGCACACAAAAACGAAATTCCTGTAAATTGGTGGGACAGAGAAGTAGTCTTTTCTATTTTTTATTTGTAAATTAGAATGGCAAGTTCACCGACGGTTTAGCAATTTTTCTATTAGCGATGGTTTTGAGGAGATGTATCTCCCTTCAGCGTTACGCTTTCACGTATGCTATTTCGGCTGCAGCTTGTCAGCGTATTGGTCAAGAGAACTACTGGATTATAATTTATTATTCTTAATACGAGCCTACCGAAAAGGACAGAAACATCGTCCAATCTTCTGCTATAAAATGTAATTTATAAAACAAAATCGGAAAAAGAAAATGAAGAGACATATTTTTAGATGAAAGTTATGCAAGTGTGGCTCAATTTCTGCATAAAAAGCAGGCTAGATTCTTTCAGCTCCTACGTCAGAGTGTTTCGCGCTTGTATTCTATTATAATTCGGCTAGGGTCCTGGCCTAATCCTACGCTTACCGATACGTGCTGGTTCCACCTGAAGATGCGGGCTACATAGTGCACTCTCGGTTGTTAGTGTCACGCTTGTAGGTCATGATCTAAACGGAAAGGTGTACGTATCGCGATAATGTCCTATCGGCGTCCGTGTAGAGTCGTACAGCGATTCGATACGCTTCCCGTCCTCTGTAGCTGCGCACATTTAGTGAATACAAGAAAATTCTTCATCCTGCCACCAGTCACAGGTACATTTGAATCGGGTAGGTTGACGAGCATTCACAAGGTGTGGAGTTTAAAATTATATGAGTATTTTGAATGGAGAGTAATACTTTTTTACAATCCAATCAGCACTTATCATTTATTCGCATGCGTACTTATGAGCTAAAGCTATCAGTGTACTGTGTGTTTCCACTTTGTATGGTAATTTTTTTAAAGATTGGGATTAACCCAGTACATATGTACTGTATTATGGAATTTCCTTCGCGTGACTACTTCGGACGGATACGGTTATTAATAGCCGGATAATTATGATGGGTTCAAACCACACCAGCAGAAGCAGCTGTTCTAAAAACCGTCAAGCAAAAAAGAGCTTTGTCGTTTGTCTTACCGGTATCAAATCTCCAAAGCCTATTCGGATCTCCAGCTGCACCGGATAGCTTAGGATCTCCAGCTGCACTGGATTATTAATAAGTACATCTCAAAACTGCGAAATTACCTCTTCATTTTGTTAACAGTAAATAATCGAATGGTATAAGGATGTAGTTGTAGAAAAGCTGTCCATAAATTTACGTATTATACAGGTAGCAAACAAAGAGGAGTATCCGCATCAGACTTACAGCATTTACATGTACACTACGTACTGCGTCCAGAACAAGTATAATACAAAACACCCTTTTGAAGTTTTTAAAAGTACCCCCTGAAAATGAAGGAGATTCTACCAGGGGAGACCCATCAAAGCGGATGATTAGAGATAACCTTGGGATGTGAAAAAGATTGCCCAATCTTAATGCACATACAAAAAAGTCAGTATAATCGCCACTCCCCTTTTCAATTTCTCAGGATTTCCTCTTTTCGGGCACGATTTTGAAAATTCCCCTGTGGGCATATTAATAACCAGCTTGATTACAGCGTTCGTAGAATCATACGAAGCGTCTCAAATCGTATGCACCATTTTAGCCGGTTTTTCAAAGCCAAGCGAAAGATGCCGTCGCATCTTCTCCAAATGCCGCATTATTAAGAGCTCCACCAGTTTGAAATCTTCGAGACCACAAGCACCGGTAGTTCCTGCCCCCACCTATCCGGATATGTGTATTCGCCAAGTCCTGATCCTATTCGCCCTCCTTGCTAGTTGCACCATTTCAATCGATGCAGAACAGCGGGTCCTCGTAAGCGAGCAGCGCACTGTGGACCGTGCGAGATCGTTGCGTGCCGCCGAGTCCACCAACAATGAAGAACGAATAATGACAGAAATCATCAAGCGTCTTAGAACGGCAACCTGGATCGAGACCGGGAGAACTGATGACTACGTCAAGACAACGCTAAGACTTGACAATCTCTCCGGGGCTGCGCTTAAATCTGCCCCAAATTACATGTACTATGAGCATTTTTTGAACGCTCTTGAAGGACGAATATTGGAGGTATGGCTGTCCAAGGGAGTTCCCACGAAAAACGTATGGGCAACGTACAAACTGGACGATATTCCTACAGCCCAACTCAATGACAACGATGGTTTCAAGACATATCTGCGCTATGCGATAATGGAGGATAACAAAATTTTCAAACTGAAAAGCAATGATCAGCCCGTAGCGATCGACTATAGCGGCACTCCAGCGGAGTTGAAAGCTAAGGTGGATATGTGGGTCTCGTTAAAGCGTCCAAACTATTATGTCAGCAGGATGCTGGATCTGGATCGCAGATCCATAAACACCTTCAGACGTAGTCCAAAGTTTCAGCTATACGAGATGTTTCAGATGAAAACTTGGGCGGCGAAAGGTTATCCAACGAATTATTTCTGGAAAGACCACAGGTTGCATGAGGTCCCGCAACAACAACTGCAGAACAATGGCCTCTACAAGAAGTTTGTGCGTTACGCGATGATGGTAGATGACGAGAGCTTTAAAAATGGGAAGACGGTCAAGATCACGGCTGACGAATCCAAGGCGGAGATCAGCACAAAAGTGACGATTTGGGCCTCCAAGGATAGACCTTTCGAGTACGTCAAAAAAGTGCTGGGACTGAGAGGTGCCGCAGACACGACTAATGCAAACTACAAGTACTTCGAGGATTTTTTGTTGCAGACAAATAGACCGAAGAAAAGTAAATAAAGCCAGCTCATACTTACCAGTTTACATTAAATGTACTTACCAGTCTACATGAAACGATGTTTTCTGCTATTGTATTGAAGCAGCTACTGCTTCTGATTCAGCACCTTTACAATATTTAGCACCTTGTACAGGAAAACAGGCTGCTATTTTTTTGATGATTTATCTTGCATCTCCGAACAAGTGCATTGGACAGCGATTTCTCCTGACATCCAAACACAATTCTACTAGTAGAACTATGTTTTAATGAATCGACGAACAGTAAATGCCCTGACATCCGTTTGAGATTTCAGGGCTCGGACACGTCGCTGCTTGAGTCCATTTGTGTCAAAGACCAGCATTTGTCGGGTCGGCTGTCAGCACAGTAGCGCTATCGCAGCAAACCTCGGCGAGCCCTGCAGTCGCGTACATTCCCGATTGCCACACCATAAAGGATGACACATCTCCCGACAAATGCACCGGCACAAAATCGATCTCTCCCGACGATTTATCCTCCCCGATGGCGTATCTGCCTTGACATTCCAGCCCAGCGTATAACACAATTCTCGAGTCCGAAGGGATGCTAGACCATACTGCTGATCGACTTTTGCTGTTCCAGCAGTCGGAAAGAGAGTAGCACCTTTGCGCTGTGCTGAAGGAAAACCTCTTGGATGGATCTCTGGGTTTCCCACCGCTGAAGATGCGCACACGCCATGCGGGACACTCTGCTCCGAGAATTATGATCATCACGATAGAGAGGCAAATGGATGACATGGTGTTGGGGCGGAGAAACCCCAGTTGCGGGAGCTATCCGCGGTGGTGTGTGTACATGTTGACCCCTCACGTTATTTCTGGTCAAAAATGATTCATTTCATCCGAAGATTCTGGCTATCGGTGCTCCAGAATTTCAATCCTGTGGCTTACACTGTTACATTTACTTTTTTTCGGCCAAGAAAGCATGCGCAACCAGCTCTAATGCGGTTGCTCGCTCTATCAGCATGAAATAAATACAAATTAGTCAGGGCAAGTGGCTCAATTTCCCCATAATGCTGTTGTACCTTCCGGGTCGATACAGAAACAGCGCTGGAGAAACGATTTGGCTTCGGACGACAAATGCTCTGGTAGCAGAGGTGGCGCTGTAGCCATCGCGATGGTATACATAGCGGCAAGGCCATTATGACAGTTTGGCCACGGATGCTTAGCTGTAGCCATCTCGATAACTGTTGCACCGATACTCCAAATATCAGCTTTGTACCCATGACCGATTTGTTTGACGACCTCAGGCGCCATCCATGGGATGGATCCTCGAATCGAACGCAAAGATTCCTCTAAGCTTGTGGTCAACATCTGCGGAATCTGCTTCGAACAGCCGAAATCGGCCAGTTTGGACACTCCTTGCTCGTTTACCAACACGTTCGCACCTGGTCGTTGGAAATCGAATTATTAGAGATGCATTAAACGTTCAGTTTTGACCCTTGTGTTTTCGTTCCAACCTTTGATATCCCGGTGAATTATACCCATSTCGTGAAGATACGCAACGCCCTGTACGATCTGCCGAGTAAAAATCCGAATGAGATCTTCACTAAAAGCGTCGAACTGCTTGAGCATACTACACATCTCAAGTTCCACATTAAGTGTCGGGCGGAGAGAGAGACACACACACACACCACACGTGACACGCACCTGGCTATGGACCCACCAGGAACATACTCCATGAATATATAAAAATGATTTTCGGATCGATAGCTTCCCTTGTAGCTAAGACACGCACAGATAAAAGCACATTAGGTCGCACCCCTTTATAACCAAGGAAGACGTAAAAAACCGTGCTCAACAGACCGAACAATGTGCTTGTGGCTGAGATTATTCATGAGCGAGATTTCCTCGCCAAGCTTTTGCATCTGAGTGACCTGATCGTCATTAGGACGCGAATGAATTTCAATCTCCTTCAACGCAAACAGTTCCCCCGTCGCAATATTCAGTCCTTTGTAAACCTGTCGAAATAAAAAAAAAGTTGACCTTAATGACTTCTGCCTAATAGCATAAAAACAAAACAAGTAATGATCACGAACCTTCCCGAATGTTCCTTCACCAATGAGTTCGCCTCGTTTCCACTGTGTGATGGGGTTGCTGACTTCAACTGACGAGCTGGTATCAGTTTCTGTTTCCAATTGCTGGTTGTTTGTCGGCGGTGTGGTCCGTTGGCGAAGTGATACTTCTGGTAAGGAGGAAAATGCTGCTCCTGCACCTGGGCGTCTGATGCGCGGTGATGTCGATCTTGTAAGAGACATGGACTTGGGCATATCTAGCACCGACAAAGCTGCTAAGTGAGGATGTGTCACATGTAGCTGGGAAACCATTTCGCTAGGCGTCGGCGTATCTTTCGCATGTCTGGAGTTGGAGCTTCCTTTAGTCTCGAGAGACTCATTTCCAGTTGATAACGATGAAGAAACCAAGGGAGGGCTATGCGTGGGTTTCGACTCTCGGGTACTGGAACAGGAGAAAATGATGGCATCCGGCAAACTTTTGCTCTTGGCTGCTTTACCCCAAAGACCAAAACTGGACACGGCCCTGTTTTCGATCTGTGCTTCTTGTTCTTGAATCACTTGCTCGAAAACTTCTGAATTATTGAACGAGGTTTCTTCATTTGGGTTCCAATTGATCTTCTCAGTTTCATCACTACTGTCTTCTCGCTGCTGTTGGTCCTCTCTACGGGCTGTATGCCTCCTTGGACGTTCCGGTCTCTCACAAAGCTCGGCGAGTCCTACGGGATCCGTCGACGATGTTACTCGCTCGAGGTCTTTCAACACATGTGCGGAGATAGAAAAACTGCTACTGCGTTGTAAAATTCTGTCATTGTTGCTGGTTGGCTCCCTGTTGTTATACTCGCTGCTGTGTAGACGCTCTCGTCTCTCTGGTCTCCTCCCGGAAGGTGCTCGAGGTGTTAGCGCATTCGGTGCTGACAATAAAGCTCCGTCAGCTAGCGAGAGCTGTTTGAGTCGAAGATTCCGTCCACTCGAGCTGCGATGTAGCTCAACGACCGGAGATTGACGTAGCGCGTCCACTGCTCGATGACTACGTGGCGGTGTGTTGTCCTGTGGCATAAACGAAGTTGGTTCCTGGTCCGAGATCGAGGACGAAGCCTCTGGCATCGCAACCCTTCGCATCTCGCTGGAATTGTTGGATTCTGCATCGACCGAGTCGTCGTGGAGGGTCGGTTCAATTGCAGCGGATACTTTGATCCTGTCCTCGCTCGATAGTGCTGCATATTGAGAGATATCTTTACCAGTATCCATTTGAGCGCCACGGGATCGCACCTCAGAGTCGATATCGACTTGTAGCGATATACTAGACTAATCCAGCTGCAAACAGACGACCAGCCTGCTGCTTGAAGTGATCTGGGCGGGCGCGTGCCGTTTCGGTCTACTGCAGCAGCGAGTGAACGCTCGACTCGATTTGACACGCAACTCGACAATGGCGCTGTGGCGAACTCAAATCTCGGTGGTCTCTAAACCAAGTTGGTTTAGTCAATGCTGGTTTACATACAGGACATGTCGCCTTAACCGATTGATTTTTGCCAAATGTCGGCAAACCAGTCTAATTTTATTAGGTAAACGGCATTAGTGTGCGTGTGTGCTGTTGCCG

>Contig_65

CTTTAAGTGTTTGCTAGTGGTCAGAAAAGTGTATGCCAGCGGTCAGTTCGAGGCATAAGTGTTTGGTCGTGTCAAATTATGTCTACCGTTCACCTAAAAGTGTTTATTTTTGGCCAGAAAAGCGTATGCTCTCGTACCAGACTCCCTATTTACTAAAAAAAATGAAAAAAAAAATTGATTAGGAGGGTGTGGACGAGAAAAAAAAACGTACTTACGTAAAACAAAATCGTACCTATGGGGAAAGTGGTGAGTTCATGCTGTTATGTTTCGGCTGAATGATAACAATCGGATGCCTACGACAAAAAGACGGCTACAGTGTTTCTTATCACTAATCAGCTAGCGCTCATTTGATTTTTTGGTCTTAAGATTGAACTTTTGATTAAATGTGGCCCTTTATTTCCGGACAGAGGAAGTCATTAATTTTGCCCGCCCATCCTGCTTATATATACGGGACGGAGGAAGTACCAATACATTATTGGCCATTTGCAACCCGGGTTGTATTTCTTGAAATCCGAAGCTACGATTGGCTGTCTTCTCGGTATCAGCCAGATCTGAGGTCGATACCGTGCCTAGATGTGGAGTGCCGTTTGCAACTATCGTTGCATGTACGGTAGCGTGAACGGTCGACTTTTGCAACCCTAGTATACAAATTTCAAGTCAAGTTACCTATAGTGAAGCTAAGTCACGGTTGCAAACGAGAGGGTACACAGCTCGTATACAAAAACTAAGTCATGGTTGCAACGTGAAAGATTCAGTTTGAAAATACAATCCACCACAATGAGCACTAGCCGCTACAAGGCAGAGCTCGTAAAGTTCATGTCGTTCAAGGACGACAAGGAGTACACTGCTAGCCACGAATTCACGCCAGCGGACCTCCTCAGTATCACGCCTGGACTGCTGTGCCGCTGGATGAACACGCGGGCCTACGGAGATTCAGAGCCAAGCGAAGACATGAGGCCTGTTCACCTTCGGTCGAGCACGCTGGAGTTCGCCAAGAAAGCCATCTCAGCGTACATGCCTAGGATCAACGCACCATGGGACCCTGTGGCCATGCAAGGCAATCCAACACGCTCCGATGATGTCAACAAGCTCATAAAAAGAGTCAAGCGCTTTGAAGTTCGTCGGGAAGGTGCTGAGTCAAAAGCTCGTCGCTCTTTTGAATTTGATGAATTCATGAACGTGTTGACGCTGGTAAGATCGCTGCATTCACGCTCTGATGAACAACTCATGGTTAGCAGTGTTTTGACACTGCAGTGGCATATTGTTGCTCGCATCGACGACATGATGAAGCTTCAATTTAACAATTTCACTCACAACACTCAGTACCCGTCTACTATTCTATGTCAAATGCGATGGTCGAAAAATATATCCGAAGAGAGAGACGCTCCGGAGCAGATTGTGGTTGGTAGTATGGATCCCAGAATGTGTCCCCTGCTCAATCTTGCAGTATACATCGAGGCAACGGTGAATGTGGCAAGATCTTCTTTCTTATTTGGAAATCCAAACGATAAAGATCGAGTGGTGAGGCGTTTTCTAGCTGATACAATTAAAAAATCGGAATTTAAGTCGTTGAAGACGGGAAAGCTGGGAACGCACAGCTTCCGTAAAGGTGCTGCTACTTATGCGACTCGTAGTGGTGTATCTAAAGACTTTGTCAATCGGCGAGGACGGTGGAGAACTCGCAAAGGCGTCGTCGACGTGTATATCGACAACACTCAGCCTTATCCGGACGCATGCACCGCCGCAGTCCTTGCTGGTCCAGCTGGACCTTGCTTTTACTCGCTGAAAGAGGGCATGCGGTGTGTCACTACACCACTTCTCGTCGACGAGATTGCTCCAACAATTAAACAGGTCATGGGAGAGCCAATAGCAAAAACATTGGCACAGGTGTTACTGTGGGCTGCGCTGGAGACGGATTCCAGCTTTAATTATTGTCTTCTTCCGGAAAAGCTGAAAAAAAGAATTTTACGGGCTTACATTAACGCCGGTGGAAGTACGAATTTGAATCCGATTCAAAGACAAGAATTTTATGTCTTGGGGGACGGGTCTCAGCTCAATCTCGTCGTTATTGACAAAACGCAAGAAGTGGAATCTGGTGTTGGTAGTATTGTTTCTGCAAGAAGTGCATTGGTAGCGGGCTCCAATGGACAGGGGACTCAACGTGAAATTGCTGCTGTACAATCTCAAATAGCGTCTGGCCGTCGCTACATGGCAGAGGTTATGAATGAAGTACTGCGGTCCCGAAGCGAGTCACATCGAGAGATGCAAAAGATACAAGCTATCTTGAGACGGATAGCCATGCAGCCGTTCACACCTCGTACTACTGATGGGCACGGAGTACCGTATCCACCAACAAACACTGCTCAAGGTGGATTTCGTGGACAAAACGCGGCACGTCTTTCAAAGAGACCTAAAGACCTGTATGAACTTTGGCACGAGTATCAGCTCGGATCCGGTGGGCTAAAACCAGCCAAGGAGTTTACATCCATTGAGCGAGGCGCAAACAAGTTCGCCTACTCCAGGCGGAAAGTCTTCTGGGATGTTATTTCTCAATTGGTTCGCTCTGGACACACTAGCGACTCTGCAATAGATAGAGTGTATCAAACGTACGGTAGGAATCTCTCTGTGTCTAGTATTTTAGTTAAACTTCGTACAGATCGTAGACGTGGAGGACATCCAAGTCTGCGATTGTAGCACTTCAACACGGTTCAATTCAGCCAATCAAACCTTATCCTTCGAAATATTAGCCTATCAAAACTATGGTTGCAATCCAAGTTGCATAAATGACTAATATTTATACAGTACTGTACGAAGTAATCACTGAGTACAGCGGGACACTTGCCGTAGTCACAAATCTTGTTTGAACGTGCCAACAGATCTATCCAGCTGTATTTTAATTAATAATGGCCGCACACCGCTCACTCTGATATTTCCCCGTTCCAGCACTCTTATGACATGCAGTGAGCAATTTACTTCGGAGCGAACCCCGGACACACCCTGATGATGCGACTCTACCTTACTGCGCTGTTAAGTGCAATTTCAGCTCTACTAGCGCCGGGTGGAAGTGCCCCAGTGTCTGCTCTTCCAGACTTTCCGGCTGGCTACTTACCATGTAATGAACTACGTACATTAACAAACGCACCTGAAGAATCTCCATCCCACAGGCGATTGAGAATTTCCGATACACATGATGACGAAGACAGAATAAAAAGTATCAGCATTGAAAAACTATCGGGGTTGATTAAGACTGGAGTATCGAGGATACATGGATACCTATATTTAGGACCGTCAGCAACTAGAGAACAACCAGCAGATGAGATTCTTCGAATGTTCAAGCTTGAGGATGGAATAGAGAAGGCCTTGGCTAGTTCTAACTTGAAGACCATGGAAACTTATGTGAAGGAACTGCGCACCAAGAACCGAAAGAGCACGACGTCAGTGCTTGGAATACTCACGAATCACTACGGGGACGACGCAGTAGCCAGTGCACTTGTGACTGCACCGCATAATACCATTATGAAAGACATGGAGGATACGATATGGCGATTACGAAACACACAGCTTTCAGCTTGGCTGAGTAGCGACAAGTCTGTCGACGATGTTTTCAACCTGCTAAAGCTCCGTCAAGATGGCTACCTAGCTCTCGCCAGTCCAAAGTTGGAGGTGCTGGACGACTACATAAAGCTGATTATCCGCTCCAAATCCAGCCAAGAAACGTTGCGTGATGTGTTAACGAGGGGATTTGGAGAGCGGAGATTGGCCAGACTGCTAGTCCGTGCGAAGCAAGATGACCGAACAAAAGAACTGGCGACGGCACTGCAAAATGCGATTTTAAACAAGTGGGTTACAGACAAGTTGCAGCCGGTCAACGTCCTACAACGACTGAGATTGGATAGAGGTGTCACCAAAGCTATGACAGACTTGAACCGAGACACTTTGACGAGGTACATCTCGCTGTTTAAAACACATAATCCAAGCAGTAAAACGTCATTCATTGGTACGCTTTCCGCGCATTATGGAGACGATGCAGTTGCGAAAGCACTCGTGACGGCGTCGTCGGATGCGAGTACAAAAGAAGGCGCGATTCAGCTACGGAGTGAGCAGCTGACTGACTGGCTGAACAACGAGAAGACTGTCGACGAAGTTTTCAAGCTGCTAAAGCTTCGCGATGACGGAGAAGTCGGTCTAATTAGTCATAAGTTGGAGGCTCTAAAAGATTACATCAAGCTATTCAACCGCGAAAGAACAGGAGATGAGACTTTACTCAAGACGTTGACGACTGGATTCGGCGGAGAAAGTGGATTCTCGAACATTCTACTAGCAGCAAAGGCCGATCGACGTACAAACACAGTGGCTATGTCATTGCAAAGCGAGCTACTTCATCAGTGGCTTAAGAGCGGATTGCAGCCGGGAAGCGTCTTGAAGAAGCTCAAATTGGACCGTGGGATAACAGAAGCACTCTCCGATGGAAACATCCACACTTTGACAGCATACATTTCGTTGTATAGCACACAGAATCCAAGTAATGCAGTATCGCTGATTAAGATACTATCCGCGCATTATGGAGACGATGTTGCCAAGGCACTTGCCATGGACGATTTTGCCACGACTGAGCTGGCGTCCAATCTGCTGACACAGCAGTTGCAGCTGTGGCTGAAATCTGTTGGAGACGTTTTCGCGATACTGAATGTTGGACACCTCGATTTCTTGTCCATGAAGAGTCAGAAGTTGCAGATTTTGGACAGCTACTTGAAGATGTATAACGCCAAAAATCCGCTTGACGCCAAGAGTATGTTCGCGGTAGTAAGAAAGGGCTTTGGCGGTGATGCCGGGCTTGCACGTGTGATTGGTAAGGCGCTTGTAACCTCGCAAAATGAGCCGAAGATGGCTCTCAAATACCAGAATGAGCTATTCAACCAGTGGTTCAATAGAAACATTGAGCCCAAGAACGTTTACGTAGAGGTCCTCAAGATCAAGAAGCGCTCTGCAGACTTTACAGCAAAGGGGGTCGCTAAACGATACAAGAACTATTACAAGAAACGGGTGGGGGAGGTTATAACCTTTAACAATCCAAGGCGGTCTTAAGAAAAAAAAAGCGTTATTAGATGTTTCAATCCTAATTCCAACGGAGCGTGCATAATCTGCATTGTTTTACGATCAGCGTGGAAATTAAATTATGTTTGCAAGTTGGGAGGAGTCGACGTCCTGTTAGCCAAAATTGCTAAAAATGGAAGAGACAGACGTTCGTATGACGCGTATCTTCACTACCAAACCTTTTCGGATGAATGTCTTAATCATATCATGTAAAACTGTCTTGACTGAGAAAAAGTGACGATAAAAAGCGCTTCTGTCGCTTGTATCCTATGCAAAATACGGTTAACCGTGAAGAATTGCGGTGCGCCACCAATAACGTGTGTTTTACCGCCGCGCCTTTTTCCTGCTGACCATGCCCAAAACCATCTTGCGCCGCAGCGGCTTATCTGTCGGCAGAAAATTCGATGGTTAAGTGGCAAGATATCGTACTTAAAAAAATCATATTACCGGTAGTCAGAATCACATGAACGTAAGCCACTCTAAAAGCCTACAGCTCATGGTCTCTTCCAGCTATTCGATGATCGGTGGGCTGGGATGAATAGTTGGTATCAGCGCAAAATAAAATGGAGTAGGGTCTTCGCTTGTGACCACTCCGGTCTACAAACGCCTTCAATCGTTGAAAGCCCCGAAACGACGAGGAACTATCTTCTCGTTGTAGTATACTGTGTATCGGCTTACAATCATATCATCCAAATGACTAGCAGAAGCCTCTTCAACATCGAGGAATCTGGAGTAAATGCTTTTTGGTTCTGCCTTGCTGCGGAACCAGCGCTTAAACAGCAACCTTTCGTACTCAGCACCAAAAGAGACGAGGGAGTCGATGTTGTGAACGCCTATCGACTCAATTGCACCAAAAACCGCGCGTGCAAATTTGCCTTCGCCACCAAATCCATCGCTTAGCACCGTGAGAATGTCGGTCTTGTATCCGGGATTAATGACGTTGATCCTCTTAACGTACTGCTCCAAGAGATGGAGCTTCGTACTTGCCACGGATACAAACTGGTTCTTGTTCGGGATGTTAAGGTTTAACTTAAGCAGTCTGAAGATGGTGTCGGGCAGGCCTCTGCGATTTGAATTATCATATTGAATAATATCGCATATTGGGAATGCAATTATGTATTGAGCAGCGAGTAAAATTGCAAGCATTTTTTAGCATTTTATCAGTTATTCTAGGTTAATACTGTCCACCAAAACTACTGGTTGCCACCACCTCAAAAGAGACTTTACGAGCCTTGTGCAATCTCCTCAGCTCATTCGACCTTTTGCAGTCATCCCAACAACTCTTCAGCCCCCTTTCGCCCAAGTACCCCATGAAATTTACAAACAAGGACATCTAAACGCGTATCTGGCTGATCATGAACCCATGTTGGCCAAGATACACGCGCTCATGAAACACCTGAGCACCATCAAGTGTCGTGCTGCCCTTCGCAAGGTGACCTCTCTAGCGCCCGTCATGCCCAACGCGACTCGTTGGTCGAGTACCTACAGCAGTACGACAAGATTTGTAGCGCTCTTCTCGCGTTGTACCACGCTACGGTGGCTAAGCATGACATCGCGCGCTTTCTACTGACGCCAGAGGAAACCGAAGCCGCTCGCTTCCTTCTCAAGTCGCTGCACGAGCTGAATGAGGTGAGTAAGACGCTACAAGACTCAACTCTAACAGTGGTGGGTGCACGACGTGCCTTTGATGCAGTGTTGCGCAAGTACCCCCGCATGAAGACCCGGCTTGCAAGTGACGCCTCCGTCGTGAACAATCCCGAACTGGAGAGCGGCATCGTGAAGATCATTGGCGGTGTCGGCTGAATGCACGCGAACAAGCGGCGTGTATTCATCTCAAGCGCAGCAGCGACGACACTGTGGTAAACCCAGCAGTTTCCACGTCATTTCTCGCGTCGGCCTTCAAGAAGGCCCCTGTGGCGCGCTCCCCATCGCAATACTTTCCTCTCGAGTGGGTTCCACCCACGTCGAACGAGTGCGAGCGTTTCTTCAGTCAAGCCAAGCTGGTTCTGACCGACCTAAGGAAAGCCATGGACCCTAACACTCTTGAACTGCTTATGTTTCTATCCTACAACAAGAAGGATTTAGTGTTAAAGCTATCAGGCAGAGTATGGGTAGTCAACTTCGGGAATAATGCCATTATTATTGTCTTGGCGTAATAGCAGCCGAATATATTGAGTATGGCATATTGGGGAACCCTGGTGTCGGGAGATTTGCCGCTTTTCAACCACGCATCCATTTGCTCTCTTAAGCTCTAAAGCCGTCGCCTTCGTGGCCTCATGAGACCTCGCAGCGCTGAGCGTCTCCGCCACTTCACCGTCCCCATAATTCCTGATAAATGTCTCGATCAATGACGTTTTGGTACGGGGATCCTTGCGTTGTACATCAAGATAAAAAGTGTGATAAGTTGGGTCCAGCAGAGCTTCATCCATGTACTCGTTCAGTTTGAGCATCTTCAGCACATCTACAGGTCGCGACTTACTCGAAAGCCATTTATTCAGTAGCGCGTTCTCCATCGCATTCGCCAGCTCACTCGTCCGGGGATCTTTCTTCGCACGAATAAGCAGATTGGCCAGGTTGTGCTCCCCGTCGAAACCTTTCGTCAAGACTTGAATCAACTTTACTTGTCCTGATTTTTCACGGTTGAACATCTTCATGTAGTAATCCAATGCTTCTAGCTTCGGACTTCCGAGAGCACTGTATCCGTCTTTCTGTAGCTTTAACAGCTTGAATACGTCGTCGACCGATTCATCATTCTTCAGCCAACCCGACAGCTGAGCATTTCGCAGCTGCAGAATTGTTTGAAGCTCACTTTCACTCTTTTTCGTCCCTCTACGCGGATACGAGTACCGCCGCCACTGCATCATCTCCATAGTATACCGAAAACACTCCAAGTACCGACATTTTGTTCTTTCGTTTGTATTTGTTGACTGACCTTAGATAAGTTTCCATTCTCTTGACATCAGGACTACTCAGAGCCCTGTCCAAGCCGTTTTCTAGCCTGAATAGCT

>Contig_67

TTTTTTCTGGCAAAAAGGTTTTGTAGACACGTTGGGGTTCCAGTATTGAATGGAAACCAATACTTTTGATTGCTACAGGGAAAATACAGCTCATTTACAAAGAGCCAGGGCAGAGTGCACTGATTTGAAAACTGTTTATAGAGAAGTAAAATCTGCGACTTTTTTTAATCAAGAGGGATTACTTATTGGGGTTATCACGGTTAAAAAATATCTTTGACTGGCCAATCAAATTAAATCTTCGCGTTGCCGTTTTTTTTTTTCAGCAGCGAAGATTTACTATGTTAAGTGGAAAACCATGCCAGAAGGTTTGATTTTGGTGGAGCAAAATATTAAAAGTTTCAGCCCTAGAACATGAATAATAATAGTGGCTCTCAATTGCATGGTTTTCTGTCCAATCAGGCTCACGGAACGTTGACATAATGCTAACCTGCAGATGAGTTGAAAAACAAACCTTTTTGAGTGGTCGCAGGTAACGGCTACCGACTTCCACTCGGCTACCATAACCGACTCCCAGTTTGCAGGGTCAGTGCAGGTGTGAAGATACAGGAATCCTATTGGCTGGTGAAACAGAACAAAACCCGGGTTATTAATTCTCGGAACACCAAAACCGTCAAATCGAGAAACGAAATGTAAAGGTTGCATCCCAATTTACATGATTACCGTTCTTTGTAAAAAAATCTCGTCATGTTTTCCAAAAACTCCACATGTTCGCGAAGTGTTCTGCCACGCAATTAAAGGGATATGGGTCTACAAAGAAGGAAATTAGGCTATTGATTATGGTACGTTTAGTATTAATAGTAATCCCTACACCTGCCGCCTCTGCAGCCATCGCAGCTTGCAGCTGCTCCGCTCTGATCTCAGATAGAGCTCTGCCTGCTCCATCACCTGCTTGCGTTCGGCTGCCTCAATCTCCTTGCGGAAGTTCAAAAACTGCGCCTTGTCCGGGTAAAACCCCAACGCCCTATCAACGACCTCGTTCGCTTCGTTAAAGCGACGCTCCATGAGCCGCAGCTTGGCCATCGCGTACAGCGCCTTGGTGGACGTCTCCTCTACAGCCAATGCCTTGGCTGCGTACTCGTTGATTAGCGATCGATCCTTCATTTTGATGCCACAGATCGCGACGTTATTGCACAGCGTCACGTATCTTTCTTGCATGTCTAGATCGTCCCGCTCCGCCGCAGGGACAGTTTTGCCGTAGTATTCACTCTGCTGGTGCTTTAGCACGGCCTCCAGGCTCTTTAAGAAGGCGTTCTTTGCCGCATAATAGTTCTTTTCCTTGAATTTCGACTTGCCATCGCTTTGCGCTCGTCCGCCTCCTCCAGAAACTCAGGAGGCGTCACATCGTCCGGATTCTGCAGCGTCATGGCTGCAAGACGCTCTTCTGCTGCTCGAGAATACCACTGGTCCAGGTCTTTACCCTGCGCGTGCGAATGCGGCTCAGAGTCCCGCATCTGTAAGAGTGTTTGTCGCACTTGTGGGGACAGAGATTAAATTATTGGTTCCTGAAGTTTAGTACTTAAAGTAGCAAACAAGCCTAAGATGATTTTACCTTTCACCTCTCCCTTGTAATAGGGATTCCGAATTACCTAGAATAACTGTTATGAGGTGCTTAATTATGGGAATGTCACTATTGTGTATCATATCCGAAGAAGGCAAGCAAGGACACTTGCTGTTTGCACCCTTCCTTCCTGCTTAGCTCATATCGTAGCTATCTCTGCATCGATCCAAGTTTTTGATCACCTCTACCGACACACCCGTTGAATCTGCTCAAATAATTAGCTTTGCGACCCACTTTAGGTGTTTCATAATATTCGTAAGAATTGAGCTCTGCATAACCATTATCTGCTTCACTGAGCATGAGCATGATGTGAGCTGACTTCGATTCGACTGGCGTCTTGATGGCACCAGCTTTGAAAAATGAAGAAAATCGCAAGGAATGTACTTGTAATGGATGATAGCGTGCTGGCTTCTGTAAATAGACCATTTTGAAGGTACACATAGCTGAGCCACCTCACAACAAGAATACCCTTATGTAGGCATAAAGTGTTGGTTGGTTCCCGGCGCTACGACTACGTAAGGGCTCACCTGTATTTTTTATCGTGCTGGACTGTACACCTAGAAGTTAGTAATCATGTGTACCGGTACAGTATGCTTAACTGTAAGTATCATTCTGGATATTCGCCTAATAATGTGTTCTCCAAATAAATGGTTACCTTCTAGTATGTTCTTCTTGGGTGGAGACAGCTACACCAAGAGGTCGTTATCTACCGAGTGAGGTCGATGATCGACAAAGTGTTCAATGTGAACACGTACAAACTGAGTATTAGGACCAGCGTAAGTCTGAAAACCGCATAAAAAAAGGTCACTAATTGCTCCAGTTACACAAAAGAGCGTCCTCGATGATGCCGACCTCTGTCAATTTAGAGATACAAGCCCGGCTCCTATTACAGTAATACTCTTAATAAGGGAGCCGAATATACCGGGCTCTCGACTTGAAAATATAAAAGCGTGCTCCGGCTCGGTTCGTCCATCCCATGATGATTACACACTGTGCTTCGTTTAAAAGGTACTACAGAAGTGCTATTACATGTGCCATAATAATACCCACCGGTATTAAATTGATACCGGTGTGTATTCTTTACTATTACATGTAATAGGTAAGAACTGTACATGTATTTGATTCCAAGATTTAAGATGCGATTGTGGAGATTGTGAAACAAGTGACGACCTCCCGGAGCCTGTCAACTTCACAATTTGCTGTCCAACGCTCAAGTATGCGATTCTATTCGGTGTTGCTGACTATTGTGACTCTCATTGCCAGTACTTATGATGCAAAAGTAAACGCTTCAGGCATCCAGGCTATTGCTGTCAGTAGCATTTCCCACGATGCTCCTGCTGCGAGGATGCTACGAGCTGACCATGCCGATGAGAGAGGAATAAGCGTACCCAGTGCCTCAAAGATCGTCGAATGGATGCTTACTCCGAGAAAGCTCACATTTCTCGAAAACCGCAAGGTCCAGAAGTGGGTAGACAAACAGAAGACGCAGGAGTATGTCTTTACGAAGCTGGGCCTTAACAGCGGACTTGATAAGGCTCTCTCCAACCCGAAGCTCCATGTTTATGCTGCCTACATCGATCGTTTCAACGTGAAAAACCCCTCAAACAAAGTGGCATTACTCGACAAGTTTAGTGAAAAGTACACAGACGAAGGGGTAGCCAAGATGGTAGAAATGGGAATACGATCCTCAAACTTGGAGACGGAGAATTTCGCGTCGAGACTGTGGAGGGAACTGCTGAATAAGTGGATGGGCAATGCCGAATCTGCCGAGGGAGTTTTCAAGATTCTAAAGCTTGACGAAGTGGGAGGTGGCATTTTCGCGACGCCGCTGTTTAATACTTGGTACGCTTTCATAAAAGAGGGTTATACGCGGCAGGCAGAGGATGTCGTGCTTCGAGTCTTGTCGGACAGGTATGGCTATGACGGGCTATCGAGGATTTTCTTCCGTGGGCAGCGTAACTTTGATCTTGTGGGCGATCTCCCCATCAAATTGGAGACAAGAATGGTGAATAATTGGCTGAACAAAGATGTATCTCCTGACAAAGTGTTCAAGCTGTTGAAGCTGGATGAGGGACTGGATAAGCTTCTGACCAACTCGAATATGCAGGTGTGGGAGAGCTACATGATGAAATATAATTTGATGCCCGATGTGGAGCCAACGACGATGATGCAGACGATCACGCGTTTCTACAATTTCAAAGAATTGTCATCTATGCTCGAGAACGCGAAAATGGTGCCTGAACTGAATAAAGTTGCAGAGAGATGGCAACATGAACTACGTGTTCATTATTTGAGAGCCCCCAAGATGAAGAAAGAAGGATGAAAACGTCGAGCAGAGTTGTACTGAGGGTTAGCTTGCTTTCCCTGCCGCCAAGGCATGACTCAAATACGGGTTAACTAACAAAGACAAGATAATGCATGTACAGCAGATCCGTTGTGCTGGAGAGGGGAGTAAGAACGGAAGAAGATGATACTTCAGTACGTCCTAAACGTCCTACTGCTACTGTAGTATTTGTCTAAATAAAACGATTTATTTTTGAGGTCCAATTACCAGTTATGCTTTTAAGTAAGAATCCTCGTATGTGTGATTGGTCAATGTCCCTTTTGAGATCTATATGATTGGTTGAAATATGTACAAGATGATTTGTATGCATTTAATTATAATTTGTCCTAGCGCTGCGAATGAAGAAGGCTAACCGCGCTTTCGTTGAAGCAGTACAACAGAAGTTGTCACGACTTTAGAGACCTACTATTTTTTTTTCGGACCGGACAAGTATGTCGATAGCTGCATGAAAATCTCATATTAAAGACAAGTTTAAATATTTAAAAAAATAGGACATTCTAAACTCGCTTTGGTCCCGAGCCTCAGCATGCCTTTTCAGCAGGTGATCACACGCAGCGTAAGTCTAGACAAACGGATCAGTGAGGGACTATTAAAAACATTACAGCTGGCGTGCTTACTCATTTGGTGATAGTAGGGATCAACTAGATCGTACGCAGGTAAAAAAAAATATTTTATCCGAGTCTAAATTCGCACTATGCCCAATTTTGCACACACTTGACAAGTGTACGATTCAGCTAACCGGTCCGTTGGACCGGAAGAGGAACAAGAGGTTTTCGACGCGCAGAAGAAACAACGGCATTTACCACGTCCCGGAGCAGAATGAAGAGGAGCACGGTTACCCAGGACTTCGAGTCCCTGCACAGCGAAGATCGAGCCAGGCCCAGCATTATGACTCTTGACCCCCTGACGTCCATGACGCGCTCCGAGGAACTCCGCAACCAGGATGTACGGCTCGAGTGCCCGCCTAAGCTGGATGACGGCGAGTGGGACGGCTTCATTTACTCGCGGCTTCAGTAGCCTCGTCATATCAAGCCCATCCACCCAAGCGCCTTTGAGCTCGATGATTCTTCCTGACGCCCCGGTTGGTTGGGTGAGCTGATGCTCGATGCTCCATAATTTGATGCCGTGCCAACATGCCTCGATGCCTGGCAGCCACACCGGTGATTATCCCATTCCCCCGCATCGGATGACCGAGGACCTCAGCCAGGAGCTATAAGACCGATCTCGTCGGTTACTGTTCTTGTTTATCCGGTTCAGAGTGTGTTAAATATCCTGCGAGGTACGAGCTTACCACGAAGCTCATCTGGACGTCTATCTCGTCGGACAAGTTGTTGCCTATTTCCATTCGCGGGCTGCCAGCTGTAGTACGCCGACCGGTGCTGGCCTGCAGCGAGCTTGTGACTTCATGTGTATTGCGGATCTGCGCTCTCGGGTGGATATACCAGCGAATCATGTGGCATTCCTTCCACCTCTGAATGGGGCCTCCGAATAACCCGTGACGAGATCAGCGTCGGCAGGTGTCACGGCGTTTCCGTTGATGCCCAGCCATCACGACGCCTAACGCCCTTTTGATCTTGATGGTCGAATTAATTATATCGGACAAGCCGGAACCTGAATTAATTCGGTTTTACTAACATTGGAACCTTTATCCCGCCATCAACTATTAACTTTAGTGAAGGAGAACTTCGTGTAATTTCTGAATAGGATACACATTTGTCCTCTGAATTACCCTATCCAGTACATGAGCCAGCTCGATCCTCAACTCGCATGGATGATGAGGTTAACATCGATTGAGCGTCATATTTCAACCTTCTTGCTATCGGCCTATCTCGTAGCCAGCCCCTTTCCGGTGGAACACCCGACTTTTCTAGATTCGGCGACCGTACTGCCATAGAGGTCTTCCTGTGAACGGCCCCTTCCGTCCAGACGGTGATCGAGAACACAGAGCCACGCCCTTTGCTCGAATCTGTAACATCGCTCGAGCCGACACCTAGTGTTAGTGTAACGGACGCGAGCTCTGGCGAACCTGTCTCTACGGTCGTCCGTTTTGACCGCGGCAGTACAACGGGCCCTACACAGACCAAGTTTCCATGTCTACTTCTACCCTGAATCCGACGGATGAACACCTTGAAGCAATTGCAGCGCTAGTAATAGCCACCCAAGCGGACCTCTTTTGGCAGGATGCAGCTCAACAAGCATTCCAGGAGCAGCTCCTGCAGCGCCTGGCGCTACGCAACGATGTACCATCGCCTAGACTTACCGAAAACCAGGTGTCCGATATCCGAAACAACGGTTTGTAAAACACAACTACTATTAATAGTCTTCTGCTCGTCTCCCAGGTGTCACGCGGTAAGGTTGGGCTGTCCGGGTCGTGTATCCTGGTAAGATAGGTCACTACCTCAGCTCCAAGTTTTGCGAAAGAGTGAGCGTGTTTGCAACTGCGAAAGTTGCATCGCTTTTGAATCTGCAACCGCAGGCACAGCGCCTTGTCCTCGTGTCTGGAAATCTATCGGATGATGTTGTCAGGAACCGACGACGATTGCTGACACCGTGAACGCGAACCCCGCTGCAGACCACCACGGAGTCGAGAGCTAGCAGGTCGATTTGAGGTGTGGTCGCTTCTCAAAGAAAGTGGGGTTTCAACTTGAGCCCACGTCGACAACAGGCCTCGAGCAATGATATGTTGAATCAGGCGAATATGGTCAGGTAAATGCGGAGCTATACTTGCGCGAGTTTAGTATTTGGCAGCGCACCGCGGGTTGATCTAAAAAAAAACAAATGCGTAAAACGTGCGAATGTAGGTTCGCCATGGCGCGTACAAGGCGTCGTGGGGCGTCATCGAGCACCCGATAATTGTAATCGACGTGGGCTGCCGCAAATACGCTCACCACTGGCTCAGGATACCATTCCGTGGTCACTTCCTCGAAGGCAGTGTCATTGTTGACGTAAGTCATCGAAGGACTTGATTAGGCTACCAGGGGGGGGGGGGGCAGACGAATACGTGGAGCGAATGTGAAGACGGTGCGCATGTCGTCATCCCGGGCCGGCGGAGGAAGGATGTGATCTAACGTCTCCCTTTGAAATTCACGGCAAATATTATTTTGGCCGCCACTTTCGTGACGTGGTACTGACCGCGTAGCGTTTCATGATGGTGGCTAGTAGAGGCGCTGACAACCGCCAAAATGACAACAGGTTAGATTCAGTAACTCACCCAAGGGTTGCGGCCCAGCTCACGCTGTCTGGCGAAGGCCGAAGCCCAGCGGTTTCAACAAGCTTCACTGCTGAGTGTGAATTCCGGCTGAAGATGAAGAAGCGCGAATTCATGCTGTCTCGGAACACACGATCAGTAGGTGGCTCCGGACCACGATGGTATGATGGAGAAGTCAGCGGCACCTCCGGTCGGTGAAGATAGGAATATCCATGCGAAAGGAGGTGAAAGATTTGGTCAGAAGTGGCTCTCTCGCCAGGAGAGTGGGTTCGTATAGATTAAACAAGCGCATGACTATCGAATCTCTCTGACCCGAAACTCTCCCTGTCCCTGATGCGACAATTCAAGTACTGGAACATCCGATCGATCGTAAGCAGGACCGTTGTTGGCGCCCCAGTTGTGGCGAGGAGGCGCCCGTTGATGACGTTGCTCTTGAGGTACATCAATGAATCTTGCGGCCGCTGGACCTGCTGCGGAATCGGTGAGAATGAGAGCTTTTTGGTCGACTTCGTGAGCGAGAGGCATAGTTTCGTGATCTCGGGTTTGGGCTAAGACTCTAGTCTCATGGAGCTCTACCGCTCTTAGACGCACAGTGACCGTGTTCGAGGTAAACGACCGCGCGACCCTTGCTTCTGATATCGGAAACCTGGTTCCCGGTAAGTCTAGGCGATGGTACATCGTTTCGTAGCGCCAGACGCTACAGTACCCGCTCCTGGAATGCCTGATGGGCTACTGTCTGCTGAGAGAGGGTCGATTGGATGGCTCTCGCTAGCGCTGCAGTTGTTTCAAGGTCTTCATCCCGCGGATTCAGGGTATAAGTAGAAATGGAAACTTGGTCTGTTGTCCCGTACCCGGGAGCGAAGGAATCATTCCAGGAGCTGTGCGTCATGACGGTATGACCCGAGGTCCTGTGGCTATTCCTCGCCCGGCCGTCTCGTCAGGATGGTTCCCGTTAAGCGCGATGGCTCGCGTGCCCCTGGCCGCGCCCGTTAGAGTGGCTCTATAGGGCGATCCGGGAACGCTGATTCTCAGTCAACGTGGGACTAAGACACCTAGCGCCATCGGGTCCCTTCCCAGAGCTCCCTGTTGTCCACGAGGGCGGACGGCAGGTGAGGTGTACTCTTCGTTCAAGTG

>Contig_68

ACACTCTCCCCCACTAAGATGAGAACATTCCGTTTTCGAAACTCTCCTCAGCGAGGTCACGTCTTGTTAAGACGGCGGAGGTTACTACACCTCACACCAGCGTTGTACAGCTCCGTGAGCTCACAAACGCCTACGCATCGTTGATGCCCAACTTCTCATCGTCGGCTCACGGCCCAATGCTCCAAGCGCAGCTCGCATCTTGCTCTCAAGCGGTCTCAGACCTACGCTCGCGCTCTGCGCCTACTCCAAGAGCTGCAAACCGCTTAGAGCAATGCTCTTGACTCATTCGAGTCGTCGACAACAGCTCAGCGGTTGAACACTCGCTTCACAAGCGATAGGTCGGTGGTTCGAACCCACTAGGGCACAGCCCTCTCGTTCCATGAACGATTGTTCACGCTTGGACAAGCTTAAGGGCAACACCCTTTTAGCTCAATGGTTGAGCGTTGGCTTTGAGAGCCACAGGTCGGTGGTTCGAACCCACCAGGGCACAGCCCTCTCGTTCCATGAACTGTTGTCCTTGAATGGACAAGCTTAAGGGCAACGCCCTTTCAGCTCAGTGGTCGAGCGCTGGCTTTGAAAGCCACAGGTCGGTGGTTCAAATCCACCCGGGCGCAGCCCTCTCATACCATGAGTAGTCCGCTCTCGGACTGACGTACGGGCATCGCCCTTGTAGCTCAATGGTAGAGCTCCGGCCTGTCAAGCCAAAGGCCAGTGGTTCGATCCCTGTGCGGGACACCACTTTCGCTGTTACAGCGCATCACACATTTGGATCATGCTCATCGAGCAAGTTGTTTCAAAACAACTATGCGTCTATCGAGACGCTTGTGCCTGAACCTTGTTCAGCACAACACGAAGCACCTGCTTCCTTCGGAACCATGTCCCTAACACTACGAGGTCTCTCCACCTCCTGCTCCCGCTGCGAGCACCGCTCACACGTACGAGCTGGCTGCTGCAGCCACTGGTCAGAGCCTGAGCTCAACTACTGACCTCATCGCTCCCCACTCTAGTGGGAGGCGCCCAGCGCATGGCGACGCTGTCGCACGCCATGGTGGCGCTTCGGCACATGATGTGCCCGGTCGTAAGCGTCAACGACGTCAGGGCAATCTTGCCGGAGGATAACCTCAAACTCCCATGAGTTCTGTGAGTGAGGGTAACCCCTCCACTTCACTAAGTACTGGGTACGACCCTGGCGTCTACGCCACGCCACAAGGCGTTCCACGTGAAAGTGGAGCTCACCATGCTCATCAAGCAGAGCAGGAGGAGGCCTTGCCTCAGCTGGGCTCTCGCGGGGAGAGTCAGCACTTGCCGGAACGGCGTCTGCGTCCATACGACGCCGGCGTCCACGACGCGAACGCTCACCGCCCATCGGTCGATCTCGAAGAGATGATCCACCTTGTGGATAGGTTAGAGCCACCACTGTTGACTCACAACCAGGTCTCGGACCTTGAGCATCCAGCTGCTCGGCTGAACGTTCAGCTTGCCTTGCTCCTGCATCTCGAGCACTTCGCTCCGCGACCTGTGGCTCTGCCACGTGCTGTTGATCAGCCATCTGTGGATCCACCACTTGCTATCGTTCAGCCTCCCGTGGCTCTACCACCTGCTGCTCTGCAGCTGTCCGTGGCCCAGCCACCGTTGGTGCCAACGCCTCCGCGCTCACCTGAGCAGGGTCTTGGTATGGCTTAAGCAAGCCGACATAGAAGACCGGATGCGTGCGCATCTTCTTTGGAAGGTTGAGCGTATACGCTAGGCCCTTCCTGACCACGACCTTGAACGGTCCTATGAACCGAGGTCTTAACTTCGTCTTGAAGACTGCAGACACTGCATGAGTAGGTAGGTTTTTAGCGTTTAATAAAACTTGGTCACCGACCTCRAACCTCTCAACATTGCTTCTGCCCTTGGCATCTGCATGTTCCTTCTGCGTGTCTTGGCTTTCAGCCATAGCGTCGCGTACTTGACGCATTACGCTCAGTCTCAAAGAGACAAAGTCATCGACTTGCTTGCGAACCGCNAACAGGGCTGACATCAGCTAGCCGCTCGGCTATCCCTCTCCCACCAAGCCCTGAGCCACTGCGTGGCGGCGTCAGTGGGACGCGAGGGTTARCCAAGCCGTTCACATAGAACGGAGTGTAGCCTGTCGAGGCGTGCACTGCATTATTTAATGCGAACTCAACGAGTGGGAGCATCTGGCTCCAGCGTTTTGGCGCCTCAGCGCATACGCTTCGGAGGGTATCCTCCACAACGCGATTCACGCGTTCGGTTTGACCATCGGTCTGCGGATGGTCCGCCGTGGACATGTCYAAACGAGTGCCGAGCACTCTGAAGAGGRACGTCCAGAACTTCGCGGTGAAGCGCGGATCTCGATCCGAGACAATTGCCTCAGGCAAGCCATGTTGGCGAAAGACTCGGTCTAAAAACAGCAAAGCTGTACCTTCACCATCAATGGTGTCCGGCACTGCCGCTAAATGAGCCATCTTGCTCAATCGGTCTACAAAGACCACTATACCTGTGTTTTCCCGCCTTATCCTTGGGAAGGCCAAACACAAAGTCCATACTCATGGACTGCCAGCATCCCTTGGGGACAGGCAAACTCGCCAATGGCGCTGCCGTGCTCGGCGCGGACTTTGTCCGTTGACATGTCTCACACGTGCGTACATAGGTGCCGACCCACTTATACATCTTGGGCCACCAGTACATCGAGCTCACCGAGCCATAGGTCTTCTCCCGACCNTAAGTGCCCGCCCACGGGCGTATCATGAACCTCATAGAGGATTCGATACTTTAGATCCTCATCATGAGGAACGACTACACGAGGGTTATCCTCAGCGTCGGTGCTGTAGTACAGCAGGCCGCCATCGAGTGAGTATCGATGCAGCCTCGCGCGTAAGCGCCCAGACAATTCCTTGTCAGAGTCTTTAAACTCTTCGCTCCCAAGAGCCCGTAGCAGCGCTACGCACATGTCGTCTTGTGCATACGCTCCGCGNNTATCAAGTCAGGCACTGACGACGTCACTGTCGTGACATGAGCTAGCTCGTAGTCCGGCCTGCGCGAGAGCGCATCGGCCACTACATTAAGCCGCCCCGGCTTGTACTTCACCTCGAAGTTATACTCGGCAAAGAAGGAAAGCCATCTTGCCATTCTCTGAGAGAGATGAGGCGACTGTGTCGCAGTGCGTAACGACGCATGATCAGTAAAGATCACAAAAGGCTTAGAGCCTAGCAGGTGTACCCTGAATTTGACTAGAGCATACTTCATTGCAAGTAGCTCTTTGTCATGAACTGGATAGTTCTTCTCTGCAGCTTTGAGCTGGCGAGACTCAAACGCGATCACGCGTTCACGCCCTTCGGCGTCTGCTTGTAGTAAAGCACAACCGATTGCAAAATCGGAGGCGTCACAGACGACGCTAAACGGACTATCCGGGTCCGGTAGCGCAAGGATCGGAGCCTGGAGAAGGCTCTCCTTCACAGCCTCAAAGGCTCCGTCGTGCTCGACGTTCCAGCACCATGGTGCGTCCTTCTTAAGGAGATCTGATAATGGCCGAGCCATCTCCGCGTAGTTCGCACTGTACTTGTGCAAGTAATTGGCTAAGCCTAGCCACTTGCGTAAGTCKTTTTGGTTCTTGGGAACCGGCCAGTCCACGATGGCCTTGACTTTGGCAGGATCTGCCCGTAAGCCACGCTTCCCAATGAAGCACCCGAGGAAAGGTATCTCCTCCGCGCCAAAGACGCACTTGTCCAAGTTGGCATACAACTTATTAGTGCGCATGCACTCGAGCACCGCTCGCAAGTGGTCAATGTGGTTATCCACGTCTGACTTTCCGTGCTCGGCACGACTATGGACGAATATATCGTCAAAGTACGTTTGAGCGTACGCCCGGTGGGGCCTAAACAGCTGCGTCACAAGACGATTGAACGTTGCTGGTGCGTTCGACAGCCCCTGGGGCATCACCAGCCACTCCCAGAGCATACCGCTCGGAGTACTCACCGCTGTCAGCGGAACGTCGCTCGCTCTCATGAGCAATTGGTAGTAGCCATCGACCAAGTCGAGCGCGCTGTACAGCGTACAGCCTACCATGTTGTTCTGCAGAACATCCTTGCGAGGAATCGGCGTTTGCGCCGGGATAGTGGCCGCATTTAGCTTGTTAAAAGCATGAACAATGCGCCACTTGCCGTTCGGCTTTCTGACACAGAACGTGGGTGTCGAGTGCGGAGACTTACTCTCACGCACCATCCCTGCCTCGTGCTTCGCACGGAAGAATGCGTCAATGACGTCACACTGCTCTCTCGGCAAAGGCCATTGCCTTGTTACGCAATATTTGGTTCCAGGAACCAGGTCAATCTCATGACACACGCCTCGATCCGGAGGCAGGCCCGTCGGTGGGTCTTTTGAGACAACGTCTCCATACTCCTTCACCACCGGGTAGAAGGGATCCGAAGGATCTTTGAGGATCGCCGATCCACTTCTTGCGCTCAGCGCCTTCTTGGTGTCCTCTAGGACCGCGTCGTCCTCGACGGATGAGGAGTTCAACTCCTCCTCAGGTCCCAGCAAGACCACCTCGGCTAGCTCGCCTGCCTTTAAGGCTTCGCCGAACTCGGCAATGGACATCTCGTCCAGCTCTAGCAGAGCATCTGCAGCGGGCAGCGTATCAAGGCTGATATCGCCTTCTACCTCCCCCGTCACCTCCGTTCACGAGTGTATACAGCTGTTCACGACAATCACGTGGAGCATCACCGCTCGTCACGTTGGACACATCGTCCGACGTCGAAGACGTCACAGACGCCCTGCGTCTTCTTCTCCTCTCCCTCCGCGACCGAGCCCGGCCGGATGAGGGCGGTGTGGCGACATCTCCGTCACCACCACCGGGTGCAGCACCATCACTCCTCGAAGAGGATACAGTGTCTCGCAAGACACTCGGCTGAAAGCCAAGAGGTTCTTGACCTTCACACAGTGAACTAGGTGTCACTTCACCTGCATCTCCTGGGCCACGCCCATGATGCCTCGACTCGCAGTCGACCGCGACCCTCGGGTCACGCAGCGGTAGGTCATCGACCAGATTCATACCACTCAGTGGATTACGCGCCACTCCGCGCTCGTCCTCACCAGGACCCAGCAGTTGGTCATTGACCTGACCTGCACCGCTCCGCGGATAACGTGCCGCACCACGTACGCCCTCAGGGCAAACAGCAACCTCGTTGCTAACAATTTCGGACATACCAATGTCCAGCACTACGGCCGCCTCGGCCTCGTGCTCGCGCCAAAAGCGCTTCTGCTTCCTAGCAGAGGTGGGTTCATGACTCACCAAAGCTCCACTAGGAGCAGGGTGCGACGCACCCAGCGTTTTCGACTTCCAGTCGATCCATGGCTCATGACGTTCCAGCCATGCCATGCCAAGGATGAGGTCATACCTCGCATCCAGGTCTAAGACCAAACAGCGCTCCACGCTATCGAAGTTCTCAAACTTCACTCCAAGGTCGACAGAGACCTTCGACACAGTTACCAGTGTCCCCGTCGCGAGACGGACAGAGATCGTATCTCTCGTTGGCACTTCCAGTGCCTCAGCATACAGCTGACTCCCTTCCAGGGTGGAGCGACGGGCGTAGTTCCCCGAGGCTCCCGAGTCAATCAAGACTCTCCATGGCTTTGCAAAGCCCTTCACGTTCGCTTGAACGACCAGCAAGCCCGGCTTGCACACACTCTTCTCGTAAGAGAGCTCAGCTCTCTTCAAGGCTGAGCCCCGTTGTCCTCTACCTCTTCGAGGCACAACAGAACTCAGTTCTTCCCCAGTAGGCCGCCGCGCGCCTACTGGGAGTTGCCGTTTCCCGGCGATGACCCGGAGGGCTGGCTCGCCGGAGCCTCGCGCTTCTTGCGCAGAGGGCAATAGGCCCTCAAGTGCTTAGTGCTATTGCACACAAAGCATCGGCGGATGCCGGCGCGCTGCTCCGCAGCAAGAAGCTCCGCTTCTTCGTCTTCAGCATAGCTGAGGTCCATCGGCTCTGGACCGCTCGAAGAGCTTGCGCTACCAGCGCTCCAACCAGGTCGCGCCGACCTGAAGTTGTGCTCGGCATTTAATGCCACATTCACCGCCTCTTCGAAAGAGTTAGGGTGTACACGGAACACTTCCGTTCGAGCTGCGCTCACACGAAGTCCCTCCATGAACACGGTCACGCTGACCAGTTATGGAAGAGGGTCCGCCGCCATTCCGGCGATAAGAGTTCTCAACTCTTGGACATAGTCCAATAACTCCTTCTTACCCTGGCGGGTAGCAAGGAACTTCGACCGGATACGGTACGCCTGATTAGGCGGCGCGAACACGCGCGAAAGCTGTTGCTTCAGCTGATCCCAAGTGGGAAATGCAGCGTCAACAGACGTGCCGCACGTTAGTGCCCATTCGCGAGCGCGACCTCCAAGCCTGGAGATAGCCAGCGCCACGCGCTGATGCTCAGACTGGAGCAGGGCCGATCCCATGGCCATCTCGACCTCACGGGTCCAGAGAAGGAGATTCTCTCCATCCTTCCCCTCGAAGGTTTTTACGCTCACCATGAGCGGTTTCAGTCTCATAGACTCTTCACGCGTCATCGACGCCGCCGCCGTTGCGGCGCTCATCTTCTCTTGGACATGCTCCAAGAGCGCGTTCTCGTAGCGGGAGAACGATTCCAGCCTGGCAGTGACCGCCTCTGGACCTTGCGATGCAAGATGAGCCACGCCCTCCGGGCCAAGCAAGGACATAAGCTTAGTAAGCGCGTCCTGCTGCGCCGGGGGCAAATTCGCGAACACGGGGTGTTCCATCCTCGCAGAGGATATAGTCAACCGACTCAGGAGCGGGATTGACTACAAGTGCTACCAAGTGCAGCGGGGCTGCCGTAGCTTTAGCGTCAGAGGAAGACGTTCGACTGAAAGAGTCACTAATACTAAGTTCCTTATCCAAAGGACACTTACAGCCCAAGACCAGGGCTCAAAGTTAATACCTTAACTTTTATGCTTCAAAACCAGAAGTGTTTTATTTACTATTATTGCGGCCAAGCCGCCTAGGGGTAAATGTATCTCTTTAACTATCTAGCATAGTTAAACCTAATCAAATGCATAACGTGTGGCTTTCAGCCAACGTACTGAGAGACCTTGTTAACTCTCCAACTTATGTACTGAGAGTTTGTTAACTCTCCGGGTACTTCGTACCCCTGTGTGCTCCACACCATGTAACCCCGTTACACTTCCTTATGTGGTAGTCAGAAGCGTGGGAACAGGGCTAGCAGCGGTCGAGCTACCAAAGTGGGAAAGATAGAGCGAGTTGCAGCGTGGCTATCGCGATGGCAATCACCGAGATGAAGAAGGGCAACCCTGAGAATTATGTGTATCTTAATAGTCGCTTACAATACGAGAAGGACGAGGCCAAAAGAATGAGAGAGCACGAGTTAAAACTGGAACAGCAGCGTCAAGAAGCTGAGCGCGCACGTGATGAGCGTATGCAAGCTGCTGAACGTGACCGCGAGAAGTTTATGTTGCAGCTACTGGAAATCGCTCTCGGACGTGCAAAAGACAAACAGTAACGGATTATAAACATACGACACGTTTTGTCATTCTTAATTTGCGTTAAGGTAGCTGTCTACTGATGGTGGAAGTAAATTAAAGTATATAGATATCTGTTTTCCTGTTGGTTGAAGACATGCATGAAGGTTGGTTAGTAGCACTCCAACAACGAAAAGCTTTCCAGCCGCGGGCTGTCGGGTTCGCAGCTTTTTATCCCAATTCAGGAATGACCATAGCATCTTGACACGCCCGAATCCCCACTCTACGGCCTCGCGAACACTGCTCATACGTGAATTGAAAAGTTTCATAGCTCCATTTGATCCAGGATTTTCGAATGGACAGCAAACGTACTGGCTGCAGCCATACGCCGGATCTCCGTAAATTACTTTTTCATTGAAAAGCATGGATTCATCATTTTTAAAGACATCCAAAATACCACTCATGCTTAACATCGTCGAATCATGCCGCCCGCCTTCCACGGGACCGAATATGCTGACAATTTTTCCATCTGGAGCTGTAACAGCTTGCCAGTTGAAGCAGTGGCGTCTCGGATGACCATTATACAGAGATCTTTGTAGGTTCTCGTATGGTGACGTCGGATTCCTACGAGCGCTTGGTCTGGATACATACTCCTTAGTTCCATCCACAAAACCCCAGCAACGTGCTAACGGGGAGCCCTTGGCTCTAATTGCGTCACAATACCTCTCAATACGGGCTGCAACATTACCATGGTTGAAATACAGGAGAGCCTTGTATTTGTTGTACAACGTATCCGCTAAGTGGCGACAGACGCGCGTGTGCGCCCCAGGTGATCTGCCGAGCTCATTAGCTACTGTATACAGCATCGATGGCTCCTTCAGCCTTCTGCACAGCATAGCAAGCGCTTCAACGGTCGGGACAGCGTCACGCTCTGGGGTAATAATGACTGGTTTCGGTAAATGCATTTTGGTCGCTAGCAATCGTAGCTGAGGCAGCGTAAAATTGAAGCGTGTCCGTGCCGTCGCGTCGTCCATGGAGGGGTCGAAGTGGTTGCTTGTGTTGATCCGAGGAGGTGGGTCTCGAAGACGGAAGCAATGACTGGCGATGAGGAAGTGCATTGTGCGCAGGAAGCGCTTCCGATTACCCCGTGCAGCAAGCAACAAACAGCTAATGATGACAAGTGCTTGCTCCGCTGTGAGCTTCGGTGCTATGAAGACCATTGACGAGGCNTGGATGGTGCTAGCTGTGAAGACGAAAGTGAGCTCAAATCCGAGTTGAGCAAACCGGCCAGTTAGCTCAAAAGCAATTCCGTTTGAGTATCGTATTTGAGAGGGCTCACCCTAGAAAATCAAAAGAAGACTGAAACTCAACAAAAAACAGGTTGCGAAAGGTCTCAATACAGTCGTGCACCTCCAATTCGGCAGGGCGGACCATATCAACAGTGCCAGGGCATAAGTGCACAACGTAAAGCAAAAACCAAACGCAAATCCAGTGCAATGATATTTGTTTTGTTAATTCAGTACAAAATTTACTTCAATCTTCTGCATAATCTTGGCGTCTGACGCGCGAATCGCCGGCGCTCAACAGCGCTTCCGGTCGCGATAGGCATCCGGCAAGGCGGCAACTTCCGCCGACCGCTTAAGAATACTCTGGACGGTAGTGCGCTTTACTGCTTGGGGGGTCTTAAAGCGCTCCGTTGCCCAACGAGCAAGTTCGGAGTGAGAGCATGCAGGATCTGACTTGTGCTTCTCACAAAGACTGCTATTGTGCTAGAGTTATGGACATGTAGGAGGAAGGGCGATGTCCCATTGTCTTTGGCGGTAAAAATCAAAGTGTGTGTTTGTATGAAGACGTCCGATCCAAAATTTAAAATCAGATCGATGCGGCGTGTCATCCCTCCGCGAGGAGGGGAAACCGTCGAGGTTAATTGCTAGGGTGGTGATGGGCAAAGGTAAGGGAAAATGCCAATTAAGGGCCGTTCAAAGCAACCTTCTCTTGTTTTGATTGTATCTTTTGATCTAGTAAAAAGCAAAATATGGCCTTGGCATCAATCAAAAGCTGACACAATTGTATATTTCCCTGGCTATTGAATTGTTTTACATCAGCTATGGTGCGTTCAGTAGGACAAATTAAAACAGTGCATAAAGTGTGACGGCCATGCCAATTAAGGGCCGCTTGATTTCACTATGGGTTGCTAAGTAATCTCTTATATGATACCTTAGTAGTGGCCAATGAAAATAGATAAGCCTGAAATTCCGAATTGCCTATTGGCTAACCGAATCCGGCCCTTAACTGGTCACCGGCCCCTAATTGGCATTTTCCCTTAATTGCGCATCCGATTTGCGTGATCATAAAATCTGGCAGGGTATCGACTGATACCGGTGCCAAATTGGAGGTGCGACTGTATTATATTTCACTTCATGCTTCAGTGTGTGTGCCAAACACATTATTGCGTAGCCACAATGTGACGCAATTGCGAACCACAGGAGTGCTTGACAAGCACTACTTTGCTATCATGGTATTCGGATCAATGCGCCACGCTGTTCAGCACCTTCGCCAGTCGGCTAGGAACACTTCCACCCGACGTGCAACTGTATCTGCTTCCTCCTCCTTTTCCTGCGCTCCACGTGCTCTGGTGCAGCAAGCCTGTGTCAATGCTGCGCCACTCGTTGCCCAGCCGCAACACATGAAAGCGGACCCAACGGGTATCGTGGCCGGCATCGCCATCGCGGGTGCCTTGATTGGCGTGGGTAAGATGTGGTGGGACGCGTCGTCGTCTAGTACGGTGGAGCCACCGGCCTTCCAGGAGATCCCGCAAGCAGAGATCGCGCTCTTCTTCACGGAACTCACGGCTGCTGTCAAGGACCTCTTCGTACGCGTGCTGTGTCTGCTCTTAAGTGGTATTGATCGAGTACTTACACTTATTTTATGCCTCGTAATTGACAGAAACAATTACCGGAGATTGAGAACGCTGTACGCAAGTACCTGAAGGACAACAACCACGAGCTGAGTGACGCCGAATTTAAGCAAGCTATTCTGTCGCAGCTGTATCAGATGATGGAAGGCATCGAGCAACAGATCGTTGCCAAGCGCCTGTGGAGCCGTCAGAGCCTCGAGTTTGCACTTGAGAAGTACGCACAGGACCCGGAGGTACTCAAGCTCCAGGACAACCTGAACACCATCATGCGATCGTAAGTTTACACCGATTCTGTGTGGAGTGCTCGGGCGTTGCAGATTTCGAAACACTAATAGGATTCTCACTACCCGCTACTTGGCTGCTTCTTCCCGCTCAGTGTGTTCCCTGCTCCGGAGCCCGTGGAGGTTCCTGAGGATCTCACTGCCGACAAGACACTGGTGATTTTGAAGGAAATGGTGAGAGGCATGGAGAAGGCCATGGCAGACATGCTTGCGCACGCTCGGGCGGAGGGCATCACCGATGTGCAAAAGGCTATGGAGGAATTCCAGTACTTGTACATGGAGCACGTGGAGCAGATGACACAGACTCAGATGAAGACCCAGGGCATATCGCAGGAGGTAAGCACAGCTTTTCTTTTCCGACAGTTCGAGCTGCACCGTGGTTGACTGACGTGCATCTACTGTGTGATCCATTGTTCATTACAGATTTTAACGGCTGCTTTGCAGAAGTACCACACCGAAAGCGAGCAGTTCCGGCACCAAGTGGAGCAGATCTACGCTCAGCAGGCGAAAGCGTACGTTACTGGAAGATGCACACGTCCGCATGATAGAAACGATTCTAACAATTTGTCGTGTGAACAGCTTCCAGAAGATGGGTCTGCCCGTCGAGACCCCATAAAAGAGTCCTTGTTGCATTTGCTCGAAGAGAGATGGCTAATACGTCCAAGCTCTTGTGTGCTGAAGAGTTTTGCCGAAAGCTTCGTGTTTTATTTCTCGCGTTATGTTAGAAAGACCCAGTAAAGACGACAATGAAACACGCGCTATATCAAGATCATGAGATCTATTACTTGTCAATAAAGTTGGAATTGCCATCGTCATCAAAACCTGTGCTGATAAAGTCTAATAGAACAACCTTCGCACCTGCAATACACAACAATTAACGCAACCTTAAAAAGCAGTCCGATTTTGAAGAAGCAGCAAGAGTTGTCGATCTTGTTGATTTCGCTTAGGTCACTCCACGTGTTGCCACGCGAGGATCATCGTCCACCACAATAATGCCAGAATTTCAGCAATCCCCACGAGGGAACATAGCCAGAAAGCGTCACCCAGCAGCAAATTACTTGCCGCCAAGGATGCCGACCGTGCGACCACGACGGCCGGTGGTCTTGGTGTGCTGGCCGCGCACCTTGAGGCCCCACCAGTGACGCAGACCACGGTGAGCGTAGATCTTCTTGAGACGCTCGTAGTCCTCGCGCCACTTGCCCTGCAGGTTGTTGGCCACGACCTGCGAGTGCTTGCCGGTCTTGAAGTCCTTCTGTCTGTTCAGGAACCACACCGGGATCTTGAACTGCAGCGGGTTTTGGATGATGGCCACGACGCGCTCGATCTCGTCGTTGGTCAGCTCACCGGCACGCTTGGTGACGTCCACCTCGGCCTTCTTGCAGACCATGATGGAGAAACGGCGGCCCACACCCTGGATGGCGGTCAGAGCGACCCACACCTTACGGCGACCATCAATGTTGGTGTTAAGAATACGCAGAATGTGCTGGAAGTCCGGCCCGATGACAAGCGACTGAGTGGCAGCAATGAAAAAAGGTTAGCAACTTGGTTCATTTATGCTGCAAACTCGTACTGCCAGAGCTGCCAAATTGAGACAGCGAGGGAGAAGCACGAGTCGCATTGGCTTTGAAGGCATCCATTTGGTACTCGCGACTGCATCACTAGATATCCGAGGAGTTCCTGTTACTGCGCTGTTGGTTGTTCGATCCCTGCAGTTGCTAGCTCGCTTGCCCTCCCCCCTTGCTGTACTCATTGGTCTCTCCCTAATATAGGCATGGAGCAGGCCACTCACCATGGTTAACGAATGCTTTTGCTTCTGGCAGTGGGAACAACGCTGGGTGTGGCTGGTTGGCTCAAAACTCCTGGTCTCTTGAATCCATTATGGTCATGCAAATGACTGGCAGTCGGGACTCGGCGTCACGCACAGCCAGTAAAAAAACTCCTTTTGCTTTAAGCCAATCAGCGGTAAATACTTTCTCATTTTAACCCTATCAGACGAAATCAGCAATACTCTAAAGCTGTACCCATTCTATTACACAGGATATGATGTATGATAGTACTCCAGTACCGTACCGCTCTGAATACAAGGACAAACAGAGCTCTCTTTTCGAAGCATCACTAAGATCCCCCCTTAGCCAAAGATTTCAGAGAGAGAATGAGCGACTTCCGCCGCACGCAGAACACCTTCGGGGACGCCTTCCCTACCGCAGATCAGCTACATGGTGGCTTCAACTTCGACAACTGCCGCCGCAATGAGCTCTTGTTCAGCCAGTCGGGCGGTAAGCAGAAGCTGAAGGCCACTAAGACAGGCACTACCATCGTCGGCGTCGTCTACAAGGCAAGGCTGTGGCAAGCGCAATGGTTATGATTGTTCTCGAATGATATATGCTTACTAATTGCCATTGGTATAATGCAGGACGGTGTGGTGCTGGGCGCCGACACGCGCTCGACGGGCGGCTCCATCGTGATGGACAAGAACTGCGAGAAGATCCACTACATCGCGCCCAATATCTACTGCTGTGGCGCTGGCACAGCTGCCGATACGGAAAACACCACAGCTCTTATTTCATCGCAGTTGGAGCTGCACCGCCTGGTTCGTCGGACTCGTTGGAGTGATAATTGCTGCAATTTTTTCTGACAAGTGTTTTGTCGATTGCAGAACACGGACACGCAGTCGCGCGTGGTGACGGCAATGACGCTACTGAAGCGGATGTTGTTCCAGTACCAGGGTCATGTCTCGGCTGCACTAGTGCTTGGTGGTGTGGACATTACGGGCCCGCACCTCTACACCATTTACCCTCACGGAAGGTGCGCGCACAGTCTCGGTGCATGTGGAGCTTCTAAAAAAGTGATGACTAAACTGAATTTTACTAATGAAATTTAACAGCACGGACAAGCTTCCGTTTGTGACGATGGGGTCGGGTAGTCTGGCCGCCATGTCGGTGTTCGAGCACGGCTACAAGGACGACATGACTGTATGATGTTGAGAAGGACGCGGGTAGTTTTGTGCAATGTCTGACATGATTTCATTCTGAGTTTCCAGGAGGACGATGCTAAGAAACTGGTGCAGGAAGCAATTCTGGCTGGTATTTTCAATGATCTGGGCTCTGGCAGCAACGTGGACGTGACGACAATCAAGAAGGTCAACGGCAAGGTGGAAGTGGTCAAGGAGTTTAACTGTATCAAGCCCAACGAGGTGTCGGAGCTGCGGTCGCAGATTAACCGGGACATCGTCACGCACATCCCGCGCGGAGCTACTCGTACGTTCAATTGCATGCACTTAACACCAGTTATGTTTACTAATTCCTGGGTGTGTTGTGCTACTTGCAGATGTTCTAAGCTCCAAGACTGAGCTGTTCCCTGCCAGCATTGTCGTCGAGGAGGCCACGCCCATGGAACTGTAGACGCAGGTTGTTGGCTTACTGGCATTCTGACGCGATACGTGACCTGAAATGGCTAAATTGCGAAAGAAAATGCACCTTTGAAAACGAATATCTGCGTTGCAAATTCATCCCTATAGTGGACCGCACTTTAGTCCTTGTGCACTGGCATGACCATATCCGCTGTCAACTGCAGTTTNCTCTACAATATCTGTGGGGATAGCAAACGTCGGATGAATCTGTCGGATGCCTTGATACTCGGGGTGGTTCTGCTTTTCACGGTCAAAGTAGCCGAGC

>Contig_69

TACAAATTTCTATTCAGCCAAAATGACATATGTTTTATTGTATAAAGGTTTTTGTATTGGGCCGATGGTGGCCGTAAATTGTGTCCAGCCTGACAAATCTCTTTTTTGTATGTGAGCCTCCCTATTAAGTGCAAAACCGCGAGCGTTAGCACATCCTTGCTATTTTGAAGCATGTTTTGCAACTTGTGTGAAAACCGCACCGTCAAATATTAAAAATTGACTGAGTTATGAACACTCGACGAGGTGGCCGTATAATTGTGTCCAGGAGTGTAATAGTAGTAATAGTTAACAACTAATGCGGTTGAATGAGATTCAGATTAACTCGGGAAATTTAATGGGTGAGGGCAATCAGTTTAACTCGCGCGAGCTAATTTGAATCGAGGTTCACCTCATCTTAACTCGCGCGAGTTGATTTAAGTCGAGGACATTAACGCTAATGTGAGAATCGGTGAAATCATCAAAATGGTCGCATCATTGACTTTTAATTCGTTTTTGCGTGACGAACTTGAGGTCGATACGAGCTTACATCTAGCTTGTTCTTCATGTACTCGACAGGGGTTTGATCTACTTATAAATGGTAAAAAGTAGGTGGCTTCTTCTTCTTGTTTTGTTTCGACAGAAAGTTGAGATACGTAGAGTACTTCATGTACTCGTCCGTTTGCATGAGCCGCTGGAGAACGGTGTTCGAGCTTTGAGCACGTACAACATCGTCAGCAATGCCTAAGTGCTTAGCAAAATCGTCCAAAGAATTCCCCGCTTCAAATAACCGCTTCATCTCGGCTTTAGATAGAGACCTCTCCTCATCGGGTTCCTGATCCTCCACAGGGGTCTGGTGAGACCGCAATTGACGTTGGTGCTGCTCATTTGGTGAAGAGAGCATAGCTTGGTTCGAGTTCACGAGCGCGTTGCTAGAAACGAAGAGAGTTGCCATCGTGGCCGCGAACACGTAAGGAAGACGCATGTGTATAGCGCAGCTTGAGAGGTGGAGAGCAAATCTCAAAGTGAGTGGCTTTGAAAACTACTCCACGGGCNGGGGGGGGGGGCTCTTGTGGAGGAGGCTATCGTAGTAATCGACACCCTGCACTTACAATCCATTTGTTTTACCTTTAGTATACAGTACAATGCACTGTACATAAGGTACAACATAGCGAGCATGGGAGATATCAAGTGAGACCTGCTGTGAATTCACATTTAGGTAGAGGCTGAGCCTCTGATGCTACCCAAAATCGTAGCCTGTTTTCTACTCATGTCGTACGCAGGGATCTTTTGATTCGATCAGTGATAAGGTTCAGTTTTTTTATATTCAGAATTTTTCGAAGGTAATCCTGCTATCCCTTAGCTACTGTAAATGGTTTTCCAAACATTAAGCTAAGATGTAGTTTCCTACGAAACTGGCGTGCATAACTGATGATGTAGATTCCCCTTTTGTACGACCACCCGGAACTTTGTTCAGTCAAGTAGATATCAACGCCACGACTAATACTTTGAAGTATTATGGTGACACAGAAGTCCCCTTCAGTAAGCATAAAGGTCGTCTGATTAATTTGCATATGGAATCCACAGCGATCTTGTGCTCTTGTCAAAAGTACCTCAATCCAAATGATCCGATATAAAGGAGTATATTAAGTACATGCTACTCTTATTCAAGGGACTTCTTTACCTCACTTTCGGTACTGTAGATTTAGCTAAACCCGCCTGTGCCTGCATTTGGAAATATATCCAGTTGATTAAACATTTGCAAACAACTTAAGTTCAATATGGGCGATCTGTGAATAAATTTAGGGGTCTGTGATTTTCGCCTCCCATCCGATTACCCTTAGCTTCTATTCGTTTTATTTAGCGGCAAAATTGATGAAAACCGCAATACCGGTAGCGGCTTACATCGTCCATCTTGGTTGTTGACGCACTCAGACGGTTGGTGTGACCAATCAATCGCTTCGTATTTCAAAAGAGATGGTTTGATTAGTCACGGGGATTCGACTCTAAGTAATCGGACTGCCTTAGTGATACTCAATTGTGCTTTGGAAATGGAGTGCCAGCGTGGTCAGCGCATAAACCCGGGCGAACAATAAAACACGTAAATGTATTAATGCATGTAGGTCGAAGCAAATTTACGCAACACTGCCAAAACGGATGTCGATATTTAGGAACCACTAAATCCAGAACTTCATGCTTTTCATTAGAATTGTGAAAAAAGAGTACATATTTCGTACTGCGTACATTACATAAAAGTGAGCGTTTCAGGGTCTGTCGTGTGTCAAAGCTTGATATTTTTATGGCCCAATCTATCCTTCGAGTCAGGTTTGAGGTCGGCTACGAGTCAGAGTACACATCCGAGGTTCTTACACTACTGTAGTTTCGGATACGATCAGCCGTCACCTGGATTATCTTTATAATACTTCGGTCAAATAGCAACCCCCACACCCGAGTGATACTTACATGCGCAAGGTCTATCCAGCGTTGAAGCCAGTTGAAGCGAAGCATCCTCCTAGCGTATTGACAATCTAACAAGCAGCAAGAGTCAATCTTCGGATCTGTCGAAGTAAGCATCTCCGATGTGATGATGACTATCTAGTGGAATATAACTGTACTATTTCATATAGAGAAATCGTATATAACTGGACTGGTTGGTGGAGAGTAACTCCCCTCCAGAACACCAGACTCTATAATTCATCACTTCTTCTTTTACCAAGTCCACCACAAGCTCTTCTCCTTTTACCGAGCCAGAGGACAGATGACGGATAGGAGGCGAGCTGGACGCGGCACTCCAAAATGTTATAAGCCCGACGCTTCCTACCAATAAGGGCGGCTCAAACGCAGAGCGTCACCTGGCTTAACAGGGCATGAACTCGACGCCGACAAGAGGACGCACGGGAGTCTACCATGTGCCACCGGCACCTCAAGGCCATACTGCCCTCTATGCCAGCTAACGTGCTGCGCACGTTAAGCAGGACGTCTTCCCCGCGATATTGAGCTAGAGGGATATCTCCCTAGACATCAAACCATACGCACTGCGTATTGATCCCGCTATAAGCGGCGCATTTGGGTTGTAAATGCGCCCACAATGATAAGCATTGCATATTGAGCACGCTATAAGCGGAGTACGTAGGCAGAAATGTCGCCCACGCATTGGCCCGCTACAAGCGGAGCATACGGGCTGTAATATCGCCCACTATAATACGCACTGCGTAACGAGCCTGCTATCAGCAGAGCATACAGGCTGTATTATCGGCCGATAATGTTGGGATTACCATACATGTATTACTATCTCACTTCTACTACAAACAATAATCCAGTATACTACCATTGAGTGTAAGGATAAAAGCAAGCCAGCGTGCCTCTGCATATTCAGGCAAAGTGCCATTTACTGTATGATCCAACAATAAAAAGTTTGATTCAAAAATTAAAGAAGATAATCCAAAGTAGGCAAGCCAACGATCCACTGCCATCGATGTTGCTGCATACTTGTATTGTTCACGAAGAAACATAGAGAAGAGAAATCAAAGTCCAGGCAAGCCACTGAGCCGCTGTACAATCCTAAAGAAAAAGAAATAAAGAGTGAAAATACACTACACACACGAAAACGTCAACTTGACAATGCAAACACAAATGATGCCTACTGCCCAATTCAACCAAGTGAAAAAAAACTGTATTGTACTCTGAGTTAAACGACCTCAAGAAACTAATCTGAAAAGATAAGCCAAATAAAAATGAGGAACTGGAAAATAACTTAGTCAAGGTGAAAATGAAAGGCAAGAACAAAAATGTACAAGCCTCTGCCGTAAAATGTCTCGACAGATAATGAATGGCATACTACCAAGACATTCAGCTTCCTGAACAGTAAAGATGTGCTAACAATGCCTCCATCTACGCTTACCGAGCAATAACACTTTTTTTGCATGAGCAATGTCTCTGTCAATTGACTAAATAGTGTGCATGCTTCTCACTACTTCGAAGTATAATAAAATCCGGAATACAACTCGAAGAGCCACTAGGGAGAACAATGCCCCAAAGATGTGAGCTATAATAGGTAAAATGTGAGTGAAGTGTGTTCCTACTGAGAATGTTGCTTTGGTGCAAGCATTGTTACCTAGCTGCTCAATACATATTTATGCTACATTCACAAAAGAAATATAATGAACAAAAACAAATATGCAATTTGCATGATGCAAATACAAAATTGCAAATTTCACAAAGACTGCCTAGCTTGTACGACAAAAAAAAACGCTGAGAGAGAAATACTTTAACAAATGTATGAAACTGGGATGCATCAATGCCAAAAACTATTCATGCATTCGCCAAACTTTAACATTTATTGTTACTGGCAAAAGTCTCAAACATTTCCATAACCGTACCAAAAGAATGAATAAAAAATAAAAATTATTCAGCACTTTATGACTACAAGACAAGCCTACAAAAGCCTCTGTCTACAGAAAGCAAACTATAATTTTACTGCATCAATTTCATCTACAATGAATTCGATAGTTTGCAAATCTATCGCTAGCGCTTGTGAAGTAAAATAAAAATTATTATAATTCCAAACGAGAAACAGCCTCTAGGGAGAAAAAGAACCCCAACGACTAGAGCCTGCGCACTTACTGCAAATATGATGCGTACGCTACCGATGACACTGTTTGATGTTATTTGTGCTTTTAAAAGATGTAGCCTTTCTTAAAAAATATTTGAAAACTATAAGCTCTCAATAACAATAGTCAGAACAATTTTTCAATGACTGCAACACCTATATCAACCATTTCATGAAATTGGGAGGCAATTCTATAAGACCTCAGCAGCGAAAAACAAAAACCAAACCACACATTGCGAAGAAATCCACATATGAAAGAGCAAAACAATGGCAGTTTAAAAAATGAACAAAGCTAAAACGCTTACACATTATATATAAAGATAAATACGATGAACAGGTGTATCCTCAAAGGTTTCCTGAGTAGCCAACAACAGAGCGAACAGGTTTTGATCCTTAAAATTAAAGGTTTCCCTGAGGTAGCTTCTACAAATTCGTAAAGCTAAAACGATAACAGCAACAATTCAGCGAACAGGTTTTGATCCTTAAAATTAAAGGTTTCCCTGAAGTAGCTGCTACAAATTCGTATAACTAAAACGATAACAACAACAATCACTACGGTGAGCAGGTTTAATCCTCTAAGGTTTTCCTGAGTAGCCAAAATAACAATGAAAACAAGAAATCAGAACAGTTATTGCCAAATCAAAACGGCACAAGGTGACAAATCTGACAATGAAAGTCAAAGGAAAAAGATCATCAATATCAAAACAAGATCTACTTCTTTTTAATGCTCATGTACGTACAGGACTGTTATAAACAGCCACCTGTGGTACATTAAATACTATCCGACAGTTAGAGCATCATACACATTTCTCGATGTGGGAAGCTGTCGAAGTAAGCATCTCCGATGTGATGATGAATATCTAGTGACATATAACTGTACTATTTCATATAGAGAAATCGTATATAACTGGACTGGTTGGAACTTCCCTCCAGAACACCAGACTCTATAATTCATCACTTCTTCTTTTACCAAGTCCACCACAAGCTCTTCTCCTTTTACCAAGTCCAACACAAGCGTCTTCTCCTTCTACACTCAAGACTTCCTCTCCACTATGGCCACCCTACTTTTGTCCTCACTCCGTACTGTACTGCCCTAACAGCTCTGCGCAAATCATCCCTCCAACAGGATCCATGCCATTAATAAGAATGATACAGTAATGCTAGTTTAGAGAGCGGGAGCTTTTTTTCACCGCCCCTGTCTGTGTAAACTTAAAGCTGGGATACCCGTACGGACGGGGCGGGCCGGTGGAAGCTTTCCGGTAGAACCCGTTCACTTATCACTTCTCAATTTTCTTGTGGCCAACAACTCACCTACGAACCTGATTATGAAGTCCCTCCACGCTGTCAATTTGGTCTTACTCCTGCTGCTTGCATGTTTTGCTCCTGCCCCCGCTACAAGGGAATTGAATCTGAGGGCCGCCGCTAGCGATTCAACTCGCGTTGTCGACAACGCCACTACTGAGCGGCTTCTAAGAGCCCACAGTAGTGGCAAGGAAGAACAAAAAGAGGAAGAGGAAAGGGCAATCTCGATAAATTTTCCAAGTCTGGAGAAGATCTTAAAAAACGTTACGTCAGGCAAATCTACGGAGCTGCAAGGAATGCTTAAGGCTGACGAGGCCCTTGGGAGTGCTTTCAAGACGCTAAAACTTAGTACAATGCGGATTGGCAAGGATGATACCAAGATGGTGGCAAAATTTCTGTCAAGCCGCAATTTCAAGATTTGGTTCCAGCACGCCGTCAAGATCAACAAAGATGATCCCTATGGCGAGATGCTTAAAGCACTCACAAATGTCTTTGGTGAGAAAAATGTGGCGATGATGATCCTAGTCGGGAACCTGTCCAGAAACTCGCGCGACGTCGCAAAGAAGTAAGAAAAGGCCCAGTTCTACAAGTGGTACTTCGTCGATAAGTACAAGACAGCAGATGAGGTTTTCACGAACGTGCTGAAAGCTGATCGAAATAGAATTCATGGGTATGGTCGGGAGAAAGAAATTTGGGGAGATTACGCGAAGTACGTCACGACCACAGTGATGAAATATTGATAAACTAAGTTTCCAGCACTTCTCTTGTTTTGGGCTAACATATATACTATACGTAATCCACACTATATAGTGGATTACGTATGAATCAATGTATGGGTTTTAAATCCAGTTTAGAATCCTTTTATACTCATACTATGTATTAATACTAGTTTAAAGTAGAAGTAAAATGTTTCTAGTTCATATTGATATGATGTTAGTAGATATTGAGTATTTCAATGCTTTTGACCAGCCTGACGCTCAATTTATAATAAATAGAGGTCGTTTTATTAGATCGAGGTCGATAAAAATACAAATCGATCAATCTTTAACCCCTTGGTTCCCGGCGAAATGTACCGAAAACGAGATAAATAATGTCGTAACTTACGACATGTCCATGTCACTACTTGTTTGTATGTGGCATATGTTCGACGTGCGTATAGGTGTATAAGTTTTTGACAGGTTGCTCTCTCAGAAGTCGTAAGTTATACTACTTAGTAGCTAGCAGAAAACACTGTATGTCGTAAGTTGCCCCACATTAGGAAGTACAGCGGGAAGATCTGCTGCACATCGGGGAAAAAGTGGTGCACAGAGCTGTTTTATGAATCCAGATCAGTAGAATAGACAGAACAAAAAGTACAGATGCTCAAGGAAATGTGTATTACGACATGACAAGCGCCCACACTCTTTTGATGTCACGCTGGCAAGCCTACTCTTGTTCACTCTCATGTTCATCACCGCCGTCTTCCTCATCTTCACGAAGTCGCAACTCTCGACGGGTCTTCTTGGGCACACCTACCTTCTTCCCCGGGCGTCGCAAGACAACTCTCTTTCCCAGTGTTGCAGGAATGTTTTGGCCGCAGTCGAAGTCGTCGTGCCAGATCTCAAAGCACGTTTTAGCTACACCCTTGTACTGACGTCGTATCTTGCTGCACAGCCAGCACTTTGCATCGTCAATAGAACAGCATTCGCAAAAGTAAGTTGTAGCAAACGACTTCTTCTTTCGATCCGTGCGCAACAGGGCACACACCTTGCAAGATCGCTGGCGACGCTTCTGAATGCCCGTAACGGTCACCCAGTCGTCGGCCTGCTCGACAGCATGTGTCAGACGTAGTTGGGGGCGCTTCCGCTTCTGGCTGCTAGGTGTCGGTGTTGCAACAATACCAGCGAAGTCCTCCACTTTCAGCTGCAGTAATTGGTTTTGCAGGACTCCGAACCATTCTCCTCGCTTCATCACCGGCGTTCCAGCGATACGTGCAGCCTCTTTGTGCGAAATAAACGCATTTACAAGTGCCAAATCCACGAAGCCYAAAAAAAGGCTTTTGTAGTATTTCTTGAACTTCGTAGACTTCTGCAAAGAATACTTTTGTAGGCGGAGTTGATCATGCACATCAACTCCGCCCATCCAACGTTGATAGTCGGTTACAGCCGCCGGGCAGGGCACGGTAATTGTACCAACGCGCTTGACGTTTCGTGTAATAGTCGTCTCTGTCATTGCCGAGCCTGTGCACAGGTAATGCACAGGTTTCCGGTCCCACCAGTGAAATGCAACCATATTTGGAATGGCGACAGAACGAGAAAACGTGAACATGCCTCGGGGAACGCTTGCTGGACGTAACTTGCGTTTCTCTTTGACATTTGCATCATATCCAAGCCGGTTGGTCATTATCGTGCCTATCACGTACACTTGCTTGCCCAGTAGCTCGATAGCAAGTAGAATTGACGAATAGAAGCGGTCGATAACTACTGCGTGCCATGGAAGCCGGGTGTTGGGACCAAGCACTGTCTTCAAATTTCTAACGACTGCAGCCGCACCGGTCTTATGGTCAAATGTTGAATGCTCTCCATCTCTCCTTTTGCCCGCATACAGCTCAAATCTAGTAACATACGACATAATTGGTGAGAAACCAATCTAATGTCATAACTTACGACTTAAGTAAAAAAAAAAAACCTGTGGCAGTACGCGGTCCTTGAATCACACACCATAAACATTTTCGAGCCATACCGATGAGACTTATCGGGCATGAACATTCTAGTCGTGTTTCTTTTAGAAGTAGAAGGCAGAACACCCTCGTCAAAAGCGAACACTGCCGGGAGGGTCCACGCAATCAAGAAACGATTCTGCATTGTCTCACCATTCGCAACTTCCAGAGCCTGTCTCGCGTACGCTACGATGCCCTCAACAGTCAAGCCAAGCAGCAACGTGAGGACGCGTTACGTTCAATGGAGTGGTCTCCAGTCACTCACGAGTTTGAGGAGGGTGTCGAAGCGTACTCTGGTCTGAATATGGAAGAGGCACGACCGGTCGCTGAGCTACTAAACGTTTGCCACTCTTCACTACTTACATTCTTTTACTTTATGCCCAAGTCGCTATGGGTCAAGATAGCTGCAGAGACGAATCGGTATGGTCTTCAGCAAGTCACCAGACGTGCAGAAAGAATTCATGCAAAGCAACACGATCGGAGAAAGGAGACGGTTAAGCAGATTAGTCGGCGTTTGAAGGCAAAGCCGGGATACGAGACGCACGAGATCTTACACGTGATAGGTCTCCTGATTGCGCGCATGCTCTGCCCGCAAAAGCGACGTTTTGCCGCTCACTGGTCGATGGTGGAGGATGGGGCCGTTCCTGCAGGTAATTTTGGAAGGTTCATGGGCCGGAATCGATGTCAAGACATGTTGCGCGACTTGCACTTTGTGGACAACGAGGCGCTGCCTACTTGAAGTGAGTCAATGAACGCTGCGTCCATTGGCACAGCGTCATCTTCTGACAGCTCATCCAATTCTTCAGGTACTTCACAATCATTGCCATCATCGTCATCATTGTCTTCTTCAATGTCGTCATCACTGTCTCCAGAGCTAAAGTTCTCGTAGTCACTCAGGTCTTCGTCGTCAGTCACAAAATTTACATCCTTCTTGACAGGATGACGGGGGCGTAGGACAGGACAGGTCGGCGTAGCTGGCACACGAACAGGTTGGGAAGCGTGCTTGCGTACGGGTTGGGCGTCAGACTCACGTGTAGGTTGGGCGACGGGCACAGTGACAGGTAGGGCGGTTGGCACAGTTATAGGCTGTGCGGGCACTGGACTGTCAGGCGGGGAAGCAGCGCAACATCCGACTGAGCGTCACTCTCGGGTCCAGATGACAGAAACTGCAGGTTTGTCGCTGTGTCTCGAAGCTCTCCGGGCGACAAATTAAAAGCCCGAGTAACTGCTGCCTGTGAAAGCTCGACTTCAGAGCTATTGCAGCTGCTTGGGGTGCCAAACAGATCATTCATGGTCTGCTGTGACAGCTCAGCACTTGTGTCGATCTGCGACGCACGTACGTCCTCTAAACAATTATCGGCGGCGTCTTTACTGGAAGTGACGGCGTCTTCAGTAGCAGAAGCAGCGGCGCTCTCGTCATCGTCGGCTACTACGTCAACAGTGACCTCATCGTCTGCAGCGTTGGCTTCCTCTGCGCTCTCCGGTTCAGCCAATATTCCTGTCTCTAATGCATACGCCACCACAGCCTCCTCTCCAACTAACACCTTTGAACCATCTGCACTTACGTACTTCCCTTTAGTTTGTATCCCGGTTGGTCGCTTGTACTTCCATCCCACAGCTCGAAGTTGTCGCCAAAAGTGGCCAAAGTCAATGTTGGGCGCCACCGCAGCAACGGCGGCTGTTTTGGTTCGTTTTCCAGGCCGGATGCGAGCCATCGAGCAAATTCTGTAATGAGCTGTGGGCCACCGAGTGGCAACTGGATACTTTCCCGCGTATCCCATCTTTTCCGCGCGTCCCTGATCCCTGGCTCGTTTTTGGATTTTTCTCATTTACCGGTTGAACTTTATCGATTTCAAATGACTTGATTAAGTGAAGGATTTCCATTTTTGAGCCATCTTCTAATGTTATTCCAATTATGTCGTAAGTTACGACATAAACAAAGAGTCATGTCGTAAGTTACGACGTCGGGAACCAAGGGGTTAATATTATATTTTGTAAGGATCCTTCTGTATTCGGGCATGTCCACTGCGCTCAGAAAAAATACAAAACACCATTTCGAAGTTTTCACAAGTACGAGTACCTCGAAGTACCCCTTGAAAAATGAAGGAAATTCTACCAATCAAAAGCGGATGCAGGTTGATTACCTTGGGATGTGAAAAAGTGTGCTAAATCCTGATGCACTTACAAAATGTCAGTATAATGCCGCCCCCCTTTTCTTTTCGGTCAAAGGGGCACAATTTTGAAAATACCCCCTGCGGACATGCCTGAGAATAGTAATCTTTGAGTGCCCTACAAACACCCCTACACTCCCCAGACCGACACCTGTGAATACTTTAGGGAAGACCAGGATTGTACACAGCAGAATTCAATTAGCTCTTCCTCCTTCTCAAAAACAATGTCCCATTACGATGATTCAACGCGTATCGGAACCTGATGGTAGCTGTCTAGGAGATGATTTATAATTTCCCGCCTTGTGATCGACGATTACGATACAACTAAAAGAGCGGGAGTATTTTTAACCGTTATTCTCTTCAAAGCTGGGATACTGGTACATCCGGGATGGGCCGGTGGAATCTTTCCGACAGAACCCGTTACAGTCATCATTTCTCAATATTCTTTTGGCCAAGAACTGACCTAACGAGCCTCATTATGAAGTATCCCTACGCTGTCTGTTTGGTCTTTCTCCTACTGTTTGCATATTTTGCTCCGGTCCCAGCTACAAGGGAATTGAATCTGAGTGCCCACACGCGCAGTTCAACTCGCGTTGTCGACAGCGTCACTACCAAGAGGCTTCTAAAGGGCCCACAGTAGTGGCAAGGAAGAATGCACTCAGAACAAGAAGAACAAAGGGGTATTTCGATAAATGTTCCAAGCTTGGAGAAAATCTCAAAATATTTACGTCATCCAAAACTACGGAGCTGAAGGAAAGCTTATGGCCGACGAGGCACTTGATAGTGCTTTCGACAACGCTAAAACTCAGTAATATGCGGATTAGTAGTCATGACTTTGTCGAGACCACAAGATGGTGGCCAAGTTGTTGTCAAGCCGAAACTTCAAGGTTTGGTCCCAGCACGCCGTCAAGATCAACAAGGAGGACCCCTATGGCGCGATGCTAACAACACTCACAAATGTCTTTGGTGAGAAAAATGTGGCAATAATGATCCTAGTCGGGAAACTGTCCAGAAATTCAGCGCGACGGGTCGCAAAGAAGTTGGAAAAGGCGCAGTTCTACAAGTGGTATGTCGTCGATAAGTACAAGACAGCAGATGAGGTTTTCACGAACGTGCTCAACGCTGATCGTAACACAATTCATGGGTATGCTCGGGAGAAAGCAATTTGGGGAGAGTATTTCAATACATCATGGATACAGTGATGAAGTATTGATAAAAACAAGTTTCCTGCACTTGTTTGGGTACCTGTCTGTATAGAAAGAGCCCTTACGTATGAATTGATGTATG

>Contig_70

CCGTTCCGCAACGAAACGGGCTCCAAGACCACCATGAAAGGAGACAAGGAGATCATGGAGGCAAGTTTCGCGAAATCAAAGCGGATCCTAGCCGCTAAAGCGATTACGGCGCTTAAATCCAAGCTGGACTAACTAGAAACAGCCTCSGAAAAGAGCGGAGGGAGTATGCTAGAAACGATTTTGCTACTTCGCGAAGAGACCGAGCGGAAGGCGGAAATCCGCCGTGCGGAAGAGGATCAACGACGTCGTGACGATGCTGCAACCCAGGAGGCACGTCGCCTCGCGGACAAGACGGAAGCTGAAGAGCACCGTCGTCAAGACAAGATCGAGATGGACGAACGAGCTCGCCGTGACAAGGAAGAAGCCAGAGCTCGCACGCAGGAGCTCATCTTGCTCACCACGAGCATCAACAAGAAGCCCTAAGGGCCATCGGAAACAGAAGAAGGCGCCAAAAGACCGGCACCTTCGAAGTGACGTCGTTGTTACCTGTAGGATCTCTACAACGCATTAAAACATAATTTTGGATGTCAAATTACTAGCATGGTCACGTATCGAAACAAAATAAAATCCAAAAACCTGAGAAGGTTTACTAAGAAGCAGATTTCAGTCTAGATACCAATCATGCTCAAAGTAAAGTAAACAAATCGGCCTAACAAAGCTACCCCTTCATCCATAACACCCGAGAAAGTCGTACGTATTTGCGAGATCCCAACAGTCCGTACCCGGTAGTTGGTAAGGCGAAACAAATTGTCCAGCCTCATACCGCGAAGCTGTGGATCGTATGGGAGCGGCAAACTAAGCCGGGGGTATACCTTCTGCATACTCCCCATGCCCCATTCGGCTGCTTGACGCACCGAAGTTATAGCATTATGAAGGGTGCGTGCTTGACTGCGCAGGGCAGGGTGAATCCGCTCTAAGTCGCCGTCCTTCAGCGGCGTTAAGATGCGTCCGACCATCGCTGTAGAGCACGGAAAGGCAGAATCGAACACGACATTATTTCTCGCATCTGAGCACAACGCCGGGTCCAGGAGCTTCGACCAAAATTCCATGGAGGTGTCCGAGTCGTTCCAGGACCCGGGACAGTTGTTCTTGCACCAGATTACACAGCCATCAGCCGCGAAGCAAATGACCCCCGTAACCAATACTGTATGCAGCCACCCGCTGTACATCGCGTTCTGTAGATCAGCGTTGGATGGCTGCTGCATCTACTTTGATAGCATTGGTAAAATATTCGAATTAATACACTTTCTAAATGCAAACATGTACGCACTCGTAGGTTTTTACCGTCGATAAACCCGAACGTGTGATGAAGAAGGGTTTAACGGGCTTCTACCAGCCTCGACAGCTTGACTTGATGGCCTGGTGACGGCCAGGATATGCGCGCAGGAGCGAAATCATGTAGCGCCTTGCTCAACGACTCCTCCACTTTCCGTAGTGTCCTCGATAGCGTGCTGGGAGGTGCTCCAAAGAGCATACAGAGCGTCGTTCGCTCCATCGAGCTAGTGTAGAGCACTAATACTAAGCCCAACACCTGTTGGAGGTTACGGAACTTTGGCGGGCGTCCACGTACTCTTGGCCCCGGTAAACTGTAGAATTGTGAAAAACGCTGTAAGAGCTGAGAGAAAGCAGACCTGTTGGTACATGGCCTGTCAGCATCCAAGTCAAGAACAGCATCCAGACGAGCAATAATAGGCGACCTCGTTAAACTGGTGGCGTTAAGAAAGTTTAGATCACTCCCGTTCTCGTATAGAGCCATCCAGGACGCGTTGCTCGGGGAATCCAAACAATACCCGGTCAAATAGTGGCGGCTGCGCATGGCGGCTCGCCAGGCCTCCTCGATTAGGAGGCCGAAGACCTCTCGGCGCACTTCGTGCAGCTCCTGCCTCTCCCGGTCGATGGCCTCGGCCACAACAAGCATTGCTTCCGTGTCTTCGCTCTCCATGCTTTCCCGGACGTGCGTAAATTGCGAAGTGACGTACAAGAAGAGCTTCCCCTGCCTGTGTCCCATTCGAAGTGTCGGGTACGCTTCCATTTAATGAGTTCGGTCTGTGTAAGATCTAGGTAGAGACATATAAACAGGACAGAGGTCTTTGAGATATGCCTCATTTATGGTATTTGTGGTGGACGTTTTGATACTTAAGCTCAGCATACATGTAAAGTATGACAGGCTTATCTGCTGCAAATATGACACGTGCTTATGCTATTTATGGAAGGCACCCACTACTGGCACAATACCTGATACGAAACTTTAAGGACAAGAAGAACTAGTAATTAATTTGATGTCAGTAAACAGCAACCAGCAGCGTTTATATAATTCTTCCTGCATTGAGAGCACATGCGATTTGTATGTGTTCTCGATGCAGTCGAAGTCGGCACTGAAATATTATGTACCAAAACTGTTACTAGTCTAGTTAAGATTTAGTCGGTCACAGGTCCATCAACAAGTTGGTTGCCGAAAATACAAAACGACAGTAATCCATCTAAACGCTATCATGGTATCTGAAAATAATCAATATTATACGTGTTTTGGAGAATGCGATGCTCCATTGCATTTTCACAAGTATTCCTGACATAGAGTCGAAGTTAGTGGAGGTCTACACGTGTATACATGTAATTGAGGCTGCACGTAGGTGAAGAAGCGTTGAATGACCTGGTGTTTCTCAGTTTAGCAAATGATTACTTATCCTGGAGTCCAGGAGAAACTTTCTTACTTAAATCCATATAGACGGTTTCAGTATGATAGGCCTGTGTGGCTCCACATTAGACCTGAAAACGCACAGCAAAGTACATCTGCATGTTATGCTTCTGTAAGCCGGCATGCTTCCTCACCTGGCGCATTATACAGACTTTCTAACTCAACCAACGACTAAAGACGACCTGGGTAAGACGATGTGCTTATGCCTGATAGAGAAGTCGTCCACGGGTTAAATCAGGTGTGAAAGGTCTTACTCAATTATGCATTTCGGTGTCTCGCAAGCCGTTCTCAGCAGTGTTTTTGCATATTATGTCCTGTTATTGGAGGAACCAATGTATTTCTGAAAAACGGTTTGTACTTTCCACCGTCGAGACTACGTGGGGACTCTTACGCCGACTGCTTTCAAGTAGTCAGTGTACATTTCCAATGTCTTAGCCGTCAGGGCATCGTCGACATCTGGCATTTCTTTCAGTATCTTGGCCAGTTCGTCGGGACTCTTGTTCGCATCGTCAGCCTGCTTGAACAGATTATTCAGCTTGTCATCTATAATAGTCGAAATCCTCTTGAGCTCCCCAAAGTCGTCAATTTTCTCGAGATCCGAAAACGAGCTCTTCTTCATCAGCTTGTTAACAAAGTTGTTAGTCATCGCTTGCTTAACAAGCTCCAGAAAGTTTATCTCTTCGTTGTCATCTTCGTCGATGCTGCCGCCTCTGAGAAACCGGGACTGGTCGCTTACACTTTGGTCAGCGCCAACGAAGTGAATGAAGCCCAGTGACAGTACACCGGTTAGCGCAACATCGTTAGCGAAGTAGGGGGTAGAGGTGACGTGGCGGGCGAATAGGGTCGACGCAGCGGCCAGCAAGACGTAAGTTAGGCGCATTGCTGGTGTCTGGATGCTTTAGGTGTCGAAAGCGAGTCAAATTGCGAAGTGAGAATTGGTATCCAAACCACACAAGAAACGCGTGGCCAGAATTCCTATAACAAAGTGGTTTGGGATGTGTATTATCTGTCGATGGTGAGAAATAGAGCAATTCGGAAAGAGGTATGTTGTTTATAAAGAATGTTGTATGGTGGTCGTATTGTACAGAACGCGAACAATTATACATCCGGGTAATTAGGATTCCCTCTCTTCAGTCAAAAGGGACCGTTTTGAAAATACCGTCTGCGGGCATGCCTGCAAATTAAGCTAGAATTACGAAATCCAAATGGTTATACTTAATTCTTGTAAGGGCATCAAGATTAAAGGTAATCTCTTATAACAACATATTAATACATCCGCTTTGATAGGGCATAATTTCCTTCATTTAAGAAGGAGTACCTTTGAGCGCTTCAAAAGGTATTTGTCTCTTTTCTGAACGCAGTTCCCTGCGGGCATGCCTGCTGATCCAAAACGTTTTCTGCGACCCGTAGTAATCACAAATAGTTCAGGAAAGATTACCAGGATATTAGAAATATGTAGTTTTAACCGTACCGGGCGACGTCCAACCACCTCATTTCTCATTCCACAATTTATCCACCAGTCCACAAGAAAAATTCCGGCCATGCGTCTTTTCAACTTCACTGTTGCGGCTCTCGCGGCTGTCCTCATTGCAAGTGGCGCAGCAGTGTCAAAGACTGATCAGACCTCACGTGTTCTCGCCCGGAATGACAAGCGGTTTCTCCGAATCTACCAAGCGATGGATGGCAAAAACAAAATTACGTAATACGACAATGAAGAGAGAAATATTAACTTATTCAGCGCATTGAAACTGTCTGATATGCAGCACGATGGAATTTTTCGATTCCTCATGTTTGGAAAATGGAAAAGCCAAGTATACTCCGCGGATGACATCGCAGAGCACATTCCGGCGAGCCTCCTCGAGAAATACCAAGCGTACAGGAGTATCCATGGCTAAGAGGCGGTTTCAAGCTACCAGAGAATAGAGCTCGGCCACAACGACACTCGAATGAGGTCGCGATACTGGCACCCGCCTTGATGCTATCGAAAATATGTCTCGGGCGCGGTATTGCAGAGGAAAGACTTTACTCAAATAAAACTCGTGTATGGTTCGTTAGACTTTCTCAAACGACCACTGCATTTCTTGTCCGACTCCAACGGAGGCCCACCCACTCTTAAAATCGGTAATTTGTACGTAGTAATACGTTAGCCTGGACGCTCGGACGCTCATCTGGAGAGTGTGGCCGTTTGACAGAGCTCCAATTAGTAGAGTGTTTTTGCGCTAGGTTTGAGAGAAACTAGTAGACCAGAGGTTCCAAGAAAATAATACTACTAGAAATATTTTATAATATTTCCGTGAAACAACATATTACGAATCGGTGAAACTTAAATTTTATAGGCAATATTTCCAAATTTCGTAGAAATATTGGGAAGACTGTACAGTTCACGACTCACATTAATAGTACCAGCTGCCCTCATACGTACCAGATACCTTCTACTGACATTCATTCCTCCTACGTCAAACTGCGTGGAGAGAATATTCTCTCAGGCTTTCTCATCATCCCACTCGGATTAAATTAAGATGCTGCTCTTTTTTAAGACAACAGGCGCTTTTGGTCGGCGAAAACGGTAAGTAAAGTTGTGATTCGTCGATCCCCGTAGTGCTTTTTGCCGAATATAATATTGATATTTTATTGTGGAAGTTTTTTTTTATAATTGACTGCTCGACGACCTTGAATCCCAAACAATTATCTGTTTGCGTTGATCTCTTAATAACATGATAGTTGAACAAACTGGACCCCTGCTTATGTAGCCCTAACGCAGGCGTACACATCCAAGGCTCTATTTAAGAGCTTGAAAGTATCAAGGGAGACTGGGTGATGGATTCATCTTCTGGGCTCCCGATTATATCATGGCGCTATTAATAATTTCTTACGAAATCATTCATGTATTTATTTTGACTTCATTTATGAAATTAATAGCTTTTTTGCTAATAGCGATCTCTTATAATTTAAGAAAATCATTATGTACGAGCCACATTGCATCTTAACTTACATACATCGAGAGTTCCCAAACCAACTACGCGCACAAGCCGGTGAAACGATCACTTTTCATTTTGGACAAACTACAAACGATAAGCACGAAAGCAGTTCAGACTGTCGATGAAAATGGTGTTACTTACCTCTGATTGTGCTCTTATTTATCACTTGATCACTGTAGATCCCATAATTTACGGTTTGTCGCAAGCTGCCTCATGCGAGGGACAAACGTGCGAGGATTTCTGATATAACAATGACATAATCAGGCAGACTCTCTTCCGGTTAGCTCTCAGTCTTACATTTCCCACCTTCTACTCAAGTGGAAGCCAAGTCAATAAAGAAACTTATCATCACTTTAAGTGCTCTTGTCTACCGTTCCGGCCACTGAACGAGACCGTACTCTCGAGCGATGAAGATCGACGTTGGAAACACCACCATAATTTTCCATGTACTACTTTGCGCTGTGGGGGGACGATGCAATGCCATAATCGGGCCCGCTGCAGGTTATAACGTCGAGGTGGCATTGACCAGGATCCAGGGGCAGCATCATAAGCAAAGTCAACGTGTAAGCGTCTCGCTATTGCAGTGACAACGGAGATCAGGATAATTTTGTAACAAGTCTCATGACGCCTGATTGCCGGCGGGTCGTAGCGCTTGACAGTGCTGCGGTGCTCTGTCACGGGCAGAGTTCGACTAGATACAATACACAACACCTGTAGGCCACCGCTAAGTAGCCGGAACGGACTCGTTCCTATTTATTGACTCGTTCTTACATACTTATTTTCGTCCAAATCATCCTTCGTCTCATAGTCCTTCTTCTAGTACTCGTCCTTTCGGTTGTTGTCATCGAGCTCGTACCCGTCCAACTTGCTCTTCGTACCGTACATCGTACTCGTCCAGCTTGCGCTAACCACCAAACTCGTGTTGACTCAACTCATAACTCGTCACTTTCACTACAAAGACTTCTCGTTCTTGTTCGCTCCGCTATAGGGCAGCGACGTCTCATCACGGGTACATGTCGATTCACCACGCGTCCGTCATTCCCCAACAGCTCATCTCATTTGGTGCGTCTTACTGCTCAGTCCCTCTCAAAGGCTGTGCCTCACTGCACAGACTTCTCAACATCATATGCTTCGTCACTCACGACAAAGACTTCTCGGCCGTCATAACTAAGTCACGCAGCTCCCCATAATTCAGCT

>Contig_72

ACGGATTTCCACCGTTCCATAGAGCGTGACAGCTTGTTGGCGACCTCAGAGAGCGGCATGCTCCGATCCACAGCCCCCCGTATCCACTGAAAATGATGGGGCGTCTCCTCGGATACGAGGCTATAGTCCTAAGGAACTTCCGCGCTGCCGGACAGAGAGGCGCCATAATGGGCCTTGAAGCCTTCGTGCTTGAGTTTCAGGTGGGCCATGAGCCAGAGTTGGCGAGCTGTTTGCGCACACTACCGCACTGCTTGCAGCGGAAGTGACCCTCGCCGAGGGGCTCGTAGAAGTAAGCGCAGATGTCTTTGTCGGAGGGCATCGTTCTTGAGGGAGGGTGCTTTTGGAAGTCGGAATAATAGAGTGAGCAGTCCACTTGCAAGTTGCTTATTTGTGGACTCTGAGGTAATGATTGGCTATCAGATTTTTAACTAAGAGCTAGTATAGGCAATACAGACATCAAGACATGGCGCTGAGGCGGCCGCTGACGTCCTAATTGCTTCGTACGGCACCTGAAGTTTCTATGAGCCTACTTTTGTAAATTATACTGGAAAAATTGGAACGATATCATATTCAAGATTATAAAAAGGTTGCAACAATCCGTTTTTGTTTTAAGAAACTGATTTTAGTGATACAGAAAAAATTTCAGACCCTGGTTTTTGTATTCTAATGTATCCTAGGTAGCCCAGTCGGCCATCCTCTAGGTTCATCTTGTACGGTACAGTATAAGGTTTAGCTCAGATGAAACTCGTGAGATTACCCAGACATCACATTTGCCCTTCAGAACCATGAAGCCGTCGCTGGTTTTGAAACCTGCACGCAGCTTGTAGCGCAGCCTCGTGGGGCCTTGATAAATCTCGTCGTGCATCTACTGGGTCCGTGTCAGTCAGTGTCCCACATACTGGAGTCTCTACAAAGTGCTTGACGTTTCCTGTGATCGTTCTTATTCTCGATATTATGAGCTGGGTCTCGCGTCCACGGGGTCGACACGAACATCTTATCCTTGGCTAACCGCCAGGTTACACCGCTGTACGCACCACGATTCACCAACGTTTTTGCTACCAAAAAATGTTTCTCTTTGAAAGGGCCACACCTTAAGTGGGGGTTTCTCGTTGGCAAGCCTCCATCCAATCGCAGGGCAGCTAGAGGTTAGGGACGCCTGGGTCGACAGGCTGTCCCATGGAGTTAGGAAACCACCATCAGGCCGACAGACTGTGATTGATGAAGCAATGCATTAGGTGATTTTACCCAGAACAACTCAATAGACTTTGGGTTTGTTAGGAAGCGCCAGCTTCCCTCGTAGTGTGTTTTATCACTAGACCCACTACAAAGAATTCTTTAGTTGCGAAGGTCTCGCGACTGAGCTAAGATGAACATGACCAGTGATATTAGAAGCCAAGGTGACGATGCAAGGGAGAATCGCAGCACGGAGAGAGCGCATACCGTTAAAGCTATTATTTAAACGGAAGCGACGACCACGTCATGCACGACCAGAGTTCCGCAGCCAATTTTCGCCACCGTTTCTGCTTCAAAGGTAACGTCTACGTCGAGAGATGCCCGGATAGAGTGGCTNAAAGCTCCGTAAGGAGTACGAAGAGGCTATGAAAGAGCGATGCAAGGATGGGAAGGAGGACTTTGATGCAGTGCTGCGAAGTGTTTAAAAGCTCGTTCGATGTCGATTTGCTGACTACGCTGTGCGAGGCGAATTGGGGTGTTTCCAAGAGCAGTCTTACCGATGAAATTCTGCTAGAGCAGATTCACGCAATCACGGACAGCTATCAGAACCAAGTTTTGCCCCCTGTCAATGAACTTTTCGCCGTTGACTGAAAATGAACATGACTAACTCCGACATCCAGTCTCGCGTGATTGATTATTTCTCTCGTGCAACTCGTTGATCAACAAGTATGGTTTTACTAGTTTTTTCGATGGTGACAAGGGAGCTAAGAAGAAATATAAGCTTTTGGTTAATTCGTTGCCGGAAGATCTGAAAGTGAAAGTGAAGAACGAGATCTATAATCGTTGCCCAGAGGCAAGCACCAGTGTATTACGACTATCGAAACTTATCAACCAGCAGGCTCTCGAGCAAGTCATCGGGGACCGTGCCTTGAAGAGGATCGAAGGTGCAAAGCGCAAGCCGGTGCCGAGTGAGCAGCACCGAGACTTTCAGAGCAAGAAGCGTTCATTTAAGGGCCAGCAACACCAATCCGAAAAGCAGCCGAAGAAATTCACCTCGCGGGAGGTTTAAACTGTTGTTGGAAGAAATGGTCAAACATCCAAGAAAGGAGTCCCACAGAAAGGATGCAGTAGTGCGCACTATTTGAGTACATGCCCCACCGCTACCCAAGACGACCGAGATCACATTGCGGCTCAACGGGACAAGAAAGGTGGGATGCTTCCCGCGGGGAGGCACCGCTCGCCTCCGACGATTAGCTGAATGCCTGCCAACACAGACCCGCTCGGTCGTGTTGGAAGACGCCTATACGGTGCCTTTCTGTGTGGACAGTGGTGTGGACAGGTCCGGAATGAGCATGAACGTACCGGTATATGAAGATTTTGTGCGAGTTTGTCCTGAAGCTCAAATGGTGAAACTTGAAGTGCCGTTCACTTGCAAAGGAGCTGCTGGAGACCTTATTGAAGTTAAAATGACTGTGAACTCGTAGTTTAAACTACGGACAGTGGTTGGTAGCGTTCGAATCACCGAGCCAGTTGAGTGCCTTATCATTCCTGGTGACGCGACCAAGGTCTTGCTGGGTAACGACGTGCTGACCATGCTTGGCATTGACGTGAACGAGCAGCTCGATTTGCTAGTGGCCAATGCTGTCCAAGGTAGCAAAGACGATGAATTTTATGATATGAATGAACCCGAGATCGGTACGAATGTGAAGCTGAGTGATGAGATTCGTGTCGCCTTGGAAAAATTAATGGAAAAGAAAAAAAGGGTTTTCCAAAGGAACTGTTGCCTCAGTTACGGCGTATTGCTACCCGTTTCGATATCTGGAAACTGAGGCACGAGTGCCGCCGATGACAATTCGTCTCAAAAACTGGTGCCAAGCCATATCGCTACAAGGCAAGGCGACATAATATAACAAGAAACTGATTTTGTGTTTGTGTCAGCTATTTTTCTCCGTTGTGCCAGCAACGTCGAGTAAGTTTCTGTCCCAAATGTACCATAAATCGGACAAATACGCGCCACAAAAAAAAAATGCGATTTCGGGAAGCACAATAAGATATCAAAACAGTATATGCTAGATATAATACTGCTTGCTCCGCATGGCTTCATTCAATATCTACACCCCCTCTTCCCAGTGCGGCATCGTCTCACACAGATGCATGGCAAACCGCAAGAGTACACTGCTACTGCTTCGAAAACGCACGCCTAACTTCTCTTTGAGCAGGGAATGGCTGTGTTTGAAATAAATGCGAAAGAAGCATCTGCGGCACTTTTCCACGAAGTGGCTATCTAAGCCACATTTTAAGCTCAGTGTAGCAAGCAAACTTAAGAAAAATTAATAGCTAACAGCTAATGTGGATTAATGAGATTCAGATTAACTCGCGAGAGTTAATTTGAGGTGAGGGCAATCAGTTCAACTCGCGCGAGTTAATATAAGTCGAGGACATTAACGCTAATGTGAGTATCAGTGAAATCAACAAAATGGTCGCATCATTGCCTTTTAATTCTTTTTTGCGTTACGAACTTAAGATCGACACGAGCTCACATCTAGCTTGTTCTTCATGAACTCGACAGAATTTGCTCTGCTTATGATCCGTAGATAAGAGGTGGCCTCTTCTTCTTGTTTTGTTGTGACACAAAGTTGAGATACGTAGCGTACTTCATGTACTCGTCCGTTCGCATGAGCTGCTGGAGAACGGTACTCGAGGTTCGAGCACCTACAACATCGTCAGCAATGCCCAAGTACTTAGCAAAATCGTCCACAGAATATCCCGCTTTAAAAGACTGCCTCATCTCGGTAATAGATAGAGACCTCTCCTAATCAGGTTCCTGATCCTCCACAGGGGTATGGTGAGACCGCAATCGACGTTAGTGCTGCTCATTTGGTGAAGGGAGCATCGCTTGGTTTGAGTTCACGAGCGCGCTGCTAGAAACGAAGAGAGTTGCCACCGTGGCTGCGAACACGTAAGGAAGACGCATGTGTATATCGCAGCCTGAGAGGTGGAGAGCAAATTTCAAAGTGAGTGGCTTTGAATACTACTCCACGGGCGGGGGGGGGCTCTTGTGGAGGAGGCTATTAATATCGTAGTAGCACTTACAATCCATATGTTTTACCTTTAGTGTACAGTACAATGCACTGTATATACGGTTACAACATAGCGACCATGGGAGATATTACCGTAAATGAGACCTGCTGTGAATTCACACTCTGATGCTACCCAAAATCGTAGCCTGTTTTCTACTCATACGACGTACGCATTGATCTTTTAATGCGATCAGTGATAAGGTTCTCAGTTTTTTTTATTTTCAGAATTTTTCGAAGCTAATCCTGCTATCTCTTAACTACTGTAAATGTTTATTCTTCGAGCCTTACACATTAAGCTAAGATATAGTTTCCTGTGAAACTGGCGTGCATAAGTGATGATGTAAATTTCCCTTTTGTACAAAATACACCCGGAACTTTGTTCAGTCAAGTAGATATCAACGCCACGACTAATACTTTGAAGTATTATGGTGACACAGAAGTCCTTTTCAGTAAGCGTAAAGGTCGTCGGATTAATTTGCATAGGGAATCCACAGCGATCTTGTGCTCTTGTCAAAAGTACCCCGATCCAAATGATCCGATATAAAGGGGTATATTAAGTAGATGCTACTCTTATTCAGGGGGACTTCTCTACCTCAGTTTCGGTACTGTAGATTTAGCTGTAACAGGAAACCCGCCTGTGCCTGTATTTGGAAAAATATCCAGTTGATTAAACATTTGCAAACAGCTTAAGTTCAATATGGGCGATCTGTGAATAAATTTAGGCCGTGTTCAGCTTGCAGGGGCAAATCCAGCTTGTTCGAAAATAAGCTAGAGCTGTAGAACAGTCGTTTTGTTAAGAAAAATAAAGATAAAATAAGTGATCGTGTTCCATCAGAAAAAGACTGGATTCGAAGGCGCAAAGCAGGACGATTCAAAATTCAACCCAAAGCTTAATTTTGAAACCAGTCGTTTGGAGCTATCCTGCTGTATGTCATTTTTTGTGTTTCTTAGCGGAATAAAGCTGGTTGAGACATTCCAGCTCTATAATACCAATTACAGCTGGTTTTAGCTTAAATCGAACACGGCCTTAGGGGTCTGTGATTTTCGCCTCCCATCCGATTACCCTTAGCTTCTGTTCGTTTTATTTAGCGGCATAATTGATAAAAACCACAATACCGATAGCGGTTGCATCGTCCATCTTGGTTGTTGACGCACTTAGACGGATGGTGTGACCAATCAATCGCTTCGTCCCCCACACCCGAGCGATACTTACATGCGCAAGGTCTATCCAGCGTTGAACTAGTTTAGAGAGCGGTAGCTTTTTTTTCACCGTCCCTGTCTGTGTAGCTTAAGAGCTGGGATACCCGTACGAACGGGGTGGACCGGTGGAAGGTTTCCGGTAGAACCCGTTCACTTATCACTTCTCAATTGTCTTGTGGCCAACAACTCACCTACGAACCTGATGATGAAGTCCCTCTACGCTGTCAATTTGGTCTTACTCCTGCTGCTTGCATTTTTTGCTCCTGCCCCCGCTACAAGGGAATTGAATATGAGGGCCGCCCCTAGCGATTCAACTCGCGTTGTCGACTACGCCACGACTGAGAGGCTTCTAAGGGCCCACAGTAGTGACAAGGAAGAACAAAAAGAAGAAGAGGAAAGGGCAATTTCGATAAATTTTTCAAGCCTGGAGAAAATCTTTAAAAAAGTTACGTCAGCCAAAACTACGGAGCTGCAAGGAATGCTTAAGGCTGACGAGGCCCTTGGGAGTGCTTTCAAGACGCTAAAACTTGGTACAATGCGGATTGGCAAGGATGGCTCTGTCGATCCCAAGATGGTGGCAAAATTTCTGTCAAGTCGCAATTTCAAGATTTGGTCCCAGCACGCCGTCAAGATCAACAAAGATGATCCCTATGGCGAGATGCTTAAAGCACTCACAAATGTCTTTGGTGAGAAAAATGTGGCGATGATGATCCTAGTCGGGAACCTGTCCAGAAACTCGCGCGACGTCGCAAAGAAGTTAGAAAAGGCCCAGTTCTACAAGTGGTACTTCGTTGATAAGTACAAGACAGCAGATGAGGTTTTCACGAACGTGCTGAAAGCTGATCGAAATAGAATTCATGGGTATGGTCGGGAGAAAGAAATTTGGGGAGATTACGCGAAGTACGTCACGACCACAGTGATGAAATATTGATAAACCAAGTTTCCAGCACTTCTCTTGTTTTGGGTTAACATATAGTGGATTACGTATGAATCAATGTATGGGTTTTAAATCCAGTTTAGAATCCTTTTATACTCATACTATGTAGTAATACTACCTCCGTTCTGAAAAAGATAACCATTTCAAATTATATTACAGCCTTCCCAACAAAATACTACCTCCGTTCTGAAAAAGATAACCATTTCAAATTATATTACAGCCTACCCAAAACTATACCCCCTCTATGCCGATCACTTTTTGGATTTCGAATCTCCCGCGCTTTCCCGACCAGACTGGCTACCGCTCCTTTGAAAGCTGTCACCTGGGGGAGATCACAGACAGTGAGCGCCAGGCCGTCTGGGAGTCCTTACTGCTTCGCAGCGTGGGAGGAGTGCTCAAACATGGAGAGATCGTTCGTGTTGCAGCGTTCTTCCGTGTGGGCCGCCAAGTTGTCGAACGAATTTGGAGGCGTGGAATTAATTCGATGGGAGATCGAGTAGCAGCCGTGGTCAAGTCAAGGAAGAGCGCATGTGGTAGGAAGAAGGTGAATCGAGCTGAGCTGTGCGAGAGAATCGCTGCAGTTCCTGTCAATGAGCGCGAAAACCAGCGTCTGCTTCAGCGCGCGTCTGCTATTTGGCTCAGCAGCTGATCTAGGAAGGCTACATACGGCGTGCGCTCAGACAGACCCGTCCACTACTTACACCCACGCACAAGTTTGCGCGCCTCCGGCACAAGGACAAGCAAGTACGAGCTGGCACTCTCCCTAAGTCACTGCCTTGTGACGCTATCTCACTCGCCAATGCTAACGCGTACATGCTGCTTTTTGCAGCGAGCAGCCCCCCCACCGCCTACCGCTGAAGTTCTAGAAGCCCCACTGAGCGCCCCTTCATAATGTGGCGATATAGTTGCCGTCTTAGATCCGTGTTTGTATCTGTAGTTGCACGGCGGAGCATGAACGCCTGTGCCTTGCTTAGTTTCTCACTGTCAGCTCTCGGTCATAAAGCTAGCTAGCCAGCCTCTCACAAATTAATAAAGGGTCAAATGAACGAGGCGGAGCGCTGCGCAAAAAAAAAACAAATGGTTATCTTTTTCCGAGCGGAGGTAGTAGTAGTACTACTGTACTACTACGTAGTACTACATGTACTACTAGTAGTAGTAGCAATTGAAGAAGCGATACTTAAGTAATGGAGACGTTCTCGTTCGGCCTAGAGGTCTACTACATCATACCAGAAGATGAATCCCGTTAAGATAGCACTTTAGACTTGTTCTTCTTCGTCCGAGTCGCCCTCGTTTTCGCTTACGATTTCGTTTCTACTACTACAGTAGTAGTACCATGTAAATATGTTCTGCTCTCTTTTCGACACCAGTCTAACGTGAGAGTATCGATGCGGTTGGATTTGTCTCTAAGAGCGCATCCAACGGATCGTAGCAGCGGGCAATACTCCGTCACTGCCTCCCAGAATGTATCTCCGTACATTTTGTTTTTTATAATGTATCTGTGAGTTGGATGCAGTCGACATCGACCCAAAGGCACATTAGTGCAGGACATGCCTTCAGCACAGCACGAGCTTCAGTAGCCAAGACGCCATACCTCGGAGGATGTTAGAATGCACCTTAATTGGTCAAATATGTAGCCAATAGAAAAGGTTCAAACATCCTACTTCTTAAGGAAACTTAATAAGTAAACACATTGCCGAGGGATGTTAATGGCGAGGAGGACGCGTGGCGCGTACGCTTGAAAAGGCCTCCTCACTTTGAAACTGACGGCCTATTACATAGAGCTTAATATTTCAGATACGTACGGAGACTAAGGCTCATGGTTAAAGGACACAATAGGAGTGCCTTTCCATGGGGAGGACTACACCATGTGGCGAGACAAGTGCTAGCGCACATAGAGACGCTGTATGAGAAATACCAGCGAGGGTTGTTGGAAAAGGACCAGTTTGAGGCTACGGTTGTCATGATGGACTTCCTTGAGGGTACGCCTGAGAAACCCATCATTTCGGTGGAGGGAAATATTTCTCAAAAGGAGGCGAAGGCCAGGCGTTGGCGCCACCAACACTGGACTAGGGCTCGGTCCGAGCTGTTGAACTTATTCAACCAAGCGTTGCCTAACGTTTTCATGAGTGGGCTGCCGGATCAAGTGTCCAGAATGAATCCCTGTGACATATGGAAGGAGCTCGAGCAAAAGTACGGGCTCGGGGATGCAGGAGGCGTAATCGAGCTGCGCCGCCGGTGGGAGCGTCTTCTCGCAGCGAACTGGACCAATCTGGGGGCCCTATTCGCCCAGTTAAAGATGCTGCGTAACGACATTAACAGGAAGATGAGGGGTCTGGTTGGCAAGGACATGGTGACCGAGACCTGGCTGTGCATGGAAGTGTTGGCGTTACTACCTAGCGAGTTTTGGGGAGGCACAATCATCATGACTGAAGAGTGGTTTTCTATTGAAAACGTGGAGACGAGTCTTCGCCGCGTTTTCGGTGATCGATCGAGGAAGGAAGCCACGATGCTGACTGATAAGAGACGCCCAGTGACTGTCAATGCGGCAAAAAAGTTTACGGGGAAGAAAAGGAACCAAGGACGATCTCTGAAGTGGCAGTAAGCGAGTGTTATTACTGCTTTGAAGATGGCCACTGAAAGAAGGCCTGCCCAGTGTTAGCGGCTGACCGCAGCCCGAAAAGGGCTGGAGGCAAGCTGCTTCGTTCAAACATA

>Contig_73

ACTTCGTTGGTACGTTCTGCAGACAGCTCGGTATATATACTCACAGTACTGAAAGGTATGTGCAGGTGAACAAGAACTCATGACCATTTGGACGTCGTCAGGGTTGCTGGAGCTCTCACCTTTGTCATCACTCCCCAGCAAGGATCCCCATGGGAAAAAATGCGGCGTATGAGCTGGCCCGCCGTCGTGCGCCGGTTGAAGAAGTGCTGCCAGCGGAAGAACAAGCGGCACCAACTTTGGAGGTGACGCCTACTCCTCCATCTCCTCCTTCGAGTTTCCCTTCGCGTACTTCAGCTTCTCCACCGTCGACTCAAGCTCAAGCAATTCAGCCAGATACAGAACAAGAAGGTGGGAGCACCACTACTAGTATGGAGGATCCGGAATACGATGCACGCTCGGGTCATGAGCGTGCCGACGAGTCGAAGCGTGAAAGCGACGATTTCAACGGGATTGATGAGTTTGATGGAGAGAACTTCATGGATGCGCTACGTGGGGAGAAGCTGTTCGGGTCAGATGCTGTAGATGATGTGAATGTGTGCGATGAAGTGAAGATGACAACAACAGTATCGGTTGTGTCTTCGATGATGAGCTGGATGTGCCCGTGTCGCCACGAGAGGTCTACGAAAGCGAAGTAACTACTGAAAACGTCTCTAGAGCACAGATNGTACTACGTATGGATAGTAGGTAGGAAGGAGAAGACNAAACGCACTGGTGTTATAAGAACAATTTAATAAGCTAATATGTGCAAACGTGAACAGGTTTTCATACGCATCATGGGCACTGAGGTTGGCCTTCTTAAATACTTGCAACCTCAACATTTGACAGGAGCACTGGTTGCAACCAAATCTCCTTCCCTATCATGTCCCGGACACGATACCCCATGCGCTGGATCACTTGCGCGCTCTCAACTCTTTGGGCCTTTTCAATGAGATCCTTGGCAAACACATTTTGGCTGCTTTTGGCTGCGGTGCGGGCTTGTCACATGTTTGTGATCTGGATTGCCGTCGCCTTGGCGAAACCTGTGCAGCGTAGCGCTCTCTGTTGAGCAAGCTGACGGAGAAGTCGCTGGCCAATGTGCTCTTCGAGCGGAGCACACGAAYYTTAAATGAGCACATGGTTTTGCTGGAACACACGTAGATATTCGCTGCTCCCCCAGACAGCTTCGAGCTCAGCCCGGCCTGTTTGTCCTGTACAAACGCGAAACCCTTTATGTGTAGCGTTGCGTCTTGTCGAGTGGTGAGCCACATACTAATGGAGGGCGAAATGGAGACCGGGAGGCTACTCCACCGTCACGCACCCCGACATCACGCTGGAGGAAATTGAAAATGGAGACTTGTGATCTGATTTTTCGAATACCAGGTCCCAAAATTTAATTGCATTTAATGTAATCAGCCAGCCAGGATCTGGTAAATGTAATTACGCTTGACGATGCCTAATCAWCCAGTATCAGGATTGAACAATTTACCTACATATAAGGCACGAGGTTTAAGATAGGGATGTCATGGTCACCAAGTTGCAAATCATCGGAGTAAAACCCTAGACAGAGCCCTGAGTTTGGAAATTCAAGCAAAAATAATTAGTCAAGGGTGTAAGTCGTAGACTGAACCATGCTATGGCGTGCCGGTGCAGATTGGGTTCAGCTTGTCCCGCTTCCGCAAATCAAACGAAAGCACAGTGTATGACTCGATCATCAATGCCCCTTAACCCTTTTAGAGCGGAGACCTGCCAGGGCCTTCAAAAATGTGACCATTAAGATTACCGTATTTCGCAATTTCGAATAATGATTTATGGCTATTTACTATTGTGATGTCATTCTGGTCGAGTTAGAATTCCAACTGGGACTCTTGGAAAACAAAAGACAGGGCACGACAGCAGGGACTGGCAAACGGTCTATAAATGTATATTTAGCCAAACGTATGCTTACGTAAGAGGCCCTTCCAGGGACTTTAAGGCTTAGGTCCTAAAGGTCCATACACTGTGTTACCCGATTTTGGTAATTTGCTACGTCACACTACACTGTATGAGTGTTCCGCTACACTATATGAGTGGAAGTGGGTACAGTACTTTCATTAAGTTTCATCAGACTCTCTCCTCAATTGTTTTACCAGGCAGATATATAAACAGAGAGTCATGTATTCAGCAGCCGCCAAACAATGGGTTACGTCTCCCTACAATCGAAAACTTTAGAGCGTCGAAGACAGGTTATTAGTATTTTAACTAGTAATAGTCTGATTGAAATGGTAAAAGGTTGCTATAATAGAAAAGAAACACGGTATCGATACGAGACGTTGGTGCGGAAGGCTAGAGCGAGGCAGGCATGGCCGCCATTTAAGAATTTAAAAAAAGTACAGTCGAGAATCYCTTGTGATTACGCTTTCGAGCTGCGAATCGAAGTCAGTTGGTTTCCCGTCATCATATCATGTTTCGTCACGAGACTGTCGTGGCATTTTACCCAGTTCGAGCTGCAAACATATCATGAGATAAGCAATTTGGTCTGGTCCGTGGTCGGACCCTTACTGACGGACCGTCAGTGACGGTACACATTGAGTCCGGTGATGACCAAAATACTGCTGGTACAGAAGTTGGCAATGAACACTTTCGTGCTGTGTTTTCTTATGGCTGTAGGTCACCTGGATCCGTTGGTGCAGGAGTGTAGCTAGAAATGTTTTCACCCGGCCAATTTCCTGGTTGGAGATTGGAACCTTTTTTTCTTCATATTCTAAAGTAATCGAACTCCTGCTCAAGTCCTATACTCCAGCTCTATCTCTAGAATCAACAACGGTTACATTGCGTCTTTGGGACAGGCGAACTACGCGGAGAGCCTCCGAGCACCTGTGACGCACGGTGTGAGTGCTCACGCACTGCTGTCAATTGACGCTCGATCAATATGCTAAAAGCGCCTCTTTTTTTGTCTTTTGGGTTTTAGCTACATGTAAATCGGTGTGGACGACGTCGACATCTATAGCTTCAGCACAGATTCGGCATTGATAAAGTTTTTATCGTGGATTTCAGTCTCTTCTTTCTTGAGCCTTTTGTTTAGCCTCTAAATATTCGATTCGAATCAGCTTTGCAGACCAATTGGCGTCGGACCAGTATTCCGCTCGGAGCGTTAAAAACTGAATGGTTAATAATCAATGATTATCTATTGCTGCTACGTCAACGTTCRCTGTACTATTCGCACTTAAAATGGAGAAGTATCAATGATATCTCGATCAACATTCAGCATCAAAAAGAAAATTTTACGAGCCCTCGCAGGTGTCGACGTCGAAGCTGCTAAAGCTTGCGAGGATTTATGAAAACATTTCGTGAGCTAACGAAGTCGCGTCCCTGCTCTCTTACTTCAATTGAAATATTTGCTCATACAATTGGTGATGAAACGCCACCAAAGAAGATCTTTTAAAATTTACCTCGTACACGAGCAGACGCATAGCAGTTAATGAGGCTTCAATAGAAAATGAAAATATTTGAAAAAGACGAGGCCAGCTTTAATCAACATCGTTAATTTTTAGCTTTGGGTGTCATTTTCTTGTGAGATGAGCRCCATGCGGTAGAATGAACTTACGAAATCATCGCTGTACGTTCGCAGCAGCATCAAGAGCAGCATTAATTTTGTTTTTGCCGAATAACGTTTTCACTCGCTCCACCGTGCCCTCCCCACCTCGCTGTGCTGCATACCACAAGGTGTACTCCTTTAGCGTGCCGTAAAGTGGATCACTCTTTGTCACCAGCGGTGACACCCAACGATTTTGGAAATCTTTCGGTGTCGATTTCGCGTATAATCCGTGAAACACCTGAGCTTGCATACTTTCGGCGAGCTTCTCCAAATCCGGGATATTCTTGATCGATTGGAGACGTTCAACCAAACTCGGAATCGACCAATGCCTCAAAAAGAGCTCATTCATCTGGGATACGGGAAACGAATCGACCCCCATCGCTGTCTTGTACATCTCCGTGTATCTGAGCCATTGAATAAGATACTGGTCTTGGAAACCGTTTGGGGGAAGTACCAAGTTGAACAGCTCTCTGGGGTTTTGCCGGGAATGCAGCCACGCTCCGTTCATCAATGTTGTGCATGGATGGATACGTTTCAAACAAGTGCGTCTGCAGCCGATCAACAAGACCCTTCATGCCGTCGACTCGGCGGAGGGATTGAAGCAGCTCCAATACCTCTTCATCCGGTTTTGCATTTCGTAGCAAGGCGGCAAATATGGCGTCTGAGAACGAGGTTTCTCCCATTTTGCTTCTGTACCTAAGTACATACTGAAGCCAGCTCATGAACTCTGTGCTGTCGTCAAGCATCCCGACAGTCTCGGAGAAACGGATTCGCGTGAAGAAGTCTTCGGGTTTAACCTGCATTTTTAGCGCCAACTTGTACGTCAGCTTCTCCGTAAACGCCGATCTTCTTTTGCCTTGAGCTCGTTCCTCTTTATCTTGGTCGCCAAAACGCGTTTCATTCGTCGGGGGTGTAGACCTAAATGATCTGCTCGCTGTAGGTGCTGCGGCGGCTACATGAGGCATAGCCCTTAAGTTGGGAAAAGATTGTCCATAGCAAGTATTGTTTCGAGCAGCAAAGATAAGTAGCCAAGCGTCAAAATGAGACATCGCAAACTCATCGTGGGTGGTTGCTTCCTGGGTAACAAATTGCAGAGTGAAGACTGTCCCTTGAGAATGTCTGGTCGTGACCGCAATCGGTTGAATCAAGCTGAACTTCAATCTTCAATTCCTCCCTGCATTTTGTTTGTAATGTAAATCATTGCTATCCAGAATACAATTAACATCCAAGTTTTTGTCTGAAGGCAAACTTTGATTCGGGCAGCAACGATAAGAAGTAGCCCACGCCTCGCGATAGGACAGTGTAAACTCATGTTGGGCTGTCTGCGTAATTGTAAACGGGTTTTTTAAGCCAACATGGACTGATACGAAACTTCAACGATTCGTATTTTATTTTAAAAGTATAATATTTCAGCCGATACATTTAAAATCCAAATCGNTTTTTTTTTTTTTTTTTNGTGAAGCCGGCAACTCTGAGGCCTGCTTTGCAACCGCAGGAGATGCATCGAAGTACTAAGGAATATACGAAGTATACTTGCATTACTATGTGTCAGTCCGAGTACGATACAGTACTGCACCCCGTTTTCAAAATCTGGTTTATTTGCATTCAGGGTCGCTCTTAACCACGCCATGCCATGCCATGGCACAAAAAGCTGTGATCCGATTGGATGAGCGCTTGCTAGGGCTAGAGTTTAAAGTAAATAAAATGACAGGAAGTGCCACGGATGGCATTCGCAAGTGTCATGGYAAGAGGCAATTGCCATGTGCCATGCTACTTTTATTTTCATTAATGTATGTTCTAAAAATAGTTTTCATGATGGCACAGTGGCAGGGCAATTCTATTCGTAACCACGCCATGCCATGGCTCAATTTCTAGTGCCATGGCATGGCACACGTGGCAGTGGTTAAGAGTCACCCTCAGGATCATCACATCCGGAAGTTTATCAAAGGTCCAAGGGCGCGAAAACGGGGTGTAGCTACTGTCTGCTCTACCAGTAGCGCATTGACAGAACGTTTTATCGCAAATGAATATCATTGGTTCAGATTTAGCTTCAATATCAGGATAGATTRGACAGTGGTTGATGTTCCTCAAACTGCAGAGCTTCTTCATGCAGCGCGCGACTTTGTCTAATCACCTGTTTTTCCGCTTTACAGGCTATATCGTTTTTATCGACTTCTTCAGACCGGCATTTTCAACTTATATTTTGACATATTAAACTACTTCGAAGTAGATATTTGCACTTTTTCAGATAAAAAGGGGTAAAGCCTCCATAACCCTGTATTTATAGATGGTGCTCGAGGTTAAAGATAGGGATGTCATGGTCACCAAGTCGCAAATCATCGGAGTAAAACCCTAGACAGAGCCCTGAGTTGGGGAGTTCAAGCAAGAATAATTAGTCAAGGGTGTAAGTCGTAGACTGAACCATGCTATGGCGGTGCCAGCCCAGATTGGGTTCAGCTTGTCTCGCTTCCGCAAATCGAACGAAAGCACAGTGTATGGCTCGATTATCAATGTTCCTTAGCACCTCTTGTACAATGCGTATATACTGTGATACCCGATTATGGTAATTTGCTACGTCACACAATATGAGTAGAAGTGGGTATAGTACGTTCATTCAGACAGTAAAGTTTGCTTTAAAAGATAGTCTTGTATTCAGTTTATGGGCTACGCTCGAATGTTTTAGAGCGTCGAAGACAGGTTATTAGTATTTCAACAAGTAATAGCCTGTTTGAAAAGGTAAAAGGTTTCTATAATAGAAGAGAAACACGGTGCCAATACGAAAAGTTGGTGCGGAAGGCCTGAGCGAGGCAGGCGTGGCCGCCATTTAAGGATTTTNTAAAACAATTCAGTCGAGAATCCCTTGTGCGATTACGCTTTCGAGCTTCGATTCGAAGTCAGTCGGTTTTCCGTCATCATATCATGTCTCGTCACGAGACTGTCGTGGCATTTTACCTGCGCACAAATCATGAGATAAGAAATTTGGTCTGGTCGGTAGTCGCACCTTTACTGACGACTACAAAGTAGTACTGTAAGCAGCTCTGTGCAGTATGCTGGTCTCCCAAGCGTCGTAGCAATACAGGTCTATAAATTGAGCATGTCCTACACTTTCATTACGGACCGGATTCCGTAAATGCTACCAGATTCAGTTTCCAATTTTCCCGCGTGAGTCTGCGTTATCATATATTGGTTCCATGCTCTCCTGGTGACTGTGTTTGCATTCCTCGCCAGCACTGATTCTCTGTCAGCAGCAGCACATTCCGGACAAAGGAAAACAGGCTTGACGAAATCGTCAACCACGGCGATCGTTTCAAACGCAAAGAGATCACTGAGATTCAACGACAAGGAAAATGGTGTGGTTGGCGGTGAAGATATTATCGAGGGGAGAGGCGGTGGCAAGCTGTCCGACTACTTTGGGAGGTTTGGTGGCATGAAAAGTAAAATGTTACAGTGGGCTCGGGCAGGCAAGTCCGACGATTTCGTGATTGAGAATTTAGACCTTAAAGGTCTATCGGTACATACGCTCAAGAGTAACAAGAACTACAAGCATAATAAGCAGTTTCAAGAGGCTCTCTTGGATATCTCGCTCAAGAAAATGACCTCAACTTCTGAAATTTGGAGGCGAATGGGATTCGAAAAACTCAAGACAATCGACGACGTATATGCCGCACACGCGACGGATGCATTCCAATTGTACATGCGATACGCTAGACATTTTGACGACGCTGCGCTTAAGAATAACGTCAAACACAAAACGCCCATACCAGTGATCAGCGACGACGTAACTTATGCAGAGGCGACGGCAAGAATATCAAGATGGAGGTTGGACGACAGACCTGCCGATTACGTCAAAGCGGCCTTAAGACTGGACAACCTGTCGCCCGCAGCGCTCCTAGAGCGCCAATACTTCGACTTATACGTCATTTTTTTGAAAAGCAAGACTAATCGCATGTTCAGTGCGGGAGAGTCCAAGGAAAAAGTTGAATCATTCGTCAAGACGGCACTAAATCTCAACAGTATGTCGCCAGAAGACATCCCAACAACTATCGGCAAGTACTACAGTTACCTTTTGGTACCAAAGGCCCGAACTCTTGATTGAGTGAAGATTGGATGAAAAAAGTGGCACACTGCTCGTGCGTTTGCATTCTTGAACACTGCGCAATGGTAAGATCAGCGCAGGGTTTCGCGAAAAACAAGTTACTCGGCACTTAGCTCATTTAAAACAGACGAAATCATTTTTCGTGCTGAAACAAATGCACCTTAAGAAAAATTGCGGCGCTCCGCCTCGTTTAATAACCGGAATAAACACGAGGGCGATTTGCGCAAAGCTTTTTGTCAGGAACTTGCTTGATTTGAGGGGATTTAGTCTTCTCTTTGGTAACAGACGCAAAATTTAGTGTTTAGACTGCGTGCGTACTTGTTCCGCCTCTTGAGTTTAGATGCAAGTGTGGGAGGATCAGTTTAGAAACAAAGTTGACAAATACCTGGGATCGGTGAAAATAAAATTGAAACCAAATACTACGGTATTGGTCCATTGATTATTGAACCTGGTCGGAAAATTAAATAAAATATTGTATTGGATTTAGAAAAATCCGTTATTCCGTATYGATAAAAACAATATTAAGTACTGCAATTATTGTCAGGAATTGGTAGGGTCAGAGCGGTGCAGTGGTTTGGGATGACCCTACCAATCCACTGCACAGGGTTCAAGTCCCACACCTGTAAGAGAAGCTGGTGGCTACCACCGGGACTTTTGTCCGGGCTTAGGGTACTCTAGCAAACAAAAAATGCGCCGGCGACGGCAGGCAGTCCACCAGCGGATATGAATTTTGTATCTGAAAGTTTTATACCTGCTATATTTAATGATAAGACATATTATCACTAATAAGAGGTTATCCTTCTTGARKGACTTTCATCTACTCTGCGTCTTCATCGGCCGACTCATGGAACCCCAACAAAGAAGTGAAGTCTCAGAGCTCACGGTTAGCGCGTAAGAACACCAACATCTCGAAGTTAGCTGGCAACAGACTGGAGCGTAGAGGGTTGAGCACCAACTTGCACTGCGAGAAGAGGCGCTCACAGCGATTGCTGGTTGAAGGAACCTTTTGTAGCACGTCAATGTACTTGGTTCCAGCGGCTCGGCGAGGTCTCTTCGCTTGACGAAGCGTTTCCGTCGCGAAATCTGTTTTCTTCTTCGTGTTCGGTGCTGCTGGTTCGCGATCAGCAGGGCCAGCAAAACGAGCCACAGCTTCTTCTTCATCGGCGCTTAACGGTCGATCCGACAGCAGCTTGACAACCGCATTCTCAAAGGCCGGCGAGTGGACGATTCGAGCCGATGCACTAAGGTGGTGTGCCGTCCCTGGGTACTTGGAAATGACAGCGGCGAAAAGGAGTCGCACATCGGCAAGGTTACCTTCTTCAGCCTGTAGCTTGACACAGACGCTGTCTAGCGCCTCAAGCTTAGTGACAAGCTGGACAATCTGCCGATGGGTGCTTGGTCGGGGTAGGAGGTCCTCCACAGCTGTCACCATTTTGATAGCGTCCCGGATCTCCACATAGCGAACCAGCATCTGGTAAGTGGATGACCAGCGCGTAGCGTTTGCCTTCAGCGGCTTGTACTTCGTAAACCGAGCCAGGTCCGCTGCATTGTTGGCGTGCCGCAGCTGAATACACAGCTTTGGACTTGGTCGATCAGCGGCTTGTAGTCTTCAAGGTACCGGCCGACAGCGAGGTTGAAGCGATGACTTGCGCATCCTACGAGCGGTACTCCCATGCGCGTAGCGACGGCTTGGTTTGTAGGGCAGTTGTCAGCAACCATGAACAGGACCATACTGCGATCCTTCTTGTAGACATCCAGCACCGCATCAATCATGTCGATGTGAGCATCAGCGGTCTGGCCGTGCTCGGCAGAAGACAGCCCGAGCAAAACTTCATGTAGCGCCCCGTCCTTCGTGAAGACACCGTAGATAGCGATGAAATGGTAGGTCCCTGAAGTCCAGCCGTCAAACATGAGGTCAAACTGGTTGCCAATGTCTGCCGCAATACGCGTTCCCACCTTGCCCGCCACGTGACGCATGTACTTGACCAACGTCTTCGAAGACACTGGCTTGATTGCAGCCAGCGCACGCGTCATGGCGTCGTCCACCACACTCAGGGGCATGTTCCGGACGAGAACCCACTCCATCCACTTGAAGATCTCCATAGTGCGCGGGTCAATGAAGCCGTGTGCTTCGAGGGACTGGCCGAACGATCGGTGGCTCTCGTCGTAAGTCTTCTTGTAGCCGGGGTGGCTCGTTGCGAGGTGGTTGATGAGATTTGTGTAGCCAGTCCCCGGCGTCTGCTTGCGAGCCTTGCCGCACTGCTTGCAGGAAAACTGCCCGTTGCCGCAGTCCGTGAAGAAAAAGCTGGAGATGGCGCGAGAAGTTGACATGGCACTGGCGAAATGAGAAATGAGAGGCACGAAAGCTCAGCCTGATGCCTCCGGCAAACTCACACAAACCATTCGTTTTGATTGCATGCTTGACAACCACTACTTTACTCTCTGTTGCTTTTTACGTTGGGTAAAAATTTAATATAAT

>Contig_74

TGGCCAAGACGACGGCAATTGCACGGCGAACTGGGCTGGAGCGGCTTGGCTCACGTCGAGGAGATGGCCAAACTCGTTGGACGGGATATGTGGTTTCTTGGGCTTGCTGACGACGAGAGCCTTCTTCGCGGACGCCTTAGGAGGGGGGTCGTCACTGCCGGCCTTGCGTTTACCATGCGCTGGCGACTTGGGCGACTTGGATGGCTTCTTAGAAGGCGACTTGGACGGCTTCTTAGGGCGACTTGGACTGCTTGCTGGACAAAGTTGTCGACGCCGAAGCGGAGCTGAAGAGGGAGCGGAGTCTGGCGGCGCGGGGATGGCAACGACGATTCCTTGGGGCGAAGCGCCGGTCCAACCATTCAGGATGTCCTCGGCGAGCTGGTCCAAGAACTGAAGACCTGACGACATGCTGTTAGCGCCCAGACGCGCGCGCAGCTCGGTCCAAAACGTTTGCTGGAAGGCCCGCGCGGTGTCCCCAGTGGTGCCCTGCTCCTCATAGTTGAGGGTGGCGTTGATGAACGACCGGAAGCGCCTCAGCTCGGCGGCGGAGAGTCGCGTGGACATGGCGAGATGGCAAAATGCGAAATGAGAGAAATTTGGAAGCTGTAAAGACTCGCGTCTTTCTCTTGATGGCTGCCAAGAGGCACGCGAGAAGTTTCGACAGTTGAGACGTGGCGTTGATTGAGGGACGCAATGATGCTTGGTGACTTGGGCGGACAAGGTGTCGGTTGAAGAGCCGTCCTAACGGCTAACGGTCTGGAGAAGCTGCGGTCGTGACGGCGTCACTTGACGATTAGTGGCGATGTTGGGAGTGTTTGTGGAATGGGCGGAACTGGTGAACGTGGTGACGGAGATTGAAGTGGAGGACGAGATTAGGGTCGTTGGCAGCGCTGTAATGGGGATCGTGATTTGGATCGCAACGACTCCTCTTCGGAACTTGTCGTGGGCGTTCCTCGACGGGACGATCGAGAGCGTACGTGCCATCTACTTCTTCTGGTACACGTGCTGTGACCGCTACCTGGTGGCGGCCCACCATGCCGCTGCTGCGCCTCCTCCGGAGAACGCGTGCGCGCTAGTGAAGCTGCTGTTTTTGGTTTCTATCGCGGTGCTACAGGACTACGAGTCACGGCTTGGAATTACAGGTCGTATGTCTTAAGTGGTGTGAAGCGTTCTACCATTTGTGCTGCACATACGTTCCTGATGCGAGCCTACTGCAATGGAGCACGTCCTGCCGGTACGCACGACCGCGAGCGAAGCTGTGGTAGCTGTTGTATATATAGTTGGAGATTTTATCGTTGGGAGCTACTTGCTGACGTCTCTACTCCTGTCGATCCTAATCTAATTGCCCCGCCTGGTGTACGATAGTGTCGATGGAGTGCGTGTGGCTGGATGGGGGTGGCGTTGGTTGTTTGGTGGTGGACGTCTCCCTGGAGCTCGATATCGGCTGCTGCTACTGGAATTTGGCTGTGTTAGTGGTCGGCTGCTATAGTTACGCGATGTGCTATTACGAAGAGCTCAAGCCGAGTGCCGCGGACCAGAATGACAGGGCTGTGAGACGCAAGTGGGAAGGACTTGCTGGGCGCATCGGGCGTCGTGAGGAAAGAGAGCGTGCTAGTGTGGAGCGACTGCGAGGTACACAAGCCGAGGGAACGTCGCCTGGAGCAAGGAACGAGAGTCAAACTAAGAGTGGGACGTCGCGTGGGAACGTTTCAGATTTGGCTGTGGAGCACACCTTGCGACAGGCTCTGAGAGATGTGGCTCGGTGAGGACTCCGAGCATCAGCTGAGGAGAATTGTCACGATGTACAGTTAGATGGTGGATTAACGATGCTGGCTGACTGCCTGCAGAAGGTCTACCCACTCCAGACTCTCCAGACCACGTCCAAGCGAGCGGGACCTACGGCCAAGACAGTGACGTTCCAAGTGTGAAGTCGTGAAGCAGCTGGTCGTGGCGGGAATTAGAGCTGGACCGCCACCCACGAGGGATCTGCTGAAAGGTAAGGAGCTCTAAAACTCTTAGCGAGGGGAAATGAGGAGATCAGTCGTGGATTTCCTTCTAAGAAACAACACAAGAATATATCAGCTTTTTCATTACTATACCAGTAGTGTCGCTATTACTACTACCTCCTACTTCGGCCCAAGATACACGACCCCGATTAGAGGAGGACCGCGACAATTTGCAAAAGCCCATTGCCAGACCGCCAGCCTCCAGCTTTTAATATTATTAACAGCCGTTTTATTTATTTTTTCTTTCTCCCTACGAGGGCGGCTGGCTTACTATTGGTTTTACAGAATTATAATGTGCGTCAGTTATAGGAGGCACAAGAAAGGGCCAACAAGCCGGTCCAAAAAAACTTTGTTTTTAAGTCTATTACGAAGGCGACAGATCAAATTGACCTTTTTTCTGGCACTTGGAAAATGTCAACCCTTTCTTGTGCCTCCTATAACTGACGCACATTATAATTCTGTAAAACCGAAACAAAAATAAAAAACGGTTGTTAATATTATTAATAGTTGGAGGCTGGCGGTCTGGCAATGAGCTTTTGCTTCGTCTGCAACAGCTGCACCGCTACCAAAATCATAATCGACTCGTGTATGATAAGAAAACCTACGGACTTTAAAGTGCAGTGGAAAAGTAGAATTCGTGCTCACTTTGCATCTTACCGGTACTGCCAAGCGCACTTGTCTACAATATTTCTATGGGTGTCATCCAGCCGTATAAAGAGCGTTTACAGCGGTCCGCTTTTGGCTACTGTTACTCTGTAGATCTGAGTTAGATTGGTGTTAGGAACAGCCAATGTGAGGGTGGAGGGTACTGATGTGGACCTGAAGAATAACATCCGACTTGATCGGCGGTTGAGGATGCTGATTGCTGATTCTAACTACAGCCCCGAAGACAGAGGCGTCTCCAGTATAAAAGAGCTGGCTCAAACAATTAGTGTGGATAAACTTGCTAAGAGTATAAAGACCTTGGTGGCACCTGACAGCCAGACCAAGATCGCCAAACGCTTTAATAAGATCAAGGGTAAAGGCGTAGAGTCCAATGTGCTCTTGAGCGACAAAATTTGATGACTGGGCTAGCTACGTCCTCCTCAAGGTACTCAAGGGTGAGCAGGCAGCAGCAGACAAAGCGATATTCGCGACGCTGGCAACGCACTATGGTGATGTCTTCCTGCCACACCTGTTGGGGGCAAAGCGAATGGGTAAACACTCGATAGTCAGTCGGTTGGAAACGATTCAGCTTAAAAACTGGATGGGCGATGGAAAAAGTGTGGACGACGTCTACAGAATACTAAAACTCGACGAAGAAGGCCAAAATATTCTCAAGAGTCCAGCCTTAAGCACGTGGATGAGCTATGCATCGAAGCTGAAGGAAGATCCGCTCAACATGCTACTGCTCAAACTACGAGCACAATACGACGACGCAGTAGTACTAAAAATGATTGCGATGTCAAAGGATAAATCCGATGACGACACGTTTAGGAAACTTGAAGGTGCACTGATGGGGAAATGGCGACAAGACGGTAAAACTGCGGACGATGTGTTCCGGCTGCTGAAGCTGGATGAGGAAGGTTCAGATCTTCTAAGAAATCCGTTGCTAAAAGCTTGGGTGTCTTACGCGGATGGTCGGCTCAGAGCCGACCCGTATACGCATATGATTTCAGTTCTCAGACAAAAAAAGTGTCAATGATGCGAAGTTAGCGTGGATGATCGGTACAGCAAAGCAAGACCCCAGTGCAACGTGGATTGCCGGCAACATGGAGTCTAGGCTGATCAAACAATGGTCGGGCGAGAGAAAAAGTGGTGAGGACGTCTTCACGCTGATCGGACTCCATAAGGAGAGAGACCAGTTGTTTAAGAGCCCMGTATGGAGTACTTGGGAAGCCTACCTGAAAAAAAATTGAAAAAGAACCGTACAAAGCCATGTACGCAGTATTGGGGGCACGATTCGACGATTCACAGTTGGCGGCTCTTGTTTCGAGYTCGAAAGAGGCCGAGAAAGTTATTCTGAACGTTTGGAGCGCGAATGGAAAAGCCGCAGACGAAGTTTTTGACCTCCTGAAGCTGAGCGAGCAGGGAAGCAAGATGTTCGAGAGTCCGGTATTACGCACCTGGGTCACCTATGTCGACWAGTTGAACAAACTCAGGCAATACCCAGATGAGTTTGAAGCTGTCAAGCTGTCGGAAAAACGACTAGGCAGCTCTCCCCTGGCACTTGAACTCGCTTATTACAAGTATAAGGTCTATACTGCAAGTGCACAACAAGTGATCAAGAGGTTGCAAGCAATGCAATTCCAAAATTGGCTGGCCCAACACGAAAATCGATTCACCCCCGTTGAAGCTATGGCATTATCGATCCATCCAGCGGTGAAATCAGAATTCCTTCACTACTTCACAGGTATGTTGGAACACGGTGGCTTCGAAGCAACTCGCAAGCACGCTGTCAGATAAGAAACCGTGCTGATTCGAGCGGTGTAGGCAATATGGTTTGGTCGTTAATGCCATGAACCAGGCCAATTCCTGAAGAACAATTCCTTATGTAGGCTGTCATCTGTGGCCAATGTCGCGAGAGTAAGGCTGATACTTCCTGGCTGCTTAAACAATGCTTCAGTAATCGGTAAGCAGTACTGCATGCACGTATTTGATCTTTGTTTATCGTACTGCTGTAGTAATTATTAAACTTCTTGCGTAGTAGTATTATAAAACGCTACTGATATACTATAGGCTAAGGAATAATGTACTATTAATAAGTAGCGTATTCTTCCGAACAATAACAATACTGTGGACCGGCAGCTTTAAGTTAAGTCGTGAGATGTAGTGGCCTATCCGGACGAGTTTTGCACCGACTACCGCTGCGGCGTCTACACTCCACGCTGTCACTGGTGGCATCGAGCTGATCATGCCGAGTAGTTGAAGCTTATCGTCGTCTATGACTGCTGGCTAATACATGTAGAGAGGCGTCCTCGGATAAATGCTCGATTATTTTTGAGGATTTGCAATTATGAGCGCCCGACGACATGATAGGCTGGAACAAAACAATGTTACTCTAGTCGTACGAAGCCGTGTTTCACTGAGAAGCTCTGCATTACACTACTATGTGTAGGGTGGGTGCTTCACTATTGGCTTCACCGTATTGTAACGGACGTCAGTAAGGAGCGTCACATTCTCAGTGCCAGAAAAAAAGTACTTGTCAATTTGATCTGCTCAAACTTTACCAGGATATTAGTTCCTATGCGCTCCTTGTTGCCGCACTTTACAGTATACTATTCCTGCAGTCATGTTTACCGTAGTAAATACGCGTCGCACTGCGGCCACAAGAAGGATATTTAGCAGCCGCGGTAAATCTGTTCGGGCAACATTCACGTGATTAAATTACTTCGGCTACCGGTAGTCGTAATTTACGGGATGTTGGGTAGTATCACTGATGCAATCATTGACTAAAGTCAATGTGTTGTTTCTGATACAAGCTGAAATGACACGTTGTGCAGGCTCAGACAGTAAACCCATTATATTGGGTATGACAATCCAATGTAGATCTTGTACTCATGTATCAAGTGCTGTAGTAAATTTATTTAAGTTTTGAGCTTGAAGCGTTAGTTTGGGATCTGTTTAGAGTAGTTTTGGGTCAGAATCACTCGCCCCCTCAACTCGGGATATAATCCTGAGAGCTCGATACGATACGATGTTGCCAAATGGGCGGGATGACCTCCAAAGGCCTGCACGCTCTGCTTTTATCTAATATTCCCTTGAGCATAAGCGACTAGGAGTTTGGACTCGCATCTGAAAATGAGGACAACACGCACTTTAAGGATGGGTCTAACGTCGCAGGTCAGGTCTGGTTAAACTGAACATTTTCATTCATTCAGCTCTGAACATGGATCCAAAAATGGTTGTCAGCACCTTCAATTGGGTACATGTAATCTAATCTATCTGACACGGCTGAAAGTGGTTGGCGAAAGCTTTGTTTGACGCAATCGCCCGTAACATTTGCTCGTCTGTCTAGAGCTCACTCACTTTAGCTAAACCTAAAGAGCTTTCAAACTAAAAATGCCGACTTCCAACGTTGCCTATCGTTTCGGTGCACAAATTCGACGGAGCCCTTGAAAGACAATGAAGTCCTTATCGAACTCCGTGGAGCTGCTCTCAACTACCTTGAGATCTCGTCATCGCCAACTCCACCTACCCCAGTTCATTGACGGAGCACGTCGATCCATGCTCCGATGGGGCTAAAGTAGCAACAGTACTGGTCGACAGCATTCTAGTGGGTGACCGTGTCATCACCAACTTTGACGTTGCTAGCTTAAACAACCCCGCTCTTAGCGATGGATCGCATTTAGGAGGTACATTAGGTGGTATGTTGCGTCAGTACGCTACGGTGCCAGCTCAAGCTGCCCCAAGGACTGTAAGCTAGACTTCGTGCAGCTCGCCTTTCTCGTGTGTGTGACTAGTCTAACTCCACTGCGTTCTGGTGGTTCTCCGAGGCACGGGTGGCGTCTCCATCTTCGGCTTGCGGCTACTTCAGCCTCGGACGAGAACTCAAGTTCGTGAAGGTAAACGCGTCGACTTCGTGATTGAGACTGGAGGCTCTGGTAGAGCATCAGCGTCATTAAACTGGGTGGTCATCGGCTTCCTGTCTCGCGCAAGCCCGACGTCGCAGGCATGGCGTTAGGCAATTGATGTATCATTAGCATTCAGATCGGCAGCAAGCAACTAACGGAAGAGTTGGTACGTGTGGTCACAAGCCAAAAACATCAAGCCGCATTTTTGCAAGACGTTCGGGCTCGACGAGGAAGAACTATACAAACAGCGTTTTAAGTATCTGGAGATAGCTAGTCACATTGGAAAGATCAGCAACACCATCATAGACTCATAAAGCCCCTGAAGCTTTCCTCTTCTAAGTCGATTACACGCATCATTTTTCGCTTATAAATCCTCATCGTCGAGCCCAAGGAAGTCGGCAAGTCCACCATTGGACAGTCCGTTCTCGGTTTACTTTTCTTGCTCACGTCTATTCTGCTTCCTAGAAGGCTAACTGATTTGCCAAGCGCACGGATCGTATTAGCAGTCAGGAACACTGCCCATCGGTGTGATGATCCTCGAGCATTGTGGG

>Contig_76

TTACTAAGTCGTACAGCTCACCCAATATCTTAACAGATAGTTGGGTATAAAATAAATGATTTTTCTCGAGGGTCTTAATTGAGAGTTGGTAGAATTTTCTGGAGATTTGTTGCTTGTCTCACGCGGGCAGTAATTTAGCTTATATTGGAAGCTGTATTAGGCTGTTTTCGTACTATTTGTCTCCGTAGACAGCAATGTCGTTAGTGTATCGCTGAGAACCTGTTTACTGACTGTTAGCTAGAGAGTCACGGAAGTAGCTGACATGCTGGTGGAGTTAGACTGGTTCATCGTCAATTTCGGAGCAGTTTGTCTTTCTCGTGTGTGTTTTCGTTTTGATGTGAGCCGTTTGTGTTTCGATCTAACGATTTGATCCAGAAATAGTGTACATTGTAAACTGGGGATCCTCACCCAATCTTGCTTAAGCCTAAGGCTTGCCGTGCCTTCGGTGGTCTATTACATTGTAATACTCCAGAGTTTTAATATTTCGTTTGGTTGGATGTCGTTAATGTCAACAGGCTTGTCGTTATGCGTGTCGGATCGCAACCAAATGCTGCACTATCCATGCGCTGGTTATCGGGATCTGCGTTATATCATTGGATTTGTTATGCCTGGTCCTTTTTGACGAGAGACCGGTCAATGTGTTGTTAGCATATTGAGCTGCAAAACAGAATATTGCAAGACGCTTCAACCACGTGGATTTACGTAACGTTTATGATGCCAGGTGAAGAATTGGTGCCTGTAGTTACATGTATCTGCTTCCATCGGCTACATGCAAGTTCATTCATTCATAATTACAATACAGTCGTGTACTATTGGGATGACATTTTTGTAACGCCGAAAACTACGACTGTACTGATGCATGAGCGCTAAGGTTCACTTACTACGAAGTCTTAAGGAACAGAAGCCAATAGATAACGGACTCGAAGGCAAACCAGATCTTTATTGGAGACAACATACAACACTGTGCAGCCCAATCATTTTGTAAAAAGTGAACTAAGGAGAAGCAGCAGCAAATTGCTTAGACGACGCAATACACAAGTCATTAAGCGTCATTATAACGTACGACTGAATGCATATCTTAAAAGAAAAACTCACTTACCGTCTTGCTTTTCTATGGAGTGACAGTTATGAATCTGGAAAGTAGGATGCGTAGATCCGTTTTTCTACACTTAAAGCATGTTTTCATGTCATCTTGAATTATTAAAACTAATATGTTGTAAAGCAGTTGCAAATATCTGCCCCCGTACAGCAGCAGCGTTATCGGATGTCCTAGGAGACTACTAAACGTTCTATAATACTCGAAGTGGTCTAGCTCCTTCAATAAGCAAGCGATAGTCAAAAGCGTAGTAGCGGAGCAATTACATGCTGAATGAAAAAAAAATGCTTACTTACTTGAATTTCGTGAATACACCGAATTGCGCGAGTTATTGTGAATCGTCACTGGGCTTACATTAAATTGCGACAGTAGCGTCAACAGGTTTGTCGTTATGCCTGTTGGATAGTCAGTAAGTTCTCAACTATGGATGCGCAGCGCATCATGCGCTGGTCACCATGGACAGTGATTGGGGCTGGTCCATAATGACAAGAAACCGGTCAATGTGTTCTGCTGATATGGAAGAAAATTCCTATCAGCTCCTTTACATTAAATGATGTTAATATGAATAATCCAGAGAGTTCTTTATCACTTTAAAGAACGATTTACTATTGTAATAGTAAAAACCACAAAAGATACAATCACAAGCGAATGAGGCTAAAGGAGGCGCTGTCTAGAAATTAAAGAGTGAAATTGATTGAGTTAGAATCCTAGTACAGTACCCCCTTCTACATCTAACATGCAGCCCCTACTGTAGGGCCTCCCAACTACGGGCAGCCAGCGTTTTAGAAGCTAGCATTTATATCGAACAATATTTTTCTTGAAGGAAGATGGGTACTCGCACCTATCGCACTCTTCTTTATAGCCAGTGACCTTTTGTGCGGGGTCCCGTACAGCCTTAGTGGTAACAATGTCCAATTTAGGTCGCTGGAACAGGAAATTGCAAGTGTTTCTTAGCTCGCTCGTTTATTTCATTGATAATGAAGCATGTGCTTAGAACAGACAGGTAGTCATTTAATTCTACCGCAGTCTATCGTCGTTTTACATGTTTATTGAGGCTCTGGTGCAGGCGACACATCTCTTATGGTACGTTAGATGGAACAGAAGAAGCACTGCATGGGGTGAACATCTCTAGCTGTATTAGACTACTGGTCGCTCAAACATCGTCACCTACAGCCTTGAATGTGGATTTCTTGACGATGGCAATGCTTATTTACTTGTGCGTTTACAGCGCTTGTAGACTCACTAAGCTTAACATGGTCTTGCACTTGTCGCAGATACATTATACGAACTCGTTTACCCAATCAGCTACACGAAATACCATCTCGTACAGTTTACCAGCGTTTTATCGTGTTGGGTCTACATTCTTCGTGTGATATTGCTGCGCTTAGCCAAGGTGTAAAAAAGTTTTATAAGCTCATCAACTACTACGTCTTCAACAGCAAGACACCATCATCGTCATGTTTAAAGCAACGCTGCAGCCCAGGTAACTGCTGTATTCAATAGTTACGAAAGAAGATTGTTCACAATGAGCTTGTAGTCATCCGGTTACTTGTTGTGGTAGTACCAGCTGCGGTACAGCATACGGAGATTGTCGTACTCCTCGTCAGGAATTATGTTACCGAGTTTGACAGGGTTGTAGCCGTATGCTTCCAATCTCTTAAAATAATTCAAGAGGCTTCCACTCACATTGTTGGCAGTTCGCTTCTGGGCTTTGTTTACATACACCGTTAGCTTGTCGACGTTGAAGAGGTTGGCAGCATTCGCCCTTTCCTCTTCATCGTCATCGTCGCGATAATCGTTGCTGTCGAAGCGCAAAAGCCTCTTCTCGCGCTCAGCACCAAGGTGAACTGAAACTTCTGGTGATGCATACTTCGCGATATTGGGCATGGGCTCGGAGGCTTCGGCTGCAGCATTAGCGCTTGCGATGATAGTGGTCACAGTAGCGACTACTAGATAGACGCAGGACAGACGCATTGGTGAACGGCGGTGGCTCTTGATCCGAAATGAAGACAGTTGGGGCACAAAGTGATGGCTTTCGTACGACGCAGATTGCGGAGTGAAATTGTTAGCTGCTTTCGTGTGCTCCAGCTACTTCGATGTCGAGTGATATCAATATTACAAAGTACAGGTGTCACAACGCAAGCCAGGTCTCTGTATGGGACAGTATGTGAAAAAGGCAGGGCATTGATTAGATACAGGTTACGATATCCTGAATATCCACTCGGTTTCTAAATCGGGTTATATATATTGCTTGTCTGGTCCTCTCTTTTAGACTGCTATGCTTTCTGTTCAGTAGTATATTAAACCACTTGAAAACTTCAAAAATTGGGAAGCGTTCGTCAGAAGCACGTTTCCCTGAACTATAATGTTACTGGTCGTAACGCTACACTTCACCGTCGTTGACTGTAAGCGATAGTTGGGTATAAAATAAATGATTTTTCTCGAGGGTCTTAATTGAGAGTTGGTAGAATTTTCTGGAGATTTGTTGCTTGTCTCACGCGGGCAGTAATTTAGCTTATATTGGAAGCTGTATTAGACTGTTTTCGTACCATTTGTCTCCGTAGACAGCAATGTCGTTAGTGTATCGCTGAGAACCTGTTTACTGACTGTTAGCTAGAGAGTCACGGAAGTAGCTGACATGCTGGTGGAGTTAGACTGGTTCATCGTCAATTTCGGAGCAGTTTGTCTTTCTCGTGTGTGTTTTCGTTTTGATGTGAGCCGTTTGTGTTTCGATCTAACGATTTGATCCAGAAATAGTGTACATTGTAAACTGGGGATCCTCACCCAATCTTGCTTAAGCCTAAGGCTTGCCGTGCCTTCGGTGGTCTATTACATTGTAATACTCCAGAGTTTTAATATTTCGTTTGGTTGGATGTCGTTAATGTCAACAGGCTTGTCGTTATGCGTGTCGGATCGCAACCAAATGCTGCACTATCCATGCGCTGGTTATCGGGATCTGCGTTATATCATTGGATTTGTTATGCCTGGTCCTTTTTGACGAGAGACCGGTCAATGTGTTGTTAGCATATTGAGCTGCAAAACAGAATATTGCAAGACGCTTCAACCACGTGGATTTACGTAACGTTTATGATGCCAGGTGAAGAATTGGTGCCTGTAGTTACATGTATCTGCTTCCATCGGCTACATGCAAGTTCATTCATTCATACTTACAATACAGTCGTGTATTATTGGGATGACACTTTTGTAACGCTGAAAACTACGACTGTACTGATGCATTGAGCGCTAAGGTTCACTTACTACGAAGTCTTAAGGAACAGAAGCCAATAGATAACGGACTCGAAGGCAAACCAGATCTTTATTGGAGACAACATACAACACTGTGCAGCCCAATCATTTTGTAAAAAGTGAACTAAGGAGAAGCAGCAGCAAATTGCTTAGACGACGCAATACACAAGTCATTAAGCGTCATTATAACGTACGACTGAATGCATATCTTACAAGAAAAACTCACTTACCGTCTTGCTTTTCTATGGAGTGACAGTTATGAATCTGGAAAGTAGGATGCGTAGATCCGTTTTTCTACACTTAAAGCATGTTTTCATGTCATCTTGAATTATTAAAACTAATATGTTGTAAAGCAGTTGCAAATATCTGCCCCCGTACAGCAGCAGCGTTATCGGATGTCCTAGGAGACTGCTAAACGTTCTATAATACTCGAAGTGGTCTAGCTCCTTCAATAAGCAAGCGATAGTCAAAAGCGTAGTAGCGGAGCAATTACATGCTGAATGAAAAAAAATGCTTACTTACTTGAATTTCGTGAATACACCGAATTGCGCGAGTTATTGTGAATCGTCACTGGGCTTACATTAAATTGCGACAGTAGCGTCAACAGGTTTGTCGTTATGCCTGTTGGATAGTCAGTAAGTTCTCAACTATGGATGCGCAGCGCATCATGCGCTGGTCACCATGGACAGTGATTGGGGCTGGTCCATAATGACAAGAAACCGGTCAATGTGTTCTGCTGATATGGAAGAAAATTCCTATCAGCTCCTTTACATTAAATGATGTTAATATGGATAATCCAGAGAGTTCTTTATCACTTTAAAGAACGATTTACTATTGTAATAGTAAAAACCACAAAAGATACAATCACAAGCGAATGAGGCTAAAGGAGGCGCTGTCTAGAAATTAAAGAGTGAAATTGATTGAGTTAGAATCCTAGTACAGTACCCCCTTCTACATCTAACATGCAGCCCCTACTGTAGGGCCTCCCAACTACGGGCAGCCAGCGTTTTAGAAGCTAGCATTTATATCGAACAATATTTTTCTTGAAGGAAGATGGATACTCGCACCTATCGCACTCTTCTTTATAGCCAGTGACCTTTTGTGCAGGGTCCCGTACAGCCTTAGTGGTAACAATGTCCAATTTAGGTCGCTGGAACAGGAAATTGCAAGTGTTTCTTAGCTCGCTCGGTTTATTTCATTGATAATGAAGCATGTGCTTAGAACAGACAGGTAGTCATTTAATTCTACCGCAGTCTATCGTCGTTTTACATGTTTATTGAGGCTCTGGTGCAGGCGACACATCTCTTATGGTACGTTAGATGGAACAGAAGAAGCACTGCATGGGGTGAACATCTCTAGCTGTATTAGACTACTGGTCGCTCAAACATCGTCACCTACAGCCTTGAATGTGGATTTCTTGACGATGGCAATGCTTATTTACTTGTGCGTTTACAGCGCTTGTAGACTCACTAAGCTTAACATGGTCTTGCACTTGTCGCAGATACATTATACGAACTCGTTTACCCAATCAGCTACACGAAATACCATCTCGTACAGTTTACCAGCGTTTTATCGTGTTGGGTCTACATTCTTCGTGTGATATTGCTGCGCTTAGCCAAGGTGTAAAAAAGTTTTATAAGCTCATCAACTACTACGTCTTCAACAGCAAGACACCATCATCGTCATGTTTAAAGCAACGCTGCAGCCCAGGTAACTGCTGTATTCAATAGTTACGAAAGATGATTGTTCACAATGAGCTTGTAGTCATCCGGTTACTTGTTGTGGTAGTACCAGCTGCGGTACAGCATACGGAGATTGTCGTACTCCTCGTCAGGAATTCTGTTACCGAGTTTGACAGGGCTGTAGCCGTATGCTTCCAATCTCTTAAAATAATTCAAGAGGCTTCCACTCACATTGTTGGCAGTTCGCTTCTGGGCTTTGTTTACATACACCGTTAGCTTGTCGACGTTGAAGAGGTTGGCAGCATTCGCCCTTTCCTCTTCATCGTCATCGTCGCGATAATCGTTGCTGTCGAAGCGCAAAAGCCTCTTCTCGCGCTCAGCACCAAGGTGAACTGAAACTTCTGGTGATGCATACTTCGCGATATTGGGCATGGGCTCGGAGGCTTCGGCTGCAGCATTAGCGCTTGCGATGATAGTGGTCACAGTAGCGACTACTAGATAGACGCAGGACAGACGCATTGGTGAACGGCGGTGGCTCTTGATCCGAAATGAAGACAGTTGGGGCAAAAAGTGATGGCTTTCGTACGACGCAGATTGCAGAGTGAAATTGTTAGCTGCTTTCGTGTGCTCCAGCTACTTCGATGTCGAGTGATATCAATATTACAAAGTACAGGTGTCACAACGCAAGCCAGGTCTCTGTATGGGACAGTATGTGAAAAAGGCAGGGCATTGATTAGATACAGGTTACGATATCCTGAATATCCACTCGGTTTCTAAATCGGGTTATATATATTGCTTGTCTGGTCCTCTCTTTTAGACTGCTATGCTTTCTGTTCAGTAGTATATTAAACCACTTGAAAACTTCAAAAATTGGGAAGCGTTCGTCAGAAGCACGTTTCCCTGAACTATAATGTTACTGGTCGTAACGCTACACTTCACCGTCGTTGACTGTAAGCGATAGTTGGGTATAAAATAAATGATTTTTCTCGAGGGTCTTAATTGAGAGTTGGTAGAATTTTCTGGAGATTTGTTGCTTGTCTCACGCGGGCAGTAATTTAGCTTATATTGGAAGCTGTATTAGACTGTTTTCGTACCATTTGTCTCCGTAGACAGCAATGTCGTTAGTGTATCGCTGAGAACCTGTTTACTGACTGTTAGCTAGAGAGTCACGGAAGTAGCTGACATGCTGGTGGAGTTAGACTGGTTCATCGTCAATTTCGGAGCAGTTTGTCTTTCTCGTGTGTGTTTTCGTTTTGATGTGAGCCGTTTGTGTTTCGATCTAACGATTTGATCCAGAAATAGTGTACATTGTAAACTGGGGATCCTCACCCAATCTTGCTTAAGCCTAAGGCTTGCCGTGCCTTCGGTGGTCTATTACATTGTAATACTCCAGAGTTTTAATATTTCGTTTGGTTGGATGTCGTTAATGTCAACAGGCTTGTCGTTATGCGTGTCGGATCGCAACCAAATGCTGCACTATCCATGCGCTGGTTATCGGGATCTGCGTTATATCATTGGATTTGTTATGCCTGGTCCTTTTTGACGAGAGACCGGTCAATGTGTTGTTAGCATATTGAGCTGCAAAACAGAATATTGCAAGACGCTTCAACCACGTGGATTTACGTAACGTTTATGATGCCAGGTGAAGAATTGGTGCCTGTAGTTACATGTATCTGCTTCCATCGGCTACATGCAAGTTCATTCATTCATACTTACAATACAGTCGTGTATTATTGGGATGACACTTTTGTAACGCTGAAAACTACGACTGTACTGATGCATTGAGCGCTAAGGTTCACTTACTACGAAGTCTTAAGGAACAGAAGCCAATAGATAACGGACTCGAAGGCAAACCAGATCTTTATTGGAGACAACATACAACACTGTGCAGCCCAATCATTTTGTAAAAAGTGAACTAAGGAGAAGCAGCAGCAAATTGCTTAGACGACGCAATACACAAGTCATTAAGCGTCATTATAACGTACGACTGAATGCATATCTTACAAGAAAAACTCACTTACCGTCTTGCTTTTCTATGGAGTGACAGTTATGAATCTGGAAAGTAGGATGCGTAGATCCGTTTTTCTACACTTAAAGCATGTTTTCATGTCATCTTGAATTATTAAAACTAATATGTTGTAAAGCAGTTGCAAATATCTGCCCCCGTACAGCAGCAGCGTTATCGGATGTCCTAGGAGACTGCTAAACGTTCTATAATACTCGAAGTGGTCTAGCTCCTTCAATAAGCAAGCGATAGTCAAAAGCGTAGTAGCGGAGCAATTACATGCTGAATGAAAAAAAAATGCTTACTTACTTGAATTTCGTGAATACACCGAATTGCGCGAGTTATTGTGAATCGTCACTGGGCTTACATTAAATTGCGACAGTAGCGTCAACAGGTTTGTCGTTATGCCTGTTGGATAGTCAGTAAGTTCTCAACTATGGATGCGCAGCGCATCATGCGCTGGTCACCATGGACAGTGATTGGGGCTGGTCCATAATGACAAGAAACCGGTCAATGTGTTCTGCTGATATGGAAGAAAATTCCTATCAGCTCCTTTACATTAAATGATGTTAATATGGATAATCCAGAGAGTTCTTTATCACTTTAAAGAACGATTTACTATTGTAATAGTAAAAACCACAAAAGATACAATCACAAGCGAATGAGGCTAAAGGAGGCGCTGTCTAGAAATTAAAGAGTGAAATTGATTGAGTTAGAATCCTAGTACAGTACCCCCTTCTACATCTAACATGCAGCCCCTACTGTAGGGCCTCCCAACTACGGGCAGCCAGCGTTTTAGAAGCTAGCATTTATATCGAACAATATTTTTCTTGAAGGAAGATGGGTACTCGCACCTATCGCACTCTTCTTTATAGCCAGTGACCTTTTGTGCGGGGTCCCGTACAGCCTTAGTGGTAACAATGTCCAATTTAGGTCGCTGGAACAGGAAATTGCAAGTGTTTCTTAGCTCGCTCGGTTTATTTCATTGATAATGAAGCATGTGCTTAGAACAGACAGGTAGTCATTTAATTCTACCGCAGTCTATCGTCGTTTTACATGTTTATTGAGGCTCTGGTGCAGGCGACACATCTCTTATGGTACGTTAGATGGAACAGAAGAAGCACTGCATGGGGTGAACATCTCTAGCTGTATTAGACTACTGGTCGCTCAAACATCGTCACCTACAGCCTTGAATGTGGATTTCTTGACGATGGCAATGCTTATTTACTTGTGCGTTTACAGCGCTTGTAGACTCACTAAGCTTAACATGGTCTTGCACTTGTCGCAGATACATTATACGAACTCGTTTACCCAATCAGCTACACGAAATACCGTCTCGTACAGTTTACCAGCGTTTTATCGTGTTGGGTCTACATTCTTCGTGTGATATTGCTGCGCTTAGCCAAGGTGTAAAAAAGTTTTATAAGCTCATCAACTACTACGTCTTCAACAGCAAGACACCATCATCGTCATGTTTAAAGCAACGCTGCAGCCCAGGTAACTGCTGTATTCAATAGTTACGAAAGATGATTGTTCACAATGAGCTTGTAGTCATCCGGTTACTTGTTGTGGTAGTACCAGCTGCGGTACAGCATACGGAGATTGTCGTACTCCTCGTCAGGAATTCTGTTACCGAGTTTGACAGGGCTGTAGCCGTATGCTTCCAATCTCTTAAAATAATTCAAGAGGCTTCCACTCACATTGTTGGCAGTTCGCTTCTGGGCTTTGTTTACATACACCGTTAGCTTGTCGACGTTGAAGAGGTTGGCAGCATTCGCCCTTTCCTCTTCATCGTCATCGTCGCGATAATCGTTGCTGTCGAAGCGCAAAAGCCTCTTCTCGCGCTCAGCACCAAGGTGAACTGAAACTTCTGGTGATGCATACTTCGCGATATTGGGCATGGGCTCGGAGGCTTCGGCTGCAGCATTAGCGCTTGCGATGATAGTGGTCACAGTAGCGACTACTAGATAGACGCAGGACAGACGCATTGGTGAACGGCGGTGGCTCTTGATCCGAAATGAAGACAGTTGGGGCACAAAGTGATGGCTTTCGTACGACGCAGATTGCGGAGTGAAATTGTTAGCTGCTTTCGTGTGCTCAGCTACTTCGATGTCTAGTGATATCAATATTACAAAGTACAGGTGTCACAACGCAAGCCAGGTCTCTGTATGGGACAGTATGTGAAAAAGGCAGGGCATTGATTAGATACAGGTTACGATATCCTGAATATCCACTCGGTTTCTAAATCGGTTTATATATATTGCTTGTCTGGTCCTCTCTTTTAGACTGCTATGCTTTCTGTTCAGTAGTATATTAAACCACTTGAAAACTTCAAAAATTGGGAAGCGTTCGTCAGAAGCACGTTTCCCTGAACTATAATGTTACTGGTCGTAACGCTACACTTCACCGTCGTTGACTGTAAGCGATAGTTGGGTATAAAATAAATGATTTTTCTCGAGGGTCTTAATTGAGAGTTGGTAGAATTTTCTGGAGATTTGTTGCTTGTCTCACGCGGGCAGTAATTTAGCTTATATTGGAAGCTGTATTAGGCTGTTTTCGTACTATTTGTCTCCGTAGACAGCAATGTCGTTAGTGTATCGCTGAGAGCCTGTTTACTGACTGTTAGCTAGAGAGTCACGGAAGTAGCTGACATGCTGGTGGAGTTCGACTGGTTCATCGTCAATTTCGGAGCAGTTTGTCTTTCTCGTGTGTGTTTTCGTTTTGATGTGAGCCGTTTGTGTTTCGATCTAACGATTTGATCCAGAAATAGTGTACATTGTAAACTGGGGATCCTCACCCAATCTTGCTTAAGCCTAAGGCTTGCCGTGCCTTCGGTGGTCTATTACATTGTAATACTCCAGAGTTTTAATATTTCGTTTGGTTGGATGTCGTTAATGTCAACAGGCTTGTCGTTATGCGTGTCGGATCGCAACCAAATGCTGCACTATCCATGCGCTGGTTATCGGGATCTGCGTTATATCATTGGATTTGTTATGCCTGGTCCTTTTTGACGAGAGACCGGTCAATGTGTTGCTAGCATATTGAGCTGCAAAACAGAATATTGCAAGACGCTTCAACCACGTGGATTTACGTAACGTTTATGATGCCAGGTGAAGAATTGGTGCCTGTAGTTACATGTATCTGCTTCCATCGGCTACATGCAAGTTCATTCATTCATACTTACAATACAATCGTGTATTATTGGGATGACACTTTTGTAACGCTGAAAACTACGACTGTACTGATGCATGAGCGCTAAGGTTCACTTACTACGAAGTCTTAAGGAACAGAAGCCAATAGATAACGGACTCGAAGGCAAACCAGATCTTTATTGGAGACAACATACAACACTGTGCAGCCCAATCATTTTGTAAAAAGTGAACTAAGGAGAAGCAGCAGCAAATTGCTTAGACGACGCAATACACAAGTCATTGAGCGTTATTATAACGTACGACTGAATGCATATCTTACAAGAAAAACTCACTTACCGTCTTGCTTTTCTATGGAGTGACAGTTATGAATCTGGAAAGTAGGATGCGTAGATCCGTTTTTCTACACTTAAAGCATGTTTTCATGTCATCTTGAATTATTAAAACTAATATGTTGTAAAGCAGTTGCAAATATCTGCCCCCGTACAGCAGCAGCGTTATCGGATGTCCTAGGAGACTGCTAAACGTTCTATAATACTCGAAGTGGTCTAGCTCCTTCAATAAGCAAGCGATAGTCAAAAGCGTAGTAGCGGAGCAATTACATGCTGAATGAAAAAAAAATGCTTACTTACTTGAATTTCGTGAATACACCGAATTGCGCGAGTTATTGTGAATCGTCACTGGGCTTACATTAAATTGCGACAGTAGCGTCAACAGGTTTGTCGTTATGCCTGTTGGATAGTCAGTAAGTTCTCAACTATGGATGCGCAGCGCATCATGCGCTGGTCACCATGGACAGTGATTGGGGCTGGTCCATAATGACAAGAAACCGGTCAATGTGTTCTGCTGATATGGAAGAAAATTCCTATCAGCTCCTTTACATTAAATGATGTTAATATGGATAATCCAGAGAGTTCTTTATCACTTTAAAGAACGATTTACTATTGTAATAGTAAAAACCACAAAAGATACAATCACAAGCGAATGAGGCTAAAGGAGGCGCTGTCTAGAAATTAAAGAGTGAAATTGATTGAGTTAGAATCCTAGTACAGTACCCCCTTCTACATCTAACATGCAGCCCCTACTGTAGGGCCTCCCAACTACGGGCAGCCAGCGTTTTAGAAGCTAGCATTTATATCCGGAGTCGTTACTACAACTTTACCACAATTACACGATAGGTGGTTTA

>Contig_77

GGTTACCGAGCTGGGAGTAATGTTCAGAAGGTCAGCAACGTGCTTGACGCATTCTCCCCAAAGAACCTGCGGTAGGTTACCATCAATGAGGAAAGCCCTGGTTCGTTCAAGTAGTGTACGCATGCGCCTCTCTGCAACTTTATTTGCTGGGGCGTGTACGCATTTGTAAATTGTGCGTGCGTATTGTACTTCTGAAAAATCATTTTTCCAAGTTTCTCATATTCTCGCTTTGAAGATGTCTAACTTCTTCATCGTGTATGCTCTCATTGAACTGGAGTTCGCCTACACCAGCGCGAAAAAACGTTTTGTGCTTTCTTGCGAAAATCTTCATAGCATTCGTACAGCTCAGACTTTCGCTTGATGATGTAGATGAACATGTACCCGTGAGTAGTCATCTATAAAGGTCAGAAAATATTGACACCCCGAGAGTGTGGGGACGGGTAATTGACCAGCCATATCAGAATGAGCTTTTTCAAGAGGGAACTTCGCCCGTTCAACGGTACGTTTATTGAATGGGTTACGATGGGCCTTGGCGTATGTGCAGGAAGAGCAAAAGTAGTCTTTCTTGGAATCAAAGTCCTTCAGGCTCATGTCCATGACAGTGTTGGCTTTAGCCAGATCCTGCATAATGCGAAAGTTAGGATGACCCATTCGTTTATGCCAAAGTAAAACATTATCAGGCTTACCTGAAGTGACCACATGGGCTACCTGACTTGAGGTTGCCGGGCTCGCTTGAAACTGGTAGAGTCGAGTCCCTTCACCAATTTTGGCTTCAAATTTGGTTCGGCTAGCGAAGAACAGAACGCACTTCTTTGCGTTGGGGAAGTTGATTTTGAAATCATCTTCGACGGCTTGGCGCACGGAGAGTAGGTTGAACTTCAGCTGGGGCGCTTATAGCACGTTGGCCAAGGATATTTCTCGTTCCTTTCCCTTCGTGTCCTTGATTGTCATCTTGACATCACCAGTTCCTTTTATCGGAATCTGGTGATTGCCGCCGACAGTGATTGACTTGCCAGCACTTGGAATCAGCTTCTCAAACCACTTCGGATTGGACGTAGCGTGGCGAGTGCAGCCTGAGTCGACAGTCCACACTGGGTCATGCTCCGCATCTTGGGCGGTGAGACTTACAGCCTCTGTTTCTTGTCTGAGGTCCAGAGTGGTGATGGCTATGATTCCATACTCGTTGTCGGAGTCGGTGCCCTTGCGGCGACCGTGATATTTATTGTGACTCTTGGATTTTCCAGATGTATTCTTCTGGTTGCCGTTCTGGCTTCTATTTCGATTGCTGTTCCGCTTGCTCTTGTAGTCATTCCGATTTTGGTTTCGTGACTTGCCGCCGAAACCGTCCTTGTAAGGATGCTGTCGAGCTTGATTGGACGAAGGTTTCAGCTTAAAGTTGGCGGGAAGCACCGTTCCTGCCTTTACCTGACCATTTTGTAGGTGGCGCTGTAGAACATAGCAGTCGACAGTATCGTGGTTGGTGCGCAGGCAGTATGTACAGCTCATCGTTGAGATGAGAGCCTTGCTCTCTTGATAGGGAGTTTGAACCAAGGTTGGTTCAGGTGCTAAAGCCTGTAGGGCCTGCTCGTTTCGGGTTTCCCTCGATTCGGGTGTTCCTTGTTTGAGCGTGTAGCGGTTTTTCGCCAGTTCATGCTGCACCTTGGCCTCGATGTGACGCTTTAGCTCTTCGTAGGGAATGAATTTCCTGCTACCCTTCCAGACCGATAGTTGCTGCTTCCATTTTACAGGTAGCGCATGGTACAAGTAGAGTGACTTCTGTTCGTCACTGAGCACACTGTTTGTGGCATCGGAGGCAACCTTCATGGCAGATTCGATGTCAATTATGAACCCCGTAAGGTCTGAACCTTCTTCATACCTCAGTGCCATCAGGTATGATAGCACATGATACGGGCCGCCGTGGATGGCAGCACCTTCGTACTTGCTACAGATCGTCTGAAAGATCTCAAACGCAGTAGTCTCGTCCTTGACCATGAGAACATGTTGGTCATCGATGGTCTTGATAAGGAAAGCTTTTGCTTTTGCCTCGTTGAGTCGAAGCTTTTTCGAGCTCAGCCTCTGGCTTTTAGCCTTCAGCTTCTCTGCTCGCTTCAGTTCATCCCGCTTCTGAGCAGTGAATGACTGGATAACGGGGGGATTCTCCTGCCCCATTTCCACATCGCCGTCGTCTCCGGCTGTGGGTGCATCCGAACTTGCGGAGCTGGCATCAGAGGAGGACTCGCTCGGTGAGGAGCCCACCATTTCGTCGGCCTTGGGAGCACCGGCAGCATCAAGCGCGGCGGTCATGTCGTTCATGTCGGACAGTGCCGGGTTGAGTTCTTCATCGGAGTCGAACTCGTAGTCGGAGTCACCGGTGTAGTCGATACGCTCGACGTAGCCGAGAAGGTTCTTTCCTTCGAGGGCGGCAGTGACGCGGGTCTTCCAGATGATGAAGTTCCGGCCATTGAGGCGCGGAAACTCGCGGTCGTCAGCGGTGGAGGCTTGCGTTGGGCTCATAACCTATTGAAGGTGAGGCGAGGAAGGTGGAGGAGCTTGAGTAGAAGGAAGAAAAAAGAGATGTGATTGATTTTTATGAGTCTGTCTTCAGAGAGTTTCCTGGCACCATTGATTTATCTTTCCTGTAAAAGGTGATGTATCGACTTATGCAGGATTGGGTCCAGAACATATTTTGTATGGTAAGACATTTTGTACAATAACTTGTACGCTATGTCACCAATTAGGACACATCATTGACACATCCTATTTTTTGTGTTCGCTAAAAAATATAGAGTGCTTTGGATGGCTCTCTAGCGGGATGGGATGCCTGCGATTTCGCGAATCCGCAGGACTTCCAACCCGGTGTTATAGACAGGTGGATCGTGTCGGTGGTGTGTCACACACACTGTGTGCTGCTGTGGAATAGACGATTGGACTTGAGATAAGCCCAAGACCGTGCCACACAACGTCCCAGTGGGACGAGAAGGGGTGTCAACCTTACATGTCATCCCAATATCCCGCAAACTAGATCGCTGATTAGTCCATCCCTCTTTTGGGACGATATAAGACTGAGCCTAACGCCTCTCCCTGCGAGAACGGGTGCAGTACAGAACGCTACTGCGACATTCGCATTACATTAAAAATACAGACAGTACATTTTCTTTTAATACCGGTACGGATGCACCAGTACGTTTTATTAATAGAGAGAGCAAGTTCGAATCGAGCTTCGATGAACATGTATTTGTGATGAAAAAGGACGCAATAATTACATAAAGACTCTTTATTGTTTACGCCTCTTTATTAAAGAGGCGTAAACAGTTTTAAAAACAGCTTTCAGATACGGTGTTATTAATAAGCAGATTTTATTTTTTGAAATCTGTCAGTGGAATTTGGAAGCATTGCCTCTACTCCTGAACATGTCCACAAACTGGAAGTCGTGAGCTCTGGTTTTGGCGATGAGGCCAAGAGAAAGTGAACCGAAGATCTTATCCGAAGATTACCAGATGGTATTGCAGTACACCTATTGCAGGTACCGGTACATGTACATATTTTCACTACAGTAAGACGGGTGTCACATTAATTACTGAGGGGTGCCTCACTCCGCAATTGGACATCTCGAGTAATCACCATGACTATCAAGCCCAAGCGCAAGTTGTTTTTTTATCAAGTACTGCTGGTGACTGTAGCTCTATTGATCTGTGCCAGTAAGGCATTGACGGCCATCGATTCCAAGAATCTCCCCACCCTCGACAGAATCAGAGCCGACCGGCGATTGAGGGCGACGGTAGATGGTTACGACTACAACTACGAGTCCGAAGACAGAGCCTTCACCGGCATTACGAAGCTAAAAGAATTTGCTCAGGCTGGGACGAAGAAGCTGCAGAAGGCCGTTGATACTGCCAAGACGAAGCTGACGTCCAAGCCCACTATTGACCAGCGCTTTAAGCAGTTCAAAGTCGATCAAGTCGAGTCCAACGTTTTCGAAAGCATGCAATTTAATGCCTGGGCCAAGTCTGTTGCAAAAACGACCAAGAACAACCAGGACGCCACCGACGCTGCAATGCTCGCAACGTTGGCGACTCATTACGGCGATGAGACTCTTGCCCGTATGCTAGATGCCGCAAAACAGGTGTCAAGTACAAAATCGACGGCCACTAGACTGGAAAATGCGCAGATAAGCAAGTGGGTGGATGACGGGGACGCACAACTCAACAAATGGCTTGCTGATGGAGTAAGTGCGGACAGCGTTTATAAGCTTCTGCGGATTGATGCTGAAGGCAGTAATCTACTGAAAAGCCCCAAGGTGAACATGTGGATGAGCTACCTGACAAAGTTGAACAAGGACCCATACGACGTCCTACTTTACAAGGTGAGAGCGCACTATGACGACGTGGGACTAGCGAAAATGTTTGTTCTGTCCAAGAAAGACTCTTCAACAAAAGTGCTGGCCGAGAAGCTCGAGACGCTGCAGCTCGAGAAGTGGATGAACAATAAAAACAGCGCGGCTGACGTCTTCCGGATCCTGAAGTTAAACCAAGAAAGCACGACACTTCTAAAAAATCCAGTGCTAACCACATGGGTCGCATACGTTGAAAAGCTGCAAAAGAATCCCTACGAAATGCTGTTTTCAGCGATCAAGGCAAAGGGCTTCGACGACGTAGAGTTGGCGAGGCTCATCACCGCAGCAAAGCAAGACCTCCATACAGGGACTGTTGTCGCGAAACTGGAGAAAGTGCAGCTCCAGAAATGGGCCACGGATGGGAAGACCAGTGGAGACCTCTTTAAGTACCTCGGACTGTACAAAGCGGGTGACAAGTTTCTTGATAGTCCAGTGCTGAACAATTGGTTCTCTTACATGGAAATGCTGAGGAAGGACCCCTACACGATGCTAGTCCATACAATCAGGAAGTCAGGCTTGGACGAAGTAGACTTGGCGAGGCTTGTTAACAAGGCCAAGCAAGACACCAATTCGAAAACCATGGCTGCGAATGTGGAGAAGATGCAACTTGGTAAATGGTCGGTAGATTCGAAAACCAGCGACGATGTCTTTAAACTCCTTCGACTCGACAAAGAAGGAGACAAGGTGTTCGAGAGCCCAGTGTGGAGCACTTGGACCGCATATCTGAACAAGGTGGAAATTGACCCTGACGCAGATCTGGTCATGTACACAGTACTGAGGAACAAATTTGGTGACGAAGGGTTGGCAAATCTGGTTGCGAAAGCGAAGCAAGTAGCGAACACCAAAGAGACCGCTGAGAAGCTGCAGCTGGAGATTTGGCGGGTCGGTCAGAAAAGCTCAGATGACATTTTCAATCTTCTCAAGTTGAACGAAATGGGTACAAAGCTGTTCGAAAACCCGGGGCCATTAAGGACGTGGATAGCCTATGTGAACAGGGTGAATAGCTTCAAGAGAAATAGAGTGAAGGTGTTCCAGCCGATCATTCAATTGGAGAAACGCTTTGGAGAAGAGGAGCTAGCGGTGCTGTTGGTCAACTCGAAAGCGAAACACTATTTGACCAAGGCTGGTATAGCCGAAGATTTGCAAGAGTGGCAATTCAAGAAATGGATGGTTCACAAGACAAACGTCGATAAGATGTTCCCTTTTGAAGACCATACCAGCATACGAATTAAATGGGAGTACAAGCAGTTCTACAAGGAGAATGCCGATTCATTGATCATTTAGACACGATACAAGGCAAAGTAGTTCTCTTAGCGAAAAGTGGTTCACCGCTAGCTTGACAGTACTTTAGAAAGGATTCCAGGTTCGGATAATAAACGGGATCCATTAGGATAGATAGTTTTAGATTGAACAAAATGAACCCATATGTGTTACTGCTGTAAACCATTGTTTTCTAAGATAGCAAAATCCGATCCTACTAAGCTGGCGGACGTGGCGCCGACAGGGGCGTAGGATACGATTTGAAATGGAGCGAACAAAACATGAAAGGTGCTAATTATAGTTGGCTAAGTAGATAGTTTTAGATTGAACAAAAAGAACTCATACGTGTTACTGCTGTAAGCCAGAGTTGCTTAGATAGCAAAATCCGATACTACATAATGGGTGGACGTGTCGCCGACAAGGGCGTAGGATACGATTTTAAATTGAGCGGTAAGACAACAACAAAACACGAGAGGTGTTAATTATAGTTGGCTAAGTACATGTAGATTTCATAAAAAAAGCTAATAAATTTGGATCCTAAATGACATTAACCATCCTGGAATCATCGCGTCTTGCGACGCATTAACCAGATGCTTTAATCATAACTGGCACAGATTAGAGTGTCTAATGATTCATTCTAAATCAGCACAAAATGAGCGCTAAATGTATTAAAATGAGCAAAAATTGAATATTTGTTGAGTTTGGTATGAGAAGTGATCAATCTTTAGCTACTTACAAGATTAAACATGCAGTACAAGCGGGTAAGTAGTGCAAGCTGACACGCCATTACTATCTTTTGCGCTGATGTGAGCTAAGTTTCATGGCTCAGAAAGTACGACGCTAAACTAAACATATTGTGTAGCTTTTCTTATAAATGTATTTGGAGTGCAGTGGTTTAGGGTGGAAGATGAAGATACAATGATAGCCAACAATGATAAAGAGGTTGTAGTAAGAAGCTTGAATCAGGATCATTAGAATAATTATGCTTAATCAGGTAAGCATGATGCTGACCCAGATTACTTTATGAAATTAAAAATATCGAGGCGGCATAGGGGGCCTAACTTTTGTAATACTTATCCCGCCGAAGCGTAGCGGAGGCGGGAAGGCCACAACAAGTCTAACTAAACCTAAACTACCGCTGCGTAGCGGCGGGTAGCAGTGTAAATAACGCCACAACATCCAAAATTTAGAGTTATTAAAACAGCATTAAATTGCGTGTGCACCACACACGGGTGTGTTTCTTACACTAAACCGTGTGGAACACATTTTTTCACACTTAGCAAATATTTCAAAGATCCAACATCGCAGCAAGGATGGACGGAATGTCGCTCGCTCGGGAGCTGGTGCGCGCGCCCATCTACGAGCTCATGGACATGGAGAACACGCTAGAAAAGATCGCGCAAGCCTCGAGAAGATACTAATAGCTCAGATGGAGTTACTGTCACGCATTGAGGTGAGCGAGGAGAGCATCTACGTGCTGGCGTCTGAGATTGCCAACGCTGGTGCGAGTCATGACGCCCACCTATTCTTCGGAGTTTTGCTAGAAATGGCAGTTAACTGATTGGATTGGTAGTACAGTAGTATGCTAACGGTTTTGTATTAACGGATAGCGCAGCTACAGCTACCTACTTCAGTTGCTTGAGCAGTTTCTCATGAATAGACTTCGTCTTCTCTGCAGTGCGACGGGCCATACGGCTCTTTCTCGATTTACAGAGCATTTGCAGCGGGGTGCCGTAGCTTTAGCGTCAGAGGAAGACGTTCGACTCGAAGAGTCACTAATACTAAGTTCCTTATCCAAAGGAACTTACAGCCCACGACCAGGGCTCAAAGTTAATACCTTAACTTGTATGCTTCAAAACCAGAAGTGTTTTATGTACTATTATTGCGGCCAAGCCGCCTAGGGGTAAATGTGTCTGTTTAACTATCTAGCATAGTTAAGCCTAATCAAATGCATAACGTGTGGCTTTCAGCTAACGTACTGAGAGACTATGTTAACTTTCCGCCTTGTCTACTTAGATTTTGTTAACGCTACGGGAACTCCGTACCCCTGTGTGTATCACACCATGTAACCCCGCTACAGTATGAACCACAGCATTCGGAGGCGCATAGCTTCCAAATCCCGCTCGGAAATGTTTCTGCATTGCCAGCGAAGATTCACGGTCACATAGCTGTAAGGCTGGGCCACAACAAACACGCCACAGCTTGAGTAGTCGGGCTGCTGTGGAACTTCAATCCACTCAATAGGGTTCATGCATACTT

>Contig_78

GTGTCTGATACAGGCCGTCGCGGTCAAGGGGTTAAAACTGGAGCAGCAGCCGTCGTGCGGAGCTTGAAATTTGCCCTCACTCCAGCGTGGCACCACCCGTGGCATGTTGTCGTCATTGACCGGTATTACTCGTCCGTAATACTTGCTGTTGAGCTACTAAAGATGCAAATCTATGTGGTTGGGACTATTCAGACGAACCGCCTGGGCTTCAACAAGAAGACCCTGTCGAAATCAAGATACGCCCTGCAACCATCCCCCGCGGTCCATTCGTGTATTCGCGTTCGGTCACCGTTCCCAGCATGGTTTCGTACCTCTGGTAGGATCGCAAAGCAGTATACTATTTGTGTACTGGCTCCGTTTTGATGCCATCCCTAATTGAGAGGAAGGTCAAGCGAGTTGGCGCGATATAGGTCGGGTGGCCTTCGGCGGTAAGCGACTACCAGAACTGGATGGGCGGAGTCGATCGCCTTGATCAACTCCGCCTCCAGTCATACTCGCTTCAGATGTCGACGCGGTTTTCTCAAGTATTACAAAAGCCTTTTTCTTGACGTCCTTGATCTCGCGCTGGTAAACGCGTACCTCTTTCACAAAGAGGCGGCCAAGGTGAATCAAACGGTAGCTACGAGGCGTTCAGAGTGGTTTTCGGTCCTTCCAAACCAGCTCCAACAGCTAAAGGCCAAAGATTTTGCGGGGATAGAGGTGACACCACCTCCGTCAAGTCACAAGCGACGGCGCGCACCAGTTCGGCCTACCCATAGCGGTCAGTAGTCTAAAGATTGGGTCACCGTTAGCGGCGTCCAAAAACGCCGTCAGCGATCTTGTAAGGTCTGCGCGCTCTTGCGTACAGACAAGAGGTAGTCGTTTGCAACGCCTTATTATTGCGTGCGCTGCTCTATCGGCAGTGCAAAGTGCTGGTTTTGCAACAGGATTCGCCGAGACTACAAGGAAGTTGCCATAACCTGTTTTGAGATCTGGCACGACGACTTTCAGGCCGGCCAAGTGACCCCCACCTCCAACTTAGAAACGTGAGTTGTGCTGCGTCGACCGGGCCAAAGTGCTGGAAGTGCAAGAAAACGAAGCGGCGGGAACGCCATCTTCGCCTTGATCAAGGCAGCGACAGCAGAAACAGTGGCGACGATGATAATGATGGCAACGATGATTAGTTTAGGCTGCGTATCAATTGATGAGTGGTTAGTTTCGTGTGGTTAAATGCAACATTTCTTGAGCTGACGTACCTTCTTCCTTGCGCTTTCTGAAATGAAAGCCCTGGCATTGCATCATCTTAGCCATCTATTGAATGGATTAGTAGATGGTTACGACATTTCTTGGCCTCTGTTGCTTCTCAATGTCGTATGAGCTACGGCATTGAATCGGAAACCTGCTGAAAGCTTATATGCCAATCTCGATAGCAAACACATGGTCAATACAAACACCAAGAGCATATGAATGGTGTATTTTACGACATACGCTTACATGTCTTTGGCGAATGCCGTTCGGAGCCAGTGTGTTAAGAAAAGGCAAACGTAAGAAATGAGCGCCCATAATTCGACTCTAAACAGGGCGTCTGTTTAGCCACTTACTTTTTTCTTGTCCAGTTAACATGGTTAAGCACTTTGAACCAACCGCCTAGATGGCGTCACAATTGCTGGCACAACGTCAGCTTCCTGTGTTGCAAGAACATGTATAAAGTACTGTGTCAGTTACGTGTACAATACAATTTGTATTTAATCTTCTTTTCTATTGGATGTATGTTTTTTGTCGAAGCGATTTGATTTTCATCTTTACAGCCGAGGAAACTGGCAACCCCGCTTAAATGAGGACATTTTTCAACGCATGGTCATTTGTGACCCAAAGTAGGCAGCGCATTTTAGGAGTGTCTTTATTGATAAATAGGACATATCGCTGAAAAGTAATGCAGACTCAAATTTAGGCCTGCCAAAAGTGGCACGTAACTGGCACAATACAGTAGCTGATACGGAACTTTAAGGACAAGGAAAACTAGTAATTAATTTGATGTCAGTAAACAGCAACCAGCAGCGTTAATATAATTCATCCTGCATCGAGAACACATACGATTTGTATGTGTTCTCTATGCAGTCGAAGTCGGTACTGAAATATTATGTACCAAAACCTGTAACTAGTCCAATTAAGATTTAGTCTGTCACAGGCCCACCAACAAGTATATTGCCGAAAATACAAAACGACAGTAATCCATCTAAACGCTATCATGTTATTCAATATTTTAAGTGTTTTGGAGAATGCGATGCTCCATTGCATTTTCACAAGTATTCCTGACATAGAGTCGAAATTAGTGGAGGTCTACACGTGTATACATGTAATTGGGGCTGCACGTAGGCGAAGAAGCGTTGAATGACCTGGTGTTTCACAGTCTAGCAAATGATTACTTATCCATGAGTCCAGGAGAAACTTTCTAACTTAAATCCATATAGGTAGACGATTTCAGTATGATAGGCCTGGGTGCCTCCACATTAGACCTGGAAACGCACAGCAAAGTACATCTGCATGTTATGCTTCTGTAAGCCGGCATGCTTCCTCACCTGGTGCATTATACGGATTTTAATAGCTCAAACACGATGTGCTGCTATGCCTGATAGAGAAGTCGTCCACAGGTTAAATCAGGTGTGAAAGGTCTTACTCAATTATGCATTTCTGTGTCTCGCAAGCCGTTCTCAGCAGTGTTTTTGCATTTTATGTCCTGTTATTGTAGGGACCAATGTATTTCTGAAAAACGGTTTGTACTTTCCACCGTCGAGACTACGTGGGGACTCTTACGCCGACCGTTTTCAAGTAGTCAGTGTACATTTCCAATGTCTTAGCCGTCAGGGCATCGTCGGCATCTGGCATTTCTTTCAGTATCTTGGCCAGGTCGTCGGGACTCTTGTTCGCATCGTCAGCCTGCTTAAACAGATTATTCAGCTTGTTTTCTATAACAGTCGAAATCCTCTTGAGCTCCCGCAAGTCGTCAATTTTCTCGAGATCCGAAAACGACTCCTGCTTTATCATCTTGTTAACAAAGCTGTTAGTCATCGCTAGCTTAACAAGCTCCAGAAAGTTTCTCTCTTCGTTGTCATCTTCGTCGATGTTGCCGCCTCTGAGAAACCGGGACTGGTCGCTTACACTTTGGTCAGCGCCAACGAAGTGAATGAAGCCCAGTGACAGTACACCAGTTAGCGCAACATCATTAGCGAAGTATGGGGTAGAGATGACGTGGCGGGCGAATAGAATCGACGCAGCGGCCAGCAAGACGTAAGTTAGGCGCATTGCTGGTGTCTGGATGCTTTAGGTGTCGAAAGCGAGTCAAATTGCGAAGTGAGAATTGGCATCCAAACCACACAACAAACGTGTGGTCAGAATTCCTATAACACAGTGGTTTGGGATGTGTATTATCTGTCGATGGTGAGAAAAAGAGCAATTCAGAAAGAGGTATGTTGTTTATAAAGAATGTTGTATGGTGGTCGTATTGTACCTTGACAAATAGAGTGCAAAAACAATTATACATCCGGGTAATTAGGATTCCCTCTCTTCAGTCAAAAGGGACCGTTTTGAAAATACCGTCTGCGGACATGCCTGCAGATTAGGCTAGGATTACGAAATCCAAATGGTTATACTTAATTCTTGTAAGGGCATCAAGATTAAAGGTAATCTCTTATAACAACATATTAATACATCCGCTTTGATAGGGCATAATTTCCTTCATTTAAGAAGGAGTACCTTTGAGCGCTTCAAAAGGTATTTGTCTCTTTTCTGAACGCAGTTCCCTGCGGGCATGCCTGCTAATCCAAAAAGTTTTCTGCGACCCGTAGTAATCACAAATAGTTCAGGAAAGATTACCAGGATATTAGAAATATGTAGTTTTAACCGTACCGGGCGACGTCCAACCACCTCATTTCTCATTCCACAATTTATCCACCAGTCCACAAGAAAAATTCCGGCCATGCGTCTTGTCAACTTCACTGTTGCGGTTCTCGCGACTATCCTCATTGCAAGTGGCGCAGCAGTGTCAAAGACCGATCAGACCTCACGTGTTCTCGCCCGGAATGACAAGCGGTTTCTCCGAATCTACCAAGCGATGGATAGCAAAAACAAAATTACGTGATACGACAATGAAGAGAGGAATCTTAACTTATTCAGCGCATTGAAACTGTCTGATATGCAGCACGATGAAATTTTTCGATTCCTCATGTTTGGAAAATGGAAAAGCCGTGGATACTCCGCGGATGACATCGCAGAGCACATTCCGGCGAGTCTCCTCGAGAAATATCAAGCGTACAGGAGTATCCATGGCTAAGAGGCGGTTTCAAGCTACCAGAGCTTAAAGCTCGGCCACAACGTCACTCGAATGAGGTCGCGATACTGGCACTCGCCTTGATGCTATCAAAAATATGTCTCGGGCGCAGTAATGCAGAAGACAAACTTTACTCAAATAAAACTCGTGTATGGTTCGTTAGACGTTCTTTCAAACGACCACTGCATTTCTTATCCCATTTCAACGGAGGCCCACCCATTCTTAAAAAAGGTAATTTGTACGTAGTAATAATTTATCCTGGACGCTCGGACGCTGATCTGGAGAGTGTGGCCGTTTTACAAAGCTCCATTTAGTAGAGTGTTTTTGCGCCAGGTTTGAGAGAAACTACAGACAACGACGGAGAAATCCAACAGACACAAAAATCCGACTAGTGATTGTGAGCACACCGCTTTCCCATAGGGATATTTACCTCCAGACTCCACACAACGAACGCACACTCTCCCATTGGCTAAAATACGTCCGTTTCAAAAGTTGTTTTTAGGAAGATAGGAGTACGTAGCTAGGCACAACATACAGGAATTCGCGCTGGAGAAGTAAAATGCCGATTGACTTATTGGGAGAGTATGGCTAGAGCGATATTTCCCTTCACATTGCTTTCGCAAATGCTGATATTGCTGGGAAATTAATCAATGACTTTGCACTGAATACAAACAAGTCTGTACGGCTCGAGAACAACCATGGAAACTACAGGAAATGGGTTTGTTCTGCGAGGTGCTGTCAGTGGTTCGTTGTCTTATCACGGAAACGAGTGAGATTAGAAACGAAGAGTAAACGTAAGCGACCGGTGACCAAGCTCAGCTTTGTTCCAGACAACTTTTGGTACACTTCGAAATTGAATCTTCAACATGCTCAATGCTGTTGTAGCACGGCCAACCCAAATAGAGGTCAAATCGAGCGCTTGGACGGATTTCGAGCGGCCATTTTGGAGGGCCATAGTTCGTCAAAGACTCGAGTTATCAACAATTTGGAGTCTGTCGAGCACAGCAATGTTTCGAAGAAACATTCTGTGCTTTACCGAGCTATCAGCTCTGCTTTGAGTTGGTTGGAAAGCGATACAAACGAAGAATTCAAGGCGCTCCCAGCATACATGGCGTTGTTCGCAGAACAAAACGAAGGTTCGCGTGTGTGTTGCCAGCTCGATGATTCGGGTCGTTTCTATCGTTCTTTTTTTCAATTGGTCCAACTTTGAAAGTTCAGGACTCACTTCTTCCTGTGTGGAAGTGTGACGGAACCCACATGAAACACGAAAAGTATAATGGCGTTTGTTTGACACTAGTTGGAAAGGACGGCAACAAGAGAATTATACCTGTTGCTGTCGCGTACGTTCACAAAGAGACCATTAAAAAATTTGTTTGGTTTTTTGCAAACTGCATTGCAGCAGGAGTATTGCTACATGACCGACCGACCTTCTCGGATCATGGGGAACAACTTGGCGCTCAGGAAGAGCTCCAACGGTTAGGCTTACACGTGCATCTCAAGTTTTGTGCAGTTCACATTCGGTTTAACACCGTCGACAAATTCAAGAAGGTCGATGTAAAAAAAGACAATGTCAATCAGCACATAATGCGCTTGCAATCGGCTCGCACAGTACGTGAATATGAACGGAACATCGAAACTTTGAAGGAGATGTTTCCGGTTGGAATACCGTCCGGTAGTGGTGGAAAACCCACGGAGGAATTTGTGTGGACGTACCTGCAGGAGATTCACCCGACTAGCTGGACAGTCTTGGGCAACAGTGATTTGAGCGATGGTGAAACTGCGTGGCTCAAGGAGAATTGGAAAGATACGTCTTCTTATGGATTCGGCCTTCCTCTTTTTGGTGTGCGTTCAACCAGCGGAGTAGAAGGTGATAACAACGGCTTATCTTTCAAGCGAGCCCGCAACAACCTTGTGTTTAAAGCTTTGAGAGCTTACTGCTTGCGAGCTGTAGAGAATCACCATGCTTTGTTAGAGGCTGCACAAAGTTGGGTTGCTGAGCAATTTGACATCACACCACATGCTCAGAAGCTATTTGACGCCGAACAGAAGCATGTTGCGAAGCTTACAGTGTTGCCGTCTCAAATCGGCTGCTTTTATGTATTCGACCCATTTGACCGCAGCGATGTTGCTTCAGCGACGAAAATGTTTGAGACGAACATGATTTCGGGAAAGTGTCGGCCGTGCCGCGTTTCGGAACAATTAGAAATTCCGTGTCGCCATATCCAGGCTATTCTCTTTGACTTGGAGAGACGCAATCCTGGCAAGATTCCTGTGC

>Contig_80

GACTCATGCTGGTGCTCGGCAAATTTGAAACTGAACACAGATGAGATTTGATTGGCTGATCTAGGATTCAAAGGAGCGGTCCTCCATCTAGAGGTGGAGAACAACGCGATCTCACCTGGAAACATTATCCATCTCCAGCCCCAGGTGGAGAGGGCACACTACCACACCCTAAAAGCTGGTTTAAAGTGATGGTAGCTTACACATAATTCTCCTACTGGTGTATCGTAGGGCTGTATCTCTTTTGTATTAAAGCAGTAAGTACAGTATTTTTACTACATGTACTTTTAACCCGTTTTTACTACACGTTGTAAGCCTTTAAAATGAGTATCCGTCAGAAATGTAATCTTTTTCACTGCGTAGGTACTTTTTCATAGTGATCAGCTGGTCTGCGGTTCGTCACAAACCACATCAAAACTAGGCACAAGCTGGGTGGTCTCGTAAATCCATGCACACTCGTGCTTGATAAGTAAAGCAGACTGTTAACTTCAGTCGCCGTTCTTCTCCGAACGCAATACCAAGTCAACATGGCATCAACTAATCTTCTCTCTTTCTCAAAACAAGCTCTTTAATGATTACCGTGTACCTTGTTCCTCACCTGATCGTTTAAGATGCACTCAATCCCGTCGTTAGTCATAGCGTCGATATACTGCTTTTATTCTTCTTCAGAGGATTCCTATCCCAAAATGTTCTTTGGCCTGTAAGACAGTTGATTGTTTGTGTTCGTTTAAAAAAAACTACTAATAATACAATTTTATTAATTTTAGAAATATAGTATGAAGTAACATTTAGCGTTGCTGCTGGGACAGACAAGCAATAAAAGAATGCAGGCGAGAGAGACGGCAACTGCTTCGTGCGCATCGAGAAAAAACGCCATACCGCCAAAATAAGTTGGTTCAAGCGAAAGCGACAAGAATCGCTATTGTGCCAACTTGCTGAGCGTTTTCTGGAAAAGGTTGCGTAGACGAGTAATCAGCTGGAGATATTACGACCCTGTGATAACTATAAGCATCCAAAGCTTGCGCTAGCTGATTACCACTGCTGCGGATGCCAACCCCGGTGCCTTTGTCTTCAATAATGAGCGGCTTCTCGCATTGTACTACATGTACCAAGGGGATTGTTTAGGCCCAGATGACTATCCTTGCTATTAACACAGTGGGGCCTCGACTTATAAGGAAAACGGCTCCCCTTCCGGCACCGGAGCGGTTGAAATCTTTATATGTTTCAGCAGCCAGTCAAATCAAATCTTCACTTTTTAACTGCATATCAAATCTTCAGTTTTTTACTTGTGAAAAATGGCTGCAGTACTCTGATTGGTTGATATATCAATGGCACTGAAGGCTCTTCCTTCTACGCTATAGTTCTAACATGCAAAGATGCCCACCGACATGCCGGAAGTAGGTATTTGTAGTGACCGATTTTTCACCAATTACGTCAAATCTTCACATCTCAGAGGAAGGTGAGCGCTGAAAAAGATGGGGGAGGTAGGTTTGATCTGATTGGCTGCTGAAATCTCACTTCTTTTCATGATAACCACTAATGATACGAGTACTATTTTCTGCCAATGCAAATATCGCTCTCACTTCCGGTATCGTGGTCCGACTATCTCCTTGAATTTGATATATTATGTTAACGTAAAAAGTTACAAGCTCAGCTGTGAGGCTAAGATAATGACAGCTTCTACGTCTACATCGGGTCCGTAGCTGGATTGCTCGGTTGATTTACTTCTCTCATTTACGGCATTAAAAGTAATGCCTGTGTGGCGAAAACAGTTCTCGATAGTACGCTGATCCACAGCATATGCCTTCCTTTCAATCTCAACACCGTCCTTGATCTTGTCGGAAAACCCACCGCAACTGCTTCTTTCGGTACTCCAGTTTAAATGATGCAATGATGCCAGCATCCATAGGTTGTAGGAACGCAGTAGTGTTGGGCGGAAGAAACTGCTGTTCACTATCTTGGAGGCGTCTTGAAGGATATCAGAAACAGTAGTTTGCGCCGGCGCTCGCTTCAGCTTGAACGTCGCTTTTGCCCATGCCGCCAGCTCTTCTTGCGTCATGCTGGGGCAGTCGCTCGCCTTCTGGGTGAAATCGCGCTTCTGTTTTGTCTTCATCCAGGCTCCCATGGGTATGGGCCGACGTAAATTACGCACTGTTCGACATTAACACGGTTCTGCTCTTAAGAATGACTGGAGCTGAGCATTTCCCGCCAGAAACTCCCCCCTTAAAACAACTGCATCGCTTTGCTCAGGGTATTAATTTATACCAGTACCAAAATAGAGAGCGATGTTTGTACTACACCTGTCGAAAACGGCAGTCAAACTCGTAACTTAAAAATTGGCAAATAGGAAAATTACGGTAGGAGCCCCTAATCAGTATTCAATGTTGAAAAAACTGCAATCAACCTCGTACAAGATTTGCGCGACTGGTGGACAATCTCCCTAGGGGAGGCGCGAAGCACGTCAAGATGACGCCTGAAGCTAAGGTCCTCCTGGAGGAGTACCTGAATGATAACTGTACCTATACGCTGGACACTATGAGGACAATGTTGTTTCTGGCCTGTGGCGTTAAGGTGGACACGTCGACGATTAGCCGGCACCTGAATGGCATGCTGTTTACCGTACAACAAGTGAGAGTCGAGCCCACAGCGTGTAATAGCGATAGCAACTGAGAAACGACGTGTGTTCGCCGTGAAATTAAAGGAGCATCAAGACGCAGGTAGCTTTGGCGCGAGTCAAACAGAGCCTATCGTTTTGGCGCGAACCAGTCGCGCAAATCTTGTACCAGGTTGATTGCAGTTTTTCCAACATTGAATATTGATTAGGGACTCTTACCGTAATTTTCTTATTTGCCAATTTTTGAGACGAGTTTGACTGCCGTTTTCTAAGAGGTTGAGTGCACAAAGGTGTAGTAGTCAAGGGTTGTGAAGTCCCTTCTCGCTCAAGAAGCACCGCTACTCATTTCGCAAAGTGCGACTTAAAGCTCTGTACCCTTCAATTTTCCATATCATGCGCCTGGCGGTAATTGGTATTAGCTTCGCGTCCTGCAGTGTAGCAGAGTTCGACCAAACCAAGATATTGATGAATGAATTACCAGCTCACTCTCACGTTTCAGCCAGAGGAATATTTCTTCGCACGCCTCAAGAGAATGAAGCAGTGGCTGAGAAGCGAGCCCCGAATTTTAACTTGGCTGAACTAAAGAAGGGGAGACACGCTAAACAATTGGCCGAAGATCTCATGGGCAATCCTCGGTTGCCGAAAGCTGCATTCCAGTGGTGGGAACATAACCAGTATTCTTTGTCTAAAATTGATGAGTTCTTGAAACGGGCGAGTCGCAAGGCTAACGGCAAGAATTTCGATGAGATCTACAATGGATACTTGCTGCATCGGGGGTATGCCGGAGTCTTGTTGTCGGTAGCTAGCTTTCGAGCTGACTTGCTATTACTACTACGCTTATGTGAGCACTTGGAGATTATACAAAATGTTGTTCTTAATTACACTAATGTCAGCACTTTAAGATGATAAGAAATGACGTAGTGCTGCTGGTTGCGCTCATACCACGTACAGTACTAGGGGCACGTAGAAACAGTCTTGACAGCCTTTGGAAGTCTTCTTATTGTGAAGTACTAATACGAAGTACTACGTAAATGCTCGCTATATTTGGCGCACTAACTCTCTGTAGTGCGATCTCGTCAGCAAGACGAATTAGAGAAAAAAACTGGTCGAGCTCTGTCACCGGCATGAGATGACAACTCAAAGCCAACCAGCTTAAATTTAATAAAAGCCAAACCTAAATGTAACTCCGTCAGACATCCTGAATCCAATGGCTGAACAGAATTTGTGCATCTATATACTAAAAAAAATACTTAAATTATTATCTGAATCCAATCTAATATTTTATTCGTAGATATCGTCCGATCTTCTATGTCTGAGGGGATACCTTAAGCGAGCAAGCAAATTTAACTGTAAAGTCATTTTACTACTCCCGTACCGGCAACAACACTATCGTAAAATACATGCCTGCGTATAGATTGGTACGTTTCATTTAGCAAAAAAAACAAACCGAAAAAAACGCTTTTTAGTTTAGTATTTTCAGCCTGAAACGTATTTGAACCAATAGAATGATTGTAAGGTTTCTAACTATCTAAGTCCACCGTCAAAAAGCAAATTTTCTTTTCACTCATGCTGAAGATTCCGCTACCATTATCTTCTCTCCTTTCATTTGCCAGACAAACGCTGCTCCAATGTCTGAGCAATCCAATAGTTTCTGTCGTCTTGTTTCGCTGTTGCGATCATCTTCCCGAGCCCCGCATCATCAATACCAGCCTTTTTGAACCACGAGACCAGCAGCTCATAAGGGTCGTTTCTTTTCATCTTCCACACATAATGAAACCAAATCTCCAAAATCGGATTTTTTAAAAGAGTAGTGCCTGCGTCATTCAATTTCAGCAGCTGGAACACCTCCATCTCAGTAATGTGATTCTTTCGCCAAGCAGTGCTCAGTTCCTTCTCGAGCTGCCCGCTAATGCGTTTGTTGACACCGCTCTGTGATGAAACAATCATTTTCGCCACGGTTACGTCGTCGTATTGCTCGCGTAGCTTTAAAAGCAATGCCTCGATGTAATCGGTACTTGGACTCTTTACATAACTCAGCCATGCCGGTAACCCCGGATTATTCAAGACGTTTTGGCCTCCTGCGTCAAGCTGGARAATAGCGTAAACATCATCCGCACTTTTTCCACTTTCCTTCCAACTTTTCTGCTGAACTCCTTTTAGCTGATATACCACCGTGTCTCTGCTTGTTCGCTTCGCAGTAGCCAGCATGCGCGCCAGTTCGTCATCGCCATAGTGAGCCGCCATCGTCTTGAAAATTGCTCGATCTGCTGGCAAGCGTCCTCTCTTGCAGATTTTGGCCACATAGTGCGCCCAGTCAGTAAATTCGCTACTCGAGAATACGTCGGTTTTTACTTGTTTGACCCGAAGCACTTCAAAGCGGTTATTGACCTTGAGCTGCTTGTCTAGACTAAATGTCAGTTTAGCGTTGTGAGCTAGATCTTTCAACACCTTCACAACAGAGAATACTCTGTCTTCCGAGGCGTAGTAGGCATCAGCTGTCGTCGTCCTCAAACGTCGATAGGCTTGGATGTCAGCCAAATCAGCTCCAGCCTTGGCATCAGCTCCCTTAGTGATTGCAAGGAAGAACAATACAGCAATCGAAGTCAAGAGGGCGCCCCAGCGAGCACTACTTAAACGCTTGAACGTGATCATTATCCGTGGCTTCTGCGCTTTTTGAATCAAAGTAAGAGCAGTTCACTACTTATGTGATTGGCGACGCTCGGCCATTAATCCAGCAGCCTATCAAATTGATTTAAATTTCGTGCCGTCGCGCTTTATGTGCTTGGCGACCAATATACATGTAATTTTGAACATGTAATATAGTTTTTCTATGCAGGTATCTTTTATGCTGGCAGAAAGGCTAGACCACCTCAAGAATTTGACTGTCGCCAATCACATAAGGAACACTGTTCACCGCTGGGTCTTGTTTATGATGTGCGGTGTTCAGTACTGTACGGTACTATTAAGTAAGATAGAGACAGAACATGCCAAACACATGTATTTACTGTTTCAGCACACAGCAAATCATTTGATTTGACTTATGTATACTTTTGAACTTCTGAGTGTCGCAATAAGACATAATCGACGATTAGAGGTCACAGCAGAAAAGAGACCAGCCGCGCTTCGCTTGGCTGCCCCTTCGGCGCCCCCGCGGTTCGCTGCGCTCGCAATATGGAGATAGAATGTTCTGGAAAGTGTAGTGCTCGGCTCGCTGGTGAGCTCAGCGCTGGGCCCGCCTCCGCAAATACCGCGCGGCCCTGCTTCGCGGGCTCTCCGCGCGGGTTTGCTGCGGCTACGCGCCTCGCACCCGCTCACCTTAGTTGGCGCCGCGGCTGTGGCACCCACGTCGCCCAGCCCCGTGTGTGTTATCCCAGCGATCTCCTTTTTTCTCTAAGATACAGTGCTATCATAATGCGGACATGTTGAAGTTATACTTCGCAGTCGGTACAATGTACTGTACATGTACCGGTAGTATCAAATTTAGGGATAGGGCTACGACGCATGATTTTGATCGAGATATTTTCATAAATTAGTACATGTAAATTCTTTCAAATATTTTGCATGCTAATATACATCCAAAAATTATCCATTTCGACGGCTACAGGCTGTAACGAAACTAAAAGCCTATTCCAATCTTCAGCAAATCATCTTTACAGTTCTATATTGAAAATTGTAAAGGCCGTAATCTATTTCGATCCCAATTGTCCGCGCTTACACCTGCAAGGTGGTCTAAAAGGGCGGAGTCTTGGTTCGATCGTAAATACACGCCGGCCCAAATTAAGGAGAAATTGACTAGTGTGGGCGGTGACATGAGCAACAAGAACGGAAGGAAGTACTACCTTGTTCATGAAGAAGTATAATGCGGCGAATCCGCGGCCGTGATTGGTGCATAGCGCTTATGTGACGATTGAGACTGAAGATAAGGCACAGTTTAACCTCTTACCATTACGCATTTTCACTTGTCAACCACAACTTTTGTGTACTTCTTCTAACATCGAAATAAATGCATGTGGGTGGCTAAGGTAGCTCCACATGTCACTCACATGGCATGCCAGAGTACGACCCTTATTTCAGTCCCAACGTGATACTGAGCACGAAGGGGGGGGGGGTCCCCAGAAACCCACACACAAATGGCTAGCACTATCCTGGATAGATAGAGCTGGTGGATATTTTATTTTCCCAATTGATGGAAAATTTCGTTAAAAATACAACATTGCATTGTGTTCATTCAGAATTCCCTTTGCTAGATTGTTACTGTACCGGTTAAGCAGTGCAAAAGCGGCATCATGTCCAACTGCTCGACGACGCAGTTTGAGCATGCGGGGGTGTTTGTGGGCAACTCGCCGTCTTACTTGAAGATCATGGACTCGTGGGACCAGCAGTTGAGTGAAAATGGCTACAGCCGACTCAAGTGTGGCAGCGGCACAAGGAGCACCGGTACGTGCGGCATCATCGGTCCATTGTCCGTCTACCTTCAGCTGTAGGTGAGCTCGGAAAAAACTAACGTCAATGTGTTATTAGTCTACGCCCCCTCGCTTCTTCTAAGCGATGTTGGTAGAGGGGAGGACGCAATGGCTGAAGGGCTTAGGAAATCACGTTCGAGGCCTTCTGCAAGTGGTGCAGCGCAAGGATCCAAGAGAGTCACTACTCGAAGGAAAAACTCGTGGAAGAGATTTTCCGGTTGGTGGACGCTGATGGCGGCAGGATTATCTCGATGGTCGAGTTCGTGTCCATTTTTAAGACCTTGGGCCAAGCGCTAGACTATGACGACGGGCGTGAGGTGGTTTCCCAGATGGACCGCAATGGCGACGGTAAGATCGACCTCGAAGAGTTCCTTAGGCAAAGTAAAATATACACGAAGGATGGAGCTTTTTTTACTTGCCATGGTATATAGCGACTTGTGCTCATTTTCCGAAACCCTAATGAAACTATTATTTAAATTATTTTATTTTGACAAATAAACTATTAAGTAAATAGTACTTTTAAACACTTATTTATATTTTTTAAATTTTCGGTTTTAGGGCGAATACTGAAAATATATGAAGTAAAAAAAGCGTTTCAAAAATAAATTTAGCATAACACTTTATTGCCTTTACGAAGTAGTACACATCTCCCGACTGGAGTGCCCGATTCTTAATTTTAACCCCTCTGTTACCAAGGTCGTATCTTACGACTTCACAAAACTACAAAGTCGTAAGATACGACTTTCGCAACACCAACAATAGCAAATGGCTCGATAAAAATGTTCCATTACTTTAACCAAGTGTAATTTCTGCTTTAAAAGGTATAAGGTCACACAGGTCAAACTTGCATGTGCACAAAACGGGCGGTGGAAAGGGTTATGCGGGAAATGCTGATCCGTACCCAGTGATGAGTGGCTCCACGGTTCCAGACGTGCTCATTTGGAAATTGGAGCTCTGTCACGCAAGTGGATCGACGGCGAGGTTGCGAACAATTACGGTGACAAAAGATG

>Contig_82

TCCAGCGCTTGTCAACGTACGACAACTCGCACTGACAAACCTCCAAGATCAACAGCATCACTGGAAGTAGCACAGCAGGTTCACGCTCTTTGTGGTCAAGAAGAACACCCACACACGTGTTGATCTCTTCCATTATCGACGCGTCTGGCTTCCCCGACTGCATCGACACCTCATAATAGTTAGCCATCGCTTTCATTACCAGCTCAAATACCGATCATACTCACACTGAGCTGCAGTTTCCGTCGTATAAACAGCGCCAGCACCCTGGTAAATGATACACGGCGTTCTACCGCGTTTGTCGGTGACGCCATTTGCCAGAACTTGGCCTGAGCAGCCAGGCTGATCTCTCCTCCTTCGTCGTAGCAGAACGTCTCTTCAAGTTCCTCTCCAACCGGGTTGTTGACAGCCTTCGGTCGAGCCAAAAGCACTTCATGCTGCTTTCCAACTGACCCTACCGCCGTCGTCGCTTTGCTATCATACGACGAGAACCCGTCGTCATCCTGGTCATAACTCAGCAATTCGGCAAGCCACCGCTCGCCTCGGAAGCGCTCCACTACTCGGTTCGACAGCAGCAGCTTCAAAACTTGATCTATGATCTCTTCTGATACGTTTCCACCGCTCTGGTTCTTGGCCGCTTTGACGGCTTTATCCAGACCGGGATTCACTCCAAAGTACGCGTCAACCTGAACTACCAAGCGGAACTGGTTGACGAACACGTTCAGTTGCTTCTCCGACGCCAACGAACAGCTCGGCAACAATTGCTTTACCACCCGAGAGCTCGAAATGTACGGGGACAACAGAGTCTGCTTTATACCCTCAGGAGGATCCTCCCAGTCCACAAAAGATCTCCAGATTTGATCAAGTCCGGCATTGCTGATGCCTTCACGGCTTCCAGTACGTCGAAGCTGCTCGCAGACTAGATCCACAAGAACCATGCACGCTAATCGCTGCAGTCGAGCAATTGGGGGAGTGTAAAAGTGGTAAAGTAGCAGCTCCAGACCCACGTACTGCTCAGCAGCTGCCACTGGCAGTCCGCAGTTCCACACCACGTGATCTTTGGTCTCCAACTGGCCAGTGACTTTGAGTGCGTACATCGTCGCTACAACCAGCTCCATCGCCTTCAGTTGAAGCTCCGAGCCAATCGTATCGCGGTTGCTAGCCTCCGCCACTAGAAGCAGAAGTGTGCGCTCATCCAGAGACCTTAAGCGCTCGGAAGAATAATTGCCGTTCGCTTTTTTGACGAAGAACTCCAAGCATCCAACCGCTTGGTCGACCCAGTCAACTTCAATATCAAATTTGACAACAGACTCGGAGGCTACTGACGTTGATACCACCACCGCCTTGGCTAACATTTCTCGTAGCAACTTGAACAAATTCACCTCCAATTGTTGCGCTCGAGTCGTCAGCGAGGCCTGCTGTGTCGATAATATCGCTGTAAGTGAAGCAACACTTGTGTTCAAGAACAATGTGAGCGCTAGACGTCGCCACTGCGGTGACGGAAGAACTGCTAGTTCCAGCAGTTTAACTCTCACTCCTGATGCGTCTAAATCTCCACCTTCACTGGTAAGACACGCTTGAGCCAGCACATTCACTGTAGCTTGAGTGAGTACAGCTGCGTACTCTTGTTCCCGTAAGGTTAGCAGACTTTTGTCGTACAGATCATGAAGGTCTCGGAAAATCTCGTCCAGCTCATCCCATGCCGGGTACTGCGTCATTTGGTTATTCAGACGCGGTGGTTTGGATAATTCAGCCACTGAGGGATGATTGGGATGTAGAGCGCGCTCCGTAGCGTCCAGTTCTCGACTCAACTCATAAAGATTGGTCACAGCAAGAGTCTGGAGTTCGCTCCCGACTCGCCGACTTTTGGAAGATAAAGAGTGAGTATTTGTTGGACAAGTGGCCCCATGAGAGCGTCGAAAGAGACGAGAGAGCAGGTCGTCCGAATCTTCCTCATGTAGAACCATCTTAATCTCGCAGCGTTATTGAGAAGTCGGCCAACTGAAGATCAAAAGTCTATAAACGACAAAACTGGTTACCGTCGTTTCAGACGCGCAAAAAATTGCAATGGACACGGAAGCCACTCCGTTCATCTCTGCGCGCGAGTGTGACGCATTGAACGATGAAAACGTGACACCACTACCAGCGTCGCAGCCGCATGTCGTATTCGTAAGTTTATCTTTTCAGGAAATTACTCTATTTGACTTATTTGTGTGTTTTATTATTTGCAGCGACTTCGACGAGAAGACGAAAAAGTGCCGTGGATTGTCGAGTTGGTTTTCTTGCTTTTCAACGCGCAGGTAGGTAGGTCAGCCTTCGCTTGGGGTTCAATATGCGGTGCGTTCGAGATGTACTAAGCGCCTATTGTGTTTTTTTAGGGCGACATCTTGCAGGTCGCCCGTGGGGCCAACACTCCAGACACTTCAACAACCGGTCACCGCACAGCTGGTAGCCATGCTGTTGCGTATTTTGGCGAAGAAAACTTCCGAGCCACACGAGCCAAGTTGAAAGAGACCGACGAGCGGGAAGTCGTTCGGTTTTCCATGACCAAAGTGCATCCATTCGTCGAAGTCGTGGGCTGTCTAGTCACGTATGCGGAGGACGAAATCGCTCGCTCTCCCCAACTCAACGCGTTCTCTCTCACGTGCGATCTCCACGCTCTTGACAGCTCTCAGGCCGAAGCAGAAGCGCAAGCGCTCGCCGCTGGTGGCAAGAGATTCGTGTTCAATGACAGTGGAGAACACACGCGTTTATTGAATATTTATCACGACCCGAAGACATTGAGAAGACACATTGGCAAACAGACGAACGTGGTGATATTGTGCAAACTCTTTCGACGTCACGGGAAACACCACGACTGGGCTTTCCATGCAGCTACGGAGGATCGAAGCAACGTGGTCGTACGGAGCGCTGTGACAGCGTCTCTGGTTGAAACCATGCAGATTTTCCTGCTAGATCTCATGCCGGATATCAAAATCCCGAATCGCAACCCGCTGTCAAGTGTTGCCGGGATCTGTGCAGCTCTCACTTGCAACGAGTTTCTGGGTATTGAGCGTCACTTTCCCAAAGCAGGACTGTCCAAAGTGGATTTTACTCGCTTGTTACTGTGGGAATTGATGCGCGCTCGACCGAGTTTGATGCGGCATCTAAATCGCGCTTCTGCTCTTGTTGGACTGTTGTTCGAGATGTTTGAGCAGATCGACATTAACGGCGATTCCGTCGTGGATTGGGACGAATTCACGTCGTTTTGTATCGCGTTGGGACTTGTGGCCACTCGAGTTCCTCATGACGAGAACGATCACGATCAAGGACAAAGTCATGGAGATAACCACGACGCTCCACCTCGAAACATGATAGTGTATCGCCATGAACCTCTGAGTAGTGGAACTGCAACTCGGTAAGGTTTTTATTTTATCTTACTTTACTTTTGCCAACAAACAACTTATTGTACACCACAGGACTTTTCCTTATCAGATCAACAAGCTTAAAAACTTCCCGCAACTCAAACGCCTTGCAGTAATCGAACACAAAACTCCTCGAATCCTCGTACGTGAGAATGTTAACATTTATTTCTTTTTTTTCTTACATTATTTTTTTCATGAATTCAAGTTTTTCGATCTGGATATGAGCTTCCTCCACGAACTCAATTGCAGTGAGAAACTCGCAGCCGAGAAGAAAGCAGGAGAGAAACAGCAAGACGTATTAGACTCGTTGGAAGTTCTGGACGCAGAGCATATTCCATCTCGAAATGCTCTAGCGGTGGCAAGCAGCGACTTGTGCATTTCATTGTGGTCCATTATCGACGCTACTGTGGGCTCGTACGTCTTCAATGGGAAACTGGCGGGACGTTTCCCCGCTCTGTTCGTCAAGTGGTGTCCTCCTCCTCTCAAGCGATTGTTCGTTGCTGGGGGTTCGACAGATCACGTTCAGCTCTGGGACTTAGAAGTGCCCATGCACGCTGGAGCTGCTCCGCCGCCTCCGCCGATGCTGCTGCCTCGCAGTCACACGGAGCGGATTGCCGCTTGCTTAGATCTACCGGAGACTCCGTACGTCGCGACGGCCTCTTTTGACCACACGATCACGATCTGGGAGACTGTGGCAGGAGCCAATTCCGCTCTGGGGGGCCCATCTGCTGTGCTTACAGTCTCGTTCGTGCTTCGAGGACATCAGCAAGCCGTTTTGACACTGGACTCGGCTCATAATCTCTTGCTAAGCTCAGGATTTGAGTACCAAGCGTACTGTTGGGGGATTTCGGGACGAGTTCTAAAGACTAAGCTAGGAGGTCATCACCATTGCCTAATGGGGGCCAAGTTTGTGTCCACCTCTGCATCTGGACCTTGTCTTGTAGTCACGGGTGATCAAAGTGGACATTTCAAGCTCTGGGATATCACTCGATGCGCCAAAGGATTGTCGGGGAACCACTTGTCGATCATGTTGCAGACTTTTGAGCTTCACACGCCGAATCTCTGTCGCTTCCGGATGTTTATGACGTCGTCAAGTGGAGCTACTGGACCGGAATCGCAGCATGACGAGCCGGGAGCTGAATCGACTGAGAGTCCGGCGTGCGATATCGTAACGGGAAATCTGCGTCTCTATCGCTTCAGTGCGTTCACCCAGTCGGTGGGATCTCAGGACGACGCAGCAGTCGACGAATCGGCGGGTTCAGCTCCCACGAGGTTCATCGTGTTCAATGCCGTGGCCAACACTTTCGTTGGTGCAGTTGAGAATCGTATTACGGTGTGGAATGCAAATTCTGGCGCGAAAATTGAGGAACCAGTGACAATCCGAGACGCCGAAGTCTGCGCGATCACCTTCGATAGTCCTCGCGAAAGAAAACTTTTCGTGGCGACCAACGTACGTTGAGAGAGTTGCTAACCTAACGAGTGTTGCGTGCAACTAACTCTGCGAAAACTATGGCCTCAGGATGGTGCAATTCGGCTTTACAACCCTGTGACTGGAGCTCTACTGCAGGTAGGATATCTCCATTCTTACGCCGCACCTCTTTGATAATTTGTACCTGAAACATGCGGCTAGAAACTGGTAGTGCACGACGGCACTGTTACCTCTCTTGTGTTCTGCTCGCATGCCAATGTACGTGACCGTCATTTATTAAGGAAAACCGAGCTTTTGATCGTCATTAGTGGTATAATTCTTGCTCTAGTGCTTGATTTCGACTGGAGACGATCGTATGGTCTGCGTAGTGGACTCGCCACCTGGAAAGTTGAAACTGGAAGTGATCCACTACGTGGAAAAGGCTCACAACTCCAGTATCACGTGTTGCGCTTGCTCACCGCCACCAGCGACCGAGACACACACGTCGAGGTTCATTGCCACAGCGGACGATGCTGGCGGAGTGCAAGTGCACGACCTTCACCACATCACGTTCCAGTTCCGCTGTGCTACCGTCCACACTCGAGAGATCCGAGCTCTGCATTTCCCAGCTGCCACACCGGGTTTCCTCGTCTCGGGGGATGTCAGTGGTGTCATCTTCGTTTGGCCAACAATTGGAGTTCGAACTTCGATCGCGCAACCTCTTATGAGGCTCAAGATGCAAGAAACCACACCGTCAAGACCTGTATCGAGCAACGTAGAGTTCGATGGGATCACAGCCATATGCTCCACTACGTCACACGACTCTCACTCCATGCTCTACGTTGGCGTCGAAACGGGGCAAATTTTTGCATGGGATCTTCATTCTCTTCCTGTGAAAACCCAGTCGGAATCTACAAGACGCTCAGCCATTCGACGTCGCTCATCTGTTTCCAAAGGTGAAATTTCAGTGTTGGAAAGTGGAGGCAAAAGTGCCCCGTTGCCGGTGATTCTGTCGACCAAGTCGTGGATGGCACACCAAGCAGGTGTTTTATCAGTGCAAAGTGTGTCTTGGCCTGGTGAGTTGCTCTCGTTGGGTGCAGACGGCATGGTTAAGATCTGGGATCGCACTGCAACTTGTGTCGGCCACATTTTAACCACAGCAGAACAGAGCTCGGTCTCCGCTTCCGCGTGGAAATTCATACGTCGCGACCAGACCCCAGGAGGCCATCAGAACGATACTTTTGAGCGAATTGCTCGTGAGGTTACTGCCAAGCACCAGCGTCGACTGAAAAAGGAGCTCAGTCGACAGCGAAAATCAGGACAGCAACCTCAAAATGAGCATACAGTTTCTGACAATTCCCTCTCAACTTCTTCGTCACTTTTAGAGCTGGACTCGCCAGCAAAATCACCACCGGGACTTCAATTTACAGGAAATGCACATTTTCCTGATGCGTCGATAGCGATGACCAACGCAGCAAACGCCCTAGTGGCACTTGTGCCGTTCTCCGTGACATCCGTGACATCGGGAGTGCATCAAGGAATTTTTGGACCGGAGGAGGCGCAACATCTCCGAGCCATTGCGAAAAACTCCAAGGCTTTCCTATTAGATGCATCGGACAAACGGAAGCGTGTCGCAGCTCTTGCGCCGTTATTTGCTTCACCTGAAGAAATGATGAGAGCGCGTGCAAAAGCGAAGGCAAAAGCTCGAGCAACGATGAACAACAGTCCGCAGATTTTGGATTTTCCTTCTCGAGCGCTAACAAATTATCCGCTGGAACTCGAACGCCGCGCTGCTACCAACGCTAAAGCTGCAGCAAGCGCTTTACGCGCTTTAGACCCAGAGCCTTCACAATTTCTACGTGAAAAACTGCAGGATGCTGCGTCATTAATTGTGAAATCGACACCGAAAAAACCTTTGCAACGTCTGATAGCAACTCCAGAAATTGACATTAAAATGAGTGCAAGTGTGGTGATACTAGCGAGGAATGTGTCATTACCGAAGCTGACGCAGCCACCCAGCCAAGATCTTGATGACCCTCCACCCACGTTGAGCACCAGTGCGTCTGCACCTGTTCTACATGTAGTTGCAACAGACCCGCCTTCAATGGAAAGTCCTGAAAAGAAAAATCGCAGTAGCAATATCGAGCGCAAGTTGAAGTTGTGTCAGAAGATCGTGGCAAATGTTTGTCTCATGAGCTCCAAGCCCAAAGTGTCCAAAGTGCGCAATGAAAAAGAAAAACCCGGTTCTCCAGCGTCAAATTTGCGTCAGCAGACTTTGACACTAGCGATAGCACAAGGAAAAAACCCTTTCGGACCACACTACACGGTGAAACAAGTTGCACAGCTCGCCATCCGACTTGCACGACTAGATGAAGACGGCTCCGGAGATCTAGATCAACACGAGTGGAAGCAGTTGGCAGAGTTTTGCGGTCTCGACAGCAACGCAACAAGTATTGACACCCTTTTCCACTCTCTCGACCGCGATTCGGATGGCACAATTAGCGTCCGTGAACTGCTTCCTTCTCTCGTAAGTGGACAAAGTTCTTTATTTTGCATGAGTTTTGTATTTTTTGTTAAGACTTTGGTTCTCACAATTTTTTTTTGTGTTTTGCTCTGTAGTTCCGGCAGGCGTCTACCGAACAGCTCCAGCAAATGCGAGCGTTCATTCAAACCCGGATGATAGAGCTCCGTGGAGCGAGACAGTAGCGGGATGAGGAGGTTTGGGAGACTACTGCACCGCTGCCGTGGAAGCGGCAATACAATCCGGTGAATCGGCCACGAGAATGTCGATGGGAACACGGTGCAGCTCGCCATTTGCATCGTGAACCAAAACTGGCACGTCCAGGAAGCCAAACTCGAAAAAATAGGCTCGCGCACGTGCAAAAGGCGCGTGGAGGATGCCAAACTGGTCCACTTGCATTAAGAACTGGGGCTTGACGGAGTTTTGCTTCGCTTCGACGAAGGGAATGAGCTTGGAAACGGCGTCCAGCGAGCTGCCATATTCCAGCGTCTTGTGTTGCCATGTGGCACGCACTTCGCCATCGGAGAACTTCGTACCAGCGGCATATCTCAACGGGAACAGATCGTCTCGTGCATCTTTCGCTGTTGCTGCCAGTGCCTTGAGATAAACTTCCTGTTCATCTGGTGTCATCCGCGAAAGTTGCAACTTGACAAATTTTGTTGCATTTGTTGCAATTTCACTCTCCTGTTGCAAATCCGTGGCAAACCGTGAGCAAAATTTCCGCGCGAACGGCAACACTTGAGAGCGCGGCACTCGGTCGTAGAGTGACGGGAATGGGTACAGCTCCGCCAATGTGGTGACGCACGGGTGGCACTTGGTGGCTCGTGACACGAACGGCGTGCTGTCCTCGATCAACGGACTGGACAGCAAAAACTCGCGGCAAAGCGTTCCAGGTGTTAAACACTGAGGCGAATCCGACACGATGTAGATTTTGCGTACTCGACGCGCTGGCAAAAGTTGCAATTGCGCCATGGCGGCACGTTCGGCGCATGTGGCCCCTCCGATATTTGCACATTCGGCGTTCGCACCAATCACGAACTTGTCGGTTTGGCCCTTGAGATCTTCGTACGAGAAGAGCGCCAATACTCGAAAGCCCGAGAGTAACGGGGCAGTGGTGAAGTGCTGGTCACGACGACCTTCCAGTAGCGTAAGATACTGCTCGTCTTCGGGTGTGAGGACGGCGTCGCGCATTTGTTGACGTAAAGGGGGGATTGGAGGTTTGGCGTGGGGGGTCGTGGCGAGTAGGCGCGACTTGGCAAATGCAGTAGTGACGGTGGATTTTGTGCAGGTTGCAGTGTTGGTAAGCATTGCAGTGGTGACATGTGCCGCCGTGAATGCGAGTGGAAGTGCATAGATATGGCTGCAACTTGAAGGTGCAGACGAATAGAGCCATTCGTGCTGACGAAGCACCAAGGCATCCGCTCGATTAGAGCGATGTAGACACTTCTTTTTAGAACCTGTAGTGTCAAATTACGGAGAATGGGGAGTACATGTAGTTGCCGAACACTGACATTTGGTTGGTTGTACGGTGGTGAGCACGCTGAGCCTAAAAATAGAAACAAGAGCTTCTACTCGGCAACAGCAAGCTCAAAGTTATCATAAATGGCGACGTAAACAACAAGCCGGACCAAAACGACCTCCACGTGGGTAACGTGGATCCGGCGCGTAGCACACCAACACGGTGACGCTTTATATATTCATATTTCTGCTCTACGATCGCAACATCCGTCACAGTTTGGACTCAGTTCCCGTAAAAACGTCGAAGCAAACTCATTCGCGTGGATTCCAGGAATTGAATAACACACGCTTGGCTGTCAGCAGTAGCATCGGGGCTGGGGGGAGAAGTAGAGGCAGCCAGTGTTACAGCAACGGGGGCTGTGTCTGCTTCGCCGTCAAGTAAATTCACTTGCAAGTCACTCATGCAGTTTGTGAGGAAGAACGCCGCAGTAAATTGCAGTCGCAATACCAAAATTCGTAGTGGCGCTTGGTCATTTAACTGCTCCTGCAGCATGAAAAATACCTGCGAGAAGTCCACCGCATCGTACTCCAACACTACAGACCAAAAGAGCGTCAGCAGCAGACATTAGCTCGAGATTTCCCGCTTCAACGTTGTACGCTAACTCACCGATGACGTGGCGACGAAGCTCTGCAATAAAATCTTTGCGCCTCCTCCACTGTTGCAAAAACATCTAAGTACACATTTTGCAATTAGCAACAGCAAAGCGCCTCCATTGTCACTCAACACAACTGTAATTTACCTTTTTGCACGAGGCAACGAGCTCTACCGAAGATTGCGCTTTACTGGACAGCGGGGCTTGCGTCACTTGACTGGACAGCAGCATTCTAGATGGCCATTCAACAAAGAGTGGCCATACACCATCAGCGTTTCTCTCTTCATCAATAGGATATCCAATGAAAATTGTCAATCTGTCGGTTGTGGCGGAGAAATTCGGGGCATGGAAGCCGTCAGGTATGTACACTGAGTCTAGATCCTCTGCCTCAATCAAGCGTTTTGGGTCCCATTGAGGTTTTTCTCCAAGCAATGGCAACGCAAACAGCACGTAATTGCCGATCAATTCACTTCGCAGAACCAGAATCTCCAGTTCAGAATGATGTTGCAACATTTCGTACTCGAACTTCGCCCTCTGCGACGTTACAGACTGCAGCGTCTGCAACAACAAAAAGACACAGACTCATAAGCGAATGACGATTGACAGTGGCAATGCCTTCCCACTTTTCATCTGCTGACTCACCTGCACTTGCTGTTCCCGGAATGCGTCAACCAATTGCTTGACCACATCTTCCAACACTTTTGTCAACTCGTCGCCAGTCAATGTTGGCAGTTCACCGAGGTCCGCCACATTCAGATCGAGTTTTTCAACTTTTTGCAACGCTTTCAGTTCACTTCCATTTGCCACGTCAACGGCCACTTCATTTTCGTTTTGTTGCAGATACACACTCGAAGACTTGACTGCAAAAGATTGCCAAAACACACAATGACAATAGTAAATAAAACAACACTGAAACATTTTTGTTGTTGTCCATTGACAGTCTTGACGGCCATCTGTACAACCTTGGCGAAGACAAACGTCTCTGCTCGTCCAGATATCCACGCGCACTGCCAGCCATGCGTTGCGTGCGTACAGAGCCTCCACATAGCCTGCAAACAGCGGCGTCTTACGGTCTACGCTGGCTGCTGAGTCGACGCCGGTCCGAATGGCGCCAAAGGAGTCCACTGCCACGAGCTCGGCGACTCGCACGCGACGCCGGCATCGCCGCGTCAGCTCGAGTTCCATACGTCAATTGATAATAAATCGCCACATAGATTGCTATTCCTGCAAGCTAATTCAACTCAACCATGTGTTTTAGAATCCCCCTCTCACCTGCGATTCACTTGACGTTCGTATCGAATCCAATCGCGATAGACGGGTACTAGATCGGAAGAGCACACGTCTGAACTCCCAGTCACCCAGCTGGATCTCGTATGCCGTCTTCTGCTTGACAACACTTGGCCACCATCTTGGTCTCGACAAAGTCTGACT

>Contig_83

ATACGGAGTTGTATTTGTGAAGTTAGTACGAGTCAGCGTAATTACTGGAGCATTCGTTCCGATAGACTACCATATTCGCTAAGTGCAGATCAAAGCCAAATACTACCTTGAATCTAGCGATATACCAATCTAAAATACGAATTTGCTACGAATTTGAGAAATTGAACAATATTCTGTCATATTCCAAGTAAGTAATTTTGAGCCCTATTGACCGCTCGGGACCAACCTCCAAATCAATCCTGTAAATCGGCCCTTTTTGCCGTTTTGAAATGGTAATTCGGTAACAGATAAAACAAATCGATGCCTGCCTCTTCCCATCTCATTTTTACGCGTGGTTTTTTATGCTCCGACCATAGGTTTTAAACGGATGGACTGCAAAGCACAATACAGTAAGTAACCTACCGGTATCCTATCGTAGGCACAGTACAGTACTGCACCCCGTTTTCGCGGGGGTGGACCATATAGGATTTGCAAATAAATCAGATTTTTGCTTACAAAGTTACGTTCATTTGGATTCAGGATCATCACATCCAGACGTTTATCAAAGGTCCAAGGGCGCGAAAGCGGGGTGCAGCACTGTACCGGTACTTAAGAACATGAAGTGTTTAGGAAGGCTCTCGCAAACATGGCAGCTGGGCTGCCGTACAGTACCGGTAGCCTACAATTTGTAGAATCGTGCAATGTTTTCGGCTTCGCAGATTGCTTGGCAAGTAATCATTTTCAAGGATTTCCTCATACAGTTCGAAACAGAAGTTTGATCGGCTGTTTCATACCGGAGGAATCCTTGACAAACTTGTTAACTTTTAAACGCACTTGATTTGAATGTTTATCATTTTCTTTGAATCTACTTCGATATTGAGCACCTTTGCAGGATTCTTAGCCTCTGTAATATACAGTACATTTTTTTCTGCCGAATTCGTAAGGTGGAAACAATAAAGACTGCGACGTTGAAAAATTCGTTCAGGTCTCACGTAGATTCAAATGAATTGTGTTATCTAAACTCTTGTATTCGGTCTTTTAAAAATATGATTGTGCGCGTTTACAAGTAGACGACACTTGTCTCGCATCTTTCTTTACAGTGCCGCAGGCACGCATTACTTCTTCAAGGTACATTGCTGGTCGGATTAGGCTGTACAGTACATGCTTAAAGTACATGATGTACTGTATCTATTTTAAACAGGCCTGGGATCGGGCTGAGCCTATAAGACTGAGCTTTTATCCCGGCCAAAAAGGCTTGGACAAAAATGGTAATATATTTTATCTTAAATTAGAGGAATGTAACTCCGCAACCGTAGAGCCGATTTCGTTGTGTGTCCCCTTAATAGATAGCTTGGTGTCTATTTATTCTCGTAAGAACTTCACAGCATCTAATAATGCTCACTACTTTTCAAATGGTCGCCGGTCTGCTGCTGAGAAGTACTTGGTTGTGAAGACCTACGAGTTCCTCCGCCAAAAGAAGGCCTCTGCACCGCATCTATGGAAAGGAGGAGTTCGTTATCATGTCAAAGAGTGTTTAGGCTTTGCTACGGGCACCATTTCAGCTATATGGGCGCACTGGGAGCAGCACCACGACAAGAATTTCACCTCGGTAGGCTGTACTTCATATCATTTCAGTTATTATCGATATTTATCATTTCAGTGGTCGACAAATTAATTATGACAGATGGAAGGTCCAAAACCATCTGGGAGGCCTCCAGAAATACGAGACAAAATATCTGCAGCAAGGTGATGAATGCGAAATTGCATCGGAACCAGAGGGCAGCAACGAGGACACGGAAGAGGATGACGCCGTCGATGACAATGAAGCTGTAGCCGATTAGGAATATGAACGGGTGACCCGTTGTATGTTCTTTACACTCTAGATATTCTTTTGACGGGTTCTTTTTAACAAGTTTATAATTTTCTAACAATCGTCAAATTGCGGTAATATTTGGAATTTATCCATCCGGTTCTGAAAATCCCCACCTGTTATCCCGGCTCTGCCAATGAATATGCATGCATGTTAGTACGTGCCAAGCCCACAATTGCAAACAATTCTGCAAGATATAGAGCCTTTTTTAAACGGCCGATTGGGAGATCAATGGTTGCAGATACGCAAAAATAGACCTCAAAATCGGAATCTACGCCCGATTTTACCCCTTATATCTAACTATCGTTATGGGTTTTGTGAAGGGAAGGGTTACCTTTTTTGGACCAAAGTTTGTCCAAGGTTTGGTGGCCGGGATAATAGGACGATATAAGACTGAGTCACTGACTCAGTCCAAGCTGACCCAGCCTCTCACTGAGAGGCTGAGAGTCTCAGTCACTGAAAGGCTGAGTCAATGAACCCAGGCTTGGTCGACAACTTGAACTCCACCCTATTTTCAGCTGAGCAAATTATTAGATTTTAATGAAAGCCGAAGAAGATAGCGCTAGCTCCTTTCCTTGTAGGAGGTGCTGATGAAGTCCAGATGTCAGCTACCCAACAGTAGAACACTGCATCGAAGAGCCACTGCAATATTTGCTACAAACTTTTATGAGCTTAACACTTAACTAGCATTTTGACAGCTAAAAAGAAAATTGAAGATGTAAATTAGTACTAAGTCCAAGCAATGGCTATTGGTGCGGATCCTCTAGAGCACATTATGAAAGTTTACGGAGCTGCTCAAAAGAGTTTTAAATGGACTCGCGTAGTACCAAACATGAACGTGAAGTTAAGAAGGTCATAACTCTGTAAATTTAGGAGCGTATTCAAAGAACATAATATGTTCTTTGAATACGCTACTAAAAAAACAGTACGGTATATTATACAGTACTGTTTTTTTTAAACTTTAAATACATGAGCCGGTACAGGCATGTACTTTGAATACTGTACGTGTTCAGAAAACGTTGCGGGACGAGTACAATGATGACGATGCTTCTGCAACACATGTATTCCCAGCAGACATGGCTTCACAGCAGTGTCACTGGCTTTGGCACTATAATACCAGGCTGGTAGAAGCCATAGTTATGGATATGGCTGGTTACCAGCCATATCCATAGCTATGGCTTCGACCCAGTTATCAATTTCCTCACCATACACCCATCGAGCGTTCTTGCACGTACTGACTACCGGTAGCTTTACGCTAGGAAGTTTGTCATGTCAACTGCGAGGACCCAGCCCTCCGTCACGAAAATACTGGCTACAGTAATTATCCTGGCATGCATCAACGTGGTCTCCACAGCTTCGACGTCCAAGCTGGTCAGAATAGGGAGTTTTGCGGCCAATTCTGACCTGACCGAGGTTTCTCATCGACGCTTCTTGCGAACGTCCGTTGCCGAAGAGAGAAAACTTGCTTTAAGCTTTCCATGGCTTGGACAAGCCGTAAGTGGTACACAGTCCTGGGCAGCGACGTTGCTTCAGACACTCCAACAAAAGTGGTCACAGATGAGAATGAAATCTCCGAATGATATGTTCAAGAAGCTGAAACTTGACAATACGGGCGACCAACTGTTCAGCAGCCCAAGTTTTTCCAAGTGGCTCAGCTACGTCAGGACAAACAGTAAGACAAACCCCGACATGGCAATCTTTTCGACATTGGCATATCATTACAGCGACGAAGCTTTAGTTAAATTGCTTGATGCAGCCAAGAAGGTCGACAGCACAAAAGTTCTCGCTACTAAACTGGAGGGCCTTCAGTTCACAAATTGGGTCCACGCTAGGGAATCCCCTGAATACGTTTTCAAGGTTCTGGCACTCGATCGGATGGGGTCAAACACCTTTACCAGTCCTCAGTTTTCCAAATGGCTGTCATATATGAATAAAGCCGAGACGAGCGACCCGGAGATGGCCATTTACAGAGTATTGGGAACATACCATAGCGATGACGTCCTGGTAAAGATGTTCGCTGCTGCAAAACAAGCTGAAAGTACACGAGCCCTTGCCTCATCACTGGAAAAAAGTTCAATTTGAAAACTGGGCTCGCGGTGGTGAATCTCCTAGCCACGTTTTCAAAGCTTTGGCGCTTGATCAGATGGATACACAAATTTTTGCAAGCCCGCAGTTTTCTAAGTGGACTTCATTTGTTTCTAAAGCAAACACGAAGAACCCGGATGTAGCCATGTACACAACACTGGGAACCTTCTACAGCGACGATATTCTGGCGAAGATGTTTGCAGCAGGTAAACAAGTCGACAGCACGAAAGGTCTTGCCACTAGACTAGAAGGAATCCAGCTGGCAAACTGGGAGAACGCTGGTAAATCAGCTGAGAGCGCCTTTAAAACGCTGAAGCTCGACACTTTGCCTGGGAGCCAGCTATTCGAGAGTCAGTTCATCAATACGTGGGCCTCTTTTGTGACCAGGACGCACAAGGATCCAGATGCAATTATGGTTGCGCTGTTAAAAGATCGATATGGTGATGAGACTCTCGCGAAGATGATTGCGGCGGCCACCAAGACTGAAAGGACGGAGAAGCTAGCTGTGGATTTACGCTCTGCACAGTTTAAGACGTGGTTCAGCCAAGGCAAAACCCCCGAGAATGTCAACACTCTGTTCAAGGTAGCAGCTAATTCTGACGACCTGACGAAGAAAATATCACGAGAATACGATATATTCTTTAGCAAAAGCAAGGTGGCTTTCAATAGACCGGCGAACAGACCTGCACGGAATGGGATTTATATAGCAGGATAATCTCTTTCAGAATAAACTCCAAAAGTTTCAAACTAAGTGTTTTAATTGAGAACTTAATTCTTTAACATGGGAAATCTACACGACGAGAAATCATGGTACCGAAATATGGGTACGTAGTAAATTAAGCCTTTTTTGGCATTGGATGGTCAAATATTTCGATAAGTTAATCAGTGAAAAAAACTGAAACCAAGAAAACATTTGACTAGCTACCCCGACAACGACTGAGGCCTATAATAAGGTACCAGACCCCCCCACGTTGCATAGATTTTCATACTGTTATCCGCTTAAGAAAATCTTAGCGAAACGAGCGACTTTAAGCGAGCTTAAATTTGTCTGACCTCCGAAAACTGCTTCTTTTCGCCTGGTTGGGAAAGTTTTCTTATGCTATTAAGAAGTGGCCTGCGCAAGTACAACATTCTCTTACTCGTTTTAATTAAAAATTGAGGGCATTTCTTACTTGCGCAGGCCTGTTAGTACTACTGCTACAAGCATTAATAATAATATGTACTAGTGCTCTGAAGCTAACGTTTTGGGTCGTACAACTAGTTTGACCTGATATTAGTTAAGATCGAAATTATTTATCCATCTCACTGTGGGTGTACAAACCAATACGACAAAAGTTTTTAGACTTTCTTTTTTAGCCTCACAGCTGTATCAAATCGACGGCGCTGAAAGTTCTTAGATACAGTGCTGGCACCAGCTACTGTATAGGAACTATACCATTTATTAGATTTTGGCCACCACAATTCTTCAAATGTGATCAAAATTGGCCAATCGACGCGTCGGCGAAAAATACCGCCCTGGCGTGGTGGCATGTCAGCCGGATTACGGCCGTTGATCAAGCTATGGTCACGACAGCGTGCTGGCATGAACCAATGGCGAGAGTGAAAGCTGTAAGGCACGCGTCATAGTGCCTTTCGATGAAGAGGAGGCTTACGCTACTGCGCTGCATTGGACTTGGAGCACGCTGCATGAATGACGAGTGGACACGGGCACGTGTCCTCGACTGCATAATCGCTGAGCCTGGATGAAGACACAAGGAGAGTTAGTTCAGGTCAGCGAGTTTGTTTGTTTGCTTTTCTTTCGTTAATAAACTTTCCTCATCGGAAGAAAACCCCGGGTCTCGCGACAAAGTAGCTTTGCGCTCTATGAAGTGTGAGTTTGTTGATCAACCTTTCTGGACGCAGTCTACCGTCCAGATCGTCATCTCCTCCACGCGCTACTGGATATTGCGCGCCCAAGTGCGATCGGCTTCGACTGACTTAAGCTCGACTTAAGTTCGGTTGATGTGTTAAGTTTGAACAACTTCAATATGATGGGCTTCCCTGTTGACGCTGACGGAGACGTAGAGATGTCAATCCCTCAACCGATCTTTGAGGTCATTAAGGCTCCAGAGCTGACCAGCTGGGAGCACGCTGCTCTGATTGAGTGCCATCGTGAGTGGGAGCGTTACGTGGAAAAGATTCGCCACCGATGCTCCACCACGGGTGAGACGTACGACAATGTTGTTGCAACTGTCAGGGGCTCGGTGAGACGGCAGACGCTTAATAACCTGGCTAAGTACGTGCTCAAGAAGCCTATTGCGTTGGTAACCGACGCGGACATCATGAGTGTTGTCGAAGCACGCTGTCGTAAGCTCAAAAACGAGTTCGTTCCTGACGTCTCGTCGCTGTTCCGTGCGAGCCTAAGGATGAACATGACAATTGATGACTGCGATGCACTCATCTTCTGCTACTATAAAGATTTTAACGGGATTGTGGAGGACAATGGGCTACAAGGGCTTATTGGGAACGAGAATGAAGCGGACGCAGGCTACAAGAGTAGAATGAAGGCCTGTTGCCGACTAGTGGTTGAAAACTTGCAGCCCCCTGTCCTCAAGGCTCAGATCGGTCGCCGTATCGATTTAGAGAGGCGTGACTGCAAATCTGATGATGTCGCCCTTTTCGACCTGATTCTGGAGCATGCAAAGGTACAACAGCGGTTTCATCGAATTTCACAGGATTATGCGGGAAAGCAGGATTCTAAAACTATCAAGCCAGAAAAGAAACCACAGCGTGGTGCTCCGACCAAGCCCACGTCAGCACTCTCGCCGGCGCCCACGACGACCACTACTGCGACGGGACCTCGCCCTACGCGCTCTCCTCCTCGTGATGGCTGTCTGTTCTGCAAGGGAGAACACTGGCTTAATGACTGCCCCACAGGCACGGACGCACAGCGCGAAGAGGCCGTGAAGAAGTTTCGCGCGGCTAAAGAGCGGCGTTCGGGGCCGGTGCGGTCAAAGGCCGCCAGCTATGCGACGCCTGCCGGCTCTGTACGAATCAACGAGCTCCTAGATGTTCCCTATACACCCGATACCGGCGCTGACAAGAGCGTGGTTCCGGAGAAAATAATGGCTTTACTCCTGGCAGTGCAATCTACGCTAGAGACTACCTCACTAAGTACGCCTATTGAAACTGTTATGGCTGACGGCAGGGTCCAATTGTTCAAACAAGAGGTGAAATTGGATCTCGAGCTAACTACAGTGGCCAGGTTGGTCTCACTGCGCTCAGTACCGTGCTGGGTTCTATCTGGTGAAGGTGATGAGTTTCTATTAGGCCGGAATGTCCTGAAGGGGCTCGGCATCGACGTGGAGCAACAGCTCGCTCAACTGGCTGGATCCCCACTGCCGGAGGCACAACCTGACGAATTTCCTGTAGGTGCTGAGTTTTCGGGCCTAAATAGACCAATCGACTCGCCGGAATCTCTTCTCGACCGAGCGGTGGCCAATGGGCTGCCGAGTGAACATGGCGGTACTGTTAGTGACTTGCTGGATGAGTTTTCAGACGTCTGGCGTGATGCAGTTGGTCCTGATCCCCCTGCCAATGTGGAGCCTCTACGCGTGTCGCTGAAGGTGGACGCAACGCCCTACAGGAGCCCTCCACGCAAGTATGCGCCTTTGCAAGCCCAGTTCATCCGTGATTATGTGCAATATTTGGTTGATAATGGGCTGGTAGAGCAGAATAACCCATCTCGCTGGGCAAGTGCTGTAGTGCCCGTGCGCAAGCGGGGGACCAAGGACGAGTTCCGGGTGACCATTGACTATAGGGTCATCAACAGTATGACAGTCCCCATTACTGGTACGATGCCTAGTGTCGCCACTACGACCGACACCTTCAACGGTAAGAAGTTCTTCGGACGTTTTGACTTCACTAAGGGATTCTGGCAATTACCTCCACATGAAGAAAGTCGAGAGATATTTTCTTTTATTACGCCCCGATGGTGTATTTACACCTGATCGAGTCCACCAAGGAGCGATAGACTCCGCCTTGCATTTCCAGAGCCAAGTCCAGACCGAACTAGCCCCGCTAATACCACATTCGGCCCTTGTGTGGGCCGACGACGTAATTTTATTCGCACCAACCAATACATGACTTCCTACAGACGCTTCGAAAGTTCTTCGAGATCGTCAGTGCAGCCAACTTCAAGCTCAACATGCTCAAGTCGTCGCTGTTCGAGCTAGAGATCAAGTGGTGTGGCAAAACTATGTTCAAGCGACGGAATTCGCCATGATCATGCGCGCGTGGACGCTTTGGTGGAACTTCCGCTCCCTGCAACTGTGGCAGATCTGCAGCGGTTTGTATTTGCAACGAACTGGTTGCATGACTCACTTCTGGACTACGCACGCACTGTGGCTCCGCTTCATGGCAAATTGGAGGCCGAAAAGAAGCGGATCGGAAGACGGAACCGAAATGCATTACAAGTGGCTACATCATGGGATAGTGACGACTGTGGCTCCGCTTCATGACAAGTTGGAGGCCGAAAAGAAGCGGATCAGAAGACGGAACCGAAACGCATTACAAGTGGCTTCATCATGGGATAGGCTACCTATGAGAATGTACTTTCGTTGGTGAGAGACTCTGCGCTGATGGCGCACCATGATCCGTACGCTGAACTGTGCGTCTTCACAGATGCGTCGCTTTCCGGGTTCGGTATCGTGGTAACACAAGTCA

>Contig_84

CACATCTACTGAACATGCTGCGGGGTGAAGCCCTGTTGCACCTGGTGACTACACTAAGTATTTTTCGACAAAAATGCTGGGAATGGCCTTGTCGTATGAGAAGCTGTACGGACGGAAGCCGCCCCGAGTATCATGTGGTCCTAGGACTGCATTCCGCATGTGCGAGTACTACCTGAGTCAAGACAGAGAAAGAAGAAGCTTGAGCCATGGGCACAAATATGTATATTGCTCAATTACAGTCACTCTACGATGGGCTACAAATTTCTAGAAAATCGACCTGTCGGGTCAAGACTACACAGAGTTAAATTTTCCGTTTTCACGTGGAGCTCGCAGTGGATGGGACCTACGGCGTACCTGGAAACGTTACACGTGCTGCCGGAGAAGCCGCGTAGACGCGTCAAGCGGTTCGAGACTCGCTTCGTCATTCACAGAATCAAGCAACAGTTTGGCATCAACTACAGGGAAACACAAGTACCCGTGATTCGCTTCGAGAATATTCGTGCGGTCATCTACTTCGCTGTGCAGCAAAGAGATACCCCAGTACGACGTGAAGACGGCGGTTCTGTACGGAGATTTCGACGAGATAACAACATGGAGCGGCCGACCGGGCTCCAAGCTAATGGACCGAGCACTATATGCGAGCGCTTAAAGATCCGGTATGGACTGAAACAGGCGTTCAATGTTTGAAACCGAGCTCTACATGCGAAGCTGGTTACACTGGGCTTTGAGCGTACAGACGTATAGACATAGACTATGGACTGTATGCGTTGAAGGGGGAGGACTGCTGTTGACTGTCTACGTGGATGACCTACTGCTAATGGGACCCCAAGACCTGTGCGTCATAATTGCAGCTACACTACAAGAATCCTTCGAGGTGAATACGATGGGTACCGTCGAGTACCGGTTTGGTGTCGAGATCCTGATCAACAGACCAAATGGACAGATTGTCTACTGTCAAAATAAATATGTGGTGGAAGTCTTGAAGCACTCCAGATGTCGGATTGCCACGGAGGTGCAACTCCTGAGGCAACTGTTTTGGCTTAGGCCACTGTACCTGCGATGAAGGAACATATGCCGTACCGTGTGCTGGCGCGCTTCAGTCGCTTCGAGGTCTGACATTGCTTATACCACGAGACATACTGGTAGGTTTTTGGCGTCTTACGACCCCATACGCTACGCGCAAGCTAAGCGGATACTTTGGTATCGAAATGCGACGTGTGGTTACAGATCGGTGATCGGCGTACCTGAAGAAACTAGTTCCAATGTACGATGCTACTCAGATGCGGATTGTGCGAATGATGAACGGACGAAGTAGAAGTGGCTATGTGAACACGCTTGACGGTAATTTGATATCATACGTGTCGAGAAAGCAGGAGATCAAATCGCTGAGTACGTGCGAAACGGAGTATGTTTCGCTGGTTAAAGCCTGTTGTGGCTTGCGGATCTGTGTAAAGAGCTGTCCGGGGAGCGTTCAGTGTCATTATGGCTGGGTGCCAATGAGGGGGCAATCCCGCTGTCCACCAAGCATATAAAGCACTTCAAGCCTAAGCACATCAACAATAAGAACAATATGCTGAGACTAAACGTGGAGCTTAAGCTGCTCACCACACAGCACACTGAGACCGAACGGATGATCGCGGACATCACGACCAAGCCACTTGCGCTGGTGAAGCTCACTCGCTTTCGTCATGCGATGGAGGTGTCTCCTCGGACGATACTACGGCTGCTGTTGCTGCGGCTACTGCGGAGACTATGTCTCCCACTATGACTGCTAATGCTGCAGTGCGAACGGATTAGAGAGAGACTGAACTCTGTCACACAGTGAAGGACTAGAACAAATGAGAAAAGGCAAAGCGACCGCGATGGCTAAGCGTTACAGCAACCGGATGTTTAAGTTTAATGGTGTGCTGAAAAGGCCGGGCATGTTGTACCTGGATTGCGTGGTTGTACTACGACCCAACAAGCGCTTCATCTGTGCTGACAGCGGGCGCTGAACATCTAGACTACGACTACGAATGCGAAGGCTACTACGATGGATGATGGGCGGTGCTAACTGTGATGTGCTCTTTGTTAGACGGACTACTGTGCTGCTGGACTGCAACTTCAGCCTATGGGGTAGCGACTTTAGCCTGCGTGTCACGTGCGGACGCTTGCGAAAACATACGGAGTTTACTATGATCACTGTCCAAGCGCACTCACTGTGTTTACGTATCTCTTGTGATTGCTCAATTTGCTAGTGCTATTTAAGTGTAAGCCTGCTCTAATGACATCTACTCTAAGAAGCGACCTACGCTTGTAGTCCAGTGACGCGCTCTGCGTCGCCTCATACTTGAGGATTTTTGTTTTAGACTTTTTTAAGGTACGTCGCAGCTGTAGCGCGGTCTGTTCGGCAACGCCAGCACAGTGCCCTTCATGCATGTGCTGTGACCATAGTGTGCGTTTTTATTGGGGTTAAATTTCTTGTTGTTTGTATGCTACTGGAATTGATTTGGCTTTTGTNAAAAAAAAACCGCTAGATCGGTGAAAAATCAGCCGGTGACCGTATGTGTTGTTTCCATGGGAAACCGGTAGAAAACTGTTTCGACACCTGTACCTTTTGTTTTAGTATACTTAAGGTATGTTTACGCAATGCATAAATTTCCAATTTTAATTTACTATAAAAATGGTAAATTTAGTGATAAGCTTAAGCTGAAGAGAATAAAAACGCAAGATGCTACCAAGGTTTCGTCTGCTGATGTCTTTCTGTTGTATAGCATGACGTACTTGTCCAAAGTGTCCACGTTTCGGCTGATAACAGCTCCAATACCAGCGCTCTTAAAATCTAGAACGTTGAAAACTTGGTCCACGGACATGTCCTTTTTCAACCAGCCCTCAAGCTGTTGGTTCTGCAAACGTTTAGCGAGCATATTGTCATCCTTAACTGCAGACACAATCGCCTTTGCCACATCAGAGTCACCGTACTTCTTCGTGAGCGTCCCGAGCAGCGTCGCCTGATTGTTTCCGTTCTTGAGGTTGAAATTGATGATATACTCATCAAGCCTTCTCAGTTCTGTGCTTTTCAAAGCCTTACCCACATCGTTATCAAGATTTAGCAGCTTCATGACACTTGCTGGATCCAGATTTTCGCCTTGCCATTGTTTAAGCTTCTTTAGCTCGACGGCCTTCGCATGTGTAAAGGCGTTGTATTGTGCTCCGTCAAGAATTCTTCTCAAATTGCTCTCACCGCCAAACCCCTTCGTCAAGGTCTTGAGCAAAGTTTCCTGGCCGTTTTTCTCGCTGTTGATCTTCACAATGTAATCATCCAGTACTTCCAACTTTCGACTTGTGAGCGCCATATGCCCGTCTTCACGAAGCTTGAGCAAAGAGAAAACTGTATCGACGGATTTACCACCGTCCACCCAAGCACTCAGTTGTTCAGCTCGCAGCTTCTTTGCCAGCGTCACCACCTCAGCTGAAGACTGTCCCTGCGTTTGAACCTTTACTAACGCTCTCGCCAAAGCATCGTCTCCGTAGCGGGTCGTGAGGGTCCCGACCAGTGAGATTGCCTTGTCCGGATTCTTCTTGTTAAACTTCGTGATGTACTGATTCAAAGTCTCCATTTTAGAATTTTTCAAAGCAGCTGCCACCTCGTCCCCAAGTTTTAAGCTGTTCAAAACGTCGACTCCACTACTTTTCTTAAGCAAAAACACCTTCAGCTTAGCGGAGTCGACAGCCTTCGATGTACTGGTCACAATAGAATTCAAGGCGTTACCAATAGGAGCTCTCTCTTCGTCATTCCCGTTGTCGTAGGCCCTCAAGAGCCTTTGAACCGGAGCACTGTCTTTCTCATCAAGATAACCGCGGGTGAACCTCATCGAGGTTGTATCCTCTGCGGCGCACGCTAGAAATGCGGCAACAACCAGCAATACGACTGAAAAGAGACGCATCATCGCAAGGGACTTCTGCTGAGTTCTGTAGAAATAGTCCTGGTGTTTGAGGAACGAATTGCGAAATGAAAGCGACTCGACCGAGGCCATGAGGCGCCAACTTGGCGCTCATTCAAAATGGATACCACGAACTTTCCATGTAACTAGCCAGATTATCCCGTGTCAGTTGTAGATCATGAGCTTTGCTAGGACTGTTTAATACTTGAAAGCAGTTGTCATATCGTAAACAAATTGAGAATGGAACTCGGGCCACTTTTAGAATTTCAAGCATCAACTTTAAGATGATCGTCATAGTCATCGAGAGGTGAAGGATAACATCTTCAAGCAGGTGAATGTAACTCAGCGTATCGCCCACTCGCTGGACCTCCCGAGTAAATGCGCGCAGTCGCGATCAAAAACCGAAAGAGAGTAGAAAGCTACACTTCATTTTATGAAGTATTGATCGGTGCCCAATGGAGGTTATAAAATAGAAAATCTCTAATAAAGGCCACTGAAAGAGCATGAGACAGATTGAGAGAGCTGGGCTGCAGCCCAGTTCTTTTAATGCTGCAGTGCAGCATTAAATGAGAAAGTTTCTGTTGCATTAATGTCATGTATGGTGAGTTGGACACGTATTAGCCTCACGCAAAGCAGGTCATGAAACGATTGGATTGACGCGCGCCCACCAAGTGCACCATCGATTGGGGTGCCTACACCTTCCAGTACGTCAGAAAGAAGCGCTTCAGTTAGACTATTTACTTTCAGCTACGTGGGTCCTATGCTATTTGACGCTGGATTTAAATGCTTTAAAAAAATAGGGAGATCGGGATACAATACCGGTAGTAAGAGCGAGGTGTGAAAACCATGCGAAAGGTCACACCCAAAAAATGCGACGTGCAAAAACAACATTTTTTTTAGGAGAGCTATTTTAGAAATGGCAGTCTAAAAAATTAAATTCAAAATCTATACCAGTGGAAAAGTAGCAAACAACCAAAAATGCCAGGCCAGTCAGAAATAGCGTACTCCGCAGACATGCTCGCATTTCTACTGTACCATAACTAGCTGTTTGTGGTGTTAAGATACTTTAAAAATATTAAACACCAGATACAAAAATGTAGGTGCTGTTAGAGTCGACTCGACTCTAAAGGTGTGTACTCGACTGAGTCGAGTACACACCTTTGTACTTTAGATACTTGAGCATTACACCGATCTGTCTTATCGACTTTCATTTTAATATGCTTATTTGTAACAAATAGATAATATAAGCCTTCGAGGCAACGTTCACGTTTTATCTTCGAGCTATGTTCTATTTTACTTCATTTAATCGGTTGATACCGAAACGAGCACTGCATAACCCCACCAACCTGGCGTTCTTCTGCTTAAGCTAATACGCTCCGAATTTTTAAAATATTTTTTTATTATGCATAAGTCATAGAGGTTCAGTACCCGATGAGATATCGTTGCCATTTGCCAAGTGCGTTGCACGGACAAATGTGTCGTCGCACTTAAGAAGATGACTACTGACTATGGTGCTTACTGGTTTGTACCTCAATGTTGAGGTTCGCTACTTGATATTTTGTTTGTCGCAATCTACTCAAAATGGTTTTACCTATACGATGAGCAACACGCACAAACAATGCACTTAACGTCAATTAGCCTAATATCCAAGACATGAAGTTGACGTAGTTAATGATTTAAATCCTAAAACGCATGTAGCGAAGAATCTAGACTGTATGTTATACTGGAACGTACGGAGGTTAAAGGGAACATCTTCACCAAGATTAAGCTAATCGTACGGCATGTTAGCAACATCACTGCAGTCATCTGTGTGCGTGCAATTACTCCGACTTAATTTGTTTTAAGCAAGTAGTAGCGTTTCCCTATAATTATGGCTGANCCCCCCCCCTTTACACACAGTGATATGGACGACATTTTCTTCTCAAGACACAAAGCATTGTACTACAAGTTCATTTTAACTAGCACCTATTGTCAAAGTCCAGATGCTTTGTTTTAAAATGATATCTGTGTCGTTGCCCTAGCAGTTTTAAACGGTTTCGTCCACCGTTTGAAGCGAGATTTCTATAGCAGCCTTATTGTAACCAAACTATTTACATGAAGAATGAGGTGCTGTTACGTCGTGGCAACCAGTGTTCGCAGACTGTACTATTAAAAATATCTGTTTAGCTAACTTTTTGTTACTGTGTTGTATGTGTAGCTAATATGTTAGAGAAGTATTAATAGTAGCATCTTAGTTTCTAAATTTGGACATGGTTATATATAGGCCATTTCTAAAATGGTTAGCAACTTTATGTAGTACGTAATTTTTGGGTGTGGTCTTTCGCATTGTTTTCACGAACTCCATAGATTTCCTCAGCACGTGAACAATAACATTCTCGCCTTAATTCCCCTCATTCCCCTCAGCCTTCTGGCTCAATGGCCCTCACCGTATACCAAGTACATAATATGTACGATAATCGTCTATCGTTTGACAGAACGTGATACCGGATCAGTTTTAATATTACGTACTTGAAGTATACGGCAGAATGATGGCCTTTTTTTGATCTGACTGTAGCATACAGTGCATGTACGAATTGTAGTACGAGGGGAAATGCAAGTACAGTATCGATCAGCACCCACTTTAGCTGATCGGCTATTGCCACGGATTTAAGGCGTGGTCGTGTTCTTGGCTAAAAACGTTTTGTTACTGTAACTCATGAGGGTGTTTTCGTTTAAATCTAATACCGGTAAAAACTAGACTCACAGTCGTGCCAATAACATTAAAACGCTCACGATTGCGCTGTGCTACACTCTTTTAAATACCTGTAATGACCAGTGTATTAATAGTGTACACTGCGTACGCAGTGCATCTGGTCTGCATGCACTGTACATCAATACCAGGGATGGCATATTCCCAGGTGCTTGCCCATTTTCCCAGGGAATCCTGTAGG

>Contig_86
[truncated: 5,185,945 more chars]
