# Supplementary material for: Identification of Avramr1 from Phytophthora infestans using long read and cDNA pathogen‐enrichment sequencing (PenSeq)
Source: Mol Plant Pathol. 2020 Sep 15;21(11):1502–12. doi: 10.1111/mpp.12987 (PMC7548994; doi:10.1111/mpp.12987)
Supplement: Supplementary file 9 — NOTES S4 PacBio PenSeq contigs of US23 [file MPP-21-1502-s009.docx]

>Contig_6

ATAGTACCATTAATGGTGTGTTCTATCCTAAGAGGAGGACGAAGAGTTATTATTCAACCCATAGCTAACGGATTCTACTGACGCAAGAACACGTCTGTGCGGGTCCAAAATTGGGAAGCACGAGGAATATCTCGATACAGAGGAGGAGTTGAATCTCGCTCTCTTATTTCCTTCACATCGCAATTTACCAGCATCCTGCAAGCTCCCCCCCCTCCCCCCAACTTTCAGAGCTCCACGTGTCTCAAACACCTGAAACATGACGAAATCTACTTCGCGACAGAAGACCAACAACGCCAACACTAGCGCTGGAAAGAACGCGACCACCGCCCCCACAACACCTGCGAACCCGGCCAGCACTAAAATTACCAACAAAAACACGAACGTCACCCAGCATGCCAACAAGAACACGGGCAAAATGTTCGCACTCACTGAGGTGACTACGGCAAGCGTGCAACAAGCTCGCCAGATCCGCAAGACGGACTTCGAGCGCTTCCAGAAATTCCACAACTTCTTCATGGCTAATGAAGAACCGACTGCACGTGTGATAAACGACAAGGCTAACGCCAAGCGTGAGATAGTGGACAAGTGGATTGGTGGCGCTGTGAGCCCGAAGACGCTCGCGTGTATCCAGGAGATCCAGGAGACTCAATCGGGACTTCTGGAACTGCTCATCGTGTCAGCAGCACTCACCAAATACGGCGAGAAGGAGCTGGCTCGTAGAGACAGTCTAGGCGAGATGGACTACGTGGTAGGTGCTCGCTTCGGCACGAATGGGCTGCGCTCGAAGCAGCACGCACCACACAGTGTAGGCTTGAATTTCGTGCTGCGGTTGGTGCTCGGTCTGGCACCGGAGAAGCGTAGCTCGTGTGAAGTCTTACGTGAGATCCTGGGTCACGAGAGCAACGGACGCCTTGCATTAATCCCAACAGAAAGTGGTCTGCAGCCGTATTCTGATGCTACTGCTTCATACTCGGATGACTGGGCCGAGCTTAAGCCAGATCAGCTCCGTCAGATGGCAAGTCAGACGCAGTCCGACAAGTTCCAGGACACCATGCAGCAGCTCAGTGGTGAGCACCTGTACGATGCGATCCGTACACAGCTGAAGCAGACTGGCGGTGCGTCAGTGTGGGATGCTACTAAGGACAAGTCTGCACTCAAGGTGAAGAACATCCGTCCGCGCATGGGGTCGGCTGACAAGAATGCAAAGTCGAAAAAGGAGGCCGATCGTCAGCAGAAGCAGCCTGTGAAGATAAATGAAGCTTCCCAAGTGAAGAAACATCTTCCGGTTGAGGTTGAGAAGGTGACGCTGGTTCCTCCACCGGCCCCGGTGATCATGAAATCGACTACCGATGCAGCTACCAAGAAAGAGGGTGGAAAGAAGAAGGGGAAGAAGGGGAAGAAAGCGGACGCGGAGAAGAATGTCGCCCCTGTTAAGGACGCCACTGCGGTGAAGAACATTGCGACTACCAGCCAGAAAGCTGGTGCCATCAAGAGCAGCGCGCGTGACGGTCGGGCGGTTGTCTCCAAGGCCCTCTAGACGTGCATATTTGGTGATGCACTCATCTTTCATCTCAAGAGATTTATTAATAAACTTTTTTTAAATATAATTTGTTTTCTCGTAACGAGACCTGCACAGTATCGTACCGTATTGTAAAACCCGTGAAATCGAAAGAAAGAATCAGTGCACATTTCTCTCAGGTTTCACGAATACACTTTCATTCTTGAATTTGCATCCAGATGCATCTCTTCTCTCTGACAGCCGTAGCTTTCGTCATCGCCAGTTTATCTGTCGACGCATCAGTCGCGAAAGATCCACGAGGGCACGCTCCCAACAGGACTGAAGTCGATACCGTAAATGCGAGTTCAAGCACGAGGCTTTTGCGAAAAAATAGTACTGTTGATCTAGTCGGCGAGGAGAGAGCACCCAGCGTCGTAGAAAATATCAAGGCGTTGGTCAAGTCTTCAGCGGTGACTCCAGCGAAGCTTCAGCAATGGCTAGACGAGCGACTACCTGCGGGGCTAGTGTTCAAGAACATGAACCTTGACGAACCAAAGATCTTCTCTTTGTTGCATGAACCCAACTTTGTTAAGTGGGTCCAGTACGCCGACGACTTGAGTGCCAAGTCATCTCATAAAGAATCGTCAGTGATCTCCACCCTGACGTCATTGCACGGCGACAAAGTTGTCTACGACACAATTCAAGCTGCTAAAAAGTATCCACAGCTGAGTGAACTCGCCCTTAAATTGGAAAAGGACCAGATACGCTTCTGGATTGCCACTCGAAAAGACCCGTCGGTGTTTTTTGAAGCCCTCAACCTTAACTGGGTAGGGACATCCATCTTCTCAAAACCTGAATTCTCCGCTTGGCTCAAGTACGTGGACGATGTAAACGCAAGACATCCCAAGAAAACCCCATTTTCGATTATTCCTACGCTCAAGCAACATGTAGCTCAAAGTGACGAAGCCGACACAGACGTACTTCTTAAACTGATTGCGAACGGGAAAGCAACGGCTGAAACCAAAACTGTCGCCAACAAGGTAGATAGTGCATTGTTTGACTTCTGGCTCAGCAAGCGAGAAACACCCGACAAAGTTATGGACGCGTTCAAACATGGCAGTACAGCTCAAGCTTTCTTGGGGAGTTCACGGTGGAAAGAGTGGGAACGGTACTTGAGCGTTTACAACGCGAGATACCCTGAAAAGAAGACCACCGTGATAGAAACGTTAACGCGGAAGTACGGAGATGCACAATTACTCGACACGCTTATCACCGCGAGCTCGAAAGGTGAGACGAAAACGCTTGCAGCCAAGCTGCAGGCACAGCAGTTCGATAGGTGGATGAGCCTTAAAGAGTCTCCTCTCGACGTCTACAACAGGCTACGGCCTTCATATGGGGATAGGAGCTTCTTCGACGAGCCACAACTCAATGTGTGGGTCTCGTACATGAATGTGTTCGTCGACAAGAACCCCAGCAAGGTGGACAAAATGTTCTTGGAGTTAGGTGATACCTTTGGGAACATGCATCTCTTTCGAGTCCTCGGAGAAGCCAAAAAGTTCCCCAACATGGAAAGCACTACAGCCAAGCTGCAGATGGAGAAGGCTTCGACTCTTTTTGCCAGCGGAAAATCCCCGGAGGATATATTCAGGGTGCTAGCGCTTGACAAGGTCGGAAATGATATACTCAGCAACACGCTGTTCCACAAGTGGCTGGCATATCTGCAGAAATTCAACAAAGAGCACGAACAGCCAAGGATCGTGGTTTGACATGCTACGTATTATTTACCAAATGTTCGGTCTCGAAAGGATTATCGAGACGGGAAGAAAAAATCCAATCACAAGAGGGATGGCTGAAAAAGTGGAGGATGCGTATCACAACTACTGGTTGGATATTAAGATGGAGCCTAAGACAGCCTTCCGCTCCCTGCATCTCGACGAAAGCGGTGAGAAGCTCCTTGCCGACCCAAAATTCAACACGTGGGTGCAGTACCTGAAAACCTTCATCGACCGATATCCTAATGAGAAGACGACAGTCATCGACGGGCTCAGGGATAACTATCATGACATAGCTCTACTCCGAATGTTTTCAGCCGCGAAGAATGATCCCAGCACGGAGAAACTCGCTACTGATCTACAGAGTGCGCTAATCCTCAAGTGGCAAGATGCGAAGAAGACACCAGAAGAACTAAAGAGAGTGTTTGTTGGTGTGCCAACCTCTGGTGAAATAATCGATCGGTACGACAAGCTAATATCGGCGACGAGAGCCACCTTATAGTGTATTTGACCGAAGCGGTTATCGTGGAGAGCGAGAAGGAATCTAATGTAGCCCTTGATTTTGACTTTTTGGTAAGCTTTGAGTCGCATGAGCAGAACTGGTTCCTTCTGACTCGACGATACTACAGGAGGTGGCAGTGGTTACCGATCTCCATCTTCGATCGACGTGCCCTCCAGCCACTCGAAATATACAAGCAGAAGCGGCTATCAATTGTGATTTTCTCTGGGTGGAACCTCGTAGAAGTGCGTCCTTTAATTCTTGGGGGGGGCATAAATGCCAAGTTGTACCTGTGTTTTCATAATGGACCTTGCATATACAGTCTCCGTGTACGATTACGCGCCGTAAGCTACCAACTTCACGTAATATTTTACACTACGGCTGTGGGGGCATTCTTGATCGTCGAGCAACTCACGATAACAATTCCACGAAAGCTGGCAATTAATATCCTATGTATCATCCTTGTATGAACAATAACAGTTAGCTTTCAGAGCCATCAAACGTCAAATACTGTACGCAGGGCTATTCTGTGATGACATAAAGCTAAATCTTAATTTGAATAAATTTACATTGTGAAGTCCTCGTTAATATTCGTTTTGAGCTGCTGCCATCATATGCCGCGTATATTACCGATATAATCGAGTTACGTCTGTTGGTGCTGTAGTCGAGGCCATATTCGTACTGTGCCGCCAGTCGTGTGACTAGCAGCAGGGCTGCTCAAAATTAATCAATAGACCAAACATTACTATTTTGAAACTAATAGCACTCATAATTGTGGGTTTGCTACAATAAAAACTTTCCCTAATTGTTTTGGATTGAATTGGGATTTGAAGGGCGCTTAAAGTCGTCGTTCGTACAATCAAAAAGTGAATGTTGCTTTATTTTTTTAAATGTCAATCGTATTATACTTCATTTGCTGCTCTCTAACGAAAAAAGACATCATGTGCATATAAATTACATTCGCAATGGACTGACAGCGTTTATCTGATATTTGGCGAGCTATGAACGTGCGCAACTACAAGTAGCTGTACTAGATTACATTTCTTACACGTAAAAAAGTACCACCTTCTGCCATTAGCTGTGTAGCTTATTTTGTATTTGCCGACGTTTATAGACGCTAAAAATGGCAACCCGTTCTATAAATCGATGTACCAGCATGCGGTGCCTGATAAATTCACTCCTACTTGATTGTACAAAAGACTATACGAGCGAGGTTGGTGAAATTCCCACTCGATGATAAATTTAATTGTAGATATGCTTGATTTTCTGCTATTTGTTTAGACCTCTTGGTAAAAAAAATAAACGTGGTACATGTATTAATAATGTCACGGCCCATGATAGCCAATAGGTCGCTCATACATGTACCAAGAAAACGTACAGACTCTTAAGAGTCAGGAAGTGTTTCTGTGCATTTTTTGTAGATATGTCTGGTCAGGGCTAGGTTTAAAGAGGCAAGGATTTGGGAAGGTGAATTCGATCTTTTAAACTTAAGTTTTAATCCTGCATTTTCAGGCTAGAGGCAGGTGTAATTAGGCGCAAGTCGAAGCTAGAGTAGACCTACCATGCGTTTGTACGTATAGCTGGAATCACAAGACTCTTGGCGTTTATCGGGATTATAACTACCGTGACACCCATCCCATCGGGGACGGGAAGCAGTACGGTGTTTTTTTTTCACTCATTGACGTGACCTGGTCCTGGCCTGGCCCCGGACGATGATGTATATGAGCAAAAAAGAAGAAACAATTTATCCCAATCAACCGTAATCAATCATATGTTCAGTACGGGTACCCGAAGAAGTCCAGAATTAAGTTAAAAGGTTTTTAAATTAAAACAAGCACTTTTCAATCTGTCTTGATATCAAAGTTAAACGATTAATAAAACAAATGCTCTTATTGTAAAAAAGCTCAGTCAAATCGCTACTAGCAGAGTAGACAGCATCGTTGTCGTGCTTGGGCTGCCACATATTAACGTCTTTTCGTATATTCCGTTCTGTTTGTCCTTGGCGCTTTGAAGCCCCTCCTGTGCCTGCTTTGCAGCCGCAGCATCATCCYACTGCTTACATGGAAAAGTATACCCCTCATAGTATGAGGGGTATACTTTTTGCGAGCAAGAGCTACAATCCATCAAACATAACACCGAAACACTCGCCCATCTCCTCGGCGATGGTGTTGCCGACACGTTCAGCGACTCGTTGCATGTAGGTCTTCAGCGTCTCCGCTGAGGTAGGCTTCATGCGAACTAACTGTCTCGTTAGTGGGTTCTCAACCTCGCACAGAGGGAGATTCCGCTCGACAATCCAGCGTAGCCAGTCGTACATGTGAGTCGTCGTTTCGTCCACGAATCCAAAAACCTCGAGGGAGGTCAGGTTTCGGCGCTGAAACTCAGCATACTCTTCGCCGTGAGTGGGATGCTTCGAGTTCAGGTGAGAGATCAGGTTTGTGTAGCCTGGGCGCTGGGCTTGCTTGCGTGGCTGTTCGCAGATGTTGCACCGGTAGAGCCCAGGTTCGAGAACGGTGTAGAAGAAAGCGGCGAGTTACTGGGACGACATGATGTAAATTAAGAATGACCGTCTTCTCAAAGATTGAAAGTAAACTAGGCGCTGAAAAATGGCTGGTGGAGAGGAATGGGTGGCTGCAAGCCACTCGAACGGTAAGCCTTTATATTTGTATTATTCCGTCCGTATCACACTATATTATTATCCCGAAAAAACAATAATCCGTAGTATAATAAAAATAGGTAGTAATTGCGGTACTGATATTATTTTCTTGGAGGCCTGCTCATACATGTCTAATTAGAGATAAGGGCGAGAAAGGTCTCTACAGACGTGGTAAACACAAACCTCTCTAGTTGGTTTGACAGCGTTGCGTTAACTGCTACGAACCGTCGAAAGTGGCTGAGGTCGTTCTGAATATCGGGAGTCTTCGCCTTCAGACTATCATAACTCACTGCAAAGTCTACAGCTACGCTACTATTGTTATTATTTACTTTTATTAATACATGTATTTATAACTGTGCTGTAAAGTAGCTGTGACAATTGAATAACGAAATCCTTCATAAGCAACCGTAATCAATCATATGTTCAGCATCTGAATTTAAGAAGCTTCGTTTAAAAAAAAGGCTGGCGATACAGCCCATGTTTTTAAAAGGGTTAGGTATCGAAACAATGGTGATCATGGATACATTTCCAGGTGCCATCCATGTTTTCTGGGAATAAATGACGAAGGTCCGGTATTTTATCTTATACATGTTTCACATGTTTTGTAACATGTCCAAACATGTTATGAGTACGACGTAAAAAAGTAAGAGTGAAGAGGATTCTGTGTTGTCGGCGACACCTCACCCCCACATTCGTCATGGGTTGAAACTACCTTACGTAATGTATCCATCTCAGGTGTCTGATACTAAAACATGT

>Contig_8

TACCGTCTGCTTCTTCTGGTATACTTGTAGTGACCGATTCGTCACTATCGTACGTGGAGCCAACGCTCAAGAGCTGCACCCTAGAGAATATGCCTGCACTTTGGTCACGCTGATGTTCATGGCTGTGTTACGAAAGGATGAACCCCGCTTCGGCATTTTCGGACTGTTGTGTCTCAGATGGTGTGAGGCGTGGCACCACTCGATCTGCCTCTTCGTTCCAGATGTGCTGCAACTTGTGTGGACGTCGTGCTGTCGTTACACGAAGGCGATAGCTGAGGCTTCGGTAGCCTCGATCGATCGAGTGGGCGAGTACCTGATGGGCGTGTGTCTGGTGGGACAACTGTTATTGTCGCTTGTGTTTTACCTGCCTAGAGTATTCTATGAAGGCGTGGAAGGTGCCCTATGTGGTTGGATGGGGCGTTGCCTTAGCTACTCTAGTGGTGGACGTGATATTGGAATTCTCGGTCGCGAGTCTCATTGGGAACGTGTTCGTATTGGTACTGGGCTGTTATAGCCACGTTACGAGGTACTACTGGGCGTCGAGGGTGAAGACTGTCGACGGCTCTGGAATTGAGGCCGCACAAAAGGGTTGGGCTAAGCTTAAAAGTCGGAAGGAGCTACGAGAAAGGAGAGAGAGGGCTAGCCTCGACCATTTGCGTGCAGGTCCTAATGATACGACCGCTGAGGCGCAAGGAAATTCGGATTCGACGTCGATTGGAACGAGGTCGATCGTTCCAGATCTGCCGGTCGACTCGGCGATTGGACCTGCTGGGAAAGATGAGGCTCGGCGAGGACTCCGAGCGGCAGTCGGGGAGAAATGTCACGGTCTTCGGCTAGAGGAAGGACTCGCTATTCTGGACGACTGCCTACGGCAATTTAGCGAGCGGCGTAAGAAGAGTGTCAGGCCCGATCAGTCTCGTTCCCCGTCGCCGACTACCTCGTCCCTTGTCTAAAATGGAACGAAGGAGTTCAGACAGTGGTACGCTGACTCCGTTTACGAACGACAACTAGAGGTACTGGTGCTGGTAGGAGGTACTTTGGCAAGGTAGAGGGCTGGTGAGAGAGCTCTCAGTCGTGAATTTCCTTCTTTTACGAGAAGTGCTCAAGAAAAACAAATAAGATACAAGTATATTTTACACATCTAATACGTCTAAGTTCCTACGTCGGCTTTCACAAGATACACGACCCCGACAAGAGAAGGAGACCGCGACAATTGGCGATGGGCGGAATAAGAGCAGACAGTCATTGCAGACCACTGGACCAGTCGCCTGCAAAGACTGTCTGCCCTTATTCCAGTGACTGAAGGCAACCGACACTATAGTGGCTATTAATAGACTTCCTGATATTTGTAGGCGTCGCACAGCTTACGGATTGATGAGTATTATACTGTAGGAGGTGAATTAACTTAWGAATCGGGCATTACTATGGACTCGGAGTACATGTACCAGTCTTGCCACATCACACTTTAGACATTATCTTTATGATACGACTGGCTTAGATTCATTAAGGTTTGCGTACAATGCCAAGTAATGTTTGCAATTTTGAATTTTGTGCCAAGAGTCAGTTTTTAACCAATTACTACCGTTCCGAAGAATGGAGATCGTATCACTGGATTAAATAGGATTGCGGTGTCTACCCCATAGACGTCATCATTAGCTACATGTGATAGTACTGTACCGGTACAGTAGGAGCAACATACCGGTACCTAAATACCTGCTGCTCGAGAAATTATTTTTACAGCGGTAGACATTTATGTAATGACGTCGCGGAGGGATACCAATCATACGGTTAGAAAATGCTGGTGCGTTATGCGAACCTAATTTTTCGGCGACAGTATAAGTTTTTTTACTGGCCTCTGGGTACCTTTTCTGCTGGATTATGGTAGTGGCTTTACCATAAACCAATAGAAAAAGTCTGCCTATCCCATTACTTGTCGAACTGTATACACCTTTAAAATACAGTACAGTACAGTATATATTAATAGTATGGTTTGCTTCGCATGAACAAGTGACTCGGAACGTAATAGGTGGTTATAATAGGATAGTATGGACTTAGTTTCGTGAGCATTTATGTGCTGGTGTTCTTTTCATTTCTAACGACTCGACGAAACACATCGTTCATTTATCGCGAATGCCCACTGGGCATGCATAGGTACAGCCTGTCATATGAGTGCTCGGGGGAGGGGGCGGGGTTAATCCGTTCCGTGTCACTTCACGTTTTGTTCACCTGTACGCAAGACGATGGGCTGCCGGTATGCTGTGCTCGCATTAGCTGTAGCTTATTTCGCAGGCTCGATCGCAGCCAACGATTCGCAGATTGTCGCTGTYAAAGGCCCTGCTTCGATTCGATTTACACCTGCAATCCATGTCGTCCGTGGAAGGTTCTTGAGGGCTGCGAACACCGCTGACGAGCGCAACGAGGACAGGGGAATCAATCTCAAGTCAATGCCTGGCTTCGAAAAAATAGCAAGTCTGTTCACGAAGAAGAACACGCCAGGCCCGCTCTTAAGTTGGTTCGAAAAGAAGAAGTCACCGGACTACGTATTTCTTAAATTAAAGATTAACAAGGGCAAGCAACAGCTTTTTGACCACCCCGACTGGAACGTCTGGGTTCAGTACACGACCAGTGTGGTGAAGTCGGATCCGGAAGAAGCAATGATCGCCGCACTGAGGACACACTACACTGACGATATTCTGTCAAAGCTTCTCGAGTCGGCCAAGAACGTCCCGAAGACTAGTGGACTTGCCACCAAAATGCAAATGGAGCATTGGGTGGCCAGCAAGACACCGAGCCAAATGTTTCAGTTCCTTCGGCTTGATAAGGTCCGCAACGGAGTCCTCGACGACCCGACGCTTTCCATCTGGATTAACTACATGAAGCTGTACAATTCCAAGCCAGTGAACAAGAAGCAGCAAGTGACTTTAGTCAGCATGCTGACAACACACTACAAAGATCGAGGAGTGCTGGACATCATTGAAGCGGCGAAGAAAGTCCCGAAAACGGCTCCCGCTGCGAGACAATTGGAAATGGAACAAATCCAGTTTTGGTTAAAGAATGGCAAGTCACCGGACGAGCTACTCACGGTTTTGTCCCTCGATAAGGCCGGAAACCAGCTCCTCGCCAGTCCGCGATTCAAATTTTGGTCCAAGTACGTCGACAACTACAACAGAGACTTCCCCGACGAGGCTACAACCGTGATGGCGACTTTGCGGAACCAGCTCGGCGACGAAGACATCACGCCAATTCTAATAGCTGCAGGGAAAGTACCAAGCACCGAAAAGGCCGCCGCTAAACTGCAGGCCGAGCAGTTCAAAAGCTGGCTACGTGAAAACGAAGATCCGGCGAAAGTTTTCCAGCTGCTAAAGCTCGACAATTCAGCGGATGATCTTCTGGGCAGTCCACAGTTTAAACTTTGGGGGAAGTACGTGGAAGATCTCAACTTGAAGCCAGAACACAACGACCTTCAAGTCTCCATTATCACCATCTTGCGGAAAAACTATGGCGACGATGTGCTGGGGAACATGGTACTTGCTGGGAAGAAGGCTCCTAGTACGAGCTTTATGGCCCGACGACTTGAGGACGAGCTCTACAAGGGATGGATTGCCGCAGGCTCGTCACCCGACGGTGTCTTCAAGCACCTGAAATTCGACAAGGCGGGAGAAAACGTGATCCAAAGCCCGCTCTGGGGCCTGTATACAAAGTTTTTGGAGCACTATTACAAGTCGTTCCCGACGCCAATGATGTCGGCACTTGCGAAAGGCTACGATGGAGATGCGCTCGCGAAACTTCTCATTGCAGCGGAGAAAATCCCGACTTCGAACACGCTGGCAACGAAGCTACAAACTGGCCAAATTCAACGTTGGCTGGATGATAAGGACCAGCCAGGAAAGATATTCAAGGCGCTCTTGCTTGACGACATGGCGGACGACATTCTCACCAGCCCGCTGTTCAATACCTGGACAAGGTACTTGGATGAGTTCAACAAGAAGTTCCCTGATGAGAAAGTGTCCATGACGGACACGTTCCGCACCAGCCTGGACGATGAAACTTTGAAAAGTTTACTCATCACAGCAAAGGAACTTCCCGACATGAAAACGCTTTCGACCAAGCTACAGACAGTCCAGATTGAGCGTTGGTTGGCGAGTAAAACCTCTCCGGAGGATGCTTTCGCAGTACTCGCACTTAATAAAGCAGGGGGCAACGTTCTGTCGAAGCCATTGCTGAACACGTGGGCGGCGTATCTGGAGAGTTTCAACGCCAAGTTCCCAAGATCGCGAGTTTCAATGATTGACACATTTAGAGAATTTTTCGGCGACAAAGCGTTACTGACTACGCTAGCTGCGGCGAAGGAGGTTGAAAGCACCAAGAAAGTGGCGACGAGTTTGCAGGATTCGCTCCTTTCGAAGTGGGTACTGGCTAAGAAACCCCCAAGCGGCGTAGCTAAGCTGGTGGGGACGGATGAGGCGGGCGCGAAGCTACTGAAAACCTACACCACCAAGTATATGGAAAGATACGGGCAGTGATTCTCTAAGCTCGCAGCTAGAACGTAGGGTGAACCATAACTTACCGATATTAGACTTGCAACCACGAGCTTTGTTATTTAAAACGGTTGCTGATCTGTAAAATGGCAACTCAGATTTCAACAAAGGATAAATGCCAGCGCAATTTTAGATACATCCTAAAACTAGTTGGTCAAGGTTCACAATACCGGTTACTGTGTACTTATCAATGGGCCACTCCTCATTTGAGTGACAAGAAACCCCAAACATGAGCCGAGATCATCGTCTGTCACCCTCTTTATCCTCTGTTCTGTAGATGTTTCTTTTGACTAACCTCTGATCTCCCGCCATGCAGGTGACAAGGGCCGTAGCGAGCGCAGACGACACGCTAGGACGTCGTCAAGCCACAGATCCAGTCGTAGAGACGAAGAACGTTTGTCTAGACATGGGTCAAAACTGCCGAAGGGAGCTAAAAAGATCACAAAAGAGGACTATTTTCTGTGCCAAAAGCAGTTTCGCGTGTGGCTGGCACAATCCAGGTACGTGGCAGAGTCGGCGATTCTGCGACTGAGACCAAAAATGGATCTCATTTGTGATGATTATTTTGCAACGATAGGAACAAATATGTCGATGACCTGTCGACGGACGAGGCCATGGAGCTGTTCACGGACGAGTTTGCGAAGAAATGGAACCGTGGGAAGCTGTCCAAGATGTTCTACCAAGGCCTCCCCGACGCTGTGGTGGAGCAGACGAAGCGGACGCGTCATCAATGGGGTTTTGTGTCTAAACTGGGAGACAGGGAGAAGTTTGAGTTGGCAACAGCCAAAGATTCGGTGGATGTAGCCACAAAGAAGAAGAACTTGCTGGTGTCTGACGAGAAAGCGAAGGGGGGAAGAGAAGAAGAAGAAGGAGACAGGAGCAGGTCTAGACGTCGAGCTAAAGAGAACGGAGAGGGCGATAGAGAAGGAGACAGAAAACGTCGCAAGCTGGAGCGTAGAAGAGATCGAGAGTACCGAGACGTGGTGATGGATGAACTCGCGCCCAGAGCGACGGGAAGAGAAGCGCAGATCGAGAAGAGACGCCAAGTTGGAGACAAACTGCACGGAGCCGCGAGAGACCGGGAAGATACGCGAGATGGACTTGATCTAAGTGACGCCTTCTTGATGGGCGGAGGTGGAGGTGATGACGATCTGAAGCGCCGCATGGCTCAGAGGGATGTGGCACGACGTCGCAAACAGGAAGAGCAGCAGGACAAACTAGCGGGTCTGAAGGTACGAGGCTTTTTTGGAGAATATTGTAACGTGGGTTGCCGAGTTGATGAGGTTGTGCTTGATCGTGCTGCTAGGCGAAGGAGTCGGCGCGTATGGACAAGTTTTTGGAAGATATGGGCCTTGCTGGACCCAACGCGAATGGTGGCAAGCCCATGACCATTGCGCCTCGTCGATAGACACTCGCTGCATGGATTCTCGAAGGACGTAAAACCAAGGAAAATATCATCTGTGTAGTATTAGATACGCGGTAGTGTGGGTTGTCAGTAGCTTCGCGCTCGCTTCATCCGCCCCAAGCTGTTTGCGTACGACTGCTGGGATTTTCTTTGCAATAATCCATGTGTTGATCAGTGAGCTCTCAAAATCGGTGGCGATGTTCTTGGTGCTTTGAACCTCCTTCGCTGGGATCAGCATCTTCACGAGAGCTTTGTCTCCAAAGAATTCCCGGAAAGTGTCAACCATTGACACTTTCGATCTTGGGAACTCGGCGTTGAACTCCTCCAAATACTTGGCCCAAGTATTTAGCAACGGGCTGGACAGAACGTCGTCCCCCCGATCATCAAGCTTTTTTTAATATATCCGCCGGGGTCTTTTTACTCGTCAGCCAACGCTGGACCTGCTCACTCTGCAGCTTGGTCGTAAGCGTTTCCATCGTTGGAATCTCCTTTGTTTTGACCAACATACCCGCGATCGATTTGTCATCAAAGCGCTTCCGCAACGTATCGATCATCGAGACTTTGTCTTCCGGGAATTTGGCGTTGAACTCATCCAAGTACGTGGCCCAAGTGTTGAATAGCGGACTGGTGAGAGCGTCATCTGTTGCCTCGTTAAGCTTGAGGGCCATGAAGATCTTGGTCGGCCTGTAGCTTTGTAGCGAGCTTCTCCGTTTGTGGCACTTTCTTCGCTGCAATGAGAAGTGTTGCCAGTGCGTCGTCTTCGTAGTTTCTCGCAAATGCTGATATCATCGAAGTTTGCCTATCTGGAAACACTTCCTTGTGGTGCTTTGTATACATGCTCCAGAGAGGGCTGGCAAAAGCCTTTTCACCTGCCTCGTTCAGCTTGAGATTCTTAAAGACGACGTCTGGAGTATTCCCGTGCAGTATCCATCCTTTGAAGAGCTCGTCTTCCACTCGCTGAGCCATGTCCTTCGTGCCAGGAACCTTCATCCCCGCAAGAATCATTTTCGCCAGCACGTCGTCGTCATAGTTGTCCCGTAGAATGTTCCTGTAGCAATAACTATCCATCATTTCGGTACCAGAGGTAGTAATAAGCGAGACTTGGCGGTCGTTGTTTTCCGCCTTCAGGTTGAGATTCTCCACATAAAATATTATTTGCCCCAGTACTTGAGCTGAGGGTTGACTAGAAAATCATCGCCCGCCTTGTCGAGTTGCAGCAATTGAAGACCTTATTTTTCTCCCCATTCAATCAACCCACCTATGGAAACTTCGAGCACCCTCCAAAAGGCTTTAATTCCACCACAAAAGGTGTACATTTCTTAAGCTATAAGCTAGACAGCTTATAAAAAGAGCGGTTTGCATCAGATGACCGCAGGTATTAATCTATGACAGGAGAAGTTGGTCCAACTAATAGTACCGATTAGCAATTTCTTCAAACTTCGAGAAAACGATTTTTGTTCTTCAAAATGATTTTTGGGGCTCACTCCATATTAAGCTCAGCTTCTACTTCACGTACAACCGAGTTTAAACATCAAATGTGAACGCGTCGGCCTCGCACCTCTGTCGCTGGAATCATGCTAGATTTATTGATATGGAGAAAAGCATTCCGTCTTGCTTCTCTCGCATAGAGCTTGGCTCTCGTTGTTGACTTGCGCACTTGTCTCGCTAGAACAGCAGCTGATTGGTATTCTCCGAGAACTCCGTCACGCGCTCATCTTCGGGCAGCGCGCCTAACGCCAAATAGCCGGCGTACTTC

>Contig_9

TCAAAGACAAGAATTTTATGTCTTGGGGACGGGTCTCAGCTCAATCTCGTCGTTATTGACAAAACGCAAGAAGTGGAATCTGGTGTTGGTAGTATTGTTTCTGCAAGAAGTGCATTGGTAGCGGGCTCCAATGGACAGGGGACTCAACGTGAAATTGCTGCTGTACAATCTCAAATAGCGTCTGGCCGTCGCTACATGGCAGAGGTTATGAATGAAGTACTGCGGTCCCGAAGCGAGTCACATCGAGAGATGCAAAAGATACAAGCTATCTTGAGACGGATAGCCATGCAGCCGTTCACACCTCGTACTACTGATGGGCACGGAGTACCGTATCCACCAACAAACACTGCTCAAGGTGGATTTCGTGGACAAAACGCGGCACGTCTTTCAAAGAGACCTAAAGACCTGTATGAACTTTGGCACGAGTATCAGCTCGGATCCGGTGGGCTAAAACCAGCCAAGGAGTTTACATCCATTGAGCGAGGCGCAAACAAGTTCGCCTACTCCAGGCGGAAAGTCTTCTGGGATGTTATTTCTCAATTGGTTCGCTCTGGACACACTAGCGACTCTGCAATAGATAGAGTGTATCAAACGTACGGTAGGAATCTCTCTGTGTCTAGTATTTTAGTTAAACTTCGTACAGATCGTAGACGTGGAGGACATCCAAGTCTGCGATTGTAGCACTTCAACACGGTTCAATTCAGCCAATCAAACCTTATCCTTCGAAATATTAGCCTATCAAAACTATGGTTGCAATCCAAGTTGCATAAATGACTAATATTTATACATTATTGGCCATTTGCAACCCGGGTTGTATTTCTTGAAATCCGAAGCTACGATTGGCTGTCTTCTCGGTATCAGCCAGATCTGAGGTCGATACCGTGCCTAGATGTGGAGTGCCGTTTGCAACTATCGTTGCATGTACGGTAGCGTGAACGGTCGACTTTTGCAACCCTAGTATACAAATTTCAAGTCAAGTTACCTATAGTGAAGCTAAGTCACGGTTGCAAACGAGAGGGTACACAGCTCGTATACAAAAACTAAGTCATGGTTGCAACGTGAAAGATTCAGTTTGAAAATACAATCCACCACAATGAGCACTAGCCGCTACAAGGCAGAGCTCGTAAAGTTCATGTCGTTCAAGGACGACAAGGAGTACACTGCTAGCCACGAATTCACGCCAGCGGACCTCCTCAGTATCACGCCTGGACTGCTGTGCCGCTGGATGAACACGCGGGCCTACGGAGATTCAGAGCCAAGCGAAGACATGAGGCCTGTTCACCTTCGGTCGAGCACGCTGGAGTTCGCCAAGAAAGCCATCTCAGCGTACATGCCTAGGATCAACGCACCATGGGACCCCGTGGCCATGCAAGGCAATCCAACACGCTCCGATGATGTCAACAAGCTCATAAAAAGAGTCAAGCGCTTTGAAGTTCGTCGGGAAGGTGCTGAGTCAAAAGCTCGTCGCTCTTTTGAATTTGATGAATTCATGAACGTGTTGACGCTGGTAAGATCGCTGCATTCACGCTCTGATGAACAACTCATGGTTAGCAGTGTTTTGACACTGCAGTGGCATATTGTTGCTCGCATCGACGACATGATGAAGCTTCAATTTAACAATTTCACTCACAACACTCAGTACCCGTCTACTATTCTATGTCAAATGCGATGGTCGAAAAATATATCCGAAGAGAGAGACGCTCCGGAGCAGATTGTGGTTGGTAGTATGGATCCCAGAATGTGTCCCCTGCTCAATCTTGCAGTATACATCGAGGCAACGGTGAATGTGGCAAGATCTTCTTTCTTATTTGGAAATCCAAACGATAAAGATCGAGTGGTGAGGCGTTTTCTAGCTGATACAATTAAAAAATCGGAATTTAAGTCGTTGAAGACGGGAAAGCTGGGAACGCACAGCTTCCGTAAAGGTGCTGCTACTTATGCGACTCGTAGTGGTGTATCTAAAGACTTTGTCAATCGGCGAGGACGGTGGAGAACTCGCAAAGGCGTCGTCGACGTGTATATCGACAACACTCAGCCTTATCCGGACGCATGCACCGCCGCAGTCCTTGCTGGTCCAGCTGGACCTTGCTTTTACTCGCTGAAAGAGGGCATGCGGTGTGTCACTACACCACTTCTCGTCGACGAGATTGCTCCAACAATTAAACAGGTCATGGGAGAGCCAATAGCAAAAACATTGGCACAGGTGTTACTGTGGGCTGCGCTGGAGACGGATTCCAGCTTTAATTATTGTCTTCTTCCGGAAAAGCTGAAAAAAAGAATTTTACGGGCTTACATTAACGCCGGTGGAAGTACGAATTTGAATCCGATTCAAAGACAAGAATTTTATGTCTTGGGGGACGGGTCTCAGCTCAATCTCGTCGTTATTGACAAAACGCAAGAAGTGGAATCTGGTGTTGGTAGTATTGTTTCTGCAAGAAGTGCATTGGTAGCGGGCTCCAATGGACAGGGGACTCAACGTGAAATTGCTGCTGTACAATCTCAAATAGCGTCTGGCCGTCGCTACATGGCAGAGGTTATGAATGAAGTACTGCGGTCCCGAAGCGAGTCACATCGAGAGATGCAAAAGATACAAGCTATCTTGAGACGGATAGCCATGCAGCCGTTCACACCTCGTACTACTGATGGGCACGGAGTACCGTATCCACCAACAAACACTGCTCAAGGTGGATTTCGTGGACAAAACGCGGCACGTCTTTCAAAGAGACCTAAAGACCTGTATGAACTTTGGCACGAGTATCAGCTCGGATCCGGTGGGCTAAAACCAGCCAAGGAGTTTACATCCATTGAGCGAGGCGCAAACAAGTTCGCCTACTCCAGGCGGAAAGTCTTCTGGGATGTTATTTCTCAATTGGTTCGCTCTGGACACACTAGCGACTCTGCAATAGATAGAGTGTATCAAACGTACGGTAGGAATCTCTCTGTGTCTAGTATTTTAGTTAAACTTCGTACAGATCGTAGACGTGGAGGACATCCAAGTCTGCGATTGTAGCACTTCAACACGGTTCAATTCAGCCAATCAAACCTTATCCTTCGAAATATTAGCCTATCAAAACTATGGTTGCAATCCAAGTTGCATAAATGACTAATATTTATACAGTACTGTACGAAGTAATCACTGAGTACAGCGGGACACTTGCCGTAGTCACAAATCTTGTTTGAACGTGCCAACAGATCTATCCAGCTGTATTTTAATTAATAATGGCCGCACACCGCTCACTCTGATATTTCCCCGTTCCAGCACTCTTATGACATGCAGTGAGCAATTTACTTCGGAGCGAACCCCGGACACACCCTGATGATGCGACTCTACCTTACTGCGCTGTTAAGTGCAATTTCAGCTCTACTAGCGCCGGGTGGAAGTGCCCCAGTGTCTGCTCTTCCAGACTTTCCGGCTGGCTACTTACCATGTAATGAACTACGTACATTAACAAACGCACCTGAAGAATCTCCATCCCACAGGCGATTGAGAATTTCCGATACACATGATGACGAAGACAGAATAAAAAGTATCAGCATTGAAAAACTATCGGGGTTGATTAAGACTGGAGTATCGAGGATACATGGATACCTATATTTAGGACCGTCAGCAACTAGAGAACAACCAGCAGATGAGATTCTTCGAATGTTCAAGCTTGAGGATGGAATAGAGAAGGCCTTGGCTAGTTCTAACTTGAAGACCATGGAAACTTATGTGAAGGAACTGCGCACCAAGAACCGAAAGAGCACGACGTCAGTGCTTGGAATACTCACGAATCACTACGGGGACGACGCAGTAGCCAGTGCACTTGTGACTGCACCGCATAATACCATTATGAAAGACATGGAGGATACGATATGGCGATTACGAAACACACAGCTTTCAGCTTGGCTGAGTAGCGACAAGTCTGTCGACGATGTTTTCAACCTGCTAAAGCTCCGTCAAGATGGCTACCTAGCTCTCGCCAGTCCAAAGTTGGAGGTGCTGGACGACTACATAAAGCTGATTATCCGCTCCAAATCCAGCCAAGAAACGTTGCGTGATGTGTTAACGAGGGGATTTGGAGAGCGGNAGATTGGCCAGACTGCTAGTCCGTGCGAAGCAAGATGACCGAACAAAAGAACTGGCGACGGCACTGCAAAATGCGATTTTAAACAAGTGGGTTACAGACAAGTTGCAGCCGGTCAACGTCCTACAACGACTGAGATTGGATAGAGGTGTCACCAAAGCTATGACAGACTTGAACCGAGACACTTTGACGAGGTACATCTCGCTGTTTAAAACACATAATCCAAGCAGTAAAACGTCATTCATTGGTACGCTTTCCGCGCATTATGGAGACGATGCAGTTGCGAAAGCACTCGTGACGGCGTCGTCGGATGCGAGTACAAAAGAAGGCGCGATTCAGCTACGGAGTGAGCAGCTGACTGACTGGCTGAACAACGAGAAGACTGTCGACGAAGTTTTCAAGCTGCTAAAGCTTCGCGATGACGGAGAAGTCGGTCTAATTAGTCATAAGTTGGAGGCTCTAAAAGATTACATCAAGCTATTCAACCGCGAAAGAACAGGAGATGAGACTTTACTCAAGACGTTGACGACTGGATTCGGCGGAGAAAGTGGATTCTCGAACATTTTACTAGCAGCAAAGGCCGATCGACGTACAAACACAGTGGCTATGTCATTGCAAAGCGAGCTACTTCATCAGTGGCTTAAGAGCGGATTGCAGCCGGGAAGCGTCTTGAAGAAGCTCAAATTGGACCGTGGGATAACAGAAGCACTCTCCGATGGAAACATCCACACTTTGACAGCATACATTTCGTTGTATAGCACACAGAATCCAAGTAATGCAGTATCGCTGATTAAGATACTATCCGCGCATTATGGAGACGATGTTGCCAAGGCACTTGCCATGGACGATTTTGCCACGACTGAGCTGGCGTCCAATCTGCTGACACAGCAGTTGCAGCTGTGGCTGAAATCTGTTGGAGACGTTTTCGCGATACTGAATGTTGGACACCTCGATTTCTTGTCCATGAAGAGTCAGAAGTTGCAGATTTTGGACAGCTACTTGAAGATGTATAACGCCAAAAATCCGCTTGACGCCAAGAGTATGTTCGCGGTAGTAAGAAAGGGCTTTGGCGGTGATGCCGGGCTTGCACGTGTGATTGGTAAGGCGCTTGTAACCTCGCAAAATGAGCCGAAGATGGCTCTCAAATACCAGAATGAGCTATTCAACCAGTGGTTCAATAGAAACATTGAGCCCAAGAACGTTTACGTAGAGGTCCTCAAGATCAAGAAGCGCTCTGCAGACTTTACAGCAAAGGGGGTCGCTAAACGATACAAGAACTATTACAAGAAACGGGTGGGGGAGGTTATAACCTTTAACAATCCAAGGCGGTCTTAAGAAAAAAAAAGCGTTATTAGATGTTTCAATCCTAATTCCAACGGAGCGTGCATAATCTGCATTGTTTTACGATCAGCGTGGAAATTAAATTATGTTTGCAAGTTGGGAGGAGTCGACGTCCTGTTAGCCAAAATTGCTAAAAATGGAAGAGACAGACGTTCGTATGACGCGTATCTTCACTACCAAACCTTTTCGGATGAATGTCTTAATCATATCATGTAAAACTGTCTTGACTGAGAAAAAGTGACGATAAAAAGCGCTTCTGTCGCTTGTATCCTATGCAAAATACGGTTAACCGTGAAGAATTGCGGTGCGCCACCAATAACGTGTGTTTTACCGCCGCGCCTTTTTCCTGCTGACCATGCCCAAAACCATCTTGCGCCGCAGCGGCTTATCTGTCGGCAGAAAATTCGATGGTTAAGTGGCAAGATATCGTACTTAAAAAAATCATATTACCGGTAGTCAGAATCACATGAACGTAAGCCACTCTAAAAGCCTACAGCTCATGGTCTCTTCCAGCTATTCGATGATCGGTGGGCTGGGATGAATAGTTGGTATCAGCGCAAAATAAAATGGAGTAGGGTCTTCGCTTGTGACCACTCCGGTCTACAAACGCCTTCAATCGTTGAAAGCCCCGAAACGACGAGGAACTATCTTCTCGTTGTAGTATACTGTGTATCGGCTTACAATCATATCATCCAAATGACTAGCAGAAGCCTCTTCAACATCGAGGAATCTGGAGTAAATGCTTTTTGGTTCTGCCTTGCTGCGGAACCAGCGCTTAAACAGCAACCTTTCGTACTCAGCACCAAAAGAGACGAGGGAGTCGATGTTGTGAACGCCTATCGACTCAATTGCACCAAAAACCGCGCGTGCAAATTTGCCTTCGCCACCAAATCCATCGCTTAGCACCGTGAGAATGTCGGTCTTGTATCCGGGATTAATGACGTTGATCCTCTTAACGTACTGCTCCAAGAGATGGAGCTTCGTACTTGCCACGGATACAAACTGGTTCTTGTTCGGGATGTTAAGGTTTAACTTAAGCAGTCTGAAGATGGTGTCGGGCAGGCCTCTGCGATTTGAATTATCATATTGAATAATATCGCATATTGGGAATGCAATTATGTATTGAGCAGCGAGTAAAATTGCAAGCATTTTTTAGCATTTTATCAGTTATTCTAGGTTAATACTGTCCACCAAAACTACTGGTTGCCACCACCTCAAAAGAGACTTTACGAGCCTTGTGCAATCTCCTCAGCTCATTCGACCTTTTGCAGTCATCCCAACAACTCTTCAGCCCCCTTTCGCCCAAGTACCCCATGAAATTTACAAACAAGGACATCTAAACGCGTATCTGGCTGATCATGAACCCATGTTGGCCAAGATACACGCGCTCATGAAACACCTGAGCACCATCAAGTGTCGTGCTGCCCTTCGCAAGGTGACCTCTCTAGCGCCCGTCATGCCCAACGCGACTCGTTGGTCGAGTACCTACAGCAGTACGACAAGATTTGTAGCGCTCTTCTCGCGTTGTACCACGCTACGGTGGCTAAGCATGACATCGCGCGCTTTCTACTGACGCCAGAGGAAACCGAAGCCGCTCGCTTCCTTCTCAAGTCGCTGCACGAGCTGAATGAGGTGAGTAAGACGCTACAAGACTCAACTCTAACAGTGGTGGGTGCACGACGTGCCTTTGATGCAGTGTTGCGCAAGTACCCCCGCATGAAGACCCGGCTTGCAAGTGACGCCTCCGTCGTGAACAATCCCGAACTGGAGAGCGGCATCGTGAAGATCATTGGCGGTGTCGGCTGAATGCACGCGAACAAGCGGCGTGTATTCATCTCAAGCGCAGCAGCGACGACACTGTGGTAAACCCAGCAGTTTCCACGTCATTTCTCGCGTCGGCCTTCAAGAAGGCCCCTGTGGCGCGCTCCCCATCGCAATACTTTCCTCTCGAGTGGGTTCCACCCACGTCGAACGAGTGCGAGCGTTTCTTCAGTCAAGCCAAGCTGGTTCTGACCGACCTAAGGAAAGCCATGGACCCTAACACTCTTGAACTGCTTATGTTTCTATCCTACAACAAGAAGGATTTAGTGTTAAAGCTATCAGGCAGAGTATGGGTAGTCAACTTCGGGAATAATGCCATTATTATTGTCTTGGCGTAATAGCAGCCGAATATATTGAGTATGGCATATTGGGGAACCCTGGTGTCGGGAGATTTGCCGCTTTTCAACCACGCATCCATTTGCTCTCTTAAGCTCTAAAGCCGTCGCCTTCGTGGCCTCATGAGACCTCGCAGCGCTGAGCGTCTCCGCCACTTCACCGTCCCCATAATTCCTGATAAATGTCTCGATCAATGACGTTTTGGTACGGGGATCCTTGCGTTGTACATCAAGATAAAAAGTGTGATAAGTTGGGTCCAGCAGAGCTTCATCCATGTACTCGTTCAGTTTGAGCATCTTCAGCACATCTACAGGTCGCGACTTACTCGAAAGCCATTTATTCAGTAGCGCGTTCTCCATCGCATTCGCCAGCTCACTCGTCCGGGGATCTTTCTTCGCACGAATAAGCAGATTGGCCAGGTTGTGCTCCCCGTCGAAACCTTTCGTCAAGACTTGAATCAACTTTACTTGTCCTGATTTTTCACGGTTGAACATCTTCATGTAGTAATCCAATGCTTCTAGCTTCGGACTTCCGAGAGCACTGTATCCGTCTTTCTGTAGCTTTAACAGCTTGAATACGTCGTCGACCGATTCATCATTCTTCAGCCAACCCGACAGCTGAGCATTTCGCAGCTGCAGAATTGTTTTGAAGCTCACTTTCACTCTTTTTCGTCCTCTACGCGGATACGAGTACCGCCGCCACTGCATCATCTCCATAGTATACCGAAAACACTCCAAGTACCGACATTTTGTTCTTTCGTTTGTATTTGTTGACTGACCTTAGATAAGTTTCCATTCTCTTGACATCAGGACTACTCAGAGCCCTGTCCAAGCCGTTTTCTAGCCTGAATAGCTTGAGGATATCTTCTGCGCTCTGGCTGATTGGTAACCAGTACCCCAAGTTCAAATTTTCACCAATCTTTACTGCTCCCGTCTTGATCAGCCCTGCCAGTTTTCCGATCTCACCGCTTATCATCCTGTCTTCTTTTTCTTCGCGGAATTTCTCAAAAGTCGTCTCGCAGACGAATCTTCTGGCTTGATTGAACGCATTTGAGGTAAGTTCAAACCCTGCGCTTCCGAGACGACAGTCGTAAAACCCCAATTGGCACATACCAGCAAAATTGAAGCACCACACAATAGCGGTTTCAAGCAGAGCCACATGGTTACAGGCTTGAAAATTTGCGACTGGTTAACCCCCAGGAGAGCAAGCACTTTCGACCATTCTACAAATATTTTTGTCTCGTCCATGGAAGTGATACGAGTTCTTTTCTGTAATCAACGCTTGTTGCAGAATGGCGTCAGAAGAACATCTGTTTACGACAACACATCAGATTTTTAAGATCTAAAACCGACCACAGTACAAGTAATTTATTTTAAACGACGAGGCGAAAAGAATCTACATTTTATCGCAGAAACTGAATTTAAAGTACATACATGTCACAGACGTGTGTTTTTACACTTAATTGTGTATCACAGGATTTTTCACATAAAATGCGTGTACCACGTACACTAGCACAGTTTGGGACTTTCGTGAAGATCTTGTGGTCTTGCAGAATGGGTAGGAGAACGCTGGCACGAGAGCTCGTTCACAGTAGGCTGGAGCACCTCGCCGATGTGAAGAAAAACCTAGTAAGGGTGCGAGTGAATCTGGATAAGATGTTGGCTGTTTAACTGGAGATTCAAGCTGCTATTGAGGAGGATGAAGAGCGCAGCTACGAAATAGCAGCACGTCTAGGAGTCGGAGGGTCTCAATTTAGTGGCGTTCTATCATTCGAAGTGTTGCTGGAGATGGCAATTAACAGGCTGAAAGCTTAGCATACACGTTCAGCTGTATTAAATTTTTAATACAGGTCTGATTTCCTACTTGAGCTCGTCCTTGTAGGCTCTCATGAATCTTACTTACTTTATCAGCAGTAGCATTGGACGCTTTGCGCTCATTCGAATTACACGATGGTCCATAGCATCCGTAAGCGCATTACCTTCACATCGTTCTTTGAGACGTTGTACTTCTGCCGTTCCACGTTTCCAGTAAGGTAATTGTGTGCCTGGGCTACCACCAAGACACCACAGCTAGCAAAGTCGGGCTGCTGTGGGGCATCAACCCACTCAATTGGGTCGAAAACAACTTTGCTTCATGGCATCGAAGCCTAATGCCACCGCGCTACAAAGTCCACTAGCCCTTCTTTTACCTTGGTTTTGGTGCCATTCTCCTCCAAACTATAGCTTTCATTCCACACTGTCTCCATTTCGTCATGGTATTCGTCGTCAAAAAGAGGTTCATACATGTAAGCATGCGCATGTAGCGGGATTGTAGCTC

>Contig_11

CAACTGTTTATAACTGCGGCAAATCTACAGCCCAAGATTTCTACACTATGATTTATATATGATTTGGCCCATGGCTGACCATGTTTGTAATTTAAAATACTTTTTCGTTTTAAATACAGGCGGGAAAGCCAGTCATTTGTCTGACACGCGTCACGAGAAGGCACACCGCTCGCCACTGCTGCCCTGCGCGTCCTGGCAAGACCCCGAGACCCCCGCCAGCCGACAGCGGCCCCTCTGCTCCCCCCCCACGTCTGCCCGCAGGTACGCATAATCTATTATGCTCCATCATCTTCATTGACGCGTACAGGACGCAAACTAACTCTTCCTGCTGCTGAATCATGTTGTGTTGAGCTGTAGAGGCGTCTCATCCGCCCTAGAGCCTGATTCCTGGCCCCTAATCGTCTATGTGTTCGCTTTAAAGGCTCCGAAGTAGAGAAACCTCGAAGAGGTGCAAATATGCTTGTTGATTGGATATGGATGCAGCTTTTGCTGATGAAATGCCTGCAGCTCGCCCAGAATCGCTCACACGCGCAATACATCACCGTGAAGCAGTTGCGAAGCACGACCAGAGTTCTCTACGAAACGGTATGACACATCTAATACGATGTTACCATGCGATGCATGTTCGACAACTAACTCTTTAGCTGACTCCTTGTATACTAATGTCACTTCGTAACCACTGCATTACTGTCACTTGGTACTCGCTGGAGTTTCCGTTCAGGTATGTTTGATCCATGACCTTCGTGGCGATGATAGATAGTCAGACTAAATAGGTCTTCCATGTTTGCTCCATGTTTCTCCCATGTATTGATGTTACAGCATCATGGTTCTATACCGCCAGCATGTTAAGAGCCCTGTACATGGAAGTATTGTCAAGTAGCAGTAGCGACGCGGACAATCTGCTCGATGACGCTGAGCTGCAAGTATCTTCTGGGAGTGATGATGACCGAGGCTGTGCTTGATGGCGTACCAAATGCTCAGAACACCGATGATATTGCGGGGGTCAACCCGAGACGTTGGTGATGATAAGACTGACAACAAGTTCTTCGGACCGTGGTGGACTTTAGCGATGCGAGTAAAGGTGAGCAGGACTAAGATGCACCACTAACACGATGGATGCATACACATTGCGCGTTTCACTATATTATACTAAGTAGTTCTGGATCATGCAAGATAAGAGGGGAGAGCTGACCGACACCCCAGCTATTCTCTCACTCCTCAAATTATATATCGTCCTGGAAATATGTTTTACCATAGTTATTAATGGCAAATACATGGACATCATTTATGACACGTGGTAGTATTGCTCATGCAGTATGAGGGTCCGACAGGCGAAAATCGCGTTCAGTCTTCAATTATCGGGTAGTGGTGCGAACATGTTCTGGCCGTGACCCAACATGGGGAATACATGTTCAAAATAAAATACCCCACTCCGTAATATACTTCGGGGGAATCATGGAGAAATTATAGTAATCATGGGCAAAATCTATGATTACTGTTCCCTAATCCTAATGAAAAACATGGGCTGTAGCACTGGGCTTATAATTGTATGGTCTACCTGCCCTTAACATATGGTTTAATATCGCTAAATATTGATTGAACTGCGACAAAAACCTAACGGCCTATAGATTGGAAAGTGTTTGAGCAGATATTACTCTACCATGATTGATGTTAAAGCAGACTTAAAATATGTCCGTGTGCGCCCGGACTGGGGCTATGAGTTGGTGCGCTATTGGGGGCCTTACAAAACCTGTATAAAAAGTTTAATCATTGGGGTTAGGGTGGATTAAAAGGTGATTAAAACTTAGTGATAAAATGGAAATTTTTGATGAGACCAAAAAGTCGTCTCATCAAACTTCGCGTTTAATCACCTTTTATCCTCTTTAATCACGCACCCTAATTTAGCAGCTGATAAATAAGTGACTTGCGTATTGTATATGATTAAGCGACTCCCCTAACTAGCAAAAATTTTGAAATTGAAGATGAGATCTTCGACAGCTTCAAAGGCGCGAAAATTCGCGCCCACTAAGGATCGTTTCATTTACGAATTTGCTGCCTGCCGCATAAGGCTCGTCGAGACGTCGGCGTTGCATGGTTGCGGTACGTACCATCGTAGATCTGGATCATCCATCATATGTGCTAACCTTATGAAGCAGCTGTGACGAGGCTTCACTACAGGCCGGTGAGTGCGTATTGCTGTGCGTTTCTTTTATACTGATACATTATTTTTCCGTACTATTTGGTCTTCAGGCCGAGCGAGATCACTACCGAATTCTTTGATGAGTTGGCCCCCTCAAATTTCACCGAAGAGTTGCCCCCCCACCCGGATTTCCTATACTAAATGATGGGCAACATTTGAAGAGACTTCCTCAATGAGTTGGNCCCCCCCCACCCGATTAACGTCCAATCACAGACTTGCAGCCGACACTGGTCAGAAGATCCGTGCAGTTTTAACCAATGATATGCATGTGTGTCTGAAGGGTCTTCATCGATGAGTTGGCCCCCTTTGCTGATGTACCACGGGGGGCCAACTCATCGAGGAATTCGGTAGATGTGGTGATAAAGATTCCTGTCCTGGCAGCAAACTCCGAGAAGAGAAGGCAGAGGCGCGTCCTGCTGATCAACACGAGGGCGGTGAGTTGCCGGGGTTCGGAGAACACGAGAAGACCCTGGTTTATCCGTCAATGTTACTATGATCGACAAGAGAAGCCCTTACTCGCGCAGTGCTACCGTTGAGGGTGAGCACAAGAACAACGACGCTCGACACCTGGTGGTGGTGCTGGAGCCGCCGTGGCCGACCGCCTGCTGGATTGTATACTGGAATGATGTAGAATTGGGAGAATCGCTCCAAGAGCTGCTGAAACGCGCCCCTGGCACACATAAATAAATAAAAAAAAATAACAGGTTAAAACAAATCATTGCTCTGGCACATACAAACATCGTCGTAAGTTACTACCTTGATAGGCTAGTTTTGTTGAGCATATTCTACTCGGTGCCCCGACGCCAAAAATCCATCCAATTCGACTATTCGGGGTGTTTTAGACAGTCGAGGGTCACGTAATACCGCATGAGCAGCAACCGTTCCCACTCTACACGCCTCCTCTCTTCATGCAGCCGTTTACGAAGCCGTTTAACACACTTTTCGAGATTGTGTCGCGTTTGCGACGTCGCGCATAGCGCGAGAAGAACGCTGGCGCCGCGCATGGGCCCCTCTCTCGTTTGAATCAGGCGAGAGAAAGCCGTGAGTTGCAGGGAAGGGGTCGCTGGCAGCGCGCCATCGTGGTGGTATCTTAAAAAATAGACAAATTTATCAGTTTTTTGTCCCATTTAAATTAGTAAAGATCGTCCGTTAATAATGAATCAATATGTTAGATACATCAACCTCACTCTATTCGGAACGAAATAAATTTTAATGTAGGGTTTTGGCAGAACGGTTTTGATAAAACCGGTATAAAAACGCATCACCGTGATAAAAGTGTGATTAAAGAAGGGGTCATCAAAACTTTGTGATAAAACCGTTTTATACAGGTTTTGGAAGTTCTCGAGCAATCGATCAAAACCAGAAACCGTCATATTAGAGAGGTAAATCATATTGTTGTAAAACTCTGAAATAAATCTCAGAGAATACTTTTTATTAAGGTATTCTCTTTAAAAAAAGTGACAAGCGTTAAAAAAGTATAAATTCTTTTCAGCTTCAAAAATAGATTTACTGTGTGCTCGTTGATATGATTTCCTTAGTTCATTTTCTTAGCCTTGTAAGTCTACATGTAGCTTTAGCAAACCTTAAACAAATCCAGGCCGAAGGCGCGACTTTGTTTGCAAGAGAAAACCATAGCAGCAACGCCGCCGAAGCCCCGCGGGAGGTAGAGGGGCCAGGGTTCGCACTCTGAGGTTTTGAGCTTAGTTTACAGGGTGCTCAAGGATTTATACACGTGGTATTATAGGACGCTACCTGTGTTTACGCTCCATGTGCGAAGCACGCGGGCTTTATTAATAACCCCGACCCATGATAAAGGTATTAATAGAGCTGTATCTGGTGTTTATGATCCGTGTGCGAATACACTGCCAGGATAAACAGTTAAAGCTCCTAATTCGAGGAGTAGGTGTAACATTGTTAGAAGTGTTCTACCTCTACTAAGCCATAAACTCCTCGGCGCCCTATTAGTACAACGGCGATCCACCATTAGCCTTGTAGAAGTCATAGAAGCCGAGGACGACGCCGTTGTTTCTTATGTCACGCGGTTGCTCGGCTACCAGTGCAGCGACTCTTCCTGGATCCAACCTCTTTTGGTTCATCCACTGATCGAACTGCTGGTTCCGCAAACTGAAAAGACGCTCTACAGAATGTCCTTTCATCTCGGCTTGGATCTTAGCATGGCTAAGCATTCGTGCGAGGTCCACGTAATCGAAGCGTTTTTCGAGCTCCGTAAAAAGAGCGAATTTATCTGGATGTTTCTCGTACTTGTTGAGTCTTTCAACATACAAGATCCAAGCGTCAAACATCGGGGCCTCGAACAGGTCATCACCTGTTTTGTCAAGTTGAAAGAACTTAAAAATATCATCCGCAGATTTCCCTGGGCTCCGCCACATTTCCGCCGCTAATTTTGAAGCCAGGGCTCTCGTTAATACACTTGTTTTTGCTTGAGCAATCATTTTTGCGAGGATCTCGTCGCTGTAGTGCTTCTTCATCACTGAGAACACCAAGGCATCAGTATTTGTATAGTCAACTAACTTCACGTAGGACACCCAAGTATTAAACACTGAGCTCTCTAGGAGTTCTTCACCCTCCTTGCTTAGTCCAAGATGTCTGAAAATGTCATCTGCCGTGTCCCCCCTTCCATGCCATTTACTGAGCTGTAACTTTTGTAGAGCAGTAACATTGATCTTCACGTTTTTGTCCCTCGACGCCGCGACTAACACTCTGGCTAACTCTGCGTCGCTAAATCGATTCTGCAGCTCCAAGAATAAAACTTTGTTGGGATCTTTGTTGCGTTGTCGAATGTACGACAACCACGTGGGCAGAGCCGCGCTCCGCAAAAAACTTTCTCCCTTGTCTGCGTTTAGTCGGAGAAGCTTGGCGACGTCACCTGTCGTTTTACCATCCCTCAGCCAGGCTGTAAGCAGTGCATTCTCCAGATTACGAACCACACTACTTGTAAGGCTATCGCCCTTTGCTGCAATTAGCATGTGAGCTAGTGCTTCTTCATCATTGGTTTTTGTCAACTTGAGCAGCAACAAATTGTACGGATCCTCGTTATCAAGCTTTTTGACAAACGAAACCCACGTATTCAACAACGGATTTCTCAGAAGACCCTCCTTGTCCGCGTCCAGCTTTAAAAGCTTGTAGATGTCCTCCTCCGTTTTTCTGTTTGTGACCCAATTGGCTATCTGCATCGATTCAATTTTCTTGGCTGTCCTTATCGCCGCTGCGGTTGGCTGTGCTTCTGATACCAGTTTGGCCAGAATGTCATCGCCGTGGTGACGTACAAGTGTAGAGAATATCGCTGCTTGGCCTTTTTCAGGGTTCTTCTTGTAGGATTTCATCACGGATGCAGCCCACTTCTGATACGGAGTAGACGCAAACAGCTTCGATTCCACCTCCCAGACCCCGAGACTCGCAAACAGTTTGTCGGTAGCTCTTTGATTGTTGGATGTAAGAATTTCCATGGCTCTTGTTGAAAGTTTTTTCGTTGGCTGCGAATTAATCAGCTTTTGAAGCACCGTAGCAGCTGACAGCTTAACTCCAATCGCCCTCGCCTCGGGGCTGCCGTCTTCCTGTTCAGCTGTCCACTTGCTCTCACGCAAGTATCGGTGGATCTGACCGCCCACCTCGCGATCCAAAGCATCATTTCTGGCGTCGATTACGCTGGATGCCGTAGCCACCTTAGATCCACTGTGGATGCATACCAGCAGAGCGAGGGCCGCCAGCAAGATGAAGTAGCTTAAGCGTGGTGTTAAGGCAAGTTTGAGGGGCATCGCACCGACAAAAACGAAAAGTGAGGGCTTCGTAGCAAATTGCTGAATGAAAAGCTCACTGAGGCTTCGGCTTAATTTAAATACCGGTACTCTCGTCCATCAAATCATTACGCTGTGTAGAGTGGGCTGGGGGCATCAGCCACTACAGTGGGCGGCAGTACTGATCTTCGGATTGGCAGTGGCAGGGTTGCTGCTGCCAACCATCGAGTGATGATTGAAGTTCAGTATTGCCGGCCCGAGCTGGCAATATATACAGGGTATGCCAAGTATACATAGTATGTATACACAGAGTACAGACAAAGAGTCAGTTAGTCAGTCTAGAACTTGCTTAGTGGAGAGCAATACACTAGGCAGAAGCTTTAGGGAGTGCCAGCTCCCCCAGTTGTGTGTTACTTCCACGACCTACTACAAAGTTAACTTTAGTGCCCGGGTCAGTGAAGAGGTTCAAGATCCAAGATGGCGTGTGGCGGACGACGAAGGTGGTGCCAAAGTCATGGGGTCGGAGGGCGTGAGCGTTCCGGACACTGAGGATGCTGAGTCCGAGTGCGTCGAGGTGTCTAAGGTGTCGGAGGGCGCGATCGTCTTGGACCCAAGAACGTGAGTCCGACGGCGTGAGTACAAGGACGCTGGAGCGTTGGAAGCCGAGGACGGGGATGCCAAGGTCAAGTACGTGAAGGCGTCCAACGCGCTCGTCGGGACTCAAACAACAAGCCCCGGGAAGCGCAAGAATCGGCAGGCATCTGAGGCGTGACGGTTGAGAAGCGCGAGTGTGCCGACACCGGAACATCGTGGGTGCCGTCGAGGAAACAGTGTCGGGAAGGCGCGAGTGTGCTGGATGCTGGAGCGTCATGCCCGGGTGGATGCCAAGGTCGGGTCGTGTCAGTGCTTGTGGGCGCGGGTGTGCCAGGCATTTGATCATCGAGCTCGGAGGCGTCAATTCCAAGGTACGTGGTGTCAGAGAGTTCCTGACACCTGAGCATCTCACCCTGGGGATGTTGTGGAGGTGCGTGTGTGCCTGCCATTAGAGGTCACGAGCATGGCAGTGTCTAGAAGCGCGAGTGCGTGGACATGAAAGTGCAAGACCAAGGAGAAGATCATACCACGTGGCTGGATGAGTCTTTAAGAAGAGGGTCTGTAGTGGGCTGGGGGCATGTAGCCTCATCCAGCCACTACAGTGGGCGGCAGTACTGATCTACGGATTGGCAGTGGCAGGGTTGCTGCTGCCTACCAATCGAGTGATGATTGAAGTTCAGTATTGCCGGCCCGAGCTGGCAATATATACAGGGTATGTATTGCCAAGTATACATAGTATGTATACATAGAGTACAGACAAAGAGTCATTTAGTCAGTCCCGTCCAAACAGAGAATAATGTCAAGCAAAGTGGACAATATGTTTTTGATTATTACAAATATCTGATTACGGGATCAAAATTACCAGTGAATCAGAGAATAATCTCATTCAAACCGAGACGACACAGTGAGCTACTTCCCATAGACCATGTCTTTCATCTCCTCGGCCGCGTTCACCGCATCGCGAGCGTGAAGCTCCATCTTCGTCACTAACGTAGGTGTTATATACTTCATACTCGATTCTGCAGCCTTCTCTAAGAAGCGCATAGTGCGCTCCTTGATCGTTATTGGGGTGCCGTCTTCTTCCACCATGTTACTCCGGTCGCAGATCTCTTCGCGGTACAAGGCCAGGTATCCCTTGATTCGAGCTTTTAAAACGGAGAAGCAACCCTCAATCGGGTTGCACATCGGTGAGTAGGGTCCGAGTCGTAGGAGTACGAGGTCGTCACGTGCTTCGACACGTTCTTCGGTTTGGCGGTGAGCGGGGGCATTATCCAGGACAATAATGACCTTCTTCCCAGCGAAGCTGTCATGAAACACGGAGGATGCCTTAACCGTCCGGTAGATTTCCTCAATGAAAGCTGCATTCTGCTCCATTCGGATGCTTCCCCTCTCCAGACGGTGGAGAACCACACCCATGGCCGAGCTTACAGCGCACTGGACTTGCAAGTTTGCACCCTTGGACGGCGGCATCACGAGCGTTGCACGCTCACCTCTCCGTGCACGTCCTCGACCTCGCGTACAGTAAACGTTGTAGTTAGTCTCATCAAAATAGATTATACAGTCACCGTTCTTTTGATGGTCCTTCAGCGATTTTGCAAACTTCTGCCGCTTGGTCTTGTTGATCTCGTTGTTGCATGTCATGGGCTCTATGCGAGTTTGTTTGACGGTGAACAGCATGCCAAGCAAGTGGCGGCTGATCGTTGAAGTGGAGACCCGCACGTTCGTATCTAAAAAGAGCATCGTGCGCATAGCTTCCAGGGTGAAGGTGCAATTGTCGTCAAGATATTCCTCCAGCTTGGACTTTGCTTCTGGTGTCATCTTCACAGCTCTACCACGAGCTCCACCGCGCGGTAGATCTTCTACGCGTTCTGTGGCTACAGTGCGGTATGCCACCGCACGCGATATGCCGTTATTGGCGGCCACCTGAAGCCAGTCAGCGCGTCCCGCGCGATGGGCAGCTAGGACACGGAGCTTCTCAGCGGGGGAGTGCTTGCGGAGGAGTGGTTGGAAGGGCATTCGTCTGAAGAGGTGAATTGAAAAGTGAACTGCTTCAGGCGATGGTTTGCTTCAGGCGAGGTTTTCGCGCGCATTTTGGCGCCAACGCACCCTCACCACCCCTTCACGAATGATTTCCAGCCATCGCCTTTCCTTGTCTCGCTTTGGATAAGATCTTTCTCTGTTTGTTTGTTGATTACTTTTACTAAAGTTAAAATTTGTACTATAAAAACCAAATTGTCCACTTTGCTTGAAATTATTCTCTGTTTGGACGGGCAAAGGTGTGGAACTTGCTTAGTGGAGAGCAATACACTCGGCAGAAGCTTTAGGGAGTGCCAGCTACCCCAGTTGTGTGTTACTTCCACGACCTAGTAACACGCTGGTATAANCCCCTGCGCACTTCAAAGTGAATTATCTCTACCGGTATTCACATGTCAACGCATAATGATACAAATATAAAAAAGTAAAATATTCCACTAGATAGTCAACATCACATCGGAGACGCTTACTCCAACAAAAATGATGCTGAAACGCCCATGTTCACGCTGGCATGGGCTATTCAATTATCGTTTTCTCCGTAAGATTGGATATAGACCTACAGCAATGCTATAAGACATTTCCAGTGTATAATGTAAAGGTCGTGGCAATAAAATTGTTTCCCTACACTATTTACAGTTTTTACGGTGCATGTATCCACACCCTGGCTCTTTAGATTTAAACAAGGATGGCGTTTACACCACGTCAACCACTGTCTTTATAGCTGTACTATTTCATAAAGAGACATATATATAACTGGACTGGTGTTGGAGAATAACTACTCTCCCGACCAACAGACTCTCTAATTCATCACTTTTCCTTTTACCGTCTTCTCATTGACTTCTTC

>Contig_13

AAATTACCGAACAACACAGCAAGCTGCTGCGTTGCTCTAAACCACCTATCGTGTAATTGTGGTAAAGTTGTAGTAACGACTCCGGATATAAATGCTAGCTTCTAAAACGCTGGCTGCCCGTAGTTGGGAGGCCCTACAGTAGGGGCTGCATGTTAGATGTAGAAGGGGGTACTGTACTAGGATTCTAACTCAATCAATTTCACTCTTTAATTTCTAGACAGCGCCTCCTTTAGTTGTGATTGTATCTTTTGTGGTTTTTACTATTACAATAGTAAATCGTTCTTTAAAGTGATAAAGAACTCTCTGGATTATCCATATTAACATCATTTAATGTAAAGGAGCTGATAGGAATTTTCTTCCATATCAGCAGAACACATTGACCGGTTTCTTGTCATTATGGACCAGCCCCAATCACTGTCCATGGTGACCAGCGCATGATGCGCTGCGCATCCATAGTTGAGAACTTACTGACTATCCAACAGGCATAACGACAAACCTGTTGACGCTACTGTCGCAATTTAATGTAAGCCCAGTGACGATTCACAATAACTCGCGCAATTCGGTGTATTCACGAAATTCAAGTAAGTAAGCANTTTTTTTTTTCATTCAGCATGTAATTGCTCCGCTACTACGCTTTTGACTATCGCTTGCTTATTGAAGGAGCTAGACCACTTCGAGTATTATAGAACGTTTAGCAGTCTCCTAGGACATCCGATAACGCTGCTGCTGTACGGGGGCAGATATTTGCAACTGCTTTACAACATATTAGTTTTAATAATTCAAGATGACATGAAAACATGCTTTAAGTGTAGAAAAACGGATCTACGCATCCTACTTTCCAGATTCATAACTGTCACTCCATAGAAAAGCAAGACGGTAAGTGAGTTTTTCTTGTAAGATATGCATTCAGTCGTACGTTATAATAACGCTCAATGACTTGTGTATTGCGTCGTCTAAGCAATTTGCTGCTGCTTCTCCTTAGTTCACTTTTTACAAAATGATTGGGCTGCACAGTGTTGTATGTTGTCTCCAATAAAGATCTGGTTTGCCTTCGAGTCCGTTATCTATTGGCTTCTGTTCCTTAAGACTTCGTAGTAAGTGAACCTTAGCGCTCATGCATCAGTACAGTCGTAGTTTTCAGCGTTACAAAAGTGTCATCCCAATAATACACGATTGTATTGTAAGTATGAATGAATGAACTTGCATGTAGCCGATGGAAGCAGATACATGTAACTACAGGCACCAATTCTTCACCTGGCATCATAAACGTTACGTAAATCCACGTGGTTGAAGCGTCTTGCAATATTCTGTTTTGCAGCTCAATATGCTAGCAACACATTGACCGGTCTCTCGTCAAAAAGGACCAGGCATAACAAATCCAATGATATAACGCAGATCCCGATAACCAGCGCATGGATAGTGCAGCATTTGGTTGCGATCCGACACGCATAACGACAAGCCTGTTGACATTAACGACATCCAACCAAACGAAATATTAAAACTCTGGAGTATTACAATGTAATAGACCACCGAAGGCACGGCAAGCCTTAGGCTTAAGCAAGATTGGGTGAGGATCCCCAGTTTACAATGTACACTATTTCTGGATCAAATCGTTAGATCGAAACACAAACGGCTCACATCAAAACGAAAACACACACGAGAAAGACAAACTGCTCCGAAATTGACGATGAACCAGTCGAACTCCACCAGCATGTCAGCTACTTCCGTGACTCTCTAGCTAACAGTCAGTAAACAGGCTCTCAGCGATACACTAACGACATTGCTGTCTACGGAGACAAATAGTACGAAAACAGCCTAATACAGCTTCCAATATAAGCTAAATTACTGCCCGCGTGAGACAAGCAACAAATCTCCAGAAAATTCTACCAACTCTCAATTAAGACCCTCGAGAAAAATCATTTATTTTATACCCAACTATCGCTTACAGTCAACGACGGTGAAGTGTAGCGTTACGACCAGTAACATTATAGTTCAGGGAAACGTGCTTCTGACGAACGCTTCCCAATTTTTGAAGTTTTCAAGTGGTTTAATATACTACTGAACAGAAAGCATAGCAGTCTAAAAGAGAGGACCAGACAAGCAATATATATAAACCGATTTAGAAACCGAGTGGATATTCAGGATATCGTAACCTGTATCTAATCAATGCCCTGCCTTTTTCACATACTGTTCCATACAGAGACCTGGCTTGCGTTGTGACACCTGTACTTTGTAATATTGATATCACTAGACATCGAAGTAGCTGAGCACACGAAAGCAGCTAACAATTTCACTCCGCAATCTGCGTCGTACGAAAGCCATCACTTTGTGCCCCAACTGTCTTCATTTCGGATCAAGAGCCACCGCCGTTCACCAATGCGTCTGTCCTGCGTCTATCTAGTAGTCGCTACTGTGACCACTATCATCGCAAGCGCTAATGCTGCAGCCGAAGCCTCCGAGCCCATGCCCAATATCGCGAAGTATGCATCACCAGAAGTTTCAGTTCACCTTGGTGCTGAGCGCGAGAAGAGGCTTTTGCGCTTCGACAGCAGCGATTATCGCGACGATGACGATGAAGAGGAAAGGGCGAATGCTGCCAACCTCTTCAACGTCGACAAGCTAACGGTGTATGTAAACAAAGCCCAGAAGCGAACTGCCAACAATGTGAGTGGAAGCCTCTTGAATTATTTTAAGAGATTGGAAGCATACGGCTACAGCCCTGTCAAACTCRGTAACAGAATTCCTGACGAGGAGTACGACAATCTCCGTATGCTGTACCGCAGCTGGTACTACCACAACAAGTAACCGGATGACTACAAGCTCATTGTGAACAATCATCTTTCGTAACTATTGAATACAGCAGTTACCTGGGCTGCAGCGTTGCTTTAAACATGACGATGATGGTGTCTTGCTGTTGAAGACGTAGTAGKYGATGAGCTTATAAAACTTTTTTACACCTTGGCTAAGCGCAGCAATATCACACGAAGAATGTAGACCCAACACGATAAAACGCTGGTAAACTGTACGAGAYGGTATTTCGTGTAGCTGATTGGGTAAACGAGTTCGTATAATGTATCTGCGACAAGTGCAAGACCATGTTAAGCTTAGTGAGTCTACAAGCGCTGTAAACGCACAAGTAAATAAGCATYGCCATCGTCAAGAAATCCACATTCAAGGCTGTAGGTGACGATGTTTGAGCGACCAGTAGTCTAATACAGCTAGAGATGTTCACCCCATGCAGTGCTTCTTCTGTTCCATCTAACGTACCATAAGARATGTGTCGCCTGCACCAGAGCCTCAATAAACATGTAWAACGACGATAGACTGCGGTAGAATTAAATGACTRCCTGTCTGTTCTAAGCACATGCTTCATTATCAATGAAATAAACCGAGCGAGCTAAGAAACACTTGCAATTTCCTGTTCCAGCGRCCTAAATTGGACATTGTTACCACTAAGGCTGTACGGGACCCCGCACAAAAGGTCACTGGCTATAAAGAAGAGTGCGATAGGTGCGAGTACCCATCTTCCTTCAAGAAAAATATTGTTCGATATAAATGCTAGCTTCTAAAACGCTGGCTGCCCGTAGTTGGGAGGCCCTACAGTAGGGGCTGCATGTTAGATGTAGAAGGGGGTACTGTACTAGGATTCTAACTCAATCAATTTCACTCTTTAATTTCTAGACAGCGCCTCCTTTAGCCTCATTCGCTTGTGATTGTATCTTTTGTGGTTTTTACTATTACAATAGTAAATCGTTCTTTAAAGTGATAAAGAACTCTCTGGATTATCCATATTAACATCATTTAATGTAAAGGAGCTGATAGGAATTTTCTTCCATATCAGCAGAACACATTGACCGGTTTCTTGTCATTATGGACCAGCCCCAATCACTGTCCATGGTGACCAGCGCATGATGCGCTGCGCATCCATAGTTGAGAACTTACTGACTATCCAACAGGCATAACGACAAACCTGTTGACGCTACTGTCGCAATTTAATGTAAGCCCAGTGACGATTCACAATAACTCGCGCAATTCGGTGTATTCACGAAATTCAAGTAAGTAAGCATTTTTTTTTCATTCAGCATGTAATTGCTCCGCTACTACGCTTTTGACTATCGCTTGCTTATTGAAGGAGCTAGACCACTTCGAGTATTATAGAACGTTTAGCAGTCTCCTAGGACATCCGATAACGCTGCTGCTGTACGGGGGCAGATATTTGCAACTGCTTTACAACATATTAGTTTTAATAATTCAAGATGACATGAAAACATGCTTTAAGTGTAGAAAAACGGATCTACGCATCCTACTTTCCAGATTCATAACTGTCACTCCATAGAAAAGCAAGACGGTAAGTGAGTTTTTCTTGTAAGATATGCATTCAGTCGTACGTTATAATGACGCTTAATGACTTGTGTATTGCGTCGTCTAAGCAATTTGCTGCTGCTTCTCCTTAGTTCACTTTTTACAAAATGATTGGGCTGCACAGTGTTGTATGTTGTCTCCAATAAAGATCTGGTTTGCCTTCGAGTCCGTTATCTATTGGCTTCTGTTCCTTAAGACTTCGTAGTAAGTGAACCTTAGCGCTCAATGCATCAGTACAGTCGTAGTTTTCAGCGTTACAAAAGTGTCATCCCAATAATACACGACTGTATTGTAAGTATGAATGAATGAACTTGCATGTAGCCGATGGAAGCAGATACATGTAACTACAGGCACCAATTCTTCACCTGGCATCATAAACGTTACGTAAATCCACGTGGTTGAAGCGTCTTGCAATATTCTGTTTTGCAGCTCAATATGCTAACAACACATTGACCGGTCTCTCGTCAAAAAGGACCAGGCATAACAAATCCAATGATATAACGCAGATCCCGATAACCAGCGCATGGATAGTGCAGCATTTGGTTGCGATCCGACACGCATAACGACAAGCCTGTTGACATTAACGACATCCAACCAAACGAAATATTAAAACTCTGGAGTATTACAATGTAATAGACCACCGAAGGCACGGCAAGCCTTAGGCTTAAGCAAGATTGGGTGAGGATCCCCAGTTTACAATGTACACTATTTCTGGATCAAATCGTTAGATCGAAACACAAACGGCTCACATCAAAACGAAAACACACACGAGAAAGACAAACTGCTCCGAAATTGACGATGAACCAGTCTAACTCCACCAGCATGTCAGCTACTTCCGTGACTCTCTAGCTAACAGTCAGTAAACAGGTTCTCAGCGATACACTAACGACATTGCTGTCTACGGAGACAAATGGTACGAAAACAGCCTAATACAGCTTCCAATATAAGCTAAATTACTGCCCGCGTGAGACAAGCAACAAATCTCCAGAAAATTCTACCAACTCTCAATTAAGACCCTCGAGAAAAATCATTTATTTTATACCCAACTATCGCTTACAGTCAACGACGGTGAAGTGTAGCGTTACGACCAGTAACATTATAGTTCAGGGAAACGTGCTTCTGACGAACGCTTCCCAATTTTTGAAGTTTTCAAGTGGTTTAATATACTACTGAACAGAAAGCATAGCAGTCTAAAAGAGAGGACCAGACAAGCAATATATATAACCCGATTTAGAAACCGAGTGGATATTCAGGATATCGTAACCTGTATCTAATCAATGCCCTGCCTTTTTCACATACTGTCCCATACAGAGACCTGGCTTGCGTTGTGACACCTGTACTTTGTAATATTGATATCATTAGACATCGAAGTAGCTGGAGCACACGAAAGCAGCTAACAATTTCACTCTGCAATCTGCGTCGTACGAAAGCCATCACTTTGTGCCCCAACTGTCTTCATTTCGGATCAAGAGCCACCGCCGTTCACCAATGCGTCTGTCCTGCGTCTATCTAGTAGTCGCTACTGTGACCACTATCATCGCAAGCGCTAATGCTGCAGCCGAAGCCTCCGAGCCCATGCCCAATATCGCGAAGTATGCATCACCAGAAGTTTCAGTTCACCTTGGTGCTGAGCGCGAGAAGAGGCTTTTGCGCTTCGACAGCAACGATTATCGCGACGATGACGATGAAGAGGAAAGGGCGAATGCTGCCAACCTCTTCAACGTCGACAAGCTAACGGTGTATGTAAACAAAGCCCAGAAGCGAACTGCCAACAATGTGAGTGGAAGCCTCTTGAATTATTTTAAGAGATTGGAAGCATACGGCTACAGCCCTGTCAAACTCGGTAACAGAATTCCTGACGAGGAGTACGACAATCTCCGTATGCTGTACCGCAGCTGGTACTACCACAACAAGTAACCGGATGACTACAAGCTCATTGTGAACAATCATCTTTCGTAACTATTGAATACAGCAGTTACCTGGGCTGCAGCGTTGCTTTAAACATGACGATGATGGTGTCTTGCTGTTGAAGACGTAGTAGGCGATGAGCTTATAAAACTTTTTTACACCTTGGCTAAGCGCAGCAATATCACACGAAGAATGTAGACCCAACACGATAAAACGCTGGTAAACTGTACGAGATGGTATTTCGTGTAGCTGATTGGGTAAACGAGTTCGTATAATGTATCTGCGACAAGTGCAAGACCATGTTAAGCTTAGTGAGTCTACAAGCGCTGTAAACGCACAAGTAAATAAGCATTGCCATCGTCAAGAAATCCACATTCAAGGCTGTAGGTGACGATGTTTGAGCGACCAGTAGTCTAATACAGCTAGAGATGTTCACCCCATGCAGTGCTTCTTCTGTTCCATCTAACGTACCATAAGAGATGTGTCGCCTGCACCAGAGCCTCAATAAACATGTAAAACGACGATAGACTGCGGTAGAATTAAATGACTACCTGTCTGTTCTAAGCACATGCTTCATTATCAATGAAATAAACCGAGCGAGCTAAGAAACACTTGCAATTTCCTGTTCCAGCGACCTAAATTGGACATTGTTACCACTAAGGCTGTACGGGACCCCGCACAAAAGGTCACTGGCTATAAAGAAGAGTGCGATAGGTGCGAGTATCCATCTTCCTTCAAGAAAAATATTGTTCGATATAAATGCTAGCTTCTAAAACGCTGGCTGCCCGTTGTTGGGAGGCCCTACAGTAGGGGCTGCATGTTAGATGTAGAAGGGGGTACTGTACTAGGATTCTAACTCAATCAATTTCACTCTTTAATTTCTAGACAGCGCCTCCTTTAGCCTCATTCGCTTGTGATTGTATCTTTTGTGGTTTTTACTATTACAATAGTAAATCGTTCTTTAAAGTGATAAAGAACTCTCTGGATTATCCATATTAACATCATTTAATGTAAAGGAGCTGATAGGAATTTTCTTCCATATCAGCAGAACACATTGACCGGTTTCTTGTCATTATGGACCAGCCCAATCACTGTCCATGGTGACCAGCGCATGATGCGCTGCGCATCCATAGTTGAGAACTTACTGACTATCCAACAGGCATAACGACAAACCTGTTGACGCTACTGTCGCAATTTAATGTAAGCCCAGTGACGATTCACAATAACTCGCGCAATTCGGTGTATTCACGAAATTCAAGTAAGTAAGCATTTTTTTTTCATTCAGCATGTAATTGCTCCGCTACTACGCTTTTGACTATCGCTTGCTTATTGAAGGAGCTAGACCACTTCGAGTATTATAGAACGTTTAGCAGTCTCCTAGGACATCCGATAACGCTGCTGCTGTACGGGGGCAGATATTTGCAACTGCTTTACAACATATTAGTTTTAATAATTCAAGATGACATGAAAACATGCTTTAAGTGTAGAAAAACGGATCTACGCATCCTACTTTCCAGATTCATAACTGTCACTCCATAGAAAAGCAAGACGGTAAGTGAGTTTTTCTTGTAAGATATGCATTCAGTCGTACGTTATAATGACGCTTAATGACTTGTGTATTGCGTCGTCTAAGCAATTTGCTGCTGCTTCTCCTTAGTTCACTTTTTACAAAATGATTGGGCTGCACAGTGTTGTATGTTGTCTCCAATAAAGATCTGGTTTGCCTTCGAGTCCGTTATCTATTGGCTTCTGTTCCTTAAGACTTCGCAGTAAGTGAACCTTAGCGCTCATGCATCAGTACAGTCGTAGTTTTCGGCGTTACAAAAGTGTCATCCCAATAATACACGATTGTATTGTAAGTATGAATGAATGAACTTGCATGTAGCCGATGGAAGCAGATACATGTAACTACAGGCACCAATTCTTCACCTGGCATCATAAACGTTACGTAAATCCACGTGGTTGAAGCGTCTTGCAATATTCTGTTTTGCAGCTCAATATGCTAACAACACATTGACCGGTCTCTCGTCAAAAAGGACCAGGCATAACAAATCCAATGATATAACGCAGATCCCGATAACCAGCGCATGGATAGTGCAGCATTTGGTTGCGATCCGACACGCATAACGACAAGCCTGTTGACATTAACGACATCCAACCAAACGAAATATTAAAACTCTGGAGTATTACAATGTAATAGACCACCGAAGGCACGGCAAGCCTTAGGCTTAAGCAAGATTGGGTGAGGATCCCCAGTTTACAATGTACACTATTTCTGGATCAAATCGTTAGATCGAAACACAAACGGCTCACATCAAAACGAAAACACACACGAGAAAGACAAACTGCTCCGAAATTGACGATGAACCAGTCTAACTCCACCAGCATGTCAGCTACTTCCGTGACTCTCTAGCTAACAGTCAGTAAACAGGTTCTCAGCGATACACTAACGACATTGCTGTCTACGGAGACAAATAGTACGAAAACAGCCTAATACAGCTTCCAATATAAGCTAAATTACTGCCCGCGTGAGACAAGCAACAAATCTCCAGAAAATTCTACCAACTCTCAATTAAGACCCTCGAGAAAAATCATTTATTTTATACCCAACTATCGCTTACAGTCAACGACGGTGAAGTGTAGCGTTACGACCAGTAACATTATAGTTCAGGGAAACGTGCTTCTGACGAACGCTTCCAAATTTTTGAAGTTTTCAAGTGGTTTAATATACTACTGAACAGAAAGCATAGCAGTCTAAAAGAGAGGACCAGACAAGCAATATATATAAACCGATTTAGAAACCGAGTGGATATTCAGGATATCGTAACCTGTATCTAATCAATGCCCTGCCTTTTTCACATACTGTCCCATACAGAGACCTGGCTTGCGTTGTGACACCTGTACTTTGTAATATTGATATCACTAGACATCGAAGTAGCTGAGCACACGAAAGCAGCTAACAATTTCACTCCGCAATCTGCGTCGTACGAAAGCCATCACTTTGTGCCCCAACTGTCTTCATTTCGGATCAAGAGCCACCGCCGTTCACCAATGCGTCTGTCCTGCGTCTATCTAGTAGTCGCTACTGTGACCACTATCATCGCAAGCGCTAATGCTGCAGCCGAAGCCTCCGAGCCCATGCCCAATATCGCGAAGTATGCATCACCAGAAGTTTCAGTTCACCTTGGTGCTGAGCGCGAGAAGAGGCTTTTGCGCTTCGACAGCAACGATTATCGCGACGATGACGATGAAGAGGAAAGGGCGAATGCTGCCAACCTCTTCAACGTCGACAAGCTAACGGTGTATGTAAACAAAGCCCAGAAGCGAACTGCCAACAATGTGAGTGGAAGCCTCTTGAATTATTTTAAGAGATTGGAAGCATACGGCTACAACCCTGTCAAACTCGGTAACATAATTCCTGACGAGGAGTACGACAATCTCCGTATGCTGTACCGCAGCTGGTACTACCACAACAAGTAACCGGATGACTACAAGCTCATTGTGAACAATCTTCTTTCGTAACTATTGAATACAGCAGTTACCTGGGCTGCAGCGTTGCTTTAAACATGACGATGATGGTGTCTTGCTGTTGAAGACGTAGTAGTTGATGAGCTTATAAAACTTTTTTACACCTTGGCTAAGCGCAGCAATATCACACGAAGAATGTAGACCCAACACGATAAAACGCTGGTAAACTGTACGAGATGGTATTTCGTGTAGCTGATTGGGTAAACGAGTTCGTATAATGTATCTGCGACAAGTGCAAGACCATGTTAAGCTTAGTGAGTCTACAAGCGCTGTAAACGCACAAGTAAATAAGCATTGCCATCGTCAAGAAATCCACATTCAAGGCTGTAGGTGACGATGTTTGAGCGACCAGTAGTCTAATACAGCTAGAGATGTTCACCCCATGCAGTGCTTCTTCTGTTCCATCTAACGTACCATAAGAGATGTGTCGCCTGCACCAGAGCCTCAATAAACATGTAAAACGACGATAGACTGCGGTAGAATTAAATGACTACCTGTCTGTTCTAAGCACATGCTTCATTATCAATGAAATAAACCGAGCGAGCTAAGAAACACTTGCAATTTCCTGTTCCAGCGACCTAAATTGGACATTGTTACCACTAAGGCTGTACGGGACCCCGCACAAAAGGTCACTGGCTATAAAGAAGAGTGCGATAGGTGCGAGTATCCATCTTCCTTCAAGAAAAATATTGTTCGATATAAATGCTAGCTTCTAAAACGCTGGCTGCCCGTAGTTGGGAGGCCCTACAGTAGGGGCTGCATGTTAGATGTAGAAGGGGGTACTGTACTAGGATTCTAACTCAATCAATTTCACTCTTTAATTTCTAGACAGCGCCTCCTTTAGCCTCATTCGCTTGTGATTGTATCTTTTGTGGTTTTTACTATTACAATAGTAAATCGTTCTTTAAAGTGATAAAGAACTCTCTGGATTATTCATATTAACATCATTTAATGTAAAGGAGCTGATAGGAATTTTCTTCCATATCAGCAGAACACATTGACCGGTTTCTTGTCATTATGGACCAGCCCCAATCACTGTCCATGGTGACCAGCGCATGATGCGCTGCGCATCCATAGTTGAGAACTTACTGACTATCCAACAGGCATAACGACAAACCTGTTGACGCTACTGTCGCAATTTAATGTAAGCCCAGTGACGATTCACAATAACTCGCGCAATTCGGTGTATTCACGAAATTCAAGTAAGTAAGCATTTTTTTTTCATTCAGCATGTAATTGCTCCGCTACTACGCTTTTGACTATCGCTTGCTTATTGAAGGAGCTAGACCACTTCGAGTATTATAGAACGTTTAGCAGTCTCCTAGGACATCCGATAACGCTGCTGCTGTACGGGGGCAGATATTTGCAACTGCTTTACAACATATTAGTTTTAATAATTCAAGATGACATGAAAACATGCTTTAAGTGTAGAAAAACGGATCTACGCATCCTACTTTCCAGATTCATAACTGTCACTCCATAGAAAAGCAAGACGGTAAGTGAGTTTTTCTTGTAAGATATGCATTCAGTCGTACGTTATAATGACGCTTAATGACTTGTGTATTGCGTCGTCTAAGCAATTTGCTGCTGCTTCTCCTTAGTTCACTTTTTACAAAATGATTGGGCTGCACAGTGTTGTATGTTGTCTCCAATAAAGATCTGGTTTGCCTTCGAGTCCGTTATCTATTGGCTTCTGTTCCTTAAGACTTCGTAGTAAGTGAACCTTAGCGCTCATGCATCAGTACAGTCGTAGTTTTCGGCGTTACAAAARTGTCATCCCAATAGTACACGACTGTATTGTAATTATGAATGAATGAACTTGCATGTAGCCGATGGAAGCAGATACATGTAACTACAGGCACCAATTCTTCACCTGGCATCATAAACGTTACGTAAATCCACGTGGTTGAAGCGTCTTGCAATATTCTGTTTTGCAGCTCAATATGCTAACAACACATTGACCGGTCTCTCGTCAAAAAGGACCAGGCATAACAAATCCAATGATATAACGCAGATCCCGATAACCAGCGCATGGATAGTGCAGCATTTGGTTGCGATCCGACACGCATAACGACAAGCCTGTTGACATTAACGACATCCAACCAAACGAAATATTAAAACTCTGGAGTATTACAATGTAATAGACCACCGAAGGCACGGCAAGCCTTAGGCTTAAGCAAGATTGGGTGAGGATCCCCAGTTTACAATGTACACTATTTCTGGATCAAATCGTTAGATCGAAACACAAACGGCTCACATCAAAACGAAAACACACACGAGAAAGACAAACTGCTCCGAAATTGACGATGAACCAGTCTAACTCCACCAGCATGTCAGCTACTTCCGTGACTCTCTAGCTAACAGTCAGTAAACAGGTTCTCAGCGATACACTAACGACATTGCTGTCTACGGAGACAAATAGTACGAAAACAGCCTAATACAGCTTCCAATATAAGCTAAATTACTGCCCGCGTGAGACAAGCAACAAATCTCCAGAAAATTCTACCAACTCTCAATTAAGACCCTCGAGAAAAATCATTTATTTTATACCCAACTATCGCTTACAGTCAACGACGGTGAAGTGTAGCGTTACGACCAGTAACATTATAGTTCAGGGAAACGTGCTTCTGACGAACGCTTCCCAATTTTTGAAGTTTTCAAGTGGTTTAATATACTACTGAACAGAAAGCATAGCAGTCTAAAAGAGAGGACCAGACAAGCAATATATATAAACCGATTTAGAAACCGAGTGGATATTCAGGATATCGTAACCTGTATCTAATCAATGCCCTGCCTTTTTCACATACTGTCCCATACAGAGACCTGGCTTGCGTTGTGACACCTGTACTTTGTAATATTGATATCATTAGACATCGAAGTAGCTGAGCACACGAAAGCAGCTAACAATTTCACTCTGCAATCTGCGTCGTACGAAAGCCATCACTTTGTGCCCCAACTGTCTTCATTTCGGATCAAGAGCCACCGCCGTTCACCAATGCGTCTGTCCTGCGTCTATCTAGTAGTCGCTACTGTGACCACTATCATCGCAAGCGCTAATGCTGCAGCCGAAGCCTCCGAGCCCATGCCCAATATCGCGAAGTATGCATCACCAGAAGTTTCAGTTCACCTTGGTGCTGAGCGCGAGAAGAGGCTTTTGCGCTTCGACAGCAACGATTATCGCGACGATGACGATGAAGAGGAAAGGGCGAATGCTGCCAACCTCTTCAACGTCGACAAGCTAACGGTGTATGTAAACAAAGCCCAGAAGCGAACTGCCAACAATGTGAGTGGAAGCCTCTTGAATTATTTTAAGAGATTGGAAGCATACGGCTACAACCCTGTCAAACTCGGTAACATAATTCCTGACGAGGAGTACGACAATCTCCGTATGCTGTACCGCAGCTGGTACTACCACAACAAGTAACCGGATGACTACAAGCTCATTGTGAACAATCATCTTCTCCACTGGATGGTTTGGCTTCGTAACTATTGAATACAGCAGTTACCTCGGCTGCAGCGTTGCTTTATACATGATGATGATGGTGTCTTGCTGTTGAAGACGTAGTAGGCGATGAGCTTATAAAACTTTTTTACACCTTGGCTAAGCGCAGCAATATCACACGAAGAATGTAGACCCAACACGATAAAACGCTGGTAAACTGTACGAGATGGTATTTCGTGTAGCTGATTGGGTAAACGAGTTCGTATAATGTATCTGCGACAAGTGCAAGACCATGTTAAGCTTAGTGAGTCTACAAGCGCTGTAAACGCACAAGTAAATAAGCATTGCCATCGTCAAGAAATCCACATTCAAGGCTGTAGGTGACGATGTTTGAGCGACCAGTAGTCTAATACAGCTAGAGATGTTCACCCCATGCAGTGCTTCTTCTGTTCCATCTAACGTACCATAAGAGATGTGTCGCCTGCACCAGAGCCTCAATAAACATGTAAAACGACGATAGACTGCGGTAGAATTAAATGACTACCTGTCTGTTCTAAGCACATGCTTCATTATCAATGAAATAAACCGAGCGAGCTAAGAAACACTTGCAATTTCCTGTTCCAGCGACCTAAATTGGACATTGTTACCACTAAGGCTGTACGGGACCCCGCACAAAAGGTCACTGGCTATAAAGAAGAGTGCGATAGGTGCGAGTACCCATCTTCCTTCAAGAAAAATATTGTTCGATATAAATGCTAGCTTCTAAAACGCTGGCTGCCCGTAGTTGGGAGGCCCTACAGTAGGGGCTGCATGTTAGATGTAGAAGGGGGTACTGTACTAGGATTCTAACTCAATCAATTTCACTCTTTAATTTCTAGACAGCGCCTCCTTTAGCCTCATTCGCTTGTGATTGTATCTTTTGTGGTTTTTACTATTACAATAGTAAATCGTTCTTTAAAGTGATAAAGAACTCTCTGGATTATCCATATTAACATCATTTAATGTAAAGGAGCTGATAGGAATTTTCTTCCATATCAGCAGAACACATTGACCGGTTTCTTGTCATTATGGACCAGCCCCAATCACTGTCCATGGTGACCAGCGCATGATGCGCTGCACATCCATAGTTGAGAACTTACTGACTATCCAACAGGCATAACGACAAACCTGTTGACGCTACTGTCGCAATTTAATGTAAGCCCAGTGACGATTCACAATAACTCGCGCAATTCGGTGTATTCACGAAATTCAAGTAAGTAAGCATTTTTTTTTCATTCAGCATGTAATTGCTCCGCTACTACGCTTTTGACTATCGCTTGCTTATTGAAGGAGCTAGACCACTTCGAGTATTATAGAACGTTTAGCAGTCTCCTAGGACATCCGATAACGCTGCTGCTGTACGGGGGCAGATATTTGCAACTGCTTTACAACATATTAGTTTTAATAATTCAAGATGACATGAAAACATGCTTTAAGTGTAGAAAAACGGATCTACGCATCCTACTTTCCAGATTCATAACTGTCACTCCATAGAAAAGCAAGACGGTAAGTGAGTTTTTCTTGTAAGATATGCATTCAGTCGTACGTTATAATGACGCTTAATGACTTGTGTATTGCGTCGTCTAAGCAATTTGCTGCTGCTTCTCCTTAGTTCACTTTTTACAAAATGATTGGGCTGCACAGTGTTGTATGTTGTCTCCAATAAAGATCTGGTTTGCCTTCGAGTCCGTTATCTATTGGCTTCTGTTCCTTAAGACTTCGTAGTAAGTGAACCTTAGCGCTCATGCATCAGTACAGTCGTAGTTTTCGGCGTTACAAAAGTGTCATCCCAATARTACACGAYTGTATTGTAAKTATGAATGAATGAACTTGCATGTAGCCGATGGAAGCAGATACATGTAACTACAGGCACCAATTCTTCACCTGGCATCATAAACGTTACGTAAATCCACGTGGTTGAAGCGTCTTGCAATATTCTGTTTTGCAGCTCAATATGCTAACAACACATTGACCGGTCTCTCGTCAAAAAGGACCAGGCATAACAAATCCAATGATATAACGCAGATCCCGATAACCAGCGCATGGATAGTGCAGCATTTGGTTGCGATCCGACACGCATAACGACAAGCCTGTTGACATTAACGACATCCAACCAAACGAAATATTAAAACTCTGGAGTATTACAATGTAATAGACCACCGAAGGCACGGCAAGCCTTAGGCTTAAGCAAGATTGGGTGAGGATCCCCAGTTTACAATGTACACTATTTCTGGATCAAATCGTTAGATCGAAACACAAACGGCTCACATCAAAACGAAAACACACACGAGAAAGACAAACTGCTCCGAAATTGACGATGAACCAGTCTAACTCCACCAGCATGTCAGCTACTTCCGTGACTCTCTAGCTAACAGTCAGTAAACAGGTTCTCAGCGATACACTAACGACATTGCTGTCTACGGAGACAAATAGTACGAAAACAGCCTAATACAGCTTCCAATATAAGCTAAATTACTGCCCGCGTGAGACAAGCAACAAATCTCCAGAAAATTCTACCAACTCTCAATTAAGACCCTCGAGAAAAATCATTTATTTTATACCCAACTATCGCTTACAGTCAACGACGGTGAAGTGTAGCGTTACGACCAGTAACATTATAGTTCAGGGAAACGTGCTTCTGACGAACGCTTCCCAATTTTTGAAGTTTTCAAGTGGTTTAATATACTACTGAACAGAAAGCATAGCAGTCTAAAAGAGAGGACCAGACAAGCAATATATATAAACCGATTTAGAAACCGAGTGGATATTCAGGATATCGTAACCTGTATCTAATCAATGCCCTGCCTTTTTCACATACTGTCCCATACAGAGACCTGGCTTGCGTTGTGACACCTGTACTTTGTAATATTGATATCATTAGACATCGAAGTAGCTGAGCACACGAAAGCAGCTAACAATTTCACTCTGCAATCTGCGTCGTACGAAAGCCATCACTTTGTGCCCCAACTGTCTTCATTTCGGATCAAGAGCCACCGCCGTTCACCAATGCGTCTGTCCTGCGTCTATCTAGTAGTCGCTACTGTGACCACTATCATCGCAAGCGCTAATGCTGCAGCCGAAGCCTCCGAGCCCATGCCCAATATCGCGAAGTATGCATCACCAGAAGTTTCAGTTCACCTTGGTGCTGAGCGCGAGAAGAGGCTTTTGCGCTTCGACAGCAACGATTATCGCGACGATGACGATGAAGAGGAAAGGGCGAATGCTGCCAACCTCTTCAACGTCGACAAGCTAACGGTGTATGTAAACAAAGCCCAGAAGCGAACTGCCAACAATGTGAGTGGAAGCCTCTTGAATTATTTTAAGAGATTGGAAGCATACGGCTACAACCCTGTCAAACTCGGTAACATAATTCCTGACGAGGAGTACGACAATCTCCGTATGCTGTACCGCAGCTGGTACTACCACAACAAGTAACCGGATGACTACAAGCTCATTGTGAACAATCATCTTCTCCACTGGATGGTTTGGCTTCGTAACTATTGAATACAGCAGTTACCTCGGCTGCAGCGTTGCTTTATACATGATGATGATGGTGTCTTGCTGTTGAAGACGTAGTAGGCGATGAGCTTATAAAACTTTTTTACACCTTGGCTAAGCGCAGCAATATCACACGAAGAATGTAGACCCAACACGATAAAACGCTGGTAAACTGTACGAGATGGTATTTCGTGTAGCTGATTGGGTAAACGAGTTCGTATAATGTATCTGCGACAAGTGCAAGACCATGTTAAGCTTAGTGAGTCTACAAGCGCTGTAAACGCACAAGTAAATAAGCATTGCCATCGTCAAGAAATCCACATTCAAGGCTGTAGGTGACGATGTTTGAGCGACCAGTAGTCTAATACAGCTAGAGATGTTCACCCCATGCAGTGCTTCTTCTGTTCCATCTAACGTACCATAAGAGATGTGTCGCCTGCACCAGAGCCTCAATAAACATGTAAAACGACGATAGACTGCGGTAGAATTAAATGACTACCTGTCTGTTCTAAGCACATGCTTCATTATCAATGAAATAAACCGAGCGAGCTAAGAAACACTTGCAATTTCCTGTTCCAGCGACCTAAATTGGACATTGTTACCACTAAGGCTGTACGGGACCCCGCACAAAAGGTCACTGGCTATAAAGAAGAGTGCGATAGGTGCGAGTACCCATCTTCCTTCAAGAAAAATATTGTTCGATATAAATGCTAGCTTCTAAAACGCTGGCTGCCCGTAGTTGGGAGGCCCTACAGTAGGGGCTGCATGTTAGATGTAGAAGGGGGTACTGTACTAGGATTCTAACTCAATCAATTTCACTCTTTAATTTCTAGACAGCGCCTCCTTTAGCCTCATTCGCTTGTGATTGTATCTTTTGTGGTTTTTACTATTACAATAGTAAATCGTTCTTTAAAGTGATAAAGAACTCTCTGGATTATCCATATTAACATCATTTAATGTAAAGGAGCTGATAGGAATTTTCTTCCATATCAGCAGAACACATTGACCGGTTTCTTGTCATTATGGACCAGCCCCAATCACTGTCCATGGTGACCAGCGCATGATGCGCTGCACATCCATAGTTGAGAACTTACTGACTATCCAACAGGCATAGCGACAAACCTGTTGACGCTACTGTCGCAATTTAATGTAAGCCCAGTGACGATTCACAATAACTCGCGCAATTCGGTGTATTCACGAAATTCAAGTAAGTAAGCATTTTTTTTTCATTCAGCATGTAATTGCTCCGCTACTACGCTTTTGACTATCGCTTGCTTATTGAAGGAGCTAGACCACTTCGAGTATTATAGAACGTTTAGCAGTCTCCTAGGACATCCGATAACGCTGCTGCTGTACGGGGGCAGATATTTGCAACTGCTTTACAACATATTAGTTTTAATAATTCAAGATGACATGAAAACATGCTTTAAGTGTAGAAAAACGGATCTACGCATCCTACTTTCCAGATTCATAACTGTCACTCCATAGAAAAGCAAGACGGTAAGTGAGTTTTTCTTGTAAGATATGCATTCAGTCGTACGTTATAATGACGCTTAATGACTTGTGTATTGCGTCGTCTAAGCAATTTGCTGCTGCTTCTCCTTAGTTCACTTTTTACAAAATGATTGGGCTGCACAGTGTTGTATGTTGTCTCCAATAAAGATCTGGTTTGCCTTCGAGTCCGTTATCTATTGGCTTCTGTTCCTTAAGACTTCGTAGTAAGTGAACCTTAGCGCTCATGCATCAGTACAGTCGTAGTTTTCGGCGTTACAAAAGTGTCATCCCAATAATACACGATTGTATTGTAAGTATGAATGAATGAACTTGCATGTAGCCGATGGAAGCAGATACATGTAACTACAGGCACCAATTCTTCACCTGGCATCATAAACGTTACGTAAATCCACGTGGTTGAAGCGTCTTGCAATATTCTGTTTTGCAGCTCAATATGCTAACAACACATTGACCGGTCTCTCGTCAAAAAGGACCAGGCATAACAAATCCAATGATATAACGCAGATCCCGATAACCAGCGCATGGATAGTGCAGCATTTGGTTGCGATCCGACACGCATAACGACAAGCCTGTTGACATTAACGACATCCAACCAAACGAAATATTAAAACTCTGGAGTATTACAATGTAATAGACCACCGAAGGCACGGCAAGCCTTAGGCTTAAGCAAGATTGGGTGAGGATCCCCAGTTTACAATGTACACTATTTCTGGATCAAATCGTTAGATCGAAACACAAACGGCTCACATCAAAACGAAAACACACACGAGAAAGACAAACTGCTCCGAAATTGACGATGAACCAGTCTAACTCCACCAGCATGTCAGCTACTTCCGTGACTCTCTAGCTAACAGTCAGTAAACAGGTTCTCAGCGATACACTAACGACATTGCTGTCTACGGAGACAAATAGTACGAAAACAGCCTAATACAGCTTCCAATATAAGCTAAATTACTGCCCGCGTGAGACAAGCAACAAATCTCCAGAAAATTCTACCAACTCTCAATTAAGACCCTCGAGAAAAATCATTTATTTTATACCCAACTATCGCTTACAGTCAACGACGGTGAAGTGTAGCGTTACGACCAGTAACATTATAGTTCAGGGAAACGTGCTTCTGACGAACGCTTCCCAATTTTTGAAGTTTTCAAGTGGTTTAATATACTACTGAACAGAAAGCATAGCAGTCTAAAAGAGAGGACCAGACAAGCAATATATATAAACCGATTTAGAAACCGAGTGGATATTCAGGATATCGTAACCTGTATCTAATCAATGCCCTGCCTTTTTCACATACTGTTCCATACAGAGACCTGGCTTGCGTTGTGACACCTGTACTTTGTAATATTGATATCACTAGACATCGAAGTAGCTGAGCACACGAAAGCAGCTAACAATTTCACTCCGCAATCTGCGTCGTACGAAAGCCATCACTTTGTGCCCCAACTGTCTTCATTTCGGATCAAGAGCCACCGCCGTTCACCAATGCGTCTGTCCTGCGTCTATCTAGTAGTCGCTACTGTGACCACTATCATCGCAAGCGCTAATGCTGCAGCCGAAGCCTCCGAGCCCATGCCCAATATCGCGAAGTATGCATCACCAGAAGTTTCAGTTCACCTTGGTGCTGAGCGCGAGAAGAGGCTTTTGCGCTTCGACAGCAACGATTATCGCGACGATGACGATGAAGAGGAAAGGGCGAATGCTGCCAACCTCTTCAACGTCGACAAGCTAACGGTGTATGTAAACAAAGCCCAGAAGCGAACTGCCAACAATGTGAGTGGAAGCCTCTTGAATTATTTTAAGAGATTGGAAGCATACGGCTACAACCCTGTCAAACTCGGTAACATAATTCCTGACGAGGAGTACGACAATCTCCGTATGCTGTACCGCAGCTGGTACTACCACAACAAGTAACCGGATGACTACAAGCTCATTGTGAACAATCATCTTCTCCACTGGATGGTTTGGTAGTAAGGGAAATAACAAGATTTTATTCGCTTTCTGCTACTGTCCAGAATACCAGCGCAATTACGTAACAGGGTTATGAGCCCAACGATGCGGAACAGAGGAAACGGAGACTCAGAAGCCAAGAAAGCGGAGGTAGAGTAGAATAAAGATGGTCGTCCAGTGAATTGGAATGGACGCAATTGGCCTCTTTACAAGCGCACCATGATGAGATACCTGACTTGCTACAACGTAGAGGACAATGGCTGGCGTTTGGACGACATCTGTGACGGTAGTGTTGAGTACCGAAACACCAATGGTAACGATGAGGAGCAACGATTTATGCGGCAAGACCAGTTACTCGCCAAGTTGATTGCAAGTTCACTATCGTGCACCCTCGCCCAGCAAGTAATGCGGTTTGACTACGGGTCGGACATGTGGAACTACTTGGCTACGCGCTTCGAAGGGCGTGAAAACGAGATGACTACACTGTACACACAACGTGCCGTTCGTCAGAAGCTCGAAAGTGCATCATGTCGGCCAGGTGCAGACGTTGAGAACCACTTACTGTACATGATGGGGCTACGCGAACAGTTGGTTGTACTGGATGCAGACGTTAGTGACTCTTGGATGGTCGACTTCATGGTTCGATCTGTGTCGCAGCTGCCATTTTACAGAGAGTTGAGAACTCTCATGCTAACTGGAGGCCTCCACACGATGAAGACACCAGATGAAGTGAAGTCAATGATCCTCGTTCTGGACAAGGAGGAGCTAGTGGATAAACAATCCCAAGTTCGAATTAATCCCGATGTACGAGATGAAGCTGGAGGCGGTCGGCAAAGTGGCCGCAGTAACGGTGGCAAGCCGATGCCAAAGTACCAGAATCGTAACAAGGACAAGTCTACCATAAACAGCGGTGGCAGTCAGAAGTACTCGGGGTACTTCAAGAGAAGAACCGACGAGCAGCAGCGGCAGTATGAAGAAGATCGAGAGAACAAGAGCTGCTTTGGCTGTCACCAGCCGGGGCACACTCGAAGAAACTGCCCAGAAGAGATGAAGCCGGAGCCGAAGAGCCAAAATGGCAGTAGTGTCGGTAACGTCCGCAATCATGCCAACTTCACTCATTCGTCGGGGGTGGCTAGCCGCGAACCACAAGGACCTGCGCAAAAGCAGGAGNGGACGCGCCTACTCACGAGAAGCTTCACAGTCGAAGAAAGGCACTGCCAGGTACCAACAGAAGCTTGAATACCAGCCGGAGACTTGGGTGTTCGACAATGGAGCAACACAGCATTTGGCCGGAGATAAGAGATACTTTGTCAACTACCGTGATCTAACGCTAGAAGAACAAGATAGAGCTACAGTTCATGGCTACAACGGCAAGAGTACTCCAATCGGCATGGGATCAATTGACTTATGGGTCAATGTTAACGACAATCCAGTAGTTCTTCGAGTCGAGAACGTATACTATTCGCCACAACGGACTAATTTGCTCTCCCAGTCGGCGGCAACGGAGCAAGGATTCCAAACCTCATATGACGACTCGACGAGAGAATACACGTTGAATATGAACGGTGAAGTAGCACTTAAGGTGCAAGTACAACCGTCCAAGCTGTGGACGTTCACAGCAGACAACTCATTCTTACCAGGGAAAAAGTCGAAAGAAAACCAGTCAGCGCCAAAGACAATGATCAACTACGCAATTAGTGATGGCGTCGCAGACTTGCAGAGCTGGCACGAGCGACTGGGCCACATCTGCCCTCGATTTGTGAAGCAAATGGCCGATCAAAACTTGGTCGAAGGGATGATGTTAAGAAAGAGGGACTTTGACATGTGTGAAGCCTGTCAACTTGGAAAACAACGCGCAAAGACACCATTGAAACAACTAGATCGTGGAGTGAAACGACGAAACCAGTTAGTGTTTGCCGATCTGCTATTTCCGCCAAGTCACTACAATTGTACGCGTTTCAAAGCAGTATTGGTGATAATGGACGCTCACACGAGGTTTACAACGATTTATCCGGTGCAGACCAAGCAGAAGGAAGAAAATTAATGCACTGATGAAGCGATATGTCGTTTGGGCTGAAAGGCAATCACCAGAGTGGAAGGTGAATGAGATCATTACAGACGGCGATGGTGAGTTCGAGAATGGACAGATCACAACATGGTACCAGGAACACGGCATAATACATACCGTCATACCACCAAAAACATCTCGGCTGAACATGGTGGAGCGTACCCATCAGACGATAATTGGTATGATGAAGACAATGATGAAGGAATCGGGCTTCCCGACGTCATTTTGGATCGAAGCATTGTATTATGCAGTGTATCTTAAGAATCGATCACTCTCAAGTTCAATTAACTGCACGCCGTACGAAGGAATGTGGGGAAGACGCCCAGATATTCACCACATTAGGAAATTCGGAGCTCTAGTCTACGCACACACGAAGGTTGGGCCGTCACGTCACAAATTTGCCGACAACTGCAAGATTGGATTTGTGCTCGGCTATCGTGATGGAGTTCTTGGATGCAAAGTCTATTTTCCTGCAGAAGGT

>Contig_15

AGGATCAGCGCTTGAAGCAGCAGTTGCTGAACAAAGAGCGAGAAAGAGGGGGAAATATTGTCAACTTTACTGAAGAGGACTATGTGCTACGCTCCCGAGTTGATGAAAAAAGCGGAAGCAAACGCCTGGTAATTTGGGTCGGACCTTATCGTATTGTGCGAGCAGATGCACACTCTTTCCTCGCTCAACATCTGATCACGGGTACAGAGCTGGATGTCCAGGCTCAAATTTTACGCGGATTCCAGTTTTAGCGTCACTGAAGAGCTTTTGGAGCACATTTCGTCTCAAGGTGTTATTCTCGCTGTGGATAAGCTCAAAGGAATCGGTGGAATAGTAGCATTAATGACTTCGAAATTCTCGTGCAGTGGAAGGACTTGAGTCAATCGAGGCCTCGTACGAGCCGCTCACCAACCTAGCTCGTGATGTTGCAACATTGATACAACAATACGTTAGCACCGCTGACCAAGATTTGCAAGAACACTGGCAGCGAGTGACCCGTGTGGAATTGCAGCAGCCAAAAGCTGCCGAGGCCTCAACAACTGGAAGGCTTGGACGATGCAATAGACGTCGCAAAGCCAACCGCAATGGACAACCTTCAACGCGCCCGGTCGCCCCTGGTATTTCGGCTGTCGAATCTCAAGAGCCTAACAGAGGTGGTTCAGCTGATGCAGTGCTTCAAACATTAGTAAACCGACCTGGGTTTGAAAACCAACAATCAAATTTGTCTACGACTGATACCCCTTCTCAGCAGCACAGGATCGACAAAGAAGTCGACTATCTTCAGGGCAGCGCTTATCTCCAACGGATGCCCGCCGATGGAGATCGTGTCGGTCGATGTACGTGCTCGCGGACTGCTGGTGCCGACCAATCCGCAGCCAGAACCCAGCAGGATGCAGCACATTCCCGCAGACACTGAAGGAGAGGAACGGGTTTACTAGACTCACAGGAGGCCAGGCAGTACCTACATGGTGCTTGATATGACTGCTGACTGGGTGTTTAGTAGAGAAGGCGTACTTCAGCGGCGAAAACTTCGCTTTAGGGACAGTGGAGCACATGGTCACCACGTGTGCTCCTCGCTCCGAGTAGTCGATGACCAATGTAGGAACCACCCCGAGGCTGGCTTCTCCATCGCTCACGTAGCGATTCCTGGGAGGCGCGTGTTCGTGTCCTCGATCGGGATGTTATTAATGCGGCAGGCGCTCTCGATCTGGAACGGCCATTCGCGTAAGTCCTCTCCCCTCTTGCCGGTGAACTTGGGTAGCTTGGTCGGCACTCGCGTGCGGTCTGCCATAACTTGATTAAGCCGGTCGATCTCAACTTGCAGCTGGGCGTTCGTCGCCATGGGCTCCGTTTGTCAGGTCACTGGGACCAATAGGTGCTACCAGTATTTCAAAAGGAACCTGCTTTTAGGGAGGGCGCCAAAAAAAGGGAAACTGCCGCCTCCACAGTTTAAGACGGATGTAGAAGCTTCCACAGTCGCATGTGTTCAATTTAATAGCATCTTACTATGTCGTATTAGATCACAGTTATTTCTAGACTACATATAATACTAATCACTCACATTATTGATACAGTATCTGTGTCATGTGTGCAGCAGTGGTGTTACCTACGGCTCTCGTCGATACAGAGAGCCCAAAGTATGCACTTATTGACATTCGAAACGCTCTTTTTTGAAACGATCACTGCATTTGATTGTAAATTTCTTGGTAGTCAATTTAATTTTTGCGAAGACTAAAAAAGCGGTCTTATTGTCAGATGTCACAAACTTTCTTCTCTTCTCACAGATTTTGCGTGAATGGCGAAGATGACACTCAGCTCTGGCGAGGTGAGAAGAGCATTTACTGTCACCATTGACACCGTGGAAATCCAGTCCTATGAGAAGAGATCAAGTCGCAAAGCGAAGGCATTTAGACTCCAGCATGTCTCTTGCAGCTGCTCTCGGTAAAGAAACCGAGGGTATAACAAAGTCGTGCTTCACCCGAAGAAGCTCTGAAAACGCGGATATTGCAAGTGCAACAGACTGGTCATATTTTAAATCATAATCTCAGATTTTACTCGATCCCGACGATGATGTTCGCTTGCTGATTCTACATTTAATGTAAGTACAGCATGAGGCCAAGTTGGTGAGGAGCCACATGCGAAGCAACCTTTTCGACCTTCGCTTGCTCGTTTTGGCCTACGGCGCTTATCGTCGGATAAGGGTACGTACAAGTAGCAGGTATCGTATTTTTATGGCCTTGCGCCCAAAACTACCCTTATGCCCGCATTTACCCACCATTCCGACAGGTATTTCTTCGTGTATTATTCGTGTAACATCCAATTTATAATAGGCGCCGAAGGATTCTGAAAACGCTGTGATAGAGAAATTAAAGCGCTATAATAGATGGGGTGTGGCCTAAAATTCCAGTCTGCGCCTCCACAGTTTCTGCCGCGGTCCTAAACGAAGTCCTCAAACACCAGTGCGTAGTAGGCGCGGTGTGGTGGATTGCTGAATCAAGCAAGCTTCCTCCATCTGTAGTGACCCTGGAGTACACGAGGCGGGCCAGGTACATCTTGGTCAGTGACGTTTCCTCACGTCATTTATATAACAGATTCTTTTAGCTAGCATCCAGTTGTCCTTGTGGATTTACTTTAGGCCTTGTATGTCCCCTAACTGCACCTCTATGTGCTTGAGAAAGAAGGGCAGCCTCAAGTTGTTCCTGACTATCACTCTACATTTCACCAGTAACCAGAATAGCAGTTGCACACTAGGCTATCCGTTCCGGCAGGTTCGCCACATGCCTTGGCGGTGTTTCCCCTGTTTCAACTCCGGGAACCAGTACACGCGGAATGTTAATGTGTTCAAGGCGCCCAAAAGTGAATTAAGCAAGTAATTAATAGCGCCCAGCGAAATGGCTGTTGCGTACATACAAGTATCAATGTCCGGCCCAGGCTTAAGCCAAGCATGTAGACTAAACGATCCAACGACTGCTTGGCAATGGCAGGATCCTCTAAATAATTAAGTTCTCTTAGACACGAGCCTTGACTAAATGCCCTCAGCGCGTGTGCCAAATCCATACCAGAATTCAATTTCTTCAAATTGCCAACTATTGACTAAATTCTCAGAATTTTATCCGTGAAAATATTTAAATCGTCCTAGAGTATTTCTCTGCACTTTGAAACTCAAAGGTTCTTAGCAGATGCTTTGATGCAGACTCATCTGAATTGCACATCAGCAGCACCATCACGACATTTTTGATGATGTTGGCATTTGAGCACTAATCAATGCCGTAATGATTACAAGCGAGGTCCGTAATCTGGCATCCTGATAGCGTTAAAATTCTGTGAATACAGTGGCTGATTCTATTTCCTTAGACAAAGGGCTCATCCAATTCAACATAAATTAAAATTCTCCGAGCTCTACTTGATTGCATTCAGAAAAAAGGCGTTTGGCAAATCTGCCTGATTCATTTATGTTTACGGTAAAACGACTTTGAAAGACCCCTGAGTCATGGCCCATGATACACAGCAATATCACAATTTCACTTTCCTGTAGCCCAACACTTATTTAAACTGTAAAGCCACTATGCGCTTCTGCTTCGTTCTGATCATTTTCCTAGCTGGAATTTGTAAGTGTCTCTTGCTCAATTGGAGTGGCAATACGTTAAAGGAACGAAGCCACACCAGCACCAATTCTCTCCCCAGTCCACCTTTCATCTCAACAATTAAGACCCCAAAAAGGTTCTTAAGATCCTACGACGCACCTAAGCAAGACAACATCGGTCATGATACGGACGAAAGAGCTGGAATTTCTGGGATAGCCATGATTGACGATCTTGCGTACAAGTGGGCGTTAAAGAATACGAGGGATCCAATGGATGCATTCCAGCGCTTACATGTTGTGAAAACTGGCGGCAAATTGGAAGGCAACAAGGAATTCATTCGGTGGCTCCAGTACGTAAATCGATACAAGGCGACACGACGAGTCAAGTTCGGTGAGGATGAGCTGCTCAGCCTGCTAATGAAACGAGAGCAGAAGAAGAACTCGTGTCCCTGTTCCAATCACTTCGACAATACCCGGACATTACAAAGATGGCTAACGATATGCAAGCGTCCATGATTTTGAGCTCTGCGTCTAGTCACAGACTGATCAATGAGGCATGGTTAATGTCCCGAGAAACTCCCGGCGAAGTTTTCAAAATCTTGCGACTTGGAGATAACAGCATCAGTCGGCTAGACAATAACCCCCTCTTTATTCAGTGGCTCAGATATGTTACGATGTACAGGGCTGTACACGGAGGTGACCCATTTGCGAATTTGGAAGCACTATTTAAGCGTTTCTCGACTGTACCACAATTTGGAACTCTCATTCAATCGTTGCAGAACATCCCAGATTTGGAGAAACTCGCACTAAGCTTACAGACCCACCTCTATCGGAAATGGATGATCGAGATCCAGCTTACCCCATCTGAGCTCTTGGGTCTTCTAGAGACAACCAGAGTCGCGAGAAGTGATTTTAAATACCGCAATTTGGAAGCTTACACCATGTACTTCGCTGAAAGCCGAGGTGGTACGCCTTTGTTGAATAAATTGAAAACGCTGTTCACGGATGGCGATCCCTACGCAGCACTGTCTGCCGCTTCGAGCGCCTAGCAAGGAACTTCACCCTTTCATTTAATACAACGTTTAAAAAGATACAGTCATTTTGCTTAAATTAATGATTCTTAGACGGAATTCGTCGGACATCCGTCAGTCATCGCTATTTTCATGAAGTTTGATAGACTACCTAGAGAACGTCATACAGTCCGTGTTGAGTTTGGCAAATGCTTGCCGTGTCTTGCTGGCATTATTCATTTTCACAAATGGTTCTTAAAAGTTGACACAACAGTGCTCGCACATTGAAAAGCTATGACAGACAACTCAATATACACGTACGAGACATCTAAGATATCAGTATGCTATTTGATATCATAGCTAAGCACACTCAATCTGATAGTTGCTCTTCATAGCGGTAATGTGCGCCGATGACAGCGCGGCGCGCTGACACTGCTCGCCACTGAGCCATTTAGGCCATGGAGGACGACTGCCGGAAGGCGTCCACAAGCTAAGGGAAAAACAGTTTTTTTTTTTCAGAAAATGTCAGACAGAATGTCCGACGCGAACCAATCAGATGGGTACGATGTCCGACAAATTTTGAGGAATGCTCATGAGAACCAGCCTGCTGAAATATAGGATAAATCACGCATATAGGAGATAATTTGGGTGAAGGAATTACAATTGAGTTGCCGTGTAGCCTTTTACATCCAGAATATGAATTTAAACGCATTTACTAAAAGAAGTTTATACCCCTACTATGAGCTGAAAAGCTGGCTCTGGCGGGGCGTCACTCGTCCGTTAAGTTGTATTATCAACCTCAACATGTGATGCAGGCTCAATGCAAGGCGACCCAATTGTCTACGTAGATTTGCGGCACGTGTATCGCGCAATCCTAAACACTTTCAGCGTAAGACGATTTATAGTTCTACACTCAGTCTTCTATTTTAAAAGAGGTCAGTTTACGAAGGGCGCGGATGGTATTCGAGTGCACGTTGAGGGGTTTTCGAGTGCAGACATTTTTGCCACGAAATGGCGGAATGCCGAGTGCAGATAACACATGGCGATATCGTCGGCTCTATACTTGTCTTGTCCGGTTTATGCTTGTTTGAATTTACTACACTGTCCTTGGGGCCGGATGCAGAGCTCACCAGGATGTAAGAGGAGAAAAATACATAAATTTGTGAGAAGAAAACATGAATGAGCCTTGGAACCTGGTTTACTTCCTACACAAGGTACGCTGAGCTCCGCTAGTCCAGTTCTACGCCCACATGCTCAATGCCAACTGTCACTCCTGATCGTCTTAGCTGTCCAAGTACTGATCGTCGTCGGCGTCACGGTCGACACTGCGGGGATGCGATTCGTGTGCGCTTGCGTGGGCGGCAGCACTCTTCGTAACATCGAGGCCAGTACGTCTTGATTCTACGATCAGCTGCACCAATTCCTGGCACTCTAGGAAGACTTGCTTGCCTCCCTCGAAGTCGACAAGCCATCTATATCGCGACTTGCGCGCTTTGCATCGTTCATGAACCGAGATGCGTTGGTACCGTCCTCCGGTCGATGGACGACTCGGGCTATGCTCTCCCGTGGAGCTCTCCACCATTGCCCTGAACACAATGAGAATTGCACACGGTAGGATAGATGAGTATGAAGTAGAAGCAACTTAGAGTATTACGGTGAACGTACGGCGAGCGTTGAATTTGCTCTTATCGTGTTGACGGAGAAGCGCTTGAACGAAAGGAACTCTTACACTGTGGGTTGGCGTAGCTGGCGAGCGAAGTTGGCGATGGAAGCACACTAACCCTAGCTCGTGGATCGCTTTGTAGACGAACGCCTGCTTATCGTTGCTGCAACATGTAGCTTACATTAGCTACGTCTAAACTAATGAAGGCAGAGCCTTGACGCATCTTCTTCCGTGCTCAAGCCTTAACCTCAGCAGTGCACTCGGTCATTCCCTCAAGTTGAACAAGGGACACTCGGTTTCACACCTTTCGAACAAAAAACTAACGGTGCACTCGATTTCCTNAAAAAAAAAAGCACTCGAAAGTAATATCGTTCCGTTTACGAATGTTACCTCTTGGGAAGTATTAAGGAAATACTCATCACAAGACTTTTAAAATATAATAATGGACACCTTTTTGGAGAGGTGGAATATAAATTCTCTTCTGAATTGCACATCGACAGAACGGCAACGACACTTTTGTTGACGCTCATTTCTTAGCAAGTGAGTAATCGAAGTCCGTAATCTTCAAACTAAAAAACGTAATCAGTAATAAGAGTGGGATTTAAAGTAGCTCTTCACTATTGAGAAGAAATGTTCTTCAATGTCGTGCTGATCACATTCATCGTACGGATTTCGCTTTGCTCCTCGTTCAACTCATTGATAAGCACCAACCAGCTCAAGGGCCTGAGCCACACCAGGTCAACGTCCATCCCTGCAACGAAGAGATCGTATTCCACAACAAGGTTTCTAAGGTTCAGCGACGCATCTAAGCACGATGATATTGATGATAATAGCAAAGAGAGAGCAGGGGTTTCTGGGATAGCATGGCTTGGTGATCTGGCATCCAAGTGGGCGCTGAAGAACACGAGGAATCCGATGCAAATTTTCAAGCTTTTACGTACTGTGAAAACTGGCGGTAAGCTGGAGGGTGACAAGGAATTTGTTTGGTGGCTTCTGTACGTGAATCGATACAGAGCTAAGTTACAAGACAAGGCCTCGTTCAGTGACGACAAGCTATTTGATTTGGTGCGGAAACTGAATTCGGAAGAGTAACTGGTGTCTCTGTTTCAATCGCTTCGACATTATCCGGACATCAAGAATATCGCCGATGATATGCAGGCGTACCTGATCTTGAGCTCGGCGTCTAGTCACTGATGAATAATGAGGCATGGTTAAAATTCCGAGAAACCCCCGAAGAAGTTTTCAATATCTTGCGGCTTGAGGATGAACCTCTGTACGCTCTCGACGGTAATCCCCTGTTTATTCAGTGGCTCAGATACATTAAAGCATACAGAGCTGTGAATGGAGGCCACTCGTTCACAGACGTGCATGTGTTCGACTTTTTACATGAGTTTGCTTCTTTGCCGCGATTTGGAATATTTCTTCAGTCGTTAAAGGACATCCCAGGTTTGGAGAAACTCGCAAAAAAGTTTACAAACGCAGGGTTGAGGTCGATTGGCTCACCCCATCGCAGCTCGAAAAAATATTTGGCTCACCGTATCCAATCAACTTCGCGGAACTCCCAAAGAGTGATGCCAGGTACCGCAATTTGGAAAGCTTCACGGTGTATTTCGGTGAGTACTGGGAAGGAACAGCATTATCGGATAAAGTGACAATATTGTTCGCCAAGAACGACCTATACGCTGCAATTCGAGCCGCTTCAAAAGGTTAAAGATTCCTGACTCTAGCTGGCCGAAAGTGTTAGGGATTAATAACCCATCGCAAGATTAGCGAAGTTTTCTATTGCAGTCCAACAATTGCTACGATACTTACTTCGGCGTGCGCAGCCATCTGGAGAGCCAAGTGTGAGGTTATCTAAGATATTTAACATTGGTAGGAACCCTTTTTACAGCACCAAAAAAATGAACCGCACTATTGTTTTTCTTCTGATGAAAAACATGTATCTTCGTTCCATTTTTCATTGCGTCTGTATTACACCGGATTTTAAATGGCTTCACAAGAAGCTTACAGACTTTGTGAGCTTATTGCCTAAATCCGGGATATCCTGTATCAATTGAATGGATACATGTAACCCAAAGCACGGCAAGGTCACAAAAAGCTCGTATGATGGAGTGTTGTCCGAGGAAAATTAAACCCCGTCCCGACACGAAAACACCGCAAAAAAACAGCTCTTTGCAAACGCAATTACTTTAGATATTATCAAAGTGATCCAGCATTAAAAAATGAAGTACAATTATTGCAACTGTGCAGTTGTTTACCGAATGACTGTGGCGCTTGAATTATTATTCACTTTCAGTTTGGCCAGAAATCCTTAACACCACGCTTCAATTTCCATGCTGACCATCTTCCTTTCGGGAGATGTTTCTGTTTCTCGTTTAGCTTGACAGCTCACGGACCCGGGTCACAACGGAATAGGAACAGGTATGTCGATCGACTTCTAGTAAATCTAGCTGCATGCATTACTAAAAAGCTGTCGAACGGCTGCAATTGCAACGTCAAGATCTCTGTTGTGGAACAATGATTTTACTTTATCCTGCAAAGCATTTCCACTATGTTGCTCTGCGACGTACATGGTGTAAGCTTCCACATTATCTGGAATCATTCGTCGACAGCTCGCGACATTCATGTGGCAGATTGACTGCCGTTTTCAAAATAGAAAATAGCTTCTCAGAAGTTCTCACGGCGCTTGCGTCCAGCATAACCAGCGACAATATCTTCAACGATGGCTGCGCATGACCCACGGTGAAATTGTTGGGCGCAAAAGCATCGTCGGGTACACGTAATTCCGTCAAATACCTTTTTTATACTTCAAGTAATTATGGAAACTTCTTTATCCCACACCATACAAACGTCAAGTAAAACTCAACCTCACCAGCCTCTGGCGTCATTATCAGTCTGACCATGGAGAAGGATCTTCGCGACGCATATGCAGCACTCAAGCTGCTTCGGGCAGACCTTGTGAAGACCCGCCGCGATAAACGTGCTCTAGAGGCCTCACTGACCCACCTGCAGACCCACGGGCCGCCTCCCAGCGCTGCACAAGCTCGCGAGAGTCGCGAGACGCAACAGATGCAACACGAGGACGCCAATGCGCAATTATGGCGTCTCGCTGCAATTTACGAAAGTCGATTGGCGGAAATGGAAATACAATTATTGCACAATAATTGTCAAAAGAAGGAGGCAACACCCGAAGTTGAAGACAATCAACAAGAAGTCGAGGAAGTGGAGAAGCTCGCGCTGCTTCACAAGCTCCACAGTCTGACCGCCACCGTCGAGCAGCAGACGCAGACGATGCTGGCGCAACAGGCAGCTTTTGCGCTGCAGAAGGGCGAGCTGGAGACGACGCTGGAGGACACGCAGCACCAGCTCCAGACAGAGAAGAGCAGGGCGACTGATGCGCTATTAGAACAACAAGCAGCTAAAGAGAGGTATGAGTTCTTGGAGACTCAAGTGGAGATCTTGAAGCAGGACAAGAAGACGTTGGAGGAGGAGAACTATACACTCCACAAGAACCTAGCGACACATGCCCAGACGAGTAGAATATTGCAACAACAAGTGCAGGAGAAGGACGAGGAGCTAACTCTGCACGAGAAAACTATTCAAGAACAAGAAGAGAGACAAGAACGATACATAACGATGCTGAGAGAGTTGGAGAACACGTGTGAGACGTTTAAGACCCACGATGCGGCTTCTGAGGCCAAACGAAGTGCTGAAACGAAGGAGTATGAAGCGAAACTAGCAGGTATTCAAGAAACGTACACATTCCAGGTGGCAAAGCTGGAAAGTGAGCTGAGAACGATTAAAAAACAACTGACAGCAGCGACAACTAGCCGAGCGATCGAATTGAAAGAGCACGACGTCAAGTTTCAAGCGGTTTCGGACAAATTGAGGCAACAGGAGCAACAGGCGACGACACTGCGAGACTTGGAGACTAATTTCGCGGACGTGCAGGCCAAACTCACTATAGCGGAGACCAAATTGGCTGATGGAGTGAAGCAATACGAGCAACAACTTGCTCAGGCCTCGCAGAAGCTACTTGATCAAGAACAAGACCACGAACAGCACGTTAGTTCTCTACGTGACGTGGAAAACGAGCTGGCACGAGTTCAGACGCAACTCACGGATACGGAGGCCAAGTTACAGCTAAACGTGACCATATTCGACGAAAAACTCGCGCAGACTTGCCAACTTGCTGCTGAACATGAAAAGACAGCTCGAGTGCTAGCTATGGAGAAGAAGGCGCTTCAACAAGACGTTCGTGAAGCTCGACAGACATCGAAAGTCAAGACCGAAGAGCTTCTTCATTTCCAGTCGATGATGAAGCAGAAAACTGGGGACCATTCGCAGCGTTTAAGCCAGTATCAAGCGCGTTGTGAGCGTCTAGAGACTCAACTATTATCATCTGAAGACCACAAGGTGTACGAAAGCAATCCAAGCGGTGAGTTGAGAGCTTTCCATGGTCTTAACGACGCCGAAATCATCGCGCTGCCCTCTTCCAAGGCGTGGGTTATATTACACTCTGCAATAACCAAGCTGGAAGACTTCTTCCCATATCTTGAAGCACTGAGTAGTGCATTACAAGACGTGTTAGCCTTGTGCAAGAGCCATGCGACTTTTCTTCCAAACGCTATGTGAGCGTCCTCGAAGACAAGGACAGTTAGCGACAAGACCCAGCCCGGTCTTGGTTATGGCGTTAAAGCTGGTACGCTTTGCTGTCGTGTTGAAGACTCAGGTCCAGCAAGACGACGCTGTTGTGACTTTAAAAGCAGTTCAAGGTTTCCGTAAACGCGTGCTGGATGCTCTCGCGCAATGGTACGAGTGTGGCGTGGATGCTTGCGACCAAAGTGGAAATGGGTCCATGCCCACACCCACTTTTACTACCACTTCTCGAGAGACAGCACTCATTTTACAGAATTGGACCAGTGACCGGACAAAGCAGTTGGGAGTGAGACGCTGGTTGGCACGGATGGAAGCGTATCCTGGCGTCCCGCCACTTCGAGGAGCGTCGTCAAACCGTGTTCTGGAACTCCCTGCGGAGGGTTGTACACTGGAGCTGGAAGATATGACGCCGGAGGTGAAAGATGCGTTTCTGTTGCTCCTGATACCGATCTTGAAGCAGAACCGAGCGCTACATGTGCGTGTGTTTACACGTTATACTGAAAACCGCGGAAGCACATCAAGTTTGTGATGGTGGTACTGGGAAAGTGTGGGCCATGCGGATCCATGTCCAGAGTGCTGTCACTCTACAAACCAGGAAGTCTCGCCCAAACTTC

>Contig_18

TGCAGCAACGACGACCAGTTTCTCCGTTTCGACTTTACTCAGCCTTTGCTGCTCACGGCTCACTTCGAATCGAAGCTGGAGAGGAAGAGCTACACGATCTTTCGTAGACGACGATGAGCGTTTTGGTCCTGCAGTGAATATTTGGGGCGTATTCTCACTCACGACGTCATCGAAGCGCAAAGAGAAAGACAAGTCCACGCTGCCTGACCCGCTGTCGTCTGTTTTGTACCTCCAGGTCACGACTATTTGTCGTGATGCTCAGGGTGAAGCCTGGATCTTTGGCCATGTACTAGCGCACCGTAAGCACTCGGGATCGAGGCGTGAGGAAGGTGACGAGTATGAAGTTGTGCCAGACAAAGATCGAGGGTTAGTGTTTGCACTAGCAAGTCAAGTCGTGGGCTCTGCGTCGATTCATTATTGCTCCAAGACCGTTTTCTATCGGAAACACCACGCAAAGGATGAGGAAAGTGAAGCGATAACTTGCACGACGTCAAGCAAAAGAGGCAACACGTTTCCGCGGAATCTGTTTTGTCGACCTAGTTATTCGTCACAATTGACTCAAGAGGAGTAAGCAAGTTACGTTTTGAAGTTGAAGTGTCATGATCTGATAACCAGCGTGACAAAACAGTAGATATCCATCGATTGTGGTTGACATTTTGGGCATTTTCTTGTCTTAACATGGATGCACTACGACCCCTACTGGTTGTGTTCAAGGCTGAGGGTGGGGACTGCAGTTCATTTGCAAATTTGCTTCCTTCTAGCAACATCCCTTCAACACGTCAAGATGAACACTGCCTCTTCTGCTCCTTCTCGCCTCTGTGGCTGCTACCGATGCCGCTGTTCCTCTTGTCCCGACGGATCGGATGGCTCAGTTTCTCCAGGAGAAGAGCGGTCTTGAGTCGGAGCTGAACACGTGGAAACAGAGCGATGCTGGACAAGTATGCCAAGGACCATGGCTTCCTTCCTATTCCGACGTCGAGAGATGTCGGTGGTCAAGCAGACCGAGGAATTGCGTAGTCGGTTCTTCCTGACCAAGTTGATGATTCAAGAGGCATCAAAGGTGACCAACCCGGAGGCTATTTTCTCCACTGATACACGCCCCGTTTACGCTGATGACGGACCAAGAGTTTGTCAAGTTCATCGGCAACTCCTATCAACGCGGCTCTGGGGTCCTCAAGAGCCACTTCGTTCGTTGATGAAGTCCCGTCCAACTCGAGTGATGCTTCCAGCACAGATAAGGACTGGACCACGTCTGAATGTGTAGTTCCGGTCAAGAACCAAGGCCAGTGTGGTTCCTGCTGGGCCTTCGCAGCTGTGGCAGCTCTCGAGAGCGCCATTTGTCTGTCTGGACAGCCTCTGACGCCCCTGTCGGAGCAAACAAGTGGTGGATTGCGACGAGGCTTCGTACGCTTGTCAGGGAGGCTTCCCTGGCGATGCGTTGACTTTCATTCAACAGTCAGGCGGTTGTCTGTACTGAAGAGGCTTACCCGTATGTATCCGGTGACTCCGGGCGACCGCGATACTTGCAAGTCATCTTGCACTCGTGAAGCTGTGACTATCCGCAAGGTGGTGGGCGTTCCTGAGAGTGACGCTGGGTTGGTCCAAGCAATCAACACTCAACCAGTTGCTGTGGGTGTCGCAGCAGGAAACCCCACGTGGAAGCAGTACAAAAGCGGGAATTGTGTCGTCCTGCACGACGTCAGAGCTCGACCACGCTGTGCTGGCAGTGGGCTACTCACCGTCCTACTTCAAGATCAAGAACTCGTGGAGCACGCAGTGGGGCGAGGAAGGCTACATGCGACTCAAGCGTGGTGCCGGCACTAGYAGCGCCGGCACGTGCGGCATCATCGGCCCCAAGTCCGTCTACCCTCAACTGTAGACGAATGGAATGTAGCCAACTTCAACGTATGCGTTCCTGACCACACGTTTTTAACCGCTAAAACCATCATTCTTGTTTTGGATTTTTTATCCTGACTTTTGTAAGAGCGATCTTGAAGAGACGCGATCTTTGCCAAACGGTCTCTTACCATGTATACACATTTGCCATTCGTCGCTTAGGATGGCGACTAACTCACGCCCCGCATGGCGCTGGGCATCGCTGCGACTAGTGAGTTTACTAGTGCTGCTAGTGGCTCTGGTGGTCGCCCAGGTGACTGAAAACCCGCCTTCAGAGCATAAGGGAGCCCCAGAGCACGAAGCTAAAGAAGGAGAAGGCGAGGGGGAGCCGGACAGTGGTCGAGTGGGCGTCGTGGCGGCAGTGACCATCTCGACGCTTGTAGCCATCTCTATCTTGTTCGAAGTCTGTACAGAGGAGCTGCGGGAACACACAGACGAGCTCAACATGCCGTTCGTCAACACGGTCTTCGGAGAGCTCACGACGCTAGGTTTTATCGGTTTACTGCTCTTTGTTGTCACCAAGATCGAGGTGCTACCGTGGCTCTCCAGGGCTATACTAGGAGGCAGTGCGGAGCTGCAGGAGATCATCGAGAAGCTGCACATGGCGTTGTTTCTCTTTATCGTCATCTTCTTGGTGCTCTGTCTCGGATTGCTACGTCTCGGGATGCACGTGCAGCACGAGTGGCGCGAGTTTGAGCGCAGTTGCGCTGACATTCCCTCTGTCCTCTCAGAGTACGCTCTCGCTACGGAGCCTCCTAAGACGTGGATCCATCGTTTGTCGTGGCGTCGAGCGACCACAGCAAGGAAAGCCCAGCGAGAAGTGGTCTACCTTGCTCTACGACGACGATTCATGGACTACCGCTCCAATCATCCGGATGAAGAAACAGCACGACTCTTAGCAAAGGAATTCCAGCTTCAAGGAGACGATTCTCGGTTCCCATTCAATGAATATCTCTCTATCATCTCTGGCGAAGTCATGGGCCGACTGATTCAGATCGACATGGCTACATGGCTCGCCCTCGACGTCGTCTTGGTGGTTCTTCTTGGTCTCTGCTGGCACGCAGGACCTCGCGGAGAAGTCGCGATCCTCCTGATCGCTGGCTTTTCCCTCATTGCCCTGAACGATTTCGTGTACCGACGAGTGAATGCGATGCGGTGTTTACTGACACCAACGCGACTACAGCACGACGCAGAGAGACTGCGTCGTAAGGCTGCGTGGCGTTGCCAGCACGGGCTATCGCCGCTCGCAGAGCCAGGAACCTGTCCTCATCCCAATGAGAAAACATGGCTACTAGTCGATGCAGAGGAAGCCGGACGCGACTATGGCGACCCGGACGGATGGGTCCCTCCCTACGTGGATCTGCTCCCCAACGGCGGACGGGATCTCCCCGAGAAGGAACTTAAACGACGTCAACGAAGCCTCATCGGTGCTGGATGGGGCAATGGAGTCGTCCTGGCACTCTTCTCTACCAGGATGGTGTTCCTGCTCACAGCGCTGCATCTCTCAACGTTCTTACTGCGTGAGACGTACCAGATCTCGGAGCTTTTCGGTGACCACCCCATCTTCGTAGCTCTACTCTGTTTCCTCTTCTTAGTTCCTAGTATCGCAGTGCCCTTCATGTCCGCACGCATCGCACGCGACGGCCTCTTAGCATTCAACGTGGAGCACATGAAAGTGTCCCAAGTCATCGTCCAAGTGACGCGGCTACTGCGTGCTCGACAGACGTTGAGAACGCTCCGCTTTGTAGCTGAAATGAAGATTCATCTTCGAGAAAATGTCCGGCGCAATCACGAACAATTGAGGGAAAATATCACCTTGATGCCGGAGGTTCCCATGCCCGTCAGTCGTCGACGTTCCAGTATCATGCCTATCGACGCTAGCGCAACATTCAAAGCTGCTCGTCGTCGCTCCTCGACGCATCTCGACCCGACCGTCACTGCCGGAGTGCTGCGCTCTATCCAGCAAGTCCCTGTCCCGCTCCAAGTGTCTCCAAAGGCGACAATCGAGCCGCCGTTGTCTCCGTTGGCAGCATACCTTACACCGAGAGAGAAACGAGGAGACGCGTACCGTCGAGAGATGGAGAGACGGGAAATTCACACGATTTTCTGTCTCTTTGACGTGGACGGCTCCGGGTCGGTATCGAGAGACGAGATGGCCAGTCTCTTACTAGCTATTACCCACGATCTGGACGATATGCAGCTTAACCGGTTAATGACGGATCTGGTAGCGGAGGAACTGGAGGGCGACAGTGATGCAGTTAAAGAAGAGGTAATGGTTGAAGGGCCTCAGGAAATCACGTTCGAGGCCTTCTACAAGTGGTGCAGCGCGAGGATCCAAGAGAGTCGCCACTCGAAAGAAGAACTCGTGGAAGAGATTTTCCGGATGGTGGACGCCGATGGCAGCGGAACTATCTCAGTGGACGAGTTCGTGTCCATTTTTAAGACGTTGGGCCAAGCGCTAGACCATGACGACGTGCGCGAACTGGTTTACCAGATGGACCGCAATGGCGACGGCAAGATCGACCTCGAAGAGTTCAGTAAGATGCTACAGAAGCACGAGGTGTAGCTATACGAAATTAATACAGTTACCAGTATTAGGTTCGTATCGATAGAACGTATTACAGTTTTTAGTTACTGTTCGCCCCCAGCGATATTTCCTGGCGTTCGCGGACAAGAAGCAATACAGGATCTCAGTTTAGTTAGTTAGCTTTTACTTCACGTACATGAACGTCAAGCTTACATGACCGTATTTCGGAATTTTGCAATGAAAATGATCACATTGTTATCGGTTCTTCATGGTCGATGCGTCCCTCTCCACCTCTCATCGCGTTAGGCGAAAAGCTCGTCTAATATGCACCGGTCTCAAACAGGAACTTTAGAATGACTGGAGCTGAGCATATCGACATGGCATATTCGGTGACGCTTTCCAAATCCCGATCCCCCGCTTCAGCCCTCGTTAAAAAATAATTGCTACATGTAGGAAAAATGATTGTAGGGTATCTAAACTATCTAAGTCCAAGTGTAGCCGTCAAAAAGCAAATTTTCTTTTTACTCATGCTGAAGATTCCGCTACCATTATCTTCTCTCCTTTCATTTGCCAGACAAACGCTGCTCCAATGTCTGAGCAATCCAATAGTTTCTGTCGTCTTGTTTCGCTGTTGCGATCATCTTCCCGAGCCCCGCATCATCAATACCAGCCTTTTTGAACCACGAGACCAGCAGCTCATAAGGGTCGTTTCTTTTCATCTTCCACACATAATGAAACCAAATCTCCAAAATCGGATTTTTTAAAAGAGTAGTGCCTGCGTCATTCAATTTCAGCAGCTGGAACACCTCCATCTCAGTAATGTGATTCTTTCGCCAAGCAGTGCTCAGTTCCTTCTCGAGCTGCCCGCTAATGCGTTTGTTGACACCGCTCTGTGATGAAACAATCATTTTCGCCACGGTTACGTCGTCGTATTGCTCGCGTAGCTTTAAAAGCAATGCCTCGATGTAATCGGTACTTGGACTCTTTACATAACTCAGCCATGCCGGTAACCCCGGATTATTCAAGACGTTTTGGCCTCCTGCGTCAAGCTGGAGAATAGCGTAAACATCATCCGCACTTTTTCCACTTTCCTTCCAACTTTTCTGCTGAATTTCTTTTAGCTGATATACCACCGTGTCTCTGCTTGTTCGCTTCGCAGTAGCCAGCATGCGCGCCAGTTCGTCATCGCCATAGTGAGCCGCCATCGTCTTAAAAATTGCTCGATCTGCTGGCAAGCGTCCTCTCTTGCAGATTTTGGCCACATAGTGCGCCCAGTCAGTAAATTCGCTACTCGAGAATACGTCGGTTTTTACTTGTTTGACCCGAAGCACTTCAAAGCGGTTATTGACCTTGAGCTGCTTGTCTAGACTAAATGTCAGTTTAGCGTTGTGAGCTAGATCTTTCAACGCCTTCACAACAGAGAATACTCTGTCTTCCGAGGCGTAGTAGGCATCAGCTGTCGTCGTCCTCAAACGTCGATAGGCTTGGATGTCAGCCAAATCAGCTCCAGCCTTGGCATCAGCTCCCTTAGTGATTGCAAGGAAGAACAATACAGCAATCGAAGTCAAGAGGGCGCCCCAGCGAGCACTACTTAAACGCTTGAACGTGATCATTATCCGTGGCTTCTGCGCTTTATGAATCAAAGTAAGAGCAGTTCACTACTTATGTGATTGGCGACGCTCGGCCATTAATCCAGCAGCCTATCAAATTGATTTAAATTTCGTGTCGTCGCGCTTTATGTGCTTGGCGACCAATATACATGTAATTTTGAAACATGTAATATAGTTTTTCTATGCAGGTATCTTTTATGCTGGCAGAAAGCTCAAGAATTTGACTGTCGCCAATCACATAAGGAACACTGTTCACCGCTGGGTCTTGTTTATGATGTGCGGTGTTCAGTACTGTACGGTACTATTAAGTAAGATACAGACAGAACATGCCAAACAAACACATGTATTTACTGCTTCAGCACACTTGATTTGACTTATGTATACTTTTGAACTTCTGAGTGTCGCAATAAGACATAATCGACGATTAGAGGTCACAGCAGAAAAGAGACCAGCCGCGCTTCGCTTGGCTGCCCCTTCGGCGCCCCCGCGGTTCGCTGCGCTCGCAATATGGAGATAGAATGTTCTGGAAAGTGTAGTGCTCGGCTCGCTGGTGAGCTCAGCGCTGGGCCCGCCTCCGCAAATACCGCGCGGCCCTGCTTCGCGGGCTCTCCGCGCGGGTTTGCTGCGGCTACGCGCCTCGCACCCGCTCACCTTAGTTGGCGCCGCGGCTATGGCACCCACGTCGCCCAGCCCCGTGTGTGTTATCCCAGCGATGGACGTGTTTCTCCTTTTTTCTCTAAGATACAGTGCTATCATAATGCGGACATGTTGAAGTTATACTTCGCAGTCGGTACAATGTACTGTACATGTACCGGTAGTATCAAATTTAGGGATAGGGCTACGACGCATGATTTTGATCGAGATATTTTCATAAATTAGTACATGTAAATTCTTTCAAATATTTTGCATGCTAATATACATCCAAAAATTATCCATTTCGACGGCTACAGTCTGTAACGAAACTAAAAGCCTATTCCAATCTTCAGCAAATCATCTTTACAGTTCTATATTGAAAATTGTAAAGGCCGTAATCTATTTCGATCCCAATTGTCCGCGCTTACACTTGTCTTTGGTATTGTTAGTGCATCTTCCGTCGATAATATCGACGGAAGGTATGGCCCAGTGATATATATATAGTACACATATAATGTGTAGGCTATTTGGGCGTACGCTAGCTATTTCCGGGTAAATTGTGCGTGGTGAATTCCGTGCAAGAAGGTGCTCTTCGGCTGGTCGGTCGGGAACTTTTGTATCTTTTTGAAACTGCCTCCTCGGTCGACCATAAGAAGCTGGGGTTTTCCGCCAATCACGAATCGCGGAAAATTATTCAGATACAGCTGAGAATTGTTCGTTACCTATGTCAAAACAAGGATGGTGGGATAGGTTTTAATATTAGCCTAAGTAAAAAAAGTATATATTTTGAAAAGAATTACTTTTTTTAGCTTAGTTCAGTTATTAAAACCTCTAGTTGTCCCATTTTTTAAGAGAGTGCAGTCCAGACGACCGACCAAATTTTGAAGGCTAATATATTCACCCTCACTGATCTAATTGAATCGTCAGTCGTATTTTGAGCCCCCAAAACAATTTATAAGCCTACCTCCGAATTTTACAACGAAATTTAATTTCATTTGGACAGGCAAGATAAATCCGGGCTAATTTGGCGGTCAAACGATGTCAGATGTCTACAATTATGCCTACATTTTGTAGAACAAGAGTGAGTGAGGACCTTGTTGCACGAAAATGGTGTTAAAAGGTCATTTAGTCGAGCATGGTCTAAATAAATGATTATTTATTTATTATATTGTTTAATATATTAAAAAATAAATTAAAGTGTAGCCGTAACTTTTAACTTTTAAAATTTTTGTGGCTTCTAAAGACGAACGACTGTGAAATATATTCGTTCTCATTGAGGGTAGCCAACTACGATCATAAGAGGGCACAAACGTGATCCAAAGCTTACTGTAAGTGCAAATGCAAAAAGGTCAATGAAGAAGACGTAAGGCACATTACGGTGCCAAGATTCCTGTTTATTCCCGAGCTATGTGGTCACTCGAATTATTCGAACATAGCGTATGTACCCGAAGATAGTGTGGTGTAGTGATTTCTCCGTGGTTGCGGATTATCACCCGTCGACAAAGACGTGACACCGCCCAAGTTGTTCAAGCGACGAACCCTTGGTCGGGTGATGTACGGAGACGCCAAGGTGCAAGGGACTCGACGGCGTCACCTCCGAGTTTTCAACAAATATCGGATACGGCCCTATGTCACTGAATCCAGGCTGTCCTTATTATTAATAATGGACTGGCCGGATTCGCATTCAGCGGACCATCTACGGTACGAACCCTCAGTGGCGGTTGGGATGAGTTCGCGGAATACTCAGCGACCTTTGGAAGACACGTTAACCGTCTGGGACAGGGCGCGGACCGTTAAAGATGCTCATGCGACAGTGCAGACGACGCTTGGCATGCAAGAAGATCCATAACGGATCATTGTTCACTTGATATTTGTATACTTGATTAGGACTTCTTATTAGGAATAGACCCGAAAGGGTAAGCTTAAATACAGAACAGAATTTCCACTCAAGCACACAGAGCGACGTCCGGCTTCAAGGTAAGCGTCAGGTCAAGCTTGTTTGAGTGTGTTATGCCTGTCGGTGAACTCGAAGCTCTGTAGCGAGGGTTCATCCGGCAAGGCCAAGAGACTTTGTGTATTGGCCGATTATTATCGCGAGGTTTTACGTGACGGCTTATTGTTGCCTATTTGCACGACTGGGAGTGCTTGAATTGCCTCCCTTTTATTTCTTCCAGCCGAGTGTTGAACACTCGGTGATCTCGTAA

>Contig_20

CCAATGGCATGACAGCTTTTCAGCACACGCTAGTCGTGTGCTGATGCACCTACTTATGTAAAGAAATCTACCTACTTATATAATGGAGGGGCCCATCGCCTCCACGGTCATAGATGCTTTGCCATCACCACTTTCATTCTCCACGTCCACGTCCTCATCCTCATTCTCCACCTCCATGTCTTTGCCATTATTCTCAACATCCACATCCACGTCATCATTCTTTACATCCACGTCATCATTCTTTACATCCACGCCATCATCATCAACCTCCACTTTCAATTCACCCTCGCTCCCCACGTCATTTCCAGATACGGTTTCTATATCTCTCTGTGCAACCGACCCTTCTTCAGCGCTTGGCGTCTCCGTATCACCAGCTCGCGTGATGTCTGAGAGGCCGGGGCCTCTGCAGCGGGGAGAACCGTCCCGAGTGCGCATGTCTCCTTCCTCGTCCTCGACATCTTCTCACGTCTAGCCACAGAAACCAAAATGAAAGACAATCGCGTAATTTATCTTGATCGGAAATAGCGCAGAACGGAAGTAAATTTATCTTACAGACATTTCCCGAATTAAACAATCCTGGCCTCCAACGGTTCCCTTTAAAAATTGGTTGTCGTTAATCACAGAAGGAATACGGTACTGTACATGCGCGCCTGTTTTTAATAGGTCACTTTAATTATTTCTCAAAACGATCACNAAAAAAAAACAATTGGGTGCCTTTTCGTGTTTTGAGCACTCCCAAGAAAAAGTAGAAAACGAGCTCTTGATTTTTATCTGGAATGGACGCCTCCGGAGACGCGCCCGCTTTAAGCGAGCAGGAGCTGCTGGCATCGCTTCCTCCTCCTCCCTCGGCTCTCCCTGACTACCCAGGAAAAGCCGACGCCACCGACAACGAGCACAGTGCATCGAATGTCACCAATGAAGAGACTCCGGAGCGCACATCCTCTAACTCCAAGCTGCCTGGATCCGCCGCCAAAGCTTCATCCGCTGCGCGTTCCATGGCCTCTAAGCTCGCAAAAAGCGGGCGTAAAGTACTGTCGCCGCTAGGCCACAGTAGTAAGAGCAACACAACAGCCACATCGTCTCCTTCCTCTACATCGACCCAAAGCGAGGCGGGGACGACCTCTCCGAGCTCAATCGACGCCGCCATCGCCGATCCTTTGGGCTCTGTGGCCTCTGCTGCCGGCTCTTTGACCTCAGGCCTCACATCGTCGCTAGCCAGCGCGTCATCGTCCTTCGGATCGCTACTATTCGCGTCCCAAAGCTCCAGTGTAAGCAACGCTGACGACGTGGAAGATCGCTCGAGTAGCGCTGACAAAGCAGCTGAAGAGGCTGCAGCGGTTGCTGCAGAGGCTGCAGCGGTTGCTGCAGAGGCCGCAGCAGTCCTACGACAAGTGACAGCTCAGCAGAGAATGAGGATCCTGGAGGATTTAGTGCAACACCGTCGCTCGGACTGGAACTACCTTAAAGCGATGCATGAAGGCTCCAACTACTGGTTAAATGTGGCTCTACTAAGAGAGCAGCAAGTGATGAATCATGTGGGGTACAAGCAAAGTATTCGACGTGGTGCGCAGTTTTTCTACTTGGGGATCGGATTAGGGCGATTGGTGGGGGAATCGACCCACCCGGAACTACTGGCTATGGACTGCTGCCAATTACTGGAAGAACTGGAATTTTACTTCTCGTCCTCGACGGTGCAAGGAATGGTAAGTAAAAAACCANTTTTTTTTTTAATTGTAGTGAGGAGATATTAACGTTATGGTTATATAGAAAATGATGGTGGCGACGTCAAGCACGCTACACGAGCCTCTGGACACGGAGAATAGTCCGCAATACTCAGCCGATGAGCCATTCCGGCCCACTATGCACAAGTGGAACCAGCGGCCAGTTTATCGACGACTATTGACGCCTCCGATTGTAGGNTTTTTTTTTATTGGAATATTTATTTATGAGTATTAATATTCTGCGTGTTTTATAGCCTTTTCCACTGGACTACCGAGAGATTTTGCTGTCTTTGTGCGATATTCTAGCGCTCATTTACAGCAAACTCGTCGAGGATAACAGTGCGTCTGAGAACTTAAACCTCTTCCAGTCGATCATTCGCTTCGATGATCGCATTAAGGTACTTTCTAAGAGTCGACGGCGTTCAACGGTTCCCGTTTGCTAAGATTTTTGTTGTTGTTGTTTTTTGTGTGCTGCTGACTTAGAAACTTTTCATTGACCCTGTGAAGAAAGAGTTTTCAGCCGTAGCGTCTCAAGTAATGGCAGAGGAGATGCGACTCGTTCGCAAGGCCTACGCGTGTGGAAGTCGGGACGGAGAACCTCTAGCAGCGAGTCCGACGGAAAGTATTGAAACCAATGCCGCCACTGTGGATGGGAATTAGGTACTTTAATAATATAGTCGGTTGGGATTGATACATGTAATGAAGTGCCTCGCTCGACCATGTATCGGGGAGCCGCATTTTATTAGTACAGTATTATTGAGAGGAATCAGTATTAGGAGCTGGACCCGATTTACCAGGTCGTTGTTGCCGTTCCTTAACCACAAAGTGGCTCAATTTTTGCCGGTTTGCTCAAGTGGTGTGGCATCAGCTACCAACGGCGATGGCATCTATCAAATTTCTTCACCCAACACTACACACCTTCGTCCACTGAGGGGTACAATGCGCTTGATCACATGCAACAATTAGAAACACCCTGGAGCAAGAACAGCTACTCAAAGTTAGATTCAGAGCTCACAAAGACTGGTTGGTAGGAAAAAAAGGTAAGACAAAACGTCACTGTTGCATTTACATGCAATGCGACGTTTGCCTCTGTGAATTTCCGACCAAATTTGCCGTTTTGGTAATATACGTATTTCGTACTTAATGATCTCTTTTAGGTAAGTCAGATTTGCGACTTTCTAAATGATCGTGTGAGATTAGTCACGCGATCAGTGATGATCAGGATTTCGACATTATTAATATAGAAGTGTTAGCGAATGCGAATCTCCTTATTATTAGCTGCCATTTTAATAATTGGTGCACCTCGGTCATTTGGCGATTCATCAAGAGCAAGTTGCTCTTAACAACCATGGGCATCGCGGGTTTAATGCTCGTTACTGTTGTAGCCTTGTTAGCCGGAGCACTTGCTCAAGAACATACTACTCAAGCGTACTCGGTGAAAAGAACTTCCATTGTCTCCAAGTCTCACATCAGCATAGCGACGGAAAGACAGTTAAGATCCTTCCAAACATCGACTTTACGTGAAGATGGCGAGGACAGAGTCAATGTACCCTGGTTATCAAAGGTCGACGATCTGATACAATCCATGTTCAAGTCCAGTTTGTCACCTGATAAAGTGCAAATCGGAACGTGGGTGCAGTCCAAGGTGCACCCTAAAGACCTGTTTAGCACCTTACGTCTTGGAGAGAGCGCAGCCAAGCTTGACGACAATCCGAATCTTCTTCAATGGTTCAGATTCGTAGCGCCTTACCAAACTAAACATGGCGAGAAGGCGATTTTGAATTTGGATGTCTACTACTGTTACTAGAGACTCACAGCAACCAGGAACTGATGACACTACTGAAATCTCTAAAGAAAACTCCAGGGCTGAGTAAGTTTGCGTCGAATATCCAGGAGTCACTATCTGGAGCGTGGGTGTCCAAAACACTACTGAAAGAAACAAGTTCAAAGACAGCCTTCGAAGTGCTACGCCTAAAAGAAGCTGGCTCGAAGTTGGATGACACGCCCGTTTTCCACCAATGGTTGAAGTACGTGGAGGCGTACAGAGCGAAGAGAGGGACGGTTATGTTCGGAGACATTGATATGTTCTCGTTATTGCAAAAGACAATGCCGGAACGTGAATTGACAACCCTTTTCTATTCACTTCGGAAGGTCCCGAACATGAAGAATAACGCTGAAACCATGCAGAGATTTATGTTTTGGAAATTTAAAACCAGTCGCAAAACTATGAATGACGTTTGGCTAAAGTTTCGAGAGCCACCCGAGGAAATGTTCAAGGTCTTACGTGTGGCGGAAAGTCGGGCCAGAGACGACAGTGACATGATTCAGTGGCTCAGGTATACCCAACTGTTTCGTAACCGCATTAAGAAGAGTGCGTTTTCGGATGAGCAAACCTTGCAATTCTTGACGAAGTCCGACCCATTGAAATCAGATTGAATATTTGCAACACAGTTTCAATCTCTTAAGGAAGTTCCAGATTTGAAGAAACAAGCGGAAAACATGCAGTCTTCCCTATTCCAGAATTGGATAAGTGCGAAGATAAATCCAGATGAGGCTTCAAAATGGCTGGCAAGTCCTTTCAACGAAAAAGTTTTGAACCTGCCGAAGGATGATCCCAGATACGGCACTTGGGAGAGTTATATGCTTTTCTATGCTGAAAACAAGGGCGGACAACCTTTGCTTCAGAAAGTGAGAGCATATTTCAAAATCGATGACCCTGTCGATGCTCTGGCAGCAGTTATGAAGTCTAGATGAACACTAGATTTGATGGCGTTTCATTCAACGAAATAAAAACGGGGTGCCTTTGAAAACACGGTACATTTAAATTTGGATTTCGTGATTTTAAAGCATTTTTTAATTATTATTATTTCAGCATGATTTCGCACAGCGCGGCACTCGCGATTAAATGCTCTTTTTAAGGAGAGTACAATCGATGGCTGGCTGGTAAGCTCGGTTTTGAACCCGCCTACACAAAATCTCGCGCGGGTTTGCCACACTCCCTCCCGGTCGCCAGAAATAAAACTACATAATTAATTCGATACTTGTTTTTGGACAGTTTACCGGTATTGAGACAACGTTAATCTTCCTAAGCGATTTGGGCATTATTTGGACAAGTTGATGACGCTATTTGAAAATGAACTAATCAACTCGTTGGAACGTTGATGTGCCGCTGCTTTAAACCGTCACGATGAAGGTGCTAGACGTACCTTCATACGCCGCCAATGCACCAAGCAGGTTTTTCGAATACCCGCGGTGCACGAAGCGATACGTGCCTGCCACACTAGTGCGTCCACCTTTGCGGATGTTCCACTCACAAGTGTTCTTGCTCTCAGCAATGAGATAGCGCTCCCAGCGGTAGCGCAAATCCCAATGCGCATCGGTCATGACGGTTGTATACGCGCCATTCGATCCAAGTTTCTGCACGTCACAGAACGATGAGACCAGCGTGAGTGCGTTCTTCGGGTGCGCGCCGGCGAATACCGCTGAAGCGACACTCCCCACGGAGTACGAACTCGACGGTTGTGTGCGTACATCACTAAAGGAGCGCAGAAGCGGAGCTGAGTCCATCACCACACCAGTCTGCAACGTGATGAGCGAGTTGCGGTTGATCTGCAACGGTGTGGGACCCACGTCCAGCGGTACGGACGAATCAGCGACGGACGTGGCGACTCTAGTGAGCTCCTGTTGCACTGCAGCCAGCTGGTTGGGACCGAAAAGCGTCGACGCGCCTTCGTAGTGCTGCGTACGGTATTCCTCTTTGGTGGTCATATACTGCGCGTAAGCATTGCTGATGGCAGCCAACTCAACCTCCGTGACTCCAGCACCTGTAAGTGCCTTCTTGACTGTGCTTCGGATACGTCTTCCGGTAGTCTCAAACGTGGTGACTGCGATAGCGAATTGGCCGATCTTAGCGATCTGTACGGGTAGAATGTTGAGTACCCACGGCACAGGATCCATGAGTCCGACTGCCAGTAGTGGCACTTTGTTGACGTTCTGGCAGTCCTTCACCCATTGTGGTGCCACCACCGAGGGTCCGGAAGAGTGCGTTGGCCTTACGGTTTCCCTCTGTGAACATACTGAGCACTCGACCGTCTTCTCTGCCAGCAGCAAAGTTCTGTCCGATGACCGCAGGACACGTTCTATTAGCGTACGGGTCTGCTGTGTTTGGTGTGGCGCCGGAGAGTTTTACATTAGAGAAGTCGACGTAAGAAAGATTAGCGACGGTGGAGCCATGGACGAGCTCAGAGGTTCCTTTGATCAGCGATGACAGCGTATCGTACTGCCGTTTGCCCATAATCTCGGCAGACTCGATCGTTGTATTGCCCTCGCCACTAAAGGTCCCGTCTCCATTATCGATGAGGTTCGGACTCACGTCACCAGCGTTAGTAATGCCGATCCCAACGATGACGTCGTCTAGTTCATCTTCAAGAAGGAGCTCGGCGTATCCCTTGTTGTCTCCACTGATGAGAAGGTTTTGTACACTAAGACTGGTAGGATGGACGGGGTAGAACGCCAGCACACCTCGAAGCTTACCGGAACTACTGAAGAAATGTAGTGCCCGCATTGTCGAGTCGACGTCACTGCTGTATTGTGCACGTTCAGACGACGGGTTGGCCAGGTAGGCCTCGGGAGAGCGGTTCTTGACTCCTTTAGAGACTTCTCCCTTGTTCCATCGGATGGTACCGGACTGGATGGAGTTGTGTGCAGCGTCAATGGCGCTCACGATACCACTGACGATCTTGTCGAAGTTTTCACTGACGTATCCAAAGCTGGAGACGTCGTAGAGGAAATATCTTGCGGTCCCTCCTGGCCCCGCGTGGGTGTGCGTAGCATGTAGAATGACGTTCTGTTCGGTGTAGAGTCCCTTGTATTTGGTAGCGAGCTGAGCTAGAACTTCTTGATGTACTAGTTGCATGACGGAGTGCAAGTCGCAGTGGACAAACATGACACGCTTCCTGGTAATAGCGTCTTGGATGAGGAATGCTCGGGCGTACAGACAATTAAGGATCCCAGCGGAGCTTTCGTCGGAGCTTGCGAACCCCATCATTACCACTTGGGCTGCAGGTCCAGTGATGTCGCTCTTGCCCACACCGATGTTATACGTCGCTGTAGCGTGTCCGGGGAGCTCGATAAGCAACAGGATAAACAGCAACGACTGGAGAAGTCGGAAGGACGACATCGTAGTCTTGCCTTGATGAGTCGATGAAGCGGTTTGAGCACCAGGCGGCACGTTTGAAAATGGCCAAGGCTTTAGTTGCCATCCGGTATCGCTTCTTGATGAGCTGTTCCGGTGGAGAGAGGGCGCATTGCAGTATCGCTTGTTAAACCGTCCTAGTATACCCCAACATGACTCATCGAGTGGCTCATACTCATCATGTTGAAGTGGACTGATCATAGTTCACCATCATGGCTCCGTTGAAAGACATTTCTTTTTTTGCTTTGCACGCTTACAGTTGGACCGAAATGATTAGTCCGACGTAAATGCAACGTAGCACGTTGCATCATCGTTCGGAAAGGTACTCATAACCTCCACTTCAATGTACTCATCCGTCGCCCTGCTCAGATTACTCCTTATAACGATAATAATTTGTCTTTACCGGTTCTCCACAGTTAACTTTTTCCTTGTTCGACGTCGCTGGATAGCTTGTGAGATCCGAAGCATCTGAACACCTTTCAGCCAACGTGTTCGAGACCGTGTGCGCGATGCTAACTCGTAAAAGTACTGAACTTTGCCGATCAACAGCAGCCTCTCTCCTTCATCATCGTCATCATTTAGTTCACTAGCGCCAACCGCACTTAAGCGACGTTGACGTTGACGACGAAGTGCAGCAGAGAGAAACCACATGAGCTTGCAATCCCAGAGGAAAATAGTCCAGAGACGCGAGACCAAGGCTGACAAAGTGCTCACTTCGACACGACGACCGAAGATAACTCGATCCACAACTGCAATTTTGGGTACAATCCAGCGGTCCAAAGCGAAGAGCTCCAGCGACAATATAACCAGTCCCATAAGCTCGAAAGTCAGCACGGATAGCATAAGAACAGAGCGTCGAAAAGCCAATAAACGCATCGTACTCGGAGGTAAAGCGTCCAGCGTAATGGCCCACTGCATCCAAAGCCACGCGCCCCACACCCACACAACACGACGATCCCAAGTCAAAATTGACTCAAAGCTAACGACGCTACTGTGAGCTTCGTGGACAAAAGAGGAAGTTAAACGACGAGTATAATCTTTTGAGTAGTTGCCGTTGATATGAAGCAAAGAATA

>Contig_23

AACAATCGTAAACTAGTGTGACTTATTAAGGTGAGACATTAATTAGTCTAAGTGTGACTAATTCTACCACACCTTAAATAGTCAGAGTGCATAATTAACCACACTCATACTAATTTAGGTGAGCAGAGTCTGCAGCACCTCACATAAATTAGTATGAGTGAGACTAAATACCGTAGCTACTGTACTAGTAATACAATTTTATTAATTTTAGAAATATTGTATGAAGTAACATTCAGCGTTGCTGCTGGGACAGACAAGCAATAAAAGAATGCAGCCGAGAGAGACGGCAACTGCTTCATGCGCATCGAGAAAAAACGCCATACCGCCAAAATAAGTGGGTTCAAGCGAAAGCGACAAGAATCGCTATTGTGCCAACTTGCTGAGCGTTTTCTGGAAAAGGTTGCGTAGACGAGTAATCAGCTGGAGATATTATGACCTTGTGATAACTATAAGCATCCAAAGCTTGCGCTAGCTGATACCACTGCTGCGGATGCCAACCCCGGTGCCTTTGTCTTCAATAATGAGCGGCTTATCGCATTGTACTACATGTACCAAGGGGATTGTTTAGGCCCAGATGACTATCCTTGCTATTAACACAGTGGGGCCTCGACTTATAAGGAAACGGCTCCCCTTCCGGCACCGGAGCGGTTGAAATCTTTATATGTTTCAGCAGCCAGTCAAATCAAATCTTCACTTTTTAACTGTATATCAAATCTTCACTTTTTAACTGTGAAAAATGGCTGCAGTACTCTGATTGGTTGATATATCAATGGCACTGAAGGCTCTTCCTTCTACGCTATAGTTCTAACATGCAAAGATGCCCACCGACATACCGGAAGTAGGTATTTGTAGTGACCGATTTTTCACCAATTACGTCAAATCTTCTTATCTCAGAGGAAGGTGAGCGCTGAAAAAGATGGGGGAGGTAGGTTTGATCTGATTGGCTGCTGAAATCTCACTTCTTTTCATGATAACCACTAATGATACGAGTACTATTTTCTGCCAATGCAAATATCGCTCTCACTTCCGGTATCGTGGTCCGACTATCTCCTTGAATTTGATATAGTAAAAAGTTACAAGCTCAGCTGTGAGGCTAAGATAATGACAGCTTCTACGTCTACATCGGGCCCGTAGCTAAATTGCTCGGTTGATTTACTTCTCTCATTTACGGCATTAAAAGTAATGCCTGTGTGGCGAAAACAGTTCTCGATAGTACGCTGATCCACAGCATATGCCTTCCTTTCAATCTCAACACCGTCCTTGATCTTGTCGAAAACCCACCGCAACTGCTTCTTTCGGTACTCCAGTTTAAATGATGCAATGATGCCAGCATCCATAGGTTGTAGGAACGCAGTAGTGTTGGGCGGAAGAAACTGCTGTTCACTATCTTGGAGGCGTCTTGAAGGATATCAGAAACAGTAGTTTGCGCCGGCACTCGCTTCAGCTTGAACGTCGCTTTTGCCCATGCCGCCAGCTCTTCTTGCGTCATGCTGGGGCAGTCGCTCGCCTTCTGGGTGAAATCGCGCTTCTGTTTTGTCTTCATCCAGGCTCCCATGGGTATGGGCCGACGTAAATTACGCACTGTTCGACATTAACACGGTTCTGCTCCTTAGAATGACTGGAGCTGAGCATTTCCCGCCAGAAACTCCCCCCTTAAAACAGCTGCATCGCTTTGCTCAGGGTATTAATTTATACAAGTACCGAAATAGAGAGCGATGTTTGTACTACACCTGTCGAAAACGGCAGTCAAACTCGTAACTTAAAAATTGGCAAATAGGAAAATTACGGTAGGAGCCCCTAATCAGTTTCATATTCAATGTTGAAAAAACTGCAATCAACCTCGTACAAGATTTGCGCGACTGGTGGACAATCTCCCTAGGGAAGGCGCGAAGCACGTCAAGATGACGCCTGAAGCTAAGGTCCTCCTGGAGGAGTACCTGAATGATAACTGTACCTATACGCTGGACACTATGAGGACAATGTTGTTTCTGGCCTGTGGCGTTAAGGTGGACACGTCGACGATTAGCCGGCACCTGAATGGCATGCTGTTTACCGTACAACAAGTGAGAGTCGAGCCCACAGCGTGTAATAGCGATAGCAACTGAGAAACGACGTGTGTTCGCCGTGAAATTAAAGGAGCATCAAGACGCAGGTAGCTTTGGCGCGAGTCAAACAGAGCCTATCGTTTTGGCGCGAACCAGTCGCGCAAATCTTGTACATGTACCAGGTTGATTGCAGTTTTTTCAACATTGAATATTAATTAGGGACTCTTACCGTAATTTTCCTATTTGCCAATTTTTGAGACGAGTTTGACTGCCGTTTTCTAAGAGGTTGGGTGCACAAAGGTGTAGTAGTCAAGGGTTGTGAAGTCCCTTCTCGCTCAAGAAGCACCGCTACTCATTTCGCAGAGTGCGACTTAAAGCTCTGTACCCTTCAATTTTCCATACCATGCGCCTGGCAAGTATGGTGTTCACTCCGGTAATTGCTATTAGCTTCGCGTCCTGCAGTGTAGCAGAGTTCGACCAAACCAAGATATTGATGAATGAATTACCAGCTCACTCTCACGTTTCAGCCAGAGGAATATTTCTTCGCACGCCTCAAGAGAATGAAGCAGTGGCTGAGAAGCGAGCCCCGAATTTTAACTTGGCTGAACTAAAGAAGGGAGACACGCTAAACAATTGGCCGAAGATCTCATGGGCAATCCTCGGTTGCCGAAAGCTGCATTCCAGTGGTGGGAACATAACCAGTATTCTTTGTCTAAAATTGATGAGTTCTTGAAACGGGCGAGTCGCAAGGCTAACGGCAAGAATTTCGATGAGATCTACAATGGATACTTGCTGCATCGGGGGTATGCCGGAGTCTTGTTGTCGGTAGCTAGCTTTCGAGCTGACTTGCTATTACTACTACGCTTATGTGAGCACTTGGAGATTATACAAAATGTTGTTCTTAATTACACTAATGCCAGCACTTTAAGATGATAAGAAATGACGTAACCACGTACTAGGGGCACGTAGAAACAGTCTTGACAGCCTTTGGAAGTCTTCTTATTGTGAAGTACTAATACGAAGTACTACGTAAATGCTCGCTATATTTGGCGCACTAACTCTCTGTAGTGCGATCTCGTCAGCAAGACGAATTAGAGAAGAAACTGGTCGAGCTCTGTCACCGGCATGAGATGACAACTCAAAGCCAACCAGCTTAAATTTAATAAAAGCCAAACCTAAATGTAACTCCGTCAGAAATCCTGAATCCAATGGCTGAACAGAATTTGTGCATCTATATACTAAAAAAATACTTAAATTATTATCTGAATCCAATCTAATATTTTATTCGTAGATATCGTCCGATCTTCTATGTCTGAGGGGATACCTTAAGCGAGCAAGCAAATTTAACTGTAAAGTCATTTTACTACTCCCGTACCGGCAACAACACTATCGTAAAATACATGCCTGCGTATAGATTGGTACGTTTCATTTNNNAGCAAAAAAAAAACCCGTTAGTATTTTCAGCCTGAAACGTATTTGAACCAATAGAATGATTGTAAGGTATCTAACTATCTAAGTCCAAGTGTAGCCGTCAAAAAGAAAATTTTCTTTTTACTCATGCTGAAGATTCCGCTACCATTATCTTCTCTCCTTTCATTTGCCAGACAAACGCTGCTCCAATGTCTGAGCAATCCAATAGTTTCTGTCGTCTTGTTTCGCTGTTGCGATCATCTTCCCGAGCCCCGCATCATCAATACCAGCCTTTTTGAACCACGAGACCAGCAGCTCATAAGGGTCGTTTCTTTTCATCTTCCACACATAATGAAACCAAATCTCCAAAATCGGATTTTTTAAAAGAGTAGTGCCTGCGTCATTCAATTTCAGCAGCTGGAACACCTCCATCTCAGTAATGTGATTCTTTCGCCAAGCAGTGCTCAGTTCCTTCTCGAGCTGCCCGCTAATGCGTTTGTTGACACCGCTCTGTGATGAAACAATCATTTTCGCCACGGTTACGTCGTCGTATTGCTCGCGTAGCTTTAAAAGCAATGCCTCGATGTAATCGGTACTTGGACTCTTTACATAACTCAGCCATGCCGGTAACCCCGGATTATTCAAGACGTTTTGGCCTCCTGCGTCAAGCTGGAGAATAGCGTAAACATCATCCGCACTTTTTCCACTTTCCTTCCAACTTTTCTGCTGAATTTCTTTTAGCTGATCTACCACCGTGTCTCTGCTTGTTCGCTTCGCAGTAGCCAGCATGCGCGCCAGTTCGTCATCGCCATAGTGAGCCGCCATCGTCTTGAAAATTGCTCGATCTGCTGGCAAGCGTCCTCTCTTGCAGATTTTGGCCACATAGTGCGCCCAGTCAGTAAATTCGCTACTCGAGAATACGTCGGTTTTTACTTGTTTGACCCGAAGCACTTCAAAGCGGTTATTGACCTTGAGCTGCTTGTCTAGACTAAATGTCAGTTTCGCGTTGTGAGCTAGATCTTTCAACGCCTTCACAACAGAGAATACTCTGTCTTCCGAGGCGTAGTAGGCATCAGCTGTCGTCGTCCTCAAACGTCGATAGGCTTGGATGTCAGCCAAATCAGCTCCAGCCTTGGCATCAGCTCCCTTAGTGATTGCAAGGAAGAACAATACAGCAATCGAAGTCAAGAGGGCGCCCCAGCGAGCACTACTTAAACGCTTGAACGTGATCATTATCCGTGGCTTCTGCGCTTTATGAATCAAAGTAAGAGCAGTTCACTACTTATGTGATTGGCGACGCTCGGCCATTAATCCAGCAGCCTATCAAATTGATTTAAATTTCGTGCCGTCGCGCTTTATGTGCTTGGCGACCAATATACATGTAATTTTGAAACATGTAATATAGTTTTTCTATGCAGGTATCTTTTATGCTGGCAGAAAGGCTAGACCACCTCAAGAATTTGACTGTCGCCAATCACATAAGGAACACTGTTCACCGCTGGGTCTTGTTTATGATGTGCGGTGTTCAGTACTGTACGGTACTATTAAGTAAGATAGAGACAGAACATGCCAAACACATGTATTTACTGTTTCAGCACACAGCAAATCATTTGATTTGACTTATGTATACTGAGTGTCGCAATAAGACATAATCGACGATTAGAGGTCACAGCAGAAAAGAGACCAGCCGCGCTTCGCTTGGCTGCCCCTTCGGCGCCCCCGCGGTTCGCTGCGCTCGCAATATGGAGATAGAATGTTCTGGAAAGTGTAGTGCTCGGCTCGCTGGTGAGCTCAGCGCTGGGCCCGCCTCGCACCCGCTCACCTTAGTTGGCGCCGCGGCTATGGCACCCACGTAGCCCAGCCCCGTGTGTGTTATCCCAGCGATGGACGCGTTTCTCCTTTTTTCTCTAAGATACAGTGCTATCATAATGCGGACATGTTGAAGTTATACTTCGCAGTCGGTACAATGTACTGTACATGTACCGGTAGTATCAAATTTAGGGATAGGGCTACGACGCATGATTTTGATCGAGATATTTTCATAAATTAGTACATGTAAATTCTTTCAAATATTTTGCATGCTAATATACATCCAAAAATTATCCATTTCGACGGCTACAGTCTGTAACGAAACTAAAAGCCTATTCCAATCTTCAGCAAATCATCTTTACAGTTCTATATTGAAAATTGTAAAGGCCGTAATCTATTTCGATCCCAATTGTCCGCGCTTACACTTGTCTGCTTGCCGCATAGACTGCAAGGTGGTCTAAAAGGGCGGAGTCTTGGCTCGATCGTAAATACACGCCGGCCCAAATTAAGGAGAAATTGACTAGTGTGGGCGGTGACATGAGCAACAAGAACGGAAGGAAGTACTACCTTGTTCATGAAGAAGTATAATGCGGCGAATCCGCGGCCGTGATTGGTGCATAGCGCTTCTGTGACGATTGAGACTGAAGATAAGGCACAGTTTAACCTCTTACCTTTACGCATTTTCACTTGTCAACCACAACTTTTGTGTACTTCTTATAACATCGAAATAAATGCATGTGGGTGGCTAAGGTAGCTCCACATGTCATGCCAGAGTACGACCCTTATTTCAGTCCCAACGTGATACTGAGCACGAAGGGGGGGGTCCCCAGAAACCCACACACAAATGGCTAGCACTATCCTGGATAGATAGAGCTGGTGGATATTTTATTTTCCCAATTGATGGAAAATTTCGTTAAAAATACAACATTGCATTGTGTTCATTCAGAATTCCCTTTGCTAGATTGTTACTGTACCGGTTAAGCAGTGCAAAAGCGGCATCATGTCCAACTGCTCGACGACGCAGTTTGAACATGCGGGGGTGTTTGTGGGCAACTCGCCGTCTTACTTGAAGATCATGGACTCGTGGGACCAGCAGTTGAGTGAAAATGGCTACAGCCGACTCAAGTGTGGCAGCGGCACAAGGAGCACCGGTACGTGCGGCATCATCGGTCCATTGTCCGTCTACCTTCAGCTGTAGGTGAGCTCGAAAAAAACTAACGTCAATGTGTTATTAGTCTACGCCCCCTCGCTTCTTCTAAGCGATGTTGGTAGAGGGGAGGACGCAATGGCTGAAGGGCTTAGGAAATCACGTTCGAGGCCTTCTGCAAGTGGTGCAGCGCAAGGATCCAAGAGAGTCACTACTCGAAGGAAAAACTCGTGGAAGAGATTTTCCGGTTGGTGGACGCTGATGGCGGCAGGATTATCTCGATGGTCGAGTTCGTGTCCATTTTTAAGACCTTGGGCCAAGCGCTAGACTATGACGACGGGCGTGAGGTGGTTTCCCAGATGGACCGCAATGGCGACGGTAAGATCGACCTCGAAGAGCTCTTTACGCAAAGTAAAATATACACGAAGGATGGAGCTTTTTTTACTTGCCATGGTATATAGCGACTTGTGCTCATTTTCCGAAACCCTAATGAATCCTTTATTTAAATTATTTTATTTTGACAAATAAACTATTAAGTAAATAGTACTTTTAAACACTTATTTATATTTTTTAAATTTTCGGTTTTAGGGCGAATACTGAAAATATATGAAGTAAAAAAGCGTTTCAAAAATAAATTTAGCATAACACTTTATTGCCTTTACGAAGTAGTATGTCGTCTTTTGCTCGGAAATTTCTCCAAATTCACTTTCGGGTCTCCCGCCTCTCTTGCGCTTACCAAGTCCGCCTGAATCTAGCACTAGACACGACCACATGGCCAAAACGCACTATTTCTCCAGCATGAATTCCAAATTTAAAGCACATACGCGCACGCCAGTCGTACGTGAGGTTCGCCTTATTAGAGATCTTCAGAAACACCGTCAACTAAAATCACGTCGTCTAATCGATACTGTTATTTGCTGCGTGTATTTCAGACATAGCAGCAAGTTTAAAATCAAGTTGCTGTATCGATTGATATATACAAAGCGCTGGATTGACA

>Contig_25

GAATCGGCTCTGGTTGTGCTTGTTATTGCTGAAGTTTAAGAGAGAGAGAGAGAGACGAGCGTTGCTGTCTCCCACGAGAGAGACTAGGCCCAGGAATGAAGAAGAATGCGGTGCTGCTGCGTGAAGGTGGCTCTTATCCTTACGCAAGTCTTTTCATAGTCTCTTACACGATGTTATAAGAACGTGTCACTTAGAAGAAGAGCAAGATGCACTAAGAAGTAATTGGAAATTTGTATGTGTGTGCGTGACTTGCCTGTGCGCTCTACTCGAGCTTTGTTTTGAACCTGAGTTAGAATAAAGGTGAGGGTGGATCAGCCATGGCCGCCTCGATCAGTGACCTACTGCGTACGCTAAAATAATACGAACTGCGAGGCATTTTAAACATTGCCGAGACAGCTTCGTTGTTCTTCTTGCGAGCCTCGCCAGAAAGCACGTGCAACACAGACGGTTGCATCCAATATTTATTGGGATGTCAAGAAAGTTGGAGGTTGAAAAAGAATACTGCAGAACAGACTACTTCGGACGTGCTGCACAATACTGACTTTTCATAATTAAGAACCCCAGAGCAGTCATTTGTGGTAGTTTTGTATTTCATATTACGAGGCTTGTAAACTGCTTGACTTCGGAGCCCGAAATTTTATTCCTCTCGGAGGGTATTCCAAGAGTATGATTTTTTGCCGAAGTCCATAAAAGTAATGATTTCTCATATTTAGGAGCGTCCAGTCGGTCTACAGAGAGCGCCTCAAAGGGAAGAAATTGACAAGTAAAGCTGCTTCTTAAGAAGCGCGACAGCACGGCAATGGCAAACTACCTCGACTTTGCCGGATTTCAAGCTACGTCGATCATGAATTCACCGAAAGGATTGCCAGTATTTTTTGAAATGTATGCATACAAAGCTAATGAAAGTCGGTTTCGATCATAAAAAAGTACCGGGCATTCTTCCAGTGGTCAATTTTCTGAGGGTAGTGCTTCATCCTCGGGCTAACACGTTTTGAGGACAGCACTATAGAGATTATTTATCGAGTGTGAGGACCACTCTACTTGTAAATAGTCAAGTGATACAACACAACGGTGTCGCGTCCTTGATGCGAAGCATGCTACCTACACTGCTTCAAATACATCGTCGATCGAAAGTGCAAACGTCCTATATAATCTAACTTTTCATGTGCTCTAGCAAACATTTGTTTTAGATCCAAAATGAATCTGTCGCACTTTTTGATCATCATATAAAATGATAGAGCATCATTTATGGAAGGGACCTGTAGTGCTTCAGTATGACGTTGTCCAAAGTTGCGGTGTTAGTGCGATCAGAACTATTGTGACCAGCGAGCTCGCAAATATTTTTTCAGTCTGTCCTGAATGGGTCATTCAAGTACTGTGATAAATGATTTGGTTACAACCAAGCAAAATCCCAGAATTAGGCAACCCAAGCACGTCTTACGACGAGTTAAAATTATTATATCCAGTGGTACGATAGTTGGGAGCACACATTATGTGGTAAAAAGTTTGATTTATGTATGTTCAGCATGGTGCTGATGCCACTTCGTCTGATTCCTTAGCTATGGTGTGGAAAAGCACACGATGTTTAACCTGACCGTACATCAATCTCTTCTTGGAGACCATAAGTTCAACAGCTCGCCAGCGCCGTGTGCGTTATACCGAAGCGCCACCCAAGTCCTATACCCGTCATCGGCCTTTAATATTTCATCCAGGATTGCCTTAAGCAATGATCTGAGGTCTGCATTCTCCACCATGTATTTAATCTTAGCCATTAGTTCACCAAGACCTCGCTCCTCCGCTCCGGTTTCTCCATCCTTTCCGTGTACTCGTAAATATCTTTTCGGGGGTCCCAGCGTCGCTGCGCACTTCGTCAAGCGTGTGGACGAGCTCTGGCGATCCCATTGCTGACAGCGTGATTGGATTGAAGTCTCCAGTCGCGTTACAGATCGCAAAGTAGAGGGTAGCCATCGCAACAGACAGCATGAATGAGATACGCATGCTGCGAGAGAATATTTGTGGAACTAAAGCGCTTTAACCTTGGGCGAAACGAATAATGTCATCACTTTTCACCCAGACCGGTGGTGGTGCGCGTCTAGATTTCGGTGCGGTATTCACTACGATCCGCGATACGTACCGACAGCAACCCACATATTTGTATTACACTTGGAAAACAGTAATCGCAGGACCTGTTTATCGAGGACTGCACCACCGATAATTTTTGATGTGGCAAAGCACGATTCCTGGGCTTTCCGCCCGAAGCTACTTTCTCTACGCTCCAATGCTATACTGTACCACAGACTGTACAGCAATGTACGTAGTCCGATGATGTACACGTTGCCCTTATTTGACTACTACAATTCCAGACGTGCCGAGTGCCGACAGCAAAATGTGGCACGAGCCCTCCGCAGCAACAAGAAATAAGGCGTACGTCTGAGATCAATTGGCAATTTGTTTATGCACGTGAGCGCCTTCTGTGGAACGTTGTGTGATCATTTCGGGTGGTGGTGGTGATAGTAGGGGGGGGGGTACTGGATCTGGACTGCATATACCTACTGTACATGTATTGGCATCGCTATTTATTACTTTGAAAGTTCGCACGTTGTCAATGATGTGTCCTAATTGGTGACATAGCGTACAAGTTATTGTACAGAATGTCTTACCATACAAACTATGTTCTGGACCCAATCCTGCATAAGTCGATACATCACCTTTTACAGGAAAGATAAATCAATGGTGCCAGGAAACTCTCTGAAGACAGACTCATAAAATTCAATCACATCTCTTTTTTCTTCCTTCTACTCAAGCTCCTCCACCTTCCTCGCCTCACCTTCAATAGGTTATGAGCTCAACGCAAGCCTCCACCGCTGACGACCGCGAGTTTCCGCGCCTCAATGGCCGGAACTTCATCATCTGGAAGACCCGCGTCACTGTCGCCCTCGAAGGAAAGAACCTTCTCGGCTACGTCGAGCGTATCGACTACACCGGTGACTCCGACTACGAGTCGACTCCGATGAAGAGCTCAACCCGGCACTGTCCGACATGAACGACATGTCCGCCGCGCTTGATGCTGCCGGTGCTCCCAAGGCCGACGAAATAGTGGGCTCCTCACCGAGCGAGTCCTCCTCTGATGCCAGCTCCGCAAGTTCGGATGCACCCACAGCCGGAGACGACGGCGATGTGGAAATGGGACAGGAGAATCCCCCCGTTATCCAGTCATTCACTGCTCAGAAGCGGGATGAACTGAAGCGAGCAGAGAAGCTGAGGGCTAAAAGCCAGAGGCTGAGCTCGAAAAAGCTTCGACTCAACGAGGCAAAAGCAAAGCTTTCCTTATCAAGACCATCGATGACCAACATGTTCTCATGGTCAAGGACAAGACTACTGCGTTTGAGATCTTCCAGACGATCTGTAGCAAGTACGAAGGTGCTGCCATCCACGGCGACCCGTATCATGTGCTATCATACCTGATGGCACTGAGGTATGAAGATCGGAAGAGCGTCGTGTAGGGAAAGAGTGTAGCCAATGGAATCTCGGTGGTCGCCGTATCATTCAAGCAGAAGACGGCATACGAGATTCCATTGGGTGACTGGAGTTCAGACGTGTGCTCTTCCGATCTGAACGGGAGATTGATGTCGGTGCTTACGCGACTGGACAATTCGACTTTCGCAGACTCCACCGCCTCTAGAATGCGATAACTGGCCATGTAGTCTACTGACAGATCAATTCCGTGATCCTGCTCAAACTTTTCTACCACCGAACGGAACAGAAGCTCGTTCACTTGGTGGCCTGAAATCGCATGAGTCGACAGCGTGTGAAGTAGCTTGAATCCGCCACTACGACGTTCCTTCTTGAGCAACGACATCGTCGATACGTAGCCTCCAATATCGAGCACTGCAATTGGACCTTTCGTCAACAATGCTTCAGCGTCTTGCAGCTCCTGAATCGCACACTCCTCCGCCGCATGAAGAGCAGCCACAGGCTCGTCAATTGCTGCAAATACAGTAAATCCCGCGCTGTCCGCCACTTTTTCATACGCTGCGCGCTCGTGCTCATTTGTCCCCGCGGGGACTGCAAGGACAGCCGGGAAGTTTTCAATTGGATCTTCTCCTAACGAAGACGCTGCGGTGCTATGCAAATGGTCCAACATGATCTCAGTAGCCCACTAAATAGTGTGCAAAAGTCAGTGTNCTAACCTTCAACCACGAGAAATTTGTCAACCCACCTCAGGGCTGTAGGTCTTCCCATCTAGCTCTATCTCCAGCGNTCTCTCCACGTTGATGCACCGTATACGGCAGCTTGTTGATAAGCTCGTCTCTCTCTTTAGTGGCAGGTCCAAGAAGGAGCCTTGTTGCAGTCGCTGTGTGGCCTGGTTTTCGTCCCTGTAGTGCCTTGGCAATTGCTCCGATCGAGACGCTGCCATTGTCCACCGCCACCNGCCGCTGGAGTCGCTCGCAATCCATCTGCGCTCTCGATCACCCGCGCACCATGCGCCGAATCCAGCAACGAGGCCGTCGCCACACGTAAATTTGATGATCCCACATCTACTCCTAAAACCTGCTGCATTTTACTTAAGGGTGACGATCCTTCCTCTGTCCCTTCACCCTCATCCCCCTCGCCCGGTAATCCCGCGCGTCGTCTTCGCACTCGCTCCTGGGCTCGTAGGATGTAGCGCGTACCCAAGAATAGACCCGCGCATGTAAGCCCCACGGGGACAAGCCATGGTTGCGGCTGTGTCGCGTGGAAGTGGCGCAAGGCTGTCTGGTGCCTCAGCGACGTCTTGAGTCCCGGAAGTACGACTCGCATGTCTGTTGGTTTCCAAGTGAGCTCATTATGGGAGCTACGAAGCAATTTGATTAGCAGAAAATCCGCAAGTTGAAGACACATGGCTACATTTTGGGTCAGAGTCCAAGTCGAGGCAACAAGTCTGCCGCTTGCACGAGTTTCGACGTGTTGGTCACAGTAGGGAAGATCTCAGCAGGTGCAGCATATTTGCTTTCAGATTTTGCGCGGAGTCTGGGTAATTCGTTCGCGTCGCGGCGACTTTCGAGTGGGATGCCTTCGATTGTGCGTTGGTGACGTTGTTTGTGGCCATACAATGACTTGGACGGTGAATGACGGAGACTATCGCTATTTCCTCGCTTAGCACTGGGCTGAAGACTGAGCTGTTTCCATTTTTCCTTCAAATGCATCCGTCGATCGATCTTCGAAAGCTTCTCCGAGTAGTAGAGGTATTTGTCGGCATCGGCTGTGAGCTGCTTGGTGTTGAACATCGACTTGGTCTGCTCTATCCGTCGTCTCATTTTCTCGTTTTCCTTTGCGATTCGCTTTTGTTCGCGCTGCCATTGAGAGTAGTTTGATGGACTTTGGATGCCCAGATGCTCCTGGGTGTGATGTGAGACGTCAAACTGCTTCGGGAGCTTTTTGTGGATGCGTTCGAGTCGATCTAGTAACAGTTGGTTGTGCTGAGCGATTTCGATCTCTCTGTCTGCTCAAATGGAGTTAAGGGGTCTCTAATGTCGAGATTAGGAACTTGATAAGTGATTCACCAACGCGCACCTTGCTCTTGCTGAAGCTTTTTCGCATTATTGACGGTGCGTATGCCTGCTTCCGTGTCTATGACCGAGTGCGCATGTTTGATTCGCCGTTGATTTCGGACTCTATTCTCCTCAAAGTAAGCCAAGGTTAGAATGTTGTCGTCGCCAAACTCATATTCTGCCGAATATGCCAGCGACCGTCGTGGCTTCGTCATAAAGCTGACAACGCTCAAATTGTGCGAGTATTGTCAAGGATAGCAAACCTATTTCGAGGCATACTGGGATAAAACTCTTATGAATCTGTATCGTCACAAGTTCTAGGGTGCATCAGGCTGGCTCTATCAGGAACTCTTTGCACGCTTGCCCTTCTTCGCTGTAGTTGCGGATTTCTTTGGGTTTTCTTCGTCATTACTACGCTCAATATCTTCGTCATCTCCGACCTGTTTAGCTTTGTTCTTGCCTCGACCTCTCTTCTTCGACTTCGACGTCGTTGTGGGATCTGTTCCAGAGATGCCAAGAAGCAACAAATGACGAGTGGATGGAGAAGCAGCAACGAGATCGATGCGCTCTTCCTCCGGGATAAACGCGTACATCGCCCCATCCGTGATGGAAGATTTTGTACGTTTCCACACTGTCAGCGTGTCAATGCTGGAGTGCCATGAAGGCAGTGGCACCTGCAGCACCTTGTGGTACACAGTCGCCAAGTGAGTTTGGAACTCTTCAGACGATGTTCGCCCCCTGTACCATCAACACTGCGTTAAAAAAGGCTTCAGCTTCACGAACAATGAAAACTGCACTCACCAAGCGCCAGGAAGGCAAACAGTTTGGCCGAATAACTCTCCGACATGGATCACAGTATCCCCTGCGTAATTACAGAGGCTTGCCATCGCCATTGACTCGTGGCTGTCTTCGAAATCGTCCGGATAGCACAGGAATAACGCGCGGTCCGCGTGCTTACGTAAGTCCTGCTCATTGTCGCAGTAGGGAATGATCAGTATAATCGAGCATTGGATCTGTACAGTGTCACCGTGTGTCATGCACCTTAGGTGTTCCCTTGAGCACATCCGTCCAGTAGACCTGCTCCACTTCCACCTCTTCTTCGTCCTCGCTCTCCTCTTCAATTTCTTCCTCTTCCTCATCAGGCTCTTCATCTGCTTCTTCGCCACCGCTGCCCTGTGTCTCCTCGGCATCGCCATGACTCTCTTCGCCACTCGTAGCAGCAGTTTTACCATTCTCCTTCTCTTGATCGTCACCCTCTTGTTCCTTCTCCTCTTCTTCTCCAGCAACGTGCAGATCATAGGCGACAACATCCACGCCACGTAGCTGCAACAGGCGAGCCCAGTATCCGCTACCTGCTCCCATCTCTACAATCTACCAGCACAACTGTCAATTAAGCCATGGCACCCAAGACGTGACTGCAGCTCACCGGTCCATAATGTTTGATAATCTGCAGCGCACGCTCATCAGGAATTGCCCACGAGTATTTCTACGTATTAACATTGATTTACAACTGAAGGTTAGCGCCGAAAATCTCAAAGAAATGCAGTCCAGTGAAGCTCGCACCATCGCCACGCTCAGGTCCAGCGCCGCATACATCTCGGAGCGCGCTTCATCGCTCAGCTCCGGGTCAAAAACCGCTGTGAGATCTCCTGGGTGTTCACGCACGAACTTGCGGTAGAAATCCAGGTACGGGTTGCTGGTAGGTGTCAGCTCCAGCTCTTCCGAGTCCGCCATGCTCTTTGACTGGCAGGTGGGCTGCACAATACAGCTTTTGATTGGACAAAATTGATTCAAATTCTTAACTACCAATCCGATGCGTTAAACATGGCTACACTTTCATGCAAGTTGCACTGCAACGAGCTCAACATCACGGATAATCTTGAAGCACTGGAGCCTATGCCGATAGCAGTGGCTGGTTCTCACCCTCGTATCGCATGGGAGGCGATACGCTCATGAAGCTGCACGAGGACAGCGATCGGTAACGCATATTGACACGCGTGCCACAGAAGAACACGACAAGTAGCAGCACAGCGAGACAGATGCCGAATGCCGTGAACGTACTGCAGATTTTCAGACCAATCGGCAGGGACCCACCAGCAACGGACACGACAGCCACCAATACAGCATACGGCAAGATGCTTCGTATGTGGTTTTCCAGCGGCACATGCGCGGAAAGAGACGCCAAGATGGACGTATCGGCGATCGGTGAGCACGTGTTGCCGAACACGGAGCCGCCCAGGATGCTACCGAAGCATTGACGCAGGTTCTCAGCGTCACCGCCACTAATAGACCACGATAGCGGCGCGAGAATGGGGAACATGATGGCCATTGTGCCAAACGCACTGCCAACTGCGAACGACACGAGGTAGCAAAGGAGACACGCGATGGCCGGCAAGAACTGCTTCGGGATCGAATCTCCAACCACCGAAGCGATGTACGGAGCCGTGCTAACTTCACCAATTACCGAGCCAAGAGCCCACGCGAGTGTGAGAATCAATGTCGGGTCCAGGATATCCTTAACGCCTTCCATCCAAGCCTTGGTAGCTTCGTTCAGTGTGACGATACGCTGCAACAACAACAGCGTGACTGCAATAACCCATCCAGCAGCAGATGCACGGATCAGCGACGACACTGAGTCGCAGTGGCTTAACGCATCCAGAATCCCGAAGGAGCCATTGGGGTCTTGGCTGTACAAACTGTCGAAGCCGTCGAAAATCATCCCCACGAAGGTCAGCACGACGATACTGAGGAACGGCACCACAGCATTCTGCCACCGCAGAGGTTTAGTTGTGTCAGGTTCCAACGGAGCGAGTTCAGGTTTGGGGCTGTCCATCGGTCCCGGATACACTGGAGTCATGGAACCGTCGTTGATCGGCGATGCAGCGGCGTCCTTCTCGAACTGCACCATTGGGCCGAAGTCCTTCTCGCAAATGATCGTGATGAAAATAAAAGCGATGAAGAGGATCGGGAAGAAACGGTAGTGCAGACACGAAAGACACGTGACGAACGGGTCCCAGTCGAGATTCAGATCGCGGAGCTGTGCGGCGACGTAGCCGATTTCTACACCAATCCATGCGCTTACAGGAGCTGCAAACACAATCATTAAATGTTAGCAAAAAACTTAGTTAGGTACTACAGCAGCAACGGTAGGTCTGATCAAGGTTCTTACACATGGATGGCAAGCAGACGCCGACAGTGTGAATAATAGCCGCAAACTTCTCGCGACTGACACCCGTCTGACTCAGCACCTGACGTAGCGAGCTTCCAACAATCAGGATACACGAGTAGTCATCGAAGAAGATGACAAGACACAATAGCCAGGTCGACAGTTGCATACGCAGAGACGACGTCATAAACTTCTTGGCAACCAGCGCTAAACCGTGGCCGCCTCCACCCTTCTGAACCACGCCAATAGTCCCACCAAGAACAATCGTGAATAGCAATACACCAGCGTGATCGTCCACTGTAAACGCATTTACCCAGTACTGGTCGAATGTGCGCAGGAAAGCCGTGAACGGGTCTCCATTTGAGACGATGATGGCGCCACACCAGATACCGGCCAGAAGCGATACTGTTACTTGGCCCAGCACAGCCGACATTACCAGAGTCACCAGTGGTGGTAGCAGTGATACCCACCCCGGTGACACAACTACTACGGTAGTGTGAGTAGCACGGAACAGCAGCACATCGTCGAATTGCACCTCATCGAGTTGTACCGTCGACGTAAGCAGCACCGAGGTGTTGGCGATTGATTCATTCACTTCAGGCGATTGCGTTAGCGACTCAATCTTCTGTTCCTCCCACACGGTTGTAGTGAGGTTGTGCTCTCCATAAGAGTCCAACTGCAACTGCTTGATAGGGATCGACAGCTGAGACCTGAAATTGCCATCGACATCGAGCGTCTTGACGGTGCCATTGGTCACAACAGACCCGTCCGAATCCTCTACGCGATACCACAAATGCTTCGGGTATACAGACCCTATAGAAATGTTAGTTGTAATGCATTGCAAGCCCGCAACGGAGACTGTACAAAATCACGAACCATTCTTGAGCTCCGGCAGCTCGATCGACGCCTCAAAGTCTACCTGCTGCAGGATCACAGTCGGCGTCTGCAGCGCGATAGAGCTGGTGTGGACACGGTGTGCATTGCTCAGGCCAGCAGCCACAGCCGCCGCTCCAAGGAGAGGCAAGAAGCCCATGGAGATCGATGCCAATCACACAGCGAGACTGGACTGCGGCAGACGCCGTCCACAGCTGACTGTGTACCAAAGCAAAATTTGTTGTGTCCAATTCATTTCTGAATCGAGTAATAGTACAGTGGCTCTTCCGCTAAATACACGAACTTCAACGAGAATCTTTAAACTTTTTAACGTTATTACATTTGGAAGCGGAATACTACTACAGTAGTAGTTACGTACTACTAGTATTAGGCTGCGAGCGCCCGCGTGACTCTGGGATTCGCTCGGCGTAGCATTGGCATTAGGTTGGTCCACCGGAACATCTGCGAGAGGATGCAAGTGGAGAACCACACAGTCACAAGCAGGCAAATTGACGCCTCCGAGCTAAAGAAGCTAAAGAGCTGGGCCTTCTCGTACAATGGGGCAGTCAGCTGCCCAAATACTGAGATAATAAAACTGAGAGATAAAGCGTAAGCAAGCCAAAGTTAGTCAGTCAAGGGTCCATTTCAACGAAACACTGAGAAGAACGACGTACAGATTGTGGGTGGAGTGGTAAACGACGCTGGACCAAATGACGTAGCTGGAGTTACCCTGGATCCAGCACATAATGATGCGGGAGCCCACGAGTAACAGCGTAAATATTGCCAGTACGTAGAGTAGGTGGAAGTCCCGCGTTTCAGCGACAGAGTAACCAGAGCCCTTGGGCACCACGTCCAGCGCGTCGGCGATGATCATGGGCCAGTGCCACAGCGCCCATATGCACCCAGTAATAGCCGACGCGAGGATGGTCGAATGGCTGAAAACGATCTCCAGCGCTGCAGTATATAAAAAATGTCCATTCTTTGTAAAAACAAATCTTCTAGAGTCGCAATCAAAGAAAAACGTTACCTGGAAAAAGTGATCCGCTCCAGCCAATTTCCTGAATCATTGCGAAGAAAAACCAGCTTGTACCTACTCCATGAGCAATCAACACAGGCAATATCAAATCAATAAAGGTATAGCGTGTACGATGACCGTCGATGAAGGCGAATGACGTCACCTAAAGGTCGGAGACCCATCGAAAGCCCGACGTCGTATCGCTGCGGCGGGACCGGATCCCAAAAGATCCCAAGGAAGCTGATGTAAAACACGTAGAAAACAAGTCCAGAGTCGAGCGCTTCTACATCATTGAAGCTGAATACCATCTCCAGTTTCCGCACGAAGGTGCGGTTGAACTGCATGCACAATAAATGTCAAAAGATCATCGAATAAAACTCTTCTAACCGAGTTGACGCACCGGGTTGACCATCCTTGTCGCGGCAGTAAATGAAAATGCAGCCAATTCACACAGGACTGGGATCGCGTAGCTAAGCAGTGCAAGTCGTAATGTGGATCTTGATGAGTGGGAACTCCTGAATAGGTGATACAGCGGCGGGATGTTCCAATGTGGTGGCGTGTTGCAGGCCTGGAGCCTCAGTTTCGCAACTGTTATAGCCATAACAAAATTATAGCGACATTGTCAGTATCACATCCATTTGCTTCCGTTCATGTCCACTTCCAACGTACTGTTCCGTAGAGCGAGCGATGCTACCGCTGGACTGATAAACCAAAGGCAGCGGAAGACGAACACGGTGGGTGTGTTAGGCGAGATTCCCCACTGTTTGCAGAACACCAAGCCCGTGAGCGAGAGGACCACCATTGAGTAAAGCGACACGAGGTAGGCGATGACTGCGTCGCGATAGGCCCACATGACGTCGGCCGAGAACATGCCGTGGAAGAAATAGTTGGACGAGCCGCGGTCGTTGCGGTGGTGATACTGGTGCGCCGGTTGCACAAGCGGATAATACTCATCCCATATATTCTTGCCATCACGCCCACACGATTTGGAGGTTGAAGTCGTCACAGGACCAGCAGGCACCGGGCCCACAAGCGCACTTGCGGCAGGGCGGGTCTCGGGCGGAGGCCCCGGCTCCAGGTCCACAATCGTGCCGTAGTTGGTGGTAGACGTAGAGCTAGGCACACTACTCATGACGTTGCTGCTGGTGGCGCTGCTGCCGCGACTTCGCTTGCCCATAGCCGAGCAAGCCTTAGCTGCCTGTTCCAGTTGAAGTGTGTAAGGCGGCTGAAGGTTGGGGCCGCTGAAGTCGACGAGAGATGACCGATTGATGGGGTCACCGCGCTCACCCGACCAATTCTATCTTGGACCTATGAAGAGACCGCCCGCTGGTAGCTTACACTATTTGCTTCACCAAAAAAGTGTGGCAGCATTTTTGGCTTGGACATTTAGGAACGACCTTCGCGCGACATNAAAAAAATAGACACCTTGCAAAGTGTGCAAATTTTGTACACTTTGAAAAAAAGGCTGCCATGCTTTTGAAAAGGGTTAGGTGTCAAAACAAAAGGGATGCATGTTTTCTCAAAATAAATTACGAACATTTCTTACAGGATCTGTAATTTATTCGGTACATG

>Contig_27

AAATCAAACCTTCTCTTGTAACAGACTTGTAAACATTGACCCATGTTTTGATAACGGGTTGACTTTGCTTTGACCCAAAGGCATTTAGAATAGTCATATGTTCCCTGATTATCTAAAGTTGGGTCAATGTTGACCCAAACTAATCTTAATTGGGTCACGATTGATTCTTGTTGCTCGACCTGGGTCAATTGTGACGGGCCTTTCTTTTGGGTAGTTACATACTCTCCAGATACCATGTCTATCACGAGAGCCTTCCGACGAGGGACCTCTTAGCGATCCCCCGGAAGGGTCGGTCGGGTGGAAACACAAGCGCGGATGAGCCGTCCGGCACCAGAAAGCACCGCCGTGTGGTGGTTAAGAGCGCCTCTTGCAAAAAGCAGGCGAAGGTTCTTGCGTCGATTGATATGCGAAGCGATCCATAACGGATCGTAGCTCACTTGATATTAGTATACATGTTTAGGACTACTTATTAGGAATAGACCCGAAAGGGCAATACTAACACAGAACAGGATTTTCACTCAGGTGCACAAGCGACGTCCGGCTTCGAGGTAATCGTCTCGTCAAGTTTACTTGTATGTGTTATGCCTGTCGGTAGACTCGAAGCTTACTAGCGAGGATTTTTTCGGCAAGGCCAAGAGACTGTCTGTGTTTTTGACCGATTATATATCGGAAGGTTTACGTGACGGCTTTATTTTGCGTATTTGTACGACTGGAATTGCGTGTTGCATTCCCTCTTATTCTTCCAACGGAGTTCTAAACACTAACTGCCCTTGTTAAAGACTTGCCCGTGCCTGGTACCGTTCTGCTGTTGGGGACCACGAGGATCCAAAACACCTGCTTGCTTTGCTCGTGCTATTGTGTTATCATTTTCACTGGCTGGCCTATATAAGCTATTCGCAAGGTCCAATCTGCCCTGCTGCTTGGGCATATTTTGGGTCTTCAGGTCCGCTCGATATATCGCTTGCCGTTCTGGTAGACGTATTGGTACGGGAACAACCTATCTACACTTGTAGCTAGAGTTCACGAGGGATTTCCAACACCACAGTGTTTTCTATTATGATGTCTCCATTGGGGTGTCCACTAGTATAGGCAACGTATCCGCTGTAACTCGCACCGTTGACACCGATGCACGGGTCGTAGACGAGCTGGCGAAGTGCGTAGGCAGCGATCCGTCGTGTTGCGCGAAGTCACATTGTTCGTAGCGCTCAAAGTAAATGAAGCTTGGTCGTCTACCAGCTCTGGCTCCTGCTACCAGGTGATGTCTCCATCGAACTCGGAGTTGTCAGATCCGCCGCACTACTCGGTGGTAAGGTCGCAGTCGCTGCTGATGTGGCCTGCATTGGTGTTGCGGGTCGTCGGAACCGTCGTTATGGTCATATTCGTGGTCATGGGGGTGTACACCGTCATGCGATAGAGGAAGGCAGTGAACGGTCCATCGCGCTGGTCCTGCTCCAAGTTAACATCGACGTCTTGTGTTGCCGTGGTGGGGGGAGCACACGTCAACGCTTGTAATCAATTAGCGGCTCGGTCGGCTCGGTCGTCTGCATGGGTTTTGCTGCAGATCCAAAGCGAGGAGGCCCACGAAGACGAGAGCGACGTGCACGCCACGGTCCGAAGGAGCCAGTGTTGTTAGTGGCTGGGTCTGGTGGAAGGTTACGACCTGTTACCGCTGGCCCTTCTCCAGCTACCGGCGTGTCTTGAAATGGTATATGCGGGGTCAGACGTACAGGACTGGGAGTGCTCTGCGTAGTTTCGCAGTGGAGGCTGTGAGTTTTTACCGCTGCTCTATGAGTGAATGACGCGAAAGATGACCGTGGAGGTGGTGTCGCGGTGCTGTCGACGTCTGTACGCGCGCAGGTCTGCCAGTTGCGGCGCTGTCGCATCGCTCAGCTTTTTGTACGCATAAACGGTGCGCCGTCCGCCCTGTTGATTGGTTGCGAAGCAAGACTAATTTGCGCGAACAGTGTAAGTGCCAAAAGCAAAAGAACACTGAAGCCACCAAACCACGAATAGCTTTCTAGCGTTGTTGTGAAGCCTTTTAGCAATCAGGTTAAGTTTCTAGGAAAAGGGTGGAGGTGGGGGCGGCGCACGCGCTCACCCCCGCTGACAAAAATTCTACATGTGGAATTACTCAAAAGAGCATTCCTGAACCCTTACAGCACCGGAGTGCAAGGGGCCTGCTCCGGCTCATGTCAACGCCCTCATTGAGCAACGTCAAGCACCAGTCAGGTAAGTATGATTTGAGATATACTATTCCGGGTAATACTATAACTCTTAGCAGACCTCATGTACATATTCTAATTAATATTATCCCAATATAATTCTCATTATAAACGCTTACAGCTAATAATAACAAAACTCTTCATAAAAAATGACAAATGAGACTTGGTCTTAGAAAAGCTACGATGTACTAAAGCTGCGCAACAAGAACATTACTGTAGATCCATAAATCTACCCTTCTCTTCTATCAACTATAGCATAGTAGACTTGGCCTTGCTCTTTTTCTTCTTAGTGAGCTTCTTGACAGCCTTCTTGTGCTTCTTAGTCGCGAATCCTTCGTCCTCACTCTTCGCAACTGTCTTCTTGTCCACAAAACCGTCCCGAAGGTACGTTTTCAGCGCAAGGACCGTATCCTCCAACTGCTTGTGCGTATGCAGCGCCGAGATAAAGAAGCGCAGACGACATTTCCCTTCCTCCACCGCAGGGTACACGATCGGCTTGACATTGATCTTGTGCACCGCCAAGAACTCGGACGCTTTGGCAGTCGCAATAGTACTTCCGATCATCACCACGACCACTGGAGCACCACGGAAAGTGGTCTCCCCCATAGGAATGTTGTGTTCCTTACACAGATCATAGAAACATTTACTACGCTCCTGTAGAGTAGTCGTACGTGACGGACTCTGTGTCATCAGCTGCAACGATTTCAACGCAGCAGAGCCACACGCCGGCGCTAAGCCCACCGAGAACACGAAGCCTCCAGCACAATGCTTGAGGTATTTAACCAGCGCCTTCGAGCCAAGGATGAAGCCTCCAACGGACCCCAAGGCCTTGCTCATCGTACCCATACGTACATCAATGTCTTTCGGGTCCACCTCGGTGTGTTCACAGATACCGCGCCCAGTACCGCCCATCGTACCGAACGAATGCGCCTCGTCCATGAACAGTAACGCCTTGTACTTTTTCTTGATACGAATCATCTCTCGGACATCCGGGACGTCACCGTCCATGCTGTAGACCCCTTCAACGACAATAAGAACACGTCGATACTTGGTACGAAGCTTCGACAACATACGTTCCAGAGCCTTCGTGTCATTGTGCGGGAACGGCAAGATAGTGGCGCCACTGAGACGTTGGCCACTCACACACGAGTCGTGATTAAGCGCGTCACAGAGAATCAGATCACCTTTAGACACCAACGCATCGATCGTCGTGACATTGGACACCCAGCCGCCCACGAAGAGAACACTGGCTTCCGCCTTAAAGAACGTACACAGCGCCGTCTCCAAGTCCACATTCACCTGCGTCTGACCCACAATAGGCGACGAACTCATCGTCGTACCGTACTCGTCGATTCCTGCTTTTGAAGCCGCAGCAACCTCGGGGTCCGACGCGTTGCCGAGATAGTTGTACGTGTTGAAGTTCGTCATGCGACGCTTCGCCGGCGTCAGCGTCTCCAGAAACGGTACACGTAGTCCGGCACTCTCGAACTCTTTCATTTGTCCAAAAAGGCCCTGAACTTCGGGGAAATTTTCGATTTCAAAGCAACTCGAGTCGATCTCGGCAACTGTCGTGGCCGTCTGAGCACCAGCTTGGTCGTCACCTTCCTGGTCGCCCGCGAGGTCTCCCGTCGCCAATCCGGGGGCATTGGCGAGCTTCAACGGCGTGTCGTATTGGAAAAACGCCGAAGGAGACACAATGCAACCCAGAAACTCTCCTAGATCCGACGATAATCCCACAATCGCCACGGAATCCATACCAAACATGGCCCACGGTGTGTTTGGGTCGATCTCGGCAGCATTCGCAGTTGCTTTCTGGCCGTCAGACGACTCTCCTGTGGGCATCTCCATCTCCTGCGCCACATGTTCTAATAGCCACGCGAGGATCTCGTCCGACGTCTTCACCGCTCCAGAAGACGTAGGAATAGGCTGTTCCTTCGGCTTTCCAGCGCTAGAAGACAAACCTGCCTTCGCTCTGTACTCGAATAAAGGCTTAGCAAGTGTGCCATCCAAGAAATGAGCCTTGGAGGCACTTCGTTGGATCTTGCCACTCGTCGTCTTCGGGATCGTCTTCTGACGCAACAGAACGATCGCTTCGCACTTCAATTGGTGCTCCGACAGGACCGTCTTGATGATCTCCCGGCAAATCTCCTCGAGTGTCTCCTGCGAGGATCCGTTCTTGACCTCGGCGACCACGACAAGCGCTTCCTCGTCACTTTTTTCGATCGAAAAGGCCGCAGTGCATCCGGGACGAACATTTTCGTGCGCGTGTTCAACAGATGCTTCCACATCCTGAGGACATACATTCCGACCACGAATGATGATCAGGTCTTTGAGTCGACCTGTCACAAACAACTCGCCTTTACGAAGGAAACCCATGTCGCCAGTGCGTAAATACGTGTTGGTCGCGTCCTTCTCGTGTGCCACTTGTGCACGGAACATCTCCTGTGTATACTCCGGTCGGTTCCAGTAGCCAATTGCCACTGATGGTCCCTGCACCCATACTTCTCCGACTTGTAATTCGTCCAATGGCTGCTTGGTTTCGGGATCCACAATGGCGACGCTAAATGTGGGCATGGCTTTACCACAACCCACAAGTTGCATCACTTCGGGTTTCTTTCCCTTCTTGTTGGAGCTCACAACAGCAACTTTACGTTGCGTTTCCAACACGCGCTTGTTAACGTCCAATAGCGTTGGCTGCTGAGGCGGCTCTTGGCCCGTACACACCAGAGTGACTTCCGCCAATCCGTAGCCACAATTGAAAGTATTCGGATCGAATCCGGAGCCACTAAACTTGGACGTAAAGGCCTCCAGTGACTCGCGACGAATGGGTTCAGCCGCACAGATTGTCTGTTTGAGAGAACTCAGATCCATCTCTGCCGCCTGCTTGTCGCTAGTCTTTCTAGCTGCCAACGCATAGCCGAAATTCGGGGCACACACATGCGTAGCCTTGTATTTACTTGCAGTGCGCATCCACAACGCCGGGTCTTTAATGAAACTAATAGGAGACATACTAACACACCGCGCAGCTGTGACGCATGGAGTTATGATGAATCCGACCAGTCCCATGTCGTGGTAACTCGGCAGCCAACTGACCATCGTGTCGGTGGGTTCAATGCTCTCCCACGTCTTCAGTTGCGCTCGTAAGTTACCGTGAGAGATCATCACAGCCTTGGGAGCTGACGTGGACCCCGAGCTGTATTGAAAGAACGCCACGTCATTGGACGTTAATGACAGAGCATCTTCTTCGTCGTATTGCGCAACTAACGAGTCGGGAAGACTATTCGTAGTGATCCACTGCAAGTTGGCGGGCCACGACGTCCTGGACGTGGAGAAGTAGCCTTTCACTGTAGCCATCTTACTGGCAAGATGATACGTCGTGTTGGTGAGGACCACGGCAGCCCCCGAGTCCTCCACCAGACGGTTAAACTTAGGTAGATCTTTGGCCAGAGTGCCAGGGTAAGGGGGGTACACGGGAATACCCACGACGCCGGCGTATAGGCAGCCCCAGAAGGCCAGAGCGAAGTCTAGACCTGGGGGGAAGCACAGCACCACCCGGTCACCCTTGACCACGTGCGCGTCACGTTGGAGCGCTGCGGCCACTTTGCGAGCCGCACGGTCCACATCTTCGAAGCTTAAGTTGACGGTCTCACGGCCCAAATCGTCCAGGAAGGTGTAGACGAGCTTGTGACGCCACTTGGTGGACGCAGCACGACGCTTAAGTTCCCCAACCACGGGCTCCGTGTTGATGGCGACGCCGTTAGCAACAACCACCGAATGTTTCGGTTGTGCGGCATCAAGCTTCTTGTAGCGACGCTTCTTGGGCGCGGGGGTTGAGGACTCGTCGGTCTCACGCTGGCGCTTTTTGTAGTGTACACCCAAGGCCGCGACAGACGCAGCGGCAGCTAATGATGCTGCTGCCACCACCTCAACGGCCACGTCGCCCAGCAGGGAGGACATTAGGTCTGAAAGGCGTAGGGTGACTGGCGGACTGTGAGGGATCGGTGTAAAATACACACGATGTGGTACACTTAATTATGTCTTTTTAGCCAATGGGAAAGGAGCTGATCCCTTTATTTCCCTGTCTATTCACACTGCAGATTGGGAGGGGTATGTAGCCATGTGTCATAGGCACTTCAACATCGTTTTATAATCCTGTCCTCTCACCGCTTGTGCAGACGACTGAACGTCGGCATGTTGCCGCTACGAGCAGCTGGGGGTTGTGACGGCATGTGTCCTGGTCTTGACTGAGTCGATAATCGTGGCAAGGTGGCGCTGTTGCCAACCACCAAGCCTGGTCCACGCATCATGGCACTGAGTGGCGCTGCATTTCGTTGGATTTGGGGCAACTTGTTGGCGACCTGCCCAGCCACAGGACCCGACGTCGGTCCTCCACGTCGACTTAGTGGCTGCCGAGTTGACATCGACCTGACTGGAGCTGTAGAGCGAGCGAATGGCAGTCTGGTTCCGCCCAAGCCAGAGGTTTGTCGAGTATTAGCTCCAATTACCGCGCTCCGAGCAAAAGATCGCTGGTTTCGGGTGGCAAGTGTTGTTGTCGTACCAAAACGACCCGTAGATCTACTGTTAGTTGTGACTGGTAGACGCTGGAAAGCCTCTTTCCGATTCGTATACCCTTTCTTGATGTCATCTAGCTTTTGTAGATGGGTGTTCGCAATACGCTTCCCAGATGCAGCACCCACGGGGGCTCTTACACGCGGTTGTGGGGGTAGTCGCTGCTTCTGGTTTCTTTCCGTTGTTGACTTTGCCGATGCACCAGCCCGTACGTGCGATGTGGTGGCCTTCCGTTGGTTGGCAGCAGCGCGCTTCTTCTCAAGCCATCCTTCAATGACGACGTCCTGTTTGCGAGCTTGTCGTTGCGCTTCCTTCGCCTTCTCGTTCTTAAGGAATGTCCGTCGTTCACGTTCCGTCATGCGTCGAAGTTGACGCTGCTTGTTCTCAGGGAGACTCGTAAGGAACGTGTCTGTCTTCGATCTGGTCCTGTTCCTCGTTCCAGTATTGCTATTAAGCGCTCGCTTCTTCGCGTTCGCCGCTGCTGTCGACCCCTGTGCTCGTCGTGTTGACGTCACTGTTGTTTTATCTCGTGCACGCATTTTAGACGGCCCGGTGACAGCATACACCGTTCTGGAGGGCTCATGCAGCCCACCAGTAGCTCTCCTCTTAGCAAACCGGAGACCGCTACCAGTGGCGGATTGCTTGGACCCTGCTGTTCTACTTGAGGTGTTACCACCTCGTGCGTTGTCAAGATCATCCACTGCTAAGCCAGCATCTTCCAGATGCCTAATACGCTGCTCCAGCTCCATCAGTTTGTCAAGACTGGTGACTACGTTTGCGTTGTCTTGTGCAGCTTTGCCTCCTGCAGCCATCGCAGCGAGGTGATACACACTATTACTATTCCCACGGTCGCGTTTGTTAGGTGAGCTGGTTTCCTCGTCTTGATTCGATCCCTTCGCCTTTGACCGACTGGAATCGTTCCCACTGGCTTGAAGCTCCAGCATCTTTTCGCGATATTGTTGACGTCTTCGCTGCATATCCTCGCGTTGTGTCATCGACTCTTTGAGTTTTCGAAGATGCTGGATGTGGCGCTGTACATCGTCGTCGAACTTGGCCTCGTCACGTTGGCCTTTGGCTTCCAAAGTGCGGAAAAACTCCGTGTTTACCTGCAAAGGCTTAAGTTGCTCTTCTTCGATAATCTGCTGGGCACGCTGCGCACGCTCTTCGCGCTCACCCTGAGTGAGCGCACTGGCGAATTGCGAGTCTTCCTGTTTGATCTTATTGGTCTTGATGATGGCAGCCTTGAGAGCTGCCTCGTTCGCTAGTTTGTGCCAATTTAGCAACTTCATTTCTTGCTGTAGTTCTTCTGTTTGTGAGATCGACATGGCGCTCGTCGCTCGTGGCTTCATCTTTCGGGTTGTATTCGCAGTAGAAATTGATGACTGTCGGGTCGCTTTCCCACTCTCATTTGGAGCTTCAAGACGCTCGTCAGCTTGACCATCGCCACTCTGGTATTCGTCTAGCTCACGCACCCAACCAGAGTAGTCTGCAGCTACCGACATGGCTCCGGTTGCCGCAACAGCGAGAAAAGCTTAACGAAAGAGTGTAAGGTACAAACACAGCGCCAGACTTACGTGGAAAGACATCGCTGTTTTTAGCCGAGGGTCTAACATGCAAGTGTAAATAGCCATCCAATCGCTTCGAGCTGCCATGAAGAAGAGGGTTACCCCAGGGTTACCCACCCGTCCACGTGGATTATTAAATAACCCATCCACCCCGTTTACCGGCTACTCATAAAAGAAACAATACTTGTATTCAAGCGACGTACCAAGCTACAGTGGTAGCTCAGTGCTACTATAGTAGTACTACGCATACTCGGCACTCACTCATGGACGCTGAGTCGCGCATTACGCCTACGTGGACGCTCAGTTCGCAGCTTGCGTTGTCGCCCCCGCGTAGACTGGTACATCCAAATAGAATCAATAGTTTCTCGCTCAAACTCACGAGCCGCTTGCCACGGAATGTTGGCTACAGGCTCCCAGGTCAGTTCATGGTATTCTCCCCACTGCACCAGATAGAATGTAGCCTCATTAATCCAGCGCTTAGCCCACACCTTCTTGAGAGGCATACTCGTTCTCTGCAGTGCCACCACGTCTCGTAGCAAACAATCCTCCACCACGCCGTCTACGACTGGCCGAATCTCCGTGTCTTCCTCATCACCACTAGACGGGTCTACATGCTCGTGTTGCAATCGCTTCCTCCAAAACGAGCCGTCGAGGCACCTACCGTCTTACCCCGCATATAAGGCATATGTGTTCTAAATAAATTCGGGATTCGCTCGACATCTAGCTTAAACCTAATATTCTTGAGAGCTCCCAACCAACCACCCTTCTGAACAAACTTGTCGGTGCTGCTGTTACTTGGCTTTAATTCTCTCATTTAATAATTTACACAGTCATGAGTGCTGAGCGACGTAGCTATAGTATCCACGAGAAACTCGCTGTTCTAGCAGACTCGGATAGGCGTGAAAGGTCACGGCTTTGCTGCTCTTAGCAAGAAGCACAGCGTGGCGGTTTTGAACCCACGCCGATCGCGTAGGAGGGTCAAGCCTAAACGACCAAGCCAGATTCCAGGACCCACAAACTTGATTCTTCAAAGTCAAACAATTTGTTCGTTGTACAACACATAGGCTTTCTAAACGAATATAACACACAAGCAGCGTTGTATTCATTTACAGCACCTATGCCTTATATGCGGGGTAAGACGGTAGGTCTTTATTGAATTGCGAATTGAATTGCTCTTTCTGTGTATTAAACACAATTTCCTAATAGTGGACTAGACACTGTTTTTTGTAAGTGAATAGGGCTACAAAAAACCATTATCCAACCACCCCAGCAACCTCTTCGACCCACCCAATGTCTTGAGAAAACCCGGGTTTTCCAGAACACTGACCACCAATAGATTACTGTATCATGTCCCGGGAACCACTCGTCGTTTTCTAATTACGTACCAGTACAAT

>Contig_29

GTCTGTCACCCTTGATTTAGTTGGGTACGGTAGTATCGCGCTCCCCAGAGAGCTCCGATTCTACCCTTTTATCGTGTTCCAATGCTCGGTGATCTCCCGTTTATTGTCTGCCGTGGCTTCATGCTTGGTCGGTCTACTGTGCTCCGTATTTTACCGTGGTAATATGGGAGAATTCCTGTCTGTGCACTCTAAGCGTTTCGTATTCTACCGTGGCTTCGAAGGTGCGTGCTCTTTCGTAGCCCGTGCTTTTCAACGTTTTGGGGTGCCAATTGTATTCCGTCATCTGTACGCTCCCGTCATGTTCTTGCCTTTCCAAGTTGTCATGGTAGTGTGCTCGTCCGTGGATCGTGCTTATCAGAATCATTAGGTGCCTTCATTGATTTAGTTCTCTTTCATGGTTCGTGGCGTCCTTCATGTCCTTCGTTCTATCGCATCATGTACAGTCTAGCTCGGTGCTCTCCGTGTTTCGTATTCTTATTTGATCATGCGCCTCTACGCTAATTAAATCACAAACACTGCTCCCAGTGCACTGCATATACGCTACGTATCGTGTTGGAAAACGCGCACATTCTGTAAGGGTCGCACACTACGCGCAAATACAACGAAACACGCTCTTTTTTTCGCGGCGGAGGAGTGGTGTGTTTGGGTAGGTAGTGTGTGGAGCCATTATAGTCGTATTTGCAAGCAATTCATATTTGTAATAAAATTGTGTTTCTTAAGCGTTAGAAATAATCAATATTTTAATTTAATTTAGCACTGATTTAATAATTATTAGTACGACTACTACAAATAATTTGAAAGCAAAATATGAAAGAAATTTAGAATAGTACTACGCAAAACAATCTTGTAAATAAAATTATGAAAAACGTGCTTCAAATCTATAAATATCAAATTTGCTAGCTATACGTACTTAAATGACTATTTAAGACGAATCGTGCGTCACCAGGTCCTACAATTTTTACTACCAGCTCCTACGTCAATATAGTACGTGTATGCACGTCGGCGAAGCCTACCAATGCAAACTAACCCGCACACGCGCAGCGCCCTTTGCGTGGCCTGGCTGAAAATGGTGCAAAATGAAATTTTAACGGTGTATTTAAGTATGATGTGTAGACCACATATAATATGTACGCCACATATTGCAATTTTAGCAAAACGACGCCGCCTTTGCTACGCTCGGCAGCTATGTAACTCTAAACAGACGGACACCAGGGACCTTTTGTCAAAAGGTCCCTGATCAAAAGGCTTTTAATGCTTTTTTGATACACTGTATGTGCTGCCATTTCTGGGAGGTGCAACAGAGCTTCAGAATTTCAGGTGAAAACATATGAGGATTTTAAGAAGGTCAATCTGGCTCGGATTTGACTTTCTCTTTCCTTTACTGTACATGTACTGTACATGGACAATAAAAAAAGTACTTCACATCATGCAAAAATGACGCCAACTACTTTTGTAAATTGCACGTGTTGCTGTGCATAAGTAAATCCCCAGATAACGGCTCATCACCATCAAAATCCTCAGGAAGTCGCTTGCATCTTCAGAATCTGCTCCGCAAATGCAGTCGCCACGCTCCTGCGATAAAAAGATTCTCTTACCTGGAGAGTGCTGTGTTCGGCTCGCAGGAAAGCGGCGCTGGGCCCGCCTTTGCAAATACCGCACGGCCCCTGCTTCGCGGGCACCCCGAGCGGGTTTGCTCGCATCCGCTCTCATAAGATGGTCTTCTCCCTTTTGATTTGCACTCAATTTCACCCTGTCCTTCTCCAGCCGAGCACCATAGTCACTTGCCTTATAAAATTCTACTAGTAGATACAAATATAAATACATGAGCACTAGTACATGTAGCTCTGCTGTCATCACCTCAGTCGAGAGGGGCTTTACCGTAGTCTCCTTCACTTAAGCTTCGTAAAACACTTACAAGAGAGCCATTCGTGGTAGGATTTAAAACAGGATCTGAGTGTGCAAAAAAGTCAATGCGGCGCACATGTACGGTTCCGGTACATGTAGTTTTTTTTTAATAACCAGTGATGCAGTACAGTACTTGTACAGTGTCTCTTGCTCCCTTCATCATTTGGCGAACACCACAGAATTACTCGGATCATGAGGCTTTACTCGGCTGCGCTGCTATCTACGATTGCAGCCTTCCTGTCGTCGTCCTCGGTGGCTGGGGCGACTGCTGACTTACAAACGACGAATTGTATTCGTACACTGTCCGATGCGCCTGCTAATAGTGTGACGAAAAGGCGATTGAGAGCATCCGAGACAGGGACAAACGACAACGAAGACAGAGTGTTGAACGCTGCTATTGAAAAGCTCACGGGTCTGGCGAAGGCGGGGGCCTTGAAGATCAGTAACATGGAGTGGAAGTTTATGCTGACGGGGGAAGGTGGGGCTGATAAGATTTTAAAGTGGTTTGACCTCGATAGAGGAATGAAGAGGGCTCTGGCCAGTCCGAACTTGAAGGTTTTGGAAAGCTACGTGAGAGCAATGAACGGCAAAAACAAAATATCAGTTATCGGAATATTCTCGACGCATTATGGAGACGACCTCGTCGCAAAGTCTCTCGTGACCATGGAGAGCAAGGCTAAGACGCCAGAAGCGGTGAATACTATAAAGAATTTACGCAAGGATCAACTATCGGCCTGGATGAATAGTGAGAGGTCTGTCGACGATGTTTTCAACCTGCTAAAGCTTCGTGAAGATGGCTACAAAGCTCTTGCCAGTCCGAAGATGGAGGTATTGGATGACTACATGAAAATGGTGATCCAGACTAAAGCAGGCAAGGAAACGTTGCTCCAGACCTTAACTAAGGGCTTTGATGGCGAAGAGAAGTTGGCGAGGCTGCTGGTGCGTGCGAAGGAGCATTCAAAGTCGAAGGAACTGGCGACGGCCTTGCAGAACGCACTCGTGAAGAAGTGGATCGAAGCGGACAACATGACACCGGATAGTGTCGCTCATATGCTTCAACTGGATCGTAATCTGGATGCGCTAGTGAACCCAAACGTACACACTTTGGGGGCGTTTATCTCGGTTTACAACGCCAGGAATCCAGCCAGCAAGGCGTCGTTGATTGGGAGGTTCACTACGCAGTACGGGGACGATGTGGTGGCACTGGATCTGGTGTATGCGAGATCTAAATCTGCGAAAAGACCTGTGGCCATATTTATGCAGCAGCAACAGTTCCAGGCCTGGCAAAAGAGCAAGAAATCGGCGGTTGATGTTTTCAAGCAATTGGATATTACACCCACGGACTTCGAACCCGTAGTGAGCCCAAAGATGGAGGTACTGAGCGGATATATCAACGCTCTTAATGCCGCCAATCGAGACAAAACCGATATGATCACGGTGCTCATCCACGGGGCCGACGGGGAAGGCCCTCTTGCACGTGGGGTCGTTACGGCTCTTTGGAATGCAGCGTCACAGGATCGGGTTAAAGCCGTCATCTCCACAGCAGCTGAGTACGAAAAGTTGCTGCATAAGCGGTGGTATAGGAGCAAGATTGAGCCGCCAAGAATTTATACGGATATTCTCAAAGTACAGGAGACTTCTGCTATCGGTCTGGACCAGCTGATCGTAGCCCGATACGCGAGCTACTACAGCGATAAGATCGCTGCTGCTCGTGCTACTCCTTCGATGGAGAATGCCATTCGTCCTAGGCGCTCTTAGTTGCCTCGACCAGTATGTCGGCTGGAAAGGTGGAGTCAAACATGTTTTGCTCATGTAAGGTAGCAATGCCGTCGGCGATCTTGCGTTAGACTCTGTGCGACACTGCTGCCTTCTACTGCCTAATTGTAGTTAGTAATATCCAAGCGATCGTTCAACATGTAGCCGAAAGCCAACAACGCTGAGAAAATAAATTGGATAATCGATAAGTGAATACTACGACGAAAAGAAAGAAGCTTCTCGTCATCACTTTTGAATCCCTCGACGCTAAATCAGCCGTTGAATTTCGGCTTTGCGGCGAGTTCGATTTGGTTCATTCGTATCGGAAGAAAATATTGCCCAGACCAGACCTTTGTCATCTGAGTAGTACTTGCTCACCATATCACTGTATTGCTACATATCAATAATAAAAAACAAAATATGCGATACTGTCCTATTGGTTCGGGCTAATACCGGTAGTACTATTTTGCTTATTTGTGTTAATCCCGCACAGGCCCTTCAAGAGCAAGCATGCTGCTGCCCTATCGACACCTATTTTTCGTACTATACGAGAGCCTCCTTAGCGGGTCCCTTGCATAAAAATATGGTCATCTGACATGCATTAATAAAAATTAACTCATATCGTAAGTTTTTTTTGCTAATCTATGAAGAATGTTTTGGTGAAACGTTATTGCAAATTAATGATTCTACATCTGGTCCGAAATGATGGATAATATGCTACAGGATGGTATATGAAAAATAGGTTGAAAGCAACTCGTTTTGTTTTGTTAAATCGATCAGACCATCTTTAACAGGTATTACTTATTCTTAGACTCAAAGAAGATGAGACTTAGACGGCCAGCCTCACTAAGCTATGTCTTGGCTCTGTCGTACTTCTTTCGCATTCTAGTACTTGAGGAAGAAGACCTCTCCTCCGCAGCTTATCTTTTCTAAGAAGTGGCAAGGTAGATTAATTCCCACCCTGTTGTTGAAGCACGCATAGCATACAGCTCTGCAAAGTCAGCAAAATGTTGTCCAGTGTGTCGGCACTCATGTCTCCGTACGCATCTTGAGCAGCCTTGATGAGATTGTCGATTTCACGGATGGGTATGCGGTGTTGGATGCTTTGATATTCATTGGAGACCAAGGTCCAGCACGTTAAGGTCCGGTGAATTGGCAGGCTGGCACAGCTGACGGAAGCACCACCCACCTTCACACCCCGCATCAGCAATGTCAGGGTGTTGGAAAGACACGTGGGTCTTAGCGTTATCCTGTTGGATAAGGATAGGGCGCCGTCGGTCTCCACAAGGCCGACGCTTCTTAATAGCAGGAGTAGCCAATCGAATAAGAAAGTCCTAGTAAACAGCTCGATTCACCACTTCAATATTGCACGTACACACAGATCCTTTGGGGCGGTTACAGCCGCTTATCTAGGTTATGTACGCCTACGTCGGTTGCCACAGTCCCAGCTTTTCGTTTCAACTTACAATGCGAGTCATATCTAATTACACAGAGTTTAAAGGTTAATTTGTTTGTATAACATATAAAAAGTAATGGGTATTACCTCGGTCGGGCAACGTCTGTCAGGAACATGGTCTTCGGAATAAACGTCTTGTGCGGTGTGGCGGGGTTTCGCCAGGAAACAGTAAGTAGCTTCGCTTATCCCTGTCCTCAAAGAACCTTTTTTCATCTACGTGGACCACGTCGTCCAGCGGCTCGAACTCAAGCGTGTCGTCGTTAGTAAAGGTGAGCACATGCTCCACCCTATCCAGCTTGTTTTTTGGTGTCAATGTCGACCGGATGCACGCAACCTTCTTTACCAGCTCGCCCGCAGCCACAAGCAAGCGTATGATGCGTCGCGAAACGCCAGTTAGCCCGGCAGCTCGACGTTCAACCGAGCGATCTTTTATCGGAATCTCTTGCAGTCGCGATGCATTTCGGCCTGCCCTTCCTCTTTCTACCGGAGCTCTCCTTGATACAGCTTTGCCACTCCCCTCCTTCTACCCCTGCGGTGACGCTGATGCAATAGCGGGTCCAGATTTTGCTGATAGTGGACGGGCTGCGCTTAAAGTCTCTGGCGACGGCCGCCATTGCTCAATGAGGTAGATCTCCGTCAATACTGAGTTTTAGGAGCTCGTCGATGATGCAAAGGCGCTGGTAATCGGTCTGGTTGGTCATAGAGAAGTCGAAGAGCACCGATAGAGCGTAGTGCTAGCTGAGCGAGTACTTGGAAAATGAGGAAGAAGGTGGACAGCTATTCAAATAATTGTAGCTCGATCCCCAGTACGCTTTCAGCGCCAATCAAACCACCAGCCAATAGCTGGCAAATACACGCTCCAGTTGAACCAAATAGATTTTGATAACAATGGCTGGAATTGGTTTTAATTACATTTAAATTAAATATCCGTCATTTTGGATCAGAGGTAGTACGTCGTTTTATACGACAAAAAAAGAACTTTAGTTTCGGATAATAACGTAAGGCTTTGAATAGCGAATATCTGGATGAAATATGAATAAATGGCAAGTGAATTACCGATCGTTATTCCAGTACTCTATCCAGTGGAATAACGCGACCCTTCCATTATACTGGAATATATATTTCTGAACGAAGAAAATTATTATTCCAAACCATGAGTTATTTTCTAATGAATGCGTGACAAGACGTTATGCATTATATAAGTGGCTAAAAGAAAGGCATTYTTTCTTCAATCCAGCATATCCCCTTGTTCGGAGTGACATTACCCCCCGCCGTACTTACAGAAGTTTTGCCACCCGGTTTTGAAGAGTTCTCCTCTTTTTTTCTAACCATGTTGTATTTTAGAAAGAATTGATAAATATACAACATGCTAAAAACAAATTACTCTTGGGTTTTGTATTGAATACCAGAAGTAAGGGACCGCTAAAGCAGCCCGGGAAGATCTCCAGACAAGTGATATTTTGATTGCAAACTTCCTAGAAGTTTCTGTTCCTCCACGTTGTCAAATACACCCACGCTTCGGTGTCGAGCTCAAACTGGGTATATCCTATACATGTTTGTAGTGGTCCGTTTTATTAAAAATGAAGCACTGCACACAAATTGTTTGGCTATGTATGTTGTCAACATTGATCGCAATCAGAATGCTCTGAAACAAGTTAGCTATCTTGGGGTCAGCCTGGCTATTACACCATAACCCAGTAGCGCTGCCCTCTCCAGCACTACCACCATTACTGTTGGTCACATCGTTGGCCTTTCCACTACTGCACTCTCCCGCTGCCGCCTCGCTCCCCACAGCTGTCCTCTTCGCCACGATCGGGGCTACCCTGTCCGATGCCATCGCCGCGGCGCTTCTTTCCGAGTCTGCGCATTTGGATGTTTTGACCAG

>Contig_33

TCCCTTTCAGTCTGCATTTGCGTATTTCTCTTCCTCGCCTCAAGTTGGAGGTTCACCCTCCGCCATGACCTCCGACTGCTCCAGCGCCACTTACGAGCACCTGTTCCGCTCTCTAGCGCGTCATTCGCGTCTGTTCCCATTGCGCAGCTCCGACGTAACGTTATTGGCCACACCCACAGACTTCTACCAGCAGCTAGTGCACAATATACAGCTCGCAGAGACACGAATCTCCATCTCCTCGCTGTATCTCGGCACGGGTCAATTGGAGAGCGATTTGGTGGGCGCACTAGCCACGAGACTAAAGAACGACCGCATCTCCAGGTGCAGATCGTACTGGACTATTCTCGCGGCCAACGCGGCGGTGTCACGGCCAGTTCCGTCACCATGTTAGCTCCATTATTGAAGCAATTTCCGAACAACGTGGAGCTATTTCTATTCCGCGTACCACAACTCTCAGGACTCAAAGCCAAACTACCTCCGCMATTTAATGAGACGCTGGGCGTCTCTCACGCTAAAGTGTATTTAGTGGACGACACTCTCGTGCTTAGTGGCGCAAATTTGAGCAACGATTACTTCACCAATAGACAGGATCGATACGTGCAATTGACAAACTGCGGGGCGTTGGCACAGTTCTACCATCAGTTCGTACAACTGGTTACCGGTTTTTCGTATAGAGTCAAACTGGAATCGTTGACATCCGCCAAGAGCGACTACAAGTTGTTGGCACCTCAACTGGCTCACGACTCGGAGGCCGCAAAGACGGCAATGCGACGCGATCTCGAGAAGCTAGTGGACCCAAGTCAGTATCAGCAAGATAAAGACGACGCACGCACCGATGCGTGGGCGTTTCCAACGCTGCAATTTACACCAATCTCCATGGACCACGATGAGCGTGTGCTAAGTGAGTTTGTGCGGCAATTACCGCGTGGTTCGCAGCTCCAAATTGCGTCGGGATACTTGAATTTTCCTCCGTTTCTAAGTGGATTATTGGAACACTGTCAAGCTGGTTTAGACGTGATTTCTGCTGCCCCACGAGCGAACGGCTTCTACGACGCACGTGGCGTGAAAGGAGCGTTGCCTATGGCTTATTCTCTGATCGAGCAAGACTTTTTTGAACGCACTCTTAGCCGTAAGTATCCAACAGTGCTACGGGAATTCAACCGACCAAACTGGACTTTTCACGGCAAAGGCATGACGAGTCGAACCACTCCTCGTCGCTGTCGACCCCCTCTTCACTATCACTCGCCCCTCCTCCGTGGCGCGGCGGTCGCACCGCCGCTTTGACGCGCAGGTCGGGCGCCATGGCTTCCTGGCTGCTGCTGATTTGCCCCCGCTGCAAAAATGAGTGGGGGGGGGAAAGTATACGCTACACGAGGTATACCCGCAAGCCGGATGCGGATCTCAAGCCCTCCAGCACCGTCCCATGTGCACGAGGTGGGTGCAGCACTCACTCCCTTGATATTAATGCTTCTGATAAAAGTACAGAAAACTTAATTGTTGTCGCTTACCGTAAAATGCGGCCGGAGTGAGACAAACGGGCGGAATGCAACAGCATCAATAATTACATGTATTGATGAACACCCCTCTTGCTTAAATACCTGTAGTGGGTCTCGATAACTCGAACACACACGAGGGAAGCTGGCGCTTCCTGTTAACCTTGGTTGTATTGCTTGCTAGCCGGTAGATTTCAATCTGACTAAACTAAGGGAACGTTATGTATAGAGCTCACTAGTAGTCCTCGTACATGTACTTCTATGCCAGCTTGGCCTCACGTTCTGCGCGGCCCAGTGCAGTGGACAAGGCTTGGACTCAACCAACCACTACAATAAGTAGTACTTAAGATTAAAAGGATTATTGAACGTCCTGTTCATATTTAATGTAGTTGAAGCATTCGTGAGATGGCAACATTTCCCAGCGTTTTCTAAACGCGTTGGGTCCATGCCTGCACGCATGTAAGCGCGGTCCATAAGGAGGGATGTCACAGCTGTCAGTCCTGTACTAAACCTCATAAGATTTTTTTCTTTTAAATTAGGAATTTAAGCAAAAAATGTTGTCTCACTATTGTTAGCAAGCTAACCTTCAAGTGGGCCCACGGCCCAAACCTCCACGTCCCATAAAAAATAATATTTGTTGTGTGTTTCCATAAATTTACAGCTCTCCAATTTCCAAAAATAAATAATCTTGTGAGGAAATAGCTTATTGTTMGATAATGTTATTGACAATGCTGTTTTCGGAGGCCCTGCGGGATAAGGTGTACAATCCCTCAATTACCAATGCTAGCGTGGCCTCACGTTCTGTGCGGCCCAGTGCGTTGGACTGCGCCAGCCACTACATGTACAGGCACCCGATTCTGGATAATAGCACTTGAAAAGTAATAAAGTGTAGTGAGTCACAGTGTCCGAGCACAAGTCTTACGGCCTATTTTATATATAGGCCGTAAGACATGTGCAAGCTAGGCTATAAATTCAATGGGTTTATCATAAACTGTAGTATTGTCTAGAAGTTCAGCCATAGAAAAGCATTGACGGGGCGGGTAACCTGCACCAGAATAATACGGTCTCGTATTCAAGTTGCTTTATTTCTTCTGTCGCTACACAAACACTGAAAAATGGGACAAAGAGGGGAGTTCAAAACGAACTTCAGCTGTTGAACTACTTGTAGCTCTCTACTAGCGACAATCGTGTGGTCTTCGGGTGAGGCGTGCGTAAAATTCTACTTCTTCGTGGGTATTGTTTGGCGGTAATTGTCGTAACGCTTGACGATCATACTGACGTCATCGCCCGACTTGCCAAGCGAAGCGAAGTATTCTCGGACTTTGTCGGGGTGCATTTTCTTTCTGACCCAAGAGGGGAACGCAGCTGTAAACAGAAATTCATCGCTTTCGAATTGTTTGAACCACTTGTCAACGGCAGCTTTCCTTTTAGCCGCATCCTGGGCCTTCAGTGCTGCCTTTTGAGACGCCTTAAGTGCTTGTGCGTTTTTAAAGGCGCTTGTACCCGGAACAATCTTCTTAATCGACGCGAAGTTAATTGTCCTTTCTTCTTGGTTGCCGGTTTCCACAGGCTGTGGCACAGCATTCAACCGCAACAATCTTGCTGTTTCGTGAGTACCTTCACTGCTCTTGGGAGGTCCGAGTGCTGCCGCGGAGACTCGTCCATCAGGAATGATCGCAAATGCTACAGCCACCAAAACAAACGCTGCCAGTCGCATAGTTGGTGGAAAAGAGTAGTGGTGGGGTTAGTACCTTCGGGTAGAATTGCGAATGAAGTAAATGAAGGAAAAGGTGGATCACAAAGAGGCGAAACGCTCAAACACCTGGATCGAAAGAGCCAGTAAACAGTCCGTGTGCTGTACCAATTCGTATTACAACTGGCATGACACATCTAAGGATAATACATGTAGTACATAGTATGAACTACATTCAATTGACATTAAATGTGTCGTCTTGATATTAAATAGGCACTCGTTATTTTGCCCGACTGAGGTAACATAGCATCGCATTTTATACGTGTACAAATAGTAGAGTACGGTTAAATTGGAGTAAAGGTAGGACACTAGCGCGTTTGCACTCGTCTGCGGACTTATAGCCGTGGGCCCGCCCCTCTCTTCACTTGCTGCGCTGCGCTCGGGGGGCGCCGCTGCGCGGCCTGCCCCACCCCGGCCTCTAAGTCCTCGACTCGGCAAGGCGCTGTGTCTGGGATTAGAAATTAATTCCGAGCAAGTGTTGCCAAGACCGTCGGCTAAGTACATTTTATGGATGTTGGGGGGTATAATTGTATAGTCGGTTAGCCAGTCGGGCAAAATAGACAGAACATGCCGCCCCCGCGGTTCGCTGCGCTCGCAATGGATAGAATATTCTGGAAAGTGTTGTGCTCGGCTCGCTGGTGAGCTCAGCGCTGGGCCCGCCTCGCACCCGCTCACCTTAGTTGGCGCCGCGGCTATGGCAACCACGTCGCCCAGCCCCGTGTGTGTTGTCCCAGCGATGGACGTGTGTTTATCCTATTTTCTCGGCAAAATACTCACTTCCTATTAAATAGCATGAAGAAGTATGAATTCGTATTAATAAATAGCAAGAGCTGGCGACACATCTAAGGATATTCGTAATATCACAATACAAATGGAAATCTCTTCTGAAAATCGTCCAGTACTGTACTTTAATAAACCGGTACAATGTACAGTACTGCACCCCGTTTTCGAGTCCCTGGACCTTTAATAAACTTCCGGATTTGATGATCTAAAATAAAACAAAACTCAGCTGATTAAACTAAAATTTGATTTATTCGCAAAATTTATATAGTACACCCCCGCGAAACGAAGTGCAGTACTGTACCCTGCAGAAGTTCTACTTAGATTGGGAGCTCGAGGAGCCCAAATCTAAAATAGAGGTATTTGATGTATTCAAAAATGATCCAAAGGTCTCGCCTGTAGACCCTCATAAAATTGCAATGTCGTGAGCTGGGCCAAGAGAATGCGGTATGCCCCGCTCAAAAAATTACAACGGAGATGTATTGTAATATATCACGAAGCTATATTACGCGTCAGCTTTGCTAGTCGGTGGCTTAAGGTAGAGTAATACATCGACCACCCTAAATTTGGAGCTTTCCTTGATTTTCTTTTTGCAAAATGCATTTGGGTATATCATATTGGCCCTTTTCTAATTTTGGGTACCTCGAGCTCCATTGTTTTTCCCGTGGATTAAAAGCAGTTTAGGAGAGCCTACCTAATGTCATATCCCTTTCAGTCTGCATTTGCGCATTTCTCTTCCTCACGAATTGGAGCCTCAAGTTGGAGGTTCACCCTCCGCCATGACCTCCAACTGCTCCAGCACCACTTACGAGCACCTGTTCCGCTCTCTAGCGCGTCATTCGCGTCTGTTCCCATTGCGCAGCTCCGATGTAACGTTATTGGCCACCCCCACAGACTTCTACCAGCAGCTAGTGCACAATATACAGCTCGCAGAGACACGAATCTCCATCTCCTCGCTGTATCTCGGCACGGGTCAATTGGAGAGCGATTTGGTGGGCGCACTAGCCACGAGACTAAAAGAACGACCGCATCTCCAGGTGCAGATCGTACTGGACTATTCTCGCGGCCAACGCGGCGGTGTCACGGCCAGTTCCGTCACCATGTTAGCTCCATTATTGAAGCAATTTCCGAACAACGTGGAGCTATTTCTATTCCGCGTACCACAACTCTCAGGACTCAAAGCCAAACTACCTCCGCCATTTAATGAGACGCTGGGCGTCTCTCACGCTAAAGTGTATTTAGTGGACGACACTCTCGTGCTTAGTGGCGCAAATTTGAGCAACGATTACTTCACCAATAGACAGGATCGATACGTGCAATTGACAAACTGCGGGGCGTTGGCACAGTTCTACCATCAGTTCGTACAACTGGTTACCGGTTTTTCGTATAGAGTCAAACTGGAATCGTTGACATCCGCCAAGAGCGACTACAAGTTGTTGGCACCTCAACTGGCTCACGACTCGGAGGCCGCAAAGACGGCAATGCGACGCGATCTCGAGAAGCTAGTGGACCCAAGTCAGTATCAGCAAGATAAAGACGACGCACGCACCGATGCGTGGGCGTTTCCAACGCTGCAATTTACACCAATCTCCATGGACCACGATGAGCGTGTGCTAAGTGAGTTTGTGCGGCAATTACCGCGTGGTTCGCAGCTCCAAATTGCGTCGGGATACTTGAATTTTCCTCCGTTTCTAAGTGGATTATTGGAACACTGTCAAGCTGGTTTAGACGTGATTTCTGCTGCCCCACGAGCGAACGGCTTCTACGACGCACGTGGCGTGAAAGGAGCGTTGCCTATGGCTTATTCTCTGATCGAGCAAGACTTTTTTGAACGCACTCTTAGCCGTAAGTATCCAACAGTGCTACGGGAATTCAACCGACCAAACTGGACTTTTCACGGCAAAGGCATGACGAGTCGAACCACTCCTCGTCGCTGTCGACCCCCTCTTCACTATCACTCGCCCCTCCTCCGTGGCGCGGCGGTCGCACCGCCGCTTTGACGCGCAGGTCGGGCGCCATGGCTTCCTGGCTGCTGCTGATTTGCCCCCGCTGCAAAAATGAGTGGGGGGGGGAAAGTATACGCTACACGAGGTATACCCGCAAGCCGGATGCGGATCTCAAGCCCTCCAGCACCGTCCCATGTGCACGAGGTGGGTGCAGCACTCACTCCCTTGATATTAATGCTTCTGATAAAAGTACAGAAAACTTAATTGTTGTCGCTTACCGTAAAATGCGGCCGGAGTGAGACAAACGGGCGGAATGCAACAGCATCAATAATTACATGTATTGATGAACACCCCTCTTGCTTAAATACCTGTAGTGGGTCTCGATAACTCGAACACACACGAGGGAAGCTGGCGCTTCCTGTTAACCTTGGTTGTATTGCTTGCTAGCCGGTAGATTTCAATCTGACTAAACTAAGGGAACGTTATGTATAGAGCTCACTAGTAGTCCTCGTACATGTACTTCTATGCCAGCTTGGCCTCACGTTCTGCGCGGCCCAGTGCAGTGGACAAGGCTTGGACTCAACCAACCACTACAATAAGTAGTACTTAAGATTAAAAGGATTATTGAACGTCCTGTTCATATTTAATGTAGTTGAAGCATTCGTGAGATGGCAACATTTCCCAGCGTTTTCTAAACGCGTTGGGTCCATGCCTGCACGCATGTAAGCGCGGTCCATAAGGAGGGATGTCACAGCTGTCAGTCCTGTACTAAACCTCATAAGATTTTTTTCTTTTAAATTAGGAATTTAAGCAAAAAATGTTGTCTCACTATTGTTAGCAAGCTAACCTTCAAGTGGGCCCACGGCCCAAACCTCCACGTCCCATAAAAAATAATATTTGTTGTGTGTTTCCATAAATTTACAGCTCTCCAATTTCCAAAAATAAATAATCTTGTGAGGAAATAGCTTATTGTTAGATAATGTTATTGACAATGCTGTTTTCGGAGGCCCTGCGGGATAAGGTGTACAATCCCTCAATTACCAATGCTAGCGTGGCCTCACGTTCTGTGCGGCCCAGTGCGTTGGACTGCGCCAGCCACTACATGTACAGGCACCCGATTCTGGATAATAGCACTTGAAAAGTAATAAAGTGTAGTGAGTCACAGTGTCCGAGCACAAGTCTTACGGCCTATTTTATATATAGGCCGTAAGACATGTGCAAGCTAGGCTATAAATTCAATGGGTTTATCATAAACTGTAGTATTGTCTAGAAGTTCAGCCATAGAAAAGCATTGACGGGGCGGGTAACCTGCACCAGAATAATACGGTCTCGTATTCAAGTTGCTTTATTTCTTCTGTCGCTACACAAACACTGAAAAATGGGACAAAGAGGGGAGTTCAAAACGAACTTCAGCTGTTGAACTACTTGTAGCTCTCTACTAGCGACAATCGTGTGGTCTTCGGGTGAGGCGTGCGTAAAATTCTACTTCTTCGTGGGTATTGTTTGGCGGTAATTGTCGTAACGCTTGACGATCATACTGACGTCATCGCCCGACTTGCCAAGCGAAGCGAAGTATTCTCGGACTTTGTCGGGGTGCATTTTCTTTCTGACCCAAGAGGGGAACGCAGCTGTAAACAGAAATTCATCGCTTTCGAATTGTTTGAACCACTTGTCAACGGCAGCTTTCCTTTTAGCCGCATCCTGGGCCTTCAGTGCTGCCTTTTGAGACGCCTTAAGTGCTTGTGCGTTTTTAAAGGCGCTTGTACCCGGAACAATCTTCTTAATCGACGCGAAGTTAATTGTCCTTTCTTCTTGGTTGCCGGTTTCCACAGGCTGTGGCACAGCATTCAACCGCAACAATCTTGCTGTTTCGTGAGTACCTTCACTGCTCTTGGGAGGTCCGAGTGCTGCCGCGGAGACTCGTCCATCAGGAATGATCGCAAATGCTACAGCCACCAAAACAAACGCTGCCAGTCGCATAGTTGGTGGAAAAGAGTAGTGGTGGGGTTAGTACCTTCGGGTAGAATTGCGAATGAAGTAAATGAAGGAAAAGGTGGATCACAAAGAGGCGAAACGCTCAAACACCTGGATCGAAAGAGCCAGTAAACAGTCCGTGTGCTGTACCAATTCGTATTACAACTGGCATGACACATCTAAGGATAATACATGTAGTACATAGTATGAACTACATTCAATTGACATTAAATGTGTCGTCTTGATATTAAATAGGCACTCGTTATTTTGCCCGACTGAGGTAACATAGCATCGCATTTTATACGTGTACAAATAGTAGAGTACGGTTAAATTGGAGTAAAGGTAGGACACTAGCGCGTTTGCACTCGTCTGCGGACTTATAGCCGTGGGCCCGCCCCTCTCTTCACTTGCTGCGCTGCGCTCGGGGGGCGCCGCTGCGCGGCCTGCCCCACCCCGGCCTCTAAGTCCTCGACTCGGCAAGGCGCTGTGTCTGGGATTAGAAATTAATTCCGAGCAAGTGTTGCCAAGACCGTCGGCTAAGTACATTTTATGGATGTTGGGGGGTATAATTGTATAGTCGGTTAGCCAGTCGGGCAAAATAGACAGAACATGCCGCCCCCGCGGTTCGCTGCGCTCGCAATGGATAGAATATTCTGGAAAGTGTTGTGCTCGGCTCGCTGGTGAGCTCAGCGCTGGGCCCGCCTCGCACCCGCTCACCTTAGTTGGCGCCGCGGCTATGGCAACCACCCCGTGTGTGTTGTCCCAGCGATGGACGTGTGTTTATCCTATTTTCTCGGCAAAATACTCACTTCCTATTAAATAGCATGAAGAAGTATGAATTCGTATTAATAAATAGCAAGAGCTGGCGACACATCTAAGGATATTCGTAATATCACAATACAAATGGAAATCTCTTCTGAAAATCGTCCAGTACTGTACTTTAATAAACCGGTACAATGTACAGTACTGCACCCCGTTTTCGAGTCCCTGGACCTTTAATAAACTTCCGGATTTGATGATCTAAAATAAAACAAAACTCAGCTGATTAAACTAAAATTTGATTTATTCGCAAAATTTATATAGTACACCCCCGCGAAACGAAGTGCAGTACTGTACCCTGCAGAAGTTCTACTTAGATTGGGAGCTCGAGGAGCCCAAATCTAAAATAGAGGTATTTGATGTATTCAAAAATGATCCAAAGGTCTCGCCTGTAGACCCTCATAAAATTGCAATGTCGTGAGCTGGGCCAAGAGAATGCGGTATGCCCCGCTCAAAAAATTACAACGGAGATGTATTGTAATATATCACGAAGCTATATTACGCGTCAGCTTTGCTAGTCGGTGGCTTAAGGTAGAGTAATACATCGACCACCCTAAATTTGGAGCTTTCCTTGATTTTCTTTTTGCAAAATGCATTTGGGTATATCATATTGGCCCTTTTCTAATTTTGGGTACCTCGAGCTCCATTGTTTTTCCCGTGGATTAAAAGCAGTTTAGGAGAGCCTACCTAATGTCATATCCCTTTCAGTCTGCATTTGCGCATTTCTCTTCCTCACGAATTGGAGCCTCAAGTTGGAGGTTCACCCTCCGCCATGACCTCCAACTGCTCCAGCACCACTTACGAGCACCTGTTCCGCTCTCTAGCGCGTCATTCGCGTCTGTTCCCATTGCGCAGCTCCGATGTAACGTTATTGGCCACCCCCACAGACTTCTACCAGCAGCTAGTGCACAATATACAGCTCGCAGAGACACGAATCTCCATCTCCTCGCTGTATCTCGGCACGGGTCAATTGGAGAGCGATTTGGTGGGCGCACTAGCCACGAGACTAAAAGAACGACCGCATCTCCAGGTGCAGATCGTACTGGACTATTCTCGCGGCCAACGCGGCGGTGTCACGGCCAGTTCCGTCACCATGTTAGCTCCATTATTGAAGCAATTTCCGAACAACGTGGAGCTATTTCTATTCCGCGTACCACAACTCTCAGGACTCAAAGCCAAACTACCTCCGCAATTTAATGAGACGCTGGGCGTCTCTCACGCTAAAGTGTATTTAGTGGACGACACTCTCGTGCTTAGTGGCGCAAATTTGAGCAACGATTACTTCACCAATAGACAGGATCGATACGTGCAATTGACAAACTGCGGGGCGTTGGCACAGTTCTACCATCAGTTCGTACAACTGGTTACCGGTTTTTCGTATAGAGTCAAACTGGAATCGTTGACATCCGCCAAGAGCGACTACAAGTTGTTGGCACCTCAACTGGCTCACGACTCGGAGGCCGCAAAGACGGCAATGCGACGCGATCTCGAGAAGCTAGTGGACCCAAGTCAGTATCAGCAAGATAAAGACGACGCACGCACCGATGCGTGGGCGTTTCCAACGCTGCAATTTACACCAATCTCCATGGACCACGATGAGCGTGTGCTAAGTGAGTTTGTGCGGCAATTACCGCGTGGTTCGCAGCTCCAAATTGCGTCGGGATACTTGAATTTTCCTCCGTTTCTAAGTGGATTATTGGAACACTGTCAAGCTGGTTTAGACGTGATTTCTGCTGCCCCACGAGCGAACGGCTTCTACGACGCACGTGGCGTGAAAGGAGCGTTGCCTATGGCTTATTCTCTGATCGAGCAAGACTTTTTTGAACGCACTCTTAGCCGTAAGTATCCAACAGTGCTACGGGAATTCAACCGACCAAACTGGACTTTTCACGGCAAAGGCATGACGAGTCGAACCACTCCTCGTCGCTGTCGACCCCCTCTTCACTATCACTCGCCCCTCCTCCGTGGCGCGGCGGTCGCACCGCCGCTTTGACGCGCAGGTCGGGCGCCATGGCTTCCTGGCTGCTGCTGATTTGCCCCCGCTGCAAAAATGAGTGGGGGGGGGAAAGTATACGCTACACGAGGTATACCCGCAAGCCGGATGCGGATCTCAAGCCCTCCAGCACCGTCCCATGTGCACGAGGTGGGTGCAGCACTCACTCCCTTGATATTAATGCTTCTGATAAAAGTACAGAAAACTTAATTGTTGTCGCTTACCGTAAAATGCGGCCGGAGTGAGACAAACGGGCGGAATGCARCAGCATCAATAATTACATGTATTGATGAACACCCCTCTTGCTTAAATACCTGTAGTGGGTCTCGATAACTCGAACACACACGAGGGAAGCTGGCGCTTCCTGTTAACCTTGGTTGTATTGCTTGCTAGCCGGTAGATTTCAATCTGACTAAACTAAGGGAACGTTATGTATAGAGCTCACTAGTAGTCCTCGTACATGTACTTCTATGCCAGCTTGGCCTCACGTTCTGCGCGGCCCAGTGCAGTGGACAAGGCTTGGACTCAACCAACCACTACAATAAGTAGTACTTAAGATTAAAAGGATTATTGAACGTCCTGTTCATATTTAATGTAGTTGAAGCATTCGTGAGATGGCAACATTTCCCAGCGTTTTCTAAACGCGTTGGGTCCATGCCTGCACGCATGTAAGCGCGGTCCATAAGGAGGGATGTCACAGCTGTCAGTCCTGTACTAAACCTCATAAGATTTTTTTCTTTTAAATTAGGAATTTAAGCAAAAAATGTTGTCTCACTATTGTTAGCAAGCTAACCTTCAAGTGGGCCCACGGCCCAAACCTCCACGTCCCATAAAAAATAATATTTGTTGTGTGTTTCCATAAATTTACAGCTCTCCAATTTCCAAAAATAAATAATCTTGTGAGGAAATAGCTTATTGTTAGATAATGTTATTGACAATGCTGTTTTCGGAGGCCCTGCGGGATAAGGTGTACAATCCCTCAATTACCAATGCTAGCGTGGCCTCACGTTCTGTGCGGCCCAGTGCGTTGGACTGCGCCAGCCACTACATGTACAGGCACCCGATTCTGGATAATAGCACTTGAAAAGTAATAAAGTGTAGTGAGTCACAGTGTCCGAGCACAAGTCTTACGGCCTATTTTATATATAGGCCGTAAGACATGTGCAAGCTAGGCTATAAATTCAATGGGTTTATCATAAACTGTAGTATTGTCTAGAAGTTCAGCCATAGAAAAGCATTGACGGGGCGGGTAACCTGCACCAGAATAATACGGTCTCGTATTCAAGTTGCTTTATTTCTTCTGTCGCTACACAAACACTGAAAAATGGGACAAAGAGGGGAGTTCAAAACGAACTTCAGCTGTTGAACTACTTGTAGCTCTCTACTAGCGACAATCGTGTGGTCTTCGGGTGAGGCGTGCGTAAAATTCTACTTCTTCGTGGGTATTGTTTGGCGGTAATTGTCGTAACGCTTGACGATCATACTGACGTCATCGCCCGACTTGCCAAGCGAAGCGAAGTATTCTCGGACTTTGTCGGGGTGCATTTTCTTTCTGACCCAAGAGGGGAACGCAGCTGTAAACAGAAATTCATCGCTTTCGAATTGTTTGAACCACTTGTCAACGGCAGCTTTCCTTTTAGCCGCATCCTGGGCCTTCAGTGCTGCCTTTTGAGACGCCTTAAGTGCTTGTGCGTTTTTAAAGGCGCTTGTACCCGGAACAATCTTCTTAATCGACGCGAAGTTAATTGTCCTTTCTTCTTGGTTGCCGGTTTCCACAGGCTGTGGCACAGCATTCAACCGCAACAATCTTGCTGTTTCGTGAGTACCTTCACTGCTCTTGGGAGGTCCGAGTGCTGCCGCGGAGACTCGTCCATCAGGAATGATCGCAAATGCTACAGCCACCAAAACAAACGCTGCCAGTCGCATAGTTGGTGGAAAAGAGTAGTGGTGGGGTTAGTACCTTCGGGTAGAATTGCGAATGAAGTAAATGAAGGAAAAGGTGGATCACAAAGAGGCGAAACGCTCAAACACCTGGATCGAAAGAGCCAGTAAACAGTCCGTGTGCTGTACCAATTCGTATTACAACTGGCATGACACATCTAAGGATAATACATGTAGTACATAGTATGAACTACATTCAATTGACATTAAATGTGTCGTCTTGATATTAAATAGGCACTCGCTATTTTGCCCGACTGAGGTAACATAGCATCGCATTTTATACGTGTACAAATAGTAGAGTACGGTTAAATTGGAGTAAAGGTAGGACACTAGCGCGTTTGCACTCGTCTGCGGACTTATAGCCGTGGGCCCGCCCCTCTCTTCACTTGCTGCGCTGCGCTCGGGGGGCGCCGCTGCGCGGCCTGCCCCACCCCGGCCTCTAAGTCCTCGACTCGGCAAGGCGCTGTGTCTGGGATTAGAAATTAATTCCGAGCGAGTGTTGCCAAGACCGTCGGCTAAGGACATTTTATGGATGTTGGGGGGTATAATTGTATAGTCGGTTAGCCAGTCGGGCAAAATAGACAGAACATGCCGCCCCCGCGGTTCGCTGCGCTCGCAATGGATAGAATATTCTGGAAAGTGTTGTGCTCGGCTCGCTGGTGAGCTCAGCGCTGGGCCCGCCTCGCACCCGCTCACCTTAGTTGGCGCCGCGGCTATGGCAACCACGTCGCCCAGCCCCGTGTGTGTTGTCCCAGCGATGGACGTGTGTTTATCCTATTTTCTCGGCAAAATACTCACTTCCTATTAAATAGCATGAAGAAGTATGAATTCGTATTAATAACTGGCGACACATCTAAGGATATTCGTAATATCACAATACAAATGGAAATCTCTTCTGAAAATCGTCCAGTACTGTACTTTAATAAACCGGTACAATGTACAGTACTGCACCCCGTTTTCGAGTCCCTGGACCTTTAATAAACTTCCGGATTTGATGATCTAAAATAAAACAAAACTCAGCTGATTAAACTAAAATTTGATTTATTCGCAAAATTTATATAGTACACCCCCGCGAAACGAAGTGCAGTACTGTACCCTGCAGAAGTTCTACTTAGATTGGGAGCTCGAGGAGCCCAAATCTAAAATAGAGGTATTTGATGTATTCAAAAATGATCCAAAGGTCTCGCCTGTAGACCCTCATAAAATTGCAATGTCGTGAGCTGGGCCAAGAGAATGCGGTATGCCCCGCTCAAAAAATTACAACGGAGATGTATTGTAATATATCACGAAGCTATATTACGCGTCAGCTTTGCTAGTCGGTGGCTTAAGGTAGAGTAATACATCGACCACGAGCTTTCCTTGATTTTCTTTTTGCAAAATGCATTTGGGTACATCATATTGGCCCTTTTCTAATTTTGGGTACCTCGAGCTCCATTGTTTTTCCCGTGGATTAAAAGCAGTTTAGGAGAGCCTACCTAATGTCATATCCCTTTCAGTCTGCATTTGCGCATTTCTCTTCCTCACGAATTGGAGCCTCAAGTTGGAGGTTCACCCTCCGCCATGACCTCCAACTGCTCCAGCACCACTTACGAGCACCTGTTCCGCTCTCTAGCGCGTCATTCGCGTCTGTTCCCATTGCGCAGCTCCGACGTAACGTTATTGGCCACACCCACAGACTTCTACCAGCAGCTAGTGCACAATATACAGCTCGCAGAGACACGAATCTCCATCTCCTCGCTGTATCTCGGCACGGGTCAATTGGAGAGCGATTTGGTGGGCGCACTAGCCACGAGACTAAAAGAACGACCGCATCTCCAAGTGCAGATCGTACTGGACTATTCTCGCGGCCAACGCGGCGGTGTCACGGCCAGTTCCGTCACCATGTTAGCTCCATTATTGAAGCAATTTCCGAACAACGTGGAGCTATTTCTATTCCGCGTACCACAACTCTCAGGACTCAAAGCCAAACTACCTCCGCAATTTAATGAGACGCTGGGCGTCTCTCACGCTAAAGTGTATTTAGTGGACGACACTCTCGTGCTTAGTGGCGCAAATTTGAGCAACGATTACTTCACCAATAGACAGGATCGATACGTGCAATTGACAAACTGTGGGGCGTTGGCACAGTTCTACCATCAGTTCGTACAACTGGTTACCGGTTTTTCGTATAGAGTCAAACTGGAATCGTTGACATCCGCCAAGAGCGACTACAAGTTGTTGGCACCTCAACTGGCTCACGACTCGGAGGCCGCAAAGACGGCAATGCGACGCGATCTCGAGAAGCTAGTGGACCCAAGTCAGTATCAGCAAGATAAAGACGACGCACGCACCGATGCGTGGGCGTTTCCAACGCTGCAATTTACACCAATCTCCATGGACCACGATGAGCGTGTGCTAAGTGAGTTTGTGCGGCAATTACCGCGTGGTTCGCAGCTTCAAATTGCGTCGGGATACTTGAATTTTCCTCCGTTTCTAAGTGAATTATTGGAACACTGTCAAGCTGGTTTAGACGTGATTTCTGCTGCCCCACGAGCGAACGGCTTCTACGACGCACGTGGCGTGAAAGGAGCGTTGCCTATGGCTTATTCTCTGATCGAGCAAGACTTTTTTGAACGCACTCTTAGCCGTGAGTATCCAACAGTGCTACGGGAATTCAACCGACCAAACTGGACTTTTCACGGCAAAGGCATGACGAGTCGAACCACTCCTCGTCGCTGTCGACCCCCTCTTCACTATCACTCGCCCCTCCTCCGTGGCGCGGCGGTCGCACCGCCGCTTTGACGCGCAGGTCGGGCGCCATCTGCTGCTGATTTGCCCCCGCTGCAAAAATGAGTGGGGGGGGGGAAAGTATACGCTACACGAGGTATACCCGCAAGCCGGATGCGGATCTCAAGCCCTCCAGCACCGTCCCATGTGCACGAGGTGGGTGCAGCACTCACTCCCTTGATATTAATGCTTCTGATAAAAGTACAGAAAACTTAATTGTTGTCGCTTACCGTAAAATGCGGCCGGAGTGAGACAAACGGGCGGAATGCAACAGCATCAATAATTACATGTATTGATGAACACCCCTCTTGCTTAAATACCTGTAGTGGGTCTCGATAACTCGAACACACACGAGGGAAGCTGGCGCTTCCTGTTAACCTTGGTTGTATTGCTTGCTAGCCGGTAGATTTCAATCTGACTAAACTAAGGGAACGTTATGTATAGAGCTCGCTAGTAGTCCTCGTACATGTACTTCTATGCCAGCTTGGCCTCACGTTCTGTGCGGCCCAGTGCAGTGGACAAGGCTTGGACTCAACCAACCACTACAATAAGTAGTACTTAAGATTAAAAGGATTATTGAACGTCCTGTTCATATTTAATGTAGTTGAAGCATTCGTGAGATGGCAACATTTCCCAGCGTTTTCTAAACGCGTTGGGTCCATGCCTGCACGCATGTAAGCGCGGTCCATAAGGAGGGATGTCACAGCTGTCAGTCCTGTACTAAACCTCATAAGATTTTTTTCTTTTAAATTAGGAATTTAAGCAAAAAATGTTGTCTCACTATTGTTAGCAAGCTAACCTTCAAGTGGGCCCACGGCCCAAACCTCCACGTCCCATAAAAAATAATATTTGTTGTGTGTTTCCATAAATTTACAGCTCTCCAATTTCCAAAAATAAATAATCTTGTGAGGAAATAGCTTATTGTTCGATAATGTTATTGACAATGCTGTTTTCGGAGGCCCTGCGGGATAAGGTGTACAATCCCTCAATTACCAATGCTAGCGTGGCCTCACGTTCTGTGCGGCCCAGTGCGTTGGACTGCGCCAGCCACTACATGTACAGGCACCCGATTCTGGATAATAGCACTTGAAAAGTAATAAAGTGTAGTGAGTCACAGTGTCCGAGCACAAGTCTTACGGCCTATTTTATATATAGGCCGTAAGACATGTGCAAGCTAGGCTATAAATTCAATGGGTTTATCATAAACTGTAGTATTGTCTAGAAGTTCAGCCATAGAAAAGCATTGACGGGGCGGGTAACCTGCACCAGAATAATACGGTCTCGTATTCAAGTTGCTTTATTTCTTCTGTCGCTACACAAACACTGAAAAATGGGACAAAGAGGGGAGTTCAAAACGAACTTCAGCTGTTGAACTACTTGTAGCTCTCTACTAGCGACAATCGTGTGGTCTTCGGGTGAGGCGTGCGTAAAATTCTACTTCTTCGTGGGTATTGTTTGGCGGTAATTGTCGTAACGCTTGACGATCATACTGACGTCATCGCCCGACTTGCCAAGCGAAGCGAAGTATTCTCGGACTTTGTCGGGGTGCATTTTCTTTCTGACCCAAGAGGGGAACGCAGCTGTAAACAGAAATTCATCGCTTTCGAATTGTTTGAACCACTTGTCAACGGCAGCTTTCCTTTTAGCCGCATCCTGGGCCTTCAGTGCTGCCTTTTGAGACGCCTTAAGTGCTTGTGCGTTTTTAAAGGCGCTTGTACCCGGAACAATCTTCTTAATCGACGCGAAGTTAATTGTCCTTTCTTCTTGGTTGCCGGTTTCCACAGGCTGTGGCACAGCATTCAACCGCAACAATCTTGCTGTTTCGTGAGTACCTTCACTGCTCTTGGGAGGTCCGAGTGCTGCCGCGGAGACTCGTCCATCAGGAATGATCGCAAATGCTACAGCCACCAAAACAAACGCTGCCAGTCGCATAGTTGGTGGAAAAGAGTAGTGGTGGGGTTAGTACCTTCGGGTAGAATTGCGAATGAAGTAAATGAAGGAAAAGGTGGATCACAAAGAGGCGAAACGCTCAAACACCTGGATCGAAAGAGCCAGTAAACAGTCCGTGTGCTGTACCAATTCGTATTACAACTGGCATGACACATCTAAGGATAATACATGTAGTACATAGTATGAACTACATTCAATTGACATTAAATGTGTCGTCTTGATATTAAATAGGCACTCGTTATTTTGCCCGACTGAGGTAACATAGCATCGCATTTTATACGTGTACAAATAGTAGAGTACGGTTAAATTGGAGTAAAGGTAGGACACTAGCGCGTTTGCACTCGTCTGCGGACTTATAGCCGTGGGCCCGCCCCTCTCTTCACTTGCTGCGCTGCGCTCGGGGGGCGCCGCTGCGCGGCCTGCCCCACCCCGGCCTCTAAGTCCTCGACTCGGCAAGGCGCTGTGTCTGGGATTAGAAATTAATTCCGAGCAAGTGTTGCCAAGACCGTCGGCTAAGTACATTTTATGGATGTTGGGGGGTATAATTGTATAGTCGGTTAGCCAGTCGGGCAAAATAGACAGAACATGCCGCCCCCGCGGTTCGCTGCGCTCGCAATGGATAGAATATTCTGGAAAGTGTTGTGCTCGGCTCGCTGGTGAGCTCAGCGCTGGGCCCGCCTCGCACCCGCTCACCTTAGTTGGCGCCGCGGCTATGGCAACCACGTCGCCCAGCCCCGTGTGTGTTGTCCCAGCGATGGACGTGTGTTTATCCTATTTTCTCGGCAAAATACTCACTTCCTATTAAATAGCATGAAGAAGTATGAATTCGTATTAATAAATAGCAAGAGCTGGCGACACATCTAAGGATATTCGTAATATCACAATACAAATGGAAATCTCTTCTGAAAATCGTCCAGTACTGTACTTTAATAAACCGGTACAATGTACAGTACTGCACCCCGTTTTCGAGTCCCTGGACCTTTAATAAACTTCCGGATTTGATGATCTAAAATAAAACAAAACTCAGCTGATTAAACTAAAATTTGATTTATTCGCAAAATTTATATAGTACACCCCCGCGAAACGAAGTGCAGTACTGTACCCTGCAGAAGTTCTACTTAGATTGGGAGCTCGAGGAGCCCAAATCTAAAATAGAGGTATTTGATGTATTCAAAAATGATCCAAAGGTCTCGCCTGTAGACCCTCATAAAATTGCAATGTCGTGAGCTGGGCCAAGAGAATGCGGTATGCCCCGCTCAAAAAATTACAACGGAGATGTATTGTAATATATCACGAAGCTATATTACGCGTCAGCTTTGCTAGTCGGTGGCTTAAGGTAGAGTAATACATCGACCACCCTAAATTTGGAGCTTTCCTTGATTTTCTTTTTGCAAAATGCATTTGGGTACATCATATTGGCCCTTTTCTAATTTTGGGTACCTCGAGCTCCATTGTTTTTCCCGTGGATTAAAAGCAGTTTAGGAGAGCCTACCTAATGTCATATCCCTTTCAGTCTGCATTTGCGCATTTCTCTTCCTCACGAATTGGAGCCTCAAGTTGGAGGTTCACCCTCCGCCATGACCTCCAACTGCTCCAGCTGTTAGACCAGAATGAAGCGTCGAACCCGATTGGGCAACGGAGTGGTGACAGCAGCAGCATCGACGACTAAGACTTCGCAAGATGGCAAAGCGACTTGTTGAAGTTCGACACGAGAGGTGCATTGAAGATTCGTGGCGACAAGCACAAGCGCAGAAGGGAGCGTGCTGGGCGGCCTGACAGGTGAAGCGCCCCTGGCAAGTGCATGCAGTGTAGTTAGTTTTTCTTTTCAGAGGACTCCCAATCAATCACTTTTCAACTCACCTTCTAGTGCGCACTTCCTCTCCTCCACCACTCGACCACCAAGTTTCCTCTGCGGCCAAGTTTCAACAGGTTATGATGTCCTCAAAATCGGCCGCGTCCTACACGACTAAGCTGAAGCTGTTCAACGGGACTGGGTTCCCAGCGTGGGGCGCCCAGGTTAAGCTGGTACTGGAGATCAAGGGGCTGTGGGATGCAATCCAGCTACCCATGCCCACGGAGCAAGATCTGAAGGTCGAGAAGGATCCAGCAGCGGCTGGGTCGGAATCGTCATCGGCGTCCGGGACGCCATCGGCTGCGGCAGCATCAGTGGCTGCTGTGGCACAAGTGAAGGAAGCAGTGGGAGAAACAACAACTCCGTTCCACGACCTCATGATCAGGCAAAAGATGGCCTCATCAATCATCCTCGCGGCGCTCGATGAAAAGATTGCCGCAGAAGTGTACCTGCTGGACCATCCGATGGCAATTTKGAGACACCTTCGCATGACGTACAACGTCAAATGCAGTGCTAGCGTTGGAGCTGCCAAGCGCGAGTATATCGGGCTGTACCTGGACGGTGACAATTCGATGATCGACCACATCAAGAACACGAGGCGTGTGATCGACGAGCTGCAGGAGCAACGTGTGGTTCTGTCTGATGAAGAGAAAAGGCAGAATTTTATGCAGAGTCTGGGACCTGCTTGGAATGGCTTCGTTGGCGTGCTCGAGTCTTGCGCGACATTCGAGCTCATGATCCAGCGTTGCCAGGCTGAAGCGATCCGTCGTGATCAGCAAAAGAATCGACGCTCGACGGCTGGATCGGGCAGTGGTAAAGGTGCTGCTGCGTTCAGTGCAGAACAAACGGCTGGCAAGCAGAAGGCGGGCAAGCCGAAGAAGAAACGTGACATGAGCAGGGTGAAGTGCTACAAGTGTGGCAAGATGGGACACTTCGCTCGAGACTGCAAGACTGATGAATCACCGTCGAAGGCGGAAGCTGCCAGTGTCGCATTCACTGTGGACGACGCGACCGATGACAACAAGAGAGGGTGGATCGTGGACAGCGGAGCCACGAGCCACATGACGGGGCACTTTGACAATCTCATCAATGTGCGTGAGTTGACGGAACCTCGTGTACTGACGGTAGCATCCGGCGACAACTTGGTGGCGACTGCGGTTGGTCAAGCACCGCTGGAAAAGGACGGACGAGAAGTCTGTGTCCTGCAGGAAGTACTGTTCGTCAAGGGACTGGCACGCAACCTGATTTCAGTAGCTGCTGCATCACGTCGCGGTATGACGGTGGAGTTCAAGGGCGCGTCGTGTATCATTCGCTCGCCATCTGGAGTGTCAGTTCTGGCGTCGCGAGAGGGTCAAGCAATGTATGTGGTGGATGCCACTAGTGTAGCTGTTGAGCAGGCGGCGATGATGACGGCCGAAACGCCGCATCTTGAAACCTGGCACAAGCGACTTGGACATCTCAATTCTCGCAGCGTTCACCAACTGCTTGAGGACCTGAAAGTGCCATGGCACGGAACCGTGAATCAATCGTGTCCGACCTGTGTGAGTGGGAAACTCGCGCAGAAACCATTTGCGGTTAAGGGAAGGCGCGTGCTGAAAAACAACCAAATGCTGTTGGCCATCGACTACGTAGGTCCGATGCAGGTCACAGCTCGGGAAGGATATACTGGTTTGGCCAATATCGTTGTGGAGCCCTTTCACTTGGAAATGGCGTACCCGCTGCGAGAGAAGAGCTCTCGTGCTCAGCTGGATGCAGTCAAGGACTGTATTGCAAAGTTGAAGGCCTATGCGCCGGAGTATCGTGTGGCGTTCATTAAGTCCGATAACGCTGCAGAGTACGTGGGTGGCGAGTTTGCTGCGTACTGCAGCAAGTTCGAGATTGTGCAGGAATTCTCGACACCCTACAGTCCCCAACAGAACGGAAAGGTGGAGCGTAGCAATCGCGTCATAGTAGAGATGGCTCGTACGCTGATGCAGGGAGCAAATCTACCCAATGCCTACTGGGCTGATGCAGTCGTGTGTGCGGCGTACATCAGGAACCGCTGCCCGACTAAGGTACTAGACGGTAAAACCCCGATGGAAGCTTTTCTTGGCTCGCCGCCCGACATCAGCAATCTGCGTGTGTTTGGATGCAAGGTGCAGGTTTTGGTGCCGAAGGAGAACAGGAAGAAGCTGGACTCGAAGACTCGGAATGGGATTTTTGTTGGCTACGCTACCGGTGGCGCTTATCTCGTGCACATTCCGGATCGTGGCACTGGAGAGACTATAACAGCGCGCACAGTCGTCTTCTACGAGGATCAGTTCTTACCACCAAGAGATGAAGAAGAAGTTAAAATCTACACGCAGCTCGATCTAGCTCAGCAAGAGTTAGTTAATCGCATGGAGGGTCAACTTCGGTTGGAGGATGTTGAAGAGGAGAAGGAAGACGTGGTCATGAGATCTACTGCCAATCGGTCAATTGACTCTGAAGCAACGGAAACTCCTTGACAACCCCCGTCTGGTCCGGAACCAGTGGGAGTTGTGCGACAGCAAGCTCGTCTTCGAGGAGATCCCCGTGTGGAAAAAGCAATTCCTCTTCCGCGTCGCTCGAGCAGAATCCCGAAGCCCAGTCAGCGACGTCTGAATTATGAGGAAAGTCATCTTTCACTGGAACAAGTGTGCAATATGGTAGAGGAACGACTGGCGCCTGCACAGGAAGATGGTACAAGCATTTCCGATGGCTTTATATATCGTCAACTGACCCCGAGTGACCTAACTAACTGGGCACTAGGTGTGGAACCCATTAACTTGGATGAAGTAAATGCTTCGGAGGATCGGAACGAGTGGGAGCAGGCAATGGACGACGAAGTCCAGAGTCTTGTGGACAATGGAACATTCGTGGAGGCCCAGCTACCATCCGGTCGATCAGCGATAAAGTCGAAGTGGGTGTTTAAGAAGAAAACCAACGCTGACGGAAGTCTGGACAAATACAAGGCGCGTCTTGTGGCAAAAGGTTTCAGCCAGCACGCCGGTGAAGATTACAGTGAAACGTTCAGTCCTGTGATACGACATAGCACACTTCGACTCGTGTTGGTCATTGTGGTAATCAAGCGCATGAAGAGATTGCAGCTGGACGTGAAGACCGCCTTCTTGAACTCCAATCTGGACGAGGAGATCTACCTGGAACCAGCCGAAGGATATCGCAGGTGCAAAGGCTGTGTATGGCGCTTGCTGAAGGCTCTGTATGGGCTAAAACAAGCCTCTAGGTCGTGGTACGAACACTTGTGCGCTTTTCTGAATGCGCAGGGTTTTCGTCAAGGCCAGGCGGATTACTGTCTATTCGTGAAAGGCGCTGCGGAAGACTTGGTGATTGTTCTCATCTACGTTGATGACATCTTGATTTTCGCCATGCGAGACGAAGACATTGTATCTTTCAAGGCAGCAATGGAGGCTGCGTTCGAGGTGAACCATTTCGAGGATGTTAATTTTTTCTTGGGCCTCGAGCTGACCTGGTCACCCAGTGGTGACGCAGTTTGTGTTGGCCAGCGAAAATATTCTCACACAATTCTGGAGCGATTTGGTATGGAGAAGACAAGATCTGCAGCTACACCAATGGAAGAGCGATACCGAGATAAGCTCTTCCTCGACGAGGATATTTGTGAATTCAAGCCTCGACCTGCCATCGGTGCACTGCTGTATTTGTCGGTCATCAGTAGACCAGACTTGGCTACTGCGATTCGCCTACTTGCTCAAGAGACCGAGCGACCTACACGTGCTGTGGAAGACGGGATTAACCGTGTGTTCCGATATCTCAACGGTACGCGTGATTTTGGACTGGAATTCAAGCAACGTGATGAAAGCGGACTTGTCGTGTATTGTGATGCTGCTTTTTCGGTGGAACGTGAGTCGCATTCGTCTACTGGCTTTGCCATATTCTATTGTGGCAACCTCGTGGAGTGGGGCTCGAAGAAACAATCAATGGTGACGCTGTCCTCCACAGAAGCGGAGTATATTGCAATGGGGACAGGCGTTCAGGAGTGTATCGGGCTCAACATGGTGCTGAAGGATTTGAGGATGGAAGCTGCCGAGATTCTTGTGTTCGAAGACAACCAAGGTGCTCAACATCTCGCTGAAAGCAAAGCAGTAACTCAGCGATCGCGTCATATCAACACTAAGTATCACTGGTTGCGACAAAAGGTTAAGGAGCATGAAATCCGCATCCAGTACTGCCCCACATCAGAAATGGTTGCCGACCATTTTACCAAGCCTCTTGGGACCATCAAGTTCAAGTATTTTCGCGATGAACTCGGAGTTCGCCGAGTGGGAGTGTTAGACCAGAATGAAGCGTCGAACCCGATTGGGCAACGGAGTGGTGACAGCAGCAGCATCGACGACTAAGACTTCGCAAGATGGCAAAGCGACTTGTTGAAGTTCGACACGAGAGGTGCATTGAAGATTCGTGGCGACAAGCACAAGCGCAGAAGGGAGCGTGCTGGGCGGCCTGACAGGTGAAGCGCCCCTGGCAAGTGCATGCAGTGTAGTTAGTTTTTCTTTTCAGAGGACTCCCAATCAATCACTTTTCAACTCACCTTCTAGTGCGCACTTCCTCTCCTCCACCACTCGACCACCAACCTTCCTCTGCGGCCAAGTTTCAACACCAGCACCACTTACGAGCACCTGTTCCGCTCTCTAGCGCGTCATTCGCGTCTGTTCCCATTGCGCAGCTCCGATGTAACGTTATTGGCCACCCCCACAGACTTCTACCAGCAGCTAGTGCACAATATACAGCTCGCAGAGACACGAATCTCCATCTCCTCGCTGTATCTCGGCACGGGTCAATTGGAGAGCGATTTGGTGGGCGCACTAGCCACGAGACTAAAAGAACGACCGCATCTCCAGGTGCAGATCGTACTGGACTATTCTCGCGGCCAACGCGGCGGTGTCACGGCCAGTTCCGTCACCATGTTAGCTCCATTATTGAAGCAATTTCCGAACAACGTGGAGCTATTTCTATTCCGCGTACCACAACTCTCAGGACTCAAAGCCAAACTACCTCCGCAATTTAATGAGACGCTGGGCGTCTCTCACGCTAAAGTGTATTTAGTGGACGACACTCTCGTGCTTAGTGGCGCAAATTTGAGCAACGATTACTTCACCAATAGACAGGATCGATACGTGCAATTGACAAACTGCGGGGCGTTGGCACAGTTCTACCATCAGTTCGTACAACTGGTTACCGGTTTTTCGTATAGAGTCAAACTGGAATCGTTGACATCCGCCAAGAGCGACTACAAGTTGTTGGCACCTCAACTGGCTCACGACTCGGAGGCCGCAAAGACGGCAATGCGACGCGATCTCGAGAAGCTAGTGGACCCAAGTCAGTATCAGCAAGATAAAGACGACGCACGCACCGATGCGTGGGCGTTTCCAACGCTGCAATTTACACCAATCTCCATGGACCACGATGAGCGTGTGCTAAGTGAGTTTGTGCGGCAATTACCGCGTGGTTCGCAGCTCCAAATTGCGTCGGGATACTTGAATTTTCCTCCGTTTCTAAGTGAATTATTGGAACACTGTCAAGCTGGTTTAGACGTGATTTCTGCTGCCCCACGAGCGAACGGCTTCTACGACGCACGTGGCGTGAAAGGAGCGTTGCCTATGGCTTATTCTCTGATCGAGCAAGACTTTTTTGAACGCACTCTTAGCCGTGAGTATCCAACAGTGCTACGGGAATTCAACCGACCAAACTGGACTTTTCACGGCAAAGGCATGACGAGTCGAACCACTCCTCGTCGCTGTCGACCCCCTCTTCACTATCACTCGCCCCTCCTCCGTGGTGCGGCGGTCGCACCGCCGCTTTGACGCGCAGGTCGGGCGCCATCTGCTGCTGATTTGCCCCCGCTGCAAAAATGAGTGGGGGGGGGGAAAGTATACGCTACACGAGGTATACCCGCAAGCCGGATGCGGATCTCAAGCCCTCCAGCACCGTCCCATGTGCACGAGGTGGGTGCAGCACTCACTCCCTTGATATTAATGCTTCTGATAAAAGTACAGAAAACTTAATTGTTGTCGCTTACCGTAAAATGCGGCCGGAGTGAGACAAACGGGCGGAATGCAACAGCATCAATAATTACATGTATTGATGAACACCCCTCTTGCTTAAATACCTGTAGTGGGTCTCGATAACTCGAACACACACGAGGGAAGCTGGCGCTTCCTGTTAACCTTGGTTGTATTGCTTGCTAGCCGGTAGATTTCAATCTGACTAAACTAAGGGAACGTTATGTATAGAGCTCACTAGTAGTCCTCGTACATGTACTTCTATGCCAGCTTGGCCTCACGTTCTGTGCGGCCCAGTGCAGTGGACAAGGCTTGGACTCAACCAACCACTACAATAAGTAGTACTTAAGATTAAAAGGATTATTGAACGTCCTGTTCATATTTAATGTAGTTGAAGCATTCGTGAGATGGCAACATTTCCCAGCGTTTTCTAAACGCGTTGGGTCCATGCCTGCACGCATGTAAGCGCGGTCCATAAGGAGGGATGTCACAGCTGTCAGTCCTGTACTAAACCTCATAAGATTTTTTTCTTTTAAATTAGGAATTTAAGCAAAAAATGTTGTCTCACTATTGTTAGCAAGCTAACCTTCAAGTGGGCCCACGGCCCAAACCTCCACGTCCCATAAAAAATAATATTTGTTGTGTGTTTCCATAAATTTACAGCTCTCCAATTTCCAAAAATAAATAATCTTGTGAGGAAATAGCTTATTGTTCGATAATGTTATTGACAATGCTGTTTTCGGAGGCCCTGCGGGATAAGGTGTACAATCCCTCAATTACCAATGCTAGCGTGGCCTCACGTTCTGTGCGGCCCAGTGCGTTGGACTGCGCCAGCCACTACATGTACAGGCACCCGATTCTGGATAATAGCACTTGAAAAGTAATAAAGTGTAGTGAGTCACAGTGTCCGAGCACAAGTCTTACGGCCTATTTTATATATAGGCCGTAAGACATGTGCAAGCTAGGCTATAAATTCAATGGGTTTATCATAAACTGTAGTATTGTCTAGAAGTTCAGCCATAGAAAAGCATTGACGGGGCGGGTAACCTGCACCAGAATAATACGGTCTCGTATTCAAGTTGCTTTATTTCTTCTGTCGCTACACAAACACTGAAAAATGGGACAAAGAGGGGAGTTCAAAACGAACTTCAGCTGTTGAACTACTTGTAGCTCTCTACTAGCGACAATCGTGTGGTCTTCGGGTGAGGCGTGCGTAAAATTCTACTTCTTCGTGGGTATTGTTTGGCGGTAATTGTCGTAACGCTTGACGATCATACTGACGTCATCGCCCGACTTGCCAAGCGAAGCGAAGTATTCTCGGACTTTGTCGGGGTGCATTTTCTTTCCGGCCCAAGAGGGGAGCGCAGCTGTAAACAGGAATTCATCGCTTCCGAACTGTTTGAACCACTTGTCAACGGCAGCTTTCCTTTTAGCCGCATCCTGGGCCTTCAGTGCTGCCTTTTGAGACGCCTTAAGTGCTTGTGCGTTTTTAAAGGCGCTTGTGCCCGGAACAATCTTCTTAATCGACGCGAAGTTAATTGTCCTTTCTTCTTGGTTGCCGGTTTCCACAGGCTGTGGCACAGCATTCAACCGCAACAATCTTGCTGTTTCGTGAGTACCTTCACTGCTCTTGGGAGGTCCGAGTGCTGCCGCGGAGACTCGTCCATCAGGAATGATCGCAAATGCTACAGCCACCAAAACAAAAGCTGCCAGTCGCATAGTTGGTGGAAAAGAGTAGTGGTGGGGTTAGTACCTTCGGGTAGAATTGCGAATGAAGTAAATAAAGGAAAAGGTGGATCACAAAGAGGCGAAACGCTCAAGCACCTGGATCGAAAGAGCCAGTAAACAGTCCGTGTGCTGTACCAATTCGTATTACAAACATCTAAGGATAATACATGTAGTACATAGTATGTACTGCATTCAATAGACATTTAATATCCTTCGATGTGTCGTCTTGATATTAAATAGGCACTCGTTATTTTGCCCGACTGAGGTAACATAGCATCGCATTTTATACGTGTACAAATAGTATAGTACGGTTAAATTGGGGTAAAGGTAGGACACTAGCGCGTTTACACTCGTCTGCGGACTTATAGCCGTGGGCCCGCCCCTCTCTTCACTTGCTGCGCTGCGCTCGGGGGGCGCCGCTGCGCGGCCTGCCCCACCCCGGCCTCTAAGTCCTCGACTCGGCAATGCGCTGTGTCTGGGATTAGAAATTAATTCCGAGCGAGTGTTGCCAAGACCGTCGCTAAGGCCATTTTATGCATGTTGTGGGGGTATAATTGTATAGTCGGTTAGCCAGTCGGGCAAAATCCTCACTTCCTATTAAATAGCATGAAGAAGTATGTATTCGTATTAGTTACTACTAGTAATAGCAAGGGCTTGCGACACATCTAAGGATATTCGTAATATCATAATAAAAATGCAAACCTCTTCTGAAAATCGTCCAGTACTGTACGATAATAAACCAGTACAATGTACAGTACTGCATTCCGTTTCCGAGTCCTTGGACCTTTGATAAACTTCCGGATTTGATGATCCAAAATAAAACAAAACTCAGCTGATTAAGCTAAAATGTTATTTATTCGCAAAATTTATATAATACACCCCCGCGAAAACGGAGTGCAGTACTGTACATGTCTTTGCCCTGCAGAAGTTCTACTTAGATTGGGAGCTCGAGGAGCCCAAATTTAAAAGGGAGTGAGTGCTGCACCCACCTCGTGCACATGGGACAGTGCTGGAGGGCTTGAGATCCGCATCCGGCTTGCGGGTATACCTCGTGTAGCGTATACTTCCCCCCCCACTCATTTTTGCAGCGGGGGCAAATCAGCAGCAGCCAGGAAGCCATGGCGCCCGACCTGCGCGTCAAAGCGGCGGTGCGACCGCCGCGCCACGGAGGAGGGGCGAGTGATAGTGAAGAGGGGGTCGACAGCGACGAGGAGTGGTTCGACTCGTCAGCGGAGTCGTCGGGTACCGGCAGCTCCACATCCAAGCAGCGTCGATCGGATGTTTCCGACAGTCTCCAGACAATCCAGAGCGGTGAGCAGCCAGTGGTGATAGCTCCGTCACTCGAGAAGAAAGAGTTTAATTCCTGGGGTGCCTTAGACGCGTATCTAAAAGTCTATTCAGCCGAGACATACCAGGTAGCGTTTGATGTACTGCATATGGTAGCAGGAGCCGTATCGCATTATCTAACTGGTTTGTGCGTACAATTGCTGCGTACAGAGCTTCCGCGTTCGCACTAACAACAAGGTCGTGACGAGAAATAAGAAGATTCGAGACTCCGGGTCTACGAAGCCGCTTGTGCCCGAGGGGTGGACTCATTATTCGAAGACTTTCGTCTGCACCCACGCAGGGAAGTACAAGCCGCGTGGACAAGGCAAGAGAAAGCGGCAAGAGTCGAGAGCTCTCGAGTGTGACGCGCAGGNTGTGTGACGCTCAGTCGGGCTCAGTCGGGCTCAGTCGGGCTCAGTTGGGCTCAGTTGGGCTCAGTTGGGCTCGGATGGACTGAAATGGATTTGAATGGTGCTGTTGGTTGGCCATGCGTGTGCTCACGTACACTTGTATCACTACGTAGATCAATGCCTGCGTACAAGTGACCGACTCGGCTGCAGCAGTTCCCACTTTTGTGCTGCGAATTACTGTTGCCCGTTTGGAGCATAACCATCCGCTCTCCAGACATACTTTCGACCATTATCCACACAGCCGGACAGCTGTTAAGTCTGAGCTGGCCGGGACTGTTAGCGAGCTTGTCAAAGCCGGAGCCAAGAAGAAACGCATCCTGCAGTTCATCCACGAGAATTCTAGCTGCTACCCAACGAGCCAGGACGTACACAATCTGGTGCGCAAGCTGAAGAAACAGACCCATACAGCTCAGACATCTGCAAAACGATTGAGGCAATGGATGACTGAGCTCACGCAAGAGCTAGGGAATGTTGGAGGCATTTTTGTCGACTCAATCCATGATAAGGTATGAACACTATTGCTGTTACTGTGTAGCCGTCGCTTGTTTAACTTTCTATGCTGATGTTAGACTGTAGCTACATGTATAACACTGCAGACAAAGCATATGCGGGAGCTCTTCGACCATTTCCCAGAGGTCGTGATGATCGACGCCACGCACGGTACAAACTTGTCAAAGTACAAGGTTTTCTCGATTATGGCACACGACGCATTTGGGAAGGGCCAGTTCGTACAGCATGCTGTTCTCCAGAACGAGCGTAACCAGACGCTTCTGACGGCCCTGGAGCAGTTCAAGCGCTACAATCCGGCGTGGACGCACATCAAATGCATACTGATCGACAAAGATTTCGGCGAGATCGGCGTGTTGAAGAAAACGTTTCCGGACGCGGCGTTGCTGTTGTGCCGGTTTCACGTGTTGAAATATCTGCGAGAGCAGATAGCATCAAAAGACTATGGGTTCAATGCTTGGCAGAAGCAGCAACTGAACGGATTGATCAACTTGCTACCATCTGAGAAAGCTTAGGTCTGGGGATGTTGTGTTGGGCACTGGTCAGACGGAGATGGGCACGGATGGTGTCCAGTTGGGCATCAGTCGGGCGGAGTTGGGCGCGGATGGCGAACCGCGTGAAGCGT

>Contig_34

TGAAGCGGCTTCTTTTACTTCTCGGACAATCTGACCCAAAGTCCAGCAGTCTGTGCGGGCGAGGTGCTTGCGAATATGACCAAAGCCACGGTCACATGAATTCTTAGTGTGTCCTTTCACGAAGAACTTATAATCCACGCTCTCAAAAACCTCCATGTGGGCTTGTGCTAGCAGAAACTTGATCACGTAGTTATTTTTATTCTGGCCAGAGCAGTTGTCAGCGTATATCACCAATCGTTTTTTGCCAGCAGGAACCAACACATTGTCGATAAAGTGCTGCAGCGTGGAGCTGATTTGCTCGCTTCCCTTCCCACCGACAGACTCATCGTAGATGTAGTTCGGTTGCTTGCCTTCATTCTCGTAGTATATCCCAAACACACTGACAGAGACCAACGAGCAGAAGTACCATTGCGACGGCGTAGATGTGACGGACGGAGCAGTCAGATTCTGAGAGTAATCCATAACGATTACTGCGACGTCCTTGCCCTCTGTTGTCTTTACGAGAGCCTTATCCCTGTTATATTCCTGTCTACAAATACACCAGCAAAATAAAATGATATTAGTGTCCACCTTGGAAATTGCTAGTAAAAAAAATGATACTCATATCCCAGATCCAAAATTACCTCGAATCCTGATTTCCTCCACGTATGTGTGCATTTCATCATAGATCGCATCCCACGTGAGCGTCGGCGGTAGCAAAGTGTACATCTCAGAGCTGTATTACTTTTTCACTGTGCCATTCGCCGTCTTCTGCAGCCGAACACGCACTGGTACTACTTCCCCGACCTCCGCAGAGAACTCTTTAAACCACTTCACCATCCACACGAGATCGATGACAGAAGCGTGCTTGTTCAGTGTGTTCCCGTGCGCCTTAGCTGCAATGTTTCCTTCGCGCACACGCTTCTTGTATCGCTGGATAGTCAAAGGAGTCACCCCGAGACATTCTGCAAACATGGTCCGGCAAACTCTCCCAACCAGTGGCAGGTAGTAGTGAAATTTCTCGCGTTTTCCGTCTCCTCGGTGCCTCTCCACAGTATCCGTCTGCATGAGAACGCCCAGCATCGTGTCAACTTCTCTGATCTGGTCATCTGTGAGATTGAACACACGATCCACTCAAGTTCTTGGGCTTTTCCCTGAAGACATCGGCGATCACACTTGTCGGCCTGAATTAGACCCGCCACTGTTTGTTGCAGACTCACCTCAATCAAATTGATACTTATGTCCTCCTCCTCTTCAGCTGCGTCGCTTACACCACTCTCACCAGTGCTTGCCTCCTCGTCTTCCTCTTCATCTTCATTGTCGTCACTCCAATCTTCGTCGTCACTGTCGTCAGCTTCCTCATCGCTCTCCTGTTCGTCTTCACTCCCTTCATCTGCTTCATCGCTGGCAGGCGACCCTTCCTGCTGGGGTACTGTAGCGGAGGGCAAAGCGACACGAGGAGCAGTAGGTTGAGCGATACTTGTTAGGTCGGCGGTGGCCGTGTTTGTGTGCGCCCTGGTGTCGGCTGGTTGCTTCGTGGCACGGTGGGGGCCGTGCTCTCCCGCATTGACCAGGACAGGGGGAGCAGGGGGCGGCACTGGAACAGCGACGGCAGGAGCGGGTGCCGTCGTGTTGCTTTCGGTCTCGGGGCACTGCATTCTCGAGGCTGGAGTGCGCTGTTCGGGAATGATACACCTGCTTCAAATTTCTAAAAAACTTTGACGCTTGTTTTTTCAGCTACTCGATAAAGTGCGAAAACACGTGGGCGATCTTTAGACGCGAATATCTCGATAACTCCAAATGGTTGATTAGTATCATTATGTCTCGCGCAGTCTCATATTCGGTCCTTTACAAAAAACTATGTAAAATAAAAATGTGTGATTACGGCTATTGCTCGACGTTGTTGCCACAATACCGGTTTCCCAAGCGAACGGCCGTGTGCGCAGCTTGTTGTAGGTTTTGGGTTGCTACACAAAATTAGTAGCTAAAACTTAAGATTTAGGTAAATTCACTGATACTTAAGTTGTTTATTTTAGCCAATAAAAAAGTGGACACGGCGGAATTCGAACCCGCGTCCAGTATTCTCGTGATCAAACCAACTGCCACATCCAGCAGCTAACCGGAATAGTTTGTTGAGCATCCGAGGTGACGGYCCCGGCCTACTTCAACGACTCGCAGCGTCAGGCGACTACAGATGCTGGAGCTATTGCTGGTTTGAACGTGCTGCGTATCATTAATGAGCCGACGGCTGCTGCGATCGCCTATGGGCTCGACAAGAAAGGAGGCGAGCACAACGTGCTGATCTTCGATCTCGGAGGCGGCACGTTCGACGTCTCACTGCTAACTATTGAAGACGGGATCTTCGAGGTCAAGGCGACGGCGGGTGACACGCACTTGGGCGGTGAGGACTTCGACAACCGTCTGGTCGAGTTCTTCGTGACCGAGTTCAAGCGTAAGCACCGCAAGGATATGACGTCCAACCAGCGAGCTCTGCGTCGTCTTCGTACGGCGTGCGAACGCGCCAAGCGGACGCTGTTCACTTCGGCGCAGGCGTACATCGAGATCGACTCGCTGTATGACGGCGTGGACTTCAACTCGACCATCACGCGCGCGCGGTTCGAAGACTTGTGCAGCGATTATTTCCGCAAGACCATGGATCCTGTTGCGCAGGTTTTACGAGACTCTAAACTGTCCAAGAACCAGGTGAACGAGATCGTCCTGGTCGGAGGCTCGACGCGCATCCCGAAGGTGCAGCAGTTGCTGAAGGATTTCTTCAACGGGTAGGAGCTGTTCAAGTCCATCAACCCGGACGAAGCCGTCGCGTACGGCGCCACCGTGCAGGCTGCGATTCTGAGTGGCAACGAGTCGTCTTCGAAGCTGCAGGACCTGTTGCTACTGGACGTCGCGCCGTTGTCGCTGGGCCTGGAGACAGCCGGTGGCGTCATGACTAAGCTCATCTTCGTCAGCAGCCACTTCGAGTGCCTTAAACTTCATCATTGGCGAGGTGACAGGACAGCCGGCTTTGCGCATTGAATTTATCCACAAGACGATAGCACTCTCAGCCTCACTGGAAAGTATCGTTCCTTGTTCACTACACCGAAGATTCTTGTGATGGCCATGTCCACCTTCGCAATCGGCTTGTATCTGCGCTGCCTGCTTTTTCCACTTCGATATCTGATTTTGCTTGCGCTTTCTGTCTGAGCTAGATAAGGGCGGAAAGAGGTGAGCTATTGCTTCATTCACAGTATGCTTAGATTCCATATATTGTAGTATGGTACGCTTATGAGCATAGTCTACAGCAATACGAGTAAACCTCTTTGGCCTTCTGCCTGTTCCAGATGAGCGTGGCCTCCCCCTGTCCTAAGCGAAGTGCGAATTTTTATTTTAGCCAATCACATTTCTTCATTTAAGAATTATGTCCACAGCTAAAAATACTCTCACAACCAGTTAATTATGAGATTGTTCCTGCCACACATTTGGCTTTGTCACATGCTGAACACACCATAGGGAAGGCCGAAAGAGAAAAACAAGTACGAGCAAATCCTAAGTCAAATCACTATGTATATGAGATTCGTCAAAACCTCTAGAGAATTATTCAAATCGTCCATTAAGATCTTCTAAAAGAAAAATAGAAAATCCAGACTCGGTCAAATTTAATATCCTTCTCTCCCCAATAAATTAATAAATATTGCTATCATGACCACAAGAGTCCTAATCTGGAAATCGAGTAGCTTAGTTTGTGCGTCGCAAAATTTAAATGAGTGGTGTTTATTTTTCTCGCATTTGATGTATGGTTAAAACAGATATACGAAAAACGAATTGTTTAACAGAGATAAACGCGACATACAGCAATCAGTTTAAAAACCGTGTGGAAACTTTGGTTTCGTTTCTTGAAATATTAAAAATTCTATCGCCCATGTTTTTGAAAGAAAAACCAACCAAATCAAATTTTGTCATCACAATTCGAGTCAAACGCTCGCCGACGGCGCACCATCTTCGACAAGCGCTCATGTAGCCTGGTGTTTGTACTGTATTACCCCTTCATATCTGGGATACAAATGTTGGAGGGATGATTTGCGCAGAGTTGTTAGGGCAGTACAGAACGGAGTGAGGACAAAAGTAGGCAGCCATAGTGGAGAGGAAGTCTTGAGTGTAGAAGGAGAAGACGCTTGTGTTGGACTTGGTAAAAGGAGAAGACACTTGTGTTGGACTTGGTAAAAGAAGAGGTGATGAATTATAGAGTCTGGTGTTCTGGTGGGAGTTACTCTCCACCAACCAGTCCAGTTATGTACGATTTCTCTACATGAAATAGTACAGTTATATTCCACTAGATAGTTATCATCACATCGGAGATGCTTACTTCGACAACCGATAATCTAAAGTGCACTGGTGCAGAAAATTGACAATTACAAAGTTAACAAATAAAGGCACGAGTTGCAGTGCCCGGCTAAGAATCAACGACCTTAGTAAACCCAAGACAACGGCTCATCACCATCACAATCCTTAAGATAAAGAGGACGGAAGTCACTTGCATCTTTATGGTTTGCTCCGCTACGCGTGGCAAATGCAGAGCTGCGCTCCTCCGATAAAAAGATTCTCTTAGCTGGAGAGTGCACCGCTCGACTCGCTGGAAAGCTTGGTGCTGGGCCCGCCTCCGGNAAAAAACTCACGGCCTTGCTTCGCGGGCACTCCGCGCGGGTTTGCTGCGGCTACAGTTGGTCTTCTTCGCTTTGATTTTAACTCATTTTCACCCCCTCCTCCCGCAGCCGGGCACGATAGTCACTTTCAATAATAAATGCATTCTGGAGGTTGCACCCGGACATGAAAAAACATGTGCTGGGGAGTGCAATGGGTCCGTTTTCTGAGCTGAAAAGTATTAGGAAAGGCGCGGCGCTCCCTAGATTATCTACAGTATCTATTAGATTACCGGTACAGTACATGTANCCCCCTTTATCTGGCGTTTGACAAATCATGGTTCCCTTTCTTACGGTACTGAGTACCGGTACCGGTACAAGAAAACTATACGGATTACGGTGGTGGAAGGTACAATCAGCCTGCAAGAAATGAGGACCAGTAGTCTACTGGCAGTAGACAAATTTTCACAAAGCTGCTAGACCCAGGGTCACGAATATGCACGCGTACTCGGCGGCTGTACTGATGGGTCTCCTAATGGTTGCCGAGGGTGCCTATGTATCTACGACCGCTTGTGCACGCTCGGCGCCGAATCCCACGATCCAACGCTATGAGCCTGAAAGCATCCCAACCAGATTGGCTCCCAATAGGCTACTGAGAGAACCTGAGACAACCGAAGCGAGCAACGAGGACCGAGTTGTAAGTATCCATGCTGGCATTGAAAAACTCTCGGACCTGATAAAGACTGGAGTATCAAAGGTTCATGGATACCTGAACTTGGGACCGTCAGCAACCAGAGACCAACCGGCAGAKGAAATTCTTCGCATCTACAAGCTAGATGATGGAATCGAAAAGGCTCTGGTCAGTCCAAACTTGAAGGCTATGGAAAGCCACGTGAAGGAGTTAAGCACGAAAAACCGAAAGAGCGAAGCGTCGGTGATTGGAATTCTCACGAGCCATTACGGGGACGATGCAGTGGCGAAAGCCCTTGTGACTGCACAGAAAACTGTACAATCGGATGATGATGTGAAAACGATATGGCGATTGCGAAACGCGCAGCTTTCCAGCTGGTTTAGCAGCGATAAATCTGTCGATGATGTGTTCACTTTGTTGAAGCTTCGACATGATGATTACTTAGCCCTTGCCAGTCCGAAGATGGAGGTCTTGGATGATTATATGAAGCTGATCAACCGCGTGACATCAGGTCAAGAGACTTTGCTAAACGTGTTAACAAAGGGCTTCGGTGGAGAACAGACCATGGCAAAACTACTTCTTCGTGGGAAGGAAGAGCCCCAGACCCGCGAGCTGGCGACGGCGTTGCAAAATGCGCTGCTGAACAAGTGGGTCACTGATAAATTTCAACCGGAAAGTGTCTTGAAGAAGCTCAAACTGGACCGAGACCTGATGAACGCTCTCTCGGATCCAACCCGACACACTTTGACTAGTTACATCGCGGTCTTCAACACTAGGAATCCAGGGAAAAAGGCATCGTTTATCGGCACACTTTCTGCTCATTACGGAGATGAGATGGTGGCGAATGTACTCATCGCGGCCTCGAGGAACGGCAACACAAGGAGAATGGCCAACCAACTGCGAACCGATCAGTTGTCAGACTGGTTGAACAACCAAAAGTCTGCTGACGAAGTCTTCAGCCTTCTAAAGCTGCGGGCAGATCTTCCGAACATAGATGGAGCTCTCGCCAGTGGCAAATTGAAGTTACTGGAGGACTACATCAAGCTATTTAACCGTGAGAAGGCTGGAGACGAAACGTTGCTCAAAACGTTAACAACCGGCTTCGATGGAGAAAGTAACTTGGCAAAAGCGTTGCTGACTGCTGAGATCAACCCACACTCGAATAAAATGGTTGTTAAGTTACAGGGTGAGCTGTTGAACCAATGGCTCCTGAAGGGATTGAAGCCGGAAAGTGTCCTCAAGAATCTTGGACTGGACCGTGGGATGAAAGAGGTACTATCAGACCCGAATCGGCACTTTTTGACCAAGTACATATGGGAGTACAACTCAAGAAATCGATTCGACAGAACGTCATTGATTTGGACTCTTTCTGCGCATTACGGAGACGACGTGGTAGCGAGAGCTCTTGCTGTTGCGAAGGGCGACTCTGGCCTGGCACGGACGGCTGCAATTCTGCAGAGGCAACAGCTGGAAGGGTGGCTCAGTAGTGGGAAATCTGCAGACGATGTCTTCACGCTACTGAGAATTGGAGCCGACGATTTCTTACCCTTGAATAGCCAGAATTTGGAGACACTGGAAGATTTTGTCTGGCTATTGAACCTCAAAAATCGCCGCATCCAGACGAATATATTCACAGTGGTAGAAAACAAATTCGGTGGAGATGTTCAGCTGGCACGAGCGGTCGTTAAAGCCCTGAATGAAGCAGACGAGAGAGGGCTGCGCGATTCTGTCGGTATTGCGTCAAGATACCGGAGCAAGCTGTTTGGTCGATGGTTCGACAAAAGTATCGAGCCAAAAGATGTTTACGCGATGATCCTCAAAGTAAACGGAGCTTCTGCAGATGCTTTGGAGAAGTCGATTGTTTCCCGATACACGGCATTCTACAAAAAGAGGCTGGCTAAGGCTTTCACCTTCGACAGTCCTAGACGGCTGTAGCTTTGCTTTTTAACCAATTTAAAAAATATCGCCTGTCATGCTCCTATACTTGGAGAAACAATGGAGAGCTCGACGTGCCTTCTTTTAAGAAGTACCTTGTAAGTACCCAAACGGTAATCCAAGGGTCTCTATGTAGATCCTGCAATCATGCTAACCGGGCTAATAGAATACACTATGCATAAGGCGTTCCGCCAAAATGCCAGGAAGTTAACCAGATCGATGCACTGTCAAAGCTACATAGCTACTATTGCTTGTAGTTGTCGCTCAGCCACACAAGTACTTTCGTCAATTGACACATTTAACTTTTCATGTGTCCTCGTTCAATTGCACGGGCTCGTACACTGAGGCTAGACCACATTCATGAACGCCTGCATTTGCGCTAGAGTGAGCTCCTGTTGCTTCATTTATACGTTATTATGCTACTTTATGCCGCCGACTAGCAAAGCTGAGGCGTAATAATAAATATGGCAACAACACAATACATAGCACATATAAAGTGCTTTTTTCGTGTATGCCTAGAGCTCTGTACTACATCATGAGGTTGCTTGTGCAGATAGCGCTTGTCCACAGTCTTCACCAAATCCTACTAGTCACTACATGTATTCCAAAGCAATCTCTTACAAAAAACTATGGTGATTGCGGAAACAATCGACGAAAACAAAAGAGTTGAGTTTTACGGTGATGTATATAGTAAACACAATTGCGAATACATGAGGTAGATAGGTACTGTAGTACTTGACAAAGTTACTCCAAGCCCCACAGTTCGACAGCCAGCTTCACTCTACCCATGCGACTCTCCTCGACTGGGCTGCTAGCAATTACAGCTCTTGTTGCATCTAGCGGCATTGCCTCGATGTCTACTATCGCCAACATGGCGGTGCCTGACAAACTGTATAATCTGTCTAGTACAATTTCCACCAGGTCGTTGAGAGATTCTGAGCCAAAAGGCGGAAGAAACGAGGACCGAATGAATGCCGGTATCGAAAAACTCTGGGGCCTGATCAAAGCGGGGACATCCAAGATCAAGGAGTACGGGCAGTTCGGGACACTAGAGTGGTTTAGCAGAAAGCTACGAACGTGTGACGCCCGGTTTTTTTTGTGAGGATTGCCAGAGACGAGCGTCACACGTTCGTTCAAGTGATGTAGCTACGACTGATAAGCTTTCTACAAGTCGTATACAAATGTATATCTATGCACTTTAACATCCGAATACTGTATACTTGAGTACATTTTGTGCCCATTATAGCACTGCCGTTGGTTGCGCACGGGTCGGGCGATGCCACGCACCGTGCTATCAATGAAGCCGATACACCTGTCGATAGGACTGCCGCGTGCAAGAAGCTTGCTGCACTTAGCGTAGATTAGGTCCACCATGAAGCGAAAGATCAGGCACAGCGCACTGGGCGACCTGCCGAACATCACAGCAAGATCCCCGTACCGTCCAGGGTACGCGAGCTGGCGCAGCAACACACAGAACCCCTCGACTTCACCAACGCGAAGGCGGATCGGTAGCTTGTAGTCTTCCTCAAGCCGCAGCGCCTTGTGCAGCCGAATTACGTCACGCTTCCAGAATCGGAAATTAAAACGACAGTCTCCGTCGCTTAATGCGTTGAAATTGAAGTTGCCCTTCAGTGTGAGGACTGGTCGTAGAATAGGAGATCTGGCGAAGAGCACAGCGGCGTCCTCGACGAGGACGAGGTTCAGAGCGGTCGCTGCGAGGACTAAGCTCTCCTCGAGCATCTTGACTGGCTTGTGTGCTTTGCAAATGTAGTATACACCAAGGACGGATAAGAGCTCGCGTTCGCTCTGCAAAATGCTTCGCTGGCAAACCAACGTGTGCGGCAGTGTGATTAGGGTTGTGTGATTGGCGCTAGTCTACAACTCACACGAGTTCCTGCTGCTAAACCACTCTACTGGGATCTAAAGACCAACCTGCAGCGGAGATTTTGAAATTTTTCAATCTTGCGGATGGTATGGACAATGCTCTGGCTAGTTCCAACTTGAAAGTTATGGAAAACTACGTGAAGAATCTAAATTCGAAAAACCGAAACAGCAAGGCATCGATGATCGGGGTTTTCACGACGCACTACGGAGACGAAGCTGTGGCGAGCGCTCTAGTGGCTGCGGAGAAAAACGCGAAAACGACAGAAGCAGCTAACACGATAAAGCAGTTGCGAAAGGATCAATTATCAGCTTGGCTGACCAGTAAAGTATCCATCGACGATGTCTTCCATTTGCTCAAATTTCGCGATGATGGCTACGCAGTTCTTGCTAGCTCAAAGATGGAAGTACTGGACGACTACATGAGGCTATTTAATCGAGAGAAATCAGGCCATGAAACTCTGTTGGACGTGCTTACGAAAGGTTACGTTGGGGAAGGATATCTGACGAAGGTGCTTGAGCGTGGGAAGAAAGATGCGAGTACGAGTGAGTTGGCGACTGCGCTGGAGAACGCGCTTTTTAACAAGTGGTCAGTGGAAAATTTACGGTCGGAAGATGTCCTGAAAAAGCTAGGACTCGGCCGCGACATGAAGAAGGTTCTCTTCGATTCAAATCGAGACATTCTAACGAGGTACATTTCCATGTACAATGCGAAGAACGCAGAGAGCATGACGTCGTTGATTCAAACTCTTTCGACCCATTATGGAGACGGCATCGTTGCAGGCACATTGGTGATTGCGTCGTGGGATAAAAACACAGAGACACTCGCGAGACAGCTACGGAGTGACCAACTAACTGAGTGGTTGACGAGTCAGACATCTGTCGCCGAAGTTTTTCCGCGACTAAAGCTCGGGGATGACCGCTACCCGGATCTCATCGATCCAAAGGTGGAGGTACTGGACGACTATATTAAACTGTTTAACCGAGAGAAATCAGGTCAAGAAACGTGCTCAATGCCTTTACAGAGGGCTTCGGTGGAGACGACATTTTTTTGAGAGTGCTGGCGGCTTGGAAGGAAGAACCTCACACCCGCGATCTTGGAACTGCATTGCAGGACACCCTTTTCAAAAAGTGGGTCGCTGATGATTTGCAACCGGTAACTGCACTGAAGAAGCTCAGACTGGATCGTGACATGATGAGCGTATTATCGGATCCAAACCGACACACTTTAGCCAGGTACATATCAATGTACAATGAATGGAATCCGACCAGCAAGGCCTCGTTGGTTGGAGCGCTTTCCTCTCATTATGGAGATGGTGTTGTGGCTTACATCAGGGCATGGGAAATACAATACCCATTGTTATTTTGCGTGAATCACATCCGTAATAAAGACTGCAATACCTGGCAACACAATAATAATCACTGCAATAATCACAATATATTGCTATCACTTAGTCTTCGATGCAATCGAGAAGGGTGGAGCAGTTCCACATACGGCGATTAGCACGAAGAAACATGGCCGTTTCAAAATTAGCTGGTAAAGTAGATGAACGTAGCGATGTAAAAACAGGCTTACACTCAGAGAAAAGTCTCTCGCACGTGTTGCTTGTCGGAGGAGCGCAGAGCAGGAGAGGATCATACTCGACAGACGCCTGCGATGCAAGCCGCGGCTTCTTGGCTTGCCGGAGGATGGAGGTCGCGAAGTCCGCCGCAGTCGGCACTTCCTCTTGTGCGGTAGACGGAGATAAAATAAAACTGTCGACTGCATGTAATTCATCCGCGGAGAGGGGTAGGTCGTTTTGCAGCTTYACTATTGCAGACTCGAAATGGGGCGAGTGAACAATCTCAGCTGTTGGCGCTAAGTAGTTAGCCATGACAGGATACTTAGCAGCACACGCATCAAACAACAGGCGCACMTCGGCAAGCGTGCATGACTCCGCTTGGATTTTCACACACACACTGTCCAGCTCACGAAGCTTGTCCAATACACCTCTAACGCGGCGGTGCGCATGTCCACGAGGCACAAACTCTTCAACAGCCTCCACCGTCAGGATCGYATCACGGATCTTGCAATATCGGTCAATCATCTGGAATGTAGACGACCAGCGCGTAGTGTTTGCCTTAACAGGCTTGTAGTTGGTCGCCTTTGCCAGTGCAGTAGCATTTTTGACATGACGCAGCTGAATCATCAGGTTTTGAATCAGATCTATCTGAGTCTGATAATCCTGTAGAAATCGATTAACCGCGAGGTTAAATCGATGACTTGCACAGCCAATGAGGGGGACGMCCATTCGTGTAGCCATACTCTRATTGGTTGCACAATTGTCCCCCACAACATAGCGCACCATCTGCAGGTTCTTCTCGTACACGCTCAGTACGGTGGATATATGATCCAGGTGGGCTTCAGCTGTCTGGCCGTCTTCCATTGGCGAAAGCGCAAGAAGGCGCTGGTGGCGCACACCATTGTAGACGTAGATCGCGTAGATGCCGAGGAAGTGAAGTGAGTGACTAGTCCAACCGTCGAACATTAAAGCAAAGGAAGTTCCCATCTCCTTTGCAATGATTGCTCCAACGGCTAGAGCCACATGACGCATGTACCGCTTCAGTGTCTTGGTAGTCGTAGGCTTCATTGTAACTACAGCACGCGTAGCCTTGTTGTCCACTTCAGTGATTGGTAGATTTCTCTCGATCACCCATACCATCCATTGGTAGATATTGCGCGTCACATCGTCCACAAAACCAAACGTGGTAAGTGCTGGCGTCTGCGTGGCATGAAGTTCTGCAAACCCAGCAGCATACCCAGCGTGCTTGCTGTTAAGGTGGCTGAGGAGGTTGCTGTACCCCGTGCCGGGCGTCTGCTTCCGAGGAAGCCCACAGGATTTACACTCAAAGCGACCTTCACCGAGGTCGCTGAAGTAAAATGCGCAGAGTTGACGGGAGGTCATCGTGGGTAGCGTGGTGGGTAGCGTGGCTAAGAGTGGTGGCTTGTTCTCTAAAGAGAAAATTGAGAAGTGGGGAGTGTGTGAATCAAAGCGCGCAGAGTGTATTTGCAAGGTAGTAGAGAACTGTTGGTTGTCAGCGCTCCTGGAAATGGTTGGAAGCGTGTAAAATATTGTATTATTGTGTACAGTTATGTATTATATTTATCTTGGAAAAATCAATAGTAAGTTATTCCGTAAATATAATACCCGTAATATTGTTATTATACTATTTTTCCCATGCCCTGTCTACTACGTGGAGAAGTATCGGGCTCACGACACCTACTAGGACGGCAGGACGCCCGCCTTGTTGCAACAAACAATGCTTGACGAAGGCGTGGTAATGCTGCAGCACAAGCTTCGAATCATTCCAGGCTTGGAGGAACATGTCGATAAAGTTGCTGTTTTTGGCGTCTTCCACCAGTCGCAAAACAATGAACAAGTTGTGGCATGAACTTCATGAACCGCCCGAGGAAGTTTTCACAGTTTTACGACTGGCAGAAATCAGAATAGTGGGCAGTGATTCCAAGCTATTCCATTGGTTCAGGTACATTAAACTATTCAGAGATCACAACAAGAAGCATGTATTTTCCGAAGAGCAAACCCTTAATCTCTTATTAAAGGGCAACACCAAGGAGCGATCAGAGGGAAACTTGCGACACTGTTCGAATCGCTCAAGGATCGTATTCCTGATTTGAGGACATTCGTTGGAAATATGCAAATCCGTTGATGCCAGGACTGATTAAAACGGAAGCTAGATTGAAACTTTGTATCGACTAAACTGTCAAATTATGCCGGTGGTCAGCTGAAACACGATCCTCCGTACAAGACTTGGGCTTCACGTTGCCAAAACCAAGGGTGGAGATACGCTGGTGAAAAAAGTGAAGGTACTTCTCAGCAACAACAATCTACGTGGTGCAGTTGCTGCACTTCCGAAATCAGAAAGATCCGACAGTAAAGATGCCTTGAAACTTTAGTTTCTCGACTAACTCATGATTTTGGAATAGCAATTGTCAGAAAGGGGGTTCAAGCCTGGTCCAAAAATATATTCCAGTGACAATGCGACCCTTCGAAATTCCACTTGAAATAAATAATCAAA

>Contig_35

AGATCCTGGCGCGGTTGAGGATGCCTTGCGCAGTCAATGTCGATCTTCCTGTACAGCGGTCGACTGCTTTCCAGTACTATTAATACTCAGGTGGCGTGCTTACGGGTGCGAGCTGCGATAAGACATGGGGACTTGGAGTGGGTTTGGTGAGAGGAAAGATAGCATTCTGTATTGGCGCATTAAGAAGCAGTGGGGATACTTCATGGGGTGAAGATGGTTACGTTCGTGTCCAGCGCAGCTTCTTCGGAGACAGCAAGATGTATGCTGTGTCGAGCTGTACGCGACCTGGCCTACCTTTGACGTCTCGGCAGCCCTACTTAATCTCCAACACCAATACATCCGCTCCTCCCGTTACCTCCACCCCCTTCGTCACAGCTGCAACGCGTTTAAGCTCTCCCGGTCACGACAGTGGCGCCATCAACTAGCCCGGGCAAAGACACACCTTCAGCAACAGAAACCGTGGTGGATCAAACTGCCAGCCCAAGTGCGGCATTGGGCTATGAGCTCCACCATCACGAAGGTTAGCGTTGATCAAGTGACAATCGCCGCATCAGGTTCGATCGCGCAAATGAGGCAGTGAACACGGACGCAGCCGATGACTCACTCGATTCGACGGATACGCCTGCAGCAACTGCGATTATCCAAGTGTTGCTGGTGATGCGTTCTACACCGCAACACGCACGGGCAACTGGGAATATGCTTCCGATACTAGAGATTTTCATAATGGAATTGACCATGCCGTACTTAGAGCTGCTGGCAACGACCGCAGCATTCACTCCCTGTGATAAAGCCGACTGCCCAGCCTCAGTCTTGGCGCCCAAAACGCCACGTCTGACGCTCGACTTCGACGATCTTGATGCTGTCGTTCTCGGGACAGGAGACCGAGATGACCTCGCCGCCAGATTGGCTGGGTGGCTCCCTCCACTTCTGTATGAGCAGTCTCGCGGACCATCAACACAGCGAGCGAGTCGTGAAGACGGTACCATCCGGCGAGGCACCGACGTAGTCGTTCTCTTTGTCCATTGGGGCGACGACCAGCTCTAGCAGCTTGAGTATCATGCCGCGAACACACTGGCGTTGTAAGTGCGGCAGGCGTTGAGCTGAAGGCGCTGTGATTTATTAATATAAGATATGAATTAACGTATCGCCCTTAGTGCGCTTGACATTTAAATCGTAGGGTTGAAACGAGTTATTTCATCCTAGTATTTTGCCCGACTGGCTAACCGACTATACAATTGTACTCCCAACATGCATAAAATGGACTTAGCCGACGGTCTTGGCAACACTATTAATAGCTTGGACTTAATATCTAATCCCAGACACAGCGCCTTGCTAAGTCGAGGACCTGGAGGCTGGGTGGGGCAGGCCGCGCAGCGGCGCCCCCCGAGCGCAGCGCAGCAAGCAAGCAAAGAGAGGGGCGGGCCCACGCTGCAGACGGCGCTAGTGTCCTACCTTTGCTCCAATTTAACCGTACTACACAATCTGTACACGTATAAACGTCGATGATATGTTACCTCAGTCGGGCAAAATAACGAGTGCCTTAATCCTTTCGACATGGGTTTAGGCCAATAGACCGGCCCGGCCATTGGCCTGCGGCCATAGCCCGTGGCCTTTGCTAACACTGCTAGCACTGCTAGGATGGCTCCGACGCCGTCCGTAGGTGTTCATGAAGCTGCACGCTGCGTTTGCATGTACAGAAGTCCCTCAAAGTAAGCGTAGGGGGGACCGAGACACGATCTTAGAGGGTGATAAAACAGATGTTTAAAAAGTACTGATATTTAGTTAAGAAGCGAGGCGATCTCGACAGAATGCCTGCGTGCCGCTAGCAGTTCCGAATGGTTGTGGGCGACATCTCGTCGTAGGCCACCTTACTTCAACAAAGAGCGGTGTAGATATCTAGAGCCAGTGTCAGCCGATGTTTGGGGTCTTTGCGTCCAGAGATCACCTTCCTCTGCGTGAGAGTGTACTTAGCCTCGCTCCGAAAGAAGAACGAGGTCTCATCCACGTTGCAGATCTCGGACCGGGCGTACGAGTCCGTCAGCTTTCGTAGTTTCACACGTCCGGCTTCAGCTGCCTCTTGGTCAACTGACGCAGCTTCGCCGTGCTTCCGCTTCTTGGAGATTCCGTGACGTTCTTGCAATCTATACAGCCAGCCCTTAGAGAGCTTCAACGTGGTGTTCTCGCCATACGCGTTCACGCGAAGAGCAATGGCCTTTATTACCTTGCCTGTGATAGCCTCCACCTTGCCGTCCATGGCCTTAAAGACCTTCATAATGCGAGCGTCCGTCTCCTGGATTTGGGGGCTGACGAGCTGCTTGCGCTGTAGCTGACAGGGCCCCATAGCGGCCCACTTAAACTCCCCAGCGATAACATTGCGAGCGGCATCGCGTGAAAGAGAATTTTCCAGCTTGAACGCACGTGTTGCCCACGCTGCGAGCTGTGTGTTGGACAGTTTGCCTTTGGCGCGCATATCCTTCGCATACTTGCACATGGCGATCTTCTGCTCCATATTAAGGCGGATCCGGCGCGGCATGGGGCTGGCGGTCATGGTCGCTTGGAAGAGTGATAGTGTGCATGGAGGGAGGCGTACGTAGCGGGAGCATGAGTTACCTCGTACATTTAATCTCCATTCTCAGTCTTTCAATGGTTTAGTTAGCGAAGAGGCCGGACCGGTCTGGAAATGATGGGAGATCTTTCCCGCCGCTGCAGTTCATCCAAAATCAGTGCATTTTTGTGATTGGCCAGTTGAACCTATTTGTACCGTAGCAGATATGGCGGGGTATTACGTAAGTAGGTGACGCTTACTTTGAGGGACTTCTGTACTATTATAGGATGAATTGATTCTGTCGCAACATCTTTGTATCAATGACTGTCGCATTAATTTGCAAGTTCTACTTCTGGCGGCCAGTCGTTTTGCAATCCGATTTAAAATCCAGAGCTGTTCTGGGCGATTGCACGCCTTTGTTGCACATAAGTATCCGTATCCATTGCCATGCATCAAGTAGTGTTCATGATTGCAAGACGACGTGAGTCGTTTATGAACAAAGTCGTAAACATAACCATTTTCGGACCAGACTAACCTTTCTTTGCATTTATGTTTACCGGCGTACGTAAGCGTAAAGCATTTTATTAATACCAATGTACTGTACATTTCATTGCACAGTACCGTACCGTATTGTAAACCCTCAGGTTGCACGAAAACACTTTCATTCTTGAATTTGCATCCCGATGCATCTCTTCTTTCTGACAGCCGTAGCTTTCGTCATTACCAGTGTATCTGTCGACGCATCAGTCGCGAAAGATCCACGAGGACACGCTCCCAACAGGACTGAAGTCGATACCGTAAACGCGAGTTCAAGCACGAGGCTTTTACGAAAAAATAGTACTGTTGATCTAGTCGGCGAGGAGAGAGCACCCAGCATCGTAGAAAATATCAAGGCGTTGGTCAAGTCTTCAGCGGTGACTCCAGCGAAGCTTCAGCAATGGCTAGACGAGCGACTACCTGCGGGGCTAGTGTTCAAGAACATGAACCTTGACGAACCAAATATCTTCTCTTTGTTGCATGAACCCAACTTTGCTAAGTGGGTTCAGTACGCCGACGACTTGAGTGCCAAGTCATCTCATAAAGAATCTTCAGTGATCTCCACCCTGACATCATTGCACGGCGACAAAGTTGTCTACGACACAATTCAAGCTGCTAAACTGTATCCACAACTGAGTGAACTCGCCCTTAAATTGGAAAAGGACCAGATACGCTTCTGGATTGCCACTCGAAAAGACCCCTCGGTGGTTTTTGAGGCCCTCAACCTTAACTGGGCAGGGATATCCATCTTCCCAAAACCTGAATTTTCCGCTTGGCTCAAGTACGTGGACGATGTAAACGCAAGACATCCCAAGGAAGCCCCATTGTCGATTATTCCTACGCTCAAGCAACGTTTTTCTCGAGGTGACGAAGCCGGCACAGACGTACTCCTTAAACTGATTGCGAACGGGAAAGCAACGACAGAGGCCAAAACTGTCGCCAACAAGGTAGAGAGTGCACTGTTTGACTTCTGGCTCAACAGTCGAGAAACGCCCGACAAAGTTATGGATGCGTTCAAATATGGCACTACGACTCAAGCTTTCTTGGGGAGTCCACGGTGGAAAGAGTGGGAAAGGTACTTGAGCGCTTACAATGCGAGATACCCTGAAAAGAAGGCCACAGCGATAGAAACGTTAACGCGGAAGTATGGAGATGCACAATTACTCGACACGCTTATCGGCGCGAGCTCGAAAGGTGAGACGAAAACTCTTGCAGCCAAGTTGCAGGCACAGCAGTTCGATAGGTGGATGAACCTTAAAGAGTCTCCCCTCGACGTCTACAACAGGCTACGGTCTTCATATGGGGATACCGCCTTCTTCAACGAGCCGCAACTCAATGTGTGGGTCTCCTACATGAATGTGTTCGTCGACAAGAACCCCAGCAAGGTGGACAAAATGTTCTTGGAGTTAGGTGACACCTTTGGGGACATGCGTCTCTTTCGAGTCCTTGGAGAAGCCAAAAAGTTCCCCAATTTGGAAAGCACTGCAACCAAGCTGCAGATGGAGAAGGCTTCGACTCTTTTTGCCAGCGGAAAATCCCCGGAGGGTATATTCAAGGTGCTAGCACTTGACAATGTCGGAGATGATATTCTCAGCAACACGCTGTTCCACAAGTGGCTGGCATATCTGCAGAAATTCAACAAAGAGCACCCAAACAATCAAGAATCGTGGTTTGACATGCTCCGTATTAGTTACCAACCGTTCGGCGTCGAAAGGATTATCGAGACAGGAAGGAAAAATCCACTCACAAGATTGATGGCTGAAAAAGTGGAGAATGCGTATCACAACTACTGGTTGGATATTAAGATGGAGCCTAAGACAGCCTTCCGCTCCCTGCATCTCGACGAAAGCGGTGAGAAGCTCCTTGCCGACCCAAAATTCAACACGTGGGTGCAGTACCTGAAAACCTTCAACGACCGATATCCTAATGAGAAGACGACAGTCATCGACGGGCTCAGGGATAACTCTCATGACATAGCTCTACTCCGAATGTTTTCAGCCGCGAAGAATGATCCCAGCACGGAGAAACTCGCTACTGATCTACAGAGTGCGCTAATCCTCAAGTGGCAAGATGCGAAGAAGACACCAGAAGAACTAAAGAGAGTGTTTGTTGGTGTGCCAGCCGCTGATGAAATGCTCGACCGGTACATCAAGCTACTGGCGGTGGCGTCATCTACGCCATAATCTATTCCACAGAGTAGAGCTATCGAATGTGATTCTTGATAATTGCAATTGAATAGATGCTGCATTTTTACGCGTATCAGGTCTTTAAAGCGGTTTCAGTATCGCCATATAATTGTGCATGAACGTGCTCGACACTAAGAACTCCAGCTGCAAATTGTCCTTTGAGTTAGATGATACCTCTGAGGTCATGCAGCTGTTCAAAATGGCAAGACGTTCGTGTCTTTCGGATCCTTGAAGCAGCCACCAAGTAATTTGACATTTAAAATATTGAAATGAAGCTGCAGTGGGAGAAGGTCCAAAGTGTTGTTCCTATTAATAGATGTGAAGGAAACGCAAAAAAACTTTAAAAAAAATCCTTTGCCGACACATGTTGGCAACAGGGAACAATTTCACAAGGTAGAAGCTGTAAGCAATGTAGGAAACTTTTACTGTACCTGAGCGAGAAAAAATATTTGGTGTCAAATTCAATGAGCCACTCGCATGCAAATTTTCTGTTATTTTAAAAAGAAACTTTATTGGGCCTACATCGTACGTAAGTGGATTATCACTAAAAGAAGTGCTTTCAATGATTCTGCTTAGGTTTTTGTGTATCATAAAATTACCGGTATAGAAAGAATAAATATAGGTATTCCAATTGGCTGAGCCTTACATGTAATACTTTCTGATAAGTAATGGTCTAACAAAGCCTTCATATAAAACATAAATGCAGCATAATTAGCATTTATGTTTTATATGAAGTCTTTGTTTCACACCTAATCTGCAAAAGAAGGAGCCCGAGCGACTTCTATTACAGTTANCCCCCCCCCGTACTCATATGTTGTAAACGACAATATGTACATAAATAGAGCGACTCGTCTATCTCCACATGGGATGCACAGGTGTAGCAGAAATATTTACAAAAATCGAGCAAAAATGACAAAGACGGCCTAGGCAGGAAAGCCAGCTTGTTGCTAAGTACGAATAGTTTTGTGTCTGTGGAGTCTCCCCCTCATTCGAAAAAATAAATAAAACGATTATGGCGCCGCGAAGGGAGACGCGCCGCAAGGCTGAAGCGTTCCGAACGCGCCTTGAGGGAAAGAGTACTGCCGACAGGCACCGCCTGCTGGCAGAGCACCGAGCGTTTTTGAGTGGTGGGAGGCAAAATGATGCCGACTTCGGAGACGAGACGAGGGACACGAACGCTGAGGGGCCGGAGCGCGTACCCGCGTCAGGAATACAGCGCCCTCCCACGTGAAAGACCGCCTCATACATCGAGGCCGCAAAGCAGCGCATGGCGGACCGCCTGGCTGAAAAAGCCGCGGCGAAGAAGAAGATGGGTGCGAAGAAGAAGGGCGGTCCGTCCTGCAAGACCAAGACATCAACGCGAAAGAGGTATGTAGTGTTCATGAAGTATATACAACGTGTGAGGACTGAACTGATGTTATATTCATGCTATATTATCCTAGCCCGGTCAAACAGAAGATATCATAGCGACAGCACTCTGCCCTAACTCGAAAAAAAAGCTTAGGAGTCCAGGGCGCTGGTGCGAGCTGAATCAGCAGCCGCACTTCGATACGCTTCGAAAGCGAAAAAGCGAGAGCGCAGGAACGACGAGGAGCCGACACACGTAAACGCGCCCCTTGCTGATCTGAAAGAAAAAGATCAGTGGAGACTGCTGGCTTGTCGGAGACGACGACTGCCACGGGTGCAGAAAAACGCAAAAAGAGCGACACGACAAGGTTAGTCACGCGATCATGGAGCATATACTACTTGTGTTGACTAACATAATGTTGATTTTTTTGTGCATTTCAATTTGCAGCCTCTCGTCCCCGGCGCCGGGTCGGCGAGGACTCCGGTTCTCACGTGGTGGATACCACCGCGGAAGTAGATGACTTAAACACCTCTGATGAGTGGTCGGTGGGCCTACCTGAGCAATCACAACAACCTGTTGTAACGAATGCTTCTACAGCGCAAGTACAGGCTCCTGACTCTCCAGATGGGTAAGTTGCTATATCATGTTCTATGTAGGACATATTTGAAAACGATTATGTTGTATATTAACGCATTACTATGTGAAATGTTATAGAAGCAACACTGTCGAATCCGCCGAGCACGGACCAGATGAGGCTTCGCGCGCGGATGCTACTTCTGACGACGAGGCACTGAATTCCGAGCTTGATGGATGGGACGGTGCTGACTCAGACGAGTGGTATATCGAAACGGAGACGAATTTGAAGACGCTGTCAGGTGTCATTGATGCGACTGCATCGCTAACACCAGAGGGTGATTATGAAGAACCTGAAGAGAAGCCTGACCAAACTCCTTCAGATGAAAGTGATGGAGCTTTGAATGGAGCGAGTTGCTACAGAGAGCAATCGATACTACAGCCAGCATTTGAACGAGCGCGTGGATACAATGCATTAAAAACGCTTCGGTGAAGGTGAGGATACGTCCAGGGATGAAGTACTGCTCGGTGAAACAAAAAGACAAATCAAGGCGAAGGAAGTATTGCATTGCATTGGATTATTTGTGGCGAGAATGCTTTGCCCTCACAAGCGACGATTCGCTGATCAGTGGGCCAAGCCTGATGTTGGTGCTATCCCAAAATGGACGTTCGGGCAGTACACGTCAAAGGCGCGTTTCGGTAGGATAATGCAGAGCCTGTACTTTACGGACAACGCCGACTCGAGGGCGGAGACGGATCGAGCCTGGAAGGTGCGATTTGTGGTGGACACGCTGCAACAGCAGGTACCACACCCCGCCTGTCATGTCGTTCGATGAAGTCATGATCTCGTCACGTAGCCGCAACAA

>Contig_40

TGAATGCAAACTCTTTGTGAGTGATGGTTTTTGTTGATATTTAGAAATTTTCCCTGTAGCCGATTTGTGCGATACAGGATATAGTGCCATATGTTAGAACCACCATTTTAACTCTTACCCTGTACTGCACAGAGTAGATAACTGACAGAGATTGTGAGCTTGGAAAAACTCCGCAAATAAAAAACCTAAGTCATTTCTTACCCTGTAGAATACAGGGTAATTTCCGCCCGAAAACGCGGGCCGAGAACGTCATGGTGCCGTTGTGGTAAATTGCGGCGGCATTGTTTGTGTCAGGAGAGAGAATTGAAAAGTGAAGGTGGATGTAAGAATTCATTGTGCTGATGCAAAAATATGGAATCGACTCGGTACTACGCTGAAGATCAAGTTAAACAAGTGGAAGCATGATTCGAACGCATGCTTTCACCTTCTTTAATGGGTTGTTGTGCTTATGGCGGAAGGGTCGGTAACTACTACGTGATGCCAGGCTCATACTACCCACATCATATCATTAGCGAAAACCCTCCCAGCGTGAGCAACAAAAGAAAAAAAAATAGATTCTCTTCTAGATGTATGTTTTCGTTTTGAGGCATCATGACAGTTTGGATTTTGGAAATATCCATTCTGTTGTCTAACCCTTTTTTATACTACTTAGCCTAGGTCATTTCTATTAAAGAAAAGTGGCCTTGGACTCGGTTTAACCCTTTAAGCCCTGAGGTTTTAGAGACCCTCCGAGGGCCTCTAAAAATGCAAATCACGAGCATCTATTGGAGCAAAATAGTATACGAAAGGTCACGTTGGGATGCCAAGTCGGGCAGAAGCCCTCACGGGACCTCTTAGAAGGCCAATCAGACGTGTCGCGAAGGTACGACCAGGGTGGGCGACGTCCTTCCCCCCCTTGCGCGATCAGAATCATTCTTGCATTTCAAGATCTGACTTGACGAGTAACATGGACATCGAGTTATTGCTTTCAGAATGGGAACCTGAAGGGAACGATCGCCACGGGTCACAAGGAAATGAAAACGACAGCGAAGAAGAAAGCAAAGGCGACGATGCTGAATGCGTCGGTAGGGATGATGGCGCAGTCGGCGGCACGATGATCGGTCCTCGAGTCGACCCAGACATTCTCCGAGTTGGCCAGCGCTACATTGATGGGCTGGAGGCTGAAGGTGGTATCACCTTGCTCCAAGAGGAGAAGGCTATGCGTGCATTCCGGGAAAAAGGCATACTGGGTCTATTTGCGCTGTTCTTCACACGTAAGTTGCGCAACACACTACTGCAGTGGATCAACCCTCGTCTTCGTGAAAACGGTACACCAGACATCACACTTCGGGAGCTGAACGCGTATATTGGCCTTGAGATTGCCACAAGCCTCTGCCCTCTCAACCGTCTTCGGGATTATTGGTCTACAAATGAGCTCTATGGCCACCCTCTTTTCCAGTCGACAATGCAACGAGACCTGTTCCTTGGAATCCGAGCAGCGATTACGCTGCACCCAACGGATACGGTGCCAGAGGAAGTGAAACAGCGTGATCCTTTGTGGCATTGCCGCAGTATACTTAACAACATTTTGAAGAAGTGTGCTTTATTGGCGAGCCGTGCGATCTCGAGCTACCTAAGCTGGTCATCCGAGAACCTGCGTCTGGTATCAGAAAGAGAAGACGCATTAGCCCAGCAGTCTAACCTCCGCTTACATCAATAACATATTCCCTTTCGCTTCAATCATGCCAATGAGCTCGAATCTCTCGCATAACACACTCGGGGACCCGAACCATACGCGTCTTCACTAAACTTCCGAACTTGAGATACACGTAGAGCTGGCAAACAGCCTTCCGGACCTTCTTCATGCCACTGGCATCGACATTGTTGCATAGCTCGTCACCACGCTCTACTAGGAGCTCGTGCATTGCATCCCAGTCGCACGGCGTACCGCCACACAGACCACAGATGTCACTGCTGCTGTCTGAGCTGTTGTCGTCGCTTTCTTCTTGGCTGCTCATCTCACTATGTCACCCCTCTGTGTAGCCAAAACGGGTCTACCACGTGTCTGCAGCTGTGAGCTCGATTGGTCGATTTTCTGTATTTATGATAAACGATTGGCGGTCCTGAAGACCTCCATAGGGACTCTAAGAAAAGTTCAGGTCTTAAAGGGTTAAAAGTCTTCTTCACACAGGTAGCATCATTGTTGTCTACTAAGTAGCATGTCTTATTTTTTAATTTCGTAATAGAACAGCGACCAAGGTTATATTTGACAGCATGAATTCATGGATATCTCGATATTCGAAAAAGTCGCGAAAGTGTACTTTACTTATTCCACTCATATCACCAATTATCACATACGTACTACTTTTTGGCTTAGTAAATCTTACAATGTGGGTGGAGCCAATCGAAACAGCATAGTAGATAAGAGCTACCACCAGAACAATCACGAGTTGATCGGGTCTCATGTCGACAGAGAACACGCAGCACGCGGGGCGCCAACGACCGCGGACCTATCGTATTCTTAAAAAATCGTATATGAGTACAGAGTCGAACGGATTGGTGGGGGCAGCCCTGTATGCTACAATGCTAAAGCTTCCCAGCATATTTCAATAGTCTACTCAAATGGACAACCGTTTCAAGTAGTTGTATGGATTGACCTTGGGAATACCCACCGAATTAAGAGATGGAAAAATACAAAATCACTCTGATCAAGGAACTTTACTCGCCCGAATCCAGAGAGCCCGAAAATGCAATACTTAAATTAATACATGGATGCCTGAGAAAATTAATAATTTAATTATTTGCAAAACTTCGCAAGTCACGATTGTAGTTCAGAAACATTATCATCTCGAGCCTGGCTGGAGACATTCGCTTCCGTAGATCAGACAGCACCAGCTTAGCGAGAGAGAAGAAGCGCTCACACTCGTTAGACGTTGGCGGGATGTACGCGACGTCGACGTAGCGCAATTGCGTTTCTTGAACGCCGCTCTCACAATAGAACGCTCTCGGCGGCTCTTGAAGACGACCGTTCGAGATCAGGCGACCTAAATTCAGCGCAGGCTAATTTTTCCGCGACGTTCAGCGCACCCCCACGCTGTAACTTGACTAGACCTTACTCAAAATTGGGGTTGTTCCCGTTGTGGACAGCCATAAATTAATAATTAAATTAAAAAATTAGGATTAATTTTTACGCATATTATTTTTTCGGGTTCCCTGCCCAAATACATAGGCCTGGTATTTGTAGAAAAGCGCCCGGAATGTCGGGTTTTGGTCAACCCTCAGCTTCTTGTAAACGGCATCGTCGGACAATTTCGAGGTGTATTTCCATTTGTAAAACTTCTTTGTCCGATAGCTTATGTCGGTCAGCATCTCCGTGATTTTGTCGAGCTTGAACCGCTCTTCATCAACGTTATCGTTGTTGAGCGTACTATGACGTATGTGACCTCGTAAAACTCGACCATGGCCACTTGGATGATCATGACCAGGATCCAAGCTTGCATCCAAGCCATTGGCAATCTCTGTGGGTGCTGAGAGAGCGTCCTGCGCCACGAAACCCAACGCTGCCGCGACCACGAAAGCCAAAGACAGCTTCATGATCTGATGTAGAAAGTCAGTGGCTTGAGAGCTGGATGAAGGAATGCAGAATGGCACGAAAAGAAATTCAACGCTTTCCAGAAAAGTCTCAACGGACAATCCTTCTGCGGCTCATAATGACCATTCTATCAAGTTATAAACGGGACTCCGATTTCTTGTGATACAGACGTACCAGCGACTGGGCTGCATTCATGTTAGTGCGACGAGGAAGGGAATTATCCTCCGCAGCCGTTTTTAGTTCATTGTAAATCCTTTTAGAATTTTTGAGCTATCCATGTAGTTTAAGACTCCAGCTACAAGTTGAAGACTACAGATCACATGATCTTCCACGTATCGACACCGCGGCATGCAGCGTTCGGCATCCAGGATGACAAGATACCCTGTAACGCAGGCCTCGGACAATACAATACGAAATTGTACTGCAAGACATCGAGTCCCATAATAATAACGGGAATATGATGAAGTACAATAATAATAACGGGGCCAATATAAGTATATTGTGGCCTCTAACTACCAAGTCTTCCTACTCATTGGCGACGAGCGAAGTCGCGTTCCACATGTCGCGGTTCACGCGAAGAAATGCTAACATCTCAAAGTTTGCAGAAAGCATGCATGACCGCTGAGGCGTCAGAATGAGCTTGCACTGCGAAAACAGTCTTTCAACTGTGTTCGAGGTGGGCGGCACCACCTTTGCCAGCTCATGAAAGCTCACACCCGCAGCACCAGCCGCTCGCATCTGCTTCCCCCCTCGTAGAATTGCGCTGGCGTAATCGCTCGTCGAGCGCTCCTTCCTCTTACCAGTTGCAGCATCTGGCTCCACCACCAATCGCTGGACTACGCGCGACTCAGACGCAGTGAGCTTCGCATTGTTGCAAATCTTGACCAAAGCTGCCTCAAAGGCCGGCGCATGGACGATCTTCGCCGCCGGACGCAGATGGCTCGCCATAACTGGGTAGTCGTCAACAACCTGGTCAAACAAAACACGCACGTCCGCCATGCTGGTGTCCATGCGCTGGAGTGTCTTGCAGACACTATCGAGTTCTTTTAGGTGCTCTAGCAGGCCGAGAAGCTTCTTGTGGGCCGCACCTTTTGGGATGAACTCGTCAACAGCATCACCCTGCCGAATAGAATCTCGAATTTTCTTATAGCGGTCCACCATTGCGAATGTCGAAGACCAACAGGTAACATTCCGTTTGACAGGTTTCAGGTCCGTGAATTTTGATAGTTCCGCAGCATTGTTCACGTGTCGTAGCTGCACCATCAGACTGTTAACTGCAGCGAGCTCCGGCTCATAAGCAGCTAAGTAGCGATTCACCGCAAGGTTGTACCGGTGACTTGCACAGCCAACCAGTGGCAGCTCCAACAACGTAGCGATCCGCTGATTGGTCGCGCAATTGTCGGCTACCAGAAAAGCCACCATAGAGATGTCCTTGTTATACAGCGCCAGCACGCGCTTGAACATCTCGATGTGCACTTCAGCATCTTAGCTGCCGTCCTCCAGCGGTGACAGCGACAGCAGTCGCTCACGGCGCTTGCCGTCTGATTAGCAAACACCGTAGATACCGACGTAGTGCGTGGTACCGTGCGTCCATCCATCTCACATGACACCAAACAGCTTGCCCAACTCGCTTTCGATCAGCACACCAACGCTCTCGGAGCACTTCTTCATGTCTTCCTTCAACGTCTTGGACGAAACGGGCTTCCAGGACGACATGGACCTTGTGAGGTGGTTGTCGACCTCGGAGACGGGCATGTGCCTCTCTACGACCCACTGAAGCCACTGATAGCGATGCTGAGTCGCCTCGGACACGAACCCAAAGTTGTACAGAGAAGCGTCCGTAGCCGCAGATGCGCTGTAGGTCTCGTTGAAGTCGGGGTGCGTCAGGTTGAGGTGGGACATCAGGTTCGAGTACCCGGTACCGGTTTGCTGCTTGCGGTAAGAACCGCAAAGCTGACAGCGGAAGTCGCCTTGGTCCTTGTCTTCGTAGAAGAATGTGCAGATATCACGGTTCGGAGCCATCGTGGTCAAATGAAAAGTGAGGGAGTGCTGTGAGGCTTCGGCTCGTGCTCGTGTCTGTGCCTGCCAGCCAACCACTTGAACGATTGGCGCGAAGAGCTAGTTGCCTCTAACATATTGGTATAAACCGGTATTGTACTGATATTATTGGATCAATATCATATTATATTGACTTTGGACCGATCCAATAATAATAATTKTAGCCGGATAATACAGTAATTACGGGATTGATATTATTTTTTCCCACCCCTGCTGTAACGCCACACGTGATGACAAACTTCGTGTCCAACCAATGCAGCTGAAGACAACCAGTACCATGTAGTCTAGCTGCATGTAGTAGGAACTCACTAAGTGAGGTCCTACTACAGAAAGTGAGTCACTTTGTATTTACTCAGCAATACAATAACGGCTAACTTTCAGGGAGCACCGTCGACTCCCTTCTCCGTGTCTTCGTTTCTTGACGAACACCACACAATGACACGACATCATCTTTGTTGGTGTAACTACAAACTTATGTACACGGAGATTGAGTAAGAACCAGGGATTTTCACATGTCGAGTAGCGGGGGATAGCGTGAGTGCTTTCACAAGTACATGTAGGGTAACGGAAGATGGCGTTAATGTCTTCACGATTGCCTTCACACTCCCGGCAATTTTTTCTAATACACCCAAAAGGCACTGTAGGCTACAGCGCCAGCTATAGTGTAGCTTAGGGAGTATACAGCCGCTTAGCGGATAGAGTGAAAGCCTTACGAACATACCCCGAGACACGTGTTTATGACATGTTCAGCTCCCATAAATGATACTCTATCATTTAATATGTCGTGATAGGTGATTAAAAATAGAGGTTTCTTGATGTCCCGTATAATACTGAATACCTATTTGCCAGTACACCGTATAGTATACGGCGTATTGGCTGATAGGTTTCAGTAAGAACTTTAATATTTCTGCCTAGGTTAATATATGATAAATTTGGCGCGTCTTCGTTATTTTTAAAACAAATTCAAAGATCTATGAAGCGTCTTTTGCTCGGGGTAGTAGCTATCGGGTATTACTGTAAAAATCCTAATATTTGAAGCTGATATCTCGATATTTGGAAAAGTCGCGAAAGTGTACTTTACTTATTCCACTCATATCACCAATTATCAGATACGTACTATTNTTTTGGCTTAATAAATCTTACAATGTGGGTGGAGCCAATCGAAACAGCATAGTAGATAAGAGCTACCACCAGAACAATCACGAGTTGATCGGGTCTCATGTCGACAGAGAACACGTAGCACGCGGGGCGCCAACGACCGCGGACCTATCGTATTCTTAAAAAAATCGTATATGAGTACAGAGTCGAGCAGATTGGTGGGGGCAGCCCTGTATGCTACAATGCTAAAGCTTCCCAGACTATTTCAATGGTCTACTCAATGTCACGGCCTACCGCTAGATGGTGAATTAACGATGCTGGCTGACTGCCTGCACAAGGTCTATTCGCTCCAGACTCTCCGCACCACGGCCAAGCAAGCGGGACCTACGGCCAAGTCAGTGACGTTCCAAGTGTGAGATCGACAAGCAGCTATTCGTGGCGGGAATTGGGGCTGGACCGCCCCATGCAAGGGATCTACTGAAAGGTAAGGAGCCTTTCACTCTTAGCTAAGCGGAGTGAGGCGTTAAGTCGTGAATTTTCCTTCTAGCAAAGAACACAAGAATACATCAAGGTTAACAGTACTATTTAAGTAGTGTCGCTTATACAACTACCTCTTACGCCGGCCCAAGATACACGACCCCGATTAGAGGAAGATCGCGACAATATGATGGAGAAACAGCGGAGCTTGTAGCCGAAAATCGTAGGAGTACAAGCGTGGAGAGAAGCCCTGGCAGCGGAGCGCCAAGGGAGAGGGAGAAAGGAGTGGGAAAAGGATGAGCGCTCATGGATGGGCAAATGAAAGACTTGGAGCCGGTGAAGGGTTAGGGGGATAAGCTCCACCGTTGGAAACGGAGCCCGTGGAGATTTTCGAGGAGGACGAGGAAGCGCGCGCTGAGAGAGCTGCTGGCGGCGGCACGCTGCAGAGACCTCAAGGAAGGAAACGCCAGGGAGATGGCGAGGAACGTGGCACGCTGCAGCGAGCTGATCCCGAAATTGGAGGGTACGAGATACGAACGGTCCCTTTGGAAGGCGAGGGCACGTTCGGTGTTACGACGCCGTCTCCGGGAGCAAGGAGGACGGTTGGGTCTGTGTCAGGGACGTCTATTGGTGGACAGATGTCCAGCACGATCCTAGAGCAGTGAGCACGTTAGCCGACGCCCAGCGAAGGTGCGACACCGGCGCGGACGATGACGGGTACGCCCTTGGAAGCATCGGTGATGGACTCGGGAAGTCGCCCCAAGGTGGAGTTCGCCTTGGATCGGACTCATTCCTCGTTACAACCGGTAGACAGCGCTGGAGGAGACGTGCCAATGGTCACGTCAGCGTATGGCCGACCCAGTAGCAGCGTACCGAGCCCACAACGAAGCCGACGTGCCCCAGGGACTGCACCTGTTGCTGCTGGGACACCAACGCAATCGTACGTACCTAGTAGAAGTCGTGGAACGACGCCTGTTCAATGAAAGAGGACGTCAACGAGGGGAAGTGGAGCGCCGACGACGGACCCAGCCTTACCGGCACTCATTATGGACAGTATCGCCCAAATGCTACAGAGGGCTGCGATGATGAACGGAACGCCGATGCCGACACCAACATCTCAAGCTACGGGACAAGTACCACGTACCGTGACGACGCCGACCCAGTTTCAAAATATTAGCATTTGTACAGTAATACCCGATAGCTACAGTACTACCCTGTTCAAAAGACGCTACCGTACATAGTTCTTTGAAACTGTCTAAAAATATCATAGAGGTGCCAAATCTTATCATATAATAACTAAGGCAGAAAACATTAGCTGAATCCTTATCAACACGAAAAACCTCGATTTTTGATCACCTATCACGATATATAAAACGAGAGAGCAATCATTTATGGGAGCTGAACCTTCTCGTATGTTCGGGAGGCTTTCACTCTATCCGCTAAGCGGCTGTATACTCCCTAAGCTACACTATAGCTGGCGCTGTAGCCTACAGCGCCTTTTGGGTGTATTAGAAAAAATTGCCGGGAGTGTGAAGGCAATCGTGAAGACATTAACGCCATCTTCCGTTACCCTACATGTACTTGTGAAAGCACTCACGCTATCCCCCGCTACTCGACATGTGAAAATCCCTGGTTCTTACTCAATCTCCGTGTACATAAGTTTGTAGTTACACCAACAAAGATGATGTCGTGTCATTGTGTGGTGTTCGTCAAGAAACGAAGACACGGAGAAGGGAGCCGACGGTGCTCCCTGAACGTTAGCCGCTATTGTATTGCTGAGTAAATACAAAATGACTCACTTTCTGTAGTAGGACCTCACTTAGTGAGTTCCTACTACATGCAGCTAGACTACATGGTACTGGTTGTCCTCAGCTGCATTGGTTGGACACGAAGGTTGTCATCACGTGTGGCGTTACAGGGTATCTTGTCATCCTGGATGCCGAACGCTGCATGCCGCGGTGTCGATACGTGGAAGATCATGTGATCTGTAGTCTTCAACTTGTAGCTGGAGTCTTAAACTATAATACATGGATAGCTCAAAARTTCTAAAAGGATTTACAATGAACTAAAAACGGCTGCGGAGGATAATTCCCTTCCTCGGCGCACTAACATGAATGCAGCCCAGTCGCTGGTACGTCTGTATCACAAGAAATCGGAGTCCCGTTTATAACTTGATAGGATGGTCATTATGAGCCGCAGAAGGATTGTCCGTTGAGACTTTTCTGGAAAGCGTTGAATTTCTTTTCGTGCTTCATTCTGCATTCCTTCATCCAGCTCTCAAGCCACTGACTTTCTACATCAGATCATGAAGCTGTCTTTGGGTTTTGTGGTCGCGGCAGCGTTGGGTTTCGTGGCGCAGGACGCTCTCTCAGCACCCACAGAGATTGCCAATGTCTTGGATGCAAGATTGGATCCTGGTCATGATCATCCAAGTGGCCATGGTCGAGTTTTACGAGGTCACAAACGTCATAGTGCGCTCAACAACGATAACATTGATGAAGAGCGGTTCAAGATCGACAAAATCACGGAGATGCTGACCGACATAAGCTATCGGACAAAGAAGTTTTACAAATGGAAATACACCTCGAAATTGTCCGACGATGCCGTTTACAAGAAAGCTGAGGGTTGACGAAAACCCGACATTCCGGACGCTTTTCTACAAATACCAAGACTATTGATTCGGTCGAGTAAAGTTCCTTGATCAGGGTGATTTTGTATTTTTCTTCCTCTTAAATCGGTGGGTGTTCCCAAGGTCAATCCATACAACTACTTGAAACGGTTGTCCATTTGAGTAGACTACTGTATTGAAATATGCTGGGAAGCTTTAGCATTGTAGCATACAGGGCTGCCCCCACCAATCCGTTCGACTCTGTACTCACATACGATTTTTGAAGAATACGATAGGTCCGCGGTCCATTGGCTAGCAAGTTCACTTCTACCCGGAGATCCACTCGGTTTAAGTGCTTTAAAGACAGATCTCCAGTTCCGTAACACAAAGATCGGTGTGACATCACGCACAGAAGCGACAGAGACAGCGAAGAAGGTTCTGGGTCTGATGGACGTGACGCTGCGCAGTGAAGATGGTACGGAAGTGGCCTAGACGACTCTTAACCCCTCTGTTACCAACGTCGTAAGTTACGACTTTGAGTAGGGCGAAAGTCGTAACTTACGACGTAATTCGAGTCTTCAAACAGAAAATGGCTCGAAAAAAGAGGACACGATACTTTAACCAAGTCAAAGTTTGGAGGCAAATTATATAAGCTGAAAGAGGTCGATTCGGCAAATGTAAAAGAAAGAATGGGGGAAGGGTAGATGTGCTGCACCGAACAGAATGATGAGTGGCCGAGAGCGCCGACTCGAGCCACTGGTGTCTCATTCAAGAACTTGCCACCATGGCGAGAGTTCGATCAAGTACGCGGACGAAGCAATCAGCAGTTGCCAGCACGATGGACGTGCGTGCCATCGACTTCAAGCATCTCTGGCGGCAGCTACGCGCAGTGGGATGGACGTCGAAGAGACCTTCCGGTGGACTTGCGAAGGAGTGGACGTACACGTCGCCGGACGGGAAGTCGCTCACTCGTGCGAAGCTCGTATATGGTTATGTGCTTCATTTGTCTAATTTATATCACTTGTCTWGGGGAGGATGCTGTGGTGGCTTATGCAATCGAGACTGGGCTTATTGCTGAAGAGAGTGGCGACAGTGAGAGGGAGGTGGGCAGTGATGAGGTCGTGCCGGTGGACGTTACAGTGACGGCGTCACAGATAGACACATCCATCGCGCTGTCGGCCAACACCTTATCGGCGATGTTTGGCACGGACAGTGACCCCCAGTCGGAAGCGGAGGATCAGTTGGAGCAGTCGGATCGAGAGATGGTCGTGGGGGCGTTCCAGCGGCTCCTGTCAGGCGCCGAGTCGAGCAATTCGGACGACAACGATGATGGTTGCGCCAGTGTGGAGGAGGAAGGCGCAAGTGTGTGTGGAGGTGAGACTGTGGAGGATGACTGCCGTGTGGCACTGCCGGTAACCGACGTGAATGTTATGGGCGATGGAGATCTTTCGGATGAATACGAGGCCGTCAATTCTAGTGGCAGCGACAGCAGCGACAATAGTGGCGACGATATCATCGTGAGGCGTGAGTACCCCGACGACACACTTGAGGACGATGAAGACGTTGCTCTGATGGACGACGCATTTGTCGAGGCGTTAGGTGGCAAATTAACACTGGAGTCGATCGACAAGGCCGCACTCCGGAGCTTTGAGTGGAGTCCTCCTTCGTCCATCTTCGAAGCTGACAGTGGAGGGTACCCTCGCCTGTCTACGGACGTCGCCGTACCGATCAGGGAGCATCAGGACATTGCGGATTCTCCCTTGCTGCTTCTATTTTACTTCCTACCAAAATCACTGTGGGTTACCATCACCAAGGAGACAAATCGCTACAAGAAGCAGACAGTACACGCCAGAGCTAAGCGAATCCGACAGAAGCAAAGGAAACGCACTGTACCAGCCCCCGAGACTGTTAAGCAGATTGAAAGGCGATTGCGCGTCGAGGCTGCGTACGAAACTCATGAGATACTCCACGTCATTGGGCTCTTAGTGGCAAGAATGCTGAACCCAATGACGCGTCGTTTGTCTCGGCATTGGTCAATGACCGAAGACGGTGCAATACCTGCCGGAAACTTTGGCAAGTTCATGGCTCGAAACCGCTGCACGTCAATTCTTCGAGACTTCCATGTCGTGAACAACGAAGCCCCACGGATTCGAGACAAATTGTGGAAGTTACGGCCTGTTGTCGACACGTTGCAAACTCGGTTTCGATCTGGCTGGTCACTCGGCAGTAAGTTCTCGTTTGACGAAGGCGTACTACCAGCCACGTCCAAGCGCAACACCACGCGGATGTTTATGCCCGACAAGCCCCATCGATACGGAACCAAGTTGTTTATGGCTTGCGATTCTGTGAGTACATACTGCCACCGGTAAGTTTGTTTCGTTTAAAAATTATGTCGTAACTTACGGCTTGAATTGTAATACGATATTTTATTACGTCGTAACTTACTACTATAGATTCGAGGTGTACGTCGGAAAGCGTGAGACCGAGGATGGTGATCAGCAAGCAGTCGACGACAAGACGGGGGCTGCTGCAGTTATTCGAAACATGAAAGCCGTTCTGGGAGATCGATCACAAGGGTTTCGATTGGTAGTGATCGATCGATTTTACTCGTCTATCCCACTGGCTATTCAGCTTCTATCGATGGGTAGCTACGTGGTGGGAACGATCATGGTCAACAGACTGGGGTTCGACAAGCAAGTTATTGAGAAGCGTAAAACACGTCCTCGAGCGATTGAGCGTGGTACTTTTGCGTTCTCTCGCTCCACTGCTATACCGACCATGATAGCTTGCCACTGGTGGGACCGAAAGCCGGTCCACTACCTGGCCACGGGTCCGGTCATGGCAGAGGACTCTATTCGTCGTAACGTCAAGATGGTCGGGCCGTCTTGCGTCAAATGCCCAAAGTTAGTTACTGATTACCAACGCTGGATGGGCGGAGTTGATGTACATGATCAACTTCGCCTGCAGTCCTATTCAATCCAAACGGCTTTCCGTTTTAGGAAGTATTACAAGTCACTGCTCATGGGTTTCATGGACATGGCACTCGTCAATGCGTATCTCACACACAAGGAAACATGCCGGATCAAGAGGCTGGTACCAAAAGATCGTGGAGAATGGTATCTTCTACTTCATAAGCAATCGATGCAACTCAAAGCCGATGACTTCATTGAAGCTACTGCTCCTACACCTTCTCCTATGGCTCGCTCTCGCAAGCGCCGCCGACTAGACGGTCACAAACACATCCAGTTCGACGACTGGTCACCGTTTCAGGCACCCAAAGCGCCGGCAACGCTCTTGCAAAGTTATGCGCTCTTCTACGAGGCGATCGAAAAAAATCCTTTCAAACGACCTATTATTGTGAAAATCGCTCACACGCAGACGCAAAGTGCTTCCTCTGCCCCAAGTCGCGCCATAAGCTACGGAGGCGTGCGCAAAACATGCTATCAGATTTGGCACGAAGACTTTGACAGTGGGGCTTCCATCCCAGCATCGCTCGGCAAGCGAGTCGTCTTGCGACGATCAGCCAAGGCTGGTACTCGAAAACCAACTCGGCGTGAGCTGCTCTGTCAACAAGACGGCGAGGCCGACAATGAAGAGGATCAGCCAGCTCAGCAATCGGCAATCGTTTGAATGCGTTTGGACAGCACTAGTTTATGTAAATGTAGCTCTTTCTCTGTTGTACGAGGATCTCTACGGATGTGTAAATAGGCATCTGCTTCGACAAAAACAATACTTGAACGTCGTAAGTTATTGCTTTTCGTGATACGACGTGCTCATAATGTCGTAGCTCATACGACGTAGCTCAGAATATCCTCTGACAGTTTATAGCGGGAATCCAGTGTGGAAAAGACACATCCATACAATAAATACACTAAAATAAAGTCGTCACTTACGACGTAAAATAGGCGGTTCCAACACGGATCTTCTGGTAACTGAGGGGTTAAGAGACATCGCCGACTTTGTGATCATTTTACCCAGGCGGCAAGACAGCGCATTTCAAACAGATGAAGGAACAAAGCGGTGTTGCGTTTGAGGACATGCTGATCAATGACGATGATCTGGAGAACGTACACGACGTTAGTGCACTGGGAGTGGTGAGTTCGCACTGTCTCGATGGCCTCTTGGCTTCAAGCCATGGAGGTTTACTATGTAGATGATTAAAACTACCTCAGTGTTGCTGAAGGACATGT

>Contig_41

GGCTGTCTTACACCAGCATGAACACAGCGTCCGCAGGTAGCTACGCGTCGGTAGTACAGTGCTTCTAGAAGAGACCGACTGTTTTTTTCAGTTAGCTAGCCGCTGAAAACGTGTGGATGGACAAAGAATAAGTGGTCTGCCCCATAAAGACGACTAGCAATATGATAGTAGTCTATTAGAGACACTCCCAAAGATCGCGAAGGTAGAAACCGGAGCTTTAAACGTTTTGCTCAGGCTCCTGATCCCAGCTTCTGCTTAACAGGGCCATGACGCTTACACTTTTTACCAAGCGATCTTCCAAAATACTTAATGCAGAACGAGCAGCCAAAACCAGACATGTATTTGGCAGAGTATTTTAATGCGGTACTCGCCTATGTTGAACGGAACGACGAGCTGTTTTACAGCGTACAAGGCTATCGTAAAGATGTACATGTCTTCAAACCGCAGCAACATTATGAGTATTTCAAAGGAAATGAAGATTCAGGATCATTGCTTTTCGTCCCTTCTTCATGCAACCAACAACATTTTCGCGGAGGTGGCGACGTCGGAGTTGTGTCGAAAGTGNACCCACCACACTTTTGTAGACGCTATCTTGATGAAGGACATGGGTAGAATGTTGCCAAATGTACCAATAAATGGGTACAGTGGTTGAATTGAATGGACTTAGAGTAGAATAAATGGTCCAAGTCGGACATGTGCACATGTAGCGGACTATGATCGCTTAAAATTATACACCTCGGAAGTAAAACGTGCAGAAGGTAGCACTTCCTCGTGCTGCGCGGGAAACGATGAAGTGCAAATGAAACAGCCTTTGCATCAACATGATTTAATATGATGCTACATAAAAGTCGTCGATTTTTTCATCTGGACATTTAAAAAAAAAAGCTGCCAAAATATTGGACTTCGCTCTAGCATGATATTCAGCTGGTTTATGCACTTACGCAGGTCTAAATCAGTGGATATAAATCAGTATTATGGGCTACTGCAATACTACTATAAAACACAAGCGGATCCGCCCGAATAAAGCACCCTTCCGAATAAAGCCTATTTCTCTAAATATCGACAAGCTTTAAGATAAACGTGATCAGATTTTTTTAATTGTCTGACACTTGGTTCCTCAGTTTTTTTTACAACTTCCATTACAACTACAATGCTACTACTATTTATCACAAAGTAACAGCAGTAATGGGCCTGGGAAATAGTCAGAGGAACTGAGTCGTAAAAAAGGAAAAAAATCCTAATGCTTTTAAAACATAATTAATCAGTCGATCAGGCTCCTCAGAGCCCAGAAAGAAGTACAGCTTAGTACTCCCAAGTGTCACTAGATAATAGAATCGAATGGAATGACTTGAAGATGATAATTTTCCACAAAACCATTTTTGTGGTGACAAATCTTCGAGATTTAATAATTAAGTAAGCATAATTTTAACTATTCTCCGGTGTCGTGTCGAGGCCATCATTAGTCACAGAGGGCGGCTAAATATGACTTGCAAAAGCAAAATTATGTTATAGTATGGTAACGAGTGAGCTATCTCGTTCCTTTGCGCCATTCGGTTCCTCGTGAATTAGAGCAAAGAAAGCTTTTTGTCATAATTTATAGGCGATACAGTACATTTCGATCAATAGCGCGAGTCAACGTTCCGATCTTGTAACACACCTCGTAATAGCTTCTTGCTGTAGGCAACCTGATGGGGATATCTGATTGGCAATGGATGGCGTAGAAAAATCGGAGTTGCAAGCTACGGCTCAAAAACAGCAGCTACATGTATATCATTATTTTCTGTCAAAACTATTGACTATAGCAGAGGACACTACGCACGATCACATACCAGTTTTTCAGCATTATAATATCTTGATCGCATTTTCAAAGAGAGTGGTGAAAAGAAGCACTGAACAGGTGTAAACTACAGAAGGAACTCGTGTAGCCAAGCAGAAAAGACTCGATCTGTGTGAATGCTCTTGGGGTGTGCACGTCTCTAGGATAACCTTGCACAAAAGGACGATTAATTTGAACAGCTGGTTTCTGTTTTTTTTCATGATACTGTAGATGCTCTAGTAACCGTTCTGTCGTGCTAAGCAAAACCAAAACTGGTTCCCTCTTCACAATAACTACTGTGAATGACTATGTGCTTTATTATTTATAACAGCGCGCGACTTCAGTGTGTAATGAAACATGTTTCTTATTGATACGTGTACTTCAAAATTTAACTATTTTGAAAATGGTTTGCATCTTCATAACCACTATTTAGTAGTTTTACGATGCCATCCGGTTGCAAATTGCAAATTGCAGTCTGCCGGCTGTGACAACAACTCCTCGAAAGGTTTATTCTGATGCAGGATACCGGTTGGCAGCTTTAACATCGCGTTGTTTGGTTGGCCGACAAGGACGATCGTGCTTCGACATTGTGAAGCACATCCCAAGCCCTTTCATTTTCAGTCACTAAGCGCATCATTTAGTACGGTAGTGTGCTTGGACTGGCCATCTTGTTTGCGATCTCGTGTGATTACATGCTTCGTGAAAACTTAAAACGATCTGGATCGCCACCAGCCAGCGTTTACAATACATGTAGTGAAGACATTTACGGTCGATTTACACTTCGCAATCTGCTCTCGCAACCTCAAGACACCACACCGAACTCCACCACAAAAGATGCGASTTTTATTGATTCTCTTTGTCCTGATTTCATCTGCGTCGGCTACTTCCTCGGAGCACGTCGGTATCACGAACACCGAGCGATTCCCTATTGCTGCCGAAGTCCACCGGTTCCTGCGTCGACATTACCTCGAAGAGGAGGCGGACATCGAGTCCGATGATGAAGATAGAGGTGGTCTTGACAAGGTGGATGACCTGATTACGAAAGTAGACGACGCGTTGGGGATAACGGGGAAGATGGACGACGTTGCTGGTAAGCTGGGTAAAGTGCACGTAGCACCGACAACGAAGACAGCAGTCGAGAAGATGGAGCATGCAGGTCTCGTGAAGCATCTCTCCGGAAAGTATTCGGTTGCCGACAAGCTAAGCCTTACAACACTGAGACAGCTGGCGAAAGTGGACGAACAAAGGCTGAAAGATAATCGCGTGTTTGACAAGAAAACTGGCAGTGGAATGCGAAAAAAGATCGAGCCCTTTGAGGGCATGAAAATTGCTCCTCAGAAATATCTAGAAGCCCATGTAGCACGTGCTGGCCAGCTCGTCGACAAGGAGAATAATCGGCTGCTGTCTGCCGTTGTGATCGGTGATGGAGATAACGTCCTCCTCATTTCAAGCTCGAAAAAACCGAACGATTGGATTCTTCCAAAGGGAGGTTGGGACCATGGCGAGGGCATTGAGAAAGCCGCATTGCGCGAGGTTATAGAGGAAGCAGGGGTACGTCTATTTTTTTGCAATGTTTTTTTGTATATTTATAAATATTTTTTACTAATCGTCGTTCTGCAGATTCAAGCGCGTCTGAATCACGACCTAGGCAAGTTTACGTACAAGGACGGCGACAAAGGATATGGATTATTTGCCTACACCATGGATGATGTCCAACGCTTTGACGATTGGGCCGAAAGCTCCCGCTACAGAATTGATGTGAGTAGATATGCGGCAAAAAATAGTTTTTGCATTATTGGGAAGCTTACGTGCGTGTGAACCACTATAGGTCCCGATTGGCAACGCGAAAAAACTGGTTGGCAGGCGCCCAATAATGGTAAAGATTCTGGAGGCGGCGGAGAAGAAAAACGCACTAGTCAAGCGTGGAGATTTACCGAAGAGAGACCCGCAGCTCGAAAACGTCAATTTGACATGAATTAGCTGGAAAAAAAACAATGCACAAGAAGTTTCAGGGGCGCGCATCTTGGGTAGTGCATTTGTGATAACAGGCAGCCAAGTGTTAACCACAAAAAAAATAAAACATGAAAACATATTTTGGTATGATAAAAAAATAAAAACAAGTATAAGCGACCATTTTAAGGCGTATTGAGGCAAGTAGCAAAGAGAGACCCTTGATATTGCCCGAATGACAGACTTGCTACGAAATTACGGGGGCATTATTGTGGGCAAATGTATATTGTAGCTTTCGATTTCGCAAGCTGGCGGAGAAAATGCTAGTGCTTGGCATTTAGTAGCTAGTGGCCTTGGAATTATTCACGTTTGTTTGACATCGGCTACCTGCAGAAAGATCAAGTTTCTAAGTAACAAACAAATTTCAACAAATCCACATTACAAAATACTTCAATAAAGTACACCCTCTTCTATTATCTGTACCTAATTTATTTCAGAGCTAAGGAAGGATCTGTATGGGGCACGATTTTCCATGCGATTGTTTAGAAAATTATCTAAACAAATGGCTTCTTACAAGTTCCCCTACGTAAAAAGCAAGTAGCCCTGTTRGTAGTGTCAATATTATATATTATGTATTTAAACCAAAGTGTATTACTTCAAATTAATTTTGCTCCAAAGGCCACTACGGTGTGCACGAGGGAGATGATGATCGGGGCCGGATGGTGAGCTGCAGAGCGTACCCGACCTACAAGTAGAGGATATTCCCGGTCGATTTGGCAGCTGTTCGGATTTGATTCGGGTACTCGAATAATTATTTTCCTTGATTTATCAGCGTTCACAACTGCTCCAAAATGAACGCTTTCTTTATTTTTTTTCTTTCATTTTACACATTTAATGTGTTCTCGTTCTGCTGTTTCCGTCATGGGTAGGAATGGGCAACCGAAACGAGTAGCGGATTGGATTTCGGTTAATGTCGGACTTGGAGAATTAAAATTCAAAGAATTAATGCGCTCAGCTACTACGAGACTGGAGCGAATACTAAGATTACTTCACAACTGCTGGTACTGGTACTTTGGCAAGCAACATCACCACAGACGTCGCATGTTTGAAACGAGGTGTTCACGCATAAAGAGTTTTTTACGCGGAATAGAGAATGAAAGTTCTTACATGTAGCACACAACAAATCACGGATTCGTCATCATAATAGTTGCTACCGGTAGTTGCTAAAACAGAATAATGACTAACCAAAAAATGGGAGCTCATCGGTAAAGGGCGTGATTTCGATCGCCTCAGCTCGAGCAGCTGACCCACGCTAGCTTAAGAGCGACCACTTGACAACCACACTGTTCCTGACGCTGCTCAAGCAGTGCTGGATGAAAGGCTCCTATTGGACTATATGCACAAAATTCAGCATGGTGTAACCTTGAGCGAAGCTGGAGAACAACATTTAATCCTGTAATTTTCATTATAAGGGGTGTTACGTTTATTGAAGTATCTATTCATTACGATAACAGCTCATTATCTGATATTTATTATTTGTGAAGGGGTCACCCGTGCCTCCTGTTAATCTGTCAAAAAGTTTGCCGACGCTTGTGCAGAATTAGAAAACAATTAAATTTAAAGCATGTCACCCAAGTACTGGATATATTGACATAGCTCGGTGTCAAGAAACCTCCATTCCTTTTCCACTTCTCCGTCAAAGCAACAAGGAAAAGTAGCAAATGTGCGGAGCTATTTGCATCAGTGGGGTCCTTCGTTATACCATTGACGAGGAGGTATCCTATTGAAAGCTCAATCTGTTGGRCTATATAGTACGGCATGGCGGCAAGAAAGCACACTTCAAAATTCACTTTACAATCTGCGCTTGACAGACACCATAGGACGAATGATCCGAGCACTATTGGTTTTCACCTTTAGGCTGCTCTCCAGAAACGGCGGGTTTACTAAAGAGCTGATTACTGAAACTCATAAATCGCGAACATGTTTCTAATATCAAGCGACTTTGCGTCAGCATAGCGTCGATAAGGAGGTCGCAATCATGTGCGAGAATGAAGTTATCGTTGGACTTGGAGAACTGGGCCACTTGTTCGTGAATGCACTTCTGGTTTCTTTATCGCAAAGTACTTTAGATGCAAAATATGTCTGTTTATATTGGCATTGCACATGTATTCATACGAACTTGATATCTACACAGGAGAGCCTACTTCCTCTGGTGGTTGATATAACGCAGCGCCAGAGCAGCCCAGATACGGAAGAGCACAATGATTCCGATCGTGATCCCGAAGTTCCGGGCGATCTGGTGGTGCTTCATACCGAAGTATTCCTCCGTGTATCCCTTGATCGTGATGTGGCCCACCGTCTCCGGAGCATCCAACATGGGCTGACAGCCAAGCTGCGAGTTCACGTTCTCGTACGTCTGCCAAGTTTCGTTCCAAGTCGGTTCATCGTCGCAGTCCGCGAACACCAGCGCGACCAGGATGGCGATGGGGAACTTGAACGGACAGATGTCGTACAGCCACGTATAGCCCGACGGAATAGCGTACGCGGGTGGACTGAATCCGATAAACATCATTAGGACGCTATTGAACAAAATTCCGATGATCTGTGCGACTTCTTCGGACGGCATGGCGTACGCGAAGAACTGGCCCAGATACACGAACATGAGCACAAGAAGCGCCGATGCAAGCCAGAATACGACCGAGGTAGCGAAGCCAGTGAAGCCCACGAAGTAGTAGAAGATGGCAGTGAAGATCAGAGAGCTCACAAAGCAGTACGGGATCTCGGCTAGCGTCGACGCCATGAAGTACCAGAAGGCATTGTACGTCTGCGATGCACGCTCACGGTAGAATGATTCTCGCTCCAGACACGTCAACGGCATGACACTCTGGAACACTGCCATAGAGCTGAAGAATCCGGACATGAAGACCATTCCGACTCCGGAGTTGAGTCCGGAATACGATGCGTAATCGTCATTGGTCACGAAGATCAATCCGAAAAGCATGACCAGGAAGATAGACAAGTACATCCTCGTCAACGTATAGCTCGGTGTACGCCAATACATCTGGAAGAAGCGCCAAATCACAAACCTCGCTTGGGTCTTCGAGTCCGCAGCACGTTTCTTACCAAAGACCATTTCGGGAAGTTCCGGAGACGGCGTCATGATGCCTTCCTTGGCCATATTCGTCTTCAGCTGCTGGTTGTACGGACTGTTCTTAAAGTAACTCACAAAGTCCATCAAGTCTTCGGTTCCATGTCCGACACCTGCGCCAATACACTCCAACATCCACGTCGCCGGGTTGTACCCAACCGAAAGCGGAGCCACACCCGGGATGTTCTCAAAGTAATCGATCAAATTCCGGCAATTCTCGCCCAGATCTCCGTAAAACGCCGTCTGTCCACCACGTTGTAGTAACAAAAGTCGGTCGAACAGGTAGAACACTTCAGCCGAAGGTTGGTGGATCGTACAGATGATGGTCCGTCCGGAGTCGGCCACCTTCCGGACGCCGTCCATGATGATTTTTGCCGAGCGAGCGTCCAATCCGCTCGTGGGTTCATCCAGGAAAATGACACTGGGCTGTGCAGCCAGCTCCACGCCGATAGTCAAGCGCTTCATCTGCTCCACCGAGCTGCCTCGGATGATCTGGTCGGCGATATCTTCCAGTCCGAGCAACTCGATACATTCCGTAACAGAATCGTATTTTTTGGCGTCGGAAACCGAGGCATCTTGACGTAAGAAGGAGCTGAAAGTCAGGGCTTCTCGGATCGTCGCCGCTTCGGAATGCACGTCCATTTGTTCACAGTACCCCGTACAGCGTCGAATAGCCAGATCCGAAGCCTCGTAGCCGTTCAACATGATCTTTCCCGTGATTTTGCCGCCAGTTTTACGACCAGCAATCACATCCATCAGCGTCGTTTTACCCGCTCCAGTAGAGCCCATCAATGCCGTGATGGAGCCAGGAACAGCGAACCCGTCGATACCCTTAAGTAATTCGAGCTGTTCCTTGGGATTCTTCGGATTCGGAACAAAGTAGTGCAAATCTTGGAACGCGACGGTAACCGGTACGAAGTTCTGCTCACGAGCGGCAACCGGGAGACCAATTAACACGTTCCCGGTCTTCCCCTTGGGCGTCTCAGCTAGAACGTACGAGCTCTCGTCTTCAATCGACTTAACCGACACATCGACATTTTCCGGAGTCTCGTACCGGATGAACTCCATCGCTAAGTACGATAAGAACATGAAGAACACGTACACGGCGATGAGGTAGACGAAGGCGTACGCGATAATCTTCTTCTCCGTCTCCATGCCGAACAGGTCCAAATAGTACTCGCCCATAGTCATCCCGTTATACTTCGTACAGTAGTCCACATCTCCATAGACG

>Contig_44

ACGCGCCGGATGTCGTTCTGCGCTACGCCGGCCGTTGCTGGCGTCCCGCCGTTTGTCATCGCTGGCCGCCTCACAGGCAATTGCAGGAGATCACGCTATCGATGACGAGAAGTGGATCCGTCTTATGCAGCAGACCAGTCGCCTGGAGACGAACGTGTTGTTGCCTTTAAACGAAAAGCTACTGGGCCCATTGGACCGCAAGCACCGTGAGGAGAAGCTGCCGTCGCTCCCCTTCGTCTTCCTATTAGGCAATCACTCGTCCGGCAAGTCCTCCTTTATAAACTACCTGCTGCAGCGCGATGTGCAGTCCACAGGCGTGGCTCCTACAGACGACGGCTTCACCATCATCGCCCCGGGCCGCGAAGACCTGGACCAGGACGGTCCGGCGCTCGTCGGTGACCCGGACTTGGGCTTCTCCGGGCTGCGCGTATACGGCCCGGCACTCATCCAGCGCACGCAGCTCAAGGTGCGCAAGGGCATCCAGGCCAACTTCATGCTTGTAGACAGTCCCGGTATGATCGACTCGCCGCACAGTCCGCCACACCAGTCCCAGTTTAACTACACGGGACCCAGTGACCGCTTATCGTCGCAACAACAACGGAGCAACAGCTTTAAGGGACAAGACTCGGACAGAGGATACGAGTTTCCGGAGGTTGTGCGCTGGTACGCCGAGCGTGCCGATGTGATCCTGCTCTTCTTCGACCCGGACAAACCCGGCACGACCGGTGAGACTCTGTCGATCTTAACGCGCTCACTGGTAGGTATGGACCATAAGCTGCACCTCGTACTTAACAAAGTGGATCAGTTCCGTAAGATCCATGACTTCGCGCGTGCCTACGGTTCGCTGTGCTGGAACTTGAGCAAGGTCATCCCGCTCAAGGATCTCCCGCGCATCTATACTATGTGCATTCCCACGAAGGACAATCAGATGCAAGCAGCAGAAGGTCTTGGCGCGTCGATGAAGGACTTGGACGCTATGCGTGAGGAAGTGGTGAGCGAGGTTCAACGTGCGCCGGAGCGTCGTGTGGACAACCTCATCACGAACCTGTACGACTCGTCACGACTGCTCAAGATGCACGCCGAGGTCTTTGAAGATCTGCGAGCTCGTTACGCTAGTGAGAAGTGGCGTCGCTCGGCTCTCGTGGCTGCGACGTTCGTGGGAGGCAACGCACTCGCAGCTTCGGCGTTGGCGAGCGGCGTCCCCATCGAAGCTGCGGCAGGACTATCCGTTGCCAGTGTGTTGGCGTCTGGTGGTGTGGGGTGGCACAACTCGACTGTGATGACCCAACTGGAGCGCGATCTGCTGCATGAGGACTCGCTGAGTGAGCTGTTCCGTCGTCGATACGGCCGCCAGTTGGCGGAGGGAGACGAGTACGTGTTATCCTTGTGGAAGCGCGTGCTGCCGTCGCTGCAAGTGGCAGCACACACTCTTGGCTTCAGTATGATGCCGAAGCTGAAGAACTCGGAGATTGCAGCACTCGACAACATCGTGAGCAACGAAATCCCGGAGCTGCGTCGACAGTGTGCGCCTACGGACAGCAGTCTGGCGCACCAAGTTGCCAAAATGCTCCGCAGCTAGAAAAAACACGAACCAACACAATAAACCGGTCTAGGGCAGCAGACTTCTGCTCTGACCCTCTTTTTGATTTTGTCTATCATCTGGTTAAAAGCTAGTCGAACGTGATCTTTTTGCCGCTAAAGGCTGTCAGACTCACCTTCTCCTTCTCCTTTTGTTGCTGCTTTGCCAGCCACGACGGGTGAGCAGCCTCCACTGGAGCAACAGAGGCAGCACGTTGCTTCTGTGGCTCTACATCCTTCGAAGCTGCACGCGGCTTGTCATTACGCAGCTTGTGCTTGTCTGTCTTGTCGCCCTTACCTGGTTTGGCCTTCGGTTTGGCCTTCTTCGGTCGCTCGGACAGTCCGTACTTGCCTACAGACGCCGCGTTGTCTTTGTACGCCACGAAGATGCCCTTAGAGCGGTCTTCCTTGCGCTTGAGCGCCTCCTCCTTGCGAATAGCCTTCATGCGACGTGCCACCTGACCAGGTCGGTTCTTCCTCTTCTTCTTTTCGCCTAGGAACTCAGCAATATCATCGTCTGCGCCAGCCATAGTGCCATATTCGTCCATACCCATTTCCATATCATCGTCCAGTCCCCCGCGTCCAGACAGAGAGCCCAGGAACAACGAGGTGGGGGCTACACCACTGCGTCCAGTATTGCCGACCAGCGACTCGTGGCGTCCACGTTTCAACGCGCGCTTCTCATGTGCCCGCCGCTTCTTCTCGGCCTCCTTTTCCTCCTTCTCCACCAGTTTCTTGATGGCGTCCAGAAGCGGCACAATCTGCTTATGAGCCAACATGCGGTCCATCAGCTTTTCATCCAGTTCCGAGTCCGGATTGGGAGCGTCTTCCTGCGTCTCCTTCTGTTCCGTCTCCCGTCCAGCAGCAGCAGAATCTGGATCCTTTACAGTTTCGCCCTGCTCCGCACCCTGCTCACCCTCAGTCTCCTCACTGCTGTCATCTTCGCTGCCTTCCTCGTCCCATTCTTCCACCTCCTCGTGCTCATTCTCTTCGGTAGTTTGGGGCTGTGGCTTGTCCAACCCAGTCTGAGCTCTGGCTCTAGTCACCAGCTTCTGCAGATCTAGAGTCTTTAGCGCTGCATGCTCGCGCTCGAACCGTTCGATACTGTTGCGCAACTTCTGGTCTCGCTTTTGTTGCTTAGCCGCATCCTCCACAGGCTCTGCGAGCTGTTGGCGGAGCTGCTTCAGCTGCTGCACTGCCTTGCGAACCAAGAATGCTCGGACCTTCTTCGCCTCGCGCTGCAACAGCGAACAGCTCTTGTGCACCAGCTTATCGTGTGCCGAAAAAGCATCGCGCTTCCTCTTGCCTGCCATGAGGTCAGATAATGGCTGGTGTCCAGTATGAAAAGTCCCGCGTGCAGTGATTGGTTGAAAAAGATTTTTTTATGAAAGGGTATGTTGGCTACATTGTAACCCTCGTAACCCCAGCATTTCTTACCGGTATTTAGAAAGGCACTTGCTATTCAACCCGACTGGTGTGGAAGAGACAGGCTAATGGTGCCCGACATCACCCGATCAATGCCCGACCATGCCCGACACAAAATAAGTCAGTTGAGCAATCAAGCGTCCGTATACGTATATCCGGATGTGAAGACTTTGTGGTTTGAGCGTGCAAGTACGACCGGCATCATGTCATGAGCATGAGAAACTTATTCAGTAGAGCTCAGTGGGAATGGTAAGTGCAGAATCAAAATCAACTTGTTGAAAGAGTTAACTATAGACACATATATATGTATCTGTAGCGCAGATACTGTAACTCCTTCCTACTCAGCATGCACAGACCTTGGGCACCAGCGAATACCTCAAACGCTAGCAGCACATACATACCACAATTGTATGAATCTACTTGTACCCCTAGATCGCCCATGTAGTGGTGTACTCGGTATTTCCTTGGTGCGTAGTTTGGCAGTTGCATTGCCAGCTTTTCTGCGATCGCTCGGACTCTAACGACATAGCTCGAGCTCATTGGGTCATAGATGCACACATCATTCAGATTTAAGTTCACCATTACGCAGCACCAATGCGTGTTGCTGCAGCATATAGGGATCAGAATTTTCTCGTTCGTCTTGCCCGCGAAAATTGAAGCGAAGGCTCTGGTATTGACAGGACTATCTTCATTATCAATGGAACCGATAACACCTGGATCCACAATGACGACATCATTTCGTTCTCCGAACAGCTTGGACATGACGGTATTCATGGTATCGTCGCTTAGCTGTTTTTTTCCGACGAGATCTAGAATTTCTGCCTGTTTGTCAAACATAACGTTTAGTTAGAAAGTGTTGTAGTGATGGCATGATTTCGTCGACGTACCTTTGCAGACAAAAGCGGGATTGACTGCATCTTTGACAGCATATCCGTCTGTAGCACCACTTGAAACGGAGCAGGCATTGGGAGTGTGAAGTCAATGGTGTCGACCCACTTCACAGCCTTCTCGACCTGTTTTACCACCTTTATTGCCCTGTGCCACCTATTCATGATCCCAATTGTACTCGTTGAGTACGCACCAAAGCCTGGAATATCCAGCGCCACGGCACTTTCTGCTAGGCCTTTGTTCTTCGACTGCAGAGCCGAAACCTTTTTCCTGCAAATGGCAATTTATTTAGGGCGCAATATCCGCGTGATCGACTCTGCTGGTATCAAAGCGATTGTGGGTGGCAAGCTCGATATTGTTCGTGCTGTAGGTGTCTTGAACTTGAAGCCATACTCCACCATTTTGAACTTGTTCACAACTGCAGCTGACGATATGTATGTTGCATCCGAAGACAGCGCTGCATCCACGGACGCCAAGCTTAAATTCGAAGCGTACAGCTCTGAGTCTTCTTGAGCTACAATGACAGTTTTTTTTACGAGCTGCCTTCTTTGATCTGGCTGTCTGTCTTGGTAGTCCTCGCGATCTTGGAGGACTCGCTATCACAAATGGCGTGTCTTCCGTTGCTGGTGGTAGTGTGCTTAATTCAACGTCCCCGACTGTATCCAATTCTTCCCCGACTGGACCCGATTCGTCCTCGACTGGACCTGATATGCCCCCGATTGAGCCCGATTGGCCTCCGATCGAGACCGACTGGCCCGATAAATCTGCAACCTGTGTTGTTTCGACGGAGTTGCAGCTTTTGGCATGGTCAGACTCAGAGGTAGTTGGGGATACATGACTCACATTTGCTGTATCCGCATGACGAGTAAGCAGTTCTGCCGACACGTGGTGAGACGTCCGTGCAGTCGGATCGTGACCAGTAAGTAGTGGCGGCGACAGCGCATCATCGCGCGAGTTGCTAGTCGAACCGTGACGCTCAGATTCAGCTGGTGGTAAAATTGGCACACCGGGATCCTCGAATTTGCCTCTCTTGAACCGCCCAGTCACACTTTGAAGATACTGCATCGCTTCTTTATACTGCGTCATGCCGCGTCCCGCCATGGTGTCACAAATTTGGGATGCAAATTGCAGTGCTTCACGATATTTGCGGTTCGAGTCCCACGGTCTCGCCTGTGAGGATATCACATTTTTTACCTCAAAAGACTTTGGATCCGCTGTGTCAGAGACGACACTTGAATTTTCAATCGCCGCACGCACAGTAGACAACAGCCACCGCCTGTTTAACAGTAGTGTGGGGATGACTGTCTCCCGACCAAGAGCCTTTCGGAGGTAGAAAACGTGTCGACAGGGTAGCAATCTCGTGCACATGAATAGACAGGAGCACTTCCATTCGCGCTTAGTAACTGAATACTCGGCGTTCATCTCGTCCAGCGCGTCATCAGCTGTAGATATACTCTTGATAAAATACACGTCTAGGCAACCCTCATAAAACGTGTACGACGCAGATCCAATAGCATACGAATACTGCCCGAATATAAGTTCACAAGCATGCTCACTGACAATATTCGCCACCAGTGTCATCTCGCTGTCGTAATCAGGATGCTGGACGGTCACATTCTTGTATACTTCTGCAATGAACAATCGCTCTTGTAACGTTTGATAGTACATGGTCGAAGCGATGCACTCATCCACTCCCATAAACGAATCCACCCACTCTTTAAGCTGCTTCCAAGAGGCTTCTAGGCGATTGTTGGTATAATTGCCGAGTGTGACGGTATTCTGGCGCCTATGTGCACACCAGAGCTCACGACAGCTGTCCCAATTCTTTCGAAAGTACTTCACAAAAGGATGTTCATCAATGCGAGAAGAAATATCTTCCAGTTCATGATCTGTGACTTCTACACCGACTGAGCCCGACTTCACACCGACTGAGCCCGACTCCACACCTACTGAGCCCGACTCCACACCGACTACGATGCCAGATCGATCATTCGCAACTGGACCGGAATAACCAATCGACGCAAGATGCACCATGTACTGGAAGCGTTTGTCGTACTCTGCTTCCGTTTTTGCGTAGACAAGTAAGCTCACAACACCGCCAAGTTGCTCTTTCTGAGACCTCGAAAATCCATAGTCAGCTGAAGCAATCTCTTCCCGTAGGTACTTCAGCACGTGGAATTGGCAAAGTAAGACTGTCACCCCAGGAAATGCCTTTTCAAGTACGGACAGTTCTGTGAAATTCTTATCCACCAGCACACATTGAAGTCTTGACCACGCAGGATTGTTCGCTTTGAATTCCTCTATCGCTGTCAACAGGGTTTCATAACGCTCATTCTGAAGCAGCGCGTGCTGCACAAACTGGCCCTTGCCGAACGTATCGTGTGCCATGAATGAAAAAACTTTATACTTAGACCGGTTCGTCCCGTGCGTTGCGTCAATCATCAGAATTTCAGGAAATTGGTCGAATAGTGTTCGCATGTGCTTTGTTTGCAATGTGATGCAAGTCGCAACTTTCTGCGTATCCAATAGCACACAAAACAACAAATGTTAACTTCATGAATTGACATTAGACCTGATAAAAGCATTTACTCTACCTTGCCACCAACGTCGTCCGACCAACGTTCCCTGGCACATCACCGAATTGCTTCATCCACTTCTTAAGCCGCTTGTCAGTGCTTGAAGGCCCATCGCGTTGTTCGCGAGCCTTTAGTTTCTGGACGAGGTTTTGCACATCTTTTGGGGTGGGGTTACTGTCGGTGTGCTTCATTAGGTGGCTGATGATACCAGACTTCTTGACACCTTTTTTTTCGAAGTAGATCAACTGTAGTTAGCGTTGACTCGTCGAGGATAATGCGTTTCGATGGATGTGAAGAGAAAGCCAGGCTTGTCAGACGATGATTGTGCTCCAGTCGGCAACGAGTGATCCGCACAGCAAACGTCGTATTCTTTGTATCAACTACCTGGACGCAGACATTAATCTGTAGAGCATATATAACCACTGTCAGTCAGTGAACTACCCGATTGACCCCGACTGGACCCGACCAGTCCCGACCAGGCCGACATATATGTACCTGCGCCTTGCAGCCCTGTGCTCGAGTCTCTTGGCGTGGCCGCTTCGTAGCTACAGATTTGTACTTCCCTGAGTGCGTACAAACGAACGTCCTCGAATACTGTTCCCACGATGCTGGTATTTTAGCTGCTTTTGACTGTGACTTGTCAATACTGGCATTGCGCTCTGCCACGCTGGTATTAGAGCGCGCCCTGAATATCTACAAGACAGATAGCACAAAATTAGTGGGACAGCGGGGTCAGACATCCGTTCTGAATGAATCTCGCATACTTGAAACGTTTGGGCTTCATACGCAGCCAGGTACTCGTTGAAGGCCTCCCACGAGGCGAACTCCACCGTGTTGAGTGGTAATGCCACCACGCGTGTGGGGGGACCACCCTCGCTAGTGCTCCTGGTTAAATCGGTTACATTCGACACACTGAAAGCCTCGGAAGACTCTCCATCACCCGAACCAGCACCGCTCGCAGCGGGTGTTGGGAGCCAGACGTCGTCCTGGGAGTCTTCTCGGGGCGGCTCAGACTCGCTGCTGTCGCTCTCGTCGATCTCGCGCGCGACAGCCCCAGTTAGCGGCCTCTTCTGGACGGCTCGGACGCGGAGATCCGTTTCCCCCTCCTGCAGCCTTACGATCGTTACGCTGTTCGAAATTTAGTGGCTGTCAGGCTGAATGGTATACATGGTACACACTGTATACATCGTACGGATGGGAGCGCGTCAAAAAATTGTAGGGGAAGGTTAACTCAGTCGGTCAATATAGACACTCTCTACTTTAAATAGGTATTAAAATATGACAGACAGCACGTGGCAAACATGTTCGTGGGATCAAATCACTGAAGCGTTTTGATGCAAAACTTTTGAATGCACCGTCTATTAATAATGCGACTTTGATACCGTCGGATCAGCGGTGCAGTGCAATCCACTGAGTGCAATGCACTGTGCATGTATGTACCGCTCACGACATTGTGCTGTACATGTATATCTCACCATCCAGTGATTCAAGTGCACAGTACTGCAACCGATTGCTGTAATGCGGGCATTGCGTTTTGTCGCTCGCTGCGGTTGGTCTCGACCACTTCCCGTTTAGCTCCCTACCCGAAAAAAATGCGTTGTCACTGGGTCTTATACGTACTGCTGGTAGCCATCACATTCGCTGATCATGCTGACTGGAGCCCGAAAACAACCGAGATACCGGTACGTCCAATCACTCCCATGGATAGTGTCCCGAGGTTACTGCGACTGTACGAGTCTACGAGAGCTCACGAAGAAGAAAGAGGTGTAAGTGCTCCTGCAGCGGAAACAGTTGTCAACTCACTCAAATCCGCAAGCACTCAACTTGACGAATGGCTCGCAAAGGGAAAACCTACCGACGACGTTTTTAAGCTTCTTACACTCGACGTCGCCGCGGACGATCTTCTGGCCAACTCGAAGTTAACAGAGTGGATCACGTACATGAAGCTGTTCAACCAGGCAAATCCTAAAAAACAAACGTCGCTGATCAGAACCCTCACAACTCACTACGGCGACGAAGCTCTGGCCAAGATGGTCGTAGCAGCAAAGCAAGTGCCGGACACAGCGACTTTCGCCAAGCGACTACAGACCGAGCAGCTTCAGCTGTGGCTTAATCAAGGGAAATCCCCGGACGATGTGTTTACATTGTTGAAACTTGACAAGGCCGGACAGAAGGTTTTCACCCACCCTGAGATGGTCTCGTGGGCGAAATATGTGGGTGATTTCAATAAGGTGAACCCGGACAAACCAGTTACTCTGTTCTCTACGCTAGCTACGCGCTTAAATGACGAAACTCTGGTACAAATGCTCATCGCGGCGAAGAATGTTCCGAGCACCGAGAAAATTGCAGTACAAGTGCAAGCTGTCCAAACGAAGCTATGGCTGAACACTCAGAAGGAGCCGGGGGACGTCTTCAAACTGCTTCGACTTGATAAGGAAGGACATGACATCATCCAGAACCCTCTTTTTCGTGCTTGGGTCCAGTATACCGACGACTTCCGGAAGATTTACTACGGAACAGAGCTCACTACAATCGCGACGTTAACGGCGAAATATGGTGACGAAACCCTGACAAAGATGATCCTTCAAGCACTTAGCTCGCCAAGTACTGCAAACATCGCGAAACGGCTCGAAACTGAGCAGTTGAGGAACTGGTACATCCACAAATATTCGCCTCAACATGTGTTCACGACGCTCAATTTATACAGCGAAGGAGTGAACGCGTTTGACCGTCCGCTGTTTCATGTTTGGAGCAAGTACGCGACGTATTTCGGGGCTGCAGAGCCAAAATATAAACCGAGTTTTCTCACGAATTTGCTAGATGTGTATGGCGAAAAGAACCTCGTAAAAGTGATCCGAGCGGGCAGCAAGAACCCGAATACCAAGAAGATGGCTACAGAACTAGAAGATGACCTAGTCAAGGTCTGGCTGAAGGATGAGAGGATCCCGATGGACATATATGCTCTGTTGCGGCTGGAACGAGTCGAAGACCCGAAAGATCCATACAGACAGCTATACTTTAAATATTTTAAGGCATACGCACTATCTTGACTAAAAAAAAATACACCAACATATTGTTGTTACAGTCAGGTAGCTTAGGGTAGGCCGGCAAAAGCCTCAGCGTATTTTTTATAAATCAAGCTGCTCGCGTCGTGCTTTGCTGCTCCATCCACACGCAGCAGCGAGAAAACCAGAGTCGGGCTTTTCCTCTCGTCCAGCCAGCGATAGAAAAGCTTATCGAATAGTTCAGTAGCGAATTTCTTAGTGCTTAGGACTTTTTCGGCTGCAATGAGGATCTGAGCCAGTTTCTTCTCGCCGTATACCTTTATGAGCGGGGTTATATAACTCACCTTAATCCTCGGGTCCTTCAAGCTCACAAACTGCATGTAATCCTTCCAAAGAGGGAAAAGGGACCGTTCGAACACTTTCGACCCCGATTTGTCGAGTTTCAGCGTTTTAAACACGACGTCGCCAGATTTTAACGTCGCGTACCACTCGCGTTGCAGCTCGGAGCGCAGAAGGTTCGCAACGCTCTGCGTGCTCGGCGTCTTGTCCGCTGATAGACTCATTCGTGCCAACACGTCGTCGCTGTAGTGAGCTTTTAGGACCGAGATCACGGCCACTGTATTCTCTGGATTGACCATGTTAAACTCGTCCGTGTACTTGACCCAGCTCTGGAATAGAGGACTTTTGACTAGAGATATCCCGTCTTCATTAAGGTTTAACAACGTGAAGATGACGCCTGGAGATTTCTTGTTGCTGAGCCAGTTTTTGCTCTGTTCGGCTTGTATTCGGACTGCAATTTTCTCCGTCATGGGCGACTTTTTCGCTGTAATCAGCATCTTGACAAGAGTTTCTTCCTGAGTGTAGAGGCCTTTTAAATACGAAAATAACGTCACGTGTTTGTCCGGATTGGCCTCGTTGAAATCGTCCACGTACTTGGCCCACGCGACGATTTGAGGCTGCGTGAAGAGCTGATCTCCTGTTTCGTCAAGTTTCAGCAGTTTAAACACGTCGTCCACGGATTTTTTTCGACCCAGCCACAGCTGCGTTAGTTCGCTCTCCAGACGCCTAGCGGTCTTTGCTGTGGATGGCATTTGCTTGGCTGTTTCGATTAGCTTTGCCATTCCGTCGTCCCCGTAGTGGGCAGTTAAGGTCGCGATTAAACTCGTCTTTTTAGCCAATTCCGCATTGTAAAAACGTTTCATGTAGCTAAGCCAAACGGTTAAGAGGTCGTCAGCTGCGTTGTCCCGTAAGAAGAGTTTAAGCGCGTCGTCGGTTGAGACTGCGTGTTTAAACCACATTTCGAGTTGACTATCGGGCACTGACGACTTCAGGGCGTTGCGGAACATTTCGACGGCGGATCGAGTGGTATCTCTGCTTTCGTGCACACTGTTGTCTGTGCTCAATGTATCGGTGGTGAGCTCATGAAGACTGGAGGGGACTTCTTGCCATCCGGTCGCGATTGTACGCTGGTAGTTTGTGTGCGCTCCTGCGTTTACAGCGGGGAGAGCAGCGACAACCAGGACCACGACGTAAAGTGGGCGCATTACGGATTGCACCTTGCGTTTGGTCAGCGAAAGGTGGAGTGAATGAAGCAAATGATGGGCATCGCTGGCTCCATCACTGGATGGTGAGTCTTGTACATGTATTATGACTACCCGTTTTGGGCAATTTTCTAAACCCATTTACATTTACATTGTCGTAAATTGCCCAAAACGGGTAGTCAAGTCTCTTGACTCAAGAGTAAATATAATTAGAGTTACTACGTACATGTATTAATAGTACCACTACTCGCCGAGCGCCGCAGCTACGCGGCGGTAGTTTAGGTTCTTTTCTATATGTTTGTCTTTCCGCCTCCGCTTCGCTTCGGCGGGATAATTATTCCAAAAACTCTGAATCCCCATGTGCCTAAATCGACTTCTTAAAATTTAATAAATCTGGGTTCCATTCAGACCAGTCGCTACAATTTCCCAGTAAGCCGTGCAAGTATTACAGTACTACAAAAGACTGAGTCCCTAGCATCATACTTACGATGCTTATGAAGATTACTCTAGAGGTCTTGACAAAGTCTCTTTATGATTGTTAGCTTTCATTGTACCTACATCTTCCACCTAGAATCACCTGCACTTCAAGGTTTTGATGTAGAAATATTAATAAATTATCTGAGAGTGAGGTAACTGGCTGAGCTGATGAATCGTAGTACATGTACAACAGAAGCTGGTCAAAAGAACTGGCCGTAAAAAGAACGTTGTGCTAATAAACGTTGCCACCTGATCCATAAAGATAGATGAACAAAATGAACTCGTATGTGTTACTGCTGTAAGCCGTTGTTTTCTAAGAGTTGTTTAGACAGCAAAATCCGATACTACTTTACTGGCGGACGTGTCGACGACAGCGGCGTAGGATACGATTTTAAATGGAGCGGCAAGACAACAACAAAACACGAGAGGTGTGAA

>Contig_45

TCAATTTATTCCTGCTTACTGTCCCTGGATAAACGGCTCTATCGAGCGCGTTAATCGAGACGTTCTGCAAGTGATCAGAGCGATGATTCTCGAGTACAAGATTAACCATCAAGATTGAGTGTACCTGGTGCCCATGGTACAGTCAAGCCTCAATCATACTGCGATACCTTCACTTGGGAACAGCGCTCCTTTGATGTTTTTCACAGGATTGCCGTGTCCATCTCCTCTTCGTGAATTTTACATGCCCAGCAGTGGAGATTTGGTGAAGGTTCCTGTTAGTGACAAAATGGAAGATTATCTCGGTAAGTTGCGGAACAGTATTCACACCATGCACTAGGATTTAGAGGATCAGCGCTTGAAGCAGCAGTTGCTGAACAAAGAGCGAGAAAGAGGGGGAAATATTGTCAACTTTACTGAAGAGGACTATGTGTTACTCTCCCGAGTTGATGAAAAAGCGGAAGCAAACGCCTGGTAACTTGAGTCGGACCTTATCGTATTGTGCGAGCAGATGCACGCTCTTTCCTCGCTCAACATCTGATCACGGGTACAGAGCTGGATGTCCAGGCTCAAATTTTACGCGGATTCCAGTTTTAACGTCACTAAAGAGCTTTTGGAGCACATTTCGTCTCAAGGTGTTATTCTCGCTGTGGATAAGCTCAAAGGAATCGGTGGAATAGTAGCATTAATGACTTCGAAATTCTCGTGCAGTGGAAGGACTTGAGTCAATCGAGGCCTCGTACGAGCCGCTCACCAACCTAGCTCGTGATGTTGCAACATTGATACAACAATACGTTAGCACCGCTGACTAAGATTTGCAAGAACACTGGCAGCGAGTGACCCGTGTGGAATTGCAGCAGCCAAAAGCTGCCGAGGCCTCAACAATTGGAAGGCTTGGACGATGCAATAGACGTCGCAAAGCCAACCGCAATGGACAACCTTCAACGCGCCCGGTCGCCCCTGGTATTTCGGCTGTCGAATCTAAAGAGCCTAACAGAGGTGGTTCAGCTGATGCAGTGCTTCAAACATTAGTAAACCGACCTGGGTTTGAAAACCAACAATCAAATTTGTCTACGACTGATACCCCTTCTCAGCAGCACAGGATCGACAAAGAAGTCGACTATCTTCAGGGCAGCGCTTATCTCCAACGGATGCCGCCGATGGAGATCGTGTCGGTCGATGTACGTGCTCGCGGACTGCTGGTGCCGACCAATCCGCAGCCAGAACCCAGCAGGATGCAGCACATTCCCGCAGACACTGAAGGAGAGGAACGGGTTTACTAGACTCACAGGAGGCCAGGAAGTACCTACATGGTGCTTGATATGACTGCTGACTGGGTGTTTAGTAGAGAAGGCGTACTTCAGCGGCGAAAACTTCGCTTTAGGGACAGTGTGGTGGATTGGTGAATCAAGCAAGCTTCCTCCATCTGTAGTGACCCTGGAGTACACGAGGCGGGCCAGGTACATCTTGGTCAGTGACGTTTCCTCACGTCATTTATATAACAGATTCTTTTAGCTAGCAACCAGTTGTCCTTGTGGATTTACTTTAGGCCTTGTATGTCCCCTAACTGCACCTCTATGTGCTTGAGAAAGAAGGGCAGCCTCAAGTTGTTCCTGACTATCACTCTATATTTTACCAGTAACCAGAATAGCAATTGCACACTAGGCTATCCGTTCCGGCAGGTTCGCCACCTGCCTTGGCGGTGTTTTTCCTGTTTCAACTCCGGAAACCAGTACACGCGGAATGTTAATGTGTTCAAGGCGCCCAAAAGTGAATTAAGCAAGTAATTAATAGCGCCCAGCGAAATGGCTGTTGCGTACATACAAGTATCAATGTCCGGCCCAGGCTTAAGCCAAGCATGTAGACTAAACGATCCAACGACTGCTTGGCAATGGCAGGGTTCTCTAAATAATTAAGTTCTCTTAGACACGAGCCTTGACTAAATGCCCTCAGCGCGTGTGCCAAATCCATACCAGAATTCAATTTCCTCAAATTGCCAACTATTGACTAAATTCTCAGAATTTTATCCGTGAAAATATTTAAATCGTCCTAGAGTATTTCTCTGCACTTTGAAACTCAAAGGTTCTTAGCAGATGCTTTGATGCAGACTCATCTGAATTGCACATCAGCAGCACCATCACGACATTTTTGATGATGTTGGCATTTGAGCACTAATCAATGCCGTAATGATTACAAGCGAGGTCCGTAATCTGGCATCCTGATAGCGTTAAAATTCTGTGAATACAGTGCCTGATTCTATTTCCTTAGACAAAGGGCTCATCCAATTCAACATAAAATTAAAATTCTCCGAGCTCTACTTGATTGCATTCAGAAAAAAAGGCGTTTGGCAAATCTACCTGATTCATTTATGTTTACGGTAAAACGACTTTGAAAGACCCCTGAGTCATGGCCCATGATACACAGCAATATCACAATTTCACTTTCCTGTAGCCCAACACTTATTTAAACTGTAAAGCCACTATGCGCTCCTGCTTCGTTCTGATCATTTTCCTAGCTGGAATTTGTAAGTGTCTCTCGCTCAATTGGAGTGGCAATACGTTAAAGGAACGAAGCCACACCAGCACCAATTCTCTCCCCAGTCCACCTTTCATCTCAACAATTAAGACCCCAAAAAGGTTCTTAAGATCCTACGACGCACCTAAGCAAGACAACATCGGTCATGATACGGACGAAAGAGCTGGAATTTCTGGGATAGCCATGATTGACGATCTTGCGTACAAGTGGGCGTTGAAGAATACGAGGGATCCAATGGATGCATTCCAGCGCTTACATGTTGTGAAAACTGGCGGCAAATTGGAAGGCAACAAGGAATTCATTCGGTGGCTCCAGTACGTAAATCGATACAAGGCGACACGACGAGTCAAGTTCGGTGAGGATGAGCTGCTCAGCCTTCTAATGAAAACGAGAGCAGAAGAAGAACTCGTGTCCCTGTTCCAATCACTTCGACAATACCCGGACATTACGAAGATGGCTAGCGATATGCAAGCGTCCATGATTTTGAGCTCTGCGTCTAGTCACAGACTGATCAATGAGGCATGGTTAATGTCCCGAGAAACTCCCGGCGAAGTTTTCAAAATCTTGCGACTTGGCGATAACAGCATCAGTCGGCTAGAGAATAACCCCCTCTTTATTCAGTGGCTCAGATATGTTACGATGTACAGGGCTGTACACGGACGGATTTGGAAACACTATTTAAGCGTTTCTCGACTGTACCACAATTTAAATTTTTTAATTCAATCGTTGCAGAACTTCCCAGATTTGGAGAAACTCGCACTAAGCTTACAGACCCACCTCTATCGGAAATGGATGATCGAGATCCAGCTTACCCCATCTGAGCTCTTGGGTCTTCTAGAGACAACCAGAGTCGCGAGAAGTGATCCCAAATACCGCAATTTGGAAGCTTACACCATGTACTTCGCTGAAAGCCGAGGTGGTACGCCTTTGTTGAATAAATTGAAAACGCTGTTCACGGATGTCGATCCCTACGCAGCACTGTCTGCCGCTTCGAGCGCCTAGCAAGGAATTTCACCCTTTCATTTAATACAACGTTTAAAAAGATACAGTCATTTTGCTTAAATTAATGATTCTTAGACGGAATTCGTCGGACATTCGTCAGTCATCGCTATTTTCATGAAGTTTGATAGGCTACCTAGAGAACGTCATACAGTCCGTGTTGAGTTTGGCAAATGCTTGCCGTGTCTTGCTGGCATTATTCATTTTCACAAATGGTTCTTATAAGTTGACACAACAGTGCTCGCACATTGAAAAGCTATGACAGACAACTCAATATACACGTACGAGACATCTAAGATATCAGTATGCTATTTGATATCATAGCTAAGCACACTCAATCTGATAGTTGCTCTTCATAGCGGTAATGTGCGCCGATGACAGCGCGGCGCGCTGACACTGCTCGCCACTGAGCCATTTAGGCCATGGAGGACGACTGCCGGAAGGCGTCCACAAGCTAAGGGAAAAACAGNTTTTTTTTTCAGAAAATGTCAGACAGAATGTCCGACGCGAACCAATCAGATGGGTACGATGTCCGACAGATTTTGAGGAATGCTCATGAGAACCAGCCTGCTGAAATATAGGATAAATCACGCATATAGGAGATAATTTGGGTGAAGGAATTACAATTGAGTTGCCGTGTAGCCTTTTACATCCAGAATATGAATTTAAACGCATTTACTAAAAGAAGTTTATACCCCTACTATGAGCTGAAAAGCTGGCTCTGGCGGGGCGTCACTCGTCAGTTAAGTTGTATTATCAGCCTCAACATGTGATGCAGGCTCAATGCAAGGCGACCCAATTGTCTACGTAGATTTGCGGCACGTGTATCGCGCAATCCTAAACACTTTCAGCGTAAGACGATTTATAGTTCTACACTCAGTCTTCTATTTTAAAAGAGGTCAGTTTACGAAGGGCGCGGATGGTATTCGAGTGCACGTTGAGGGGTTTTCGAGTGCAGACATTTTTGCCACGAAATGGCGGAATGCCGAGTGCAGATAACACATGGCGATATCGTCGGCTCTATACTTGTCTTGTCCGGTTTATGCTTGTTTGAATTTACTACACTGTCCTTGGGGCCGGATGCAGAGCTCACCAGGATGTAAGAGGAGAAAAATACATAAATTTGTGAGAAGAAAACATGAATGAGCCTTGGATCCTGGTACTTCCTACACAAGGTACGCTGAGCTCCGCTAGTCCAGTCCTACGCCCACATGCTCAAAGCAAACTGTCACTCCTGATCGTCTTAGCTGTCCAAGTACTGATCGTCGTCGGCGTCACGGTCGACACTGCGGGGATGCGATTCGTGTGTGCTTGCGTGGGCGGCAGCACTCTTCGTAACATCGAGGCCAGTACGTCTTGATTCTACGATCAGCTGCACCAATTCCTGGCACTCTAGGAAGACTTGCTTGCCTCCCTCGAAGTCGACAAGCCATCTATATCGCGACTTGCGCGCTTTGCATCGTTTCATGAACCGAGATGCGTTGGTACCGTCCTCTGGTCGATGGACGACTCGGGCTATGCTCTCCCGTGGAGCTCTCCACCATTGCCCTGAACACAATGAGAATTGCACACGGTAGATAGATGAGTATGAAGTAGAAGCAACTTAGAGTGTTACGGTGAACGTACGGCGAGCGTTGAATTTGCTCTTATCGTGTTGACGGAGAAGCGCTTGAACGAAAGGAACTCTTACACTGTGGGCTGGCGTAGCTGGCGAGCGAAGTTGGCGATGAAAGCACACTAACCCTAGCTCGTGGATCGCTTTGTAGACGAACGCCTGCTTATCGTTGCTGCAACATGTAGCTTACATTAGCTACGTCTGAACTAATGAAGGCAGAGCCTTGACGCATCTTCTTCCGTGCTCAAGCCTTAACCTCAGCAGTGCACTCGGTCATTCCCTCAAGTTGAACAAGGGACACTCGGTTTCACACCTTTCGAACAAAAAACTAACGGTGCACTCGATTTCCTAAAAAAAAAAGCACTCGAAAGTAATATCGTTCCGTTTACGAATGTTACCTCTTGGGAAGTATTAAGGAAATACTCATCACAAGACTTTTAAAATATAATATGGACACCTTTTTGGAGAGGTGGAATATAAATTCTCATCTGAATTGCACATCGACAGAACGGCAACGACACTTTTATTGACGCTCATTTCTTAGCAAGTGAGTAATCGAAGTCCGTAATCTTAAAACTAAAAAACGTAATCAGTAATAAGAGTGGGATTTAAAGTAGCTCTTCACTATTGAAAAGTGAGGAGAAGAAATGTTCTTCAATGTCGTGCTGATCACATTCATCGTACGGATTTCGCTTTGCTCCTCGCTCAACTCATTGATAAGCACCAACCAGCTCAAGGGCCTGAGCCACACCAGGTCAACGTCCATCCCTGCAACGAAGAGATCGTATTCCACAACAAGGTTTCTAAGGTTCAGCGACGCATCTAAGCACGATGATATTGATGATAATAGCGAAGAGAGAGCAGGGGTTTCTGGGATAGCATGGCTTGGTAATCTGGCATCCAAGTGGGCGCTGAAGAACACGAGGAATCCGATGCAAATTTTCAAGCTTTTACGTACTGTGAAAACTGGCGGTAAGCTGGAGGGTGACAAGGAATTTGTTTGGTGGCTTCTGTACGTGAATCGATACAGAGCTAAGTTACAAGACAAGGCCTCGTTCAGTGACGACAAGCTATTTGATTTGGTGCGGAAACTGAATTCGGAAGAAGAACTGGTGTCTCTGTTTCAATCGCTTCGACATTATCCGGACATCAAGAATATCGCCGATGATATGCAGGCGTACCTGATCTTGAGCTCGGCGTCTAGTCACAGACTGATGAATGAGGCATGGTTAAAATTCCGAGAAACCCCCGAAGTAGTTTTCAATATCTTGCGGCTTGAGGATGAACCTCTGTACGCTCTCGACGGTAATCCCCTGTTTATTCAGTGGCTCAGATACATTAAAGCATACAGAGCTGTGAATGGAGGCGACTCGTTCACAGACGTGCATGTGTTCGACTTTTTACATGAGTTTGCTTCCTTGCCGCGATTTGGAATATTTCTTCAGTCGTTAAAGGACATCCCAGATTTGAAGAAACTCGCAAAAAGGTTTACAAACGCAGATTGGCTAACCCCATCGCAGCTCGAAAAAATATTTGGCTCACCGTATCCAATCAACTTCGCGGAACTCCCAAAGAGTGATGCCAGGTACCGCAATTTGGAAAGCTTCACGGCGTATTTCGCTGAGTACTGGGGAGGAACAGCATTATCGTGACAATATTGTTCGCCAAGAAGGACCCATACGCTGCAAGTTGAGCCGCTGCAAAAGGTTAAAGATTCCTGACTTTAGCTGGTCGAAAGTGTTAGGGATTAATATCCCATCGCAAGATTAGCGAAGTTTTCTATTGCAGTCCAACAATTGCTACGATACTTACTTCGGCGTGCGCAGCCGTCTGGAGAGCCAAGTGTGATGTTTCTAAGATATTTAACATTGGTAGGAACCCTTTTTACAGCACCAAAACTGAACCGCACTATTGTTTTTCTTCTGATGAAAAACTTCGTTTCATTTTTCATTGCGTCTACACCGGATTTTAAATGGCTTCACAAGAAGCTTACAGACTTTGTGAGCTTATTGTGAAGCCATCCTAAATCCGGGATAGATCCTGTATCAATTGAATGGATACATGTAACCCAAAGCACGGCAAGGTCACAAAAAGCTCGTATAATGGAGTGTTGTCCGAGGAAAATTAAACCCCGTTTTGACCCGAAAAACACCGCAAAAAAACGGCTCTTTGCAAAAACGCAATTACTTTAGATATTATCAAAGTGAAAAAAATGAAGTACAATTATTGCAACTGTGCAGGCTCAGAGGCTTGTTTACCGAATGACTGTGGCGCTTGAATTATTATACACTTTCAGTTTGGCCAGATCCCTTTACACCACGCTTCAATTTCCATGCTGACCATCTTCCTTTCGGGAGATGTTTATGTTTCTCGTTTATCTTGACAGCTCACGGACCCGGGTCACAACGGAATAGATGACAATGTGAACAGGTATGTCGATCGACTTCTACTGCATGCATTACTAAAAAGCTGTCGAAGCGGCTGCAATTACAACGACAAGATCTTTGTTGTGGAACAATGGTTTTACTTTATCCTGCAAAGCATTTCCACCATGTTGCTCTGCGACGTACATGGTGTAAGCTTCCACATTGCGATATCTGGAATCATTCGTCGACAGCTCGCGACATTCATAAAAATTGCAATGTGGAAGATAGACTGCCGTTGTCAAAATAGATAATAGCTTCTCAGAAGCTCTCACGGCGCTTGCGTCCAGCATAACCAGCGACAATCACTGGGCGAGAACACAAGTGTTTGGCTTCAAAGATGGCTGCGCACGTTGTAGTGCGACCCACAGTGAAATTGTTGGGCGCACAAGCATTATCGGGTGCTTCACGTTATCCCGTCAAATACCTTTTTTATAATTATGGAAACTTCTTTATCCCACACCATACAAACTTCAAGTAAAACTCAACCTCACCAGCCCTCTGGCGTCATTATCAGTCTGACCATGGAGAAGGATCTTCGCGACGCATATGCAGCACTCAAGCTGCTTCGGGCAGACCTTGTGAAGACCCGCCGCGATAAACGTGCTCTAGAGGCCTCACTGTCCCACCTGCAGACCCACGGGCCGCCTCCCAGCGCTGCACAAGCTCGCGAGAGTCGCGAGACGCAACAGATGCAACACGAGGACGCCAATGCGCAATTATGGCGTCTCGCTGCAATTTACGAAAGTCGATTGGCGGAAATGGAAATACAATTATTGCACAATAATTGTCAAAAGAAGGAGGCAACTCCCGAAGTTGAAGACAATCAACAAGAAGTCGAGGAAGTGGAGAAGCTCGCGCTGCTTCACAAGCTCCACAGTCTGACCGCCACCGTCGAGCAGCAGACGCAGACGATGCTGGCGCAACAGGCAGCTTTTGCGCTGCAGAAGGGCGAGCTGGAGACGACGCTGGAGGACACACAGCACCAGCTCCAGGCAGAGAAGAGCAGGGCGACTGATGCGCTATTAGAACAACAAGCAGCTAAAGAGAGGTATGAGTTCTTGGAGACTCAAGTGGAGATCTTGAAGCAGGACAAGAAGACGTTGGAGGAGGAGAACCATACACTCCACAAGAACCTAGCGACGCATGCCCAGACGAGTAGAATATTGCAACAACAAGTGCAGGAGAAGGACGAGGAGCTCACTGTGCACGAGAAAACTATTCAAGAACAAAAAGAGAGACAAGAACGATATATAACGACGCTGAGAGACTTGGAGAATACGTGTGAGATGTTTAAGACCCAAGATGCGGCTTCTGAGGCCAAACGAAGTGCTGAAACGAAGGAGTATGAAGCGAAACTAGCAGATATTCAAGAAACATACACGTTCAAGGTGGCCAAACTGGAAAATGAGCTGGAAACGACTCATAAACAACTCACAGCAGCGACAACTAGCCGAGCGATCGAATTGAAAGAGCAAGACGTCAAGTTTCAAGCGGTTTTGGACAAATTGAGGCAGCAGGAGCAACAGGCGACGACACTGCGAGACTTGGAGACTAATTTCGCGGACGTGCAGGCCAAACTCACGATAGCGGAGACCAAATTGGCTGATGGAGTGAAGCAATACGAGCAACAACTTGCTCAGGCCTCGCAGAAGCTACTTGTTCAAGAACAAGACCACGAACAGCACGTAAGTTCTCTGCGTGACGTGGAAAACGAGCTGGCACGAGTTCAGACGCAACTCACGGATACGGAGGCCAAGTTACAGCTAAACGTGACACTATTCGACGAAAAACTCGCGCAGACTTGCCAACTTGCTGCTGAACATGAAAAGACAGCTCGAGTGCTAGCTATGGAGAAGAAGGCGCTTCAACAAGACGTTCGTGAAGCTCGACAGACATCGAAAGTCAAGACCGAAGAGCTTCTTCATTTCCAGTCGATGATGAAGCAGAAAACTGGGGACCATTCGCAGCGTTTAAGCCAGTATCAAGCGCGTTGTGAGCGTCTAGAGACTCAACTATTATCATCTGAAGACCACAAGGTGTACGAAAGCAATCCAAGCGGTGAGTTGAGAACTTTCCATGGTCTTAACGACGCCGAAATCATCGCGCTGCCCTCTTCCAAGGCGTGGGTTATATTACACTCTGCAATAACCAAGCTGGAAGACTTCTTCCCATATCTTGAAGCACTGAGTAGTGCATTACAAGACGTGTTAGCCTTGTGCAAGAGCCATGCGACTTTTCTTCCAACGCTATGTGAGCGTTTTGAAGACAAGACAGTTAGCGACAAGACCCAGCCGGTGTTGGTTATGGCGTTAAAGCTGGTACGCTTTGCTGCCGTGTTGAAGACTCAGGTCCAGCAAGACGACGCTGTTGTGACTTCAAAAGCGGTCCAAGGTTTCCGTAAACGCGTGCTGGATGCTCTCGCGCAATGGTACGAGTGTGGCGTGGATGGTTGTGACCAAA

>Contig_47

AGCGAATGTTGAATTGACTGCCCACGACCGTTATCGTCAATGACCATCAAAGTACACAGTTGGTAGTTGTACCTAGAACACACGAGCAGATTGATATGAGATAAAGATCAATAGATGGACAATACCTAGACAATACATAGATATAACCTACCGGTTGGTCTTGTGAGTACAATCCACTAGCAGCAACTCGGGGAAGCGTACGGTCATGTCTCGCTGGTGCCTCAAAGTCATGCTCATGACACCGGAGTTGTCAGCGTCAGTCTCGTCCACAGTCGCCACGTTGCCACCCATCTCTGCAAACGCGTTAAGTGCTTGTGCGCAGGTTATCATCATCGTCGAGTCCGCCTCGAAACTCGGCCTTGACACGCTGGACAATGTTGTCCACGTCTTTCTTAACCAGGCTTTCCCCTTCGTCGAGCAGGTATTCGTAAATACGAGCGCGCTTGCGACCGTACTTCACCATTTCGCGCACGGTGGACCTTGTCTCAGAATCCTCCAACTTTCGGTTCTCTGCGTAAGTTTTGAAGTGGGCGGTACCCACGACGTGGTTGTGGTGGTAAACCCCGTTCTTCACTCGAAGCAGCCACTGGCCTTCCTCTTCCACGACTTGAACCACGAACCGAAAAGGACACCCAGTGCCACGAACAAACTGCTTCGGCCGCACACCTGTGCTCCGATCCTTCGTCCGCCACCCATGCGTACAAATATATGTGCGCTGGTACGTTGCCCACTGGGTCGGAACGTATGGTACGTCAAGACCTTCTCGTGCAGCGACAGTTTTAGCTAACCGCTTGTTCCGCAAGTCGCAGCTCACAGTAATGAAAACTCGGATGATCTGCCGCGTTGAAGATGCTTACTTTTTCAAATAGACCGCCCAGTCGGCCCAGCTTGAGTGAAGGGCTTCCGGGGCACAAACAGGCACTGGACAATCTGAAACCTAAACGCTGCATGGACGTTCCGCATTGTCGGTGCCCGACTCGTCGGAGTTCTCAGAGCCTTCTGCAGAGTTCGGAGGAGCGGAAAGAGCAGCATCTGACGCCTCTTCGCTCGAGCTAGCCTGTTGAGGGCACGTAGTTAGCAAAACCGACATACGTATGAGATCAGGTAGTTATGCAACGCACCTCTGGCTGACCTGTCGCGGTTTCTGAGGCCGGTGATGGGGCGGCGTCGGCAGTGAAGAGGGCGCGGGAGGCTCCAGTGGGGGAGTTTCAGACTTGCTATCCGACATATCGGAGCGGGGGGAGGGCGGCAACGACGTGCACAGCGACGACGGTACCGGAAGCTCCGGCTTATCGAAAGTGAAGTGAGTGGCTGTGGTAGCCCTCAAAATCGTTGAGAAGCATGGACATTTTACATGCATAATACATGTATTTTACACAACCCGTAATTTATAATTTTCAGGTCGGTAATTTTACGGCCTGGACAATACATGGATGGCCACCTGGACAATTCATATACTCAGCGCATGGACGATAGCATTACGTTAATTAATTAAGGTATCGTATATAATAAGTGGTTTTTAATGAATTTAGTAAGCGATTAATAAAATCACTACCCAGGAGGTGGTTGTATTGAGTCCGTCCTCTGGGGAGAACGGACCTGAAATGGTGTAGAATTTTGTGTTAGTTGTGGCCGTAATACCAGGCAATAGTCGCAGGACACGGACAAGAACCACCACAAACCAGTTCTGTGCATGTACGACATTGGATATGCAGCGGCTGCTGCTAATTAAATGCTTGCTGTTTCGTTACAATGTACGTACTACTATAGCCAGGATAAATTTCCCAGATATTTACTGGCCAGCCTCCTTCCCGCCAATAACATCATGACTTAACTTTCCCAAACTCTTGCCGGCGGTATAAGATACTTGGAGGAGGGGTGATGAATCTGACTGGGACGGCAATGTTCACTTCTCTTTCCTACAAATAAAACTTGGAGTCAAGCATTGCTTTGGGAGCTTAAAAAGGTGGATAAACTGAAGATTGGCACAAATATGGTCAGACGCAGGGATCTTCGGGTTTTCTTGAGAAAATCTCATTTCAACAGTCTTGAGCTACCAGTACGTCGTGAAAGTATCACATTTATAGTCTCAGATGAAGTAAAGCACAGCAACCCTCACAGTTATCCGTGTCGGTAGATGTAGCTAACGTACCAGTCATAAATTTTGCGGCGGGCTGGGTGGTCGTCGACTTTCAACTTATCCCAGATTGATGACGCAGACTTGCCATTGCCGAACCAATGCTTAAACTAAGTGGCGGCTGTACCGGCCATTAAGTCATCGATTTTCTGACCACTCAATCTTTCTTCTTCGTCAGCAGTTTCTTTGACGTCGACATCTTCCGAAGACACCGCGACTTTATGACTGCGCAGAAACCGTTTGCCTTCGACGGTGTTTCCAGCATTTGTGGGGTATGTCTGTTCAGGGAGCATCATCGCCGTGACTGACAGTTTTGTCGTTGTCTGTGCTGTGCAGTCGCCCGCAAGGAGAGAAGATACTGTCGCAGCGAGAAGAAGAATTGACGTCAATCGCATGATGTGGCGGCTTGGCGTAGGTTTGTGTATTCGAAGGCGAAATGGTGAATGAATACAGCAAGGGAACTCGTGTGGCGGGAATGCGTCGACAGGTGTGCTACCATGCCAGCCCCACGCATGTGACATGCTGCCTCTACAAATCTTTATCTGCTTTTGACCTTGGCAAAAATAGATCTAACAACTACATGTAACGTGTTCTCGGTTTTAGCTACGTTATTTTTTTGCAACACCTAGAGCGATGTTCTTACAAAGYTGTAATAAAACGATATAATAAAGTTTTAGCCTAAACTAAACCAAAAACCGATTTATGTTACGCGAGGCGAAAATTCAAAGTAGTCCAGAAAAACGGAAGCGCACGATTGCAGATGAGAATTGCTATGATCTACTCACGCGACAACTTGATCCACGACATTTCGCGCAACTACCCGAACAGAAGCTCGCAAAGCTTCGTAAAATACGAATTGAGTGTAGCCAGCAATCCAGTTCTTCCACTCGATGATGAGTGTGGCTCCCACTCACGAATTTAAAATAGCCATGCGAAGCTGTCATTATCGTACAGAGTCTGACCAATTACTGTACATGAGTTTCGCTGAAATAATCTTGAAAATGCGTAAACAACCAAAAGCACAGGCTCTCATTTTGGTTAGCTCCGAAAAGGTACGTATACGGAACTGATCAGATTCTAGATCAGATATTTATCTCACTGGAGATCTGACATGGGATCTGAAAATGATTGGTAAGTTCACGCACTTCTTCAGCCTCGAGGCTGCTCGTGCGTTGGTCAGTGGAGAAGATGAATCGAGAGGCAAACGAGGGCGCGTCGGTGGATGCCGAGGTGGAGCCTGCTGAGCCGGCTGAGGGCGCGCCGGAGGATGGCGGAGTGGAGCCAGCTGAGCCGGTCGAGGACGTCGTTAGCACTGAGTTGCTGTTCGAGCAAGCTCGACAGCAACTCGCAAATGATGTGGGGTCTGTGCCACGACGTGGTGAGCGGACGACATGCTTCTACAAGGGCTATAAGTACTGCAAGGCATGGTCGTCCTCAAGGAAAATTGTGTACAGATGCTCTAAGTTTCGCCAAGGTTGCAGAGGAAAGCTGGAGTTTACTATTGCATCGATGGCGTATGCTGCTGTAAAGCTGCACACATGCCGCATCGAAGCCATTGCCAACGTCGTCATCAACGTTGAGGACCAGATGAAAGCTCAAGCCGACCTTCTTGCGATTGAGCACGTCGCTTGGCCTGTGCGCCAAGTGTGGGAAGAATTGCGACGCCAGTTCTACTCAGCTGACAATCCCAACGTTGTTCGTGGGTTGTCAGAGCAACAAGTTGTCCGACGAGTACACCGAGCAAGAAGCGCGCATTACTCTAGCAATGTTCACGGCTCTATTGAAATTCCTCCGCTTTCATTGGCACTAGACGAGGAGCTTTCCTTTTTCCAGTTTCACTACGTCACCATTAACCGCAACGATTTGAACAAGCCTTCCCGCTTGCTTGGGTGGGCGCATCCATCTCTGGTCGCGCTACTACGCTATCATGGTACCACGTTGTTTGTGGATGGCACTTTTCGGTGTGTCCCGCCGGGTTACGCACAGTGCGTTGTGTTCATGGTCCACGACCGAGCCTCTGGAGTGTTCGTGCCGGTGTTCTACATTCTGAGCACCTCGCGTACTGGCGACGCCTACTGGGATATGATTCATTTTATCGTACAATCAACGGACCAGCAACTCGAACCGGCCGAAATTGTTTGTGACTTCGAAGCGCCACTGTTGGACGCACTACAAACACAGTTTCCGAACGCAATTGTGTTGGGGTGCTTGTTTCATCTGAAACAAGCACTCCGACGGGCTATGAAGCGCTATGCTATTCCGGAGGAAGAGTGTCTGATTGCAATGACACGTGGTGTCCTAGACACCCTGACGGTGATTGATCCTGCTCACATCGAGCGAGGTATTAAGTGGGTAAAGCGTGAGATCAAGTTGCGCTGTGCTCAAGCTGGCGTGACCTACTCTACTGAAAAATGGGCCGATTTTTGGGGGTATTTCAACCGCACTTGGCTGGAGCAGTATACCATCGATGTCTGGAACGTATTCGGCATGAACAACGAGTTGGTCGCACGAACGAATAATCCCCTGGAGAGGTTTAACAGGGAGCTTAATACACGTTTCCCAACCCCGCATCCTTCAATGGCGACATTTGTGACGGTGATCAAGGCTATATCAGCTGAGTACGTACGCCGCGTTGCTGATGTTCCTCGTGGGCGAGCCCGCCGTGTTCCTCGCGAGGTCATTCAGCTACCACAGGTTGTGGACATTCCCTCTGACGTTGACAGCGACGTGGATCCTCCACTGGAGGAACTGGAGGCAGTTGCTACACCCGCAGCGGACGGTAGTACACTCACTAGTGCTACGTTGTAGATATGTCCGTGTAGCAACCAGAAGACCTCGGTAAATGCTTCATCTTCTCCCCTTTAAAAGTGTTTTTCTTTGGTTTCAGTCTGTTCATAATTCAGGTCTCAAATCCTGATCTCTAATCCGATCAGTTCCGCATACGTACCATTTGGGAATCTACCCTCATTTTTTGATCGGCAGCTGCTATACATACTACAATCGCATCAGCCCCACCATTCGGCAATACCTTCACTTCCAAAATCTGCTCACAAGGCACAAGACCACCCCCCCTCCTCCCTTCGCCACCGGCCCCAACGCACAAGATGCGCCTTTGCTACATCCTGACGGCTGTTACTGCCATTGCAACCTTCGCTGAAGGCTCATCTGTCACGAGTCAGTCGAACGTCCTTTACCCAGGCAAGACGGACTCTGTGAGCATAATATCTACGATTGGTGGCGAGCGATTTTTGAGAACTCAAGAGAAAATGAAGGATGCCAAGATGGCCGATAGTGGAAAGGTGGATGAGGAGGAGAGGGGCATGGGACTGGAGAAGGTGCTTTCACAGAAGACATTATCTCTGAAGGAGCCTCTTTTGACAAAGAGCAAGTCGATGGTTGATCTATCCAAGATCGATGACGAGGCAGCTCTCCAATTACTCAACCAGCGCAACCATGACCTCTATAAAGAAATCGAAAAAATGGGTTATACCCCAAAATCGATGAAAGAGACGTTGATAGCTCGCAAGGGCGATGTCCTTCTGGATACTGGGAATGTTTAGCTGTTGGCCCAATACTCCAGATTTTGGAATGAAAGGCACCCACAATTGAAGAGCGGCCAAAATCTGCGGTCGTAGACTGTCGAAAAAGACGTACCAGTCGATATAGACTAAGCGTCGACTGTGAAAATTTGCTTGACAAAGTTGCAATCATGCTACAAAAGAGCGTTTTCTAGAAACGAGTAAGTACGAGAATACTACCTTTTGAAAGATGTCTTTTGGACGTTTCTTTCCTGCTTTATACCACTTTAAAATACAAACAAATAGTTCGTCCAACTAGTAACTACAATTTTTCTTGTAGTGCAAGCTTCGAAGAGGCCATCGCCACCTTTTCACGATCTTTTTGTAGCAGAACATGGAGACAACTAGAGCTTTGCGGGGGCTGAGAAAGCAGGAGTGCTTTCTTCGTTTTGAATTTGACTTACTTAAACACGTGGCTACAGTGGCAAATAATCCCCTGCGCCAGCTTCGTAGCAGCAACATCGGCTTTGTTGCCCATAATAAACAATGACATAATGTGGAGGCCGCATGAGTTTGAGCTTATTAATGCCAAATAACAGTACGAATACACTGGTCGTACCCACATTTTTTCTCGATTTCATTTTTTTTCTGATGTGCAAAACCTTGTGTGCGGACAGCGGCACAGCAATTCAAATAATACACACAGGTCATAGCTACAGCATAAGTATTATAGAGAATTGAGAGCTTTCGTTATCGACCACCTTCACGAACTTGTCGTGAGGTTACCATTTTTAGTACTACAACTTTAACGTTGTAATATTTCTGGTCAATCTGCTCATTAATCCTCCTTCAATTGGCATTGCTCAGAAGTCAGTAATTTCCCCTCAAAGTAAGTATGTTTCAATGCTTTGCGCTTAAACAACGGTCCTGGAAGTATGACCCGCGCTGAACCACTGTACCACTAGCCTTCGTCTCTTTGCAATCCAGAGCTGTTCCGATGTACGACTTTGCTATATGATGCTAAGAGCCGTGAGCTCGCATCGCTCGCGACATTGAGACAGGATTTAATGGAGAATTCGATGTTTCGCTCGCTGGTGAGCTCGGCGCTGGGCGCGCCTCCACAATGGTTTGATATAGCTACGCGTCTCTATGAATAACTAAAACAGCAACAACAGCTGTTCTAGAGCATCCATTTTGCTCAGAACTCATGTTTGACGCCTCTCCATATACTGTAGAACAGGCTGTTTAAAACGTTTTCAACGAAACTCTCGTTTCCGCAGATGATGTTGTCACATCGACAGTATTTACTCTGACCAAATAGCGGGGATACATGGCATTGGAGCTACCAGTGGTAACCTTTTTAATTTAGCACGCTGGTGGGCCCGCCGATTTGCAGTTCTTAGCTCTTTTATACATAGTAACTTTTTGGACATTATATCCAGACTTCTTACCAGGATCGCAGACCCTCGGAAGACCTGTACGAGAATATACTTCAGCTAATATACAACTGTATGTAGAACGGTCTGGACATTTCTAAAAATACGACGAGATGTCATGATGATCCCAAACTCCTCCTCCGTGTTGCAGCTTTCCATCACATGGTGTCCAAAACGATAGTTTCATTTCAGTATTCAGCTGGCCCTTAGAGCAAATCATGCTTCATCGGTTTATCGTCTGAAGTCCGAGGACGCAAATAATATTTTTTGTGGTGCTGCTCCGATTACGTGCCACTCGAGAAGACTGGCAAAGCTTTCAACCTTTCGTTGCGTAGCTAATAGACACCTCAAAATAGCCTTCCTGCTTTTCTCATTTGACAAATTCGTGAAAAGCAGAGGTTGGTCCGCATATTTTCGTTTTATAATTTGAAGCTCCTTGATAGATCCAGAATATGGAGTGAGTGCAGCATCAACTTCACCTTGAATGCTGTCTGTATTCCAGATCGCTTGCGGCGTGTTTGGAGCTCTCCGTGCTGACCGCTCTGTTTATTCCCATTGTCCGTGTAACAGGGCTGCACTACATGTAACCACTTGATTACGAAGAACGTTGGTGTGCGATACAACAATAATTACATTTGGCAGACTACTGCTCTATATGCATTTTTGGTTCTCTCATTGCTTAAAACTCAACATCTTCAACCAATTATAGTAGTAGGATTCTGTCACATTTGTTGGCAAATACCTTTCATTTCAATTATCACATAAGGAGGAAAGCCCTAGCAAACAAATAAACAATTCTAAAGTTTACGGTTAATCGGCTTTGCTTTCCTGATTTGAGTAAAATCTATACGTTCGGAGCGAATCTAAGAGAAGCGCACCTTAACGTTTCCTCACGCGTGCGCTAACGTTTAAGCCGAAGTAGTGGGCGCCGAATAGGCCCATTACAGTCCGTAGCTGCGGAATCGAGACAGATAATTCGAAAGAAACGAGTACAGGCCTGGTGCTCAACACAGCGATCGACTCTAGCTCCCGTTGTTACGTATGAGGGCATCGCTGTATGCAACGTAAGACTGGAGCACAATGAACGCTCTCGAAACGCTTTCAAGGGGTAATACGCACTCACACTTGGGGCGTGTTGATGTCTTGGGGACAGGGTAGGACCTGGTGGCCATCTGTACTTGTCGCAATCTCCAAGCTAGACAGAGAGCTATATAACAAAGACTTTAATGAATTTAGTGAGTAACTTGAAGTGAAGAAGGACCGAATGAGGGAGCTGAAGTTGTTGCCTGTCGCTTATTGTGCAAAACCTAAAGTGATGAGAATTGTCTGCAGCAATCTGGCTGAATTGATCAAAACAACCTATCCTACTCCATGAAACCCTGTTAGCCAATGAATTTGTCTAACACATGCGCACCAACGCCAAGCTCCTTTCCCGCGTTGCGCAAGCGCTCACAGGCAGCTTAGACGTAAGGACAATTCAGTACCCCCCTCCGCAGTACGAGCCTCAGTGCCACTGCATTGCCTTTGACCCTGCGCGTTGCCTTGCTCCAAGCTTCCATGATCCTGTGACTTCGTTAACTCACGCCTAGGTGTCTGCTATCGTTGCATCACTTCTCGGTCATTGACGGCTACACTTCGTGCAGACTTCTGTGAATTACGTGGGCACCATGAAGGTTTAGCCTGCTACATTTTCTTTGTGCTAAAGCGCTGACACCGGGTGTTACCCTACCGACTTAATATCCCCGGATATACTCAAACTGCTCATGAGGCAGACCGTTGGTCATCGTGTAAGCCTTATTCTCCTTTCTCGCGACGTACTGCAGCACCACCTCATCGAGCTGCACGTACACCCGGATGAAGTGGTATCTCACATCGATGTGCTTCACCCTGGTGTGTTGGGCAGGCTTTTCCACGATGCCAATGCAGGCTTAGTTATCTTCTTGCACCACTGTCCCCGCCTGCTCATATCGAAGCTCCTTTGAAAGCTGCCGTAGAAACAACACTTCTGTGGTGCAGTGCGCGAGTTCCTCATATTCTGCATCTGCAGTCGACTGCGCCACAATCCTTTGCTTCCGCGAGCTCCATGTAGCAGTACAGTTACCGATCGTCATCACATATCCTGAAACAGACCGACGATCATCATCACAGGCAGCATAATCCGAGTCCATGAACGCTCTCAGCGTGACAGATTTACCGCTATACTTCAAGCCATGGCCACGAGAGGTATCGAATGCAGCGCGTAACATCTTCCCTGTGCGCTTCACCGTACCTTAACAGACATTTGCTCACGTCTTGGATAGCGAAGGCAATGTTGGGACTACTGCCAATCATTAGATCCATAAGACTGCCAACAGCTTGTCGAAACGGCTTCTTCAGCGTCTTGGTCACGGTTTCTTCACTGCTTGGCGACTGCTGCTTCGATAGCTTCTTACCCACAGCTGCTGGTGTGGCAACACCAGCACAATGCTCCATATTAAACTTCTTCAAAACAGCTGTAAGCTGATCAATAGCTTCCGTGCACGCAGAGCCCGAGTAACCTTGGGTCCCAAAGTAAAGCTCACTTATCCAAGATCGCTTGCTTTAAATGCTGCACTGATAGATCGCTTCAGCCCCACCATGACCTCCACTGACTTGGACAGTAGCAATAAGTCGTCGACGTAGATTGCAATTACCTCAAGCTCGTCGTATGTTCTTCTCACGTAAATACAGGACTCACAGGCTAGCTTTTCAAAGCCTTCACGAACGAGAAACAATGTGAGCGCCTTGTGCCAGGCGCGTGGGGCCTGTTTAAGGCTTTACAAACGCTTCTGTAGCTTGCACACTACCTTCGACTTCCATCCCTTGTGGCCGTTTCAAGTACACCTCGTCTTGAAGCTGTCCGTATAGAAATGCAGCCTTGAGGTCCATTTGGTGGCACTTCAGGTCCCCGATGCATGTGATAGCAAGAAGAACACGAAGTGCTTCGAGCCGAGCCACGGGGACAAAATCTCATCAAAGTCAATGAATTTCACTTGCTGGCACCCAATTAATATCAGCCGTGCGTTGTATATCTCAGCATTGCCGTCGCTTTTGTACTTGAGCTTGACCAATCACCAGCACGAGAGTGCACGCTTTCCGGGGGGGGGNAAGAGGCACCAAAGTCCAAGTTTTGTTAGATTTTAATGATGCCATCTCTTTGTTGATTGCCTCTTTCCACTCTCGTGCATCGGGTCTAGCCACTTCTTCCTGGTACGGTTTCGGCTCCGATGACACCACCCGTTGTGATATTACCCGTAAATGCCTTGGCATTAATGTTCTCAAGCGCGACCAACGCTGCTGCCTCCTGCTGCTTACGTACGACGTCCAGTATTGGATTACGTGCTTGAGGTCACTTGGTAAGCACTTGAGGTGAGATTCCATTAACCACCGTACACGTCACTGAATCGTACTCGCGGGTGTTAGTCGCAGCTGCTCTAAGCGACAGGTCGAGGGGCATGAAAAGTGTTGGACTACTCGAGTGGCAGCGATCTGGTGTCATGTCCCGTTTTATTTGATGAGAAATTTTTGCCTGCTGCGGAAGACTGCGTCGCCAAAACTTTCGTTCGCTCAGCATGTCGACTCTAACTTCTACACGCGCTCAGCAGCAAGTGAAATGATCTGCTCTGTTACAGCCAGCAAGCTAACATCTCAAGAGGTTAAGTTACTGGAAGAGGTTGTGAGCACCTCTGCTGGGGTAAATATCCCACAAAACGCCAGCCTCTGCTGTTTTTTTTTAACTTGTACACCGCCCATGCCCTGTCGTGCTGAAGAAGGCGTCGCCTTTGTCGGTAA

>Contig_49

CTTCGTGCACGCCTTCAAGGCGCTCGTGGACGCGGAGACGCCCATTCTGGACCAAATGGAGCGTCTGCAGCTGGAAGCGCCCGAGAGACGAGGCAACATTTTCCGCTGTGCGTCGTGCAAAGCGGCCTTTCTGGACTTCTACGAGAGCGACGGCTACCTCTGGCACTGCGACTGCCCCACGTGTGTGGAGCCCGACAAGAAGATTTTCTACCGACAGCGGNAAACTGGTGCAGATGATGAACGAGATGGAGCTCGAGAAGCAACTCAAGAAGGAGAAAAAGGGACAAGGCAAAGCTGCAACAGCCAAAACGTCGACGTCGACGCCTCGTGGGACCAATTTATCGTCCCGAAGTCGACGCACAAGAGGCGGCAGCGCTGCGTTGGACGAGCAGGTTGTCGCCTCTCGAGCGTCTCTAGAAGCTGTGGAGAAGGAGAGCAACGACGAGAAGACGAAGGAGCTGAAAGTGGACGAAGGTGGACAGGAAGCCGAGACGCTGCGTCTCCCAGCTGAGACTGAAGACGCTTTTGCGCTGTCCGGCGACGCGCTGGTTCAAGCTGNTGCGCGTGGCACACGACGACAGGAGCGGGGCCTGGACGTTCCCAGTCATGAGCTCGCGCAGCTCGAGTCTCCACGCGTCTGGCATCATGAAAACGGGCACTTGCCGGTGGTTTGTCAAGAAAGTGGCCTCGGTGCAGTGCGACTGCTGCCACCGACTGTTTCGCTTCCCAGAGTTTGTGCACCACACGGACAGCGCGTTAGTGCGAGACGCCAAGTGCGCGGACGAAGACCCGATGCCGTTTCTATTCGTCGAGCATTGCGACAACACGCAGCATTCGCCTCTCGAGGAGTTCCAGGCTGCGCTACGCAGTTGGGTGGGGCGTCAGAGTGCGAGCAACACGCCGACTAAGGCGCCTAACAGTCGGAACTTAAATCCGGACGCACGCATTGACACACCGACGACGCCAGAGCCAGAAGTGGACGCAGCCATGTCCAGACTACGCACGCTGGCGCTGTTCAAGCGTCGGAAGCACCGGAGCGACAAAGTGTCTACAGCCGCGCGTCTGACTGACCAAGAGCCGATGGATTTCGTGGCTCAAGTCGTGTGTCTGTCGCCAAAGTACGTCATGAACATGGCGGACGGAGCTCTTGCCGACCGTGTGGTGCGGTCCAGAACAGCCGTTCCGGACGGCTCGTTCCCGCGCAAAGCCGGCTGGCTGACGTTCAATAGCAACGGCCTCAAGGCGCGCCAGATCACGTGCGTCTGCTGCGAGAAGAGATTCGTCTGCGAAGAGTTCGTCAACCACGCTGGCATCTCGCAGACGGAGCTGAAGAAGACGCCCCGCAAACTTTTGTACGTCGTGGAGCGTCAGGACGAGTCGGCGTTGATGCCGTACATGACGTTTGCAAAAGACCTGGAAGCTGCAGCCACCAATAAGGTGCTGGATGCGCTGCTCGATGGGCTGCAGCCGCCACCTCCGTCCCCTCGACCACTCGAGCTATGACAAGACTAAGGATTAAGATTTTACTTTAAGAAATGCACACTGACTTTGGGTTGTGTACAGCCGGTTTCGTACCAAAGTCTACGAAAGCATTTTTTGTTCTTTCACTAGGGTGTTTATTAAGAAAAAAAAAGAAGTTGAATTGACTGGTAATTATAACGAAGCGGGCTCTATAGAATGCAAGAGCGCACCCAAAAACACTGCTACTTCCGATGCTGTGCGCGTTGGGCGTTTGGATTCCTCTCGTTGAATATCTTAATATATATTCGTCTTCTTCACCAGTCATCGATAACTTTTAAAAGCGCAGAAACATCGGACTTACTTGGTCACTTGGGCAATGACCGTCTACTGTAGGCCAACGGACTTCTCCATCGATTCAACTTTTCTTTTTTGCAAACTTGTCGGCACTTACAAGATCTTTTGACACTTCCGCTTCTGTACATACATGTAAGCATCTGGAGCACCGGCGCTTCGGGATTTTCAAAATGAACTGGGAGGTATACGACGAGAAAATCCTCAAACGACCGCTCTTAAACGCATCAGCTACATTTTCCTAGCTCTAGTCTCGTCGGGCTGGAGCTTATTCTTTAACCATTTCTTGACAACGGCGGCCATTTTCTCGAAACTGGTACTTGGACTGTGTCTTACAGCGGGTAATCCTGATCGATTTAAAACCCTCGATTAATAAAACGGCCTCTATTTATTAAAAATCGTGCATCAGGCTAAGTGAAAAGGACTAAAGTCCACTTTTTTTAAAAAAAATACTTCTGTACACCTCATACTGCACTACACCCGGGCAAGACCATCTCGTTAGGAGAAAAATTAATATCGCAAGTCGCTACGGAGACGATGCCGTGATACAAGTACCGAACGAGAATAGGGACAAATTTAAAAAATACGGCGTTGACAAACCTAGATTCAAGGCTATAATACGGGATGGACATTCCATCTGCAAAGCGCAAGTAGTTAATGCGGTACAACCGTGGTCGAAAAGTGCTATTGAACGAAGTAATAGGAAACATTGGTGCAAGAATATTACCGGTATTGCTATCTGTAGGAATAGATATCATTGGTACCAGAATATTACCGGTATTATTTTTGTTTGTTTTCAATGATCCGCTTTTTTGTGTAACACAAATTGTGTTCGTTGCCAGCGAATCGTCGATACTGTACAGGTAAATCATGATCGGAGGTACCATCCCAAATCGATCTTGCTACCCTGGCTCCGTACATGAACCAGTACCGGTACCATACACATGAAATGATTATGGTGCGGAACCAGGGTATTCAAAGCAAGATAGATTTGGAATACATGTACCAGGTACAGTAACGAGCCTCATTTCTTGAAAGATTCTATGGCCGGGCCATAGCATCTTTCAAGAAAAAGTCCTACGGTACTACAACGTCTGGCTGGCTCTCAACTTCTGGTCCTATCTGAAAACCTTACCAACCATTGGTTGGTACAGTAAGGTTTTCAATTATTTATTGTATTAAAGCATCATTTTGTTGGGTTTTGATTTGCTTTTAGGCATACTTCATCTGGTACAGCTGGTCGTAGTTTTCATACATCTTCCTGATCGCATTGTCCTTCGACGTCCCCTCCACACGAAGCCATTGATAAACATTGGTGGGGCTTTCTCTGTTGTACATCCAACGGAGCGTGAAAGCATCCAGTAAGTCAGTAGCCATTGTCTTCGTGCTGGAGACCTCGCTTGCCTCAACGAGCATCTTTGCTAGAACACCATCGTCTTTGTAGTGCTCCTTCAAGATCGCGAGCATGTTCACTTTCTGCTTCGGTTTTCTTCGCCTGAAGTAGTCGACGTACTGCCTCCAAGTATCGAGAATTGGACTATCAAAAAGCCTTTCCCCTGTTTTGTCGAGCTGCAGTCGTTTGAAGACGTAACTCGGGGCATTTCCTTGCTTATACCAGTTTTCAAGTAGCTCACGCTGCAATTGACTCGCCAAGCTCTCTGTGCTCGGGTTCATGCTCGCTAGAGCAAGTATACTTGCCAGCTTATCATTCGGGTAATTCTTCGTCAAGGTCGAAATCACGGGGAATGTTTCCTTGTGATACATCAAATTGTAGTAGTCCGTGTATTTCGTCCATGCAACAAAGAGCGGGCTCTCCAGGAGAGCATCTCCGCCCATGTCCAGCTTTAGCAGCTTGAAGATAGCGTTGGGGGATTTCTGTATTGACAGCCACGCGTTCGTCAGGTCAGCCTGGACTCGCACTGCAATTTTCTCCGTAGCTGGAACCTTCTGCGCAGCGATCAGCATATCCACCAGAGTTTGCTCGTCGTATTTCTTTAACATTGAAAACAGAGTCGTCTTTTGCTCAGGGTGAGCCTTGTTGAAAGCGTCCACATACTTCGCCCAAGTAACCACCTGAGGCTGCTTGAACAGCTTATCTCCCGCGTTGTTAAGCTTCAGTAGCTTGAACACGACGTCTGGGGACTTCTCGTAAGCGATCCAACGTTGAGTTTGCTCGGTCTGTAATCGCTTCGCGATAGTCGCTGTGGCTGGGACCTGCTTCGCTGCTTCGATTATCTTGGCTACACCGTCGTCGCCGTAATGCGAAGTGAGAGTCTTGATTAATGTTGTTTGCTGCTTCGGGTTCTCCGTATTAAATTTTTTCATGAACTTGATCCAAGCTTGCAACTGAGGGCTTGCTAAGAGAGTTTCGGCTGCATCATCGAGTGTGAGCAGCTTGAAGACGTCATCTGCGTTCTTTCCTCCATTAAGCCACGACATGAGCTGTTCATTGGTAGTTGACTTCAGCGAGTTGGATAGTGATTCTACACTGACACCGGATACCCCCCTCTCCTCGTCTAAGTCGTAGTATGAGGTCTTGCCCGCCCTCAAAAGCCTCTTGGTGGAGATGTGGTGACTATCAATACTTGAGAGGGGATTCGGCGTGACCATCGAGTATTTCGCTAGACACGAAGTTGAGAGTGAGATGGCAGCCAGAAGCAGCAGTCGCGTCTTGAGATGTAACATGTCTGCGCTCGAGCGCAAGGAATGGGAAATGCGAAGCTAGAGAGAGAGAGAGACACCGCGACCACAAAACAATTGTCTCTGACAGAAACTGTAGACAGTATGGACAGAAACTGGAGACAGTATGGACAGTACATTCTGGACAGAAACTGTAGACAGTATGGATATCACAGTACATTCTATACCGGTATTTGAACTGTGTATCTTTTCACGGTATAAAGAATGGCTCATTGTTTAATTCGTTTTAACCACTTGATTTAAGAATGGTTTCGCATCTCAAGGCCAATCAAAAAACGACTTAAGTTTCTTGTTACCGATGCAGCGTAAAATGAAAAGTGAGAATCAAACTAAACTAGCCCTCTACATCCTTCCTGCACTGCCTACTGCAACACTCGAATTCTAGAAGATTGGAATGACTCCGAATGTTCGATGACACAGCAAGTTCCCTTTATGTGCACCACACAATCCTGTGACGTGGTGCAGACTTCAACACTCCCCCTCAACTTGCTCTTCTTGCACTTGGTTGTTCACAACTCCGCTCGATCTCACGAGAGCAATAAACTGAGGAGTCGGTAGGGGCTTCGTCATGTAGTCGGCCAGCTGGAGCTTGGATGGCACGTGCTCGAACTTAATTGTCTGCTTTTGAACGTGATCACGGACGAAGTGTACACGAAGATCAATGTGTTTGGCACGAGGAGTGTACCCGCTGTTGGTAGCCATGCTTATAGCAGCCTTGTTGTCAACTAGCACCGTCGTAGCGCTATGGATCTTGACTGTCATCTCTGTCAGTAGCTGCCGCGCCCATAACACCTCTTGAACCGTGACGGCGACTGCCATATACTCAGCCTCAGCTGATGACAGCGCGACTGTAGCTTGTTTCTTGGCCTTGAACAAAACTGGTCCACCACAAATTTGAACCAGCACTCTAGAAGTTGAACGGCGCGTAGCTAAATCACTGCCCCAGTCAGCATCGCTAAATGCGACCATTTTAAACTCGGTAGCACGCATGTACTTGATTCCAGTATAAGAGGTCCCCTTGAGGTAACGCAGCACTCGCACCGCAGCTCTCCAGTGCATTTCGCACGGATTTTCAAGATGCTGAGAAAGAATACTGACAGCGATGCAAATATCGGGTCTAGTTGCATTTGCAACGTACAGTAACGAGCCGACAAGCTCCCGATACGGGGTTTTTGAGTCCAAACGAGGGTGTTCGTCACTGGCACGTAAGTCTTGACCGACTACACTTGGGTTTCGAACTGGGTATGCACTCGCTTGCCCAAAACGCTCAAGCATCCGCAAAATATACTGCGACTGGCCCACGAACAGCCCCCCCTTCTTTAGGTCATAGTGAAGCTCCATCCCAAGGATGTATTTTGCATCTCCTAGTGACTTCAGCCGGAAATGCGTACTTAAATTCGCAGCAATACTTGACGCCTCCTCTGCTTTAGCACAACCAATAAGCAGATCGTCCAAGTACAAAACGACAAACACCGGACCGTGTGAGCCAACACGAACAAAAAGACACGGCTCTGATCTGCACTGCTTGAATTCCATTGCGAGAAACACTTGACGAATCGTCTTGTGCCAAACTGCTGCTGCCTGTTTTAAGCCGTAAAGGCTGCGACGTAAACGACATACCATGCCATCTTGAGCTCGAATCCCTTCCGGTGGCACCATGTACACTACTTCTTCTAGATTGCCGTTTAAGAATGCTGTCTCCACATCGTATTGCTTCACCACGAAACCAAGCTGGCAACAAACCGCCAAAAATATGCGTACAGTGTTCAAATTTGCAACAGGCGAATAGGTCGACGTGTAATCCACCAAACGTCTGAAAACAACCGAGAGCAACCAGTCTCGCCTTGTATCGGATAATGTTGCCATGTTCATCTCTTTTGAGTGCAAACACCCACTTATGGCCAATGACTCGCACACCTGGGGGGCGATACACCAAGTCCCAAGTGTGGTTTCTCGCATGAGCACGAATTTCAGCACGGCAAGCCTCCTTCCACTTGGCGGCATCAGGTGACTTCATCGCCTCCATAAACGAATTGGGAACTGACTCAACAGCAGAGAGGGCGATTTCGTATCCATCATCAATTCTTGGCCGTTTAGAGTCAGGTTCAGCAGTACTTTCATCCGACGAGTAGCCGTTTCCACGTTCATTGTCTCCATTTTCCAGTAGTAGCGACGCAGCATCATCTTGACTGTTYCGGGCTTGGCTCGGAGGTAACGCCAGATACCTGGACGGTGTTCTCGAGCTGGCTGACGGAGGTCCAGCCTCTAGAAGGCGGGGTTGGAACTGATTCGAAATACGAGCTTG

>Contig_53

GCCATCCTGCTTTTTTACTTGCGTCTGCACGCAGAGTCCACTATTTGGTCGCTGGCAGTAGTCAAAAGTACACTGTACCTTTTGAACAGACGTCTGTTTGTTCCTTCAAAATCCAATGGACACTTTAGACACTTTTAAACATGTCCGCTGTAAAGACGTCGTATCTTTCCACTGCCAGCACTGCGCGGTCATCCGAACATCGACCTAATGACAGAAAGCACTCGTATGCACGGAACCAGTGCGGAGCAATAATTGATGTCTGGGCACTACAGATTCTGCCAATCCCTATTTACTGTTGTGCGACGTGCGCATCGGGCGTTCCTCTGCGGGCTTGTACCAAAAGAAGAATTTCAAGTTTCAAACTGGTCATAAAGGATGAACAACAACGCCATATTTGGTGAGCCGCAGCACCTTAATCCATAAGTGGGTGTCGAAGGGGTGTATTGTTTTACGTCGTTTGCATTTATGGACGTCGAGGTGTAAATCTTCAAATATAACTGACGTCGACAGCTCTACTGAGAGAAGACCTCGTACGGATTCGGTGGAGTACTAGCTGAATTATGGGGAGCTGCGTGACTTAGTTATGACGGCCGAGAAGTCTTTGTCGTGAGTGACGAAGCATATGATGTTGAGAAGTCTGTGCAGTGAGGCACAGCCTTTGAGAGGGACTGAGCAGTAAGACGCACCAAATGAGATGAGCTGTTGGGGAATGACGGACGCGTGGTGAATCGACATGTACCCGTGATGAGACGTCGCTGCCCTATAGCGGAGCGAACAAGAACGAGAAGTCTTTGTAGTGAAAGTGACGAGTTATGAGTTGAGTCAACACGAGTTTGGTGGTTAGCGCAAGCTGGACGAGTACGATGTACGGTACGAAGAGCAAGTTGGACGGGTACGAGCTCGATGACAACAACCGAAAGGACGAGTACTAGAAGAAGGACTATGAGACGAAGGATGATTTGGACGAAAATAAGTATGTAAGAACGAGTCAATAAATAGGAACGAGTCCGTTCCGGCTACTTAGCGGTGGCCTACAGGTGTTGTGTATTGTATCTAGTCGAACCTCTGCCCGTGACAGAGCACCGCAGCACTGTCAAGCGCTACGACCCGCCGGCAATCAGGCGTCATGAGACTTGTTACAAAATTATCCTGATCTCCGTTGTCACTGCAATAGCGAGACGCTTACACGTTGACTTTGCTTATGATGCTGCCCCTGGATCCTGGTCAATGCCANCCTCGACGTTATAACCTGCAGCGGGCCCGATTATGGCATTGCATCGTCCCCCCACAGCGCAAAGTAGTACATGGAAAATTATGGTGGTGTTTCCAACGTCGATCTTCATCGCTCGAGAGTACGGTCTCGTTCAGTGGCCGGAACGGTAGACAAGAGCACTTAAAGTGATGATAAGTTTCTTTATTGACTTGGCTTCCACTTGAGTAGAAGGTGGGAAATGTAAGACTGAGAGCTAACCGGAAGAGAGTCTGCCTGATTATGTCATTGTTATATCAGAAATCCTCGCACGTTTGTCCCTCGCATGAGGCAGCTTGCGACAAACCGTAAATTATGGGATCTACAGTGATCAAGTGATAAATAAGAGCACAATCAGAGGTAAGTAACACCATTTTCATYGACAGTCTGAACTGCTTTCGTGCTTATCGTTTGTAGTTTGTCCAAAATGAAAAGTGATCGTTTCACCGGCTTGTGCGCGTAGTTGGTTTGGGAACTCTCGATGTATGTAAGTTAAGATGCAATGTGGCTCGTACATAATGATTTTCTTAAATTATAAGAGATCGCTATTAGCAAAAAAGCTATTAATTTCATAAATGAAGTCAAAATAAATACATGAATGATTTCGTAAGAAATTATTAATAGCGCCATGATATAATCGGGAGCCCAGAAGATGAATCCATCACCCAGTCTCCCTTGATACTTTCAAGCTCTTAAATAGAGCCTTGGATGTGTACGCMTGCGTTAGGGCTACATAAGCAGGGGTCCAGTTTGTTCAACTATCATGTTATTAAGAGATCAACGCAAACAGATAATTGTTTGGGATTCAAGGTCGTCGAGCAGTCAATTATAAAAAAAAACTTCCACAATAAAATATCAATATTATATTCGGCAAAAAGCACTACGGGGATCGACGAATCACAACTTTACTTACCGTTTTCGCCGACCAAAAGCGCCTGTTGTCTTAAAAAAGAGCAGCATCTTAATTTAATCCGAGTGGGATGATGAGAAAGCCTGAGAGAATATTCTCTCCACGCAGTTTGACGTAGGAGGAATGAATGTCAGTAGAAGGTATCTGGTACGTATGAGGGCAGCTAGTACTATTAATGTGAGTCGTGAACTGTACAGTCTTCCCAATATTTCTACGAAATTTGGAAATATTGCCTATAAAATTTAAGTTTCACCGATTCGTAATATGTTGTTTCACGGAAATATTATAAAATATTTCTAGTAGTATTATTTTCTTGGAACCTCTGGTCTACTAGTTTCTCTCAAACCTAGCGCAAAAACACTCTACTAATTGGAGCTCTGTCAAACGGCCACACTCTCCAGATGAGCGTCCGAGCGTCCAGGCTAACGTATTACTACGTACAAATTACCGATTTTAAGAGTGGGTGGGCCTCCGTTGGAGTCGGACAAGAAATGCAGTGGTCGTTTGAGAAAGTCTAACGAACCATACACGAGTTTTATTTGAGTAAAGTCTTTCCTCTGCAATACCGCGCCCGAGACATATTTTCGATAGCATCAAGGCGGGTGCCAGTATCGCGACCTCATTCGAGTGTCGTTGTGGCCGAGCTCTATTCTCTGGTAGCTTGAAACCGCCTCTTAGCCATGGATACTCCTGTACGCTTGGTATTTCTCGAGGAGGCTCGCCGGAATGTGCTCTGCGATGTCATCCGCGGAGTATACTTGGCTTTTCCATTTTCCAAACATGAGGAATCGAAAAATTCCATCGTGCTGCATATCAGACAGTTTCAATGCGCTGAATAAGTTAATATTTCTCTCTTCATTGTCGTATTACGTAATTTTGTTTTTGCCATCCATCGCTTGGTAGATTCGGAGAAACCGCTTGTCATTCCGGGCGAGAACACGTGAGGTCTGATCAGTCTTTGACACTGCTGCGCCACTTGCAATGAGGACAGCCGCGAGAGCCGCAACAGTGAAGTTGAAAAGACGCATGGCCGGAATTTTTCTTGTGGACTGGTGGATAAATTGTGGAATGAGAAATGAGGTGGTTGGACGTCGCCCGGTACGGTTCAAAACTACATATTTCTAATATCCTGGTAATCTTTCCTGAACTATTTGTGATTACTACGGGTCGCAGAAAACGTTTTGGATCAGCAGGCATGCCCGCAGGGAACTGCGTTCAGAAAAGAGACAAATACCTTTTGAAGCGCTCAAAGGTACTCCTTCTTAAATGAAGGAAATTATGCCCTATCAAAGCGGATGTATTAATATGTTGTTATAAGAGATTACCTTTAATCTTGATGCCCTTACAAGAATTAAGTATAACCATTTGGATTTCGTAATTCTAGCTTAATTTGCAGGCATGCCCGCAGACGGTATTTTCAAAACGGTCCCTTTTGACTGAAGAGAGGGAATCCTAATTACCCGGATGTATAATTGTTCGCGTTCTGTACAATACGACCACCATACAACATTCTTTATAAACAACATACCTCTTTCCGAATTGCTCTATTTCTCACCATCGACAGATAATACACATCCCAAACCACTTTGTTATAGGAATTCTGGCCACGCGTTTCTTGTGTGGTTTGGATACCAATTCTCACTTCGCAATTTGACTCGCTTTCGACACCTAAAGCATCCAGACACCAGCAATGCGCCTAACTTACGTCTTGCTGGCCGCTGCGTCGACCCTATTCGCCCGCCACGTCACCTCTACCCCCTACTTCGCTAACGATGTTGCGCTAACCGGTGTACTGTCACTGGGCTTCATTCACTTCGTTGGCGCTGACCAAAGTGTAAGCGACCAGTCCCGGTTTCTCAGAGGCGGCAGCATCGACGAAGATGACAACGAAGAGATAAACTTTCTGGAGCTTGTTAAGCAAGCGATGACTAACAACTTTGTTAACAAGCTGATGAAGAAGAGCTCGTTTTCGGATCTCGAGAAAATTGACGACTTTGGGGAGCTCAAGAGGATTTCGACTATTATAGATGACAAGCTGAATAATCTGTTCAAGCAGGCTGACGATGCGAACAAGAGTCCCGACGAACTGGCCAAGATACTGAAAGAAATGCCAGATGTCGACGATGCCCTGACGGCTAAGACATTGGAAATGTACACTGACTACTTGAAAGCAGTCGGCGTAAGAGTCCCCACGTAGTCTCGACGGTGGAAAGTACAAACCGTTTTTCAGAAATACATTGGTTCCTCCAATAACAGGACATAATATGCAAAAACACTGCTGAGAACGGCTTGCGAGACACCGAAATGCATAATTGAGTAAGACCTTTCACACCTGATTTAACCCGTGGACGACTTCTCTATCAGGCATAAGCACATCGTCTTACCCAGGTCGTCTTTAGTCGTTGGTTGAGTTAGAAAGTCTGTATAATGCGCCAGGTGAGGAAGCATGCCGGCTTACAGAAGCATAACATGCAGATGTACTTTGCTGTGCGTTTTCAGGTCTAATGTGGAGCCACACAGGCCTATCATACTGAAACCGTCTATATGGATTTAAGTAAGAAAGTTTCTCCTGGACTCCAGGATAAGTAATCATTTGCTAAACTGAGAAACACCAGGTCATTCAACGCTTCTTCACCTACGTGCAGCCTCAATTACATGTATACACGTGTAGACCTCCACTAACTTCGACTCTATGTCAGGAATACTTGTGAAAATGCAATGGAGCATCGCATTCTCCAAAACACGTATAATATTGATTATTTTCAGATACCATGATAGCGTTTAGATGGATTACTGTCGTTTTGTATTTTCGGCAACCAACTTGTTGATGGACCTGTGACCGACTAAATCTTAACTAGACTAGTAACAGTTTTGGTACATAATATTTCAGTGCCGACTTCGACTGCATCGAGAACACATACAAATCGCATGTGCTCTCAATGCAGGAAGAATTATATAAACGCTGCTGGTTGCTGTTTACTGACATCAAATTAATTACTAGTTCTTCTTGTCCTTAAAGTTTCGTATCAGGTATTGTGCCAGTAGTGGGTGCCTTCCATAAATAGCATAAGCACGTGTCATATTTGCAGCAGATAAGCCTGTCATACTTTACATGTATGCTGAGCTTAAGTATCAAAACGTCCACCACAAATACCATAAATGAGGCATATCTCAAAGACCTCTGTCCTGTTTATATGTCTCTACCTAGATCTTACACAGACCGAACTCATTAAATGGAAGCGTACCCGACACTTCGAATGGGACACAGGCAGGGGAAGCTCTTCTTGTACGTCACTTCGCAATTTACGCACGTCCGGGAAAGCATGGAGAGCGAAGACACGGAAGCAATGCTTGTTGTGGCCGAGGCCATCGACCGGGAGAGGCAGGAGCTGCACGAAGTGCGCCGAGAGGTCTTCGGCCTCCTAATCGAGGAGGCCTGGCGAGCCGCCATGCGCAGCCGCCACTATTTGACCGGGTATTGTTTGGATTCCCCGAGCAACGCGTCCTGGATGGCTCTATACGAGAACGGGAGTGATCTAAACTTTCTTAACGCCACCAGTTTAACGAGGTCGCCTATTATTGCTCGTCTGGATGCTGTTCTTGACTTGGATGCTGACAGGCCATGTACCAACAGGTCTGCTTTCTCTCAGCTCTTACAGCGTTTTTCACAATTCTACAGTTTACCGGGGCCAAGAGTACGTGGACGCCCGCCAAAGTTCCGTAACCTCCAACAGGTGTTGGGCTTAGTATTAGTGCTCTACACTAGCTCGATGGAGCGAACGACGCTCTGTATGCTCTTTGGAGCACCTCCCAGCACGCTATCGAGGACACTACGGAAAGTGGAGGAGTCGTTGAGCAAGGCGCTACATGATTTCGCTCCTGCGCGCATATCCTGGCCGTCACCAGGCCATCAAGTCAAGCTGTCGAGGCTGGTAGAAGCCCGTTAAACCCTTCTTCATCACACGTTCGGGTTTATCGACGGTAAAAACCTACGAGTGCGTACATGTTTGCATTTAGAAAGTGTATTAATTCGAATATTTTACCAATGCTATCAAAGTAGATGCAGCAGCCATCCAACGCTGATCTACAGAACGCGATGTACAGCGGGTGGCTGCATACAGTATTGGTTACGGGGGTCATTTGCTTCGCGGCTGATGGCTGTGTAATCTGGTGCAAGAACAACTGTCCCGGGTCCTGGAACGACTCGGACACCTCCATGGAATTTTGGTCGAAGCTCCTGGACCCGGCGTTGTGCTCAGATGCGAGAAATAATGTCGTGTTCGATTCTGCCTTTCCGTGCTCTACAGCGATGGTCGGACGCATCTTAACGCCGCTGAAGGACGGCGACTTAGAGCGGATTCACCCTGCCCTGCGCAGTCAAGCACGCACCCTTCATAATGCTATAACTTCGGTGCGTCAAGCAGCCGAATGGGGCATGGGGAGTATGCAGAAGGTATACCCCCGGCTTAGTTTGCCGCTCCCATACGATCCACAGCTTCGCGGTATGAGGCTGGACAATTTGTTTCGCCTTACCAACTACCGGGTACGGACTGTTGGGATCTCGCAAATACGTACGACTTTCTCGGGTGTTATGGATGAAGGGGTAGCTTTGTTAGGCCGATTTGTTTACTTTACTTTGAGCATGATTGGTATCTAGACTGAAATCTGCTTCTTAGTAAACCTTCTCAGGTTTTTGGATTTTATTTTGTTTCGATACGTGACCATGCTAGTAATTTGACATCCAAAATTATGTTTTAATGCGTTGTAGAGATCCTACAGGTAACAACGACGTCACTTCGAAGGTGCCGGTCTTTTGGCGCCTTCTTCTGTTTCCGATGGCCCTTAGGGCTTCTTGTTGATGCTCGTGGTGAGCAAGATGAGCTCCTGCGTGCGAGCTCTGGCTTCTTCCTTGTCACGGCGAGCTCGTTCGTCCATCTCGATCTTGTCTTGACGACGGTGCTCTTCAGCTTCCGTCTTGTCCGCGAGGCGACGTGCCTCCTGGGTTGCAGCATCGTCACGACGTCGTTGATCCTCTTCCGCACGGCGGATTTCCGCCTTCCGCTCGGTCTCTTCGCGAAGTAGCAAAATCGTTTCTAGCATACTCCCTCCGCTCTTTTTCGAGGCTGTTTCTAGTTAGTCCAGCTTGGATTTAAGCGCCGTAATCGCTTTAGCGGCTAGGATCCGCTTTGATTTCGCGAAACTTGCCTCCATGATCTCCTTGTCTCCTTTCATGGTGGTCTTGGAGCCCGTTTCGTTGCGGAACGGGGTTAGATCCACTCCTCCAAGTCGATTTGAGGCAGTCTGGAATTCAGCAGATCGGTTTTGGGCCACAGACGTTTTCGTCTGAATTTCAGTCGTCTTAGAGAGCTTTGTGGGCCCCCCGAGCCTTCGCTCGAGTCTTAGGTCCGGTCCGCGGCGATGCTGAAGTGCGTACGGTGCCGATATTCTCGTTCCGATCTTCTGAAGTAAAATGGGAGGAAAGCATATCTTGGAATCCCCCAGAGGTCGGCGCGTTTTCGGTGCCCTGAAAGCTCCCATCTACATTCACCTGACCGTCAGAGCTCTCAGCCACAACACCGAGCCCCACAGACTCTAAACAATTGCCGCCAACAGCAAATAAACCGTCATCAAGGCTCGACGTCGAAAGCGAAATCCTCTCTGAAGTCTTCTTCCTCGTTGTCGGCCCCGTCATCCA

>Contig_54

GCCACGTAGTTGTAGGCCGCTTTGTCGGGGTTGCGCGAGGGCTCCGTCTTAACCACGAAAAAAGGGTCATACTGTTTTCCAGTCGAATCTGCCAGNAAACATGCCTGTCAGGCGTTCCTTGTCCTTTCCACCACATCTGACCCACACTGTATTGACACCTTTTCTGTCCACCGTCTGCTTGGGCAGATACTCGAAGCAAATGCCGCTTTGGTCTGCATTGTAGAATTTATCTACACCAAGATCATTTTTTGCTTGCTCTACCTCTTGCCAAAAGTTGTTGGCAGCGTCTTCCATAGCAGCAGGGGTTTTCTGGCCTTGACGCGTTTTTGTTCTCAAAGACAAGCCATGACGAGACAGAAACCCCTGCTGCCAGCACCAACTCCCAGTGAAAACACCATCTGCTACGCCTTCGTCACGGGCAACTTCCTGAGCTTGAAGTTGAAGCATTAGACGGGACACAGGGATACCCTCACCACGTAGCGAATTAATCCACTCAACAATGCTTTCTTCTCCAGACTTGCTGATTGTTGTTGCTGTTCCTGGAGCTCTGTAAGACCTCAAATTTGCCGTAGCGGTGGAGGCAACCATGGCTTCAATGTGTGTACGCTGCTTCGACCACTCGTTGATCCGCTTGCGCTTTGATACGCGTTTTGCTGGGATGAGTTGGCAGTAAAATCTATCGAGAGTAGCATCCATGTCATCATTGTTGTCACGAAGGAAATTTATTACGTCCAGCTTTTTAGTGTAGCTCTCGCACTCGCGTTTGTAGCCGTTTGGCTTTCGCCCAGCGCCACCAGGAGCGCGGCTCCGCCCTCTTGCCATGCTGGCACTTGCAGGCTCTGAATGTGAAAGGCTGGTTGTCTGCTTGCTAGTAGTTTCACCGCGGCTTTATCCTGTGCATAAATAGTAGAGTGGGTAAAAAAAGGTAGATCCGCATTGACAAATGCTCCATAGTAAGGTTTTTTCAAATAGCTACATCATTATGATTGGCTAATTCTGGCGTCCGGATGGACAAAAGGTCCGGATGCACAAATGTTTTACAGTATTACAGTACAGAATGATACAAGTTATTTTTAGCCTTTTAATAAATTAATTTATTAAGAGTACGCCATGGAACAAATCCCTCGATCTGGTCCGATACTTAAAAACAATCAGCAATTTGTTTAATCAGGTGTAAGAAGTTTATTTTCAAATTAGGCATGAATACTTTATGCGAGTGGCAACAAACTTTTCCACTATACATTACATAGTGAAAAAGTTTAATATACACCTCTAGGTCCGACAAACACTTGAATATGGTATAGTCGTGACACACAATTTATCCTACAGCCAAATATACTTTTAAGCTGCAGAGGAGGCTTTTACGAGGACTTACGAGGTGTACAGTAGATGCAGATACGATAGAGTCTATCATAGACTGGTGAAGCCTCCCAATCACAAAATGATCCTAATACCCTAGGACGCAATTCGACGGTGTTACAGCCACACTTTTGTAACATTTGCTGCGTAGAGCTGCCTTGTCCACAAGACAAAGTACGTGCTTATTTTAATTAAAAGGCACTCGTTATTTCGCCCGACTGAGGTAATATAGCATCGACTTTTATACGTGTACAGATAGTATAGTACGGTTAAATTGGAGTAAAGGTAGGACACTAGCGTGGTCATTATATCCAATCAAAACTATGGCGCATTTTTCCGTAGCATCTCTTAAAAGTACACTATGGAGGATACTGGTCAGTGCTGGCGTATTTTTCCGTGGCAAGCGACGCCAAGTAAGCGTGGCGTATCAGGACNGGGGGGTACTGGCTGTCTAGTTATGAGGGAAGCAAAAATGGCGGTTCGCTACTCGCTTTTTATATCCAGGTGTATCTCCGTGAATTGGGCATCACTCGTTAACTTTCGAAATGGGTAATTAATCAAGTCCAATGGGTCAATTCTTTCTTCTTGAAATAAAGGACTGTAGTGCAGTGCATGTATTTTTGCTCTGATTATCTCCGTCAATTGGAGTAAACTGGAGCGCATTCCGCAATTCTTGAACCAGCCCTCAGTAGCCACCTGTTGATATTAATAGAGATGCGTTTCTTCCCAGCCGTACTCCTGGCAACTGGGGGTGTTCTCCTGACAAATGCTACGTGTGCGTCCTTCGACTCGGAGTTTGAGCTAGCCACCGCCGACCATCCGACTGTGTTCCGTTCCCTCGCCTACCACCATAACAGCGTCGCTTCTAAACGGCTGTTGCGACGTTACGATGTTGACAACGAAGGGAGAACCGTCGGGGGCGGCGCCAAGATCAAGAGCCTTTGGTTGAAGGTCCGTGCGTATTTACTAAATCGTAAGAAAGATGAGGCTGACATGGCAGCGAAATTACAACTCGGCGGTATTGACCACGCCTTGTCAAGCTCAAAACTGGAGCAATTGACCAAGGAAGTCCAAGTGTTTAACAAAAAGTCCATGGCCAAAGTCACGGTGATTGGGACGCTCAGGACCCTCTACGGAGATATCGATCTGGCAAAGGGGCTCAAGGCTGCCGAAAGGGAGGCGTCGCCCACACTTCTAGAACAACTCAAAGCGTTGAGGCAGGACCTACAGTCGAAGTGGCTGAACCGCGGCATCTCCGCGGACGTTATTTTCAAGCAACTGGGGATTCGCGAAGAAAAGTATCAAATGTTTTTCAGTGGGAAACTGGACATTCTGGAAGCCTATATCAAACTCATCAACCAGAACAAAAAGAAGGGCGATCCGGTATCTCTGGTGAGCATCTTGAGTAAGGGATTTGGCGGGGAGGACAAGCTGGTGGCGCTCGTGACTTCTGCAAAAAAAAGTAGCATGACGGGGAAAAAAGCCGACGAATTGGAGACTGCTTTACTCAATAAGTGGCTGAGAGAAGATACCTTACCGAAAGACGTCTTTGTATGGCTGAAGCTTTCCGACGACGTGGACGACGCTTTCTCCCCTCAAAACTTGAACAAGTTCGCAGCGTACATCGACAATTTTAACACGAGGAGGCCCAATCATCAACAGTCAGCGATTGCTATCTACACGAGCAGTTTTGGAGACGCTGCTGTCGTAAACAAGCTCATCTCGGCAGTGGACGACGGGGCGACAAGAAGTATCGCGAACAAGCTACAGGAGGCGCAGTTTGAGAGCTGGGTCAGCCGCAGATTGGGTTTTGGCCAAGTCGAAACGATACTGAAGATTGACAGCTCTGGCGATGCAGTAGTTACTCGCCGAAAGCTAGGCTTGTTGGTCAAGTATATTACGCAAATGATGGATGGAGATGAACGTTTAATCAGGACGTTGACGGAGCAGCTTGGAGGGAGAGACAAACTGGCGTTGGTGTTGGAGAAAGCAAGTGAGTCCACGGCCGCCTCTGCGCTGCAGAAGAAGCAATTTGCGTCATTGAAAGACGAACGTATTACACCGGAAGTCATCGTCTCCTTTTTATTTAAAAAGGCTCAAACAACTACGACAGCCGAGAAGGCGATTGTGGCCAAGTTCAATCTGTTCTTCATTGAGACAAGTGGGTGATGGAGCGAAGGCGTTCAGGATAGAGTATCTTACTGGGTCATAGGAAGTATACAGGACTACGTAAATTTCACGCATACTTAAGCACACGGAAATGTAGTGTTCATGTAGCACTGCTACCTTTGTTATTGGTTCTCTTTTACTTCAAGTATAAGACTTACTATTAATCGTTTTAATACATGTACCTTTAAAACCCATTCACATGTAAAATAACCAAAACCCAGTAGAGGATCGTTGCATTTTTCTATGAGCTGTCTGTTGTAGCTCTCCACACTATGAATCTCGTGGGGCCACCAACGGCAAAGGCTCTTTTTGTAGCGGTCAGCATCGTAAGGCGACAGGATATATCGCTCGAGTGTCCAGACAAGCTTTATTTCGAGCCTGGCAGGAGCGATGCAATGCTTGAGCGATTCCATTGAATACCTTTTCCAGCTAAGGGGTTTGCAGCAAATGCGTCATTTGGTTATACGGTGTCGTACAGAGCAGTAACATGTGCTTCGTTTTAAACATCCCAGTTTGTACATAACGAAGTTGTGTTTTACGTAGACGTAAACACTGGAAAATGTTTTGGTCAGTGCTGGCAATCGCTACTCGTGATTGTTCAGCACGTTTGTTGACGCGTCCAAATGCTTATGCCAAATGCCAGCATCACGCAGCGGAGAAGGAAGAAGTCTCACGCCCTGCAGCTAAATCAGCGCGGGTGGCGGATCATCTCACTCTTCAAAACATTCTCAGCTTCACAAGCTCACCAACACCCCCCCCCTCACCCTCACTTCAACCATGGCCTCCACCAAGAAGACAGCTCTCGTCACCGGCAGCACGCGCGGTATTTTGTCGAGCACTACGTCAAGGCTGGCTGGAACGTCATTGGCACGGCTCGCGCCAATAGCAACACGGAAAAGGTACAAAAAAATGTTGCTAAGAGACGTTGGGGTTATCAGTACTAGTTGCTCATCTTTACTGTCATTGTGCAGCTGAAGTCGCTTGCCCCGTTCAAGATCATTGCAATGGATACGAGCGACGAGGTCTCCATCCTCGAGGCAGCTCGTCAGCTGGAGGGGCAGCCCATCGATCTGCTCATCAACAATTCCGGTATTGGTATCCCAAGCGAGTTCGATACTGGCACCAAGGACGCCCTTATGCGCCAATTCGAGGTGAATGCCGTCGGGCCATTCCTCGTGACCAGATCTTTGCTACCCAACCTGGAGCTGGCAGCAAAGGACAATGGTAGTGCCTTCGTTGTGCAGCTCTCGTCATTCCTCGGCAGCATCGGCAGCTACACAAATGACACTGTCGATTTCTCCAAGCAGGCTGGCTACGGCTACTCGTCCTCCAAGACTGCGCTCAACATGATCACGCGAGGGCTTGCGTTCGACCTGCGCTCAAGTGGCGTCGTCGTCGTGTCGGTGCATCCAGGATACGTGGACACGGACATGACCCAGGGCAAGGCGACGCTGAAGCCAGCGGATAGTGTGGCGGCCATGACCGGCCTCATCGCCAAGCTTGGCTCTCAAAGTACGGGCAAGTTCTTCAACGTGGACCCGCAGATCCCCGTGGTGGAGCTGCCGTGGTAACTTGGTATGGGTTACTGCGTGAGACAGAAAAATACGAGTACGGTAGCTGCTAGGACGTTGGAGTTCTGGCTAGGTTACATTCGTGCGTCTCACCCGAAACAGGCTTAAAATTCATTAGTATTTCTTTCAACCATGTTTTAATTTACAACAGTGAAAGAGCCATGATCAAGGAGTCCTAGATCAAAGCAGTCGTCACACCTAGACTAGGACAGCGATTTGATAAAAAGTTGCTCATCAACTTCCAGAAGGATTATTACTGTGGGATAAGTTCTATTGCTGTGACGGGGTCACACAGCACCTTGCACAGAGAAGTGCTTAAGCGTATTAACTACCCGAGTGAACGTTAGTCCACGTGTTCCCGGCCTCAAAGGGAACGGTTTGCAGAGTAGATTTAAGACTGATGTATTTATAAGTAAGATAAGCTAGGAAGAGAATGCATTTGTACGAAGAGGCTTCCCTTGTATCCCTTCCAGTTGTTACGAAAAAGATGAGGAGGGCGCCTCGCGGGCTTGACATGCCTGCTGGCACGGTAGGGCAGGAGCTTGCTGCAGTGAAGGTTTTCAATGAATTCCTGGTATCAACAGATATAAGCAGTGACGCCCTAGACAAACTGCACTCCTCGTTATCCAATGCAACTAACATGCCGATGCAAAACTTCTACTCTTTACTAACAGCCTTTGGAATCTTTTTACAGACAAAGAAAAGTGGAAAAGCTCGTGCTGCTGATGAATTCCTAGCAAAGGCAACAGCCTTGGGCTACTTCTCTCAAATTATGAACCTATTACGCGAACGATACAGTGGATCTCTCTCTCGGATGCCAAGCGAGTTGCTAGAATTTAAGACCAAATGGCTAGTGCTATCGAAGGACGGAATCTCCGTTCCAATGTGCAGAGCAATGGTTTTCCGGGCTGTACTTTATCGGATCTCTGTGTGTTGGTGGAACATCTTATTGTTCATGCTGATGCGACCACGAGAATCAAGTGTGTTCATGAAGCAGCTATGTTAGCTATGATGTGGCATACATTTGAACGAGCCATCGATACGTGTTTCGCCCGGAAGCAACAAATTTCGATCTCTGCATCTGGAGAGCTGTTTTCGCATATCGCTCGCTTGAAGACATCAGTTGTCCAAGGTGTATCTATTTACAAGTCCGCAGAGAGATGACAACAGTGCATGTTGCATGCGTTTGGTATGCTATTTATCTGCTACGATGATCCATCCGAATACCTCTTCCCACTAGTGCCCCGTTGCGCGGTGTCGGGCCTTCCAGGAGGCCACACGTACACACAAGAAGAAGCTGTGATCTTTTGGGAGAGCTTGCATGACAACATTGAAAAGGAGACCCAGCCGCACCTAAACGGGAGAGAAAGCGGCCAAACATAGCGATTCATATTACCAAAGTCATCCGCGATTACATCCGAAACATGTCTCCCAATGTGCACCAGACCGTGACGCCGAACATGAGCAGCCACTCGCTGCGTCGAGGTGCCGCTGCGTACGCAAATGCGTCCCCGAAGTTGAAAATCCAGTGGATATCGACTAGAGATGCT

>Contig_58

GCTAAAAGGTGCCCCCGAAGTCGCTGAAAGTCTAACTCGGATCGCGAGAAGTGTAAATCGTACACCCCACGTATTAAGCTAGCCGATATAACCGATGGGAAAGCTCTTAATCGCCTTGTGAATGTTCTCGACAACATGGAAAAAGCACATAAGGAATGTAAATCCCTGCGTCTGGCCAAACACCGAGTTGGGTGCGTTATACTGGGCTTGGTCTGCGTCTGCCATTGCGTATCGAACTACCAGATTCTTGTGCGTAATAAAGTAGAATACGCGCTGCAGTGACAGTGGGTTTCTTGAGAAACCACAAACAATGCCACTAGATGAAAAACTCGCGCACGATCAGCCACACCAACGACAATAACCGGGTAACCACGGTGATTCATATTGTACGTGGCATCTACGTGCAAGATGAAGCCGTCCGGTGGCATCAACAGCTGCATCATCAAGCCCTTCGTCGACATTTCCACAATAAACGGTCGTTCGTCGGAGCAGTCCCCCACCTCAGGGAGTCCCTGAGCATCAGCCTCTCAGCAAAACGTGAATGCCTGGTCCTTATCTTCCCTTCCGCTGTAATTCCTCGCGTGCACCCATTTCCGAATGTCGTCTACTCTATCATGGTTGTCAAGATAGGTGCGACTGTAGTGGTTTACAAAGTTCTGGACGACGCTCAAATCTGGAAGGTCCTCAAGCGGGGTGGAGAACTTGCGAGACATCCCGTGTCGGATCCGCAACGGCCGGATGTTATCGTCGGCCTTCTCTCTGCAATACGTCTTCTGTGCAGTAGTTAGCTTCTTCTTCTTCGGCGAAGACACCTCAGTAGCGTGCGCACCATAGTCGTAGATAGAAACGCGCAGCGTTGCCTGGCATGAAAGCAGCTTGCCTCGCCAGGTGCAGGGCGACGACTGGCCCTCGCCGCATAATGAAGAGCTACACTCTAGCAGTATATTGCGCATCTTGTGCCGCTCCGCTCTCATACACACAGTGCACGCCATGACGTGATTCTTAACAACGTCGAAGGACTTCACCCCCTCCAGAAACGCGTCTACGACGGACTCCTCGGCTGCGAAGGCCTTCTGCTGCCACGGGATACGCTTTGGCATTTGGATGAGTGTCAGACGGCCACCATGCAGGTTCTGGAAGGCCACCCGTTGAGTAGTGGTCCACACACTTTCTGCCGACGCACGCTGTGTCACGCTCACGATGCCTCCCTATGCCAAGATCTCATTGGCTTCTCCCTATCCTCTGCCTTCAACTCGATGCACAGCCACAGAACGGCCACAGATTTCATTGACCCCACTTTTAATTGTGTTGCTACCGTAACGTGCTGTGTTCCCCCACTTTTGAGGTGCTGCCGCAGATCTAAGGTGCCGAAACGCAGTGTAGTGTGAAATTGACGCGTTTTCTCTAGAAAAATCGATTTGTACTTTTTTAGACCTCGATTTGATAAAACGACCTCTATKTATTAAAAATCGAGCTTCAGGCTTAGTAAAAAGGACAAAAGCATTGAAATGCACAATAATACAATACCTCCTTATTAATAGGGGTACAGTACTGCATCCCGTTATCGCGGAGGTGGATCATATAGTATTTGCAAATAAATCTGATTTTAGCTGAGCAAACCTTTGTTCTTTTGGATTCAGGATCATCACATCCGGAGGTTTATCGAACGTCCAAGGGCGCGAAAACGGGGTGCAGTACTGTACATGTAACGGGAATCCCGCCTGTGCCTGCATTTCAATATACTGTACAGTACTGACAGTACTACTGTATATCCAGTTTATCTAAAGATTTGCAGTCTTCATACCGGTAAAGTTTACCGTAAACAGCTTGAGCTCAATAAGGACGATCTGCGAATAAGTTGTGATCATCGTCTCCTACATTGAGTCCAATACCGGTAAGGGCGATCTGTGAAAACGTGTGGGGGTTCTGTGATTACATGAGTCCAATACCGGTAAGGGGATCTGTGAAAACGTTCGGGGGTTCTGTGATTACATGTATTGTTACATGTACATGTACCGGCAACTGTAACGATTTGACAAAAAAAAATCTGAGCCGGTGTAGTATTAGGATAAGGTGGTGCATGTACAGGTACGACGGGACTCGAAGAGTGCAGACTTCCGAATGATGGTTCGATTATACATGTACAGTACTGGGATTCAGTGCAGTTTGCTACAGTATTGAAGGTGAGTACTCGTGGTACTGTACATGTACATGGAGTCCGATTACATTTCGCTCATTTGCGATCATGAACATTTCATTTCACGAAGTTCCTAAAAACAATTAAGACCTCCACTGTGGAGATCTTGACAATGGAGTCCACAACATTCACCGGAACTGCTCTTGTAGCACAGTAGGTCTATGGTGTATTTAATGTATGTGGTACACGTACATGTACAGTGTCTAGCGCACTATTCTTTATCTTTATCACCGGCGACCTCTTCTGTCCGAGCCCCGCATATTCTTTAAATGCATAAATAGTACGATAGGTACAGTATATGGATACCCCCGGTCCAATGCACCGGGTGCATGTATCATGGACCATAGTACCAGTGGGGGCCCCTGGTACCATGATTCATGGTACATGTACCTGGGGCCCCCACTGGTACTATGATAAAACGGATGGACATTACATCTGCAAAGCACAATAAATGTACTGTCATAGTGGACCGGTATTTTAGGTACAGACTTGTGTGCGGGCCGGGCCAGGGACCTGGGGCCTCCAGGGGGCACAGACTTTATATCTAAAAGGCTGTACAGTCCCTGGCCCGGCCCGCACACCTGTACTACCGGTCCGATATGACAGTACATTTATAATACAGGCTGTACTACAGGCTGTACTGGTATTAGTACATGTACAGCCTTAGTTTTGAGCTCGTTGGTAACAACTTGATCAGGAGTGTTTCTTACCAGTAGGCTACTATTAAAAATTATACTACTGGCAGTAGCAGAATCGGAGACTTGTGGTATGAAGACTTATTAAGAATTACTTAACAGCACGTTAGACGTTCCGTGTGATGATTGCGCTTTTGGTTTCTAACGTCATTTTGGTTGAGTTTTCTCTGCTTGGTGCAGGCGACTGTGAACGCAATAAGTGTACCTTGTGTGGACAGTATGTGATGATGAACAACTCTACTGTAGCTGTTCTACCGTCACCTAAAGCTCTGGGTTAGATTTTCTTGTGGACGTCAAGGTTCGATTACTTTTGCCGCACAAGCTGTGCGTTGACAACGCTGAACAGATCCAGCAAGATACGAACTATAATTCGCCTAAGTGGGGGTTGGTGTGAGCTTTAGGTTATTAGTGGAAGTCCATCAATTGCTACCGTATGGATAGCAATTGAGGGACGTCCACAATAAATTCCATGGTCATGCATTTCTCACATAATCTGTAGTGTCTGTGACTTCTACAGAATGACTCGTAGACAGCGAGCCGTAGCCCCATCTTGGATATCATCATGGACTGTTTGTGGTAGCTCGTGAGAATGCCCATCTGCCCACCCAGTGCAGTACTGACAGTACAGTAGGTAAAAGCTTTAGTAGGCGATCTACTATTGCTTTTGAAGCCACCACCTAACTGCTTTCTTCCTAGAACTCAAGCCCCAAGAAGACTCAAGCGGAATGGGCTTAATGCACCGACTATTGCTGCTTGCGACGTTAGCCCTGTTGTGTACGCTTGCAAAAGCTGCCGGATTCGACCACGCCAAGGTTCCAAGGACTGTCGAAAGAGGTGGCGGTGCAAGACAACTGCGCACGGCCACGATGAGCGACGACGAAGCTAGAGGGTGGAAATTGCGGTCATTTTTCGAGTCCTATCTTAAAGACCGAAAAATCAAGAATTGGATCAAGGGCAAAGTTACTGACGACTTTGTCCTGAGCGAGCTAAAACTCGTGAGATTGCCTGGGAAGAACCTGGCGGACGACCCAAATTTCAAGCTCTTTCAAAAGTTTAAGATTGACGGCTGGCTCGAGGAAAAGGCTACTACAACGAAAGCCTGGGAAAACCTTGGCTTGAATTTACTCCCAATTGATCGAGTGAGCAAAACCGACATGTTCAAGACCTATACGCAGTATGTGATGGCGATTAACAAGAAGGCAAGCGAACTCGATATTGACAAGTTGGAGGGGCTGTGAAGTGGAGGATCGCCTCAGAAATTGATGGCCAAAGCAATGATATTGAAGAATTTGGGCAGAAATGTTCTAGAGCGCAGAGCCATGCTTGGTGCACATGTTGTGGTACCAGTTTAAGGTTCGTAGCAGACGATGGCTGCATGGGAATGAAGCACATGGGTATTAAAAGTACTGTCCTTTCCCACTTAAAACATTGTTCTTAAGAAGCATGCATCGTGTCATCTACTACATGTACATATAGTAGTAAAAATCAAGCCTTTTGCAGCCATTTATTTAGTTCAAATACTTGCTGCTCGGCGTTTGCGAATCTAAGCTGCGTATGCACATGTACCGGTAAATTTTGCATTGCATTGTCAGACGAGGGCATGGTGAAAAGTGAGCTAAACATCAGGCCTACGTTACATGATGAGAGCGACAAATGCAACCAATCTGCTTTGGGCAATGATTCAGCCGGCAAGATGATTCCTTTGGTCGGACGCGCTCGGGCATCGTATGCTGGCGGCGTTTGTGTCTGGCAAGAGTTAAGATATCGCAGTTCTCACTTGGGCCGATAAAATGACCGCGGTTAGCAAGAGAACCGAGTAGTACATTACCGGTAACAGAACTATGAGCGCATGTTATTATTTCTTCGGATTATTGTAATATATGCCTACCAATTTTGCCGTTTTGGTCCTGTTCGGTGAGTTACTCGCTGACTAATTTACAGTTATTCAAGCCGATATTAGCTACTACAGTAGACGCCCCCCCCCTTCGCGCTCACGGTTCGCTCTGCTCGCGATTTAAAGATTGGGTGTACTGGAATGCTCGGTGTCGAATAAAGTGTCCTAGATTTAGACCTGTTTTTATTTGAGATTTTGAGCGATTACCTGCACCAGAGCCAATACCCGTATTTCGCATGTAGAGAGATGCACCAAATGCTCACATAGCAAATCAAACTCGGATAAATACATATTCAAAAGACACAATCAAATTATACAGGAACCTTTTTTTCCTTTAGAATTTTTCCTTTAGAAAAAGTGTATTTACACGACGGCTACGTCAGCACTCACGTTGCCATCCTCCTCTGCGGTGAGCCATCTATCAAAATCGTGATCTTCGTCAGTACTTAACCCTACTAGCGATAGTCGCTGCAACTCCTCCACCAAAGACGCAGCCGCAAGGCAGTCGGTGTCGAGACGCAAGTCGCACTTCTTGTACCCATTTTGAATGGTTTCTGCACTCAGAGCTTCCCAACTGCTGTACACCCATCGGCACATCCCCGCGCGTCCAGGCGGCGCTAGCTTGAACTTTGATCCCTCTTCTCTTGGCTTTTGCAGCTGTTCTTGCAAATTTCGAGCCAGCACCTACGTAGTCCGCTTTTGAAAGGATGATATCATGCTACGTCTGCCGGTTGACAGACAGACGTAGCATTTGGTGGCACCTTCATCAACACGACGTTGATTGAAGCAGCATAGGCAAAAACCTCTGCGGTCCAATGACCGCTAAAATCGTCCCAGAGGAGAAGCACCGGCTCCTCTTGAAACGATCGGCCGCCAAAGTGGAACTTCAGAAACTTCAGCGATAGATAACTATTCCACCAGCCTAAGTAAAAAATAATTAGAATCAATACAACTATTAATAAATCGCTTTCAAAACACATACCTTTTGCATTCGTGTATATTTGCAATTTGGTCTCTTCTTGAAGGCGCTTGATCTCCGGCCACGTTCTAACACCAAATCCGAAGCGCTTTTCGATATTTTCGGCATGAGTCCTCTTAATCTTGGATGGATTCATCTTGAAGATGACGAACGGGGTGTATTTAGTCCCGTCAGAGTCTCCCAGTAGCATTACTGTAGCTCTTTCTTTAGAGGCTCCAGCACACTTCACCCAAACGGTTTTGGCCCCGCGTTTACTGATAGTTCTCTTCACAGGCAAATATTCAAAGAAAGCAGCTAAATAAAAAGTTAAATTAAAGCGTTTTTTAATAAAATCAAGTAAAAATAAAAATAAAAACGAACCTGTTTGGTCTGCATTAAAAATGCGCTTGATACCCAGCTGACGAGCCTTCTTCTCAACTTCAATAGCAAATGCTTCTTTTACCTTGTCAGCATCCGAGGGCCTGACTTGCCCTTGACGAGTAGGTACACGCATACTCAGGCGGTGTCGAAGTCTAAAGCCCTTAATCCACTTATCTGAAGCTCTAAACCCGTTGATGCCAGCTTCCATGGCAGTCTGACGCGCTTGAATCGCTAGCATTCGAGGCGATACTGGTACACCCTCTTCCCTTAAGCAGTTCACCCACTCCACAATCTCTAATTCTACCTCCTTACTCAGAATTGTTCCTACGCCTACGCTCCTTACCTATTGATAGCCACCTTTATTCTTCTTCAATGCTGACCTTAGCTTTGTTTCCTCCCGTCGCCAGCGCTGAATCATAGTGCGCTTTGAGTTGTAAAAATCAGGATTTAGATCGTGCCAAAACTGATCAATTGTATGACGCATTTTAGGAAGAGGAGCATTTTCCCAAAACGACAGAATTTGTAGCTTCTTCTGCACCGTGATGCCCTTGACCGTGTGTTTGGTCTGGTGGCGTCCCTTACCACCGACACGTGGTCTGCCCACTGGGCGAGCCTTCGTGCGTGACATGGCAAATGACCGACTGTCGGCGCAAATTTTGCCGCAAAGACTTGGTTGGCTTAGGGATGGTGACTCTCTCTACATGCGAGTTCCTCAATTCAAGATAGCCGAATGTGATTGGTCAATATCTAGGTGCATCTCATATGAGGGTGCAGCTCTCTACATGCGAAATACGCCTGTGTATAACAAAGCGTACTTAACGTACATGTAGTTTTTTTAGGACAAGTTTCTCCTAAAGACGCGTTAAGTACGAAATATAAAAGGACATGTTTAGGACGTTCTGAATGCTCGGCTCGTTCGGCGCTACGCAAATACCGCTCCCGCGCGAATCTGCTTCAGCTATGGGTGCTTGCACGCTCGCGCTTAGACAACGCAATATAGCTACTTATTCGTTAAAATTGCTTTGGACAATTTGGACAAATTTTGGCAAGCTTGTTTCTCTCAGTGTTCGGTTATTAGCCAGCAGTTAAAAACTTTACCTCGAAGCTTTTACATGAGGGGTGAGCAGTACGGATAGACGTGACCACAGTACAATTTGAGGTGGAAGTG

>Contig_59

CATTCTCTCCTACCACCTCATATATTGAGAGTGACCCTGCATACGCTCCGTAATTGTGCGACGGGAAGGTATCTCGTAAATACCGTTGATGGTTTGAATCAAGGTCTCAAAAAATCGAAGTTCTTTGTCTTCCGCGACGCTCAATGGACGCAGTCGCTTGGCAATCTAGTCAAAAAAGGCTCGTGTAGCCATTTCTTCTTCTGCTCGGTCGACCCGCTTTGTTCGTTTGGTAGGAGAGAAAATCACTGCACCAGATGTCCGCTTACTTGTACCAGCCAGCGCGTTATTAACCCTTCTTGCAGCCTCCGCTTGCCCTATCCTGACAAGCTCTTCTGGGTGATTTTGGTTCATGTGCTTTCGGACGCTAGCACTACTGCCAGCTCTAAAGTTAAACATGATTCTGCATTTCAGGCAATATGCCAGTTCTGCATCCTATAATGCTCACTTTGCTTTCCCAGCGGGCAACTGAGCACTTGGAGCCGCGAGCATTATAAACGTCCAGTACTCTTCCTTGTGTACGCGAGGCAGCTTGAAAAGAGGAGGGGGAGGCGACGGAGAAGGCGGGGTTGACGGTGTGTTATCTTCCATGGTGACACACTCAGCAACACAGCGTACGGATAACACTTGACGACAGATTGAAGTCGTGTTCACCAGCACTGGAACGTAGAAATTCTCTCGTAGTAGGATGTCTTGACAGTACGCTCTCGAGCCCTAGATCAGATAGAGAAACTGCGATCGACGCGGCGGGGGAGATGAGATTCGCCCAGGACGGTGGACTCGACGAAAGCACTGTTCACAGTGTCGTACAGGCTGCTGAAATTGAGTACTGAAGTCAGAAGGAGTACTTGTACGCAATACTTCAAGTGGCGGCATCAAACACTCGCCTCGTGTCCGTCAGCCGCTGGCTTTTGTTTTAATCAAAACGAAGGGATTTGTTTAAATCACGTGATGATGTCTTGATAACGTTAACTTCACGTAAGGGGTATTTCGGACCGGACCTATTTACATTTTTTACGCCCCCATCACTAACCGGTATGCATCGGTCAAGACAAGACAAGACGTATACAGTATGCTGACATCAGCTACGTAATACAGTTCCCTACTGATTATCAGTAGTCGAGCATGTACTTTACTGACCTCTTAAATCTTATAAGACTAACAGAATGTCAGATTACTAAAACCAATAGTCGATACTGAAAAAACTATTAGTCTAAATAGTACTGTATAGTAATATATACTAAGCGCTAAAAGGAATAAGTCAGGCTTACTCACCCAATAGCGATCACGCGGGCCCAAAGGAAGTATTATCTCAAAAATCGTGAGAGGGGATTAGAAAATCAAAGGAAGTACAGTACTACATACCGTTTTCGCGCCCCTGGACCTTTAATAAATTTCCGAATGTGATGATCCTGAATCCAAATCAACAACCATTCGTATGCTAGAGTCTGATTTATTTGCAAATCCTATATGGTCCACCCCGGCGAAAACAGGGTGCAGAACTGTACGATAACGAACATCGGGAAAAAACAAGCACTTATTAAAAATGGTACCAAATCAGTGATCTAAAAAAATTTGAATTTGAAGTAAGTTAAAAAAATTTATAGTAAGTAATCTGATACATGATCTTGTTTATATAGTAAGATTTTCTGGAACTATATTGCCGACGATATAAAGCGATCATGTTTGCCTATGTAGCTTGAAGGAACTAAGCGGCTACGCCTTAGGCCTGTTAAATGAGGTTAAGCTACTGCGTGAAGGTACGTGGACACACTGGTAAAATGGTGTATATAGGAAGCCAAACCCATCCCTTGGAAACCGCGGTGGAGCGAGACCGCGGTAATTTATTTTTAGTTTTCTGCCACAGCAGCATTTTGCTCCCGCGATGGAGACGCCCTAAATGATATATTTTGAAACACCTATTTTGAATTGAGCCTTTTTGTTTTGATAATTCGCTGAGCTAATATATTACCAGGTTTTATTAAATAAATTTTCAGGATGCTAGCTCTGATATAAATTTCAGGGAGTTGTACGTCCAAGTGTTTTTAGCACCTTTCGCAAATACTCTTGAATGAACCCAGCCTATGCTTTCATTGCGTTGGACACTTGCACCCGCACTCGGCCAATGACATCGGCTGTCTTATGTCGATTGGTTCATGACACGATATGACTATTTAGTGTAATCAATGGCTATCGTATTACTAAAGCGAAGGTTACCTTTTTCCGTTTAATAAAAAAACCACATCTTATCACGACATAAAGGTATTGTACATCAGTACTATACCTTTATAGAAATAAAACATTCCTGTTTAGAAGATCAAAGCTTCAACCACAGTCTTTGCCATAACCCAATCAGAGTCATGATGGCCAGCTCCGAGCACGCGCTCGAGGCTCTACCTCCTGGCTCCACCGACCCCTCTGCAAACCCACATCGCCCACGCTTCTCCTGGTCCGTCTTCTGGTCGTACACTGGCCCCGGTTGGCTCATGAGCATGGCCTACTTGGACCCTGGAAACCTCGAAGCAGATCTACAAAGTGGGGCCTACACACGCTACCAATTGCTCTACGTTGTTCTACTCAGCGCCATGTTCGGAGGCCTCTACCAGATCTTAGCTGCCAGATTGGGGGCCTGTACCGGTCGTCACTTGGCTCAATTATGTCGCTCCGAGTATCCACCACTCGTCTCTTTCGGACTCTGGATCATGACGGAGCTCGCCATCATCGGCAGCGACATCCAGGAGGTACTGGGATCGGCTGTAGCCTTCAAGCTTCTATTTGGGTTACCTCTATGGCTCGGTTGTCTGCTTACAGGACTCGATACCTTCACCTTCCTGGCCCTACACCGAGCGGGAGACTTCAAAACTCGATATATCGAGATGTTCTTCTTGCTGCTCATCGCCACCATGTGCGTCTGCTTCTTCGCCGACTTCACCATGAGTGATCCAGACGGAATAGAGATTATAAATGGGATAGTAGAGCCCAGAATGGACAAACAGAACACCATGCAGGCGGTAGCGATGCTGGGGGCCATCATCATGCCACATAATATTTTTCTTCACTCAGCATTGGTCCAAGAGAGGACAATTAATACGAGATCTCCGACAAAAGTAAAGGAAGCCAACTACTATTTCGGATTGGAAGCAGCATGGCGCTGTTCGTCAGCTTCTTAATCAACGCGGCGGTGGTTTGTGCCTTTGCCAGCAGCTTCTTCAGCAGCCAGTGTTATGCGCTCAGTAGTAATCCTATTTCGAGTTTGTATGGGAGAGATATCCAGACGTCGTGTATTCCAGCAAAGGCAGCGTTGAGCTCGGGGAGTACGATTTACGATGCTTTCACGGGCAATGTCTGTGTGTTTGGTGGCGAGAGTGTGTTTAAAGGGGATATGACCGCGGATATTATCAATACAGTGACAAAATGCACACCGTGTTACGTGGATGGACGTGGAGATAGGTTTGATACGGTATCGTTTGGGCTGGGGCCGACAGCTGGATATTGCCAAGAGATTGGTCTAGCACAGGCTGGCGAGGCAGTACGTGAAGCACTTGGTGGGTATGCGAAGGTAGTGTGGGCCATTGGCTTACTTGCGTCAGGTCAGGCTTCGACCATGACTGGTACTTACGCTGGTCAGTTCGTCATGGAGGGATTTTTGGATATCCGGATTGCAGCTTGGAAGCGTGTGGCGCTTACGCGAGGAGTGGCATTGATCCCAGCGATTCTCGTGGCGCTTATCAGTCAAGATCGACAATTCCAGAGCGACCGCTTTAACGAGCTCCTTAATGTACTCCAGAGTGTTCAGTTGCTTTTCGCACTTCTGCCGTTGCTCGCATTTACCACAAGCACGAGGCTGATGGGGCTACTTTTTGTAAATAATAGGTGGATTGGAGCTCTACTCGTCGTGGGCACGGCGTTGCTTTGTGGCGTGAATTACACGCTCGTCTACGATATTCTCATCAAGAACCTCCCGGAAAAGTTATCAACGGCAGCTTGGATTGGTCTGGTCGTCGTGGCTGGGTTATATATGACGCTGCTGCTGTACTTGCTGCTAGTATACCCGTCCGAAACAGTCCCATCACACTATGTCGTCATTCCGTCTGCAGTGGACTCTGAAGGCTTATCTGAAGAAAATTGCAAGTCTACAGATATTGCTGAGGAACGCGTACCGGAGGATGAAGGTGTCGTATGATGCAAGGACAGCGAAGCTGATGATACGGTTAATAATAAAATATCATCTTGAAAGTCAAATTCTCGTTGATGGTTTCCCCCTTAAGTAATCTAGTCTCGATCACGTACAAGCGCTAGACCTCGAGCTCGCAGCTCTTGCTCCCATCTACATATTGCAACATAACAGTAAGCAAAATACATTTAAAAAATACTGATACGTACTCTTGGTAGCAGGTTTCATAGTACTTTTGGAGTCGCGCTTGTCGGATCTTGAGTACCTCTTTGTCCAACAACTCTCGCTCTTCATTAAGACTACAATGCAAAAAAGTATTAAAGGCCATATATTAAACTAAATAGCCTCGGCAGACATTCATGTATTATCCACGAACCGTTTGTTCTGCTGGGTACGAGCACCACTGCCCGATAGGCGATTTTGCTTTTCTTCCCAAAGAGCTCTACTCGCGTTAGTTTCGTTGTTATAGGTGTATTTCCGTGCCTGGTGTAGCTCTTCCTCGCGAGCGATAAAGCGTGTGACGTTCAGTCCCGACGGCTTCTTCACTGCATGGGGTCGGTCGATCGCTCGAGATGGAATTCCAGCAGGACGGTTGCGGTCCACGGGCATGACTCGTCTTCAATAGTTGCCTTCTAGATGCTAATTTTTAGTGCTGTGTCTGTCTACGCGGCATTGCCAAAAATAGCATCTAGAAGTTTCAGCTTTAAGTTACATTTTGGTGAAGCACTAAAGCTCTGGATTTTTATTGAGAACGATGCTCACCAGGTCATTCGCTACGTATTTGGATACCATTCACTTGTCTCCAGCTTGAGGCTAGTACTTGCTGCTGCTGAATGGGCAAGTGAACTTCATTGGTGTCTTACCGATTACACTTTTATTTGTCACCATCAAAAACGACCCTTATCAATAACGCGACACTGATGCGAAGTGGAGACCGAAGCTTTCAACAAGACAATGTGTTGAAGACAGTCAGAACCATGTGAGTAAAAATGATTATGTTGCTCGGGTGGTAGATCTACTTATTCACAGAACCAACGAAACTCTACTTGAAATGCTCTGCGAAATAGTAGTGAAGTTGCAATAAGAGCTTCTACAAGTTTCGTATTTGTGAAGTGCGTATAATATTAAAGTAGTACACAGCTTGTCTCTGTATCAACGCACCATGCTCTTCATTGGTGCCGTTAGTGATTCTACATTTACAGTGCTATTTCTTACTACAGTACATGTATTACTATTAAAGTACTACTGTGGTACTCGATTTTTTCGGCGCAAAGTCATCAAGTGTAGAGTAAAGCATGCAGGGTTAGTCGGTTCCGTAAATCTCATTCAAATTCTGCTCAGCGCATGGTATCGTCGATCACTGTTCATGTCTAAGTGTCATAGGGTGGAATAATACGTCCTTAAATGTTTCGTACTTAACGTTGTTTTTAGGACAAAAAGTAACAGCTAGGACGCCGTCCCGGCTAATCCGCTCGAATGCTGGGGGGGGGGGGGTTGTGGAAGTTGTGGTAGCGCACCACAAGCGAAGCAATATCGAAAGGATTTTTGTGAAACTGTAGGATTGCCCACGCGACATCACGCTTTCTATAACCCATACTCTTCCAAACTGCGAATGCCTTGAAGAGCTCGTCGTCGTTGCTTACTGTTGCGAATTATACTGATCTACACTATGGCACTTGAAGTTTGTATTGTGTGTGACAATGGGTACAGTGAGAAGGATTGGAGCGAGGGGGCTCAGTTGAGAATTTGCAGTTGAGAATTTGCTGCTAATATACCCAGTGGCTAAAGGTGGCTTTTGCCGCCCAGTGTGTGTCTGTTCTTCCTACTCTGCGTGTGCGTCGAGGAGGGATTTACAGTTCTCAACTGGTTATGAGCCCAACGGCCGCAACGTGGAAGACGACACTTGATTAGTCGTTATGAGCACTGATAGTAAAGATGGAGAAGAGAAGAATAAGATGAAGACGGCAAAAGAGGATAAGATCATGCACCAGCCGTTTAATGGAGAGAGCTTTGAGGTTTGGATGATGAGCAGCGACCAAGAAAAGAATGAGAAGGACATGAAGAACGATGAGATGGGAGACGACAGGGAGATGCAGCAGAAGAATAGAGAGGCTTTGCGAAGCTGGTTGTTGGGCAGTGAAATAGTTGTGCTTGTGAGTGTAAGAGCGTACCAGCGGGCCACGATGTGGTTGAACGCACTGGGTAAAACAACAGTGATTAAGCAAGCGTGATGGCCTCGAGTGGAGTGCTTAACAGCCTCTAGACGAAGAGCTTTTCGTGTTTGGCTTGGGCTCATGTTGTGGGAGTTGCTAGCGGACCACAAAGAGATGTGGTTGAACGCGTTAAGCAGTGAGAAGGCACAAAAAAAGAGTCTCAACTCAAATGCCAATTGAAGTGAGATGAGGCATAGTGAGCTGGTGAGGCCAATTTTTGCGATTTGAGAGTTTTGATGAGATTGAGAAAGAGTGTTGAGCTGGTGGGGCTGCGTGAGTTGTGCTGAAGATGTTGCCGGCAACAACGAAGACTGGGGATAATACAACAGCGAGTATATGGCAGACAAGATGGAACTGTAAAGAAGTTGAGAAGAGAGATGGTTGGTGCATATCCCATGATGATGAGGCATATTGAGCTGTGAAGCCAATGGTGCGTGTTGAGAGAGATGGTCGAACTGGTGGGGCCATAATAAGCGTGAGTGGATGAAAAGAGAGAAATGTCAGCAGGCCACCTACATGGGGGTTGAACGTGCTGTACATGTTGAGAGTGGTGCTGACTTATTATGAATACCTCAAACTAAGTTCCTAACAGCCTTGGTAAGAAGCGTTTTCCGAGCTGTTGAGCTGAGCCAAAGAAAGAGAGAATGATTGAGGGTTGTATTGATGAAGGTCACGAAGTCTCAGAGTGTGAGGTGATAGTTGTGGATCAGTAGGTGGGGCTATTGCGAATTATTCTGATCTACACTATGGCACTTGAAGCTTGTATTGTGTGAGACAATGGGCACAGTGAGAAGGATTGGAGCGAGGGGGCTCAGTTGAGAATTTGCAGTTGAGAATTTGCTGCTAATATACCCAGTGGCTAAAGGTGGCTTTTGCCGCTCAGTGTGTGTCTGTTCTTCCTACTCTGTGTGTGCGTCTAGGAGGGATTTACAGCTCTCAACTCTTACCATGGCTTTATACCTGTCGGCATTGCGTGCCACTCCCTTTCGATTTTCTTGGCGGCAGTCAAATTAACACCTCGAGGAAGTTTGGAAAAGTCGTTTGCTCTCTCTTGGTTGTCATAGTCATCCTTATGCGAGCGAAGGAATCTGATGTCGTTCGTGTTTTTACCAAGGGGTGGGGTCACGTCGATAGGCGACCTCAGTTTTGTCACTCGGTCGGTTTTAGCTGCTGCAACTGCGTTGGTACCTGTAAAGATGATAATAACAGCCCCAAGAAAAACAGTAAGCTGGCGCATACTGCGTTTTCACTTGTGCGTCTTCAAAGTACAGGTACTATGAAGATTACCGAATGAAACGATTATTAGGACATCAATTCATATATTTCACAGGTCACGAAGTGTCACAGTGACAGTTACTCACAGGGTAATCATCCTGGCCACAGATATTACAGTCCGTAATCACTTTGGTCAGGTTGTCCCCCAGAGTGGTTAACGCTCCATTTTGGTGGTGCAACACTCCGTGCCAGGTTAGATCGGCCACCAACGTTGGTGGCCCTGACTCGTCCAATTTTAAATAAGGTAAGTTATTCATACAAATATCGTTTGAGTGTGCGTGGGTCAATTTTAAGGCNNAGAGACTATTGCCACAAGGGGAAGAATGGGTGTTATGAGACTGCAAATCTCCAAAAGTGGAGGACTTGGCGTGAATTCTATCACCCTCCTAAACGCAAACCAGGGCTTCCTGGGCATTAACCCTATAGGGCCGAGCGAGGTTTAGCCATGCTTGGTCCTGGGTTTAGGACCGCGTGCTTCGCACACGGATCTTAAGCCGCAGGCATGGCCGAGCGAGTCGACTTTCACCCCCTCCTCCCTATTTCGTACTTTATGGGGAAAACCCGTCGGCTTACAAATCCCAAGTAGCTCATATTGTGGTAATAGTCAAGCTCCCTGAGACATACTTCAAAGTACTACGTACAGCGAGGTATTTCGCTAGATACTATCCTTACCGTACGTTATACTTGACTTTAGTAAGGACATGGTGCCAGATTTAATAATGCGCAGTCGCTGTTTGCTGGCCTTGCAAATTGCAGCGCATTTAACTGCGACACACTGAAAGGCTGGGACCAGGGATGGCATATCCCTAGTTCTGCTGAGAATTGAGTGGCATTTGAGCTTTATTGTACCAGTATCTTTGCAGTAGTCGCATAGAAGCTGTGAAGAAGTCCTGCTTTCTCTTGCTTAAAGTAATTAGGTTATCGGTATCAAATCGGTGAATACCGATTCGCTGAGAATTGGTCACCGGGAATTGAGAATCAGACGAGACTGAGAACAAACTCAAAAACCCGCCGTTGCAACAGAGAAATCCGCCACACTATAAAACACGCCATCAAAAGCTGAAGTTCGGTATTCAAGTCAAATATTTTAATCAGTAGCATTGCTGAATTTACTGAATACATCTAAGCTTTATCCAATAGTTTGCTCTTTCCATTGACTGGACATCCATGCCTCCTTCACAGCAGAGGCATTTTCGCTCGTCATTTGCAAAATGACGCCTCAGTGCTCATACCTAGTATTCAAAAAGCGAGAAGCTATCGCTAAAGCCAAAGAACTCGGTGTTTTACCAGCCGCGAAGCAGCTAAATATACCCCGTCGCACGTTGCGCGACAGGGTAGACAATAAAGAAAGCATCGATAGCTTTAGCAGTTACCACACAAGCAAGACTTTGAAAGGACAAGGAGCTAAGGGCATTATACCTTTTACACATGACCTGATTACGTTTATGAAAGATGTACGGCGCGAGGAGGAATTAAGAGAGCCATATGGTTGCCGGGTTTGAGTTATCCTATCGTCTAATGTCCATTTATGTTTTTTACTAGTATTTATGTACAGGGCTCATGATTCAATACATGAAAGTACAATAACCACATTGGTTGCACTAGTATATTGCTACTAAAGATAGCCTTAAACAAGCTTTGAGCGCTCTAATGAGGCTCTGCCAGCGATTTACAGCACGGTTATATTTTCACTTTAAATTTTAATATTGACTAAATTAACTCTCCTAAAACATTTTTTAGCTACGGGTTAACTACGCAGAAGCCTCAACACACTAAGAAGAATGAAGATGCGATGGTGGAAACGCACTGGCAGTTTGCTCTAAAGAGTTGGGATGAGTTTGAATCATTTCCACTGAGCGCTATCTACAATGTTGACGAGACCGCAGTCTATTTTGACATGCCACCTCTCAAAATTTGGGCTGAGCGAGAAAGGAAAGGATCGGCCGGCGTAAAACGCACGCAGAAGCACTCGGACCGACTGACGGCATGCTCGTGCTGATGGTAAGATAGTTTTTACGCTGTACTGTACAAATCTGAATCAATTTAAATTTTAGCTTATTTATTTTTTGTTAGGTAAAAGACTTCCCATTCTTTTTATAATTCGCGCGATGCCCGGCGGTTCAGTCGAAAGAGACGAGNTTTTTTTTTGCCACGTATTCAGCTGAGCATATCTACACAGTTCAAGAAAATGGGTAGATGGACCCAAGAGTTTGGTCGTTTTATATCTGCGAGCTGCTACGTTACGAAATTGACGCCCCATCAGTTCTTATTCTGGACAGCTTTGATTGTAATGTATCAAAGCATTGATGTAGTGGCTGAAGCGACGTCAGCTTTGGTTTGTCAGCTACCCGTCAACAGTACTGCTGTCTGTCAACCCTTGGACGTCGGGGTCATGGGGTCTCTCAAGAAAAAAAATAACGGCTCTCTGGGTGGCCGAGACCCGCAAACCAAAAAAGGAAGGGAAGTGTGCTAAATGCGGGTTGGTTGAAGGCTGCGCGTGCAAAAAGAAGTGCGAGTGCCAAGGAGAGTGTGTGTGCTGCTCTAAGTTGCGGGCTTGGAGACGATTGCATCTGTTGGGATCAGTGTCAGTGCTTGTTCGGTGGTGAATGGCTATGCAACGAGGAGTGCGTGTGTATTATCAACGAAATCGATGAGATGCCACAACACGTTGCCATTGACGTGACAACGCAGGAGGAGCAGAGGAAGGCCGCCGGGAAGCGTAGATGGCTACACCAAACAACTGCAGTCGAAAAGAGACGCTCTACCATTGCGAGAACGATAAAGGCTTGGGATGATTTGAAGACGGATGTCATAGTAAAAAGTTCTAAAAAAGTAATACCACAAAATCCCATTGTAAAACTCTAATATAAAATCTTCTTCTTACTAATCTTATGAAAAGAGTTTGTGTTCTGTACCGAAGCTCTGGGTTTTAGCCAATTAGACGAATGGGTGGATTTTTTGTCTCTGATGGCAGCAACTGATTATATGGAGGATGTTATTCAGATAGGGGCGGATTTCTCCGTCGTTGTCTGTAACCCTGCTGGGATAGCTCAAATCTCGTTAGTTTCCTGAAGCCGATAACTGAAGTCAG

>Contig_61

CAGTTGCGTCTCTACACGTTGAAGAAGTGCTGGAGCGAACGGAATCTGCGATCGTGGATTATTCGGATTCAGATATTTGTCGTAGATTTTCTTGGCTGAACGCAGGACGATCTGGTAGCTCGGCAGTCTTCGGTAGTGTTCAATCTCCTCCCACAACATTAGATTCTCGGAACAATGTTGAAAATCCAGAAACTTCCGGAAGGCTTGCATACATCCTGGATATGTTAGAAAAACATCCAAGTGGCACAGCTCGAGCTTTCCTGCTTTTTGCTGCTTGGTCGACTGTGGCCAAGTGGGGATCGGCAGGCGTAGCTTTACTATCCCGACGTAGATCCTTGTAGTAATTTGACTTGACGAAGCGAGGCAGCGAGTCCTGCAGCATCCCGTGACGTACCCGGTTCGCGATCTTCTTGAACATATCCACGCTTGGATTCGCGAGTCGCGTGAAAATCTCTTCTCGCATCGTTTTACTCATATCCACCTGTAGTCGAGCACTGGGGATAATGTACTTGCGGTAGATTTTCTTAGCGGAATGTTGGAGAAACTCGATACCTGGTAGCAGCTTAAAATCTTCCACTTCTTCGCAGAAGGCAAGCAAAGAATCCACGCCTCGTCGAGTCATGAAGAGCTTTAGATATTTCTTGGCGAATGGGTCGCCCAACAGCAGCTTCAGCTCCTGCTCAGGCGTGAGAGTCTGCTCCTCTTCGAGTTTTTGAATGGATTTTCGTCGAGCTGCCCCTTGGATAGAATCTGGAACTGAGATAGGGATGAGCTTCTCGACCGGATAATGCATGGCCGCGTCGATGAGCTGGAGCTCTGCCAAGCCTTCCAGTTTCTTTTGCACCGCCTCGCTCTCTTCGAAGCGATTGCCGCGTCGTGCCAGATGTTTCGCGTCCGGTTGGACCTCTTTCGAATGATCCAGATATTCCTGGGAGCGACAGAATCCAGGCCAGATGTCGTCGCTAATCCGGTTAAATACGCCTATCTGGACCTCATAGAATAACTTTGGCACGAGAGCTCCTTTGTTCTCCACCACGTCGTGGATTTCTTTCAGCGCGTCTTTGTCTCCCAGCCCGACAATAAAGTTCTTGGACGATGGAGAGCAGTAGCGATCGTAGATTTTTCGCGTCTTGTTGACCACGAACGACAGATTCGGCAATCTCTTGCAATCTTCGACTTCCAGGTAGAAAAGCAGATTTTCCAGCGCCAATGCTTCCATACAGAACGTCTTGAGGAAGCGCAGCTTAGCTGGATGTGCTAGAATACCCAACAGCGAATAATCCTCAGCATCTTCCATATTATCCGTGATGTTGGCAGCCACCGTTCCTGTAGCCGAAGCAGCAGCTAACAAAGTAGGTCCATTGCTCGAGCCAATGTTAGATGACGCGCCAGCGTTAGGAACGACCGGAGCGCAGAAATCCTGCATCTTACGGAACAATTTACTGTCTCGGAATCGCGGATACACGGATTTTTCCAGCGCGATATAGCAGATTTGCTGTGCACCGGCGTAGATTCCAGCGGAGATATTATCTCCTTCGAGGGCTTTCTCGATATCCTGCAGCATCTGCGCCGTGACACACACAGGCGAAGGCGCTTCAGGGGACAAAAACTTGTCGTAAATCTTGCGTAGTCGACGGTGCGTGTACGAATGGGACGGAAGGTGCTGCAGTTCGTCGATTTCGACCCAAAATCGGAGCATATCCGCTCCGCTGGGCACTTTGTTGGCTTCTGCGTACTTGAGGATCATCTTGAGCGTTGTTGGGTCAGTTAATACGTCAGTTGTGAACACAGGAGCGAGGTTTTGCACCAGATCAAGTGCAGCCCGAGACGAACCCACAGCCCGTCCAAAGCCTTTTTCAACAGTTGCTCGGAGGATTCCCTGCTCGTCGATGTGCGTTTCGACTTCATGGTCGTCATGGAATGGATTCAATTGGACGTCGTCGTCGTCGGGTATCGTCTTGGCTAGTGCGATACTGGTCTCGATAGTCGATTCTTTGCTTTCGGACGAGGTGGTCACTGCAGCTAACGTAGCAGCATCCAGCAACGAGCTGGAACGTTTCTCTAGATCTCCAGACTAAAACAATAAAAGAGAGGTGAGTTGTGGACAACCAAGTAGCCGATGATAAAAAACACGTAGATTTTTTTACCTCAGATATTGCTAAATCTTGGACGAGAAAGGATTCCAGACTCTGTGACGTGTGGAGGAGTGGGTGGGTGCCTCCTAAACGGGTGGCAACGATATCTCGAAGACTGGAGAAAATTTCTTCCTTCGAGTCACCCGCAGCAACTCTCCGCTCATATTCCTCTTTCGTCTGCAACAATAATGTTAGAATACTAGTTAAAAAAATAAAGTTTACAAGAACACAAACCTCTCTAAAAACCACGGACGCGTCGGGGTCGTCCATGGACGCAGCTGCTCCCATGGCTACCGAGTCCTCGTCAACGCACGACGCTCGTCGAGCGCTTGGCTTGAAGAAAGTGGAAGCCTGCACCGACTCAAGCCGAGATTGAATCTAATTTCGGCGCTACGAAACTTATCAAGCCTCCCCAGAGCACTGGGCTTTCTCAAGATTAGTTAGTAAAAAGATTACTAAGTAGTAATAGTACTTCAGTAATACTTTCTTTGTATTAACTTTTATATTAGTACTATTTAATTCGAGTTCCTAAGTACTCACTCCAGAACATTCTAATGGGTGAATTCTTGGCTTCACGTTCCACATGCAGTGCACTGTAGACGCGTGATGGGTCCATCTTGGCTGCTTGCCAGAGCTCGAGCAGCTCCTTCTGCAACTTAATGGCGATATCCTTAGTACTCGGAGCTTTCTCCGCCGCAAGGAGCAAGGCCGTCAGGTCTCTGTGACCATAGATCGATACCAGCATCTTCACTGGATCGTCCTTGAACGTGGGGTTCACCTTTTTGAACATGGAAATGTAGCTCGTCCACATAGAGTACAGCGGACTTTCAAACACCTTTGGGTCCATTTTGCCGAGGTTCAGACGCAAAAAGACCCGCACGGGTGTCGCTCCAGTGAGGGACCATTCGCGTAGTAGCTCAGCATGCAGACGTTTCCCCATTTTCGCCGTTTTCGCCACTTTACTCGCCTCAATGAACAATTTCGCCAGCGTTTCGTCACTGTGGGTTTTTCTCAACGTGGTCAGTGCAGAGCGATATGTCCCTAGGTTTTTCTTGCGGTAGTCGTCCGTGTATTTTTCCCATGCATTAAAAATCGGGTGACTAAGGAAGTTGGTGCCCAATTTCTTCAATTGTAACATTTCGAACACCTCTCCCGGCGTTTTGTTACTTTTCAGCCAAAGTTTCGTTTGTTCCGCCTGAATTCGAGTCGCGGTTTGCCCCACGGAGCCCCCTTTGGTCCTCGCCACGACGAGCATTTGAGCCAGCGCATCCTCCTTGTATCGTGTCCGCAAGGTGGACAATAAGGTCGTTGTACTCGTCGGGTTGGCCTCATTGAAAGCGTCCACATACCTCACCCACGTGTTCAGTTGAGGCTGGTCAAACAGCCGAGAGGTCGCGGTATTGAGCTTCAGTAGGACGAAGATGTCGTCCGGATGTATCTTGACGCTAAGCCAATGTCGACTCTGCTCTCTCTGGATACTCGAAGCAAGACGTTTCGTCGTAGGCGTCTTCCTAGCTGCTTCGAGCATCGTTGACGCAGCTTCGTCACCGAATCGAGCGGTGAGCGACGCGAGTAGGCTCATTGGCTGCGTTGGATTCTCCTGGTTGAACACCTTGATGTACGTGGCCCAAGCGCTTAGCATGGAGCCGTTGAGGAGCTCGTCAGCCGTGTTGCCCAATGTGAGCAATCGGAAGGTTTCGTCAGTGATTAAACCTCTTTTTAGCCAATTCTGGAGCATTTCAGTCTTAAATCGGGGATCCACCTTGTTGAATAACGCAATGTACTTGGTCAAAACTGGGGCTACAGACGTTTGGCCGGGCGTGCGAAGACCTAGCAGACGTATGACGTCTGTCGGGGTCTTCATTTCGTTAAACCAGGTGCGTAACAACGCCGTTTCCACGTTTTTACCAGCCTCCGCAGTTTTCGCGTTTTTACCAGCCACAATGATCATCTTGGCTAATACATCGTCGGTGTACAATTTCCTCAATGTCGCGATGGTAGAAAACTGTGCTCCAGGGTTAGCCTTGCTGAAATTGTCCATATACTGTAGCCAAGTGTTGACTGGAGGCTGATCGAAGAAAAGCGACTTGGCGGTATTGAGTTTCAGCAGCGTAAAAACGTCGTCCGGGGTTTTACCAAGCGTCATCCAGTGCTGAATCTGCTCCGCTTGAACTCGATTGGCGACGTCCTTTGTCGTGGGCACACTCTTGGCTGTTTCAACCATCGTCGACACAGCTTCGTCACCGAATCGAGCGGTGAGTGTCGCGAGTAAACTCAGTTGCTGCGTAGGATTCTCCTGGTTTAACACCTTGATGTACGTGGCCCAAGCGCTTAGCAACGAGCCGTTGAGAAACTCGTCAGCCGCGTTGCCCAGCGTTAGCATTCGGAACGTCTCGTCAGTGATCAACCCCTTCTTCAGCCAGTCCTTGAGCATGTCGGTCTTGAATTTGGGATCCACTTTTTGGAATAAATCGTCGTATTTCGTCCATATAGACGCGAAGAATTTTTGACTCTGGCCAGTCCCGCGAGCATTTAACAGCCTTAGGATATCTGTCGGGGTCTTCATTTCGTTAAACCAGGTGCGTAACAACGCCGTTTCCACGTTTTTACCAGCCTCCGCAGTTTTCGCGTTTTTACCAGCCACAATGATCATCTTGGCTAATACATCGTCGGTGTACAATTTCCTCAATGTCGCGATGGTAGAGAACTGTGCTTCAGGTTTAGCCTTGCTGAAATCGTCCATATACTGTAGCCAAGTGTTGACTGGAGGCTGATCGAATAAAAGCGACTTGGCGGTATTGAGTTTCAGCAGCGTAAAAACGTCGTCCGGGGTTTTACCAAGCGTCATCCAGTTCTGGATCTGCTTGGCTTGCATTTTAGTAGCGACGTCCTCCGTCTTCGGGACCCTTTTAGCTGTTTCAATCATCGTGGACAGAGCTTCGTCTCCGAAGCGAGCGGTGAGCGCTGAGATCGTCTTCATCTTCTCTGTAGGGTTCTCTTTATTGAAGACTTTAATGTAGCTGACCCAAGCGCTCAATAGCGAACCGCTAAGTAAATCGTCGGCCTTGTGACCGAGTGATAACAGGCCAACGGCTTCGTCGGAGAGCAGACCTTTCTTGAGCCACCCCTGCAGCATCTCGACATTGGGGGTACTGATCCCTCTATCCTCGGTATTGTGAGCCCTTAGAAACCTCTTCGTATCGTGCCGTGTTAGACATGGTGTGGTCAAGTGGGACTTCGCAGCTGAGGAGACAGTATCGGATATTGCCATCAAGACAACTGTCAGTAGTATTGCCCTGGGGACTCGCATGGCGGCACCTGGGGAGCTCACTCCGAGCTTCGCTTGGCTCAGAGAGAGAATGACGAAGTGACGCTGTGGCAACCATCTATTCGATGTGGTACGGTTCGCTTCTGAGACGAAAACGAGGCTAAGATACAGAAAAATGATGGGATGAATGATCACTGCGGGCGATAATTATTTTTAAAAGGTTTATCTTATTTCTTAAAAAATTACCGCTCCATTTGCTGTTTTATCTCGGGGACCGATTTAAAAAAAGGGACAGCGGCTACGGTAATTGGGTCCGATTTAGCACATTTTGTTTCAAGATAGGAACTCGATGATCAGTACAGTACTGCACCTCGTTTTCGCGGGGGTAGACCATATAGGATTTGCAAATTATATCAGATTTTAGCAAACGCCCATTCTTTCGGATACTTGTCTTGTAGTCTTCCTTCGTCTCGCACATTGGCATCACTAGAGTGTTTGTGAAGAGCTCGCGAATGAAATGCTGCGCTCCTGCGAAAAATCTCAGCGTCATGCGTGCTCCCACCGGTGTGCTCCATGCAATTAATCGCGACACCGTTCGGCAGCACCGACACCTCCATTTTGTAGCCATTCAAGTGGTGTTTGCCACTGTAATAGCGTAAGATCTCGCTCATGTTACCGCTGGGTTTGTTATCATGCTGAAAGGTAACGTCCGTAGCATATCGAGCATACGGAAAGTCCTTAAATGCATGCCCTGATCGTACAATCTTCCCCAGTGTCCACTGATCATTTGCTTTCTCTACGTACATTTCGTAGAGAATAGGAGACAGTATATCCATAAACTTGCGGATCATTTTCTGAAAGGTGGATGGCGGGATTCTGAAAACTCGAGCAACGGTGTCCCTTTTTCCAAGATGTTTGAGCGAACTCAGTGTCATAAAGAAAACGTCGCGCGCAGCGTAGCGGCATTTTTTACCGCGGCCCACGCTCCATTGCCCCTGCAAATGAGGGTACACAGCTGCCCAAACATGGTCGAGCTCCTCTGGCGCAAAGTTGGTGAGCTTGTGCACCCCATCGGGCCCTTCAGCTTGTAAGCAGAAGTCAAAGACGGACGGCAAAGTTCCCACGCCGGAGCTGTCCCCTTCGTCATTGTCGTCTCTGCGGCTATGTACGACGATTTGAGCGACCTTTGCCTTGCGACGTTCCTCCCGCTGAAATAACTTGGCAGTGGAGGCACGAAAGTCGGCCTCGTTGAGCGTCTCAGCGGCGCGGTCTTGGCGGTCTTGGGAGCGTACCATGGTGGCTTTATATGTAGATAGAAAATTCGGAAATGACCGAGGAACTGCATTTTACATCTTGCATTATCTCAGCAAACAGATAGGGCGAATTATGGCTACTATCTCTTCGCATGCAAACTATGAAAATATATAGCGATCAAATCATCAGATGTTTCCTATCCACATGTGTAAACTACATTCCAGAATTGTACTACAGCGTTCCAGAATTCTTGAATGGTACTGTTACACATACTCTGTATGTACGAATGAGCTCTAATATTTCGTTCATTTGGATTCAGGATCATCATATCCGGAAGTTTATCATAGGCCCATGGGCGCGAAAACGGGGTGCAGTACTGTACTAGAATATATCGGGGTAACGGCAAGTTCGGTATAGGAGTTATTTATGAGGAAAAATACAATAAACAAATGCGTACTGTACATGTAGGGCGATTTCGGTGATACATGTACTGTATGATCGTGGCATTGGTAGATTAGCAGGTTGCAGATCTTACTACCCAATCGGGTTCACGGAACGTTGACAGAATGCTCTACTGTATTGTGCCGTGGGCAGGGATTGTCAGTAAGGCAGCAAATGGTAAAATCCAGCAGTATTATATTTCTACAAACGACAATAGTAATATGTAGTTTAAAAATCTTCTTCAATTTATGTGCTGAGTACTTCAGTAACTTACGTCGTCTTCGAGTAGTACTAAAACTTCTACTGGTAGTAAATCAATTCGGACGATAATCATCTTAGGGCAGCGCTGTCGTTCGTTTAAAAGGTGTTGTGGAACGACATTCTTTTCTTTTGAAAAATTTGAGCGCCAAAGCCGTAGTGTAACCGAGCTGCACATCAGTCCTCATTTGATTTTGACATCGACACCAACACGTCCAAGATAAAGTGCGCAATAACCAGCTGAAGCAACCCCGAGATCAGTGCTTCGTGCTTCGGTTGACTCAGGTGCGTCGAGCAATTTTGCTCGACTGGAAGGTCTCCCGAAGCTAAACTACGAGGAGTTGAAACACCTCGAAATGTGTTGGAAGTGCGGTTGGCAGCAGTTGCCACCGTAAGAACGGAGAAGCGCGTAAAGCGCGCGCGCTTCTCGTACAAACAACCGAGTGTTTGTGGAAAATATTATCGTCCTAGAGCTGGGCGAAAAGTTTGACTTAGTATTGGGCATGCCGTGACTCGCACGGCATGATACAGTGATCAGCTGGGAGAAGTATACACTTGTACACAATCGGACGCAATGCGATAAAGAGCAATGGCCCTGTCAGTGTCGCGCATGCACCGAGAGGTGCATCCGTTGAGGCAGCACCTTACGATGCAGGCTCCGGTGCCCATTTACAAGTCGCAACGACTGAGGCAGTTGCTGGGTCTGTCCCAAATCGGAAATCAGATTTGAGACACGTTCATACTTCAATGACCGGACCAACTCAGGGCGTTGATATGAGGGAACCTCAAGTCACAGCCTGGCCACGAGAGAAGATACAACCAAAAAAGATTGTAGCACATTTATTTAAAAATAGTCAGCTCTTTCATTTTTAATGCAATTAGCTTAAAACCGCTACAAGCTCTTTTTAAATTGTTTAGAAGACTCCTAGAAAAGCGGTAGGCCGCATTCGTTGTTGCAACTTCCGAAATATGGGCCGCGTTAAACCTCTCATCCCGCAGGAAAGGCACAGGATAGTGAAGGCACATGAATATTTTACATCAGAAGAAAAGCTGGACGATCCGGAGGCGCTGGGACCCGGGACCGTTTAGTAGCCTGTCTCGACGCTGGTGCGGCGACGGTTACTCGTGTAATAGCACATTGGAACAAGAATCCCGGAGCGA

>Contig_63

TCAGATGTTTTGTTCCCCGAGTGATGATGCAGTTGAGCTGTCGCAGACTGCAGTGAGTAAAACATTCAATCTCTCCCCAGGCGATCTTCAAGACTATGGAGCGCGCGATTTAGCTGCTGGCCTGCATCTGATTTCATAGGCTTCAGGATTAGAGAGCGGGGGCGATCTGAACCATGAAGAAGCAGTAGCCTCTAAACTCAACGGCGAATACGAACACCTTTACCAAGCAAACAAGATGTGAATGTTTTACTTGATGGGGTGAAGTCAAGTGACTATGAGAACTATAGCACGGGTGATAGCAACGACGACGGCTTAAGTGATGGCGACAGCGACCTCGGCGCCGGTGCGCGTGATGAAGATGACGTTTTCCTGCCCGACAGTGACACATATGACATGGACCGCGCATTCATTGCGTCACTGCAAGTGGGCAATAACACACTTAGCAGAGGTGCTGTAAAGGAAAGAGAGCAGAGCACTACGGGATATGCAGTGGATATCAATGTCATCGCAGTACGAAACGGATGCATCTGCGTATTCTGGCCTGGGCACCGAAGCAGCCAGGCCTGTGGCCGAGCCGCTGGATCTATAAAGGTCGCAAATCCTCACCTTCTTTATTTCATGCCAAAATCGCTGTGGATTTCGATTGTGTCTGTGACGAATCGCTATGGCTTACAGCAAGTTGATGAGTGCGCCCGTAAGATGCATGATTGATAGAGTGACCGCCGGGAGGAGACTATCAAGCAAAGTACGCGTCGTTTAAAGGCTAAACCAGTATACGCTACCCACGAAATTCTTCACGTCGTTGGGCTGCTTGTTGCACAGACGCTCTGTCCACAGAAGAGGCGTTTCAGTGCGCACTGAAAAATGGTGGAAGATAGGGCTCTCCCTGCTTGTCTTATGGGGCGGTACATGGCGCGCGACAGGTGCCAGAGTACCATGAAATACCTGCACCTCTTTGACAACACAATTGAGCCCGGCAACAATAAACGCTAGAAGCTGCTCTTCGTAGCTGACAATATCCAAGAGCGCTTTCTGGTTGAATGGTCTCTTCCTGCTGCCTTCTCGTTTGATAAGGGTGTTTTGCCGGTCACGACGAAGCGCAACCCGACCGGCATGTTCATGGCCGAAAAGCCTCACCGCTACGGGTCCAAAATCTATATGGTATGCGACGCACACACAGCTTACTGCCGCAGAACTTTTTGTATGTCGTAGCTTTCTGCTTTGAGTTTGTCTGACAGCGCTTAATGTCGTAAGTCTGTAGATTTGAAGTTTTTGTCGGCAAGCATGTATCTGCTGATGGAGCGGATAACTCAGTCTACAAGACTAGTGCCTCAGCCGCCGTAGGGAACGTAAAGGTCGTCCTCTGTCAGTCTCGGCACACGTAGCATACCGTTGTGATCGACCGCTACTACCCGTCCGTGCCCCTTGCAATTGAGCTCCTGCGTTTGAAGGTGTATGTTATTGGCACGATCATGACCGACCGCCTTGGGTTCAACAAGAATTTCAAGACTACAGGGAAGACACGCCCAGGTAGTTACCCCACGGTACATTCTCATTCTCGTGCTCTATCGCAATTCCGACAATGACTTCGTGCGTTTAGTGGGACCGGAAGCCCGTATATTGTCTCTGCACGAGCTCCGGTATAACGGCTTCGACAATTAAGCGAAAGATAAAGCGTATCGGTGTGGTCCGAGTGCAGTGCCCCTTTGCTGTGAGCGACTACGTGAACTGGATGAGCGGAGTTGATGTCCCCGATCGACTAGTCGTTTTCGCTGCAAATGTCAACGAGGTTTTCGAAGTATTNAAAAAAAACTGTCTTAGGCTACCCGGACATGGGCATAGTAAACGCGTATATGACCACAACGGGGGGGGCTAAGATCAAGGGGATGATTGTAATGAAACGCTCAAAGCGACTTTTCGTTCTCCAAAATCTCTTACTTGAGCTCAAAGCCGAAGATTTTGCAGGAGTAGAGGCAACGCCGCCCGCAGCGTGCCAGAAGTGTCAGAGCATTCCAGTTCGACTCACGCACGCACGCCAGCAGTCCGAAGCCTGGGTCATTGTGACTGGGGTCCAGAAGCGCCGGCAGAGATCGTGCAAGATCTGCGCCCTTGCGTACTGGAAAGAAGAAGGCATTCGCAACTACGTTCTTTTGCAAACGATGCTTGGTTGACAATGCCAGGTGTTGGCTGTGCAACCGGCTTCGACGGGAACACAAGCGCATAGCTAAGACATGCTTCGACATCTGGCATAATGACTTCGACGCCTGCCAATCCATTCCACTAAAACCGGGGAAGCGCATCGTGCTCCGTCGACCCGGCCAGAAAGCTGTTCCAAGAAAGAAGAGTCGACGAGAACTTCGGCTTGGGTGCGATGGCGGCACCTACAGCGGCAACGCCGGAAACAGCGAGAATGGTAGCGCTGACGGGTGAGGGTGATCTGCGTGTCAGTTGATAAGTGATTTGTTTGAAATGCACAGTTTCTTGAACCAACGCACCTTCTTTCTGGCATTTCTTGAAGTGAGTGGACAGTCGGTGAAGCAATTAAACCATATGTTGGTGGCACAAACGACATGGAGCAGTCAGACAGTCGCTCAATGCCGTCAGAGTTACGACATTAAAAAGGAATCCTTTTGCGAACTAATATAGCTAGCCTCAGGCTAAACGGGCACTCATACAAATACGAAGCGACATCGCTATGCCGTATCTTACGACATGCGTTACGCGTTCTAGGTAAGTACCGTCCGGACGCAAGGTGTTAATGAGTCATAATTAAATTTGCTTTAATTTAAGAACAAAATTAAAAAAAAAGTCTGTTTTAGCTGTCTTAAAAGTCTGTTATAGCACTATAATAAAGAGAGAGAAAAAGTGTATCTACAAAACAATTAATAATTACATATCCACATGTAATAACACCGGGAGCCCAATTTTGATTTCGATTCTAATTTAAGCTGCTACTTAAGCCACCAGTCTGGTGACATCTACTGTATAGAGCAACCTGGCTAACACAAGTCATCTTTGATGTCGATCCATGGCTAGCAGAGCATGTCCGTAATCAAGCCACTTCCAACTTTTGAAAACATCTATTGTATGTACTGTACCCATCGCTTGATAGATTACAAATCCAAGTGCACCAACTATTAATACTTATTAAAAAAAGTTGCTGTGTCTCTTATTGCCTACTGTAAATCCCCGCATGATGATTATCATGCTTTCAACTCCCACTTTCCGTTTCACGATCAACTCCAGAGCACGCTTTCGACCACTTCAAGAATTAATCCTAGAACCAACCAGAGCTACCACGCCGTTGTGTTGACGTTGGTTGCACTACTGGTGTGTGTCAGTGCCGGATCCATTCCCTCCGAGTTGAACAGTATGAAAATCTCTGCCAACAGCTTCGACACCCGTGTCAGACGGTATTTGAGAGCACCAGACCTCACCACCGAAGATCGCGCATTCGATGCTCTAGGTCTCACGAAGCTTAAAGATGTAGCTACAACTGGAACTCAGAAGCTGCAAAAGCTGGCGAGCAACGCAAAGACCAAGATGACTTCGAACAACCAGCAGGCCACGGACAAGCTGTTTAAGAAATTAAAGGTCGGCAAAGTAGAGCAGAACATCTTTGAGAGCTCGCAGCTCAAACAATGGGCCGCGTCTGTGGCTAAGTCGTATAAGAAAAACCCCGAGGCTGGAGACTTTGCCATGGTCTCGACTCTTTCGTCTCGCTACGGTGATGATGTTCTCGCGAGCATGGTGGTTACTGCAAAGAGCTCCCAGACGATTGATCCTAGCTTGGAGAATAAACTTATGTCCTCATTGCTGACTAAATGGCAGACTGAAGCACGGACTACAGACGACGTGTTCAAGCTTCTGAAACTAGATATTGACGAGGTGAATCTACTGAAGAACCCCGTGCTGAGCACTTGGATTTCTTACGGCCACAGGGTCGGAGGTAAATCGCCGTACGAAGAGCTATTCACCAAGCTGATTGCACGCTACGATGATGAGGGACTAGCGAAGATGATTATTACTGCTAAAGATGACAAGGTCGTGTCGTTTATCAGGACTGACATGACGAATAAACTGTTCGAAAAATGGCAAAGCAGTGGGAAAACCGCCACGGACGCTTCAAATATTCTCAAGCTAAACGAGGAAGGAGCTTCTCTTCTGAAGAATCCAACGCTCCCGATTTGGATCTCCTACGTCTACTCGCTCAAGCAGAATCCTTATGAGCTGCTACTACTGAAGTTTAAGGCACACCACACTGACGCTTCCGTGGCAAGGGTGATTGCTTCTGCGAAGTCGGATCAAAACTCGATAATCATTGTCCAGAACATGCAGAGAACACAGATCGAGAGTTGGCTCAAGGCCGGAAAAAGTGACGAAGAGCTGTTCAAGCTCCTGGAGCTTAACAAGGCAGGAGACCAAGTGCTTGAAGATCCGCTTTGGAGAACTTGGGTCGCATATCTGAAAAACTTGAAAGGGGACGCTGACAATAGAATGTATTCGGTACTAAAGACACAACTTGGTGAGGAGCAATTGACGAAGATTATTACCAGAGCCAAGACGGTCGGTAGAACCAAGGCAACCGCAACGAAATTGGAGCTGCAGGTTTGGGAGCCAACGGTCGAAGCAGACGATGTTTTATGATCTCTTGAAACTGGACATTAAGAAAAGCGACGTAGTTGAAAGCGTCGCTTTCAAAACGTGGTACACCTACATGTAGACGATGAAACAATACAATGAAAAAGCAGTCATCACAAAGATGGAGAAACACTTCGGCGAAGTTCGTTTGGCTTAGATGCTTGCAGCTTCAAAGCGGACTGCGACTGACGAACCTACGAAACGGTTTCTTCGTGATTTACAGCGGAGCCAATTCTCTAAGTGGGAGGGTAAAAAAAAGAACCCCAAAACCTTTAGCAGCATGCTAAGTCCAAGTGATCGCAACTCTGAAAGCGACAGAGTTATGAGCGATTTCAATACTTTTTTCGGTGAAGGGATATTCGACATTCTGCGCATGGGTCGACAGTAGCTGGTAGATTTAGAGGTAGGAAGCACACCCCAAATTTCTTCGATCCTAGAATATCCGCTATGTTTTCTTAAAATTAATTAAACCACTGATCTTGATATCGCTCGACAGTTTTTAGCAACTTGTGTTTGAATCGAAACGTATCACTACTAATAGGTTTGGGGCATACATGCAATATAGATACATGTACAGTCAGAATTTCTGCGAAGAACTACCAGACCCCTATCTTCTGGCTACAGTTTTTGTTGTATTTTACTGTACTATAATACAGTATTTTATCCAACATTTGTTTAAGTCTTAAAACGAATAAGCAAAATAAATGAACATAGCTCTAATCATTCAGATACCGTAAAACTTATGTGCATTCGGACCGCTCATAATTTATCCTAGACTACTTTCTAGCCAATGAACACCGCCCAAAACTGGCGTTTTCAATCAACAATCAGAGTACACAGAATACTTGTTTTTCCTTGGTAGCTCTTAACTGTCGAACGGCCGTTTGCACATAAGTTTTACTGTACACTCAGACGATTATTCATTGGTCATGTTCTGCTCTGTTTTTGCAGGGCCTTTCAAAAAAAGATCTCAATCGCATGATAATTTCATCAAACAAGTAATATCAGATTATTTAGTAGCAACGTGATTGAAACACTTGAACGGACTTCACTCTAATCGAAGCTCGTGTCAACTCAGAGAATCTTTGCATAAGTTTACTTTCAAAATAAGCTAAAACGTACCTTTAAACCTAGCCCTTTCAAAATTGGCCAAAAACACATATACCAGGTAATGAAATGTACCTCATGATTTTTTCAAATATTTAGCAAGAAATCTTATTCCCTAAGATACATGTATCAATCGAGCCGAAATGATCTACCATTATTTGGATATTAACGAAATTAGATTGGCTAGATTAATAGGCAACAGGGTGCCTTCCTAACCCTACATAAAACGGTAAAATCACACATCTTTTTAATCACCTTTTAAGCCAGTTTTTGTCAGGCCCACTTTAAAAACGTCTTGCCAAACCCACAGATAAGAATCTAGTCTGTCCCTACCTTGAAGCTGACAAAATGCTACGTTTTCATATTCACCGTGGATAAGGACACATTTAAAATGAACAAAATAACTGTAAAACGTCTCTATATTAAAGGATATGGAGGTGTCAGATCCCCCCCCCTCCCAGCAACCTGTGTCAGTGGCGATGATTAGTCACTATGGCATGCTTATAGCGCTTTGACCGGCGACCCAGACACGTTGCAAATTGGAAGGAGCTCTATCGCTTTCGTAAGTATTATTAATTCTTTTCGAAGTAACGCCAACTTGTGGCGAGAAGATTCGAGAGATACACGTACGGGAAAAAGGAGCCATTGAGGCTTACCACGGTACAGCTTTATTGCCTACTACTTGACTTATTAATCTTTACCATCAGCTCTCTCTCTCTCTCGACGTCTGATTTCTTGACGCACATAATAGTAAAAATTTTGGTTTCATATTTTAAAACCGGTTAAAGAAAGAATGCGCATCGAAGCAAACCGAACAGTCAGAGAACAAATGTATGATGTTTAGCGCATCCATCGGTACCATCATACGATAGAAACAACGTACCAGCTCCGATTCCATCAGCAATTGGTATCGCATCCTCGAACGTCATCAACAAAGGCAGCCGCAAAAACACTCAGGAAGCAGCCGCAAGATATGCCCTCCAATGGTTAAGAGCAACCTTTACAGATGAAGCTGTCCTATTCAACTTCAAAAGAGCCCACTTCGCAGTTTTAAGTACACAAATAACAGCGCTTTCAAACGCAAAATGGGGCTCCGTCGTGGAATGCGCTTGGTAACAATTTCGTTTCTGCTCTATTTAGGTTCGGCTTTGATGATGGCTAGTGCTGGACTCATGGCTCCCAGTGTGAATCACGTTCGTACCATCGGCGCACTAGGGCGCCAACAGTAATGCCTCAGAAGATAGAGTTAGTCCCAGTATTACGAAGATGACAGAGCTGGTTCCAAAGATTTGCTTTCAGAAGCTACGAGACTGCGCGAAATCAATCTTCAAAACGGATAACCAGCTCACCATGGCTACTCCAAGCTTTCCGAAGTTGAGGCTTGATAAAGCAAAGTCCGATTTCTTCTTGAGTGAAGCATTCGATGAATGGGCTCGTCATGTACTCAAAAATCTAGAACAATAGTCAGTCCTCTGCTGACGATGCGATGTTCAACACGTTTGGGTCTCACTACGGCGACGATGATCTAGCACGCATGCTGACTACTGCAAAGCCGACGAGTGAAAACTCGATTGTCACGCGGTTGAGAGAGATGGAAAACTGCAGGGAAAAGTATGAATGTAGTAATACATACGATCAAAACCTATTGAAGACTTCAGCATTGGGCATGTGGATTAACAATGCCTTCAACACGCTTGAGAAACCAGTAGACGAAATTGTGAGAGCACTTAAAGCACACAACGACGATGTACTCGTAGCGAAATTGATGGCTCTGTCAAGAAACGACGTCAGCAGCGGCATGGCTTGGAAATTGGAGAGGTCGCTGACGGAAACGTGGCAAAGAAACGAGAGTGTAACTGGAGTGTTTTAGCTACTCAGATTCAACGAAGAAGGCACGTCGCGTTTT

>Contig_65

CCAAGTACGACTTCAATTTTTGGTAGCTCTCGAGTAAATCTCCATGCAATTTTTCGTCCAGGTCCTGCTTGATCCTAGAAATCGAAGATTTGCTGACATTGATGCCCTTCCCTTTAAAAAACTTCTGCACAGCTTTCCCTGAAAGAGTTGAAGTTGAGTTGGCATGAGCTGGCAGAATTTCACGTGCTACTACAGCCGATAGTTGAGCTTCACGGACAGACCGGTTCCTTTTCGCCTTTTTTTCACGAGGGATATTTCCCTCCGCGAGTGGTTTCTGAGTAGATCCAATGTTTTTGAGATGGTTGTGGGCGAAGTTGGCTTGAGTAATCTTCCATACCCCATTCTTTCCGTGTACATTGATGAAGAATGGGCAGCTTAGATCTTTTGCCGGGGTAACTTTATCAAAGAACTCCATGCCATTTAGTTTTTTGCAGACGTACTTCGCGTTTCCTGAGTTGTTTGGCTTCACACTCGAAGATTGTACAATCAACTGGAATCCACATACCGTTGCAAAATCGTCACATGTTGCCCTTGCTGCTTTTTGTGTTGGAAACTCAGTGTCTGCAATATTGGTTCGCGTCGCGTATACTCCCCCATACTTTTGGCTGGAGCGGTCTGAGGTATCAACTTTTGCGTCTTCCCTCAAAACACCCATGCTTATAGCTGTAGCGATGTCAGAAAGATCTACCACGAAGGGAGAACTCCCATAATTCGGAGGCGGCTTTCGAGGCATGGTACGCTTAGGGCAAAAGCGGTTTTGCTGAAGGCAGCAGAAATGTAGCTATGATTAGAGTTAGGTACGGATCGAACTTAATCATTTTCTACTGATTGCCGTCTTTTTTGCGCCGCGTTGGGGACAGATCAGTAGTTTTCTACTGACCGATGGTATTTCCTCGTCCATGTGCAGTAGCGTAGTTAGAGACGGGTCCTGCTGCACTTCAACATAACGCTCTAAAAGCCGCTTGCACGTCAAACCAATGCGAAATGGGTTGCCTTGAGACCCCGTGCCAAGAGCAGGCTCCACAACGCCGTCCACAGCTTTTGTGTCGCACAGTATTAGCAATGCACCTGGTAGGAATGTGAACGCTCGCTGCCGTACATTCGCTGACGGCAAATCTCCAGCACGGGGTCCATACTATCCGGTCGGTTTTTACGCCTCCATCCTTTCTGATAGTTTTGGACCTGTGCAAGCTGTGGGGTAGGTCCGCCGATACGCCCATCCTTAATATCACGACACAAGAATGCGTACAGTGCCTGCGCTACCATAGACGGAGAGTCGCTTAGCTTGACATCTAGATAGGACTTCATCTCGGCGGTCAGCTTAGATGCAATGGGGGATGGTGGAGCATCGTCGGCCATCGTATGACCCCCTTGCTCGAATATAATTTTTCCATCACTTCCTTGACACTCCAAAAACTTATAGCGACAAGTGCATGTTTGTGTTGTGGCTTTGCAGCGCTGTGATTTGCATTTAGTTACGATATACCGCATTTCATGCCATGTTACAGCCAGCTCATCTTCGTAGCTCTCAACGTGATCAATTGTACAGTGGTGTAGCTCACTTGTAGGCCGTGAAAAGCTCGGCAATCCTGCCAGAAAGTCATCAAAAAGGACAGGATCGCCAGATTTCCACACTTCATGCCACGGAAGACGTCCTGTACGGCCACCGTGATCATTCTCCATTGTATCGATGCTCCACTCACAATGCTGTTAATTTTTAAATTTCTTAGATGCCGGGAAGCTAAGCTCTTAACTATTTTAATAGTAGTGGCTAGCGATATCTATTTGTGGAGAGAAAAGATTTTTGCGAAACGACACTTATTTTTGGTGTGGTGTACTGCTCTTTCTTTTGGTGACCCTTTCTGCGTCTGTGGCGCTTCACATCCTATTTTTTGTGTTCGCTAAAAAATATAGAGTGGGATGGGATGCCTGCGATTTCGCGAATCCGCAGGACTTCCAACCCGGTGTTATAGACAGGTGGATCGTGTCGGCGGTGTGTCACACACACTGTGTGCTGCTGTGGAATAGACGATTGGACTTGCGATAAGCCCAAGACCGTGCCACACAACGTCCCAGTGGGACGAGAAGGGGTGTCAACCTTACATGTCATCCCAATATCCCGCAAACTAGATCGCTGATTAGTCCATTGGGACGATATAAGACTGAGCCTAACGCCTCTCCCTGCGAGAACGGGTGCAGTACAGAACGCTACTGCGACATTCGCATTACATTAAAAATACAGACAGTACATTTTCTTTTAATACCGGTACGGATGCACCAGTACGTTATATTAATAGAGAGAGCAAGTTCGAATCGAGCTTCGATGAACATGTATTTGTGATGAAAAAGGACGCAATAATTACATAAAGAGGCGTAAACAGTTTTAAAAACAGTTTTCAGATACGGTGTTAAGCAGATTTTATTTTATTGAAATCTGTCAGTGGAATTTGGAAGCATTGCCTCTACTCCTGAACACGTCACCCACAAACTGGAAGTCGTGAGCTCTGGTTTGGCGATGAGGCCAAGAGAAAGTGAACCGAAGATCTTATCCGAAGATAACCGGACAAACATGTCCAGTTCAGATTACCAGATGGTATTGCAGTACACCTATTGCAGGTACCGGTACATGTACATATTTTCACTACAGTACAGTATGCTTCCTGCACAAGACAGGTGTCACATTAATTACTGAGGGGTGCCTCACTCCGCAATTGGACATCTCGAGTAATCACCATGACTATCAAGCCCAAGCGCAAGTTGTTTTTTTATCAAGTACTGCTGGTGACTGTAGCTCTATTGATCTGTGCCAGTAAGGCATTGACGGCCATCGATTCCAAGAATCTCCCCACCCTCGACAGAATCAGAGCCGACCGGCGATTGAGGGCGACGGTAGATGGTTACGACTACAACTACGAGTCCGAAGACAGAGCCTTCACCGGCATTACGAAGCTAAAAGAATTTGCTCAGGCTGGGACGAAGAAGCTGCAGAAGGCCGTTGATACTGCCAAGACGAAGCTGACGTCCAAGCCCACTATTGACCAGCGCTTTAAGCAGTTCAAAGTCGATCAAGTCGAGTCCAACGTTTTCGAAAGCATGCAATTTAATGCCTGGGCCAAGTCTGTTGCAAAAACGACCAAGAACAACCAGGACGCCACCGACGCTGCAATGCTCGCAACGTTGGCGACTCATTACGGCGATGAGACTCTTGCCCGTATGCTAGATGCCGCAAAACAGGTGTCAAGTACAAAATCGACGGCCACTAGACTGGAAAATGCGCAGATAAGCAAGTGGGTGGATGACGGGGACGCACAACTCAACAAATGGCTTGCTGATAGAGTAAGTGCGGACAGCGTTTATAAGCTTCTGCGGATTGATGCTGAAGGCAGTAATCTACTGAAAAGCCCCAAGGTGAACATGTGGATGAGCTACCTGACAAAGTTGAACAAGGACCCATACGACGTCCTACTTTACAAGGTGAGAGCGCACTATGACGACGTGGGACTAGCGAAAATGTTTGTTCTGTCCAAGAAAGACTCTTCAACAAAAGTGCTGGCCGAGAAGCTCGAGACACTGCAGCTCGAGAAGTGGATGAACAATAAAAACAGCGCGGCTGACGTCTTCTGGATCCTGAAGTTAAACCAAGAAAGCACGACACTTCTAAAAAATCCAGTGCTAACCACATGGGTCGCATACGTTGAAAAGCTGCAAAAGAATCCCTACGAAATGCTGTTTTCAGCGATCAAGGCAAAGGGCTTCGACGACGTAGAGTTGGCGAGGCTCATCACCGCAGCAAAGCAAGACCTCCATACAGGGACTGTTGTCGCGAAACTGGAGAAAGTGCAGCTCCAGAAATGGGCCACGGATGGGAAGACCAGTGGAGACCTCTTTAAGTACCTCGGACTGTACAAAGCGGGTGACAAGTTTCTTGATAGTCCAGTGCTGAACAATTGGTTCTCTTACATGGAAATGCTGAGGAAGGACCCCTACACGATGCTAGTCCATACAATCAGGAAGTCAGGCTTGGACGAAGTAGACTTGGCGAGGCTTGTTAACAAGGCCAAGCAAGACACCAATTCGAAAACCATGGCTGCGAATGTGGAGAAGATGCAACTTGGTAAATGGTCGGTAGATTCGAAAACCAGCGACGATGTCTTTAAACTCCTTCGACTCGACAAAGAAGGAGACAAGGTGTTCGAGAGCCCAGTGTGGAGCACTTGGACCGCATATCTGAACAAGGTGGAAATTGACCCTGACGCAGATCTGGTCATGTACACAGTACTGAGGAACAAATTTGGTGACGAAGGGTTGGCAAATCTGGTTGCGAAAGCGAAGCAAGTAGCGAACACCAAAGAGACCGCTGAGAAGCTGCAGCTGGAGATTTGGCGGGTCGGTCAGAAAAGCTCAGATGACATTTTCAATCTTCTCAAGTTGAACGAAATGGGTACAAAGCTGTTCGAAAACCCGGGGCCATTAAGGACGTGGATAGCCTATGTGAACAGGGTGAATAGCTTCAAGAGAAATAGAGTGAAGGTGTTCCAGCCGATCATTCAATTGGAGAAACGCTTTGGAGAAGAGGAGCTAGCGGTGCTGTTGGTCAACTCGAAAGCGAAACACTATTTGACCAAGGCTGGTATAGCCGAAGATTTGCAAGAGTGGCAATTCAAGAAATGGATGGTTCACAAGACAAACGTCGATAAGATGTTCCCTTTTGAAGACCATACCAGCATACGAATTAAATGGGAGTACAAGCAGTTCTACAAGGAGAATGCCGATTCATTGATCATTTAGACACGATACAAGGCAAAGTAGTTCTCTTAGCGAAAAGTGGTTCACCGCTAGCTTGACAGTACTTTAGAAAGGATTCCAGGTCGTCGGATAATAAACTGATACTTCGAAGTACATTCTAGTACAGCAGTAGTAGAAGTACGTACAGTACACGTCTTTACTACGTATAATTTTTCAATTACTACAGTAATACATCAGTTTAGAAGAAGTGTACTTGAAGTAGTATTTTATTAATACCAGGCAGGTATTTCCAGCTAATGCCTGGCTTGTAAAATTTGTGAGAAGAATGTATCAATAATACATAAGCTGGAATCAACTGATGACGGAGACATGAATGTTATGTAAAGAAGAGCGCTCTAGATAACCGTGTATCATTAATATTAAAATAGAGTACACGTCTTAATACTTTTACATTGACAGCTGTATTAGGAGAAGGTCGAAGTGCGTGGTGGCAGTGGGTGTTTAAACGTCTGTTCCTCAAAAGACTGTCTATAATACATGTAGACTTTTTTAATAAAATACTACTTCTGGTACAGCTATACTGGTGTCTGACTATAGACACAGTTGACGTCTAAACAGTTAATACAGTTTTTACCGGTGTAAAAACTGTATTTACGCGGTGCGTTGCGTAATTATCCTTCATTTTGGTAATTTGATATAATTGAAGTAGCAAAAATGCTCATCCACCCATTCCACTATCCGAAGGCGGCGCCATTTTAATACTACGAAGTATCATATTTACTATTTTAATAATGCATGTTACTTTTAATATTTGAGTGCTAAACTGAGGGCTTGCTTCCAATACAGCAAGGTGCTTTGCAGACACGTAGGAAGCGTATTTTCGAATCCATACTTTAAATTTAAGTAATACTTTGTACTCAGTAGTTTCTGCGAGTTACATTGACTAAAATATAACTACTATTGTTTTATTTTAAAATATTAGCACTAATAAGTTATCTGAGTGGGTTTTAACATGTTTTAAAATGAGGTACTGCTAAATCGTACATCAGCAGTAGTAAGAACAATGCTGTGCTACAAGTAATACAGTACGTTGTCACTCGATCCATAAAGATAGATTGAACAAAATGAACCCATATGTGTTACTGCTGTAAACCATTGTTTTCTAAGATAGCAAAATCCGATCCTACTAAGCTGGCGGACGTGGTGCCGACAGGGGCGTAGGATACGATTTGAAATGGAGCGAACAAAACATGAAAGGTGCTAATTATAGCTGGCTAAGTAGATAGTTTTAGATTGAACAAAAAGAACTCATACGTGTTACTGCTGTAAGCCAGAGTTGCTTAGATAGCAAAATCCGATACTACATAATGGGTGGACGTGTCGCCGACAAGGGCGTAGGATACGATTTTAAATTGAGCGGTAAGACAACAACAAAACACGAGAGGTGTTAATTATAGTTGGCTAAGTACATGTAGATTTCATAAAAAAAGCTAATAAATTTGGATCCTAAATGACATTAACCATCCTGGAATCATCGCGTCTTGCGACGCATTAACCAGATGCTTTAATCATAACTGGCACAGATTAGAGTGTCTAATGATTCATTCTAAATCAGCACAAAATGAGCGCTAAATGTATTAAAATGAACAAAAATTGAATATTTGTTGAGTTTGGTATGAGAAGTGATCAATCTTTAGCTACTTACAAGATTAAACATGCAGTACAGTAGTGCAAGCTGACACGCCATTACTATCTTTTGCGCTGATGTGAGCTAAGTTTCATGGCTCAGAAAGTACGACGCTAAACTAAACATATTGTGTAGCTTTTCTTATAAATGTATTTGGAGTGCAGTGGTTTAGGGTGGAGAAGATGAAGATACAATGATAGCCAACAATGATAAAGAGGTTGTAGTAAGAAGCTTGAATCAGGATCATTAGAATAATTATGCTTAATCAGGTAAGCATGATGCTGACCCAGATTACTTTATGAAATTAAAAAATATCGAGGCGGCATAGGGGGCCTAACTTTTGTAATACTTATCCCGCCGAAGCGTAGCGGAGGCGGGAAGGCCACAACAAGTCTAACTAAACCTAAACTACCGCTGCGTAGCGGCGGGTAGCAGTGTACAACATCCAATATTTAGAGTTATTAAAACAGCATTAAATTGCGTGTGCACCACACACGGGTGTGTTTTCTTACACTAAATCGTGTGGAACACATTTTTTCACACTTAGCAAATATTTCAAAGATCCAACATCGCAGCAAGGATGGACGGAATGTCGCTCGCTCGGGAGCTGGTGCGCGCGCCCATCTACGAGCTCATGGACATGGAGAACACGCTAGAAAAGATCGCGCAAGCCTCGAGAAGATACTAATAGCTCAGATGGAGTTACTGTCACGCATTGAGGTGAGCGAGGAGAGCATCTACGTGCTGGCGTCTGAGATTGCCAACGCTGGTGCGAGTCATGACGCCCACCTATTCTTCGGAGTTTGCTAGAAATGGCAATTTACTGATTGGATTGGTAGTACAGTAGTATGCTAACGGTTTTGTATTAACGGATAGCGCAGCTACAGCTACCTACTTCAGTTTGCTTGAGCAGTTTCTCATGAATAGACTTCGTCTTCTCTGCAGTGCGACGGGCCATACGGCTCTTTCTCGATTTACAGAGCATTTGCAGCGGGGTGCCGTAGCTTTAGCGTCAGAGGAAGACGTTCGACTCAAAGAGTCACTAATACTAAGTTCCTT

>Contig_67

GAAACGCCATTTCCAGTTCTCCACGTAGCATCTCGGCTGGATAATCTTCGTGAACCGTGGCCCACGTCCCCACTAGGGCCATAAAAATCCGAGAATCCAGGAAATCCGGCATCACGACGTTGCGCATGTGGTCGTAGCACTCGTTCTCGACCTCATCGAACGTCGTTCGACTCGCCACTTCCAGTTGCGTCTCTACACGTTGAAGAAGTGCTGGAGCCAACGGAATCTGCGATCGTGGATTATTCGGATTCAGATATTTGTCGTAGATTTTCTTGGCTGAACGCAGGACGATCTGGTAGCTCGGCAGTCTTCGGTAGTGTTCAATCTCCTCCCACAACATTAGATTCTCGGAACAATGTTGAAAATCCAGAAACTTCCGGAAGGCTTGCATACATCCTGGATATGTTAGAAAGACATCCAAGTGGCACAGCTCGAGCTTTCCTGCTTTTGCTGCTTGGTCCACTGTGGCCAAGTGGGGATCGGCAGGCGTAGCTTTACTATCCCGACGTAGATCCTTGTAGTAATTTGACTTGACGAAGCGAGGCAGCGAGTCCTGCAGCATCCCGTGACGTACCCGGTTCGCGATCTTCTTGAACATATCCACGCTTGGATTCGCGAGTCGCGTAAAAATCTCTTCTCGCATCGTTTTACTCATATCCACCTGTAGTCGAGCACTGGGGATAATGTACTTGCGGTAGATTTTCTTAGCGGAATGTTGGAGAAACTCGATACCTGGTAGCAGCTTAAAATCTTCCACTTCTTCGCAGAAGGCAAGCAAAGAATCCACGCCTCGTCGAGTCATGAAGAGCTTTAGATATTTCTTGGCGAATGGGTCGCCCAACAGCAGCTTCAGCTCCTGCTCAGGCGTGAGAGTCTGCTCCTCTTCGAGTTTTTGAATGGATTTTCGTCGAGCTGCCCCTTGGATAGAATCTGGAACTGAGATAGGGATGAGCTTCTCGACCGGATAATGCATGGCCGCGTCGATGAGCTGGAGCTCTGCCAAGCCTTCCAGTTTCTTTTGCACCGCCTCGCTCTCTTCGAAGCGATTGCCGCGTCGTGCCAGATGTTTCGCGTCCGGTTGGACCTCTTTCGAATGATCCAGATATTCCTGGGAGCGACAGAACCCAGGCCAGATATCGTCGCTAATCCGGTTAAATACGCCTATCTGGACCTCGTAGAATAACTTTGGCACGAGAGCTCCTTTGTTCTCCACCACGTCGTGGATTTCTTTCAGCGCGTCTTTGTCTCCCAGCCCGACAATAAAGTTCTTGGACGATGGAGAGCAGTAGCGATCGTAGATTTTTCGCGTCTTGTTGACCACGAACGACAGATTCGGCAATCTCTTGCAATCTTCGACTTCCAGGTAGAAAAGCAGATTTTCCAGCGCCAATGCTTCCATACAGAACGTCTTGAGGAAGCGCAGCTTAGCTGGATGTGCTAGAATACCCAACAGCGAATAATCCTCAGCATCTTCCATATTATCCGTGATGTTGGCAGCCACCGTTCCTGTAGCCGAAGCAGCAGCTAACAAAGTAGGTCCATTGCTCGAGCCAATGTTAGATGACGCGCCAGCGTTAGGAACGACCGGAGCGCAGAAATCCTGCATCTTACGGAACAATTTGCTGTCTCGGAATCGCGGATACACGGATTTTTCCAGCGCGATATAGCAGATTTGCTGTGCACCGGCGTAGATGCCAGCGGAGATATTATCTCCCTCGAGGGCTTTCTCGATATCCTGCAGCATCTGCGCCGTGACACACACAGGCGAAGGCGCTTCAGGGGACAAAAACTTGTCGTAAATCTTGCGTAGTCGACGGTGCGTGTACGAATGGGACGGAAGGTGCTGCAGTTCGTCGATTTCGACCCAAAATCGGAGCATATCCGCTCCGCTGGGCACTTTGTTGGCTTCTGCGTACTTGAGGATCATCTTGAGCGTTGTTGGGTCAGTTAGTACGTCAGTTGTAAATACAGGAGCGAGGTTTTGCACCAGATCAAGTGCAGCCCGAGACGAACCCACAGCCCGTCCAAAGCCTTTTTCAACAGTTGCTCGGAGGATTCCCTGCTCATCGATGTGCGTTTCGACTTCATGGTCGTCATGGAATGGATTCAATTGGACGTCGTCGTCGTCGGGTATCGTCTTGGCTAGTGCGATACTGGTCTCGATAGTCGATTCTTTGCTCTCGGACGAGGTCGTTACTGCAGCTAACGTAGCAGCATCCAGCAACGAGCTGGAACGTTTCTCTAGATCTCCAGACTAAAACAATAAAAGAGAGGTGAGTTGTGGACAACCAAGTAGCCGATGATAAAAAACACGTAGATTTTTTTACCTCAGATATTGCTAAATCTTGGACGAGAAAGGATTCCAGACTCTGTGACGTGTGGAGGAGTGGGTGTGTGCCTCCTAAACGGGTGGCAACGATATCTCGAAGACTGGAGAAAATTTCTTCCTTCGAGTCACCCGCAGCAGCTCTCCGCTCATATTCCTCTTTCGTCTGCAACAATAATGTTAGAATACTAGCTAAAAAAATAAAGTTTACAAGAATACAAACCTCTCTAAAAACCACGGACGCGTCGGGGTCGTCCATGGACGCAGCTGCTCCCATGGCTACCGAGTCCTCGTCAACGCACGACGCTCGTCGAGCGCTTGGCTTGAAGAAAGTGGAAGCCTGCACCGACTCAAGCCGAGATTGAATCTAATTTCGGCGCTACGAAACTTATCAAGCCTCCCCAGAGCACTGGGCTTTCTCAAGATTAGTTAGTAAAAAGATTACTAAGTAGTAATAGTACTTCAGTAATACTTTCTTTGTATTAATTTTTATATTAGTACTATTTAATTCGAGTTCCTAAAGGCCTTGACGTACTAGTACTCACTCCAGAACATTCTAATGGGTGAATTCTTGGCTTCACGTTCCACATGCAGTGCACTGTAGACGCGTGATGGGTCCATCTTGGCTGCTTGCCAGAGCTCGAGCAGCTCCTTCTGCAACTTAATGGCGATATCCTTAGTACTCGGAGCTTTCTCCGCCGCAAGGAGCAAGGCCGTCAGGTCTCTGTGACCATAGATCGATACCAGCATCTTCACTGGATCGTCCTTGAACGTGGGGTTCACCTTTTTGAACATAGAAATGTAGCTCGTCCACATAGAGTACAGCGGACTTTCAAACACCTTTGGGTCCATTTTGCCGAGGTTCAGACGCAAAAAGACCCGCACGGGTGTCGCTCCAGTGAGGGACCATTCGCGTAGTAGCTCAGCATGCAGACGTTTCCCCATTTTCGCCGTTTTCGCCACTTTACTCGCCTCAATGAACAATTTCGCCAGCGTTTCGTCACTGTGGGTTTTTCTCAACGTGGTCAGTGCAGAGCGATATGTCCCTAGGTTTTTCTTGCGGTAGTCGTCCGTGTATTTCACCCATGCATTGAAAATTGGGTGACTAAGGAAGTTGGTGCCCAATTTCTTCAATTGTAACATTTCGAACACCTCTCCCGGCGTTTTGTTACTTTTCAGCCAAAGTTTCGTTTGTTCCGCCTGAATTCGAGTCGCGGTTTGCCCCACGGAGCCCCCTTTGGTCCTCGCCACGACGAGCATTTGAGCCAACGTGTCCTCCTTGTATCGTGTCCGCAAGGTGGACAATAACGTCGTTGTACTCGTCGGGTTGGCCTCATTGAAAGCGTCCACATACCTCACCCACGTGTTCAGTTGAGGCTGGTCAAACAGCCGAGAGGTCGCGGTATTGAGCTTCAGTAGGACGAAGATGTCGTCCGGATGTTTCTTGACGCTGAGCCAATGTCGACTCTGCTCTCTCTGGATACTCGAAGCAAGACGTTTCGTCGTAGGCGTCTTCCTAGCTGCTTCGAGCATCGTTGACGCAGCTTCGTCACCGAATCGAGCGGTGAGCGACGCGAGTAGGCTCATTGGCTGCGTTGGATTCTCCTGGTTGAACACCTTGATGTACGTGGCCCAAGCGCTTAGCATGGAGCCGTTGAGGAGCTCGTCAGCCGCGTTGCCCAATGTGAGCAATCGGAAGGTTTCGTCAGTGATTAAACCTCTTTTTAGCCAATTCTGGAGCATTTCAGTCTTAAATCGGGGATCCACCTTGTTGAATAACGCAATGTACTTGGTCAAAACTGGGGCTACAGACGTTTGGCCGGGCGTGCGAAGACCTAGCAGACGTATGACGTCTGTCGGGGTCTTCATTTCGTTAAACCAGGTGCGTAACAACGCCGTTTCCACGTTTTTACCAGCCTCCGCAGTTTTCGCGTTTTTACCAGCCACAATGATCATCTTGGCTAATACATCGTCGGTGTACAATTTCCTCAATGTCGCGATGGTAGAGAACTGTGCTCCAGGGTTAGCCTTGCTGAAATTGTCCATATACTGTAGCCAAGTGTTGACTGGAGGCTGATCGAAGAAAAGCGACTTGGCGGTATTGAGTTTCAGCAGCGTAAAAACGTCGTCCGGGGTTTTACCAAGCGTCATCCAGTGCTGAATCTGCTCCGCTTGAACTCGATTGGCGACGTCCTTTGTCGTGGGCACTCTCTTGGCTGTTTCAACCATCGTCGACACAGCTTCGTCACCGAATCGAGCGGTGAGTGTCGCGAGTAAACTCAGTTGCTGCGTAGGATTCTCCTGGTTAAACACCTTGATGTACGTGGCCCAAGCGCTTAGCAACGAGCCGTTGAGAAACTCGTCAGCCGCGTTGCCCAGCGTTAGCATTCGGAACGTCTCGTCAGTGATCAACCCCTTCTTCAGCCAGTCCTTGAGCATGTCGGTCTTGAATTTGGGATCCACTTTTTGGAATAAATCGTCGTATTTCGTCCATATAGACGCGAAGAATTTTTGACTCTGCCCAGTCCCGCGAGCATTTAACAGCCTTAGGATATCTGTCGGGGTCTTCATTTCGTTAAACCAGGTGCGTAACAACGCCGTTTCCACGTTTTTACCAGCCTCCGCAGTTTTCGCGTTCTTACCAGCCACAATGATCATCTTGGCTAATACATCGTCGGTGTACAATTTCCTCAATGTCGTGATCGTAGAGAACTGTGCTTCAGGTTTAGCCTTGCTGAAATCGTCCATATACTGTAGCCAAGTGTTGACTGGAGGCTGATCGAATAAAAGCGACTTGGCGGTATTGAGTTTCAGCAGCGTAAAAACGTCGTCCGGGGTTTTACCAAGCGTCATCCAGTTCTGGATCTGCTTGGCTTGCATTTTAGTAGCGACGTCCTCCGTCTTCGGGACCCTTTTAGCTGTTTCAATCATCGTGGACAGAGCTTCGTCTCCGAAGCGAGCGGTGAGCGCCGAGATCGTCTTCATCTTCTCTGTAGGGTGTTCTTTATTGAAGACTTTTATGTAGCTGACCCAAGCGCTCAATAGCGAACCGCTAAGTAAATCGTCGGCCTTGTGACCGAGTGATAACAGGCCAACGGCTTCGTCGGAGAGCAGACCTTTCTTGAGCCACCCCTGCAGCATCTCGACATTGGGGGTACTGATCCCTCTATCCTCGGTATTGTGAGCCCTCAGAAACCTCTTCGTATCGTGCCGTGTTAGACATGGTGTGGTCAAGTGGGACTTCGCAGCTGAGGAGACAGTATCGGATATTGCCATCAAGACAACTGTCAGTAGTATGGCCCTGGGGACTCGCATGGCGGCACCTGGGGAGCTCACTCCGAGCTTCGCTTGGCTCAGAGAGAGAATGACGAAGTGACGCTGTGGCAACCATCTATTCGATGTGGTACGGTTCGCTTCTGAGACGAAAACGAGGCTAAGATACCGAAAAATGATGGGATGTATGATCACTGCGGGCGATAATTATTTTTAAAAGGTTTATCTTATTTCTTAAAAAATTACCGCTCCATTTGCTGTTTTATCTCGGGGACCGACTGACAGCGGCTACGGTAATTGGGTCCGATTTAGCACATTTTGTTTCAAGATAGGAACTCGATGATCAGTACAGTACTGCACCTCGTTTTCGCGGGGGTAGACCATATAGGATTTGCAAATTAAATCAGATTTTAGCAAACGCCCATTCTTTCGGATACTTGTCTTGTAGTCTTCCTTCGTCTCGCACATTGGCATCACTAGAGTGTTTGTGAAGAGCTCGCGAATGAAATGCTGCGCTCCTGCGAAAAATCTCAGCGTCATGCGTGCTCCCACCGGTGTGCTCCATGCAATTAATCGCGACACCGTTCGGCAGCACCGACACCTCCATTTTGTAGCCATTCAAGTGGTGTTTGCCACTGTAATAGCGTAAGATCTCGCTCATGTTACCGCTGGGTTTGTTAGCATGCTGAAAGGTAACGTCCGTAGCATATCGAGCATACGGAAAGTCCTTAAATGCATGCCCTGATCGTACAATCTTCCCCAGTGTCCACTGATCATTCGCTTTCTCTACGTACATTTCGTAGAGAATAGGAGACAGCATATCCATAAACTTGCGGATCATTTTCTGAAAGGTGGATGGCGGGATTCTGAAAACTCGAGCAACGGTGTCCCTTTTTCCAAGATGTTTGAGCGAACTCAGTGTCATAAAGAAAACGTCGCGCGCAGCGTAGCGGCATTTTTTACCGCGGCCCACGTTCCATTGCCCCTGCAAATGAGGGTACACAGCTGCCCAAACATGGTCGAGCTCCTCTGGCGCAAAGTTGGTGAGCTTGAGCACCCCATCGGGCCCTTCAGCTTGTAAGCAGAAGTCAAAGACGGACGGCAAAGCTCCCACGCCGGAGCTGTCCCCTTCGTCATTGTCGTCTCTGCGGCTATGTACGACGATTTGAGCGACCTCTGCCTTGCGACGTTCCTCCCGCTGAAATAACTCGGCAGTGGAGGCACGAAAGTCGGCCTCGTTGAGCGTCTCAGCGGCGCGGTCTTGGCGGTCTTGGGAGCGTACCATAGTGGCTTTATATGTAGATAGAAAATTCGGAAATGACCGAGGAACTGCATTTTACATCTTGCATTATCTCAGCAAACAGATAGGGCGAATTATGGCTACTATCTCTTCGCATGCAAACTATGAAAATATATAGCGATCAAATCATCAGATGTTTCCTATCCACATGTGTAAACTCCATTCCAGAATTGTACTACAGCGTTCCAGAATTCTTGAATGGTACTGTTACACATACTCTGTATGTACGAATGAGCTCTAATATTTCGTTCATTTGGATTCAGGATCATCACATCCGGAAGTTTATCATAGGCCCATGGGCGCGAAAACGGGGTGCAGTACTGTACTAGAATATATCGGGGTAACGGCAAGTTTGGTATAGGAGTTATTTATGAGGAAAAATACAATAAACAAATGCGTACTGTACATGTAGGGCGATTTCGGTGATACATGTACTGTATGATCGTGGCATTGGTAGATTAGCAGGTTGCAGATCTTACTACCCAATCGGGTTCACGGAACGTTGACAGAATGCTCTACTGTATTGTGCCGTGGGCAGGGATTGTCAGTAAGGCAGCAAATGGTAAAATCCAGCAGTATTATATTTCTACAAACGACAATAGTAATATGTAGTTTAAAAATCTTCTTCAATTTATGTGCTGAGTACTTCAGTAACTTACGTCGTCTTCGAGTAGTACTAAAACTTCTACTGGTAGTAAAACAATTCGGACGATAATCATCTTAGTGCAGCGCTGTCGTTCGTTTAAAAAGATGTTGTGGAACGACATTCTTTTCTTTTGAAAAACTTGAGCGCCAAAGCCGTAGTGTAACCGAGCTGCACATCAGTCCTCATTTGATTTTGACATCGACATCAACACGTCCAAGATAAAGTGCGCAATAACCAGCTGAAGCAACCCCGAGATCAGTGCTTCGTGTTTTGGTTGACTCAGGTGCGTCGAGCAATTTTGCTCGACTGGAAAGTCTCCCGAAGCTAAACTACGAGGAGCTGAAACACCTCGAAATGTGTTGGAAGTGCGGTTGGCAGCAGGTGCCACCGTAAGAACGGAGAAGCGCGTAAAGCGCGCGCGCTTCTCGTACAAACACCGAGTGTTTGTGGAAAATATTATCGTCCTAGAGCTGGGCGAAAAGTTTGACTTAGTATTGGGCATGCCGTGACTCGCACGGCATGATACAGTGATCAGCTGGGAGAAGTATACACTTGTACACAATCGGACGCAATGCGACAAAGAGCAATGGCCCTGCCAGTGTCGCGCATGCACCGCGAGGTGCATCCGTTGAGGCAGCACCTTACGATGCAGGCTCCGGTGCCCATTTACAAGTCGCAACGACTGAGGCAGTTGTTGGGTCTGTCCCAAATCGGAAATCAGATTTGAGACACGTTCATACTTCAATGACCGGACCAACTCAGGGCGTTGATATGAGGGAACCTCAAGTCACAGGCAATGTTGTCCTGGCCACGAGAGAAGATACAACCAAAAAAAGATTGTAGCACATTTATTTAAAAATAGTTAGCTCTTTCATTTTTAATGCAATTAGCTTGAAACCGCTACAAGCTCTTTTTAAATTGTTTAGAAGACTCCTAGAAAAGCGGTAGGCCGCATTCGTTGTTGCAACTTCCGAAATATGGGCCCGCGTTAAACCTCTCATCCCGCAGGAAAGGCACAGGATAGTGAAGGCACATGAAAATTTTAC

>Contig_68

CTTCAAGCTGTGGAGGCCTCGATGAAAGTAGCAGGTGTAGCTTATGAACATGATGGTTTGACTCAAAAGAAAGCGGGATCGGATGCGGTCGAAACACAACTTGATTCGGTCGAGATGATCGAAGCGACCTATCATTATTCCGCAACACAAGAAGCAACCCATGACTCAACTGAAGAAAATCATGATCAAATTGGCGGTGGAGCCCAGCGTAAGCCTAAACGCCGTGTCGTATATGTAGAGATGCGGCGCCGAGAGCGTGCCGACGTTGTTGTTCTGTCATCAGAAGAGAAATACTGCTATGCAAAAGCTGTGTTAGAACCTGTGATGGAACATCTAGCGTAATTGTCGAGTCCAGCATTCTACTCCGCCCTGAAGGCCTGGAAAACAATAGTTAACAAAGGTCTACACGATGATGGATCAGCCGAAGAGCAACATCAGACGCTACAACAGTGTCAGATGAAGACGGATCAGGAGAAGACTTGGATGCTATTGACACTACGTCTGACATAACACCAGCAGATCTCATTGAAACTATGAATTTCATTCGTGAAATGGAGAAACGTGAGTTCGATCTGCAAAAACAACCTACAAGGGCCGGGTGATCGCAGAGAAGTGAGCTTGGTAGCTCAACCTCACGGAATGAAACAGTTCCATCCAGTGACATTGACTTCGCCACTTTTTTACTTCGGCGAATCCTGGCGTGACGCCCTGATTAACGAAGTATTACCCGACAAAAGCCGCGGTCACGGTAAAATCTACGGAAGCACCACCCAGTGTGAAAAGCGATTACCCCCAAAAGCTGCAATAAATGCAGAAGCGACCAAGAAGATCGAAAAAAGTTGGAACACAAGGAGGTCGTCTGTAAGTGCTACGTATTCCAAAACCCAAACCACAAAGCAACCAGAAAAAAAAACACTGAAGCAAGCTCGGCTGGAAAAACTCGCAAAGCCTAATAAGCTAGCCGTTATTACGCTACCGGACAAACATGTGCCATCTGTGTCTCGCGTCATCGTGTGGGCAACAAATACTTCCGACCGGAATCATGTCAGCGAGATCTTGGCAGGTTACCCAGCGATCTTGGACGACGATTTCATGAATGCACGTGTTGCACACAGCTGTCGAGAGTCTGTGTCTCCCAACGACTACGTCTATAACTTTGTGATTCCTAAACCGCTAGTAATCAAGCTGAAAGCGTTCATTGAAGCAGAACGGAAGAAGCGACCCAGGTCGAAGTGTTTCAACCCCGTGGTTGAGCACCAKGACAGCAACACCGAAGCAATTATTGCATATTTTCCTGGTGGAACGCCACGATTTACAAGGTACGCTTTTGTTGTGTTGCTTGTTGTGTTGCCCGGTGTGTTGCTCGGTGTGTTGATATGACTGACTTGCTTCTACAATGTTGTTGGCTGCTGTACAAATCCGTTACAACAGTGAAGCCGTCTTCAAAATGTCGGAGTTTTACAACGTGGTAAAAACAGCTAGCGCATGGAGAGCTGGCATGGAATGGCTGCAAACGACAATATCCTTGCAAACCCGAGTTGTTTAAAGCGGAGACTGATTCGGACGACATATTCTTACTGTCGGCGGGGAAAAACATCAGGAGCTGGCAAACGAAGTGGTGGTACAGCTAGAGGGTGCTTGTTTAAGTTCTAATTTCCGACTATCCAGTGGTGAAGGCGCCGTTAAGGTCGACACGCTCGTTGGAATGCTTGCGCGTGACATAATGCTGTCTGATGGCATTATCAATTTCAGTGTTCGATGCATCTGTGATGCGCTGGGAGATTGTTACGCATTGGATTCATTTTCTCCAACTATGGGCTGTCCGAAACCACCACAGTCACGGATTTTAAGCTTCCACTACCTTGTGTTGCCTCTGCATTTAAGCAACATCCATTGGGGTGTTGTCATTGTCGCTATTGCTTACAGAAGATAAGATCCGTGCTTTACACCCTACTACTACGAGCCAATGTGCGGATCTTCGTATAGCGATACGATGGAAGCGACTTACACGTCTACGGTTGTGCCATTTTTGAAAGAGTGGCATGATAAAACCATGCCGAATGAAGATTACCCAGTGGAGAACCGTAAAGTATGGTTAATGTCGCCAAAGCAGCCCGATGGAACTTCGTGTGGTGTCTTGACTATAGCCCAAGTGTTTTCCGTGTTAAAGGGCAGCTTCCCGCTTGCACAAGGTGTCGTTACAAAGGACGATATCGCAATTATGCGGCTACGTATCATGTGGAGGATAGTTATGCAACCAGACGTTAACACTGGAGCAAACCAAATTGCTAAGGAGATAGAGGCGGCTGACTTGGAGTTACTTGCGACCATAAAACATTAGCACGAGCAGTTGCCTTCGCCAAATGGTATCGACTTTTTAAGTAGTTACGTTGCCTCGTCTTCTTCCTGTGCGTTTTGTATTATGCTGATTTAAATATGCTCTAATAGAGTTTCGCAGCACTGTACGAGAGCCACTGAGAACGACATCACGTAGCCTGTTTCATGTGTAGGTATTCCAGCAGCGGCGGCAAGGCGGATAGTCTGTTCCTACTCGTGAGCATCATCAGAGCCAACTCGTCTCGTTCATTCATAAGCTCTAGATAATACTGTGAGATTTTCTCCAGCTTTTCTTCTATCAGCTTGATATTCTGGAGGCGAGAGCGCAGTATTTGCTTGCAAACCATAGCCTTTCGAGTGCGCGAGAGAGTGGTGCCTTCATTACTTCGTGACTGTAGCGTTNGGGGGGGGGGGGCAGTGGCGGTGGCAGTTGATCGGAGCAAATGCCGTCGAAGTGTTTGCCGCTGCCTTGGTCCATTTTTTGTGTGCGCCAACAGCCGCTATTCATCTTGGGCAACTGTGGTATTTGTCAAATGGCTTATGGAACTTTCACAGTTCCGGTATTTTATTTCTCAAAAGAAGAAAGCCCTAAAATAAATTACGCCTTGTGTTGCTTCTGGGGTTGCTTCTTGTGTACGGGAGGCTAATAGGTTGCTGCAAGACTGTCGTTTTGTCGTAATGAGGTTGCAGTAACGGGACTCAGAAAATCTTGTCCAACGTTCAATGACGAGTACAATAACTATCCTATTTGGAACGTCACCATCGTCCAGGAGCAAACCAAAGGCGAAGTTTGGAGTCTTGACTCTTTCAGTAGTAATTGGGGGAGTCGGACATTGCAACAAAAAAAAGGGCTCAAAATTTTCTCCGGGTTACGCTTTTTGACAATATCCTCSAAGTAAGCCACCTCCGGAGGTGCCTTATGGGCTAGCCCTTTTATYTCCTAAATAATTCTCCTTCCCATGGAAATGAAGGAATGTGCGCGCCTGTGTCGGCGCCCCAGCAGTGTATGTGCGGGTCGTTGCTTTAAAATGCAAATAGTCTCTTTTAAGTGCGAATTTGAGCTTACTCAGTAAATGTGCACTCCCCCGCTTCAAATTGGATACGTCTATGCTTATTTTTTTGGACCATTTTTTAATGTCGACTCCCCCTAATAATATATATCACAAGCGAGCTGATAATCGAAGTACCACGCTTGATAGTGTACAAACGGAGTCTATTTACGGTAGGCTCGAAGTGTATGCAGCATTAAAGCGTTTGTAGTTTTCGACGATTTCCTTTTCGAGCTGTGTGGTTCGTTGACTGGTAGCGCTTCCGGGTACAGTTCTAGACGAAACTAGGAGGTGCGCGACAGCACTTGGCGTCTTCTCCTCACTTGCCCACTTGCCAAACTGCAAGATCTGGAAATCATTAGCCATTTTCTTAGAGCTGTCGATCAACAAACCCTTGTGGGCAATGCGAGCCAGATCTCCGTCCTTATAGTGCTTGGACAGTGTTGATAGCAAGGTCGTGGATTCGTCTGCGAAGTCCTTGTTAAATCGCTTCATGAAGGTGATCCACGTGCTCAACTGTGTCCTAGCGAAAAGGTCGTCCCCTGCGAGATGGAGTTTAAGCAGTTTAAACACTTCATCGGTCGACTGTCCTGTGGCCATCCAATTGTTCACTTGTGCGGCCTCAAGTTTATTCGCAATGCTCTTCGTCTTGCGGCTCATCTTCGCCGCCGCGAGCATCTCAGCTAATACGTCGTCACCGTAGCGAAGCGTTAACACGGAGGCCATTGTTTGTTCCGGATTGGTCTTGTCGAATTTGCTCACATAAGTAACCCAAGTTTTCCATCGTGGGTTCTTGAACAAATTGGACCCTGCTTCGTGAAGTTTCAGTAGGTTGAAGACTTCTTTCGGTGACTTTTTCGTTATTAACCACACTGGAAGTTGGGCGTACCCCGTGAGCTTTGATGTCACAGACTTAGCTGCGTTTGTTAATGCGGACGTTGGGAGCGCTCGACCTTCTTTGTCGTCTTCGATTGTCAGCGCAGCAGCTCGAAGCCGTCTCCTGGTGAGACCGCGGGAATCGCGTATGGCTGTCGTCGACGCCGCAGCTGCCGATAATACCGCCGCAAGAATCAAAATCGCGAAGAATGTTCGCCTCATTTACCTAATTTAAATTCCGAAGTGGTGCTGATGCACAGGACAGGTACGAATTGTTGGTGCACTGCTGAGGTTGCATGACTGTACCGGTACTGTAAATCCAGTTCGAGCCCGGAAGCATAAATTTTGATGTGCTAGTACATGTACAGAGACATGAAATCCGCTCAGTTTTACTCACTATTAGTACATGAAGAGTATACCCCTGAAACATCCACCACATGGAGGTTAAAGCCTCGCTTGTATCTAGTCATGATCGGGACTAGGTTTGCATCCGCGTGTTCCGCACAGGCTCTGAAACCGCAGGCCGCCTGCCAGGCGTGCTCGCTGCGCTCGCATTAGATACCCTGCGCGCGTGGTTCAAGTTTGGTTTTATTAATAACCCTTCGGTGTGGGGTCTAGCAAAAAAGCTTCACTTGCTACACGTATATTACTTCAAAGCGCTGCATTACGATGATTCATGTATTATTAATAGCAGGGTGCACTAATACACGTACAACACATTGTGCTGCAGCAGAGTGCTGTACCTCGTCAGTGGTGAATCGTGATCATCTCAAATTGAGATACAATTGAGCCATCGAATATTGATAATTATAAACACATTAGGGTACGGTACTGTTTCTGTAATTTTTAGCAAAGCAGAAGAGTCTAGTAACGCGGGAACCAACCTAGCAAAGGGTATTCTTGGCTCGGCCAGGTGTATTTCCGAAATGGTACACTGTACATTTACAGAGCACGCTACCATCCATTCCATTCCTTGCGTTTGCGGTCCTCTCGGGTCTCCCCCATTCTTCGATTTTCCAAGCTATTGCCACCAAAGGAATCTAAGTCAACCCGCTACATGCTCACCAATGTTCAGTGTTGTGCCGCAATATCATTCAGTTTGTATGCAGAGAAATGAAGTAAAATGATAGGTCAATAATGGTGTAGCTCTCTGCTATTGGTTGCGATGGCAGCAACTCGTCCTAGCCATTCAAGAAGAAAGAGGCTNGCGTGCCCCATCACTAAGATAGGTGAAGCCGAGGGATGTAATGGAGAGATGGCCGATGCCATCTCACTTCCAAAACTGACGGCCTAGATCAACAACTACCTACAGCTGTACGCAACATTTCAAGTACTCCGATGTCCACCGATGCTAAGTCTGGCGGTGGACTCGAGAAGTTTAAGGGCAAGTCGTACACAATGTGGAAAGACAAGCTTTTGACGCACGTCAATCAACTCGACTATCAGTACCAACGCAAGCAGCTGGAAAAGGGCCAGCCTGAAGCGAAGGTGCTCATGGCGGACTTCCTGCGGGGATGTCCGGACAAGCCACCATCACCGACCAATACTATGGATGAGCAAGAGGCGCTCTCTATCAGATGGGACATGATGCACTGGATGAGAGGAAGAGGAGACCTTCAGAACCTCCTCAACCAAGTCCTCCCAGACTTCTTCCTAAACACGTTGCCGGACGTGGTGTCCTCAATGGACCCCAGCGAAGTGATTCGGCTCCTGGAAAAGGATTTCGGTCAGGGTGACGCGGCTGGCCTCATCGACTTGATGCGATCCTGGGCTAAGTTGACACGCGGCCCATGGCGCGATTTAAGGTCGCTGTTCGCGCAACTGAAGAAGGCTAAGAACGAAATCAATAGAAAGACAAAGAAGCTGTTCGATGAGGAAATGGTGACAGAGTCCTGGGTGTGTGTCGAGGTCCTCTCACAGCTGCCTAGCGAGTTCTGGGCCTCTTCGATCTCGCTGAAGAAGGGCGATTTCAATATCGATCAGGTCGAGAGCGCACTGCGCAAGATATTTGGAGACAAGTCCAAGAAAGAAGTGGGTCTTATGGATAAGTCCCACCCGATCACCATCAACAACGTGCGGGTGAATCGTGGGCAAAAGCGAAAAATGGGTGGCAATGAGGGTGGCAAGTGCTTCTACTGCCTACAGACGGGACACTTCAAGACGAATTGCCCGACTATGGCTGCCGACAGGGACCCCAACCGTTCAGGTGGACCCCTCTTCCGCACCGACGTTAACACGGCACCAGGTGCAAAGAAGGCCAAAAAGGGCAGGACGACCGCTATCAACACGATCACAGCGGTTGTGAAGGACGGCAAGACGCACGTCAACAAGGACGGAAAGACGCTGTTGGAGGAGTGCATGGAGGATGAGGCTATGGACGACATTGAGAGTTTAAACCCAGCCCTCCATGAGGATTTTGAGGACATGGGTAACTCAACCCAGACCCCAAATGAGGCTCTCCAAGATATGGACCAACTCGAAGATGAGGTACGTAAACAAAATGCATCGTACTTAAAAACGTTAAAGCGGTTAACCGTAACCGATGATATGTGGGTGGTCGACACTGGTGCAGGCCGAGCTATAACATCAGACAGAAGCTGGTTTACTGGGAAGCTCCGACCTGGCCAGAATACCGTGTTCACCTATGGTAATGGAACTGTTTCACACAGCTCTCTTAATGGATCAATAAAACTTAGCGTTTTGACTCCAAAAGGAAGATTAAGTGATATATCATTGACAGATATTTCGTTTGACCGACAGTGTGATAGCAACTTACTCAGTTCATATTACCTTGCTCGGCACGGTTATAGACACCTACAGTCTAAATCAGGTGATTTTCTGTTCTTTTTAGGAAAGAACTTTAAATTGCTGTTTGCAGCTGTGGCAATAGGCGAAGTCTACTACTTACCGAGCGCGAAACCAGCTAGAAAGAGTGCTTTTAATGCTCAACTAAGCAAAGTTGGAGACATACTAAAGGAATGGCATCTGAGATTAGGCCATGTGGGCAAAGAACGCCTAATTAGAAGCATGAGCAATCAGAAACTAAAGGGGTTACCTAACCTCTCTTACTCAGAACTCAAGAAAGTTTCATTCTTTGTAGTACATGCGCCTCAATGAAGGATCGCAGAATGTCCTACAGGAATTTGATAGGAAACAAATCAACAGAGCCATTGCACACTCTGCA

>Contig_70

ACCACAAGATACCATCACTAGCTTACACTATAGTGTGGAGGAAGCTCGCAATAGTAAAACCAAGTCTTAGGTTTGTGTCAAATTCAACTCTGATTTATTTAGGACCAGGGCGGCTACCCCTGGCCTCGTGTGAACCACTCAAACACATGTTTATACCTGATTGGTCAATAAGTGAGTCTGGCTACTCACTATCAACCAATCAGATGATGACACCTTGGCACACGCTACCCGTGTGCCACTTTACCTACTTATGTAATAACTAAAGAAATATCTACCTACTTATGTAATTGGAGTTGGGCCCATTACAGACAGCGTGCTATTCGCTTTATTTTTGTGACTTTATAACAGCGACCAGCAAGAAAATAACGCCGCGTGTGGGCTCATATTTGGGTTAGTTTTCGGAGTTTGTAGGTTTATTAGCGAAATTTTATAAAAATTACGGTAAATGCTCATTTCGATATGGCGATCCTATACATTGTGTGTAGCACTCGCTATTAATAATAGGTGCTCAATTACATAAGGTTTTACTGCTTGTTAAGTCGATATGTATCAAATCAGAGGGAAATGTCATCAAAACACTGGAAGAAACACGGTAACCAGCCAATGAGATCATTGTAATTTTTTGTGTATTGCACACAAAAACGAAATTCCTGTAAATTGGTGGGACAGAGAAGTAGTCTTTTCTATTTTTTATTTGTAAATTAGAATGGCAAGTTCACCGACGGTTTAGCAATTTTTCTATTAGCGATGGTTTTGAGGAGATGTATCTCCCTTCAGCGTTACGCTTTCACGTATGCTATTTCGGCTGCAGCTTGTCAGCGTATTGGTCAAGAGAACTACTGGATTATAATTTATTATTCTTAATACGAGCCTACCGAAAAGGACAGAAACATCGTCCAATCTTCTGCTATAAAATGTAATTTATAAAACAAAATCGGAAAAAGAAAATGAAGAGACATATTTTTAGATGAAAGTTATGCAAGTGTGGCTCAATTTCTGCATAAAAAGCAGGCTAGATTCTTTCAGCTCCTACGTCMGAGTGTTTCGCGCTTGTATTCTATTATATTTCGGCTAGGGTCCTGGCCTAATCCTACGCTTACCGATACGTGCTGGTTCCACCTGAAGATGCGGGCTACATAGTGCACTCTCGGTTGTTAGTGTCACGCTTGTAGGTCATGATCTAAACGGAAAGGTGTACGTATCGCGATAATGTCCTATCGGCGTCCGTGTAGAGTCGTACAGCGATTCGATACGCTTCCCGTCCTCTGTAGCTGCGCACATTTAGTGAATACAAGAAAATTCTTCATCCTGCCACCAGTCACAGGTACATTTGAATCGGGTAGGTTGACGAGCATTCACAAGGTGTGGAGTTTAAAATTATATGAGTATTTTGAATGGAGAGTAATACTTTTTTACAATCCAATCAGCACTTATCATTTATTCGCATGCGTACTTATGAGCTAAAGCTATCAGTGTACTGTGTGTTTCCACTTTGTATGGTAATTTTTTTAAAGATTGGGATTAACCCAGTACATATGTACTGTATTATGGAATTTCCTTCGCGTGACTACTTCGGACGGATACGGTTATTAATAGCCGGATAATTATGATGGGTTCAAACCACACCAGCAGAAGCAGCTGTTCTAAAAACCGTCAAGCAAAAAAGAGCTTTGTCGTTTGTCTTACCGGTATCAAATCTCCAAAGCCTATTCGGATCTCCAGCTGCACCGGATAGCTTAGGATCTCCAGCTGCACTGGATTATTAATAAGTACATCTCAAAACTGCGAAATTACCTCTTCATTTTGTTAACAGTAAATAATCGAATGGTATAAGGATGTAGTTGTAGAAAAGCTGTCCATAAATTTACGTATTATACAGGTAGCAAACAAAGAGGAGTATCCGCATCAGACTTACAGCATTTACATGTACACTACGTACTGCGTCCAGAACAAGTATAATACAAAACACCCTTTTGAAGTTTTTAAAAGTACCCCCTGAAAATGAAGGAGATTCTACCAGGGGAGACCCATCAAAGCGGATGATTAGAGATAACCTTGGGATGTGAAAAAGATTGCCCAATCTTAATGCACATACAAAAAAGTCAGTATAATCGCCACTCCCCTTTTCAATTTCTCAGGATTTCCTCTTTTCGGGCACGATTTTGAAAATTCCCCTGTGGGCATATTAATAACCAGCTTGATTACAGCGTTCGTAGAATCATACGAAGCGTCTCAAATCGTATGCACCATTTTAGCCGGTTTTTCAAAGCCAAGCGAAAGATGCCGTCGCATCTTCTCCAAATGCCGCATTATTAAGAGCTCCACCAGTTTGAAATCTTCGAGACCACAAGCACCGGTAGTTCCTGCCCCCACCTATCCGGATATGTGTATTCGCCAAGTCCTGATCCTATTCGCCCTCCTTGCTAGTTGCACCATTTCAATCGATGCAGAACAGCGGGTCCTCGTAAGCGAGCAGCGCACTGTGGACCGTGCGAGATCGTTGCGTGCCGCCGAGTCCACCAACAATGAAGAACGAATAATGACAGAAATCATCAAGCGTCTTAGAACGGCAACCTGGATCGAGACCGGGAGAACTGATGACTACGTCAAGACAACGCTAAGACTTGACAATCTCTCCGGGGCTGCGCTTAAATCTGCCCCAAATTACATGTACTATGAGCATTTTTTGAACGCTCTTGAAGGACGAATATTGGAGGTATGGCTGTCCAAGGGAGTTCCCACGAAAAACGTATGGGCAACGTACAAACTGGACGATATTCCTACAGCCCAACTCAATGACAACGATGGTTTCAAGACATATCTGCGCTATGCGATAATGGAGGATAACAAAATTTTCAAACTGAAAAGCAATGATCAGCCCGTAGCGATCGACTATAGCGGCACTCCAGCGGAGTTGAAAGCTAAGGTGGATATGTGGGTCTCGTTAAAGCGTCCAAACTATTATGTCAGCAGGATGCTGGATCTGGATCGCAGATCCATAAACACCTTCAGACGTAGTCCAAAGTTTCAGCTATACGAGATGTTTCAGATGAAAACTTGGGCGGCGAAAGGTTATCCAACGAATTATTTCTGGAAAGACCACAGGTTGCATGAGGTCCCGCAACAACAACTGCAGAACAATGGCCTCTACAAGAAGTTTGTGCGTTACGCGATGATGGTAGATGACGAGAGCTTTAAAAATGGGAAGACGGTCAAGATCACGGCTGACGAATCCAAGGCGGAGATCAGCACAAAAGTGACGATTTGGGCCTCCAAGGATAGACCTTTCGAGTACGTCAAAAAAGTGCTGGGACTGAGAGGTGCCGCAGACACGACTAATGCAAACTACAAGTACTTCGAGGATTTTTTGTTGCAGACAAATAGACCGAAGAAAAGTAAATAAAGCCAGCTCATACTTACCAGTTTACATTAAATGTACTTACCAGTCTACATGAAACGATGTTTTCTGCTATTGTATTGAAGCAGCTACTGCTTCTGATTCAGCACCTTTACAATATTTAGCACCTTGTACAGGAAAACAGGCTGCTATTTTTTTGATGATTTATCTTGCATCTCCGAACAAGTGCATTGGACAGCGATTTCTCCTGACATCCAAACACAATTCTACTAGTAGAACTATGTTTTAATGAATCGACGAACAGTAAATGCCCTGACATCCGTTTGAGATTTCAGGGCTCGGACACGTCGCTGCTTGAGTCCATTTGTGTCAAAGACCAGCATTTGTCGGGTCGGCTGTCAGCACAGTAGCGCTATCGCAGCAAACCTCGGCGAGCCCTGCAGTCGCGTACATTCCCGATTGCCACACCATAAAGGATGACACATCTCCCGACAAATGCACCGGCACAAAATCGATCTCTCCCGACGATTTATCCTCCCCGATGGCGTATCTGCCTTGACATTCCAGCCCAGCGTATAACACAATTCTCGAGTCCGAAGGGATGCTAGACCATACTGCTGATCGACTTTTGCTGTTCCAGCAGTCGGAAAGAGAGTAGCACCTTTGCGCTGTGCTGAAGGAAAACCTCTTGGATGGATCTCTGGGTTTCCCACCGCTGAAGATGCGCACACGCCATGCGGGACACTCTGCTCCGAGAATTATGATCATCACGATAGAGAGGCAAATGGATGACATGGTGTTGGGGCGGAGAAACCCCAGTTGCGGGAGCTATCCGCGGTGGTGTGTGTACATGTTGACCCCTCACGTTATTTCTGGTCAAAAATGATTCATTTCATCCGAAGATTCTGGCTATCGGTGCTCCAGAATTTCAATCCTGTGGCTTACACTGTTACATTTACTTTTTTTCGGCCAAGAAAGCATGCGCAACCAGCTCTAATGCGGTTGCTCGCTCTATCAGCATGAAATAAATACAAATTAGTCAGGGCAAGTGGCTCAATTTCCCCATAATGCTGTTGTACCTTCCGGGTCGATACAGAAACAGCGCTGGAGAAACGATTTGGCTTCGGACGACAAATGCTCTGGTAGCAGAGGTGGCGCTGTAGCCATCGCGATGGTATACATAGCGGCAAGGCCATTATGACAGTTTGGCCACGGATGCTTAGCTGTAGCCATCTCGATAACTGTTGCACCGATACTCCAAATATCAGCTTTGTACCCATGACCGATTTGTTTGACGACCTCAGGCGCCATCCATGGGATGGATCCTCGAATCGAACGCAAAGATTCCTCTAAGCTTGTGGTCAACATCTGCGGAATCTGCTTCGAACAGCCGAAATCGGCCAGTTTGGACACTCCTTGCTCGTTTACCAACACGTTCGCACCTGGTCGTTGGAAATCGAATTATTAGAGATGCATTAAACGTTCAGTTTTGACCCTTGTGTTTTCGTTCCAACCTTTGATATCCCGGTGAATTATACCCATCTCGTGAAGATACGCAACGCCCTGTACGATCTGCCGAGTAAAAATCCGAATGAGATCTTCACTAAAAGCGTCGAACTGCTTGAGCATACTACACATCTCAAGTTCCACATTAAGTGTCGGGCGGAGAGAGAGACACACACACACACCACACGTGACACGCACCTGGCTATGGACCCACCAGGAACATACTCCATGAATATATAAAAATGATTTTCGGATCGATAGCTTCCCTTGTAGCTAAGACACGCACAGATAAAAGCACATTAGGTCGCACCCCTTTATAACCAAGGAAGACGTAAAAAACCGTGCTCAACAGACCGAACAATGTGCTTGTGGCTGAGATTATTCATGAGCGAGATTTCCTCGCCAAGCTTTTGCATCTGAGTGACCTGATCGTCATTAGGACGCGAATGAATTTCAATCTCCTTCAACGCAAACAGTTCCCCCGTCGCAATATTCAGTCCTTTGTAAACCTGTCGAAATAAAAAAAAAGTTGACCTTAATGACTTCTGCCTAATAGCATAAAAACAAAACAAGTAATGATCACGAACCTTCCCGAATGTTCCTTCACCAATGAGTTCGCCTCGTTTCCACTGTGTGATGGGGTTGCTGACTTCAACTGACGAGCTGGTATCAGTTTCTGTTTCCAATTGCTGGTTGTTTGTCGGCGGTGTGGTCCGTTGGCGAAGTGATACTTCTGGTAAGGAGGAAAATGCTGCTCCTGCACCTGGGCGTCTGATGCGCGGTGATGTCGATCTTGTAAGAGACATGGACTTGGGCATATCTAGCACCGACAAAGCTGCTAAGTGAGGATGTGTCACATGTAGCTGGGAAACCATTTCGCTAGGCGTCGGCGTATCTTTCGCATGTCTGGAGTTGGAGCTTCCTTTAGTCTCGAGAGACTCATTTCCAGTTGATAACGATGAAGAAACCAAGGGAGGGCTATGCGTGGGTTTCGACTCTCGGGTACTGGAACAGGAGAAAATGATGGCATCCGGCAAACTTTTGCTCTTGGCTGCTTTTACCCCAAAGACCAAAACTGGACACGGCCCTGTTTTCGATCTGTGCTTCTTGTTCTTGAATCACTTGCTCGAAAACTTCTGAATTATGAACGAGGTTTCTTCATTTGGGTTCCAATTGATCTTCTCAGTTTCATCACTACTGTCTTCTCGCTGCTGTTGGTCCTCTCTACGGGCTGTATGCCTCCTTGGACGTTCCGGTCTCTCACAAAGCTCGGCGAGTCCTACGGGATC

>Contig_71

GGACAGTTAGAATGAGTATTTAGATAATGATGTTAATGCCAGTGAGCCTACGAGTACGTGGGAAGTGTCCACTGGTTAGATAGGCGGCTCCACAAATAAGGATAGGTGAAGCCGGCATGAGAAACACATACGTAGCGAATCAGTAAAATTAAAACAGCCTGCACAGGGAAAGATCTTTGCAGGCGGCATCGCTCGCGCGATGGTGGTCTAAGTGCTAATGGAAGCTCACTTCTCCTTTTTCCTACGACTGAGCGCATCAGTCTATAAGAGCATTAAGAGAATAGATAAAAGCGTTAAGCGCTAAGCTTTAGCTTAAGCTGACCATTCAGCACGCGAGCACCCCGCGCAAATTTGCTGCGGCTACGCAACAGCGCACCCGCTCACCTCCATCGGGAGGCCGTTGTTGCGGCAGCTTCAATTAGCTTGTATCAGTATCTTTTAGTGCAATTAGTGCATTTTAGAGCATATTTAGGTTTGTTTTCTCCAGCCCCGTGCGCTTTCCGTATTGTCTGCACCAAGCCGGAGTATGGCAACCAAAGTTGATGCTGCGGTTGCGAAATAGCCACAGGAGGTGCTTTGTCACTACGAGAGTTGCAACACACAATATAATACGACGAACACATAAATTGTTAACAAGCTCGCTAAAGTACTATATTTTGAGCTAGTACTAATGGATTCTACCAAGTAATATACTACGTGCGGTATACTATTCCATTACAATACCTCAAATTGCGCTAGGATACATACACTACAATATAATGGAGAGGCTTCTGGCCAATTAAAATCAACCCCCCTACATAAAATTATCTGTTTGAAGAGATAATCGTCGAGTTTGATTTACTTACAAGAGAGCTTTCCGAGACCAAACGCAGTACGACTTGATGCTGAAGAAACTATCTGCAGCACTCTAATATTTTGCCAAACCGAAAACCCCCCAGAAAATCCAGTGCTCCGAATAGGAAACGCAGCTGGAAAACACGAGATCGATTTCTAACTGCAGACAATGAAGTTCCTTGCGAAAGAGAACACTCAATCAATATCAAAGAGCTAAATATTCTGATCTGGCTTATGTTTGGGTCGAAACCGAGCTCAAAAATGGTCGAGAAATTCATGAACGCGTCAGATAGGGATCTCTCAACGTGATGGAGTGGGTTTCGTATGGTCTTGAGGCCAATAAACACACCGGAAGCCAATCATTCGCCAACCGGATCCACCTTCTCTTCGCAACGTCAGACGTCTGCCAGACTAACTCTACCAGAAGTTCAACTTGGTCTAGTTTCCATTTTATAGACTATTTAGACCCTATAAACGCGCCGCCTAAACCAGAATCGACCTGGGATCAACTGAGAAAACGCGGCATAATTCTCAATCATTTCAAGTCTCGCCACGACCTGGTCAAGCCACGCGACGTATCGAGTGGCACGAGCTCCGACAATTTCTTGGGTACCTTACATTTAAGCACACGCTCTAACAAGTTAGATTATATCCACTTTCTTGAAATAGACCAAAATAAAAAAAAAACTGAAAAATGCAGGTAGAATCTGATATAAAATGCTGCAGGTGAAGTTGCCTATGACACCTTCTTTTTTTTGCCAGATATTAGGCCCAGATCTTGAGTAGCGTGTCCCAGCTGCCTGTGCACAGTGCCTGACCTGCCGGGTTAACTCCGAGGCAAGACACACGGTTCTCGTGTCCCGCGAGCTGGTAGATATGCGCTCCGGAAGTGCTGAGCACGTCCCAGCAGTAGCAATTGTAGTCGTCGTAGCCCGCGAAGAGGAAGCGACCCGACTTAGAGAAGCTCACGGACGTGATGCCGCATAGAATCTTGTCGTTACTGAAGTTGTTGAGCTCTCCATACGCTCGAAGATCAAAGAGACGGCAACTCGAGTCGTCCGAGCCGGTACCCAGAGCGTTGCCACTCGGGAAGAAATCGACCGAGTTAATATCCGACTCGTGGCCCTGGAACGTGTGCGTGGTCTTGCCCGTTCTGATATCCCAGACCTTGGCGGTCGAATCACACGAGCCCGAAATAAACATACTGGGGTTATGTGGGTTAATGCTCACGGACATGACGTCGCCCGAATGTTCGCGGAATGTGGTCTTTACTTCGCCACTTTCGACGTCCCACAAGATGCAGTTGGAGTCCCCCGAGCTAGTGACAATGTTCGCTTCGTCGACGAATCGGCAACAGCTCAGGTAGCCATCATGGGCGGCTAGTTCCTTGGTGGCGCGCATCACCTGCGCCTGTGATAGGTGGAAAATGCTGCACAAGTTGTCCAACCCGCCACAGGCGACCATGTTGCGCTGCTTCTGCTCGAAGGCACAGGTCATGACCCAACTGGAGCGTAGAGGGATGGCCTGGATCTTGTTGGTGGTCTGAGCGTTCCACACGATGAGCTTGCCGTCCTGTGACGCCGACACAAGACTCGAGCTGTCTCCGCCCCATTGCATGGCGTAGATCTTGCCAAAGTGGCCTTTCAATAGTCGTCGACATTTGGGTGGGGCCAGGATCGCCTTGGCGCCGGAGCTGGCATTGGCGCTCTGGAAGCCGCCATCACTCTTGGCTTCACGCGTCTTCTCAATCGTCTCCTTGAGGCTCTCGCATTTCTTCTTGAGCTCCGCAGCGTCGCCCATGGCCTTCGAGATGAGGCGGCGTACTGGGGAATGCGACTGTTTCTAACTGCGTGTCGCGACGCTTGCGAGAATATCTTCCACGAAATAACGAGATGTGATTGGAGGGGAAAATGTGAAAGAAGAACGAAGATTGGCTGAGAACTCAAATGGGATGTAGCCATATGAAACCAACGTTGAAGTGGGTACATGGCTAATACTGTAGCAATAATATTTTACCGTTCCGGCTCATACAGTATTCAATATCTACAATTTGGTTTATTTTACCATTTCAAATATTTTACCCACTCTTTTGAGCTACATGCAGAATACCTTAGCTTTCTGATATTAAAAGGTAATACTGTACATTGCTACGCTCATTTCTCTCGAGCACCGACCGTATAACTCCCATACGCCATGAGAACACTAACGCCGAGAAGCNAAAAAAAAAAGAGGGTTTTCAGGGAGAGCAGAAAGTGCCATGCAAGCCTCTTTTCAGTACTTAAGAGTACATCAAACTCGAGTTTAGCCAGTAAAGTCTAGAGAAATAAATGCACTTTTGATAGCAATAAACTGTATTTGCCTATCTACAGTATAAAACCAAACAATTCTAAATTGCTATCGCTTGATCTTATTAGCCAGCTCATGCTGCCATTTCGCTGCAATACTCGCAGTTGCAGGAACGTTGATCGCTTTCTCGAGCATAGCGGACAGATCCTTGACACCGTAGAATGTCAGGACCGTGCTTATCGCCGGAGGAACCTTCTCAGGTGTTTGTGAGCTCAGCTTCATCCTGTAGCTCTCCCACATTCTCAAGTTGGGGTTAATCAGAAGACCGTCCAGTCCTCTATTCAGCTTCAATAGCTCGAAAACTTGGTTCGATGATTTGCTTTCTGTCAGCCACCGTTTTACCATAATGTTCTCCAAATCCATAGGAAGCTTCTTAAAAAGATCTATCCTTTGCTCTCCAAGTATAAAAATCTTTGATAGGCCGTCGTAACCATAATGTCCAACCAACACATCCAGCATAACATCTTCGGGTGCTCGATCATAACTGCTTTTGATATGGACATACCATGTATTCAACTGCGGAGACGCGAAGAGGTCATATCCCGCTTCGTCAAGCTTTAGAATCTTGAAAACATTCTCCGCGGGTTCTTCACTGTTATGCCACTTACCTAACAGATCTCCCCGCAATAATGATGAGACTTTTTCCATTTTGGGGTTTTTCAACCCCAGTTCAACCATCTTCGCCACATCGTCCTCACCGTAGGTCTTACTGAGCATGTCGAGCATTGAAACCTGCTCCTTGGGTTTTTCCTTGTTAAATATATCAAGGTAGCGTGCGTACAGATAAAGCTTTGAGTTGGTGACTTCCTTGTTCATCGGCGGAGTCGTGAAGATTTTATATGCCCCCCTGTCCAGCTTTAGCAGCGTGAAAATCTCGTCTGCGGTTTTCCCCGACGTGACCCAACTAGTTGCGAGCTCCGTCCGCAGTCTCGATGTAAATTTCATAGTAGTTGCGTCGCTTGTTCCCATTCCAAGAAGTTTGATTACACCGTCAGTTCCATAAGTCGTCGTGAACATGTCAAGCATTGAGACCTTATTCGTGGAGTGTTGCTTGTTGAACCGGTCGATATAGGCTGCGTAGAGATGTAGCTTCGGGTTGGTGAGAATCCCATCCAGCCCGCCGCTAAGACCTAGCTTGTTAAAGACGTATTCCGAGTTCTTCTGCTTGTACATCCACTTTTCGATCCTGATTTTTTCGGCTAAATTGAGCTTCTTCGCCGCTGCCACCTTGGGTGAAAACAACCATTCGACAAACTTTGATGGTGTGGGAGCGTTGAAGCTGATTGCTCGCTGTTCGCTGTCCTCCGTTCTGTCACTTCGTAGCTTCCTGGTAGGAGCAGCATCTCGAGGGTTGTTTCTTAAAAAATCGACTGGCAGGCCGACATTTGCTTCCATAGCCGCGGATAAAGCAGAAGTTCCTGCAAATAGGGTAGCCAGTAATACCACGAAGAATCGCATGCTCCGCGCGTTGGGTGGCAAATTACAATTTGAGAAGCTCGCTATAAAAGCAACATAAAGCAAACAACTGCTCGCTCGACAATCCCAACAATCCCATCTTGCATCGAAGTACAGTTCCATTTCTGAGGTTCTGCAGGCACAAGCAGTGATTGTGCCTGCGTAAAATCTATAGTGTGAATAGAGCTATTAATAGCAGTTTACATGTTAGTTATCAAATTTTATTTAATAATTAAAAAAAAACATTTGTATTGATATTATTTAAATTTTGAGCAGCCCAAAAAAAATCAACAGTACATGCATCGTGGAATGGCCTAGAAATCCAGCTGAACTATCACAAGACAAAATATCTTCTATAGATACGCAATTAACGTTATTCGCAAGGATCAAGCTCACAGTCTCTACCATCATTTAATCAAAGGAAAGACTAGGAATAGGAGTCCAGTATCGCTAAATCTGCTGGCAAATCGGTGATGAAACGCGAATACGATTATTAATAATAGTACGCCCAGTATGATGCTGATGAAAGGTGTAACGTAAGATGATGATCAAAGGTGTAACGTATAATGATGATGAAAGGTACAGCCTGAATTACCTACAACATTTATTTCTTTTTTGTGTGAAGCTGCCTGTGTACACTGAAAACCAAAACTTTGTAATACGTCCTTTGATATGTGATGCGGAAGCCTTAAGTCCTGGTTGTAGTTTCGAAGAAGAGTCGGTATTGTTTTACGCATGTCGGGTATCGTCTTGCCGCCAGATACTACTACGTAGTACATTTATTAGTACTAGCGCTACTTGTATTGTATTAATCTTGATCATCACAAATACTCTTTTTATTATTTATTACAACAGCTGGAGCTCGGAAAAGCGGTACAAAAAGTTAAATACCGTCGTAAATGTGAACTTGTACCGGTTTTGAGTGCAAAACTGTCCTTTTTTTCGTAAATTTGAGCTTGTTTTGGTTTTGAGTGCAAAACTGTCATTTTTTTTGCGATTGTGAGCTTTTACCGGTTTTGAGCTTAAAACTGTCATATTTTCGGCGACATCTGATTCGCTGGTAAAACCGCACGTCGATCGAATTGCACTGTCTGATTCGCCGCGATGAATCGCTCATACCGGTTCATCTTTGTCACATTTCATCACTTCAATTGGGAATCAAAACGCCTTCTTCGTTCCTTTCGACACTCATGGGGTTTTCCTTCATGGAGCTGCCCGTCAACCAGCGGGTCGTCGTCATCTCCATCCTAATTCTTCTGTTCGGGCTTTTCTTTGGTCTCTCGCTCGACGCCAATGCGCACATCCCCAAGCCGTGGAACCGCGTCTCCAGCATCATTGGCTGGATCTATTTCTTCTGCTGGAGCGTGAGCTTCTACCCGCAAGTGTTCCTGAACCGCCAACGCCGCAGCGTCGTGGGGTTGTCGCTGGACTACACGGTGCTCAATATGCTGGGCTTCACGTGCTACTCCATCTTTAACGTGGCCTTCTACTACAGCGAGAGCGTTCAAGAGCAGTACATGCGGCGCCACGACGGCCACCGCAACGCCGTGGAGCTCAACGACGTCTTCTTCTCTCTGCATGCGGCGGTTCTGGTGGCCGTATCGCTCTTCCAGTGCGCAATATACCCGCGAGGCGGACAGGTGGTCAGCAAGCCCACGATGTTGTGGACCGGCGCGACTATCGTCGCCGCTGTTGTCTTCGGGCTTGCAGTCTTGTTCACGGGAAATAACGAAGATTCGCTCATCAATACGTTGAACGTATTGTATCTGCTGAGCTACGTGAAGCTCATGACGACGCTGGTGAAGTGTCTCCCGCAGATTGCGCTGAATTACCAGCGCAAGTCGACGGTGGGGTGGACAATCTGGAACGTGCTGCTGGATATTGCTGGAGGGTTGCTGTCCATTGGGCAGCAGCTGCTGGACTCTGCAGCGACGAACGACTGGACAGCCATGACGGGGGACCCAGTCAAGTTCTCGCTGGGCTTTGTGAGTATTATTGTGGACGTGGTGTTCATCTTGCAGCACTATGTGTTTTACGCCGACAACAACAACTTTATGCTGCATGGTGGAGAGACCAAGCCTTTCTTGCCCAAATAAGTCTAAGAAAAAAAGAAGTGGTTAAAACAAAATTCGCCATGGAGGCGTTATTTTGTGTCTAGTTCGAAAAGCTAACGTAAAAATGAAGCGAAATATTCAACATGGTGACATTGAGCGTGACAAGTGAAACAGCCACCAGCGTAAGAGTTGGCTCGCCCTGYATCATTTTTAGTTGGGCTGCTTGGGAAAAGCTGCCGCCCAACCCATTGCCTCAACATCCTCCCACAATGCCGTACACGCTGKGCTTCCGCAATTCCCTTGATCTGATCAATAGAGAGTGACAAAAGCATGTCTCGATATCGCCTGAAACTGAGCTTCGCTGCACTTTGTCGCGAACTAAAATCATCCTCGATTAGAAATCTTTTAGCGACTTGTATTAATTTCTACACAACTGAAGAGAAAACGAAGTAAGCAAGTCCGGAGAGGAGGTCATTGCGCCAGCACCCATCCATTCAAGCTGCACATGCACTGTAAAGAGCTCCATTATTGCAGCTGTCGAAGCTGGTTACCGTTGATTTTTAGATAAAGTTTTTACCCGAACGGCTCAAATTATTGTTTTTTTTTTTTTCACTCTAAATCTTCTGCAGAACATGCCCACCCGCTAACTATCAGTACGTTTGATCTAGAACAACATGACGGAGAGAGTCACGCCGACGTATCCGACGATGGAGACTGTGATCGTTGAACTCGAGCTGATGGACGAAGCTTCATCCACTTCTACTTCGGCGTCAGTAGAGCTGCTTGGCGATCCACTGCTGCCACAAGCTTCCGTGACGGGTTCAAAAATGTCCTTTTGCATCGAGGTCCCCGTGCTGGGAATTGTGCAGTCGCCTAGACCCATTGTGCGCACATCGTCGTATAATGCCATGCAAGGGCCGCTTGCGCAGAACAACTTGACTGATCGCTAGATAGAGCAGCCAAACTCAGAATCTCCACGAATTCCATGGCTTCGCGCATGGATCCACATGTGAGTTGCTCGCCACCGCACTTATTTGCTCAATGGTACAGTCGTCTGCGCTAACCGAGCTGGCGATGAAG

>Contig_72

GCTACGGAGGGGGCTACCGTCACTGTCGCTGGTAGAGTGGGTGACATCGTCATAGTTGGATACCTATTGACGACGCTGCTTTTATCTATCCTGATCTACGTTCCACGGCTGGTATATGAAGGCACTGAAGGGGTGCACCATGGCTAGATGGGCGTGGCTTTGGTAGTGCTGATTGTCTTCATGGAATTCTCTACATGGTGTTACTACTGGAACACCTCGCTGCTGATGGTCGGCTGCTACAGTCATCTGATTCGCAACTATTGAGGGCTTCGTGCTGATAAGGAACAAGGGCCAAGAGTGAGAGCCAAGCAAGGCTGGTATAGGCTCGCTGGGCGGATTGGGCGACGCGAAGAGCGCGAGCGTGTCAGCGTGGCCCGGTTGCGGGACATCAAGGACCAATGGAACGCCTTCTCCGGTCGTGAACGCGAGTCCTGTACACATTGGAACGCCTCTGGGTTTCGTTCCCACTGAAGTGGCTGACAGCAACAGCTTTACATTTACGTGATAAACCTAAGTTCCTACGTCAGCCTGCTAGATACACGACCCCGACCTAAGGAGGATTACTGCGACACTACTATTCCGATTTTGTTACCTTTCTGGCTTCAGTTGCTATCTTCAATTATCGACTTTAGCTAATCGGCGTGTTCCTCATGATGCAAGACTTGGCTACGTCGACGTTGATTGGCGAATATTGAGGTTAGGTAGAGCTATTTACCGCGCTGTCAGTTCAAATTAGGGAGTTATGGTCGTGGCTGCTCGCGCGGAGGCTGCCGGCGTTGCGGGGTTTTGCAGCGCGCTAGACGACTTCAAGGCGTGGGAAGGATCACCTGCGCCTGCTCCGTCCGTCCGCCAATTGGTACTGCTCTAAGGTGTGTAGCTGTTGAGATTTTGGTGTTAGAAGAATTATATTTTTGTTTGTCAAGTGTAGAACTGAGAATATGTTGCTTGTAGATCTGCGACTGGGGACGAAGCGACGAATTTTGGCTACTGTTCACCTTCGGAGCCAAAGCTTAGCGTGTTTATCTACCAGGTTTGCAGGGATTTAGTAATGAGGTGATGCCGAACCGTCTAAGCAGAGCTGTCAATGGCAGGTGACAAGCGATAGAGATCTGCTGGCGTGAAGGTCTCGTTCTTCGCACAATTGGTGCGTGGCAAGTGCGATCGTCGTGTCACTGCGGTGCTCTTCTGATGGCCACAACGGGGGAGCTGCGGCTCGTATGCGGCGAAGAGGAAGGCTCTGGTGGGTGTTGCGTTAGTGATGAGGCAGTTGAGAGCATTAGTGGTGACGTACGTGCTGTAGGGATGTGGCGTCGTTGACGATGATCGAGCAGCACAAGAAGCACAAGGCGGAGGTGACGGCTGTGACGGTGTCTGCTACACTGGACGCGATTTATGTCATTGCAGGTGATCGACAAGGACAGATCAGTGCGACGCGTGCGTGACGCTTGTTTGTTCTTGTGCATGTTGATTGGTCCTGTTGTTATCGTGCCTTGTTACGTGTAGAGGAAAAGTGACTGTGTTCATGCCAGTCTCAGGTGACGGGATCCATTCAATGGCGATTTCGCCGCATGACAAAGCGCTGGTAGCTGTAGCATACAGGAGTGGAGTCTTGTGTCTCGTGGATGCTGCTCGAGGTTCTATACGCCATTGCCTTGATGGTCATATTCAAAAAGTCCAATGTGTTGCGTGGACGTCAACGAACAGCTCTATCACTGTTGACCAGGACAATTGCGAATCGCAATCGTCGTGGCACCATCGCAATTTGCGATGGTGCCGAATCATATACCTCCGCTCGGAAAAAGATAGCCATTTGTTTTTTTTTGCGCAGCGCTCCGCCTCGTTCATTTGAGCGTTATTAATTTGTGAGAGGCTGGCTAGCTAGCTTTATGACCGAGAGCTGACAGTGAGAAACTAAGCAAGGCACAGGCGTTCATGCTCCGCCGTGCAACTACATATACAAAAACGGATCTAAGACGGCAACTACATCGCCACATTCTGAAGGGGCGCTCAGTGGGGCTTCTGGAACTTCAGCGGTAGGCGGTGGGGTGCTGCTCGCTGCAAGAAGCAGCATGTACGCGTTAGCATTGATAGCGTCACAAGGCAGTGACGTAGGGAGAGTGCCAGCTCGTACTTGCTTGTCCTTGTGCCGGAGGCGCGCCAACTTGTGCGTGCGTGTAAGTAGTGGACGGGTCAGCGCACGCCGTATGTAGCCTTCCTTGATCAGCTGCTGAACCAGGTAGCAGCTCATGTTGGACGCGCGCTAAAGCAGACGCTGGTTTTCGCGCTCATTGACAGGAACTGCAGCGATTCTCTCGCCCAGCGCAGCTCGATCCACCTTCTTCCTACCACATGCGCTCTTCCTTGACTTGACCACGGCTGCTACTCGATCTCCCATCGAATTGATTCCACGCCTCCAAATTCGTTCGTCAACTTGGCGGCCCACACGGAAGTACGCTGCAGCCCGAACGATCTCTCCATGCTTGAGCACTCCTCCTACGCTGCGAAGCAGTAAGGTCTCCCGAACGGCCTGGCGCTCACTGTCTGTGATCTCCCCCAGGTGCCAGCTTCAAAATGAAAGCTAAAAGTGATCGGCATAGAGGGGGTAATTTTTGGGTAGGCTGTAATATAATTTGAAATGGTTATCTTNTTTTTTTTTTTTTTTTTTTTTCAGAACGGAGGTAGTACAGTACTGCACCCCGTTTTCGCGCCCTTGAACCTTTGATATATTTCTGGATGTGATGATCCTGAATACAAATTAATAATATTAGACCTAAATGAAGAAAGGTTTGTATTCTAAAATCGAATTTATTTGCAAATCCTATACCGCCAAAACGGAGTGCAGTACTGTATGTCAGATCGGGGAGTGTAACTGGCTTCTTCATCCAGGGATAAAACGATTAAGGTCTGGAGGATTACGTCAGGTGGTCTTGTCGGTACCGTATTGACTCAATACAATCAGCCTGATTGTATTGAGTCAATACTATCAGGGGATGTAGGTTTTTGCCAGCTGGGAATTGAGGGAACTGAGTGAGAATTGAGCTTTTTGACAGTAGTCGCATAGATGCGGCGTGCTTTATCTTGGTTAAAGCTAACTGGTTATCCAAATCAAATCGGTGAATACATGTATTGGCAATTTTAAAGGCGACCCTTCCAGGCCCTACCTGTATACGAGCCGTAAGAAATTCTTCCGATTCGAACCGGCCCATTAGAGGCAGGAGTCGTGACGATCGAACAAACACTAGCCGATATTTGAAGCAGTTTTACATTTACTGTAGTATTAATTAAGGACGCACGCTCGCTTCGCCGCTTTTGTAGGCTCTTTCTTTATACAAACGAGTATGATAAAGACAGGAAGTTGACGTCAATTTCGGTTAAAAAGGTTTTTGCAAAAGCTTACAGCGCGGGTAACCAAGGTGATCATGCTGTTCAGTGGCAGTACAGCGGTTAACAAATTACTTGTAGTTTTGTATTATAGTTGCTACCGGTATAAGTAAGCGTACGAGTAATACTTACGGCAGTGTATCGCCACTCAGTGTCAGCTCGTCTTCATCGTGGGAAATAGCGTTAGATTGAGAGGGTGACTGTAAAATGCACATTATAGGTGTTCGAGCCCATTTTTAAAGGTACTGCAGGTTGTTATAGACGCTCTACGGTAAAATGCCACTTTAAGTCATTTGGATCAGTTTAAGGCGCACCAATAGCAGTCGTAGATGCATCTGATTGGTTATTTTTAGAGCGGCGCTCGCCTCCACGAATACCTGTCAGTTTTGATCTGGCTTCCAACCATGAAGTGTCGCACAACGTATAGCAGCAAGAAGAAGATGGAATTGGTGAGGTCTATTCGCGAGGGAGGCAACATTGAAGCTATTGGCGAGGAGAGCGGTGTTCCAGCTCGAAACTTCTTCCGGTGGGTTAGAGATGACGTTAGTTGGCGTGAGAACGGGAATAATGGCCCGTACACCTTCAGCAAACGGCGCGGACCTGATCCCACTCTGTCCAAGGAAGCCGAAAAAAGTATTCCAACGGCACACACTTACTGCAGCCATTGGATACTGCCGTGTTTTCTTCGTTCAAAAATACGCTCCAAGATCGTATTGATGCTATTATTCGAGCTAGCGGGATTAATACTATCAGCAAGGAAAAGGCTCTTCAAATGGCCGCACTAGCGTGGACAAGTTGCAATTTCAACAGCAATATCTCATCTGGATTTCAGAGCTGCGGCATTTTTCCGTTAAGTCTTGAAAAAATGCACGTGAAGCTACTCAATTTCCAACACAACGGAGTTTCTGCTACCGTCAAAAAGGCGTCTTGGCTGCGATACAAGGAGACACTACAGCACGATATCTTGCTTCTGCATGCTCGCCAACAAAAGCAGAGTAGGAAAAGAAAAACCAAGAAAGTGGCTGGTAGATTGCTAACGCGTGAGATGCTTCAGCAACCTGACCAGTTCTCCCAACGAAACGCGCCGAAGCGAACAAGAAAATCGCAGAGATACCGAATTTCTACAAAAACAACAGCGAGTGGTATGCACCACCAGGACCAGGAGCTTCATCAAAAGCAATACCACGATCACAACCACGAGAACGAGCACAAACACGAAGAGGAACAAGGCATGGAGAGCGAATTGGTGCTAGTGCAGCAGCAGTCAGAAGTTGCTAGCAGTGATACGGTTCTGTTCGAGGTGAGCTTCTAAATACTCCGGATTTTAATACACCATCTATTTTGATCCAAGTCAAGGTTATAATTTTACGTGGTTCTAATAGGAATTTACAGAAATTCAACAGTACGTGCACGATCGTACATAAAAGTCTCTTGTCCATACTCAAGTCAAGTTTAAAATTGTCTTAAACTGGCACACGACGAAAAAAGATGGTAATTTCAAACCGCCACTGATGGCCGAATAGTACTCAATTAATGTGTTGCGCTTAACGAAAGACGAGATTCTGATAATTCCAGTCGTGGTTGTTTTATTCTCATCTTCAATGTCCGTTGAAAATTACGAGATTTTAAAGTTGGTCATCTGTCACTGACTTAAACTGGCATTTTACCTTATGATGTTGTGCTGCTGTCGAGAGTACTAATCTGCCTCAATAAAGAGCACTGAAAAGCTGATAACCTTCAACATATTATGCGACGTAGTAGCTATCTCCACTCATTTCATGTAATCTTGAGTTGTTTCTGAGAAGAAAAATATGCTCTTTGTTGCGGCAAGTTACTGAACTACTTCGAAGTAAATGACAATTAGGAATTTTACTGAATCCTTATCGCCAATCTGTGACATTCGATCCGTATGCTTCCCGTAATCCTATCCTGTGATGCATGCAAATTACAATGTGCCGATTTCTCGTGGCCACTGTCTTTCAGACGCCTCCTTACTTCGCATTTGCTACCCTTGAAGATATTCATGTTCAGCGCCACTTTGGTTGTCCAATGCTCCTTTTTGAGATGGATTCTTCTATCCATAGTCGCTGCTCTAGCGTGCACTCCGGCGACTTCGGTATCGATGAAGGTCGCTCACAGCACGCAAAACACCATCGCGCGGGATTTGGACTTCGCTCTCCAGCGATTCTTGAGGTCTGAGGATATCAAAAGACCGGGGGAGGAAATCGGCAATTTGGAAGAGAGGGCGCCGGCGATAATTGCCTCGGAAACGCCACTGATAAGAAAATTCGTCCAGAATATTAAGCTTCGATTGGGAAAGCAGTCTCCAGGCGACGCGTTTAAAACTCTTGAGCTGGACCACCTGGGTACAAACTTGTTTAAAAGTCCCGATTTTTCCAAATGGGTGAAATTTGTCACAAAAGGAAACAAGAACGATCCAGATATGGCAATTTACACAACACTGGCAGCTCGCTACCCCGACGAAACATTAGTGACGATGTTAGCTGCAGCTAAGAAAATCGACAGCACCAAAGATATTGCCATGAAACTGGAGGGGGCACAGGTTATGAATTGGATCGCTAAGAAAAGAAGTCCCGACGACGTTTTCAAGATTCTGGCCCTTGATCAAATAGGAGAAATGGGTATAACGAGCCATGCGTTCTCGAGGTGGACTGACTTCATCGGTAAAGCGAGCACGGAGAAACTGGAAGTGGTCGTTTACAAATCATTGAGAGCACGTTACAGTGACCAAGCGTTGGCAAAGATGATAGCTGCAGCTAACGAAGTTGGCAGCACCAAAGCTCTAGCTACAAAATTGGAGGGGGTCCAGCAAACGAGGTGGAAGGACTCGCATGCTTCGGTAGACTACGTGTTCAAGACCGTAAAGCTCGATGAGACTCGTACAAAAATATTCGAGAGTCCGCTTCTAAGCGCCTGGACAAACTACGTAGCCAGCATCCAACCCGACAACCCGAATGGTATAATACTCGCGAAGCTGAGCTCACAGTTCAAAGGGTTTTCAACGCTTCCGAAGATGATTGAAGCGGCGACCAAGGTCTCAAGCACGAAGAAGCTGGCAAACGAACTACGGTCTGTGCAGTTTAAGAATTGGTTGACGCAGGGGCTAACCCCCAAAAAGGTAACCAAAGATTTAGGTGTAACCAACACCAACGGTTTGGACAAGAAGCTCTCGAATGATTACAGAAAATTCTATCGGAAGGCCATAACGAAAGCGATGAATTGAACATGATTCTCTGTAATCCAAGCGTCACGTGACCTTGCTCAGCAGGTGGCAGTACACTTAAAGAAAAATAATGCAGACATGCTTCGCACTATTGTAGAATATCATAATGCCGCTGTGCTCTGGATTTTACAATGAATAAAAACTGTTAATCCACACTTCCTGGATACATGTCAATGTCTCATTTTGTGAATGACGGCACCAGAGACTACTGGTAAGTGCTTTTGAATTTTAAAATACAGTATCCATGAACAGATAATGTACGTTTCATAGCTAAACTTTAGCACCTGTAAATGTTTAAATCTGTTTAAGAAGGTAATGCGATTTGAGCGAGGTTATTGCGGTTGTGAGCTTTTGAAAGAAACAATCTTTCAAAAGCTCACAGCCGTAATTTCTCAGCGAGCTCACCTGCTGCGGCGATCACCACCCGATGACACTATTCGTCGCTGCAGCGGTGGGTTATCTTCGCTACTCATCTACGTGATACGTCTTGAGGTGACGCAGGGCGCCCTGGACTGCTCTCTAAGTGTAGGTCACTTCTTAACGTAGGTGCCATTAGAATTGACTTACACTAAAGTAGTAAAGCAATCAAAAAGAGGTACATAAACACAGTGAGTGCCGGGCAAAAGGCCACGACAGGGCTGTAGTACTGGTGTGGGCTTGCTCTTTTCTCTGGGGTTCGCTGAACCGCAAATTGAGTTGTGTGAGAGAGAGAGAGCAAGCCTCACGCAAGCCAGTACGCGTCTCCGCAAGTTCTATTCGATACATACAATAATATAAACCACACATTACCTAAGTCAGTGTCGTCGTGAGCAGCACGGCGGCACGGTCGCTCTTGTCCATCGATCATAGTAAACTTGGTATGACAGCACGCATCTGCACGTGGCACGTCGGTAAGTGCGCAGCCGCAGGCTGCAGCAACAGGCGTAGGATGTAGGATCGGCTGTCAGTGTCAATGCCAGGCACTGACAGTCCACTTGTATGTACTGCTGGTACCGGCCTCTTCACACAGGGCTGGCATCTCACATTTCCACTTGAACTCGGCAATACAACACCTTTATTGACGCGATCACAGACATGACTTTACAGTTTGAAGTAGCTGCCCCATAGATCAAAGTCATAGACCAGCAGCACAGTAGTCCCAACAGCAAATAGCATGCAACAGTGAACACCGTCCTCATCCATAGCAGTAGCCTTCACATCCGTACTCAGAGCGTCCGCTGTCAACCCAGGTGTAGCACGTGTAGTATCGTAGTAGAACTACGCAAGACAGGTGCAACCTTTTTTGGCTTTTCCCAGCACACCATCGCACTCAAAACACACTGTAGCTGTCACGCTTGTCCAACGCAATCGCTCGCCCTTCTCGCATTTGTCCTATTCCTTTCGAATTGCAGCAATAGCAGTCGAAGCAGGAGACGCAGTCTCCGCAGTAGCCGCAGCAATAGCAGCCGTAGTAGATGCCGCAGGCTCCGTAGCGGTAGTCTTATCATCCGAGGAAACCACTGGCAACACCTTCATCGCTTGACGAAAGCGAGTGAGCTTCACCACCGCAAGTGGCTTGGTCATGATGTCCGCGATCATCTGCTCGGTCCCAATGTGCTACGTGGTGAGCCGTTTAAGCTCCACGTTTCGTCTCAGCATATGATACTTGTTGTCTATGTGTTTAGACTTGAAGTGCTTTCCAGGCTTGGTGGTCAACGAGATTGCTCCCTCGTTATCACCCAACAGTAATGGTACTGGCTGCTTCCAGGCCAGTTCCTCACACAGACCCGCATTCCACAGCACGTCCTTGGTGGCTTCAGCCATCGCCACATATTCCGCTTCGCACGTACTCAGGGAGTTAATCTCCTGTTTTCTTGATGCATACGTTATCACGTTACCGTCAAGTGTGTTCACATAGCCACGGATGCTTCAGATGTCCACAGGATCATTTGCATAGTCGGCACCTGAGTAGCAGCGTACATTTGCAGTAGCTCCCTCAGGTACGTCCATCACCAAACCGTAGTCACACGTAGCTTTTAGATACCTAAGTACCCTCTTAGCTTGCGCGTAGTGTGTGTGGTCGTAAGACGCCAGGAACTTGCCAAGATGTCTCGTTGCATGCGCAATGTCAGGCCTTGACGCATTCACTAGGTACTGAAGTGTACCCACTAGCTCACGGTACGGCAGATAGTCCGTTGTCGCAGGTACAGTAGCCTTAGATAGTACAGTAGCCTCAGGAGTAGTACATCCGTTGCAATCCGACATGTGGAACCGCTTAAGTTCTTCCAGCACATATTGTTTCTGACAGTATACAATCTGTCGGCTTGGTCTGTCGATCAAGATCTCGACACCCAGCAGGCACTTGACAATGCCCATCGTAGTCAGCTCGAAGGTTTCTTGCAGTACAGCTGCAATCTTGGCGCACAGGTCTCGGGGTCCCATTAGTAGTAGGTCATCGACGTAGACGGTCAATAGCAGCTTAACTTCACCCTCTTCCTTTAACGCATACAGCCGATAGTCTGAGTCTGTGCGCTCAAAGCCCAAAGTCACCAACTTCGCATGTAGAGTCCGGTTCCAATCATTAGGCGCCTGATTCAATCAATAGAGGCTCTTCAATAGCTCGCATACAACGCTTGGTCCATCAACTTGGAACCCAGGCGGCTGCTCCATGTATATCATCTCGTCGAGATCTCCGTACAGGAACGCTGTCTTCACGTCGTCTTCCATCCTCGCTGCGCAGCGAAGTCAATGGCCGCACGAATAGTTTCGAAGCGAATCACCGGTGCATATGTTTCACTGTAGTTGATGCTGAACTGTTGCTTGAATCCGTGGATTACGAGACGAGCTTTGAACCGCTTGACATGTCCACGCTCATCTTGTTCACCGCAAAGACCCATCGACATGTAATCACCTTGACACCTTTCGCCTTTCTCCGCGGTACAAGTCACCAGGTCTTGCGCTTCTTCAGCGACGCCAGCTCTGCCAGCATCGCGGCCCGCCACTGGGTCCAGAGCTTACTCTTCCGTGCCTGCTTGTAGGTTGTAGGAATGTCCACTCCATGCCATGCGCTATCACTTGGCCCACCACGTAGTCGCTCAATCGTATGTTTGCCTTCTGCGGCCTTCTCGGTCGCTTCAGACGTCCATGCCATGCGCTATCACTTGGCCCACCACGTAGTCGCTCAATCGTACGTTTGCCTTCTGCGGCCTTCTCGGTCGCTTCAGACATGGCTCGGCTGGAGGTTCTTGGATTGCAAATCCAGTGTCTTCCTTCACGGTTTCTTTCACGCCTTGCGCTTGCGTCTCTTTGTCACCTTCACCGGTTCAGACCCCTTCTCAGGTGCGACCTGAGGGTCAGCAGACTTCTCCACATCTTGCTGTTCGACGCTCGACGAGTCATCGTGCTCTGCACTCCCGCTGGACGTTTGTTCTACGTCCA

>Contig_74

TTTACGACTTGCACAACCATACCTACGCTTCCTATGCAACATGACATGTGCACGTACGCGTCTTAGGCAATATGAACACCTTCGACACCTTCATACATGCACAGCGCGCGCTCCGCTTAAGTACGACACCAACTTGTCATATACATCATGCGTTTCCTCCCGTCACCTTTCATCACCTCCATATTCACACCCATGCGCCGTCACCTTCCTCGCGCCATCACCTTCACCATTTTCACCTCCATATCCGCGCTTCCTCCGCCGTCACCTTCCGCGTACGCGTCCATATTCGCTGCGTTTCCTCGCGCTTCCTGCCTGTTATTAATCCAAGCCAAAAACATCTCCAAACCCATACAATTAGAACAGCAAACAACTTCGCATTTTTTCACAAAGTCCTCAGCTTCATTTTGCATACTTTATCCTAGTCTAATTCACGCTATTCCGCTCTCTTTAACATACTTAAACTAAGTCTAATTCACGCTACTCTGCTCTCTCTAACATACTTAAACCAAGTCTAATTCGCGCTATTCTGCTCTCTTTAACATACTTAAACCAAGTCTAATTCACGCTACTCTGCTCTCTCTAACATACTTAAACCAAGTCTAATTCGCGCTATTCTGCTCTCTTTAACATACTTAAACCAAGTCTAATTCACGCTATTTCGCTCTCTTTTAACATACTTAAACCAAGTCTATTTCATGCTTTTCCGCTCTCTTTAACATACTTAAACCAAGTCTAATTCACGCTATTTCGCTCTCTTTTAACATACTTAAACCAAGTCTATTTCATGCTATTCCGCTCTCTTTAACATACTTAAACCAAGTCTAATTCACGCTATTTCGCTATCTTTTAACATAGTTAAACCAAGTCTATTTCATGCTATTCCGCTCTCTTTAACATACTTAAACCAAGTCTAATTCACGCTATTTCGCTCTCTTTTAACATAGTTAAACCAAGTCTATTTCATGCTATTCCGCTCTCTTTAACATACTTAAACCAAGTCTAATTCACGCTATTTCGCTATCTTTTAACATAGTTAAACCAAGTCTATTTCATGCTATTCCGCTCTCTTTAACATACTTAAACCAAGTCTAATTCACGCTATTTCGCTCTCTTTTAACATACTTAAACCAAGTCTATTTCATGCTATTCCGCTCTCTTTAACATAGTTAAACCAAGTCTATTTCATGCTATTCGCTCTCTTTTAACATACTTAAACCAAGTCTAATTTTACGCCCTGCCGTCTAGCTTGCCCTGTTAAACCCCCATACTAAAATCTCCGATTTTGGCTCAGAAATCATTAGCGCTGGCACTTAGCGATTTTCACGCAAAGTGTTTCTCGCTTTCTGCCTGTTATAAACCCGATACTAAGGTCGCCGAATTTGGCTCGGAAAACAATAGCAGCGGCACTTAGCGATTTTTGCGAACAAACCTCACTAAGCCAAAACAAAGACTTTGACGATCTTAAGCTTAACAGTATTAACTCTCTACCAGCACTTTGACCGGTTACTTTCGCCGCCCCATAGCGAAAGTAAATCACTTTAACGCAGCCTTATTTCGACTGCTTAACTTTATAGTTTTAACATTGCGCGTTTGCAATTGGGGGGAGAAATGTTCATTTCTAAACAAATCAATAGAACAAGGGCTAAATCTCAGCAGATCGTAGTACAAGACTACTCTCATGCTTACAACACCCCGTTCTGTAGTTAAGTCGTCTACAAAGGATTTATCTTTCCCAATATTTGAAATTACAAATCAGCCTACTCTCCACCGCCAGTACTTGACAGGCAGCGGAGTGCGTTGCAATTGTATGATGTTGGGTGTCCAGAAGACACCTATCGCTATTCGCGGCACATCGGGAAAGGTGATTATCGTCTATTCCAGCATGGATTCTGACTTAGAGGCGTTCAGCCATAATCCTACAGATGGTAGCTTGGCGGCACTAGCTTTTCAGCTAACCGCAGTTACCAATTATCTGAACCAACGGTTCCTCTCGTACTGGGTTGGATTACTGTTTAGACAACGAACTCATCAGTAGGGTAAAACTAACCTGTCTCACGACGGTCTAAACCCAGCTCACGTTCCCTATTAGTGGGTGAACAATCCAACAATTGGCGAGTTCTACTTCGCAATGATAGGAAGAGCCGACATCGAAGAATCAAAAAGCAACGTCGCTATGGACGCTTGGCTGCCACAAGCCAGTTATCCCTGTGGTAACTTTTCTGACACCTCTAGCTTAAAATTCAAAAGAACTAAAGGATCGATAGGCCACGCTTTCACGGTTTGTATTCATACTGAAAATCAAAATCAAGTGAGCTTTTACCCTTTTGTTCTACATGAGATTTCTGTTCTCATTGAGCTCACCTTAGGACACCTGTGTTATCATTTAACAGATGTGCCGCCCCAGCCAAACTCCCCNANACCGGTCAGGTGGTGCAGTGGTTAAACACTGGGCTTCTGATCGGGAGGTTGCGGGTTCAAACCCCGGCGCGTCGGAAAATTCCGATTCGCTTAAGTACGAATTCGTGGCCGCAAAACCGACTGGGCCATAGGGTCGCAGCCGGTAGTTGATGTGAAGCAAGCGTGCACAAGGTACGCCACGGAGACGCAAATCCTGTAAGTTATTGATAGCAGGTCTTAGGTAGATAAGGATTGTAATAAAGAATCTACCTTGGGTAGAAGGAATTTACCCAGCCAAACTCCCCACCTGACGATGTCTTCCACGTAGCTCACCCAGGCAAAAGCCCAAGTTTACAACTAGAACTGAGCGACCGCGAGGCCACACAGATGCTACATCATGGTATAAGTAAAACAACATTGAGAGTAGTGGTATTTCACAGACGGCAGAGCCTCCCACTTATTCTACACCTCCCAAGTCATTTCACAACGTCAGACTAGAGTCAAGCTCAACAGGGTCTTCTTTCCCCGCTGATTATTCCAAGCCCGTTCCCTTGGCTGTGGGTTCGCTAGACAGTAGATAGGGACAGTGGGAATCTCATTAATCCATTCATGCGCGTCACTAGTTAGATGACGAGGCATTTGGCTACCTTAAGAGAGTCATAGTTACTCCCGCCGTTTACCCGCGCTTGGTTGAATCTCTTCACTTTGACATTCAGAGCACTGGGCAGAAATCACATTGTGTCAACACCGTCTCCGGCCATCACAATGCTTTGTTTTAATTAAACAGTCGGATTCCCCTTGTCCGCTCCAGTTCTGAGCCGGTTGTTCAACGCACTAAGGAAACAGCAGCCAGCCCGAAAGCCAACTACCTTTCTCTCCGGCACAACAAGAGCAGCCCGACCGCCAGGCCGCATCCGGTCCCCGAAAGGTCCAGACGCAGCCCAACGTAGGCCGCCACAAGTTCTCCAAAAAGACCCCTAGGCCCAACCCTCAGAGCCAATCCTTTTCCCGAAGTTACGGATCTATTTTGCCGACTTCCCTTATCTACATTCTTCTATCAACTAGAGGCTGCTAACCTTGGAGACCTGATGCGGTTATGAGTACGAACGAGGGTGCGAATAAATCTCTAGCCAGGATTTTCAAGGGCTGTCGTGGGCGCGCAGGACACTTCAAAAAGTAAAGTGCTTTGCCAAGGCATCCTCCTTATCGCCGGATAATCCGTTTCCAAGGTAGGATATGCTTGTTAAAAAGAAAAGAGAACTCTTCCCTGGGCACACGCTAGCGTCTCCTGGGTCGGTTGTGTTACCACATATTATCCACGTCTCGGTTCAGGAATATTAACCTGATTCCCTTTCGATACACGAGGCTGCATACAAAACGCAAGGACGAACCCCACGCCTCAAACAGCCCAGCTTTCAAACGGAATTATCCTATCTCTTAGGATCGACTCACCCGTGTCCAATTACTGATCACACGGAACCTTTCTCCACTTCAGTCTTCAAAGTTCGCATTTGAATATTTGCTACTACCACCAAGATCTGCACTAAAGGCCGTTTCACTCAGGCTCACGCCACGAGCTTCTTCACGACCTTCACGCCCTCCTACTCATTAACGCGTACATTATAAATGCTATCGCGCTAACGGCAAAGTATAAGTAGCCCGCTTTAGCGCCATCCATTTTCAGGGCTAGTTCATTCGGCAGGTGAGTTGTTACACACTCCTTAGCGGATTCCGACTTCCATGGCCACCGTCCTGCTGTCTAAATGAACCAACACCTTTTATGGTATCTAGGTGAGCGAGCATTTTGGCACTTTAACTTTGCGTTCGGTTCATCCCGCATCGCCAGACGAGCTTACCCCGTATGGCCCACTAGCAACTTGATATTCACATCCACAAGTTCAATTAAGAAACCTGCAGGTCTTACAGATTTAAAGTTTGAGAATAGGTCGAGGAAGTTTCTTCCCCGAATCCTCTAATCATTCGCTTTACCTCATAAAACTATCGTAAATAAGTTGCTGCTATCCTGAGGGAAATTTCGGAGGGAACCAGCTACTAGATGGTTCGATTAGTCTTTCGCCCCTATACCCAAGTTTGACGATCGATTTGCACGTCAGAATCGCTACGAGCTTCCACCAGAGTTTCCCCTGGCTTCACCCTACTCAGGCATAGTTCACCATCTTTCGGGTACCAACATATGTGCTCAAACTCAAATCTTTCACCACGAAGGTTCATGATCGGTCGATAGTGCCACACCGCAGACCGAAGCCCGCAGCATTTCCTACCTCAGACAACTTACGCTGCCCTTCACTTTCATTTCGCGCTGGGGTTTCCACACCCTAACACTTGCACACATGTTAGACTCCTTGGTCCGTGTTTCAAGACGGGTCAAATCGCTCCATTTCGTCAACGTCCCGAACGGCAACAAGTTAAAACCCACATCAATCCACACACAACCACACACGCATAGCAAGCTATCCGCACACGGCGCACACAGAGACACAGGCCCACTCAAAAACACGTTGTAGGCACCTCAGTCCCAACCGCGACTACTCGTCCACCAAGATATAACAGACAAACGCAAGCGTAAGCCCTACCTCCTCGGCGGCCATTTCCCGCAGCATACGAACTGACCTTGACGTCCCACCGCAGCACAGGGCACCAGCAAGCACACACAGCACACACGCCGAGCGCAGCCAAAAAAGCCGCTGCCACAGCACACGCACGCACACACTCGCCAATGAAATATACCACGGATTATAGACACTGGAAACGATTCGGTTCCCTTTCAGCAGTTTCAGGTACTCTTTAACTCTCTTTTCAAAGTTCTTTTCATCTTTCCCTCACGGTACTTGTTCGCTATCGGTCTCGCACCAATATTTAGCTTTAGATGGAATTTACCACCTACTTTGCGCTGCATTCCCAAACAACGCGACTCAAAGAAAACGGATCGTACGCACGAGCAACTCAGGGATGAACGGGAGTATCACCCTCCATGCTGTCCTCTTCCAAGGAACTTGCCCCAAGCGCCCACACTGATCACGCCTCTATAGACTACAATTCGCCGCGCAAAACTTGCACGGAGATTTTAAGCTTGAGCTCTTCCCGCTTCACTCGCCGTTACTAGGGGAATCCTTGTTAGTTTCTTTTCCTCCGCTTATTAATATGCTTAAGTTCAGCGGGTAATCTTGCCTGATATCAGGTCCAATTGAGATGCATACCGAAGTACACAACATTTCCCAAATGGATCGACCCTCGGCAGCCGAAGCCGCCACTCTACTTCGCAACAGCAAAGCCGATTCAAATGCCAAGCTAAAGAGCCATGGTTCACCAGTCCATCACGCCACAGCAGGAAAATCACCCAATAAGCGCATTGTTCAGCCGAAGCCAACCATACCACGAATCGAGCATTTCTCCATTAACGCCGCAGCAGACAAACCGGTCGCCAACTCGCTACAATAGCAGCGTCCACAACCAGCAATGCCACCACTTTTGGAGCAAAGAGAAGTACAGTTTAGTACATTTAAAAGGACTCGCAGTCGGTCCGAAAACCAACCGCAAGACACTTCACATCTGGCATCTCCTCCACCGACTACACGGAAGGAAGAAAGCCAAGTTTGTTGTACGGACACTGATACAGGCATACTTCCAGGACTAACCCGGAAGTGCAATATGCGTTCAAAATTTCGATGACTCACTGAATCCTGCAATTCGCATTACGTATCGCAGTTCGCAGCGTTCTTCATCGATGTGCGAGCCTAGACATCCACTGCTGAAAGTTGCTATCTAGTTAAAAGCAGAGACTTTCGTCCCCACAGTATAATCAGTATTAAGTAAAGGGTTTAAAATAAAAAAGCTACTAGCTCAGACCGAAGTCCAAACGCTCGCCTTTTGATAGGGCTCGCCCAGCAGTAGCCGCCAGCAATAAAGCCAGCAGCCGCCGCCAAGTAAGACCCCCAACTATTGGGTTGAAACGGTTCACGTGGAAAGTTTTTAGGTGTGGTAATGATCCTTCCGCAGGTTCACCTACGGAAACCTTGTTACGACTTCACCTTCCTCTAAATGGCGAGGTTTAAAAAAGTTCCCATCAATCCACTCGCAATAAAGCAAACAGACTCACGGTCCCAATTTTTCACCGAGTCATTCAATCGGTAGGTGCGACGGGCGGTGTGTACAAAGGGCAGGGACGTAATCAATGCAAGCTGATGACTTGCGTTTACTAGGAATTCCTCGTTCAAGACTGAAAATTGCAACAGTCTATCCCTAGCACGATGCGCATTCACAAGATTACCCAGACCTATCGATCAAGGTTGTATACTCGTTGAACGCATCAGTGTAGCGCGCGTGCGGCCCAGAACATCTAAGGGCATCACAGACCTGTTATTGCCTCCAACTTCCTTTGGTTTGATTACCCAAAAGTCCCTCTAAGAAGTCCACAAACCTACCAAAAATGGTAAGCGGAACTATTTAGCACGCGGAGGTCTCGTTCGTTAACGGAATTAACCAGACAAATCACTCCACCAACTAAGAACGGCCATGCACCACCACCCATAGAATCAAGAAAGAGCTCTCAATCTGTCAATCCTTACTATGTCTGGACCTGGTAAGTTTTCCCGTGTTGAGTCAAATTAAGCCGCAGGCTCCACTCCTGGTGGTGCCCTTCCGTCAATTCCTTTAAGTTTCAGCCTTGCGACCATACTCCCCCCGGAACCCAAAGACTTTGATTTCTCATACGGTGCTGACAAGGTCATTTAAAGTAAACGACTGCCAATCCCGAGTCGGCATAGTTTATGGTTAAGACTACGATGGTATCTAATCATCTTCGATCCCCTAACTTTCGTTCTTGATTAATGAAAACATCCTTGGTAAATGCTTTCGCCTAAGCTCATCTTTCAGCGATCCAAGAATTTCACCTCTGACGCTGAAATATGAATACCCCCAACTGTCCCTATTAATCATTACCCTGGTGTGCAAACCAACAAAATAGACCACCAAGGCCGTATCTTATTATTCCATGCTAATGTATTCAAATGAGCAAACGCCTGCTTTAAACACTCTAATTTTTTCACAGTAAACGATGCAAGCCCACCAACCCACCAACTTAATGGCAGAAAGGCAGCCTCACAAAAAATGCCTCGAATATCCATAAGTACACACTCCCAGAGGGAGCGGACCGGACATCCAAAACAGAAATCCAACTACGAGCTTTTTAACTGCAACAACTTTAATATACGCTATTGGAGCTGGAATTACCGCGGCTGCTGGCACCAGACTTGCCCTCCAATTGATCCTCGTTAAGGGATTTAAATTGTTCTCATTCCAATTGCCCGACTCGAAGAGCCAGAGCATTGTTATTTATTGTCACTACCNTCCCTGTGTCAGGATTGGGTAATTTACGCGCCTGCTGCCTTCCTTGGATGTGGTAGCCGTTTCTAAGGCTCCCTCTCCGGAATCAAACCCTAATTCCCCGTTACCCGTTAATGCCATGGTAGGCCCATATCCTACCATCCAAAGCTGATAGGGCAGAAACTCAATCGATTTATCGCGCAAAAGCGCGATCCGCACAGTTATTATGACTCAACACAATACCAGCCCGAAGACTGCATTGGTTTCAATCTAATAAATGCTACCCGCCCGACAAGCAGTTGGGTATTGATGCATGTATTAGCTCTAGAATTACTACGGGTATCCAAGTAGTAAGGTACTATCGAGTAGACTATAACTGATATAATGAGCCATTCGCAGTTTCACAGTACAAAATTGTTTATACTTAGACATGCATGGCTTAATCTTTGAGACAAGCGTATGACTACTGGCAGGATCAACCAGGTTTATATCCTCCATACTAAAGAAGACGACAAGTCGCCCCCAGCACAAAACAACAACACAGAGGCCAGCCGCAAACAGCCCCGCAACCGCCGAAACAGTCGCTAGGCCACTCGCAGCTTTTCTCAAAGCCGCCATTAATAGCCGAGAATCNTTTTGCACAGAAAGACAAAAATATGATCCTCACTTCAACAGTTCCACCTAAAAATAGGTAGATCAAGAAGCGAATAATAGAGCACGACGCAGCATACTTTGGGTTGTAGTCCCCTCCGCACGCCGCATAACTAGCCCCAGATCAAAAACGGTTCTACCCGAAATTGAAACTTTCCACCGGAAAAATCCAGCGCAAAMCGCACCACAGAAGGTATCGCATAATGACACACAATCCGAAAGATCGGAAGAGCGTCGTGTAGGGAAAGAGTGTAGCCAATGGAATCTCGGTGGTCGCCGTATCATTCAAGCAGAAGACGGCATACGAGATCCAGCTGGCTACACTCTTTCCCTACACGACGCTCTTCCGATCTCATAATGGAATTGACCATGCCGTACTTAGAGCTGCTGGCAACGACCGCAGCATTCACTCCCTGTGATAAAGCCGACTGCCCAGCCTCAGTCTTGGCGCCCAAAACGCCACGTCTGACGCTCGACTTCGACGATCTTGATGCTGTCGTTCTCGGGACAGGAGACCGAGATGACCTCGCCGCCAGATTGGCTGGGTGGCTCCCTCCACTTCTGTATGAGCAGTCTCGCGGACCATCAACACAGCGAGCGAGTCGTGAAGACGGTACCATCCGGCGAGGTACCGACGTAGTCGTTCTCTTTGTCCATTGGGGCGACGACCAGCTCTAGCAGCTTGAGTATCATGCCGCGAACACACTGGCGTTGTAAGTGCGGCAGGCGTTGAGCTGAAGGCGCTGTGATTTATTAATATAAGATATGAATTAACGTATCGCCCTTAGTGCGCTTGACATTTAAATCGTAGGGTTGAAACGAGTTATTTCATCCTAGTATTTTGCCCGACTGGCTAACCGACTATACAATTGTACTCCCAACATGCATAAAATGGACTTAGCCGACGGTCTTGGCAACACTATTAATAGCTTGGACTTAATATCTAATCCCAGACACAGCGCCTTGCTAAGTCGAGGACCTGGAGGCTGGGTGGGGCAGGCCGCGCAGCGGCGCCCCCCGAGCGCAGCGCAGCAAGCAAGCAAAGAGAGGGGCGGGCCCACGCTGCAGACGGCGCTAGTGTCCTACCTTTGCTCCAATTTAACCGTACTACACAATCTGTACACGTATAAACGTCGATGATATGTTACCTCAGTCGGGCAAAATAACGAGTGCCTTAATCCTTTCGACATGGGTTTAGGCCAATAGACAGGCCCGGCCATTGGCCTGCGGCCATAGCCCGTGGCCTTTGCTAACACTGCTAGCACTGCTAGGATGGCTCCGACGCCGTCCGTAGGTGTTCATGAAGCTGCACGCTGCGTTTGCATGTACAGAAGTCCCTCAAAGTAAGCGTAGGGGGGACCGAGACACGATCTTAGAGGGTGATAAAACAGATGTTTAAAAAGTACTGATATTTAGTTAAGAAGCGAGGCGATCTCGACAGAATGCCTGCGTGCCGCTAGCAGTTCCGAATGGTTGTGGGCGACATCTCGTCGTAGGCCACCTTACTTCAACAAAGAGCGGTGTAGATATCTAGAGCCAGTGTCAGCCGATGTTTGGGGTCTTTGCGTCCAGAGATCACCTTCCTCTGCGTGAGAGTGTACTTAGCCTCGCTCCGAAAGAAGAACGAGGTCTCATCCACGTTGCAGATCTCGGACCGGGCGTACGAGTCCGTCAGCTTTCGTAGTTTCACACGTCCGGCTTCAGCTGCCTCTTGGTCAACTGACGCAGCTTCGCCGTGCTTCCGCTTCTTGGAGATTCCGTGACGTTCTTGCAATCTATACAGCCAGCCCTTAGAGAGCTTCAACGTGGTGTTCTCGCCATACGCGTTCACGCGAAGAGCAATGGCCTTTATTACCTTGCCTGTGATAGCCTCCACCTTGCCGTCCATGGCCTTAAAGACCTTCATAATGCGAGCGTCCGTCTCCTGGATTTGGGGGCTGACGAGCTGCTTGCGCTGTAGCTGACAGGGCCCCATAGCGGCCCACTTAAACTCCCCAGCGATAACATTGCGAGCGGCATCGCGTGAAAGAGAATTTTCCAGCTTGAACGCACGTGTTGCCCACGCTGCGAGCTGTGTGTTGGACAGTTTGCCTTTGGCGCGCATATCCTTCGCATACTTGCACATGGCGATCTTCTGCTCCATATTAAGGCGGATCCGGCGCGGCATGGGGCTGGCGGTCATGGTCGCTTGGAAGAGTGATAGTGTGCATGGAGGGAGGCGTACGTAGCGGGAGCATGAGTTACCTCGTACATTTAATCTCCATTCTCAGTCTTTCAATGGTTTAGTTAGCGAAGAGGCCGGACCGGTCTGGAAATGATGGGAGATCTTTCCCGCCGCTGCAGTTCATCCAAAATCAGTGCATTTTTGTGATTGGCCAGTTGAACCTATTTGTACCGTAGCAGATATGGCGGGGTATTACGTAAGTAGGTGACGCTTACTTTGAGGGACTTCTGTACTATTATAGGATGAATTGATTCTGTCGCAACATCTTTGTATCAATGACTGTCGCATTAATTTGCAAGTTCTACTTCTGGCGGCCAGTCGTTTTGCAATCCGATTTAAAATCCAGAGCTGTTCTGGGCGATTGCACGCCTTTGTTGCACATAAGTATCCGTATCCATTGCCATGCATCAAGTAGTGTTCATGATTGCAAGACGACGTGAGTCGTTTATGAACAAAGTCGTAAACATAACCATTTTCGGACCAGACTAACCTTTCTTTGCATTTATGTTTACCGGCGTACGTAAGCGTAAAGCATTTTATTAATACCAATGTACTGTACATTTCATTGCACAGTACCGTACCGTATTGTAAACCCTCAGGTTGCACGAAAACACTTTCATTCTTGAATTTGCATCCCGATGCATCTCTTCTTTCTGACAGCCGTAGCTTTCGTCATTACCAGTGTATCTGTCGACGCATCAGTCGCGAAAGATCCACGAGGACACGCTCCCAACAGGACTGAAGTCGATACCGTAAACGCGAGTTCAAGCACGAGGCTTTTACGAAAAAATAGTACTGTTGATCTAGTCGGCGAGGAGAGAGCACCCAGCATCGTAGAAAATATCAAGGCGTTGGTCAAGTCTTCAGCGGTGACTCCAGCGAAGCTTCAGCAATGGCTAGACGAGCGACTACCTGCGGGGCTAGTGTTCAAGAACATGAACCTTGACGAACCAAATATCTTCTCTTTGTTGCATGAACCCAACTTTGCTAAGTGGGTTCAGTACGCCGACGACTTGAGTGCCAAGTCATCTCATAAAGAATCTTCAGTGATCTCCACCCTGACATCATTGCACGGCGACAAAGTTGTCTACGACACAATTCAAGCTGCTAAACTGTATCCACAACTGAGTGAACTCGCCCTTAAATTGGAAAAGGACCAGATACGCTTCTGGATTGCCACTCGAAAAGACCCCTCGGTGGTTTTTGAGGCCCTCAACCTTAACTGGGCAGGGATATCCATCTTCCCAAAACCTGAATTTTCCGCTTGGCTCAAGTACGTGGACGATGTAAACGCAAGACATCCCAAGGAAGCCCCATTGTCGATTATTCCTACGCTCAAGCAACGTTTTTCTCGAGGTGACGAAGCCGGCACAGACGTACTCCTTAAACTGATTGCGAACGGGAAAGCAACGACAGAGGCCAAAACTGTCGCCAACAAGGTAGAGAGTGCACTGTTTGACTTCTGGCTCAACAGTCGAGAAACGCCCGACAAAGTTATGGATGCGTTCAAATATGGCACTACGACTCAAGCTTTCTTGGGGAGTCCACGGTGGAAAGAGTGGGAAAGGTACTTGAGCGCTTACAATGCGAGATACCCTGAAAAGAAGGCCACAGCGATAGAAACGTTAACGCGGAAGTATGGAGATGCACAATTACTCGACACGCTTATCGGCGCGAGCTCGAAAGGTGAGACGAAAACTCTTGCAGCCAAGTTGCAGGCACAGCAGTTCGATAGGTGGATGAACCTTAAAGAGTCTCCCCTCGACGTCTACAACAGGCTACGGTCTTCATATGGGGATACCGCCTTCTTCAACGAGCCGCAACTCAATGTGTGGGTCTCCTACATGAATGTGTTCGTCGACAAGAACCCCAGCAAGGTGGACAAAATGTTCTTGGAGTTAGGTGACACCTTTGGGGACATGCGTCTCTTTCGAGTCCTTGGAGAAGCCAAAAAGTTCCCCAATTTGGAAAGCACTGCAACCAAGCTGCAGATGGAGAAGGCTTCGACTCTTTTTGCCAGCGGAAAATCCCCGGAGGGTATATTCAAGGTGCTAGCACTTGACAATGTCGGAGATGATATTCTCAGCAACACGCTGTTCCACAAGTGGCTGGCATATCTGCAGAAATTCAACAAAGAGCACCCAAACAATCAAGAATCGTGGTTTGACATGCTCCGTATTAGTTACCAACCGTTCGGCGTCGAAAGGATTATCGAGACAGGAAGGAAAAATCCACTCACAAGATTGATGGCTGAAAAAGTGGAGAATGCGTATCACAACTACTGGTTGGATATTAAGATGGAGCCTAAGACAGCCTTCCGCTCCCTGCATCTCGACGAAAGCGGTGAGAAGCTCCTTGCCGACCCAAAATTCAACACGTGGGTGCAGTACCTGAAAACCTTCAACGACCGATATCCTAATGAGAAGACGACAGTCATCGACGGGCTCAGGGATAACTCTCATGACATAGCTCTACTCCGAATGTTTTCAGCCGCGAAGAATGATCCCAGCACGGAGAAACTCGCTACTGATCTACAGAGTGCGCTAATCCTCAAGTGGCAAGATGCGAAGAAGACACCAGAAGAACTAAAGAGAGTGTTTGTTGGTGTGCCAGCCGCTGATGAAATGCTCGACCGGTACATCAAGCTACTGGCGGTGGCGTCATCTACGCCATAATCTATTCCACAGAGTAGAGCTATCGAATGTGATTCTTGATAATTGCAATTGAATAGATGCTGCATTTTTACGCGTATCAGGTCTTTAAAGCGGTTTCAGTATCGCCATATAATTGTGCATGAACGTGCTCGACACTAAGAACTCCAGCTGCAAATTGTCCTTTGAGTTAGATGATACCTCTGAGGTCATGCAGCTGTTCAAAATGGCAAGACGTTCGTGTCTTTCGGATCCTTGAAGCAGCCACCAAGTAATTTGACATTTAAAATATTGAAATGAAGCTGCAGTGGGAGAAGGTCCAAAGTGTTGTTCCTATTAATAGATGTGAAGGAAACGCAAAAAAACTTTAATTGCCGACACATGTTGGCAACAGGGAACAATTTCACAAGGTAGAAGCTGTAAGCAATGTAGGAAACTTTTACTGTACCTGAGCGAGAAAAAATATTTGGTGTCAAATTCAATGAGCCACTCGCATGCAAATTTTCTGTTATTTTAAAAAGAAACTTTATTGGGCCTACATCGTACGTAAGTGGATTATCACTAAAAGAAGTGCTTTCAATGATTCTGCTTAGGTTTTTGTGTATCATAAAATTACCGGTATAGAAAGAATAAATATAGGTATTCCAATTGGCTGAGCCTTACATGTAATACTTTCTGATAAGTAATGGTCTAACAAAGCCTTCATATAAAACATAAATGCAGCATAATTAGCATTTATGTTTTATATGAAGTCTTTGTTTCACACCTAATCTGCAAAAGAAGGAGCCCGAGCGACTTCTATTACAGTTACCCCCCCCCGTACTCATATGTTGTAAACGACAATATGTACATAAATAGAGCGACTCGTCTATCTCCACATGGGATGCACAGGTGTAGCAGAAATATTTACAAAAATCGAGCAAAAATGACAAAGACGGCCTAGGCAGGAAAGCCAGCTTGTTGCTAAGTACGAATAGTTTTGTGTCTGTGGAGTCTCCCCCTCATTCGAAAAAATAAATAAAACGATTATGGCGCCGCGAAGGGAGACGCGCCGCAAGGCTGAAGCGTTCCGAACGCGCCTTGAGGGAAAGAGTACTGCCGACAGGCACCGCCTGCTGGCAGAGCACCGAGCGTTTTTGAGTGGTGGGAGGCAAAATGATGCCGACTTCGGAGACGAGACGAGGGACACGAACGCTGAGGGGCCGGAGCGCGTACCCGCGTCAGGAATACAGCGCCCTCCCACGTGAAAGACCGCCTCATACATCGAGGCCGCAAAGCAGCGCATGGCGGACCGCCTGGCTGAAAAAGCCGCGGCGAAGAAGAAGATGGGTGCGAAGAAGAAGGGCGGTCCGTCCTGCAAGACCAAGACATCAACGCGAAAGAGGTATGTAGTGTTCATGAAGTATATACAACGTGTGAGGACTGAACTGATGTTATATTCATGCTATATTATCCTAGCCCGGTCAAACAGAAGATATCATAGCGACAGCACTCTGCCCTAACTCGAAAAAAAAGCTTAGGAGTCCAGGGCGCTGGTGCGAGCTGAATCAGCAGCCGCACTTCGATACGCTTCGAAAGCGAAAAAGCGAGAGCGCAGGAACGACGAGGAGCCGACACACGTAAACGCGCCCCTTGCTGATCTGAAAGAAAAAGATCAGTGGAGACTGCTGGCTTGTCGGAGACGACGACTGCCACGGGTGCAGAAAAACGCAAAAAGAGCGACACGACAAGGTTAGTCACGCGATCATGGAGCATATACTACTTGTGTTGACTAACATAATGTTGATTTTTTTGTGCATTTCAATTTGCAGCCTCTCGTCCCCGGCGCCGGGTCGGCGAGGACTCCGGTTCTCACGTGGTGGATACCACCGCGGAAGTAGATGACTTAAACACCTCTGATGAGTGGTCGGTGGGCCTACCTGAGCAATCACAACAACCTGTTGTAACGAATGCTTCTACAGCGCAAGTACAGGCTCCTGACTCTCCAGATGGGTAAGTTGCTATATCATGTTCTATGTAGGACATATTTGAAAACGATTATGTTGTATATTAACGCATTACTATGTGAAATGTTATAGAAGCAACACTGTCGAATCCGCCGAGCACGGACCAGATGAGGCTTCGCGCGCGGATGCTACTTCTGACGACGAGGCACTGAATTCCGAGCTTGATGGATGGGACGGTGCTGACTCAGACGAGTGGTATATCGAAACGGAGACGAATTTGAAGACGCTGTCAGGTGTCATTGATGCGACTGCATCGCTAACACAGAGGGTGATTATGAAGAACCTGAAGAGAAGCCTGACCAAACTCCTTCAGATGAAAGTGATGGAGCTTTGAATGGAGCGAGTTGCTACAGAGAGCAATCGATACTACAGCCAGCATTTGAACGAGCGCGTGGATA

>Contig_75

AATTCAGAAAAGACGGATACCAGAGGTCGTTTGGGTTATTGTGAAGACCGATTTGTTTGTCTACATGTACTTGGATTGTAACAACCGATTTAGGATCTGAATAAGTCCGATTAAAAACGAAACACGATAATACCTTACTTCGGTTTAGACTTCTCTATTGTCCTCTATATTACAAAGTGAAGTACAAGATGATCAGAACCGATCTAAGATAAATTCGACCGGGAAGCGAATTAAAAAAAGCAAATTGCCTGAATATTTTATGTTGTTGCGGGGTGTCGAAACTCTGTCCTAAAAATGTTTAAAACACACCTTTGAAAGTTGTGGTAAGGATGGTATATTACAACGACAACTTTTAGGACATTCGTTCATCTAAACATCAAACCCATTTACAATTGTAATGCAATTAGAAGATACGTTCTTTCTTGGTAGGACATTGGCTATCGTTTGCGATTCACGCTCGCGGTCGCTCGCCCTCACCCGATATAATTCGCTCATCGTCCACCGAACAAGGCAACAAAGTAGCGCCAAATGTGCTATGTGAAAGTGCTGGCTCGATTGCAATGATGATCCCATCAAATGCCAGTAACAAAAACCAAATCTTTAAATATTTGGACACATTAGAGTTATTAAGGGCAAGTGCCGAGTATACCACCAAATCTGCTCAGCAATACAAGGTCATTAAACAAGCCACGACGATCTAATTTGAAGCCTAGTCGGAGGGGCGGAGGGTACGCTGCTGATCCAATCGCCACGTATGAGAATCACAGGAATAAACCGGATCATTTGGCTTCGAGCAGTATGTGCTCGCTGCTAGAAACAACTAGAGCCTAAGAATCGTAAGCCCCCCCCCCAAGCCCACTACATGTACACCCCGAGTTAAGACGCTGCTCGACCCATCGATGGCTTCTTGTTAGAGTGTGAAGATGTTCGTGTTGGGCCAATCTCACGTACGTACTTGGAGGCCCCACGAGCTACACAACCGGATGTAACGTAGTACAGTAATCGCTGCCAGACAACCTACATTGCGGTAAAACCAACACCTTTGCACAATCGTCGACGGCCAAACTCAAACTCAATCCGATCCTATCGCTCGCACTGGCGACGTTCAGATTCATGGGCCCTCCGTTTTACAGATTACAATCAACGTGGTGGAATGTCGAGCTGCCTTCGGCTTACCGATCAGCAGCCTTCTCGCTAACGGCGTGTAATGTGTGCAATCGATGAGGATTGATTGTAAATTAAAAGTAATGAGGTTGTCATAACGGATACACATCTCGGAATAAAATCCTCCTTTTACTCCTGGGGGTAGGAATTAAAGCAATTCTAGTGGACAATCTGGATAACGAGGGCTGGACACCTAATGATTGCTATCTTCCATAGTCACGAGAATGTGGTAGAATGTCGGTTATATTCTGGAGCAGACGTTGATTCCCAACGTCCAAACGGATCCACTGCGCTTTACATGGCTTCGGCATTTCTCCATGCTTGAATCACTTCTCGAGAAGGGAGCGGATGTTGAGAAATGGACCAGTGGCGGATGGAACCCGCTTATTATCGCTGCGGGAGCAGGTCACGTGGATGTGGTGACCTGACTGCTGAAAAGAGGGGCTAACATTGACGCACATGGCTCTCATGGAACTACTGCGTTGCTGTTGGCATCGGAATATGGTCACGCCGATACTGCGATGCTTCTTCTTGAAAACGAAGCGTCCGTCGATGCAGGCGATGGAGATGGCGACAATTCTCTGCTACTGGCAGCTGGATCAGGGCTTGATAATGTGGTAAGGGAGCTTATCGATGCCAGTGGATTTTTATTAACAACGAAGGCGACAGTGCACTGATCAGAGCGTCGGAGGGAGACCACGGCAATGTAGTTTTGATGCTTCTGAAGAATGGAGCCAGCGTTGATCAAAAGACGCACTAAGTGGCGCTGGCAAGAATCGAAGTTGCAAGGTTTTATTGAAGCCACAGATACTGGAAACGATGACAATAAGAACAATTAGCAATGCCGCCCGCGTACAGGATTAAGACCAAGTGATTGTATACAAGAGTAATACTTGCGTATAGTATAATACATGATGAGATTCGTTAAGATGACGGACATATCCACGCTTAAAATTTCGTTAAACATTATTTTTAAAGGTTAAGGGTTCGACTTTCTTCGTCTAGAGCAGCTACTTTGAAGTATTCAGCTGAGCGACAAAAACTTTATCAGGTTGATTCTAGATCTATGCCCACCAGATTGTAACCTCTCCTGGAAATATATGAGAGCCTACACGTTCAATTATATACTACAGTAACCTAATTGGCTACAACAACTTAAACGAAAAAGCACACTTCACAACTGTCTTTCAAACTTCTCTTCATTGATGTAATAAATGTCCAATATGTAGACGACAAACTGACACTTGCAGCGATTCGACATCTGAGCAAATAGACGTCGTCACTTCTGCAGAGACTTCGAGGCCGACGACGACAGTGGTGACTTTAGCCGCCCAAACGCTTTGAGAGGCGGCAGCATATTGCCAGATGACGTCGCCGGAGACACAGAAAGACTTGAATCTGGTGTCATCACCACTGACGCTAAGCGGTGTAATGAAGAAAGTCCCACTCCACCGAGGCGATAATTCGGACTGGGCGATAAAGCACTTGAAGGAGAAGCCATCAGCGACGGAAGCACTCGTGGAGTTATCATAAATGGAGTTCTGTTGTCGTCTGATATCGGAGATTCTCTGTTTGATACTGGAACGTGGGGAGGAGAGTGTTGTGGCTGGACCAATACACTCGCAGCTACGAGCTCCGAATGCGAATAGCCCGTCGGGTGTTGCTGAATAAACGGTCGATGGCCAACACGCGGAGTTTGCACTCTCCTCTGAGCCATAGGAACCTCTTCAGCTCGAAATTTGGAGCTTAGGTACAATCCAACAGGACTCTGAATCGATGGTAGCTTCATCAACGGAGTCGTCACGGCCGTAGCATTTGCTGTAGAGTAAAAGTTCCCGCTGATCCCTGACAATCCACTCGAAGTAGGAGCGCGAGAGGGTGTTCGGGTTTCAGCTTCCTCGCTCGTCTTGCGGTGTTTAATACTCGACTCCTCCCTTGGACGTGACTGCGTCGCATCCTCATCCGACAAACTGTCCCGTCGTCTCTTCCTTGAAAACTTTTTAGCGTCGAATTTGCGTTGGTGCTGGTTCTGCTTGGATCGGTGCTCCTCACACAAATTATGAGGTTTGCCATTGCGTTTAATAGCTCGTTCATTCTCGCATTTGCGGGACTGGTACATACATTTGCCACGATAGGGACCAGGACGACGGGTCTCGGATAACTGGTTAGAAGACGTAGCAATACTGACGAGGTCGACCTTGGCTGCGTCCGTAGATTTATCCGCTTTCTTCTGAACGTTCCTGCGTTTCTTGATACCATCTGAGGTGATGCTATCGGAGACTTTGGTCTCTTCCACTGAACTCGGCGGCTGCATTATAGATCGCGACTGGGCTGCAGCAGACTCCGACGTTCGACGTGAAGAGATGATTGCCGAGTGACGCCCAACCTGATACGGGGAGGGTGTAAGTGAACGATCGCACGACAGCGTCGTACATGCACAAAGTCCACCGTTGCTCCGAAATTGACCCATAATAATGGTACATGTACAGGGCAATTACGGCTATTAATCGGTTTAAATTAATCCGATAACCTTGTGTTTGTATGTGGAAAACTGGGCTATATCTTTTTTTCTTACGTTATTAATAGATGCCAGATTTTCGAAGCGAAATGGCGGGCGATCTGATTGGTTGAAGAAAGGCCAAATTTCGAATGTTGTCTGGCTGTAGAACGTCATATGTGTTGCATAATCCGGTCCATGGATCAGAACTATTATACTTCGAGGTACATTTTTGGCCCAAAATGTCAATGGTTATTATAGTTACCGGTAGGGAATGAAGAGGCGAAGCCGTTGGCAAATGCGGTTGCAACGCCATCGACTCCTCCCAGTTCTTTTTCAGCTCTCATTTGAGCCTTCTTCGACGTTGGGAATGGTACAAGCTATACAGATCATCAGAAAAGCTCATCATACGGAGTCGCCGGCGGAACGACATTCGACTCGTCTAATTCTTAAACTGTAAGTGTTTTGATTTAACCGTGTACCGCTACCACACAACAGCCAGCCACTCTGTTGCCTCTTCTCCAAAGTGCAAGTGCGTGTATCGGATCCCTCCGCTGGCGTCACGCGCTGGCTTCTCTGCTAAGCTTCTTAAAACTTTTAAGTTAAAAGCAGCCCGCTTTAGAAAAGCAATCCCACATCGAGAAGATTTTTTACCATTGGTAAACTGATCGTCGTGCTCAAAACTATTAGCTTAAAGTTTACTTAAAAGCTTACCAATGCGTTTTTACGAGACGACCTGTACACATGTCACAGCTGTGTGGGGACCACGCTTCAAGATTCTCTGCGGTATTATTTCGGCCTTTACAGCAGCGAAAGAAATTCACACGTATAGCCTACACCTACTGAATCCAGCTCTGACAACCACTTCTCGTTAACCCCATTGACAATTGCGGCAAAGTCACAATACGCTTCCAGTACAGTAAATGTACTTTCGAATGTTTGTGCCGTTGTTTTCTTCACATTCGCTCCCATTGAGCCGTCACAAACGCTCGATGGCTCGTCAAGTTGACGGTCAACAGCATGCGAGTCCGCCACGCACGATTCGACGCGCCGACATTTACTCTGCTCAAAATAAAACATAACCAGTGCGTTTTTGGACCAACACCGAAAAGGGTAACAGTGTTACCGTACCGGTAACACTGTTTGATTATTAAAACAAGTAAGATTATTTTACTTCAAAATTAGTCCTTCTAAATTAGATCTAGGAAGACAAAACATACAAAATGCATCTCATTTGACTAATTACTTTTTATCTTGCTTAGAGTGTAAAAACATACTGTAAACTTTGAGCGTCGATACAAAATATTGTACCTTTTTTTGTTAGGTAGTGACCAAATAATACATACTATTATTTGTAACACAAAGTGTTTGATCACAGAAGTGTATTACAGCGAGCAATGTCAGCCTATTATTCGCATTCGAGTCACTTCTTCTCCACGGAGCACAGTGAAATACGCTCATATTTAAGCTTTTTAAGCGGCGGTCAGTGCAGCGTAAGGTTCATTGTCGGCAAACAGCTTCTTCACTTGCTCCAATAGCGCTGCTCCTCCCCTCTGTTCAGCGAAGTGCATGGTGTATGCTTGAAAATTTTGATACCTGGGGTCCCTCTTCGGTATTATTCTGAAATTAAGTGAGTATGGGACCCCTAGTTGTCTTGCGAGTTCATTTGGGTCTACGTTATGATCCCTCATCCATTTGCGATAGAGATGTGTTTGCATGCCTTTTAGAAGATCGTCTAAGTCCGGTGTGTCCTTGATCGGTTGAAAGAGATTTCCGAACTTGAACTCATTTGTGAATGGCTTTCTCTTCTGCAAAAATCTGAGTGTATCCAAGTCCGAAAATGACTCACTTTCAATCGAAGCCCTGTGTGCTTTGATGTATCTCAGCCAATGAATAAACAGAGAGCTGCTGCCGAGACTCTGTAAGGTCTCATCTCCGAGACGTAAGATGTCGAAAACCTGTTGGGGCGTTTCTCTGGCCTTTAGCCAAGCCTCATTCGTCAGTTTGTGGCTCGTAGCGGAGATCAATATCATGTTTGACTGCATCTTGTCGGCAATATCTTTGAAGTTGGGGTATCCTCGAAGTGATTGAAATAGTGTCACCAGCTCAGCTTCCGGTTTCGTTTTCCTCAGTAAGTCAAACAATTTGCCATCGCTGAACCATCCCCCCCCTCCGCGCTTAACTCTGTATTGCTTCACGTACAAAAGCCATCTCAGAAATCCCTTGTCGCCTTCCAACTTTCCACCAGTTTTGGCAGCGCGTAACCCCTTAAATATATCAATGGGGTTCGTCTCCACTTTCAAAGCGAGTTTGTAAGCTAGATCGTCGAGCTTTGTCATTCCTGGAAGTCCAGCTCGCTCTCCTGCGAGTTTGTCTTGTACGTCTTCATGGTCCTGGTGAGTTGTGGTGGGCAGCCTTAACAACCTTGTCGAGGTGTTTGGAGGCTCAGATGCATTAAGAATATTTGTTTTGGTGAGAGGATTGGTGTTGGCCACGCCGTCAAGCATTTTCGCGCTTGTACTGAGGAAATACGTAAGGCTGATTCCGAGCAACAGCAAGAAACGGGAGCGCATGTCAATTTTGTGGTAGTGATATGGGGGCTCTGGCAAATTGGCAAGTGAGATATATATCCAGTTATTTAGGTGGTTGAACATGCGGAAATGGTATCGCACCTAGAGCAATGAAAAAAATAATAGTGTGCTGGATTTGGCAAATTAAAAGAACAGCGTTTTGTAACATTACAAAACTGAATGTGCGCAAGTTAGTTTCGCAAAAAATAATCAAAATCGGAATGCCATGCTCTCTGAGGTATTTTTTAACTGACTTGTTTATACTAAAACACTTCACGCCGTCGAAACAAAGAAAAAAGAATTCGCAATGATCTCAACAGCGTATCCACTGACGACAGCTACTGTATATATAAAGTAAAAATCTTAGTGAGCAGCTCCACGACGAAGTACCAAAAAAATTACTTTTTGGCTGCATTCACTGTTTAAGTCGAATGCACACATATTCAGTCATTTTTTCTTTTGCCCTTTTTCTAGAGCAGATTAGTCCCAAAGGCAAGCGAACGCTGGAACTCATCATAGCTGGATCGCTAAAGTCGAATGCACACATGCTCAGTCATTTTTCTCTTGCCAGCAGATTTGTCCCAAAGGCAAAGCGAACCCGCTGGAACTCATCATAACTAGATCGTATTTAATAAAGCGCTACTTTTACAATGATGCTCTTCCCGTAACCTCATCTCTATCTATTACACTCAGCGACAGCCACCAACGTTGGAGGTCGTAGTTCTGGTAGGAAAACGGGATTAGTGTACAGTACACTGCAATTCATGTGGAATAGCTAGCGAGAACACACATACGACATTTGTATGGGGATGCAGCTGTGGCTAGCGTGCTCGGCAACTATTAATAGCAGGGAAACCGAAATATAAATATATAATATTTGTTGACTCATGTATCTGTAACGTATTTCGCAAAATCTAGATCCGTGGTGATATATTTTTCGAGCTAGCCCCAACAGTGATCGTCCCTGATATATTTGGGTTTTTCATCAGTTTTGCTGCGTGCTATTTTGGGGACCGATTTAAATAAAGCAGGCAAACGTTTGGGTTTCCGGGTCCGAGTTAACAATATCTGATCCGAATGATCAGTACGACGTCATTTCTAGTCGATCATACCTTGCGGGTACCTTGAACGACTACGGCGAGTTTGGGATATGAGTTTTTATAGTAGGAAATATACATTAACCGAATATCACGATTGCAGAGTATAACGGTGACATAATTGCCAATGGTAGCTAGATTACGCATAATCGCTGATTCGAGGTGTCTTAGCGAATGGTTTTAAGTGGGTCACTTATCCGCCCAATGGCAGCAATTCTGGCATATCCTTCACAATTATAAGTGTCTCTGGGACATACCCAACCCGAGATAGTCACCATACATTACCCTTGCTCTTATTTCGGAGACTGTCAGCAAATTGGGACCTATACATTCGATCTCGGACCATCTGTTTCGTAAATAGATGAAAACCCACTATATCATACCGCAATGAACACCGTTTTCAGAAATGATTTACAGCCAGCACACAAGCCCCTGTGACGCCACGCCATGTAACTGTGTCGAAGGTCTCTGTCGACCTTGATGTGAAGTTCGAGAGCTTCGATTGCGTGGAGCGCTGTTTGGTTGTAGACCTGGATGCGAGAAATGACCTCATCCATGGATTGGCTGGAACGCCATGAGCCGTGGATCGACTGGAAGTCGAAAACGCTGGGTGCTTCGCACCCTGCTCTAGTGGAGCTTTGGTCATGAGCCCACATCAGCTAGGAATTTTGGCGCGAGCACGAGACCGAGGCGAGTGCTGGACATTGGTATGTCGGGAATTGCTAGCAACGAGGTTGCTCTTTCCCCTTAGGGCGTACGTGGTGCGGCACACTATCCGCTGAGCGGTGCAGGTCAGGTTTATGACCAGCTGCAGGGTCATGGTGAGGACGAACGCGGAGTGGCGAGTAATTCACTGAGTGGTACAAATCTGGTCGGTGGGTTTGCCGTTGCGTGGCCCTAGGGCCGCGGTCGACTGCGAGCCGAGGCATCGTGGGCGCGGACCAGGAGATGCAGGTAAGCCCTAGCTCATTGTGTGAAGGTCAAGGACCTCTAGGCTCTCAGCCGAGTGTCTTGCGAGACATTGTATCCTCTTCGAGGAGTGACAATGTTGCTGCACCCGGTGGCGGTGATGGAGATGCTGCCACACCGCCCTCATCCGGTCGGGCTCGGTCGCGGTGGGAGCGAGAGAAGAAGACGCAGGGCGTTTGTGACGTCTTTGACGTCGGACGAGGTGTCCAACGTGACGAGCGGTGAGGCTCCACGTGATTGCGGTGAGCAGCTG

>Contig_77

TGTTTCAGCCCATTTGATAAGATAACACTCAAAATCATTCACCCAGCGTTTTACGTTCACTTTTTCGACGTCAAATTCATCATTCTCTTGCTGGCAAATAGACCACCACCGCACATCAATCGAATTTCTGGCTTTTCGGTGCTTGGTGGGCGGTTCGTAGTGTTGGAATTGTTAGTGAGCCATTAGAAAGAGTCGTCGAGCCTGCGCTCCATGTTTCTGACGAGTTTTCTTCCTCTTATTAGCGGGTGGCATTGTAAAGAAACACTTCTCCTCTCTAAATATGACTTTAGATTGGCCTAATTTCACTAAATTTTTCTATTTTTATTTCTGAATTAGAAATCGCACATTCACGTCGGTTAAGCAATTTTTCTATTCACGATGGTTTGTAGATACTGTATGCATCTCCTCCTTCAACTTTACGCTTTCATGTATGCAATTTCGGCTCAGCGTATCGGTCAAGAGAACTGCTGGAGTAGATTTTATTAGCTCTTAATACCGGTATGAGCCTGCCGAAAAGGACCGAAACATCTGGAGAAGGACGGTGCTTTCAAATCTTTAAATTAAGGCAAAAGTAGTATTAATTATTAAACTTCATGCTCTAATTATATACTATACAGAACAAGCATTTACAAGGCGTGGAGTATTAAAAAAATATATATATGGAGAGTAATGGGAAAATCTTACTTTTTTACAATCCAATTAGCACCACAAATGCGAACTTATGAGCTAAAGCTATCTGTACTTTGTAAGGTTACTTTTTTTAAAGATTAGAATTAATCCAGTACATATGTACTGCATTATGGAATTTCCTCCTTTTTGCTGTGGTATCCTCTCCAGTCCAACTGTACCGTAAACATCGGTGTGGACATGTAGTACATTTCCAATTTTCGGTACGTTTTTTATTTGAGAACATCCCATGTAAAGGTACGATGAGATTATCATATGTGATTTGCACTATCTGACGCTTCCACAGGAACGTCTGCAACACATACAAACGACCACTTCGGTATTATTCAGTAATCTTTTGACTTGGAATACAGAAGTCCCCCTCAACGTGGCCGTCTGATAAATTGTCAGAGGAACATCATTGCTATTAAGAGATTTGTCCAATGTATCTTCAATCAATGTGATCCGATATAAGGGGGTATTTGACGTGCAATCTACGCTTATTAAGGGGGATTCATCACTCTAAAGGAAGAGAGAGTACTGTACATACAGATTGCACGCTATTAATACAATGTACTTCGAAGTACGTGGTACACGAACAGATTTCTCTAAAAGTCTTATAAAACATGATGAAAGACTGCCGAGGTGGATCGTCGTATGTGCGGTACATTTACTGTACTGTACAAAATCAGTACTTGTAGCCTACAATGGACAGAACATGCCAAACACATGCTTCAGCACACAGCAAACCATTTGACTTCTGTATTTGTTTTACCTTCTGAGTGTCGCAATAAAACATAATCGACGATTAGAGGTCACAGCAGAAAAGGGACCACCTGGTCTTCGCTTGGCCCGCGGTCGAGATAGAATGAACTGGAACGTGTAGTGCTCGGCTCGCTGGTGAGCTCAGCGCTGAGTCCGCCTCCGCAAATACCGCGCGGCCCAGCTTCGCGGGCTCCCCGTGCGGGTTTGCTGCGGCTACGCGCCTCGCAACCGCTCAACTAAGCGGCGCCGCGCCGCGGCCGAAACCACGTCGCCCAGCCCCGTGTGTATTATCCCGCGTGTTTCTACTAATTTCTGCCTACAATGTACAGTCGTGCACCTCCAATTCGGCAGGGACAACGTAAAGCAAAAAACAAACGCAAATCCAGTGCGCTGATACTTTATTTGTTAATTCAGTACAAAATTTACGCCAATCTTTTGCATAATCTTGGCGTTAGACGCGCGAATCGCGGGCTCACAACAGCGCTTCCGGTCGCGATAGGCGTCCGGCAAGGCGGCAACTTCCGCCAAGCTAAAGAAAACTCTGGACGGTAGTGCGCTTTACTGCTTGGGGGGTCTTAAAGCGCTCCGTTGCCCAACGAGCAAGTTCGGAGTGAGAGCATGCAGATCTGACTTGTGCTTCTCGCACAAAGACTGCTTTTGTGGGACATGTAGGAGGAAGGGCGATGTCCCATTGTCTTTGGCGGTAAAAATCAAAGTGTGTGTTTGTATGAAGACGTCCGATCCAAAATTTAAAATCAGATCGATGTGGCGTGTCATCCCTAGGCGAGGAGGGAAAAACCGTTGAGGTTAATTGCTAGGGTGGTGATGGGCAAAGGTAGTTGCGCATCCGATTCGCGTAATCATTAAATCTGGCAGGGTATCGACTGATACCGGTGCCGAGTTGGAGGTGCACGACTGTAGGTGGTACATGTACAGTACGTATATGTACGGTCAAAAAGCTTTGATCTATCTCAAGCTTTGAAAAGGAAGCTCACAAAATATCAAAATGATGCATTAACCACCCGTTGTAGGCTACAATGTAGCATACATGTCGACGGCGTCTCCACTTCGGAAAAAGATGCGGTGGTGCCGTGTCGCGCTCGTTGCGTTGATTTCCCTGTTTCTTCCCAGCAGCGACGTTCTTTCGGAGGCCAAGGTATCGACGGCAGCGTCGCGCGCTACCTTGATATCTGCTAACCCCGTCGTCGCTGCGACTGCCCGTAGAAGGCATTTAAGGACGGTAAACTGGGACAACACAAGGGGTTCCTCCTTCCAGGACATCGATCGCCGTGATGGCATCAATGGCGACTTCGAGACTGACACGCCCCACGCGAACGACCGCGAGGAGCGGGCAGGGAACGGAGCAGTTCTTGAGAAACTCCAGTCCCTCAAAGCATTTGAAAAACTCAAATCCTTTAGACCGATGAAGTCATTCTCGCGTTCAATGACCAAAGCTACGAAATCTTTTCGTGAACATGTTACTCCGCTGTTAGCGACAAGCGCGAAGGTGGATGTATGGCGCAAGAACAAGAAGTCAGTGTCCTTCGTCAGGAACGCGCTGGGGCTGGACAACCTGGAGGGTAGCGCGCTCACCGGAGCCACGAATTTCAGGTTCTACGACGACTTCGTGATCTCCCAGATACCAGTCTGGAAGAAGAACGAGTGGACTCCCAACCAAGTTGCCACTGAGTTGGGTATTTATGGGCTGCAAGGAGCTGCGCTTACGTCCAATCCTAATTTCAAGTACTATAGCCAATTTATGGAGCAGCAAGCATTGCTGTGGGCTAAAAATGATCTTGACATCGACAATATCTTGGTTCGGCTAGAGCTGAACACAGTGCAATTAAAAGATCGCCCACAAGCTGAAAACTTCAAGTTCTTCGAGGAATTCGTGGCTGGTCTAATGAGGTCCTGGATGCAGAAAGACACACCTCTGGCCGACGTCATGGTGAAGCTGAAGCTGGATAAGTTGACTGGAAACGCGCTCTTGAGCCACCCGAACTACAAGTACTACAAAAACTACGTGAAAAACAACCTGAAAGAGTGGGCTGCCGATCTGAAATCCTATAATTTCGCTACGGAGAAGCTGGGCATGAAGAAGGGTCTGTCCGGGAACAAGCTGAAAGAACATCCGAATTATGTCTTCTTGGAGAAACTTGGAACACATGCGAACGAGTACCGAGAAAAGTTGTGGCTACAGCGACATGTCACGTCTTTTGATGCGTGGAACAGGCTTGGAGTAAGCCGAGTACATCCAACATTGCGTTTCAAGTCGAGCACGTTCCATCTCTACGAGAATTACGTCAACCTGGTGGACGATACAATGATTCAGTTGATAAACAAGGGCGAAACTAAACTGCCAAACCTGGTCAACAAAGAGGCATCTGGTCGAGAACTAAACGAGAAGGCGCTTATTTGGGCCAAAAAGGAGAGACCGGAGTGGTACGTCAAGTTTTCCTTGGGCTTGGACGGCTTGGACGAAACTGCACTGAAAGAAGCCACCAACTACAAATACTACCAGCGCTATCTACAGCACACGCACTGAAGCCGTCGTTGACGTTGAATAAAGACTGAATAAATGAAGAAGTCACGAGCCAGTAATGTTTTTTGCAGCTTTCTGAGGTACAAGCAGTTTTAATTTCTGTTTGTAGCATCTCCACCGGTCAACAAAAACTTTGACAAGAAAAACTCAACAAATTATCGAGGCTGCAGGCGTTAATACTACCCAGTGCACTTGTTTGGAAATGTGCGTTATTTTCCGTGCAGAGGTTGTAACAAGTCACTTACTACAGTAGTAATTCTGAAACTAAGTCGACGTACATTGCATGGAATATCTTGGAATATTCGGAGCTGACGTTTTACTCACTTACCGGTGCTACCTTGCACTATGGAAAGGAAAGTTGCTGACAAATATTTTTTGCACAATCATGGCTCGCGCTTGCATTTACTTCAGGTTATTATGCTATGTTCTTCGAATTTAGAAAGCGCTCGTAGAATTTTTAGTTGACTGCTCTTTTCAGTGCTACGTCATCCAGTCCATCGAAGCCCAACGAAAACTTGACGTGCCACTCCGGTCTTTTGTTAGCGATCCAGATAAGTGCTTTTTTTGGTTAGCTCTCGCACAGACGCGCTGTCACTGATCAATTTTGGCAGTTTTCTCCCTACGTAAAATGCTATCGTCAAGCAGAGTGACATACTTCTCGCTGGCCTTGAACATGTCTGAGGTTCTGCTCAACCTCACTCTTTTCCCACGCAGCGTAAGACATGACACCTTGTGTTAGCCACTCCTTTTCTCACTATGCATCCGTGTGCGTCCTCAGTTTCTCCAAGAAGATATTATTCGGGTGATGTTTCAGGAATTGCCCTCGAGAATTCTTCAAACCCAACTTGTCCTCAGAGGATCTCCGATCGGCGGCCCAGGCTTTCAAATGGATTTTGAAGTTGTTTTCGTAGAATTTGTAGTCCGGGTGCTACAACAATTCAGCTTGTCCAGCTTAAACTTAGTCATGACTTCGGTCATTTGTAATTCTTTGTCCTTCCACGACCTCATCACACCTTCCACGAACTCTTCATAGTGAGGATCAGAATGGCAACTGTTGAATAACATAGTCATCGTAGAACTTGAATTGGCTTGAGTACACGCAACATCGTCCAGCTTATCCGGTCGCAGTTCCTTCTTGACGACTGAGACCGACCTCTTGTTCTTGTGCCCGACTTGTAATTTAAACGTCAGTGATAATGCTTGGGCATTTTCTGGACTAAAAGAGTTGCTGGCTTTCGACAGAGCCCGCTTATGTGCTTTCAAAGACTGAAATTTCTCAAGTGTCTTGAGTTCTCTCCTCTCGACATCTTCTAGTTGTCTTGTCTACTTCCTGTTCATATCGAACGCTGGTCTTATCCGACTCGTCCACCCTCAGAAATCTTCGTTTCGCGTTGTCAGTGACGATGGCATTGGTAGATATCAAGGTTGCGGGCGAAGACACGGCCGACGTTTTGGTCGCCAAGTGACCATCGCTGCTAGCAAGGAAGCAAATTGACATGACGAGAATATGACACCACCGCATCTTTTTCGAAGTGATAATTTGGCTGTGCTACTAATCATGTTTTTTAAATTGGTGACCTGACAAGTCAGATGATCAATAGTAGGACTTCGGAGTCCATAGAATATATCACAGTACTGTTGTATTAGTCGGGCCGCGTTTCGCGGTCTCCTATCGACTTCCTTGAAAAGTTACACCAAGCAGCAGAACACAATATCTGTTTAATCTGACCCGTGTTGAGGTTAGGGCCCGTCACAGGGACCGCCTCTCCATCGTGCTCTCTATAAACTAATAATGAAGAGACACGGTATTTTTGACTCTAATAAATAATGTACTAAAATGTCTGGAACATTTTAGTACGATGAATGGTAGCGGCAATTCTAGACTATGTTTCTGAATGCCGCATACTAATTTCTTGTTAGTAGCCCGCTACTTTCTTAAACATTAATCTTACTTCTTCATTTACCGAGTCCACCACATGCTCTTCTTCTTTTACCAAGTCCACCACAAGCGTCTTCTCCTTCTACACTCAAGACTTCCTCTCCACTATGGCCGCCTAGACCACTCTACTTTTGTCCTCACTCCGTACTGTACTACCCTAACAACTCTGCGCAAATCATCCCTCCAACAGGTTATCAGCCCAACGCTTCGCCAGGGTACTGACGAGCTGAAAGCGGCAAGACGATGCGCCTCCAAGCCCCAAGGTTCATGAAGGAGATCGGAGAGAAAATCGCCCGACGGACACCAACTCGCGTGAGGCCACGTTGCCAACTTGGCTCCAGCCACTGTGCTGTAGCACAGCATGCGTAGTTATTCGCCATGCTGGGAGGTCATGAAACCCCCGCGCCAGCCATGGAGCTGTAGCGCCGCACGAGACACATACATTCCGTGCCACACCGGCCATTGCCGAGTTGTGAGGCCATGATGCCAGACCAGCCATAGCGCTGTACTATTGATGCTGGTTGAAAAGCCGTACTAGAAGGCCACAGTGCCTTGTGCTCTACGTACATGTACGCGTACACGTACACGTACACGTACACGTACACGTACACGTACACGTACACATACACGTACATGCATTTGATGATGTACAAGTACATGTAGTACATATTTAAGGCGAAGAACTAAATACCGCCCTCACGAACCAGCAGCATTGCTGTAGTGTCACGCCATACTGGGAGGCCAAATGCCCCACTTGCGAGCTATAAAGCTGTGCTTAAAATATTCCAGCTACAAGCTGTGTATTTACTACTAGTATATGTGTACTTAATGAGATACCATTTTAAATATGTACATCTCGAGCTACAAGGCCGAATGGCACAGCTAAAACCTCGCTTAATGCAAAGCTGCAGCAGTCTACTAAATGCAGATCTACGGCTTGTTGAGAACAGGTAATCCACTACATTTTGGACCTGTAAAATAAAAATATACCTTAAAAAAACAAGAAAGAGATAAAATGAAAATGGGCAATTGTCCATACAAACATGTGTCTCGTATTTAACCAAGTTAAGAAATACGCCCAGAAATGTATGAAACTGGGAGGCAACAACCGAAACAATTCATTCAATTCGCCAAACTTAAAAAAAAATTATTGCGGCAAAAGTTTCAAACATTTCCAGCATAACAGTACCAAAAGAATGAGAAAAAAAATTCAACACTTTATGACTACAAGACAAGCTACAGAAGCCTCTGTCTACAGAAAGCAAACTATAGTTTTACTGCATCAATTCCATCTACAATGAATTCGATAGTTTGCAAAATTTATCGCTAGCGCTTGCGAAGTAAAAATAAAATTATAAACTTCGAAACGAGAAACAACTCGATGAGCCTCTAGGGAGAGAAATAACCCCAACGACGAGAGCCTGCACACTTACTGCAAATATGATGCGTACGCTACCGATGACACTGTTTGATGTTATTTATGCTTTTAAAAGCTGTAGCCTTTCTTCAAAAATATGTGAAAACTATAAGCTCTCAATAACAATAGTCAGAACAAATTGAAAATGACTGCAGCACCTATATCAACCATTTCATGAAATTGGGAGGCAATTCTATAAGACCTCAGCAGCGNAAAAACAAAAAACAAACCACACAGTGTGAAGAAATATCCACATATGAAAGAGCAAAACAATGGCAGTTTAAGAAATGAACAAGCTAAAACGCTTACACAAATATATAAAGATAAATACGGTGATCAGGTGTATCCTCAAATGTTTCCTGAGTAGCCAACAACAGAGCGAACAGGTTTTGATCCTTAAAATTAAAGGGTTTCCCTGAGGTAGCTTCTACAAATTCGTAAAGCTAAACGATAACAGCAACAATTCAGTGAAACAGGTTTTGATCCTTAAAATTAAAGGTCTCCCTGAAGTAGCTGCTACAAATTCGTATAACTAAAACGATAGCAACAACAATCACTACGGTGAACAGGTTTAATCCTCTAAGGTTTTCCTGAGTAGCCAAAATAACAATGAAAACAAGAAATCAGCACAGTTATTGCCAAAGCAAAACAGCACAAGGCGACAAATCTGACAATGAAAGTCAAAGGAAAAAGATCATCAATATCAAAACAAGATCTACTTCTTTGTAAATGCTCATGTACGTACAGGGACTGTTATAACAGCCACCTGTGGTAACTATCCGACAGATAGAGCATCATACACATTCCCTCGATGTGGGAGGCTGTCGAAGTAAGCATCTCCGATGTGATGATGACTATCTAGTGAAATATAACTGTACTATTTCATATAGAGAAATCGTAGTCGAAGGTAGCTAGCTTTGGGGCAGCCATTGTAATGAGATGCACGGCGATAGTAGGGTCCATCCGGGCCACCTGCGCCGACCGAGACATGATCCTCAAAGCTGGCGTTAAATAACTGAGCCTCGAGCCAATCTGCCTGGTCTCCACAAGTATCCTGAATGACGCCGACTGAGCGAGAATGAAGGCGTTGCAATACCCAAAGCTGGCTGTTGAAGCACCGAAATTTGTTGGTGTCGTTGGCCTTGACGTGCGTGTCCTTGACACGTGCGTGCACCTGGTGCTGGTGACAGTAGAATGTAAATTGTGATGTGAGACTACGAGATCAGCTCGAGTGAGCCCCCGAGCTGCTGATTGATCCTATACCAGCGCGCCGGACCATAGAATAAGACCTTATGCCGTCCCAGCATCATCTGCGTTTGTCGTCTCTACTAATTCAAGGCTGTCCGTCATGTGGGCCGATTCTCTTGGCATTGAGGTTCTCTAAGTTAGGCATACACGCTCCGATAAGATGGCCACTTGTTGTAACTGCTATTAACTTCAATACAAGTGGTACTCTCAAAACCTGTCACTGTCAAAACTAATTCGTTTTGAACTTTCGAGTCATTTTTCAGTCTGCCACCTGGTGCACAGGTGGCCAAATGGCGATTTGGACTCCACCTCCCCAGAAAAACTGCCCTGGCGAGTAGAGTCCAAATCCGGTCTCCGTTTGGCTGTAGGGCCTCTAAGAAACGCAAAAAACACGAATTCGTGTAGCAATTTTGTAAATTGACTGAACACTTTTAGCAGGGTTTTCGTAGACCAATCAAAACACGTGTATTATATTATTCAATGACGTTCCCTGATGTCGATCCAGGAAGGCTGTTGAGGTTAGGGCCCGTCACAGGGACCGCCTCTCCATCGTGCTCTCTATAAACTAATAATGAAGTAGACACGGTATTTTTGACTCTAATAAATAATGTACTAAAATGTCTGGAACATTTTAGTACGATGAATGGTAGCGGCAATTCTAGACTATGTTTCTGAATGCCGCATACTAATTTCTTGTTAGTAGCCCCGCTACTTTCTTAACCTTAATCTTACTTCTTCATTTTACCCCGAGTCCACCACATGCTCTTCTTCTTTTACCAAGTCCACCACAAGCGTCTTCTCCTTCTACACTACAAGACTTTCCCTCTCCACTAGACCACTCTACTTTTGTCCTCCACTCCGTACTGTACTACCCTATTGACAACTCTGCGCAAATCATCCCTCCAACAACCCGGAAGAGTCTCACTGTGAGGACTAATCATACGATTCCATCGGACGCAAAAATCTGCTCCCTCAAATATCGGTGAGTACAGATGCGTAGGTTTTGTCGTCCAACTGCGGACAGTGTTAGTGTGATGCACTCCTCCTGCCGATTGAGGCTCTGTAATTGGTTATCCAATGGAATGCGGATAAGAAACCGCTTTGAAAATCCGCTTTCGCTGAGATCTCAAATTTAAATGAATACTCGAAGGCAGCCAAGTGAGTCAAAAGCTGCTCTCGAGTACTTTGTTCCTGCTTTTAATTGCGCGAGCAGCTGCTCGTCCGACTCGGAGGCGGCTCGGAGCTGATTTGGTGAGTAAACACAAATACCATTTTCAAAATGAACTACATGTATTAATACTCCGTCAATCAAATATTCTCTCCGGATAAGGCTTACGTGTATAGAAATTAATCGAGCTAGTGCGGAATGATACACATTGTACCCTGTTGTTGAGTGTACAGTTCAGTAAGGACCCCCAAGCTTGGTTGCCAAGAACAATGCTCGGTGGAAGTTTAGCTGATTTGCAAGCCCACCGACAGTGTCTATTAATACCGCTATACGGTATCAAAGGGGCGTTTTGCCTCTGTTCTAAGACATTTTAAGCTTGGTGTGGCTAGCTTAAATAGGTAATTGGATCAGAACAAGCTTTTTATGTACCATACATTCAGTTACTGTACTGAATGTATGATTCACAAAAAAGCTGCGTACAGGCAAGTTGGCAATCAAAACATGCCCTGTTTCCCTTGCTTGATACAACAGTATGTACACTTTATGGTATCGCGTTCACCTGTTTGCATTTAGTAAAACATTTCGAACCGAGCGTTTTCTTACAATTGGTCAGCACTGAGACCGGTCGTTTACGGTACATACCGTAGCAAGCCTCGCCTCAGTACGAAAGGTGTAGTAATTATAATAAAGTTTCCACTTCTATTAAATGTTGCCGGTAAGACAGAAGTTAACAAAATGCAAAATAAATCTGACGAGATTTTCAAACAAAGGCTACGATACCGGTAGATAGCATACAGTACACCATTTTCCAGATCAAATAAAAGAATCTCTCCCTCAAAAATGAGAAAAACACAGCTCAAATTGAGAAAGCCCGCAATCGCTATGTAATTATACCTCACTACAGTCACCTTTCAGTACTTAATAATATGTGTCACACCTATCGACTCTAACCGTGCTTCAAATCTCACCCCTCAAAAAACGATTAAGACGGGGCATTTCGTCCGTCTCTTTTGGATTTTCTTTTTTGATCTTGGGATTTCCTTTTTTGAACTAGAGGGATGTCTGTTAGGTATGGCTACATGTTCCTGGAATATAATGGATGGTGTTTTTACGATGTAATCTACCACTCCGTATTAAAATGAAGCTCCATACATGCAGTTTTGGCTGCATTAAAACAGATGTCATTTTTAAAAGGGCTTGATTTAGTAAGAGGGAAGAACGGAAGTTGCGGCAGTAAATGAACAAAACCAAGACAATAAAGCTCATTAATAGTTTTGTATTGCGATAAATAGGATGACTTATGGCGGTATGATAATTCCGCTTTTTGACCGCCAATACTTATTGCCAGATATAAATAGGAGCAATTTAAAAACGATTGCAACCCATATCGACAGGTACAATGCTGTCCTATTCTACATCAAACATGGGGACCAGAAATGACCCAATTACTCGCCAAATCATCGTAATGGGACTATCATTTTTTTGAGACCTGGATAGTGGTACAAATAGCTGAGGCCACGTGCGACGAGCAGACTGGGTTTTTCGATACTTAATTATACCGTAAACCGTCAGGTCCTGCACACTTGCGACGCCAGTATTTTTGTGCTTCAATACGTAATTTCGCGAACATGCTAAGCCAAGCCAAAGCGAGAGCTTTCCATATTTCAGTGCTTATTTTGCTGAGGAACTAAACAAAAAAATTGTGATCGCGTGATAGCTTTAGTGGTATGGATAATATCAATTATGAAAACAACCAGGTGATTAAAACTATGTTTATAAAGTGTTCGTCAATGTTAAAAGAAGCGTTTTTTTTAAAAGTCGGGGTCGGGTTGCTGTCTTTGATATTGGAAATACTCAAAAAAAAATGTTGGCTCAGATTCGCTACTGAAATAAGTGATTGTAATATCTAGCAAAGCGATTTTTCTTTCTCAGTGAAAACACTCGGATCTCAAGCTTTGCCCCTCGATACTAAGACGAGGACGCGTAGCCGCAGCAAACCCGCGCGGCTACGCGTCCTCGTCTTAGTTGGCGCCATAGCTGCGTCGCCCCTCCCCGGAAGGTGCTATTATGCGTTTGAGGTGGCTGCACCACCGTTTCGAACGGTGTTTTTGGTATTTTGTCCTTTTTGCCCACTTTGGAAAATTATGCGGTGGTATCACGTGCTTCTCGTCGCGGCGGCATTCCGTCCAAGCCACCAGCGACGCTACCCCCCAACGTCCACACTAGTCTCACCCCCATTTCATCGTCGCGGAGAATACCAAGCCAAGATTTTTAAGATCTGAAGACTTATGTTACGGGGACCGGCAGTCGTTCAAGAACGGTGAGAGCAAGAATGGCGTTAGTGGCAATTCCGGACGAGAGGGGCAGGGGGCACTCGAAAAGCTTACGTCGTCCAAGACCTTCAAG

>Contig_79

TAAGTGGTAATACTAGTAAGCTGGTAAAATCATCAGCACAGCTGGCGATCTCGAGCCAATCCAAAACAATATGTTTCGCAAAGGCAACGCGTAACCATAAGTCCTATCAGCGCCTAAAATATACCAACTGCATGCAATGAGCACGCTAAATTAGTGAGGACTTGAAGGTGCCACACTTTCCTTTTCGACGGGTGGCTGGGAACGTTCAAGAAACGACATACAGAAGTCACCCTTAACTAGCGTAGGGGGTCAGCTGATAAATTTGCATAGGGAAATCAAAGCGATCTGGTGATTTGTCTGTAATTTCTTCAATTCCTATGATCAAGCGGTATATGAAGTGCAAGCTACGTTTAACAGGAGGACTTCTGTATACGAACGAATCAGGTTGTGTATTGACGAAGTGCTGTTGTGCCTTTCTCCTGTGCTCGACCGATACAAGGTACTAAACTTATACATGTAGGACGTTTTAATTATACTGAGACGGGACTGTTTCAGGAATGGCCGCAGATTTACACCAGATATAATTCATTATATTTGGTCCGGTAAGCTTGGAGCGTCCACGTTCTCAACACAGTATAGATACTGGAACCACCGAGATGTCTATTCTGAAACAAAAAGCCAACAAGTTACATTTATAGCAATATTTTATATTGCTAATATATAATCACCTGAAAATTAATGGTTTTACGAGTTGAAATAATAATTATATTTAGATCAAACCTGCTCCTGAGTTTCATGCACGGTTATTATAAGATGGTGTGGGGAACGCAAACTAGACGTATAAATAATAACGCTTAATATATTGTGTAGTCTTTGACGAACTTCGAGCAGAGAATACGGTGTTACATTTTCTTGGGGCCAAAGTTCAGGTCCTTTATCAATGAGCTGACGAGCACTTTACTGTTTATATAAACTGACTGCCTAACATGGACGGCTGCGCAACTTAGTTAGCAACCAATTTAGCTGGGTTTTGCGACTTCAAAGAACTGAGTTACTGTATATTGTTGGAGGACACTGTAAAAGTGCTGGCAAACAGCGCTGGTTTGCGTGATGTTGGAGGACGGCAAACTTTTTATGGTCCTAAGTTGCTAAAATGACAAACATCGCTGGTTGGGCAACTTTGTTTAGTTGGCGTAATGTTGCAGCACAACATGCTTTTAACTTGCTACAATAACAAACACCGCTAGTTGCGCATCTTTGTCAAATAAATGAACACCTGAACATGTTCAGAGACAGTACCGAGACGATTAAAGTATTTTTATGAGCAATGGATTTCAAAAATACTCACTTCATAGTTTTAATTTAAATTTATAAGCTAACTTTGAGCATGCAAAAAAAAAGGAGAAATAACTAAGGCAGGATATTATGGGTAAATATAGCTTGACGATGCTGGAGATAACATATTTTGAATTTTCGCACAACCTCGAATACTCTGAGCGGTAAAGCAAACCTTTCAAACCTCCACCTACAAAAAACAAGTCACAAACCAATCGCGCATCTTGGTATAACATTTTGACATGTGACTCACGGTATATGCCATTCAGAATAATATGATATTGTTCATGGTCAGTCAGCTCATCGCCCTCCTCGACCATGCGCTTTGCCGAGGAACAATATATATCGGGTTACAGCGTTTGCTCGCTCGCTCGCATCGAACAGCCAGAGACTCGCTCTCGTAGCCGCTCTCAATCTACTTCGGTGTTGTTTTGAGCTGAACTGTATTACAAAAAACGATCGTTTATTTGGTCCCAAAATGCCTCTATATCGTGCAAAAAAGATTTATATTCGGGACATTTGGGATGTATAGTTGGCTCCAGTTAAGAGCTACTACTACTACTAAGTCGTAAATTAGAGCTGCCGCCACAGGCAACATTCTTCTGTGTTTTTGTGACACCCACCGGGCATTAATTATAAGAGGTCAAACGATCAAGTTTCGGTACGGAGTGGTCCATTAATTTATCGTTTAAAGATAGAATGCCACCATATTTTTTCTGTATGACCGTGTGTGTTTGGTGGTGGTGGTTGANGGGGGGGGGGGCTAGATGATACCGTAGCCGTGAGGAAGGAAAAAATTGGTCACGCAAGAGGCGACGGCTGAGTGGCTTTTGAATCTAGCACCAAAGATAATGGAAGAACAAATGAAGTAGACCTGGGTCTTCATTATGCGGTCGAGTGCTACGGTAGCTGATCAAATTAGGCCACATCACGCCCTATTCCCTAAAGAATGCCTGAATTACCCACGTTGTGACTGCTGGTGTTAGCGAGCCAGCGGGTACCTTGACGACCGTCGGCGACGATTAGTTCTTAGTCCTTCATGATCGTCTCTCTCATTTTTTCGAGCCACGCCTGTAGCTTCATCGTTTCGAGGATAGACTTCTTGCGTTTTGCAGCTTCTTTTGCCTTTTTCACGGCTTCAACGGGATCCAGCTTCTTCGCAGCTTCCAAAATAGCGTTACCGATGGTCCTCTCTTCCTCACTGTCATCTCGATCGTTGTCGACACGCCGAAGGAATCTGCCGCCATCCAAAGGGGCTGACGCGACAACGTCAAACGACGCCCCATTCTCGGTAATTGGCTTGGGCTCCGTGACAGCAGGAAGCGCAGAGGTACCGAGGTGGAGAGTGGCTGCAGTCATCGTCGCTAGGATGTATGTCAGGCGCATAGTTACTGTTAGTTTTGAAGGGCTAAGACGAGAGTGTCGTTCGCTAAAGTGAGTCAGTTGTGGAATGCCAAGAGAATCGAGGAATGGAGCTCTCGGAGTTTATCGAGTTGGTTTGTCGAGAGCACTTGAGCTGACTCAAATACAGCGTTTCCAAGGCCAGCGTGCAATCGTCGATTGAGGAGAGTGGTCATCGATTGCCAATCGATGACCACTCTCCTGCCATTTTCACAAAAAAAGGCAAATATTTTAGGATTTGAAATCATTTGGTTCTATAATCAAATCAAACCTTTTGCTATAATTACACACTCGCAACGATTTTTGGAGCGGCCATCAAGAACATTCGCAATGGTGGCATACATACGTACGAGGAAAAAGAAGTCTAAGCACAAACCCACGAGTTTTTCGTGTCTAGACCATGATGCTAGATCACAGCTGTGATCTTGTGTTGGCTGTGAAAATCCGCGGCACTTTAAAATTGCTTATCCATAAAAATACTACAACCCCCCCCCCCCCCTCGTGTGTTGCACCCGAGGATGGGTACTGTATTACTTAAAATTGCAGTGGGCAAAGAACTATGTGCAGTAAACTTGCTATGCTCATCAAGATACAGTACCTCCGTATGTGATAGTACCGGTATGGAGCTTTAGTTGTTTACATTGGGAGCAGCAATACTCTGGATCAAATCTGAATTAATGACGAATGCAGTTACGAAGGGATGATTAAATACCTAATTCCAGAGAGTCTATGGTTTAGGTCAGTACAGATGTAGCCCATTTATTAAAAAATTACAGTTTACGGTGATCTAAATGGGCTAAATTCTGAAGTATTAAGTTCATGTATTTTATTCATCAAACACAACTGCATGATCAAGAGGCGTAAACAGCCAGATACGTAAGCAAGGGAAGAAGGAGAAGATGTGGCGTAGATGTGGCGTGCGAAAATAATACGTGTAGGAAAGTGAATCTGTCCATCAATTTGCATATCCAGTGTTCAACGGGTTGTAGAGTGGTTGTGTGTATATACTGTATATCCACAACTCACTTGAGATTTTTTTTACCAAGTGAACTTTGGCAATCAAAAGTGTCTGAGTTTTAATACACGACATAATAACTGTAGCATTTAAGTACCACTATTGTCTTATTAATTGCCCACAAGAACTTAACCCATGCAAATGTTTCGAATCGTAGCCACACACACATGCCTTGTCAAAATCGGTGGTGCGATGCTCACCCTCTTGAGTTGCTATTAATACTGTTGCGACAATGCCGCCTGTTTTACAATAGATCACCGTGTTGTTGTAGCGATCTTACTTGCGTTTCAATTTCGCTCGAATAGACGTGGGGCGGCAACACATTTTAGATAATTTTAAAACAGAGTAACCCCCTCATGCAGCTGCTGGAGGCCCTCGATCGTTTGTGCCCATTTAATCGGCCACAGAAATTAAACCTAAGCAGCAGAGAAGATTCGCATCCTGCGCGAATATAGACAAACTCAAGAAGCACCAGTAAATACATAATAAAATCTACGCAAATGAATGTTTCAGACCTGTCGACGTGCGTTAGATGATCTTTAAGGGACATTGACCGATCAGAAACACAATAACGCCAACATTTAGAAATGAATAGACGTGACAAAAAACCAGCATATCATGCCAGTCTACGCATTGTACGCTTATGCGTGCCGCTGAGACCGCAAGAACACAGTGCACCGCCGCTACTACAGGGGAGCGGCTTGCTTACGTGTTAAACCGATCGAGTTACGGCTGCGTGTTTTATCCAGCATTAAGTTTGCTCAATAGTTTTCATCCTTTACGGTATACTATTAGTTGCTACGGAAAAGCATGCTATAATTTTTTTGGATGAAACGAACACGAGAGATTGGCAATTTCCAAAGAAGAAGCGCATTTTTTAAGCGAAAGATGATACAAAACCTAGCAACATCTGAAATGCCACTTTGGACAAAGGGTCAAGGTACGACCAATACAATATGCGGAAATAGGTAGAGAGCTATTGCAAGCCAGAGTATGACCATCGACAGAGAATACACATCCCAAACCAGTTTGCTCTAAGAATTCTGGCCACGCGTTTGCTGTGCGGTTTAGACCTCAATTTCTCACTTCGCATTTTGACTCGCTATCTACACCTAAAGCATCCAGACACCAACAATGCGCCTAAATTACGTCTTACTGGCCGCTGCCTCGACCCTACTCGCCCGCCACGTCACCTCCACCCCGTACTCCGCTAATGATGTTGCGCTAACTGGTGTAATGTCGCTGGGCTTCATTCATTTTGTTGGCGCTGACCAAAGTGTAAGCGACCAGTCCCGGTTCCTGAGAGGCGGCAACATCGACGAATATGACAACGAAGAGAGAAGTTTTGTGGAGGTTGTTAAGCAAGCGATGATTAACAACTTTGTTAACAAGCTGATGAAGAAGAACTCGTTTTCGGATATCGAGAAAATTGACGATCTTGGGAAGCTCAAAAGGATTTCGGCTGCTGCAGATGACCAATTGAATTCGGCGTTCAAGCTGGCTGACGATGCGAAAATGAGTCCCGACGACCTGGCCAAGGTGCTGAAAGAAATGCCAGGTGCCGACGATGCCCTGACGGGTAAGGCAAAGGAAATGTACACTGAGTACTTGAAAACAGTTGGCGGAAGAGTGCACACGTAGTCTCGACGGCGAAAAGTACAAACCGTTTTTTAAAAAATGCATTGGTCCCTACAATAACAGGACATACATGTAATAGCAAAAACACTACTGAAAACGGCGTGCGCCACACAGAAATGCATAATTCAGGAAGACCTTTCCTACCTGATATAACCCGTGGACGACTTCTCTGTCAGGCACAGCAGCTTATCGTGTTACTCAGGTCGTTCGTTGAGTTAAAAATCGATATCAGGCGCCAGGTGAGGAAGTATGCCGGCTAACAGAAGCATAAAATACAGATGTACTTCGCTGTGCGTTTCCAGGTCTAACGTGGAGGCACACAGGCATATCATACTAAAGCTGTCTAAATGGGTTTAAGTAAGAAAGTATCTCCTGGACTCATGGACTCGTGGATAAGTAATCATTTGTTAAACTGCGGAACACCAGGTCATTCAATGCTTCTTCACCTACATGTACCTCAATTACATGTATACATGTGTAGACCTCCACTAATTTAGACTCCATGTCAGGAATACGTGTGAAAATGCAATGGAGCATCGCACTCTCCAAAAACGCATAAAAATGATCATCATTTTCCGATAACACGATCAGGGGTTAATGGTAACTCCAAAATCGGTGAAGTTAATATTGTGAAAATATCACCTGTAGACAGTTAATAAAAAAAATATCACCTAATATTTTCTTCTGAGACAATATGATATTATTAAATATCAACTTAATATTAGCTAATGCGATATTATATTTTTAAAGATTGCGTAGTCGGGATAAATTTACCAATCAGATGTCGGTAACTAGCTGAATTATTACTAATCAACCGTGAGGGAGGGACAATTCTGAGAGTGACGGCAAGGGTTGGCGTCGTGTGTTGGCACTGCTGAGCGCATTAATCTAGCGCCACAGGCAGCAGGTTTATTAGACGCACGATGTGGACGAGGAGATCGGTAGAGAACAAGAACAAATGTCTTGATTTCGGTCTATCAATCAAAGTTAAACACCTGCAAGCGTTTGCAGTTTTCCATCTCGATCGCCTTTGTCTGTGTCACAGTGAAATACTAGAGCAAATACTCGTCAGTCTTAAAGCCTACTGCTTTTCTAGAACCTGATCGAAAAAGTTATTTGGCTCCGCTCGGTTCACAGAGGGTAAGGTGGTAAGAAACGTACTTAAGTGGGAAAATATCATAAAATATCAAGTGTATCTAATATTTTTTATTTCTCCGAGTTAATATTAAAATACTATGCTAACTTTTGCAAAGAAGAGTTAATATTAATATTTAATATCGAAAATAGTACTATTACTATCAAAAATATCAAACGATATTTGGTTACCATTAGCCCTAGAGGTTAGATGGATTACGGTCTTTTTGTATTTTCGGGCAACAAACTTGTGGTGGACCATTGTGACAGATTAACTTTTTTTGGACTAGTTATAATTTTGGTACATAAATATATTTTAGTTCTGACTTCGACTGCATCAACAACACATACAGATCGTATGTGTTCTCGATGCAGGAATGCACTAGCTGCTCAAGTTCCGGGACATTTCCTTGAAAGTACTAGATGATTTATAAATATTTACCGACTAATATATAGGCCTTATTAATCCGTCGAAACCGGGTGCTGCATAATGCCGGAATACAATTTCAGTTTAACGACTTTTAGACTTAAAGTTTGTATGACGTGTTTTTTCGCTCGCCATAAGGCATACAATTTTTAAGACAACCAAAAATGTATGTTTCTGTGTGGGTCTTTTTTTTCTCAAGAGGTTAAACGGGGCAACATTTGTCTACCGGTAAATCTAAAGATCAACTTTTCGATGTTTGTGAGAATCTAGATGATTTGTTTCACCTACCGGAGGTAACGGGCTGATTCCACTCAGTGAGTGGTGCACAGCCAACGACGATCACCGCCTTCTCTCATTCGAGATTTTACGCCCCGAGGGCCGAAGACGCAGGAGGATGGTGGGAGTCCGACGCAAAGTGCTACTAGACTGCGAAGTTACCGCACGCGACTTATCTTTCAAGTCGGTGTGGAGAGAGCTGAAGAAGGACGGGTGGACACGAAAGCACCTTCGGGACGCAGTCTCGATGACCGCTACTTTTATATGCCTCCCGGTAAAAACGTGAAGGGGGGCGAAGGCGTCGATTACTTCCGTGGCGAACAAGCTGTATTGGAGCATTATGCAAAGGGTGTGTGTATAACTTGCAGTTGAATAGCTCCTTGCTATCTCTAATACCAAGTTTGTTGCTTGTAGAGCTGCGACGCCATGCGGTAGGCGTCCAACCCAGTGTTCCTGGAGATGATCAACTAGCTGCCGCAGCTATCGTACGTGAAAACTACGCTGTTGATATCGAAGCGGCAGAAGCAAGCGCTCGAGCTCAGGCTACTTCTCAACGTGTCCCCACCAACTAGCCAACTAACGGTGTGCCTCCCGTCCCCGTACGTGAATCTACTACCAGGCCACATTCACCATCACAGCGAGCGCCGACGCGTCGCTCTTTGATGAATACTTCAAGTCCTATGGCAAACTGCCCAGTTAGAAGTACCTAGCCAACGTCTCCGCTCACCCCGACGTCACCACACGTTCAAGAGGCCAACTGTTCATCTGAAGAAGCAGAAGAATCTGATAATATCAGCGGCAGCAACTCCACACATCCAGCACAAACTCAAACCATAAACGGTATGCTGCATTGTATACTGCATGCTATGCTGAATACATTGCAGTATAATGTACTGCATAGTGCTCTGCATATTACGCTGCATTATATACTGCATAGTATATTATGCTAATCCGAATACTGACATATTATTATGTGCTGTTTTTAGATGATGCTAATGAAGACAGAGAAGATTCTGCTCTTGGTAGCAAGCTGCTTGCCGATAGCACCGACGATTTGAACGTGGTTATTGATCACGCCATTGAACATCAGTTTGGAAAGTTGGATTCGGGTGCTGAGGCGGAAACCGATGGACATGGAGACTGGAGAGTGTGTATCGGGTGAAGAGGCGGGAATTTATTGTGCATCTGTCGACATTGGTGATTATCTGGCCGCGACGGAAGACGAAATCACTGCAGAGGTTCATTTCGCGGAGAATTTTCTTGATATTTTGGTGGAGAAAACGAAGTTCTCGCTGGAAACCTAAAGAACGCTGTCCTGCGTGAGATGTCCGCTATTGACTGGGAGAATGTTATTGAACCTGATACTGCTGAGTACATGCAGGGCCCGTACGACCCCGTCGACAATGCTTCAAGCTACCCTGGCCTTCGCCAAGAGTACTCGGGTCCCACTGCAGATGTTTTGC

>Contig_83

ATCCTGTGCCTTTCCTGCGGGATGAGAGGTTTAACGCGGCCCATATTTCGGAAGTTGCAACAACGAATGCGGCCTACCGCTTTTCTAGGAGTCTTCTAAAACAATTTAAAAAGAGCTTGTAGCGGTTTCAAGCTAATTGCATTAAAAATGAAAGAGCTAACTATTTTTAAATAAATGTGCTACAATCTTTTTTTGGTTGTATCTTCTCTCGTGGCCAGGACAACATTGCCTGTGACTTGAGGTTCCCTCATATCAACGCCCTGAGTTGGTCCGGTCATTGAAGTATGAACGTGTCTCAAATCTGATTTCCGATTTGGGGACAGACCCAACAACTGCCTTCAGTCGTTGCGACTTGTAAATGGGCACCGGAGCCTGCATCGTAAGGTTGCTGCCTCAACGGATGCACCTCGCGGTGCATGCGCGACACTGGCAGGGCCATTGCTCTTTGTCGCATTGCGTCCGATTGTGTACAAGTGTATACTTCTCCCAGCTGATCACTGTATCATGCCGTGCGAGTCACGGCATGCCCAATACTAAGTCAAACTTTTCGCCCAGCTCTAGGACGATAATATTTTCCACAAACACTCGGTGTTTGTACGAGAANNGCGCGCGCGCTTTACGCGCTTCTCCGTTCTTACGGTGGCACCTGCTGCCAACCGCACTTCCAACACATTTCGAGGTGTTTCAGCTCCTCGTAGTTTAGCTTCGGGAGACTTTCCAGTCGAGCAAAATTGCTCGACGCACCTGAGTCAACCAAAGCACGAAGCACTGATCTCGGGGTTGCTTCAGCTGGTTATTGCGCACTTTATCTTGGACGTGTTGATGTCGATGTCAAAATCAAATGAGGACTGATGTGCAGCTCGGTTACACTACGGCTTTGGCGCTCAAGTTTTTCAAAAGAAAAGAATGTCGTTCCACAACATCTTTTAAACGAACGACAGCGCTGCACTAAGATGATTATCGTCCGAATTGTTTTACTACCAGTAGAAGTTTTAGTACTACTCGAAGACGACGTAAGTTACTGAAGTACTCAGCACATAAATTGAAGAAGATTTTTAAACTACATATTACTATTGTCGTTTGTAGAAATATAATACTGCTGGATTTTACCATTTGCTGCCTTACTGACAATCCCTGCCCACGGCACAATACAGTAGAGCATTCTGTCAACGTTCCGTGAACCCGATTGGGTAGTAAGATCTGCAACCTGCTAATCTACCAATGCCACGATCATACAGTACATGTATCACCGAAATCGCCCTACATGTACAGTACGCATTTGTTTATTGTATTTTTCCTCATAAATAACTCCTATACCAAACTTGCCGTTACCCCGATATATTCTAGTACAGTACTGCACCCCGTTTTCGCGCCCATGGGCCTATGATAAACTTCCGGATGTGATGATCCTGAATCCAAATGAACGAAATATTAGAGCTCATTCGTACATACAGAGTATGTGTAACAGTACCATTCAAGAATTCTGGAACGCTGTAGTACAATTCTGGAATGGAGTTTACACATGTGGATAGGAAACATCTGATGATTTGATCGCTATATATTTTCATAGTTTGCATGCGAAGAGATAGTAGCCATAATTCGCCCTATCTGTTTGCTGAGATAATGCAAGATGTAAAATGCAGTTCCTCGGTCATTTCCGAATTTTCTATCTACATATAAAGCCACTATGGTACGCTCCCAAGACCGCCAAGACCGCGCCGCTGAGACGCTCAACGAGGCCGACTTTCGTGCCTCCACTGCCGAGTTATTTTAGCGGGAGGAACGTCGCAAGGCAGAGGTCGCTCAAATCGTCGTACATAGCCGCAGAGACGACAATGACGAAGGGGACAGCTCCGGCGTGGGAGCTTTGCCGTCCGTCTTTGACTTCTGCTTACAAGCTGAAGGGCCCGATGGGGTGCTCAAGCTCACCAACTTTGCGCCAGAGGAGCTCGACCATGTTTGGGCAGCTGTGTACCCTCATTTGCAGGGGCAATGGAACGTGGGCCGCGGTAAAAAATGCCGCTACGCTGCGCGCGACGTTTTCTTTATGACACTGAGTTCGCTCAAACATCTTGGAAAAAGGGACACCGTTGCTCGAGTTTTCAGAATCCCGCCATCCACCTTTCAGAAAATGATCCGCAAGTTTATGGATATGCTGTCTCCTATTCTCTACGAAATGTACGTAGAGAAAGCGAATGATCAGTGGACACTGGGGAAGATTGTACGATCAGGGCATGCATTTAAGGACTTTCCGTATGCTCGATATGCTACGGACGTTACCTTTCAGCATGCTAACAAACCCAGCGGTAACATGAGCGAGATCTTACGCTATTACAGTGGCAAACACCACTTGAATGGCTACAAAATGGAGGTGTCGGTGCTGCCGAACGGTGTCGCGATTAATTGCATGGAGCACACCGGTGGGAGCACGCATGACGCTGAGATTTTTCGCAGGAGCGCAGCATTTCATTCGCGAGCTCTTCACAAACACTCTAGTGATGCCAATGTGCGAGACGAAGGAAGACTACAAGACAAGTATCCGAAAGAATGGGCGTTTGCTAAAATCTGATTTAATTTGCAAATCCTATATGGTCTACCCCCGCGAAAACGAGGTGCAGTACTGTACTGATCATCGAGTTCCTATCTTGAAACAAAATGTGCTAAATCGGACCCAATTACCGTAGCCGCTGTCAGTCGGTCCCCGAGATAAAACAGCAAATGGAGCGGTAATTTTTTAAGAAATAAGATAAACCTTTTAAAAATAATTATCGCCCGCAGTGATCATACATCCCATCATTTTTCGGTATCTTAGCCTCGTTTTCGTCTCAGAAGCGAACCGTACCACATCGAATAGATGGTTGCCACAGCGTCACTTCGTCATTCTCTCTCTGAGCCAAGCGAAGCTCGGAGTGAGCTCCCCAGGTGCCGCCATGCGAGTCCCCAGGGCCATACTACTGACAGTTGTCTTGATGGCAATATCCGATACTGTCTCCTCAGCTGCGAAGTCCCACTTGACCACACCATGTCTAACACGGCACGATACGAAGAGGTTTCTAAGGGCTCACAATACCGAGGATAGAGGGATCAGTACCCCCAATGTCGAGATGCTGCAGGGGTGGCTCAAGAAAGGTCTGCTCTCCGACGAAGCCGTTGGCCTGTTATCACTCGGTCACAAGGCCGACGATTTACTTAGCGGTTCGCTATTGAGCGCTTGGGTCAGCTACATAAAAGTCTTCAATAAAGAACACCCTACAGAGAAGATGAAGACGATCTCGGCGCTCACCGCTCGCTTCGGAGACGAAGCTCTGTCCACGATGATTGAAACAGCTAAAAGGGTCCCGAAGACGGAGGACGTCGCTACTAAAATGCAAGCCAAGCAGATCCAGAACTGGATGACGCTTGGTAAAACCCCGGACGACGTTTTTACGCTGCTGAAACTCAATACCGCCAAGTCGCTTTTATTCGATCAGCCTCCAGTCAACACTTGGCTACAGTATATGGACGATTTCAGCAAGGCTAAACCTGAAGCACAGTTCTCTACGATCACGACATTGAGGAAATTGTACACCGACGATGTATTAGCCAAGATGATCATTGTGGCTGGTAAGAACGCGAAAACTGCGGAGGCTGGTAAAAACGTGGAAACGGCGTTGTTACGCACCTGGTTTAACGAAATGAAGACCCCGACAGATATCCTAAGGCTGTTAAATGCTCGCGAGACTGGGCAGAGTCGAAAATTCTTCGCGTCTATATGGACGAAATACGACGATTTATTCCAAAAAGTGGATCCCAAATTCAAGACCGACATGCTCAAGGACTGGCTGAAGAAGGGGTTGATCACTGACGAGACGTTCCGAATGCTAACGCTGGGCAACGCGGCTGACGAGTTCCTCAACGGCTCGTTGCTAAGCGCTTGGGCCACGTACATCAAGGTGTTTAACCAGGAGAGTCCTACGCAGCAACTGAGTTTACTCGCGACACTCACCGCTCGATTCGGTGACGAAGCTGTGTCGACGATGGTTGAAACAGCCAAGAGAGTGCCCACGACAAAGGACGTCGCCAATCGAGTTCAAGCGGAGCAGATTCAGCACTGGATGACGCTTGGTAAAACCCCGGACGACGTTTTTACGCTGCTGAAACTCAATACCGCCAAGTCGCTTTTCTTCGATCAGCCTCCAGTCAACACTTGGCTACAGTATATGGACAATTTCAGCAAGGCTAACCCTGGAGCACAGTTCTCTACCATCGCGACATTGAGGAAATTGTACACCGACGATGTATTAGCCAAGATGATCATTGTGGCTGGTAAAAACGCGAAAACTGCGGAGGCTGGTAAAAACGTGGAAACGGCGTTGTTACGCACCTGGTTTAACGAAATGAAGACCCCGACAGACGTCATACGTCTGCTAGGTCTTCGCACGCCCGGCCAAACGTCTGTAGCCCCAGTTTTGACCAAGTACATTGCGTTATTCAACAAGGTGGATCCCCGATTTAAGACTGAAATGCTCCAGAATTGGCTAAAAAGAGGTTTAATCACTGACGAAACCTTCCGACTGCTCACATTGGGCAACGCGGCTGACGAGCTCCTCAACGGCTCCATGCTAAGCGCTTGGGCCACGTACATCAAGGTGTTCAACCAGGAGAATCCAACGCAGCCAATGAGCCTACTCGCGTCGCTCACCGCTCGATTCGGTGACGAAGCTGCGTCAACGATGCTCGAAGCAGCTAGGAAGACGCCTACGACGAAACGTCTTGCTTCGAGTATCCAGAGAGAGCAGAGTCGACATTGGCTCAGCGTCAAGAAACATCCGGACGACATCTTCGTCCTACTGAAGCTCAATACCGCGACCTCTCGGCTGTTTGACCAGCCTCAACTGAACACGTGGGTGAGGTATGTGGACGCTTTCAATGAGGCCAACCCGACGAGTACAACGACGTTATTGTCCACCTTGCGGACACGATACAAGGAGGACACGTTGGCTCAAATGCTCGTCGTGGCGAGGACCAAAGGGGGCTCCGTGGGGCAAACCGCGACTCGAATTCAGGCGGAACAAACGAAACTTTGGCTGAAAAGTAACAAAACGCCGGGAGAGGTGTTCGAAATGTTACAATTGAAGAAATTGGGCACCAACTTCCTTAGTCACCCAATTTTCAATGCATGGGTGAAATACACGGACGACTACCGCAAGAAAAACCTAGGGACATATCGCTCTGCACTGACCACGTTGAGAAAAACCCACAGTGACGAAACGCTGGCGAAATTGTTCATTGAGGCGAGTAAAGTGGCGAAAACGGCGAAAATGGGGAAACGTCTGCATGCTGAGCTACTACGCGAATGGTCCCTCACTGGAGCGACACCCGTGCGGGTCTTTTTGCGTCTGAACCTCGGCAAAATGGACCCAAAGGTGTTTGAAAGTCCGCTGTACTCTATGTGGACGAGCTACATTTCCATGTTCAAAAAGGTGAACCCCACGTTCAAGGACGATCCAGTGAAGATGCTGGTATCGATCTATGGTCACAGAGACCTGACGGCCTTGCTCCTTGCGGCGGAGAAAGCTCCGAGTACTAAGGATATCGCCATTAAGTTGCAGAAGGAGCTGCTCGAGCTCTGGCAAGCAGCCAAGATGGACCCATCACGCGTCTACAGTGCACTGCATGTGGAACGTGAAGCCAAGAATTCACCCATTAGAATGTTCTGGAGTGAGTACGTCAAGGCCTTTAGGAACTCGAATTAAATAGTACTAATATAAAAATTAATACAAAGAAAGTATTACTGAAGTACTATTACTACTTAGTAATCTTTTTACTAACTAATCTTGAGAAAGCCCAGTGCTCTGGGGAGGCTTGATAAGTTTCGTAGCGCCGAAATTAGATTCAATCTCGGCTTGAGTCGGTGTAGGCTTCCACTTTCTTCAAGCCAAGCGCTCGACGAGCGTCGTGCGTTGACGAGGACTCGGTAGCCATGGGAGCAGCTGCGTCCATGGACGACCCCGACGCGTCCGTGGTTTTTAGAGAGGTTTGTATTCTTGTAAACTTTATTTTTTTAGCTAGTATTCTAACATTATTGTTGCAGACGAAAGAGGAATATGAGCGGAGAGCTGCTGCGGGTGACTCGAAGGAAGAAATTTTCTCCAGTCTTCGAGATATCGTTGCCACCCGTTTAGGAGGCACACACCCACTCCTCCACACGTCACAGAGTCTGGAATCCTTTCTCGTCCAAGATTTAGCAATATCTGAGGTAAAAAAATCTACGTGTTTTTTATCATCGGCTACTTGGTTGTCCACAACTCACCTCTCTTTTATTGTTTTAGTCTGGAGATCTAGAGAAACGTTCCAGCTCGTTGCTGGATGCTGCTACGTTAGCTGCAGTAACGACCTCGTCCGAGAGCAAAGAATCGACTATCGAGACCAGTATCGCACTAGCCAAGACGATACCCGACGACGACGACGTCCAATTGAATCCATTCCATGACGACCATGAAGTCGAAACGCACATCGATGAGCAGGGAATCCTCCGAGCAACTGTTGAAAAAGGCTTTGGACGGGCTGTGGGTTCGTCTCGGGCTGCACTTGATCTGGTGCAAAACCTCGCTCCTGTATTTACAACTGACGTACTAACTGACCCAACAACGCTCAAGATGATCCTCAAGTACGCAGAAGCCAACAAAGTGCCCAGCGGAGCGGATATGCTCCGATTTTGGGTCGAAATCGACGAACTGCAGCACCTTCCGTCCCATTCGTACACGCACCGTCGACTACGCAAGATTTACGACAAGTTTTTGTCCCCTGAAGCGCCTTCGCCTGTGTGTGTCACGGCGCAGATGCTGCAGGATATCGAGAAAGCCCTCGAGGGAGATAATATCTCCGCTGGCATCTACGCCGGTGCACAGCAAATCTGCTATATCGCGCTGGAAAAATCCGTGTATCCGCGATTCCGAGACAGCAAATTGTTCCGTAAGATGCAGGATTTCTGCGCTCCGGTCGTTCCTAACGCTGGCGCGTCATCTAACATTGGCTCGAGCAATGGACCTACTTTGTTAGCTGCTGCTTCGGCTACAGGAACGGTGGCTGCCAACATCACGGATAATATGGAAGATGCTGAGGATTATTCGCTGTTGGGTATTCTAGCACATCCAGCTAAGCTGCGCTTCCTCAAGACGTTCTGTATGGAAGCATTGGCGCTGGAAAATCTGCTTTTCTACCTGGAAGTCGAAGATTGCAAGAGATTGCCGAATCTGTCGTTCGTGGTCAACAAGACGCGAAAAATCTACGATCGCTACTGCTCTCCATCGTCCAAGAACTTTATTGTCGGGCTGGGAGACAAAGACGCGCTGAAAGAAATCCACGACGTGGTGGAGAACAAAGGAGCTCTCGTGCCAAAGTTATTCTACGAGGTCCAGATAGGCGTATTTAACCGGATTAGCGACGATATCTGGCCTGGGTTCTGTCGCTCCCAGGAATATCTGGATCATTCGAAAGAGGTCCAACCGGACGCGAAACATCTGGCACGACGCGGCAATCGCTTCGAAGAGAGCGAGGCGGTGCAAAAGAAACTGGAAGGCTTGGCAGAGCTCCAGCTCATCGACGCGGCCATGCATTATCCGGTCGAGAAGCTCATCCCTATCTCAGTTCCAGATTCTATCCAAGGGGCAGCTCGACGAAAATCCATTCAAAAACTCGAAGAGGAGCAGACTCTCACGCCTGAGCAGGAGCTGAAGCTGCTGTTGGGCGACCCATTCGMCAAGAAATATCTAAAGCTCTTCATGACTCGACGAGGCGTGGATTCTTTGCTTGCCTTCTGCGAAGAAGTGGAAGATTTTAAGCTGCTACCAGGTATCGAGTTTCTCCAACATTCCGCTAAGAAAATCTACCGCAAGTACATTATCCCCAGTGCTCGACTACAGGTGGATATGAGTNAAAACGATGCGAGAAGAGATTTTTACGCGACTCGCGAATCCAAGCGTGGATATGTTCAAGAAGATCGCGAACCGGGTACGTCACGGGATGCTGCAGGACTCGCTGCCTCGCTTCGTCAAGTCAAATTACTACAAGGATCTACGTCGGGATAGTAAAGCTACGCCTGCCGATCCCCACTTGGCCACAGTCGACCAAGCAGCAAAAGCAGGAAAGCTCGAGCTGTGCCACTTGGATGTCTTTCTAACATATCCAGGATGTATGCAAGCCTTCCGGAAGTTTCTGGATTTTCA

>Contig_84

GAAACCCACCACCTCTTGACCAGGTCCAGAGTCTGGTCCAGAGTCTGGAGGCTCCAGACTTAAGCCAACAGAGCAGGGTTCGCCAGTGTATGTGGAGGAGTTGGCAAGTACAGTACTGCTCCCCGTTTTCGCGCCCTTGAACCTTTGATAAGCTTTCGGATGTAATGATCCTGAATCCAAATGATCTGTTGTTTGTACGCCAAGATATGATTTATTTTCAAATCCTATATGGACCCCCCCCGCAAAAACGGGGTGCAGTACTGTATGGGGCTTTGTAATCCGAAACATTTTCAAATCACTGGTTCGTATTCAAAACCGGGCCCTGAGGTCCTGAATTTGTTGTAGGGCTTCGATTACGATCCAGCATTTAATGTAAATGAGTCTAAAAGTCGAAAAAGCCCTGGCCAGCCACTACAGTACATCGCACTGAGGTTCGAAGCCGAGCACTTATTCATGTCAGGTTATCCTTTAAAATCAATGCTTCTGCAGCGATAAGGCGTTCATACAGCCTCTGCACGTTACAGAGTACTTCGCATTATTGCGCATAAATAAAGGCATAAAATGGCGTAGATTTGATTTTTGATTTTGACGCAAAACGTTTTTTAGTTTTTAGGTCATCCTTGAATTTGATATTCCCTGTGTGCCAATCTGGTTACGAACCAGGTTGAAATTCTTCGAAGTACTGTACAGTGCCAATTTTATCTGAAGCTCTATCATCACCAACGGCCTGTTTGGCTTGGTCCACACTTCCTAATTTATGCTCAGCAACACATAGACACCGGCAGCAATACCAACAAGCTCAGTCATACCCCTTTGCTATGGCGTCTTACTCGCAGCAACACTTGTAGCTGGGAATAATGCGAAACCTGCAACAATTTGAAAACTTCGTCGCTATTACTTAAAACAAAACAGCTTGGATTTGCTTCATATACCTTTCTAAGCTGTTAAGGAAAAAAAAACTTTCACTTTATTTCACGTGTTGAGTGTACGGCGCAAGGCCGTAAAAAACTTTTTTACTTACGAGAAACTGGAGAATATGAGAGCACTTCATTTTAAACTTGAATTGCCAGTACCTGCTTCTCTGTGGACCCTAGTGCTACATGAGTAGTACGTCCAGCCCAATAAGGATTAATAATACTAATCAGTAGTACGTACAGCCCAATAAGTTTTGATATTTTTCATCCCTTACGTACTACTCATGTAGCACTACCAGGATAAATATGCTCGTACTCCATGACGCCAAGATTTAAATGGTTAATATCACTTTGCTTGCGTAAGCAAAAGCTGTTGCGGCAGGACTAGGGATATCTCCCTCGTGCATTTATTATATGATAAACGGCGGTGGGCTGCAAACATCTCTACGGAGCAGTAACCGAAACCATACGGTTAGCTCCGAAAAGGTACGTATACGGAACTGATCAGATTCTAGATCAGATATTTATCTTACTGGAGATCTGACATGGGATCTGAAAATGATTGGTAAGTTCACGCACTTCTTCAGCCTCGAGGCTGCTCGTGCGTTGGTCAGTGGAGAAGATGAATCGAGAGGCAAACGAGGGCGCGTCGGTAGATGCCGAGGTGGAGCCTGCTGAGCCGGCTGCGGGCGCGCCGGAGGATGGCGAAGTGGAGCCAGCTGAGCCGGTCGAGGACGTCGTTAGCACTGAGTTGCTGTTCGAGCAAGCTCGACAGCAACTCGCAAATGATGTGGGGTCTGTGCCACGACGTGGTGAGCGGACGACATGCTTCTACAAGGGCTATAAGTACTGAAAGGCATGGTCGTCCTCAAGGAAAATTGTGTACAGATGCTCTACCAAGGTTGCAGAGGAAAGCTGGAGTTTACTATTGCATCGATGGCGTATGCTGCTGTAAAGCTGCACACATGCCGCACTGAAGCCATTGCCAACGTCGTCATCAACGTTGAGGACCAGATGAAAGCTCAAGCCGACCTTCTTGCGATTGAGCACGTCGCTTGGCCTGTGCGCCAAGTGTGGGAAGAACTGCGACGCCAGTTCTACTCAGCTGACAATCCCAACGTTGTTCGTGGGTTGTCAGAGCAACAAGTTGTCCGACGAGTACACCGAGCAAGAAGCGCGCATTACTCTAGCAATGTTCACGGCTCTATTGAAATTCCACCGCTTTCATTGGCACTAGACGAGGAGCTTTCCTTTTTCCAGTTTCACTACGTCACCATTAACCGCAACGATTTGAACAAGCCTTCCCGCTTGCTTGGGTGGGCGCATCCATCTCTGGTCGCGCTACTACGCTATCATGGTACCACGTTGTTTGTGGATGGCACTTTTCGGTGTGTCCCACAGGGTTACGCACAGTGCGTTGTGTTCATGGTCCACGACCGAGCCTCTGGAGTGTTCGTGCCGGTGTTCTACATTCTGAGCACCTCGCGTACTAACGACGCCTACGGGGATATGATTCATTTGATCGTACAATCAACGGACCAGCAACTCGAACCGGCCGAAATTGTTTGTGACTTCGAAGCACCACTGTTGGACGCACTACAAACACAGTTTYCGAACGCAATTGTGTTGGGGTGCTTGTTTCATCTGAAACAAGCACTCCGACGGGCTATGAAGCGCTATGCTATTCCGGAGGAAGAGTGTCTGATTGCAATGACACGTGGTGTCCTAGACACCCTGACGGTGATTGATCCTGCTCACATCGAGCGAGGTATTAAGTGGGTAAAGCGTGAGATCAAGTTGCGCTATGCTCAAGCTGGCGTGACCTACTCTACTGAAAAATGGGCCGATTTTTGGGGGTATTTCAACCGCACTTGGCTGGAGCAGTATACCCTCGATGTCTGGAACGTATTCGGCATGAACAACGAGTTGGTCGCACGAACGAATAATCCCCTGGAGAGGTTTAACAGGGAGCTTAATACACGTTTCCCAACCCCGCATCCTTCAATGGCGACATTTGTGACGGTGATCAAGGCTATATCAGCTGAGTACGTACGCCGCGTTGCTGATGTTCCTCGTGGGCGAGCCCGCCGTGTTCCTCGCGAGGTCATTCAGCTACCACAGGTTGTGGACATTCCCTCTGACGTTGACAGCGACGTGGATCCTCCACTGGAGGAATCGGAGGCAGTTGCTACACCCGCAGTGGACGGTAGTACACTCACTAGTGCTACGTTGTAGATATGTCCGTGTAGCAACCAGAAGACCTCGGTAAATGCTTCATCTTCTCCCCTTTAAAAGTGTTTTTCTTTGGTTTCAGTCTGTTCATAATTCAGGTCTCAAATCCTGATCTTTAATCCGATCAGTTCCGCATACGTACCATTTGGAAATCAACCAACCATACACTGTTTTGCATGGATTTAAGTGCGATGCATGCTCCAGTACCTGACTACGTGAGGCGTAGTTTTGAAGAAATGAGTTTTATCAAAAGTAGAGAAATTTTAAATTGCAAATTCACTTTTTTATTGACCGTAAGCTTAAATCGATCTCCAATTTCAGTTTACCTTGCTCAATAAACATCAAAAGGATCATCAATTCCCTAATAGTCCCCACTGAATCTTTGTGACACCCTTACTTGCCATCCTTCAGTGCCATGAGGGCTTTCAAGGCCTCTTTCTCTTTTTCAGTAAGATCTTTGACAGCATTGCCGGCCTGTTTCACTTTTTTGACGGAGGCTTTCGATAACTTCTGTATGGCTTCGGTGGCATCTGATCTCTTGATGACATCAACGATGCTGCCAAAACCTCGATCTTCTTCATTGGTCGTCGAGTCATCATCTTCCGGGCCTGCGACTCGAAGAAATCTCTTCCGGGAAACAGTATTGGTCATATCGCTGGCTGCGAAGTCGGGAGAGACCTTCGACAAGAGCTGAGATTCATCGGCATTCGTGAATGCTGCGACGGCACTGCAGCGAGCTAAGACGGCGACGACAAGAACGATGTAGAATGTACTGCGCATGAGTCGGATGGGGCTACGAGGGCACTTGTGGTATTGAGGGCGAAAAGTCAAGTGACTGCCGGCGATCTTTCGTTTGGTCACTGGTCCAGTGGTTAATACACGAACATTGAGGGTTCATCATCATGAAGTCACGATGGGGACGTACGGCAATCATAACCGTCGGTTACAGTAAACACGAAAGCACCATTAGCAAATAGTATACTTTAAAAAAGTGAGCACTACGCCCTGAAGGGGGTAAATACGAGTGCAAATCAAAACGGAGAAGACAAACCAGGGCGAGCGGATGCCAGGTCGCAAGAAGGCCCGTGTCGCATTTGCGCAGGCGGGCCCACCGCCGATCCTTCTAGCGAGCCNGGACATTGCTGTCTCAAGGTTGAGATAATCTTTTTAGTGGAGGAGCGCAGCGATTTGTCGACGACACACCCGCTAGGTATTTTCGGTTTTTGATGTCTAAACAACAAAGTTGCTGTCTTCACAACTTGAAAGMAAACAATGATCAAGTTCTATTCTTTAGCACATATGAGCTCTTTCTGTTCATTCTGAAACTATCTACAGTTTAATTTGGCAGTTCAGCCTGTTAGGTCACAGAAATCCACTCAGGTAACNTTTTTTTTTTAGCAGAAAAACAATTGGAGCTATTTTTTCAGTCGATAATTGAAGCGTAAGTAAAGCATAATGCGACCAGATAGCCTTGCAATCTGCATATGTACAGTGTGAACCAGCTTAATCTGAATTTTGGAATTTGGAGGTACATAATTTGCTCTATTTTGACTCAAAATTTTTGAGAGATCTTTATAGTGATAAACATGATTACGAGCACCAAACACGATTGTGAGCACCAAACACGATTGTGAGCACCATAATCATGTCAAAATGTGATTGCTCGAACTAGAGTCGCTGACAGCGGGAATGGACACACAGCGAGTCACTTTGTGTCCATTTCCTGCTGCTCCTGGCGGTACAGACGCGCCAATTCGTCTCTCAAGTCTCGTAGATTACGCTGGGTCACCCAACCTGGGTGTGGACCAGTCGCTGGGAAAGCGCGGCTTTGGGGTGTCACGATCCGCCAGGAGTTGTCGGCTCTCATCACGCAGCATTTGCGTCGCGATATGGAGCGTGTCGTCAATTACGACTCGTCATGCACTTTGTTGCATGATGGAGAGGAGAGCAGACGAGGCTCTTTTCTGGTCGTTGCCAACGACTCGTTGGACAGGTCATTTGGGCCAAATTTTAGCGAGGAGCGTGGCTGGTTGGGCGAGCGCGCACGGGGCGACTTGATTTACGTACTCTATGTTCGTGCATGGAGTTGCTTTTTGAATTATGAAACGCAAGATTTATCGTCGTTTCGANGCGTAATGAAATCGACATGGCAGAAAGTCAGTGAATACTCTTTATAAAAACAGCGATAATTATATATATTGTTCTTACAGAATAAACACATTTGGGGGGGCGAATATCGTACTTAAAAGGAATTTAAACTATTACAGTAAAAGTAATTCAAATTTTAAACATAGGATGTCCGTTTTAGCGTAAGACGGACATCCTATGTTTTTCAACAAAATTATATAAAACATGAAGGCGTAAGTATGAAATGACCCAGAATGGATTCAGTGAAACCTGCATTCGTAGGACGGTCGTCTTACGCTTAAAGATCGACTCCTCTTGGAGAAATGTTTTGGCACTCATTTTGCGACGCAAACATGGAGACCGTCAGGCATAACTTCACTCCTGCACCTGGTTCCCGCTGCCGAATTACCCAGTACATGTACTAGTGTAGCGTCAGCCCCTGTGCGAAATGCATAGCACAATCAGCAAGAGAAACTGTTAATTTCTTGAGGATAACTGTGCCGCTAAAGGCGTCTCGCTTTATGTCAAACCTTTATAAAAATGTACTCAATGTTTAAAGCTGTAAATTCACAGTTCGACGGCATCTAATTAATCTTGAACGATCTTGTTTTAGCTCTAAAGTAATACTATTGTATTAGTAAATGAAATATATACACTGTACTGTAGTAGTTTTAATTCACGAAATAGTAAAAAAGCATTAAGATTTATAAATTCCTGTTTATGACGATTTTACAATGATTTCCGTAAATGCACTACCTGCTCAACTCTCTGGTATTATTAATTGATTGCAAGATAAGGCGTGGACACTTAAACAAAAGTCGTTCTGTTTCCTCCTCTTACAAACCCTAATGTCTACATAAAGTTGATCCTTGCCGAGTTTGGGACGGACGGTGTTGAGCTGCTTAATCAACGAAAGGTCCGCGAACTTTGGGATCTTGAATAAAGCCGAAAATTGGCCAAGCTGGGGAGTCGGGATCGCCCTCTCTTCGCCCTTGTCACCCAACTCAGTACTTCCTGTAAGATAGCGGTGGCCATCGTTTACGCGTTCGTAAGGTGATCCAATCACCAAGTTTGAAGTCTCGACAGTTTTATCAGCACTTACAATTGCTTGCCAGCGCCAGTAGGAAAAACTGTATTCCGCGCATGATTGATTCCAAGTTGCACCTGTCCTGTTCGGGTCGAAATCGTGCGTGCGATGACTGTGGAATTAGTTTAAGAGCCACATTCTGCCGGTTTGCTCCCCTGTCGACTAATCGTCTGAATCAGTTGGAGTAGGATGAGTTTAAATAGGGTATAGCTACAAGCTGCTTGTGATTGCCTCAAGATGTGGATAGGATCTAAAAATTCAGATGTATTTAAAATCGCAACCCTTTGAGGTTTCACATTTGAGACAAATAACAAAATCAGAAAAGTACGCAAATTCCACTAAAAAAACTACTGTATCTGAGTTATTAAGTTCGTATCCAGACTTTGAAGCACCAAGTCACTTCGTGAACATGGCCTTCGCGGCAAAACATATACGTTGAACATGACCGAGGTCAACCTAGAATTTATTGAGCTTTTTTTTACCCTTACACGAACGAAGAGGCAAAATGCCCCTATTGATTTGTCCACGGTACTATCGCTACGACTATAATATTACAATTGAAAGTAAATGTTATGTTAGCAAAATTAAAAGGGATAGTAAGGTACTACCCAGCTAAATGATTGTTAAAAAAAGATATTATTACTACAGCAATTTCAATGAAATCGTGGCTGTCGCTCGCATGCATGTTAGCTCGGCACAACATCTATTTGCATGTTTTGCTGTGCTTGTTCGCAATCATCAAGTGTTCGCCACTGAGATCAATGCATCCAGCTCAACACTGGCGCTTTTCTAGTTTCTCGCTCTGCTCTCAGAAACGCTCATCGCGCTCACTGTTGCTGCGGTGAAAGTAGTCGATCGTTTGATCAAGGAGTACTTAAGCATTTATGAGCTTCGTCCTGGCTTCTTCTTTTGAGCCTTGAGGCTAGCAAACCCACCAACGCACCAACGACGTCTGCATACGCGCTCCAGCAGCCTTTGTCAAAGCTTACTATCATTACTTGCAACGCTTGGTACCAAATCGACTCATGAGAGCAAAGCGTGTAAGTGACTTGAACCAACTATGGATGCTACACATACACTACAAAGAATGATACTTATATCAAATTTTATACAAAACTCAAAATGTTATGATAAGTTTGTTAATCTCGTTGAGGAAAAGCTACTACAATTTCCGTATTCTACTACTAGTTCACGATGTAGCGAAACGTTCTGGACCATTTCCTTATATATCCTTTGGACCTCCCTTCGAATGACTTCTTCAAGCATGACAGGCCGGCGCAAGCGAAGCTTAGAGAGTGCACTTGTAGACTCAAGCAAGCGCACGAGACCTAGGCGAGGTTCAGCGGCGTGTGGAGCAGAG

>Contig_88
[truncated: 3,884,629 more chars]
