# Supplementary material for: Identification of Avramr1 from Phytophthora infestans using long read and cDNA pathogen‐enrichment sequencing (PenSeq)
Source: Mol Plant Pathol. 2020 Sep 15;21(11):1502–12. doi: 10.1111/mpp.12987 (PMC7548994; doi:10.1111/mpp.12987)
Supplement: Supplementary file 10 — NOTES S5 Protein sequences of Avramr1 homologs [file MPP-21-1502-s010.docx]

>Avramr1-1B1

MRLSTIILAVSAVTLVAGVNALCEAANVDQAMVMPLTYANSVSEDLNNADGKRYLRSSENEERLGGANMFNIKKLEDALNDTTYAKTLFRRWKRSGVNEDTITTKFKNMQISLDENALELIQSYRIWLNAHATKTNPKLFDRAKIKKALEDGTYANVLYGRWKRYGFESDDVFKRFQRMGVNKDDNLYQVYKNYVTWLNVHHPLKKTTLTTPEAFLFYPSRIQRAKSDPAFAERLFSKWKSSGLDEGPVYKKLWDMGLKKDNAIYKLYTDYVLWLEKHFPLPAKATN

>Avramr1-1B2

MRLSTIILAVSAVTLVAGVNALCEAANVDQAMVMPLTYANSVSEDLNNADGKRYLRSSENEERLGGANMFNIKKLEDALNDTTYAKTLFRRWKRSGVNEDTITTKFKNMQISLDENALELIQSYRIWLNAHATKTNPKLFDRAKIKKALEDGTYANVLYGRWKRYGFESDDVFKRFQRMGVNKDDNLYQVYKNYVTWLNVHHPLKKTTLTTPEAFLFYPSRIQRAKSDPAFAERLFSKWKSSGLDEGPVYKKLWDMGLKKDNAIYKLYTDYVLWLEKHFPLPAKATN

>Avramr1-1D1

MRLSNIILVVNALTLVAGDNALCGAAAADQAMVMPLTYTKHVSEDLSNADGKRYLRSSDDEERLGGANTFHIKKLEDAFIDTTYAKTLFRRWKRSGVNEDTITTKFKNLQISLDENALELIQSYRIWLNAHATKTNPKLFDRAKIKKALEDGTYANVLYGRWKRYGFESDDVFKRFQRMGVNKDDNLYQVYKNYVTWLNVHHPLKKTTLTTPEAFLFYPSRIQRAKSDRAFAEKLFSKWKSSGLDEGPVYKKLSGMGLAKDKTTYKLYTDYVLWLDKHFPLPPKATN

>Avramr1-1D2

MRLSNIILVVNALTLVAGDNALCGAAAADQAMVMPLTYTKHVSEDLSNADGKRYLRSSDDEERLGGANTFHIKKLEDAFIDTTYAKTLFRRWKRSGVNEDTITTKFKNLQISLDENALELIQSYRIWLNAHATKTNPKLFDRAKIKKALEDGTYANVLYGRWKRYGFESDDVFKRFQRMGVNKDDNLYQVYKNYVTWLNVHHPLKKTTLTTPEAFLFYPSRIQRAKSDRAFAEKLFSKWKSSGLDEGPVYKKLSGMGLAKDKTTYKLYTDYVLWLDKHFPLPPKATN

>Avramr1-6B1

MRLSTIILAVSAVTLVAGVNALCEAANVDQAMVMPLTYANSVSEDLNNADGKRYLRSSENEERLGGANMFNIKKLEDALNDTTYAKTLFRRWKRSGVNEDTITTKFKNMQISLDENALELIQSYRIWLNAHATKTNPKLFDRAKIKKALEDGTYANVLYGRWKRYGFESDDVFKRFQRMGVNKDDNLYQVYKNYVTWLNVHHPLKKTTLTTPEAFLFYPSRIQRAKSDPAFAERLFSKWKSSGLDEGPVYKKLWDMGLKKDNAIYKLYTDYVLWLEKHFPLPAKATN

>Avramr1-6C1

MRLSNIILVVNALTLVAGDNALCGAAAADQAMVMPLTYTKYVSEDLSNADGKRYLRSSDDEERLGGANTFHIKKLDDAFIDTTYAKTLFRRWKRSGVDEDTVTTKFKNMQISMDENALELIQSYRIWLNAHATKTNPKLFDRAKIKKALEDGTYANVLYGRWKRYGFESDDVFKRFQRMGVNKDDNLYQVYKNYVTWLNVHDPLKKTTLTTPEAFLFYPSRIQRAKLDRAFAERLFSKWKSSGLDEGPVYKKLSGMGLAKDKPTYKLYTDYILWLDKHFPLPPEATN

>Avramr1-6D1

MRLSNIILVVNALTLVAGDNALCGAAAADQAMVMPLTYTKHVSEDLSNADGKRYLRSSDDEERLGGANTFHIKKLEDAFIDTTYAKTLFRRWKRSGVNEDTITTKFKNLQISLDENALELIQSYRIWLNAHATKTNPKLFDRAKIKKALEDGTYANVLYGRWKRYGFESDDVFKRFQRMGVNKDDNLYQVYKNYVTWLNVHHPLKKTTLTTPEAFLFYPSRIQRAKSDRAFAEKLFSKWKSSGLDEGPVYKKLSGMGLAKDKTTYKLYTDYVLWLDKHFPLPPKATN

>Avramr1-13A1

MRLSTIILAVSAVTLVAGVNALCEAANVDQAMVMPLTYANSVSEDLNNADGKRYLRSSENEERLGGANMFNIKKLEDALNDTTYAKTLFRRWKRSGVNEDTITTKFKNLQISLDENALELIQSYRIWLNAHATKTNPKLFDRAKIKKALEDGTYANVLYGRWKRYGFESDDVFKRFQRMGVNKDDNLYQVYKNYVTWLNVHHPLKKSTLTTPEAFLFYPSRIQRAKSDPAFAETLFAKWKSSGLDEGPVYKKLWDMGLKKDNAIYKLYTDYVLWLDKHFPLPAKATN

>Avramr1-13A2

MRLSTIILAVSAVTLVAGVNALCEAANVDQAMVMPLTYANSVSEDLNNADGKRYLRSSENEERLGGANMFNIKKLEDALNDTTYAKTLFRRWKRSGVNEDTITTKFKNLQISLDENALELIQSYRIWLNAHATKTNPKLFDRAKIKKALEDGTYANVLYGRWKRYGFESDDVFKRFQRMGVNKDDNLYQVYKNYVTWLNVHHPLKKSTLTTPEAFLFYPSRIQRAKSDPAFAETLFAKWKSSGLDEGPVYKKLWDMGLKKDNAIYKLYTDYVLWLDKHFPLPAKATN

>Avramr1-13B1

MRLSTIILAVSAVTLVAGVNALCEAANVDQAMVMPLTYANSVSEDLNNADGKRYLRSSENEERLGGANMFNIKKLEDALNDTTYAKTLFRRWKRSGVNEDTITTKFKNMQISLDENALELIQSYRIWLNAHATKTNPKLFDRAKIKKALEDGTYANVLYGRWKRYGFESDDVFKRFQRMGVNKDDNLYQVYKNYVTWLNVHHPLKKTTLTTPEAFLFYPSRIQRAKSDPAFAERLFSKWKSSGLDEGPVYKKLWDMGLKKDNAIYKLYTDYVLWLEKHFPLPAKATN

>Avramr1-13C1

MRLSNIILVVNALTLVAGDNALCEAAAADQAMVMPLTYTKYVSEDLSNADGKRYLRSSDDEERLGGANTFHIKKLEDALNDTTYAKTLFRRWKRSGVDEDTVTTKFKSMQISMDENALELIQSYRIWLNAHATKTNPKLFDIAKIKKALEDGTYAHVLYGRWKRYGFESDDVFKRFQRMGVNKDDNLYQVYKNYVTWLNVHDPLKKTTLTTPEAFLF

>Avramr1-13C2

MRLSNIILVVNALTLVAGDNALCGAAAADQAMVMPLTYTKYVSEDLSNADGKRYLRSSDDEERLGGANTFHIKKLDDAFIDTTYAKTLFRRWKRSGVDEDTVTTKFKNMQISMDENALELIQSYRIWLNAHATKTNPKLFDRAKIKKALEDGTYANVLYGRWKRYGFESDDVFKRFQRMGVNKDDNLYQVYKNYVTWLNVHDPLKKTTLTTPEAFLFYPSRIQRAKLDRAFAERLFSKWKSSGLDEGPVYKKLSGMGLAKDKPTYKLYTDYILWLDKHFPLPPEATN

>Avramr1-13D1

MRLSNIILVVNALTLVAGDNALCGAAAADQAMVMPLTYTKHVSEDLSNADGKRYLRSSDDEERLGGANTFHIKKLEDAFIDTTYAKTLFRRWKRSGVNEDTITTKFKNLQISLDENALELIQSYRIWLNAHATKTNPKLFDRAKIKKALEDGTYANVLYGRWKRYGFESDDVFKRFQRMGVNKDDNLYQVYKNYVTWLNVHHPLKKTTLTTPEAFLFYPSRIQRAKSDRAFAEKLFSKWKSSGLDEGPVYKKLSGMGLAKDKTTYKLYTDYVLWLDKHFPLPPKATN

>Avramr1-23A1

MRLSTIILAVSAVTLVAGVNALCEAANVDQAMVMPLTYANSVSEDLNNADGKRYLRSSENEERLGGANMFNIKKLEDALNDTTYAKTLFRRWKRSGVNEDTITTKFKNLQISLDENALELIQSYRIWLNAHATKTNPKLFDRAKIKKALEDGTYANVLYGRWKRYGFESDDVFKRFQRMGVNKDDNLYQVYKNYVTWLNVHHPLKKSTLTTPEAFLFYPSRIQRAKSDPAFAETLFAKWKSSGLDEGPVYKKLWDMGLKKDNAIYKLYTDYVLWLDKHFPLPAKATN

>Avramr1-23B1

MRLSTIILAVSAVTLVAGVNALCEAANVDQAMVMPLTYANSVSEXLNNADGKRYLRSSENEERLGGANMFNIKKLEDALNDTTYAKTLFRRWKRSGVNEDTITTKFKNMQISLDENALELIQSYRIWLNAHATKTNPKLFDRAKIKKALEDGTYANVLYGRWKRYGFESDDVFKRFQRMGVNKDDNLYQVYKNYVTWLNVHHPLKKTTLTTPEAFLFYPSRIQRAKSDPAFAERLFSKWKSSGLDEGPVYKKLWDMGLKKDNAIYKLYTDYVLWLEKHFPLPAKATN

>Avramr1-23B2

MRLSTIILAVSAVTLVAGVNALCEAANVDQAMVMPLTYANSVSEDLNNADGKRYLRSSENEERLGGANMFNIKKLEDALNDTTYAKTLFRRWKRSGVNEDTITTKFKNMQISLDENALELIQSYRIWLNAHATKTNPKLFDRAKIKKALEDGTYANVLYGRWKRYGFESDDVFKRFQRMGVNKDDNLYQVYKNYVTWLNVHHPLKKTTLTTPEAFLFYPSRIQRAKSDPAFAERLFSKWKSSGLDEGPVYKKLWDMGLKKDNAIYKLYTDYVLWLEKHFPLPAKATN

>Avramr1-23C1

MRLSNIILVVNALTLVAGDNALCGAAAADQAMVMPLTYTKYVSEDLSNADGKRYLRSSDDEERLGGANTFHIKKLDDAFIDTTYAKTLFRRWKRSGVDEDTVTTKFKNMQISMDENALELIQSYRIWLNAHATKTNPKLFDRAKIKKALEDGTYANVLYGRWKRYGFESDDVFKRFQRMGVNKDDNLYQVYKNYVTWLNVHDPLKKTTLTTPEAFLFYPSRIQRAKLDRAFAERLFSKWKSSGLDEGPVYKKLSGMGLAKDKPTYKLYTDYILWLDKHFPLPPEATN

>Avramr1-23D1

MRLSNIILVVNALTLVAGDNALCGAAAADQAMVMPLTYTKHVSEDLSNADGKRYLRSSDDEERLGGANTFHIKKLEDAFIDTTYAKTLFRRWKRSGVNEDTITTKFKNLQISLDENALELIQSYRIWLNAHATKTNPKLFDRAKIKKALEDGTYANVLYGRWKRYGFESDDVFKRFQRMGVNKDDNLYQVYKNYVTWLNVHHPLKKTTLTTPEAFLFYPSRIQRAKSDRAFAEKLFSKWKSSGLDEGPVYKKLSGMGLAKDKTTYKLYTDYVLWLDKHFPLPPKATN

>Avramr1-23D2

MRLSNIILVVNALTLVAGDNALCGAAAADQAMVMPLTYTKHVSEDLSNADGKRYLRSSDDEERLGGANTFHIKKLEDAFIDTTYAKTLFRRWKRSGVNEDTITTKFKNLQISLDENALELIQSYRIWLNAHATKTNPKLFDRAKIKKALEDGTYANVLYGRWKRYGFESDDVFKRFQRMGVNKDDNLYQVYKNYVTWLNVHHPLKKTTLTTPEAFLFYPSRIQRAKSDRAFAEKLFSKWKSSGLDEGPVYKKLSGMGLAKDKTTYKLYTDYVLWLDKHFPLPPKATN

>Avramr1_(PITG_07569)

MRLSTIILAVSAVTLVAGVNALCEAANVDQAMVMPLTYANSVSEDLNNADGKRYLRSSENEERLGGANMFNIKKLEDALNDTTYAKTLFRRWKRSGVNEDTITTKFKNLQISLDENALELIQSYRIWLNAHATKTNPKLFDRAKIKKALEDGTYANVLYGRWKRYGFESDDVFKRFQRMGVNKDDNLYQVYKNYVTWLNVHHPLKKSTLTTPEAFLFYPSRIQRAKSDPAFAETLFAKWKSSGLDEGPVYKKLWDMGLKKDNAIYKLYTDYVLWLDKHFPLPAKATN

>Avramr1L_(PITG_07566)

MRLSNIILVVNALTLVAGDNALCEAAAADQAMVMPLTYTKYVSEDLSNADGKRYLRSSDDEERLGGANTFHIKKLEDALNDTTYAKTLFRRWKRSGVDEDTVTTKFKSMQISMDENALELIQSYRIWLNAHATKTNPKLFDIAKIKKALEDGTYAHVLYGRWKRYGFESDDVFKRFQRMGVNKDDNLYQVYKNYVTWLNVHDPLKKTTLTTPEAFLF

>PcAvramr1

MRLSVILLAVSALALLENGSALSASVDVDEGTMVSKMAPAEAELLRVGLSNGDKKRFLRNHDDEERLAGANMFNIKKLEEALNNAGYANTLFQRWKRHGVDEAKVSRKFKNMGISTDTNAQELAQSYRTWLSAHAANIDPKLFDKAMIYGTLEDVTRAKELFGKWKSYGLESDNVFKKFQTMGVRNDNDLYKVYLNYVAWLNTHHPLKETKLTSAQEFLFHPSRIKRAKSDSAFAENLFAKWKDSGLDELPVYNRLRKMGLGKDNAIYDMYRNYVFWLDKHFPLPAIATT

>PpAvramr1

MFDIKKLDDALNDAGYANTLFQRWKSHDFDDAKVLEKLKSMGISINEDAEWLLQSYRTYLNSHSLKGDPKLLFDRAKIDEALTNGKSTNALFGKWKSYGYESDDVFKKFQTMGVRTDDSLYNVYLKYLAWLNTHHPLKYTKPTTADEFLFDSTRIKNAISDSVFAEKLFAKWKGSGLDERPVYDKLWKMGLKNDDDLYQLYRSYVFWLDEHFPLPAKASTP
